# Substrate-Directed Enantioselective Aziridination of Alkenyl Alcohols Controlled by a Chiral Cation

**DOI:** 10.1021/jacs.3c00693

**Published:** 2023-03-24

**Authors:** Alexander Fanourakis, Nicholas J. Hodson, Arthur R. Lit, Robert J. Phipps

**Affiliations:** Yusuf Hamied Department of Chemistry, University of Cambridge, Lensfield Road, Cambridge CB2 1EW, U.K.

## Abstract

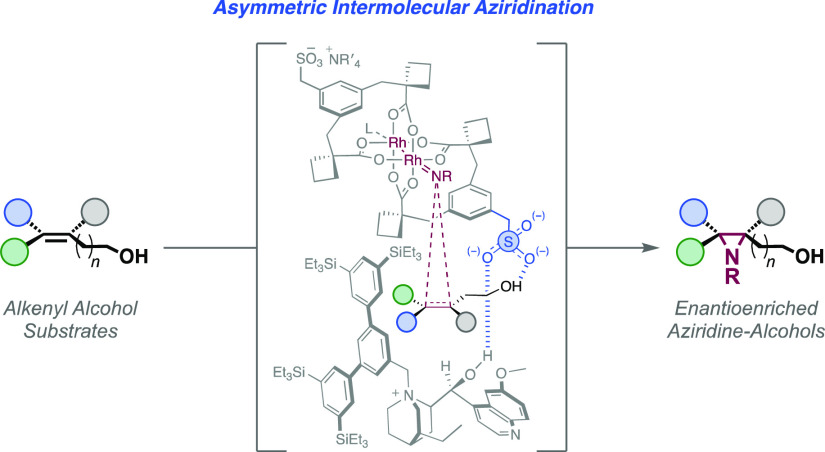

Alkene aziridination is a highly versatile transformation for the construction of chiral nitrogen-containing compounds. Inspired by the success of analogous substrate-directed epoxidations, we report an enantioselective aziridination of alkenyl alcohols, which enables asymmetric nitrene transfer to alkenes with varied substitution patterns, including those not covered by the current protocols. We believe that our method is effective because it is substrate-directed, exploiting a network of attractive non-covalent interactions between the substrate, an achiral dianionic rhodium(II,II) tetracarboxylate dimer, and its two associated cinchona alkaloid-derived cations. It is these cations that provide a defined chiral pocket in which the aziridination can occur. In addition to a thorough evaluation of compatible alkene classes, we advance a practical mnemonic to predict reaction outcome and disclose a range of post-functionalization protocols that highlight the unique synthetic potential of the enantioenriched aziridine-alcohol products.

## 1 Introduction

**Figure 1:**
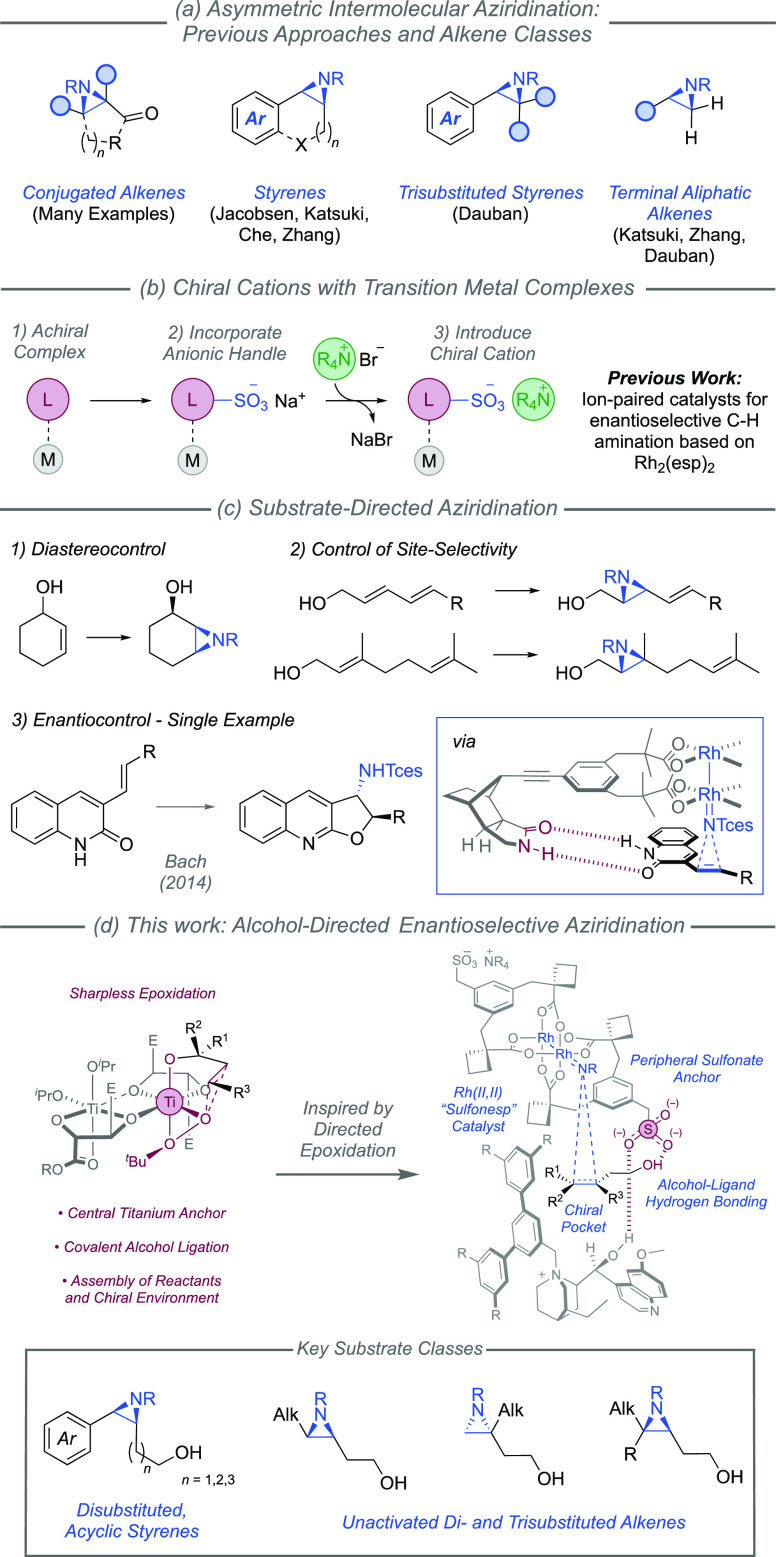
(a–d) Enantioselective intermolecular alkene aziridination: previous work and our approach.

Oxygen and nitrogen are the two elements most commonly found in small molecules after carbon and hydrogen, and methods for their introduction are of fundamental importance. For oxygen, alkene epoxidation is one of the most widely explored, largely due to the remarkable versatility of the epoxide for further synthetic elaboration. A powerful array of catalytic asymmetric protocols exists for this transformation, and as a result, retrosynthesis to a chiral epoxide is frequently a key disconnection when planning an asymmetric synthesis.^1^ Given the similar prevalence of nitrogen atoms in molecules of societal importance, alkene aziridination has the potential to be equally enabling,^2^ and great advances have been made in intermolecular asymmetric aziridination over the past three decades (Figure 1a).^3^ Olefins conjugated with electron-withdrawing groups were the first to be subjected to the asymmetric transformation, as in Evans’ aziridination of cinnamate esters using copper-bis(oxazoline) complexes.^4^ More generally, alkenes conjugated with carbonyl groups have since proven amenable to a variety of asymmetric organocatalytic methods.^3d^ Electron-neutral alkenes are typically aziridinated using transition metal nitrene chemistry,^5^ and a number of enantioselective systems have been reported.^3e,6^ Styrenes are the substrates of choice when benchmarking new catalytic asymmetric aziridinations, and key examples include systems reported by Jacobsen^7^ and Katsuki^8^ for styrene aziridination alongside the later protocols disclosed by Che^9^ and Zhang^10^ employing metal porphyrin systems. Enzymatic catalysts refined through rounds of directed evolution have also been successfully applied to styrene aziridination and offer the added advantage of furnishing products containing unprotected nitrogen atoms in certain cases.^11^ Beyond styrenes, a handful of the methods described above have been applied to terminal aliphatic alkenes with high levels of enantioselectivity.^8d,9c,10b^ Most recently, Darses, Sircoglou, Dauban, and co-workers disclosed a highly enantioselective intermolecular aziridination using rhodium(II,II) paddlewheel complexes bearing chiral carboxylate ligands.^12^ This system is very well matched to challenging trisubstituted styrenes as well as terminal aliphatic alkenes and arguably constitutes the state-of-the-art for intermolecular asymmetric alkene aziridination. Despite these advances, there remain significant gaps in the scope of alkenes that can be accommodated by the present methods. Although terminal styrenes are well explored, any substitution on the alkene tends to be limited to cyclic systems, with a few notable exceptions.^8d,12^ In particular, systems for the functionalization of unactivated alkenes that are di- or trisubstituted are very rare and represent a foremost challenge. For aziridination to be considered a viable and general disconnection in asymmetric synthesis, conceptually novel catalytic approaches capable of expanding reaction scope, particularly with respect to the underrepresented alkene classes, are needed.

We recently developed an unconventional approach for the control of enantioselectivity in transition metal catalysis; in comparison to a more traditional strategy in which chirality is embedded within the ligand architecture, ours locates it “off-complex” on a cinchona alkaloid-derived chiral cation, ion-paired with an achiral transition metal complex that has been rendered anionic through the attachment of a sulfonate group (Figure 1b).^13−15^ Our first proof-of-concept study achieved asymmetric, desymmetrizing C(*sp*^2^)–H borylation using iridium catalysis, and we further validated the approach on C(*sp*^3^)–H amination, wherein a chiral, ion-paired version of Du Bois’ Rh_2_(esp)_2_ catalyst^16^ mediated selective nitrenoid C–H insertion at a prochiral methylene.^17^ We speculated that our ion-paired Rh “Sulfonesp” catalysts developed for that work might also be capable of discriminating between prochiral alkene faces. In our previous report, we believe that hydrogen bonding interactions between a functional group on the substrate and the anionic sulfonate group on the ligand are key and that in this case could allow us to develop a substrate-directed asymmetric aziridination reaction with the potential for complementarity with existing approaches. The advantages of substrate-directed catalysis^18^ are evident in the field of enantioselective epoxidation where the Sharpless asymmetric epoxidation (SAE) occupies a foremost position.^19^ In that process, the caveat of requiring a specific functional group is compensated by the fact that terminal alcohols are ubiquitous, and the resulting reaction is highly effective and general. Substrate-directed strategies have barely been explored for enantioselective aziridination, although they have been used to exert control over diastereoselectivity^20^ as well as site-selectivity (Figure 1c, upper).^20a,21^ To the best of our knowledge, the only substrate-directed aziridination to control enantioselectivity is that developed by Zhong and Bach in which a chiral lactam motif^22^ is appended to a Rh_2_(esp)_2_-derived complex (Figure 1c, lower).^23^ In this system, dual hydrogen bonding interactions with a quinoline-containing substrate present the alkene to the Rh-nitrenoid in a chiral environment. Clearly an important demonstration, it nevertheless necessitates specific substrate types. In this article, we describe the development of a primary alcohol-directed enantioselective aziridination using our “chiral cation” approach. We systematically explore the relationship between alkene substitution and enantioselectivity and use our data to develop a straightforward predictive mnemonic that we envisage will allow users to apply this reaction with confidence (Figure 1d).

## 2 Results and Discussion

### 2.1 Reaction Optimization

We began by evaluating the aziridination of bishomoallylic alcohol **1a** using similar catalysts and reaction conditions to those employed in our previously reported C–H amination (Table 1). If the reactions were analyzed immediately after workup, it was possible to observe and isolate the aziridine product **3a**. However, when the crude reaction mixtures were left in solution, 5-*exo* cyclization to **4a** became apparent. As a result, during optimization, a combined yield of (**3a** + **4a**) was recorded. Following ^1^H NMR analysis, the enantiomeric excess (*ee*) was then determined from **4a** after full cyclization of **3a**. From the outset, we decided to use pentafluoroiodosobenzene (C_6_F_5_IO) as an oxidant instead of iodosobenzene (PhIO) due to the poor solubility of the latter at low temperatures. Initially, we kept the achiral, anionic catalyst scaffold constant (Rh_2_(**A**)_2_) and varied the associated chiral cation, comparing dihydroquinine (DHQ) and dihydroquinidine (DHQD)-derived cores quaternized with the same bulky benzyl group that had proven optimal previously (Table 1, entries 1 and 2).^17^ The DHQD core of **C2** gave a small but significant selectivity increase over the DHQ core of **C1** (+68% *ee* vs −57% *ee*). We then observed by chance that when a sample of catalyst Rh_2_(**A**)_2_·(**C2**)_2_ that had been previously dissolved in pyridine-*d*_5_ for nuclear magnetic resonance (NMR) characterization was used in the reaction, the *ee* was noticeably improved. We deduced that after evaporation of the solvent, two molecules of pyridine-*d*_5_ must have remained coordinated to the axial Rh sites. On repeating this operation using non-deuterated pyridine we discovered that the “pyridine solvate” complex once again gave improved enantioselectivity (Table 1, entry 2 vs entry 3). We believe that this effect is related to the observation, noted in our previous work, that in solution the quinoline motif in the cation can bind reversibly to the axial Rh sites. It seems plausible that the pyridine affects this covalent interaction by competitively binding at the same axial sites with a resulting small but significant effect on *ee* outcome. Diffusion Ordered Spectroscopy (DOSY) NMR studies in pyridine-*d*_5_ (see Supplementary Information) suggest that upon displacement from the axial positions, the chiral cations still remain associated with the anionic dimer, now presumably through ion-pairing interactions alone. We further note that although pyridine is a strong axial ligand, it should dissociate from at least one of the axial sites under the reaction conditions to enable nitrenoid formation.^24^ The precise origin of the improved selectivity is unclear at present, but others have also noted how minor changes to the axial coordination environment can significantly affect *ee* in asymmetric Rh(II,II)-catalyzed processes.^25^ The addition of 2 mol % of exogenous pyridine to the reaction also improves the selectivity when using the non-solvated Rh dimers, but the best results are obtained using the pre-formed pyridine solvate (see Supplementary Information). During optimization, we observed that substoichiometric amounts of weak acid also had a small beneficial effect on *ee*. We found it convenient to utilize C_6_F_5_I(OTFA)_2_ (“TFA-Ox”) as an easily measurable source of trifluoroacetic acid in solution, and 10 mol % of this in combination with non-solvated Rh_2_(**A**)_2_·(**C2**)_2_ gave a 12% improvement in *ee* (Table 1, entry 2 vs entry 4). In control experiments, the addition of substoichiometric amounts of TFA was found to have the same effect, supporting this acid-release hypothesis (see Supplementary Information). When we combined the use of the pyridine-solvated catalyst with the “TFA-Ox” additive, we obtained 90% *ee*, higher than that for either modification alone, demonstrating that the effects reinforce each other (Table 1, entry 5). We tentatively ascribe the acid additive effect to subtle modulation of the p*K*_a_ of the solution, an effect that, combined with pyridine solvation, could influence the extent of axial re-binding of the quaternized alkaloid via its quinoline nitrogen atom and impact *ee*. Modifications to the achiral dimer scaffolds at the cyclizing groups adjacent to the carbonyls were next evaluated (**B**-**E**, Table 1, entries 6–9). Cyclobutyl scaffold **B** was marginally better than **A**, and as the steric bulk of the cyclized alkyl groups increased further, the *ee* decreased. We then once again turned our attention to the cation and tested a slightly larger DHQD-derived cation **C3** in which the −^*t*^Bu groups on the periphery were replaced with −SiEt_3_ groups (Table 1, entries 10–12). **C3** was paired with the most promising achiral scaffolds, and this identified the optimum catalyst as Rh_2_(**B**)_2_·(**C3**)_2_·(Pyr)_2_ (Table 1, entry 11).

**Table 1:**
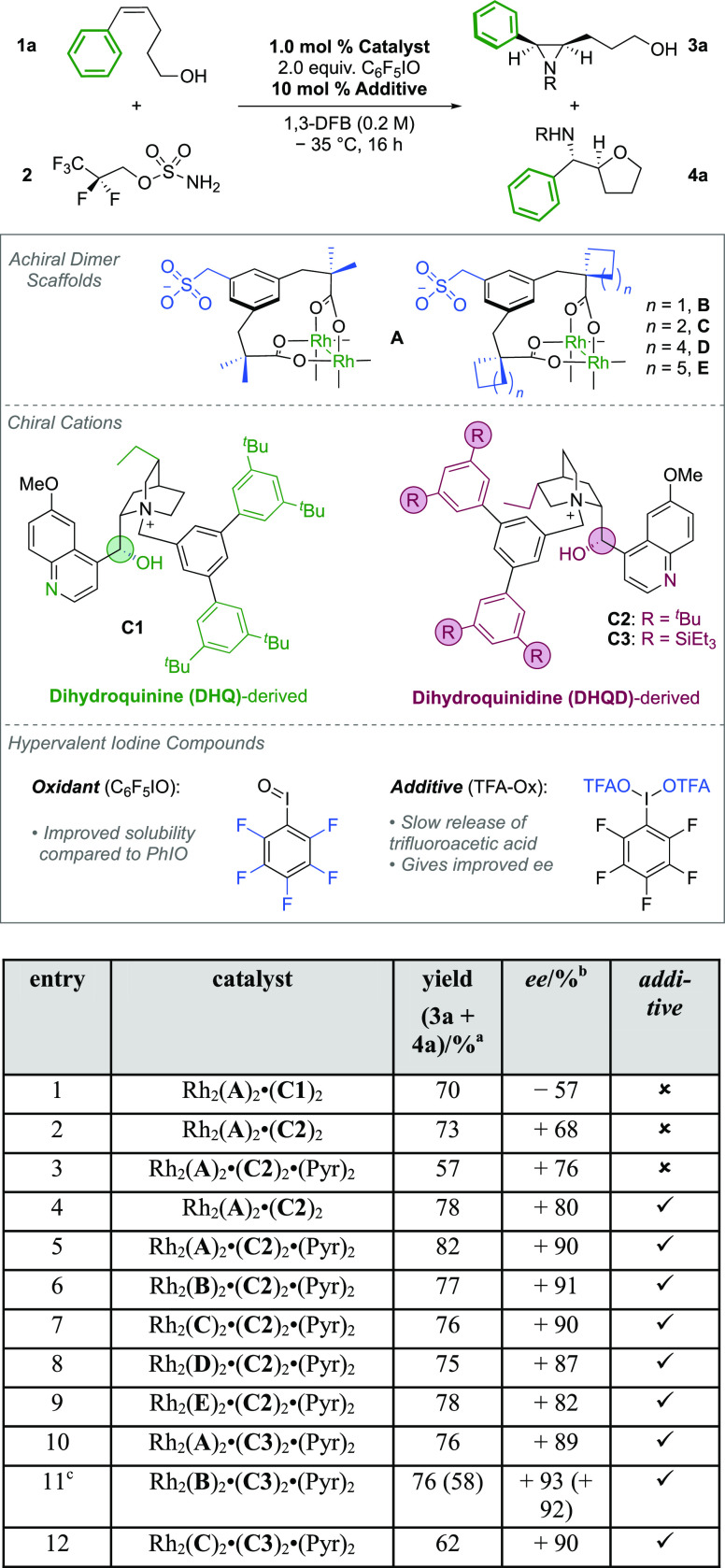
Optimization of the Aziridination Reaction

a Reactions performed on a 0.1 mmol scale with respect to **1a** and using 1.2 equivalents of **2**. Yields determined by ^1^H NMR with reference to a 1,2-dimethoxyethane internal standard.

b ee determined by chiral SFC analysis of the crude reaction mixture following full conversion of (**3a** + **4a**) to **4a** by heating in MeCN for 2 h.

c 2 mol % of the additive was used. Data in parentheses correspond to the isolated sample of **4a** following full conversion of (**3a** + **4a**) to **4a**. DFB = Difluorobenzene. The second strapped dicarboxylate ligand is omitted from the achiral dimer scaffolds for clarity.

### 2.2 Scope of Styrenyl Alcohols

**Figure 2:**
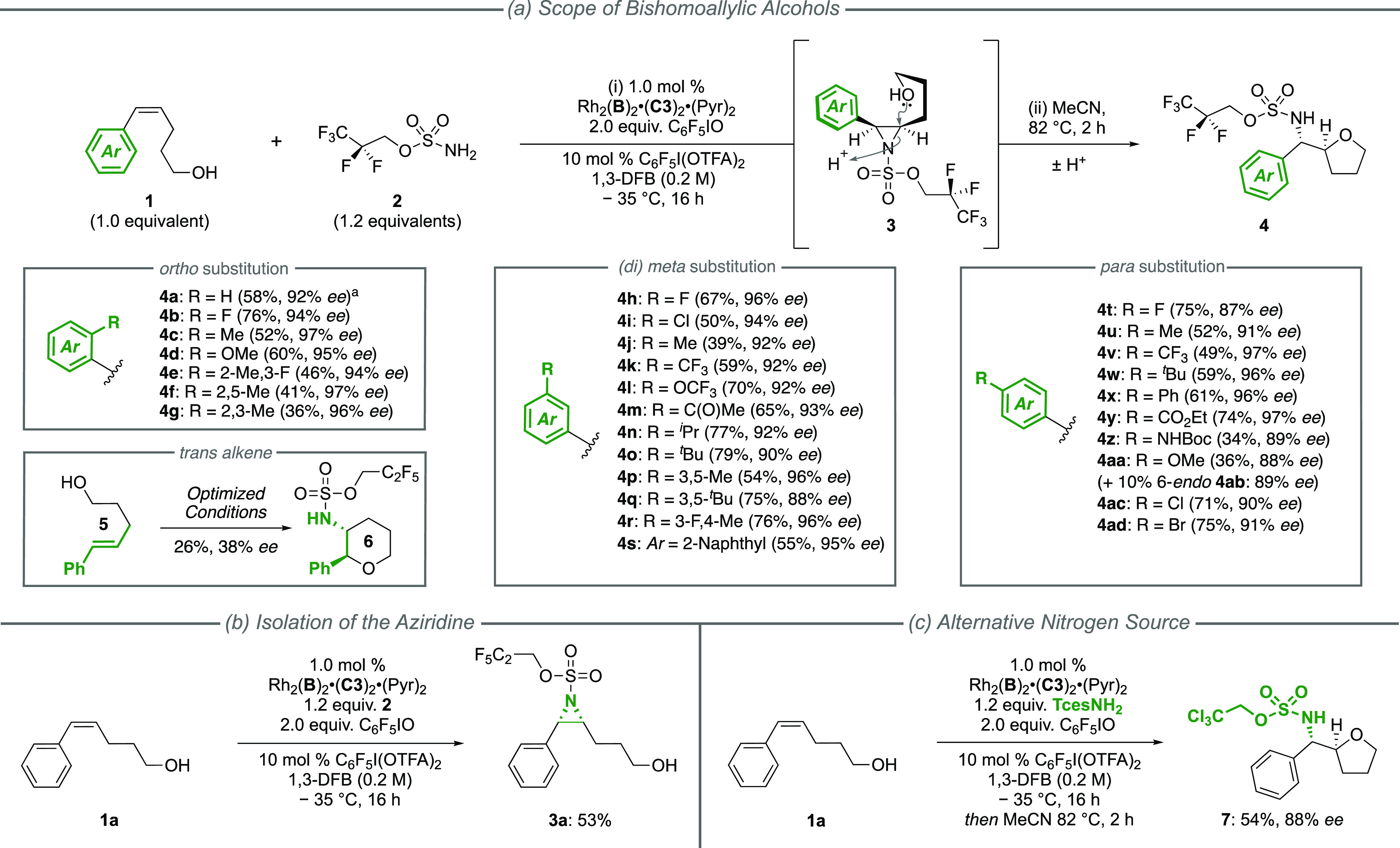
(a–c) Asymmetric aziridination of bishomoallylic styrenyl alcohols. ^a^Performed using 2 mol % of C_6_F_5_I(OTFA)_2_.

With 92% *ee* secured for the optimization substrate, we next evaluated the reaction scope of *cis*-bishomoallylic styrenyl alcohols (Figure 2a). Given the lability of the intermediate aziridines, we found it convenient to perform a telescoped aziridine opening to isolate the aminoetherification products **4**.^5b,26^ The tolerance toward arene substituents with a range of steric and electronic demands at different ring positions was remarkably high, and almost all ring substitutions, of which many were tested, delivered products with >90% *ee* in moderate to excellent yields. Reduced yields were observed in a few cases, such as with bulky *ortho*-substituents (**4e**-**g**) or alternatively with electron-donating *para*-substituents where a competing cyclization mode was accessible (**4z**-**aa**). The use of a *cis*-alkene as the substrate proved crucial as the corresponding *trans*-isomer **5** gave only 38% *ee* under the same conditions and in much reduced yield (interestingly, in this case, the aziridine obtained cyclized spontaneously to afford the 6-*endo* product **6**, Figure 2a).^27^ If isolation of the aziridine itself is required, this is possible provided that purification is performed without delay (Figure 2b, **3a**). In terms of the nitrogen source, the perfluorinated aminating agent **2**, previously reported by Sigman, Du Bois, and co-workers for benzylic C–H amination, gave the highest enantioselectivity,^28^ and following aziridine ring opening, deprotection of this group can be achieved using a two-step procedure (Scheme 9).^17^ However, if alternative deprotection conditions are required, the more common *N*-trichloroethylsulfamate ester (TcesNH_2_), which is deprotected under mild reductive conditions by treatment with Zn, can be used with only a minor decrease in performance (Figure 2c, **7**).^5a^

**Scheme 1:**
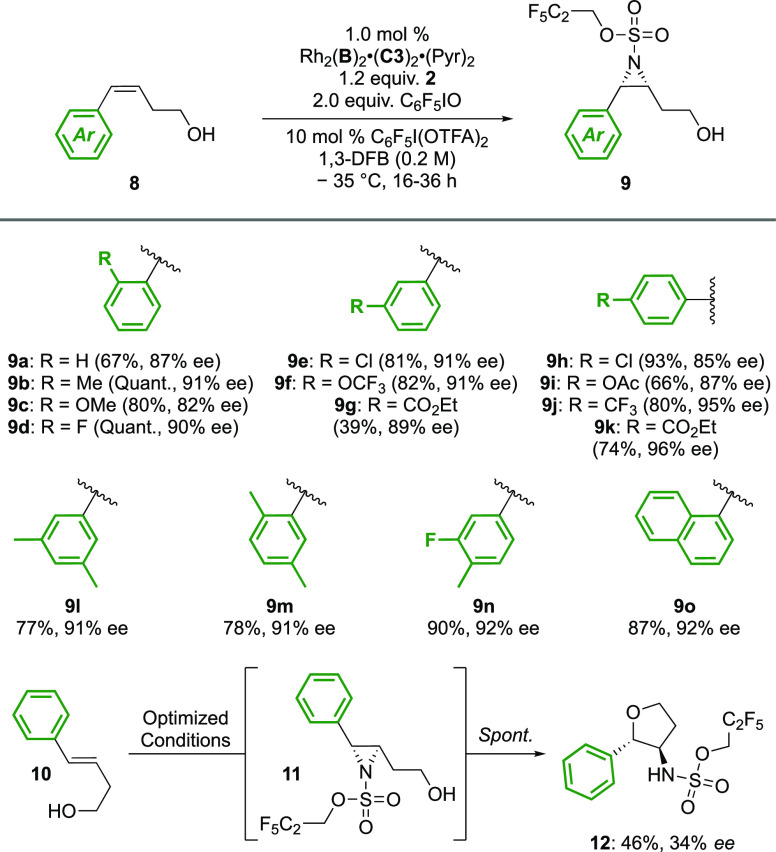
Asymmetric Aziridination of Homoallylic Styrenyl Alcohols

**Scheme 2:**
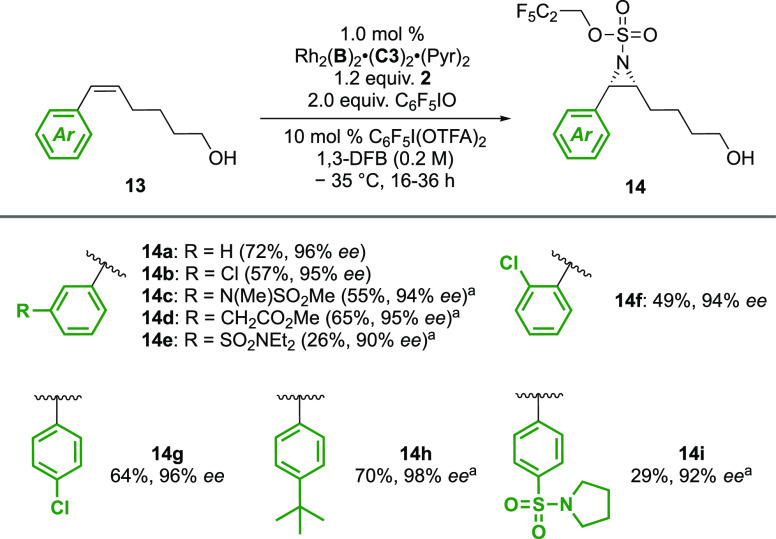
Asymmetric Aziridination of Trishomoallylic Styrenyl Alcohols Performed using 2 mol % catalyst.

We next investigated the effect of modulating the alkyl chain length between the olefin and the terminal alcohol. We were pleased to find that homoallylic alcohols, one methylene shorter, worked well, with no need for amendment of the catalyst structure or reaction conditions (Scheme 1). For this substrate class, the aziridines were readily isolated, without cyclization. Once again, a *trans*-Ph alkene (**10**) performed poorly with only 34% *ee* obtained in the cyclized **12**, further emphasizing the requirement for *cis*-styrenyl substrates to fit effectively in the catalyst pocket. Extension of the chain was equally well tolerated, and trishomoallylic alcohols (**13**) gave outstanding selectivities for a range of substitution patterns (Scheme 2). An effort was made to include ring substitutions not featured previously, and while aromatic rings bearing electron-withdrawing sulfonamide functional groups (**14e** and **14i**) afforded products with noticeably reduced yields due to suspected competing C–H amination adjacent to the nitrogen atom, high enantioselectivity was maintained in the aziridine products.

**Figure 3:**
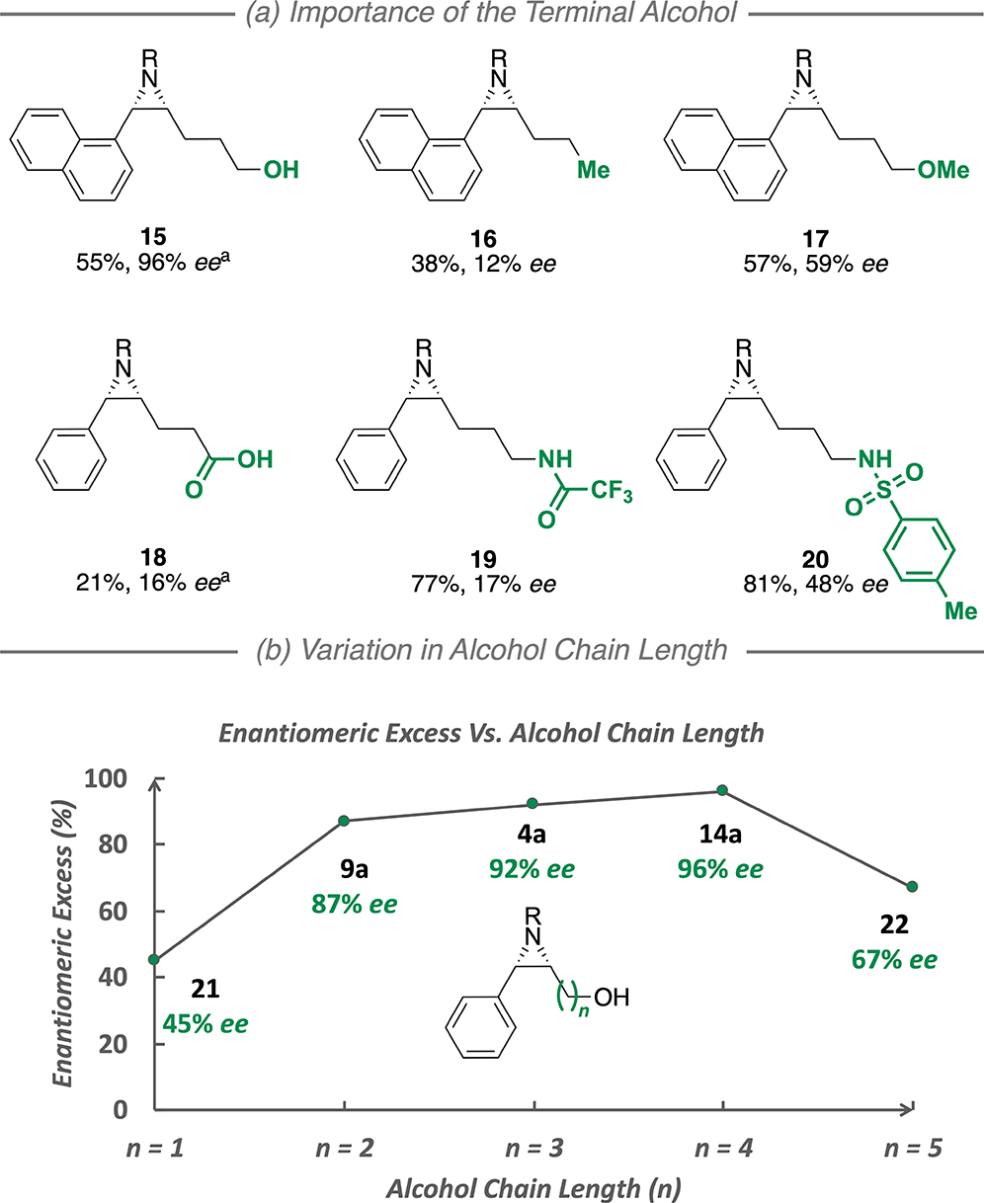
(a, b) Control experiments and limits of chain length variation. ^a^Data correspond to the cyclized product. R = −SO_2_OCH_2_C_2_F_5_. All results were obtained using Rh_2_(**B**)_2_·(**C3**)_2_·(Pyr)_2_.

In a control experiment to provide support for the substrate-directed nature of the reaction, we completely removed the terminal hydroxyl group from a representative substrate and observed a dramatic decrease from 96 to 12% *ee* (Figure 3a, **15** vs **16**). Intriguingly, methylation of the hydroxyl group resulted in a far less severe decrease (96 to 59% *ee*), with the sense of enantioinduction being retained (Figure 3a, **15** vs **17**). This suggests that an interaction with the terminal alcohol is the main organizing element between the substrates and the catalyst, but different sets of attractive interactions may be possible for other substrates given the many feasible interaction points on the chiral cation. This further raises the possibility that these catalysts may have the ability to recognize other ubiquitous functional groups, potentially enhancing reaction generality.

We have also tested analogues of optimization substrate **1a** in which the alcohol was replaced with other functional groups that could potentially interact with the sulfonate through hydrogen bonding: carboxylic acid (**18**), trifluoroacetamide (**19**), and sulfonamide^29^ (**20**), but all of these gave reduced selectivities (16, 17, and 48% *ee*, respectively). In terms of the terminal alcohol substrate series, further chain contraction and extension to encompass allylic (**21**) and tetrahomoallylic (**22**) chain lengths resulted in decreases to 45 and 67% *ee*, respectively (Figure 3b), placing these substrates outside the effective operating range of Rh_2_(**B**)_2_·(**C3**)_2_·(Pyr)_2_. Absolute stereochemistry was determined for homoallylic and bishomoallylic styrenyl aziridines by derivatization and comparison with literature reports, with that for the trishomoallylic class assigned by analogy (see Supplementary Information for full details).

### 2.3 Application to Unactivated Alkene Substrates

**Scheme 3:**
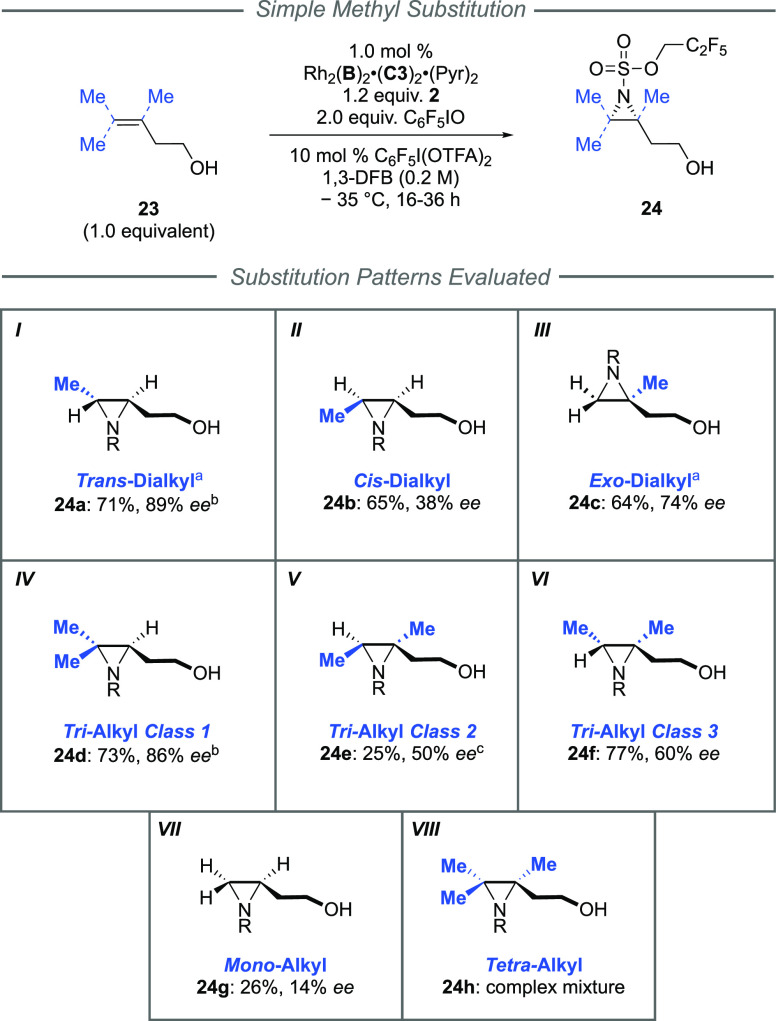
Systematic Investigation of Simple Aliphatic Homoallylic Alcohol Substitution Patterns Depicted absolute stereochemistry determined for this substrate class, and absolute stereochemistry for the other substrate classes was assigned by tentative analogy. ^b^Data correspond to the cyclized product. ^c^Yield estimated from ^1^H NMR with reference to an internal standard.

We next turned our attention to unactivated alkenes for which, to the best of our knowledge, effective intermolecular asymmetric aziridination protocols are absent in the literature for all but terminal, monosubstituted examples. At the outset, it was unclear whether these alkenes would be sufficiently reactive toward nitrene transfer and whether the high enantioselectivities would be maintained in the absence of an aromatic ring. In order to obtain as complete a picture as possible of the capabilities of our catalytic system, we decided to carry out a systematic study on homoallylic alcohols in which all permutations of methyl substitution on the alkene were evaluated (Scheme 3). This study encompassed three disubstituted isomers (***I**−**III***) and three trisubstituted isomers (***IV***–***VI***) along with unsubstituted mono-alkyl (***VII***) and fully substituted tetra-alkyl examples (***VIII***). Poor reactivity and enantioselectivity were observed for the simplest monosubstituted homoallylic alcohol (***VII***, **24g**). In contrast, simply incorporating a methyl substituent at the *trans* position led to a dramatic improvement with good yield and high enantioselectivity for the resulting aziridine (***I***, **24a**: 71% yield, 89% *ee*). The corresponding *cis*-isomer gave good yield, but the *ee* was poor, suggesting a mismatch with the chiral pocket (***II***, **24b**: 65% yield, 38% *ee*). For the final disubstituted isomer, moving the methyl to give 1,1-disubstitution led to encouraging selectivity (***III***, **24c**: 74% *ee*), which could be later improved with larger substituents (see Scheme 5). Turning to the trisubstituted alkenes (***IV**−**VI***), we were pleased to find that the first class examined, incorporating a prenyl group, evidently fit very well into the chiral pocket (***IV***, **24d**: 86% *ee*). However, transposing either one of the two methyl groups of **23d** to the same carbon to which the alcohol chain is attached led to significant reduction in *ee* (***V*** and ***VI***). Tetrasubstituted alkenes were not tolerated, leading to a complex mixture of products arising from allylic amination, aziridination, and both (***VIII***). The absolute stereochemistry of the products from categories ***I*** and ***III*** was determined by derivatization and comparison with commercially available enantiopure compounds or reliable sources in the literature (see Supplementary Information). Products from the *trans*-dialkyl class (***I***) were consistent with nitrenoid delivery to the same alkene face, as drawn, as that for the previous styrenyl classes. Intriguingly, the aziridine **24c** derived from a 1,1-disubstituted alkene (***III***) arose from aziridination of the opposite alkene face, relative to the positioning of the alcohol as drawn.

**Scheme 4:**
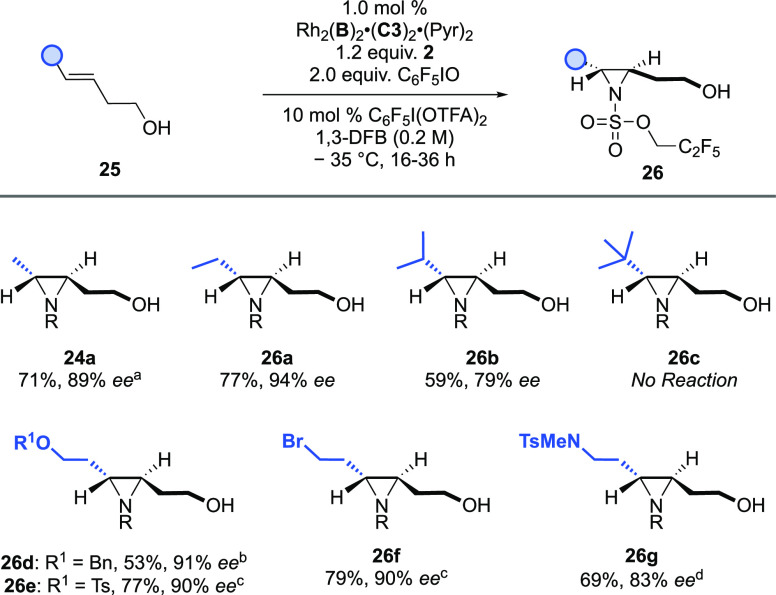
Asymmetric Aziridination of *trans-*Dialkyl Alkenyl Alcohols Data correspond to the cyclized product. ^b^Reaction time = 48 h. ^c^3 mol % catalyst. ^d^2 mol % catalyst.

**Scheme 5:**
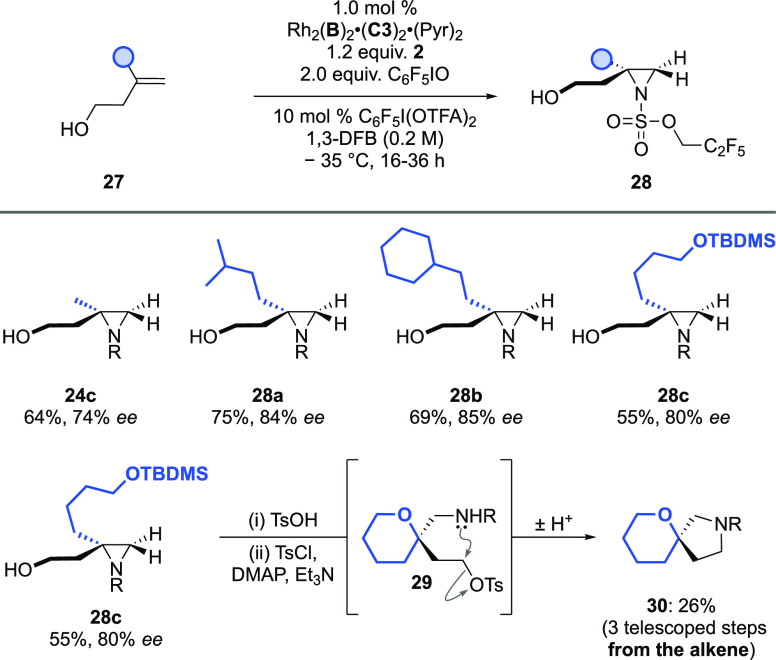
Asymmetric Aziridination of *exo-*Dialkyl Alkenyl Alcohols

For the three alkene substitution patterns that showed the best compatibility with our system (***I****trans-*dialkyl, ***III**exo-*dialkyl, and ***IV*** tri-alkyl Class 1), we probed their constraints with further examples of each. For the *trans-*dialkyl substrates (Scheme 4), a systematic increase in steric demand from Me to ^*t*^Bu (**24a**, **26a**–**26c**) demonstrated that small to medium *trans*-substituents are well tolerated, whereas large groups inhibit the reaction, presumably by preventing nitrenoid approach, as evidenced by the lack of formation of **26c**. We tested a series of substrates bearing useful functionality such as a protected alcohol (**25d**), a protected amine (**25g**), and leaving groups (**25e**, **25f**) at the second alkyl terminus. In all cases (**26d**–**26g**), good to excellent outcomes were observed affording versatile and densely functionalized enantioenriched molecules. We next examined the *exo*-dialkyl alkene class (***III***) and noted an improvement in enantioselectivity upon replacement of the methyl group of evaluation substrate **23c** with larger groups (Scheme 5, **28a**–**28c**). We also demonstrate how the aziridine product of one of these (**28c**) can be readily transformed into an enantioenriched spirocycle (**30**) in a three-step procedure telescoped directly from the alkene.

**Scheme 6:**
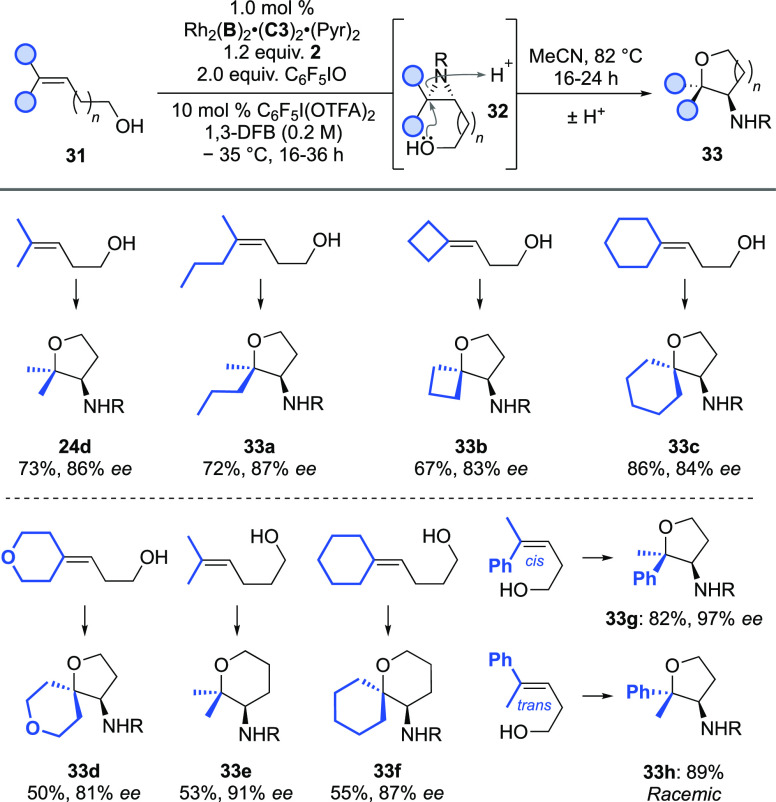
Asymmetric Aziridination of Tri-alkyl Class 1 Alkenyl Alcohols R = −SO_2_OCH_2_C_2_F_5_.

Finally, we explored the tri-alkyl class 1 alkenes (***IV***) (Scheme 6). In these cases, the aziridines **32** were visible in analysis of the crude reaction but cyclized readily to the corresponding tetrahydrofurans/tetrahydropyrans. As a result, prior to purification for analysis, we promoted their full cyclization by heating in MeCN. Excellent results were obtained for dimethyl (**24d**), non-symmetrical dialkyl (**33a**), and several cyclic dialkyl examples (**33b**–**33d**). Longer chain bishomoallylic substrates also performed excellently (**33e** and **33f**). In a number of these examples, linking together the alkyl groups to form a ring allows facile access to spirocyclic scaffolds, generating building blocks of potential pharmaceutical interest.^30^ Both ring sizes can be easily varied, and the products contain a nitrogen exit vector for further functionalization (**33b**–**33f**). When replacing one of the alkyl groups with a phenyl ring, to intersect with the styrenyl substrate class, we observed a striking divergence between selectivity outcomes for the *cis*, **33g** (97% *ee*), and *trans*, **33h** (racemic), isomers. This correlates with our previous observations related to alkene geometry for the disubstituted styrenyl substrates, but the effect is clearly accentuated by the presence of the extra methyl group (see later discussion).

**Scheme 7:**
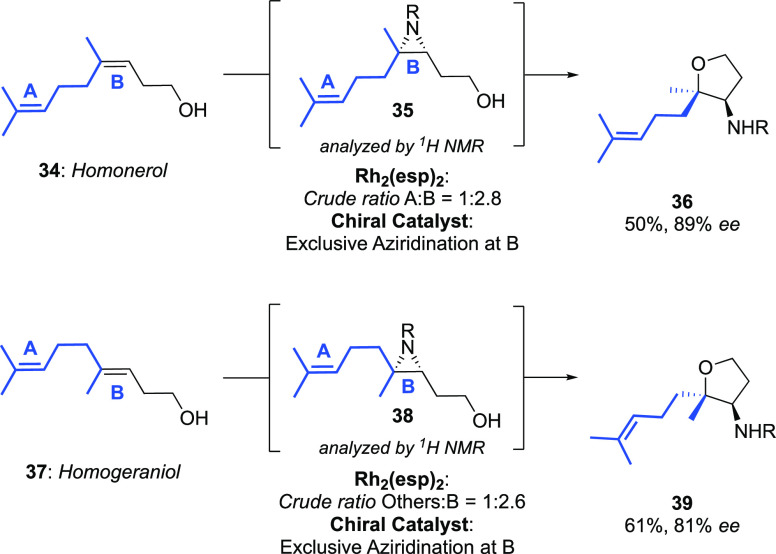
Investigating Simultaneous Catalyst Control over Site-Selectivity and Enantioselectivity R = −SO_2_OCH_2_C_2_F_5_.

In addition to controlling enantioselectivity, our chiral catalyst can simultaneously influence site-selectivity, a hallmark of substrate-directed catalysis (Scheme 7).^18,31^ For the homologated terpene homonerol, aziridination with high *ee* was observed exclusively at the alkene proximal to the alcohol (**36**: 89% *ee* after cyclization) with the distal alkene remaining untouched. The same was true with homogeraniol (**39**: 81% *ee*), and both results contrast sharply to those obtained with Rh_2_(esp)_2_, in which significant functionalization at both alkenes was observed.

### 2.4 Access to Antipodes and Predictive Mnemonic

**Figure 4:**
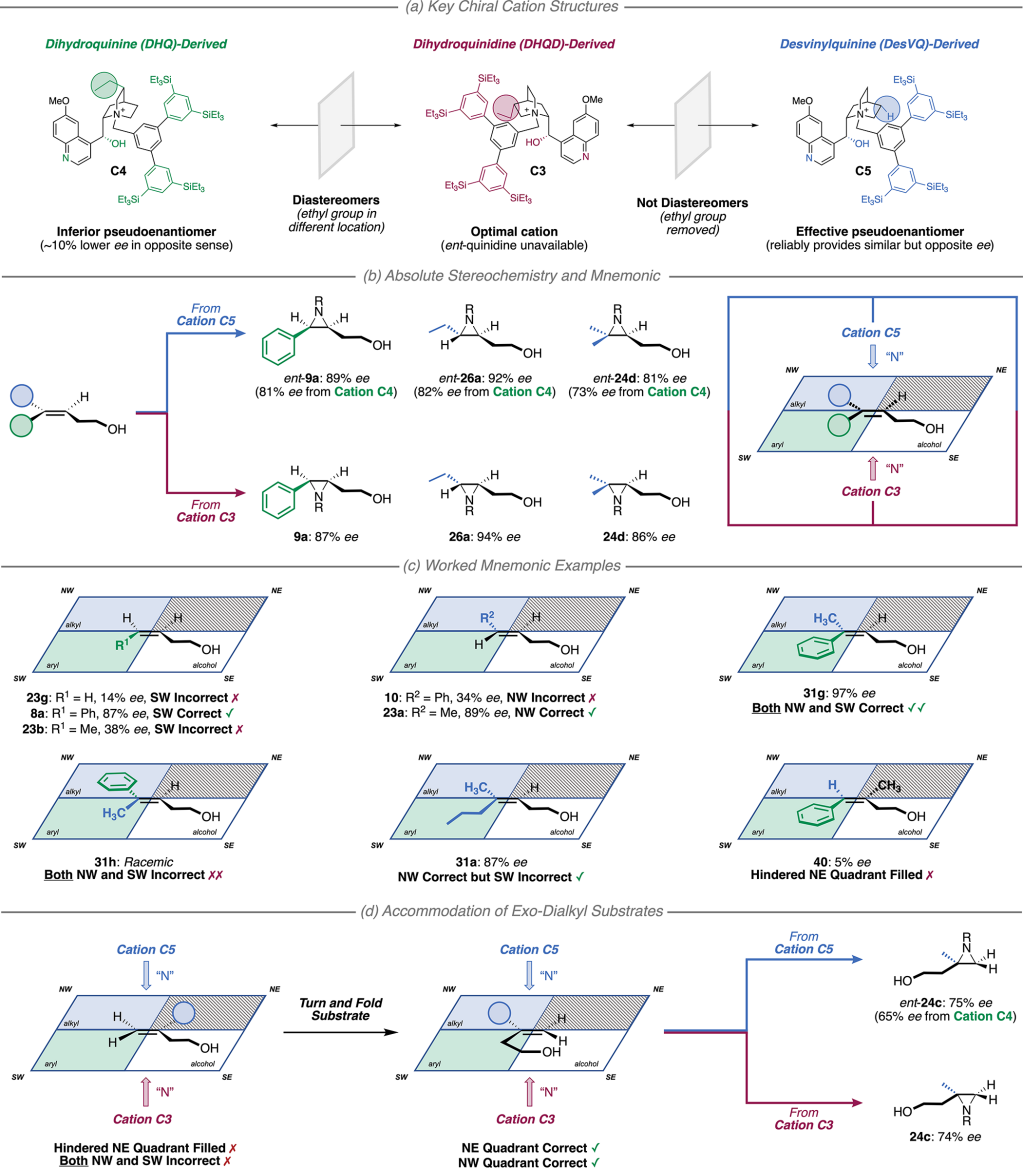
(a–d) Studies to access both enantiomers and proposal of a mnemonic to predict reaction outcome. The results in panel c were all obtained using Rh_2_(**B**)_2_·(**C3**)_2_·(Pyr)_2_. R = −SO_2_OCH_2_C_2_F_5_.

At this stage, we set out to achieve two further objectives. The first was to secure access to the opposite aziridine enantiomer for the key substrate classes. During optimization, we found that the pseudoenantiomeric chiral cation **C1** derived from DHQ gave the opposite stereochemical outcome to **C2**, derived from DHQD. This was as expected but unfortunately delivered around 10% lower *ee* (Table 1). A similar underperformance was observed in the aziridination of the other substrate classes when using the slightly larger DHQ-derived **C4** (Figure 4a) in place of the optimal **C3**. Other examples of “uneven efficiency” with cinchona alkaloid-derived catalysts have been noted previously and if not overcome can limit the practical application of an asymmetric reaction.^32^ Following Deng’s precedent, we found that removal of the quinuclidine-located ethyl group on DHQ was key to accessing an effective pseudoenantiomer.^32^ A *desvinylquinine* (DesVQ)-derived cation **C5** afforded the aziridines in very similar but opposite *ee* compared with the DHQD-derived catalyst (Figure 4a), and this was the case across all key alkene classes (Figure 4b).

The second objective was to translate the findings obtained from our substrate evaluation into a mnemonic to assist in prediction of whether a given alkene is suitable for the reaction and if so, the absolute configuration of the product for a given cation. Taking inspiration from both the SAE (alcohol-directed) and the Sharpless asymmetric dihydroxylation (utilizing a cinchona alkaloid-derived chiral pocket), we superimpose the substrate on a parallelogram, in our case based on three attractive quadrants and one repulsive (Figure 4b, right).^19b,33^ First, the terminal hydroxyl group should be placed in the south-east (SE) quadrant. Second, the north-east (NE) quadrant can only accommodate a hydrogen atom. Third, the north-west (NW) quadrant should be filled with a small/flexible alkyl group *or* the south-west (SW) quadrant should be filled with an aromatic group. In terms of the absolute stereochemistry of the aziridines, cation **C3** delivers nitrogen from the bottom face of the parallelogram and cation **C5** from the top face. This device rationalizes the successful outcomes and helps also to explain some of the poorer results (Figure 4c). For example, the aziridine arising from a simple homoallyl alcohol (**23g**) is obtained in low *ee* since neither the NW nor SW quadrants are filled correctly. However, upon satisfying the SW by adding a *cis*-Ph group, the selectivity is greatly improved (**8a**, 87% *ee*). With a *cis*-methyl substituent, the *ee* is poor (**23b**, 38% *ee*) since the aliphatic methyl is not matched to the aromatic quadrant. The same reasoning holds true for the substituent in the NW: a phenyl ring in this position is mismatched (**10**, 34% *ee*), while a methyl group is matched (**23a**, 89% *ee*). The impact of these two quadrants seems cumulative: substrate **31g**, which satisfies both the NW and SW simultaneously, results in a greater selectivity (97% *ee*) than either the matched NW (**23a**, 89% *ee*) or SW (**8a**, 87% *ee*) component in isolation. Conversely, alkene **31h**, which violates both NW and SW together, results in a racemate. It appears that provided the NW quadrant is filled correctly, the nature of the group occupying SW is unimportant (e.g., **31a**). Finally, control experiments demonstrate the intolerance of the repulsive NE quadrant to any group larger than a hydrogen atom if either the NW or SW is filled, as evidenced by comparison of **8a** and **40** (87% vs 5% *ee*). An interesting situation arises with the *exo-*dialkyl substrates (Scheme 5), which appear, at first sight, to seriously contravene the mnemonic; these substrates give moderate to high *ee* but fill neither the attractive NW nor SW quadrants while simultaneously filling the repulsive NE (Figure 4d, left). Crucially however, we established during the scope exploration that this class of substrate gives rise to the opposite enantiomer of the product compared to that which would be predicted by the binding mode in Figure 4d, left (see Supplementary Information). In order to avoid the sole alkene substituent occupying the repulsive quadrant, we propose that the *exo*-dialkyl substrates adopt a different conformation within the parallelogram compared to the other lead substrate classes, resulting in an effective “flipping” of the alkene and nitrogen delivery to the “opposite” face (Figure 4d, center). In practice, we suspect that the binding of the *exo*-dialkyl alkenes within the catalyst itself approximates somewhat to that of the *trans-*dialkyls. As for the other substrate classes, chiral cations **C3** and **C5** deliver equal and opposite enantiomers of aziridine with **C4**, again affording diminished selectivity (Figure 4d, right).

### 2.5 Transformation of the Aziridine-Alcohol Products

**Scheme 8:**
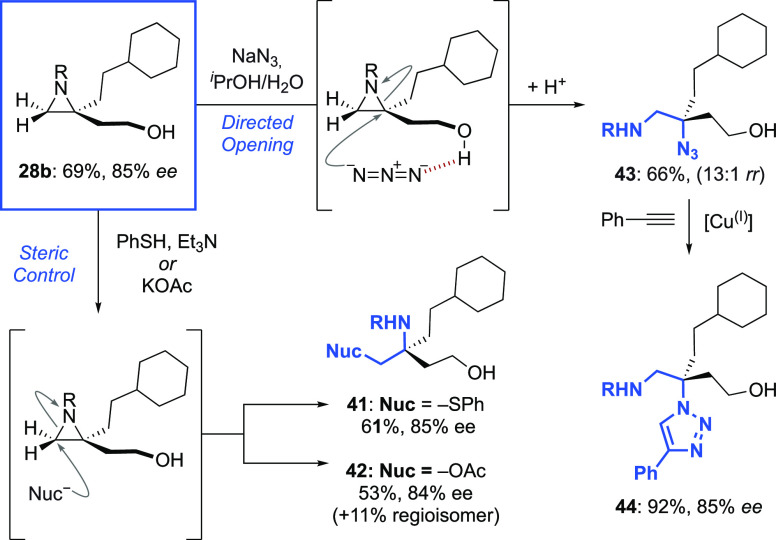
Nucleophilic Ring Openings of an *exo-*Dialkyl Aziridine Product R = −SO_2_OCH_2_C_2_F_5_.

As with any substrate-directed reaction, the requirement for a specific functional group (in our case, a primary alcohol) could be viewed as a potential limitation.^18a,18b^ Below, we demonstrate that the unique clustering of functionality found in the aziridine-alcohol products allows access to many useful motifs in addition to those, such as spirocycles, that have been discussed already. In particular, the presence of the alcohol can allow for discrimination between the two ends of the aziridine, which are in some instances sterically and electronically very similar. It is also well known that aziridines are versatile building blocks, amenable to a range of post-functionalization protocols, which have been extensively discussed elsewhere.^34^ We first turned our attention to the derivatization of the aziridines obtained from the *exo*-dialkyl alkenes (Scheme 8). Product **28b** could be ring-opened under steric control with nucleophiles predominantly attacking at the less hindered terminus, affording enantioenriched protected amines (**41** and **42**), equipped with functional groups primed for further transformations. In contrast to acetate or thiophenolate, sodium azide preferred to attack almost exclusively at the more hindered tertiary position, potentially as a result of a guiding hydrogen bond with the terminal alcohol to afford **43**, which contains two orthogonally protected amines and which could be converted to triazole **44**. We next chose to focus on reactions in which the terminal hydroxyl group was involved to a greater degree using styrenyl aziridines of varying chain lengths (Scheme 9). Hydrogenation led to selective cleavage of the benzylic C–N bond with full enantiospecificity across all three chain lengths (**45**–**47**). Crucially, *N*-deprotection is facile, as illustrated by deprotection/reprotection with a Cbz group (**48**). These hydrogenation products offer valuable complementarity to molecules typically obtained from asymmetric intermolecular benzylic C(*sp*^3^)–H aminations, since the new C–N bond is formed one carbon further away from the aromatic ring.^17^ Mitsunobu cyclization of the hydrogenation products then provides ready access to enantioenriched pyrrolidine and azetidine heterocycles (**49**, **50**), and the *N*-sulfonyl azetidine could be ring-opened with thiophenolate in tandem with *N*-deprotection (**51**). We also investigated the ability of the terminal hydroxyl group to selectively deliver reagents to the proximal and less electronically activated carbon of these aziridines.^35^ This started with a directed reduction of **9a** using Red-Al in which a proposed pre-complexation between the alcohol and the reducing agent results in excellent regioselectivity for the formation of protected benzylic amine **52**. Similarly, in situ reaction of the aziridine alcohols with either Cl_3_CCN or Cbz-Cl in the presence of base led to the formation of cyclic products **53** and **54** through intramolecular S_*N*_2 attack of the trichloroacetimidate and carbonate intermediates onto the aziridine. The regioselectivity of the ring openings was excellent, and given the precedented, mild hydrolysis protocols for the resulting heterocycles, these constitute formal enantioselective, diastereoselective, and regioselective alkene diamination and oxyamination reactions, respectively.^36^

## 3 Conclusions

**Scheme 9:**
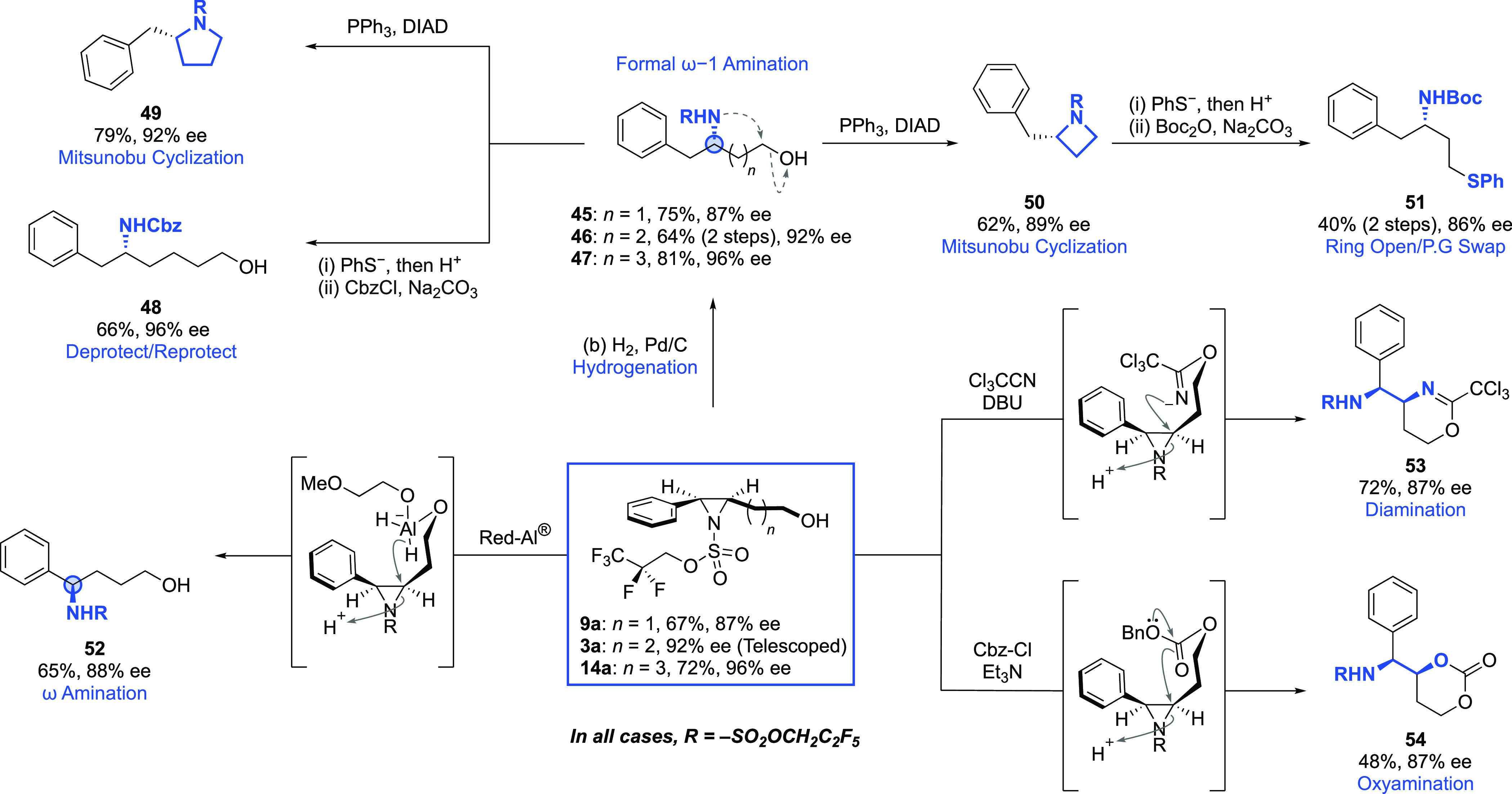
Post-Functionalization of the Styrenyl Aziridines with Focus on Participation of the Primary Alcohol

## Supporting Information Available

Additional optimization, full experimental details, and characterization data for compounds (PDF)
