# Supplementary material for: Substrate-Directed Enantioselective Aziridination of Alkenyl Alcohols Controlled by a Chiral Cation
Source: J Am Chem Soc. 2023 Mar 24;145(13):7516–27. doi: 10.1021/jacs.3c00693 (PMC10080694; doi:10.1021/jacs.3c00693)
Supplement: Supplementary file 1 — ja3c00693_si_001.pdf [file ja3c00693_si_001.pdf]

Substrate-Directed Enantioselective Aziridination of Alkenyl Alcohols Controlled by a Chiral Cation

Alexander Fanourakis, Nicholas J. Hodson, Arthur R. Lit and Robert J. Phipps*

Yusuf Hamied Department of Chemistry, University of Cambridge, Lensfield Road, Cambridge, CB2 1EW, United Kingdom.

Corresponding Author

*rjp71@cam.ac.uk

TABLE OF CONTENTS

General Experimental....S4

General Protocols....S7

Note on Substrate and Product Stabilities....S14

Further Investigation of the Role of Axial Pyridine Ligation....S15

Further Investigation of the Acidic Additive....S17

Synthesis of Chiral Cation Bromide Salts....S20

Synthesis of Chiral Rh(II,II) Tetracarboxylate Dimers....S31

Synthesis of Bishomoallylic Styrenyl Alcohols....S42

Synthesis of the Aminating Agent and Oxidants....S73

Synthesis of Homoallylic Styrenyl Alcohols....S76

Synthesis of Trishomoallylic Styrenyl Alcohols....S94

Synthesis of Control Substrates....S113

Synthesis of Substrates Bearing Alternative Hydrogen Bond-Donors....S117

Synthesis of Allylic and Tetrahomoallylic Styrenyl Alcohols....S124

Synthesis of Simple Aliphatic Alkenyl Alcohols....S128

Synthesis of *Trans*-Dialkyl Alkenyl Alcohols....S133

Synthesis of *Exo*-Dialkyl Alkenyl Alcohols....S140

Synthesis of *Tri*-Alkyl Class I Alkenyl Alcohols....S143

Synthesis of a Further Mnemonic Test Substrate....S160

Characterisation of Bishomoallylic Styrenyl Aziridines....S163

Characterisation of Homoallylic Styrenyl Aziridines....S206

Characterisation of Trishomoallylic Styrenyl Aziridines....S228

Characterisation of Control Substrate Aziridines....S240

Characterisation of Aziridines Obtained from Substrates Bearing Alternative Hydrogen Bond-Donors....S246

Characterisation of Allylic and Tetrahomoallylic Styrenyl Aziridines....S251

Characterisation of Simple Aliphatic Aziridines....S254

Characterisation of *Trans*-Dialkyl Aziridines....S269

Characterisation of *Exo*-Dialkyl Aziridines....S282

Characterisation of *Tri*-Alkyl Class I Aziridines....S293

Characterisation of a Further Mnemonic Test Aziridine..S315

Characterisation of Post-Functionalisation Products..S317

Determination of the Absolute Stereochemistry of the Aziridine Products...................S339

Synthesis and Testing of Unsuccessful Substrates..S358

Chiral SFC, HPLC and GC Traces..S364

NMR Spectra...S476

Diffusion Ordered Spectroscopy (DOSY) Studies of $Rh_2(B)_2$•$(C3)_2$ S1190

References S1200

General Experimental

Reaction Setup, Solvents and Reagents: Excluding the enantioselective intermolecular aziridination, reactions were carried out under an inert argon or nitrogen atmosphere using standard Schlenk-septa techniques in heat gun-dried glassware. The enantioselective intermolecular aziridinations were conducted under an atmosphere of air with minimal precaution taken against moisture. Reactions performed in 4.0 mL crimp-top vials that required cooling were placed in a Polar Bear Cub (by Cambridge Reactor Design) featuring a deep-welled heating block. All reagents were used as supplied from commercial sources without further purification unless otherwise stated. CuI was purified according to the protocol reported by Kauffman and Fang.[1] NaOH was finely ground using a pestle and mortar. Tetrahydrofuran, Et_2O, MeOH, MeCN, CH_2Cl_2, *n*-hexane and toluene were purified by distillation on site under inert atmosphere *via* the following processes: Tetrahydrofuran and Et_2O were pre-dried over sodium wire then distilled from calcium hydride and lithium aluminium hydride. MeOH, MeCN, CH_2Cl_2, *n*-hexane and toluene were distilled from calcium hydride. 1,3-Difluorobenzene was purchased from Fluorochem and was used as received. In the text below, “Petrol” refers to petroleum ether 40–60 °C.

Chiral HPLC Analysis: Performed on either a Shimadzu XR-LC system with DAICEL CHIRALPAK AD-H (4.6 x 250 mm, 5.0 μm) or IC columns (4.6 x 250 mm, 3.0 μm) or a Waters ARC system with YMC CHIRAL ART SA, SC or SJ columns (4.6 x 250 mm, 3.0 μm) in a mixed solvent system of hexane and ^{i}PrOH.

Chiral SFC Analysis: Performed on a Waters ACQUITY UPC2 System with YMC CHIRAL ART SA, SB, SC, or SJ columns (4.6 x 250 mm, 3.0 μm), or DAICEL CHIRALPAK IG, IH or IE columns (4.6 x 250 mm, 3.0 μm) in a mixed solvent system of supercritical CO_2 and MeOH or ^{i}PrOH. A system backpressure of 138 bar was used in all cases.

Chiral GC Analysis: Performed on an Agilent 6890N Flame Ionisation Detector Gas Chromatograph System equipped with a CP7502 – CP-Chirasil-DEX CB column (25 m x 0.25 mm x 0.25 µm).

Chromatography: Analytical thin-layer chromatography was performed using precoated Merck glass backed silica gel plates (Silica gel 60 F254). Visualisation was by ultraviolet fluorescence (λ = 254 and 365 nm) and/or staining with potassium permanganate ($KMnO_4$) or Ceric Ammonium Molybdate (CAM). Flash column chromatography was performed using silica gel 60 (pore size: 60 Å, mesh: 40-63 μm) from Material Harvest® or Fluorochem.

High Resolution Mass Spectrometry (HRMS): Recorded on a Waters Micromass LCT Premier, a Waters Xevo G2-S QTOF, a Waters Synapt G2-Si, a Waters Vion IMS QTOF and AGILENT 6230 LC/TOF at the Department of Chemistry at the University of Cambridge. The ionisation method is noted as either positive or negative electrospray ionisation (+/–ESI). Measured values are reported to 4 decimal places and are within ± 5 ppm of the calculated value. The calculated values are based on the most abundant isotope unless otherwise stated in the chemical formula. For ions bearing more than a single unit of charge, the masses reported as 'found' and 'required' are the mass/charge ratios.

IR Spectroscopy: Recorded on a PerkinElmer Spectrum One FT-IR spectrophotometer sampling accessory, a Bruker ALPHA FT-IR Spectrometer, or a Bruker ALPHA II FT-IR Spectrometer canning from 4000-400 cm^{-1}. IR absorption maxima (ν_{max}) are reported in wavenumbers (cm^{-1}) of weak (w), medium (m) or strong (s) intensity and of broad (br) peak shape where appropriate.

NMR Spectroscopy: ^{1}H NMR spectra were recorded on 700 MHz TXO Cryoprobe, 600 MHz Bruker Avance DRX-600, 500 MHz Bruker DCH Cryoprobe, 400 MHz Bruker DPX-400 Dual, 400 MHz Avance III HD, 400 MHz Avance III HD Smart Probe or 400 MHz Neo Prodigy spectrometers. Chemical shifts are reported in parts per million (ppm) and the spectra are calibrated to the resonance resulting from incomplete deuteration of the solvent ($CDCl_3$: 7.26 ppm; CD_3CN: 1.94 ppm, p; $(CD_3)_2CO$: 2.05 ppm, p; D_2O: 4.79 ppm; $(CD_3)_2SO$: 2.50 ppm, p; $C\mathit{D}_3OD$: 3.31 ppm, p; C_5D_5N (H–C–N): 8.74 ppm).[2,3] ^{13}C NMR spectra were recorded on the same spectrometers with complete proton decoupling. ^{13}C NMR experiments of compounds containing poly-fluorination were performed using a UDEFT sequence to increase the signal:noise ratio for ^{13}C signals of carbon nuclei along the poly-fluorinated

chain.[4] Chemical shifts are reported in ppm with the solvent resonance as the internal standard ($^{13}CDCl_3$: 77.16 ppm, t; $^{13}CD_3CN$: 1.32 ppm, sept; $(^{13}CD_3)_2CO$: 29.84 ppm, sept; $(^{13}CD_3)_2SO$: 39.52 ppm, sept; $^{13}CD_3OD$: 49.00 ppm, sept; $^{13}C_5D_5N$ (C–N): 150.35 ppm, t).[2,3] ^{19}F NMR spectra were recorded on 400 MHz Avance III HD or 400 MHz Avance III HD Smart Probe spectrometers. Chemical shifts are reported in ppm with $CFCl_3$ as the external standard ($CFCl_3$: 0.00 ppm). Data are reported as follows: chemical shift *δ*, multiplicity (s = singlet, d = doublet, t = triplet, q = quartet, p = pentet, sext = sextet, sept = septet, br = broad, m = multiplet or combinations thereof (^{13}C and all other nuclides except ^{1}H are singlets unless otherwise stated)), coupling constants *J*, number of nuclides (signals for all other nuclides except ^{1}H refer to one nuclide unless otherwise stated), assignment. ^{1}H NMR spectra are assigned as fully as possible, using ^{1}H-^{1}H COSY, ^{1}H-^{1}H NOESY, DEPT-135, ^{1}H-^{13}C HSQC and ^{1}H-^{13}C HMBC where appropriate to facilitate structural determination. Assignments either follow the numbering system shown on the structures or are described unambiguously. ^{1}H NMR signals are reported in ppm to 2 decimal places and all other nuclide signals are typically reported to 1 decimal place. Coupling constants are reported in Hz to a maximum of 3 significant figures. For cinchona alkaloid-derived compounds the appearance and chemical shifts of the peaks in the NMR spectra can vary significantly depending on sample concentration and other factors. For spectra acquired in C_5D_5N the residual water peak is often visible at approximately 4.9 ppm in the ^{1}H NMR spectrum.

Optical Rotations: Measured in spectrophotometric grade $CHCl_3$ or C_5H_5N or in LiChrosolv® hypergrade CH_3CN for LC-MS on a Perkin Elmer 343 Polarimeter using a sodium lamp (λ = 589 nm, D-line). $[\alpha]_D$ values are reported at the stated temperature, with concentration in g /100mL.

Naming and Numbering of compounds: Systematic names were generated by the computer program ChemDraw according to the guidelines specified by the IUPAC. However, the numbering on the structures does not correspond to the systematic name.

General Protocols

General protocol for Nickel-catalysed ring opening of 3,4-Dihydropyran (**GP1**)

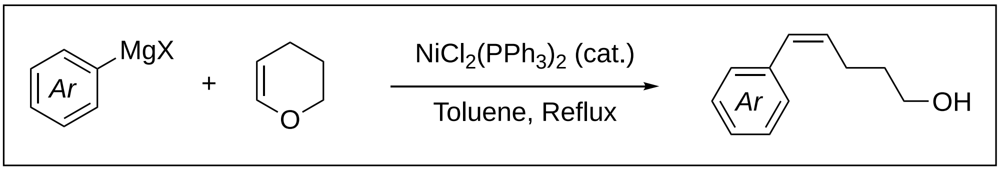

A variant of the protocol described by Brown and co-workers was employed.[5]

Preparation of the Grignard reagent stock solution: A heat gun-dried round-bottomed flask fitted with a reflux condenser was charged with magnesium turnings (370 mg, 15.2 mmol) and three small iodine crystals. A solution of the appropriate chloride/bromide/iodide (15.0 mmol) in dry Et_2O (10 mL) was added dropwise and the reaction mixture was maintained at a gentle reflux either through cooling with an ice bath or through gentle warming. Transfer of the chloride/bromide/iodide was made quantitative with an additional 5 mL of Et_2O. Following this, the stock solution of Grignard reagent was maintained at a gentle reflux for 1 hour.

Ring Opening: Following this, a heat gun-dried three-necked round bottom flask equipped with a large stir bar was charged with $NiCl_2(PPh_3)_2$ (98 mg, 0.15 mmol) and three times evacuated and backfilled with N_2. After the addition of toluene (30 mL), the Grignard reagent (0.15 mL, 0.15 mmol) was added to the stirring mixture. After stirring for 15 minutes, the remaining Grignard reagent (9.85 mL, 9.85 mmol) was added followed by 3,4-dihydropyran (1.37 mL, 15.0 mmol) The resulting mixture was refluxed overnight. The reaction was allowed to cool to room temperature and then quenched with a saturated aqueous solution of NH_4Cl (30 mL). After extracting with Et_2O (3 x 100 mL), the organic layer was dried ($MgSO_4$), filtered and concentrated. Purification by flash column chromatography afforded the title compounds.

General protocol for Nickel-catalysed ring opening of 2,3-Dihydrofuran (**GP2**)

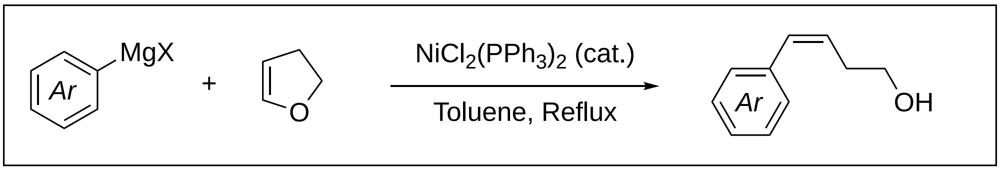

A variant of the protocol described by Brown and co-workers was employed.[5]

Preparation of the Grignard reagent stock solution: A heat gun-dried round-bottomed flask fitted with a reflux condenser was charged with magnesium turnings (370 mg, 15.2 mmol) and three small iodine crystals. A solution of the appropriate chloride/bromide/iodide (15.0 mmol) in dry Et_2O (10 mL) was added dropwise and the reaction mixture was maintained at a gentle reflux either through cooling with an ice bath or through gentle warming. Transfer of the chloride/bromide/iodide was made quantitative with an additional 5 mL of Et_2O. Following this, the stock solution of Grignard reagent was maintained at a gentle reflux for 1 hour.

Ring Opening: Following this, a heat gun-dried three-necked round bottom flask equipped with a large stir bar was charged with $NiCl_2(PPh_3)_2$ (98 mg, 0.15 mmol) and three times evacuated and backfilled with N_2. After the addition of toluene (30 mL), the Grignard reagent (0.15 mL, 0.15 mmol) was added to the stirring mixture. After stirring for 15 minutes, the remaining Grignard reagent (9.85 mL, 9.85 mmol) was added followed by 2,3-dihydrofuran (1.13 mL, 15.0 mmol) The resulting mixture was refluxed overnight. The reaction was allowed to cool to room temperature and then quenched with a saturated aqueous solution of NH_4Cl (30 mL). After extracting with Et_2O (3 x 100 mL), the organic layer was dried ($MgSO_4$), filtered and concentrated. Purification by flash column chromatography afforded the title compounds.

General protocol for the synthesis of *cis*-aryl styrenes *via* Suzuki coupling (**GP3**)

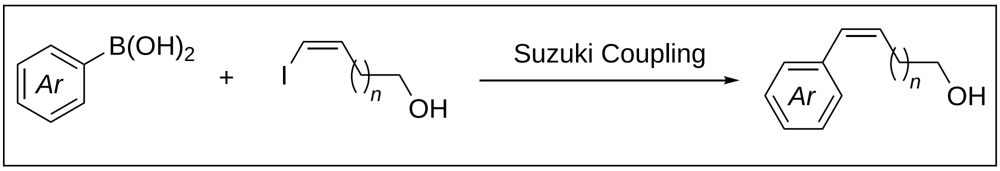

A microwave vial was charged with cesium carbonate (2.0 equiv.), arylboronic acid (2.0 equiv.) and Pd(dppf)Cl_2•CH_2Cl_2 (2.0 mol %) and placed under a nitrogen atmosphere. Vinyl iodide ((*Z*)-5-iodopent-4-en-1-ol or (*Z*)-4-iodobut-3-en-1-ol, 1.0 equiv.) in MeCN (0.15 M) was added and the reaction mixture was heated at 50 °C overnight. After cooling to room temperature, the reaction mixture was diluted in EtOAc and filtered over Celite®, eluting with further EtOAc. The filtrate was collected, the solvent removed under reduced pressure and purification of the crude residue by flash column chromatography afforded the title compounds.

<u>First general protocol for Sonogashira reactions (aryl iodides) (**GP4**)</u>

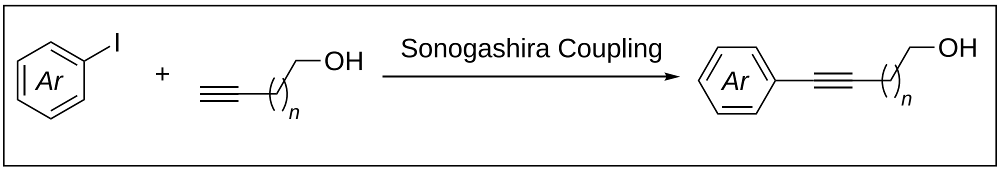

To a solution of aryl iodide (5.0 mmol, 1.0 equiv.) in MeCN (20 mL) at room temperature was added Et_3N (2.7 equiv.). Following this, $(Ph_3P)_2PdCl_2$ (4.5 mol %) and freshly-prepared CuI (9.1 mol %) were added. The resulting mixture was stirred at room temperature for 20 minutes following which the alkyne (1.2 equiv.) was added and stirring continued at room temperature overnight. The solvent was then removed under reduced pressure. Purification of the crude reside by flash column chromatography afforded the title compounds.

Second general protocol for Sonogashira reactions (aryl bromides) (**GP5**)

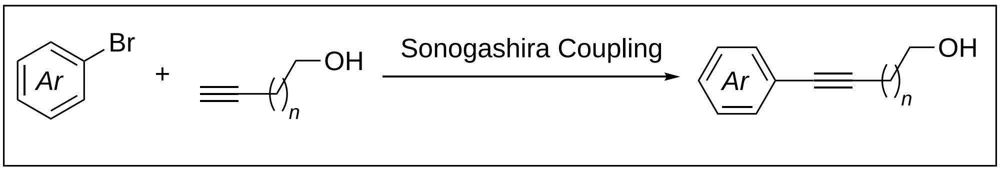

A crimp-top vial was charged with the aryl bromide (5.0 mmol), $(Ph_3P)_2PdCl_2$ (4 mol %) and freshly-prepared CuI (6.5 mol %). The vial was sealed and then evacuated and backfilled with N_2 three times, following which THF (12 mL) and ${}^{i}Pr_2NH$ (7.1 mL) were added. The reaction mixture was stirred for 15 minutes following which the alkyne (7.5 mmol) was added and the reaction heated at 65 °C overnight. The reaction mixture was then allowed to cool, diluted with CH_2Cl_2 (100 mL) and the organic layer washed successively with saturated aqueous NH_4Cl (30 mL) and saturated aqueous $NaHCO_3$ (30 mL). The organic layer was then dried ($MgSO_4$) and the solvent removed under reduced pressure. Purification by flash column chromatography afforded the title compounds.

General protocol for Nickel-catalysed alkyne semi-reduction (**GP6**)

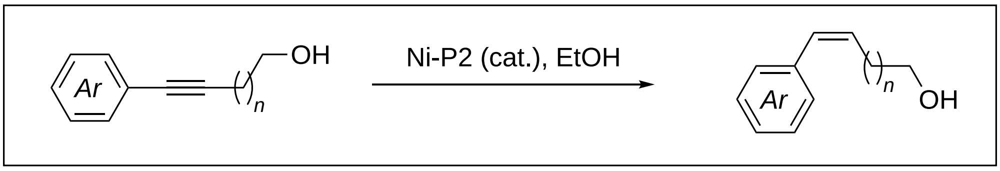

A crimp-top vial was charged with $Ni(OAc)_2{\bullet}4H_2O$ (124 mg, 0.5 mmol) and EtOH (5.2 mL). The reaction flask was then capped with a fresh suba seal following which it was evacuated until the solvent started to boil and backfilled with a balloon of hydrogen gas. This process was repeated a further two times following which the reaction mixture was left under an atmosphere of hydrogen gas. A solution of $NaBH_4$ (18.9 mg, 0.5 mmol) in EtOH (1.3 mL) was then added upon which the reaction mixture turned black and the reaction was stirred for 1 hour. Following this, ethylenediamine (0.12 mL, 1.7 mmol) was added, followed by a solution

of the alkyne (2.5 mmol) in EtOH (5.0 mL). The reaction mixture was then stirred at room temperature for a further 10 hours. Following this it was filtered through Celite®, eluting with a large volume of EtOAc. The volatiles were then removed under reduced pressure. Purification of the crude residue by flash column chromatography afforded the title compounds.

General protocol for intermolecular Wittig reactions (**GP7**)

Note: To obtain reproducible results, it is essential to use freshly-prepared and thoroughly-dried phosphonium salts and freshly-distilled ketones.

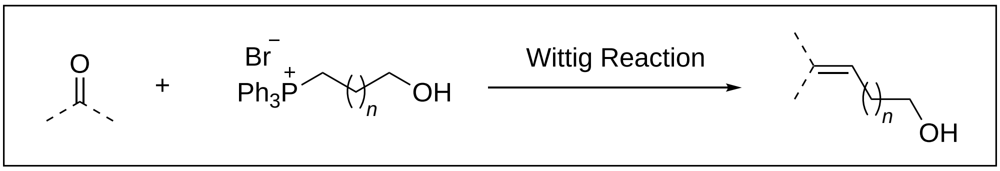

To a solution of the alcohol phosphonium salt (5.8 mmol, 1.2 equiv.) in THF (40 mL) at – 10 °C was added *n*-BuLi (7.2 mL of a 1.6 M solution in hexane, 11.5 mmol, 2.4 equiv.) *via* syringe pump over 15 minutes. The resulting solution was stirred at – 10 °C for an hour following which a solution of the ketone (4.8 mmol, 1.0 equiv.) in THF (10 mL) was added *via* syringe pump over 15 minutes. Transfer of the ketone was made quantitative with THF (5 mL). The reaction mixture was slowly allowed to warm to room temperature overnight, following which it was quenched with water (50 mL) and the THF removed under reduced pressure. The aqueous layer was extracted with Et_2O (3 x 50 mL) and the combined organic fractions were dried ($MgSO_4$) and the solvent removed under reduced pressure. Purification by flash column chromatography afforded the title compounds.

General protocol for the synthesis of *Exo*-dialkyl alkenyl alcohols (**GP8**)

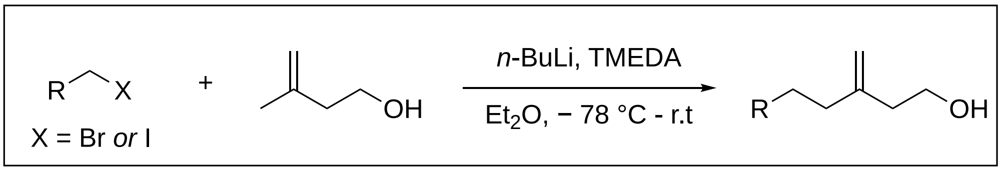

The protocol described by Chong and co-workers was employed.[6] To a solution of TMEDA (5.2 equiv.) in Et_2O (1.73 M) was added *n*-BuLi (4.0 equiv., 1.6 M in hexanes) dropwise at 0 ˚C. The resultant solution was allowed to stir at room temperature for 1 hour before cooling it to 0 ˚C. 2-methyl-1-buten-4-ol (2.0 equiv.) was added dropwise at 0 ˚C and the resultant mixture was stirred at room temperature for 6 hours to give the desired dianion as a hetereogeneous yellow suspension. The suspension was cooled to – 78 ˚C, followed by the dropwise addition of a solution of the appropriate electrophile (1.0 equiv.) in Et_2O (1.67 M). The reaction mixture was slowly warmed to room temperature overnight and stirred at room temperature for an additional 6 hours. The reaction was quenched with sat. NH_4Cl solution and the layers were separated. The aqueous layer was extracted with Et_2O, and the combined organic layers dried ($MgSO_4$) and concentrated *in vacuo*. The alkene product was purified via silica flash column chromatography.

General protocol for intermolecular aziridination reactions (**GP9**)

Under an atmosphere of air, a 4.0 mL crimp-top vial was charged with the substrate (0.1 mmol, 1.0 equiv.) and Rh_2(**B**)$_2$•(**C3**)$_2$•(Pyr)$_2$ (1.0 mol %). A solution of 2,2,3,3-pentafluoropropyl sulfamate (**2**) (0.12 mmol, 1.2 equiv.) in 1,3-difluorobenzene (0.5 mL, 0.2 M) was then added *via* syringe. The vial was then cooled to – 35 °C over 10 minutes. Following this, pentafluoroiodsobenzene (PFIOB) (0.2 mmol, 2.0 equiv.) and (perfluorophenyl)-λ^3-iodanediyl bis(2,2,2-trifluoroacetate) (TFA-Ox) (10 mol %) were added together in a single portion. The vial was then capped and the reaction mixture stirred at the same temperature for 14 hours. Sat. aq. thiourea (1.5 mL) and $CHCl_3$ (1.5 mL) were then added and the biphasic mixture was stirred vigorously for 5 minutes at – 35 °C. The mixture was then allowed to warm to room temperature and stirred for a further 15 minutes. The layers were separated and the

aqueous layer extracted with $CHCl_3$ (3 x 2 mL). The combined organic layers were dried ($MgSO_4$) and the aziridine products purified by flash column chromatography.

Cyclisation Protocol: In some cases, we chose to perform intramolecular ring-opening of the aziridine with the pendant alcohol in order to aid subsequent analysis. In such cases the combined organic layers were filtered ($MgSO_4$), concentrated and re-dissolved in MeCN (3.5 mL). The solution was then transferred to a crimp-top vial and heated at 82 °C for 2-24 hours. Following this, the MeCN was removed under reduced pressure. Purification of the crude residue by flash column chromatography afforded the title compounds.

Note on intermolecular amination reactions performed using $Rh_2(esp)_2$: In order to obtain racemic SFC/HPLC/GC traces, racemic intermolecular amination reactions were run alongside the corresponding chiral ones. The racemic reactions were performed according to **GP9** with the following procedural modification: $Rh_2(esp)_2$ was used as the catalyst at 2 mol % loading and the 10 mol % of (perfluorophenyl)-λ^3-iodanediyl bis(2,2,2-trifluoroacetate) (TFA-OX) was omitted.

Note on Substrate and Product Stabilities

We have found that the some of the alkenyl alcohol substrates will decompose if left on the benchtop over extended periods. As a result, we recommend that these substrates are used fresh in the subsequent aziridination reaction in order to obtain optimal results. Likewise, the aziridine products are generally stable to isolation and characterisation but will decompose if left on the benchtop over extended periods.

Further Investigation of the Role of Axial Pyridine Ligation

Supplementary Table 1: External axial ligand study

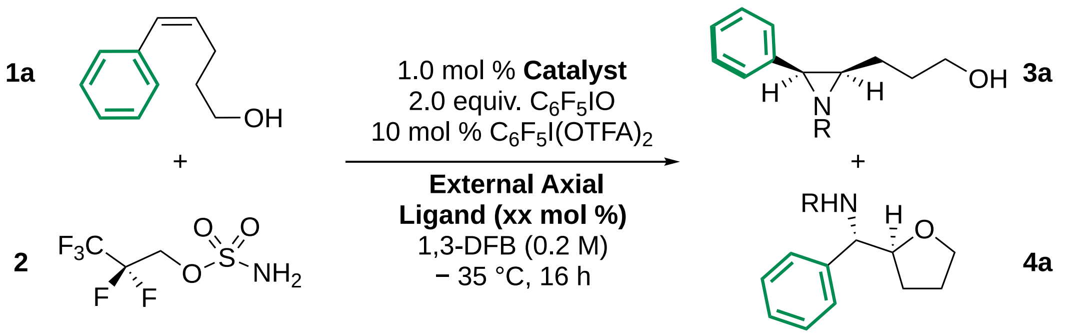

Entry	Catalyst	External Axial Ligand (mol %)	Yield (3a + 4a)/%[a]	*ee*/%[b]
1	Rh_2(**B**)$_2$•(**C3**)$_2$	None	80	+ 82
2	Rh_2(**B**)$_2$•(**C3**)$_2$	Pyridine (2 mol %)	85	+ 88
3	Rh_2(**B**)$_2$•(**C3**)$_2$	2-Cl Pyridine (2 mol %)	79	+ 84
4	Rh_2(**B**)$_2$•(**C3**)$_2$•(Pyr)$_2$	None	76 (58)	+ 93 (+92)

[a]Reactions performed on 0.1 mmol scale with respect to **1a** and using 1.2 equivalents of **2**. Yields determined by ^{1}H NMR with reference to a 1,2-dimethoxyethane internal standard. Data in parentheses corresponds to the isolated sample of **4a** following full conversion of (**3a** + **4a**) to **4a**.[b]*ee* determined by chiral SFC analysis of the crude reaction mixture following full conversion of (**3a**+**4a**) to **4a** by heating in MeCN for 2 hours.

Using the desolvated catalyst (in which it is assumed that the chiral cations occupy the axial sites of the dimer) under the optimised conditions leads to product formation in 80% yield and 82% *ee* (**Supplementary Table 1**, entry 1). If external axial ligands are added at 2 mol % loading (with the aim of displacing the chiral cations from the axial dimer sites) the selectivity is improved (**Supplementary Table 1,** entries 2 and 3), particularly with pyridine. The best

selectivity of all (93% *ee*) is obtained when the pre-formed pyridine solvate is used (**Supplementary Table 1**, entry 4) as per the optimised reaction conditions.

Further Investigation of the Acidic Additive

Supplementary Table 2: Acid additive loading study

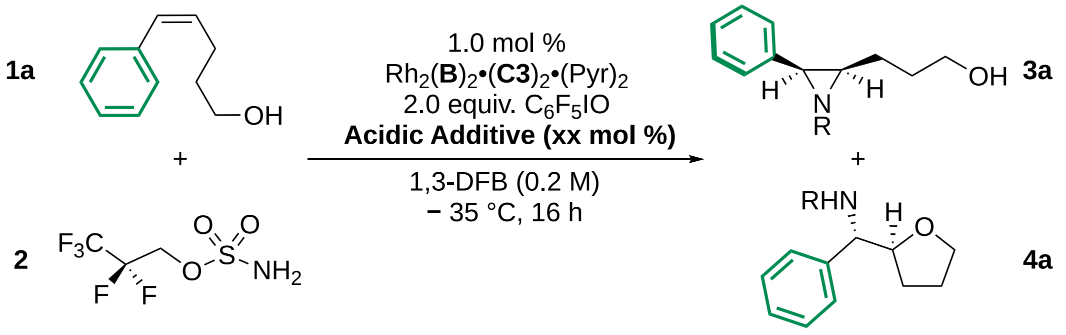

Entry	Acidic Additive (mol %)	Yield (3a + 4a)/%[a]	*ee*/%[b]	Comment
1	None	65	+ 74	
2	TFA-Ox (2 mol %)	76 (58)	+ 93 (+ 92)	
3	TFA-Ox (5 mol %)	67	+ 93	
4	TFA-Ox (10 mol %)	76	+ 93	
5	TFA-Ox (20 mol %)	77	+ 93	
6	TFA-Ox (50 mol %)	65	+ 90	
7	TFA-Ox (200 mol %.)	14	+ 33	C_6F_5IO Omitted
8	TFA (20 mol %)	80	+ 93	

[a]Reactions performed on 0.1 mmol scale with respect to **1a** and using 1.2 equivalents of **2**. Yields determined by ^1H NMR with reference to a 1,2-dimethoxyethane internal standard. Data in parentheses corresponds to the isolated sample of **4a** following full conversion of (**3a**+**4a**) to **4a**. [b]*ee* determined by chiral SFC analysis of the crude reaction mixture following full conversion of (**3a**+**4a**) to **4a** by heating in MeCN for 2 hours.

The results of the additive loading study demonstrate that between 2 mol % and 20 mol % loading the beneficial effect of the additive on enantioselectivity is maintained within the limits of instrumental error (**Supplementary Table 2**, entries 2-5). However, when the additive loading is increased beyond 20 mol %, the combined yield and enantioselectivity start to decrease (**Supplementary Table 2**, entry 6). When the stoichiometric C_6F_5IO oxidant is

omitted and 200 mol % of TFA-Ox used as both the oxidant and as the source of acid a drastic decrease in reaction performance is observed (**Supplementary Table 2**, entry 7). When 20 mol % of TFA was used as the additive in place of 10 mol % TFA-Ox (1.0 equivalent of TFA-Ox releases 2.0 equivalents of TFA), the results were identical within the limits of instrumental error (**Supplementary Table 2**, entries 4 and 8). During the substrate scope, an additive loading of 10 mol % TFA-Ox was chosen.

Supplementary Table 3: Investigation of other acidic additives

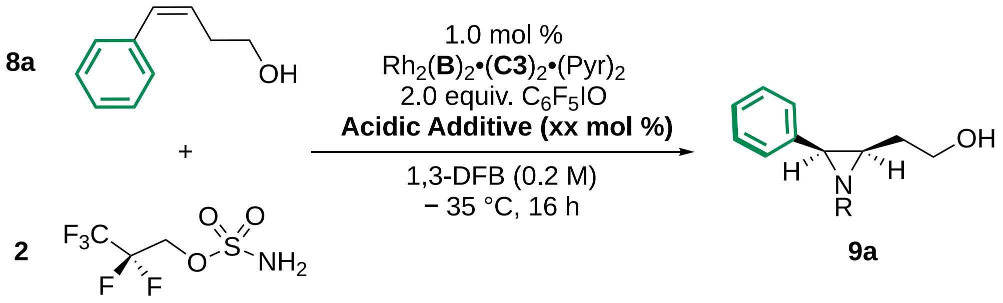

Entry	Acidic Additive (mol %)	Yield (9a)/%[a]	*ee*/%[b]
1	TFA-Ox (10 mol %)	(67)	(+ 87)
2	$PhI(OPiv)_2$ (10 mol %)	74	+ 70
3	PhI(OTs)(OH) (20 mol %)	71	+ 70
4	$C_6H_5CO_2H$ (20 mol %)	79	+ 79
5	$C_6F_5CO_2H$ (20 mol %)	71	+ 86
6	$F_3C(CF_2)_8CO_2H$ (20 mol %)	66	+ 86
7	$(C_6H_5O)_2P(O)OH$ (20 mol %)	73	+ 72
8	2,4,6-^{i}Pr-$C_6H_2SO_3H$ (20 mol %)	72	+ 80
9	$TsOH•H_2O$ (20 mol %)	68	+ 81
10	$C_6F_5SO_3H$ (20 mol %)	62	+ 75

[a]Reactions performed on 0.1 mmol scale with respect to **8a** and using 1.2 equivalents of **2**. Yields determined by ^{1}H NMR with reference to a 1,2-dimethoxyethane internal standard. Data in parentheses corresponds to the isolated sample. [b]*ee* determined by chiral SFC analysis of the crude reaction mixture.

In a separate experiment the nature of the acid additive was investigated on the aziridination of the homoallylic substrate **8a**. The results demonstrate that the best enantioselectivities are obtained using fluorinated carboxylic acids (**Supplementary Table 3**, entries 1,5 and 6).

Synthesis of Chiral Cation Bromide Salts

We are very grateful to Dr Georgi R. Genov for elucidating the synthetic route to the chiral cation shown below and for an initial gift of dihydroquinine-derived chiral cation and chiral cation precursor.

The syntheses of cations **C1** *and* **C2** *have been reported previously.*[7]

1,3-bis(triethylsilyl)benzene

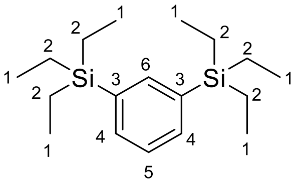

To a stirred solution of 1,3-dibromobenzene (6 mL, 50 mmol, 1.0 equiv.) in THF (100 mL) at – 78 ˚C was added *n*-BuLi (32.5 mL, 1.6 M in hexanes, 52 mmol, 1.04 equiv.) dropwise under an inert atmosphere of nitrogen. The reaction mixture was allowed to stir at – 78 ˚C for 30 min before the dropwise addition of chlorotriethylsilane (8.5 mL, 50.6 mmol, 1.01 equiv.). The reaction mixture was allowed to warm to room temperature overnight. The reaction mixture was subsequently cooled to – 78 ˚C before the dropwise addition of *n*-BuLi (37.5 mL, 1.6 M in hexanes, 60 mmol, 1.20 equiv.). The reaction mixture was allowed to stir for 30 min before the dropwise addition of chlorotriethylsilane (9.65 mL, 57.5 mmol, 1.15 equiv.). The reaction mixture was allowed to warm to room temperature overnight and quenched with saturated aqueous NH_4Cl. The aqueous phase was extracted with EtOAc and the combined organic phases were dried over $MgSO_4$ and concentrated *in vacuo*. Purification by flash column chromatography (SiO_2, Petrol) afforded the title compound as a colourless oil (12.2 g, 39.8 mmol, 80%).

***R_f* value** = 0.64 (Petrol);

^{1}H NMR (400 MHz, $CDCl_3$) δ 7.62 (d, J = 0.7 Hz, 1H, H-6), 7.49 (dd, J = 7.3, 1.1 Hz, 2H, H-4), 7.35-7.31 (m, 1H, H-5), 0.98 (t, J = 7.8 Hz, 18H, H-1), 0.83-0.77 (m, 12H, H-2) ppm;
^{13}C NMR (101 MHz, $CDCl_3$) δ 140.3 (C-6), 136.3 (C-3), 134.7 (C-4), 127.1 (C-5), 7.6 (C-1), 3.5 (C-2) ppm;

The spectroscopic data is in agreement with that reported in the literature.[8]

(5-(4,4,5,5-tetramethyl-1,3,2-dioxaborolan-2-yl)-1,3-phenylene)bis(triethylsilane)

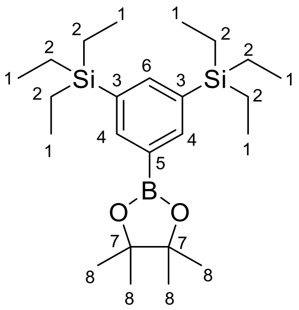

A heatgun-dried 100 mL round-bottomed flask charged with $[Ir(OMe)(COD)]_2$ (113.4 mg, 0.17 mmol, 1.5 mol%), 4,4-di-tert-butyl-2,2-dipyridyl (87.2 mg, 0.32 mmol, 2.8 mol%) and B_2pin_2 (5.51 g, 21.7 mmol, 2.0 equiv.), was evacuated and back-filled thrice with argon. Argon-sparged THF (30 mL) was added to the reaction mixture to give a dark-red solution, which was heated at 50 ˚C for 1.5 hours and cooled to room temperature. Two heatgun-dried vials, each charged with 1,3-bis(triethylsilyl)benzene (1.53 g, 5.0 mmol, 1.0 equiv.), were evacuated and back-filled with argon thrice. The dark-red stock solution (15 mL) was added to each vial and heated at 50 ˚C overnight. The reaction mixtures were combined and concentrated *in vacuo*. Purification by flash column chromatography (SiO_2, 0-5% v/v EtOAc in Petrol) afforded the title compound as a yellow oil (3.68 g, 8.48 mmol, 85%) that solidified on standing to give a white amorphous solid.

***R_f* value** = 0.52 (5% v/v EtOAc in Petrol);

^{1}H NMR (400 MHz, $CDCl_3$) δ 7.91 (d, J = 1.2 Hz, 2H, H-4), 7.71 (t, J = 1.3 Hz, 1H, H-6), 1.34 (s, 12H, H-8), 0.97 (t, J = 7.7 Hz, 18H, H-1), 0.84-0.78 (m, 12H, H-2) ppm;

^{13}C NMR (101 MHz, $CDCl_3$) δ 143.3 (C-6), 141.2 (C-4), 135.1 (C-3), 83.7 (C-7), 25.0 (C-8), 7.6 (C-1), 3.5 (C-2) ppm;

Note: Carbon C-5 was not observed due to quadrupolar relaxation;

HRMS (ESI) *The compound did not ionise*.

(3,3'',5,5''-tetrakis(triethylsilyl)-[1,1':3',1''-terphenyl]-5'-yl)methanol

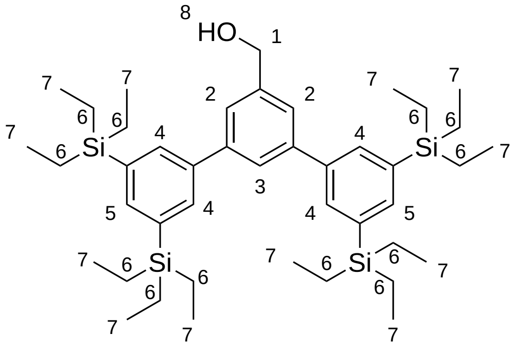

To a round-bottomed flask charged with 3,5-dibromobenzaldehyde (441 mg, 1.7 mmol, 1.0 equiv.), (5-(4,4,5,5-tetramethyl-1,3,2-dioxaborolan-2-yl)-1,3-phenylene)bis(triethylsilane) (1.66 g, 3.8 mmol, 2.3 equiv.), and [1,1'-bis(diphenylphosphino)ferrocene] dichloropalladium(II) (1:1 complex with CH_2Cl_2, 41 mg, 0.050 mmol, 3.0 mol %) under an argon atmosphere was added degassed 1,4-Dioxane (20 mL). Na_2CO_3 (1.8 mL of a 2.0 M aqueous solution, 3.7 mmol, 2.2 equiv.) was subsequently added, and the resulting mixture stirred under reflux for 16 hours. The reaction mixture was allowed to cool to room temperature and the majority of the 1,4-Dioxane was removed under a stream of compressed air. The resulting slurry was extracted with CH_2Cl_2 and the combined organic fractions were dried ($MgSO_4$) and the solvent removed under reduced pressure. MeOH (30 mL) was then added, along with a

small amount of 1,4-Dioxane to aid solvation and the resulting solution cooled to 0 °C, following which $NaBH_4$ (129 mg, 3.4 mmol, 2.0 equiv.) was added portionwise. Monitoring of the reaction by TLC indicated sluggish conversion of the aldehyde to the alcohol so $LiBH_4$ (37.0 mg, 1.7 mmol, 1.0 equiv.) was added and the reaction allowed to warm to room temperature over 2 hours. The reaction was subsequently quenched *via* addition of 3 M HCl and the volatiles were removed under reduced pressure. The aqueous layer was extracted with CH_2Cl_2 (2 x 50 mL) and the combined organic extracts were washed with 3 M HCl (30 mL), dried ($MgSO_4$) and the solvent removed under reduced pressure. Purification by flash column chromatography (SiO_2, 0-20% v/v EtOAc in Petrol) afforded the title compound as an amorphous white solid (873 mg, 1.2 mmol, 72%).

***R_f* value** = 0.37 (5% v/v EtOAc in Hexane);

^{1}H NMR (500 MHz, $CDCl_3$) δ 7.73 (d, *J* = 1.1 Hz, 4H, H-4), 7.72 (t, *J* = 1.7 Hz, 1H, H-3), 7.63 (t, *J* = 1.1 Hz, 2H, H-5), 7.57 (d, *J* = 1.6 Hz, 2H, H-2), 4.87 (d, *J* = 6.0 Hz, 2H, H-1), 1.78 (t, *J* = 7.8 Hz, 1H, H-8) 1.01 (t, *J* = 7.8 Hz, 36H, H-7), 0.82-0.88 (m, 24H, H-6);

^{13}C NMR (126 MHz, $CDCl_3$) δ 143.2, 141.9, 139.6, 139.4, 137.0, 133.7, 126.3, 124.9, 65.8, 7.6, 3.6 ppm;

IR (film, cm^{-1}): ν_{max} 3300 (br, w), 2952 (s), 2908 (m), 2874 (s), 1599 (w), 1573 (w), 1457 (m), 1415 (m), 1372 (m), 1236 (m), 1181 (w), 1140 (m), 1004 (s), 973 (m), 897 (w), 861 (m), 799 (s), 750 (m), 715 (s), 687 (s), 647 (m), 594 (m), 581 (m), 447 (w), 417 (s);

HRMS (+ESI) *m/z* Found $[M–OH]^+$ 699.4628, $[C_{43}H_{71}Si_4]^+$ requires 699.4627, (δ = – 0.1 ppm).

(5'-(bromomethyl)-[1,1':3',1''-terphenyl]-3,3'',5,5''-tetrayl)tetrakis(triethylsilane)

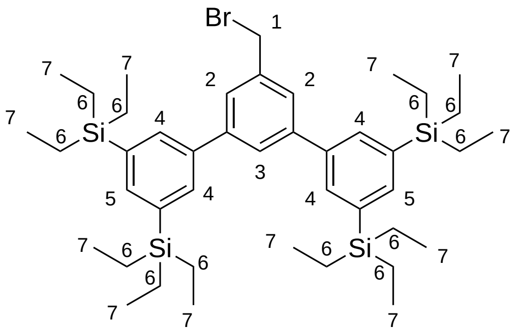

A round-bottomed flask was charged with (3,3'',5,5''-tetrakis(triethylsilyl)-[1,1':3',1''-terphenyl]-5'-yl)methanol (861 mg, 1.2 mmol), CBr_4 (597 mg, 1.8 mmol) and then evacuated and backfilled with nitrogen three times. Following this, CH_2Cl_2 (30 mL) was added and the resulting solution cooled to 0 °C. A solution of PPh_3 (477 mg, 1.8 mmol) in CH_2Cl_2 (8 mL) was added dropwise *via* syringe pump and the reaction mixture was allowed to slowly warm to room temperature and stirred for 36 hours. The solvent was removed under reduced pressure, and purification by flash column chromatography (SiO_2, 0-5% v/v Et_2O in Petrol) afforded the title compound as a white amorphous solid (888 mg, 1.1 mmol, 95%).

***R$_f$* value** = 0.30 (100% Hexane);

^{1}H NMR (400 MHz, $CDCl_3$) *δ* 7.70-7.71 (m, 5H, H-3, H-4), 7.64 (t, *J* = 0.9 Hz, 2H, H-5), 7.57 (d, *J* = 1.5 Hz, 2H, H-2), 4.67 (s, 2H, H-1), 1.01 (t, *J* = 7.8 Hz, 36H, H-7), 0.82-0.88 (m, 24H, H-6) ppm;

^{13}C NMR (101 MHz, $CDCl_3$) *δ* 143.4, 139.7, 139.1, 138.7, 137.1, 133.6, 127.0, 126.8, 33.9, 7.6, 3.6 ppm;

IR (film, cm^{-1}): ν_{max} 2952 (s), 2908 (m), 2873 (s), 1598 (w), 1573 (w), 1457 (m), 1415 (m), 1372 (m), 1331 (w), 1236 (m), 1215 (m), 1141 (m), 1004 (s), 973 (m), 906 (w), 865 (w), 799 (s), 715 (s), 688 (s), 646 (m), 580 (m), 446 (w), 417 (m);

HRMS (ESI) *The compound did not ionise.*

(1S,2R,4S,5R)-5-ethyl-2-((S)-hydroxy(6-methoxyquinolin-4-yl)methyl)-1-((3,3'',5,5''-tetrakis(triethylsilyl)-[1,1':3',1''-terphenyl]-5'-yl)methyl)quinuclidin-1-ium bromide (**C3•Br**)

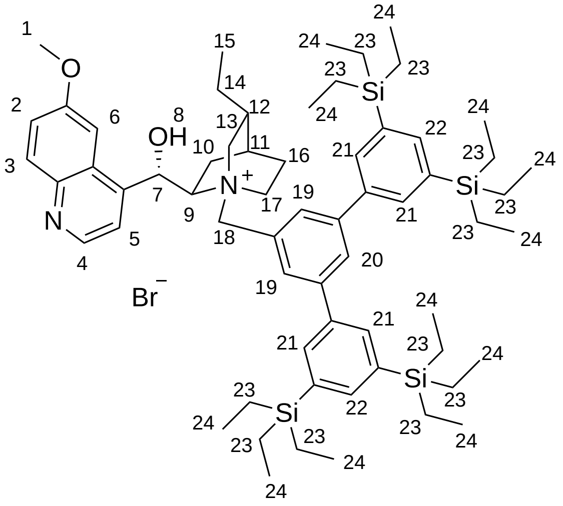

The protocol described by Phipps and co-workers was employed.[9] A crimp-top vial was charged with dihydroquinidine[9] (195 mg, 0.60 mmol) and (5'-(bromomethyl)-[1,1':3',1''-terphenyl]-3,3'',5,5''-tetrayl)tetrakis(triethylsilane) (468 mg, 0.60 mmol). The vial was sealed and evacuated and backfilled with nitrogen three times, following which dry THF (15 mL) was added. The reaction mixture was then heated at 75 °C overnight following which it was allowed to cool and the THF removed under reduced pressure. The crude residue was taken up in CH_2Cl_2 (20 mL) and washed twice with water (2 x 10 mL). The organic layer was dried ($MgSO_4$) and the solvent removed under reduced pressure. Purification by flash column chromatography (SiO_2, 0-10% v/v MeOH in CH_2Cl_2) afforded the title compound as an off-white solid (310 mg, 0.28 mmol, 47%).

***R**$_f$* **value** = 0.68 (10% v/v MeOH in CH_2Cl_2);

^{1}H NMR (500 MHz, $CDCl_3$) *δ* 8.74 (d, *J* = 4.6 Hz, 1H, H-4), 8.09 (d, *J* = 9.3 Hz, 1H, H-3), 7.88-7.89 (m, 4H, H-5, H-19, H-20), 7.74 (d, *J* = 1.0 Hz, 4H, H-21), 7.69 (t, *J* = 1.0 Hz, 2H, H-22), 7.38 (dd, *J* = 9.2 Hz, 2.6 Hz, 1H, H-2), 7.32-7.34 (m, 2H, H-6, H-8), 6.74 (d, *J* = 5.6 Hz, 1H, H-7), 6.43 (d, *J* = 12.4 Hz, 1H, H-18a), 4.74 (d, *J* = 12.3 Hz, 1H, H-18b), 4.52-4.56 (m, 1H, H-13a), 3.98 (s, 3H, H-1), 3.78 (t, *J* = 9.0 Hz, 1H, H-9), 3.71 (t, *J* = 11.1 Hz, 1H, H-13b), 3.55 (br s, 1H, H-17a), 3.14 (q, *J* = 10.3 Hz, 1H, H-17b), 2.53 (t, *J* = 11.6 Hz, 1H, H-10a), 1.92 (br s, 1H, H-11), 1.78-1.81

(m, 2H, H-16a, H-16b), 1.57-1.73 (m, 3H, H-12, H-14a, H-14b), 1.01-1.04 (m, 37H, H-10b, H-24), 0.92 (t, *J* = 7.1 Hz, 3H, H-15), 0.84-0.89 (m, 24H, H-23) ppm;

^{13}C NMR (126 MHz, $CDCl_3$) δ 158.3, 147.3*, 144.3†, 144.1, 143.5†, 140.2, 138.3, 137.5, 133.7, 131.8*, 131.0, 129.2, 128.1, 126.1, 121.1, 120.9, 102.3, 69.8, 64.9, 64.7, 57.8, 56.2, 56.1, 36.4, 24.9, 24.8, 24.5, 21.5, 11.6, 7.7, 3.6 ppm;

*Weak signal but clearly visible in the ^{1}H-^{13}C HSQC spectrum.

†Weak signal but clearly visible in the ^{1}H-^{13}C HMBC spectrum.

IR (film, cm^{-1}): ν_{max} 2953 (s), 2909 (m), 2874 (s), 1621 (m), 1598 (w), 1508 (m), 1459 (m), 1416 (m), 1374 (m), 1329 (m), 1142 (m), 1006 (m), 886 (m), 800 (m), 720 (s), 690 (w);

$[\boldsymbol{\alpha}]_{\mathbf{D}}^{\mathbf{25.0}}$ = + 74.3 (c. 0.9, $CHCl_3$);

HRMS (+ESI) *m/z* For cation found 1025.6608, $[C_{63}H_{97}N_2O_2Si_4]^+$ requires 1025.6622, (δ = – 1.4 ppm).

(1S,2S,4S,5R)-5-ethyl-2-((R)-hydroxy(6-methoxyquinolin-4-yl)methyl)-1-((3,3'',5,5''-tetrakis(triethylsilyl)-[1,1':3',1''-terphenyl]-5'-yl)methyl)quinuclidin-1-ium bromide (**C4•Br**)

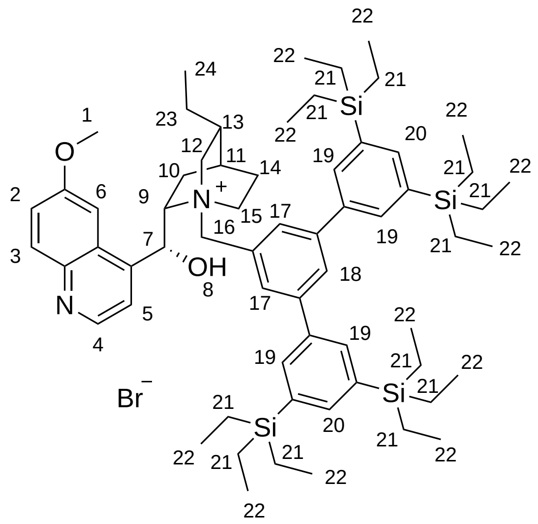

Prepared by Dr Georgi R. Genov for which we are very grateful. The protocol described by Phipps and co-workers was employed.[9] A crimp-top vial was charged with dihydroquinine[9] (652 mg, 2.0 mmol) and (5'-(bromomethyl)-[1,1':3',1''-terphenyl]-3,3'',5,5''-tetrayl)tetrakis(triethylsilane) (1.56 g, 2.0 mmol). The vial was sealed and evacuated and backfilled with nitrogen three times, following which dry THF (15 mL) was added. The reaction mixture was then heated at 80 °C overnight following which it was allowed to cool and the THF removed under reduced pressure. The crude residue was taken up in CH_2Cl_2 (20 mL) and washed twice with water (2 x 10 mL). The organic layer was dried ($MgSO_4$) and the solvent removed under reduced pressure. Purification by flash column chromatography (SiO_2, 1-5% v/v MeOH in CH_2Cl_2) afforded the title compound as an off-pink solid (1.67 g, 1.5 mmol, 75%).

***R_f* value** = 0.37 (8% v/v MeOH in CH_2Cl_2);

^{1}H NMR (500 MHz, $CDCl_3$) δ 8.80 (d, J = 4.5 Hz, 1H, H-4), 8.10 (d, J = 9.3 Hz, 1H, H-3), 8.00 (s, 2H, H-17), 7.90 (t, J = 1.5 Hz, 1H, H-18), 7.83 (d, J = 4.6 Hz, 1H, H-5), 7.77 (d, J = 1.0 Hz, 4H, H-19), 7.68 (t, J = 1.0 Hz, 2H, H-20), 7.42 (dd, J = 9.3 Hz, 2.6 Hz, 1H, H-2), 7.24 (d, J = 2.6 Hz, 1H, H-6), 6.90 (br s, 2H, H-7, H-8), 6.80 (d, J = 12.1 Hz, 1H, H-16a), 5.25-5.30 (m, 1H, H-15a), 4.29 (d, J = 12.0 Hz, 1H, H-16b), 3.97 (s, 3H, H-1), 3.77 (dd, J = 12.3 Hz, 10.9 Hz, 1H, H-12a), 3.60 (dd, J = 10.3 Hz, 6.4 Hz, 1H, H-9), 3.20 (q, J = 9.9 Hz, 1H, H-15b), 2.61-2.66 (m, 1H, H-12b), 2.39-2.45 (m, 2H, H-10a, H-14a), 2.05 (br s, 1H, H-11), 1.80-1.86 (m, 1H, H-13), 1.67-1.69 (m,

1H, H-14b)*, 1.46-1.51 (m, 1H, H-10b), 1.27-1.35 (m, 2H, H-23a, H-23b), 1.02 (t, *J* = 7.8 Hz, 36H, H-22), 0.83-0.89 (m, 27H, H-21, H-24) ppm;

*Overlapped with residual water peak

^{13}C NMR (126 MHz, $CDCl_3$) *δ* 158.2, 148.1, 144.4, 144.0, 143.6, 140.2, 138.2, 137.6, 133.7, 132.7, 131.0, 129.3, 127.8, 125.9, 120.9, 120.7, 101.9, 71.7, 65.0, 64.1, 63.2, 56.2, 51.2, 36.4, 26.6, 25.4, 24.4, 21.3, 11.4, 7.7, 3.6 ppm;

IR (film, cm^{-1}): ν_{max} 2952 (s), 2909 (m), 2874 (m), 1621 (w), 1598 (w), 1509 (w), 1458 (w), 1416 (w), 1374 (w), 1328 (w), 1239 (m), 1143 (m), 1006 (m), 974 (w), 906 (w), 866 (w), 826 (w), 800 (m), 719 (s), 690 (m), 647 (w);

$[\boldsymbol{\alpha}]_{\mathbf{D}}^{\mathbf{25.0}}$ = – 108.8 (c. 0.4, $CHCl_3$);

HRMS (+ESI) *m/z* For cation found 1025.6605, $[C_{63}H_{97}N_2O_2Si_4]^+$ requires 1025.6622, (*δ* = – 1.7 ppm).

(1R,2S,4R)-2-((R)-hydroxy(6-methoxyquinolin-4-yl)methyl)-1-((3,3'',5,5''-tetrakis(triethylsilyl)-[1,1':3',1''-terphenyl]-5'-yl)methyl)quinuclidin-1-ium bromide (**C5•Br**)

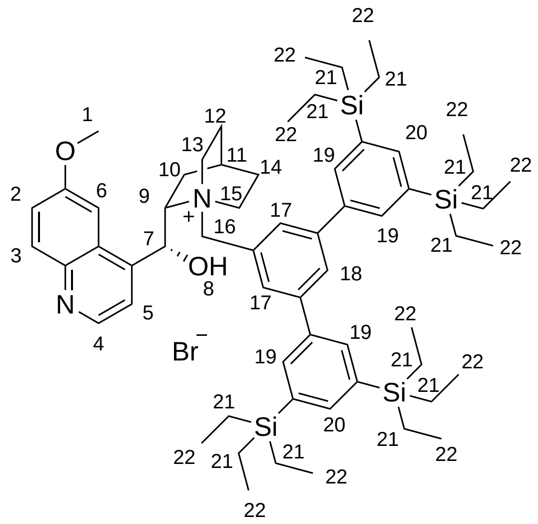

The protocol described by Phipps and co-workers was employed.[9] A crimp-top vial was charged with desvinylquinine[9] (167 mg, 0.56 mmol) and (5'-(bromomethyl)-[1,1':3',1''-terphenyl]-3,3'',5,5''-tetrayl)tetrakis(triethylsilane) (437 mg, 0.56 mmol). The vial was sealed and evacuated and backfilled with nitrogen three times, following which dry THF (15 mL) was added. The reaction mixture was then heated at 75 °C overnight following which it was allowed to cool and the THF removed under reduced pressure. The crude residue was taken up in CH_2Cl_2 (20 mL) and washed twice with water (2 x 10 mL). The organic layer was dried ($MgSO_4$) and the solvent removed under reduced pressure. Purification by flash column chromatography (SiO_2, 0-10% v/v MeOH in CH_2Cl_2) afforded the title compound as an off-white solid (314 mg, 0.29 mmol, 52%).

R

$\boldsymbol{R_f}$ **value** = 0.46 (10% v/v MeOH in CH_2Cl_2);

^{1}H NMR (500 MHz, $CDCl_3$) δ 8.73 (d, *J* = 4.6 Hz, 1H, H-4), 8.12 (d, *J* = 9.2 Hz, 1H, H-3), 7.97 (br s, 2H, H-17), 7.89-7.90 (m, 2H, H-5, H-18), 7.77 (d, *J* = 1.0 Hz, 4H, H-19), 7.68 (br s, 2H, H-20), 7.39 (dd, *J* = 9.3 Hz, 2.5 Hz, 1H, H-2), 7.35 (br s, 1H, H-6), 7.19 (d, *J* = 6.0 Hz, 1H, H-8), 6.82 (d, *J* = 6.1 Hz, 1H, H-7), 6.55 (d, *J* = 12.2 Hz, 1H, H-16a), 5.10 (t, *J* = 11.6 Hz, 1H, H-15a), 4.56 (d, *J* = 12.1 Hz, 1H, H-16b), 4.01 (s, 3H, H-1), 3.91 (t, *J* = 8.8 Hz, 1H, H-9), 3.48 (br s, 1H, H-13a), 3.35-3.41 (m, 2H, H-13b, H-15b), 2.43-2.47 (m, 1H, H-10a), 2.20-2.23 (m, 1H, H-14a), 2.13 (br s, 1H, H-11), 1.83 (br s, 2H, H-12a, H-12b), 1.70 (t, *J* = 11.1 Hz, 1H, H-14b), 1.35 (t, *J* = 11.3 Hz, 1H, H-10b), 1.02 (t, *J* = 8.0 Hz, 36H, H-22), 0.84-0.89 (m, 24H, H-21) ppm;

^{13}C NMR (126 MHz, $CDCl_3$) δ 158.5, 146.5*, 145.3†, 144.0, 142.7†, 140.2, 138.3, 137.5, 133.7, 131.13, 130.99, 129.3, 127.9, 126.3, 121.7, 121.0, 102.4, 70.4, 64.8, 64.1, 57.8, 56.3, 51.4, 26.1, 24.1, 24.0, 20.6, 7.6, 3.6 ppm;

*Weak signal but clearly visible in the ^{1}H-^{13}C HSQC spectrum.

†Weak signal but clearly visible in the ^{1}H-^{13}C HMBC spectrum.

IR (neat, cm^{-1}): ν_{max} 2950 (m), 2908 (m), 2873 (m), 1509 (w), 1457 (w), 1415 (w), 1373 (w), 1238 (m), 1141 (w), 1004 (m), 963 (w), 898 (w), 886 (m), 828 (w), 798 (m), 717 (s), 688 (s), 646 (m), 580 (m), 415 (m);

$[\boldsymbol{\alpha}]_{\mathbf{D}}^{\mathbf{25.0}}$ = – 71.1 (c. 1.0, $CHCl_3$);

HRMS (+ESI) *m/z* For cation found 997.6334, $[C_{61}H_{93}N_2O_2Si_4]^+$ requires 997.6309, (δ = + 2.5 ppm).

Synthesis of Chiral Rh(II,II) Tetracarboxylate Dimers

The syntheses of catalysts Rh_2(**A**)$_2$•(**C1**)$_2$, Rh_2(**A**)$_2$•(**C2**)$_2$, Rh_2(**B**)$_2$•(**C2**)$_2$, Rh_2(**C**)$_2$•(**C2**)$_2$, Rh_2(**D**)$_2$•(**C2**)$_2$ and Rh_2(**E**)$_2$•(**C2**)$_2$ *have been reported previously.*[9]

Bis[rhodium (1S,2R,4S,5R)-5-ethyl-2-((S)-hydroxy(6-methoxyquinolin-4-yl)methyl)-1-((3,3'',5,5''-tetrakis(triethylsilyl)-[1,1':3',1''-terphenyl]-5'-yl)methyl)quinuclidin-1-ium (3,5-bis(2-carboxy-2-methylpropyl)benzenesulfonate)] (Rh_2(**A**)$_2$•(**C3**)$_2$)

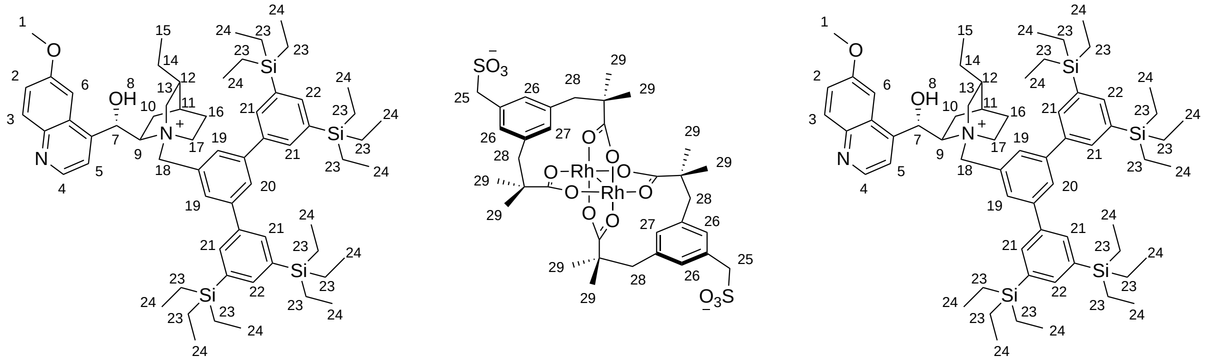

A sinter funnel was loosely packed with a pad of Amberlite® IRC120 H hydrogen form beads (approximately 6 cm column height) and equilibrated by flushing through with three column volumes of MeOH. Following this, a solution of Bis[rhodium tetrabutylammonium (3,5-bis(2-carboxy-2-methylpropyl)phenyl)methanesulfonate)][9] (58.5 mg, 0.041 mmol) in MeOH (4 mL) was loaded onto the pad and eluted with light suction. The eluent was then recycled through the pad a further four times. Following this, the eluent was evaporated to dryness and taken up in water (10 mL). Drops of 10% NaOH (aq.) were added until the solution was basic. A 3:1 mixture of $CHCl_3$/^{i}PrOH (20 mL) was added, followed by (1S,2R,4S,5R)-5-ethyl-2-((S)-hydroxy(6-methoxyquinolin-4-yl)methyl)-1-((3,3'',5,5''-tetrakis(triethylsilyl)-[1,1':3',1''-terphenyl]-5'-yl)methyl)quinuclidin-1-ium bromide (**C3•Br**) (81.8 mg, 0.074 mmol). The biphasic mixture was stirred rapidly overnight during which the blue colour of the aqueous phase disappeared and a deep red colour was observed to move into the organic phase. The

layers were separated and the aqueous phase extracted with a 3:1 mixture of $CHCl_3$/^{i}PrOH (3 x 20 mL). The organic fractions were dried ($MgSO_4$) and the solvent removed under reduced pressure. Desolvation of the catalyst was achieved by heating at 90 °C overnight under vacuum to afford the title compound as a brown powder (102.7 mg, 0.034 mmol, 84%).

Formation of the pyridine solvate is achieved by taking a portion of the desolvated material and dissolving it fully in pyridine to give a bright-pink solution. The excess pyridine is then removed under a stream of compressed air and the oily residue dried thoroughly under vacuum to a free-flowing pink powder. Following this the solvated catalyst is ready for use.

^{1}H NMR (500 MHz, C_5D_5N) δ 9.07 (d, *J* = 4.5 Hz, 2H, H-4), 8.68 (br s, 2H, H-8), 8.61 (s, 4H, H-19), 8.37 (d, *J* = 9.2 Hz, 2H, H-3), 8.28 (br s, 10H, H-20, H-21), 8.05 (d, *J* = 4.4 Hz, 2H, H-5), 8.00 (s, 4H, H-22), 7.92 (d, *J* = 1.7 Hz, 2H, H-6), 7.55 (dd, *J* = 9.2 Hz, 2.5 Hz, 2H, H-2), 7.16-7.17 (m, 6H, H-26, H-27), 7.06 (br s, 2H, H-7), 6.02 (d, *J* = 12.0 Hz, 2H, H-18a), 5.52 (d, *J* = 12.3 Hz, 2H, H-18b), 4.52 (t, *J* = 9.2 Hz, 2H, H-13a), 4.36-4.45 (m, 6H, H-17a, H-25a, H-25b), 4.23 (t, *J* = 9.2 Hz, 2H, H-9), 4.00 (s, 6H, H-1), 3.79 (t, *J* = 11.0 Hz, 2H, H-13b), 3.30 (q, *J* = 10.1 Hz, 2H, H-17b), 2.62 (t, *J* = 11.3 Hz, 2H, H-10a), 2.50 (d, *J* = 12.3 Hz, 4H, H-28a), 2.41 (d, *J* = 12.3 Hz, 4H, H-28b), 1.67 (br s, 1H, H-11), 1.58 (q, *J* = 10.3 Hz, 2H, H-16a), 1.39-1.48 (m, 8H, H-12, H-14a, H-14b, H-16b), 1.09-1.12 (m, 74H, H-10b, H-24), 0.98-1.03 (m, 72H, H-23, H-29a, H-29b), 0.77 (t, *J* = 7.1 Hz, 6H, H-15) ppm;

^{13}C NMR (126 MHz, C_5D_5N) δ 197.2, 159.0, 148.5, 145.7, 144.9, 144.3, 140.8, 139.9, 138.7, 138.1, 134.8, 134.7, 132.9, 132.6, 131.2, 130.8, 130.1, 129.2, 127.4, 122.1, 121.8, 103.6, 69.3, 66.5, 64.0, 59.4, 57.5, 56.8, 56.5, 47.5, 46.3, 36.4, 26.48-26.49 (m), 25.5, 25.1, 25.0, 21.9, 11.9, 8.3, 4.2 ppm;

IR (neat, cm^{-1}): ν_{max} 2951 (m), 2909 (m), 2873 (m), 1586 (m), 1456 (m), 1406 (m), 1374 (m), 1240 (m), 1225 (m), 1170 (m), 1141 (m), 1033 (m), 1003 (m), 973 (w), 865 (m), 798 (m), 718 (s), 688 (s), 647 (m), 629 (m), 602 (m), 570 (m), 529 (m), 501 (w), 448 (m), 415 (m);

$[\boldsymbol{\alpha}]_{\mathbf{D}}^{\mathbf{25.0}}$ = + 57.0 (c. 0.1, $CHCl_3$);

HRMS (+ESI) *m/z* For cation found 1025.6604, $[C_{63}H_{97}N_2O_2Si_4]^+$ requires 1025.6622, (δ = – 1.8 ppm).

HRMS (−ESI) *m/z* For *monoanion* found, 945.0183, $[C_{34}H_{42}O_{14}Rh_2S_2+H]^-$ requires 945.0210, (δ = – 2.9 ppm).

Bis[rhodium (1S,2R,4S,5R)-5-ethyl-2-((S)-hydroxy(6-methoxyquinolin-4-yl)methyl)-1-((3,3'',5,5''-tetrakis(triethylsilyl)-[1,1':3',1''-terphenyl]-5'-yl)methyl)quinuclidin-1-ium (3,5-bis((1-carboxycyclobutyl)methyl)phenyl)methanesulfonate)] (Rh_2(**B**)$_2$•(**C3**)$_2$)

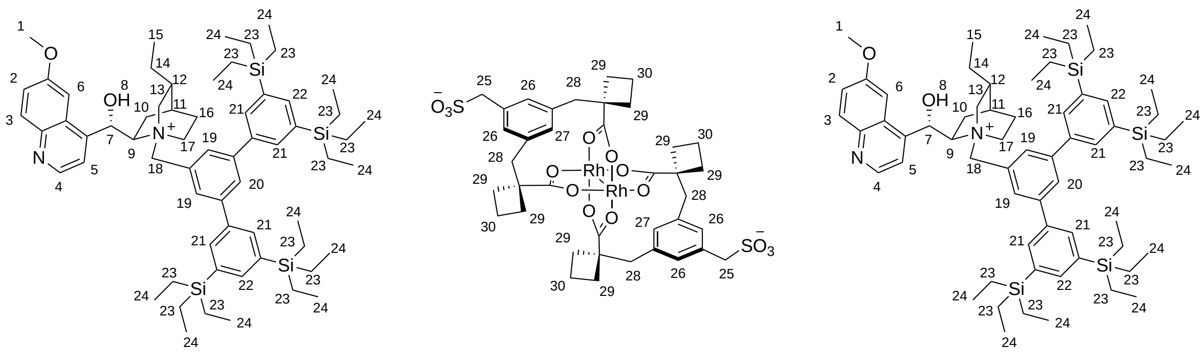

A sinter funnel was loosely packed with a pad of Amberlite® IRC120 H hydrogen form beads (approximately 6 cm column height) and equilibrated by flushing through with three column volumes of MeOH. Following this, a solution of Bis[rhodium tetrabutylammonium (3,5-bis((1-carboxycyclobutyl)methyl)phenyl)methanesulfonate)][9] (60.5 mg, 0.041 mmol) in MeOH (4 mL) was loaded onto the pad and eluted with light suction. The eluent was then recycled through the pad a further four times. Following this, the eluent was evaporated to dryness and taken up in water (10 mL). Drops of 10% NaOH (aq.) were added until the solution was basic. A 3:1 mixture of $CHCl_3$/^{i}PrOH (20 mL) was added, followed by (1*S*,2*R*,4*S*,5*R*)-5-ethyl-2-((*S*)-hydroxy(6-methoxyquinolin-4-yl)methyl)-1-((3,3'',5,5''-tetrakis(triethylsilyl)-[1,1':3',1''-terphenyl]-5'-yl)methyl)quinuclidin-1-ium bromide (**C3•Br**) (81.8 mg, 0.074 mmol). The biphasic mixture was stirred rapidly overnight during which the blue colour of the aqueous phase disappeared and a deep red colour was observed to move into the organic phase. The

layers were separated and the aqueous phase extracted with a 3:1 mixture of $CHCl_3$/^{i}PrOH (3 x 20 mL). The organic fractions were dried ($MgSO_4$) and the solvent removed under reduced pressure. Desolvation of the catalyst was achieved by heating at 90 °C overnight under vacuum to afford the title compound as a brown powder (101.4 mg, 0.033 mmol, 81%).

Formation of the pyridine solvate is achieved by taking a portion of the desolvated material and dissolving it fully in pyridine to give a bright-pink solution. The excess pyridine is then removed under a stream of compressed air and the oily residue dried thoroughly under vacuum to a free-flowing pink powder. Following this the solvated catalyst is ready for use. The data presented below is for the desolvated catalyst apart from the specific rotation which corresponds to the pyridine solvate.

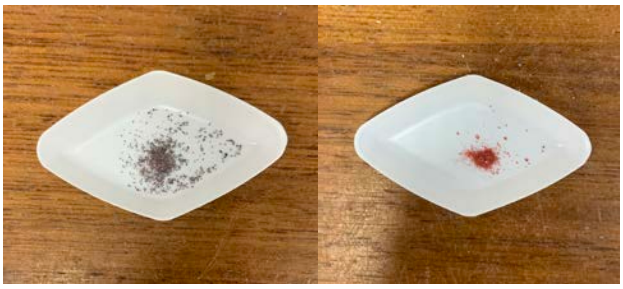

Figure S1. ***Appearance of desolvated (left) and pyridine-solvated chiral catalyst (right).***

^{1}H NMR (500 MHz, C_5D_5N) δ 9.11 (d, J = 4.4 Hz, 2H, H-4), 8.61 (br s, 4H, H-19), 8.56-8.58 (m, 2H, H-8), 8.42 (d, J = 9.2 Hz, 2H, H-3), 8.27-8.28 (m, 10H, H-20, H-21), 8.06 (d, J = 4.2 Hz, 2H, H-5), 8.00 (s, 4H, H-22), 7.87 (br s, 2H, H-6), 7.59 (s, 2H, H-2)*, 7.20 (s, 4H, H-26), 7.10 (br s, 4H, H-7, H-27), 6.15 (d, J = 12.3 Hz, 2H, H-18a), 5.47 (d, J = 12.3 Hz, 2H, H-18b), 4.52 (t, J = 8.9 Hz, 2H, H-13a), 4.40 (t, J = 10.7 Hz, 2H, H-17a), 4.18-4.21 (m, 6H, H-9, H-25a, H-25b), 3.99 (s, 6H, H-1), 3.80 (t, J = 10.6 Hz, 2H, H-13b), 3.36 (q, J = 10.2 Hz, 2H, H-17b), 2.80 (d, J = 12.5 Hz, 4H, H-28a), 2.73 (d, J = 12.5 Hz, 4H, H-28b), 2.65 (t, J = 11.6 Hz, 2H, H-10a), 2.30-2.33 (m, 8H, H-29a), 1.60-1.80 (m, 20H, H-11, H-16a, H-29b, H-30a, H-30b) 1.46-1.50 (m, 8H, H-12, H-14a, H-14b, H-16b), 1.09-1.12 (m, 74H, H-10b, H-24), 0.98-1.03 (m, 48H, H-23), 0.76 (t, J = 7.1 Hz, 6H, H-15) ppm;

*Obscured by the residual solvent peak but clearly visible in the ^{1}H-^{1}H COSY.

^{13}C NMR (126 MHz, C_5D_5N) δ 196.5, 159.0, 148.5, 145.7, 145.0, 144.3, 140.8, 139.9, 138.4, 138.1, 135.4, 134.9, 132.9, 132.6, 130.7, 130.5, 129.9, 129.3, 127.4, 122.1, 121.8, 103.5, 69.6, 66.2, 64.3, 59.3, 57.6, 56.7, 56.5, 52.0, 44.4, 36.5, 31.2, 25.5, 25.2, 24.9, 21.8, 16.2, 11.9, 8.3, 4.2 ppm;

IR (neat, cm^{-1}): ν_{max} 2951 (s), 2909 (m), 2873 (s), 1582 (s), 1458 (m), 1406 (s), 1374 (m), 1225 (m), 1173 (m), 1034 (s), 1017 (s), 1003 (s), 973 (w), 888 (w), 886 (m), 798 (s), 758 (s), 716 (s), 686 (m), 647 (m), 600 (m), 581 (m), 563 (m), 528 (s), 454 (w), 414 (s);

$[\boldsymbol{\alpha}]_{\mathbf{D}}^{\mathbf{25.0}}$ = + 52.6 (c. 0.08, $CHCl_3$) (acquired as the pyridine solvate);

HRMS (+ESI) *m/z* For cation found 1025.6631, $[C_{63}H_{97}N_2O_2Si_4]^+$ requires 1025.6622, (δ = + 0.9 ppm).

HRMS (−ESI) *m/z* For dianion found, 496.0062 $[C_{38}H_{42}O_{14}Rh_2S_2]^{2-}$ requires 496.0069, (δ = – 1.4 ppm).

Bis[rhodium (1S,2S,4S,5R)-5-ethyl-2-((R)-hydroxy(6-methoxyquinolin-4-yl)methyl)-1-((3,3'',5,5''-tetrakis(triethylsilyl)-[1,1':3',1''-terphenyl]-5'-yl)methyl)quinuclidin-1-ium (3,5-bis((1-carboxycyclobutyl)methyl)phenyl)methanesulfonate)] (Rh_2(**B**)$_2$•(**C4**)$_2$)

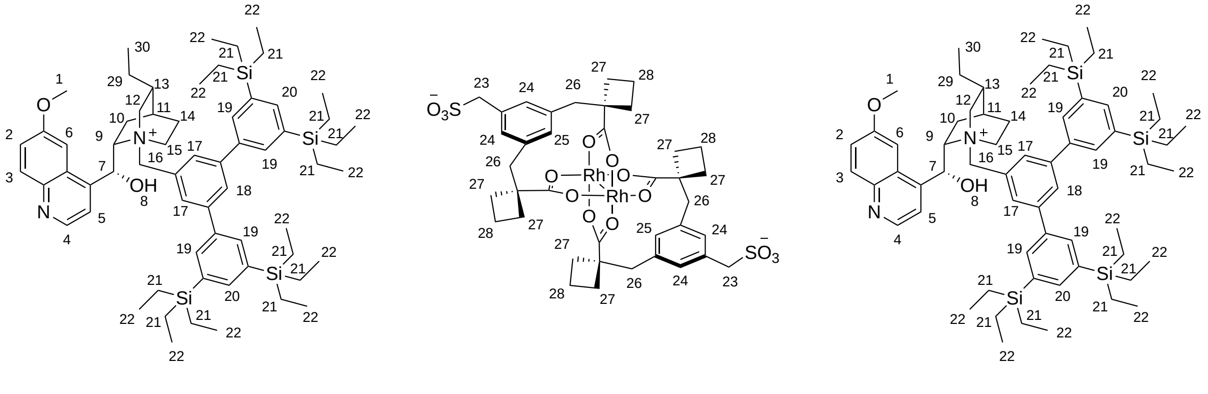

A sinter funnel was loosely packed with a pad of Amberlite® IRC120 H hydrogen form beads (approximately 6 cm column height) and equilibrated by flushing through with three column volumes of MeOH. Following this, a solution of Bis[rhodium tetrabutylammonium (3,5-bis((1-carboxycyclobutyl)methyl)phenyl)methanesulfonate)][9] (60.5 mg, 0.041 mmol) in MeOH (4 mL) was loaded onto the pad and eluted with light suction. The eluent was then recycled through the pad a further four times. Following this, the eluent was evaporated to dryness and taken up in water (10 mL). Drops of 10% NaOH (aq.) were added until the solution was basic. A 3:1 mixture of $CHCl_3$/^{i}PrOH (20 mL) was added, followed by (1*S*,2*S*,4*S*,5*R*)-5-ethyl-2-((*R*)-hydroxy(6-methoxyquinolin-4-yl)methyl)-1-((3,3'',5,5''-tetrakis(triethylsilyl)-[1,1':3',1''-terphenyl]-5'-yl)methyl)quinuclidin-1-ium bromide* (**C4•Br**) (81.8 mg, 0.074 mmol). The biphasic mixture was stirred rapidly overnight during which the blue colour of the aqueous phase disappeared and a deep red colour was observed to move into the organic phase. The layers were separated and the aqueous phase extracted with a 3:1 mixture of $CHCl_3$/^{i}PrOH (3 x 20 mL). The organic fractions were dried ($MgSO_4$) and the solvent removed under reduced pressure. Desolvation of the catalyst was achieved by heating at 90 °C overnight under vacuum to afford the title compound as a brown powder (109 mg, 0.035 mmol, 87%).

**Obtained as a gift from Dr Georgi R. Genov for which we are very grateful*

Formation of the pyridine solvate is achieved by taking a portion of the desolvated material and dissolving it fully in pyridine to give a bright-pink solution. The excess pyridine is then removed under a stream of compressed air and the oily residue dried thoroughly under vacuum to a free-flowing pink powder. Following this the solvated catalyst is ready for use.

^{1}H NMR (500 MHz, C_5D_5N) δ 9.18 (d, *J* = 4.5 Hz, 2H, H-4), 8.57 (br s, 4H, H-17), 8.44 (d, *J* = 9.1 Hz, 2H, H-3), 8.28 (br s, 8H, H-19), 8.25 (t, *J* = 1.4 Hz, 2H, H-18), 8.01 (t, *J* = 0.9 Hz, 4H, H-20), 7.95 (d, *J* = 4.5 Hz, 2H, H-5), 7.88 (br s, 2H, H-8), 7.54-7.58 (m, 4H, H-2, H-6), 7.22-7.25 (m, 2H, H-7)*, 7.17 (br s, 4H, H-24), 6.97 (d, *J* = 12.2 Hz, 2H, H-16a), 6.89 (br s, 2H, H-25), 4.81 (d, *J* = 12.1 Hz, 2H, H-16b), 4.76 (t, *J* = 10.1 Hz, 2H, H-15a), 4.21 (s, 4H, H-23a, H-23b), 3.98 (t, *J* = 11.4 Hz, 2H, H-12a), 3.83 (dd, *J* = 10.2 Hz, 5.4 Hz, 2H, H-9), 3.77 (s, 6H, H-1), 3.36-3.40 (m, 2H, H-

12b), 3.01-3.16 (m, 2H, H-15b), 2.67-2.72 (m, 8H, H-26a, H-26b), 2.46-2.48 (m, 2H, H-10a), 2.25-2.33 (m, 10H, H-14a, H-27a), 1.70-1.78 (m, 14H, H-27b, H-28a, H-11), 1.50-1.61 (m, 8H, H-13, H-10b, H-28b), 1.30-1.36 (m, 2H, H-14b), 1.15-1.24 (m, 4H, H-29a, H-29b), 1.10 (t, J = 7.6 Hz, 72H, H-22), 0.98-1.03 (m, 48H, H-21), 0.73 (t, J = 7.3 Hz, 6H, H-30) ppm;

*Partially obscured by the residual solvent peak but clearly visible in the ^{1}H-^{13}C HSQC spectrum.

^{13}C NMR (126 MHz, C_5D_5N) δ 196.4, 158.9, 148.5, 145.8, 145.6, 144.6, 140.9, 140.0, 138.3, 138.2, 134.9, 134.8, 132.9, 132.6, 130.5, 130.2, 129.9, 129.5, 127.0, 122.1, 121.5, 102.6, 72.0, 65.0, 63.8, 62.9, 59.1, 56.1, 51.9, 51.4, 44.3, 36.4, 31.1-31.2 (m), 26.4, 26.0, 25.5, 20.8, 16.2, 11.7, 8.2, 4.2 ppm;

IR (neat, cm^{-1}): ν_{max} 2951 (m), 2933 (m), 2910 (m), 2873 (m), 1622 (w), 1579 (m), 1512 (w), 1456 (m), 1405 (m), 1373 (w), 1223 (w), 1174 (w), 1142 (w), 1034 (m), 1005 (m), 973 (w), 899 (w), 865 (m), 830 (w), 798 (m), 716 (s), 646 (m), 559 (m), 521 (m), 473 (w), 415 (w);

$[\boldsymbol{\alpha}]_{\mathbf{D}}^{\mathbf{25.0}}$ = – 61.8 (c. 0.1, $CHCl_3$);

HRMS (+ESI) *m/z* For cation found 1025.6660, $[C_{63}H_{97}N_2O_2Si_4]^+$ requires 1025.6622, (δ = + 3.7 ppm).

HRMS (−ESI) *m/z* For dianion found, 496.0063, $[C_{38}H_{42}O_{14}Rh_2S_2]^{2-}$ requires 496.0069, (δ = – 1.2 ppm).

Bis[rhodium (1R,2S,4R)-2-((R)-hydroxy(6-methoxyquinolin-4-yl)methyl)-1-((3,3'',5,5''-tetrakis(triethylsilyl)-[1,1':3',1''-terphenyl]-5'-yl)methyl)quinuclidin-1-ium (3,5-bis((1-carboxycyclobutyl)methyl)phenyl)methanesulfonate)] (Rh_2(**B**)$_2$•(**C5**)$_2$)

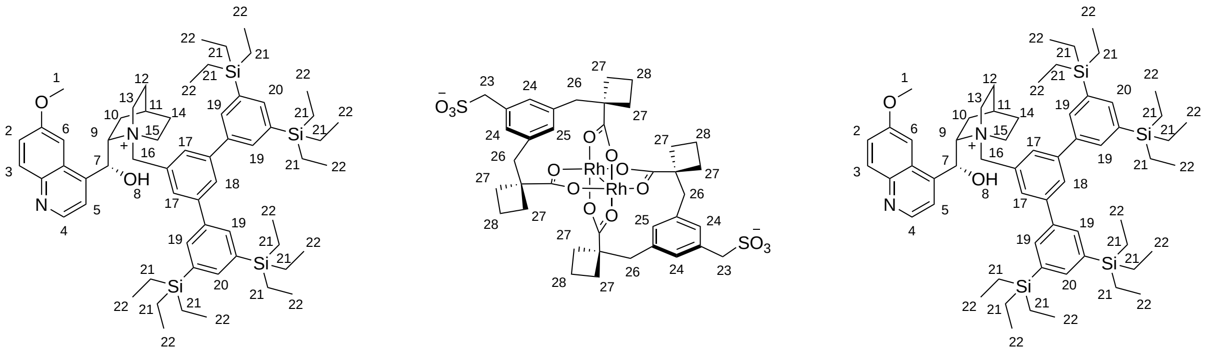

A sinter funnel was loosely packed with a pad of Amberlite® IRC120 H hydrogen form beads (approximately 6 cm column height) and equilibrated by flushing through with three column volumes of MeOH. Following this, a solution of Bis[rhodium tetrabutylammonium (3,5-bis((1-carboxycyclobutyl)methyl)phenyl)methanesulfonate)][9] (60.5 mg, 0.041 mmol) in MeOH (4 mL) was loaded onto the pad and eluted with light suction. The eluent was then recycled through the pad a further four times. Following this, the eluent was evaporated to dryness and taken up in water (10 mL). Drops of 10% NaOH (aq.) were added until the solution was basic. A 3:1 mixture of $CHCl_3$/^{i}PrOH (20 mL) was added, followed by (1*R*,2*S*,4*R*)-2-((*R*)-hydroxy(6-methoxyquinolin-4-yl)methyl)-1-((3,3'',5,5''-tetrakis(triethylsilyl)-[1,1':3',1''-terphenyl]-5'-yl)methyl)quinuclidin-1-ium bromide (**C5•Br**) (79.8 mg, 0.074 mmol). The biphasic mixture was stirred rapidly overnight during which the blue colour of the aqueous phase disappeared and a deep red colour was observed to move into the organic phase. The layers were separated and the aqueous phase extracted with a 3:1 mixture of $CHCl_3$/^{i}PrOH (3 x 20 mL). The organic fractions were dried ($MgSO_4$) and the solvent removed under reduced pressure. Desolvation of the catalyst was achieved by heating at 90 °C overnight under vacuum to afford the title compound as a brown powder (110.3 mg, 0.037 mmol, 90%).

Formation of the pyridine solvate is achieved by taking a portion of the desolvated material and dissolving it fully in pyridine to give a bright-pink solution. The excess pyridine is then removed under a stream of compressed air and the oily residue dried thoroughly under vacuum to a free-flowing pink powder. Following this the solvated catalyst is ready for use.

^{1}H NMR (500 MHz, C_5D_5N) δ 9.11 (d, J = 4.5 Hz, 2H, H-4), 8.53 (br s, 4H, H-17), 8.43 (d, J = 9.2 Hz, 2H, H-3), 8.29 (s, 8H, H-19), 8.21 (t, J = 1.5 Hz, 2H, H-18), 8.08 (d, J = 5.5 Hz, 2H, H-8), 8.01

(s, 4H, H-20), 7.93 (d, *J* = 4.5 Hz, 2H, H-5), 7.62 (d, *J* = 2.4 Hz, 2H, H-6), 7.57-7.58 (m, 2H, H-2)*, 7.16-7.19 (m, 6H, H-7, H-24), 6.97 (br s, 2H, H-25), 6.64 (d, *J* = 12.3 Hz, 2H, H-16a), 4.97-5.02 (m, 2H, H-16b)†, 4.71 (t, *J* = 10.0 Hz, 2H, H-15a), 4.21 (s, 4H, H-23a, H-23b), 3.94-3.99 (m, 4H, H-9, H-13a), 3.88 (s, 6H, H-1), 3.57 (dt, *J* = 11.6 Hz, 5.5 Hz, 2H, H-13b), 3.24-3.30 (m, 2H, H-15b), 2.70-2.75 (m, 8H, H-26a, H-26b), 2.50-2.53 (m, 2H, H-10a), 2.28-2.30 (m, 8H, H-27a), 2.00-2.04 (m, 2H, H-14a), 1.87 (br s, 2H, H-11), 1.70-1.80 (m, 12H, H-27b, H-28a), 1.56-1.65 (m, 6H, H-28b, H-12a), 1.48-1.56 (m, 2H, H-12b), 1.33-1.38 (m, 2H, H-14b), 1.24-1.28 (m, 2H, H-10b), 1.11 (t, *J* = 7.7 Hz, 72H, H-22), 0.99-1.04 (m, 48H, H-21) ppm;

*Partially obscured by residual solvent peak but clearly visible in the ^{1}H-^{1}H COSY.

†Partially obscured by residual water peak but clearly visible in the ^{1}H-^{1}H COSY.

^{13}C NMR (126 MHz, C_5D_5N) δ 196.5, 158.9, 148.5, 145.6, 145.5, 144.3, 140.8, 140.1, 138.3, 138.2, 135.0, 134.9, 132.9, 132.6, 130.5, 130.4, 129.9, 129.6, 127.0, 122.1, 121.5, 103.0, 70.8, 64.9, 64.5, 59.1, 57.5, 56.3, 51.9, 51.6, 44.3, 31.2, 25.9, 24.6, 24.3, 21.5, 16.2, 8.3, 4.2 ppm;

IR (neat, cm^{-1}): ν_{max} 2950 (m), 2907 (m), 2872 (m), 1621 (w), 1570 (m), 1512 (w), 1458 (w), 1405 (m), 1373 (w), 1221 (m), 1173 (m), 1140 (m), 1034 (m), 1011 (m), 973 (w), 897 (w), 865 (w), 829 (w), 798 (m), 716 (s), 688 (w), 647 (w), 559 (w), 522 (w), 415 (w);

$[\boldsymbol{\alpha}]_{\mathbf{D}}^{\mathbf{25.0}}$ = – 47.7 (c. 0.1, $CHCl_3$);

HRMS (+ESI) *m/z* For cation found 997.6334, $[C_{61}H_{93}N_2O_2Si_4]^+$ requires 997.6309, (δ = + 2.5 ppm).

HRMS (−ESI) *m/z* For dianion found, 496.0063, $[C_{38}H_{42}O_{14}Rh_2S_2]^{2-}$ requires 496.0069, (δ = – 1.2 ppm).

Bis[rhodium (1S,2R,4S,5R)-5-ethyl-2-((S)-hydroxy(6-methoxyquinolin-4-yl)methyl)-1-((3,3'',5,5''-tetrakis(triethylsilyl)-[1,1':3',1''-terphenyl]-5'-yl)methyl)quinuclidin-1-ium (3,5-bis((1-carboxycyclopentyl)methyl)phenyl)methanesulfonate)] (Rh_2(**C**)$_2$•(**C3**)$_2$)

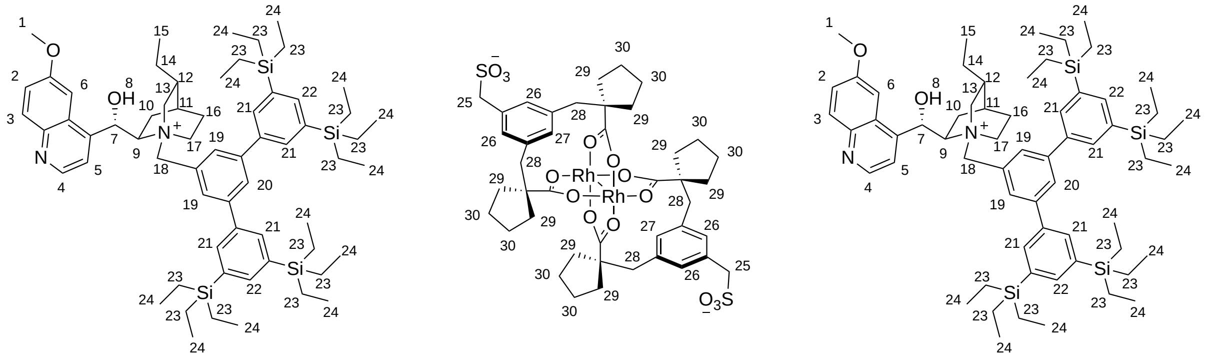

A sinter funnel was loosely packed with a pad of Amberlite® IRC120 H hydrogen form beads (approximately 6 cm column height) and equilibrated by flushing through with three column volumes of MeOH. Following this, a solution of Bis[rhodium tetrabutylammonium (3,5-bis((1-carboxycyclopentyl)methyl)phenyl)methanesulfonate)][9] (62.8 mg, 0.041 mmol) in MeOH (4 mL) was loaded onto the pad and eluted with light suction. The eluent was then recycled through the pad a further four times. Following this, the eluent was evaporated to dryness and taken up in water (10 mL). Drops of 10% NaOH (aq.) were added until the solution was basic. A 3:1 mixture of CHCl3/^{i}PrOH (20 mL) was added, followed by (1*S*,2*R*,4*S*,5*R*)-5-ethyl-2-((*S*)-hydroxy(6-methoxyquinolin-4-yl)methyl)-1-((3,3'',5,5''-tetrakis(triethylsilyl)-[1,1':3',1''-terphenyl]-5'-yl)methyl)quinuclidin-1-ium bromide (**C3•Br**) (81.8 mg, 0.074 mmol). The biphasic mixture was stirred rapidly overnight during which the blue colour of the aqueous phase disappeared and a deep red colour was observed to move into the organic phase. The layers were separated and the aqueous phase extracted with a 3:1 mixture of CHCl3/^{i}PrOH (3 x 20 mL). The organic fractions were dried ($MgSO_4$) and the solvent removed under reduced pressure. Desolvation of the catalyst was achieved by heating at 90 °C overnight under vacuum to afford the title compound as a brown powder (99.0 mg, 0.032 mmol, 78%).

Formation of the pyridine solvate is achieved by taking a portion of the desolvated material and dissolving it fully in pyridine to give a bright-pink solution. The excess pyridine is then

removed under a stream of compressed air and the oily residue dried thoroughly under vacuum to a free-flowing pink powder. Following this the solvated catalyst is ready for use.

^{1}H NMR (500 MHz, C_5D_5N) δ 9.07 (d, *J* = 4.4 Hz, 2H, H-4), 8.65 (d, *J* = 3.8 Hz, 2H, H-8), 8.61 (s, 4H, H-19), 8.38 (d, *J* = 9.2 Hz, 2H, H-3), 8.27-8.28 (m, 10H, H-20, H-21), 8.05 (d, *J* = 4.3 Hz, 2H, H-5), 8.00 (s, 4H, H-22), 7.91 (s, 2H, H-6), 7.56 (dd, *J* = 9.3 Hz, 2.6 Hz, 2H, H-2), 7.18 (br s, 4H, H-26), 7.16 (br s, 2H, H-27), 7.02 (br s, 2H, H-7), 6.01 (d, *J* = 12.3 Hz, 2H, H-18a), 5.47 (d, *J* = 12.2 Hz, 2H, H-18b), 4.52 (t, *J* = 9.4 Hz, 2H, H-13a), 4.42 (t, *J* = 11.3 Hz, 2H, H-17a), 4.32-4.38 (m, 4H, H-25a, H-25b), 4.21 (t, *J* = 9.2 Hz, 2H, H-9), 4.00 (s, 6H, H-1), 3.79 (t, *J* = 10.8 Hz, 2H, H-13b), 3.30 (q, *J* = 10.1 Hz, 2H, H-17b), 2.59-2.63 (m, 6H, H-10a, H-28a), 2.53 (d, *J* = 12.5 Hz, 4H, H-28b), 1.98-2.00 (m, 8H, H-29a), 1.67 (br s, 2H, H-11), 1.59 (q, *J* = 10.4 Hz, 2H, H-16a), 1.32-1.48 (m, 32H, H-12, H-14a, H-14b, H-16b, H-29b, H-30a, H-30b), 1.07-1.12 (m, 74H, H-10b, H-24), 0.98-1.03 (m, 48H, H-23), 0.76 (t, *J* = 7.2 Hz, 6H, H-15) ppm;

^{13}C NMR (126 MHz, C_5D_5N) δ 197.2, 159.0, 148.5, 145.7, 145.0, 144.3, 140.8, 139.9, 139.2, 138.1, 135.2, 134.8, 132.9, 132.6, 130.8, 130.5, 130.1, 129.2, 127.4, 122.1, 121.8, 103.7, 69.4, 66.5, 64.1, 59.4, 58.6, 57.5, 56.8, 56.5, 45.5, 37.0, 36.4, 25.5, 25.3-25.4 (m), 25.1, 25.0, 21.9, 11.9, 8.3, 4.2 ppm;

IR (neat, cm^{-1}): ν_{max} 2952 (m), 2908 (m), 2873 (m), 1622 (w), 1579 (m), 1511 (w), 1454 (w), 1401 (m), 1373 (w), 1240 (m), 1173 (m), 1141 (m), 1034 (m), 1003 (m), 865 (w), 799 (m), 717 (s), 646 (w), 562 (w), 518 (w), 472 (w), 441 (w), 416 (w);

$[\boldsymbol{\alpha}]_{\mathbf{D}}^{\mathbf{25.0}}$ = + 33.9 (c. 0.1, $CHCl_3$);

HRMS (+ESI) *m/z* For cation found 1025.6639, $[C_{63}H_{97}N_2O_2Si_4]^+$ requires 1025.6622, (δ = + 1.7 ppm).

HRMS (−ESI) *m/z* For dianion found, 524.0380 $[C_{42}H_{50}O_{14}Rh_2S_2]^{2-}$ requires 524.0382, (δ = – 0.3 ppm).

Synthesis of Bishomoallylic Styrenyl Alcohols

5-iodopent-4-yn-1-ol

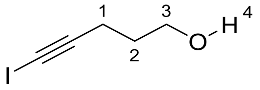

The protocol described by Denmark and co-workers was employed.[10] To a round-bottomed flask charged with 4-pentyn-1-ol (1.68 g, 20.0 mmol) under a nitrogen atmosphere was added MeOH (20.0 mL). After cooling the flask to 0 °C, KOH (freshly ground) (2.81 g, 50.0 mmol) in H_2O (6.0 mL) was added and after 10 minutes, iodine (6.60 g, 26.0 mmol) was added in one portion and the reaction mixture was warmed to room temperature over 3 hours. The reaction mixture was diluted in Et_2O (50 mL) and H_2O (20 mL), the layers were separated and the aqueous layer extracted with further Et_2O. The combined organic layers were concentrated under reduced pressure, re-dissolved in CH_2Cl_2, washed with brine, dried over $MgSO_4$ and concentrated under reduced pressure. Purification by flash column chromatography (SiO_2, 0-7% Acetone in $CHCl_3$) afforded the title compound as a pale-yellow oil (2.11 g, 10.0 mmol, 50%).

***R$_f$* value** = 0.20 (20% v/v Acetone in Hexane);

^{1}H NMR (400 MHz, $CDCl_3$) δ 3.72 (t, *J* = 6.2 Hz, 2H, H-3), 2.48 (t, *J* = 7.0 Hz, 2H, H-1), 1.88 (br s, 1H, H-4), 1.75 (p, *J* = 6.7 Hz, 2H, H-2) ppm;

^{13}C NMR (101 MHz, $CDCl_3$) δ 93.9, 61.5, 31.2, 17.4, − 6.3 ppm.

The spectroscopic data is in agreement with that reported in the literature.[10]

(Z)-5-iodopent-4-en-1-ol

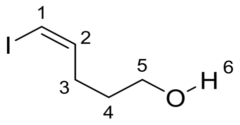

A variant of the protocol described by Denmark and co-workers was employed.[10] An oven-dried 3-necked round-bottomed flask charged with 5-iodopent-4-yn-1-ol (1.33 g, 6.33 mmol) was placed under a nitrogen atmosphere. MeOH (18.0 mL), pyridine (3.1 mL) and potassium azodicarboxylate (737 mg, 3.80 mmol) were added, followed by the dropwise addition of acetic acid (2.44 mL) by syringe pump over 8 hours. During the course of the addition, the remaining portions of potassium azodicarboxylate (4 × 737 mg, giving 19.0 mmol total) were added after 1 hour, 2 hours, 4 hours and 6 hours respectively. After complete addition of the acid, the reaction mixture was quenched with slow addition of aqueous 1 M HCl solution (40 mL). Et_2O (50 mL) was added, the layers were separated, the aqueous layer extracted with further Et_2O and the combined organic layers were washed with aqueous 1 M HCl solution, saturated aqueous $NaHCO_3$ solution and brine. The resulting organic layer was dried over $MgSO_4$, concentrated under reduced pressure and re-dissolved in Et_2O (10 mL) and H_2O (3 mL). *n*-Butylamine (2 mL) was added and the mixture was stirred at room temperature overnight. Et_2O was added and the resulting mixture was washed with aqueous 1 M HCl solution, brine, dried over $MgSO_4$, concentrated under reduced pressure and purified by flash column chromatography (SiO_2, 0-20% Acetone in Hexane) to afford the title compound as a colourless oil (400 mg, 1.89 mmol, 30%).

***R**$_f$* **value** = 0.23 (20% v/v Acetone in Hexane);

^{1}H NMR (400 MHz, $CDCl_3$) δ 6.25-6.16 (m, 2H, H-1, H-2), 3.67 (t, *J* = 6.4 Hz, 2H, H-5), 2.24 (q, *J* = 6.6 Hz, 2H, H-3), 1.70 (p, *J* = 6.7 Hz, 2H, H-4), 1.58 (br s, 1H, H-6) ppm;

^{13}C NMR (101 MHz, $CDCl_3$) δ 140.7, 83.2, 62.2, 31.3, 31.0 ppm.

The spectroscopic data is in agreement with that reported in the literature.[10]

Note: potassium azodicarboxylate was prepared as follows: To a 0 °C solution of KOH (2.88 g, 51.3 mmol) in H_2O (7.2 mL) was added azodicarbonamide in small portions over the period of 2 hours (5 × 233 mg, giving 10.0 mmol in total). After stirring for an additional 2 hours, the bright-yellow solid was filtered off and washed with ice-cold MeOH (10 × 1 mL). Additional product was isolated via precipitation from the filtrate and re-filtration. Drying the solid under high vacuum afforded the desired product, which was stored in the freezer before use.

(Z)-5-phenylpent-4-en-1-ol (**1a**)

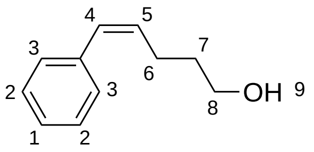

Prepared according to **GP1** on a 10 mmol scale with respect to commercial PhMgBr (1.0 M in THF). Purification by flash column chromatography (SiO_2, 0-30% v/v EtOAc in Petrol) afforded the title compound as a colourless oil (663 mg, 4.1 mmol, 41%).

***R_f* value** = 0.28 (30% v/v EtOAc in Hexane);

^{1}H NMR (400 MHz, $CDCl_3$) δ 7.32-7.36 (m, 2H, H-2), 7.28-7.30 (m, 2H, H-3), 7.23 (tt, J = 7.1 Hz, 1.5 Hz, 1H, H-1), 6.46 (dt, J = 11.6 Hz, 1.9 Hz, 1H, H-4), 5.68 (dt, J = 11.6 Hz, 7.3 Hz, 1H, H-5), 3.67 (t, J = 6.5 Hz, 2H, H-8), 2.43 (qd, J = 7.4 Hz, 1.8 Hz, 2H, H-6), 1.79-1.67 (m, 2H, H-7), 1.39 (s, 1H, H-9) ppm;

^{13}C NMR (101 MHz, $CDCl_3$) δ 137.6, 132.2, 129.6, 128.9, 128.3, 126.7, 62.6, 33.0, 25.0 ppm.

The spectroscopic data is in agreement with that reported in the literature.[11]

(Z)-5-(2-fluorophenyl)pent-4-en-1-ol (**1b**)

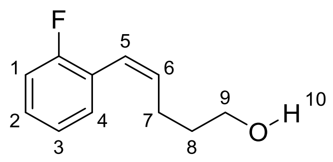

Prepared according to **GP3** on a 1.08 mmol scale with respect to (*Z*)-5-iodopent-4-en-1-ol. 2-Fluorophenylboronic acid was used as the arylboronic acid. Purification by flash column chromatography (SiO_2, 0-4% v/v Acetone in $CHCl_3$) afforded the title compound as a colourless oil (90 mg, 0.50 mmol, 46%).

***R*$_f$ value** = 0.44 (10% v/v Acetone in CHCl3);

^{1}H NMR (400 MHz, $CDCl_3$) δ 7.29 (td, *J* = 7.6, 1.8 Hz, 1H, H-4), 7.22 (m, 1H, H-2), 7.10 (td, *J* = 7.5, 1.1 Hz, 1H, H-3), 7.04 (m, 1H, H-1), 6.46 (d, *J* = 11.5 Hz, 1H, H-5), 5.80 (dt, *J* = 11.5, 7.3, Hz, 1H, H-6), 3.65 (t, *J* = 6.5 Hz, 2H, H-9), 2.32 (qd, *J* = 7.6, 1.2, Hz, 2H, H-7), 1.71 (p, *J* = 7.6 Hz, 2H, H-8), 1.44 (br s, 1H, H-10) ppm;

^{13}C NMR (101 MHz, $CDCl_3$) δ 160.2 (d, $J_{C\text{-}F}$ = 247 Hz), 134.3 (d, $J_{C\text{-}F}$ = 1.1 Hz), 130.6 (d, $J_{C\text{-}F}$ = 3.7 Hz), 128.6 (d, $J_{C\text{-}F}$ = 8.2 Hz), 125.1 (d, $J_{C\text{-}F}$ = 14.8 Hz), 123.7 (d, $J_{C\text{-}F}$ = 3.6 Hz), 122.2 (d, $J_{C\text{-}F}$ = 3.2 Hz), 115.5 (d, $J_{C\text{-}F}$ = 22.3 Hz), 62.5, 32.6, 25.3 (d, $J_{C\text{-}F}$ = 1.1 Hz) ppm;

^{19}F NMR (376 MHz, $CDCl_3$) δ − 115.3 (s) ppm;

HRMS (+ESI) *m/z* found $[M{-}OH]^+$ 163.0924, $[C_{11}H_{12}F]^+$ requires 163.0918, (δ = + 3.7 ppm).

(Z)-5-(o-tolyl)pent-4-en-1-ol (**1c**)

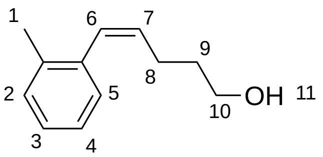

Prepared according to **GP1** on a 10 mmol scale with respect to 2-bromotoluene. Purification by flash column chromatography (SiO_2, 0-24% v/v EtOAc in Petrol) afforded the title compound as a pale-yellow oil (977 mg, 5.5 mmol, 55%).

***R_f* value** = 0.54 (30% v/v EtOAc in Hexane);

^{1}H NMR (400 MHz, $CDCl_3$) δ 7.14-7.19 (m, 4H, H-2, H-3, H-4, H-5), 6.48 (d, J = 11.4 Hz, 1H, H-6), 5.73 (dt, J = 11.4 Hz, 7.4 Hz, 1H, H-7), 3.62 (t, J = 5.7 Hz, 2H, H-10), 2.21-2.27 (m, 5H, H-1, H-8), 1.63-1.70 (m, 3H, H-9, H-11) ppm;

^{13}C NMR (101 MHz, $CDCl_3$) δ 136.7, 136.4, 131.9, 130.0, 129.1, 128.8, 127.0, 125.5, 62.6, 32.8, 24.7, 20.0 ppm.

The spectroscopic data is in agreement with that reported in the literature.[12]

(Z)-5-(2-methoxyphenyl)pent-4-en-1-ol (**1d**)

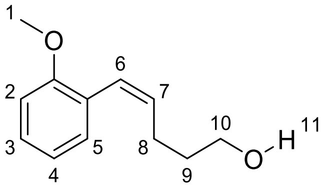

Prepared according to **GP3** on a 0.865 mmol scale with respect to (*Z*)-5-iodopent-4-en-1-ol. (2-Methoxyphenyl)boronic acid was used as the arylboronic acid. Purification by flash column chromatography (SiO_2, 0-8% v/v Acetone in $CHCl_3$) afforded the title compound as a colourless oil (122 mg, 0.635 mmol, 73%).

***R_f* value** = 0.33 (10% v/v Acetone in CHCl3);

^{1}H NMR (400 MHz, $CDCl_3$) *δ* 7.28-7.22 (m, 2H, H-3, H-5), 6.97-6.87 (m, 2H, H-2, H-4), 6.56 (dt, *J* = 11.5, 1.7 Hz, 1H, H-6), 5.75 (dt, *J* = 11.5, 7.3 Hz, 1H, H-7), 3.85 (s, 3H, H-1), 3.65 (t, *J* = 6.5 Hz, 2H, H-10), 2.34 (qd, *J* = 7.3, 1.8 Hz, 2H, H-8), 1.71 (p, *J* = 7.1 Hz, 2H, H-9), 1.56 (br s, 1H, H-11) ppm;

^{13}C NMR (101 MHz, $CDCl_3$) *δ* 157.0, 132.2, 130.1, 128.3, 126.4, 125.1, 120.2, 110.6, 62.6, 55.5, 32.8, 25.1 ppm;

HRMS (+ESI) *m/z* found $[M{-}OH]^+$ 175.1125, $[C_{12}H_{15}O]^+$ requires 175.1117, (*δ* = + 4.6 ppm).

(Z)-5-(3-fluoro-2-methylphenyl)pent-4-en-1-ol (**1e**)

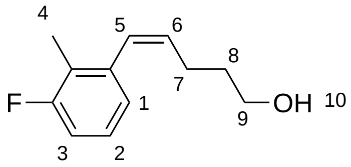

Prepared according to **GP1** on a 10 mmol scale with respect to 2-bromo-6-fluorotoluene. Purification by flash column chromatography (SiO_2, 0-6% v/v Et_2O in CH_2Cl_2) afforded the title compound as a pale-yellow oil (1.21 g, 6.2 mmol, 62%).

***R_f* value** = 0.45 (5% v/v Et_2O in CH_2Cl_2);

^{1}H NMR (400 MHz, $CDCl_3$) *δ* 7.10 (q, *J* = 7.0 Hz, 1H, H-2), 6.89-6.95 (m, 2H, H-1, H-3), 6.43 (d, *J* = 11.4 Hz, 1H, H-5), 5.73-5.80 (m, 1H, H-6), 3.61 (t, *J* = 6.3 Hz, 2H, H-9), 2.16-2.23 (m, 5H, H-4, H-7), 1.63-1.69 (m, 2H, H-8), 1.29 (s, 1H, H-10) ppm;

^{13}C NMR (101 MHz, $CDCl_3$) δ 161.5 (d, J_{C-F} = 243.4 Hz), 139.1 (d, J_{C-F} = 4.6 Hz), 133.0, 127.8 (d, J_{C-F} = 3.0 Hz), 126.2 (d, J_{C-F} = 9.0 Hz), 124.7 (d, J_{C-F} = 3.0 Hz), 123.4 (d, J_{C-F} = 16.1 Hz), 113.5 (d, J_{C-F} = 23.1 Hz), 62.5, 32.7, 24.8, 11.3 (d, J_{C-F} = 5.2 Hz) ppm;

^{19}F NMR (471 MHz, $CDCl_3$) δ – 117.9 (s) ppm;

HRMS (+ESI) *m/z* Found $[M–OH]^+$ 177.1081, $[C_{12}H_{14}F]^+$ requires 177.1074, (δ = + 4.0 ppm).

(Z)-5-(2,5-dimethylphenyl)pent-4-en-1-ol (**1f**)

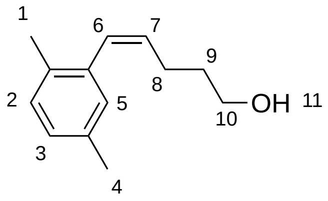

Prepared according to **GP1** on a 10 mmol scale with respect to 2-bromo-1,4-dimethylbenzene. Purification by flash column chromatography (SiO_2, 0-30% v/v EtOAc in Petrol) afforded the title compound as a yellow oil (776 mg, 4.1 mmol, 41%).

***R_f* value** = 0.53 (30% v/v EtOAc in Hexane);

^{1}H NMR (400 MHz, $CDCl_3$) δ 7.07 (d, *J* = 7.4 Hz, 1H, H-2), 6.96-6.98 (m, 2H, H-3, H-5), 6.45 (d, *J* = 11.4 Hz, 1H, H-6), 5.70 (dt, *J* = 11.4 Hz, 7.4 Hz, 1H, H-7), 3.62 (t, *J* = 5.8 Hz, 2H, H-10), 2.31 (s, 3H, H-4), 2.21-2.27 (m, 5H, H-1, H-8), 1.63-1.70 (m, 2H, H-9), 1.21 (s, 1H, H-11) ppm;

^{13}C NMR (101 MHz, $CDCl_3$) δ 136.5, 134.8, 133.2, 131.7, 129.9, 129.7, 129.0, 127.7, 62.6, 32.8, 24.7, 21.2, 19.5 ppm;

HRMS (+ESI) *m/z* Found $[M–OH]^+$ 173.1332, $[C_{13}H_{17}]^+$ requires 173.1325, (δ = + 4.0 ppm).

(Z)-5-(2,3-dimethylphenyl)pent-4-en-1-ol (**1g**)

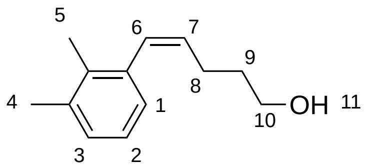

Prepared according to **GP1** on a 10 mmol scale with respect to 1-iodo-2,3-dimethylbenzene. Purification by flash column chromatography (SiO_2, 0-30% v/v EtOAc in Petrol) afforded the title compound as a dark-yellow oil (240 mg, 1.3 mmol, 13%).

***R_f* value** = 0.50 (30% v/v EtOAc in Hexane);

^{1}H NMR (400 MHz, $CDCl_3$) δ 7.04-7.07 (m, 2H, H-2, H-3), 6.99-7.04 (m, 1H, H-1), 6.52 (d, *J* = 11.4 Hz, 1H, H-6), 5.72 (dt, *J* = 11.4 Hz, 7.4 Hz, 1H, H-7), 3.60 (t, *J* = 6.4 Hz, 2H, H-10), 2.29 (s, 3H, H-4), 2.16-2.22 (m, 5H, H-5, H-8), 1.62-1.69 (m, 2H, H-9), 1.23 (br s, 1H, H-11) ppm;

^{13}C NMR (101 MHz, $CDCl_3$) δ 136.83, 136.82, 134.8, 131.7, 129.8, 128.6, 127.0, 125.1, 62.6, 32.7, 24.7, 20.6, 16.1 ppm;

HRMS (+ESI) *m/z* Found $[M–OH]^+$ 173.1333, $[C_{13}H_{17}]^+$ requires 173.1325, (δ = + 4.6 ppm).

(Z)-5-(3-fluorophenyl)pent-4-en-1-ol (**1h**)

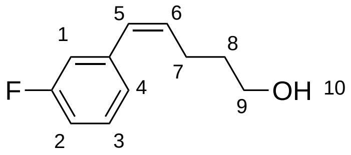

Prepared according to **GP1** on a 10 mmol scale with respect to 1-bromo-3-fluorobenzene. Purification by two consecutive flash column chromatography procedures (SiO_2, 0-35% v/v EtOAc in Petrol followed by 0-10% v/v Et_2O in CH_2Cl_2) afforded the title compound as a pale-yellow oil (246 mg, 1.4 mmol, 14%).

***Rf* value** = 0.38 (30% v/v EtOAc in Hexane);

^{1}H NMR (400 MHz, $CDCl_3$) δ 7.26-7.31 (m, 1H, H-3), 7.04 (d, *J* = 7.3 Hz, 1H, H-4), 6.98 (d, *J* = 10.1 Hz, 1H, H-1), 6.92 (td, *J* = 8.5 Hz, 2.5 Hz, 1H, H-2), 6.41 (d, *J* = 11.7 Hz, 1H, H-5), 5.71 (dt, *J* = 11.7 Hz, 7.3 Hz, 1H, H-6), 3.68 (q, *J* = 5.8 Hz, 2H, H-9), 2.42 (qd, *J* = 7.4 Hz, 1.7 Hz, 2H, H-7), 1.69-1.76 (m, 2H, H-8), 1.28 (t, *J* = 5.1 Hz, 1H, H-10) ppm;

^{13}C NMR (101 MHz, $CDCl_3$) δ 162.8 (d, $J_{C\text{-}F}$ = 245.1 Hz), 139.8 (d, $J_{C\text{-}F}$ = 7.7 Hz), 133.4 (br, s), 129.7 (d, *J* = 8.4 Hz), 128.6 (d, $J_{C\text{-}F}$ = 2.2 Hz), 124.6 (d, $J_{C\text{-}F}$ = 2.6 Hz), 115.5 (d, $J_{C\text{-}F}$ = 21.2 Hz), 113.6 (d, $J_{C\text{-}F}$ = 21.2 Hz), 62.5, 32.8, 25.0 ppm;

^{19}F NMR (376 MHz, $CDCl_3$) δ – 114.6 (s) ppm;

HRMS (+ESI) *m/z* Found $[M–OH]^+$ 163.0925, $[C_{11}H_{12}F]^+$ requires 163.0918, (δ = + 4.3 ppm).

(Z)-5-(3-chlorophenyl)pent-4-en-1-ol (**1i**)

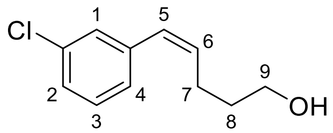

Prepared according to a variant of the protocol described by You and co-workers.[13] A microwave vial was charged with cesium carbonate (543 mg, 1.67 mmol), 3-chlorophenylboronic acid (195 mg, 1.25 mmol), $Pd(PPh_3)_4$ (10 mg, 1 mol %) and (*Z*)-5-iodopent-4-en-1-ol (177 mg, 0.833 mmol) and placed under a nitrogen atmosphere. MeCN

(sparged with argon for 10 minutes) (6.0 mL) was added and the reaction mixture was heated at 50 °C overnight. After cooling to room temperature, the reaction mixture was diluted in EtOAc (10 mL) and filtered over Celite®, eluting with further EtOAc. The filtrate was collected, the solvent removed under reduced pressure and purification by flash column chromatography (SiO_2, 0-4% v/v Acetone in $CHCl_3$) afforded the title compound as a colourless oil (29 mg, 0.15 mmol, 18%).

***R_f* value** = 0.38 (10% v/v acetone in $CHCl_3$);

^{1}H NMR (400 MHz, $CDCl_3$) δ 7.27-7.24 (m, 2H, H-1, H-3), 7.20 (m, 1H, H-2), 7.15 (dt, J = 7.4, 1.3 Hz, 1H, H-4), 6.38 (dt, J = 11.7, 1.8 Hz, 1H, H-5), 5.17 (dt, J = 11.6, 7.4 Hz, 1H, H-6), 3.68 (q, J = 6.0 Hz, 2H, H-9), 2.40 (qd, J = 7.4, 1.7 Hz, 2H, H-7), 1.73 (p, J = 6.7 Hz, 2H, H-8) ppm;

^{13}C NMR (101 MHz, $CDCl_3$) δ 139.4, 134.2, 133.6, 129.6, 128.8, 128.4, 127.0, 126.8, 62.5, 32.8, 25.0 ppm;

HRMS (+ESI) *m/z* found $[M+Na]^+$ 219.0551, $[C_{11}H_{13}ClNaO]^+$ requires 219.0547, (δ = + 1.7 ppm).

(Z)-5-(m-tolyl)pent-4-en-1-ol (**1j**)

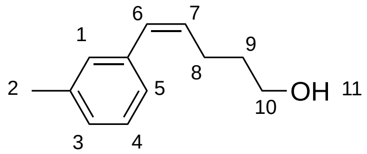

Prepared according to **GP1** on a 10 mmol scale with respect to 3-bromotoluene. Purification by flash column chromatography (SiO_2, 0-26% v/v EtOAc in Petrol) afforded the title compound as a brown oil (830 mg, 4.7 mmol, 47%).

***R_f* value** = 0.40 (30% v/v EtOAc in Hexane);

^{1}H NMR (400 MHz, $CDCl_3$) δ 7.23 (t, J = 7.8 Hz, 1H, H-4), 7.09-7.11 (m, 2H, H-1, H-3), 7.05 (d, J = 7.6 Hz, 1H, H-5), 6.43 (d, J = 11.6 Hz, 1H, H-6), 5.65 (dt, J = 11.6 Hz, 7.3 Hz, 1H, H-7), 3.67 (q, J = 5.5 Hz, 2H, H-10), 2.43 (qd, J = 7.4 Hz, 1.8 Hz, 2H, H-8), 2.35 (s, 3H, H-2), 1.69-1.76 (m, 2H, H-9), 1.30 (t, J = 4.4 Hz, 1H, H-11) ppm;

^{13}C NMR (101 MHz, $CDCl_3$) δ 137.9, 137.6, 132.0, 129.7, 129.6, 128.2, 127.5, 125.9, 62.6, 33.0, 25.0, 21.6 ppm.

The spectroscopic data is in agreement with that reported in the literature.[12]

(Z)-5-(3-(trifluoromethyl)phenyl)pent-4-en-1-ol (**1k**)

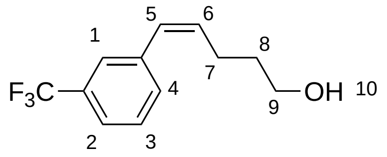

Prepared according to **GP1** on a 10 mmol scale with respect to 3-bromobenzotrifluoride. Purification by flash column chromatography (SiO_2, 0-30% v/v EtOAc in Petrol) afforded the title compound as a yellow oil (1.01 g, 4.4 mmol, 44%).

***R$_f$* value** = 0.28 (20% v/v EtOAc in Hexane);

^{1}H NMR (400 MHz, $CDCl_3$) δ 7.51 (br s, 1H, H-1), 7.42-7.49 (m, 3H, H-2, H-3, H-4), 6.46 (dt, J = 11.7 Hz, 1.7 Hz, 1H, H-5), 5.77 (dt, J = 11.6 Hz, 7.3 Hz, 1H, H-6), 3.67 (t, J = 6.5 Hz, 2H, H-9), 2.40 (qd, J = 7.3 Hz, 1.8 Hz, 2H, H-7), 1.70-1.77 (m, 2H, H-8), 1.40 (s, 1H, H-10) ppm;

^{13}C NMR (101 MHz, $CDCl_3$) δ 138.3, 133.9, 132.0 (q, J_{C-F} = 1.3 Hz), 130.7 (q, J_{C-F} = 32.0 Hz), 128.8, 128.3, 125.5 (q, J_{C-F} = 3.8 Hz), 124.3 (q, J_{C-F} = 272.3 Hz), 123.4 (q, J_{C-F} = 3.8 Hz), 62.4, 32.8, 25.0 ppm;

^{19}**F NMR** (376 MHz, $CDCl_3$) δ – 62.7 ppm;

HRMS (ESI) *The compound did not ionise.*

5-(3-(trifluoromethoxy)phenyl)pent-4-yn-1-ol

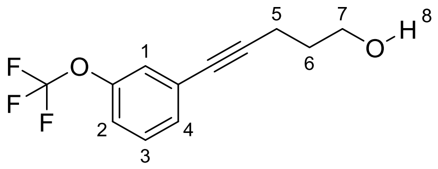

Prepared according to **GP4** on a 5.00 mmol scale with respect to 1-iodo-3-(trifluoromethoxy)benzene. Purification by flash column chromatography (SiO_2, 0-25% v/v Acetone in Petrol) afforded the title compound as a dark-orange oil (1.18 g, 4.84 mmol, 97%).

***R$_f$* value =** 0.24 (20% v/v Acetone in Petrol);

^{1}**H NMR** (400 MHz, $CDCl_3$) δ 7.32-7.27 (m, 2H, H-3, H-4), 7.24 (br s, 1H, H-1), 7.12 (m, 1H, H-2), 3.81 (t, *J* = 6.1 Hz, 2H, H-7), 2.54 (t, *J* = 7.0 Hz, 2H, H-5), 1.86 (p, *J* = 6.6 Hz, 2H, H-6), 1.73 (br s, 1H, H-8) ppm;

^{13}**C NMR** (101 MHz, $CDCl_3$) δ 149.1 (q, $J_{C\text{-}F}$ = 1.9 Hz), 130.1, 129.7, 125.8, 124.1 (q, $J_{C\text{-}F}$ = 0.9 Hz), 120.6 (q, $J_{C\text{-}F}$ = 257 Hz), 120.5 (q, $J_{C\text{-}F}$ = 0.8 Hz), 91.2, 79.8, 61.7, 31.4, 16.0 ppm;

^{19}**F NMR** (471 MHz, $CDCl_3$) δ – 58.8 (s) ppm;

HRMS (+ESI) *m/z* found $[M+H]^+$ 245.0792, $[C_{12}H_{12}F_3O_2]^+$ requires 245.0784, (δ = + 3.3 ppm).

(Z)-5-(3-(trifluoromethoxy)phenyl)pent-4-en-1-ol (**1l**)

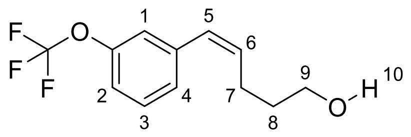

Prepared according to **GP6** on a 2.00 mmol scale with respect to 5-(3-(trifluoromethoxy)phenyl)pent-4-yn-1-ol. All other solvent and reagent amounts were scaled proportionally. Purification by flash column chromatography (SiO_2, 0-20% v/v acetone in petrol) afforded the title compound as a colourless oil (385 mg, 1.57 mmol, 79%).

***R_f* value** = 0.20 (20% v/v Acetone in Petrol);

^{1}H NMR (400 MHz, $CDCl_3$) δ 7.34 (t, *J* = 8.0 Hz, 1H, H-3), 7.20 (d, *J* = 7.8 Hz, 1H, H-4), 7.13 (s, 1H, H-1), 7.08 (d, *J* = 8.0 Hz, 1H, H-2), 6.41 (dt, *J* = 11.7, 1.9 Hz, 1H, H-5), 5.74 (dt, *J* = 11.6, 7.4 Hz, 1H, H-6), 3.66 (t, *J* = 6.5 Hz, 2H, H-9), 2.40 (qd, *J* = 7.5, 1.6 Hz, 2H, H-7), 1.72 (p, *J* = 7.1 Hz, 2H, H-8), 1.62 (br s, 1H, H-10) ppm;

^{13}C NMR (101 MHz, $CDCl_3$) δ 149.3 (q, $J_{C\text{-}F}$ = 1.8 Hz), 139.6, 133.8, 129.6, 128.2, 127.2, 121.2 (q, $J_{C\text{-}F}$ = 0.8 Hz), 120.7 (q, $J_{C\text{-}F}$ = 257 Hz), 119.1 (q, $J_{C\text{-}F}$ = 0.9 Hz), 62.4, 32.8, 25.0 ppm;

^{19}F NMR (376 MHz, $CDCl_3$) δ − 58.7 (s) ppm;

HRMS (−ESI) *m/z* found $[M-H]^-$ 245.0787, $[C_{12}H_{12}F_3O_2]^-$ requires 245.0795, (δ = − 3.3 ppm).

1-(3-(5-hydroxypent-1-yn-1-yl)phenyl)ethan-1-one

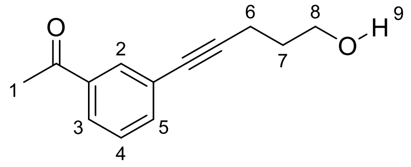

Prepared according to **GP4** on a 5.00 mmol scale with respect to 3-iodoacetophenone. Purification by flash column chromatography (SiO_2, 0-20% v/v Acetone in Petrol) afforded the title compound as a dark-orange oil (998 mg, 4.94 mmol, 99%).

***R_f* value** = 0.08 (20% v/v Acetone in Petrol);

^{1}H NMR (400 MHz, $CDCl_3$) δ 7.95 (m, 1H, H-2), 7.85 (dt, *J* = 7.8, 1.3 Hz, 1H, H-3), 7.56 (dt, *J* = 7.8, 1.2 Hz, 1H, H-5), 7.37 (t, *J* = 7.7 Hz, 1H, H-4), 3.82 (br t, *J* = 6.0 Hz, 2H, H-8), 2.58 (s, 3H, H-1), 2.55 (t, *J* = 7.0 Hz, 2H, H-6), 1.87 (p, *J* = 6.5 Hz, 2H, H-7), 1.71 (br s, 1H, H-9) ppm;

^{13}C NMR (101 MHz, $CDCl_3$) δ 197.7, 137.2, 136.0, 131.7, 128.7, 127.4, 124.5, 90.8, 80.3, 61.8, 31.4, 26.8, 16.1 ppm;

HRMS (+ESI) *m/z* found $[M+H]^+$ 203.1076, $[C_{13}H_{15}O_2]^+$ requires 203.1067, (δ = + 4.4 ppm).

(Z)-1-(3-(5-hydroxypent-1-en-1-yl)phenyl)ethan-1-one (**1m**)

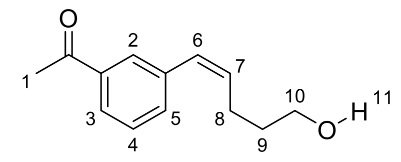

Prepared according to **GP6** on a 2.00 mmol scale with respect to 1-(3-(5-hydroxypent-1-yn-1-yl)phenyl)ethan-1-one. All other reagent and solvent amounts were scaled proportionally. Purification by flash column chromatography (SiO_2, 0-20% v/v Acetone in Petrol) afforded the title compound as a colourless oil (27 mg, 0.132 mmol, 7%).

***R_f* value** = 0.08 (20% v/v Acetone in Petrol);

^{1}H NMR (500 MHz, $CDCl_3$) δ 7.87 (br t, J = 1.7 Hz, 1H, H-2), 7.82 (dt, J = 7.6, 1.4 Hz, 1H, H-3), 7.48 (dt, J = 7.7, 1.4 Hz, 1H, H-5), 7.43 (t, J = 7.7 Hz, 1H, H-4), 6.49 (dt, J = 11.6, 1.7 Hz, 1H, H-6), 5.77 (dt, J = 11.6, 7.4 Hz, 1H, H-7), 3.69 (q, J = 5.5 Hz, 2H, H-10), 2.61 (s, 3H, H-1), 2.43 (qd, J = 7.5, 1.7 Hz, 2H, H-8), 1.75 (p, J = 6.6 Hz, 2H, H-9), 1.32 (br t, J = 5.0 Hz, 1H, H-11) ppm;

^{13}C NMR (126 MHz, $CDCl_3$) δ 198.4, 138.1, 137.2, 133.5, 133.4, 128.74, 128.65, 128.61, 126.7, 62.5, 32.8, 26.9, 25.0 ppm;

HRMS (+ESI) *m/z* found $[M+H]^+$ 205.1232, $[C_{13}H_{17}O_2]^+$ requires 205.1223, (δ = + 4.4 ppm).

(Z)-5-(3-isopropylphenyl)pent-4-en-1-ol (**1n**)

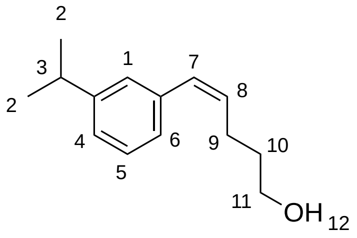

Prepared according to **GP1** on a 10 mmol scale with respect to 1-Bromo-3-isopropylbenzene. Purification by flash column chromatography (SiO_2, 0-24% v/v EtOAc in Petrol) afforded the title compound as a yellow oil (619 mg, 3.0 mmol, 30%).

***R_f* value** = 0.25 (20% v/v EtOAc in Hexane);

^{1}H NMR (400 MHz, $CDCl_3$) δ 7.26 (t, J = 7.6 Hz, 1H, H-5), 7.09-7.13 (m, 3H, H-1, H-4, H-6), 6.46 (d, J = 11.6 Hz, 1H, H-7), 5.66 (dt, J = 11.6 Hz, 7.3 Hz, 1H, H-8), 3.68 (t, J = 6.5 Hz, 2H, H-11), 2.90 (sept, J = 6.9 Hz, 1H, H-3), 2.43 (qd, J = 7.4 Hz, 1.8 Hz, 2H, H-9), 1.70-1.77 (m, 2H, H-10), 1.39 (s, 1H, H-12), 1.26 (d, J = 6.9 Hz, 6H, H-2) ppm;

^{13}C NMR (101 MHz, $CDCl_3$) δ 148.9, 137.6, 131.9, 129.9, 128.3, 127.1, 126.3, 124.9, 62.6, 34.2, 33.0, 25.1, 24.1 ppm;

HRMS (+ESI) *m/z* Found $[M–OH]^+$ 187.1488, $[C_{14}H_{19}]^+$ requires 187.1481, (δ = + 3.7 ppm).

(Z)-5-(3-(tert-butyl)phenyl)pent-4-en-1-ol (**1o**)

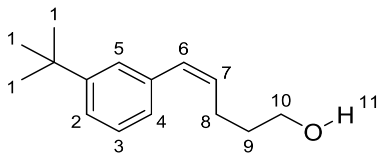

Prepared according to **GP1** on a 10.0 mmol scale with respect to 1-bromo-3-*tert*-butylbenzene. Purification by flash column chromatography (SiO_2, 0-20% v/v EtOAc in Hexane) afforded the title compound as a dark-brown oil (600 mg, 2.75 mmol, 28%).

***R$_f$* value** = 0.27 (30% v/v EtOAc in Hexane);

^{1}H NMR (400 MHz, $CDCl_3$) δ 7.31-7.27 (m, 3H, H-2, H-3, H-5), 7.13 (m, 1H, H-4), 6.48 (dt, *J* = 11.8, 1.8 Hz, 1H, H-6), 5.68 (dt, *J* = 11.7, 7.3 Hz, 1H, H-7), 3.68 (t, *J* = 6.6 Hz, 2H, H-10), 2.44 (qd, *J* = 7.4, 1.7 Hz, 2H, H-8), 1.74 (p, *J* = 6.7 Hz, 2H, H-9), 1.42 (br s, 1H, H-11), 1.34 (s, 9H, H-1) ppm;

^{13}C NMR (101 MHz, $CDCl_3$) δ 151.1, 137.2, 131.8, 130.1, 128.0, 126.0, 125.9, 123.8, 62.6, 34.7, 33.0, 31.5, 25.1 ppm;

HRMS (+ESI) *m/z* found $[M–{}^tBuOH]^+$ 145.1016, $[C_{11}H_{13}]^+$ requires 145.1012, (δ = + 2.8 ppm).

(Z)-5-(3,5-dimethylphenyl)pent-4-en-1-ol (**1p**)

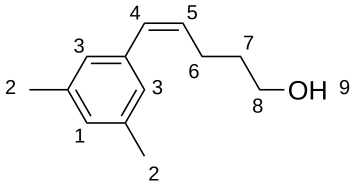

Prepared according to **GP1** on a 10 mmol scale with respect to 1-bromo-3,5-dimethylbenzene. Purification by flash column chromatography (SiO_2, 0-25% v/v EtOAc in Petrol) afforded the title compound as a colourless oil (348 mg, 1.8 mmol, 18%).

***R**_f* **value** = 0.44 (30% v/v EtOAc in Hexane);

^{1}H NMR (400 MHz, $CDCl_3$) *δ* 6.91 (br s, 2H, H-3), 6.88 (br s, 1H, H-1), 6.40 (d, *J* = 11.6 Hz, 1H, H-4), 5.63 (dt, *J* = 11.6 Hz, 7.3 Hz, 1H, H-5), 3.67 (q, *J* = 5.7 Hz, 2H, H-8), 2.43 (qd, *J* = 7.4 Hz, 1.8 Hz, 2H, H-6), 2.32 (s, 6H, H-2), 1.69-1.76 (m, 2H, H-7), 1.30 (t, *J* = 4.7 Hz, 1H, H-9) ppm;

^{13}C NMR (101 MHz, $CDCl_3$) *δ* 137.8, 137.6, 131.8, 129.8, 128.5, 126.7, 62.6, 33.0, 25.0, 21.5 ppm;

HRMS (+ESI) *m/z* Found $[M–OH]^+$ 173.1332, $[C_{13}H_{17}]^+$ requires 173.1325, (*δ* = + 4.0 ppm).

(Z)-5-(3,5-di-tert-butylphenyl)pent-4-en-1-ol (**1q**)

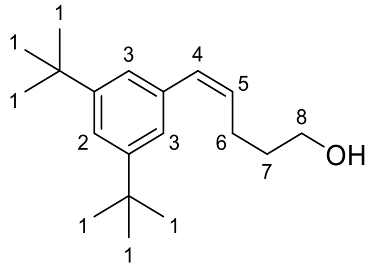

Prepared according to **GP1** on a 10.0 mmol scale with respect to 1-bromo-3,5-di-*tert*-butylbenzene. Purification by flash column chromatography (SiO_2, 0-20% v/v EtOAc in Hexane) afforded the title compound as a pale-yellow oil (220 mg, 0.802 mmol, 8%).

***R_f* value** = 0.40 (30% v/v EtOAc in Hexane);

^{1}H NMR (400 MHz, $CDCl_3$) δ 7.32 (t, *J* = 1.8 Hz, 1H, H-2), 7.15 (d, *J* = 1.8 Hz, 2H, H-3), 6.49 (dt, *J* = 11.6, 1.7 Hz, 1H H-4), 5.67 (dt, *J* = 11.6, 7.3 Hz, 1H, H-5), 3.69 (t, *J* = 6.5 Hz, 2H, H-8), 2.46 (qd, *J* = 7.4, 1.7 Hz, 2H, H-6), 1.76 (p, *J* = 7.1 Hz, 2H, H-7), 1.35 (s, 18H, H-1) ppm;

^{13}C NMR (101 MHz, $CDCl_3$) δ 150.6, 136.7, 131.4, 130.6, 123.1, 120.9, 62.6, 34.9, 33.1, 31.6, 25.1 ppm;

HRMS (+ESI) *m/z* found $[M-(^tBuOH)]^+$ 145.1018, $[C_{11}H_{13}]^+$ requires 145.1012, (δ = + 4.1 ppm).

(Z)-5-(3-fluoro-4-methylphenyl)pent-4-en-1-ol (**1r**)

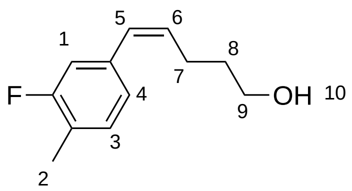

Prepared according to **GP1** on a 10 mmol scale with respect to 4-bromo-2-fluoro-1-methylbenzene. Purification by flash column chromatography (SiO_2, 0-6% v/v Et_2O in CH_2Cl_2) afforded the title compound as a yellow oil (358 mg, 1.8 mmol, 18%).

***R_f* value** = 0.40 (5% v/v Et_2O in CH_2Cl_2);

^{1}H NMR (400 MHz, $CDCl_3$) δ 7.12 (t, *J* = 7.9 Hz, 1H, H-3), 6.95 (br s, 1H, H-4), 6.93-6.94 (m, 1H, H-1), 6.37 (d, *J* = 11.6 Hz, 1H, H-5), 5.66 (dt, *J* = 11.6 Hz, 7.3 Hz, 1H, H-6), 3.67 (t, *J* = 6.3 Hz, 2H,

H-9), 2.41 (qd, J = 7.4 Hz, 1.8 Hz, 2H, H-7), 2.26 (d, J = 1.6 Hz, 3H, H-2), 1.68-1.75 (m, 2H, H-8), 1.43 (s, 1H, H-10) ppm;

^{13}C NMR (101 MHz, $CDCl_3$) δ 161.2 (d, J_{C-F} = 244.0 Hz), 137.1 (d, J_{C-F} = 7.7 Hz), 132.6, 131.2 (d, J_{C-F} = 5.6 Hz), 128.5 (d, J_{C-F} = 2.1 Hz), 124.4 (d, J_{C-F} = 3.2 Hz), 123.2 (d, J_{C-F} = 17.3 Hz), 115.2 (d, J_{C-F} = 22.4 Hz), 62.5, 32.9, 25.0, 14.4 (d, J_{C-F} = 3.5 Hz) ppm;

^{19}F NMR (376 MHz, $CDCl_3$) δ – 118.0 (s) ppm;

HRMS (+ESI) *m/z* Found $[M–OH]^+$ 177.1081, $[C_{12}H_{14}F]^+$ requires 177.1074, (δ = + 4.0 ppm).

(Z)-5-(naphthalen-2-yl)pent-4-en-1-ol (**1s**)

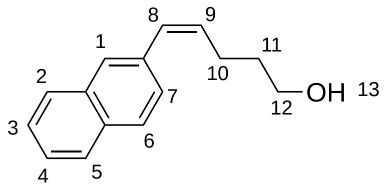

Prepared according to **GP1** on a 10 mmol scale with respect to 2-bromonaphthalene. Purification by flash column chromatography (SiO_2, 0-5% v/v Et_2O in CH_2Cl_2) afforded the title compound as an off-white solid (221 mg, 1.0 mmol, 10%).

***R_f* value** = 0.51 (5% v/v Et_2O in CH_2Cl_2);

^{1}H NMR (500 MHz, $CDCl_3$) δ 7.79-7.82 (m, 3H, H-2, H-5, H-6), 7.73 (br s, 1H, H-1), 7.42-7.49 (m, 3H, H-3, H-4, H-7), 6.61 (d, J = 11.6 Hz, 1H, H-8), 5.76 (dt, J = 11.6 Hz, 7.3 Hz, 1H, H-9), 3.69 (t, J = 6.5 Hz, 2H, H-12), 2.52 (qd, J = 7.3 Hz, 1.8 Hz, 2H, H-10), 1.74-1.79 (m, 2H, H-11) ppm;

^{13}C NMR (125 MHz, $CDCl_3$) δ 135.2, 133.5, 132.6, 132.2, 129.7, 128.1, 127.8, 127.7, 127.5, 127.3, 126.2, 125.9, 62.6, 33.0, 25.1 ppm;

The spectroscopic data is in agreement with that reported in the literature.[12]

(Z)-5-(4-fluorophenyl)pent-4-en-1-ol (**1t**)

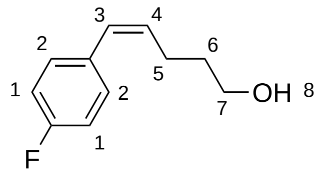

Prepared according to **GP1** on a 10 mmol scale with respect to 1-bromo-4-fluorobenzene. Purification by two consecutive flash column chromatography procedures (SiO_2, 0-30% v/v EtOAc in Petrol followed by 0-10% v/v Et_2O in CH_2Cl_2) afforded the title compound as a pale-yellow oil (169 mg, 0.94 mmol, 9%).

***R_f* value** = 0.34 (30% v/v EtOAc in Hexane);

^{1}H NMR (400 MHz, $CDCl_3$) δ 7.21-7.25 (m, 2H, H-2), 6.98-7.03 (m, 2H, H-1), 6.39 (d, *J* = 11.6 Hz, 1H, H-3), 5.64 (dt, *J* = 11.5 Hz, 7.3 Hz, 1H, H-4), 3.66 (q, *J* = 5.2 Hz, 2H, H-7), 2.42 (qd, *J* = 7.4 Hz, 1.7 Hz, 2H, H-5), 1.69-1.76 (m, 2H, H-6), 1.30 (br s, 1H, H-8) ppm;

^{13}C NMR (101 MHz, $CDCl_3$) δ 161.6 (d, $J_{C\text{-}F}$ = 245.9 Hz), 133.6 (d, $J_{C\text{-}F}$ = 3.4 Hz), 132.1 (br s), 130.4 (d, $J_{C\text{-}F}$ = 7.8 Hz), 128.5, 115.2 (d, $J_{C\text{-}F}$ = 21.3 Hz), 62.5, 32.9, 24.9 ppm;

^{19}F NMR (376 MHz, $CDCl_3$) δ – 116.6 (s) ppm;

The spectroscopic data is in agreement with that reported in the literature.[12]

(Z)-5-(p-tolyl)pent-4-en-1-ol (**1u**)

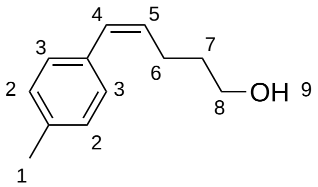

Prepared according to **GP1** on a 10 mmol scale with respect to 4-bromotoluene. Purification by flash column chromatography (SiO_2, 0-30% v/v EtOAc in Petrol) afforded the title compound as a yellow oil (911 mg, 5.2 mmol, 52%).

***R_f* value** = 0.42 (30% v/v EtOAc in Hexane);

^{1}H NMR (400 MHz, $CDCl_3$) δ 7.19 (d, *J* = 8.2 Hz, 2H, H-3), 7.14 (d, *J* = 8.1 Hz, 2H, H-2), 6.42 (d, *J* = 11.6 Hz, 1H, H-4), 5.62 (dt, *J* = 11.6 Hz, 7.3 Hz, 1H, H-5), 3.67 (t, *J* = 5.7 Hz, 2H, H-8), 2.42 (qd, *J* = 7.4 Hz, 1.8 Hz, 2H, H-6), 2.35 (s, 3H, H-1), 1.69-1.76 (m, 2H, H-7), 1.29 (br s, 1H H-9) ppm;

^{13}C NMR (101 MHz, $CDCl_3$) δ 136.5, 134.7, 131.4, 129.5, 129.0, 128.9, 62.6, 33.0, 25.0, 21.3 ppm.

The spectroscopic data is in agreement with that reported in the literature.[12]

(Z)-5-(4-(trifluoromethyl)phenyl)pent-4-en-1-ol (**1v**)

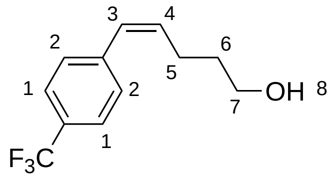

Prepared according to **GP1** on a 10 mmol scale with respect to 4-bromobenzotrifluoride. Purification by two consecutive flash column chromatography procedures (SiO_2, 0-5% v/v Et_2O in CH_2Cl_2 followed by 0-20% v/v Acetone in Petrol) afforded the title compound as a yellow oil (48 mg, 0.21 mmol, 2%).

***R_f* value** = 0.49 (20% v/v Acetone in Petrol);

^{1}H NMR (400 MHz, $CDCl_3$) δ 7.58 (d, J = 8.2 Hz, 2H, H-1), 7.38 (d, J = 8.1 Hz, 2H, H-2), 6.47 (d, J = 11.6 Hz, 1H, H-3), 5.79 (dt, J = 11.7 Hz, 7.2 Hz, 1H, H-4), 3.68 (t, J = 6.4 Hz, 2H, H-7), 2.42 (qd, J = 7.3 Hz, 1.8 Hz, 2H, H-5), 1.70-1.77 (m, 2H, H-6), 1.26 (s, 1H, H-8) ppm;

^{13}C NMR (101 MHz, $CDCl_3$) δ 141.2, 134.3, 129.1, 128.7 (q, J_{C-F} = 32.3 Hz), 128.4, 125.3 (q, J_{C-F} = 4.0 Hz), 124.4 (q, J_{C-F} = 271.7 Hz), 62.5, 32.8, 25.1 ppm;

^{19}F NMR (376 MHz, $CDCl_3$) δ – 62.5 (s) ppm;

HRMS (ESI) *The compound did not ionise.*

(Z)-5-(4-(tert-butyl)phenyl)pent-4-en-1-ol (**1w**)

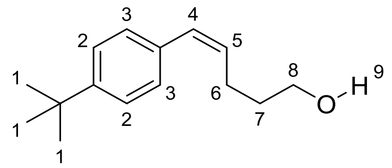

Prepared according to **GP1** on a 10.0 mmol scale with respect to 1-bromo-4-*tert*-butylbenzene. Purification by flash column chromatography (SiO_2, 0-24% v/v EtOAc in Hexane) afforded the title compound as a yellow oil (390 mg, 1.79 mmol, 18%).

***R_f* value** = 0.33 (30% v/v EtOAc in Hexane);

^{1}**H NMR** (400 MHz, $CDCl_3$) *δ* 7.37 (m, 2H, H-2), 7.25 (m, 2H, H-3), 6.44 (dt, *J* = 11.5, 1.6 Hz, 1H, H-4), 5.64 (dt, *J* = 11.7, 7.3 Hz, 1H, H-5), 3.68 (t, *J* = 6.5 Hz, 2H, H-8), 2.46 (qd, *J* = 7.3, 1.9 Hz, 2H, H-6), 1.73 (p, *J* = 6.7 Hz, 2H, H-7), 1.55 (br s, 1H, H-9), 1.34 (s, 9H, H-1) ppm;

^{13}**C NMR** (101 MHz, $CDCl_3$) *δ* 149.7, 134.8, 131.5, 129.4, 128.6, 125.2, 62.6, 34.6, 33.0, 31.4, 25.1 ppm;

HRMS (+ESI) *m/z* found $[M{-}^{t}BuOH]^+$ 145.1019, $[C_{11}H_{13}]^+$ requires 145.1012, (*δ* = + 4.8 ppm).

(Z)-5-([1,1'-biphenyl]-4-yl)pent-4-en-1-ol (**1x**)

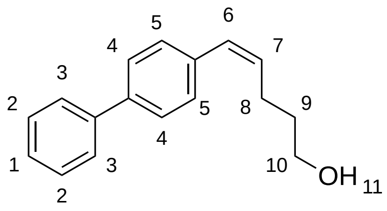

Prepared according to **GP1** on a 10 mmol scale with respect to 4-bromobiphenyl. In this case the following procedural modification was used: THF was used in place of Et_2O. Purification by flash column chromatography (SiO_2, 0-24% v/v EtOAc in Petrol) afforded the title compound as a white amorphous solid (439 mg, 1.8 mmol, 18%).

***R$_f$* value** = 0.17 (20% v/v EtOAc in Hexane);

^{1}**H NMR** (400 MHz, $CDCl_3$) *δ* 7.57-7.63 (m, 4H, H-3, H-4), 7.45 (t, *J* = 7.3 Hz, 2H, H-2), 7.38 (d, *J* = 8.0 Hz, 2H, H-5), 7.35 (tt, *J* = 7.3 Hz, 1.2 Hz, 1H, H-1), 6.49 (d, *J* = 11.6 Hz, 1H, H-6), 5.71 (dt, *J* = 11.6 Hz, 7.3 Hz, 1H, H-7), 3.70 (t, *J* = 6.5 Hz, 2H, H-10), 2.49 (qd, *J* = 7.4 Hz, 1.8 Hz, 2H, H-8), 1.73-1.80 (m, 2H, H-9), 1.42 (s, 1H, H-11) ppm;

^{13}C NMR (101 MHz, $CDCl_3$) δ 140.9, 139.5, 136.7, 132.4, 129.3, 129.2, 128.9, 127.4, 127.1, 127.0, 62.6, 33.0, 25.2 ppm;

HRMS (+ESI) *m/z* Found $[M–OH]^+$ 221.1331, $[C_{17}H_{17}]^+$ requires 221.1325, (δ = + 2.7 ppm).

Ethyl (Z)-4-(5-hydroxypent-1-en-1-yl)benzoate (**1y**)

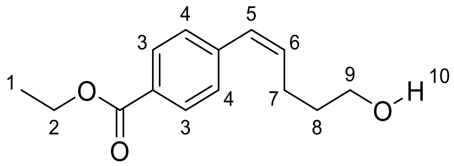

Prepared according to **GP3** on a 0.865 mmol scale with respect to (*Z*)-5-iodopent-4-en-1-ol. 4-(Ethoxycarbonyl)phenylboronic acid was used as the arylboronic acid. Purification by flash column chromatography (SiO_2, 0-15% v/v Acetone in $CHCl_3$) afforded the title compound as a colourless oil (148 mg, 0.632 mmol, 73%).

***R$_f$* value** = 0.32 (10% v/v Acetone in $CHCl_3$);

^{1}H NMR (400 MHz, $CDCl_3$) δ 7.99 (d, *J* = 8.2 Hz, 2H, H-3), 7.33 (d, *J* = 8.2 Hz, 2H, H-4), 6.46 (dt, *J* = 11.7, 1.8 Hz, 1H, H-5), 5.77 (dt, *J* = 11.7, 7.3 Hz, 1H, H-6), 4.36 (q, *J* = 7.2 Hz, 2H, H-2), 3.66 (t, *J* = 6.5 Hz, 2H, H-9), 2.42 (qd, *J* = 7.4, 1.8 Hz, 2H, H-7), 1.72 (p, *J* = 6.5 Hz, 2H, H-8), 1.53 (br s, 1H, H-10), 1.38 (t, *J* = 7.1 Hz, 3H, H-1) ppm;

^{13}C NMR (101 MHz, $CDCl_3$) δ 166.6, 142.2, 134.3, 129.6, 128.8, 128.7, 128.6, 62.4, 61.0, 32.8, 25.2, 14.5 ppm;

HRMS (−ESI) *m/z* found $[M−H]^−$ 233.1180, $[C_{14}H_{17}O_3]^−$ requires 233.1183, (δ = − 1.4 ppm).

Tert-butyl (Z)-(4-(5-hydroxypent-1-en-1-yl)phenyl)carbamate (**1z**)

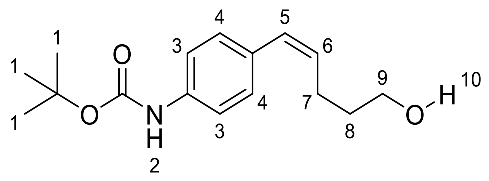

Prepared according to **GP3** on a 0.865 mmol scale with respect to (*Z*)-5-iodopent-4-en-1-ol. 4-(N-Boc-amino)phenylboronic acid was used as the arylboronic acid. Purification by flash column chromatography (SiO_2, 0-15% v/v Acetone in $CHCl_3$) afforded the title compound as a pale-yellow oil (185 mg, 0.667 mmol, 77%).

R

$\boldsymbol{R_f}$ **value** = 0.32 (10% v/v Acetone in $CHCl_3$);

^{1}**H NMR** (400 MHz, $CDCl_3$) δ 7.31 (d, *J* = 8.5 Hz, 2H, H-3), 7.20 (d, *J* = 8.6 Hz, 2H, H-4), 6.55 (br s, 1H, H-2), 6.37 (dt, *J* = 11.5, 1.6 Hz, 1H, H-5), 5.60 (td, *J* = 11.6, 7.2 Hz, 1H, H-6), 3.65 (br t, *J* = 5.7 Hz, 2H, H-9), 2.40 (qd, *J* = 7.3, 1.8 Hz, 2H, H-7), 1.71 (m, 2H, H-8), 1.51 (s, 9H, H-1), 1.44 (br s, 1H, H-10) ppm;

^{13}**C NMR** (101 MHz, $CDCl_3$) δ 152.9, 136.9, 132.5, 131.3, 129.5, 129.0, 118.4, 80.7, 62.6, 33.0, 28.5, 25.0 ppm;

HRMS (−ESI) *m/z* found $[M{-}H]^-$ 276.1605, $[C_{16}H_{22}NO_3]^-$ requires 276.1605, (δ = − 0.1 ppm).

5-(4-methoxyphenyl)pent-4-yn-1-ol

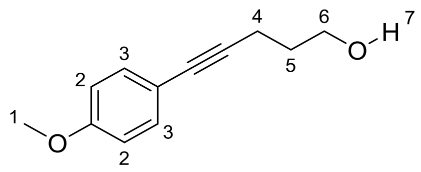

Prepared according to **GP4** on a 5.00 mmol scale with respect to 4-iodoanisole. Purification by flash column chromatography (SiO_2, 0-40% v/v EtOAc in Petrol) afforded the title compound as an orange solid (932 mg, 4.91 mmol, 98%).

***R_f* value =** 0.15 (30% v/v EtOAc in Petrol);

^{1}H NMR (400 MHz, $CDCl_3$) δ 7.32 (m, 2H, H-3), 6.81 (m, 2H, H-2), 3.82 (q, *J* = 5.6 Hz, 2H, H-6), 3.80 (s, 3H, H-1), 2.52 (t, *J* = 6.9 Hz, 2H, H-4), 1.86 (p, *J* = 6.5 Hz, 2H, H-5), 1.51 (t, *J* = 5.3 Hz, 1H, H-7) ppm;

^{13}C NMR (101 MHz, $CDCl_3$) δ 159.3, 133.0, 116.0, 114.0, 87.8, 81.0, 62.1, 55.4, 31.6, 16.2 ppm;

HRMS (+ESI) *m/z* found $[M+H]^+$ 191.1073, $[C_{12}H_{15}O_2]^+$ requires 191.1067, (δ = + 3.1 ppm).

(Z)-5-(4-methoxyphenyl)pent-4-en-1-ol (**1aa**)

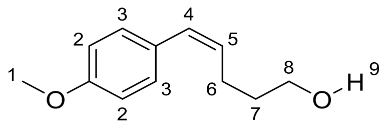

Prepared according to **GP6** on a 2.23 mmol scale with respect to 5-(4-methoxyphenyl)pent-4-yn-1-ol. Purification by flash column chromatography (SiO_2, 0-30% v/v Acetone in Petrol) afforded the title compound as a colourless oil (140 mg, 0.729 mmol, 33%).

***R_f* value** = 0.24 (20% v/v Acetone in Petrol);

^{1}H NMR (400 MHz, $CDCl_3$) δ 7.23 (m, 2H, H-3), 6.87 (m, 2H, H-2), 6.39 (dt, *J* = 11.7, 1.8 Hz, 1H, H-4), 5.57 (dt, *J* = 11.6, 7.3 Hz, 1H, H-5), 3.81 (s, 3H, H-1), 3.67 (t, *J* = 6.4 Hz, 2H, H-8), 2.42 (qd, *J* = 7.2, 1.8 Hz, 2H, H-6), 1.72 (p, *J* = 7.0 Hz, 2H, H-7), 1.40 (br s, 1H, H-9) ppm;

^{13}C NMR (101 MHz, $CDCl_3$) δ 158.4, 130.6, 130.3, 130.1, 129.0, 113.7, 62.6, 55.4, 33.0, 25.0 ppm;

HRMS (+ESI) *m/z* found $[M{-}OH]^+$ 175.1125, $[C_{12}H_{15}O]^+$ requires 175.1117, (δ = + 4.6 ppm).

5-(4-chlorophenyl)pent-4-yn-1-ol

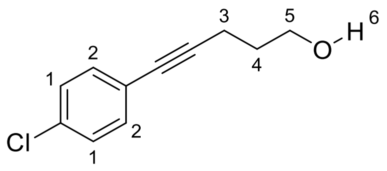

Prepared according to **GP4** on a 5.00 mmol scale with respect to 1-chloro-4-iodobenzene. Purification by flash column chromatography (SiO_2, 0-30% v/v Acetone in Petrol) afforded the title compound as a dark-orange oil (930 mg, 4.79 mmol, 96%).

***R_f* value =** 0.17 (20% v/v Acetone in Petrol);

^{1}H NMR (400 MHz, $CDCl_3$) δ 7.31 (m, 2H, H-2), 7.25 (m, 2H, H-1), 3.81 (t, *J* = 5.9, 2H, H-5), 2.53 (t, *J* = 7.0 Hz, 2H, H-3), 1.86 (p, *J* = 6.6 Hz, 2H, H-4), 1.50 (br s, 1H, H-6) ppm;

^{13}C NMR (101 MHz, $CDCl_3$) δ 133.7, 132.9, 128.6, 122.4, 90.6, 80.2, 61.8, 31.4, 16.1 ppm;

HRMS (+ESI) *m/z* found $[M+H]^+$ 195.0564, $[C_{11}H_{12}ClO]^+$ requires 195.0571, (δ = − 3.7 ppm).

(Z)-5-(4-chlorophenyl)pent-4-en-1-ol (**1ac**)

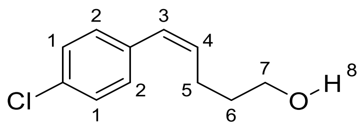

Prepared according to **GP6** on a 2.00 mmol scale with respect to 5-(4-chlorophenyl)pent-4-yn-1-ol. Purification by flash column chromatography (SiO_2, 0-20% v/v Acetone in Petrol) afforded the title compound as an off-white solid (193 mg, 0.995 mmol, 50%).

***R_f* value** = 0.19 (20% v/v Acetone in Petrol);

^{1}H NMR (400 MHz, $CDCl_3$) *δ* 7.29 (m, 2H, H-1), 7.20 (m, 2H, H-2), 6.38 (d, *J* = 11.8 Hz, 1H, H-3), 5.68 (m, 1H, H-4), 3.64 (q, *J* = 4.7 Hz, 2H, H-7), 2.38 (p, *J* = 6.5 Hz, 2H, H-5), 1.76-1.59 (m, 3H, H-6, H-8) ppm;

^{13}C NMR (101 MHz, $CDCl_3$) *δ* 136.0, 132.8, 132.4, 130.1, 128.43, 128.38, 62.4, 32.8, 25.0 ppm;

HRMS (+ESI) *m/z* found $[M{-}OH]^+$ 179.0618, $[C_{11}H_{12}Cl]^+$ requires 179.0622, (*δ* = − 2.2 ppm);

5-(4-bromophenyl)pent-4-yn-1-ol

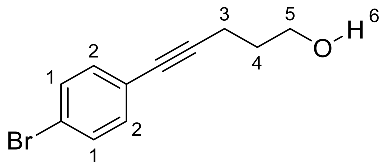

Prepared according to **GP4** on a 5.00 mmol scale with respect to 1-bromo-4-iodobenzene. Purification by flash column chromatography (SiO_2, 0-40% v/v Acetone in Petrol) afforded the title compound as a light-brown solid (990 mg, 4.16 mmol, 83%).

***R_f* value** = 0.16 (20% v/v Acetone in Petrol);

^{1}H NMR (400 MHz, $CDCl_3$) δ 7.40 (m, 2H, H-1), 7.25 (m, 2H, H-2), 3.81 (q, J = 6.1 Hz, 2H, H-5), 2.52 (t, J = 7.1 Hz, 2H, H-3), 1.85 (p, J = 6.3 Hz, 2H, H-4), 1.55 (t, J = 5.3 Hz, 1H, H-6) ppm;

^{13}C NMR (101 MHz, $CDCl_3$) δ 133.2, 131.6, 122.8, 121.9, 90.8, 80.3, 61.9, 31.4, 16.1 ppm;

HRMS (+ESI) *m/z* found $[M+H]^+$ 239.0073, $[C_{11}H_{12}BrO]^+$ requires 239.0066, (δ = + 2.9 ppm).

(Z)-5-(4-bromophenyl)pent-4-en-1-ol (**1ad**)

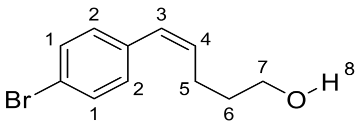

Prepared according to **GP6** on a 2.00 mmol scale with respect to 5-(4-bromophenyl)pent-4-yn-1-ol. Purification by flash column chromatography (SiO_2, 0-20% v/v Acetone in Petrol) afforded the title compound as a white solid (398 mg, 1.66 mmol, 83%).

***R_f* value** = 0.19 (20% v/v Acetone in Petrol);

^{1}H NMR (400 MHz, $CDCl_3$) δ 7.44 (m, 2H, H-1), 7.14 (m, 2H, H-2), 6.36 (dt, J = 11.6, 1.8 Hz, 1H, H-3), 5.69 (dt, J = 11.6, 7.3 Hz, 1H, H-4), 3.64 (t, J = 6.3 Hz, 2H, H-7), 2.37 (qd, J = 7.5, 1.4 Hz, 2H, H-5), 1.79-1.64 (m, 3H, H-6, H-8) ppm;

^{13}C NMR (101 MHz, $CDCl_3$) δ 136.4, 133.0, 131.4, 130.4, 128.4, 120.5, 62.4, 32.8, 25.0 ppm;

HRMS (−ESI) *m/z* found $[M-H]^-$ 239.0078, $[C_{11}H_{12}BrO]^-$ requires 239.0077, (δ = + 0.3 ppm).

5-phenylpent-4-yn-1-ol

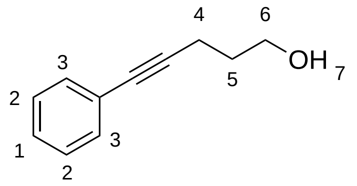

Prepared according to **GP4** on a 10.0 mmol scale with respect to iodobenzene and using 4-pentyn-1-ol as the alkyne. All other reagent and solvent amounts were scaled proportionally. Purification by flash column chromatography (SiO_2, 0-30% v/v EtOAc in Petrol) afforded the title compound as a golden oil (1.31 g, 8.2 mmol, 82%).

***R_f* value** = 0.30 (30% v/v EtOAc in Hexane);

^{1}H NMR (400 MHz, $CDCl_3$) *δ* 7.36-7.40 (m, 2H, H-3), 7.25-7.29 (m, 3H, H-1, H-2), 3.81 (t, *J* = 6.2 Hz, 2H, H-6), 2.53 (t, *J* = 6.9 Hz, 2H, H-4), 1.82-1.89 (m, 2H, H-5), 1.67 (s, 1H, H-7) ppm;

^{13}C NMR (101 MHz, $CDCl_3$) *δ* 131.7, 128.3, 127.8, 123.8, 89.4, 81.3, 61.9, 31.5, 16.1 ppm.

The spectroscopic data is in agreement with that reported in the literature.[14]

(E)-5-phenylpent-4-en-1-ol (**5**)

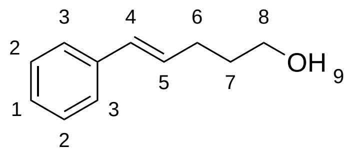

Prepared according to the protocol described by Floreancig and co-workers.[15] To a solution of 5-phenylpent-4-yn-1-ol (961 mg, 6.0 mmol) in THF (10 mL), was added $LiAlH_4$ (21.0 mL of a 1.0 M Solution in Et_2O, 21.0 mmol), The mixture was stirred at 0 °C for 20 mins and then slowly warmed to reflux and stirred at reflux overnight. The reaction was then allowed to cool to room temperature and quenched through the careful portion-wise addition of

sodium sulfate decahydrate. After the effervescence had ceased, the quenched mixture was filtered, eluting with plenty of EtOAc. The filtrate was collected and the solvent removed under reduced pressure. Purification by flash column chromatography (SiO_2, 0-30% v/v EtOAc in Petrol) afforded the title compound as a colourless oil contaminated with approximately 13% of the over-reduced alkane (507 mg, 3.1 mmol, 52%).

R

R_f value = 0.36 (30% v/v EtOAc in Hexane);

^{1}H NMR (700 MHz, $CDCl_3$) δ 7.35 (d, J = 7.6 Hz, 2H, H-3), 7.30 (t, J = 7.6 Hz, 2H, H-2), 7.20 (t, J = 7.3 Hz, 1H, H-1), 6.43 (d, J = 15.8 Hz, 1H, H-4), 6.24 (dt, J = 15.8 Hz, 7.0 Hz, 1H, H-5), 3.71 (t, J = 6.3 Hz, 2H, H-8), 2.32 (q, J = 7.2 Hz, 2H, H-6), 1.76 (p, J = 6.9 Hz, 2H, H-7), 1.44 (br s, 1H, H-9) ppm;

^{13}C NMR (176 MHz, $CDCl_3$) δ 137.8, 130.5, 130.2, 128.6, 127.1, 126.1, 62.5, 32.4, 29.4 ppm;

The spectroscopic data is in agreement with that reported in the literature.[16]

Synthesis of the Aminating Agent and Oxidants

2,2,3,3,3-pentafluoropropyl sulfamate (**2**)

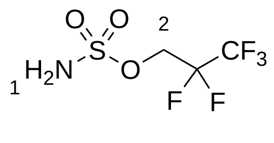

Prepared according to the protocol described by Du Bois, Sigman and co-workers.[17] To a stirred solution of chlorosulfonyl isocyanate (2.03 mL, 23.3 mmol, 2.20 equiv.) and MeCN (6.0 mL) at 0 °C was added dropwise formic acid (0.89 mL, 23.7 mmol, 2.24 equiv.). The reaction mixture was allowed to stir at 0 °C for 30 minutes following which it was diluted with MeCN (10 mL) and allowed to warm to room temperature and stirred for a further 30 minutes. The reaction mixture was then cooled to 0 °C and a solution of 2,2,3,3-pentafluoropropan-1-ol (1.0 mL, 10.0 mmol, 1.00 equiv.) in dry *N*,*N*-dimethylacetamide (8 mL) was added dropwise. Transfer of the alcohol was made quantitative with dry *N*,*N*-dimethylacetamide (2.0 mL). The reaction mixture was allowed to warm to room temperature and stirred for a further 48 hours. Water (20 mL) and EtOAc (40 mL) were then added and the layers separated. The aqueous layer was extracted with EtOAc (2 x 40 mL) and the combined organic layers were washed with water (3 x 20 mL), 10% w/v aq. LiCl (2 x 20 mL) and brine (2 x 20 mL), dried ($MgSO_4$) and the solvent removed under reduced pressure. Purification by flash column chromatography (SiO_2, 35% v/v EtOAc in Hexane) afforded the title compound as a pale-yellow oil (2.09 g, 9.1 mmol, 91%).

^{1}H NMR (400 MHz, $(CD_3)_2CO$) δ 7.21 (br s, 2H, H-1), 4.71 (tq, J = 13.0 Hz, 1.0 Hz, 2H, H-2) ppm;

^{13}C NMR (101 MHz, $(CD_3)_2CO$) δ 119.4 (qt, J_{C-F} = 285. 5 Hz, 34.8 Hz), 113.0 (tq, J_{C-F} = 254.8 Hz, 38.0 Hz), 64.4 (t, J_{C-F} = 28.1 Hz) ppm;

^{19}F NMR (376 MHz, $(CD_3)_2CO$) δ – 85.3 (s), – 125.2 (s) ppm.

The spectroscopic data is in agreement with that reported in the literature.[17]

Synthesis of Hypervalent Iodine Additive and Oxidant: TFA-OX and PFIOB

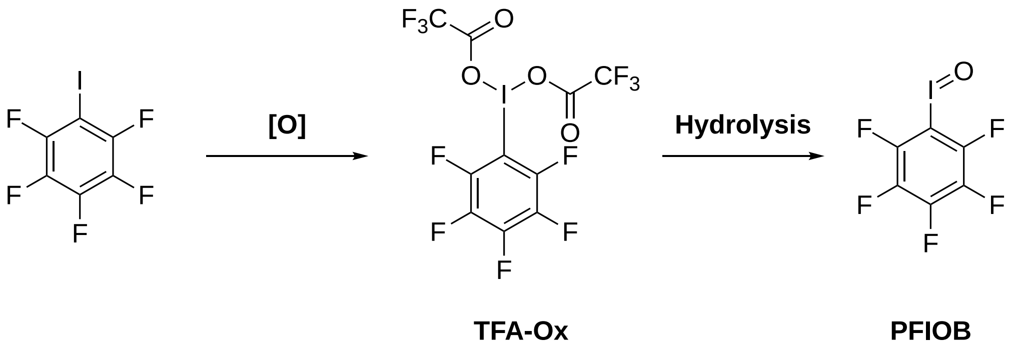

TFA-Ox was prepared according to a variant of the protocol described by Zhdankin and co-workers.[18] PFIOB was prepared according to a variant of the protocol described by Goldberg and co-workers.[19] To a solution of iodopentafluorobenzene (8.82 g, 30.0 mmol) in trifluoroacetic acid (90 mL) and $CHCl_3$ (30 mL) was added Oxone® (27.7 g, 45.0 mmol) under air. After stirring at room temperature for 4 hours, the reaction mixture was concentrated under reduced pressure, diluted in $CHCl_3$ and the insoluble inorganic salts were removed by filtration. Liberal washing of the salts with $CHCl_3$ and concentration of the filtrate under reduced pressure afforded the crude TFA-Ox. To obtain the purified TFA-Ox, the crude TFA-Ox is washed with plenty of ice-cold hexane on a filter and dried under high vacuum to afford the desired product as a white solid (6.73 g, 12.9 mmol, 43%).

^{13}C NMR (126 MHz, MeOD) δ 161.8 (q, $J_{C\text{-}F}$ = 38.7 Hz), 146.7 (m), 146.4 (m), 138.4 (m), 116.2 (q, $J_{C\text{-}F}$ = 288.7 Hz), 99.7 (td, $J_{C\text{-}F}$ = 27.6, 3.5 Hz) ppm;

^{19}F NMR (376 MHz, MeOD) δ – 77.3, – 124.5 (m), – 147.1 (m), – 159.4 (m) ppm.

The ^{19}F NMR data is in agreement with that reported in the literature.[19]

To obtain pentafluoroiodosobenzene (PFIOB), the TFA-Ox (4.36 g, 8.39 mmol) is suspended in saturated aqueous $NaHCO_3$ solution (75 mL) and stirred overnight under air. The remaining solids were isolated by filtration, washed with ice-cold H_2O (20 mL), ice-cold $CHCl_3$ (20 mL), ice-cold Et_2O (20 mL) and dried under high vacuum to afford the desired product as a white solid (1.65 g, 5.32 mmol, 63%).

^{13}C NMR (126 MHz, MeOD) δ 145.8 (m), 145.5 (m), 138.3 (m), 101.1 (t, $J_{C\text{-}F}$ = 29.1 Hz) ppm;

^{19}F NMR (376 MHz, MeOD) δ – 125.2 (m), – 150.0 (tt, J = 19.6, 3.7 Hz), – 159.9 (m) ppm

The ^{19}F NMR data is in agreement with that reported in the literature.[19]

Synthesis of Homoallylic Styrenyl Alcohols

(Z)-4-iodobut-3-en-1-ol

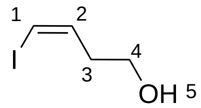

A three-necked round bottomed flask was charged with potassium azodicarboxylate (11.6 g, 60.0 mmol) and the flask evacuated and backfilled with nitrogen three times. Following this a solution of 4-iodobut-3-yn-1-ol* (3.9 g, 20.0 mmol) in MeOH (10.0 mL) was added. Transfer of the substrate was made quantitative with MeOH (5.0 mL). Pyridine (9.8 mL, 120 mmol) was added followed by the dropwise addition of AcOH (7.7 mL, 135 mmol) *via* syringe pump over 5 hours. The reaction mixture was then quenched with 1 M HCl (100 mL) and extracted with Et_2O (3 x 100 mL). The combined organics were successively washed with 1 M HCl (aq), saturated aqueous $NaHCO_3$, brine and then dried ($MgSO_4$). The solvent was then evaporated to afford a pale-yellow residue which was dissolved in *n*-butylamine (5.0 mL) and stirred at room temperature overnight. The crude mixture was then diluted with Et_2O, washed twice with 1 M HCl (aq) and brine and then dried ($MgSO_4$). The solvent was removed under reduced pressure. Purification by flash column chromatography (SiO_2, 0-18% v/v Acetone in Petrol) afforded the title compound as a pale-yellow oil (912 mg, 4.6 mmol, 23%). *Note: The title compound is volatile and should be stored in the freezer.*

***R_f* value** = 0.36 (20% v/v Acetone in Hexane);

^{1}H NMR (400 MHz, $CDCl_3$) δ 6.36 (dt, *J* = 7.5 Hz, 1.3 Hz, 1H, H-1), 6.25-6.31 (m, 1H, H-2), 3.75 (t, *J* = 6.4 Hz, 2H, H-4), 2.43 (qd, *J* = 6.5 Hz, 1.3 Hz, 2H, H-3), 1.50 (s, 1H, H-5) ppm;

^{13}C NMR (101 MHz, $CDCl_3$) δ 137.8, 84.9, 61.1, 38.2 ppm;

The spectroscopic data is in agreement with that reported in the literature.[20]

**The 4-iodobut-3-yn-1-ol was prepared following the procedure for 5-iodopent-4-yn-1-ol outlined above but using 3-butyn-1-ol in place of 4-pentyn-1-ol. In this instance we elected to take the freshly-prepared 4-iodobut-3-yn-1-ol directly to the diimide reduction without any purification fearing loss of material due to the suspected high volatility of the iodo compound.*

(Z)-4-phenylbut-3-en-1-ol (**8a**)

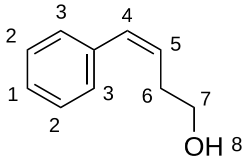

Prepared according to **GP2** on a 20 mmol scale with respect to bromobenzene. All other reagent and solvent amounts were scaled proportionally. Purification by flash column chromatography (SiO_2, 0-24% v/v EtOAc in Petrol) afforded the title compound as a colourless oil (1.59 g, 10.7 mmol, 54%).

***R$_f$* value** = 0.41 (20% v/v Acetone in Petrol);

^{1}H NMR (700 MHz, $CDCl_3$) δ 7.33-7.35 (m, 2H, H-2), 7.30-7.31 (m, 2H, H-3), 7.24 (tt, J = 7.2 Hz, 1.3 Hz, 1H, H-1), 6.59 (d, J = 11.7 Hz, 1H, H-4), 5.70 (dt, J = 11.7 Hz, 7.4 Hz, 1H, H-5), 3.76 (t, J = 6.5 Hz, 2H, H-7), 2.62 (qd, J = 6.5 Hz, 1.8 Hz, 2H, H-6), 1.37 (s, 1H, H-8) ppm;

^{13}C NMR (101 MHz, $CDCl_3$) δ 137.3, 131.8, 128.9, 128.4*, 127.0, 62.7, 32.1 ppm;

*^{1}H-^{13}C HMBC shows that this signal corresponds to two ^{13}C environments.

The spectroscopic data is in agreement with that reported in the literature.[21]

(Z)-4-(o-tolyl)but-3-en-1-ol (**8b**)

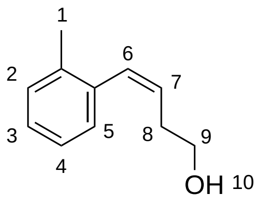

Prepared according to **GP2** on a 10 mmol scale with respect to 2-bromotoluene. Purification by flash column chromatography (SiO_2, 0-24% v/v EtOAc in Petrol) afforded the title compound as a pale-yellow oil (812 mg, 5.0 mmol, 50%).

***R$_f$* value** = 0.30 (20% v/v EtOAc in Hexane);

^{1}H NMR (500 MHz, $CDCl_3$) δ 7.14-7.22 (m, 4H, H-2, H-3, H-4, H-5), 6.61 (d, J = 11.5 Hz, 1H, H-6), 5.76 (dt, J = 11.5 Hz, 7.4 Hz, 1H, H-7), 3.68 (t, J = 6.5 Hz, 2H, H-9), 2.44 (qd, J = 6.6 Hz, 1.7 Hz, 2H, H-8), 2.27 (s, 3H, H-1), 1.58 (s, 1H, H-10) ppm;

^{13}C NMR (126 MHz, $CDCl_3$) δ 136.43, 136.36, 131.0, 130.0, 129.1, 128.3, 127.2, 125.5, 62.6, 32.0, 20.0 ppm;

HRMS (+ESI) *m/z* Found $[M–OH]^+$ 145.1017, $[C_{11}H_{13}]^+$ requires 145.1012, (δ = + 3.4 ppm).

(Z)-4-(2-methoxyphenyl)but-3-en-1-ol (**8c**)

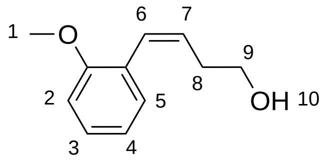

Prepared according to **GP3** on a 0.9 mmol scale with respect to (*Z*)-4-iodobut-3-en-1-ol. (2-Methoxyphenyl)boronic acid was used as the arylboronic acid. Purification by flash column chromatography (SiO_2, 0-10% v/v Acetone in $CHCl_3$) afforded the title compound as an orange oil (130 mg, 0.73 mmol, 81%).

***R**$_f$* **value** = 0.43 (10% v/v Acetone in $CHCl_3$);

^{1}H NMR (400 MHz, $CDCl_3$) δ 7.23-7.27 (m, 2H, H-3, H-5), 6.93 (td, *J* = 7.2 Hz, 0.6 Hz, 1H, H-4), 6.88-6.90 (m, 1H, H-2), 6.67 (d, *J* = 11.6 Hz, 1H, H-6), 5.74 (dt, *J* = 11.5 Hz, 7.4 Hz, 1H, H-7), 3.83 (s, 3H, H-1), 3.71 (q, *J* = 6.0 Hz, 2H, H-9), 2.51 (qd, *J* = 6.5 Hz, 1.7 Hz, 2H, H-8), 1.58 (t, *J* = 5.4 Hz, 1H, H-10) ppm;

^{13}C NMR (101 MHz, $CDCl_3$) δ 157.0, 130.1, 128.50, 128.47, 127.7, 126.1, 120.3, 110.6, 62.5, 55.5, 32.2 ppm;

The spectroscopic data is in agreement with that reported in the literature.[22]

(Z)-4-(2-fluorophenyl)but-3-en-1-ol (**8d**)

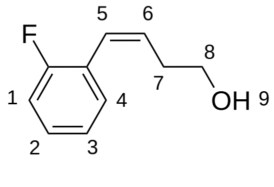

Prepared according to **GP3** on a 0.9 mmol scale with respect to (*Z*)-4-iodobut-3-en-1-ol. 2-Fluorophenylboronic acid was used as the arylboronic acid. Purification by flash column chromatography (SiO_2, 0-10% v/v Acetone in $CHCl_3$) afforded the title compound as an orange oil (106 mg, 0.64 mmol, 64%).

***R**$_f$* **value** = 0.57 (10% v/v Acetone in $CHCl_3$);

^{1}**H NMR** (500 MHz, $CDCl_3$) δ 7.32 (td, *J* = 7.6 Hz, 1.6 Hz, 1H, H-4), 7.21-7.26 (m, 1H, H-2), 7.10 (td, *J* = 7.5 Hz, 1.1 Hz, 1H, H-3), 7.05 (ddd, *J* = 9.9 Hz, 8.2 Hz, 1.1 Hz, 1H, H-1), 6.58 (dd, *J* = 11.6 Hz, 0.7 Hz, 1H, H-5), 5.83 (dt, *J* = 11.6 Hz, 7.5 Hz, 1H, H-6), 3.74 (q, *J* = 5.6 Hz, 2H, H-8), 2.49-2.53 (m, 2H, H-7), 1.49 (s, 1H, H-9) ppm;

^{13}**C NMR** (126 MHz, $CDCl_3$) δ 160.2 (d, J_{C-F} = 246.8 Hz), 130.6-130.7*(m), 128.8 (d, J_{C-F} = 8.2 Hz), 124.9 (d, J_{C-F} = 14.8 Hz), 124.5 (d, J_{C-F} = 2.7 Hz), 123.8 (d, J_{C-F} = 3.2 Hz), 115.6 (d, J_{C-F} = 22.3 Hz), 62.4, 32.4 ppm;

*^{1}H-^{13}C HMBC shows that this signal corresponds to two ^{13}C environments.

^{19}**F NMR** (376 MHz, $CDCl_3$) δ – 116.2 (s) ppm;

HRMS (+ESI) *m/z* Found $[M–OH]^+$ 149.0765, $[C_{10}H_{10}F]^+$ requires 149.0761, (δ = + 2.7 ppm).

4-(3-chlorophenyl)but-3-yn-1-ol

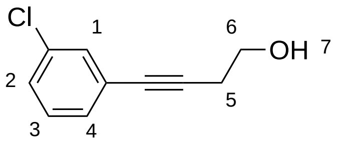

Prepared according to **GP4** using 1-chloro-3-iodobenzene as the aryl iodide and butyn-3-ol as the alkyne. Purification by flash column chromatography (SiO_2, 0-23% v/v Acetone in Petrol) afforded the title compound as a brown oil (891 mg, 4.9 mmol, 99%).

***R$_f$* value** = 0.29 (20% v/v Acetone in $CHCl_3$);

^{1}**H NMR** (400 MHz, $CDCl_3$) δ 7.40 (t, *J* = 1.7 Hz, 1H, H-1), 7.19-7.30 (m, 3H, H-2, H-3, H-4), 3.81 (q, *J* = 5.8 Hz, 2H, H-6), 2.68 (t, *J* = 6.3 Hz, 2H, H-5), 1.91 (t, *J* = 5.3 Hz, 1H, H-7) ppm;

^{13}C NMR (101 MHz, $CDCl_3$) δ 134.2, 131.7, 129.92, 129.60, 128.4, 125.2, 88.0, 81.2, 61.2, 23.9 ppm.

The spectroscopic data is in agreement with that reported in the literature.[23]

(Z)-4-(3-chlorophenyl)but-3-en-1-ol (**8e**)

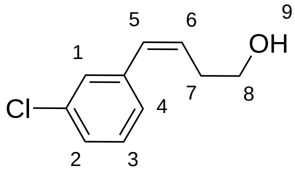

Prepared according to **GP6** using 4-(3-chlorophenyl)but-3-yn-1-ol as the alkyne. Purification by flash column chromatography (SiO_2, 0-20% v/v Acetone in Petrol) afforded the title compound as a yellow oil (386 mg, 2.1 mmol, 85%).

***R_f* value** = 0.25 (20% v/v Acetone in Hexane);

^{1}H NMR (400 MHz, $CDCl_3$) δ 7.28 (br s, 1H, H-1), 7.26 (t, *J* = 7.8 Hz, 1H, H-3), 7.21 (dt, *J* = 8.2 Hz, 1.8 Hz, 1H, H-2), 7.18 (dt, *J* = 7.4 Hz, 1.4 Hz, 1H, H-4), 6.51 (d, *J* = 11.7 Hz, 1H, H-5), 5.75 (dt, *J* = 11.6 Hz, 7.4 Hz, 1H, H-6), 3.75 (t, *J* = 6.4 Hz, 2H, H-8), 2.58 (qd, *J* = 6.5 Hz, 1.8 Hz, 2H, H-7), 1.52 (s, 1H, H-9) ppm;

^{13}C NMR (101 MHz, $CDCl_3$) δ 139.1, 134.2, 130.4, 129.9, 129.6, 128.8, 127.00, 126.99, 62.4, 32.0 ppm;

HRMS (+ESI) *m/z* Found $[M{-}OH]^+$ 165.0471, $[C_{10}H_{10}Cl]^+$ requires 165.0466, (δ = + 3.0 ppm).

4-(3-(trifluoromethoxy)phenyl)but-3-yn-1-ol

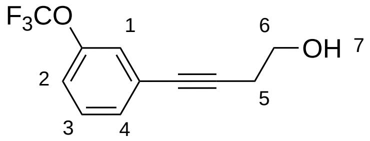

Prepared according to **GP4** using 1-iodo-3-(trifluoromethoxy)benzene as the aryl iodide and butyn-3-ol as the alkyne. Purification by flash column chromatography (SiO_2, 0-23% v/v Acetone in Petrol) afforded the title compound as a brown oil (1.12 g, 4.9 mmol, 97%).

***R_f* value** = 0.34 (20% v/v Acetone in Hexane);

^{1}H NMR (400 MHz, $CDCl_3$) δ 7.26-7.34 (m, 3H, H-1, H-3, H-4), 7.15 (d, J = 7.2 Hz, 1H, H-2), 3.83 (q, J = 6.1 Hz, 2H, H-6), 2.69 (t, J = 6.3 Hz, 2H, H-5), 1.87 (t, J = 6.1 Hz, 1H, H-7) ppm;

^{13}C NMR (101 MHz, $CDCl_3$) δ 149.1 (q, $J_{C\text{-}F}$ = 1.9 Hz), 130.2, 129.8, 125.4, 124.2 (q, $J_{C\text{-}F}$ = 1.0 Hz), 120.8 (q, $J_{C\text{-}F}$ = 0.9 Hz), 120.5 (q, $J_{C\text{-}F}$ = 257.5 Hz), 88.2, 81.1, 61.2, 23.9 ppm;

^{19}F NMR (376 MHz, $CDCl_3$) δ – 58.8 (s) ppm;

HRMS (+ESI) *m/z* Found $[M+H]^+$ 231.0634, $[C_{11}H_{10}F_3O_2]^+$ requires 231.0627, (δ = + 3.0 ppm).

(Z)-4-(3-(trifluoromethoxy)phenyl)but-3-en-1-ol (**8f**)

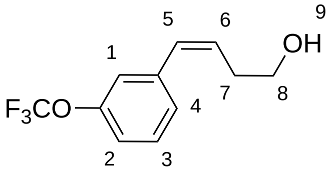

Prepared according to **GP6** using 4-(3-(trifluoromethoxy)phenyl)but-3-yn-1-ol as the alkyne. Purification by flash column chromatography (SiO_2, 0-20% v/v Acetone in Petrol) afforded the title compound as a yellow oil (459 mg, 2.0 mmol, 79%).

***R**_f* **value** = 0.32 (20% v/v Acetone in Hexane);

^{1}H NMR (400 MHz, $CDCl_3$) δ 7.35 (t, *J* = 7.9 Hz, 1H, H-3), 7.23 (d, *J* = 7.8 Hz, 1H, H-4), 7.16 (br s, 1H, H-1), 7.08-7.10 (m, 1H, H-2), 6.55 (d, *J* = 11.7 Hz, 1H, H-5), 5.78 (dt, *J* = 11.7 Hz, 7.4 Hz, 1H, H-6), 3.76 (t, *J* = 6.3 Hz, 2H, H-8), 2.59 (qd, *J* = 6.5 Hz, 1.9 Hz, 2H, H-7), 1.54 (s, 1H, H-9) ppm;

^{13}C NMR (101 MHz, $CDCl_3$) δ 149.3 (q, $J_{C\text{-}F}$ = 1.8 Hz), 139.3, 130.3, 130.2, 129.6, 127.2, 121.3 (q, $J_{C\text{-}F}$ = 0.8 Hz), 120.6 (q, $J_{C\text{-}F}$ = 257.0 Hz), 119.3 (q, $J_{C\text{-}F}$ = 0.9 Hz), 62.4, 32.0 ppm;

^{19}F NMR (376 MHz, $CDCl_3$) δ – 58.7 (s) ppm;

HRMS (+ESI) *m/z* Found $[M–OH]^+$ 215.0684, $[C_{11}H_{10}F_3O]^+$ requires 215.0678, (δ = + 2.8 ppm).

Ethyl 3-(4-hydroxybut-1-yn-1-yl)benzoate

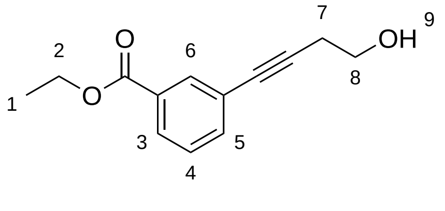

A variant of the protocol described by Beak and co-workers was employed.[24] To a stirring solution of ethyl 3-iodobenzoate (1.68 mL, 10.0 mmol) and but-3-ynol (0.95 mL, 12.5 mmol), in Et_3N (30 mL) cooled to 0 °C were added freshly-prepared CuI (38 mg, 0.2 mmol), Pd_2dba_3 (266 mg, 0.3 mmol) and Ph_3P (52.5 mg, 0.2 mmol) in sequence. The resulting mixture was stirred at 0 °C for 30 minutes and then allowed to warm to room temperature before

heating to 70 °C overnight. The reaction mixture was allowed to cool to room temperature, following which it was poured into Et_2O and washed with water (100 mL). The aqueous layer was extracted with $CHCl_3$ (3 x 100 mL) and the combined organics were washed with brine (20 mL) and dried ($MgSO_4$). Purification by flash column chromatography (SiO_2, 0-40% v/v EtOAc in Hexane) afforded the title compound as an orange oil (2.02 g, 9.3 mmol, 93%).

***R_f* value** = 0.30 (30% v/v EtOAc in hexane);

^{1}H NMR (400 MHz, $CDCl_3$) δ 8.09 (br s, 1H, H-6), 7.96 (d, *J* = 7.8 Hz, 1H, H-3), 7.58 (d, *J* = 7.5 Hz, 1H, H-5), 7.37 (t, *J* = 7.8 Hz, 1H, H-4), 4.38 (q, *J* = 7.1 Hz, 2H, H-2), 3.83 (t, *J* = 6.2 Hz, 2H, H-8), 2.71 (t, *J* = 6.2 Hz, 2H, H-7), 1.64 (br s, 1H, H-9), 1.40 (t, *J* = 7.1 Hz, 3H, H-1) ppm;

^{13}C NMR (101 MHz, $CDCl_3$) δ 166.1, 135.9, 132.9, 130.8, 129.1, 128.5, 123.9, 87.5, 81.7, 61.4, 61.3, 24.0, 14.5 ppm;

The NMR data is in agreement with that reported in the literature.[25]

Ethyl (Z)-3-(4-hydroxybut-1-en-1-yl)benzoate (**8g**)

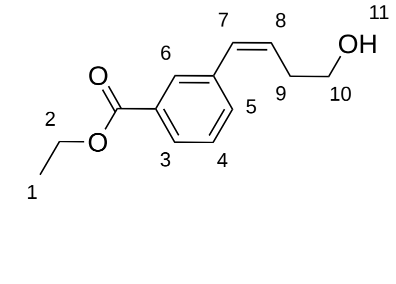

Prepared according to **GP6** using ethyl 3-(4-hydroxybut-1-yn-1-yl)benzoate as the alkyne. Purification by flash column chromatography (SiO_2, 0-8% v/v Acetone in $CHCl_3$) afforded the title compound as a yellow oil (257 mg, 1.2 mmol, 47%).

***R_f* value** = 0.37 (5% v/v Acetone in $CHCl_3$);

^{1}**H NMR** (400 MHz, $CDCl_3$) δ 7.96 (t, J = 1.5 Hz, 1H, H-6), 7.91 (dt, J = 7.7 Hz, 1.3 Hz, 1H, H-3), 7.49 (d, J = 7.7 Hz, 1H, H-5), 7.40 (t, J = 7.7 Hz, 1H, H-4), 6.60 (d, J = 11.7 Hz, 1H, H-7), 5.77 (dt, J = 11.7 Hz, 7.4 Hz, 1H, H-8), 4.38 (q, J = 7.1 Hz, 2H, H-2), 3.76 (q, J = 6.0 Hz, 2H, H-10), 2.60 (qd, J = 6.6 Hz, 1.9 Hz, 2H, H-9), 1.58 (t, J = 5.3 Hz, 1H, H-11), 1.39 (t, J = 7.1 Hz, 3H, H-1) ppm;

^{13}**C NMR** (101 MHz, $CDCl_3$) δ 166.8, 137.6, 133.1, 130.7, 130.6, 129.9, 129.7, 128.4, 128.0, 62.5, 61.2, 32.1, 14.5 ppm;

HRMS (+ESI) *m/z* Found $[M+Na]^+$ 243.0994, $[C_{13}H_{16}NaO_3]^+$ requires 243.0992, (δ = + 0.8 ppm).

4-(4-chlorophenyl)but-3-yn-1-ol

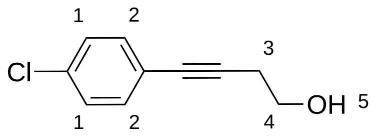

Prepared according to **GP4** using 1-chloro-4-iodobenzene as the aryl iodide and butyn-3-ol as the alkyne. Purification by flash column chromatography (SiO_2, 0-22% v/v Acetone in Petrol) afforded the title compound as a reddish amorphous solid (936 mg, quant).

***R$_f$* value** = 0.21 (20% v/v Acetone in Petrol);

^{1}**H NMR** (400 MHz, $CDCl_3$) δ 7.32-7.35 (m, 2H, H-2), 7.24-7.27 (m, 2H, H-1), 3.79-3.83 (m, 2H, H-4), 2.68 (t, J = 6.3 Hz, 2H, H-3), 1.89 (s, 1H, H-5) ppm;

^{13}**C NMR** (101 MHz, $CDCl_3$) δ 134.0, 133.0, 128.7, 122.0, 87.6, 81.5, 61.2, 23.9 ppm;

The spectroscopic data is in agreement with that reported in the literature.[26]

(Z)-4-(4-chlorophenyl)but-3-en-1-ol (**8h**)

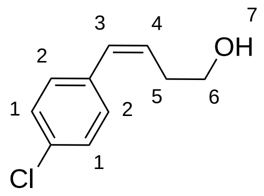

Prepared according to **GP6** using 4-(4-chlorophenyl)but-3-yn-1-ol as the alkyne. Purification by two consecutive flash column chromatography procedures (SiO_2, 0-20% v/v Acetone in Petrol followed by 0-10% v/v Acetone in $CHCl_3$) afforded the title compound as a pale-yellow oil (170 mg, 0.9 mmol, 37%).

***R_f* value** = 0.35 (20% v/v Acetone in Petrol);

^{1}H NMR (700 MHz, $CDCl_3$) δ 7.29-7.31 (m, 2H, H-1), 7.29-7.30 (m, 2H, H-2), 6.53 (d, *J* = 11.7 Hz, 1H, H-3), 5.72 (dt, *J* = 11.6 Hz, 7.4 Hz, 1H, H-4), 3.75 (q, *J* = 6.1 Hz, 1H, H-6), 2.58 (qd, *J* = 6.4 Hz, 1.7 Hz, 2H, H-5), 1.39 (t, *J* = 5.6 Hz, 1H, H-7) ppm;

^{13}C NMR (101 MHz, $CDCl_3$) δ 135.7, 132.7, 130.5, 130.1, 129.2, 128.5, 62.5, 32.0 ppm.

The spectroscopic data is in agreement with that reported in the literature.[27]

4-(4-hydroxybut-1-yn-1-yl)phenyl acetate

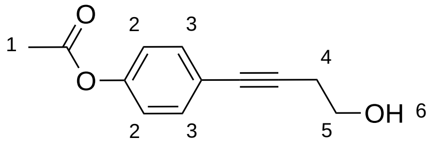

Prepared according to **GP4** using 4-iodophenyl acetate as the aryl iodide and butyn-3-ol as the alkyne. In this case the following procedural modification was used: The reaction was performed on a 2.5 mmol scale with respect to the iodide and all other reagent and solvent amounts were scaled proportionally. Purification by flash column chromatography (SiO_2, 0-30% v/v Acetone in Petrol) afforded the title compound as a brown amorphous solid (446 mg, 2.2 mmol, 87%).

***R_f* value** = 0.12 (20% v/v Acetone in Hexane);

^{1}H NMR (400 MHz, $CDCl_3$) *δ* 7.40-7.43 (m, 2H, H-3), 7.01-7.04 (m, 2H, H-2), 3.80 (q, *J* = 6.3 Hz, 2H, H-5), 2.68 (t, *J* = 6.3 Hz, 2H, H-4), 2.29 (s, 3H, H-1), 1.89 (t, *J* = 6.3 Hz, 1H, H-6) ppm;

^{13}C NMR (101 MHz, $CDCl_3$) *δ* 169.3, 150.3, 132.9, 121.7, 121.2, 86.6, 81.8, 61.3, 23.9, 21.3 ppm;

HRMS (+ESI) *m/z* Found $[M+H]^+$ 205.0867, $[C_{12}H_{13}O_3]^+$ requires 205.0859, (*δ* = + 3.9 ppm).

(Z)-4-(4-hydroxybut-1-en-1-yl)phenyl acetate (**8i**)

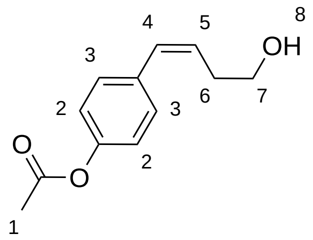

Prepared according to **GP6** using 4-(4-hydroxybut-1-yn-1-yl)phenyl acetate as the alkyne. In this case the following procedural modification was used: The reaction was performed on a 2.2 mmol scale with respect to the alkyne and all other reagent and solvent amounts were scaled proportionally. Purification by flash column chromatography (SiO_2, 0-10% v/v Acetone in $CHCl_3$) afforded the title compound as a yellow oil (106 mg, 0.5 mmol, 23%).

***R**$_f$* **value** = 0.38 (10% v/v Acetone in $CHCl_3$);

^{1}H NMR (400 MHz, $CDCl_3$) *δ* 7.29-7.32 (m, 2H, H-3), 7.03-7.07 (m, 2H, H-2), 6.54 (d, *J* = 11.7 Hz, 1H, H-4), 5.69 (dt, *J* = 11.7 Hz, 7.4 Hz, 1H, H-5), 3.74 (t, *J* = 6.2 Hz, 2H, H-7), 2.57-2.62 (m, 2H, H-6), 2.30 (s, 3H, H-1), 1.51 (s, 1H, H-8) ppm;

^{13}C NMR (101 MHz, $CDCl_3$) *δ* 169.7, 149.5, 135.1, 130.7, 129.9, 128.7, 121.4, 62.5, 32.0, 21.3 ppm;

HRMS (+ESI) *m/z* Found $[M+Na]^+$ 229.0836, $[C_{12}H_{14}NaO_3]^+$ requires 229.0835, (*δ* = + 0.4 ppm).

(Z)-4-(4-(trifluoromethyl)phenyl)but-3-en-1-ol (**8j**)

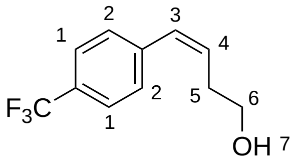

Prepared according to **GP2** on a 10 mmol scale with respect to 4-bromobenzotrifluoride. In this case the following procedural modification was used: At no point was the Grignard reagent warmed. Purification by flash column chromatography (SiO_2, 0-17% v/v Acetone in Hexane) afforded the title compound as a dark-red oil (326 mg, 1.5 mmol, 15%).

***R**$_f$* **value** = 0.55 (5% v/v Et_2O in CH_2Cl_2);

^{1}H NMR (400 MHz, $CDCl_3$) *δ* 7.58 (d, *J* = 8.2 Hz, 2H, H-1), 7.40 (d, *J* = 8.3 Hz, 2H, H-2), 6.60 (d, *J* = 11.7 Hz, 1H, H-3), 5.82 (dt, *J* = 11.7 Hz, 7.4 Hz, 1H, H-4), 3.76 (t, *J* = 6.4 Hz, 2H, H-6), 2.59 (qd, *J* = 6.5 Hz, 1.8 Hz, 2H, H-5), 1.56 (s, 1H, H-7) ppm;

^{13}**C NMR** (101 MHz, $CDCl_3$) δ 140.9 (q, J_{C-F} = 1.3 Hz), 130.7, 130.5, 129.1, 128.9 (q, J_{C-F} = 33.2 Hz), 125.3 (q, J_{C-F} = 3.8 Hz), 124.3 (q, J_{C-F} = 271.8 Hz), 62.4, 32.1 ppm;

^{19}**F NMR** (376 MHz, $CDCl_3$) δ – 62.5 (s) ppm.

The spectroscopic data is in agreement with that reported in the literature.[22]

Ethyl (Z)-4-(4-hydroxybut-1-en-1-yl)benzoate (**8k**)

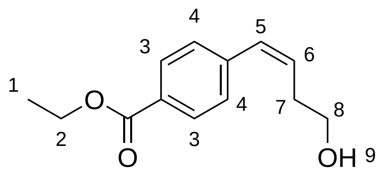

Prepared according to **GP3** on a 0.9 mmol scale with respect to (*Z*)-4-iodobut-3-en-1-ol. 4-(Ethoxycarbonyl)benzeneboronic acid was used as the arylboronic acid. Purification by flash column chromatography (SiO_2, 0-10% v/v Acetone in $CHCl_3$) afforded the title compound as a colourless oil (89.0 mg, 0.40 mmol, 45%).

***R$_f$* value** = 0.37 (10% v/v Acetone in $CHCl_3$);

^{1}**H NMR** (400 MHz, $CDCl_3$) δ 7.99-8.01 (m, 2H, H-3), 7.35-7.37 (m, 2H, H-4), 6.60 (d, *J* = 11.7 Hz, 1H, H-5), 5.81 (dt, *J* = 11.7 Hz, 7.4 Hz, 1H, H-6), 4.37 (q, *J* = 7.1 Hz, 2H, H-2), 3.76 (q, *J* = 6.0 Hz, 2H, H-8), 2.61 (qd, *J* = 6.5 Hz, 1.8 Hz, 2H, H-7), 1.50 (t, *J* = 5.3 Hz, 1H, H-9), 1.39 (t, *J* = 7.1 Hz, 3H, H-1) ppm;

^{13}**C NMR** (101 MHz, $CDCl_3$) δ 166.6, 141.9, 130.9, 130.6, 129.6, 128.84, 128.75, 62.4, 61.1, 32.2, 14.5, ppm;

HRMS (+ESI) *m/z* Found $[M+H]^+$ 221.1178, $[C_{13}H_{17}O_3]^+$ requires 221.1172, (δ = + 2.7 ppm).

(Z)-4-(3,5-dimethylphenyl)but-3-en-1-ol (**8l**)

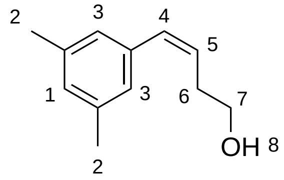

Prepared according to **GP2** on a 10 mmol scale with respect to 1-bromo-3,5-dimethylbenzene. Purification by flash column chromatography (SiO_2, 0-26% v/v EtOAc in Petrol) afforded the title compound as a yellow oil (496 mg, 2.8 mmol, 28%).

***R_f* value** = 0.32 (20% v/v EtOAc in Hexane);

^{1}H NMR (400 MHz, $CDCl_3$) δ 6.93 (s, 2H, H-3), 6.89 (s, 1H, H-1), 6.53 (d, J = 11.6 Hz, 1H, H-4), 5.65 (dt, J = 11.7 Hz, 7.4 Hz, 1H, H-5), 3.74 (t, J = 6.5 Hz, 2H, H-7), 2.63 (qd, J = 6.6 Hz, 1.8 Hz, 2H, H-6), 2.32 (s, 6H, H-2), 1.53 (s, 1H, H-8) ppm;

^{13}C NMR (101 MHz, $CDCl_3$) δ 137.8, 137.3, 131.9, 128.6, 128.0, 126.7, 62.7, 32.2, 21.5 ppm.

The spectroscopic data is in agreement with that reported in the literature.[22]

(Z)-4-(2,5-dimethylphenyl)but-3-en-1-ol (**8m**)

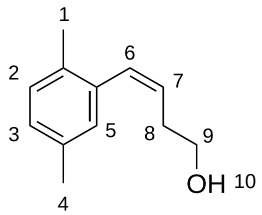

Prepared according to **GP2** on a 10 mmol scale with respect to 2-bromo-1,4-dimethylbenzene. Purification by flash column chromatography (SiO_2, 0-24% v/v EtOAc in Petrol) afforded the title compound as a pale-yellow oil (466 mg, 2.6 mmol, 26%).

***R_f* value** = 0.38 (20% v/v EtOAc in Hexane);

^{1}H NMR (400 MHz, $CDCl_3$) δ 7.07 (d, *J* = 7.6 Hz, 1H, H-2), 6.97-7.01 (m, 2H, H-3, H-5), 6.58 (d, *J* = 11.5 Hz, 1H, H-6), 5.73 (dt, *J* = 11.5 Hz, 7.4 Hz, 1H, H-7), 3.68 (t, *J* = 6.6 Hz, 2H, H-9), 2.45 (qd, *J* = 6.6 Hz, 1.7 Hz, 2H, H-8), 2.32 (s, 3H, H-4), 2.21 (s, 3H, H-1), 1.45 (s, 1H, H-10) ppm;

^{13}C NMR (101 MHz, $CDCl_3$) δ 136.3, 134.9, 133.2, 131.2, 129.9, 129.7, 128.0, 127.9, 62.6, 32.0, 21.2, 19.5 ppm;

HRMS (+ESI) *m/z* Found $[M–OH]^+$ 159.1172, $[C_{12}H_{15}]^+$ requires 159.1168, (δ = + 2.5 ppm).

(Z)-4-(3-fluoro-4-methylphenyl)but-3-en-1-ol (**8n**)

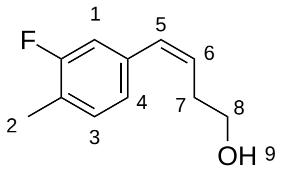

Prepared according to **GP2** on a 10 mmol scale with respect to 4-bromo-2-fluorotoluene. Purification by flash column chromatography (SiO_2, 0-24% v/v EtOAc in Petrol) afforded the title compound as a pale-yellow oil (404 mg, 2.2 mmol, 22%).

***R_f* value** = 0.36 (20% v/v EtOAc in Hexane);

^{1}H NMR (400 MHz, $CDCl_3$) δ 7.13 (t, J = 7.8 Hz, 1H, H-3), 6.95-6.98 (m, 2H, H-1, H-4), 6.50 (d, J = 11.7 Hz, 1H, H-5), 5.68 (dt, J = 11.7 Hz, 7.4 Hz, 1H, H-6), 3.74 (t, J = 6.5 Hz, 2H, H-8), 2.60 (qd, J = 6.6 Hz, 1.7 Hz, 2H, H-7), 2.26 (d, J = 1.4 Hz, 3H, H-2), 1.60 (s, 1H, H-9) ppm;

^{13}C NMR (101 MHz, $CDCl_3$) δ 161.2 (d, $J_{C\text{-}F}$ = 244.1 Hz), 136.8 (d, $J_{C\text{-}F}$ = 7.7 Hz), 131.2 (d, $J_{C\text{-}F}$ = 5.6 Hz), 130.6 (d, $J_{C\text{-}F}$ = 2.1 Hz), 128.9, 124.37 (d, $J_{C\text{-}F}$ = 3.2 Hz), 123.4 (d, $J_{C\text{-}F}$ = 17.3 Hz), 115.2 (d, $J_{C\text{-}F}$ = 22.5 Hz), 62.5, 32.1, 14.4 (d, $J_{C\text{-}F}$ = 3.5 Hz) ppm;

^{19}F NMR (376 MHz, $CDCl_3$) δ – 118.9 (s) ppm;

HRMS (+ESI) *m/z* Found $[M–OH]^+$ 163.0923, $[C_{11}H_{12}F]^+$ requires 163.0918, (δ = + 3.1 ppm).

(Z)-4-(naphthalen-1-yl)but-3-en-1-ol (**8o**)

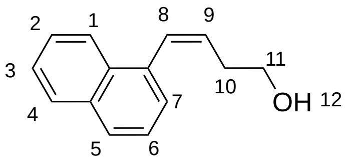

Prepared according to **GP3** on a 0.9 mmol scale with respect to (*Z*)-4-iodobut-3-en-1-ol. 1-Naphthyl boronic acid was used as the arylboronic acid. Purification by flash column chromatography (SiO_2, 0-10% v/v Acetone in $CHCl_3$) afforded the title compound as a white amorphous solid (118 mg, 0.59 mmol, 66%).

***R_f* value** = 0.58 (10% v/v Acetone in $CHCl_3$);

^{1}H NMR (400 MHz, $CDCl_3$) δ 7.98-8.02 (m, 1H, H-1), 7.84-7.89 (m, 1H, H-4), 7.78 (d, J = 8.2 Hz, 1H, H-5), 7.44-7.52 (m, 3H, H-2, H-3, H-6), 7.38 (d, J = 7.0 Hz, 1H, H-7), 7.05 (d, J = 11.5 Hz, 1H, H-8), 5.99 (dt, J = 11.5 Hz, 7.5 Hz, 1H, H-9), 3.68 (q, J = 5.9 Hz, 2H, H-11), 2.44 (qd, J = 6.6 Hz, 1.6 Hz, 2H, H-10), 1.36 (t, J = 4.9 Hz, 1H, H-12) ppm;

^{13}C NMR (101 MHz, $CDCl_3$) δ 134.5, 133.7, 132.0, 130.08, 130.05, 128.5, 127.6, 126.5, 126.1, 125.9, 125.4, 125.0, 62.6, 32.3 ppm;

HRMS (ESI) *The compound did not ionise.*

(E)-4-phenylbut-3-en-1-ol (**10**)

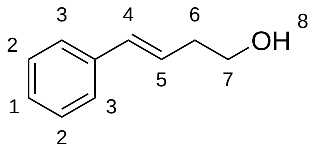

To a solution of *trans*-Styrylacetic acid (811 mg, 5.0 mmol) in THF (15 mL) at 0 °C was added $LiAlH_4$ (6.6 mL of a 1.0 M solution in Et_2O, 6.6 mmol) dropwise. The reaction mixture was stirred at the same temperature for a further hour and quenched through the careful portion-wise addition of sodium sulfate decahydrate. After the effervescence had ceased, the quenched mixture was filtered, eluting with plenty of Et_2O. The filtrate was collected and the solvent removed under reduced pressure. Purification by flash column chromatography (SiO_2, 0-30% v/v EtOAc in Petrol) afforded the title compound as a colourless oil (629 mg, 4.2 mmol, 85%).

***R_f* value** = 0.30 (30% v/v EtOAc in Petrol);

^{1}H NMR (700 MHz, $CDCl_3$) δ 7.36 (d, *J* = 7.2 Hz, 2H, H-3), 7.31 (t, *J* = 7.7 Hz, 2H, H-2), 7.22 (t, *J* = 7.3 Hz, 1H, H-1), 6.51 (d, *J* = 15.9 Hz, 1H, H-4), 6.21 (dt, *J* = 15.7 Hz, 7.1 Hz, 1H, H-5), 3.76 (t, *J* = 6.3 Hz, 2H, H-7), 2.50 (qd, *J* = 6.4 Hz, 1.3 Hz, 2H, H-6) ppm;

^{13}C NMR (101 MHz, $CDCl_3$) δ 137.4, 132.9, 128.7, 127.4, 126.5, 126.2, 62.1, 36.5 ppm;

The spectroscopic data is in agreement with that reported in the literature.[22]

Synthesis of Trishomoallylic Styrenyl Alcohols

6-phenylhex-5-yn-1-ol

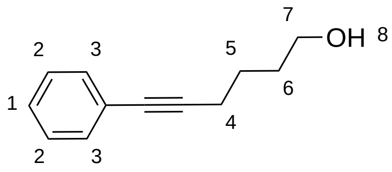

Prepared according to **GP4** using iodobenzene as the aryl iodide and hex-5-yn-1-ol as the alkyne. In this case the following procedural modification was used: The reaction was performed on 10.0 mmol scale with respect to the iodide and all other reagent and solvent and solvent amounts were scaled proportionally. Purification by flash column chromatography (SiO_2, 0-35% v/v EtOAc in Hexane) afforded the title compound as an orange oil (1.53 g, 8.8 mmol, 88%).

***R_f* value** = 0.36 (30% v/v EtOAc in Hexane);

^{1}H NMR (400 MHz, $CDCl_3$) δ 7.38-7.40 (m, 2H, H-3), 7.26-7.30 (m, 3H, H-1, H-2), 3.71 (t, J = 6.2 Hz, 2H, H-7), 2.46 (t, J = 6.7 Hz, 2H, H-4), 1.65-1.79 (m, 4H, H-5, H-6) ppm;

^{13}C NMR (101 MHz, $CDCl_3$) δ 131.6, 128.3, 127.7, 124.0, 90.0, 81.1, 62.6, 32.0, 25.1, 19.3 ppm;

The spectroscopic data is in agreement with that reported in the literature.[28]

(Z)-6-phenylhex-5-en-1-ol (**13a**)

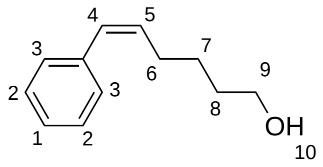

Prepared according to **GP6** using 6-phenylhex-5-yn-1-ol as the alkyne. In this case the following procedural modification was used: The reaction was performed on a 8.8 mmol scale with respect to the alkyne and all other reagent and solvent and solvent amounts were scaled proportionally. Purification by flash column chromatography (SiO_2, 0-18% v/v Acetone in Petrol) afforded the title compound as a colourless oil (1.16 g, 6.6 mmol, 75%).

R

$\boldsymbol{R_f}$ **value** = 0.32 (20% v/v Acetone in Petrol);

^{1}H NMR (400 MHz, $CDCl_3$) δ 7.31-7.35 (m, 2H, H-2), 7.26-7.28 (m, 2H, H-3), 7.20-7.24 (m, 1H, H-1), 6.44 (d, J = 11.7 Hz, 1H, H-4), 5.66 (dt, J = 11.6 Hz, 7.2 Hz, 1H, H-5), 3.64 (t, J = 6.4 Hz, 2H, H-9), 2.37 (qd, J = 7.4 Hz, 1.8 Hz, 2H, H-6), 1.49-1.65 (m, 4H, H-7, H-8), 1.38 (s, 1H, H-10) ppm;

^{13}C NMR (101 MHz, $CDCl_3$) δ 137.8, 132.8, 129.3, 128.9, 128.3, 126.7, 62.9, 32.5, 28.4, 26.2 ppm;

The spectroscopic data is in agreement with that reported in the literature.[29]

6-(3-chlorophenyl)hex-5-yn-1-ol

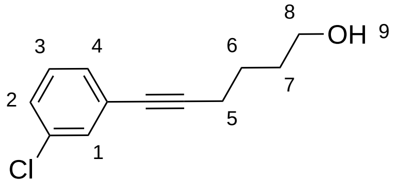

Prepared according to **GP5** using 1-bromo-3-chlorobenzene as the aryl bromide and hex-5-yn-1-ol as the alkyne. Purification by flash column chromatography (SiO_2, 0-10% v/v Acetone in $CHCl_3$) afforded the title compound as a brown oil (962 mg, 4.6 mmol, 92%).

***R_f* value** = 0.47 (10% v/v Acetone in Hexane);

^{1}H NMR (400 MHz, $CDCl_3$) δ 7.37 (t, *J* = 1.6 Hz, 1H, H-1), 7.23-7.27 (m, 2H, H-2, H-4), 7.19 (t, *J* = 7.6 Hz, 1H, H-3), 3.71 (t, *J* = 6.2 Hz, 2H, H-8), 2.45 (t, *J* = 6.7 Hz, 2H, H-5), 1.65-1.78 (m, 4H, H-6, H-7), 1.46 (s, 1H, H-9) ppm;

^{13}C NMR (101 MHz, $CDCl_3$) δ 134.1, 131.6, 129.8, 129.5, 128.0, 125.7, 91.4, 79.8, 62.5, 32.0, 25.0, 19.3 ppm.

The spectroscopic data is in agreement with that reported in the literature.[30]

(Z)-6-(3-chlorophenyl)hex-5-en-1-ol (**13b**)

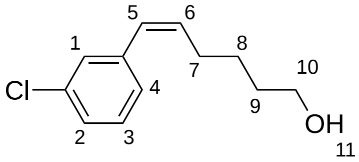

Prepared according to **GP6** using 6-(3-chlorophenyl)hex-5-yn-1-ol as the alkyne. Purification by flash column chromatography (SiO_2, 0-24% v/v Acetone in Petrol) afforded the title compound as yellow oil (455 mg, 2.2 mmol, 87%).

***R_f* value** = 0.20 (20% v/v Acetone in Petrol);

^{1}H NMR (400 MHz, $CDCl_3$) δ 7.23-7.27 (m, 2H, H-1, H-3), 7.19 (dt, *J* = 7.9 Hz, 1.4 Hz, 1H, H-2), 7.13 (dt, *J* = 7.3 Hz, 1.2 Hz, 1H, H-4), 6.36 (dt, *J* = 11.7 Hz, 1.6 Hz, 1H, H-5), 5.70 (dt, *J* = 11.6 Hz,

7.3 Hz, 1H, H-6), 3.64 (t, J = 6.2 Hz, 2H, H-10), 2.34 (qd, J = 7.4 Hz, 1.8 Hz, 2H, H-7), 1.49-1.64 (m, 4H, H-8, H-9), 1.36 (s, 1H, H-11) ppm;

^{13}C NMR (101 MHz, $CDCl_3$) δ 139.6, 134.1 (br),*129.5, 128.8, 128.0, 127.0, 126.7, 62.8, 32.4, 28.3, 26.1 ppm;

*Corresponds to two ^{13}C environments.

HRMS (+ESI) *m/z* Found [M–OH]$^+$ 193.0781, $[C_{12}H_{14}Cl]^+$ requires 193.0779, (δ = + 1.0 ppm).

N-(3-(6-hydroxyhex-1-yn-1-yl)phenyl)methanesulfonamide

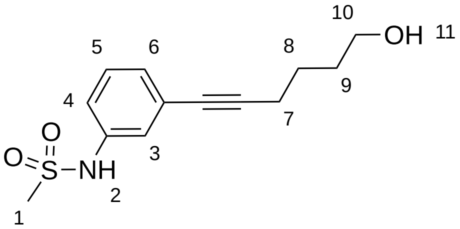

Prepared according to **GP4** using *N*-(3-iodophenyl)methanesulfonamide* as the aryl iodide and hex-5-yn-1-ol as the alkyne. In this case the following procedural modification was used: The reaction was performed on a 3.3 mmol scale with respect to the alkyne and all other reagent and solvent amounts were scaled proportionally. Purification by two consecutive flash column chromatography procedures (SiO_2, 0-30% v/v Acetone in CH_2Cl_2 followed by 0-30% v/v Acetone in $CHCl_3$) afforded the title compound as an off-white amorphous solid (735 mg, 2.7 mmol, 83%).

**Obtained as a gift from Kieran Paterson for which we are very grateful*

***R_f* value** = 0.52 (30% v/v Acetone in CH_2Cl_2);

^{1}H NMR (400 MHz, $(CD_3)_2CO$) *δ* 8.60 (br s, 1H, H-2), 7.37 (t, *J* = 1.8 Hz, 1H, H-3), 7.27-7.34 (m, 2H, H-4, H-5), 7.16 (dt, *J* = 6.8 Hz, 1.6 Hz, 1H, H-6), 3.60 (br s, 2H, H-10), 3.59 (t, *J* = 4.9 Hz, 1H, H-11), 3.00 (s, 3H, H-1), 2.43-2.47 (m, 2H, H-7), 1.66-1.70 (m, 4H, H-8, H-9) ppm;

^{13}C NMR (101 MHz, $(CD_3)_2CO$) *δ* 139.6, 130.3, 128.1, 126.1, 123.5, 120.3, 91.6, 80.9, 61.9, 39.5, 32.9, 26.0, 19.5 ppm;

HRMS (+ESI) *m/z* Found $[M+H]^+$ 268.1004, $[C_{13}H_{18}NO_3S]^+$ requires 268.1002, (*δ* = + 0.7 ppm).

N-(3-(6-hydroxyhex-1-yn-1-yl)phenyl)methanesulfonamide

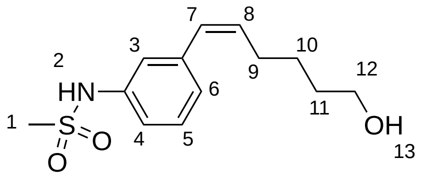

Prepared according to **GP6** using *N*-(3-(6-hydroxyhex-1-yn-1-yl)phenyl)methanesulfonamide as the alkyne. Purification by flash column chromatography (SiO_2, 0-40% v/v Acetone in Petrol) afforded the title compound as an amorphous white solid (611 mg, 2.3 mmol, 91%).

***R$_f$* value** = 0.35 (40% v/v Acetone in Hexane);

^{1}H NMR (400 MHz, $(CD_3)_2CO$) *δ* 8.55 (br s, 1H, H-2), 7.31-7.35 (m, 2H, H-3, H-5), 7.22 (ddd, *J* = 8.1 Hz, 2.1 Hz, 1.0 Hz, 1H, H-4), 7.08 (d, *J* = 7.6 Hz, 1H, H-6), 6.41 (dt, *J* = 11.9 Hz, 1.8 Hz, 1H, H-7), 5.72 (dt, *J* = 11.7 Hz, 7.5 Hz, 1H, H-8), 3.30-3.56 (m, 3H, H-12, H-13), 2.99 (s, 3H, H-1), 2.34-2.40 (m, 2H, H-9), 1.53-1.57 (m, 4H, H-10, H-11) ppm;

^{13}C NMR (101 MHz, $(CD_3)_2CO$) *δ* 139.7, 139.3, 134.4, 130.0, 129.2, 125.7, 121.1, 119.2, 62.1, 39.4, 33.3, 29.1, 26.9 ppm;

HRMS (+ESI) *m/z* Found $[M+H]^+$ 270.1165, $[C_{13}H_{20}NO_3S]^+$ requires 270.1158, (δ = + 2.6 ppm).

(Z)-N-(3-(6-hydroxyhex-1-en-1-yl)phenyl)-N-methylmethanesulfonamide (**13c**)

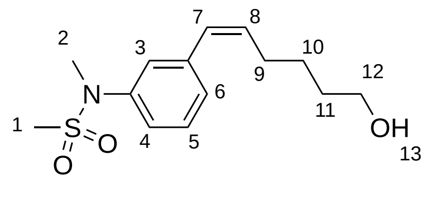

To a solution of (*Z*)-*N*-(3-(6-hydroxyhex-1-en-1-yl)phenyl)methanesulfonamide (296 mg, 1.1 mmol) and oven-dried K_2CO_3 (381 mg, 2.8 mmol) in acetone (20 mL) at room temperature was added MeI (0.14 mL, 2.2 mmol). The suspension was stirred vigorously for 48 hours following which the volatiles were evaporated under a stream of compressed air. The crude residue was taken up in CH_2Cl_2 (30 mL) and the organic layer washed with water (15 mL), brine (15 mL), dried ($MgSO_4$) following which the solvent was removed under reduced pressure. Purification by flash column chromatography (SiO_2, 0-40% v/v Acetone in Petrol) afforded the title compound as a colourless oil (284 mg, 1.0 mmol, 91%).

***R$_f$* value** = 0.54 (40% v/v Acetone in Hexane);

^{1}H NMR (400 MHz, $CDCl_3$) δ 7.34 (t, *J* = 7.8 Hz, 1H, H-5), 7.29 (t, *J* = 1.8 Hz, 1H, H-3), 7.17-7.22 (m, 2H, H-4, H-6), 6.40 (dt, *J* = 11.6 Hz, 1.6 Hz, 1H, H-7), 5.71 (dt, *J* = 11.6 Hz, 7.4 Hz, 1H, H-8), 3.62 (t, *J* = 6.0 Hz, 2H, H-12), 3.32 (s, 3H, H-2), 2.85 (s, 3H, H-1), 2.34 (qd, *J* = 7.5 Hz, 1.7 Hz, 2H, H-9), 1.49-1.63 (m, 5H, H-10, H-11, H-13) ppm;

^{13}C NMR (101 MHz, $CDCl_3$) δ 141.4, 139.1, 134.0, 129.1, 128.2, 127.9, 126.9, 123.9, 62.6, 38.2, 35.4, 32.4, 28.3, 26.0 ppm;

HRMS (+ESI) *m/z* Found $[M+H]^+$ 284.1324, $[C_{14}H_{22}NO_3S]^+$ requires 284.1315, (δ = + 3.2 ppm).

Methyl 2-(3-iodophenyl)acetate

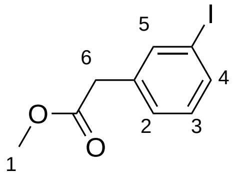

A crimp-top vial was charged with 3-Iodophenylacetic acid (1.06 g, 4.1 mmol), MeOH (17.0 mL) and *c*.H_2SO_4 (9 drops). The vial was sealed and then heated at 65 °C for 3 hours. The reaction mixture was allowed to cool following which the volatiles were removed under reduced pressure. The crude residue was taken up in Et_2O (50 mL) and washed with saturated aqueous $NaHCO_3$ (20 mL). The organic layer was dried ($MgSO_4$) and the solvent removed under reduced pressure to afford the title compound as a pale-yellow oil (1.03 g, 3.7 mmol, 92%).

***R$_f$* value** = 0.41 (10% v/v EtOAc in Hexane);

^{1}H NMR (400 MHz, $CDCl_3$) *δ* 7.64 (t, *J* = 1.6 Hz, 1H, H-5), 7.61 (d, *J* = 7.9 Hz, 1H, H-4), 7.25 (d, *J* = 6.8 Hz, 1H, H-2), 7.06 (t, *J* = 7.8 Hz, 1H, H-3), 3.70 (s, 3H, H-1), 3.57 (s, 2H, H-6) ppm;

^{13}C NMR (101 MHz, $CDCl_3$) *δ* 171.5, 138.3, 136.4, 136.3, 130.4, 128.7, 94.5, 52.3, 40.7 ppm.

The spectroscopic data is in agreement with that reported in the literature.[31]

Methyl 2-(3-(6-hydroxyhex-1-yn-1-yl)phenyl)acetate

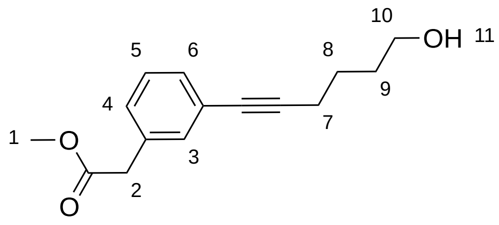

Prepared according to **GP4** using methyl 2-(3-iodophenyl)acetate as the aryl iodide and hex-5-yn -1-ol as the alkyne. In this case the following procedural modification was used: The reaction was performed on a 3.7 mmol scale with respect to the aryl iodide and all other reagent and solvent amounts were scaled proportionally Purification by flash column chromatography (SiO_2, 0-14% v/v Acetone in $CHCl_3$) afforded the title compound as a golden oil (960 mg, quant.)

***R**$_f$* **value** = 0.53 (15% v/v Acetone in $CHCl_3$);

^{1}H NMR (400 MHz, $CDCl_3$) *δ* 7.31 (br s, 1H, H-3), 7.29 (dt, *J* = 7.5 Hz, 1.4 Hz, 1H, H-6), 7.23 (t, *J* = 7.5 Hz, 1H, H-5), 7.18 (dt, *J* = 7.6 Hz, 1.6 Hz, 1H, H-4), 3.68-3.70 (m, 5H, H-1, H-10), 3.57 (s, 2H, H-2), 2.44 (t, *J* = 6.7 Hz, 2H, H-7), 1.64-1.78 (m, 4H, H-8, H-9), 1.54 (s, 1H, H-11) ppm;

^{13}C NMR (101 MHz, $CDCl_3$) *δ* 171.7, 134.0, 132.4, 130.3, 128.6, 128.5, 124.2, 90.1, 80.7, 62.4, 52.1, 40.9, 31.9, 25.0, 19.2 ppm;

HRMS (+ESI) *m/z* Found $[M+H]^+$ 247.1337, $[C_{15}H_{19}O_3]^+$ requires 247.1329, (*δ* = + 3.2 ppm).

Methyl (Z)-2-(3-(6-hydroxyhex-1-en-1-yl)phenyl)acetate (**13d**)

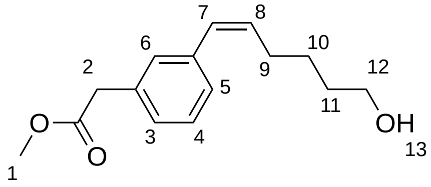

Prepared according to **GP6** using methyl 2-(3-(6-hydroxyhex-1-yn-1-yl)phenyl)acetate as the alkyne. In this case the following procedural modification was used: The reaction time was 24 hours. Purification by flash column chromatography (SiO_2, 0-25% v/v Acetone in Petrol) afforded the title compound as a colourless oil (397 mg, 1.6 mmol, 64%).

***R_f* value** = 0.38 (25% v/v Acetone in Petrol);

^{1}H NMR (400 MHz, $CDCl_3$) δ 7.28 (t, *J* = 7.6 Hz, 1H, H-4), 7.12-7.19 (m, 3H, H-3, H-5, H-6), 6.41 (d, *J* = 11.6 Hz, 1H, H-7), 5.67 (dt, *J* = 11.6 Hz, 7.4 Hz, 1H, H-8), 3.69 (s, 3H, H-1), 3.61-3.64 (m, 4H, H-2, H-12), 2.35 (qd, *J* = 7.4 Hz, 1.8 Hz, 2H, H-9), 1.49-1.64 (m, 5H, H-10, H-11, H-13) ppm;

^{13}C NMR (101 MHz, $CDCl_3$) δ 172.2, 138.1, 133.9, 133.1, 129.7, 129.0, 128.5, 127.6, 127.5, 62.7, 52.2, 41.3, 32.4, 28.3, 26.1 ppm;

HRMS (+ESI) *m/z* Found $[M+Na]^+$ 271.1312, $[C_{15}H_{20}NaO_3]^+$ requires 271.1305, (δ = + 2.6 ppm).

3-Bromo-N,N-diethylbenzenesulfonamide

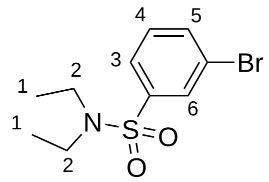

To a solution of 3-bromobenzenesulfonyl chloride (1.92 g, 7.5 mmol) in acetone (30 mL) was added diethylamine (1.6 mL, 15.0 mmol) and the reaction stirred at room temperature overnight. Following this, the volatiles were removed under reduced pressure and the crude residue was taken up in water (30 mL) and extracted with CH_2Cl_2 (3 x 50 mL). The combined organic fractions were dried ($MgSO_4$) and the solvent removed under reduced pressure.

Purification by flash column chromatography (SiO_2, 0-20% v/v Acetone in Petrol) afforded the title compound as a colourless oil (2.06 g, 94%).

***R_f* value** = 0.54 (20% v/v Acetone in Petrol);

^{1}H NMR (400 MHz, $CDCl_3$) δ 7.95 (t, J = 1.8 Hz, 1H, H-6), 7.73 (dt, J = 7.8 Hz, 1.0 Hz, 1H, H-3), 7.67 (dq, J = 8.0 Hz, 0.9 Hz, 1H, H-5), 7.37 (t, J = 7.9 Hz, 1H, H-4), 3.25 (q, J = 7.1 Hz, 4H, H-2), 1.14 (t, J = 7.1 Hz, 6H, H-1) ppm;

^{13}C NMR (101 MHz, $CDCl_3$) δ 142.5, 135.4, 130.7, 130.0, 125.6, 123.1, 42.3, 14.3 ppm.

The spectroscopic data is in agreement with that reported in the literature.[32]

N,N-diethyl-3-(6-hydroxyhex-1-yn-1-yl)benzenesulfonamide

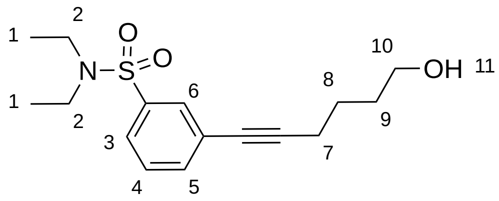

Prepared according to **GP5** using 3-bromo-*N*,*N*-diethylbenzenesulfonamide as the aryl bromide and hex-5-yn-1-ol as the alkyne. Purification by flash column chromatography (SiO_2, 0-40% v/v Acetone in Petrol) afforded the title compound as a brown oil (880 mg, 2.8 mmol, 57%).

***R_f* value** = 0.55 (30% v/v Acetone in Petrol);

^{1}H NMR (400 MHz, $CDCl_3$) δ 7.81 (t, J = 1.6 Hz, 1H, H-6), 7.68 (ddd, J = 7.9 Hz, 1.7 Hz, 1.2 Hz, 1H, H-3), 7.53 (dt, J = 7.7 Hz, 1.3 Hz, 1H, H-5), 7.40 (t, J = 7.8 Hz, 1H, H-4), 3.71 (t, J = 6.2 Hz,

2H, H-10), 3.23 (q, *J* = 7.1 Hz, 4H, H-2), 2.46 (t, *J* = 6.7 Hz, 2H, H-7), 1.66-1.78 (m, 4H, H-8, H-9), 1.13 (t, *J* = 7.1 Hz, 6H, H-1) ppm;

^{13}C NMR (101 MHz, $CDCl_3$) δ 140.7, 135.2, 130.0, 129.0, 125.9, 125.3, 92.4, 79.6, 62.5, 42.3, 32.0, 25.0, 19.3, 14.3 ppm;

HRMS (+ESI) *m/z* Found $[M+H]^+$ 310.1486, $[C_{16}H_{24}NO_3S]^+$ requires 310.1471, (δ = + 4.8 ppm).

(Z)-N,N-diethyl-3-(6-hydroxyhex-1-en-1-yl)benzenesulfonamide (**13e**)

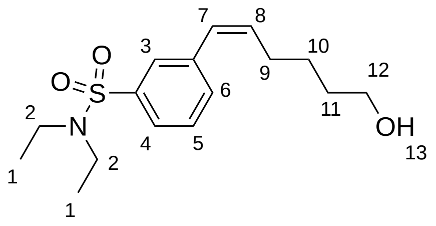

Prepared according to **GP6** *N,N*-diethyl-3-(6-hydroxyhex-1-yn-1-yl)benzenesulfonamide as the alkyne. At the end of the reaction, analysis of the crude mixture by ^{1}H NMR revealed significant over-reduction to the alkane. Careful purification by flash column chromatography (SiO_2, 0-25% v/v Acetone in Petrol) was required to separate the desired alkene and resulted in many mixed fractions which were discarded. A clean sample of alkene was obtained as a thick colourless oil (103 mg, 0.33 mmol, 13%).

***R$_f$* value** = 0.38 (30% v/v Acetone in Petrol);

^{1}H NMR (400 MHz, $CDCl_3$) δ 7.70 (br s, 1H, H-3), 7.64 (dt, *J* = 7.1 Hz, 1.8 Hz, 1H, H-4), 7.44 (t, *J* = 7.5 Hz, 1H, H-5), 7.41 (dt, *J* = 7.7 Hz, 1.5 Hz, 1H, H-6), 6.43 (dt, *J* = 11.6 Hz, 1.7 Hz, 1H, H-7), 5.77 (dt, *J* = 11.7 Hz, 7.4 Hz, 1H, H-8), 3.63 (t, *J* = 6.1 Hz, 2H, H-12), 3.24 (q, *J* = 7.1 Hz, 4H, H-2), 2.32 (qd, *J* = 7.6 Hz, 1.7 Hz, 2H, H-9), 1.50-1.63 (m, 4H, H-10, H-11), 1.13 (t, *J* = 7.1 Hz, 6H, H-1) ppm;

^{13}C NMR (101 MHz, $CDCl_3$) δ 140.3, 138.8, 134.8, 132.6, 129.0, 127.8, 127.1, 125.0, 62.6, 42.2, 32.3, 28.4, 26.0, 14.3 ppm;

HRMS (+ESI) *m/z* Found $[M+H]^+$ 312.1638, $[C_{16}H_{26}NO_3S]^+$ requires 312.1628, (δ = + 3.2 ppm).

6-(2-chlorophenyl)hex-5-yn-1-ol

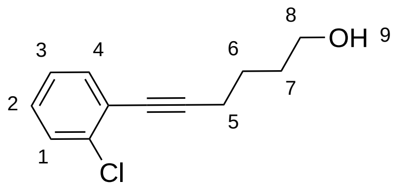

Prepared according to **GP5** using 1-bromo-2-chlorobenzene as the aryl bromide and hex-5-yn-1-ol as the alkyne. Purification by flash column chromatography (SiO_2, 0-12% v/v Acetone in $CHCl_3$) afforded the title compound as an orange oil (861 mg, 4.1 mmol, 83%). *The product was contaminated with approximately 10% of residual hex-5-yn-1-ol which was removed after the following semi-hydrogenation step.*

***R_f* value** = 0.46 (10% v/v Acetone in $CHCl_3$);

^{1}H NMR (400 MHz, $CDCl_3$) δ 7.32-7.41 (m, 1H, H-4), 7.35-7.38 (m, 1H, H-1), 7.15-7.22 (m, 2H, H-2, H-3), 3.72 (q, J = 5.8 Hz, 2H, H-8), 2.52 (t, J = 6.8 Hz, 2H, H-5), 1.67-1.83 (m, 4H, H-6, H-7), 1.39 (t, J = 5.1 Hz, 1H, H-9) ppm;

^{13}C NMR (101 MHz, $CDCl_3$) δ 135.9, 133.4, 129.3, 128.7, 126.5, 123.8, 95.7, 78.0, 62.6, 32.0, 25.0, 19.5 ppm.

The spectroscopic data is in agreement with that reported in the literature.[33]

(Z)-6-(2-chlorophenyl)hex-5-en-1-ol (**13f**)

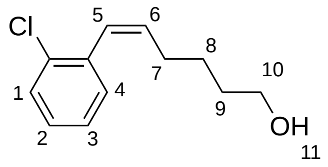

Prepared according to **GP6** using 6-(2-chlorophenyl)hex-5-yn-1-ol as the alkyne. Purification by flash column chromatography (SiO_2, 0-25% v/v Acetone in Petrol) afforded the title compound as a colourless oil (465 mg, 2.2 mmol, 88%).

***R**$_f$* **value** = 0.27 (20% v/v Acetone in Petrol);

^{1}H NMR (400 MHz, $CDCl_3$) *δ* 7.38 (dd, *J* = 7.5 Hz, 1.4 Hz, 1H, H-1), 7.27 (dd, *J* = 7.4 Hz, 2.0 Hz, 1H, H-4), 7.16-7.24 (m, 2H, H-2, H-3), 6.52 (d, *J* = 11.5 Hz, 1H, H-5), 5.79 (dt, *J* = 11.5 Hz, 7.4 Hz, 1H, H-6), 3.61 (t, *J* = 5.5 Hz, 2H, H-10), 2.23 (qd, *J* = 7.3 Hz, 1.7 Hz, 2H, H-7), 1.46-1.62 (m, 4H, H-8, H-9), 1.27 (s, 1H, H-11) ppm;

^{13}C NMR (101 MHz, $CDCl_3$) *δ* 135.9, 134.0, 133.7, 130.6, 129.5, 128.2, 126.6, 126.3, 62.8, 32.4, 28.2, 25.9 ppm;

HRMS (+ESI) *m/z* Found $[M–OH]^+$ 193.0784, $[C_{12}H_{14}Cl]^+$ requires 193.0779, (*δ* = + 2.6 ppm).

6-(4-chlorophenyl)hex-5-yn-1-ol

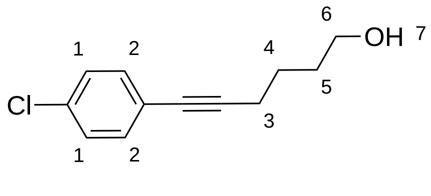

Prepared according to **GP5** using 1-bromo-4-chlorobenzene as the aryl bromide and hex-5-yn-1-ol as the alkyne. Purification by flash column chromatography (SiO_2, 0-10% v/v Acetone in $CHCl_3$) afforded the title compound as a brown amorphous solid (973 mg, 4.7 mmol, 93%).

***R$_f$* value** = 0.55 (10% v/v Acetone in $CHCl_3$);

^{1}H NMR (400 MHz, $CDCl_3$) *δ* 7.29-7.32 (m, 2H, H-2), 7.23-7.26 (m, 2H, H-1), 3.70 (t, *J* = 6.2 Hz, 2H, H-6), 2.44 (t, *J* = 6.7 Hz, 2H, H-3), 1.64-1.77 (m, 4H, H-4, H-5), 1.48 (s, 1H, H-7) ppm;

^{13}C NMR (101 MHz, $CDCl_3$) *δ* 133.6, 132.9, 128.6, 122.5, 91.1, 80.0, 62.5, 32.0, 25.0, 19.3 ppm.

The spectroscopic data is in agreement with that reported in the literature.[34]

(Z)-6-(4-chlorophenyl)hex-5-en-1-ol (**13g**)

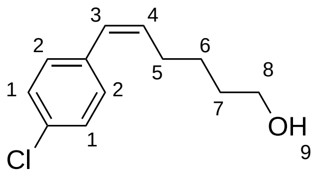

Prepared according to **GP6** using 6-(4-chlorophenyl)hex-5-yn-1-ol as the alkyne. Purification by flash column chromatography (SiO_2, 0-20% v/v Acetone in Petrol) afforded the title compound as a colourless oil (488 mg, 2.3 mmol, 93%).

***R$_f$* value** = 0.35 (20% v/v Acetone in Hexane);

^{1}H NMR (400 MHz, $CDCl_3$) *δ* 7.27-7.31 (m, 2H, H-1), 7.17-7.20 (m, 2H, H-2), 6.37 (dt, *J* = 11.6 Hz, 1.8 Hz, 1H, H-3), 5.67 (dt, *J* = 11.6 Hz, 7.3 Hz, 1H, H-4), 3.63 (t, *J* = 6.3 Hz, 2H, H-8), 2.32 (qd, *J* = 7.4 Hz, 1.8 Hz, 2H, H-5), 1.48-1.63 (m, 4H, H-6, H-7), 1.34 (s, 1H, H-9) ppm;

^{13}**C NMR** (101 MHz, $CDCl_3$) δ 136.2, 133.4, 132.3, 130.1, 128.4, 128.1, 62.8, 32.4, 28.4, 26.1 ppm;

HRMS (ESI) *The compound did not ionise.*

6-(4-(tert-butyl)phenyl)hex-5-yn-1-ol

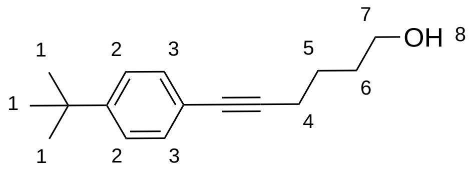

Prepared according to **GP5** using 1-bromo-4-(*tert*-butyl)benzene as the aryl bromide and hex-5-yn-1-ol as the alkyne. Purification by flash column chromatography (SiO_2, 0-10% v/v Acetone in $CHCl_3$) afforded the title compound as a thick orange oil (1.04 g, 4.5 mmol, 90%).

***R$_f$* value** = 0.50 (10% v/v Acetone in $CHCl_3$);

^{1}**H NMR** (400 MHz, $CDCl_3$) δ 7.28-7.34 (m, 4H, H-2, H-3), 3.71 (t, *J* = 6.1 Hz, 2H, H-7), 2.45 (t, *J* = 6.8 Hz, 2H, H-4), 1.65-1.79 (m, 4H, H-5, H-6), 1.44 (s, 1H, H-8), 1.30 (s, 9H, H-1) ppm;

^{13}**C NMR** (101 MHz, $CDCl_3$) δ 150.9, 131.4, 125.3, 121.0, 89.2, 81.1, 62.6, 34.8, 32.0, 31.3, 25.2, 19.3 ppm;

HRMS (+ESI) *m/z* Found $[M+H]^+$ 231.1748, $[C_{16}H_{23}O]^+$ requires 231.1743, (δ = + 2.2 ppm).

(Z)-6-(4-(tert-butyl)phenyl)hex-5-en-1-ol (**13h**)

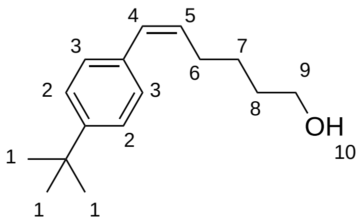

Prepared according to **GP6** using 6-(4-(*tert*-butyl)phenyl)hex-5-yn-1-ol as the alkyne. Purification by flash column chromatography (SiO_2, 0-25% v/v Acetone in Petrol) afforded the title compound as a yellow oil (434 mg, 1.9 mmol, 75%).

***R$_f$* value** = 0.32 (20% v/v Acetone in Petrol);

^{1}H NMR (400 MHz, $CDCl_3$) *δ* 7.35-7.38 (m, 2H, H-2), 7.22-7.25 (m, 2H, H-3), 6.40 (dt, *J* = 11.6 Hz, 1.7 Hz, 1H, H-4), 5.62 (dt, *J* = 11.7 Hz, 7.2 Hz, 1H, H-5), 3.65 (t, *J* = 6.4 Hz, 2H, H-9), 2.40 (qd, *J* = 7.4 Hz, 1.8 Hz, 2H, H-6), 1.49-1.66 (m, 4H, H-7, H-8), 1.33 (s, 9H, H-1) ppm;

^{13}C NMR (101 MHz, $CDCl_3$) *δ* 149.6, 134.9, 132.1, 129.0, 128.6, 125.2, 62.9, 34.6, 32.5, 31.5, 28.5, 26.3 ppm;

HRMS (+ESI) *m/z* Found $[M–C_4H_9O]^+$ 159.1175, $[C_{12}H_{15}]^+$ requires 159.1168, (*δ* = + 4.4 ppm).

1-((4-iodophenyl)sulfonyl)pyrrolidine

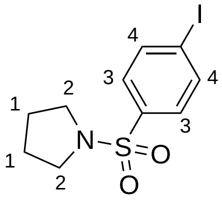

To a solution of 4-iodobenzenesulfonyl chloride (2.26 g, 7.5 mmol) in acetone (30 mL) was added pyrrolidine (1.2 mL, 15.0 mmol) and the reaction stirred at room temperature for 4

hours. Following this, the volatiles were removed under reduced pressure and the crude residue was taken up in water (30 mL) and extracted with CH_2Cl_2 (3 x 50 mL). The combined organic fractions were dried ($MgSO_4$) and the solvent removed under reduced pressure. Purification by flash column chromatography (SiO_2, 0-100% v/v EtOAc in Hexane) afforded the title compound as a white amorphous solid (2.06 g, 6.1 mmol, 81%).

***R**_f_* **value** = 0.41 (20% v/v Acetone in Hexane);

^{1}H NMR (400 MHz, $CDCl_3$) δ 7.89-7.92 (m, 2H, H-3), 7.55-7.58 (m, 2H, H-4), 3.24-3.27 (m, 4H, H-2), 1.76-1.84 (m, 4H, H-1) ppm;

^{13}C NMR (101 MHz, $CDCl_3$) δ 138.4, 136.9, 129.0, 100.0, 48.1, 25.4 ppm.

The spectroscopic data is in agreement with that reported in the literature.[35]

6-(4-(pyrrolidin-1-ylsulfonyl)phenyl)hex-5-yn-1-ol

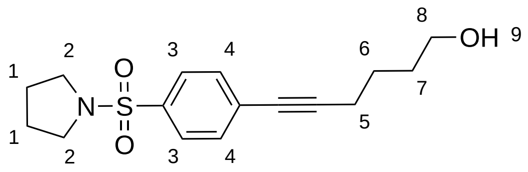

Prepared according to **GP4** using 1-((4-iodophenyl)sulfonyl)pyrrolidine as the aryl iodide and hex-5-yn-1-ol as the alkyne. Purification by flash column chromatography (SiO_2, 0-30% v/v Acetone in Petrol) afforded the title compound as a pale-orange solid (1.47 g, 4.8 mmol, 96%).

***R**_f_* **value** = 0.46 (30% v/v Acetone in Hexane);

^{1}H NMR (400 MHz, $CDCl_3$) δ 7.72-7.75 (m, 2H, H-3), 7.49-7.52 (m, 2H, H-4), 3.71 (t, J = 6.2 Hz, 2H, H-8), 3.18-3.27 (m, 4H, H-2), 2.48 (t, J = 6.7 Hz, 2H, H-5), 1.66-1.78 (m, 8H, H-1, H-6, H-7) ppm;

^{13}C NMR (101 MHz, $CDCl_3$) δ 135.7, 132.1, 128.7, 127.5, 94.1, 79.9, 62.5, 48.1, 32.0, 25.4, 24.9, 19.4 ppm;

HRMS (+ESI) *m/z* Found $[M+H]^+$ 308.1328, $[C_{16}H_{22}NO_3S]^+$ requires 308.1315, (δ = + 4.2 ppm).

(Z)-6-(4-(pyrrolidin-1-ylsulfonyl)phenyl)hex-5-en-1-ol (**13i**)

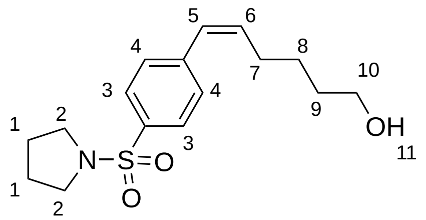

Prepared according to **GP6** using 6-(4-(pyrrolidin-1-ylsulfonyl)phenyl)hex-5-yn-1-ol as the alkyne. Purification by flash column chromatography (SiO_2, 0-35% v/v Acetone in Petrol) afforded the title compound as a yellow oil (742 mg, 2.4 mmol, 96%).

***R_f* value** = 0.58 (40% v/v Acetone in Petrol);

^{1}H NMR (400 MHz, $CDCl_3$) δ 7.76-7.79 (m, 2H, H-3), 7.39 (d, J = 8.3 Hz, 2H, H-4), 6.44 (dt, J = 11.5 Hz, 1.6 Hz, 1H, H-5), 5.80 (dt, J = 11.7 Hz, 7.3 Hz, 1H, H-6), 3.64 (t, J = 6.2 Hz, 2H, H-10), 3.24-3.27 (m, 4H, H-2), 2.36 (qd, J = 7.4 Hz, 1.8 Hz, 2H, H-7), 1.71-1.81 (m, 4H, H-1), 1.51-1.68 (m, 5H, H-8, H-9, H-11) ppm;

^{13}C NMR (101 MHz, $CDCl_3$) δ 142.2, 135.6, 134.8, 129.3, 127.8, 127.6, 62.7, 48.1, 32.4, 28.5, 26.1, 25.4 ppm;

HRMS (+ESI) *m/z* Found $[M+H]^+$ 310.1479, $[C_{16}H_{24}NO_3S]^+$ requires 310.1471, (δ = + 2.6 ppm).

Synthesis of Control Substrates

(Z)-5-(naphthalen-1-yl)pent-4-en-1-ol

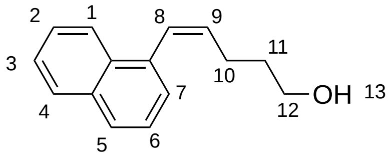

Prepared according to **GP1** on a 10 mmol scale with respect to 1-iodonaphthalene. Purification by flash column chromatography (SiO_2, 0-5% v/v Et_2O in CH_2Cl_2) afforded the title compound as a yellow oil (1.13 g, 5.3 mmol, 53%).

***R_f* value** = 0.53 (5% v/v Et_2O in CH_2Cl_2);

^{1}H NMR (400 MHz, $CDCl_3$) δ 7.98-8.02 (m, 1H, H-1), 7.84-7.88 (m, 1H, H-4), 7.77 (d, J = 8.2 Hz, 1H, H-5), 7.49-7.52 (m, 2H, H-2, H-3), 7.45 (t, J = 8.2 Hz, 1H, H-6), 7.35 (d, J = 7.0 Hz, 1H, H-7), 6.92 (d, J = 11.4 Hz, 1H, H-8), 5.96 (dt, J = 11.4 Hz, 7.4 Hz, 1H, H-9), 3.58 (t, J = 6.5 Hz, 2H, H-12), 2.24 (qd, J = 7.4 Hz, 1.6 Hz, 2H, H-10), 1.63-1.70 (m, 2H, H-11), 1.26 (br s, 1H, H-13) ppm;

^{13}C NMR (101 MHz, $CDCl_3$) δ 134.7, 133.68, 133.66, 132.0, 128.5, 127.8, 127.4, 126.4, 126.0, 125.9, 125.4, 125.1, 62.5, 32.8, 25.1 ppm;

HRMS (+ESI) *m/z* Found $[M–OH]^+$ 195.1174, $[C_{15}H_{15}]^+$ requires 195.1168, (δ = + 3.1 ppm).

(Z)-5-(naphthalen-1-yl)pent-4-en-1-yl 4-methylbenzenesulfonate

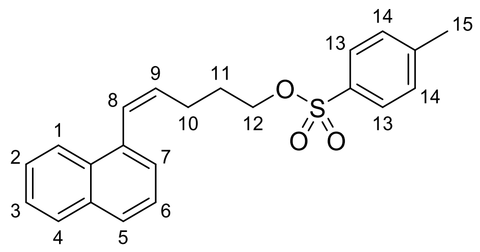

An oven-dried round-bottomed flask was charged with (*Z*)-5-(naphthalen-1-yl)pent-4-en-1-ol (509 mg, 2.40 mmol), 4-(dimethylamino)pyridine (59 mg, 0.48 mmol), *p*-toluenesulfonyl chloride (503 mg, 2.64 mmol) and placed under a nitrogen atmosphere. The flask was cooled to 0 °C, CH_2Cl_2 (12.0 mL) was added followed by dropwise addition of triethylamine (0.40 mL, 2.88 mmol). The reaction mixture was warmed to room temperature over 3 hours and diluted in Et_2O (40 mL) and H_2O (30 mL). The layers were separated, the organic layer washed with 2 M aqueous HCl solution and brine, dried over $MgSO_4$, concentrated under reduced pressure and purified by flash column chromatography (SiO_2, 10-20% EtOAc in Hexane) to afford the title compound as an off-white solid (706 mg, 1.93 mmol, 80%).

***R_f* value** = 0.32 (20% v/v EtOAc in Hexane);

^{1}H NMR (400 MHz, $CDCl_3$) *δ* 7.94 (m, 1H, H-1), 7.87 (m, 1H, H-4), 7.78 (d, *J* = 8.1 Hz, 1H, H-5), 7.69 (m, 2H, H-13), 7.53-7.47 (m, 2H, H-2, H-3), 7.44 (m, 1H, H-6), 7.29-7.23 (m, 3H, H-7, H-14), 6.91 (d, *J* = 11.5 Hz, 1H, H-8), 5.84 (dt, *J* = 11.4, 7.3 Hz, 1H, H-9), 3.97 (t, *J* = 6.6 Hz, 2H, H-12), 2.41 (s, 3H, H-15), 2.18 (qd, *J* = 7.5, 1.3 Hz, 2H, H-10), 1.75 (p, *J* = 6.7 Hz, 2H, H-11) ppm;

^{13}C NMR (101 MHz, $CDCl_3$) *δ* 144.7, 134.4, 133.6, 133.1, 132.2, 131.9, 129.9, 128.6, 128.5, 127.9, 127.5, 126.4, 126.0, 125.9, 125.4, 125.0, 70.1, 29.0, 24.7, 21.7 ppm;

HRMS (+ESI) *m/z* found $[M+Na]^+$ 389.1170, $[C_{22}H_{22}NaO_3S]^+$ requires 389.1182, (*δ* = − 3.0 ppm).

(Z)-1-(pent-1-en-1-yl)naphthalene

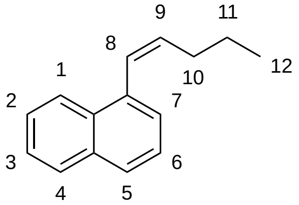

An oven-dried round-bottomed flask was charged with (*Z*)-5-(naphthalen-1-yl)pent-4-en-1-yl 4-methylbenzenesulfonate (366 mg, 1.00 mmol) and placed under a nitrogen atmosphere. The flask was cooled to 0 °C, THF (12.0 mL) was added, $LiAlH_4$ (2.4 M in THF) (0.54 mL, 1.30 mmol) was added dropwise and the reaction mixture was gradually warmed to room temperature overnight. The reaction was opened to air and quenched through addition of sodium sulfate decahydrate until effervescence had finished. The resulting residue was diluted in EtOAc (20 mL) and filtered through Celite®, eluting with further EtOAc. The filtrate was collected, the solvent removed under reduced pressure and purified by flash column chromatography (SiO_2, 100% Petrol) to afford the title compound as a colourless oil (170 mg, 0.867 mmol, 87%).

***R_f* value** = 0.36 (100% Petrol);

^{1}H NMR (400 MHz, $CDCl_3$): δ 8.04 (m, 1H, H-1), 7.87 (m, 1H, H-4), 7.78 (d, *J* = 8.2 Hz, 1H, H-5), 7.53-7.49 (m, 2H, H-2, H-3), 7.47 (dd, *J* = 8.0, 7.2 Hz, 1H, H-6), 7.36 (d, *J* = 7.0 Hz, 1H, H-7), 6.90 (d, *J* = 11.4 Hz, 1H, H-8), 5.96 (dt, *J* = 11.4, 7.3 Hz, 1H, H-9), 2.16 (qd, *J* = 7.4, 1.2 Hz, 2H, H-10), 1.45 (sext, *J* = 7.4 Hz, 2H, H-11), 0.89 (t, *J* = 7.4 Hz, 3H, H-12) ppm;

^{13}C NMR (101 MHz, $CDCl_3$): δ 135.1, 134.6, 133.7, 132.1, 128.4, 127.2, 127.1, 126.5, 125.9, 125.8, 125.4, 125.2, 30.9, 23.1, 13.9 ppm;

HRMS (ESI): *The compound did not ionise.*

(Z)-(5-methoxypent-1-en-1-yl)benzene

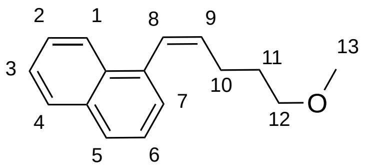

To a solution of NaH (60 wt% suspension in mineral oil, 38 mg, 1.6 mmol) in THF (5.0 mL) at 0 °C was added a solution of (*Z*)-5-(naphthalen-1-yl)pent-4-en-1-ol (162 mg, 0.8 mmol) in THF (3.0 mL) dropwise. Transfer of the alcohol was made quantitative with THF (2.0 mL) and the reaction mixture was allowed to warm to room temperature over 30 minutes. The reaction mixture was then re-cooled to 0 °C and MeI (0.15 mL, 2.4 mmol) was added, following which the reaction mixture was allowed to warm to room temperature overnight. The reaction was then quenched with water and the THF removed under reduced pressure. The aqueous layer was extracted with Et_2O and the combined organics were washed once with brine, dried ($MgSO_4$) and the solvent removed under reduced pressure. Purification by flash column chromatography (SiO_2, 0-1% v/v Acetone in $CHCl_3$) afforded the title compound as a colourless oil (103 mg, 0.46 mmol, 57%).

***R_f* value** = 0.56 (100% $CHCl_3$);

^{1}H NMR (400 MHz, $CDCl_3$) δ 7.99-8.01 (m, 1H, H-1), 7.84-7.87 (m, 1H, H-4), 7.76 (d, *J* = 8.2 Hz, 1H, H-5), 7.47-7.50 (m, 2H, H-2, H-3), 7.45 (dd, *J* = 8.0 Hz, 7.1 Hz, 1H, H-6), 7.34 (d, *J* = 7.0 Hz, 1H, H-7), 6.90 (d, *J* = 11.4 Hz, 1H, H-8), 5.94 (dt, *J* = 11.5 Hz, 7.4 Hz, 1H, H-9), 3.31 (t, *J* = 6.6 Hz, 2H, H-12), 3.24 (s, 3H, H-13), 2.23 (qd, *J* = 7.4 Hz, 1.6 Hz, 2H, H-10), 1.65-1.71 (m, 2H, H-11) ppm;

^{13}C NMR (101 MHz, $CDCl_3$) δ 134.8, 133.9, 133.7, 132.0, 128.5, 127.6, 127.3, 126.5, 125.9, 125.8, 125.4, 125.2, 72.3, 58.6, 29.8, 25.4 ppm;

HRMS (+ESI) *m/z* Found $[M–CH_3O]^+$ 195.1176, $[C_{15}H_{15}]^+$ requires 195.1168, (δ = + 4.1 ppm).

Synthesis of Substrates Bearing Alternative Hydrogen Bond-Donors

(4-ethoxy-4-oxobutyl)triphenylphosphonium bromide

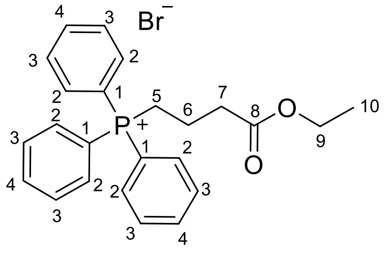

Prepared according to a variant of the protocol described by Shenvi and co-workers.[36] To a solution of triphenylphosphine (7.80 g, 29.7 mmol) in dry toluene (43 mL), was added ethyl-4-bromobutanoate (4.5 mL, 31.4 mmol) and the reaction mixture was heated to reflux for 18 hours under a nitrogen atmosphere. The reaction mixture was then cooled to room temperature, filtered and the solids washed with ice cold hexanes to give the title compound as a white amorphous solid (5.46 g, 11.9 mmol, 40%). The resultant solid was dried under vacuum at 100 ˚C for 10 h immediately prior to use.

^{1}H NMR (400 MHz, $CDCl_3$) δ 7.90-7.85 (m, 6H, H-2), 7.79-7.75 (m, 3H, H-4), 7.70-7.66 (m, 6H, H-3), 4.10-4.01 (m, 4H, H-5, H-9), 2.87 (t, J = 6.4 Hz, 2H, H-7), 1.95-1.86 (m, 2H, H-6), 1.21 (t, J = 7.1 Hz, 3H, H-10) ppm;

^{13}C NMR (101 MHz, $CDCl_3$) δ 173.3 (C-8), 135.1 (d, J_{C-P} = 3.0 Hz, C-4), 133.9 (d, J_{C-P} = 10.1 Hz, C-2), 130.5 (d, J_{C-P} = 12.6 Hz, C-3), 118.3 (d, J_{C-P} = 86.3 Hz, C-1), 60.7 (C-9), 33.4 (d, J_{C-P} = 18.5 Hz, C-7), 21.8 (d, J_{C-P} = 51.7 Hz, C-5), 18.1 (d, J_{C-P} = 3.1 Hz, C-6), 14.2 (C-10) ppm;

^{31}P NMR (162 MHz, $CDCl_3$) δ 24.4 (s) (P-1) ppm.

The spectroscopic data is in agreement with that reported in the literature.[37]

Ethyl (Z)-5-phenylpent-4-enoate

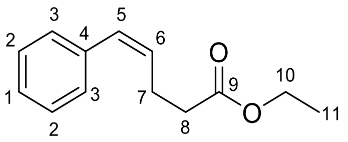

Prepared according to a variant of the protocol described by Asano, Matsubara and co-workers.[37] To a mixture of (4-ethoxy-4-oxobutyl)triphenylphosphonium bromide (5.65 g, 12.4 mmol) and KO^{t}Bu (1.55 g, 13.8 mmol) was added THF (45 mL) at – 5 °C. The reaction mixture was warmed to room temperature and stirred at this temperature for 1 hour. The reaction mixture was then re-cooled to – 5 °C and benzaldehyde (1.0 mL, 9.8 mmol) was added dropwise. The reaction mixture was warmed to room temperature and stirred at this temperature for 5 hours following which it was quenched with distilled water (30 mL) and the volatiles removed under reduced pressure . The remaining aqueous layer was extracted with diethyl ether. The combined organic layers were washed with brine, dried over $MgSO_4$ and concentrated under reduced pressure. Purification by flash column chromatography (SiO_2, 0-6% v/v EtOAc in Petrol) afforded the title compound as a colourless oil (1.47 g, 7.20 mmol, 72%).

***R$_f$* value** = 0.38 (10% v/v EtOAc in Petrol);

^{1}H NMR (400 MHz, $CDCl_3$) δ 7.36-7.32 (m, 2H, H-2), 7.29-7.27 (m, 2H, H-3), 7.25-7.21 (m, 1H, H-1), 6.47 (d, *J* = 11.4 Hz, 1H, H-5), 5.63 (dt, *J* = 11.7, 7.2 Hz, 1H, H-6), 4.13 (q, *J* = 7.2 Hz, 2H, H-10), 2.67 (qd, *J* = 7.2, 1.7 Hz, 2H, H-7), 2.43 (t, *J* = 7.5 Hz, 2H, H-8), 1.24 (t, *J* = 7.2 Hz, 3H, H-11).

^{13}C NMR (101 MHz, $CDCl_3$) δ 173.1 (C-9), 137.4 (C-4), 130.5 (C-6), 130.3 (C-5), 128.9 (C-3), 128.4 (C-2), 126.9 (C-1), 60.5 (C-10), 34.6 (C-8), 24.2 (C-7), 14.4 (C-11).

The spectroscopic data is in agreement with that reported in the literature.[37]

(Z)-5-phenylpent-4-enoic acid

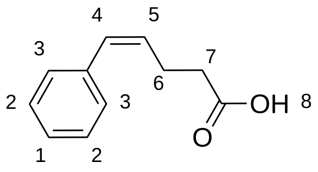

To a solution of ethyl (*Z*)-5-phenylpent-4-enoate (1.20 g, 5.9 mmol) in a 1:1 mixture of THF/H_2O (30 mL) was added LiOH•H_2O (740 mg, 30.9 mmol) and the reaction mixture was stirred at room temperature overnight. Following this the volatiles were removed under reduced pressure and the aqueous layer was washed with EtOAc (2 x 20 mL) and these first organic washings were discarded. The aqueous layer was then acidified with 3 M aqueous HCl and re-extracted with EtOAc (3 x 40 mL). These second organic extracts were combined, dried ($MgSO_4$) and the solvent removed under reduced pressure to afford the title compound as a green oil which solidifed upon standing to a yellow amorphous solid (840 mg, 4.8 mmol, 81%).

***R_f* value** = 0.33 (30% v/v EtOAc in Hexane);

^{1}H NMR (400 MHz, $CDCl_3$) δ 10.6 (br s, 1H, H-8), 7.35 (t, *J* = 7.5 Hz, 2H, H-2), 7.23-7.29 (m, 3H, H-1, H-3), 6.50 (dt, *J* = 11.6 Hz, 1.8 Hz, 1H, H-4), 5.65 (dt, *J* = 11.6 Hz, 7.1 Hz, 1H, H-5), 2.68 (qd, *J* = 7.1 Hz, 2H, H-6), 2.50 (t, *J* = 7.8 Hz, 2H, H-7) ppm;

^{13}C NMR (101 MHz, $CDCl_3$) δ 179.5, 137.2, 130.6, 130.0, 128.8, 128.4, 127.0, 34.3, 23.9 ppm.

The spectroscopic data is in agreement with that reported in the literature.[37]

(Z)-5-phenylpent-4-en-1-yl 4-methylbenzenesulfonate

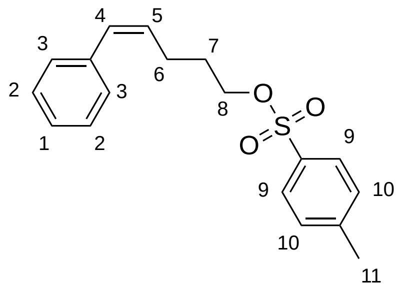

An heat gun-dried round-bottomed flask was charged with (*Z*)-5-phenylpent-4-en-1-ol (**1a**) (1.23 g, 7.6 mmol), 4-(dimethylamino)pyridine (185 mg, 1.5 mmol), *p*-toluenesulfonyl chloride (1.59 g, 8.3 mmol) and placed under a nitrogen atmosphere. CH_2Cl_2 (40 mL) was added and the flask was cooled to 0 °C. Following this, triethylamine (1.3 mL, 9.1 mmol) was added dropwise and the reaction mixture was allowed to warm to room temperature overnight. The reaction mixture was then quenched with H_2O and extracted with Et_2O. The combined organic extracts were washed with 2 M aqueous HCl solution and brine, dried over $MgSO_4$ and concentrated under reduced pressure. Purification by flash column chromatography (SiO_2, 0-20% v/v EtOAc in Hexane) afforded the title compound as a colourless oil (2.48 g, quant.).

***R_f* value** = 0.52 (20% v/v EtOAc in Hexane);

^{1}H NMR (400 MHz, $CDCl_3$) δ 7.74-7.77 (m, 2H, H-9), 7.30-7.34 (m, 4H, H-10, H-3), 7.20-7.25 (m, 3H, H-1, H-2), 6.43 (dt, *J* = 11.6 Hz, 1.8 Hz, 1H, H-4), 5.53 (dt, *J* = 11.6 Hz, 7.3 Hz, 1H, H-5), 4.05 (t, *J* = 6.5 Hz, 2H, H-8), 2.44 (s, 3H, H-11), 2.36 (qd, *J* = 7.3 Hz, 1.8 Hz, 2H, H-6), 1.77-1.83 (m, 2H, H-7) ppm;

^{13}C NMR (101 MHz, $CDCl_3$) δ 144.8, 137.3, 133.3, 130.6, 130.3, 130.0, 128.8, 128.3, 128.0, 126.9, 70.0, 29.3, 24.6, 21.8 ppm;

HRMS (+ESI) *m/z* Found $[M+Na]^+$ 339.1031, $[C_{18}H_{20}NaO_3S]^+$ requires 339.1025, (δ = + 1.8 ppm).

(Z)-2,2,2-trifluoro-N-(5-phenylpent-4-en-1-yl)acetamide

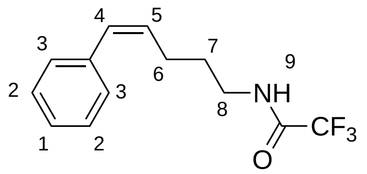

To a solution of NaH (60 wt% suspension in mineral oil, 120 mg, 3.0 mmol) in DMF (5 mL) at 0 °C was added a solution of 2,2,2-trifluoroacetamide (328 mg, 2.9 mmol) in DMF (14 mL) dropwise. The reaction mixture was stirred at the same temperature for a further 15 minutes, following which a solution of (*Z*)-5-phenylpent-4-en-1-yl 4-methylbenzenesulfonate (949 mg, 3.0 mmol) in DMF (2 mL) was added dropwise. Addition of the (*Z*)-5-phenylpent-4-en-1-yl 4-methylbenzenesulfonate was made quantitative with DMF (2 mL) following which the reaction mixture was allowed to warm to room temperature and stirred at 50 °C for 7 hours. The reaction was then quenched with water and the aqueous layer was extracted with Et_2O. The combined organic extracts were washed with water and brine, dried ($MgSO_4$) and the solvent removed under reduced pressure. Purification of the crude residue by flash column chromatography (SiO_2, 0-2.5% v/v Et_2O in $CHCl_3$) afforded the title compound as a thick, pale-yellow oil (166 mg, 0.65 mmol, 22%).

***R_f* value** = 0.60 (5% v/v Et_2O in $CHCl_3$);

^{1}H NMR (400 MHz, $CDCl_3$) δ 7.33-7.37 (m, 2H, H-2), 7.23-7.27 (m, 3H, H-1, H-3), 6.52 (d, *J* = 11.6 Hz, 1H, H-4), 6.21 (br s, 1H, H-9), 5.63 (dt, *J* = 11.6 Hz, 7.4 Hz, 1H, H-5), 3.35 (q, *J* = 6.7 Hz, 2H, H-8), 2.39 (qd, *J* = 7.3 Hz, 1.7 Hz, 2H, H-6), 1.73 (p, *J* = 7.2 Hz, 2H, H-7) ppm;

^{13}C NMR (101 MHz, $CDCl_3$) δ 157.3 (q, J_{C-F} = 36.6 Hz), 137.2, 130.8, 130.7, 128.7, 128.5, 127.1, 115.9 (q, J_{C-F} = 287.9 Hz), 39.5, 28.9, 25.4 ppm;

^{19}F NMR (376 MHz, $CDCl_3$) δ – 76.0 (s) ppm;

HRMS (–ESI): *m/z* found $[M–H]^-$ 256.0955, $[C_{13}H_{13}F_3NO]^-$ requires 256.0955, (δ = + 0.0 ppm).

(Z)-4-methyl-N-(5-phenylpent-4-en-1-yl)benzenesulfonamide

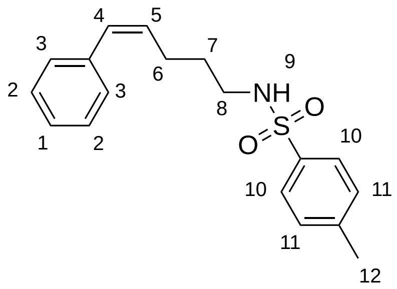

A crimp-top vial was charged with (*Z*)-5-phenylpent-4-en-1-yl 4-methylbenzenesulfonate (791 mg, 2.5 mmol), *p*-toluenesulfonamide (856 mg, 5.0 mmol) and K_2CO_3 (692 mg, 5.0 mmol). The vial was sealed and evacuated and backfilled three times with nitrogen. MeCN (15 mL) was added and the reaction mixture was heated to 90 °C for 40 hours. The vial was then allowed to cool, following which the volatiles were removed under reduced pressure. The crude residue was partitioned between H_2O (30 mL) and CH_2Cl_2 (30 mL) and the layers separated. The aqueous layer was extracted with CH_2Cl_2 (3 x 30 mL) following which the combined organic layers were washed with H_2O (30 mL), 2.5 M NaOH (aq) (30 mL) and brine (20 mL). The combined organic fractions were dried ($MgSO_4$) and the solvent removed under reduced pressure. Purification by flash column chromatography (SiO_2, 0-26% v/v Acetone in Petrol) afforded the title compound as a thick, colourless oil (458 mg, 1.5 mmol, 58%).

***R_f* value** = 0.62 (30% v/v Acetone in Petrol);

^{1}H NMR (400 MHz, $CDCl_3$) δ 7.69-7.72 (m, 2H, H-10), 7.19-7.35 (m, 7H, H-1, H-2, H-3, H-11), 6.43 (dd, *J* = 11.6 Hz, 1.6 Hz, 1H, H-4), 5.54 (dt, *J* = 11.6 Hz, 7.3 Hz, 1H, H-5), 4.46 (t, *J* = 6.2 Hz, 1H, H-9), 2.95 (q, *J* = 6.8 Hz, 2H, H-8), 2.42 (s, 3H, H-12), 2.30 (qd, *J* = 7.4 Hz, 1.7 Hz, 2H, H-6), 1.60 (p, *J* = 7.3 Hz, 2H, H-7) ppm;

^{13}C NMR (101 MHz, $CDCl_3$) δ 143.5, 137.3, 137.1, 131.1, 130.1, 129.8, 128.8, 128.4, 127.2, 126.9, 42.9, 29.9, 25.6, 21.6 ppm;

HRMS (+ESI) *m/z* Found $[M+H]^+$ 316.1363, $[C_{18}H_{22}NO_2S]^+$ requires 316.1366, (δ = – 0.9 ppm).

Synthesis of Allylic and Tetrahomoallylic Styrenyl Alcohols

3-phenylprop-2-yn-1-ol

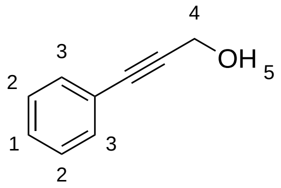

Prepared according to **GP4** using iodobenzene as the aryl iodide and propargyl alcohol as the alkyne. In this case, the following procedural modification was used: The reaction was performed on a 25 mmol scale with respect to the iodide and all other reagent and solvent amounts were scaled proportionally Purification by flash column chromatography (SiO_2, 0-10% v/v Acetone in $CHCl_3$) afforded the title compound as a brown oil (3.25 g, 24.6 mmol, 98%).

***R$_f$* value** = 0.33 (20% v/v Acetone in $CHCl_3$);

^{1}H NMR (500 MHz, $CDCl_3$) δ 7.41-7.48 (m, 2H, H-3), 7.28-7.35 (m, 3H, H-1, H-2), 4.49 (s, 2H, H-4), 1.81 (s, 1H, H-5) ppm;

^{13}C NMR (126 MHz, $CDCl_3$) δ 131.8, 128.6, 128.4, 122.6, 87.3, 85.8, 51.8 ppm;

The spectroscopic data is in agreement with that reported in the literature.[38]

(Z)-3-phenylprop-2-en-1-ol

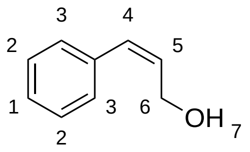

Prepared according to a variant of the protocol described by Mei and co-workers.[39] To a suspension of manganese powder (2.69 g, 49 mmol), 3-phenylprop-2-yn-1-ol (3.24 g, 24.5 mmol) and $Pd(OAc)_2$ (55 mg, 0.25 mmol) in MeCN (31 mL) was added H_2O (3.5 mL) and the reaction mixture stirred for 36 hours at room temperature. At this point analysis of the reaction mixture by ^{1}H NMR indicated low conversion of starting material. As a result a further portion of $Pd(OAc)_2$ was added (220 mg, 0.98 mmol) along with more H_2O (10 mL) and the reaction mixture was heated to 55 °C for a further 36 hours. The reaction mixture was then allowed to cool and filtered through a pad of Celite®. The filtrate was dried ($MgSO_4$) and the solvent removed under reduced pressure. Purification by flash column chromatography (SiO_2, 0-50% v/v Et_2O in Petrol) afforded the title compound as an orange oil (1.64 g, 12.2 mmol, 50%).

***R**$_f$* **value** = 0.31 (40% v/v Et_2O in Petrol);

^{1}H NMR (400 MHz, $CDCl_3$) δ 7.35 (t, *J* = 7.4 Hz, 2H, H-2), 7.27 (tt, *J* = 7.4 Hz, 1.2 Hz, 1H, H-1), 7.21 (d, *J* = 7.2 Hz, 2H, H-3), 6.58 (d, *J* = 11.7 Hz, 1H, H-4), 5.88 (dt, *J* = 11.9 Hz, 6.4 Hz, 1H, H-5), 4.45 (dd, *J* = 6.5 Hz, 1.6 Hz, 2H, H-6) ppm;

^{13}C NMR (101 MHz, $CDCl_3$) δ 136.7, 131.3, 131.2, 128.9, 128.4, 127.4, 59.9 ppm;

The spectroscopic data is in agreement with that reported in the literature.[40]

7-phenylhept-6-yn-1-ol

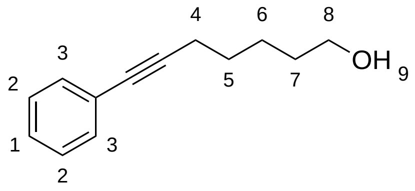

Prepared according to **GP4** using iodobenzene as the aryl iodide and hept-6-yn-1-ol as the alkyne. In this case, the following procedural modification was used: The reaction was performed on a 10 mmol scale with respect to the iodide and all other reagent and solvent amounts were scaled proportionally. Purification by flash column chromatography (SiO_2, 0-20% v/v Acetone in Petrol) afforded the title compound as a golden oil (1.99 g, quant.).

***R_f* value** = 0.30 (20% v/v Acetone in Hexane);

^{1}H NMR (400 MHz, $CDCl_3$) δ 7.37-7.40 (m, 2H, H-3), 7.23-7.30 (m, 3H, H-1, H-2), 3.67 (t, J = 6.1 Hz, 2H, H-8), 2.43 (t, J = 6.9 Hz, 2H, H-4), 1.59-1.68 (m, 4H, H-5, H-7), 1.49-1.58 (m, 2H, H-6), 1.38 (br s, 1H, H-9) ppm;

^{13}C NMR (101 MHz, $CDCl_3$) δ 131.7, 128.3, 127.7, 124.1, 90.2, 80.9, 63.0, 32.4, 28.6, 25.2, 19.5 ppm;

The spectroscopic data is in agreement with that reported in the literature.[41]

(Z)-7-phenylhept-6-en-1-ol

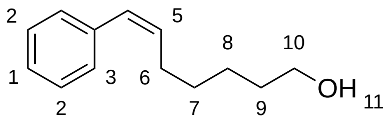

Prepared according to **GP6** using 7-phenylhept-6-yn-1-ol as the alkyne. In this case the following procedural modification was used: The reaction was performed on a 5.0 mmol scale with respect to the alkyne and all other reagent and solvent amounts were scaled proportionally. Purification by flash column chromatography (SiO_2, 0-18% v/v Acetone in Petrol) afforded the title compound as a pale-yellow oil (807 mg, 4.2 mmol, 85%).

***R_f* value** = 0.48 (20% v/v Acetone in Hexane);

^{1}H NMR (400 MHz, $CDCl_3$) δ 7.31-7.35 (m, 2H, H-2), 7.26-7.28 (m, 2H, H-3), 7.22 (tt, *J* = 7.2 Hz, 2.1 Hz, 1H, H-1), 6.42 (dt, *J* = 11.7 Hz, 1.6 Hz, H-4), 5.66 (dt, *J* = 11.7 Hz, 7.3 Hz, H-5), 3.63 (t, *J* = 6.6 Hz, 2H, H-10), 2.35 (qd, *J* = 7.2 Hz, 1.8 Hz, 2H, H-6), 1.35-1.60 (m, 7H, H-7, H-8, H-9, H-11) ppm;

^{13}C NMR (101 MHz, $CDCl_3$) δ 137.8, 133.0, 129.1, 128.9, 128.2, 126.6, 63.0, 32.7, 29.8, 28.6, 25.6 ppm;

The spectroscopic data is in agreement with that reported in the literature.[42]

Synthesis of Simple Aliphatic Alkenyl Alcohols

Note: Compounds **23b***,* **23c** *and* **23g** *were obtained from commercial sources.*

(E)-pent-3-en-1-ol (**23a**)

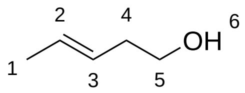

To a solution of (*E*)-pent-3-enoic acid (1.0 mL, 10.0 mmol) in Et_2O (30 mL) at 0 °C was added $LiAlH_4$ (12.0 mL of a 1.0 M solution in Et_2O, 12.0 mmol) dropwise. The reaction mixture was allowed to warm to room temperature overnight following which it was re-cooled to 0 °C and quenched through the careful portion-wise addition of sodium sulfate decahydrate. After the effervescence had ceased, the quenched mixture was filtered, eluting with plenty of Et_2O. The filtrate was collected and the solvent removed under reduced pressure. Purification by flash column chromatography (SiO_2, 0-21% v/v Acetone in Petrol) afforded the title compound as a yellow oil (46.5 mg, 0.54 mmol, 5%). *Caution: The product is very volatile and a lot was lost upon evaporation of the column fractions.*

***R_f* value** = 0.46 (20% v/v Acetone in Petrol);

^{1}H NMR (400 MHz, $CDCl_3$) δ 5.53-5.61 (m, 1H, H-2), 5.36-5.44 (m, 1H, H-3), 3.62 (t, *J* = 6.3 Hz, 2H, H-5), 2.22-2.28 (m, 2H, H-4), 1.68 (dq, *J* = 6.3 Hz, 1.2 Hz, 3H, H-1) ppm;

^{13}C NMR (101 MHz, $CDCl_3$) δ 128.7, 127.2, 62.2, 36.1, 18.2 ppm;

The spectroscopic data is in agreement with that reported in the literature.[43]

4-methylpent-3-en-1-ol (**23d**)

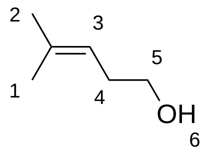

Prepared according to a variant of the protocol described by Hoveyda and co-workers.[44] To a solution of 1-bromo-2-methylprop-1-ene (0.82 mL, 8.0 mmol) in THF (10 mL) was added *t*-BuLi (9.4 mL of a 1.7 M solution in pentane, 16.0 mmol) at − 78 °C dropwise. The solution was stirred at the same temperature for a further 30 minutes following which ethylene oxide (5.4 mL of a 2.5−3.3 M solution in THF, 13.5-17.8 mmol) was added and the mixture allowed to warm to room temperature overnight. The reaction was then quenched by addition of saturated aqueous NH_4Cl (25 mL) and the THF removed under reduced pressure. The aqueous layer was extracted with Et_2O (3 x 50 mL), the combined organic layers were dried ($MgSO_4$) and the solvent removed under reduced pressure. Purification by flash column chromatography (SiO_2, 0-15% v/v Acetone in Petrol) afforded the title compound as a colourless oil (261 mg, 2.6 mmol, 33%).

***R_f* value** = 0.32 (15% v/v Acetone in Petrol);

^{1}H NMR (500 MHz, $CDCl_3$) *δ* 5.11-5.15 (m, 1H, H-3), 3.62 (q, *J* = 6.3 Hz, 2H, H-5), 2.26-2.30 (m, 2H, H-4), 1.73-1.74 (m, 3H, H-2), 1.65 (br s, 3H, H-1), 1.35 (t, *J* = 5.8 Hz, 1H, H-6) ppm;

^{13}C NMR (126 MHz, $CDCl_3$) *δ* 135.4, 120.2, 62.7, 31.8, 26.0, 18.0 ppm;

The spectroscopic data is in agreement with that reported in the literature.[45]

(Z)-3-methylpent-3-en-1-ol (**23e**)

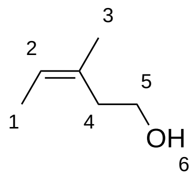

Prepared according to a variant of the protocol described by Hoveyda and co-workers.[44] To a solution of (*Z*)-2-bromobut-2-ene (0.81 mL, 8.0 mmol) in THF (10 mL) was added *t*-BuLi (9.4 mL of a 1.7 M solution in pentane, 16.0 mmol) at − 78 °C dropwise. The solution was stirred at the same temperature for a further 30 minutes following which ethylene oxide (5.4 mL of a 2.5−3.3 M solution in THF, 13.5-17.8 mmol) was added and the mixture allowed to warm to room temperature overnight. The reaction was then quenched by addition of saturated aqueous NH_4Cl (25 mL) and the THF removed under reduced pressure. The aqueous layer was extracted with Et_2O (3 x 50 mL), the combined organic layers were dried ($MgSO_4$) and the solvent removed under reduced pressure. Purification by flash column chromatography (SiO_2, 0-16% v/v Acetone in Petrol) afforded the title compound as a pale-yellow oil (162 mg, 1.6 mmol, 20%).

***R**f* **value** = 0.40 (20% v/v Acetone in Petrol);

^{1}H NMR (400 MHz, $CDCl_3$) δ 5.38-5.43 (m, 1H, H-2), 3.68 (q, *J* = 5.9 Hz, 2H, H-5), 2.33 (t, *J* = 6.7 Hz, 2H, H-4), 1.71 (pent, *J* = 1.5 Hz, 3H, H-3), 1.60-1.62 (m, 3H, H-1), 1.41 (t, *J* = 4.7 Hz, 1H, H-6) ppm;

^{13}C NMR (101 MHz, $CDCl_3$) δ 132.0, 122.5, 60.7, 34.8, 23.5, 13.6 ppm;

The spectroscopic data is in agreement with that reported in the literature.[44]

(E)-3-methylpent-3-en-1-ol (**23f**)

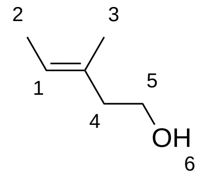

Prepared according to a variant of the protocol described by Hoveyda and co-workers.[44] To a solution of (*E*)-2-bromobut-2-ene (0.81 mL, 8.0 mmol) in THF (10 mL) was added *t*-BuLi (9.4 mL of a 1.7 M solution in pentane, 16.0 mmol) at − 78 °C dropwise. The solution was stirred at the same temperature for a further 30 minutes following which ethylene oxide (5.4 mL of a 2.5−3.3 M solution in THF, 13.5-17.8 mmol) was added and the mixture allowed to warm to room temperature overnight. The reaction was then quenched by addition of saturated aqueous NH_4Cl (25 mL) and the THF removed under reduced pressure. The aqueous layer was extracted with Et_2O (3 x 50 mL), the combined organic layers were dried ($MgSO_4$) and the solvent removed under reduced pressure. Purification by flash column chromatography (SiO_2, 0-20% v/v Acetone in Petrol) afforded the title compound as a yellow oil (82.5 mg, 0.8 mmol, 10%).

***R$_f$* value** = 0.47 (20% v/v Acetone in Petrol);

^{1}H NMR (400 MHz, $CDCl_3$) *δ* 5.29-5.36 (m, 1H, H-1), 3.66 (t, *J* = 5.8 Hz, 2H, H-5), 2.25 (t, *J* = 6.2 Hz, 2H, H-4), 1.60-1.63 (m, 6H, H-2, H-3), 1.37 (t, *J* = 5.3 Hz, 1H, H-6) ppm;

^{13}C NMR (101 MHz, $CDCl_3$) *δ* 132.1, 122.1, 60.2, 42.8, 15.5, 13.6 ppm;

The spectroscopic data is in agreement with that reported in the literature.[46]

3,4-dimethylpent-3-en-1-ol (**23h**)

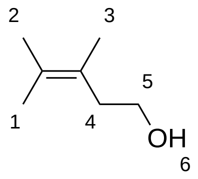

Prepared according to a variant of the protocol described by Hoveyda and co-workers.[44] To a solution of 2-bromo-3-methyl-2-butene (0.93 mL, 8.0 mmol) in THF (10 mL) was added *t*-BuLi (9.4 mL of a 1.7 M solution in pentane, 16.0 mmol) at − 78 °C dropwise. The solution was stirred at the same temperature for a further 30 minutes following which ethylene oxide (5.4 mL of a 2.5−3.3 M solution in THF, 13.5-17.8 mmol) was added and the mixture allowed to warm to room temperature overnight. The reaction was then quenched by addition of saturated aqueous NH_4Cl (25 mL) and the THF removed under reduced pressure. The aqueous layer was extracted with Et_2O (3 x 50 mL), the combined organic layers were dried ($MgSO_4$) and the solvent removed under reduced pressure. Purification by flash column chromatography (SiO_2, 0-16% v/v Acetone in Petrol) afforded the title compound as a colourless oil (225 mg, 2.0 mmol, 25%).

***R$_f$* value** = 0.44 (20% v/v Acetone in Petrol);

^{1}H NMR (400 MHz, $CDCl_3$) δ 3.64 (t, *J* = 6.7 Hz, 2H, H-5), 2.33 (t, *J* = 6.8 Hz, 2H, H-4), 1.66-1.69 (m, 9H, H-1, H-2, H-3), 1.40 (br s, 1H, H-6) ppm;

^{13}C NMR (101 MHz, $CDCl_3$) δ 127.8, 123.6, 61.0, 37.7, 20.9, 20.5, 18.5 ppm.

The spectrocopic data is in agreement with that reported in the literature.[47]

Synthesis of *Trans*-Dialkyl Alkenyl Alcohols

Note: Compound **25a** *was obtained from a commercial source.*

(E)-5-methylhex-3-en-1-ol (**25b**)

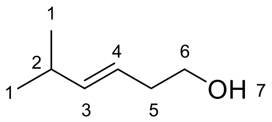

To a solution of 2,3-dihydrofuran (0.75 mL, 10.0 mmol, 1.0 equiv.) in THF (8 mL) at – 30 ˚C was added *s*-BuLi (8.25 mL, 1.3 M in pentanes, 10.7 mmol, 1.07 equiv.) dropwise. The resultant yellow solution was stirred at this temperature for 45 minutes In a separate vessel, *i*-PrMgCl (5.5 mL, 2 M in THF, 11 mmol, 1.1 equiv.) was added dropwise to a stirring suspension of $CuBr_2$•SMe_2 (1.18 g, 5.74 mmol, 0.57 equiv.) in THF (15 mL) at – 78 ˚C and the reaction mixture was stirred for an additional 30 minutes The lithiated dihydrofuran solution was subsequently added to the cuprate via cannula and the reaction mixture was warmed to – 30 ˚C and stirred at this temperature for 2.5 hours. The reaction mixture was quenched with 10% v/v ammonium hydroxide in saturated ammonium chloride (30 mL) and concentrated *in vacuo*. The aqueous layer was extracted with Et_2O and the combined organic layers dried over $MgSO_4$ and concentrated *in vacuo*. Purification by flash column chromatography (SiO_2, 0-5% v/v Et_2O in CH_2Cl_2) afforded the title compound as a colourless oil (181 mg, 1.56 mmol, 16%).

***R**$_f$* **value** = 0.39 (10% v/v Et_2O in CH_2Cl_2).

^{1}H NMR (400 MHz, $CDCl_3$) δ 5.53 (ddt, *J* = 15.5, 6.6, 1.2 Hz, 1H, H-3), 5.33 (dtd, *J* = 15.4, 7.0, 1.2 Hz, 1H, H-4), 3.61 (t, *J* = 6.3 Hz, 2H, H-6), 2.34-2.22 (m, 3H, H-2, H-5), 0.97 (d, *J* = 6.7 Hz, 6H, H-1) ppm;

^{13}C NMR (101 MHz, $CDCl_3$) δ 141.6 (C-3), 122.8 (C-4), 62.2 (C-6), 36.1 (C-5), 31.2 (C-2), 22.7 (C-1) ppm;

The spectrocopic data is in agreement with that reported in the literature.[48]

(E)-5,5-dimethylhex-3-en-1-ol: (**25c**)

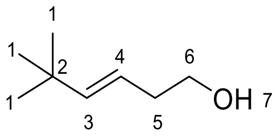

To a suspension of $CuBr_2$•SMe_2 (799 mg, 3.89 mmol, 0.39 equiv.) in Et_2O (15 mL) at – 40 ˚C was added *t*-BuLi (11 mL, 1.7 M in pentanes, 18.7 mmol, 1.87 equiv.) dropwise. The resultant black suspension was allowed to warm to – 20 ˚C, followed by the dropwise addition of 2,3-dihydrofuran (0.76 mL, 10.0 mmol, 1.0 equiv.) in Et_2O (7 mL). The reaction mixture was warmed to – 5 ˚C and stirred at this temperature for 3 hours. The reaction mixture was cooled to – 20 ˚C and quenched with water (0.75 mL). The reaction mixture was warmed to room temperature over 30 min and diluted with 10% v/v ammonium hydroxide in saturated aqueous ammonium chloride (30 mL). The layers were separated and the aqueous layer extracted with Et_2O. The organic layers were combined, dried over $MgSO_4$, filtered over Celite and concentrated *in vacuo*. Purification by flash column chromatography (SiO_2, 0-20% v/v Acetone in Petrol) afforded the title compound as a yellow oil (161 mg, 1.26 mmol, 13%).

***R_f* value** = 0.34 (10% v/v Acetone in Petrol).

^{1}H NMR (400 MHz, $CDCl_3$) δ 5.59 (dt, *J* = 15.6, 1.3 Hz, 1H, H-3), 5.28 (dt, *J* = 15.7, 6.9 Hz, 1H, H-4), 3.61 (t, *J* = 6.3 Hz, 2H, H-6), 2.25 (qd, *J* = 6.4, 1.2 Hz, 2H, H-5), 1.00 (s, 9H, H-1) ppm;

^{13}C NMR (101 MHz, $CDCl_3$) δ 145.6 (C-3), 120.3 (C-4), 62.2 (C-6), 36.2 (C-5), 33.2 (C-2), 29.8 (C-1) ppm;

HRMS (ESI) *The compound did not ionise*.

(E)-hex-3-ene-1,6-diol

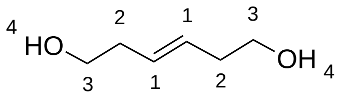

To a solution of dimethyl (*E*)-hex-3-enedioate (0.78 mL, 5.0 mmol) in THF (20 mL) at 0 °C was added $LiAlH_4$ (5.0 mL of a 2.4 M solution in THF, 12.0 mmol) dropwise. The reaction mixture was allowed to warm to room temperature overnight following which it was re-cooled to 0 °C and quenched through the careful portion-wise addition of sodium sulfate decahydrate. After the effervescence had ceased, the quenched mixture was filtered, eluting with plenty of EtOAc. The filtrate was collected and the solvent removed under reduced pressure. Purification by flash column chromatography (SiO_2, 0-10% v/v MeOH in CH_2Cl_2) afforded the title compound as a viscous colourless oil (396 mg, 3.4 mmol, 68%).

***R_f* value** = 0.52 (10% v/v MeOH in CH_2Cl_2);

^{1}H NMR (400 MHz, $CDCl_3$) δ 5.46-5.55 (m, 2H, H-1), 3.62 (t, *J* = 6.1 Hz, 4H, H-3), 2.44 (br s, 2H, H-4), 2.28 (q, *J* = 5.5 Hz, 4H, H-2) ppm;

^{13}C NMR (400 MHz, $CDCl_3$) δ 129.7, 61.8, 36.1 ppm;

The spectroscopic data is in agreement with that reported in the literature.[49]

(E)-6-(benzyloxy)hex-3-en-1-ol (**25d**)

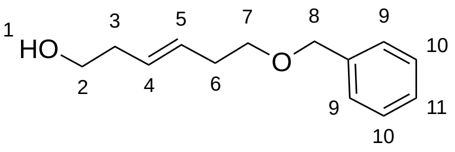

To a solution of (*E*)-hex-3-ene-1,6-diol (349 mg, 3.0 mmol) in THF (20 mL) at 0 °C was added NaH (65.6 mg of a 60% dispersion in mineral oil, 1.6 mmol) portionwise. The reaction mixture was stirred at the same temperature for a further hour, following which benzyl bromide (0.18 mL, 1.5 mmol) was added dropwise and the reaction mixture heated to reflux overnight. The reaction was then allowed to cool to room temperature and quenched with saturated aqueous NH_4Cl (20 mL). The volatiles were removed under reduced pressure and the aqueous layer was extracted with CH_2Cl_2 (3 x 40 mL). The combined organic fractions were dried ($MgSO_4$) and the solvent removed under reduced pressure. Purification by flash column chromatography (SiO_2, 0-10% v/v Acetone in $CHCl_3$) afforded the title compound as pale-yellow oil (207 mg, 1.0 mmol, 33%).

R

$\boldsymbol{R_f}$ **value** = 0.49 (10% v/v Acetone in $CHCl_3$);

^{1}H NMR (400 MHz, $CDCl_3$) δ 7.26-7.37 (m, 5H, H-9, H-10, H-11), 5.57 (dt, *J* = 15.4 Hz, 6.5 Hz, 1H, H-5), 5.48 (dt, *J* = 15.4 Hz, 6.6 Hz, 1H, H-4), 4.51 (s, 2H, H-8), 3.62 (t, *J* = 6.0 Hz, 2H, H-2), 3.50 (t, *J* = 6.7 Hz, 2H, H-7), 2.35 (q, *J* = 6.6 Hz, 2H, H-6), 2.27 (q, *J* = 6.4 Hz, 2H, H-3), 1.63 (s, 1H, H-1) ppm;

^{13}C NMR (101 MHz, $CDCl_3$) δ 138.5, 130.3, 128.5, 128.3, 127.8, 127.7, 73.0, 69.9, 62.0, 36.1, 33.3 ppm;

The spectroscopic data is in agreement with that reported in the literature.[50]

(E)-6-hydroxyhex-3-en-1-yl 4-methylbenzenesulfonate (**25e**)

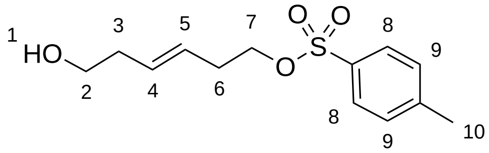

To a solution of (*E*)-hex-3-ene-1,6-diol (190 mg, 1.6 mmol), DMAP (40.3 mg, 0.33 mmol), and TsCl (347 mg, 1.8 mmol) in CH_2Cl_2 (12 mL) at 0 °C was added Et_3N (0.28 mL, 2.0 mmol) dropwise. The reaction mixture was allowed to warm to room temperature over 3 hours following which it was dried directly onto silica gel. Purification by flash column chromatography (SiO_2, 0-20% v/v Acetone in $CHCl_3$) afforded the title compound as a thick, colourless oil (192 mg, 0.71 mmol, 44%).

***R*$_f$ value** = 0.54 (15% v/v Acetone in $CHCl_3$);

^{1}H NMR (400 MHz, $CDCl_3$) δ 7.76-7.80 (m, 2H, H-8), 7.33-7.35 (m, 2H, H-9), 5.49 (dtt, *J* = 15.4, 6.7 Hz, 0.9 Hz, 1H, H-4), 5.41 (dtt, *J* = 15.4 Hz, 6.4 Hz, 1.2 Hz, 1H, H-5), 4.04 (t, *J* = 6.6 Hz, 2H, H-7), 3.61 (t, *J* = 6.2 Hz, 2H, H-2), 2.45 (s, 3H, H-10), 2.37 (qd, *J* = 6.5 Hz, 0.8 Hz, 2H, H-6), 2.24 (qd, *J* = 6.4 Hz, 0.8 Hz, 2H, H-3), 1.61 (br s, 1H, H-1) ppm;

^{13}C NMR (101 MHz, $CDCl_3$) δ 144.9, 133.3, 130.5, 130.0, 128.0, 127.3, 69.9, 61.9, 36.1, 32.3, 21.8 ppm;

HRMS (+ESI) *m/z* Found $[M+Na]^+$ 293.0828, $[C_{13}H_{18}NaO_4S]^+$ requires 293.0818, (δ = + 3.4 ppm).

(E)-6-bromohex-3-en-1-ol: (**25f**)

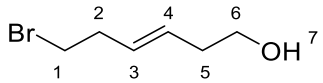

Prepared according to a variant of the protocol described by Eya and co-workers.[51] To a solution of (*E*)-3-hexene-1,6-diol (1.08 g, 9.30 mmol, 1.0 equiv.) and CBr_4 (3.35 g, 10.1 mmol, 1.1 equiv.) in Et_2O (45 mL) was added PPh_3 (2.63 g, 10.0 mmol, 1.1 equiv.) portionwise over 2.5 hours. The reaction mixture was stirred for an additional 3 hours before the addition of pentane (45 mL). The reaction mixture was filtered and the filtrate concentrated *in vacuo*. Purification by flash column chromatography (SiO_2, 0-20% v/v Acetone in Petrol) afforded the title compound as a colourless oil (730 mg, 4.08 mmol, 44%).

***R_f* value** = 0.26 (20% v/v Acetone in Petrol);

^{1}H NMR (400 MHz, $CDCl_3$) *δ* 5.57-5.47 (m, 2H, H-3, H-4), 3.65 (t, *J* = 6.2 Hz, 2H, H-6), 3.40 (t, *J* = 6.9 Hz, 2H, H-1), 2.61-2.56 (m, 2H, H-2), 2.31-2.26 (m, 2H, H-5), 1.61 (s, 1H, H-7) ppm;

^{13}C NMR (101 MHz, $CDCl_3$) *δ* 130.2 (C-3), 129.8 (C-4), 61.9 (C-6), 36.00 (C-2), 35.97 (C-5), 33.0 (C-1) ppm;

The spectroscopic data is in agreement with that reported in the literature.[51]

(E)-N-(6-hydroxyhex-3-en-1-yl)-N,4-dimethylbenzenesulfonamide: (**25g**)

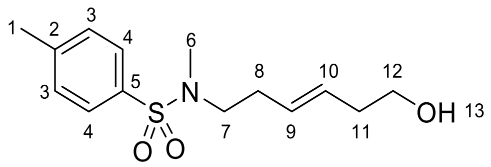

A heatgun-dried vial was charged with (*E*)-6-bromohex-3-en-1-ol (300.3 mg, 1.68 mmol, 1.0 equiv.), *N*-methyl-*p*-toluenesulfonamide (411.6 mg, 2.22 mmol, 1.3 equiv.), Cs_2CO_3 (828.6 mg, 2.54 mmol, 1.5 equiv.) and MeCN (8.5 mL). The reaction mixture was heated at 80 ˚C under

an inert atmosphere of N_2 overnight. The reaction mixture was allowed to cool to room temperature, filtered over Celite and concentrated *in vacuo*. Purification by flash column chromatography (SiO_2, 0-30% v/v Acetone in Petrol) afforded the title compound as a yellow oil (345.9 mg, 1.22 mmol, 73%).

***R_f* value** = 0.14 (30% v/v Acetone in Petrol);

^{1}H NMR (400 MHz, $CDCl_3$) δ 7.65 (d, *J* = 8.2 Hz, 2H, H-4), 7.30 (d, *J* = 8.3 Hz, 2H, H-3), 5.55-5.45 (m, 2H, H-9, H-10), 3.64 (t, *J* = 6.2 Hz, 2H, H-12), 3.05 (t, *J* = 7.1 Hz, 2H, H-7), 2.72 (s, 3H, H-6), 2.42 (s, 3H, H-1), 2.28-2.23 (m, 4H, H-8, H-11), 1.67 (s, 1H, H-13) ppm;

^{13}C NMR (101 MHz, $CDCl_3$) δ 143.4 (C-2), 134.9 (C-5), 129.8 (C-3), 129.5 (C-10), 129.4 (C-9), 127.5 (C-4), 62.0 (C-12), 50.1 (C-7), 36.1 (C-11), 34.8 (C-6), 31.2 (C-8), 21.6 (C-1) ppm;

HRMS (+ESI) *m/z* found $[M+H]^+$ 284.1323, $[C_{14}H_{22}NO_3S]^+$ requires 284.1315, (δ = +2.8 ppm).

Synthesis of *Exo*-Dialkyl Alkenyl Alcohols

6-methyl-3-methyleneheptan-1-ol (**27a**)

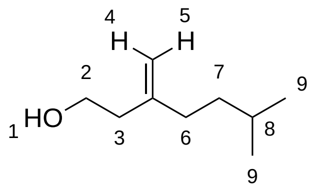

Prepared according to **GP8** using 1-iodo-2-methylpropane (0.57 mL, 5.0 mmol, 1.0 equiv.) as the electrophile. Purification by flash column chromatography (SiO_2, 0-18% v/v Acetone in Petrol) afforded the title compound as a pale-yellow oil (284 mg, 2.0 mmol, 40%).

***R_f* value** = 0.45 (20% v/v Acetone in Petrol);

^{1}H NMR (400 MHz, $CDCl_3$) δ 4.86 (q, *J* = 1.5 Hz, 1H, H-5), 4.79-4.81 (m, 1H, H-4), 3.71 (q, *J* = 5.5 Hz, 2H, H-2), 2.30 (td, *J* = 6.3 Hz, 0.7 Hz, 2H, H-3), 2.01-2.05 (m, 2H, H-6), 1.55 (sept, *J* = 6.6 Hz, 1H, H-8), 1.47 (br s, 1H, H-1), 1.29-1.35 (m, 2H, H-7), 0.89 (d, *J* = 6.6 Hz, 6H, H-9) ppm;

^{13}C NMR (101 MHz, $CDCl_3$) δ 146.7, 111.5, 60.5, 39.3, 37.1, 33.8, 27.9, 22.7 ppm;

HRMS (ESI) *The compound did not ionise*

5-cyclohexyl-3-methylenepentan-1-ol (**27b**)

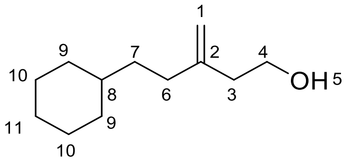

Prepared according to **GP8** using (bromomethyl)cyclohexane (0.7 mL, 5.0 mmol, 1.0 equiv.) as the electrophile. Purification by flash column chromatography (SiO_2, 0-4% v/v Et_2O in CH_2Cl_2) afforded the title compound as a colourless oil (504.5 mg, 2.77 mmol, 55%).

***R_f* value** = 0.44 (10% v/v Et_2O in CH_2Cl_2);

^{1}H NMR (400 MHz, $CDCl_3$) δ 4.85 (d, J = 1.5 Hz, 1H, H-1a), 4.79 (s, 1H, H-1b), 3.72-3.68 (m, 2H, H-4), 2.29 (td, J = 6.3, 0.7 Hz, 2H, H-3), 2.05-2.01 (m, 2H, H-6), 1.73-1.62 (m, 5H, H-9a, H-10a, H-11a), 1.52 (s, 1H, H-5), 1.34-1.29 (m, 2H, H-7), 1.27-1.09 (m, 4H, H-8, H-10b, H-11b), 0.93-0.83 (m, 2H, H-9b) ppm;

^{13}C NMR (101 MHz, $CDCl_3$) δ 146.8 (C-2), 111.4 (C-1), 60.5 (C-4), 39.4 (C-3), 37.6 (C-8), 35.7 (C-7), 33.5 (C-9), 33.2 (C-6), 26.8 (C-11), 26.5 (C-10) ppm;

The spectroscopic data is in agreement with that reported in the literature.[52]

7-((tert-butyldimethylsilyl)oxy)-3-methyleneheptan-1-ol (**27c**)

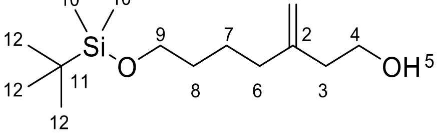

Prepared according to **GP8** using (3-bromopropoxy)-*tert*-butyldimethylsilane (1.2 mL, 5.2 mmol, 1.0 equiv.) as the electrophile. Purification by flash column chromatography (SiO_2, 0-

10% v/v Acetone in Petrol) afforded the title compound as a pale-yellow oil (536.4 mg, 2.08 mmol, 40%).

***R_f* value** = 0.35 (20% v/v Acetone in Petrol);

^{1}H NMR (400 MHz, $CDCl_3$) *δ* 4.87 (s, 1H, H-1a), 4.82 (s, 1H, H-1b), 3.71 (q, *J* = 6.2 Hz, 2H, H-4), 3.62 (t, *J* = 6.0 Hz, 2H, H-9), 2.30 (t, *J* = 6.3 Hz, 2H, H-3), 2.05 (t, *J* = 6.9 Hz, 2H, H-6), 1.56-1.47 (m, 4H, H-7, H-8), 1.43 (t, *J* = 5.72 Hz, 1H, H-5), 0.89 (s, 9H, H-12), 0.05 (s, 6H, H-10) ppm;

^{13}C NMR (101 MHz, $CDCl_3$) *δ* 146.3 (C-2), 111.9 (C-1), 63.2 (C-9), 60.6 (C-4), 39.4 (C-3), 35.8 (C-6), 32.7 (C-8), 26.2 (C-12), 24.2 (C-7), 18.6 (C-11), – 5.0 (C-10) ppm;

HRMS (+ESI) *m/z* found $[M+H]^+$ 259.2087, $[C_{14}H_{31}O_2Si]^+$ requires 259.2088, (*δ* = – 0.4 ppm)

Synthesis of *Tri*-Alkyl Class I Alkenyl Alcohols

(Z)-4-methylhept-3-en-1-ol (**31a**)

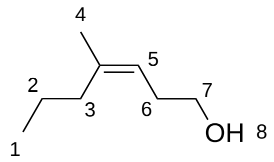

Prepared according to the protocol described by Wang and Li.[53] To a cooled solution of trimethylaluminum (8.0 mL of a 2.0 M solution in Hexane, 16.0 mmol) in CH_2Cl_2 (30 mL) at 0 °C was added 3-heptyn-1-ol (0.98 mL, 8.0 mmol) dropwise. The reaction was stirred for 20 minutes at the same temperature. The mixture was then cooled to – 45 °C using a MeCN/dry ice bath followed by the addition of a cooled (0 °C) solution of $TiCl_4$ (0.88 mL, 8.0 mmol) in CH_2Cl_2 (30 mL) over the period of 5 minutes *via* cannula. The resulting mixture was allowed to stir for 15 minutes and then quenched by the slow addition of pre-cooled (0 °C) MeOH (4.0 mL). An aqueous solution of 3 M HCl saturated with NaCl (30 mL) was then added and the mixture was stirred for another 30 minutes. The organic layer was separated and the aqueous layer was extracted with Et_2O (2 x 50 mL). The combined organic extracts were dried ($MgSO_4$) and concentrated under reduced pressure. Purification by flash column chromatography (SiO_2, 0-15% v/v EtOAc in Petrol) afforded the title compound as a pale-yellow oil (251 mg, 2.0 mmol, 25%).

***R_f* value** = 0.37 (15% v/v EtOAc in Petrol);

^{1}H NMR (400 MHz, $CDCl_3$) δ 5.13 (t, *J* = 7.3 Hz, 1H, H-5), 3.61 (t, *J* = 6.5 Hz, 2H, H-7), 2.28 (q, *J* = 6.4 Hz, 2H, H-6), 2.00-2.05 (m, 2H, H-3), 1.71 (q, *J* = 1.2 Hz, 3H, H-4), 1.36-1.46 (m, 3H, H-2, H-8), 0.89 (t, *J* = 7.4 Hz, 3H, H-1) ppm;

^{13}C NMR (101 MHz, $CDCl_3$) δ 139.4, 120.6, 62.8, 34.0, 31.5, 23.6, 21.4, 14.1 ppm;

The spectroscopic data is in agreement with that reported in the literature.[54]

(3-hydroxypropyl)triphenylphosphonium bromide

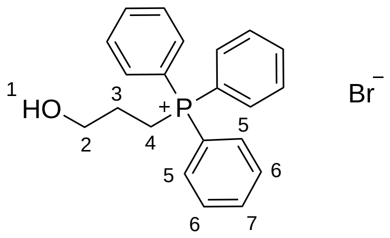

A round-bottom flask was charged with 3-bromopropan-1-ol (4.3 mL, 50 mmol) and PPh_3 (19.7 g, 75 mmol). Toluene (200 mL) was then added and the reaction mixture was heated to reflux overnight. The reaction mixture was then allowed to cool to room temperature and then cooled further in an ice bath, following which the white precipitate was collected by vacuum filtration. The precipitate was washed on the filter with several portions of ice-cold Et_2O to afford the title compound as a white powder (8.6 g, 21.4 mmol, 43%).

^{1}H NMR (400 MHz, DMSO-d$_6$) δ 7.87-7.92 (m, 3H, H-7), 7.74-7.83 (m, 12H, H-5, H-6), 4.88 (t, J = 5.3 Hz, 1H, H-1), 3.50-3.58 (m, 4H, H-2, H-4), 1.62-1.71 (m, 2H, H-3) ppm;

^{13}C NMR (101 MHz, DMSO-d$_6$) δ 134.9 (d, $J_{P\text{-}C}$ = 2.9 Hz), 133.6 (d, $J_{P\text{-}C}$ = 10.1 Hz), 130.3 (d, $J_{P\text{-}C}$ = 12.4 Hz), 118.6 (d, $J_{P\text{-}C}$ = 86.0 Hz), 60.2 (d, $J_{P\text{-}C}$ = 18.5 Hz), 25.3 (d, $J_{P\text{-}C}$ = 3.9 Hz), 17.6 (d, $J_{P\text{-}C}$ = 52.4 Hz) ppm;

^{31}P NMR (162 MHz, DMSO-d$_6$) δ 24.5 (s) ppm.

The spectroscopic data is in agreement with that reported in the literature.[55]

3-Cyclobutylidenepropan-1-ol (**31b**)

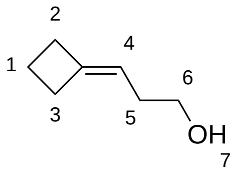

Prepared according to **GP7** using (3-hydroxypropyl)triphenylphosphonium bromide as the phosphonium salt and cyclobutanone as the ketone. In this case the following procedural modification was used: The reaction was performed on a 10 mmol scale with respect to cyclobutanone with and all other reagent and solvent amounts were scaled accordingly. Purification by flash column chromatography (SiO_2, 0-15% v/v Acetone in Petrol) afforded the title compound as a colourless oil (599 mg, 5.3 mmol, 53%).

***R_f* value** = 0.39 (20% v/v Acetone in Petrol);

^{1}H NMR (400 MHz, $CDCl_3$) δ 5.01-5.08 (m, 1H, H-4), 3.59 (q, *J* = 5.1 Hz, 2H, H-6), 2.64-2.69 (m, 4H, H-2, H-3), 2.12-2.19 (m, 2H, H-5), 1.91-1.99 (m, 2H, H-1), 1.46 (br s, 1H, H-7) ppm;

^{13}C NMR (101 MHz, $CDCl_3$) δ 144.0, 115.9, 62.4, 31.7, 31.2, 29.6, 17.1 ppm;

HRMS (ESI) *The compound did not ionise.*

3-Cyclohexylidenepropan-1-ol (**31c**)

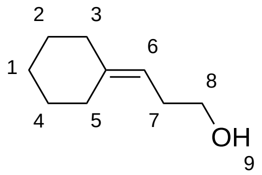

Prepared according to **GP7** using (3-hydroxypropyl)triphenylphosphonium bromide as the phosphonium salt and cyclohexanone as the ketone. Purification by flash column

chromatography (SiO_2, 0-20% v/v Acetone in Petrol) afforded the title compound as a yellow oil (364 mg, 2.6 mmol, 54%).

***Rf* value** = 0.39 (20% v/v Acetone in Petrol);

^{1}H NMR (400 MHz, $CDCl_3$) *δ* 5.06 (t, *J* = 7.4 Hz, 1H, H-6), 3.59 (t, *J* = 6.5 Hz, 2H, H-8), 2.27 (q, *J* = 6.8 Hz, 2H, H-7), 2.15 (t, *J* = 5.6 Hz, 2H, H-5), 2.09 (t, *J* = 5.3 Hz, 2H, H-3), 1.60 (br s, 1H, H-9), 1.48-1.56 (m, 6H, H-1, H-2, H-4) ppm;

^{13}C NMR (101 MHz, $CDCl_3$) *δ* 143.7, 116.6, 62.7, 37.4, 30.7, 28.9, 28.8, 28.1, 27.0 ppm;

HRMS (ESI) *The compound did not ionise.*

3-(Tetrahydro-4H-pyran-4-ylidene)propan-1-ol (**31d**)

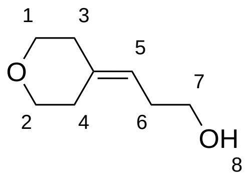

Prepared according to **GP7** using (3-hydroxypropyl)triphenylphosphonium bromide as the phosphonium salt and tetrahydro-4*H*-pyran-4-one as the ketone. Purification by flash column chromatography (SiO_2, 0-24% v/v Acetone in Petrol) afforded the title compound as a colourless oil (587 mg, 4.1 mmol, 86%).

***R_f* value** = 0.34 (25% v/v Acetone in Petrol);

^{1}H NMR (400 MHz, $CDCl_3$) *δ* 5.20 (t, *J* = 7.5 Hz, 1H, H-5), 3.61-3.69 (m, 6H, H-1, H-2, H-7), 2.27-2.32 (m, 4H, H-3, H-4), 2.24 (t, *J* = 5.2 Hz, 2H, H-6), 1.45 (br s, 1H, H-8) ppm;

^{13}C NMR (101 MHz, $CDCl_3$) δ 137.9, 118.9, 69.8, 69.0, 62.6, 37.2, 30.6, 30.0 ppm;

The spectroscopic data is in agreement with that reported in the literature.[56]

4-bromobutan-1-ol

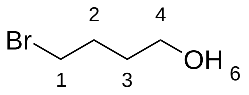

Prepared according to the protocol descibed by Aggarwal and co-workers.[57] HBr (61 mL, 542 mmol, 48% in water, 1.0 equiv.), was added drop-wise to refluxing THF (140 mL). The reaction mixture was allowed to reflux for an additional 2 hours, before it was cooled to room temperature. The reaction mixture was neutralised with saturated $NaHCO_3$ solution (400 mL), and the yellow organic layer was separated and washed with water (60 mL) and brine (60 mL). The combined aqueous layers were extracted with CH_2Cl_2 and the organic extracts were combined, dried over $MgSO_4$ and concentrated *in vacuo* to give the product (27.7 g, 181 mmol, 33%) as a pale-yellow oil, which was used without further purification.

***R$_f$* value** = 0.24 (30% v/v EtOAc in Petrol);

^{1}H NMR (400 MHz, $CDCl_3$) δ 3.71 (t, *J* = 6.4 Hz, 2H, H-4), 3.52 (br s, 1H, H-6), 3.45 (t, *J* = 6.7 Hz, 2H, H-1), 2.00-1.93 (m, 2H, H-2), 1.76-1.69 (m, 2H, H-3) ppm;

^{13}C NMR (101 MHz, $CDCl_3$) δ 62.1 (C-4), 33.7 (C-1), 31.0 (C-3), 29.3 (C-2) ppm;

The spectroscopic data is in agreement with that reported in the literature.[57]

5-methylhex-4-en-1-ol (**31e**)

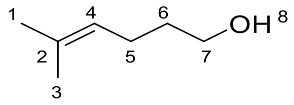

To a suspension of ethyl acetate (0.8 mL, 8.2 mmol, 2.05 equiv.) and copper (I) iodide (3.05 g, 16.0 mmol, 4.0 equiv.) in THF (30 mL) at – 110 ˚C (liquid N_2-ethanol bath) was added a solution of LDA (freshly-prepared by the addition of *n*-butyllithium (5.1 mL, 1.6 M in hexanes, 8.2 mmol, 2.05 equiv.) to a solution of diisopropylamine (1.15 mL, 8.2 mmol, 2.05 equiv.) in THF (10 mL) at 0 ˚C and stirred at this temperature for 30 min). The reaction mixture was allowed to warm to – 40 ˚C over 2 hours, followed by the dropwise addition of a solution of 3,3-dimethylallyl bromide (0.46 mL, 4.0 mmol, 1.0 equiv.) in THF (10 mL). The reaction mixture was stirred for 1 hour at – 40 ˚C, carefully quenched with saturated NH_4Cl (20 mL) and allowed to warm to room temperature. The resultant solids were filtered and washed with hexane to give a biphasic mixture, which was separated. The aqueous layer was further extracted with hexane and the combined organic layers were dried over $MgSO_4$ and concentrated *in vacuo*. The crude residue was dissolved in THF (20 mL) and cooled to 0 ˚C, followed by the dropwise addition of $LiAlH_4$ (4.8 mL, 1 M in Et_2O, 4.8 mmol, 1.2 equiv.). After stirring for 1 hour, the reaction mixture was allowed to warm to room temperature and stirred for 4 hours. The reaction mixture was cooled to 0 ˚C and carefully quenched with sodium sulfate decahydrate. The mixture was filtered over Celite and concentrated *in vacuo*. The crude product was purified via flash column chromatography (SiO_2, 0-25% v/v Acetone in Petrol) to afford the title compound as a colourless oil (177.9 mg, 1.56 mmol, 39% over two steps).

R

R_f value = 0.50 (30% v/v Acetone in Petrol);

^{1}H NMR (400 MHz, $CDCl_3$) δ 5.13 (tp, J = 7.2, 2.8, 1.4 Hz, 1H, H-4), 3.63 (t, J = 6.5 Hz, 2H, H-7), 2.06 (q, J = 7.4 Hz, 2H, H-5), 1.69 (d, J = 1.0 Hz, 3H, H-1), 1.64-1.57 (m, 5H, H-3, H-6), 1.49 (br s, 1H, H-8) ppm;

^{13}C NMR (101 MHz, $CDCl_3$) δ 132.3 (C-2), 124.0 (C-4), 62.9 (C-7), 32.9 (C-6), 25.8 (C-1), 24.5 (C-5), 17.8 (C-3) ppm;

The spectroscopic data is in agreement with that reported in the literature.[58]

(4-hydroxybutyl)-triphenylphosphonium bromide

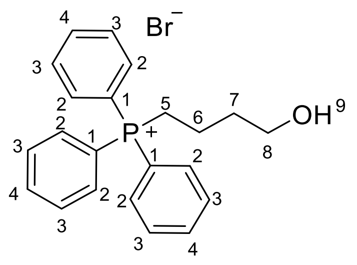

Prepared according to the protocol described by Maryanoff and co-workers.[59] A suspension of 4-bromobutan-1-ol (10.8 g, 70.6 mmol, 1.0 equiv.), triphenylphosphine (18.7 g, 71.3 mmol, 1.0 equiv.) and K_2CO_3 (9.4 g, 68.1 mmol, 0.97 equiv.) in MeCN (60 mL) was refluxed under nitrogen for 19 hours. The reaction mixture was cooled to room temperature, filtered and the solids washed with MeCN. The filtrate was treated with diethyl ether and this led to the precipitation of the product as a white amorphous solid (3.37 g, 8.11 mmol, 12%), which was used without further purification.

^{1}H NMR (400 MHz, $CDCl_3$) δ 7.83-7.66 (m, 15H, H-2, H-3, H-4), 3.75-3.69 (m, 4H, H-5, H-8), 1.82-1.79 (m, 4H, H-6, H-7);

^{13}C NMR (101 MHz, $CDCl_3$) δ 135.1 (d, J_{C-P} = 3.0 Hz, C-4), 133.8 (d, J_{C-P} = 10.0 Hz, C-2), 130.6 (d, J_{C-P} = 12.8 Hz, C-3), 118.4 (d, J_{C-P} = 86.1 Hz, C-1), 59.9 (C-8), 32.0 (d, J_{C-P} = 16.4 Hz, C-7), 22.5 (d, J_{C-P} = 50.2 Hz, C-5), 19.4 (d, J_{C-P} = 4.2 Hz, C-6);

^{31}P NMR (162 MHz, $CDCl_3$) δ 24.5 (P-1) ppm;

Data in agreement with that reported in the literature.[59]

4-cyclohexylidenebutan-1-ol (**31f**)

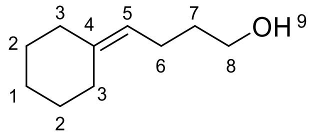

Prepared according to **GP7** using cyclohexanone (0.52 mL, 5.0 mmol, 1.0 equiv.) and (4-hydroxybutyl)-triphenylphosphonium bromide (2.73 g, 6.6 mmol, 1.3 equiv.) as the starting materials. Purification by flash column chromatography (SiO_2, 0-20% v/v EtOAc in Petrol) afforded the title compound as a yellow oil (426.8 mg, 2.77 mmol, 55%).

***R_f* value** = 0.36 (30% v/v EtOAc in Petrol).

^{1}H NMR (400 MHz, $CDCl_3$) δ 5.08 (t, *J* = 7.4 Hz, 1H, H-5), 3.64 (t, *J* = 6.5 Hz, 2H, H-8), 2.12 (t, *J* = 5.6 Hz, 2H, H-3a), 2.10-2.04 (m, 4H, H-3b, H-6), 1.60 (p, *J* = 6.9 Hz, 2H, H-7), 1.54-1.46 (m, 7H, H-1, H-2, H-9) ppm;

^{13}C NMR (101 MHz, $CDCl_3$) δ 140.6 (C-4), 120.6 (C-5), 62.9 (C-8), 37.3 (C-3b), 33.2 (C-7), 28.80 (C-3a), 28.78 (C-2a), 28.0 (C-2b), 27.0 (C-1), 23.6 (C-6) ppm;

The spectroscopic data is in agreement with that reported in the literature.[60]

(Z)-4-phenylpent-3-en-1-ol (**31g**)

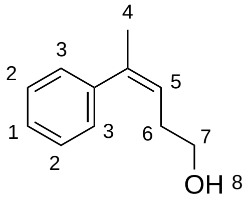

Prepared according to **GP2** on a 5 mmol scale with respect to commercial PhMgCl (2M in THF). In this case the following procedural modification was used: 5-methyl-2,3-dihydrofuran was used in place of 2,3-dihydrofuran. Purification by flash column chromatography (SiO_2, 0-18% v/v EtOAc in Petrol) afforded the title compound as a yellow oil (314 mg, 1.9 mmol, 39%).

***R_f* value** = 0.29 (20% v/v EtOAc in Petrol);

^{1}H NMR (500 MHz, $CDCl_3$) *δ* 7.32-7.35 (m, 2H, H-2), 7.25 (tt, *J* = 7.4 Hz, 1.9 Hz, 1H, H-1), 7.18-7.20 (m, 2H, H-3), 5.49 (tq, *J* = 8.8 Hz, 1.4 Hz, 1H, H-5), 3.62 (q, *J* = 5.7 Hz, 2H, H-7), 2.24-2.29 (m, 2H, H-6), 2.06 (q, *J* = 1.3 Hz, 3H, H-4), 1.28 (br s, 1H, H-8) ppm;

^{13}C NMR (126 MHz, $CDCl_3$) *δ* 141.9, 139.7, 128.3, 128.0, 126.8, 123.2, 62.9, 32.7, 25.9 ppm;

The spectroscopic data is in agreement with that reported in the literature.[61]

1-Cyclopropyl-1-phenylethan-1-ol

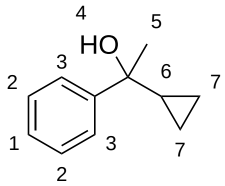

Prepared according to the protocol described by Sigman, Toste and co-workers.[62] To a solution of PhMgCl at 0 °C (6.0 mL of a 2.0 M solution in THF, 12.0 mmol) in THF (20 mL) was added cyclopropyl methyl ketone (0.94 mL, 10 mmol) dropwise. The reaction mixture was stirred for a further 2 hours at the same temperature following which it was quenched with a saturated aqueous solution of NH_4Cl (10 mL) and the THF removed under reduced pressure. The mixture was extracted with EtOAc (3 × 20 mL) and the combined organic extracts were dried ($MgSO_4$) and the solvent removed under reduced pressure. Purification

by flash column chromatography (SiO_2, 0-15% v/v EtOAc in Petrol) afforded the title compound as a colourless oil (1.27 g, 7.8 mmol, 78%).

***R_f* value** = 0.40 (10% v/v EtOAc in Petrol);

^{1}H NMR (400 MHz, $CDCl_3$) *δ* 7.53-7.56 (m, 2H, H-3), 7.33-7.37 (m, 2H, H-2), 7.24-7.28 (m, 1H, H-1), 1.61 (s, 1H, H-4), 1.50 (s, 3H, H-5), 1.22-1.30 (m, 1H, H-6), 0.38-0.57 (m, 4H, H-7) ppm;

^{13}C NMR (101 MHz, $CDCl_3$) *δ* 148.2, 128.2, 126.9, 125.3, 73.3, 28.7, 22.9, 2.1, 1.2 ppm;

The spectroscopic data is in agreement with that reported in the literature. [63]

(E)-4-phenylpent-3-en-1-ol (**31h**)

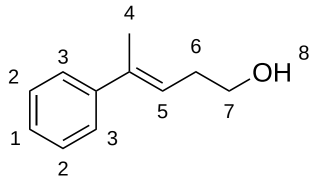

Prepared according to the protocol described by Pfaltz and co-workers.[64] To an ice cooled slurry of 1-cyclopropyl-1-phenylethan-1-ol (1.26 g, 7.8 mmol) in 1,4 dioxane (15 mL) was added 60% aqueous $HClO_4$ (2.11 mL, 19.5 mmol) dropwise over 15 minutes The reaction mixture was then allowed to stir at room temperature overnight upon which the slurry cleared to colourless solution. The reaction mixture was then diluted with water (30 mL) and extracted with EtOAc (4 × 30 mL). The combined organic extracts were washed with saturated aqueous $NaHCO_3$ (3 × 15 mL), brine (15 mL), dried ($MgSO_4$) and the volatiles removed under reduced pressure. Purification by flash column chromatography (SiO_2, 0-20% v/v Acetone in Petrol) afforded the title compound as a colourless oil (208 mg, 1.3 mmol, 16%).

***R_f* value** = 0.47 (20% v/v Acetone in Petrol);

^{1}H NMR (400 MHz, $CDCl_3$) *δ* 7.40-7.43 (m, 2H, H-3), 7.32-7.36 (m, 2H, H-2), 7.24-7.28 (m, 1H, H-1), 5.81 (tq, *J* = 7.3 Hz, 1.2 Hz, 1H, H-5), 3.78 (t, *J* = 6.6 Hz, 2H, H-7), 2.53 (q, *J* = 6.8 Hz, 2H, H-6), 2.11 (s, 3H, H-4), 1.53 (s, 1H, H-8) ppm;

^{13}C NMR (101 MHz, $CDCl_3$) *δ* 143.6, 137.9, 128.3, 127.0, 125.8, 123.8, 62.5, 32.5, 16.2 ppm;

The spectroscopic data is in agreement with that reported in the literature.[64]

(Z)-3,7-Dimethylocta-2,6-dienal (**Neral**)

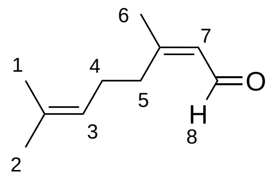

To a solution of nerol (2.6 mL, 15.0 mmol) in CH_2Cl_2 (100 mL) under an atmosphere of air was added activated MnO_2 (13.0 g, 150 mmol) portionwise and the reaction mixture stirred overnight. The solid residue was then removed by filtration through a pad of Celite®, eluting with plenty of CH_2Cl_2 and the filtrate evaporated under reduced pressure to afford the crude product. Purification by flash column chromatography (SiO_2, 0-7% v/v Acetone in Petrol) afforded the title compound as a colourless oil (1.2 g, 7.6 mmol, 51%).

***R_f* value** = 0.49 (5% v/v Acetone in Petrol);

^{1}H NMR (400 MHz, $CDCl_3$) *δ* 9.89 (d, *J* = 8.2 Hz, 1H, H-8), 5.87 (dt, *J* = 8.2 Hz, 0.5 Hz, 1H, H-7), 5.09 (tp, *J* = 8.7 Hz, 1.3 Hz, 1H, H-3), 2.58 (t, *J* = 7.5 Hz, 2H, H-5), 2.23 (q, *J* = 7.4 Hz, 2H, H-4), 1.98 (d, *J* = 1.3 Hz, 3H, H-6), 1.68 (br s, 3H, H-2), 1.59 (br s, 3H, H-1) ppm;

^{13}C NMR (126 MHz, $CDCl_3$) δ 191.0, 164.0, 133.8, 128.8, 122.4, 32.7, 27.2, 25.8, 25.2, 17.9 ppm;

The spectroscopic data is in agreement with that reported in the literature.[65]

(Z)-4,8-dimethylnona-1,3,7-triene

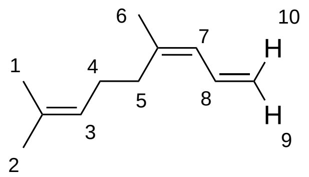

To a solution of methyltriphenylphosphonium bromide (4.04 g, 11.3 mmol) in THF (60 mL) at 0 °C was added *n*-BuLi (7.1 mL of a 1.6 M solution in hexane, 11.3 mmol) dropwise *via* syringe pump. The resulting yellow solution was allowed to warm to room temperature over 1 hour following which it was cooled to – 78 °C. A solution of neral (1.14 g, 7.5 mmol) in THF (15 mL) was added dropwise and addition of the aldehyde was made quantitative with THF (5 mL). The reaction mixture was then allowed to warm to room temperature slowly overnight following which it was quenched with saturated aqueous NH_4Cl. The aqueous layer was extracted with Et_2O, dried ($MgSO_4$) and the solvent removed under reduced pressure. Purification by flash column chromatography (SiO_2, 100% Petrol) afforded the title compound as a colourless oil (508 mg, 3.4 mmol, 45%). *Caution: The product is volatile and unstable and must be used immediately.*

***R_f* value** = 0.80 (100% Petrol);

^{1}H NMR (400 MHz, $CDCl_3$) δ 6.57 (dt, *J* = 16.7 Hz, 10.6 Hz, 1H, H-8), 5.85 (d, *J* = 11.0 Hz, 1H, H-7), 5.09-5.14 (m, 1H, H-3), 5.07 (d, *J* = 16.7 Hz, 1H, H-10), 4.95 (dd, *J* = 10.2 Hz, 1.8 Hz, 1H, H-9), 2.17-2.21 (m, 2H, H-5), 2.06-2.12 (m, 2H, H-4), 1.78 (br s, 3H, H-6), 1.68 (br s, 3H, H-2), 1.61 (br s, 3H, H-1) ppm;

^{13}C NMR (101 MHz, $CDCl_3$) δ 139.9, 133.3, 132.1, 126.5, 124.1, 114.5, 32.6, 27.0, 25.8, 23.9, 17.8 ppm;

The spectroscopic data is in agreement with that reported in the literature.[66]

(Z)-4,8-dimethylnona-3,7-dien-1-ol (**Homonerol**, **34**)

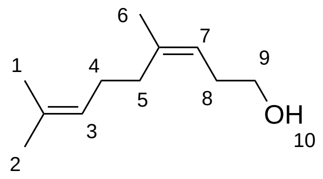

Prepared according to the protocol described by Wiemer and co-workers.[67] To a solution of (*Z*)-4,8-dimethylnona-1,3,7-triene (492 mg, 3.3 mmol) in THF (20 mL) at 0 °C was added dropwise 9-BBN (6.6 mL of a 0.5 M solution in THF, 3.3 mmol). The reaction mixture was allowed to warm to room temperature overnight following which it was cooled to – 10 °C. NaOH (2.4 mL of a 2.5 M solution in water, 6.0 mmol) and H_2O_2 (2.0 mL of a 30% w/v solution in water, 19.6 mmol) were then added dropwise and the reaction mixture was slowly allowed to warm to room temperature and then stirred at 55 °C for 1 hour. The reaction was then quenched with a saturated aqueous solution of $Na_2S_2O_3$ and the THF was removed under reduced pressure. The aqueous layer was extracted with Et_2O (2 x 50 mL), the combined organic extracts were dried ($MgSO_4$) and the solvent removed under reduced pressure. Purification by flash column chromatography (SiO_2, 0-20% v/v Acetone in Petrol) afforded the title compound as a yellow oil (236 mg, 1.4 mmol, 42%).

***R$_f$* value** = 0.55 (20% v/v Acetone in Petrol);

^{1}**H NMR** (400 MHz, $CDCl_3$) δ 5.08-5.15 (m, 2H, H-3, H-7), 3.61 (t, J = 6.5 Hz, 2H, H-9), 2.25-2.30 (m, 2H, H-8), 2.07-2.08 (m, 4H, H-4, H-5), 1.73 (q, J = 1.2 Hz, 3H, H-6), 1.68 (s, 3H, H-2), 1.61 (s, 3H, H-1) ppm;

^{13}**C NMR** (101 MHz, $CDCl_3$) δ 139.1, 132.1, 124.2, 120.9, 62.8, 32.1, 31.5, 26.7, 25.8, 23.7, 17.8 ppm;

The spectroscopic data is in agreement with that reported in the literature.[68]

(E)-3,7-dimethylocta-2,6-dienal (**Geranial**)

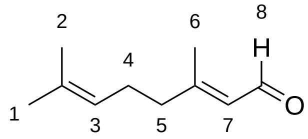

To a solution of geraniol (3.4 mL, 25.0 mmol) in CH_2Cl_2 (100 mL) under an atmosphere of air was added activated MnO_2 (17.4 g, 200 mmol) portionwise and the reaction mixture stirred overnight. The solid residue was then removed by filtration through a pad of Celite®, eluting with plenty of CH_2Cl_2 and the filtrate evaporated under reduced pressure to afford the crude product. Purification by flash column chromatography (SiO_2, 0-7% v/v Acetone in Petrol) afforded the title compound as a colourless oil (2.6 g, 16.8 mmol, 67%).

***R_f* value** = 0.49 (5% v/v Acetone in Petrol);

^{1}**H NMR** (400 MHz, $CDCl_3$) δ 9.99 (d, J = 8.1 Hz, 1H, H-8), 5.86-5.89 (m, 1H, H-7), 5.04-5.08 (m, 1H, H-3), 2.18-2.25 (m, 4H, H-4, H-5), 2.16 (d, J = 1.2 Hz, 3H, H-6), 1.68 (br s, 3H, H-1), 1.60 (br s, 3H, H-2) ppm;

^{13}**C NMR** (101 MHz, $CDCl_3$) δ 191.5, 164.0, 133.1, 127.6, 122.7, 40.7, 25.9, 25.8, 17.9, 17.7 ppm;

The spectroscopic data is in agreement with that reported in the literature.[69]

(E)-4,8-dimethylnona-1,3,7-triene

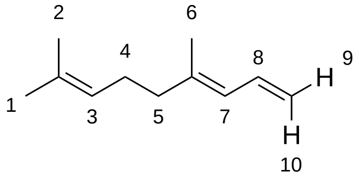

To a solution of methyltriphenylphosphonium bromide (4.54 g, 12.7 mmol) in THF (60 mL) at 0 °C was added *n*-BuLi (8.0 mL of a 1.6 M solution in hexane, 12.7 mmol) dropwise *via* syringe pump. The resulting yellow solution was allowed to warm to room temperature over 1 hour following which it was cooled to – 78 °C. A solution of geranial (1.28 g, 8.4 mmol) in THF (15 mL) was added dropwise and addition of the aldehyde was made quantitative with THF (5 mL). The reaction mixture was then allowed to warm to room temperature slowly overnight following which it was quenched with saturated aqueous NH_4Cl. The aqueous layer was extracted with Et_2O, dried ($MgSO_4$) and the solvent removed under reduced pressure. Purification by flash column chromatography (SiO_2, 100% Petrol) afforded the title compound as a colourless oil (446 mg, 3.0 mmol, 35%). *Caution: The product is volatile and unstable and must be used immediately.*

***R_f* value** = 0.80 (100% Petrol);

^{1}H NMR (400 MHz, $CDCl_3$) δ 6.58 (dt, *J* = 16.8 Hz, 10.6 Hz, 1H, H-8), 5.86 (d, *J* = 10.9 Hz, 1H, H-7), 5.07-5.12 (m, 1H, H-3), 5.10 (dd, *J* = 16.7 Hz, 2.1 Hz, 1H, H-10) 4.98 (dd, *J* = 10.2 Hz, 1.9 Hz, 1H, H-9), 2.04-2.21 (m, 4H, H-4, H-5), 1.77 (d, *J* = 0.9 Hz, 3H, H-6), 1.69 (br s, 3H, H-1), 1.61 (br s, 3H, H-2) ppm;

^{13}**C NMR** (101 MHz, $CDCl_3$) δ 139.7, 133.6, 131.9, 125.6, 124.1, 114.7, 40.0, 26.7, 25.8, 17.8, 16.8 ppm;

The spectroscopic data is in agreement with that reported in the literature.[66]

(E)-4,8-dimethylnona-3,7-dien-1-ol (**Homogeraniol**, **37**)

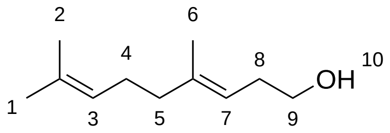

Prepared according to the protocol described by Wiemer and co-workers.[67] To a solution of (*E*)-4,8-dimethylnona-1,3,7-triene (430 mg, 2.9 mmol) in THF (20 mL) at 0 °C was added dropwise 9-BBN (5.8 mL of a 0.5 M solution in THF, 2.9 mmol). The reaction mixture was allowed to warm to room temperature overnight following which it was cooled to – 10 °C. NaOH (2.4 mL of a 2.5 M solution in water, 6.0 mmol) and H_2O_2 (2.0 mL of a 30% w/v solution in water, 19.6 mmol) were then added dropwise and the reaction mixture was slowly allowed to warm to room temperature and then stirred at 55 °C for 1 hour. The reaction was then quenched with a saturated aqueous solution of $Na_2S_2O_3$ and the THF was removed under reduced pressure. The aqueous layer was extracted with Et_2O (2 x 50 mL), the combined organic extracts were dried ($MgSO_4$) and the solvent removed under reduced pressure. Purification by two consecutive flash column chromatography procedures (SiO_2, 0-20% v/v Acetone in Petrol followed by 0-5% v/v Et_2O in CH_2Cl_2) afforded the title compound as a yellow oil (123 mg, 0.73 mmol, 25%).

***R$_f$* value** = 0.32 (10% v/v Acetone in Petrol);

^{1}**H NMR** (500 MHz, $CDCl_3$) δ 5.12 (tq, *J* = 7.3 Hz, 1.2 Hz, 1H, H-7), 5.07 (tp, *J* = 6.7 Hz, 1.3 Hz, 1H, H-3), 3.61 (t, *J* = 6.4 Hz, 2H, H-9), 2.27-2.31 (m, 2H, H-8), 2.07-2.12 (m, 2H, H-4), 2.02-2.05 (m, 2H, H-5), 1.68 (br s, 3H, H-1), 1.64 (br s, 3H, H-6), 1.60 (br s, 3H, H-2) ppm;

^{13}C NMR (101 MHz, $CDCl_3$) δ 139.1, 131.8, 124.3, 120.0, 62.5, 39.9, 31.6, 26.7, 25.8, 17.8, 16.3 ppm;

The spectroscopic data is in agreement with that reported in the literature.[70]

Synthesis of a Further Mnemonic Test Substrate

Tert-butyldimethyl((3-methylbut-3-en-1-yl)oxy)silane

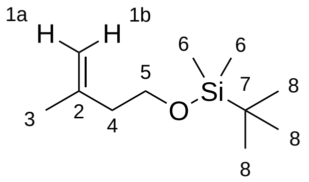

To a suspension of imidazole (6.95 g, 102 mmol, 2.1 equiv.) and TBSCl (9.14 g, 60.6 mmol, 1.2 equiv.) in CH_2Cl_2 (160 mL) was added 3-methyl-3-buten-1-ol (5.8 mL, 49.4 mmol, 1.0 equiv.) at room temperature. The reaction mixture was stirred at room temperature for 6 hours and the resultant solids were filtered off. Saturated NH_4Cl solution was added to the filtrate and the phases separated. The aqueous layer was extracted with CH_2Cl_2. The combined organic layers were dried over $MgSO_4$ and concentrated *in vacuo*. Purification with a silica plug (SiO_2, 0-5% v/v EtOAc in Petrol) afforded the title compound as a colourless oil (6.48 g, 30.2 mmol, 61%).

***Rf* value** = 0.30 (Petrol);

^{1}H NMR (400 MHz, $CDCl_3$) δ 4.75 (s, 1H, H-1a), 4.70 (s, 1H, H-1b), 3.71 (t, *J* = 7.1 Hz, 2H, H-5), 2.24 (t, *J* = 7.1 Hz, 2H, H-4), 1.74 (s, 3H, H-3), 0.89 (s, 9H, H-8), 0.05 (s, 6H, H-6) ppm;

^{13}C NMR (101 MHz, $CDCl_3$) δ 143.3 (C-2), 111.6 (C-1), 62.3 (C-5), 41.3 (C-4), 26.1 (C-8), 23.0 (C-3), 18.5 (C-7), – 5.1 (C-6) ppm;

The spectroscopic data is in agreement with that reported in the literature.[71]

(Z)-tert-butyldimethyl((3-methyl-4-phenylbut-3-en-1-yl)oxy)silane

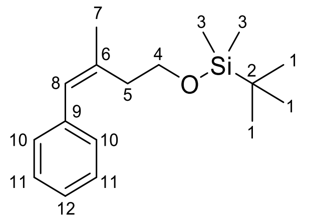

Four heatgun-dried vials were each charged with *tert*-butyldimethyl((3-methylbut-3-en-1-yl)oxy)silane (2.32 g, 10.8 mmol, 1.5 equiv.), iodobenzene (0.8 mL, 7.20 mmol, 1.0 equiv.), $Pd(OAc)_2$ (80.9 mg, 0.36 mmol, 5 mol%), tetrabutylammonium chloride (2.0 g, 7.20 mmol, 1.0 equiv.) and Na_2CO_3 (1.91 g, 18.0 mmol, 2.5 equiv.). The vials were evacuated and backfilled with nitrogen thrice before the addition of N_2-sparged DMF (10 mL). The vials were heated at 130 ˚C overnight and allowed to cool to room temperature. The reaction mixtures were combined, filtered over Celite and diluted with EtOAc. The organic layer was washed with water, 10% aqueous LiCl, brine, dried over $MgSO_4$ and concentrated *in vacuo*. Purification by flash column chromatography (SiO_2, 0-14% v/v CH_2Cl_2 in Petrol) afforded the title compound as a yellow oil (168.8 mg, 0.61 mmol, 2%).

***R_f* value** = 0.63 (25% v/v CH_2Cl_2 in Petrol);

^{1}H NMR (400 MHz, $CDCl_3$) δ 7.32-7.27 (m, 4H, H-10, H-11), 7.21-7.17 (m, 1H, H-12), 6.38 (s, 1H, H-8), 3.79 (t, *J* = 7.0 Hz, 2H, H-4), 2.48 (t, *J* = 7.1 Hz, 2H, H-5), 1.91 (d, *J* = 1.4 Hz, 3H, H-7), 0.89 (s, 9H, H-1), 0.04 (s, 6H, H-3) ppm;

^{13}C NMR (101 MHz, $CDCl_3$) δ 138.4 (C-9), 136.3 (C-6), 128.8 (C-11), 128.2 (C-10), 127.5 (C-8), 126.2 (C-12), 61.8 (C-4), 36.1 (C-5), 26.1 (C-1), 24.6 (C-7), 18.5 (C-2), – 5.2 (C-3) ppm;

HRMS (+ESI) *m/z* found $[M–C_6H_{15}OSi]^+$ 145.1008, $[C_{11}H_{13}]^+$ requires 145.1012, (δ = – 2.8 ppm).

(Z)-3-methyl-4-phenylbut-3-en-1-ol (**40**)

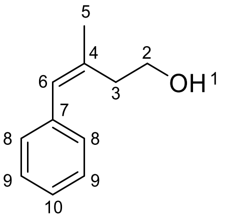

To a solution of (*Z*)-*tert*-butyldimethyl((3-methyl-4-phenylbut-3-en-1-yl)oxy)silane (134.2 mg, 0.49 mmol, 1.0 equiv.) in THF (6 mL) was added TBAF (0.55 mL, 1.0 M in THF, 0.55 mmol, 1.1 equiv.) at room temperature. The reaction was stirred at room temperature for 2 hours and concentrated under a gentle stream of nitrogen. Purification by flash column chromatography (alumina, 0-20% v/v Acetone in Petrol) afforded the title compound as a colourless oil (77.4 mg, 0.48 mmol, 97%).

***R_f* value** = 0.22 (Alumina, 20% v/v Acetone in Petrol);

^{1}H NMR (400 MHz, $CDCl_3$) *δ* 7.34-7.30 (m, 2H, H-9), 7.27-7.25 (m, 2H, H-8), 7.22-7.19 (m, 1H, H-10), 6.47 (s, 1H, H-6), 3.80 (t, *J* = 7.0 Hz, 2H, H-2), 2.55 (t, *J* = 6.8 Hz, 2H, H-3), 1.93 (d, *J* = 1.4 Hz, 3H, H-5) ppm;

^{13}C NMR (101 MHz, $CDCl_3$) *δ* 138.1 (C-7), 135.2 (C-4), 128.8 (C-9), 128.6 (C-6), 128.3 (C-8), 126.4 (C-10), 61.0 (C-2), 35.7 (C-3), 24.1 (C-5) ppm;

The spectroscopic data is in agreement with that reported in the literature.[72]

Characterisation of Bishomoallylic Styrenyl Aziridines

2,2,3,3,3-pentafluoropropyl (2R,3S)-2-(3-hydroxypropyl)-3-phenylaziridine-1-sulfonate (**3a**)

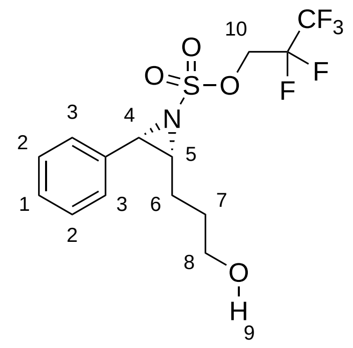

Prepared according to **GP9** using (*Z*)-5-phenylpent-4-en-1-ol (**1a**) as the starting material. Purification by flash column chromatography (SiO_2, 0-10% v/v Acetone in $CHCl_3$) afforded the title compound as a colourless oil (21 mg, 0.053 mmol, 53%).

***R_f* value** = 0.37 (10% v/v Acetone in $CHCl_3$);

^{1}H NMR (500 MHz, $CDCl_3$) δ 7.40-7.33 (m, 3H, H-1, H-2), 7.31 (m, 2H, H-3), 4.74 (m, 2H, H-10a, H-10b), 4.07 (d, J = 7.3 Hz, 1H, H-4), 3.59 (m, 2H, H-8a, H-8b), 3.21 (m, 1H, H-5), 1.65 (m, 2H, H-7a, H-7b), 1.53-1.40 (m, 3H, H-6a, H-6b, H-9) ppm;

^{13}C NMR (126 MHz, $CDCl_3$) δ 131.6, 128.8, 128.7, 127.3, 118.2 (qt, $J_{C\text{-}F}$ = 285, 34.3 Hz), 111.3 (tq, $J_{C\text{-}F}$ = 258, 38.4 Hz), 65.8 (t, $J_{C\text{-}F}$ = 28.5 Hz), 61.9, 48.7, 48.1, 29.6, 22.8 ppm;

^{19}F NMR (376 MHz, $CDCl_3$) δ − 83.5 (s), − 123.5 (s) ppm;

IR (film, cm^{-1}): ν_{max} 2961 (w), 1454 (w), 1376 (s), 1205 (s), 1181 (s), 1109 (m), 1042 (m), 1010 (m), 904 (m), 845 (m), 700 (m), 592 (m);

$[\alpha]_D^{25.0}$ = + 69.5 (c. 0.55, $CHCl_3$);

HRMS (−ESI) *m/z* found $[M+HCO_2]^-$ 434.0706, $[C_{15}H_{17}F_5NO_6S]^-$ requires = 434.0702, (*δ* = + 0.9 ppm).

Note: during the preparation of this aziridine for NMR analysis, small amounts of the cyclised product **4a** *begin to form and were observed in the NMR spectra. A clean* ^1H *spectrum can be obtained within 30 minutes of NMR sample preparation.*

2,2,3,3,3-pentafluoropropyl ((S)-phenyl((S)-tetrahydrofuran-2-yl)methyl)sulfamate (**4a**)

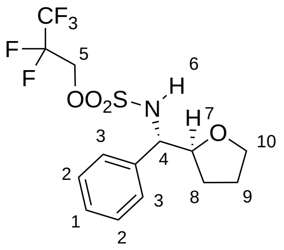

Prepared according to **GP9** using (*Z*)-5-phenylpent-4-en-1-ol (**1a**) as the starting material. In this case the following procedural modification was used: The loading of (perfluorophenyl)-λ^3-iodanediyl bis(2,2,2-trifluoroacetate) was reduced from 10 mol % to 2 mol %. Following the cyclisation protocol (2 hours), purification by flash column chromatography (SiO_2, 0-1% v/v Et_2O in CH_2Cl_2) afforded the title compound as a white amorphous solid (22.4 mg, 0.058 mmol, 58%, 92% *ee*).

***R$_f$* value** = 0.34 (20% v/v EtOAc in Hexane);

^{1}H NMR (500 MHz, $CDCl_3$) *δ* 7.31-7.38 (m, 5H, H-1, H-2, H-3), 5.68 (d, *J* = 4.9 Hz, 1H, H-6), 4.31 (t, *J* = 5.7 Hz, 1H, H-4), 4.19 (q, *J* = 12.3 Hz, 1H, H-5a), 4.03-4.13 (m, 2H, H-5b, H-7), 3.96 (q, *J* =

7.2 Hz, 1H, H-10a), 3.83 (q, J = 7.2 Hz, 1H, H-10b), 1.83-1.99 (m, 3H, H-8a, H-9a, H-9b), 1.70-1.77 (m 1H, H-8b) ppm;

^{13}C NMR (126 MHz, $CDCl_3$) δ 138.3, 129.0, 128.7, 127.6, 118.2 (qt, J_{C-F} = 286.2 Hz, 34.4 Hz), 111.3 (tq, J_{C-F} = 256.7 Hz, 38.4 Hz), 81.2, 69.0, 63.9 (t, J_{C-F} = 28.1 Hz), 62.4, 29.0, 25.7 ppm;

^{19}F NMR (376 MHz, $CDCl_3$) δ – 83.8 (s), – 123.7 (s) ppm;

IR (film, cm^{-1}): ν_{max} 3305 (w), 2960 (w), 2882 (w), 1457 (w), 1372 (m), 1257 (w), 1203 (s), 1181 (s), 1108 (m), 1048 (m), 1011 (m), 961 (w), 929 (w), 840 (w), 811 (w), 775 (w), 702 (m), 624 (w), 571 (m), 518 (w);

$[\alpha]_D^{25.0}$ = + 38.1 (c. 1.4, $CHCl_3$);

HRMS (−ESI) *m/z* Found $[M-H]^-$ 388.0646, $[C_{14}H_{15}F_5NO_4S]^-$ requires 388.0647, (δ = – 0.3 ppm);

Chiral SFC Analysis CHIRALPAK IH (CO_2:MeOH, 98:2, 2.5 mL min^{-1}, 40 °C) t_R = 4.5 (minor), 6.2 (major) minutes.

2,2,3,3,3-pentafluoropropyl ((S)-(2-fluorophenyl)((S)-tetrahydrofuran-2-yl)methyl)sulfamate (**4b**)

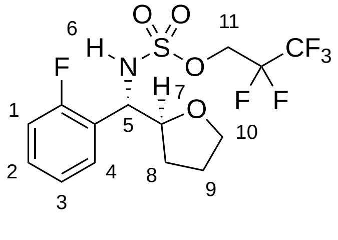

Prepared according to **GP9** using (Z)-5-(2-fluorophenyl)pent-4-en-1-ol (**1b**) as the starting material. Following the cyclisation protocol (2 hours), purification by flash column

chromatography (SiO_2, 0-2% v/v Et_2O in CH_2Cl_2) afforded the title compound as an off-white solid (31 mg, 0.076 mmol, 76%, 94% *ee*).

***R_f* value** = 0.31 (100% CH_2Cl_2);

^{1}H NMR (400 MHz, $CDCl_3$) δ 7.38 (t, *J* = 7.5 Hz, 1H, H-4), 7.31 (q, *J* = 6.7 Hz, 1H, H-2), 7.16 (t, *J* = 7.4 Hz, 1H, H-3), 7.07 (t, *J* = 9.5 Hz, 1H, H-1), 5.74 (br s, 1H, H-6), 4.65 (d, *J* = 6.2 Hz, 1H, H-5), 4.33 (m, 2H, H-11_A, H-11_B), 4.11 (q, *J* = 6.4 Hz, 1H, H-7), 3.96 (q, *J* = 7.1 Hz, 1H, H-10_A), 3.83 (q, *J* = 6.9 Hz, 1H, H-10_B), 2.04-1.84 (m, 3H, H-8_A, H-9_A, H-9_B), 1.74 (m, 1H, H-8_B) ppm;

^{13}C NMR (101 MHz, $CDCl_3$) δ 160.3 (d, J_{C-F} = 247 Hz), 130.2 (d, J_{C-F} = 8.6 Hz), 129.2 (d, J_{C-F} = 3.9 Hz), 125.4 (d, J_{C-F} = 12.8 Hz), 124.7 (d, J_{C-F} = 3.6 Hz), 118.2 (qt, J_{C-F} = 286, 34.3 Hz), 116.0 (d, J_{C-F} = 21.8 Hz), 111.3 (tq, J_{C-F} = 256, 38.5 Hz), 80.4, 68.9, 64.1 (t, J_{C-F} = 28.2 Hz), 56.7, 28.9, 25.7 ppm;

^{19}F NMR (376 MHz, $CDCl_3$) δ − 84.7, − 119.3, − 124.7 ppm;

IR (film, cm^{-1}): ν_{max} 3302 (w), 2964 (w), 1493 (m), 1457 (m), 1375 (m), 1202 (s), 1182 (s), 1108 (m), 1049 (m), 1011 (m), 759 (s);

$[\alpha]_D^{25.0}$= + 40.8 (c. 0.99, $CHCl_3$);

HRMS (−ESI) *m/z* found [M−H]$^-$ 406.0554, $[C_{14}H_{14}F_6NO_4S]^-$ requires 406.0553, (δ = + 0.2 ppm);

Chiral SFC Analysis: CHIRALPAK IH (CO_2:MeOH, 98:2, 2.5 mL min^{-1}, 40 °C) t_R = 4.4 (minor), 6.1 (major) minutes.

2,2,3,3,3-pentafluoropropyl ((S)-((S)-tetrahydrofuran-2-yl)(o-tolyl)methyl)sulfamate (**4c**)

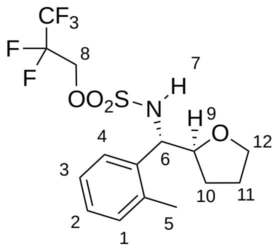

Prepared according to **GP9** using (*Z*)-5-(*o*-tolyl)pent-4-en-1-ol (**1c**) as the starting material. Following the cyclisation protocol (2 hours), purification by flash column chromatography (SiO_2, 0-2.5% v/v Et_2O in CH_2Cl_2) afforded the title compound as a colourless oil (20.8 mg, 0.052 mmol, 52%, 97% *ee*).

***R_f* value** = 0.54 (5% v/v Acetone in $CHCl_3$);

^{1}H NMR (400 MHz, $CDCl_3$) δ 7.34 (dd, *J* = 6.7 Hz, 2.1 Hz, 1H, H-4), 7.16-7.25 (m, 3H, H-1, H-2, H-3), 5.65 (d, *J* = 5.0 Hz, 1H, H-7), 4.67 (t, *J* = 6.1 Hz, 5.8 Hz, 1H, H-6), 4.15 (q, *J* = 12.2 Hz, 1H, H-8a), 3.96-4.06 (m, 3H, H-8b, H-9, H-12a), 3.80-3.86 (m, 1H, H-12b), 2.41 (s, 3H, H-5), 1.84-2.04 (m, 3H, H-10a, H-11a, H-11b), 1.65-1.73 (m, 1H, H-10b) ppm;

^{13}C NMR (126 MHz, $CDCl_3$) δ 136.7, 135.8, 131.0, 128.3, 127.0, 126.6, 118.2 (qt, $J_{C\text{-}F}$ = 285.6 Hz, 34.6 Hz), 111.3 (tq, $J_{C\text{-}F}$ = 254.4 Hz, 37.3 Hz), 81.2, 68.9, 63.9 (t, $J_{C\text{-}F}$ = 28.1 Hz), 57.6, 28.8, 25.7, 19.6 ppm;

^{19}F NMR (376 MHz, $CDCl_3$) δ – 83.8 (s), – 123.8 (s) ppm;

IR (film, cm^{-1}): ν_{max} 3303 (br, w), 2964 (w), 1463 (w), 1372 (m), 1205 (s), 1182 (s), 1109 (w), 1051 (m), 1012 (m), 963 (w), 811 (w), 761 (w), 729 (w), 624 (w), 577 (w), 518 (w);

$[\alpha]_D^{25.0}$ = + 31.4 (c. 1.0, $CHCl_3$);

HRMS (–ESI) *m/z* Found $[M-H]^-$ 402.0799, $[C_{15}H_{17}F_5NO_4S]^-$ requires 402.0804, (δ = – 1.2 ppm);

Chiral SFC Analysis CHIRALPAK IH (CO_2:MeOH, 98:2, 1.25 mL min^{-1}, 40 °C) t_R = 12.0 (minor), 14.2 (major) minutes.

2,2,3,3,3-pentafluoropropyl ((S)-(2-methoxyphenyl)((S)-tetrahydrofuran-2-yl)methyl)sulfamate (**4d**)

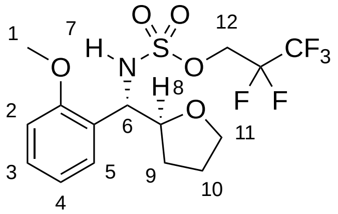

Prepared according to **GP9** using (*Z*)-5-(2-methoxyphenyl)pent-4-en-1-ol (**1d**) as the starting material. Following the cyclisation protocol (2 hours) purification by flash column chromatography (SiO_2, 0-5% v/v Acetone in $CHCl_3$) afforded the title compound as a colourless oil (25 mg, 0.060 mmol, 60%, 95% *ee*).

***R_f* value** = 0.43 (10% v/v Acetone in $CHCl_3$);

^{1}H NMR (500 MHz, $CDCl_3$) δ 7.29 (m, 1H, H-3), 7.25 (m, 1H, H-5), 6.96 (td, *J* = 7.5, 1.0 Hz, 1H, H-4), 6.90 (dd, *J* = 8.2, 0.7 Hz, 1H, H-2), 5.86 (br s, 1H, H-7), 4.57 (d, *J* = 7.1 Hz, 1H, H-6), 4.42 (q, *J* = 12.5 Hz, 1H, H-12_A), 4.26 (q, *J* = 12.5 Hz, 1H, H-12_B), 4.18 (q, *J* = 7.0 Hz, 1H, H-8), 3.92 (m, 1H, H-11_A), 3.85 (s, 3H, H-1), 3.81 (m, 1H, H-11_B), 1.95 (m, 1H, H-10_A), 1.87(m, 1H, H-10_B), 1.77 (m, 1H, H-9_A), 1.65 (m, 1H, H-9_B) ppm;

^{13}C NMR (126 MHz, $CDCl_3$) δ 156.7, 129.7, 129.1, 126.3, 121.2, 118.4 (qt, J_{C-F} = 286, 34.3 Hz), 111.4 (tq, J_{C-F} = 257, 38.5 Hz), 111.2, 80.1, 68.5, 64.1 (t, J_{C-F} = 27.8 Hz), 59.2, 55.7, 29.2, 25.8 ppm;

^{19}F NMR (471 MHz, $CDCl_3$) δ − 84.8, − 124.7 ppm;

IR (film, cm^{-1}): ν_{max} 3316 (w), 2961 (w), 1494 (m), 1463 (m), 1373 (m), 1247 (m), 1202 (s), 1182 (s), 1108 (m), 1051 (m), 1011 (m), 756 (m);

$[\alpha]_D^{25.0}$= + 39.7 (c. 1.0, $CHCl_3$);

HRMS (−ESI) *m/z* found [M−H]$^-$ 418.0749, $[C_{15}H_{18}F_5NO_5S]^-$ requires 418.0753, (δ = – 0.9 ppm);

Chiral SFC Analysis: CHIRALPAK IH (CO_2:MeOH, 98:2, 1.25 mL min^{-1}, 40 °C) t_R = 11.4 (minor), 12.8 (major) minutes.

2,2,3,3,3-pentafluoropropyl ((S)-(3-fluoro-2-methylphenyl)((S)-tetrahydrofuran-2-yl)methyl)sulfamate (**4e**)

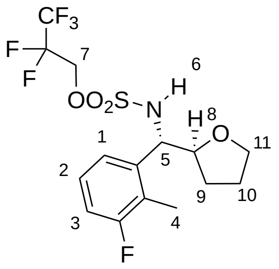

Prepared according to **GP9** using (*Z*)-5-(3-fluoro-2-methylphenyl)pent-4-en-1-ol (**1e**) as the starting material. Following the cyclisation protocol (2 hours), purification by two consecutive flash column chromatography procedures (SiO_2, 0-1% v/v Et_2O in CH_2Cl_2 followed by 0-30% v/v Acetone in Petrol) afforded the title compound as a yellow oil (19.4 mg, 0.046 mmol, 46%, 94% *ee*).

***R_f* value** = 0.65 (5% v/v Et_2O in CH_2Cl_2);

^{1}H NMR (500 MHz, $CDCl_3$) δ 7.17-7.22 (m, 1H, H-2), 7.15 (d, *J* = 7.1 Hz, 1H, H-1), 6.99 (t, *J* = 8.7 Hz, 1H, H-3), 5.67 (d, *J* = 4.6 Hz, 1H, H-6), 4.66 (t, *J* = 5.6 Hz, 1H, H-5), 4.26 (q, *J* = 12.2 Hz, 1H,

H-7a), 4.12 (q, J = 12.2 Hz, 1H, H-7b), 3.95-4.03 (m, 2H, H-8, H-11a), 3.81-3.85 (m, 1H, H-11b), 2.30 (d, J = 1.9 Hz, 3H, H-4), 1.85-2.03 (m, 3H, H-9a, H-10a, H-10b), 1.65-1.72 (m, 1H, H-9b) ppm;

^{13}C NMR (126 MHz, $CDCl_3$) δ 161.2 (d, $J_{C\text{-}F}$ = 244.6 Hz), 138.9 (d, $J_{C\text{-}F}$ = 3.2 Hz), 127.2 (d, $J_{C\text{-}F}$ = 9.0 Hz), 123.3 (d, $J_{C\text{-}F}$ = 16.7 Hz), 122.4 (d, $J_{C\text{-}F}$ = 2.6 Hz), 118.1 (qt, $J_{C\text{-}F}$ = 286.2 Hz, 34.4 Hz), 114.8 (d, $J_{C\text{-}F}$ = 23.6 Hz), 111.1 (tq, $J_{C\text{-}F}$ = 257.0 Hz, 38.4 Hz), 80.9, 68.8, 63.8 (t, $J_{C\text{-}F}$ = 28.2 Hz), 57.2, 28.7, 25.5, 10.3 (d, $J_{C\text{-}F}$ = 6.2 Hz) ppm;

^{19}F NMR (376 MHz, $CDCl_3$) δ – 83.8 (s), – 114.6 (s), – 123.7 (s) ppm;

IR (film, cm^{-1}): ν_{max} 3301 (br, w), 2961 (w), 2922 (w), 2879 (w), 1584 (w), 1468 (m), 1373 (m), 1252 (w), 1206 (s), 1183 (s), 1110 (w), 1051 (m), 1012 (m), 964 (w), 815 (w), 792 (w), 720 (w), 624 (w), 589 (w), 518 (w);

$[\alpha]_D^{25.0}$ = + 20.1 (c. 1.0, $CHCl_3$);

HRMS (−ESI) *m/z* Found $[M-H]^-$ 420.0702, $[C_{15}H_{16}F_6NO_4S]^-$ requires 420.0710, (δ = – 1.9 ppm);

Chiral SFC Analysis CHIRALPAK IH (CO_2:MeOH, 98:2, 2.5 mL min^{-1}, 40 °C) t_R = 4.3 (minor), 5.6 (major) minutes.

2,2,3,3,3-pentafluoropropyl ((S)-(2,5-dimethylphenyl)((S)-tetrahydrofuran-2-yl)methyl)sulfamate (**4f**)

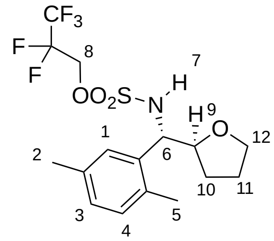

Prepared according to **GP9** using (*Z*)-5-(2,5-dimethylphenyl)pent-4-en-1-ol (**1f**) as the starting material. Following the cyclisation protocol (2 hours), purification by flash column chromatography (SiO_2, 0-1% v/v Et_2O in CH_2Cl_2) afforded the title compound as a colourless oil (17.0 mg, 0.041 mmol, 41%, 97% *ee*).

***R_f* value** = 0.70 (5% v/v Et_2O in CH_2Cl_2);

^{1}H NMR (400 MHz, $CDCl_3$) δ 7.13 (br s, 1H, H-1), 7.06 (d, *J* = 7.8 Hz, 1H, H-4), 7.01 (dd, *J* = 7.8 Hz, 1.2 Hz, 1H, H-3), 5.59 (d, *J* = 4.9 Hz, 1H, H-7), 4.63 (dd, *J* = 6.3 Hz, 5.3 Hz, 1H, H-6), 4.12 (q, *J* = 12.4 Hz, 1H, H-8a), 4.01-4.09 (m, 2H, H-8b, H-9), 3.95-4.00 (m, 1H, H-12a), 3.80-3.86 (m, 1H, H-12b), 2.35 (s, 3H, H-5), 2.30 (s, 3H, H-2), 1.82-2.03 (m, 3H, H-10a, H-11a, H-11b), 1.64-1.73 (m, 1H, H-10b) ppm;

^{13}C NMR (126 MHz, $CDCl_3$) δ 136.3, 136.1, 132.7, 130.9, 129.1, 127.5, 118.2 (qt, $J_{C\text{-}F}$ = 286.0 Hz, 34.3 Hz), 111.3 (tq, $J_{C\text{-}F}$ = 256.9 Hz, 38.5 Hz), 81.3, 68.9, 63.9 (t, $J_{C\text{-}F}$ = 27.9 Hz), 57.6, 28.8, 25.7, 21.1, 19.1 ppm;

^{19}F NMR (376 MHz, $CDCl_3$) δ – 83.8 (s), – 123.9 (s) ppm;

IR (film, cm^{-1}): ν_{max} 3302 (br, w), 2958 (w), 2927 (w), 2876 (w), 1506 (w), 1451 (w), 1372 (m), 1204 (s), 1182 (s), 1108 (m), 1055 (m), 965 (w), 812 (m), 775 (w), 623 (w), 583 (w), 517 (w);

$[\alpha]_D^{25.0}$ = + 42.8 (c. 1.2, $CHCl_3$);

HRMS (−ESI) *m/z* Found $[M-H]^-$ 416.0956, $[C_{16}H_{19}F_5NO_4S]^-$ requires 416.0960, (δ = – 1.0 ppm);

Chiral SFC Analysis CHIRALPAK IH (CO_2:MeOH, 98:2, 2.5 mL min^{-1}, 40 °C) t_R = 4.6 (minor), 5.5 (major) minutes.

2,2,3,3,3-pentafluoropropyl ((S)-(2,3-dimethylphenyl)((S)-tetrahydrofuran-2-yl)methyl)sulfamate (**4g**)

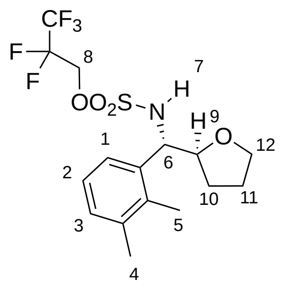

Prepared according to **GP9** using (*Z*)-5-(2,3-dimethylphenyl)pent-4-en-1-ol (**1g**) as the starting material. Following the cyclisation protocol (2 hours), purification by flash column chromatography (SiO_2, 0-1% v/v Et_2O in CH_2Cl_2) afforded the title compound as a colourless oil (15.1 mg, 0.036 mmol, 36%, 96% *ee*).

***R$_f$* value** = 0.36 (100% CH_2Cl_2);

^{1}H NMR (400 MHz, $CDCl_3$) δ 7.18-7.21 (m, 1H, H-1), 7.10-7.14 (m, 2H, H-2, H-3), 5.60 (d, *J* = 5.3 Hz, 1H, H-7), 4.75 (t, *J* = 5.8 Hz, 1H, H-6), 4.17 (q, *J* = 12.1 Hz, 1H, H-8a), 3.95-4.06 (m, 3H, H-8b, H-9, H-12a), 3.80-3.85 (m, 1H, H-12b), 2.30 (s, 3H, H-4), 2.29 (s, 3H, H-5), 1.82-2.03 (m, 3H, H-10a, H-11a, H-11b), 1.64-1.73 (m, 1H, H-10b) ppm;

^{13}C NMR (126 MHz, $CDCl_3$) δ 137.6, 136.6, 134.4, 129.9, 126.0, 124.7, 118.2 (qt, J_{C-F} = 287.7 Hz, 32.7 Hz), 111.3 (tq, J_{C-F} = 258.4 Hz, 36.4 Hz), 81.4, 68.9, 63.9 (t, J_{C-F} = 28.0 Hz), 57.8, 28.9, 25.7, 21.1, 15.0 ppm;

^{19}F NMR (376 MHz, $CDCl_3$) δ – 83.8 (s), – 123.8 (s) ppm;

IR (film, cm^{-1}): ν_{max} 3303 (br, w), 2959 (w), 2922 (w), 2880 (w), 1448 (w), 1371 (m), 1204 (s), 1183 (s), 1109 (w), 1052 (m), 1012 (m), 983 (m), 812 (w), 777 (w), 726 (w), 623 (w), 578 (w), 515 (w);

$[\boldsymbol{\alpha}]_{\mathbf{D}}^{\mathbf{25.0}}$ = + 22.4 (c. 1.0, $CHCl_3$);

HRMS (–ESI) *m/z* Found $[M-H]^-$ 416.0958, $[C_{16}H_{19}F_5NO_4S]^-$ requires 416.0960, (δ = – 0.5 ppm);

Chiral SFC Analysis CHIRALPAK IH (CO_2:MeOH, 98:2, 2.5 mL min^{-1}, 40 °C) t_R = 5.0 (minor), 6.6 (major) minutes.

2,2,3,3,3-pentafluoropropyl ((S)-(3-fluorophenyl)((S)-tetrahydrofuran-2-yl)methyl)sulfamate (**4h**)

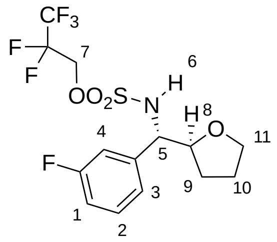

Prepared according to **GP9** using (*Z*)-5-(3-fluorophenyl)pent-4-en-1-ol (**1h**) as the starting material. Following the cyclisation protocol (2 hours), purification by flash column chromatography (SiO_2, 0-5% v/v Et_2O in CH_2Cl_2) afforded the title compound as a thick colourless oil (27.4 mg, 0.067 mmol, 67%, 96% *ee*).

***R_f* value** = 0.61 (5% v/v Et_2O in CH_2Cl_2);

^{1}H NMR (400 MHz, $CDCl_3$) δ 7.31-7.36 (m, 1H, H-2), 7.13 (d, J = 7.7 Hz, 1H, H-3), 7.09 (dt, J = 9.5 Hz, 2.1 Hz, 1H, H-4), 7.03 (tdd, J = 8.5 Hz, 2.5 Hz, 0.7 Hz, 1H, H-1), 5.68 (d, J = 5.3 Hz, 1H, H-6), 4.31-4.34 (m, 1H, H-5), 4.29 (q, J = 12.4 Hz, 1H, H-7a), 4.19 (q, J = 12.5 Hz, 1H, H-7b), 4.02 (q, J = 6.5 Hz, 1H, H-8), 3.93-3.98 (m, 1H, H-11a), 3.80-3.87 (m, 1H, H-11b), 1.86-2.03 (m, 3H, H-9a, H-10a, H-10b), 1.70-1.80 (m, 1H, H-9b) ppm;

^{13}C NMR (101 MHz, $CDCl_3$) δ 163.1 (d, J_{C-F} = 247.6 Hz), 140.9 (d, J_{C-F} = 6.9 Hz), 130.6 (d, J_{C-F} = 8.3 Hz), 123.3 (d, J_{C-F} = 2.9 Hz), 118.2 (qt, J_{C-F} = 286.3 Hz, 34.6 Hz), 115.7 (d, J_{C-F} = 21.1 Hz), 114.5 (d, J_{C-F} = 22.3 Hz), 111.3 (tq, J_{C-F} = 256.9 Hz, 39.0 Hz), 81.0, 69.1, 63.7-64.3 (m), 61.8, 29.0, 25.7 ppm;

^{19}F NMR (376 MHz, $CDCl_3$) δ – 83.7 (s), – 112.0 (s), – 123.7 (s) ppm;

IR (film, cm^{-1}): ν_{max} 3296 (w), 2964 (w), 2888 (w), 1617 (w), 1594 (w), 1490 (w), 1453 (w), 1373 (m), 1262 (w), 1205 (s), 1184 (s), 1110 (w), 1052 (m), 971 (m), 812 (m), 778 (m), 704 (w), 622 (w), 590 (w), 552 (w), 518 (w), 471 (w);

$[\alpha]_{D}^{25.0}$ = + 36.9 (c. 1.1, $CHCl_3$);

HRMS (−ESI) *m/z* Found $[M-H]^-$ 406.0553, $[C_{14}H_{14}F_6NO_4S]^-$ requires 406.0553, (δ = + 0.0 ppm);

Chiral SFC Analysis CHIRALPAK IH (CO_2:MeOH, 98:2, 2.5 mL min^{-1}, 40 °C) t_R = 3.7 (minor), 5.6 (major) minutes.

2,2,3,3,3-pentafluoropropyl ((S)-(3-chlorophenyl)((S)-tetrahydrofuran-2-yl)methyl)sulfamate (**4i**)

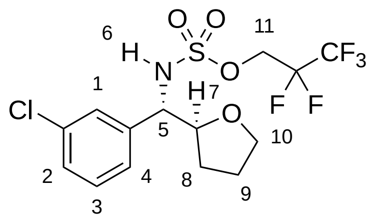

Prepared according to **GP9** using (*Z*)-5-(3-chlorophenyl)pent-4-en-1-ol (**1i**) as the starting material. Following the cyclisation protocol (2 hours) purification by flash column chromatography (SiO_2, 0-4% v/v Acetone in $CHCl_3$) afforded the title compound as a yellow oil (21 mg, 0.050 mmol, 50%, 94% *ee*).

***Rf* value** = 0.45 (10% v/v Acetone in $CHCl_3$);

^{1}H NMR (500 MHz, $CDCl_3$) δ 7.36 (m, 1H, H-1), 7.32-7.28 (m, 2H, H-2, H-3), 7.23 (m, 1H, H-4), 5.67 (d, *J* = 5.1 Hz, 1H, H-6), 4.35-4.26 (m, 2H, H-5, H-11_A), 4.21 (q, *J* = 12.2 Hz, 1H, H-11_B), 4.01 (q, *J* = 6.3 Hz, 1H, H-7), 3.95 (m, 1H, H-10_A), 3.83 (m, 1H, H-10_B), 2.02-1.85 (m, 3H, H-8_A, H-9_A, H-9_B), 1.75 (m, 1H, H-8_B) ppm;

^{13}C NMR (126 MHz, $CDCl_3$) δ 140.5, 134.9, 130.2, 128.9, 127.6, 125.8, 118.2 (qt, $J_{C\text{-}F}$ = 286, 34.3 Hz), 111.3 (tq, $J_{C\text{-}F}$ = 256, 38.3 Hz), 81.0, 69.1, 64.0 (t, $J_{C\text{-}F}$ = 28.3 Hz), 61.8, 29.0, 25.7 ppm;

^{19}F NMR (376 MHz, $CDCl_3$) δ − 84.7, − 124.6 ppm;

IR (film, cm^{-1}): ν_{max} 3310 (w), 2962 (w), 1436 (m), 1372 (m), 1204 (s), 1183 (s), 1109 (m), 1049 (m), 1011 (m);

HRMS (−ESI) *m/z* found $[M-H]^-$ 422.0251, $[C_{14}H_{14}ClF_5NO_4S]^-$ requires 422.0258, (δ = − 1.7 ppm);

$[\alpha]_D^{25.0}$= + 45.6 (c. 0.98, $CHCl_3$);

Chiral SFC Analysis: CHIRALPAK IH (CO_2:MeOH, 97.5:2.5, 2.5 mL min^{-1}, 40 °C) t_R = 4.5 (minor), 7.0 (major) minutes.

2,2,3,3,3-pentafluoropropyl ((S)-((S)-tetrahydrofuran-2-yl)(m-tolyl)methyl)sulfamate (**4j**)

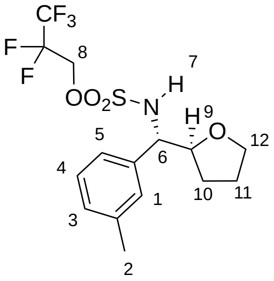

Prepared according to **GP9** using (*Z*)-5-(*m*-tolyl)pent-4-en-1-ol (**1j**) as the starting material. Following the cyclisation protocol (2 hours), purification by flash column chromatography (SiO_2, 0-3% v/v Acetone in $CHCl_3$) afforded the title compound as a colourless oil (15.6 mg, 0.039 mmol, 39%, 92% *ee*).

***R_f* value** = 0.65 (5% v/v Acetone in $CHCl_3$);

^{1}H NMR (500 MHz, $CDCl_3$) δ 7.23-7.26 (m, 1H, H-4), 7.12-7.14 (m, 3H, H-1, H-3, H-5), 5.62 (d, *J* = 5.2 Hz, 1H, H-7), 4.26 (t, *J* = 5.8 Hz, 1H, H-6), 4.18 (q, *J* = 12.2 Hz, 1H, H-8a), 4.09 (q, *J* = 12.2 Hz, 1H, H-8b), 4.02-4.06 (m, 1H, H-9), 3.92-3.98 (m, 1H, H-12a), 3.80-3.86 (m, 1H, H-12b), 2.35 (s, 3H, H-2), 1.83-2.03 (m, 3H, H-10a, H-11a, H-11b), 1.69-1.78 (m, 1H, H-10b) ppm;

^{13}C NMR (126 MHz, $CDCl_3$) δ 138.7, 138.3, 129.4, 128.8, 128.2, 124.6, 118.2 (qt, $J_{C\text{-}F}$ = 285.7 Hz, 34.7 Hz), 111.3 (tq, $J_{C\text{-}F}$ = 256.7 Hz, 38.7 Hz), 81.2, 69.0, 63.9 (t, $J_{C\text{-}F}$ = 28.0 Hz), 62.4, 29.1, 25.7, 21.5 ppm;

^{19}F NMR (376 MHz, $CDCl_3$) δ – 83.8 (s), – 123.8 (s) ppm;

IR (neat, cm^{-1}): ν_{max} 3302 (br, w), 2958 (w), 1610 (w), 1448 (m), 1370 (m), 1260 (w), 1201 (s), 1180 (s), 1108 (m), 1046 (s), 1010 (s), 962 (w), 840 (m), 776 (m), 707 (w), 667 (w), 622 (m), 580 (m), 516 (m), 453 (w);

$[\alpha]_D^{25.0}$ = + 33.7 (c. 1.0, $CHCl_3$);

HRMS (−ESI) *m/z* Found [M−H]$^-$ 402.0794, $[C_{15}H_{17}F_5NO_4S]^-$ requires 402.0804, (δ = – 2.5 ppm);

Chiral SFC Analysis CHIRALPAK IH (CO_2:MeOH, 98:2, 2.5 mL min^{-1}, 40 °C) t_R = 4.1 (minor), 5.6 (major) minutes.

2,2,3,3,3-pentafluoropropyl ((S)-((S)-tetrahydrofuran-2-yl)(3-(trifluoromethyl)phenyl)methyl)sulfamate (**4k**)

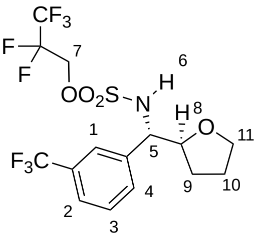

Prepared according to **GP9** using (*Z*)-5-(3-(trifluoromethyl)phenyl)pent-4-en-1-ol (**1k**) as the starting material. Following the cyclisation protocol (2 hours), purification by flash column chromatography (SiO_2, 0-1% v/v Et_2O in CH_2Cl_2) afforded the title compound as a colourless oil (27.1 mg, 0.059 mmol, 59%, 92% *ee*).

***R_f* value** = 0.62 (2% v/v Et_2O in CH_2Cl_2);

^{1}H NMR (500 MHz, $CDCl_3$) δ 7.62 (s, 1H, H-1), 7.60 (d, *J* = 8.0 Hz, 1H, H-2), 7.56 (d, *J* = 7.8 Hz, 1H, H-4), 7.50 (t, *J* = 7.7 Hz, 1H, H-3), 5.79 (s, 1H, H-6), 4.41 (d, *J* = 5.3 Hz, 1H, H-5), 4.30 (q, *J* =

12.3 Hz, 1H, H-7a), 4.19 (m, J = 12.4 Hz, 1H, H-7b), 4.02-4.05 (m, 1H, H-8), 3.95-3.99 (m, 1H, H-11a), 3.81-3.86 (m, 1H, H-11b), 1.88-2.02 (m, 3H, H-9a, H-10a, H-10b), 1.73-1.80 (m, 1H, H-9b) ppm;

^{13}C NMR (126 MHz, $CDCl_3$) δ 139.6, 131.4 (q, $J_{C\text{-}F}$ = 32.5 Hz), 131.1, 129.5, 125.5 (q, $J_{C\text{-}F}$ = 3.4 Hz), 124.2 (q, $J_{C\text{-}F}$ = 3.5 Hz), 123.3 (q, $J_{C\text{-}F}$ = 272.3 Hz), 118.2 (qt, $J_{C\text{-}F}$ = 286.1 Hz, 34.3 Hz), 111.2 (tq, $J_{C\text{-}F}$ = 256.8 Hz, 38.4 Hz), 81.0, 69.1, 63.9 (t, $J_{C\text{-}F}$ = 28.1 Hz), 61.8, 29.0, 25.7 ppm;

^{19}F NMR (376 MHz, $CDCl_3$) δ – 62.8 (s), – 83.8 (s), – 123.8 (s) ppm;

IR (neat, cm^{-1}): ν_{max} 3300 (br, w), 2963 (w), 2881 (w), 1453 (w), 1373 (w), 1329 (s), 1258 (w), 1203 (s), 1164 (s), 1125 (s), 1072 (s), 1009 (s), 962 (m), 804 (m), 776 (s), 704 (m), 683 (m), 622 (m), 564 (m), 516 (m);

$[\alpha]_D^{25.0}$ = + 60.2 (c. 1.1, $CHCl_3$);

HRMS (–ESI) *m/z* Found [M–H]$^-$ 456.0514, $[C_{15}H_{14}F_8NO_4S]^-$ requires 456.0521, (δ = – 1.5 ppm);

Chiral SFC Analysis CHIRALPAK IH (CO_2:MeOH, 98:2, 1.25 mL min^{-1}, 40 °C) t_R = 5.6 (minor), 7.8 (major) minutes.

2,2,3,3,3-pentafluoropropyl ((S)-((S)-tetrahydrofuran-2-yl)(3-(trifluoromethoxy)phenyl)methyl)sulfamate (**4l**)

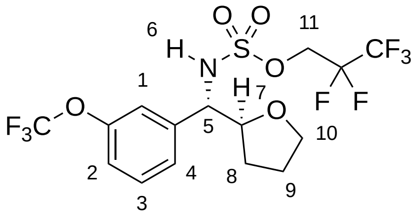

Prepared according to **GP9** using (*Z*)-5-(3-(trifluoromethoxy)phenyl)pent-4-en-1-ol (**1l**) as the starting material. Following the cyclisation protocol (2 hours) purification by flash column chromatography (SiO_2, 0-3% v/v Acetone in $CHCl_3$) afforded the title compound as a pale-yellow oil (33 mg, 0.070 mmol, 70%, 92% *ee*).

***R_f* value** = 0.60 (10% v/v Acetone in $CHCl_3$);

^{1}H NMR (500 MHz, $CDCl_3$) δ 7.40 (t, *J* = 8.0 Hz, 1H, H-3), 7.30 (d, *J* = 7.5 Hz, 1H, H-4), 7.23 (s, 1H, H-1), 7.19 (d, *J* = 8.1 Hz, 1H, H-2), 5.72 (br. s, 1H, H-6), 4.36 (d, *J* = 5.4 Hz, 1H, H-5), 4.28 (q, *J* = 12.1 Hz, 1H, H-11_A), 4.17 (q, *J* = 12.1 Hz, 1H, H-11_B), 4.02 (q, *J* = 6.4 Hz, 1H, H-7), 3.96 (m, 1H, H-10_A), 3.83 (m, 1H, H-10_B), 2.03-1.88 (m, 3H, H-8_A, H-9_A, H-9_B), 1.76 (m, 1H, H-8_B), ppm;

^{13}C NMR (126 MHz, $CDCl_3$) δ 149.7 (q, $J_{C\text{-}F}$ = 1.9 Hz), 140.9, 130.4, 126.0, 121.0 (q, $J_{C\text{-}F}$ = 0.9 Hz), 120.5 (q, $J_{C\text{-}F}$ = 258 Hz), 120.1 (q, $J_{C\text{-}F}$ = 0.7 Hz), 118.2 (qt, $J_{C\text{-}F}$ = 287, 34.5 Hz), 111.2 (tq, $J_{C\text{-}F}$ = 257, 38.4 Hz), 81.0, 69.1, 64.0 (t, $J_{C\text{-}F}$ = 28.2 Hz), 61.7, 29.0, 25.7 ppm;

^{19}F NMR (471 MHz, $CDCl_3$) δ − 58.9, − 84.8, − 124.8 ppm;

IR (film, cm^{-1}): ν_{max} 3304 (w), 2962 (w), 1452 (m), 1373 (m), 1256 (s), 1205 (s), 1179 (s), 1109 (m), 1050 (m), 1011 (m), 970 (m);

$[\alpha]_D^{25.0}$ = + 34.9 (c. 1.0, $CHCl_3$);

HRMS (−ESI) *m/z* found $[M-H]^-$ 472.0472, $[C_{15}H_{14}F_8NO_5S]^-$ requires 472.0470, (δ = + 0.3 ppm);

Chiral SFC Analysis CHIRALPAK IH (CO_2:MeOH, 98:2, 1.25 mL min^{-1}, 40 °C) t_R = 5.1 (minor), 7.0 (major) minutes.

2,2,3,3,3-pentafluoropropyl ((S)-(3-acetylphenyl)((S)-tetrahydrofuran-2-yl)methyl)sulfamate (**4m**)

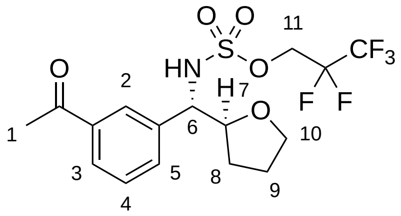

Prepared according to **GP9** using (*Z*)-1-(3-(5-hydroxypent-1-en-1-yl)phenyl)ethan-1-one (**1m**) as the starting material. Following the cyclisation protocol (2 hours) purification by flash column chromatography (SiO_2, 0-6% v/v Acetone in $CHCl_3$) afforded the title compound as a white solid (28 mg, 0.065 mmol, 65%, 93% *ee*).

***R_f* value** = 0.36 (10% v/v Acetone in $CHCl_3$);

^{1}H NMR (500 MHz, $CDCl_3$) δ 7.97 (t, *J* = 1.7 Hz, 1H, H-2), 7.91 (dt, *J* = 7.8, 1.2 Hz, 1H, H-3), 7.57 (dt, *J* = 7.8, 1.4 Hz, 1H, H-5), 7.48 (t, *J* = 7.6 Hz, 1H, H-4), 4.40 (d, *J* = 6.0 Hz, 1H, H-6), 4.32 (q, *J* = 12.0 Hz, 1H, H-11_A), 4.21 (q, *J* = 12.1 Hz, 1H, H-11_B), 4.05 (q, *J* = 6.8 Hz, 1H, H-7), 3.96 (m, 1H, H-10_A), 3.83 (m, 1H, H-10_B), 2.61 (s, 3H, H-1), 2.03-1.84 (m, 3H, H-8_A, H-9_A, H-9_B), 1.75 (m, 1H, H-8_B) ppm;

^{13}C NMR (126 MHz, $CDCl_3$) δ 197.9, 139.2, 137.7, 132.3, 129.3, 128.7, 127.0, 118.2 (qt, $J_{C\text{-}F}$ = 286, 34.5 Hz), 111.3 (tq, $J_{C\text{-}F}$ = 257, 38.7 Hz), 81.1, 69.1, 63.9 (t, $J_{C\text{-}F}$ = 28.0 Hz), 62.1, 29.0, 26.7, 25.7 ppm;

^{19}F NMR (471 MHz, $CDCl_3$) δ − 84.7, − 124.7 ppm;

IR (film, cm^{-1}): ν_{max} 3279 (br), 2964 (w), 1680 (m), 1442 (m), 1363 (m), 1278 (m), 1201 (s), 1181 (s), 1109 (m), 1049 (m), 1010 (m);

$[\alpha]_D^{25.0}$ = + 49.2 (c. 1.0, $CHCl_3$);

HRMS (−ESI) *m/z* found $[M-H]^-$ 430.0754, $[C_{16}H_{17}F_5NO_5S]^-$ requires 430.0753, (δ = + 0.3 ppm);

Chiral SFC Analysis CHIRALPAK IH (CO_2:MeOH, 97:3, 2.5 mL min^{-1}, 40 °C) t_R = 5.8 (minor), 7.0 (major) minutes.

2,2,3,3,3-pentafluoropropyl ((S)-(3-isopropylphenyl)((S)-tetrahydrofuran-2-yl)methyl)sulfamate (**4n**)

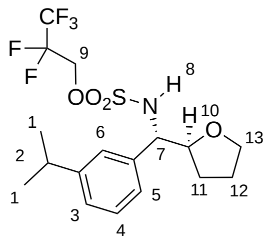

Prepared according to **GP9** using (*Z*)-5-(3-isopropylphenyl)pent-4-en-1-ol (**1n**) as the starting material. Following the cyclisation protocol (2 hours) purification by flash column chromatography (SiO_2, 0-1% v/v Et_2O in CH_2Cl_2) afforded the title compound as a yellow oil (33.2 mg, 0.077 mmol, 77%, 92% *ee*).

***Rf* value** = 0.34 (100% CH_2Cl_2);

^{1}H NMR (500 MHz, $CDCl_3$) δ 7.29 (t, *J* = 7.6 Hz, 1H, H-4), 7.15-7.20 (m, 3H, H-3, H-5, H-6), 5.70 (d, *J* = 5.6 Hz, 1H, H-8), 4.29 (t, *J* = 5.8 Hz, 1H, H-7), 3.98-4.15 (m, 3H, H-9a, H-9b, H-10), 3.93-3.98 (m, 1H, H-13a), 3.81-3.85 (m, 1H, H-13b), 2.90 (sept, *J* = 6.9 Hz, 1H, H-2), 1.84-2.01 (m, 3H, H-11a, H-12a, H-12b), 1.70-1.78 (m, 1H, H-11b), 1.24 (d, *J* = 6.9 Hz, 3H, H-1a), 1.24 (d, *J* = 6.9 Hz, 3H, H-1b) ppm;

^{13}C NMR (126 MHz, $CDCl_3$) δ 149.8, 138.4, 128.9, 126.7, 125.8, 124.9, 118.2 (qt, J_{C-F} = 286.2 Hz, 34.4 Hz), 111.3 (tq, J_{C-F} = 256.7 Hz, 38.3 Hz), 81.2, 69.0, 63.9 (t, J_{C-F} = 28.0 Hz), 62.4, 34.2, 29.1, 25.7, 24.05, 23.97 ppm;

^{19}F NMR (376 MHz, $CDCl_3$) δ – 83.8 (s), – 123.8 (s) ppm;

IR (film, cm^{-1}): ν_{max} 3296 (w), 2964 (w), 2875 (w), 1607 (w), 1448 (m), 1370 (m), 1259 (w), 1203 (s), 1182 (s), 1108 (m), 1051 (m), 1011 (m), 964 (w), 800 (w), 775 (w), 709 (w);

$[\alpha]_D^{25.0}$ = + 37.7 (c. 1.0, $CHCl_3$);

HRMS (−ESI) *m/z* found [M−H]$^-$ 430.1112, $[C_{17}H_{21}F_5NO_5S]^-$ requires 430.1117, (δ = – 1.2 ppm);

Chiral SFC Analysis CHIRALPAK IH (CO_2:MeOH, 98:2, 2.5 mL min^{-1}, 40 °C) t_R = 3.7 (minor), 5.1 (major) minutes.

2,2,3,3,3-pentafluoropropyl ((S)-(3-(tert-butyl)phenyl)((S)-tetrahydrofuran-2-yl)methyl)sulfamate (**4o**)

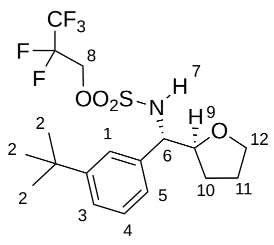

Prepared according to **GP9** using (*Z*)-5-(3-(*tert*-butyl)phenyl)pent-4-en-1-ol (**1o**) as the starting material. Following the cyclisation protocol (2 hours), purification by flash column chromatography (SiO_2, 0-1% v/v Et_2O in CH_2Cl_2) afforded the title compound as a white amorphous solid (35.1 mg, 0.079 mmol, 79%, 90% *ee*).

***R$_f$* value** = 0.32 (100% CH_2Cl_2);

^{1}H NMR (500 MHz, $CDCl_3$) δ 7.34-7.36 (m, 1H, H-3), 7.33 (t, J = 1.7 Hz, 1H, H-1), 7.30 (t, J = 7.7 Hz, 1H, H-4), 7.16 (dt, J = 7.5 Hz, 1.3 Hz, 1H, H-5), 5.72 (d, J = 5.6 Hz, 1H, H-7), 4.31 (t, J = 5.9 Hz, 1H, H-6), 3.94-4.13 (m, 4H, H-8a, H-8b, H-9, H-12a), 3.81-3.85 (m, 1H, H-12b), 1.85-2.02 (m, 3H, H-10a, H-11a, H-11b), 1.70-1.78 (m, 1H, H-10b), 1.31 (s, 9H, H-2) ppm;

^{13}C NMR (126 MHz, $CDCl_3$) δ 152.0, 138.1, 128.7, 125.7, 124.7, 124.5, 118.2 (qt, $J_{C\text{-}F}$ = 286.0 Hz, 34.4 Hz), 111.3 (tq, $J_{C\text{-}F}$ = 256.0 Hz, 38.9 Hz), 81.2, 69.0, 63.9 (t, $J_{C\text{-}F}$ = 28.2 Hz), 62.6, 34.8, 31.4, 29.1, 25.7 ppm;

^{19}F NMR (376 MHz, $CDCl_3$) δ – 83.8 (s), – 123.8 (s) ppm;

IR (neat, cm^{-1}): ν_{max} 3300 (m), 2964 (m), 2906 (w), 2877 (w), 1606 (w), 1459 (w), 1365 (m), 1262 (w), 1201 (s), 1180 (s), 1148 (m), 1112 (m), 1053 (m), 1011 (m), 971 (m), 933 (m), 800 (m), 773 (m), 711 (m), 620 (w), 561 (m), 515 (m);

$[\alpha]_D^{25.0}$ = + 45.0 (c. 1.0, $CHCl_3$);

HRMS (−ESI) *m/z* Found $[M-H]^-$ 444.1279, $[C_{18}H_{23}F_5NO_4S]^-$ requires 444.1273, (δ = + 1.4 ppm);

Chiral SFC Analysis CHIRALPAK IH (CO_2:MeOH, 98.5:1.5, 2.5 mL min^{-1}, 40 °C) t_R = 4.6 (minor), 6.5 (major) minutes.

2,2,3,3,3-pentafluoropropyl ((S)-(3,5-dimethylphenyl)((S)-tetrahydrofuran-2-yl)methyl)sulfamate (**4p**)

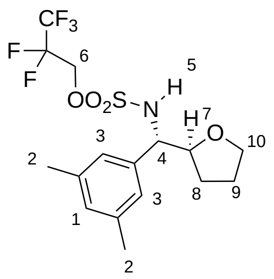

Prepared according to **GP9** using (*Z*)-5-(3,5-dimethylphenyl)pent-4-en-1-ol (**1p**) as the starting material. Following the cyclisation protocol (2 hours), purification by flash column chromatography (SiO_2, 0-1% v/v Et_2O in CH_2Cl_2) afforded the title compound as an amorphous white solid (22.7 mg, 0.054 mmol, 54%, 96% *ee*).

***R_f* value** = 0.63 (5% v/v Acetone in $CHCl_3$);

^{1}H NMR (500 MHz, $CDCl_3$) δ 6.95 (br s, 1H, H-1), 6.94 (br s, 2H, H-3), 5.61-5.67 (m, 1H, H-5), 4.13-4.24 (m, 2H, H-4, H-6a), 4.02-4.13 (m, 2H, H-6b, H-7), 3.92-3.97 (m, 1H, H-10a), 3.81-3.85 (m, 1H, H-10b), 2.30 (s, 6H, H-2), 1.83-2.01 (m, 3H, H-8a, H-9a, H-9b), 1.69-1.77 (m, 1H, H-8b) ppm;

^{13}C NMR (126 MHz, $CDCl_3$) δ 138.6, 138.2, 130.3, 125.3, 118.2 (qt, $J_{C\text{-}F}$ = 286.1 Hz, 34.3 Hz), 111.4 (tq, $J_{C\text{-}F}$ = 256.7 Hz, 38.3 Hz), 81.3, 68.9, 63.9 (t, $J_{C\text{-}F}$ = 27.8 Hz), 62.4, 29.1, 25.7, 21.4 ppm;

^{19}F NMR (376 MHz, $CDCl_3$) δ – 83.8 (s), – 123.8 (s) ppm;

IR (film, cm^{-1}): ν_{max} 3303 (br, w), 2957 (w), 1608 (w), 1447 (m), 1370 (m), 1296 (w), 1201 (s), 1181 (s), 1106 (m), 1011 (s), 972 (s), 842 (m), 810 (m), 776 (m), 709 (w), 622 (w), 584 (m), 517 (m);

$[\alpha]_D^{25.0}$ = + 35.8 (c. 1.0, $CHCl_3$);

HRMS (−ESI) *m/z* Found [M−H]$^-$ 416.0952, $[C_{16}H_{19}F_5NO_4S]^-$ requires 416.0960, (δ = – 1.9 ppm);

Chiral SFC Analysis CHIRALPAK IH (CO_2:MeOH, 98:2, 2.5 mL min^{-1}, 40 °C) t_R = 3.5 (minor), 5.1 (major) minutes.

2,2,3,3,3-pentafluoropropyl ((S)-(3,5-di-tert-butylphenyl)((S)-tetrahydrofuran-2-yl)methyl)sulfamate (**4q**)

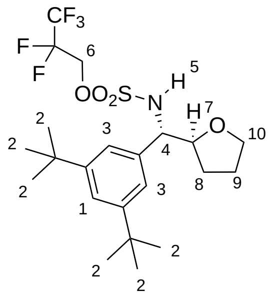

Prepared according to **GP9** using (*Z*)-5-(3,5-di-*tert*-butylphenyl)pent-4-en-1-ol (**1q**) as the starting material. Following the cyclisation protocol (2 hours), purification by flash column chromatography (SiO_2, 0-1% v/v Et_2O in CH_2Cl_2) afforded the title compound as an off-white amorphous solid (37.4 mg, 0.075 mmol, 75%, 88% *ee*).

***R_f* value** = 0.40 (100% CH_2Cl_2);

^{1}H NMR (500 MHz, $CDCl_3$) δ 7.38 (t, *J* = 1.8 Hz, 1H, H-1), 7.16 (d, *J* = 1.8 Hz, 2H, H-3), 5.65 (d, *J* = 5.8 Hz, 1H, H-5), 4.30 (t, *J* = 5.8 Hz, 1H, H-4), 4.05-4.08 (m, 1H, H-7), 3.80-3.98 (m, 4H, H-6a, H-6b, H-10a, H-10b), 1.86-2.01 (m, 3H, H-8a, H-9a, H-9b), 1.71-1.79 (m, 1H, H-8b), 1.31 (s, 18H, H-2) ppm;

^{13}C NMR (126 MHz, $CDCl_3$) δ 151.6, 137.8, 122.6, 121.8, 118.2 (qt, $J_{C\text{-}F}$ = 286.1 Hz, 34.5 Hz), 111.2 (tq, $J_{C\text{-}F}$ = 256.8 Hz, 38.3 Hz), 81.2, 68.9, 64.0 (t, $J_{C\text{-}F}$ = 27.9 Hz), 62.7, 35.0, 31.5, 29.2, 25.7 ppm;

^{19}F NMR (376 MHz, $CDCl_3$) δ – 83.8 (s), – 124.6 - – 123.1 (m)*ppm;

*Partial inequivalence leads to extreme strong coupling between these two ^{19}F nuclei.

IR (neat, cm^{-1}): ν_{max} 3287 (w), 3143 (br, w), 2954 (s), 2906 (m), 2870 (m), 1601 (w), 1462 (m), 1363 (s), 1251 (w), 1201 (s), 1184 (s), 1150 (s), 1110 (s), 1047 (s), 1011 (s), 976 (w), 925 (w), 895 (w), 838 (w), 809 (s), 775 (s), 719 (m), 668 (w), 622 (m), 581 (m), 560 (m), 516 (m);

$[\alpha]_D^{25.0}$ = + 34.3 (c. 1.2, $CHCl_3$);

HRMS (−ESI) *m/z* Found $[M-H]^-$ 500.1909, $[C_{22}H_{31}F_5NO_4S]^-$ requires 500.1899, (δ = + 2.0 ppm);

Chiral HPLC Analysis CHIRAL ART SA (Hexane:^{i}PrOH, 99:1, 1.0 mL min^{-1}, 40 °C) t_R = 7.3 (minor), 10.5 (major) minutes.

2,2,3,3,3-pentafluoropropyl ((S)-(3-fluoro-4-methylphenyl)((S)-tetrahydrofuran-2-yl)methyl)sulfamate (**4r**)

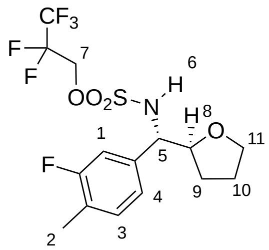

Prepared according to **GP9** using (*Z*)-5-(3-fluoro-4-methylphenyl)pent-4-en-1-ol (**1r**) as the starting material. Following the cyclisation protocol (2 hours), purification by flash column chromatography (SiO_2, 0-1% v/v Et_2O in CH_2Cl_2) afforded the title compound as a colourless oil (32.2 mg, 0.076 mmol, 76%, 96% *ee*).

***R_f* value** = 0.54 (2% v/v Et_2O in CH_2Cl_2);

^{1}H NMR (500 MHz, $CDCl_3$) δ 7.17 (t, J = 7.8 Hz, 1H, H-3), 7.00-7.03 (m, 2H, H-1, H-4), 5.64 (d, J = 5.0 Hz, 1H, H-6), 4.24-4.33 (m, 2H, H-5, H-7a), 4.19 (q, J = 12.2 Hz, 1H, H-7b), 4.00 (q, J = 6.5 Hz, 1H, H-8), 3.92-3.97 (m, 1H, H-11a), 3.80-3.85 (m, 1H, H-11b), 2.26 (d, J = 1.3 Hz, 3H, H-2), 1.84-2.02 (m, 3H, H-9a, H-10a, H-10b), 1.68-1.77 (m, 1H, H-9b) ppm;

^{13}C NMR (126 MHz, $CDCl_3$) δ 161.5 (d, $J_{C\text{-}F}$ = 245.9 Hz), 137.9 (d, $J_{C\text{-}F}$ = 6.9 Hz), 131.9 (d, $J_{C\text{-}F}$ = 5.3 Hz), 125.4 (d, $J_{C\text{-}F}$ = 17.2 Hz), 123.0 (d, $J_{C\text{-}F}$ = 2.9 Hz), 118.2 (qt, $J_{C\text{-}F}$ = 286.2 Hz, 34.4 Hz), 114.1 (d, $J_{C\text{-}F}$ = 23.3 Hz), 111.3 (tq, $J_{C\text{-}F}$ = 256.8 Hz, 38.4 Hz), 81.1, 69.0, 64.0 (t, $J_{C\text{-}F}$ = 28.1 Hz), 61.8, 29.0, 25.7, 14.4 (d, $J_{C\text{-}F}$ = 3.0 Hz) ppm;

^{19}F NMR (376 MHz, $CDCl_3$) δ – 83.8 (s), – 116.3 (s), – 123.7 (s) ppm;

IR (film, cm^{-1}): ν_{max} 3300 (br, w), 2960 (w), 2931 (w), 2883 (w), 1585 (w), 1511 (w), 1428 (m), 1372 (m), 1262 (w), 1205 (s), 1183 (s), 1111 (m), 1051 (m), 1012 (m), 969 (w), 814 (w), 777 (w), 715 (w), 623 (w), 518 (w);

$[\boldsymbol{\alpha}]_{\mathbf{D}}^{\mathbf{25.0}}$ = + 43.1 (c. 1.0, $CHCl_3$);

HRMS (−ESI) *m/z* Found [M−H]$^-$ 420.0704, $[C_{15}H_{16}F_6NO_4S]^-$ requires 420.0710, (δ = – 1.4 ppm);

Chiral SFC Analysis CHIRALPAK IE (CO_2:MeOH, 99:1, 2.5 mL min^{-1}, 40 °C) t_R = 9.5 (major), 12.9 (minor) minutes.

2,2,3,3,3-pentafluoropropyl ((S)-naphthalen-2-yl((S)-tetrahydrofuran-2-yl)methyl)sulfamate (**4s**)

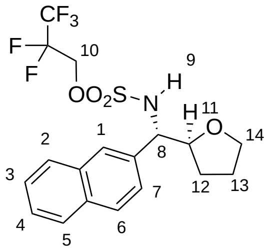

Prepared according to **GP9** using (*Z*)-5-(naphthalen-2-yl)pent-4-en-1-ol (**1s**) as the starting material. Following the cyclisation protocol (2 hours), purification by flash column chromatography (SiO_2, 0-1% v/v Et_2O in CH_2Cl_2) afforded the title compound as a white solid (24.3 mg, 0.055 mmol, 55%, 95% *ee*).

***R_f* value** = 0.42 (100% CH_2Cl_2);

^{1}H NMR (500 MHz, $CDCl_3$) δ 7.86 (d, *J* = 9.0 Hz, 1H, H-6), 7.82-7.84 (m, 2H, H-2, H-5), 7.80 (br s, 1H, H-1), 7.49-7.53 (m, 2H, H-3, H-4), 7.47 (dd, *J* = 8.6 Hz, 1.5 Hz, 1H, H-7), 5.78 (d, *J* = 5.0 Hz, 1H, H-9), 4.48 (t, *J* = 5.7 Hz, 1H, H-8), 4.24 (q, *J* = 12.3 Hz, 1H, H-10a), 4.11-4.19 (m, 2H, H-10b, H-11), 3.96-4.01 (m, 1H, H-14a), 3.83-3.88 (m, 1H, H-14b), 1.75-2.05 (m, 4H, H-12a, H-12b, H-13a, H-13b) ppm;

^{13}C NMR (126 MHz, $CDCl_3$) δ 135.6, 133.4, 133.3, 128.9, 128.1, 127.8, 127.1, 126.65, 126.60, 124.6, 118.2 (qt, $J_{C\text{-}F}$ = 286.2 Hz, 34.4 Hz), 111.3 (tq, $J_{C\text{-}F}$ = 256.8 Hz, 38.3 Hz), 81.2, 69.0, 64.0 (t, $J_{C\text{-}F}$ = 28.1 Hz), 62.7, 29.1, 25.7 ppm;

^{19}F NMR (376 MHz, $CDCl_3$) δ – 83.8 (s), – 123.7 (s) ppm;

IR (neat, cm^{-1}): ν_{max} 3334 (br, w), 2957 (w), 2875 (w), 1601 (w), 1510 (w), 1445 (m), 1361 (s), 1265 (w), 1198 (s), 1177 (s), 1156 (s), 1105 (s), 1064 (s), 1041 (s), 1013 (s), 970 (m), 911 (m), 856 (w), 815 (s), 770 (s), 747 (s), 623 (m), 572 (s), 543 (s), 516 (m), 484 (m), 452 (s);

$[\alpha]_D^{25.0}$ = + 53.2 (c. 1.1, $CHCl_3$);

HRMS (−ESI) *m/z* Found $[M-H]^-$ 438.0804, $[C_{18}H_{17}F_5NO_4S]^-$ requires 438.0804, (δ = + 0.0 ppm);

Chiral SFC Analysis CHIRALPAK IE (CO_2:MeOH, 97:3, 2.5 mL min^{-1}, 40 °C) t_R = 7.4 (major), 8.7 (minor) minutes.

2,2,3,3,3-pentafluoropropyl ((S)-(4-fluorophenyl)((S)-tetrahydrofuran-2-yl)methyl)sulfamate (**4t**)

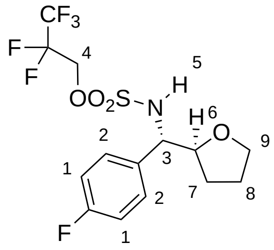

Prepared according to **GP9** using (*Z*)-5-(4-fluorophenyl)pent-4-en-1-ol (**1t**) as the starting material. Following the cyclisation protocol (2 hours), purification by flash column chromatography (SiO_2, 0-1.5% v/v Et_2O in CH_2Cl_2) afforded the title compound as a colourless oil (30.6 mg, 0.075 mmol, 75%, 87% *ee*).

***R$_f$* value** = 0.63 (5% v/v Et_2O in CH_2Cl_2);

^{1}H NMR (500 MHz, $CDCl_3$) δ 7.31-7.35 (m, 2H, H-2), 7.03-7.08 (m, 2H, H-1), 5.71 (br s, 1H, H-5), 4.30 (d, *J* = 6.2 Hz, 1H, H-3), 4.28 (q, *J* = 12.7 Hz, 1H, H-4a), 4.17 (q, *J* = 12.4 Hz, 1H, H-4b), 4.01 (q, *J* = 6.5 Hz, 1H, H-6), 3.93-3.97 (m, 1H, H-9a), 3.81-3.85 (m, 1H, H-9b), 1.84-2.01 (m, 3H, H-7a, H-8a, H-8b), 1.67-1.76 (m, 1H, H-7b) ppm;

^{13}C NMR (126 MHz, $CDCl_3$) δ 162.8 (d, $J_{C\text{-}F}$ = 247.5 Hz), 134.2 (d, $J_{C\text{-}F}$ = 2.8 Hz), 129.3 (d, $J_{C\text{-}F}$ = 8.2 Hz), 118.2 (qt, $J_{C\text{-}F}$ = 286.1 Hz, 34.9 Hz), 115.9 (d, $J_{C\text{-}F}$ = 21.7 Hz), 111.3 (tq, $J_{C\text{-}F}$ = 256.2 Hz, 38.4 Hz), 81.2, 69.0, 63.9 (t, $J_{C\text{-}F}$ = 28.2 Hz), 61.8, 29.0, 25.7 ppm;

^{19}F NMR (376 MHz, $CDCl_3$) δ – 83.7 (s), – 113.3 (s), – 123.6 (s) ppm;

IR (film, cm^{-1}): ν_{max} 3301 (br, m), 2960 (w), 2881 (w), 1607 (m), 1512 (m), 1434 (br), 1372 (m), 1203 (s), 1182 (s), 1161 (s), 1109 (m), 1051 (m), 1011 (m), 964 (m), 835 (m), 776 (w), 623 (w), 578 (w), 518 (w), 456 (w);

$[\alpha]_D^{25.0}$ = + 36.4 (c. 1.0, $CHCl_3$);

HRMS (−ESI) *m/z* Found $[M-H]^-$ 406.0548, $[C_{14}H_{14}F_6NO_4S]^-$ requires 406.0553, (δ = – 1.2 ppm);

Chiral SFC Analysis CHIRALPAK IH (CO_2:MeOH, 98:2, 2.5 mL min^{-1}, 40 °C) t_R = 4.3 (minor), 5.6 (major) minutes.

2,2,3,3,3-pentafluoropropyl ((S)-((S)-tetrahydrofuran-2-yl)(p-tolyl)methyl)sulfamate (**4u**)

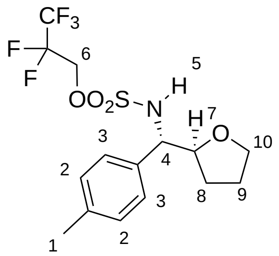

Prepared according to **GP9** using (*Z*)-5-(*p*-tolyl)pent-4-en-1-ol (**1u**) as the starting material. Following the cyclisation protocol (2 hours), purification by flash column chromatography (SiO_2, 0-1% v/v Et_2O in CH_2Cl_2) afforded the title compound as an amorphous white solid (21.0 mg, 0.052 mmol, 52%, 91% *ee*).

***R$_f$* value** = 0.53 (4% v/v Acetone in $CHCl_3$);

^{1}H NMR (500 MHz, $CDCl_3$) δ 7.22-7.24 (m, 2H, H-3), 7.16-7.18 (m, 2H, H-2), 5.64 (br s, 1H, H-5), 4.26 (d, J = 4.3 Hz, 1H, H-4), 4.18 (q, J = 12.2 Hz, 1H, H-6a), 4.09 (q, J = 12.3 Hz, 1H, H-6b), 4.03 (q, J = 6.6 Hz, 1H, H-7), 3.92-3.97 (m, 1H, H-10a), 3.80-3.86 (m, 1H, H-10b), 2.34 (s, 3H, H-1), 1.82-2.01 (m, 3H, H-8a, H-9a, H-9b), 1.68-1.75 (m, 1H, H-8b) ppm;

^{13}C NMR (126 MHz, $CDCl_3$) δ 138.6, 135.3, 129.6, 127.5, 118.2 (qt, $J_{C\text{-}F}$ = 286.2 Hz, 34.7 Hz), 111.3 (tq, $J_{C\text{-}F}$ = 257.5 Hz, 38.5 Hz), 81.3, 68.9, 63.9 (t, $J_{C\text{-}F}$ = 28.0 Hz), 62.3, 29.0, 25.7, 21.2 ppm;

^{19}F NMR (376 MHz, $CDCl_3$) δ – 84.8 (s), – 124.7 (s) ppm;

IR (film, cm^{-1}): ν_{max} 3304 (br, w), 2957 (w), 1516 (w), 1448 (w), 1372 (m), 1204 (s), 1181 (s), 1110 (m), 1051 (m), 1011 (m), 963 (w), 813 (w), 777 (w), 623 (w), 580 (w), 518 (w);

$[\boldsymbol{\alpha}]_{\mathbf{D}}^{\mathbf{25.0}}$ = + 33.3 (c. 1.0, $CHCl_3$);

HRMS (−ESI) *m/z* Found $[M-H]^-$ 402.0804, $[C_{15}H_{17}F_5NO_4S]^-$ requires 402.0804, (δ = + 0.0 ppm);

Chiral SFC Analysis CHIRALPAK IH (CO_2:MeOH, 98:2, 1.5 mL min^{-1}, 40 °C) t_R = 9.4 (minor), 13.5 (major) minutes.

2,2,3,3,3-pentafluoropropyl ((S)-((S)-tetrahydrofuran-2-yl)(4-(trifluoromethyl)phenyl)methyl)sulfamate (**4v**)

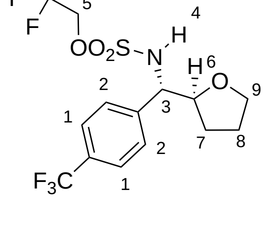

Prepared according to **GP9** using (*Z*)-5-(4-(trifluoromethyl)phenyl)pent-4-en-1-ol (**1v**) as the starting material. In this case the following procedural modification was used: 0.090 mmol of (*Z*)-5-(4-(trifluoromethyl)phenyl)pent-4-en-1-ol (**1x**) was used and all other reagent and solvent amounts were scaled proportionally. Following the cyclisation protocol (2 hours), purification by flash column chromatography (SiO_2, 0-1% v/v Et_2O in CH_2Cl_2) afforded the title compound as a colourless oil (20.2 mg, 0.044 mmol, 49%, 97% *ee*).

***R_f* value** = 0.54 (2% v/v Et_2O in CH_2Cl_2);

^{1}H NMR (500 MHz, $CDCl_3$) δ 7.63 (d, *J* = 8.2 Hz, 2H, H-1), 7.48 (d, *J* = 8.1 Hz, 2H, H-2), 5.72 (d, *J* = 5.3 Hz, 1H, H-4), 4.40 (t, *J* = 5.5 Hz, 1H, H-3), 4.30 (q, *J* = 12.3 Hz, 1H, H-5a), 4.22 (q, *J* = 12.3 Hz, 1H, H-5b), 4.03 (q, *J* = 6.4 Hz, 1H, H-6), 3.94-3.99 (m, 1H, H-9a), 3.80-3.86 (m, 1H, H-9b), 1.87-2.03 (m, 3H, H-7a, H-8a, H-8b), 1.72-1.81 (m, 1H, H-7b) ppm;

^{13}C NMR (126 MHz, $CDCl_3$) δ 142.4, 130.9 (q, $J_{C\text{-}F}$ = 32.5 Hz), 127.9, 125.9 (q, $J_{C\text{-}F}$ = 3.4 Hz), 124.0 (q, $J_{C\text{-}F}$ = 272.1 Hz), 118.2 (qt, $J_{C\text{-}F}$ = 286.0 Hz, 34.6 Hz), 111.3 (tq, $J_{C\text{-}F}$ = 256.7 Hz, 38.8 Hz), 81.0, 69.1, 64.0 (t, $J_{C\text{-}F}$ = 28.2 Hz), 61.8, 29.0, 25.7 ppm;

^{19}F NMR (376 MHz, $CDCl_3$) δ – 62.8 (s), – 83.8 (s), – 123.7 (s) ppm;

IR (neat, cm^{-1}): ν_{max} 3297 (br, w), 2959 (w), 2922 (w), 2854 (w), 1622 (w), 1424 (w), 1373 (m), 1325 (s), 1260 (w), 1205 (s), 1165 (s), 1125 (s), 1107 (s), 1067 (s), 1010 (s), 962 (m), 841 (m), 808 (m), 776 (m), 716 (w), 660 (w), 603 (m), 566 (m), 517 (m);

$[\alpha]_D^{25.0}$ = + 41.6 (c. 1.1, $CHCl_3$);

HRMS (–ESI) *m/z* Found $[M-H]^-$ 456.0516, $[C_{15}H_{14}F_8NO_4S]^-$ requires 456.0521, (δ = – 1.1 ppm);

Chiral SFC Analysis CHIRALPAK IG (CO_2:^{i}PrOH, 98:2, 2.5 mL min^{-1}, 40 °C) t_R = 3.0 (minor), 3.6 (major) minutes.

2,2,3,3,3-pentafluoropropyl ((S)-(4-(tert-butyl)phenyl)((S)-tetrahydrofuran-2-yl)methyl)sulfamate (**4w**)

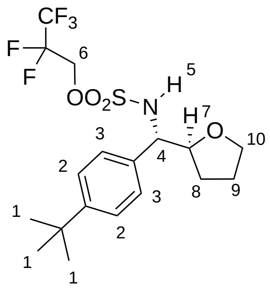

Prepared according to **GP9** using (*Z*)-5-(4-(*tert*-butyl)phenyl)pent-4-en-1-ol (**1w**) as the starting material. Following the cyclisation protocol (2 hours), purification by flash column chromatography (SiO_2, 0-1% v/v Et_2O in CH_2Cl_2) afforded the title compound as white plates (26.1 mg, 0.059 mmol, 59%, 96% *ee*).

***R$_f$* value** = 0.44 (100% CH_2Cl_2);

^{1}H NMR (500 MHz, $CDCl_3$) δ 7.36-7.39 (m, 2H, H-2), 7.25-7.28 (m, 2H, H-3), 5.68 (d, *J* = 5.4 Hz, 1H, H-5), 4.29 (t, *J* = 5.8 Hz, 1H, H-4), 4.04-4.13 (m, 2H, H-6a, H-7), 3.94-4.04 (m, 2H, H-6b, H-10a), 3.81-3.85 (m, 1H, H-10b), 1.85-2.01 (m, 3H, H-8a, H-9a, H-9b), 1.70-1.78 (m, 1H, H-8b), 1.30 (s, 9H, H-1) ppm;

^{13}C NMR (126 MHz, $CDCl_3$) δ 151.8, 135.3, 127.3, 125.9, 118.2 (qt, $J_{C\text{-}F}$ = 286.2 Hz, 34.4 Hz), 111.2 (tq, $J_{C\text{-}F}$ = 256.8 Hz, 38.3 Hz), 81.2, 69.0, 63.9 (t, $J_{C\text{-}F}$ = 28.0 Hz), 62.0, 34.7, 31.3, 29.1, 25.7 ppm;

^{19}F NMR (376 MHz, $CDCl_3$) δ – 83.8 (s), – 124.5 - – 123.1 (m)*ppm;

*Partial inequivalence leads to extreme strong coupling between these two ^{19}F nuclei.

IR (neat, cm^{-1}): ν_{max} 3300 (m), 2962 (m), 2871 (m), 1458 (m), 1364 (s), 1266 (w), 1185 (s), 1151 (s), 1104 (s), 1067 (s), 1047 (s), 1011 (s), 969 (s), 947 (s), 927 (w), 909 (w), 832 (m), 809 (m), 761 (m), 664 (w), 625 (m), 584 (s), 553 (m), 510 (w);

$[\boldsymbol{\alpha}]_{\mathbf{D}}^{\mathbf{25.0}}$ = + 40.0 (c. 1.0, $CHCl_3$);

HRMS (−ESI) *m/z* Found $[M-H]^-$ 444.1269, $[C_{18}H_{23}F_5NO_4S]^-$ requires 444.1273, (δ = – 0.9 ppm);

Chiral SFC Analysis CHIRALPAK IH (CO_2:MeOH, 98.5:1.5, 1.25 mL min^{-1}, 40 °C) t_R = 10.6 (minor), 18.5 (major) minutes.

2,2,3,3,3-pentafluoropropyl ((S)-[1,1'-biphenyl]-4-yl((S)-tetrahydrofuran-2-yl)methyl)sulfamate (**4x**)

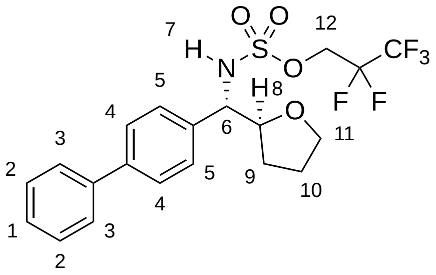

Prepared according to **GP9** using (*Z*)-5-([1,1'-biphenyl]-4-yl)pent-4-en-1-ol (**1x**) as the starting material. Following the cyclisation protocol (2 hours) purification by flash column chromatography (SiO_2, 0-1% v/v Et_2O in CH_2Cl_2) afforded the title compound as a white solid (28 mg, 0.061 mmol, 61%, 96% *ee*).

***R_f* value** = 0.45 (100% CH_2Cl_2);

^{1}H NMR (500 MHz, $CDCl_3$) δ 7.61-7.55 (m, 4H, H-3, H-4), 7.47-7.41 (m, 4H, H-2, H-5), 7.36 (tt, *J* = 7.4, 1.9 Hz, 1H, H-1), 5.73 (d, *J* = 5.3 Hz, 1H, H-7), 4.37 (t, *J* = 5.8 Hz, 1H, H-6), 4.26 (q, *J* =

12.3 Hz, 1H, H-12_A), 4.17 (q, J = 12.4 Hz, 1H, H-12_B), 4.10 (q, J = 6.4 Hz, 1H, H-8), 3.98 (m, 1H, H-11_A), 3.85 (m, 1H, H-11_B), 2.00 (m, 1H, H-10_A), 1.96-1.88 (m, 2H, H-9_A, H-10_B), 1.78 (m, 1H, H-9_B) ppm;

^{13}C NMR (126 MHz, $CDCl_3$) δ 141.7, 140.6, 137.3, 129.0, 128.0, 127.70, 127.67, 127.2, 118.2 (qt, $J_{C\text{-}F}$ = 286, 34.5 Hz), 111.3 (tq, $J_{C\text{-}F}$ = 257, 38.6 Hz), 81.3, 69.0, 64.0 (t, $J_{C\text{-}F}$ = 28.0 Hz), 62.2, 29.1, 25.7 ppm;

^{19}F NMR (376 MHz, $CDCl_3$) δ − 83.7, − 123.6 ppm;

IR (film, cm^{-1}): ν_{max} 3295 (m), 1363 (s), 1201 (s), 1183 (s), 1159 (m), 1109 (m), 1071 (m), 1041 (m) 1016 (m), 764 (m);

$[\alpha]_D^{25.0}$ = + 50.3 (c. 1.3, $CHCl_3$);

HRMS (−ESI) *m/z* found $[M{-}H]^-$ 464.0967, $[C_{20}H_{19}F_5NO_4S]^-$ requires 464.0960, (δ = + 1.5 ppm);

Chiral SFC Analysis CHIRALPAK IH (CO_2:MeOH, 96:4, 2.5 mL min^{-1}, 40 °C) t_R = 6.4 (minor), 9.0 (major) minutes.

Ethyl 4-((S)-(((2,2,3,3,3-pentafluoropropoxy)sulfonyl)amino)((S)-tetrahydrofuran-2-yl)methyl)benzoate (**4y**)

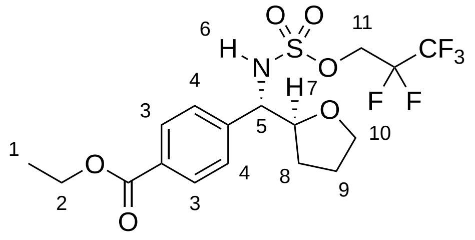

Prepared according to **GP9** using ethyl (*Z*)-4-(5-hydroxypent-1-en-1-yl)benzoate (**1y**) as the starting material. Following the cyclisation protocol (2 hours) purification by flash column chromatography (SiO_2, 0-4% v/v Acetone in $CHCl_3$) afforded the title compound as a pale-yellow oil (34 mg, 0.074 mmol, 74%, 97% *ee*).

***R_f* value** = 0.49 (5% v/v Acetone in $CHCl_3$);

^{1}H NMR (500 MHz, $CDCl_3$) δ 8.04 (d, *J* = 8.4 Hz, 2H, H-3), 7.43 (d, *J* = 8.3 Hz, 2H, H-4), 5.80 (d, *J* = 3.6 Hz, 1H, H-6), 4.40-4.36 (m, 3H, H-2, H-5), 4.31 (q, *J* = 12.4 Hz, 1H, H-11_A), 4.22 (q, *J* = 12.3 Hz, 1H, H-11_B), 4.03 (q, *J* = 6.2 Hz, 1H, H-7), 3.95 (m, 1H, H-10_A), 3.83 (m, 1H, H-10_B), 2.02-1.83 (m, 3H, H-8_A, H-9_A, H-9_B), 1.74 (m, 1H, H-8_B) 1.39 (t, *J* = 7.1 Hz, 3H, H-1) ppm;

^{13}C NMR (126 MHz, $CDCl_3$) δ 166.2, 143.2, 130.8, 130.2, 127.5, 118.2 (qt, $J_{C\text{-}F}$ = 286, 34.6 Hz), 111.3 (tq, $J_{C\text{-}F}$ = 257, 38.3 Hz), 81.0, 69.0, 64.0 (t, $J_{C\text{-}F}$ = 28.0 Hz), 62.1, 61.3, 29.0, 25.7, 14.4 ppm;

^{19}F NMR (471 MHz, $CDCl_3$) δ − 84.7, − 124.6 ppm;

IR (film, cm^{-1}): ν_{max} 3281 (w), 2963 (w), 1716 (m), 1370 (m), 1278 (s), 1202 (s), 1181 (s), 1105 (s), 1065 (m), 1010 (m), 773 (m), 575 (m);

$[\alpha]_D^{25.0}$ = + 46.9 (c. 1.3, $CHCl_3$);

HRMS (−ESI) *m/z* found [M−H]$^-$ 460.0854, $[C_{17}H_{19}F_5NO_6S]^-$ requires 460.0859, (δ = − 0.9 ppm);

Chiral SFC Analysis CHIRALPAK IH (CO_2:MeOH, 97:3, 2.5 mL min^{-1}, 40 °C) t_R = 4.4 (minor), 6.3 (major) minutes.

2,2,3,3,3-pentafluoropropyl ((S)-(4-((tert-butoxycarbonyl)amino)phenyl)((S)-tetrahydrofuran-2-yl)methyl)sulfamate (**4z**)

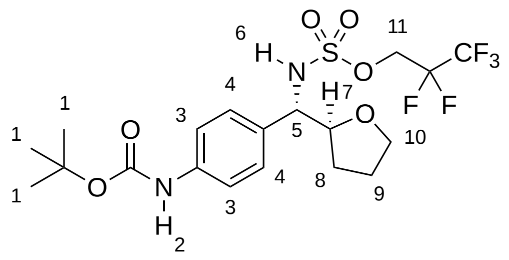

Prepared according to **GP9** using tert-butyl (*Z*)-(4-(5-hydroxypent-1-en-1-yl)phenyl)carbamate (**1z**) as the starting material. Following the cyclisation protocol (2 hours) purification by flash column chromatography (SiO_2, 0-7% v/v Acetone in $CHCl_3$) afforded the title compound as an off-white solid (17 mg, 0.034 mmol, 34%, 89% *ee*).

***Rf* value** = 0.28 (10% v/v Acetone in $CHCl_3$);

^{1}H NMR (500 MHz, $CDCl_3$) δ 7.36 (d, J = 8.7 Hz, 2H, H-3), 7.26 (m, 2H, H-4), 6.52 (br s, 1H, H-2), 5.64 (br s, 1H, H-6), 4.28-4.20 (m, 2H, H-5, H-11_A), 4.15 (q, J = 12.5 Hz, 1H, H-11_B), 4.01 (q, J = 6.7 Hz, 1H, H-7), 3.93 (m, 1H, H-10_A), 3.82 (m, 1H, H-10_B), 2.00-1.80 (m, 3H, H-8_A, H-9_A, H-9_B), 1.69 (m, 1H, H-8_B), 1.51 (s, 9H, H-1) ppm;

^{13}C NMR (101 MHz, $CDCl_3$) δ 152.8, 138.8, 132.6, 128.3, 118.8, 118.3 (qt, $J_{C\text{-}F}$ = 287, 34.0 Hz), 111.3 (tq, $J_{C\text{-}F}$ = 255, 38.0 Hz), 81.2, 81.0, 68.9, 64.0 (t, $J_{C\text{-}F}$ = 28.1 Hz), 62.1, 29.0, 28.4, 25.7 ppm;

^{19}F NMR (471 MHz, $CDCl_3$) δ − 84.7, − 124.6 ppm;

IR (film, cm^{-1}): ν_{max} 3305 (w), 2980 (w), 1700 (m), 1526 (s), 1369 (s), 1205 (s), 1158 (s), 1054 (m), 1012 (m);

$[\alpha]_D^{25.0}$ = + 36.3 (c. 1.0, $CHCl_3$);

HRMS (−ESI) *m/z* found $[M-H]^-$ 503.1274, $[C_{19}H_{24}F_5N_2O_6S]^-$ requires 503.1281, (δ = − 1.4 ppm);

Chiral SFC Analysis: CHIRALPAK IH (CO_2:MeOH, 97:3, 2.5 mL min^{-1}, 40 °C) t_R = 12.8 (minor), 17.4 (major) minutes.

2,2,3,3,3-pentafluoropropyl ((S)-(4-methoxyphenyl)((S)-tetrahydrofuran-2-yl)methyl)sulfamate (**4aa**)

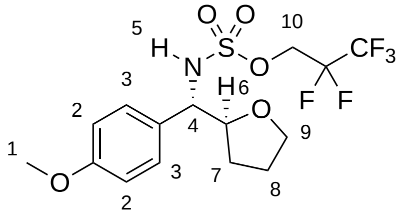

Prepared according to **GP9** using (*Z*)-5-(4-methoxyphenyl)pent-4-en-1-ol (**1aa**) as the starting material. Following the cyclisation protocol (2 hours) purification by flash column chromatography (SiO_2, 0-4% v/v Acetone in $CHCl_3$) afforded the title compound as a pale-yellow oil (15 mg, 0.036 mmol, 36%, 88% *ee*).

***R_f* value** = 0.45 (10% v/v Acetone in $CHCl_3$);

^{1}H NMR (500 MHz, $CDCl_3$) δ 7.27 (m, 2H, H-3), 6.89 (m, 2H, H-2), 5.60 (br s, 1H, H-5), 4.25 (d, *J* = 6.6 Hz, 1H, H-4), 4.20 (q, *J* = 12.5 Hz, 1H, H-10_A), 4.10 (q, *J* = 12.5 Hz, 1H, H-10_B), 4.03 (q, *J* = 6.5 Hz, 1H, H-6), 3.95 (m, 1H, H-9_A), 3.83 (m, 1H, H-9_B), 3.80 (s, 3H, H-1), 2.00-1.81 (m, 3H, H-7_A, H-8_A, H-8_B), 1.70 (m, 1H, H-7_B) ppm;

^{13}C NMR (126 MHz, $CDCl_3$) δ 159.9, 130.3, 128.8, 118.3 (qt, J_{C-F} = 286, 34.6 Hz), 114.3, 111.3 (tq, J_{C-F} = 257, 38.3 Hz), 81.3, 68.9, 63.9 (t, J_{C-F} = 28.3 Hz), 62.0, 55.5, 29.0, 25.7 ppm;

^{19}F NMR (471 MHz, $CDCl_3$) δ − 84.7, − 124.6 ppm;

IR (film, cm^{-1}): ν_{max} 3300 (w), 2960 (w), 1613 (m), 1515 (m), 1444 (m), 1371 (m), 1203 (s), 1177 (s), 1108 (m), 1034 (m), 1011 (m);

$[\alpha]_D^{25.0}$ = + 31.1 (c. 0.81, $CHCl_3$);

HRMS (−ESI) *m/z* found $[M-H]^-$ 418.0749, $[C_{15}H_{17}F_5NO_5S]^-$ requires 418.0753, (δ = – 1.0 ppm);

Chiral SFC Analysis CHIRALPAK IH (CO_2:MeOH, 98:2, 2.5 mL min^{-1}, 40 °C) t_R = 7.5 (minor), 8.4 (major) minutes.

2,2,3,3,3-pentafluoropropyl ((2R,3R)-2-(4-methoxyphenyl)tetrahydro-2H-pyran-3-yl)sulfamate (**4ab**)

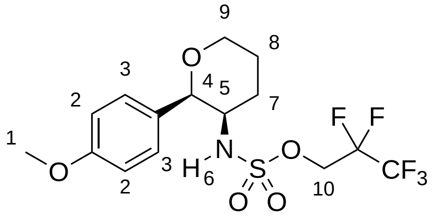

Following the protocol reported for **4aa**, **4ab** was also isolated a pale-yellow oil (4 mg, 0.01 mmol, 10%, 89% *ee*).

***R_f* value** = 0.55 (10% v/v Acetone in $CHCl_3$);

^{1}H NMR (500 MHz, $CDCl_3$) δ 7.23 (m, 2H, H-3), 6.91 (m, 2H, H-2), 4.93 (d, *J* = 7.0 Hz, 1H, H-6), 4.58 (m, 1H, H-4), 4.18 (m, 1H, H-9_A), 3.93 (q, *J* = 12.5 Hz, 1H, H-10_A), 3.78 (s, 3H, H-1), 3.71 (m, 1H, H-5), 3.66 (td, *J* = 11.7, 2.3 Hz, 1H, H-9_B), 3.19 (q, *J* = 12.5 Hz, 1H, H-10_B), 2.35 (m, 1H, H-7_A), 2.02-1.86 (m, 2H, H-7_B, H-8_A), 1.59 (m, 1H, H-8_B) ppm;

^{13}**C NMR** (126 MHz, $CDCl_3$) δ 159.4, 130.9, 126.4, 118.2 (qt, $J_{C\text{-}F}$ = 285, 34.7 Hz), 114.2, 111.2 (tq, $J_{C\text{-}F}$ = 257, 38.6 Hz), 79.7, 69.3, 63.5 (t, $J_{C\text{-}F}$ = 28.1 Hz), 55.3, 54.3, 28.9, 19.9 ppm;

^{19}**F NMR** (471 MHz, $CDCl_3$) δ − 84.6, − 124.7 (d, J = 26.6 Hz) ppm;

IR (film, cm^{-1}): ν_{max} 3298 (w), 2957 (w), 1614 (w), 1516 (m), 1372 (m), 1253 (s), 1205 (s), 1181 (s), 1108 (m), 1091 (m), 1011 (m);

$[\alpha]_D^{25.0}$ = − 42.6 (c. 0.26, $CHCl_3$);

HRMS (−ESI) *m/z* found [M−H]$^-$ 418.0763, $[C_{15}H_{17}F_5NO_5S]^-$ requires 418.0753, (δ = + 2.4 ppm);

Chiral SFC Analysis CHIRALPAK IH (CO_2:MeOH, 97:3, 2.5 mL min^{-1}, 40 °C) t_R = 3.7 (major), 6.0 (minor) minutes.

2,2,3,3,3-pentafluoropropyl ((S)-(4-chlorophenyl)((S)-tetrahydrofuran-2-yl)methyl)sulfamate (**4ac**)

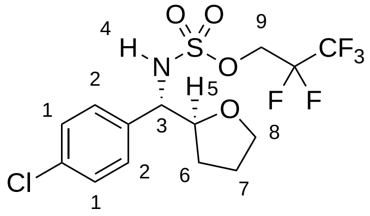

Prepared according to **GP9** using (*Z*)-5-(4-chlorophenyl)pent-4-en-1-ol (**1ac**) as the starting material. Following the cyclisation protocol (2 hours) purification by flash column chromatography (SiO_2, 0-3% v/v Acetone in $CHCl_3$) afforded the title compound as a colourless oil (30 mg, 0.071 mmol, 71%, 90% *ee*).

***R$_f$* value** = 0.56 (10% v/v Acetone in $CHCl_3$);

^{1}**H NMR** (500 MHz, $CDCl_3$) δ 7.34 (m, 2H, H-1), 7.29 (m, 2H, H-2), 5.70 (d, J = 4.5 Hz, 1H, H-4), 4.34-4.26 (m, 2H, H-3, H-9$_A$), 4.21 (q, J = 12.2 Hz, 1H, H-9$_B$), 4.00 (q, J = 6.3 Hz, 1H, H-5), 3.95 (m, 1H, H-8$_A$), 3.83 (m, 1H, H-8$_B$), 2.01-1.84 (m, 3H, H-6$_A$, H-7$_A$, H-7$_B$), 1.72 (m, 1H, H-6$_B$) ppm;

^{13}**C NMR** (126 MHz, $CDCl_3$) δ 136.9, 134.6, 129.1, 128.9, 118.2 (qt, $J_{C\text{-}F}$ = 287, 34.3 Hz), 111.3 (tq, $J_{C\text{-}F}$ =257, 38.4 Hz), 81.1, 69.0, 64.0 (t, $J_{C\text{-}F}$ = 28.3 Hz), 61.8, 29.0, 25.7 ppm;

^{19}**F NMR** (471 MHz, $CDCl_3$) δ − 84.7, − 124.6 ppm;

IR (film, cm^{-1}): ν_{max} 3297 (w), 2880 (w), 1493 (m), 1429 (m), 1371 (m), 1202 (s), 1181 (s), 1108 (m), 1091 (m), 1049 (m), 1011 (m), 963 (m), 819 (m), 575 (s);

$[\alpha]_D^{25.0}$ = + 44.4 (c. 1.1, $CHCl_3$);

HRMS (−ESI) *m/z* found $[M-H]^-$ 422.0256, $[C_{14}H_{14}ClF_5NO_4S]^-$ requires 422.0258, (δ = − 0.5 ppm);

Chiral SFC Analysis CHIRALPAK IH (CO_2:MeOH, 98:2, 2.5 mL min^{-1}, 40 °C) t_R = 6.0 (minor), 9.0 (major) minutes.

2,2,3,3,3-pentafluoropropyl ((S)-(4-bromophenyl)((S)-tetrahydrofuran-2-yl)methyl)sulfamate (**4ad**)

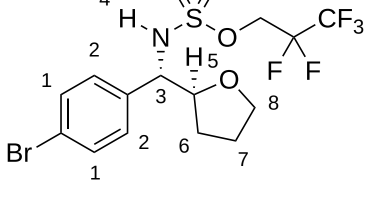

Prepared according to **GP9** using (*Z*)-5-(4-bromophenyl)pent-4-en-1-ol (**1ad**) as the starting material. Following the cyclisation protocol (2 hours) purification by flash column chromatography (SiO_2, 0-3% v/v Acetone in $CHCl_3$) afforded the title compound as an off-white solid (35 mg, 0.075 mmol, 75%, 91% *ee*).

***R_f* value** = 0.54 (10% v/v Acetone in $CHCl_3$);

^{1}H NMR (500 MHz, $CDCl_3$) δ 7.49 (m, 2H, H-1), 7.23 (m, 2H, H-2), 5.70 (br s, 1H, H-4), 4.35-4.26 (m, 2H, H-3, H-9_A), 4.21 (q, *J* = 12.2 Hz, 1H, H-9_B), 3.99 (q, *J* = 6.6 Hz, 1H, H-5), 3.94 (m, 1H, H-8_A), 3.82 (m, 1H, H-8_B), 2.01-1.83 (m, 3H, H-6_A, H-7_A, H-7_B), 1.71 (m, 1H, H-6_B) ppm;

^{13}C NMR (126 MHz, $CDCl_3$) δ 137.4, 132.1, 129.2, 122.7, 118.2 (qt, $J_{C\text{-}F}$ = 287, 34.0 Hz), 111.3 (tq, $J_{C\text{-}F}$ =257, 38.1 Hz), 81.0, 69.0, 64.0 (t, $J_{C\text{-}F}$ = 28.0 Hz), 61.9, 29.0, 25.7 ppm;

^{19}F NMR (471 MHz, $CDCl_3$) δ − 84.7, − 124.6 ppm;

IR (film, cm^{-1}): ν_{max} 3299 (w), 2881 (w), 1490 (w), 1431 (w), 1372 (m), 1204 (s), 1182 (s), 1109 (m), 1071 (m), 1010 (m), 963 (m);

$[\alpha]_D^{25.0}$ = + 42.4 (c. 1.1, $CHCl_3$);

HRMS (−ESI) *m/z* found $[M-H]^-$ 465.9749, $[C_{14}H_{14}BrF_5NO_4S]^-$ requires 465.9753, (δ = − 0.8 ppm);

Chiral SFC Analysis: CHIRALPAK IH (CO_2:MeOH, 98:2, 2.5 mL min^{-1}, 40 °C) t_R = 7.4 (minor), 11.6 (major) minutes.

2,2,3,3,3-pentafluoropropyl ((2S,3R)-2-phenyltetrahydro-2H-pyran-3-yl)sulfamate (**6**)

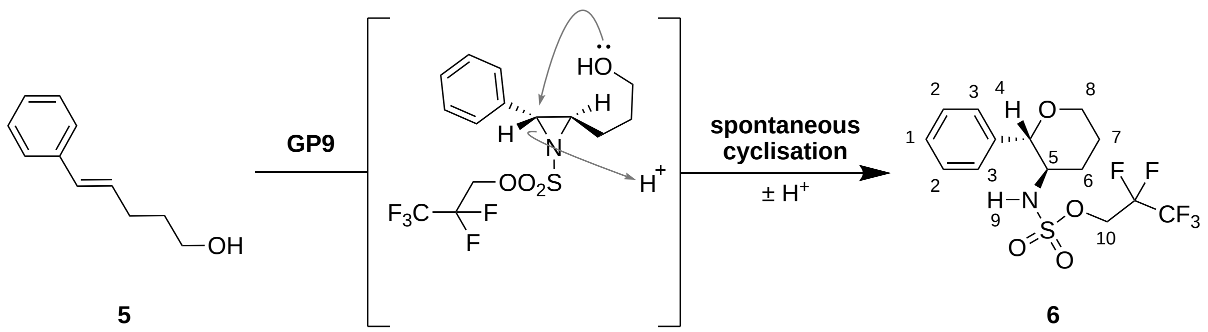

5 **6**

Prepared according to **GP9** using (*E*)-5-phenylpent-4-en-1-ol (**5**) as the starting material. The aziridine cyclised spontaneously in the reaction. Purification by flash column chromatography (SiO_2, 0-1% v/v Et_2O in CH_2Cl_2) afforded the title compound as an off-white solid (10 mg, 0.026 mmol, 26%, 38% *ee*).

***R*$_f$ value** = 0.42 (5% v/v Et_2O in CH_2Cl_2);

^{1}H NMR (500 MHz, $CDCl_3$) δ 7.42-7.33 (m, 5H, H-1, H-2, H-3), 4.40 (d, *J* = 7.6 Hz, 1H, H-9), 4.08 (m, 1H, H-8$_A$), 4.01 (d, *J* = 9.5 Hz, 1H, H-4), 3.92 (q, *J* = 12.4 Hz, 1H, H-10$_A$), 3.53 (td, *J* = 11.8, 2.6 Hz, 1H, H-8$_B$), 3.45 (m, 1H, H-5), 3.31 (q, *J* = 12.3 Hz, 1H, H-10$_B$), 2.54 (m, 1H, H-6$_A$), 1.90 (qt, *J* = 12.4, 4.2 Hz, 1H, H-7$_A$), 1.80 (m, 1H, H-7$_B$), 1.63 (m, 1H, H-6$_B$) ppm;

^{13}C NMR (101 MHz, $CDCl_3$) δ 138.6, 129.3, 129.1, 127.8, 118.2 (qt, $J_{C\text{-}F}$ = 286, 34.1 Hz), 111.2 (tq, $J_{C\text{-}F}$ = 257, 38.6 Hz), 83.5, 68.5, 63.7 (t, $J_{C\text{-}F}$ = 28.2 Hz), 56.7, 31.7, 25.6 ppm;

^{19}F NMR (376 MHz, $CDCl_3$) δ − 83.5, − 123.6 ppm;

IR (film, cm^{-1}): ν_{max} 2959 (w), 1455 (m), 1374 (m), 1261 (m), 1204 (s), 1181 (s), 1088 (m), 1037 (m), 1006 (m), 761 (m);

$[\alpha]_D^{25.0}$ = + 10.1 (c = 0.47, $CHCl_3$);

HRMS (−ESI) *m/z* found $[M{-}H]^-$ 388.0645, $[C_{14}H_{15}F_5NO_4S]^-$ requires 388.0647, (δ = − 0.7 ppm);

Chiral SFC Analysis CHIRAL ART SJ (CO_2:MeOH, 96:4, 2.5 mL min^{-1}, 40 °C) t_R = 3.4 (major), 3.8 (minor) minutes.

2,2,2-trichloroethyl ((S)-phenyl((S)-tetrahydrofuran-2-yl)methyl)sulfamate (**7**)

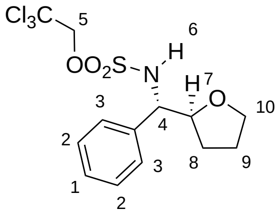

Prepared according to **GP9** using (*Z*)-5-phenylpent-4-en-1-ol (**1a**) as the starting material. In this case the following procedural modification was used: The nitrogen source was changed from 2,2,3,3-pentafluoropropyl sulfamate to 2,2,2-trichloroethyl sulfamate. Following the cyclisation protocol (3 hours), purification by flash column chromatography (SiO_2, 0-1% v/v Et_2O in CH_2Cl_2) afforded the title compound as a yellow amorphous solid (20.9 mg, 0.054 mmol, 54%, 88% *ee*).

***Rf* value** = 0.64 (100% CH_2Cl_2);

^{1}H NMR (400 MHz, $CDCl_3$) δ 7.29-7.39 (m, 5H, H-1, H-2, H-3), 5.69 (d, *J* = 5.2 Hz, 1H, H-6), 4.39 (t, *J* = 5.6 Hz, 1H, H-4), 4.33 (d, *J* = 10.7 Hz, 1H, H-5a), 4.27 (d, *J* = 10.7 Hz, 1H, H-5b), 4.06 (q, *J* = 6.5 Hz, 1H, H-7), 3.95-4.00 (m, 1H, H-10a), 3.81-3.86 (m, 1H, H-10b), 1.85-2.02 (m, 3H, H-8a, H-9a, H-9b), 1.73-1.83 (m, 1H, H-8b) ppm;

^{13}C NMR (101 MHz, $CDCl_3$) δ 138.6, 129.0, 128.6, 127.7, 93.4, 81.3, 78.2, 69.0, 62.3, 29.0, 25.7 ppm;

IR (film, cm^{-1}): ν_{max} 3297 (br, m), 2952 (m), 2874 (m), 1495 (w), 1450 (s), 1367 (s), 1258 (w), 1179 (s), 1081 (m), 1060 (m), 1018 (m), 965 (m), 923 (w), 901 (w), 855 (s), 757 (s), 724 (s), 701 (s), 630 (w), 572 (m), 537 (m);

$[\alpha]_D^{25.0}$ = + 31.7 (c. 1.0, $CHCl_3$);

HRMS (−ESI) *m/z* Found $[M-H]^-$ 385.9791, $[C_{13}H_{15}Cl_3NO_4S]^-$ requires 385.9793, (δ = − 0.5 ppm);

Chiral SFC Analysis CHIRALPAK IG (CO_2:MeOH, 90:10, 2.5 mL min^{-1}, 40 °C) t_R = 6.4 (minor), 8.5 (major) minutes.

Characterisation of Homoallylic Styrenyl Aziridines

2,2,3,3,3-pentafluoropropyl (2R,3S)-2-(2-hydroxyethyl)-3-phenylaziridine-1-sulfonate (**9a**)

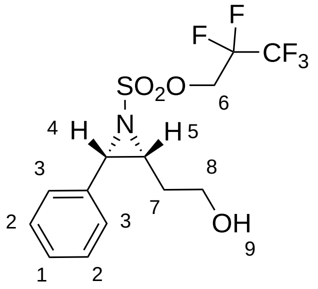

Prepared according to **GP9** using (*Z*)-4-phenylbut-3-en-1-ol (**8a**) as the starting material. Purification by flash column chromatography (SiO_2, 0-10% v/v Acetone in $CHCl_3$) afforded the title compound as a yellow oil (25.3 mg, 0.067 mmol, 67%, 87% *ee*).

***R_f* value** = 0.57 (10% v/v Acetone in $CHCl_3$);

^{1}H NMR (500 MHz, $CDCl_3$) δ 7.30-7.40 (m, 5H, H-1, H-2, H-3), 4.70-4.82 (m, 2H, H-6a, H-6b), 4.11 (d, *J* = 7.4 Hz, 1H, H-4), 3.75 (t, *J* = 5.6 Hz, 2H, H-8a, H-8b), 3.34 (ddd, *J* = 8.3 Hz, 7.6 Hz, 5.0 Hz, 1H, H-5), 1.64-1.70 (m, 2H, H-7a, H-9), 1.46-1.53 (m, 1H, H-7b) ppm;

^{13}C NMR (126 MHz, $CDCl_3$) δ 131.5, 128.8, 128.7, 127.3, 118.3 (qt, J_{C-F} = 286.3 Hz, 34.6 Hz), 111.2 (tq, J_{C-F} = 257.4 Hz, 38.3 Hz), 65.8 (t, J_{C-F} = 28.6 Hz), 60.2, 47.7, 46.4, 29.0 ppm;

^{19}F NMR (376 MHz, $CDCl_3$) δ – 83.5 (s), – 123.5 (s) ppm;

IR (film, cm^{-1}): ν_{max} 3397 (br, w), 2963 (w), 1454 (w), 1376 (s), 1205 (s), 1181 (s), 1109 (m), 1042 (m), 1007 (m), 937 (w), 849 (m), 771 (w), 700 (w), 623 (w), 591 (m);

$[\alpha]_D^{25.0}$ = + 32.4 (c. 0.3, $CHCl_3$);

HRMS (−ESI) *m/z* Found $[M+CHO_2]^-$ 420.0535, $[C_{14}H_{15}F_5NO_6S]^-$ requires 420.0546, (δ = – 2.6 ppm);

Chiral SFC Analysis CHIRAL ART SC (CO_2:MeOH, 98.5:1.5, 2.5 mL min^{-1}, 40 °C) t_R = 4.8 (major), 5.6 (minor) minutes.

The opposite enantiomer (*ent*-**9a**) was prepared as above with the following procedural modification: Rh_2(**B**)$_2$•(**C5**)$_2$•(Pyr)$_2$ was used in place of Rh_2(**B**)$_2$•(**C3**)$_2$•(Pyr)$_2$. Purification by flash column chromatography (SiO_2, 0-10% v/v acetone in $CHCl_3$) afforded 2,2,3,3,3-pentafluoropropyl (2*S*,3*R*)-2-(2-hydroxyethyl)-3-phenylaziridine-1-sulfonate (32.2 mg, 0.086 mmol, 86%, 89% *ee*). For this enantiomer, $[\alpha]_D^{25.0}$ = – 86.3 (c. 1.0, $CHCl_3$).

When Rh_2(**B**)$_2$•(**C4**)$_2$•(Pyr)$_2$ was used in place of Rh_2(**B**)$_2$•(**C5**)$_2$•(Pyr)$_2$ under identical conditions, *ent*-**9a** was obtained in 89% yield and 81% *ee*. In this case $[\alpha]_D^{26.0}$ = – 75.2 (c. 1.0, $CHCl_3$).

2,2,3,3,3-pentafluoropropyl (2R,3S)-2-(2-hydroxyethyl)-3-(o-tolyl)aziridine-1-sulfonate (**9b**)

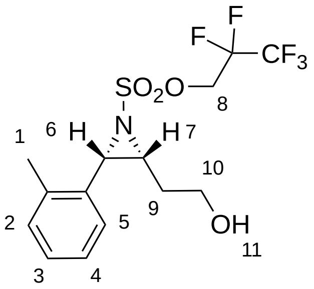

Prepared according to **GP9** using (*Z*)-4-(*o*-tolyl)but-3-en-1-ol (**8b**) as the starting material. In this case the following procedural modification was used: The reaction time was 36 hours.

Purification by flash column chromatography (SiO_2, 0-10% v/v Acetone in $CHCl_3$) afforded the title compound as a colourless oil (39.0 mg, quant., 91% *ee*).

***R_f* value** = 0.54 (10% v/v Acetone in $CHCl_3$);

^{1}H NMR (500 MHz, $CDCl_3$) δ 7.29 (d, *J* = 7.5 Hz, 1H, H-5), 7.24-7.27 (m, 1H, H-3), 7.19-7.22 (m, 2H, H-2, H-4), 4.75-4.85 (m, 2H, H-8a, H-8b), 4.09 (d, *J* = 7.3 Hz, 1H, H-6), 3.70-3.77 (m, 2H, H-10a, H-10b), 3.41 (ddd, *J* = 8.6 Hz, 7.5 Hz, 4.5 Hz, 1H, H-7), 2.36 (s, 3H, H-1), 1.69 (br s, 1H, H-11), 1.62-1.68 (m, 1H, H-9a), 1.28-1.35 (m, 1H, H-9b) ppm;

^{13}C NMR (126 MHz, $CDCl_3$) δ 136.3, 130.3, 129.9, 128.5, 127.1, 126.2, 118.3 (qt, $J_{C\text{-}F}$ = 286.2 Hz, 34.3 Hz), 111.3 (tq, $J_{C\text{-}F}$ = 257.0 Hz, 38.6 Hz), 65.8 (t, $J_{C\text{-}F}$ = 28.6 Hz), 60.2, 46.9, 46.0, 29.1, 19.0 ppm;

^{19}F NMR (376 MHz, $CDCl_3$) δ – 84.4 (s), – 124.5 (s) ppm;

IR (film, cm^{-1}): ν_{max} 3397 (br, w), 2964 (w), 1493 (w), 1461 (w), 1375 (s), 1294 (w), 1203 (s), 1179 (s), 1108 (m), 1040 (s), 1005 (s), 939 (m), 902 (w), 848 (m), 766 (m), 738 (m), 720 (w), 670 (w), 623 (w), 592 (m), 517 (w), 453 (w);

$[\boldsymbol{\alpha}]_{\mathbf{D}}^{\mathbf{25.0}}$ = + 97.5 (c. 1.0, $CHCl_3$);

HRMS (−ESI) *m/z* Found $[M{-}H]^-$ 388.0646, $[C_{14}H_{15}F_5NO_4S]^-$ requires 388.0647, (δ = – 0.3 ppm);

Chiral SFC Analysis CHIRAL ART SC (CO_2:MeOH, 98.5:1.5, 2.5 mL min^{-1}, 40 °C) t_R = 4.0 (major), 5.0 (minor) minutes.

2,2,3,3,3-pentafluoropropyl (2R,3S)-2-(2-hydroxyethyl)-3-(2-methoxyphenyl)aziridine-1-sulfonate (**9c**)

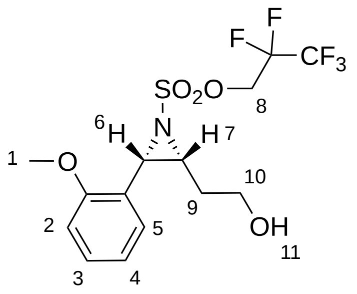

Prepared according to **GP9** using (*Z*)-4-(2-methoxyphenyl)but-3-en-1-ol (**8c**) as the starting material. In this case the following procedural modification was used: The reaction time was 36 hours. Purification by flash column chromatography (SiO_2, 0-9% v/v Acetone in $CHCl_3$) afforded the title compound as a white amorphous solid (32.3 mg, 0.080 mmol, 80%, 82% *ee*).

***R_f* value** = 0.51 (10% v/v Acetone in $CHCl_3$);

^{1}H NMR (500 MHz, $CDCl_3$) δ 7.32 (td, *J* = 8.0 Hz, 1.6 Hz, 1H, H-3), 7.25 (d, *J* = 8.9 Hz, 1H, H-5), 6.96 (t, *J* = 7.4 Hz, 1H, H-4), 6.90 (d, *J* = 8.2 Hz, 1H, H-2), 4.71-4.82 (m, 2H, H-8a, H-8b), 4.23 (d, *J* = 7.4 Hz, 1H, H-6), 3.86 (s, 3H, H-1), 3.72 (t, *J* = 5.4 Hz, 2H, H-10a, H-10b), 3.35 (td, *J* = 7.8 Hz, 5.3 Hz, 1H, H-7), 1.76 (br s, 1H, H-11), 1.61-1.67 (m, 1H, H-9a), 1.42-1.49 (m, 1H, H-9b) ppm;

^{13}C NMR (126 MHz, $CDCl_3$) δ 157.9, 129.8, 128.3, 120.6, 120.1, 118.3 (qt, $J_{C\text{-}F}$ = 286.1 Hz, 34.2 Hz), 111.3 (tq, $J_{C\text{-}F}$ = 257.0 Hz, 38.5 Hz), 110.4, 65.8 (t, $J_{C\text{-}F}$ = 28.5 Hz), 60.2, 55.5, 46.3, 45.2, 29.3 ppm;

^{19}F NMR (376 MHz, $CDCl_3$) δ – 84.4 (s), – 124.5 (s) ppm;

IR (film, cm^{-1}): ν_{max} 3399 (br, w), 2965 (w), 1605 (w), 1589 (w), 1496 (m), 1465 (w), 1440 (w), 1373 (s), 1288 (w), 1253 (m), 1202 (s), 1177 (s), 1109 (s), 1043 (s), 1005 (s), 936 (s), 903 (m), 845 (s), 755 (s), 669 (w), 623 (w), 593 (s), 517 (w), 464 (w), 418 (w);

$[\alpha]_D^{25.0}$ = + 78.9 (c. 1.6, $CHCl_3$);

HRMS (–ESI) *m/z* Found $[M+HO]^-$ 422.0709, $[C_{14}H_{17}F_5NO_6S]^-$ requires 422.0702, (δ = + 1.7 ppm);

Chiral SFC Analysis CHIRAL ART SC (CO_2:MeOH, 97.5:2.5, 1.25 mL min^{-1}, 40 °C) t_R = 6.7 (major), 7.6 (minor) minutes.

2,2,3,3,3-pentafluoropropyl (2S,3R)-2-(2-fluorophenyl)-3-(2-hydroxyethyl)aziridine-1-sulfonate (**9d**)

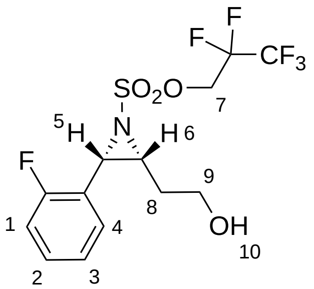

Prepared according to **GP9** using (*Z*)-4-(2-fluorophenyl)but-3-en-1-ol (**8d**) as the starting material. Purification by flash column chromatography (SiO_2, 0-4% v/v Et_2O in CH_2Cl_2) afforded the title compound as a colourless oil (39.3 mg, quant., 90% *ee*).

***R**f* **value** = 0.60 (10% v/v Acetone in $CHCl_3$);

^{1}H NMR (500 MHz, $CDCl_3$) δ 7.30-7.37 (m, 2H, H-2, H-4), 7.16 (t, *J* = 7.5 Hz, 1H, H-3), 7.10 (t, *J* = 9.2 Hz, 1H, H-1), 4.73-4.83 (m, 2H, H-7a, H-7b), 4.20 (d, *J* = 7.3 Hz, 1H, H-5), 3.76 (t, *J* = 6.0 Hz, 2H, H-9a, H-9b), 3.39 (ddd, *J* = 8.2 Hz, 7.6 Hz, 4.8 Hz, 1H, H-6), 1.90 (br s, 1H, H-10), 1.69-1.76 (m, 1H, H-8a), 1.40-1.49 (m, 1H, H-8b) ppm;

^{13}C NMR (126 MHz, $CDCl_3$) δ 161.4 (d, J_{C-F} = 247.6 Hz), 130.5 (d, J_{C-F} = 8.0 Hz), 129.0 (d, J_{C-F} = 2.6 Hz), 124.4 (d, J_{C-F} = 3.1 Hz), 119.3 (d, J_{C-F} = 13.7 Hz), 118.3 (qt, J_{C-F} = 286.2 Hz, 34.2 Hz),

115.7 (d, J_{C-F} = 20.3 Hz), 111.3 (tq, J_{C-F} = 257.3 Hz, 38.4 Hz), 65.9 (t, J_{C-F} = 28.5 Hz), 60.0, 46.0, 43.2 (d, J_{C-F} = 4.8 Hz), 29.3 ppm;

^{19}F NMR (376 MHz, $CDCl_3$) δ – 84.4 (s), – 118.7 (s), – 124.5 (s) ppm;

IR (film, cm^{-1}): ν_{max} 3300 (br, w), 2924 (w), 1588 (w), 1494 (m), 1457 (m), 1375 (s), 1202 (s), 1178 (s), 1107 (s), 1005 (s), 940 (m), 903 (w), 847 (m), 760 (s), 720 (w), 669 (w), 624 (w), 593 (m), 517 (m);

$[\alpha]_D^{25.0}$ = + 61.6 (c. 1.2, $CHCl_3$);

HRMS (–ESI) *m/z* Found $[M+CHO_2]^-$ 438.0464, $[C_{14}H_{14}F_6NO_6S]^-$ requires 438.0452, (δ = + 2.7 ppm);

Chiral SFC Analysis CHIRALPAK IG (CO_2:MeOH, 98.5:1.5, 1.5 mL min^{-1}, 40 °C) t_R = 6.8 (major), 8.5 (minor) minutes.

2,2,3,3,3-pentafluoropropyl (2S,3R)-2-(3-chlorophenyl)-3-(2-hydroxyethyl)aziridine-1-sulfonate (**9e**)

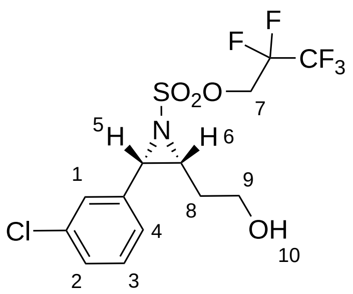

Prepared according to **GP9** using (*Z*)-4-(3-chlorophenyl)but-3-en-1-ol (**8e**) as the starting material. Purification by two consecutive flash column chromatography procedures (SiO_2, 0-

10% v/v Acetone in $CHCl_3$ followed by 0-5% v/v Et_2O in CH_2Cl_2) afforded the title compound as a colourless oil (33.2 mg, 0.081 mmol, 81%, 91% *ee*).

***R**$_f$* **value** = 0.50 (10% v/v Acetone in $CHCl_3$);

^{1}H NMR (500 MHz, $CDCl_3$) δ 7.30-7.35 (m, 3H, H-1, H-3, H-4), 7.21 (dt, *J* = 6.7 Hz, 1.7 Hz, 1H, H-2), 4.72-4.82 (m, 2H, H-7a, H-7b), 4.06 (d, *J* = 7.4 Hz, 1H, H-5), 3.73-3.80 (m, 2H, H-9a, H-9b), 3.33 (td, *J* = 8.1 Hz, 5.0 Hz, 1H, H-6), 1.64-1.70 (m, 2H, H-8a, H-10), 1.44-1.51 (m, 1H, H-8b) ppm;

^{13}C NMR (126 MHz, $CDCl_3$) δ 134.9, 133.7, 130.1, 129.0, 127.5, 125.6, 118.3 (qt, $J_{C\text{-}F}$ = 286.2 Hz, 34.2 Hz), 113.3 (tq, $J_{C\text{-}F}$ = 257.1 Hz, 38.6 Hz), 65.9 (t, $J_{C\text{-}F}$ = 28.6 Hz), 60.1, 46.8, 46.4, 29.0 ppm;

^{19}F NMR (376 MHz, $CDCl_3$) δ – 84.4 (s), – 124.5 (s) ppm;

IR (film, cm^{-1}): ν_{max} 3336 (br, w), 1601 (w), 1574 (w), 1376 (s), 1204 (s), 1181 (s), 1108 (m), 1042 (m), 1008 (s), 939 (m), 851 (m), 788 (m), 703 (w), 683 (w), 624 (w), 595 (m), 518 (m);

$[\alpha]_D^{25.0}$ = + 70.6 (c. 1.2, $CHCl_3$);

HRMS (−ESI) *m/z* Found [M−H]$^-$ 408.0103, $[C_{13}H_{12}ClF_5NO_4S]^-$ requires 408.0101, (δ = + 0.5 ppm);

Chiral SFC Analysis CHIRAL ART SB (CO_2:MeOH, 98.5:1.5, 2.5 mL min^{-1}, 40 °C) t_R = 7.6 (minor), 9.0 (major) minutes.

2,2,3,3,3-pentafluoropropyl (2R,3S)-2-(2-hydroxyethyl)-3-(3-(trifluoromethoxy)phenyl)aziridine-1-sulfonate (**9f**)

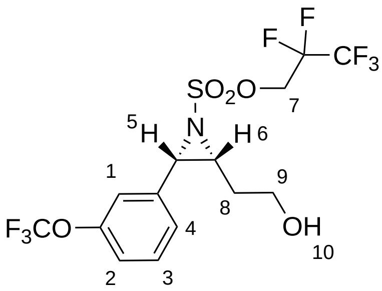

Prepared according to **GP9** using (*Z*)-4-(3-(trifluoromethoxy)phenyl)but-3-en-1-ol (**8f**) as the starting material. Purification by two consecutive flash column chromatography procedures (SiO_2, 0-10% v/v Acetone in $CHCl_3$ followed by 0-5% v/v Et_2O in CH_2Cl_2) afforded the title compound as a colourless oil (37.6 mg, 0.082 mmol, 82%, 91% *ee*).

***R_f* value** = 0.50 (10% v/v Acetone in $CHCl_3$);

^{1}H NMR (500 MHz, $CDCl_3$) δ 7.42 (t, *J* = 8.0 Hz, 1H, H-3), 7.27 (d, *J* = 7.7 Hz, 1H, H-4), 7.20-7.22 (m, 1H, H-2), 7.17 (br s, 1H, H-1), 4.71-4.82 (m, 2H, H-7a, H-7b), 4.10 (d, *J* = 7.4 Hz, 1H, H-5), 3.73-3.80 (m, 2H, H-9a, H-9b), 3.36 (ddd, *J* = 8.2 Hz, 7.5 Hz, 5.0 Hz, 1H, H-6), 1.63-1.69 (m, 2H, H-8a, H-10), 1.44-1.50 (m, 1H, H-8b) ppm;

^{13}C NMR (126 MHz, $CDCl_3$) δ 149.6, 134.1, 130.4, 125.9, 121.2, 120.5 (q, $J_{C\text{-}F}$ = 257.7 Hz), 120.5 (qt, $J_{C\text{-}F}$ = 286.7 Hz, 34.2 Hz), 119.9, 111.2 (tq, $J_{C\text{-}F}$ = 258.0 Hz, 39.1 Hz), 65.9 (t, $J_{C\text{-}F}$ = 28.5 Hz), 60.0, 46.9, 46.5, 29.0 ppm;

^{19}F NMR (376 MHz, $CDCl_3$) δ – 58.9 (s), – 84.5 (s), – 124.6 (s) ppm;

IR (film, cm^{-1}): ν_{max} 3407 (br, w), 1591 (w), 1492 (w), 1450 (w), 1379 (m), 1261 (s), 1208 (s), 1180 (s), 1109 (w), 1009 (m), 969 (w), 936 (w), 855 (w), 703 (w), 624 (w), 596 (w), 516 (w), 455 (w);

$[\alpha]_D^{25.0}$ = + 66.8 (c. 1.3, $CHCl_3$);

HRMS (−ESI) *m/z* Found $[M-H]^-$ 458.0313, $[C_{14}H_{12}F_8NO_5S]^-$ requires 458.0314, (δ = – 0.2 ppm);

Chiral HPLC Analysis CHIRALPAK IC (Hexane:^{i}PrOH, 97:3, 1.0 mL min^{-1}, 30 °C) t_R = 13.8 (major), 22.2 (minor) minutes.

Ethyl 3-((2S,3R)-3-(2-hydroxyethyl)-1-((2,2,3,3,3-pentafluoropropoxy)sulfonyl)aziridin-2-yl)benzoate (**9g**)

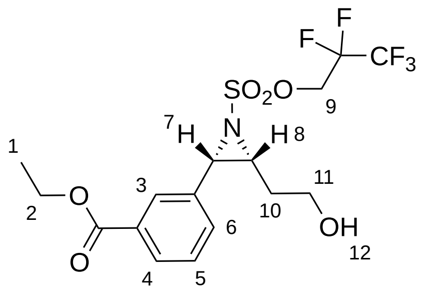

Prepared according to **GP9** using ethyl (*Z*)-3-(4-hydroxybut-1-en-1-yl)benzoate (**8g**) as the starting material. Purification by flash column chromatography (SiO_2, 0-15% v/v Acetone in $CHCl_3$) afforded the title compound as a colourless oil (17.3 mg, 0.039 mmol, 39%, 89% *ee*).

***R_f* value** = 0.41 (10% v/v Acetone in $CHCl_3$);

^{1}H NMR (500 MHz, $CDCl_3$) δ 8.03 (dt, *J* = 7.6 Hz, 1.4 Hz, 1H, H-4), 7.99 (br s, 1H, H-3), 7.51 (dt, *J* = 7.7 Hz, 1.2 Hz, 1H, H-6), 7.46 (t, *J* = 7.6 Hz, 1H, H-5), 4.73-4.84 (m, 2H, H-9a, H-9b), 4.36-4.42 (m, 2H, H-2a, H-2b), 4.14 (d, *J* = 7.4 Hz, 1H, H-7), 3.72-3.79 (m, 2H, H-11a, H-11b), 3.37 (ddd, *J* = 8.1 Hz, 7.5 Hz, 5.0 Hz, 1H, H-8), 1.63-1.69 (m, 2H, H-10a, H-12), 1.43-1.50 (m, 1H, H-10b), 1.40 (t, *J* = 7.1 Hz, 3H, H-1) ppm;

^{13}C NMR (126 MHz, $CDCl_3$) δ 166.1, 132.1, 131.7, 131.2, 129.9, 128.9, 128.4, 118.3 (qt, $J_{C\text{-}F}$ = 286.2 Hz, 34.2 Hz), 111.3 (tq, $J_{C\text{-}F}$ = 257.0 Hz, 38.7 Hz), 65.9 (t, $J_{C\text{-}F}$ = 28.5 Hz), 61.4, 60.1, 47.2, 46.4, 29.0, 14.4 ppm;

^{19}F NMR (376 MHz, $CDCl_3$) δ – 84.4 (s), – 124.5 (s) ppm;

IR (film, cm^{-1}): ν_{max} 3400 (br, w), 2970 (w), 1717 (s), 1448 (w), 1372 (s), 1287 (s), 1202 (s), 1180 (s), 1107 (s), 1008 (s), 935 (m), 851 (m), 759 (m), 699 (m), 671 (w), 623 (w), 593 (w), 517 (w);

$[\alpha]_D^{26.3}$ = + 47.2 (c. 1.0, $CHCl_3$);

HRMS (+ESI) *m/z* Found $[M+H]^+$ 448.0856, $[C_{16}H_{19}F_5NO_6S]^+$ requires 448.0848, (δ = + 1.6 ppm);

Chiral SFC Analysis CHIRALPAK IE (CO_2:MeOH, 97:3, 2.5 mL min^{-1}, 40 °C) t_R = 6.0 (major), 7.0 (minor) minutes.

2,2,3,3,3-pentafluoropropyl (2S,3R)-2-(4-chlorophenyl)-3-(2-hydroxyethyl)aziridine-1-sulfonate (**9h**)

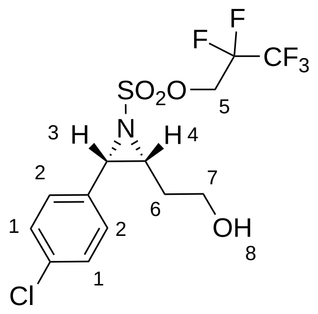

Prepared according to **GP9** using (*Z*)-4-(4-chlorophenyl)but-3-en-1-ol (**8h**) as the starting material. Purification by flash column chromatography (SiO_2, 0-10% v/v Acetone in $CHCl_3$) afforded the title compound as a yellow oil (38.1 mg, 0.093 mmol, 93%, 85% *ee*).

***R_f* value** = 0.37 (10% v/v Acetone in $CHCl_3$);

^{1}**H NMR** (500 MHz, $CDCl_3$) δ 7.35-7.37 (m, 2H, H-1), 7.24-7.27 (m, 2H, H-2), 4.70-4.81 (m, 2H, H-5a, H-5b), 4.06 (d, J = 7.3 Hz, 1H, H-3), 3.76 (t, J = 5.0 Hz, 2H, H-7a, H-7b), 3.31-3.35 (m, 1H, H-4), 1.62-1.68 (m, 1H, H-6a), 1.59 (br s, 1H, H-8), 1.44-1.51 (m, 1H, H-6b) ppm;

^{13}**C NMR** (126 MHz, $CDCl_3$) δ 134.8, 130.1, 129.0, 128.7, 118.3 (qt, $J_{C\text{-}F}$ = 286.1 Hz, 34.3 Hz), 111.2 (tq, $J_{C\text{-}F}$ = 257.1 Hz, 38.6 Hz), 65.9 (t, $J_{C\text{-}F}$ = 28.6 Hz), 60.1, 47.0, 46.4, 28.9 ppm;

^{19}**F NMR** (376 MHz, $CDCl_3$) δ – 84.4 (s), – 124.5 (s) ppm;

IR (film, cm^{-1}): ν_{max} 3400 (br, w), 2928 (w), 1494 (w), 1375 (m), 1204 (s), 1182 (s), 1107 (m), 1092 (w), 1041 (m), 1012 (s), 939 (m), 900 (w), 837 (m), 719 (w), 669 (w), 623 (w), 590 (m), 518 (w);

$[\alpha]_D^{25.0}$ = + 63.6 (c. 1.5, $CHCl_3$);

HRMS (−ESI) *m/z* Found $[M+HO]^-$ 426.0207, $[C_{13}H_{14}ClF_5NO_5S]^-$ requires 426.0207, (δ = + 0.0 ppm);

Chiral SFC Analysis CHIRAL ART SC (CO_2:MeOH, 98:2, 2.5 mL min^{-1}, 40 °C) t_R = 4.6 (major), 5.5 (minor) minutes.

4-((2S,3R)-3-(2-hydroxyethyl)-1-((2,2,3,3,3-pentafluoropropoxy)sulfonyl)aziridin-2-yl)phenyl acetate (**9i**)

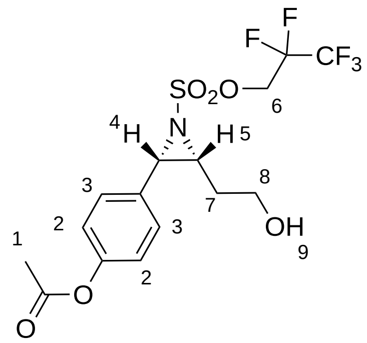

Prepared according to **GP9** using (*Z*)-4-(4-hydroxybut-1-en-1-yl)phenyl acetate (**8i**) as the starting material. Purification by flash column chromatography (SiO_2, 0-15% v/v Et_2O in CH_2Cl_2) afforded the title compound as a colourless oil (28.7 mg, 0.066 mmol, 66%, 87% *ee*).

***R_f* value** = 0.36 (12% v/v Et_2O in CH_2Cl_2);

^{1}H NMR (500 MHz, $CDCl_3$) δ 7.31-7.34 (m, 2H, H-3), 7.09-7.12 (m, 2H, H-2), 4.70-4.81 (m, 2H, H-6a, H-6b), 4.08 (d, *J* = 7.4 Hz, 1H, H-4), 3.73 (t, *J* = 5.8 Hz, 2H, H-8a, H-8b), 3.32 (ddd, *J* = 8.2 Hz, 7.6 Hz, 5.2 Hz, 1H, H-5), 2.30 (s, 3H, H-1), 1.78 (br s, 1H, H-9), 1.62-1.69 (m, 1H, H-7a), 1.46-1.52 (m, 1H, H-7b);

^{13}C NMR (126 MHz, $CDCl_3$) δ 169.5, 150.9, 129.1, 128.5, 122.0, 118.3 (qt, J_{C-F} = 286.2 Hz, 34.2 Hz), 111.2 (tq, J_{C-F} = 257.0 Hz, 38.6 Hz), 65.8 (t, J_{C-F} = 28.5 Hz), 60.1, 47.1, 46.5, 29.0, 21.2 ppm;

^{19}F NMR (376 MHz, $CDCl_3$) δ – 84.4 (s), – 124.5 (s) ppm;

IR (film, cm^{-1}): ν_{max} 3416 (br, w), 2967 (w), 1758 (s), 1610 (w), 1510 (m), 1372 (s), 1180 (br, s), 1107 (s), 1040 (s), 1007 (s), 914 (s), 850 (s), 665 (w), 623 (w), 590 (m), 518 (w);

$[\alpha]_D^{25.0}$ = + 73.3 (c. 1.1, $CHCl_3$);

HRMS (–ESI) *m/z* Found $[M+CHO_2]^-$ 478.0606, $[C_{16}H_{17}F_5NO_8S]^-$ requires 478.0601, (δ = + 1.0 ppm);

Chiral SFC Analysis CHIRAL ART SC (CO_2:MeOH, 97:3, 2.5 mL min^{-1}, 40 °C) t_R = 4.4 (major), 5.3 (minor) minutes.

2,2,3,3,3-pentafluoropropyl (2R,3S)-2-(2-hydroxyethyl)-3-(4-(trifluoromethyl)phenyl)aziridine-1-sulfonate (**9j**)

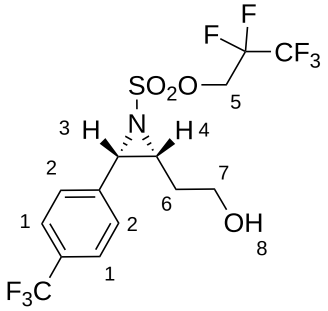

Prepared according to **GP9** using (*Z*)-4-(4-(trifluoromethyl)phenyl)but-3-en-1-ol (**8j**) as the starting material. In this case the following procedural modification was used: The reaction time was 36 hours. Purification by two consecutive flash column chromatography procedures (SiO_2, 0-10% v/v Acetone in $CHCl_3$ followed by 0-30% v/v Acetone in Petrol) afforded the title compound as a yellow oil (35.3 mg, 0.080 mmol, 80%, 95% *ee*).

***R$_f$* value** = 0.39 (10% v/v Acetone in $CHCl_3$);

^{1}H NMR (500 MHz, $CDCl_3$) δ 7.65 (d, *J* = 8.0 Hz, 2H, H-1), 7.46 (d, *J* = 8.0 Hz, 2H, H-2), 4.71-4.84 (m, 2H, H-5a, H-5b), 4.14 (d, *J* = 7.4 Hz, 1H, H-3), 3.72-3.81 (m, 2H, H-7a, H-7b), 3.35-3.41 (m, 1H, H-4), 1.61-1.69 (m, 2H, H-6a, H-8), 1.41-1.50 (m, 1H, H-6b) ppm;

^{13}C NMR (126 MHz, $CDCl_3$) δ 135.7, 131.0 (q, J_{C-F} = 32.7 Hz), 127.9, 125.8 (q, J_{C-F} = 3.4 Hz), 124.0 (q, J_{C-F} = 272.2Hz), 118.2 (qt, J_{C-F} = 286.2 Hz, 34.2 Hz), 111.2 (tq, J_{C-F} = 257.1 Hz, 38.6 Hz), 65.9 (t, J_{C-F} = 28.5 Hz), 60.0, 46.9, 46.5, 29.0 ppm;

^{19}F NMR (376 MHz, $CDCl_3$) δ – 63.7 (s), – 84.4 (s), – 124.5 (s) ppm;

IR (film, cm^{-1}): ν_{max} 3400 (br, w), 2961 (w), 1623 (w), 1377 (m), 1326 (s), 1207 (s), 1179 (s), 1128 (s), 1109 (s), 1067 (s), 1041 (s), 1016 (s), 939 (m), 900 (w), 848 (s), 654 (w), 623 (w), 603 (w), 518 (w);

$[\alpha]_D^{25.0}$ = + 71.7 (c. 1.0, $CHCl_3$);

HRMS (−ESI) *m/z* Found $[M+CHO_2]^-$ 488.0442, $[C_{15}H_{14}F_8NO_6S]^-$ requires 488.0420, (δ = + 4.5 ppm);

Chiral SFC Analysis CHIRAL ART SC (CO_2:^{i}PrOH, 98.5:1.5, 1.25 mL min^{-1}, 40 °C) t_R = 5.1 (minor), 6.0 (major) minutes.

Ethyl 4-((2S,3R)-3-(2-hydroxyethyl)-1-((2,2,3,3,3-pentafluoropropoxy)sulfonyl)aziridin-2-yl)benzoate (**9k**)

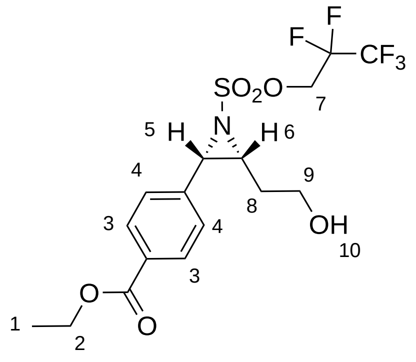

Prepared according to **GP9** using ethyl (*Z*)-4-(4-hydroxybut-1-en-1-yl)benzoate (**8k**) as the starting material. In this case the following procedural modification was used: The reaction time was 36 hours. Purification by flash column chromatography (SiO_2, 0-10% v/v Acetone in $CHCl_3$) afforded the title compound as a white amorphous solid (33.0 mg, 0.074 mmol, 74%, 96% *ee*).

***R_f* value** = 0.35 (10% v/v Acetone in $CHCl_3$);

^{1}H NMR (500 MHz, $CDCl_3$) δ 8.04-8.06 (m, 2H, H-3), 7.39 (d, J = 8.2 Hz, 2H, H-4), 4.72-4.82 (m, 2H, H-7a, H-7b), 4.38 (q, J = 7.1 Hz, 2H, H-2), 4.13 (d, J = 7.4 Hz, 1H, H-5), 3.74 (br s, 2H, H-9a, H-9b), 3.37 (td, J = 7.9 Hz, 5.1 Hz, 1H, H-6), 1.75 (br s, 1H, H-10), 1.60-1.65 (m, 1H, H-8a), 1.44-1.50 (m, 1H, H-8b), 1.39 (t, J = 7.1 Hz, 3H, H-1) ppm;

^{13}C NMR (126 MHz, $CDCl_3$) δ 166.2, 136.5, 130.9, 130.0, 127.4, 118.2 (qt, $J_{C\text{-}F}$ = 286.2 Hz, 34.2 Hz), 111.2 (qt, $J_{C\text{-}F}$ = 257.0 Hz, 38.8 Hz), 65.9 (t, $J_{C\text{-}F}$ = 28.6 Hz), 61.3, 60.0, 47.2, 46.5, 29.0, 14.4 ppm;

^{19}F NMR (376 MHz, $CDCl_3$) δ – 84.4 (s), – 124.5 (s) ppm;

IR (film, cm^{-1}): ν_{max} 3495 (br, w), 2967 (w), 1715 (s), 1615 (w), 1373 (s), 1280 (s), 1204 (s), 1181 (s), 1106 (s), 1040 (m), 1007 (s), 938 (m), 901 (w), 854 (m), 776 (w), 706 (w), 624 (w), 591 (m), 518 (w);

$[\boldsymbol{\alpha}]_{\mathbf{D}}^{\mathbf{25.0}}$ = + 84.5 (c. 1.4, $CHCl_3$);

HRMS (−ESI) *m/z* Found $[M-H]^-$ 446.0698, $[C_{16}H_{17}F_5NO_6S]^-$ requires 446.0702, (δ = – 0.9 ppm);

Chiral SFC Analysis CHIRAL ART SJ (CO_2:MeOH, 97:3, 2.5 mL min^{-1}, 40 °C) t_R = 4.1 (minor), 4.4 (major) minutes.

2,2,3,3,3-pentafluoropropyl (2S,3R)-2-(3,5-dimethylphenyl)-3-(2-hydroxyethyl)aziridine-1-sulfonate (**9l**)

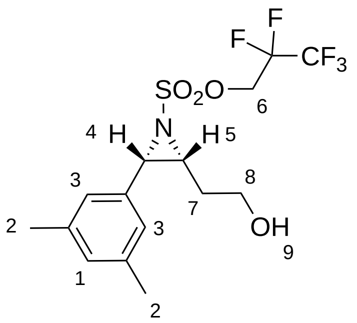

Prepared according to **GP9** using (*Z*)-4-(3,5-dimethylphenyl)but-3-en-1-ol (**8l**) as the starting material. In this case the following procedural modification was used: The reaction time was 36 hours. Purification by flash column chromatography (SiO_2, 0-10% v/v Acetone in $CHCl_3$) afforded the title compound as a colourless oil (30.9 mg, 0.077 mmol, 77%, 91% *ee*).

***R$_f$* value** = 0.60 (10% v/v Acetone in $CHCl_3$);

^{1}H NMR (500 MHz, $CDCl_3$) δ 6.97 (br s, 1H, H-1), 6.90 (br s, 2H, H-3), 4.74-4.79 (m, 2H, H-6a, H-6b), 4.04 (d, *J* = 7.4 Hz, 1H, H-4), 3.76 (t, *J* = 6.0 Hz, 2H, H-8a, H-8b), 3.29 (ddd, *J* = 8.3 Hz, 7.6 Hz, 4.9 Hz, 1H, H-5), 2.32 (s, 6H, H-2), 1.66-1.72 (m, 2H, H-7a, H-9), 1.46-1.53 (m 1H, H-7b) ppm;

^{13}C NMR (126 MHz, $CDCl_3$) δ 138.5, 131.3, 130.3, 125.0, 118.3 (qt, $J_{C\text{-}F}$ = 286.1 Hz, 34.3 Hz), 111.3 (tq, $J_{C\text{-}F}$ = 256.7 Hz, 38.6 Hz), 65.8 (t, $J_{C\text{-}F}$ = 28.6 Hz), 60.3, 47.8, 46.3, 29.1, 21.4 ppm;

^{19}F NMR (376 MHz, $CDCl_3$) δ – 84.5 (s), – 124.5 (s) ppm;

IR (film, cm^{-1}): ν_{max} 3373 (br, w), 2924 (w), 1608 (w), 1373 (s), 1298 (w), 1260 (w), 1201 (s), 1178 (s), 1108 (s), 1038 (s), 1010 (s), 939 (s), 848 (s), 788 (m), 747 (w), 708 (w), 665 (w), 624 (w), 597 (s), 573 (w), 516 (m);

$[\boldsymbol{\alpha}]_{\mathbf{D}}^{\mathbf{25.0}}$ = + 85.0 (c. 1.0, $CHCl_3$);

HRMS (–ESI) *m/z* Found $[M+CHO_2]^-$ 448.0864, $[C_{16}H_{19}F_5NO_6S]^-$ requires 448.0859, (δ = + 1.1 ppm);

Chiral SFC Analysis CHIRAL ART SC (CO_2:MeOH, 98.5:1.5, 2.5 mL min^{-1}, 40 °C) t_R = 5.0 (major), 6.1 (minor) minutes.

2,2,3,3,3-pentafluoropropyl (2S,3R)-2-(2,5-dimethylphenyl)-3-(2-hydroxyethyl)aziridine-1-sulfonate (**9m**)

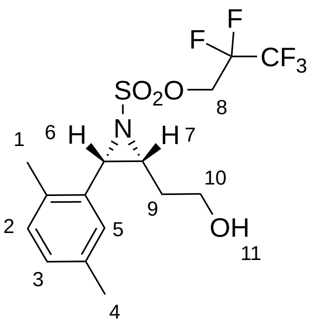

Prepared according to **GP9** using (*Z*)-4-(2,5-dimethylphenyl)but-3-en-1-ol (**8m**) as the starting material. In this case the following procedural modification was used: The reaction time was 36 hours. Purification by flash column chromatography (SiO_2, 0-10% v/v Acetone in $CHCl_3$) afforded the title compound as a white amorphous solid (31.6 mg, 0.078 mmol, 78%, 91% *ee*).

***R_f* value** = 0.57 (10% v/v Acetone in $CHCl_3$);

^{1}H NMR (500 MHz, $CDCl_3$) δ 7.04-7.08 (m, 3H, H-2, H-3, H-5), 4.75-4.86 (m, 2H, H-8a, H-8b), 4.06 (d, *J* = 7.4 Hz, 1H, H-6), 3.71-3.77 (m, 2H, H-10a, H-10b), 3.39 (ddd, *J* = 8.7 Hz, 7.4 Hz, 4.4 Hz, 1H, H-7), 2.31 (s, 3H, H-4), 2.30 (s, 3H, H-1), 1.63-1.70 (m, 2H, H-9a, H-11), 1.29-1.35 (m, 1H, H-9b) ppm;

^{13}C NMR (126 MHz, $CDCl_3$) δ 135.8, 133.1, 130.2, 129.6, 129.2, 127.7, 118.3 (qt, J_{C-F} = 286.1 Hz, 34.2 Hz), 111.3 (tq, J_{C-F} = 257.0 Hz, 38.6 Hz), 65.8 (t, J_{C-F} = 28.5 Hz), 60.2, 47.1, 45.9, 29.2, 21.1, 18.6 ppm;

^{19}F NMR (376 MHz, $CDCl_3$) δ – 84.4 (s), – 124.4 (s) ppm;

IR (film, cm^{-1}): ν_{max} 3400 (br, w), 2928 (w), 1502 (w), 1376 (s), 1205 (s), 1180 (s), 1109 (m), 1042 (s), 1009 (s), 942 (m), 859 (m), 817 (m), 726 (w), 624 (w), 593 (w), 517 (w);

$[\alpha]_D^{25.0}$ = + 89.8 (c. 1.3, $CHCl_3$);

HRMS (−ESI) *m/z* Found $[M+CHO_2]^-$ 448.0864, $[C_{16}H_{19}F_5NO_6S]^-$ requires 448.0859, (δ = + 1.1 ppm);

Chiral SFC Analysis CHIRAL ART SC (CO_2:MeOH, 98.5:1.5, 2.5 mL min^{-1}, 40 °C) t_R = 4.0 (major), 5.1 (minor) minutes.

2,2,3,3,3-pentafluoropropyl (2S,3R)-2-(3-fluoro-4-methylphenyl)-3-(2-hydroxyethyl)aziridine-1-sulfonate (**9n**)

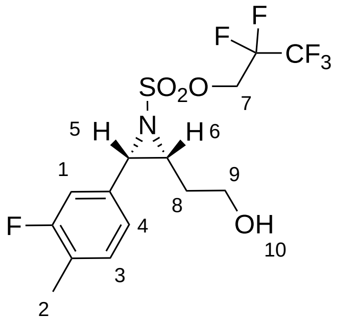

Prepared according to **GP9** using (*Z*)-4-(3-fluoro-4-methylphenyl)but-3-en-1-ol (**8n**) as the starting material. Purification by flash column chromatography (SiO_2, 0-10% v/v Acetone in $CHCl_3$) afforded the title compound as a colourless oil (36.7 mg, 0.090 mmol, 90%, 92% *ee*).

***R_f* value** = 0.48 (10% v/v Acetone in $CHCl_3$);

^{1}H NMR (500 MHz, $CDCl_3$) δ 7.18 (t, J = 7.8 Hz, 1H, H-3), 6.99 (dd, J = 7.8 Hz, 1.5 Hz, 1H, H-1), 6.96 (dd, J = 10.2 Hz, 1.4 Hz, 1H, H-4), 4.70-4.81 (m, 2H, H-7a, H-7b), 4.04 (d, J = 7.3 Hz, 1H, H-5), 3.75 (q, J = 4.6 Hz, 2H, H-9a, H-9b), 3.31 (ddd, J = 8.1 Hz, 7.6 Hz, 5.1 Hz, 1H, H-6), 2.27 (d, J = 1.7 Hz, 3H, H-2), 1.62-1.70 (m, 2H, H-8a, H-10), 1.46-1.53 (m, 1H, H-8b) ppm;

^{13}C NMR (126 MHz, $CDCl_3$) δ 161.3 (d, $J_{C\text{-}F}$ = 246.0 Hz), 131.9 (d, $J_{C\text{-}F}$ = 5.4 Hz), 131.1 (d, $J_{C\text{-}F}$ = 7.7 Hz), 125.5 (d, $J_{C\text{-}F}$ = 17.2 Hz), 122.8 (d, $J_{C\text{-}F}$ = 2.9 Hz), 118.3 (qt, $J_{C\text{-}F}$ = 286.2 Hz, 34.3 Hz), 114.1 (d, $J_{C\text{-}F}$ = 24.1 Hz), 111.3 (tq, $J_{C\text{-}F}$ = 256.2 Hz, 38.6 Hz), 65.9 (t, $J_{C\text{-}F}$ = 28.6 Hz), 60.1, 46.9, 46.5, 28.9, 14.5 (d, $J_{C\text{-}F}$ = 2.9 Hz) ppm;

^{19}F NMR (471 MHz, $CDCl_3$) δ – 84.5 (s), – 117.2 (s), – 124.5 (s) ppm;

IR (film, cm^{-1}): ν_{max} 3400 (br, w), 2967 (w), 1629 (w), 1581 (w), 1513 (w), 1425 (w), 1375 (s), 1260 (w), 1203 (s), 1178 (s), 1107 (s), 1040 (s), 1008 (s), 944 (s), 911 (m), 850 (s), 824 (s), 732 (s), 669 (w), 643 (w), 624 (m), 594 (s), 548 (w), 516 (m), 453 (w), 419 (w);

$[\alpha]_D^{25.0}$ = + 79.8 (c. 1.3, $CHCl_3$);

HRMS (–ESI) *m/z* Found $[M+HO]^-$ 424.0666, $[C_{14}H_{16}F_6NO_5S]^-$ requires 424.0659, (δ = + 1.7 ppm);

Chiral SFC Analysis CHIRAL ART SC (CO_2:^{i}PrOH, 98.5:1.5, 1.25 mL min^{-1}, 40 °C) t_R = 8.3 (major), 9.5 (minor) minutes.

2,2,3,3,3-pentafluoropropyl (2R,3S)-2-(2-hydroxyethyl)-3-(naphthalen-1-yl)aziridine-1-sulfonate (**9o**)

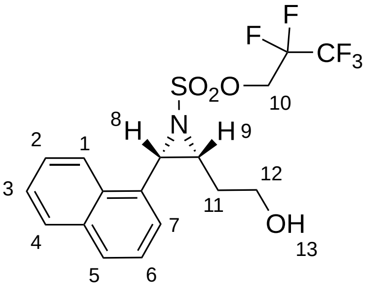

Prepared according to **GP9** using (*Z*)-4-(naphthalen-1-yl)but-3-en-1-ol (**8o**) as the starting material. In this case the following procedural modification was used: The reaction time was 36 hours. Purification by flash column chromatography (SiO_2, 0-9% v/v Acetone in $CHCl_3$) afforded the title compound as a colourless oil (37.1 mg, 0.087 mmol, 87%, 92% *ee*).

***R_f* value** = 0.53 (10% v/v Acetone in $CHCl_3$);

^{1}H NMR (500 MHz, $CDCl_3$) δ 7.99 (dd, *J* = 8.1 Hz, 1.0 Hz, 1H, H-1), 7.91 (dd, *J* = 7.6 Hz, 1.7 Hz, 1H, H-4), 7.86 (d, *J* = 8.2 Hz, 1H, H-5), 7.54-7.60 (m, 3H, H-2, H-3, H-7), 7.48 (t, *J* = 7.6 Hz, 1H, H-6), 4.86 (t, *J* = 12.2 Hz, 2H, H-10a, H-10b), 4.57 (d, *J* = 7.3 Hz, 1H, H-8), 3.65-3.71 (m, 2H, H-12a, H-12b), 3.61 (ddd, *J* = 8.5 Hz, 7.5 Hz, 4.6 Hz, 1H, H-9), 1.59-1.71 (m, 2H, H-11a, H-13), 1.25-1.33 (m, 1H, H-11b) ppm;

^{13}C NMR (126 MHz, $CDCl_3$) δ 133.5, 131.2, 129.11, 129.07, 127.5, 127.0, 126.5, 125.6, 125.4, 122.6, 118.3 (qt, $J_{C\text{-}F}$ = 286.1 Hz, 34.3 Hz), 111.3 (tq, $J_{C\text{-}F}$ = 257.2 Hz, 38.6 Hz), 65.9 (t, $J_{C\text{-}F}$ = 28.6 Hz), 60.1, 46.8, 46.5, 29.3 ppm;

^{19}F NMR (376 MHz, $CDCl_3$) δ – 84.4 (s), – 124.4 (s) ppm;

IR (film, cm^{-1}): ν_{max} 3400 (br, w), 2962 (w), 1598 (w), 1511 (w), 1374 (s), 1258 (w), 1201 (s), 1177 (s), 1107 (s), 1039 (s), 1011 (s), 993 (s), 938 (s), 900 (w), 837 (m), 800 (s), 777 (s), 721 (w), 657 (w), 624 (w), 595 (m), 576 (w), 515 (w);

$[\alpha]_D^{25.0}$ = + 100.7 (c. 1.3, $CHCl_3$);

HRMS (−ESI) *m/z* Found $[M+HO]^-$ 442.0754, $[C_{17}H_{17}F_5NO_5S]^-$ requires 442.0753, (δ = + 0.2 ppm);

Chiral SFC Analysis CHIRAL ART SC (CO_2:MeOH, 97:3, 2.5 mL min^{-1}, 40 °C) t_R = 4.4 (major), 5.5 (minor) minutes.

2,2,3,3,3-pentafluoropropyl ((2S,3R)-2-phenyltetrahydrofuran-3-yl)sulfamate (**12**)

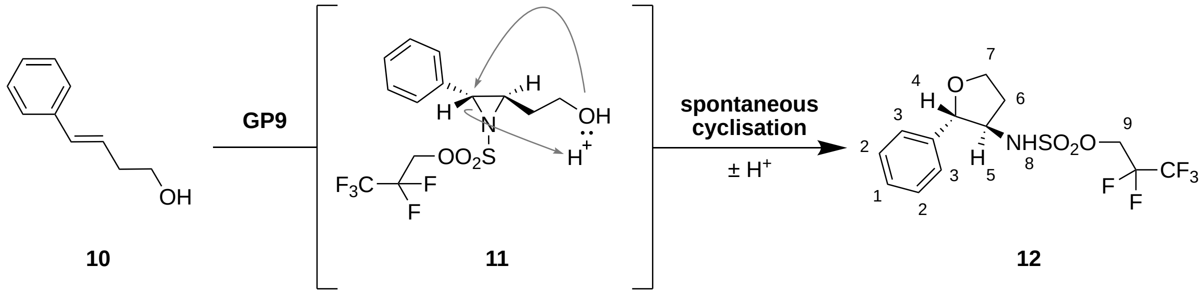

Prepared according to **GP9** using (*E*)-4-phenylbut-3-en-1-ol (**10**) as the starting material. The aziridine cyclised spontaneously during the reaction. Purification by flash column chromatography (SiO_2, 0-10% v/v Acetone in $CHCl_3$) afforded the title compound as an amorphous white solid (17.4 mg, 0.046 mmol, 46%, 34% *ee*).

***R_f* value** = 0.52 (10% v/v Acetone in $CHCl_3$);

^{1}H NMR (500 MHz, $CDCl_3$) δ 7.30-7.39 (m, 5H, H-1, H-2, H-3), 4.86 (d, *J* = 3.8 Hz, 1H, H-4), 4.40 (q, *J* = 12.2 Hz, 1H, H-9a), 4.32 (q *J* = 12.2 Hz, 1H, H-9b), 4.23 (td, *J* = 8.8 Hz, 4.5 Hz, 1H, H-7a), 4.02-4.10 (m, 2H, H-5, H-7b), 2.30-2.38 (m, 1H, H-6a), 2.00-2.06 (m, 1H, H-6b) ppm;

^{13}C NMR (126 MHz, $CDCl_3$) δ 139.2, 128.9, 128.4, 125.6, 118.3 (qt, $J_{C\text{-}F}$ = 286.2 Hz, 34.3 Hz), 111.4 (tq, $J_{C\text{-}F}$ = 256.8 Hz, 38.5 Hz), 85.1, 67.0, 64.2 (t, $J_{C\text{-}F}$ = 28.2 Hz), 62.1, 31.9 ppm;

^{19}F NMR (376 MHz, $CDCl_3$) δ – 83.5 (s), – 123.5 (s) ppm;

IR (film, cm^{-1}): ν_{max} 3302 (w), 3150 (w), 2901 (w), 1455 (w), 1377 (m), 1204 (s), 1186 (s), 1111 (m), 1047 (m), 1011 (m), 941 (w), 809 (w), 760 (w), 701 (w), 623 (w), 582 (w), 518 (w);

$[\alpha]_D^{25.0}$ = + 14.9 (c. 1.0, $CHCl_3$);

HRMS (−ESI) *m/z* Found $[M-H]^-$ 374.0480, $[C_{13}H_{13}F_5NO_4S]^-$ requires 374.0491, (δ = – 2.9 ppm);

Chiral SFC Analysis CHIRAL ART SC (CO_2:MeOH, 98:2, 1.25 mL min^{-1}, 40 °C) t_R = 8.2 (major), 9.1 (minor) minutes.

Characterisation of Trishomoallylic Styrenyl Aziridines

2,2,3,3,3-pentafluoropropyl (2R,3S)-2-(4-hydroxybutyl)-3-phenylaziridine-1-sulfonate (**14a**)

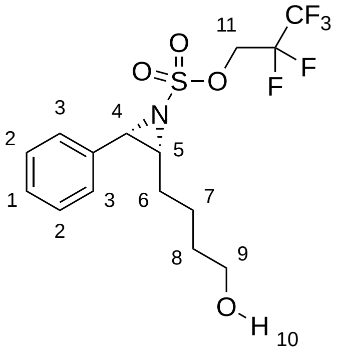

Prepared according to **GP9** using (*Z*)-6-phenylhex-5-en-1-ol (**13a**) as the starting material. Purification by flash column chromatography (SiO_2, 0-10% v/v Acetone in $CHCl_3$) afforded the title compound as a colourless oil (29 mg, 0.072 mmol, 72%, 96% ee).

***Rf* value** = 0.42 (10% v/v Acetone in $CHCl_3$);

^{1}H NMR (500 MHz, $CDCl_3$) δ 7.40-7.33 (m, 3H, H-1, H-2), 7.30 (m, 2H, H-3), 4.74 (m, 2H, H-11_A, H-11_B), 4.06 (d, *J* = 7.3 Hz, 1H, H-4), 3.54 (m, 2H, H-9_A, H-9_B), 3.15 (q, *J* = 6.9 Hz, 1H, H-5), 1.54-1.36 (m, 7H, H-6_A, H-6_B, H-7_A, H-7_B, H-8_A, H-8_B, H-10) ppm;

^{13}C NMR (126 MHz, $CDCl_3$) δ 131.7, 128.7, 128.6, 127.3, 118.3 (qt, $J_{C\text{-}F}$ = 286, 34.6 Hz), 111.3 (tq, $J_{C\text{-}F}$ = 257, 38.6 Hz), 65.7 (t, $J_{C\text{-}F}$ = 29.0 Hz), 62.4, 48.9, 48.0, 32.0, 25.8, 22.9 ppm;

^{19}F NMR (376 MHz, $CDCl_3$) δ − 83.5, − 123.5 ppm;

IR (film, cm^{-1}): ν_{max} 3362 (br), 2940 (w), 1454 (w), 1376 (s), 1205 (s), 1181 (s), 1109 (m), 1042 (m), 1004 (m), 849 (m), 592 (m);

$[\alpha]_D^{25.0}$ = + 84.4 (c. 1.7, $CHCl_3$);

HRMS (−ESI) *m/z* found $[M+HCO_2]^-$ 448.0858, $[C_{16}H_{19}F_5NO_6S]^-$ requires 448.0859, (δ = − 0.2 ppm);

Chiral SFC Analysis: CHIRAL ART SJ (CO_2:MeOH, 98:2, 2.5 mL min^{-1}, 40 °C) t_R = 3.1 (major), 3.5 (minor) minutes.

2,2,3,3,3-pentafluoropropyl (2S,3R)-2-(3-chlorophenyl)-3-(4-hydroxybutyl)aziridine-1-sulfonate (**14b**)

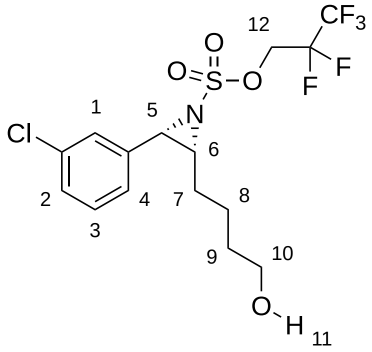

Prepared according to **GP9** using (*Z*)-6-(3-chlorophenyl)hex-5-en-1-ol (**13b**) as the starting material. Purification by flash column chromatography (SiO_2, 0-10% v/v Et_2O in CH_2Cl_2) afforded the title compound as a colourless oil (25 mg, 0.057 mmol, 57%, 95% *ee*).

***R_f* value** = 0.38 (10% v/v Et_2O in CH_2Cl_2);

^{1}H NMR (400 MHz, $CDCl_3$) δ 7.35-7.29 (m, 3H, H-1, H-2, H-3), 7.20 (dt, *J* = 6.2, 1.7 Hz, 1H, H-4), 4.75 (t, *J* = 12.2 Hz, 2H, H-12_A, H-12_B), 4.01 (d, *J* = 7.4 Hz, 1H, H-5), 3.57 (br s, 2H, H-10_A, H-10_B), 3.15 (q, *J* = 7.0 Hz, 1H, H-6), 1.56-1.41 (m, 4H, H-8_A, H-8_B, H-9_A, H-9_B), 1.38 (m, 2H, H-7_A, H-7_B), 1.25 (m, 1H, H-11) ppm;

^{13}**C NMR** (101 MHz, $CDCl_3$) δ 134.8, 133.8, 130.0, 128.9, 127.5, 125.6, 118.3 (qt, $J_{C\text{-}F}$ = 286, 34.1 Hz), 111.2 (tq, $J_{C\text{-}F}$ = 257, 39.0 Hz), 65.8 (t, $J_{C\text{-}F}$ = 28.9 Hz), 62.4, 48.8, 47.2, 31.9, 25.9, 23.0 ppm;

^{19}**F NMR** (376 MHz, $CDCl_3$) δ − 83.4, − 123.5 ppm;

IR (film, cm^{-1}): ν_{max} 2946 (w), 1601 (w), 1574 (w), 1378 (s), 1206 (s), 1181 (s), 1109 (m), 1041 (m), 1007 (m), 854 (m), 596 (m);

$[\alpha]_D^{25.0}$ = + 81.1 (c. 1.0, $CHCl_3$);

HRMS (+ESI) m/z Found $[M+H]^+$ 438.0569, $[C_{15}H_{18}ClF_5NO_4S]^+$ requires 438.0560, (δ = + 2.1 ppm);

Chiral SFC Analysis: CHIRAL ART SC (CO_2;^{i}PrOH, 97:3, 2.5 mL min^{-1}, 40 °C) t_R = 8.5 (major), 10.3 (minor) minutes.

2,2,3,3,3-pentafluoropropyl (2R,3S)-2-(4-hydroxybutyl)-3-(3-(N-methylmethylsulfonamido)phenyl)aziridine-1-sulfonate (**14c**)

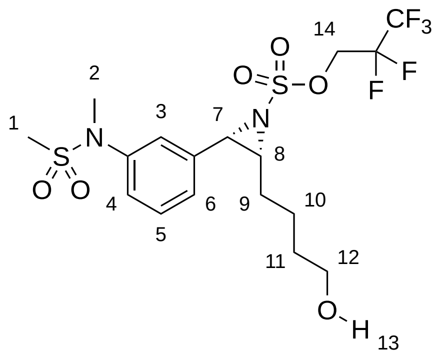

Prepared according to **GP9** using (*Z*)-*N*-(3-(6-hydroxyhex-1-en-1-yl)phenyl)-N-methylmethanesulfonamide (**13c**) as the starting material, on a 0.2 mmol scale; all other

reagent and solvent amounts were scaled proportionally. Purification by flash column chromatography (SiO_2, 0-20% v/v Acetone in $CHCl_3$) afforded the title compound as a colourless oil (55 mg, 0.11 mmol, 55%, 94% *ee*).

***R_f* value** = 0.29 (20% v/v Acetone in $CHCl_3$);

^{1}H NMR (400 MHz, $CDCl_3$) δ 7.43-7.36 (m, 2H, H-5, H-3), 7.31 (m, 1H, H-6), 7.25 (m, 1H, H-4), 4.76 (t, *J* = 12.2 Hz, 2H, H-14_A, H-14_B), 4.04 (d, *J* = 7.3 Hz, 1H, H-7), 3.52 (br t, *J* = 5.5 Hz, 2H, H-12_A, H-12_B), 3.32 (s, 3H, H-2), 3.16 (q, *J* = 6.9 Hz, 1H, H-8), 2.83 (s, 3H, H-1), 1.54 (br s, 1H, H-13), 1.52-1.32 (m, 6H, H-9_A, H-9_B, H-10_A, H-10_B, H-11_A, H-11_B) ppm;

^{13}C NMR (101 MHz, $CDCl_3$) δ 141.8, 133.3, 129.7, 126.4, 125.9, 125.1, 118.2 (qt, $J_{C\text{-}F}$ = 286, 34.6 Hz), 111.3 (tq, $J_{C\text{-}F}$ = 256, 38.9 Hz), 65.8 (t, $J_{C\text{-}F}$ = 28.7 Hz), 62.2, 48.9, 47.4, 38.0, 35.5, 31.8, 25.6, 22.8 ppm;

^{19}F NMR (376 MHz, $CDCl_3$) δ − 83.4 (s), − 123.5 (t, *J* = 12.2 Hz) ppm; *This NMR spectrum was acquired with proton coupling*;

IR (film, cm^{-1}): ν_{max} 2941 (w), 1376 (m), 1341 (s), 1204 (s), 1180 (s), 1108 (m), 1040 (m), 1008 (m), 942 (m), 807 (m), 519 (m);

$[\alpha]_D^{25.0}$ = + 65.8 (c. 0.64, $CHCl_3$);

HRMS (+ESI) *m/z* found $[M+Na]^+$ 533.0813, $[C_{17}H_{23}F_5N_2O_6S_2Na]^+$ requires 533.0810, (δ = + 0.6 ppm);

Chiral SFC Analysis: CHIRAL ART SC (CO_2:MeOH, 93:7, 2.5 mL min^{-1}, 40 °C) t_R = 6.9 (major), 7.8 (minor) minutes.

Methyl 2-(3-((2S,3R)-3-(4-hydroxybutyl)-1-((2,2,3,3,3-pentafluoropropoxy)sulfonyl)aziridin-2-yl)phenyl)acetate (**14d**)

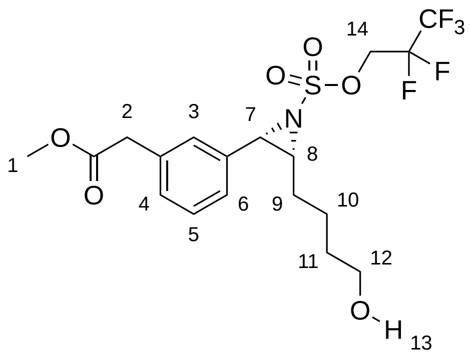

Prepared according to **GP9** using methyl (*Z*)-2-(3-(6-hydroxyhex-1-en-1-yl)phenyl)acetate (**13d**) as the starting material, on a 0.2 mmol scale; all other reagent and solvent amounts were scaled proportionally. Purification by flash column chromatography (SiO_2, 0-15% v/v Et_2O in CH_2Cl_2) afforded the title compound as a colourless oil (62 mg, 0.13 mmol, 65%, 95% *ee*).

***R_f* value** = 0.23 (10% v/v Et_2O in CH_2Cl_2);

^{1}H NMR (500 MHz, $CDCl_3$) δ 7.33 (t, *J* = 7.7 Hz, 1H, H-5), 7.26-7.20 (m, 3H, H-3, H-4, H-6), 4.75 (m, 2H, H-14_A, H-14_B), 4.04 (d, *J* = 7.2 Hz, 1H, H-7), 3.69 (s, 3H, H-1), 3.64 (2H, H-2_A, H-2_B), 3.51 (m, 2H, H-12_A, H-12_B), 3.14 (q, *J* = 7.0 Hz, 1H, H-8), 1.63 (s, 1H, H-13), 1.52-1.32 (m, 6H, H-9_A, H-9_B, H-10_A, H-10_B H-11_A, H-11_B) ppm;

^{13}C NMR (126 MHz, $CDCl_3$) δ 172.0, 134.5, 132.0, 129.6, 128.9, 128.4, 126.2, 118.3 (qt, $J_{C\text{-}F}$ = 287, 34.3 Hz), 111.2 (tq, $J_{C\text{-}F}$ = 257, 38.8 Hz), 65.8 (t, $J_{C\text{-}F}$ = 28.7 Hz), 62.2, 52.3, 48.8, 47.8, 40.9, 31.9, 25.6, 22.8 ppm;

^{19}F NMR (376 MHz, $CDCl_3$) δ − 83.5 (s), − 123.5 (t, *J* = 12.2 Hz) ppm; *This NMR spectrum was acquired with proton coupling*;

IR (film, cm^{-1}): ν_{max} 2953 (w), 1736 (s), 1438 (w), 1376 (s), 1203 (s), 1179 (s), 1108 (m), 1039 (s), 1009 (s), 848 (m), 595 (m);

$[\alpha]_D^{25.0}$ = + 75.2 (c. 1.8, $CHCl_3$);

HRMS (−ESI) *m/z* found $[M+HCO_2]^-$ 520.1081, $[C_{16}H_{19}F_5NO_6S]^-$ requires 520.1070, (δ = + 2.1 ppm);

Chiral SFC Analysis: CHIRAL ART SC (CO_2:^{i}PrOH, 95:5, 2.5 mL min^{-1}, 40 °C) t_R = 8.6 (major), 9.7 (minor) minutes.

2,2,3,3,3-pentafluoropropyl (2S,3R)-2-(3-(N,N-diethylsulfamoyl)phenyl)-3-(4-hydroxybutyl)aziridine-1-sulfonate (**14e**)

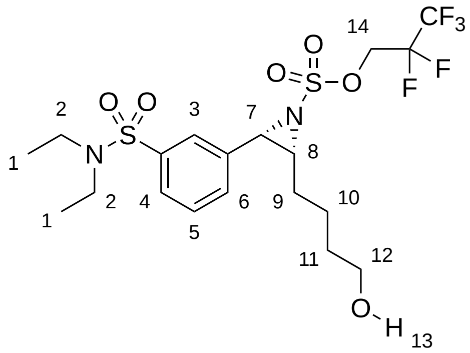

Prepared according to **GP9** using (*Z*)-*N*,*N*-diethyl-3-(6-hydroxyhex-1-en-1-yl)benzenesulfonamide (**13e**) as the starting material, on a 0.2 mmol scale; all other reagent and solvent amounts were scaled propoprtionally. In this case the catalyst loading was 2 mol % instead of 1 mol %. Purification by flash column chromatography (SiO_2, 0-15% v/v acetone in $CHCl_3$) afforded the title compound as a colourless oil (28 mg, 0.052 mmol, 26%, 90% *ee*).

***R$_f$* value** = 0.24 (10% v/v acetone in $CHCl_3$);

^{1}**H NMR** (500 MHz, $CDCl_3$) δ 7.79 (dt, J = 6.7, 2.1 Hz, 1H, H-4), 7.76 (m, 1H, H-3), 7.54-7.49 (m, 2H, H-5, H-6), 4.75 (m, 2H, H-14_A, H-14_B), 4.09 (d, J = 7.3 Hz, 1H, H-7), 3.55 (t, J = 5.8 Hz, 2H, H-12_A, H-12_B), 3.25 (q, J = 7.2 Hz, 4H, H-2_A, H-2_B), 3.19 (q, J = 7.0 Hz, 1H, H-8), 1.55-1.44 (m, 5H, H-10_A, H-10_B, H-11_A, H-11_B, H-13), 1.35 (q, J = 7.1 Hz, 2H, H-9_A, H-9_B), 1.13 (t, J = 7.2 Hz, 6H, H-1) ppm;

^{13}**C NMR** (126 MHz, $CDCl_3$) δ 141.3, 133.3, 131.2, 129.6, 127.1, 125.9, 118.2 (qt, $J_{C\text{-}F}$ = 287, 34.2 Hz), 111.2 (tq, $J_{C\text{-}F}$ = 257, 38.6 Hz), 65.9 (t, $J_{C\text{-}F}$ = 28.4 Hz), 62.3, 48.8, 47.2, 42.2, 31.8, 25.8, 23.0, 14.2 ppm;

^{19}**F NMR** (376 MHz, $CDCl_3$) δ − 83.4, − 123.5 ppm;

IR (film, cm^{-1}): ν_{max} 2939 (w), 2872 (w), 1459 (w), 1379 (m), 1353 (m), 1334 (m), 1305 (m), 1201 (s), 1181 (s), 1150 (s), 1108 (m), 1039 (m), 1013 (s), 937 (m), 848 (m), 720 (m), 581 (s);

$[\alpha]_D^{25.0}$ = + 43.2 (c. 1.1, $CHCl_3$);

HRMS (−ESI) *m/z* found $[M+HCO_2]^-$ 583.1224, $[C_{20}H_{28}F_5N_2O_8S_2]^-$ requires 583.1213, (δ = + 1.9 ppm);

Chiral SFC Analysis: CHIRAL ART SC (CO_2:^{i}PrOH, 83:17, 2.5 mL min^{-1}, 40 °C) t_R = 3.4 (minor), 3.9 (major) minutes.

2,2,3,3,3-pentafluoropropyl (2S,3R)-2-(2-chlorophenyl)-3-(4-hydroxybutyl)aziridine-1-sulfonate (**14f**)

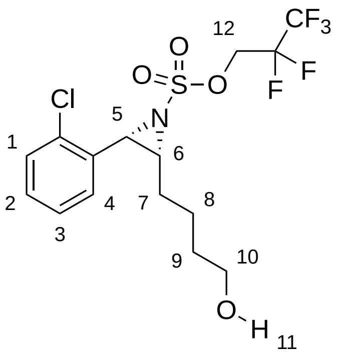

Prepared according to **GP9** using (*Z*)-6-(2-chlorophenyl)hex-5-en-1-ol (**13f**) as the starting material. Purification by flash column chromatography (SiO_2, 0-8% v/v Acetone in $CHCl_3$) afforded the title compound as a colourless oil (21 mg, 0.049 mmol, 49%, 94% *ee*).

***R_f* value** = 0.41 (10% v/v Acetone in $CHCl_3$);

^{1}H NMR (400 MHz, $CDCl_3$) δ 7.40 (m, 1H, H-1), 7.38-7.27 (m, 3H, H-2, H-3, H-4), 4.76 (t, *J* = 12.1 Hz, 2H, H-12_A, H-12_B), 4.20 (d, *J* = 7.4 Hz, 1H, H-5), 3.56 (br t, *J* = 5.6 Hz, 2H, H-10_A, H-10_B), 3.27 (m, 1H, H-6), 1.56-1.35 (m, 5H, H-7_A, H-8_A, H-8_B H-9_A, H-9_B), 1.28-1.17 (m, 2H, H-7_B, H-11) ppm;

^{13}C NMR (101 MHz, $CDCl_3$) δ 133.6, 130.1, 129.9, 129.6, 129.1, 127.0, 118.3 (qt, J_{C-F} = 286, 34.3 Hz), 111.3 (tq, J_{C-F} = 257, 38.8 Hz), 65.7 (t, J_{C-F} = 28.7 Hz), 62.5, 48.8, 46.8, 32.0, 26.1, 23.0 ppm;

^{19}F NMR (376 MHz, $CDCl_3$) δ − 83.4, − 123.4 ppm;

IR (film, cm^{-1}): ν_{max} 2940 (w), 1444 (w), 1380 (m), 1207 (s), 1181 (s), 1110 (m), 1043 (m), 1004 (m), 756 (s);

$[\alpha]_D^{25.0}$ = + 89.6 (c. 0.64, $CHCl_3$);

HRMS (−ESI) *m/z* found $[M-H]^-$ 436.0409, $[C_{15}H_{16}ClF_5NO_4S]^-$ requires 436.0414, (δ = − 1.1 ppm);

Chiral SFC Analysis: CHIRAL ART SC (CO_2;^{i}PrOH, 97:3, 2.5 mL min^{-1}, 40 °C) t_R = 6.9 (major), 7.8 (minor) minutes.

2,2,3,3,3-pentafluoropropyl (2S,3R)-2-(4-chlorophenyl)-3-(4-hydroxybutyl)aziridine-1-sulfonate (**14g**)

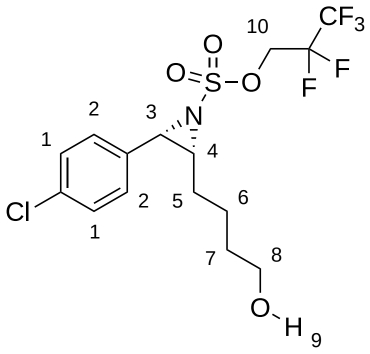

Prepared according to **GP9** using (*Z*)-6-(4-chlorophenyl)hex-5-en-1-ol (**13g**) as the starting material. Purification by flash column chromatography (SiO_2, 0-12% v/v Et_2O in CH_2Cl_2) afforded the title compound as a colourless oil (28 mg, 0.064 mmol, 64%, 96% *ee*).

***R*_f value** = 0.37 (10% v/v Et_2O in CH_2Cl_2);

^{1}H NMR (400 MHz, $CDCl_3$) δ 7.35 (m, 2H, H-1), 7.24 (m, 2H, H-2), 4.73 (m, 2H, H-10_A, H-10_B), 4.01 (d, J = 7.3 Hz, 1H, H-3), 3.57 (br t, J = 5.7 Hz, 2H, H-8_A, H-8_B), 3.14 (q, J = 6.9 Hz, 1H, H-4), 1.55-1.41 (m, 4H, H-6_A, H-6_B, H-7_A, H-7_B), 1.37 (m, 2H, H-5_A, H-5_B), 1.31 (br s, 1H, H-9) ppm;

^{13}C NMR (101 MHz, $CDCl_3$) δ 134.6, 130.2, 129.0, 128.7, 118.3 (qt, $J_{C\text{-}F}$ = 286, 34.4 Hz), 111.3 (tq, $J_{C\text{-}F}$ = 257, 38.7 Hz), 65.8 (t, $J_{C\text{-}F}$ = 28.7 Hz), 62.4, 48.8, 47.3, 31.9, 25.8, 23.0 ppm;

^{19}F NMR (376 MHz, $CDCl_3$) δ − 83.5, − 123.5 ppm;

IR (film, cm^{-1}): ν_{max} 2936 (w), 1494 (w), 1377 (s), 1205 (s), 1182 (s), 1107 (m), 1091 (m), 1042 (s), 1013 (s), 836 (m), 591 (m);

$[\alpha]_D^{25.0}$ = + 77.7 (c. 0.63, $CHCl_3$);

HRMS (−ESI) *m/z* found [M−H]$^-$ 436.0407, $[C_{15}H_{16}ClF_5NO_4S]^-$ requires 436.0414, (δ = − 1.7 ppm);

Chiral SFC Analysis: CHIRAL ART SC (CO_2:^{i}PrOH, 97:3, 2.5 mL min^{-1}, 40 °C) t_R = 8.7 (major), 10.0 (minor) minutes.

2,2,3,3,3-pentafluoropropyl (2S,3R)-2-(4-(tert-butyl)phenyl)-3-(4-hydroxybutyl)aziridine-1-sulfonate (**14h**)

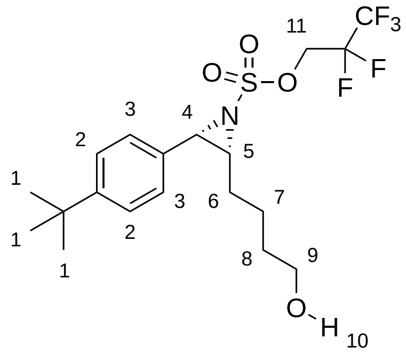

Prepared according to **GP9** using (*Z*)-6-(4-(*tert*-butyl)phenyl)hex-5-en-1-ol (**13h**) as the starting material, on a 0.2 mmol scale. Purification by flash column chromatography (SiO_2, 0-7% v/v Acetone in $CHCl_3$) afforded the title compound as a colourless oil (64 mg, 0.14 mmol, 70%, 98% *ee*).

***R$_f$* value** = 0.41 (10% v/v Acetone in $CHCl_3$);

^{1}H NMR (400 MHz, $CDCl_3$) δ 7.39 (m, 2H, H-2), 7.22 (m, 2H, H-3), 4.74 (m, 2H, H-11_A, H-11_B), 4.03 (d, J = 7.3 Hz, 1H, H-4), 3.55 (m, 2H, H-9_A, H-9_B), 3.14 (q, J = 7.0 Hz, 1H, H-5), 1.56-1.37 (m, 6H, H-6_A, H-6_B, H-7_A, H-7_B, H-8_A, H-8_B), 1.35-1.29 (m, 10H, H-1, H-10) ppm;

^{13}C NMR (101 MHz, $CDCl_3$) δ 151.7, 128.6, 127.1, 125.6, 118.3 (qt, $J_{C\text{-}F}$ = 286, 34.7 Hz), 111.3 (tq, $J_{C\text{-}F}$ = 257, 38.5 Hz), 65.7 (t, $J_{C\text{-}F}$ = 28.2 Hz), 62.4, 48.9, 48.0, 34.8, 31.9, 31.4, 25.8, 23.0 ppm;

^{19}F NMR (376 MHz, $CDCl_3$) δ − 83.5, −123.5 ppm;

IR (film, cm^{-1}): ν_{max} 2967 (m), 1378 (m), 1209 (s), 1181 (s), 1109 (w), 1045 (m), 1013 (m), 757 (s);

$[\alpha]_D^{25.0}$ = + 75.2 (c. 0.54, $CHCl_3$);

HRMS (−ESI) *m/z* found $[M-H]^-$ 458.1433, $[C_{19}H_{25}F_5NO_4S]^-$ requires 458.1430, (δ = + 0.8 ppm);

Chiral SFC Analysis: CHIRAL ART SJ (CO_2;MeOH, 98:2, 1.25 mL min^{-1}, 40 °C) t_R = 6.7 (major), 7.3 (minor) minutes.

2,2,3,3,3-pentafluoropropyl (2R,3S)-2-(4-hydroxybutyl)-3-(4-(pyrrolidin-1-ylsulfonyl)phenyl)aziridine-1-sulfonate (**14i**)

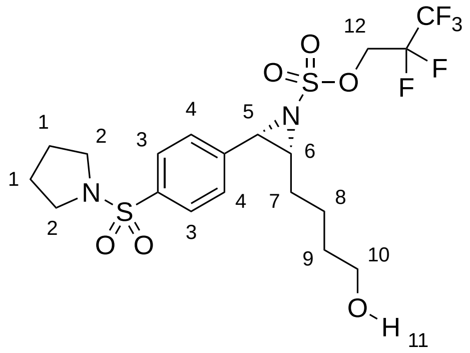

Prepared according to **GP9** using (*Z*)-6-(4-(pyrrolidin-1-ylsulfonyl)phenyl)hex-5-en-1-ol (**13i**) as the starting material, on a 0.2 mmol scale; all other reagent and solvent amounts were scaled proportionally. In this case the catalyst loading was 2 mol % instead of 1 mol %. Purification by flash column chromatography (SiO_2, 0-15% v/v Acetone in $CHCl_3$) afforded the title compound as a colourless oil (31 mg, 0.058 mmol, 29%, 92% *ee*).

***R_f* value** = 0.19 (10% v/v Acetone in $CHCl_3$);

^{1}H NMR (500 MHz, $CDCl_3$) δ 7.84 (d, *J* = 8.3 Hz, 2H, H-3), 7.48 (d, *J* = 8.1 Hz, 2H, H-4), 4.75 (m, 2H, H-12_A, H-12_B), 4.08 (d, *J* = 7.3 Hz, 1H, H-5), 3.55 (m, 2H, H-10_A, H-10_B), 3.25 (m, 4H, H-2_A, H-2_B), 3.21 (q, *J* = 6.8 Hz, 1H, H-6), 1.77 (m, 4H, H-1_A, H-1_B), 1.54-1.41 (m, 5H, H-8_A, H-8_B, H-9_A, H-9_B, H-11), 1.36 (m, 2H, H-7_A, H-7_B) ppm;

^{13}C NMR (126 MHz, $CDCl_3$) δ 137.5, 136.7, 128.1, 127.9, 118.2 (qt, $J_{C\text{-}F}$ = 286, 34.1 Hz), 111.2 (tq, $J_{C\text{-}F}$ = 257, 38.4 Hz), 65.9 (t, $J_{C\text{-}F}$ = 28.7 Hz), 62.3, 49.0, 48.1, 47.1, 31.9, 25.9, 25.4, 23.0 ppm;

^{19}F NMR (376 MHz, $CDCl_3$) δ − 84.4, − 124.4 ppm;

IR (film, cm^{-1}): ν_{max} 2948 (w), 1378 (m), 1345 (m), 1203 (s), 1180 (s), 1159 (s), 1108 (m), 1039 (m), 1004 (m), 845 (m), 617 (s), 592 (s);

$[\boldsymbol{\alpha}]_{\mathbf{D}}^{\mathbf{25.0}}$ = + 51.6 (c. 1.6, $CHCl_3$);

HRMS (−ESI) *m/z* found $[M+HCO_2]^-$ 581.1074, $[C_{20}H_{26}F_5N_2O_8S_2]^-$ requires 581.1056, (δ = + 3.1 ppm);

Chiral SFC Analysis: CHIRALPAK IG (CO_2:^{i}PrOH, 85:15, 2.5 mL min^{-1}, 40 °C) t_R = 5.8 (major), 6.3 (minor) minutes.

Characterisation of Control Substrate Aziridines

2,2,3,3,3-pentafluoropropyl ((S)-naphthalen-1-yl((S)-tetrahydrofuran-2-yl)methyl)sulfamate (**15**)

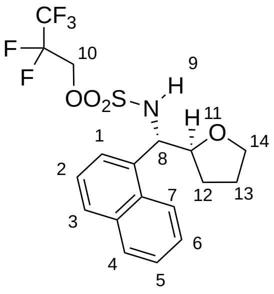

Prepared according to **GP9** using (*Z*)-5-(naphthalen-1-yl)pent-4-en-1-ol as the starting material. Following the cyclisation protocol (2 hours), purification by flash column chromatography (SiO_2, 0-1% v/v Et_2O in CH_2Cl_2) afforded the title compound as a yellow oil (24.3 mg, 0.055 mmol, 55%, 96% *ee*).

***Rf* value** = 0.32 (100% CH_2Cl_2);

^{1}H NMR (400 MHz, $CDCl_3$) δ 8.10 (d, *J* = 8.2 Hz, 1H, H-7), 7.90 (dd, *J* = 7.8 Hz, 1.1 Hz, 1H, H-4), 7.84 (d, *J* = 8.2 Hz, 1H, H-3), 7.55-7.59 (m, 2H, H-1, H-6), 7.47-7.55 (m, 2H, H-2, H-5), 5.85 (d, *J* = 5.3 Hz, 1H, H-9), 5.21 (br s, 1H, H-8), 4.28 (q, *J* = 5.3 Hz, 1H, H-11), 4.16 (q, *J* = 12.2 Hz, 1H, H-10a), 4.07 (q, *J* = 12.5 Hz, 1H, H-10b), 3.99-4.04 (m, 1H, H-14a), 3.80-3.86 (m, 1H, H-14b), 1.97-2.07 (m, 1H, H-13a), 1.86-1.96 (m, 2H, H-12a, H-13b), 1.72-1.82 (m, 1H, H-12b) ppm;

^{13}C NMR (126 MHz, $CDCl_3$) δ 134.2, 134.0, 130.8, 129.4, 129.2, 126.8, 126.0, 125.4*, 122.5, 118.1 (qt, J_{C-F} = 286.2 Hz, 34.3 Hz), 111.2 (tq, J_{C-F} = 256.8 Hz, 38.2 Hz), 81.0, 69.0, 64.0 (t, J_{C-F} = 28.1 Hz), 58.1, 29.1, 25.8 ppm;

*^{1}H-^{13}C HSQC shows that this signal corresponds to two ^{13}C environments.

^{19}F NMR (376 MHz, $CDCl_3$) *δ* – 83.8 (s), – 123.8 (s) ppm;

IR (neat, cm^{-1}): ν_{max} 3303 (br, w), 2961 (w), 2879 (w), 1599 (w), 1514 (w), 1429 (br, m), 1368 (br, m), 1260 (w), 1200 (s), 1179 (s), 1107 (s), 1048 (s), 1010 (s), 959 (s), 799 (s), 775 (s), 715 (w), 622 (m), 576 (m), 514 (m), 454 (w);

$[\alpha]_D^{25.0}$ = + 22.8 (c. 1.1, $CHCl_3$);

HRMS (−ESI) *m/z* Found [M−H]$^-$ 438.0802, $[C_{18}H_{17}F_5NO_4S]^-$ requires 438.0804, (*δ* = – 0.5 ppm);

Chiral SFC Analysis CHIRALPAK IH (CO_2:MeOH, 96:4, 2.5 mL min^{-1}, 40 °C) t_R = 5.8 (minor), 6.7 (major) minutes.

2,2,3,3,3-pentafluoropropyl (2S,3R)-2-(naphthalen-1-yl)-3-propylaziridine-1-sulfonate (**16**)

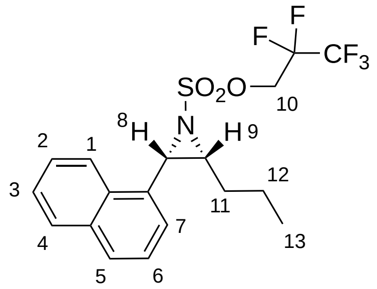

Prepared according to **GP9** using (*Z*)-1-(pent-1-en-1-yl)naphthalene as the starting material. Purification by flash column chromatography (SiO_2, 0-10% v/v EtOAc in Petrol) afforded the title compound as a pale-yellow oil (16.1 mg, 0.038 mmol, 38%, 12% *ee*).

***R$_f$* value** = 0.44 (10% v/v EtOAc in Hexane);

^{1}H NMR (500 MHz, $CDCl_3$) δ 7.98-8.00 (m, 1H, H-1), 7.91-7.92 (m, 1H, H-4), 7.86 (d, J = 8.1 Hz, 1H, H-5), 7.53-7.59 (m, 3H, H-2, H-3, H-7), 7.48 (t, J = 7.6 Hz, 1H, H-6), 4.82 (t, J = 12.2 Hz, 2H, H-10a, H-10b), 4.52 (d, J = 7.3 Hz, 1H, H-8), 3.40-3.44 (m, 1H, H-9), 1.27-1.43 (m, 3H, H-11a, H-12a, H-12b), 1.09-1.16 (m, 1H, H-11b), 0.81 (t, J = 7.3 Hz, 3H, H-13) ppm;

^{13}C NMR (126 MHz, $CDCl_3$) δ 133.5, 131.3, 129.1, 129.0, 127.8, 126.9, 126.4, 125.5, 125.4, 122.7, 118.3 (qt, J_{C-F} = 286.2 Hz, 34.5 Hz), 111.3 (tq, J_{C-F} = 258.8 Hz, 38.6 Hz), 65.8 (t, J_{C-F} = 28.7 Hz), 49.1, 47.0, 28.5, 20.2, 13.7 ppm;

^{19}F NMR (471 MHz, $CDCl_3$) δ – 84.4 (s), – 124.4 (s) ppm;

IR (film, cm^{-1}): ν_{max} 2962 (m), 2928 (m), 2874 (m), 1599 (w), 1512 (w), 1458 (w), 1379 (m), 1257 (w), 1206 (s), 1182 (s), 1110 (m), 1043 (m), 1012 (m), 987 (m), 910 (m), 835 (m), 801 (m), 657 (w), 625 (m);

$[\alpha]_D^{25.0}$ = + 14.1 (c. 1.0, $CHCl_3$);

HRMS (–ESI) *m/z* Found $[M–H]^-$ 422.0845, $[C_{18}H_{17}F_5NO_3S]^-$ requires 422.0855, (δ = – 2.4 ppm);

Chiral SFC Analysis CHIRAL ART SB (CO_2:MeOH, 99:1, 2.5 mL min^{-1}, 40 °C) t_R = 2.9 (minor), 4.1 (major) minutes.

2,2,3,3,3-pentafluoropropyl (2R,3S)-2-(3-methoxypropyl)-3-(naphthalen-1-yl)aziridine-1-sulfonate (**17**)

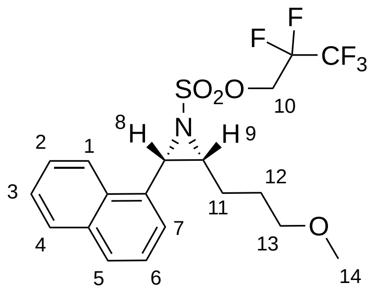

Prepared according to **GP9** using (*Z*)-1-(4-methoxybut-1-en-1-yl)naphthalene as the starting material. Purification by flash column chromatography (SiO_2, 0-18% v/v Acetone in Petrol) afforded the title compound as a yellow oil which solidified on standing to a yellow amorphous solid (25.7 mg, 0.057 mmol, 57%, 59% *ee*).

***R_f* value** = 0.46 (20% v/v Acetone in Hexane);

^{1}H NMR (500 MHz, $CDCl_3$) δ 7.99 (dd, *J* = 8.0 Hz, 1.0 Hz, 1H, H-1), 7.90-7.92 (m, 1H, H-4), 7.85 (d, *J* = 8.1 Hz, 1H, H-5), 7.53-7.59 (m, 3H, H-2, H-3, H-7), 7.48 (dd, *J* = 8.1 Hz, 7.4 Hz, 1H, H-6), 4.82 (t, *J* = 12.2 Hz, 2H, H-10a, H-10b), 4.53 (d, *J* = 7.3 Hz, 1H, H-8), 3.44-3.48 (m, 1H, H-9), 3.20-3.28 (m, 2H, H-13a, H-13b), 3.17 (s, 3H, H-14), 1.54-1.71 (m, 2H, H-11a, H-11b), 1.40-1.47 (m, 1H, H-12a), 1.18-1.26 (m, 1H, H-12b) ppm;

^{13}C NMR (126 MHz, $CDCl_3$) δ 133.5, 131.3, 129.04, 129.00, 127.7, 126.9, 126.4, 125.5, 125.4, 122.7, 118.3 (qt, J_{C-F} = 286.1 Hz, 34.2 Hz), 111.3 (tq, J_{C-F} = 257.1 Hz, 38.6 Hz), 71.6, 65.8 (t, J_{C-F} = 28.6 Hz), 58.5, 49.1, 47.2, 26.9, 23.6 ppm;

^{19}F NMR (376 MHz, $CDCl_3$) δ – 84.4 (s), – 124.4 (s) ppm;

IR (film, cm^{-1}): ν_{max} 2927 (w), 1599 (w), 1511 (w), 1451 (w), 1377 (s), 1257 (w), 1203 (s), 1179 (s), 1111 (s), 1040 (w), 1011 (m), 988 (m), 907 (m), 835 (m), 801 (m), 778 (m), 739 (w), 720 (w), 657 (w), 625 (m), 595 (m);

$[\alpha]_D^{25.0}$ = + 76.9 (c. 1.0, $CHCl_3$);

HRMS (−ESI) *m/z* Found $[M+HO]^-$ 470.1061, $[C_{19}H_{21}F_5NO_5S]^-$ requires 470.1066, (δ = − 1.1 ppm);

Chiral HPLC Analysis CHIRAL ART SC (Hexane:^{i}PrOH, 99:1, 1.25 mL min^{-1}, 40 °C) t_R = 6.9 (major), 9.0 (minor) minutes.

In order to assign the absolute stereochemistry of ***17****, we repeated the reaction using the free alcohol substrate followed by O-methylation of the intermediate aziridine alcohol (see below). This afforded the same product which was identical by NMR analysis but was obtained in 95% ee. Comparison of (i) the sign of the optical rotations for both products and (ii) the order of peak elution by chiral HPLC confirmed that the absolute stereochemistry of the –OMe aziridine is the same as the absolute stereochemistry of the aziridine obtained using the substrate with a terminal alcohol.*

2,2,3,3,3-pentafluoropropyl (2R,3S)-2-(3-methoxypropyl)-3-(naphthalen-1-yl)aziridine-1-sulfonate

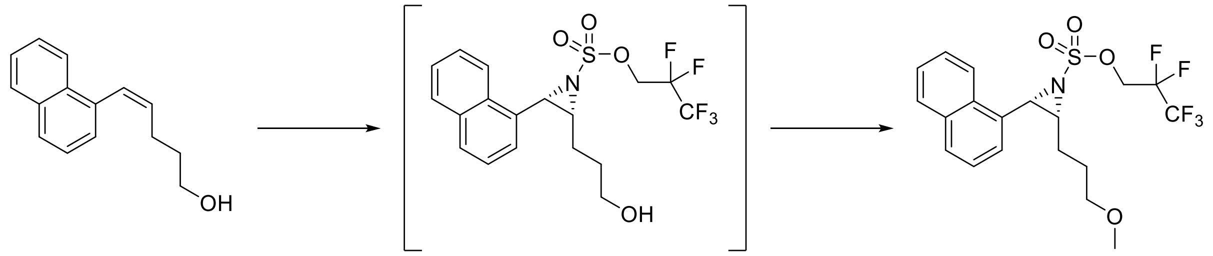

Obtained from the alcohol starting material:

The intermediate aziridine was prepared according to **GP9** on a 0.2 mmol scale using (*Z*)-5-(naphthalen-1-yl)pent-4-en-1-ol as the starting material. Purification by flash column chromatography (SiO_2, 0-12% v/v acetone in $CHCl_3$) afforded the intermediate aziridine (2,2,3,3,3-pentafluoropropyl (2*R*,3*S*)-2-(3-hydroxypropyl)-3-(naphthalen-1-yl)aziridine-1-sulfonate) as a colourless oil. The aziridine was taken forward to the subsequent methylation step immediately

A microwave vial charged with Proton-sponge® (66 mg, 0.308 mmol) and trimethyloxonium tetrafluoroborate (38 mg, 0.257 mmol) was placed under a nitrogen atmosphere following which CH_2Cl_2 (1.5 mL) was added. 2,2,3,3,3-pentafluoropropyl (2*R*,3*S*)-2-(3-hydroxypropyl)-3-(naphthalen-1-yl)aziridine-1-sulfonate (47 mg, 0.107 mmol) in CH_2Cl_2 (2.0 mL) was added at room temperature and the resulting mixture was stirred for 2 hours. The reaction was quenched with addition of saturated aqueous $NaHCO_3$ solution (2.0 mL), the organic layer separated and the aqueous extracted with CH_2Cl_2. The combined organics were washed with saturated aqueous $NaHCO_3$ solution (3 × 5 mL), dried over $MgSO_4$ and concentrated under reduced pressure. The crude was purified by flash column chromatography (SiO_2, 0-20% v/v EtOAc in hexane) to afford the title compound as an off-white solid (21 mg, 0.046 mmol, 23% *from alkene*, 95% *ee*).

The spectroscopic data matched that of **17***, synthesised through aziridination of the corresponding –OMe alkene. See above for full characterisation. However, for this more enantioenriched sample,* $[\boldsymbol{\alpha}]_{\mathbf{D}}^{\mathbf{25.0}}$ = + 124.9 (c = 1.2, $CHCl_3$).

Characterisation of Aziridines Obtained from Substrates Bearing Alternative Hydrogen Bond-Donors

2,2,3,3,3-pentafluoropropyl ((R)-((S)-5-oxotetrahydrofuran-2-yl)(phenyl)methyl)sulfamate (**18**)

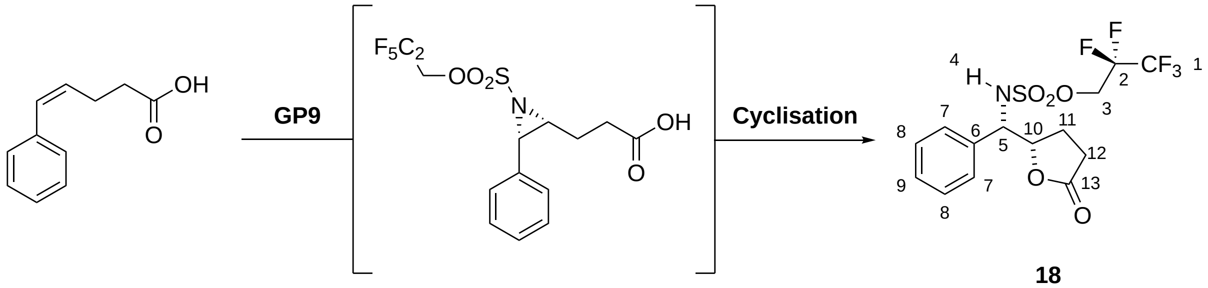

18

Prepared according to **GP9** using (*Z*)-5-phenylpent-4-enoic acid as the starting material. Following the cyclisation protocol (2 hours), the volatiles were removed under reduced pressure and the crude reaction mixture was analysed by ^{1}H NMR. This revealed only 32% conversion of the starting material along with 18% product formation. Following analysis, the volatiles were removed under reduced pressure and the crude residue was taken up in $CHCl_3$ and washed with saturated aqueous $NaHCO_3$ solution to remove the residual acid starting material. The aqueous washings were discarded and the combined organic fraction was concentrated. Purification of the crude residue by flash column chromatography (SiO_2, 0-12% v/v Acetone in $CHCl_3$) afforded the title compound as a colourless oil (8.3 mg, 0.021 mmol, 21%, 16% *ee*). Full characterisation was obtained for an authentic sample which was synthesised separately and the data is presented below for completion.

***R_f* value =** 0.26 (10% v/v Acetone in $CHCl_3$);

^{1}H NMR (500 MHz, CD_3CN) δ 7.45-7.37 (m, 5 H, H7, H8, H9), 7.03 (br s, 1 H, H4), 4.70 (ddd, J = 13.5, 8.0, 6.6 Hz, 1 H, H10), 4.55 (d, J = 6.4 Hz, 1 H, H5), 4.43 (qd, J = 12.8, 1.0 Hz, 1 H, H3a), 4.21 (qd, J = 12.8, 1.0 Hz, 1 H, H3b), 2.55-2.42 (m, 2 H, H12a, H12b), 2.09-1.96 (m, 2 H, H11a, H11b) ppm;

^{13}C NMR (126 MHz, CD_3CN) δ 177.3 (C-13), 138.4 (C-6), 129.8 (C-8), 129.5 (C-9), 128.4 (C-7), 119.1 (qt, $J_{C\text{-}F}$ = 286.4, 34.0 Hz, C-1)*, 112.6 (tq, $J_{C\text{-}F}$ = 255.0, 38.4 Hz, C-2), 82.0 (C-10), 64.6 (t, $J_{C\text{-}F}$ = 28.0 Hz, C-3), 62.7 (C-5), 28.9 (C-12), 25.5 (C-11) ppm;

*Partially obscured by the residual CD_3CN signal.

^{19}F NMR (471 MHz, CD_3CN) δ – 84.5 (F-1), – 124.3 (F-2) ppm;

IR (film, cm^{-1}): ν_{max} 3284 (br, w), 2924 (w), 1767 (s), 1458 (m), 1375 (m), 1259 (w), 1180 (s), 1154 (s), 1107 (m), 1044 (m), 1009 (s), 949 (m), 877 (w), 842 (w), 754 (s), 702 (m), 667 (w), 624 (m), 573 (m), 516 (m);

HRMS (–ESI): *m/z* found $[M–H]^-$ 402.0438, $[C_{14}H_{13}F_5NO_5S]^-$ requires 402.0440, (δ = – 0.5 ppm);

Chiral SFC analysis: CHIRALPAK IG (CO_2:MeOH = 97:3, 2.5 mL min^{-1}, 40 °C), t_R = 5.9 (minor), 6.4 (major) minutes.

2,2,3,3,3-pentafluoropropyl (2S,3R)-2-phenyl-3-(3-(2,2,2-trifluoroacetamido)propyl)aziridine-1-sulfonate (**19**)

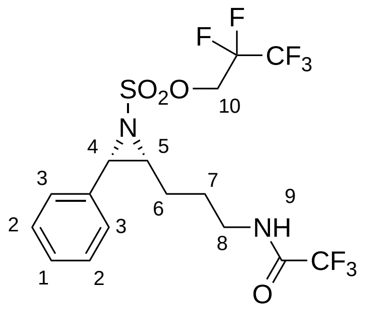

Prepared according to **GP9** using (*Z*)-2,2,2-trifluoro-*N*-(5-phenylpent-4-en-1-yl)acetamide as the starting material. Purification by flash column chromatography (SiO_2, 0-25% v/v Acetone in Petrol) afforded the title compound as a yellow oil (37.3 mg, 0.077 mmol, 77%, 17% *ee*).

***R_f* value** = 0.51 (30% v/v Acetone in Petrol);

^{1}H NMR (700 MHz, $CDCl_3$) δ 7.35-7.40 (m, 3H, H-1, H-2), 7.29 (d, *J* = 6.9 Hz, 2H, H-3), 6.23 (br s, 1H, H-9), 4.75 (sext, *J* = 12.4 Hz, 2H, H-10a, H-10b), 4.08 (d, *J* = 7.3 Hz, 1H, H-4), 3.26-3.36 (m, 2H, H-8a, H-8b), 3.16 (td, *J* = 7.5 Hz, 5.9 Hz, 1H, H-5), 1.71-1.77 (m, 1H, H-7a), 1.64-1.70 (m, 1H, H-7b), 1.35-1.45 (m, 2H, H-6a, H-6b) ppm;

^{13}C NMR (176 MHz, $CDCl_3$) δ 157.4 (q, J_{C-F} = 37.0 Hz), 131.2, 128.9*, 127.2, 118.2 (qt, J_{C-F} = 286.0 Hz, 34.5 Hz), 115.8 (q, J_{C-F} = 287.6 Hz), 111.2 (tq, J_{C-F} = 257.3 Hz, 38.4 Hz), 65.8 (t, J_{C-F} = 28.5 Hz), 48.0, 47.8, 39.0, 26.1, 23.1 ppm;

*^{1}H-^{13}C HMBC shows that this signal corresponds to two ^{13}C environments.

^{19}F NMR (376 MHz, $CDCl_3$) δ – 77.0 (s), – 84.5 (s), – 124.5 (s) ppm;

IR (film, cm^{-1}): ν_{max} 3314 (br, w), 2958 (w), 1707 (s), 1556 (m), 1499 (w), 1454 (m), 1375 (s), 1203 (s), 1176 (s), 1109 (m), 1041 (m), 1007 (s), 897 (m), 848 (m), 771 (w), 724 (w), 700 (w), 670 (w), 623 (w), 591 (m), 567 (w), 518 (w);

HRMS (+ESI) *m/z* found $[M+Na]^+$ 507.0593, $[C_{16}H_{16}F_8N_2NaO_4S]^+$ requires 507.0595, (δ = – 0.4 ppm);

Chiral HPLC analysis: CHIRAL ART SC (Hexane:^{i}PrOH, 97:3, 1.25 mL min^{-1}, 40 ˚C), t_R = 9.4 (major), 11.0 (minor) minutes.

2,2,3,3,3-pentafluoropropyl (2R,3S)-2-(3-((4-methylphenyl)sulfonamido)propyl)-3-phenylaziridine-1-sulfonate (**20**)

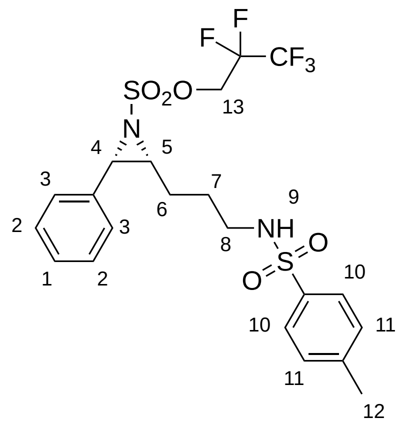

Prepared according to **GP9** using (*Z*)-4-methyl-*N*-(5-phenylpent-4-en-1-yl)benzenesulfonamide as the starting material. Purification by flash column chromatography (SiO_2, 0-10% v/v Acetone in $CHCl_3$) afforded the title compound as a pale-yellow oil (44.0 mg, 0.081 mmol, 81%, 48% *ee*).

***R*$_f$ value** = 0.68 (10% v/v Acetone in $CHCl_3$);

^{1}H NMR (700 MHz, $CDCl_3$) δ 7.68 (d, *J* = 8.2 Hz, 2H, H-10), 7.34-7.39 (m, 3H, H-1, H-2), 7.27-7.29 (m, 4H, H-3, H-11), 4.73 (sext, *J* = 12.1 Hz, 2H, H-13a, H-13b), 4.42 (t, *J* = 6.2 Hz, 1H, H-9), 4.03 (d, *J* = 7.3 Hz, 1H, H-4), 3.09 (td, *J* = 7.8 Hz, 5.8 Hz, 1H, H-5), 2.83-2.93 (m, 2H, H-8a, H-8b), 2.42 (s, 3H, H-12), 1.58-1.64 (m, 1H, H-7a), 1.51-1.57 (m, 1H, H-7b), 1.39-1.44 (m, 1H, H-6a), 1.30-1.36 (m, 1H, H-6b) ppm;

^{13}C NMR (176 MHz, $CDCl_3$) δ 143.7, 136.8, 131.3, 129.9, 128.8, 128.7, 127.3, 127.2, 118.3 (qt, $J_{C\text{-}F}$ = 286.2 Hz, 34.2 Hz), 111.2 (tq, $J_{C\text{-}F}$ = 257.4 Hz, 38.5 Hz), 65.8 (t, $J_{C\text{-}F}$ = 28.7 Hz), 48.02, 48.00, 42.3, 26.8, 23.1, 21.6 ppm;

^{19}F NMR (376 MHz, $CDCl_3$) δ – 83.4 (s), – 123.5 (s) ppm;

IR (film, cm^{-1}): ν_{max} 3286 (br, w), 2925 (w), 1599 (w), 1497 (w), 1453 (w), 1376 (m), 1326 (m), 1204 (s), 1179 (s), 1156 (s), 1107 (m), 1094 (m), 1040 (m), 1001 (m), 956 (w), 906 (m), 846 (m), 815 (m), 775 (w), 732 (m), 700 (m), 633 (m), 624 (w), 590 (w), 551 (m), 517 (m), 475 (m);

$[\alpha]_D^{25.0}$ **=** – 23.7 (c. 2.2, CH_3CN);

HRMS (+ESI) *m/z* found $[M+H]^+$ 543.1036, $[C_{21}H_{24}F_5N_2O_5S_2]^+$ requires 543.1041, (δ = – 0.9 ppm);

Chiral SFC analysis: CHIRALPAK IG (CO_2:MeOH, 89:11, 1.25 mL min^{-1}, 40 ˚C), t_R = 9.5 (major), 10.1 (minor) minutes.

Characterisation of Allylic and Tetrahomoallylic Styrenyl Aziridines

2,2,3,3,3-pentafluoropropyl (2S,3S)-2-(hydroxymethyl)-3-phenylaziridine-1-sulfonate (**21**)

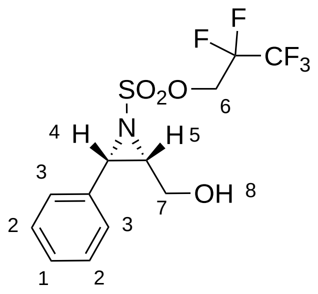

Prepared according to **GP9** using (*Z*)-3-phenylprop-2-en-1-ol as the starting material. Purification by flash column chromatography (SiO_2, 0-10% v/v Acetone in $CHCl_3$) afforded the title compound as an off-white powder (12.2 mg, 0.034 mmol, 34%, 45% *ee*).

R

R_f value = 0.51 (10% v/v Acetone in $CHCl_3$);

^{1}H NMR (400 MHz, $CDCl_3$) δ 7.33-7.38 (m, 5H, H-1, H-2, H-3), 4.73-4.86 (m, 2H, H-6a, H-6b), 4.16 (d, J = 7.3 Hz, 1H, H-4), 3.57-3.63 (m, 1H, H-7a), 3.47-3.53 (m, 1H, H-7b), 3.36 (q, J = 6.6 Hz, 1H, H-5), 1.61 (br s, 1H, H-8) ppm;

^{13}C NMR (126 MHz, $CDCl_3$) δ 131.0, 128.9*, 127.2, 118.3 (qt, J_{C-F} = 286.2 Hz, 34.3 Hz), 111.2 (tq, J_{C-F} = 257.1 Hz, 38.6 Hz), 66.0 (t, J_{C-F} = 28.6 Hz), 59.2, 48.0, 47.0 ppm;

*^{1}H-^{13}C HMBC shows that this corresponds to 2 carbon environments

^{19}F NMR (376 MHz, $CDCl_3$) δ – 84.4 (s), – 124.5 (s) ppm;

IR (film, cm^{-1}): ν_{max} 3399 (br, w), 2924 (w), 1454 (w), 1376 (m), 1205 (s), 1180 (s), 1108 (m), 1040 (m), 1007 (m), 925 (m), 857 (m), 768 (w), 738 (w), 700 (m), 625 (w), 590 (m);

$[\alpha]_D^{25.0}$ = + 27.2 (c. 1.0, $CHCl_3$);

HRMS (–ESI) *m/z* Found [M–H]$^-$ 360.0338, $[C_{12}H_{11}F_5NO_4S]^-$ requires 360.0334, (δ = + 1.1 ppm);

Chiral SFC Analysis CHIRAL ART SC (CO_2:MeOH, 98.5:1.5, 2.5 mL min^{-1}, 40 °C) t_R = 4.1 (major), 4.9 (minor) minutes.

2,2,3,3,3-pentafluoropropyl (2R,3S)-2-(5-hydroxypentyl)-3-phenylaziridine-1-sulfonate (**22**)

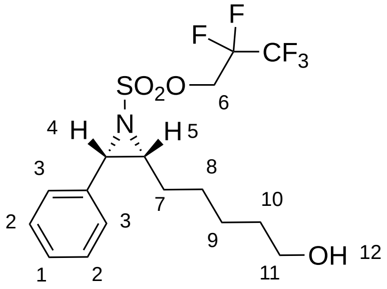

Prepared according to **GP9** using (*Z*)-7-phenylhept-6-en-1-ol as the starting material. Purification by flash column chromatography (SiO_2, 0-10% v/v Acetone in $CHCl_3$) afforded the title compound as a colourless oil (22.2 mg, 0.053 mmol, 53%, 67% *ee*).

***R$_f$* value** = 0.43 (10% v/v Acetone in $CHCl_3$);

^{1}H NMR (400 MHz, $CDCl_3$) δ 7.29-7.38 (m, 5H, H-1, H-2, H-3), 4.67-4.80 (m, 2H, H-6a, H-6b), 4.05 (d, *J* = 7.3 Hz, 1H, H-4), 3.57 (t, *J* = 6.4 Hz, 2H, H-11a, H-11b), 3.15 (q, *J* = 6.4 Hz, 1H, H-5), 1.26-1.46 (m, 9H, H-7a, H-7b, H-8a, H-8b, H-9a, H-9b, H-10a, H-10b, H-12) ppm;

^{13}C NMR (126 MHz, $CDCl_3$) δ 131.7, 128.7, 128.6, 127.3, 118.3 (qt, $J_{C\text{-}F}$ = 286.1 Hz, 34.3 Hz), 111.3 (tq, $J_{C\text{-}F}$ = 256.9 Hz, 38.6 Hz), 65.7 (t, $J_{C\text{-}F}$ = 28.7 Hz), 62.7, 48.9, 47.9, 32.4, 26.4, 26.0, 25.2 ppm;

^{19}F NMR (376 MHz, $CDCl_3$) δ – 84.4 (s), – 124.4 (s) ppm;

IR (film, cm^{-1}): ν_{max} 3349 (br, w), 2935 (w), 2863 (w), 1499 (w), 1454 (w), 1376 (m), 1204 (s), 1180 (s), 1109 (m), 1042 (m), 1008 (m), 903 (m), 849 (m), 772 (w), 738 (w), 700 (m), 671 (w), 623 (w), 592 (m), 566 (w), 518 (w);

$[\alpha]_D^{25.0}$ = + 49.5 (c. 1.0, $CHCl_3$);

HRMS (–ESI) *m/z* Found [M–H]$^-$ 416.0959, $[C_{16}H_{19}F_5NO_4S]^-$ requires 416.0960, (δ = – 0.2 ppm);

Chiral SFC Analysis CHIRAL ART SJ (CO_2:MeOH, 98:2, 2.5 mL min^{-1}, 40 °C) t_R = 3.0 (major), 3.6 (minor) minutes.

Characterisation of Simple Aliphatic Aziridines

2,2,3,3,3-pentafluoropropyl ((2S,3R)-2-methyltetrahydrofuran-3-yl)sulfamate (**24a**)

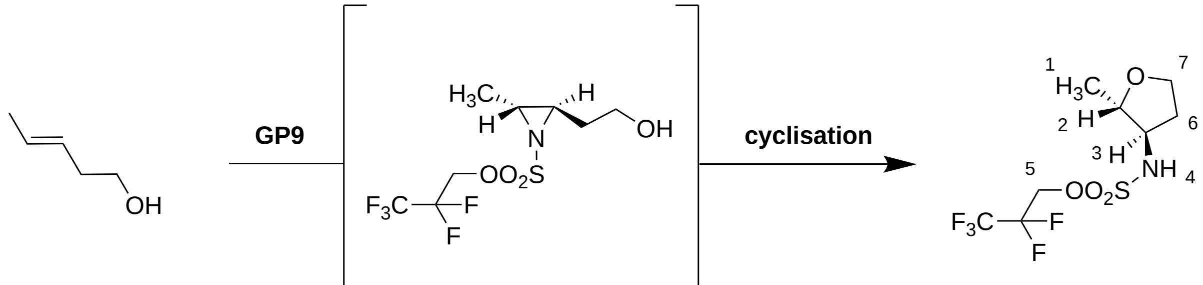

24a

Prepared according to **GP9** using (*E*)-pent-3-en-1-ol (**23a**) as the starting material. The crude aziridine was cyclised according to the cyclisation protocol (16 hours) in order to aid *ee* determination by chiral GC. Purification by flash column chromatography (SiO_2, 0-10% v/v Et_2O in CH_2Cl_2) afforded the title compound as a colourless oil (22.3 mg, 0.071 mmol, 71%, 89% *ee*).

***R_f* value** = 0.33 (10% v/v Et_2O in CH_2Cl_2);

^{1}H NMR (500 MHz, $CDCl_3$) δ 5.13 (d, J = 8.3 Hz, 1H, H-4), 4.50 (tq, J = 12.4 Hz, 0.9 Hz, 2H, H-5a, H-5b), 3.97 (td, J = 8.7 Hz, 4.5 Hz, 1H, H-7a), 3.82-3.89 (m, 2H, H-2, H-7b), 3.64-3.69 (m, 1H, H-3), 2.34-2.42 (m, 1H, H-6a), 1.91-1.97 (m, 1H, H-6b), 1.26 (d, J = 6.3 Hz, 3H, H-1) ppm;

^{13}C NMR (126 MHz, $CDCl_3$) δ 118.3 (qt, $J_{C\text{-}F}$ = 286.1 Hz, 34.4 Hz), 111.5 (tq, $J_{C\text{-}F}$ = 256.8 Hz, 38.3 Hz), 80.0, 65.8, 64.1 (t, $J_{C\text{-}F}$ = 28.3 Hz), 60.6, 32.6, 18.8 ppm;

^{19}F NMR (376 MHz, $CDCl_3$) δ – 83.5 (s), – 123.5 (s) ppm;

IR (film, cm^{-1}): ν_{max} 3299 (br, w), 3144 (br, w), 2981 (br, w), 1458 (m), 1375 (m), 1261 (w), 1202 (s), 1183 (s), 1107 (s), 1010 (s), 941 (m), 807 (m), 774 (m), 668 (w), 622 (m), 585 (m), 518 (w);

$[\alpha]_D^{25.0}$ = – 16.3 (c. 1.0, $CHCl_3$);

HRMS (–ESI) *m/z* Found [M–H]$^-$ 312.0327, $[C_8H_{11}F_5NO_4S]^-$ requires 312.0334, (δ = – 2.2 ppm);

Chiral GC Analysis CP7502 – CP-Chirasil-DEX CB column (130 °C Isothermal, 25.0 psi) t_R = 16.2 (major), 18.0 (minor) minutes.

2,2,3,3,3-pentafluoropropyl (2R,3S)-2-(2-hydroxyethyl)-3-methylaziridine-1-sulfonate (**24b**)

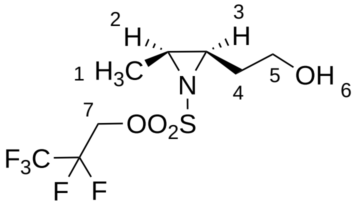

Prepared according to **GP9** using (*Z*)-pent-3-en-1-ol (**23b**) as the starting material. Purification by flash column chromatography (SiO_2, 0-10% v/v Et_2O in CH_2Cl_2) afforded the title compound as a colourless oil (20.3 mg, 0.065 mmol, 65%, 38% *ee* as determined from ring opening with thiophenolate).

***R_f* value** = 0.30 (10% v/v Et_2O in CH_2Cl_2);

^{1}H NMR (500 MHz, $CDCl_3$) δ 4.61-4.73 (m, 2H, H-7a, H-7b), 3.80-3.90 (m, 2H, H-5a, H-5b), 3.00-3.06 (m, 2H, H-2, H-3), 1.87-1.93 (m, 1H, H-4a), 1.80 (br s, 1H, H-6), 1.65-1.73 (m, 1H, H-4b), 1.31-1.34 (m, 3H, H-1) ppm;

^{13}C NMR (126 MHz, $CDCl_3$) δ 118.3 (qt, J_{C-F} = 286.1 Hz, 34.3 Hz), 111.3 (tq, J_{C-F} = 256.8 Hz, 38.5 Hz), 65.6 (t, J_{C-F} = 28.6 Hz), 60.4, 44.7, 42.6, 29.1, 12.0 ppm;

^{19}F NMR (376 MHz, $CDCl_3$) δ – 83.5 (s), – 123.7 (s) ppm;

IR (film, cm^{-1}): ν_{max} 1373 (s), 1257 (s), 1179 (s), 1108 (w), 1044 (m), 1014 (m), 998 (m), 971 (w), 920 (w), 842 (w), 798 (w), 783 (w);

$[\alpha]_D^{25.0}$ = – 1.3 (c. 1.0, $CHCl_3$);

HRMS (–ESI) *m/z* Found $[M+CHO_2]^-$ 358.0401, $[C_9H_{13}F_5NO_6S]^-$ requires 358.0389, (δ = + 3.4 ppm);

*Conversion of **24b** to the thiophenolate adduct for ee determination: 2,2,3,3,3-pentafluoropropyl ((3R,4R)-1-hydroxy-4-(phenylthio)pentan-3-yl)sulfamate and 2,2,3,3,3-pentafluoropropyl ((2S,3S)-5-hydroxy-3-(phenylthio)pentan-2-yl)sulfamate **(Inseparable mixture)***

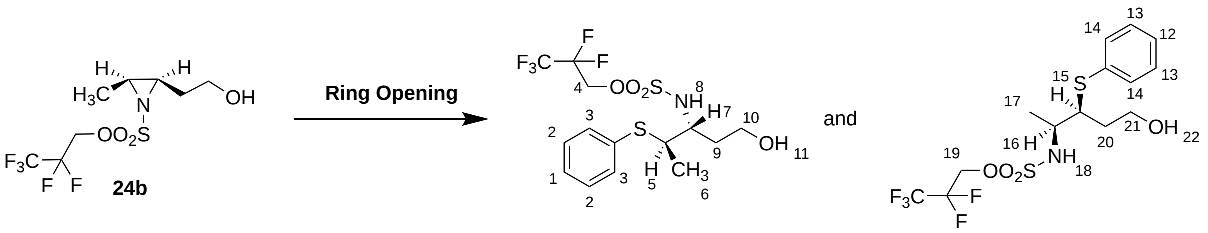

Prepared according to a variant of the protocol described by Du Bois and Guthikonda.[73] To a solution of 2,2,3,3,3-pentafluoropropyl (2*R*,3*S*)-2-(2-hydroxyethyl)-3-methylaziridine-1-sulfonate (**24b**) (20.3 mg, 0.065 mmol) in MeOH (2.5 mL) were added thiophenol (4 drops) and Et_3N (8 drops). The reaction mixture was stirred at room temperature for 2 hours, following which the volatiles were removed under a stream of compressed air. Purification by flash column chromatography (SiO_2, 0-12 % v/v Acetone in $CHCl_3$) afforded an inseparable

mixture of the title compounds as a colourless oil (23.1 mg, 0.054 mmol, 84%). The enantiomers from one set of the regioisomers were separable by chiral HPLC and indicated 38% *ee*.

***R**$_f$* **value** = 0.40 (12% v/v Et_2O in CH_2Cl_2);

^{1}H NMR (500 MHz, $CDCl_3$) δ 7.42-7.45 (m, 4H, H-3, H-14), 7.29-7.33 (m, 4H, H-2, H-13), 7.24-7.27 (m, 2H, H-1, H-12), 5.86 (d, *J* = 8.7 Hz, 1H, H-18), 5.73 (d, *J* = 6.6 Hz, 1H, H-8), 4.37-4.50 (m, 4H, H-4a, H-4b, H-19a, H-19b), 3.64-3.96 (m, 7H, H-5, H-7, H-10a, H-10b, H-16, H-21a, H-21b), 3.50-3.54 (m, 1H, H-15), 2.04-2.10 (m, 1H, H-9a), 1.96-2.03 (m, 1H, H-20a), 1.80-1.87 (m, 2H, H-20b, –OH), 1.65-1.74 (m, 2H, H-9b, –OH), 1.34 (d, *J* = 7.1 Hz, 3H, H-6), 1.31 (d, *J* = 6.7 Hz, 3H, H-17) ppm;

^{13}C NMR (126 MHz, $CDCl_3$) δ 134.6, 133.9, 132.0, 131.6, 129.4, 129.3, 127.6*, 114.6-122.0 (m)*, 109.0-114.1 (m)*, 63.7-64.3 (m)*, 60.7, 60.2, 57.8, 54.2, 51.8, 46.4, 33.6, 32.3, 17.8, 16.3 ppm;

*Corresponds to two ^{13}C environments.

^{19}F NMR (471 MHz, $CDCl_3$) δ – 83.76 (s), – 83.79 (s), – 124.3 - – 123.1 (m)*, – 123.7 (s) ppm;

*Partial inequivalence leads to extreme strong coupling between these two ^{19}F nuclei.

IR (film, cm^{-1}): ν_{max} 3547 (br, w), 3312 (br, w), 2956 (w), 1584 (w), 1440 (w), 1365 (m), 1297 (w), 1259 (w), 1204 (s), 1181 (s), 1109 (m), 1048 (m), 1007 (s), 938 (w), 807 (m), 775 (m), 748 (m), 693 (m), 623 (m), 553 (w), 517 (m);

HRMS (–ESI) *m/z* Found $[M–H]^-$ 422.0541, $[C_{14}H_{17}F_5NO_4S_2]^-$ requires 422.0525, (δ = + 3.8 ppm);

Chiral HPLC Analysis CHIRALPAK IC (Hexane:^{i}PrOH, 96:4, 1.0 mL min^{-1}, 30 °C) for Regioisomer 1 t_R = 9.2 (minor), 9.9 (major) minutes, for Regioisomer 2 t_R = 14.0 minutes (major and minor co-eluted).

2,2,3,3,3-pentafluoropropyl (S)-2-(2-hydroxyethyl)-2-methylaziridine-1-sulfonate (**24c**)

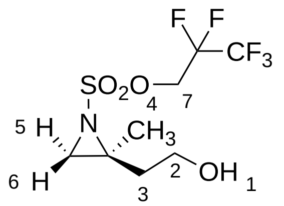

Prepared according to **GP9** using 3-methylbut-3-en-1-ol (**23c**) as the starting material. Purification by flash column chromatography (SiO_2, 0-10% v/v Et_2O in CH_2Cl_2) afforded the title compound as a colourless oil (19.9 mg, 0.064 mmol, 64%, 74% *ee* as determined from ring-opening of the aziridine with thiophenolate).

***R$_f$* value** = 0.35 (10% v/v Et_2O in CH_2Cl_2);

^{1}H NMR (500 MHz, $CDCl_3$) δ 4.60-4.71 (m, 2H, H-7a, H-7b), 3.79-3.88 (m, 2H, H-2a, H-2b), 2.66 (s, 1H, H-5), 2.58 (s, 1H, H-6), 1.98 (ddd, *J* = 14.4 Hz, 6.7 Hz, 5.5 Hz, 1H, H-3a), 1.86 (ddd, *J* = 14.4 Hz, 6.3 Hz, 5.7 Hz, 1H, H-3b), 1.63 (br s, 3H, H-4) ppm;

^{13}C NMR (126 MHz, $CDCl_3$) δ 118.3 (qt, $J_{C\text{-}F}$ = 286.1 Hz, 34.3 Hz), 111.4 (tq, $J_{C\text{-}F}$ = 256.6 Hz, 38.4 Hz), 65.5 (t, $J_{C\text{-}F}$ = 28.7 Hz), 59.2, 48.7, 42.9, 39.4, 18.7 ppm;

^{19}F NMR (376 MHz, $CDCl_3$) δ – 83.5 (s), – 123.5 (t, $J_{H\text{-}F}$ = 12.2 Hz) ppm; *This NMR spectrum was acquired with proton coupling*;

IR (film, cm^{-1}): ν_{max} 3415 (br, w), 2949 (br, w), 1371 (s), 1204 (s), 1180 (s), 1109 (m), 1042 (s), 1011 (s), 944 (m), 834 (s), 678 (w), 624 (w), 587 (w);

$[\alpha]_D^{25.0}$ = + 16.8 (c. 1.0, $CHCl_3$);

HRMS (+ESI) *m/z* Found $[M+H]^+$ 314.0491, $[C_8H_{13}F_5NO_4S]^+$ requires 314.0480, (δ = + 3.5 ppm);

The opposite enantiomer (*ent*-**24c**) was prepared as above with the following procedural modification: Rh_2(**B**)$_2$•(**C5**)$_2$•(Pyr)$_2$ was used in place of Rh_2(**B**)$_2$•(**C3**)$_2$•(Pyr)$_2$. Purification by flash column chromatography (SiO_2, 0-10% v/v Et_2O in CH_2Cl_2) afforded 2,2,3,3,3-pentafluoropropyl (*R*)-2-(2-hydroxyethyl)-2-methylaziridine-1-sulfonate (22.5 mg, 0.072 mmol, 72%, 75% *ee* as determined from ring opening with thiophenolate). For this enantiomer, $[\alpha]_D^{25.0}$ = – 21.0 (c. 1.0, $CHCl_3$).

When Rh_2(**B**)$_2$•(**C4**)$_2$•(Pyr)$_2$ was used in place of Rh_2(**B**)$_2$•(**C3**)$_2$•(Pyr)$_2$ under identical conditions, *ent*-**24c** was obtained in 63% yield and 65% *ee*. In this case $[\alpha]_D^{26.6}$ = – 16.6 (c. 1.0, $CHCl_3$).

Conversion of **24c** *to the thiophenolate adduct for ee determination: 2,2,3,3,3-pentafluoropropyl (S)-(4-hydroxy-2-methyl-1-(phenylthio)butan-2-yl)sulfamate*

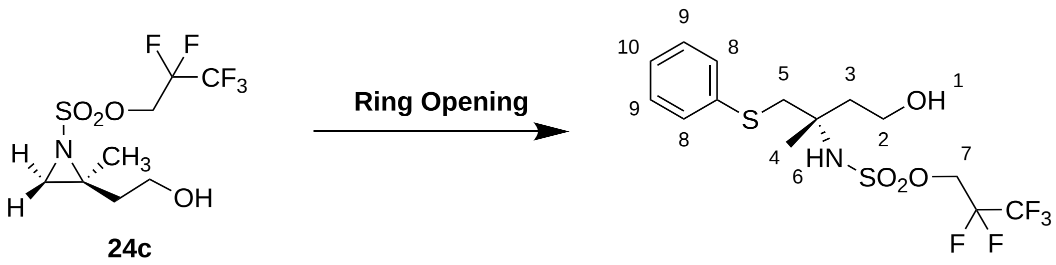

Prepared according to the protocol described by Du Bois and Guthikonda.[73] To a solution of 2,2,3,3,3-pentafluoropropyl (*S*)-2-(2-hydroxyethyl)-2-methylaziridine-1-sulfonate (**24c**) (19.2 mg, 0.061 mmol) in MeOH (2.5 mL) were added thiophenol (17.4 µL, 0.17 mmol) and Et_3N (47.7 µL, 0.34 mmol). The reaction mixture was stirred at room temperature for 2 hours, following which the volatiles were removed under a stream of compressed air. Purification

by flash column chromatography (SiO_2, 0-10 % v/v Et_2O in CH_2Cl_2) afforded the title compound as a thick colourless oil (17.3 mg, 0.041 mmol, 67%, 74% *ee*).

***R_f* value** = 0.53 (10% v/v Et_2O in CH_2Cl_2);

^{1}H NMR (500 MHz, $CDCl_3$) δ 7.38-7.40 (m, 2H, H-8), 7.30 (tt, *J* = 7.4 Hz, 1.9 Hz, 2H, H-9), 7.21 (tt, *J* = 7.4 Hz, 1.2 Hz, 1H, H-10), 6.60 (br s, 1H, H-6), 4.46 (t, *J* = 12.6 Hz, 2H, H-7a, H-7b), 3.85-3.92 (m, 2H, H-2a, H-2b), 3.50 (d, *J* = 13.3 Hz, 1H, H-5a), 3.35 (d, *J* = 13.3 Hz, 1H, H-5b), 2.05 (ddd, *J* = 15.1 Hz, 6.3 Hz, 4.1 Hz, 1H, H-3a), 1.88 (ddd, *J* = 15.1 Hz, 6.9 Hz, 4.9 Hz, 1H, H-3b), 1.52 (s, 3H, H-4) ppm;

^{13}C NMR (126 MHz, $CDCl_3$) δ 136.2, 130.1, 129.3, 126.9, 118.4 (qt, $J_{C\text{-}F}$ = 287.0 Hz, 33.5 Hz), 111.7 (tq, $J_{C\text{-}F}$ = 256.4 Hz, 38.3 Hz), 63.9 (t, $J_{C\text{-}F}$ = 28.1 Hz), 61.1, 59.5, 44.0, 40.0, 23.4 ppm;

^{19}F NMR (376 MHz, $CDCl_3$) δ – 83.5 (s), – 123.9 - – 122.5 (m)*ppm;

*Partial inequivalence leads to extreme strong coupling between these two ^{19}F nuclei.

IR (neat, cm^{-1}): ν_{max} 3555 (w), 3288 (br, w), 2934 (w), 1583 (w), 1481 (w), 1439 (w), 1365 (s), 1259 (w), 1201 (s), 1176 (s), 1108 (s), 1050 (m), 999 (s), 807 (m), 774 (m), 740 (s), 691 (m), 671 (w), 622 (m), 560 (m), 516 (m);

$[\alpha]_D^{25.0}$ = – 12.8 (c. 1.1, $CHCl_3$);

HRMS (–ESI) *m/z* Found $[M–H]^-$ 422.0517, $[C_{14}H_{17}F_5NO_4S_2]^-$ requires 422.0525, (δ = – 1.9 ppm);

Chiral SFC Analysis CHIRALPAK IE (CO_2:MeOH, 97:3, 2.5 mL min^{-1}, 40 °C) t_R = 9.2 (minor), 11.6 (major) minutes.

The opposite enantiomer was prepared as above with the following procedural modification: (*R*)-2-(2-hydroxyethyl)-2-methylaziridine-1-sulfonate (*ent*-**24c**) was used as the starting

material. Purification by flash column chromatography (SiO_2, 0-10% v/v Et_2O in CH_2Cl_2) afforded 2,2,3,3,3-pentafluoropropyl (*R*)-(4-hydroxy-2-methyl-1-(phenylthio)butan-2-yl)sulfamate (21.1 mg, 0.050 mmol, 69%, 75% *ee*). For this enantiomer, $[\boldsymbol{\alpha}]_{\mathbf{D}}^{\mathbf{25.0}}$ = + 19.8 (c. 1.0, $CHCl_3$).

2,2,3,3,3-pentafluoropropyl (R)-(2,2-dimethyltetrahydrofuran-3-yl)sulfamate (**24d**)

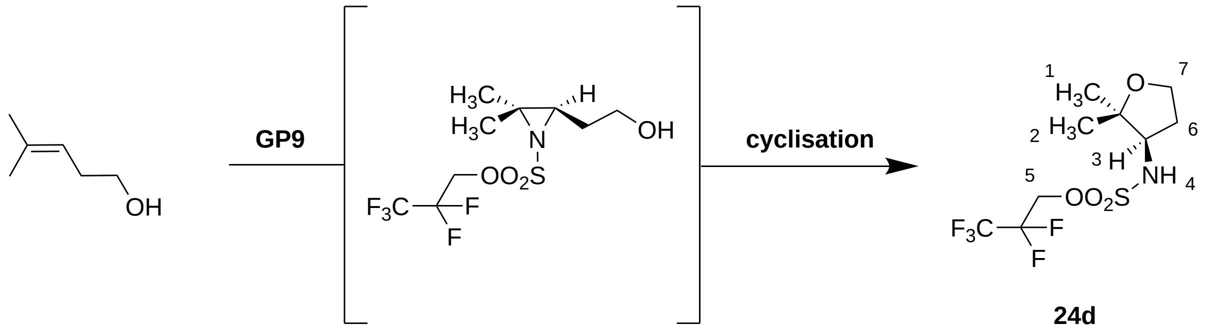

24d

Prepared according to **GP9** using 4-methylpent-3-en-1-ol (**23d**) as the starting material. Following the cyclisation protocol (2 hours), purification by flash column chromatography (SiO_2, 0-10% v/v Et_2O in CH_2Cl_2) afforded the title compound as a colourless oil (23.9 mg, 0.073 mmol, 73%, 86% *ee*).

***R_f* value** = 0.35 (10% v/v Et_2O in CH_2Cl_2);

^{1}H NMR (500 MHz, $CDCl_3$) δ 5.07 (d, J = 9.6 Hz, 1H, H-4), 4.43-4.56 (m, 2H, H-5a, H-5b), 3.91 (td, J = 8.9 Hz, 5.4 Hz, 1H, H-7a), 3.84 (td, J = 8.5 Hz, 6.8 Hz, 1H, H-7b), 3.69-3.73 (m, 1H, H-3), 2.44-2.51 (m, 1H, H-6a), 1.91-1.98 (m, 1H, H-6b), 1.27 (s, 3H, H-1), 1.23 (s, 3H, H-2) ppm;

^{13}C NMR (126 MHz, $CDCl_3$) δ 118.3 (qt, J_{C-F} = 286.0 Hz, 34.3 Hz), 111.5 (tq, J_{C-F} = 256.1 Hz, 38.9 Hz), 80.9, 64.1 (t, J_{C-F} = 28.3 Hz), 63.6, 61.9, 32.6, 25.9, 22.1 ppm;

^{19}F NMR (376 MHz, $CDCl_3$) δ – 83.5 (s), – 124.2 - – 122.8 (m)*ppm;

*Partial inequivalence leads to extreme strong coupling between these two ^{19}F nuclei.

IR (film, cm^{-1}): ν_{max} 3308 (w), 2982 (w), 1460 (m), 1373 (m), 1204 (s), 1185 (s), 1110 (m), 1040 (m), 1008 (m), 941 (m), 808 (m), 774 (m), 670 (w), 623 (w), 585 (m), 518 (w);

$[\alpha]_D^{25.0}$ = – 9.3 (c. 1.0, $CHCl_3$);

HRMS (+ESI) *m/z* Found $[M+H]^+$ 328.0642, $[C_9H_{15}F_5NO_4S]^+$ requires 328.0636, (δ = + 1.8 ppm);

Chiral GC Analysis CP7502 – CP-Chirasil-DEX CB column (120 °C Isothermal, 13.0 psi) t_R = 33.7 (major), 35.6 (minor) minutes.

The opposite enantiomer (*ent*-**24d**) was prepared as above with the following procedural modification: Rh_2(**B**)$_2$•(**C5**)$_2$•(Pyr)$_2$ was used in place of Rh_2(**B**)$_2$•(**C3**)$_2$•(Pyr)$_2$. Purification by flash column chromatography (SiO_2, 0-10% v/v Et_2O in CH_2Cl_2) afforded 2,2,3,3,3-pentafluoropropyl (*S*)-(2,2-dimethyltetrahydrofuran-3-yl)sulfamate (27.1 mg, 0.083 mmol, 83%, 81% *ee*). For this enantiomer, $[\alpha]_D^{25.0}$ = + 9.9 (c. 1.0, $CHCl_3$).

When Rh_2(**B**)$_2$•(**C4**)$_2$•(Pyr)$_2$ was used in place of Rh_2(**B**)$_2$•(**C3**)$_2$•(Pyr)$_2$ under identical conditions, *ent*-**24d** was obtained in 77% yield and 73% *ee*. In this case $[\alpha]_D^{25.4}$ = + 7.5 (c. 1.5, $CHCl_3$).

Aziridination followed by conversion of enriched **24e** *to the thiophenolate adduct for ee determination:* ***Obtained as an inseparable mixture of 2,2,3,3,3-pentafluoropropyl ((3S,4S)-1-hydroxy-3-methyl-4-(phenylthio)pentan-3-yl)sulfamate (Isomer A) and 2,2,3,3,3-pentafluoropropyl ((2R,3R)-5-hydroxy-3-(phenylthio)pentan-2-yl)sulfamate (Isomer B)***

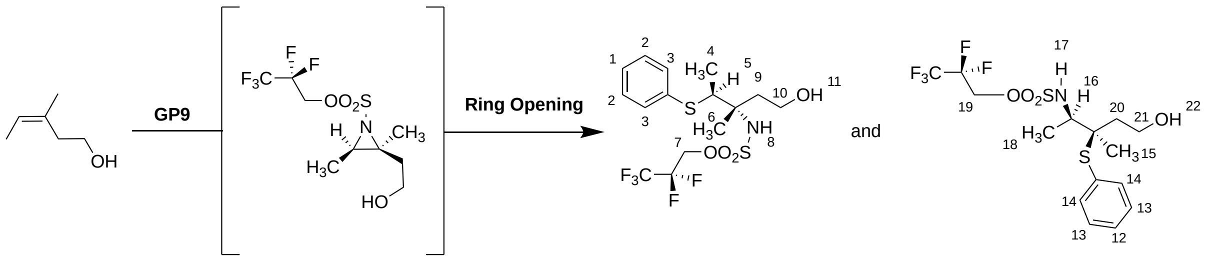

23e **24e** 25% ^{1}H NMR Yield **Isomer A** **Isomer B**

Prepared according to **GP9** using (*Z*)-3-methylpent-3-en-1-ol (**23e**) as the starting material. Isolation of the intermediate aziridine by column chromatography was attempted, however it consistently co-eluted with an impurity which we suspect arises from allylic amination. The reaction was therefore repeated and the yield of the aziridine **24e** estimated by ^{1}H NMR analysis of the crude reaction mixture with reference to an internal standard. The peak used for the yield determination was the distinctive aziridine quartet at 2.97 ppm (*J* = 5.9 Hz, 1H).

Following column chromatography (0-10% v/v Et_2O in CH_2Cl_2), the enriched sample of the aziridine (6.8 mg) was subjected to ring opening using a variant of the protocol described by Du Bois and Guthikonda.[73] The enriched aziridine (**24e**) was taken up in MeOH (1.0 mL) following which thiophenol (3 drops) and Et_3N (6 drops) were added and the reaction mixture stirred at room temperature overnight. The volatiles were removed under a stream of compressed air and purification by flash column chromatography (SiO_2, 0-10 % v/v Et_2O in CH_2Cl_2) afforded an inseparable mixture of the title compounds as a colourless oil (6.8 mg, 0.016 mmol, 16% over 2 steps). The ratio of Isomer A: Isomer B was (2:3) as determined by ^{1}H NMR and the integrals reported reflect this. Both sets of enantiomers were separable by chiral HPLC and indicated 50% *ee*.

***R_f* value** = 0.55 (10% v/v Et_2O in CH_2Cl_2);

^{1}H NMR (500 MHz, $CDCl_3$) δ 7.51-7.53 (m, 3H, H-14), 7.40-7.43 (m, 3.5H, H-3, H-12), 7.35-7.38 (m, 3H, H-13), 7.29-7.32 (m, 2H, H-2), 7.24-7.27 (m, 1H, H-1), 6.44 (d, *J* = 9.0 Hz, 1.5H, H-17), 6.33 (br s, 1H, H-8), 4.37-4.56 (m, 5H, H-7a, H-7b, H-19a, H-19b), 3.88-3.98 (m, 3.5H, H-10a, H-10b, H-21a), 3.79-3.84 (m, 1.5H, H-21b), 3.75 (q, *J* = 7.0 Hz, 1H, H-5), 3.50-3.56 (m, 1.5H, H-16), 2.08-2.14 (m, 1.5H, H-20a), 2.01-2.03 (m, 2H, H-9a, H-9b), 1.92 (br s, 1.5 H, H-22), 1.69

(ddd, *J* = 15.0 Hz, 4.9 Hz, 4.0 Hz, 2.5H, H-20b, H-11), 1.59 (s, 3H, H-6), 1.49 (d, *J* = 6.7 Hz, 4.5H, H-18), 1.43 (d, *J* = 7.0 Hz, 3H, H-4), 1.34 (s, 4.5H, H-15) ppm;

^{13}C NMR (126 MHz, $CDCl_3$) δ 137.8, 135.1, 131.9, 130.1, 129.8, 129.4, 129.2, 127.5, 114.7-121.8 (m)*, 109.1-114.1 (m)*, 63.85, 63.81 (t, $J_{C\text{-}F}$ = 28.4 Hz), 63.79 (t, $J_{C\text{-}F}$ = 28.3 Hz), 59.23, 59.19, 57.2, 55.2, 52.6, 38.3, 37.1, 23.6, 22.0, 17.3, 16.6 ppm;

*Corresponds to two ^{13}C environments

^{19}F NMR (376 MHz, $CDCl_3$) δ – 84.46 (s), – 84.50 (s), – 124.23 - – 124.21 (m)*, – 124.45 - – 124.43 (m)*ppm;

*Partial inequivalence leads to extreme strong coupling between these two ^{19}F nuclei.

IR (film, cm^{-1}): ν_{max} 3529 (w), 3300 (w), 2958 (w), 1584 (w), 1439 (m), 1356 (s), 1201 (s), 1180 (s), 1109 (s), 1044 (s), 1003 (s), 866 (w), 806 (w), 774 (m), 751 (s), 705 (m), 694 (w), 671 (m), 622 (w), 590 (m), 562 (m), 517 (m);

HRMS (–ESI) *m/z* Found [M–H]$^-$ 436.0690, $[C_{15}H_{19}F_5NO_4S_2]^-$ requires 436.0681, (δ = + 2.0 ppm);

Chiral HPLC Analysis CHIRALPAK IC (Hexane:^{i}PrOH, 97:3, 1.0 mL min^{-1}, 30 °C) for Isomer A t_R = 11.6 (minor), 14.8 (major) minutes, for Isomer B t_R = 10.3 minutes (minor), 13.3 (major).

2,2,3,3,3-pentafluoropropyl (2R,3R)-2-(2-hydroxyethyl)-2,3-dimethylaziridine-1-sulfonate (**24f**)

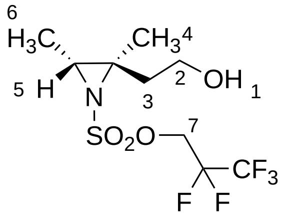

Prepared according to **GP9** using (*E*)-methylpent-3-en-1-ol (**23f**) as the starting material. Purification by flash column chromatography (SiO_2, 0-10% v/v Et_2O in CH_2Cl_2) afforded the title compound as a colourless oil (25.2 mg, 0.077 mmol, 77%, 60% *ee* as determined from ring opening with thiophenolate).

***R**$_f$* **value** = 0.37 (10% v/v Et_2O in CH_2Cl_2);

^{1}H NMR (500 MHz, $CDCl_3$) δ 4.55-4.68 (m, 2H, H-7a, H-7b), 3.88-3.94 (m, 1H, H-2a), 3.78-3.85 (m, 1H, H-2b), 3.02 (q, J = 5.9 Hz, 1H, H-5), 2.22 (dt, J = 14.2 Hz, 5.1 Hz, 1H, H-3a), 2.06-2.12 (m, 1H, H-3b), 1.66 (br s, 1H, H-1), 1.37 (s, 3H, H-4), 1.31 (d, J = 5.7 Hz, 3H, H-6) ppm;

^{13}C NMR (126 MHz, $CDCl_3$) δ 118.4 (qt, J_{C-F} = 286.1 Hz, 34.4 Hz), 111.4 (tq, J_{C-F} = 256.1 Hz, 38.5 Hz), 65.4 (t, J_{C-F} = 28.7 Hz), 59.6, 53.5, 50.0, 37.5, 17.4, 12.6 ppm;

^{19}F NMR (376 MHz, $CDCl_3$) δ – 84.5 (s), – 124.5 ppm;

IR (film, cm^{-1}): ν_{max} 3400 (br, w), 2921 (w), 1460 (w), 1369 (s), 1258 (w), 1201 (s), 1177 (s), 1107 (m), 1074 (w), 1045 (s), 1012 (s), 987 (s), 959 (m), 942 (m), 901 (w), 832 (s), 789 (m), 723 (w), 690 (m), 626 (m), 603 (m), 567 (w);

$[\alpha]_D^{25.0}$ = + 10.3 (c. 1.0, $CHCl_3$);

HRMS (–ESI) *m/z* Found [M–H]$^-$ 326.0495, $[C_9H_{13}F_5NO_4S]^-$ requires 326.0491, (δ = + 1.2 ppm);

Conversion of ***24f*** *to the thiophenolate adduct for ee determination: 2,2,3,3,3-pentafluoropropyl ((2R,3R)-5-hydroxy-3-methyl-3-(phenylthio)pentan-2-yl)sulfamate*

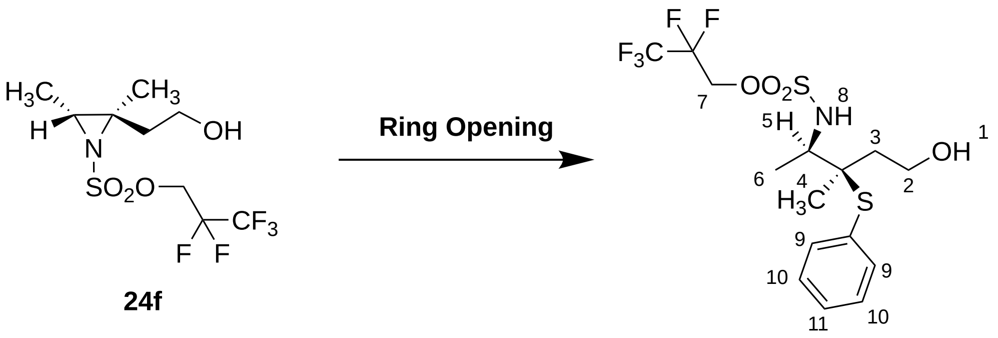

Prepared according to a variant of the protocol described by Du Bois and Guthikonda.[73] To a solution of 2,2,3,3,3-pentafluoropropyl (2*R*,3*R*)-2-(2-hydroxyethyl)-2,3-dimethylaziridine-1-sulfonate (**24f**) (25.2 mg, 0.077 mmol) in MeOH (2.5 mL) were added thiophenol (22.0 µL, 0.22 mmol) and Et_3N (60.1 µL, 0.43 mmol). The reaction mixture was stirred at 50 °C for two hours, following which the volatiles were removed under a stream of compressed air. Purification by flash column chromatography (SiO_2, 0-10 % v/v Et_2O in CH_2Cl_2) afforded the title compound as a colourless oil (5.6 mg, 0.013 mmol, 17%, 60% *ee*). *In this instance the thiophenolate attack on the aziridine was unselective and both regioisomers arising from ring opening were observed by TLC in a similar ratio. As a result, many mixed fractions were discarded in order to obtain as pure a sample as possible for a single regioisomer thus allowing for a more reliable determination of ee.*

***R_f* value** = 0.37 (10% v/v Et_2O in CH_2Cl_2);

^{1}H NMR (500 MHz, $CDCl_3$) δ 7.50-7.51 (m, 2H, H-9), 7.40 (tt, *J* = 7.3 Hz, 1.2 Hz, 1H, H-11), 7.33-7.36 (m, 2H, H-10), 6.37 (d, *J* = 8.4 Hz, 1H, H-8), 4.46-4.62 (m, 2H, H-7a, H-7b), 4.12-4.16 (m, 1H, H-2a), 3.85-3.91 (m, 1H, H-2b), 3.60-3.65 (m, 1H, H-5), 1.94 (ddd, *J* = 15.1 Hz, 7.8 Hz, 5.2 Hz, 1H, H-3a), 1.81 (dt, *J* = 15.2 Hz, 5.0 Hz, 1H, H-3b), 1.37 (d, *J* = 6.7 Hz, 3H, H-6), 1.19 (s, 3H, H-4) ppm;

^{13}C NMR (126 MHz, $CDCl_3$) δ 137.8, 130.2, 129.6, 129.1, 118.4 (qt, $J_{C\text{-}F}$ = 286.6 Hz, 34.7 Hz), 111.6 (tq, $J_{C\text{-}F}$ = 256.7 Hz, 38.2 Hz), 64.0 (t, $J_{C\text{-}F}$ = 28.2 Hz), 59.4, 58.4, 55.6, 39.0, 26.1, 16.7 ppm;

^{19}F NMR (376 MHz, $CDCl_3$) δ – 83.5 (s), – 124.1 - – 122.6 (m)*ppm;

*Partial inequivalence leads to extreme strong coupling between these two ^{19}F nuclei.

IR (film, cm^{-1}): ν_{max} 3289 (br, w), 2926 (w), 1439 (w), 1365 (m), 1260 (w), 1204 (s), 1108 (m), 1043 (m), 1010 (m), 806 (w), 774 (w), 752 (w), 695 (w), 623 (w), 583 (w), 519 (w);

$[\alpha]_D^{25.0}$ = + 5.4 (c. 0.3, $CHCl_3$);

HRMS (–ESI) *m/z* Found [M–H]$^-$ 436.0685, $[C_{15}H_{19}F_5NO_4S_2]^-$ requires 436.0681, (δ = + 0.9 ppm);

Chiral SFC Analysis CHIRALPAK IE (CO_2:MeOH, 96.5:3.5, 1.25 mL min^{-1}, 40 °C) t_R = 18.0 (minor), 21.2 (major) minutes

2,2,3,3,3-pentafluoropropyl (R)-2-(2-hydroxyethyl)aziridine-1-sulfonate (**24g**)

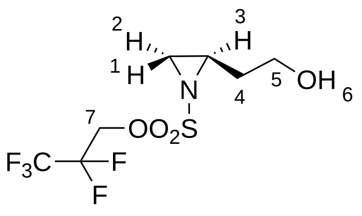

Prepared according to **GP9** using but-3-en-1-ol (**23g**) as the starting material. Purification by flash column chromatography (SiO_2, 0-10% v/v Et_2O in CH_2Cl_2) afforded the title compound as a colourless oil (7.9 mg, 0.026 mmol, 26%, 14% *ee*). *In this case, the product was contaminated*

with a small amount of inseparable phthalate plasticizer – this is most notable in the aliphatic region of the ^{13}C NMR.

^{1}H NMR (500 MHz, $CDCl_3$) δ 4.67-4.73 (m, 2H, H-7a, H-7b), 3.83 (t, J = 5.8 Hz, 2H, H-5a, H-5b), 3.01 (tt, J = 7.4 Hz, 4.7 Hz, 1H, H-3), 2.76 (dd, J = 7.1 Hz, 0.6 Hz, 1H, H-2), 2.35 (d, J = 4.8 Hz, 1H, H-1), 1.96-2.02 (m, 1H, H-4a), 1.63-1.70 (m, 1H, H-4b), 1.60 (br s, 1H, H-6) ppm;

^{13}C NMR (126 MHz, $CDCl_3$) δ 118.3 (qt, $J_{C\text{-}F}$ = 285.5 Hz, 34.2 Hz), 111.3 (tq, $J_{C\text{-}F}$ = 256.7 Hz, 38.5 Hz), 65.6 (t, $J_{C\text{-}F}$ = 28.6 Hz), 60.0, 40.0, 35.5, 33.7 ppm;

^{19}F NMR (376 MHz, $CDCl_3$) δ – 83.5 (s), – 123.6 (s) ppm;

HRMS (+ESI) *m/z* Found $[M+H]^+$ 300.0335, $[C_7H_{11}F_5NO_4S]^+$ requires 300.0323, (δ = + 4.0 ppm);

Chiral GC Analysis CP7502 – CP-Chirasil-DEX CB column (105 °C Isothermal, 25.9 psi) t_R = 50.6 (minor), 53.1 (major) minutes.

Characterisation of *Trans*-Dialkyl Aziridines

2,2,3,3,3-pentafluoropropyl (2R,3R)-2-ethyl-3-(2-hydroxyethyl)aziridine-1-sulfonate (**26a**)

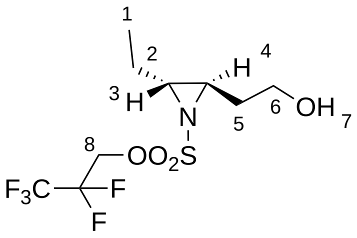

Prepared according to **GP9** using (*E*)-hex-3-en-1-ol (**25a**) as the starting material. In this case the following procedural modification was used: The reaction time was 36 hours. Purification by flash column chromatography (SiO_2, 0-12% v/v Et_2O in CH_2Cl_2) afforded the title compound as a colourless oil (25.1 mg, 0.077 mmol, 77%, 94% *ee*).

***R_f* value** = 0.34 (10% v/v Et_2O in CH_2Cl_2);

^{1}H NMR (400 MHz, $CDCl_3$) δ 4.66 (t, *J* = 12.4 Hz, 2H, H-8a, H-8b), 3.83 (t, *J* = 5.8 Hz, 2H, H-6a, H-6b), 2.88 (q, *J* = 5.8 Hz, 1H, H-4), 2.74 (q, *J* = 5.9 Hz, 1H, H-3), 1.94-2.04 (m, 2H, H-5a, H-5b), 1.67-1.88 (m, 3H, H-2a, H-2b, H-7), 1.07 (t, *J* = 7.5 Hz, 3H, H-1) ppm;

^{13}C NMR (101 MHz, $CDCl_3$) δ 118.3 (qt, J_{C-F} = 286.3 Hz, 34.0 Hz), 111.4 (tq, J_{C-F} = 257.3 Hz, 38.8 Hz), 65.6 (t, J_{C-F} = 28.4 Hz), 60.2, 51.6, 47.7, 32.5, 23.0, 11.1 ppm;

^{19}F NMR (376 MHz, $CDCl_3$) δ – 83.5 (s), – 123.6 (s) ppm;

IR (film, cm^{-1}): ν_{max} 3378 (br, w), 2973 (w), 1447 (w), 1368 (m), 1200 (s), 1176 (s), 1108 (m), 1042 (s), 1006 (s), 939 (s), 835 (s), 788 (m), 723 (w), 693 (w), 629 (m), 540 (w), 517 (w);

$[\alpha]_D^{25.0}$ = + 11.9 (c. 1.1, $CHCl_3$);

HRMS (+ESI) *m/z* Found $[M+H]^+$ 328.0646, $[C_9H_{15}F_5NO_4S]^+$ requires 328.0636, (δ = + 3.0 ppm).

Chiral GC Analysis CP7502 – CP-Chirasil-DEX CB column (115 °C Isothermal, 26.7 psi) t_R = 38.6 (aziridine, major), 37.9 (aziridine, minor), 63.6 (cyclised, major), 70.4 (cyclised, minor) minutes. *Due to the high temperature of the analysis method, the compound started to cyclise resulting in 4 peaks; the first 2 correspond to the aziridine enantiomers and the second 2 correspond to the cyclised product enantiomers. This was confirmed by comparing retention times with an authentic sample of cyclised chiral and racemic products (see below).*

The opposite enantiomer (*ent*-**26a**) was prepared as above with the following procedural modification: Rh_2(**B**)$_2$•(**C5**)$_2$•(Pyr)$_2$ was used in place of Rh_2(**B**)$_2$•(**C3**)$_2$•(Pyr)$_2$. Purification by flash column chromatography (SiO_2, 0-10% v/v Acetone in $CHCl_3$) afforded 2,2,3,3,3-pentafluoropropyl (2*S*,3*S*)-2-ethyl-3-(2-hydroxyethyl)aziridine-1-sulfonate (28.7 mg, 0.088 mmol, 88%, 92% *ee*). For this enantiomer, $[\alpha]_D^{25.0}$ = – 11.5 (c. 1.0, $CHCl_3$).

In order to assign the absolute stereochemistry, the reaction was repeated on another occasion, this time with intramolecular cyclisation of the crude aziridine followed by further functionalisation (see section: "Determination of the Absolute Stereochemistry of the Aziridine Products"). In addition, the cyclised product was also run on the GC to confirm the peak assignments above.

Intramolecular cyclisation of **26a** *for confirmation of peak assignment: 2,2,3,3,3-pentafluoropropyl ((2S,3R)-2-ethyltetrahydrofuran-3-yl)sulfamate*

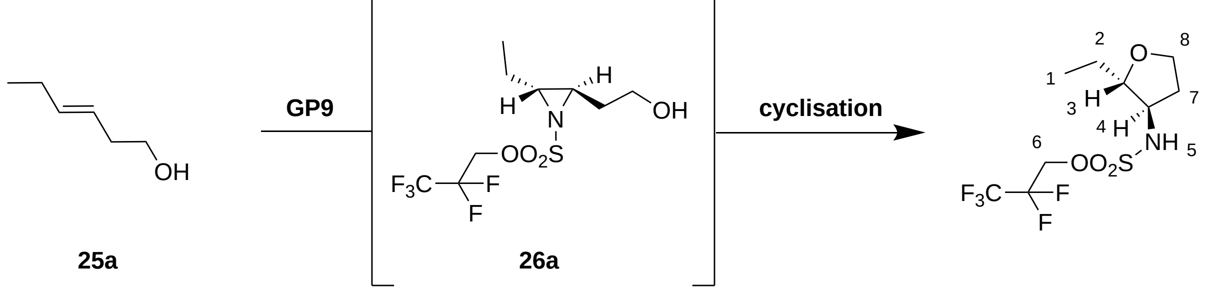

25a **26a**

Prepared according to **GP9** using (*E*)-hex-3-en-1-ol (**25a**) as the starting material. In this case the following procedural modification was used: The reaction was performed on a 0.3 mmol scale with respect to the starting material and all other reagent and solvent amounts were scaled proportionally. Following the cyclisation protocol (7 hours), purification by flash column chromatography (SiO_2, 0-10% v/v Acetone in $CHCl_3$ followed again by 0-10% v/v Acetone in $CHCl_3$) afforded the title compound as a colourless oil with some residual aziridine and sulfamate ester contaminants (40.5 mg, 0.12 mmol, 41%, 92% *ee*). *A higher yield and purity of cyclised product can be obtained by first purifying the intermediate aziridine by column chromatography and then subjecting the purified sample to cyclisation. Direct cyclisation of the crude makes the subsequent purification more difficult due to co-elution of the desired product with residual aziridine and sulfamate ester.*

***R**$_f$* **value** = 0.32 (10% v/v Acetone in $CHCl_3$);

^{1}H NMR (400 MHz, $CDCl_3$) δ 5.37 (d, *J* = 7.7 Hz, 1H, H-5), 4.49 (t, *J* = 12.4 Hz, 2H, H-6a, H-6b), 3.82-3.96 (m, 2H, H-8a, H-8b), 3.73-3.79 (m, 1H, H-4), 3.64-3.68 (m, 1H, H-3), 2.27-2.36 (m, 1H, H-7a), 1.92-2.02 (m, 1H, H-7b), 1.45-1.64 (m, 2H, H-2a, H-2b), 0.97 (t, *J* = 7.4 Hz, 3H, H-1) ppm;

^{13}C NMR (101 MHz, $CDCl_3$) δ 118.3 (qt, $J_{C\text{-}F}$ = 286.1 Hz, 34.4 Hz), 111.5 (tq, $J_{C\text{-}F}$ = 256.6 Hz, 38.4 Hz), 85.5, 65.8, 64.0 (t, $J_{C\text{-}F}$ = 28.3 Hz), 58.9, 32.8, 26.2, 10.0 ppm;

^{19}F NMR (376 MHz, $CDCl_3$) δ – 83.5 (s), – 123.5 (t, $J_{H\text{-}F}$ = 12.4 Hz) ppm; *This NMR spectrum was acquired with proton coupling*;

IR (film, cm^{-1}): ν_{max} 3291 (w), 2972 (w), 1458 (w), 1374 (m), 1205 (s), 1183 (s), 1109 (m), 941 (s), 813 (m), 774 (w), 717 (w);

$[\alpha]_D^{25.0}$ = – 17.0 (c. 1.0, $CHCl_3$);

HRMS (–ESI) *m/z* Found [M–H]$^-$ 326.0488, $[C_9H_{13}F_5NO_4S]^-$ requires 326.0491, (δ = – 0.9 ppm);

Chiral GC Analysis CP7502 – CP-Chirasil-DEX CB column (115 °C Isothermal, 26.7 psi) t_R = 51.7 (major), 58.2 (minor) minutes.

When Rh_2(**B**)$_2$•(**C4**)$_2$•(Pyr)$_2$ was used in place of Rh_2(**B**)$_2$•(**C3**)$_2$•(Pyr)$_2$ under identical conditions, the cyclised product derived from *ent*-**26a** was obtained in 52% yield and 82% *ee*. In this case $[\boldsymbol{\alpha}]_{\mathbf{D}}^{\mathbf{25.0}}$ = + 15.9 (c. 1.0, $CHCl_3$).

2,2,3,3,3-pentafluoropropyl (2R,3R)-2-(2-hydroxyethyl)-3-isopropylaziridine-1-sulfonate (**26b**)

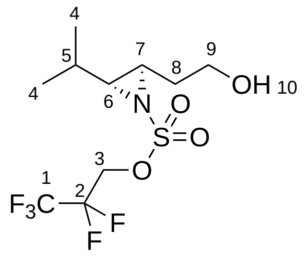

Prepared according to **GP9** using (*E*)-5-methylhex-3-en-1-ol (**25b**) (11.3 mg, 0.1 mmol, 1.0 equiv.) as the starting material. Purification by flash column chromatography (SiO_2, 0-10% v/v Acetone in $CHCl_3$) afforded the title compound as a colourless oil (20.1 mg, 0.059 mmol, 59%, 79% *ee* as determined from the cyclised adduct).

***R_f* value** = 0.29 (10% v/v Acetone in $CHCl_3$);

^{1}H NMR (500 MHz, $CDCl_3$) δ 4.72-4.61 (m, 2H, H-3a, H-3b), 3.88-3.81 (m, 2H, H-9a, H-9b), 2.89-2.85 (m, 1H, H-7), 2.62 (dd, *J* = 8.1, 4.9 Hz, 1H, H-6), 2.18-2.11 (m, 1H, H-8a), 2.02-1.95 (m, 1H, H-8b), 1.76-1.66 (m, 1H, H-5), 1.07 (d, *J* = 6.8 Hz, 3H, H-4a), 1.02 (d, *J* = 6.8 Hz, 3H, H-4b) ppm;

^{13}C NMR (126 MHz, $CDCl_3$) δ 118.6 (qt, J_{C-F} = 286.6, 34.3 Hz, C-1), 111.4 (tq, J_{C-F} = 256.9, 38.4 Hz, C-2), 65.6 (t, J_{C-F} = 28.2 Hz, C-3), 60.4 (C-9), 56.2 (C-6), 47.1 (C-7), 31.8 (C-8), 29.7 (C-5), 19.8 (C-4a), 19.4 (C-4b) ppm;

^{19}F NMR (376 MHz, $CDCl_3$) δ – 84.5, – 124.5 (d, J = 6.3 Hz) ppm;

IR (film, cm^{-1}): ν_{max} 2968 (w), 1368 (m), 1205 (s), 1177 (s), 1109 (m), 1045 (m), 1010 (s), 974 (m), 932 (m), 837 (m), 789 (w), 711 (m), 670 (w);

$[\alpha]_D^{25.2}$ = + 12.6 (c. 1.26 $CHCl_3$);

HRMS (–ESI) *m/z* found $[M+HCOO]^-$ 386.0701, $[C_{11}H_{17}F_5NO_6S]^-$ requires 386.0702, (δ = – 0.3 ppm).

Intramolecular cyclisation of **26b** *for ee determination: 2,2,3,3,3-pentafluoropropyl ((2S,3R)-2-isopropyltetrahydrofuran-3-yl)sulfamate:*

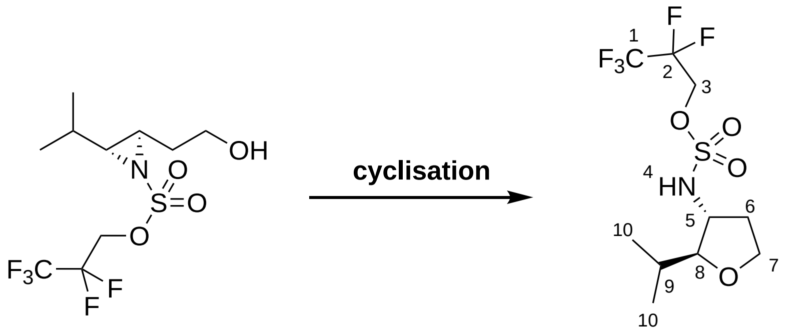

26b

To a vial charged with 2,2,3,3,3-pentafluoropropyl (2*R*,3*R*)-2-(2-hydroxyethyl)-3-isopropylaziridine-1-sulfonate (**26b**) (16.4 mg, 0.048 mmol, 1.0 equiv.) was added MeCN (2 mL). The reaction mixture was heated at 82 ˚C overnight and allowed to cool to room temperature. The reaction mixture was concentrated *in vacuo* to give the title compound (16.4 mg, 0.048 mmol, quantitative, 79% *ee*) as a colourless oil. ^{1}H NMR analysis revealed a 91% conversion to the title compound from the starting material aziridine.

***R_f* value** = 0.37 (10% v/v Acetone in $CHCl_3$).

^{1}**H NMR** (500 MHz, $CDCl_3$) δ 5.07 (d, J = 8.2 Hz, 1H, H-4), 4.49 (td, J = 12.4, 0.7 Hz, 2H, H-3a, H-3b), 3.95-3.88 (m, 2H, H-5, H-7a), 3.86-3.81 (m, 1H, H-7b), 3.41 (dd, J = 7.2, 3.6 Hz, 1H, H-8), 2.28-2.21 (m, 1H, H-6a), 2.02-1.96 (m, 1H, H-6b), 1.72 (sext, J = 6.7 Hz, 1H, H-9), 0.96 (d, J = 6.8 Hz, 3H, H-10a), 0.96 (d, J = 6.8 Hz, 3H, H-10b) ppm;

^{13}**C NMR** (126 MHz, $CDCl_3$) δ 118.3 (qt, $J_{C\text{-}F}$ = 286.3, 34.1 Hz, C-1), 111.5 (tq, $J_{C\text{-}F}$ = 256.5, 38.5 Hz, C-2), 89.6 (C-8), 66.2 (C-7), 64.0 (t, $J_{C\text{-}F}$ = 28.3 Hz, C-3), 57.4 (C-5), 33.5 (C-6), 30.9 (C-9), 18.8 (C-10a), 18.1 (C-10b) ppm;

^{19}**F NMR** (376 MHz, $CDCl_3$) δ – 84.5, – 124.4 ppm;

IR (film, cm^{-1}): ν_{max} 3301 (w), 2966 (w), 1455 (w), 1368 (m), 1260 (w), 1204 (s), 1183 (s), 1109 (m), 1047 (m), 1010 (m), 937 (w), 807 (w), 775 (w), 713 (w), 668 (w);

$[\alpha]_D^{25.2}$ = – 11.0 (c. 0.83 $CHCl_3$);

HRMS (–ESI) m/z found $[M–H]^-$ 340.0652, $[C_{10}H_{15}F_5NO_4S]^-$ requires 340.0647, (δ = +1.5 ppm);

Chiral GC Analysis CP7502 – CP-Chirasil-DEX CB column (120 °C Isothermal, 20.0 psi) t_R = 54.6 (major), 57.8 (minor) minutes.

2,2,3,3,3-pentafluoropropyl (2R,3R)-2-(2-(benzyloxy)ethyl)-3-(2-hydroxyethyl)aziridine-1-sulfonate (**26d**)

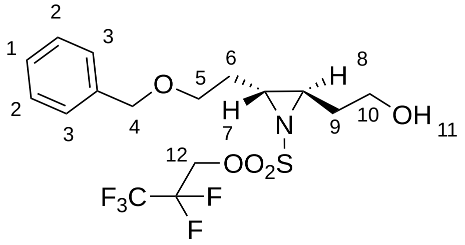

Prepared according to **GP9** using (*E*)-6-(benzyloxy)hex-3-en-1-ol (**25d**) as the starting material. In this case the following procedural modification was used: The reaction time was 48 hours. Purification by flash column chromatography (SiO_2, 0-10% v/v Et_2O in CH_2Cl_2) afforded the title compound as a colourless oil (22.8 mg, 0.053 mmol, 53%, 91% *ee*).

***R$_f$* value** = 0.37 (10% v/v Et_2O in CH_2Cl_2);

^{1}H NMR (500 MHz, $CDCl_3$) δ 7.35-7.39 (m, 2H, H-2), 7.30-7.35 (m, 3H, H-1, H-3), 4.60-4.71 (m, 2H, H-12a, H-12b), 4.56 (d, *J* = 11.8 Hz, 1H, H-4a), 4.51 (d, *J* = 11.8 Hz, 1H, H-4b), 3.62-3.75 (m, 4H, H-5a, H-5b, H-10a, H-10b), 2.90 (dt, *J* = 8.9 Hz, 4.6 Hz, 1H, H-7), 2.84 (dt, *J* = 8.5 Hz, 4.8 Hz, 1H, H-8), 2.58 (dd, *J* = 7.4 Hz, 4.9 Hz, 1H, H-11), 2.19 (dq, *J* = 14.3 Hz, 4.2 Hz, 1H, H-6a), 2.07-2.13 (m, 1H, H-9a), 1.89-1.97 (m, 1H, H-6b), 1.73-1.80 (m, 1H, H-9b) ppm;

^{13}C NMR (126 MHz, $CDCl_3$) δ 137.3, 128.7, 128.3, 128.2, 118.3 (qt, $J_{C\text{-}F}$ = 286.2 Hz, 34.3 Hz), 111.4 (tq, $J_{C\text{-}F}$ = 256.7 Hz, 38.6 Hz), 73.5, 67.3, 65.6 (t, $J_{C\text{-}F}$ = 28.5 Hz), 59.9, 48.5, 48.2, 32.8, 29.9 ppm;

^{19}F NMR (376 MHz, $CDCl_3$) δ – 84.5 (s), – 124.5 (s) ppm;

IR (film, cm^{-1}): ν_{max} 3412 (br, w), 2873 (w), 1454 (w), 1368 (s), 1258 (w), 1201 (s), 1177 (s), 1106 (s), 1043 (s), 1007 (s), 941 (s), 836 (s), 788 (w), 745 (w), 723 (m), 698 (m), 632 (m), 546 (w), 515 (w);

$[\boldsymbol{\alpha}]_{\mathbf{D}}^{\mathbf{25.0}}$ = + 4.5 (c. 1.0, $CHCl_3$);

HRMS (–ESI) *m/z* Found $[M+CHO_2]^-$ 478.0974, $[C_{17}H_{21}F_5NO_7S]^-$ requires 478.0964, (δ = + 2.1 ppm);

Chiral SFC Analysis CHIRALPAK IE (CO_2:MeOH, 98:2, 1.25 mL min^{-1}, 40 °C) t_R = 20.8 (major), 22.9 (minor) minutes.

2,2,3,3,3-pentafluoropropyl (2R,3R)-2-(2-hydroxyethyl)-3-(2-(tosyloxy)ethyl)aziridine-1-sulfonate (**26e**)

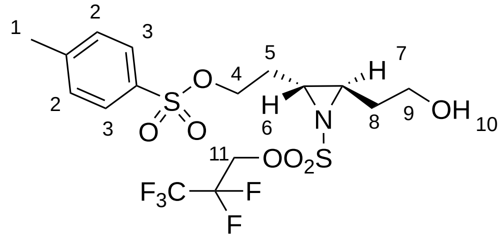

Prepared according to **GP9** using (*E*)-6-hydroxyhex-3-en-1-yl 4-methylbenzenesulfonate (**25e**) as the starting material. In this case the following procedural modification was used: The catalyst loading was 3.0 mol %. Purification by flash column chromatography (SiO_2, 0-12% v/v Et_2O in CH_2Cl_2) afforded the title compound as a pale-yellow oil (38.3 mg, 0.077 mmol, 77%, 90% *ee*).

***R_f* value** = 0.47 (20% v/v Et_2O in CH_2Cl_2);

^{1}H NMR (500 MHz, $CDCl_3$) δ 7.77-7.80 (m, 2H, H-3), 7.37 (d, *J* = 8.0 Hz, 2H, H-2), 4.59-4.70 (m, 2H, H-11a, H-11b), 4.15-4.22 (m, 2H, H-4a, H-4b), 3.79-3.86 (m, 2H, H-9a, H-9b), 2.92-2.96 (m, 1H, H-7), 2.84-2.88 (m, 1H, H-6), 2.45 (s, 3H, H-1), 2.21 (dq, *J* = 15.0 Hz, 5.1 Hz, 1H, H-5a), 1.99-2.11 (m, 2H, H-5b, H-8a), 1.83-1.90 (m, 1H, H-8b) ppm;

^{13}C NMR (126 MHz, $CDCl_3$) δ 145.5, 132.5, 130.2, 128.0, 118.3 (qt, J_{C-F} = 286.2 Hz, 34.3 Hz), 111.3 (tq, J_{C-F} = 256.8 Hz, 38.6 Hz), 67.6, 65.7 (t, J_{C-F} = 28.3 Hz), 59.8, 47.8, 46.3, 32.2, 29.3, 21.8 ppm;

^{19}F NMR (376 MHz, $CDCl_3$) δ – 84.5 (s), – 124.6 (s) ppm;

IR (film, cm^{-1}): ν_{max} 3575 (br, w), 1598 (w), 1361 (s), 1205 (s), 1176 (s), 1106 (m). 1041 (m), 1009 (m), 980 (m), 918 (m), 816 (m), 776 (w), 690 (m), 664 (w), 630 (w), 579 (w), 517 (m);

$[\alpha]_D^{25.0}$ = + 6.3 (c. 1.1, $CHCl_3$);

HRMS (−ESI) *m/z* Found $[M+CHO_2]^-$ 542.0595, $[C_{17}H_{21}F_5NO_9S_2]^-$ requires 542.0583, (δ = + 2.2 ppm);

Chiral SFC Analysis CHIRALPAK IE (CO_2:MeOH, 95:5, 2.5 mL min^{-1}, 40 °C) t_R = 8.5 (major), 9.4 (minor) minutes.

2,2,3,3,3-pentafluoropropyl (2R,3R)-2-(2-bromoethyl)-3-(2-hydroxyethyl)aziridine-1-sulfonate (**26f**)

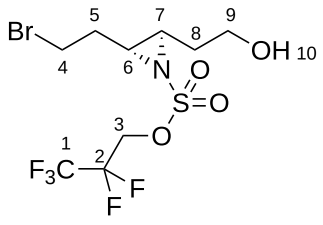

Prepared according to **GP9** using (*E*)-6-bromohex-3-en-1-ol (**25f**) (18.2 mg, 0.1 mmol, 1.0 equiv.) as the starting material. In this case the following procedural modification was used: The catalyst loading was 3.0 mol %. Purification by flash column chromatography (SiO_2, 0-10% v/v Acetone in $CHCl_3$) afforded the title compound as a colourless oil (32.2 mg, 0.079 mmol, 79%, 90% *ee* as determined following a telescoped cyclisation/displacement of the bromide with thiophenolate sequence).

***R$_f$* value** = 0.22 (10% v/v Acetone in $CHCl_3$);

^{1}H NMR (500 MHz, $CDCl_3$) δ 4.73-4.63 (m, 2H, H-3a, H-3b), 3.89-3.82 (m, 2H, H-9a, H-9b), 3.58-3.49 (m, 2H, H-4a, H-4b), 3.00-2.93 (m, 2H, H-6, H-7), 2.46 (dq, J = 14.9, 5.4 Hz, 1H, H-5a), 2.24-2.17 (m, 1H, H-5b), 2.01 (q, J = 6.0 Hz, 2H, H-8a, H-8b), 1.78 (s, 1H, H-10) ppm;

^{13}C NMR (126 MHz, $CDCl_3$) δ 118.3 (qt, J_{C-F} = 284.5, 34.2 Hz, C-1), 111.3 (tq, J_{C-F} = 256.8, 38.6 Hz, C-2), 65.7 (t, J_{C-F} = 28.6 Hz, C-3), 60.0 (C-9), 48.3 (C-6), 47.8 (C-7), 32.3 (C-5, C-8), 29.4 (C-4) ppm;

^{19}F NMR (376 MHz, $CDCl_3$) δ – 84.5, – 124.6 ppm;

IR (film, cm^{-1}): ν_{max} 3386 (br), 2966 (w), 1692 (w), 1440 (w), 1367 (m), 1258 (w), 1200 (s), 1175 (s), 1107 (m), 1041 (s), 1007 (s), 943 (m), 838 (m), 789 (m), 723 (m), 694 (m);

$[\boldsymbol{\alpha}]_{\mathbf{D}}^{\mathbf{25.0}}$ = + 17.4 (c. 1.21 $CHCl_3$);

HRMS (–ESI) m/z found $[M+HCOO]^-$ 449.9642, $[C_{10}H_{14}BrF_5NO_6S]^-$ requires 449.9651, (δ = –2.0 ppm).

Derivatisation of **26f** *for ee determination: 2,2,3,3,3-pentafluoropropyl ((2S,3R)-2-(2-(phenylthio)ethyl)tetrahydrofuran-3-yl)sulfamate*

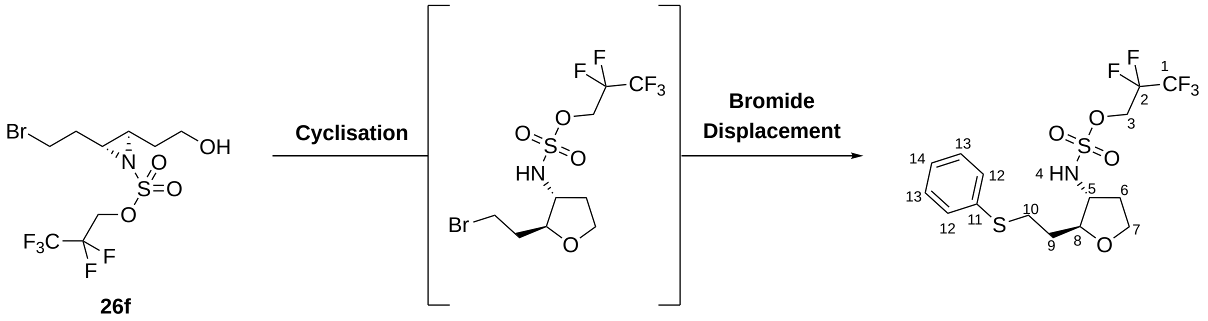

A solution of 2,2,3,3,3-pentafluoropropyl (2*R*,3*R*)-2-(2-bromoethyl)-3-(2-hydroxyethyl)aziridine-1-sulfonate (**26f**) (32.2 mg, 0.079 mmol, 1.0 equiv.) in MeCN (2 mL)

was heated at 82 ˚C overnight. The reaction mixture was allowed to cool to room temperature and concentrated under a stream of N_2. To the crude residue was added MeOH (1 mL), NEt_3 (8 drops) and thiophenol (4 drops), and the reaction mixture was allowed to stir at room temperature for 4.5 hours. The reaction mixture was concentrated under a stream of N_2. Purification by flash column chromatography (SiO_2, 0-5% v/v Acetone in $CHCl_3$) afforded the title compound as a colourless oil (7.7 mg, 0.018 mmol, 22% over 2 steps, 90% *ee*).

***R_f* value** = 0.46 (10% v/v Acetone in $CHCl_3$);

^{1}H NMR (500 MHz, $CDCl_3$) δ 7.35-7.33 (m, 2H, H-12), 7.30-7.27 (m, 2H, H-13), 7.21-7.17 (m, 1H, H-14), 5.01 (d, *J* = 8.4 Hz, 1H, H-4), 4.47 (t, *J* = 12.5 Hz, 2H, H-3a, H-3b), 3.95-3.85 (m, 3H, H-7a, H-7b, H-8), 3.76-3.71 (m, 1H, H-5), 3.12-3.06 (m, 1H, H-10a), 2.97-2.93 (m, 1H, H-10b), 2.36-2.29 (m, 1H, H-6a), 1.99-1.94 (m, 1H, H-6b), 1.93-1.84 (m, 1H, H-9a), 1.83-1.77 (m, 1H, H-9b) ppm;

^{13}C NMR (126 MHz, $CDCl_3$) δ 135.9 (C-11), 129.5 (C-12), 129.1 (C-13), 126.4 (C-14), 118.3 (qt, $J_{C\text{-}F}$ = 286.0, 34.4 Hz, C-1), 111.5 (tq, $J_{C\text{-}F}$ = 256.9, 38.2 Hz, C-2), 82.3 (C-8), 66.0 (C-7), 64.1 (t, $J_{C\text{-}F}$ = 28.3 Hz, C-3), 59.3 (C-5), 32.8 (C-9), 32.7 (C-6), 30.0 (C-10) ppm;

^{19}F NMR (376 MHz, $CDCl_3$) δ – 83.5, – 123.4 ppm;

IR (film, cm^{-1}): ν_{max} 3310 (w), 2928 (w), 1584 (w), 1481 (w), 1440 (w), 1375 (m), 1260 (w), 1205 (s), 1183 (s), 1110 (m), 1009 (m), 934 (m), 807 (w), 775 (w), 741 (m), 692 (w);

$[\alpha]_D^{25.0}$ = – 31.5 (c. 0.48 $CHCl_3$);

HRMS (–ESI) *m/z* found $[M–H]^-$ 434.0523, $[C_{15}H_{17}F_5NO_4S_2]^-$ requires 434.0525, (δ = – 0.5 ppm);

Chiral SFC analysis: CHIRALPAK IE (CO_2:MeOH, 95:5, 2.5 mL min^{-1}, 40 ˚C), t_R = 3.3 (minor), 5.3 (major) minutes.

2,2,3,3,3-pentafluoropropyl (2R,3R)-2-(2-((N,4-dimethylphenyl)sulfonamido)ethyl)-3-(2-hydroxyethyl)aziridine-1-sulfonate (**26g**)

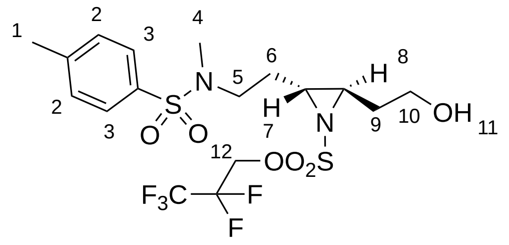

Prepared according to **GP9** using (*E*)-*N*-(6-hydroxyhex-3-en-1-yl)-*N*,4-dimethylbenzenesulfonamide (**25g**) as the starting material. In this case the following procedural modification was used: The catalyst loading was 2.0 mol %. Purification by flash column chromatography (SiO_2, 0-50% v/v EtOAc in Petrol) afforded the title compound as a colourless oil (35.4 mg, 0.069 mmol, 69%, 83% *ee*).

***R_f* value** = 0.34 (50% v/v EtOAc in Petrol);

^{1}H NMR (500 MHz, $CDCl_3$) *δ* 7.64-7.66 (m, 2H, H-3), 7.33 (d, *J* = 8.0 Hz, 2H, H-2), 4.63-4.74 (m, 2H, H-12a, H-12b), 3.88-3.92 (m, 1H, H-10a), 3.81-3.86 (m, 1H, H-10b), 3.27-3.32 (m, 1H, H-5a), 3.07 (dt, *J* = 13.5 Hz, 5.8 Hz, 1H, H-5b), 2.99-3.02 (m, 1H, H-8), 2.94 (dt, *J* = 9.9 Hz, 5.1 Hz, 1H, H-7), 2.72 (s, 3H, H-4), 2.43 (s, 3H, H-1), 2.14-2.21 (m, 2H, H-6a, H-11), 2.03-2.09 (m, 1H, H-9a), 1.89-1.97 (m, 2H, H-6b, H-9b) ppm;

^{13}C NMR (126 MHz, $CDCl_3$) *δ* 144.0, 133.7, 130.0, 127.5, 118.3 (qt, $J_{C\text{-}F}$ = 286.1 Hz, 34.3 Hz), 111.4 (tq, $J_{C\text{-}F}$ = 256.8 Hz, 38.5 Hz), 65.7 (t, $J_{C\text{-}F}$ = 28.3 Hz), 59.8, 48.0, 47.9, 47.6, 35.1, 32.7, 27.2, 21.6 ppm;

^{19}F NMR (376 MHz, $CDCl_3$) *δ* – 84.5 (s), – 124.6 (s) ppm;

IR (film, cm^{-1}): ν_{max} 3535 (br, w), 2965 (w), 2930 (w), 2884 (w), 1598 (w), 1457 (w), 1370 (m), 1337 (m), 1306 (w), 1258 (w), 1201 (s), 1177 (s), 1154 (s), 1107 (m), 1089 (m), 1040 (s), 1008 (s), 940 (m), 816 (m), 718 (s), 697 (s), 649 (m), 630 (m), 548 (s);

$[\boldsymbol{\alpha}]_{\mathbf{D}}^{\mathbf{25.0}}$ = + 2.7 (c. 1.2, $CHCl_3$);

HRMS (−ESI) *m/z* Found $[M+CHO_2]^-$ 555.0892, $[C_{18}H_{24}F_5N_2O_8S_2]^-$ requires 555.0900, (δ = − 1.4 ppm);

Chiral HPLC Analysis CHIRALPAK IC (Hexane:^{i}PrOH, 85:15, 1.0 mL min^{-1}, 30 °C) t_R = 32.0 (minor), 34.5 (major) minutes.

Characterisation of *Exo*-Dialkyl Aziridines

2,2,3,3,3-pentafluoropropyl (S)-2-(2-hydroxyethyl)-2-isopentylaziridine-1-sulfonate (**28a**)

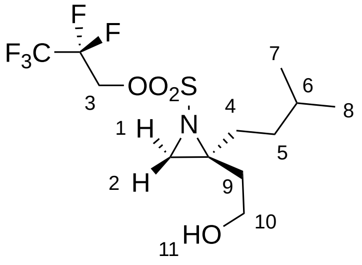

Prepared according to **GP9** using 6-methyl-3-methyleneheptan-1-ol (**27a**) as the starting material. In this case the following procedural modification was used: The reaction time was 36 hours. Purification by flash column chromatography (SiO_2, 0-8% v/v Acetone in $CHCl_3$) afforded the title compound as a colourless oil (27.8 mg, 0.075 mmol, 75%, 84% *ee* as determined from ring-opening of the aziridine with thiophenolate).

***R_f* value** = 0.46 (8% v/v Acetone in $CHCl_3$);

^{1}H NMR (500 MHz, $CDCl_3$) δ 4.64 (t, *J* = 12.2 Hz, 2H, H-3a, H-3b), 3.76-3.87 (m, 2H, H-10a, H-10b), 2.61 (s, 1H, H-2), 2.57 (s, 1H, H-1), 2.10 (ddd, *J* = 14.5 Hz, 6.9 Hz, 5.9 Hz, 1H, H-9a), 1.93 (dt, *J* = 14.5 Hz, 6.0 Hz, 1H, H-9b), 1.74-1.84 (m, 2H, H-4a, H-4b), 1.65 (br s, 1H, H-11), 1.57 (sept, *J* = 6.6 Hz, 1H, H-6), 1.25-1.42 (m, 2H, H-5a, H-5b), 0.90-0.91 (m, 6H, H-7, H-8) ppm;

^{13}C NMR (126 MHz, $CDCl_3$) δ 118.4 (qt, J_{C-F} = 286.2 Hz, 34.3 Hz), 111.4 (tq, J_{C-F} = 256.7 Hz, 38.5 Hz), 65.5 (t, J_{C-F} = 28.7 Hz), 59.2, 52.3, 42.3, 35.1, 34.9, 30.7, 28.1, 22.6, 22.4 ppm;

^{19}F NMR (376 MHz, $CDCl_3$) δ – 83.5 (s), – 123.5 (s) ppm;

IR (film, cm^{-1}): ν_{max} 2961 (w), 1468 (w), 1371 (m), 1207 (m), 1181 (m), 1109 (w), 1046 (w), 1012 (w), 905 (s), 836 (w), 729 (s), 650 (w);

$[\alpha]_D^{25.0}$ *Note: A reliable value could not be obtained for this compound: The optical rotation was very small and fluctuated in between positive and negative values. A much more stable reading was obtained with the thiophenolate adduct (see below)*;

HRMS (+ESI) *m/z* Found $[M+C_2H_4N]^+$ 411.1384, $[C_{14}H_{24}F_5N_2O_4S]^+$ requires 411.1371, (δ = + 3.2 ppm) – *Note this species is the positively charged adduct observed following (ring-opening of the aziridine with acetonitrile + protonation).*

Conversion of **28a** *to the thiophenolate adduct for ee determination: 2,2,3,3,3-pentafluoropropyl (S)-(1-hydroxy-6-methyl-3-((phenylthio)methyl)heptan-3-yl)sulfamate*.

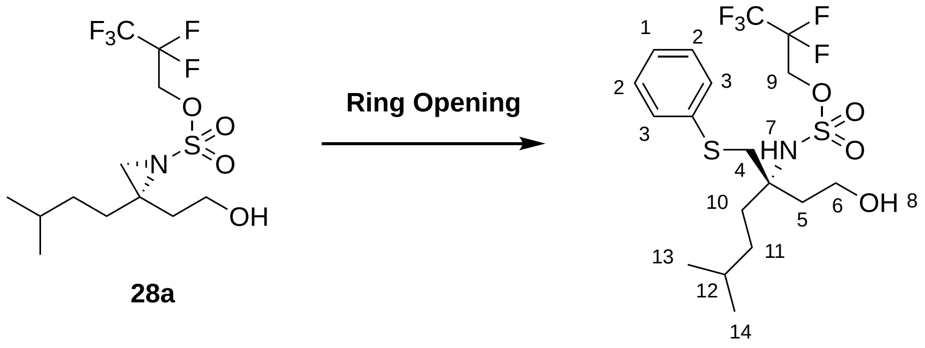

Prepared according to a variant of the protocol described by Du Bois and Guthikonda.[73] In this case the synthesis of **28a** was repeated and following chromatography of the aziridine, it was immediately subjected to a telescoped thiophenolate ring opening. To a solution of the freshly-purified aziridine (**28a**) (assumed quantitative yield from the alkene) in MeOH (2.5 mL) were added thiophenol (4 drops) and Et_3N (8 drops). The reaction mixture was stirred at room temperature for 2 hours, following which the volatiles were removed under a stream of compressed air. Purification by flash column chromatography (SiO_2, 0-10 % v/v Acetone in $CHCl_3$) afforded the title compound as a white amorphous solid (17.9 mg, 0.037 mmol, 37% over 2 steps, 84% *ee*).

***R_f* value** = 0.54 (10% v/v Acetone in $CHCl_3$);

^{1}H NMR (500 MHz, $CDCl_3$) δ 7.38-7.40 (m, 2H, H-3), 7.28-7.31 (m, 2H, H-2), 7.22 (tt, J = 6.7 Hz, 1.1 Hz, 1H, H-1), 4.50 (t, J = 12.7 Hz, 2H, H-9a, H-9b), 3.87 (t, J = 5.8 Hz, 2H, H-6a, H-6b), 3.42 (s, 2H, H-4a, H-4b), 2.06 (dt, J = 15.2 Hz, 5.4 Hz, 1H, H-5a), 1.96 (dt, J = 15.2 Hz, 6.1 Hz, 1H, H-5b), 1.81-1.91 (m, 2H, H-10a, H-10b), 1.48 (sept, J = 6.7 Hz, 1H, H-12), 1.12-1.26 (m, 2H, H-11a, H-11b), 0.85-0.88 (m, 6H, H-13, H-14) ppm;

^{13}C NMR (126 MHz, $CDCl_3$) δ 136.2, 130.3, 129.3, 126.9, 118.4 (qt, $J_{C\text{-}F}$ = 286.2 Hz, 34.4 Hz), 111.7 (tq, $J_{C\text{-}F}$ = 256.5 Hz, 38.5 Hz), 64.1, 63.9 (t, $J_{C\text{-}F}$ = 27.8 Hz), 59.2, 42.5, 36.9, 34.4, 32.5, 28.5, 22.59, 22.56 ppm;

^{19}F NMR (376 MHz, $CDCl_3$) δ – 84.5 (s), – 124.2 (s) ppm;

IR (film, cm^{-1}): ν_{max} 3553 (w), 3303 (w), 2958 (m), 1584 (w), 1468 (w), 1440 (w), 1369 (m), 1205 (s), 1179 (s), 1110 (m), 1046 (m), 1002 (s), 775 (w), 741 (w), 691 (w), 624 (w), 563 (w), 518 (w);

$[\boldsymbol{\alpha}]_{\mathbf{D}}^{\mathbf{25.0}}$ = – 3.1 (c. 1.0, $CHCl_3$);

HRMS (–ESI) *m/z* Found [M–H]$^-$ 478.1154, $[C_{18}H_{25}F_5NO_4S_2]^-$ requires 478.1151, (δ = + 0.6 ppm);

Chiral SFC Analysis CHIRAL ART SB (CO_2:^{i}PrOH, 96:4, 1.25 mL min^{-1}, 40 °C) t_R = 25.8 (minor), 30.4 (major) minutes.

2,2,3,3,3-pentafluoropropyl (S)-2-(2-cyclohexylethyl)-2-(2-hydroxyethyl)aziridine-1-sulfonate (**28b**)

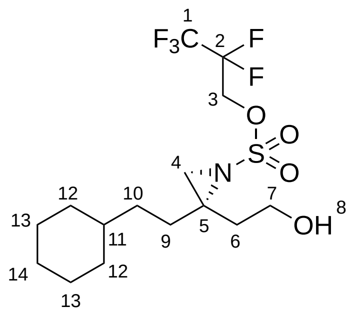

Prepared according to **GP9** using 5-cyclohexyl-3-methylenepentan-1-ol (**27b**) as the starting material. Purification by flash column chromatography (SiO_2, 0-4% v/v Acetone in $CHCl_3$) afforded the title compound as a colourless oil (28.4 mg, 0.069 mmol, 69%, 85% *ee* – determined from ring-opening of the aziridine with thiophenolate).

***R$_f$* value** = 0.35 (10% v/v Acetone in $CHCl_3$);

^{1}H NMR (500 MHz, $CDCl_3$) δ 4.64 (t, *J* = 12.2 Hz, 2H, H-3a, H-3b), 3.86-3.76 (m, 2H, H-7a, H-7b), 2.60 (s, 1H, H-4a), 2.56 (s, 1H, H-4b), 2.08 (dt, *J* = 14.5, 6.3 Hz, 1H, H-6a), 1.91 (dt, *J* = 14.6, 6.1 Hz, 1H, H-6b), 1.85-1.74 (m, 2H, H-9a, H-9b), 1.73-1.63 (m, 6H, H-8, H-12a, H-13a, H-14a), 1.42-1.35 (m, 1H, H-10a), 1.33-1.27 (m, 1H, H-10b), 1.25-1.09 (m, 4H, H-11, H-13b, H-14b), 0.92-0.84 (m, 2H, H-12b) ppm;

^{13}C NMR (126 MHz, $CDCl_3$) δ 118.3 (qt, $J_{C\text{-}F}$ = 286.4, 34.9 Hz, C-1), 111.4 (tq, $J_{C\text{-}F}$ = 256.6, 38.6 Hz, C-2), 65.5 (t, $J_{C\text{-}F}$ = 28.6 Hz, C-3), 59.2 (C-7), 52.4 (C-5), 42.3 (C-4), 37.6 (C-11) 35.1 (C-6), 33.45 (C-12a), 33.40 (C-12b), 33.2 (C-10), 30.2 (C-9), 26.6 (C-14), 26.35 (C-13a), 26.33 (C-13b) ppm;

^{19}F NMR (376 MHz, $CDCl_3$) δ – 83.5, – 123.5 ppm;

IR (film, cm^{-1}): ν_{max} 2925 (m), 2853 (w), 1450 (m), 1371 (m), 1201 (s), 1178 (s), 1106 (m), 1042 (m), 1010 (m), 945 (w), 830 (s), 722 (w), 684 (w);

$[\alpha]_D^{25.0}$ = – 2.8 (c. 0.90 $CHCl_3$);

HRMS (+ESI) *m/z* found $[M+H]^+$ 410.1423, $[C_{15}H_{25}F_5NO_4S]^+$ requires 410.1419, (δ = +1.0 ppm).

Conversion of **28b** *to the thiophenolate adduct for ee determination: 2,2,3,3,3-pentafluoropropyl (S)-(1-cyclohexyl-5-hydroxy-3-((phenylthio)methyl)pentan-3-yl)sulfamate* (**41**)

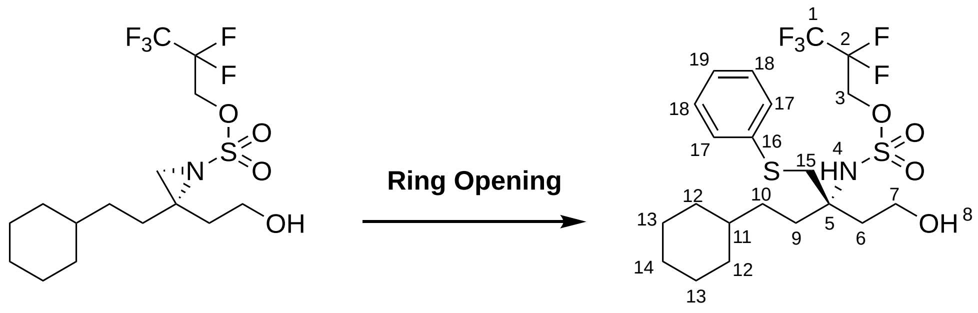

28b **41**

Prepared according to the protocol described by Du Bois and Guthikonda.[73] To a solution of 2,2,3,3,3-pentafluoropropyl 2-(2-cyclohexylethyl)-2-(2-hydroxyethyl)aziridine-1-sulfonate (**28b**) (28.4 mg, 0.069 mmol, 1.0 equiv.) in MeOH (1.0 mL) were added thiophenol (0.02 mL) and Et_3N (0.05 mL). The reaction mixture was stirred at room temperature for 30 minutes, following which the volatiles were removed under a stream of compressed air. Purification by flash column chromatography (SiO_2, 0-5% v/v Acetone in $CHCl_3$) afforded the title compound as a white amorphous solid (22.0 mg, 0.042 mmol, 61%, 85% *ee*).

***R_f* value** = 0.46 (10% v/v Acetone in $CHCl_3$);

^{1}H NMR (500 MHz, $CDCl_3$) δ 7.38 (dd, *J* = 8.2, 0.9 Hz, 2H, H-17), 7.30 (t, *J* = 7.6 Hz, 2H, H-18), 7.22 (t, *J* = 7.3 Hz, 1H, H-19), 6.15 (s, 1H, H-4), 4.50 (t, *J* = 12.8 Hz, 2H, H-3a, H-3b), 3.89-3.83 (m, 2H, H-7a, H-7b), 3.41 (s, 2H, H-15), 2.05 (dt, *J* = 15.0, 5.5 Hz, 1H, H-6a), 1.96 (dt, *J* = 15.1, 5.9 Hz, 1H, H-6b), 1.92-1.82 (m, 2H, H-9a, H-9b), 1.73-1.61 (m, 6H, H-8, H-12a, H-13a, H-14a), 1.28-1.08 (m, 6H, H-10a, H-10b, H-11, H-13b, H-14b), 0.89-0.81 (m, 2H, H-12b) ppm;

^{13}C NMR (126 MHz, $CDCl_3$) δ 136.2 (C-16), 130.3 (C-17), 129.3 (C-18), 126.9 (C-19), 118.4 (qt, J_{C-F} = 287.5, 35.0 Hz, C-1), 111.7 (tq, J_{C-F} = 256.4, 38.6 Hz, C-2), 64.1 (C-5), 63.9 (t, J_{C-F} = 27.9 Hz, C-3), 59.2 (C-7), 42.5 (C-15), 38.0 (C-11), 36.9 (C-6), 33.9 (C-9), 33.4 (C-12), 31.1 (C-10), 26.7 (C-14), 26.4 (C-13) ppm;

^{19}F NMR (376 MHz, $CDCl_3$) δ – 84.5, – 124.2 ppm;

IR (film, cm^{-1}): ν_{max} 3300 (w), 2926 (m), 2854 (w), 2192 (w), 2153 (w), 1995 (w), 1584 (2), 1449 (w), 1368 (m), 1206 (s), 1177 (s), 1110 (m), 1002 (m), 841 (w), 774 (w), 740 (w), 691 (w);

$[\alpha]_D^{25.0}$ = – 2.7 (c. 1.00 $CHCl_3$);

HRMS (–ESI) *m/z* found $[M–H]^-$ 518.1464, $[C_{21}H_{29}F_5NO_4S_2]^-$ requires 518.1464, (δ = 0.0 ppm);

Chiral SFC analysis: CHIRAL ART SB (CO_2:^{i}PrOH = 94:6, 1.25 mL min^{-1}, 40 °C), t_R = 26.0 (minor), 29.5 (major) minutes.

2,2,3,3,3-pentafluoropropyl (S)-2-(4-((tert-butyldimethylsilyl)oxy)butyl)-2-(2-hydroxyethyl)aziridine-1-sulfonate (**28c**)

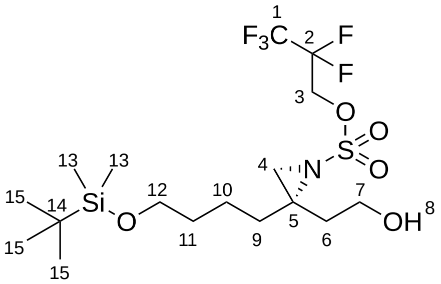

Prepared according to **GP9** using 7-((*tert*-butyldimethylsilyl)oxy)-3-methyleneheptan-1-ol (**27c**) as the starting material. Purification by flash column chromatography (SiO_2, 0-4% v/v

Acetone in $CHCl_3$) afforded the title compound as a pale-yellow oil (26.8 mg, 0.055 mmol, 55%, 80% *ee* – determined from ring-opening of the aziridine with thiophenolate).

***R**$_f$* **value** = 0.29 (10% v/v Acetone in $CHCl_3$);

^{1}H NMR (500 MHz, $CDCl_3$) δ 4.64 (t, J = 12.2 Hz, 2H, H-3a, H-3b), 3.87-3.77 (m, 2H, H-7a, H-7b), 3.63-3.60 (m, 2H, H-12a, H-12b), 2.62 (s, 1H, H-4a), 2.58 (s, 1H, H-4b), 2.10 (dt, J = 14.6, 6.7 Hz, 1H, H-6a), 1.93 (dt, J = 14.6, 6.1 Hz, 1H, H-6b), 1.86-1.76 (m, 2H, H-9a, H-9b), 1.59-1.47 (m, 4H, H-10a, H-10b, H-11a, H-11b), 0.89 (s, 9H, H-15), 0.04 (s, 6H, H-13) ppm;

^{13}C NMR (126 MHz, $CDCl_3$) δ 118.3 (qt, J_{C-F} = 286.5, 34.9 Hz, C-1), 111.4 (tq, J_{C-F} = 256.2, 38.4 Hz, C-2), 65.5 (t, J_{C-F} = 28.8 Hz, C-3), 62.6 (C-12), 59.2 (C-7), 52.0 (C-5), 42.2 (C-4), 35.1 (C-6), 32.5 (C-11), 32.4 (C-9), 26.0 (C-15), 22.2 (C-10), 18.4 (C-14), – 5.2 (C-13) ppm;

^{19}F NMR (376 MHz, $CDCl_3$) δ – 84.5, – 124.5 ppm;

IR (film, cm^{-1}): ν_{max} 2932 (w), 2859 (w), 1373 (m), 1203 (s), 1178 (s), 1104 (m), 1044 (m), 1009 (m), 941 (m), 832 (s), 775 (s), 721 (w), 681 (w);

$[\alpha]_D^{25.0}$ = – 2.2 (c. 1.3 $CHCl_3$);

HRMS (+ESI) *m/z* found $[M+H]^+$ 486.1778, $[C_{17}H_{33}F_5NO_5SSi]^+$ requires 486.1763, (δ = + 3.1 ppm).

Conversion of **28c** *to the thiophenolate adduct for ee determination: 2,2,3,3,3-pentafluoropropyl (S)-(7-((tert-butyldimethylsilyl)oxy)-1-hydroxy-3-((phenylthio)methyl)heptan-3-yl)sulfamate*

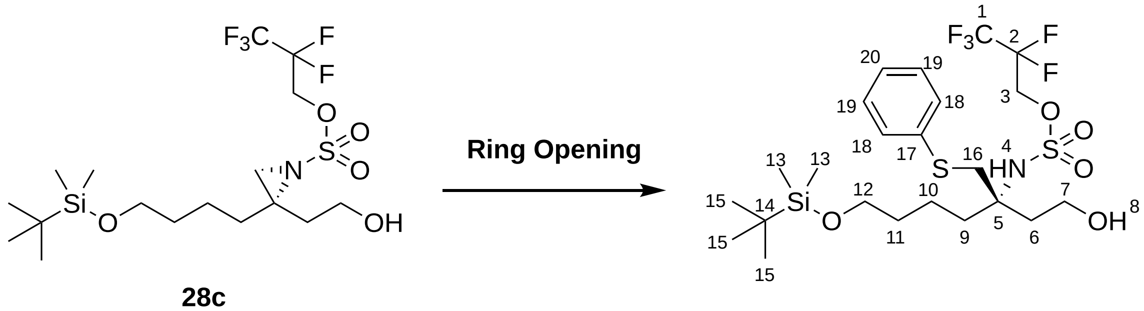

Prepared according to a variant of the protocol described by Du Bois and Guthikonda.[73] To a solution of 2,3,3,3-pentafluoropropyl 2-(4-((*tert*-butyldimethylsilyl)oxy)butyl)-2-(2-hydroxyethyl)aziridine-1-sulfonate (**28c**) (26.8 mg, 0.055 mmol, 1.0 equiv.) in MeOH (0.8 mL) were added thiophenol (0.02 mL) and Et_3N (0.05 mL). The reaction mixture was stirred at room temperature for 30 minutes, following which the volatiles were removed under a stream of compressed air. Purification by flash column chromatography (SiO_2, 0-6% v/v Acetone in $CHCl_3$) afforded the title compound as a pale-yellow oil (14.2 mg, 0.024 mmol, 43%, 80% *ee*).

***R**$_f$* **value** = 0.37 (10% v/v Acetone in $CHCl_3$);

^{1}H NMR (500 MHz, $CDCl_3$) δ 7.39-7.37 (m, 2H, H-18), 7.31-7.28 (m, 2H, H-19), 7.23-7.20 (m, 1H, H-20), 6.25 (s, 1H, H-4), 4.50 (t, J = 12.6 Hz, 2H, H-3a, H-3b), 3.89-3.84 (m, 2H, H-7a, H-7b), 3.59 (t, J = 6.2 Hz, 2H, H-12a, H-12b), 3.43 (s, 2H, H-16), 2.06 (dt, J = 15.4, 5.4 Hz, 1H, H-6a), 1.99-1.83 (m, 3H, H-6b, H9a, H-9b), 1.77-1.76 (m, 1H, H-8), 1.54-1.46 (m, 2H, H-11a, H-11b), 1.44-1.35 (m, 2H, H-10a, H-10b), 0.89 (s, 9H, H-15), 0.04 (s, 6H, H-13) ppm;

^{13}C NMR (126 MHz, $CDCl_3$) δ 136.2 (C-17), 130.2 (C-18), 129.2 (C-19), 126.9 (C-20), 118.4 (qt, J_{C-F} = 286.6, 34.4 Hz, C-1), 111.7 (tq, J_{C-F} = 255.9, 38.0 Hz, C-2), 64.0 (C-5), 63.9 (t, J_{C-F} = 28.0 Hz, C-3), 62.7 (C-12), 59.2 (C-7), 42.4 (C-16), 36.9 (C-6), 36.1 (C-9), 32.8 (C-11), 26.1 (C-15), 20.0 (C-10), 18.5 (C-14), – 5.2 (C-13) ppm;

^{19}F NMR (376 MHz, $CDCl_3$) δ – 84.4, – 124.2 ppm;

IR (film, cm^{-1}): ν_{max} 3298 (w), 2932 (m), 2859 (m), 1462 (m), 1440 (m), 1363 (m), 1256 (m), 1204 (s), 1178 (s), 1106 (s), 1048 (m), 1004 (s), 836 (s), 776 (m), 740 (m), 691 (m);

$[\alpha]_D^{25.0}$ = – 5.9 (c. 0.88 $CHCl_3$);

HRMS (+ESI) *m/z* found $[M+H]^+$ 596.1943, $[C_{23}H_{39}F_5NO_5S_2Si]^+$ requires 596.1954, (δ = – 1.8 ppm);

Chiral SFC analysis: CHIRAL ART SB (CO_2:MeOH, 96.5:3.5, 1.25 mL min^{-1}, 40 ˚C), t_R = 18.6 (minor), 21.0 (major) minutes.

2,2,3,3,3-pentafluoropropyl (R)-6-oxa-2-azaspiro[4.5]decane-2-sulfonate (**30**)

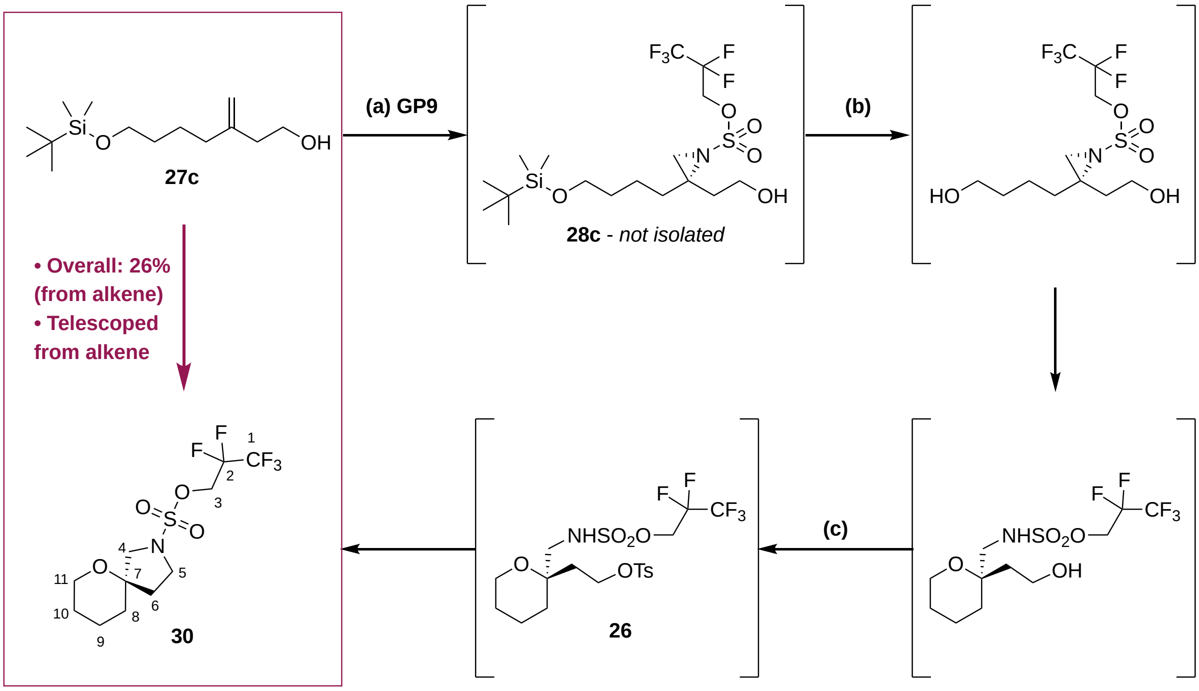

Step (a): Prepared according to **GP9** using 7-((*tert*-butyldimethylsilyl)oxy)-3-methyleneheptan-1-ol (**27c**) (26.5 mg, 0.10 mmol, 1.0 equiv.) as the starting material.

Step (b): Following work up of the aziridination step, $TsOH•H_2O$ (3.8 mg, 0.02 mmol, 0.2 equiv.) in a 1:3 mixture of $MeOH/CH_2Cl_2$ (1.5 mL) was added to the crude residue. The resultant solution was allowed to stir at room temperature overnight. The reaction mixture was concentrated under a stream of N_2 and the crude residue diluted with CH_2Cl_2 (3 mL) and saturated $NaHCO_3$ solution (1 mL). The layers were separated and the aqueous layer was extracted with CH_2Cl_2. The organic layers were passed through a short $MgSO_4$ plug and concentrated under a stream of N_2.

Step (c): To the crude residue was added DMAP (4.9 mg, 0.04 mmol, 0.4 equiv.), NEt_3 (0.02 mL, 0.14 mmol, 1.4 equiv.) and CH_2Cl_2 (1.5 mL). A stock solution of TsCl (1.5 mL, 0.075 M in CH_2Cl_2, 0.11 mmol, 1.1 equiv.) was added and the resultant reaction mixture stirred at room temperature overnight. The reaction was quenched with 1 M HCl (3 mL) and the aqueous layer extracted with CH_2Cl_2. The organic layers were passed through a short $MgSO_4$ plug and concentrated under a stream of N_2. Purification by flash column chromatography (SiO_2, 0-10% v/v Acetone in Petrol) afforded the title compound as a colourless oil (9.2 mg, 0.026 mmol, 26% from **27c**).

***R_f* value** = 0.41 (20% v/v Acetone in Petrol);

^{1}H NMR (500 MHz, $CDCl_3$) δ 4.51 (sextd, *J* = 12.2, 0.8 Hz, 2H, H-3a, H-3b), 3.74-3.62 (m, 3H, H-4a, H-11a, H-11b), 3.58-3.52 (m, 2H, H-5a, H-5b), 3.22 (d, *J* = 10.9 Hz, 1H, H-4b), 2.25-2.20 (m, 1H, H-6a), 1.83-1.77 (m, 1H, H-6b), 1.69-1.61 (m, 3H, H-8a, H-8b, H-9a), 1.60-1.56 (m, 3H, H-9b, H-10a, H-10b) ppm;

^{13}C NMR (126 MHz, $CDCl_3$) δ 118.4 (qt, $J_{C\text{-}F}$ = 285.6, 34.6 Hz, C-1), 111.6 (tq, $J_{C\text{-}F}$ = 256.4, 38.1 Hz, C-2), 80.9 (C-7), 63.9 (t, $J_{C\text{-}F}$ = 28.5 Hz, C-3), 63.0 (C-11), 56.9 (C-4), 47.8 (C-5), 35.6 (C-6), 32.4 (C-8), 25.7 (C-10), 20.9 (C-9) ppm;

^{19}F NMR (376 MHz, $CDCl_3$) δ – 83.5, – 123.6 ppm;

IR (film, cm^{-1}): ν_{max} 2945 (w), 2862 (w), 1444 (w), 1378 (m), 1254 (s), 1200 (s), 1169 (s), 1107 (m), 1082 (m), 1045 (s), 1008 (s), 944 (w), 876 (w), 806 (m), 768 (m), 668 (w);

$[\boldsymbol{\alpha}]_{\mathbf{D}}^{\mathbf{25.1}}$ **=** – 10.7 (c. 0.61 $CHCl_3$);

HRMS (–ESI) *m/z* found $[M–H]^-$ 352.0648, $[C_{11}H_{15}F_5NO_4S]^-$ requires 352.0647, (δ = +0.3 ppm).

Characterisation of *Tri*-Alkyl Class I Aziridines

2,2,3,3,3-pentafluoropropyl ((2R,3R)-2-methyl-2-propyltetrahydrofuran-3-yl)sulfamate (**33a**)

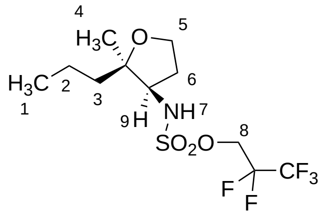

Prepared according to **GP9** using (*Z*)-4-methylhept-3-en-1-ol (**31a**) as the starting material. Following the cyclisation protocol (16 hours), purification by flash column chromatography (SiO_2, 0-10% v/v Acetone in $CHCl_3$) afforded the title compound as a colourless oil (25.7 mg, 0.072 mmol, 72%, 87% *ee*).

***R**$_f$* **value** = 0.57 (10% Acetone in $CHCl_3$);

^{1}H NMR (400 MHz, $CDCl_3$) δ 5.00 (d, *J* = 9.9 Hz, 1H, H-7), 4.50 (t, *J* = 12.5 Hz, 2H, H-8a, H-8b), 3.82-3.92 (m, 2H, H-5a, H-5b), 3.71-3.76 (m, 1H, H-9), 2.42-2.51 (m, 1H, H-6a), 1.93-2.01 (m, 1H, H-6b), 1.35-1.51 (m, 4H, H-2a, H-2b, H-3a, H-3b), 1.21 (s, 3H, H-4), 0.95 (t, *J* = 6.8 Hz, 3H, H-1) ppm;

^{13}C NMR (101 MHz, $CDCl_3$) δ 118.3 (qt, $J_{C\text{-}F}$ = 286.1 Hz, 34.3 Hz), 111.5 (tq, $J_{C\text{-}F}$ = 256.6 Hz, 38.5 Hz), 83.0, 64.0 (t, $J_{C\text{-}F}$ = 28.3 Hz), 63.5, 62.0, 37.2, 32.5, 22.8, 17.1, 14.8 ppm;

^{19}F NMR (376 MHz, $CDCl_3$) δ – 83.5 (s), – 124.2 - – 122.7 (m)*ppm;

*Partial inequivalence leads to extreme strong coupling between these two ^{19}F nuclei.

IR (film, cm^{-1}): ν_{max} 3286 (br, w), 2965 (w), 2940 (w), 2879 (w), 1459 (m), 1371 (m), 1260 (w), 1204 (s), 1185 (s), 1111 (m), 1037 (m), 1009 (m), 937 (w), 807 (w), 774 (w), 668 (w), 622 (w), 587 (w), 519 (w);

$[\alpha]_D^{25.0}$ = – 6.8 (c. 1.7, $CHCl_3$);

HRMS (–ESI) *m/z* Found [M–H]$^-$ 354.0798, $[C_{11}H_{17}F_5NO_4S]^-$ requires 354.0804, (δ = – 1.7 ppm);

Chiral GC Analysis CP7502 – CP-Chirasil-DEX CB column (125 °C Isothermal, 27.5 psi) t_R = 25.8 (major), 26.6 (minor) minutes.

2,2,3,3,3-pentafluoropropyl (R)-(5-oxaspiro[3.4]octan-8-yl)sulfamate (**33b**)

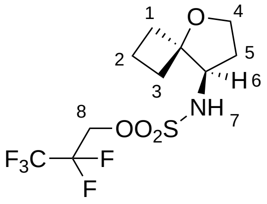

Prepared according to **GP9** using 3-cyclobutylidenepropan-1-ol (**31b**) as the starting material. Following the cyclisation protocol (16 hours), purification by flash column chromatography (SiO_2, 0-7.5% v/v Acetone in $CHCl_3$) afforded the title compound as a colourless oil (22.9 mg, 0.067 mmol, 67%, 83% *ee*).

***R$_f$* value** = 0.43 (8% v/v Acetone in $CHCl_3$);

^{1}H NMR (400 MHz, $CDCl_3$) δ 5.09 (d, *J* = 8.8 Hz, 1H, H-7), 4.51 (t, *J* = 12.4 Hz, 2H, H-8a, H-8b), 3.91-3.95 (m, 1H, H-6), 3.79-3.88 (m, 2H, H-4a, H-4b), 2.21-2.33 (m, 3H, H-3a, H-3b, H-5a),

2.03-2.12 (m, 2H, H-1a, H-5b), 1.85-1.92 (m, 1H, H-1b), 1.77-1.83 (m, 1H, H-2a), 1.59-1.69 (m, 1H, H-2b) ppm;

^{13}C NMR (126 MHz, $CDCl_3$) δ 118.4 (qt, $J_{C\text{-}F}$ = 286.1 Hz, 34.6 Hz), 111.6 (tq, $J_{C\text{-}F}$ = 256.6 Hz, 38.2 Hz), 84.8, 64.0 (t, $J_{C\text{-}F}$ = 28.3 Hz), 63.8, 59.8, 34.1, 31.1, 28.3, 12.2 ppm;

^{19}F NMR (376 MHz, $CDCl_3$) δ – 83.5 (s), – 124.2 - – 122.8 (m)*ppm;

*Partial inequivalence leads to extreme strong coupling between these two ^{19}F nuclei.

IR (film, cm^{-1}): ν_{max} 3288 (w), 3147 (w), 2988 (w), 2959 (w), 2892 (w), 1453 (m), 1370 (m), 1204 (s), 1184 (s), 1111 (m), 1009 (m), 933 (w), 808 (w), 774 (w), 666 (w), 623 (w), 586 (w), 518 (w);

$[\alpha]_D^{25.0}$ = – 29.8 (c. 1.0, $CHCl_3$);

HRMS (–ESI) *m/z* Found [M–H]$^-$ 338.0486, $[C_{10}H_{13}F_5NO_4S]^-$ requires 338.0491, (δ = – 1.5 ppm);

Chiral GC Analysis CP7502 – CP-Chirasil-DEX CB column (135 °C Isothermal, 13.6 psi) t_R = 26.2 (major), 27.8 (minor) minutes.

2,2,3,3,3-pentafluoropropyl (R)-(1-oxaspiro[4.5]decan-4-yl)sulfamate (**33c**)

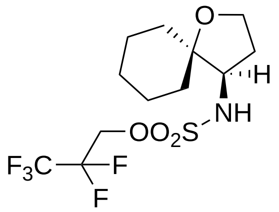

Prepared according to **GP9** using 3-cyclohexylidenepropan-1-ol (**31c**) as the starting material. Following the cyclisation protocol (16 hours), purification by flash column chromatography

(SiO_2, 0-7.5% v/v Acetone in $CHCl_3$) afforded the title compound as a yellow oil (31.6 mg, 0.086 mmol, 86%, 84% *ee* as determined from the *N*-Benzylated adduct).

***R_f* value** = 0.58 (10% v/v Acetone in $CHCl_3$);

^{1}H NMR (400 MHz, $CDCl_3$) δ 5.15 (br, s, 1H), 4.49 (t, J = 12.4 Hz, 2H) 3.91 (td, J = 8.9 Hz, 6.1 Hz, 1H), 3.82 (td, J = 8.8 Hz, 6.2 Hz, 1H), 3.72 (dd, J = 6.6 Hz, 4.8 Hz, 1H), 2.40-2.49 (m, 1H), 1.92-2.00 (m, 1H), 1.44-1.64 (m, 9H), 1.25-1.34 (m, 1H) ppm;

^{13}C NMR (101 MHz, $CDCl_3$) δ 118.3 (qt, $J_{C\text{-}F}$ = 286.1 Hz, 34.4 Hz), 111.5 (tq, $J_{C\text{-}F}$ = 256.5 Hz, 38.4 Hz), 82.7, 64.0 (t, $J_{C\text{-}F}$ = 28.3 Hz), 63.3, 61.2, 34.4, 32.6, 31.0, 25.6, 22.8, 22.4 ppm;

^{19}F NMR (376 MHz, $CDCl_3$) δ – 83.5 (s), – 124.2 - – 122.7 (m)*ppm;

*Partial inequivalence leads to extreme strong coupling between these two ^{19}F nuclei.

IR (film, cm^{-1}): ν_{max} 3291 (w), 2939 (m), 2863 (w), 1451 (m), 1371 (m), 1260 (w), 1206 (s), 1184 (s), 1157 (m), 1110 (m), 1033 (m), 1011 (m), 954 (w), 807 (w), 759 (s), 668 (w), 623 (w), 584 (w), 518 (w);

$[\alpha]_D^{25.0}$ = – 11.9 (c. 1.0, $CHCl_3$);

HRMS (–ESI) *m/z* Found $[M–H]^-$ 366.0814, $[C_{12}H_{17}F_5NO_4S]^-$ requires 366.0804, (δ = + 2.7 ppm).

N-benzylation of **33c** *for ee determination: 2,2,3,3,3-pentafluoropropyl (R)-(4-bromobenzyl)(1-oxaspiro[4.5]decan-4-yl)sulfamate*

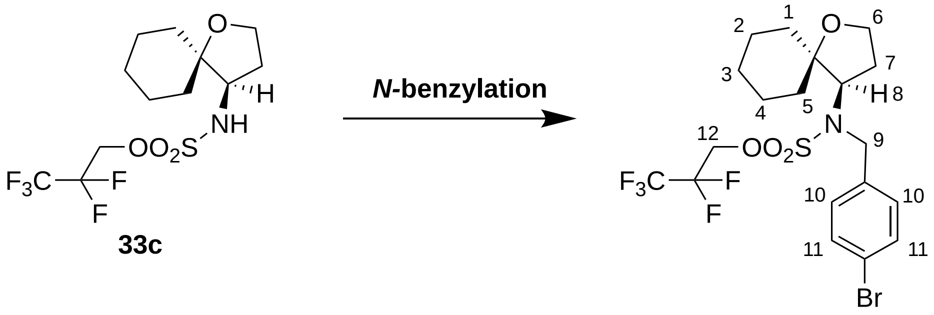

A crimp-top vial was charged with 2,2,3,3,3-pentafluoropropyl (*R*)-(1-oxaspiro[4.5]decan-4-yl)sulfamate (**33c**) (30.1 mg, 0.082 mmol), Cs_2CO_3 (60.8 mg, 0.17 mmol) and 1-bromo-4-(bromomethyl)benzene (20.5 mg, 0.082 mmol). The vial was sealed then evacuated and backfilled with nitrogen three times. MeCN (2.5 mL) was added and the reaction mixture stirred at 82 °C overnight. The solvent was removed under reduced pressure and purification by flash column chromatography (20-50% v/v CH_2Cl_2 in Petrol) afforded the title compound as a white gum (28.9 mg, 0.054 mmol, 66%, 84% *ee*).

***R_f* value** = 0.35 (50% v/v CH_2Cl_2 in Petrol);

^{1}H NMR (500 MHz, $CDCl_3$) δ 7.45-7.47 (m, 2H, H-11), 7.27 (d, J = 7.5 Hz, 2H, H-10), 4.61 (d, J = 16.9 Hz, 1H, H-9a), 4.38-4.51 (m, 3H, H-9b, H-12a, H-12b), 4.21 (dd, J = 8.8 Hz, 3.2 Hz, 1H, H-8), 3.87 (td, J = 9.2 Hz, 5.3 Hz, 1H, H-6a), 3.72 (td, J = 9.3 Hz, 6.6 Hz, 1H, H-6b), 2.35 (dtd, J = 18.2 Hz, 9.1 Hz, 5.3 Hz, 1H, H-7a), 1.98-2.05 (m, 1H, H-7b), 1.47-1.70 (m, 8H, H-1a, H-2a, H-2b, H-3a, H4a, H-4b, H-5a, H-5b), 1.26-1.36 (m, 2H, H-1b, H-3b) ppm;

^{13}C NMR (126 MHz, $CDCl_3$) δ 136.1, 131.8, 129.2, 121.8, 118.3 (qt, $J_{C\text{-}F}$ = 286.2 Hz, 34.4 Hz), 111.6 (tq, $J_{C\text{-}F}$ = 256.6 Hz, 38.4 Hz), 84.5, 66.5, 63.7 (t, $J_{C\text{-}F}$ = 28.3 Hz), 63.4, 50.3, 33.7, 31.5, 29.3, 25.6, 22.7, 22.3 ppm;

^{19}F NMR (376 MHz, $CDCl_3$) δ – 83.5 (s), – 123.5 (s) ppm;

IR (film, cm^{-1}): ν_{max} 2934 (m), 2860 (w), 1490 (w), 1450 (w), 1371 (m), 1259 (w), 1206 (s), 1180 (s), 1159 (m), 1109 (m), 1073 (w), 1025 (w), 1011 (m), 939 (w), 899 (w), 793 (w), 773 (w), 621 (w), 584 (w);

$[\alpha]_D^{25.0}$ = – 7.5 (c. 1.0, $CHCl_3$);

HRMS (+ESI) *m/z* Found $[M+H]^+$ 536.0549, $[C_{19}H_{24}BrF_5NO_4S]^+$ requires 536.0524, (δ = + 4.7 ppm);

Chiral SFC Analysis CHIRALPAK IG (CO_2:MeOH, 97:3, 1.25 mL min^{-1}, 40 °C) t_R = 8.6 (major), 20.4 (minor) minutes.

2,2,3,3,3-pentafluoropropyl (R)-(1,8-dioxaspiro[4.5]decan-4-yl)sulfamate (**33d**)

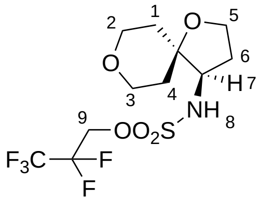

Prepared according to **GP9** using 3-(tetrahydro-4*H*-pyran-4-ylidene)propan-1-ol (**31d**) as the starting material. Following the cyclisation protocol (16 hours), purification by flash column chromatography (SiO_2, 0-20% v/v Acetone in $CHCl_3$) afforded the title compound as a colourless oil (18.3 mg, 0.050 mmol, 50%, 81% *ee* as determined from the *N*-Benzylated adduct).

^{1}H NMR (500 MHz, $CDCl_3$) δ 5.06 (d, *J* = 9.8 Hz, 1H), 4.51 (t, *J* = 12.4 Hz, 2H), 3.95 (dt, *J* = 8.9 Hz, 6.0 Hz 1H), 3.83-3.89 (m, 2H), 3.68-3.80 (m, 4H), 2.44-2.52 (m, 1H), 1.96-2.03 (m, 1H), 1.73-1.87 (m, 2H), 1.48-1.55 (m, 2H) ppm;

^{13}C NMR (126 MHz, $CDCl_3$) δ 118.3 (qt, J_{C-F} = 286.2 Hz, 34.6 Hz), 111.5 (tq, J_{C-F} = 256.1 Hz, 38.5 Hz), 79.9, 64.32, 64.30, 64.1 (t, J_{C-F} = 28.3 Hz), 63.6, 61.5, 34.4, 32.2, 31.4 ppm;

^{19}**F NMR** (376 MHz, $CDCl_3$) δ – 83.5 (s), – 124.2 - – 122.7 (m)*ppm;

*Partial inequivalence leads to extreme strong coupling between these two ^{19}F nuclei.

IR (film, cm^{-1}): ν_{max} 3287 (w), 3132 (w), 2963 (w), 1459 (m), 1371 (m), 1307 (w), 1261 (w), 1204 (s), 1184 (s), 1157 (m), 1106 (m), 1078 (w), 1040 (m), 1012 (m), 952 (m), 839 (m), 807 (m), 774 (m), 713 (w), 666 (w), 621 (w), 580 (w), 563 (w), 517 (w);

$[\alpha]_D^{25.0}$ = – 8.2 (c. 1.0, $CHCl_3$);

HRMS *Obtained for the N-benzylated adduct.*

N-benzylation of **33d** *for ee determination: 2,2,3,3,3-pentafluoropropyl (R)-(4-bromobenzyl)(1,8-dioxaspiro[4.5]decan-4-yl)sulfamate*

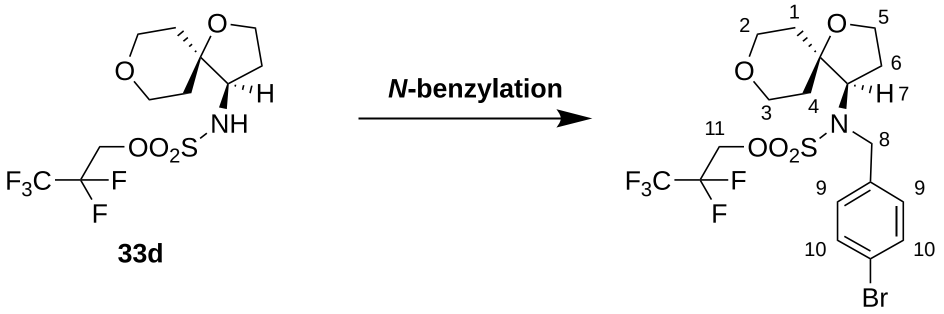

A crimp-top vial was charged with 2,2,3,3,3-pentafluoropropyl (*R*)-(1,8-dioxaspiro[4.5]decan-4-yl)sulfamate (**33d**) (18.3 mg, 0.050 mmol), Cs_2CO_3 (34.2 mg, 0.11 mmol) and 1-bromo-4-(bromomethyl)benzene (12.5 mg, 0.050 mmol). The vial was sealed then evacuated and backfilled with nitrogen three times. MeCN (2.5 mL) was added and the reaction mixture stirred at 82 °C overnight. The solvent was removed under reduced pressure and purification by flash column chromatography (SiO_2, 0-10% v/v Acetone in $CHCl_3$) afforded the title compound as a colourless oil (17.8 mg, 0.033 mmol, 66%, 81% *ee*).

***R_f* value** = 0.56 (10% v/v Acetone in $CHCl_3$);

^{1}H NMR (500 MHz, $CDCl_3$) δ 7.46-7.49 (m, 2H, H-10), 7.26-7.28 (m, 2H, H-9), 4.60 (d, J = 16.9 Hz, 1H, H-8a), 4.40-4.52 (m, 3H, H-8b, H-11a, H-11b), 4.23 (dd, J = 8.7 Hz, 3.3 Hz, 1H, H-7), 3.92 (td, J = 9.3 Hz, 5.6 Hz, 1H, H-5a), 3.84 (ddd, J = 11.3 Hz, 4.6 Hz, 3.1 Hz, 1H, H-3a), 3.65-3.78 (m, 4H, H-2a, H-2b, H-3b, H-5b), 2.35 (dtd, J = 18.2 Hz, 9.1 Hz, 5.6 Hz, 1H, H-6a), 2.04-2.11 (m, 1H, H-6b), 1.98 (ddd, J = 16.3 Hz, 11.5 Hz, 4.9 Hz, 1H, H-4a), 1.65 (ddd, J = 15.9 Hz, 11.0 Hz, 4.9 Hz, 1H, H-1a), 1.55-1.60 (m, 1H, H-4b)*, 1.50 (dq, J = 13.7 Hz, 2.7 Hz, 1H, H-1b) ppm;

*Overlapped with residual water peak so integrates to 2H

^{13}C NMR (126 MHz, $CDCl_3$) δ 135.8, 131.9, 129.3, 122.1, 118.3 (qt, J_{C-F} = 286.1 Hz, 34.4 Hz), 111.5 (tq, J_{C-F} = 256.7 Hz, 38.5 Hz), 82.0, 66.4, 64.6, 63.9, 63.8 (t, J_{C-F} = 28.3 Hz), 63.7, 50.4, 34.0, 32.2, 29.0 ppm;

^{19}F NMR (376 MHz, $CDCl_3$) δ – 84.4 (s), – 124.4 (s) ppm;

IR (film, cm^{-1}): ν_{max} 2961 (m), 2866 (m), 1490 (w), 1370 (s), 1307 (w), 1205 (s), 1180 (s), 1106 (s), 1073 (m), 1010 (s), 951 (w), 898 (m), 837 (m), 801 (m), 773 (m), 573 (w), 518 (w);

$[\boldsymbol{\alpha}]_{\mathbf{D}}^{\mathbf{25.0}}$ = – 8.3 (c. 1.0, $CHCl_3$);

HRMS (+ESI) *m/z* Found $[M+H]^+$ 538.0333, $[C_{18}H_{22}BrF_5NO_5S]^+$ requires 538.0317, (δ = + 3.0 ppm);

Chiral SFC Analysis CHIRAL ART SJ (CO_2:^{i}PrOH, 97:3, 1.25 mL min^{-1}, 40 °C) t_R = 7.9 (minor), 9.2 (major) minutes.

2,2,3,3,3-pentafluoropropyl (R)-(2,2-dimethyltetrahydro-2H-pyran-3-yl)sulfamate (**33e**)

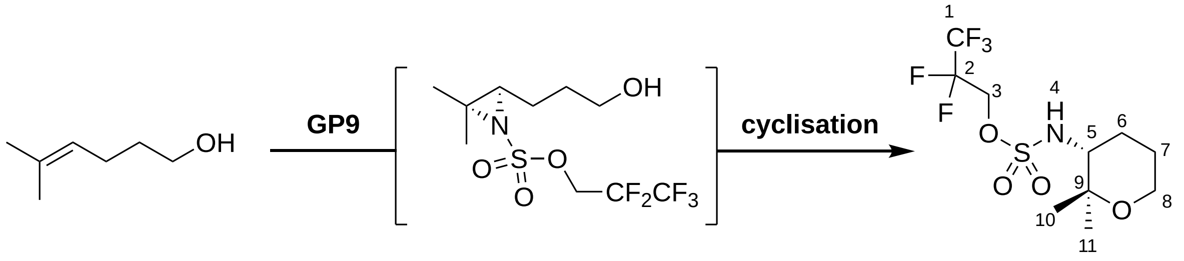

33e

Prepared according to **GP9** using 5-methylhex-4-en-1-ol (**31e**) (11.4 mg, 0.10 mmol, 1.0 equiv.) as the starting material. Following the cyclisation protocol (2 hours) purification by flash column chromatography (SiO_2, 0-5% v/v Acetone in $CHCl_3$) afforded the title compound as a colourless oil (18.2 mg, 0.053 mmol, 53%, 91% *ee*).

***R_f* value** = 0.41 (10% v/v acetone in $CHCl_3$);

^{1}H NMR (500 MHz, $CDCl_3$) δ 5.01 (d, J = 9.6 Hz, 1H, H-4), 4.49 (td, J = 12.4, 0.8 Hz, 2H, H-3a, H-3b), 3.72-3.67 (m, 1H, H-8a), 3.64-3.59 (m, 1H, H-8b), 3.36-3.32 (m, 1H, H-5), 2.07-2.01 (m, 1H, H-6a), 1.76-1.68 (m, 2H, H-6b, H-7a), 1.62-1.55 (m, 1H, H-7b), 1.33 (s, 3H, H-10), 1.21 (s, 3H, H-11) ppm;

^{13}C NMR (126 MHz, $CDCl_3$) δ 118.4 (qt, $J_{C\text{-}F}$ = 287.6, 34.1 Hz, C-1), 111.6 (tq, $J_{C\text{-}F}$ = 256.8, 38.0 Hz, C-2), 73.8 (C-9), 63.9 (t, $J_{C\text{-}F}$ = 28.8 Hz, C-3), 61.0 (C-8), 57.9 (C-5), 25.8 (C-6), 25.1 (C-10), 22.6 (C-7), 22.3 (C-11) ppm;

^{19}F NMR (376 MHz, $CDCl_3$) δ – 83.6, – 123.5 (d, J = 3.3 Hz) ppm;

IR (film, cm^{-1}): ν_{max} 3294 (w), 2955 (w), 1453 (m), 1367 (m), 1302 (w), 1259 (w), 1200 (s), 1179 (s), 1154 (s), 1109 (s), 1086 (s), 998 (s), 935 (m), 901 (m), 805 (m), 773 (m), 713 (w), 669 (m);

$[\alpha]_D^{25.0}$ = – 7.5 (c. 0.98 $CHCl_3$);

HRMS (–ESI) *m/z* found $[M–H]^-$ 340.0643, $[C_{10}H_{15}F_5NO_4S]^-$ requires 340.0647, (δ = – 1.2 ppm);

Chiral GC analysis: CP7502 – CP-Chirasil-DEX CB column (135 ˚C Isothermal, 10.0 psi) t_R = 27.7 (major), 28.6 (minor) minutes.

2,2,3,3,3-pentafluoropropyl (R)-(1-oxaspiro[5.5]undecan-5-yl)sulfamate (**33f**)

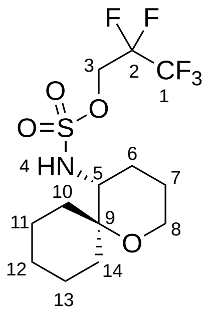

Prepared according to **GP9** using 4-cyclohexylidenebutan-1-ol (**31f**) (15.6 mg, 0.10 mmol, 1.0 equiv.) as the starting material. Following the cyclisation protocol (16 hours) purification by flash column chromatography (SiO_2, 0-4% v/v Acetone in $CHCl_3$) afforded the title compound as white plates (21.2 mg, 0.055 mmol, 55%, 87% *ee* as determined from the *N*-benzylated adduct).

***R$_f$* value** = 0.57 (10% v/v Acetone in $CHCl_3$);

^{1}H NMR (500 MHz, $CDCl_3$) δ 5.09 (d, *J* = 9.4 Hz, 1H, H-4), 4.48 (t, *J* = 12.5 Hz, 2H, H-3a, H-3b), 3.70-3.65 (m, 1H, H-8a), 3.61-3.57 (m, 1H, H-8b), 3.36-3.32 (m, 1H, H-5), 2.04-1.97 (m, 1H, H-6a), 1.96-1.87 (m, 1H, H-10a), 1.85-1.81 (m, 1H, H-14a), 1.79-1.70 (m, 2H, H-6b, H-7a), 1.61-1.46 (m, 7H, H-7b, H-10b, H-11a, H-11b, H-12a, H-13a, H13b), 1.29-1.23 (m, 2H, H-12b, H-14b) ppm;

^{13}C NMR (126 MHz, $CDCl_3$) δ 118.4 (qt, $J_{C\text{-}F}$ = 286.1, 35.1 Hz, C-1), 111.6 (tq, $J_{C\text{-}F}$ = 256.5, 38.2 Hz, C-2), 74.1 (C-9), 63.9 (t, $J_{C\text{-}F}$ = 27.7 Hz, C-3), 59.8 (C-8), 57.1 (C-5), 31.6 (C-10), 31.1 (C-14), 25.7 (C-12), 24.6 (C-6), 21.7 (C-7), 21.0 (C-11), 20.9 (C-13) ppm;

^{19}F NMR (376 MHz, $CDCl_3$) δ – 84.5, – 124.5 (d, J = 3.8 Hz) ppm;

IR (film, cm^{-1}): ν_{max} 3292 (w), 2937 (m), 2863 (w), 1447 (m), 1364 (m), 1261 (m), 1201 (s), 1180 (s), 1154 (s), 1109 (m), 1083 (m), 1008 (s), 937 (m), 890 (m), 866 (w), 805 (m), 774 (m), 712 (w), 670 (w);

$[\alpha]_D^{25.0}$ = – 8.8 (c. 1.01 $CHCl_3$);

HRMS (–ESI) *m/z* found $[M–H]^-$ 380.0953, $[C_{13}H_{19}F_5NO_4S]^-$ requires 380.0960, (δ = – 1.8 ppm).

N-Benzylation of **33f** *for ee determination: 2,2,3,3,3-pentafluoropropyl (R)-(4-bromobenzyl)(1-oxaspiro[5.5]undecan-5-yl)sulfamate*

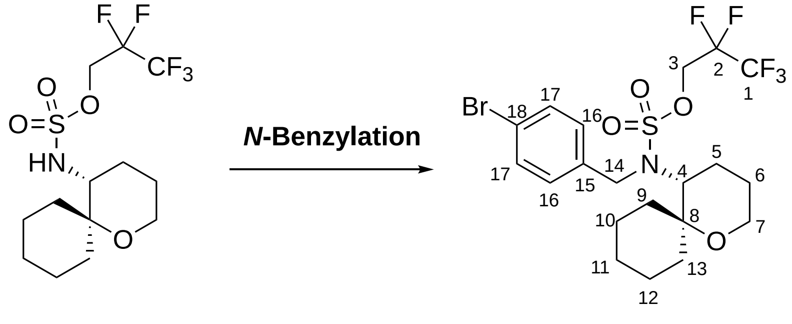

33f

A crimp-top vial was charged with 2,2,3,3,3-pentafluoropropyl (*R*)-(1-oxaspiro[5.5]undecan-5-yl)sulfamate (**33f**) (21.2 mg, 0.055 mmol, 1.0 equiv.), Cs_2CO_3 (39.1 mg, 0.12 mmol) and 1-bromo-4-(bromomethyl)benzene (15.0 mg, 0.060 mmol). The vial was sealed then evacuated and backfilled with nitrogen three times. MeCN (2.5 mL) was added and the reaction mixture stirred at 82 °C overnight. The solvent was removed under reduced pressure and purification

by flash column chromatography (SiO_2, 0-55% v/v CH_2Cl_2 in Petrol) afforded the title compound as white plates (16.0 mg, 0.029 mmol, 53%, 87% *ee*).

***R_f* value** = 0.29 (50% v/v CH_2Cl_2 in Petrol);

^{1}H NMR (500 MHz, $CDCl_3$) δ 7.48 (d, J = 8.4 Hz, 2H, H-17), 7.25 (d, J = 8.4 Hz, 2H, H-16), 4.67 (d, J = 16.7 Hz, 1H, H-14a), 4.50-4.40 (m, 2H, H-3a, H-3b), 4.36 (d, J = 18.9 Hz, 1H, H-14b), 3.75 (br s, 1H, H-4), 3.66-3.57 (m, 1H, H-7a), 3.50-3.45 (m, 1H, H-7b), 2.13-1.95 (m, 2H, H-5a, H-11a), 1.94-1.79 (m, 1H, H-5b), 1.72-1.50 (m, 7 H, H-6a, H-6b, H-9a, H-9b, H-10a, H-10b, H-13a), 1.48-1.35 (m, 2H, H-12a, H-12b), 1.19-0.97 (m, 2H, H-11b, H-13b) ppm;

^{13}C NMR (126 MHz, $CDCl_3$) δ 136.1 (C-15), 131.8 (C-17), 128.9 (C-16), 121.7 (C-18), 118.4 (qt, $J_{C\text{-}F}$ = 285.5, 34.0 Hz, C-1), 111.6 (tq, $J_{C\text{-}F}$ = 255.9, 38.5 Hz, C-2), 75.7 (C-8), 64.5 (C-4), 63.5 (t, $J_{C\text{-}F}$ = 28.5 Hz, C-3), 59.5 (C-7), 50.1 (C-14), 35.5 (C-9), 26.5 (C-11), 25.9 (C-6, C-13), 23.7 (C-5), 21.0 (C-10), 20.5 (C-12) ppm;

^{19}F NMR (376 MHz, $CDCl_3$) δ – 84.5, – 124.5 ppm;

IR (film, cm^{-1}): ν_{max} 2935 (m), 2862 (w), 1489 (m), 1448 (w), 1373 (s), 1259 (w), 1205 (s), 1180 (s), 1154 (s), 1108 (s), 1082 (m), 1010 (s), 991 (s), 962 (m), 912 (w), 897 (w), 859 (w), 843 (w), 798 (m), 670 (w);

$[\boldsymbol{\alpha}]_{\mathbf{D}}^{\mathbf{25.0}}$ **=** – 12.4 (c. 1.03 $CHCl_3$);

HRMS (–ESI) *m*/*z* found [M+HCOO]$^-$ 594.0577, $[C_{21}H_{26}BrF_5NO_6S]^-$ requires 594.0590, (δ = – 2.2 ppm);

Chiral SFC analysis: CHIRAL ART SC (CO_2:MeOH, 98:2, 1.25 mL min^{-1}, 40 ˚C), t_R = 5.4 (major), 6.9 (minor) minutes.

2,2,3,3,3-pentafluoropropyl ((2R,3R)-2-methyl-2-phenyltetrahydrofuran-3-yl)sulfamate (**33g**)

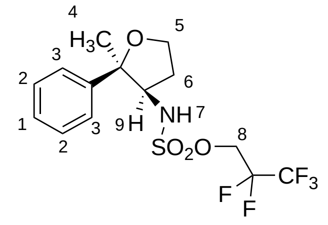

Prepared according to **GP9** using (*Z*)-4-phenylpent-3-en-1-ol (**31g**) as the starting material. In this case the following procedural modification was used: The reaction time was 36 hours. Following the cyclisation protocol (24 hours), purification by flash column chromatography (SiO_2, 0-1% v/v Et_2O in CH_2Cl_2) afforded the title compound as a colourless oil (32.0 mg, 0.082 mmol, 82%, 97% *ee*).

***Rf* value** = 0.57 (100% CH_2Cl_2);

^{1}H NMR (500 MHz, $CDCl_3$) δ 7.37-7.49 (m, 4H, H-2, H-3), 7.30-7.35 (m, 1H, H-1), 4.30 (q, *J* = 12.5 Hz, 1H, H-8a), 4.15-4.23 (m, 3H, H-5a, H-7, H-8b), 4.09-4.13 (m, 1H, H-5b), 4.02 (t, *J* = 5.1 Hz, 1H, H-9), 2.43-2.49 (m, 1H, H-6a), 2.10-2.17 (m, 1H, H-6b), 1.60 (s, 3H, H-4) ppm;

^{13}C NMR (126 MHz, $CDCl_3$) δ 141.6, 129.0, 128.1, 126.0, 118.3 (qt, J_{C-F} = 286.1 Hz, 34.4 Hz), 111.5 (tq, J_{C-F} = 256.5 Hz, 38.5 Hz), 85.0, 65.1, 63.9 (t, J_{C-F} = 28.1 Hz), 62.4, 32.7, 27.1 ppm;

^{19}F NMR (376 MHz, $CDCl_3$) δ – 83.5 (s), – 123.5 (s) ppm;

IR (film, cm^{-1}): ν_{max} 3303 (br, w), 2982 (w), 2965 (w), 2891 (w), 1447 (w), 1374 (m), 1204 (s), 1185 (s), 1109 (m), 1070 (w), 1010 (m), 942 (m), 809 (w), 766 (m), 704 (m), 669 (w), 623 (w), 592 (w), 518 (w);

$[\alpha]_D^{25.0}$ = + 1.9 (c. 1.0, $CHCl_3$);

HRMS (–ESI) *m/z* Found $[M–H]^-$ 388.0665, $[C_{14}H_{15}F_5NO_4S]^-$ requires 388.0647, (δ = + 4.6 ppm);

Chiral SFC Analysis CHIRALPAK IH (CO_2:MeOH, 98:2, 1.25 mL min^{-1}, 40 °C) t_R = 4.9 (major), 5.7 (minor) minutes.

2,2,3,3,3-pentafluoropropyl (2-methyl-2-phenyltetrahydrofuran-3-yl)sulfamate (**33h**)

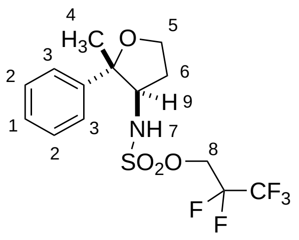

Relative Stereochemistry

Prepared according to **GP9** using (*E*)-4-phenylpent-3-en-1-ol (**31h**) as the starting material. In this case the following procedural modification was used: The reaction was performed on a 0.24 mmol scale and all other reagent and solvent amounts were scaled proportionally. Following the cyclisation protocol (24 hours), purification by flash column chromatography (SiO_2, 0-8% v/v Acetone in $CHCl_3$) afforded the title compound as a colourless oil (83.2 mg, 0.21 mmol, 89%, Racemic).

***R_f* value** = 0.57 (10% v/v Acetone in $CHCl_3$);

^{1}H NMR (400 MHz, $CDCl_3$) δ 7.34-7.37 (m, 4H, H-2, H-3), 7.26-7.30 (m, 1H, H-1), 5.24 (d, *J* = 9.6 Hz, 1H, H-7), 4.52 (t, *J* = 12.3 Hz, 2H, H-8a, H-8b), 4.24-4.29 (m, 1H, H-9), 4.05 (q, *J* = 8.2 Hz, 1H, H-5a), 3.96 (td, *J* = 9.3 Hz, 4.5 Hz, 1H, H-5b), 2.07-2.18 (m, 1H, H-6a), 1.96-2.03 (m, 1H, H-6b), 1.55 (s, 3H, H-4) ppm;

^{13}C NMR (101 MHz, $CDCl_3$) δ 144.5, 128.8, 127.6, 124.3, 118.3 (qt, J_{C-F} = 286.0 Hz, 34.4 Hz), 111.6 (tq, J_{C-F} = 256.6 Hz, 38.3 Hz), 86.5, 64.5, 64.1 (t, J_{C-F} = 28.3 Hz), 62.5, 32.4, 24.8 ppm;

^{19}F NMR (376 MHz, $CDCl_3$) δ – 83.5 (s), – 124.2 - – 122.7 (m)*ppm;

*Partial inequivalence leads to extreme strong coupling between these two ^{19}F nuclei.

IR (film, cm^{-1}): ν_{max} 3296 (br,w), 2990 (w), 2897 (w), 1446 (m), 1368 (m), 1259 (w), 1201 (s), 1179 (s), 1106 (s), 1071 (m), 1000 (s), 943 (m), 894 (w), 865 (w), 809 (m), 763 (s), 703 (s), 669 (w), 622 (w), 583 (w), 559 (w), 518 (w);

HRMS (–ESI) *m/z* Found [M–H]$^-$ 388.0643, $[C_{14}H_{15}F_5NO_4S]^-$ requires 388.0647, (δ = – 1.0 ppm);

Chiral SFC Analysis CHIRAL ART SJ (CO_2:MeOH, 98.5:1.5, 2.5 mL min^{-1}, 40 °C) t_R = 3.5, 4.2 minutes.

2,2,3,3,3-pentafluoropropyl ((2R,3R)-2-methyl-2-(4-methylpent-3-en-1-yl)tetrahydrofuran-3-yl)sulfamate (**36**)

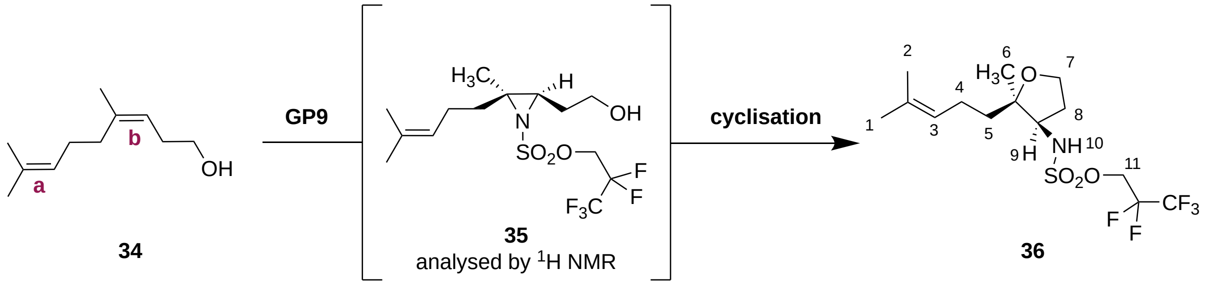

Prepared according to **GP9** using homonerol (**34**) as the starting material. Analysis by crude ^{1}H NMR prior to cyclisation revealed the following:

- In the crude ^1H NMR of the reaction mixture using Rh_2(**B**)$_2$•(**C3**)$_2$•(Pyr)$_2$, aziridination occurred exclusively at position **b** (blue spectrum, bottom).

- In the reaction using achiral catalyst $Rh_2(esp)_2$ the ratio of aziridination at the positions **a**:**b** was approximately 1:2.8 (red spectrum, middle)

- An authentic sample of the monoaziridination product at **a** was obtained from the racemic reaction (green spectrum, top) in order to confirm the assignments – the characterisation data for this product is presented below.

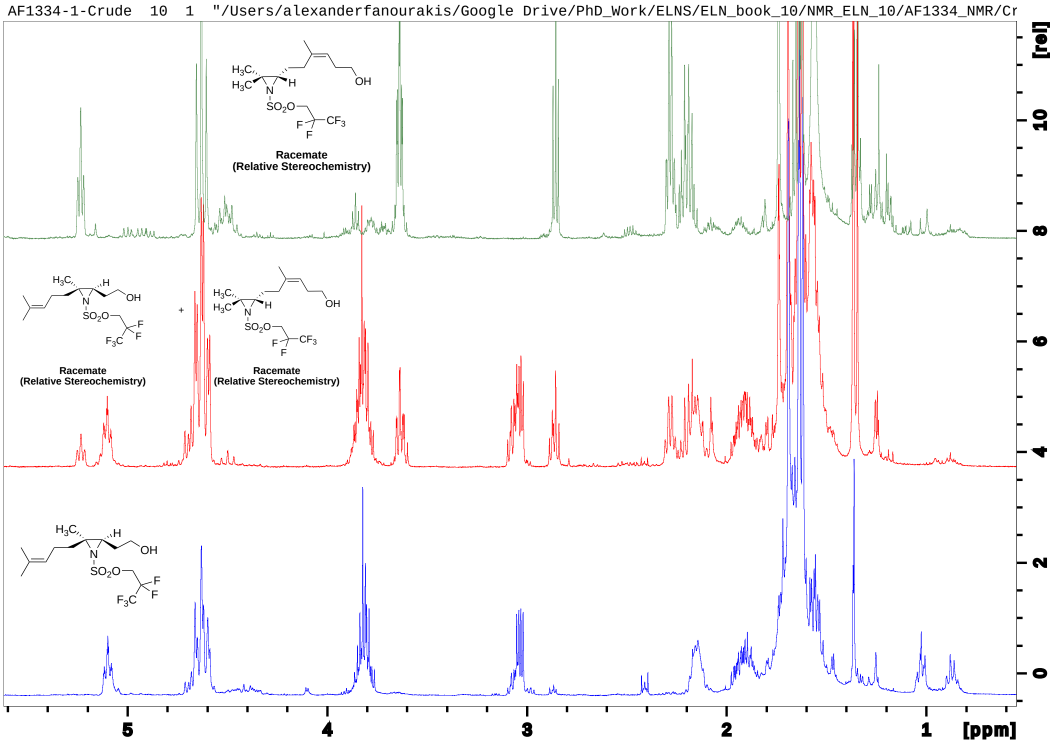

Figure S2. ^1H NMR spectra of the crude chiral reaction (blue spectrum, bottom) and crude racemic reaction (red spectrum, middle) prior to cyclisation. An isolated sample of the aziridine formed from monofunctionalisation at alkene A is shown in the green spectrum, top.

Following the cyclisation protocol (16 hours), purification by flash column chromatography (SiO_2, 0-5% v/v Acetone in $CHCl_3$) afforded the title compound as white plates (19.7 mg, 0.050 mmol, 50%, 89% *ee*).

***R_f* value** = 0.70 (5% v/v Acetone in $CHCl_3$);

^{1}H NMR (500 MHz, $CDCl_3$) δ 5.09-5.13 (m, 1H, H-3), 4.88 (d, J = 9.8 Hz, 1H, H-10), 4.50 (t, J = 12.4 Hz, 2H, H-11a, H-11b), 3.83-3.91 (m, 2H, H-7a, H-7b), 3.74 (ddd, J = 13.2 Hz, 7.5 Hz, 5.7 Hz, 1H, H-9), 2.43-2.50 (m, 1H, H-8a), 2.08 (q, J = 7.9 Hz, 2H, H-4a, H-4b), 1.93-1.99 (m, 1H, H-8b), 1.69 (d, J = 0.8 Hz, 3H, H-1), 1.62 (br s, 3H, H-2), 1.43-1.53 (m, 2H, H-5a, H-5b), 1.24 (s, 3H, H-6);

^{13}C NMR (126 MHz, $CDCl_3$) δ 132.5, 123.9, 118.3 (qt, $J_{C\text{-}F}$ = 286.2 Hz, 34.4 Hz), 111.5 (tq, $J_{C\text{-}F}$ = 257.8 Hz, 38.4 Hz), 82.5, 64.0 (t, $J_{C\text{-}F}$ = 28.3 Hz), 63.5, 62.1, 34.9, 32.4, 25.8, 22.8, 22.3, 17.7 ppm;

^{19}F NMR (376 MHz, $CDCl_3$) δ – 84.5 (s), – 125.2 - – 123.7 (m)*ppm;

*Partial inequivalence leads to extreme strong coupling between these two ^{19}F nuclei.

IR (film, cm^{-1}): ν_{max} 3288 (br, w), 2923 (m), 1455 (m), 1374 (m), 1261 (w), 1204 (s), 1185 (s), 1110 (m), 1010 (m), 935 (w), 806 (w), 774 (w), 670 (w), 622 (w), 587 (m), 518 (w);

$[\alpha]_D^{25.0}$ = – 1.1 (c. 1.0, $CHCl_3$);

HRMS (–ESI) *m/z* Found $[M–H]^-$ 394.1106, $[C_{14}H_{21}F_5NO_4S]^-$ requires 394.1117, (δ = – 2.8 ppm);

Chiral GC Analysis CP7502 – CP-Chirasil-DEX CB column (140 °C Isothermal, 27.0 psi) t_R = 38.0 (major), 38.8 (minor) minutes.

Characterisation of (2,2,3,3,3-pentafluoropropyl (R,Z)-3-(6-hydroxy-3-methylhex-3-en-1-yl)-2,2-dimethylaziridine-1-sulfonate.

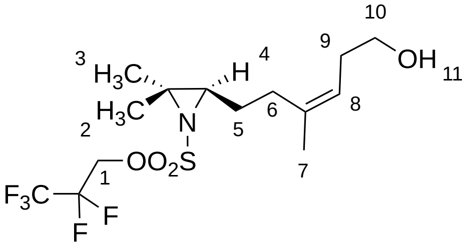

Racemate
(Relative Stereochemistry)

***Rf* value** = 0.42 (5% v/v Acetone in $CHCl_3$);

^{1}H NMR (500 MHz, $CDCl_3$) δ 5.23 (t, J = 7.4 Hz, 1H, H-8), 4.63 (t, J = 12.4 Hz, 2H, H-1a, H-1b), 3.60-3.67 (m, 2H, H-10a, H-10b), 2.85 (t, J = 6.6 Hz, 1H, H-4), 2.28 (q, J = 6.7 Hz, 2H, H-9a, H-9b), 2.15-2.24 (m, 2H, H-6a, H-6b), 1.74 (q, J = 1.2 Hz, 3H, H-7), 1.63-1.67 (m, 5H, H-3, H-5a, H-5b), 1.34 (s, 3H, H-2) ppm;

^{13}C NMR (126 MHz, $CDCl_3$) δ 136.5, 122.5, 114.8-121.8 (m), 108.9-113.8 (m), 65.2 (t, $J_{C\text{-}F}$ = 28.4 Hz), 62.4, 54.5, 51.9, 31.4, 29.0, 26.3, 23.3, 21.1, 20.4 ppm;

^{19}F NMR (376 MHz, $CDCl_3$) δ – 83.5 (s), – 124.2 - – 122.7 (m)*ppm;

*Partial inequivalence leads to extreme strong coupling between these two ^{19}F nuclei.

IR (film, cm^{-1}): ν_{max} 2967 (w), 2935 (w), 1454 (w), 1370 (s), 1259 (w), 1202 (s), 1177 (s), 1108 (s), 1043 (s), 1010 (s), 939 (m), 834 (m), 776 (w), 693 (w), 625 (w), 601 (w), 515 (w);

HRMS (+ESI) *m/z* Found $[M+H]^+$ 396.1259, $[C_{14}H_{23}F_5NO_4S]^+$ requires 396.1262, (δ = – 0.8 ppm).

2,2,3,3,3-pentafluoropropyl ((2S,3R)-2-methyl-2-(4-methylpent-3-en-1-yl)tetrahydrofuran-3-yl)sulfamate (**39**)

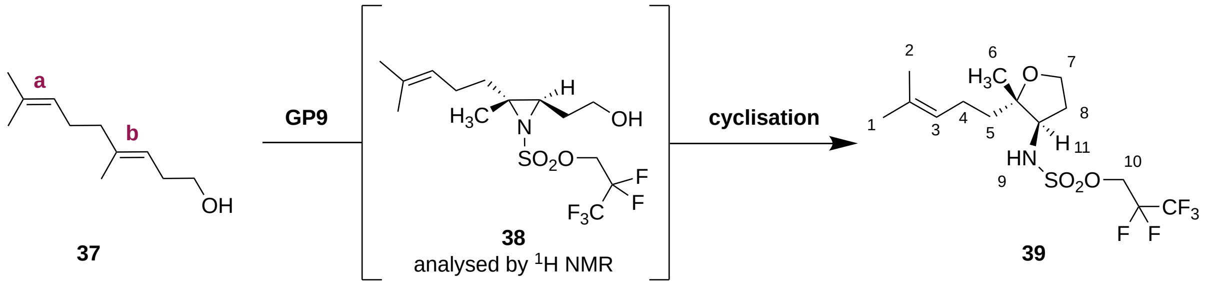

Prepared according to **GP9** using homogeraniol (**37**) as the starting material. Analysis by crude ^{1}H NMR prior to cyclisation revealed the following:

- In the crude ^{1}H NMR of the reaction mixture using Rh_2(**B**)$_2$•(**C3**)$_2$•(Pyr)$_2$, monoaziridination occurred exclusively at position **b** (blue spectrum, bottom) although there are traces of over-functionalised product visible (see peak at 3.15 ppm) which we suspect corresponds to the (proximal aziridination + allylic amination of the distal alkene) product.

- In the reaction using achiral catalyst Rh_2(esp)$_2$ the ratio of "**others" (which includes aziridination at a + residual starting material)**:**b** was approximately 1:3.2

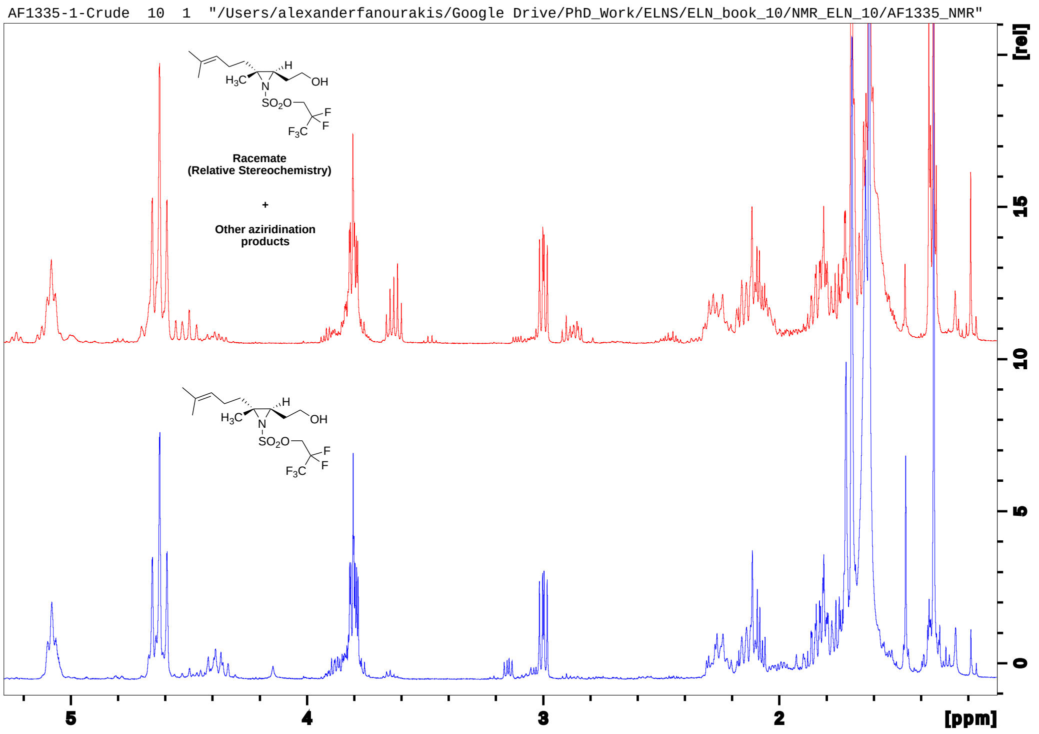

Figure S3. ^{1}H NMR spectra of the crude chiral reaction (blue spectrum, bottom) and crude racemic reaction (red spectrum, middle) prior to cyclisation.

Following the cyclisation protocol (16 hours), purification by two consecutive flash column chromatography procedures (SiO_2, 0-10% v/v Et_2O in CH_2Cl_2 followed by 0-20% v/v Acetone in Petrol) afforded the title compound as a colourless oil (24.0 mg, 0.061 mmol, 61%, 81% *ee* as determined from the *N*-benzylated adduct).

***R_f* value** = 0.33 (5% v/v Acetone in $CHCl_3$);

^{1}H NMR (500 MHz, $CDCl_3$) δ 5.08 (tt, *J* = 7.1 Hz, 1.3 Hz, 1H, H-3), 4.93-4.98 (m, 1H, H-9), 4.50 (t, *J* = 12.4 Hz, 2H, H-10a, H-10b), 3.91 (td, *J* = 8.9 Hz, 5.0 Hz, 1H, H-7a), 3.77-3.82 (m, 2H, H-7b, H-11), 2.46 (dtd, *J* = 15.7 Hz, 7.9 Hz, 5.0 Hz, 1H, H-8a), 2.05 (q, *J* = 7.8 Hz, 2H, H-4a, H-4b), 1.91-1.98 (m, 1H, H-8b), 1.68 (d, *J* = 0.8 Hz, 3H, H-1), 1.61 (s, 3H, H-2), 1.50-1.57 (m, 2H, H-5a, H-5b), 1.20 (s, 3H, H-6) ppm;

^{13}C NMR (126 MHz, $CDCl_3$) δ 132.3, 123.8, 118.3 (qt, J_{C-F} = 286.1 Hz, 34.4 Hz), 111.5 (tq, J_{C-F} = 256.5 Hz, 38.4 Hz), 82.9, 64.0 (t, J_{C-F} = 28.3 Hz), 63.8, 60.6, 38.6, 32.8, 25.8, 22.5, 20.0, 17.7 ppm;

^{19}F NMR (376 MHz, $CDCl_3$) δ – 83.5 (s), – 124.2 - – 122.7 (m)*ppm;

*Partial inequivalence leads to extreme strong coupling between these two ^{19}F nuclei.

IR (film, cm^{-1}): ν_{max} 3292 (w), 2971 (w), 2928 (w), 1454 (m), 1377 (m), 1207 (s), 1185 (s), 1158 (w), 1110 (m), 1011 (m), 944 (m), 806 (w), 774 (w), 622 (w), 584 (w), 516 (w);

$[\alpha]_D^{25.1}$ = – 7.1 (c. 1.0, $CHCl_3$);

HRMS (–ESI) *m/z* Found $[M–H]^-$ 394.1106, $[C_{14}H_{21}F_5NO_4S]^-$ requires 394.1117, (δ = – 2.8 ppm).

*N-benzylation of **39** for ee determination: 2,2,3,3,3-pentafluoropropyl (4-bromobenzyl)((2S,3R)-2-methyl-2-(4-methylpent-3-en-1-yl)tetrahydrofuran-3-yl)sulfamate*

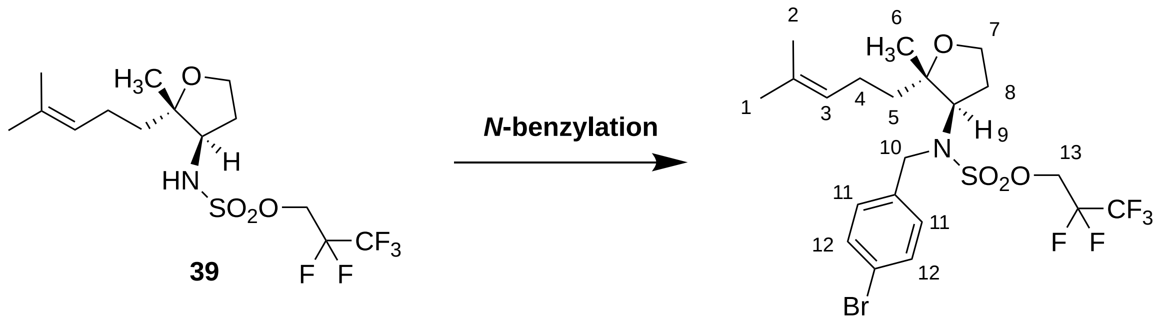

A crimp-top vial was charged with 2,2,3,3,3-pentafluoropropyl ((2*S*,3*R*)-2-methyl-2-(4-methylpent-3-en-1-yl)tetrahydrofuran-3-yl)sulfamate (**39**) (24.0 mg, 0.061 mmol), Cs_2CO_3 (39.1 mg, 0.12 mmol) and 1-bromo-4-(bromomethyl)benzene (16.2 mg, 0.065 mmol). The vial was sealed then evacuated and backfilled with nitrogen three times. MeCN (2.5 mL) was added and the reaction mixture stirred at 82 °C overnight. The solvent was removed under reduced pressure and purification by two consecutive flash column chromatography procedures (SiO_2, 0-10% v/v Acetone in Petrol followed by 0-30% v/v CH_2Cl_2 in Petrol) afforded the title compound as thick colourless oil (14.8 mg, 0.026 mmol, 43%, 81% *ee*). *In this case, the product was contaminated with a small amount of inseparable phthalate plasticizer – this is most notable in the aliphatic region of the ^{13}C NMR.*

***R_f* value** = 0.38 (10% v/v Acetone in $CHCl_3$);

^{1}H NMR (500 MHz, $CDCl_3$) δ 7.45-7.48 (m, 2H, H-12), 7.27 (d, *J* = 8.1 Hz, 2H, H-11), 5.07 (tt, *J* = 7.1 Hz, 1.4 Hz, 1H, H-3), 4.66 (d, *J* = 17.0 Hz, 1H, H-10a), 4.48 (d, *J* = 17.0 Hz, 1H, H-10b), 4.38-4.48 (m, 2H, H-13a, H-13b), 4.25 (dd, *J* = 8.9 Hz, 3.1 Hz, 1H, H-9), 3.86 (td, *J* = 9.2 Hz, 5.4 Hz, 1H, H-7a), 3.68 (td, *J* = 9.3 Hz, 6.6 Hz, 1H, H-7b), 2.36 (dtd, *J* = 18.2 Hz, 9.1 Hz, 5.3 Hz, 1H, H-8a), 1.96-2.08 (m, 3H, H-4a, H-4b, H-8b), 1.68 (d, *J* = 0.7 Hz, 3H, H-1), 1.61 (s, 3H, H-2), 1.38-1.46 (m, 2H, H-5a, H-5b), 1.32 (s, 3H, H-6) ppm;

^{13}C NMR (126 MHz, $CDCl_3$) δ 136.2, 132.3, 131.8, 129.1, 123.8, 121.9, 118.3 (qt, $J_{C\text{-}F}$ = 286.1 Hz, 34.9 Hz), 111.6 (tq, $J_{C\text{-}F}$ = 256.4 Hz, 38.8 Hz), 85.5, 65.8, 63.9, 63.8 (t, $J_{C\text{-}F}$ = 28.4 Hz), 50.2, 38.0, 29.6, 25.8, 22.4, 19.8, 17.7 ppm;

^{19}F NMR (376 MHz, $CDCl_3$) δ – 83.5 (s), – 123.5 (s) ppm;

IR (film, cm^{-1}): ν_{max} 2964 (m), 2928 (m), 2879 (m), 1490 (m), 1455 (w), 1369 (s), 1293 (w), 1260 (w), 1202 (s), 1179 (s), 1156 (s), 1108 (s), 1073 (w), 1024 (s), 1009 (s), 954 (w), 904 (w), 876 (m), 793 (m), 773 (m), 709 (w), 669 (w), 621 (w), 582 (m), 549 (m);

$[\alpha]_D^{25.0}$ = – 9.4 (c. 1.0, $CHCl_3$);

HRMS (+ESI) *m/z* Found $[M+H]^+$ 564.0857, $[C_{21}H_{28}BrF_5NO_4S]^+$ requires 564.0837, (δ = + 3.5 ppm);

Chiral SFC Analysis CHIRALPAK IG (CO_2:MeOH, 97:3, 2.5 mL min^{-1}, 40 °C) t_R = 2.9 (major), 3.7 (minor) minutes.

Characterisation of a Further Mnemonic Test Aziridine

2,2,3,3,3-pentafluoropropyl 2-(2-hydroxyethyl)-2-methyl-3-phenylaziridine-1-sulfonate:
***Obtained following aziridination of* 40**

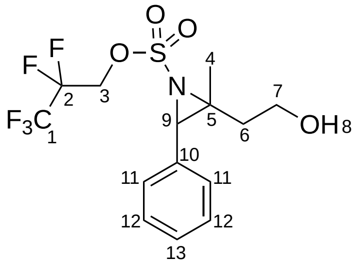

Prepared according to **GP9** using (*Z*)-3-methyl-4-phenylbut-3-en-1-ol (**40**) (16.6 mg, 0.1 mmol, 1.0 equiv.) as the starting material. Purification by flash column chromatography (SiO_2, 0-5% v/v acetone in $CHCl_3$) afforded the title compound as a yellow oil (1.1 mg, 0.00283 mmol, 3%, 5% *ee*).

***R*$_f$ value** = 0.46 (10% v/v Acetone in $CHCl_3$);

^{1}H NMR (500 MHz, $CDCl_3$) δ 7.40-7.35 (m, 3H, H-12, H-13), 7.28-7.26 (m, 2H, H-11), 4.73 (t, J = 12.2 Hz, 2H, H-3a, H-3b), 4.05 (s, 1H, H-9), 3.85-3.73 (m, 2H, H-7a, H-7b), 1.84 (s, 3H, H-4), 1.63-1.50 (m, 3H, H-6a, H-6b, H-8)*ppm;

*Partially obscured by residual water peak.

^{13}C NMR (126 MHz, $CDCl_3$) δ 132.5 (C-10), 128.8 (C-12), 128.7 (C-13), 127.1 (C-11), 65.6 (t, J_{C-F} = 28.9 Hz, C-3), 59.5 (C-7), 55.7 (C-9), 54.8 (C-5), 35.5 (C-6), 19.2 (C-4) ppm; *Note: Due to the very low sample concentration the highly-split signals due to C-1 and C-2 are only barely visible in the spectrum;*

^{19}F NMR (471 MHz, $CDCl_3$) δ – 84.4, – 124.3 (d, J = 9.4 Hz) ppm;

IR (film, cm^{-1}): ν_{max} 2173 (w), 1377 (m), 1206 (s), 1179 (s), 1009 (m), 845 (w);

HRMS (−ESI) *m/z* found $[M{-}H]^-$ 388.0640, $[C_{14}H_{15}F_5NO_4S]^-$ requires 388.0647, (δ = – 1.8 ppm);

Chiral SFC analysis: CHIRALPAK IH (CO_2:MeOH, 99:1, 1.25 mL min^{-1}, 40 ˚C), t_R = 11.9 (minor), 14.5 (major) minutes.

Characterisation of Post-Functionalisation Products

(S)-4-cyclohexyl-2-(2-hydroxyethyl)-2-(((2,2,3,3,3-pentafluoropropoxy)sulfonyl)amino)butyl acetate (**42**)

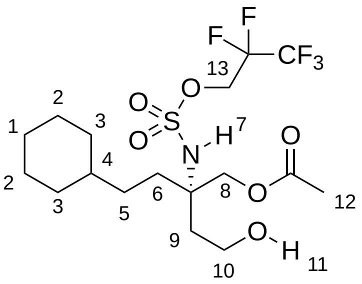

A 4.0 mL crimp-top vial was charged with 2,2,3,3,3-pentafluoropropyl (*S*)-2-(2-cyclohexylethyl)-2-(2-hydroxyethyl)aziridine-1-sulfonate (**28b**) (123 mg, 0.30 mmol) and potassium acetate (59 mg, 0.60 mmol) and placed under a nitrogen atmosphere. DMSO (4.0 mL) was added and the reaction mixture was stirred at room temperature overnight. The reaction mixture was diluted in EtOAc (10 mL) and H_2O (10 mL), the layers were separated, and the organic layer was washed with brine. The organic layer was dried over $MgSO_4$, concentrated under reduced pressure and purified by flash column chromatography (SiO_2, 0-12% v/v Acetone in $CHCl_3$) to afford the title compound as a colourless oil (75 mg, 0.16 mmol, 53%, 84% *ee* - determined following alcohol protection).

***R_f* value** = 0.29 (10% v/v Acetone in $CHCl_3$);

^{1}H NMR (500 MHz, $CDCl_3$) δ 6.20 (br s, 1H, H-7), 4.48 (t, *J* = 12.7 Hz, 2H, H-13_A, H-13_B), 4.26 (s, 2H, H-8_A, H-8_B), 3.90 (t, *J* = 5.7 Hz, 2H, H-10_A, H-10_B), 2.09 (s, 3H, H-12), 1.90 (t, *J* = 5.8 Hz, 2H, H-9_A, H-9_B), 1.84 (m, 2H, H-6_A, H-6_B), 1.77 (m, 1H, H-11), 1.73-1.60 (m, 5H, H-1_A, H-2_A, H-3_A), 1.27-1.08 (m, 6H, H-1_B, H-2_B, H-4, H-5_A, H-5_B), 0.88 (m, 2H, H-3_B) ppm;

^{13}C NMR (126 MHz, CDCl$_3$) δ 170.9, 118.3 (qt, J_{C-F} = 287, 34.2 Hz), 111.6 (tq, J_{C-F} = 257, 38.6 Hz), 66.8, 63.9 (t, J_{C-F} = 28.4 Hz), 62.5, 58.9, 38.1, 35.5, 33.4-33.3 (m), 32.2, 31.0, 26.7, 26.4, 20.9 ppm;

^{19}F NMR (376 MHz, CDCl$_3$) δ − 83.6, − 123.4 ppm;

IR (film, cm^{-1}): ν_{max} 2925 (m), 2854 (w), 1722 (m), 1450 (m), 1368 (m), 1202 (s), 1178 (s), 1109 (m), 1046 (m), 1005 (m), 775 (m);

$[\alpha]_D^{25.0}$ = + 4.6 (c. 0.99, CHCl$_3$);

HRMS (−ESI) *m/z* found [M−H]$^-$ 468.1476, $[C_{17}H_{27}F_5NO_6S]^-$ requires 468.1485, (δ = − 1.9 ppm).

Alcohol protection of **42** *for ee determination: (S)-4-((tert-butyldiphenylsilyl)oxy)-2-(2-cyclohexylethyl)-2-(((2,2,3,3,3-pentafluoropropoxy)sulfonyl)amino)butyl acetate*

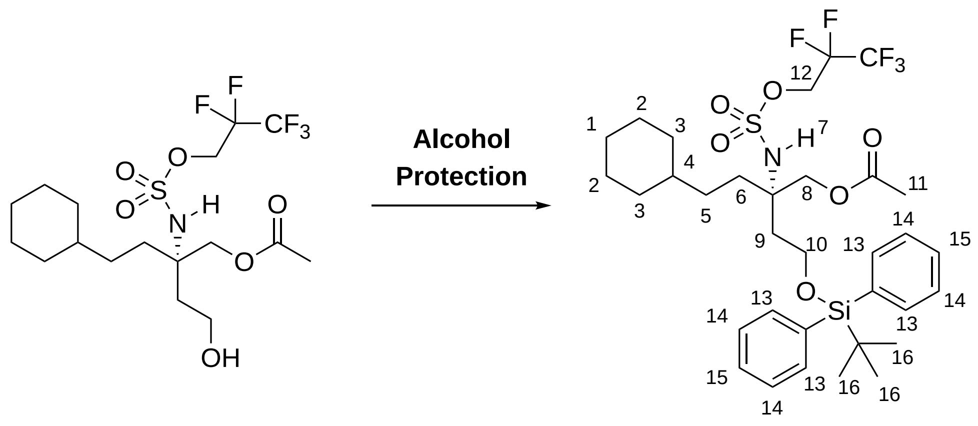

A microwave vial was charged with (*S*)-4-cyclohexyl-2-(2-hydroxyethyl)-2-(((2,2,3,3,3-pentafluoropropoxy)sulfonyl)amino)butyl acetate (**42**) (53 mg, 0.11 mmol) and DMAP (1 mg, 0.011 mmol) and placed under a nitrogen atmosphere. CH$_2$Cl$_2$ (2.0 mL) was added and the reaction mixture was cooled to 0 °C. *Tert*-butyl(chloro)diphenylsilane (32 µL, 0.12 mmol) and

triethylamine (31 µL, 0.23 mmol) were added dropwise and the vial was warmed to room temperature overnight. The reaction mixture was diluted in CH_2Cl_2 (10 mL) and H_2O (10 mL), the layers separated, and the aqueous layer extracted with CH_2Cl_2. The combined organic layers were dried over $MgSO_4$, concentrated under reduced pressure and purified by flash column chromatography (SiO_2, 0-12% v/v EtOAc in Hexane) to afford the title compound as a colourless oil (43 mg, 0.061 mmol, 55%, 84% *ee*).

***R_f* value** = 0.16 (10% v/v EtOAc in Hexane);

^{1}H NMR (500 MHz, $CDCl_3$) δ 7.66 (m, 4H, H-13), 7.47 (m, 2H, H-15), 7.42 (m, 4H, H-14), 6.70 (s, 1H, H-7), 4.45 (t, *J* = 12.7 Hz, 2H, H-12_A, H-12_B), 4.33 (d, *J* = 11.8 Hz, 1H, H-8_A), 4.25 (d, *J* = 11.8 Hz, 1H, H-8_B), 3.84 (m, 2H, H-10_A, H-10_B), 2.06 (s, 3H, H-11), 1.90-1.80 (m, 4H, H-6_A, H-6_B, H-9_A, H-9_B), 1.71-1.61 (m, 5H, H-1_A, H-2_A, H-3_A), 1.24-1.11 (m, 6H, H-1_B, H-2_B, H-4, H-5_A, H-5_B), 1.08 (s, 9H, H-16), 0.84 (m, 2H, H-3_B) ppm;

^{13}C NMR (126 MHz, $CDCl_3$) δ 170.7, 135.7, 132.4-132.3 (m), 130.3, 128.1, 118.4 (qt, J_{C-F} = 286, 34.5 Hz), 111.7 (tq, J_{C-F} = 265, 38.3 Hz), 67.0, 63.7 (t, J_{C-F} = 28.0 Hz), 62.5, 60.6, 38.1, 35.9, 33.34-33.29 (m), 32.5, 31.1, 26.8, 26.7, 26.4, 20.9, 19.0 ppm;

^{19}F NMR (376 MHz, $CDCl_3$) δ − 84.5, − 124.3 ppm;

IR (film, cm^{-1}): ν_{max} 2927 (m), 2856 (w), 1747 (m), 1449 (m), 1428 (m), 1364 (m), 1204 (s), 1181 (s), 1107 (s), 1047 (m), 822 (m), 738 (s), 702 (s), 614 (m), 504 (s);

$[\boldsymbol{\alpha}]_{\mathbf{D}}^{\mathbf{25.0}}$ = − 1.2 (c. 1.6, $CHCl_3$);

HRMS (+ESI) *m/z* found $[M+Na]^+$ 730.2614, $[C_{33}H_{46}F_5NNaO_6SSi]^+$ requires 730.2627, (δ = − 1.8 ppm);

Chiral SFC Analysis: CHIRALPAK IG (CO_2:^{i}PrOH, 96:4, 1.25 mL min^{-1}, 40 °C) t_R = 8.9 (major), 10.8 (minor) minutes.

(R)-5-cyclohexyl-3-hydroxy-3-((((2,2,3,3,3-pentafluoropropoxy)sulfonyl)amino)methyl)pentyl acetate

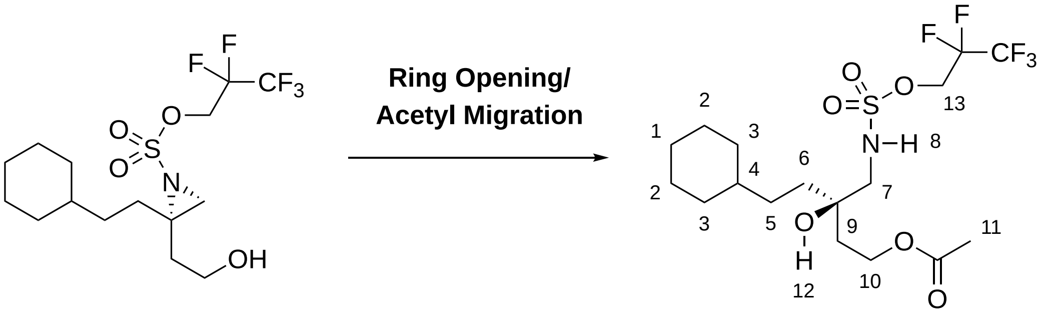

28b

Following the same protocol reported for **42**, the title compound was isolated as a colourless oil (16 mg, 0.034 mmol, 11%).

***R_f* value** = 0.37 (10% v/v Acetone in $CHCl_3$);

^{1}H NMR (400 MHz, $CDCl_3$) δ 5.35 (t, *J* = 6.3 Hz, 1H, H-8), 4.50 (tq, *J* = 12.5, 1.0 Hz, 2H, H-13_A, H-13_B), 4.20 (m, 2H, H-10_A, H-10_B), 3.15 (d, *J* = 6.1 Hz, 2H, H-7_A, H-7_B), 2.07 (s, 3H, H-11), 1.88 (m, 2H, H-9_A, H-9_B), 1.73-1.62 (m, 6H, H-1_A, H-2_A, H-3_A, H-12), 1.55 (m, 2H, H-6_A, H-6_B), 1.26-1.09 (m, 6H, H-1_B, H-2_B, H-4, H-5_A, H-5_B), 0.89 (m, 2H, H-3_B) ppm;

^{13}C NMR (126 MHz, $CDCl_3$) δ 171.3, 118.4 (qt, $J_{C\text{-}F}$ = 286, 34.2 Hz), 111.6 (tq, $J_{C\text{-}F}$ = 257, 38.3 Hz), 73.3, 64.1 (t, $J_{C\text{-}F}$ = 28.3 Hz), 60.6, 51.2, 38.1, 35.1, 34.9, 33.43-33.39 (m), 31.1, 26.7, 26.4, 21.1 ppm;

^{19}F NMR (376 MHz, $CDCl_3$) δ − 83.5, − 123.5 ppm;

IR (film, cm^{-1}): ν_{max} 2926 (m), 2854 (m), 1721 (m), 1450 (w), 1370 (m), 1204 (s), 1184 (s), 1107 (m), 1045 (m), 1010 (m);

$[\alpha]_D^{25.0}$ = + 2.0 (c. 0.99, $CHCl_3$);

HRMS (−ESI) *m/z* found $[M-H]^-$ 468.1479, $[C_{17}H_{27}F_5NO_6S]^-$ requires 468.1485, (δ = − 1.2 ppm).

(R)-3-azido-5-cyclohexyl-3-((((2,2,3,3,3-pentafluoropropoxy)sulfonyl)amino)methyl)pentyl acetate (**43**)

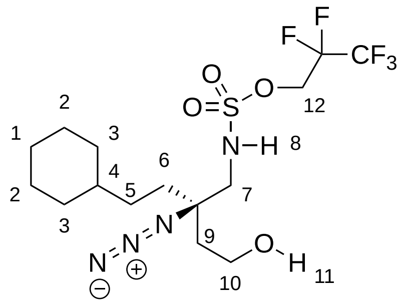

A 4.0 mL crimp-top vial was charged with 2,2,3,3,3-pentafluoropropyl (*S*)-2-(2-cyclohexylethyl)-2-(2-hydroxyethyl)aziridine-1-sulfonate (**28b**) (100 mg, 0.244 mmol) and sodium azide (32 mg, 0.49 mmol). ^{i}PrOH (1.0 mL) and H_2O (1.0 mL) were added and the reaction mixture was stirred at room temperature overnight under air. The reaction mixture was diluted in EtOAc (5 mL) and 1.0 M aqueous NaH_2PO_4 solution (5 mL), the layers were separated, and the aqueous layer was extracted with EtOAc. The organic layers were combined, dried over $MgSO_4$, concentrated under reduced pressure and purified by flash column chromatography (SiO_2, 0-7% v/v Acetone in $CHCl_3$) to afford the title compound as a colourless oil (72 mg, 0.16 mmol, 66%).

***R_f* value** = 0.37 (10% v/v Acetone in $CHCl_3$);

^{1}H NMR (500 MHz, $CDCl_3$) δ 6.01 (t, *J* = 6.8 Hz, 1H, H-8), 4.49 (td, *J* = 12.5, 0.8 Hz, 2H, H-12$_A$, H-12$_B$), 3.88 (m, 1H, H-10$_A$), 3.78 (m, 1H, H-10$_B$), 3.23 (m, 2H, H-7$_A$, H-7$_B$), 1.92 (ddd, *J* = 15.3, 7.1, 3.7 Hz, 1H, H-9$_A$), 1.84 (ddd, *J* = 15.3, 7.4, 3.6 Hz, 1H, H-9$_B$), 1.80-1.61 (m, 8H, H-1$_A$, H-2$_A$, H-3$_A$, H-6$_A$, H-6$_B$, H-11), 1.34-1.11 (m, 6H, H-1$_B$, H-2$_B$, H-4, H-5$_A$, H-5$_B$), 0.91 (m, 2H, H-3$_B$) ppm;

^{13}C NMR (126 MHz, $CDCl_3$) δ 118.4 (qt, $J_{C\text{-}F}$ = 287, 34.2 Hz), 111.6 (tq, $J_{C\text{-}F}$ = 257, 38.9 Hz), 65.0, 64.1 (t, $J_{C\text{-}F}$ = 28.4 Hz), 58.3, 49.3, 38.0, 36.7, 33.4, 33.1, 30.7, 26.7, 26.4 ppm;

^{19}F NMR (471 MHz, $CDCl_3$): δ − 84.5, − 124.5 ppm;

IR (film, cm^{-1}): ν_{max} 2925 (m), 2854 (w), 2106 (s), 1449 (m), 1369 (m), 1202 (s), 1180 (s), 1107 (m), 1042 (m), 1008 (m), 760 (m), 516 (m);

$[\alpha]_D^{25.0}$ = + 8.7 (c. 1.0, $CHCl_3$);

HRMS (−ESI) *m/z* found $[M{-}H]^-$ 451.1440, $[C_{15}H_{24}F_5N_4O_4S]^-$ requires 451.1444, (δ = − 1.0 ppm);

2,2,3,3,3-pentafluoropropyl (R)-(4-cyclohexyl-2-(2-hydroxyethyl)-2-(4-phenyl-1H-1,2,3-triazol-1-yl)butyl)sulfamate (**44**)

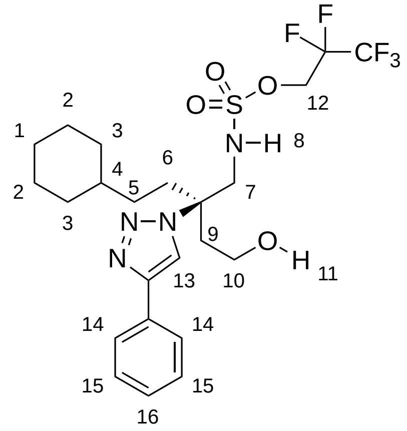

A microwave vial charged with (*R*)-3-azido-5-cyclohexyl-3-((((2,2,3,3,3-pentafluoropropoxy)sulfonyl)amino)methyl)pentyl acetate (**43**) (52 mg, 0.12 mmol) was placed under a nitrogen atmosphere. THF (1.0 mL), phenylacetylene (13 µL, 0.12 mmol), copper(II) sulfate pentahydrate (3 mg, 0.012 mmol) in H_2O (0.25 mL) and sodium ascorbate

(2 mg, 0.012 mmol) in H_2O (0.25 mL) were added. The vial was rapidly evacuated and back-filled with nitrogen twice and stirred at room temperature overnight. The reaction mixture was diluted in saturated aqueous NH_4Cl solution (10 mL) and CH_2Cl_2 (10 mL), the layers separated, and the aqueous layer extracted with CH_2Cl_2. The combined organic layers were washed with H_2O, dried over $MgSO_4$, concentrated under reduced pressure and purified by flash column chromatography (SiO_2, 0-20% v/v Acetone in $CHCl_3$) to afford the title compound as a colourless oil (61 mg, 0.11 mmol, 92%, 85% *ee*).

***R**$_f$* **value** = 0.21 (10% v/v Acetone in $CHCl_3$);

^{1}H NMR (500 MHz, $CDCl_3$) δ 8.01 (t, *J* = 6.3 Hz, 1H H-8), 7.68 (s, 1H, H-13), 7.49 (m, 2H, H-14), 7.33-7.28 (m, 3H, H-15, H-16), 4.50 (t, *J* = 12.3 Hz, 2H, H-12_A, H-12_B), 3.85 (m, 2H, H-7_A, H-7_B), 3.75 (m, 1H, H-10_A), 3.68 (m, 1H, H-10_B), 2.43 (m, 1H, H-9_A), 2.34-2.20 (m, 2H, H-9_B, H-11), 2.03 (pd, *J* = 12.1, 5.2 Hz, 2H, H-6_A, H-6_B), 1.74-1.56 (m, 5H, H-1_A, H-2_A, H-3_A), 1.25-1.05 (m, 5H, H-1_B, H-2_B, H-4, H-5_A), 0.97-0.76 (m, 3H, H-3_B, H-5_B) ppm;

^{13}C NMR (126 MHz, $CDCl_3$) δ 147.0, 129.4, 129.0, 128.5, 125.4, 118.9, 118.4 (qt, J_{C-F} = 287, 33.9 Hz), 111.6 (tq, J_{C-F} = 256, 38.1 Hz), 67.2, 64.0 (t, J_{C-F} = 28.8 Hz), 57.7, 49.9, 37.8, 36.3, 33.30-33.25 (m), 33.1, 30.1, 26.5, 26.3 ppm;

^{19}F NMR (471 MHz, $CDCl_3$) δ − 84.4, − 124.3 ppm;

IR (film, cm^{-1}): ν_{max} 2925 (m), 2853 (w), 1456 (w), 1380 (m), 1204 (s), 1184 (s), 1155 (m), 1106 (m), 1045 (m), 1009 (m), 808 (m), 760 (s), 694 (m), 516 (m);

$[\alpha]_D^{25.0}$ = + 2.1 (c. 2.6, $CHCl_3$);

HRMS (−ESI) *m/z* found $[M-H]^-$ 553.1906, $[C_{23}H_{30}F_5N_4O_4S]^-$ requires 553.1913, (δ = − 1.4 ppm);

Chiral SFC Analysis: CHIRALPAK IG (CO_2:MeOH, 92:8, 2.5 mL min^{-1}, 40 °C) t_R = 8.2 (major), 13.3 (minor) minutes.

2,2,3,3,3-pentafluoropropyl (S)-(4-hydroxy-1-phenylbutan-2-yl)sulfamate (**45**)

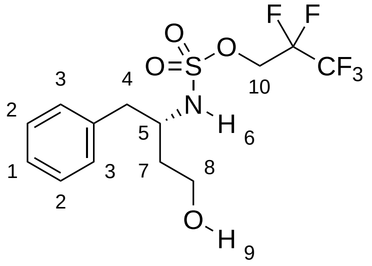

To a solution of 2,2,3,3,3-pentafluoropropyl (2*R*,3*S*)-2-(2-hydroxyethyl)-3-phenylaziridine-1-sulfonate (**9a**) (75 mg, 0.20 mmol) in EtOH (5.0 mL) was added Pd/C (10 wt % loading Pd) (10 mg). The microwave vial was then capped with a fresh suba seal following which it was evacuated and backfilled three times with hydrogen gas. The reaction mixture was allowed to stir at room temperature overnight under a balloon of hydrogen. The reaction was then filtered through Celite®, eluting with EtOAc. The filtrate was collected, the solvent removed under reduced pressure and purification by flash column chromatography (SiO_2, 0-20% v/v Acetone in $CHCl_3$) afforded the title compound as a colourless oil (55 mg, 0.15 mmol, 75%, 87% ee).

***R_f* value** = 0.24 (10% v/v Acetone in $CHCl_3$);

^{1}H NMR (500 MHz, $CDCl_3$) δ 7.33 (tt, *J* = 7.1, 1.6 Hz, 2H, H-2), 7.26 (m, 1H, H-1), 7.20 (m, 2H, H-3), 5.52 (br s, 1H, H-6), 4.33 (m, 1H, H-10_A), 4.13 (m, 1H, H-10_B), 3.97-3.86 (m, 2H, H-5, H-8_A), 3.81 (m, 1H, H-8_B), 3.02 (dd, *J* = 13.7, 6.0 Hz, 1H, H-4_A), 2.89 (dd, *J* = 13.8, 7.6 Hz, 1H, H-4_B), 1.93 (m, 1H, H-7_A), 1.81 (br. t, *J* = 4.5 Hz, 1H, H-9), 1.62 (m, 1H, H-7_B) ppm;

^{13}C NMR (101 MHz, $CDCl_3$) δ 137.0, 129.6, 128.9, 127.2, 118.3 (qt, J_{C-F} = 286, 34.4 Hz), 111.5 (tq, J_{C-F} = 257, 38.7 Hz), 63.8 (t, J_{C-F} = 28.0 Hz), 59.8, 55.5, 40.9, 35.0 ppm;

^{19}F NMR (376 MHz, $CDCl_3$) δ − 84.5, − 124.5 ppm;

IR (film, cm^{-1}): ν_{max} 3306 (w), 2928 (w), 1455 (w), 1353 (m), 1202 (s), 1177 (s), 1107 (m), 1048 (m), 1006 (s), 702 (m);

$[\alpha]_D^{25.0}$ = − 6.3 (c. 0.33, $CHCl_3$);

HRMS (−ESI) *m/z* found $[M-H]^-$ 376.0642, $[C_{13}H_{15}F_5NO_4S]^-$ requires 376.0647, (δ = − 1.3 ppm);

Chiral SFC Analysis: CHIRALPAK IG (CO_2:MeOH, 97:3, 2.5 mL min^{-1}, 40 °C) t_R = 7.1 (minor), 10.9 (major) minutes.

2,2,3,3,3-pentafluoropropyl (R)-(5-hydroxy-1-phenylpentan-2-yl)sulfamate (**46**)

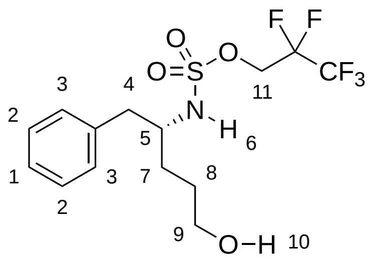

To a solution of 2,2,3,3,3-pentafluoropropyl (2*R*,3*S*)-2-(3-hydroxypropyl)-3-phenylaziridine-1-sulfonate (**3a**) (33 mg, 0.084 mmol) in EtOH (1.5 mL) was added Pd/C (10 wt % loading Pd) (5 mg). The microwave vial was then capped with a fresh suba seal following which it was evacuated and backfilled three times with hydrogen gas. The reaction mixture was allowed to stir at room temperature overnight under a balloon of hydrogen. The reaction was then filtered through Celite®, eluting with EtOAc. The filtrate was collected, the solvent removed under reduced pressure and purification by flash column chromatography (SiO_2, 0-20% v/v Acetone in $CHCl_3$) afforded the title compound as a colourless oil (25 mg, 0.064 mmol, 76%, 92% *ee*).

***R_f* value** = 0.39 (10% v/v Acetone in $CHCl_3$);

^{1}H NMR (500 MHz, $CDCl_3$) δ 7.32 (m, 2H, H-2), 7.26 (m, 1H, H-1), 7.20 (m, 2H, H-3), 5.56 (d, *J* = 8.1 Hz, 1H, H-6), 4.28 (q, *J* = 12.4 Hz, 1H, H-11_A), 4.08 (q, *J* = 12.4 Hz, 1H, H-11_B), 3.71 (m, 1H, H-5), 3.68 (t, *J* = 5.8 Hz, 2H, H-9_A, H-9_B), 2.91 (dd, *J* = 13.8, 6.3 Hz, 1H, H-4_A), 2.86 (dd, *J* = 7.0, 14.0 Hz, 1H, H-4_B), 1.84 (br s, 1H, H-10), 1.77-1.67 (m, 3H, H-7_A, H-8_A, H-8_B), 1.59 (m, 1H, H-7_B) ppm;

^{13}C NMR (126 MHz, $CDCl_3$) δ 137.1, 129.7, 128.9, 127.2, 118.3 (qt, $J_{C\text{-}F}$ = 286, 34.5 Hz), 111.5 (tq, $J_{C\text{-}F}$ = 256, 38.2 Hz), 63.7 (t, $J_{C\text{-}F}$ = 28.4 Hz), 62.6, 56.7, 41.0, 30.9, 27.9 ppm;

^{19}F NMR (376 MHz, $CDCl_3$) δ − 83.5, − 123.6 ppm;

IR (film, cm^{-1}): ν_{max} 3309 (w), 2944 (w), 1455 (w), 1366 (m), 1203 (s), 1177 (s), 1107 (m), 1048 (m), 1010 (m), 702 (m);

$[\alpha]_D^{25.0}$ = − 1.5 (c. 1.0, $CHCl_3$);

HRMS (−ESI) *m/z* found $[M-H]^-$ 390.0807, $[C_{14}H_{17}F_5NO_4S]^-$ requires 390.0804, (δ = + 0.8 ppm);

Chiral SFC Analysis: CHIRALPAK IE (CO_2:MeOH, 97:3, 1.25 mL min^{-1}, 40 °C) t_R = 15.7 (minor), 16.5 (major) minutes.

2,2,3,3,3-pentafluoropropyl (R)-(6-hydroxy-1-phenylhexan-2-yl)sulfamate (**47**)

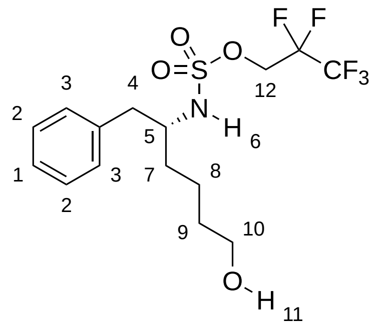

To a solution of 2,2,3,3,3-pentafluoropropyl (2*R*,3*S*)-2-(4-hydroxybutyl)-3-phenylaziridine-1-sulfonate (**14a**) (22 mg, 0.054 mmol) in EtOH (1.5 mL) was added Pd/C (10 wt % loading Pd) (3 mg). The microwave vial was then capped with a fresh suba seal following which it was evacuated and backfilled three times with hydrogen gas. The reaction mixture was then allowed to stir at room temperature overnight under a balloon of hydrogen. The reaction was then filtered through Celite®, eluting with EtOAc. The filtrate was collected, the solvent removed under reduced pressure and purification by flash column chromatography (SiO_2, 0-20% v/v Acetone in $CHCl_3$) afforded the title compound as a colourless oil (18 mg, 0.044 mmol, 81%, 96% *ee*).

***R_f* value** = 0.33 (10% v/v Acetone in $CHCl_3$);

^{1}H NMR (500 MHz, $CDCl_3$) δ 7.33 (tt, *J* = 7.5, 1.4 Hz, 2H, H-2), 7.26 (tt, *J* = 6.7, 1.4 Hz, 1H, H-1), 7.19 (m, 2H, H-3), 4.85 (d, *J* = 8.4 Hz, 1H, H-6), 4.27 (q, *J* = 12.5 Hz, 1H, H-12_A), 4.09 (q, *J* = 12.4 Hz, 1H, H-12_B), 3.69 (m, 1H, H-5), 3.64 (t, *J* = 6.0 Hz, 2H, H-10_A, H-10_B), 2.90 (dd, *J* = 13.8, 6.6 Hz, 1H, H-4_A), 2.84 (dd, *J* = 6.3, 13.7 Hz, 1H, H-4_B), 1.67 (br s, 1H, H-11), 1.63-1.45 (m, 6H, H-7_A, H-7_B, H-8_A, H-8_B, H-9_A, H-9_B) ppm;

^{13}C NMR (126 MHz, $CDCl_3$) δ 136.9, 129.7, 128.9, 127.2, 118.3 (qt, $J_{C\text{-}F}$ = 286, 34.8 Hz), 111.5 (tq, $J_{C\text{-}F}$ = 257, 38.0 Hz), 63.7 (t, $J_{C\text{-}F}$ = 28.2 Hz), 62.5, 56.9, 41.0, 33.9, 32.1, 21.8 ppm;

^{19}F NMR (376 MHz, $CDCl_3$) δ − 84.5, − 124.5 ppm;

IR (film, cm^{-1}): ν_{max} 3306 (w), 2944 (w), 1456 (w), 1369 (m), 1205 (s), 1179 (s), 1108 (m), 1011 (m);

$[\alpha]_D^{25.0}$ = – 3.9 (c. 0.89, $CHCl_3$);

HRMS (−ESI) *m/z* found $[M-H]^-$ 404.0959, $[C_{15}H_{19}F_5NO_4S]^-$ requires 404.0960, (δ = – 0.1 ppm);

Chiral SFC Analysis: CHIRALPAK IE (CO_2:MeOH, 96:4, 1.25 mL min^{-1}, 40 °C) t_R = 12.1 (minor), 12.6 (major) minutes.

Benzyl (R)-(6-hydroxy-1-phenylhexan-2-yl)carbamate (**48**)

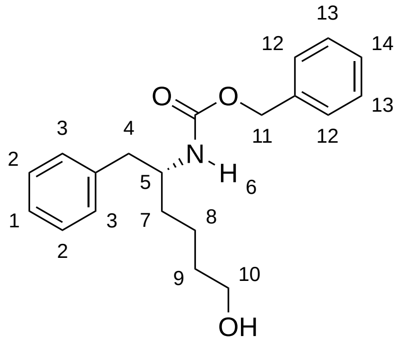

A microwave vial charged with 2,2,3,3,3-pentafluoropropyl (*R*)-(6-hydroxy-1-phenylhexan-2-yl)sulfamate (**47**) (13 mg, 0.032 mmol) and NaOH (freshly ground) (6 mg, 0.16 mmol) was placed under a nitrogen atmosphere. MeCN (0.6 mL) and thiophenol (16 µL, 0.16 mmol) were added and the reaction mixture was heated at 90 °C overnight. After cooling to room temperature, the mixture was concentrated under a stream of nitrogen and placed on a silica plug and washed with 5% v/v acetone in $CHCl_3$. These initial washings were discarded and the intermediate sulfonate salt was collected by flushing the silica plug with EtOH and concentrating the flushes under reduced pressure. The sulfonate salt was transferred to a microwave vial and placed under a nitrogen atmosphere. HCl (1.25 M in MeOH) (0.6 mL) and c.H_2SO_4 (3 drops) were added the reaction mixture was heated at 50 °C overnight. After cooling to room temperature, the mixture was concentrated under a stream of nitrogen and saturated aqueous Na_2CO_3 solution (0.5 mL), 10% aqueous NaOH solution (3 drops), benzyl

chloroformate (11 µL, 0.080 mmol) and CH_2Cl_2 (0.5 mL) were added. After stirring at room temperature overnight, the reaction mixture was diluted in CH_2Cl_2 (2 mL) and 5% aqueous $NaHCO_3$ solution (2 mL), the layers were separated, and the aqueous layer was extracted with CH_2Cl_2. The organic layers were combined, dried over $MgSO_4$, concentrated under reduced pressure and purified by flash column chromatography (SiO_2, 0-20% v/v acetone in $CHCl_3$) to afford the title compound as a white solid (7 mg, 0.021 mmol, 66%, 96% ee).

***R**_f* **value** = 0.36 (20% v/v acetone in $CHCl_3$);

^{1}H NMR (500 MHz, $CDCl_3$) *δ* 7.37-7.28 (m, 5H, H-12, H-13, H-14), 7.27 (m, 2H, H-2), 7.21 (tt, J =7.3, 2.2 Hz, 1H, H-1), 7.15 (d, J = 7.2 Hz, 2H, H-3), 5.06 (m, 2H, H-11_A, H-11_B), 4.55 (d, *J* = 8.7 Hz, 1H, H-6), 3.91 (m, 1H, H-5), 3.60 (t, *J* = 6.0 Hz, 2H, H-10_A, H-10_B), 2.79 (d, *J* = 6.1 Hz, 2H, H-4_A, H-4_B), 1.56-1.45 (m, 3H, H-7_A, H-9_A, H-9_B), 1.43-1.28 (m, 3H, H-7_B, H-8_A, H-8_B) ppm;

^{13}C NMR (126 MHz, $CDCl_3$) *δ* 156.1, 138.0, 136.8, 129.6, 128.7, 128.6, 128.21, 128.15, 126.6, 66.7, 62.8, 52.2, 41.5, 34.2, 32.5, 22.3 ppm;

IR (film, cm^{-1}): ν_{max} 3326 (m), 2933 (m), 1695 (s), 1538 (m), 1487 (m), 1455 (m), 1249 (s), 1072 (s), 1029 (m), 737 (m), 698 (s);

HRMS (+ESI) *m/z* found $[M+Na]^+$ 350.1721, $[C_{20}H_{25}NNaO_3]^+$ requires 350.1726, (*δ* = − 1.6 ppm);

$[\alpha]_D^{25.0}$ = + 13.7 (c = 0.29, $CHCl_3$);

Chiral SFC Analysis: CHIRALPAK IG (CO_2:MeOH, 85:15, 2.5 mL min^{-1}, 40 °C) t_R = 8.6 (minor), 13.1 (major) minutes.

2,2,3,3,3-pentafluoropropyl (R)-2-benzylpyrrolidine-1-sulfonate (**49**)

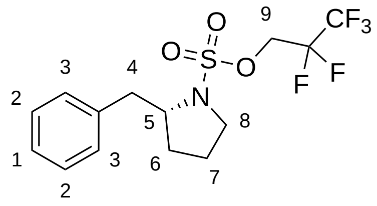

A microwave vial charged with 2,2,3,3,3-pentafluoropropyl (*R*)-(5-hydroxy-1-phenylpentan-2-yl)sulfamate (**46**) (52 mg, 0.13 mmol) and PPh_3 (52 mg, 0.20 mmol) was placed under a nitrogen atmosphere. The vial was cooled to 0 °C and THF (2.5 mL) and diisopropylazidodicarboxylate (39 µL, 0.20 mmol) were added. The reaction mixture was warmed to room temperature overnight, concentrated under reduced pressure and purified by flash column chromatography (SiO_2, 0-6% v/v EtOAc in Petrol) to afford the title compound as a colourless oil (40 mg, 0.11 mmol, 79%, 92% *ee*).

***R**$_f$* **value** = 0.26 (5% v/v EtOAc in Petrol);

^{1}H NMR (500 MHz, $CDCl_3$) δ 7.31 (tt, *J* = 7.5, 1.4 Hz, 2H, H-2), 7.24 (tt, *J* = 7.4, 1.3 Hz, 1H, H-1), 7.21 (m, 2H, H-3), 4.51 (tq, *J* = 12.2, 0.9 Hz, 2H, H-9$_A$, H-9$_B$), 4.07 (m, 1H, H-5), 3.47 (m, 1H, H-8$_A$), 3.40 (m, 1H, H-8$_B$), 3.16 (dd, *J* = 13.3, 4.1 Hz, 1H, H-4$_A$), 2.72 (dd, *J* = 13.4, 9.5 Hz, 1H, H4$_B$), 1.93-1.83 (m, 2H, H-6$_A$, H-7$_A$), 1.83-1.76 (m, 2H, H-6$_B$, H-7$_B$) ppm;

^{13}C NMR (126 MHz, $CDCl_3$) δ 137.7, 129.7, 128.7, 126.8, 118.4 (qt, $J_{C\text{-}F}$ = 287, 34.0 Hz), 111.7 (tq, $J_{C\text{-}F}$ = 256, 38.2 Hz), 63.6 (t, $J_{C\text{-}F}$ = 28.5 Hz), 63.1, 50.2, 41.2, 30.2, 24.2 ppm;

^{19}F NMR (376 MHz, $CDCl_3$) δ − 83.5, − 123.5 ppm;

IR (film, cm^{-1}): ν_{max} 2962 (w), 1454 (w), 1378 (s), 1204 (s), 1175 (s), 1108 (m), 1032 (m), 1009 (m);

$[\alpha]_D^{25.0}$ = + 21.3 (c. 0.92, $CHCl_3$);

HRMS (−ESI) *m/z* found $[M{-}H]^-$ 372.0715, $[C_{14}H_{15}F_5NO_3S]^-$ requires 372.0698, (δ = + 4.4 ppm);

Chiral SFC Analysis: CHIRALPAK IG (CO_2:MeOH, 99.5:0.5, 1.25 mL min^{-1}, 40 °C) t_R = 9.5 (minor), 12.0 (major) minutes.

2,2,3,3,3-pentafluoropropyl (S)-2-benzylazetidine-1-sulfonate (**50**)

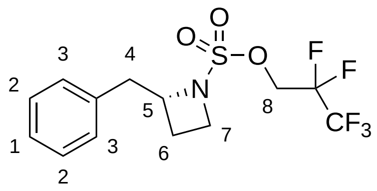

A microwave vial charged with 2,2,3,3,3-pentafluoropropyl (*S*)-(4-hydroxy-1-phenylbutan-2-yl)sulfamate (**45**) (37 mg, 0.098 mmol) and PPh_3 (39 mg, 0.15 mmol) was placed under a nitrogen atmosphere. The vial was cooled to 0 °C and THF (1.5 mL) and diisopropylazidodicarboxylate (29 µL, 0.15 mmol) were added. The reaction mixture was warmed to room temperature overnight, concentrated under reduced pressure and purified by flash column chromatography (SiO_2, 0-10% v/v EtOAc in Hexane) to afford the title compound as a colourless oil (22 mg, 0.061 mmol, 62%, 89% *ee*).

***R_f* value** = 0.42 (10% v/v EtOAc in Hexane);

^{1}H NMR (500 MHz, $CDCl_3$) δ 7.32 (tt, *J* = 6.5, 1.2 Hz, 2H, H-2), 7.26 (tt, *J* = 6.5, 1.4 Hz, 1H, H-1), 7.20 (m, 2H, H-3), 4.60 (m, 1H, H-5), 4.53 (t, *J* = 12.2 Hz, 2H, H-8_A, H-8_B), 4.00 (q, *J* = 8.4 Hz, 1H, H-7_A), 3.75 (m, 1H, H-7_B), 3.16 (dd, *J* = 13.8, 4.2 Hz, 1H, H-4_A), 3.00 (dd, *J* = 13.8, 9.2 Hz, 1H, H-4_B), 2.20 (m, 1H, H-6_A), 2.12 (m, 1H, H-6_B) ppm;

^{13}C NMR (126 MHz, $CDCl_3$) δ 135.7, 129.6, 128.8, 127.1, 118.4 (qt, J_{C-F} = 286, 35.2 Hz), 111.6 (tq, J_{C-F} = 257, 38.1 Hz), 65.9, 64.0 (t, J_{C-F} = 28.6 Hz), 49.1, 41.5, 21.8 ppm;

^{19}F NMR (376 MHz, $CDCl_3$) δ – 83.5, – 123.5 ppm;

IR (film, cm^{-1}): ν_{max} 2925 (w), 1455 (w), 1379 (s), 1206 (s), 1183 (s), 1108 (m), 1041 (m), 1008 (m);

$[\alpha]_D^{25.0}$ = + 57.3 (c. 0.86, $CHCl_3$);

HRMS (−ESI) *m/z* found [M−H]$^-$ 358.0542, $[C_{13}H_{13}F_5NO_3S]^-$ requires 358.0542, (δ = 0.0 ppm);

Chiral SFC Analysis: CHIRALPAK IG (CO_2:MeOH, 99.5:0.5, 2.5 mL min^{-1}, 40 °C) t_R = 3.2 (minor), 4.1 (major) minutes.

Tert-butyl (S)-(1-phenyl-4-(phenylthio)butan-2-yl)carbamate (**51**)

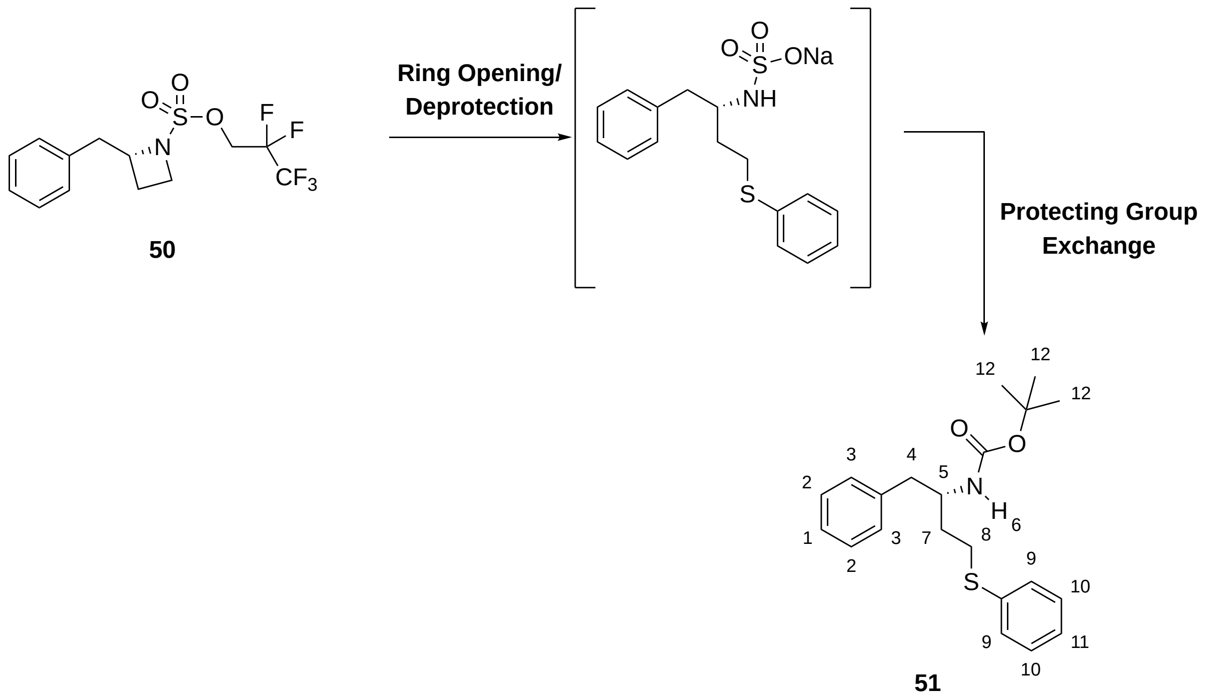

A microwave vial charged with 2,2,3,3,3-pentafluoropropyl (*S*)-2-benzylazetidine-1-sulfonate (**50**) (15 mg, 0.042 mmol) and NaOH (freshly ground) (8 mg, 0.21 mmol) was placed under a

nitrogen atmosphere. MeCN (0.8 mL) and thiophenol (21 µL, 0.21 mmol) were added and the reaction mixture was heated at 90 °C overnight. After cooling to room temperature, the mixture was concentrated under a stream of nitrogen and placed on a silica plug and washed with 5% v/v acetone in $CHCl_3$. These initial washings were discarded and the intermediate sulfonate salt was collected by flushing the silica plug with EtOH and concentrating the flushes under reduced pressure. The sulfonate salt was transferred to a microwave vial and placed under a nitrogen atmosphere. HCl (1.25 M in MeOH) (0.75 mL) and c.H_2SO_4 (3 drops) were added the reaction mixture was heated at 50 °C overnight. After cooling to room temperature, the mixture was concentrated under a stream of nitrogen and saturated aqueous Na_2CO_3 solution (0.6 mL), 10% aqueous NaOH solution (3 drops), di-*tert*-butyl dicarbonate (23 mg, 0.11 mmol) and CH_2Cl_2 (0.6 mL) were added. After stirring at room temperature for 3 hours, the reaction mixture was diluted in CH_2Cl_2 (5 mL) and 5% aqueous $NaHCO_3$ solution (5 mL), the layers were separated, and the aqueous layer was extracted with CH_2Cl_2. The organic layers were combined, dried over $MgSO_4$, concentrated under reduced pressure and purified by flash column chromatography (SiO_2, 0-20% v/v EtOAc in Hexane) to afford the title compound as a white solid (6 mg, 0.017 mmol, 40%, 86% *ee*).

***R_f* value** = 0.16 (10% v/v EtOAc in Hexane);

^{1}H NMR (500 MHz, $CDCl_3$) δ 7.29-7.25 (m, 6H, H-2, H-9, H-10), 7.23 (tt, *J* = 7.2, 2.1 Hz, 1H, H-1), 7.18 (tt, *J* = 7.3, 1.6 Hz, 1H, H-11), 7.14 (d, *J* = 7.7 Hz, 2H, H-3), 4.35 (br d, *J* = 7.7 Hz, 1H, H-6), 3.96 (m, 1H, H-5), 2.98 (m, 1H, H-8_A), 2.90 (m, 1H, H-8_B), 2.81 (m, 1H, H-4_A), 2.74 (m, 1H, H-4_B), 1.84 (m, 1H, H-7_A), 1.63 (m, 1H, H-7_B), 1.41 (s, 9H, H-12) ppm;

^{13}C NMR (126 MHz, $CDCl_3$) δ 155.6, 137.9, 136.4, 129.6, 129.5, 129.0, 128.6, 126.6, 126.2, 79.5, 51.3, 41.5, 34.1, 30.8, 28.5 ppm;

IR (film, cm^{-1}): ν_{max} 3348 (w), 2925 (m), 1698 (s), 1497 (m), 1481 (m), 1440 (m), 1390 (m), 1385 (m), 1248 (m), 1108 (s), 1046 (m), 1026 (m), 737 (m), 700 (m);

$[\boldsymbol{\alpha}]_{\mathbf{D}}^{\mathbf{25.0}}$ = + 12.7 (c. 0.41, $CHCl_3$);

HRMS (+ESI) *m/z* found [M+H] 358.1832, $[C_{21}H_{28}NO_2S]^+$ requires 358.1835, (δ = – 0.8 ppm);

Chiral SFC Analysis: CHIRALPAK IG (CO_2:MeOH, 90:10, 2.5 mL min^{-1}, 40 °C) t_R = 5.5 (minor), 6.9 (major) minutes.

2,2,3,3,3-pentafluoropropyl (R)-(4-hydroxy-1-phenylbutyl)sulfamate (**52**)

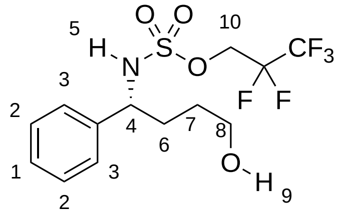

A microwave vial charged with 2,2,3,3,3-pentafluoropropyl (2*R*,3*S*)-2-(2-hydroxyethyl)-3-phenylaziridine-1-sulfonate (**9a**) (63 mg, 0.17 mmol) was placed under a nitrogen atmosphere and cooled to -78 °C. THF (1.5 mL) and Red-Al® (60 wt.% in toluene) (65 µL, 0.20 mmol) were added and the reaction mixture was gradually warmed to room temperature overnight. The reaction was opened to air and quenched through addition of sodium sulfate decahydrate until effervescence had finished. The resulting residue was diluted in EtOAc (5 mL) and filtered through Celite®, eluting with further EtOAc. The filtrate was collected, the solvent removed under reduced pressure and purification by flash column chromatography (SiO_2, 0-20% v/v Acetone in $CHCl_3$) afforded the title compound as a colourless oil (40 mg, 0.11 mmol, 65%, 88% ee).

***R_f* value** = 0.21 (10% v/v Acetone in $CHCl_3$);

^{1}H NMR (500 MHz, $CDCl_3$) δ 7.37 (m, 2H, H-2), 7.33-7.28 (m, 3H, H-1, H-3), 6.13 (d, *J* = 6.4 Hz, 1H, H-5), 4.49 (q, *J* = 6.9 Hz, 1H, H-4), 4.13 (m, 2H, H-10_A, H-10_B), 3.70 (m, 2H, H-8_A, H-8_B), 1.99 (m, 2H, H-6_A, H-6_B), 1.79 (br s, 1H, H-9), 1.62 (m, 2H, H-7_A, H-7_B) ppm;

^{13}C NMR (126 MHz, $CDCl_3$) δ 140.6, 129.1, 128.3, 126.6, 118.2 (qt, $J_{C\text{-}F}$ = 286, 34.7 Hz), 111.3 (tq, $J_{C\text{-}F}$ = 257, 38.0 Hz), 63.8 (t, $J_{C\text{-}F}$ = 28.2 Hz), 62.4, 59.3, 33.9, 28.4 ppm;

^{19}F NMR (376 MHz, $CDCl_3$) δ − 84.7, − 124.6 ppm;

IR (film, cm^{-1}): ν_{max} 3305 (w), 2924 (w), 1456 (w), 1368 (m), 1202 (s), 1178 (s), 1108 (m), 1044 (s), 1008 (s), 955 (m), 759 (s), 701 (s), 565 (m);

$[\alpha]_D^{25.0}$= + 9.6 (c. 0.75, $CHCl_3$);

HRMS (−ESI) *m/z* found $[M{-}H]^-$ 376.0642, $[C_{13}H_{15}F_5NO_4S]^-$ requires 376.0647, (δ = − 1.5 ppm);

Chiral SFC Analysis: CHIRAL ART SC (CO_2:^{i}PrOH, 96:4, 2.5 mL min^{-1}, 40 °C) t_R = 15.5 (major), 18.8 (minor) minutes.

2,2,3,3,3-pentafluoropropyl ((S)-phenyl((S)-2-(trichloromethyl)-5,6-dihydro-4H-1,3-oxazin-4-yl)methyl)sulfamate (**53**)

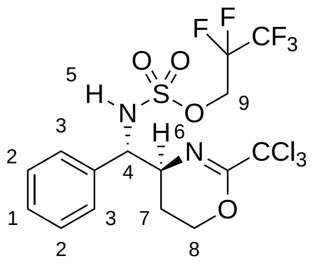

A 4.0 mL crimp-top vial was charged with 2,2,3,3,3-pentafluoropropyl (2*R*,3*S*)-2-(2-hydroxyethyl)-3-phenylaziridine-1-sulfonate (**9a**) (26 mg, 0.069 mmol). CH_2Cl_2 (1.5 mL), trichloroacetonitrile (10 µL, 0.10 mmol) and 1,8-diazabicyclo[5.4.0]undec-7-ene (1 drop) were added and the reaction mixture was stirred at room temperature overnight under air. After concentrating under a stream of nitrogen, purification by flash column chromatography (SiO_2,

0-20% v/v EtOAc in Hexane) afforded the title compound as a colourless oil (26 mg, 0.050 mmol, 72%, 87% *ee*).

***R$_f$* value** = 0.27 (20% v/v EtOAc in Hexane);

^{1}H NMR (500 MHz, $CDCl_3$) δ 7.44-7.35 (m, 5H, H-1, H-2, H-3), 6.28 (d, J = 2.9 Hz, 1H, H-5), 4.53 (dt, J = 10.9, 3.6 Hz, 1H, H-8$_A$), 4.25-4.12 (m, 4H, H-4, H-8$_B$, H-9$_A$, H-9$_B$), 3.68 (m, 1H, H-6), 1.78-1.73 (m, 2H, H-7$_A$, H-7$_B$) ppm;

^{13}C NMR (126 MHz, $CDCl_3$) δ 155.5, 137.5, 129.2, 129.1, 127.7, 118.2 (qt, $J_{C\text{-}F}$ = 286, 34.1 Hz), 111.3 (tq, $J_{C\text{-}F}$ = 257, 38.6 Hz), 92.0, 67.0, 64.0 (t, $J_{C\text{-}F}$ = 28.5 Hz), 63.4, 56.7, 24.1 ppm;

^{19}F NMR (376 MHz, $CDCl_3$) δ − 84.6, − 124.4 (br d, J = 1.5 Hz) ppm;

IR (film, cm^{-1}): ν_{max} 1673 (m), 1526 (w), 1468 (w), 1373 (m), 1203 (s), 1182 (s), 1108 (m), 1042 (m), 1009 (m), 949 (m), 822 (s), 755 (s), 702 (s), 573 (s);

$[\alpha]_D^{25.0}$= + 12.4 (c. 1.2, $CHCl_3$);

HRMS (−ESI) *m/z* found $[M-H]^-$ 516.9595, $[C_{15}H_{13}Cl_3F_5N_2O_4S]^-$ requires 516.9587, (δ = + 1.5 ppm);

Chiral SFC Analysis: CHIRAL ART SC (CO_2:MeOH, 98:2, 2.5 mL min^{-1}, 40 °C) t_R = 3.4 (minor), 5.0 (major) minutes.

2,2,3,3,3-pentafluoropropyl ((S)-((S)-2-oxo-1,3-dioxan-4-yl)(phenyl)methyl)sulfamate (**54**)

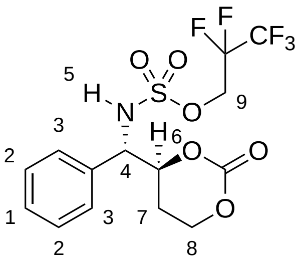

A 4.0 mL crimp-top vial was charged with 2,2,3,3,3-pentafluoropropyl (2*R*,3*S*)-2-(2-hydroxyethyl)-3-phenylaziridine-1-sulfonate (**9a**) (26 mg, 0.069 mmol). CH_2Cl_2 (1.5 mL), benzyl chloroformate (15 µL, 0.10 mmol) and triethylamine (29 µL, 0.21 mmol) were added and the reaction mixture was stirred at room temperature overnight under air. Saturated aqueous $NaHCO_3$ solution (1.0 mL) was added and the reaction mixture was stirred for 1 hour. The layers were separated, the aqueous layer extracted with $CHCl_3$ and the combined organic layers were dried over $MgSO_4$ and concentrated under reduced pressure. Purification by flash column chromatography (SiO_2, 0-4% v/v MeOH in CH_2Cl_2) afforded the title compound as a white solid (14 mg, 0.033 mmol, 48%, 87% *ee*).

***R_f* value** = 0.43 (5% v/v MeOH in CH_2Cl_2);

^{1}H NMR (400 MHz, $CDCl_3$) δ 7.43-7.37 (m, 5H, H-1, H-2, H-3), 6.68 (d, *J* = 8.0 Hz, 1H, H-5), 4.73 (dt, *J* = 11.4, 3.9 Hz, 1H, H-6), 4.57 (dd, *J* = 8.2, 4.4 Hz, 1H, H-4), 4.45 (ddd, *J* = 11.1, 5.4, 1.9 Hz, 1H, H-8_A), 4.37 (td, J = 11.7, 3.0 Hz, 1H, H-8_B), 4.29 (q, *J* = 12.3 Hz, 1H, H-9_A), 4.06 (q, *J* = 12.2 Hz, 1H, H-9_B), 2.25 (m, 1H, H-7_A), 1.96 (dq, J = 14.5, 2.5 Hz, 1H, H-7_B) ppm;

^{13}C NMR (126 MHz, $CDCl_3$) δ 148.6, 136.2, 129.40, 129.36, 127.4, 118.1 (qt, $J_{C\text{-}F}$ = 286, 34.2 Hz), 111.2 (tq, $J_{C\text{-}F}$ = 256, 38.6 Hz), 81.0, 66.9, 64.1 (t, $J_{C\text{-}F}$ = 28.2 Hz), 61.4, 24.6 ppm;

^{19}F NMR (376 MHz, $CDCl_3$) δ – 83.7, – 123.7 ppm;

IR (film, cm^{-1}): ν_{max} 1733 (s), 1459 (m), 1413 (m), 1376 (m), 1254 (m), 1201 (s), 1182 (s), 1136 (m), 1108 (m), 1045 (m), 1008 (m), 761 (m), 703 (m);

$[\alpha]_D^{25.0}$= + 43.8 (c. 0.85, $CHCl_3$);

HRMS (−ESI) *m/z* found $[M-H]^-$ 418.0405, $[C_{14}H_{13}F_5NO_6S]^-$ requires 418.0389, (δ = + 3.8 ppm);

Chiral SFC Analysis: CHIRALPAK IH (CO_2:MeOH, 95:5, 2.5 mL min^{-1}, 40 °C) t_R = 7.9 (minor), 9.3 (major) minutes.

Determination of the Absolute Stereochemistry of the Aziridine Products

1) Styrenyl Aziridines:

Benzyl (S)-(4-hydroxy-1-phenylbutan-2-yl)carbamate (**SI1**)

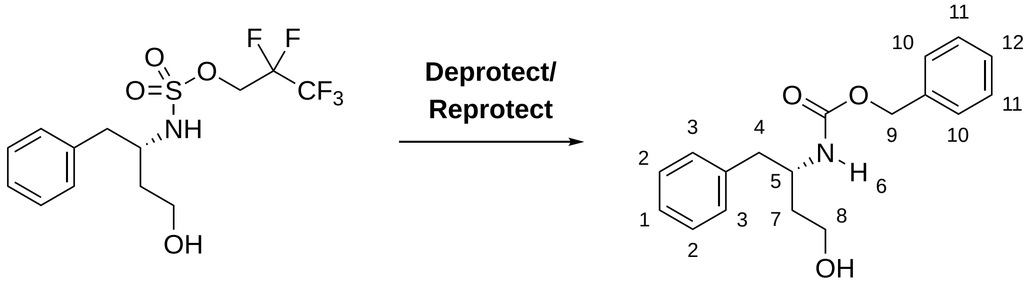

45 **SI1**

A microwave vial charged with 2,2,3,3,3-pentafluoropropyl (*S*)-(4-hydroxy-1-phenylbutan-2-yl)sulfamate (**45**) (90 mg, 0.24 mmol) and NaOH (freshly ground) (48 mg, 1.2 mmol) was placed under a nitrogen atmosphere. MeCN (4.0 mL) and thiophenol (120 µL, 1.2 mmol) were added and the reaction mixture was heated at 90 °C overnight. After cooling to room temperature, the mixture was concentrated under a stream of nitrogen and placed on a silica plug and washed with 5% v/v acetone in $CHCl_3$.These initial washings were discarded and the intermediate sulfonate salt was collected by flushing the silica plug with EtOH and concentrating the flushes under reduced pressure. The sulfonate salt was transferred to a microwave vial and placed under a nitrogen atmosphere. HCl (1.25 M in MeOH) (4.0 mL) and c.H_2SO_4 (5 drops) were added and the reaction mixture was heated at 50 °C overnight. After cooling to room temperature, the mixture was concentrated under a stream of nitrogen and saturated aqueous Na_2CO_3 solution (2.0 mL), 10% aqueous NaOH solution (3 drops), benzyl chloroformate (68 µL, 0.48 mmol) and CH_2Cl_2 (2.0 mL) were added. After stirring at room temperature overnight, the reaction mixture was diluted in CH_2Cl_2 (15 mL) and 5% aqueous $NaHCO_3$ solution (15 mL), the layers were separated, and the aqueous layer was extracted

with CH_2Cl_2. The organic layers were combined, dried over $MgSO_4$, concentrated under reduced pressure and purified by flash column chromatography (SiO_2, 0-20% v/v Acetone in $CHCl_3$) to afford the title compound as a white solid (9 mg, 0.030 mmol, 13%). *Comparison of the optical rotation of* **SI1** *with literature values enabled a determination of absolute stereochemistry at C5.*

***R_f* value** = 0.39 (20% v/v Acetone in $CHCl_3$);

^{1}H NMR (400 MHz, $CDCl_3$) δ 7.37-7.14 (m, 10H, H-1, H-2, H-3, H-10, H-11, H-12), 5.10 (d, J = 12.3 Hz, 1H, H-9$_A$), 5.05 (d, J = 12.2 Hz, 1H, H-9$_B$), 4.74 (br s, 1H, H-6), 4.16 (br s, 1H, H-5), 3.65 (m, 2H, H-8$_A$, H-8$_B$), 2.83 (m, 2H, H-4$_A$, H-4$_B$), 1.88 (m, 1H, H-7$_A$), 1.41 (m, 1H, H-7$_B$) ppm;

^{13}C NMR (101 MHz, $CDCl_3$) δ 157.2, 137.6, 136.5, 129.4, 128.72, 128.70, 128.3, 128.1, 126.8, 67.1, 59.1, 49.0, 41.6, 37.8 ppm;

IR (film, cm^{-1}): ν_{max} 3322 (w), 2948 (w), 1696 (s), 1535 (m), 1454 (m), 1259 (m), 1056 (s), 743 (m), 699 (s);

$[\alpha]_D^{25.0}$ = + 11.7 (c. 0.67, $CHCl_3$); [reported[74] $[\alpha]_D^{20.0}$ for the **opposite enantiomer** = – 10.0 (c. 1.0, $CHCl_3$)];

HRMS (+ESI) *m/z* found $[M+H]^+$ 300.1590, $[C_{18}H_{22}NO_3]^+$ requires 300.1594, (δ = − 1.3 ppm);

The spectroscopic and spectrophotometric data are in agreement with that reported in the literature.[74]

(9H-fluoren-9-yl)methyl (R)-(5-hydroxy-1-phenylpentan-2-yl)carbamate (**SI3**)

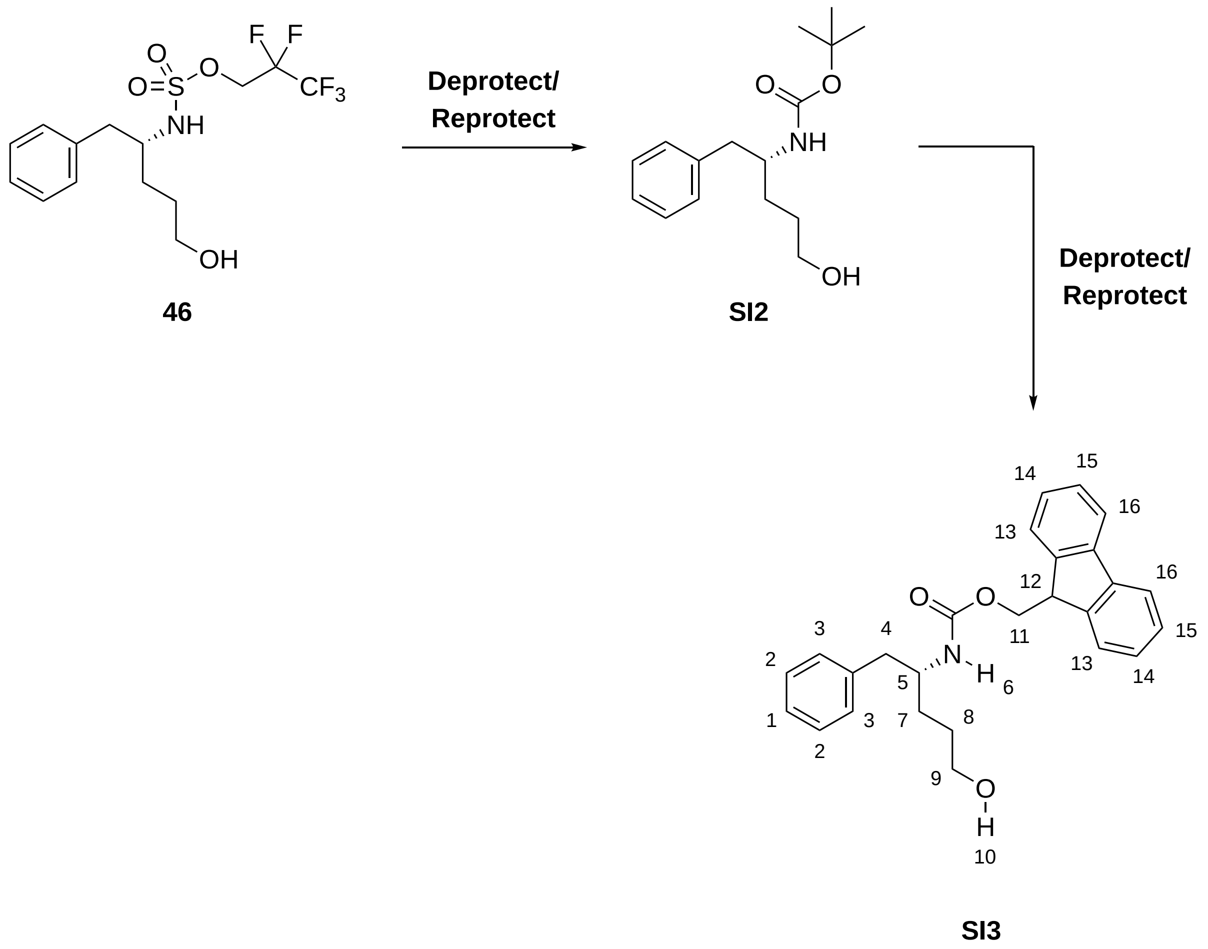

A microwave vial charged with 2,2,3,3,3-pentafluoropropyl (*R*)-(5-hydroxy-1-phenylpentan-2-yl)sulfamate (**46**) (74 mg, 0.19 mmol) and NaOH (freshly ground) (38 mg, 0.95 mmol) was placed under a nitrogen atmosphere. MeCN (3.0 mL) and thiophenol (97 µL, 0.95 mmol) were added and the reaction mixture was heated at 90 °C overnight. After cooling to room temperature, the mixture was concentrated under a stream of nitrogen and placed on a silica plug and washed with 5% v/v acetone in $CHCl_3$. These initial washings were discarded and the intermediate sulfonate salt was collected by flushing the silica plug with EtOH and concentrating the flushes under reduced pressure. The sulfonate salt was transferred to a microwave vial and placed under a nitrogen atmosphere. HCl (1.25 M in MeOH) (3.0 mL) and c.H_2SO_4 (5 drops) were added and the reaction mixture was heated at 50 °C overnight. After cooling to room temperature, the mixture was concentrated under a stream of nitrogen and saturated aqueous Na_2CO_3 solution (1.5 mL), 10% aqueous NaOH solution (3 drops), di-*tert*-butyl dicarbonate (83 mg, 0.38 mmol) and CH_2Cl_2 (1.5 mL) were added. After stirring at room temperature overnight, the reaction mixture was diluted in CH_2Cl_2 (15 mL) and 5% aqueous

$NaHCO_3$ solution (15 mL), the layers were separated, and the aqueous layer was extracted with CH_2Cl_2. The organic layers were combined, dried over $MgSO_4$, concentrated under reduced pressure and purified by flash column chromatography (SiO_2, 0-20% v/v Acetone in $CHCl_3$) to afford the intermediate Boc-protected amine (**SI2**).

At this point, the validity of the literature characterisation data for **SI2** *was questioned, and so to ensure an unambiguous stereochemical assignment, the Fmoc-protected amine* **SI3** *was synthesised according to the protocol below.*

A microwave vial charged with **SI2** (17 mg, 0.061 mmol) was placed under a nitrogen atmosphere. HCl (4.0 M in dioxane) (0.5 mL) was added and the reaction mixture was heated at 50 °C overnight. After cooling to room temperature, the mixture was concentrated under a stream of nitrogen and Na_2CO_3 (26 mg, 0.24 mmol) in H_2O (0.25 mL), 9-fluorenylmethoxycarbonyl chloride (19 mg, 0.073 mmol) and 1,4-dioxane (0.3 mL) were added. After stirring at room temperature overnight, the reaction mixture was diluted in CH_2Cl_2 (15 mL) and H_2O (15 mL), the layers were separated, and the aqueous layer was extracted with CH_2Cl_2. The organic layers were combined, dried over $MgSO_4$, concentrated under reduced pressure and purified by flash column chromatography (SiO_2, 0-20% v/v Acetone in $CHCl_3$) to afford the title compound as a white solid (12 mg, 0.030 mmol, 16%). *Comparison of the optical rotation of* **SI3** *with literature values enabled a determination of absolute stereochemistry at C5.*

***R_f* value** = 0.36 (20% v/v Acetone in $CHCl_3$);

^{1}H NMR (400 MHz, $CDCl_3$) δ 7.77 (d, J = 7.6 Hz, 2H, H-16), 7.55 (t, J = 7.9 Hz, 2H, H-15), 7.40 (t, J = 7.5 Hz, 2H, H-14), 7.31 (td, J = 7.4, 1.0 Hz, 2H, H-13), 7.27 (m, 2H, H-2), 7.21 (m, 1H, H-1), 7.15 (d, J = 7.1 Hz, 2H, H-3), 4.61 (br s, 1H, H-6), 4.45 (m, 1H, H-11_A), 4.36 (m, 1H, H-11_B), 4.18 (t, J = 6.6 Hz, 1H, H-12), 3.93 (br s, 1H, H-5), 3.63 (br t, J = 5.6 Hz, 2H, H-9_A, H-9_B), 2.79 (m, 2H, H-4_A, H-4_B), 1.68-1.38 (m, 5H, H-7_A, H-7_B, H-8_A, H-8_B, H-10) ppm;

^{13}C NMR (101 MHz, $CDCl_3$) δ 156.2, 144.1, 141.5, 137.9, 129.6, 128.6, 127.8, 127.2, 126.6, 125.2, 120.1, 66.4, 62.7, 52.0, 47.5, 41.6, 30.9, 29.1 ppm;

IR (film, cm^{-1}): ν_{max} 3323 (w), 2947 (w), 1692 (s), 1534 (s), 1450 (s), 1249 (s), 1071 (m), 758 (m), 739 (s), 701 (m);

$[\alpha]_D^{25.0}$ = + 2.3 (c. 0.75, $CHCl_3$); [reported[75] $[\alpha]_D^{23.0}$ = + 3.2 (c. 1.0, $CHCl_3$) for >99% *ee*];

HRMS (+ESI) *m/z* found $[M+H]^+$ 402.2068, $[C_{26}H_{28}NO_3]^+$ requires 402.2064, (δ = + 1.0 ppm);

The spectroscopic and spectrophotometric data are in agreement with that reported in the literature.[75]

All remaining styrenyl aziridines were tentatively assigned the same absolute stereochemistry by analogy with the two results above

2) Trans-Dialkyl Aziridines

N-((2S,3R)-2-ethyltetrahydrofuran-3-yl)-4-methylbenzenesulfonamide (**SI4**)

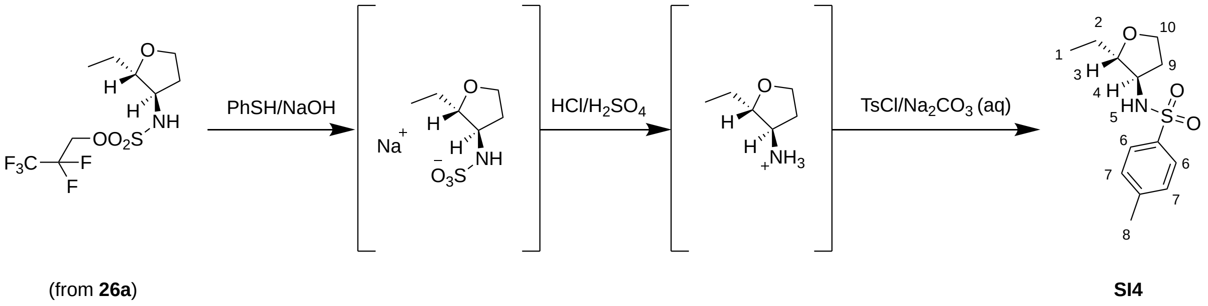

A crimp-top vial was charged with 2,2,3,3,3-pentafluoropropy ((2*S*,3*R*)-2-ethyltetrahydrofuran-3-yl)sulfamate (obtained following the cyclisation of **26a**) (40.5 mg, 0.12 mmol) and freshly-ground NaOH (24.4 mg, 0.61 mmol) following which it was sealed and then evacuated and backfilled three times with nitrogen. Following this, MeCN (3.0 mL) and thiophenol (62 µL, 0.61 mmol) were added *via* syringe and the reaction mixture stirred at 90

°C overnight. The reaction mixture was allowed to cool to room temperature and the volatiles removed under a stream of compressed air. The crude residue was taken up in $CHCl_3$ and then loaded directly onto a pre-equilibrated ($CHCl_3$) silica plug. The plug was eluted with $CHCl_3$, followed by two column volumes each of 5% v/v Acetone in $CHCl_3$ and 10% v/v Acetone in $CHCl_3$. These initial washings were then discarded, following which the plug was flushed with EtOH (5 column volumes). The ethanolic fractions were combined and the solvent removed under reduced pressure to afford the crude sodium sulfamate salt.

The crude sulfamate salt was then transferred to a crimp-top vial and the vial was three times evacuated and backfilled with nitrogen. 1.25 M HCl in MeOH (3.0 mL) was added along with 5 drops of *c*.H_2SO_4 and the reaction mixture stirred at 60 °C overnight. Following this the solvent was removed under a stream of compressed air to afford the crude ammonium salt.

To a solution of the crude ammonium salt in CH_2Cl_2 (1.0 mL) was added saturated aqueous Na_2CO_3 (1.0 mL) followed by 5 drops of 10% aqueous NaOH solution and TsCl (46.5 mg, 0.24 mmol). The biphasic mixture was stirred vigorously overnight, following which the layers were separated and the aqueous layer extracted with $CHCl_3$. The combined organic fractions were dried ($MgSO_4$) and the solvent removed under reduced pressure. Purification by flash column chromatography (SiO_2, 0-12% v/v Et_2O in CH_2Cl_2) afforded the title compound as a white amorphous solid (8.4 mg, 0.031 mmol, 26% over three steps, 89% *ee*).

***R_f* value** = 0.36 (10% v/v Acetone in $CHCl_3$);

^{1}H NMR (400 MHz, $CDCl_3$) δ 7.76 (d, *J* = 8.3 Hz, 2H, H-6), 7.32 (d, *J* = 8.1 Hz, 2H, H-7), 4.61 (d, *J* = 8.4 Hz, 1H, H-5), 3.73-3.86 (m, 2H, H-10a, H-10b), 3.47-3.55 (m, 1H, H-4), 3.39-3.43 (m, 1H, H-3), 2.44 (s, 3H, H-8), 2.01-2.08 (m, 1H, H-9a), 1.62-1.70 (m, 1H, H-9b), 1.34-1.49 (m, 2H, H-2a, H-2b), 0.83 (t, *J* = 7.4 Hz, 3H, H-1) ppm;

^{13}C NMR (101 MHz, $CDCl_3$) δ 143.8, 137.7, 130.0, 127.3, 85.5, 66.2, 57.7, 33.4, 26.2, 21.7, 10.1 ppm;

$[\boldsymbol{\alpha}]_{\mathbf{D}}^{\mathbf{25.0}}$ = – 22.5 (c. 0.8, $CHCl_3$); [reported[76] $[\boldsymbol{\alpha}]_{\mathbf{D}}^{\mathbf{20.0}}$ = – 33.1 (c. 0.8, $CHCl_3$) for >99% *ee*];

Chiral SFC Analysis CHIRAL ART SA (CO_2:MeOH, 90:10, 2.5 mL min^{-1}, 40 °C) t_R = 5.6 (minor), 10.7 (major) minutes.

The spectroscopic and spectrophotometric data are in agreement with that reported in the literature.[76]

All remaining trans-alkyl aziridines were tentatively assigned the same absolute stereochemistry by analogy with this result. The tri-alkyl aziridines were also tentatively assigned the same absolute stereochemistry by analogy with this result

3) Exo-Dialkyl Aziridines

In order to assign this class of aziridine, we first derivatised enantiopure Pregabalin to **SI9 (Figure S4)**. In a separate route, we then converted aziridine **SI12** to **SI9 (Figure S6)** and compared the optical rotations. The sign of the optical rotation for **SI9** was negative for the products obtained from both routes therefore we tentatively assign the absolute configuration for the remaining *Exo*-dialkyl aziridines as shown.

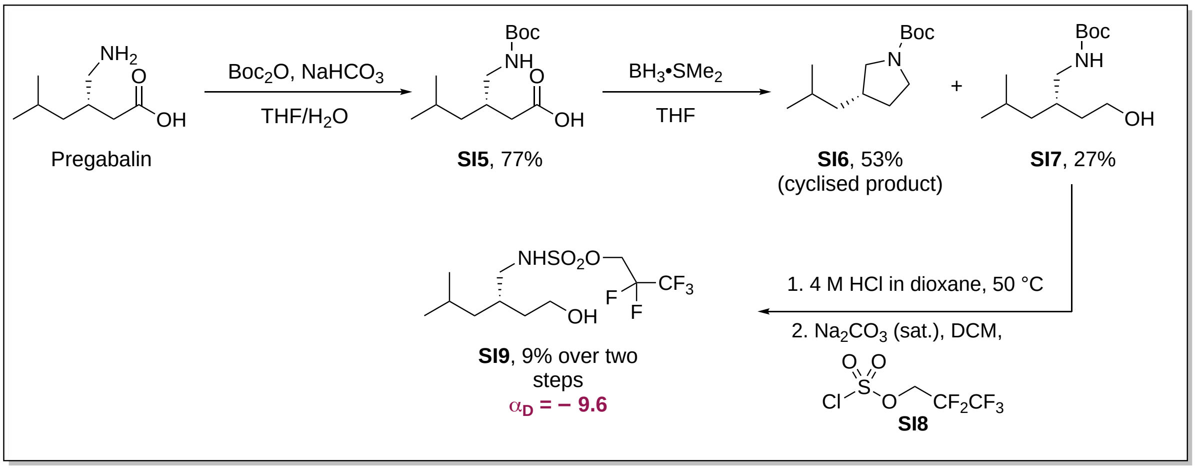

Figure S4. Synthetic route for the conversion of Pregabalin to key molecule SI9

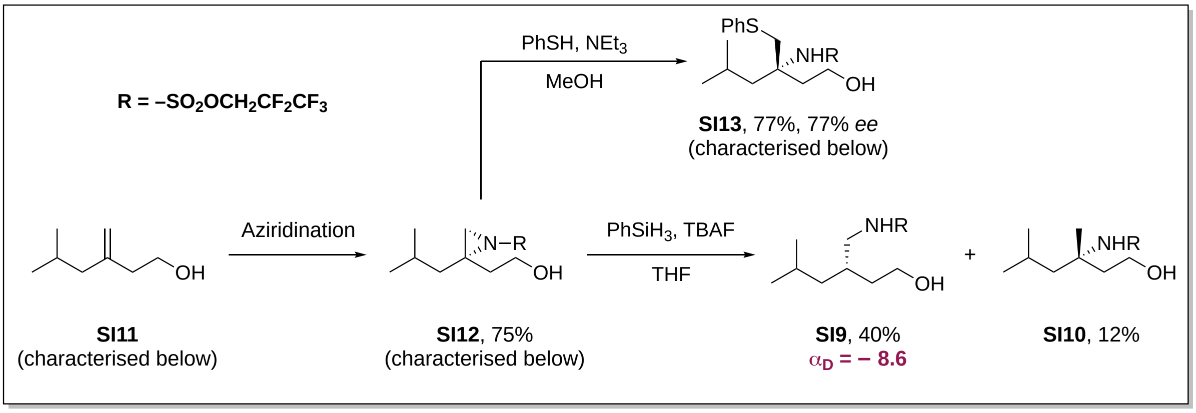

Figure S5. Synthetic route for the conversion of aziridine SI11 to key molecule SI9

(S)-3-(((tert-butoxycarbonyl)amino)methyl)-5-methylhexanoic acid (**SI5**)

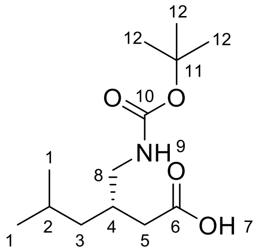

Prepared according to a variant of the protocol described by Koskinen and co-workers.[77] To a suspension of pregabalin (633.8 mg, 3.98 mmol, 1.0 equiv.) in a 1:1 mixture of THF/water (25 mL) was added $NaHCO_3$ (1.07 g. 12.7 mmol, 3.2 equiv.) portionwise at 0 ˚C. The reaction mixture was allowed to stir for 15 min before the dropwise addition of Boc_2O (1.1 mL, 4.80 mmol, 1.2 equiv.). The reaction mixture was allowed to warm to room temperature overnight. The reaction mixture was concentrated under reduced pressure and acidified with 10% citric acid solution to pH 1-2. The aqueous layer was extracted with CH_2Cl_2 and the combined organic layers were dried over $MgSO_4$. The solvent was removed *in vacuo* to give the title compound as a colourless oil (790.7 mg, 3.05 mmol, 77%), which solidfied on standing. The compound was taken forward without further purification.

***R_f* value** = 0.35 (50% v/v EtOAc in Petrol);

^{1}H NMR (400 MHz, $CDCl_3$) δ 10.73-10.53 (br, 1H, H-7), 6.26 (br, 1H, H-9), 4.77 (br, 1H, H-9, other rotamer), 3.26-3.20 (m, 1H, H-8a), 3.07-2.82 (m, 1H, H-8b), 2.36-2.09 (m, 3H, H-4, H-5a, H-5b), 1.74-1.57 (m, 1H, H-2), 1.44 (s, 9H, H-12), 1.23-1.07 (m, 2H, H-3a, H-3b), 0.89 (t, *J* = 6.7 Hz, 6H, H-1) ppm;

^{13}C NMR (101 MHz, $CDCl_3$) δ (two rotamers): 178.1 (C-6), 158.0 (C-10), 156.6 (C-10), 80.9 (C-11), 79.7 (C-11), 46.1 (C-8), 44.0 (C-8), 41.9 (C-3), 41.5 (C-3), 38.2 (C-5), 37.3 (C-5), 33.8 (C-4), 28.5 (C-12), 25.3 (C-2), 22.80 (C-1), 22.77 (C-1) ppm;

$[\alpha]_D^{25.0}$ **= – 1**0.5 (c. 1.27 $CHCl_3$) ppm;

The spectroscopic and spectrophotometric data is in agreement with that reported in the literature.[78]

Tert-butyl (S)-3-isobutylpyrrolidine-1-carboxylate (**SI6**) and *Tert-butyl (S)-(2-(2-hydroxyethyl)-4-methylpentyl)carbamate* (**SI7**)

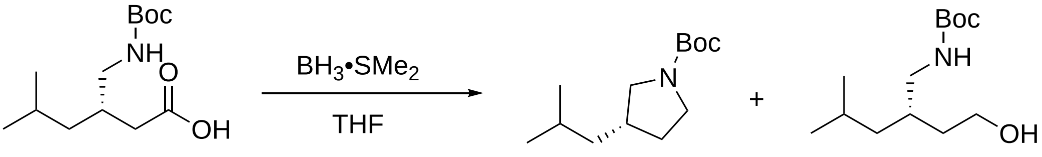

SI5 **SI6**, 53% **SI7**, 27%

To a solution of (*S*)-3-(((*tert*-butoxycarbonyl)amino)methyl)-5-methylhexanoic acid (**SI5**) (582.0 mg, 2.24 mmol, 1.0 equiv.) in THF (9 mL) was added borane dimethyl sulfide complex (1.1 mL, 11.6 mmol, 5.2 equiv.) dropwise at 0 ˚C. The reaction mixture was allowed to warm to room temperature overnight. The reaction mixture was cooled to 0 ˚C and quenched with 10% acetic acid in MeOH (10 mL). The reaction mixture was concentrated under a stream of nitrogen and diluted with water and EtOAc. The layers were separated and the aqueous layer was extracted with EtOAc. The combined organic layers were dried over $MgSO_4$ and

concentrated *in vacuo*. Purification by flash column chromatography (SiO_2, 0-50% v/v EtOAc in Petrol) afforded **SI6** as a colourless oil (271 mg, 1.19 mmol, 53%) and **SI7** as a yellow oil (148.5 mg, 0.605 mmol, 27%).

Tert-butyl (S)-3-isobutylpyrrolidine-1-carboxylate (**SI6**)

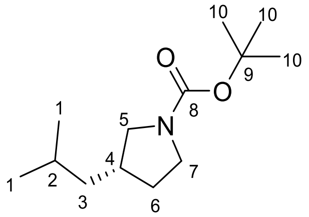

***R_f* value** = 0.50 (20% v/v EtOAc in Petrol);

^{1}H NMR (400 MHz, $CDCl_3$) δ 3.50 (dd, J = 10.5, 7.3 Hz, 1H, H-5a), 3.45-3.39 (m, 1H, H-7a), 3.24-3.18 (m, 1H, H-7b), 2.79 (dd, J = 10.5, 9.0 Hz, 1H, H-5b), 2.22-2.10 (m, 1H, H-4), 1.97-1.90 (m, 1H, H-6a), 1.56 (sep, J = 6.8 Hz, 1H, H-2), 1.47-1.37 (m, 10H, H-6b, H-10), 1.23 (t, J = 7.2 Hz, 2H, H-3a, H-3b), 0.89 (d, J = 6.6 Hz, 3H, H-1a), 0.87 (d, J = 6.6 Hz, 3H, H-1b) ppm;

^{13}C NMR (101 MHz, $CDCl_3$) δ 154.7 (C-8), 79.0 (C-9), 51.7 (C-5), 45.7 (C-7), 42.7 (C-3), 36.7 (C-4), 31.9 (C-6), 28.7 (C-10), 26.8 (C-2), 22.9 (C-1a), 22.8 (C-1b) ppm;

IR (film, cm^{-1}): ν_{max} 2957 (w), 2870 (w), 1694 (s), 1454 (w), 1399 (s), 1365 (m), 1256 (w), 1169 (m), 1141 (m), 1110 (s), 981 (w), 881 (m), 771 (m);

$[\alpha]_D^{25.0}$ **=** – 31.4 (c. 1.22 $CHCl_3$);

HRMS (+ESI) *m/z* found $[M-C_5H_8O_2+H]^+$ 128.1433, $[C_8H_{18}N]^+$ requires 128.1434, (δ = – 0.8 ppm).

Tert-butyl (S)-(2-(2-hydroxyethyl)-4-methylpentyl)carbamate (**SI7**)

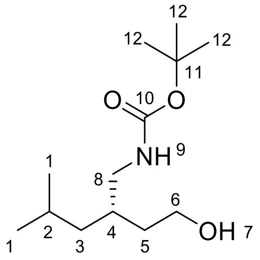

R

R_f value = 0.30 (40% v/v EtOAc in Petrol);

^{1}H NMR (400 MHz, $CDCl_3$) δ 3.77-3.64 (m, 2H, H-6a, H-6b), 3.13-3.03 (m, 2H, H-8a, H-8b), 1.75-1.61 (m, 2H, H-2, H-4), 1.59-1.47 (m, 2H, H-5a, H-5b), 1.43 (s, 9H, H-12), 1.17-1.05 (m, 2H, H-3a, H-3b), 0.87 (t, *J* = 6.3 Hz, 6H, H-1) ppm;

^{13}C NMR (101 MHz, $CDCl_3$) δ 156.6 (C-10), 79.5 (C-11), 60.9 (C-6), 44.3 (C-8), 42.1 (C-3), 34.9 (C-5), 33.7 (C-4), 28.5 (C-12), 25.4 (C-2), 23.0 (C-1a), 22.9 (C-1b) ppm;

$[\alpha]_D^{25.0}$ = + 0.6 (c. 1.07 $CHCl_3$).

The spectroscopic and spectrophotometric data are in agreement with that reported in the literature.[79]

2,2,3,3,3-pentafluoropropyl sulfurochloridate (**SI8**)

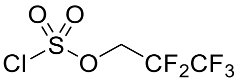

Sulfuryl chloride (0.1 mL, 1.24 mmol, 1.2 equiv.) was added dropwise to a stirred solution of 2,2,3,3,3-pentafluoro-1-propanol (0.1 mL, 1.00 mmol, 1.0 equiv.) in Et_2O (2 mL) at – 20 ˚C. The reaction mixture was warmed to room temperature and stirred for an additional 2.5 hours. The reaction was slowly quenched with water (0.3 mL) and the aqueous layer was extracted with Et_2O. The combined organic layers were dried over $MgSO_4$ and concentrated *in vacuo*. The crude material was taken forward to the next step immediately.

2,2,3,3,3-pentafluoropropyl (S)-(2-(2-hydroxyethyl)-4-methylpentyl)sulfamate (**SI9**)

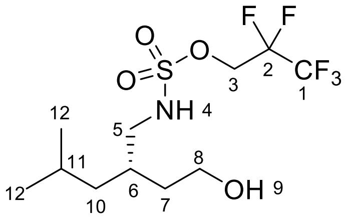

To a solution of *tert*-butyl (*S*)-(2-(2-hydroxyethyl)-4-methylpentyl)carbamate (**SI7**) (30.5 mg, 0.12 mmol, 1.0 equiv.) in 1,4-dioxane (1 mL) was added 4 M HCl in dioxane (0.63 mL, 2.52 mmol, 21 equiv.). The reaction mixture was heated at 50 ˚C for 1 hour and concentrated *in vacuo*. The crude residue was reconstituted in a biphasic mixture of CH_2Cl_2 (1.5 mL) and saturated aqueous Na_2CO_3 (1.5 mL) with 5 drops of 10% aqueous NaOH solution. A stock solution of 2,2,3,3,3-pentafluoropropyl sulfurochloridate (0.41 mL, 0.36 M in CH_2Cl_2, 0.15 mmol, 1.2 equiv.) was added to the reaction mixture, which was stirred vigorously for 42 hours. The aqueous layer was extracted with CH_2Cl_2 and the combined organic layers dried over $MgSO_4$ and concentrated *in vacuo*. Purification by flash column chromatography (SiO_2, 0-15% v/v Acetone in $CHCl_3$) afforded the title compound as a yellow oil (3.8 mg, 0.0106 mmol, 9% over two steps).

^{1}H NMR (500 MHz, $CDCl_3$) δ 6.50 (br, 1H, H-4), 4.45 (td, J = 12.6, 0.9 Hz, 2H, H-3a, H-3b), 3.84-3.80 (m, 1H, H-8a), 3.70-3.66 (m, 1H, H-8b), 3.21 (d, J = 12.8 Hz, 1H, H-5a), 2.96 (dd, J = 12.5, 8.5 Hz, 1H, H-5b), 2.10 (br, 1H, H-9), 1.86-1.79 (m, 1H, H-6), 1.71-1.65 (m, 1H, H-7a), 1.65-1.60 (m, 1H, H-11), 1.57-1.50 (m, 1H, H-7b), 1.22-1.10 (m, 2H, H-10a, H-10b), 0.89 (d, J = 6.6 Hz, 3H, H-12a), 0.88 (d, J = 6.6 Hz, 3H, H-12b). The ^{1}H NMR data is in agreement with the data obtained for fully-characterised **SI9** which was obtained from the aziridine product according to the route shown in **Figure S5** (see below).

$[\alpha]_D^{25.0}$ = – 9.6 (c. 0.35 $CHCl_3$). The optical rotation is the same sign as for product **SI9** obtained from aziridine **SI12** (see below).

5-methyl-3-methylenehexan-1-ol (**SI11**)

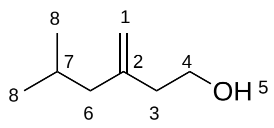

Prepared according to **GP8** using 2-iodopropane (1.0 mL, 10.0 mmol, 1.0 equiv.) as the electrophile. Purification by flash column chromatography (SiO_2, 0-3% v/v Et_2O in CH_2Cl_2) afforded the title compound as a pale-yellow oil (280.4 mg, 2.19 mmol, 22%).

***R_f* value** = 0.38 (10% v/v Et_2O in CH_2Cl_2);

^{1}H NMR (400 MHz, $CDCl_3$) δ 4.84 (s, 2H, H-1a, H-1b), 3.72-3.68 (m, 2H, H-4), 2.26 (t, J = 6.4 Hz, 2H, H-3), 1.91 (d, J = 7.2 Hz, 2H, H-6), 1.80-1.71 (m, 1H, H-7), 1.52 (br s, 1H, H-5), 0.88 (d, J = 6.6 Hz, 6H, H-8) ppm;

^{13}C NMR (101 MHz, $CDCl_3$) δ 145.1 (C-2), 113.0 (C-1), 60.5 (C-4), 45.7 (C-6), 38.9 (C-3), 26.2 (C-7), 22.6 (C-8) ppm;

HRMS (ESI) *The compound did not ionise.*

2,2,3,3,3-pentafluoropropyl (S)-2-(2-hydroxyethyl)-2-isobutylaziridine-1-sulfonate (**SI12**)

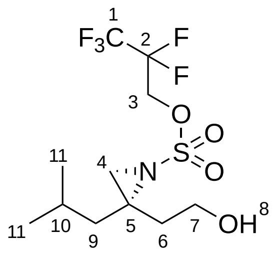

Prepared according to **GP9** using 5-methyl-3-methylenehexan-1-ol (**SI11**) (38.4 mg, 0.30 mmol, 1.0 equiv.) as the starting material. All other reagent and solvent amounts were scaled proportionally. Purification by flash column chromatography (SiO_2, 0-4% v/v Acetone in $CHCl_3$) afforded the title compound as a pale-yellow oil (79.7 mg, 0.224 mmol, 75%, 77% *ee* as determined from ring-opening of the aziridine with thiophenolate).

***R$_f$* value** = 0.36 (10% v/v Acetone in $CHCl_3$);

^{1}H NMR (500 MHz, $CDCl_3$) δ 4.69-4.59 (m, 2H, H-3a, H-3b), 3.84-3.79 (m, 2H, H-7a, H-7b), 2.60 (s, 1H, H-4a), 2.59 (s, 1H, H-4b), 2.14 (dt, *J* = 14.6, 6.1 Hz, 1H, H-6a), 1.94 (dd, *J* = 13.9, 5.2 Hz, 1H, H-9a), 1.90-1.84 (m, 1H, H-10), 1.79 (dt, *J* = 14.6, 6.5 Hz, 1H, H-6b), 1.72 (br s, 1H, H-8), 1.50 (dd, *J* = 13.7, 9.1 Hz, 1H, H-9b), 1.01 (d, *J* = 6.5 Hz, 3H, H-11a), 0.94 (d, *J* = 6.5 Hz, 3H, H-11b) ppm;

^{13}C NMR (126 MHz, $CDCl_3$) δ 118.3 (qt, $J_{C\text{-}F}$ = 286.3, 34.3 Hz, C-1), 111.4 (tq, $J_{C\text{-}F}$ = 257.5, 38.2 Hz, C-2), 65.5 (t, $J_{C\text{-}F}$ = 28.8 Hz, C-3), 59.3 (C-7), 51.1 (C-5), 42.6 (C-4), 40.8 (C-9), 35.4 (C-6), 25.8 (C-10), 23.3 (C-11a), 21.9 (C-11b) ppm;

^{19}F NMR (376 MHz, $CDCl_3$) δ – 84.5, – 124.4 ppm;

IR (film, cm^{-1}): ν_{max} 2963 (br), 1470 (w), 1371 (m), 1205 (s), 1180 (s), 1108 (m), 1045 (m), 1012 (m), 832 (m), 722 (w), 681 (w);

$[\alpha]_D^{25.3}$ = + 1.2 (c. 3.66 $CHCl_3$).

HRMS (+ESI) *m/z* found $[M+H]^+$ 356.0958, $[C_{11}H_{19}F_5NO_4S]^+$ requires 356.0949, (δ = +2.5 ppm).

Conversion of **SI12** *to the thiophenolate adduct* **SI13** *for ee determination: 2,2,3,3,3-pentafluoropropyl (S)-(1-hydroxy-5-methyl-3-((phenylthio)methyl)hexan-3-yl)sulfamate*

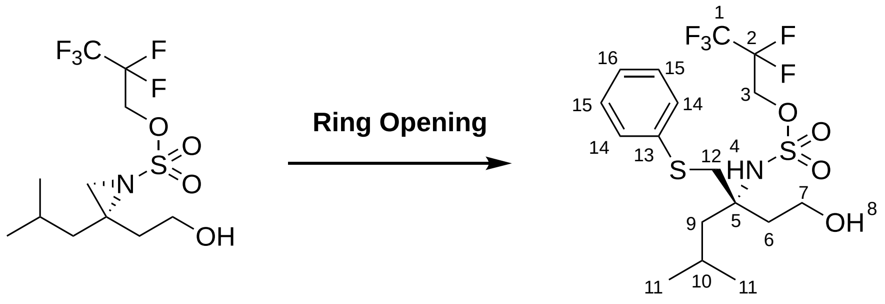

SI12 **SI13**

Prepared according to a variant of the protocol described by Du Bois and Guthikonda.[73] To a solution of 2,2,3,3,3-pentafluoropropyl (*S*)-2-(2-hydroxyethyl)-2-isobutylaziridine-1-sulfonate (**SI12**) (20.7 mg, 0.058 mmol, 1.0 equiv.) in MeOH (2.5 mL) were added thiophenol (4 drops) and Et_3N (8 drops). The reaction mixture was stirred at room temperature for 2 hours, following which the volatiles were removed under a stream of compressed air. Purification by flash column chromatography (SiO_2, 0-4% v/v Acetone in $CHCl_3$) afforded the title compound as a pale-yellow oil (20.9 mg, 0.045 mmol, 77%, 77% *ee*).

***R_f* value** = 0.37 (10% v/v Acetone in $CHCl_3$);

^{1}H NMR (500 MHz, $CDCl_3$) δ 7.38-7.36 (m, 2H, H-14), 7.31-7.28 (m, 2H, H-15), 7.23-7.20 (m, 1H, H-16), 6.32 (s, 1H, H-4), 4.50 (t, *J* = 12.6 Hz, 2H, H-3a, H-3b), 3.89 (t, *J* = 5.6 Hz, 2H, H-7a,

H-7b), 3.46 (s, 2H, H-12), 2.12-2.01 (m, 2H, H-6a, H-6b), 1.95-1.89 (m, 1H, H-9a), 1.82-1.76 (m, 2H, H-9b, H-10), 1.00 (d, J = 6.5 Hz, 3H, H-11a), 0.99 (d, J = 6.4 Hz, 3H, H-11b) ppm;

^{13}C NMR (126 MHz, $CDCl_3$) δ 136.2 (C-13), 130.0 (C-14), 129.3 (C-15), 126.8 (C-16), 118.4 (qt, $J_{C\text{-}F}$ = 286.4, 34.4 Hz, C-1), 111.7 (tq, $J_{C\text{-}F}$ = 256.9, 38.7 Hz, C-2), 64.6 (C-5), 63.8 (t, $J_{C\text{-}F}$ = 28.0 Hz, C-3), 59.4 (C-7), 44.7 (C-9), 42.7 (C-12), 37.1 (C-6), 25.0 (C-11a), 24.7 (C-11b), 23.9 (C-10) ppm;

^{19}F NMR (376 MHz, $CDCl_3$) δ – 84.4, – 124.2 ppm;

IR (film, cm^{-1}): ν_{max} 3298 (w), 2960 (w), 1584 (w), 1440 (m), 1368 (m), 1206 (s), 1181 (s), 1110 (m), 1004 (m), 775 (w), 692 (w).

$[\alpha]_D^{25.0}$ = – 3.9 (c. 1.14 $CHCl_3$).

HRMS (–ESI) *m/z* found $[M–H]^-$ 464.1007, $[C_{17}H_{23}F_5NO_4S_2]^-$ requires 464.0994, (δ = +2.8 ppm);

Chiral SFC analysis: CHIRALPAK IE (CO_2:MeOH, 97.5:2.5, 2.5 mL min^{-1}, 40 ˚C), t_R = 8.4 (minor), 9.1 (major) minutes.

2,2,3,3,3-pentafluoropropyl (S)-(2-(2-hydroxyethyl)-4-methylpentyl)sulfamate (**SI9**) and *2,2,3,3,3-pentafluoropropyl (S)-(1-hydroxy-3,5-dimethylhexan-3-yl)sulfamate* (**SI10**)

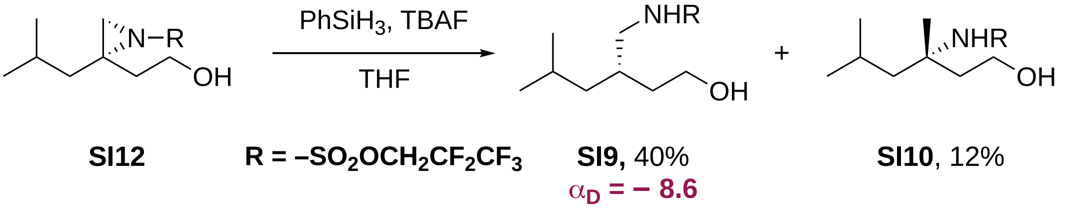

A heatgun-dried vial was charged with 2,2,3,3,3-pentafluoropropyl 2-(2-hydroxyethyl)-2-isobutylaziridine-1-sulfonate (**SI12**) (79.7 mg, 0.22 mmol, 1.0 equiv.), THF (2.2 mL) and $PhSiH_3$

(0.11 mL, 0.90 mmol, 4.0 equiv.) under an atmosphere of nitrogen. TBAF (0.45 mL, 1.0 M in THF, 0.45 mmol, 2.0 equiv.) was added dropwise and effervescence was observed. The reaction mixture was allowed to stir at room temperature overnight before diluting with THF (1.8 mL). The reaction was carefully quenched with 2 M NaOH solution (0.9 mL) and the mixture was stirred for 3 hours. The reaction mixture was concentrated under a stream of nitrogen before diluting with water. The aqueous layer was extracted with $CHCl_3$ and the combined organic layers were dried over $MgSO_4$ and concentrated *in vacuo*. Purification by flash column chromatography (SiO_2, 0-10% v/v Acetone in $CHCl_3$) afforded **SI9** as a yellow oil (31.8 mg, 0.0890 mmol, 40% yield) and **SI10** as a yellow oil (9.5 mg, 0.0266 mmol, 12%).

2,2,3,3,3-pentafluoropropyl (S)-(2-(2-hydroxyethyl)-4-methylpentyl)sulfamate (**SI9**)

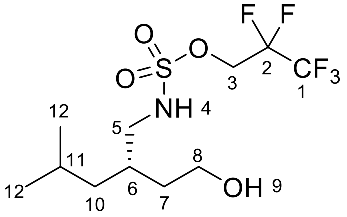

***Rf* value** = 0.43 (20% v/v Acetone in $CHCl_3$);

^{1}H NMR (500 MHz, $CDCl_3$) δ 6.50 (br, 1H, H-4), 4.45 (td, J = 12.6, 0.9 Hz, 2H, H-3a, H-3b), 3.84-3.80 (m, 1H, H-8a), 3.70-3.66 (m, 1H, H-8b), 3.21 (d, J = 12.8 Hz, 1H, H-5a), 2.96 (dd, J = 12.5, 8.5 Hz, 1H, H-5b), 2.10 (br, 1H, H-9), 1.86-1.79 (m, 1H, H-6), 1.71-1.65 (m, 1H, H-7a), 1.65-1.60 (m, 1H, H-11), 1.57-1.50 (m, 1H, H-7b), 1.22-1.10 (m, 2H, H-10a, H-10b), 0.89 (d, J = 6.6 Hz, 3H, H-12a), 0.88 (d, J = 6.6 Hz, 3H, H-12b) ppm;

^{13}C NMR (126 MHz, $CDCl_3$) δ 118.4 (qt, J_{C-F} = 286.1, 34.9 Hz, C-1), 111.6 (tq, J_{C-F} = 256.1, 38.8 Hz, C-2), 63.9 (t, J_{C-F} = 28.4 Hz, C-3), 60.9 (C-8), 48.0 (C-5), 42.2 (C-10), 35.1 (C-7), 34.4 (C-6), 25.2 (C-11), 22.7 (C-12a), 22.6 (C-12b) ppm;

^{19}F NMR (471 MHz, $CDCl_3$) δ – 83.6 (F1), – 123.5 (F2) ppm;

IR (film, cm^{-1}): ν_{max} 3332 (w), 2959 (m), 1456 (w), 1367 (m), 1262 (w), 1204 (s), 1179 (s), 1108 (m), 1045 (m), 1009 (m), 941 (w), 804 (m), 773 (m), 712 (w), 665 (m);

$[\alpha]_D^{28.0}$ = – 8.6 (c. 2.89 $CHCl_3$); [reported $[\alpha]_D^{25.0}$ = – 9.6 (c. 0.35 $CHCl_3$) from the same compound derived from pregabalin – see above];

HRMS (–ESI) *m/z* found $[M–H]^–$ 356.0958, $[C_{11}H_{19}F_5NO_4S]^–$ requires 356.0960, (δ = – 0.6 ppm).

2,2,3,3,3-pentafluoropropyl (S)-(1-hydroxy-3,5-dimethylhexan-3-yl)sulfamate (**SI10**)

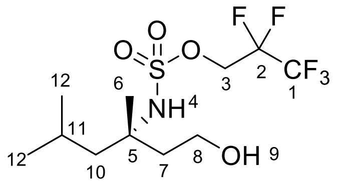

***Rf* value** = 0.43 (20% v/v Acetone in $CHCl_3$);

^{1}H NMR (500 MHz, $CDCl_3$) δ 6.26 (s, 1H, H-4), 4.46 (td, *J* = 12.6, 2.1 Hz, 2H, H-3a, H-3b), 3.94 (t, *J* = 5.7 Hz, 2H, H-8a, H-8b), 1.88 (dt, *J* = 14.9, 5.7 Hz, 1H, H-7a), 1.81 (dt, *J* = 14.9, 5.5 Hz, 1H, H-7b), 1.75-1.64 (m, 4H, H-9, H-10a, H-10b, H-11), 1.45 (s, 3H, H-6), 0.98 (d, *J* = 5.5 Hz, 6H, H-12) ppm;

^{13}C NMR (126 MHz, $CDCl_3$) δ 118.5 (qt, J_{C-F} = 285.4, 32.3 Hz, C-1), 111.8 (tq, J_{C-F} = 258.7, 38.1 Hz, C-2), 63.6 (t, J_{C-F} = 28.4 Hz, C-3), 61.2 (C-5), 59.6 (C-8), 48.4 (C-10), 41.1 (C-7), 25.00 (C-12a), 24.98 (C-12b), 24.4 (C-6), 24.2 (C-11) ppm;

^{19}F NMR (376 MHz, $CDCl_3$) δ – 84.5, – 124.2 ppm;

IR (film, cm^{-1}): v_{max} 3304 (w), 2962 (m), 1459 (w), 1367 (m), 1205 (s), 1108 (m), 1053 (m), 1005 (m), 808 (w), 774 (w), 671 (w);

$[\boldsymbol{\alpha}]_{\mathbf{D}}^{\mathbf{25.0}}$ **=** +3.0 (c. 0.79 $CHCl_3$);

HRMS (–ESI) *m/z* found $[M–H]^-$ 356.0959, $[C_{11}H_{19}F_5NO_4S]^-$ requires 356.0960, (δ = – 0.3 ppm).

Synthesis and Testing of Unsuccessful Substrates

1) Testing whether the reaction tolerates a pyridinyl-alkenol derivative given that the optimised catalyst contains two ligated pyridine molecules.

5-(pyridin-3-yl)pent-4-yn-1-ol

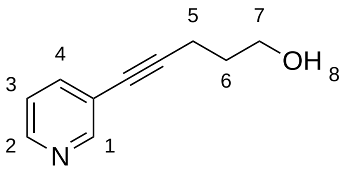

A round-bottomed flask fitted with a reflux condenser was charged with $(Ph_3P)_2PdCl_2$ (70.2 mg, 1.0 mol %) and freshly-prepared CuI (38.1 mg, 2.0 mol %). The apparatus was evacuated and backfilled with N_2 three times, following which 3-bromopyridine (0.98 mL, 10 mmol) and Et_3N (freshly distilled over CaH_2, 20 mL) were added. The reaction mixture was stirred for 15 minutes following which 4-pentyn-1-ol (1.4 mL, 15 mmol) was added and the reaction heated to reflux overnight. The reaction mixture was then allowed to cool, diluted with EtOAc (50 mL) and the organic layer was washed with water (25 mL). The aqueous layer was extracted with EtOAc and the combined organic extracts were dried ($MgSO_4$) and the solvent removed under reduced pressure. Purification by flash column chromatography (SiO_2, 0-10% v/v MeOH in CH_2Cl_2) afforded the title compound as a yellow oil (1.94 g, quant.).

***R$_f$* value** = 0.53 (10% v/v MeOH in CH_2Cl_2);

^{1}H NMR (400 MHz, $CDCl_3$) δ 8.54-8.66 (br m, 2H, H-1, H-2), 7.66 (d, *J* = 7.9 Hz, 1H, H-4), 7.23 (br s, 1H, H-3), 3.81 (t, *J* = 6.1 Hz, 2H, H-7), 2.56 (t, *J* = 7.0 Hz, 2H, H-5), 2.14 (br s, 1H, H-8), 1.83-1.90 (m, 2H, H-6) ppm;

^{13}**C NMR** (101 MHz, $CDCl_3$) δ 152.3, 148.0, 138.6, 123.3*, 121.3*, 93.4, 77.9, 61.5, 31.4, 16.1 ppm;

*Denotes a broad signal.

The spectroscopic data is in agreement with that reported in the literature.[80]

(Z)-5-(pyridin-3-yl)pent-4-en-1-ol

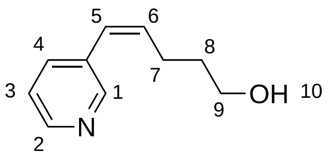

Prepared according to **GP6** using 5-(pyridin-3-yl)pent-4-yn-1-ol as the alkyne. Purification by flash column chromatography (SiO_2, 70-100% v/v EtOAc in Petrol, grading further to 0-10% v/v MeOH in EtOAc) afforded the title compound as a pale-yellow oil (234 mg, 1.4 mmol, 57%).

R*f* **value** = 0.60 (10% v/v MeOH in EtOAc);

^{1}**H NMR** (400 MHz, $CDCl_3$) δ 8.51 (d, *J* = 2.0 Hz, 1H, H-1), 8.42 (dd, *J* = 4.8 Hz, 1.5 Hz, 1H, H-2), 7.59 (dt, *J* = 7.8 Hz, 1.8 Hz, 1H, H-4), 7.24 (ddd, *J* = 7.9 Hz, 3.0 Hz, 0.5 Hz, 1H, H-3), 6.38 (dt, *J* = 11.6 Hz, 1.8 Hz, 1H, H-5), 5.81 (dt, *J* = 11.6 Hz, 7.4 Hz, 1H, H-6), 3.66 (t, *J* = 6.4 Hz, 2H, H-9), 2.40 (qd, *J* = 7.4 Hz, 1.8 Hz, 2H, H-7), 2.10 (s, 1H, H-10), 1.69-1.76 (m, 2H, H-8) ppm;

^{13}**C NMR** (101 MHz, $CDCl_3$) δ 149.9, 147.6, 135.9, 134.8, 133.3, 125.9, 123.3, 62.2, 32.8, 25.1 ppm;

HRMS (+ESI) *m/z* Found $[M+H]^+$ 164.1064, $[C_{10}H_{14}NO]^+$ requires 164.1070, (δ = – 3.6 ppm).

Attempted aziridination of (Z)-5-(pyridin-3-yl)pent-4-en-1-ol

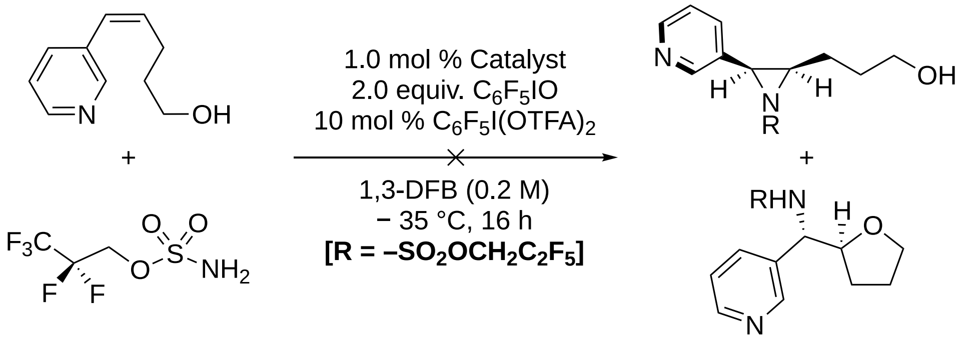

Upon subjection of (*Z*)-5-(pyridin-3-yl)pent-4-en-1-ol to **GP9** using Rh_2(**B**)$_2$•(**C3**)$_2$•(Pyr)$_2$ as the catalyst, no aziridination was observed with ^{1}H NMR analysis of the crude reaction mixture revealing only unreacted alkene and unreacted sulfamate ester. When Rh_2(**B**)$_2$•(**C3**)$_2$ was used as the catalyst under identical conditions, no aziridination was observed with ^{1}H NMR analysis of the crude reaction mixture revealing only unreacted alkene and unreacted sulfamate ester.

Conclusion: The high concentration of substrate relative to catalyst ensures that the pyridine nitrogen remains permanently bound to the axial sites and thus no rhodium nitrenoid can form.

2) Testing whether a terminal amine is tolerated in the reaction.

(Z)-N,5-diphenylpent-4-enamide

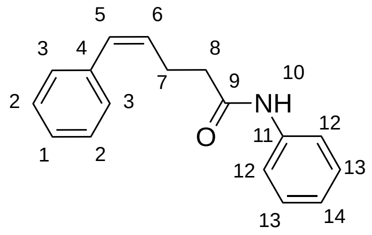

Prepared according to a variant of the protocol described by Knowles and co-workers.[81] To a heat gun-dried 50 mL round-bottomed flask, charged with anhydrous diethyl ether (10 mL), was added methyl magnesium bromide (2.0 mL, 3.0 M in diethyl ether, 6.0 mmol). To another heat-dried 25 mL round-bottomed flask, charged with anhydrous diethyl ether (10 mL), was added aniline (0.56 mL, 6.1 mmol). The aniline solution was added dropwise to the methyl magnesium bromide solution at room temperature. When evolution of gas ceased and the reaction subsided, a solution of ethyl (*Z*)-5-phenylpent-4-enoate (595 mg, 2.94 mmol, 1.0 equiv.) in diethyl ether (15 mL) was added to the reaction mixture. The reaction mixture was allowed to stir at room temperature for 2.5 hours. The reaction mixture was quenched with 1 M HCl (25 mL) and diluted with EtOAc (25 mL). The phases were separated and the aqueous layer was further extracted with EtOAc (3 x 25 mL). The combined organic layers were washed with brine, dried over $MgSO_4$ and concentrated under reduced pressure. Purification by two consecutive flash column chromatography procedures (SiO_2, 0-20% v/v Acetone in Petrol, followed by 0-30% Et_2O in Petrol) afforded the title compound as an amorphous yellow solid (282 mg, 1.1 mmol, 38%).

***R_f* value** = 0.29 (20% v/v Acetone in Petrol);

^{1}H NMR (400 MHz, $CDCl_3$) δ 7.47 (d, *J* = 8.1 Hz, 2H, H-12), 7.36-7.28 (m, 6H, H-2, H-3, H-13), 7.24-7.21 (m, 1H, H-1), 7.09 (t, *J* = 7.4 Hz, 1H, H-14), 6.52 (d, *J* = 11.5 Hz, 1H, H-5), 5.69 (dt, *J* = 11.6, 7.2 Hz, 1H, H-6), 2.78 (qd, *J* = 7.3, 1.4 Hz, 2H, H-7), 2.48 (t, *J* = 7.5 Hz, 2H, H-8) ppm;

^{13}C NMR (101 MHz, $CDCl_3$) δ 170.5 (C-9), 137.9 (C-11), 137.2 (C-4), 130.6 (C-5), 130.5 (C-6), 129.1 (C-13), 128.9 (C-3), 128.4 (C-2), 127.0 (C-1), 124.4 (C-14), 119.9 (C-12), 37.7 (C-8), 24.6 (C-7) ppm;

HRMS (–ESI): *m/z* found $[M–H]^-$ 250.1237, $[C_{17}H_{16}NO]^-$ requires 250.1237, (δ = + 0.0 ppm).

(Z)-N-(5-phenylpent-4-en-1-yl)aniline

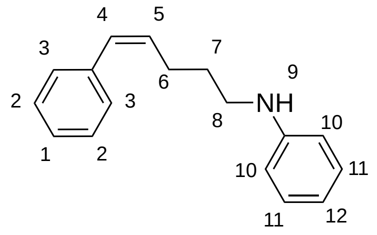

To a solution of (*Z*)-*N*,5-diphenylpent-4-enamide (196 mg, 0.74 mmol) in THF (15 mL) at 0 °C was added $LiAlH_4$ (0.77 mL of a 2.4 M solution in THF, 1.8 mmol) dropwise. The reaction mixture was stirred at the same temperature for a further 2 hours and allowed to warm to room temperature overnight. The reaction mixture was then quenched through the careful portion-wise addition of sodium sulfate decahydrate. After the effervescence had ceased, the quenched mixture was filtered, eluting with plenty of acetone. The filtrate was collected and the solvent removed under reduced pressure, following which the crude residue was taken up in EtOAc and washed with saturated aqueous $NaHCO_3$ and brine. The organic layer was dried ($MgSO_4$) and the solvent removed under reduced pressure. Purification by flash column chromatography (SiO_2, 0-12% v/v Acetone in Petrol) afforded the title compound as a yellow oil (163 mg, 0.69 mmol, 93%).

***R_f* value** = 0.49 (12% v/v Acetone in Petrol);

^{1}H NMR (400 MHz, $CDCl_3$) δ 7.33-7.37 (m, 2H, H-2), 7.22-7.29 (m, 3H, H-1, H-3), 7.14-7.19 (m, 2H, H-11), 6.69 (tt, *J* = 7.3 Hz, 1.0 Hz, 1H, H-12), 6.55-6.58 (m, 2H, H-10), 6.49 (dt, *J* = 11.6 Hz, 1.7 Hz, 1H, H-4), 5.69 (dt, *J* = 11.6 Hz, 7.3 Hz, 1H, H-5), 3.43 (br s, 1H, H-9), 3.15 (t, *J* = 7.0 Hz, 2H, H-8), 2.45 (qd, *J* = 7.4 Hz, 1.8 Hz, 2H, H-6), 1.78 (p, *J* = 7.2 Hz, 2H, H-7) ppm;

^{13}C NMR (101 MHz, $CDCl_3$) δ 148.3, 137.6, 132.0, 129.8, 129.4, 128.9, 128.4, 126.8, 117.3, 112.9, 43.4, 29.6, 26.1 ppm;

HRMS (+ESI) *m/z* Found $[M+H]^+$ 238.1588, $[C_{17}H_{20}N]^+$ requires 238.1590, (δ = – 0.8 ppm).

When this substrate was subjected to **GP9** *the reaction mixture went dark purple upon addition of the oxidant. Following the 14 hours of reaction the volatiles were removed and the reaction was analysed by ^1H NMR. This revealed almost complete consumption of the substrate and a very complex mixture of unidentified products.*

Conclusion: Amine substrates are not tolerated in the reaction.

Chiral SFC, HPLC and GC Traces

2,2,3,3,3-pentafluoropropyl ((S)-phenyl((S)-tetrahydrofuran-2-yl)methyl)sulfamate (**4a**)

Chiral SFC Analysis CHIRALPAK IH (CO_2:MeOH, 98:2, 2.5 mL min^{-1}, 40 °C, 210 nm) indicated 92% *ee*, t_R = 4.5 (minor), 6.2 (major) minutes.

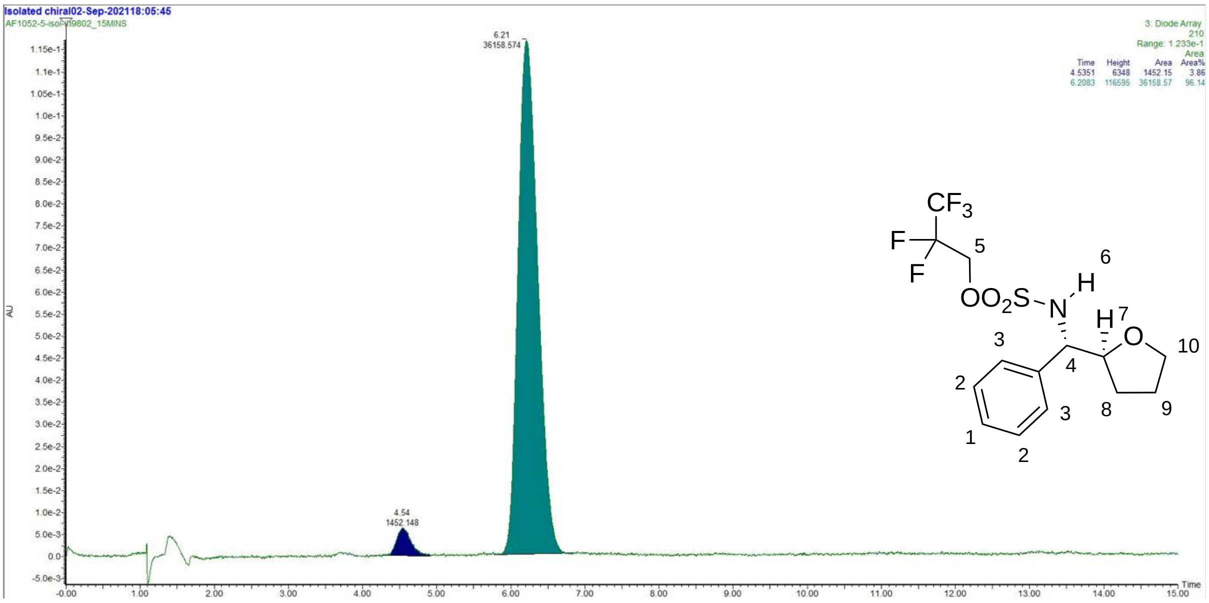

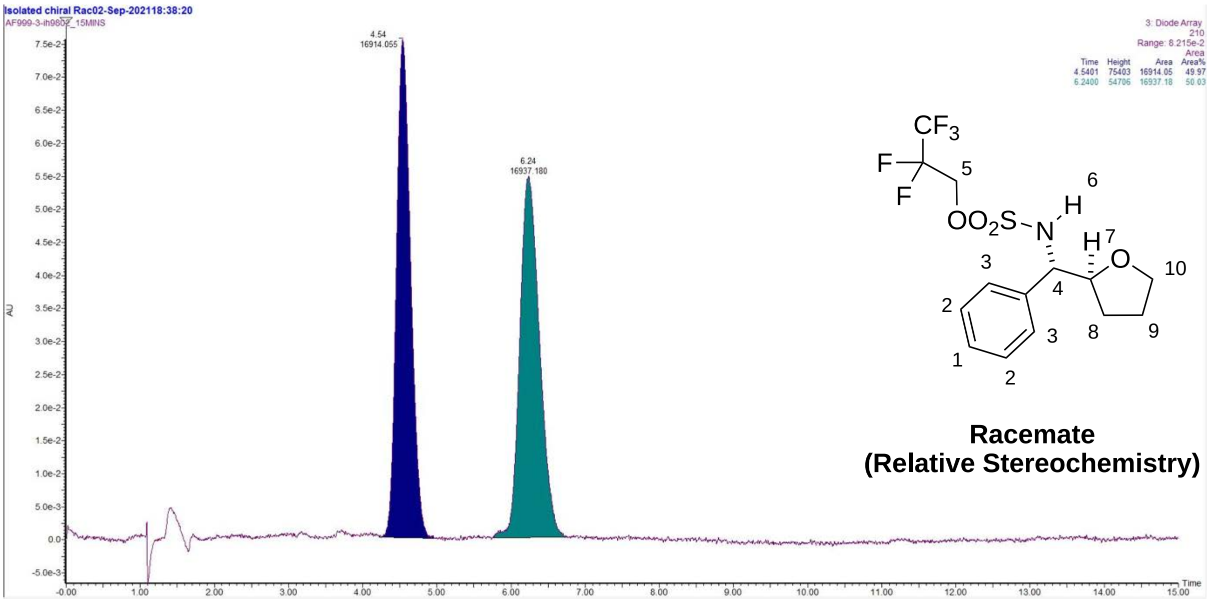

2,2,3,3,3-pentafluoropropyl ((S)-(2-fluorophenyl)((S)-tetrahydrofuran-2-yl)methyl)sulfamate (**4b**)

Chiral SFC Analysis: CHIRALPAK IH (CO_2:MeOH, 98:2, 2.5 mL min^{-1}, 40 °C, 210 nm) indicated 94% *ee*, t_R = 4.4 (minor), 6.1 (major) minutes.

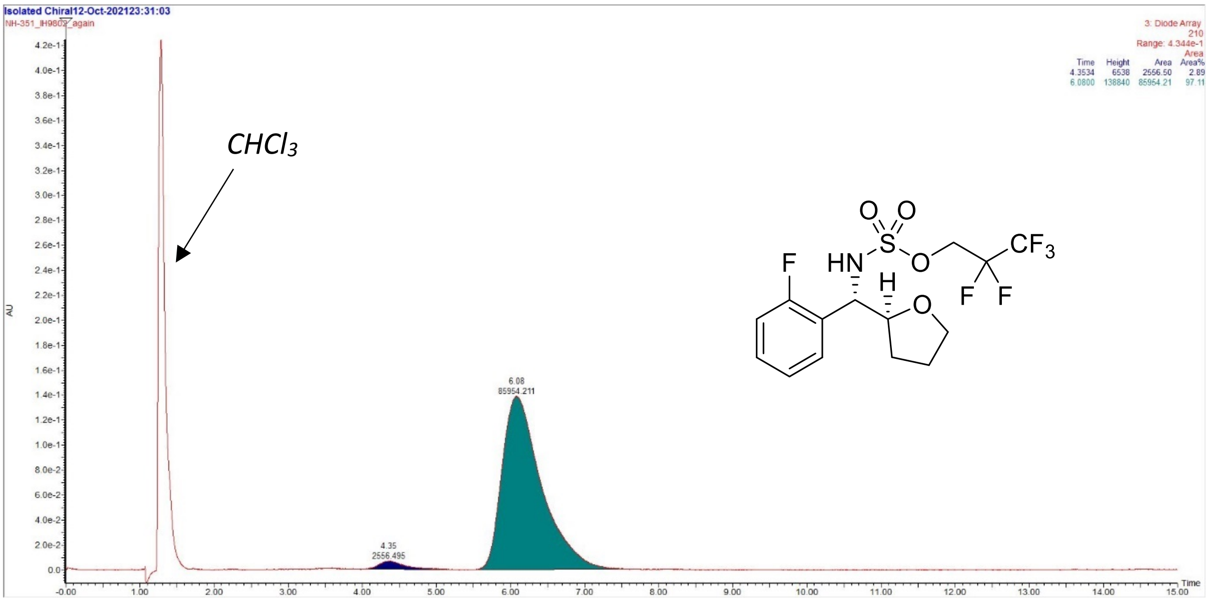

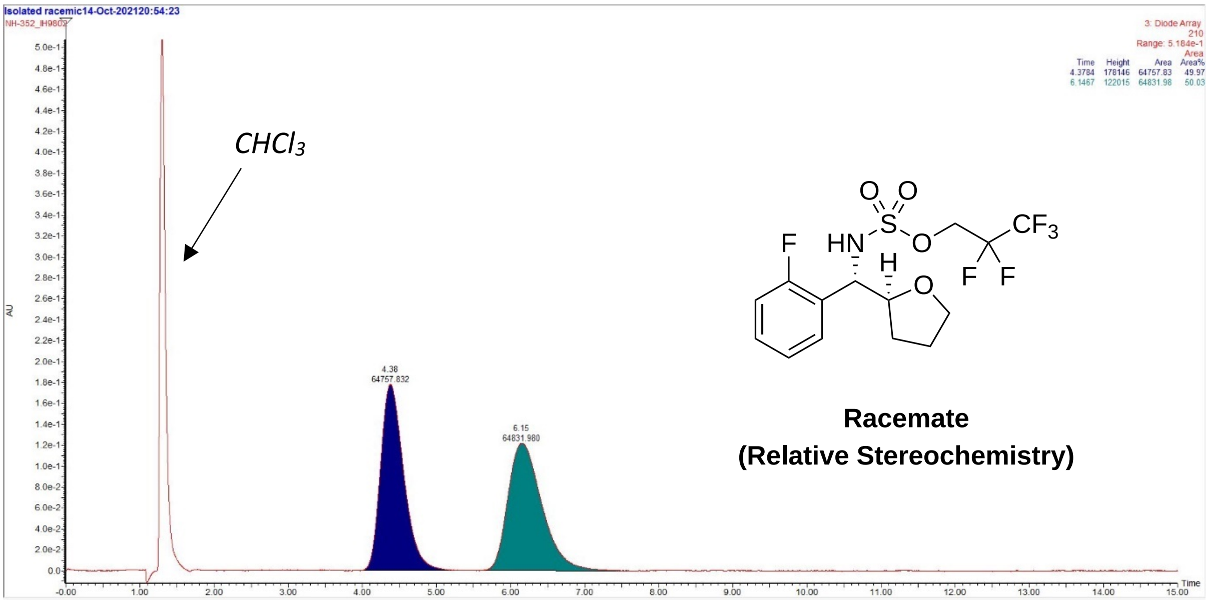

2,2,3,3,3-pentafluoropropyl ((S)-((S)-tetrahydrofuran-2-yl)(o-tolyl)methyl)sulfamate (**4c**)

Chiral SFC Analysis CHIRALPAK IH (CO_2:MeOH, 98:2, 1.25 mL min^{-1}, 40 °C, 210 nm) indicated 97% *ee*, t_R = 12.0 (minor), 14.2 (major) minutes.

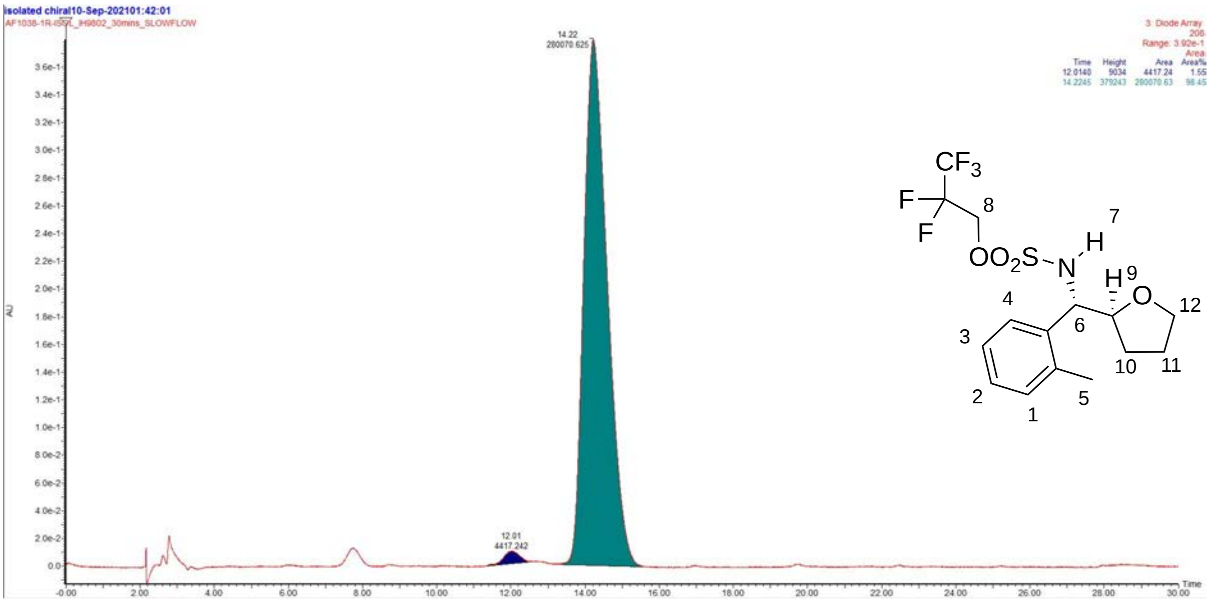

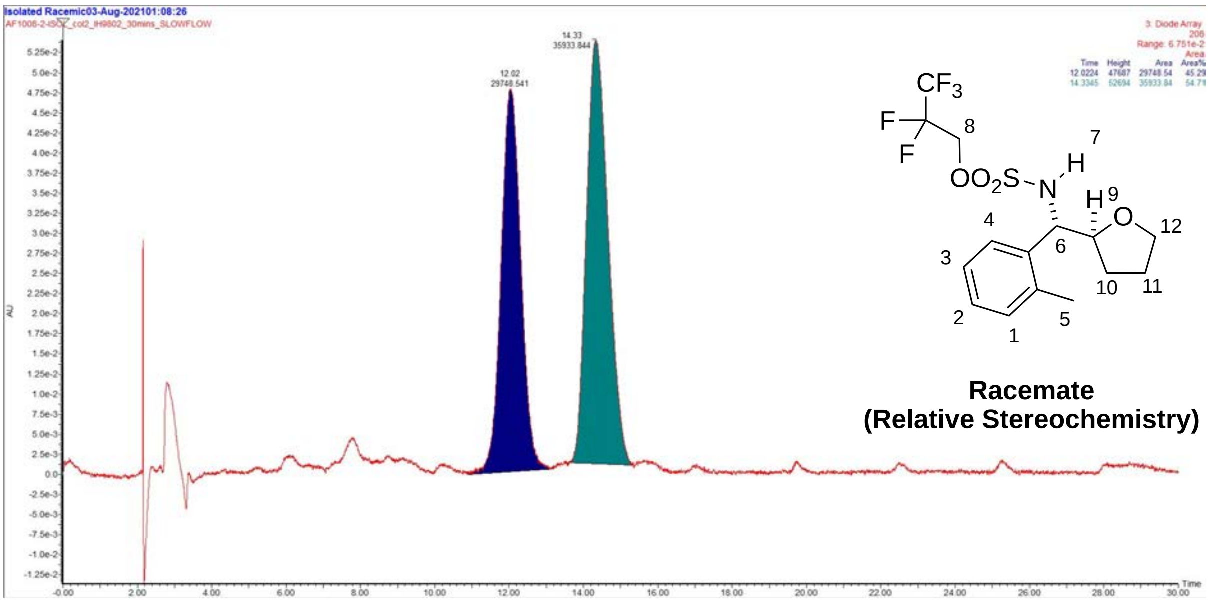

2,2,3,3,3-pentafluoropropyl ((S)-(2-methoxyphenyl)((S)-tetrahydrofuran-2-yl)methyl)sulfamate (**4d**)

Chiral SFC Analysis: CHIRALPAK IH (CO_2:MeOH, 98:2, 1.25 mL min^{-1}, 40 °C, 215 nm) indicated 95% *ee*, t_R = 11.4 (minor), 12.8 (major) minutes.

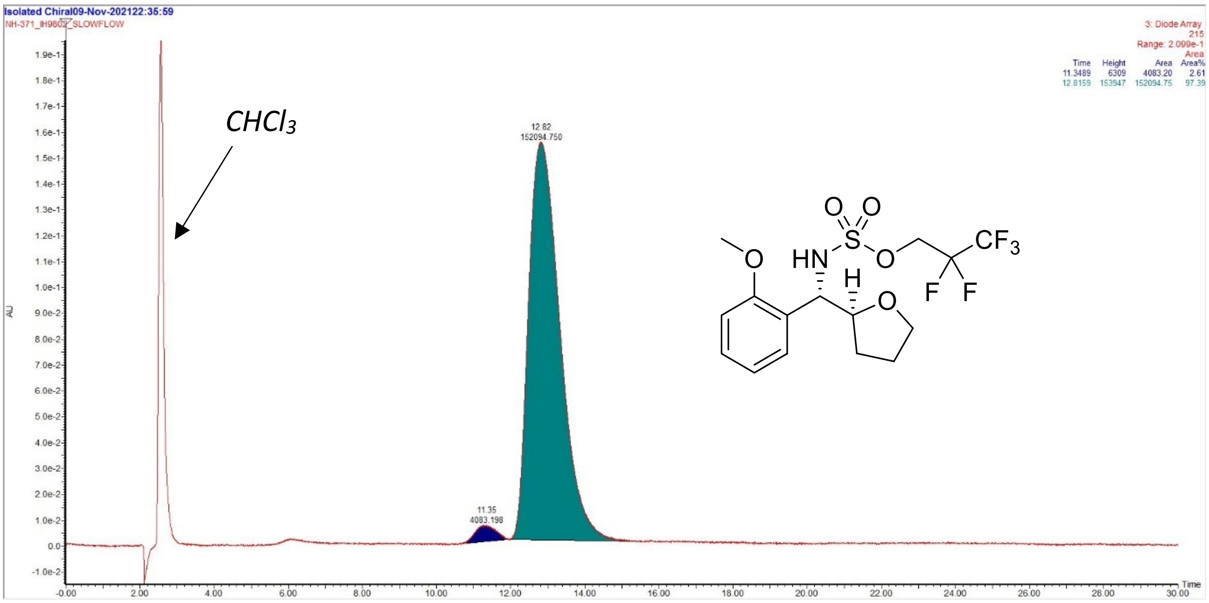

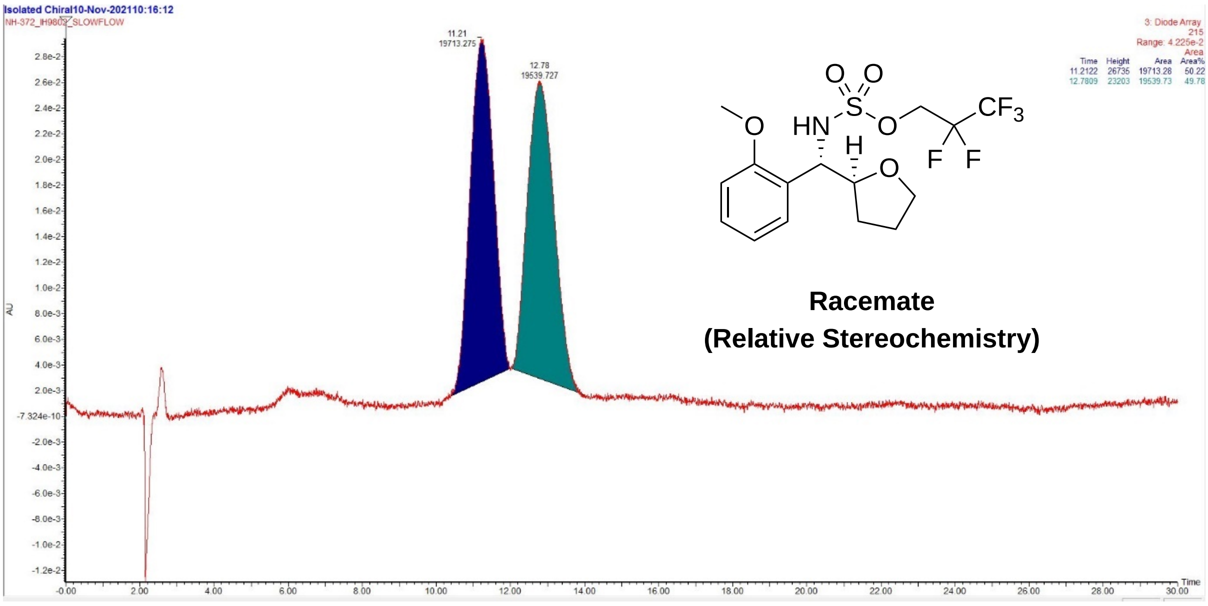

2,2,3,3,3-pentafluoropropyl ((S)-(3-fluoro-2-methylphenyl)((S)-tetrahydrofuran-2-yl)methyl)sulfamate (**4e**)

Chiral SFC Analysis CHIRALPAK IH (CO_2:MeOH, 98:2, 2.5 mL min^{-1}, 40 °C, 208 nm) indicated 94% *ee*, t_R = 4.3 (minor), 5.6 (major) minutes.

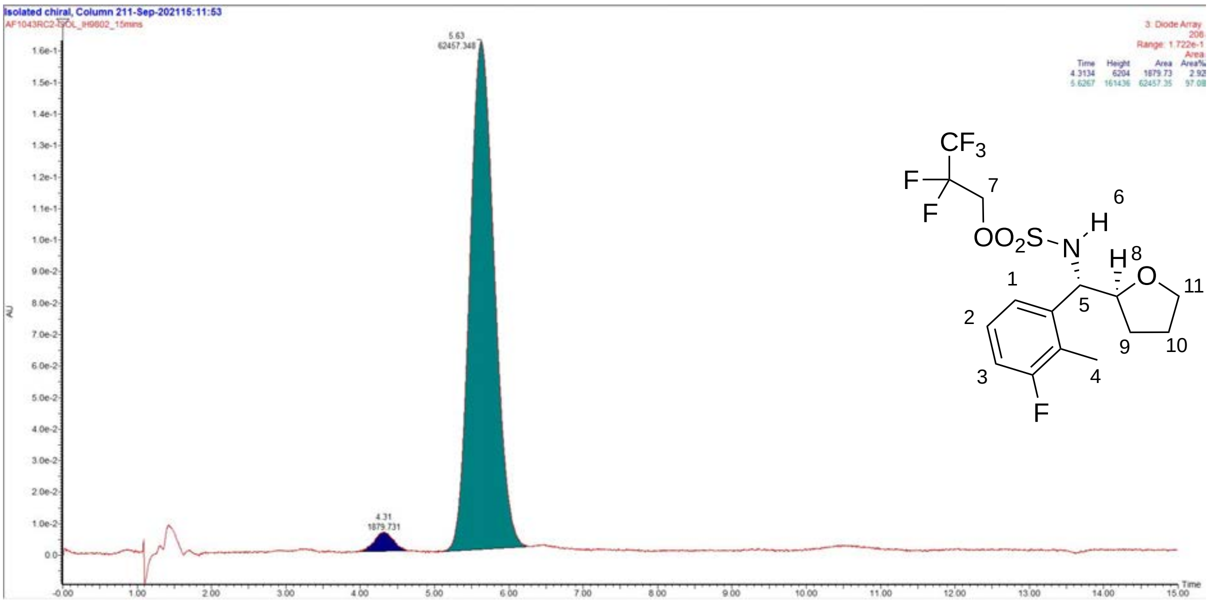

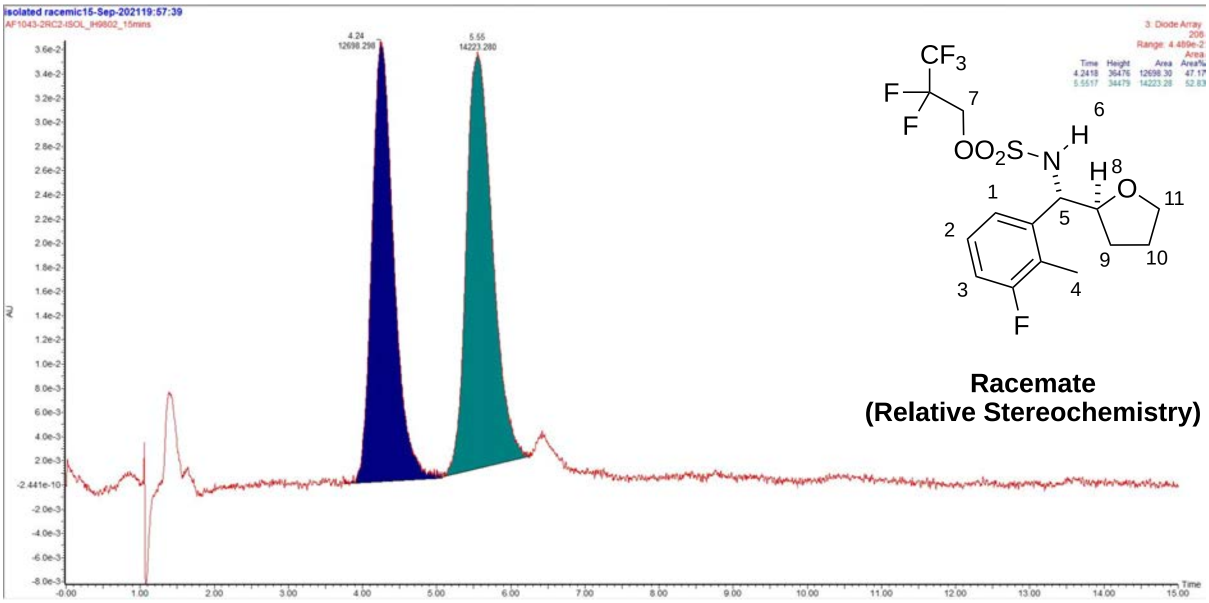

2,2,3,3,3-pentafluoropropyl ((S)-(2,5-dimethylphenyl)((S)-tetrahydrofuran-2-yl)methyl)sulfamate (**4f**)

Chiral SFC Analysis CHIRALPAK IH (CO_2:MeOH, 98:2, 2.5 mL min^{-1}, 40 °C, 212 nm) indicated 97% *ee*, t_R = 4.6 (minor), 5.5 (major) minutes.

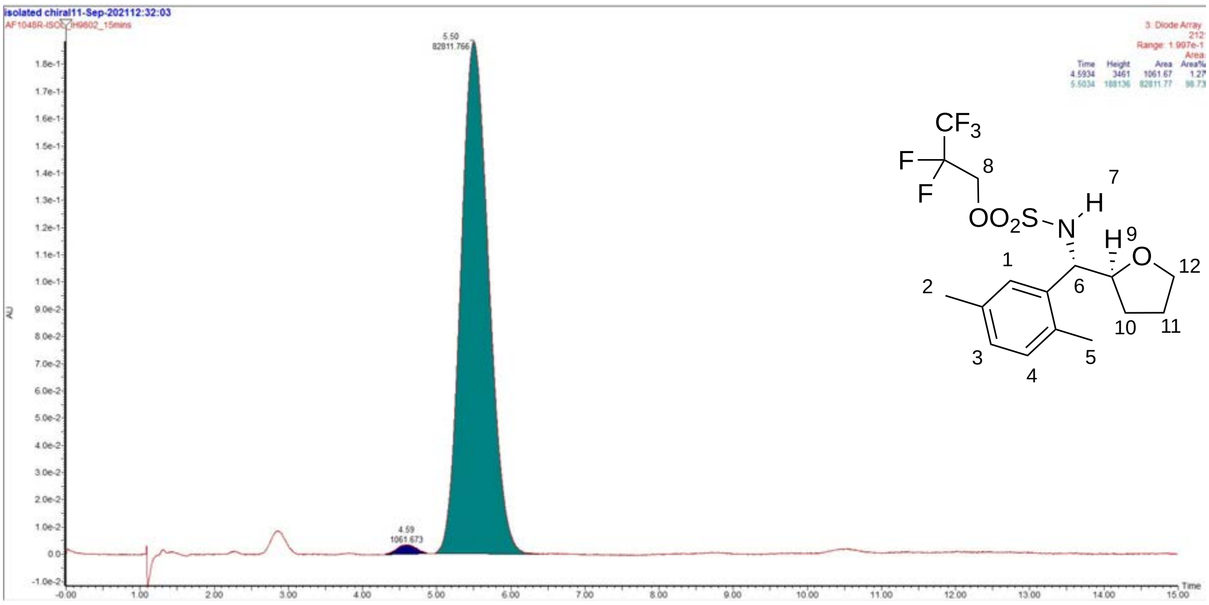

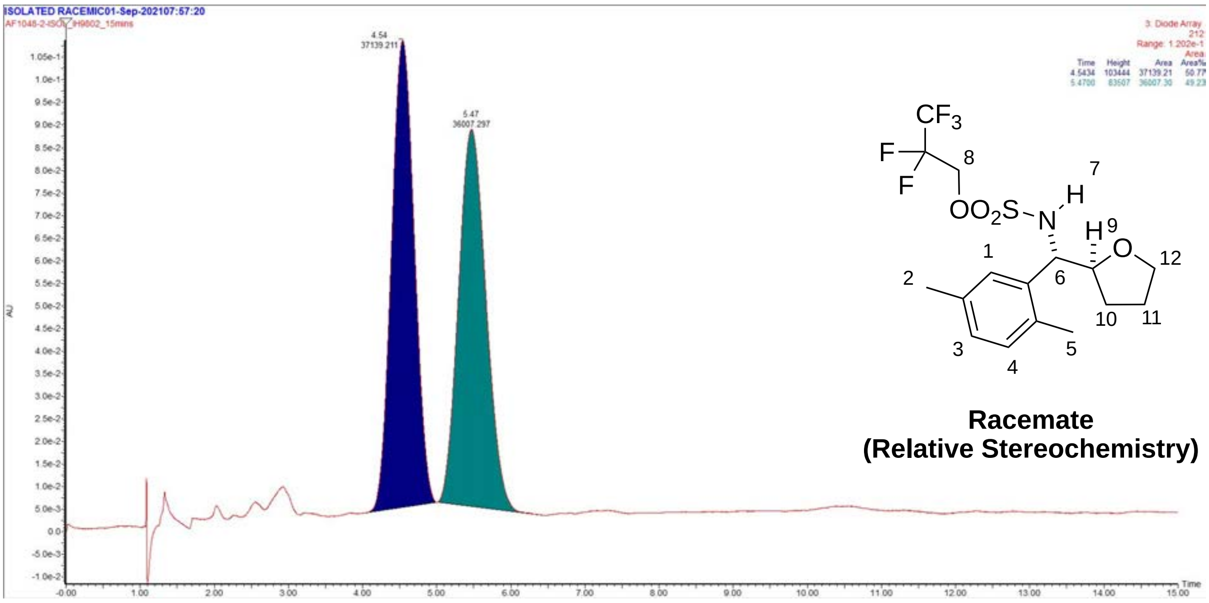

2,2,3,3,3-pentafluoropropyl ((S)-(2,3-dimethylphenyl)((S)-tetrahydrofuran-2-yl)methyl)sulfamate (**4g**)

Chiral SFC Analysis CHIRALPAK IH (CO_2:MeOH, 98:2, 2.5 mL min^{-1}, 40 °C, 210 nm) indicated 96% *ee*, t_R = 5.0 (minor), 6.6 (major) minutes.

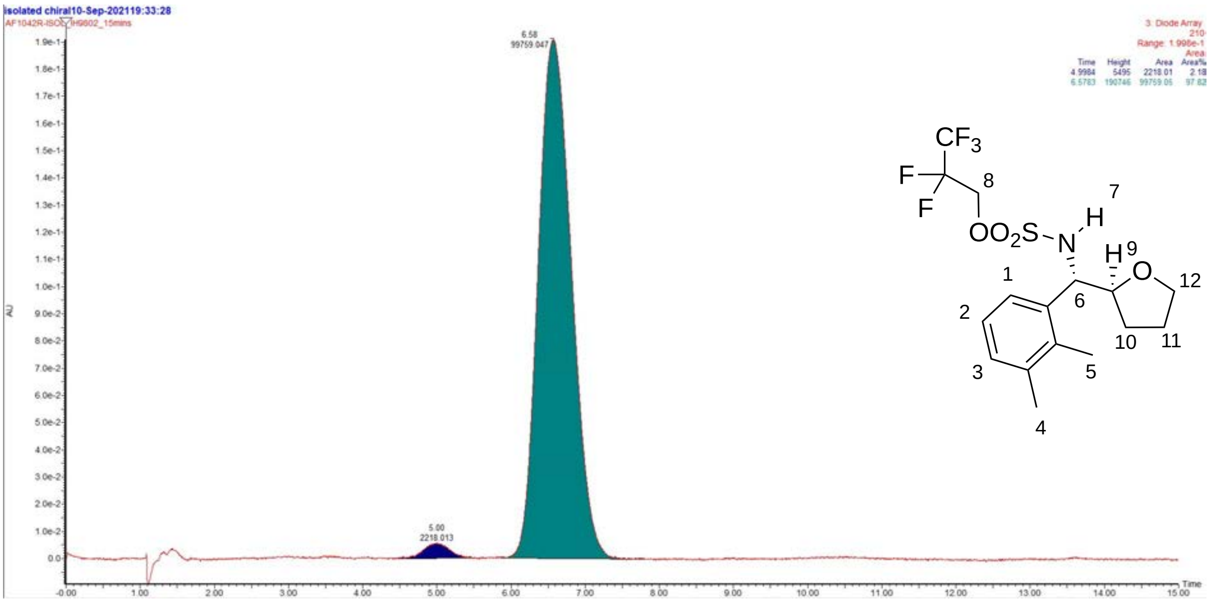

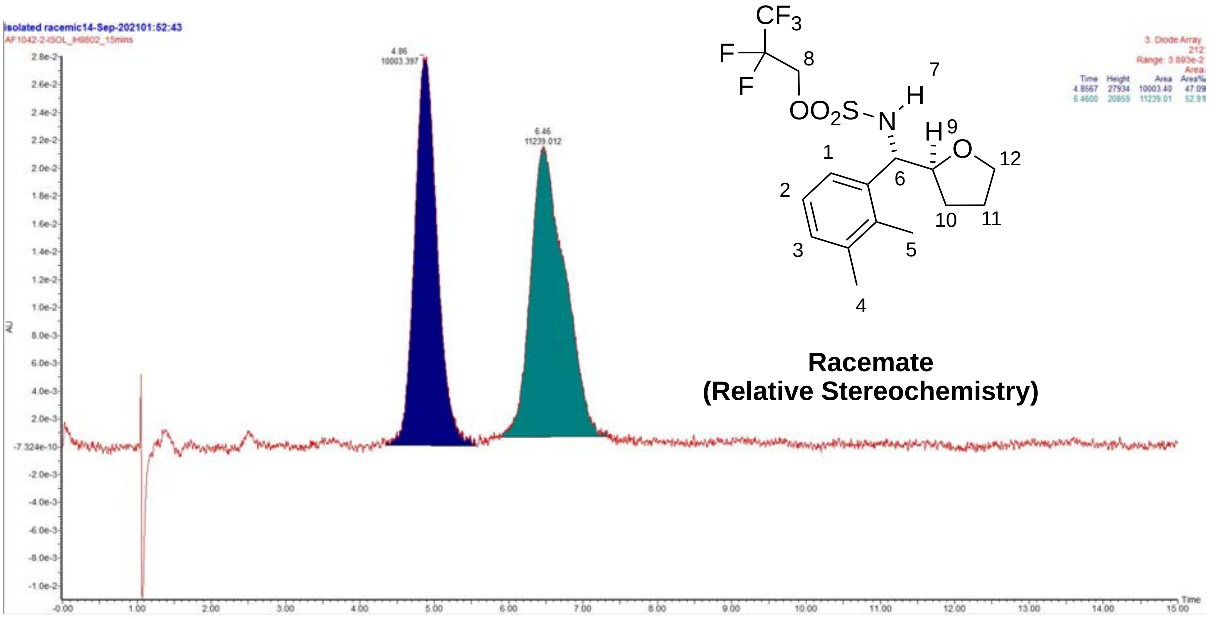

2,2,3,3,3-pentafluoropropyl ((S)-(3-fluorophenyl)((S)-tetrahydrofuran-2-yl)methyl)sulfamate (**4h**)

Chiral SFC Analysis CHIRALPAK IH (CO_2:MeOH, 98:2, 2.5 mL min^{-1}, 40 °C, 210 nm) indicated 96% *ee*, t_R = 3.7 (minor), 5.6 (major) minutes.

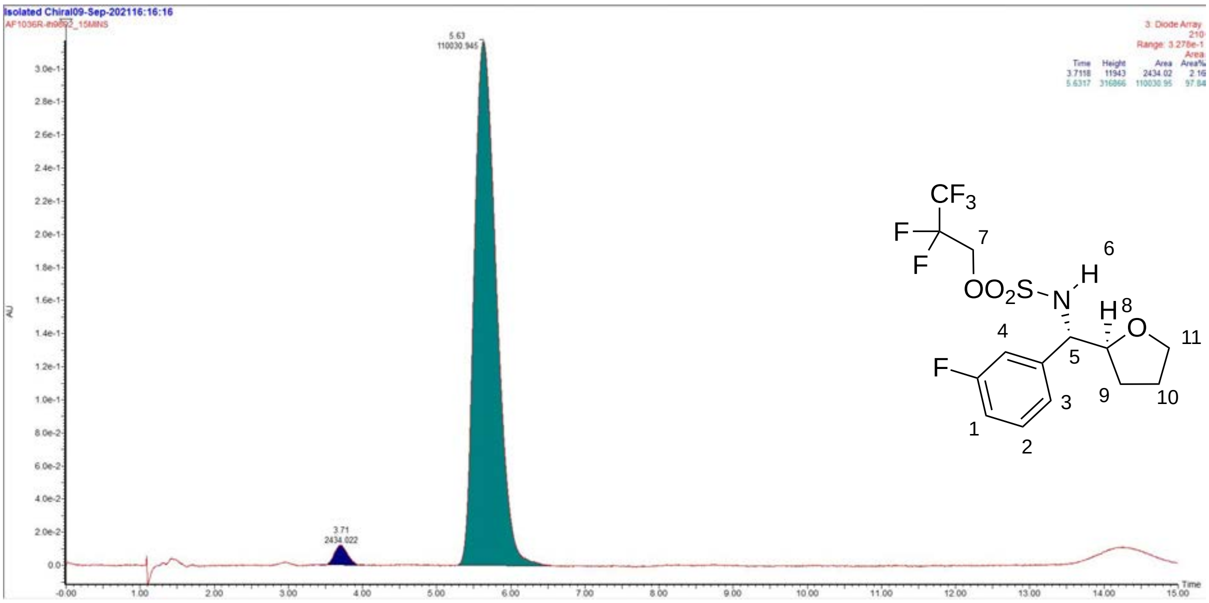

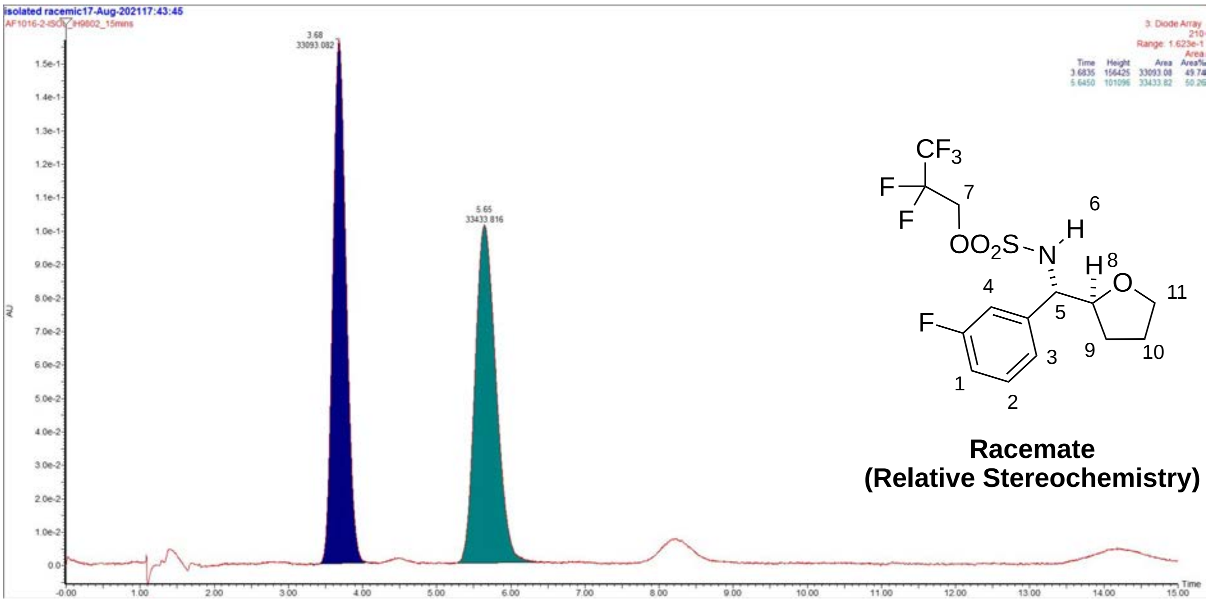

2,2,3,3,3-pentafluoropropyl ((S)-(3-chlorophenyl)((S)-tetrahydrofuran-2-yl)methyl)sulfamate (**4i**)

Chiral SFC Analysis: CHIRALPAK IH (CO_2:MeOH, 97.5:2.5, 2.5 mL min^{-1}, 40 °C, 210 nm) indicated 94% *ee*, t_R = 4.5 (minor), 7.0 (major) minutes.

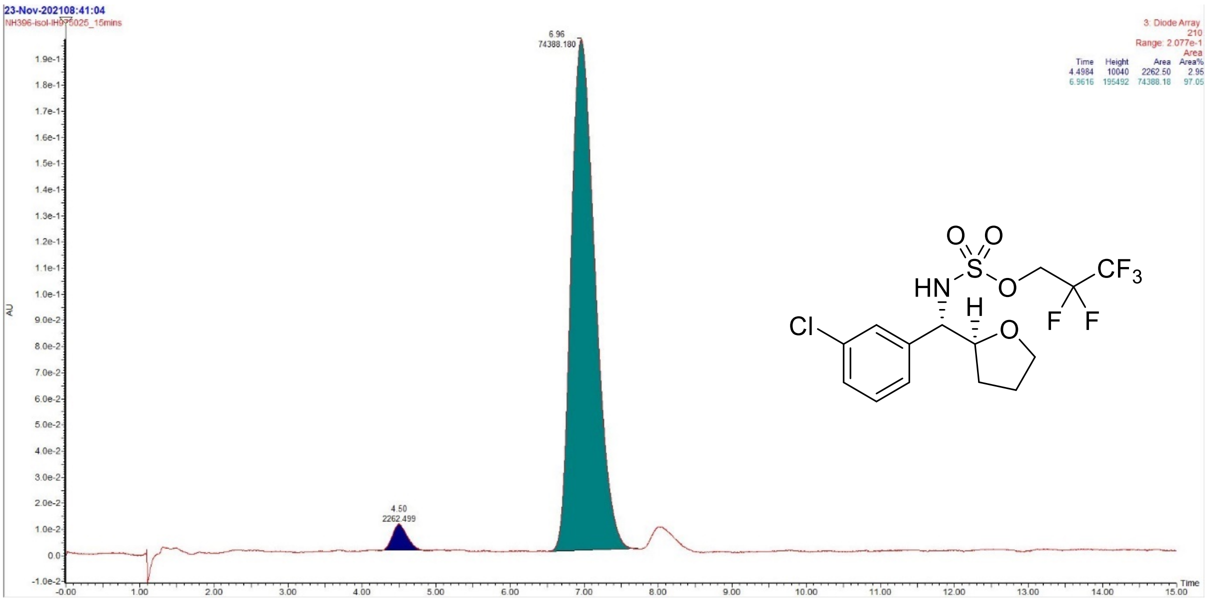

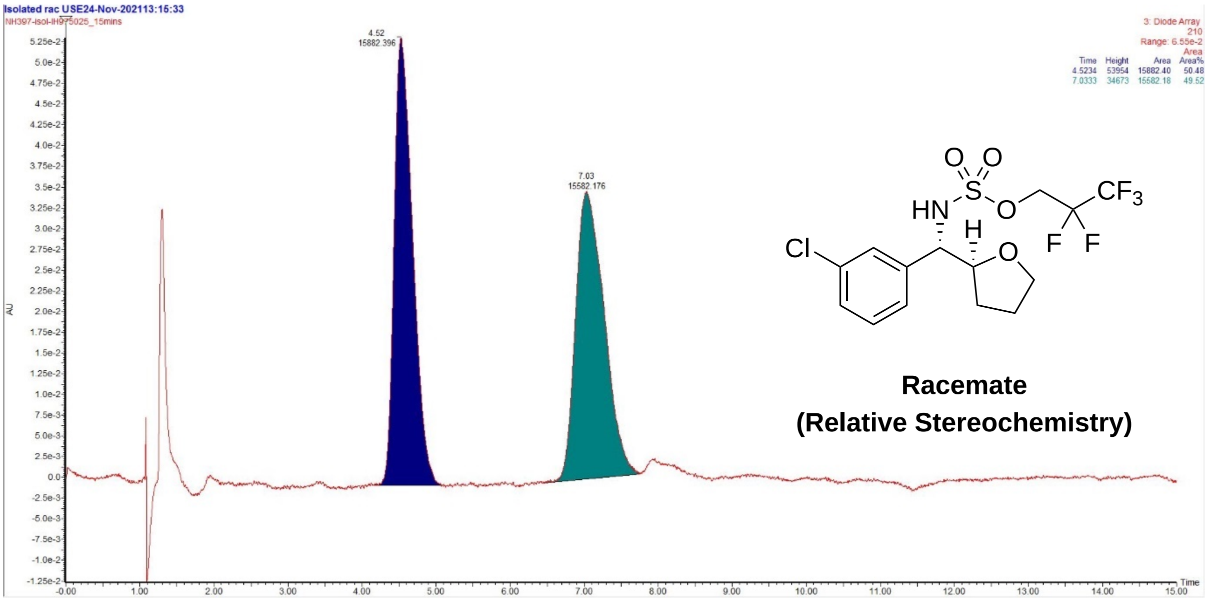

2,2,3,3,3-pentafluoropropyl ((S)-((S)-tetrahydrofuran-2-yl)(m-tolyl)methyl)sulfamate (**4j**)

Chiral SFC Analysis CHIRALPAK IH (CO_2:MeOH, 98:2, 2.5 mL min^{-1}, 40 °C, 209 nm) indicated 92% *ee*, t_R = 4.1 (minor), 5.6 (major) minutes.

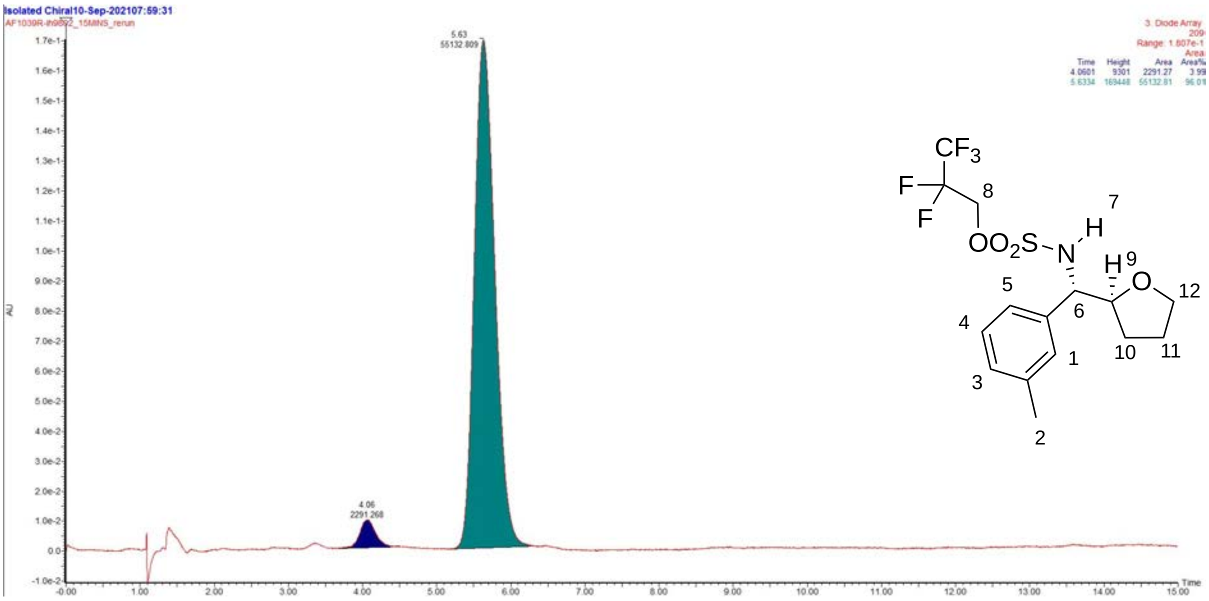

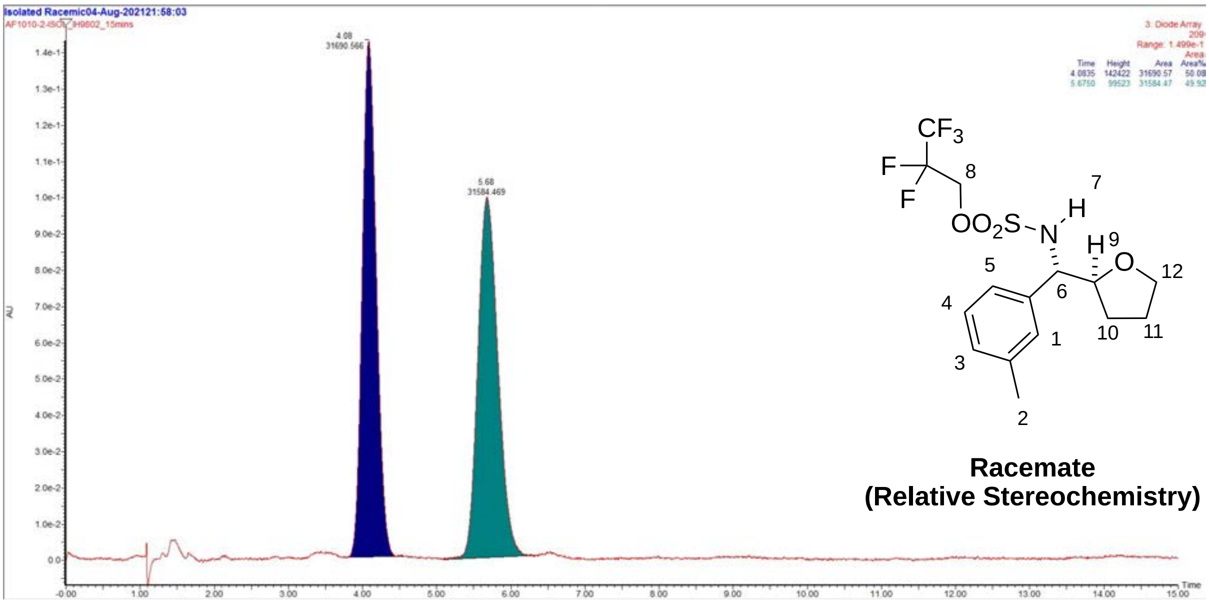

2,2,3,3,3-pentafluoropropyl ((S)-((S)-tetrahydrofuran-2-yl)(3-(trifluoromethyl)phenyl)methyl)sulfamate (**4k**)

Chiral SFC Analysis CHIRALPAK IH (CO_2:MeOH, 98:2, 1.25 mL min^{-1}, 40 °C, 210 nm) indicated 92% *ee*, t_R = 5.6 (minor), 7.8 (major) minutes.

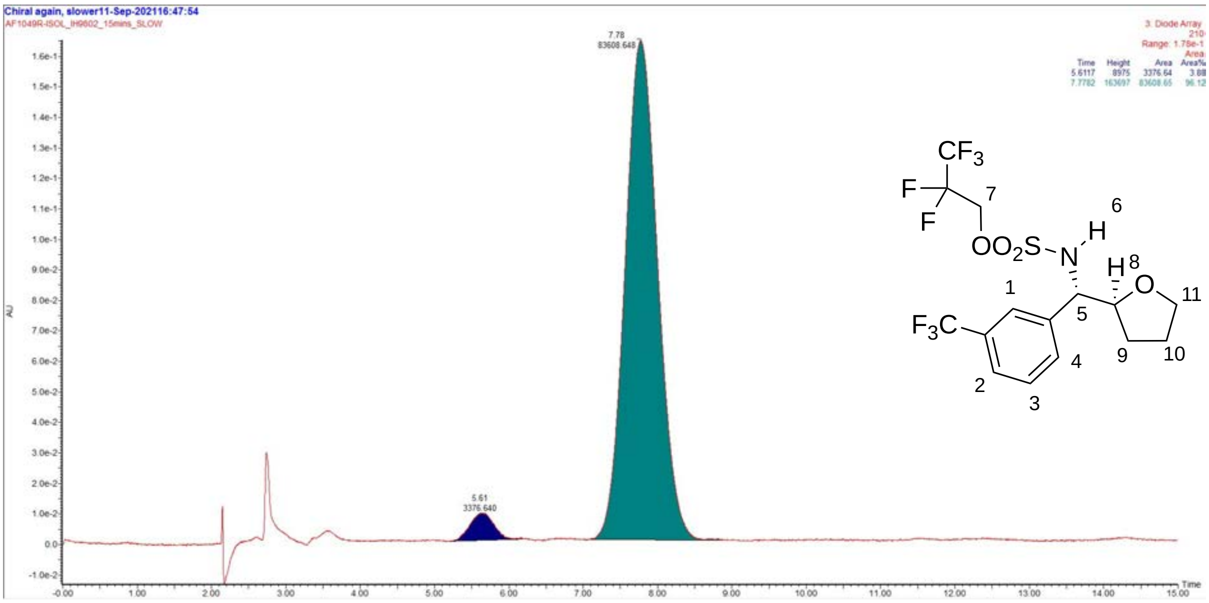

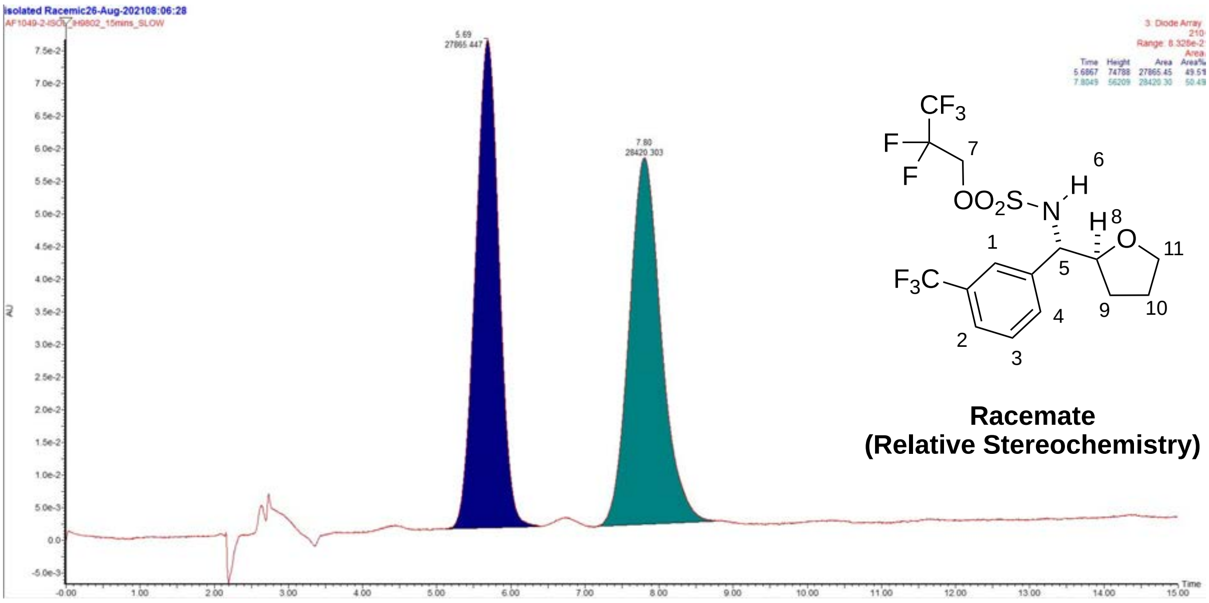

2,2,3,3,3-pentafluoropropyl ((S)-((S)-tetrahydrofuran-2-yl)(3-(trifluoromethoxy)phenyl)methyl)sulfamate (**4l**)

Chiral SFC Analysis: CHIRALPAK IH (CO_2:MeOH, 98:2, 1.25 mL min^{-1}, 40 °C, 210 nm) indicated 92% *ee*, t_R = 5.1 (minor), 7.0 (major) minutes.

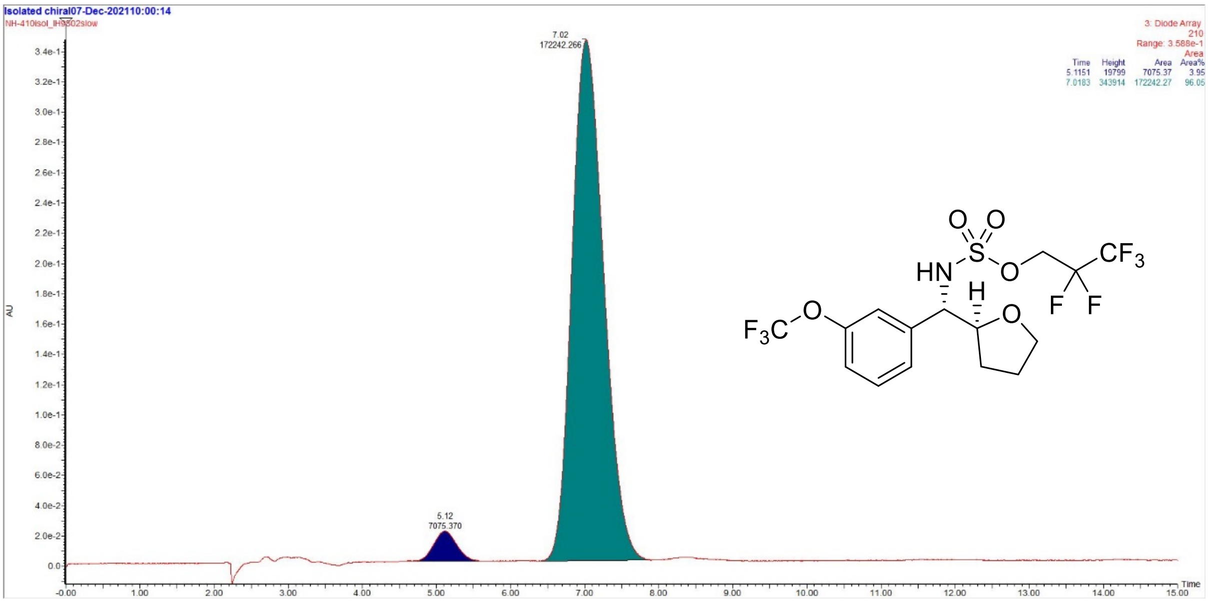

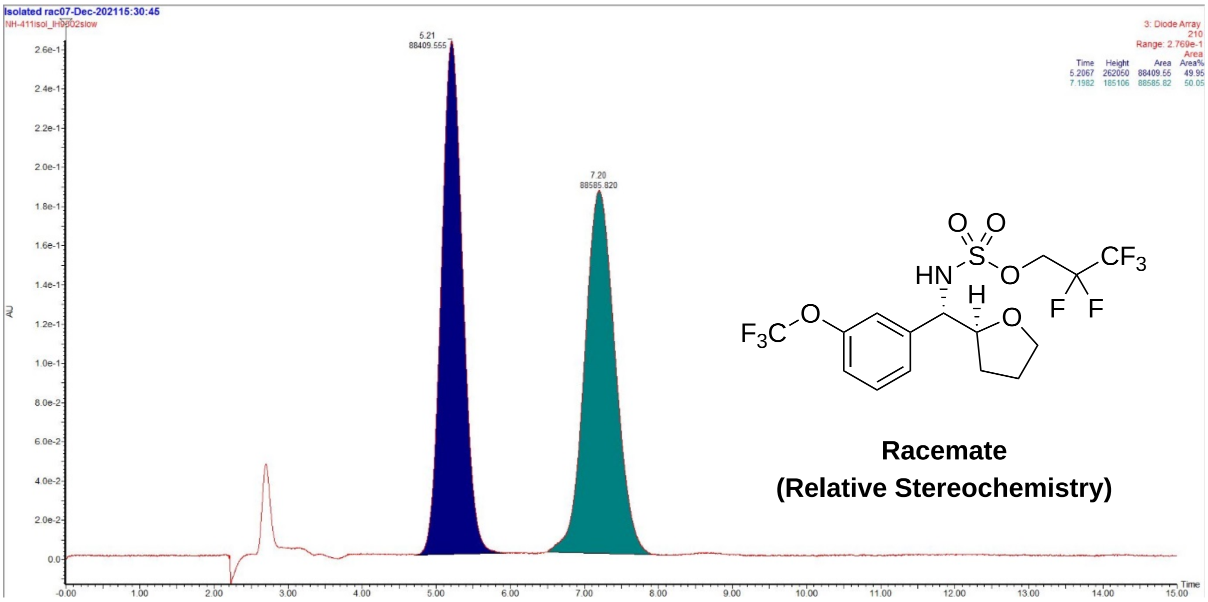

2,2,3,3,3-pentafluoropropyl ((S)-(3-acetylphenyl)((S)-tetrahydrofuran-2-yl)methyl)sulfamate (**4m**)

Chiral SFC Analysis: CHIRALPAK IH (CO_2:MeOH, 97:3, 2.5 mL min^{-1}, 40 °C, 238 nm) indicated 93% *ee*, t_R = 5.8 (minor), 7.0 (major) minutes.

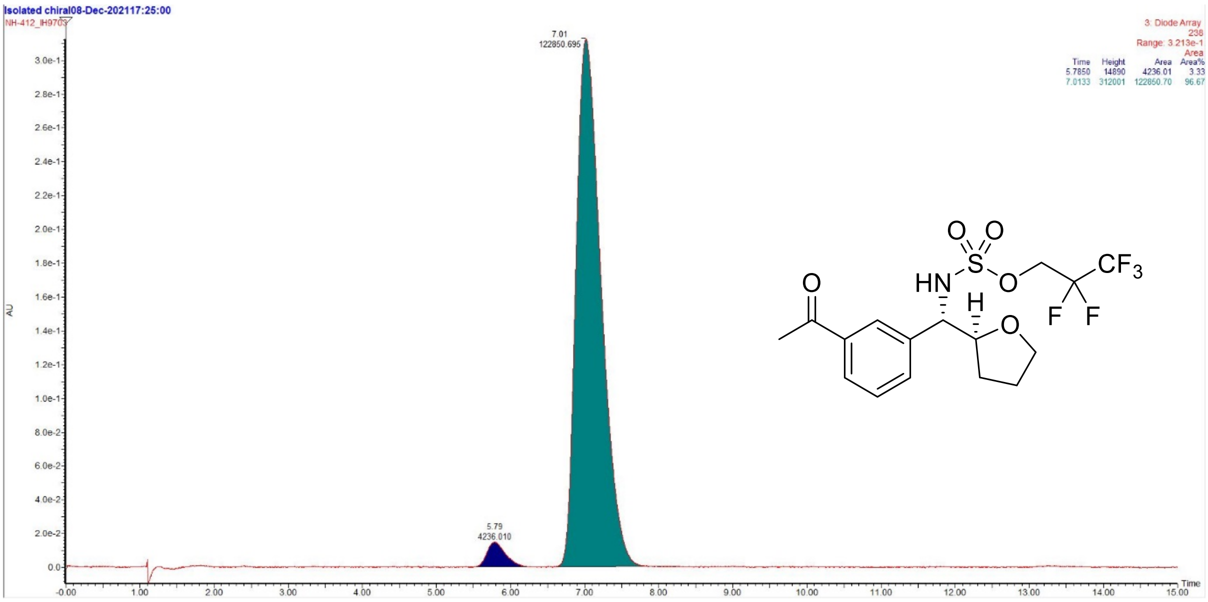

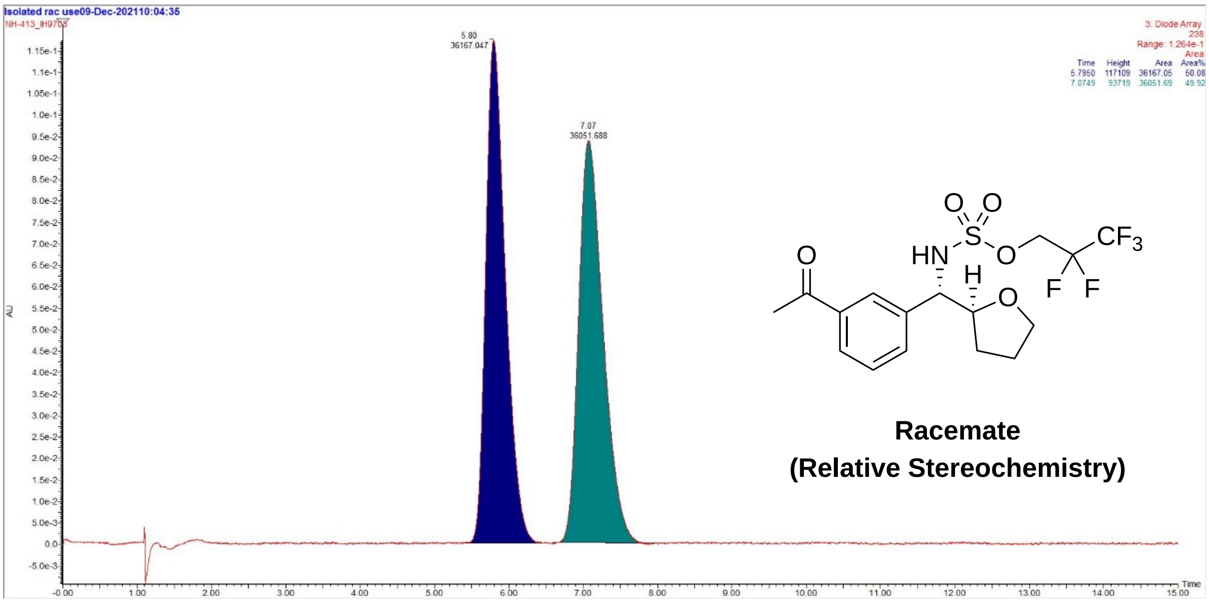

2,2,3,3,3-pentafluoropropyl ((S)-(3-isopropylphenyl)((S)-tetrahydrofuran-2-yl)methyl)sulfamate (**4n**)

Chiral SFC Analysis CHIRALPAK IH (CO_2:MeOH, 98:2, 2.5 mL min^{-1}, 40 °C, 210 nm) indicated 92% *ee*, t_R = 3.7 (minor), 5.1 (major) minutes.

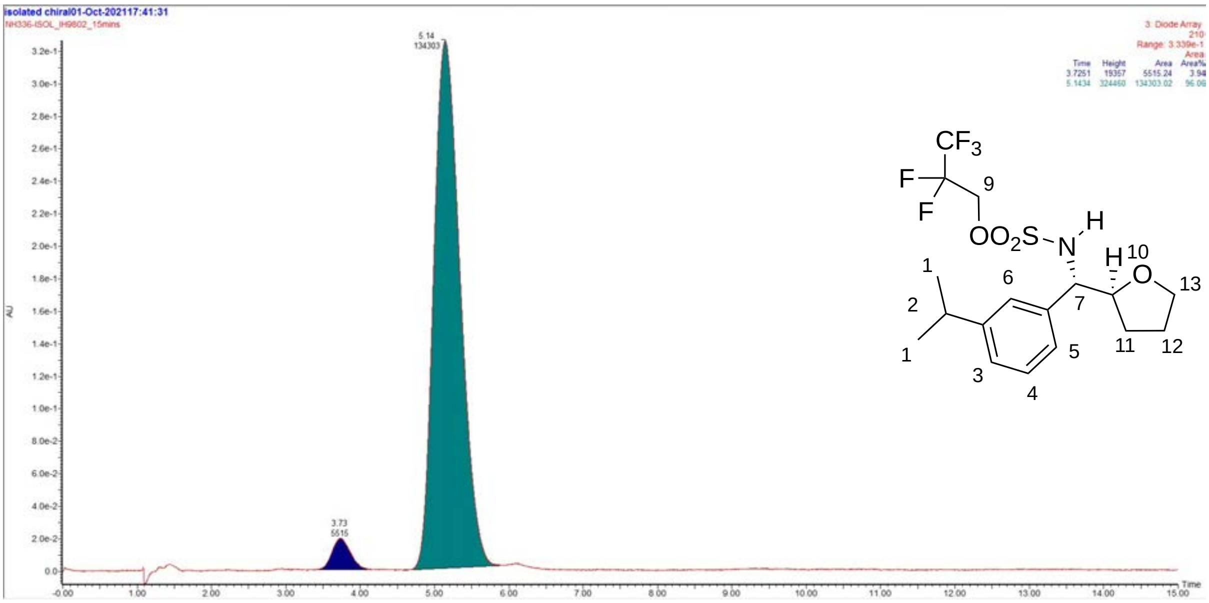

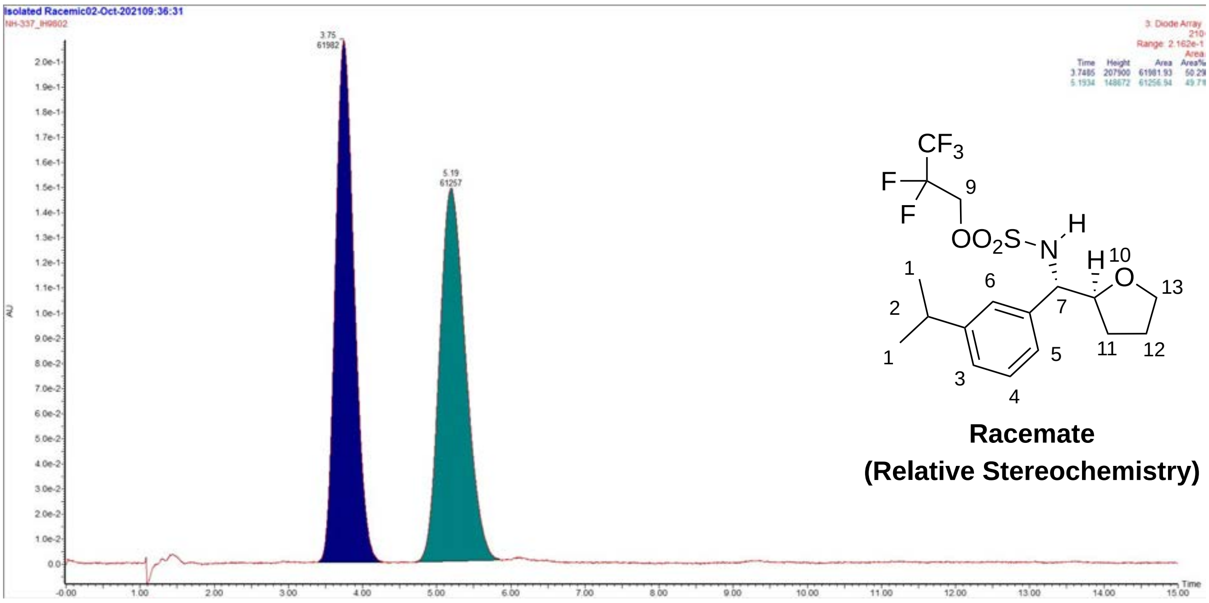

2,2,3,3,3-pentafluoropropyl ((S)-(3-(tert-butyl)phenyl)((S)-tetrahydrofuran-2-yl)methyl)sulfamate (**4o**)

Chiral SFC Analysis CHIRALPAK IH (CO_2:MeOH, 98.5:1.5, 2.5 mL min^{-1}, 40 °C, 210 nm) indicated 90% *ee*, t_R = 4.6 (minor), 6.5 (major) minutes.

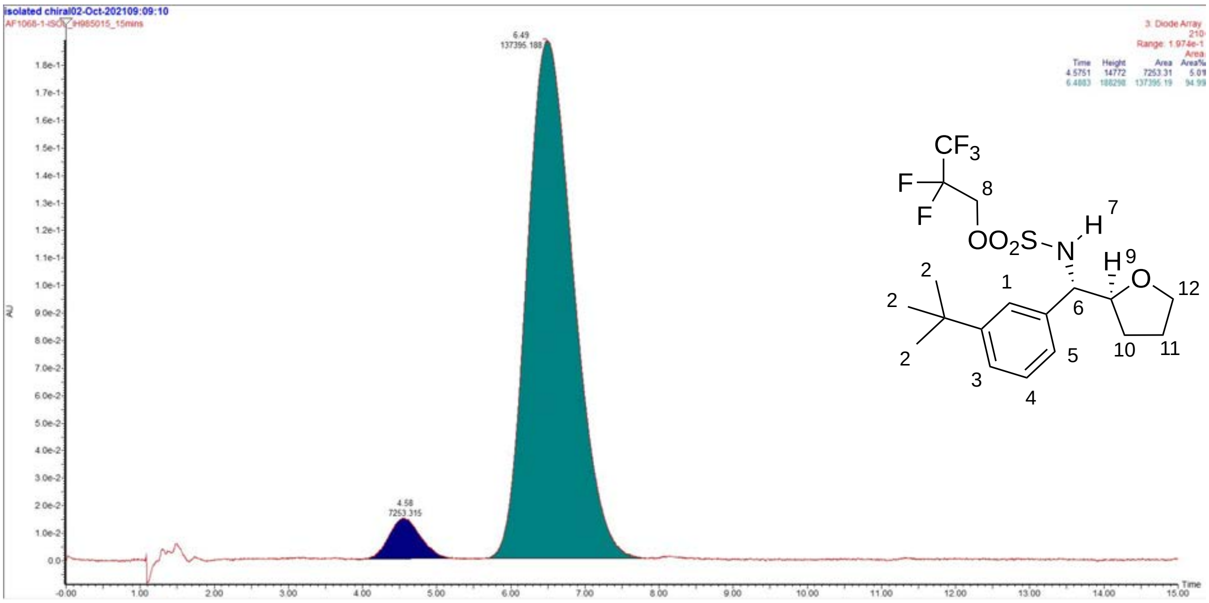

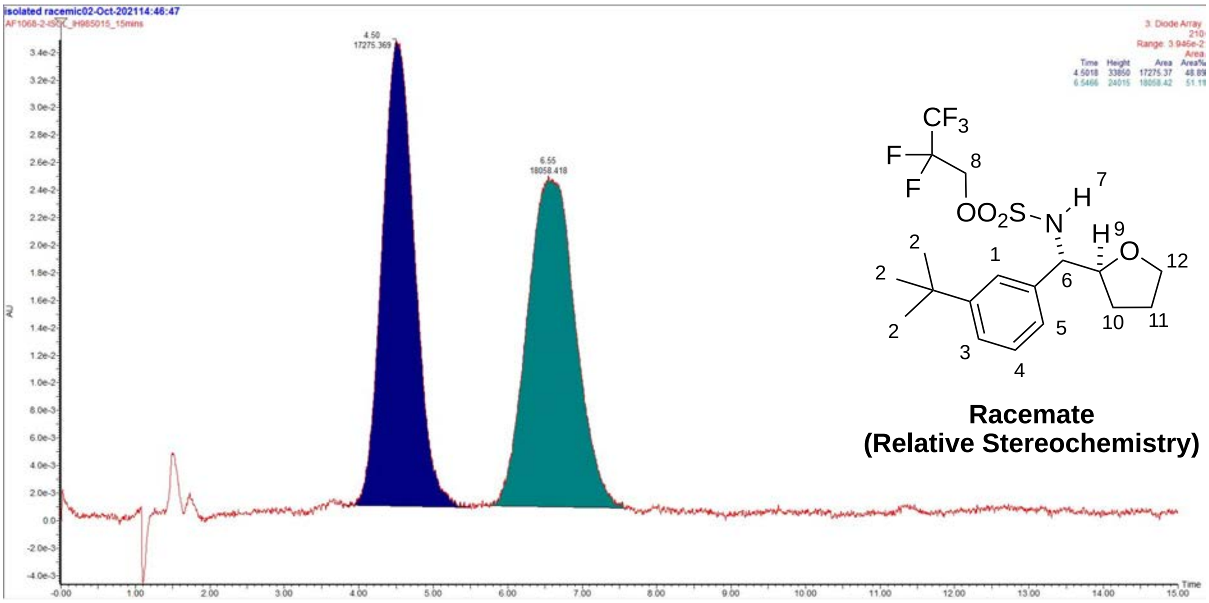

2,2,3,3,3-pentafluoropropyl ((S)-(3,5-dimethylphenyl)((S)-tetrahydrofuran-2 yl)methyl)sulfamate (**4p**)

Chiral SFC Analysis CHIRALPAK IH (CO_2:MeOH, 98:2, 2.5 mL min^{-1}, 40 °C, 212 nm) indicated 96% *ee*, t_R = 3.5 (minor), 5.1 (major) minutes.

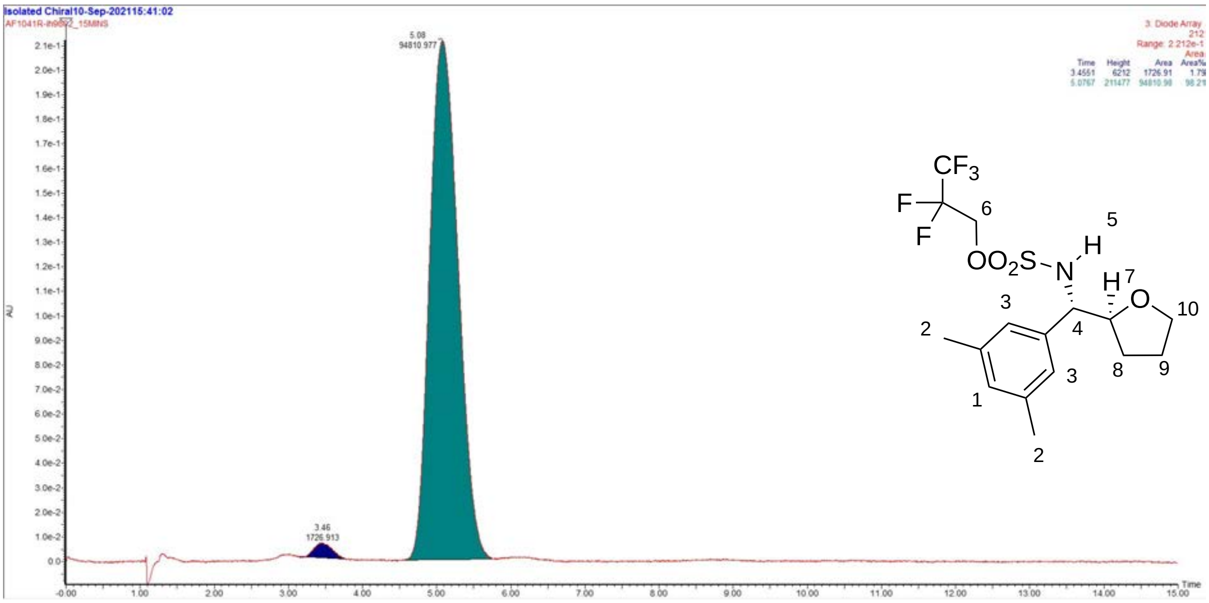

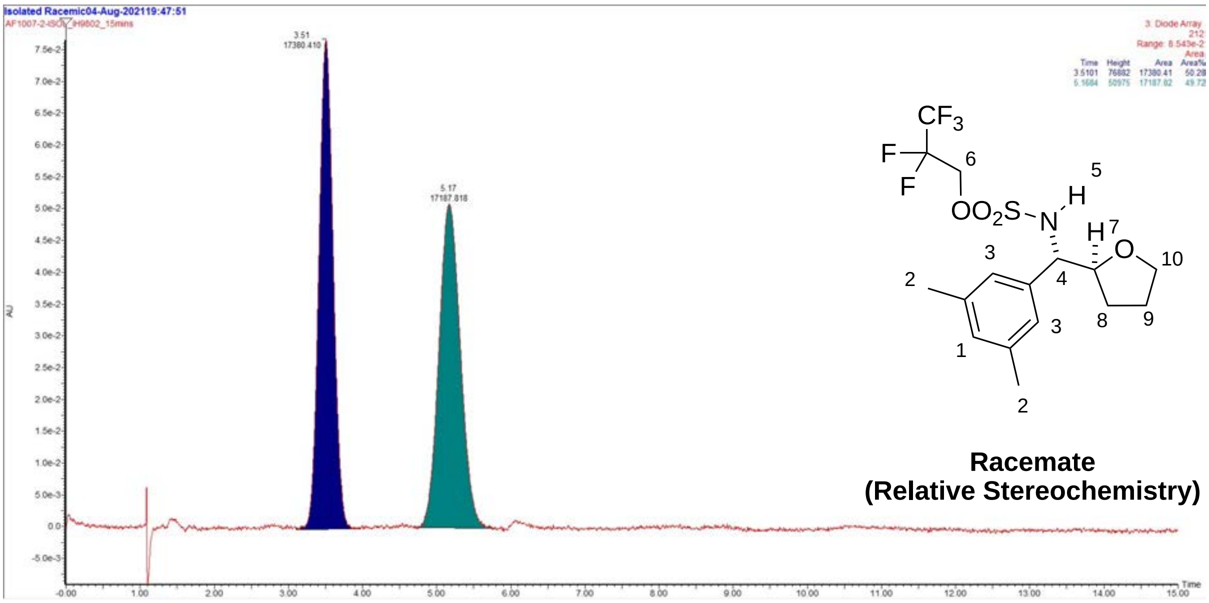

2,2,3,3,3-pentafluoropropyl ((S)-(3,5-di-tert-butylphenyl)((S)-tetrahydrofuran-2-yl)methyl)sulfamate (**4q**)

Chiral SFC Analysis CHIRAL ART SA (Hexane:^{i}PrOH, 99:1, 1.0 mL min^{-1}, 40 °C, 215 nm) indicated 88% *ee*, t_R = 7.3 (minor), 10.5 (major) minutes.

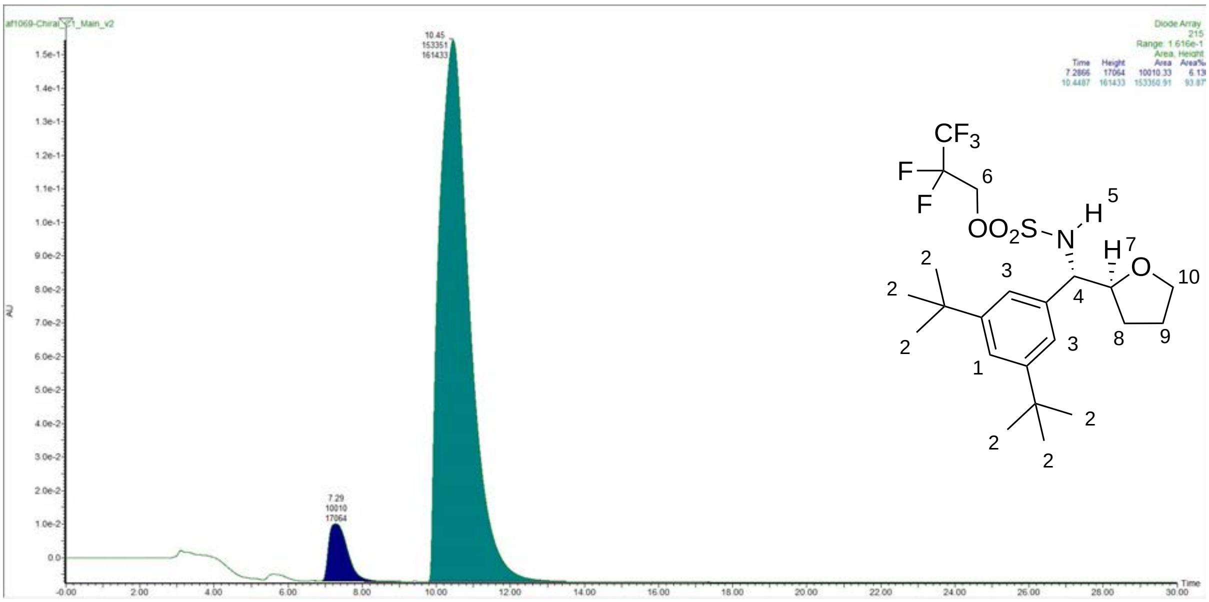

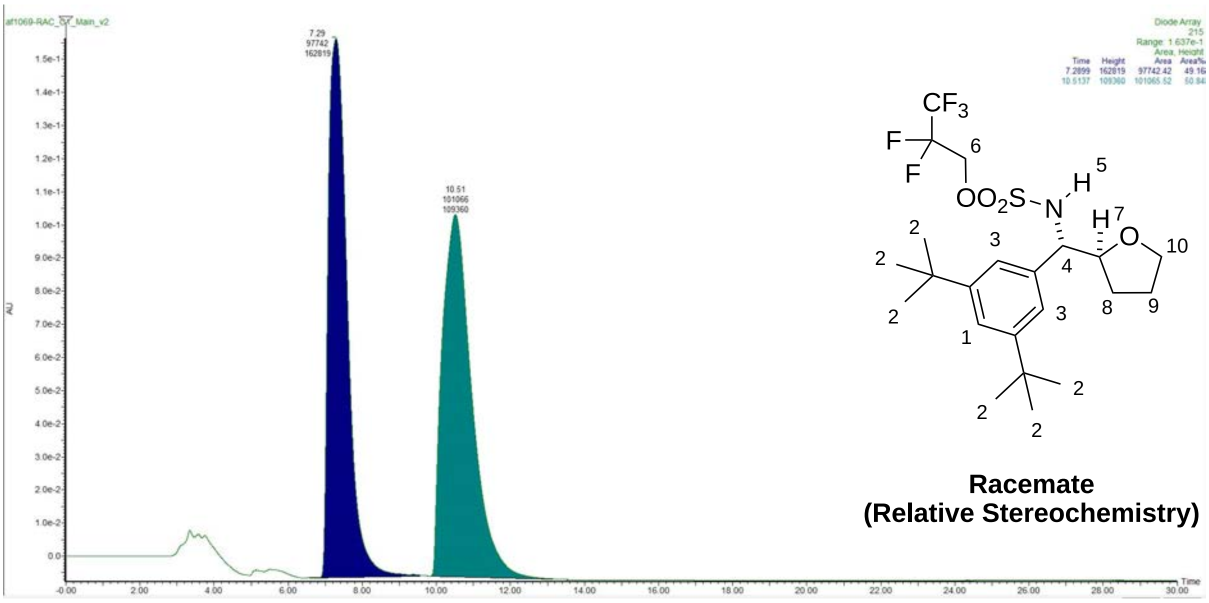

2,2,3,3,3-pentafluoropropyl ((S)-(3-fluoro-4-methylphenyl)((S)-tetrahydrofuran-2-yl)methyl)sulfamate (**4r**)

Chiral SFC Analysis CHIRALPAK IE (CO_2:MeOH, 99:1, 2.5 mL min^{-1}, 40 °C, 210 nm) indicated 96% *ee*, t_R = 9.5 (major), 12.9 (minor) minutes.

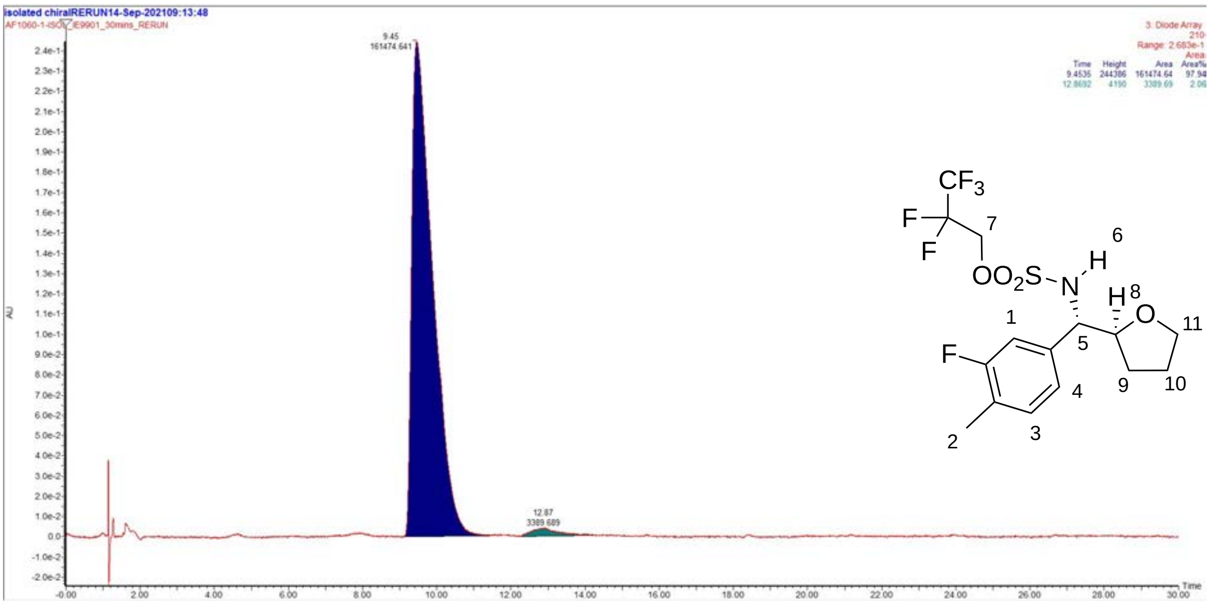

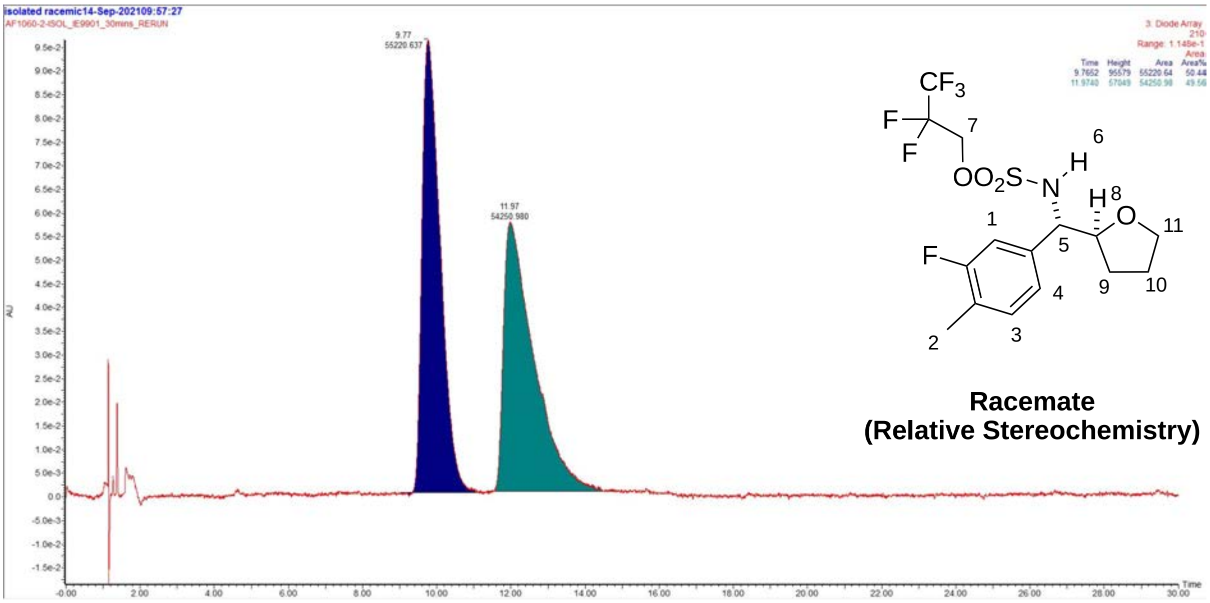

2,2,3,3,3-pentafluoropropyl ((S)-naphthalen-2-yl((S)-tetrahydrofuran-2-yl)methyl)sulfamate (**4s**)

Chiral SFC Analysis CHIRALPAK IE (CO_2:MeOH, 97:3, 2.5 mL min^{-1}, 40 °C, 220 nm) indicated 95% *ee*, t_R = 7.4 (major), 8.7 (minor) minutes.

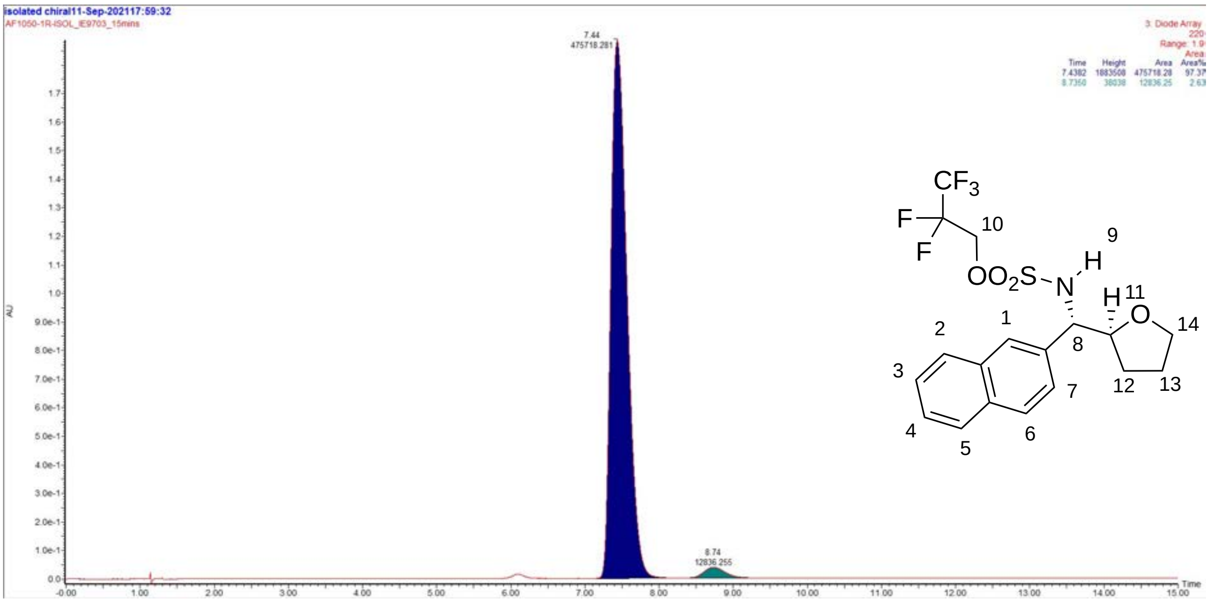

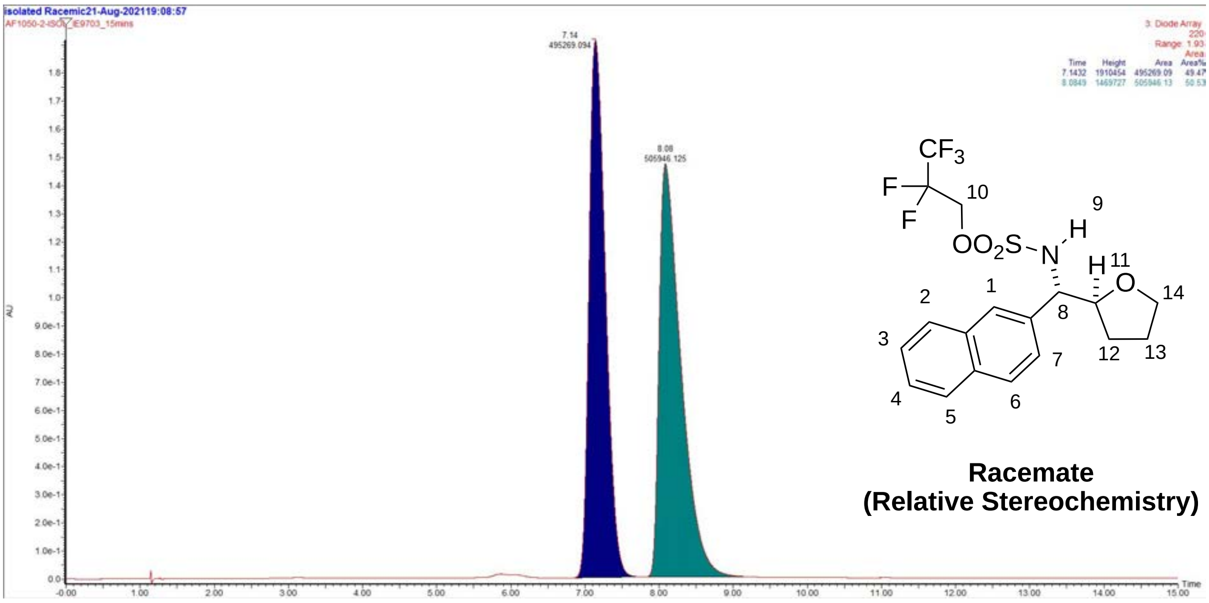

2,2,3,3,3-pentafluoropropyl ((S)-(4-fluorophenyl)((S)-tetrahydrofuran-2-yl)methyl)sulfamate (**4t**)

Chiral SFC Analysis CHIRALPAK IH (CO_2:MeOH, 98:2, 2.5 mL min^{-1}, 40 °C, 210 nm) indicated 87% *ee*, t_R = 4.3 (minor), 5.6 (major) minutes.

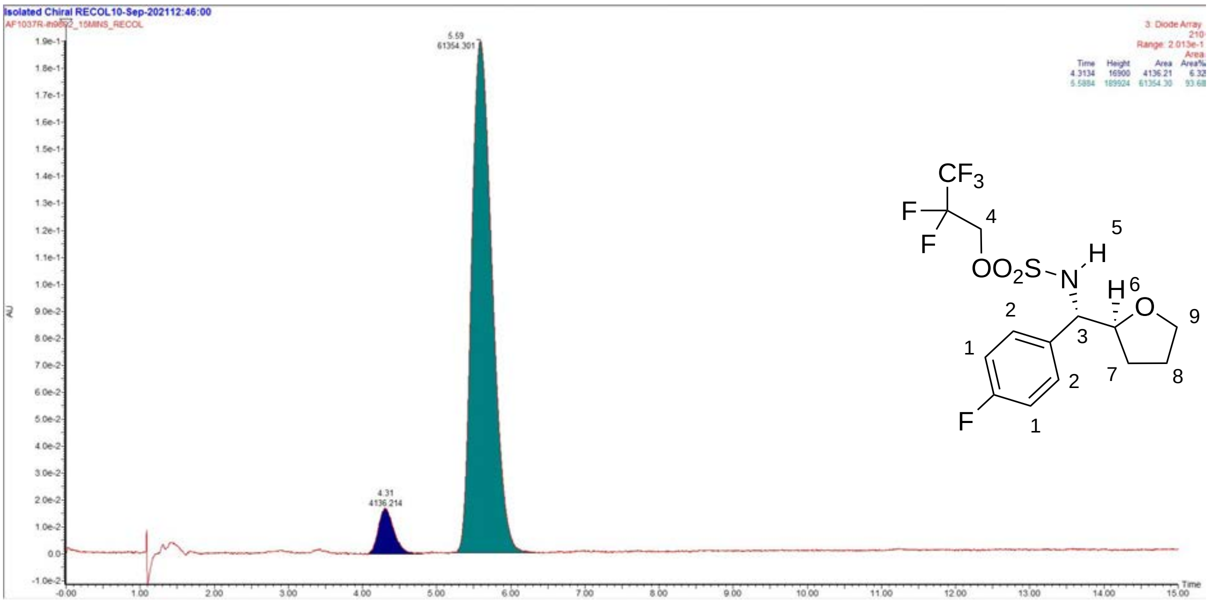

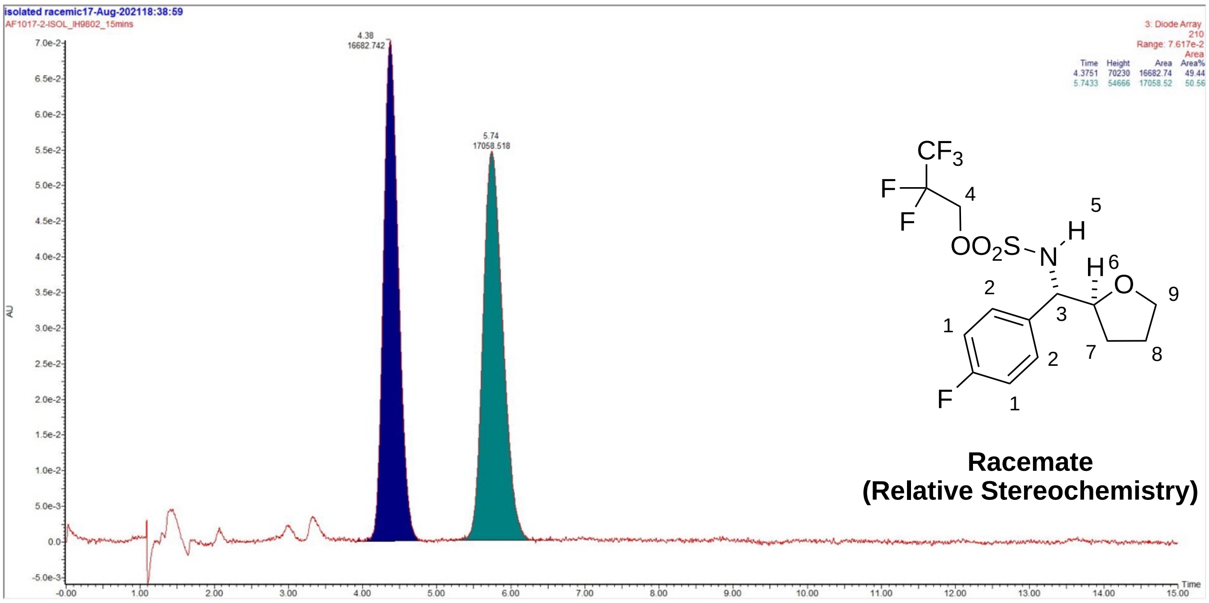

2,2,3,3,3-pentafluoropropyl ((S)-((S)-tetrahydrofuran-2-yl)(p-tolyl)methyl)sulfamate (**4u**)

Chiral SFC Analysis CHIRALPAK IH (CO_2:MeOH, 98:2, 1.5 mL min^{-1}, 40 °C, 216 nm) indicated 91% *ee*, t_R = 9.4 (minor), 13.5 (major) minutes.

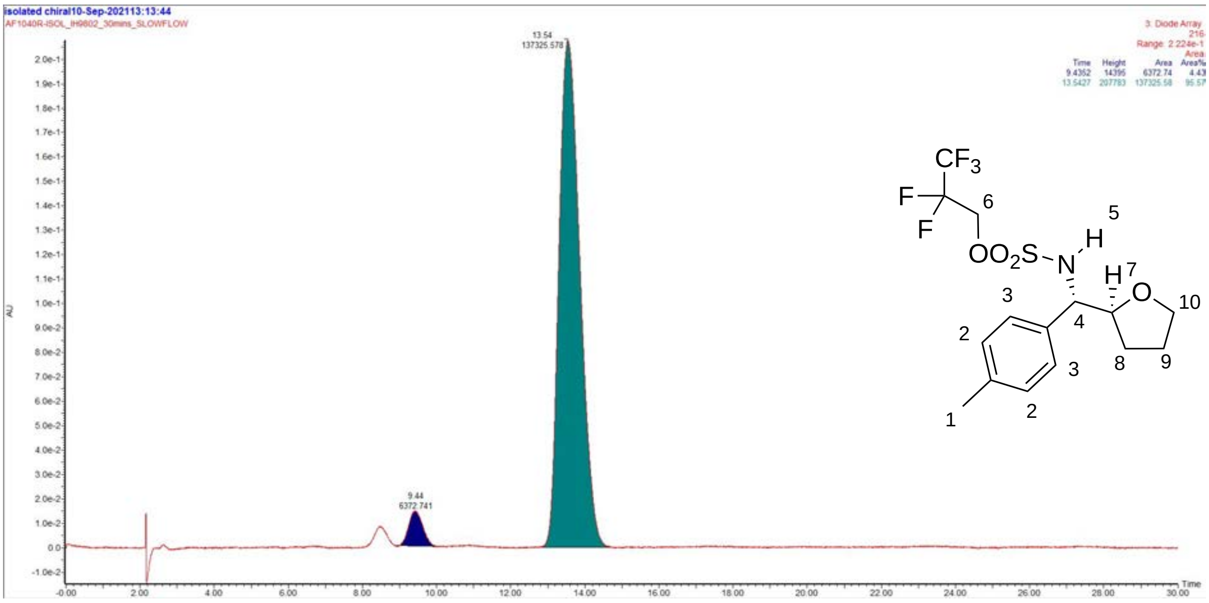

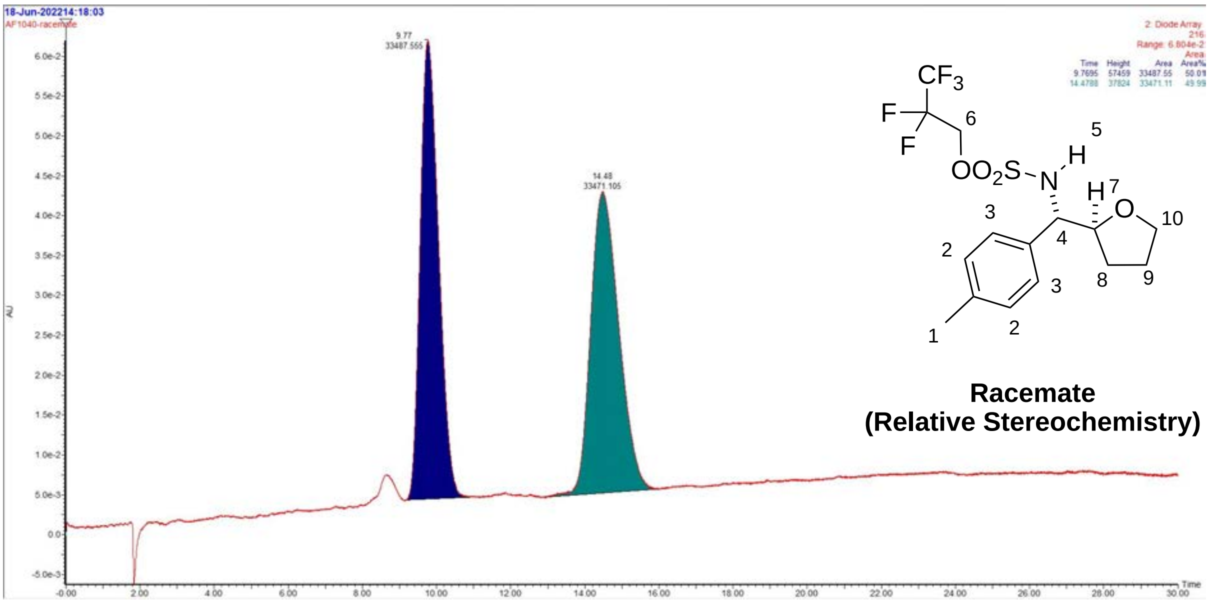

2,2,3,3,3-pentafluoropropyl ((S)-((S)-tetrahydrofuran-2-yl)(4-(trifluoromethyl)phenyl)methyl)sulfamate (**4v**)

Chiral SFC Analysis CHIRALPAK IG (CO_2: ^{i}PrOH, 98:2, 2.5 mL min^{-1}, 40 °C, 213 nm) indicated 97% *ee*, t_R = 3.0 (minor), 3.6 (major) minutes.

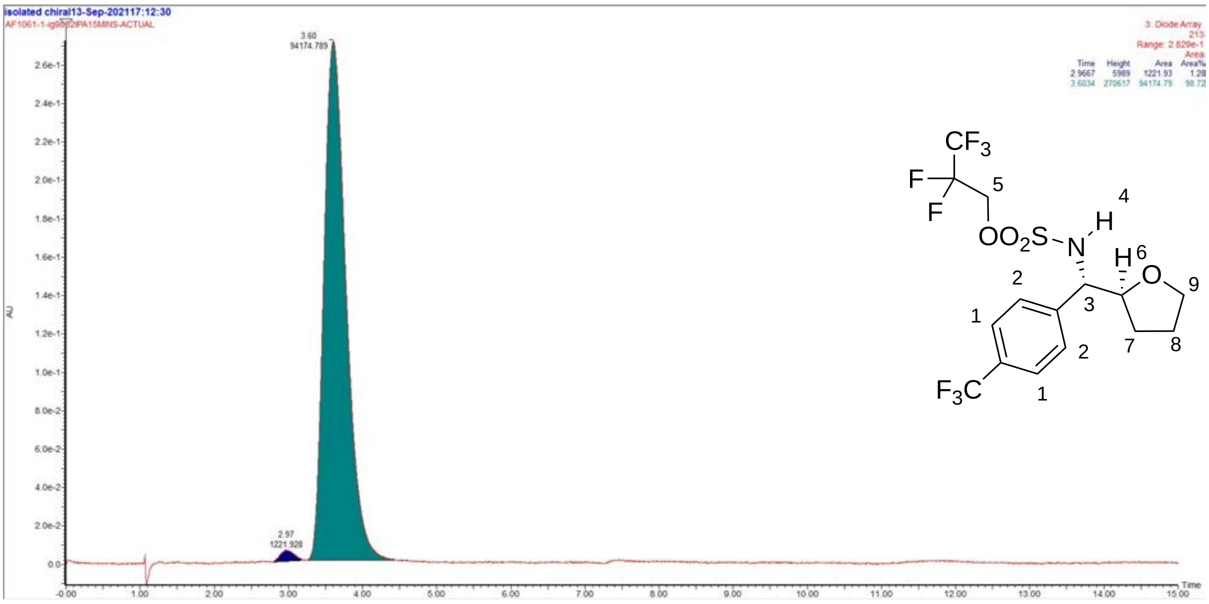

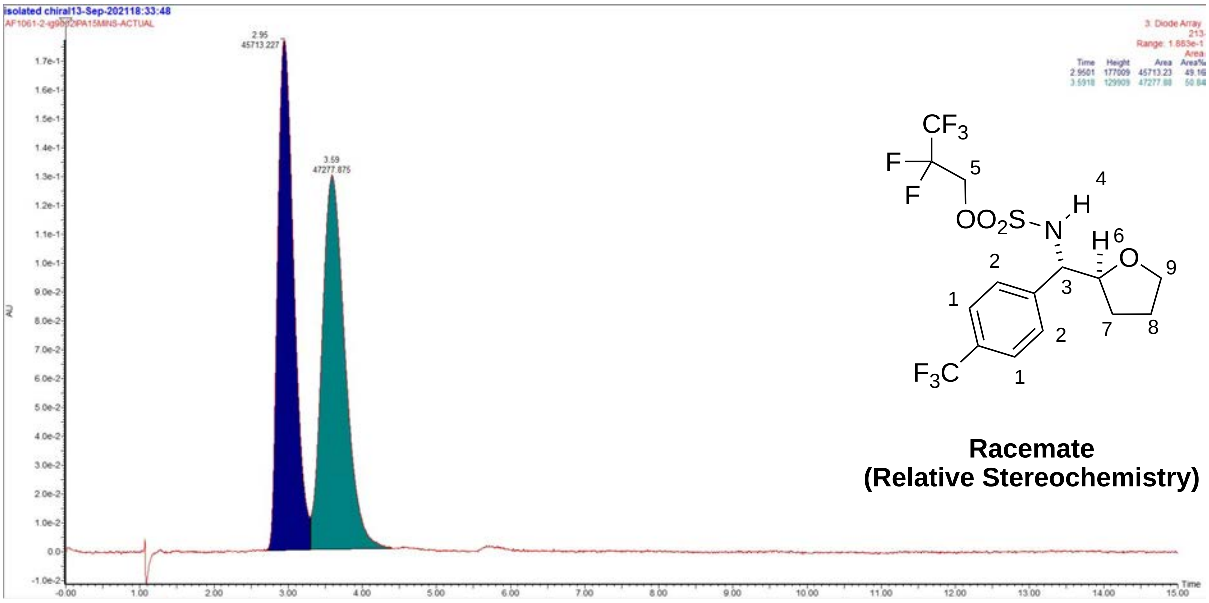

2,2,3,3,3-pentafluoropropyl ((S)-(4-(tert-butyl)phenyl)((S)-tetrahydrofuran-2-yl)methyl)sulfamate (**4w**)

Chiral SFC Analysis CHIRALPAK IH (CO_2:MeOH, 98.5:1.5, 1.25 mL min^{-1}, 40 °C, 217 nm) indicated 96% *ee*, t_R = 10.6 (minor), 18.5 (major) minutes.

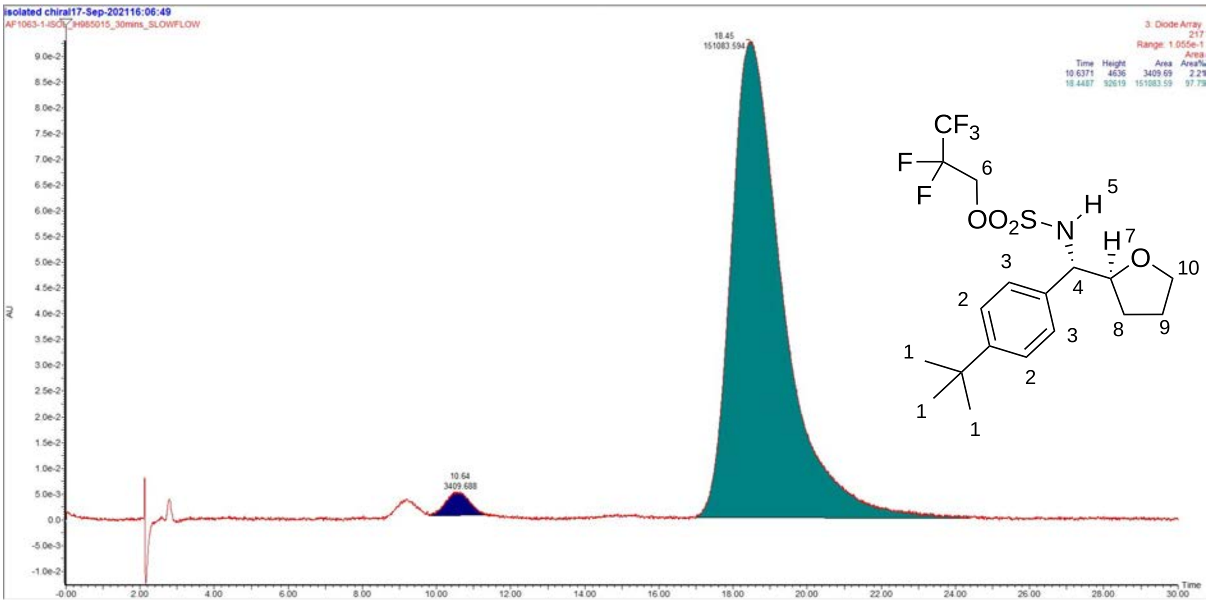

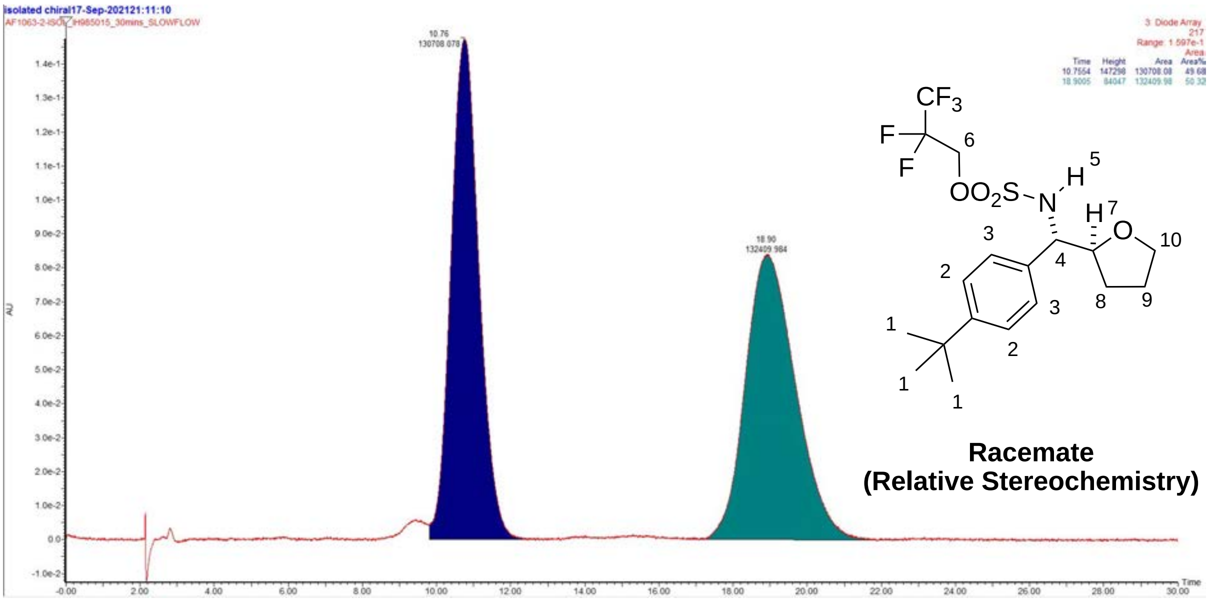

2,2,3,3,3-pentafluoropropyl ((S)-[1,1'-biphenyl]-4-yl((S)-tetrahydrofuran-2-yl)methyl)sulfamate (**4x**)

Chiral SFC Analysis: CHIRALPAK IH (CO_2:MeOH, 96:4, 2.5 mL min^{-1}, 40 °C, 248 nm) indicated 96% *ee*, t_R = 6.4 (minor), 9.0 (major) minutes.

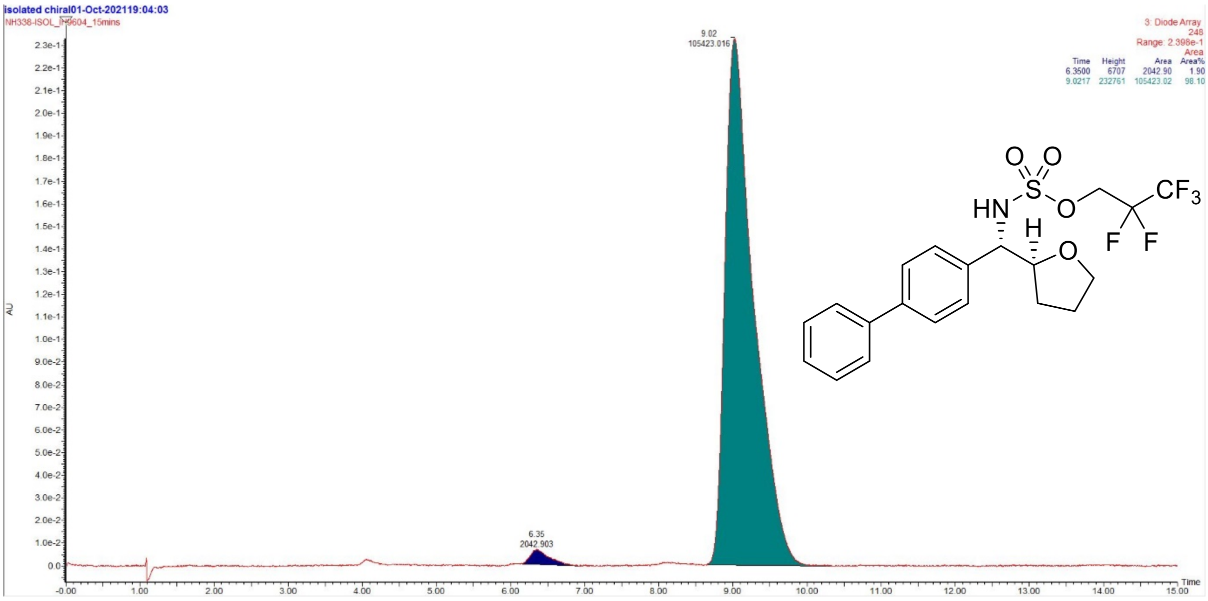

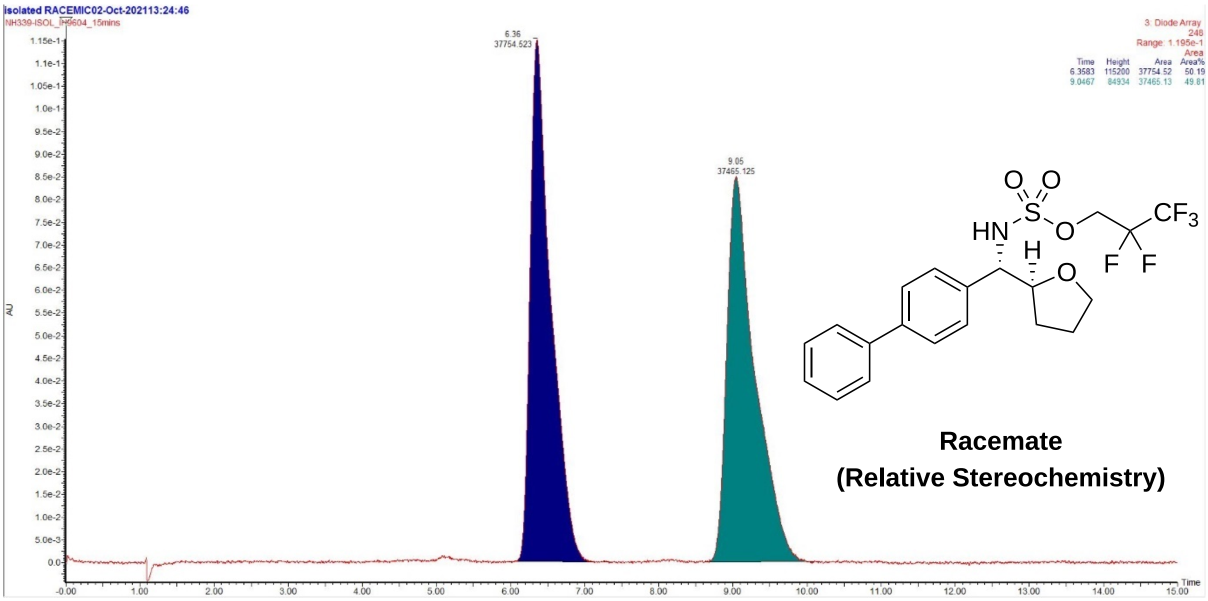

Ethyl 4-((S)-(((2,2,3,3,3-pentafluoropropoxy)sulfonyl)amino)((S)-tetrahydrofuran-2-yl)methyl)benzoate (**4y**)

Chiral SFC Analysis: CHIRALPAK IH (CO_2:MeOH, 97:3, 2.5 mL min^{-1}, 40 °C, 232 nm) indicated 97% *ee*, t_R = 4.4 (minor), 6.3 (major) minutes.

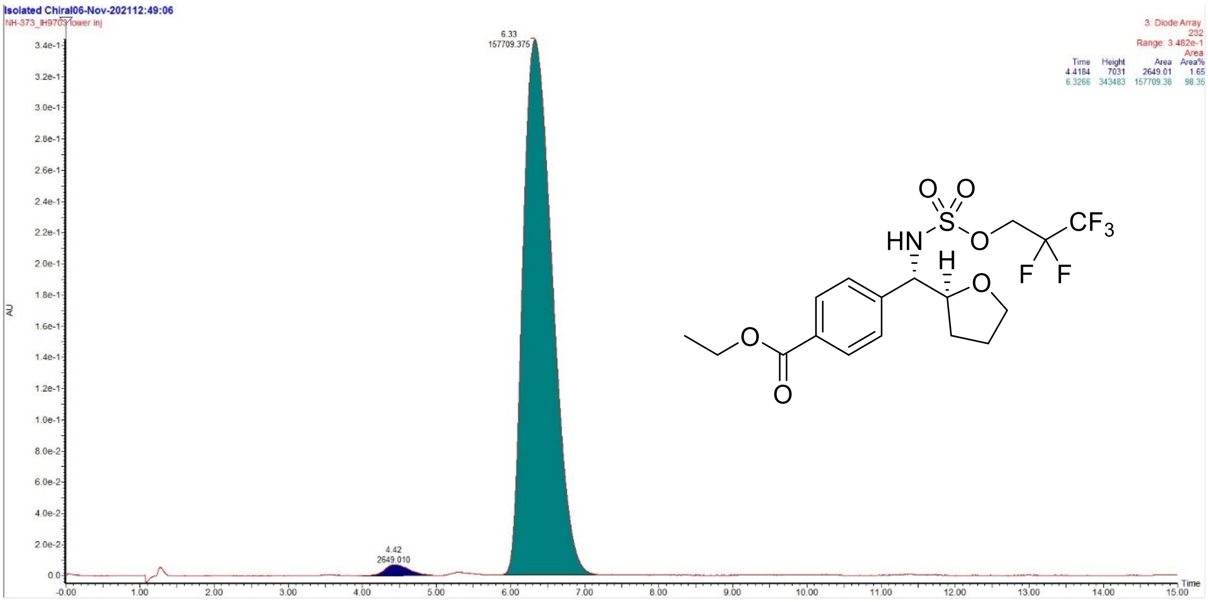

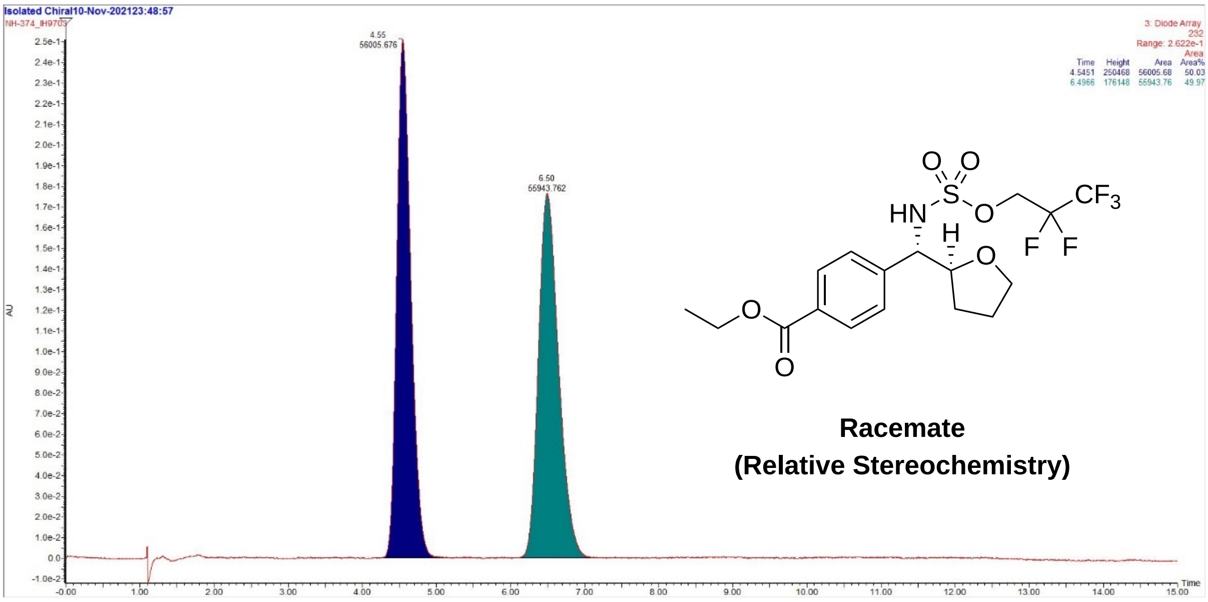

2,2,3,3,3-pentafluoropropyl ((S)-(4-((tert-butoxycarbonyl)amino)phenyl)((S)-tetrahydrofuran-2-yl)methyl)sulfamate (**4z**)

Chiral SFC Analysis: CHIRALPAK IH (CO_2:MeOH, 97:3, 2.5 mL min^{-1}, 40 °C, 239 nm) indicated 89% *ee*, t_R = 12.8 (minor), 17.4 (major) minutes

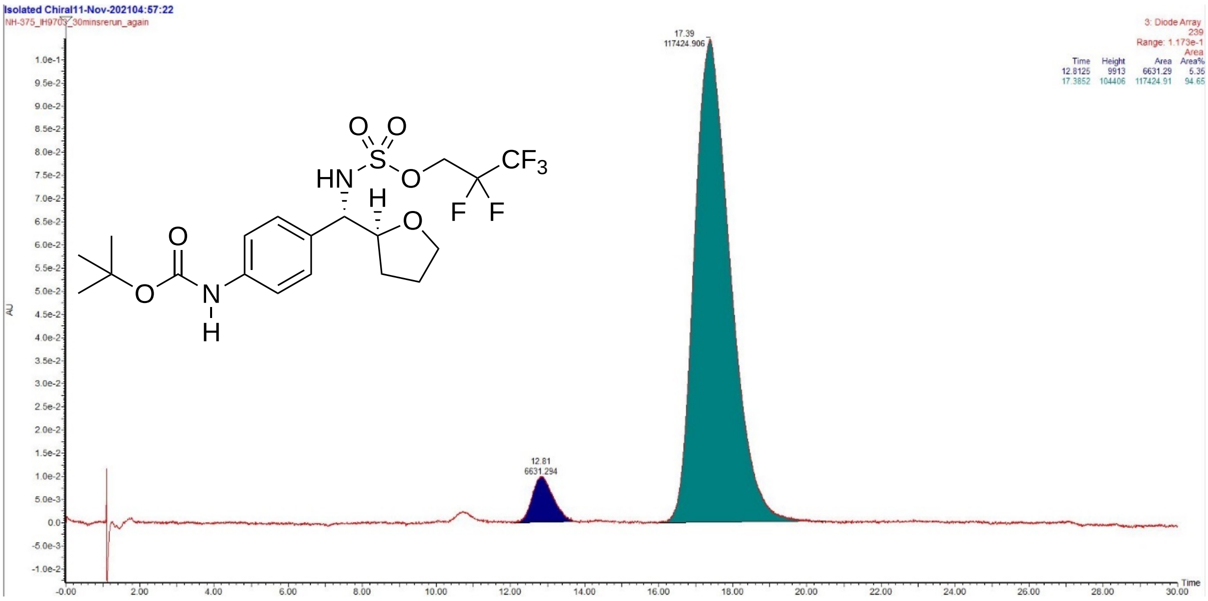

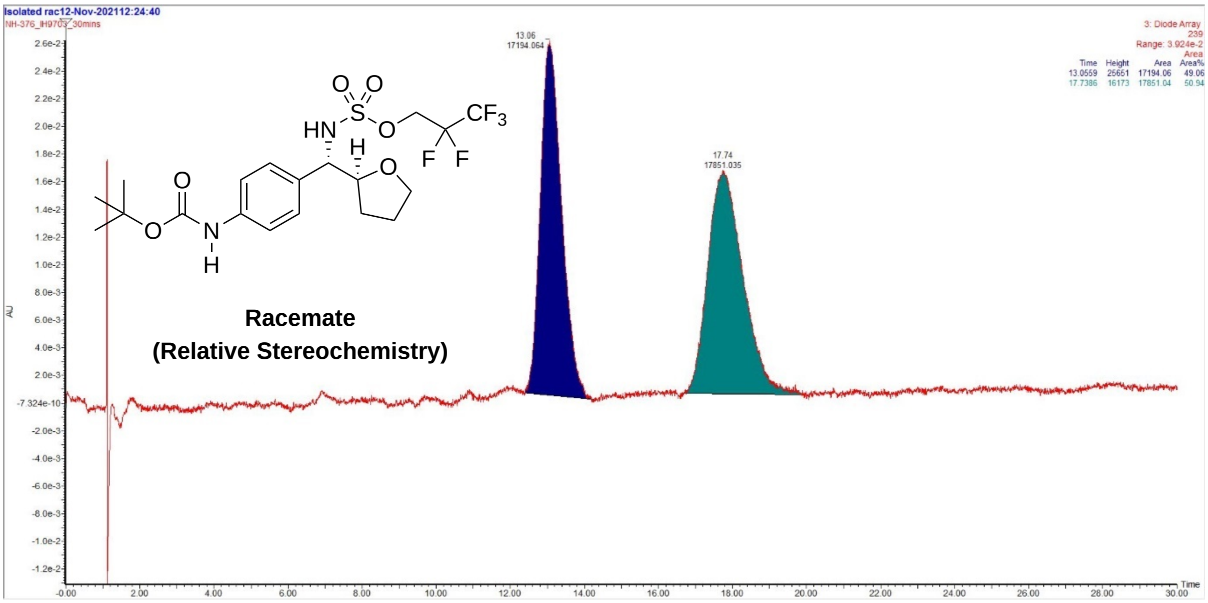

2,2,3,3,3-pentafluoropropyl ((S)-(4-methoxyphenyl)((S)-tetrahydrofuran-2-yl)methyl)sulfamate (**4aa**)

Chiral SFC Analysis: CHIRALPAK IH (CO_2:MeOH, 98:2, 2.5 mL min^{-1}, 40 °C, 224 nm) indicated 88% *ee*, t_R = 7.5 (minor), 8.4 (major) minutes.

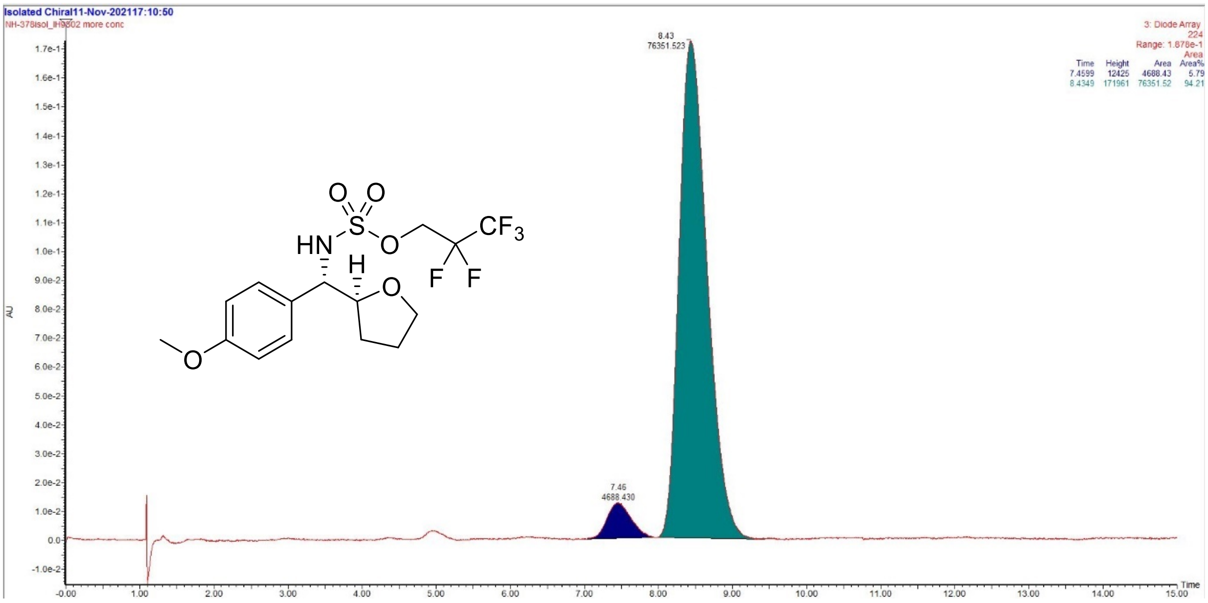

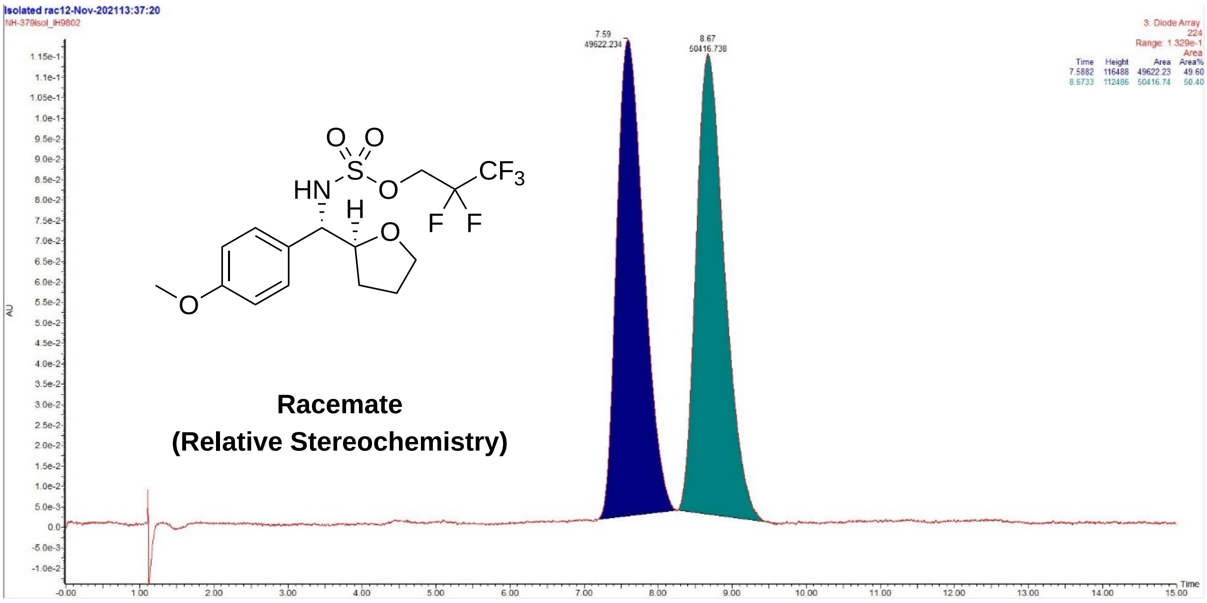

2,2,3,3,3-pentafluoropropyl ((2R,3R)-2-(4-methoxyphenyl)tetrahydro-2H-pyran-3-yl)sulfamate (**4ab**)

Chiral SFC Analysis: CHIRALPAK IH (CO_2:MeOH, 97:3, 2.5 mL min^{-1}, 40 °C, 223 nm) indicated 89% *ee*, t_R = 3.7 (major), 6.0 (minor) minutes.

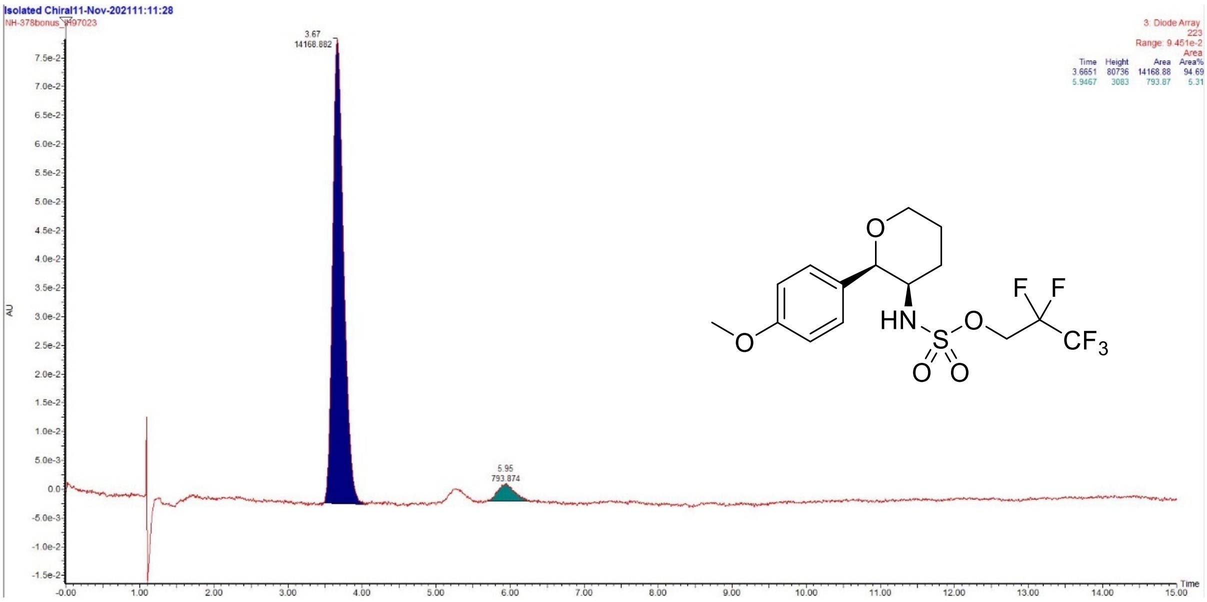

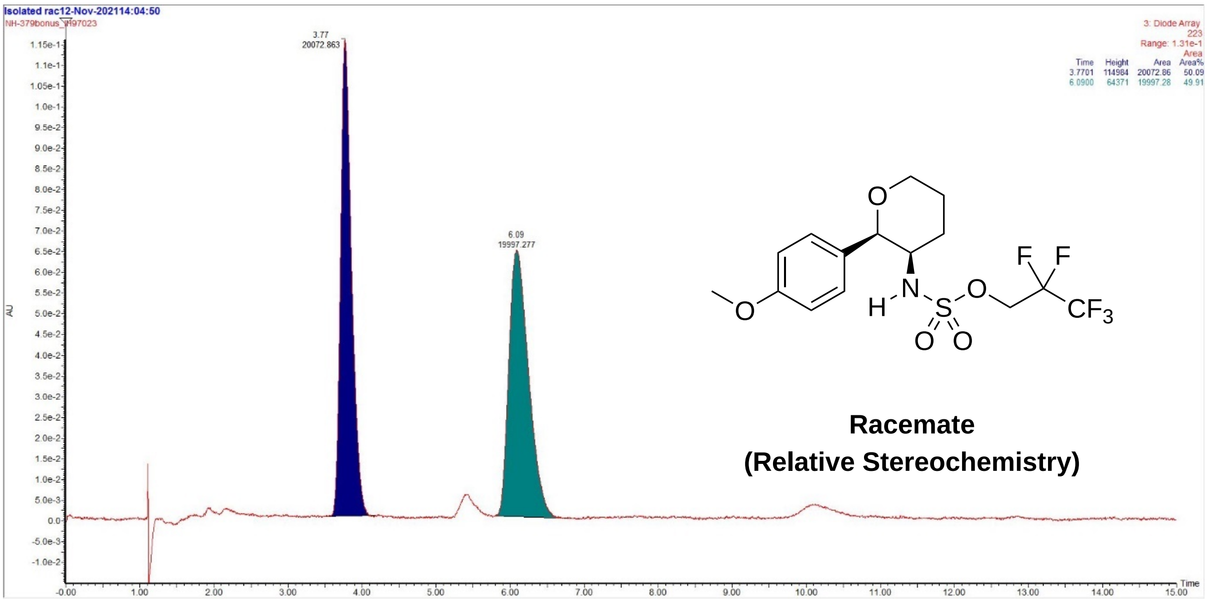

2,2,3,3,3-pentafluoropropyl ((S)-(4-chlorophenyl)((S)-tetrahydrofuran-2-yl)methyl)sulfamate (**4ac**)

Chiral SFC Analysis: CHIRALPAK IH (CO_2:MeOH, 98:2, 2.5 mL min^{-1}, 40 °C, 220 nm) indicated 90% *ee*, t_R = 6.0 (minor), 9.0 (major) minutes.

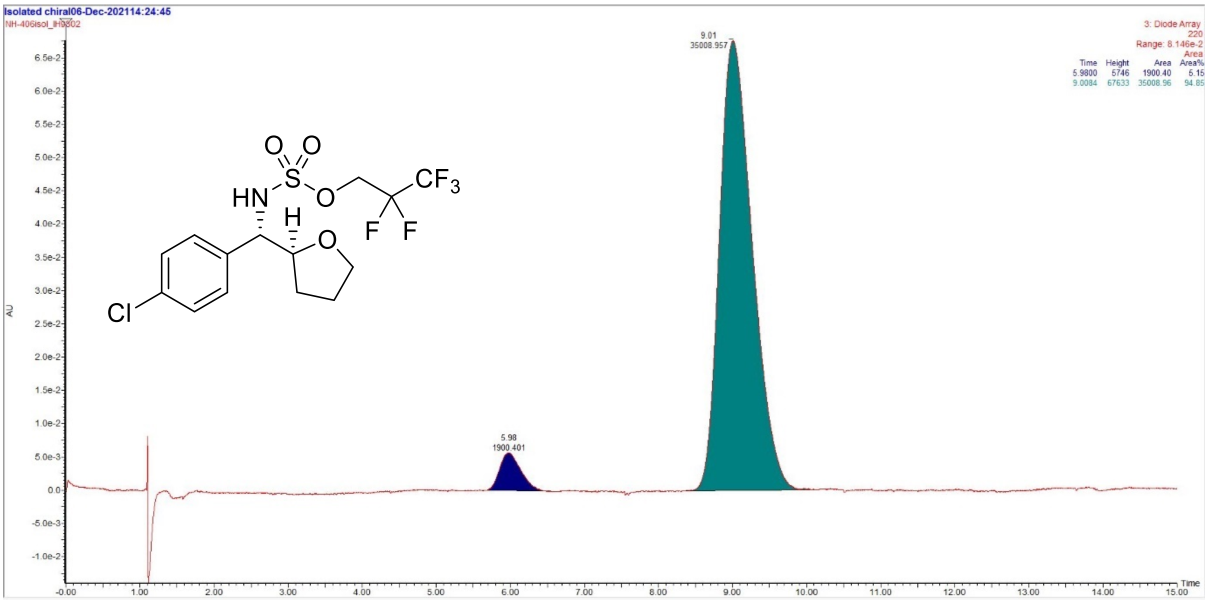

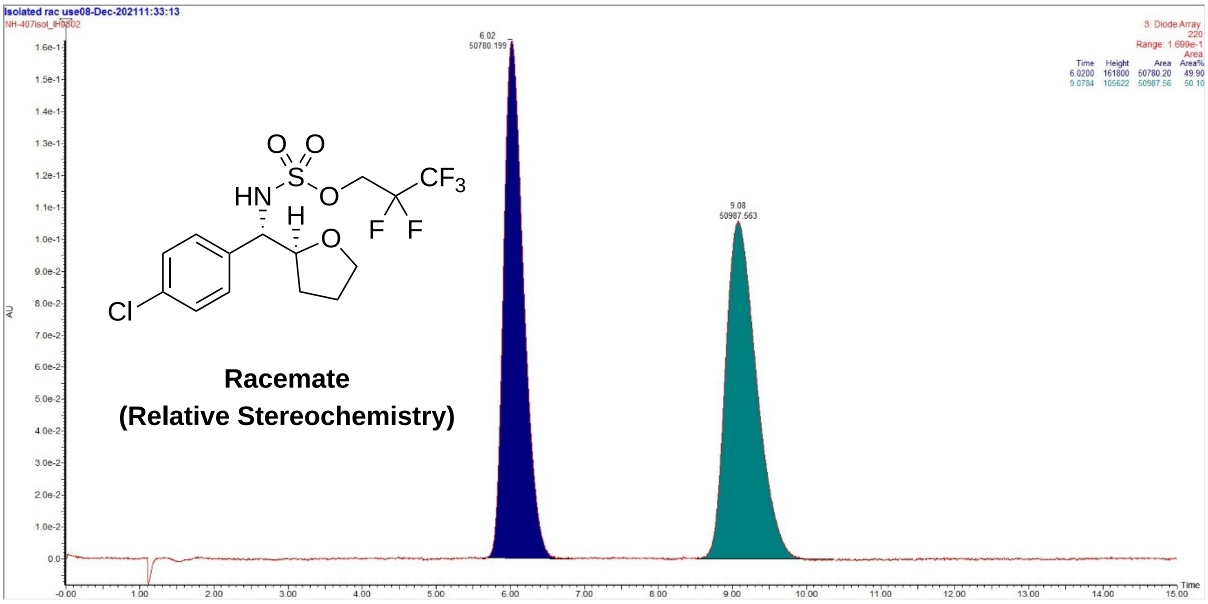

2,2,3,3,3-pentafluoropropyl ((S)-(4-bromophenyl)((S)-tetrahydrofuran-2-yl)methyl)sulfamate (**4ad**)

Chiral SFC Analysis: CHIRALPAK IH (CO_2:MeOH, 98:2, 2.5 mL min^{-1}, 40 °C, 220 nm) indicated 91% *ee*, t_R = 7.4 (minor), 11.6 (major) minutes.

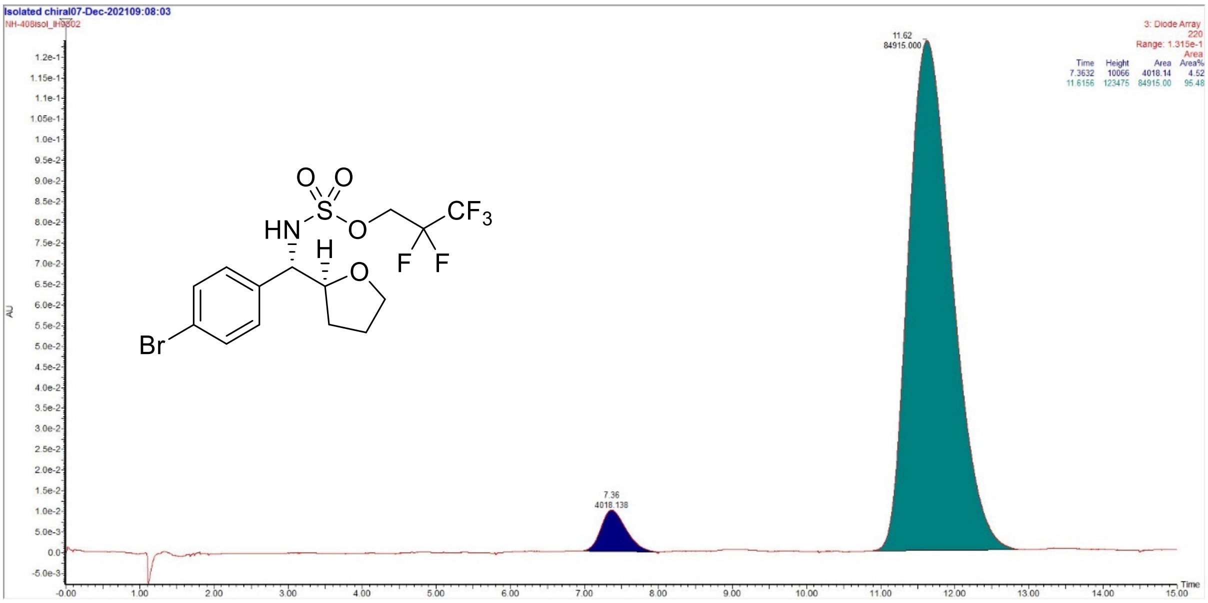

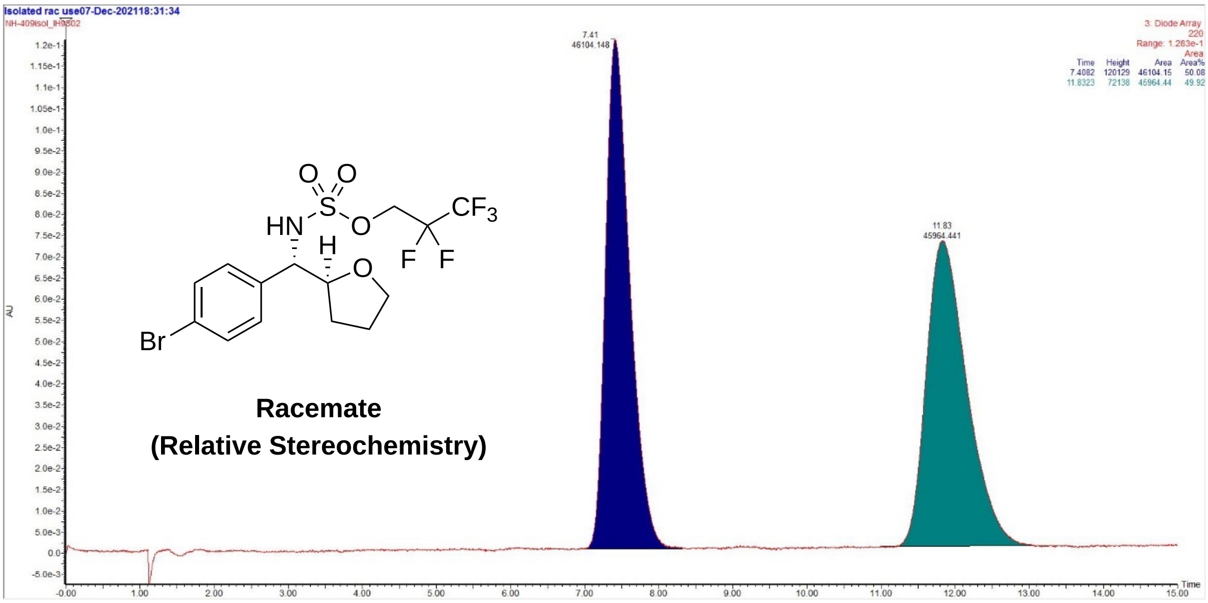

2,2,3,3,3-pentafluoropropyl ((2S,3R)-2-phenyltetrahydro-2H-pyran-3-yl)sulfamate (**6**)

Chiral SFC Analysis: CHIRAL ART SJ (CO_2:MeOH, 96:4, 2.5 mL min^{-1}, 40 °C, 210 nm) indicated 38% *ee*, t_R = 3.4 (major), 3.8 (minor) minutes.

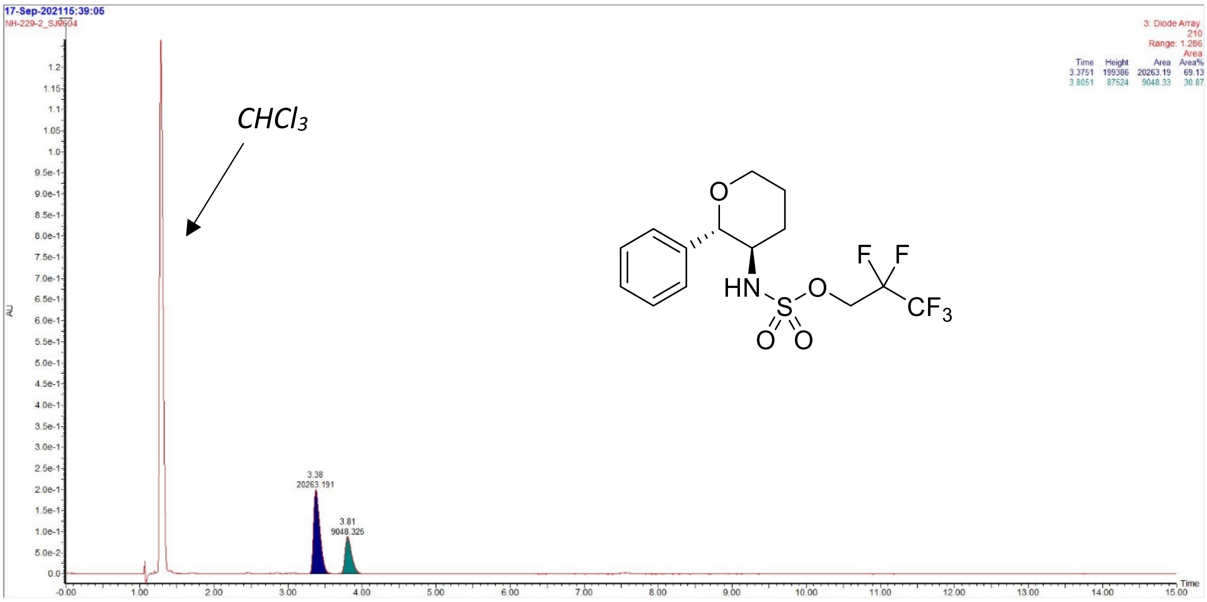

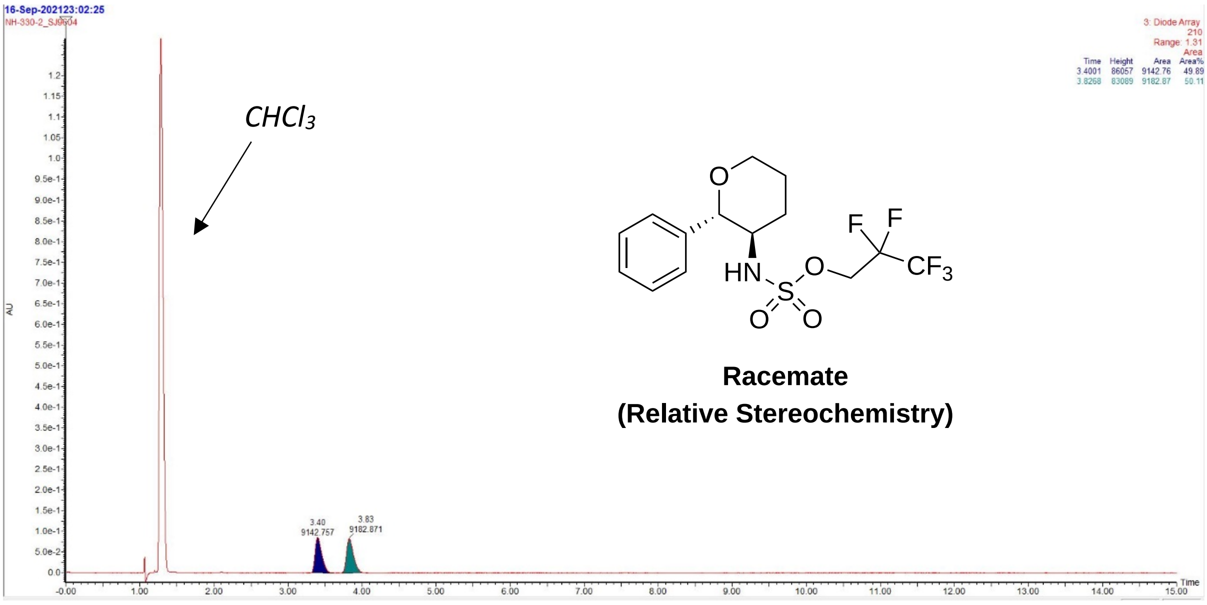

2,2,2-trichloroethyl ((S)-phenyl((S)-tetrahydrofuran-2-yl)methyl)sulfamate (**7**)

Chiral SFC Analysis CHIRALPAK IG (CO_2:MeOH, 90:10, 2.5 mL min^{-1}, 40 °C, 210 nm) indicated 88% *ee*, t_R = 6.4 (minor), 8.5 (major) minutes.

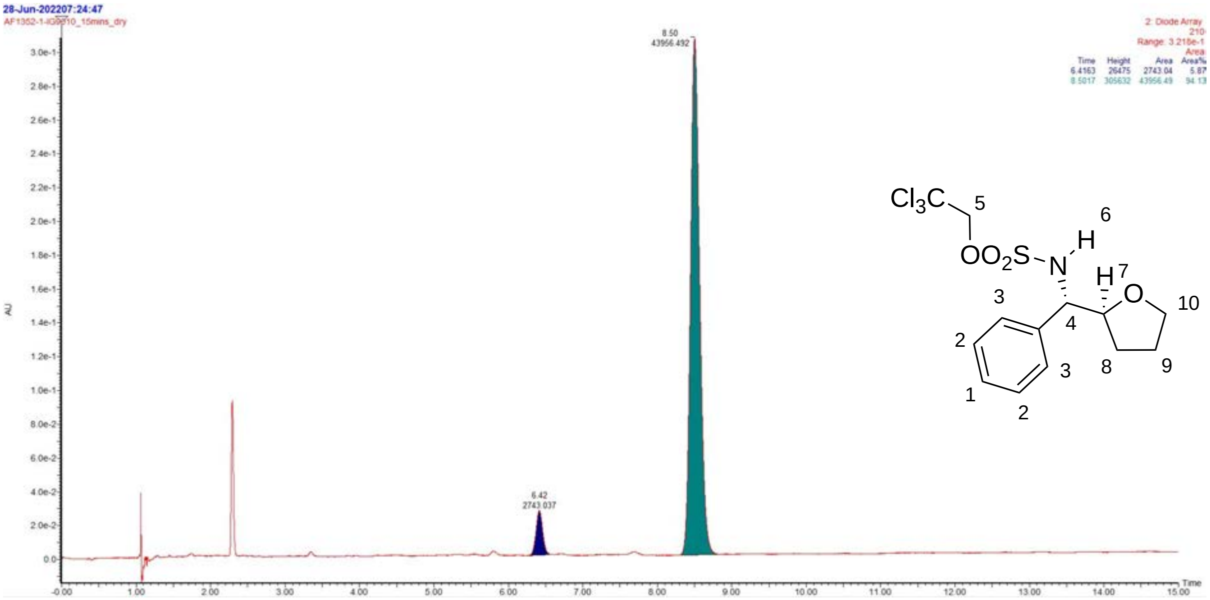

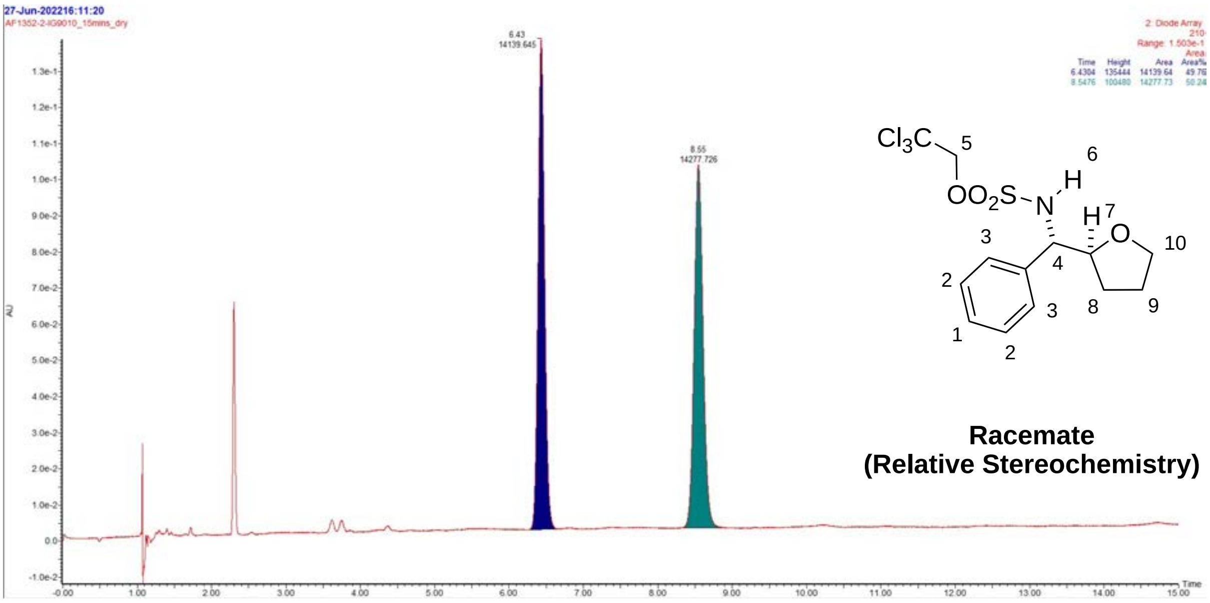

2,2,3,3,3-pentafluoropropyl (2R,3S)-2-(2-hydroxyethyl)-3-phenylaziridine-1-sulfonate (**9a**)

Chiral SFC Analysis CHIRAL ART SC (CO_2:MeOH, 98.5:1.5, 2.5 mL min^{-1}, 40 °C, 210 nm) indicated 87% *ee*, t$_R$ = 4.8 (major), 5.6 (minor) minutes.

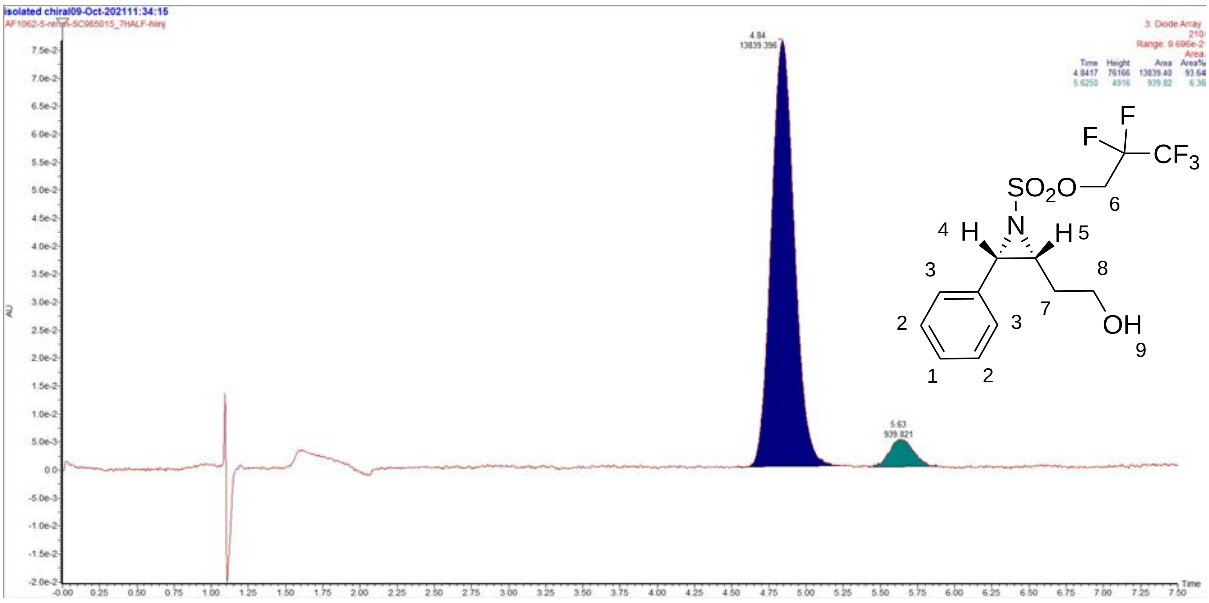

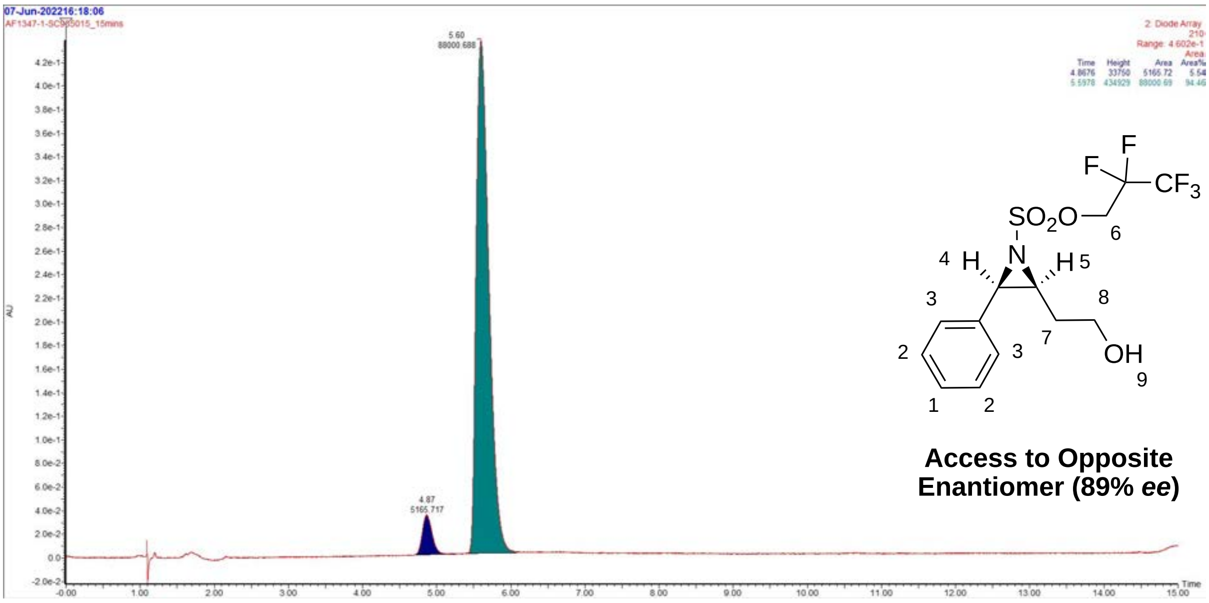

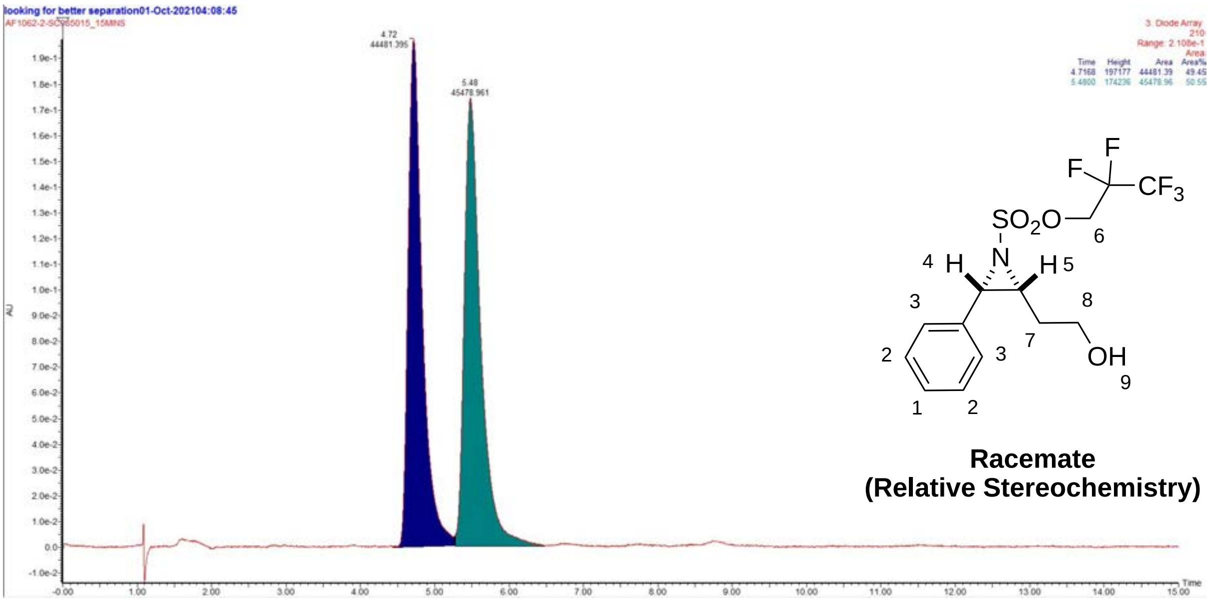
looking for better separation01-Oct-202104:08:45
AF1062-2-SC985015_15MINS
3: Diode Array
210
Range: 2.108e-1
Area
Time Height Area Area%
4.7168 197177 44481.39 49.45
5.4800 174236 45478.96 50.55
4.72
44481.395
5.48
45478.961
AU
Time
Racemate
(Relative Stereochemistry)

2,2,3,3,3-pentafluoropropyl (2R,3S)-2-(2-hydroxyethyl)-3-(o-tolyl)aziridine-1-sulfonate (**9b**)

Chiral SFC Analysis CHIRAL ART SC (CO_2:MeOH, 98.5:1.5, 2.5 mL min^{-1}, 40 °C, 208 nm) indicated 91% *ee*, t_R = 4.0 (major), 5.0 (minor) minutes.

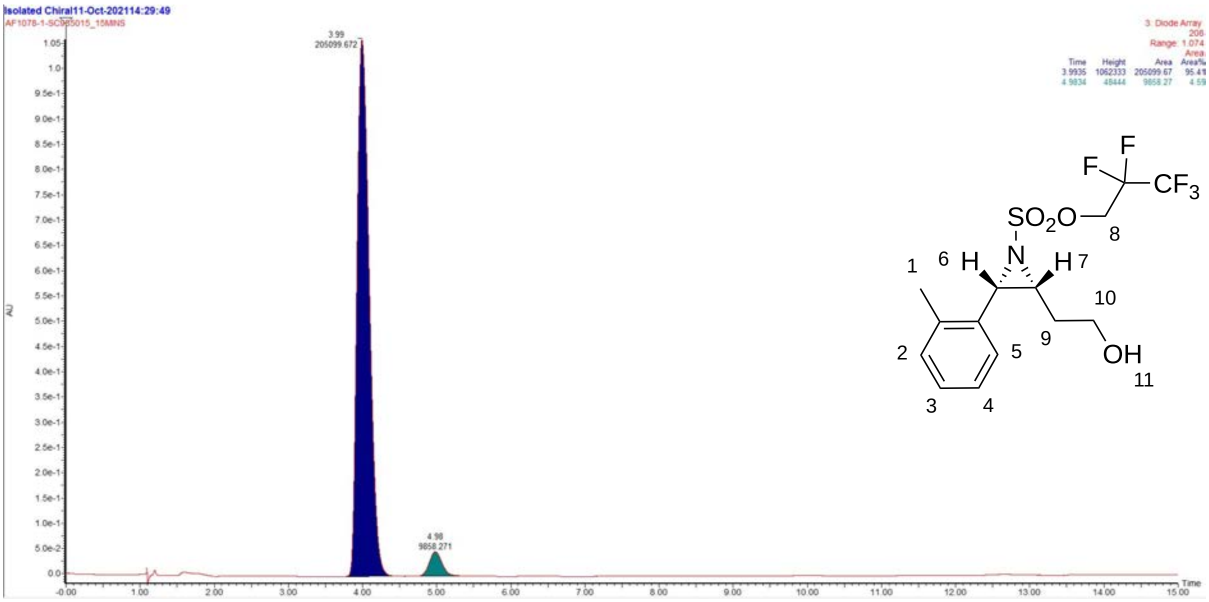

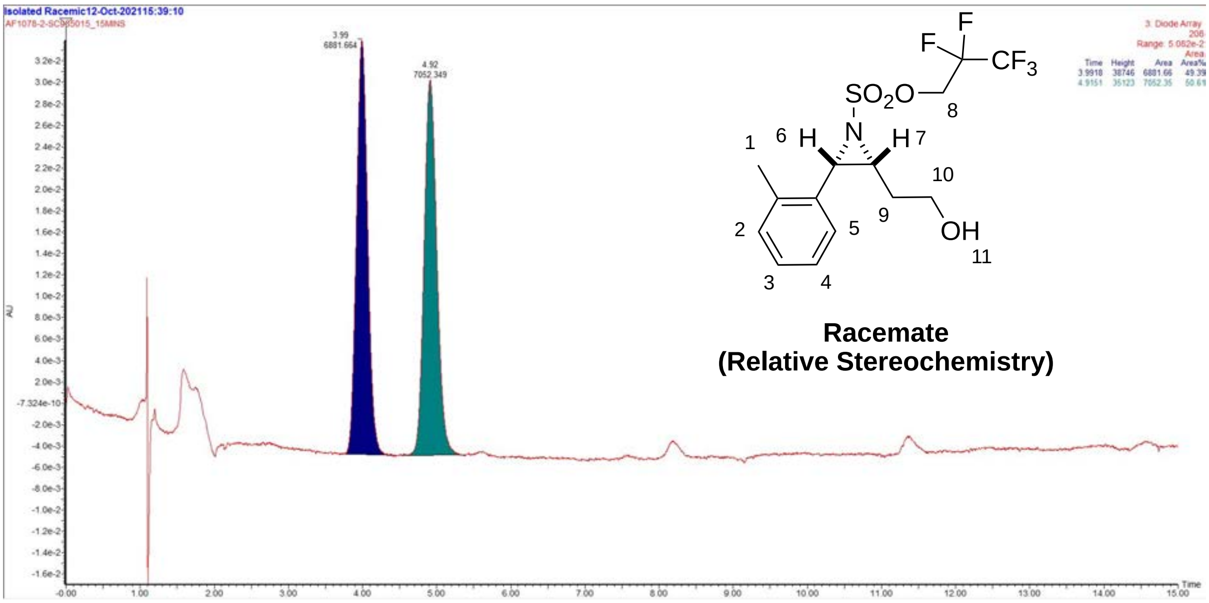

2,2,3,3,3-pentafluoropropyl (2R,3S)-2-(2-hydroxyethyl)-3-(2-methoxyphenyl)aziridine-1-sulfonate (**9c**)

Chiral SFC Analysis CHIRAL ART SC (CO_2:MeOH, 97.5:2.5, 1.25 mL min^{-1}, 40 °C, 218 nm) indicated 82% *ee*, t_R = 6.7 (major), 7.6 (minor) minutes.

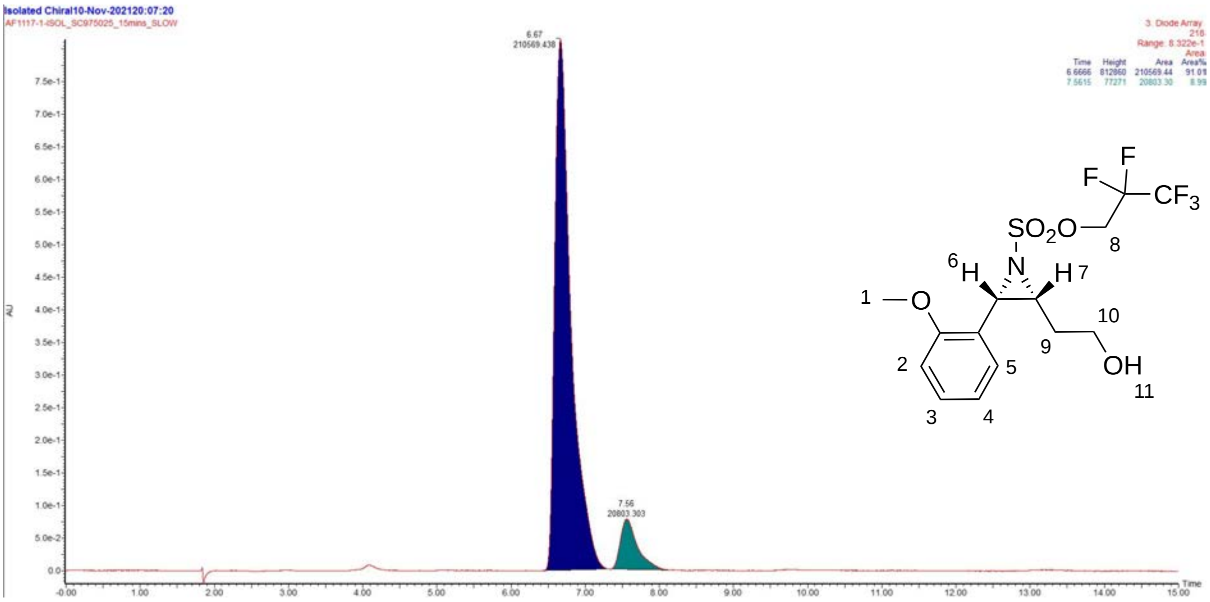

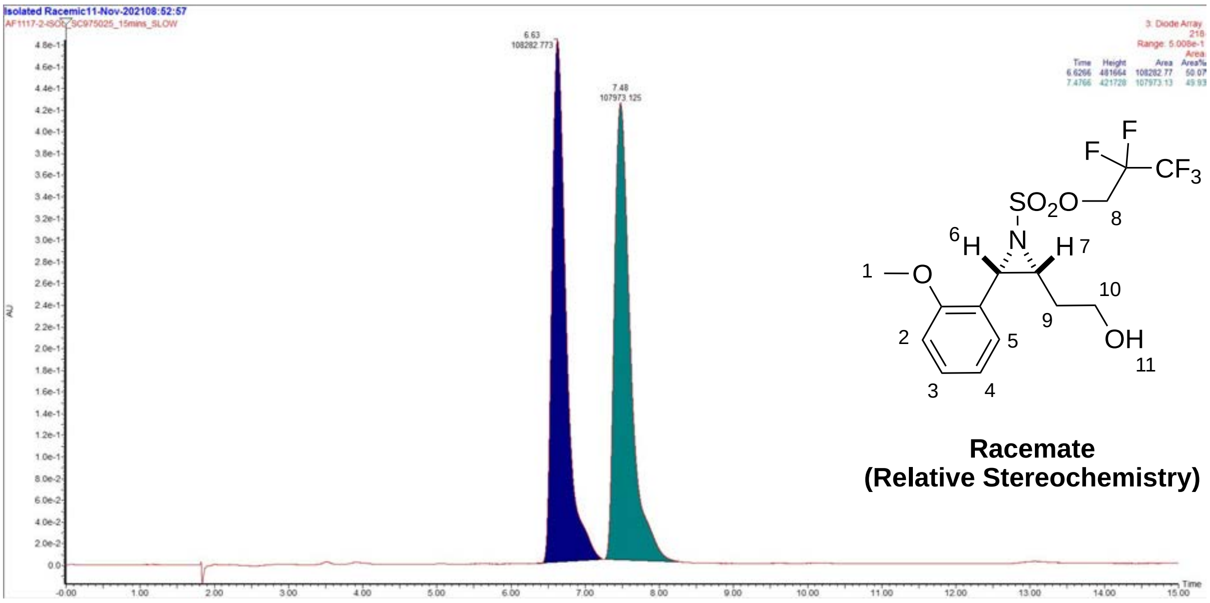

2,2,3,3,3-pentafluoropropyl (2S,3R)-2-(2-fluorophenyl)-3-(2-hydroxyethyl)aziridine-1-sulfonate (**9d**)

Chiral SFC Analysis CHIRALPAK IG (CO_2:MeOH, 98.5:1.5, 1.5 mL min^{-1}, 40 °C, 210 nm) indicated 90% *ee*, t_R = 6.8 (major), 8.5 (minor) minutes.

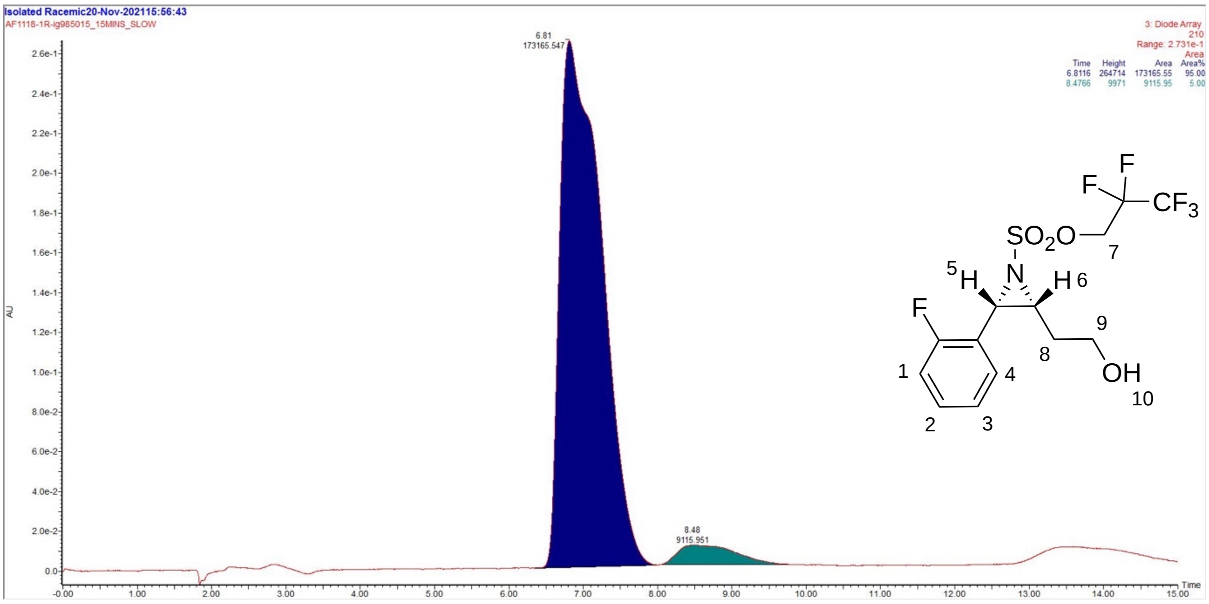

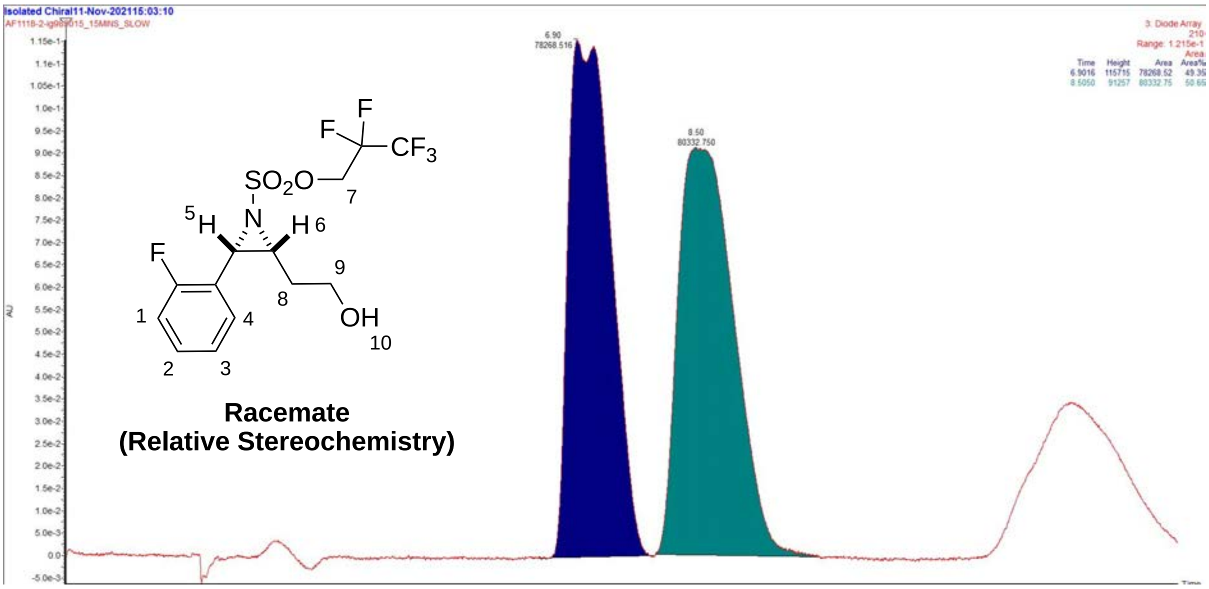

2,2,3,3,3-pentafluoropropyl (2S,3R)-2-(3-chlorophenyl)-3-(2-hydroxyethyl)aziridine-1-sulfonate (**9e**)

Chiral SFC Analysis CHIRAL ART SB (CO_2:MeOH, 98.5:1.5, 2.5 mL min^{-1}, 40 °C, 210 nm) indicated 91% *ee*, t_R = 7.6 (minor), 9.0 (major) minutes.

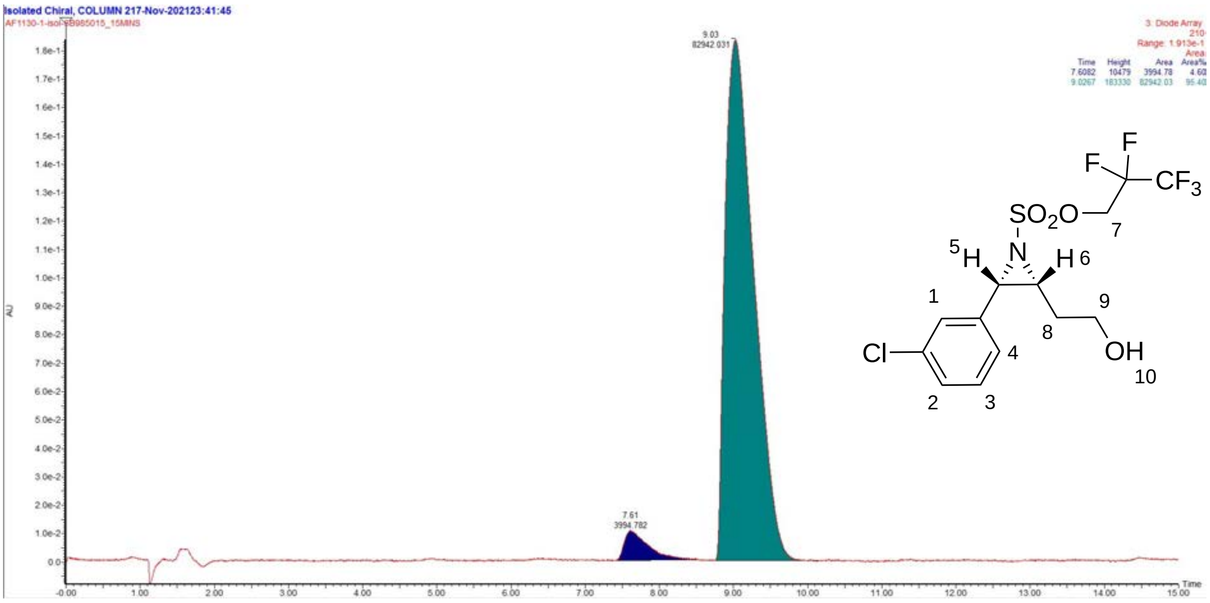

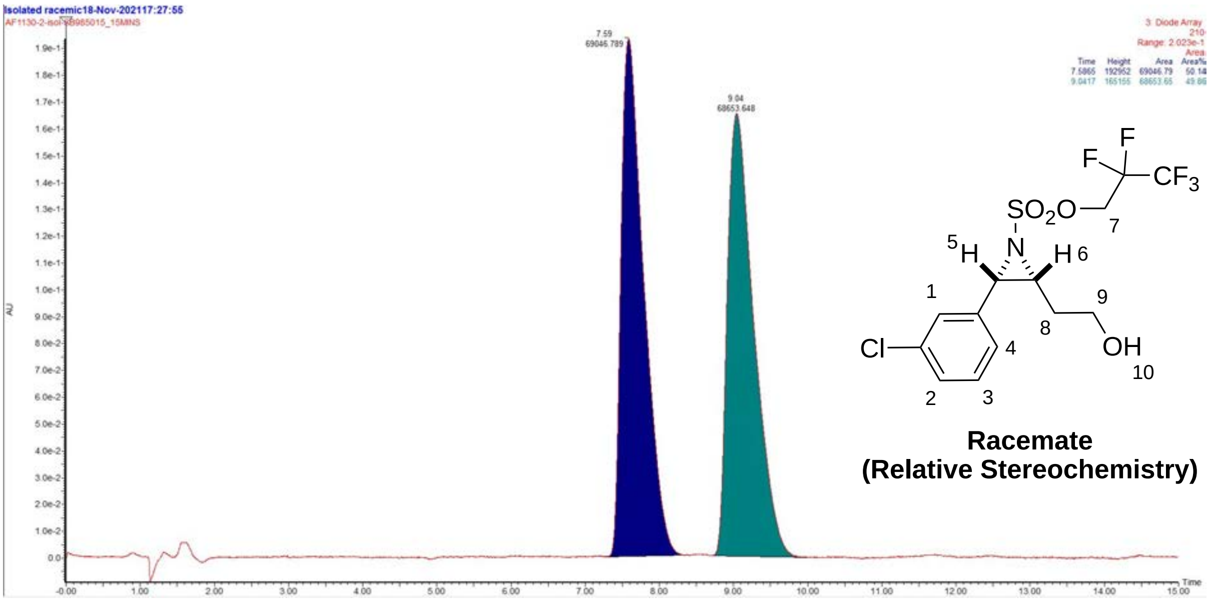

2,2,3,3,3-pentafluoropropyl (2R,3S)-2-(2-hydroxyethyl)-3-(3-(trifluoromethoxy)phenyl)aziridine-1-sulfonate (**9f**)

Chiral HPLC Analysis CHIRALPAK IC (Hexane:^{i}PrOH, 97:3, 1.0 mL min-1, 30 °C, 208 nm) indicated 91% *ee*, t_R = 13.8 (major), 22.2 (minor) minutes.

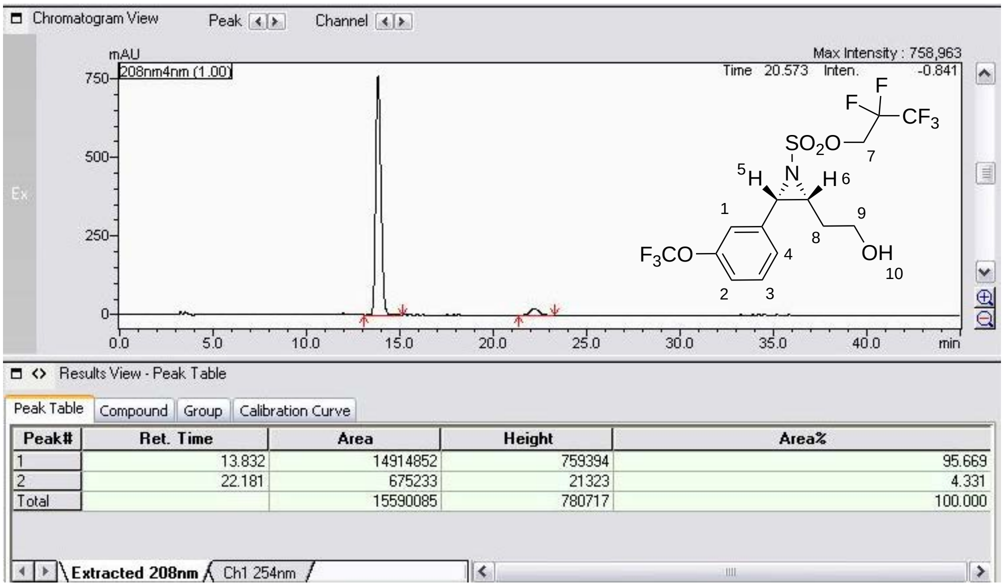

Peak#	Ret. Time	Area	Height	Area%
1	13.832	14914852	759394	95.669
2	22.181	675233	21323	4.331
Total		15590085	780717	100.000

Racemate (Relative Stereochemistry)

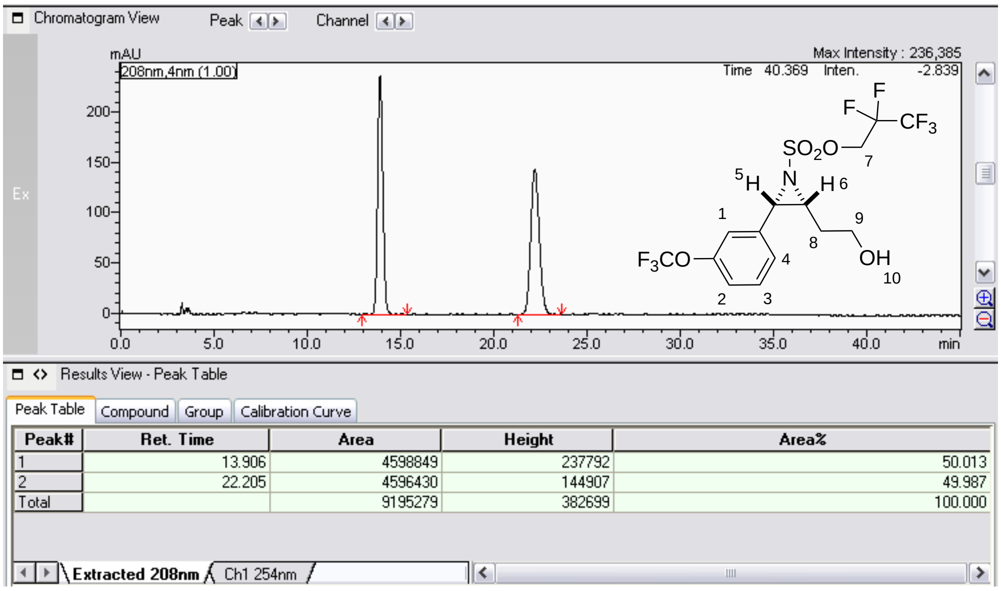

Peak#	Ret. Time	Area	Height	Area%
1	13.906	4598849	237792	50.013
2	22.205	4596430	144907	49.987
Total		9195279	382699	100.000

Ethyl 3-((2S,3R)-3-(2-hydroxyethyl)-1-((2,2,3,3,3-pentafluoropropoxy)sulfonyl)aziridin-2-yl)benzoate (**9g**)

Chiral SFC Analysis CHIRALPAK IE (CO_2:MeOH, 97:3, 2.5 mL min^{-1}, 40 °C, 226 nm) indicated 89% *ee*, t_R = 6.0 (major), 7.0 (minor) minutes

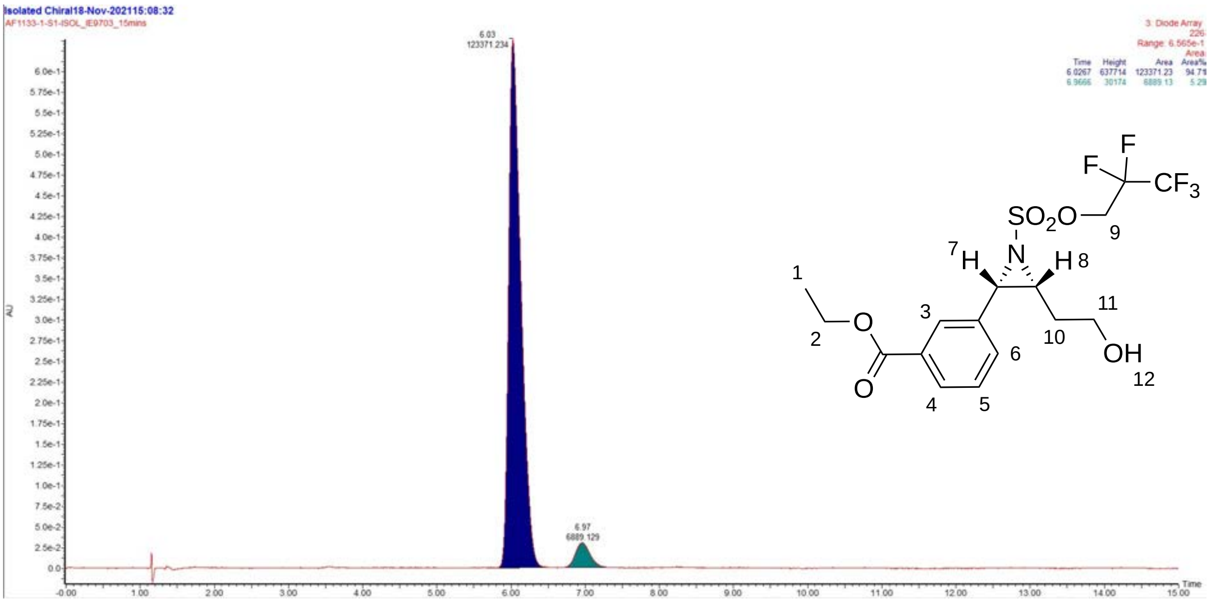

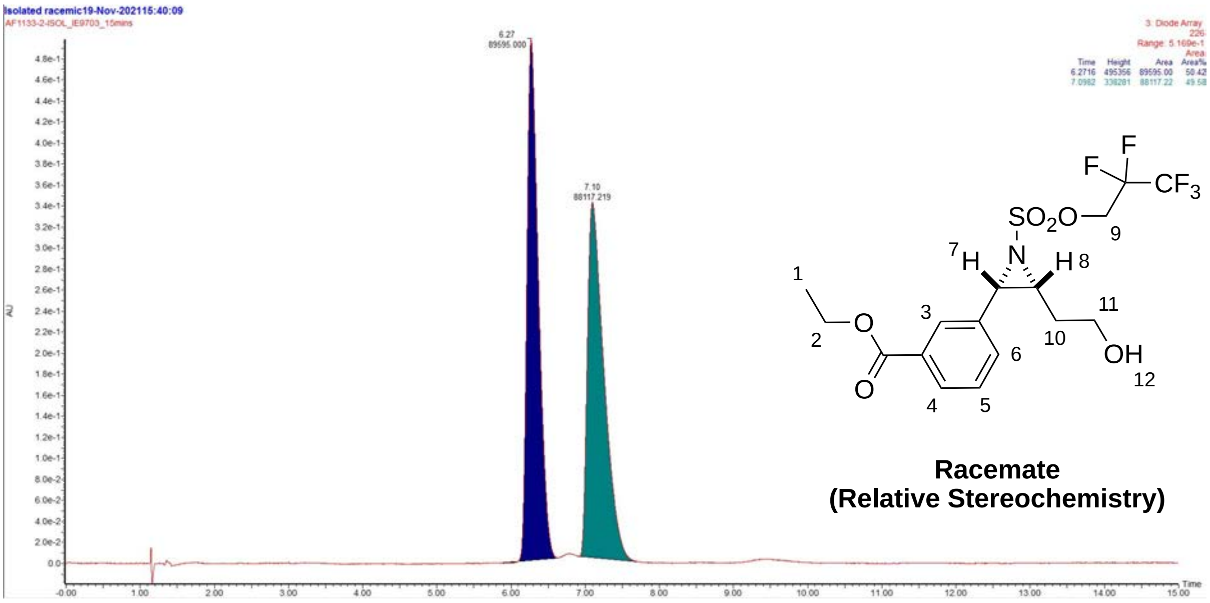

2,2,3,3,3-pentafluoropropyl (2S,3R)-2-(4-chlorophenyl)-3-(2-hydroxyethyl)aziridine-1-sulfonate (**9h**)

Chiral SFC Analysis CHIRART SC (CO_2:MeOH, 98:2, 2.5 mL min^{-1}, 40 °C, 222 nm) indicated 85% *ee*, t_R = 4.6 (major), 5.5 (minor) minutes.

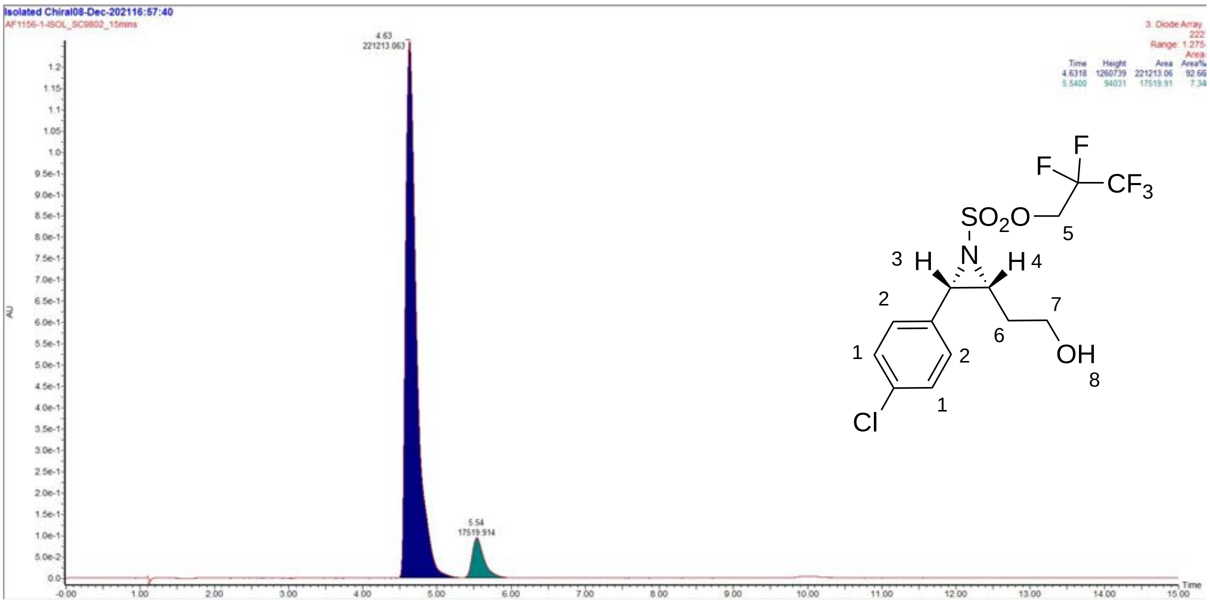

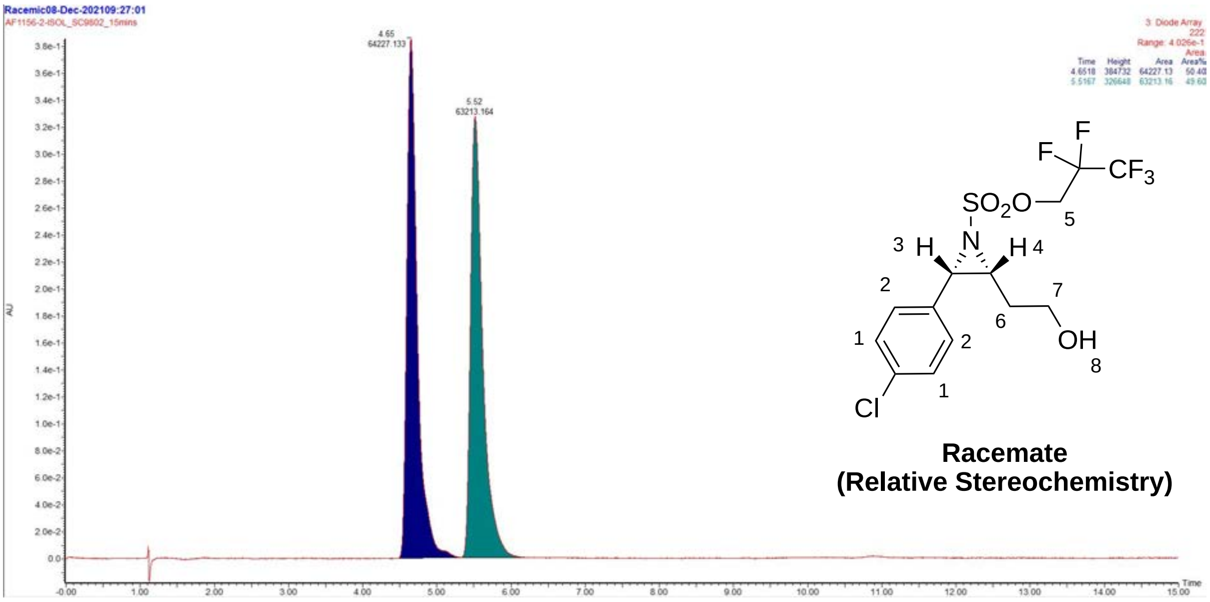

4-((2S,3R)-3-(2-hydroxyethyl)-1-((2,2,3,3,3-pentafluoropropoxy)sulfonyl)aziridin-2-yl)phenyl acetate (**9i**)

Chiral SFC Analysis CHIRAL ART SC (CO_2:MeOH, 97:3, 2.5 mL min^{-1}, 40 °C, 217 nm) indicated 87% *ee*, t_R = 4.4 (major), 5.3 (minor) minutes.

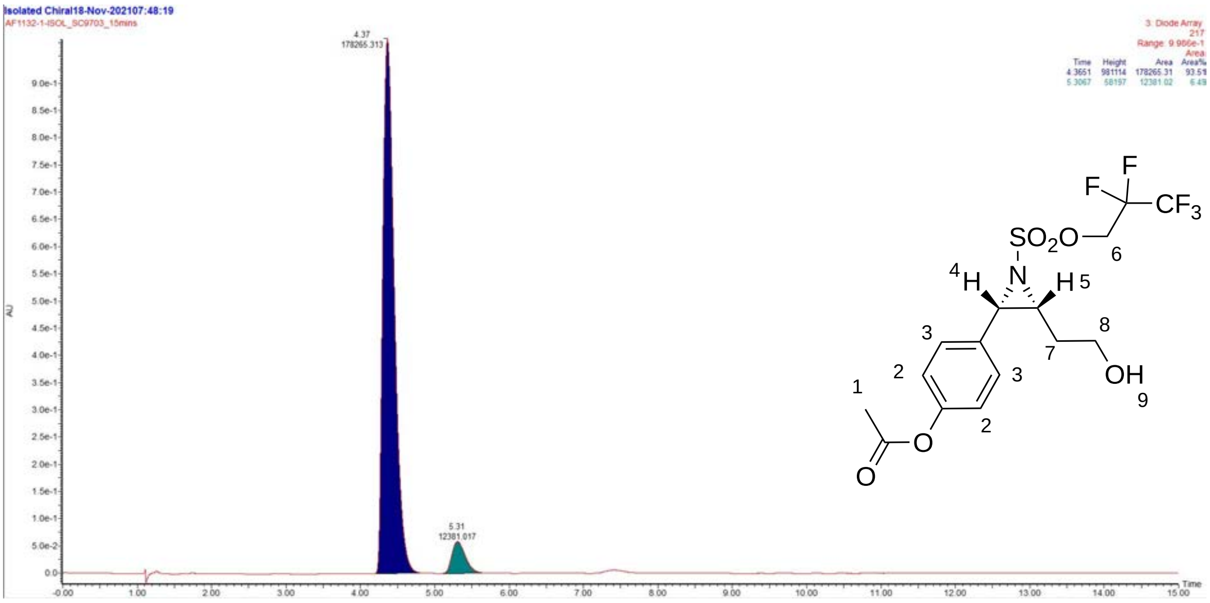

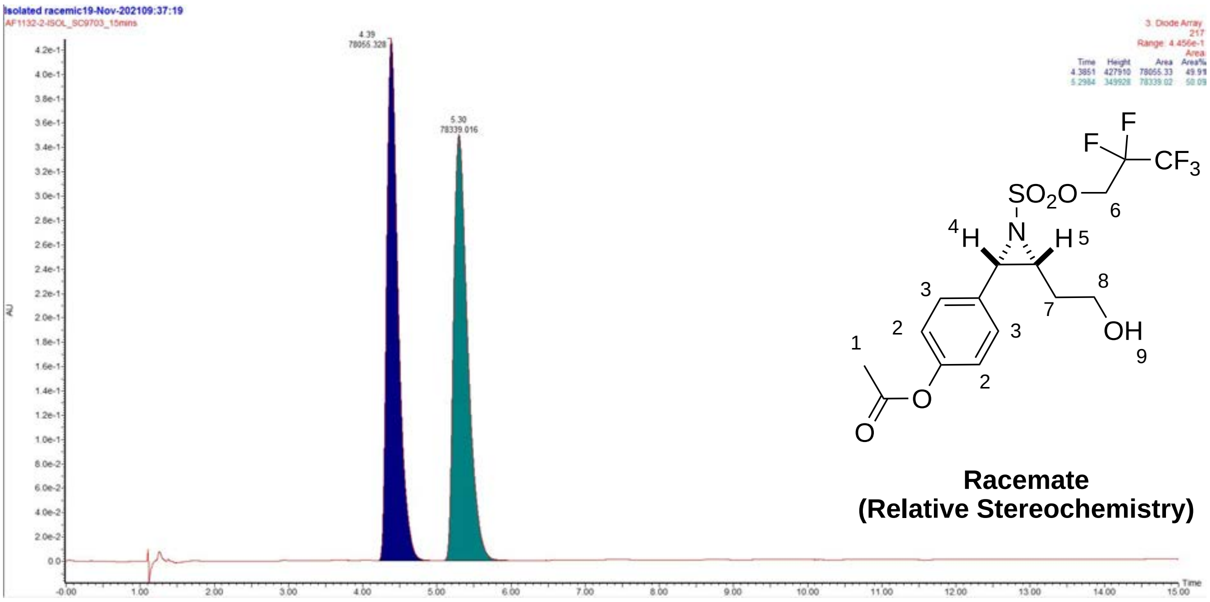

2,2,3,3,3-pentafluoropropyl (2R,3S)-2-(2-hydroxyethyl)-3-(4-(trifluoromethyl)phenyl)aziridine-1-sulfonate (**9j**)

Chiral SFC Analysis CHIRAL ART SC (CO_2:^{i}PrOH, 98.5:1.5, 1.25 mL min^{-1}, 40 °C, 215 nm) indicated 95% *ee*, t_R = 5.1 (minor), 6.0 (major) minutes.

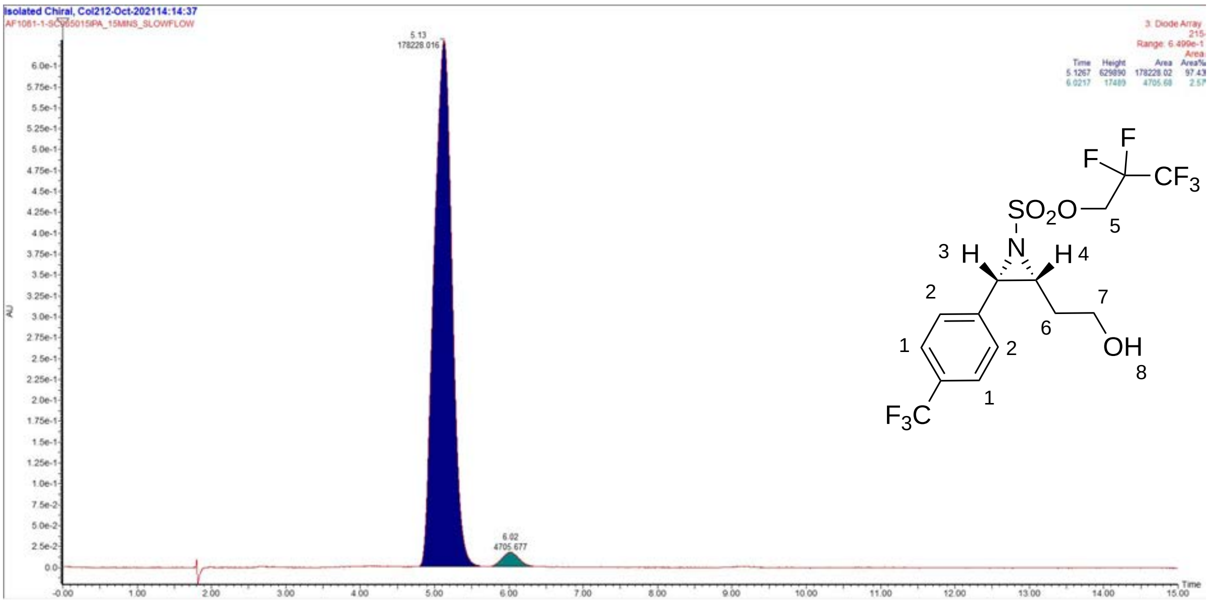

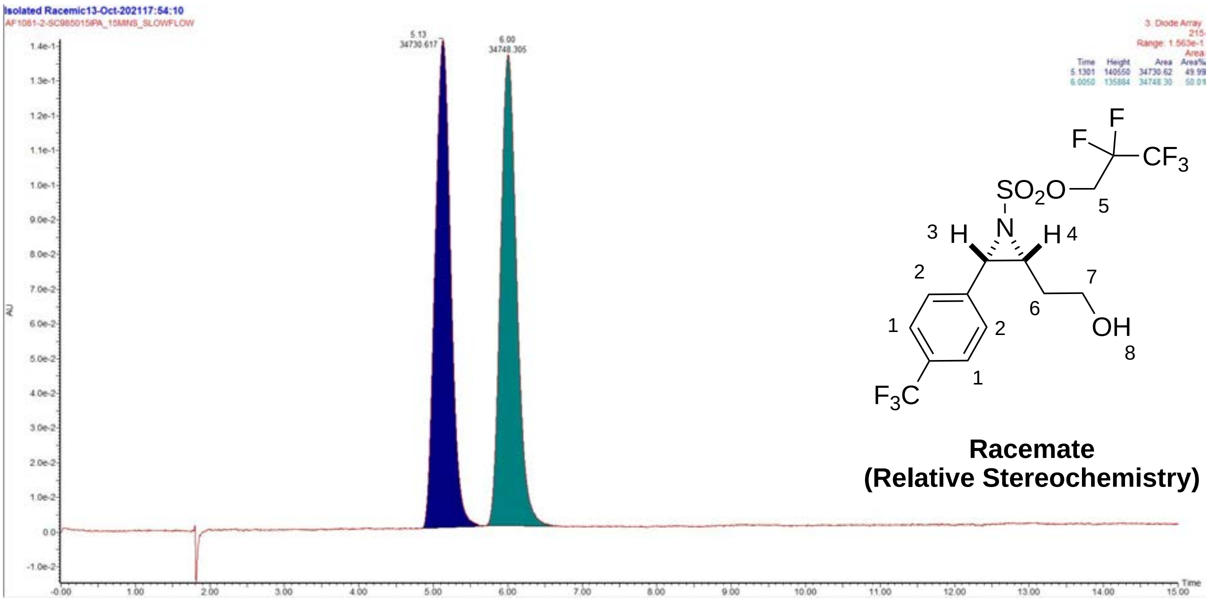

Ethyl 4-((2S,3R)-3-(2-hydroxyethyl)-1-((2,2,3,3,3-pentafluoropropoxy)sulfonyl)aziridin-2-yl)benzoate (**9k**)

Chiral SFC Analysis CHIRAL ART SJ (CO_2:MeOH, 97:3, 2.5 mL min^{-1}, 40 °C, 224 nm) indicated 96% *ee*, t_R = 4.1 (minor), 4.4 (major) minutes.

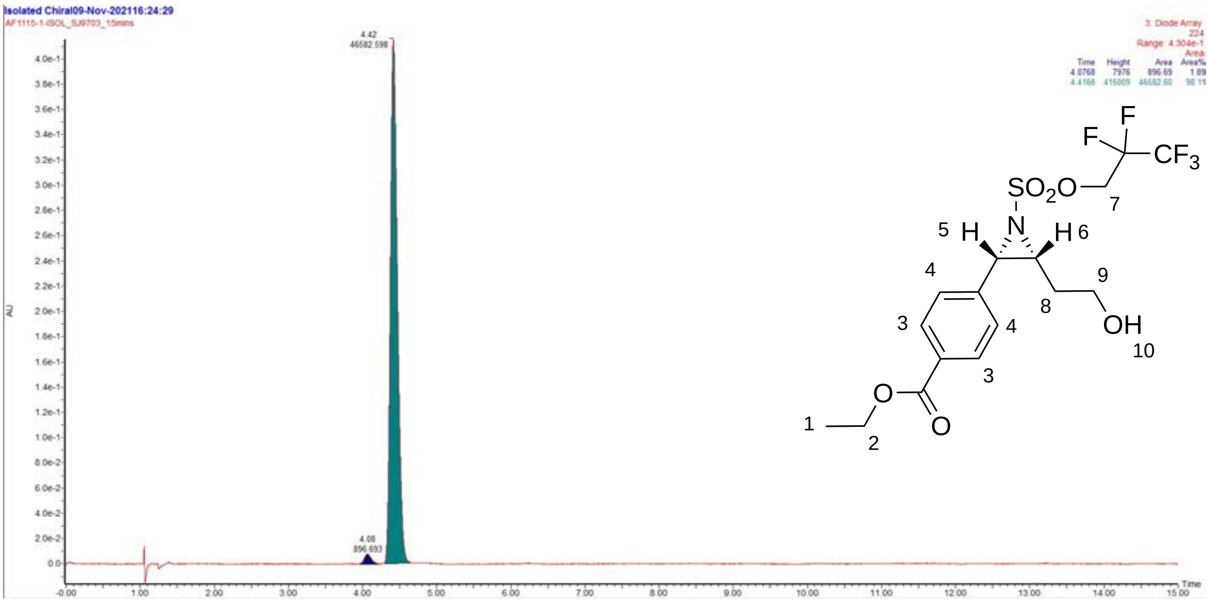

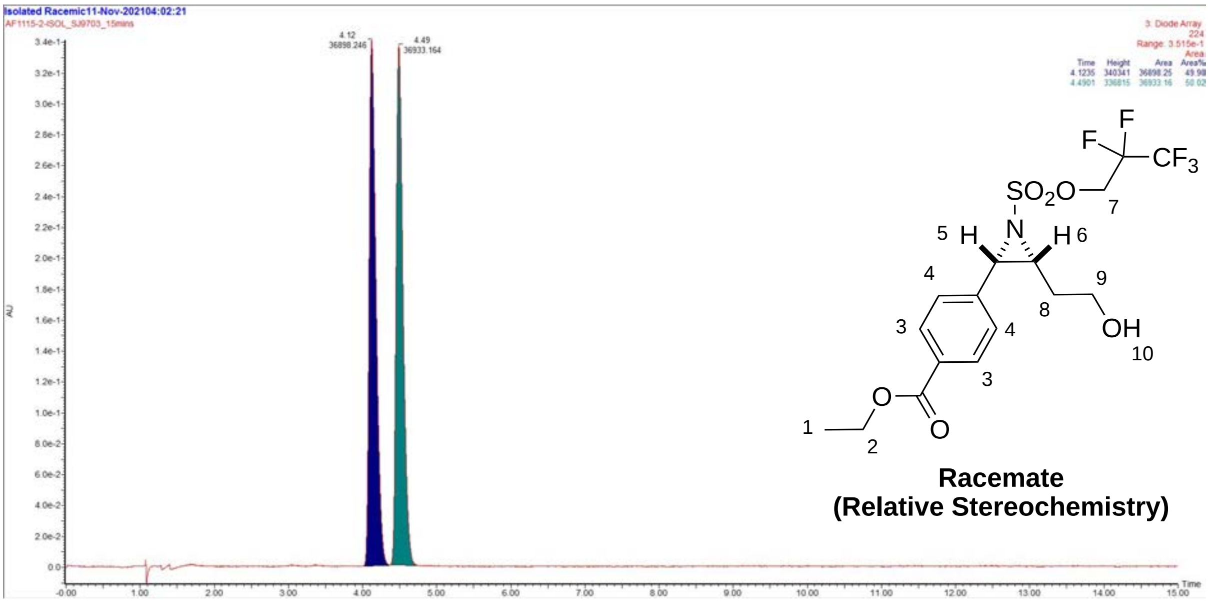

2,2,3,3,3-pentafluoropropyl (2S,3R)-2-(3,5-dimethylphenyl)-3-(2-hydroxyethyl)aziridine-1-sulfonate (**9l**)

Chiral SFC Analysis CHIRAL ART SC (CO_2:MeOH, 98.5:1.5, 2.5 mL min^{-1}, 40 °C, 210 nm) indicated 91% *ee*, t_R = 5.0 (major), 6.1 (minor) minutes.

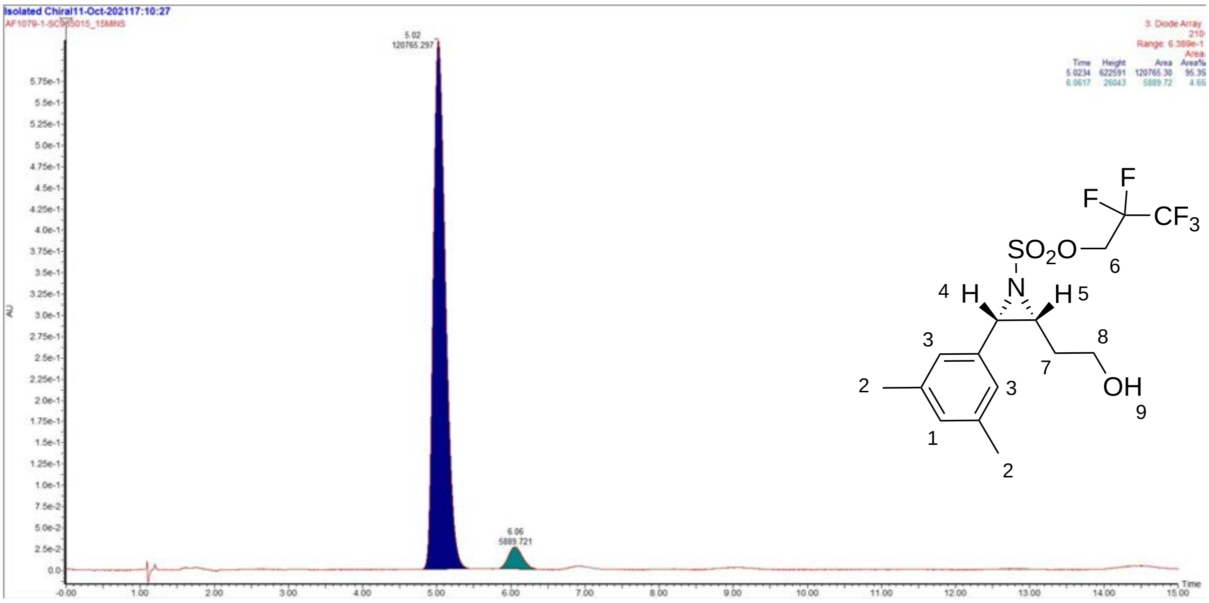

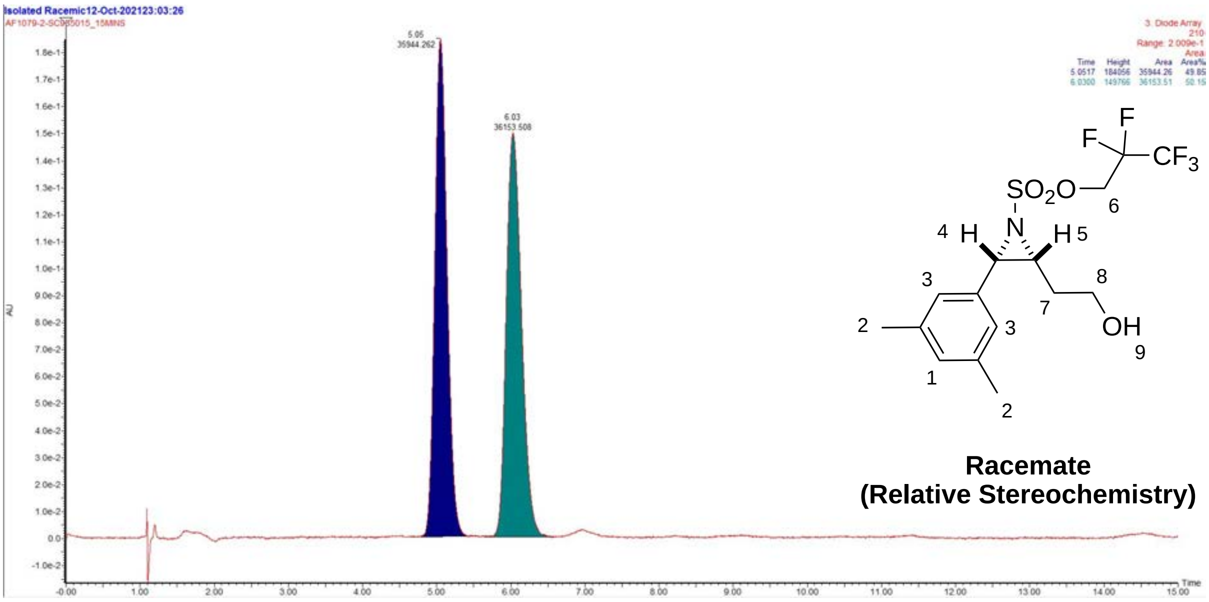

2,2,3,3,3-pentafluoropropyl (2S,3R)-2-(2,5-dimethylphenyl)-3-(2-hydroxyethyl)aziridine-1-sulfonate (**9m**)

Chiral SFC Analysis CHIRAL ART SC (CO_2:MeOH, 98.5:1.5, 2.5 mL min^{-1}, 40 °C, 215 nm) indicated 91% *ee*, t_R = 4.0 (major), 5.1 (minor) minutes.

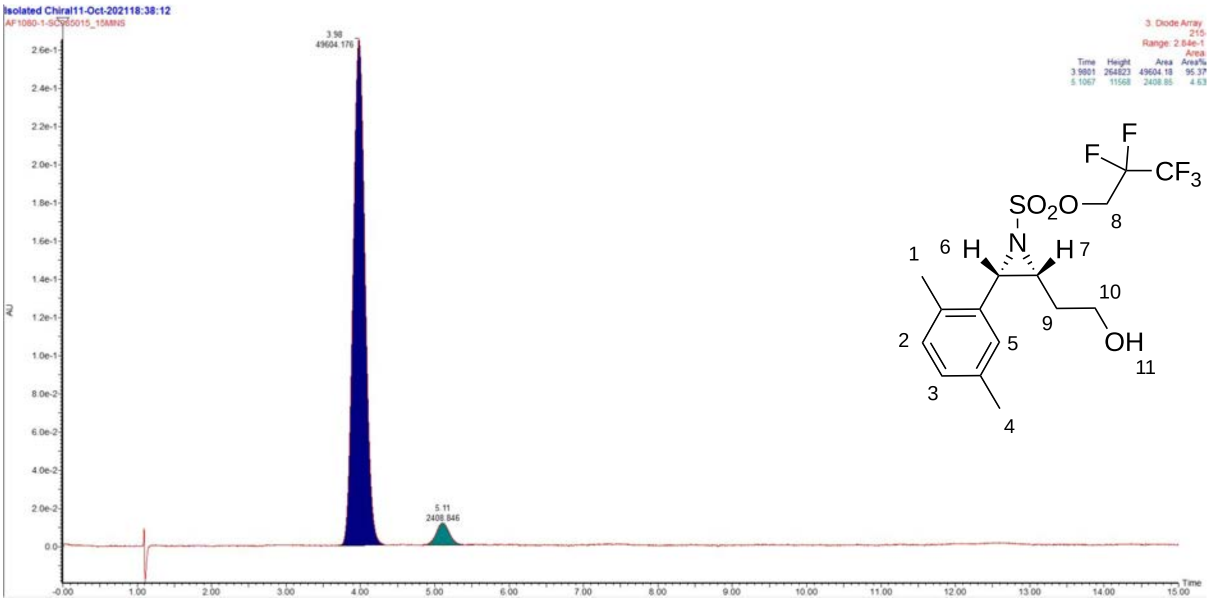

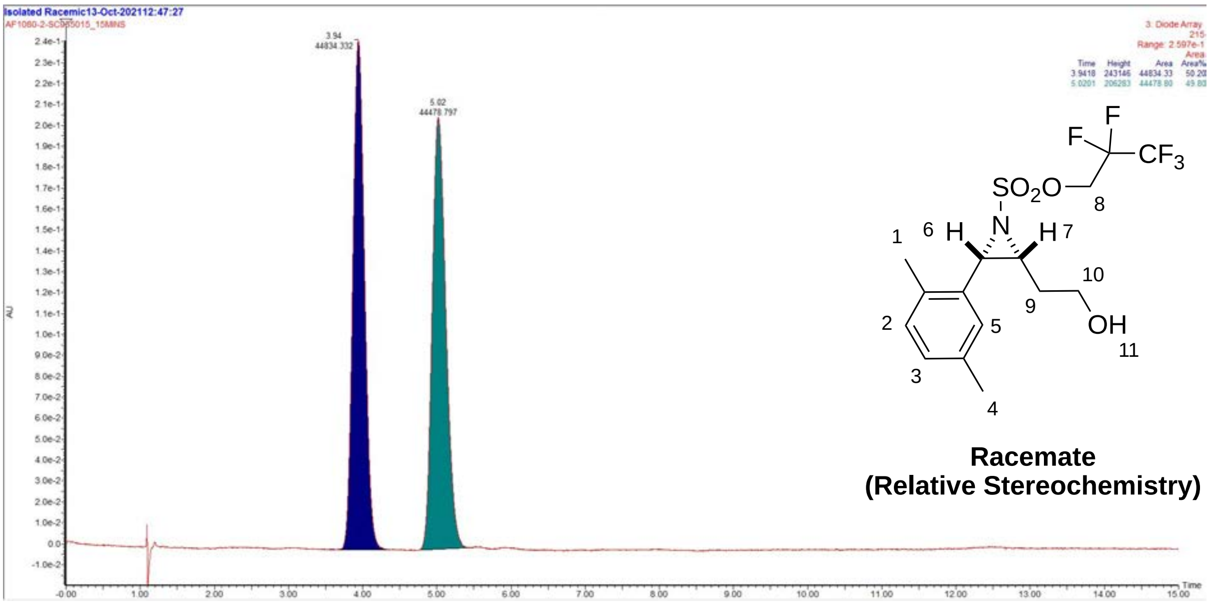

2,2,3,3,3-pentafluoropropyl (2S,3R)-2-(3-fluoro-4-methylphenyl)-3-(2-hydroxyethyl)aziridine-1-sulfonate (**9n**)

Chiral SFC Analysis CHIRAL ART SC (CO_2:^{i}PrOH, 98.5:1.5, 1.25 mL min^{-1}, 40 °C, 217 nm) indicated 92% *ee*, t_R = 8.3 (major), 9.5 (minor) minutes.

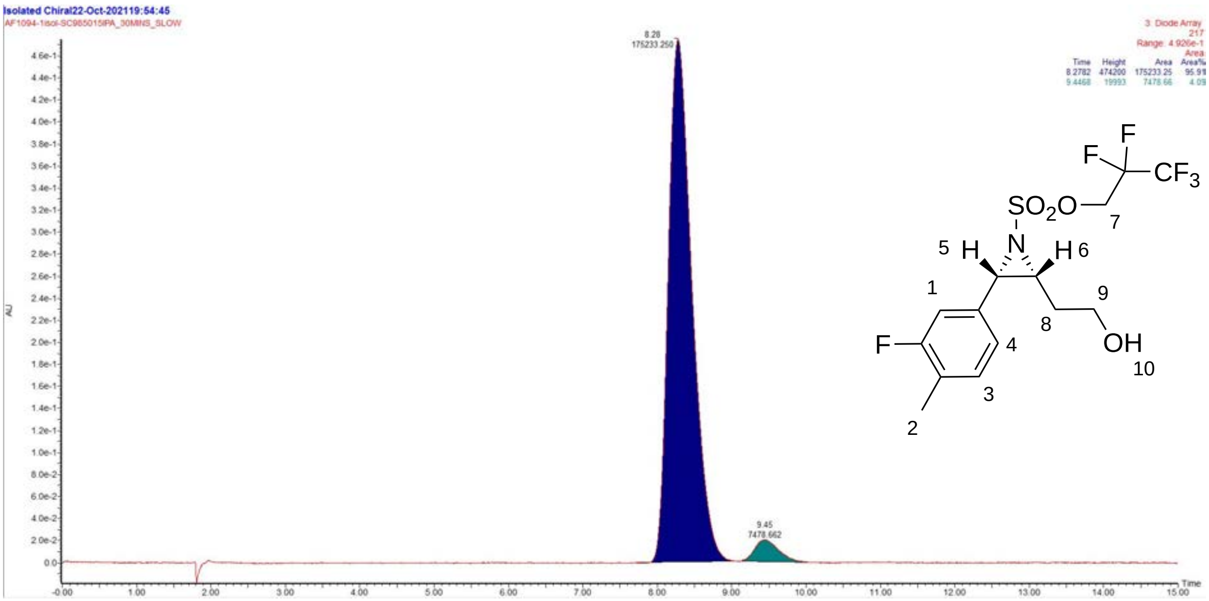

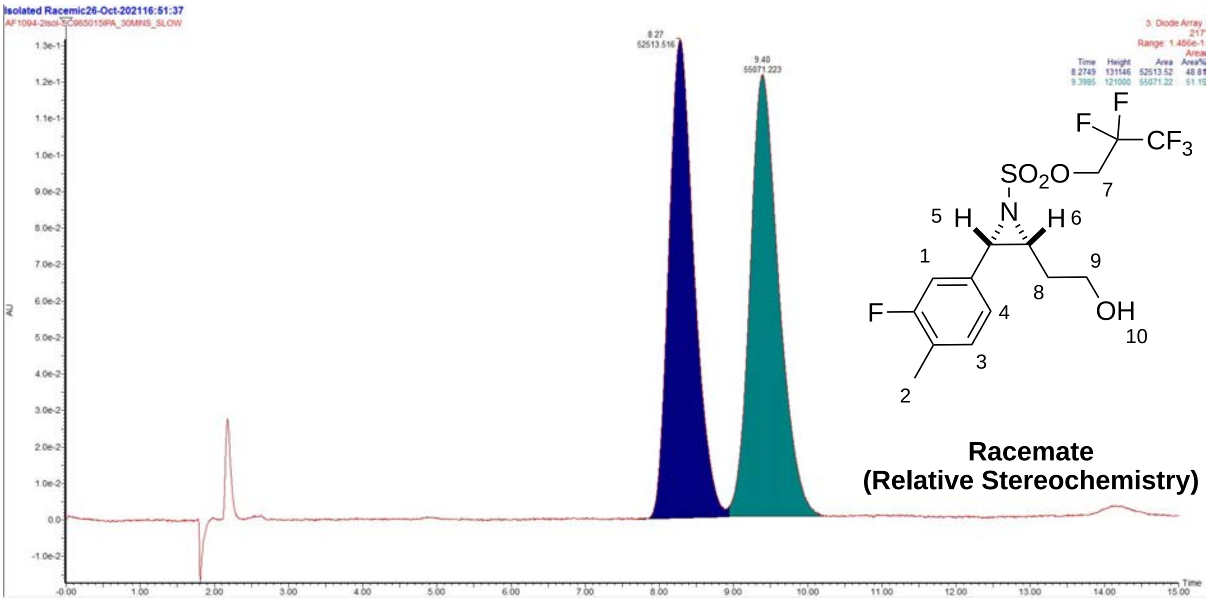

2,2,3,3,3-pentafluoropropyl (2R,3S)-2-(2-hydroxyethyl)-3-(naphthalen-1-yl)aziridine-1-sulfonate (**9o**)

Chiral SFC Analysis CHIRAL ART SC (CO_2:MeOH, 97:3, 2.5 mL min^{-1}, 40 °C, 219 nm) indicated 92% *ee*, t_R = 4.4 (major), 5.5 (minor) minutes.

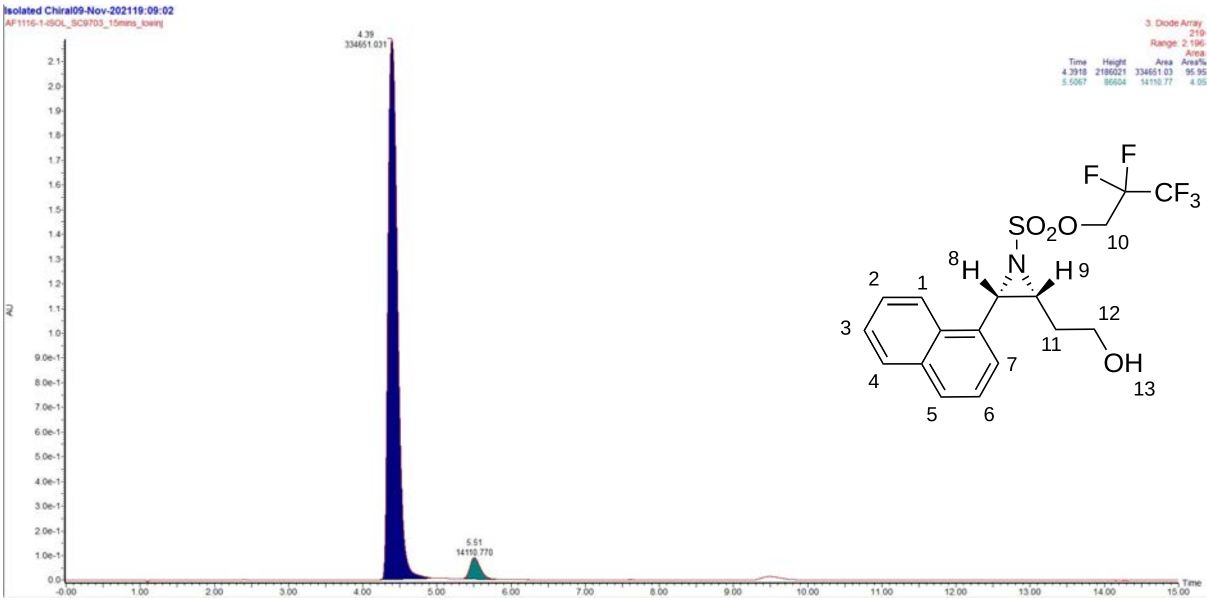

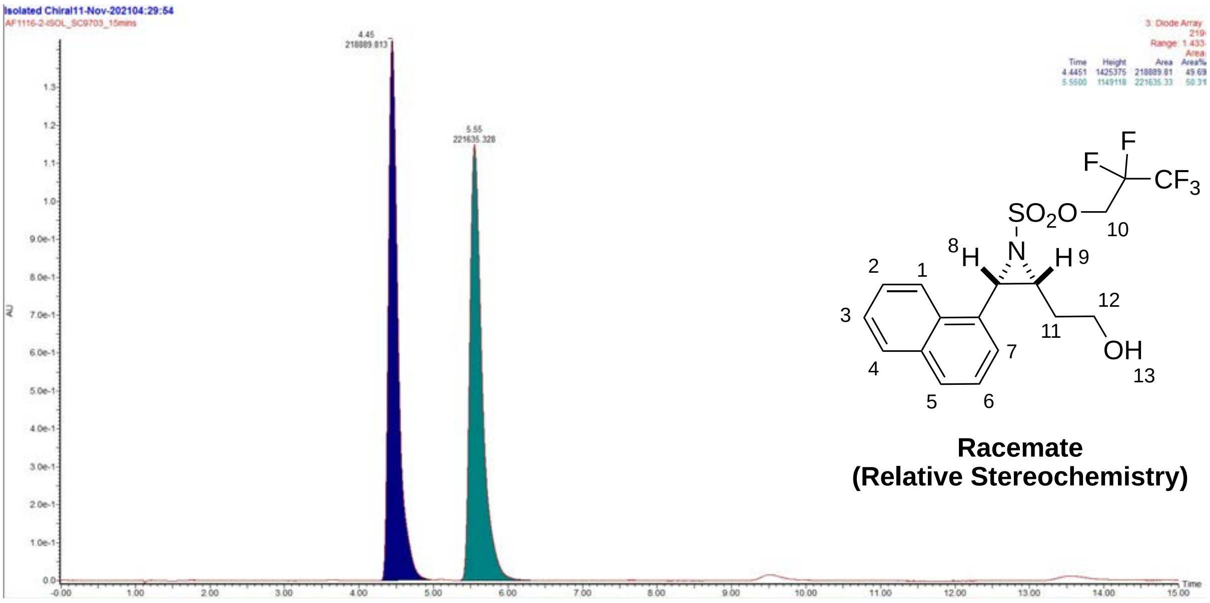

2,2,3,3,3-pentafluoropropyl ((2S,3R)-2-phenyltetrahydrofuran-3-yl)sulfamate (**12**)

Chiral SFC Analysis CHIRAL ART SC (CO_2:MeOH, 98:2, 1.25 mL min^{-1}, 40 °C, 210 nm) indicated 34% *ee*, t_R = 8.2 (major), 9.1 (minor) minutes.

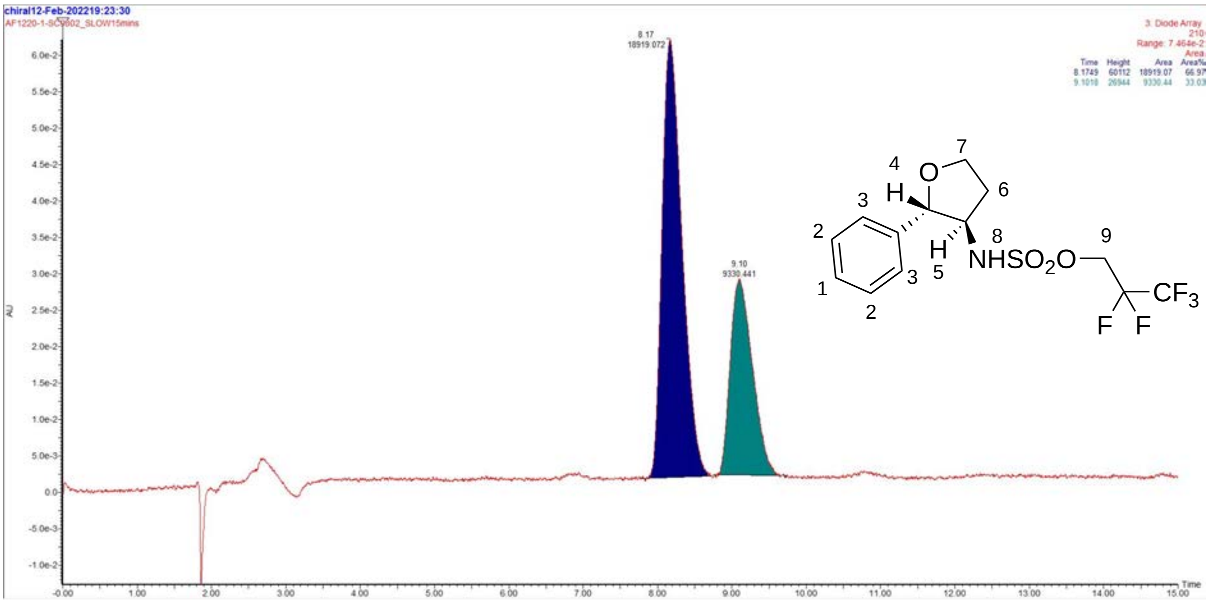

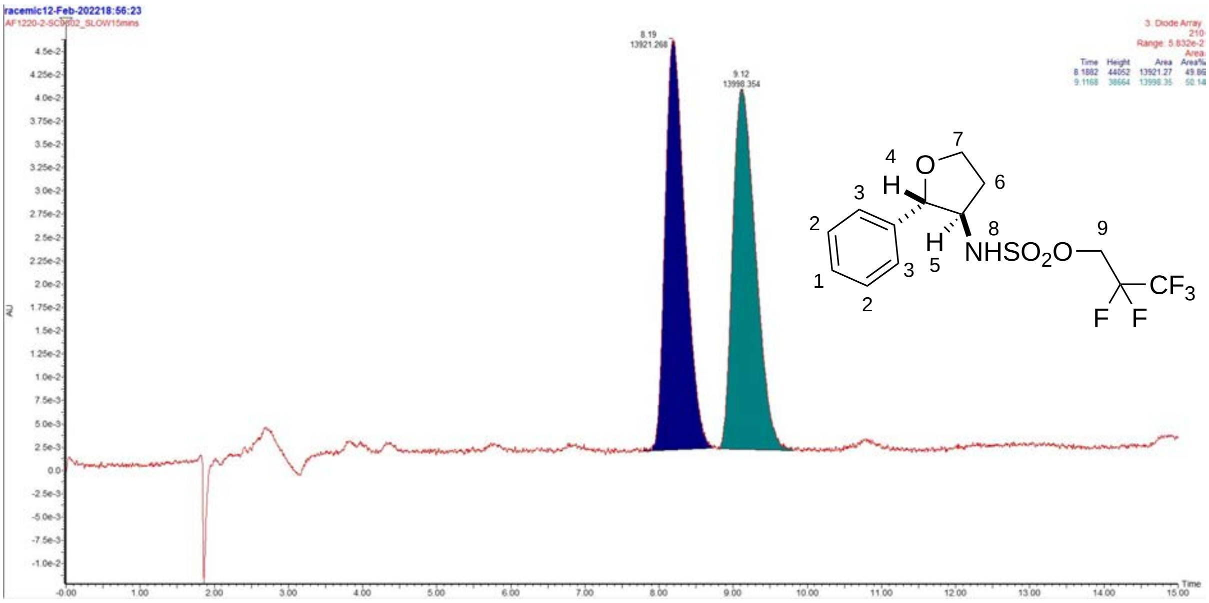

2,2,3,3,3-pentafluoropropyl (2R,3S)-2-(4-hydroxybutyl)-3-phenylaziridine-1-sulfonate (**14a**)

Chiral SFC Analysis CHIRAL ART SJ (CO_2:MeOH, 98:2, 2.5 mL min^{-1}, 40 °C, 210 nm) indicated 96% *ee*, t_R = 3.1 (major), 3.5 (minor) minutes.

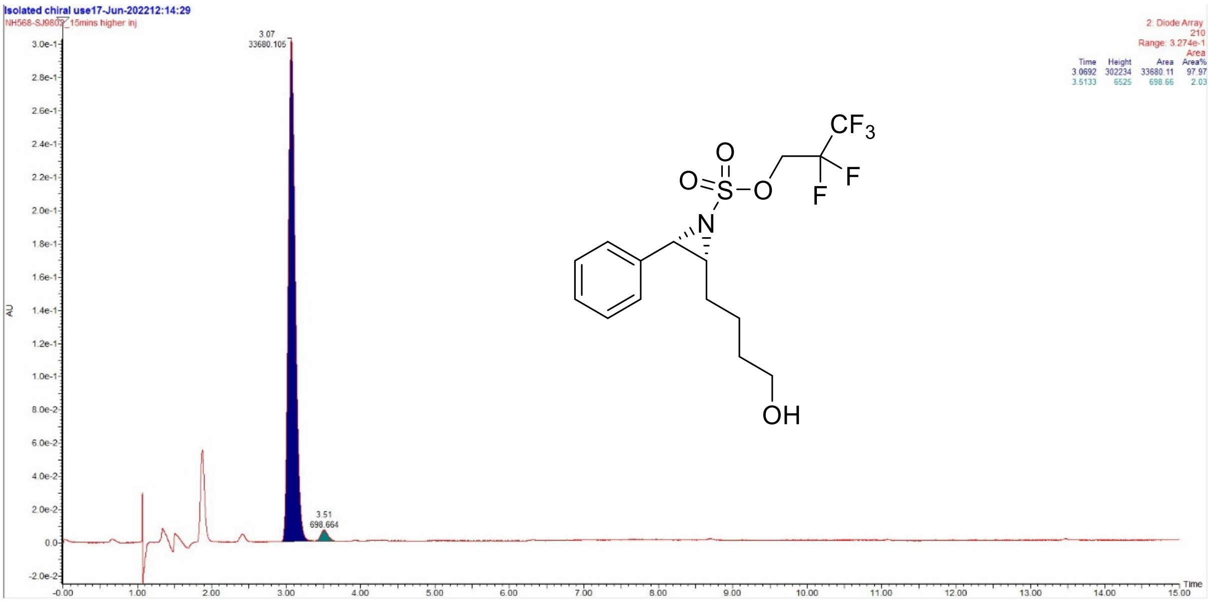

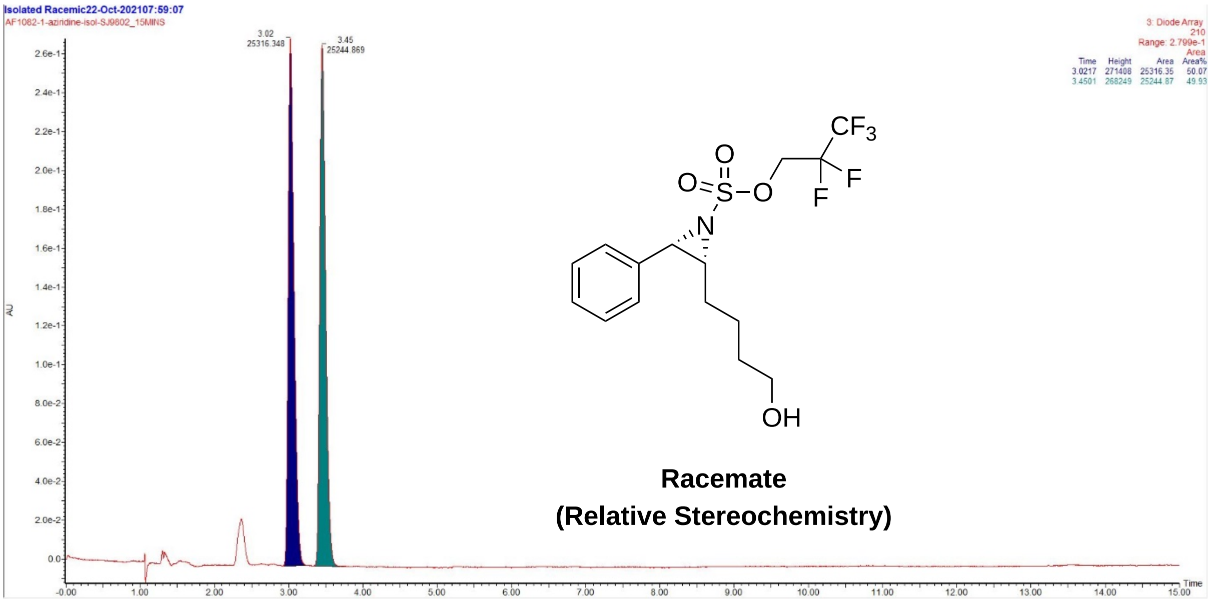

2,2,3,3,3-pentafluoropropyl (2S,3R)-2-(3-chlorophenyl)-3-(4-hydroxybutyl)aziridine-1-sulfonate (**14b**)

Chiral SFC Analysis: CHIRAL ART SC (CO_2;^{i}PrOH, 97:3, 2.5 mL min^{-1}, 40 °C, 220 nm) indicated 95% *ee*, t_R = 8.5 (major), 10.3 (minor) minutes.

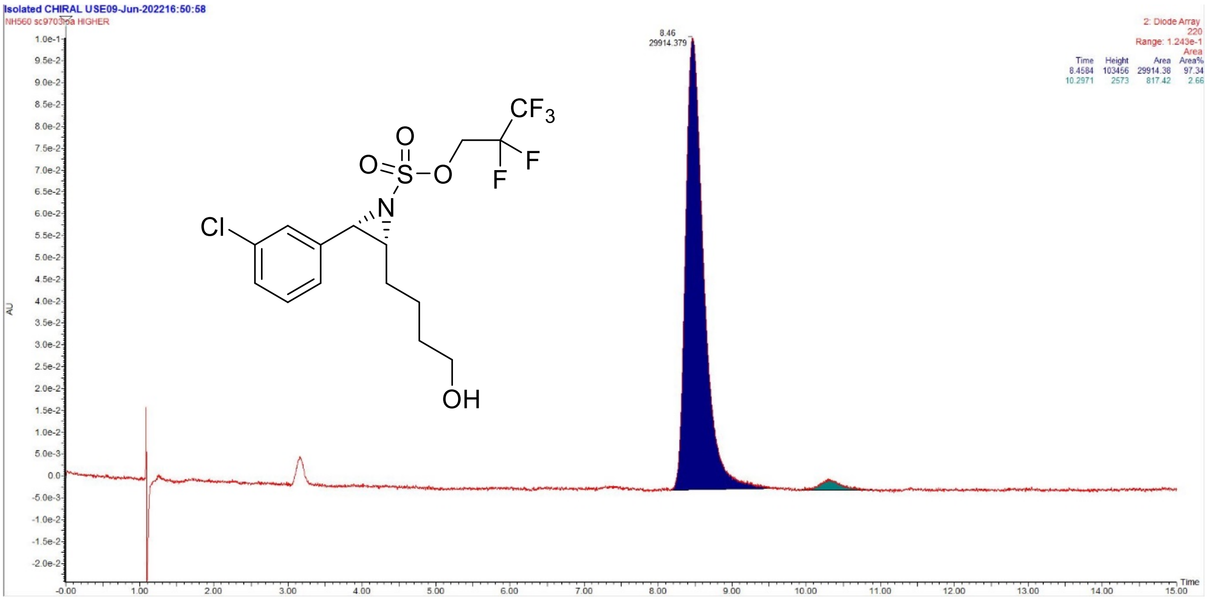

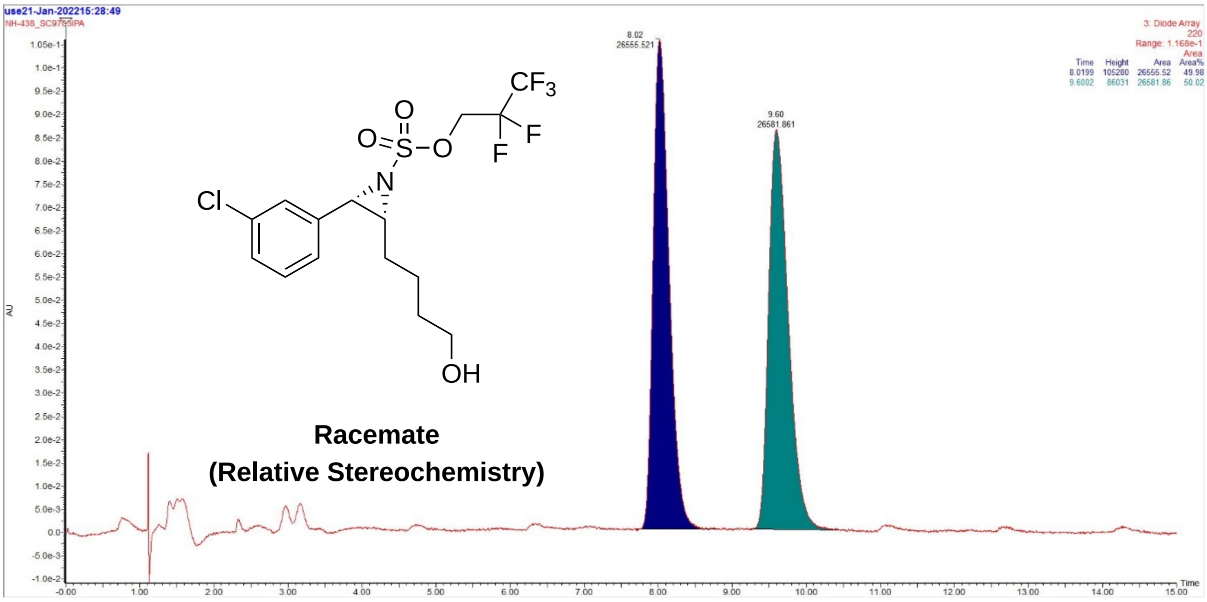

2,2,3,3,3-pentafluoropropyl (2R,3S)-2-(4-hydroxybutyl)-3-(3-(N-methylmethylsulfonamido)phenyl)aziridine-1-sulfonate (**14c**)

Chiral SFC Analysis: CHIRAL ART SC (CO_2:MeOH, 93:7, 2.5 mL min^{-1}, 40 °C, 220 nm) indicated 94% *ee*, t_R = 6.9 (major), 7.8 (minor) minutes.

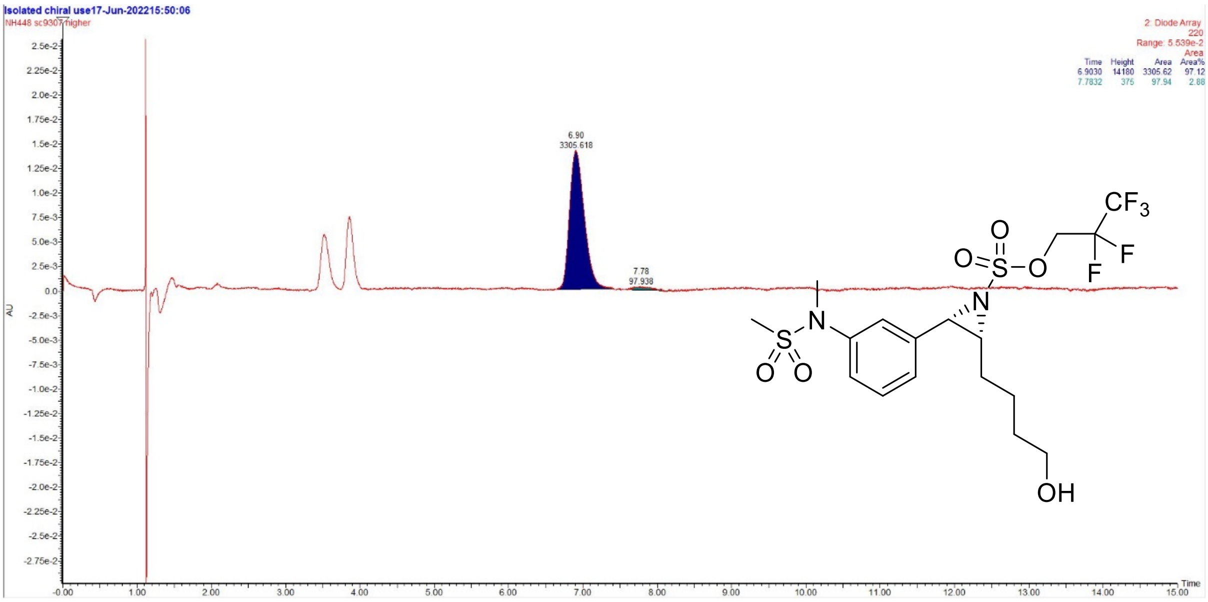

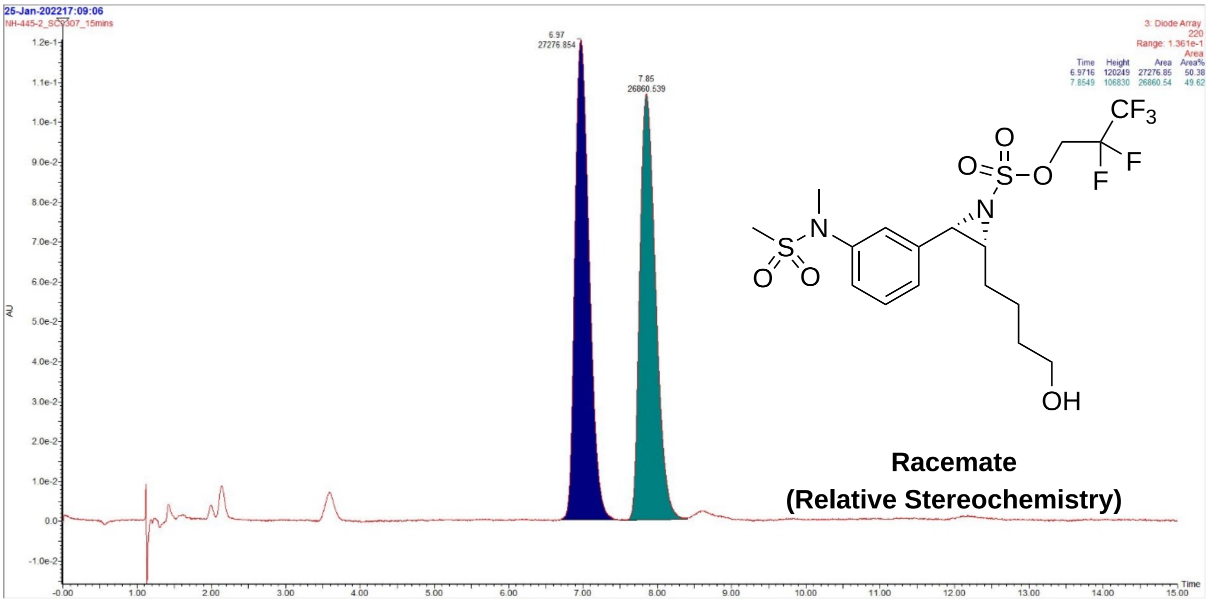

Methyl 2-(3-((2S,3R)-3-(4-hydroxybutyl)-1-((2,2,3,3,3-pentafluoropropoxy)sulfonyl)aziridin-2-yl)phenyl)acetate (**14d**)

Chiral SFC Analysis: CHIRAL ART SC (CO_2:^{i}PrOH, 95:5, 2.5 mL min^{-1}, 40 °C, 220 nm) indicated 95% *ee*, t_R = 8.6 (major), 9.7 (minor) minutes.

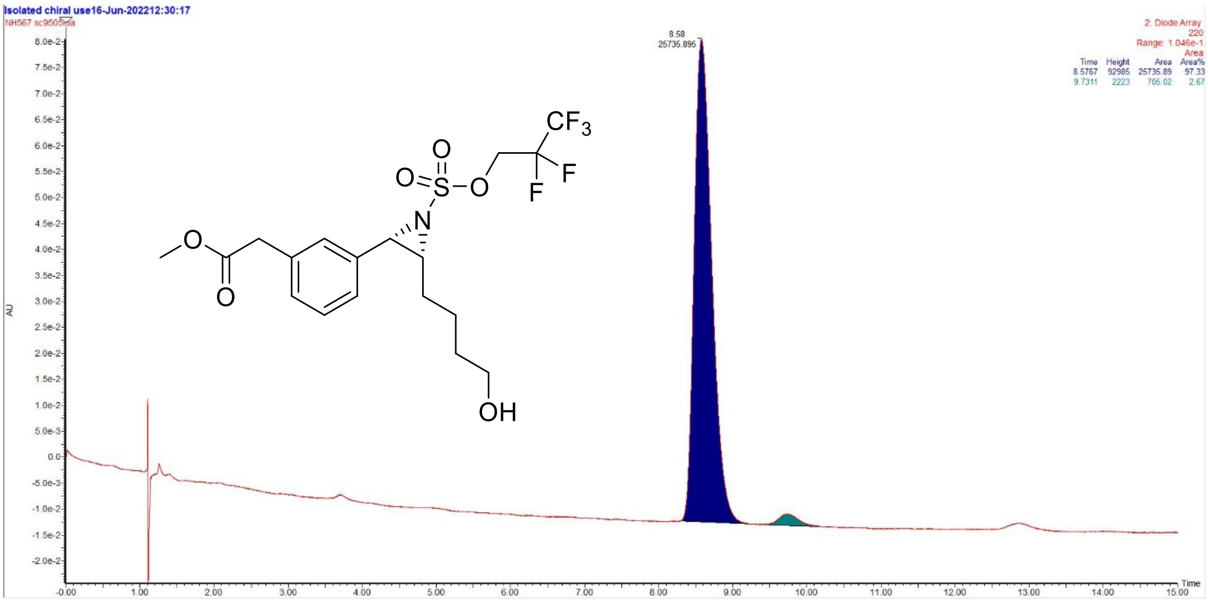

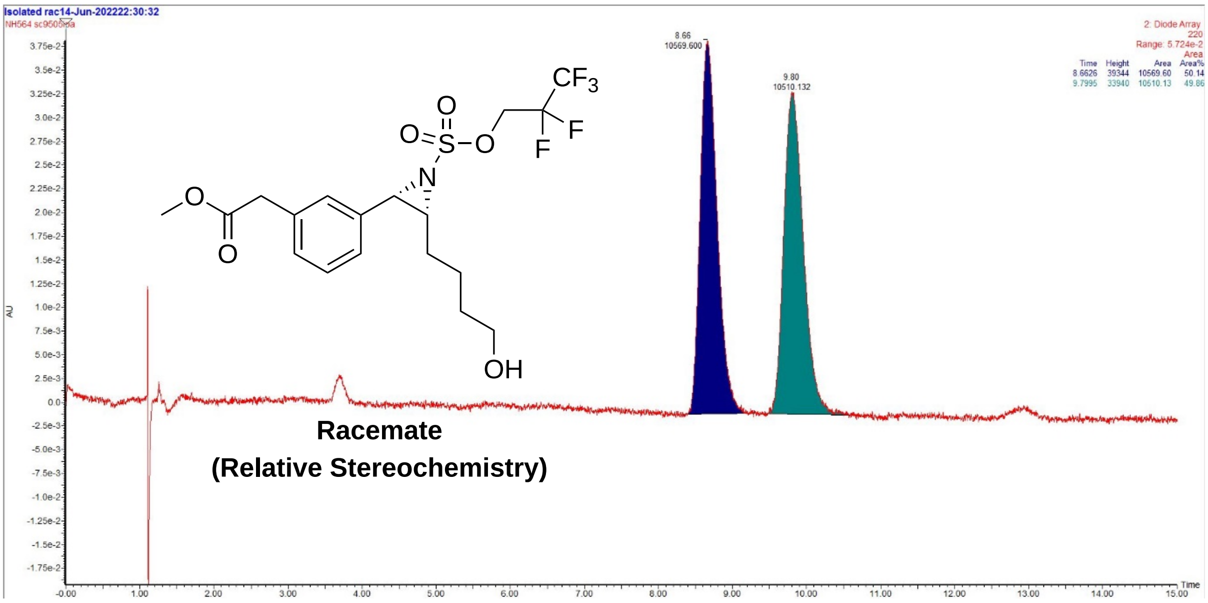

2,2,3,3,3-pentafluoropropyl (2S,3R)-2-(3-(N,N-diethylsulfamoyl)phenyl)-3-(4-hydroxybutyl)aziridine-1-sulfonate (**14e**)

Chiral SFC Analysis: CHIRAL ART SC (CO_2:^{i}PrOH, 83:17, 2.5 mL min^{-1}, 40 °C, 210 nm) indicated 90% *ee*, t_R = 3.4 (minor), 3.9 (major) minutes.

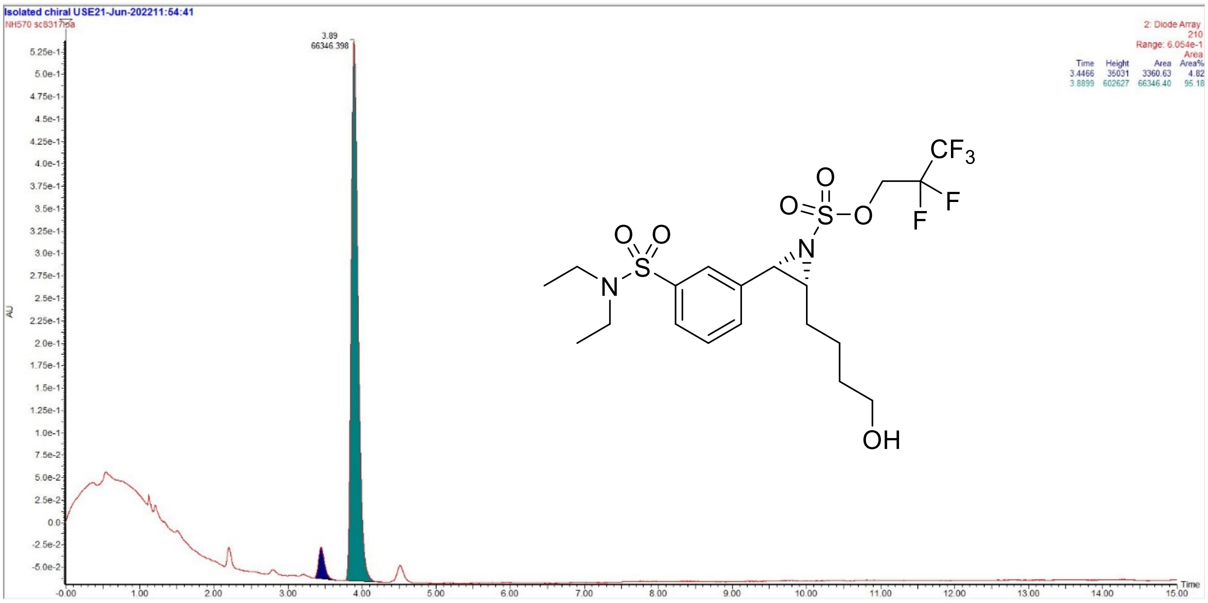

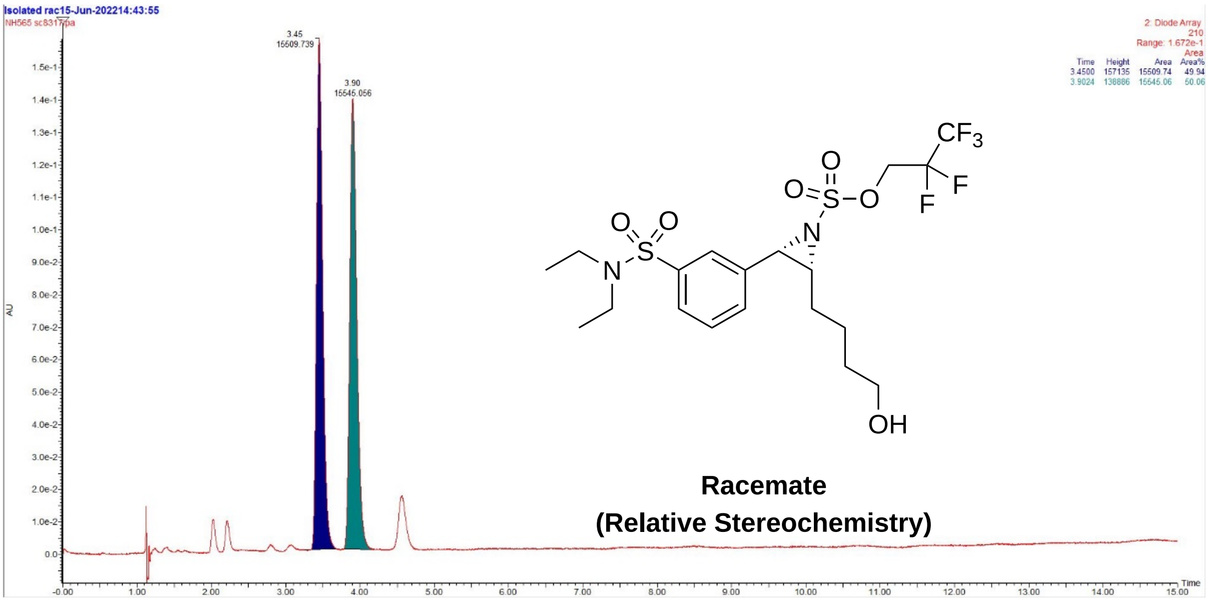

2,2,3,3,3-pentafluoropropyl (2S,3R)-2-(2-chlorophenyl)-3-(4-hydroxybutyl)aziridine-1-sulfonate (**14f**)

Chiral SFC Analysis: CHIRAL ART SC (CO_2;^{i}PrOH, 97:3, 2.5 mL min^{-1}, 40 °C, 220 nm) indicated 94% *ee*, t_R = 6.9 (major), 7.8 (minor) minutes.

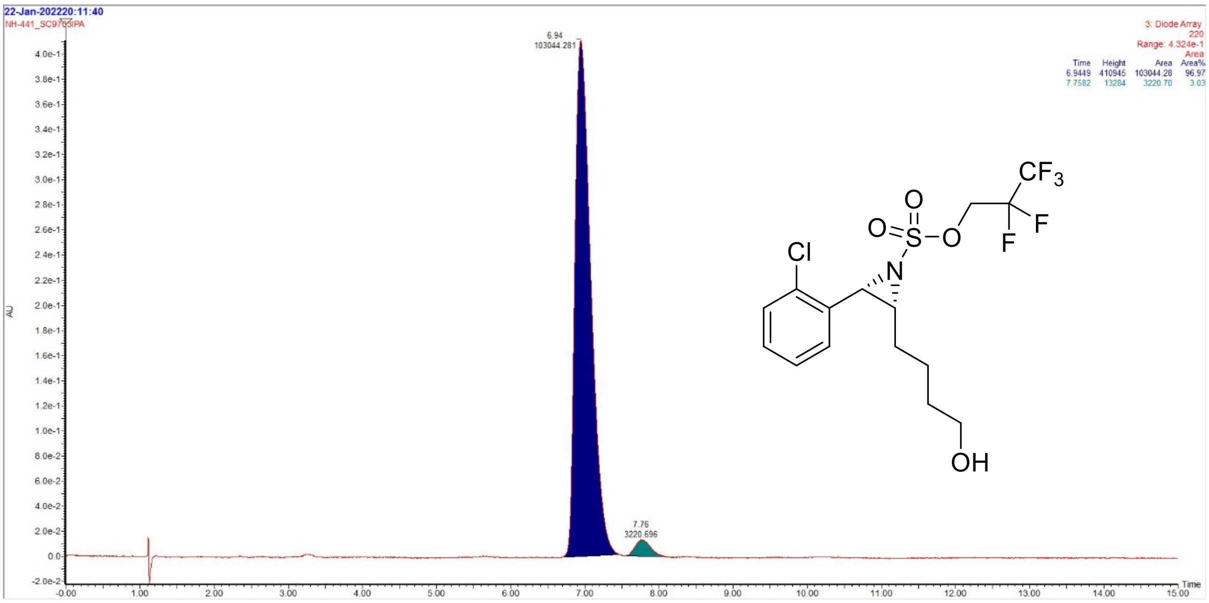

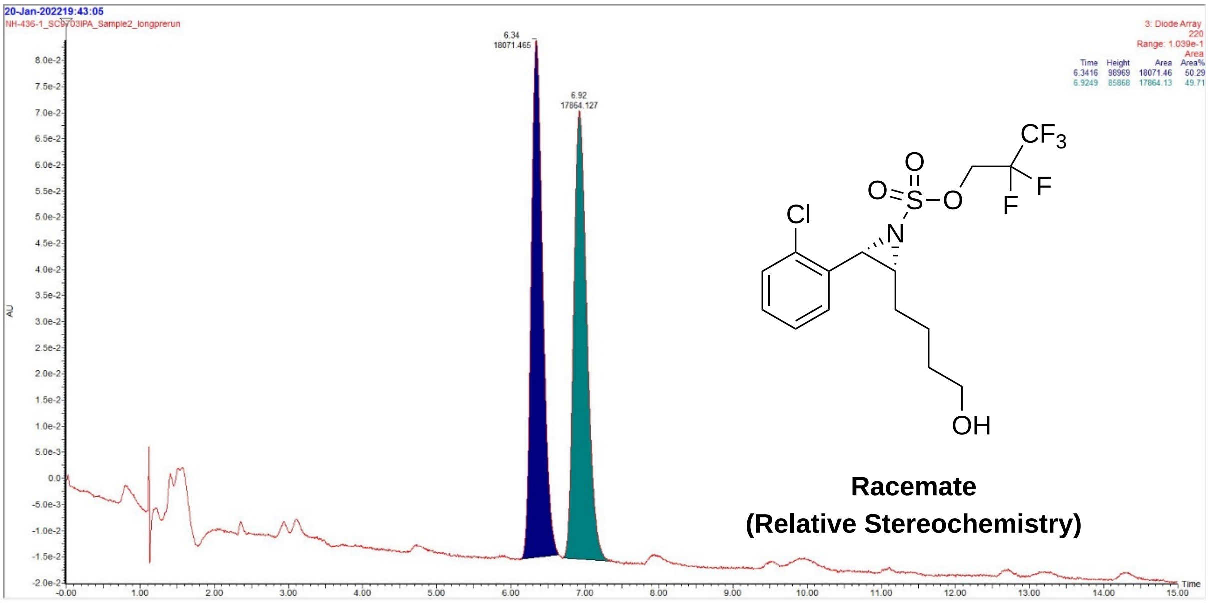

2,2,3,3,3-pentafluoropropyl (2S,3R)-2-(4-chlorophenyl)-3-(4-hydroxybutyl)aziridine-1-sulfonate (**14g**)

Chiral SFC Analysis: CHIRAL ART SC (CO_2:^{i}PrOH, 97:3, 2.5 mL min^{-1}, 40 °C, 220 nm) indicated 96% *ee*, t_R = 8.7 (major), 10.0 (minor) minutes.

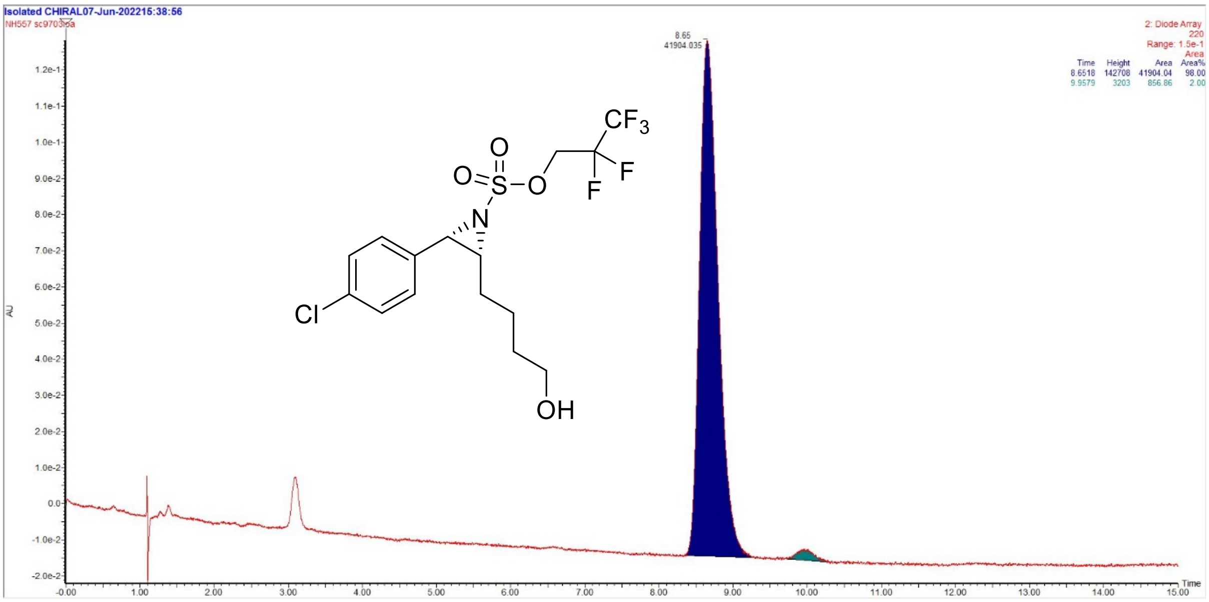

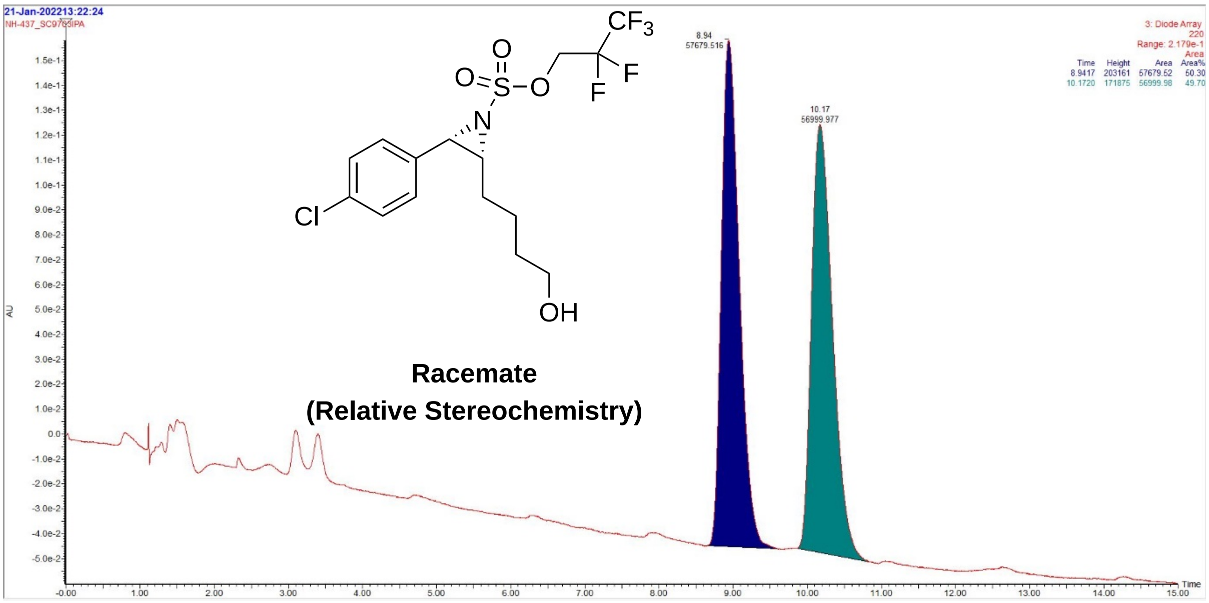

2,2,3,3,3-pentafluoropropyl (2S,3R)-2-(4-(tert-butyl)phenyl)-3-(4-hydroxybutyl)aziridine-1-sulfonate (**14h**)

Chiral SFC Analysis: CHIRAL ART SJ (CO_2;MeOH, 98:2, 1.25 mL min^{-1}, 40 °C, 220 nm) indicated 98% *ee*, t_R = 6.7 (major), 7.3 (minor) minutes.

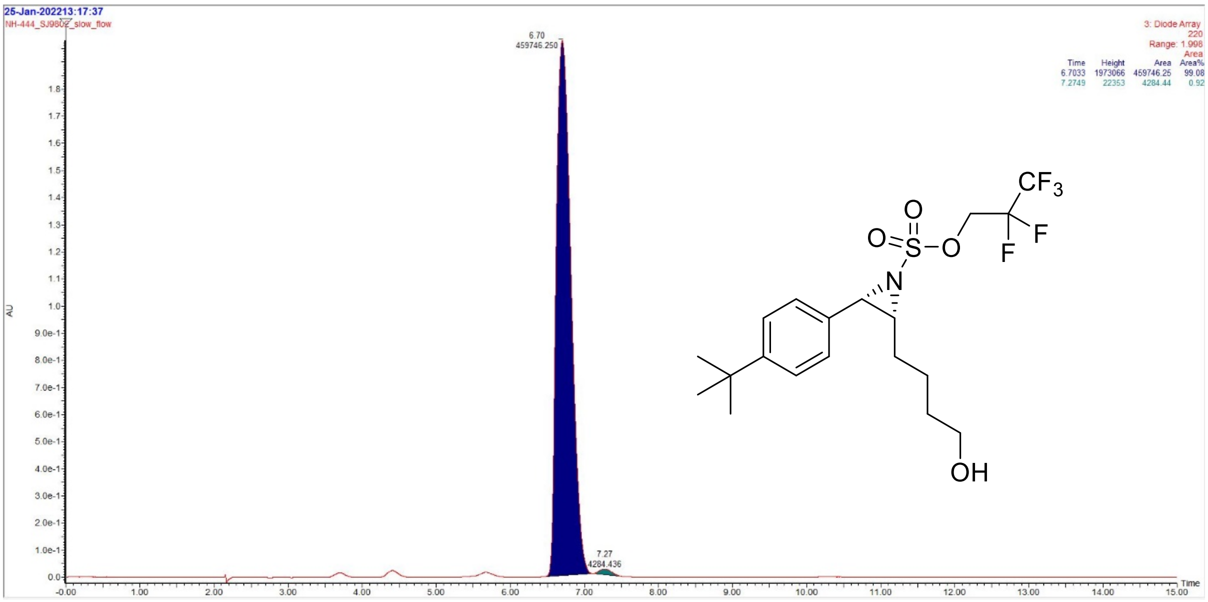

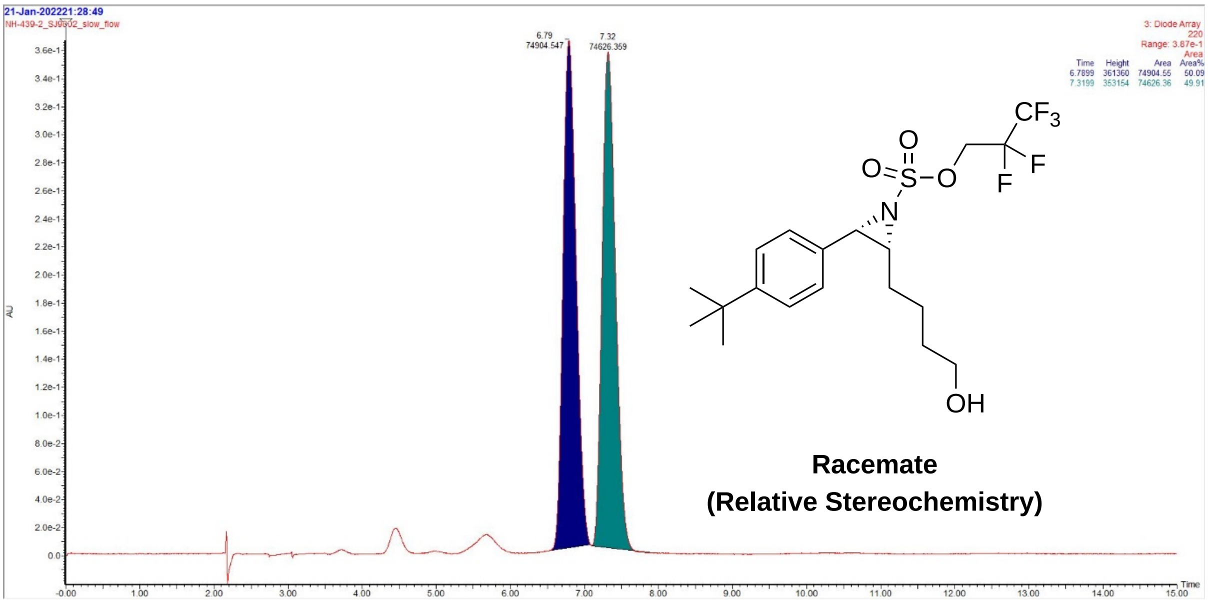

2,2,3,3,3-pentafluoropropyl (2R,3S)-2-(4-hydroxybutyl)-3-(4-(pyrrolidin-1-ylsulfonyl)phenyl)aziridine-1-sulfonate (**14i**)

Chiral SFC Analysis: CHIRALPAK IG (CO_2:^{i}PrOH, 85:15, 2.5 mL min^{-1}, 40 °C, 233 nm) indicated 92% *ee*, t_R = 5.8 (major), 6.3 (minor) minutes.

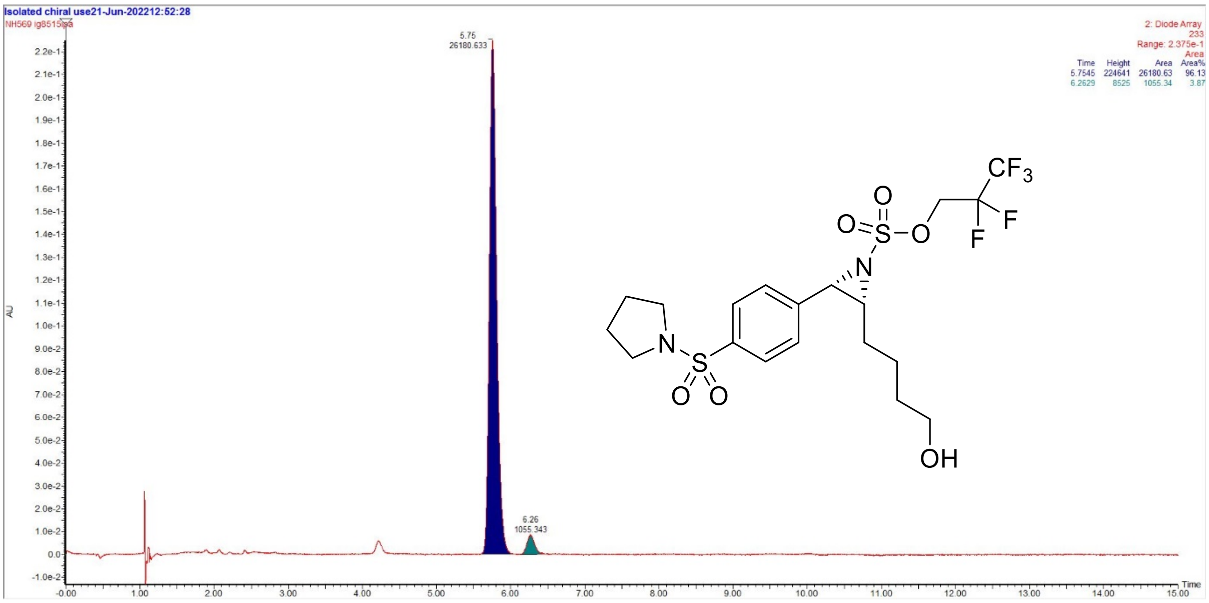

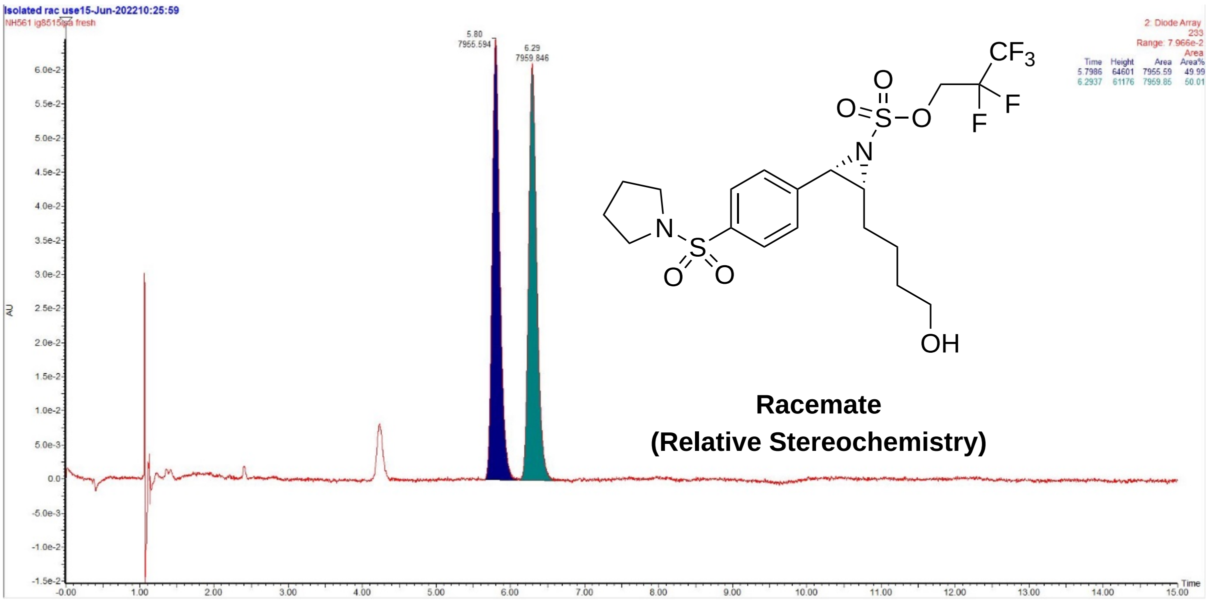

2,2,3,3,3-pentafluoropropyl ((S)-naphthalen-1-yl((S)-tetrahydrofuran-2-yl)methyl)sulfamate (**15**)

Chiral SFC Analysis CHIRALPAK IH (CO_2:MeOH, 96:4, 2.5 mL min^{-1}, 40 °C, 220 nm) indicated 96% *ee*, t_R = 5.8 (minor), 6.7 (major) minutes.

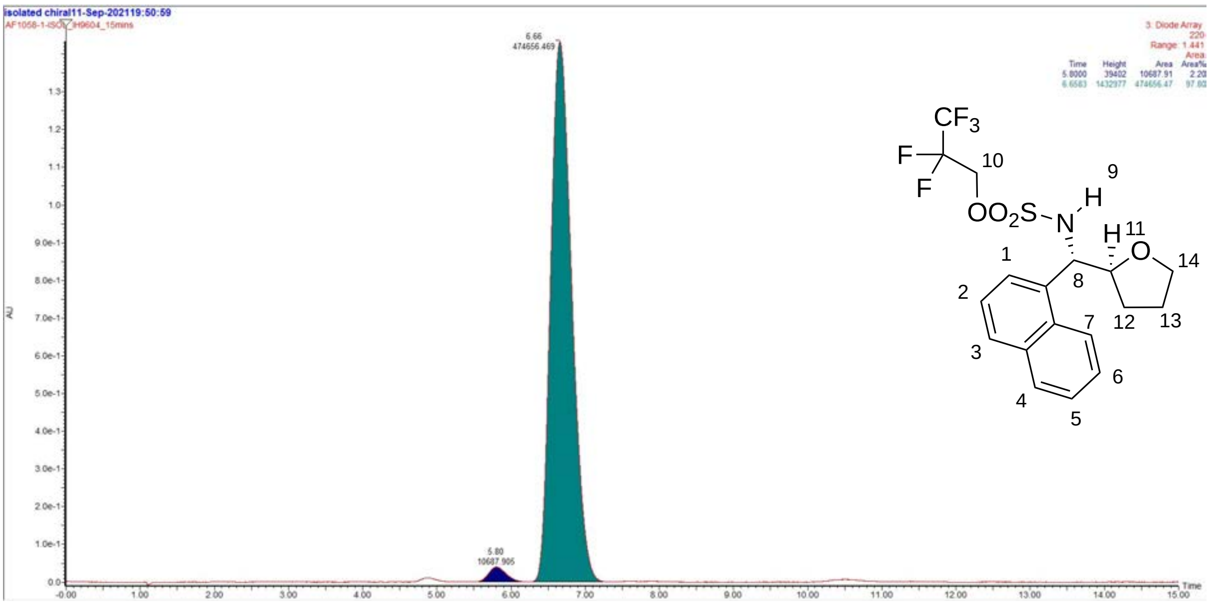

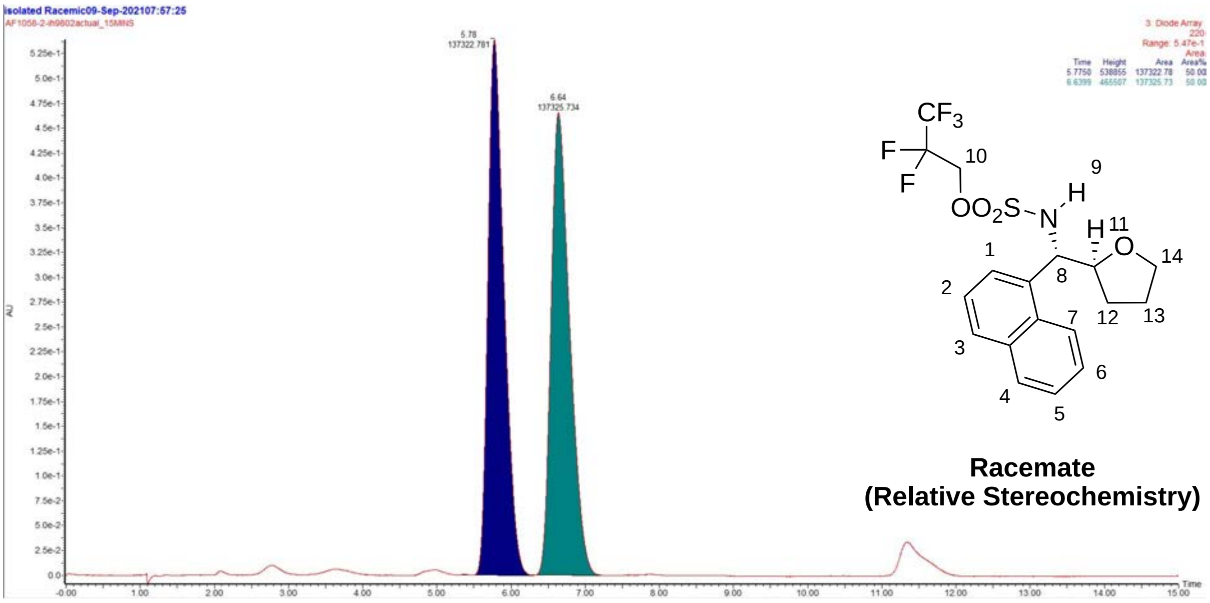

2,2,3,3,3-pentafluoropropyl (2S,3R)-2-(naphthalen-1-yl)-3-propylaziridine-1-sulfonate (**16**)

Chiral SFC Analysis CHIRAL ART SB (CO_2:MeOH, 99:1, 2.5 mL min^{-1}, 40 °C, 219 nm) indicated 12% *ee*, t_R = 2.9 (minor), 4.1 (major) minutes.

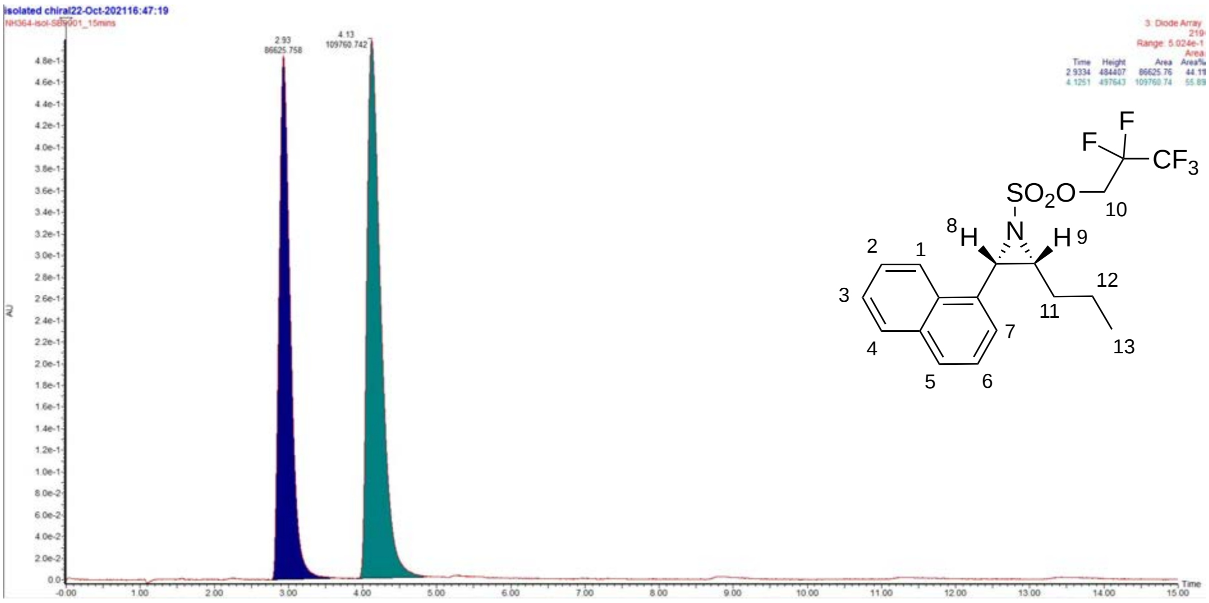

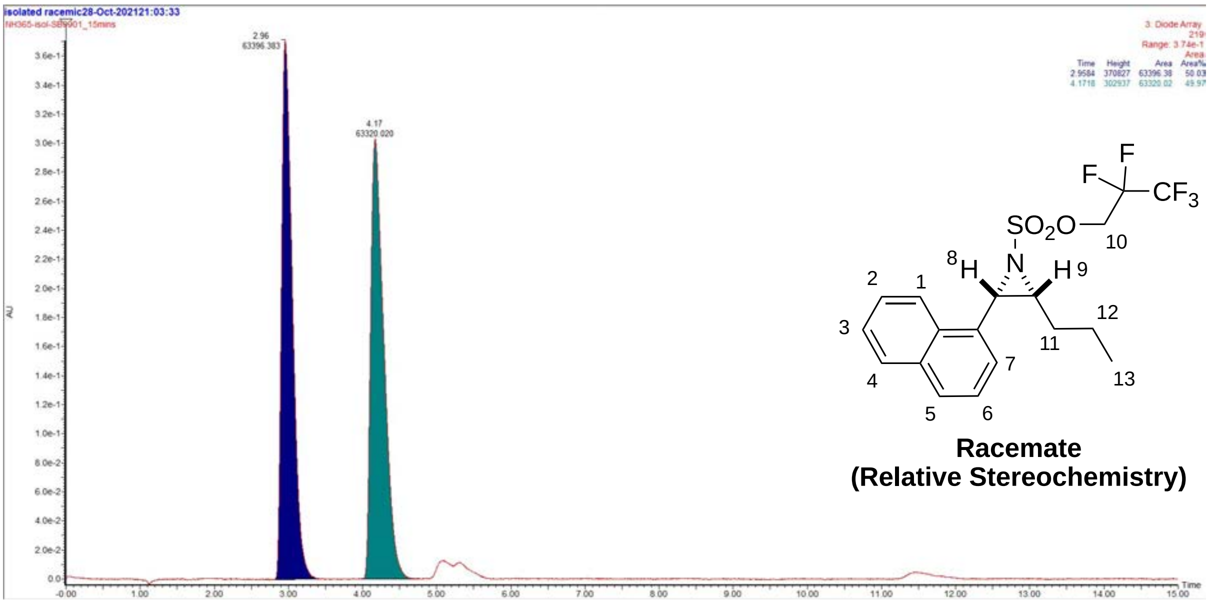

2,2,3,3,3-pentafluoropropyl (2R,3S)-2-(3-methoxypropyl)-3-(naphthalen-1-yl)aziridine-1-sulfonate (**17**)

Chiral HPLC Analysis CHIRAL ART SC (Hexane:^{i}PrOH, 99:1, 1.25 mL min^{-1}, 40 °C, 225 nm) indicated 59% *ee*, t_R = 6.9 (major), 9.0 (minor) minutes.

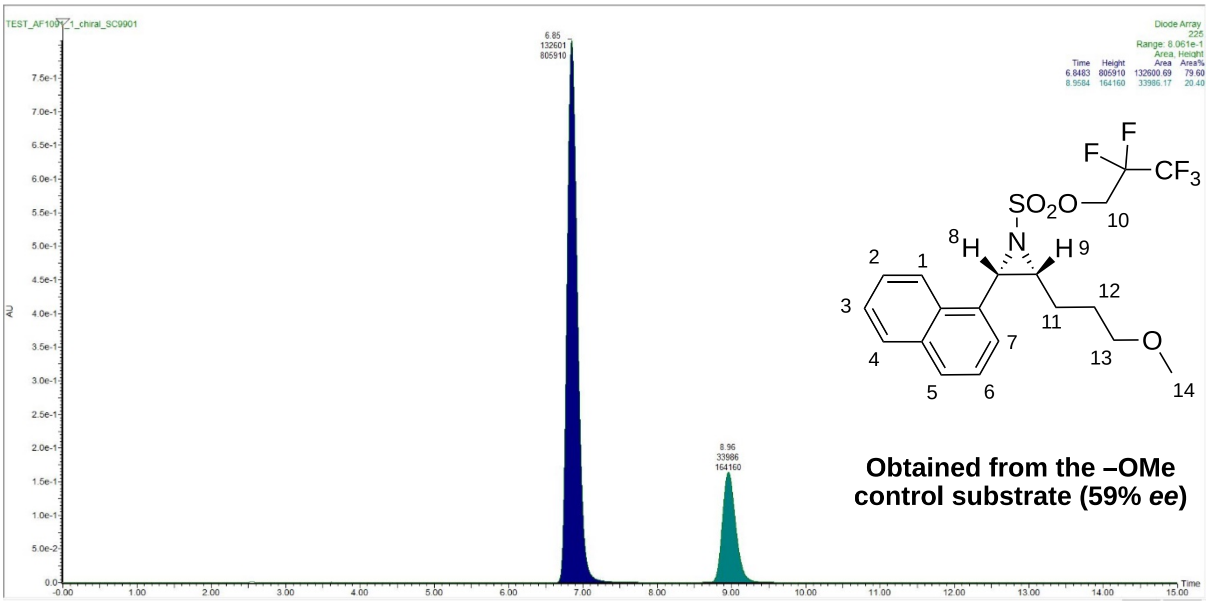

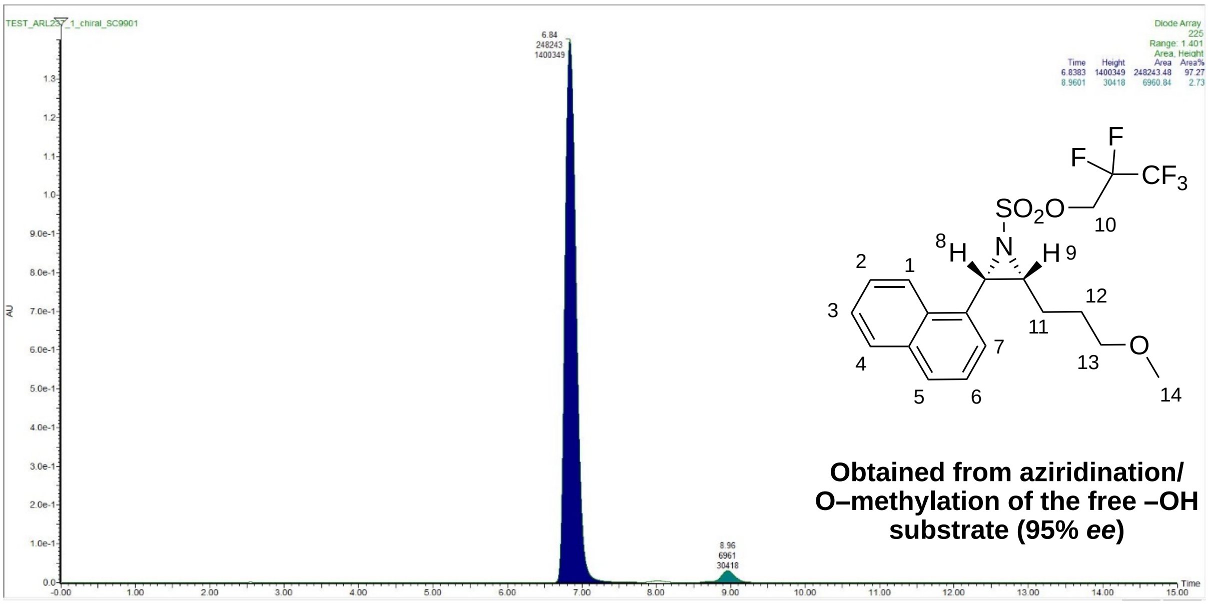

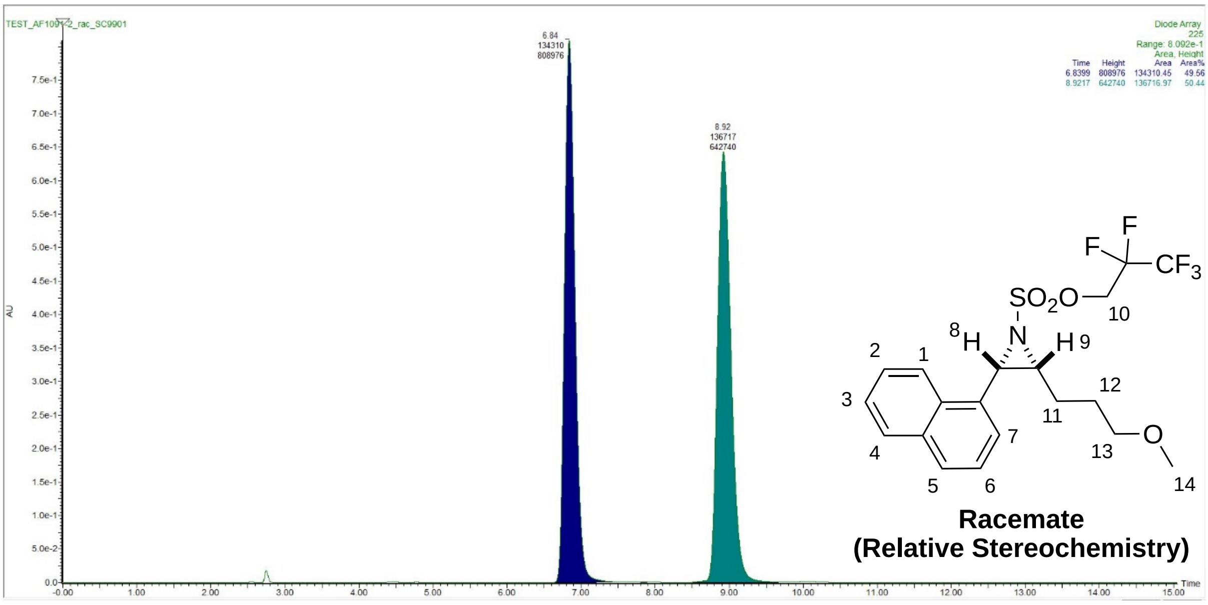

TEST_AF1091-2_rac_SC9901
Diode Array
225
Range: 8.092e-1
Area, Height
Time Height Area Area%
6.8399 808976 134310.45 49.56
8.9217 642740 136716.97 50.44
6.84
134310
808976
8.92
136717
642740
AU
Time
F
F
CF3
SO2O
10
8
H
N
H 9
2
1
3
12
11
7
O
4
13
5
6
14
Racemate
(Relative Stereochemistry)

2,2,3,3,3-pentafluoropropyl ((R)-((S)-5-oxotetrahydrofuran-2-yl)(phenyl)methyl)sulfamate(**18**)

Chiral SFC analysis: CHIRALPAK IG (CO_2:MeOH, 97:3, 2.5 mL min^{-1}, 40 ˚C, 210 nm) indicated 16% *ee*, t_R = 5.9 (minor), 6.4 (major) minutes.

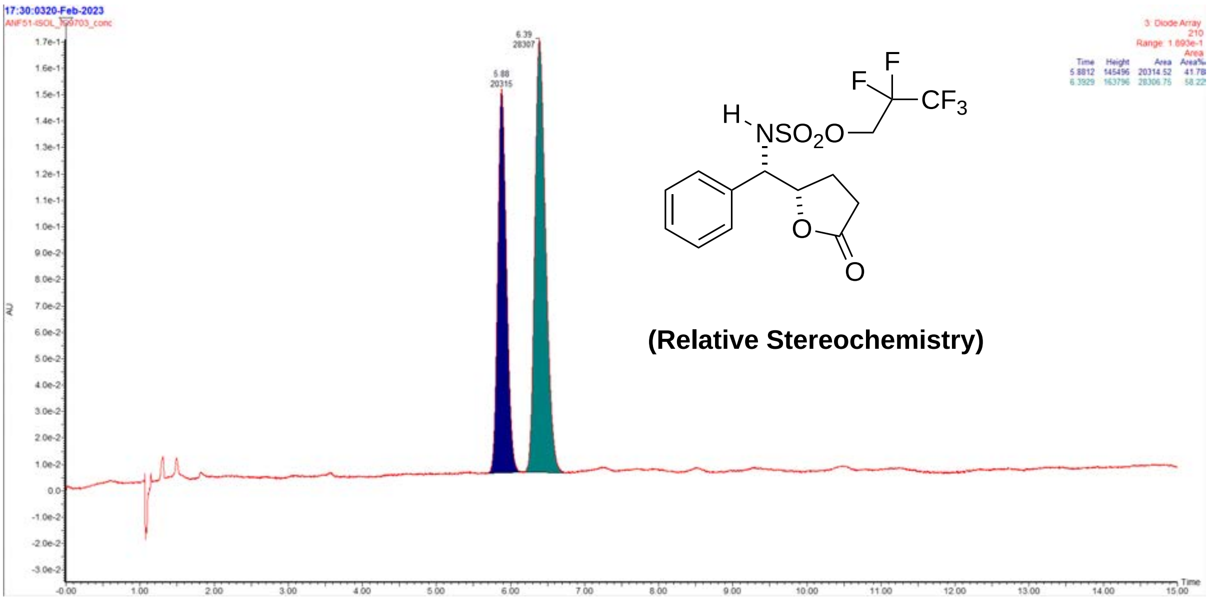

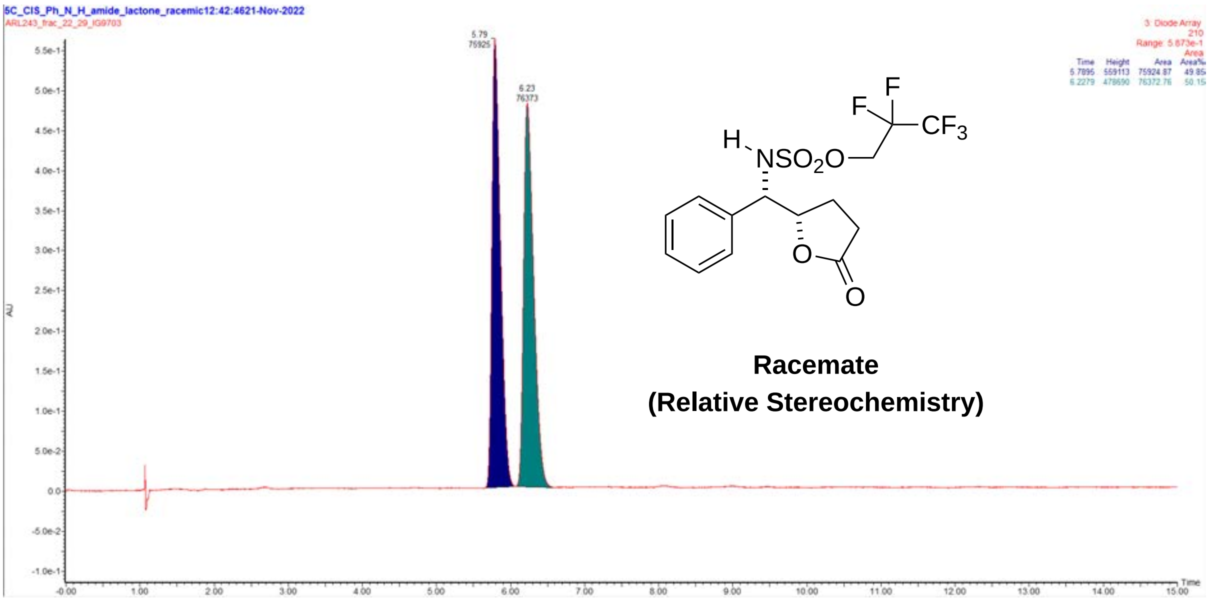

2,2,3,3,3-pentafluoropropyl (2S,3R)-2-phenyl-3-(3-(2,2,2-trifluoroacetamido)propyl)aziridine-1-sulfonate (**19**)

Chiral HPLC analysis: CHIRAL ART SC (Hexane:^{i}PrOH, 97:3, 1.25 mL min^{-1}, 40 ˚C, 207 nm) indicated 17% *ee*, t_R = 9.4 (major), 11.0 (minor) minutes.

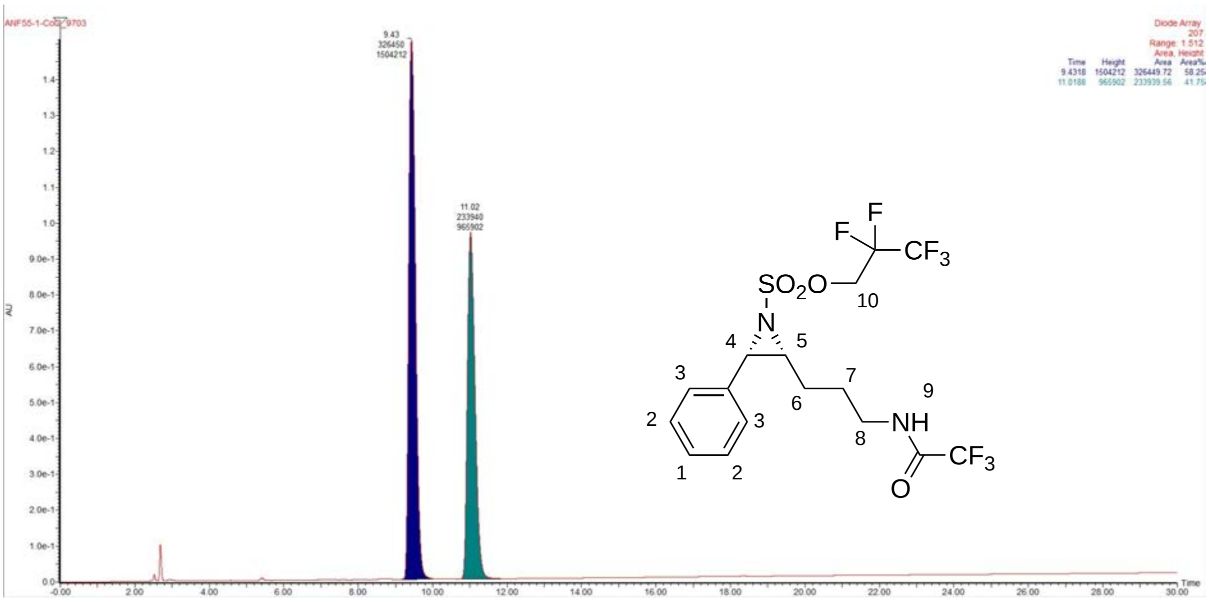

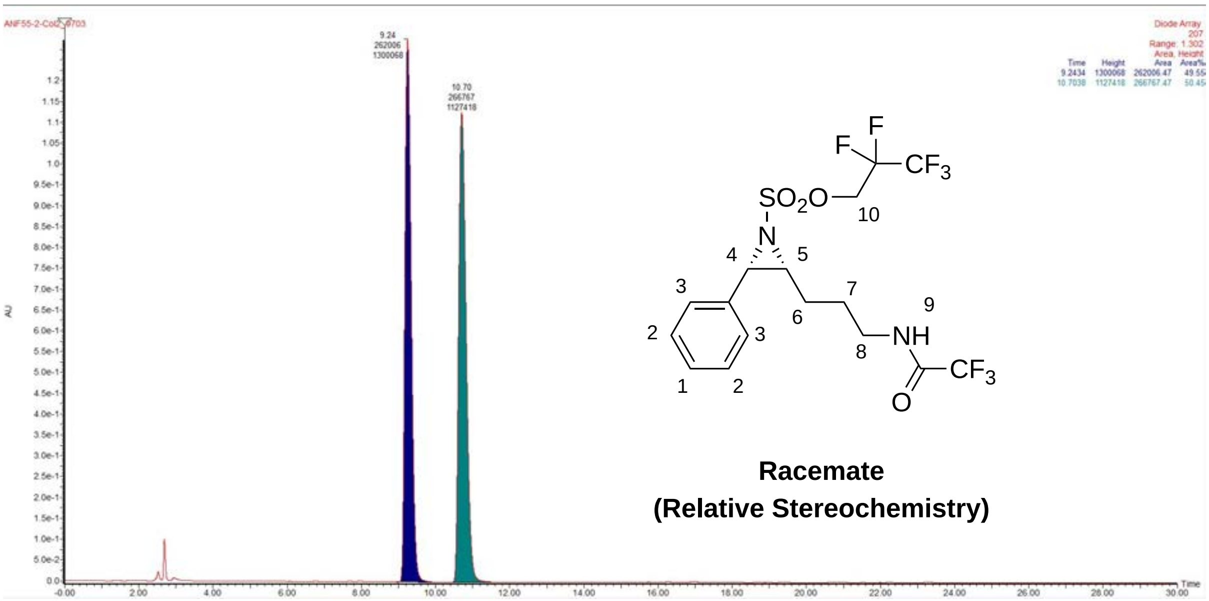

2,2,3,3,3-pentafluoropropyl (2R,3S)-2-(3-((4-methylphenyl)sulfonamido)propyl)-3-phenylaziridine-1-sulfonate (**20**)

Chiral SFC analysis: CHIRALPAK IG (CO_2:MeOH, 89:11, 2.5 mL min^{-1}, 40 ˚C, 217 nm) indicated 48% *ee*, t_R = 9.5 (major), 10.1 (minor) minutes.

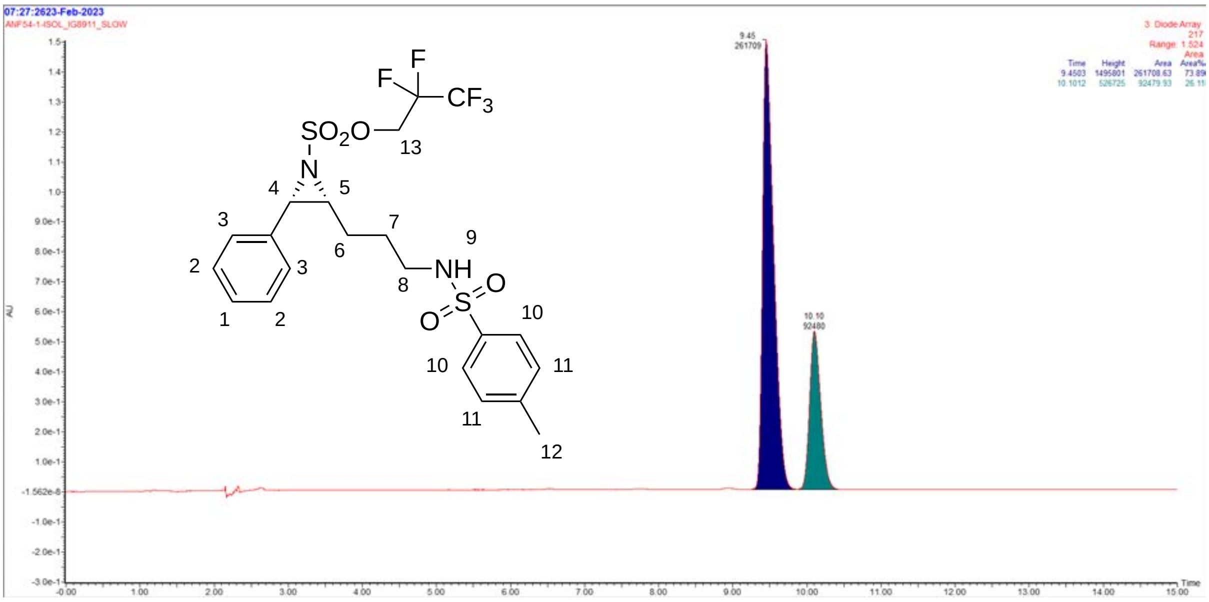

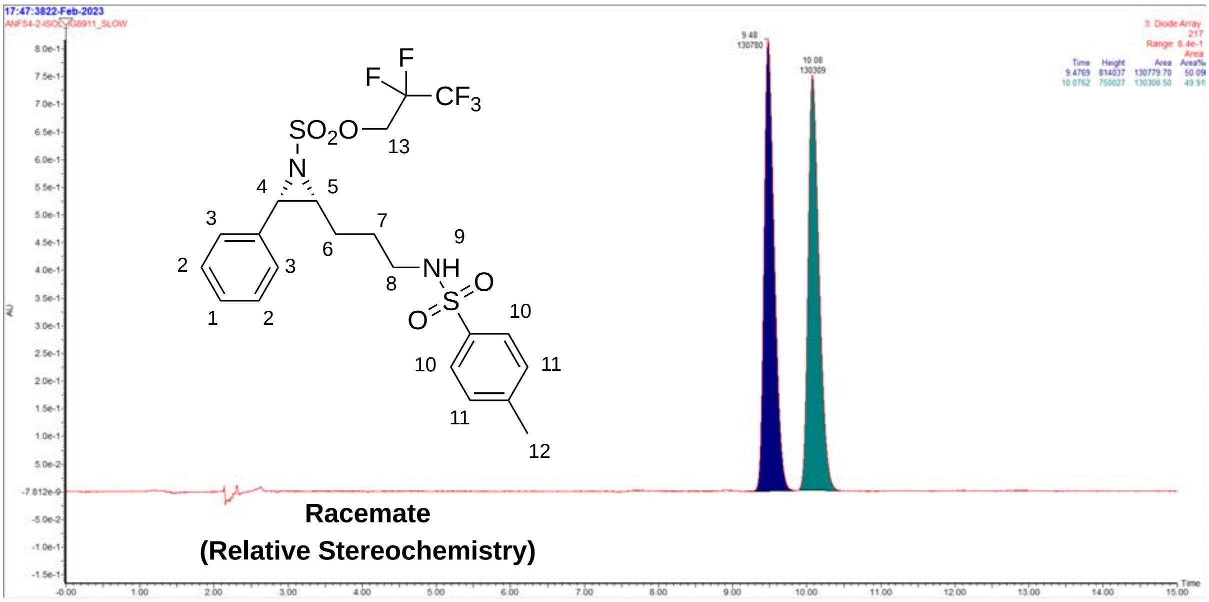

2,2,3,3,3-pentafluoropropyl (2S,3S)-2-(hydroxymethyl)-3-phenylaziridine-1-sulfonate (**21**)

Chiral SFC Analysis CHIRAL ART SC (CO_2:MeOH, 98.5:1.5, 2.5 mL min^{-1}, 40 °C, 210 nm) indicated 45% *ee*, t_R = 4.1 (major), 4.9 (minor) minutes.

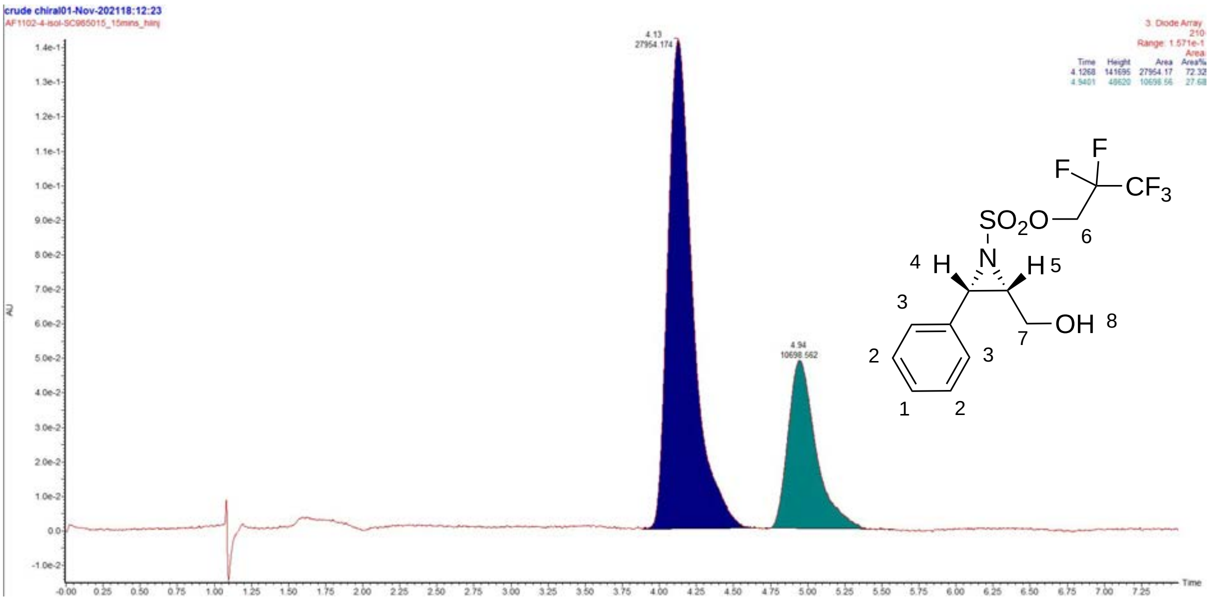

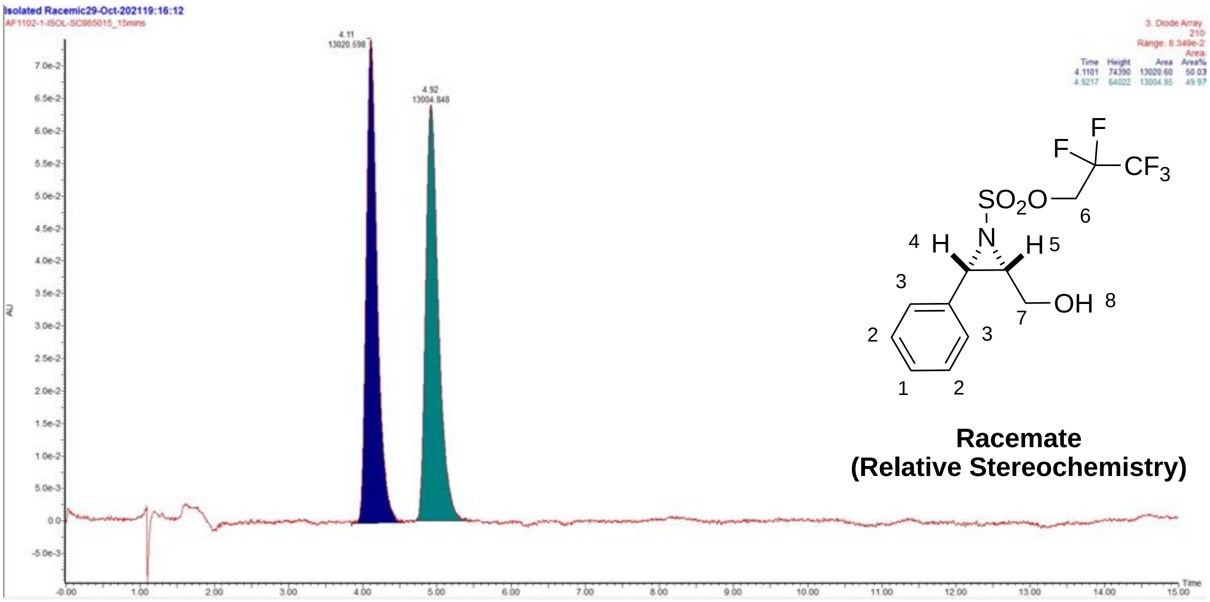

2,2,3,3,3-pentafluoropropyl (2R,3S)-2-(5-hydroxypentyl)-3-phenylaziridine-1-sulfonate (**22**)

Chiral SFC Analysis CHIRAL ART SJ (CO_2:MeOH, 98:2, 2.5 mL min^{-1}, 40 °C, 210 nm) indicated 67% ee, t_R = 3.0 (major), 3.6 (minor) minutes.

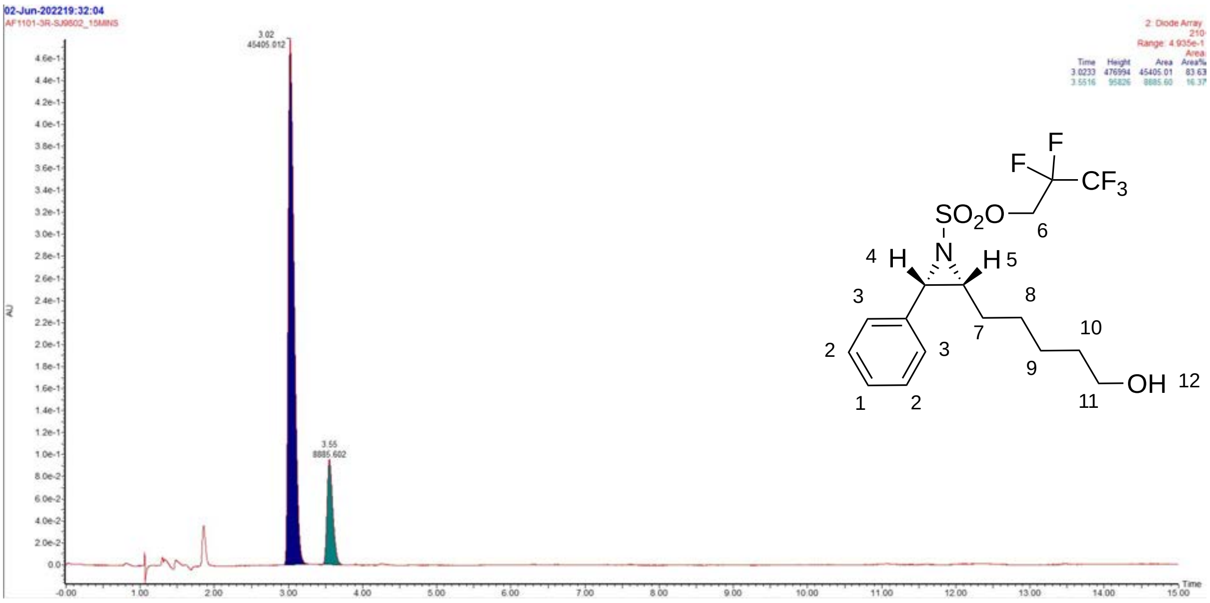

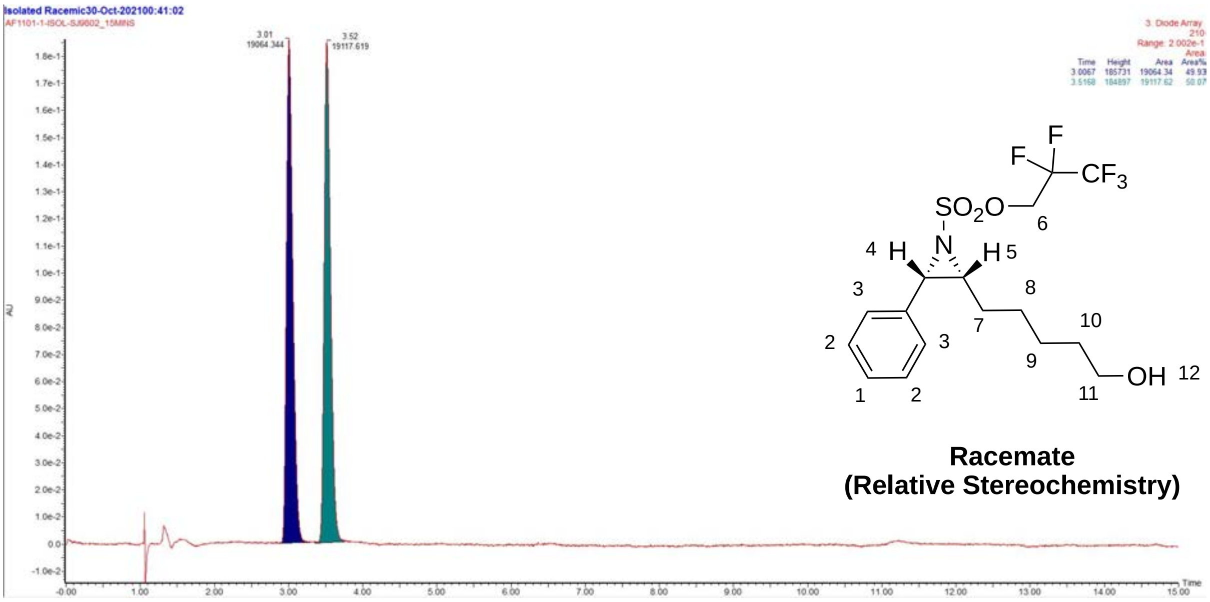

2,2,3,3,3-pentafluoropropyl ((2S,3R)-2-methyltetrahydrofuran-3-yl)sulfamate (**24a**)

Chiral GC Analysis CP7502 – CP-Chirasil-DEX CB column (130 °C Isothermal, 25.0 psi) indicated 89% *ee*, t_R = 16.2 (major), 18.0 (minor) minutes

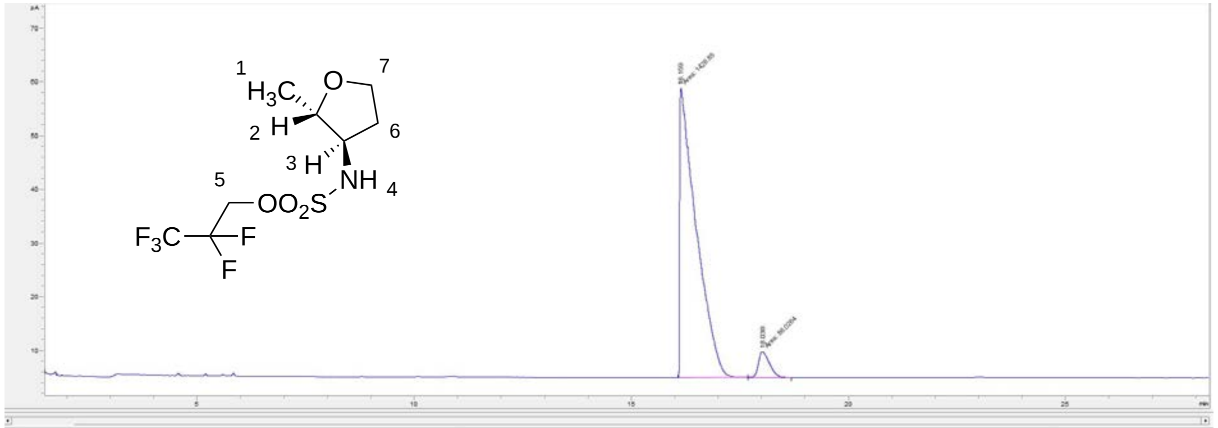

#	Time	Type	Area	Height	Width	Area%	Symmetry
1	16.159	MM	1426.8	54	0.4404	94.314	9.39E-2
2	18.038	MM	86	4.9	0.2946	5.686	0.616

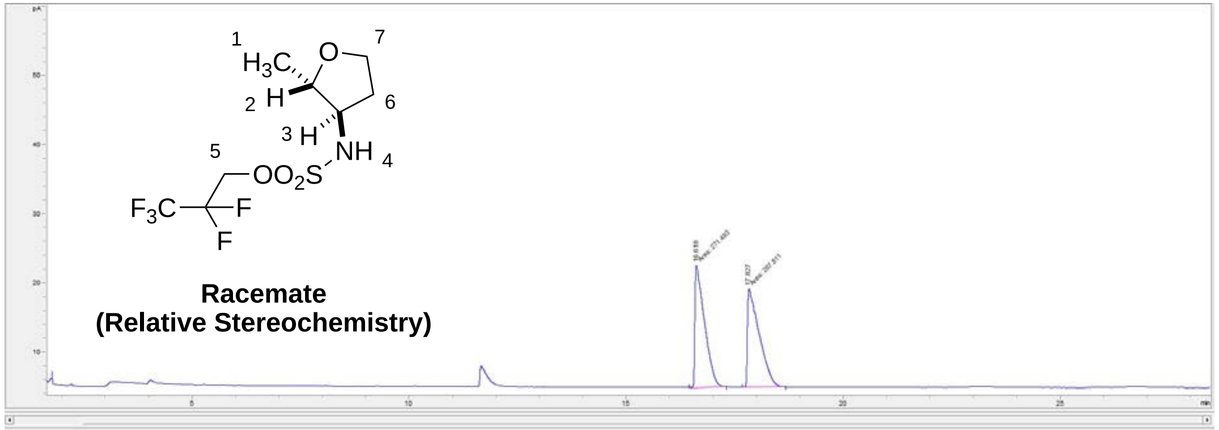

#	Time	Type	Area	Height	Width	Area%	Symmetry
1	16.618	MM	271.5	17.7	0.255	50.341	0.201
2	17.827	MM	267.8	14.2	0.3138	49.659	0.152

Mixture of 2,2,3,3,3-pentafluoropropyl ((3R,4R)-1-hydroxy-4-(phenylthio)pentan-3-yl)-λ⁴-azanesulfonate *and* *2,2,3,3,3-pentafluoropropyl ((2S,3S)-5-hydroxy-3-(phenylthio)pentan-2-yl)sulfamate:* ***Obtained following derivatisation of* 24b**

Chiral HPLC Analysis CHIRALPAK IC (Hexane:^{i}PrOH, 96:4, 1.0 mL min-1, 30 °C, 252 nm) for Regioisomer 1 t_R = 9.2 (minor), 9.9 (major) minutes indicated 38% *ee*, for Regioisomer 2 t_R = 14.0 minutes (major and minor co-eluted).

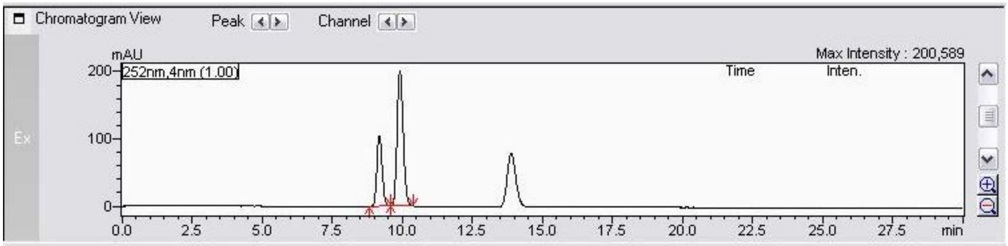

Results View - Peak Table

Peak#	Ret. Time	Area	Height	Area%
1	9.169	1363305	104365	31.137
2	9.904	3015126	200472	68.863
Total		4378431	304837	100.000

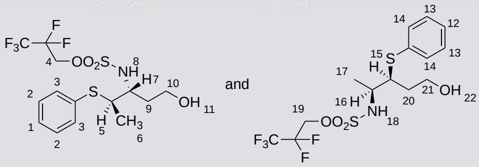

Extracted 252nm / Ch1 254nm

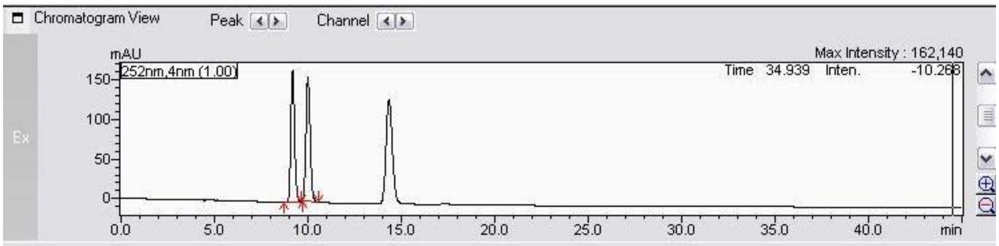

Results View - Peak Table

Peak#	Ret. Time	Area	Height	Area%
1	9.183	2327094	166339	48.589
2	10.001	2462226	158193	51.411
Total		4789320	324531	100.000

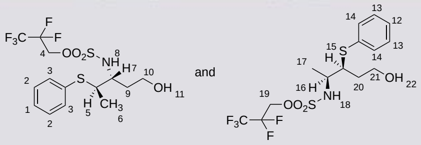

Racemate (Relative Stereochemistry) **Racemate (Relative Stereochemistry)**

Extracted 252nm / Ch1 254nm

2,2,3,3,3-pentafluoropropyl (S)-(4-hydroxy-2-methyl-1-(phenylthio)butan-2-yl)sulfamate:

***Obtained following derivatisation of* 24c**

Chiral SFC Analysis CHIRALPAK IE (CO_2:MeOH, 97:3, 2.5 mL min^{-1}, 40 °C, 251 nm) indicated 74% *ee*, t_R = 9.2 (minor), 11.6 (major) minutes.

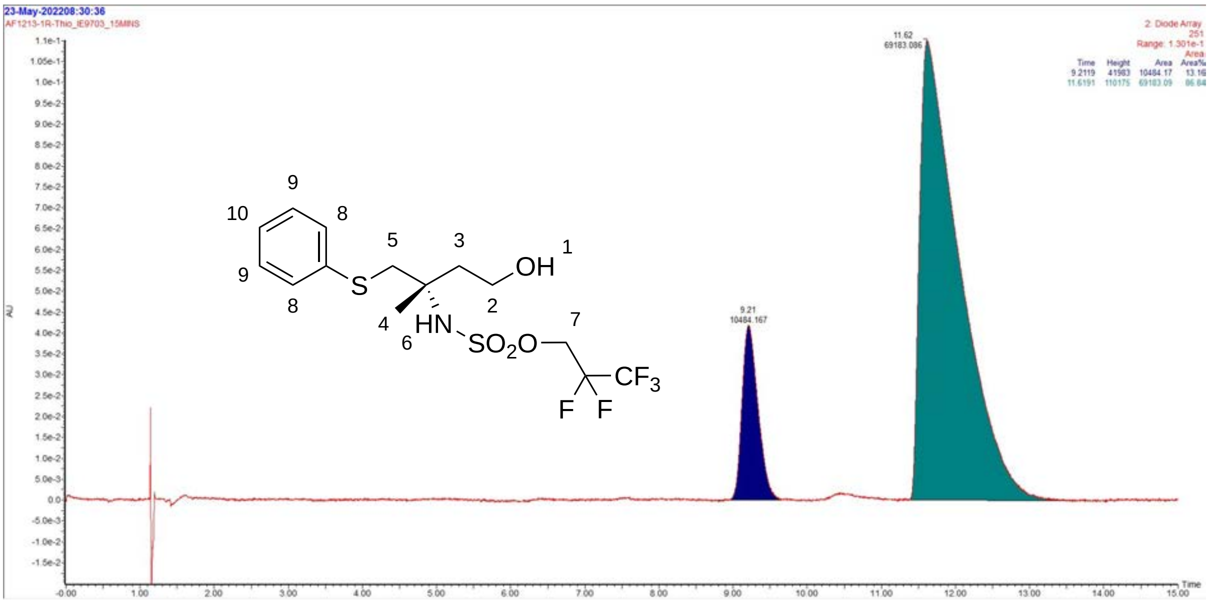

***Obtained following derivatisation of ent-*21c**

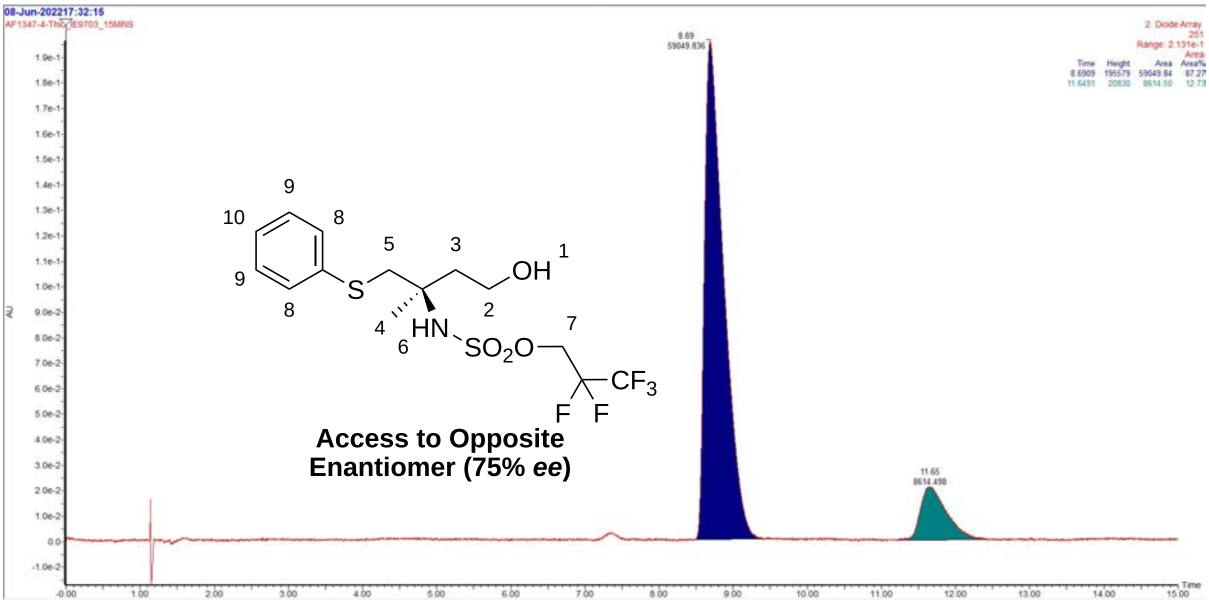

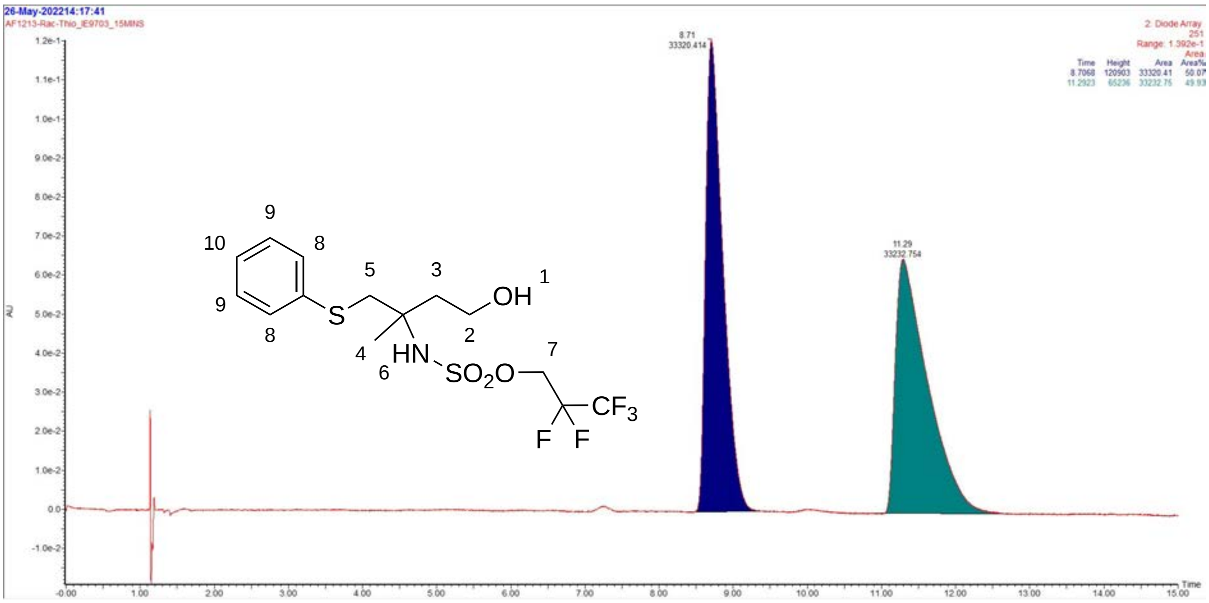

26-May-202214:17:41
AF1213-Rac-Thio_IE9703_15MINS
2: Diode Array
251
Range: 1.392e-1
Area
Time Height Area Area%
8.7068 120903 33320.41 50.07
11.2923 65236 33232.75 49.93
8.71
33320.414
11.29
33232.754
AU
Time
OH
HN
S
SO2O
CF3
F F

2,2,3,3,3-pentafluoropropyl (R)-(2,2-dimethyltetrahydrofuran-3-yl)sulfamate (**24d**)

Chiral GC Analysis CP7502 – CP-Chirasil-DEX CB column (120 °C Isothermal, 13.0 psi) indicated 86% *ee*, t_R = 33.7 (major), 35.6 (minor) minutes.

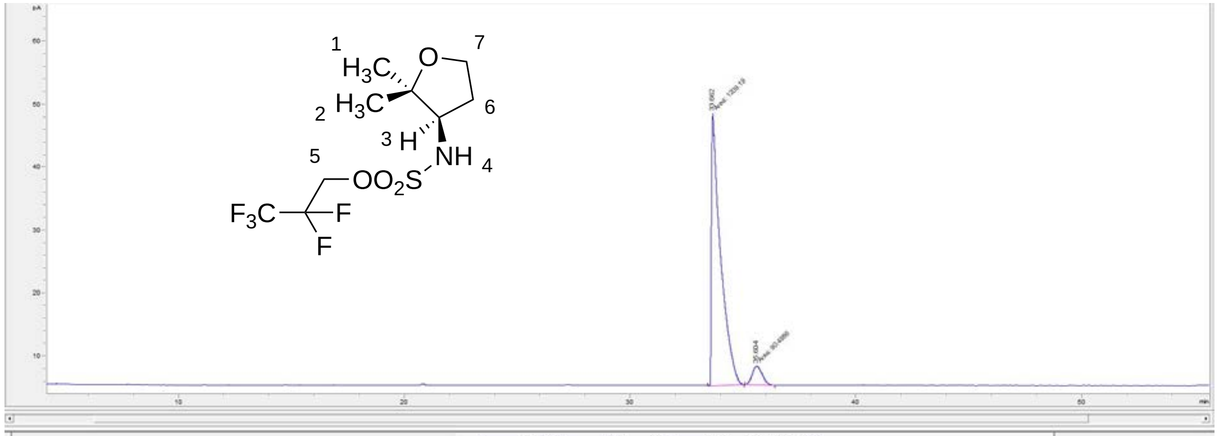

#	Time	Type	Area	Height	Width	Area%	Symmetry
1	33.662	MM	1209.2	43.2	0.4666	93.037	0.184
2	35.604	MM	90.5	3	0.5017	6.963	0.789

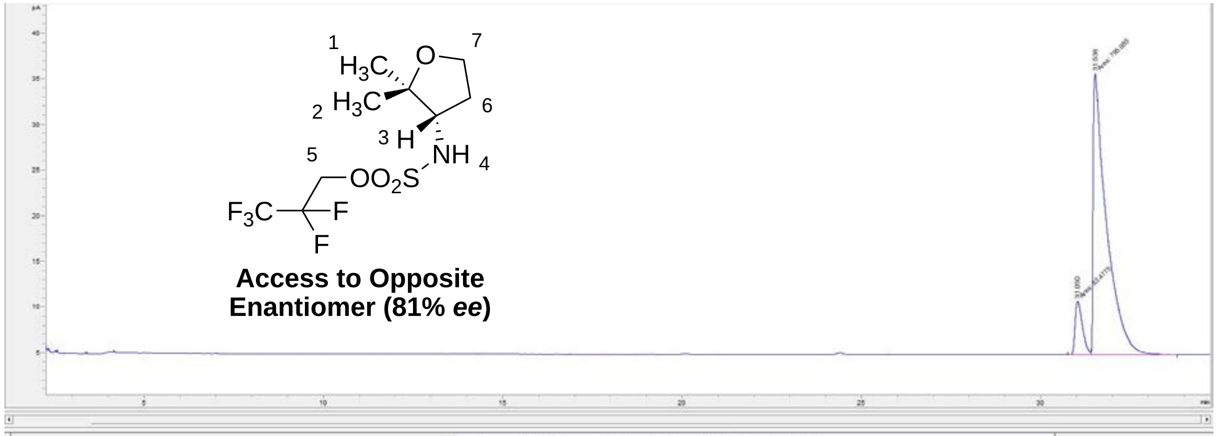

#	Time	Type	Area	Height	Width	Area%	Symmetry
1	31.05	MM	83.5	5.9	0.2369	9.492	0.555
2	31.538	MM	796	30.8	0.4313	90.508	0.184

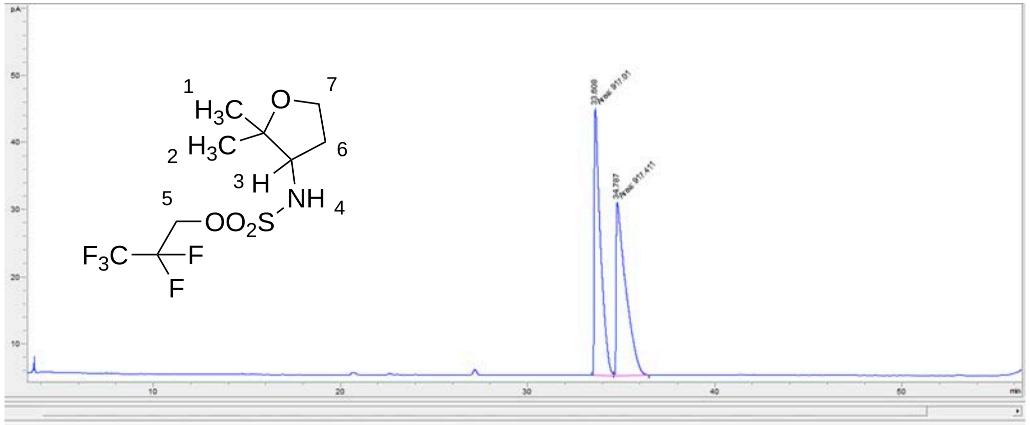

#	Time	Type	Area	Height	Width	Area%	Symmetry
1	33.608	MM	917	39.7	0.3849	49.989	0.217
2	34.787	MM	917.4	25.8	0.5935	50.011	0.147

Mixture of 2,2,3,3,3-pentafluoropropyl ((3S,4S)-1-hydroxy-3-methyl-4-(phenylthio)pentan-3-yl)sulfamate (Isomer A) and 2,2,3,3,3-pentafluoropropyl ((2R,3R)-5-hydroxy-3-(phenylthio)pentan-2-yl)sulfamate (Isomer B): ***Obtained following derivatisation of crude 24e***

Chiral HPLC Analysis CHIRALPAK IC (Hexane:^{i}PrOH, 97:3, 1.0 mL min^{-1}, 30 °C, 221 nm) for Isomer A indicated 49% *ee*, t_R = 11.6 (minor), 14.8 (major) minutes for Isomer B indicated 50% *ee*, t_R = 10.3 minutes (minor), 13.3 (major).

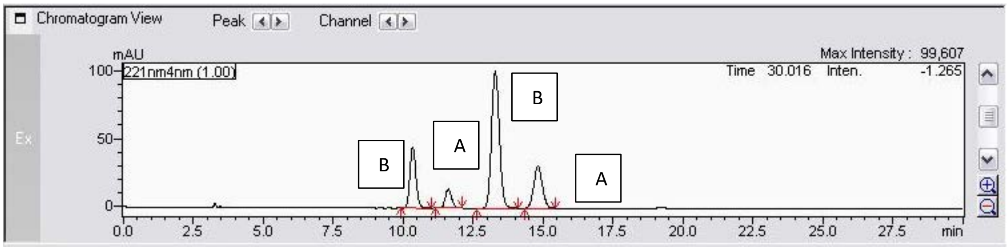

Results View - Peak Table

Peak#	Ret. Time	Area	Height	Area%
1	10.336	664653	44935	19.008
2	11.607	220604	13792	6.309
3	13.286	1954747	100716	55.904
4	14.814	656628	31083	18.779
Total		3496632	190527	100.000

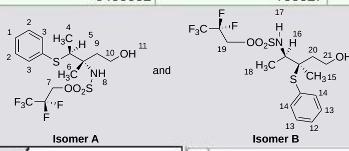

Extracted 221nm | Ch1 254nm

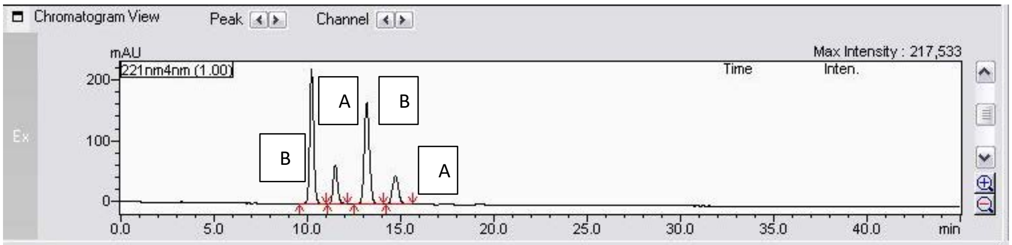

Results View - Peak Table

Peak#	Ret. Time	Area	Height	Area%
1	10.225	3240974	221191	38.179
2	11.498	1033221	63823	12.172
3	13.185	3173145	166290	37.380
4	14.719	1041455	47204	12.269
Total		8488795	498509	100.000

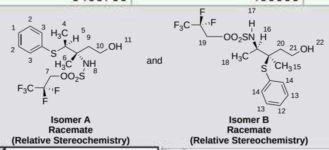

Extracted 221nm | Ch1 254nm

2,2,3,3,3-pentafluoropropyl ((2R,3R)-5-hydroxy-3-methyl-3-(phenylthio)pentan-2-yl)sulfamate: ***Obtained following derivatisation of*** **24f**

Chiral SFC Analysis CHIRALPAK IE (CO_2:MeOH, 96.5:3.5, 1.25 mL min^{-1}, 40 °C, 217 nm) indicated 60% *ee*, t_R = 18.0 (minor), 21.2 (major) minutes

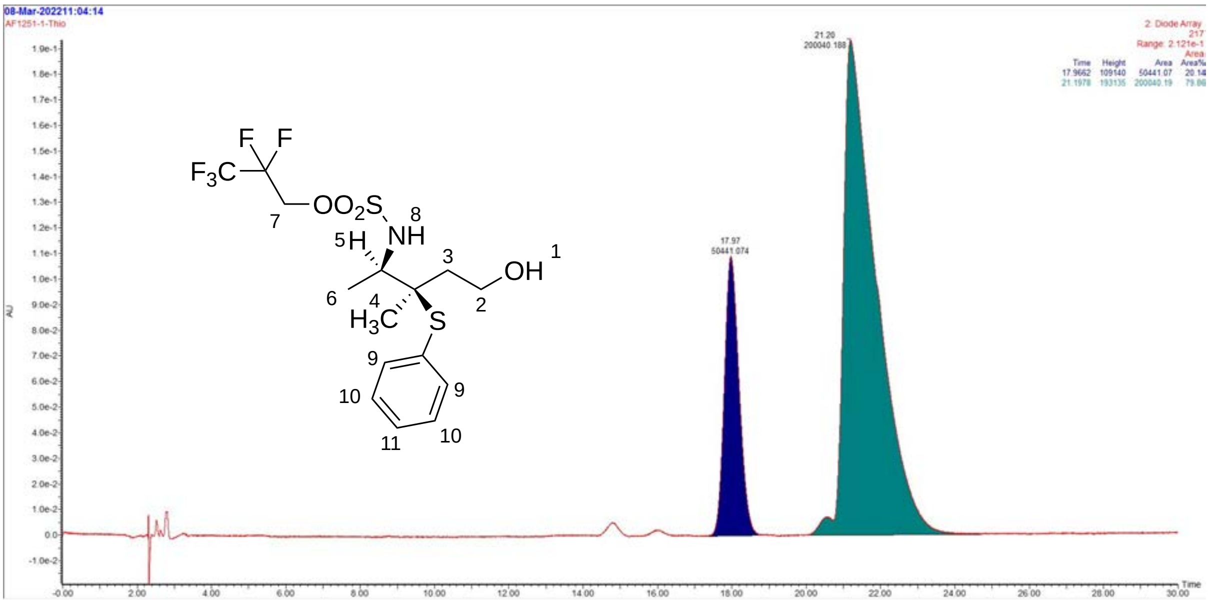

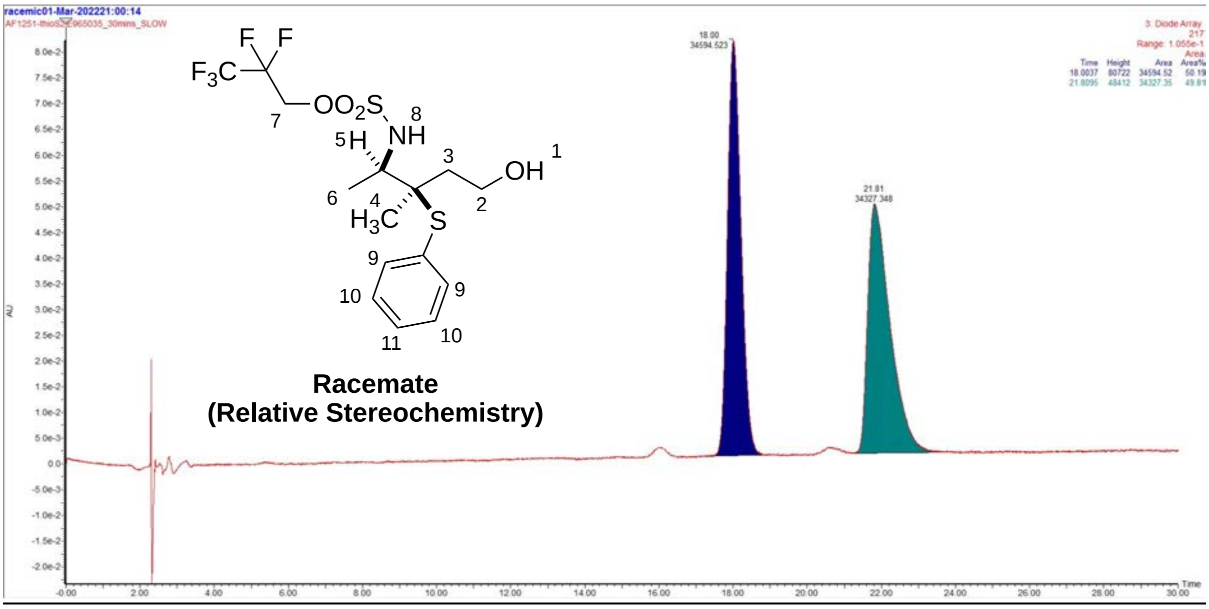

2,2,3,3,3-pentafluoropropyl (R)-2-(2-hydroxyethyl)aziridine-1-sulfonate (**24g**)

Chiral GC Analysis CP7502 – CP-Chirasil-DEX CB column (105 °C Isothermal, 25.9 psi) indicated 14% *ee*, t_R = 50.6 (minor), 53.1 (major) minutes.

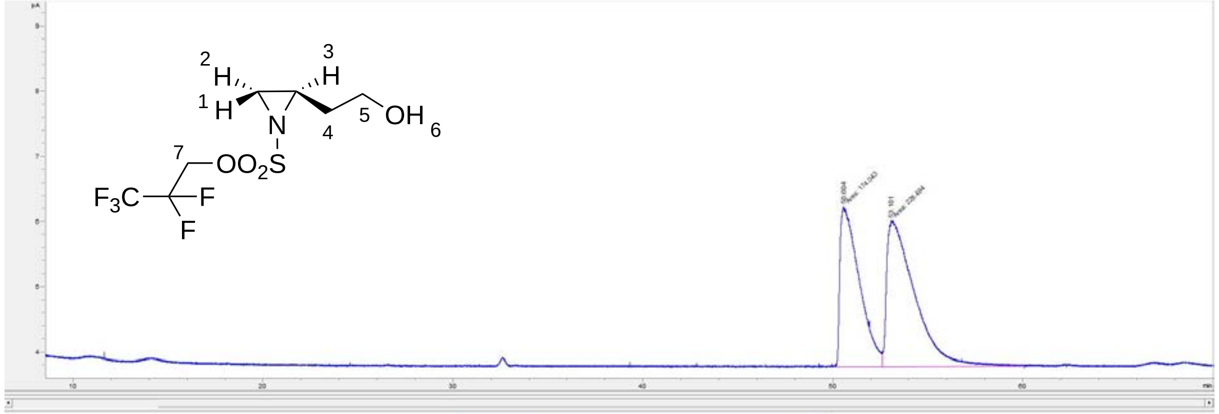

#	Time	Type	Area	Height	Width	Area%	Symmetry
1	50.604	MM	174	2.5	1.1797	43.236	0.241
2	53.101	MM	228.5	2.2	1.7002	56.764	0.191

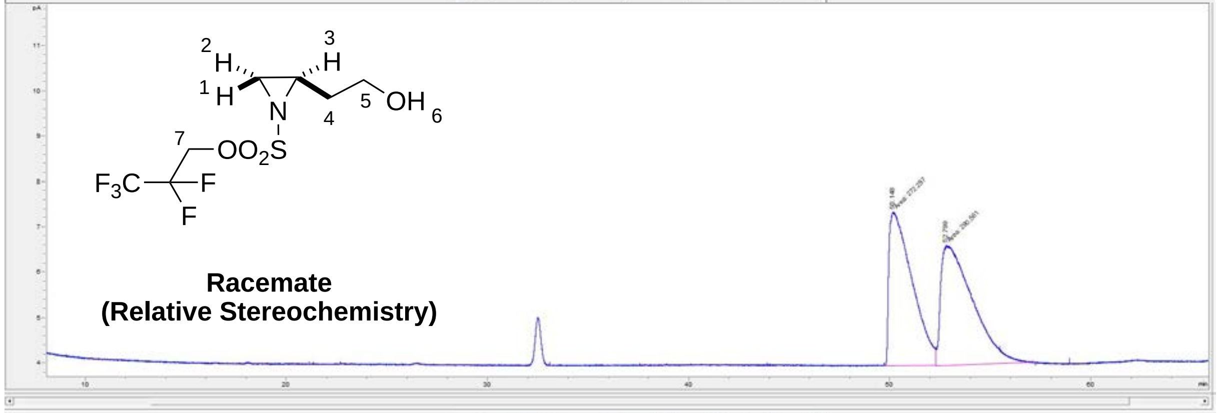

#	Time	Type	Area	Height	Width	Area%	Symmetry
1	50.148	MM	272.3	3.4	1.3361	48.374	0.207
2	52.799	MM	290.6	2.7	1.8256	51.626	0.21

2,2,3,3,3-pentafluoropropyl (2R,3R)-2-ethyl-3-(2-hydroxyethyl)aziridine-1-sulfonate (**26a**)

Chiral GC Analysis CP7502 – CP-Chirasil-DEX CB column (115 °C Isothermal, 26.7 psi) indicated 94% *ee*, t_R = 38.6 (aziridine, major), 37.9 (aziridine, minor), 63.6 (cyclised, major), 70.4 (cyclised, minor) minutes. **Note: The pure aziridine cyclises spontaneously in the instrument.**

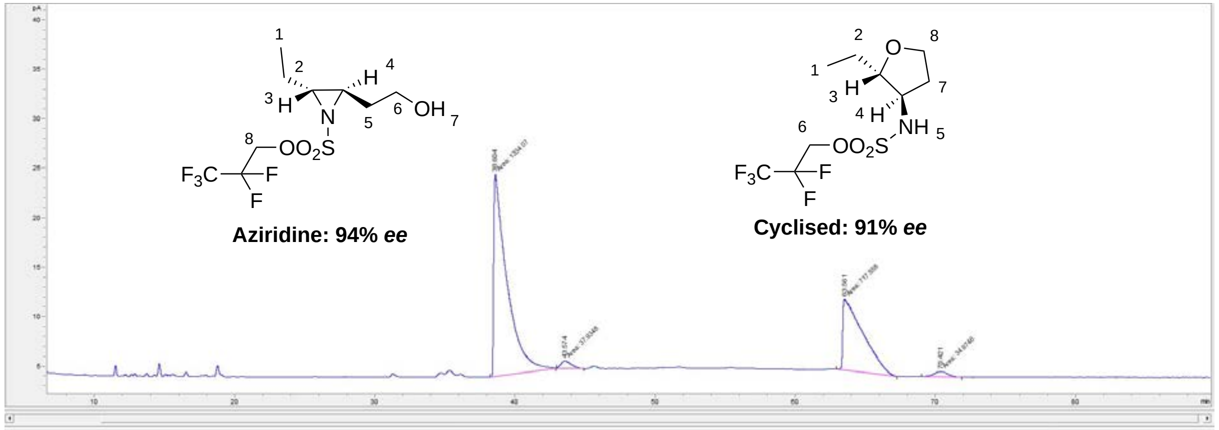

#	Time	Type	Area	Height	Width	Area%	Symmetry
1	38.604	MM	1334.1	20.4	1.0897	62.793	0.154
2	43.574	MM	37.9	7.5E-1	0.8448	1.786	0.612
3	63.561	MM	717.6	7.2	1.6654	33.775	9.62E-2
4	70.421	MM	35	5.3E-1	1.1099	1.646	0.996

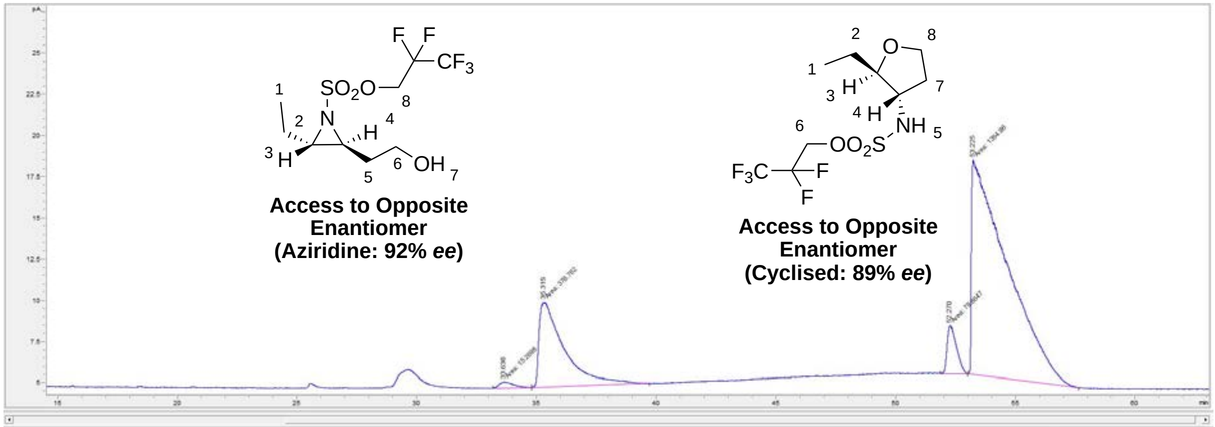

#	Time	Type	Area	Height	Width	Area%	Symmetry
1	33.638	MM	15.3	3.9E-1	0.6575	0.836	0.512
2	35.315	MM	376.8	5.2	1.2188	20.624	0.243
3	52.27	MM	79.9	2.9	0.4554	4.372	0.474
4	53.225	MM	1355	13	1.7346	74.169	4.68E-2

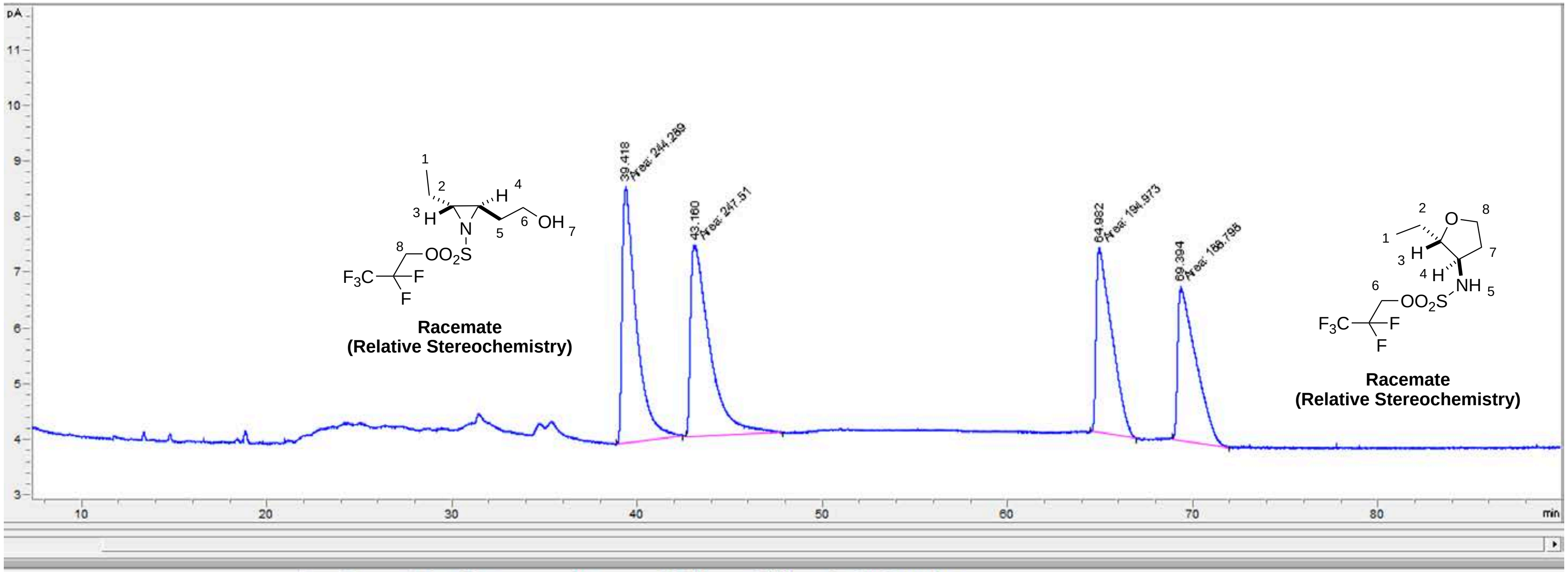

#	Time	Type	Area	Height	Width	Area%	Symmetry
1	39.418	MM	244.3	4.6	0.881	27.901	0.311
2	43.16	MM	247.5	3.4	1.2001	28.268	0.286
3	64.982	MM	195	3.3	0.9745	22.268	0.227
4	69.394	MM	188.8	2.8	1.1345	21.563	0.202

2,2,3,3,3-pentafluoropropyl ((2S,3R)-2-ethyltetrahydrofuran-3-yl)sulfamate: ***Obtained following intramolecular cyclisation of* 26a**

Chiral GC Analysis CP7502 – CP-Chirasil-DEX CB column (115 °C Isothermal, 26.7 psi) indicated 92% *ee*, t_R = 51.7 (major), 58.2 (minor) minutes.

Note the retention times have shifted slightly compared to above since these samples were run several months apart.

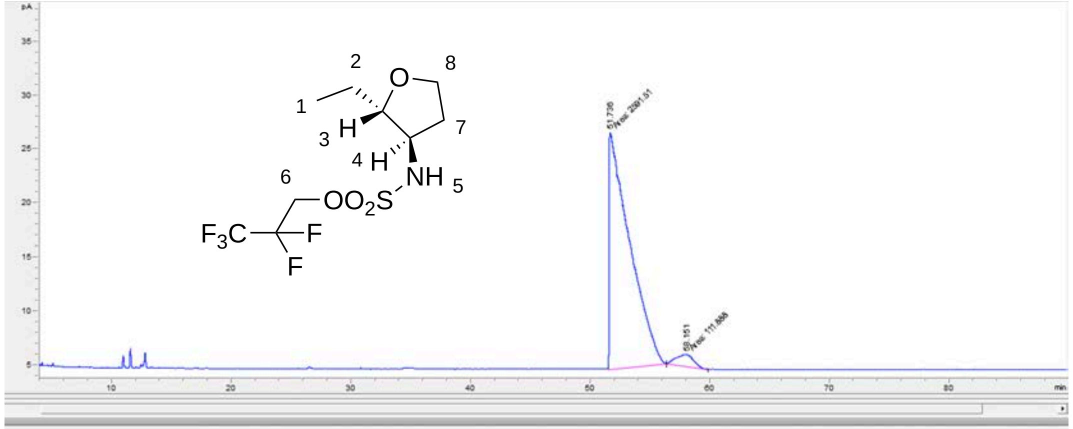

#	Time	Type	Area	Height	Width	Area%	Symmetry
1	51.736	MM	2591.5	21.9	1.9726	95.861	5.22E-2
2	58.151	MM	111.9	1.1	1.6683	4.139	1.493

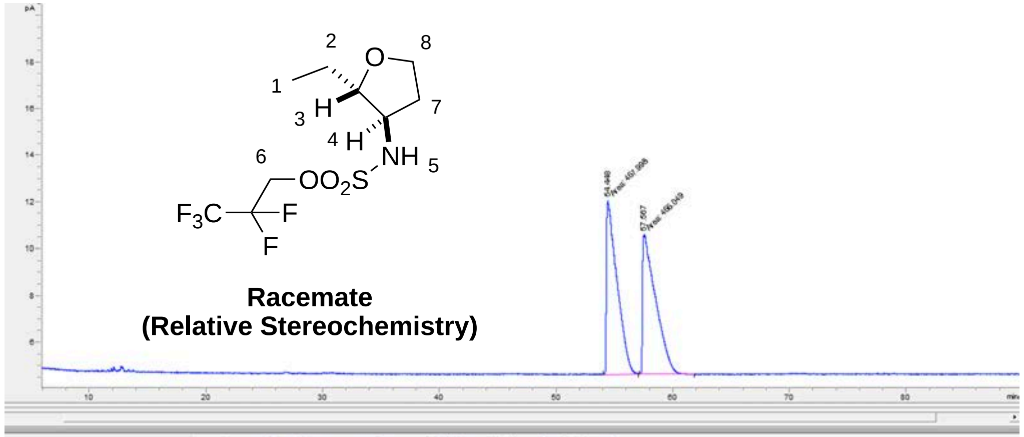

#	Time	Type	Area	Height	Width	Area%	Symmetry
1	54.448	MM	468	7.4	1.0478	50.104	0.187
2	57.567	MM	466	6	1.3027	49.896	0.172

2,2,3,3,3-pentafluoropropyl ((2S,3R)-2-isopropyltetrahydrofuran-3-yl)sulfamate: ***Obtained following intramolecular cyclisation of* 26b**

Chiral GC Analysis CP7502 – CP-Chirasil-DEX CB column (120 °C Isothermal, 20.0 psi) indicated 79% *ee*, t_R = 54.6 (major), 57.8 (minor) minutes.

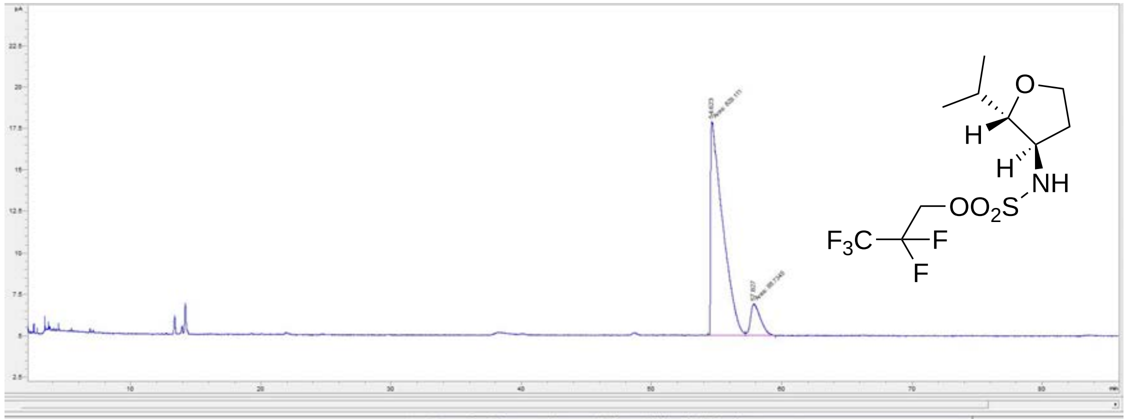

#	Time	Type	Area	Height	Width	Area%	Symmetry
1	54.623	MM	829.1	12.9	1.0711	89.359	0.114
2	57.827	MM	98.7	1.9	0.8569	10.641	0.441

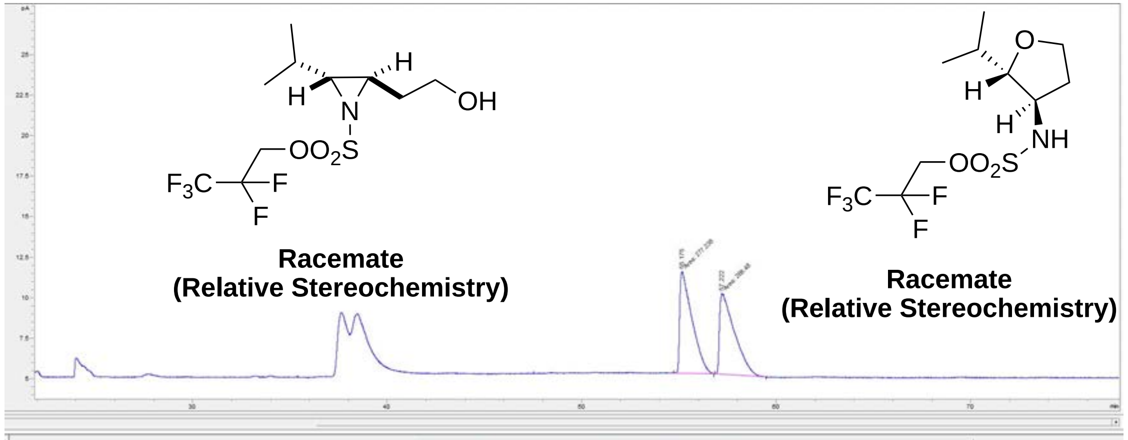

#	Time	Type	Area	Height	Width	Area%	Symmetry
1	55.175	MM	277.2	6.3	0.7365	50.802	0.273
2	57.222	MM	268.5	5	0.8966	49.198	0.217

2,2,3,3,3-pentafluoropropyl (2R,3R)-2-(2-(benzyloxy)ethyl)-3-(2-hydroxyethyl)aziridine-1-sulfonate (**26d**)

Chiral SFC Analysis CHIRALPAK IE (CO_2:MeOH, 98:2, 1.25 mL min^{-1}, 40 °C, 210 nm) indicated 91% *ee*, t_R = 20.8 (major), 22.9 (minor) minutes.

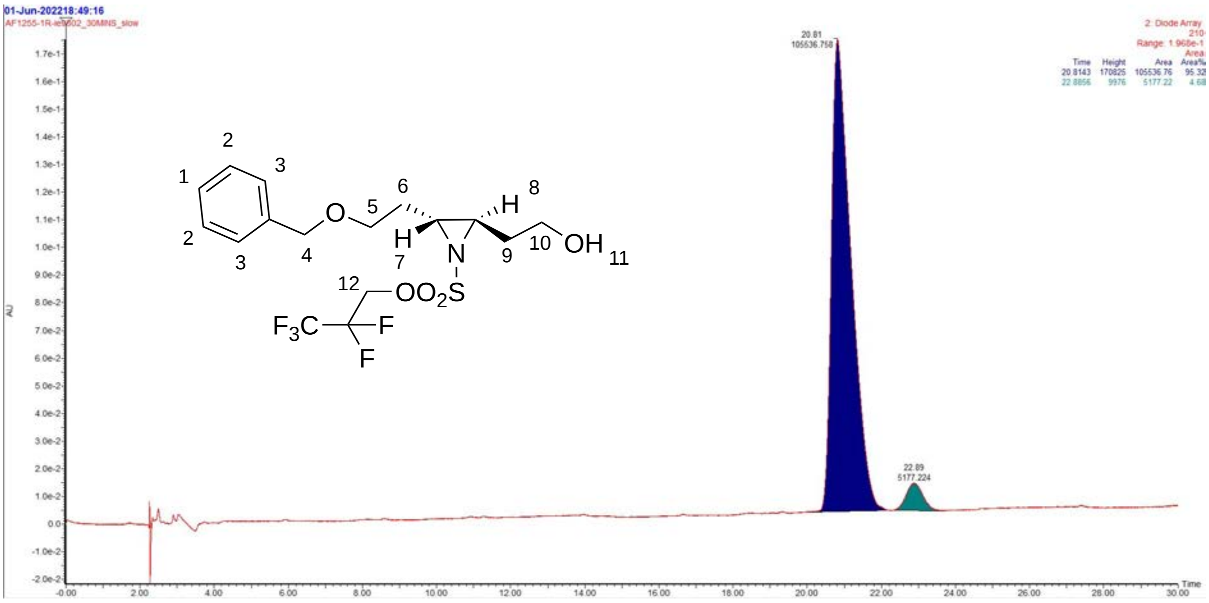

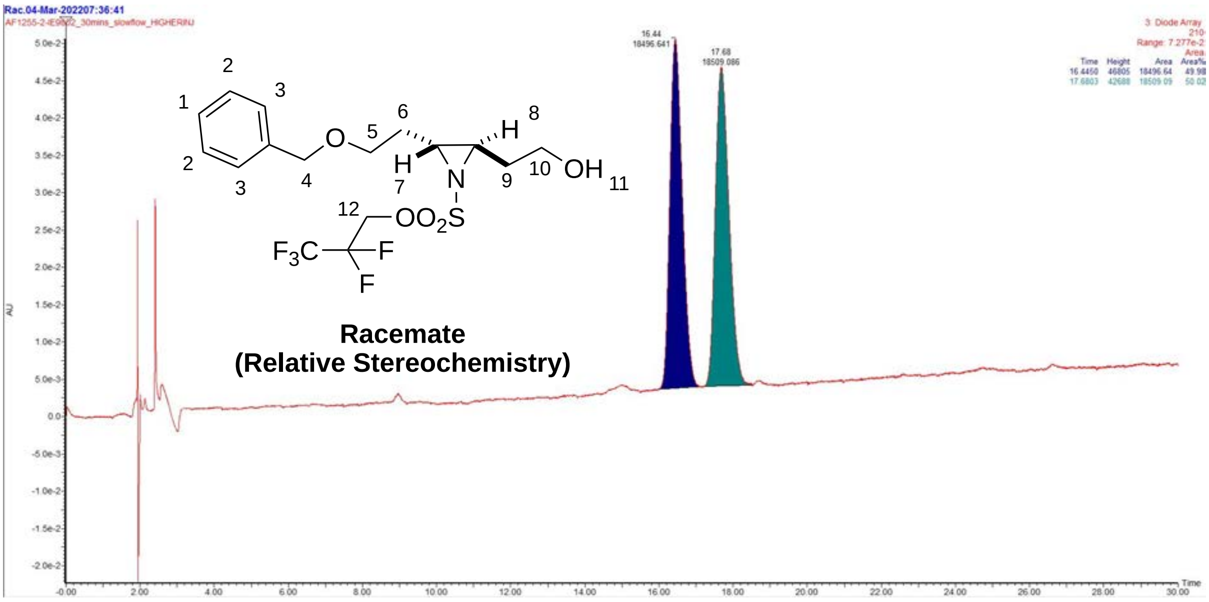

2,2,3,3,3-pentafluoropropyl (2R,3R)-2-(2-hydroxyethyl)-3-(2-(tosyloxy)ethyl)aziridine-1-sulfonate (**26e**)

Chiral SFC Analysis CHIRALPAK IE (CO_2:MeOH, 95:5, 2.5 mL min^{-1}, 40 °C, 223 nm) indicated 90% *ee*, t_R = 8.5 (major), 9.4 (minor) minutes.

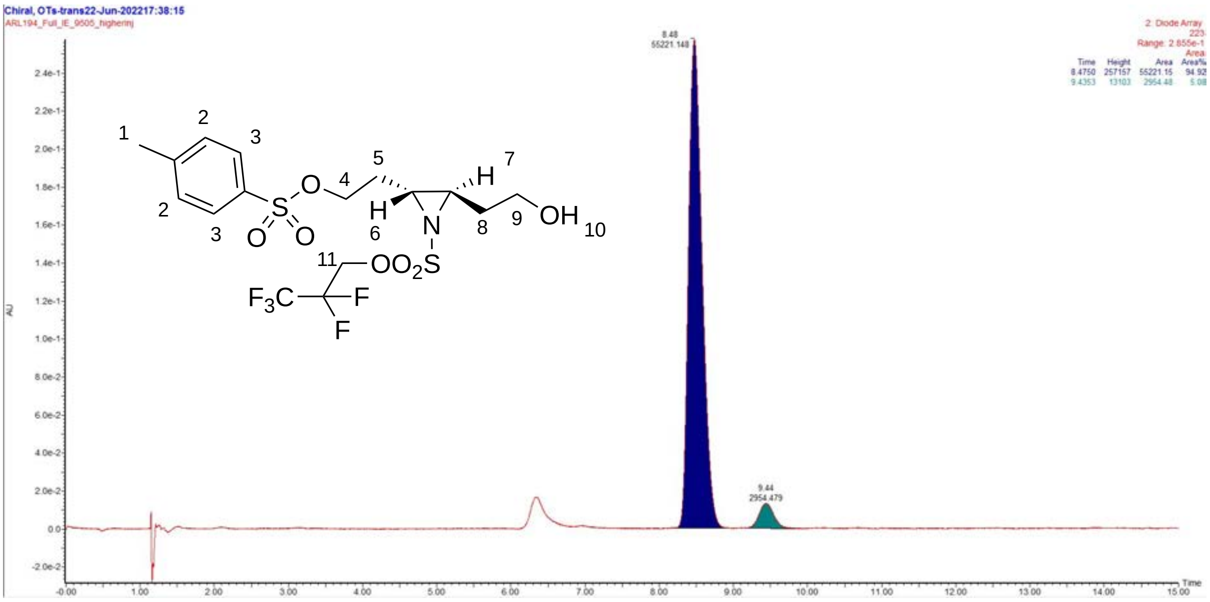

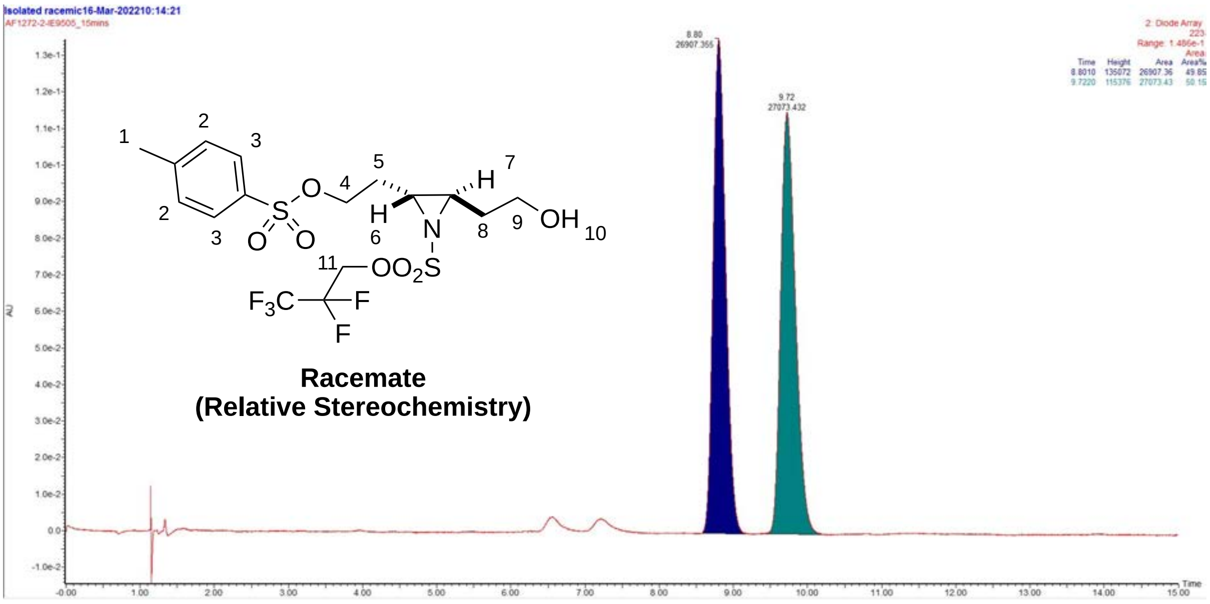

2,2,3,3,3-pentafluoropropyl ((2S,3R)-2-(2-(phenylthio)ethyl)tetrahydrofuran-3-yl)sulfamate: ***Obtained following derivatization of* 26f**

Chiral SFC analysis: CHIRALPAK IE (CO_2:MeOH, 95:5, 2.5 mL min^{-1}, 40 ˚C, 252 nm) indicated 90% *ee*, t_R = 3.3 (minor), 5.3 (major) minutes.

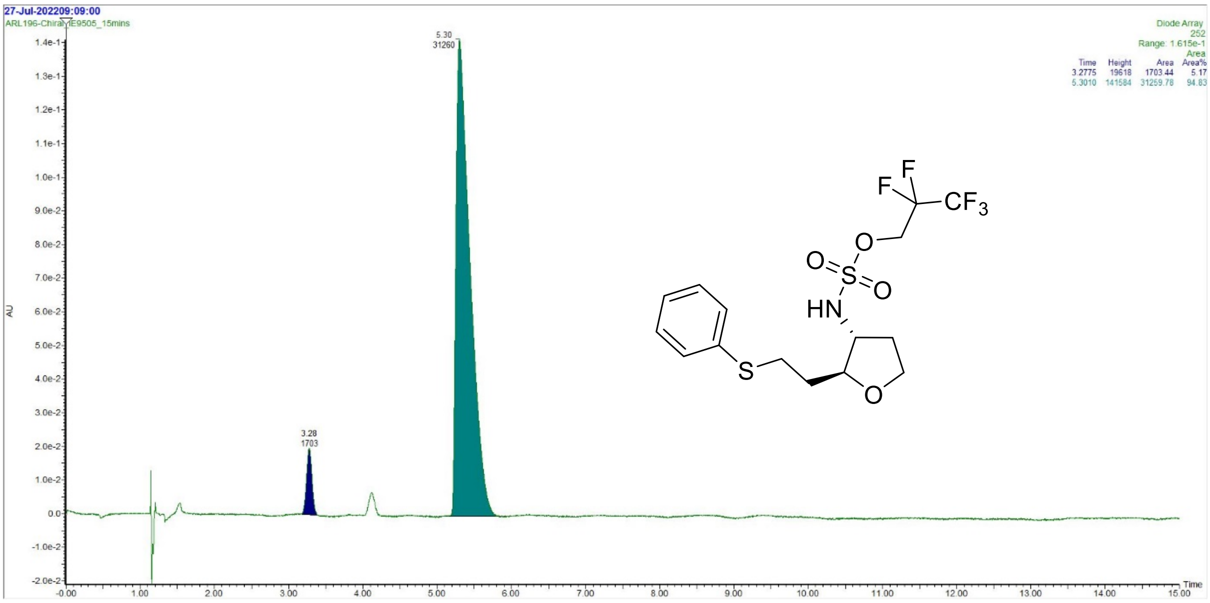

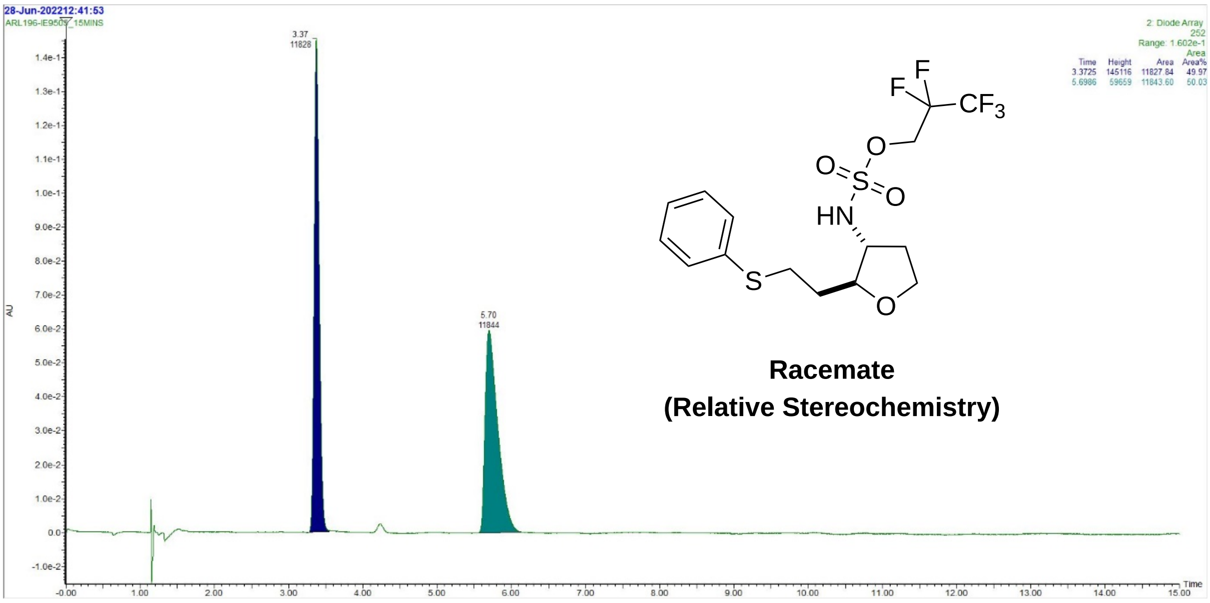

2,2,3,3,3-pentafluoropropyl (2R,3R)-2-(2-((N,4-dimethylphenyl)sulfonamido)ethyl)-3-(2-hydroxyethyl)aziridine-1-sulfonate (**26g**)

Chiral HPLC Analysis CHIRALPAK IC (Hexane:^{i}PrOH, 85:15, 1.0 mL min^{-1}, 30 °C, 226 nm) indicated 83% *ee*, t_R = 32.0 (minor), 34.5 (major) minutes.

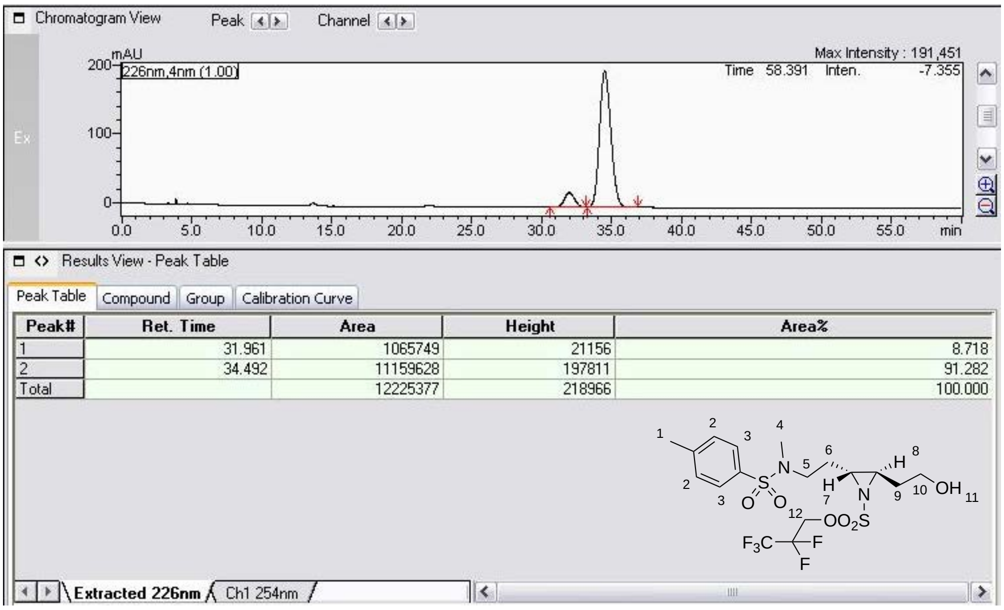

Peak#	Ret. Time	Area	Height	Area%
1	31.961	1065749	21156	8.718
2	34.492	11159628	197811	91.282
Total		12225377	218966	100.000

Racemate (Relative Stereochemistry)

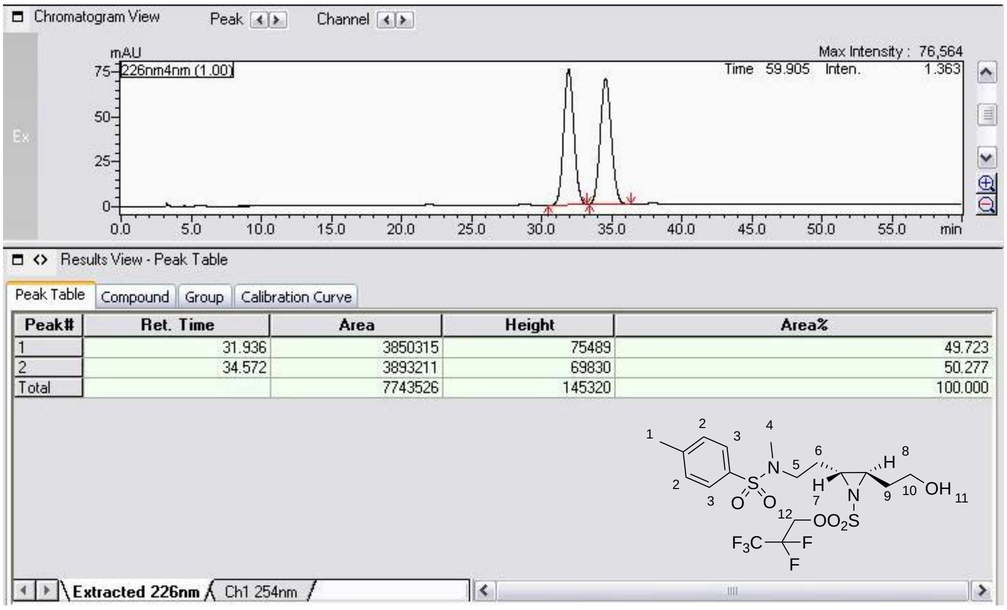

Peak#	Ret. Time	Area	Height	Area%
1	31.936	3850315	75489	49.723
2	34.572	3893211	69830	50.277
Total		7743526	145320	100.000

2,2,3,3,3-pentafluoropropyl (S)-(1-hydroxy-6-methyl-3-((phenylthio)methyl)heptan-3-yl)sulfamate: ***Obtained following derivatisation of*** **28a**

Chiral SFC Analysis CHIRAL ART SB (CO_2:^{i}PrOH, 96:4, 1.25 mL min^{-1}, 40 °C, 252 nm) indicated 84% *ee*, t_R = 25.8 (minor), 30.4 (major) minutes.

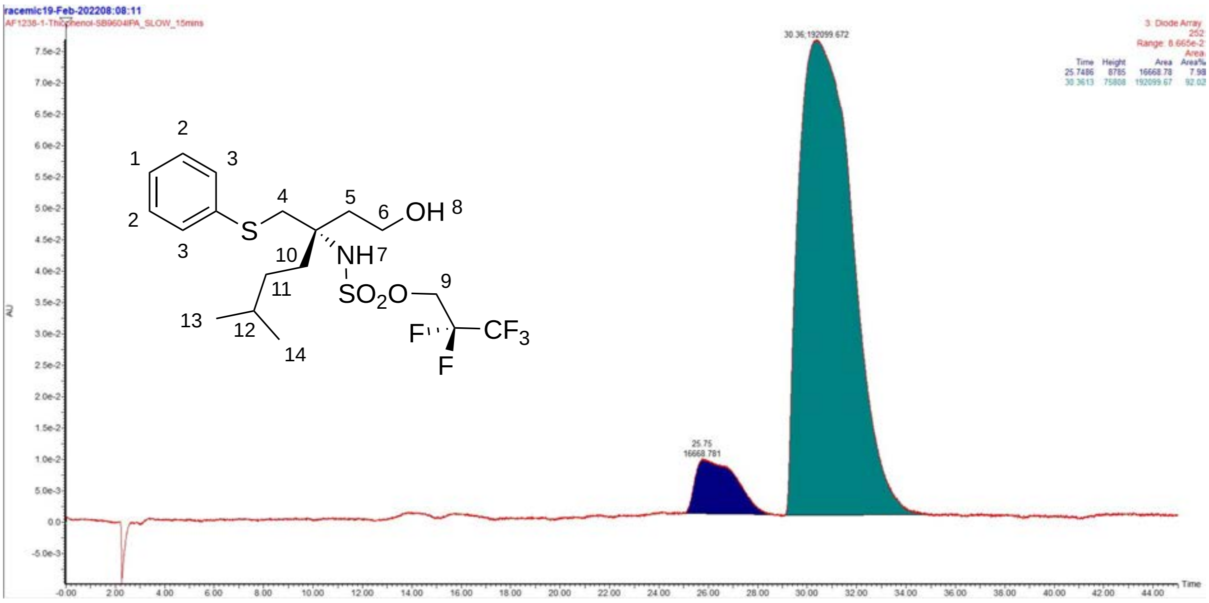

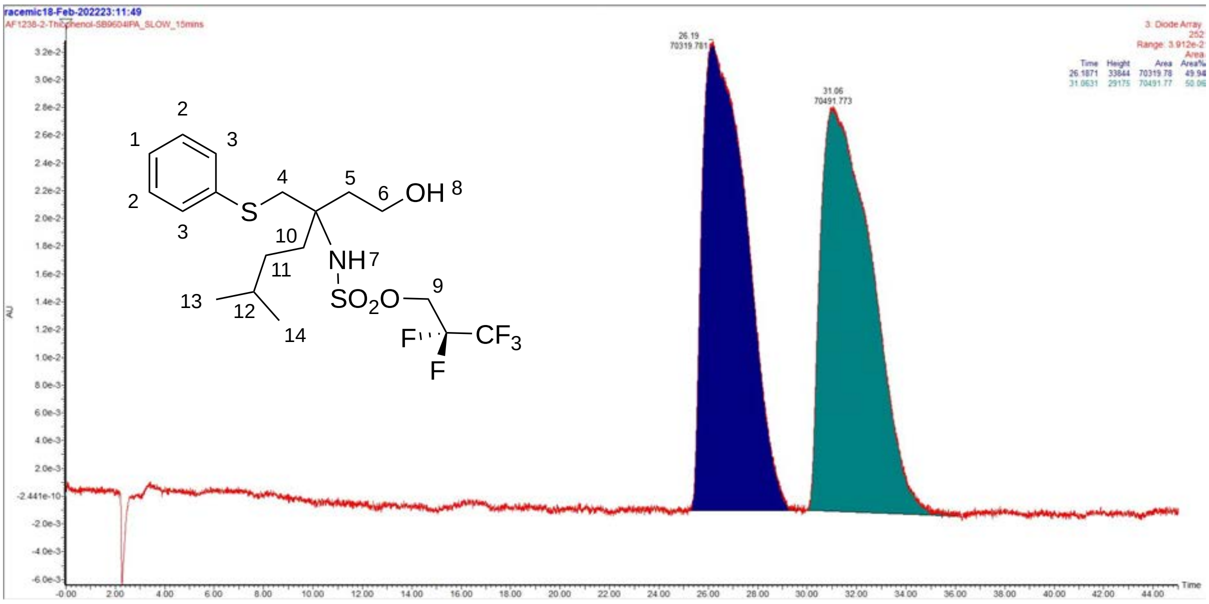

2,2,3,3,3-pentafluoropropyl (S)-(1-cyclohexyl-5-hydroxy-3-((phenylthio)methyl)pentan-3-yl)sulfamate: (**41**, ***Obtained following derivatisation of* 28b**)

Chiral SFC analysis: CHIRAL ART SB (CO_2:^{i}PrOH = 94.0:6.0, 1.25 mL min^{-1}, 40 ˚C, 250 nm) indicated 85% *ee*, t_R = 26.0 (minor), 29.5 (major) minutes.

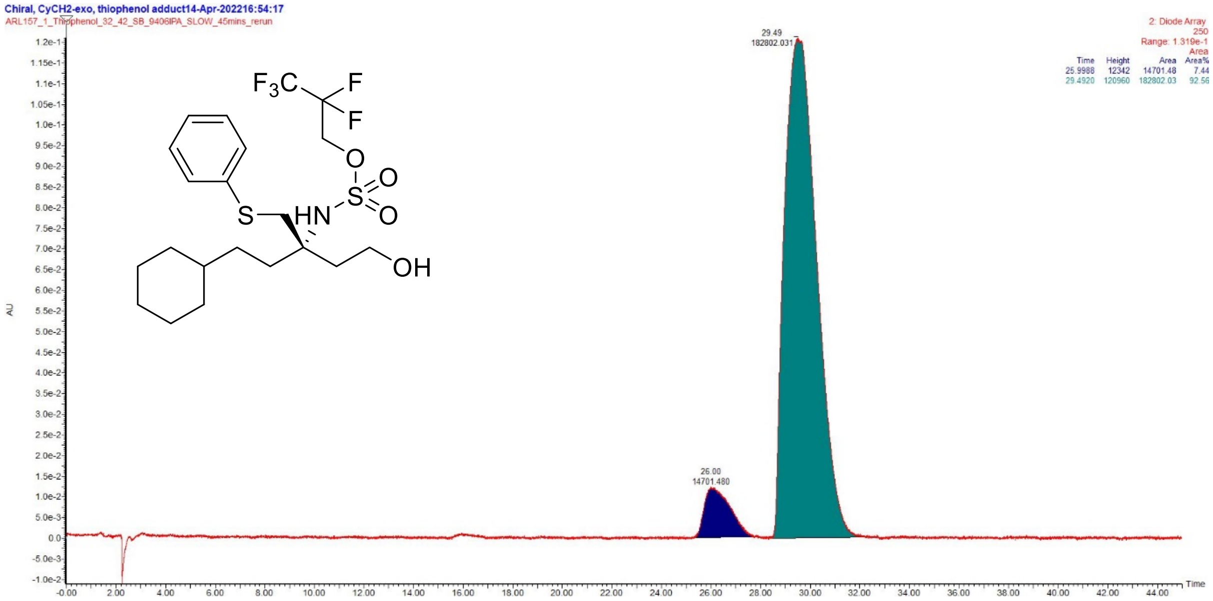

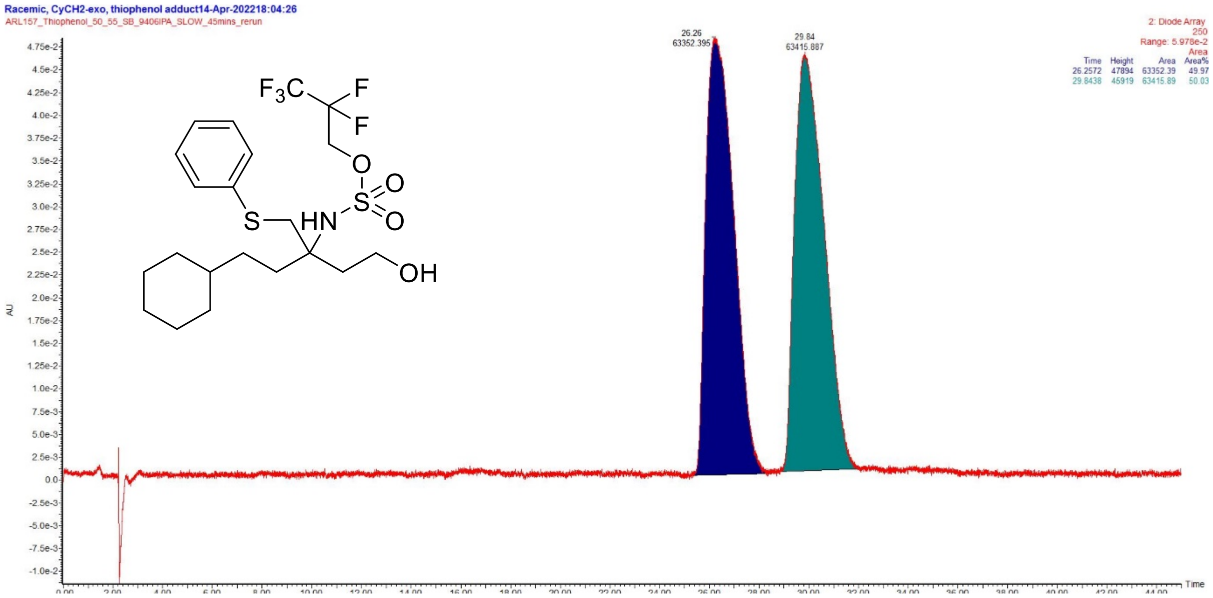

2,2,3,3,3-pentafluoropropyl (S)-(7-((tert-butyldimethylsilyl)oxy)-1-hydroxy-3-((phenylthio)methyl)heptan-3-yl)sulfamate: ***Obtained following derivatisation of* 28c**

Chiral SFC analysis: CHIRAL ART SB (CO_2:MeOH, 96.5:3.5, 1.25 mL min^{-1}, 40 ˚C, 250 nm) indicated 80% *ee*, t_R = 18.6 (minor), 21.0 (major) minutes.

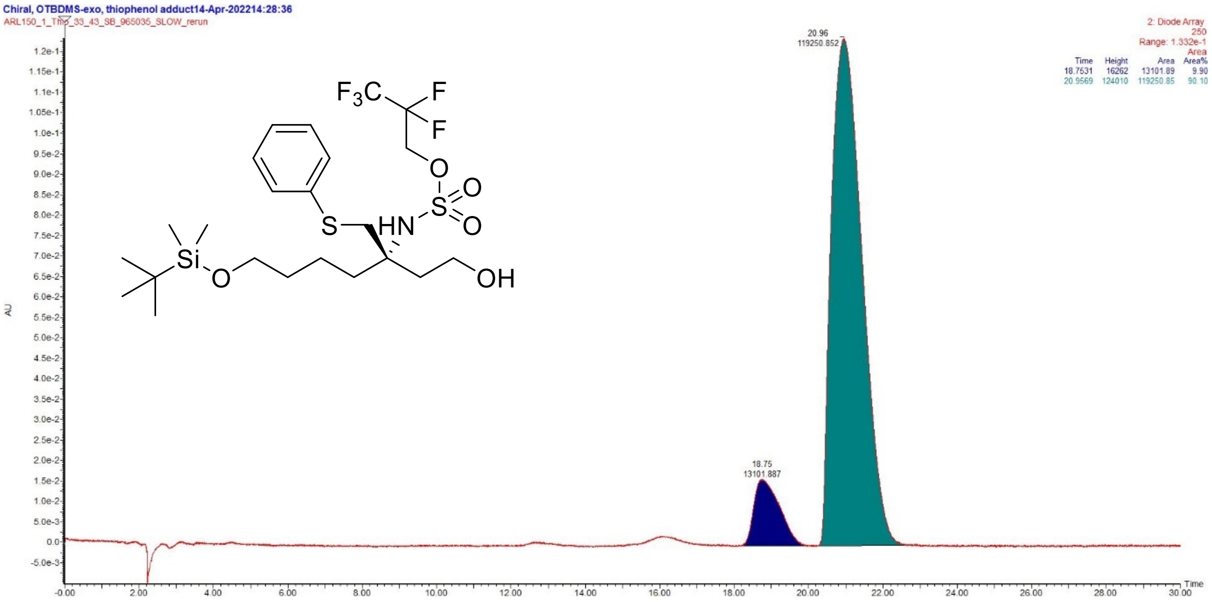

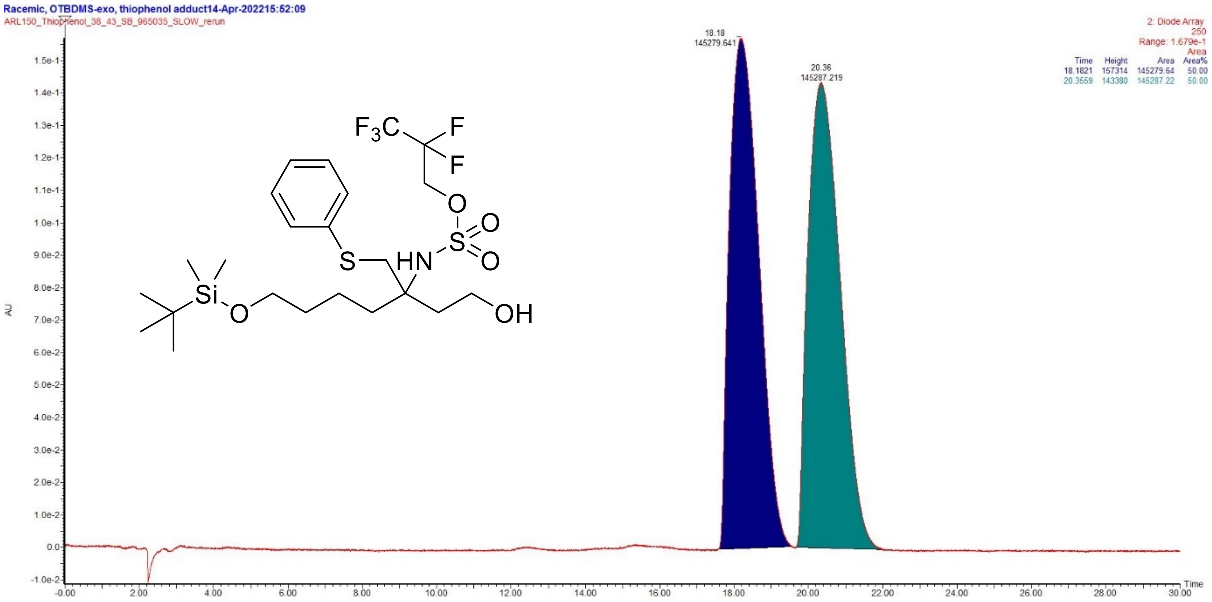

AF1225: 2,2,3,3,3-pentafluoropropyl ((2R,3R)-2-methyl-2-propyltetrahydrofuran-3-yl)sulfamate (**33a**)

Chiral GC Analysis CP7502 – CP-Chirasil-DEX CB column (125 °C Isothermal, 27.5 psi) indicated 87% *ee*, t_R = 25.8 (major), 26.6 (minor) minutes.

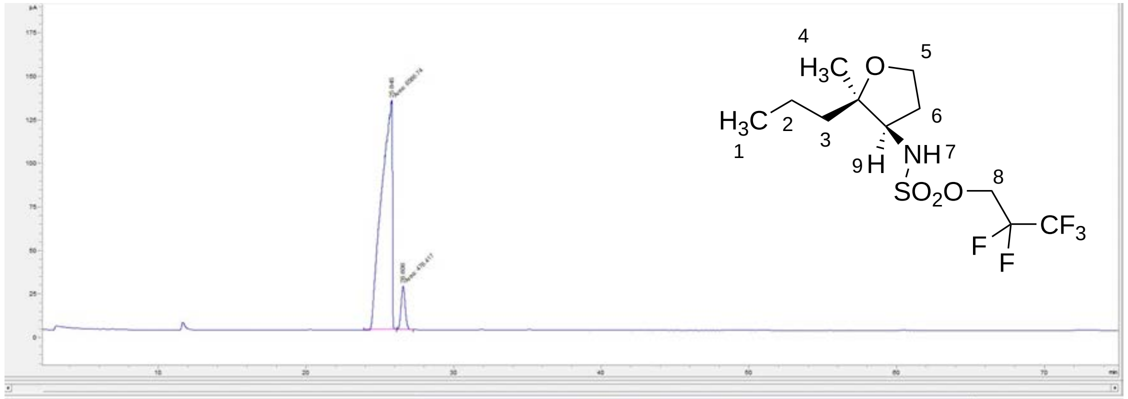

#	Time	Type	Area	Height	Width	Area%	Symmetry
1	25.845	MM	6586.7	131.1	0.8372	93.255	15.318
2	26.606	MM	476.4	24.9	0.3183	6.745	0.919

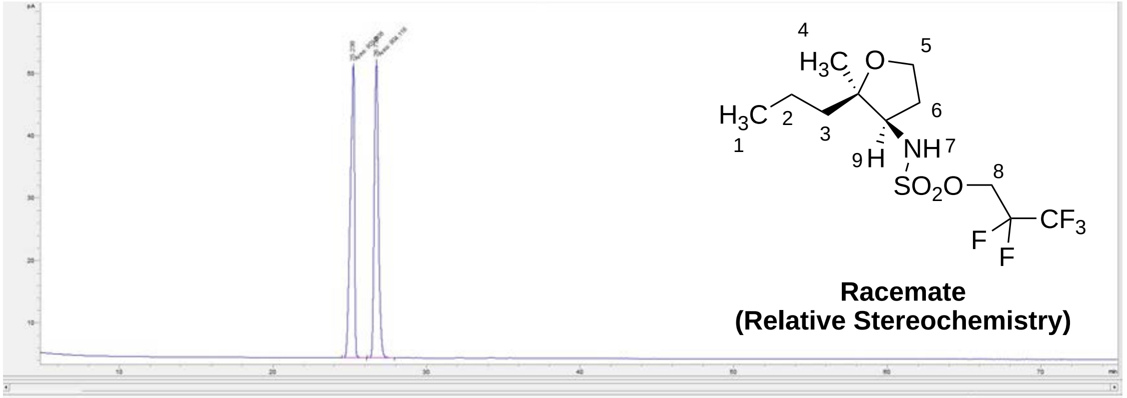

#	Time	Type	Area	Height	Width	Area%	Symmetry
1	25.238	MM	903.6	47.2	0.3188	49.986	2.786
2	26.735	MM	904.1	47.9	0.3145	50.014	1.058

2,2,3,3,3-pentafluoropropyl (R)-(5-oxaspiro[3.4]octan-8-yl)sulfamate (**33b**)

Chiral GC Analysis CP7502 – CP-Chirasil-DEX CB column (135 °C Isothermal, 13.6 psi) indicated 83% *ee*, t_R = 26.2 (major), 27.8 (minor) minutes.

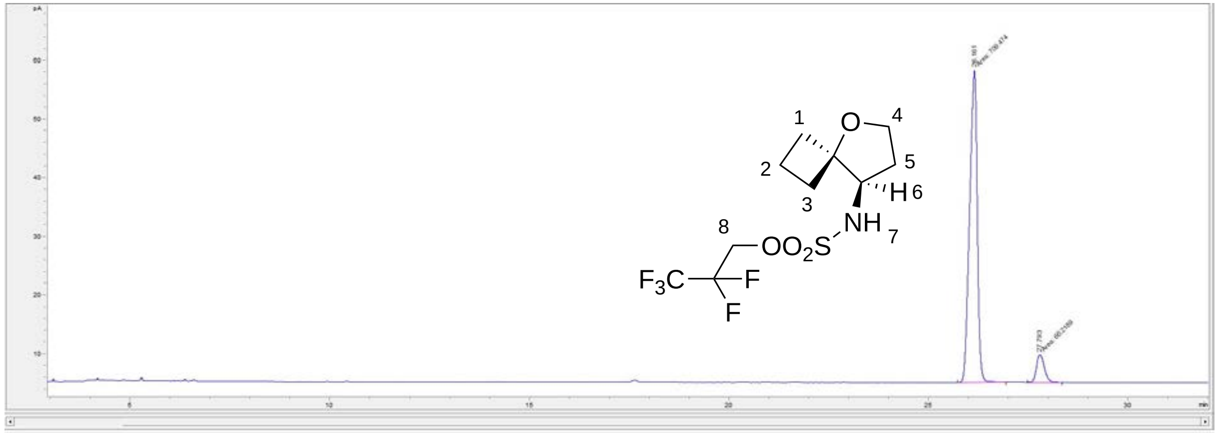

#	Time	Type	Area	Height	Width	Area%	Symmetry
1	26.161	MM	709.5	53.3	0.222	91.463	1.483
2	27.793	MM	66.2	4.8	0.2314	8.537	0.626

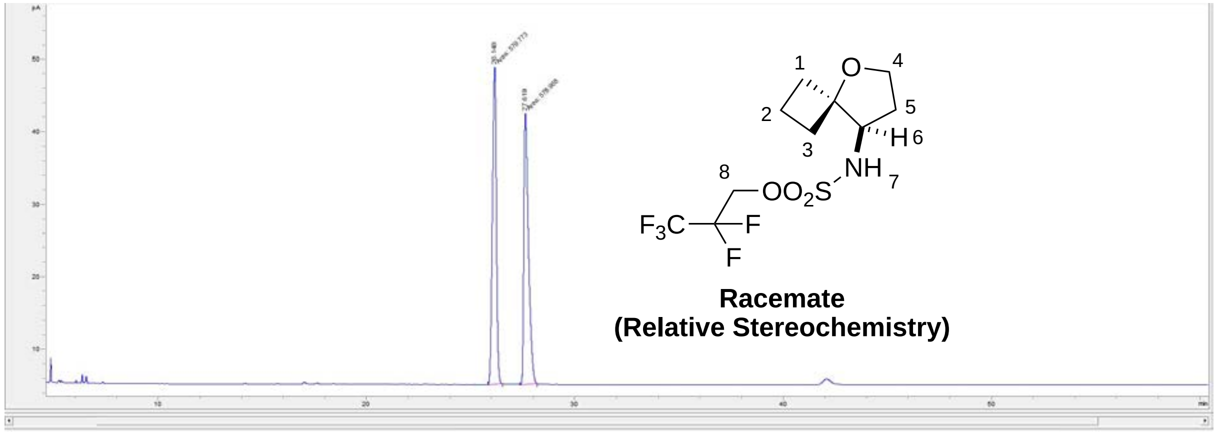

#	Time	Type	Area	Height	Width	Area%	Symmetry
1	26.148	MM	579.8	43.7	0.221	50.035	1.375
2	27.619	MM	579	37.4	0.2582	49.965	0.5

2,2,3,3,3-pentafluoropropyl (R)-(4-bromobenzyl)(1-oxaspiro[4.5]decan-4-yl)sulfamate: ***Obtained following derivatisation of* 33c**

Chiral SFC Analysis CHIRALPAK IG (CO_2:MeOH, 97:3, 1.25 mL min^{-1}, 40 °C, 221 nm) indicated 84% *ee*, t_R = 8.6 (major), 20.4 (minor) minutes.

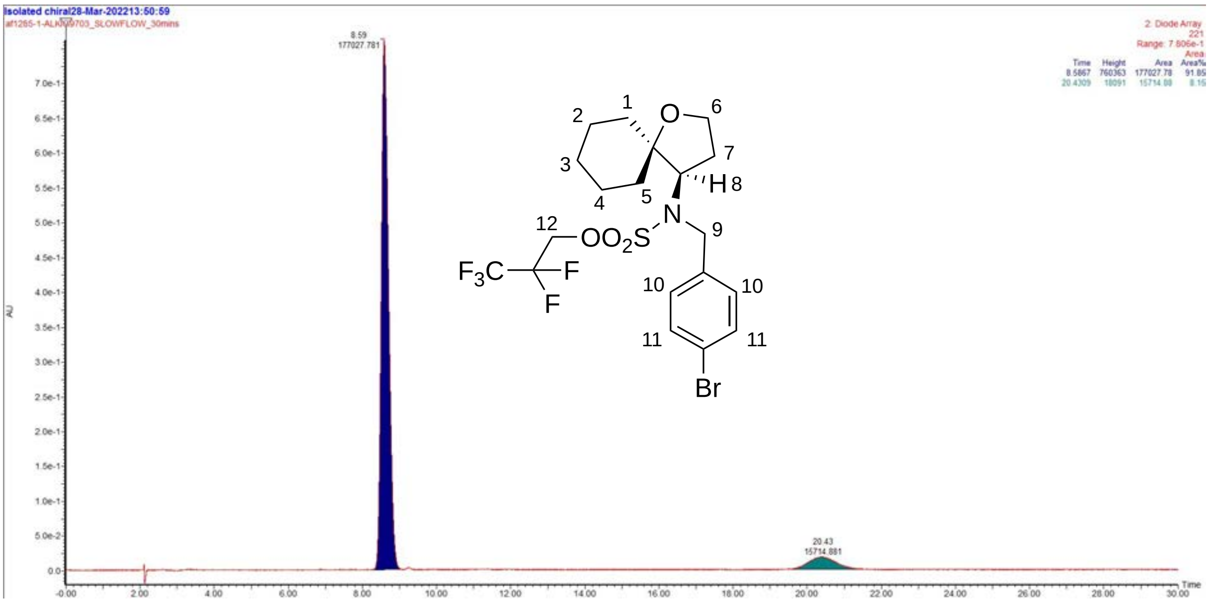

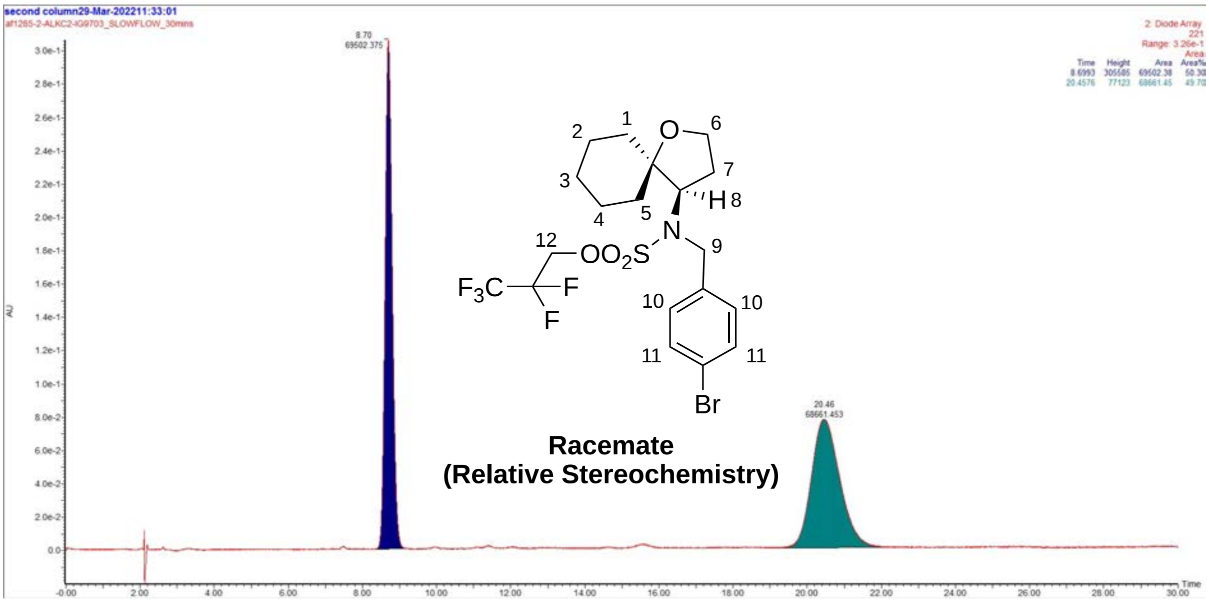

2,2,3,3,3-pentafluoropropyl (R)-(4-bromobenzyl)(1,8-dioxaspiro[4.5]decan-4-yl)sulfamate: ***Obtained following derivatisation of* 33d**

Chiral SFC Analysis CHIRAL ART SJ (CO_2:^{i}PrOH, 97:3, 1.25 mL min^{-1}, 40 °C, 221 nm) indicated 81% *ee*, t_R = 7.9 (minor), 9.2 (major) minutes.

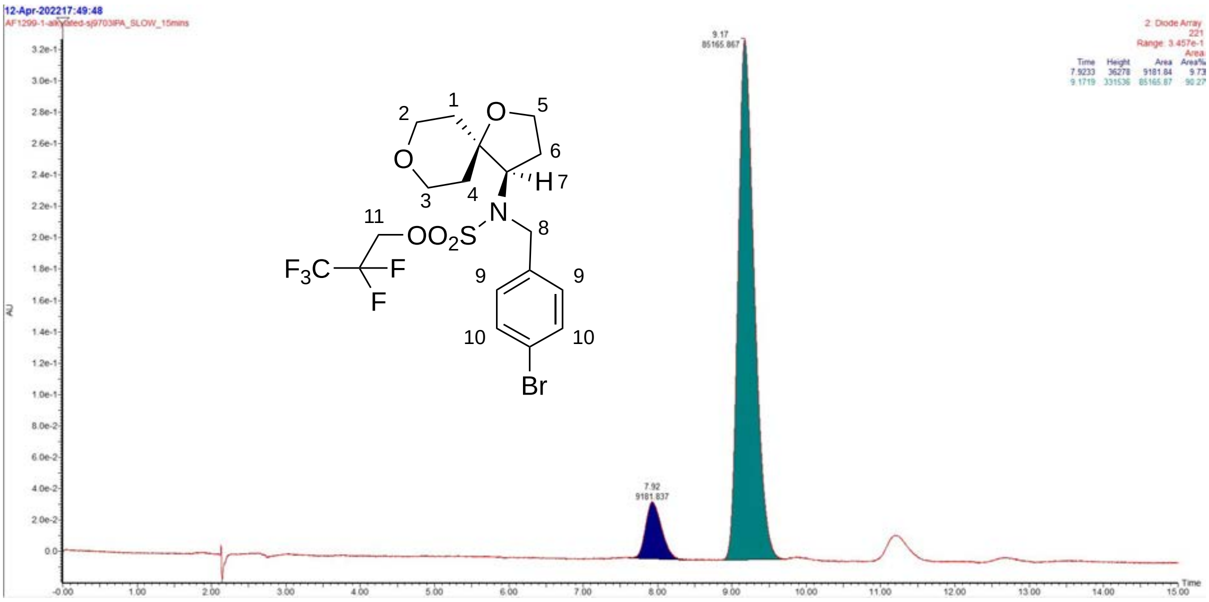

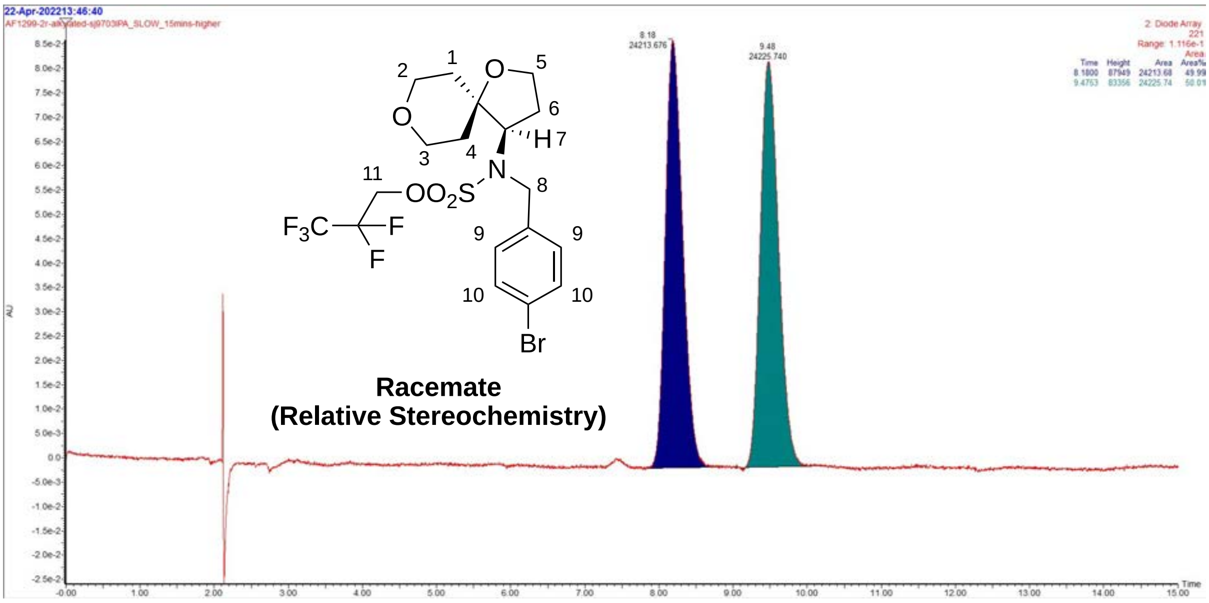

2,2,3,3,3-pentafluoropropyl (R)-(2,2-dimethyltetrahydro-2H-pyran-3-yl)sulfamate (**33e**)

Chiral GC Analysis CP7502 – CP-Chirasil-DEX CB column (135 °C Isothermal, 10.0 psi) indicated 91% *ee*, t_R = 27.7 (major), 28.6 (minor) minutes.

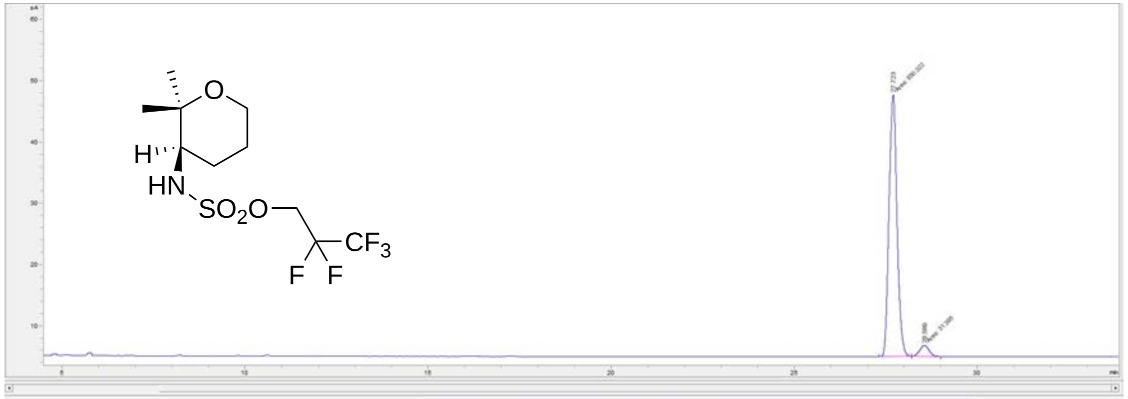

#	Time	Type	Area	Height	Width	Area%	Symmetry
1	27.723	MM	650.3	42.7	0.2539	95.395	1.144
2	28.58	MM	31.4	1.8	0.2932	4.605	1.107

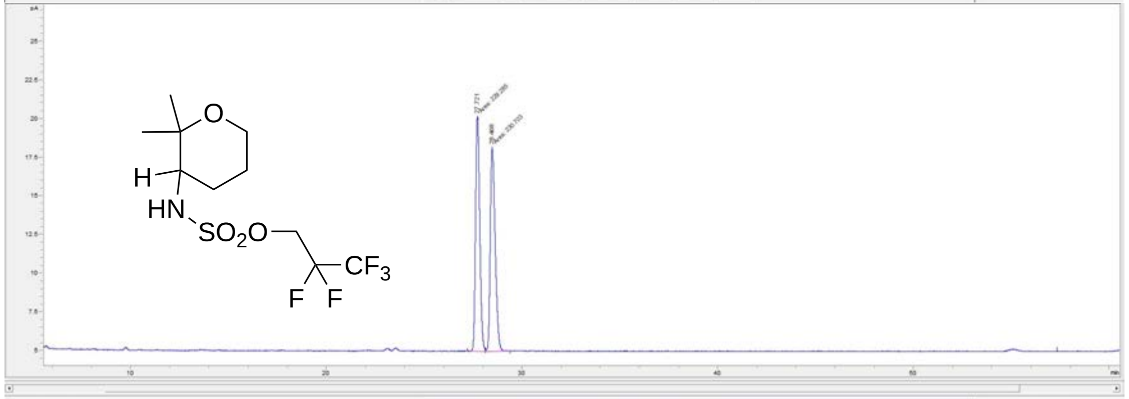

#	Time	Type	Area	Height	Width	Area%	Symmetry
1	27.721	MM	229.3	15.2	0.2511	49.846	0.867
2	28.468	MM	230.7	13.2	0.2912	50.154	0.625

2,2,3,3,3-pentafluoropropyl (R)-(4-bromobenzyl)(1-oxaspiro[5.5]undecan-5-yl)sulfamate: ***Obtained following derivatisation of* 33f**

Chiral SFC analysis: CHIRAL ART SC (CO_2:MeOH, 98:2, 1.25 mL min^{-1}, 40 ˚C, 220 nm) indicated 87% *ee*, t_R = 5.4 (major), 6.9 (minor) minutes.

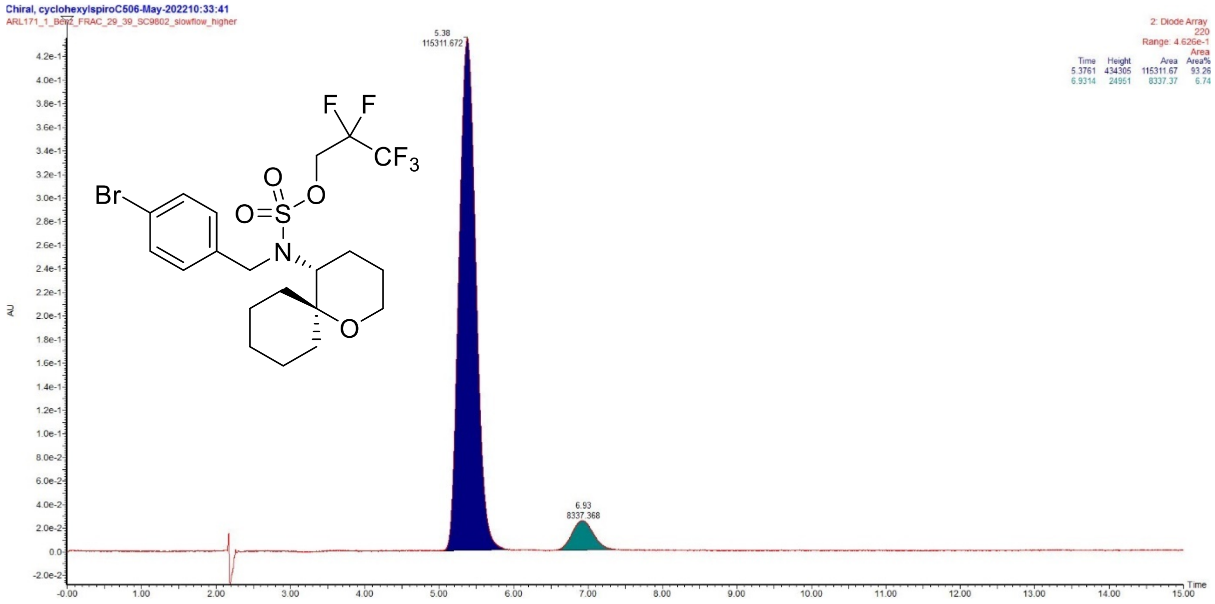

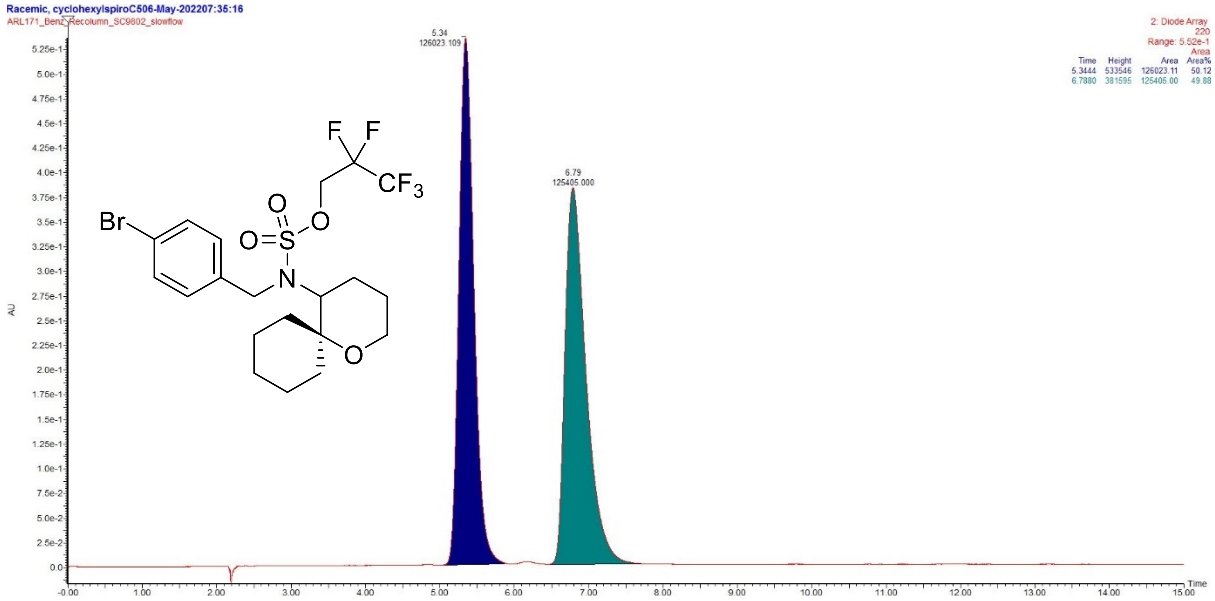

2,2,3,3,3-pentafluoropropyl ((2R,3R)-2-methyl-2-phenyltetrahydrofuran-3-yl)sulfamate (**33g**)

Chiral SFC Analysis CHIRALPAK IH (CO_2:MeOH, 98:2, 1.25 mL min^{-1}, 40 °C, 210 nm) indicated 97% *ee*, t_R = 4.9 (major), 5.7 (minor) minutes.

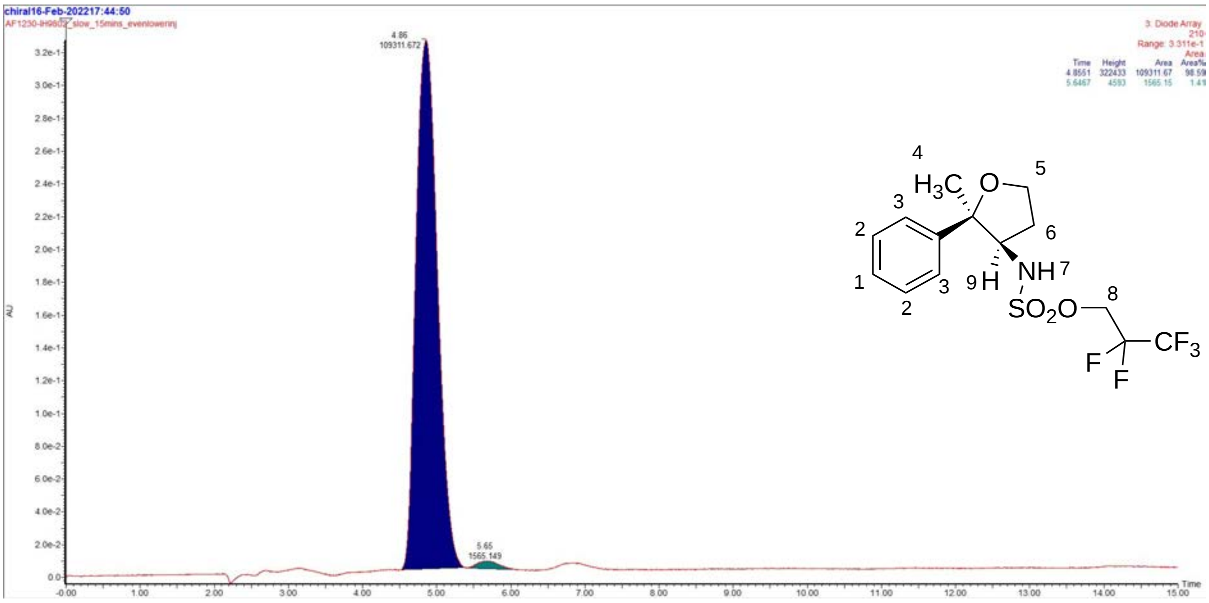

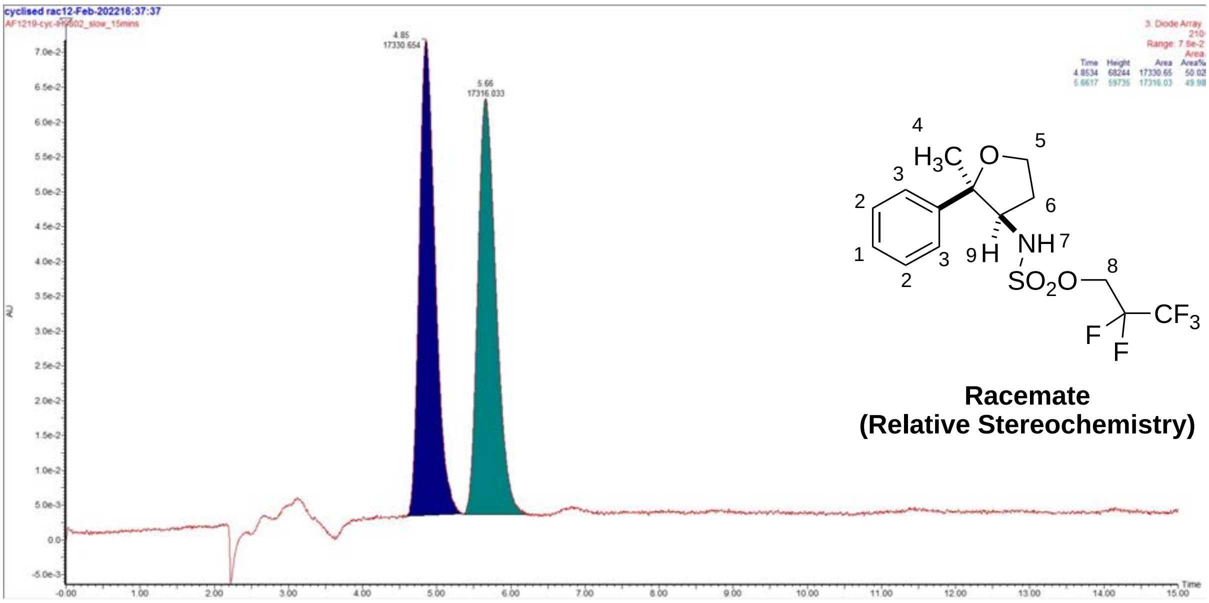

2,2,3,3,3-pentafluoropropyl (2-methyl-2-phenyltetrahydrofuran-3-yl)sulfamate (**33h**)

Chiral SFC Analysis CHIRAL ART SJ (CO_2:MeOH, 98.5:1.5, 2.5 mL min^{-1}, 40 °C, 210 nm) indicated racemic, t_R = 3.5, 4.2 minutes.

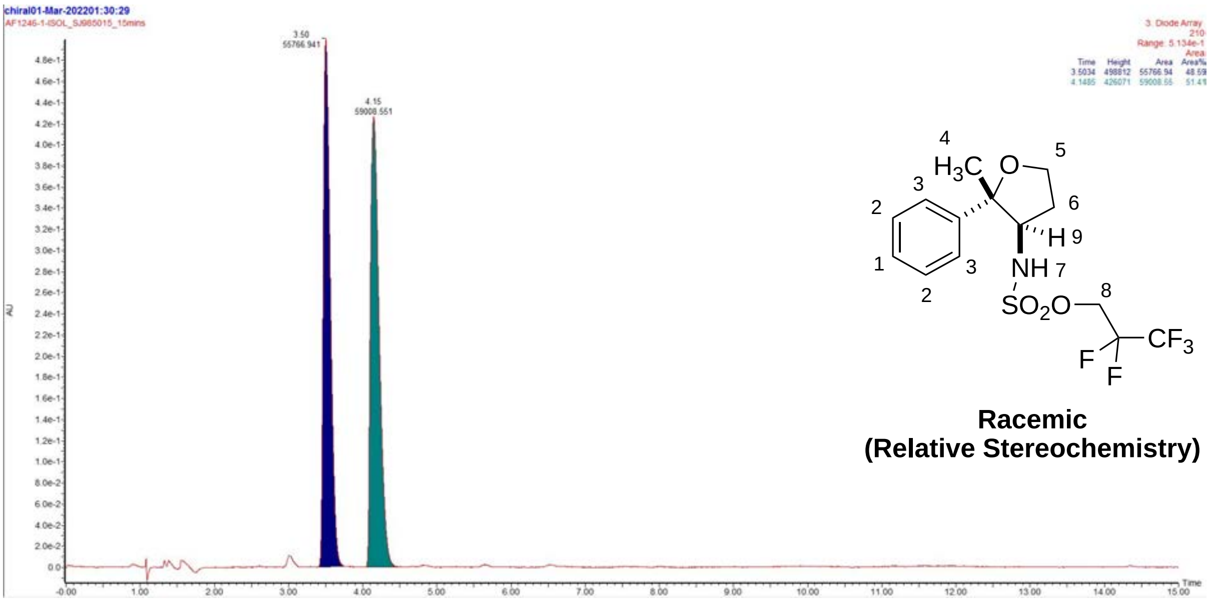

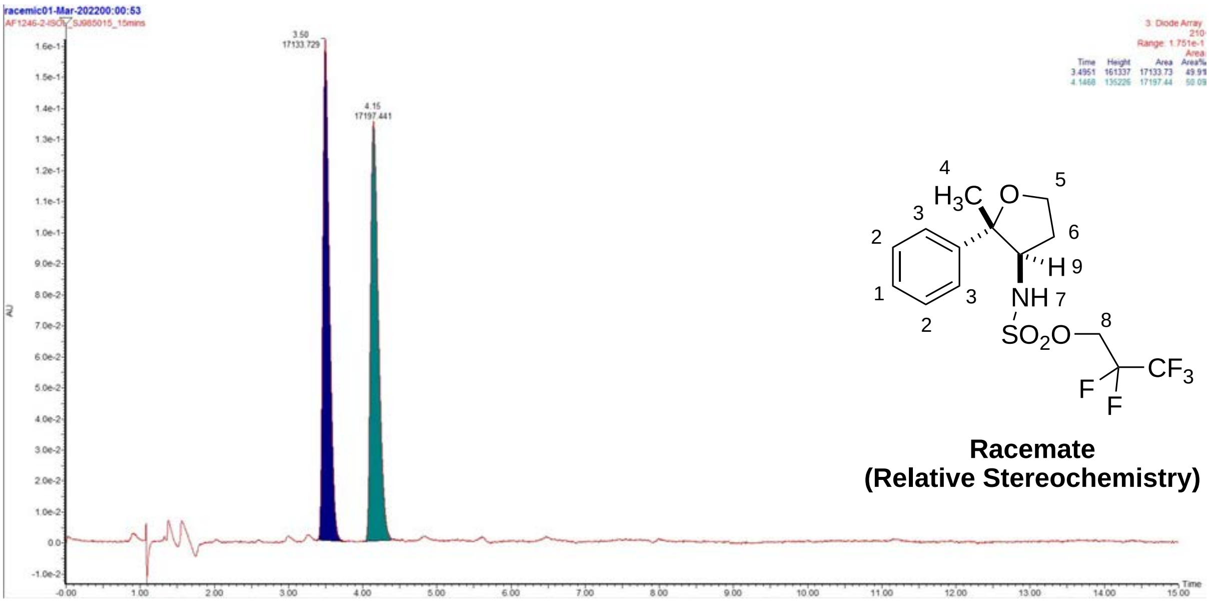

2,2,3,3,3-pentafluoropropyl ((2R,3R)-2-methyl-2-(4-methylpent-3-en-1-yl)tetrahydrofuran-3-yl)sulfamate (**36**)

Chiral GC Analysis CP7502 – CP-Chirasil-DEX CB column (140 °C Isothermal, 27.0 psi) indicated 89% *ee*, t_R = 38.0 (major), 38.8 (minor) minutes.

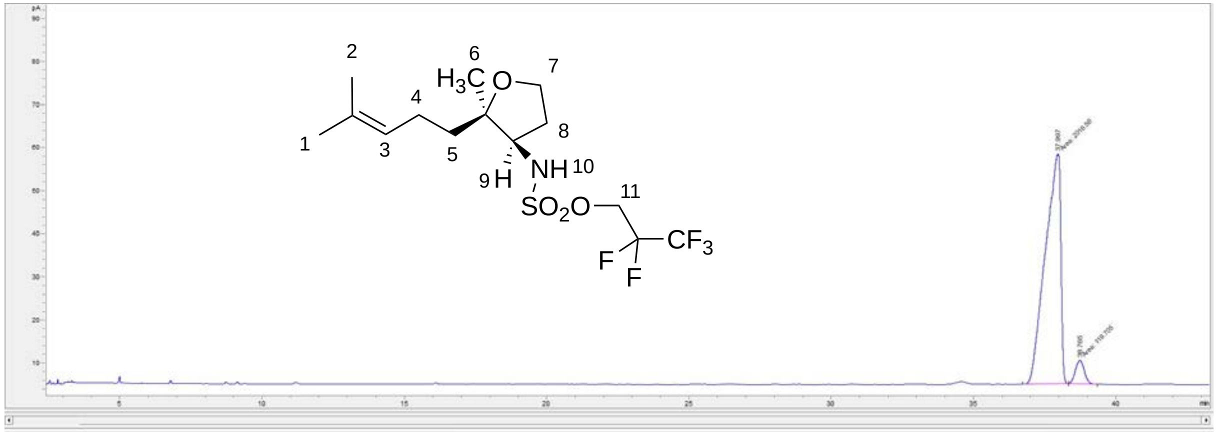

#	Time	Type	Area	Height	Width	Area%	Symmetry
1	37.997	MM	2016.6	53.4	0.6292	94.397	4.521
2	38.765	MM	119.7	5.6	0.358	5.603	1.04

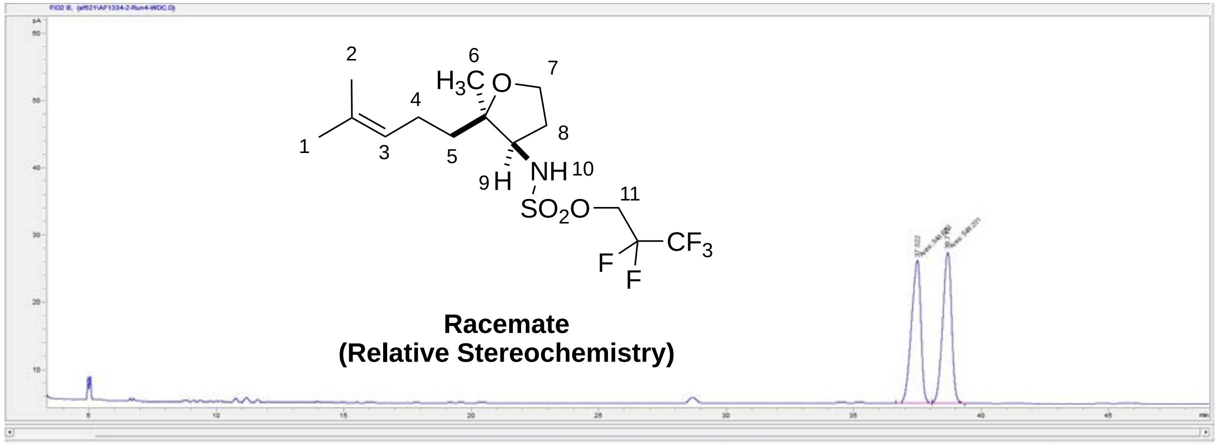

#	Time	Type	Area	Height	Width	Area%	Symmetry
1	37.522	MM	546.7	21.3	0.4271	49.931	1.664
2	38.712	MM	548.2	22.4	0.4071	50.069	1.297

2,2,3,3,3-pentafluoropropyl (4-bromobenzyl)((2S,3R)-2-methyl-2-(4-methylpent-3-en-1-yl)tetrahydrofuran-3-yl)sulfamate: ***Obtained following derivatisation of*** **39**

Chiral SFC Analysis CHIRALPAK IG (CO_2:MeOH, 97:3, 2.5 mL min^{-1}, 40 °C, 220 nm) indicated 81% *ee*, t_R = 2.9 (major), 3.7 (minor) minutes.

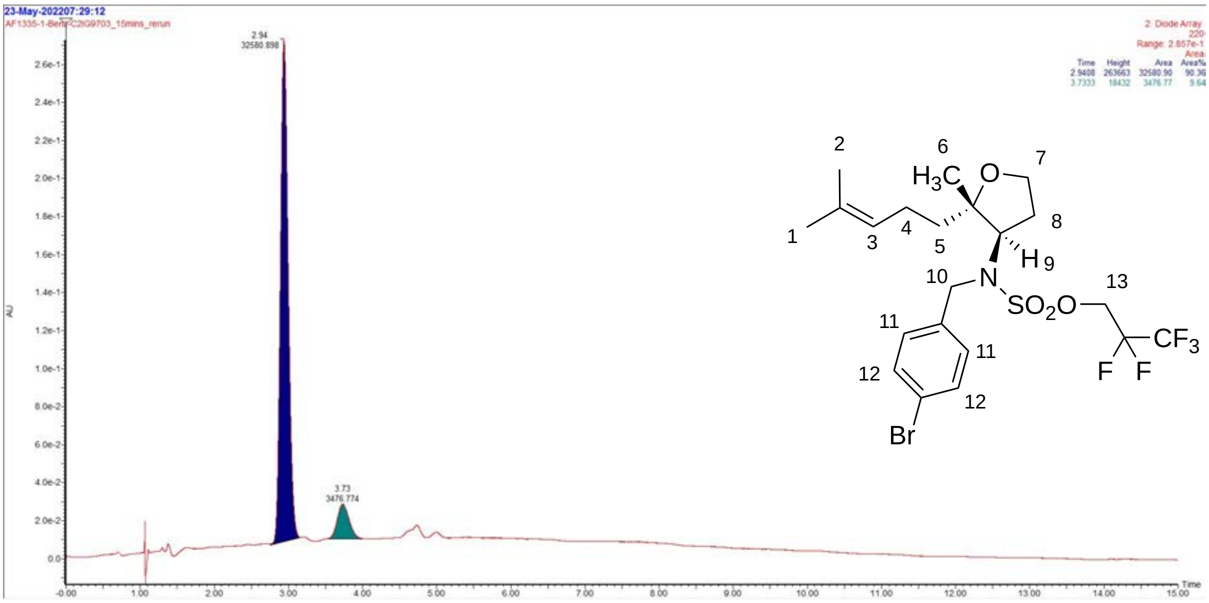

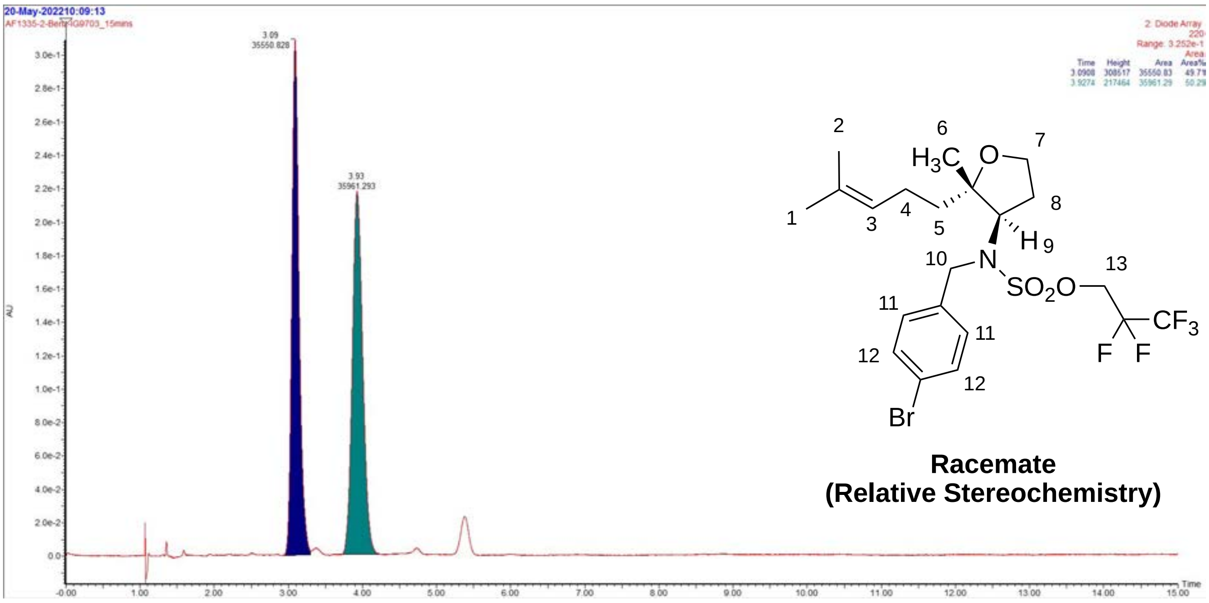

2,2,3,3,3-pentafluoropropyl 2-(2-hydroxyethyl)-2-methyl-3-phenylaziridine-1-sulfonate: ***Obtained following aziridination of*** **40**

Chiral SFC analysis: CHIRALPAK IH (CO_2:MeOH, 99:1, 1.25 mL min^{-1}, 40 ˚C, 215 nm) indicated 5% *ee*, t_R = 11.9 (minor), 14.5 (major) minutes.

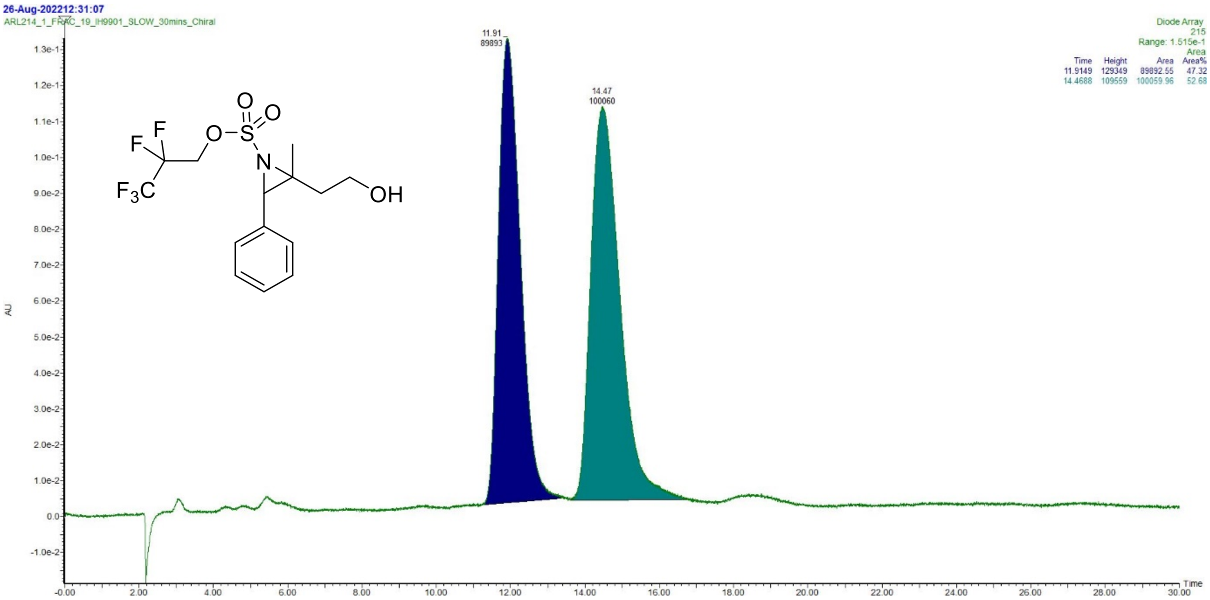

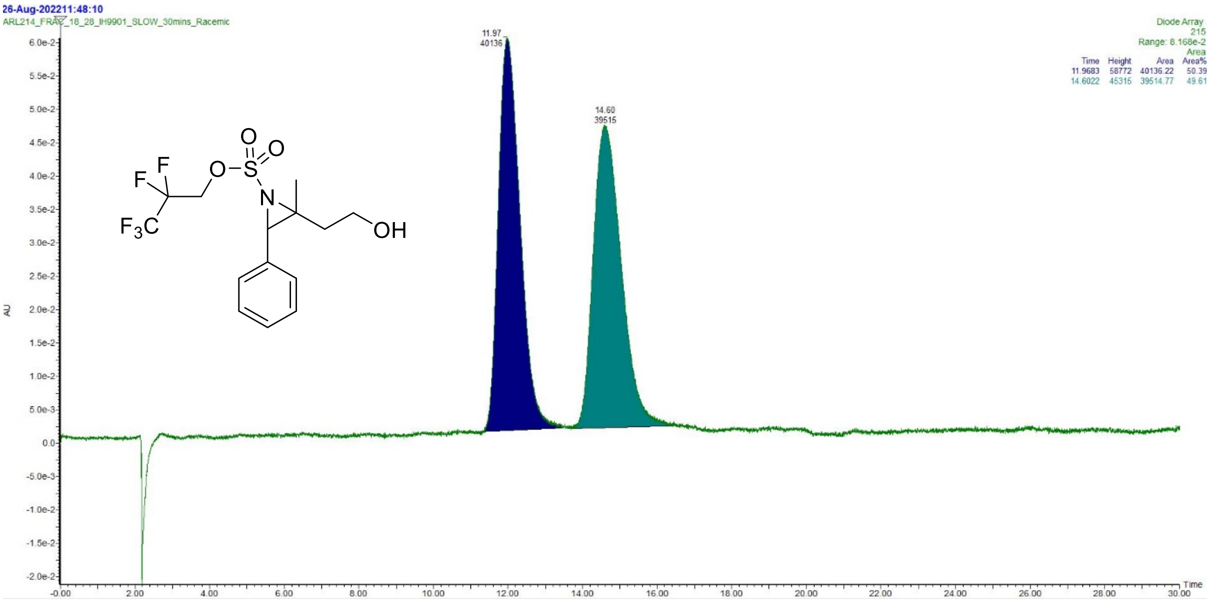

(S)-4-((tert-butyldiphenylsilyl)oxy)-2-(2-cyclohexylethyl)-2-(((2,2,3,3,3-pentafluoropropoxy)sulfonyl)amino)butyl acetate: ***Obtained following derivatisation of* 42**

Chiral SFC Analysis: CHIRALPAK IG (CO_2:^{i}PrOH, 96:4, 1.25 mL min^{-1}, 40 °C, 215 nm) indicated 84% *ee*, t_R = 8.9 (major), 10.8 (minor) minutes.

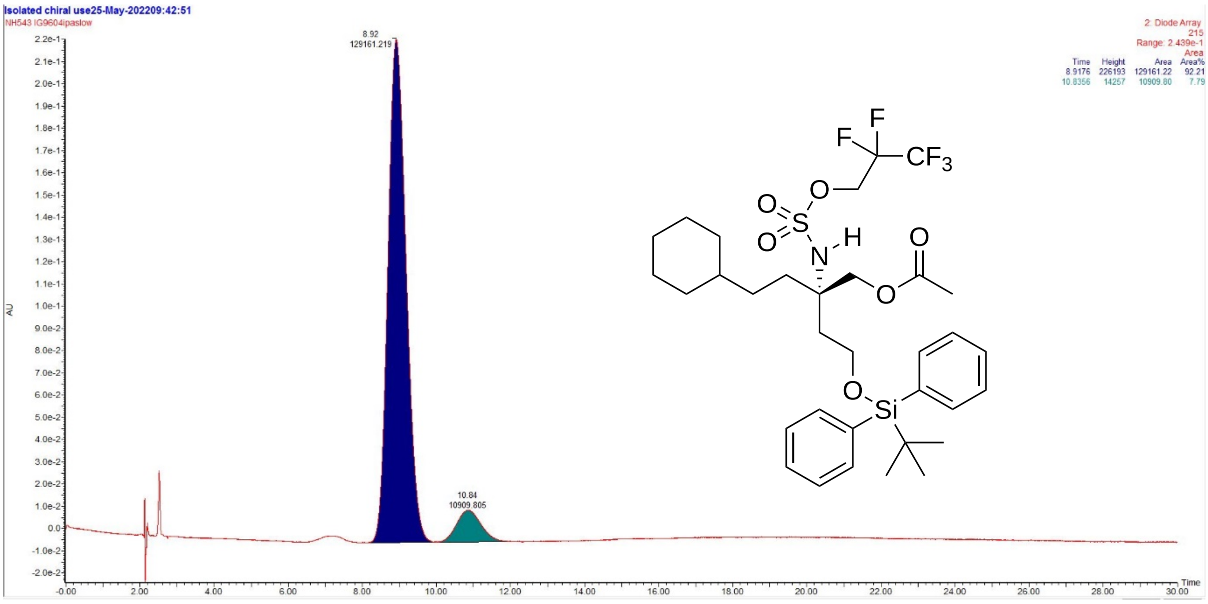

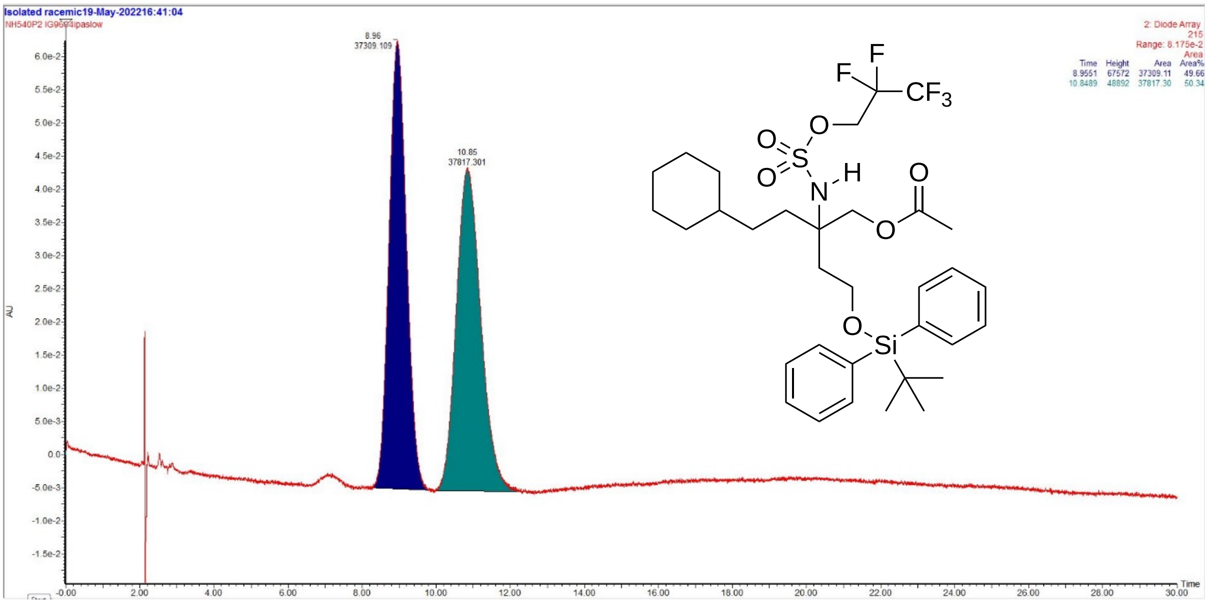

2,2,3,3,3-pentafluoropropyl (R)-(4-cyclohexyl-2-(2-hydroxyethyl)-2-(4-phenyl-1H-1,2,3-triazol-1-yl)butyl)sulfamate (**44**)

Chiral SFC Analysis: CHIRALPAK IG (CO_2:MeOH, 92:8, 2.5 mL min^{-1}, 40 °C, 243 nm) indicated 85% *ee*, t_R = 8.2 (major), 13.3 (minor) minutes.

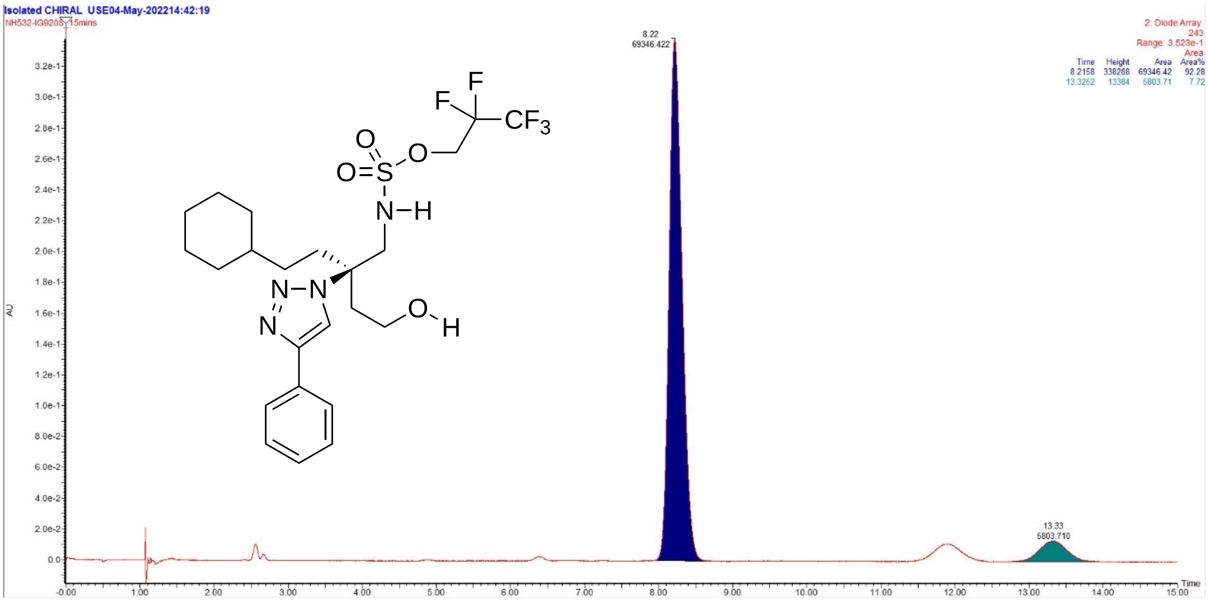

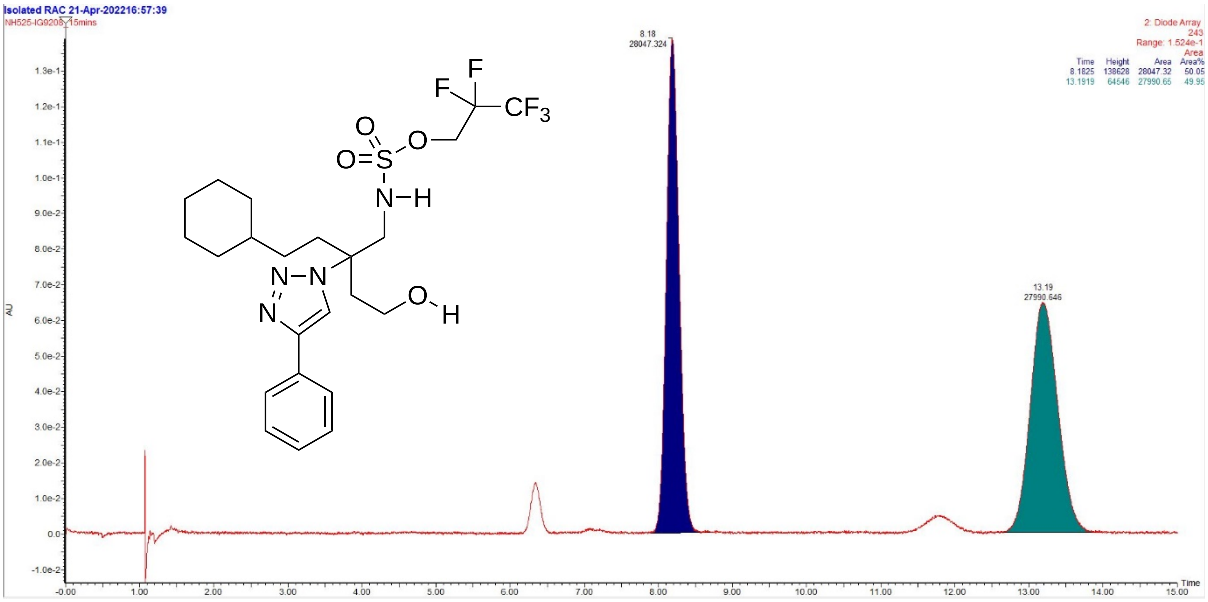

2,2,3,3,3-pentafluoropropyl (S)-(4-hydroxy-1-phenylbutan-2-yl)sulfamate (**45**)

Chiral SFC Analysis: CHIRALPAK IG (CO_2:MeOH, 97:3, 2.5 mL min^{-1}, 40 °C, 210 nm) indicated 87% *ee*, t_R = 7.1 (minor), 10.9 (major) minutes.

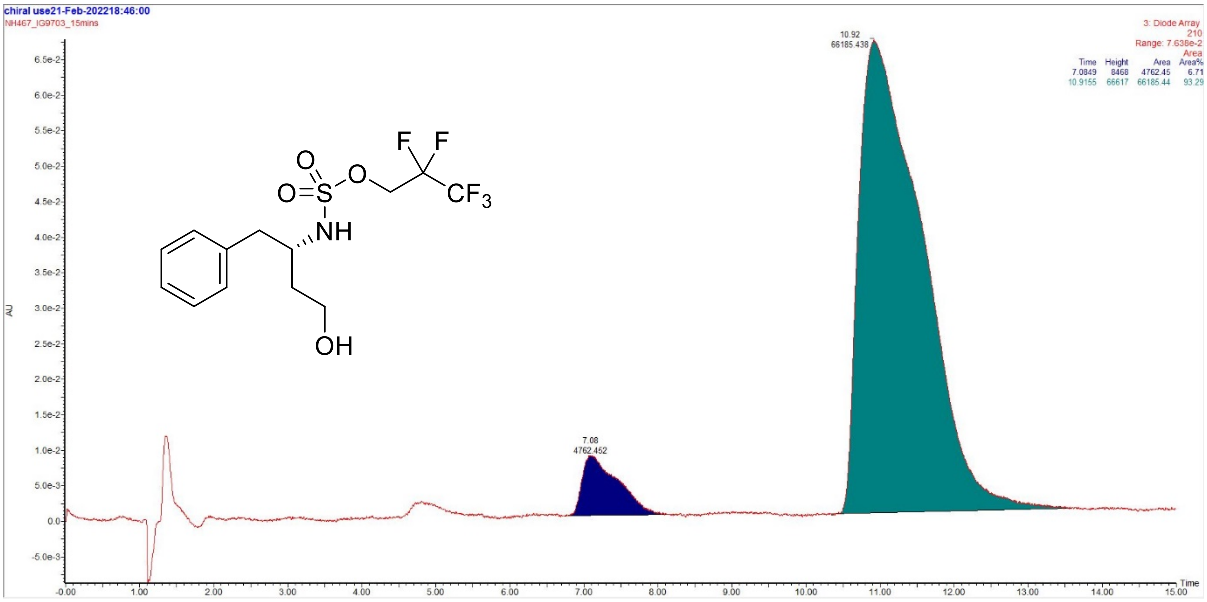

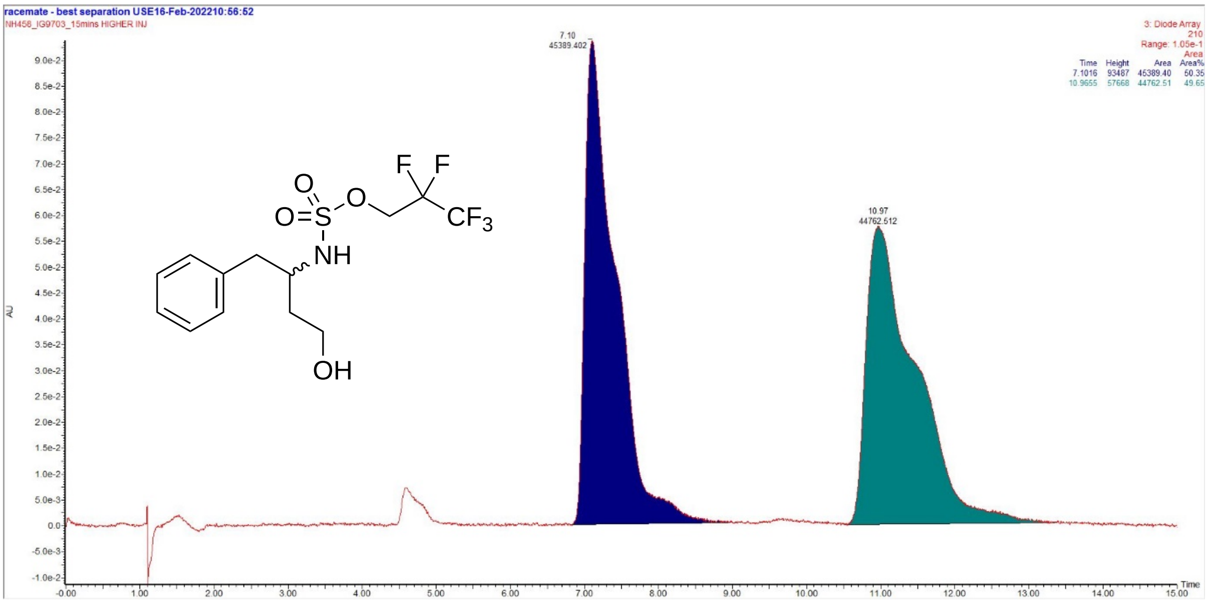

2,2,3,3,3-pentafluoropropyl (R)-(5-hydroxy-1-phenylpentan-2-yl)sulfamate (**46**)

Chiral SFC Analysis: CHIRALPAK IE (CO_2:MeOH, 97:3, 1.25 mL min^{-1}, 40 °C, 210 nm) indicated 92% *ee*, t_R = 15.7 (minor), 16.5 (major) minutes.

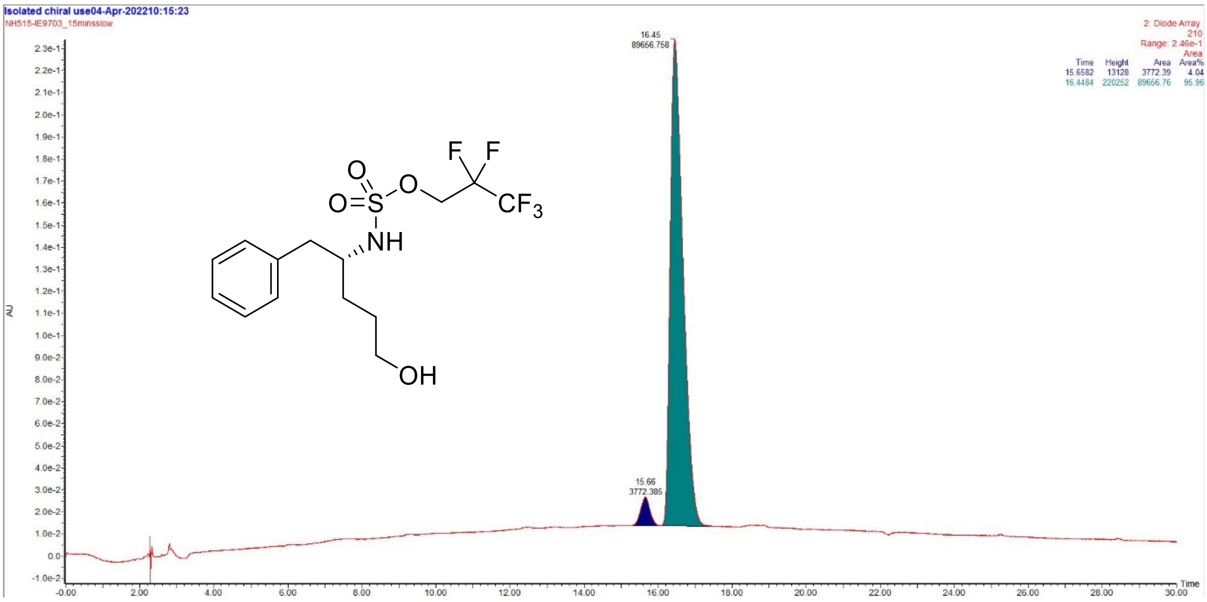

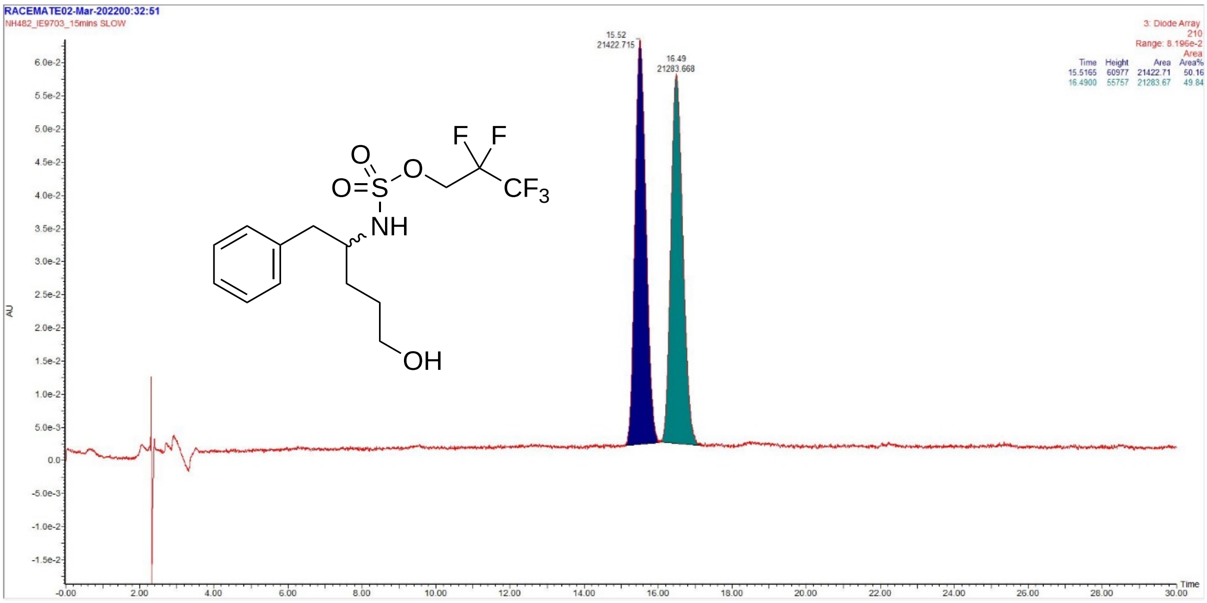

2,2,3,3,3-pentafluoropropyl (R)-(6-hydroxy-1-phenylhexan-2-yl)sulfamate (**47**)

Chiral SFC Analysis: CHIRALPAK IE (CO_2:MeOH, 96.0:4.0, 1.25 mL min^{-1}, 40 °C, 210 nm) indicated 96% *ee*, t_R = 12.1 (minor), 12.6 (major) minutes.

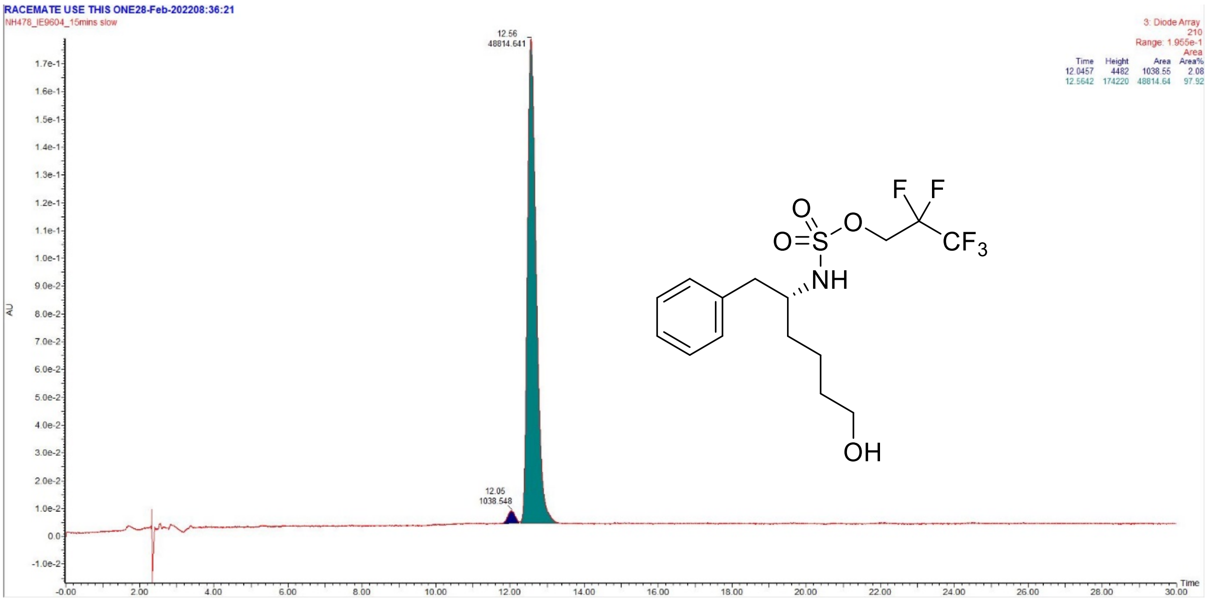

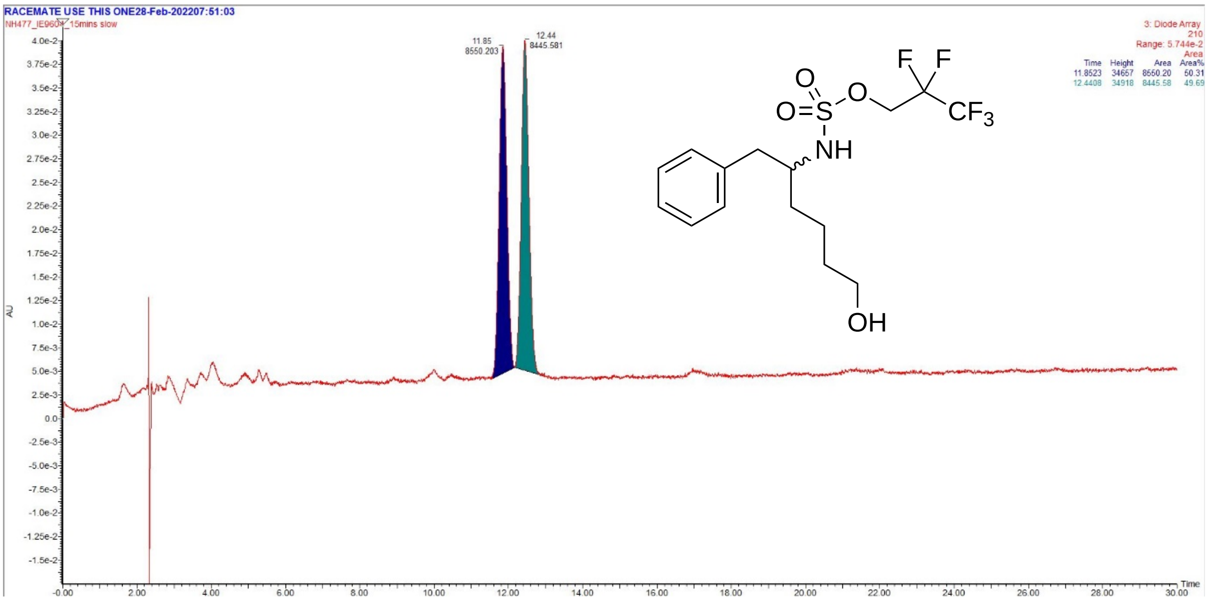

Benzyl (R)-(6-hydroxy-1-phenylhexan-2-yl)carbamate (**48**)

Chiral SFC Analysis: CHIRALPAK IG (CO_2:MeOH, 85:15, 2.5 mL min^{-1}, 40 °C, 210 nm) indicated 96% *ee*, t_R = 8.6 (minor), 13.1 (major) minutes.

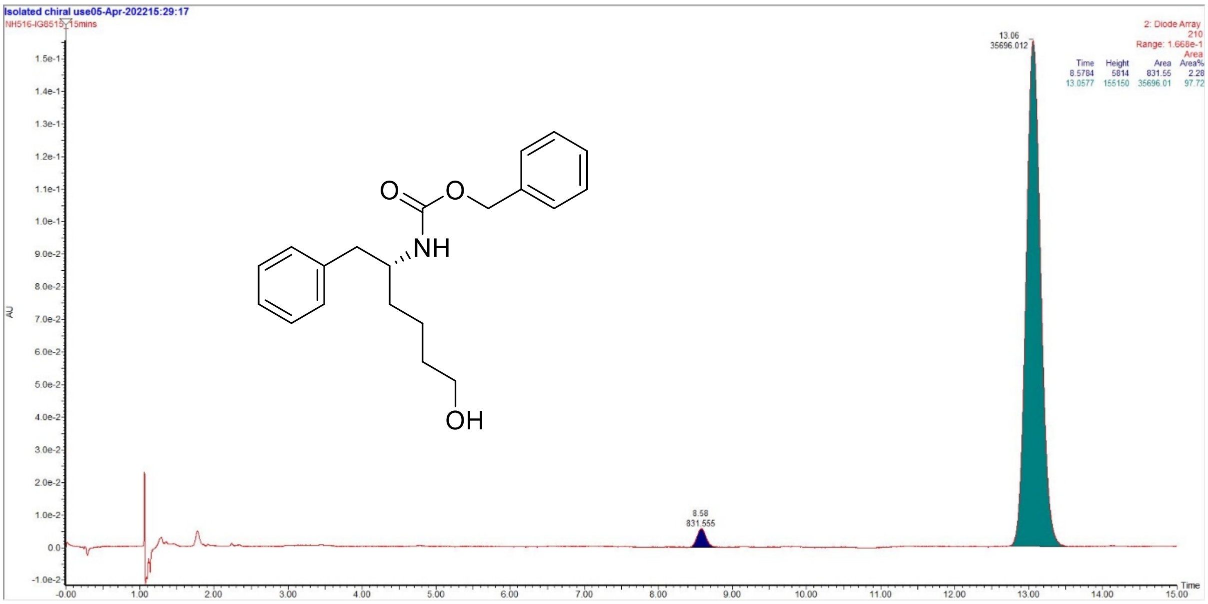

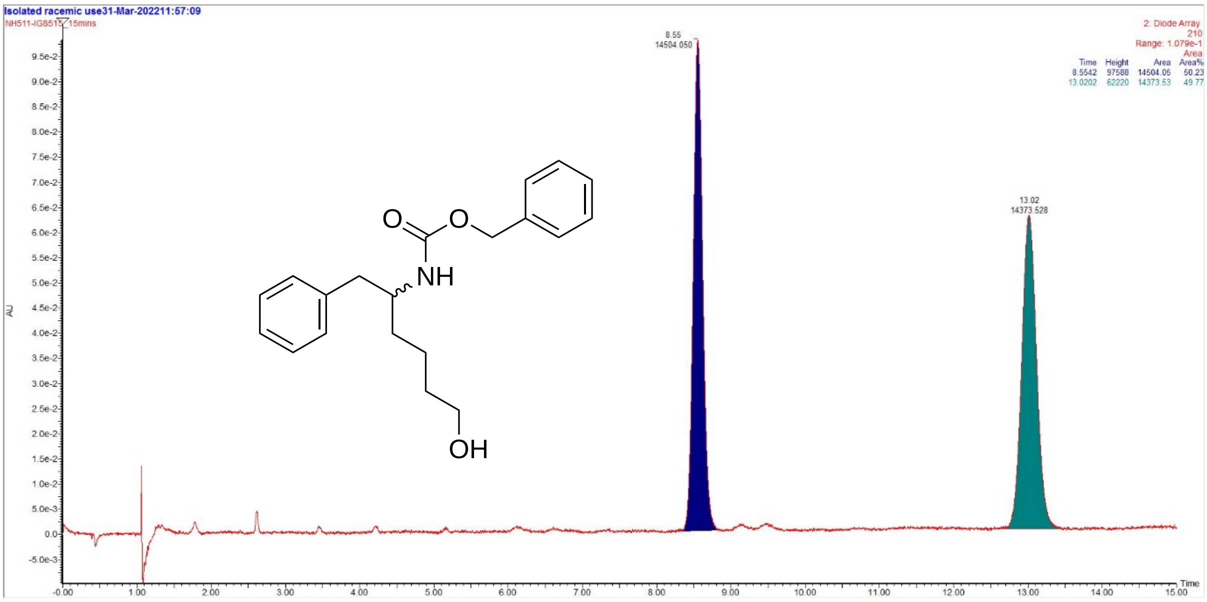

2,2,3,3,3-pentafluoropropyl (R)-2-benzylpyrrolidine-1-sulfonate (**49**)

Chiral SFC Analysis: CHIRALPAK IG (CO_2:MeOH, 99.5:0.5, 1.25 mL min^{-1}, 40 °C, 210 nm) indicated 92% *ee*, t_R = 9.5 (minor), 12.0 (major) minutes.

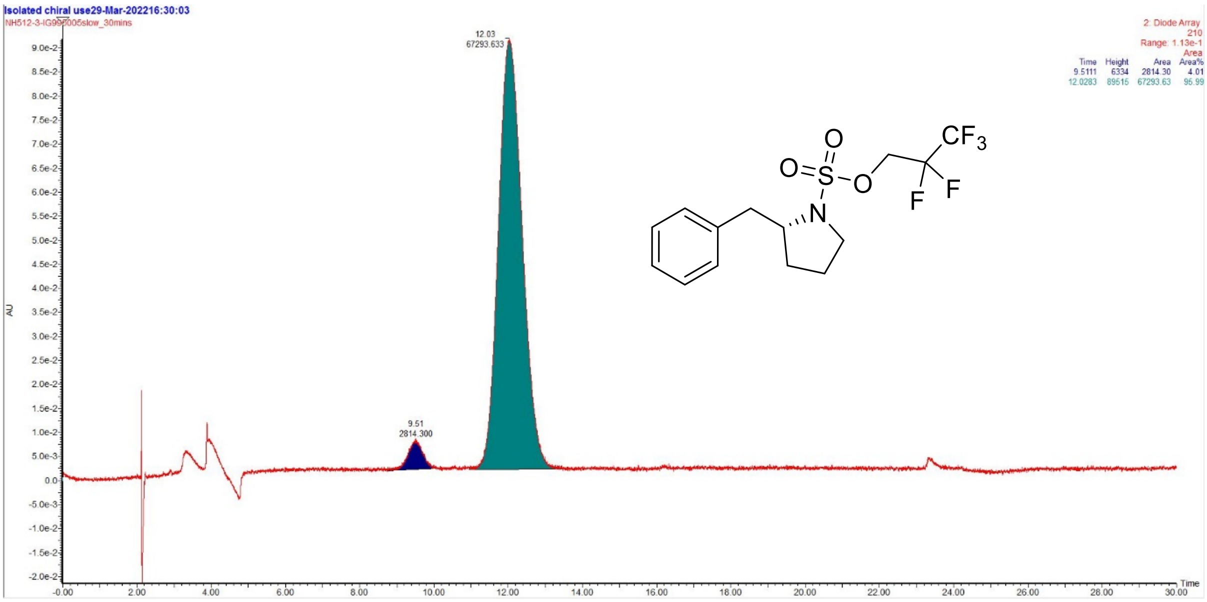

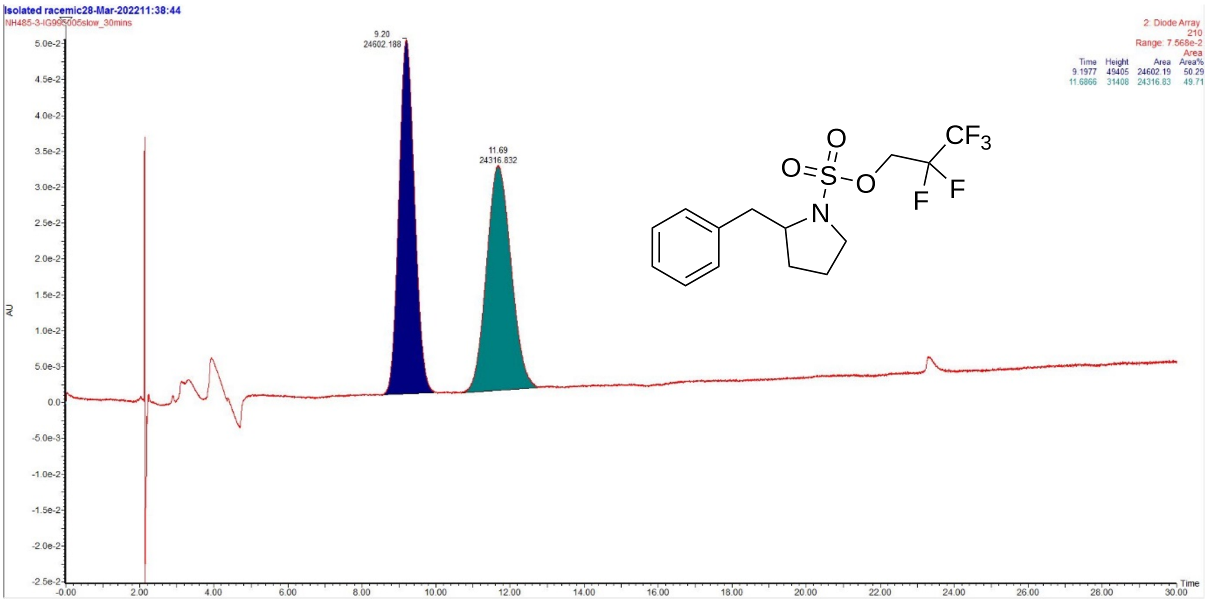

2,2,3,3,3-pentafluoropropyl (S)-2-benzylazetidine-1-sulfonate (**50**)

Chiral SFC Analysis: CHIRALPAK IG (CO_2:MeOH, 99.5:0.5, 2.5 mL min^{-1}, 40 °C, 210 nm) indicated 89% *ee*, t_R = 3.2 (minor), 4.1 (major) minutes.

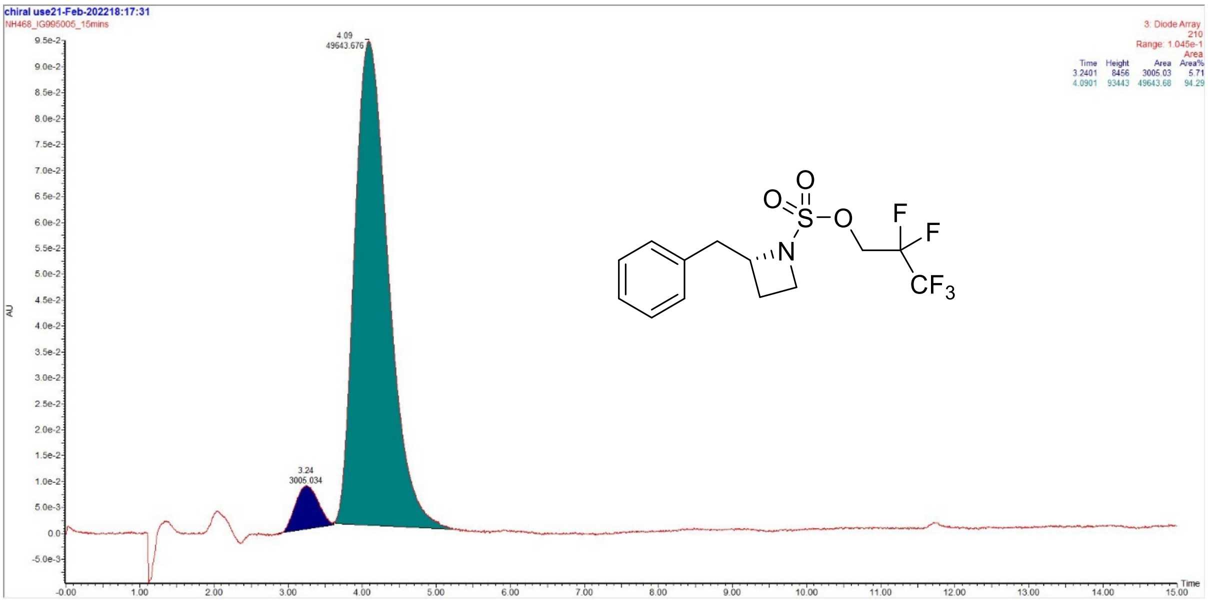

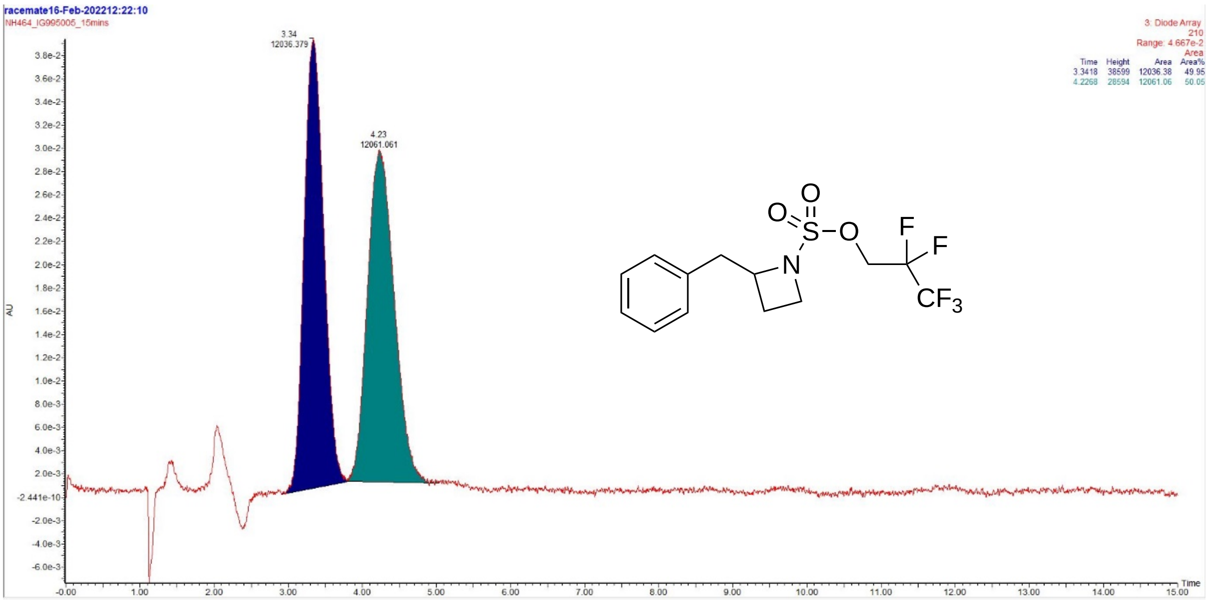

Tert-butyl (S)-(1-phenyl-4-(phenylthio)butan-2-yl)carbamate (**51**)

Chiral SFC Analysis: CHIRALPAK IG (CO_2:MeOH, 90:10, 2.5 mL min^{-1}, 40 °C, 253 nm) indicated 86% *ee*, t_R = 5.5 (minor), 6.9 (major) minutes.

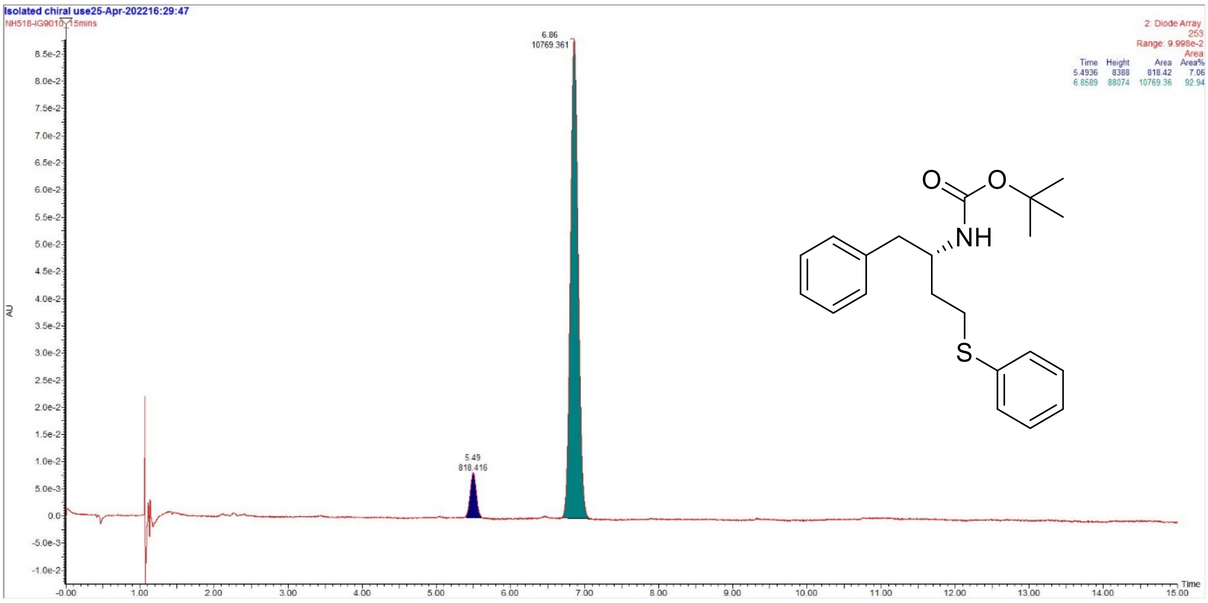

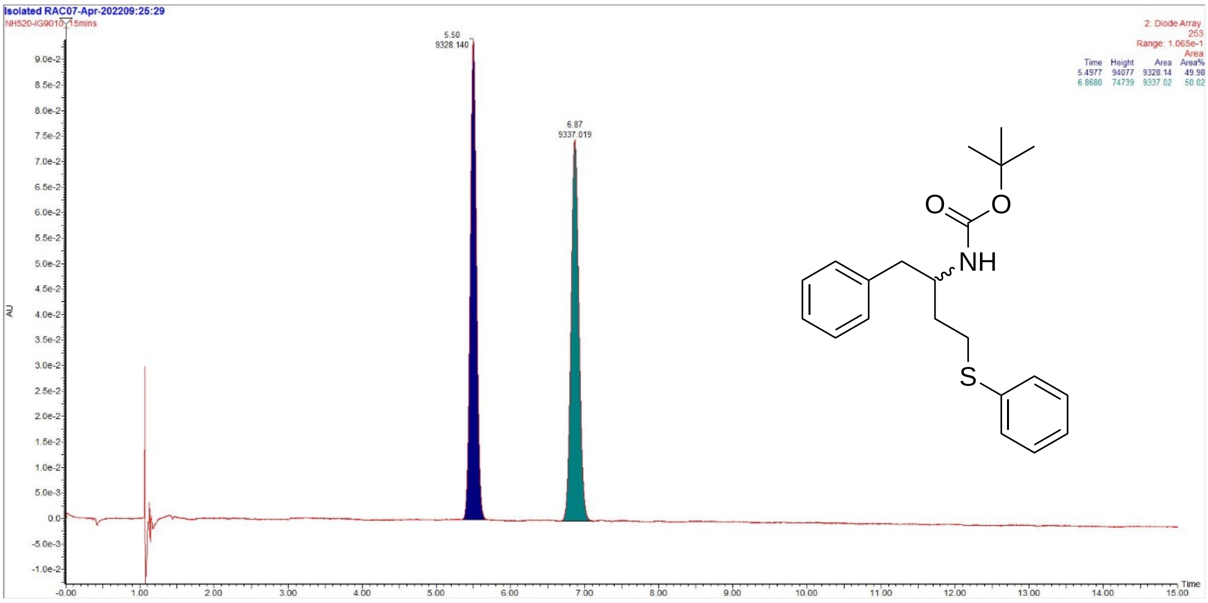

2,2,3,3,3-pentafluoropropyl (R)-(4-hydroxy-1-phenylbutyl)sulfamate (**52**)

Chiral SFC Analysis: CHIRAL ART SC (CO_2:^{i}PrOH, 96:4, 2.5 mL min^{-1}, 40 °C, 215 nm) indicated 88% *ee*, t_R = 15.5 (major), 18.8 (minor) minutes.

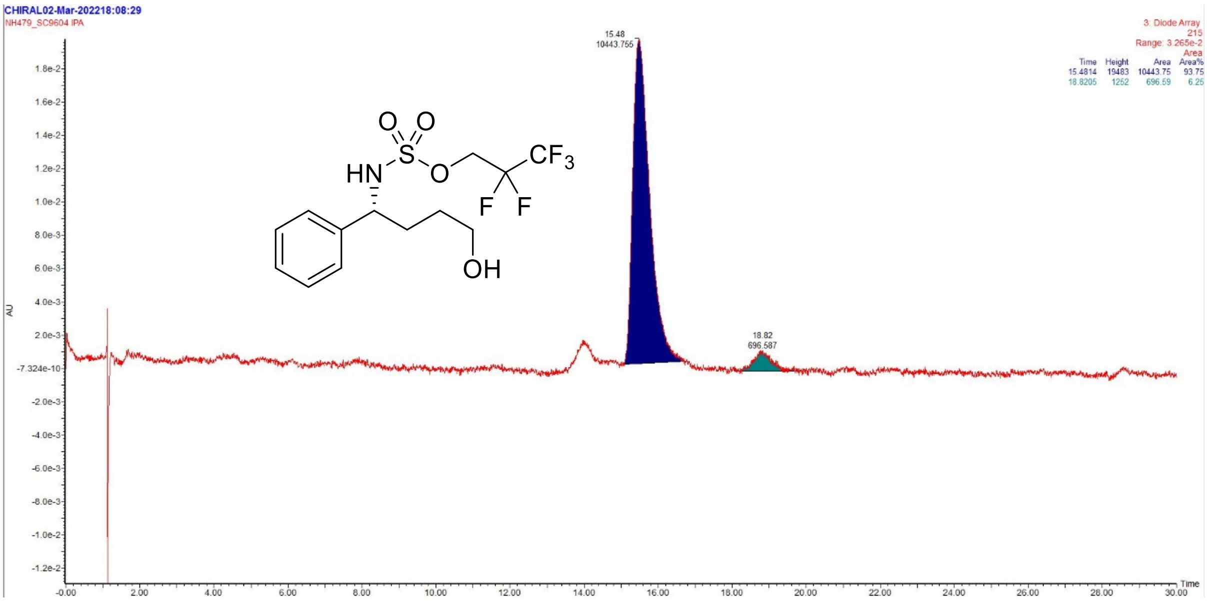

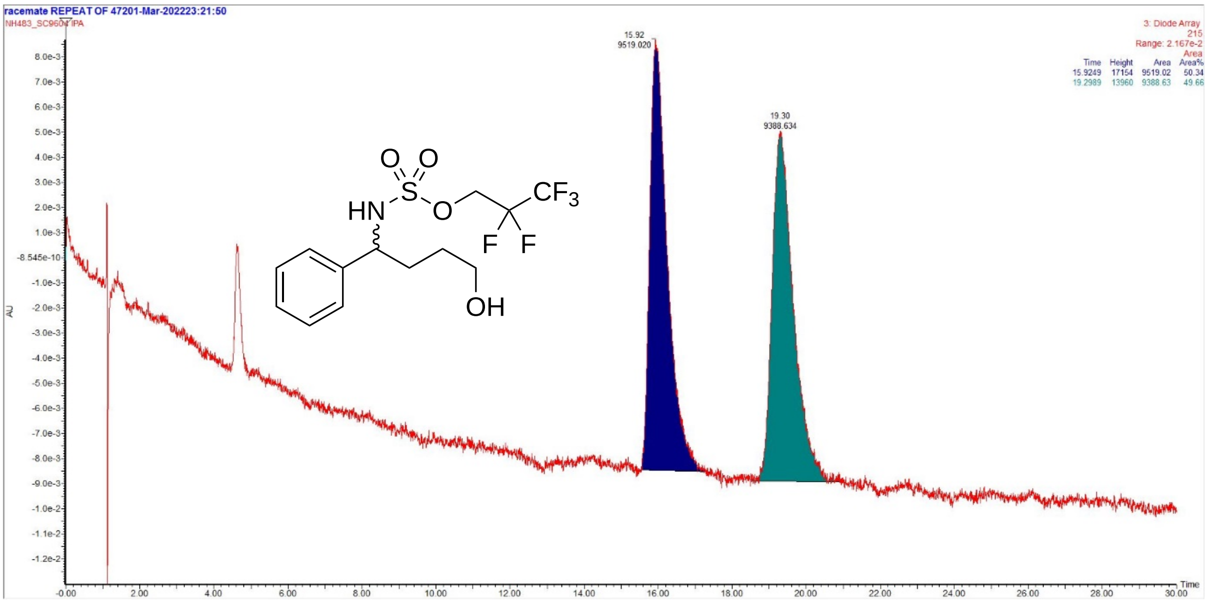

2,2,3,3,3-pentafluoropropyl ((S)-phenyl((S)-2-(trichloromethyl)-5,6-dihydro-4H-1,3-oxazin-4-yl)methyl)sulfamate (**53**)

Chiral SFC Analysis: CHIRAL ART SC (CO_2:MeOH, 98:2, 2.5 mL min^{-1}, 40 °C, 210 nm) indicated 87% *ee*, t_R = 3.4 (minor), 5.0 (major) minutes.

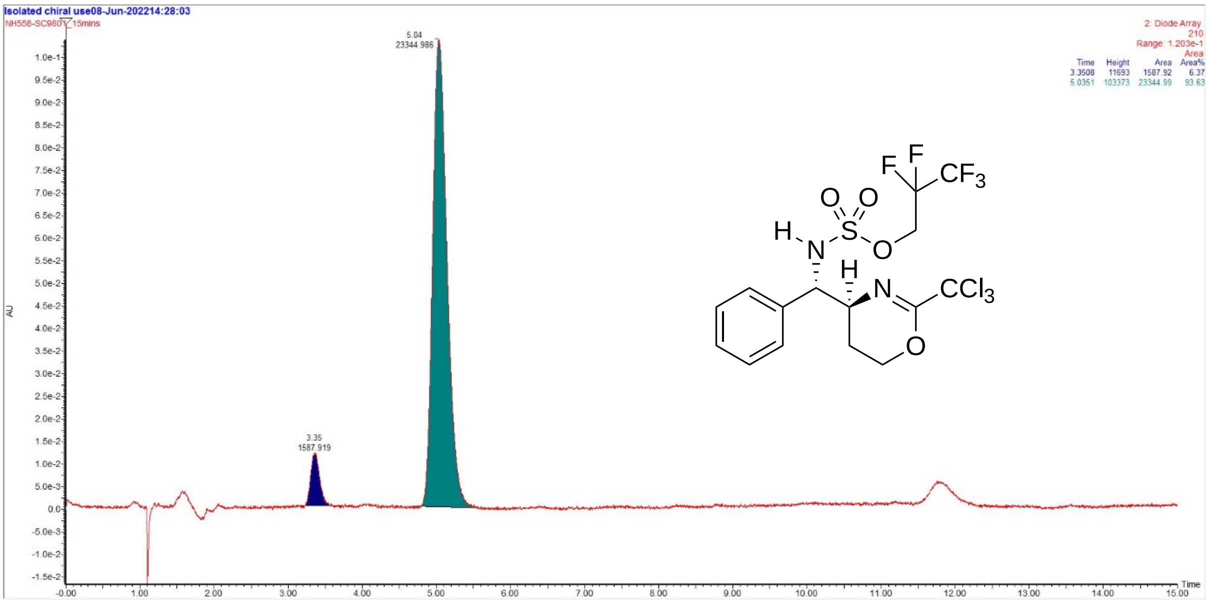

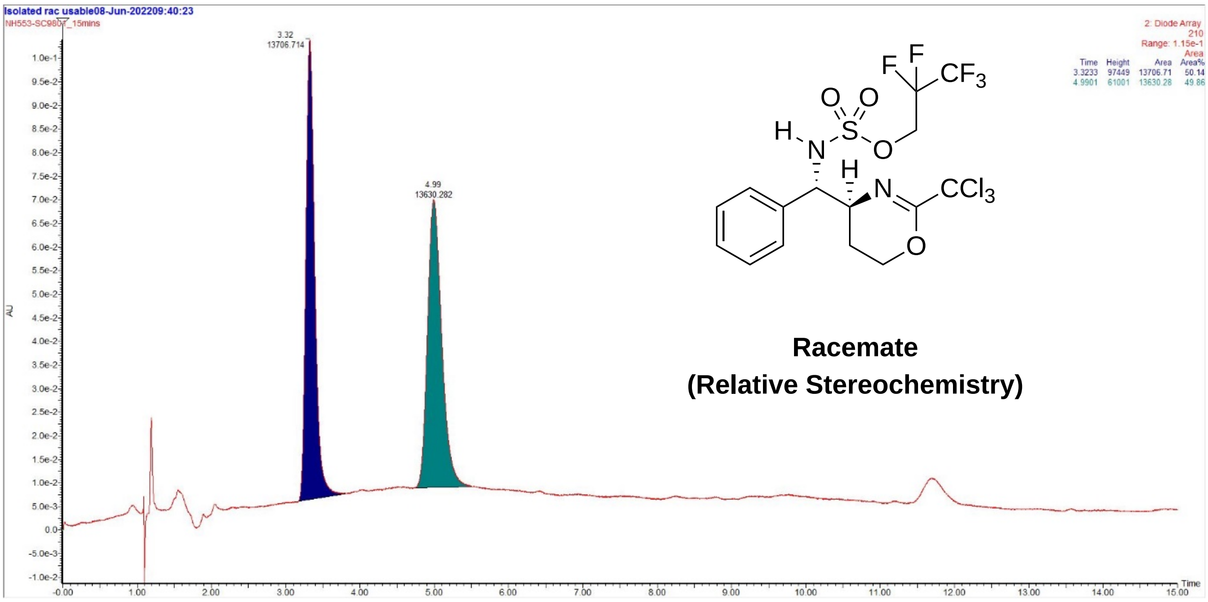

2,2,3,3,3-pentafluoropropyl ((S)-((S)-2-oxo-1,3-dioxan-4-yl)(phenyl)methyl)sulfamate (**54**)

Chiral SFC Analysis: CHIRALPAK IH (CO_2:MeOH, 95:5, 2.5 mL min^{-1}, 40 °C, 210 nm) indicated 87% *ee*, t_R = 7.9 (minor), 9.3 (major) minutes.

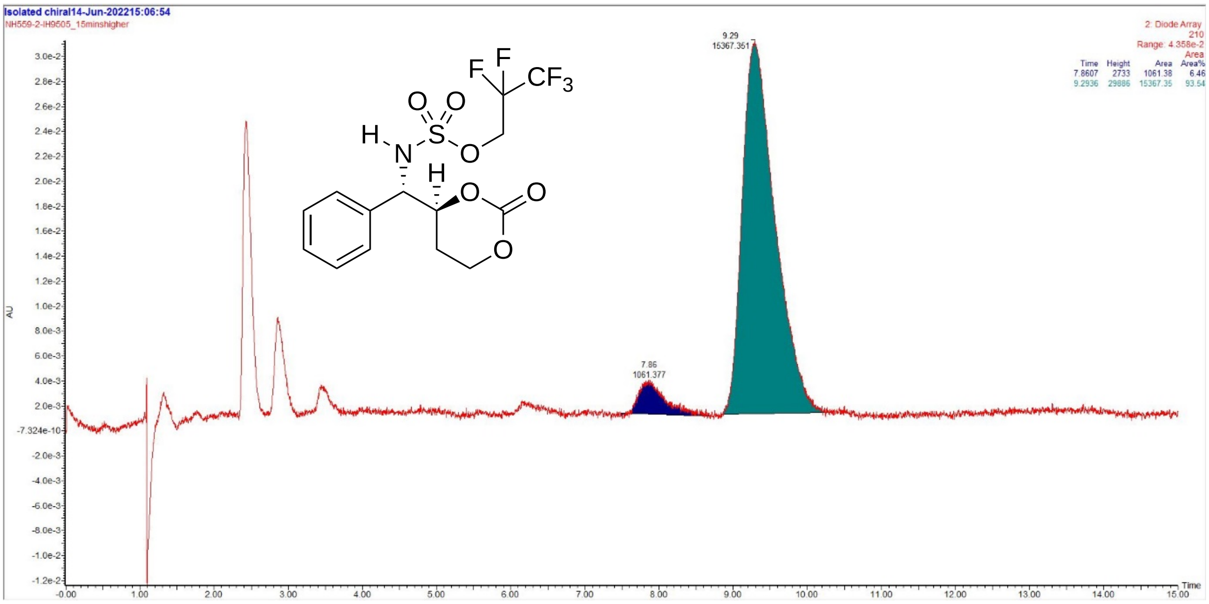

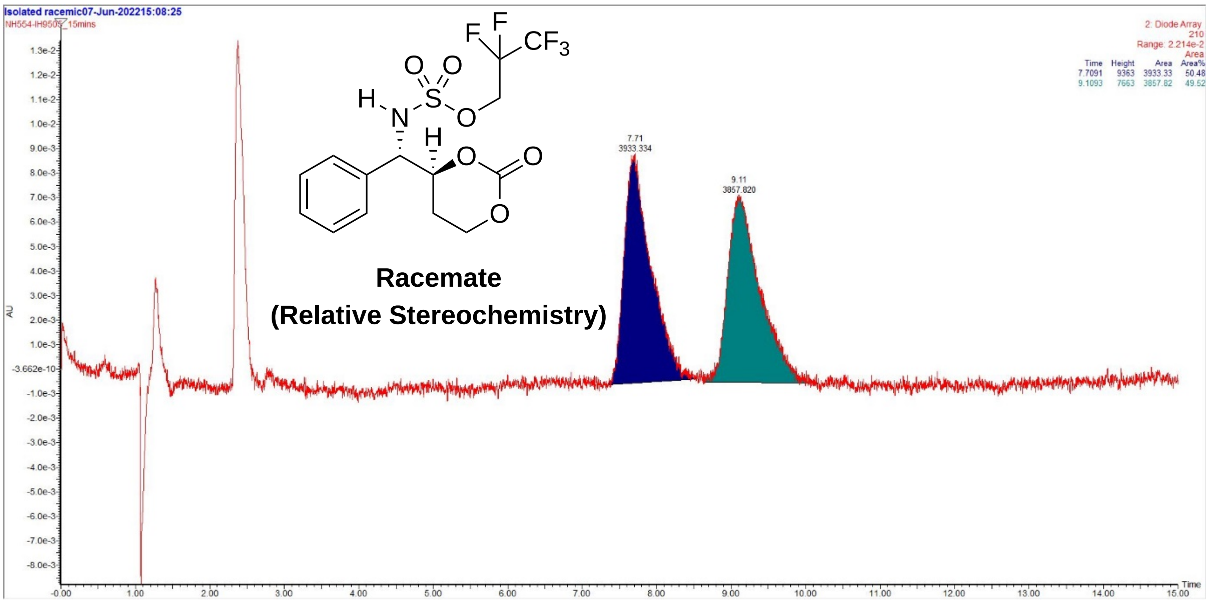

N-((2S,3R)-2-ethyltetrahydrofuran-3-yl)-4-methylbenzenesulfonamide (**SI4**)

Chiral SFC Analysis CHIRAL ART SA (CO_2:MeOH, 90:10, 2.5 mL min^{-1}, 40 °C, 227 nm) indicated 89% *ee*, t_R = 5.6 (minor), 10.7 (major) minutes.

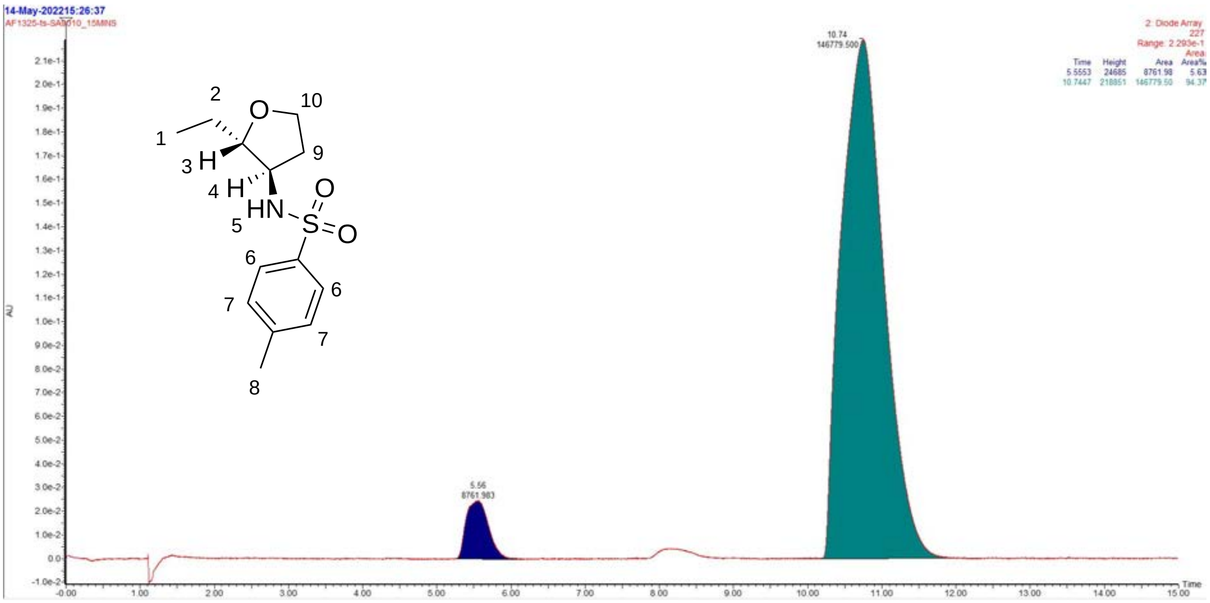

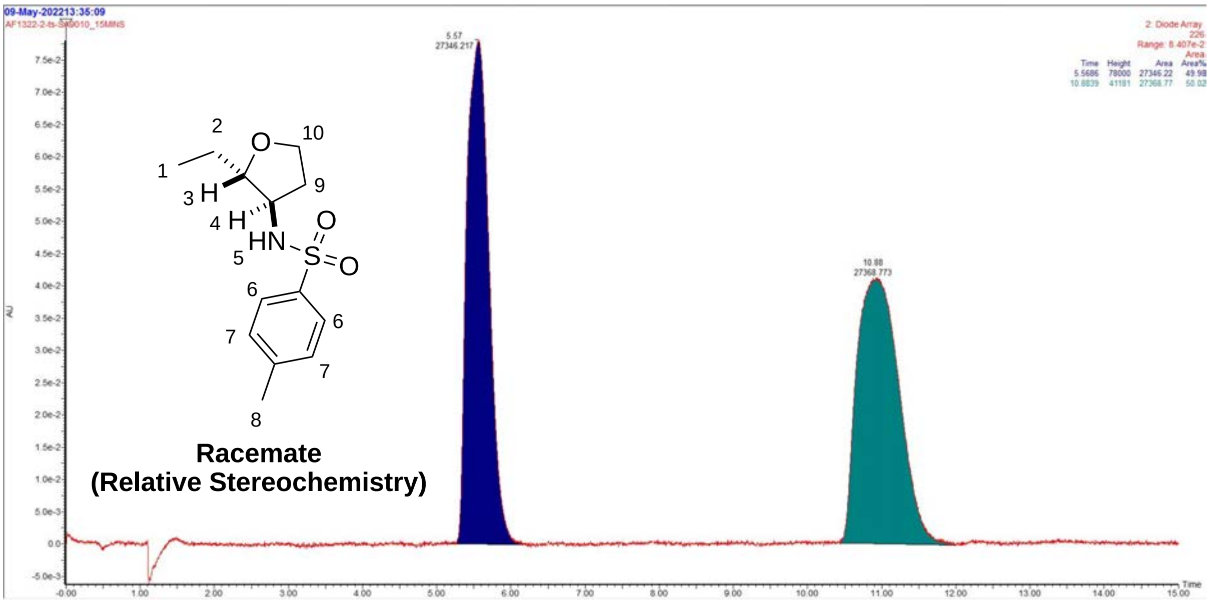

2,2,3,3,3-pentafluoropropyl (S)-(1-hydroxy-5-methyl-3-((phenylthio)methyl)hexan-3-yl)sulfamate (**SI13**)

Chiral SFC analysis: CHIRALPAK IE (CO_2:MeOH, 97.5:2.5, 2.5 mL min^{-1}, 40 ˚C, 250 nm) indicated 77% *ee*, t_R = 8.4 (minor), 9.1 (major) minutes.

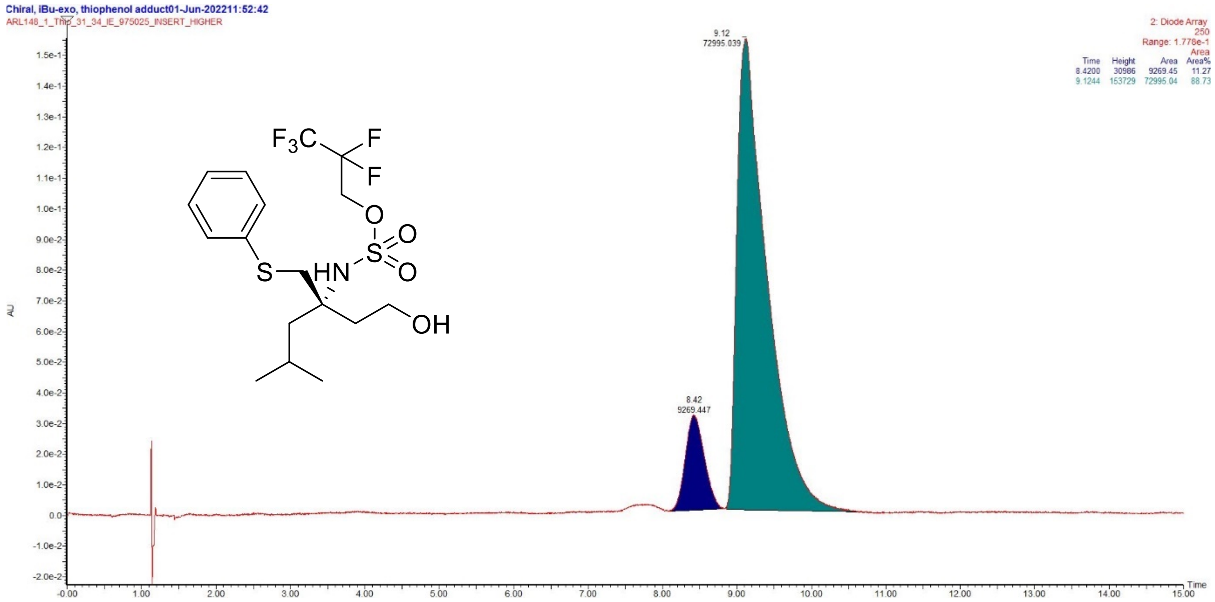

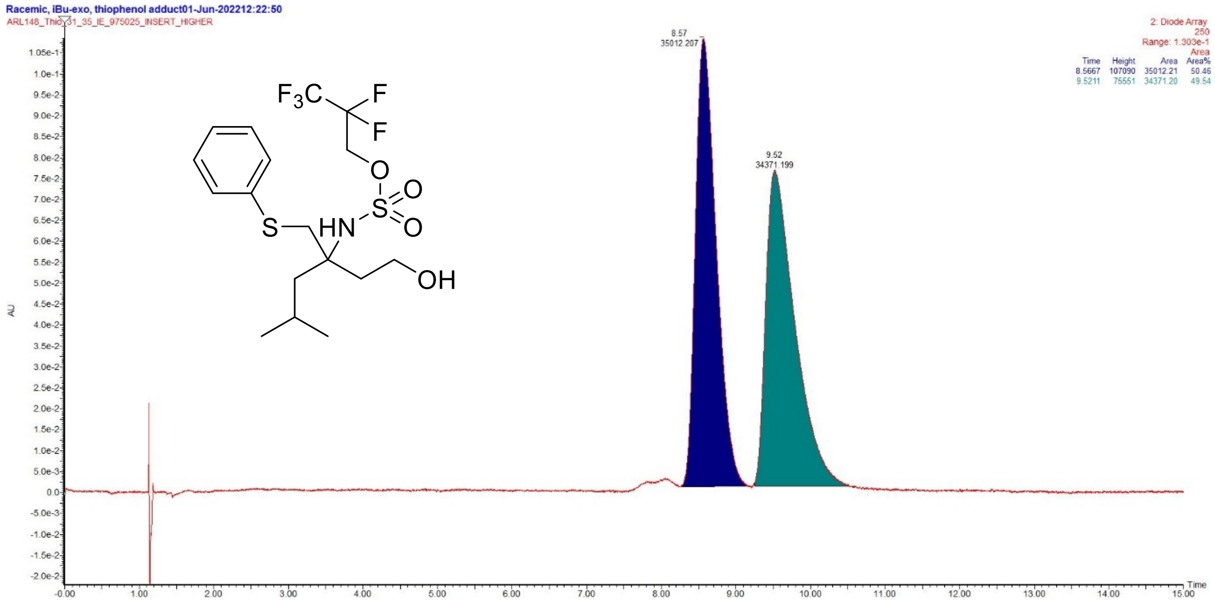

NMR Spectra

^{1}H NMR (400 MHz, $CDCl_3$) for *1,3-bis(triethylsilyl)benzene*

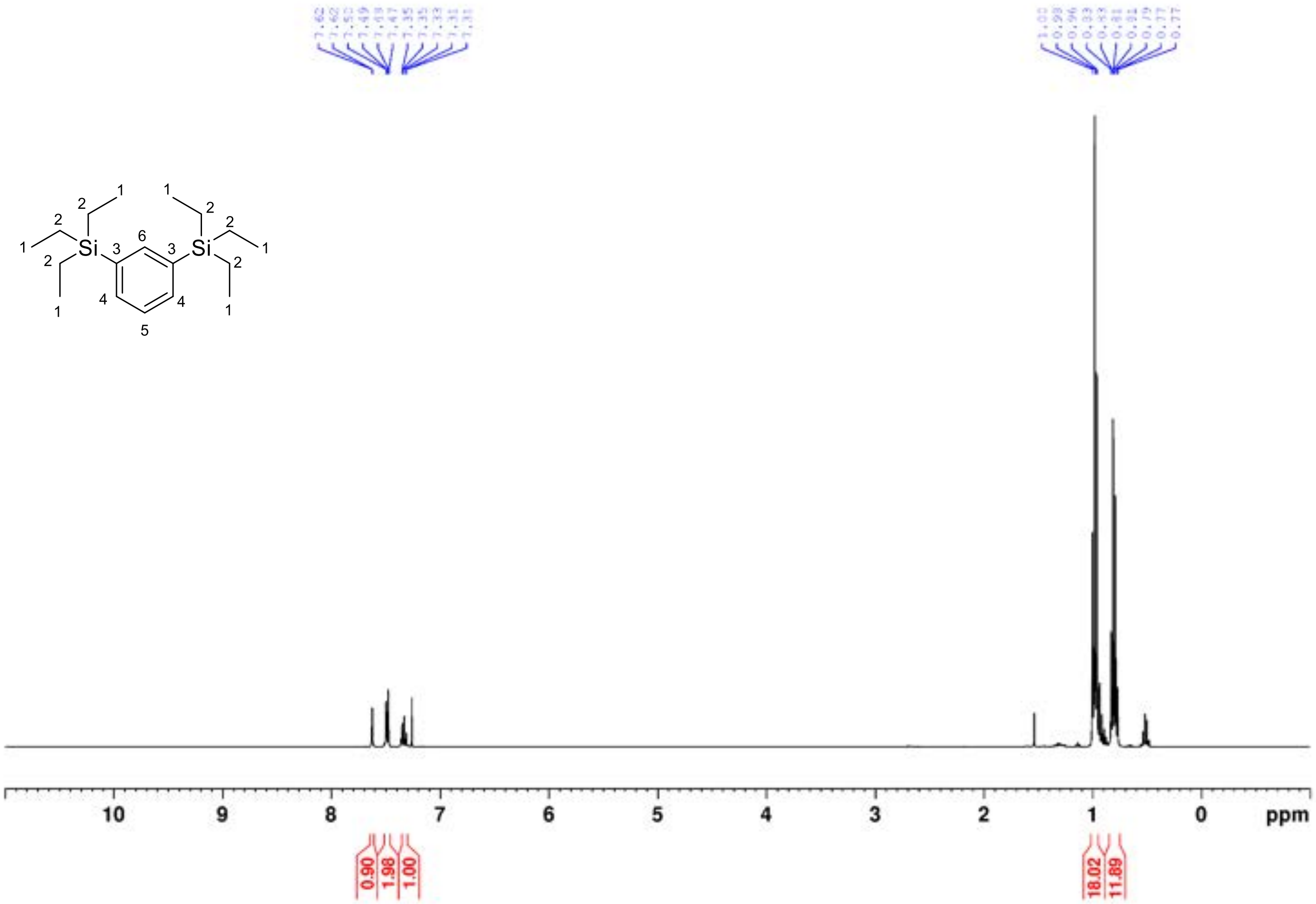

^{13}C NMR (101 MHz, CDCl$_3$) for *1,3-bis(triethylsilyl)benzene*

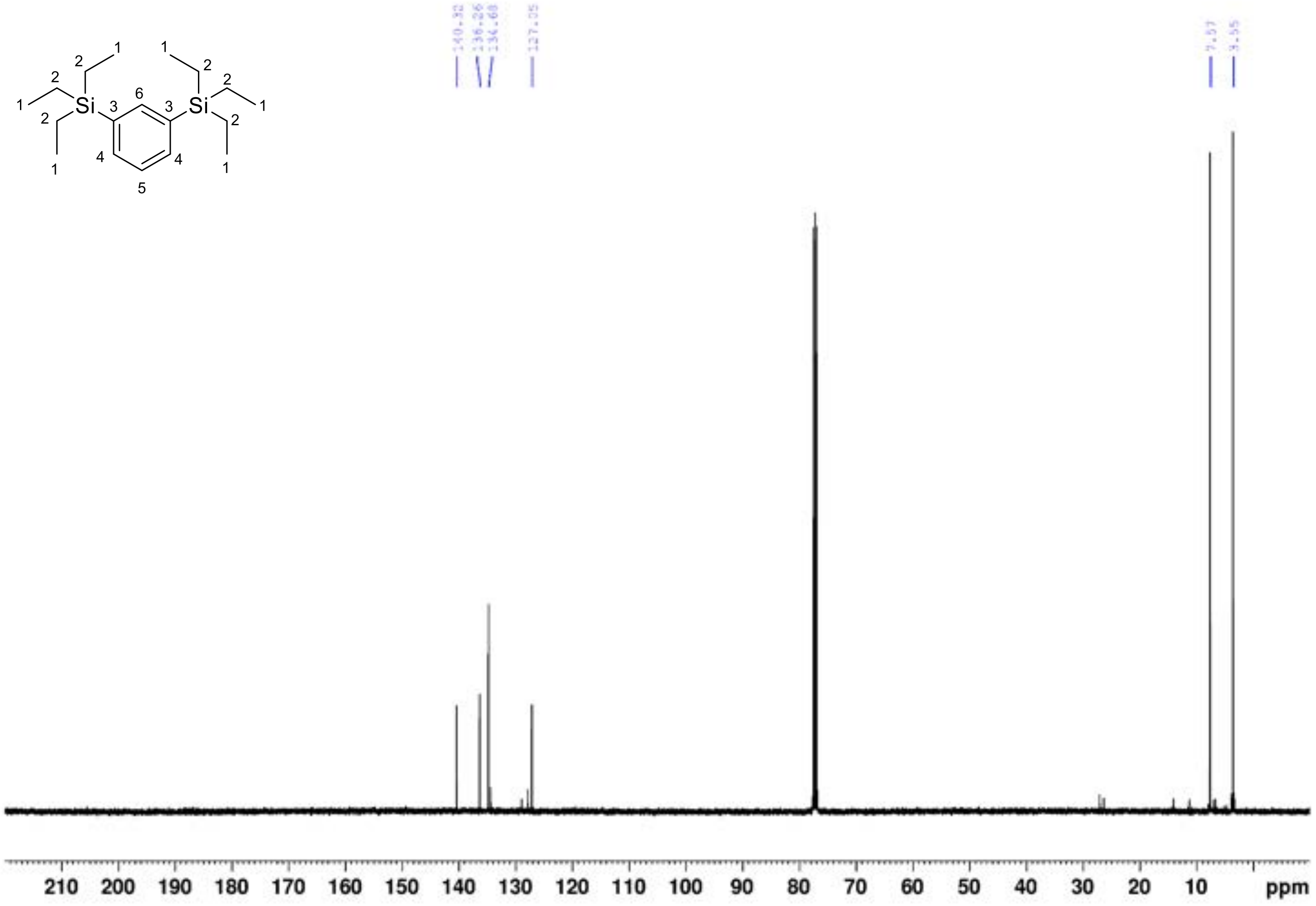

^{1}H NMR (400 MHz, $CDCl_3$) for *(5-(4,4,5,5-tetramethyl-1,3,2-dioxaborolan-2-yl)-1,3-phenylene)bis(triethylsilane)*

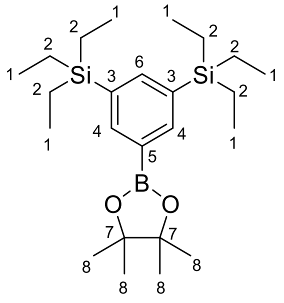

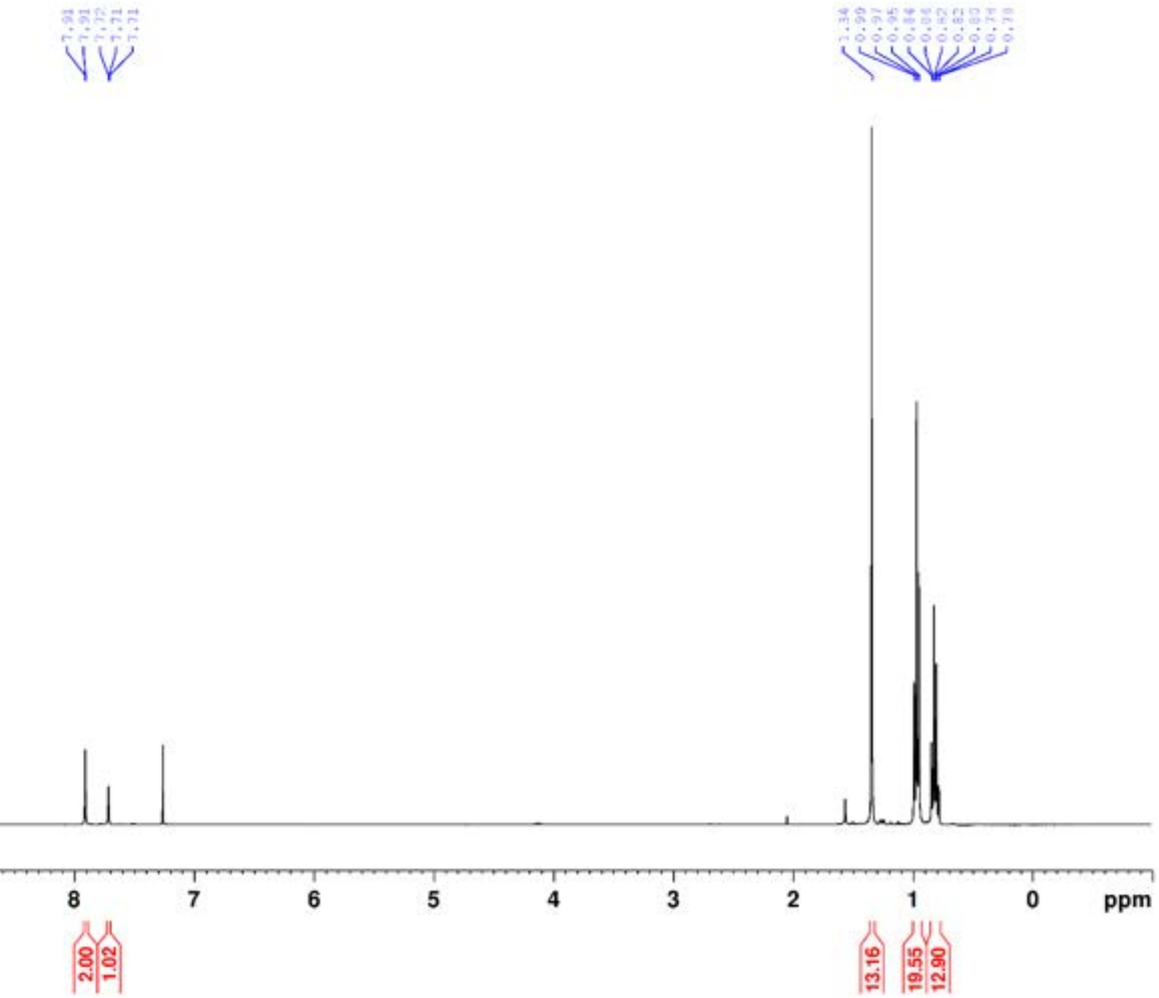

^{13}C NMR (101 MHz, CDCl$_3$) for *(5-(4,4,5,5-tetramethyl-1,3,2-dioxaborolan-2-yl)-1,3-phenylene)bis(triethylsilane)*

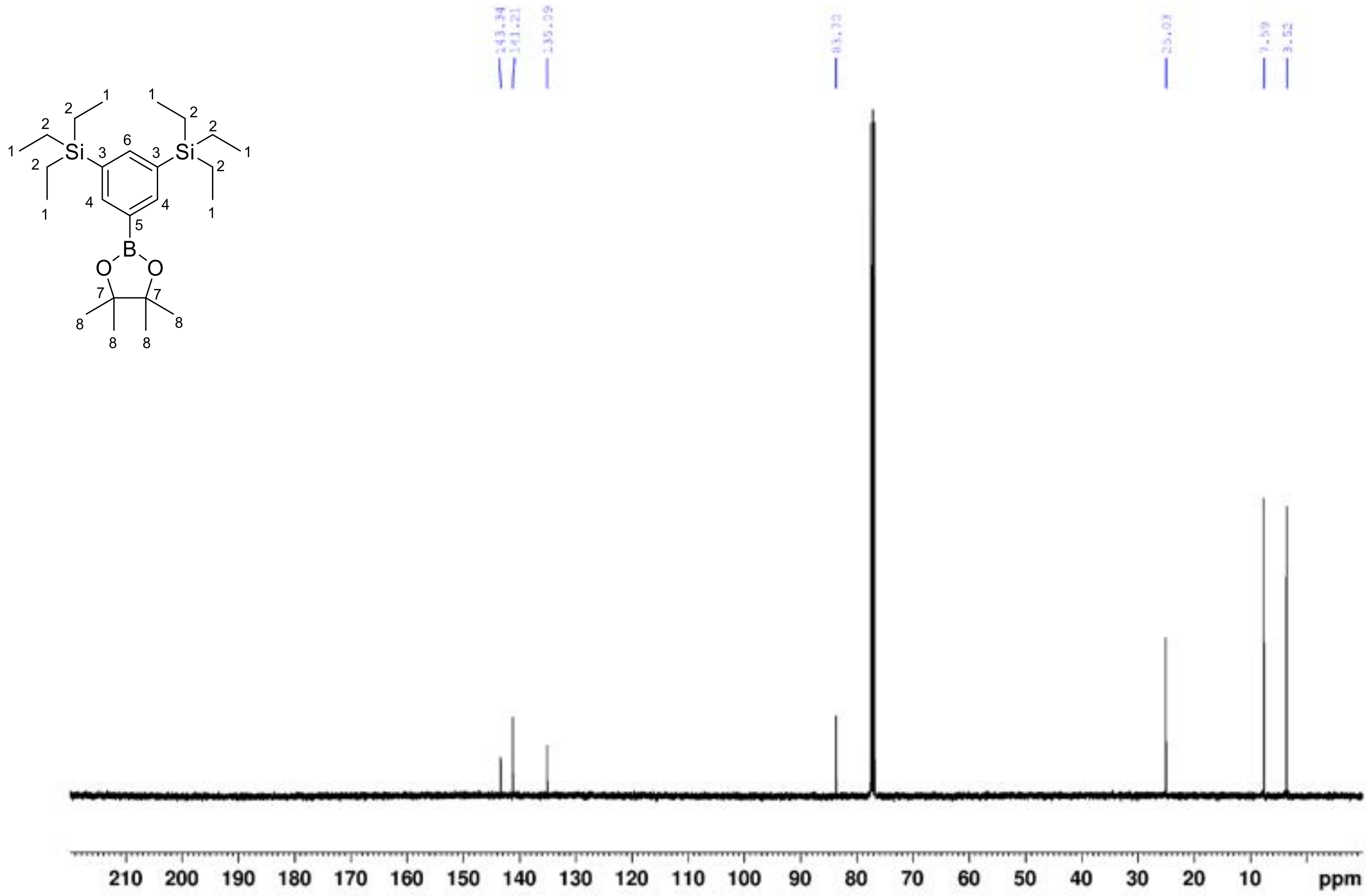

^{1}H NMR (500 MHz, $CDCl_3$) for *(3,3'',5,5''-tetrakis(triethylsilyl)-[1,1':3',1''-terphenyl]-5'-yl)methanol*

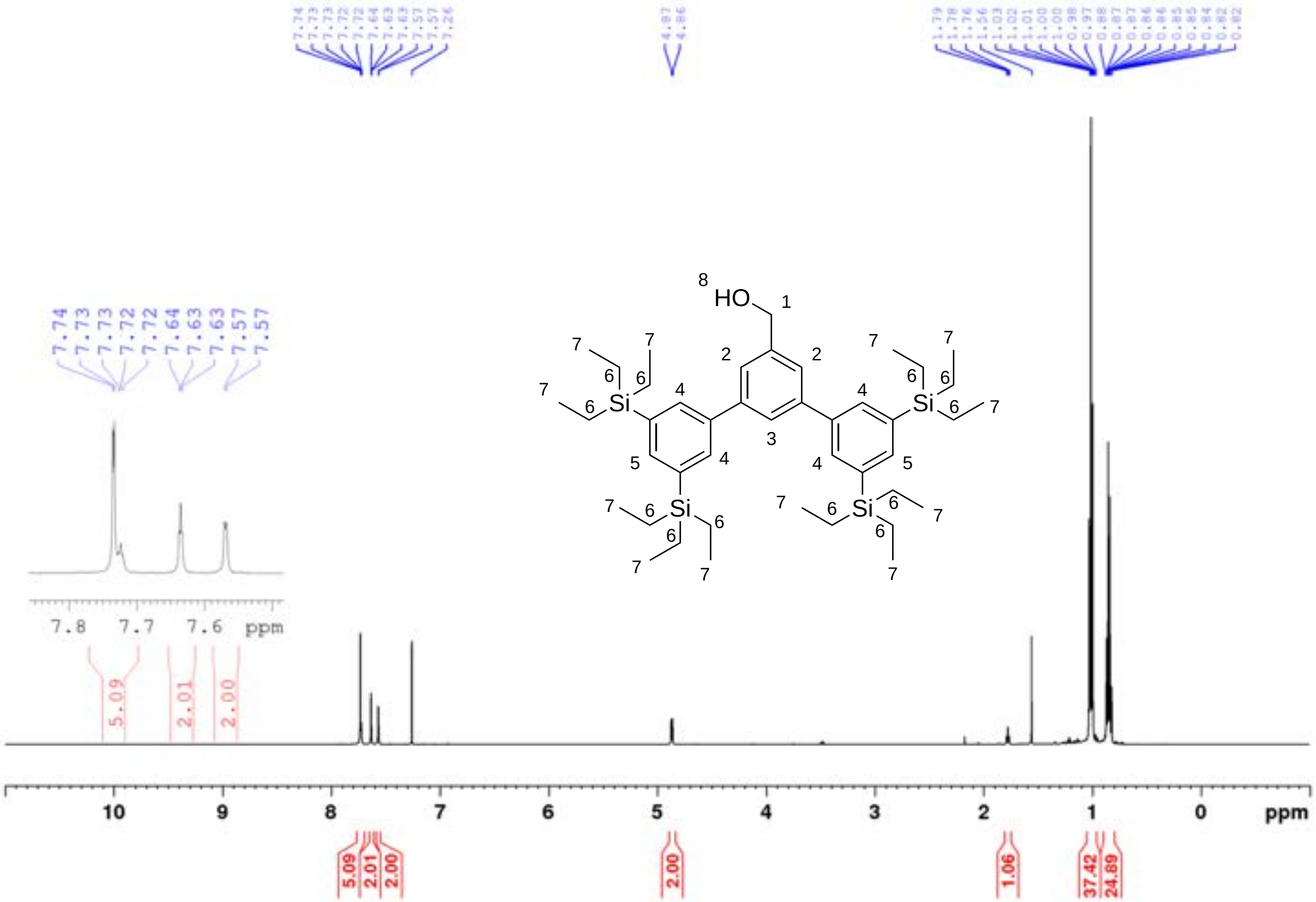

^{13}C NMR (126 MHz, $CDCl_3$) for *(3,3'',5,5''-tetrakis(triethylsilyl)-[1,1':3',1''-terphenyl]-5'-yl)methanol*

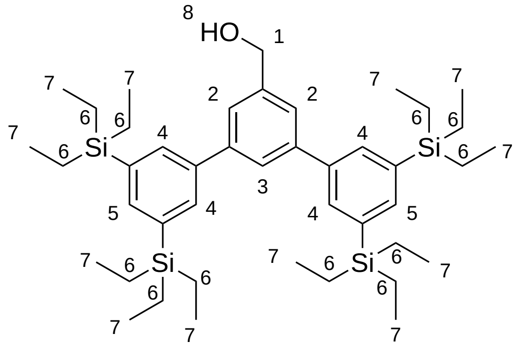

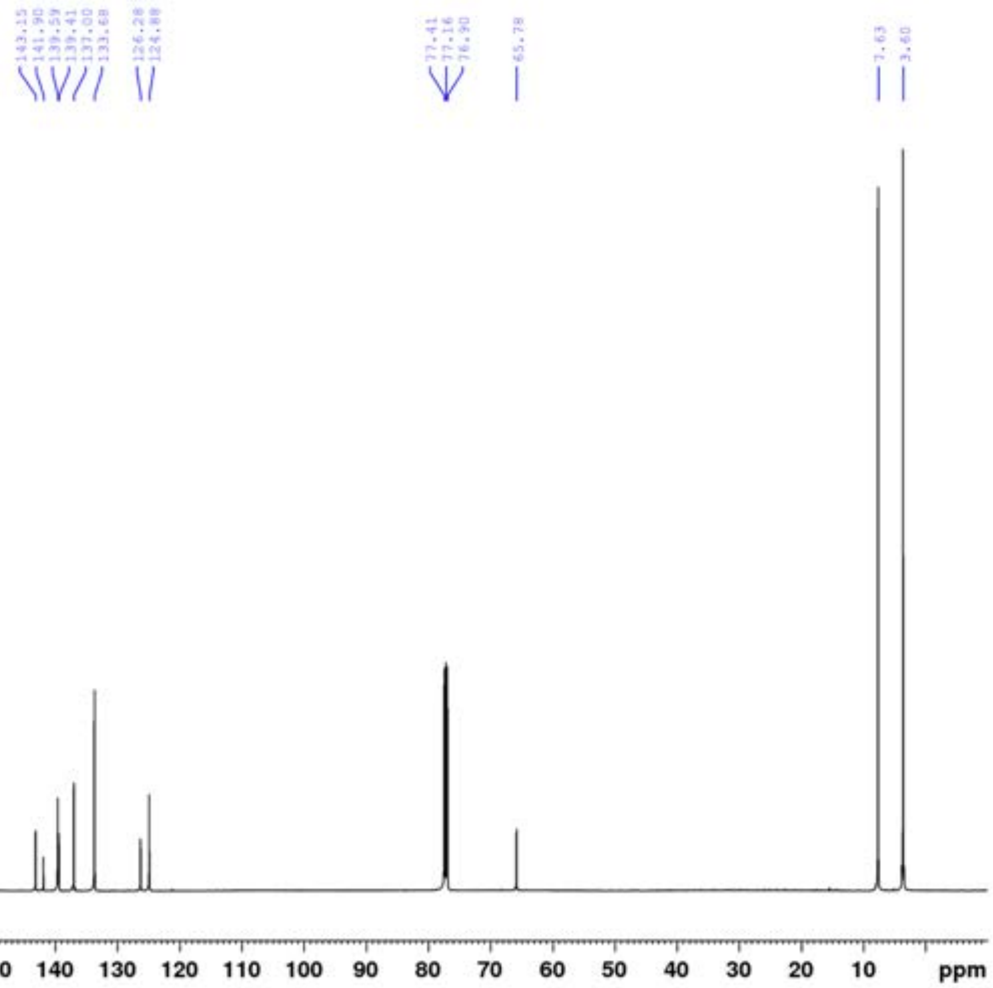

¹H NMR (400 MHz, $CDCl_3$) for *(5'-(bromomethyl)-[1,1':3',1''-terphenyl]-3,3'',5,5''-tetrayl)tetrakis(triethylsilane)*

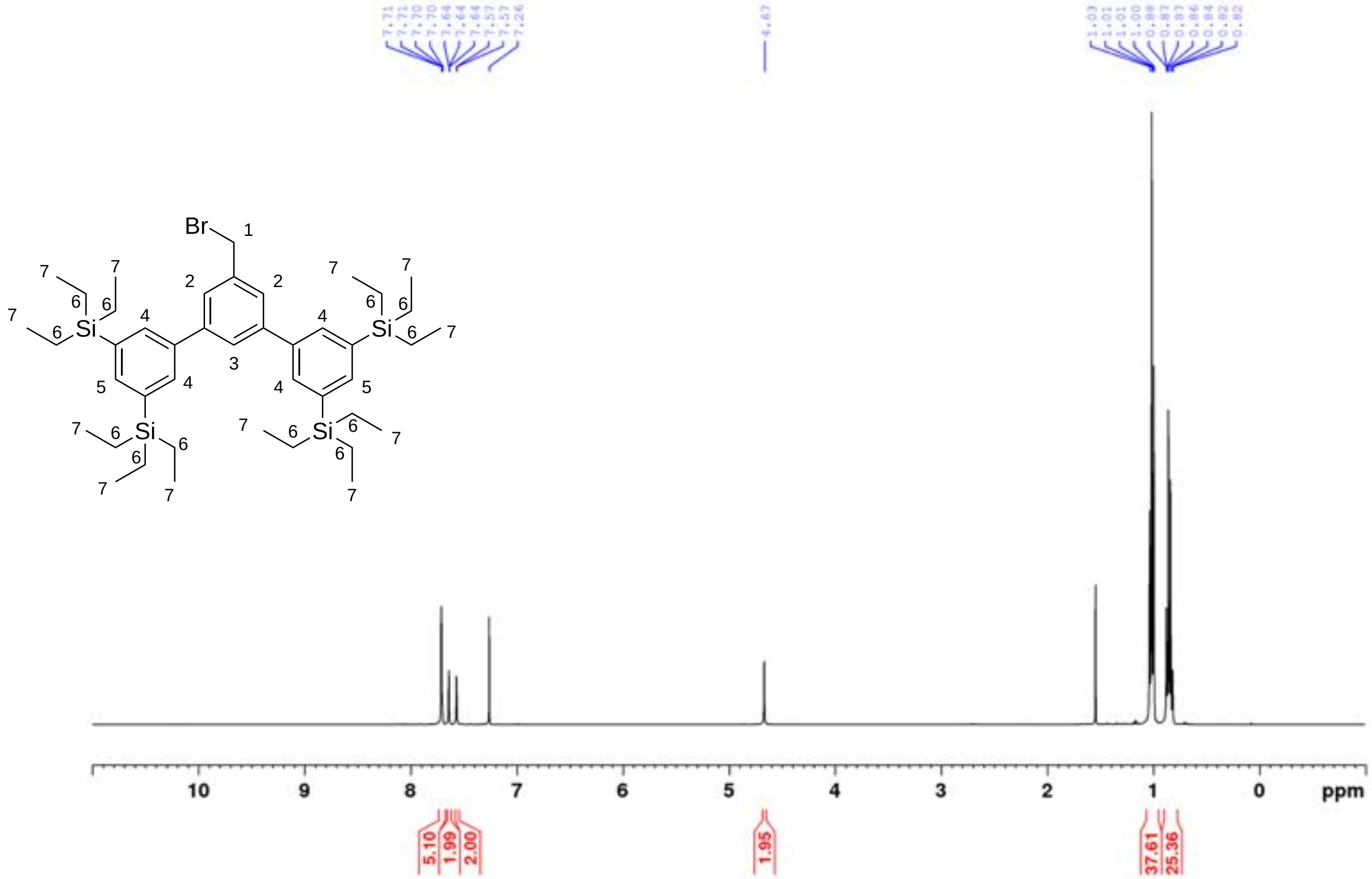

^{13}C NMR (101 MHz, $CDCl_3$) for *(5'-(bromomethyl)-[1,1':3',1''-terphenyl]-3,3'',5,5''-tetrayl)tetrakis(triethylsilane)*

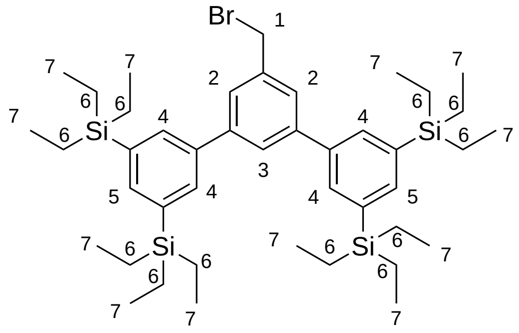

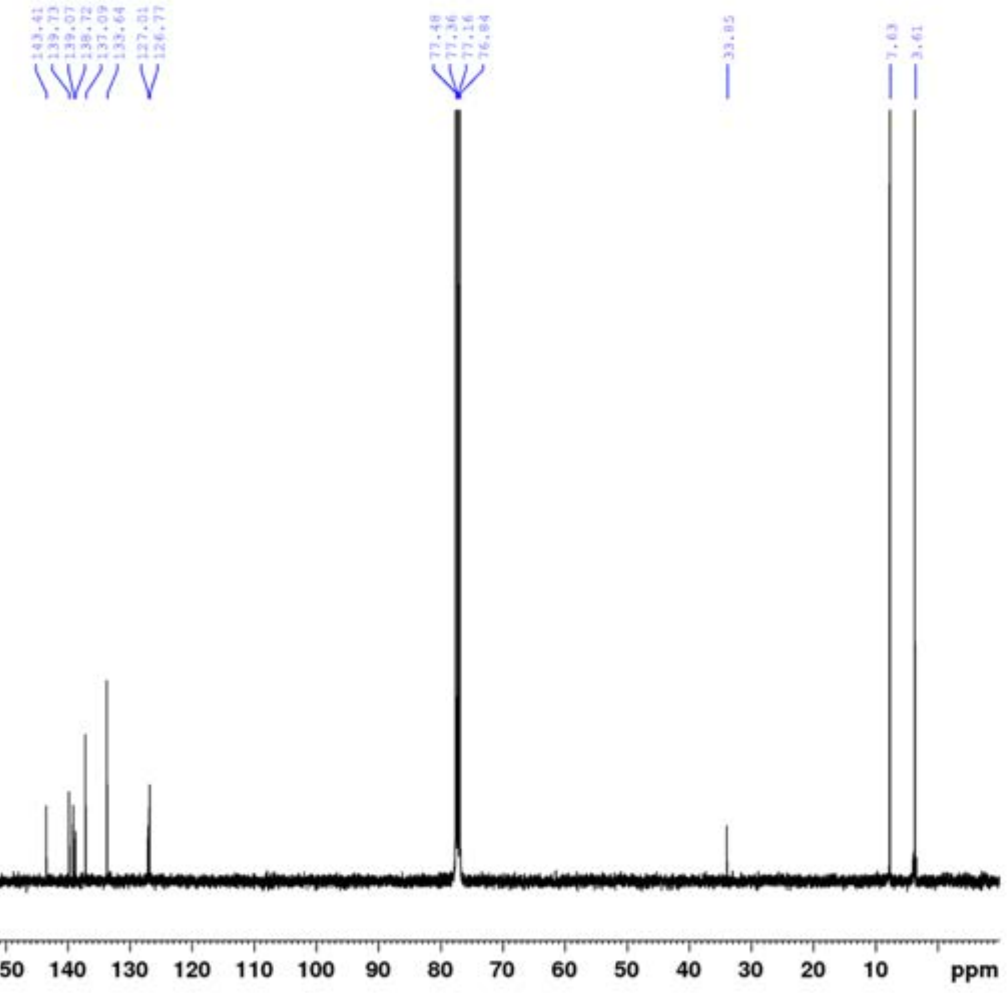

^{1}H NMR (500 MHz, $CDCl_3$) for *(1S,2R,4S,5R)-5-ethyl-2-((S)-hydroxy(6-methoxyquinolin-4-yl)methyl)-1-((3,3'',5,5''-tetrakis(triethylsilyl)-[1,1':3',1''-terphenyl]-5'-yl)methyl)quinuclidin-1-ium bromide* (**C3•Br**)

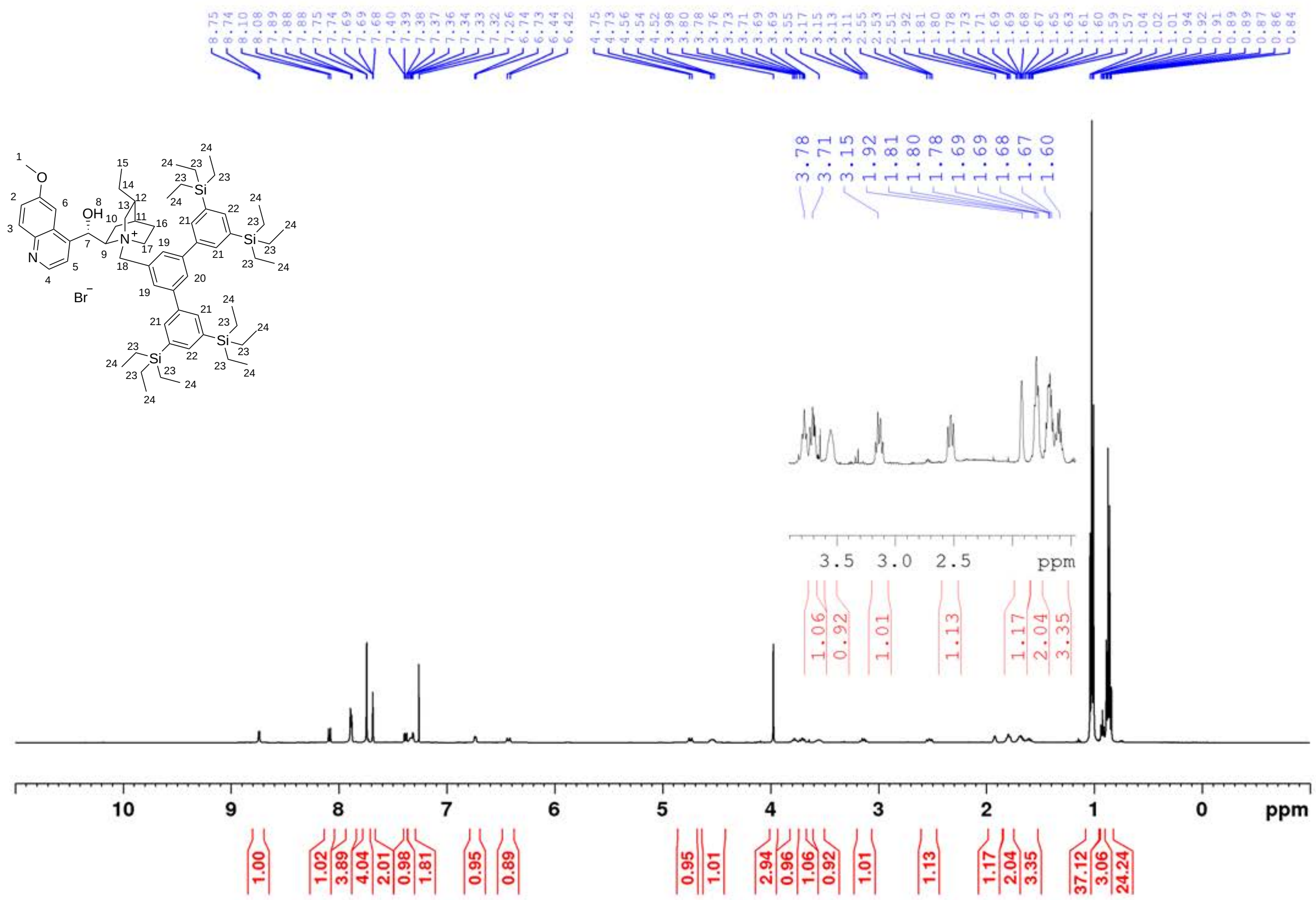

^{13}C NMR (126 MHz, $CDCl_3$) for *(1S,2R,4S,5R)-5-ethyl-2-((S)-hydroxy(6-methoxyquinolin-4-yl)methyl)-1-((3,3'',5,5''-tetrakis(triethylsilyl)-[1,1':3',1''-terphenyl]-5'-yl)methyl)quinuclidine-1-ium bromide* (**C3•Br**)

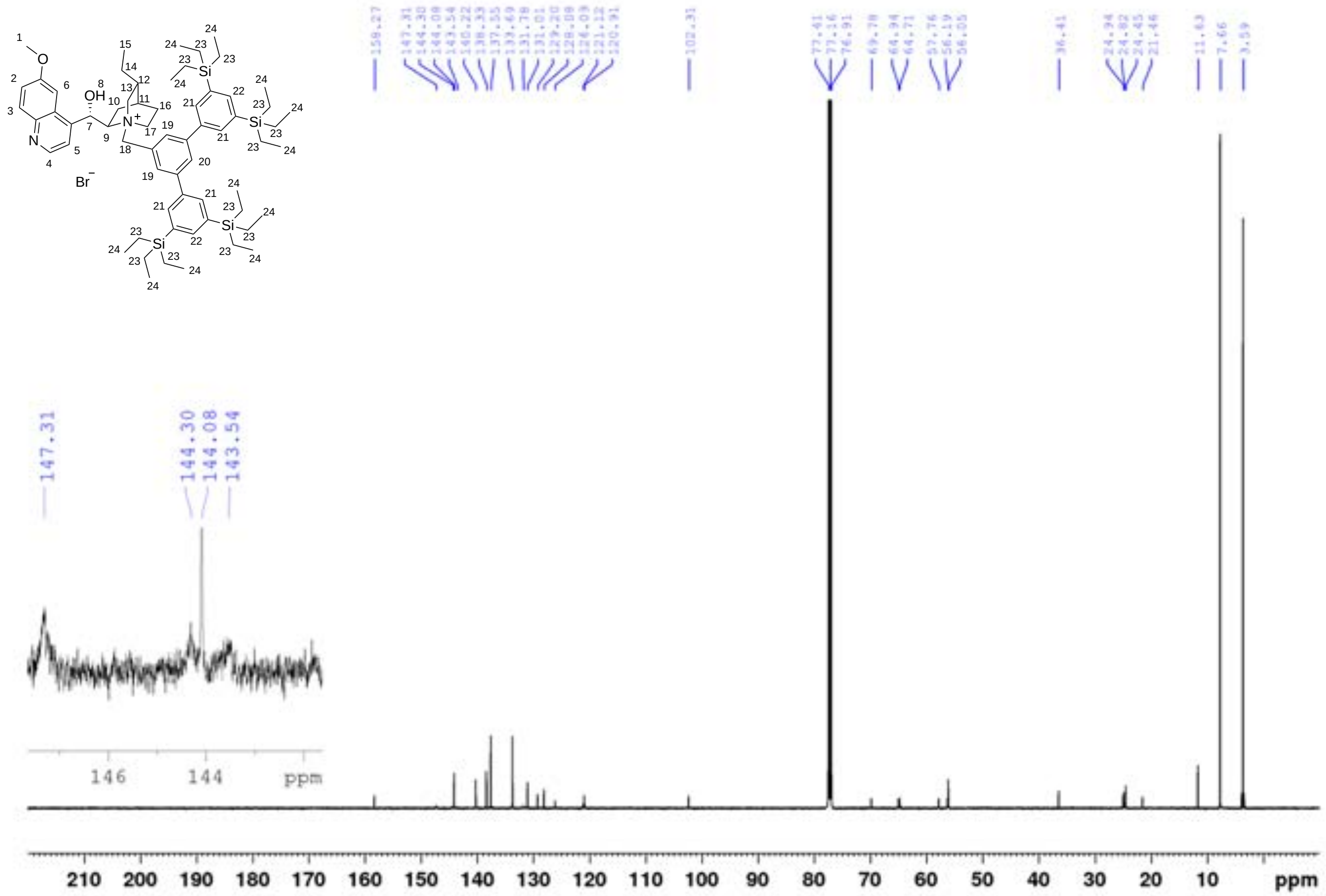

^{1}H NMR (500 MHz, $CDCl_3$) for *(1S,2S,4S,5R)-5-ethyl-2-((R)-hydroxy(6-methoxyquinolin-4-yl)methyl)-1-((3,3'',5,5''-tetrakis(triethylsilyl)-[1,1':3',1''-terphenyl]-5'-yl)methyl)quinuclidin-1-ium bromide* (**C4•Br**)

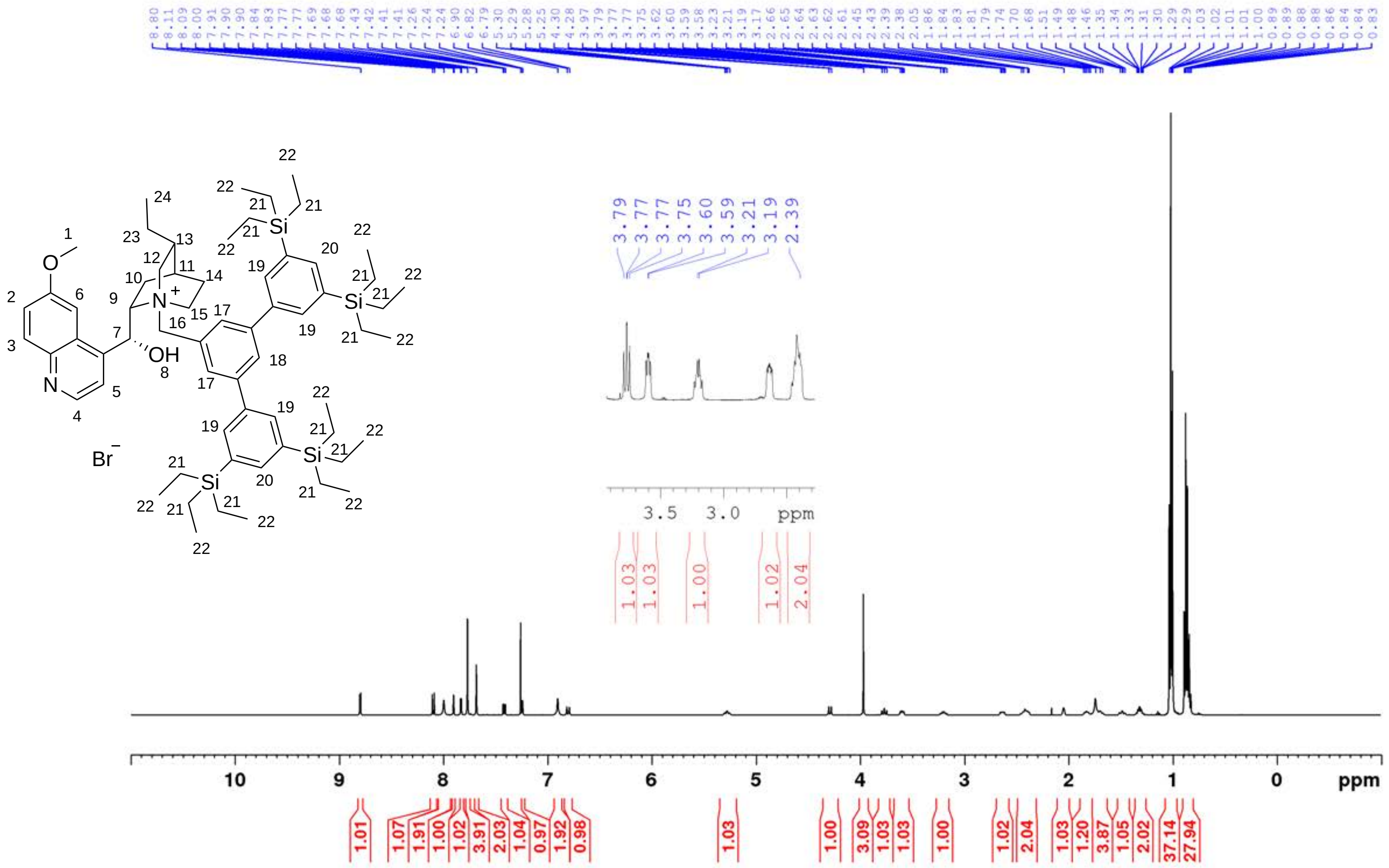

^{13}C NMR (126 MHz, $CDCl_3$) for *(1S,2S,4S,5R)-5-ethyl-2-((R)-hydroxy(6-methoxyquinolin-4-yl)methyl)-1-((3,3'',5,5''-tetrakis(triethylsilyl)-[1,1':3',1''-terphenyl]-5'-yl)methyl)quinuclidin-1-ium bromide* (**C4•Br**)

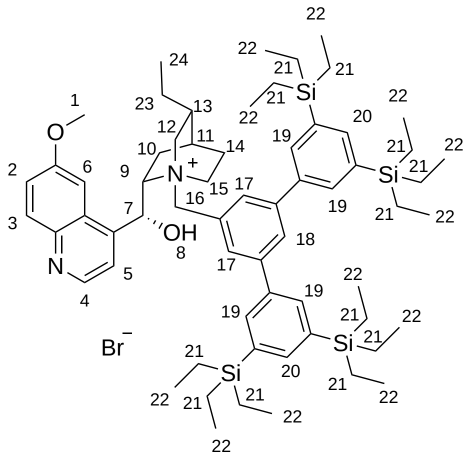

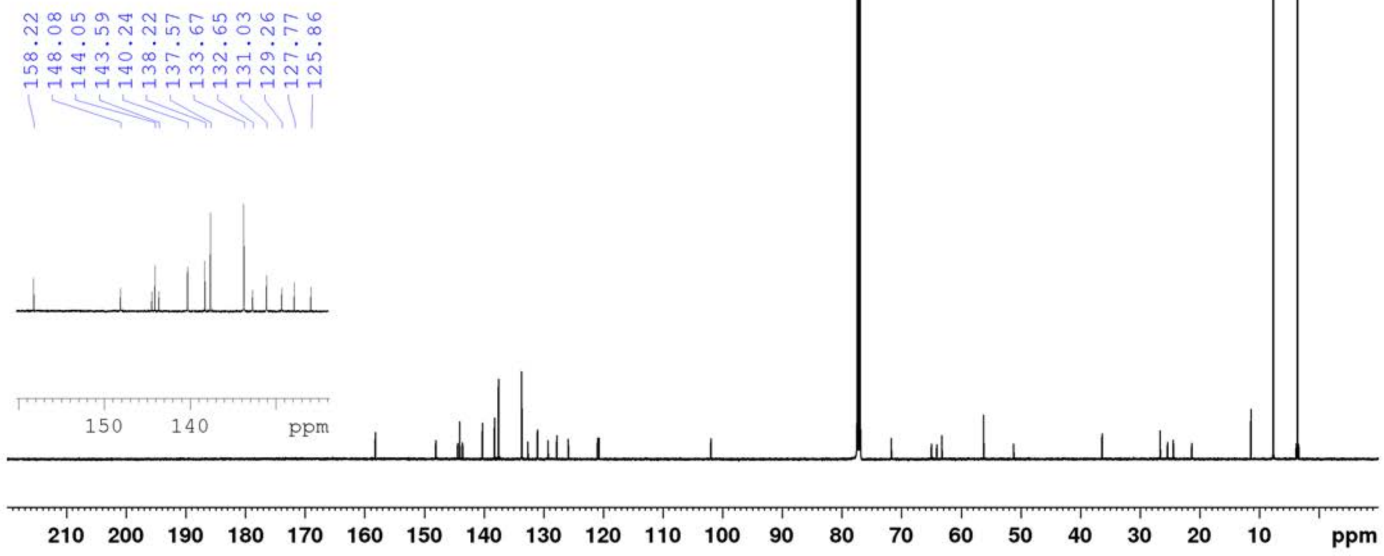

^{1}H NMR (500 MHz, $CDCl_3$) for *(1R,2S,4R)-2-((R)-hydroxy(6-methoxyquinolin-4-yl)methyl)-1-((3,3'',5,5''-tetrakis(triethylsilyl)-[1,1':3',1''-terphenyl]-5'-yl)methyl)quinuclidin-1-ium bromide* (**C5•Br**)

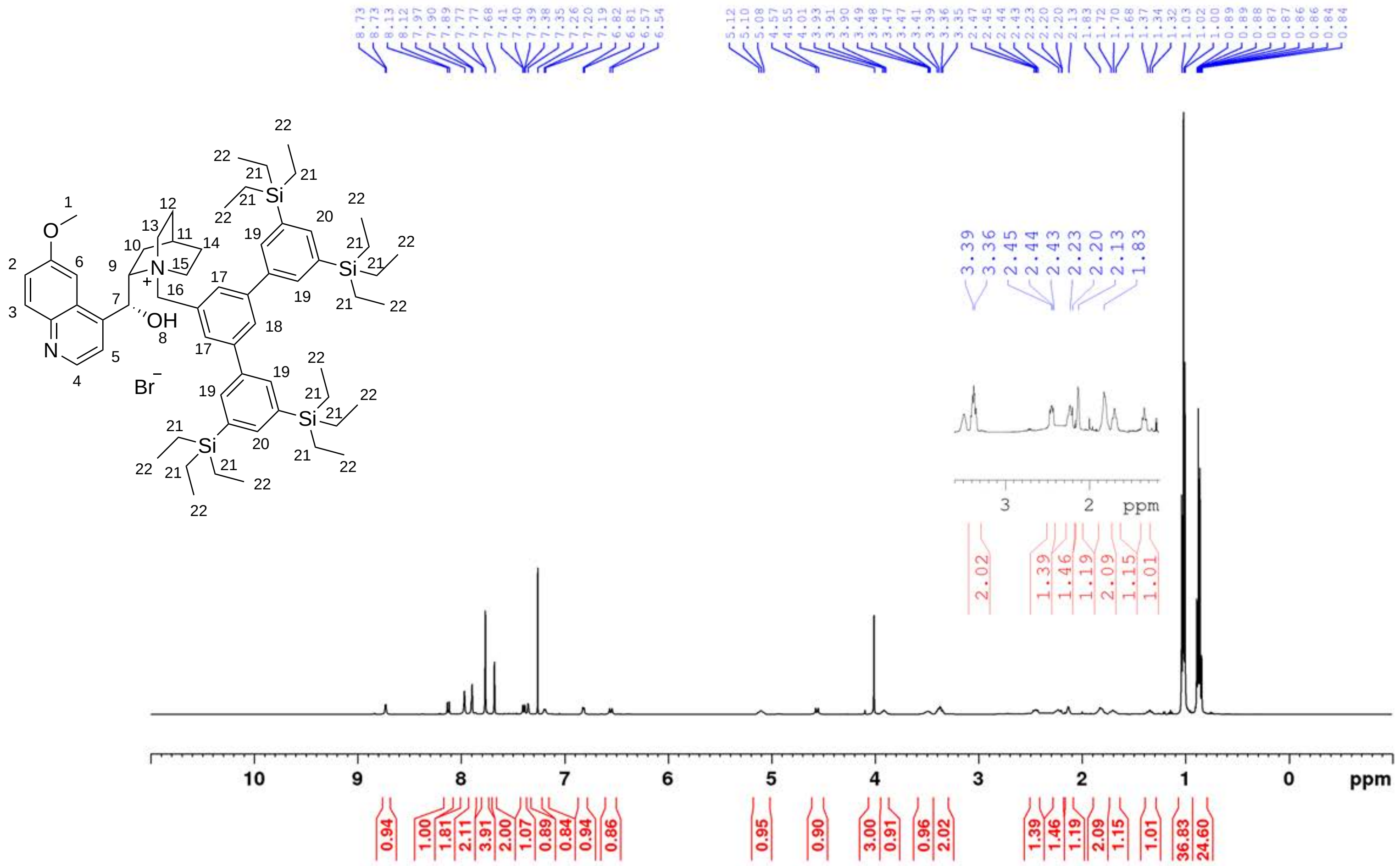

^{13}C NMR (126 MHz, $CDCl_3$) for *(1R,2S,4R)-2-((R)-hydroxy(6-methoxyquinolin-4-yl)methyl)-1-((3,3'',5,5''-tetrakis(triethylsilyl)-[1,1':3',1''-terphenyl]-5'-yl)methyl)quinuclidin-1-ium bromide* (**C5•Br**)

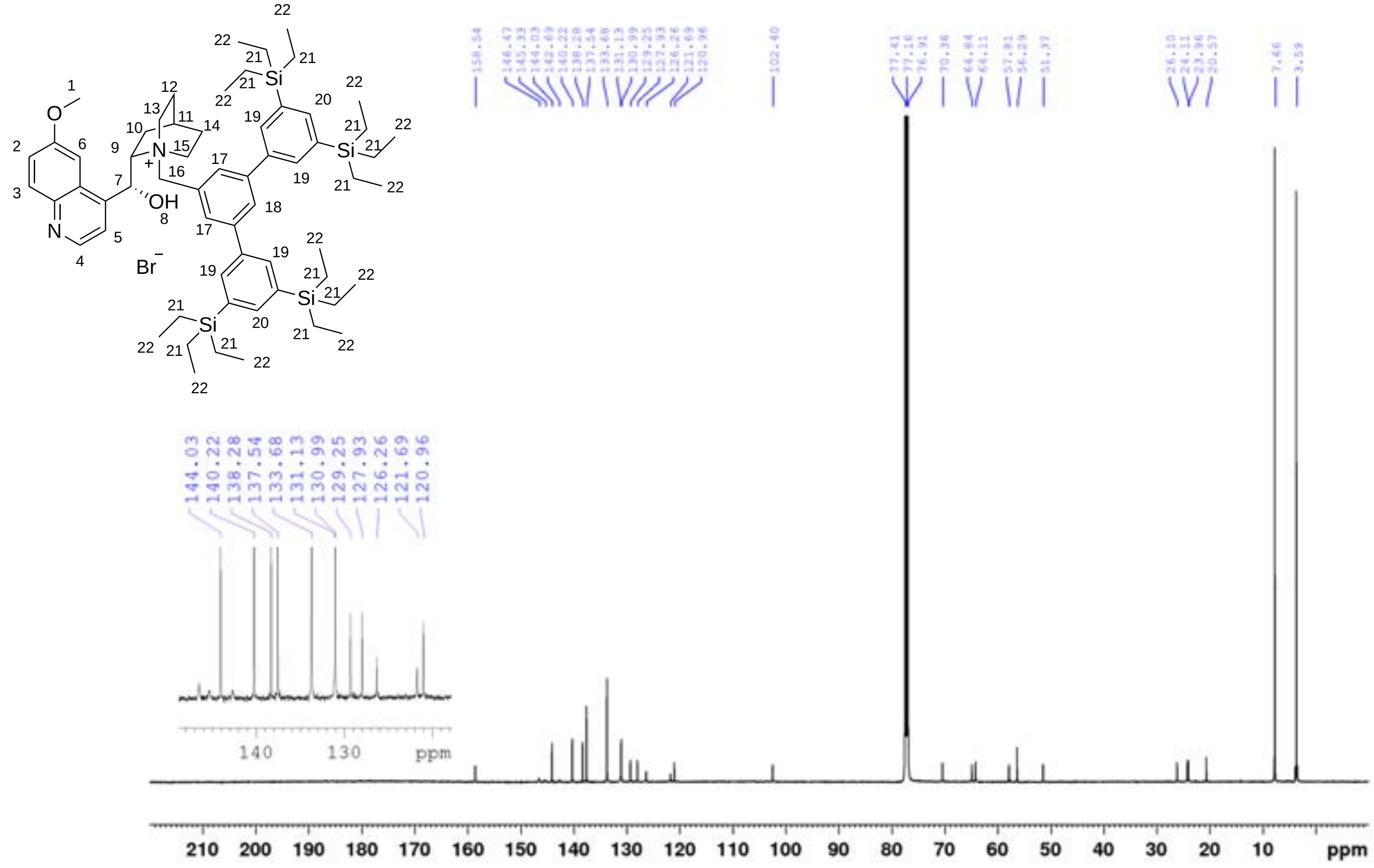

^{1}H NMR (500 MHz, C_5D_5N) for *Bis[rhodium (1S,2R,4S,5R)-5-ethyl-2-((S)-hydroxy(6-methoxyquinolin-4-yl)methyl)-1-((3,3'',5,5''-tetrakis(triethylsilyl)-[1,1':3',1''-terphenyl]-5'-yl)methyl)quinuclidin-1-ium (3,5-bis(2-carboxy-2-methylpropyl)benzenesulfonate)]* (Rh_2(**A**)$_2$•(**C3**)$_2$)

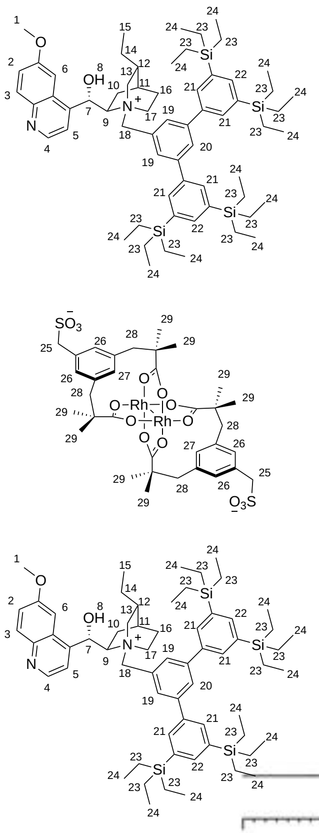

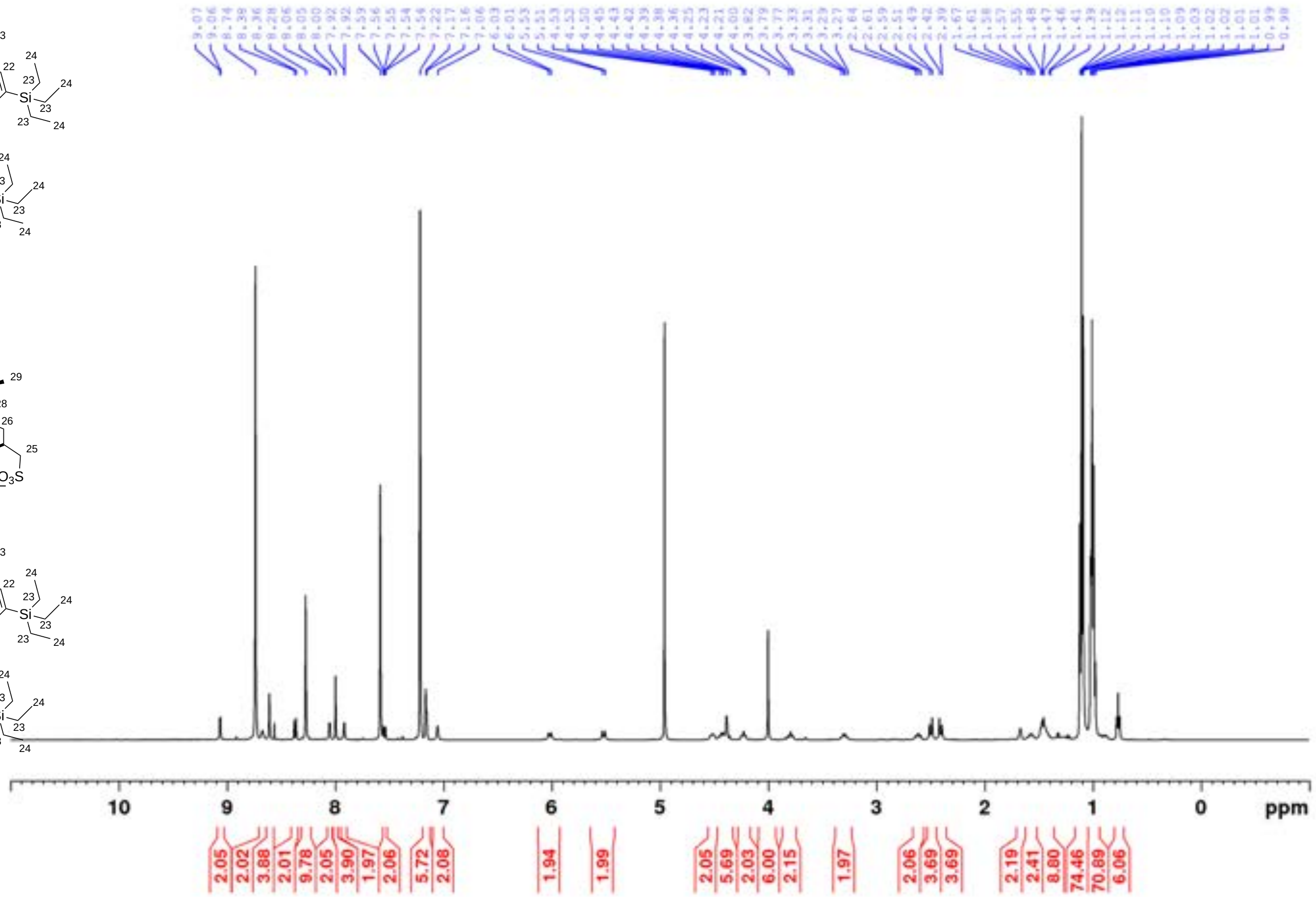

^{13}C NMR (126 MHz, C_5D_5N) for *Bis[rhodium (1S,2R,4S,5R)-5-ethyl-2-((S)-hydroxy(6-methoxyquinolin-4-yl)methyl)-1-((3,3'',5,5''-tetrakis(triethylsilyl)-[1,1':3',1''-terphenyl]-5'-yl)methyl)quinuclidin-1-ium (3,5-bis(2-carboxy-2-methylpropyl)benzenesulfonate)]* (Rh_2(**A**)$_2$•(**C3**)$_2$)

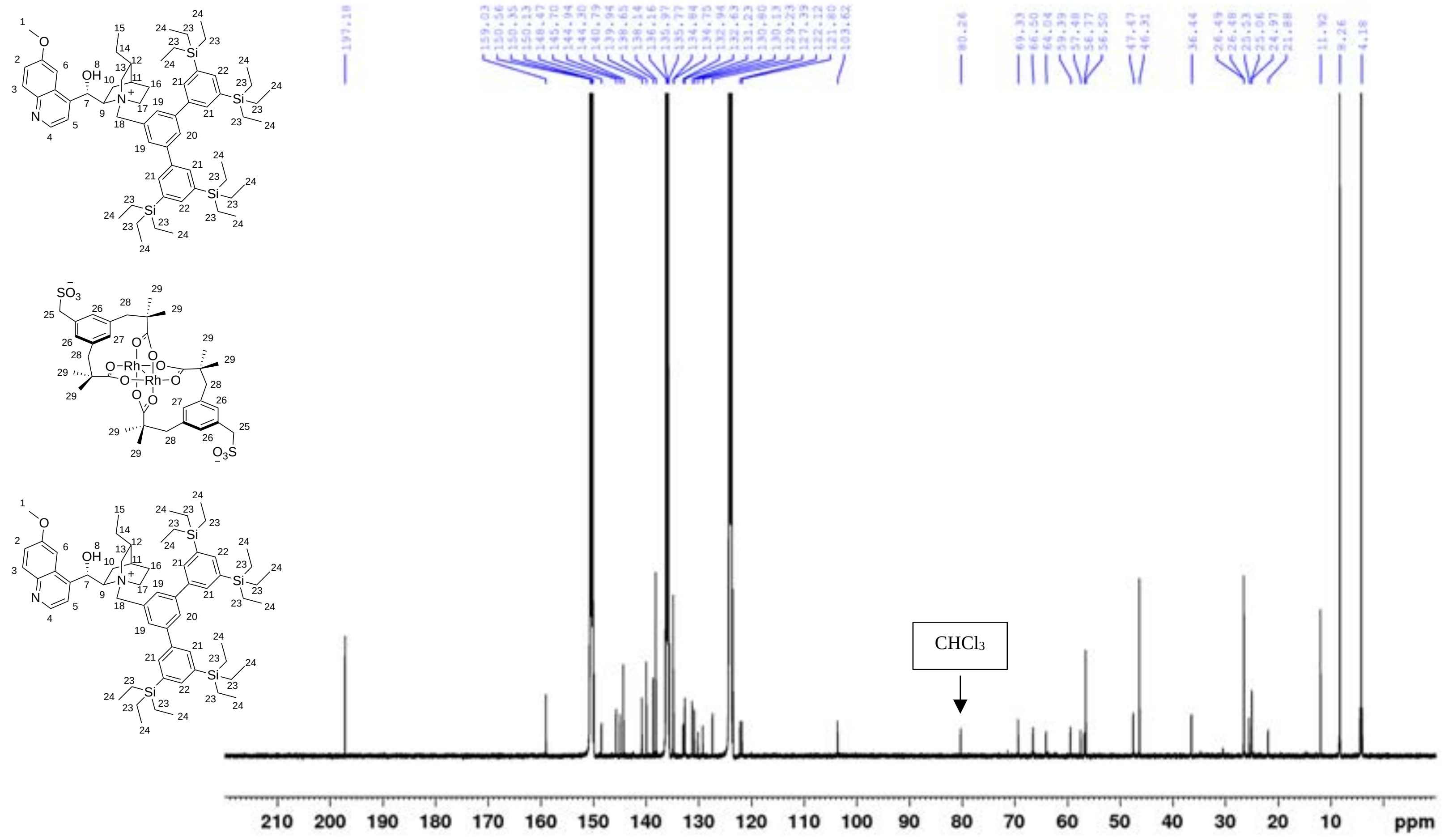

¹H NMR (500 MHz, C_5D_5N) for *Bis[rhodium (1S,2R,4S,5R)-5-ethyl-2-((S)-hydroxy(6-methoxyquinolin-4-yl)methyl)-1-((3,3'',5,5''-tetrakis(triethylsilyl)-[1,1':3',1''-terphenyl]-5'-yl)methyl)quinuclidin-1-ium (3,5-bis((1-carboxycyclobutyl)methyl)phenyl)methanesulfonate)]* (Rh_2(**B**)$_2$•(**C3**)$_2$)

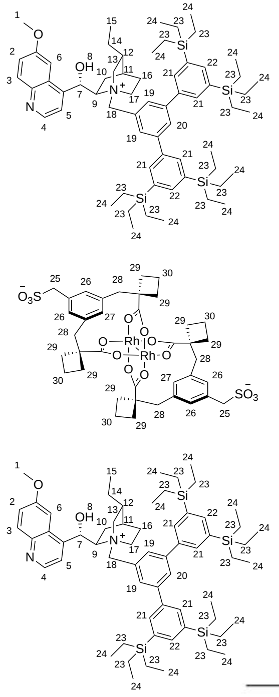

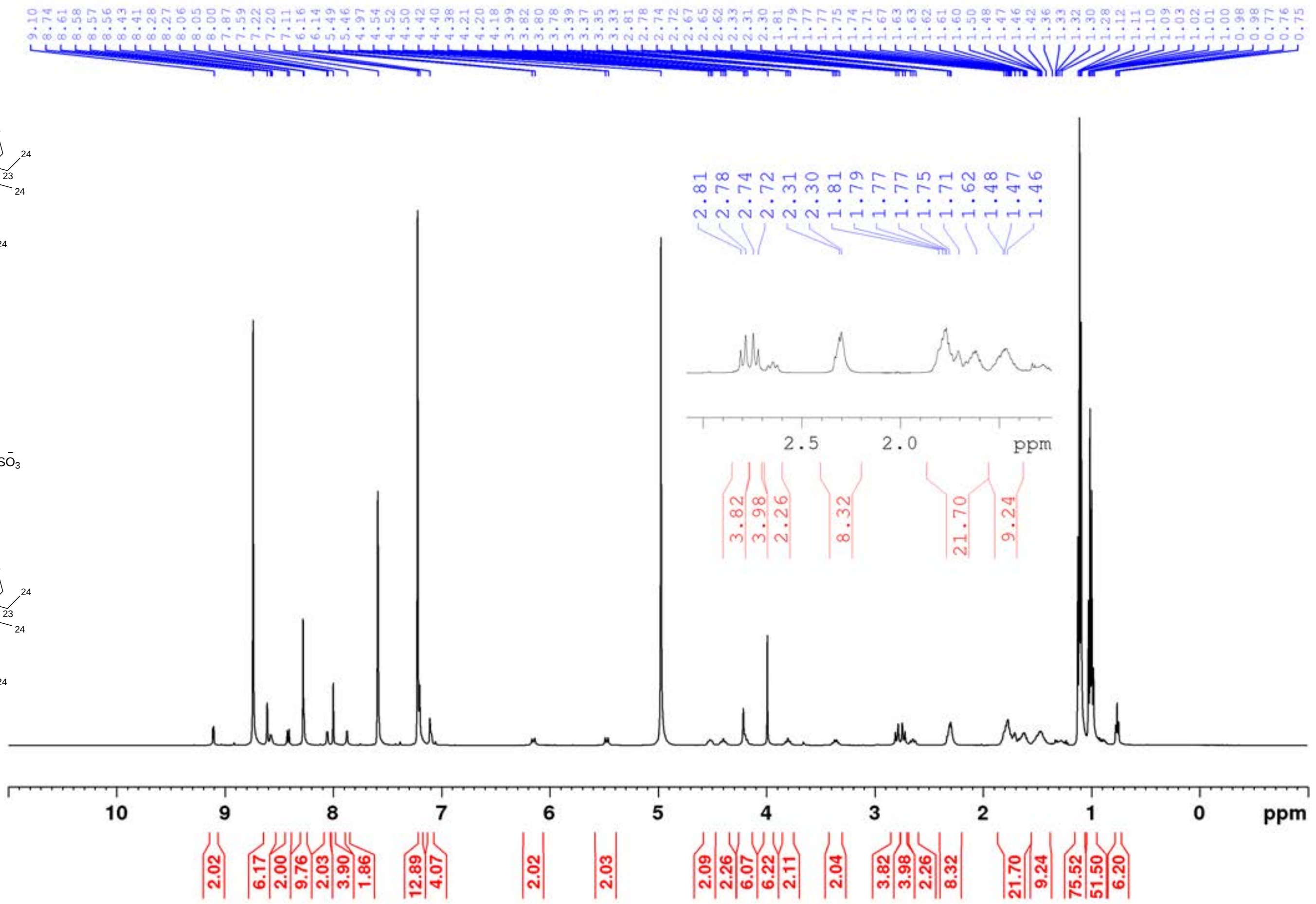

^{13}C NMR (126 MHz, C_5D_5N) for *Bis[rhodium (1S,2R,4S,5R)-5-ethyl-2-((S)-hydroxy(6-methoxyquinolin-4-yl)methyl)-1-((3,3'',5,5''-tetrakis(triethylsilyl)-[1,1':3',1''-terphenyl]-5'-yl)methyl)quinuclidin-1-ium (3,5-bis((1-carboxycyclobutyl)methyl)phenyl)methanesulfonate)]* (Rh_2(**B**)$_2$•(**C3**)$_2$)

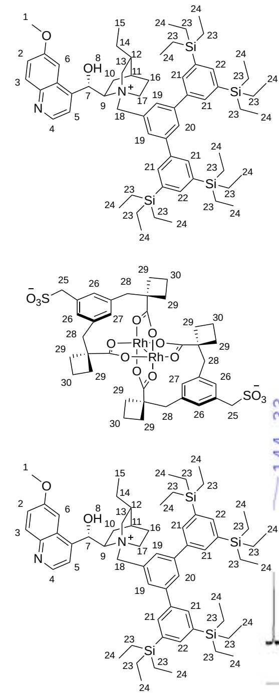

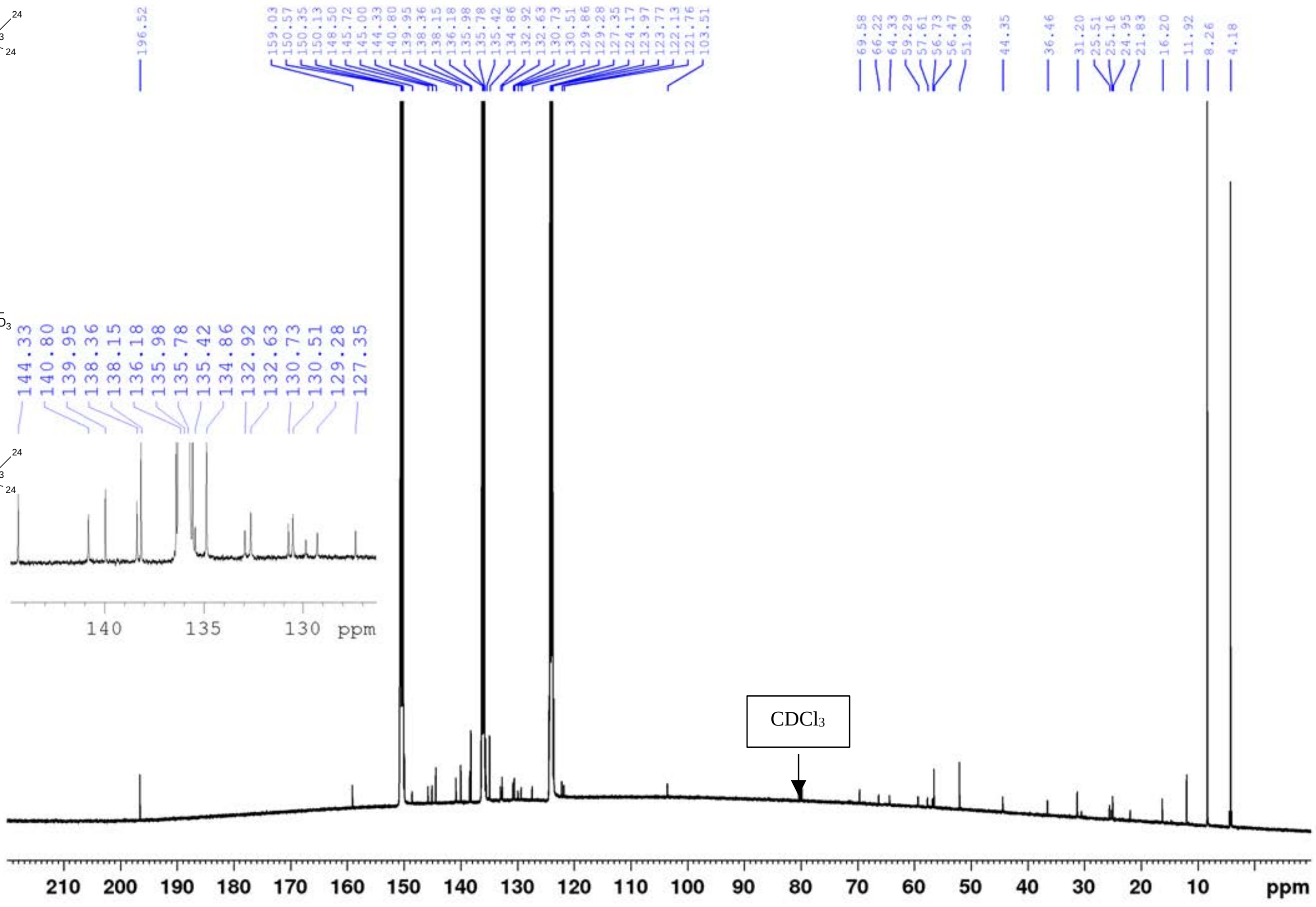

^{1}H NMR (500 MHz, C_5D_5N) for *Bis[(1S,2S,4S,5R)-5-ethyl-2-((R)-hydroxy(6-methoxyquinolin-4-yl)methyl)-1-((3,3'',5,5''-tetrakis(triethylsilyl)-[1,1':3',1''-terphenyl]-5'-yl)methyl)quinuclidin-1-ium (3,5-bis((1-carboxycyclobutyl)methyl)phenyl)methanesulfonate)]* (Rh_2(**B**)$_2$•(**C4**)$_2$)

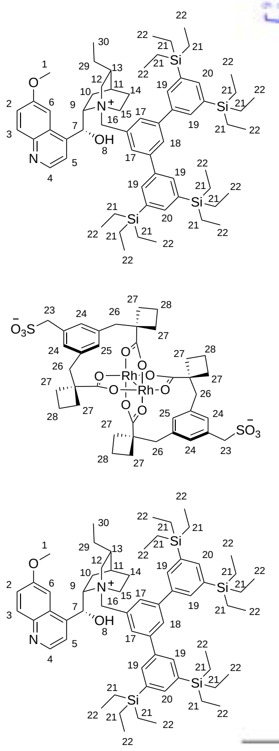

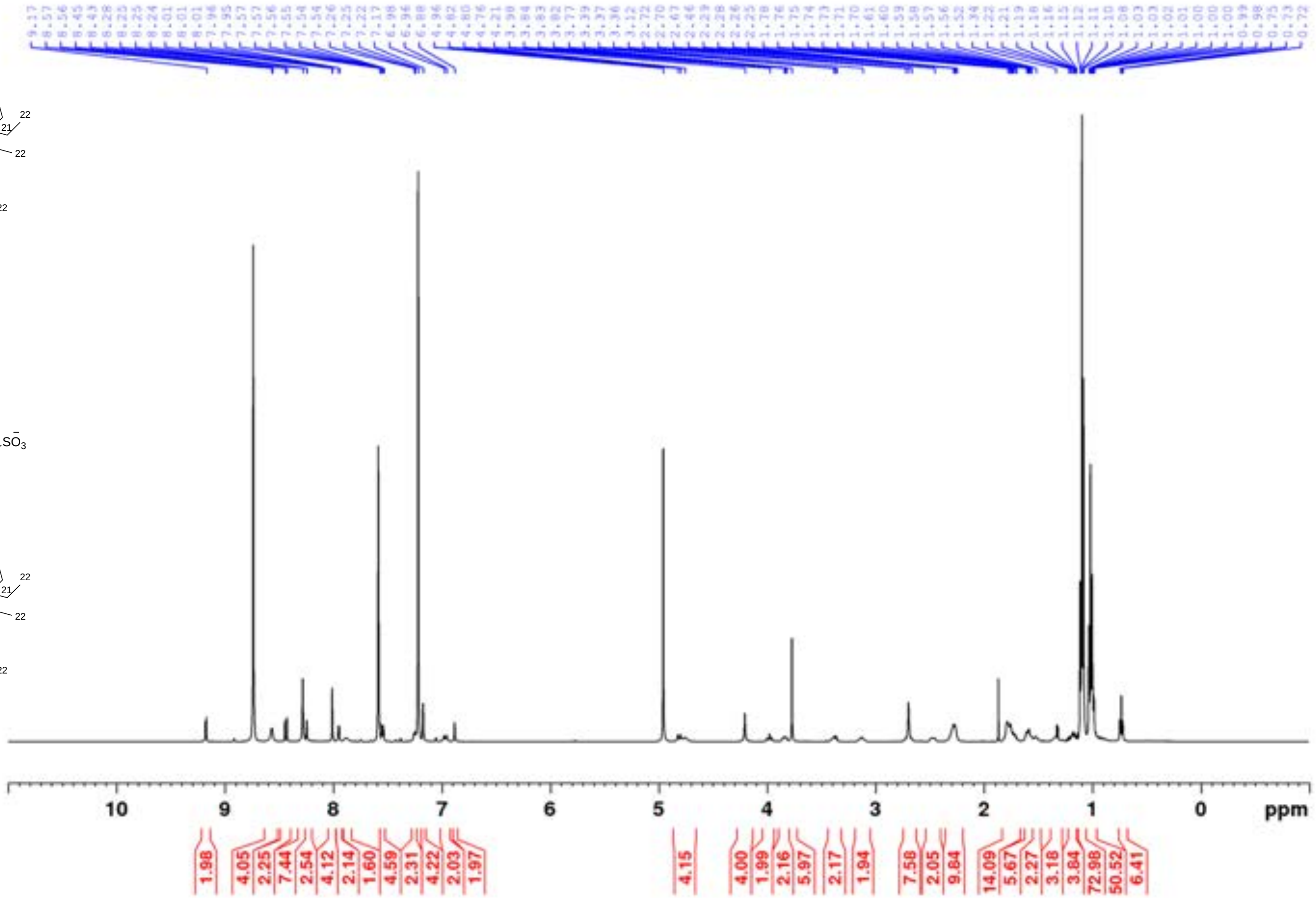

^{13}C NMR (126 MHz, C_5D_5N) for *Bis[(1S,2S,4S,5R)-5-ethyl-2-((R)-hydroxy(6-methoxyquinolin-4-yl)methyl)-1-((3,3'',5,5''-tetrakis(triethylsilyl)-[1,1':3',1''-terphenyl]-5'-yl)methyl)quinuclidin-1-ium (3,5-bis((1-carboxycyclobutyl)methyl)phenyl)methanesulfonate)]* (Rh_2(**B**)$_2$•(**C4**)$_2$)

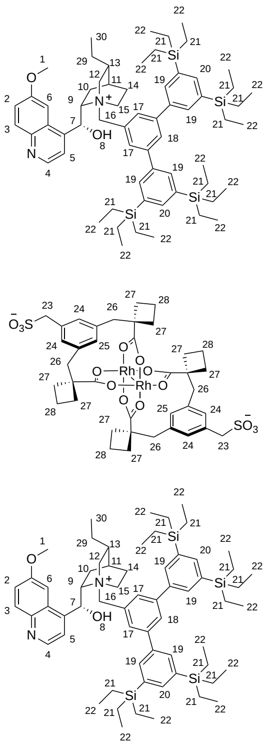

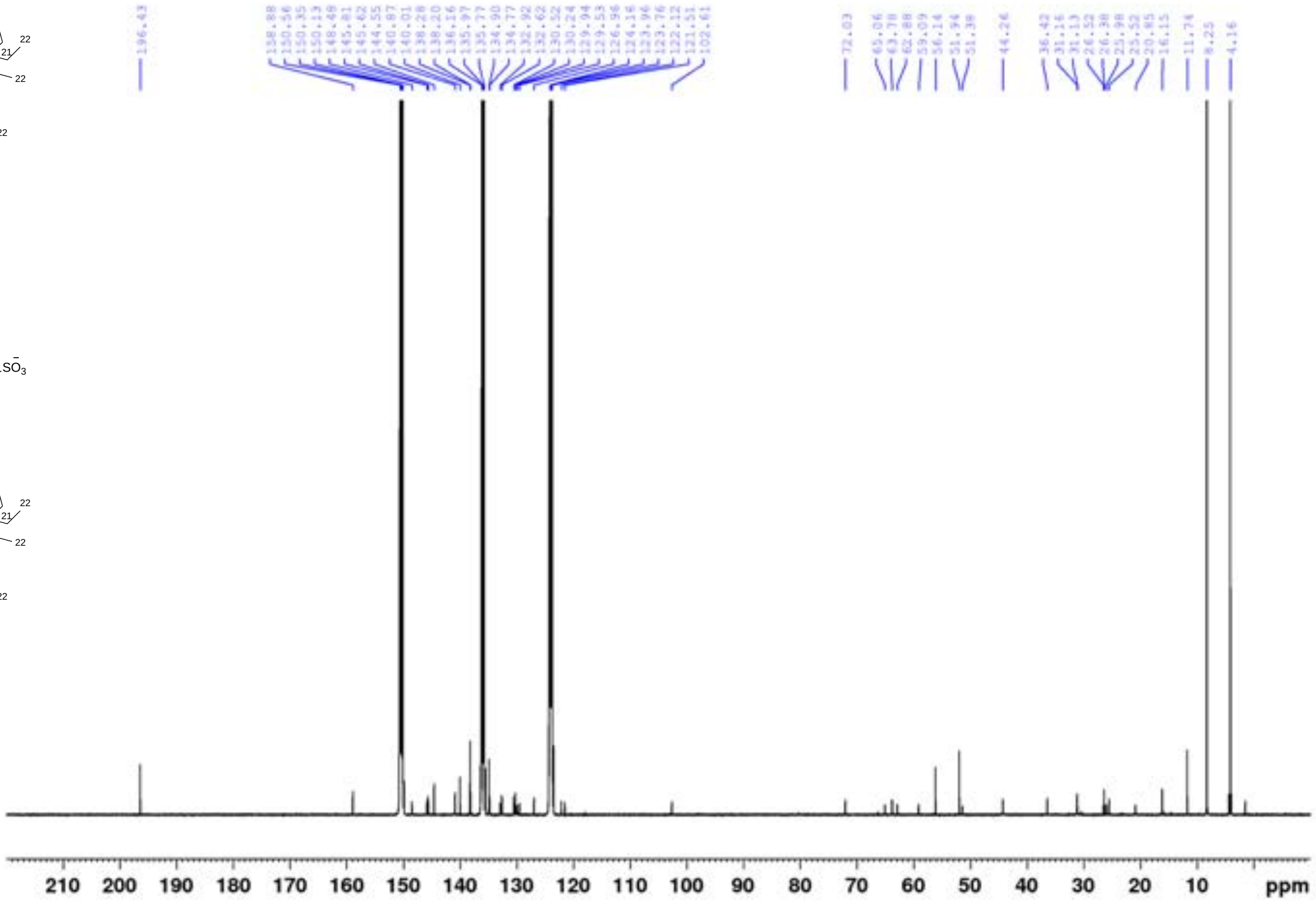

^{1}H NMR (500 MHz, C_5D_5N) for *Bis[rhodium (1R,2S,4R)-2-((R)-hydroxy(6-methoxyquinolin-4-yl)methyl)-1-((3,3'',5,5''-tetrakis(triethylsilyl)-[1,1':3',1''-terphenyl]-5'-yl)methyl)quinuclidin-1-ium (3,5-bis((1-carboxycyclobutyl)methyl)phenyl)methanesulfonate)]* (Rh_2(**B**)$_2$•(**C5**)$_2$)

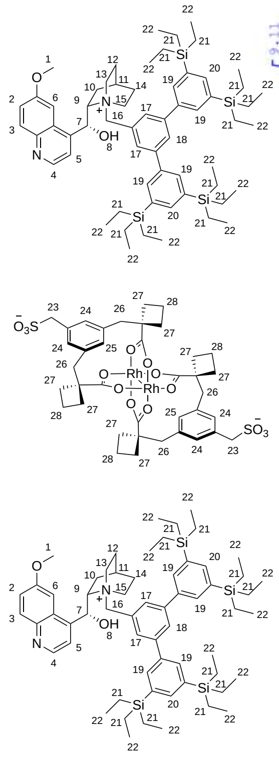

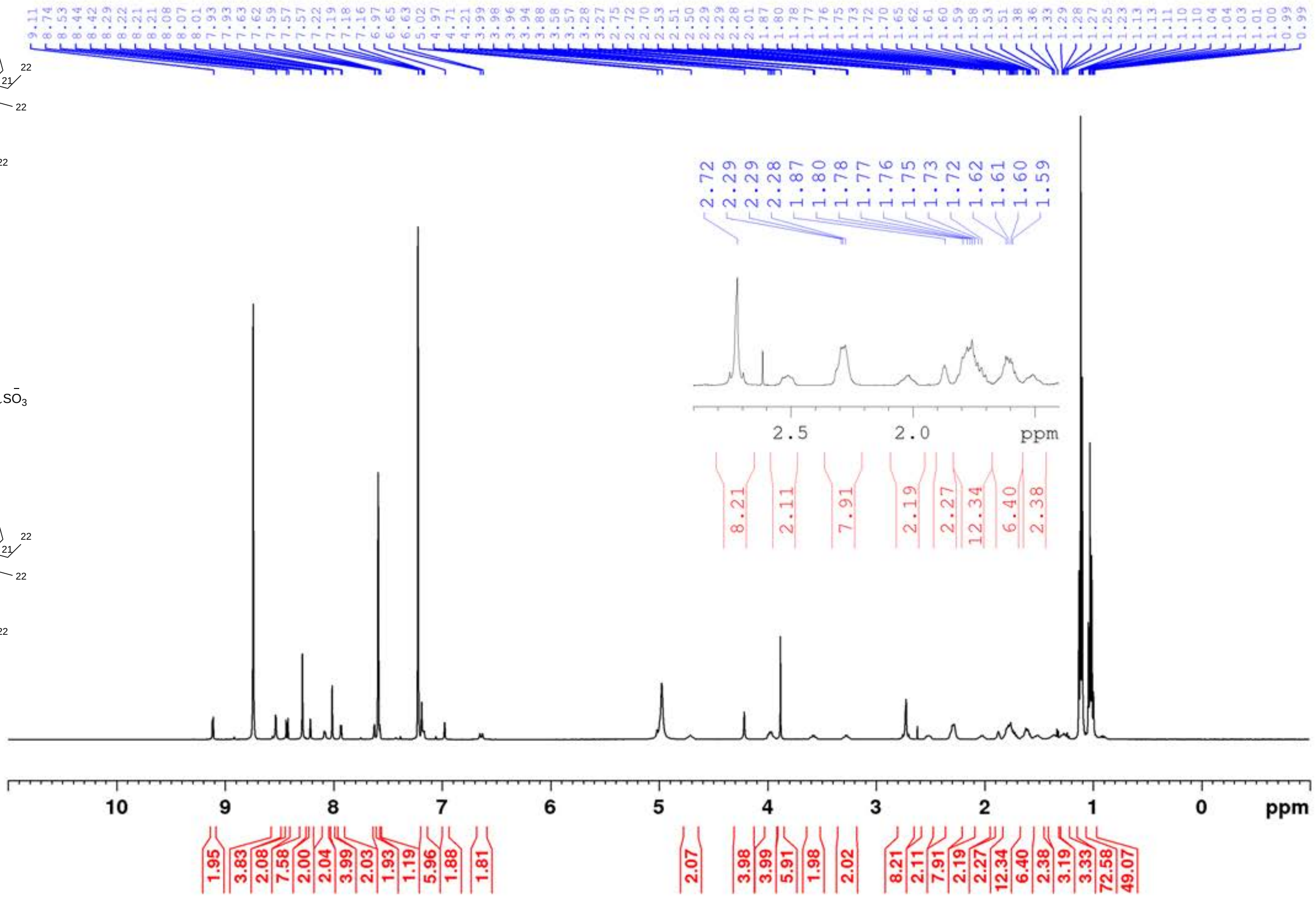

^{13}C NMR (126 MHz, C_5D_5N) for *Bis[rhodium (1R,2S,4R)-2-((R)-hydroxy(6-methoxyquinolin-4-yl)methyl)-1-((3,3'',5,5''-tetrakis(triethylsilyl)-[1,1':3',1''-terphenyl]-5'-yl)methyl)quinuclidin-1-ium (3,5-bis((1-carboxycyclobutyl)methyl)phenyl)methanesulfonate)]* (Rh_2(**B**)$_2$•(**C5**)$_2$)

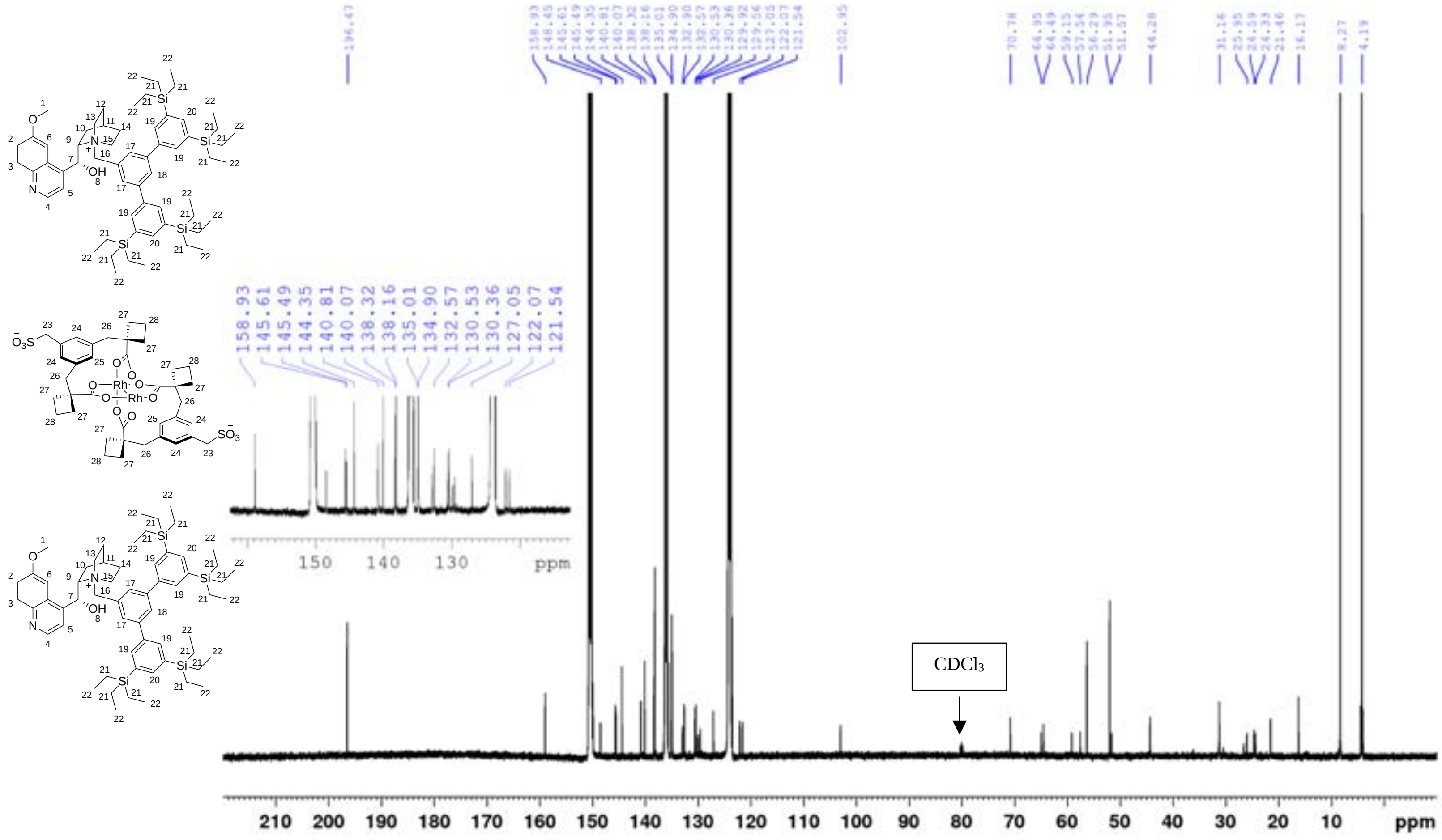

^{1}H NMR (500 MHz, C_5D_5N) *Bis[rhodium (1S,2R,4S,5R)-5-ethyl-2-((S)-hydroxy(6-methoxyquinolin-4-yl)methyl)-1-((3,3'',5,5''-tetrakis(triethylsilyl)-[1,1':3',1''-terphenyl]-5'-yl)methyl)quinuclidin-1-ium (3,5-bis((1-carboxycyclopentyl)methyl)phenyl)methanesulfonate)]* (Rh_2(**C**)$_2$•(**C3**)$_2$)

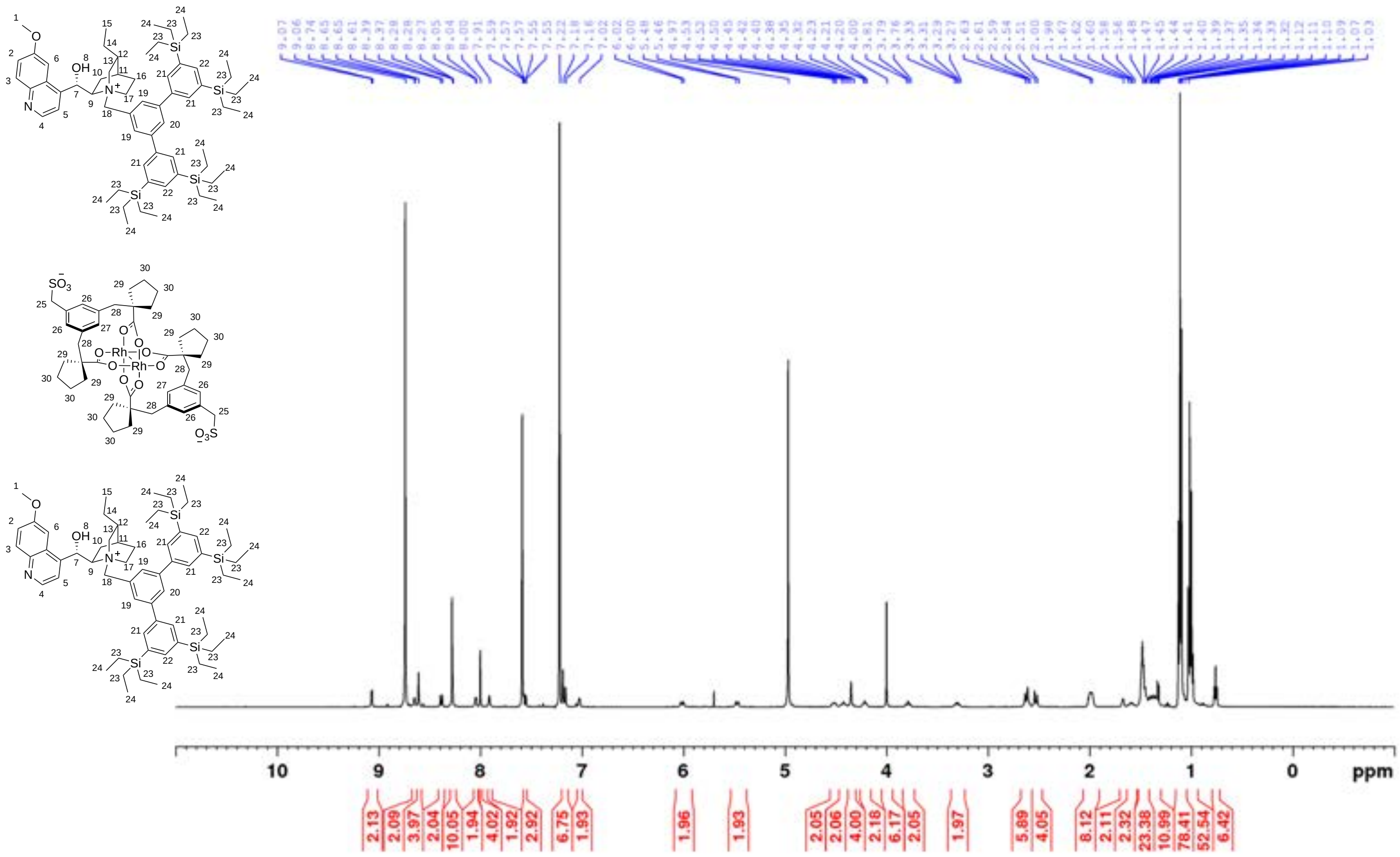

^{13}C NMR (126 MHz, C_5D_5N) for *Bis[rhodium (1S,2R,4S,5R)-5-ethyl-2-((S)-hydroxy(6-methoxyquinolin-4-yl)methyl)-1-((3,3'',5,5''-tetrakis(triethylsilyl)-[1,1':3',1''-terphenyl]-5'-yl)methyl)quinuclidin-1-ium (3,5-bis((1-carboxycyclopentyl)methyl)phenyl)methanesulfonate)]* (Rh_2(**C**)$_2$•(**C3**)$_2$)

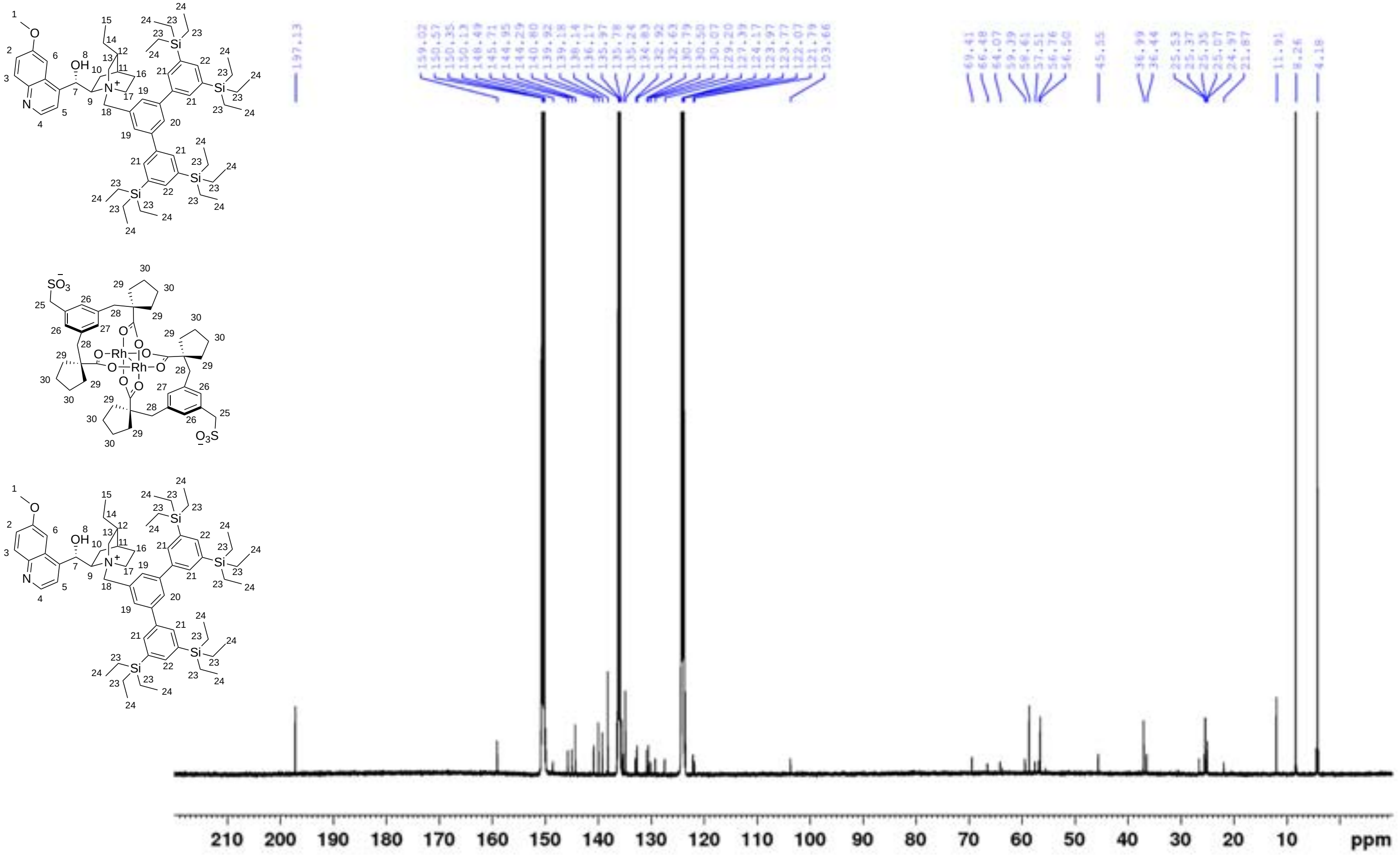

¹H NMR (400 MHz, $CDCl_3$) for *5-iodopent-4-yn-1-ol*

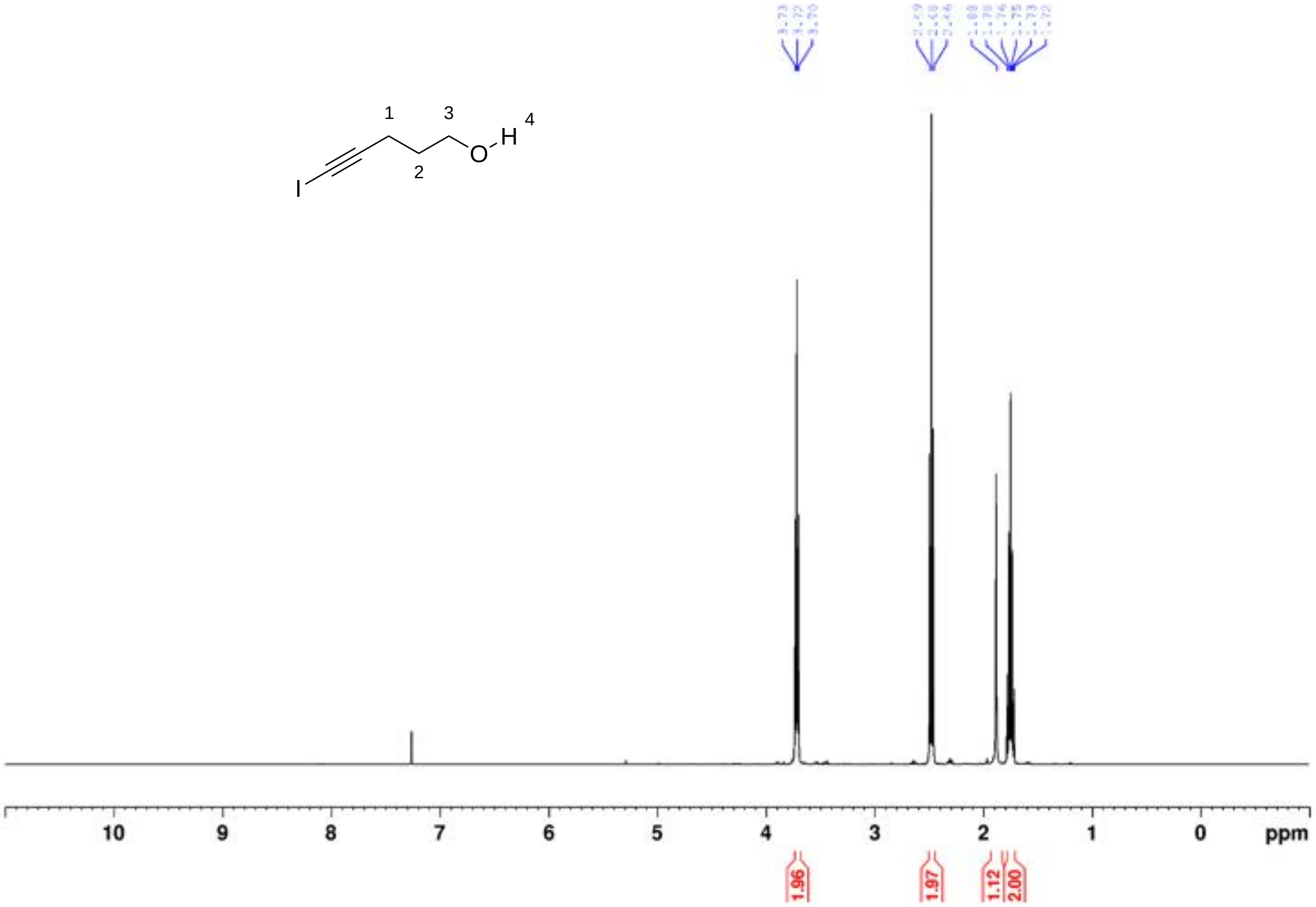

^{13}C NMR (101 MHz, $CDCl_3$) for *5-iodopent-4-yn-1-ol*

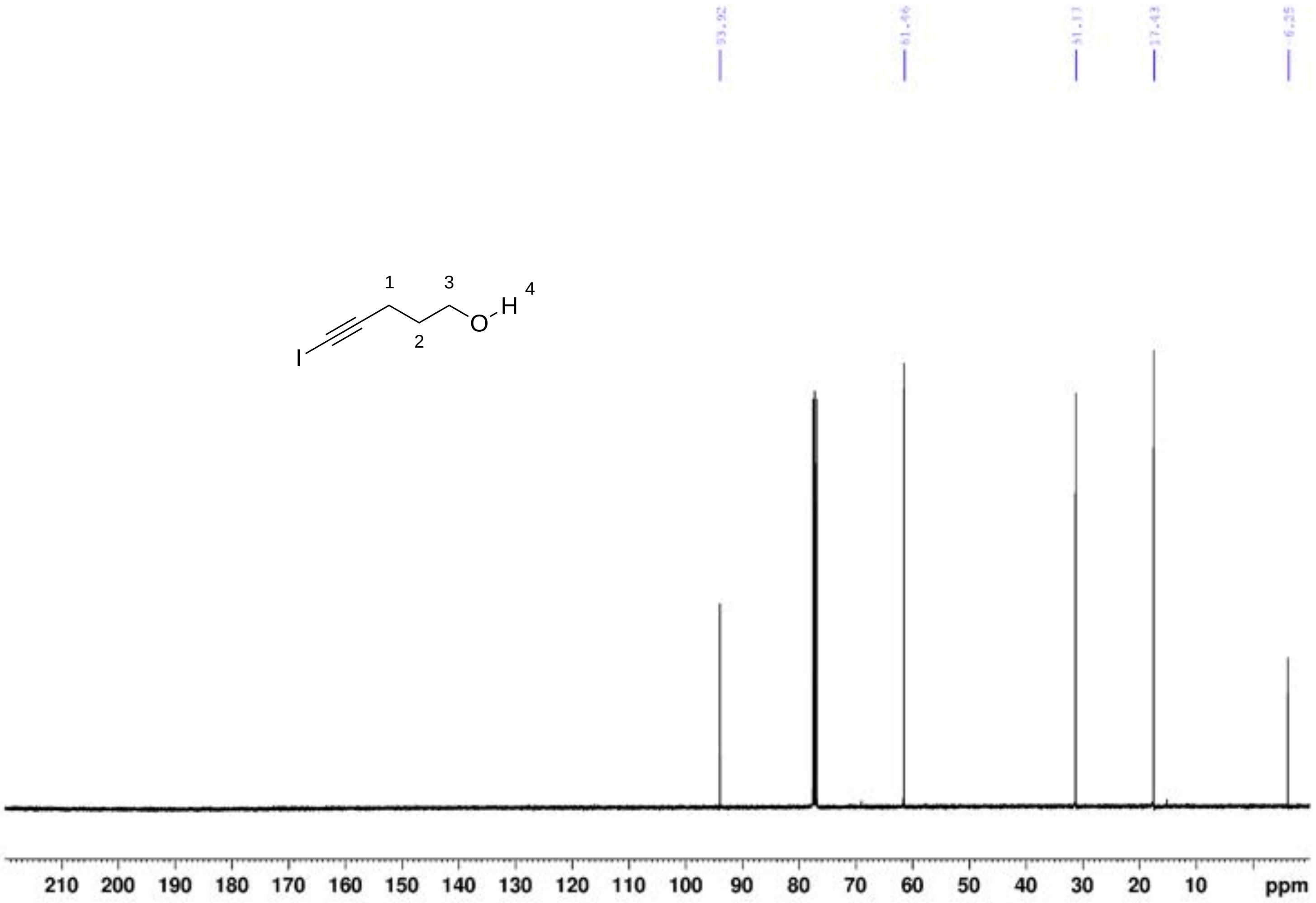

¹H NMR (400 MHz, $CDCl_3$) for *(Z)-5-iodopent-4-en-1-ol*

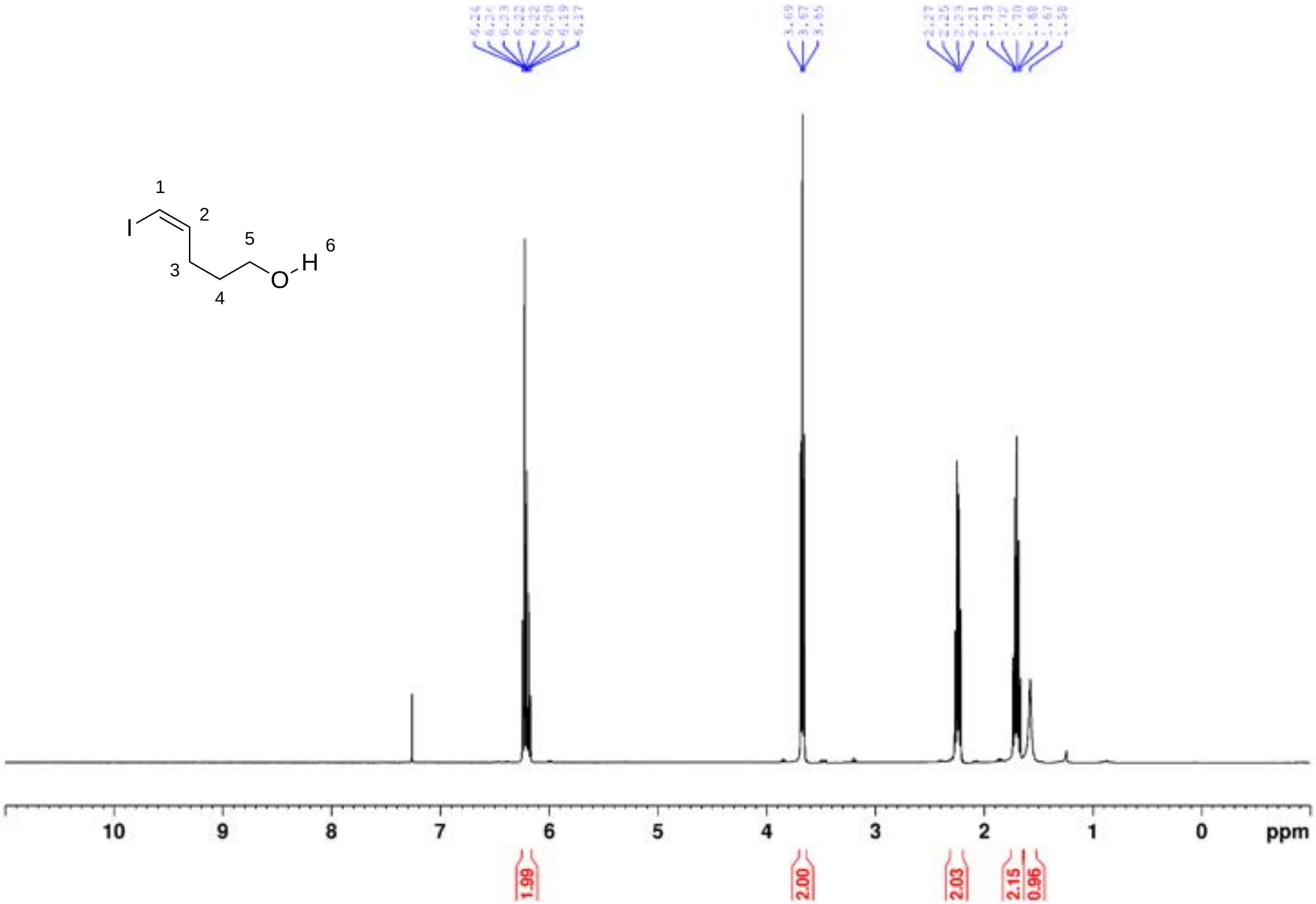

^{13}C NMR (101 MHz, $CDCl_3$) for *(Z)-5-iodopent-4-en-1-ol*

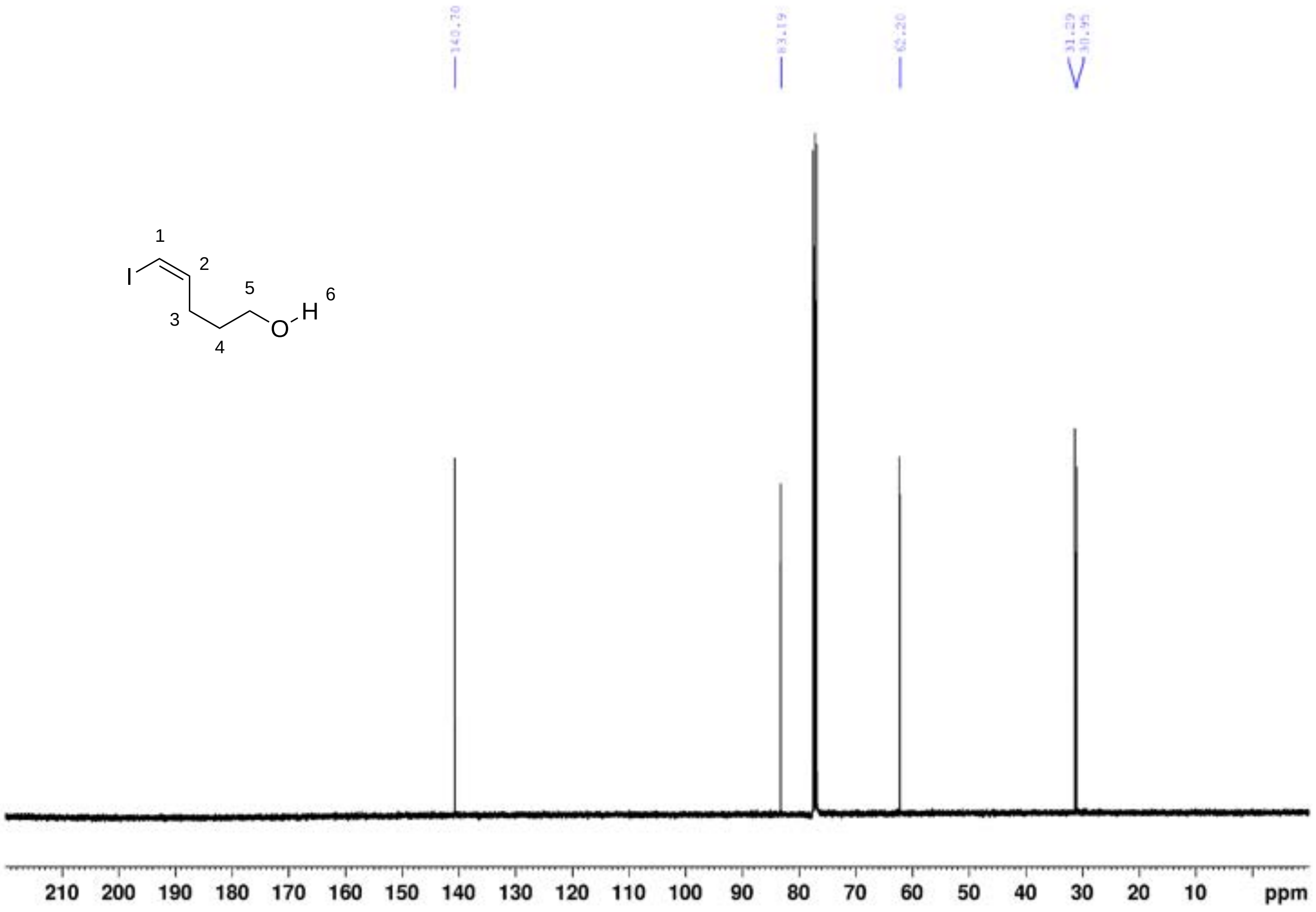

^{1}H NMR (400 MHz, $CDCl_3$) for *(Z)-5-phenylpent-4-en-1-ol* (**1a**)

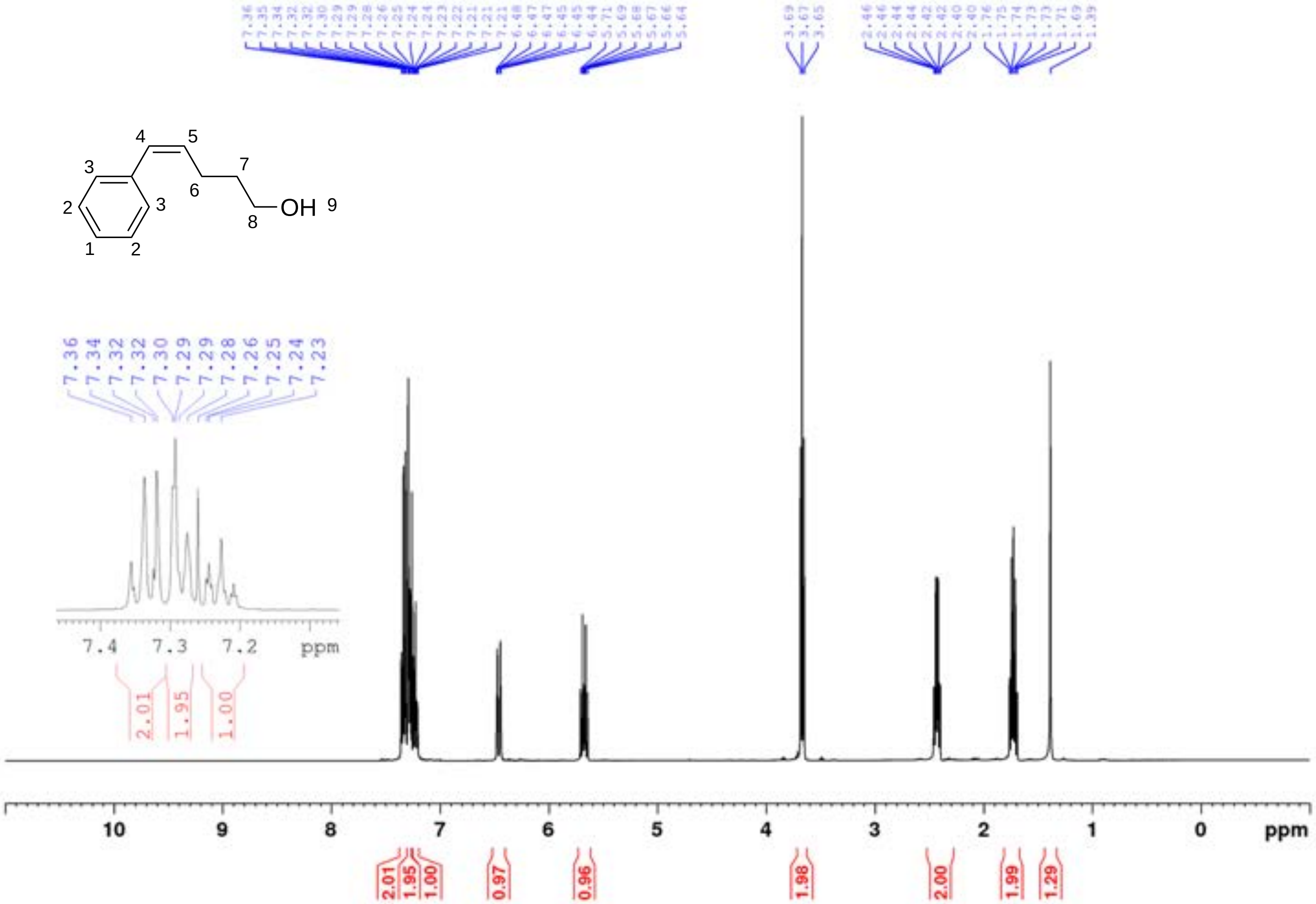

^{13}C NMR (101 MHz, $CDCl_3$) for *(Z)-5-phenylpent-4-en-1-ol* (**1a**)

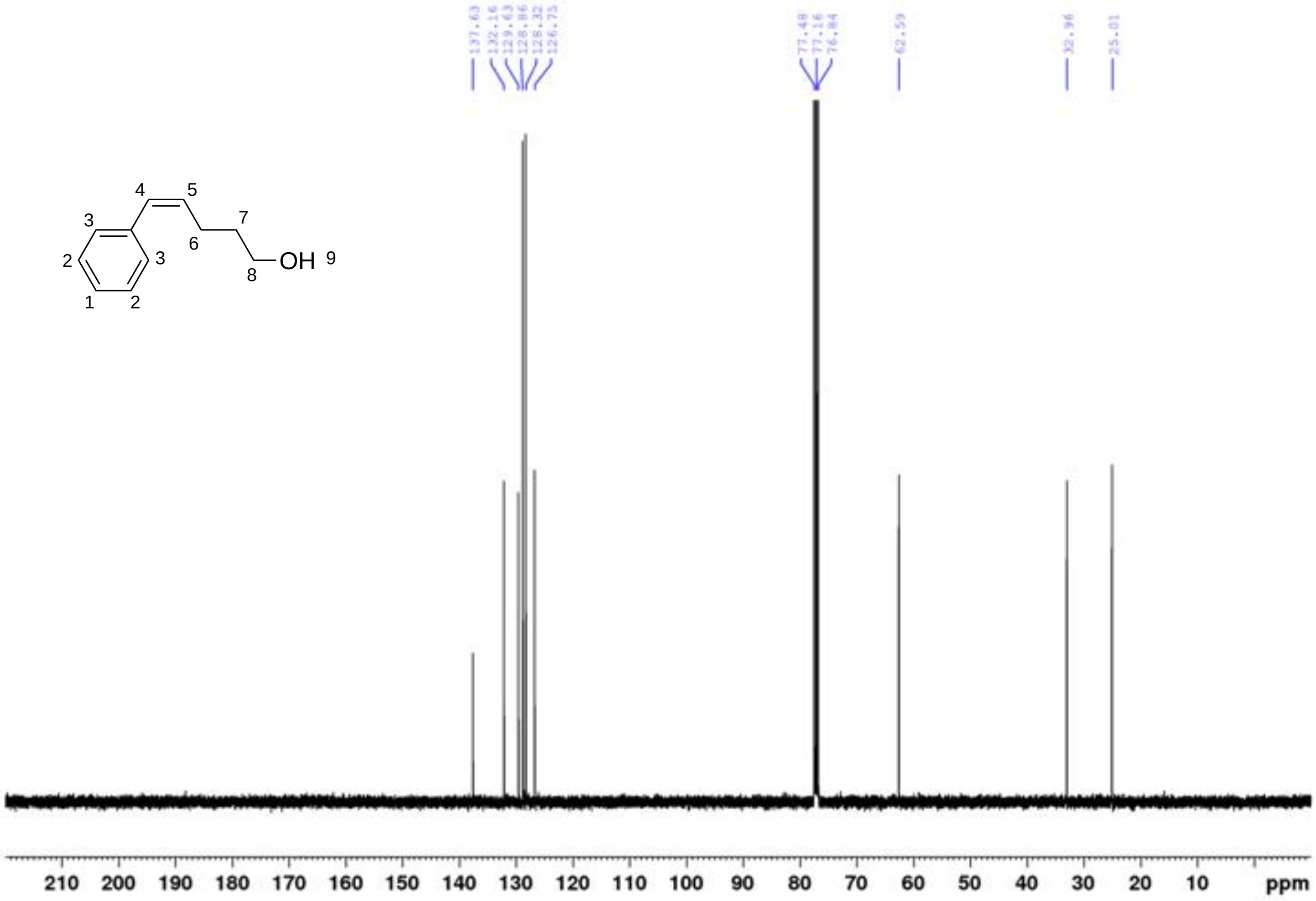

^{1}H NMR (400 MHz, $CDCl_3$) for *(Z)-5-(2-fluorophenyl)pent-4-en-1-ol* (**1b**)

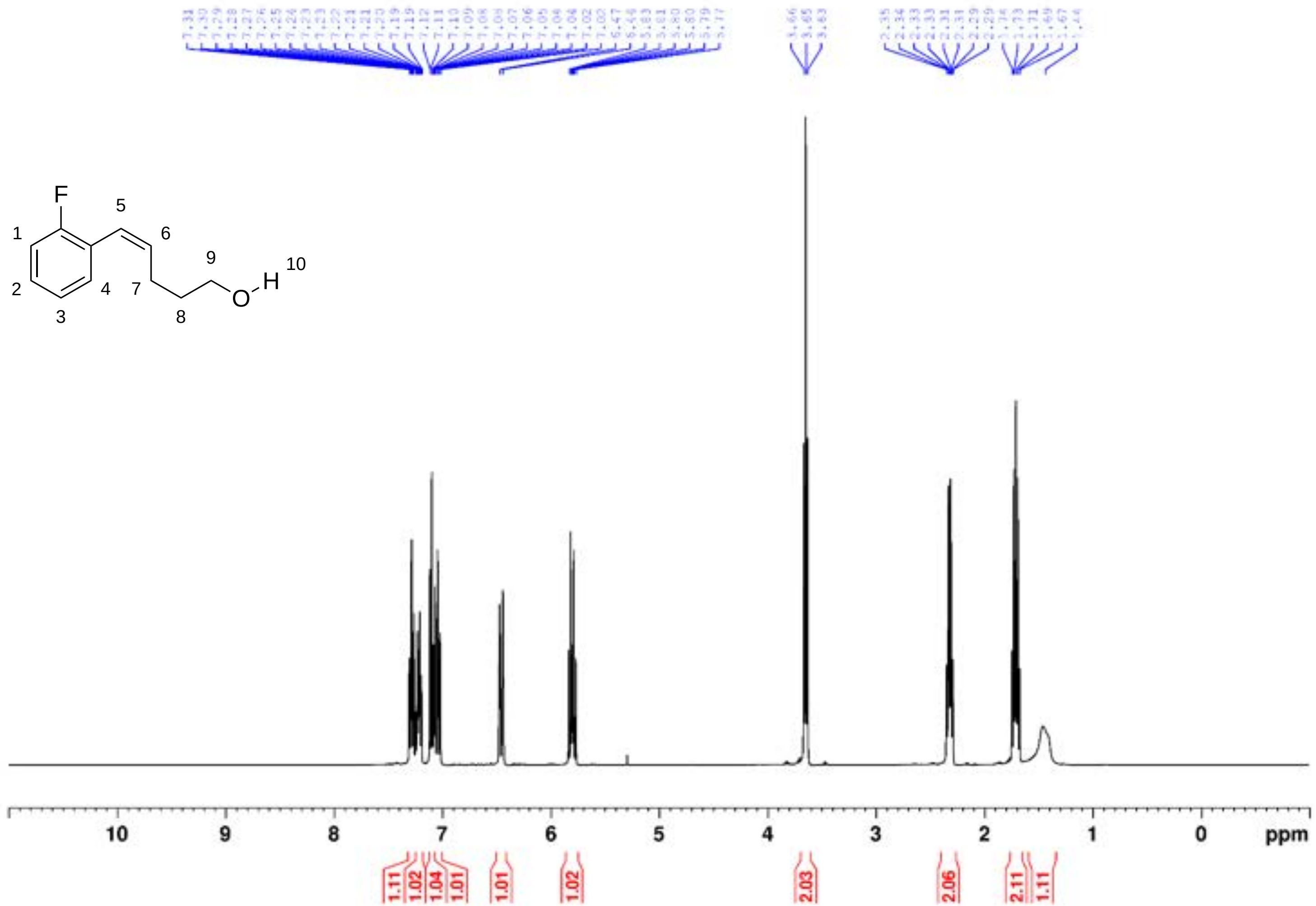

^{13}C NMR (101 MHz, $CDCl_3$) for *(Z)-5-(2-fluorophenyl)pent-4-en-1-ol* (**1b**)

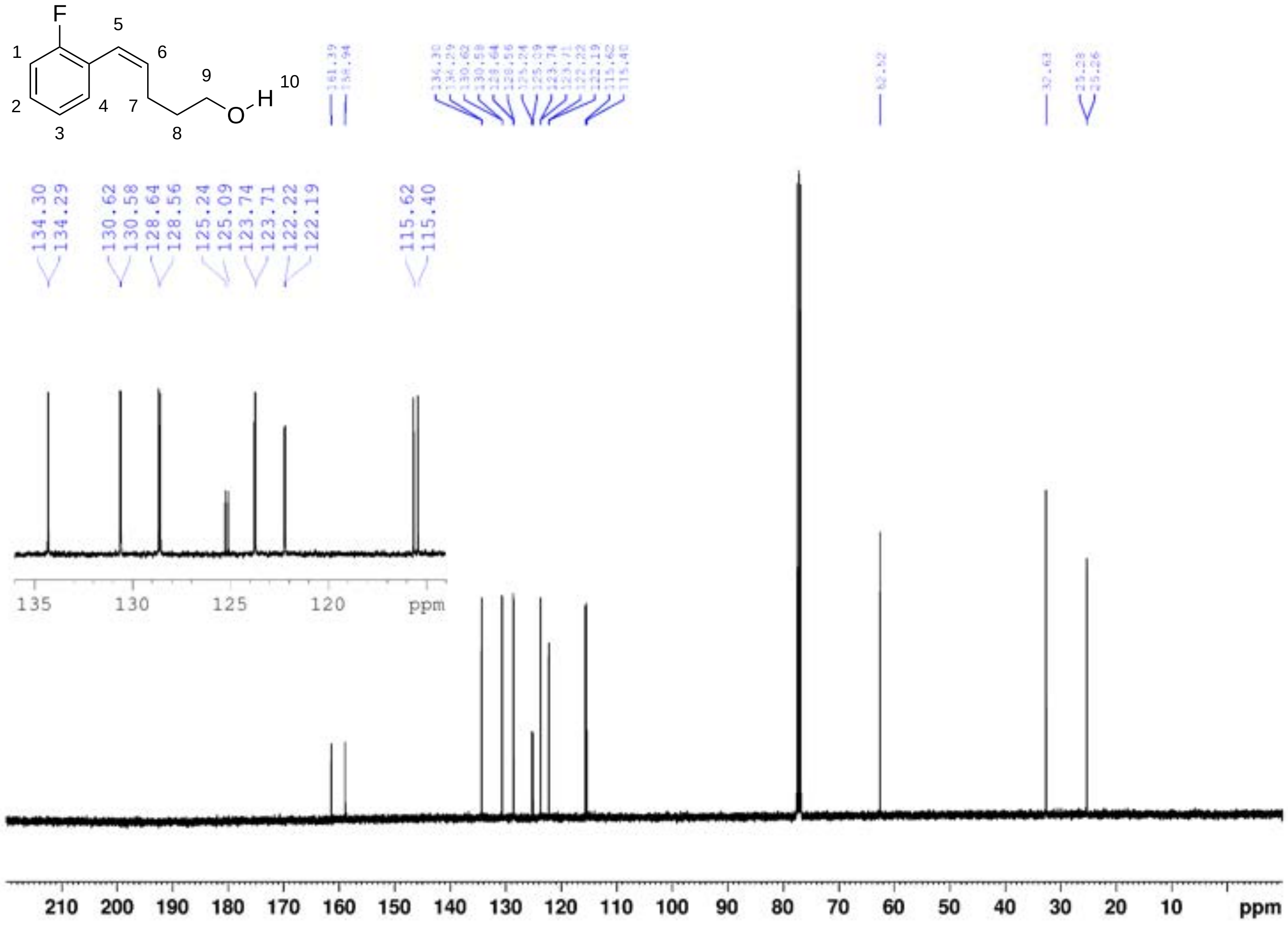

^{19}F NMR (376 MHz, $CDCl_3$) for *(Z)-5-(2-fluorophenyl)pent-4-en-1-ol* (**1b**)

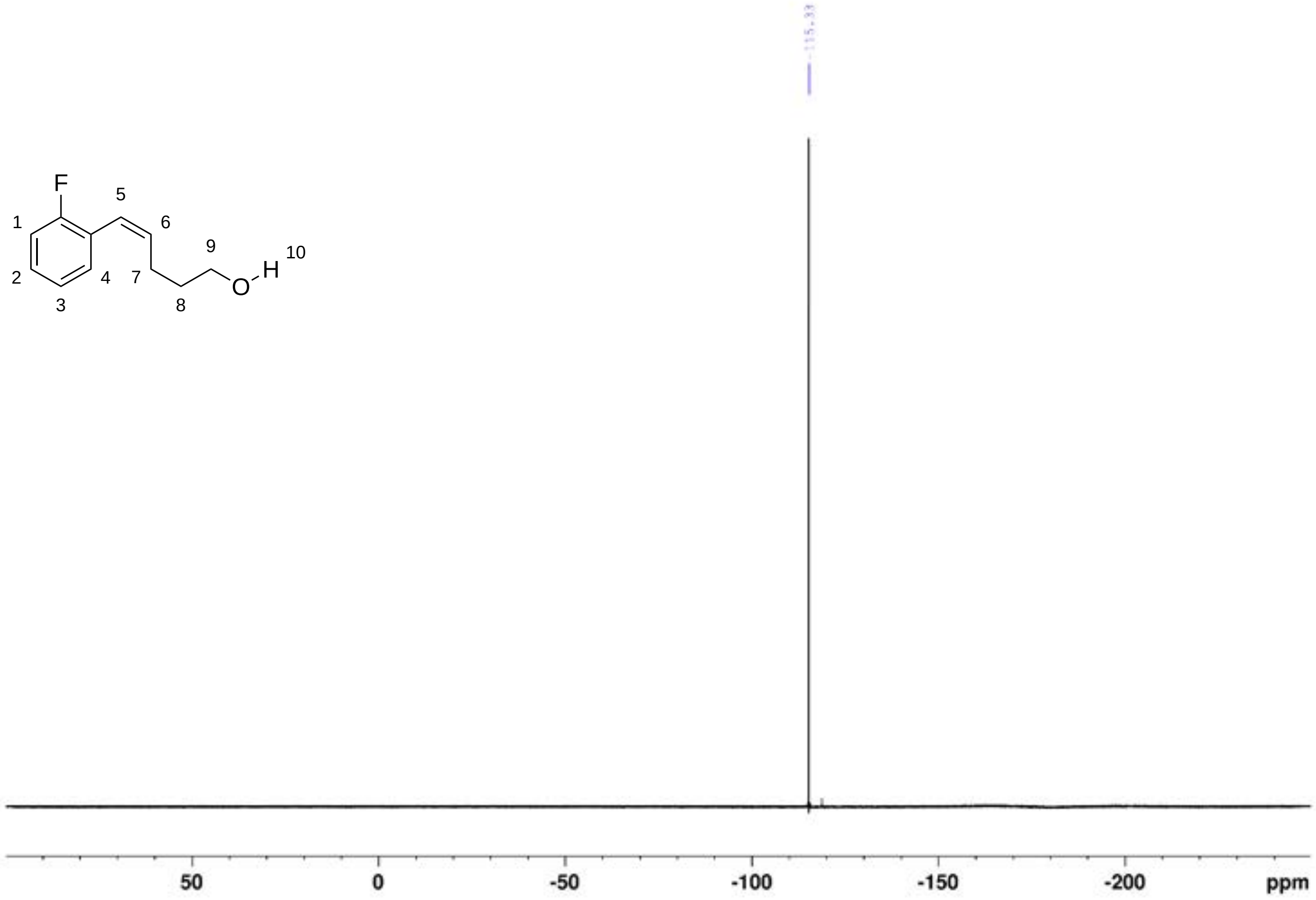

^{1}H NMR (400 MHz, $CDCl_3$) for *(Z)-5-(o-tolyl)pent-4-en-1-ol* (**1c**)

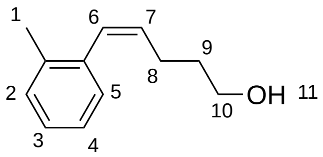

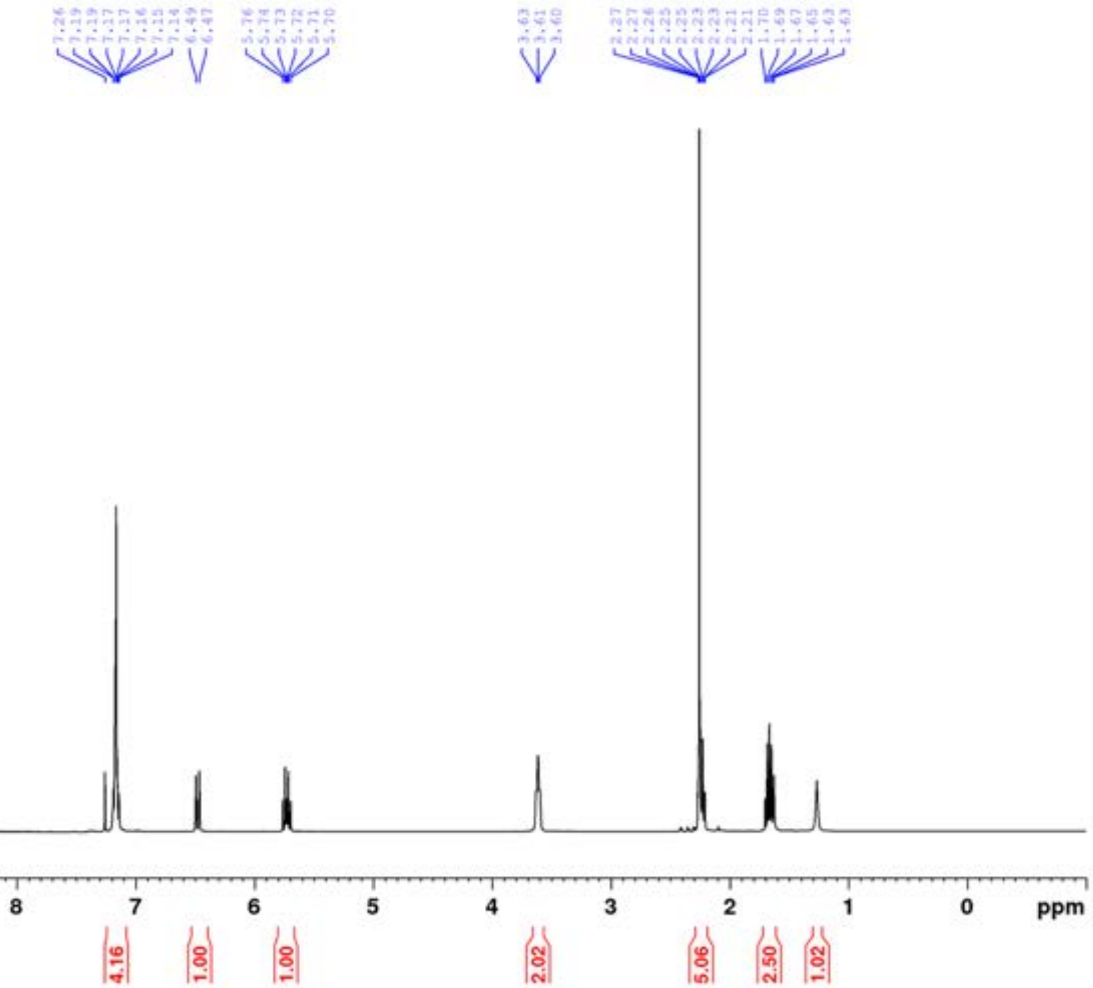

^{13}C NMR (101 MHz, $CDCl_3$) for *(Z)-5-(o-tolyl)pent-4-en-1-ol* (**1c**)

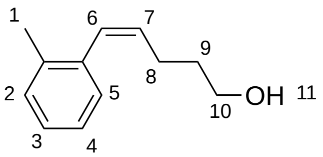

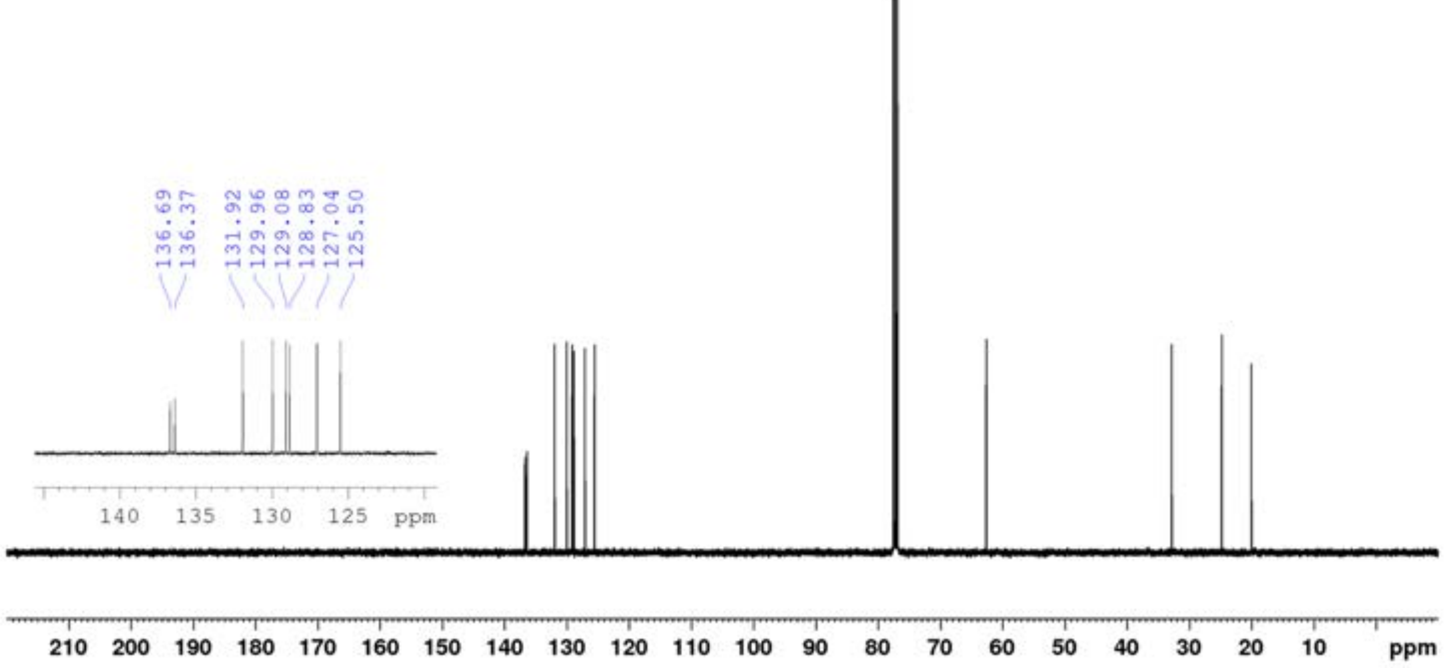

^{1}H NMR (400 MHz, $CDCl_3$) for *(Z)-5-(2-methoxyphenyl)pent-4-en-1-ol* (**1d**)

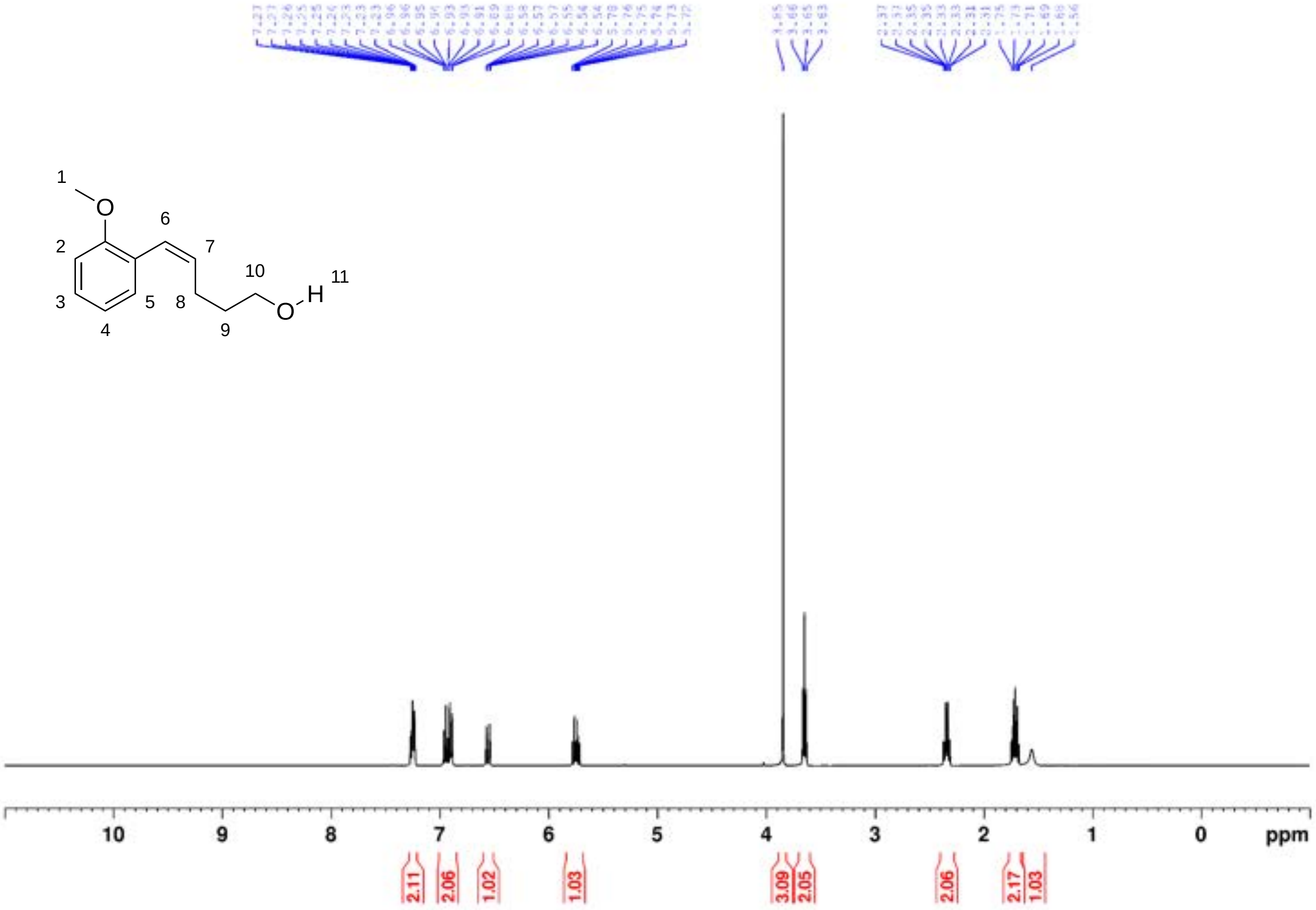

^{13}C NMR (101 MHz, $CDCl_3$) for *(Z)-5-(2-methoxyphenyl)pent-4-en-1-ol* (**1d**)

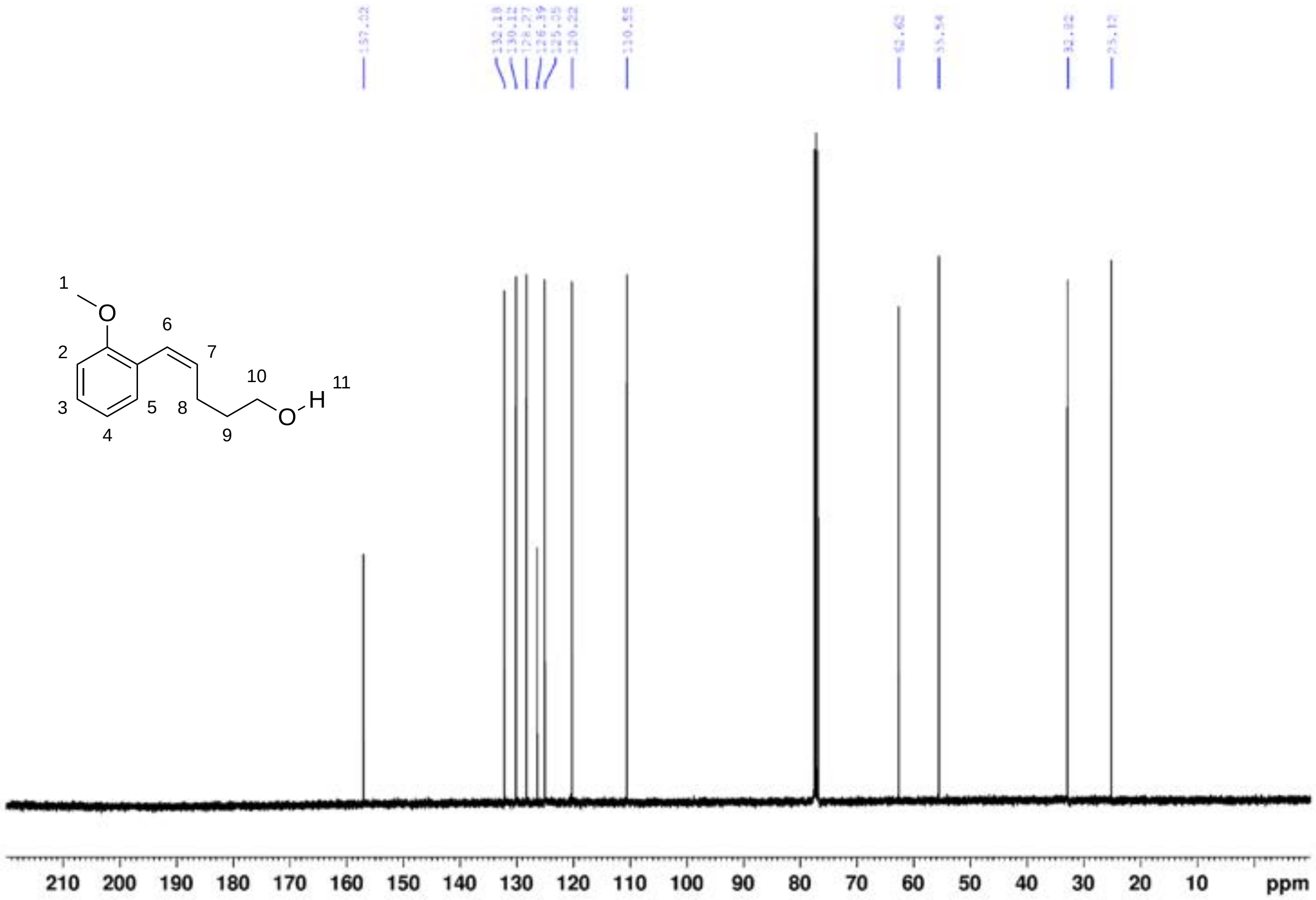

^{1}H NMR (400 MHz, $CDCl_3$) for *(Z)-5-(3-fluoro-2-methylphenyl)pent-4-en-1-ol* (**1e**)

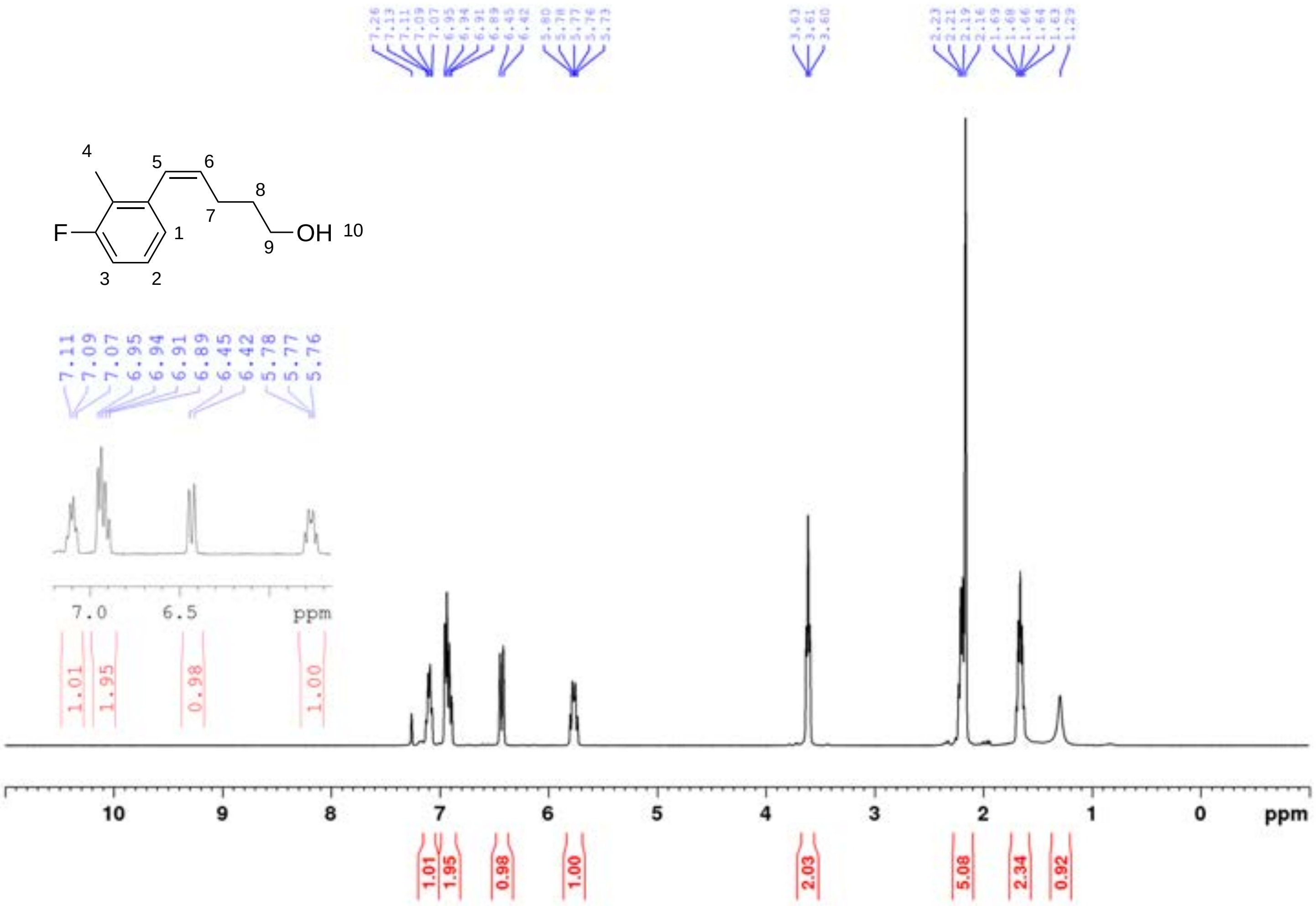

^{13}C NMR (101 MHz, $CDCl_3$) for *(Z)-5-(3-fluoro-2-methylphenyl)pent-4-en-1-ol* (**1e**)

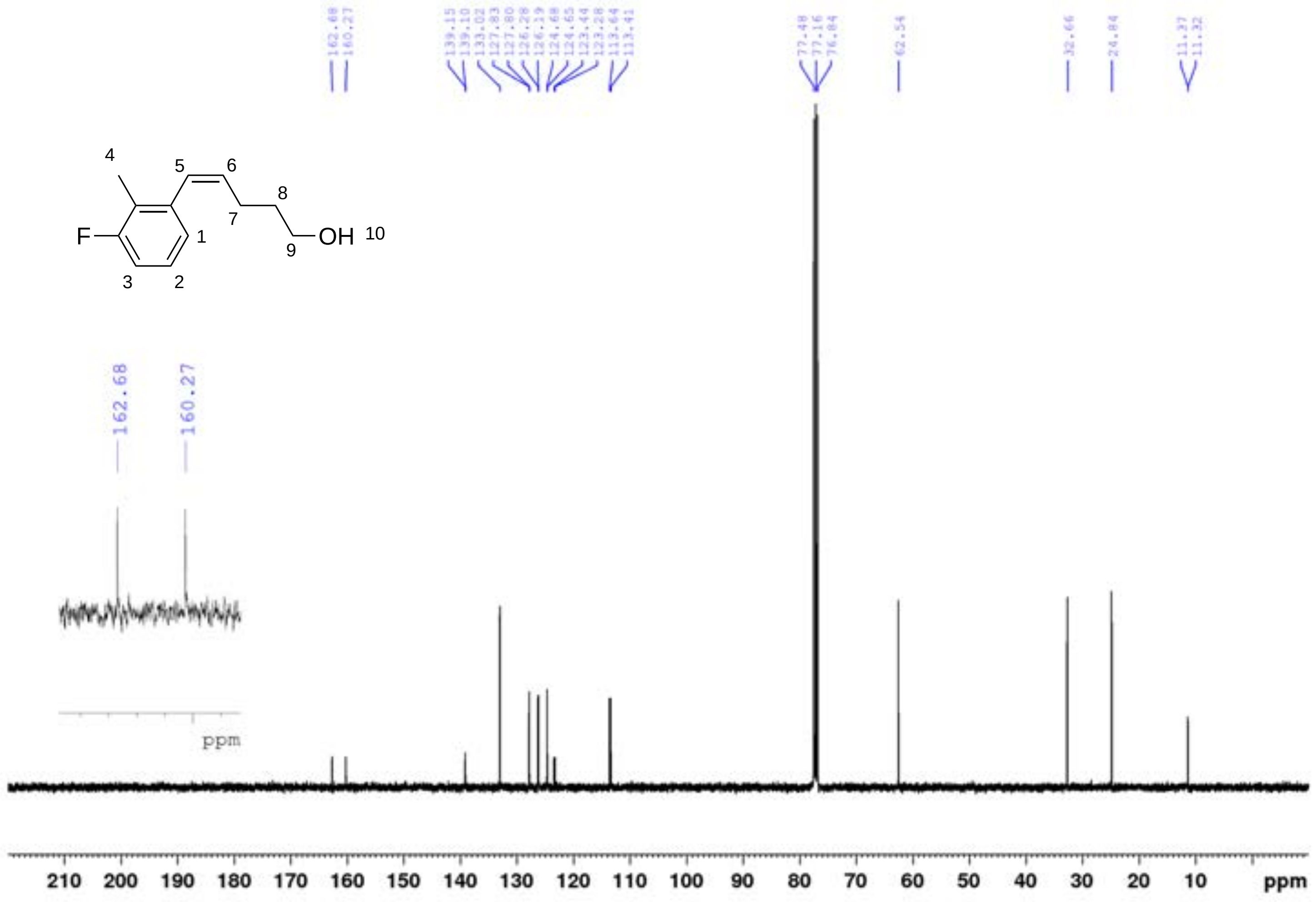

^{19}F NMR (471 MHz, $CDCl_3$) for *(Z)-5-(3-fluoro-2-methylphenyl)pent-4-en-1-ol* (**1e**)

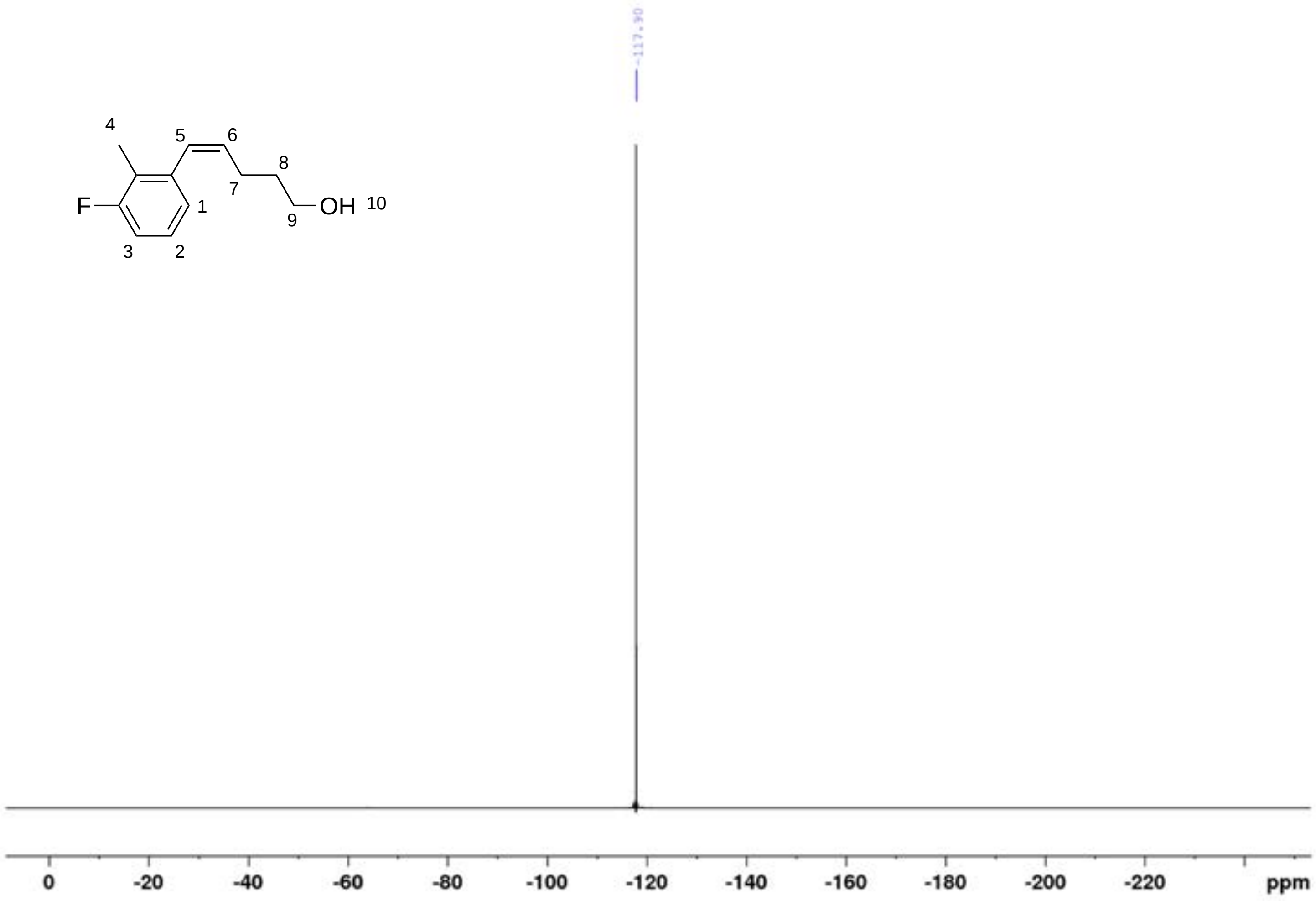

^{1}H NMR (400 MHz, $CDCl_3$) for *(Z)-5-(2,5-dimethylphenyl)pent-4-en-1-ol* (**1f**)

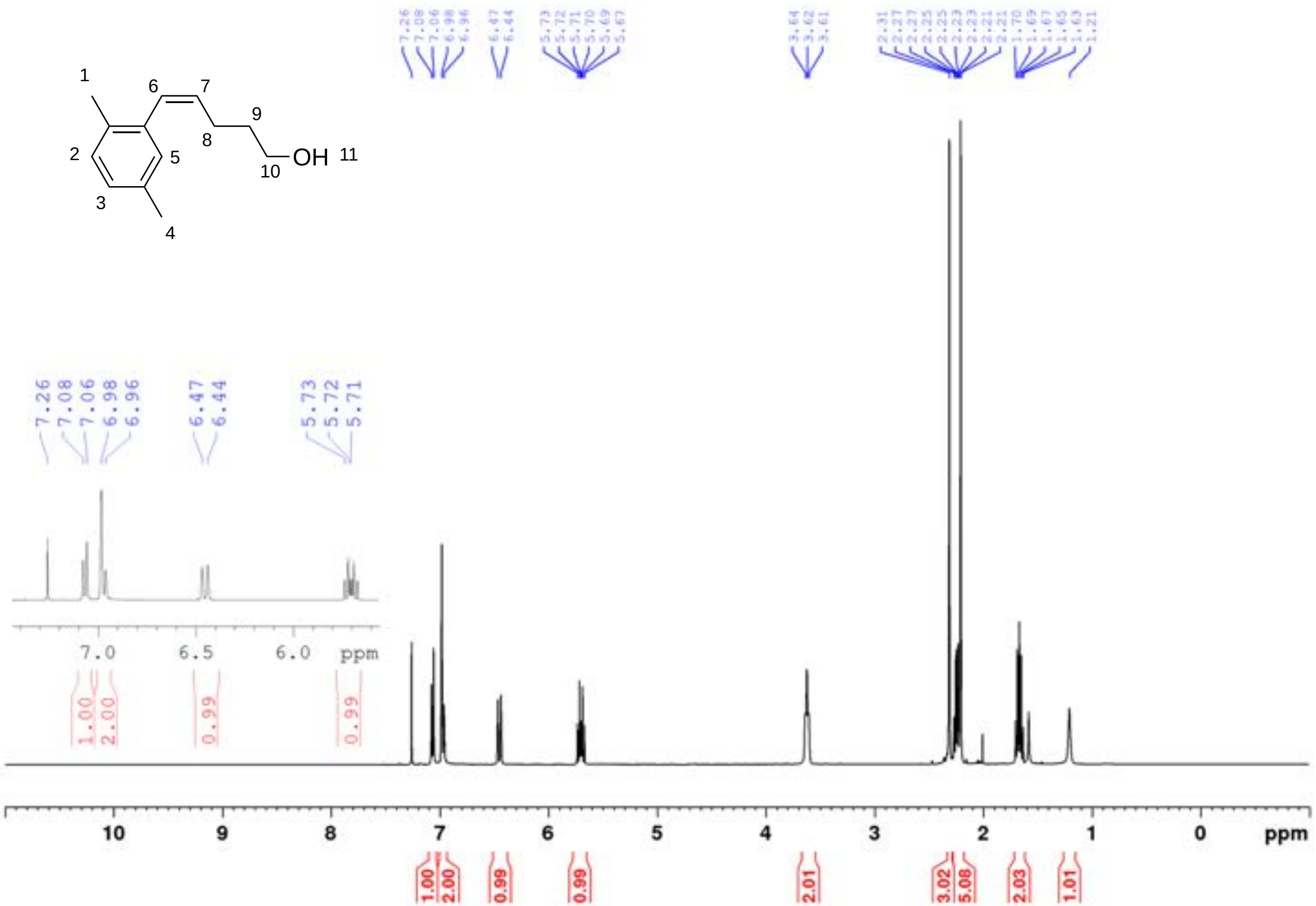

^{13}C NMR (101 MHz, $CDCl_3$) for *(Z)-5-(2,5-dimethylphenyl)pent-4-en-1-ol* (**1f**)

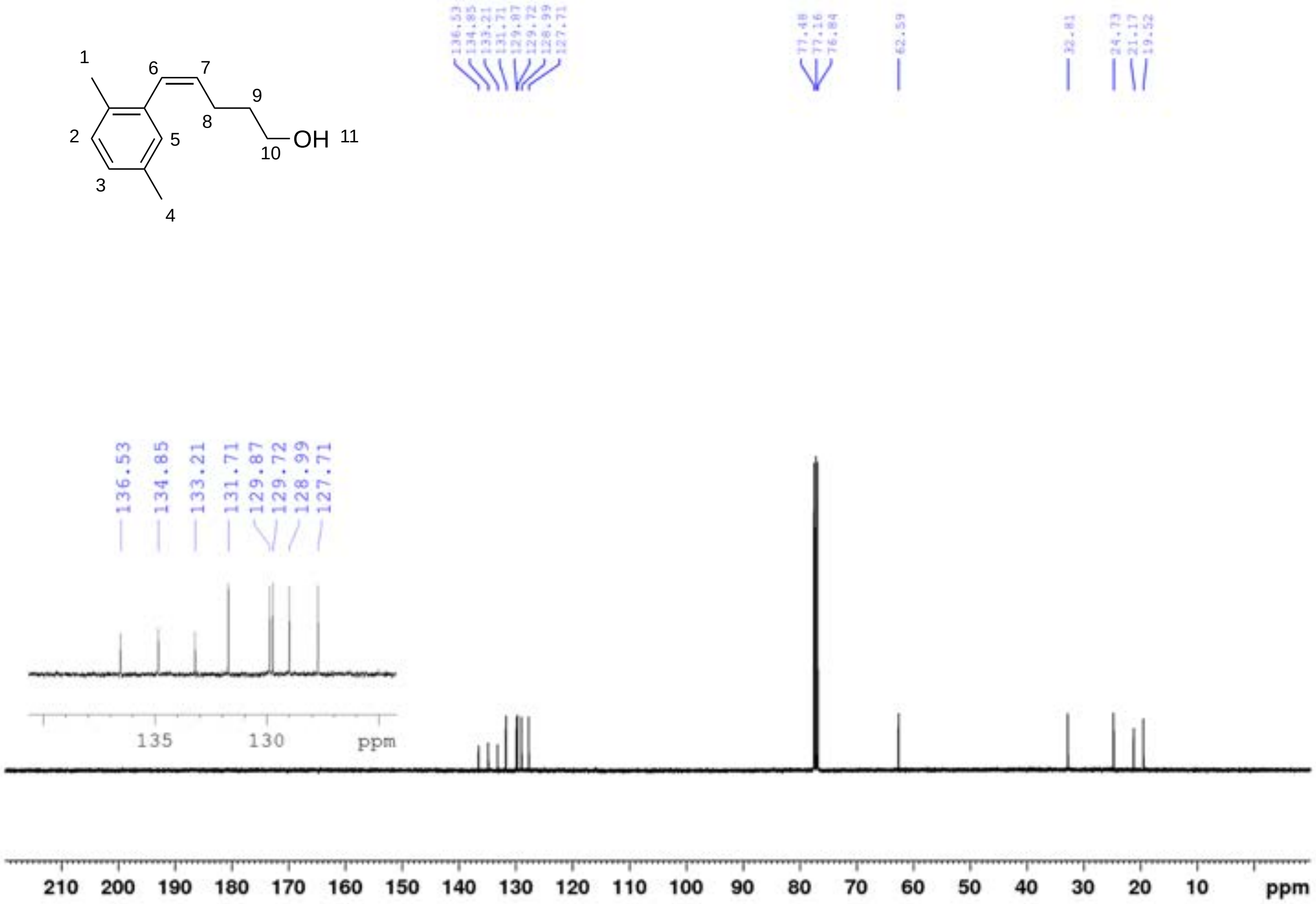

^{1}H NMR (400 MHz, $CDCl_3$) for *(Z)-5-(2,3-dimethylphenyl)pent-4-en-1-ol* (**1g**)

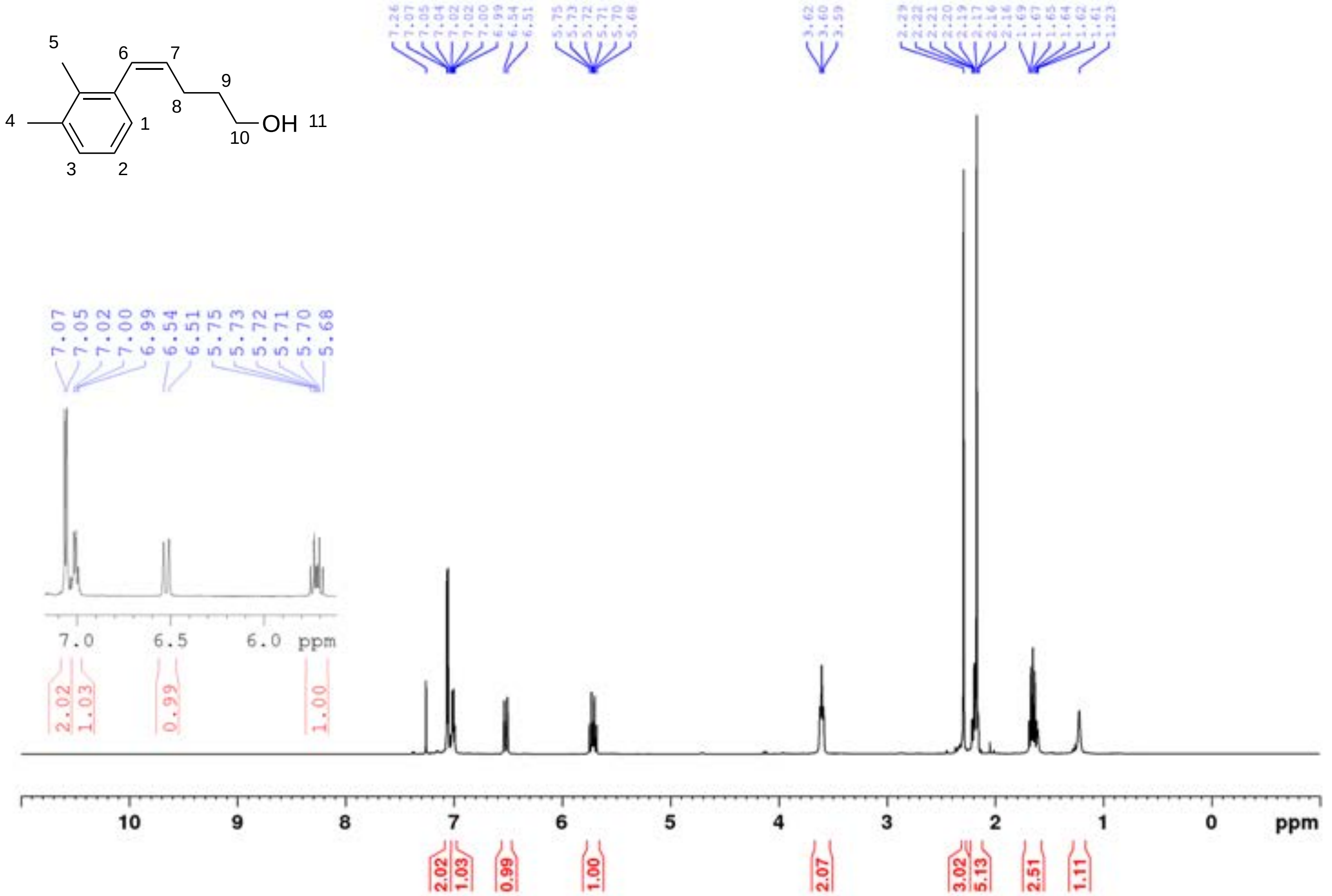

^{13}C NMR (101 MHz, $CDCl_3$) for *(Z)-5-(2,3-dimethylphenyl)pent-4-en-1-ol* (**1g**)

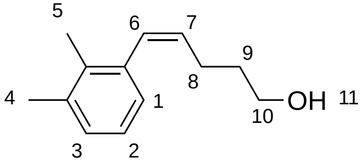

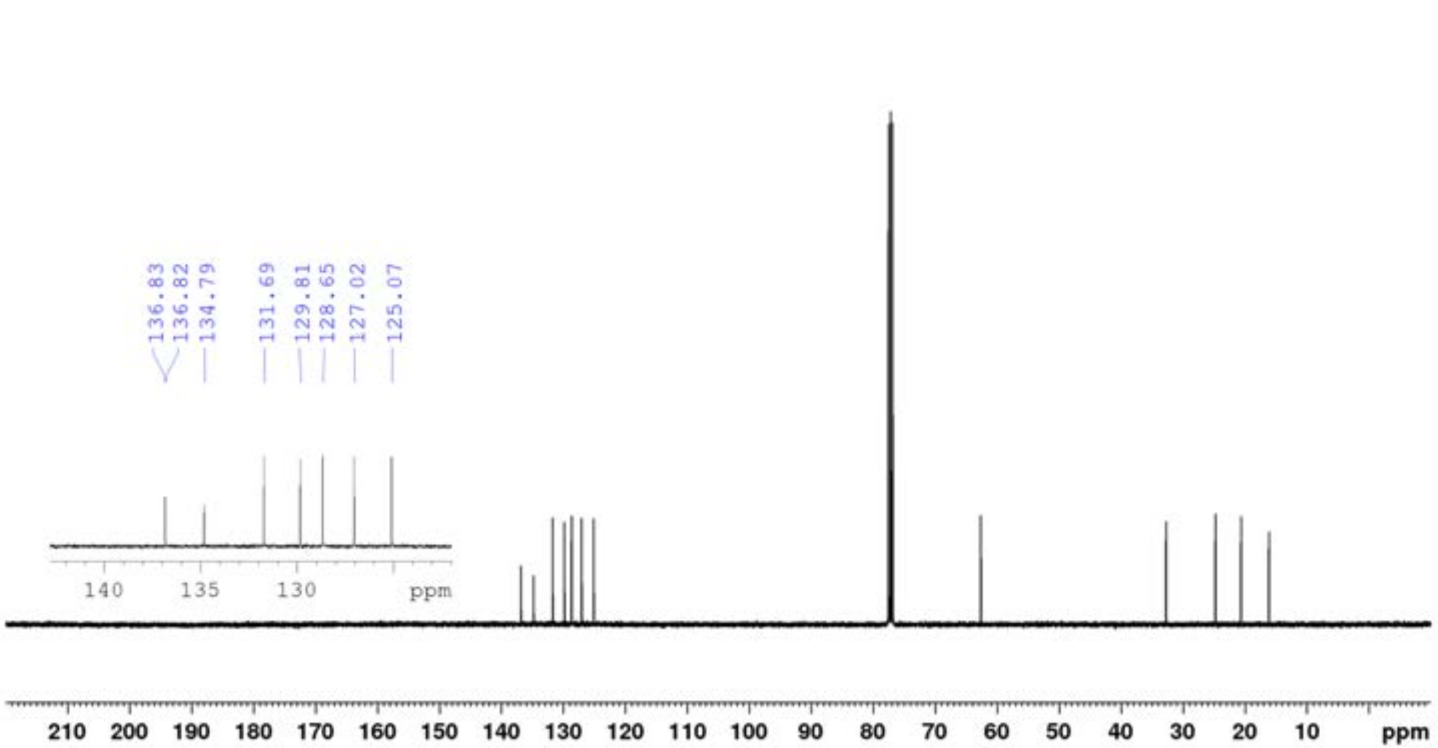

¹H NMR (400 MHz, $CDCl_3$) for *(Z)-5-(3-fluorophenyl)pent-4-en-1-ol* (**1h**)

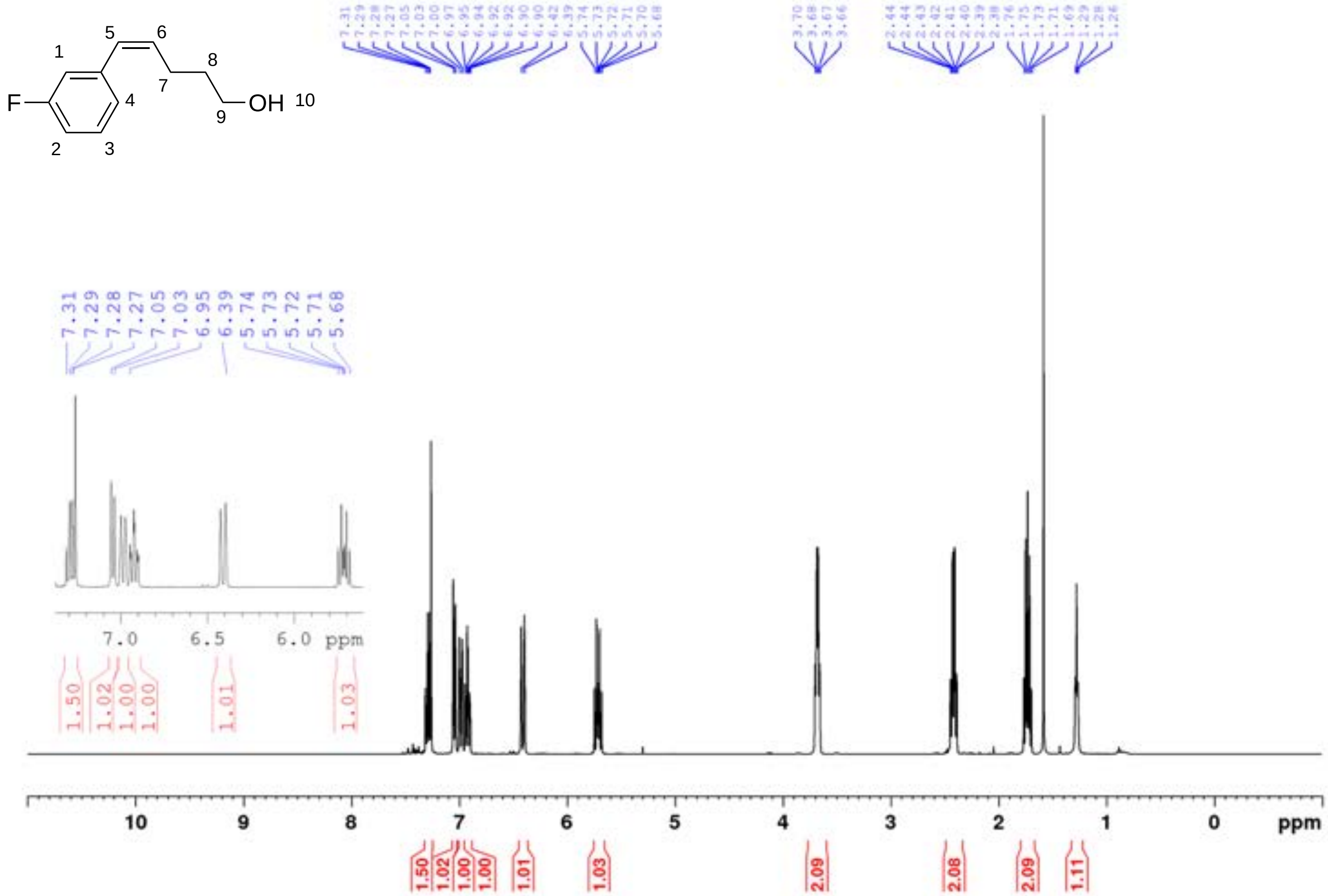

^{13}C NMR (101 MHz, $CDCl_3$) for *(Z)-5-(3-fluorophenyl)pent-4-en-1-ol* (**1h**)

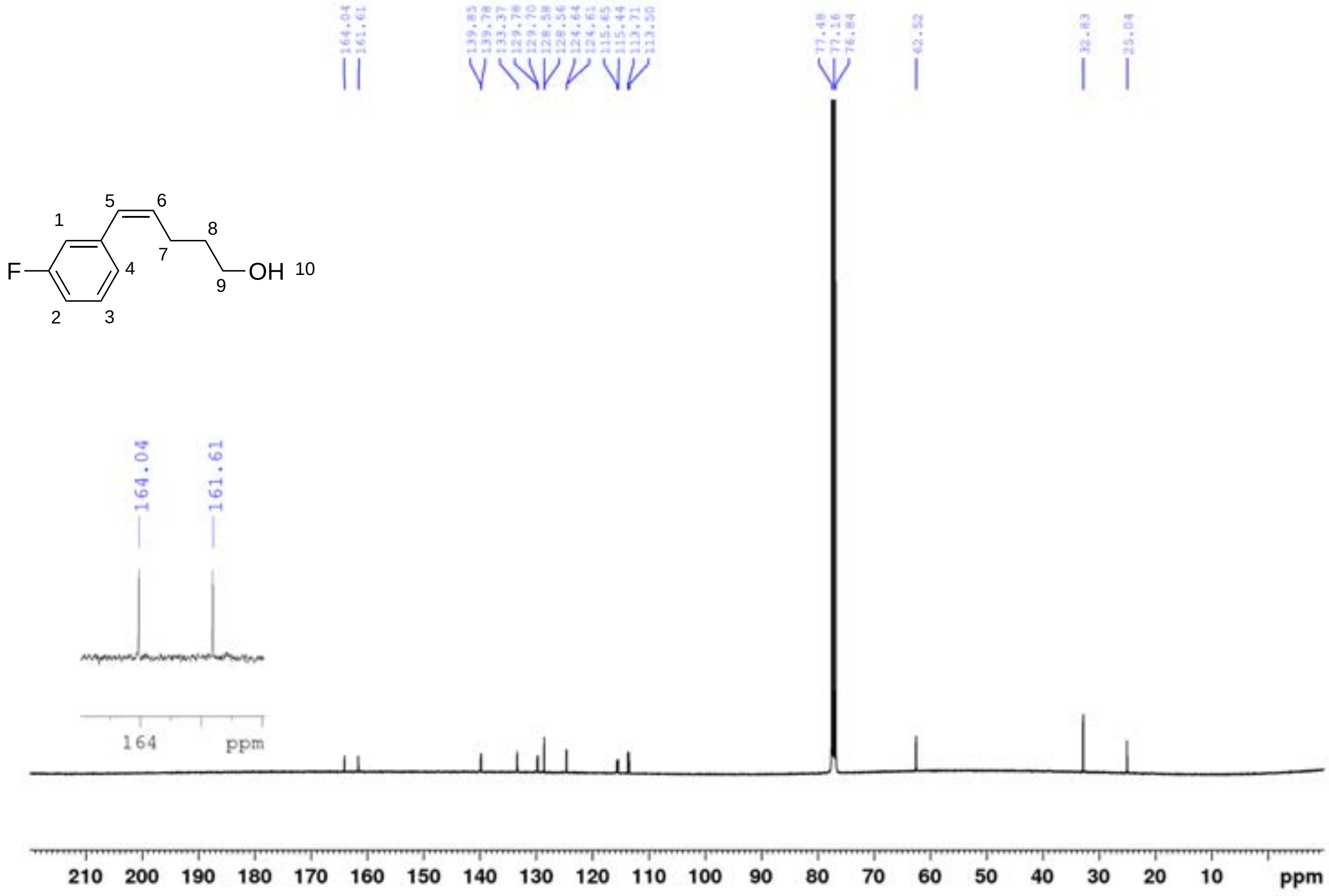

^{19}F NMR (376 MHz, $CDCl_3$) for *(Z)-5-(3-fluorophenyl)pent-4-en-1-ol* (**1h**)

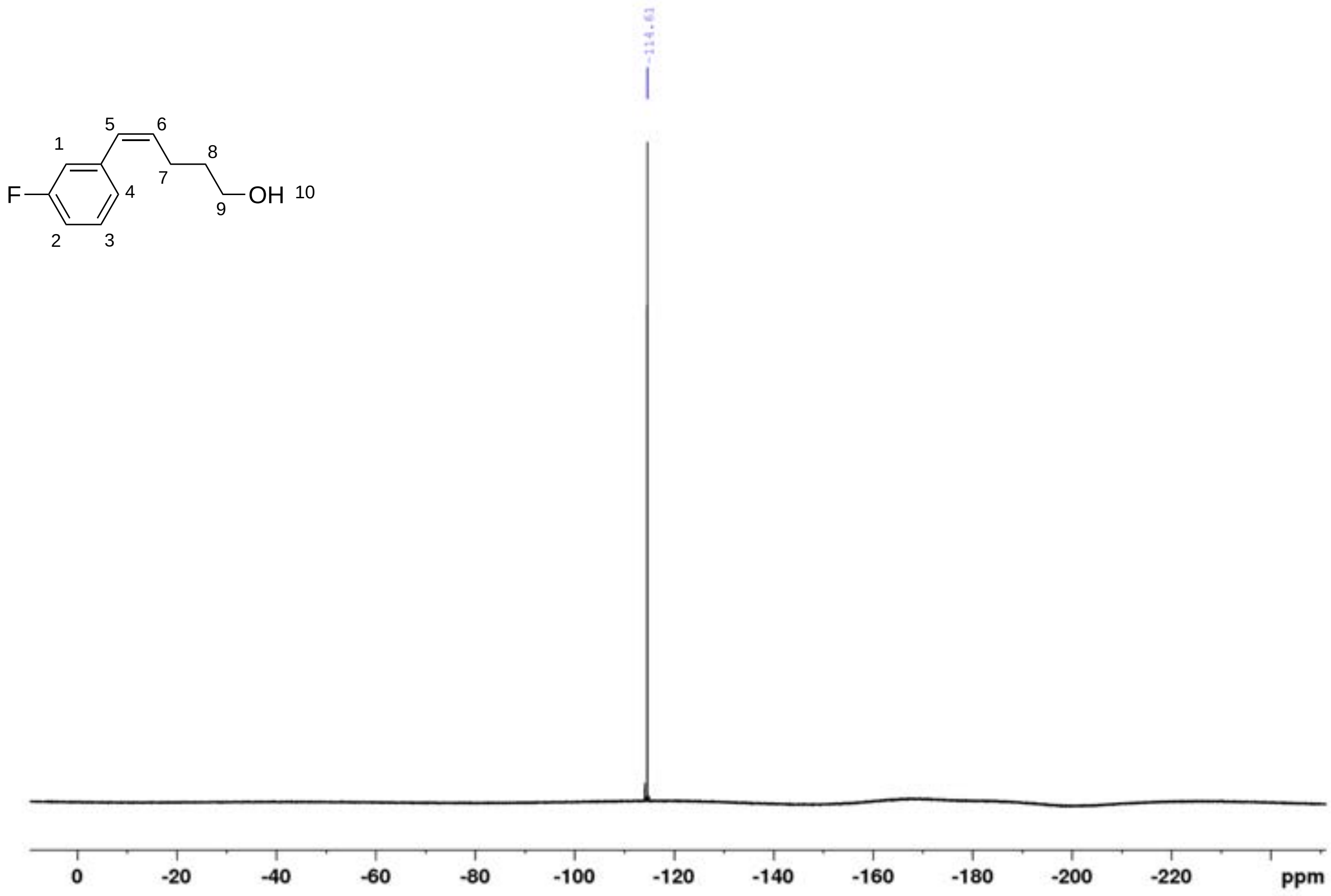

¹H NMR (400 MHz, $CDCl_3$) for *(Z)-5-(3-chlorophenyl)pent-4-en-1-ol* (**1i**)

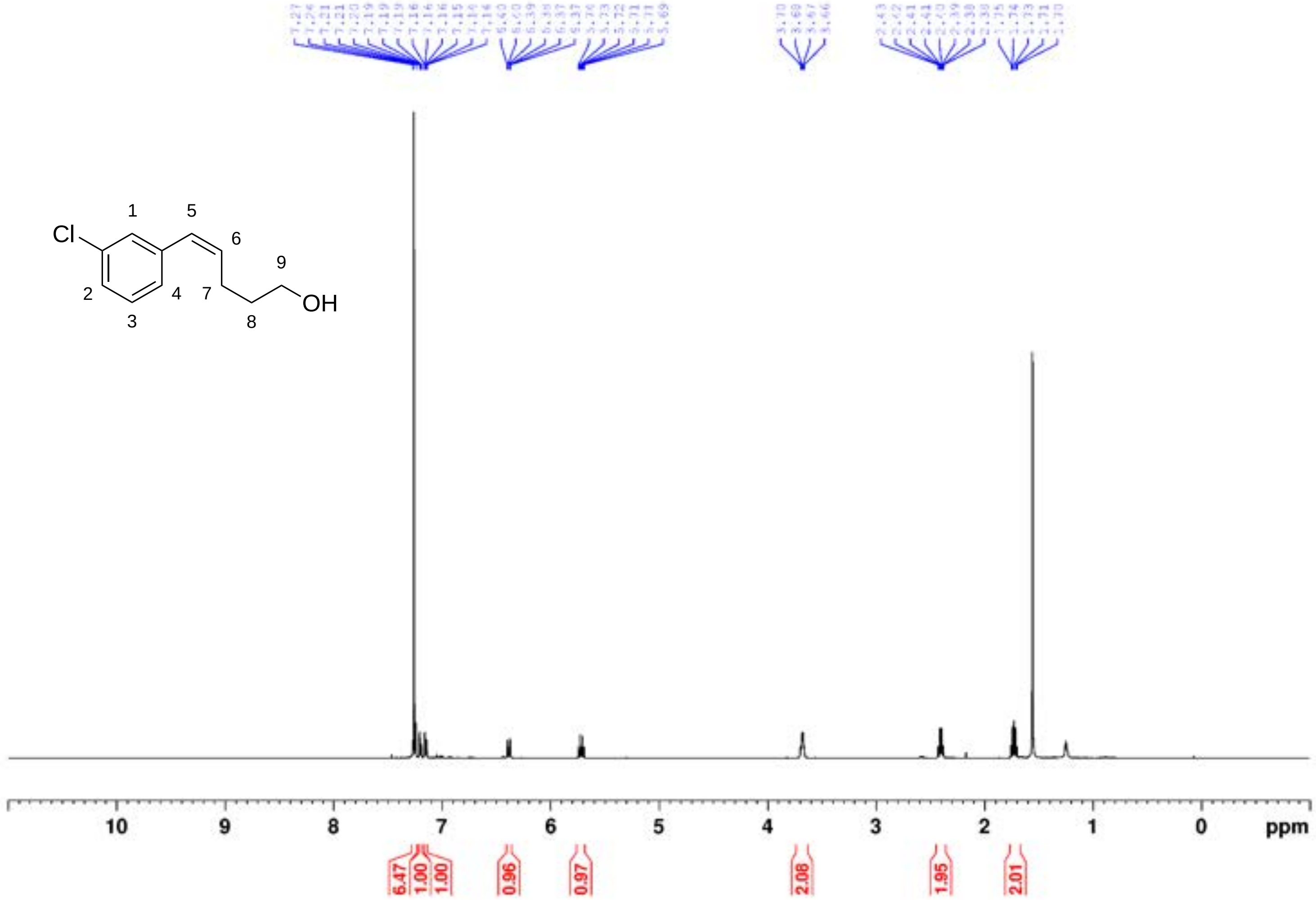

^{13}C NMR (101 MHz, $CDCl_3$) for *(Z)-5-(3-chlorophenyl)pent-4-en-1-ol* (**1i**)

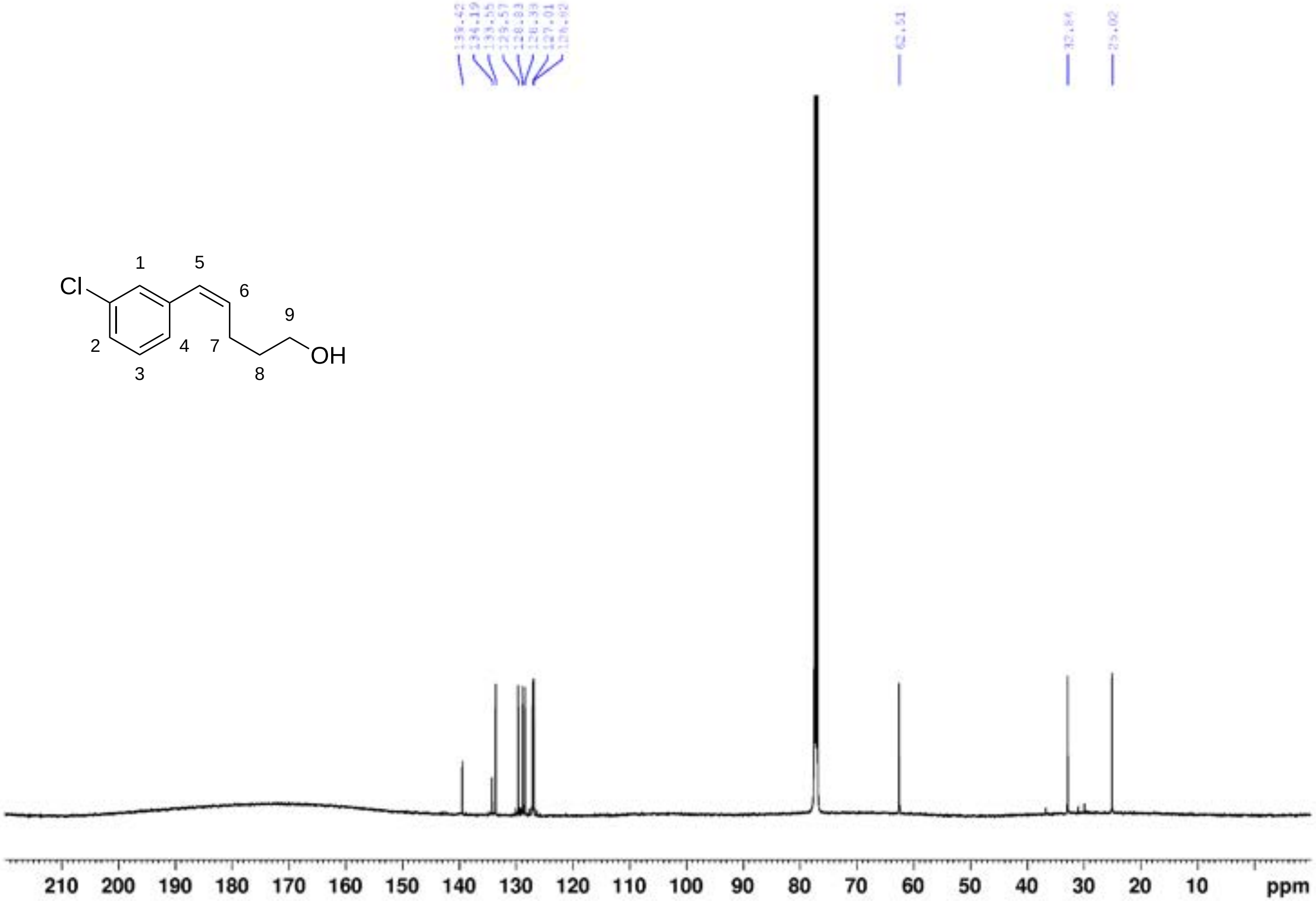

^{1}H NMR (400 MHz, $CDCl_3$) for *(Z)-5-(m-tolyl)pent-4-en-1-ol* (**1j**)

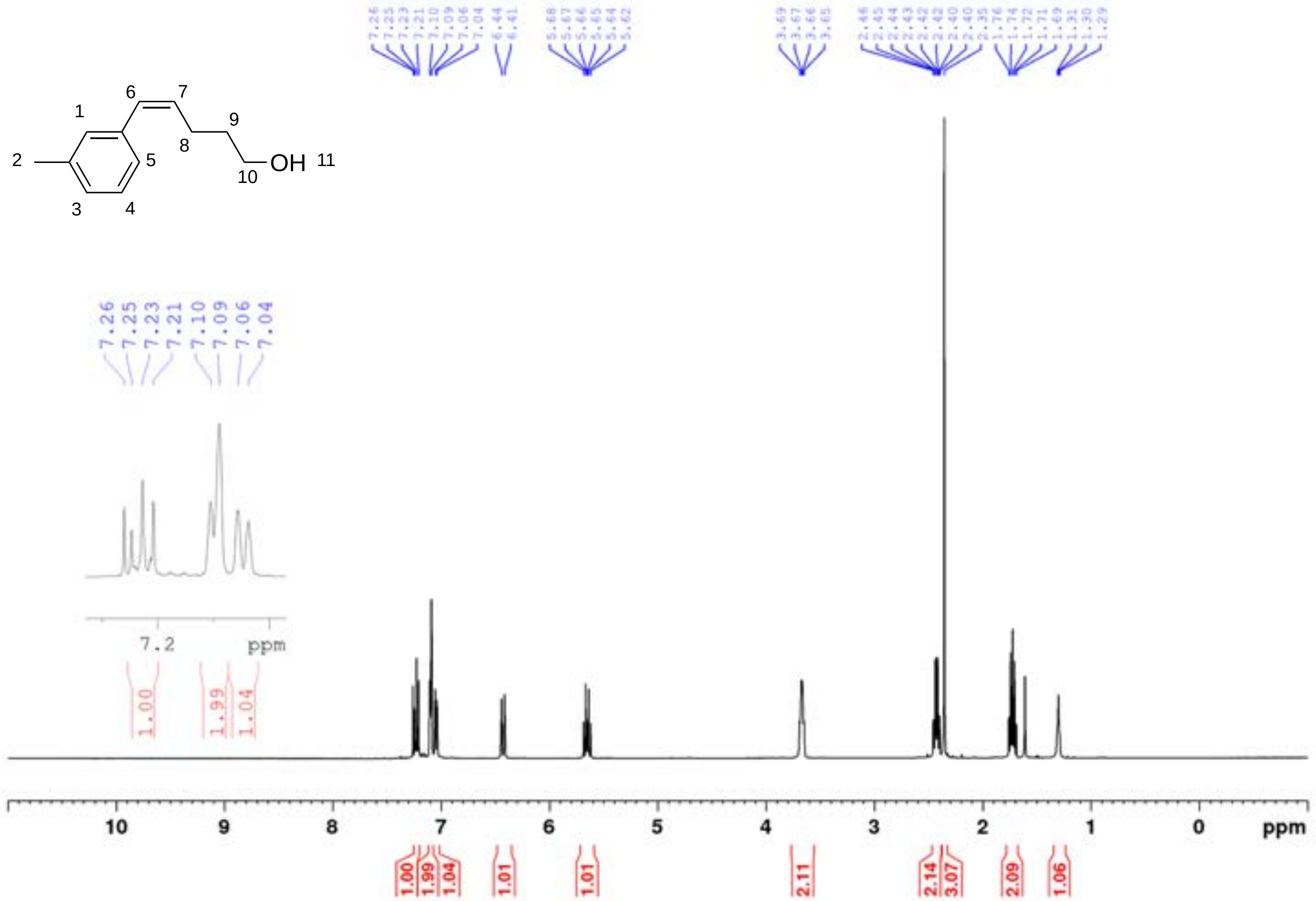

^{13}C NMR (101 MHz, $CDCl_3$) for *(Z)-5-(m-tolyl)pent-4-en-1-ol* (**1j**)

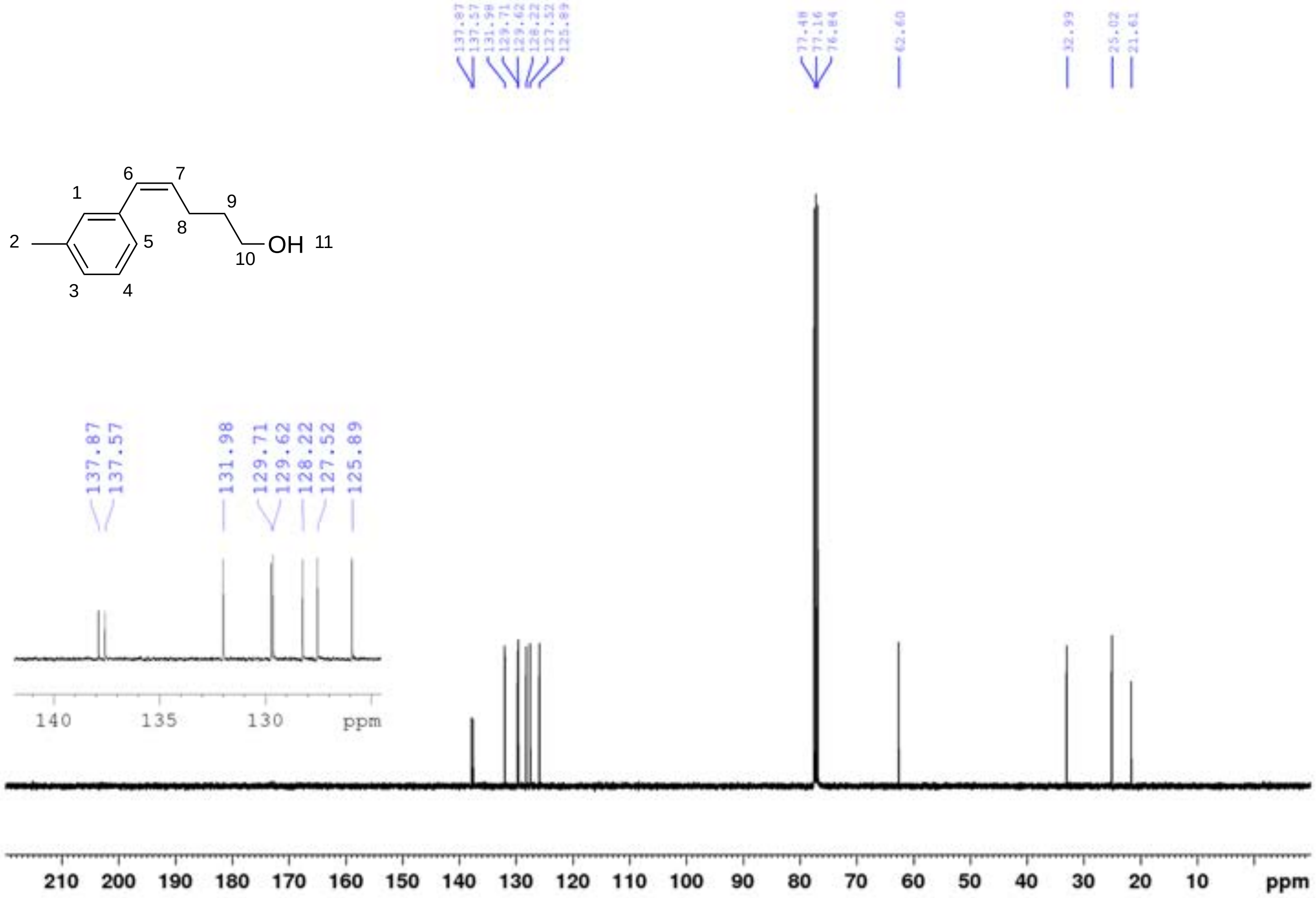

^{1}H NMR (400 MHz, $CDCl_3$) for *(Z)-5-(3-(trifluoromethyl)phenyl)pent-4-en-1-ol* (**1k**)

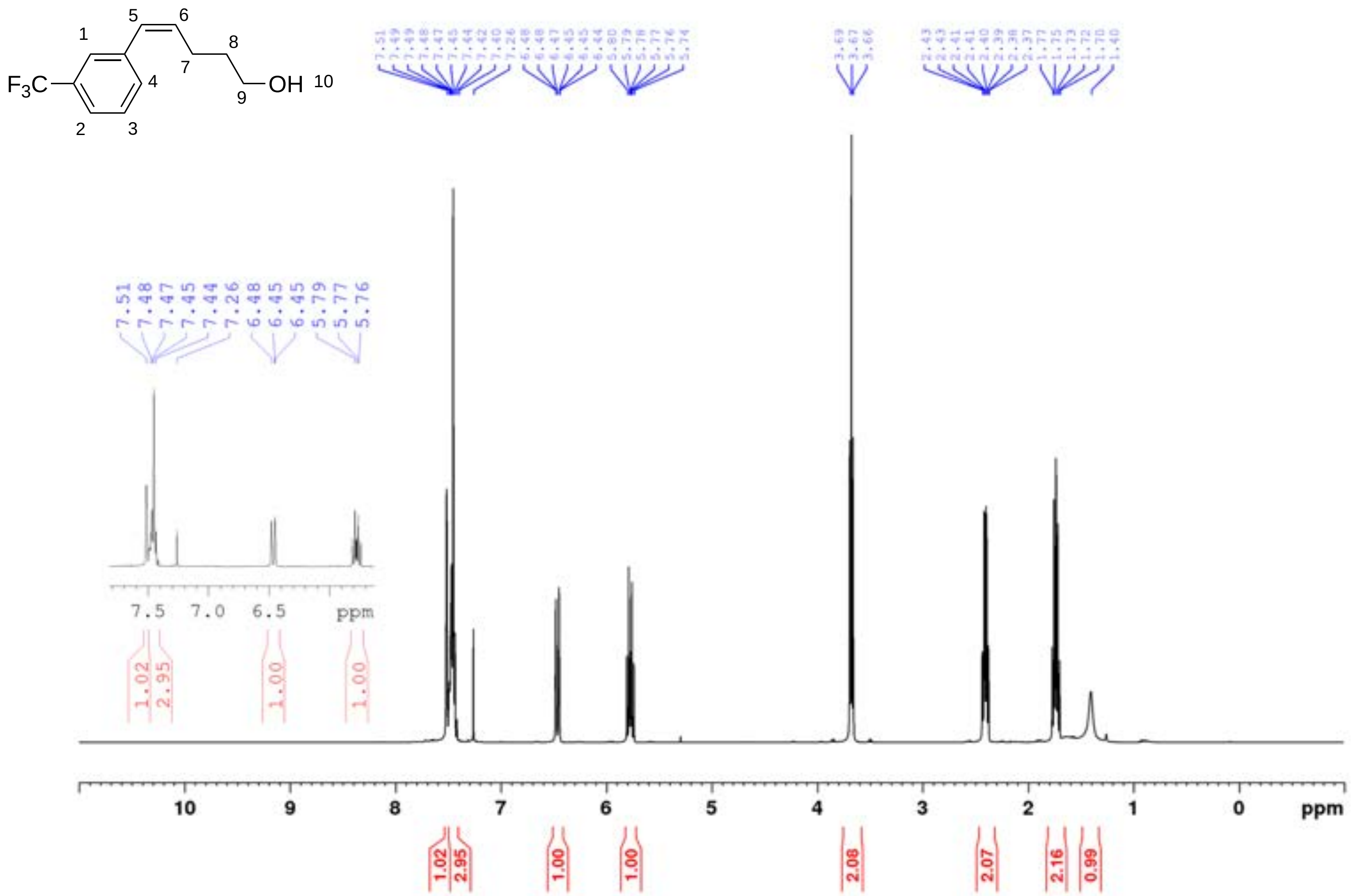

^{13}C NMR (101 MHz, $CDCl_3$) for *(Z)-5-(3-(trifluoromethyl)phenyl)pent-4-en-1-ol* (**1k**)

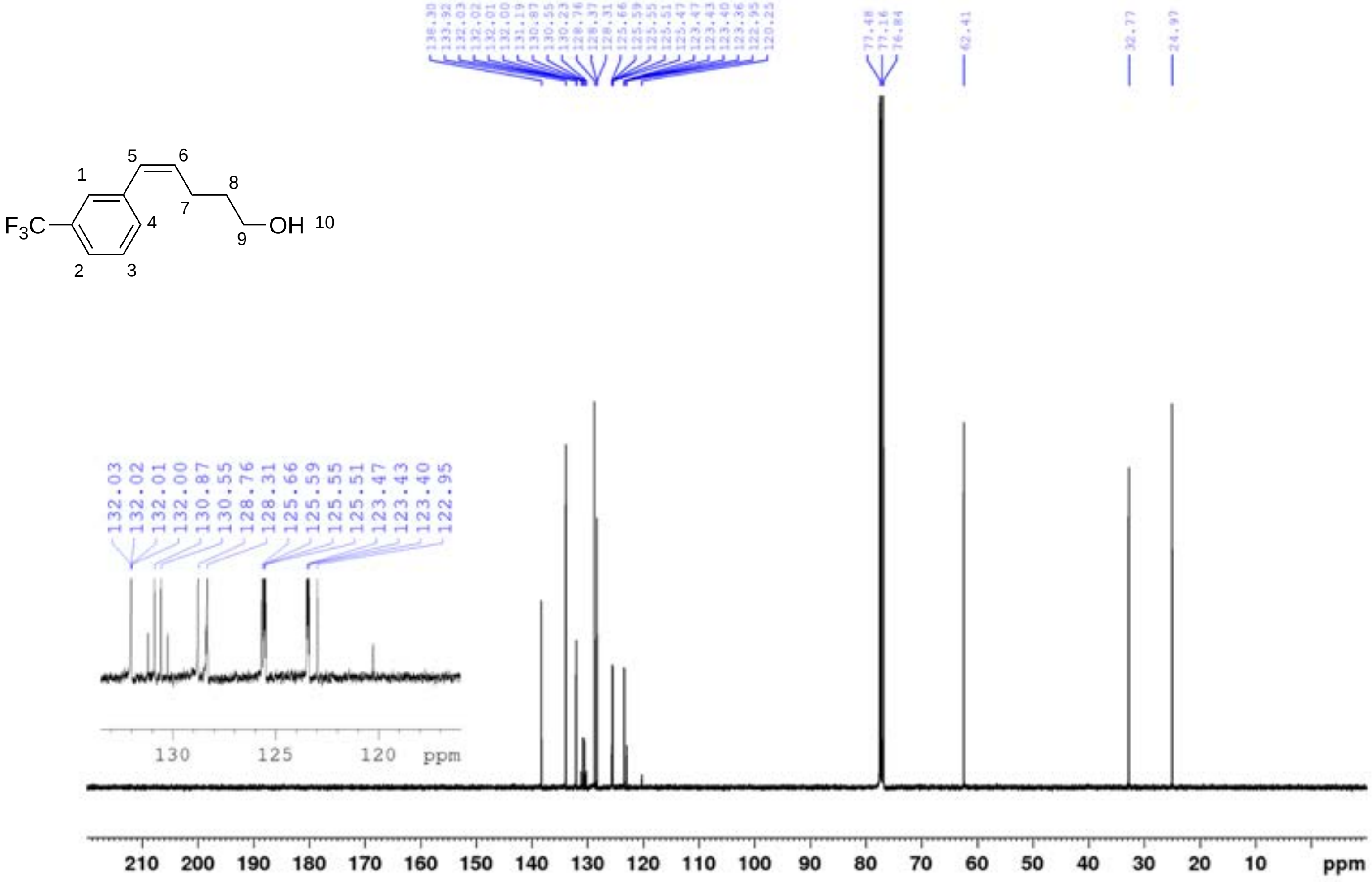

^{19}F NMR (376 MHz, $CDCl_3$) for *(Z)-5-(3-(trifluoromethyl)phenyl)pent-4-en-1-ol* (**1k**)

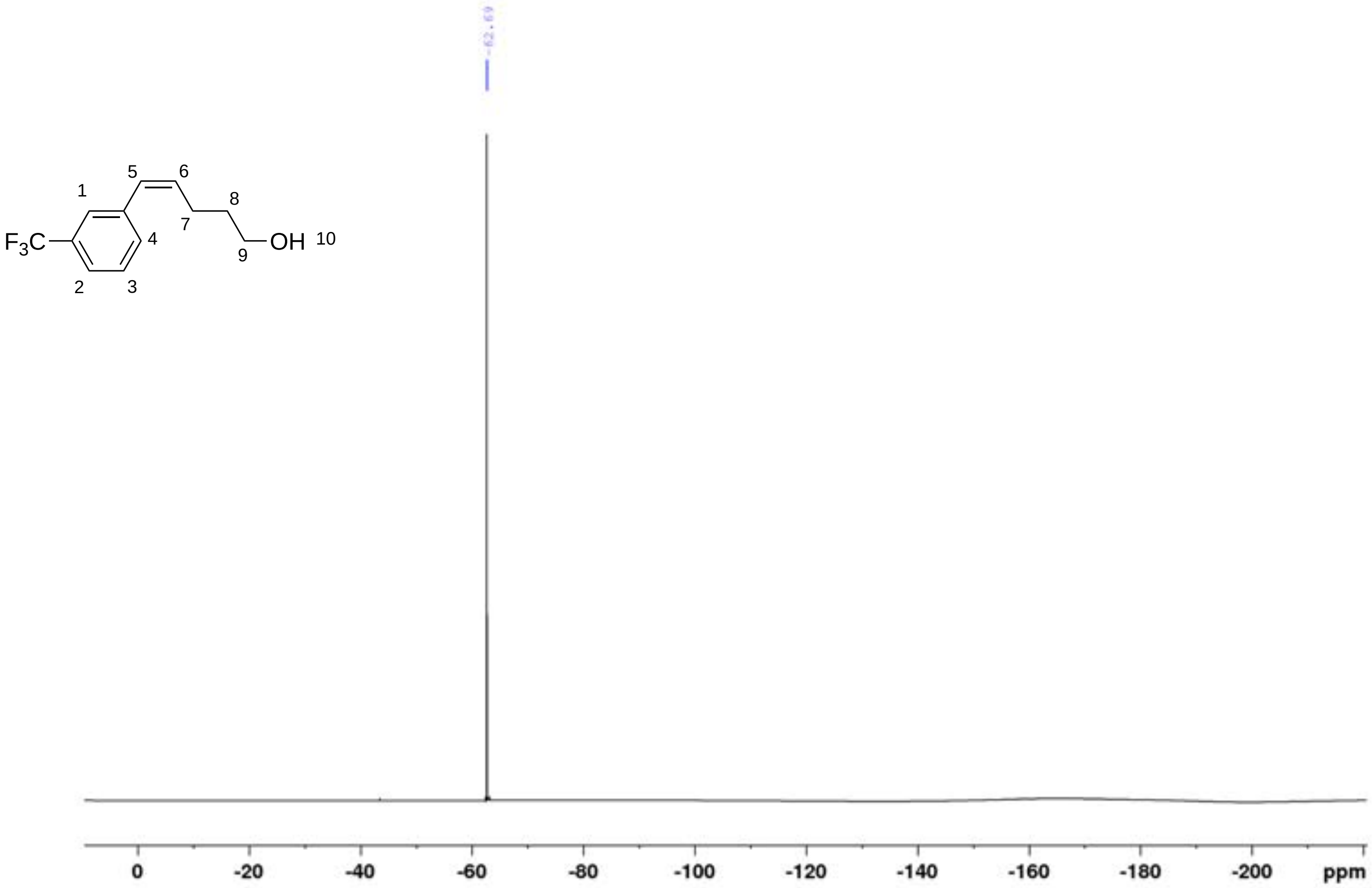

^{1}H NMR (400 MHz, $CDCl_3$) for *5-(3-(trifluoromethoxy)phenyl)pent-4-yn-1-ol*

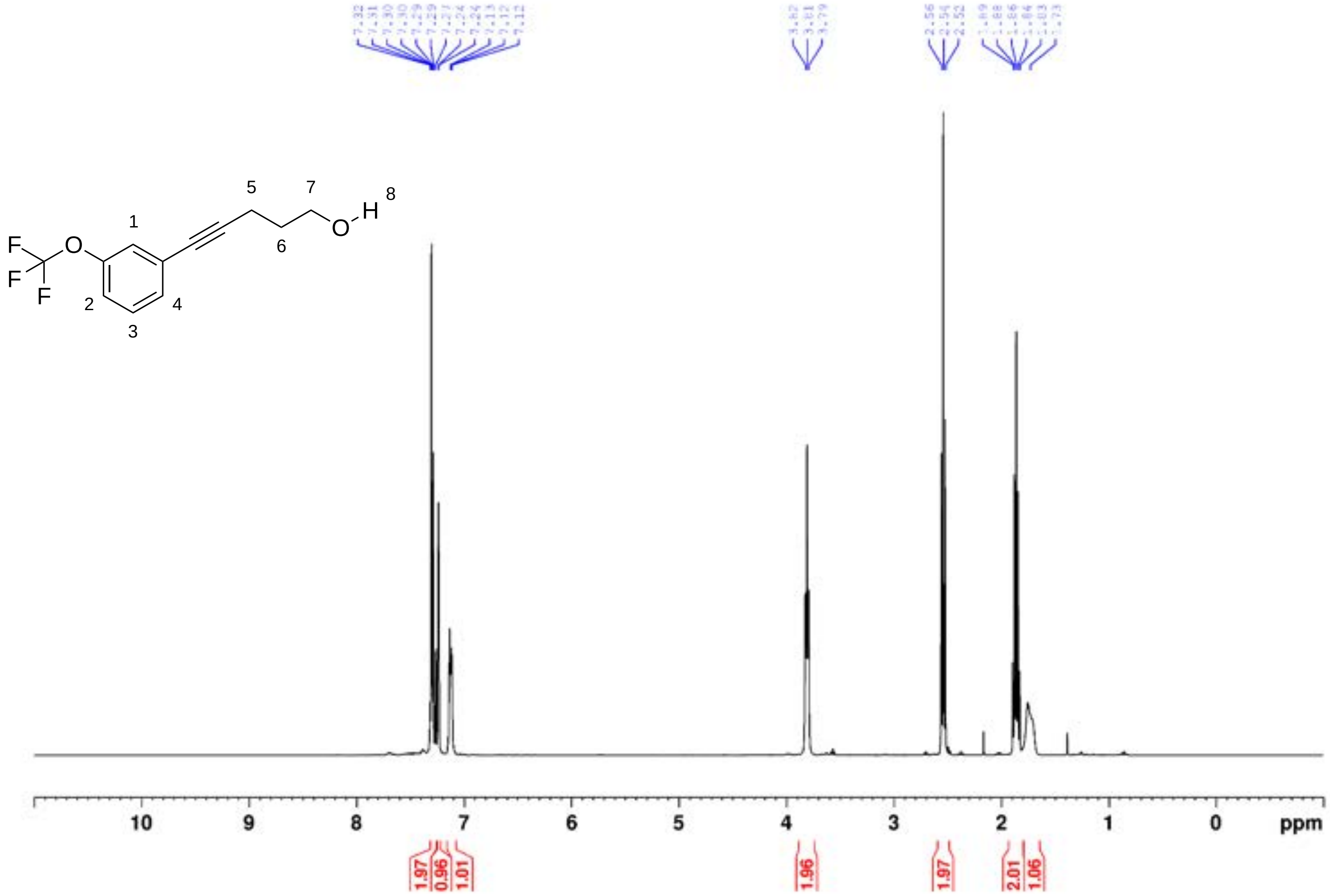

^{13}C NMR (101 MHz, $CDCl_3$) for *5-(3-(trifluoromethoxy)phenyl)pent-4-yn-1-ol*

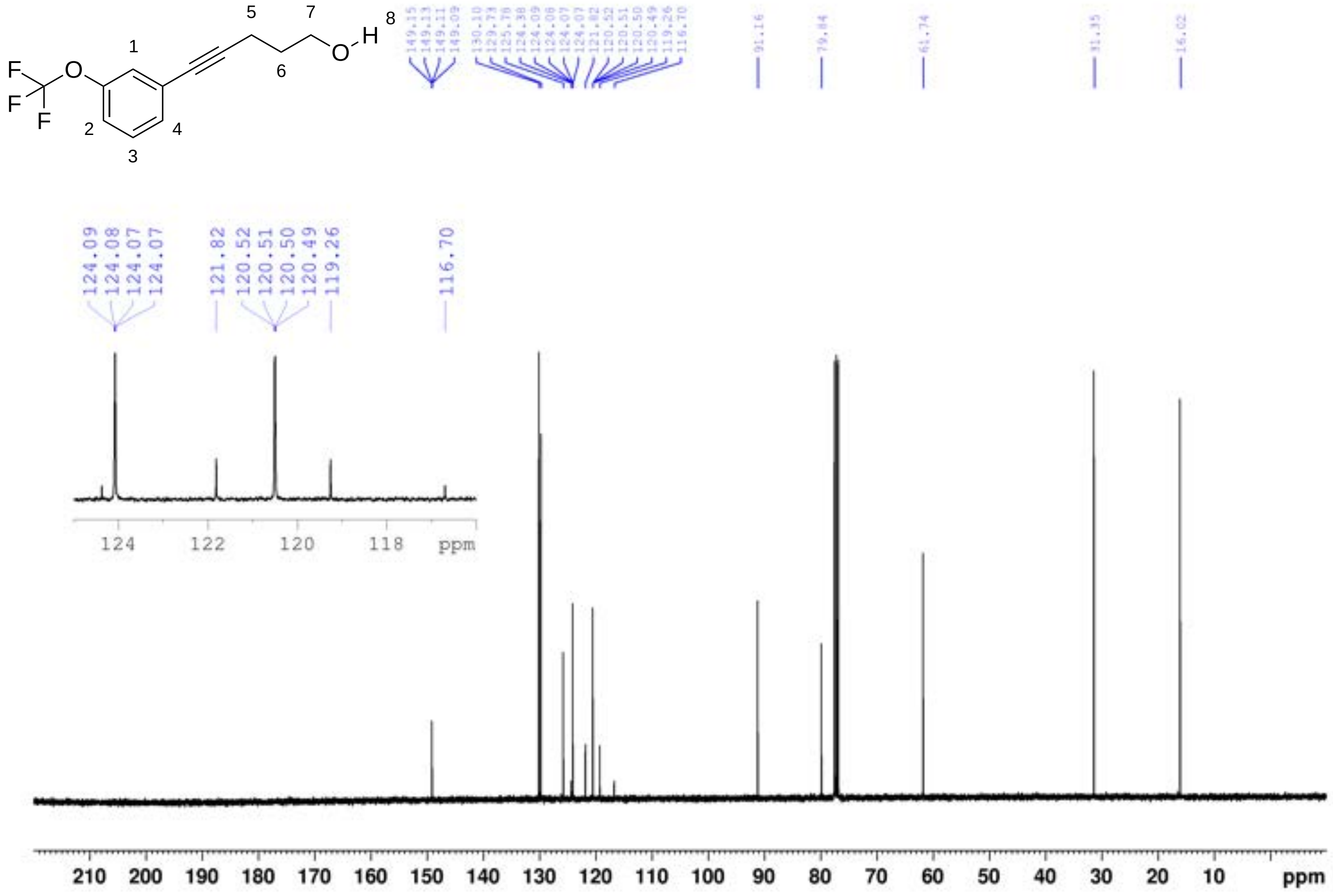

^{19}F NMR (471 MHz, $CDCl_3$) for *5-(3-(trifluoromethoxy)phenyl)pent-4-yn-1-ol*

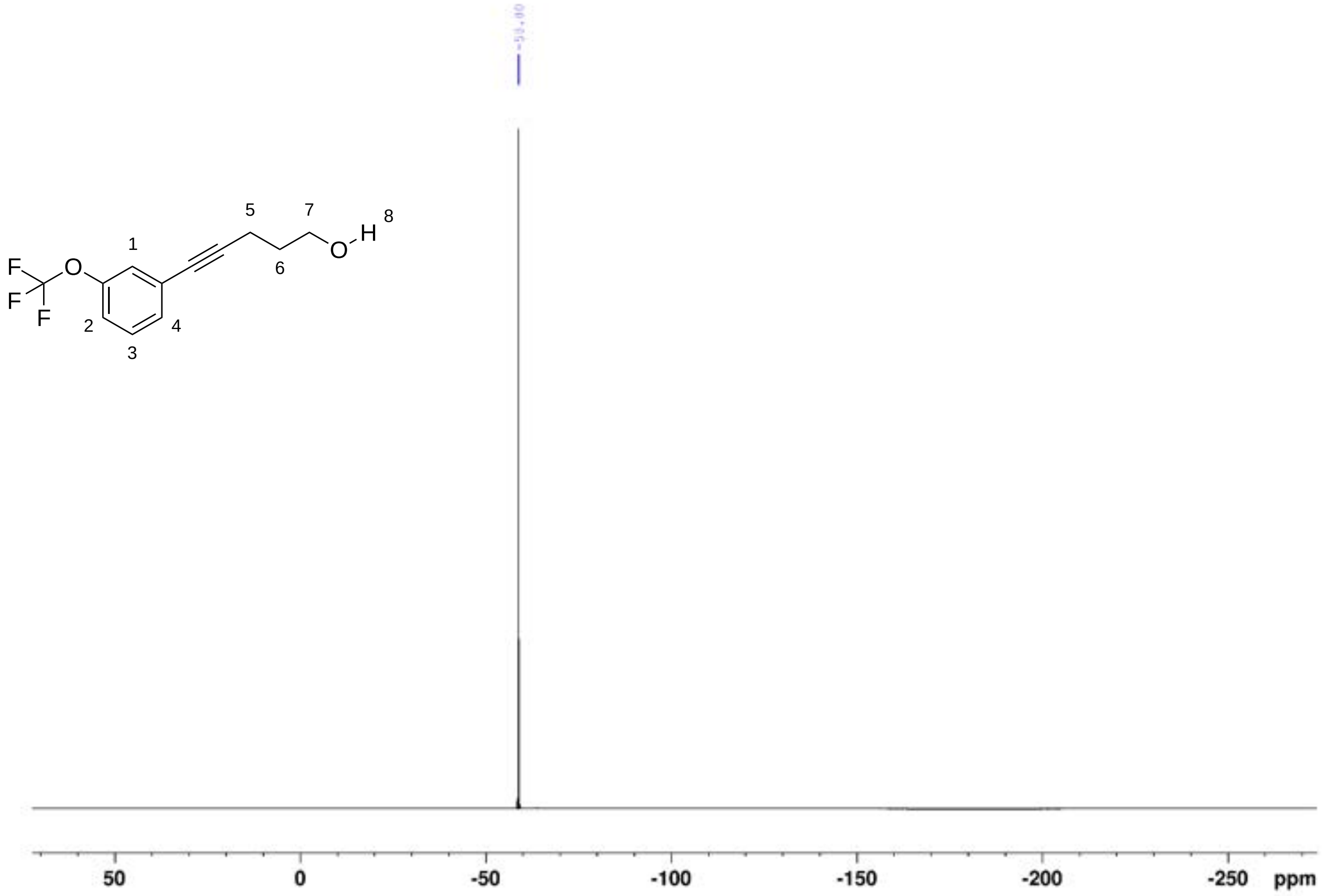

^{1}H NMR (400 MHz, $CDCl_3$) for *(Z)-5-(3-(trifluoromethoxy)phenyl)pent-4-en-1-ol* (**1l**)

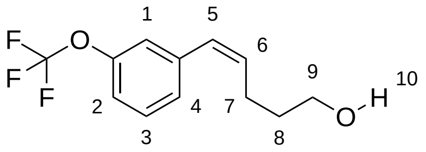

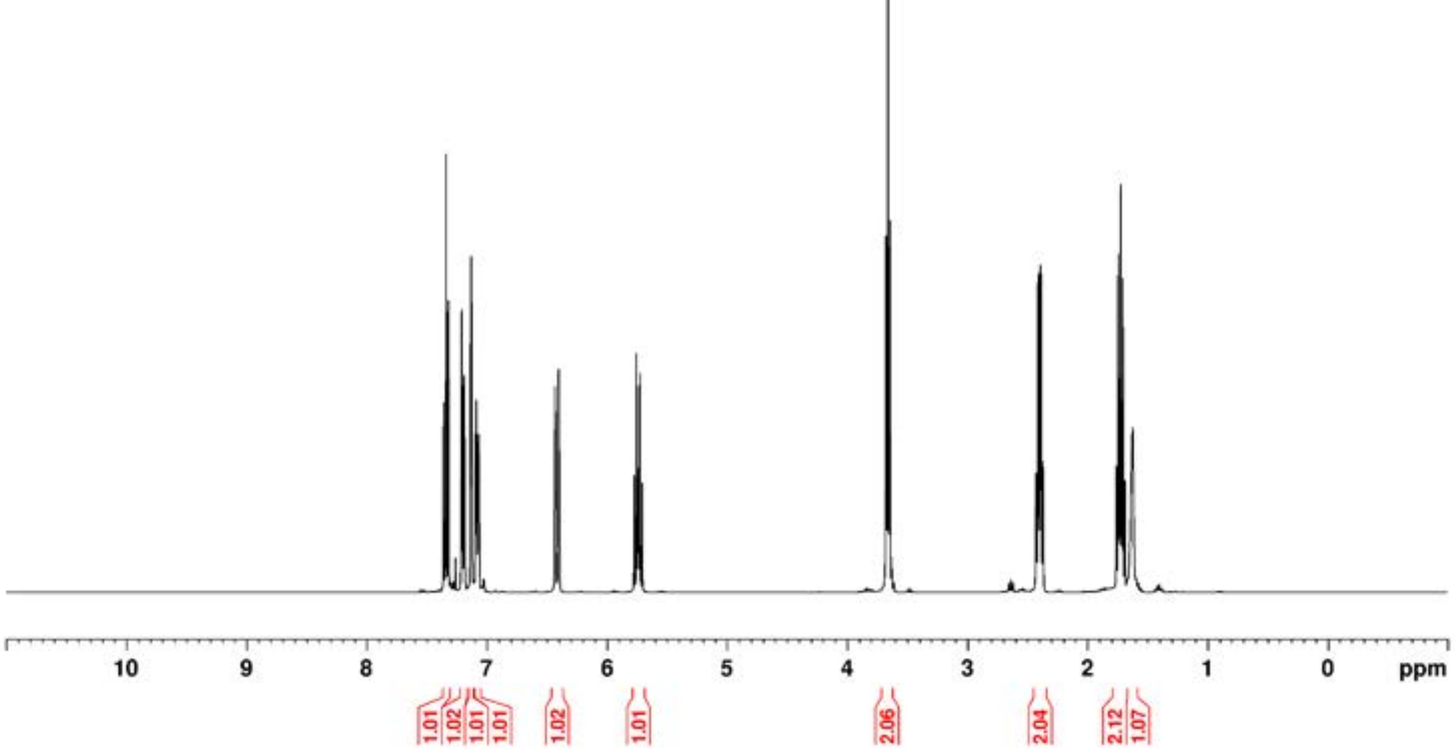

^{13}C NMR (101 MHz, $CDCl_3$) for *(Z)-5-(3-(trifluoromethoxy)phenyl)pent-4-en-1-ol* (**1l**)

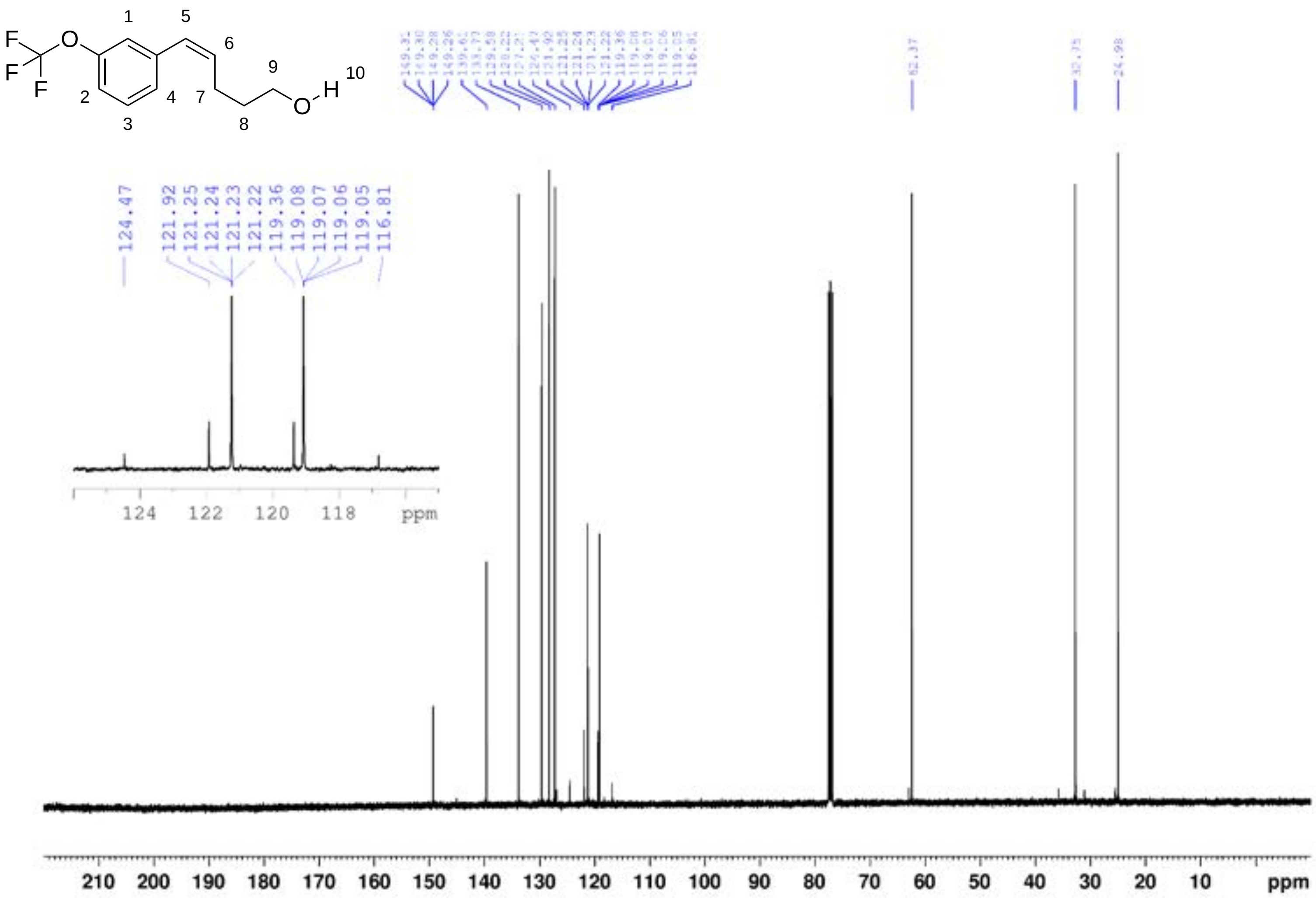

^{19}F NMR (376 MHz, $CDCl_3$) for *(Z)-5-(3-(trifluoromethoxy)phenyl)pent-4-en-1-ol* (**1l**)

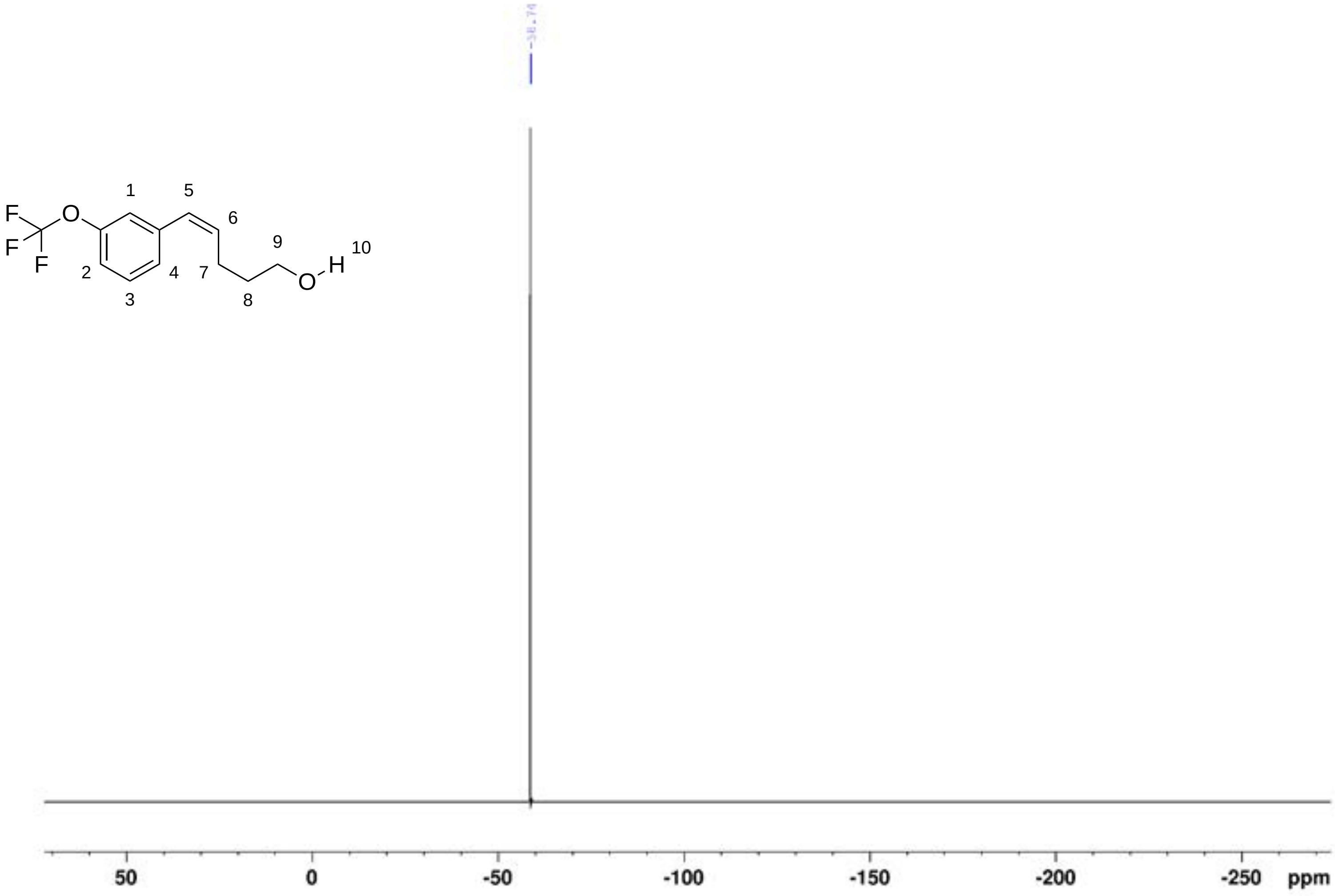

^{1}H NMR (400 MHz, $CDCl_3$) for *1-(3-(5-hydroxypent-1-yn-1-yl)phenyl)ethan-1-one*

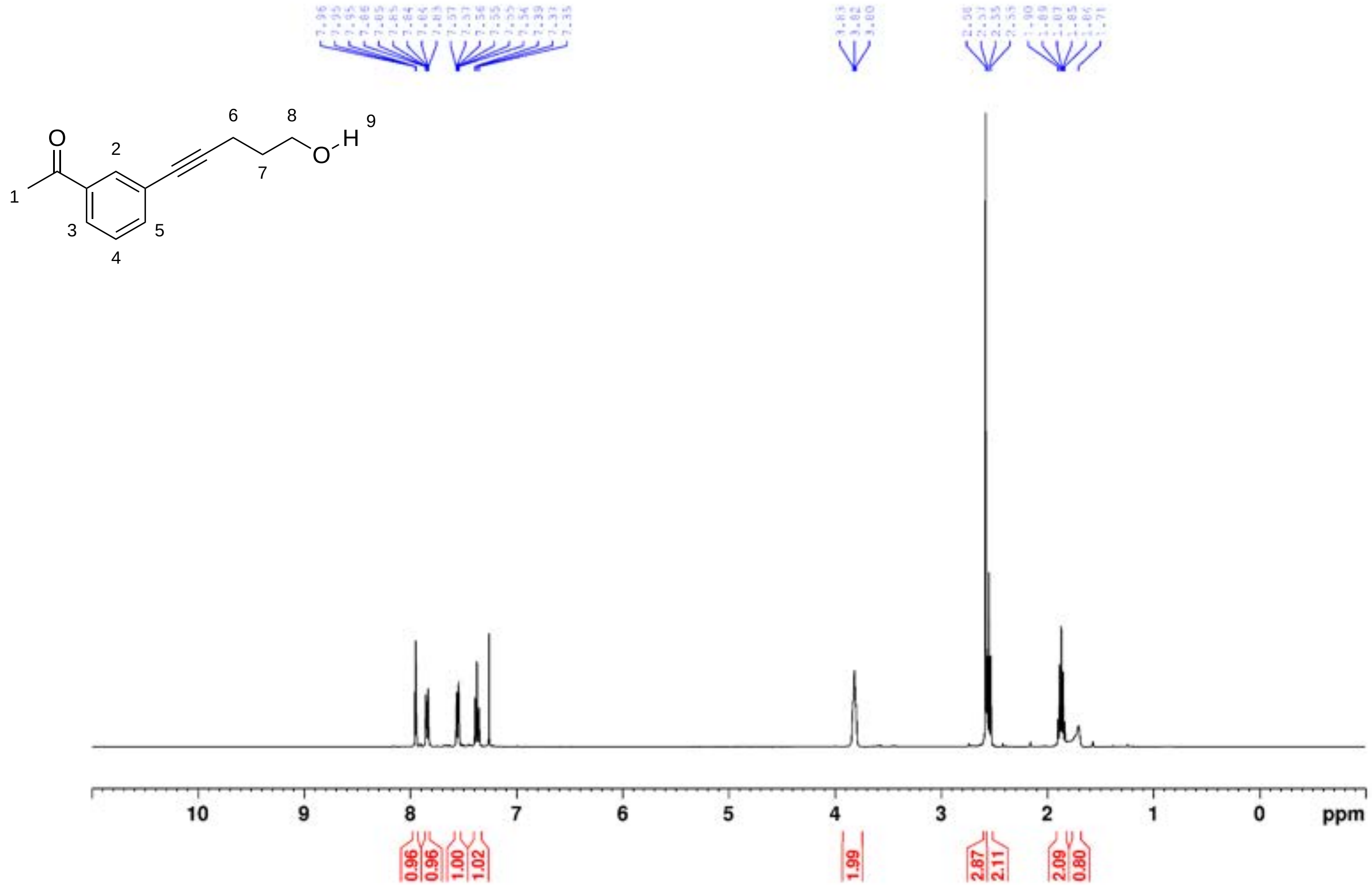

^{13}C NMR (101 MHz, $CDCl_3$) for *1-(3-(5-hydroxypent-1-yn-1-yl)phenyl)ethan-1-one*

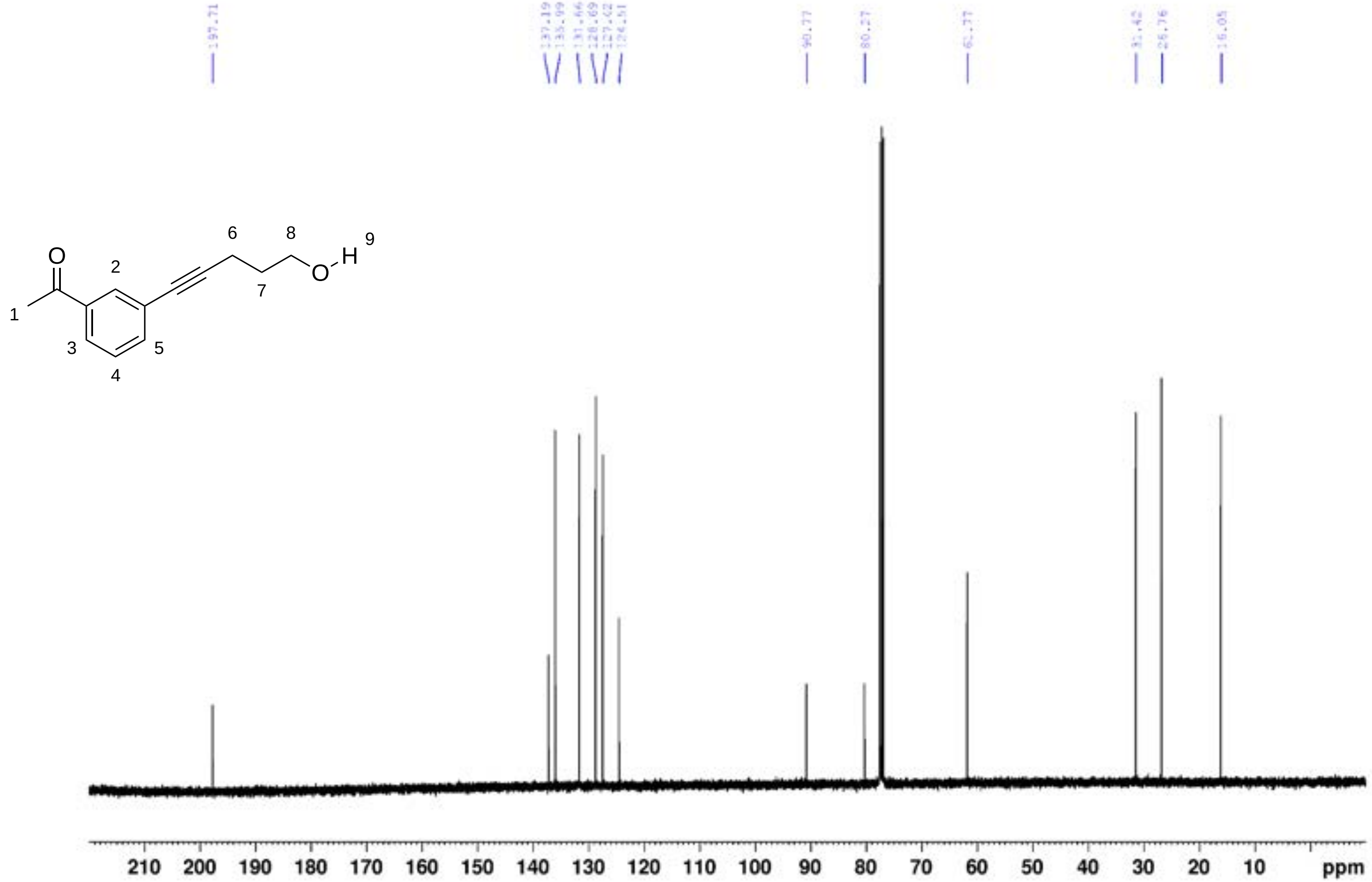

¹H NMR (500 MHz, $CDCl_3$) for *(Z)-1-(3-(5-hydroxypent-1-en-1-yl)phenyl)ethan-1-one* (**1m**)

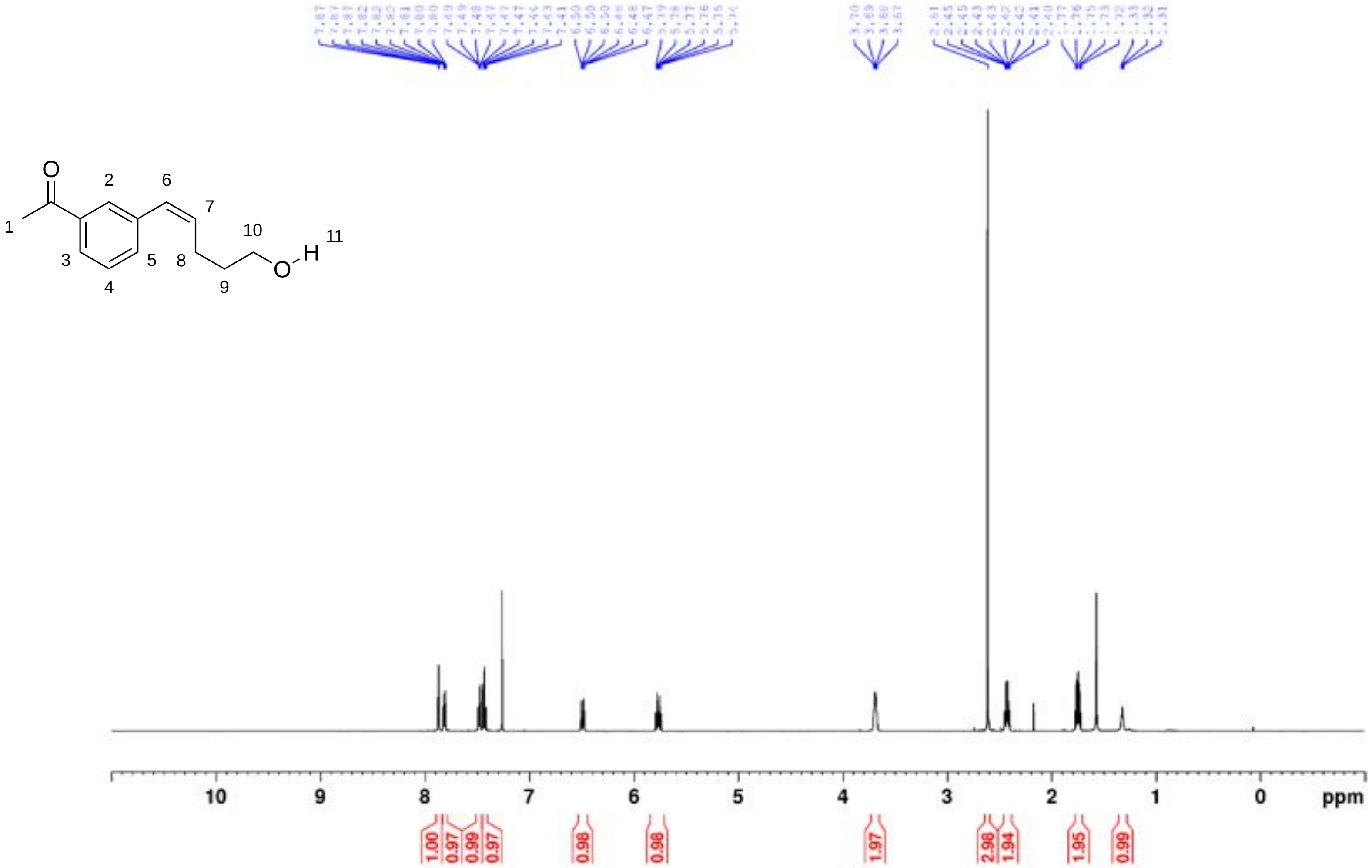

^{13}C NMR (126 MHz, $CDCl_3$) for *(Z)-1-(3-(5-hydroxypent-1-en-1-yl)phenyl)ethan-1-one* (**1m**)

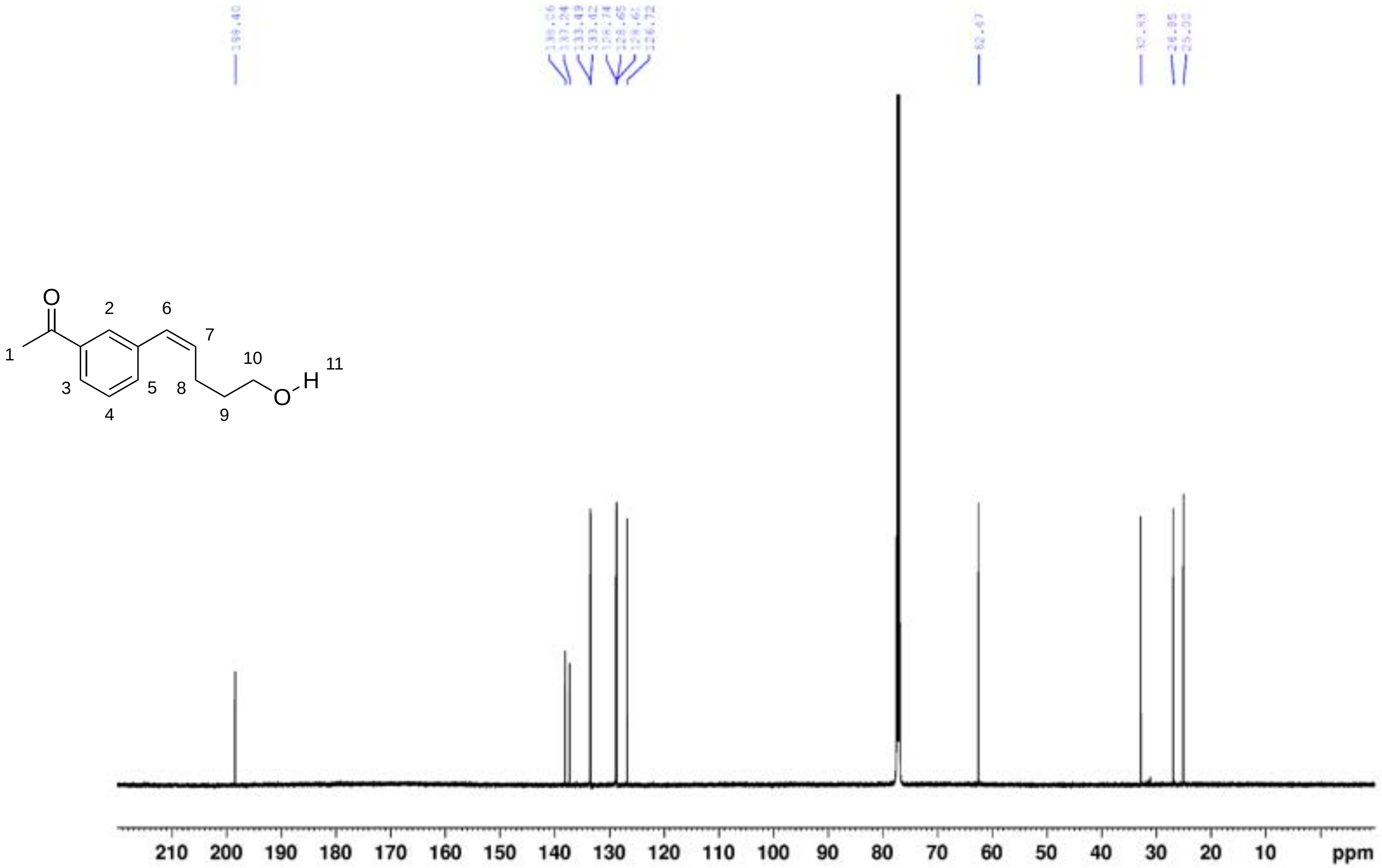

¹H NMR (400 MHz, $CDCl_3$) for *(Z)-5-(3-isopropylphenyl)pent-4-en-1-ol* (**1n**)

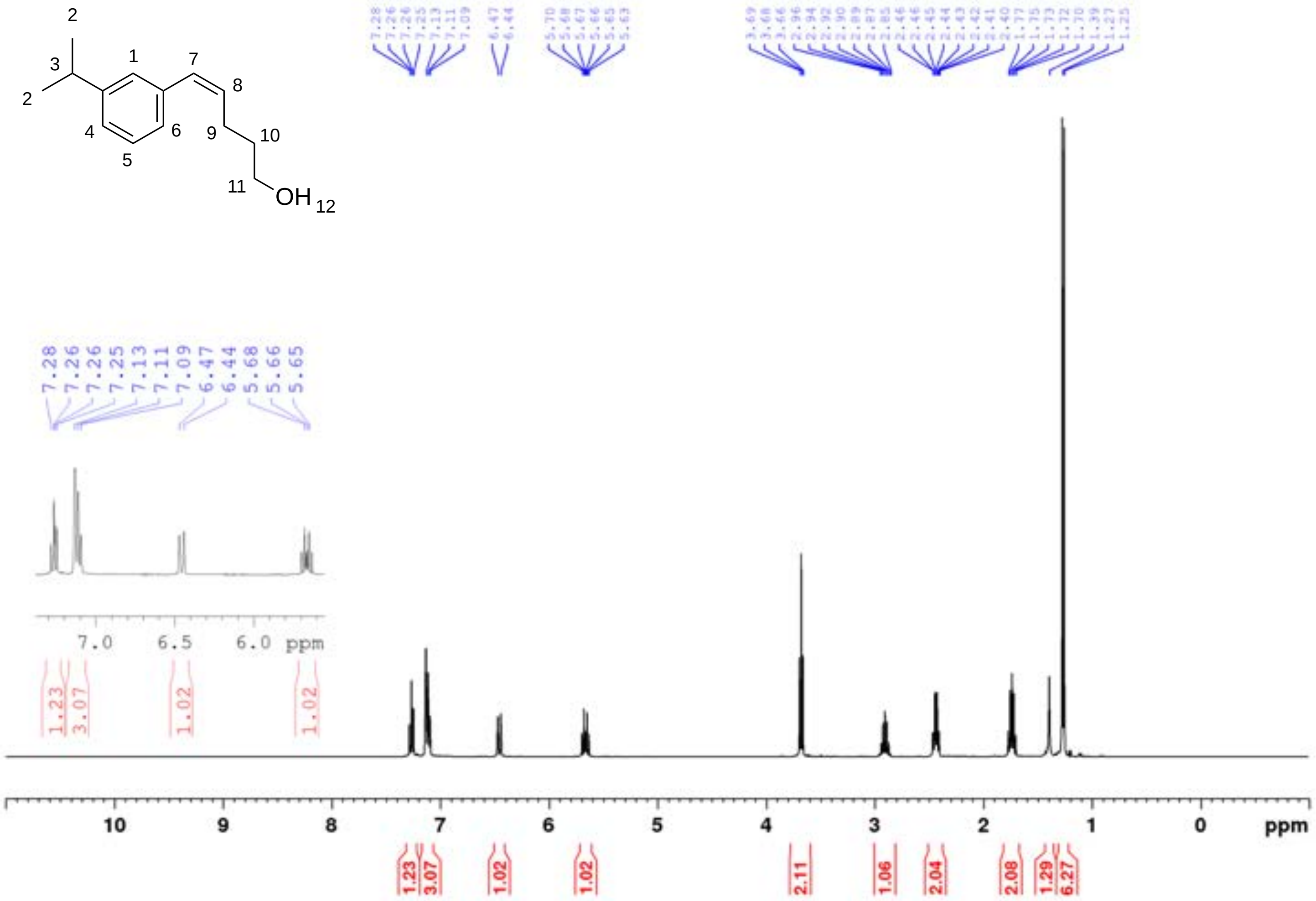

^{13}C NMR (101 MHz, $CDCl_3$) for *(Z)-5-(3-isopropylphenyl)pent-4-en-1-ol* (**1n**)

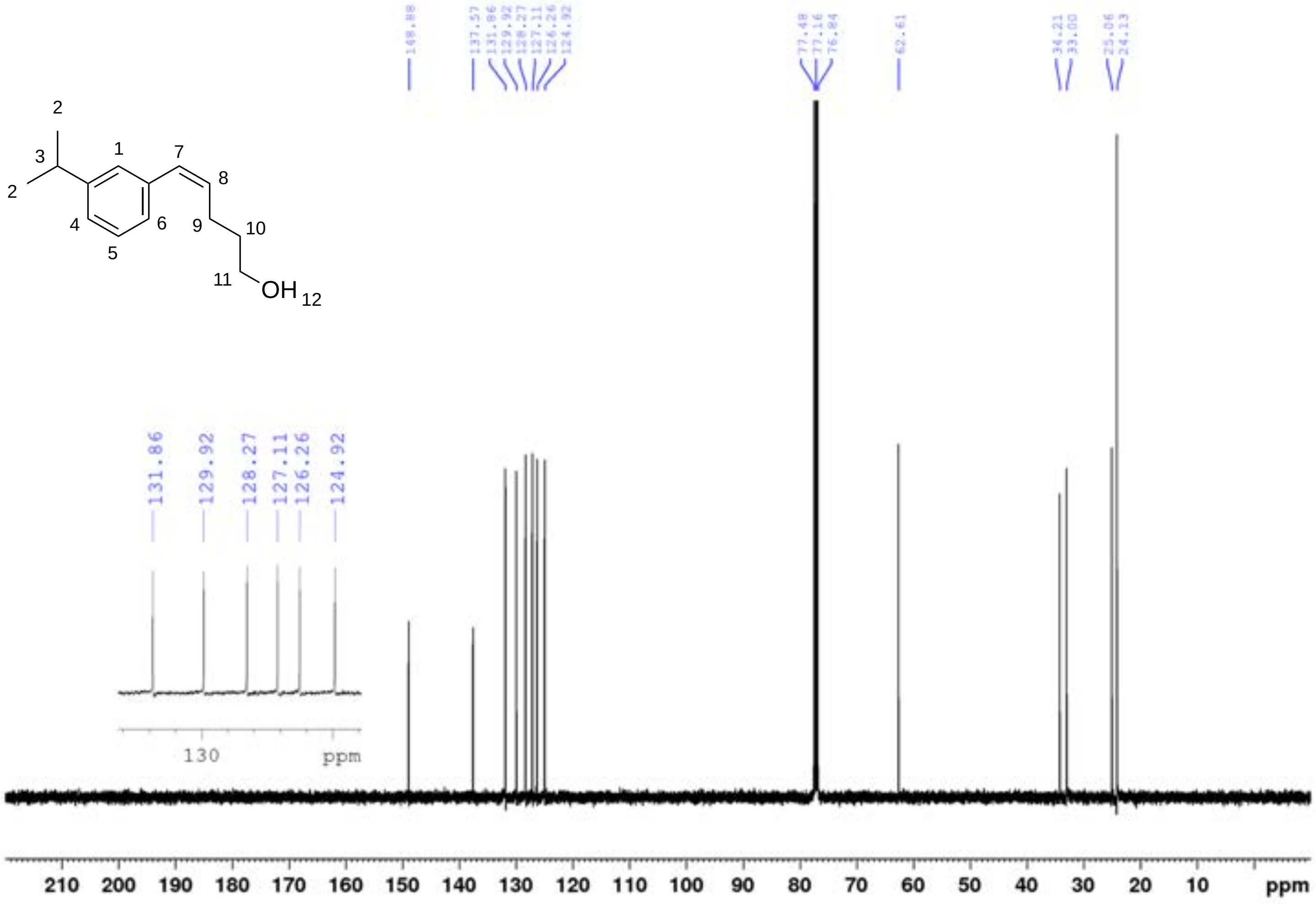

^{1}H NMR (400 MHz, $CDCl_3$) for *(Z)-5-(3-(tert-butyl)phenyl)pent-4-en-1-ol* (**1o**)

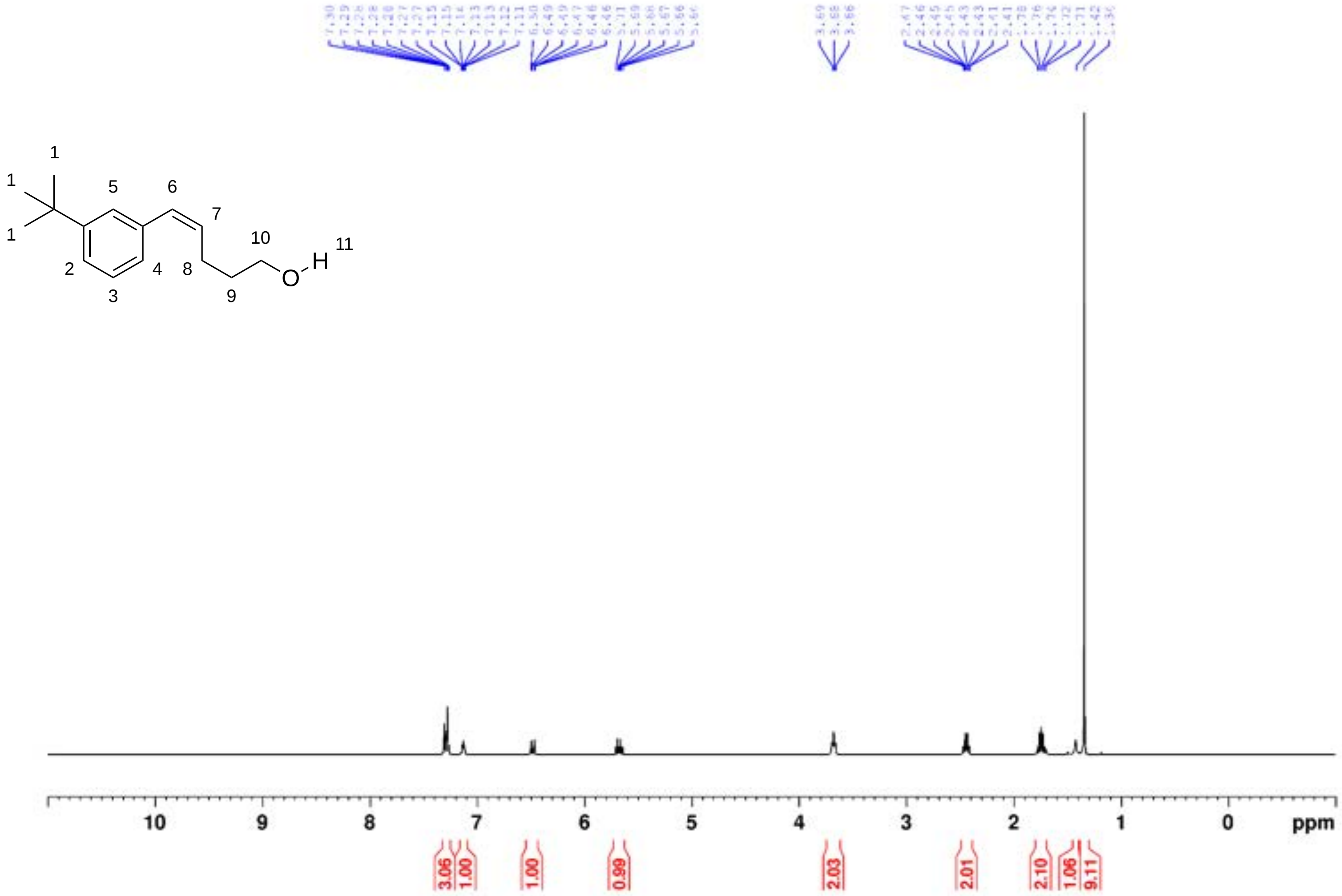

^{13}**C NMR** (101 MHz, $CDCl_3$) for *(Z)-5-(3-(tert-butyl)phenyl)pent-4-en-1-ol* (**1o**)

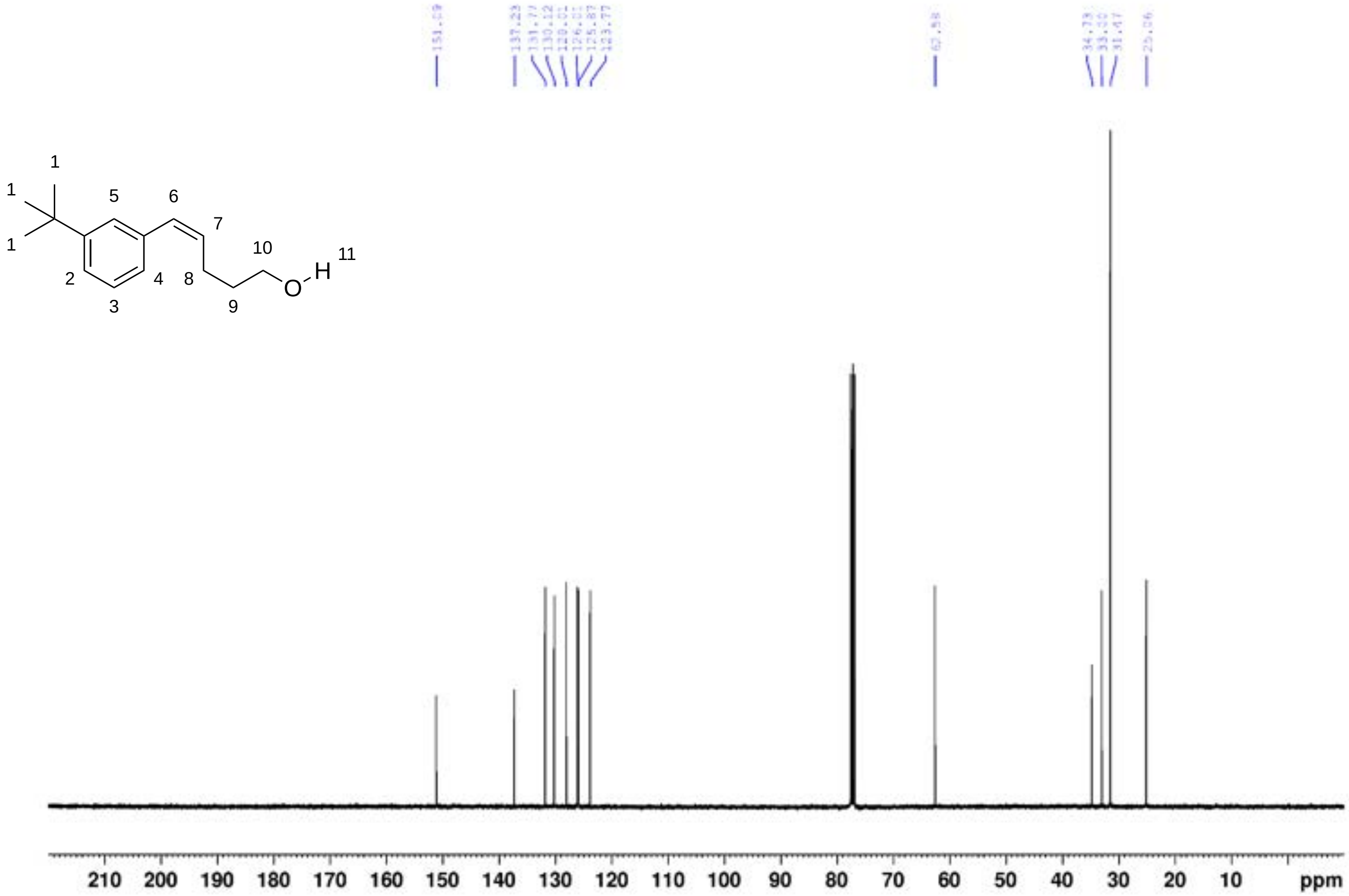

^{1}H NMR (400 MHz, $CDCl_3$) for *(Z)-5-(3,5-dimethylphenyl)pent-4-en-1-ol* (**1p**)

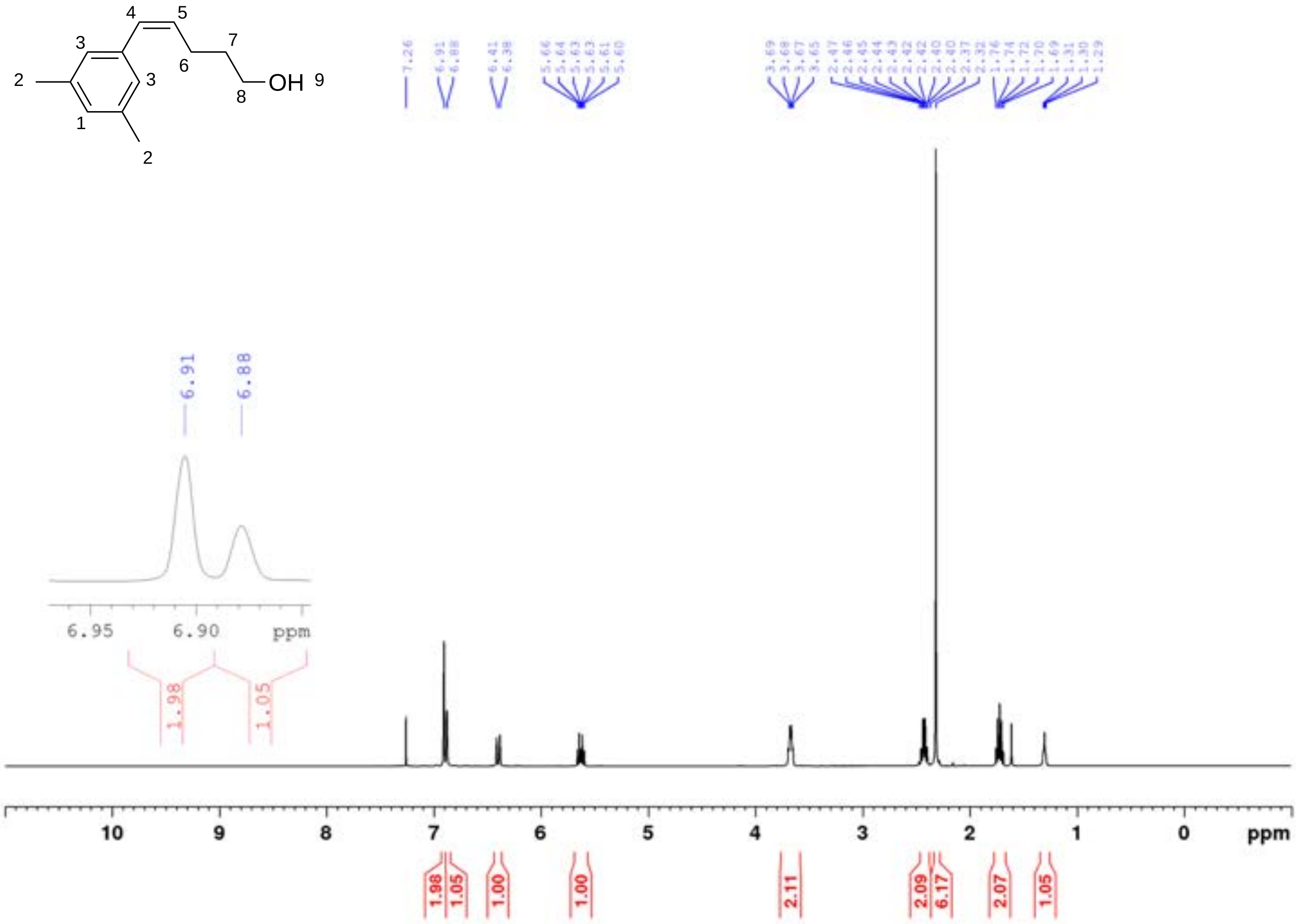

^{13}C NMR (101 MHz, $CDCl_3$) for *(Z)-5-(3,5-dimethylphenyl)pent-4-en-1-ol* (**1p**)

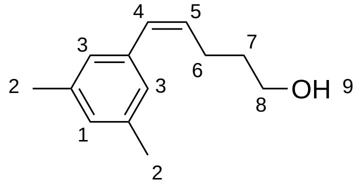

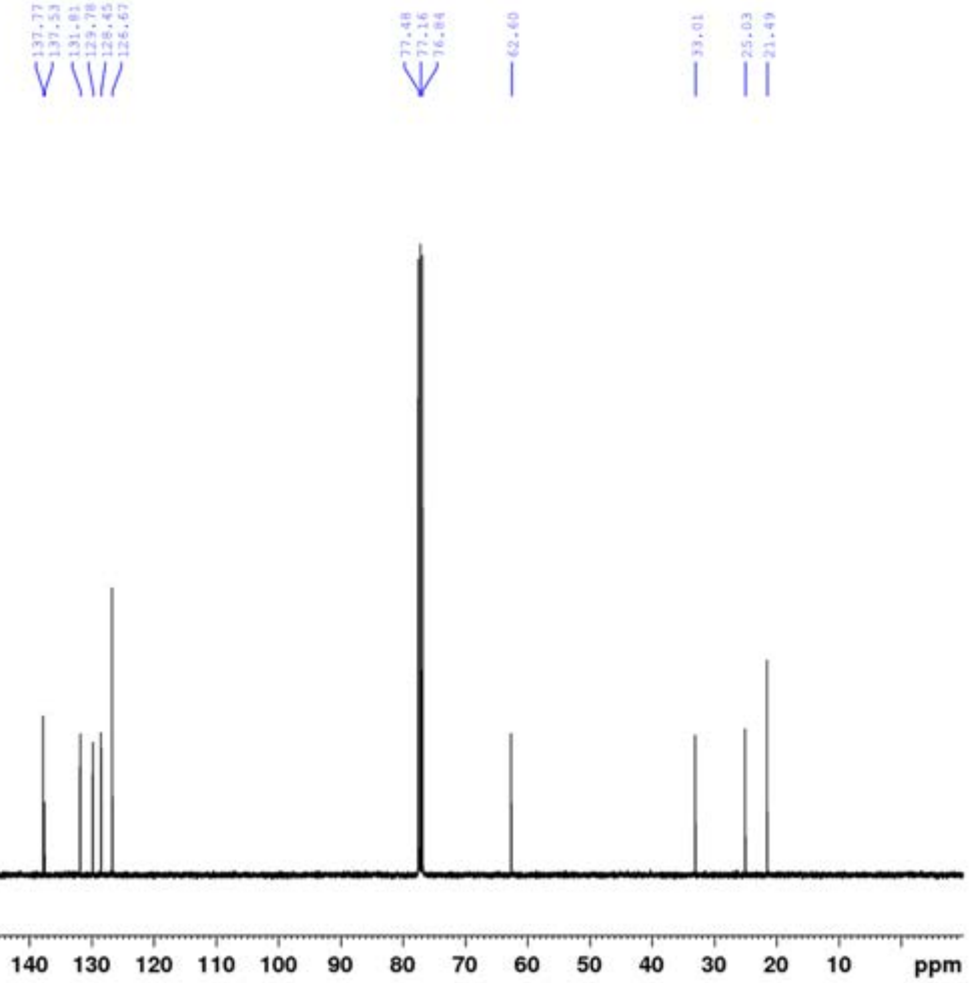

^{1}H NMR (400 MHz, $CDCl_3$) for *(Z)-5-(3,5-di-tert-butylphenyl)pent-4-en-1-ol* (**1q**)

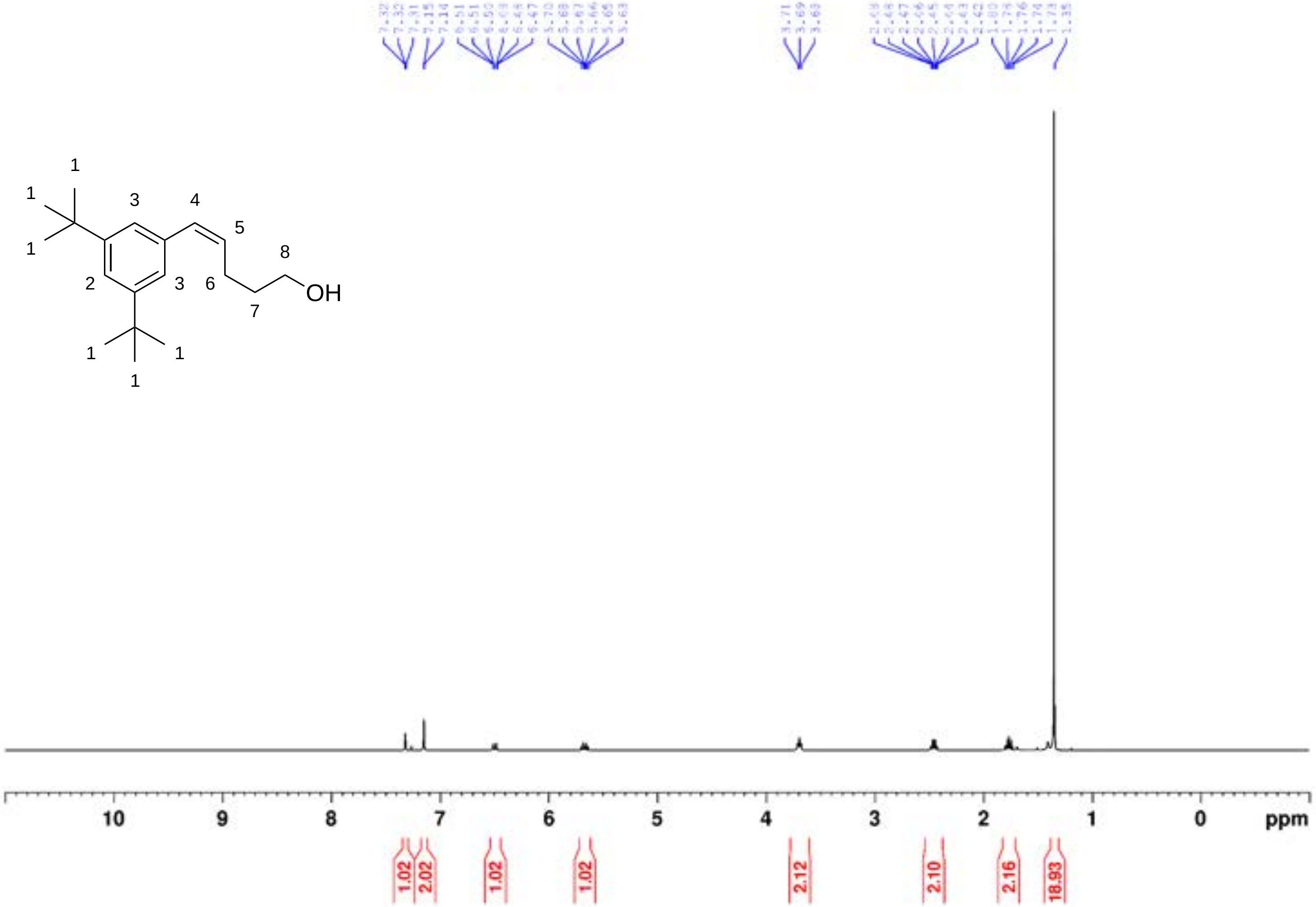

^{13}C NMR (101 MHz, $CDCl_3$) for *(Z)-5-(3,5-di-tert-butylphenyl)pent-4-en-1-ol* (**1q**)

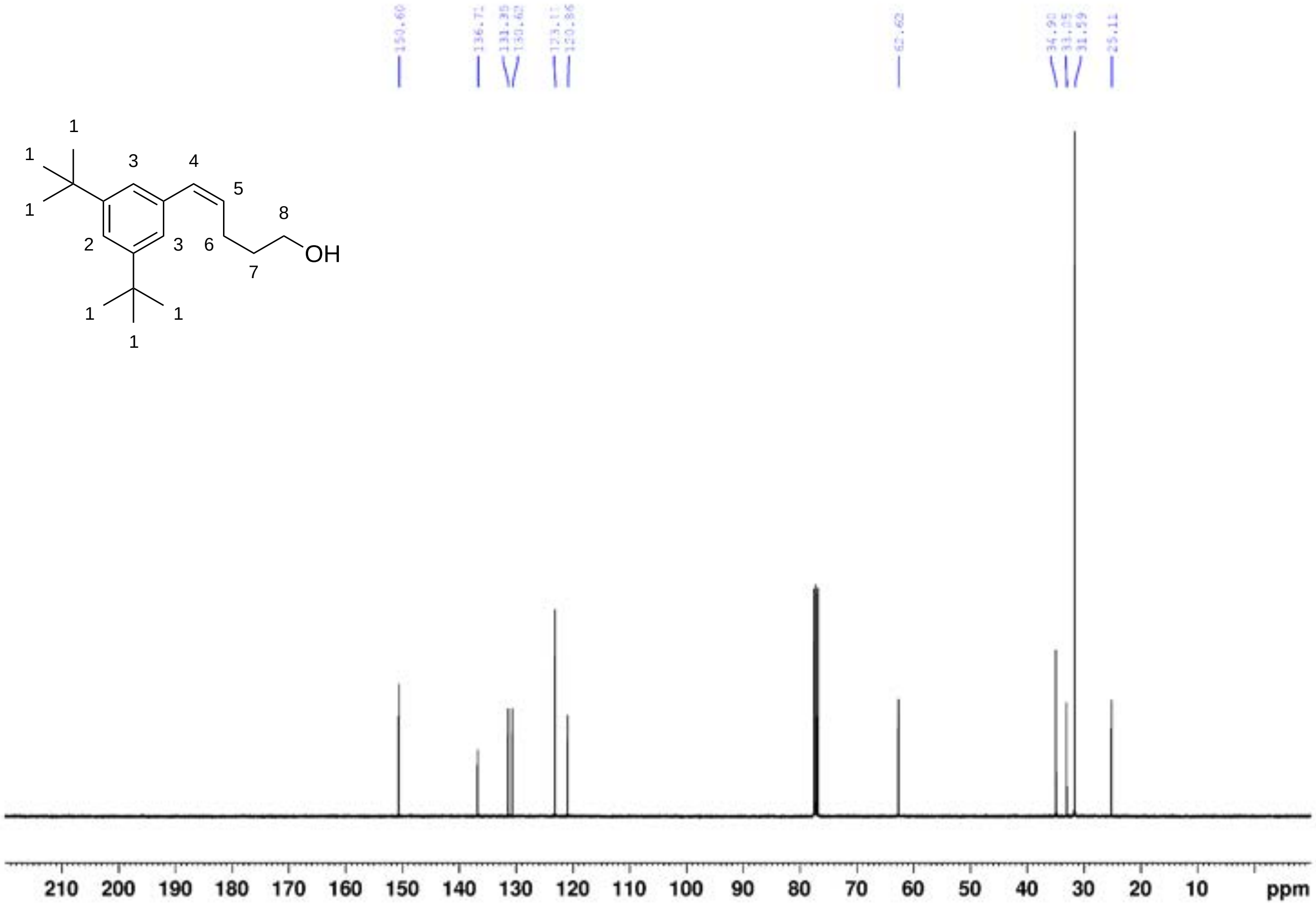

^{1}H NMR (400 MHz, $CDCl_3$) for *(Z)-5-(3-fluoro-4-methylphenyl)pent-4-en-1-ol* (**1r**)

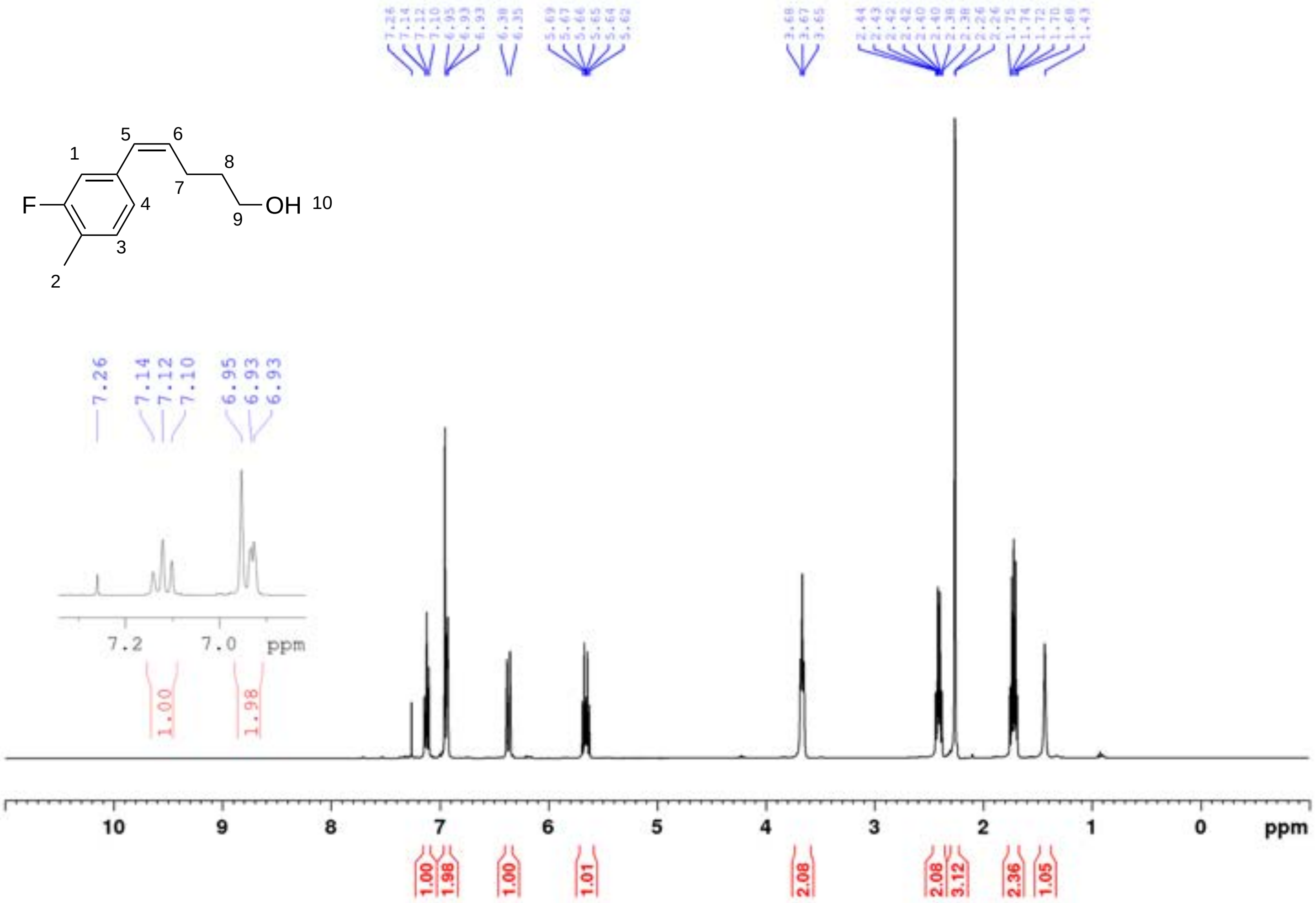

^{13}C NMR (101 MHz, $CDCl_3$) for *(Z)-5-(3-fluoro-4-methylphenyl)pent-4-en-1-ol* (**1r**)

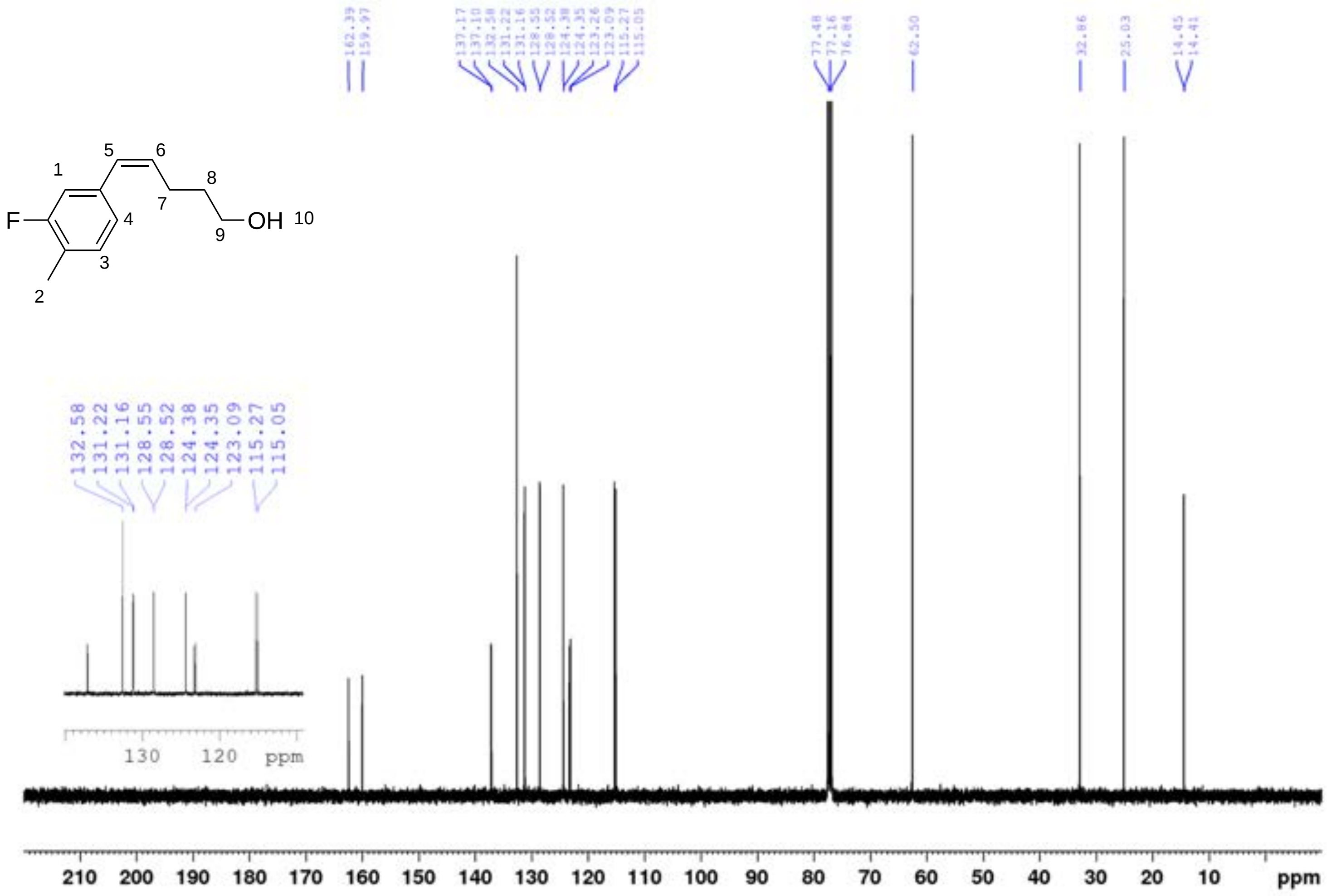

^{19}F NMR (376 MHz, $CDCl_3$) for *(Z)-5-(3-fluoro-4-methylphenyl)pent-4-en-1-ol* (**1r**)

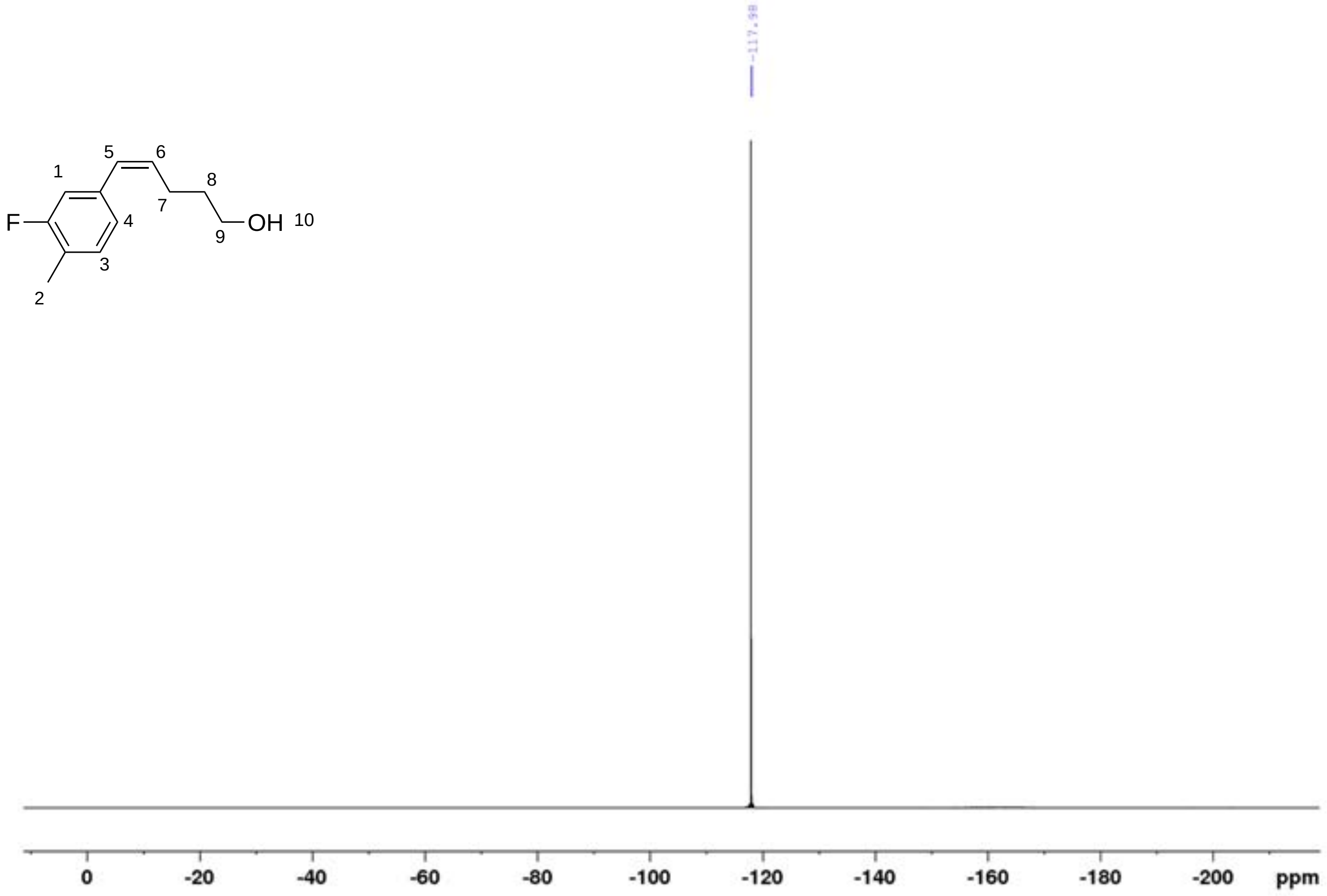

^{1}H NMR (500 MHz, $CDCl_3$) for *(Z)-5-(naphthalen-2-yl)pent-4-en-1-ol* (**1s**)

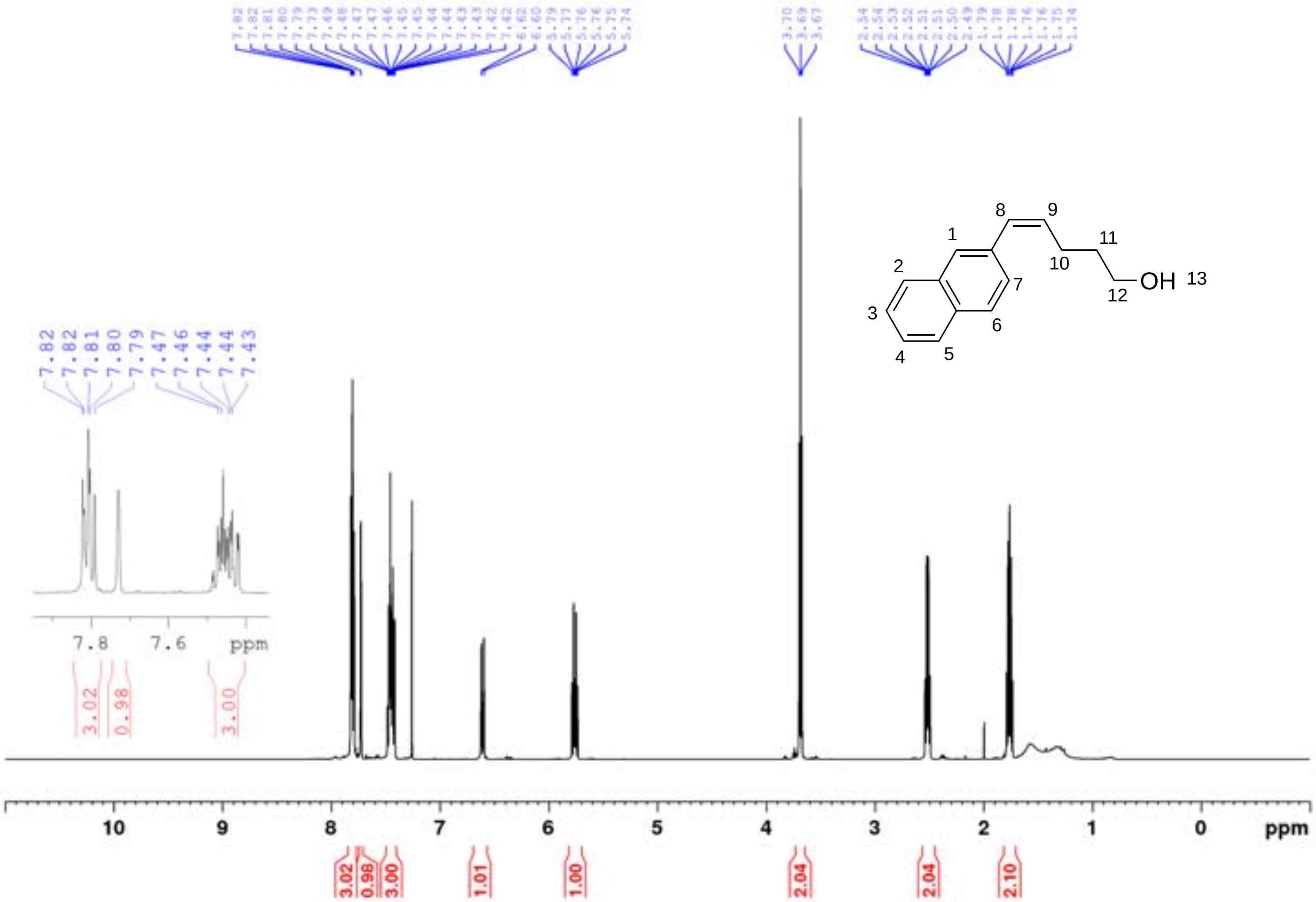

^{13}C NMR (125 MHz, $CDCl_3$) for *(Z)-5-(naphthalen-2-yl)pent-4-en-1-ol* (**1s**)

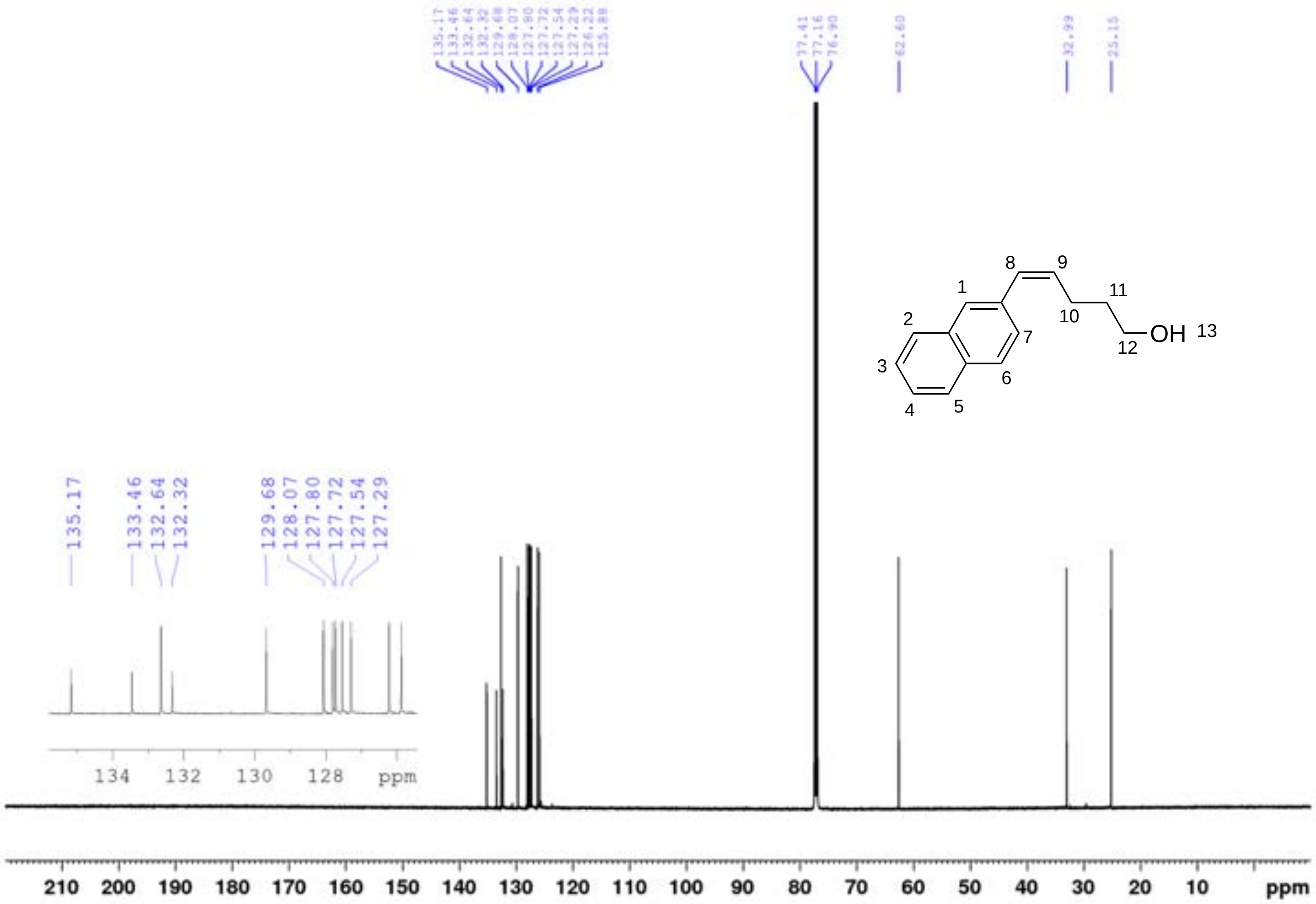

^{1}H NMR (400 MHz, $CDCl_3$) for *(Z)-5-(4-fluorophenyl)pent-4-en-1-ol* (**1t**)

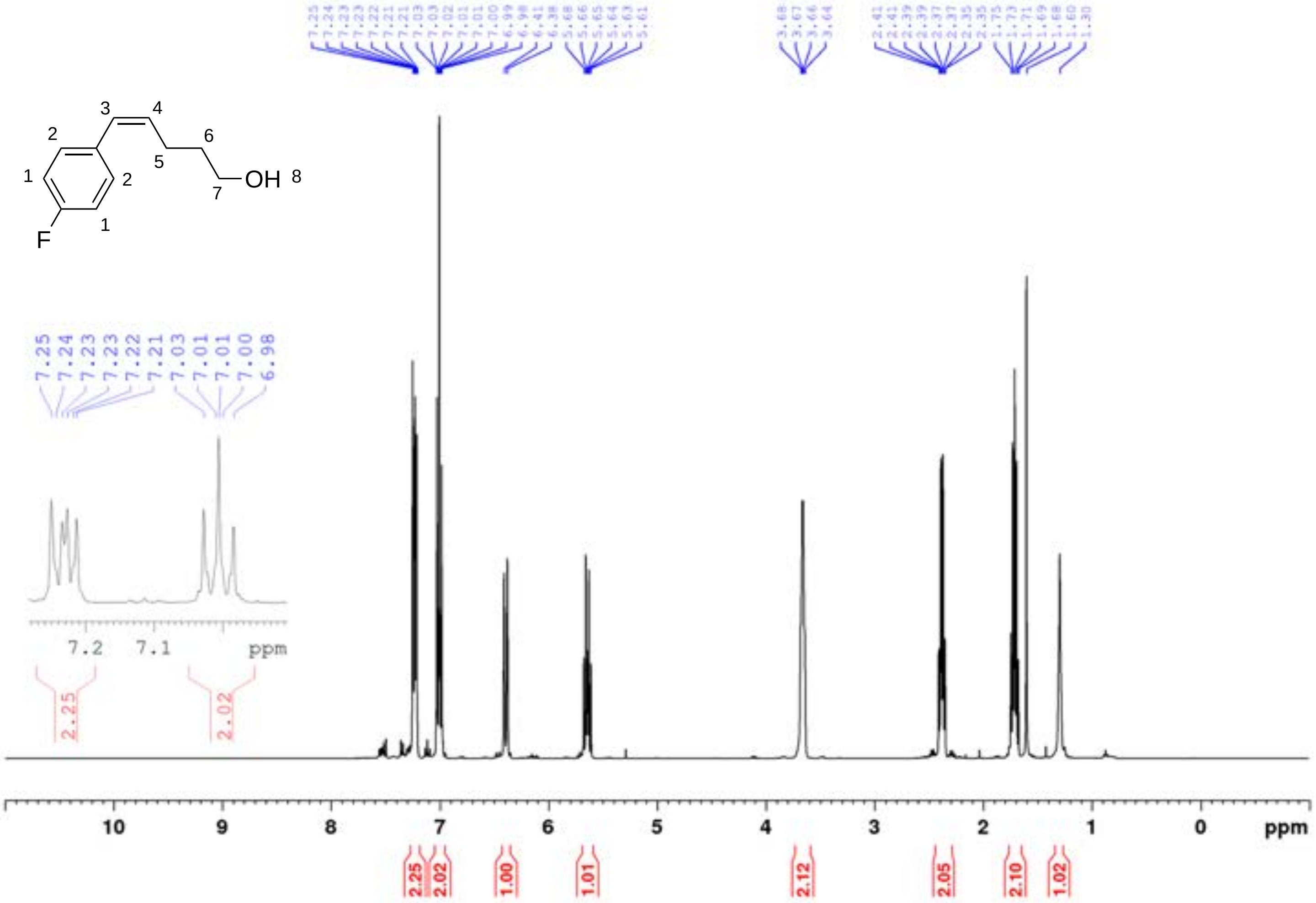

^{13}**C NMR** (101 MHz, $CDCl_3$) for *(Z)-5-(4-fluorophenyl)pent-4-en-1-ol* (**1t**)

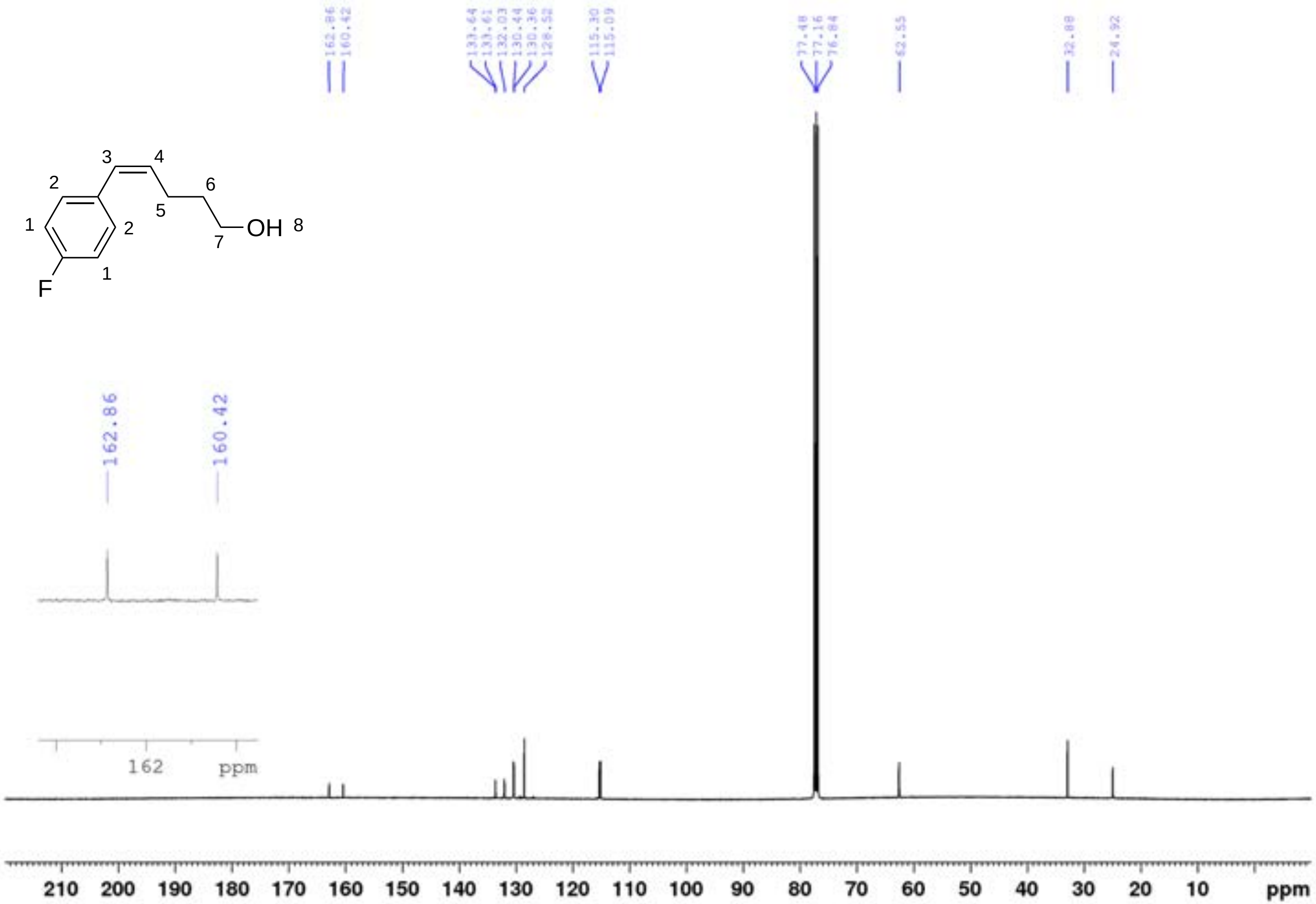

^{19}F NMR (376 MHz, $CDCl_3$) for *(Z)-5-(4-fluorophenyl)pent-4-en-1-ol* (**1t**)

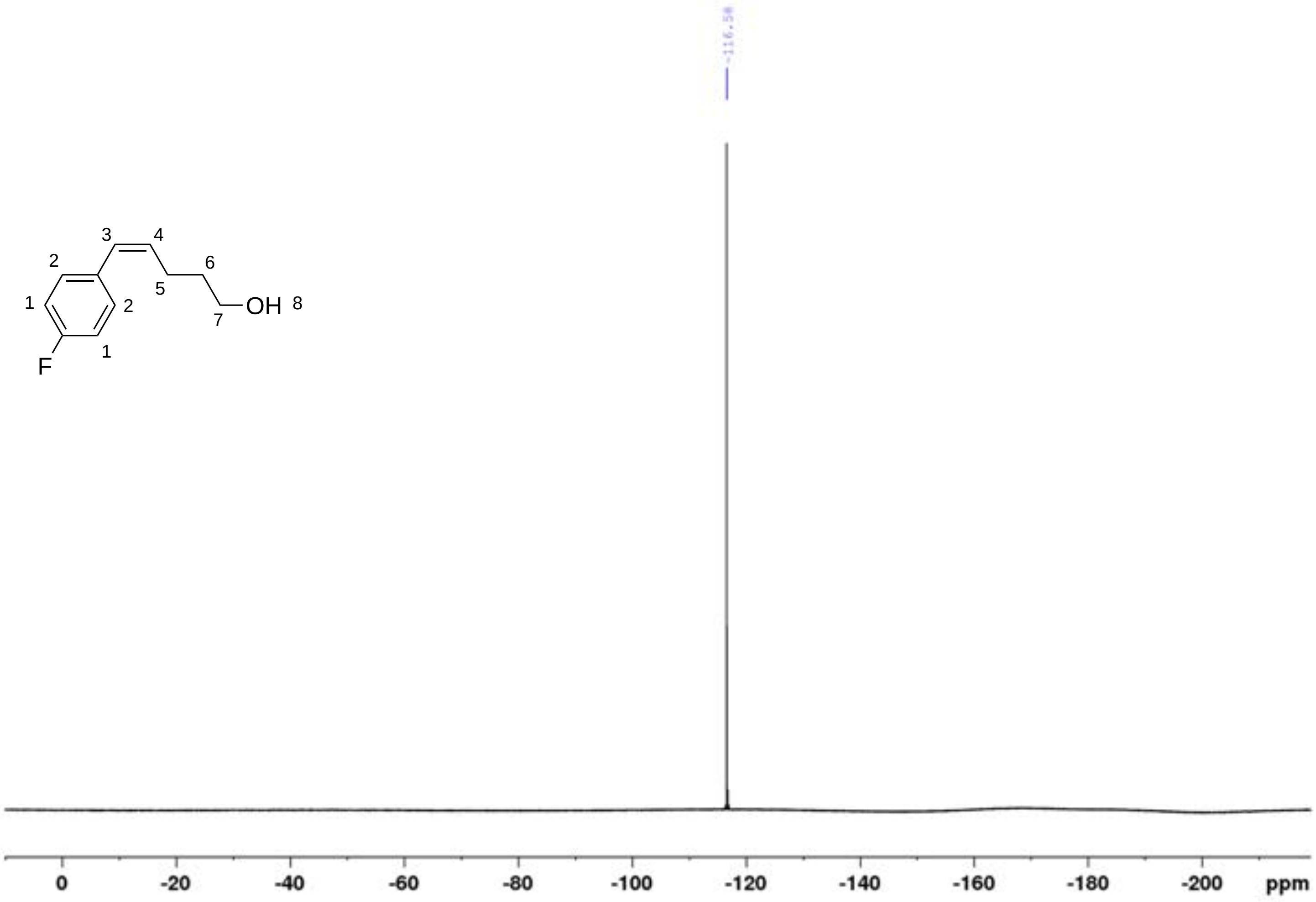

^{1}H NMR (400 MHz, $CDCl_3$) for *(Z)-5-(p-tolyl)pent-4-en-1-ol* (**1u**)

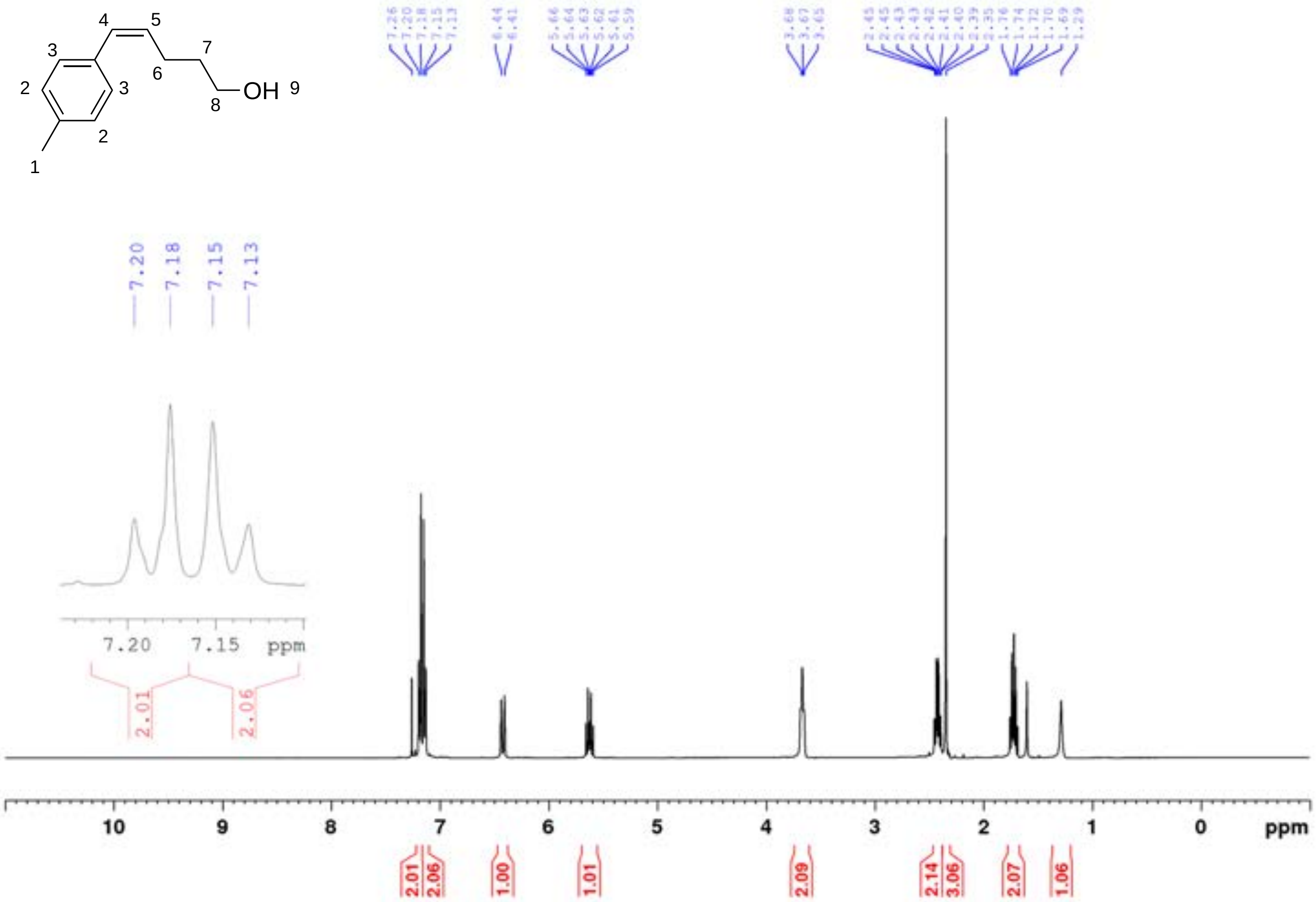

^{13}**C NMR** (101 MHz, $CDCl_3$) for *(Z)-5-(p-tolyl)pent-4-en-1-ol* (**1u**)

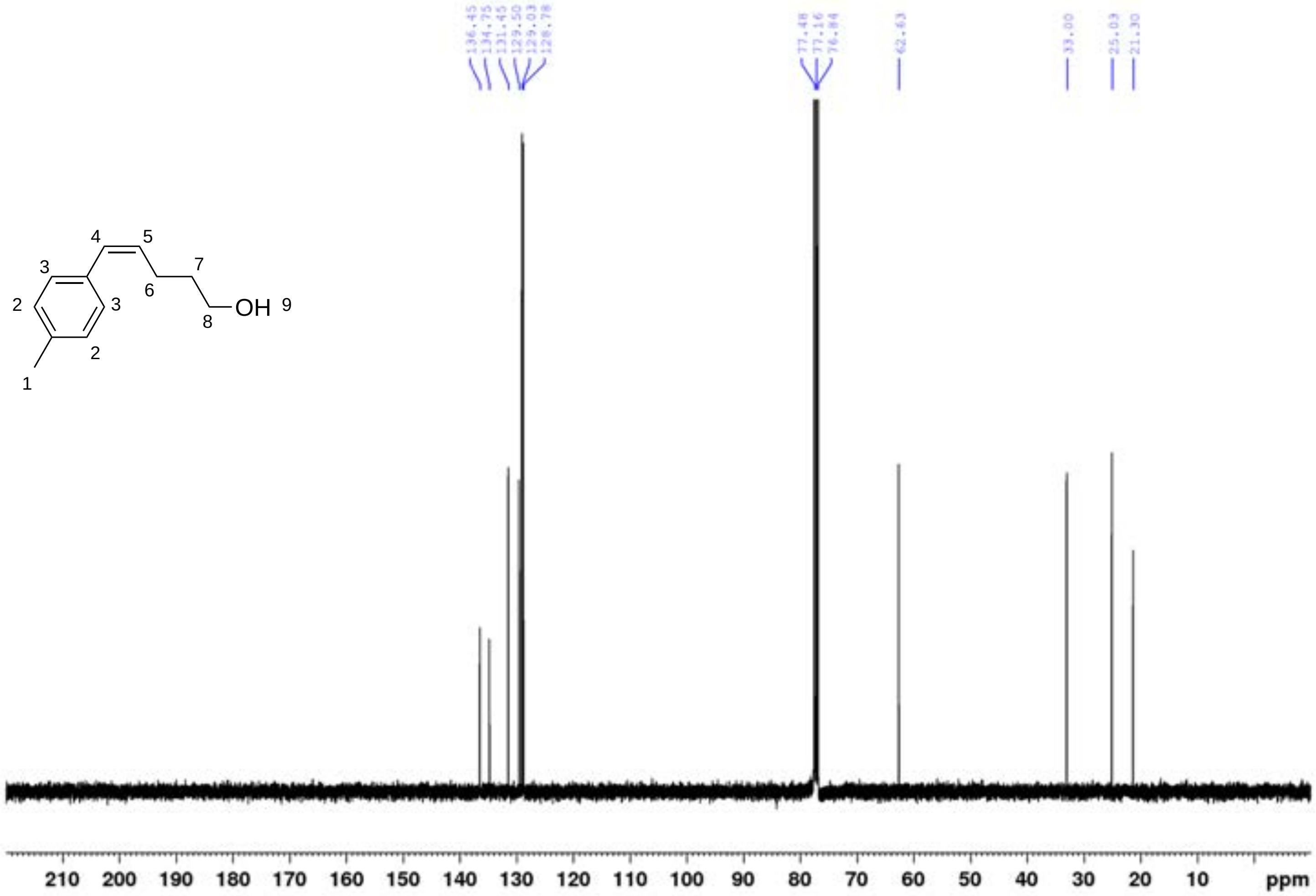

^{1}H NMR (400 MHz, $CDCl_3$) for *(Z)-5-(4-(trifluoromethyl)phenyl)pent-4-en-1-ol* (**1v**)

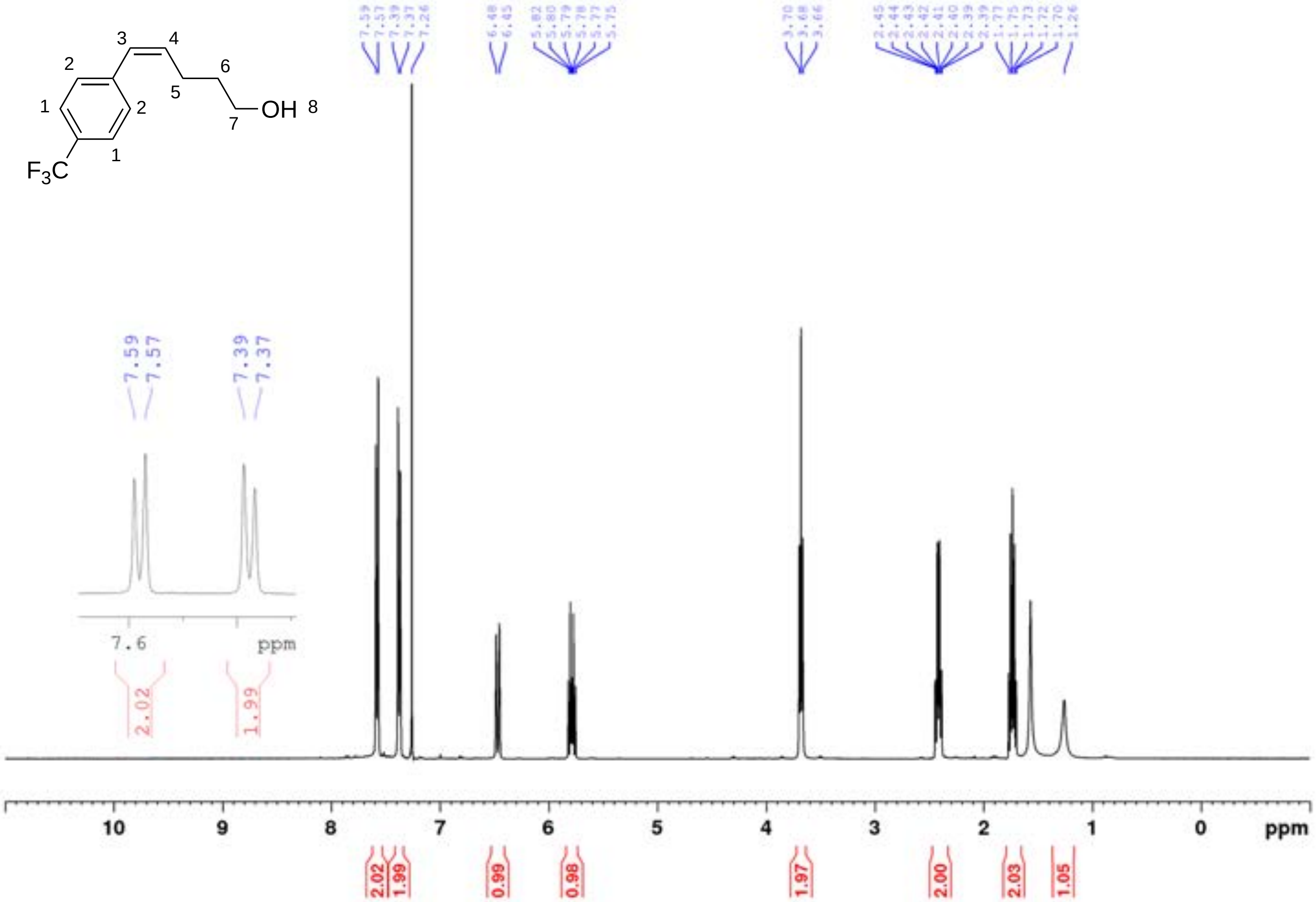

^{13}C NMR (101 MHz, $CDCl_3$) for *(Z)-5-(4-(trifluoromethyl)phenyl)pent-4-en-1-ol* (**1v**)

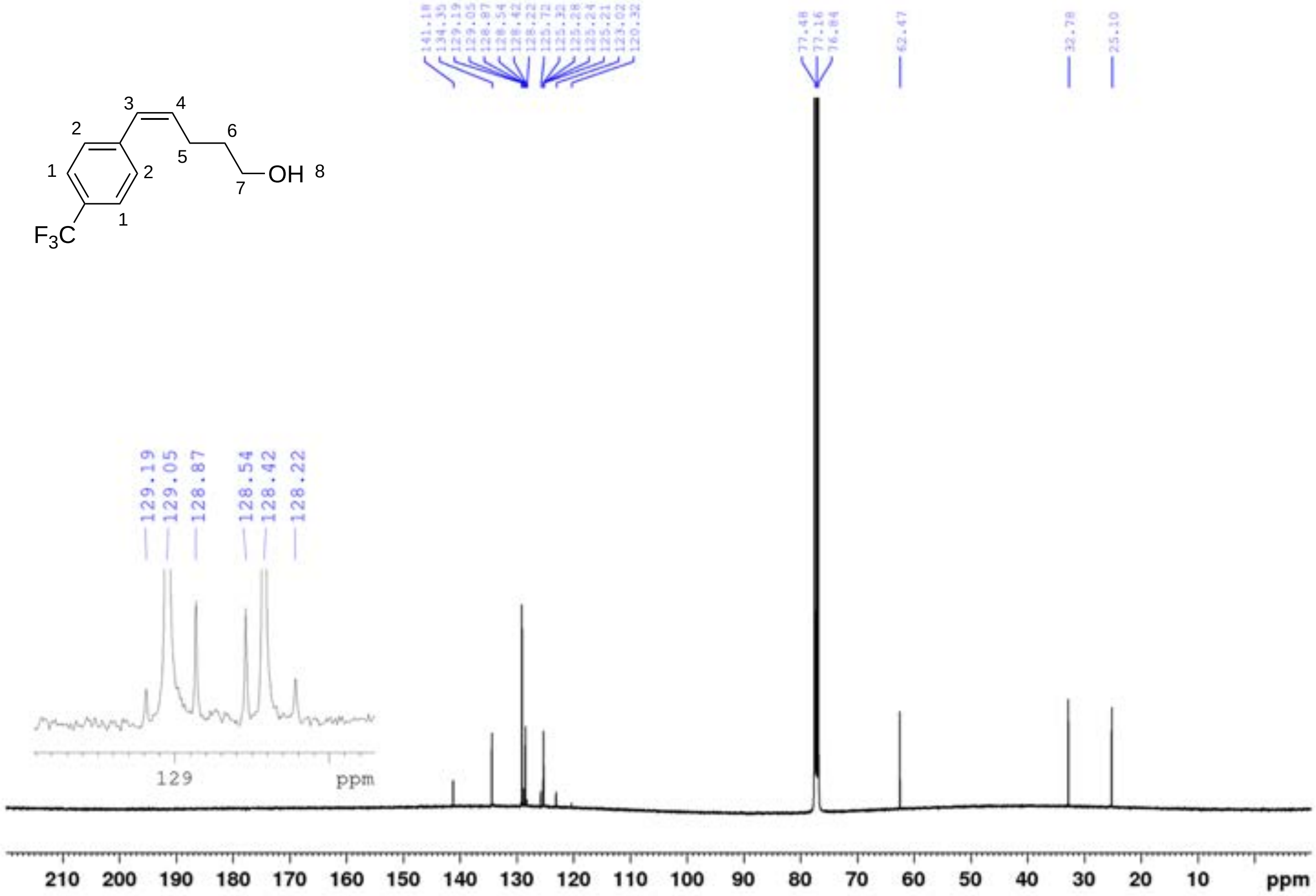

^{19}F NMR (376 MHz, $CDCl_3$) for *(Z)-5-(4-(trifluoromethyl)phenyl)pent-4-en-1-ol* (**1v**)

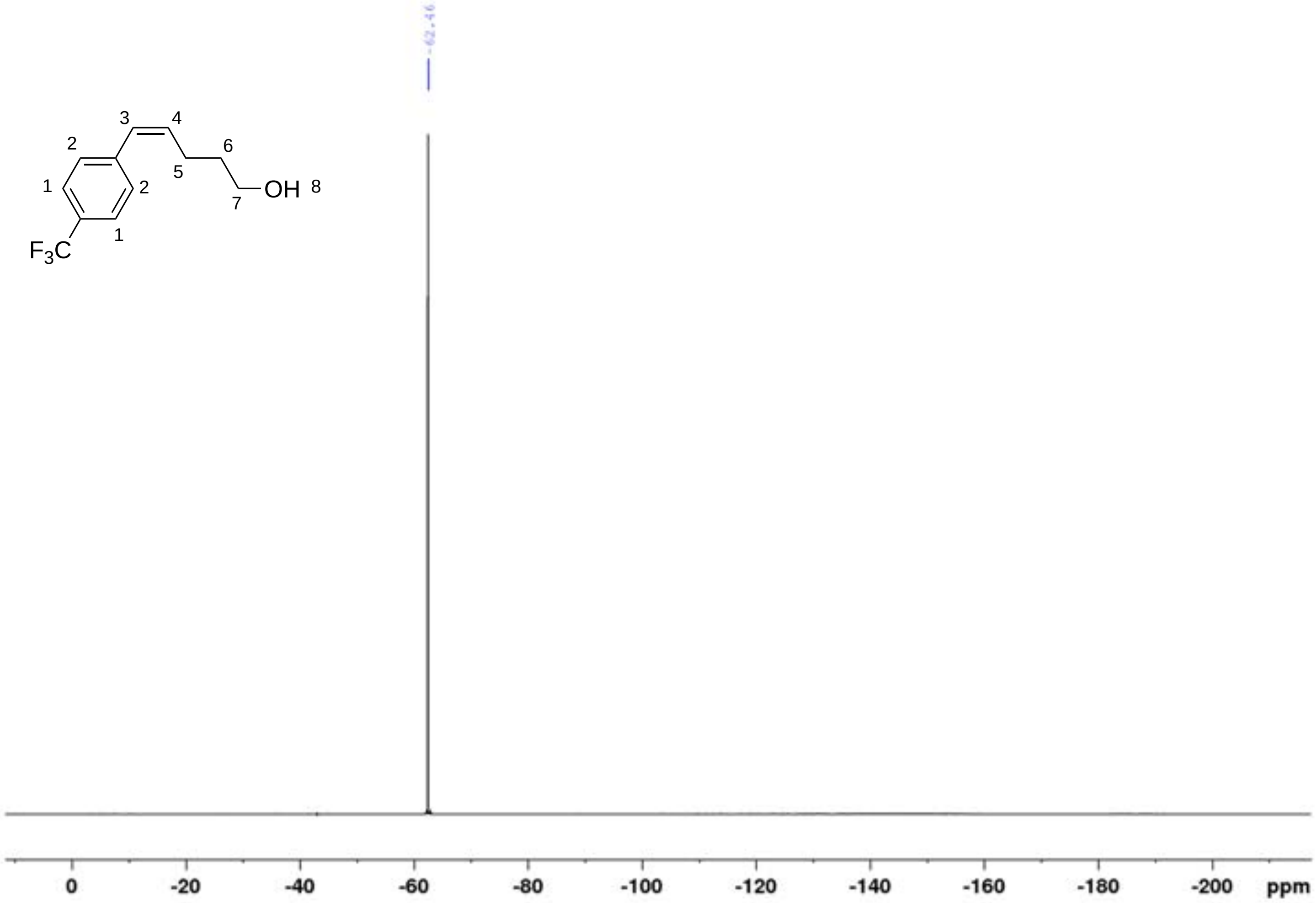

^{1}H NMR (400 MHz, $CDCl_3$) for *(Z)-5-(4-(tert-butyl)phenyl)pent-4-en-1-ol* (**1w**)

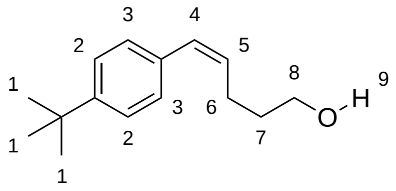

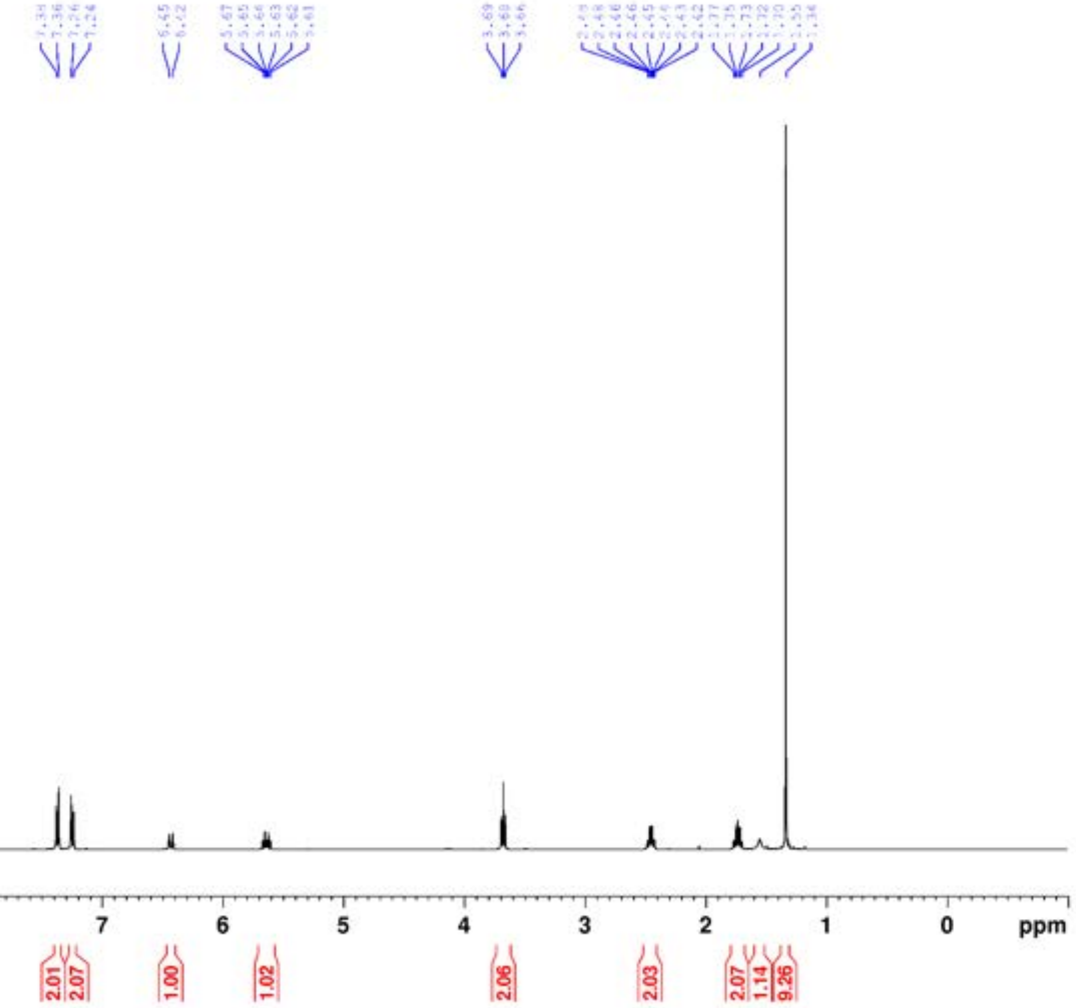

^{13}C NMR (101 MHz, $CDCl_3$) for *(Z)-5-(4-(tert-butyl)phenyl)pent-4-en-1-ol* (**1w**)

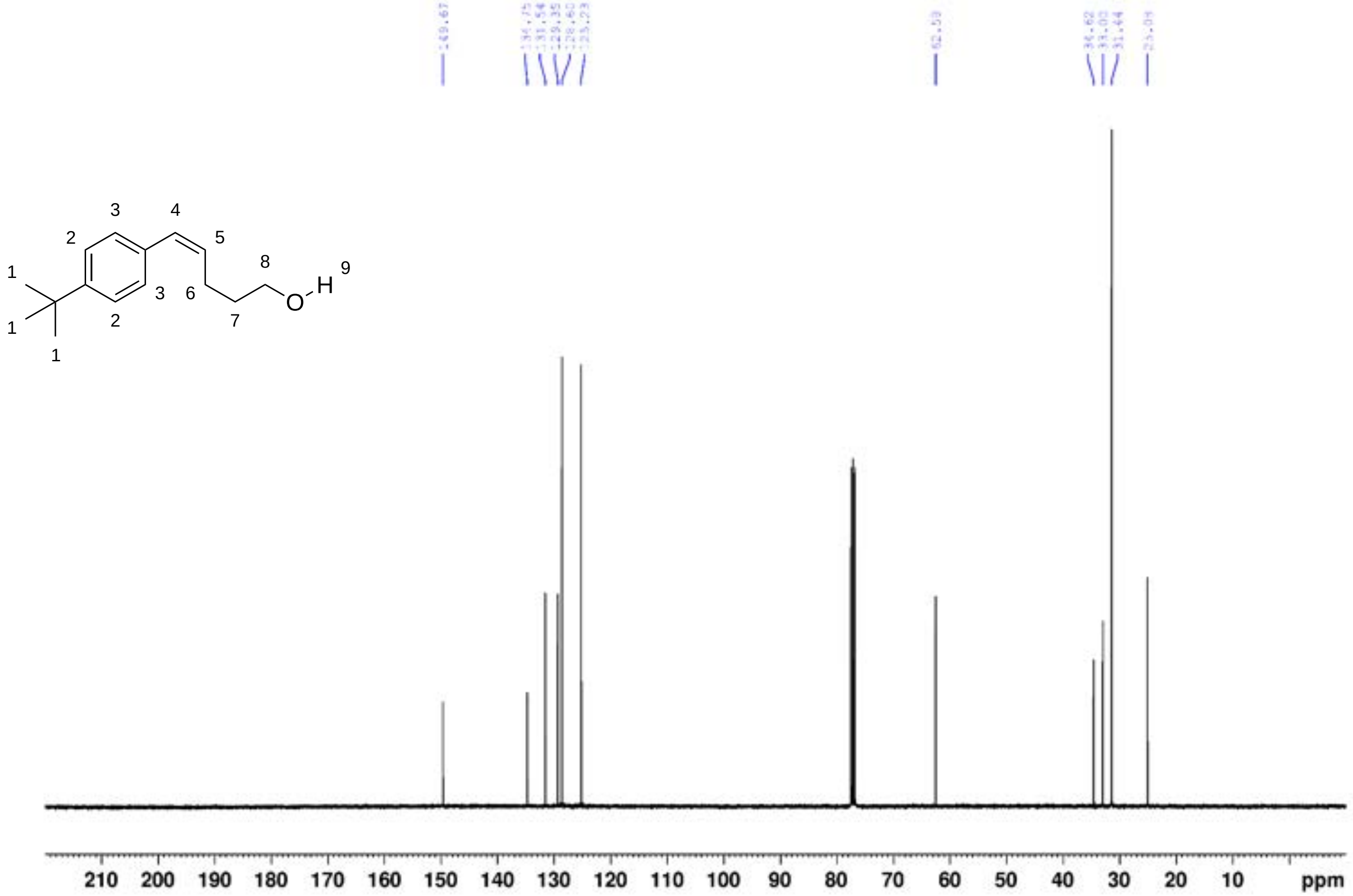

^{1}H NMR (400 MHz, $CDCl_3$) for *(Z)-5-([1,1'-biphenyl]-4-yl)pent-4-en-1-ol* (**1x**)

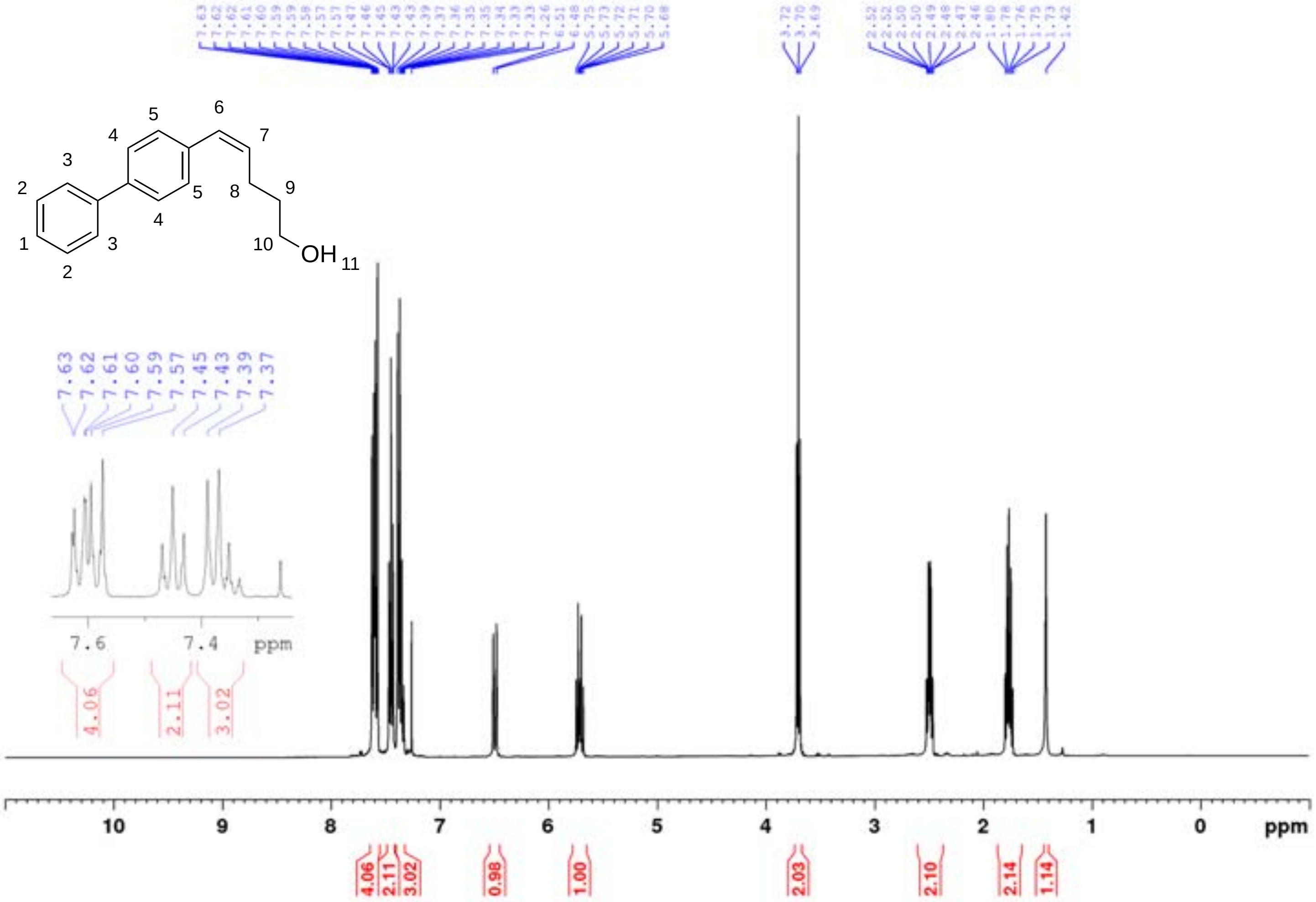

^{13}C NMR (101 MHz, $CDCl_3$) for *(Z)-5-([1,1'-biphenyl]-4-yl)pent-4-en-1-ol* (**1x**)

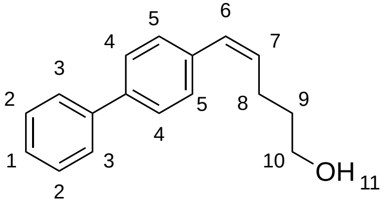

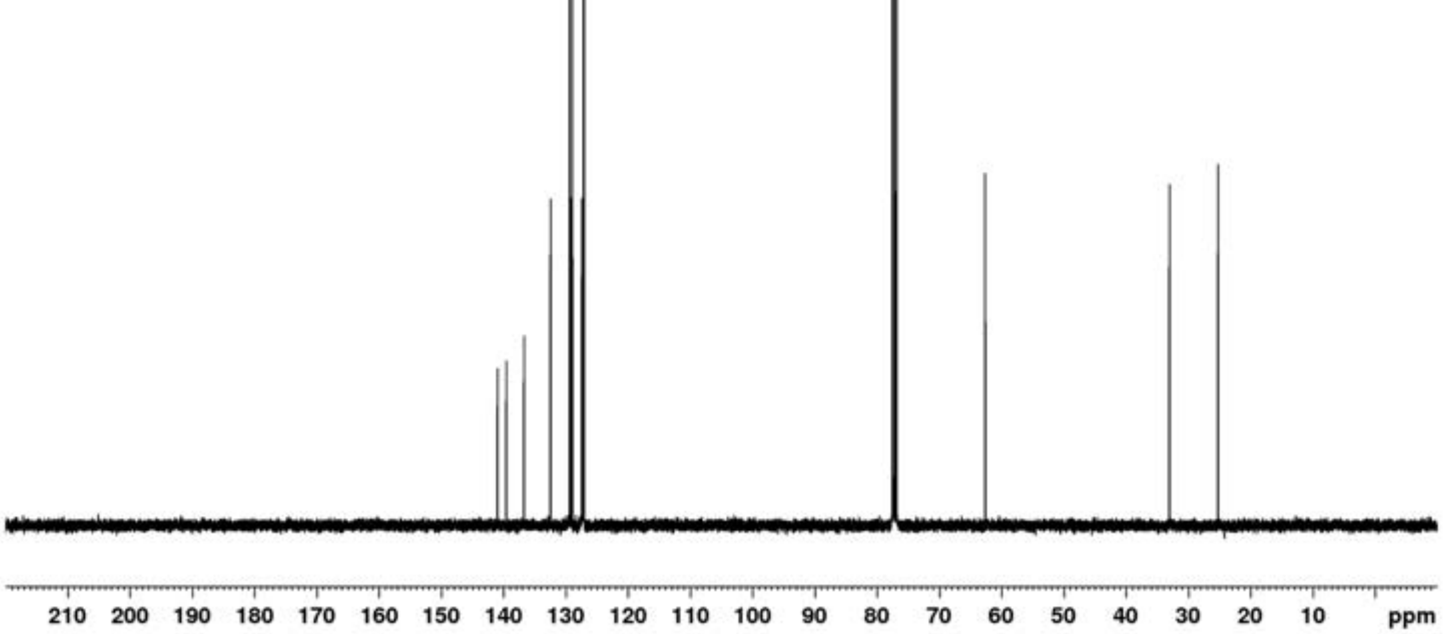

¹H NMR (400 MHz, $CDCl_3$) for *ethyl (Z)-4-(5-hydroxypent-1-en-1-yl)benzoate* (**1y**)

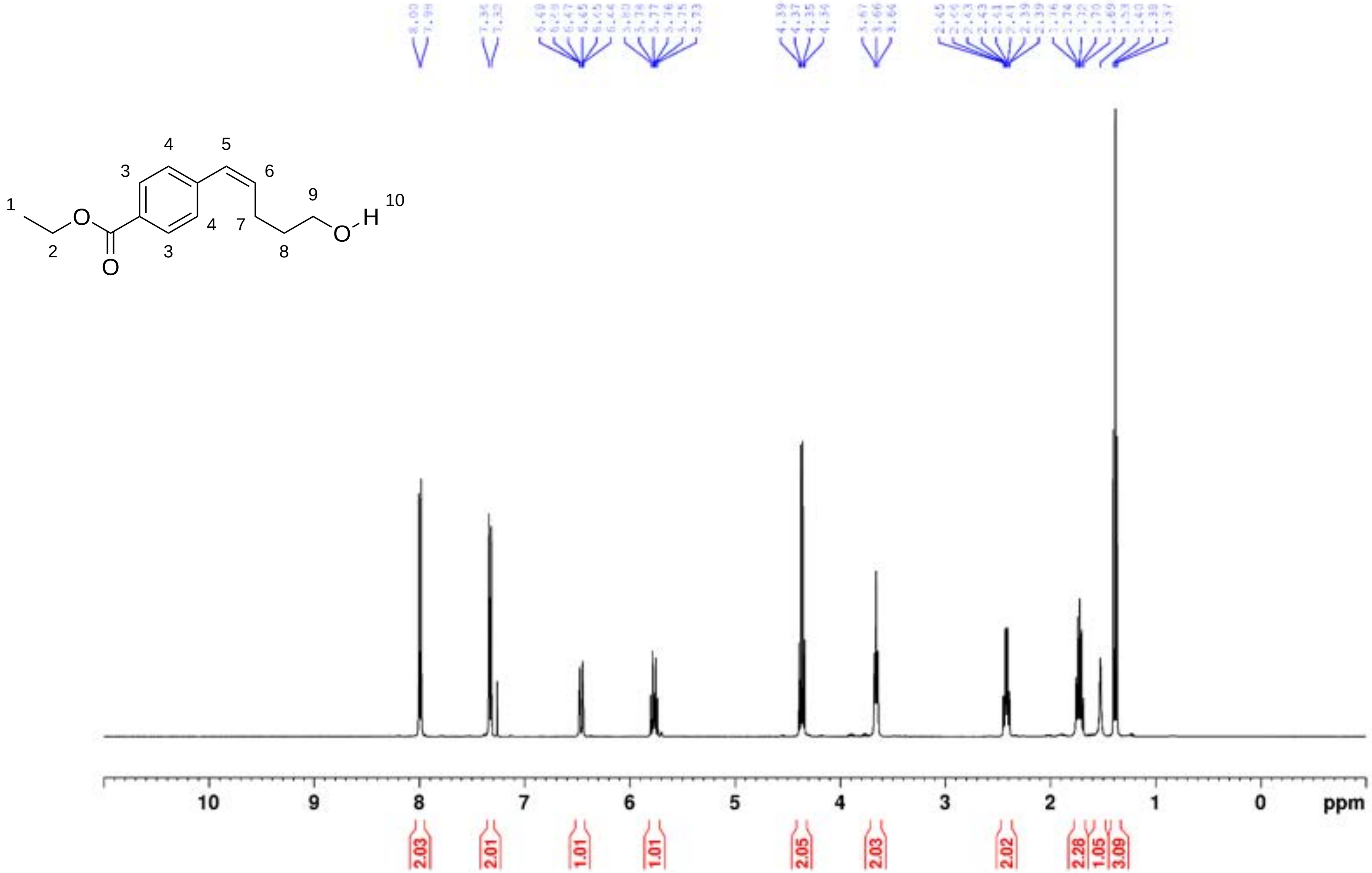

^{13}C NMR (101 MHz, $CDCl_3$) for *ethyl (Z)-4-(5-hydroxypent-1-en-1-yl)benzoate* (**1y**)

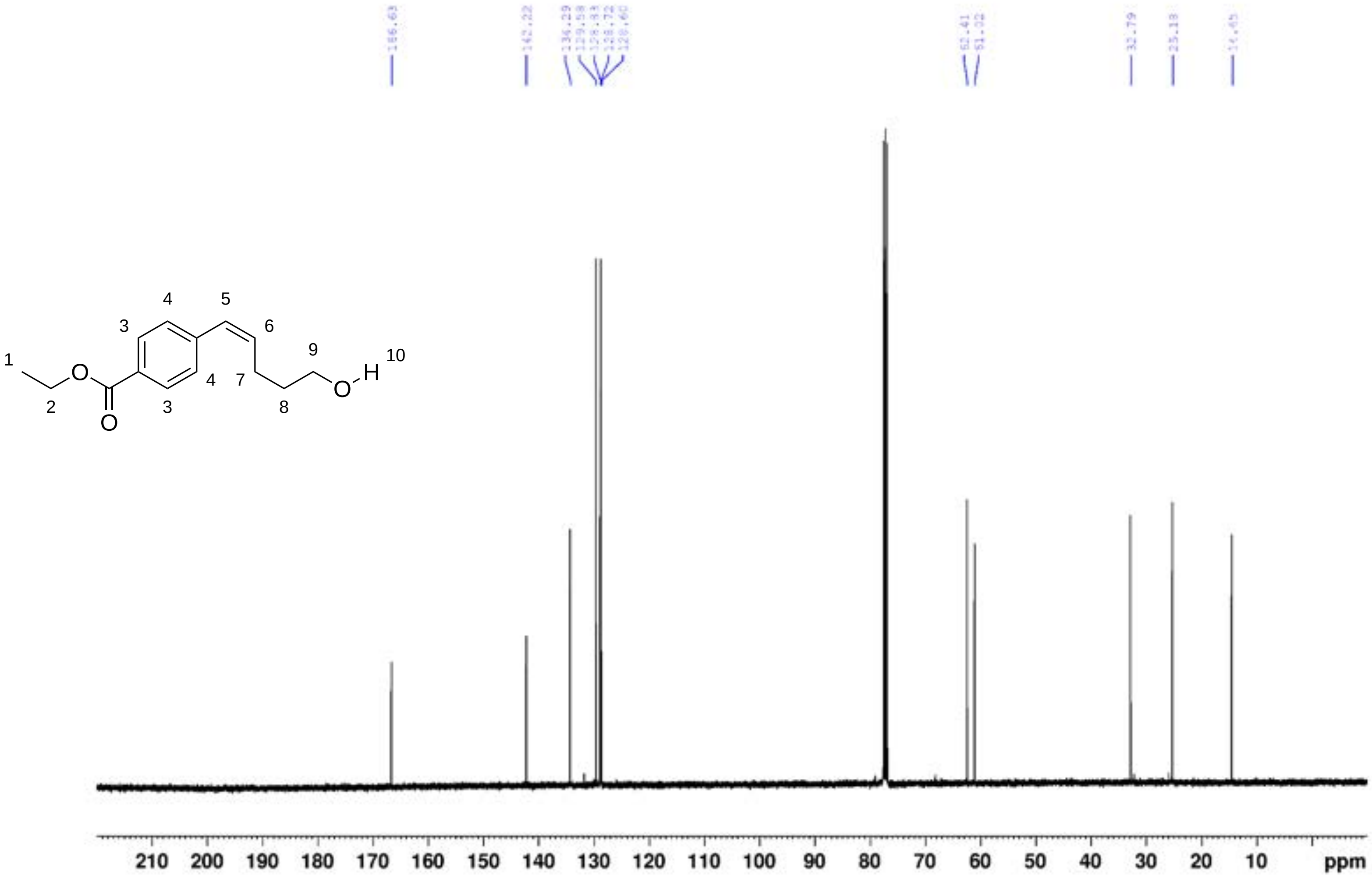

^{1}H NMR (400 MHz, $CDCl_3$) for *tert-butyl (Z)-(4-(5-hydroxypent-1-en-1-yl)phenyl)carbamate* (**1z**)

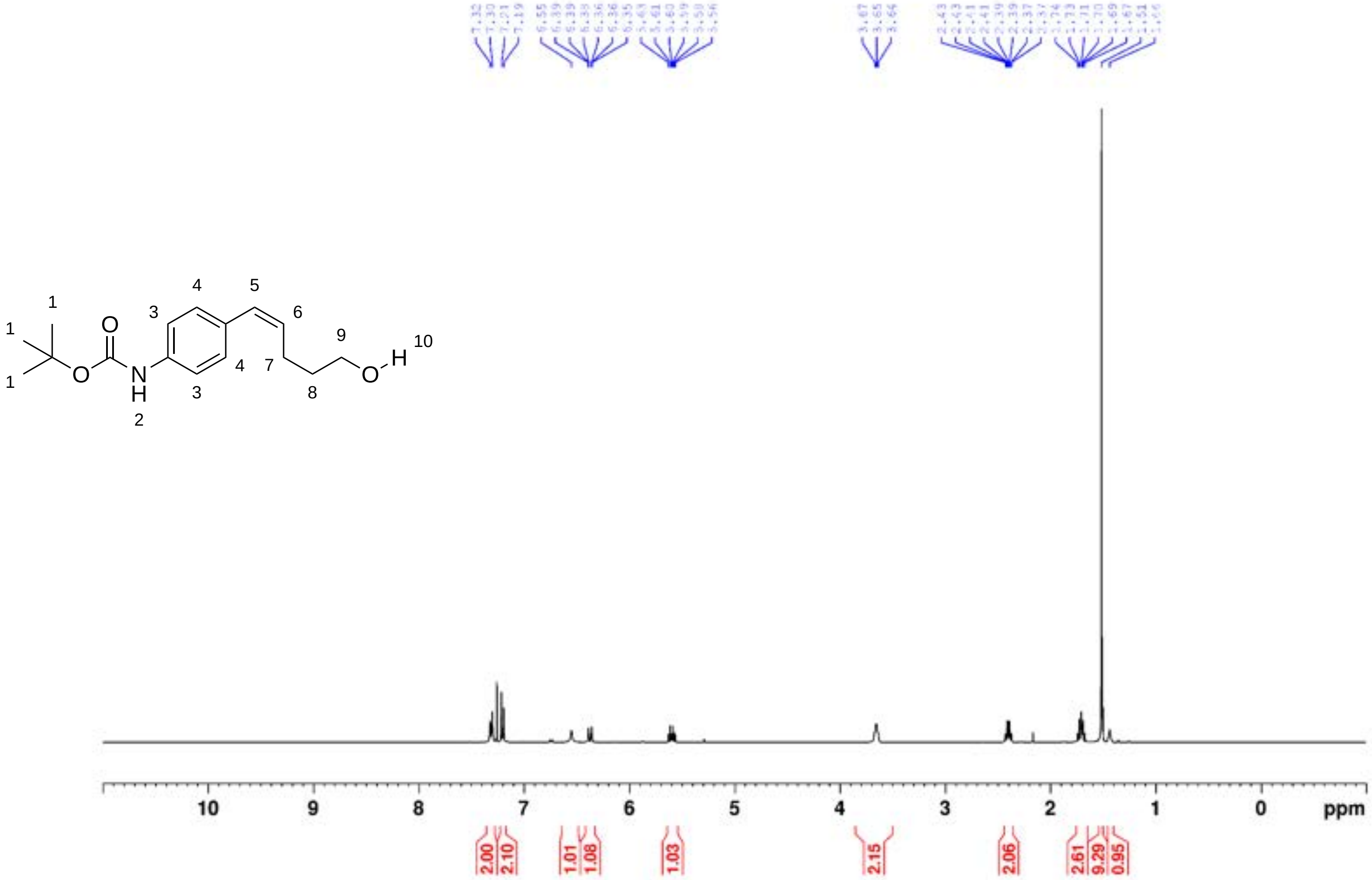

^{13}C NMR (101 MHz, $CDCl_3$) for *tert-butyl (Z)-(4-(5-hydroxypent-1-en-1-yl)phenyl)carbamate* (**1z**)

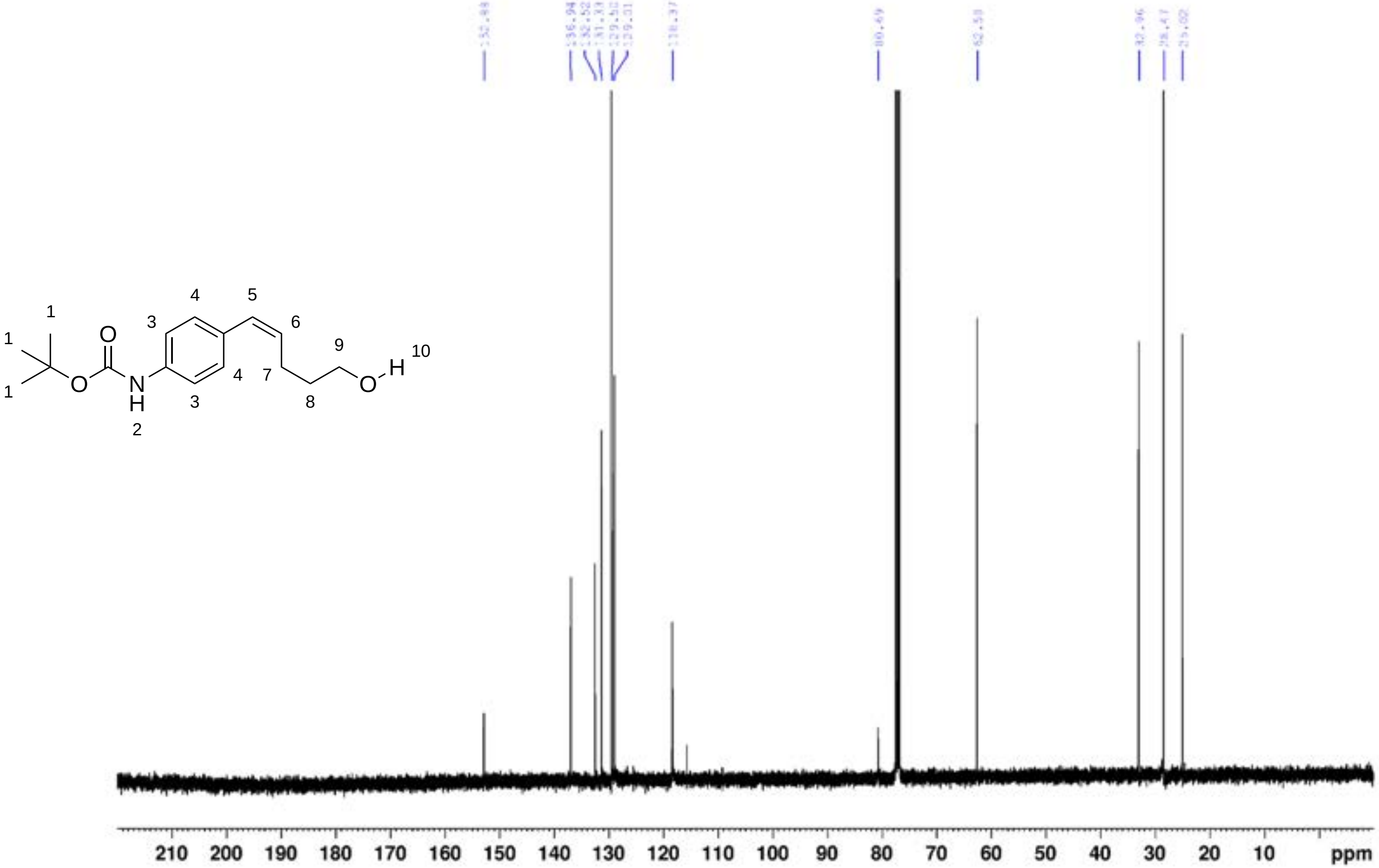

^{1}H NMR (400 MHz, $CDCl_3$) for *5-(4-methoxyphenyl)pent-4-yn-1-ol*

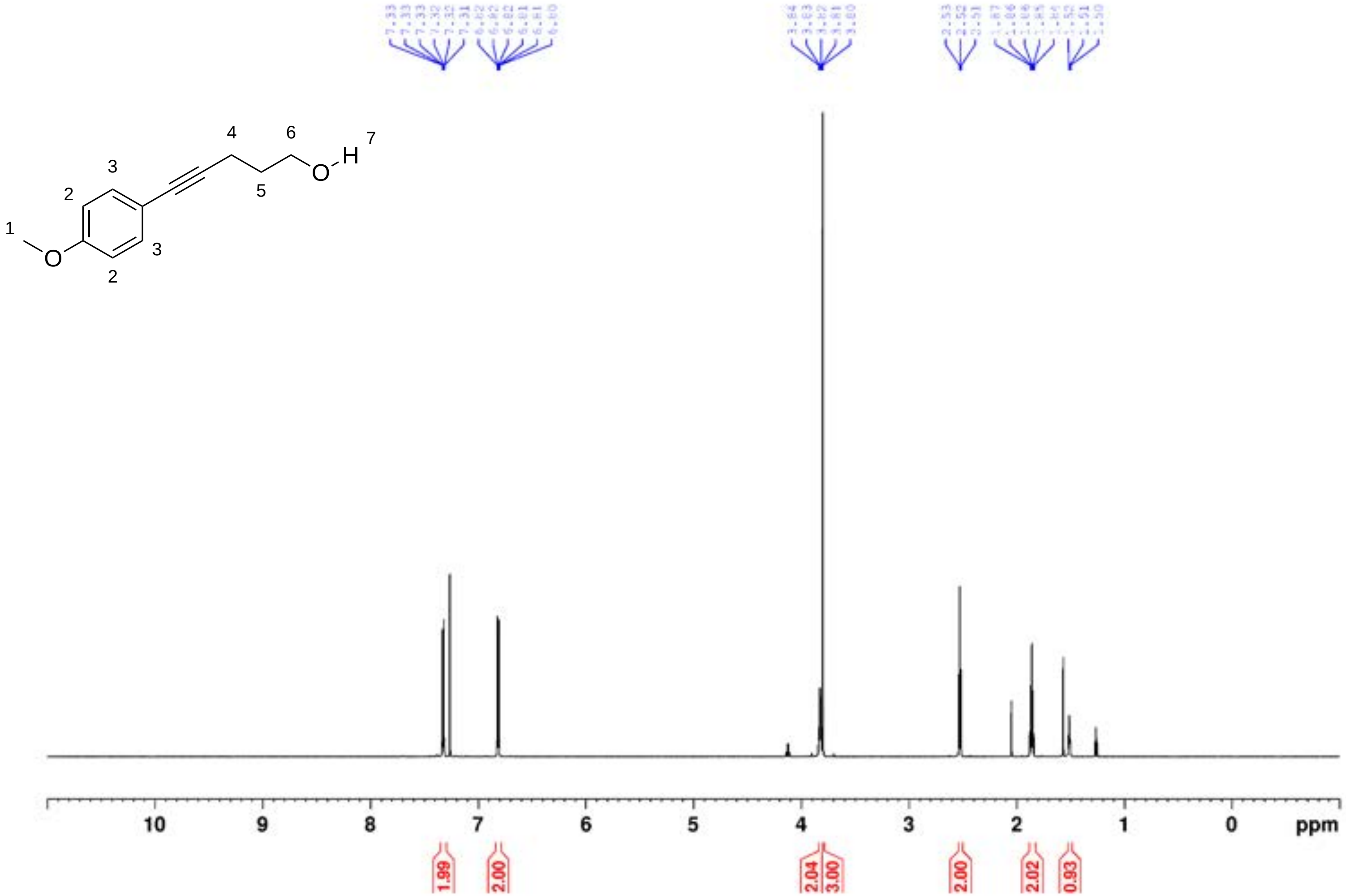

^{13}C NMR (101 MHz, $CDCl_3$) for *5-(4-methoxyphenyl)pent-4-yn-1-ol*

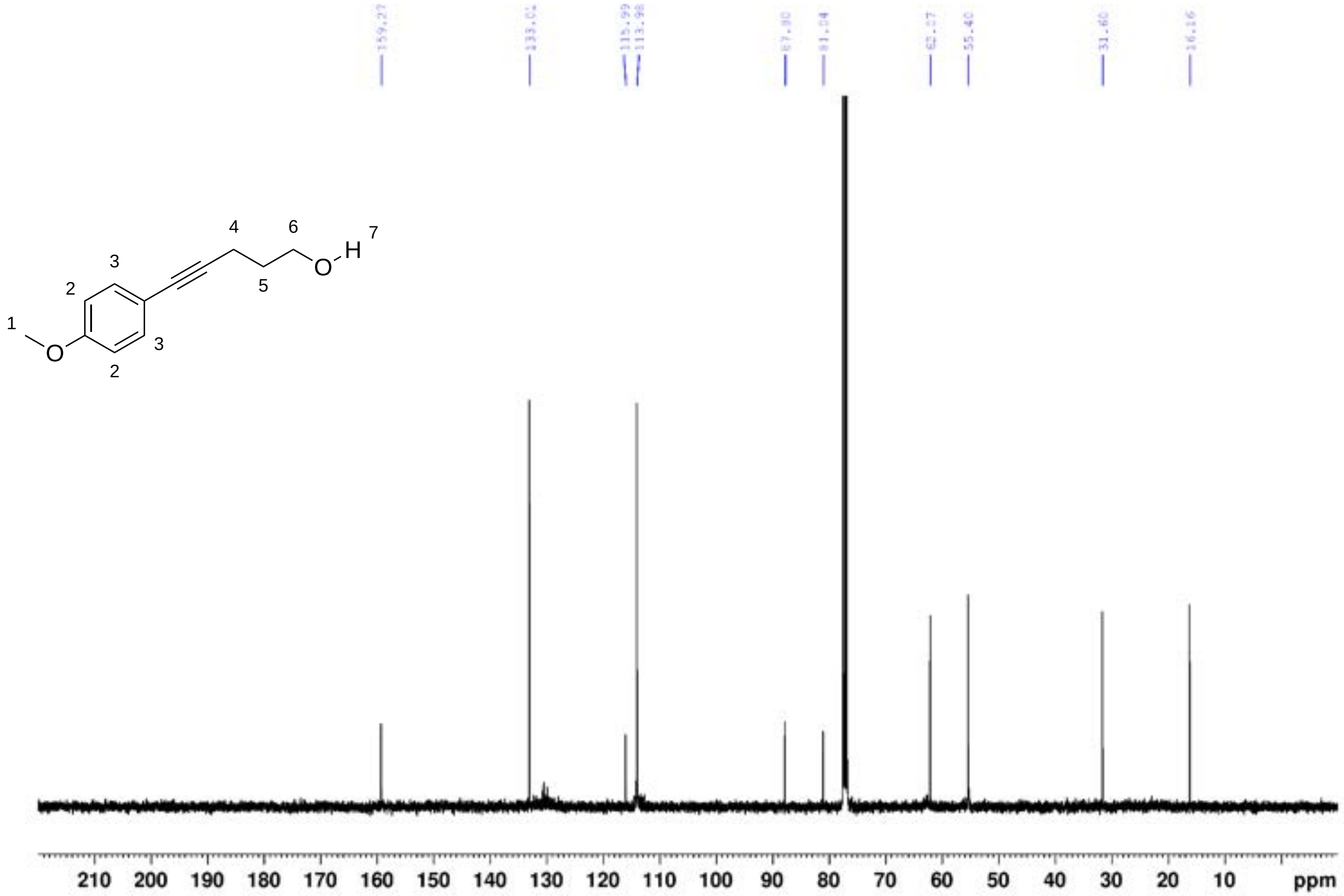

^{1}H NMR (400 MHz, $CDCl_3$) for *(Z)-5-(4-methoxyphenyl)pent-4-en-1-ol* (**1aa**)

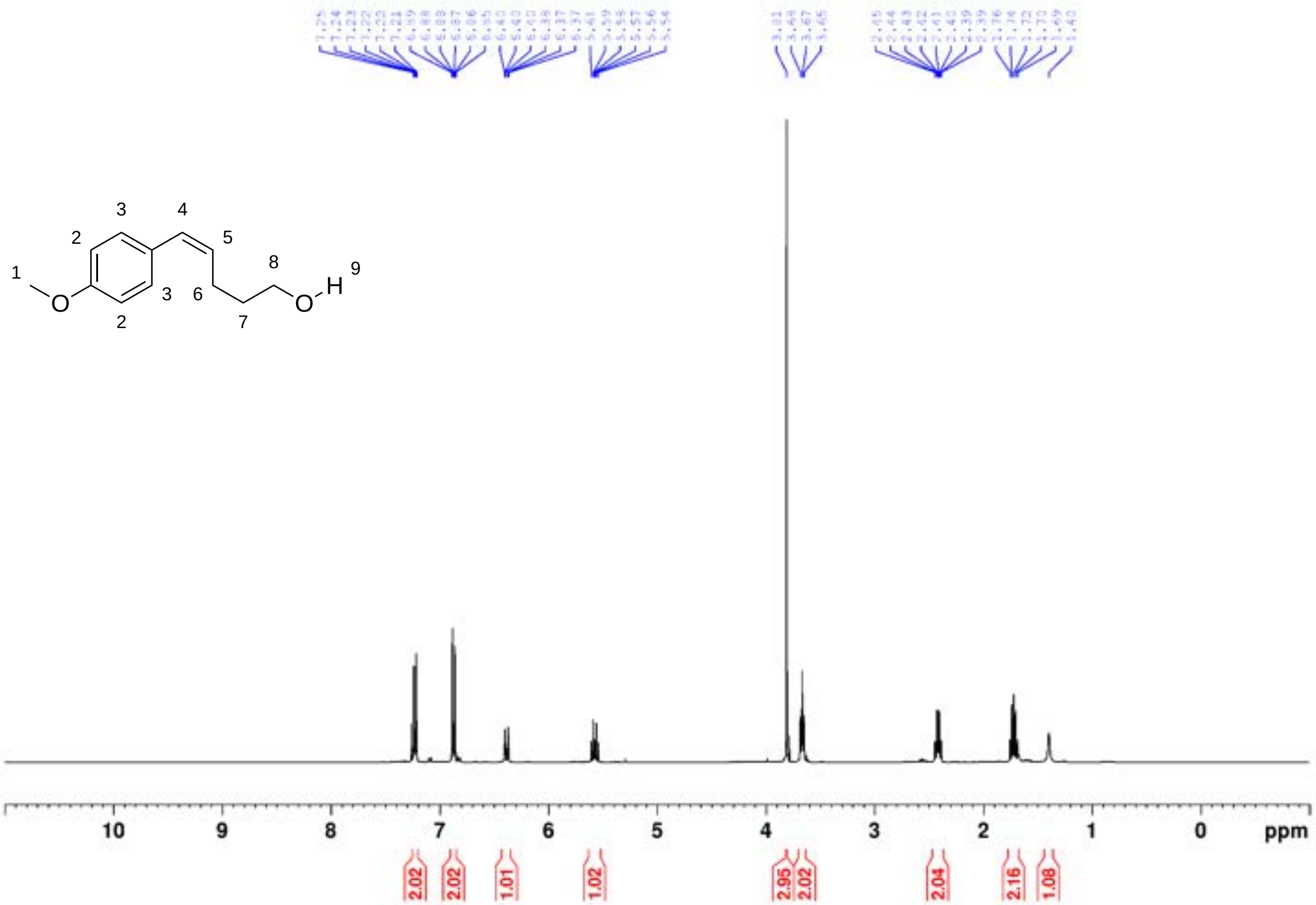

^{13}C NMR (101 MHz, $CDCl_3$) for *(Z)-5-(4-methoxyphenyl)pent-4-en-1-ol* (**1aa**)

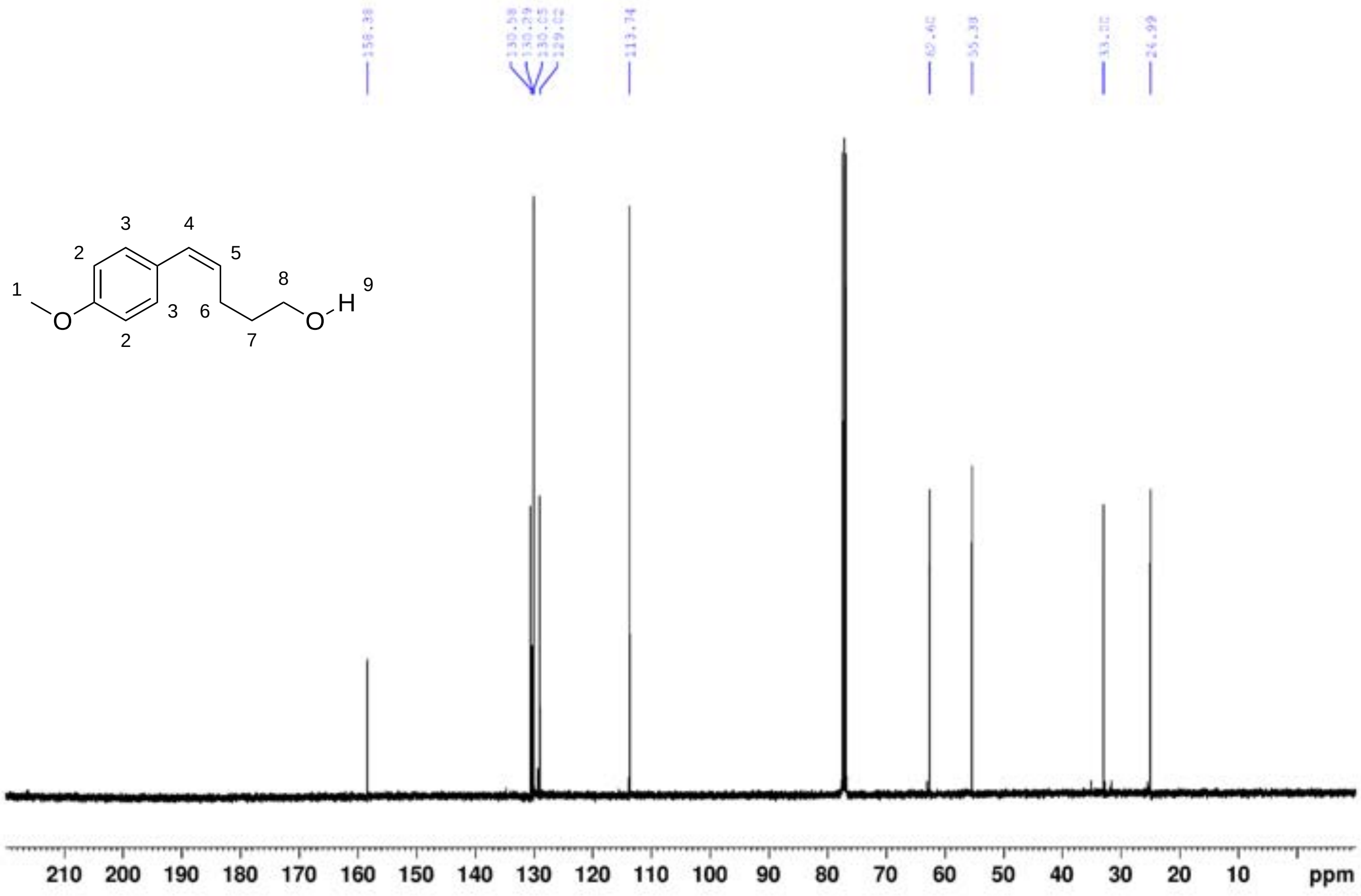

¹H NMR (400 MHz, $CDCl_3$) for *5-(4-chlorophenyl)pent-4-yn-1-ol*

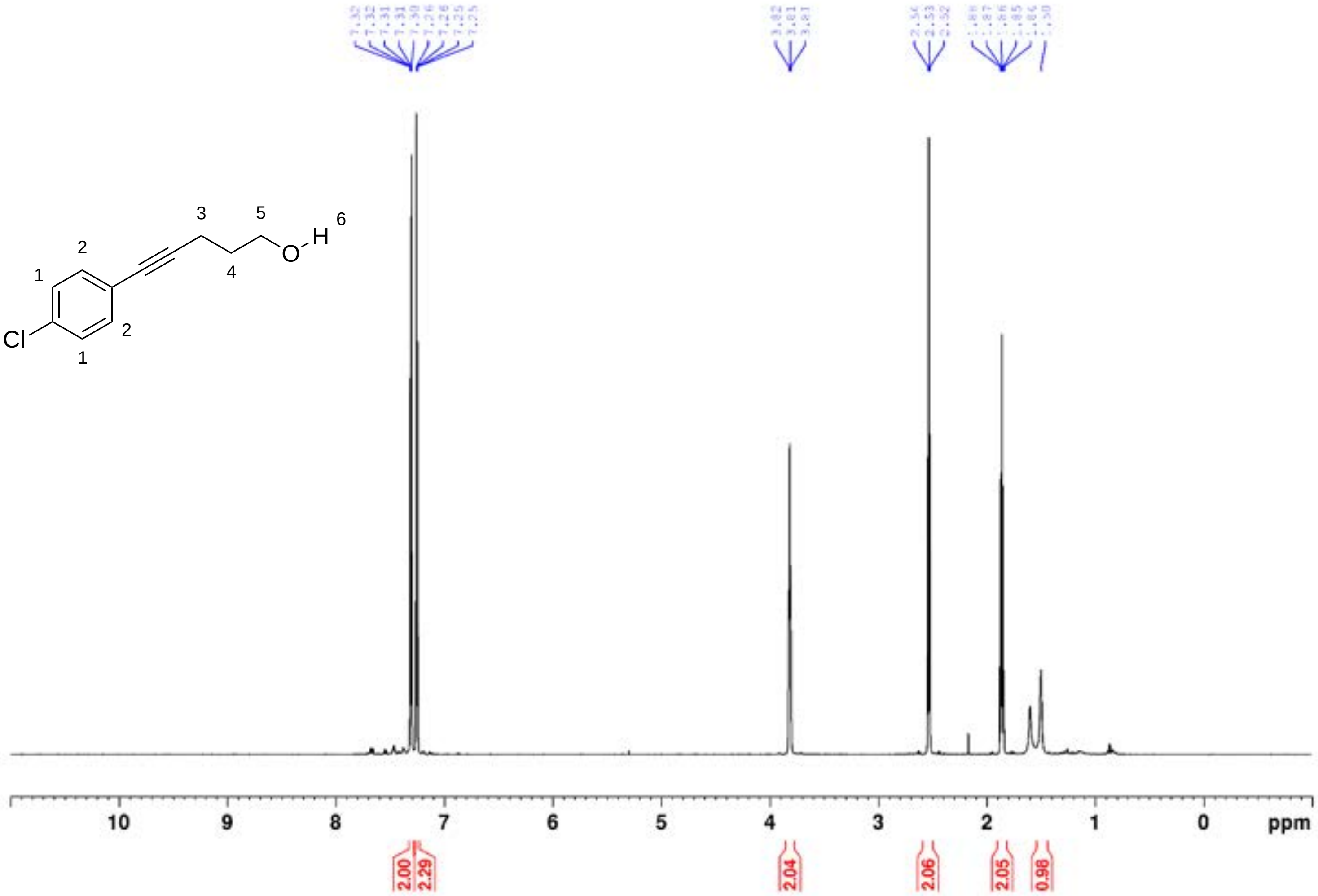

^{13}C NMR (101 MHz, $CDCl_3$) for *5-(4-chlorophenyl)pent-4-yn-1-ol*

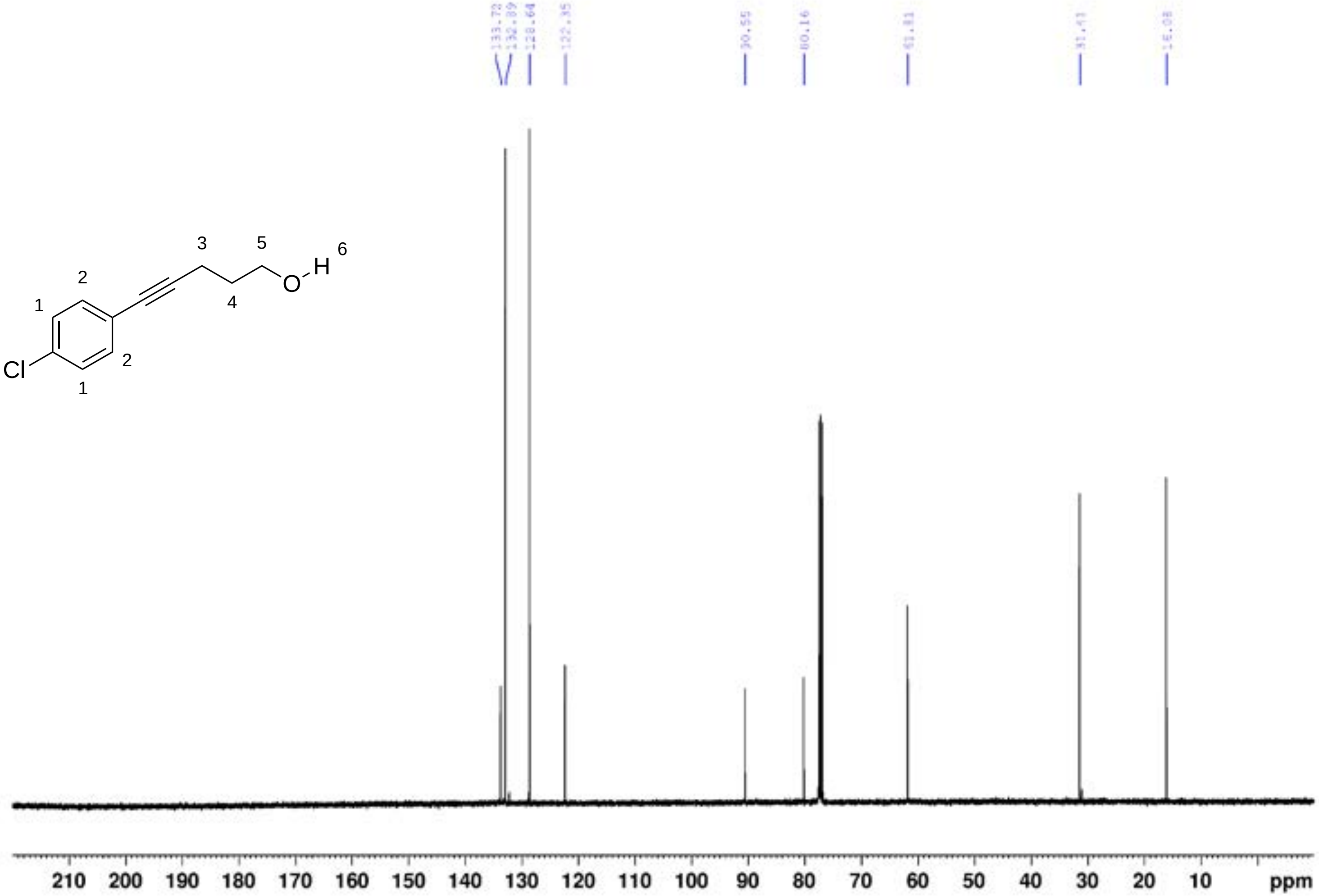

¹H NMR (400 MHz, $CDCl_3$) for *(Z)-5-(4-chlorophenyl)pent-4-en-1-ol* (**1ac**)

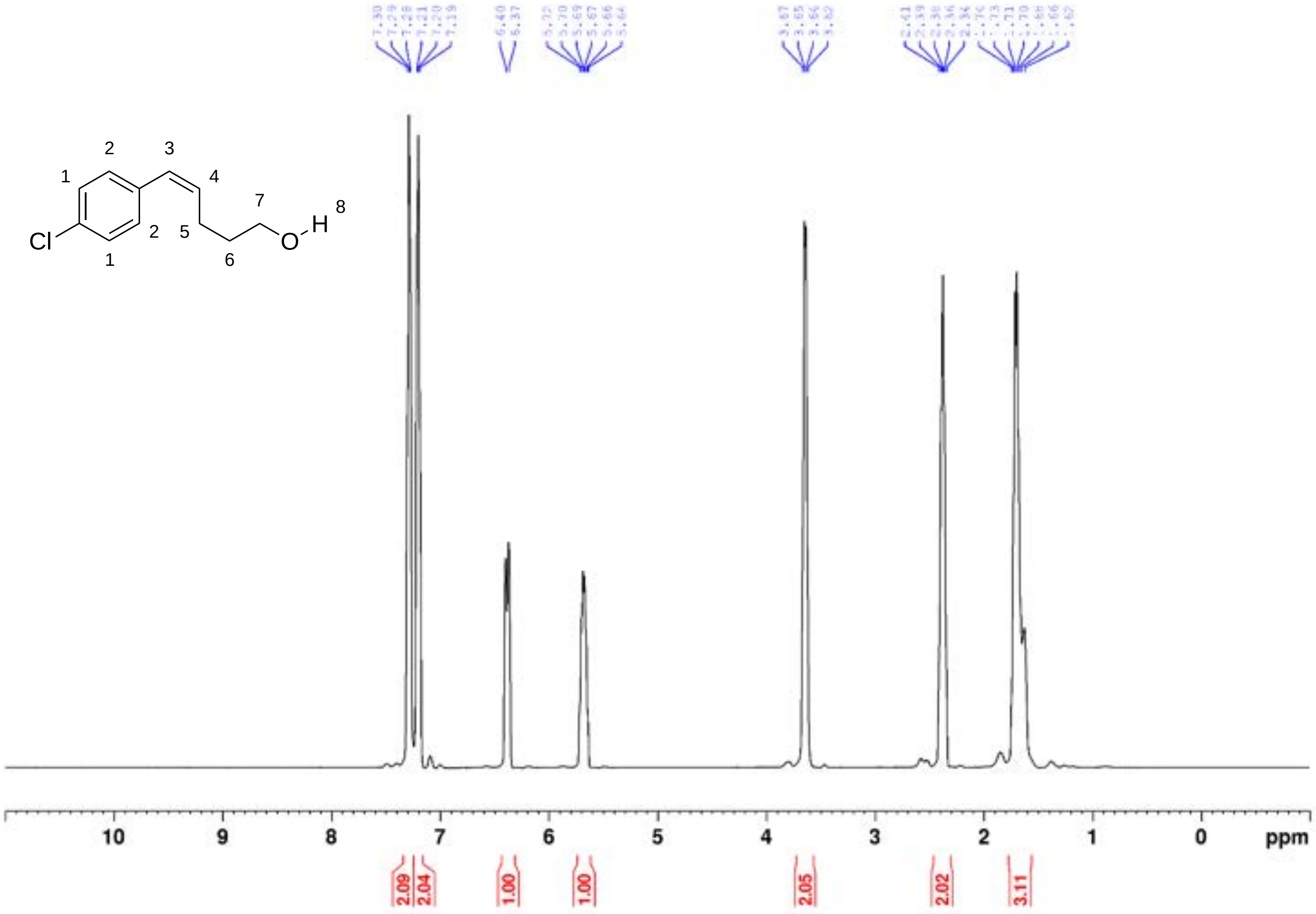

^{13}C NMR (101 MHz, $CDCl_3$) for *(Z)-5-(4-chlorophenyl)pent-4-en-1-ol* (**1ac**)

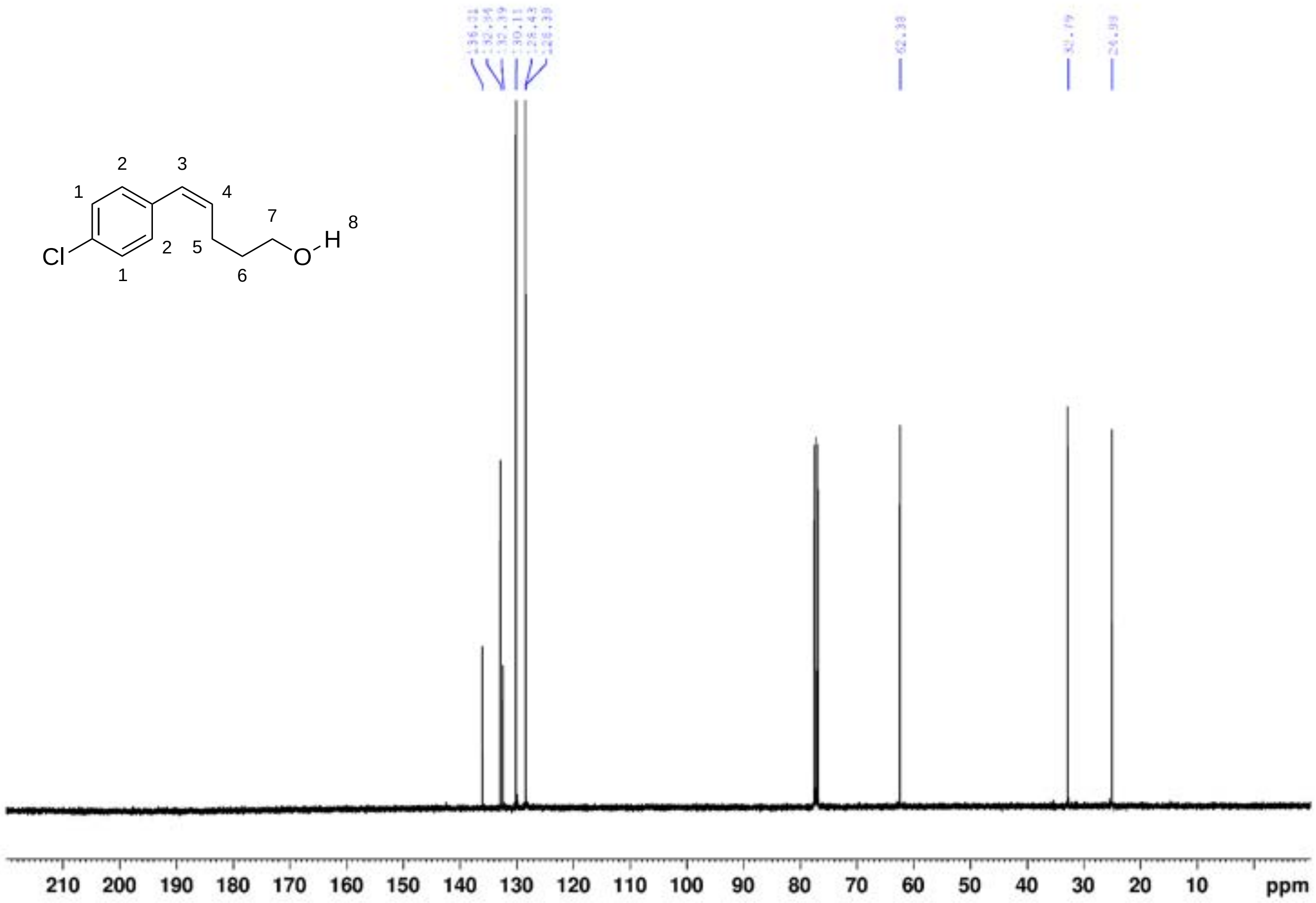

^{1}H NMR (400 MHz, $CDCl_3$) for *5-(4-bromophenyl)pent-4-yn-1-ol*

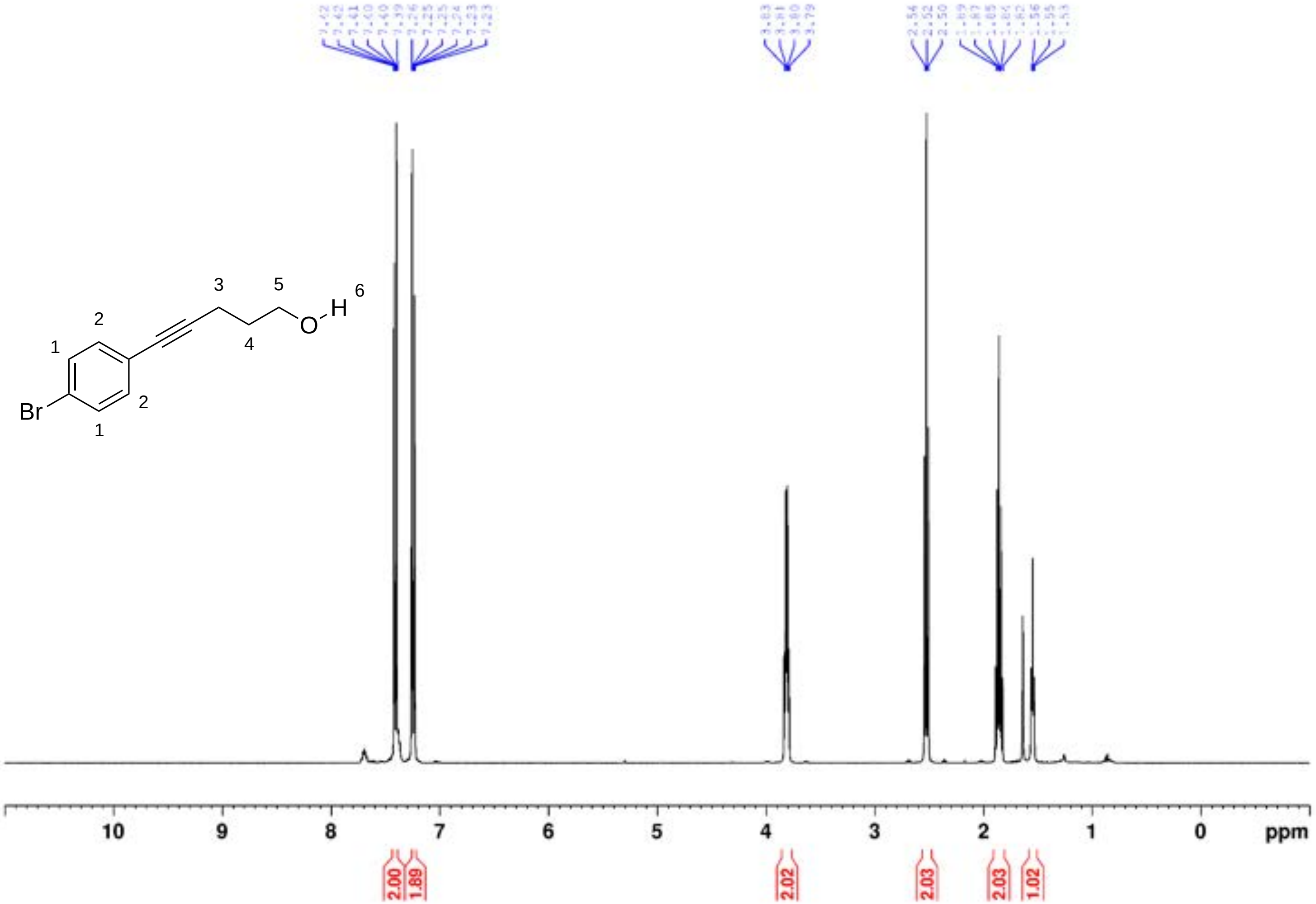

^{13}C NMR (101 MHz, $CDCl_3$) for *5-(4-bromophenyl)pent-4-yn-1-ol*

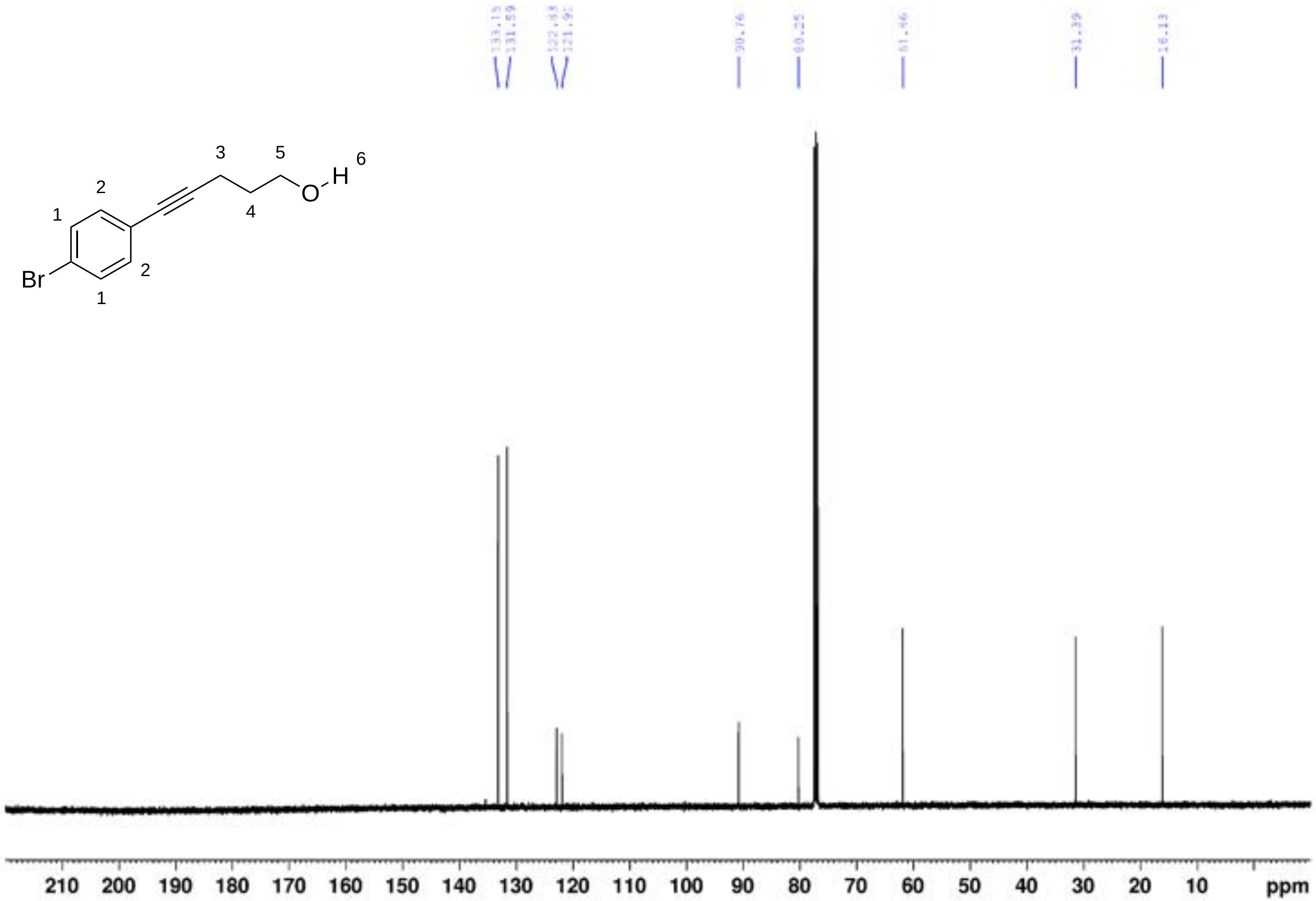

^{1}H NMR (400 MHz, $CDCl_3$) for *(Z)-5-(4-bromophenyl)pent-4-en-1-ol* (**1ad**)

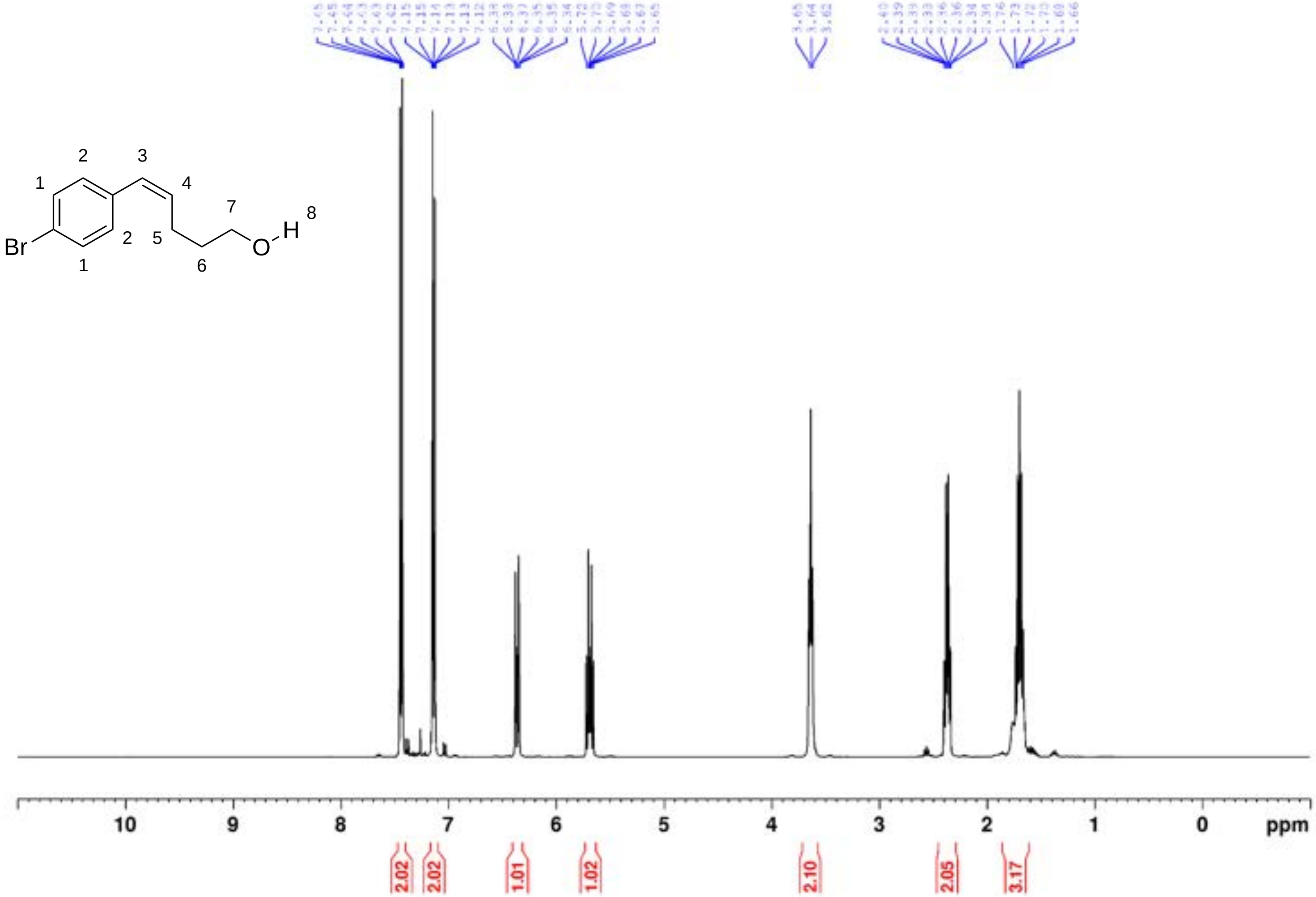

^{13}C NMR (101 MHz, $CDCl_3$) for *(Z)-5-(4-bromophenyl)pent-4-en-1-ol* (**1ad**)

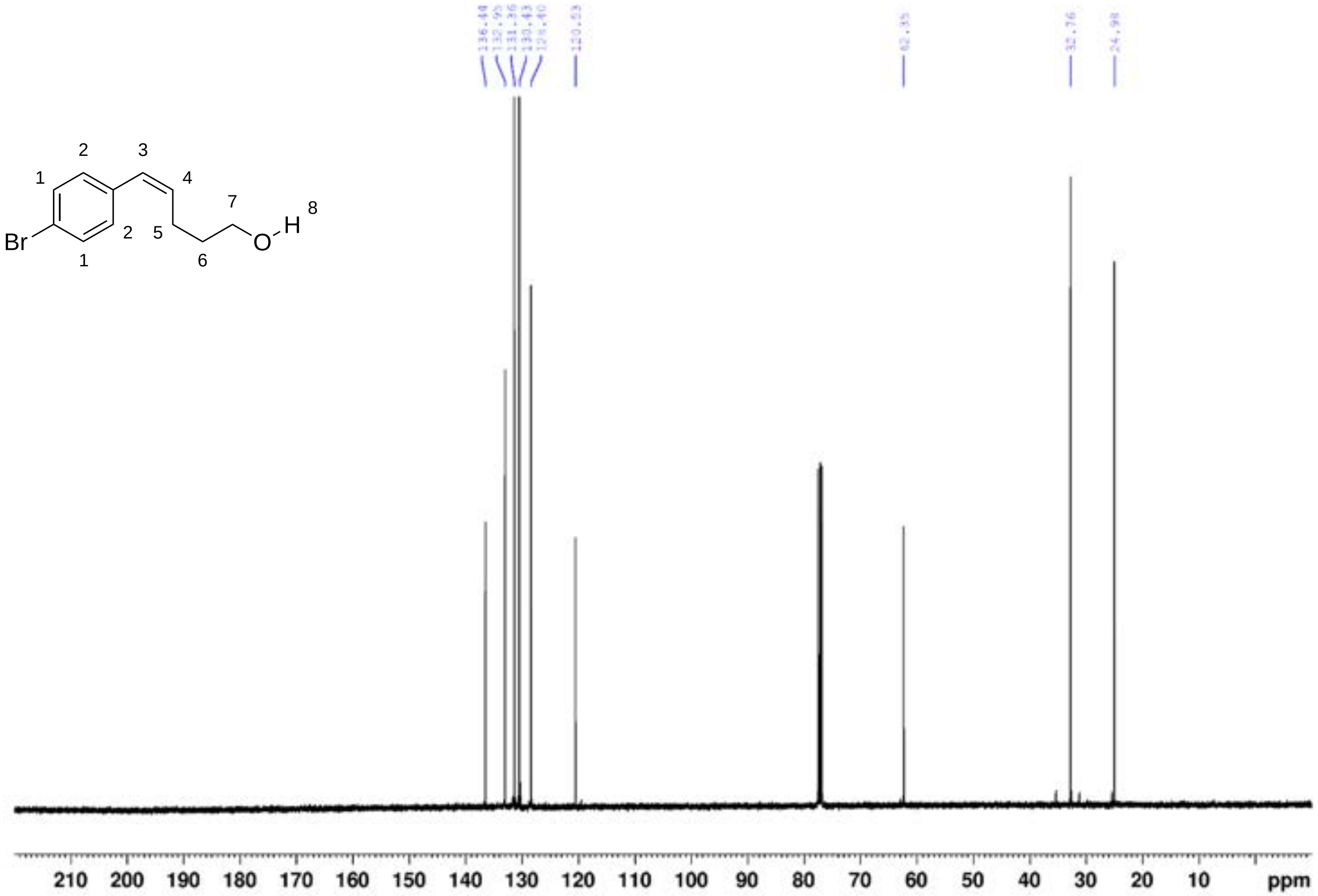

^{1}H NMR (400 MHz, $CDCl_3$) for *5-phenylpent-4-yn-1-ol*

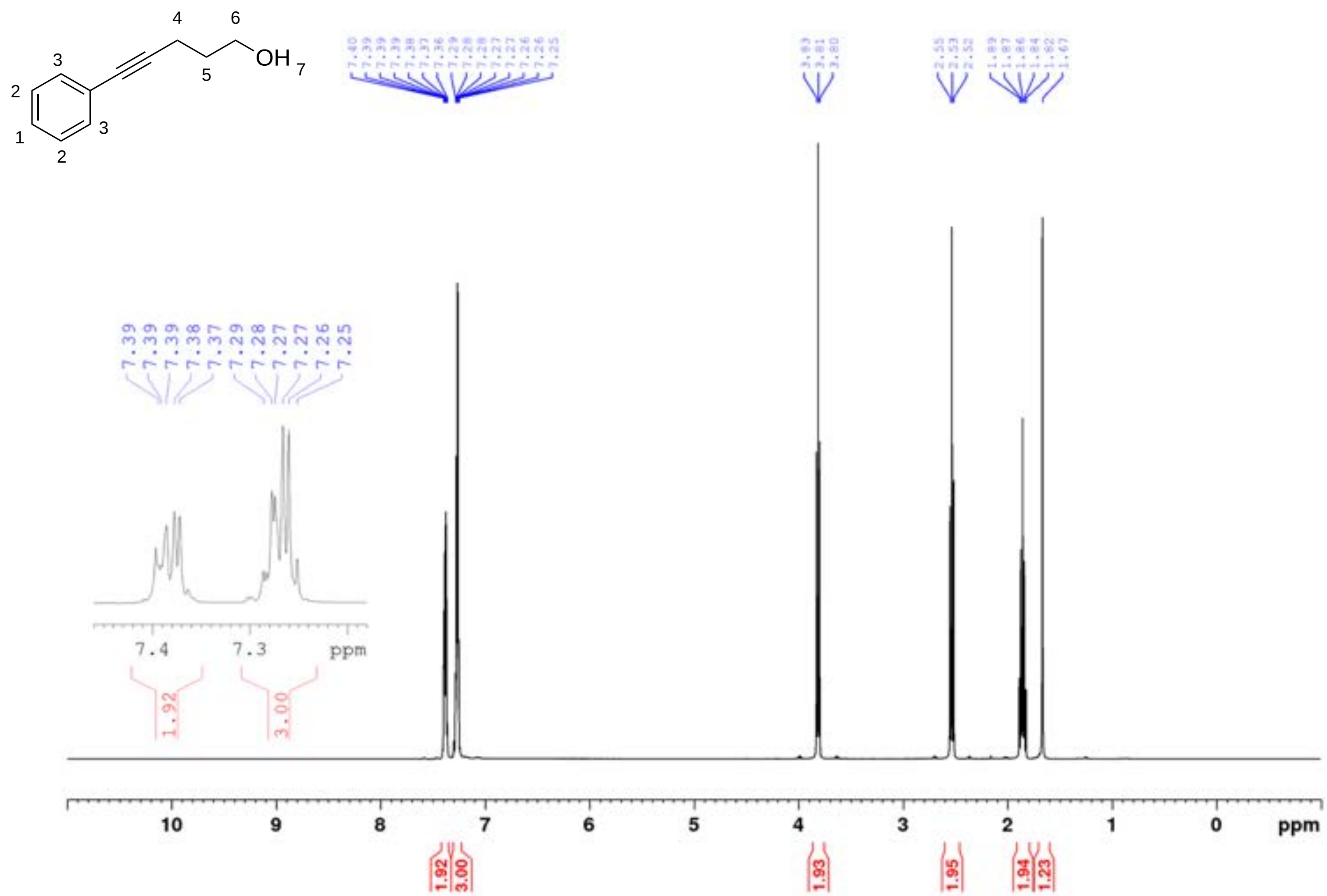

^{13}C NMR (101 MHz, $CDCl_3$)

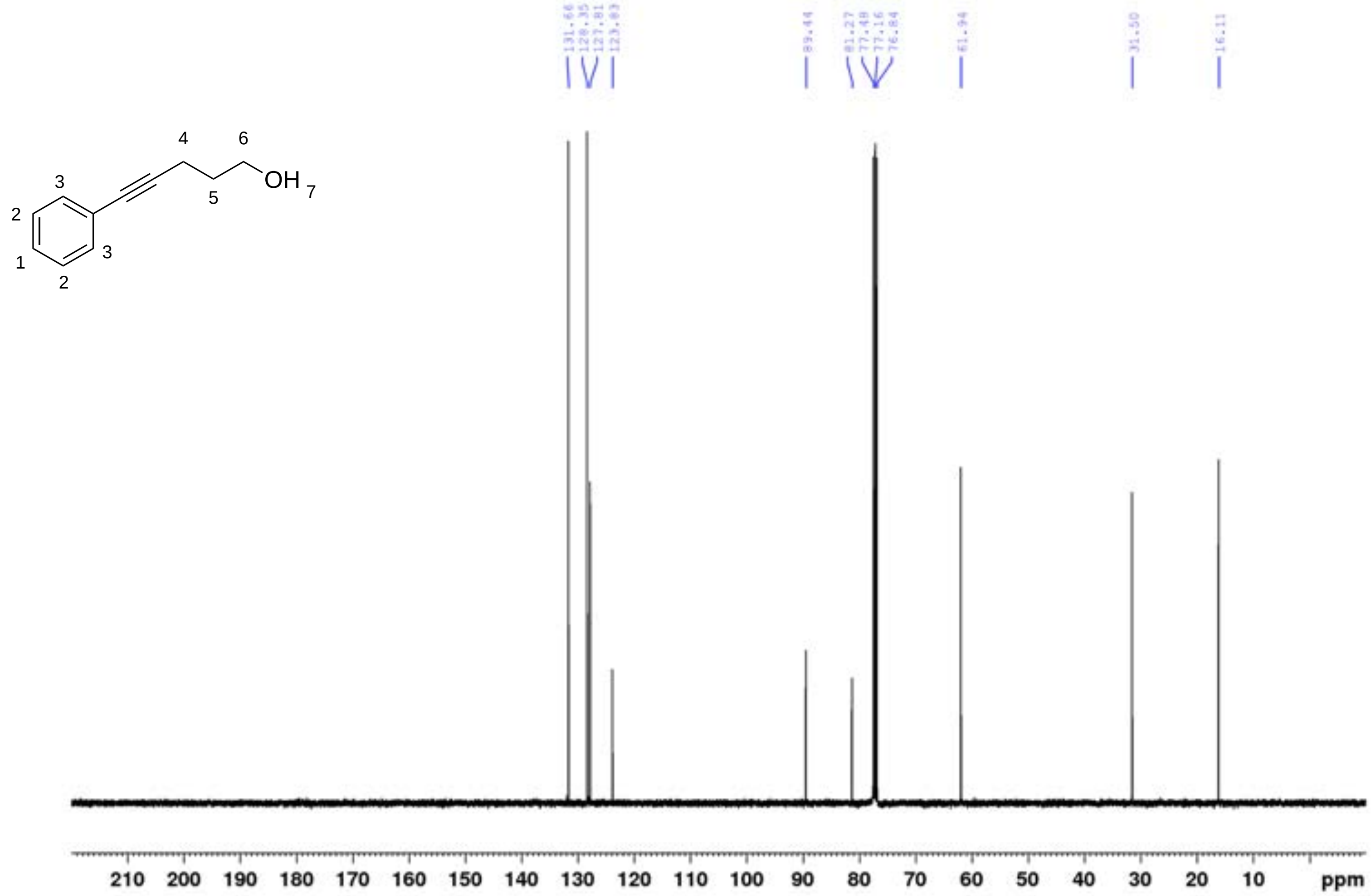

^{1}H NMR (700 MHz, $CDCl_3$) for *(E)-5-phenylpent-4-en-1-ol* (**5**)

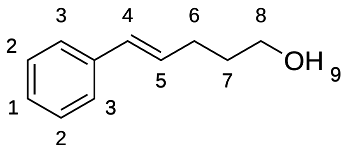

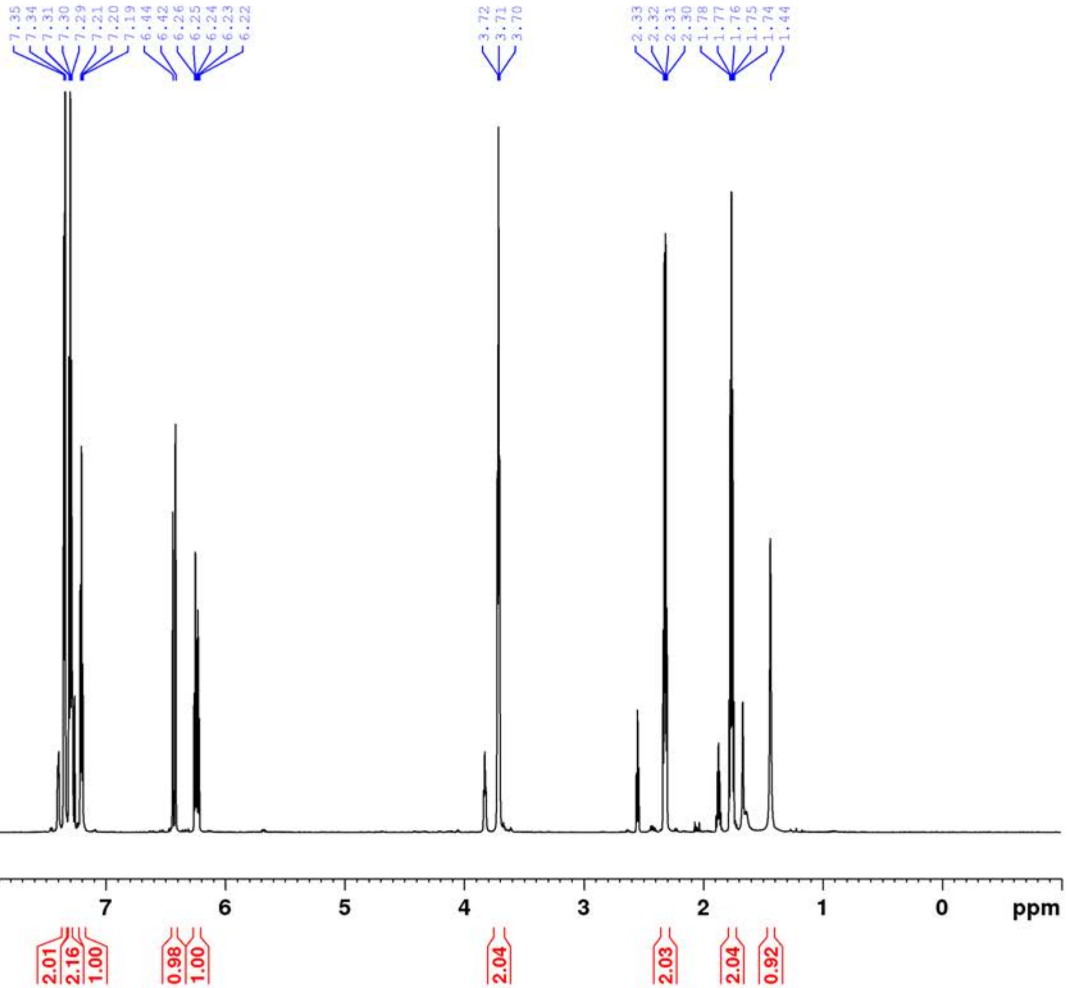

^{13}C NMR (176 MHz, $CDCl_3$) for *(E)-5-phenylpent-4-en-1-ol* (**5**)

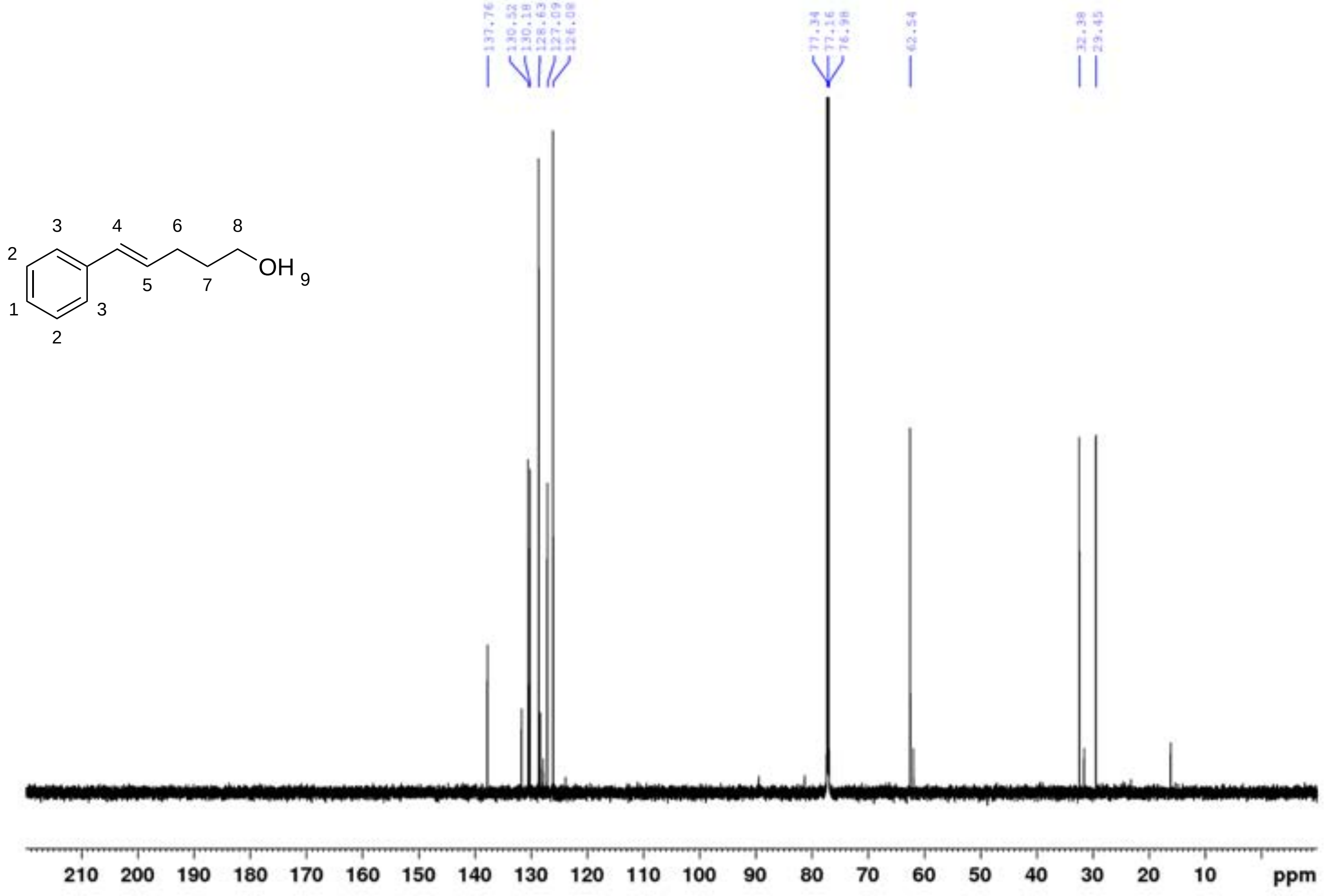

^{1}H NMR (400 MHz, $(CD_3)_2CO$) for *2,2,3,3,3-pentafluoropropyl sulfamate* (**2**)

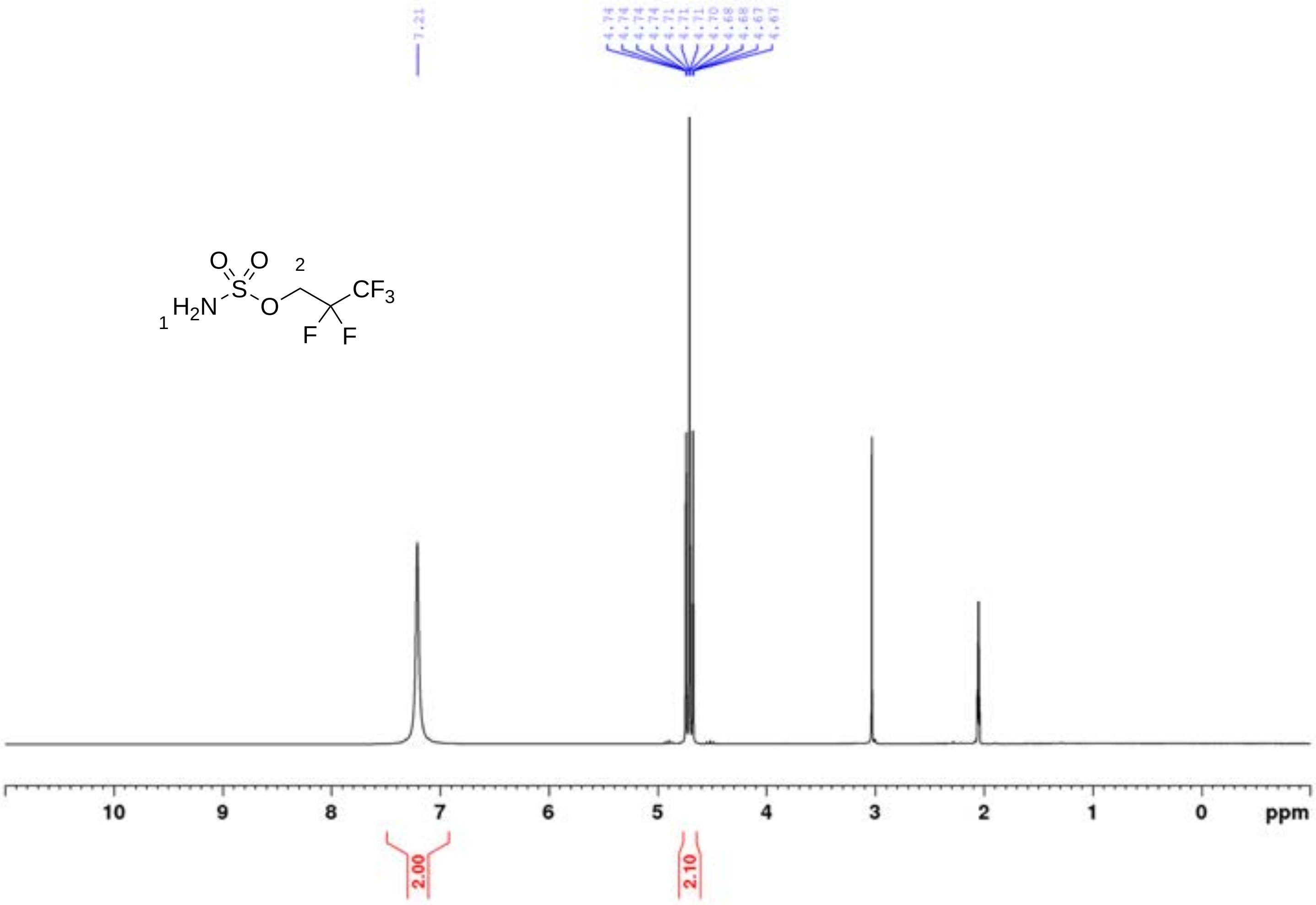

^{13}C NMR (101 MHz, $(CD_3)_2CO$) for *2,2,3,3,3-pentafluoropropyl sulfamate* (**2**)

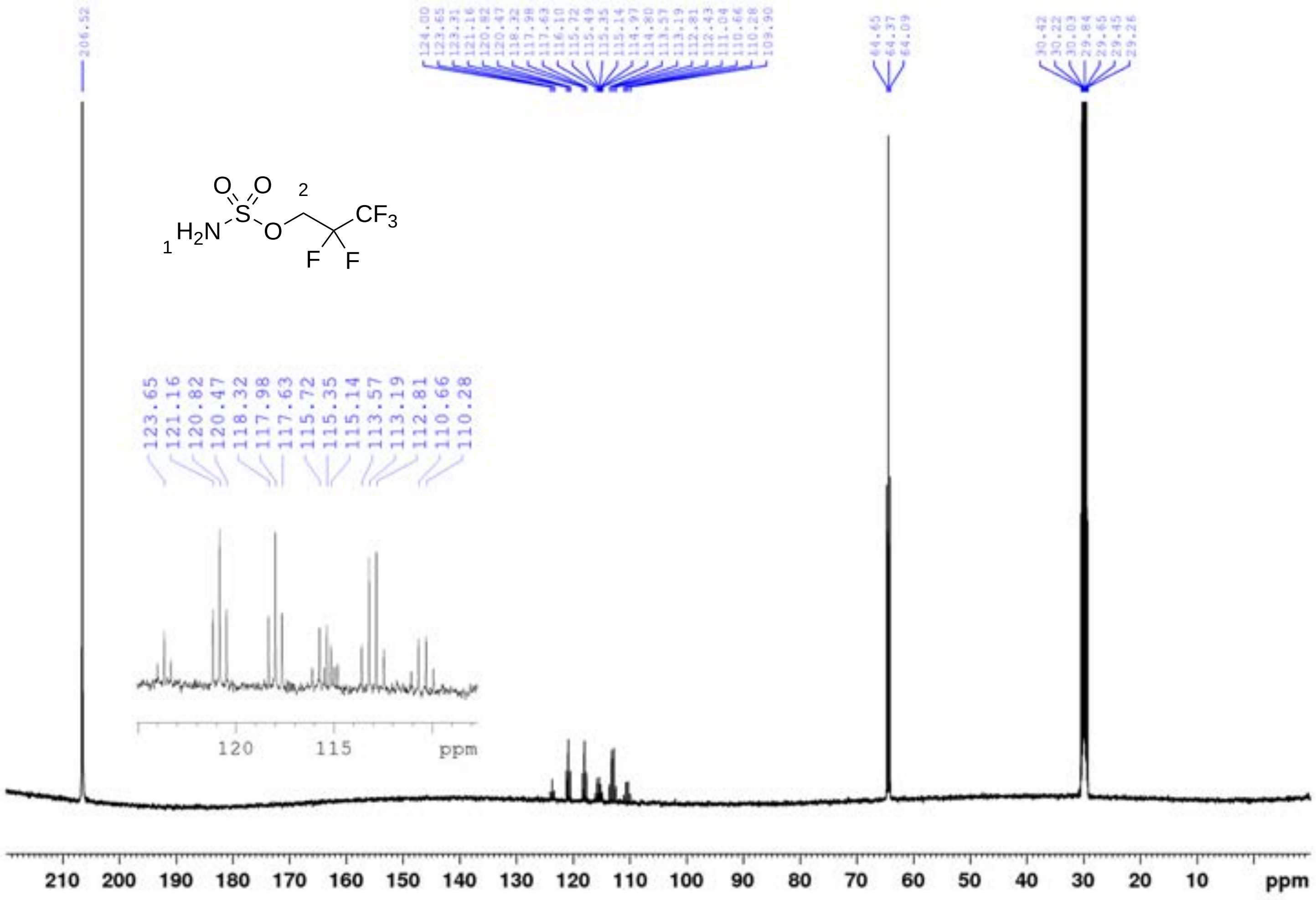

^{19}F NMR (376 MHz, $(CD_3)_2CO$) for *2,2,3,3,3-pentafluoropropyl sulfamate* (**2**)

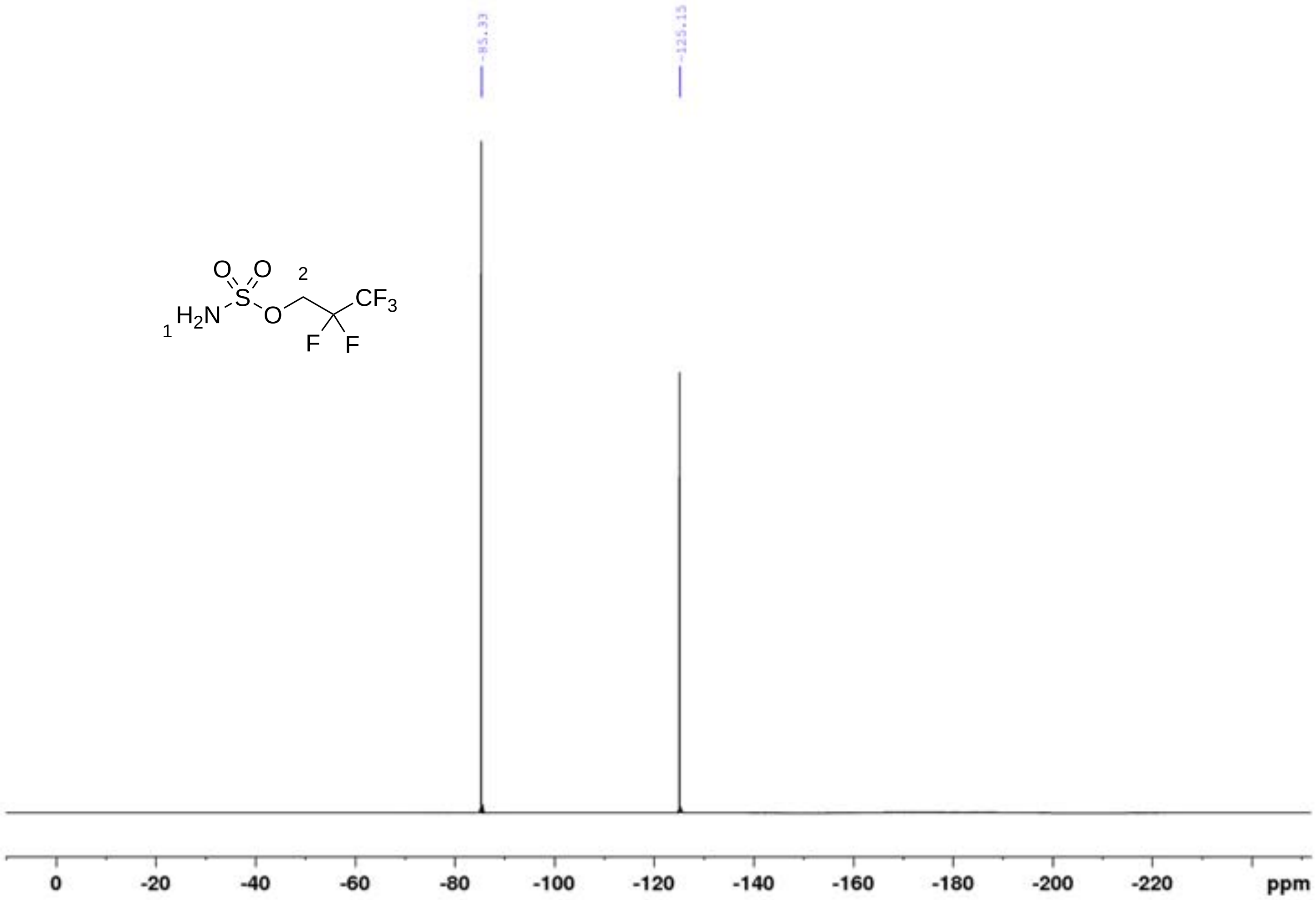

^{13}C NMR (126 MHz, MeOD) for *(perfluorophenyl)-λ^3-iodanediyl bis(2,2,2-trifluoroacetate) (TFA-Ox)*

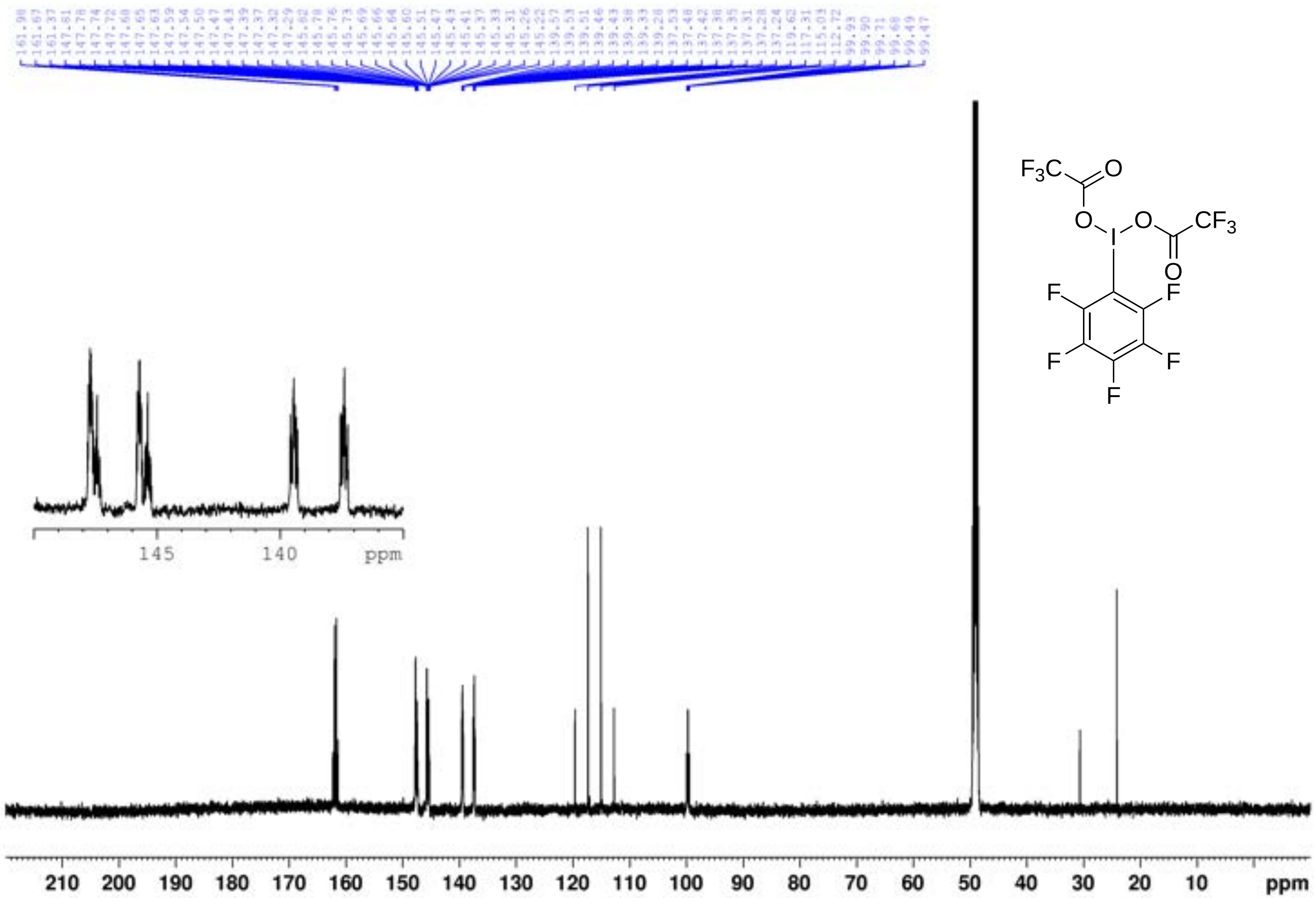

^{19}F NMR (376 MHz, MeOD) for *(perfluorophenyl)-λ^3-iodanediyl bis(2,2,2-trifluoroacetate) (TFA-Ox)*

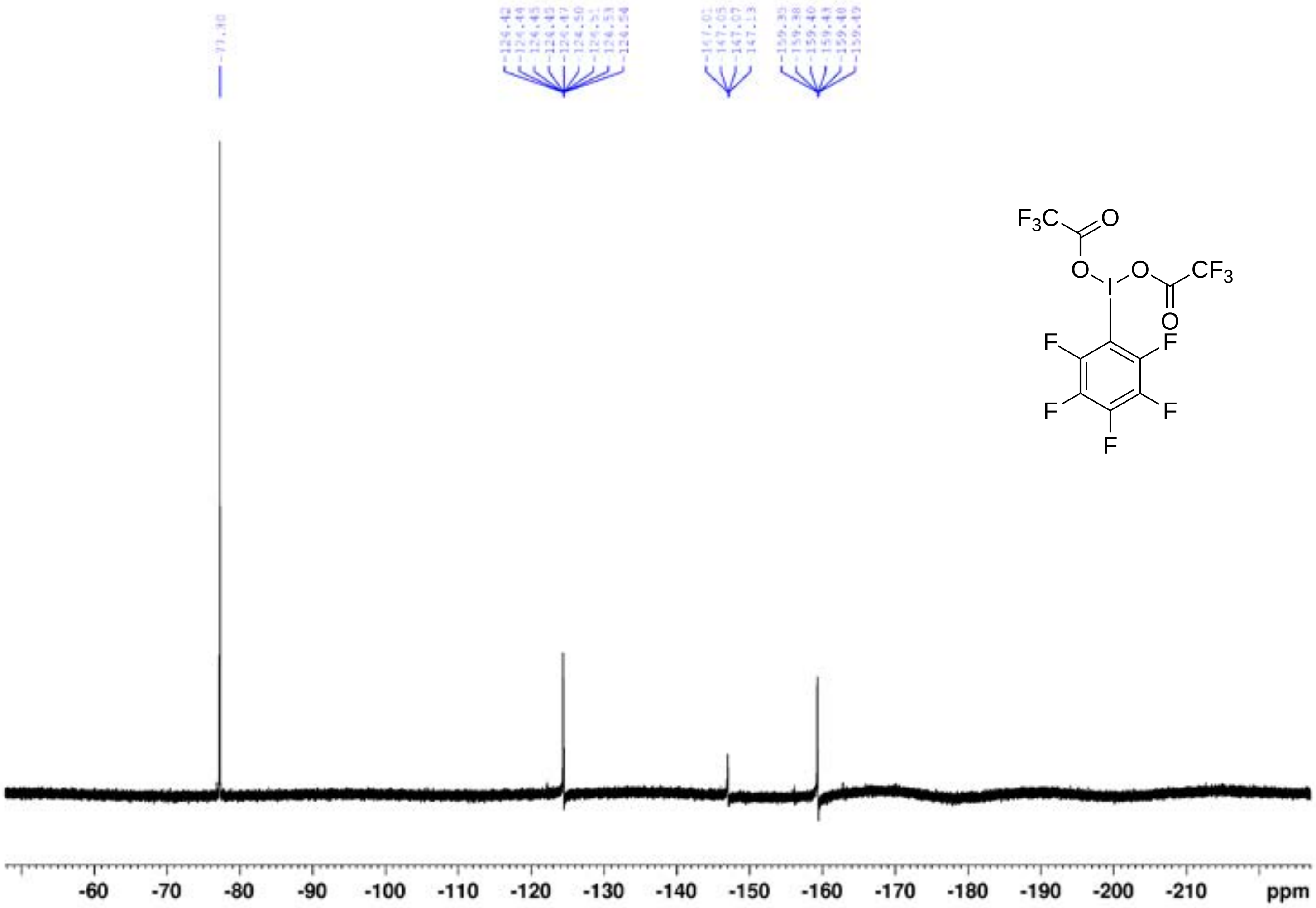

^{13}C NMR (126 MHz, MeOD) for *pentafluoroiodosobenzene (PFIOB)*

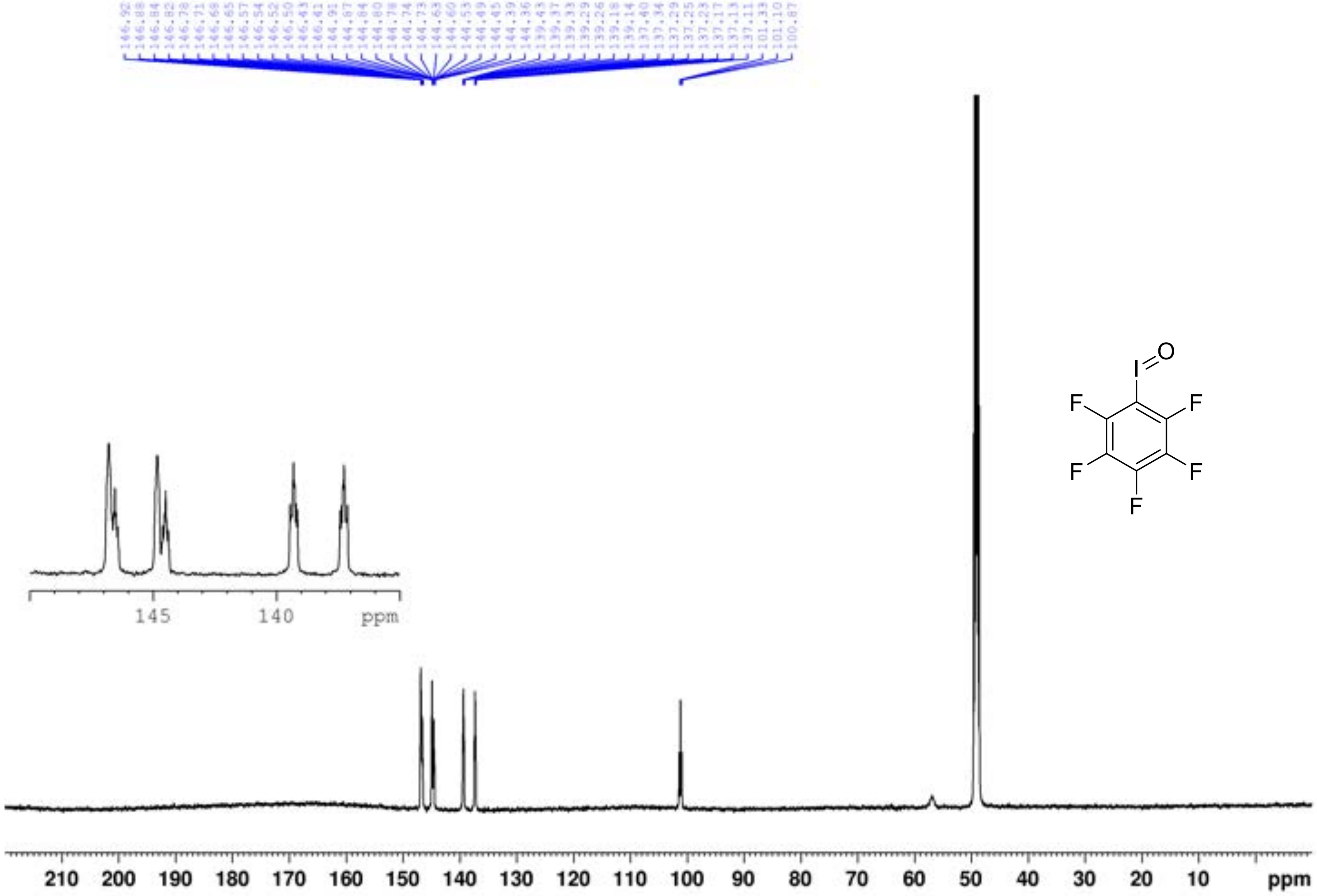

^{19}F NMR (376 MHz, MeOD) for *pentafluoroiodosobenzene (PFIOB)*

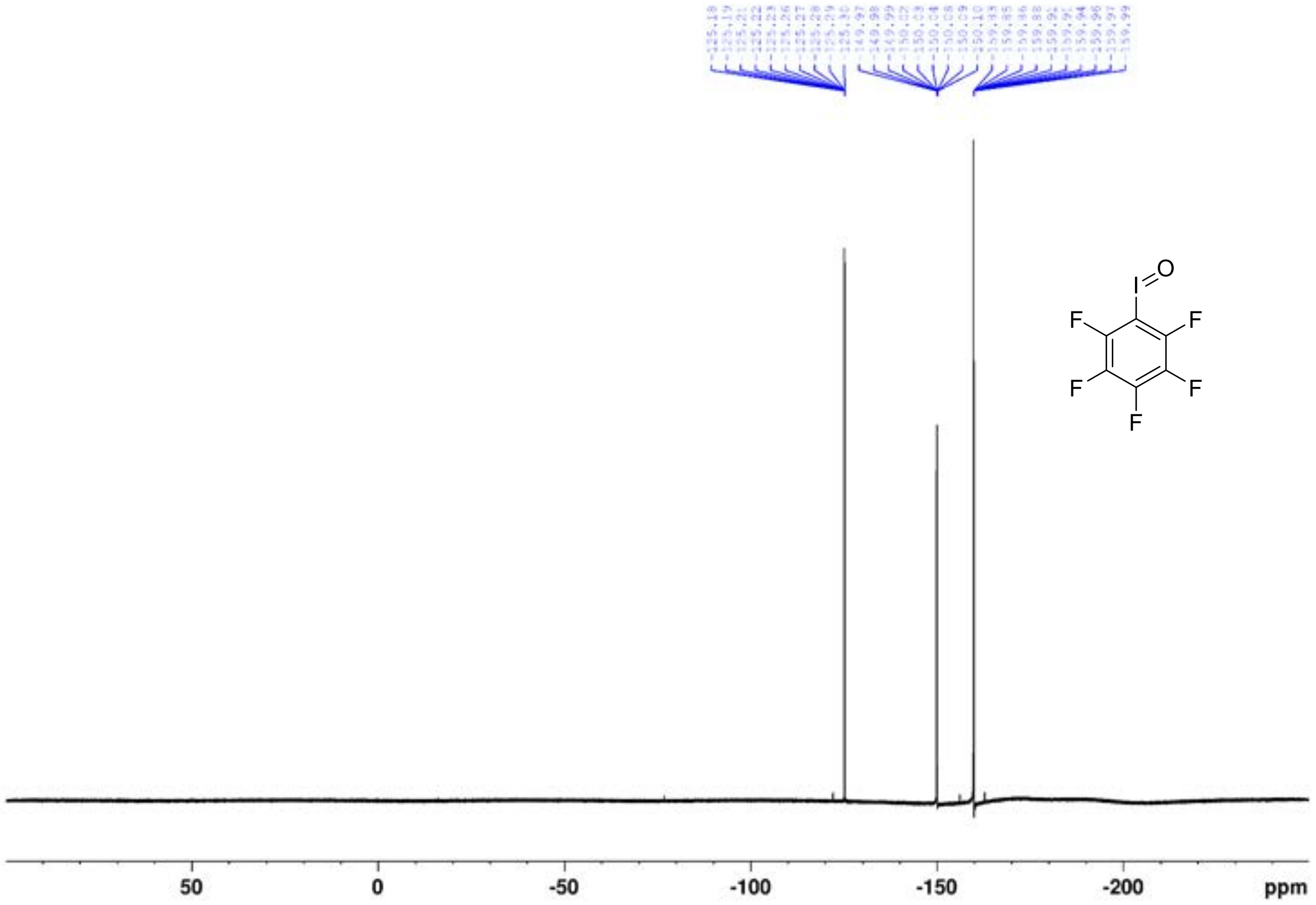

^{1}H NMR (400 MHz, $CDCl_3$) for *(Z)-4-iodobut-3-en-1-ol*

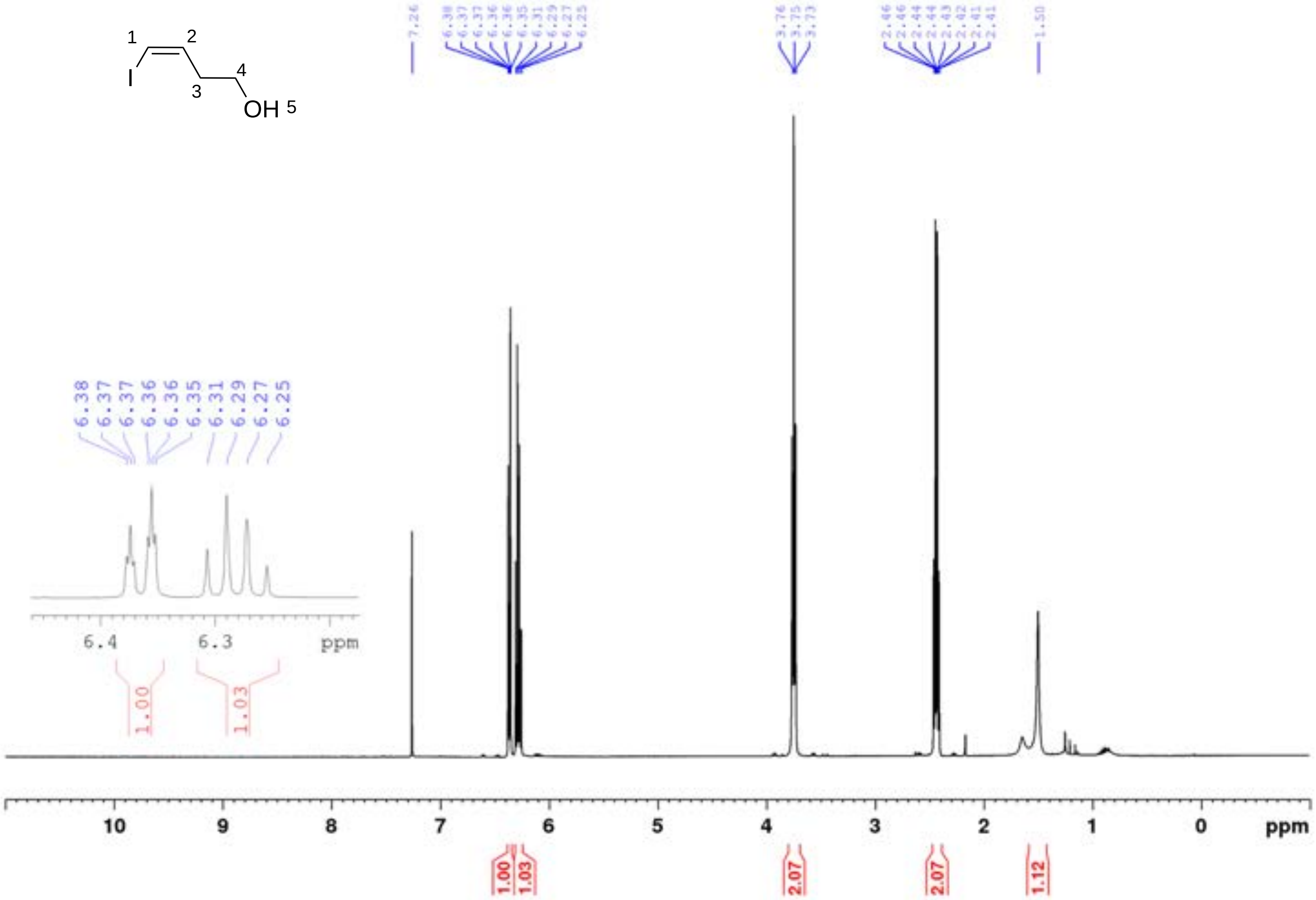

^{13}C NMR (101 MHz, $CDCl_3$) for *(Z)-4-iodobut-3-en-1-ol*

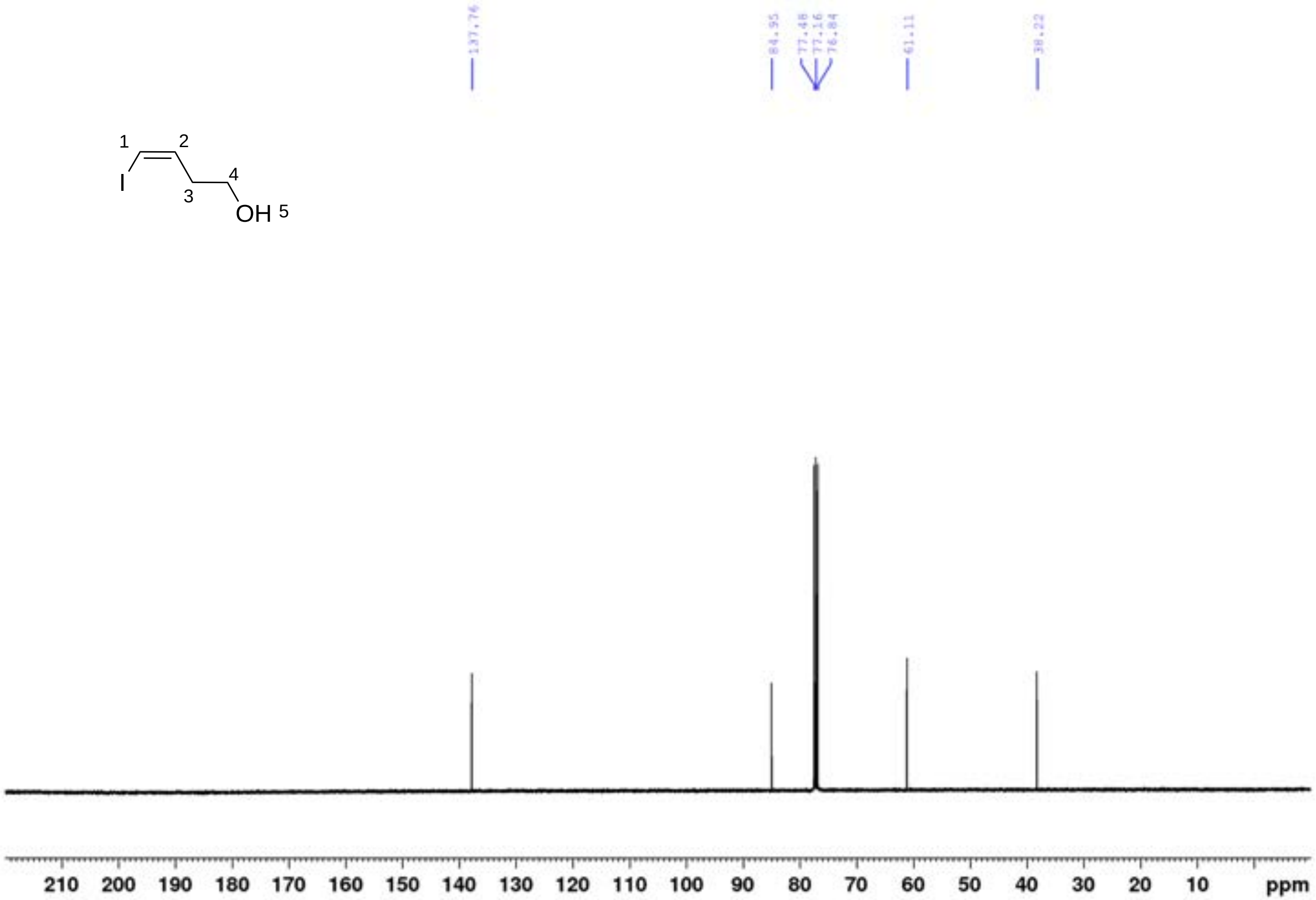

^{1}H NMR (700 MHz, $CDCl_3$) for *(Z)-4-phenylbut-3-en-1-ol* (**8a**)

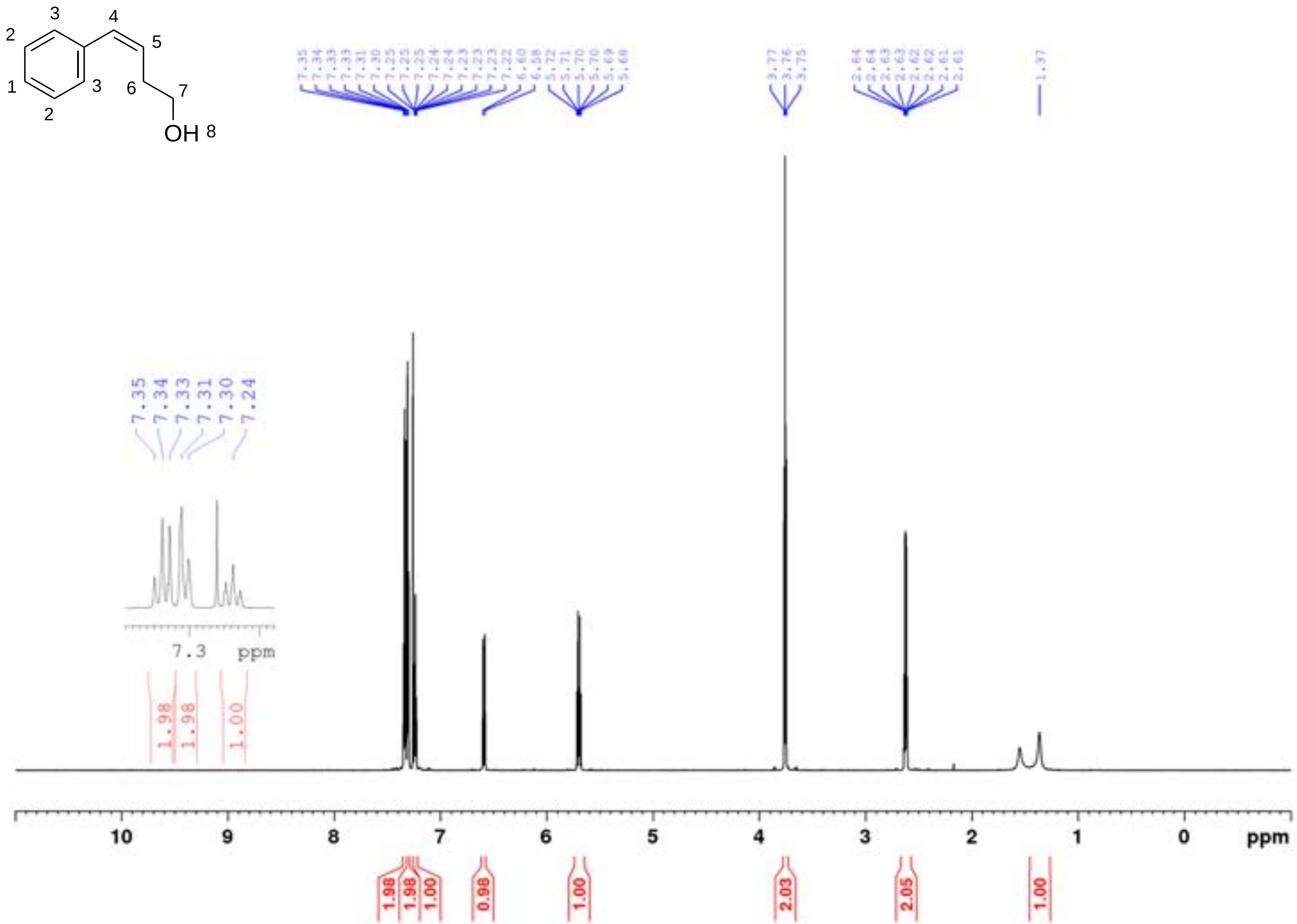

^{13}C NMR (101 MHz, $CDCl_3$) for *(Z)-4-phenylbut-3-en-1-ol* (**8a**)

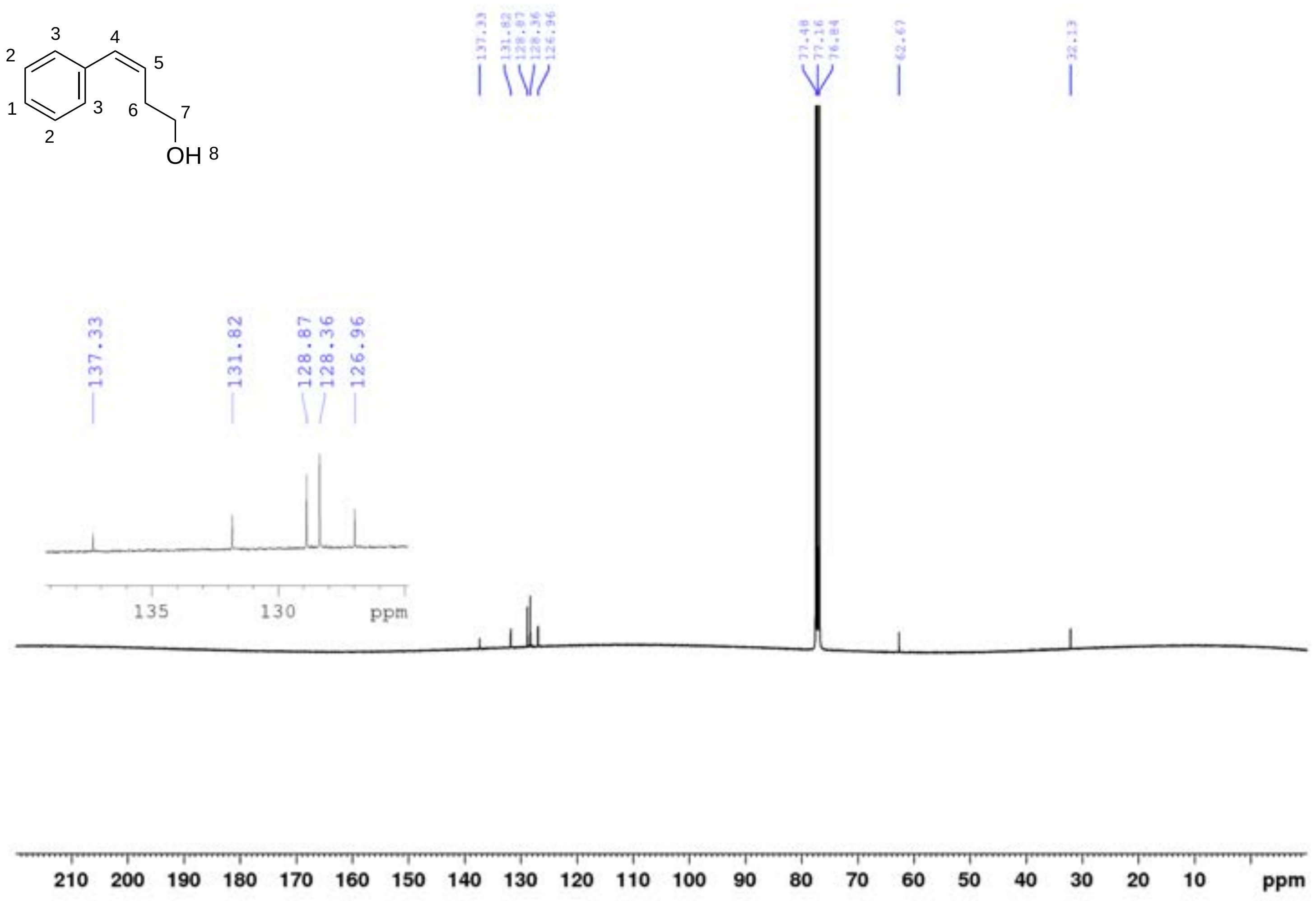

¹H NMR (500 MHz, $CDCl_3$) for *(Z)-4-(o-tolyl)but-3-en-1-ol* (**8b**)

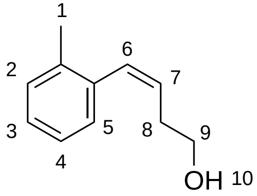

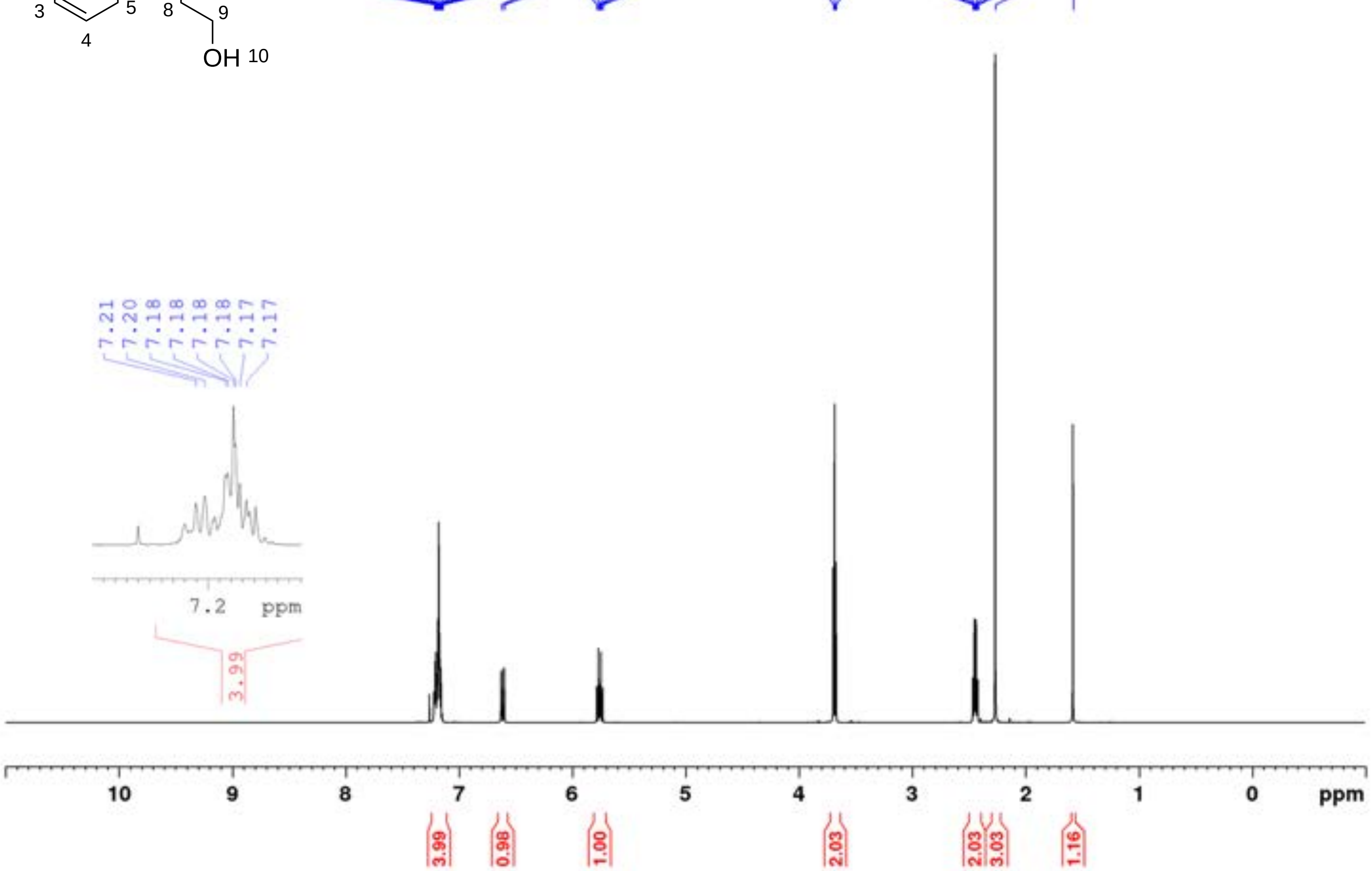

^{13}C NMR (126 MHz, $CDCl_3$) for *(Z)-4-(o-tolyl)but-3-en-1-ol* (**8b**)

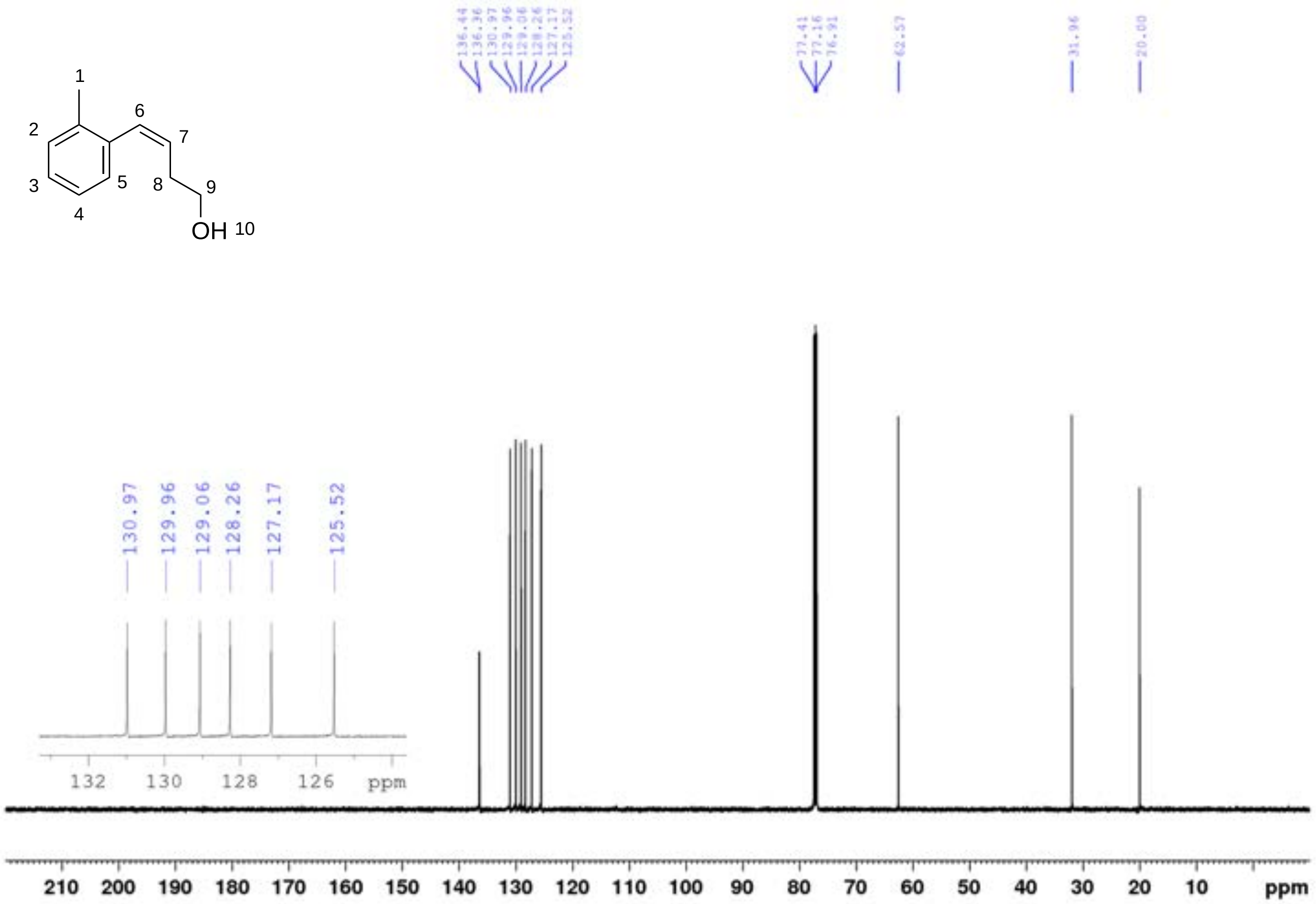

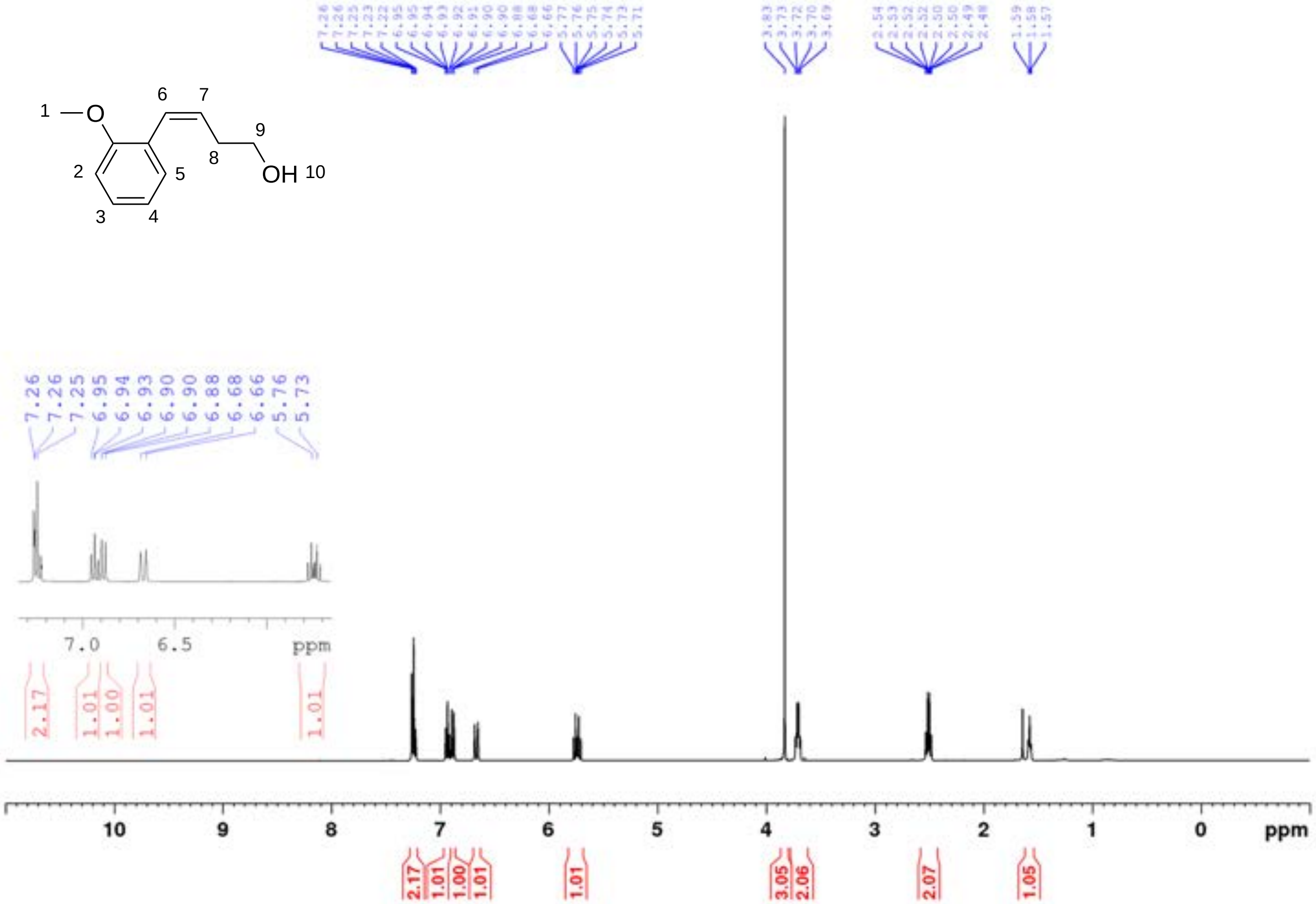

ppm

^{13}C NMR (101 MHz, $CDCl_3$) for *(Z)-4-(2-methoxyphenyl)but-3-en-1-ol* (**8c**)

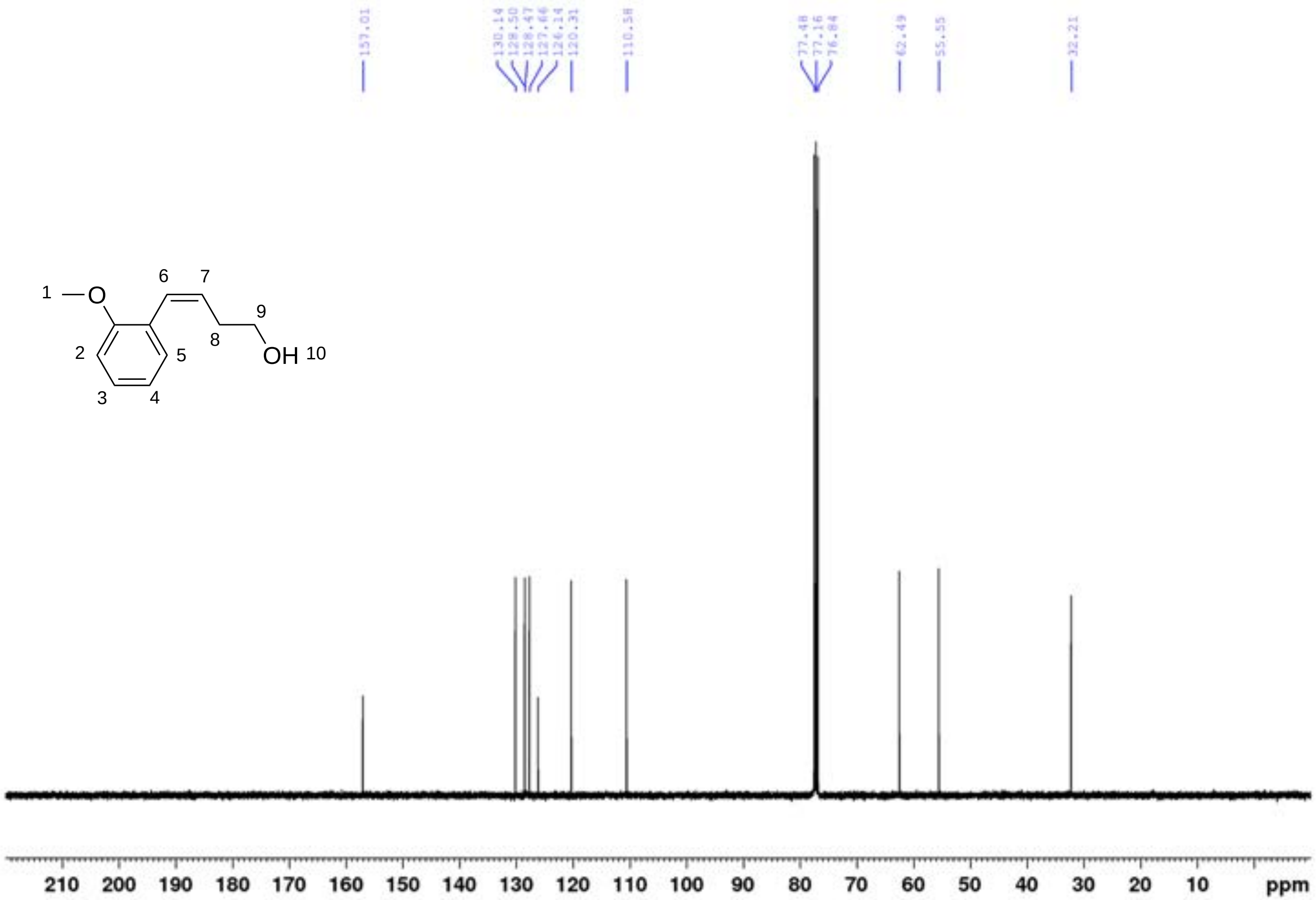

^{1}H NMR (500 MHz, $CDCl_3$) for *(Z)-4-(2-fluorophenyl)but-3-en-1-ol* (**8d**)

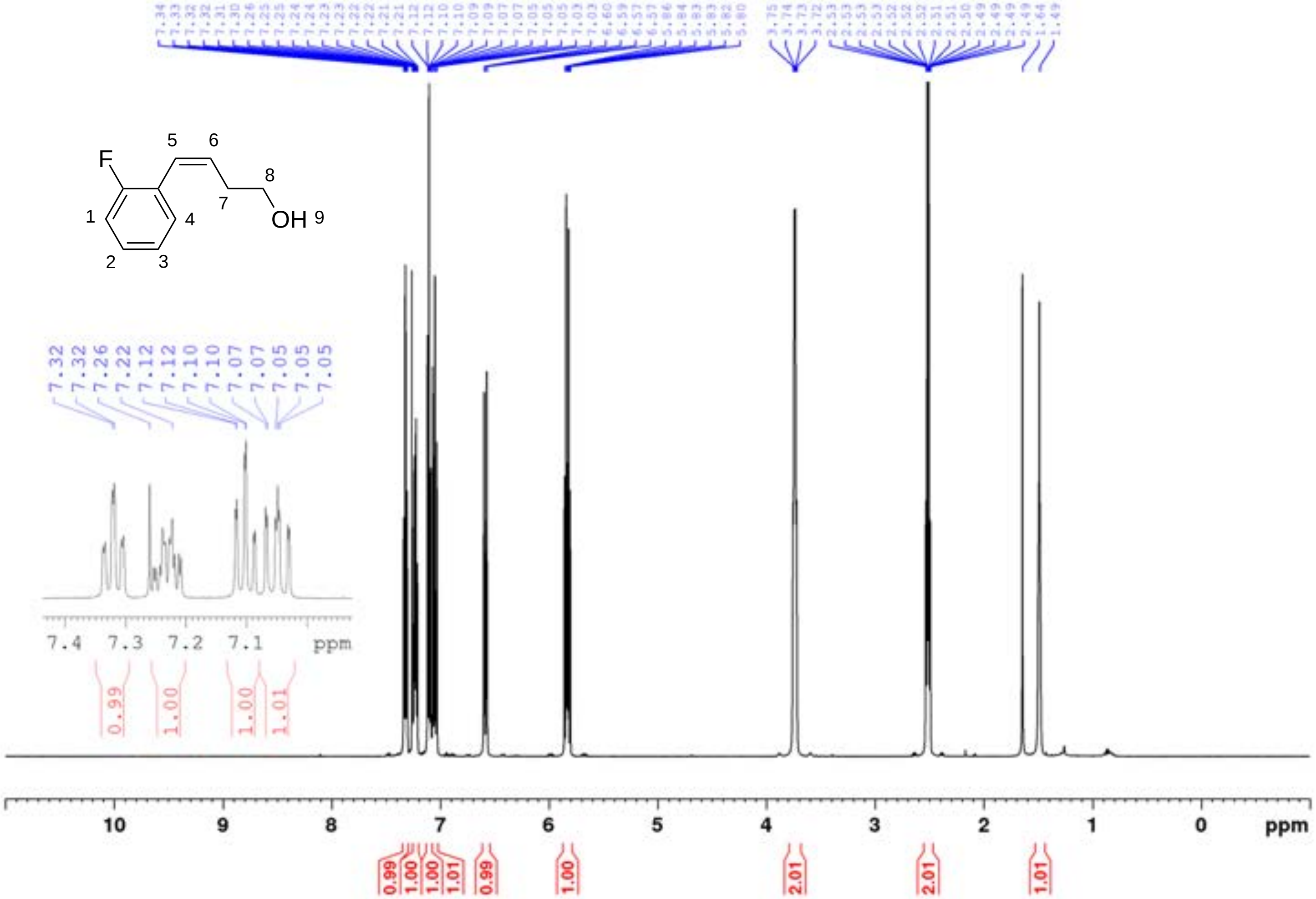

^{13}C NMR (126 MHz, $CDCl_3$) for *(Z)-4-(2-fluorophenyl)but-3-en-1-ol* (**8d**)

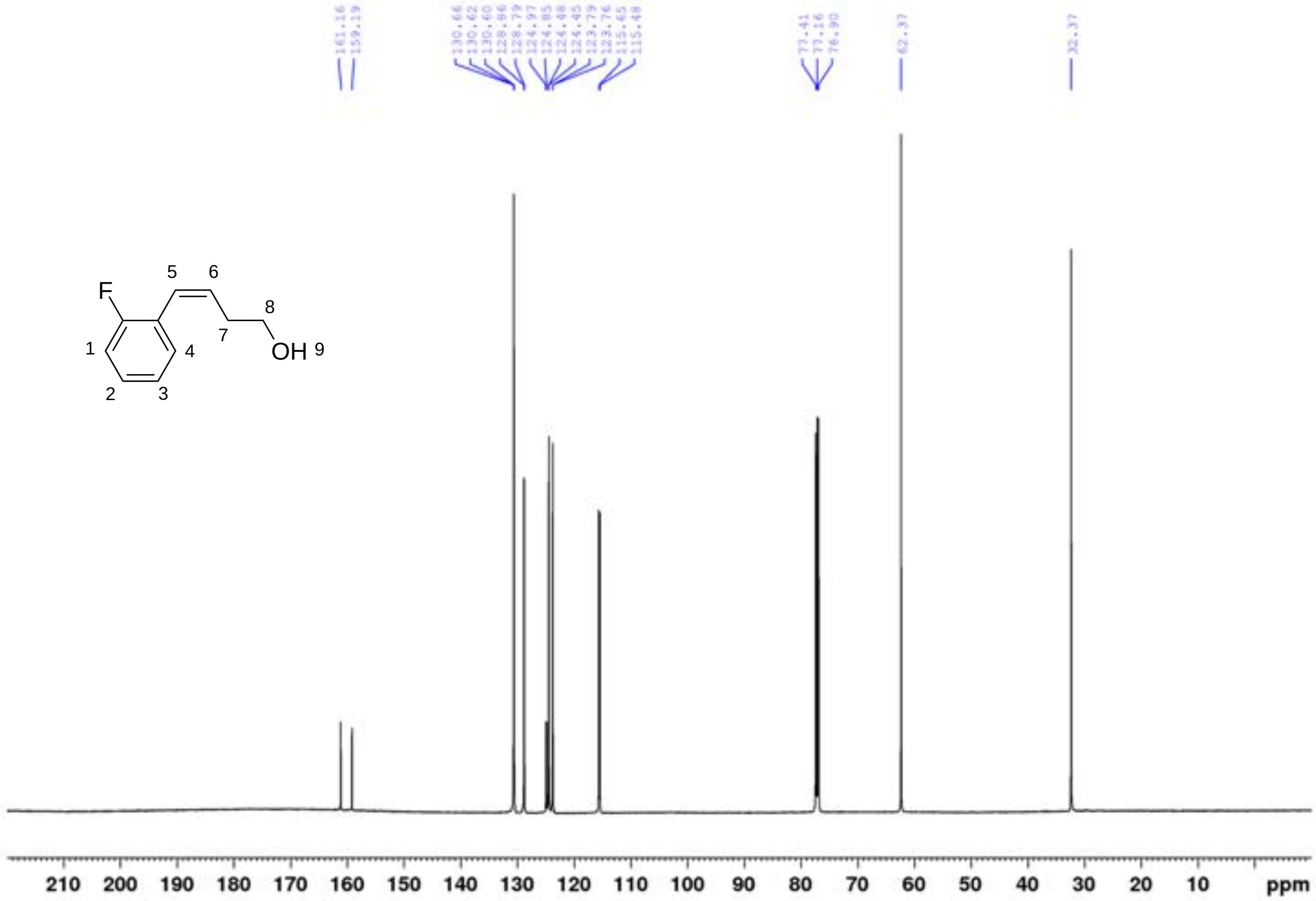

^{19}F NMR (376 MHz, $CDCl_3$) for *(Z)-4-(2-fluorophenyl)but-3-en-1-ol* (**8d**)

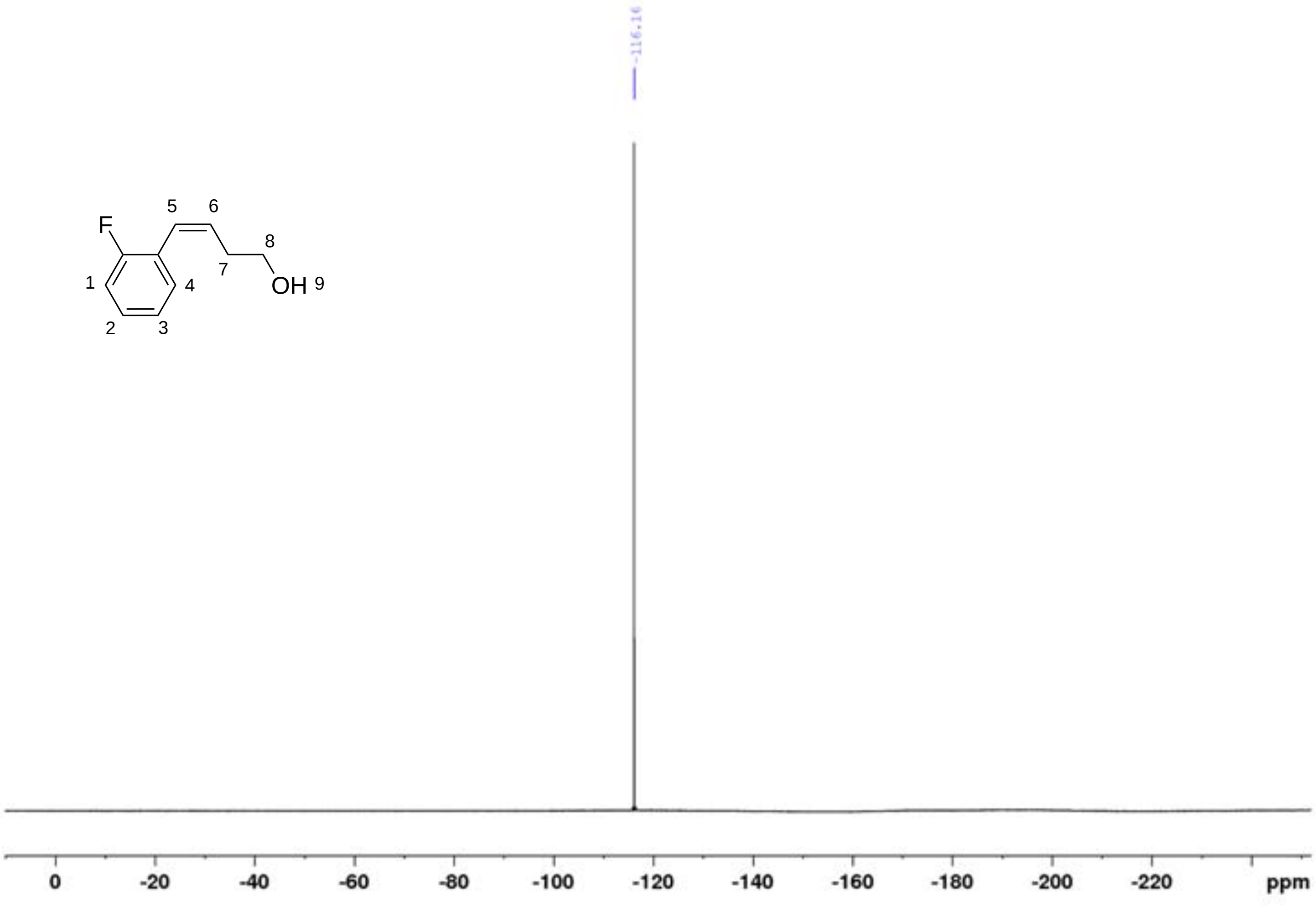

¹H NMR (400 MHz, $CDCl_3$) for *4-(3-chlorophenyl)but-3-yn-1-ol*

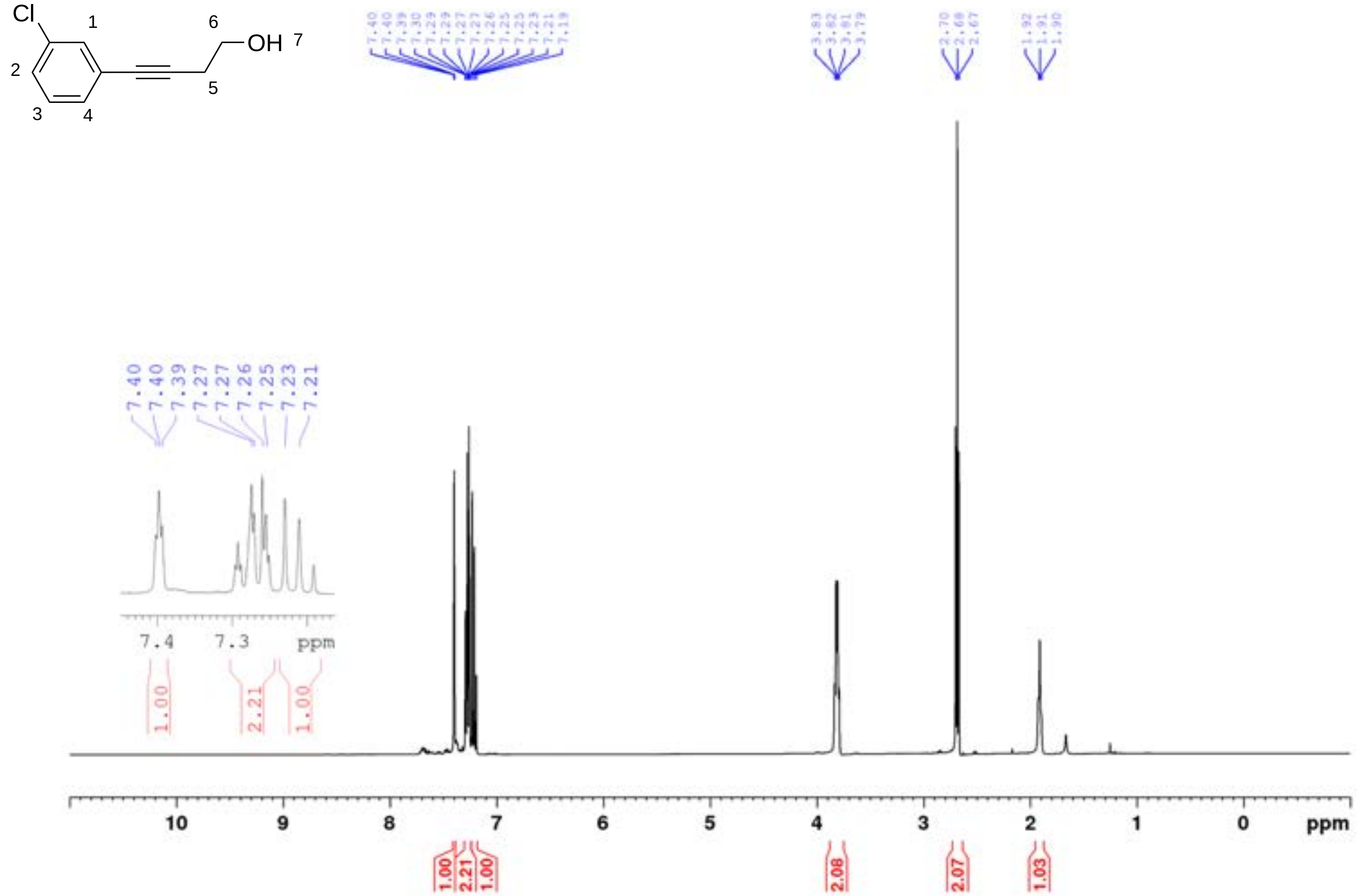

^{13}C NMR (101 MHz, $CDCl_3$) for *4-(3-chlorophenyl)but-3-yn-1-ol*

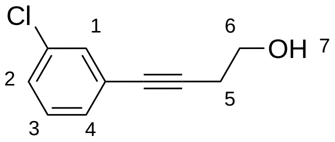

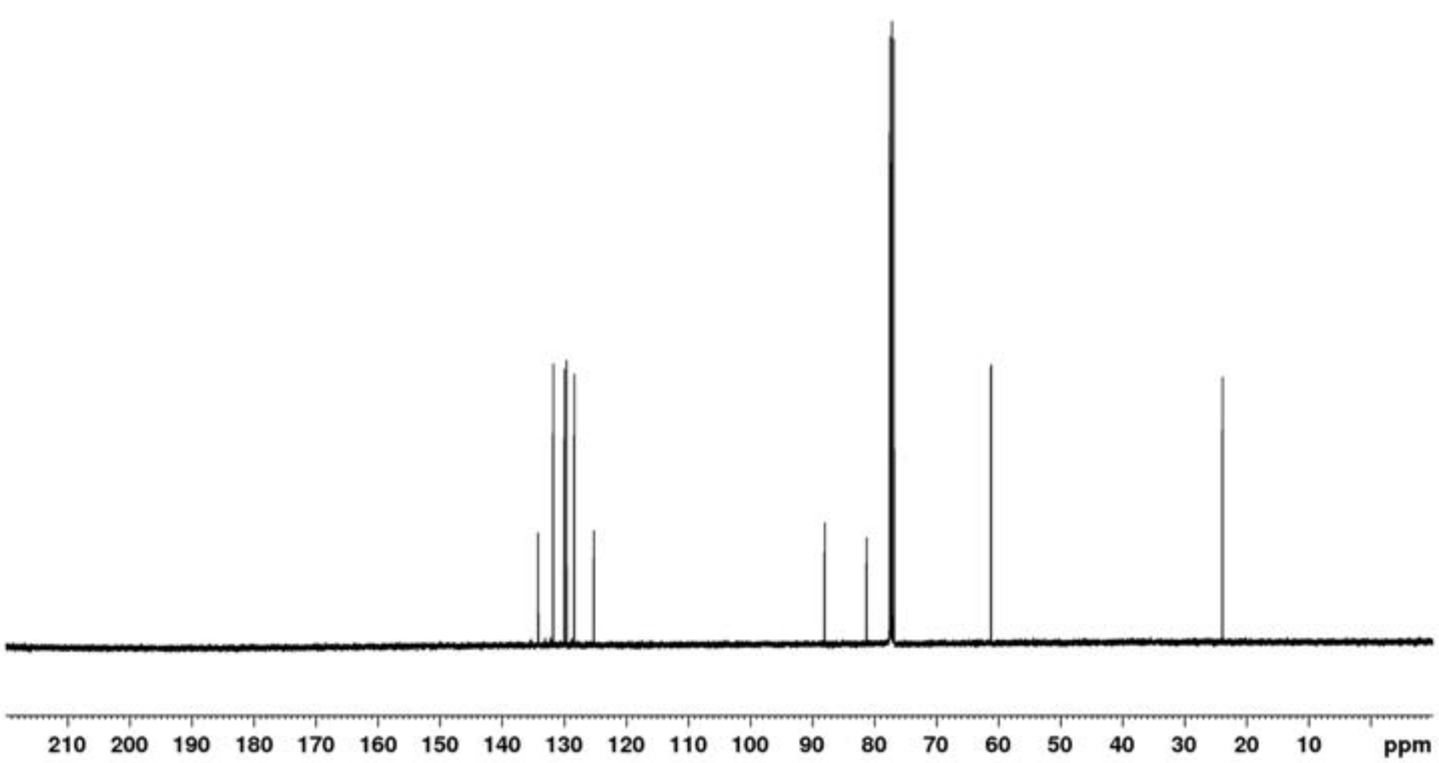

^{1}H NMR (400 MHz, $CDCl_3$) for *(Z)-4-(3-chlorophenyl)but-3-en-1-ol* (**8e**)

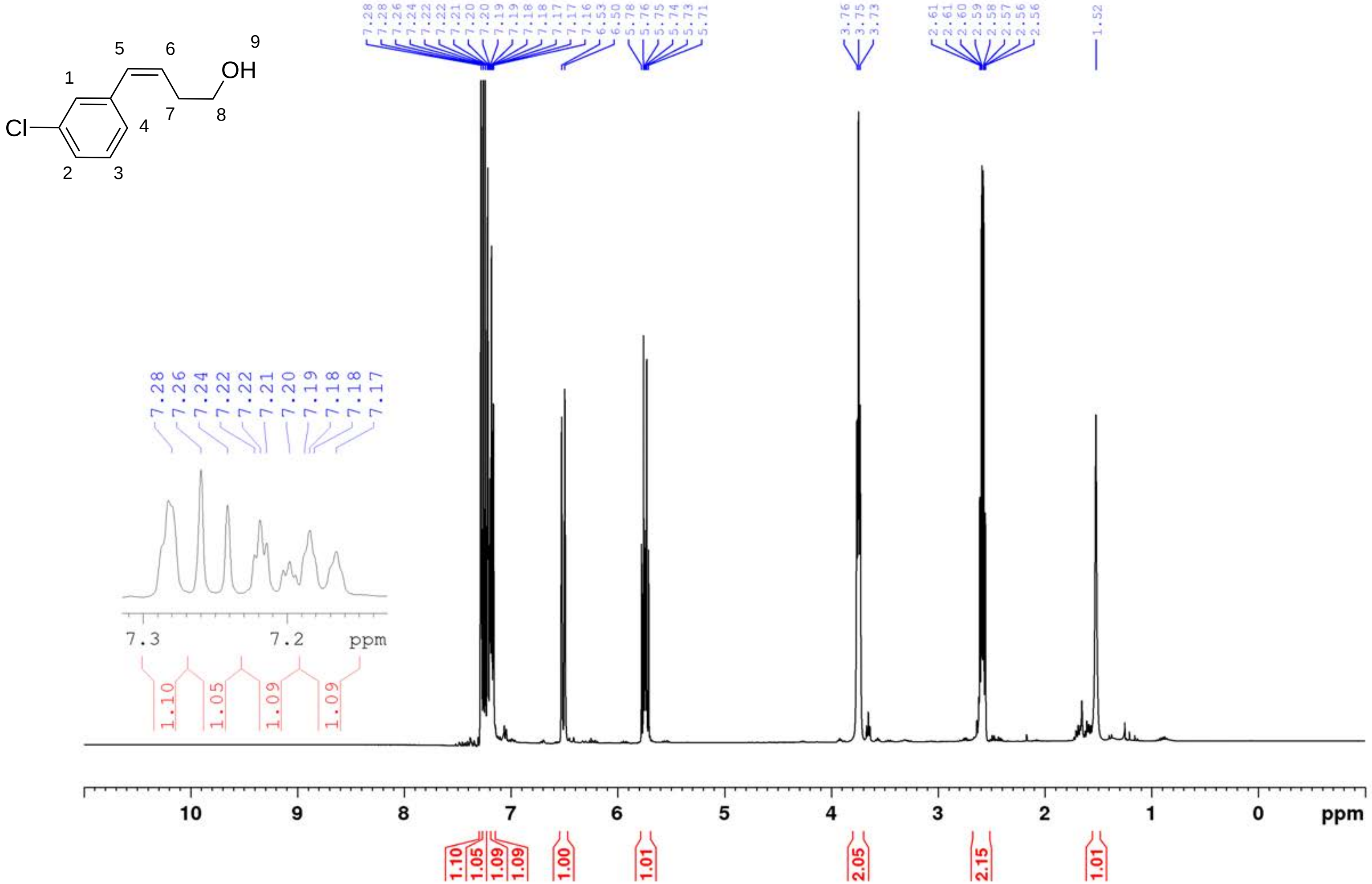

^{13}C NMR (101 MHz, $CDCl_3$) for *(Z)-4-(3-chlorophenyl)but-3-en-1-ol* (**8e**)

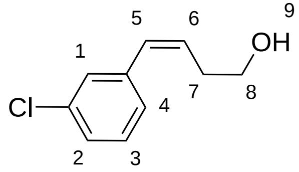

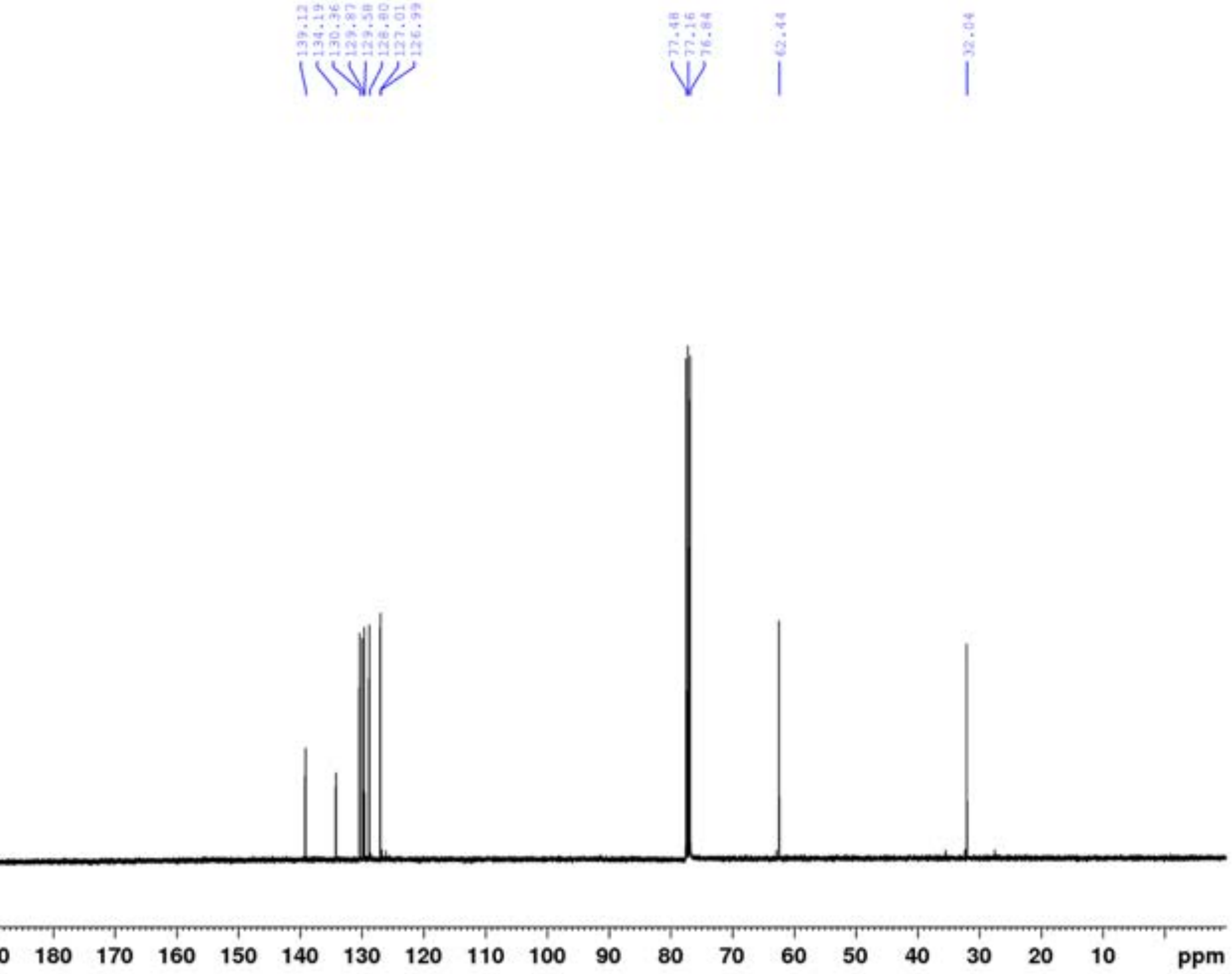

^{1}H NMR (400 MHz, $CDCl_3$) for *4-(3-(trifluoromethoxy)phenyl)but-3-yn-1-ol*

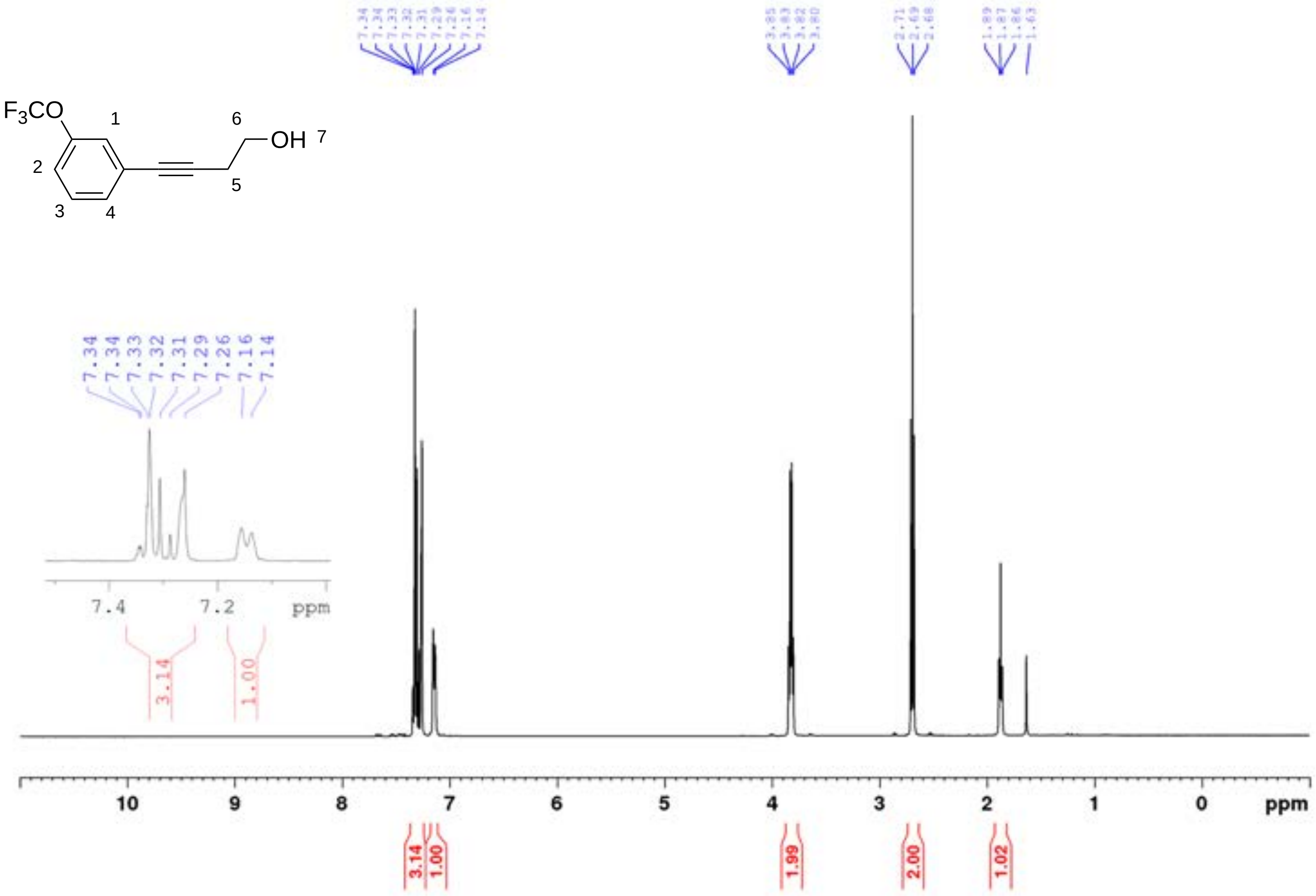

^{13}C NMR (101 MHz, $CDCl_3$) for *4-(3-(trifluoromethoxy)phenyl)but-3-yn-1-ol*

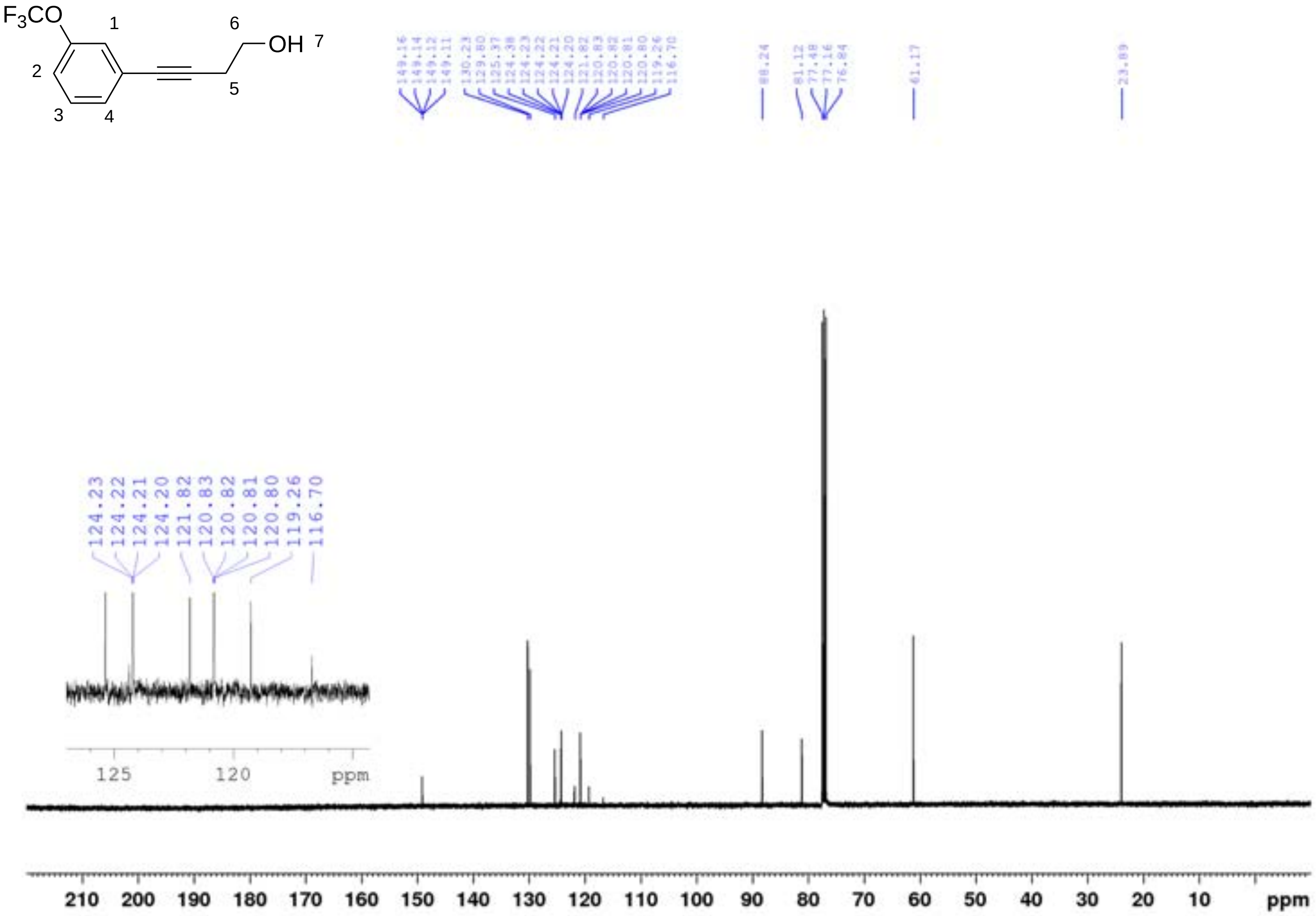

^{19}F NMR (376 MHz, $CDCl_3$) for *4-(3-(trifluoromethoxy)phenyl)but-3-yn-1-ol*

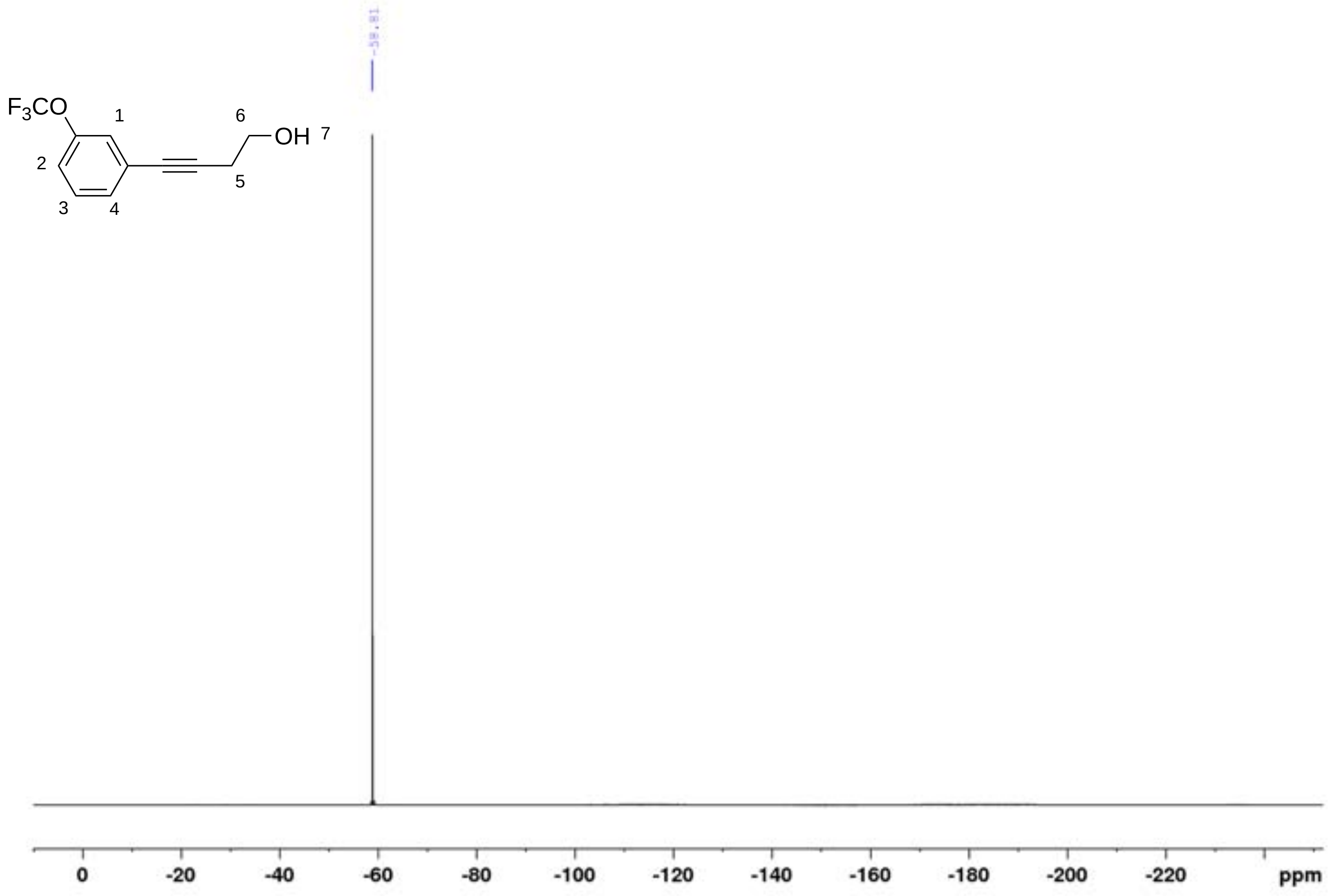

^{1}H NMR (400 MHz, $CDCl_3$) for *(Z)-4-(3-(trifluoromethoxy)phenyl)but-3-en-1-ol* (**8f**)

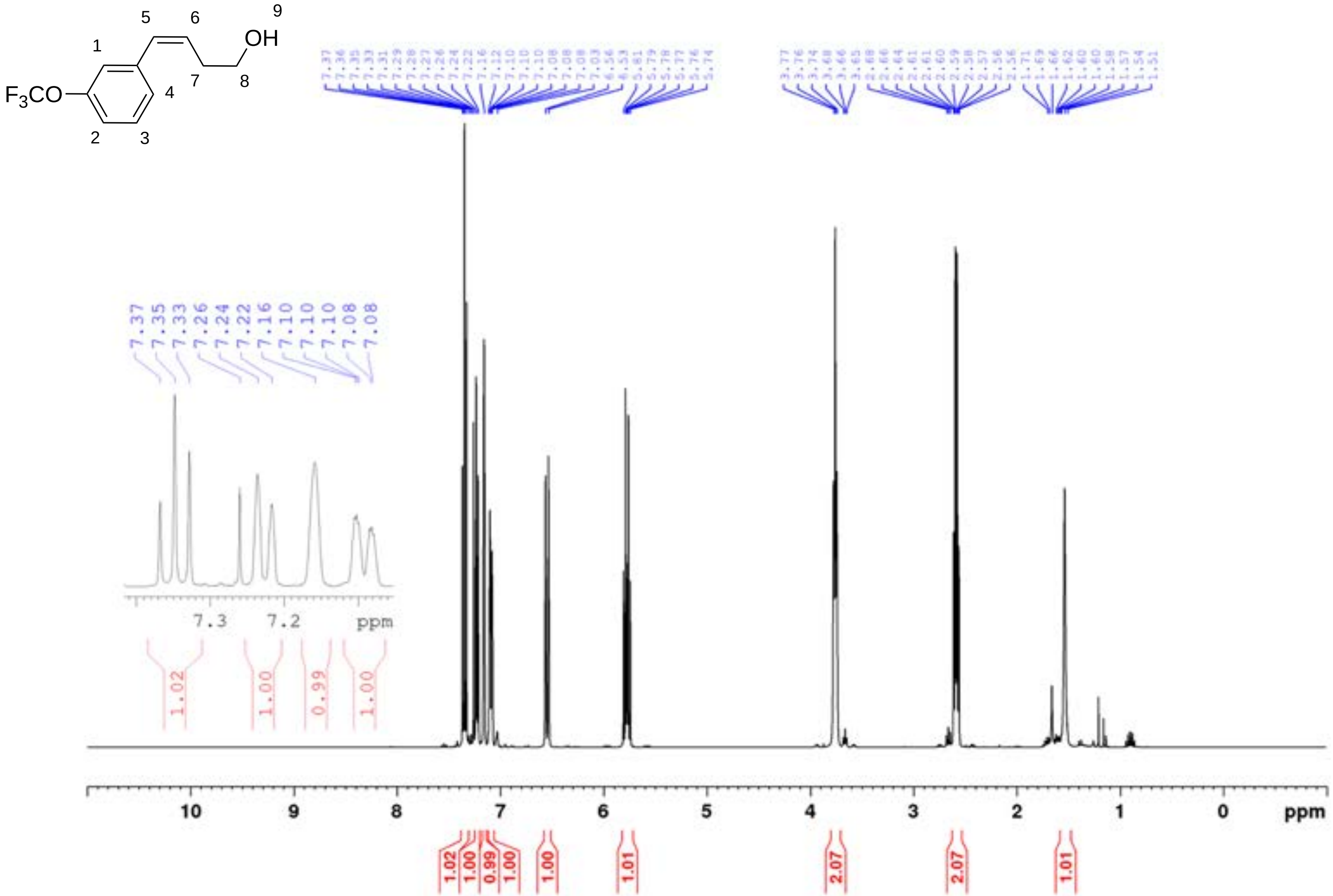

^{13}C NMR (101 MHz, $CDCl_3$) for *(Z)-4-(3-(trifluoromethoxy)phenyl)but-3-en-1-ol* (**8f**)

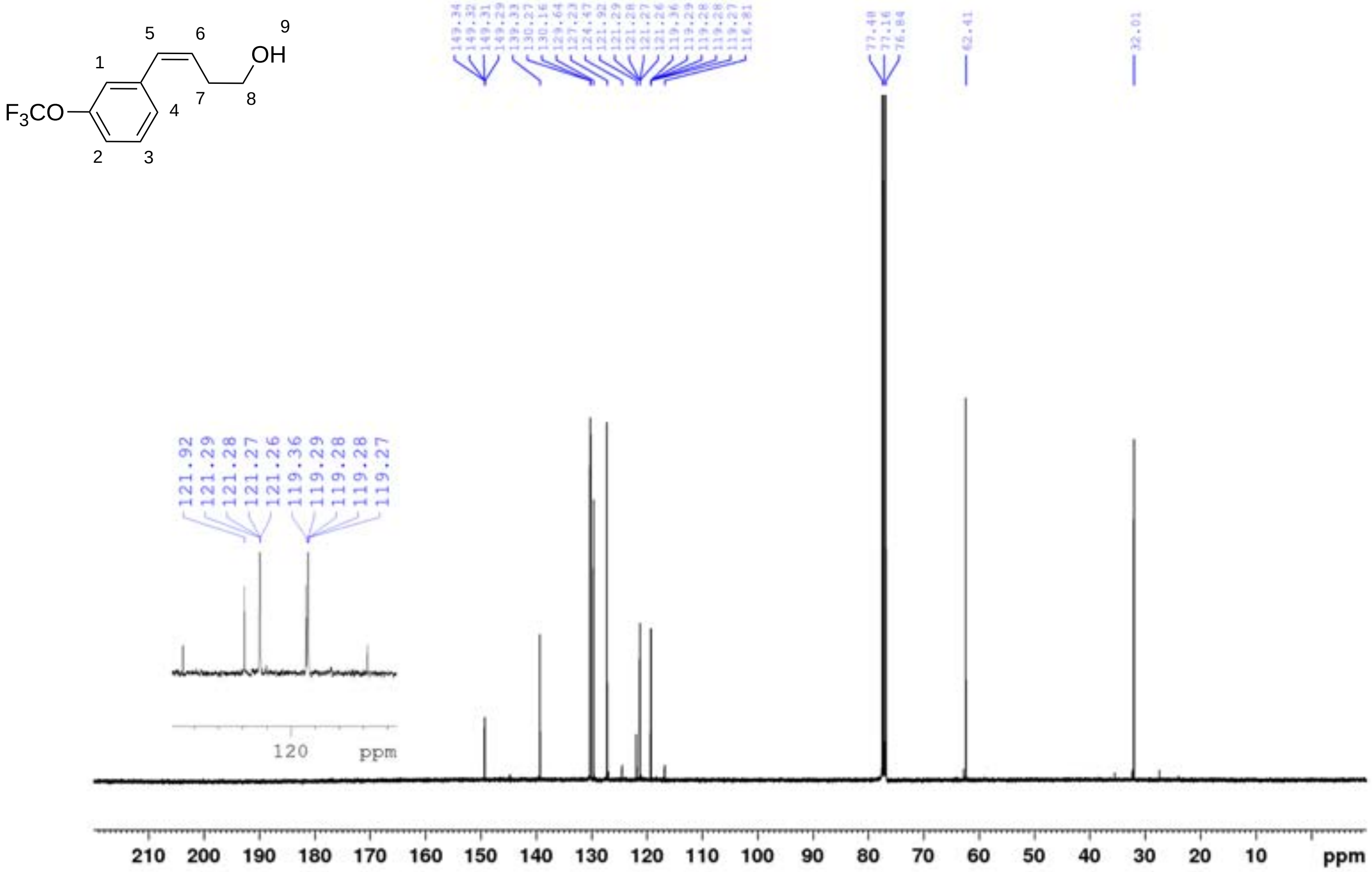

^{19}F NMR (376 MHz, $CDCl_3$) for *(Z)-4-(3-(trifluoromethoxy)phenyl)but-3-en-1-ol* (**8f**)

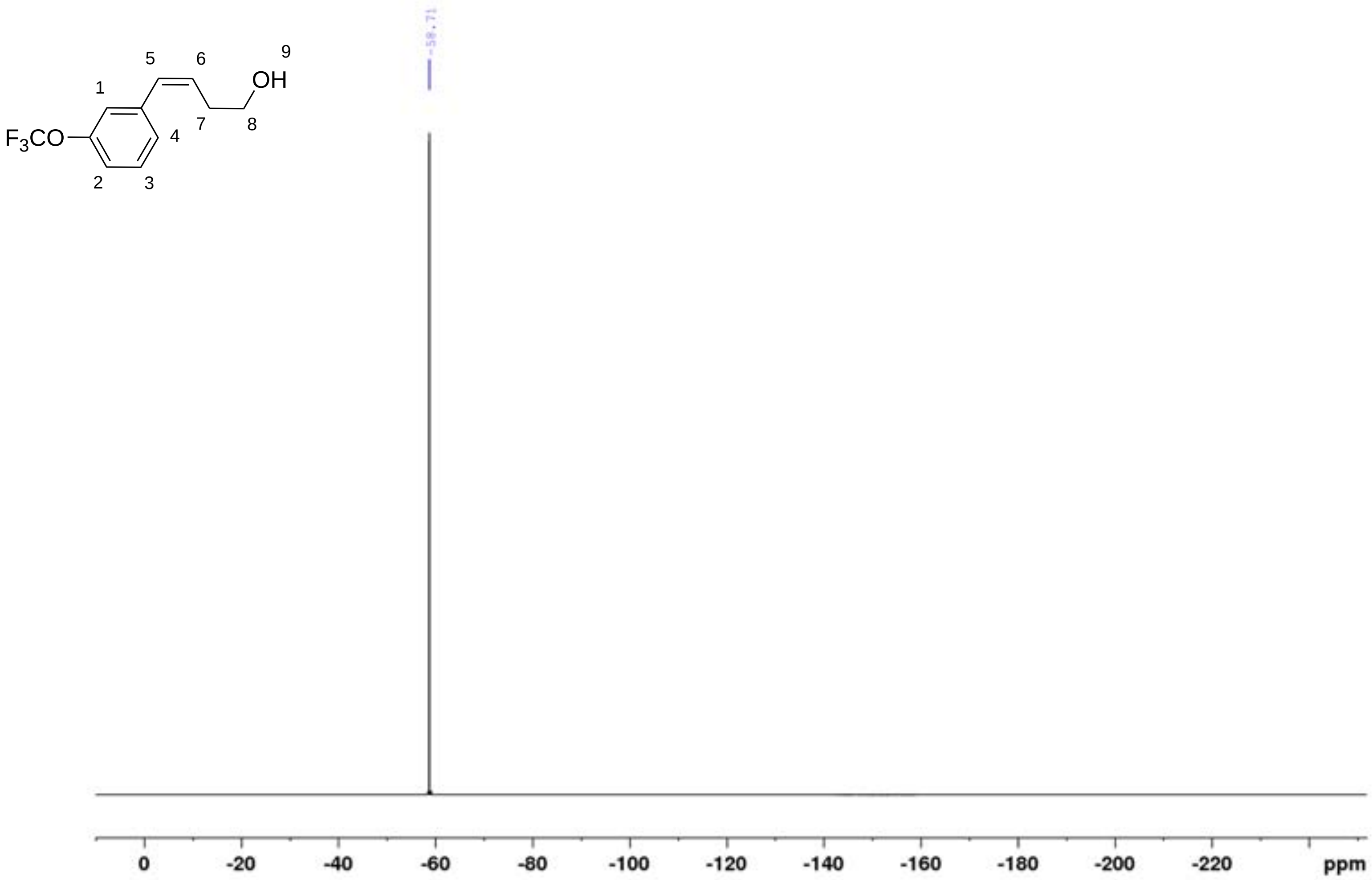

^{1}H NMR (400 MHz, $CDCl_3$) for *Ethyl 3-(4-hydroxybut-1-yn-1-yl)benzoate*

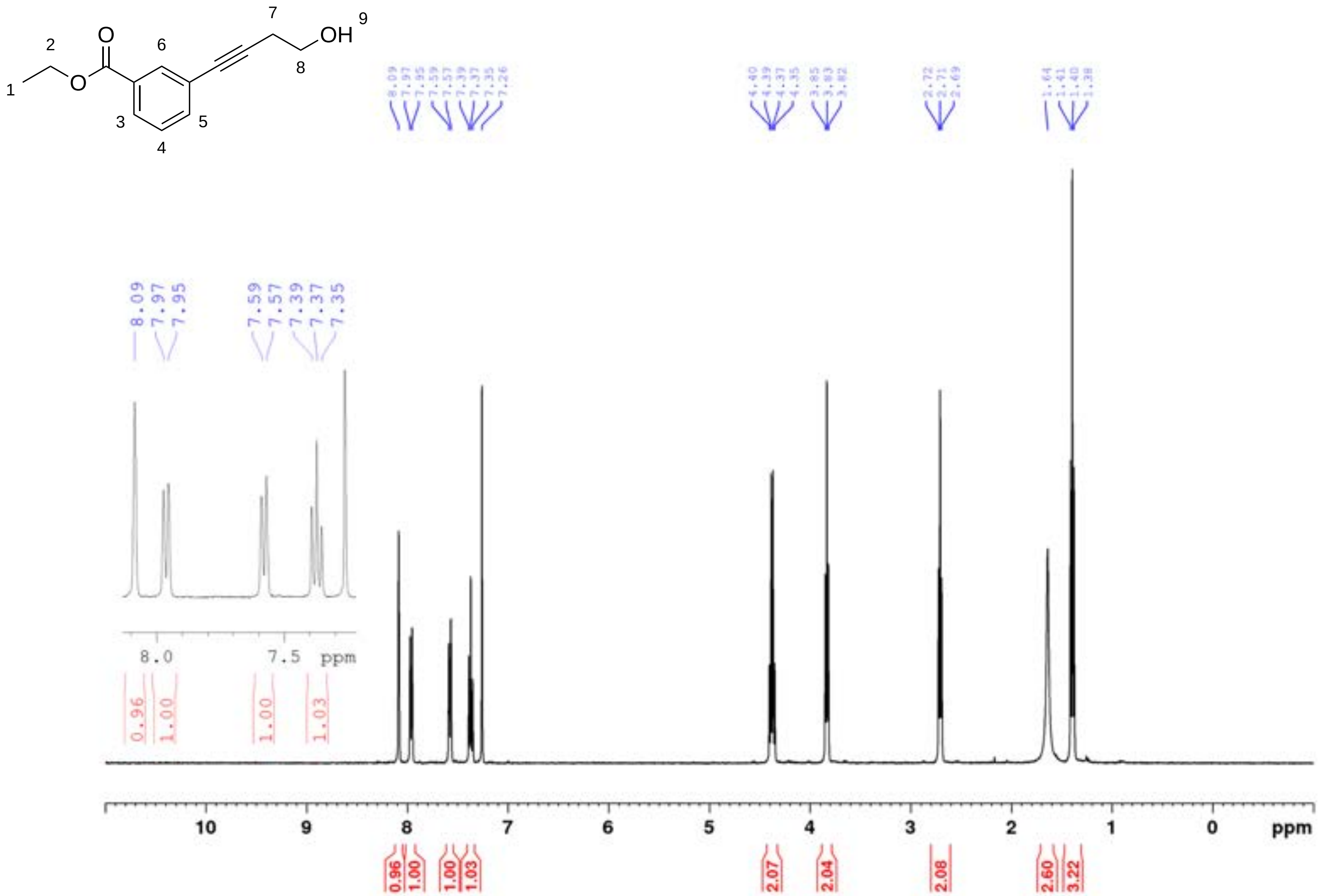

^{13}C NMR (101 MHz, $CDCl_3$) for *Ethyl 3-(4-hydroxybut-1-yn-1-yl)benzoate*

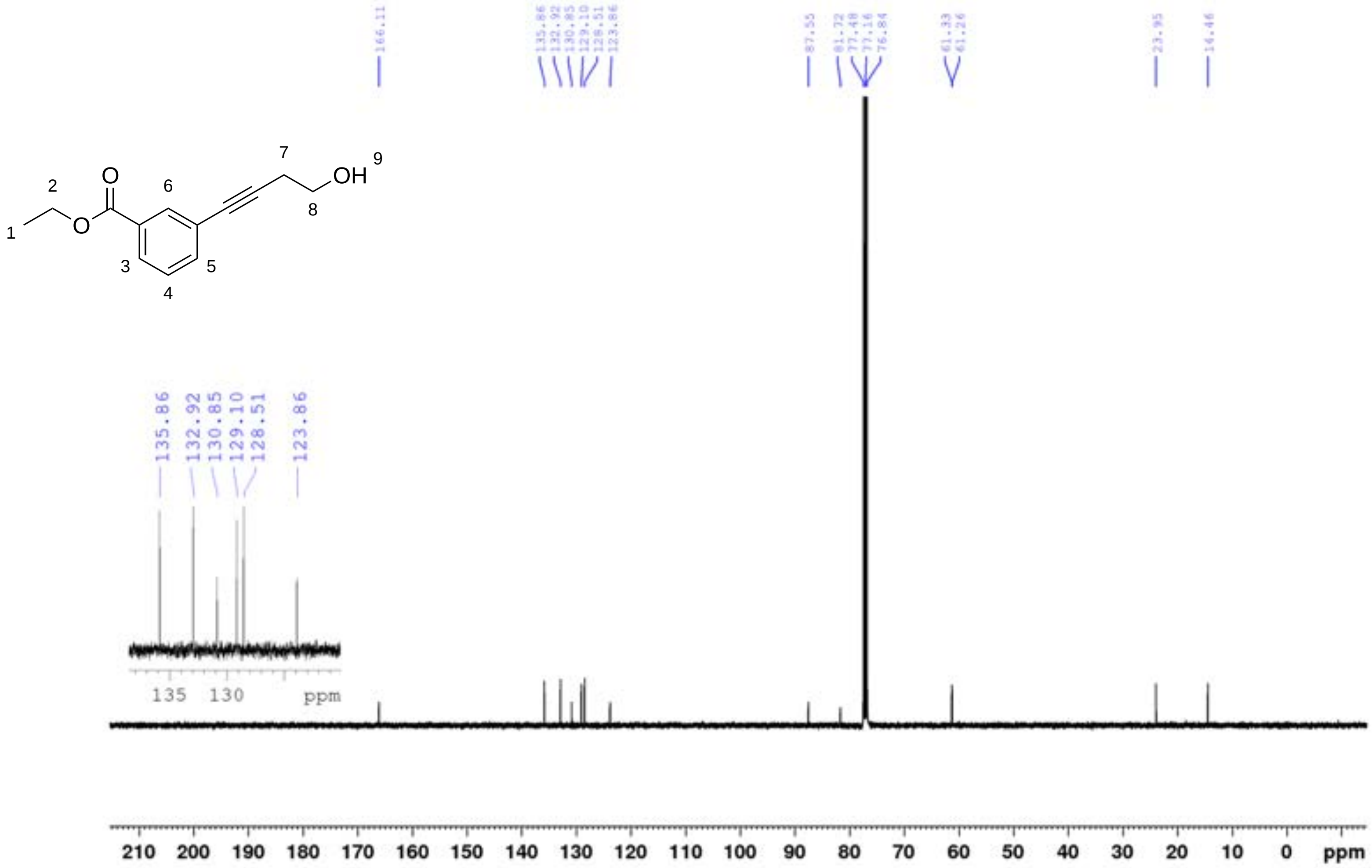

¹H NMR (400 MHz, $CDCl_3$) for *Ethyl (Z)-3-(4-hydroxybut-1-en-1-yl)benzoate* (**8g**)

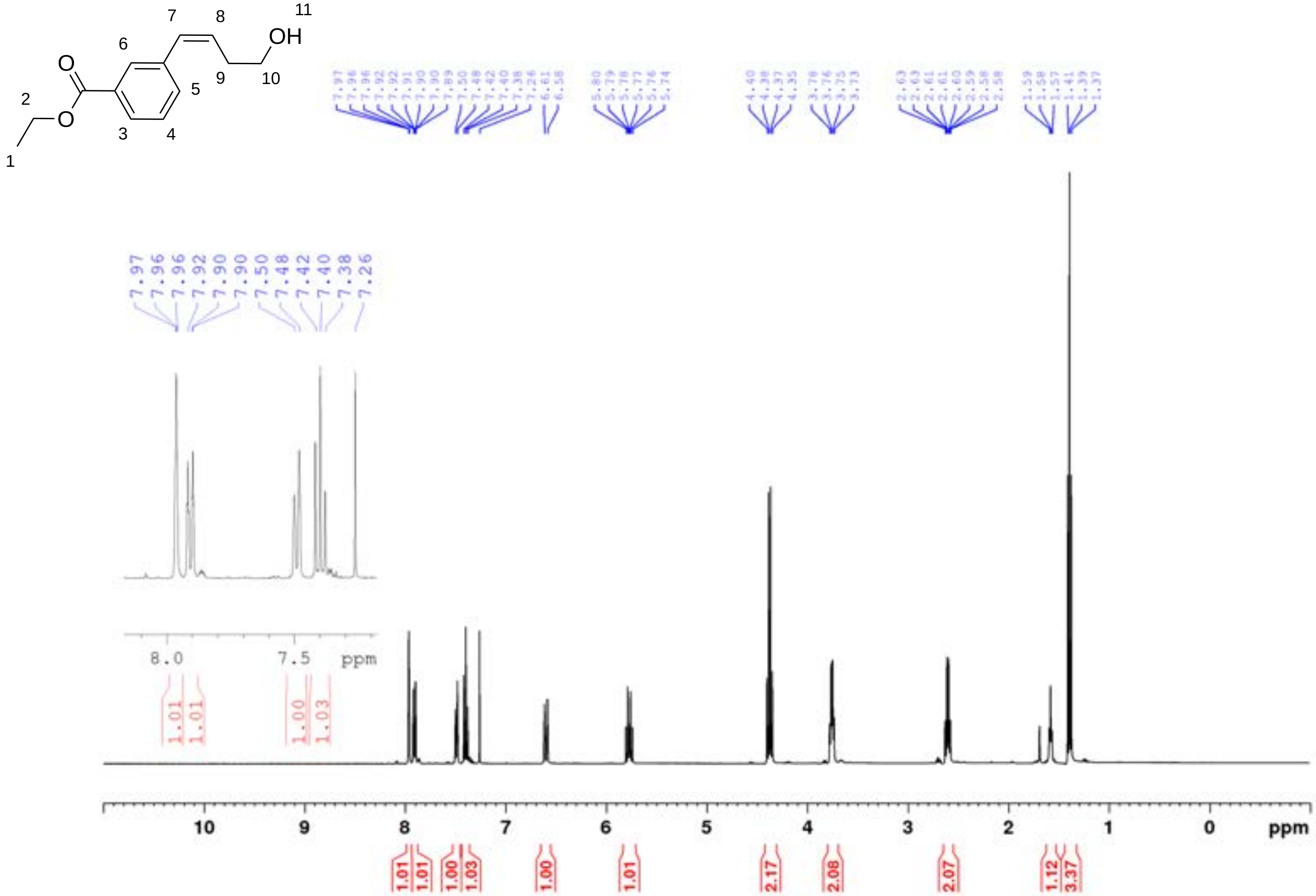

^{13}C NMR (101 MHz, $CDCl_3$) for *Ethyl (Z)-3-(4-hydroxybut-1-en-1-yl)benzoate* (**8g**)

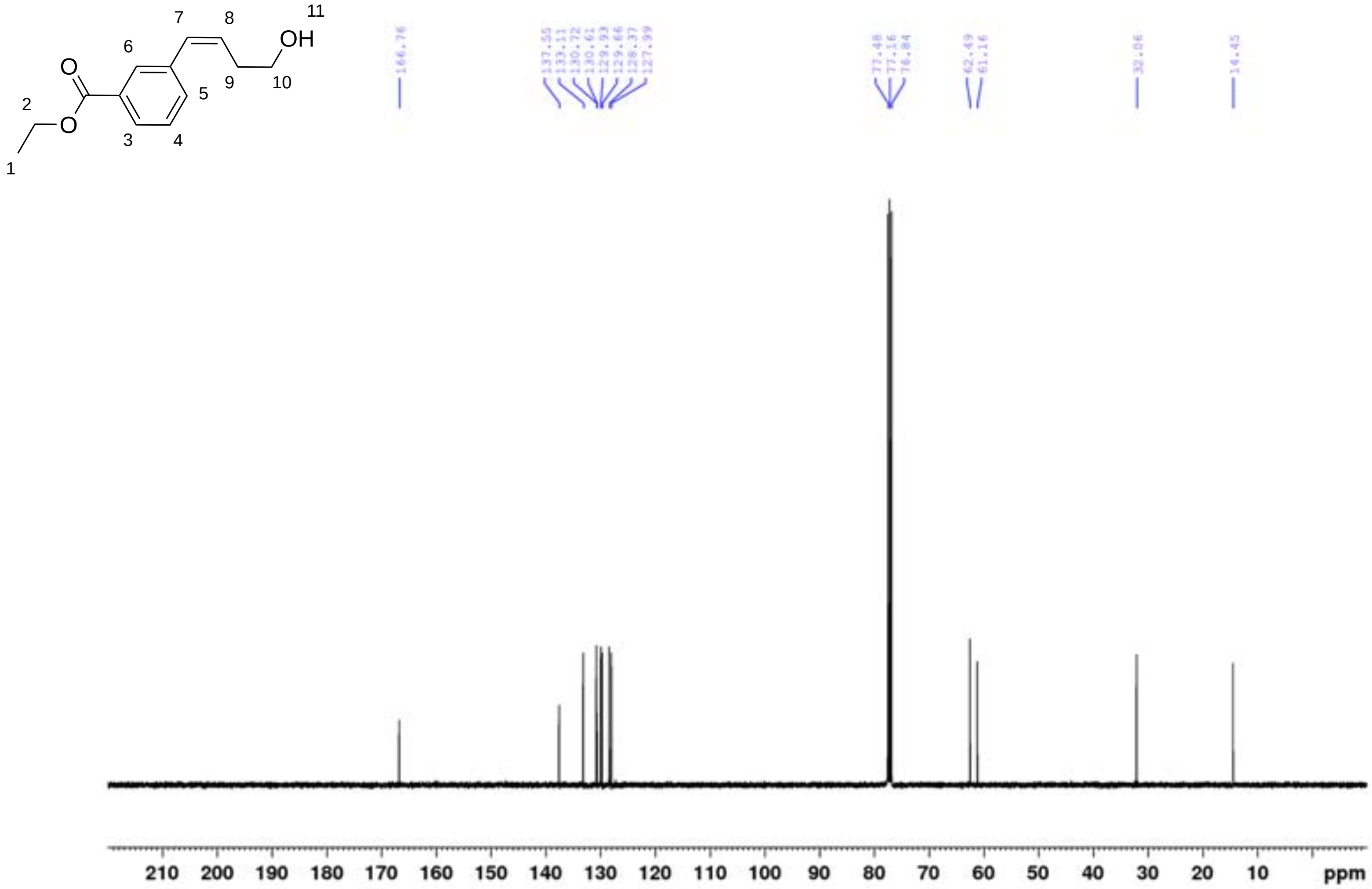

¹H NMR (400 MHz, $CDCl_3$) for *4-(4-chlorophenyl)but-3-yn-1-ol*

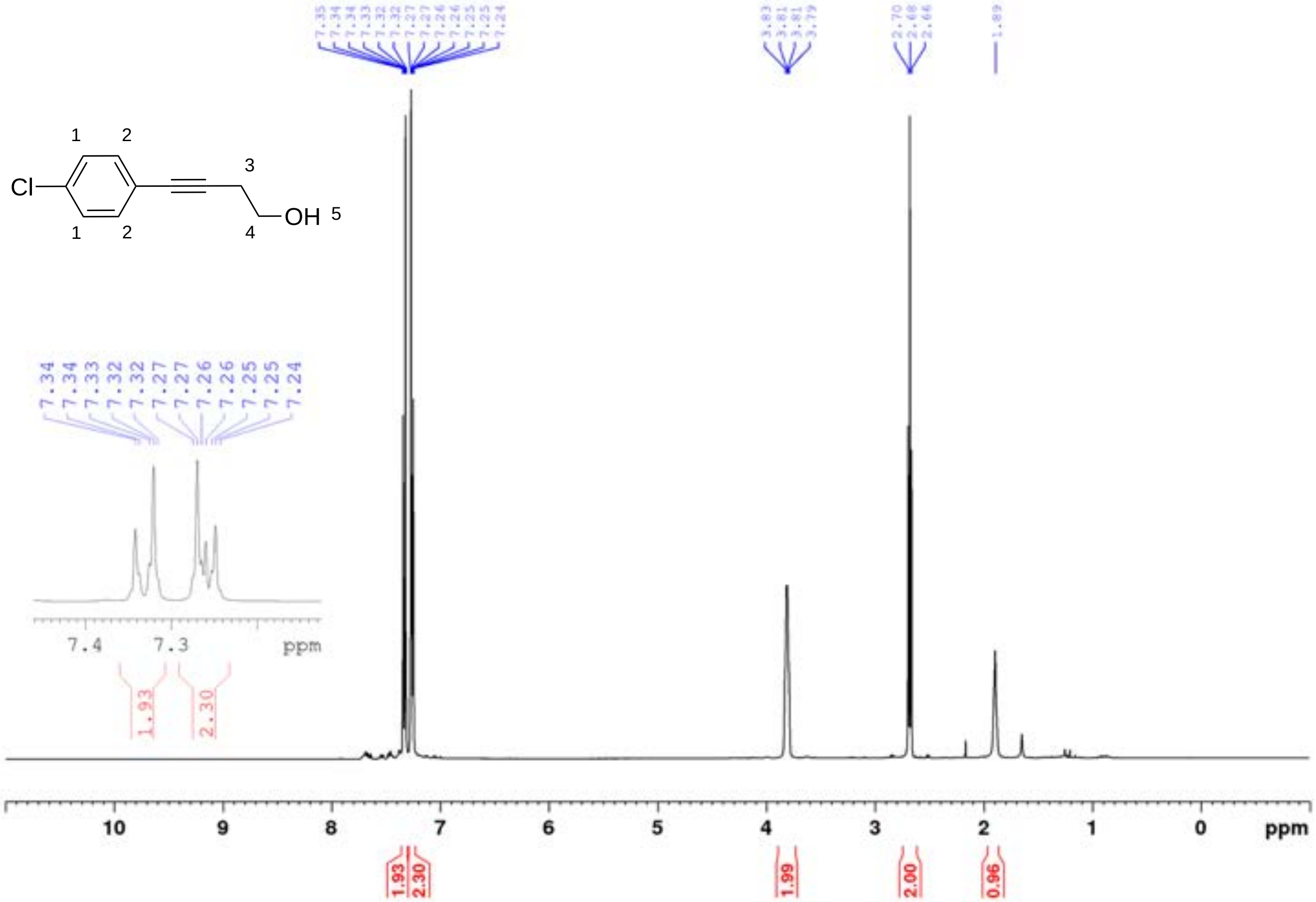

^{13}C NMR (101 MHz, $CDCl_3$) for *4-(4-chlorophenyl)but-3-yn-1-ol*

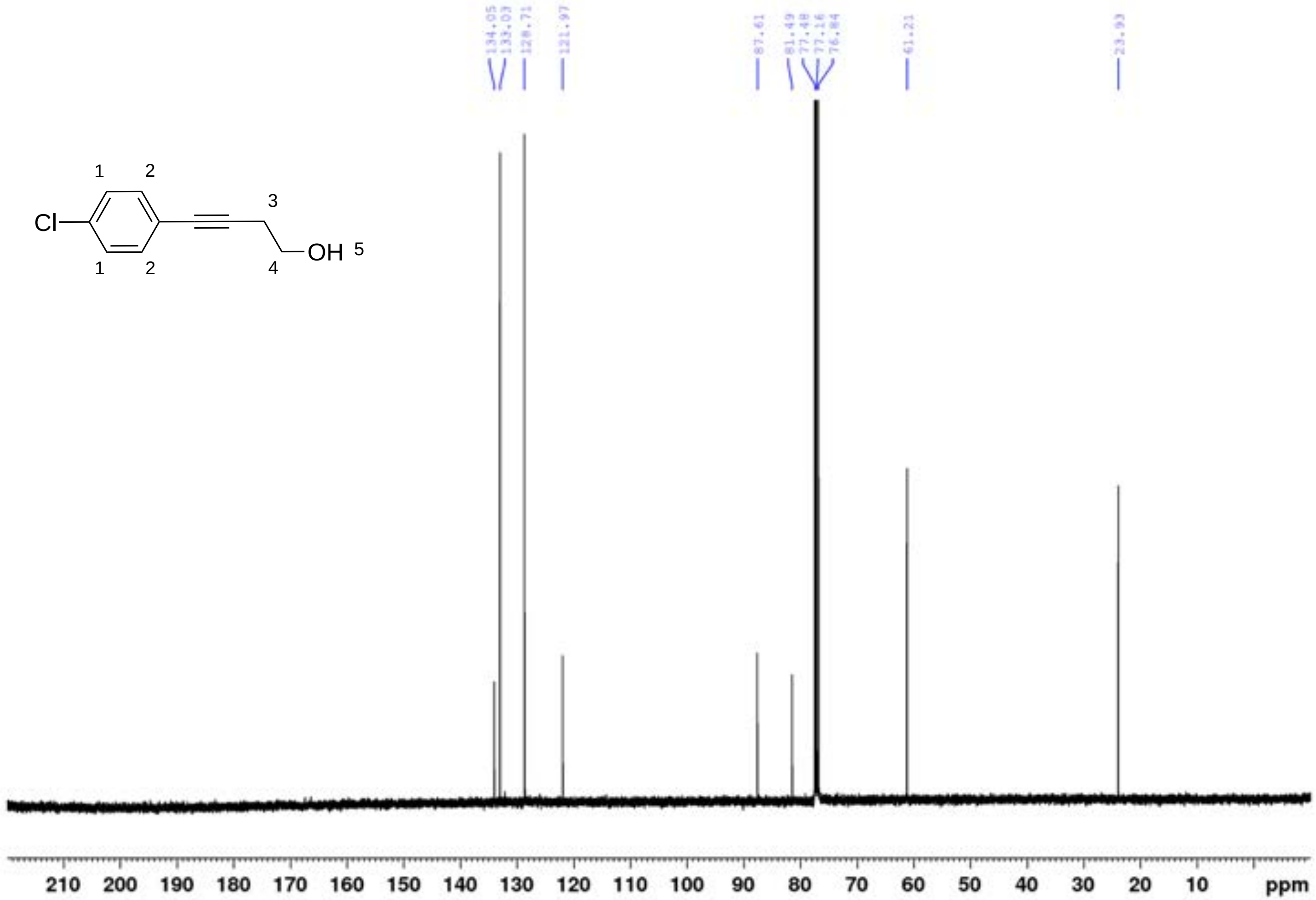

¹H NMR (700 MHz, $CDCl_3$) for *(Z)-4-(4-chlorophenyl)but-3-en-1-ol* (**8h**)

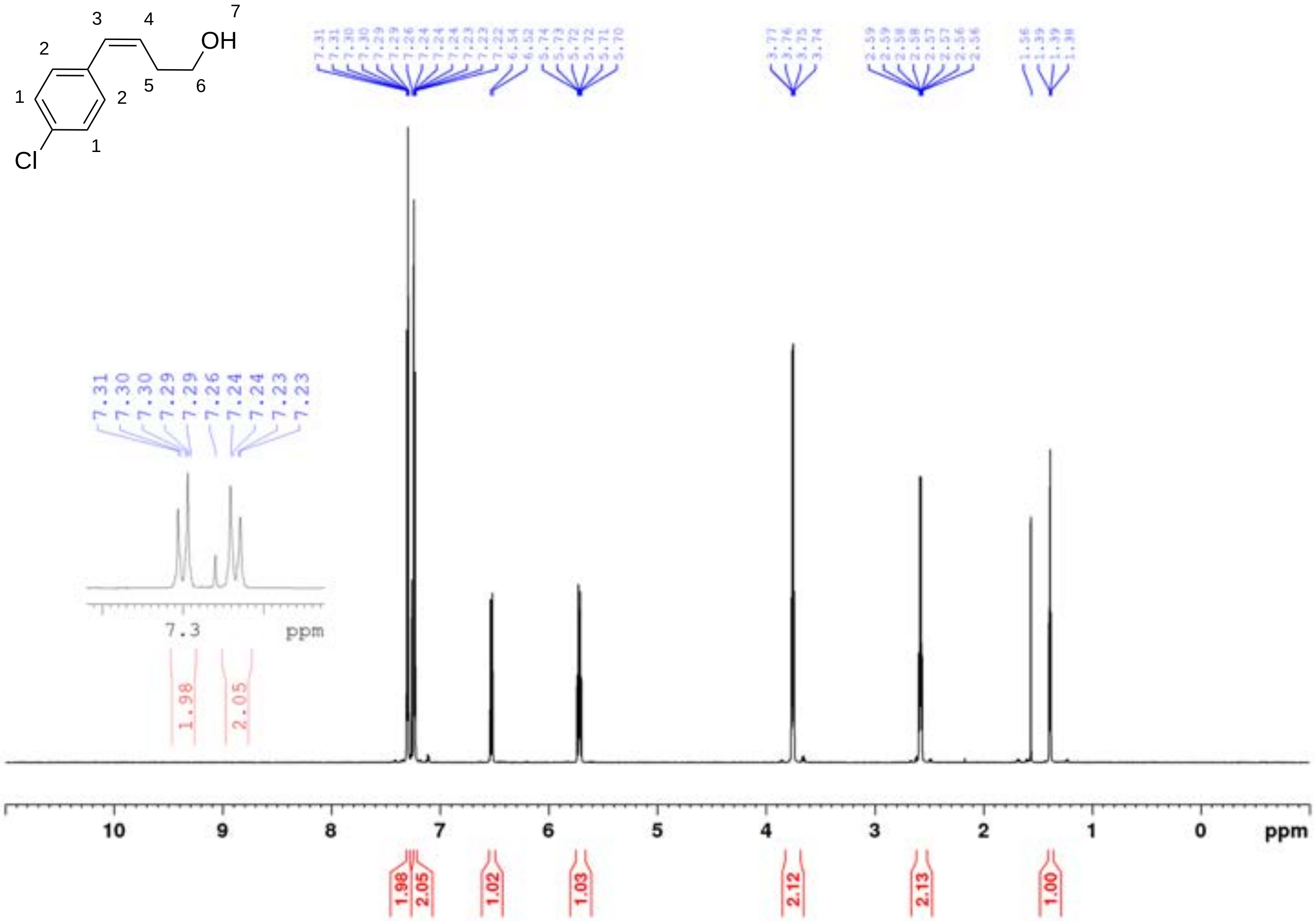

^{13}C NMR (101 MHz, $CDCl_3$) for *(Z)-4-(4-chlorophenyl)but-3-en-1-ol* (**8h**)

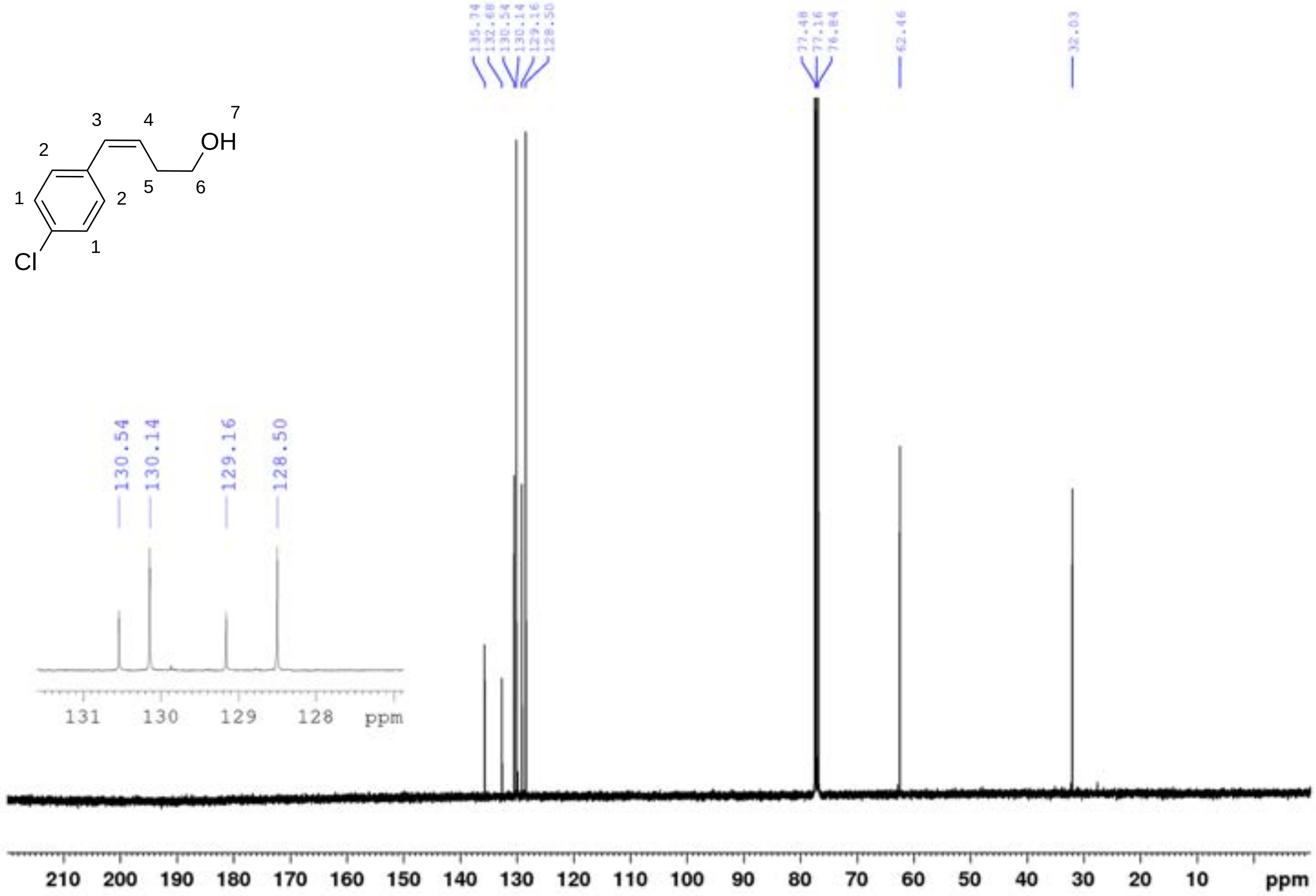

^{1}H NMR (400 MHz, $CDCl_3$) for *4-(4-hydroxybut-1-yn-1-yl)phenyl acetate*

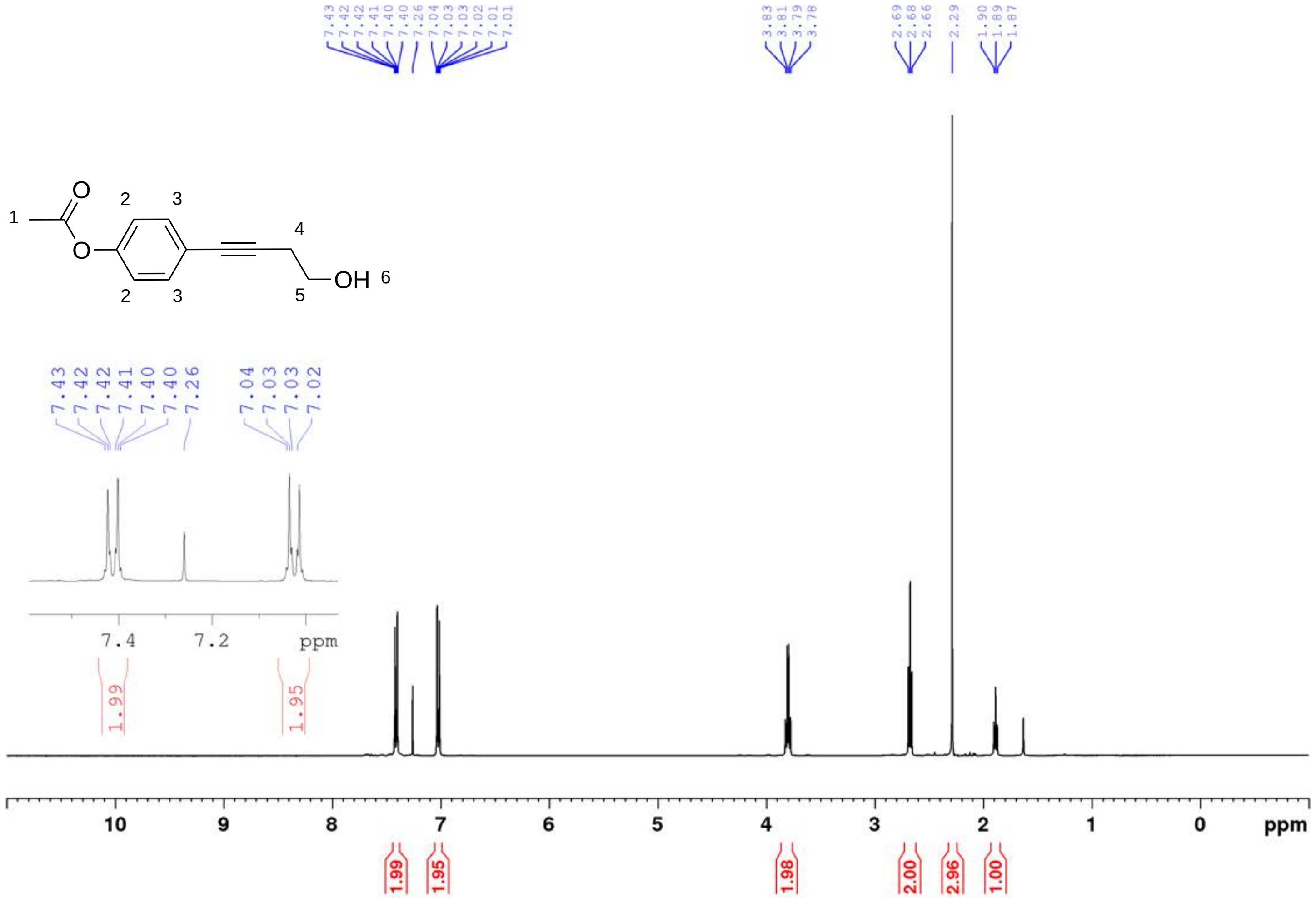

^{13}C NMR (101 MHz, $CDCl_3$) for *4-(4-hydroxybut-1-yn-1-yl)phenyl acetate*

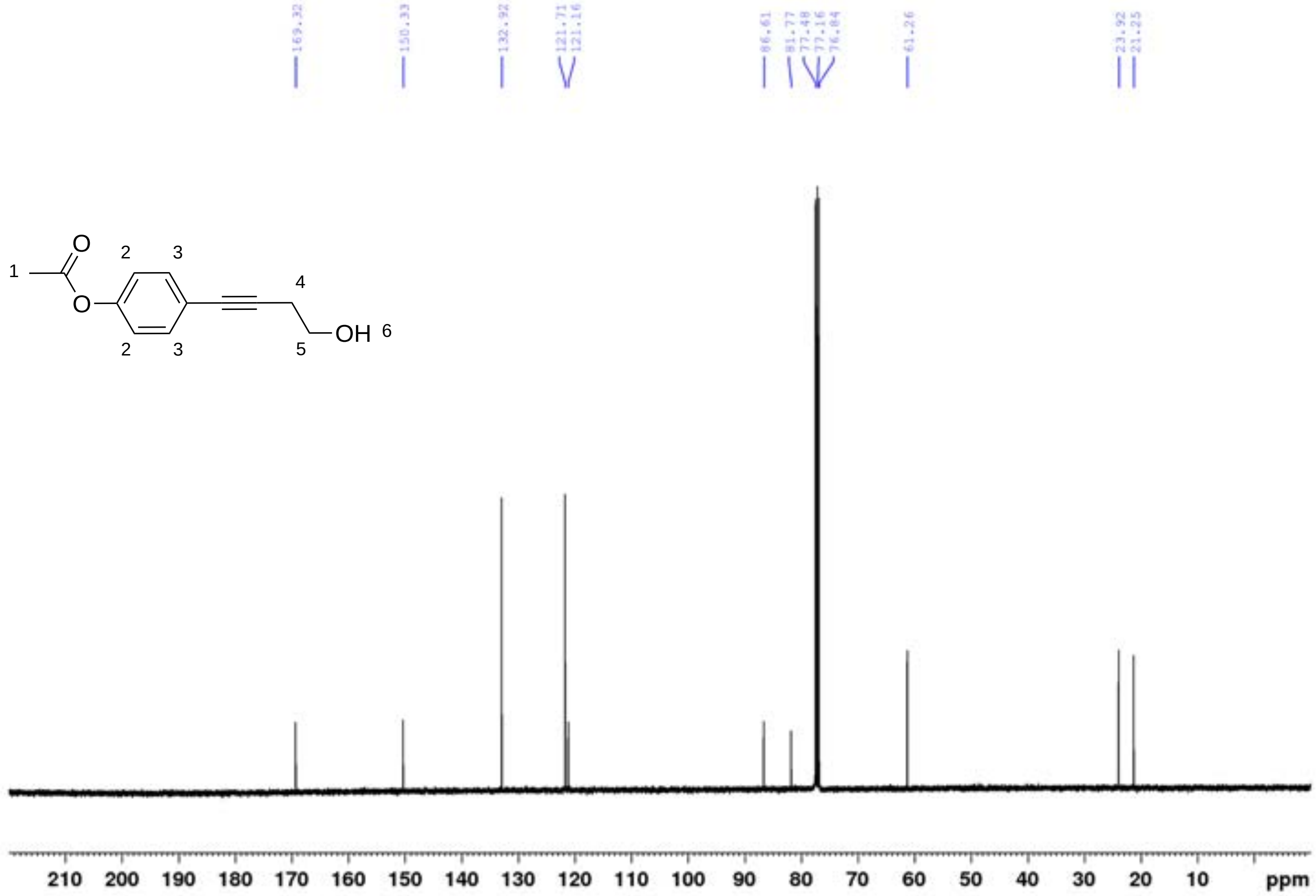

^{1}H NMR (400 MHz, $CDCl_3$) for *(Z)-4-(4-hydroxybut-1-en-1-yl)phenyl acetate* (**8i**)

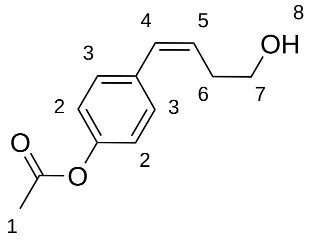

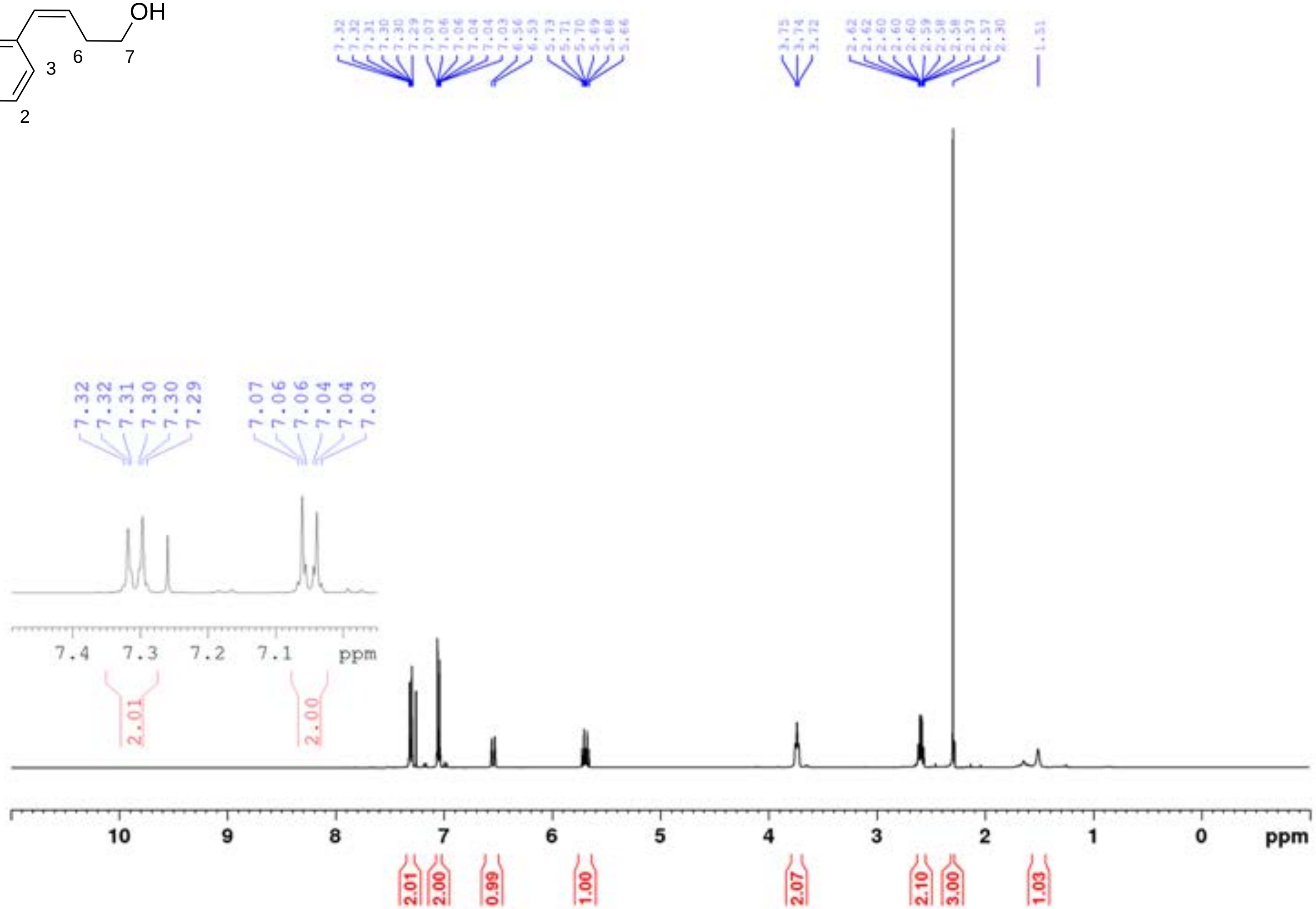

^{13}C NMR (101 MHz, $CDCl_3$) for *(Z)-4-(4-hydroxybut-1-en-1-yl)phenyl acetate* (**8i**)

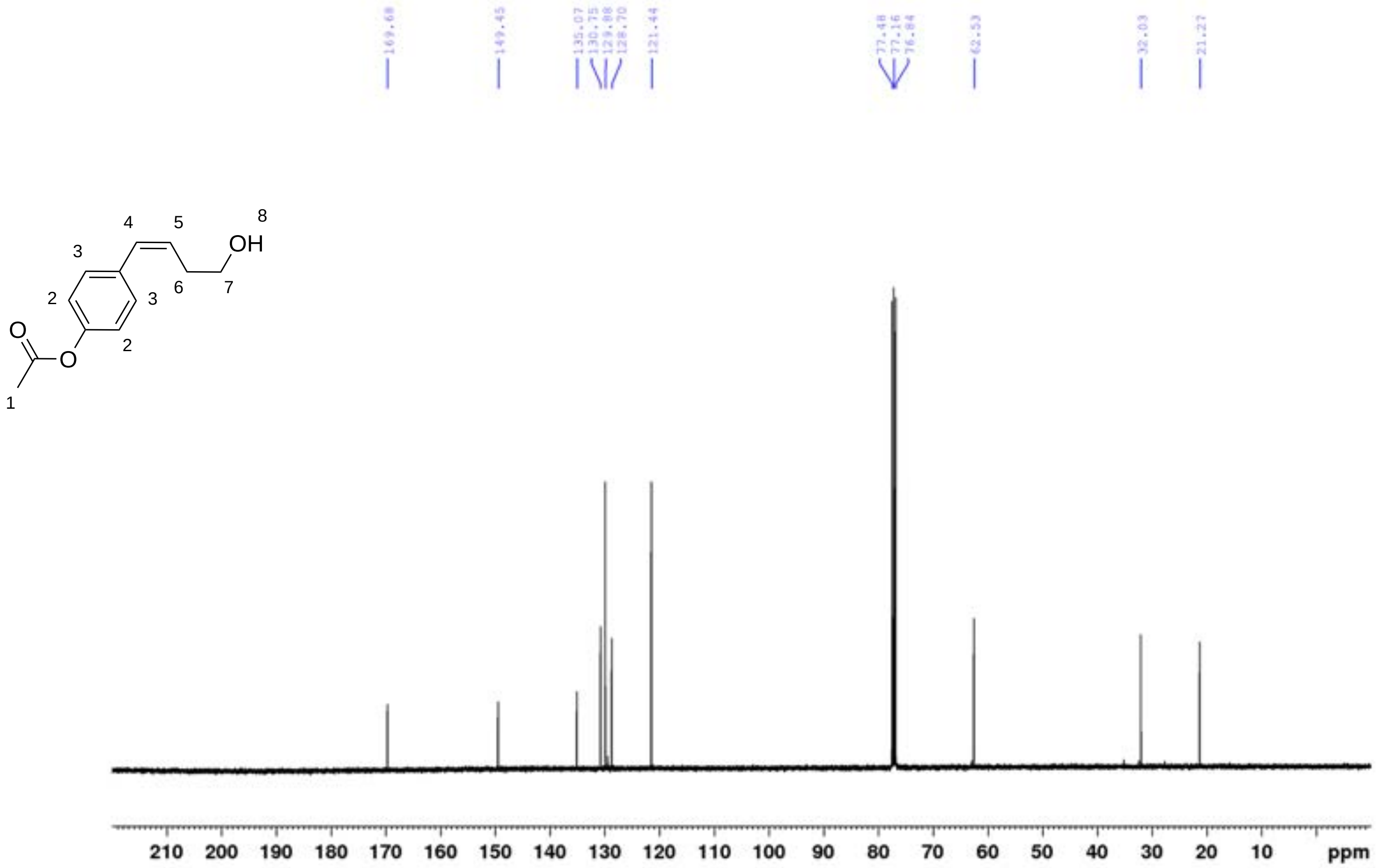

^{1}H NMR (400 MHz, $CDCl_3$) for *(Z)-4-(4-(trifluoromethyl)phenyl)but-3-en-1-ol* (**8j**)

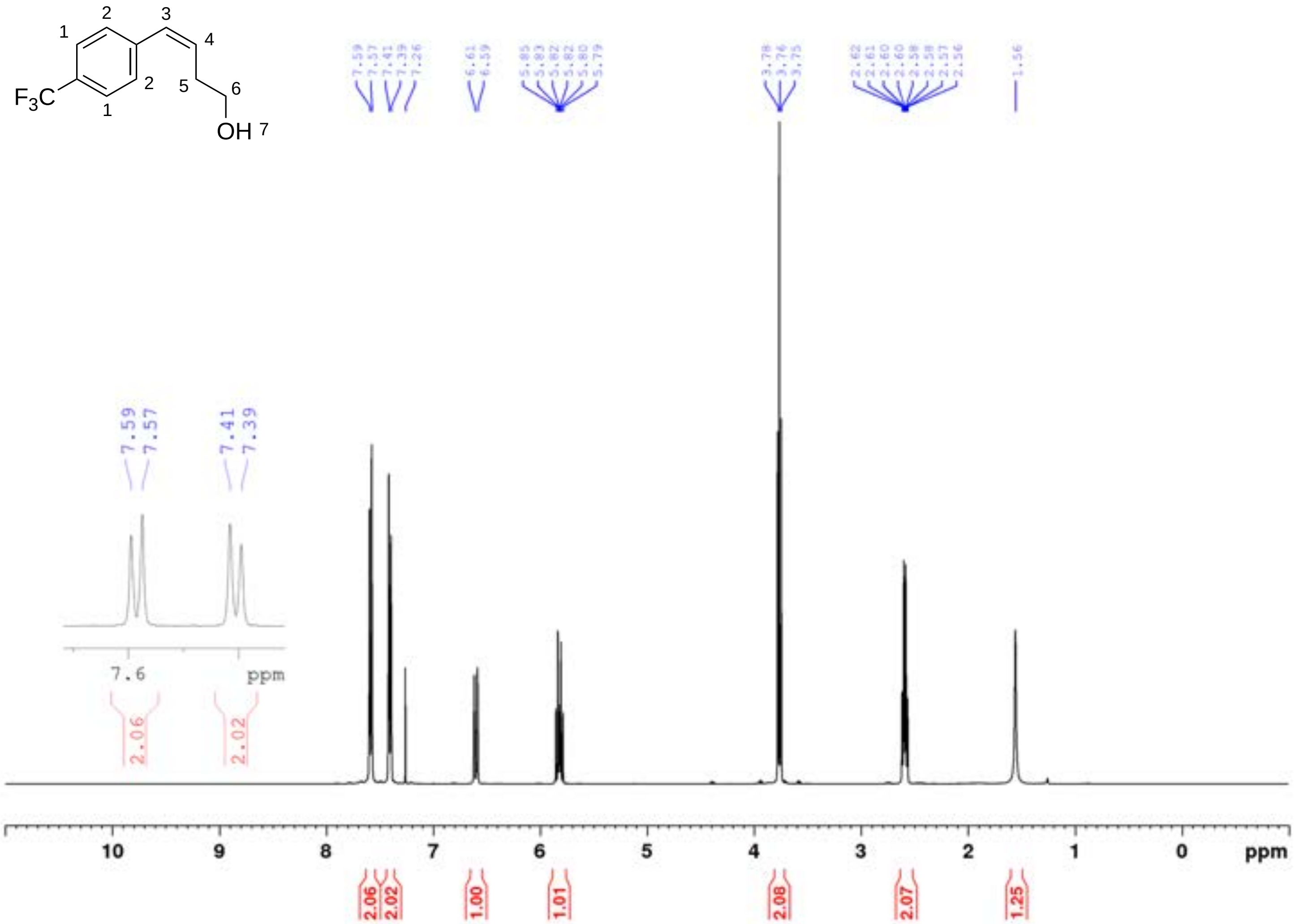

^{13}C NMR (101 MHz, $CDCl_3$) for *(Z)-4-(4-(trifluoromethyl)phenyl)but-3-en-1-ol* (**8j**)

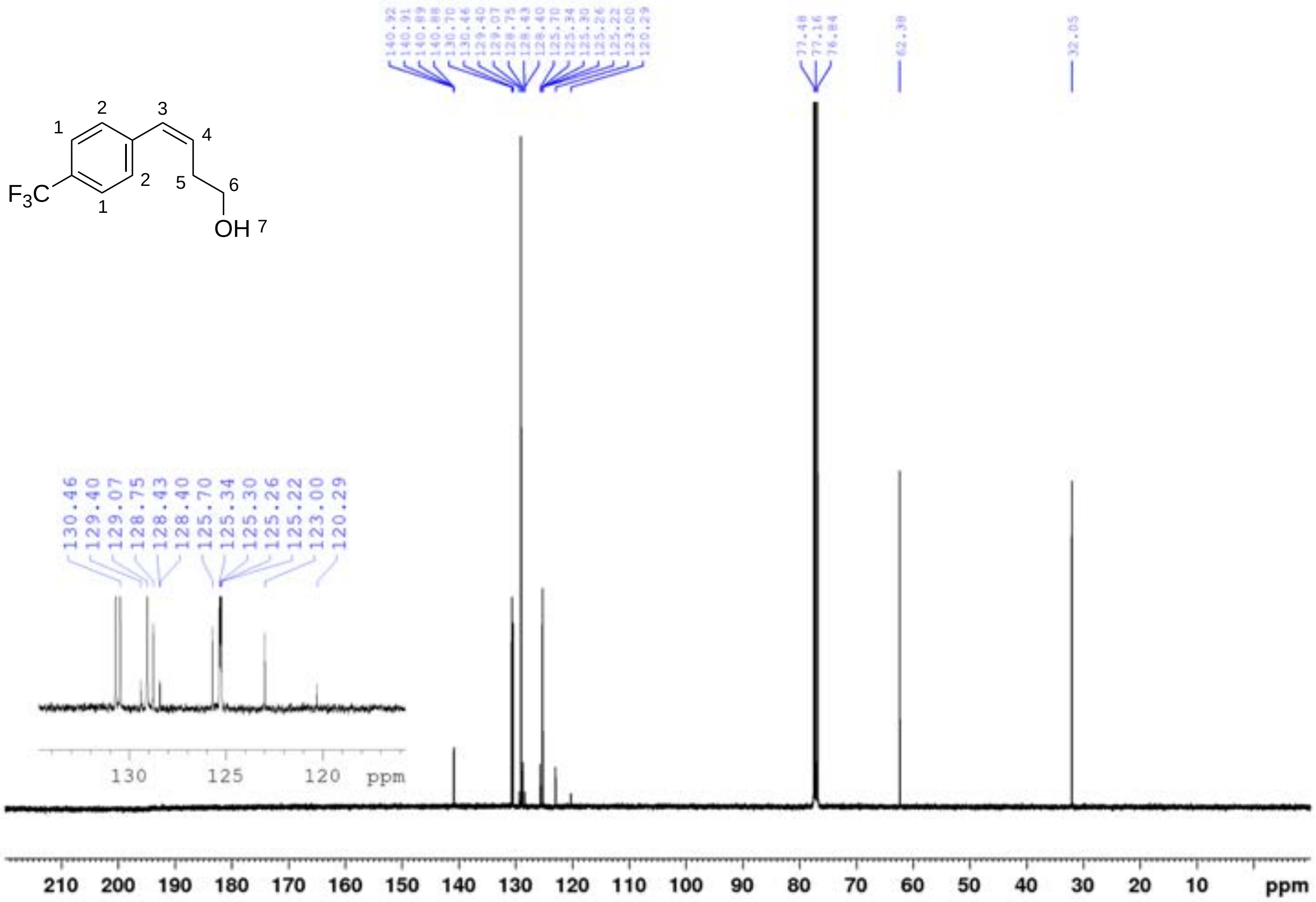

^{19}F NMR (376 MHz, $CDCl_3$) for *(Z)-4-(4-(trifluoromethyl)phenyl)but-3-en-1-ol* (**8j**)

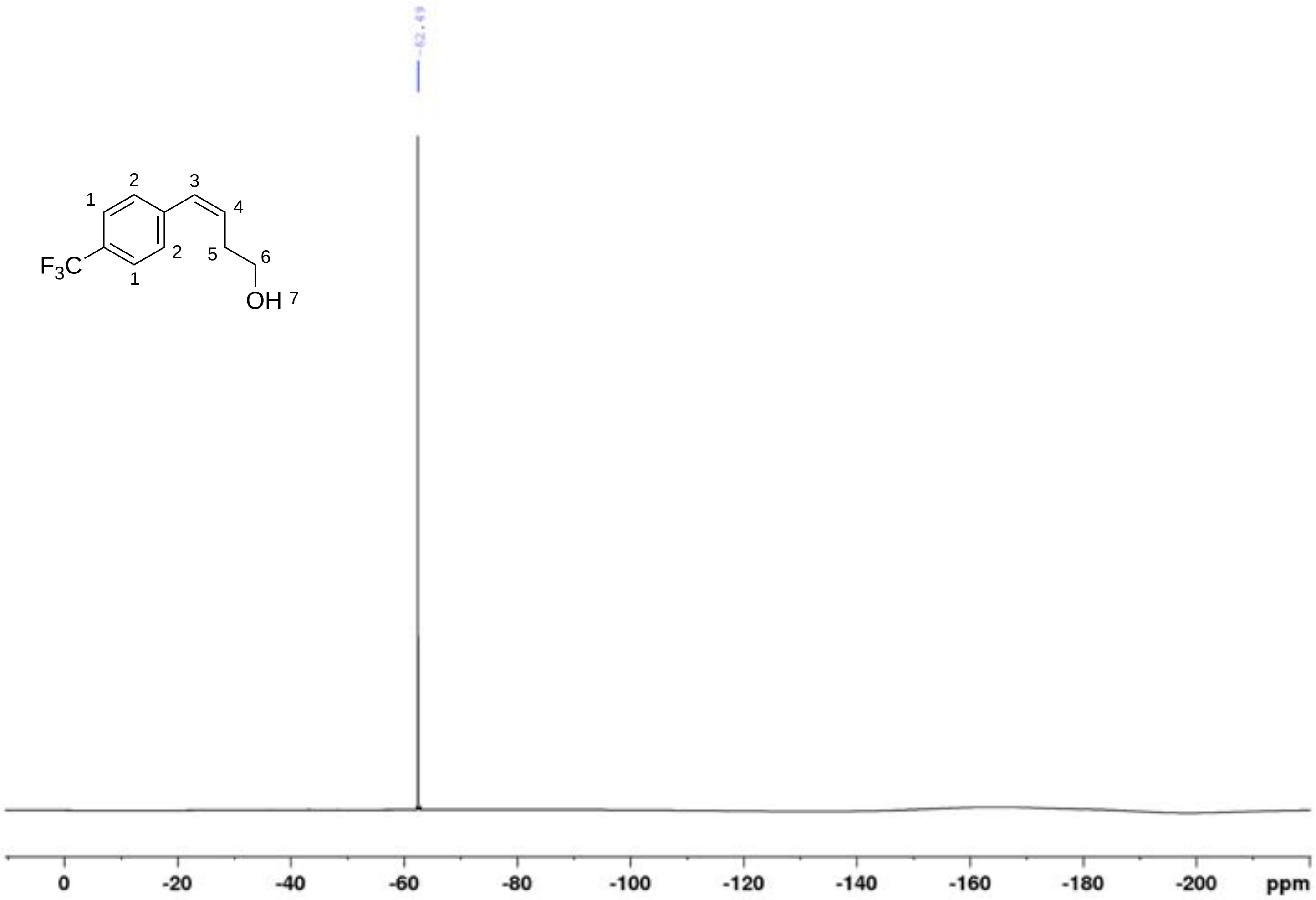

^{1}H NMR (400 MHz, $CDCl_3$) for *Ethyl (Z)-4-(4-hydroxybut-1-en-1-yl)benzoate* (**8k**)

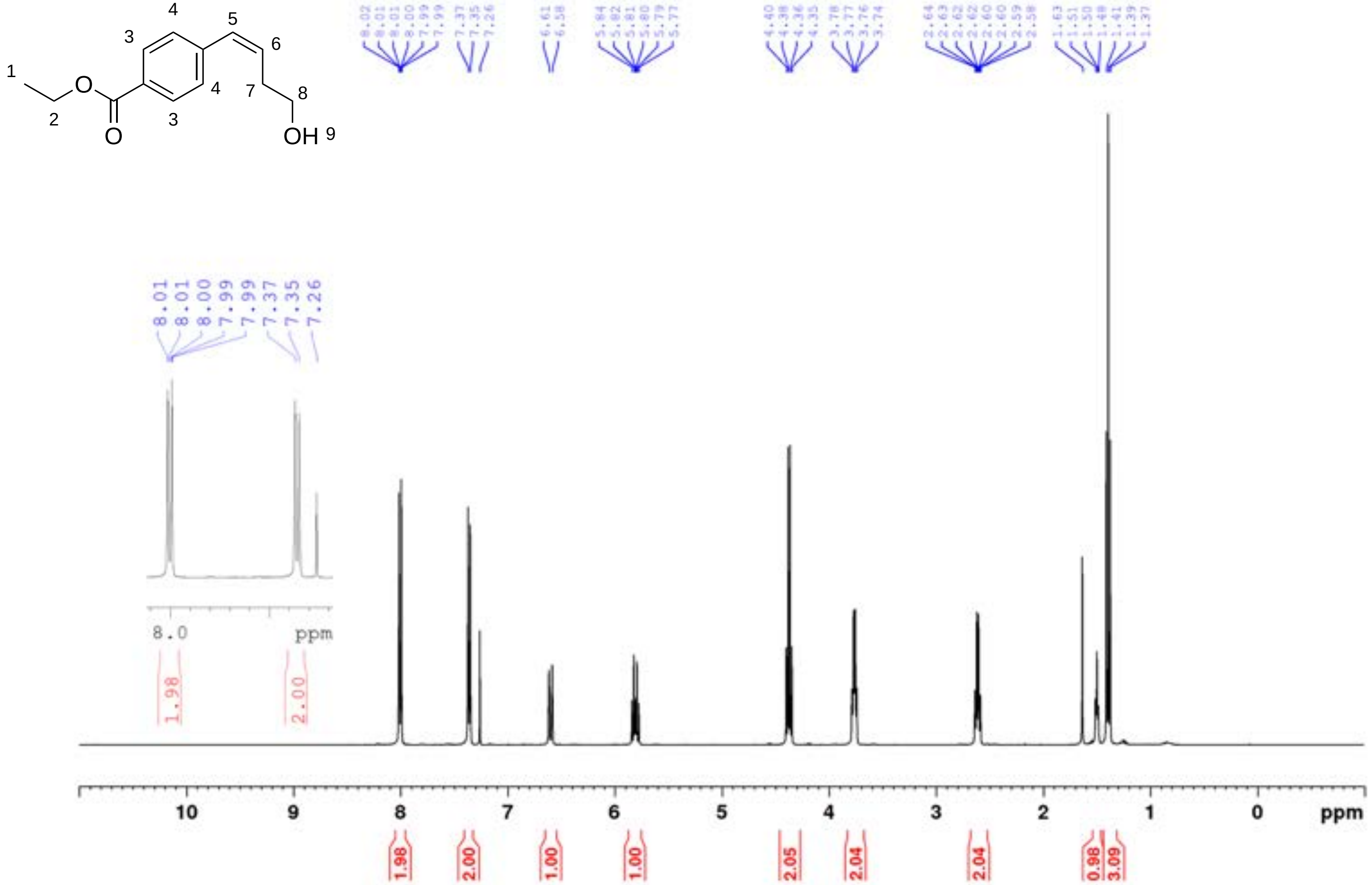

^{13}C NMR (101 MHz, $CDCl_3$) for *Ethyl (Z)-4-(4-hydroxybut-1-en-1-yl)benzoate* (**8k**)

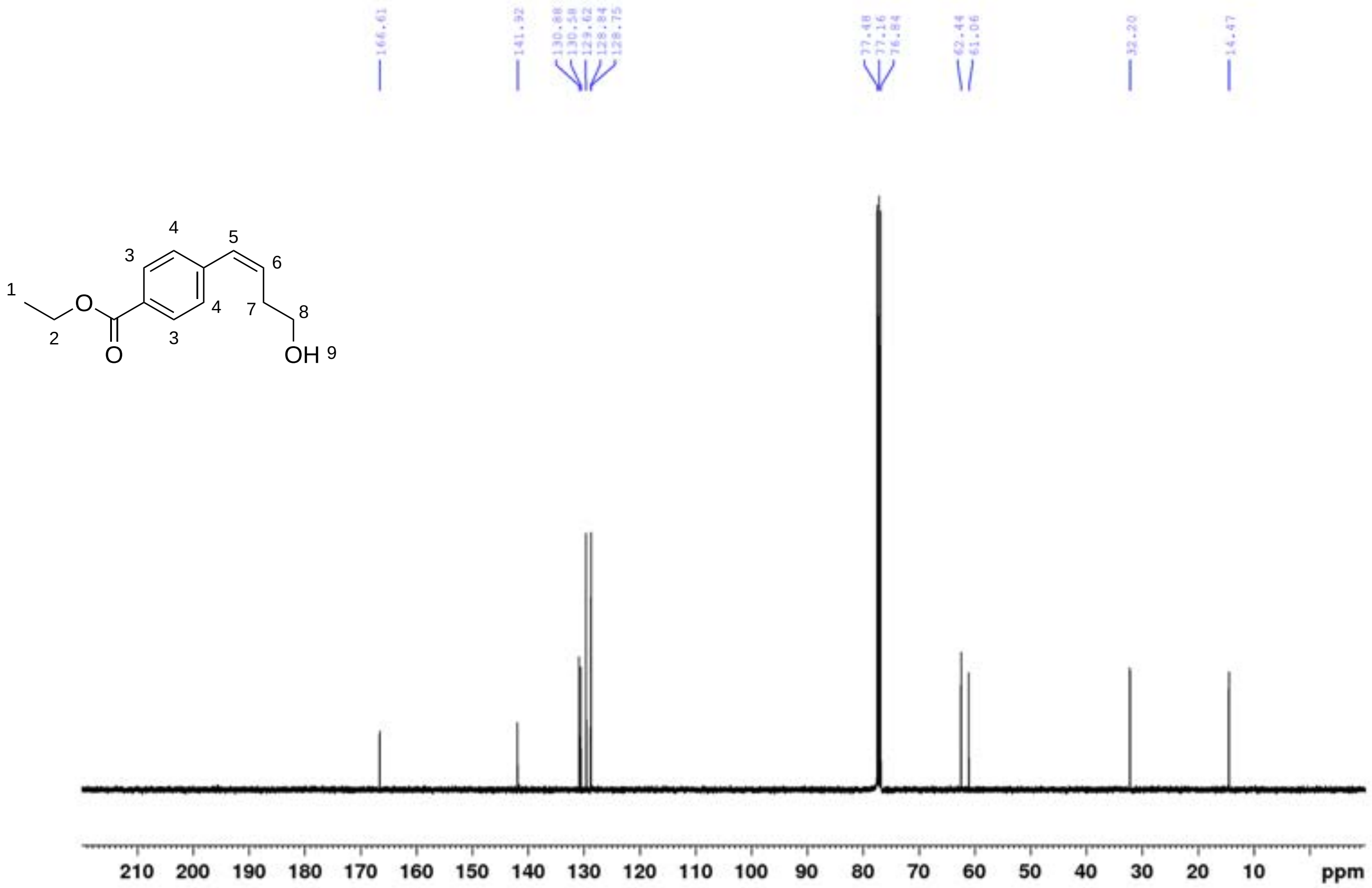

¹H NMR (400 MHz, $CDCl_3$) for *(Z)-4-(3,5-dimethylphenyl)but-3-en-1-ol* (**8l**)

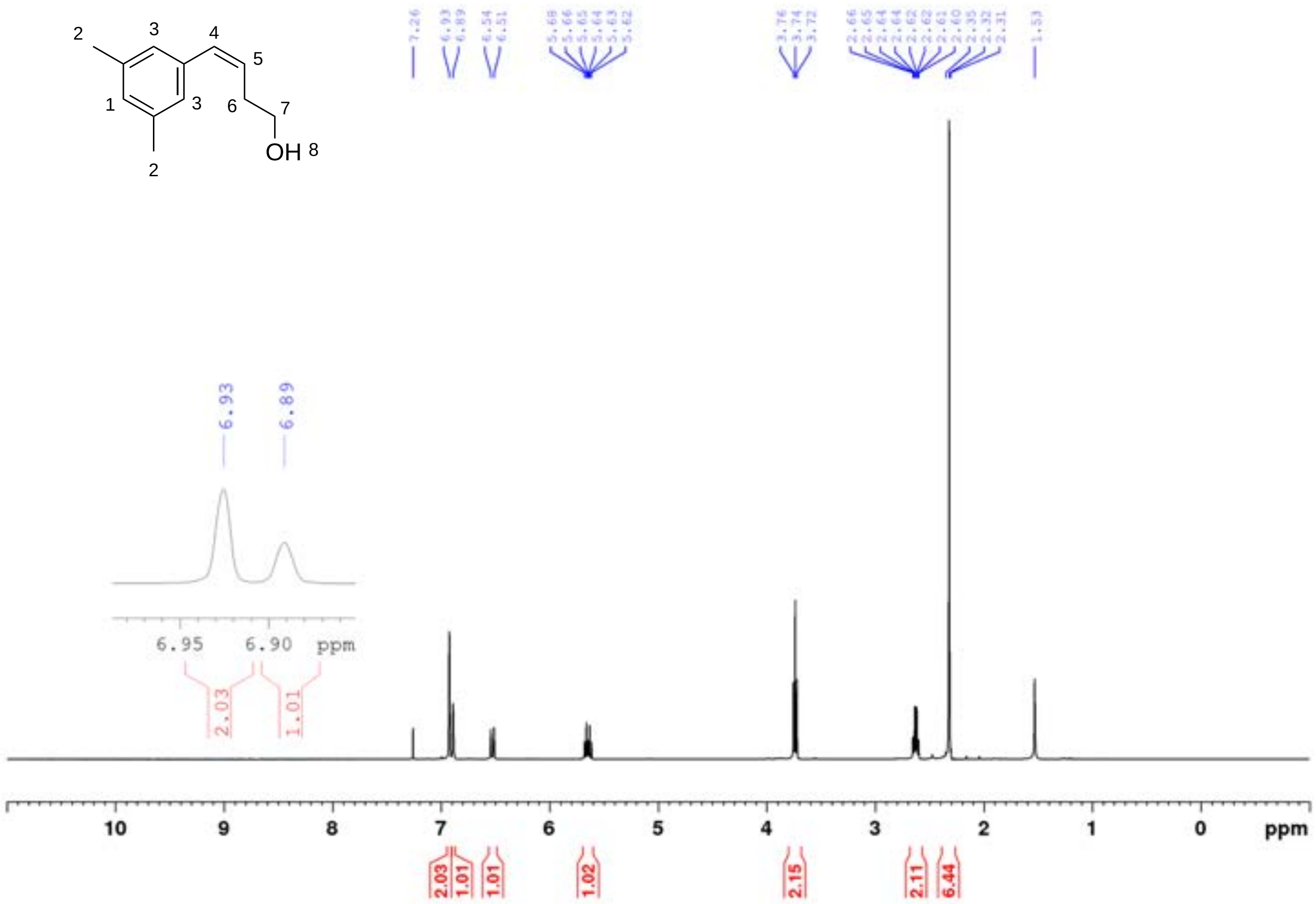

^{13}C NMR (101 MHz, $CDCl_3$) for *(Z)-4-(3,5-dimethylphenyl)but-3-en-1-ol* (**8l**)

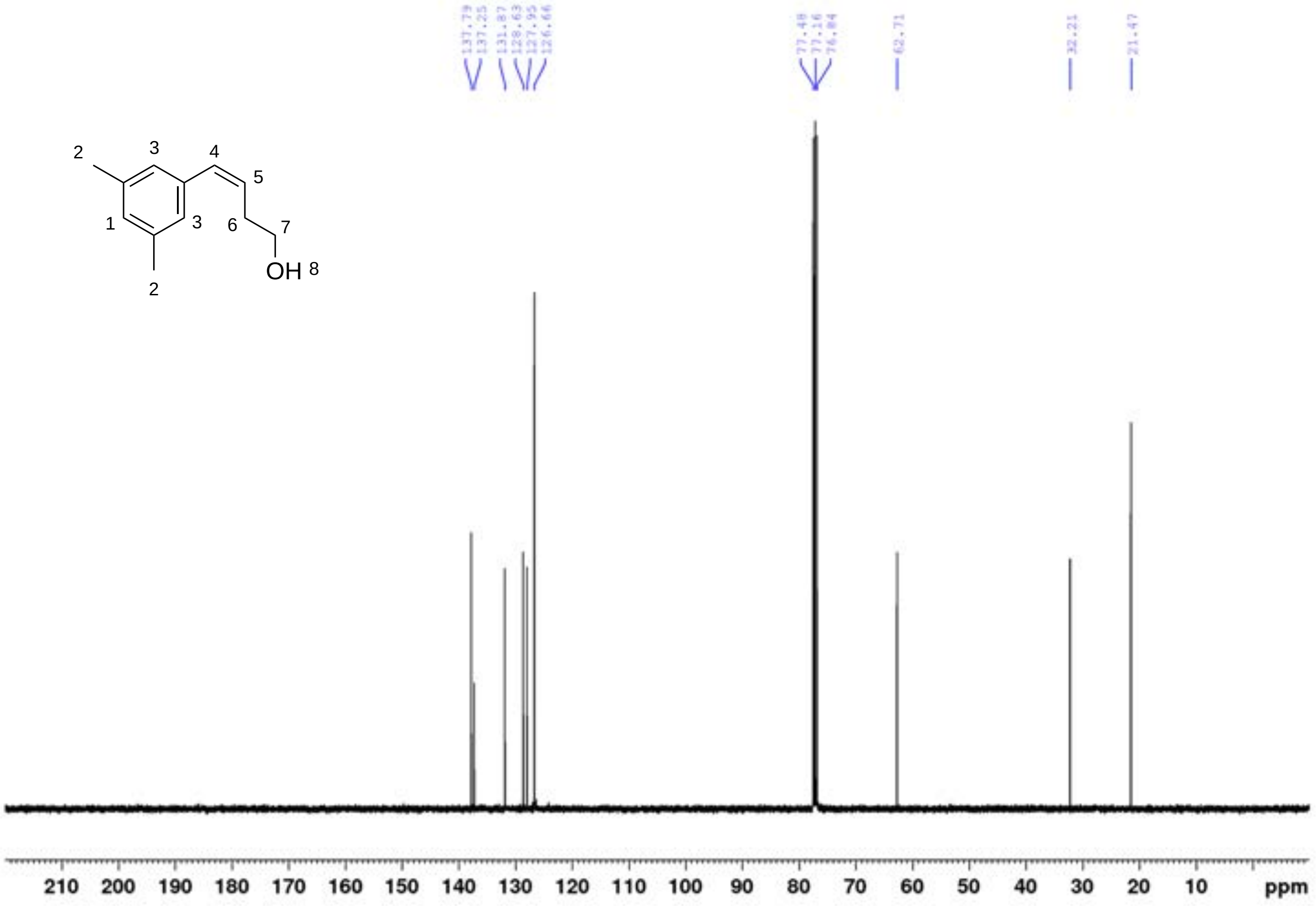

^{1}**H NMR** (400 MHz, $CDCl_3$) for *(Z)-4-(2,5-dimethylphenyl)but-3-en-1-ol* (**8m**)

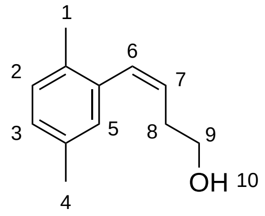

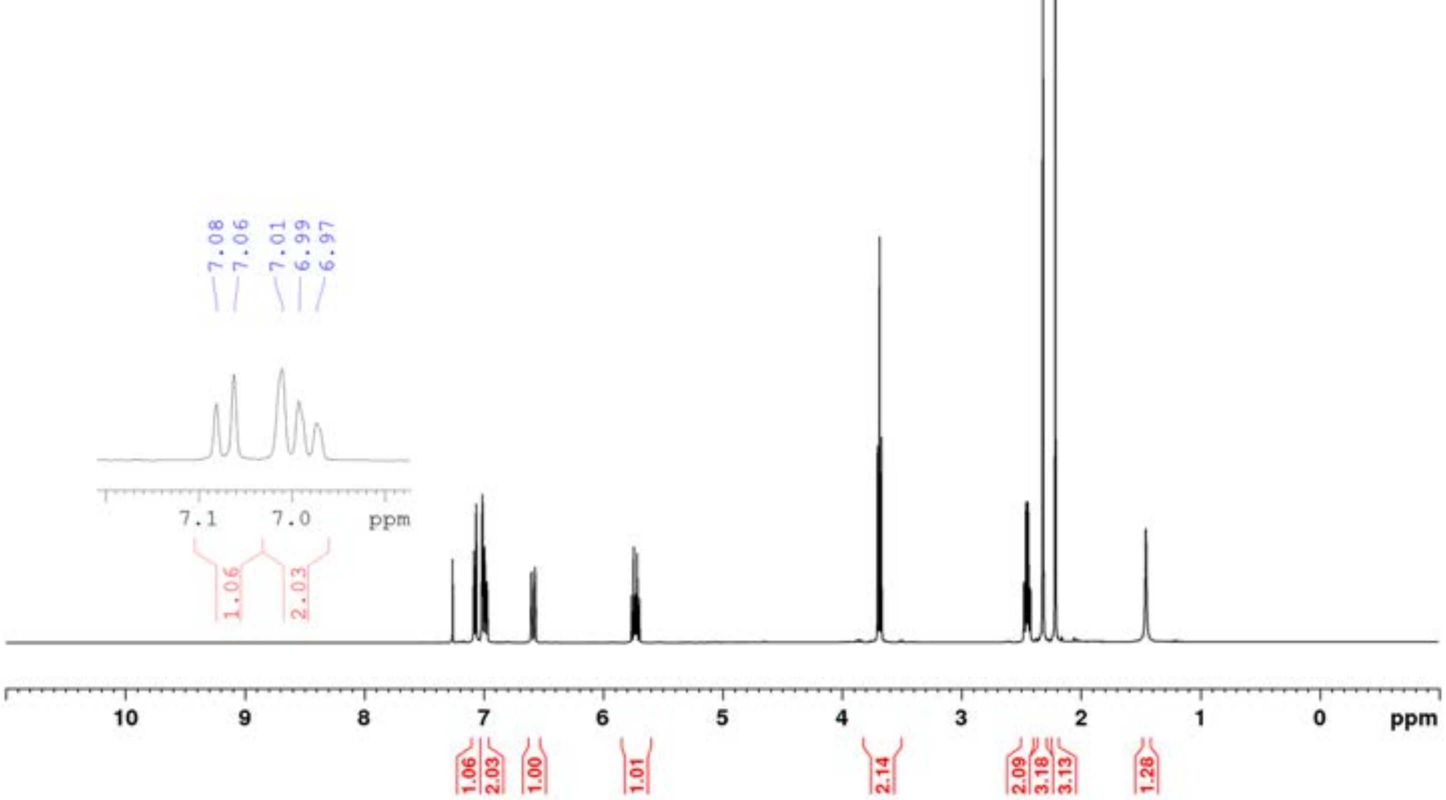

^{13}C NMR (101 MHz, $CDCl_3$) for *(Z)-4-(2,5-dimethylphenyl)but-3-en-1-ol* (**8m**)

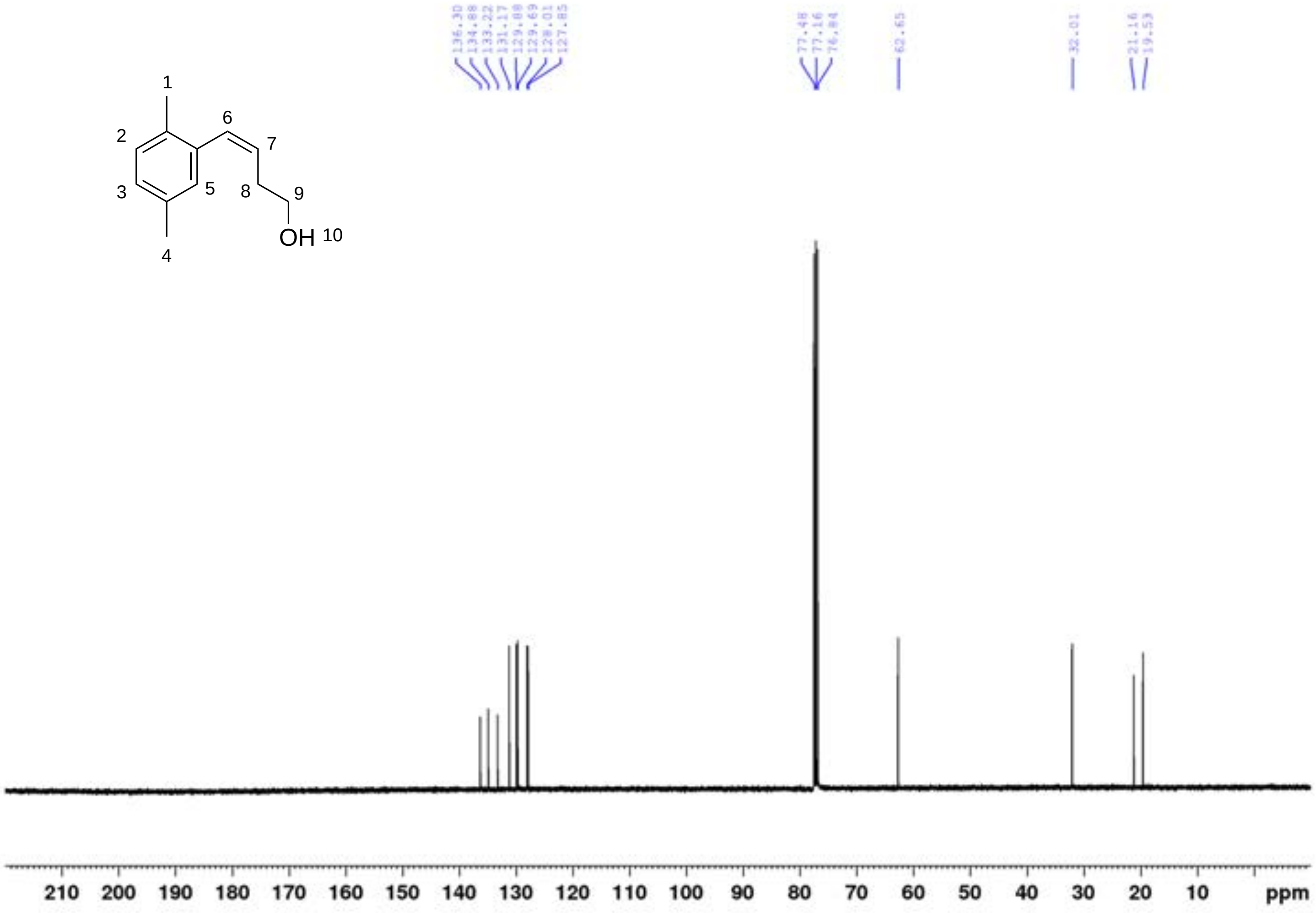

^{1}H NMR (400 MHz, $CDCl_3$) for *(Z)-4-(3-fluoro-4-methylphenyl)but-3-en-1-ol* (**8n**)

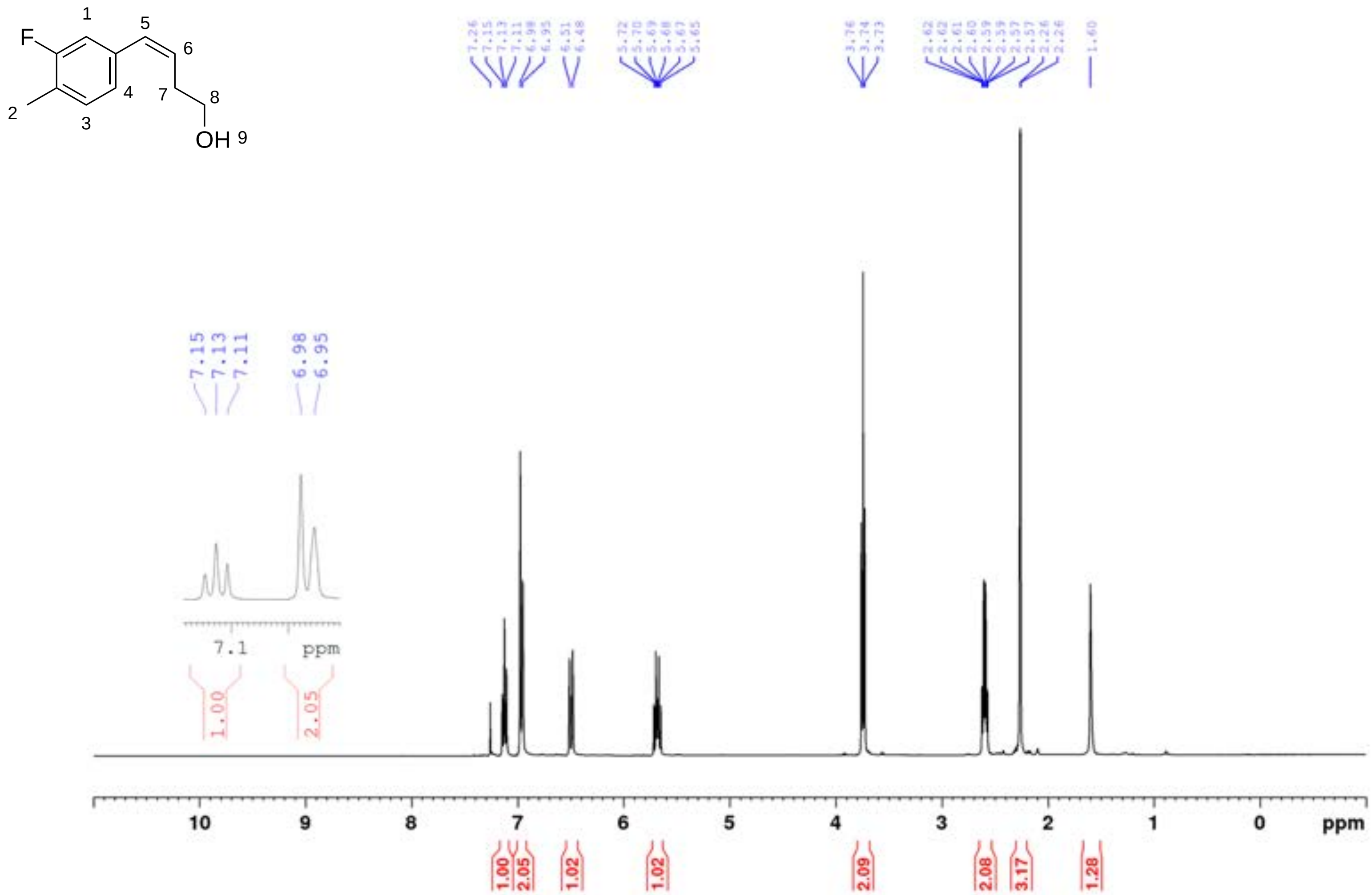

^{13}C NMR (101 MHz, $CDCl_3$) for *(Z)-4-(3-fluoro-4-methylphenyl)but-3-en-1-ol* (**8n**)

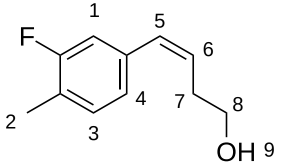

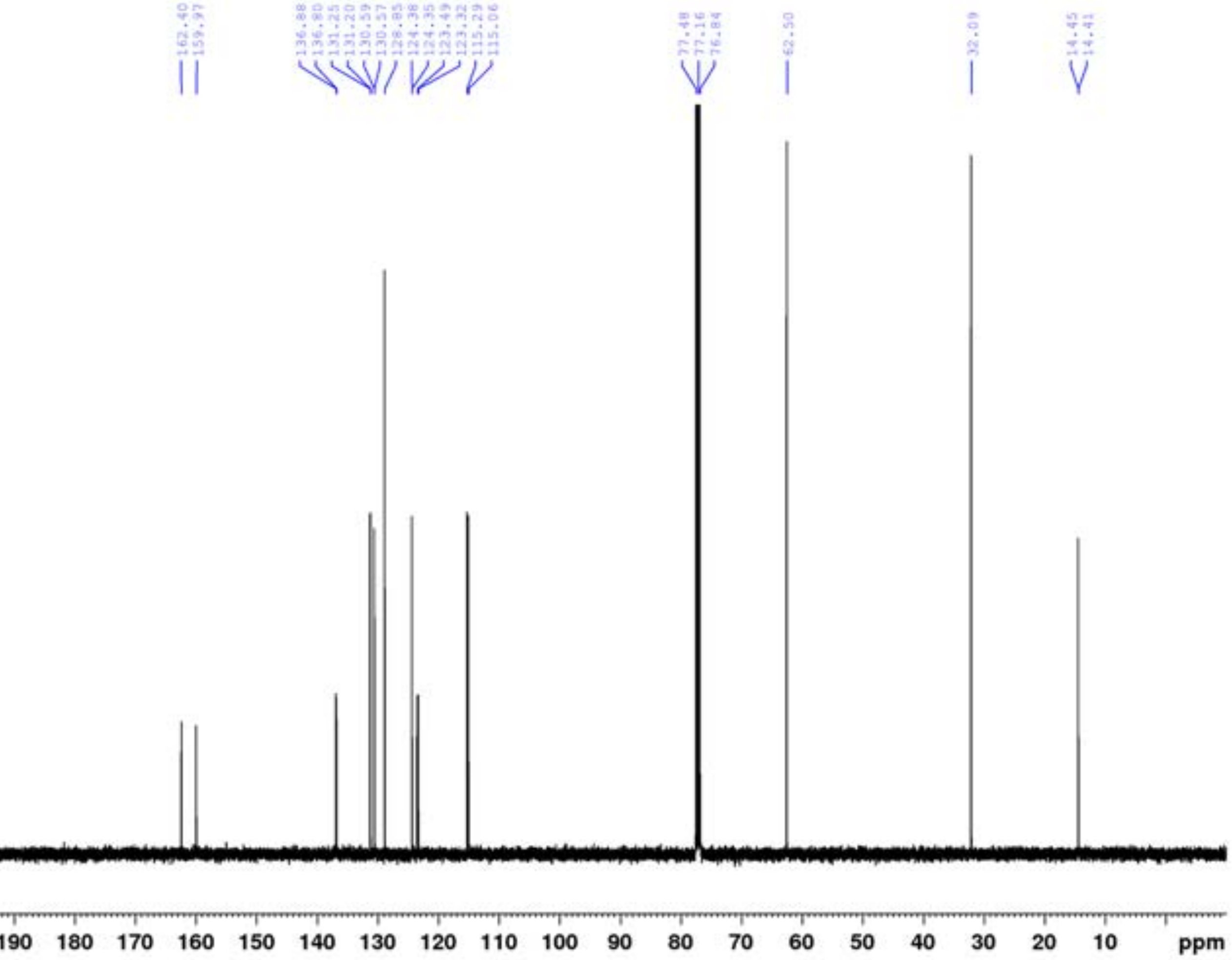

^{19}F NMR (376 MHz, $CDCl_3$) for *(Z)-4-(3-fluoro-4-methylphenyl)but-3-en-1-ol* (**8n**)

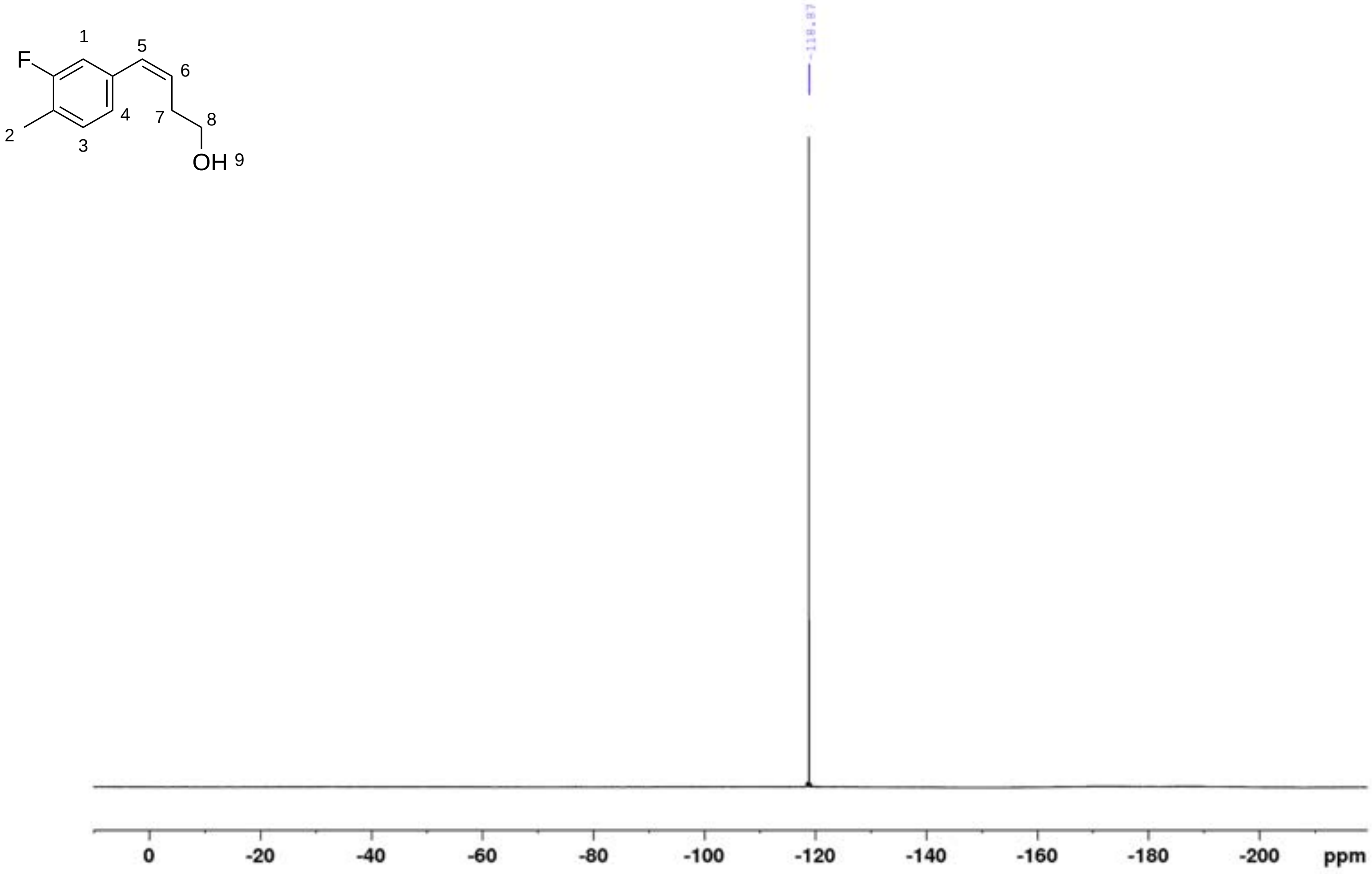

^{1}H NMR (400 MHz, $CDCl_3$) for *(Z)-4-(naphthalen-1-yl)but-3-en-1-ol* (**8o**)

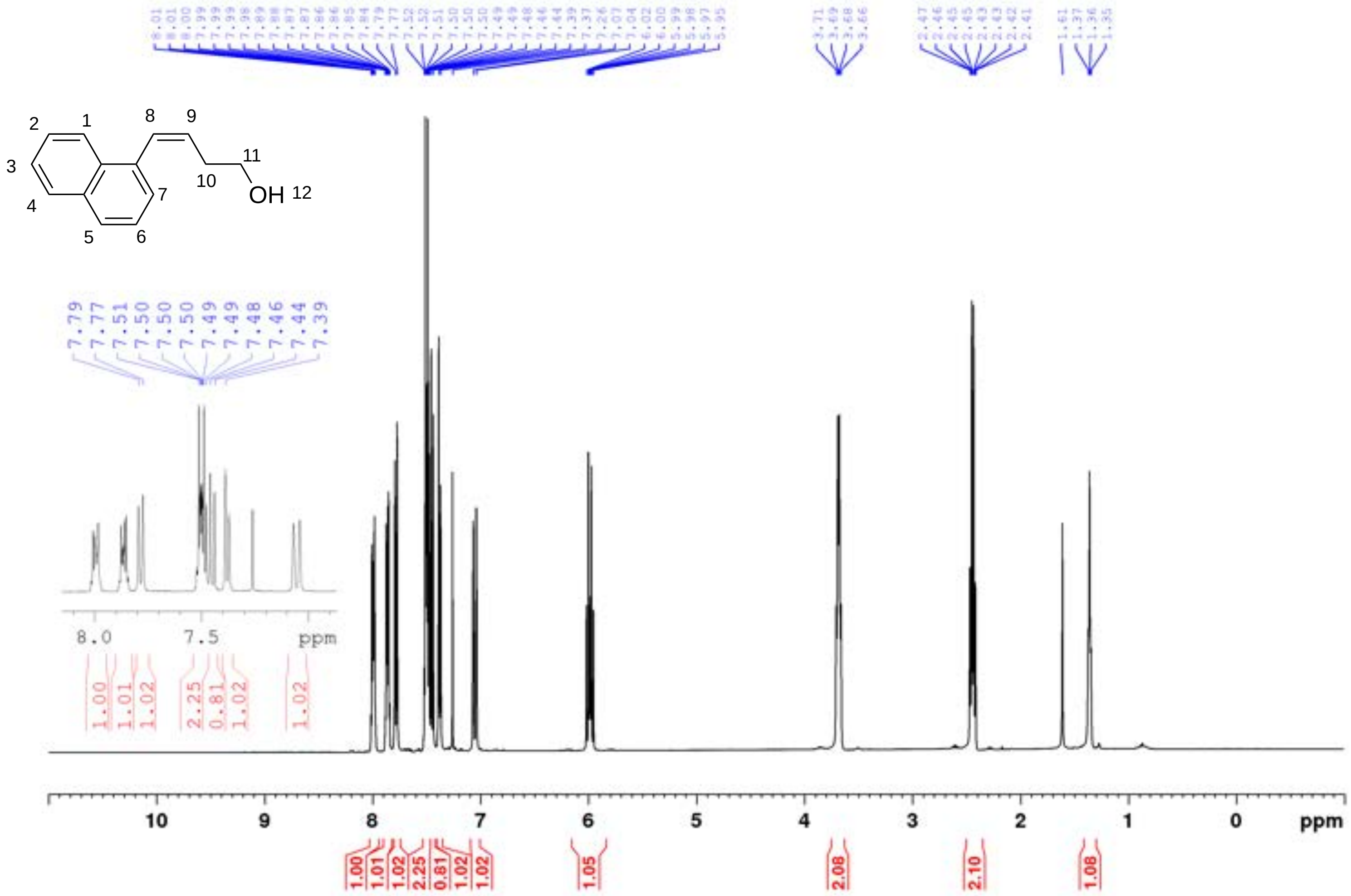

^{13}C NMR (101 MHz, $CDCl_3$) for *(Z)-4-(naphthalen-1-yl)but-3-en-1-ol* (**8o**)

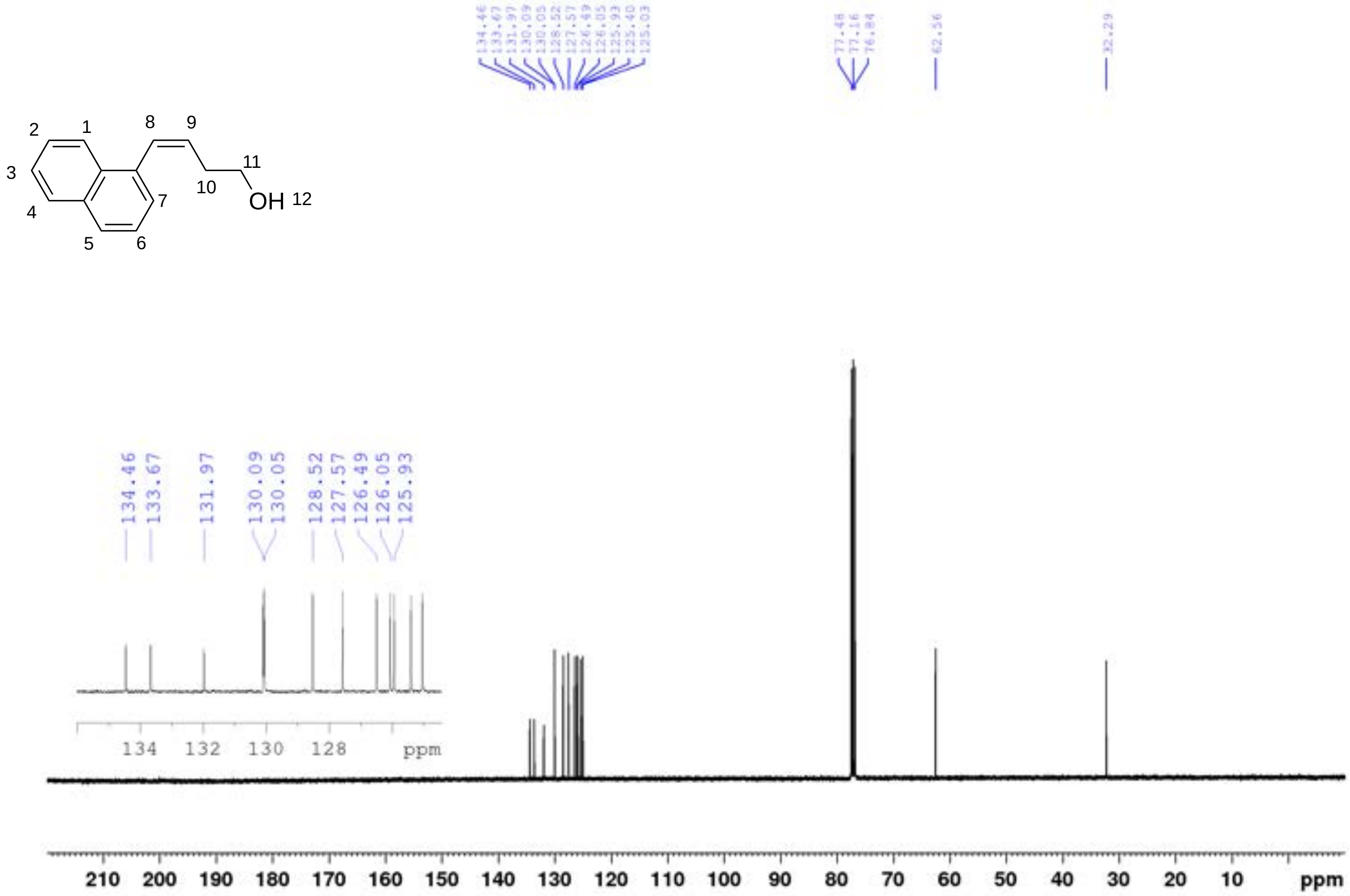

^{1}H NMR (700 MHz, $CDCl_3$) for *(E)-4-phenylbut-3-en-1-ol* (**10**)

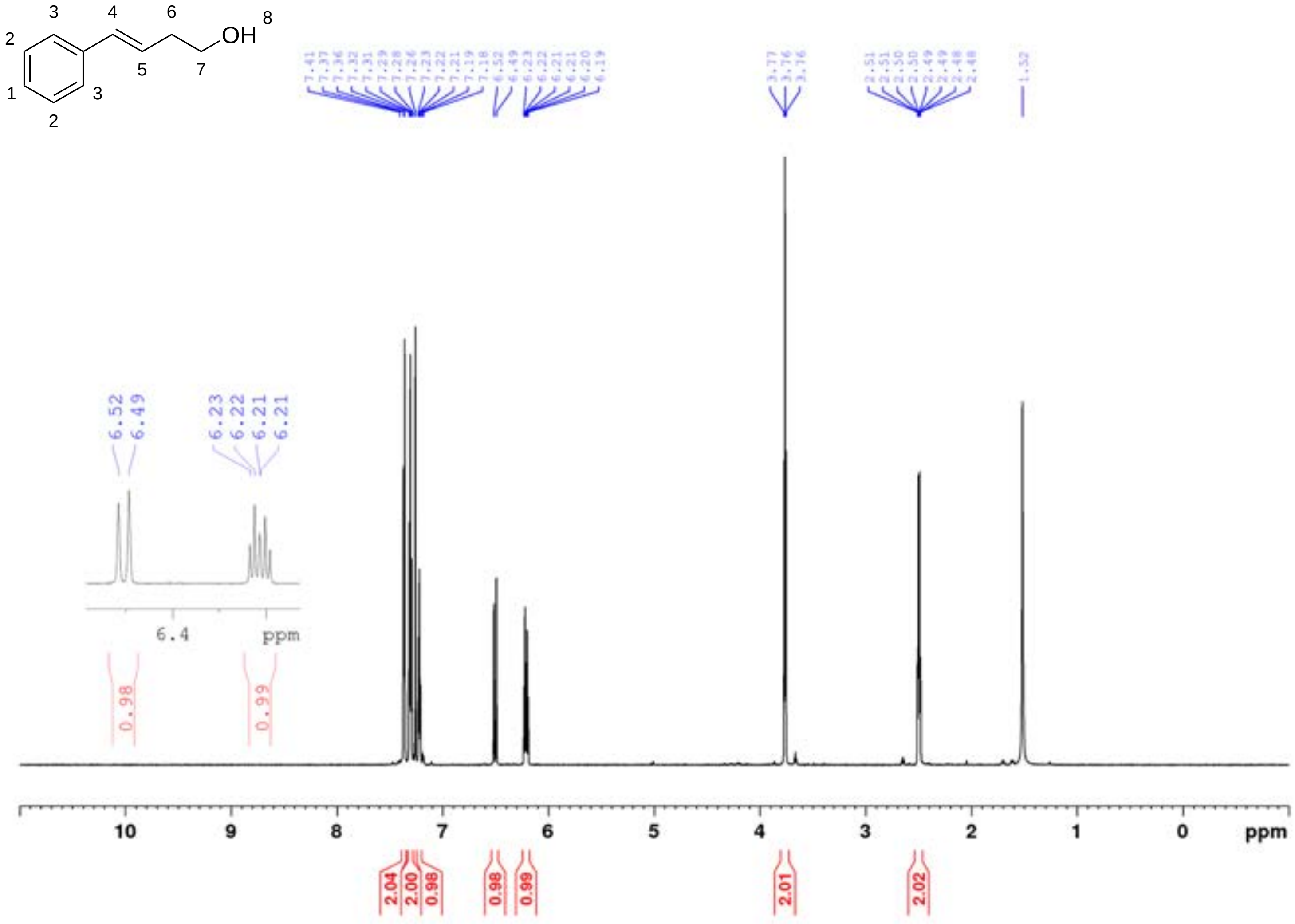

^{13}C NMR (101 MHz, $CDCl_3$) for *(E)-4-phenylbut-3-en-1-ol* (**10**)

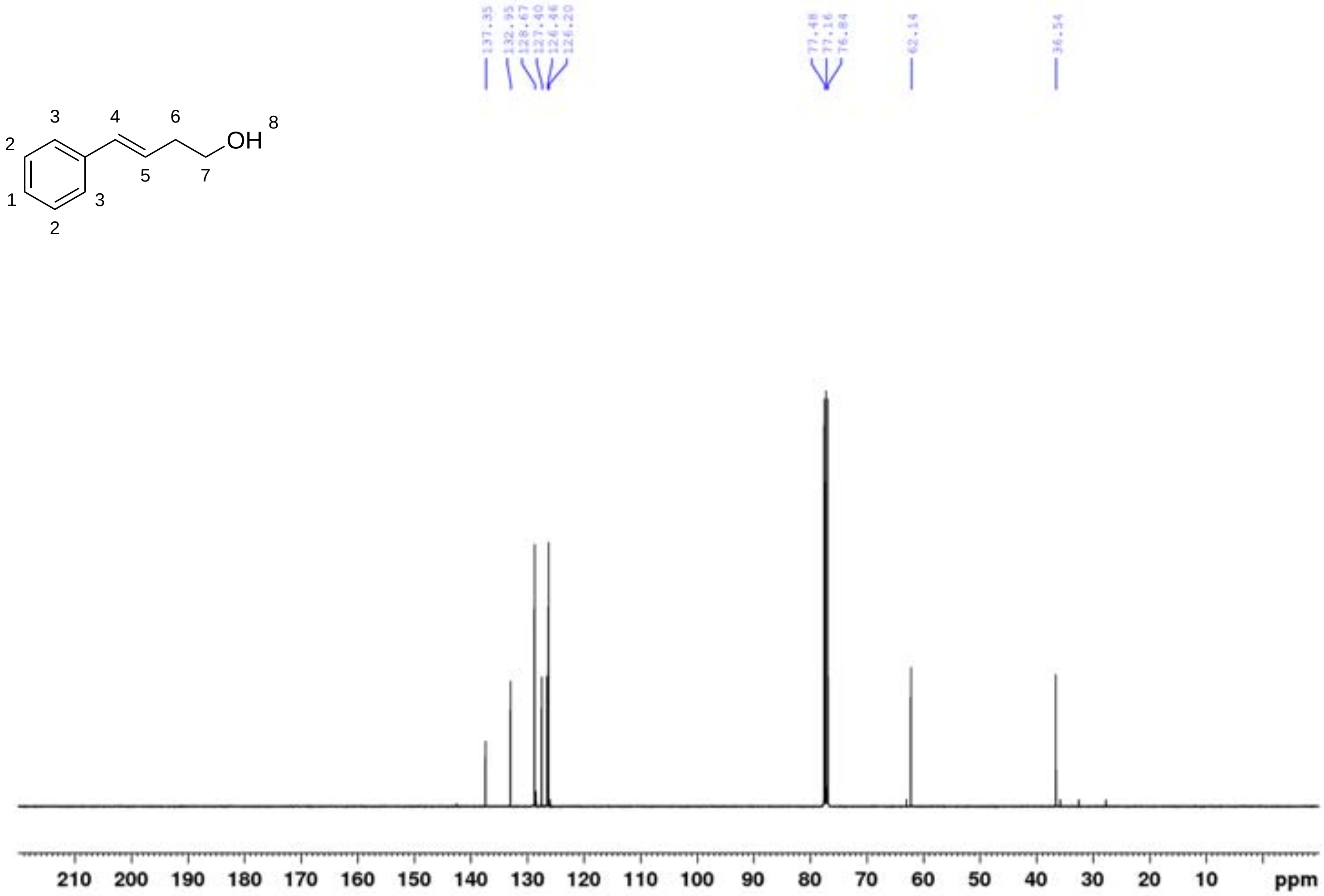

^{1}H NMR (400 MHz, $CDCl_3$) *6-phenylhex-5-yn-1-ol*

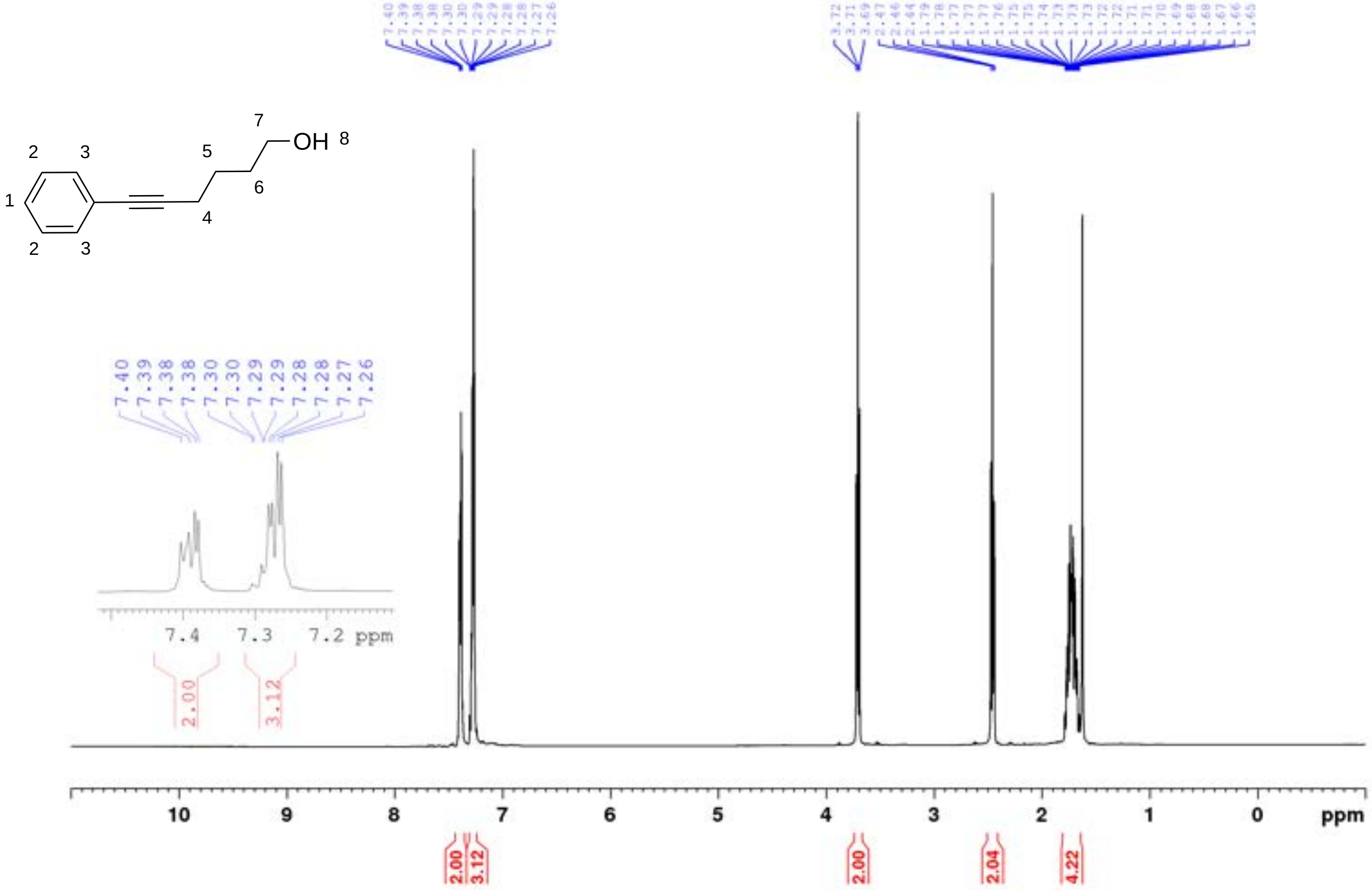

^{13}C NMR (101 MHz, $CDCl_3$) for *6-phenylhex-5-yn-1-ol*

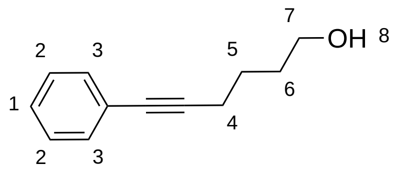

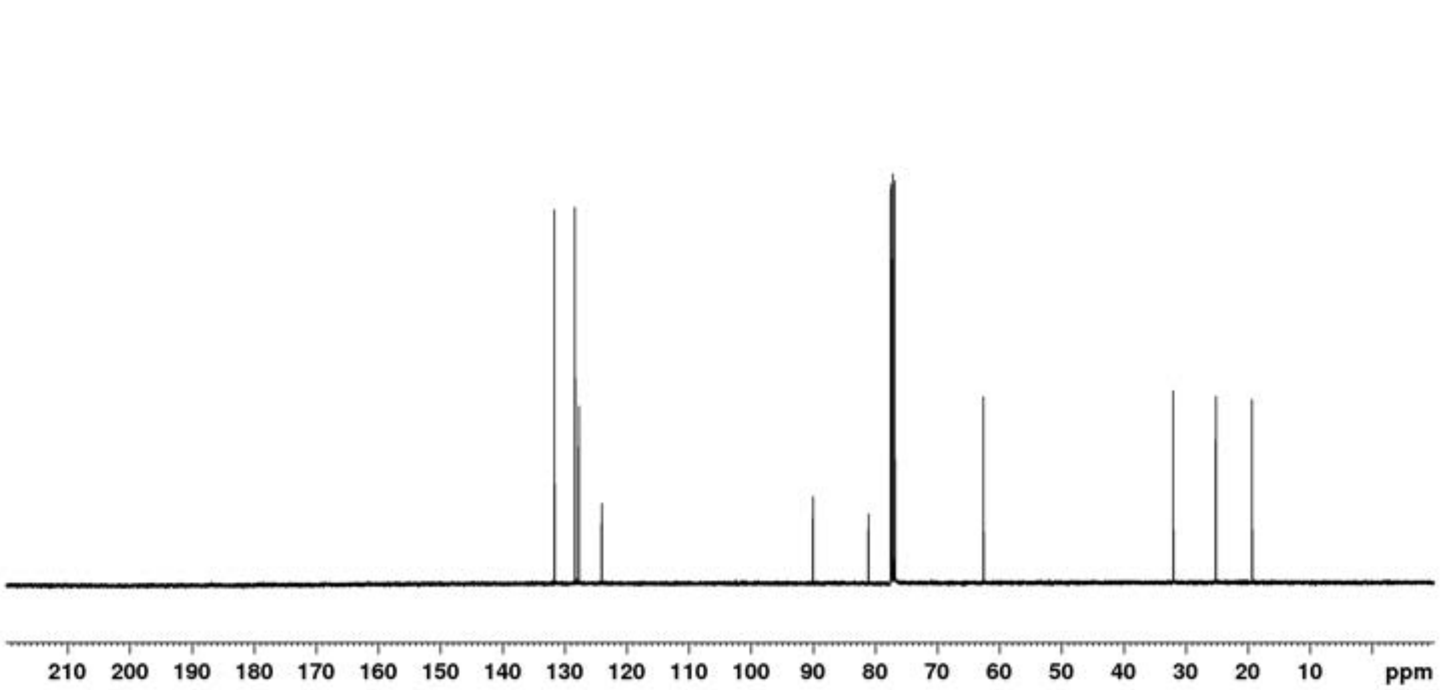

^{1}H NMR (400 MHz, $CDCl_3$) for *(Z)-6-phenylhex-5-en-1-ol* (**13a**)

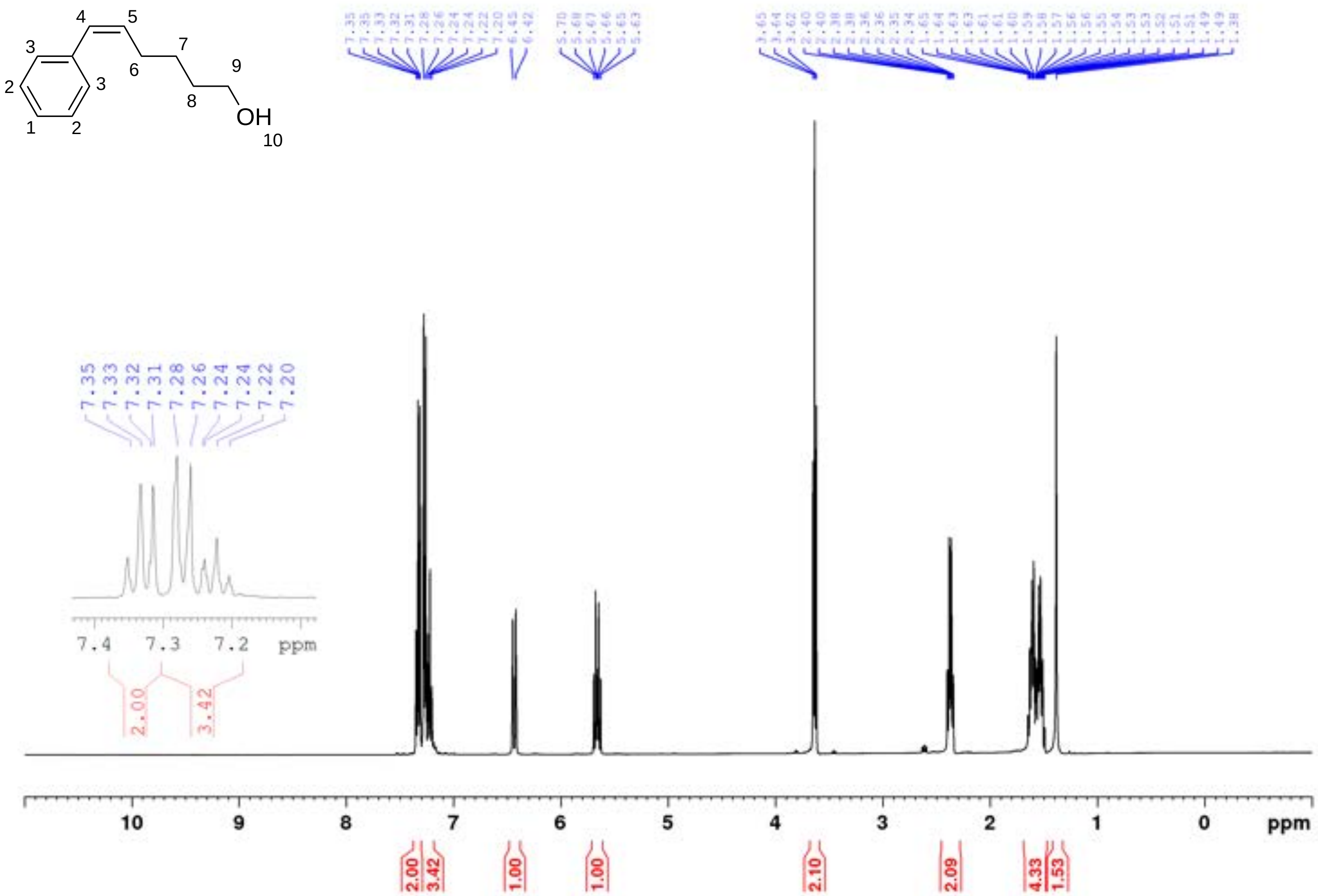

^{13}**C NMR** (101 MHz, $CDCl_3$) for *(Z)-6-phenylhex-5-en-1-ol* (**13a**)

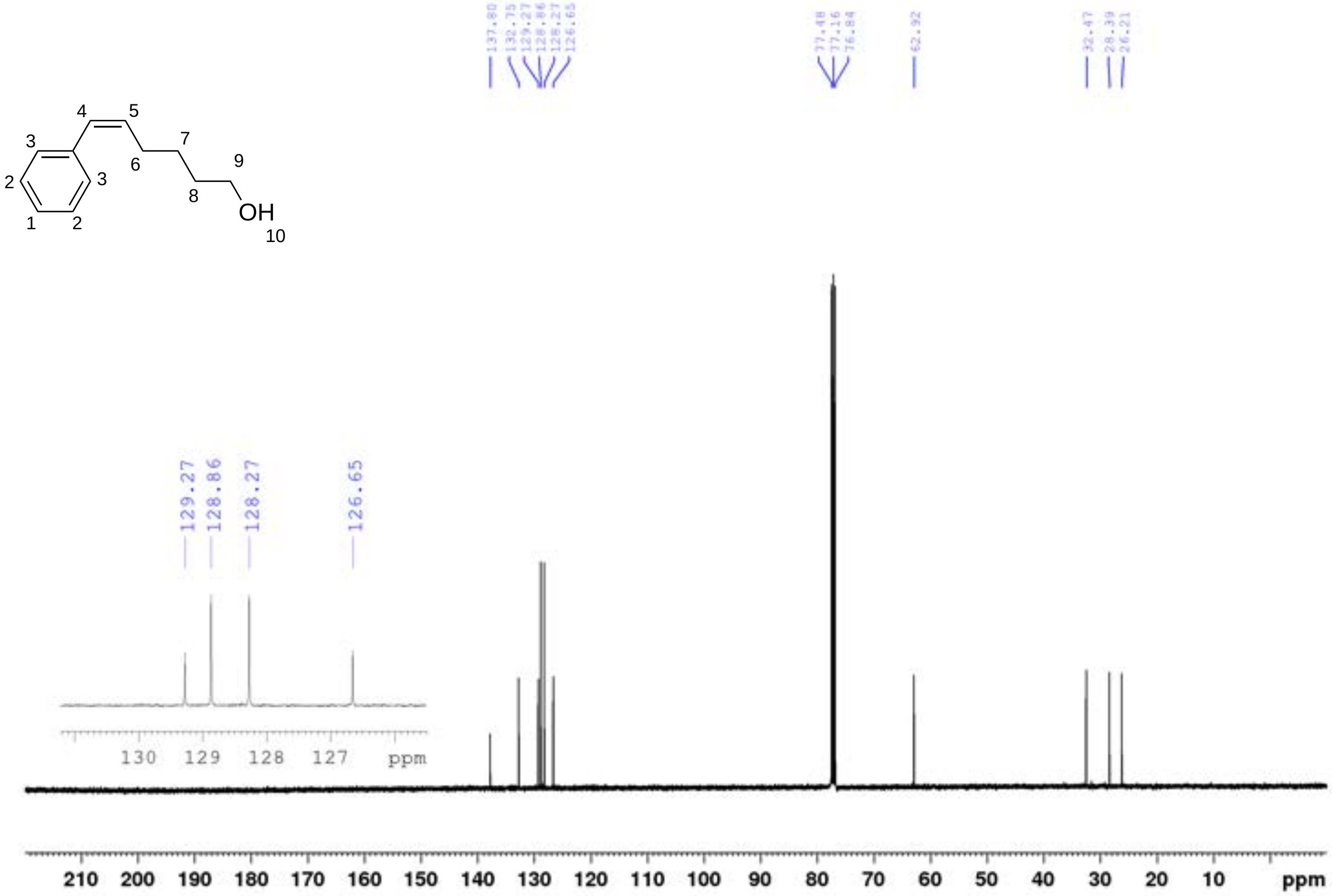

¹H NMR (400 MHz, $CDCl_3$) for *6-(3-chlorophenyl)hex-5-yn-1-ol*

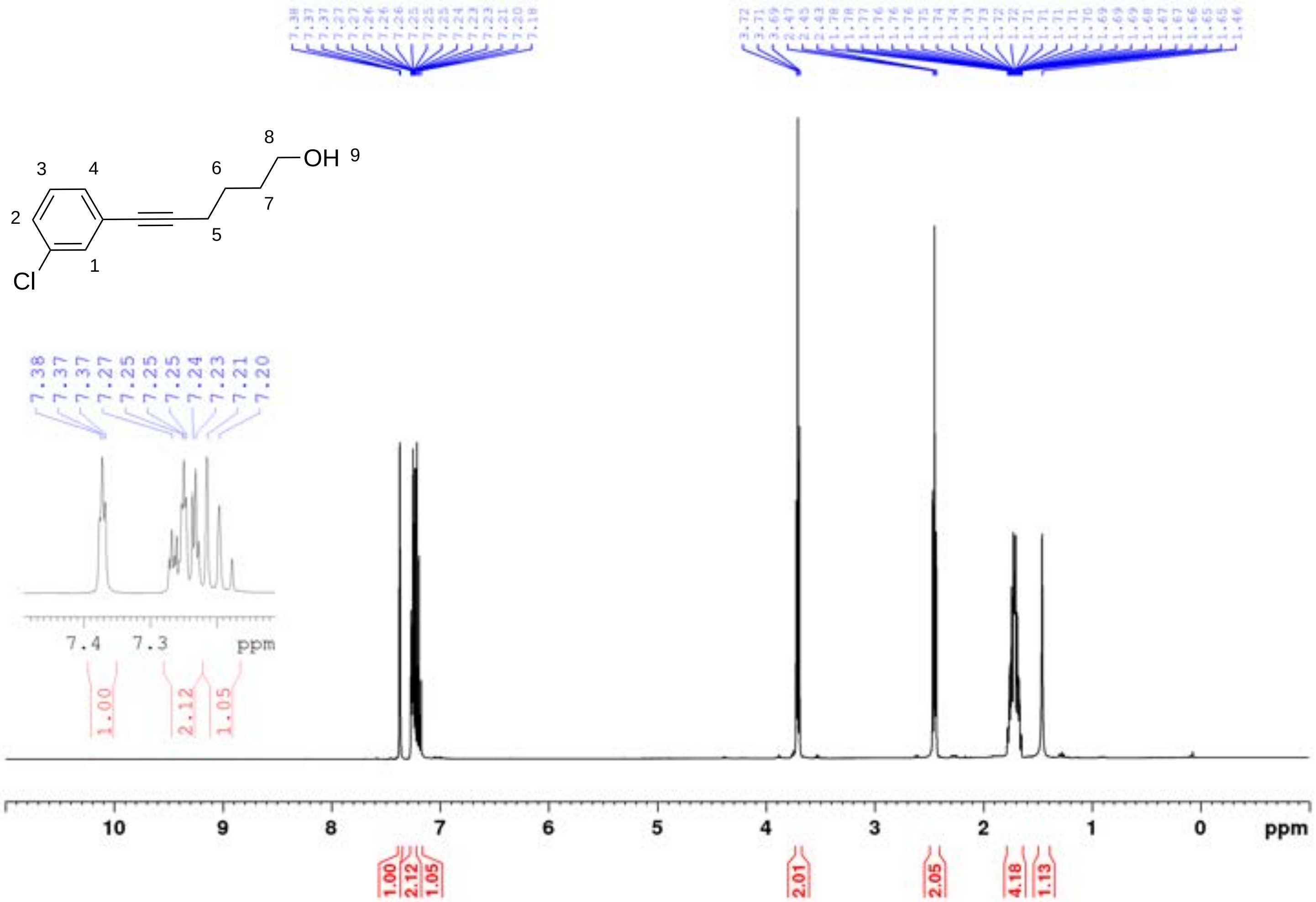

^{13}C NMR (101 MHz, $CDCl_3$) for *6-(3-chlorophenyl)hex-5-yn-1-ol*

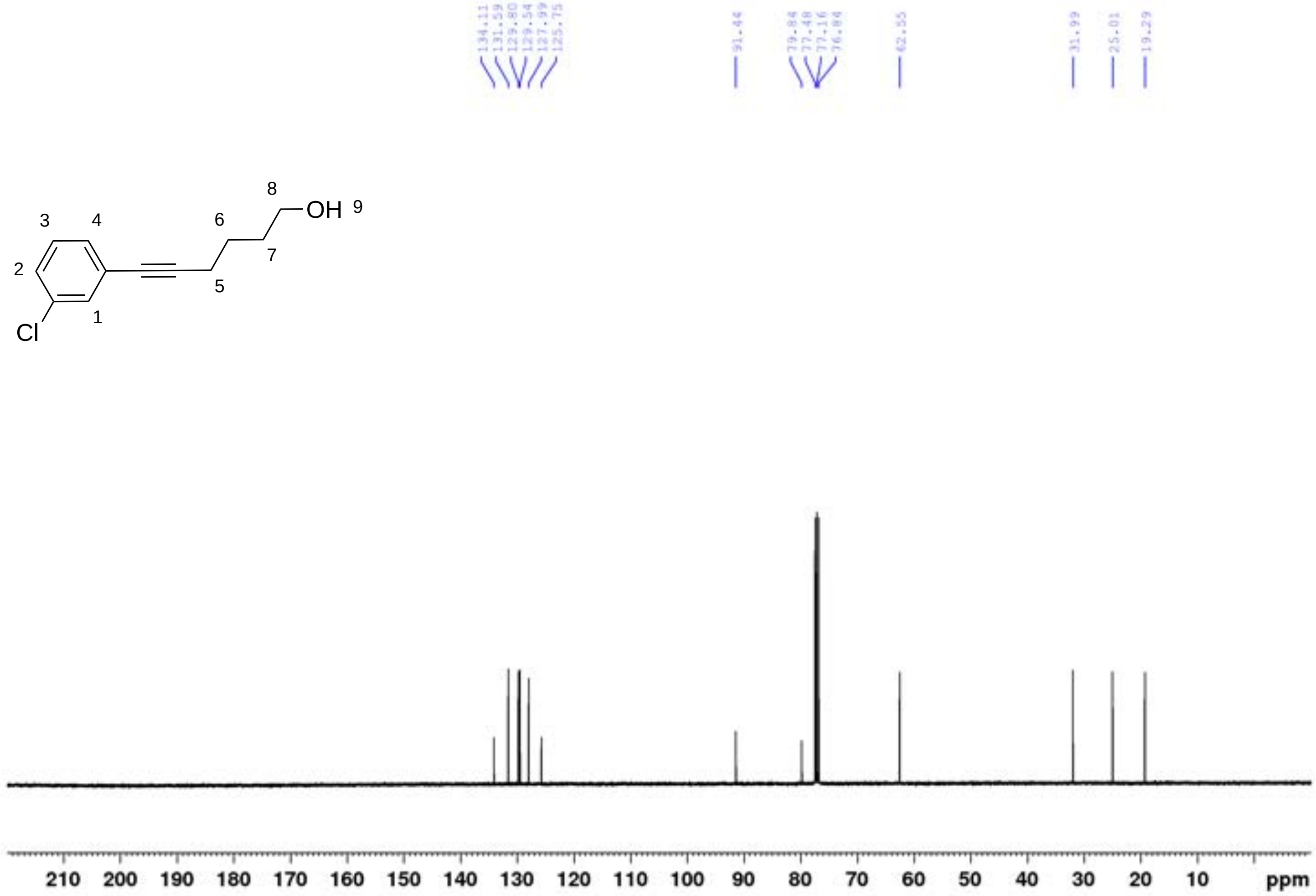

^{1}H NMR (400 MHz, $CDCl_3$) for *(Z)-6-(3-chlorophenyl)hex-5-en-1-ol* (**13b**)

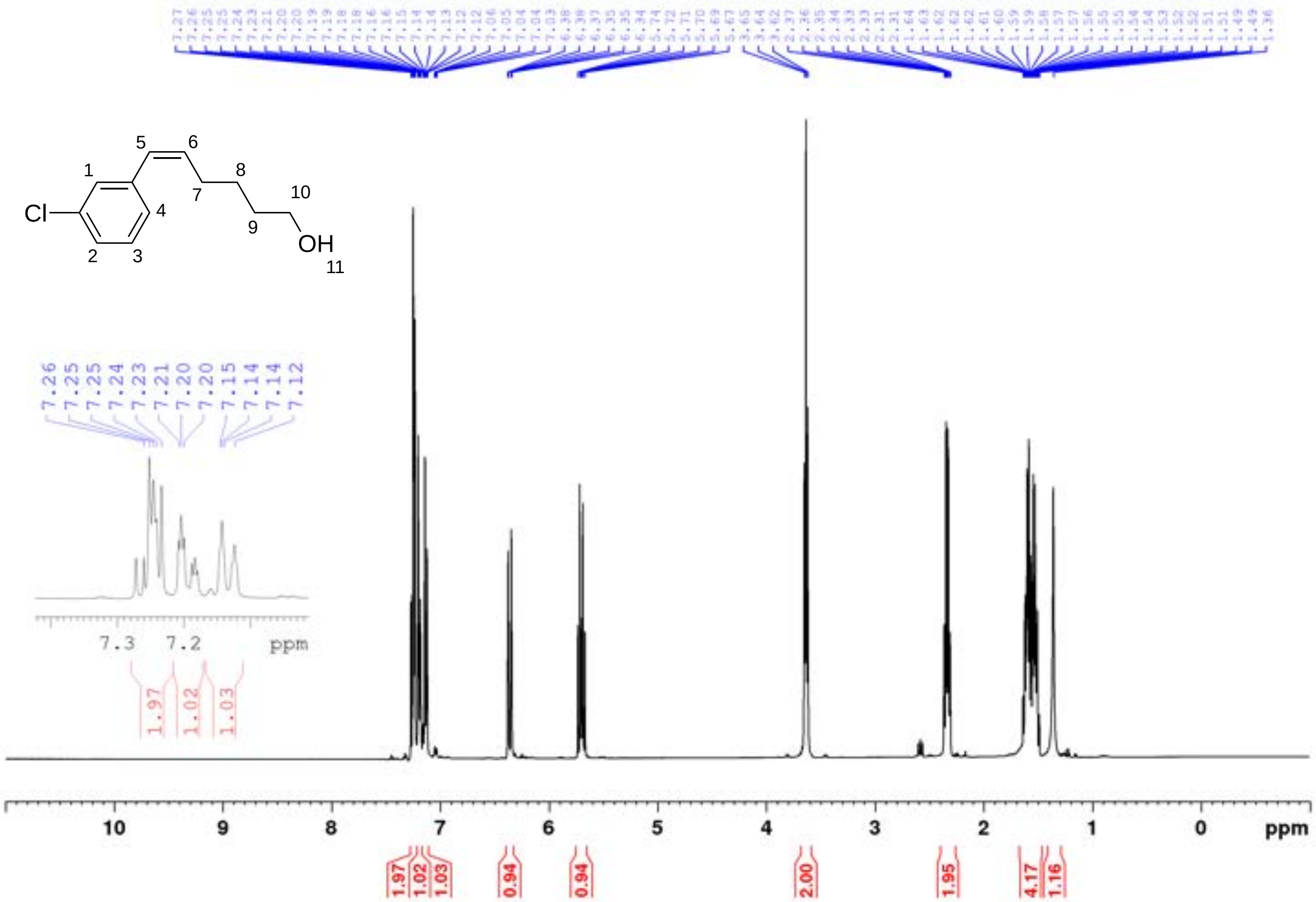

^{13}C NMR (101 MHz, $CDCl_3$) for *(Z)-6-(3-chlorophenyl)hex-5-en-1-ol* (**13b**)

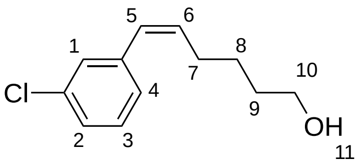

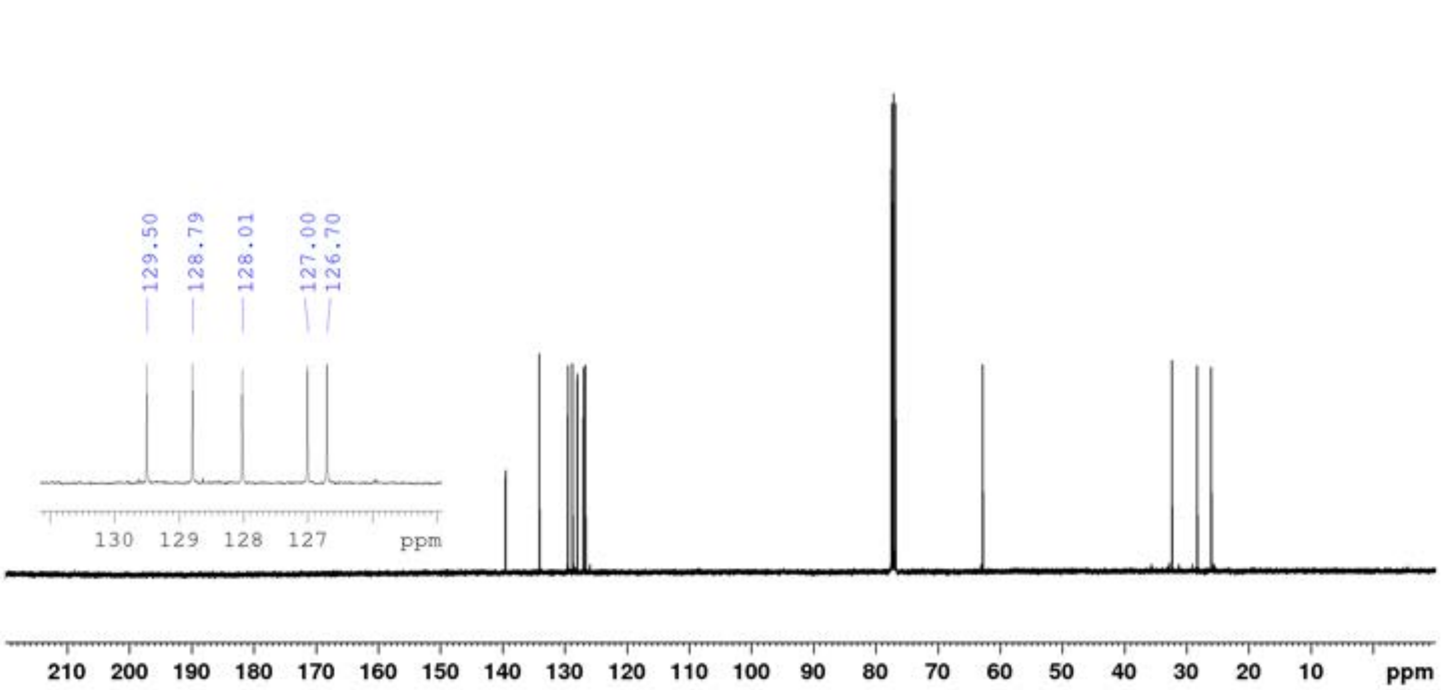

^{1}H NMR (400 MHz, $(CD_3)_2CO$) for *N-(3-(6-hydroxyhex-1-yn-1-yl)phenyl)methanesulfonamide*

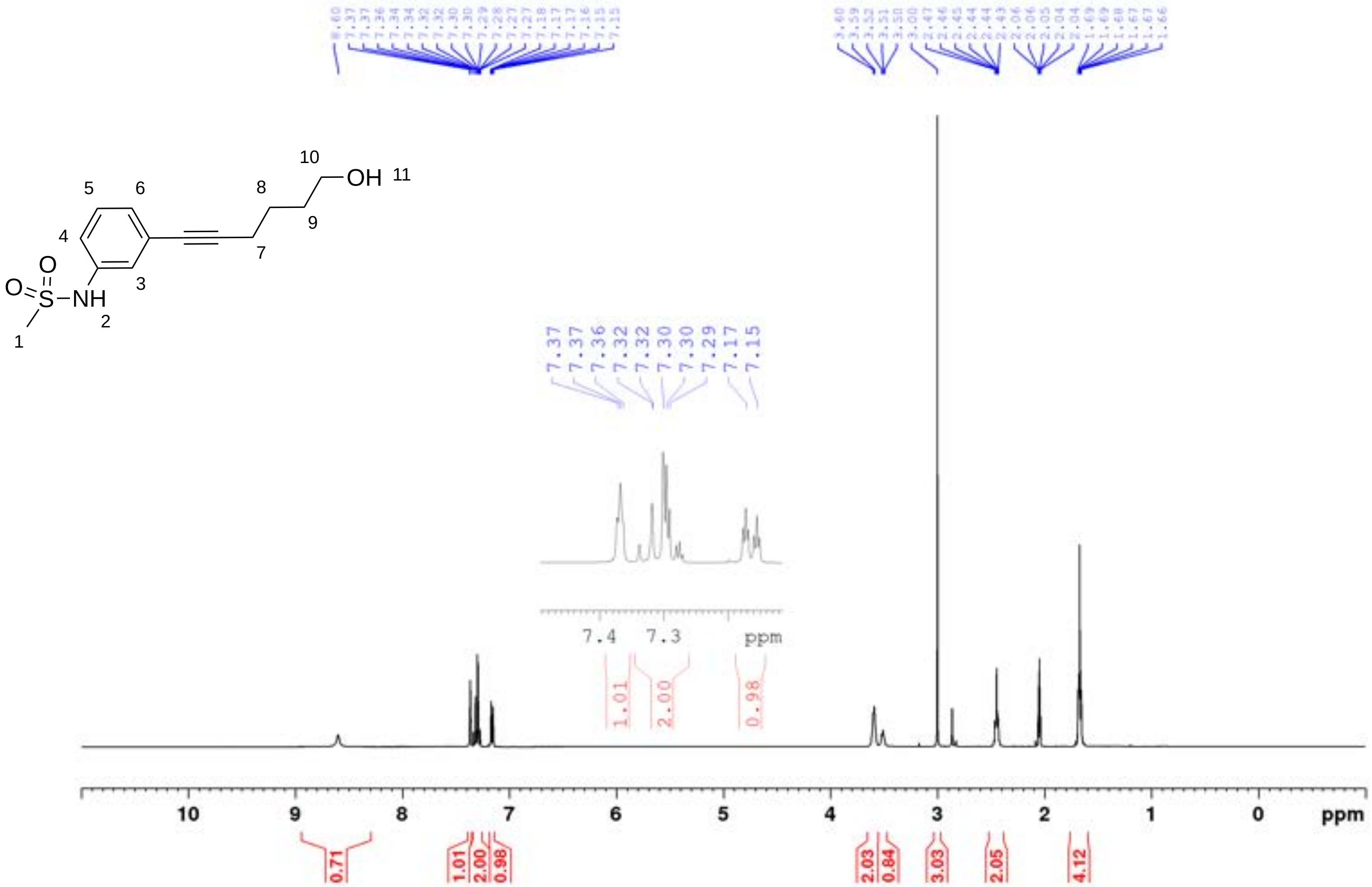

^{13}C NMR (101 MHz, $(CD_3)_2CO$) for *N-(3-(6-hydroxyhex-1-yn-1-yl)phenyl)methanesulfonamide*

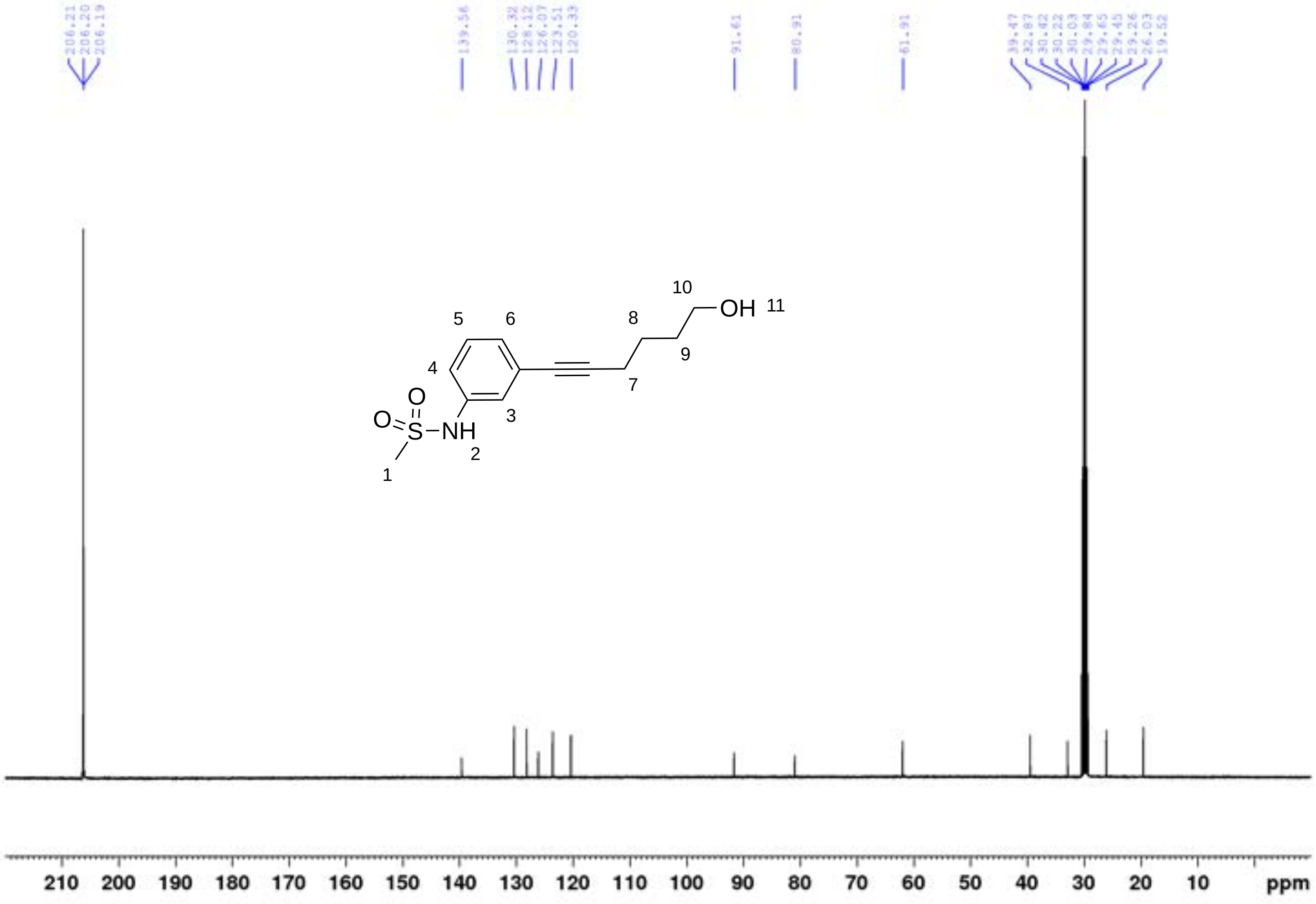

^{1}H NMR (400 MHz, $(CD_3)_2CO$) for *N-(3-(6-hydroxyhex-1-yn-1-yl)phenyl)methanesulfonamide*

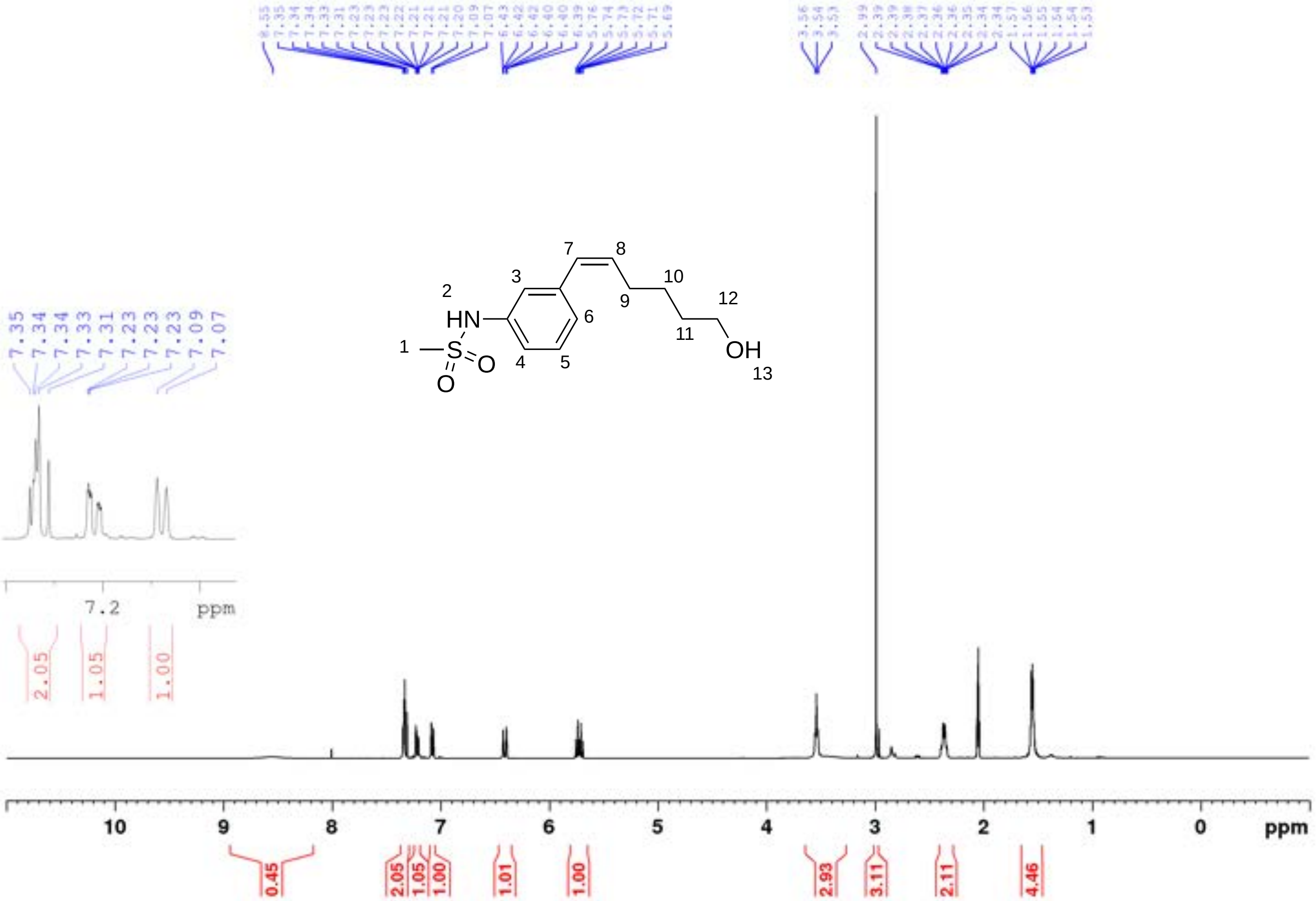

^{13}C NMR (101 MHz, $(CD_3)_2CO$) for *N-(3-(6-hydroxyhex-1-yn-1-yl)phenyl)methanesulfonamide*

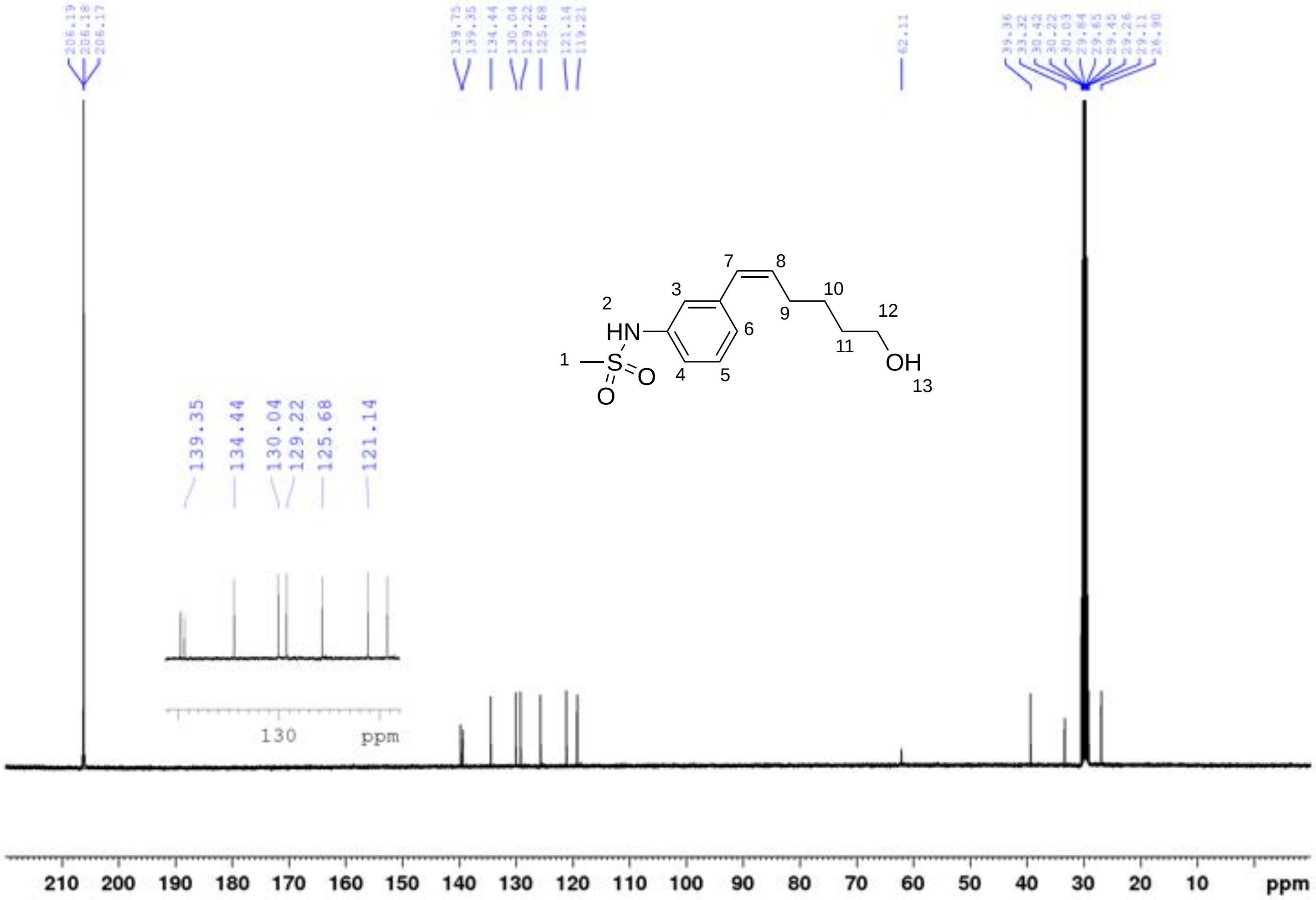

^{1}H NMR (400 MHz, $CDCl_3$) for *(Z)-N-(3-(6-hydroxyhex-1-en-1-yl)phenyl)-N-methylmethanesulfonamide* (**13c**)

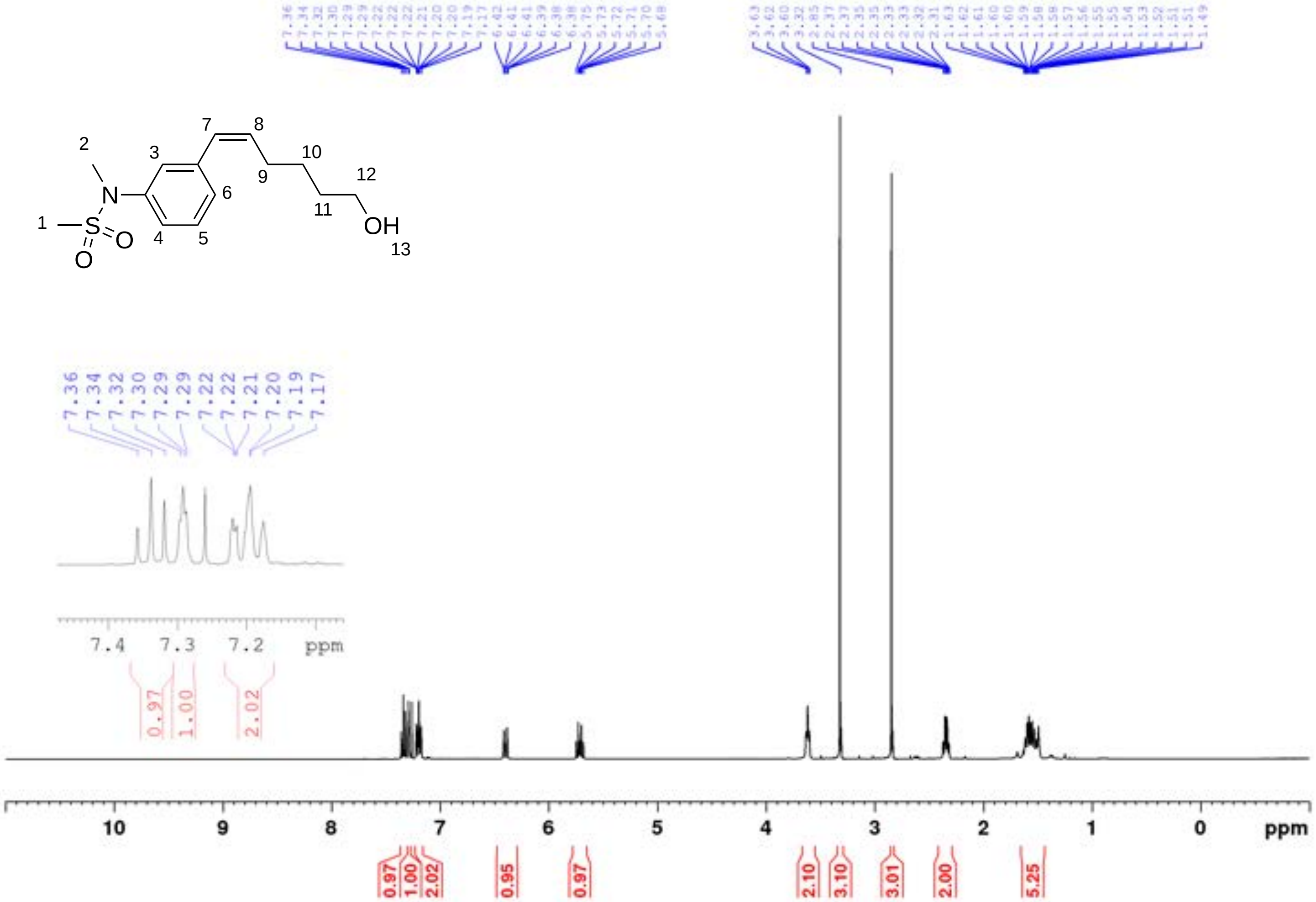

^{13}C NMR (101 MHz, $CDCl_3$) for *(Z)-N-(3-(6-hydroxyhex-1-en-1-yl)phenyl)-N-methylmethanesulfonamide* (**13c**)

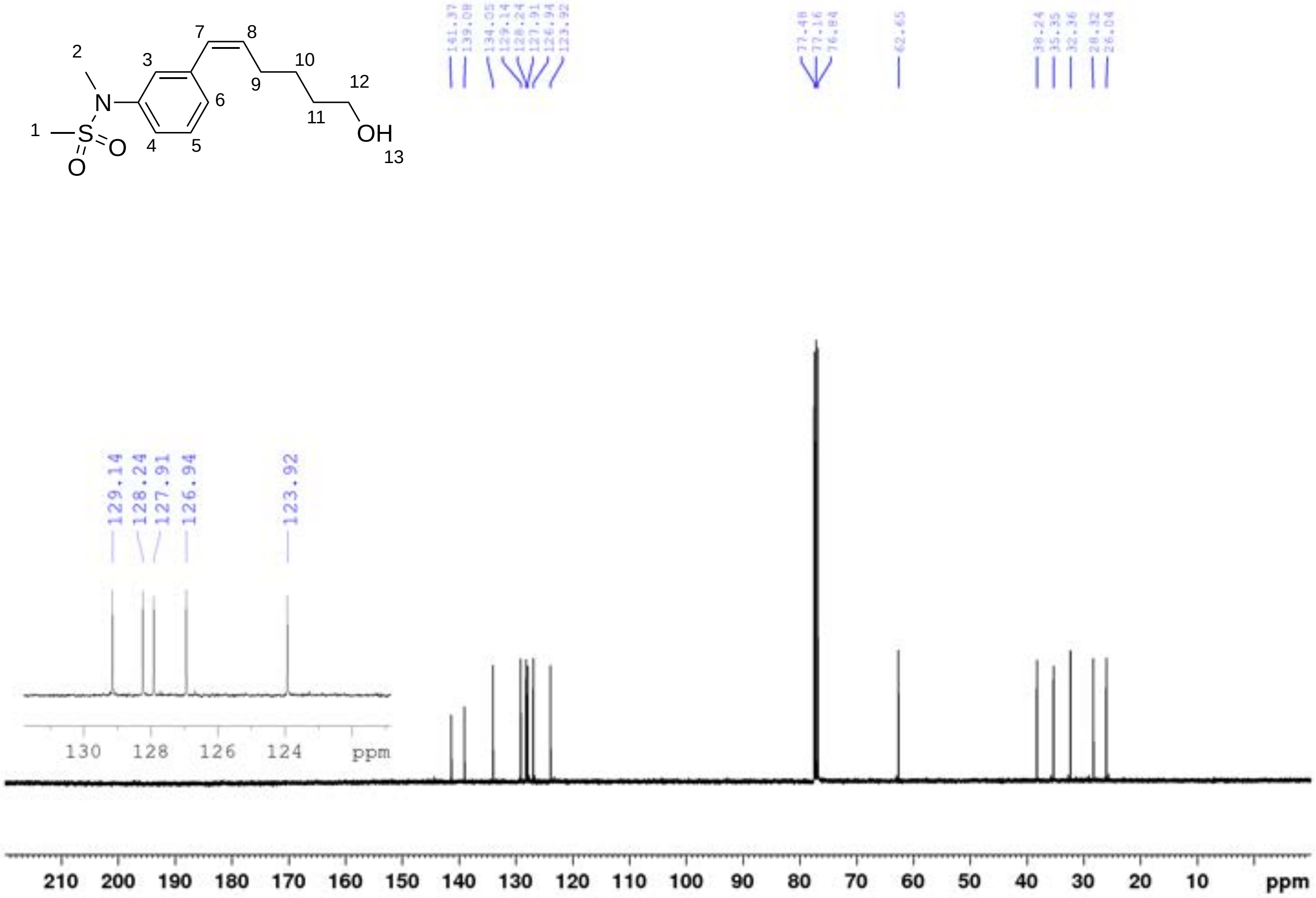

^{1}H NMR (400 MHz, $CDCl_3$) of *Methyl 2-(3-iodophenyl)acetate*

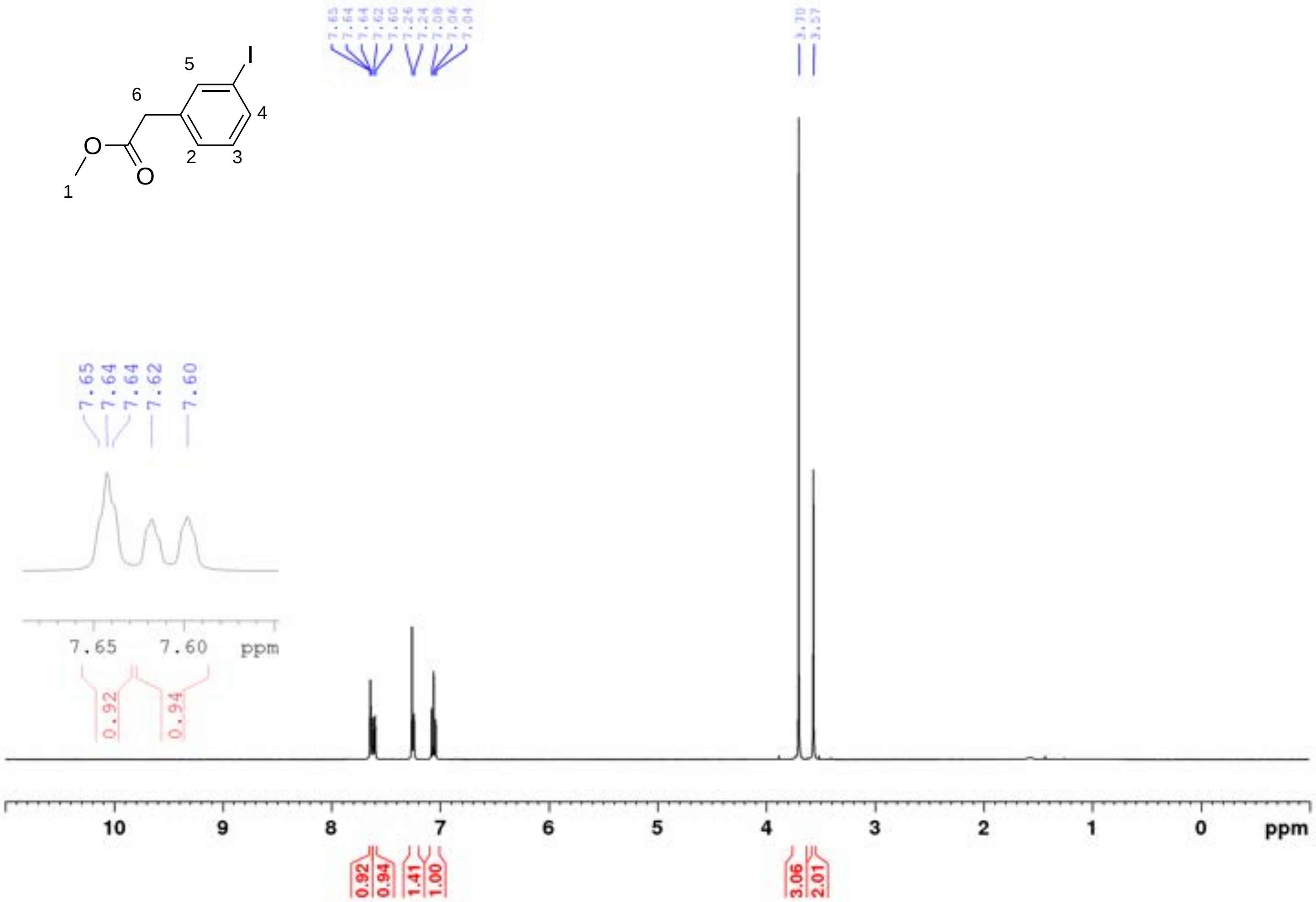

^{13}C NMR (101 MHz, $CDCl_3$) for *Methyl 2-(3-iodophenyl)acetate*

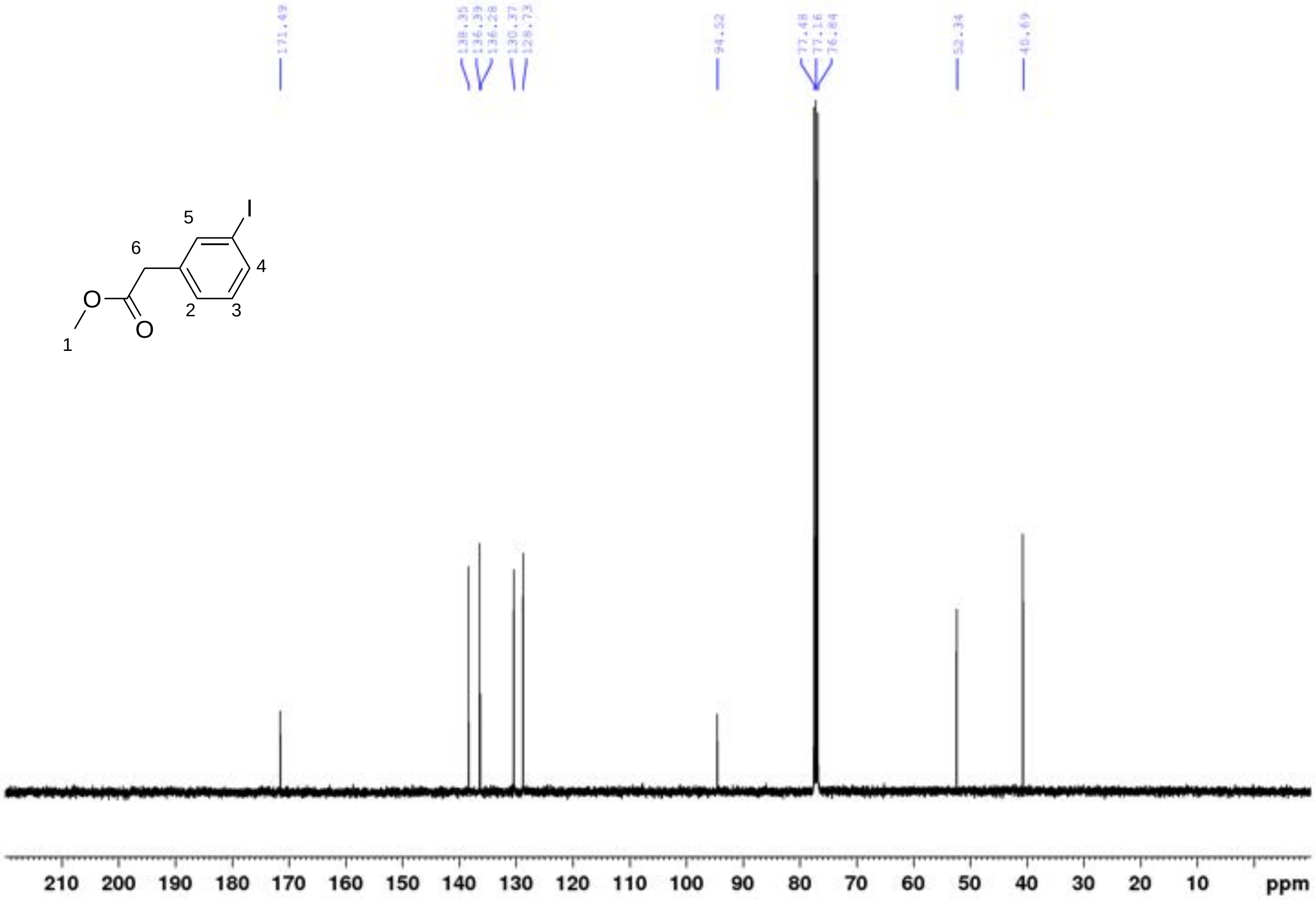

^{1}H NMR (400 MHz, $CDCl_3$) for *methyl 2-(3-(6-hydroxyhex-1-yn-1-yl)phenyl)acetate*

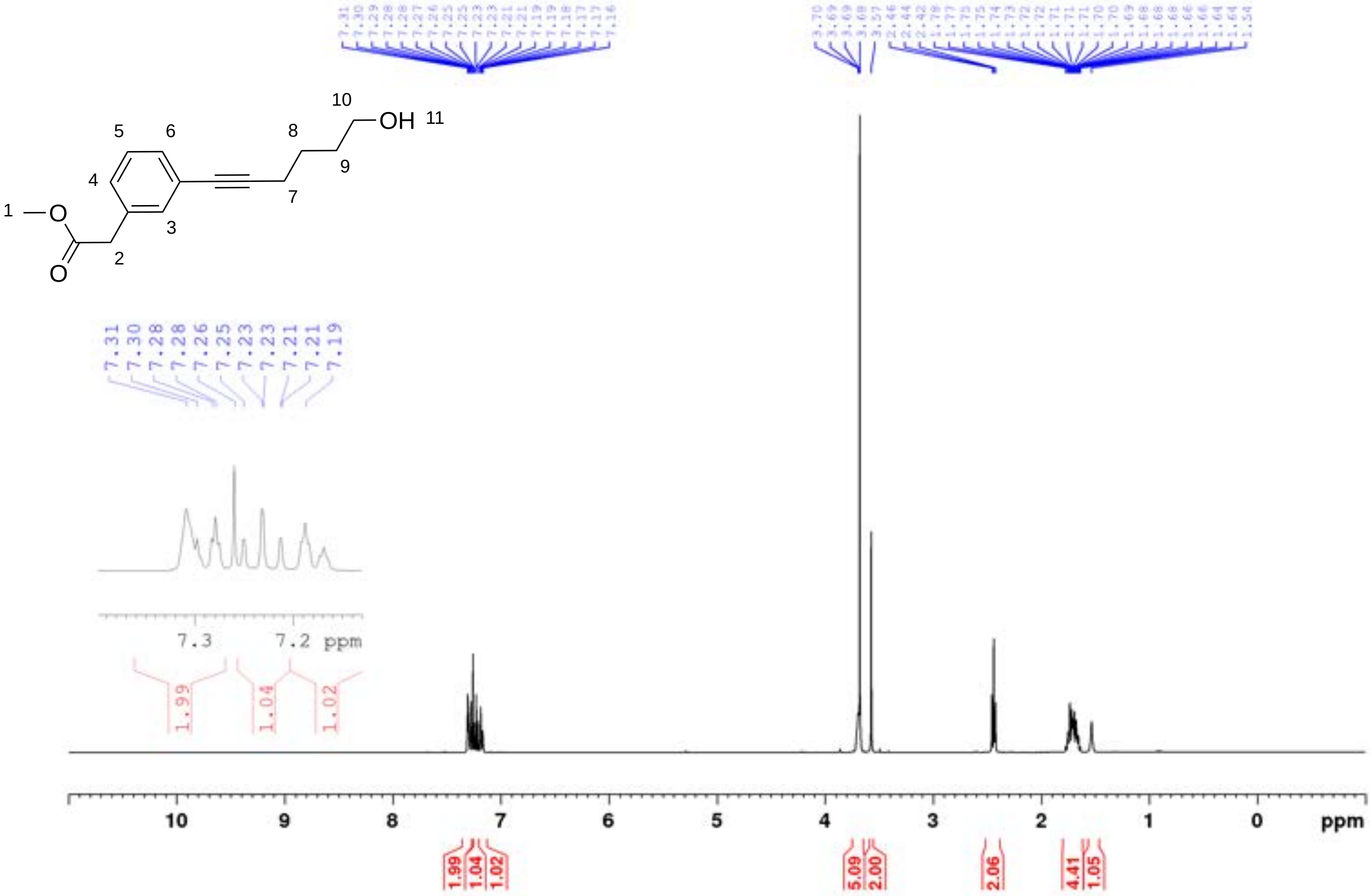

^{13}C NMR (101 MHz, $CDCl_3$) for *Methyl 2-(3-(6-hydroxyhex-1-yn-1-yl)phenyl)acetate*

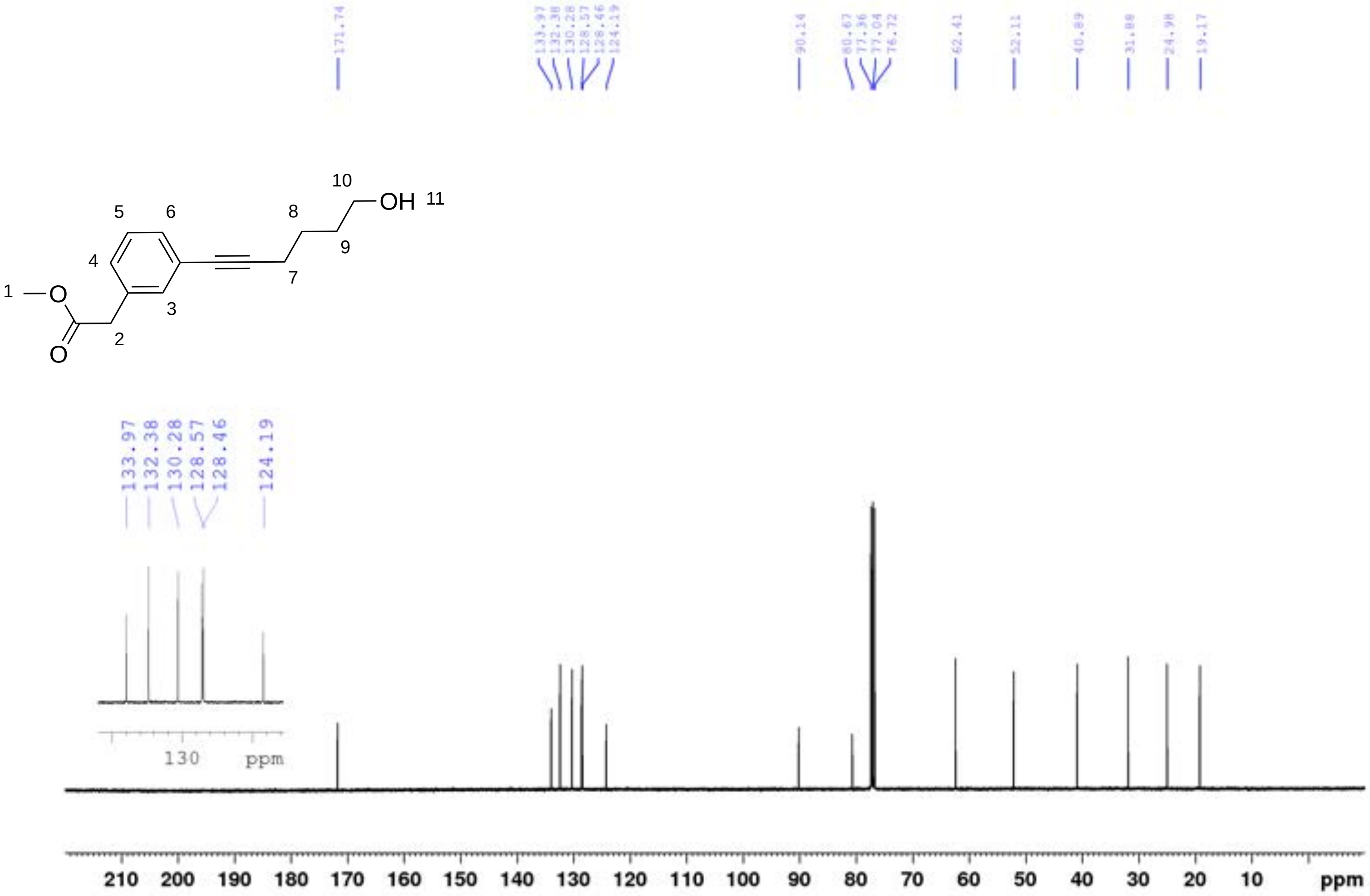

^{1}H NMR (400 MHz, $CDCl_3$) for *Methyl (Z)-2-(3-(6-hydroxyhex-1-en-1-yl)phenyl)acetate* (**13d**)

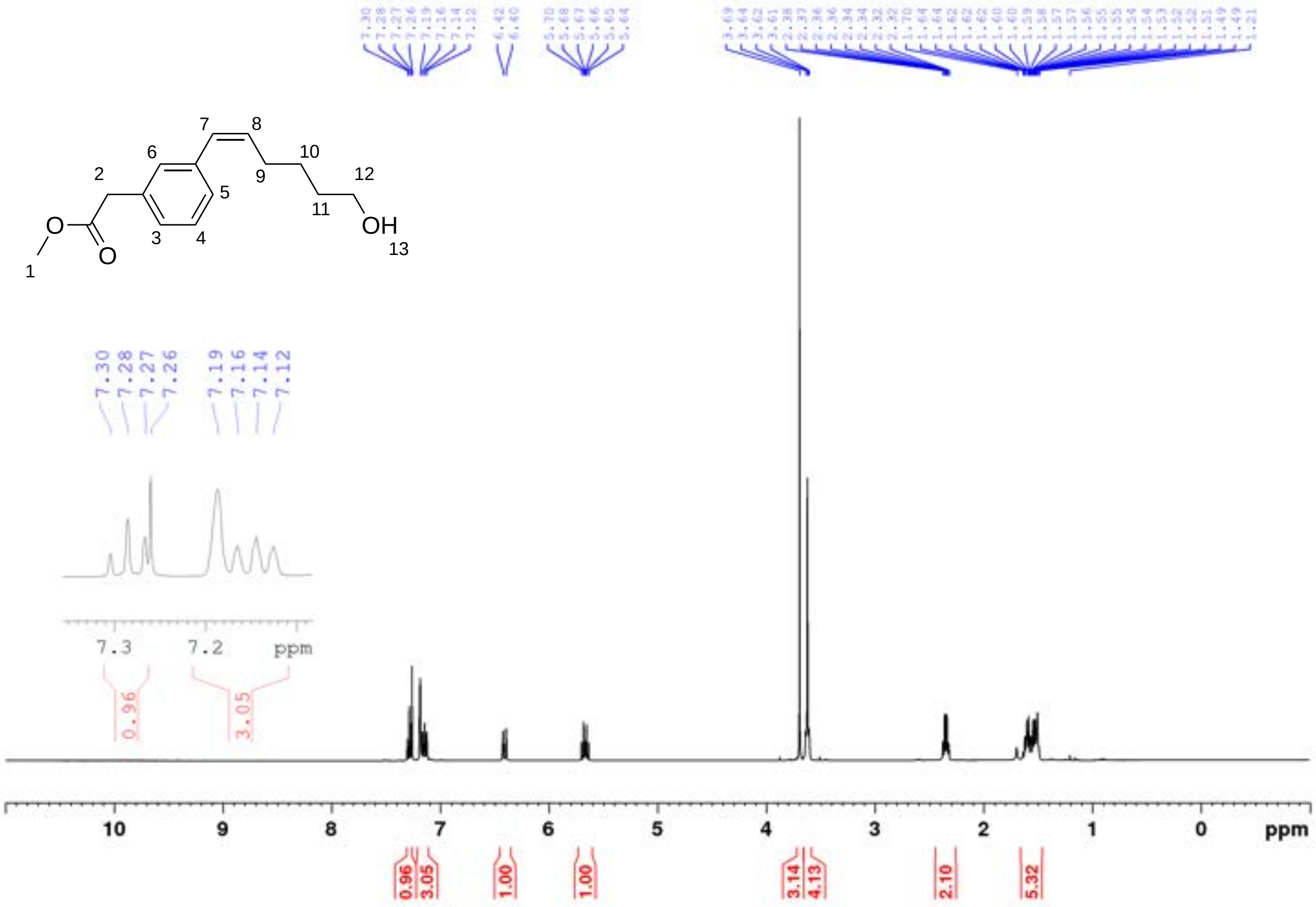

^{13}**C NMR** (101 MHz, $CDCl_3$) for *Methyl (Z)-2-(3-(6-hydroxyhex-1-en-1-yl)phenyl)acetate* (**13d**)

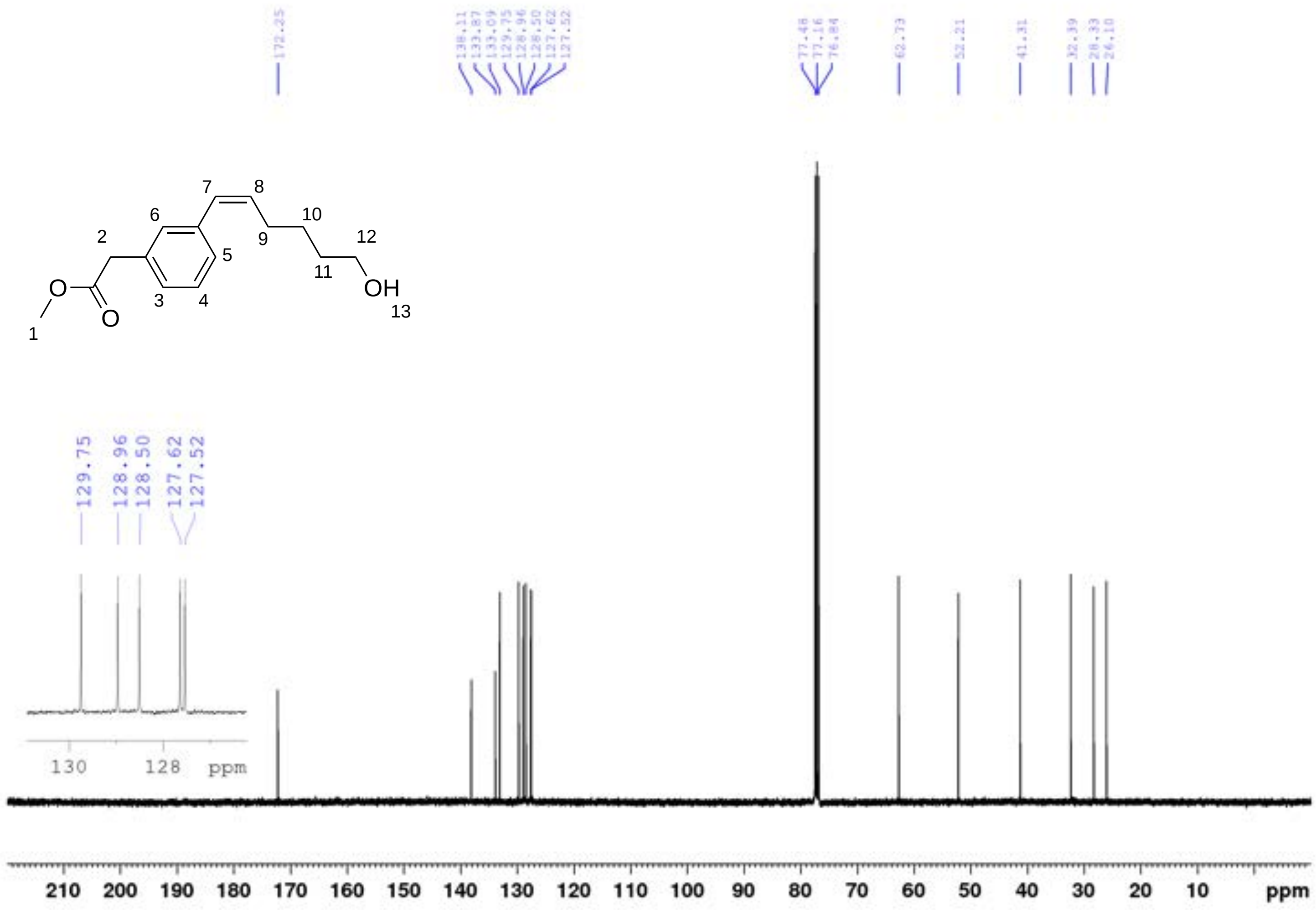

¹H NMR (400 MHz, $CDCl_3$) for *3-bromo-N,N-diethylbenzenesulfonamide*

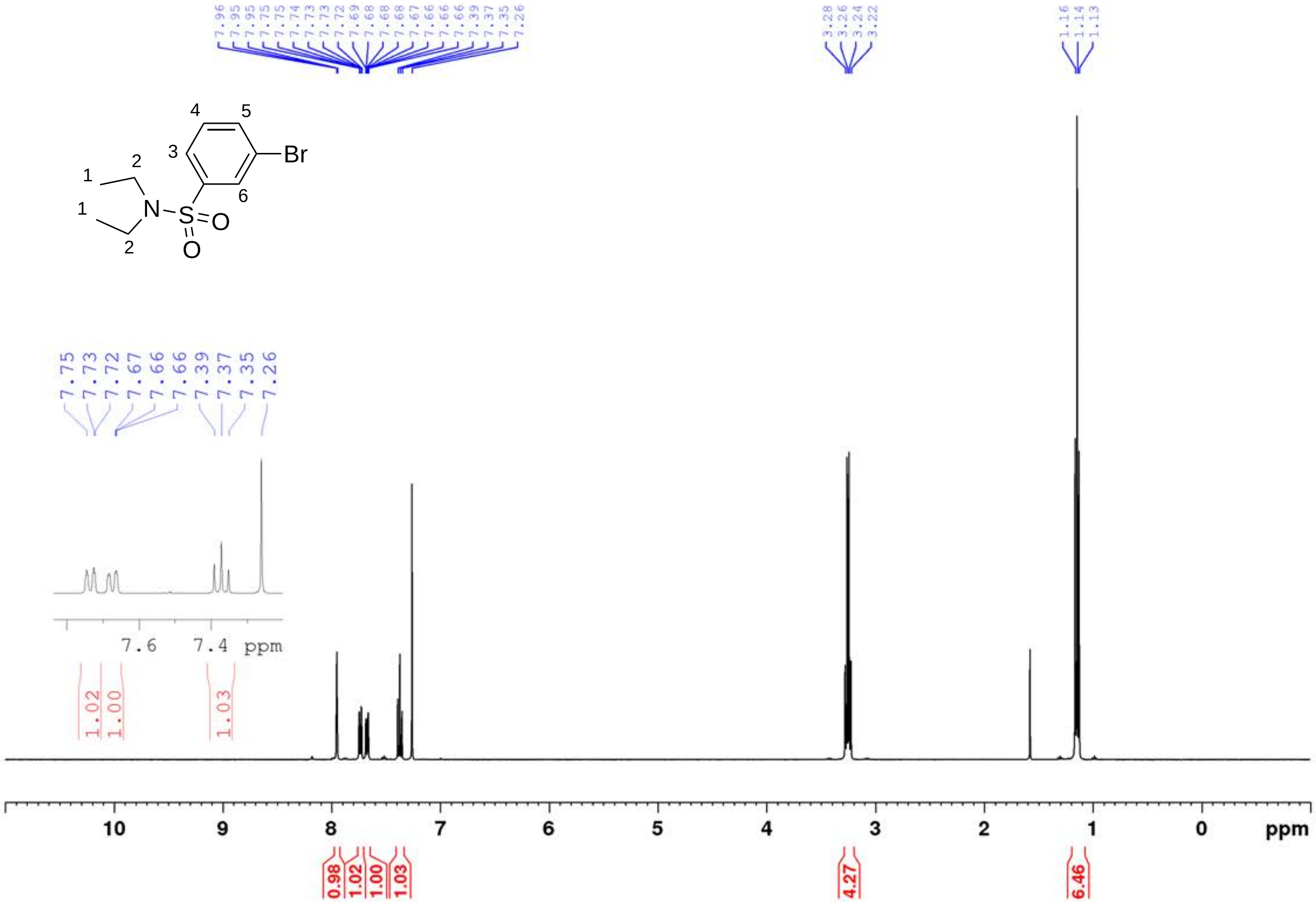

^{13}C NMR (101 MHz, $CDCl_3$) for *3-bromo-N,N-diethylbenzenesulfonamide*

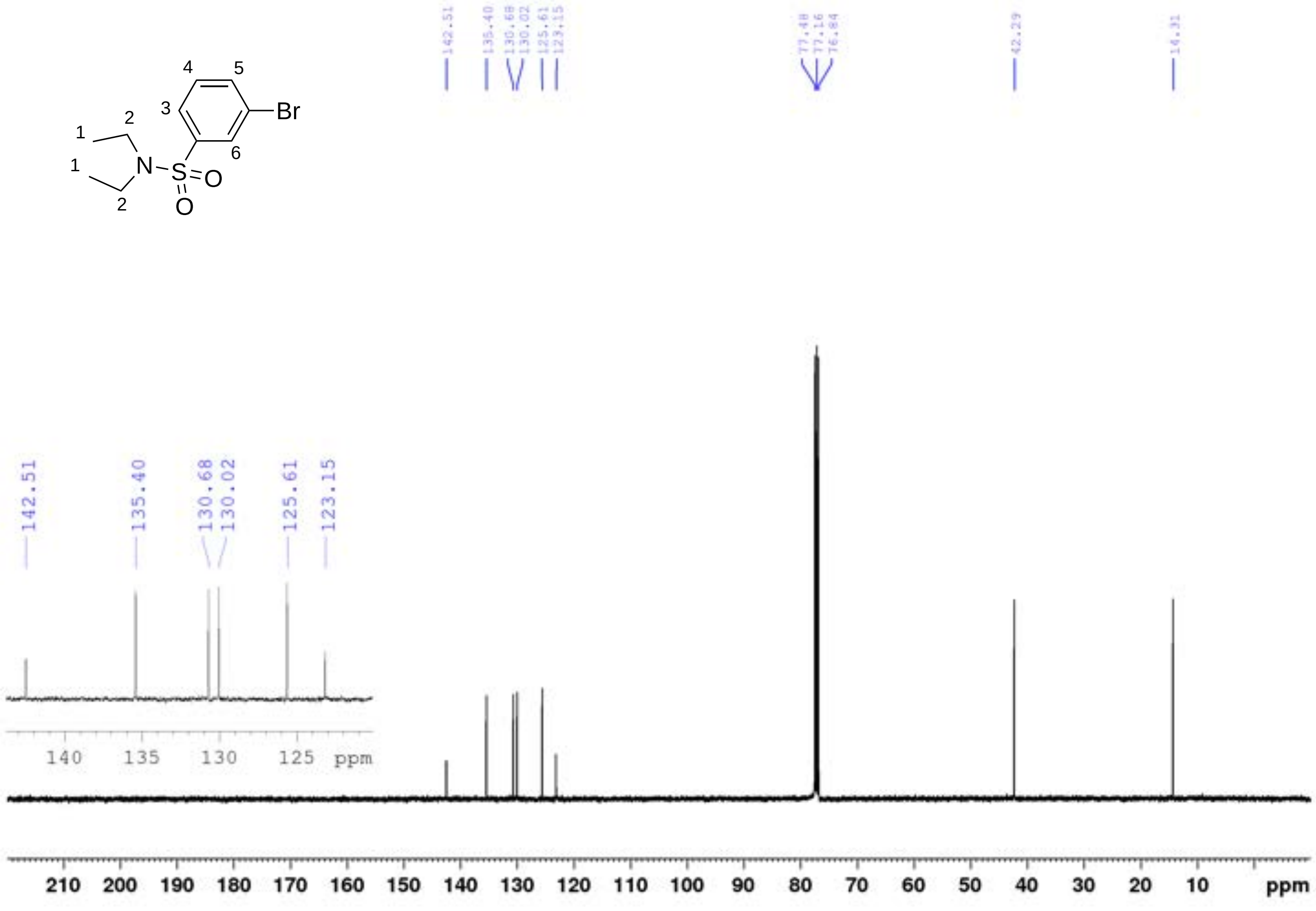

¹H NMR (400 MHz, $CDCl_3$) for *N,N-diethyl-3-(6-hydroxyhex-1-yn-1-yl)benzenesulfonamide*

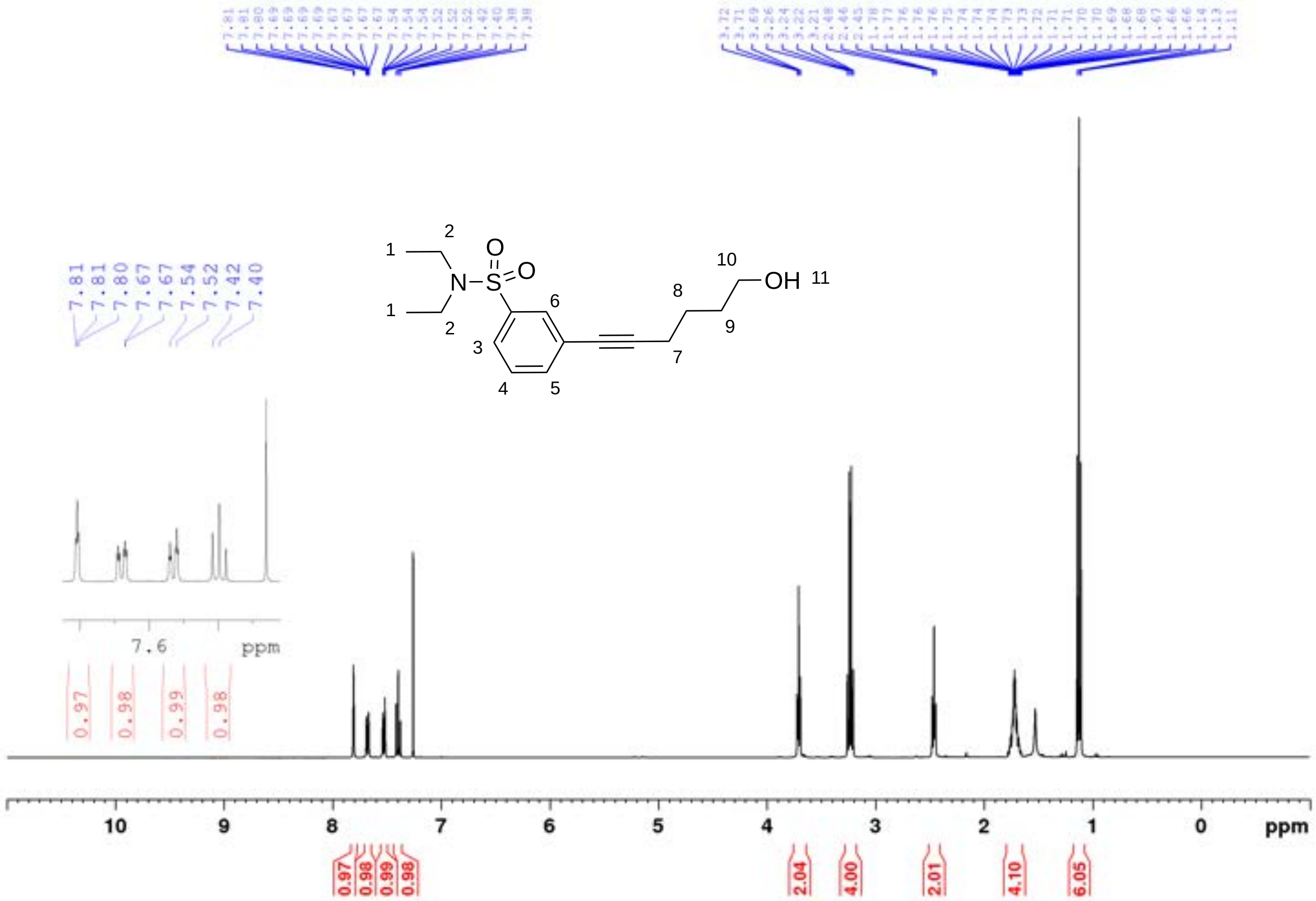

^{13}C NMR (101 MHz, $CDCl_3$) for *N,N-diethyl-3-(6-hydroxyhex-1-yn-1-yl)benzenesulfonamide*

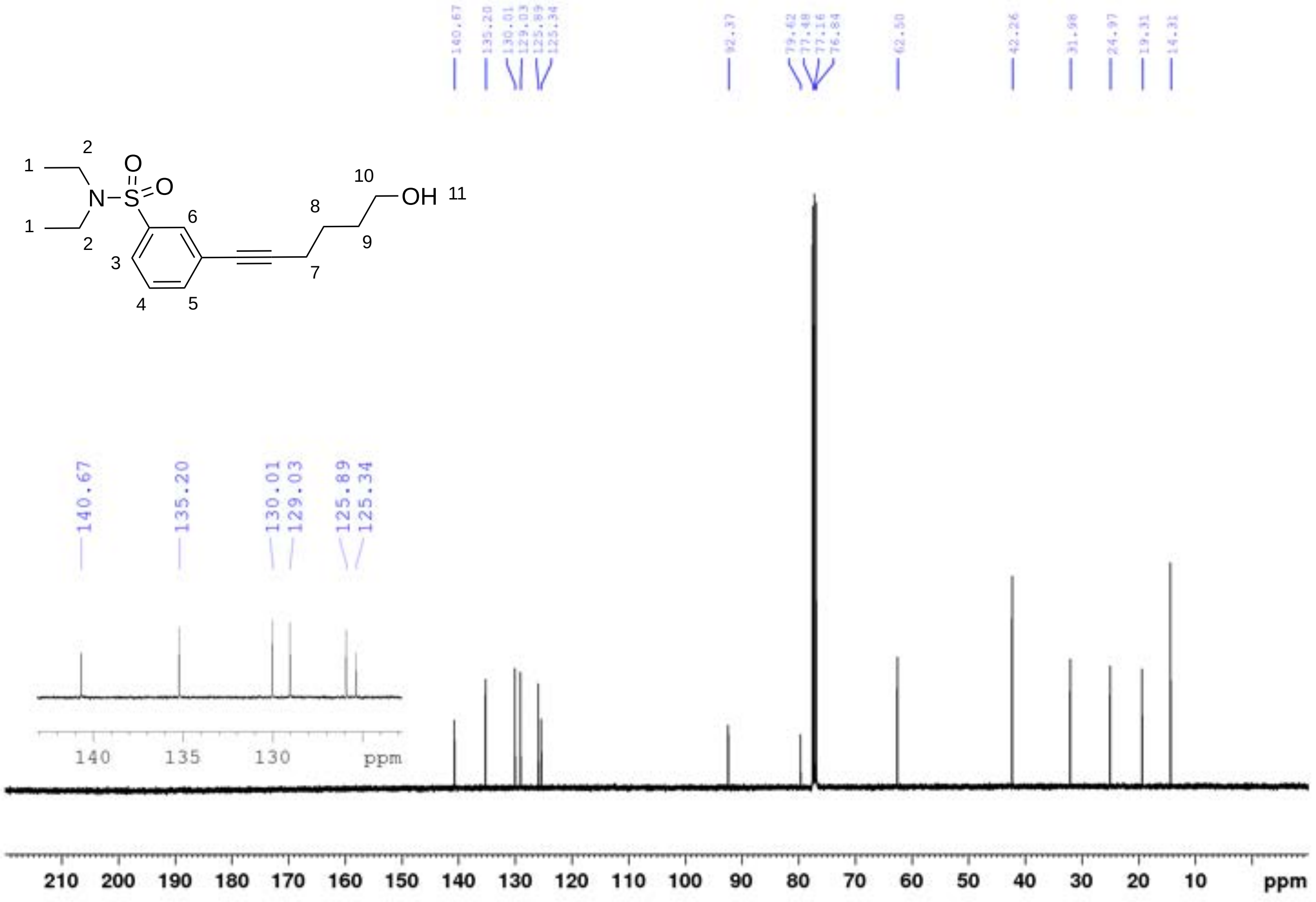

^{1}H NMR (400 MHz, $CDCl_3$) for *(Z)-N,N-diethyl-3-(6-hydroxyhex-1-en-1-yl)benzenesulfonamide* (**13e**)

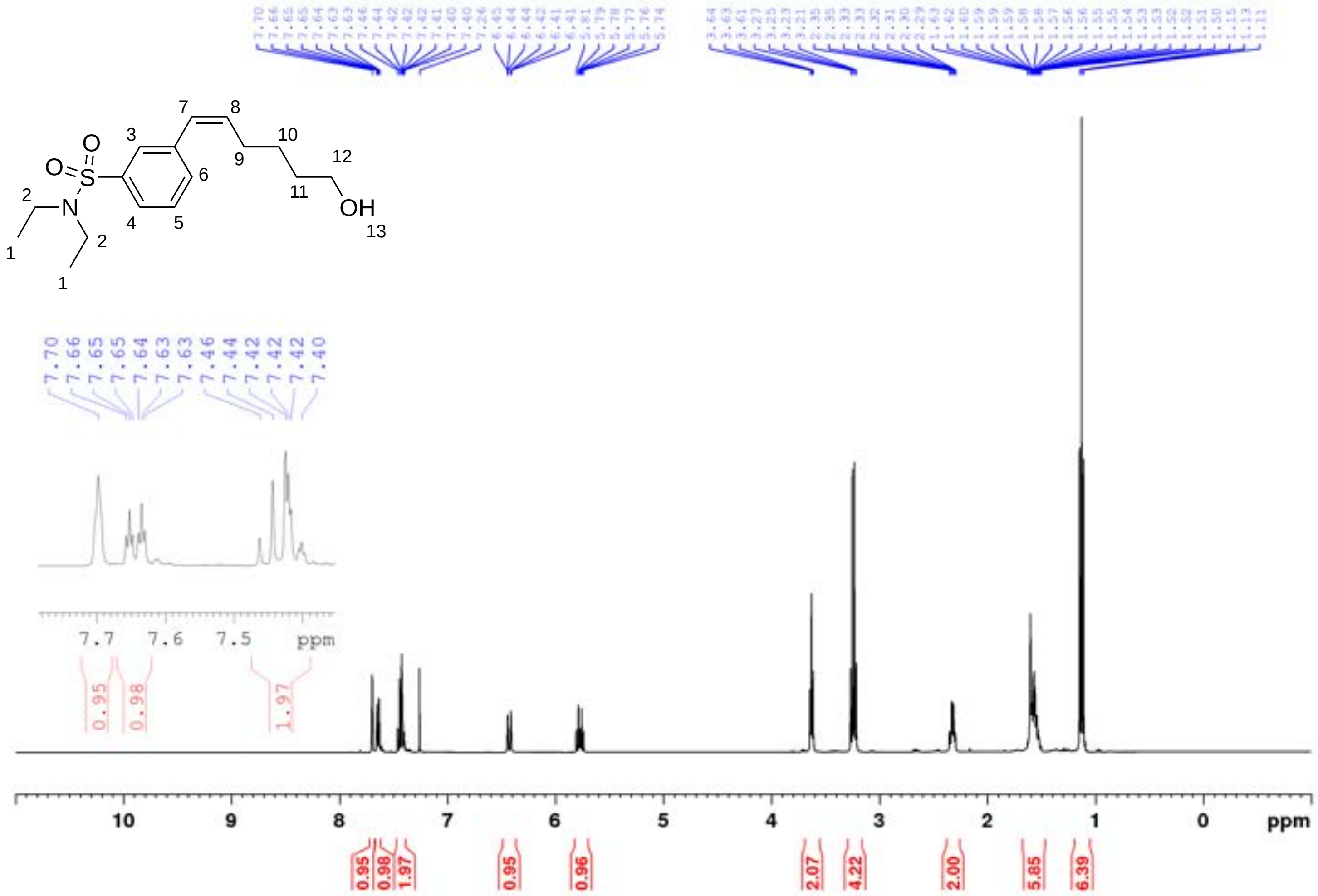

^{13}C NMR (101 MHz, $CDCl_3$) for *(Z)-N,N-diethyl-3-(6-hydroxyhex-1-en-1-yl)benzenesulfonamide* (**13e**)

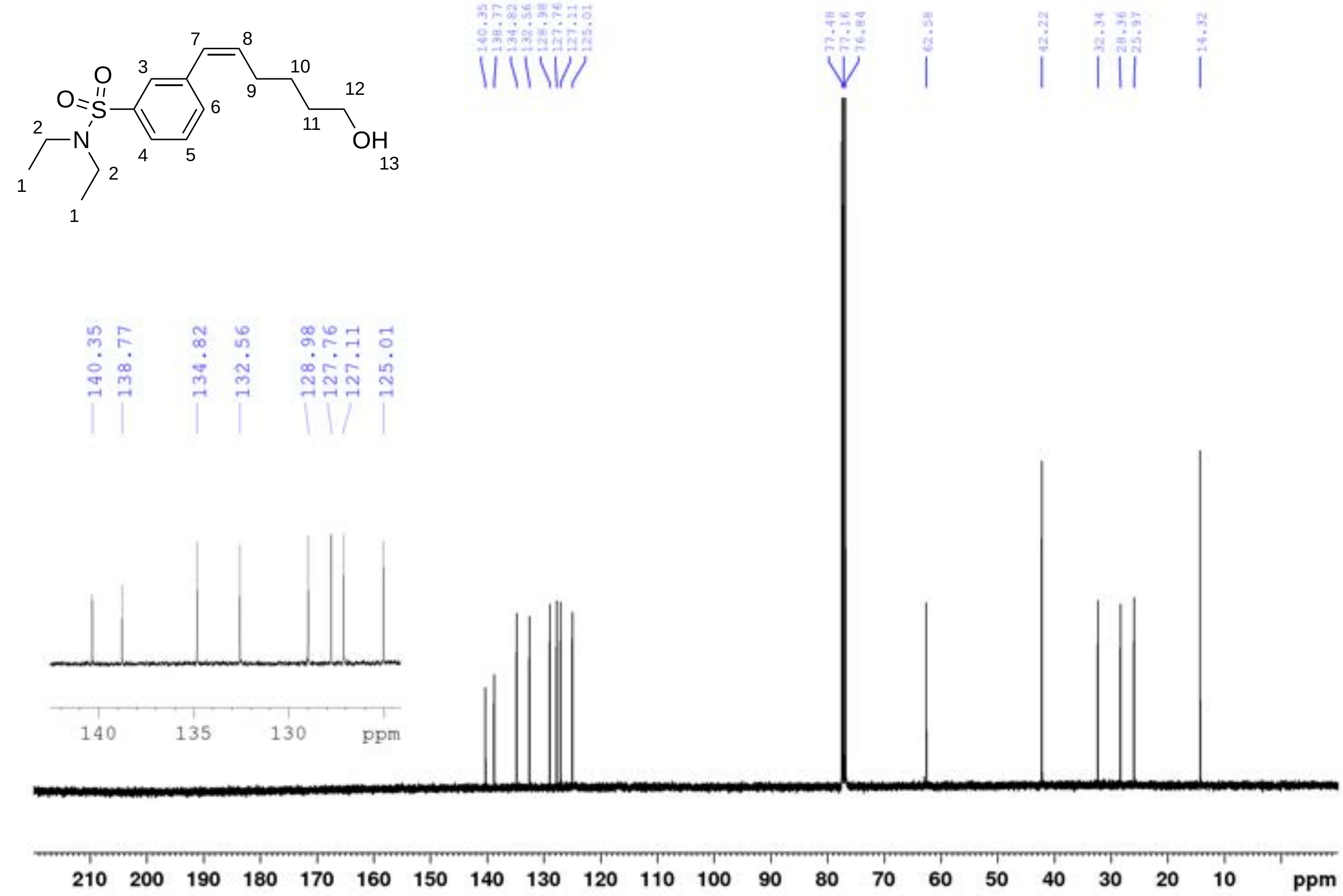

^{1}H NMR (400 MHz, $CDCl_3$) for *6-(2-chlorophenyl)hex-5-yn-1-ol*

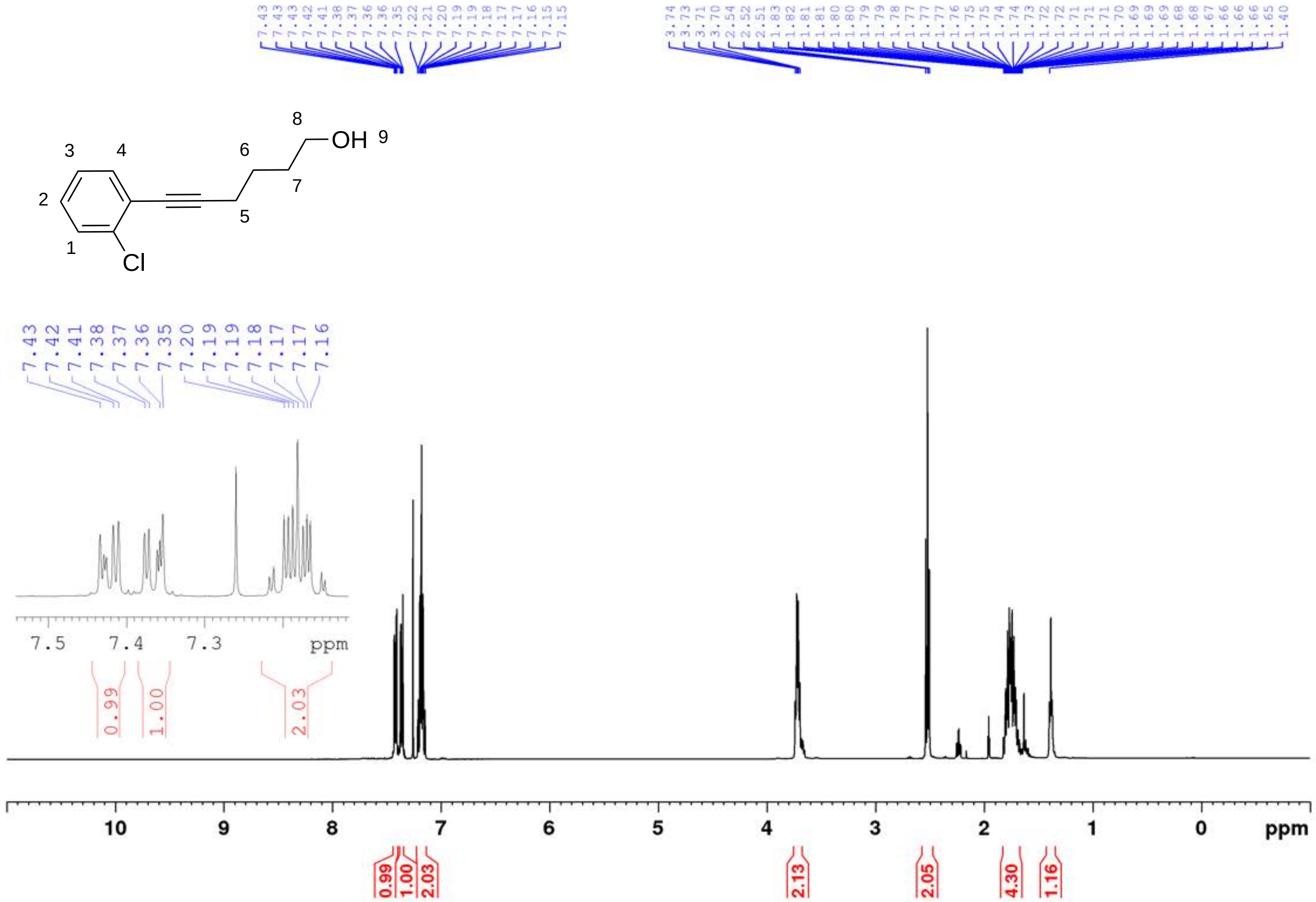

^{13}**C NMR** (101 MHz, $CDCl_3$) for *6-(2-chlorophenyl)hex-5-yn-1-ol*

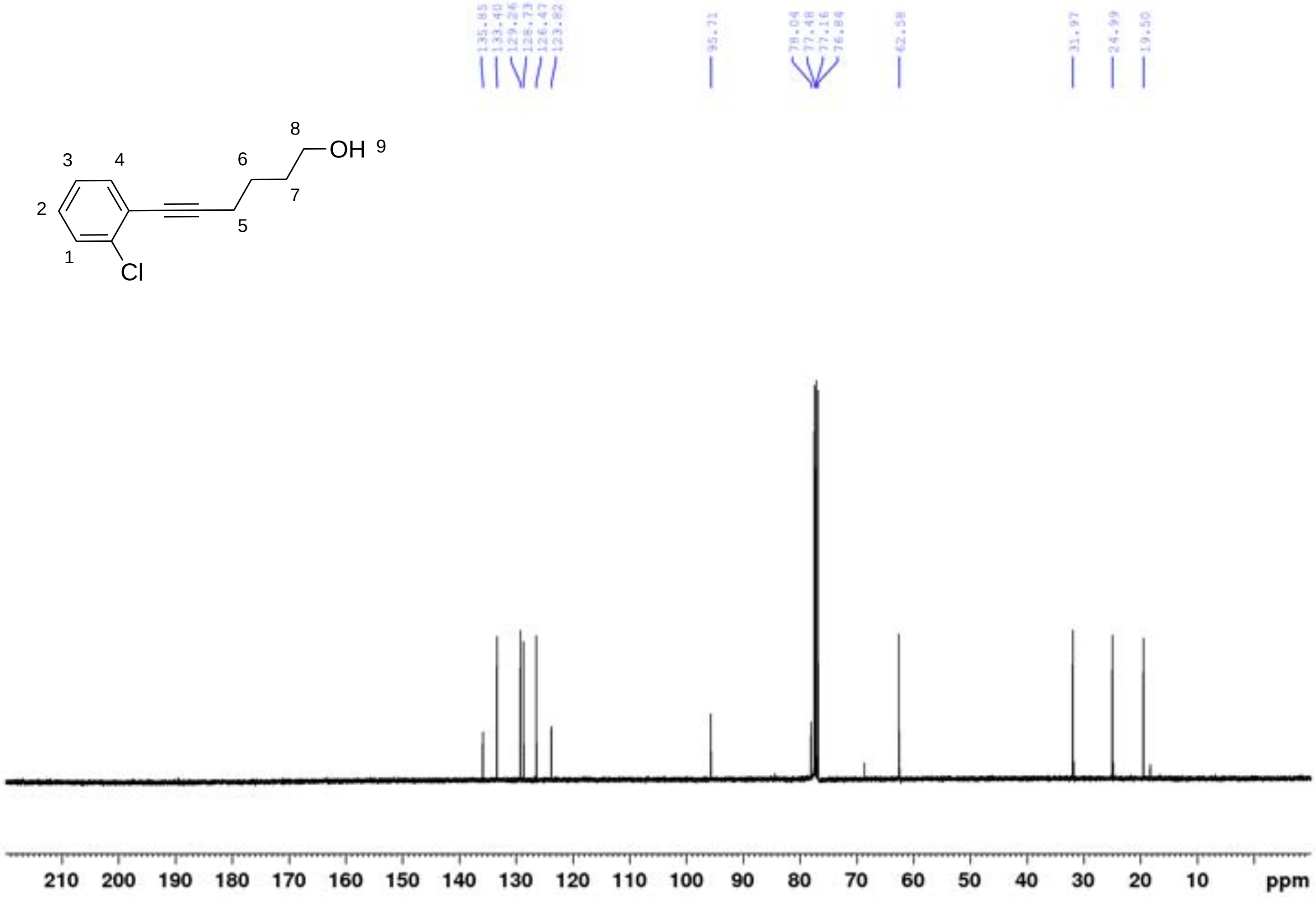

^{1}**H NMR** (400 MHz, $CDCl_3$) for *(Z)-6-(2-chlorophenyl)hex-5-en-1-ol* (**13f**)

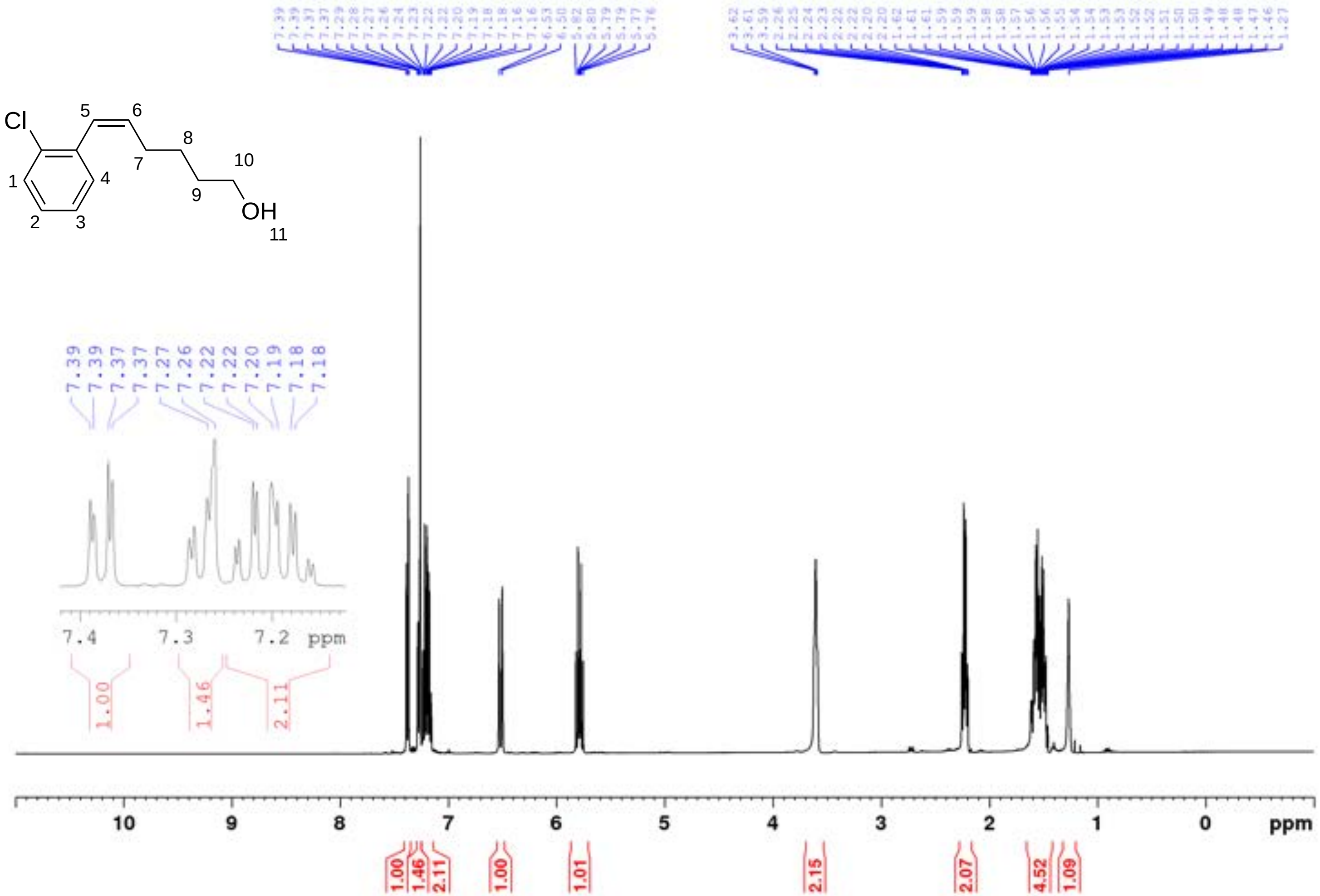

^{13}C NMR (101 MHz, $CDCl_3$) for *(Z)-6-(2-chlorophenyl)hex-5-en-1-ol* (**13f**)

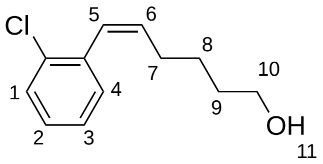

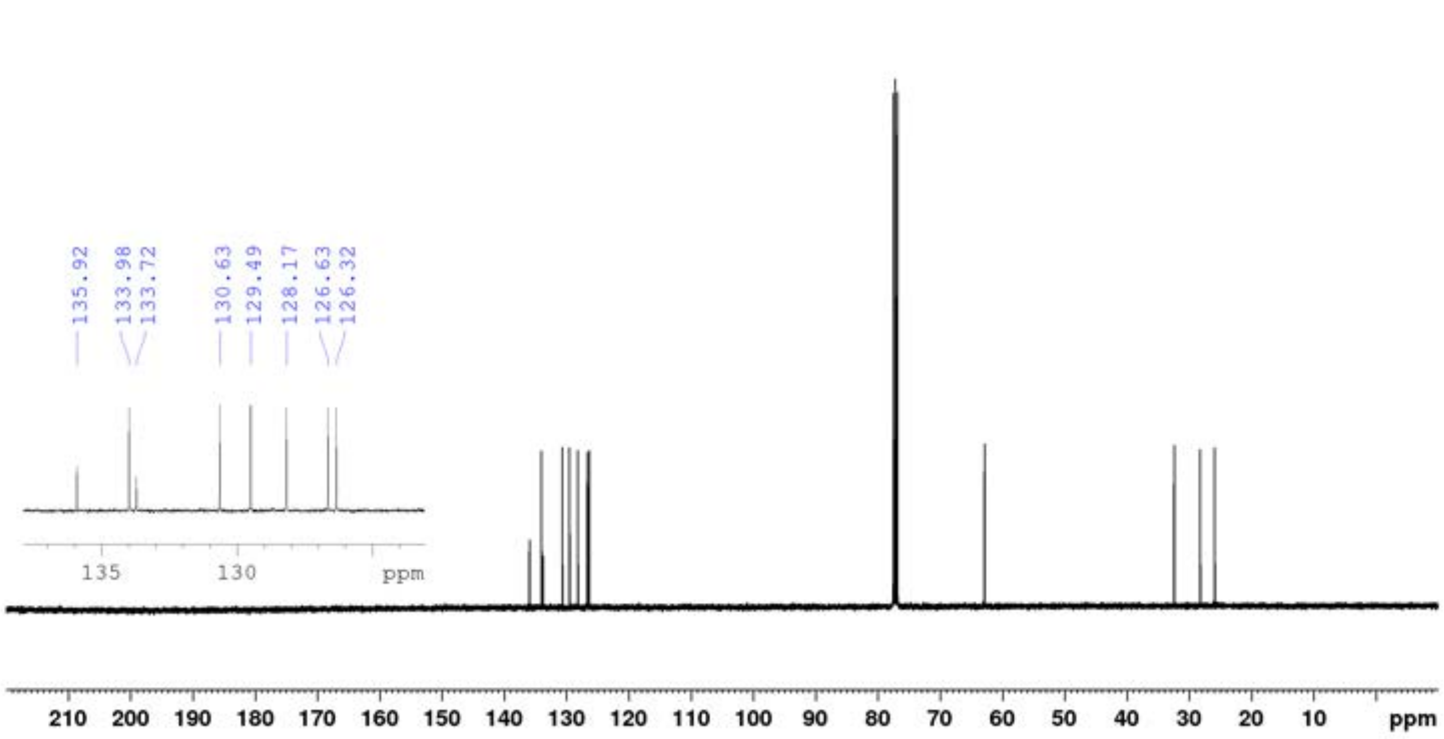

¹H NMR (400 MHz, $CDCl_3$) for *6-(4-chlorophenyl)hex-5-yn-1-ol*

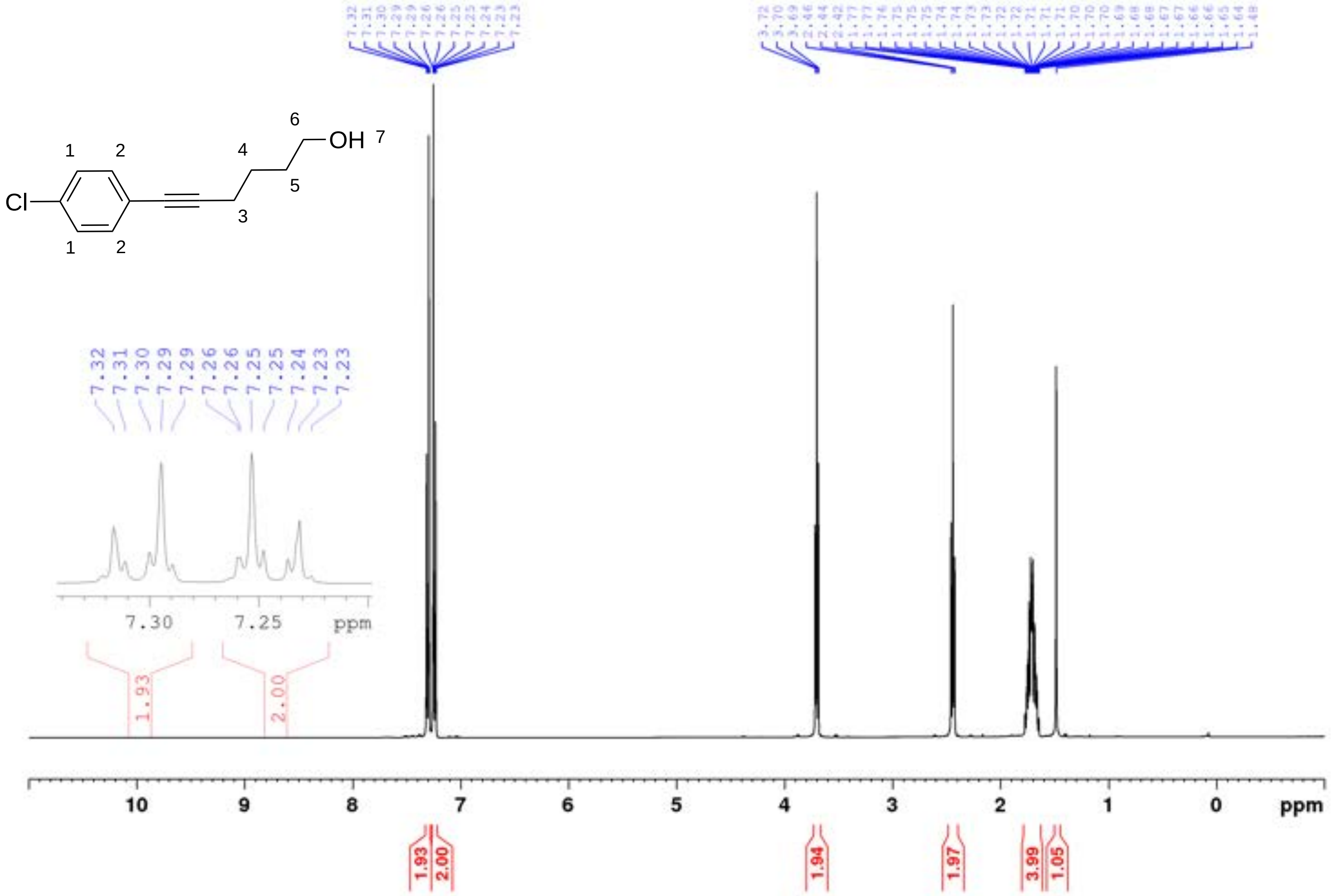

^{13}C NMR (101 MHz, $CDCl_3$) for *6-(4-chlorophenyl)hex-5-yn-1-ol*

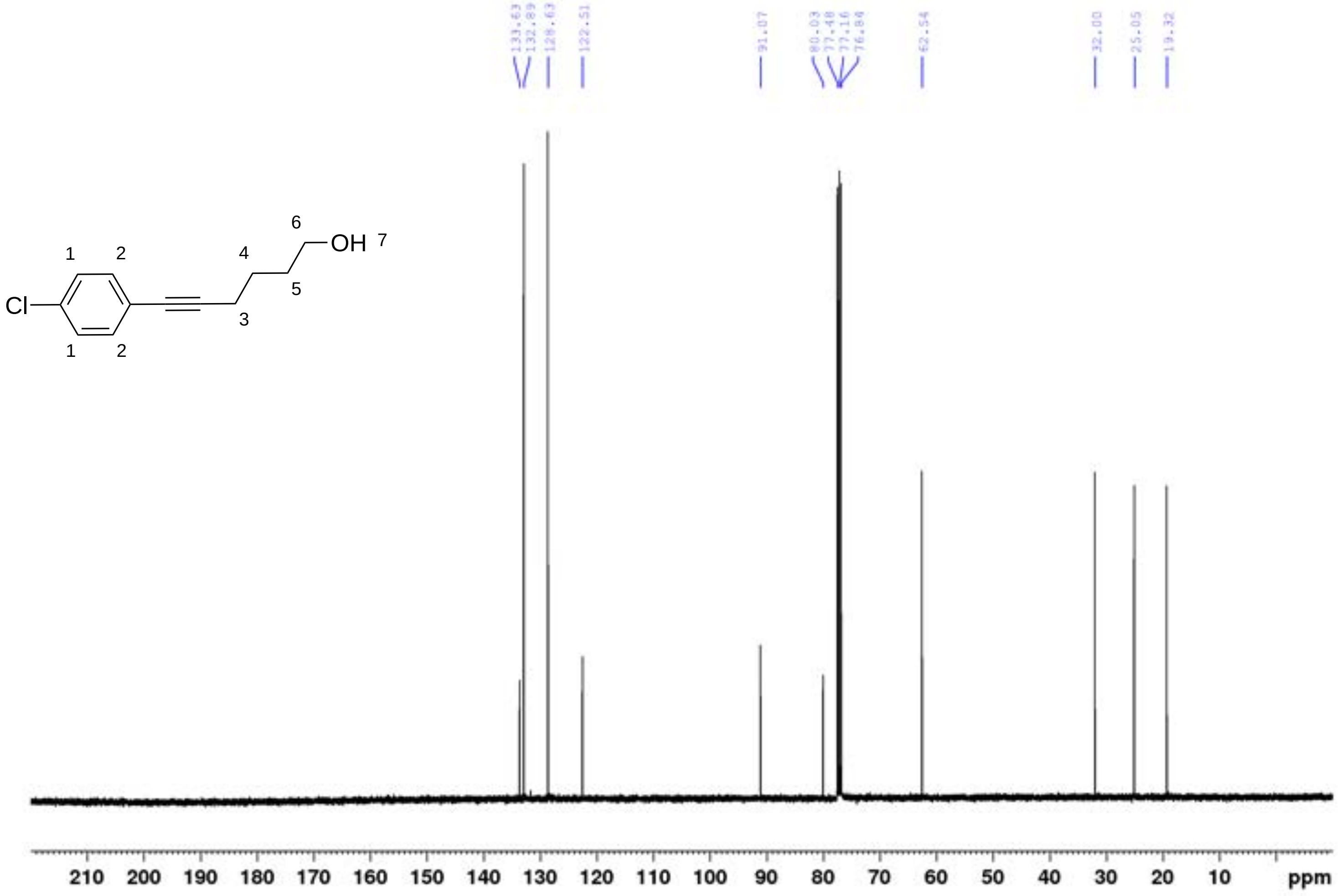

^{1}H NMR (400 MHz, $CDCl_3$) for *(Z)-6-(4-chlorophenyl)hex-5-en-1-ol* (**13g**)

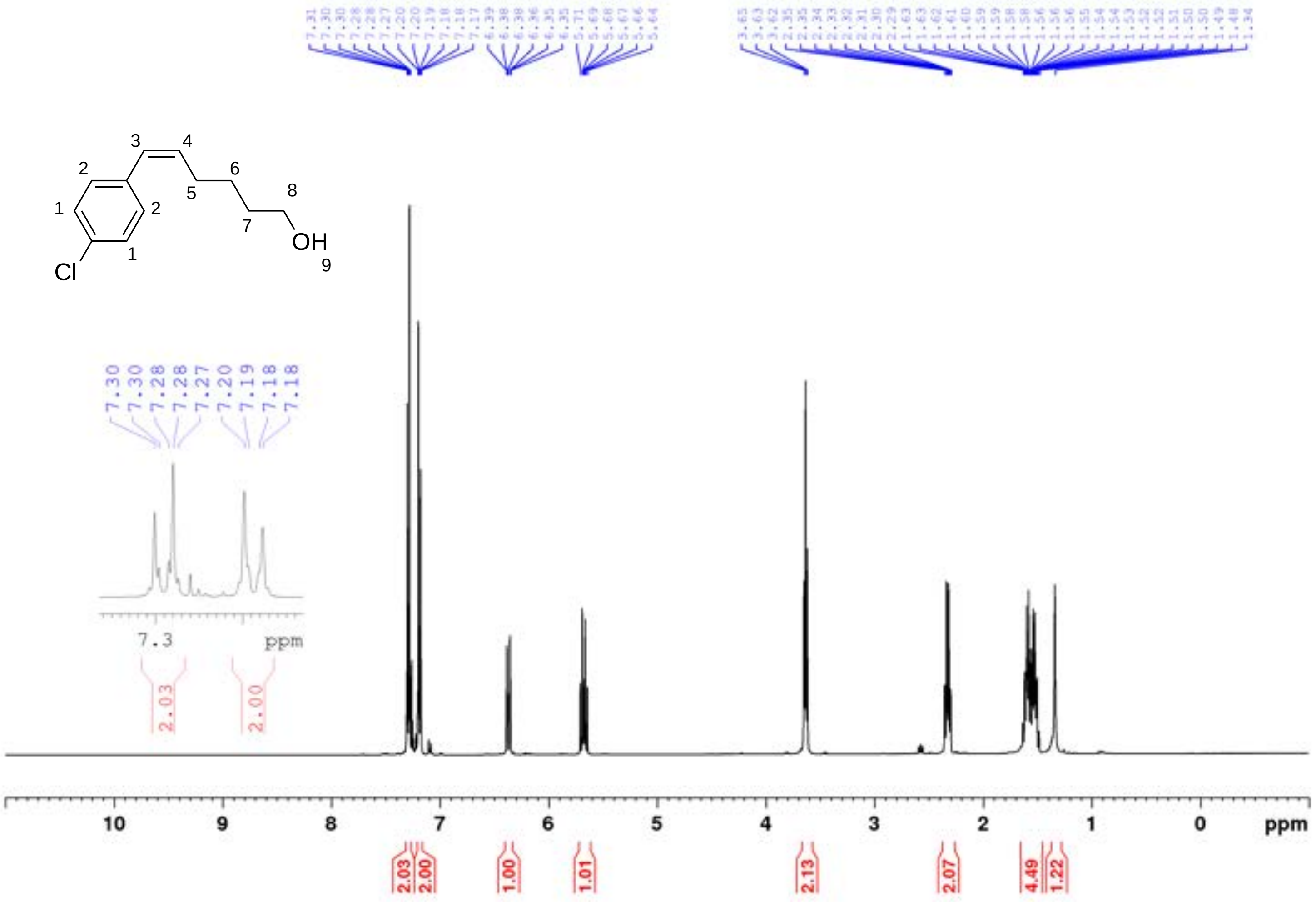

^{13}C NMR (101 MHz, $CDCl_3$) for *(Z)-6-(4-chlorophenyl)hex-5-en-1-ol* (**13g**)

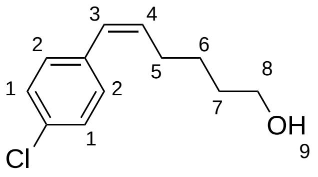

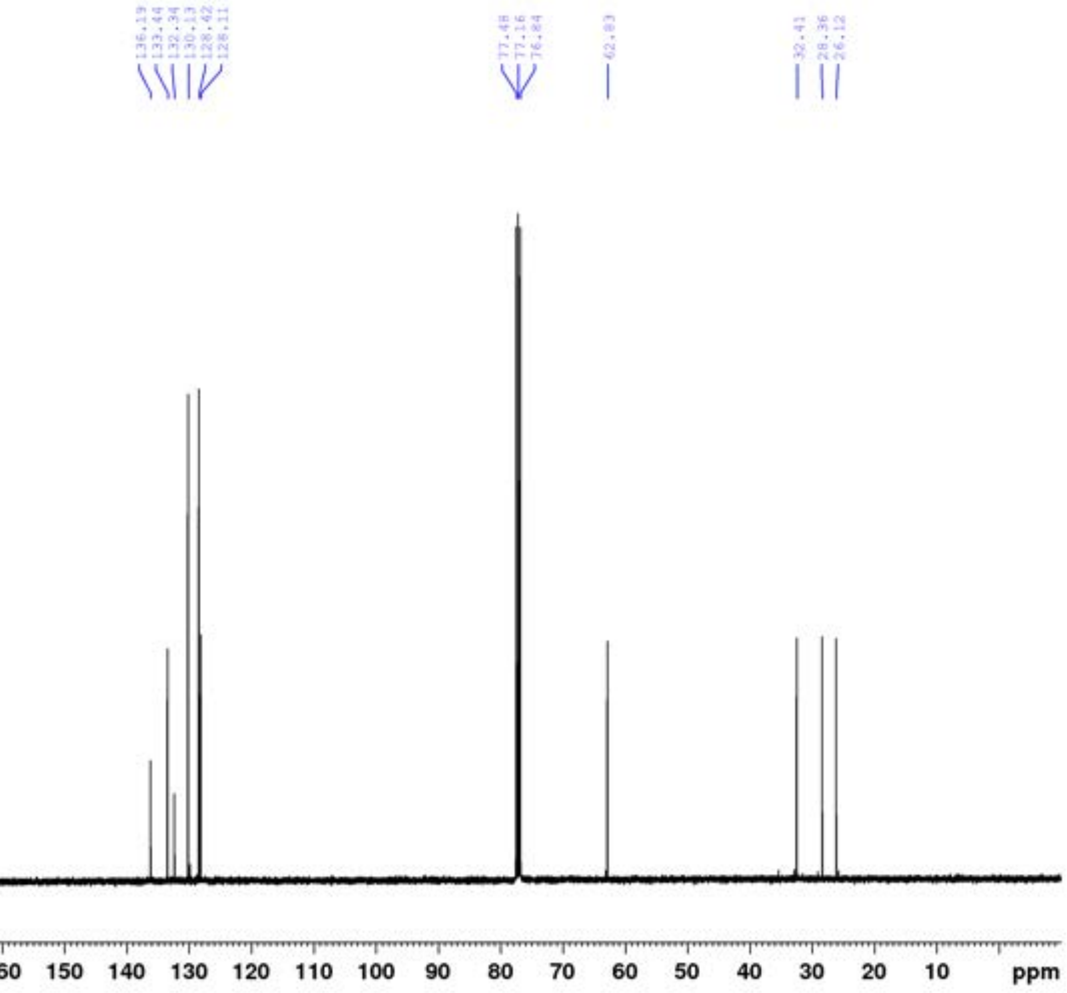

^{1}H NMR (400 MHz, $CDCl_3$) for *6-(4-(tert-butyl)phenyl)hex-5-yn-1-ol*

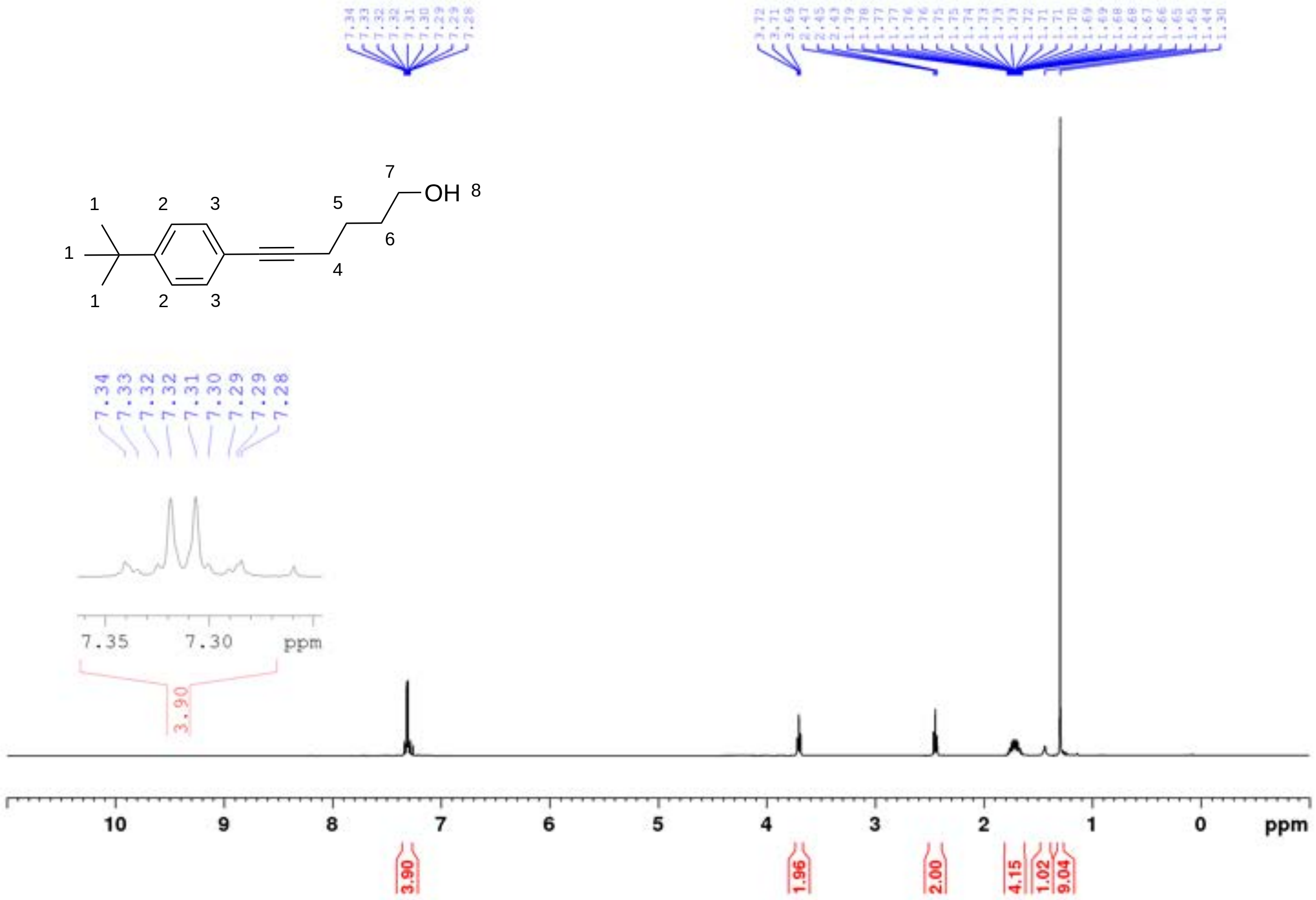

^{13}C NMR (101 MHz, $CDCl_3$) for *6-(4-(tert-butyl)phenyl)hex-5-yn-1-ol*

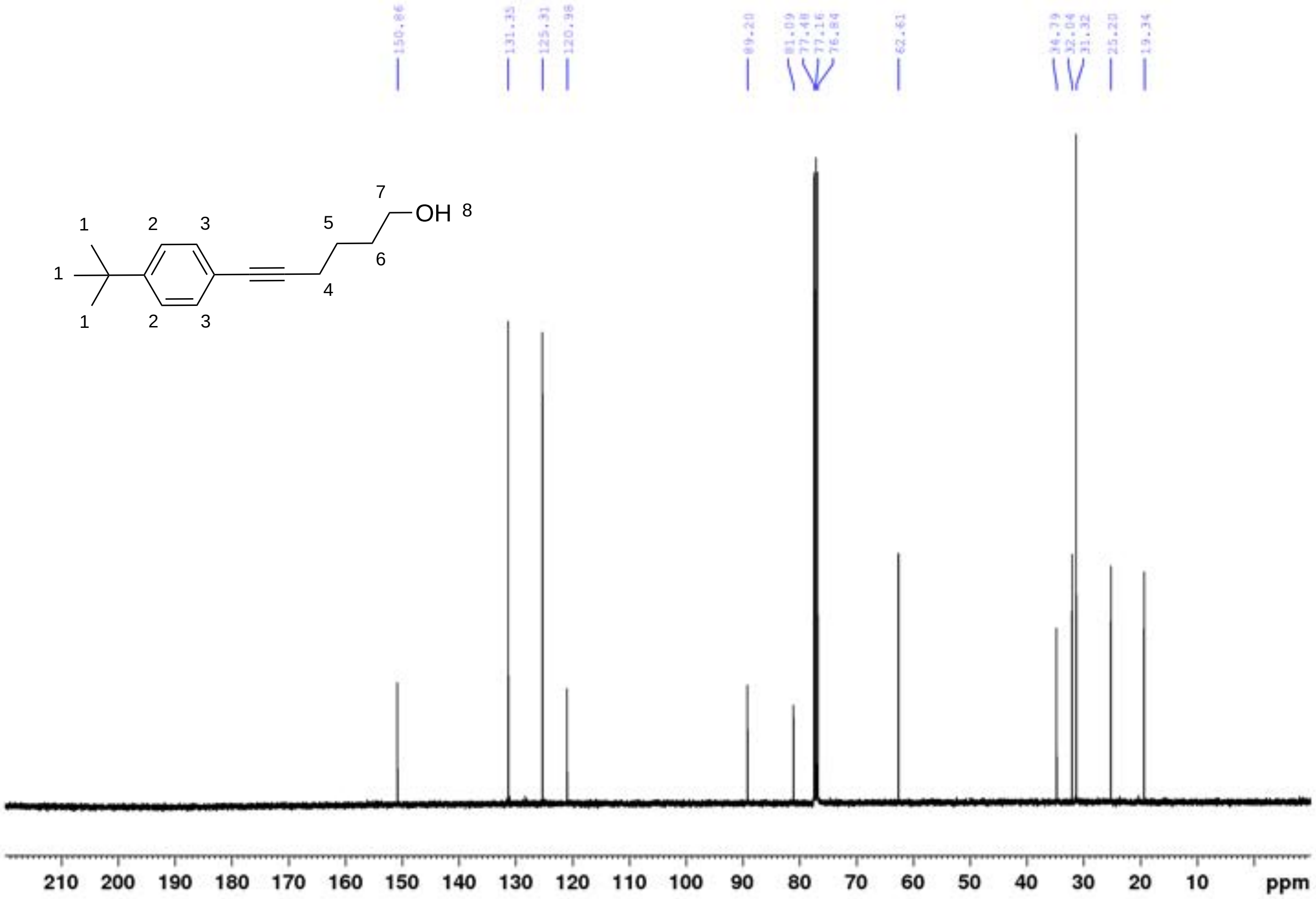

^{1}**H NMR** (400 MHz, $CDCl_3$) for *(Z)-6-(4-(tert-butyl)phenyl)hex-5-en-1-ol* (**13h**)

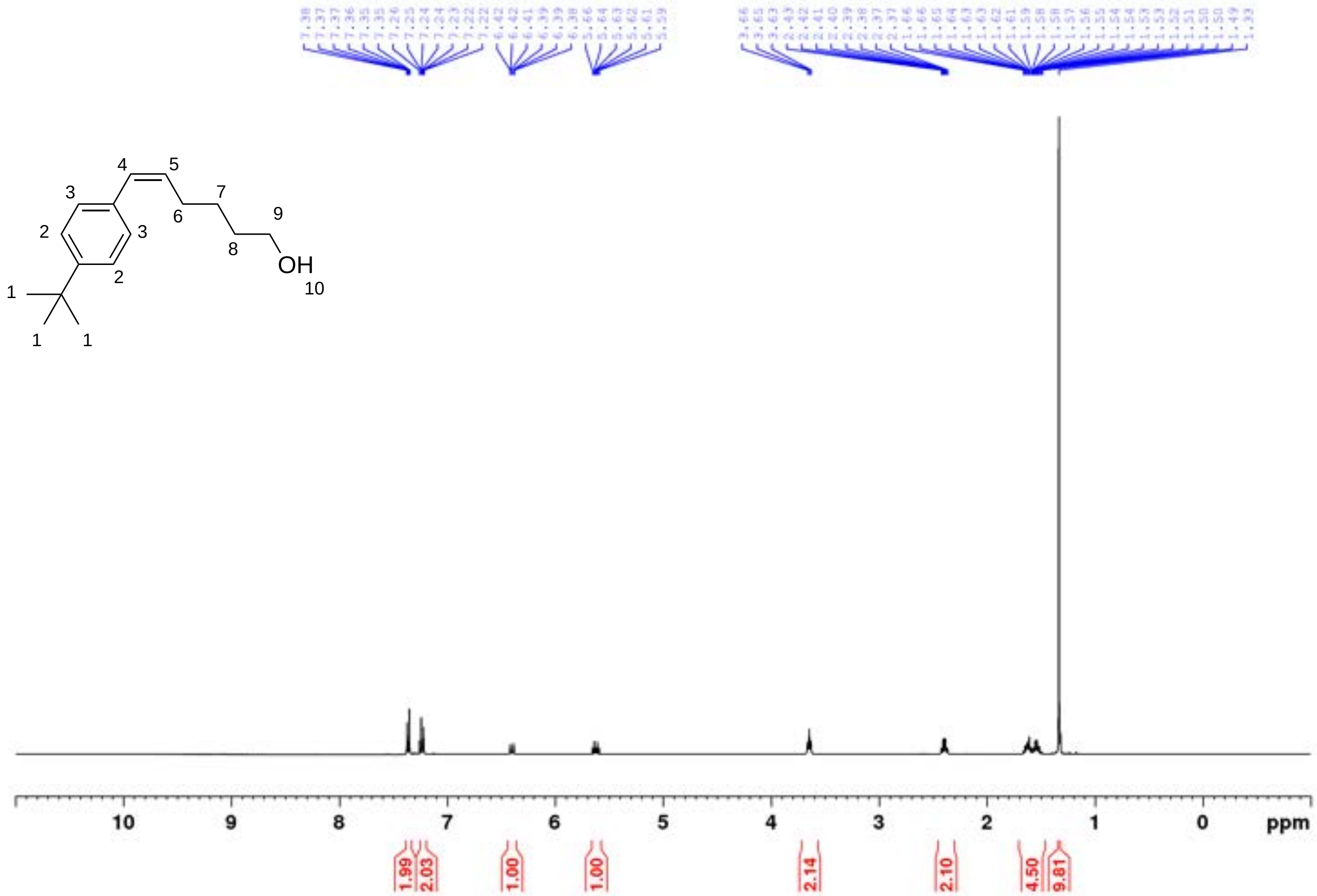

^{13}C NMR (101 MHz, $CDCl_3$) for *(Z)-6-(4-(tert-butyl)phenyl)hex-5-en-1-ol* (**13h**)

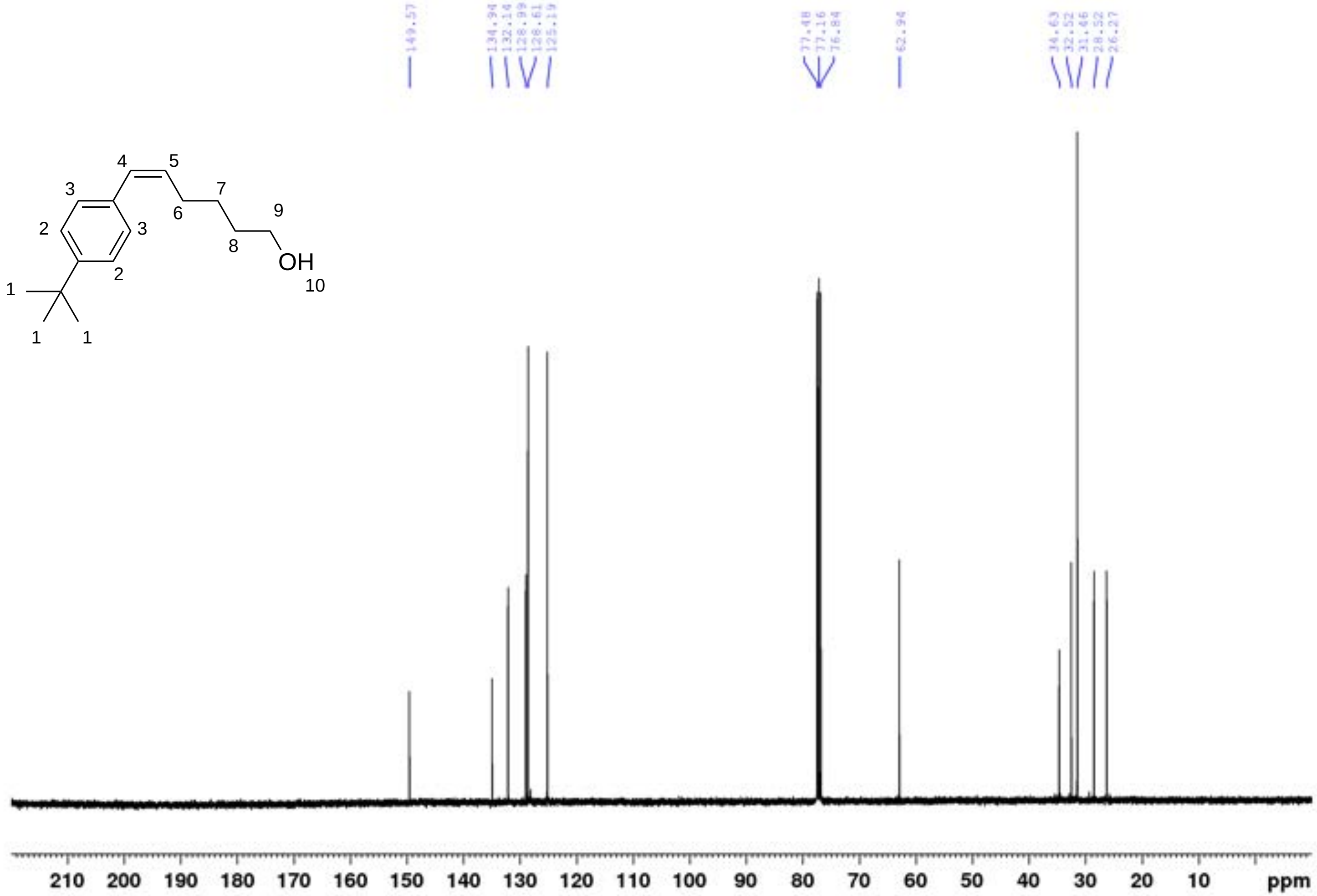

^{1}H NMR (400 MHz, $CDCl_3$) for *1-((4-iodophenyl)sulfonyl)pyrrolidine*

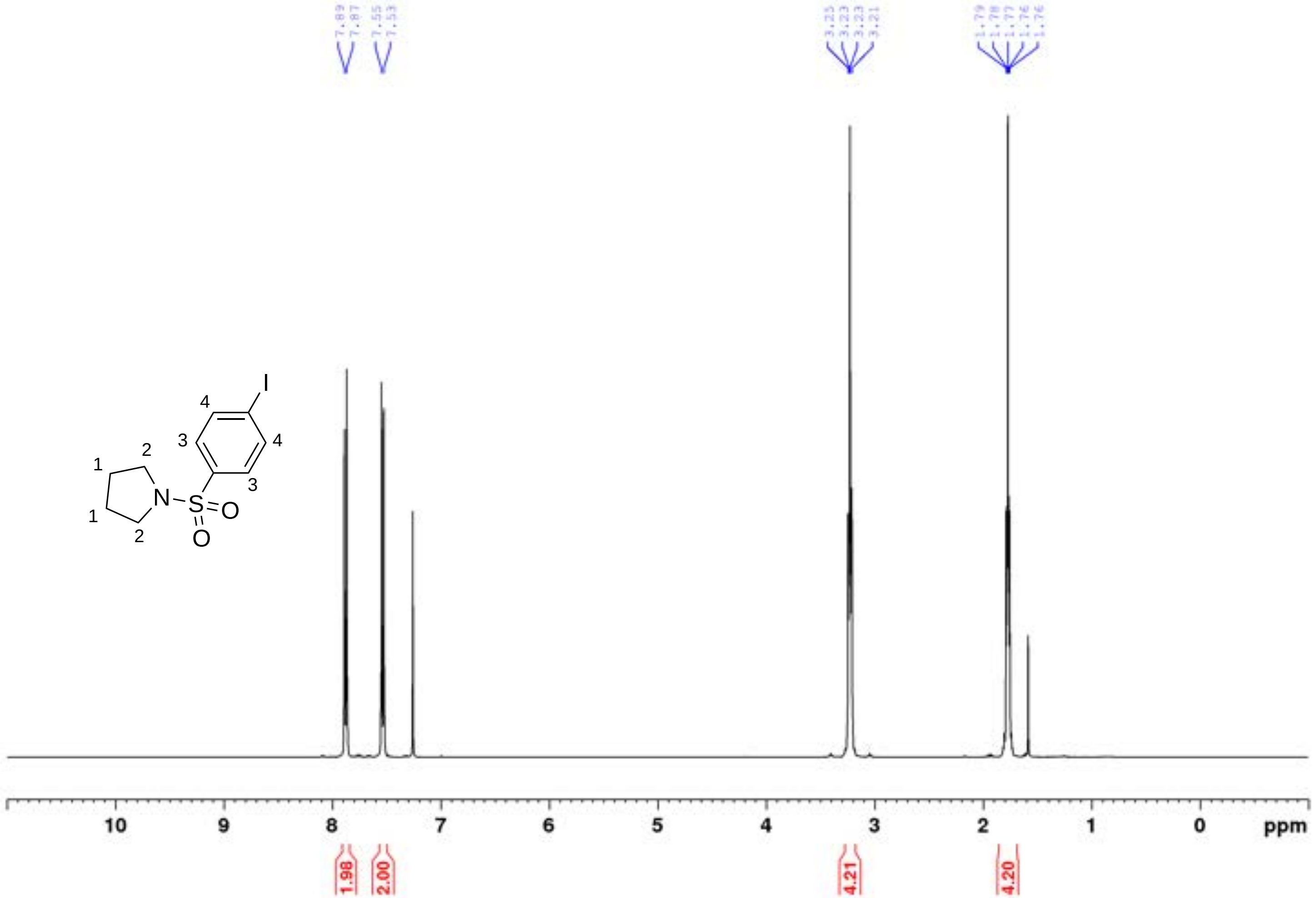

^{13}C NMR (101 MHz, $CDCl_3$) for *1-((4-iodophenyl)sulfonyl)pyrrolidine*

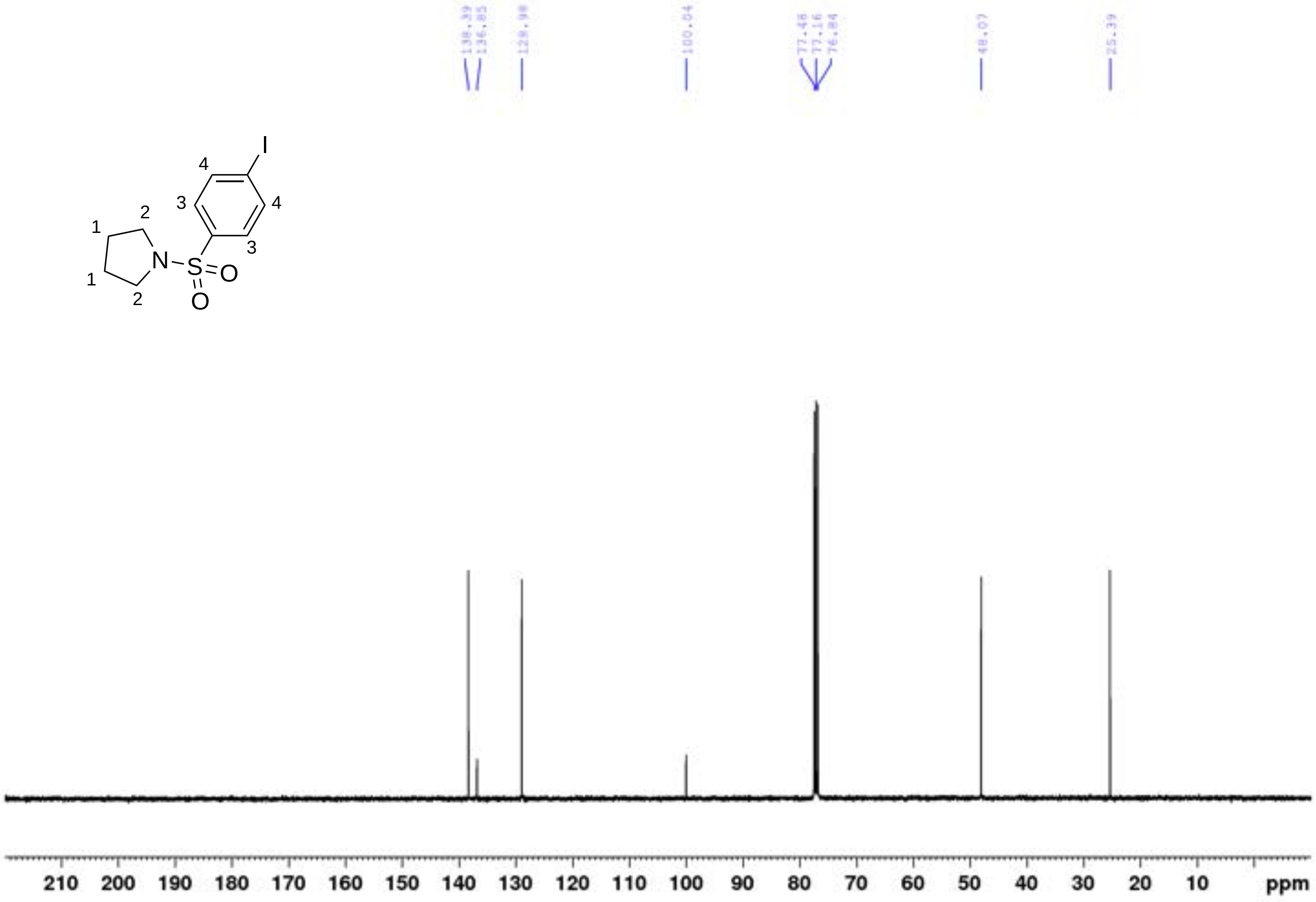

^{1}H NMR (400 MHz, $CDCl_3$) for *6-(4-(pyrrolidin-1-ylsulfonyl)phenyl)hex-5-yn-1-ol*

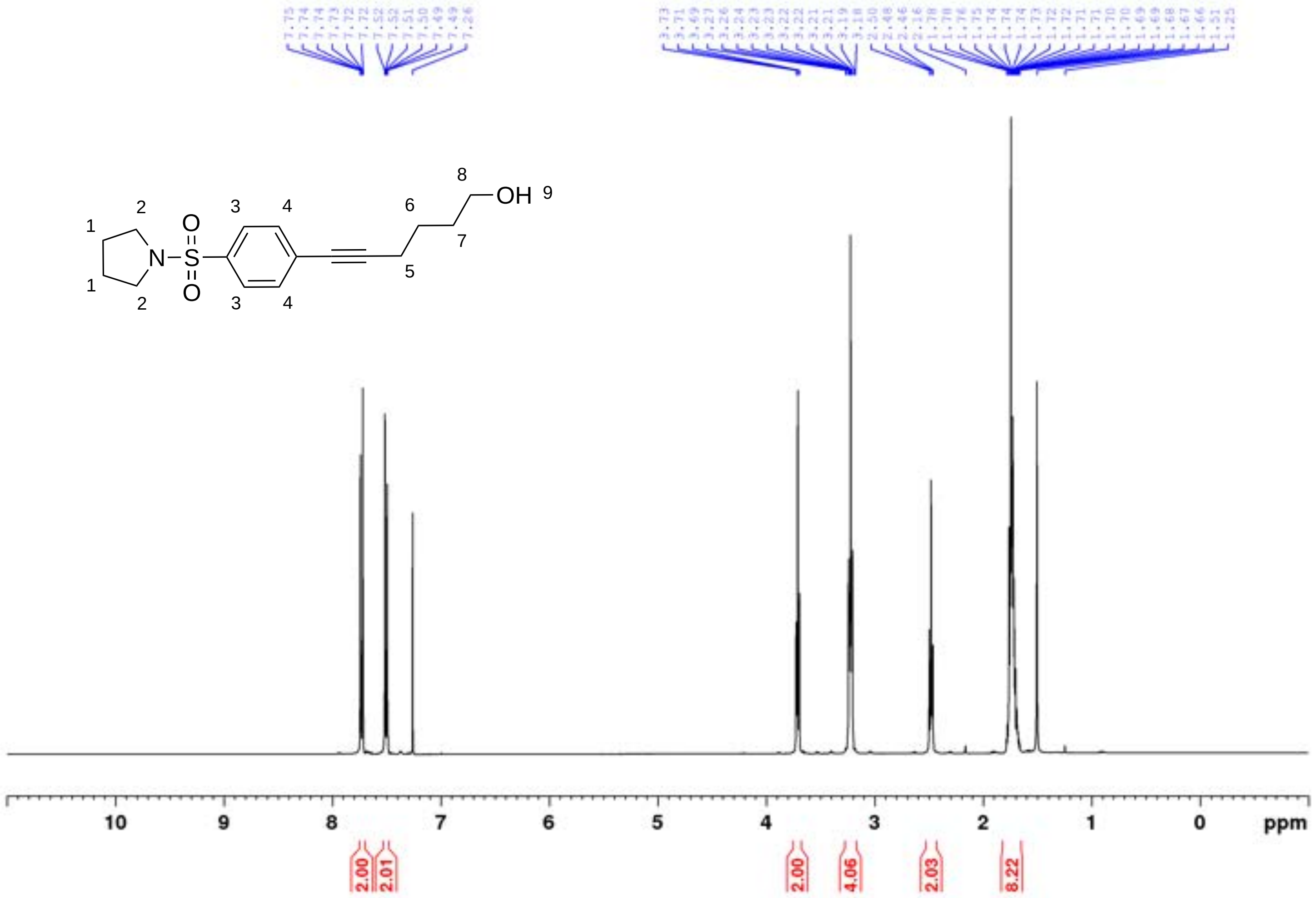

^{13}C NMR (101 MHz, $CDCl_3$) for *6-(4-(pyrrolidin-1-ylsulfonyl)phenyl)hex-5-yn-1-ol*

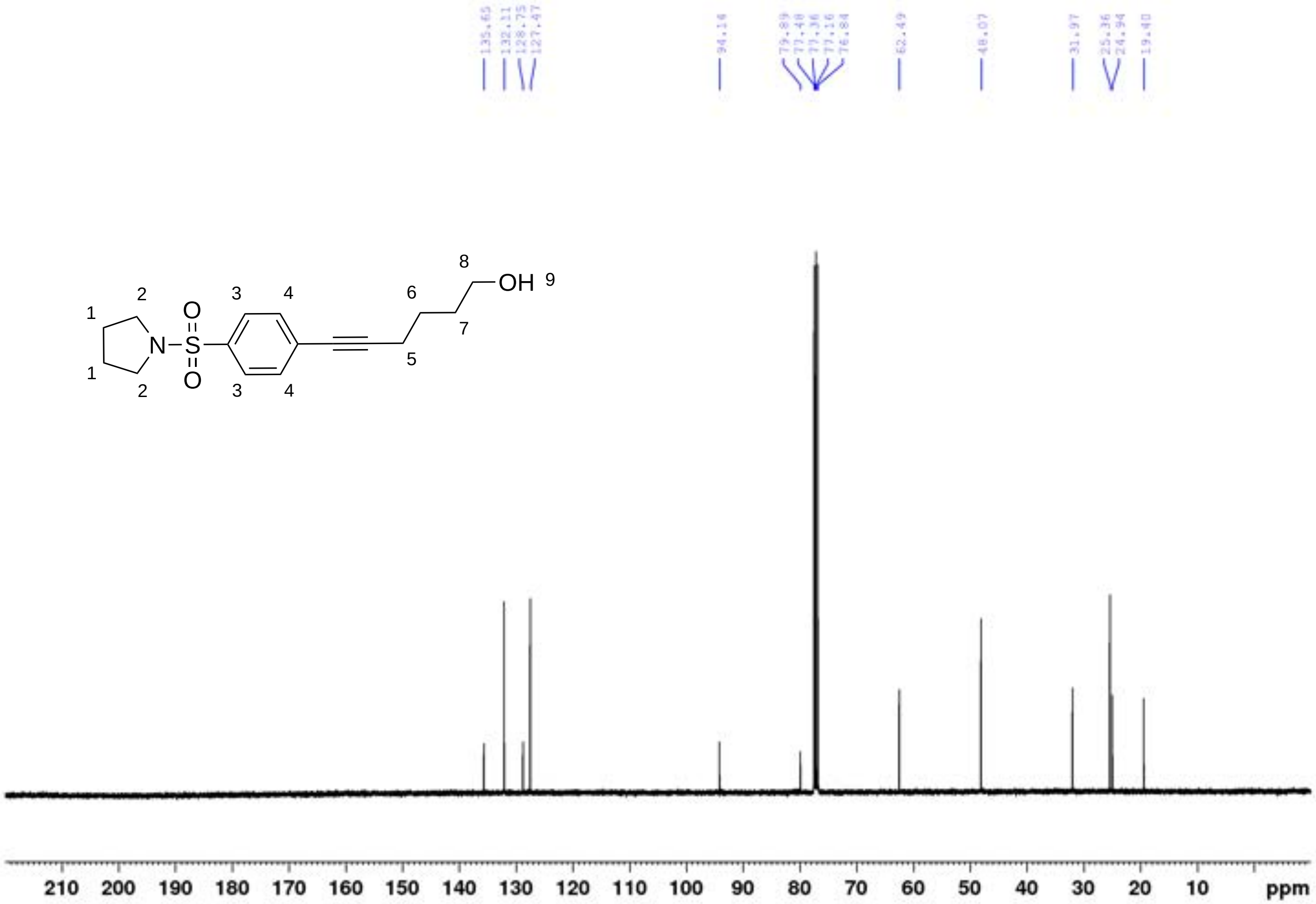

^{1}H NMR (400 MHz, $CDCl_3$) for *(Z)-6-(4-(pyrrolidin-1-ylsulfonyl)phenyl)hex-5-en-1-ol* (**13i**)

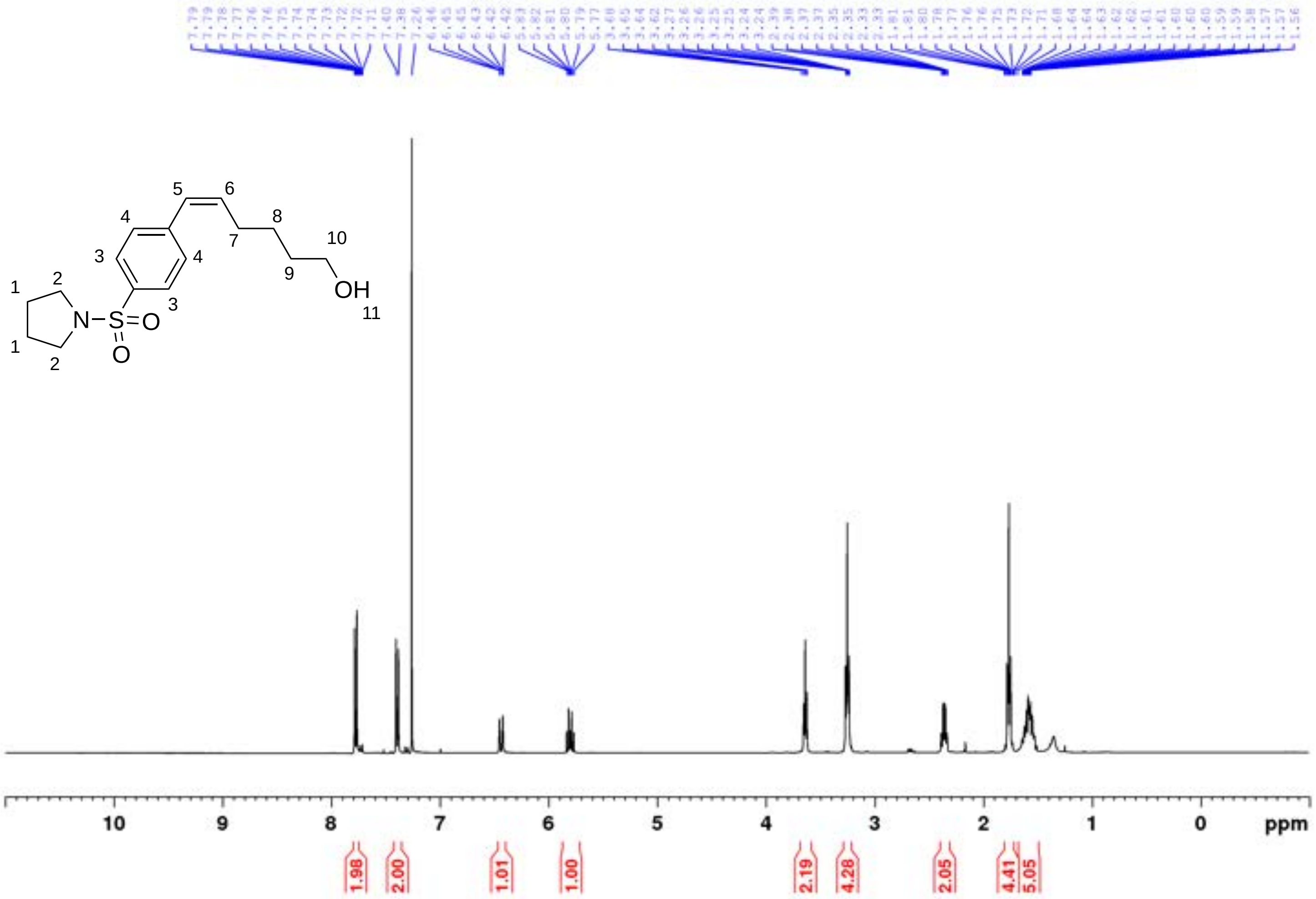

^{13}C NMR (101 MHz, $CDCl_3$) for *(Z)-6-(4-(pyrrolidin-1-ylsulfonyl)phenyl)hex-5-en-1-ol* (**13i**)

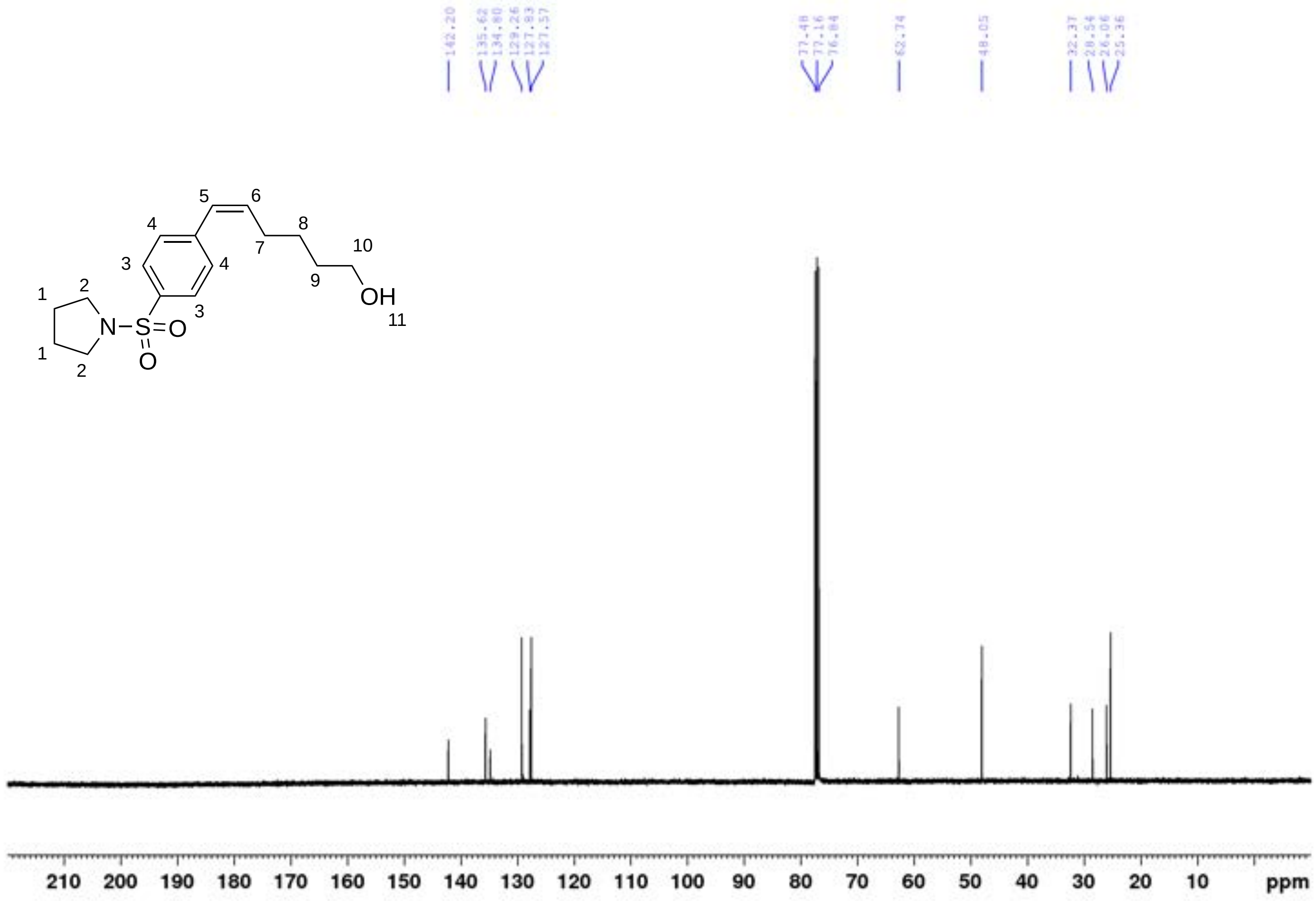

^{1}H NMR (400 MHz, $CDCl_3$) for *(Z)-5-(naphthalen-1-yl)pent-4-en-1-ol*

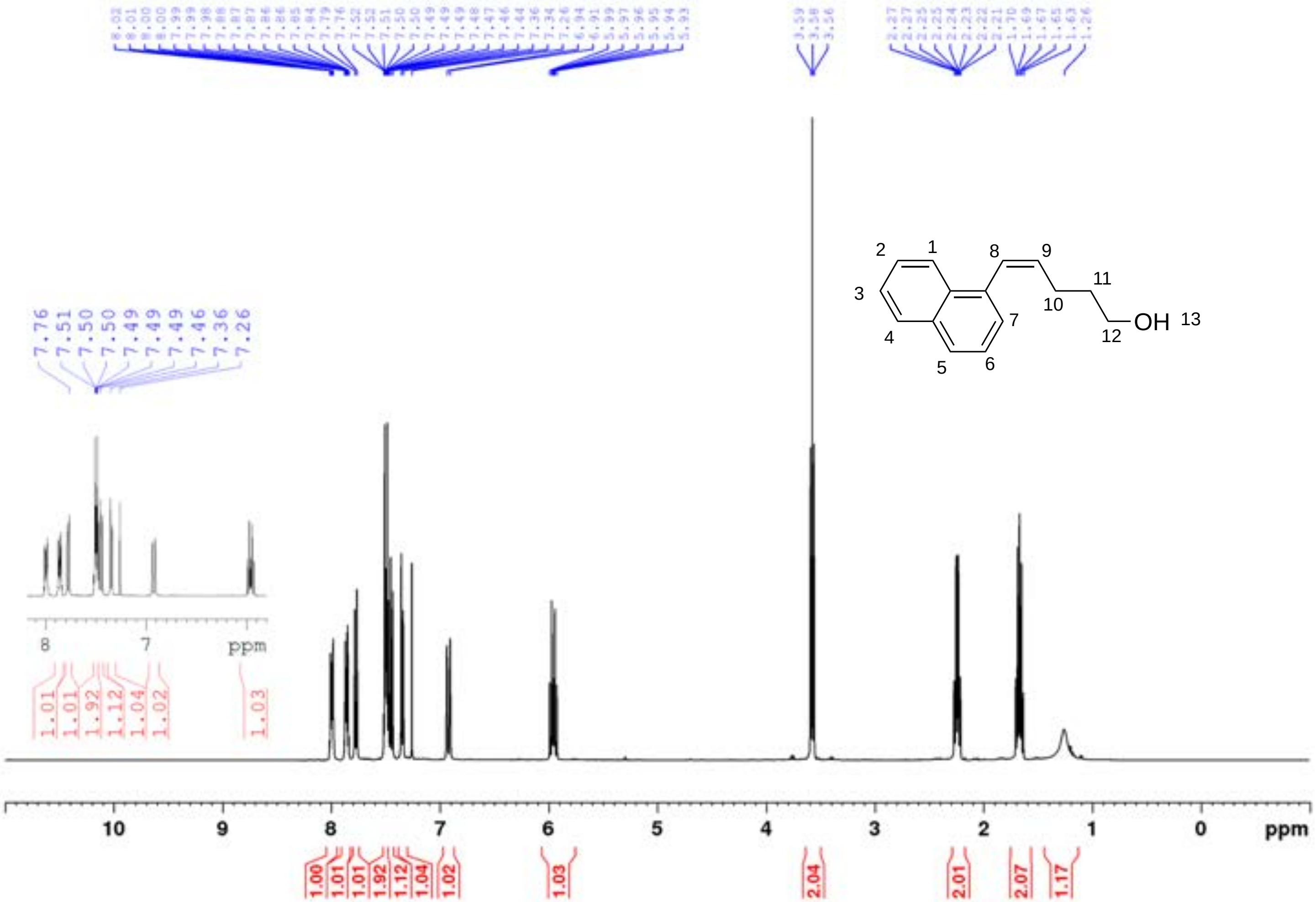

^{13}C NMR (101 MHz, $CDCl_3$) for *(Z)-5-(naphthalen-1-yl)pent-4-en-1-ol*

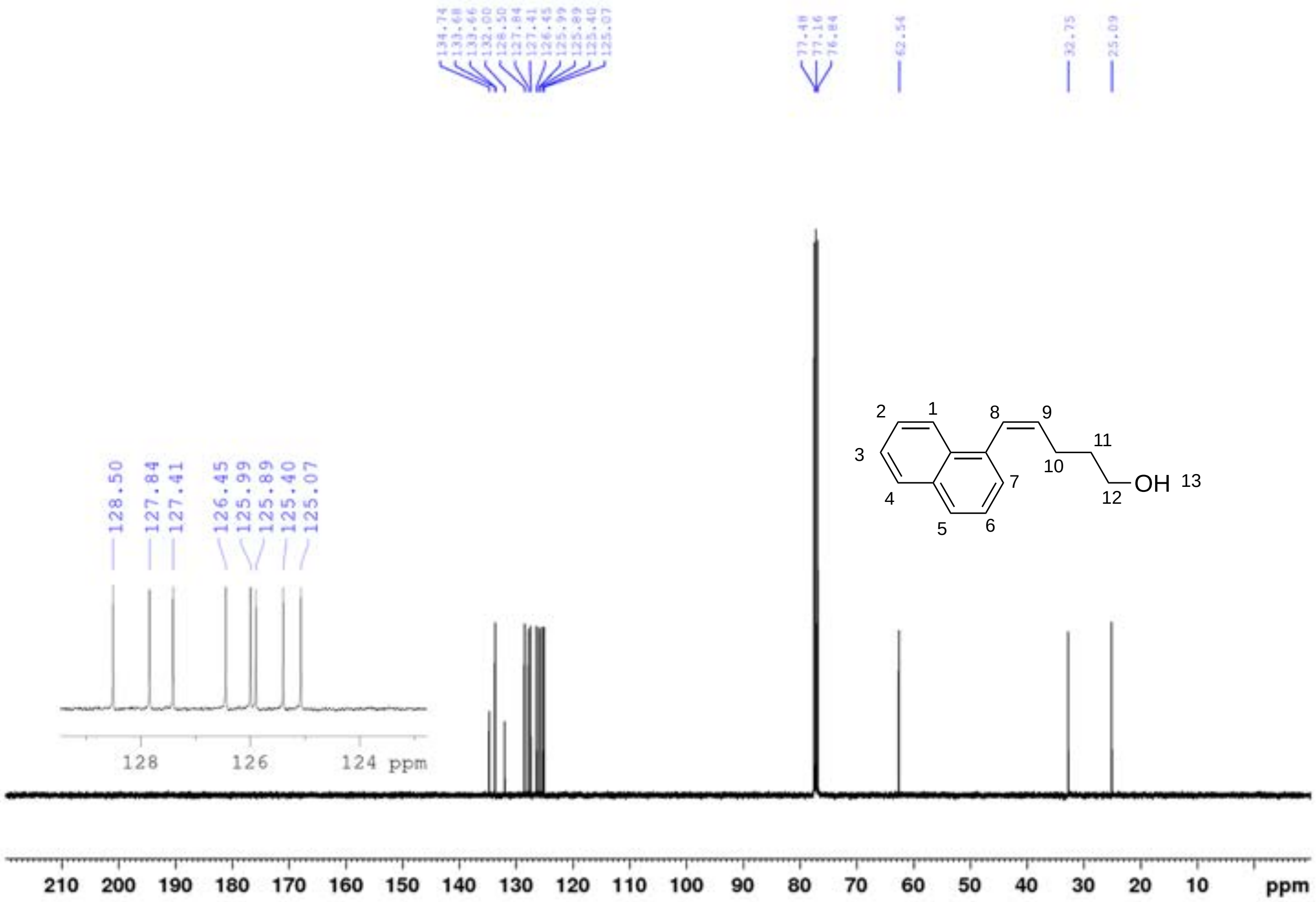

^{1}H NMR (400 MHz, $CDCl_3$) for *(Z)-5-(naphthalen-1-yl)pent-4-en-1-yl 4-methylbenzenesulfonate*

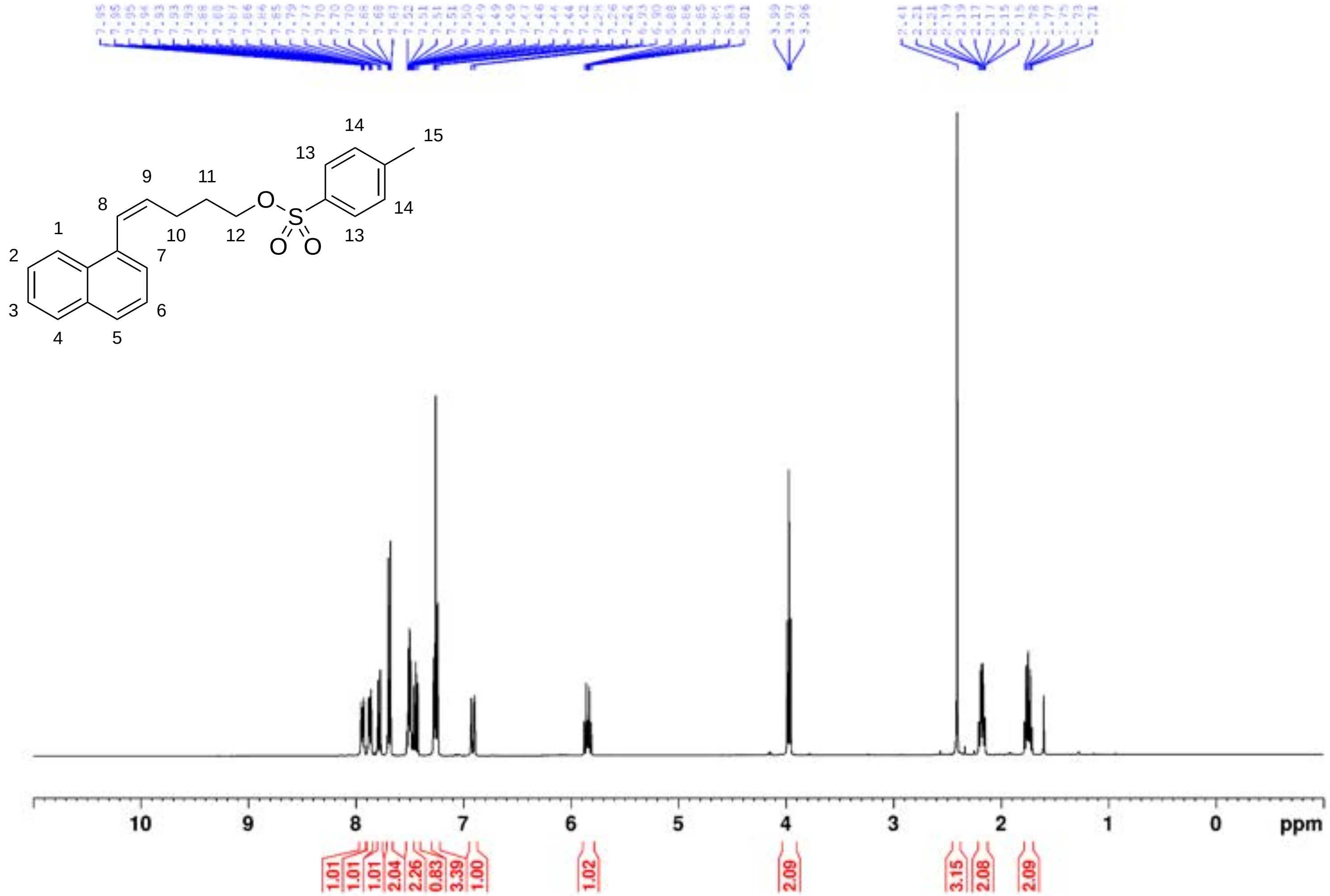

^{13}C NMR (101 MHz, $CDCl_3$) for *(Z)-5-(naphthalen-1-yl)pent-4-en-1-yl 4-methylbenzenesulfonate*

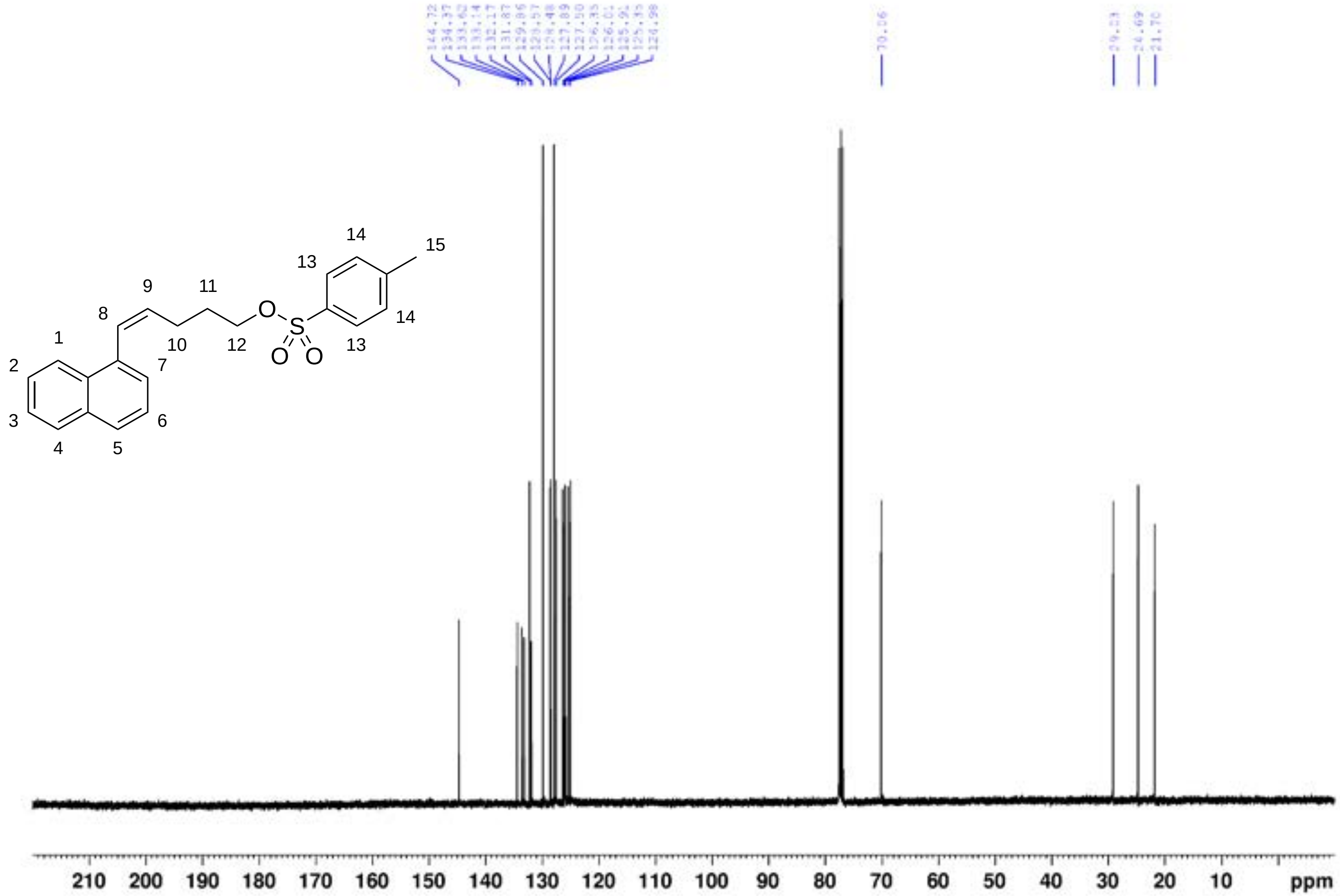

¹H NMR (400 MHz, $CDCl_3$) for *(Z)-1-(pent-1-en-1-yl)naphthalene*

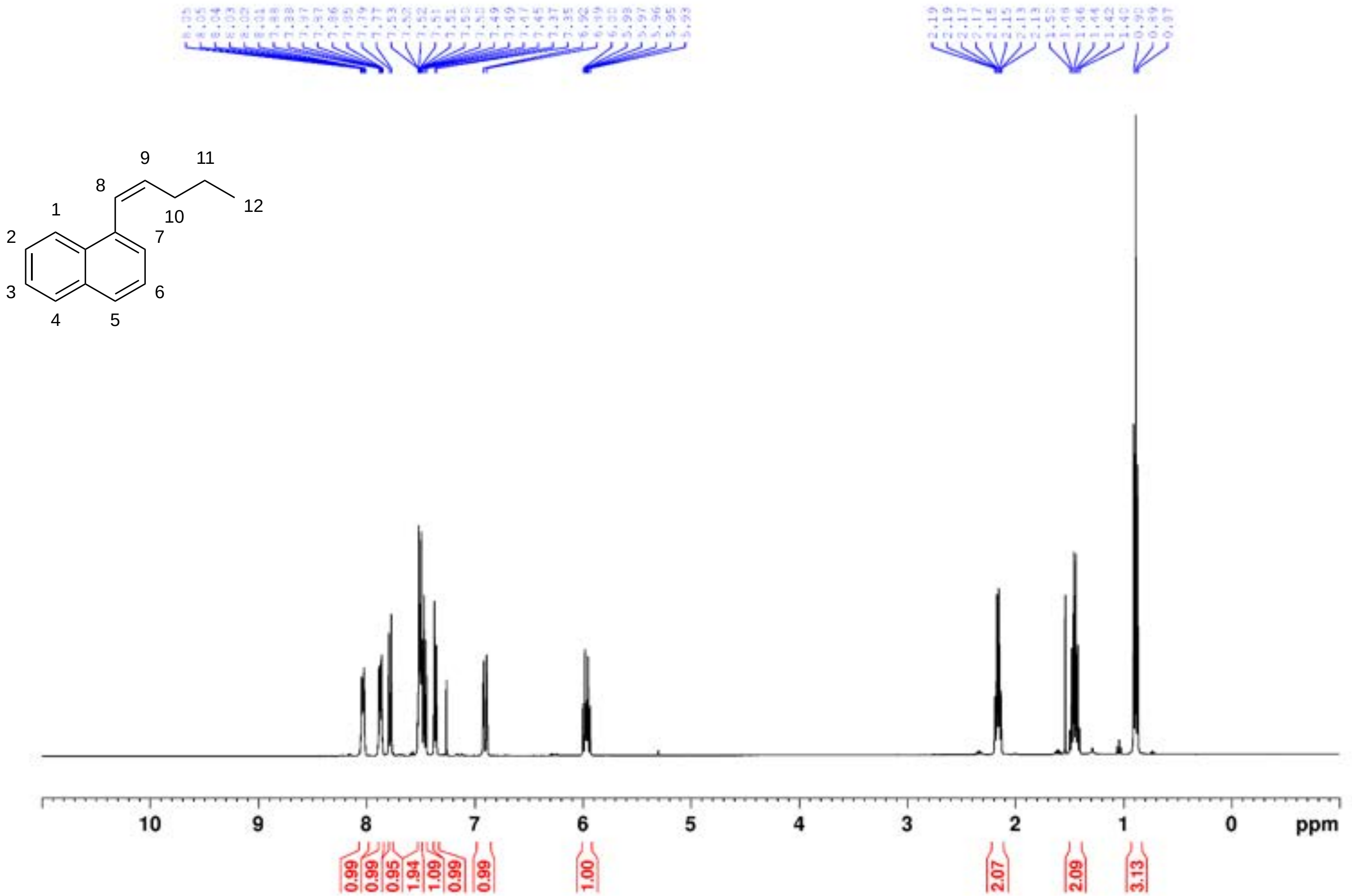

^{13}C NMR (101 MHz, $CDCl_3$) for *(Z)-1-(pent-1-en-1-yl)naphthalene*

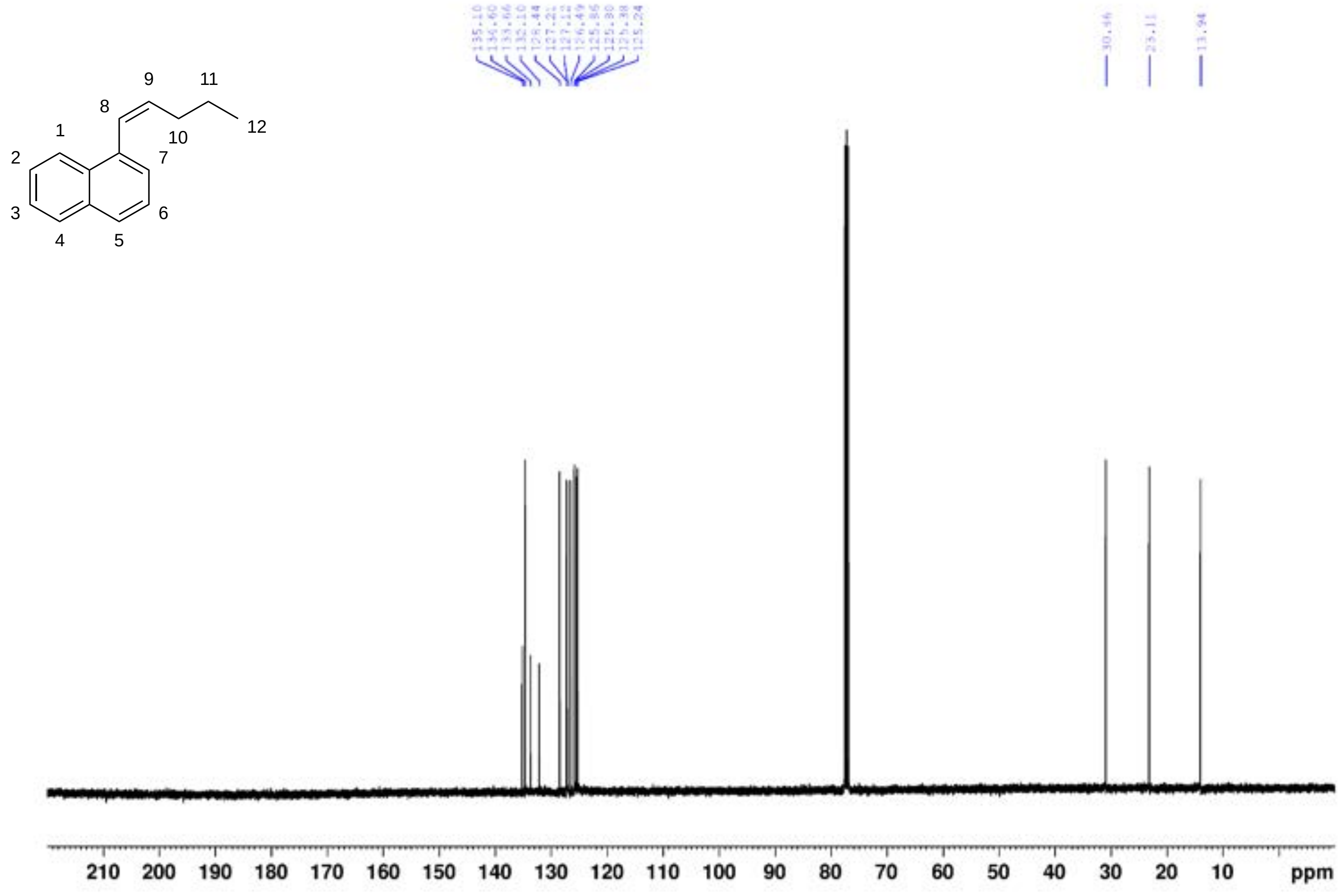

¹H NMR (400 MHz, $CDCl_3$) for *(Z)-(5-methoxypent-1-en-1-yl)benzene*

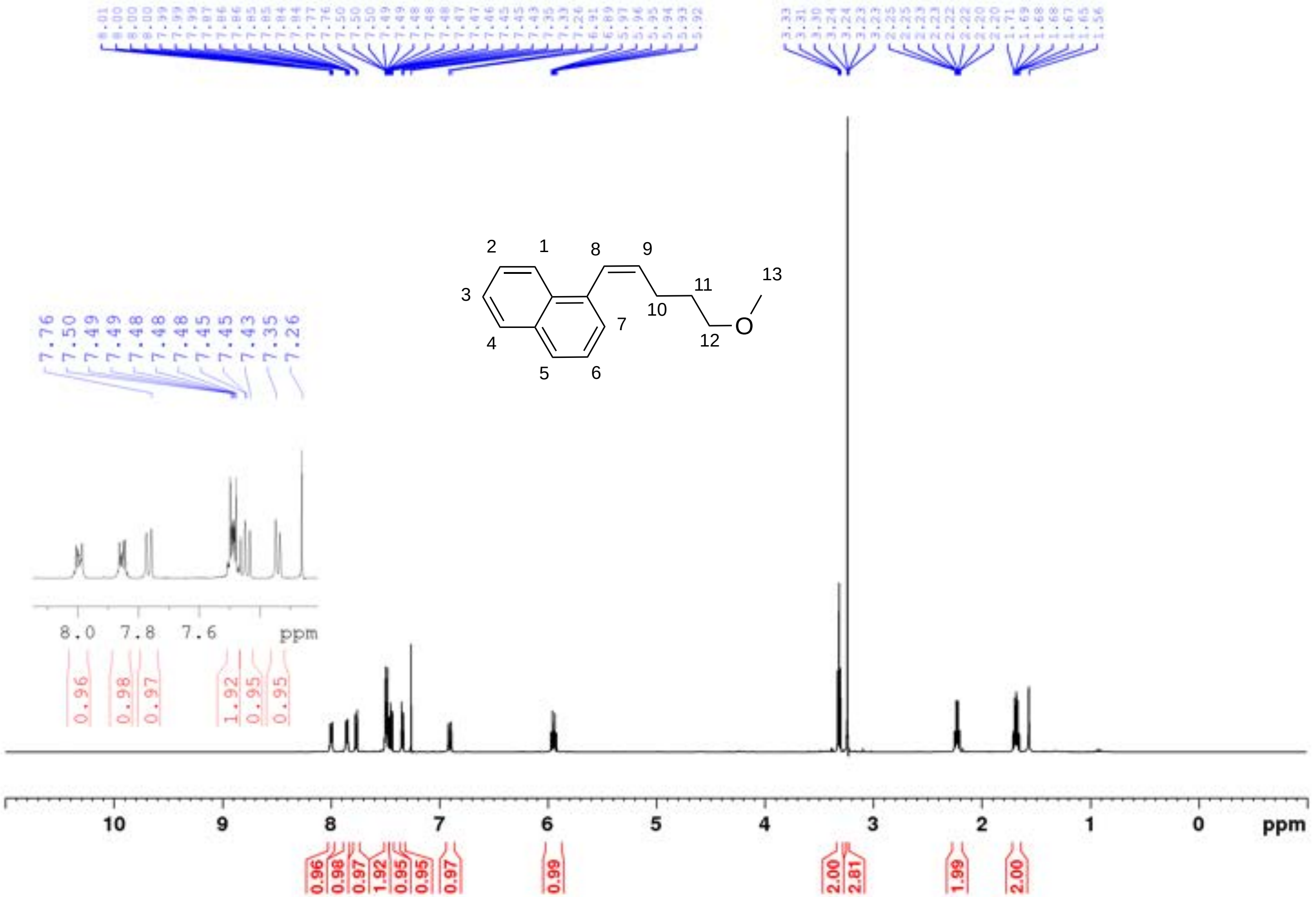

^{13}**C NMR** (101 MHz, $CDCl_3$) for *(Z)-(5-methoxypent-1-en-1-yl)benzene*

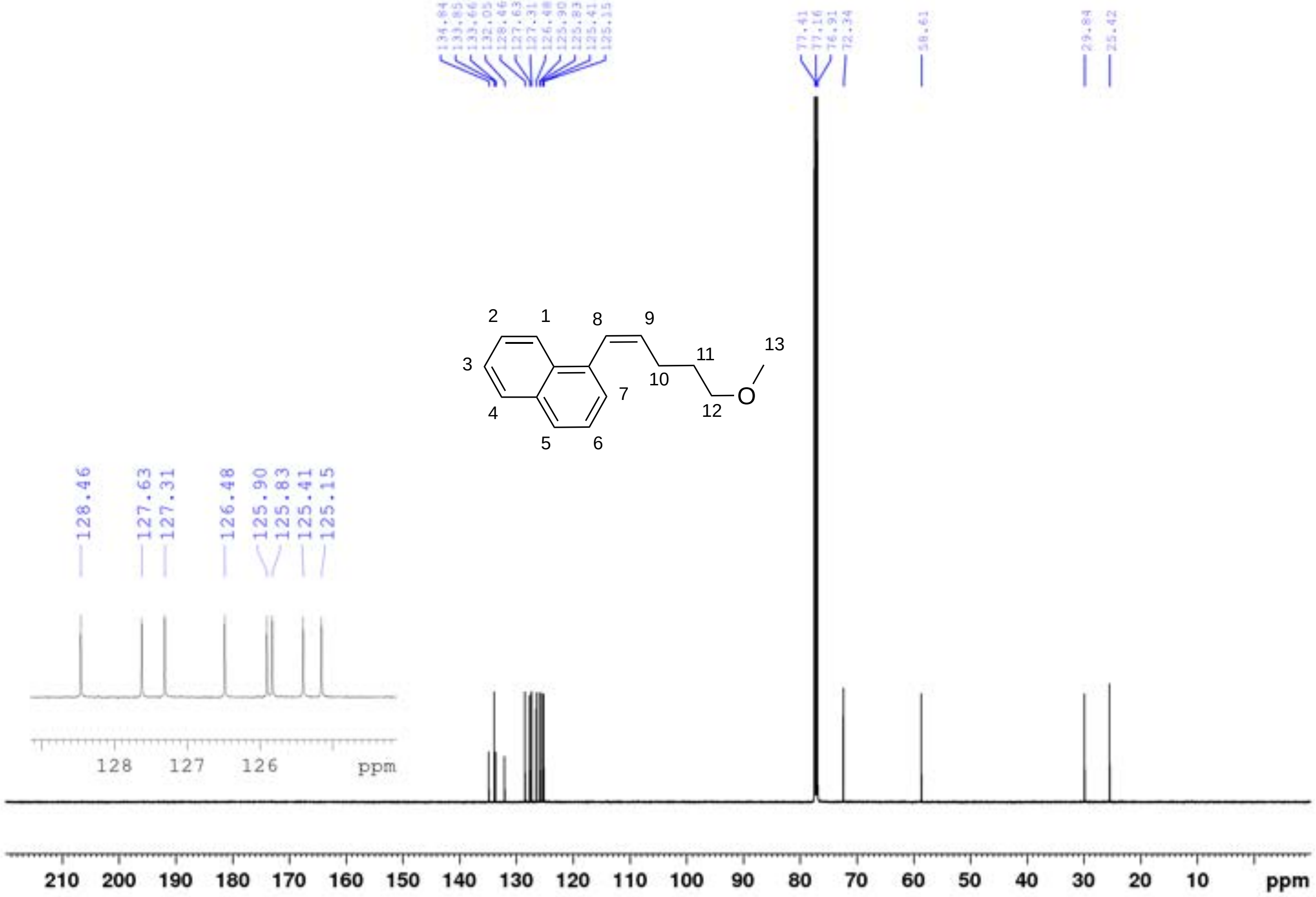

^{1}H NMR (400 MHz, CDCl$_3$) for *(4-ethoxy-4-oxobutyl)triphenylphosphonium bromide*

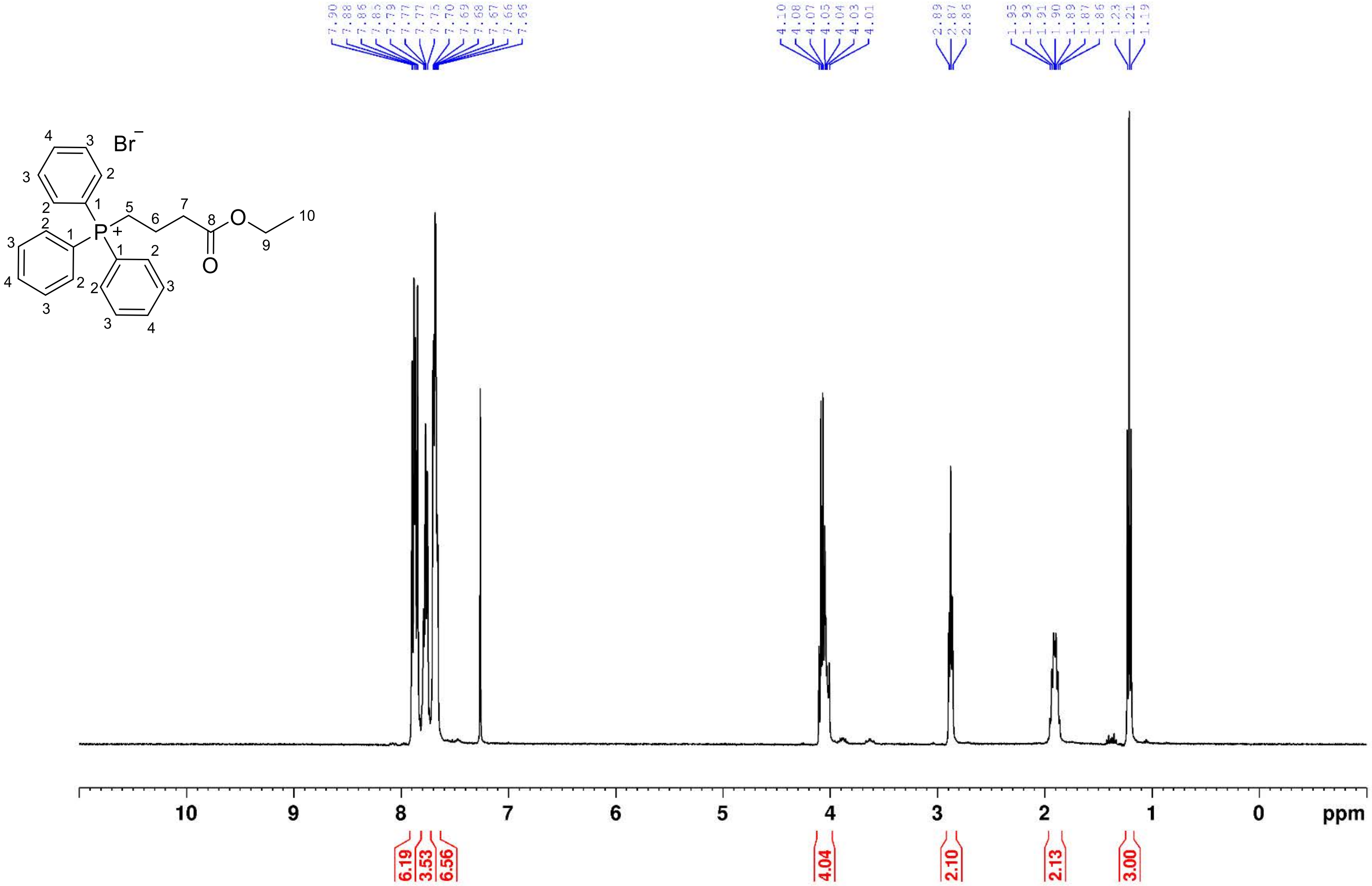

^{13}C NMR (101 MHz, CDCl$_3$) for *(4-ethoxy-4-oxobutyl)triphenylphosphonium bromide*

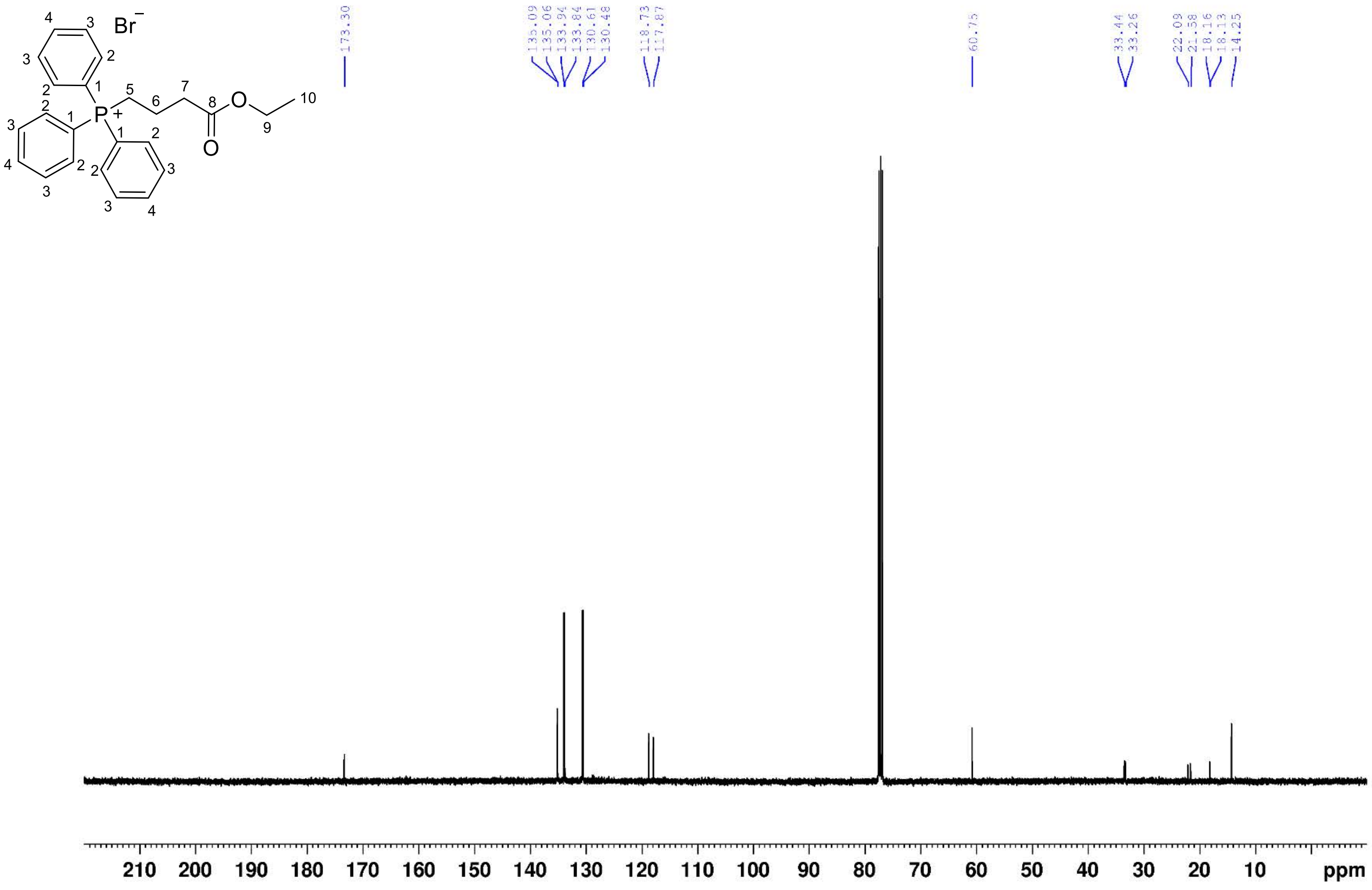

^{31}**P NMR (162 MHz, CDCl$_3$)** for *(4-ethoxy-4-oxobutyl)triphenylphosphonium bromide*

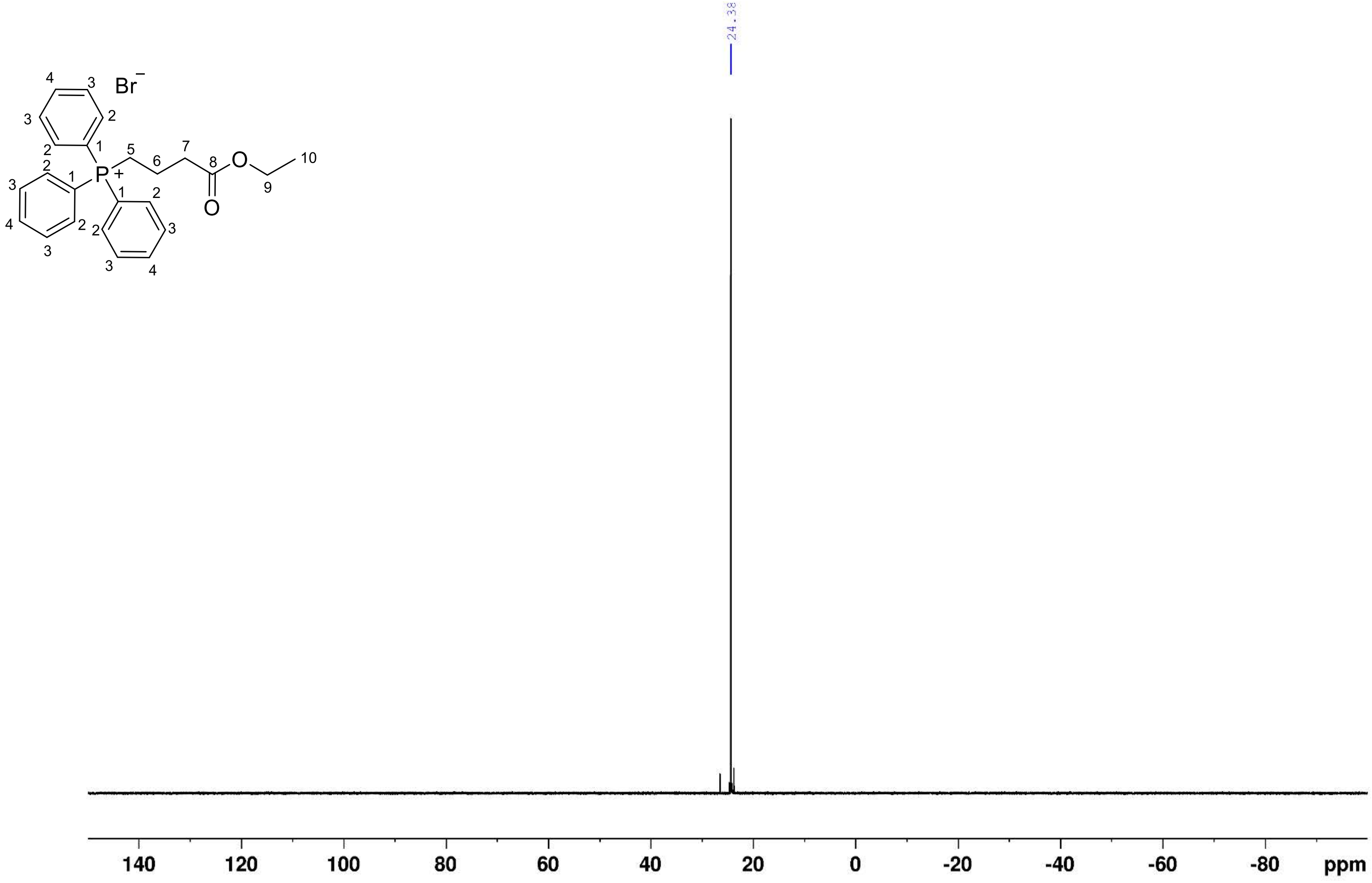

^{1}H NMR (400 MHz, CDCl$_3$) for *Ethyl (Z)-5-phenylpent-4-enoate*

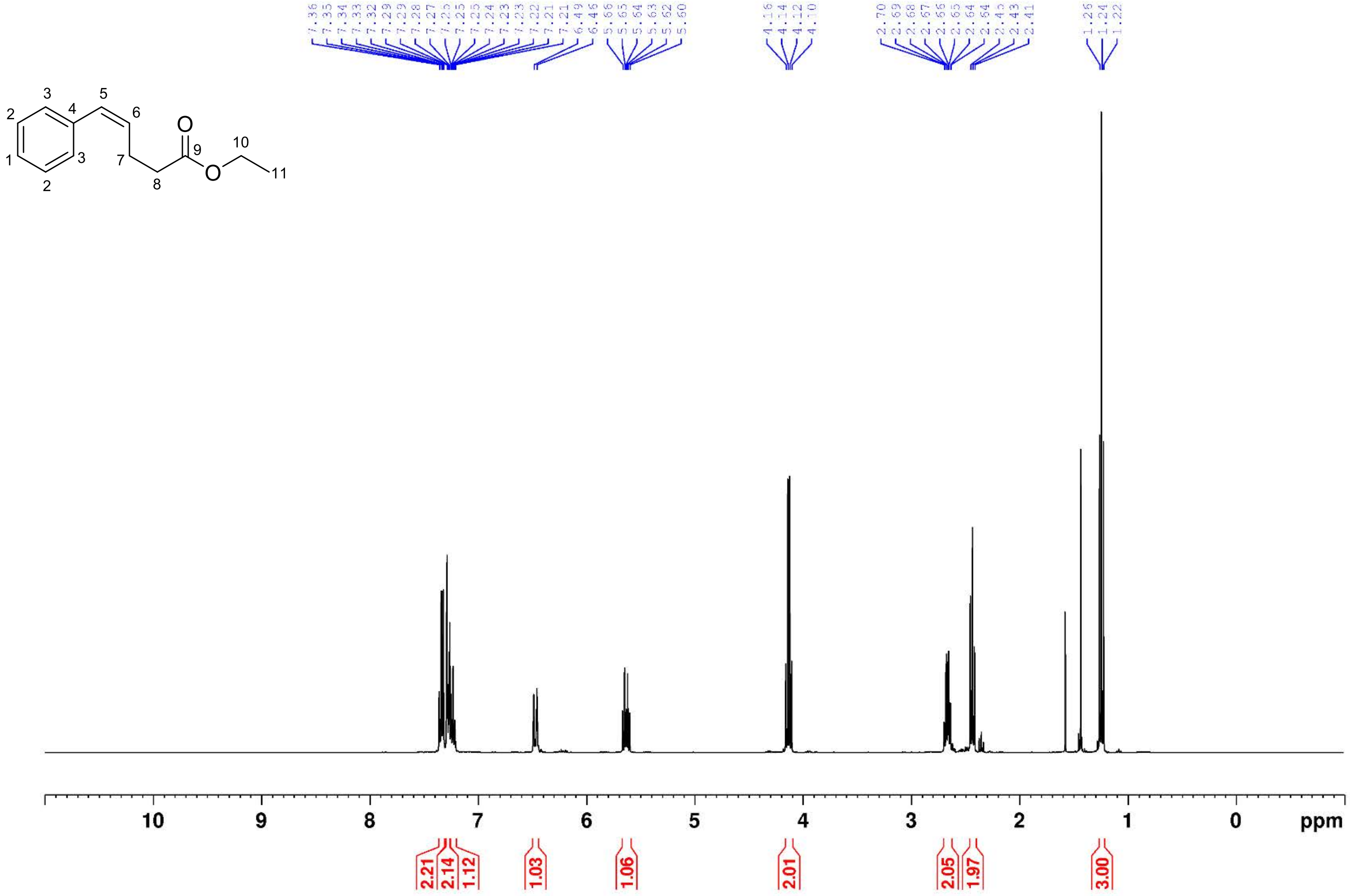

^{13}C NMR (101 MHz, CDCl$_3$) for *Ethyl (Z)-5-phenylpent-4-enoate*

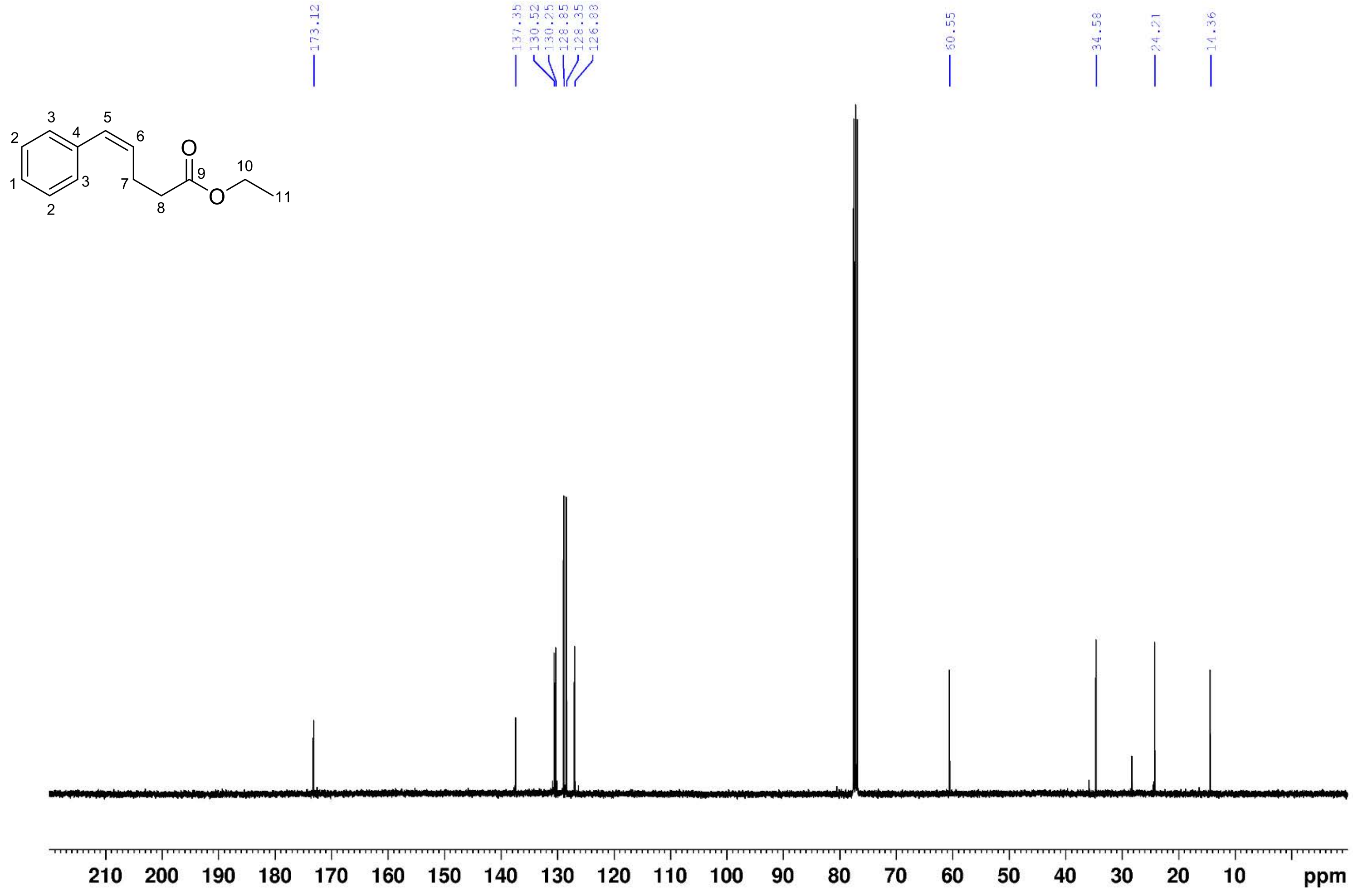

^{1}H NMR (400 MHz, $CDCl_3$) for *(Z)-5-phenylpent-4-enoic acid*

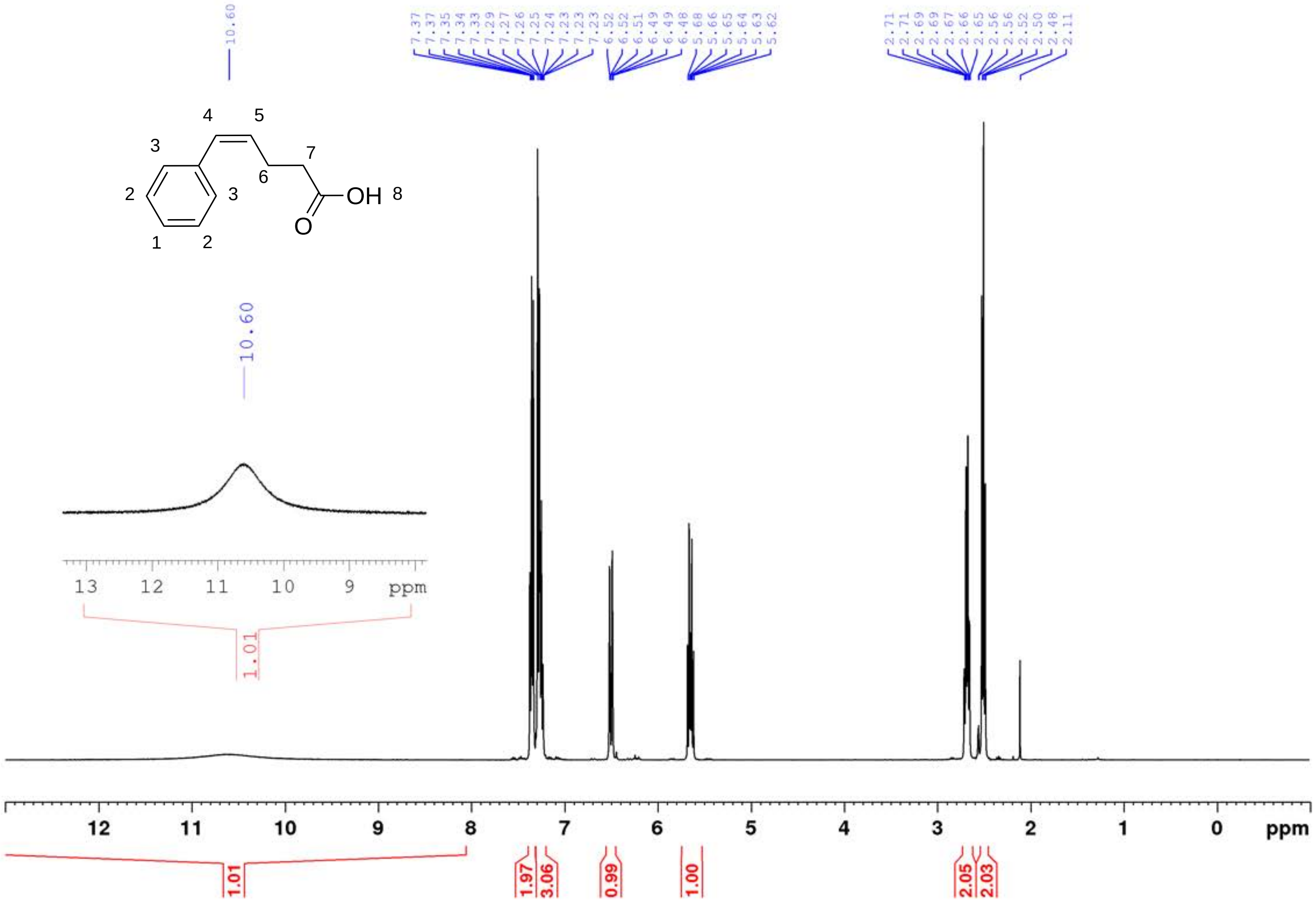

^{13}C NMR (101 MHz, $CDCl_3$) for *(Z)-5-phenylpent-4-enoic acid*

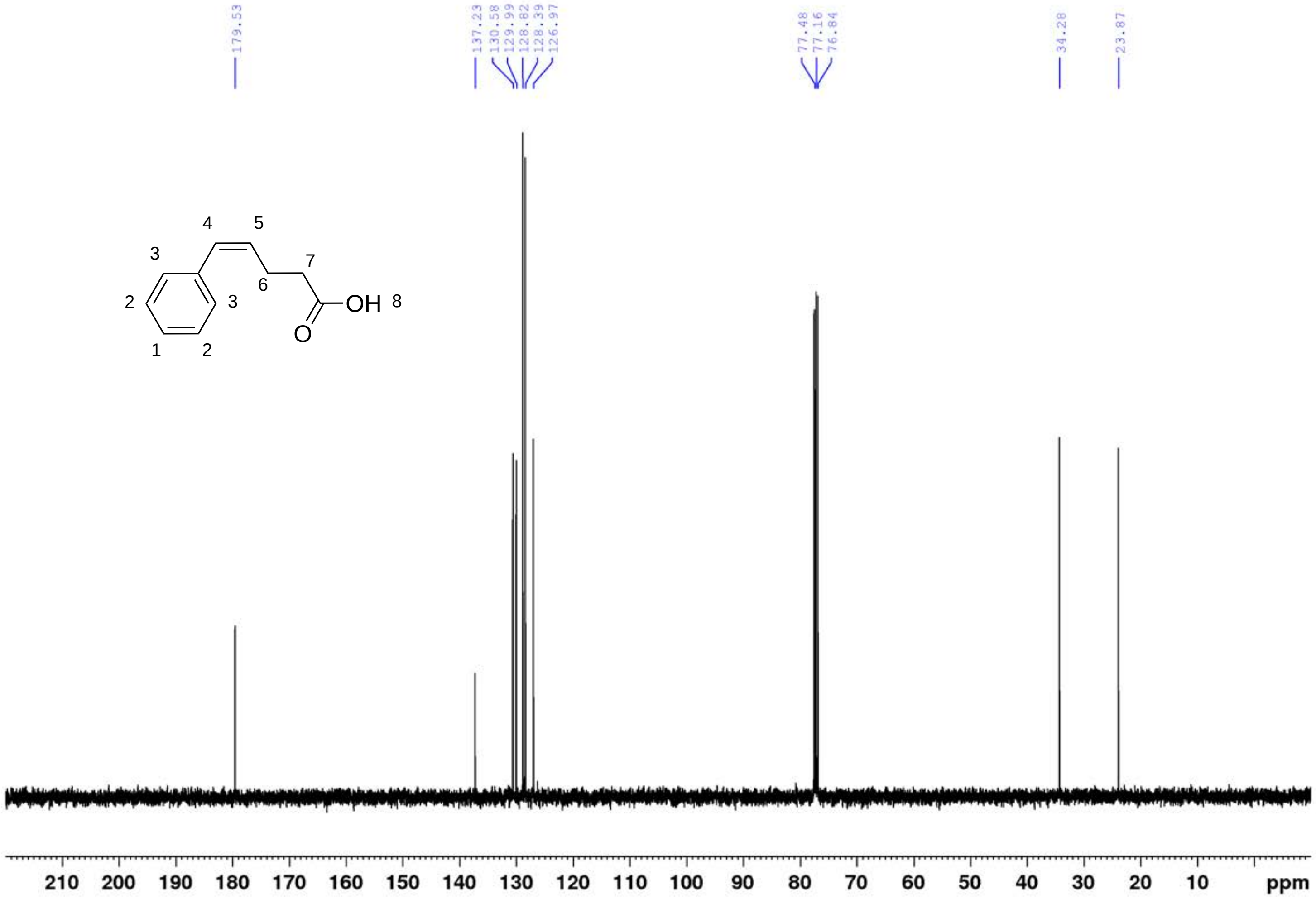

^{1}H NMR (400 MHz, $CDCl_3$) for *(Z)-5-phenylpent-4-en-1-yl 4-methylbenzenesulfonate*

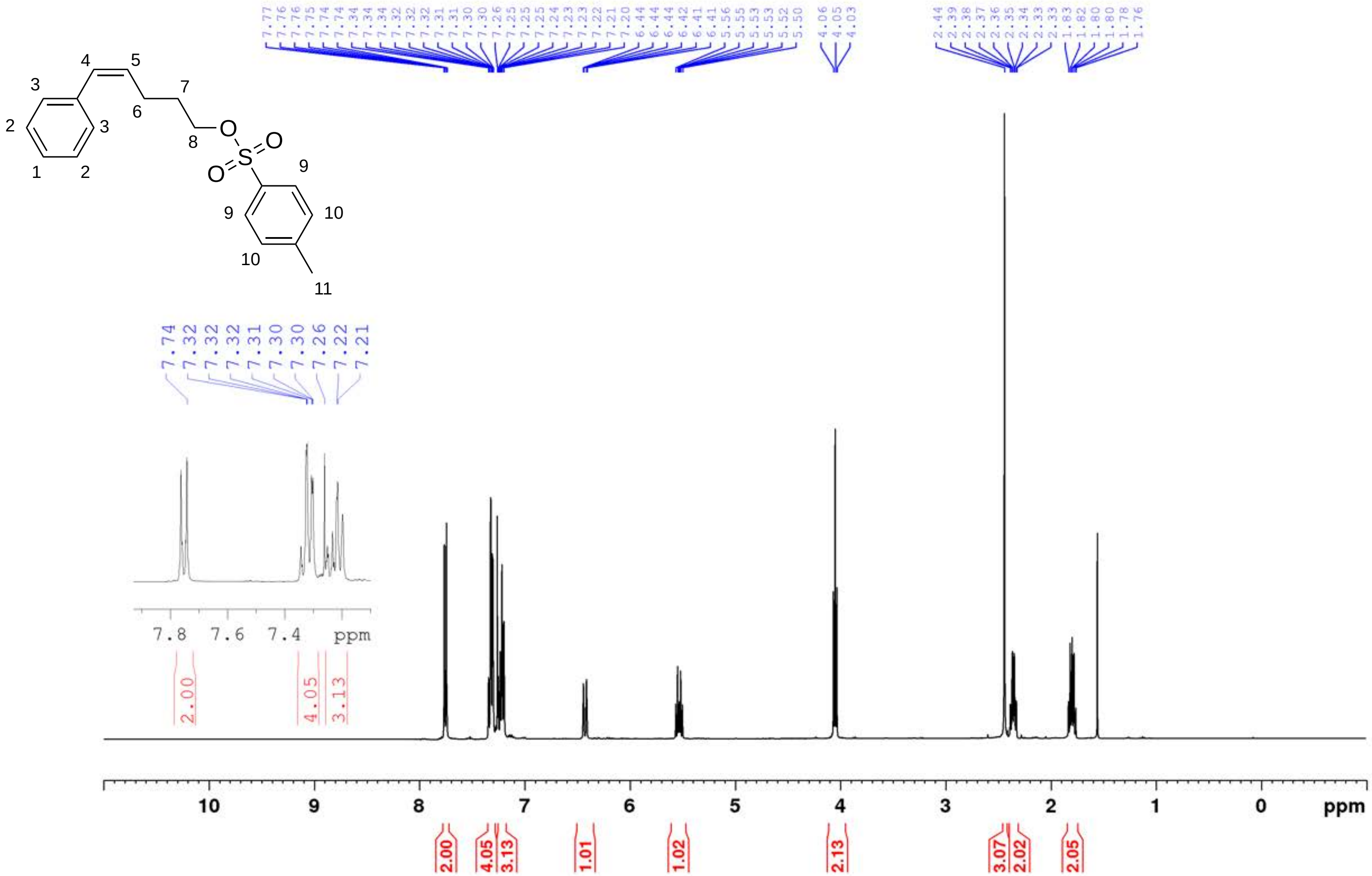

^{13}**C NMR (101 MHz, CDCl$_3$)** for *(Z)-5-phenylpent-4-en-1-yl 4-methylbenzenesulfonate*

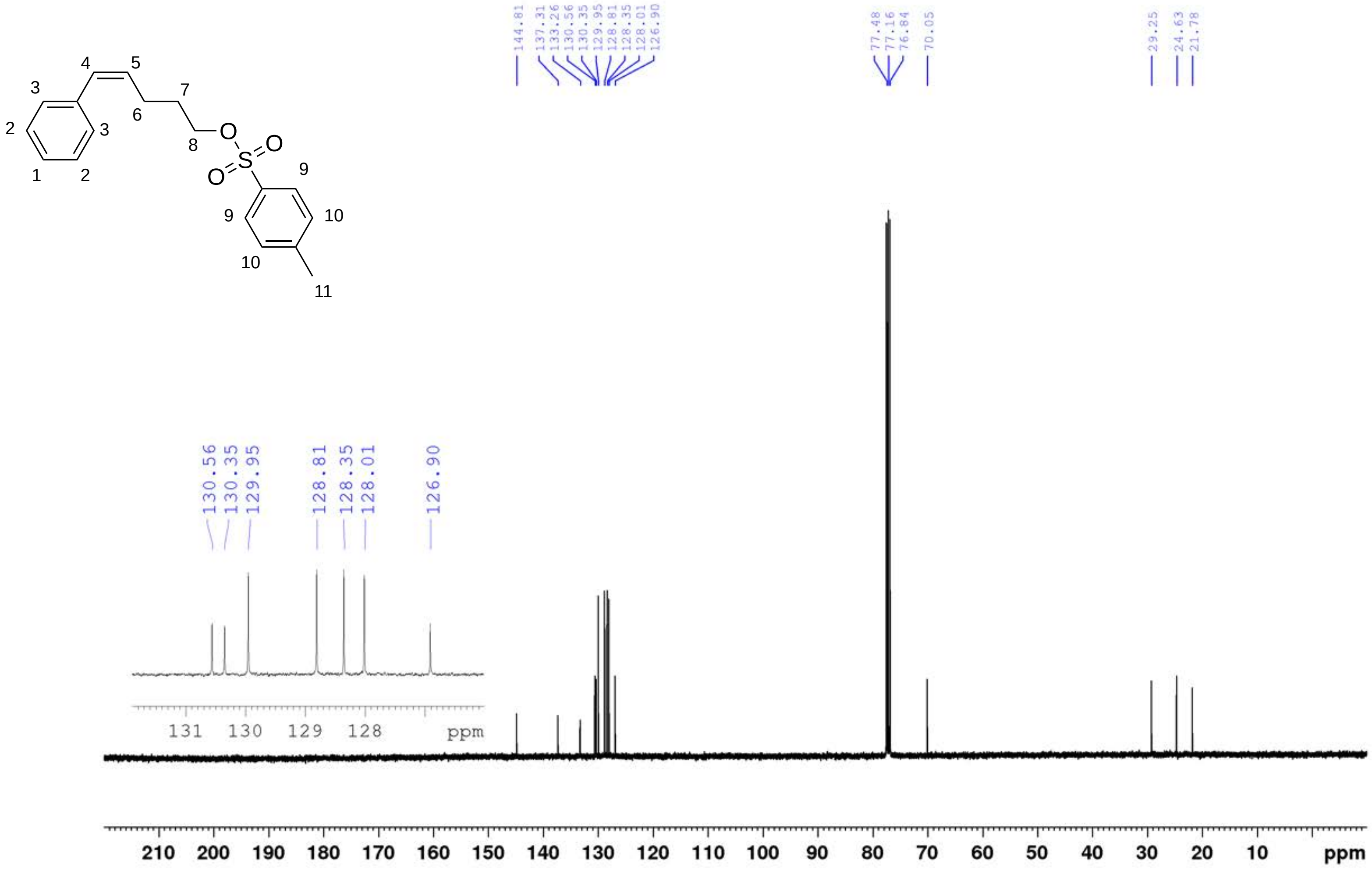

^{1}H NMR (400 MHz, $CDCl_3$) for *(Z)-2,2,2-trifluoro-N-(5-phenylpent-4-en-1-yl)acetamide*

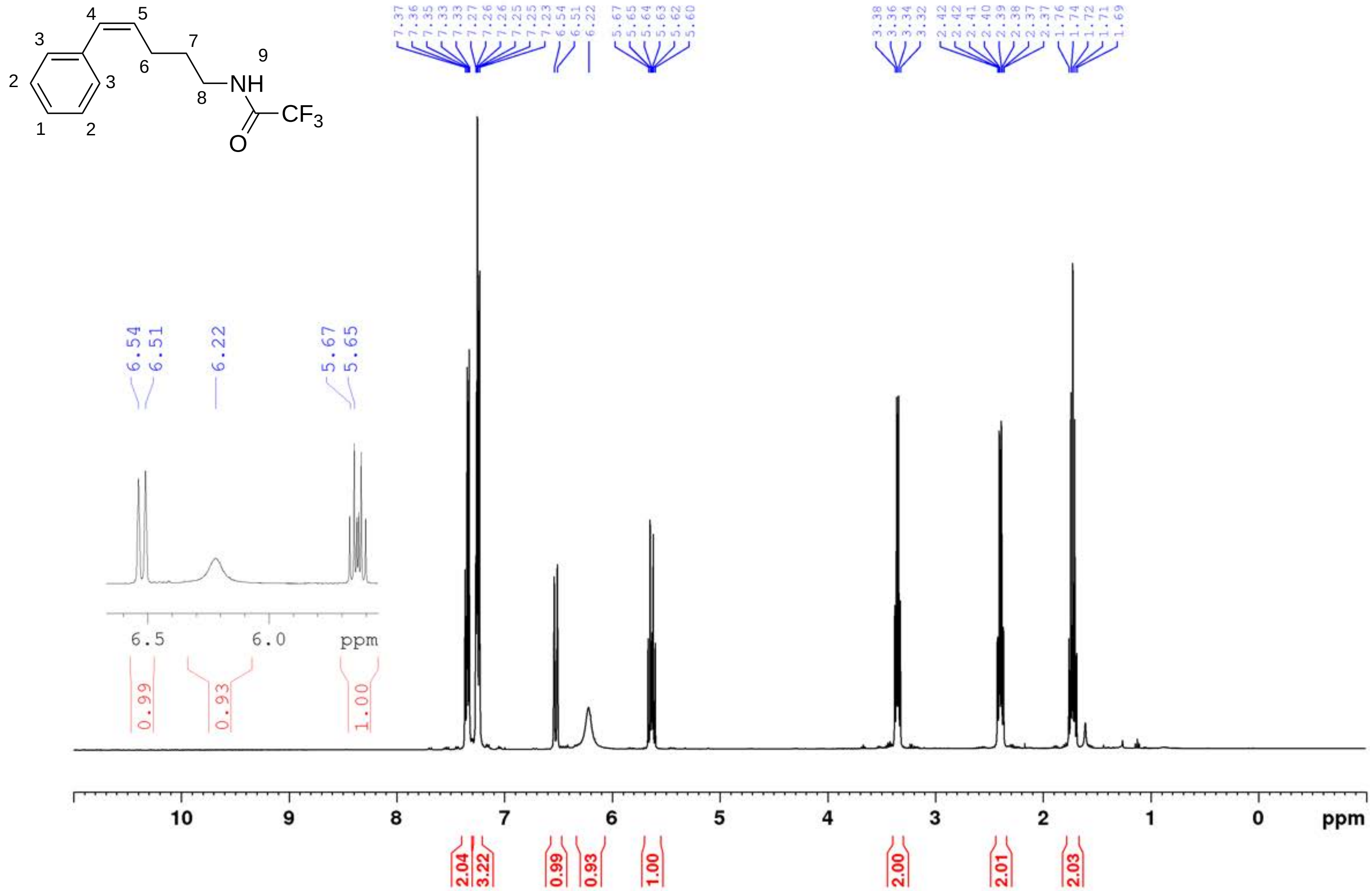

^{13}C NMR (101 MHz, CDCl$_3$) for *(Z)-2,2,2-trifluoro-N-(5-phenylpent-4-en-1-yl)acetamide*

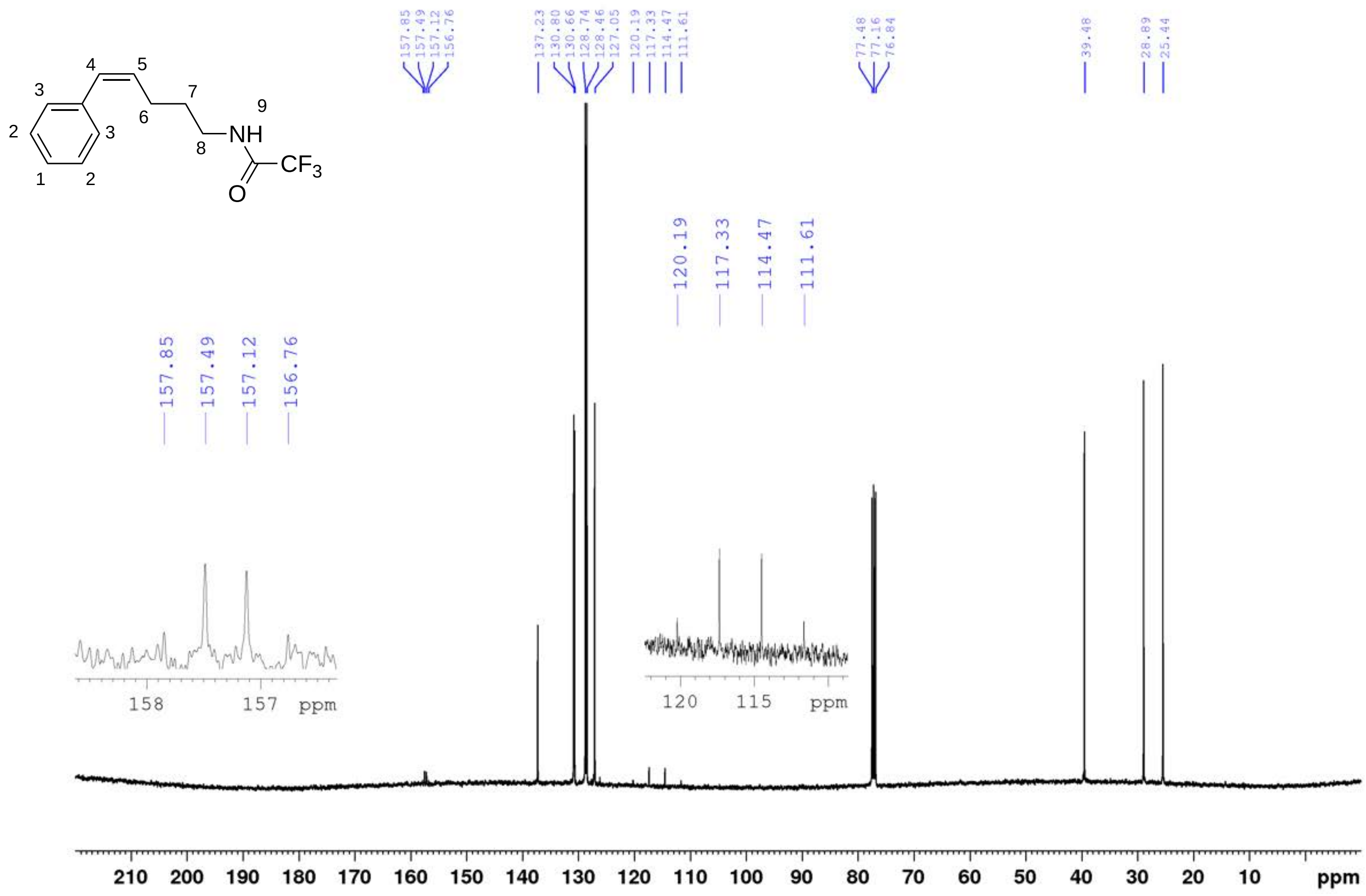

^{19}F NMR (376 MHz, CDCl$_3$) for *(Z)-2,2,2-trifluoro-N-(5-phenylpent-4-en-1-yl)acetamide*

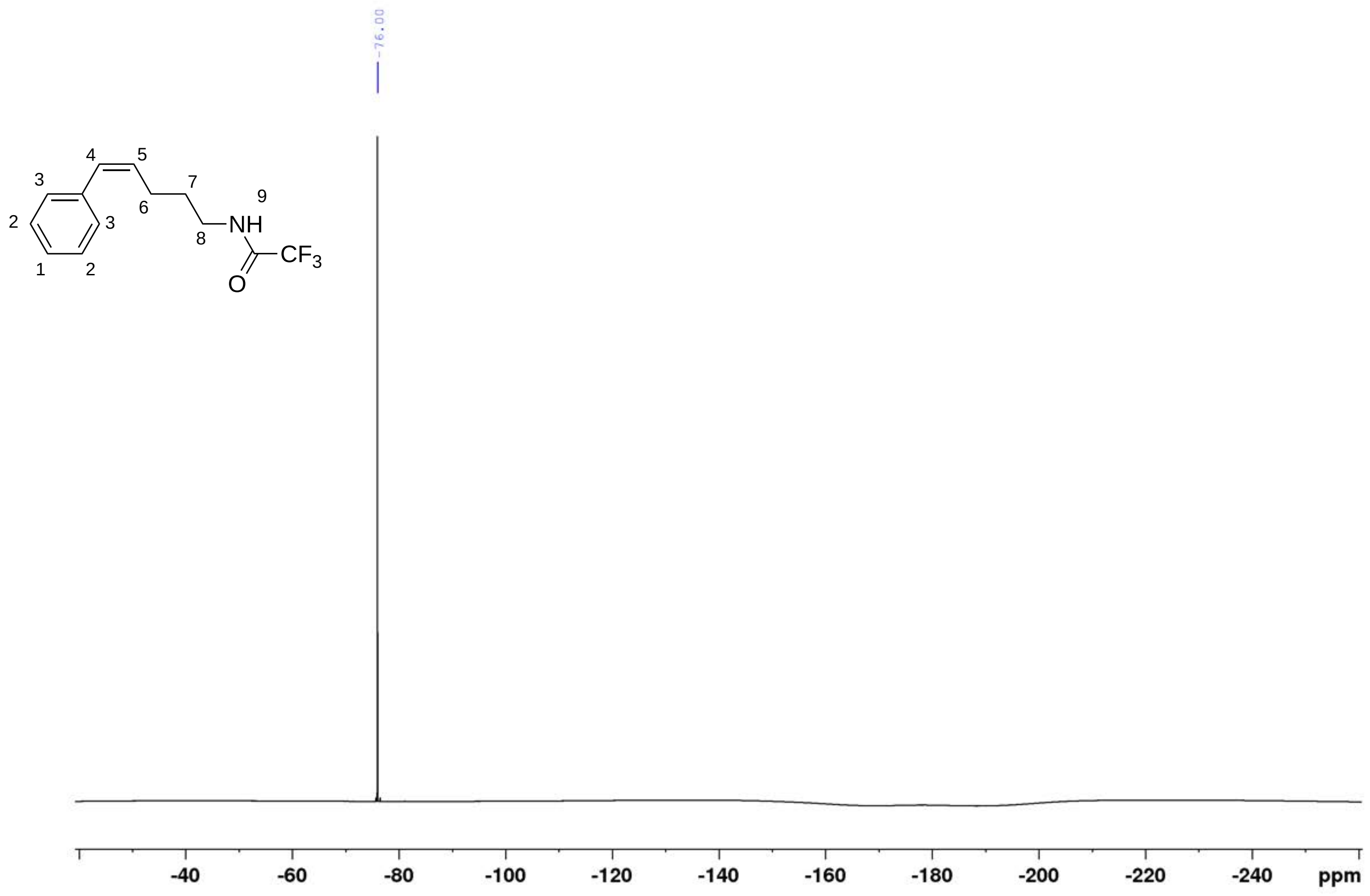

^{1}H NMR (400 MHz, CDCl$_3$) for *(Z)-4-methyl-N-(5-phenylpent-4-en-1-yl)benzenesulfonamide*

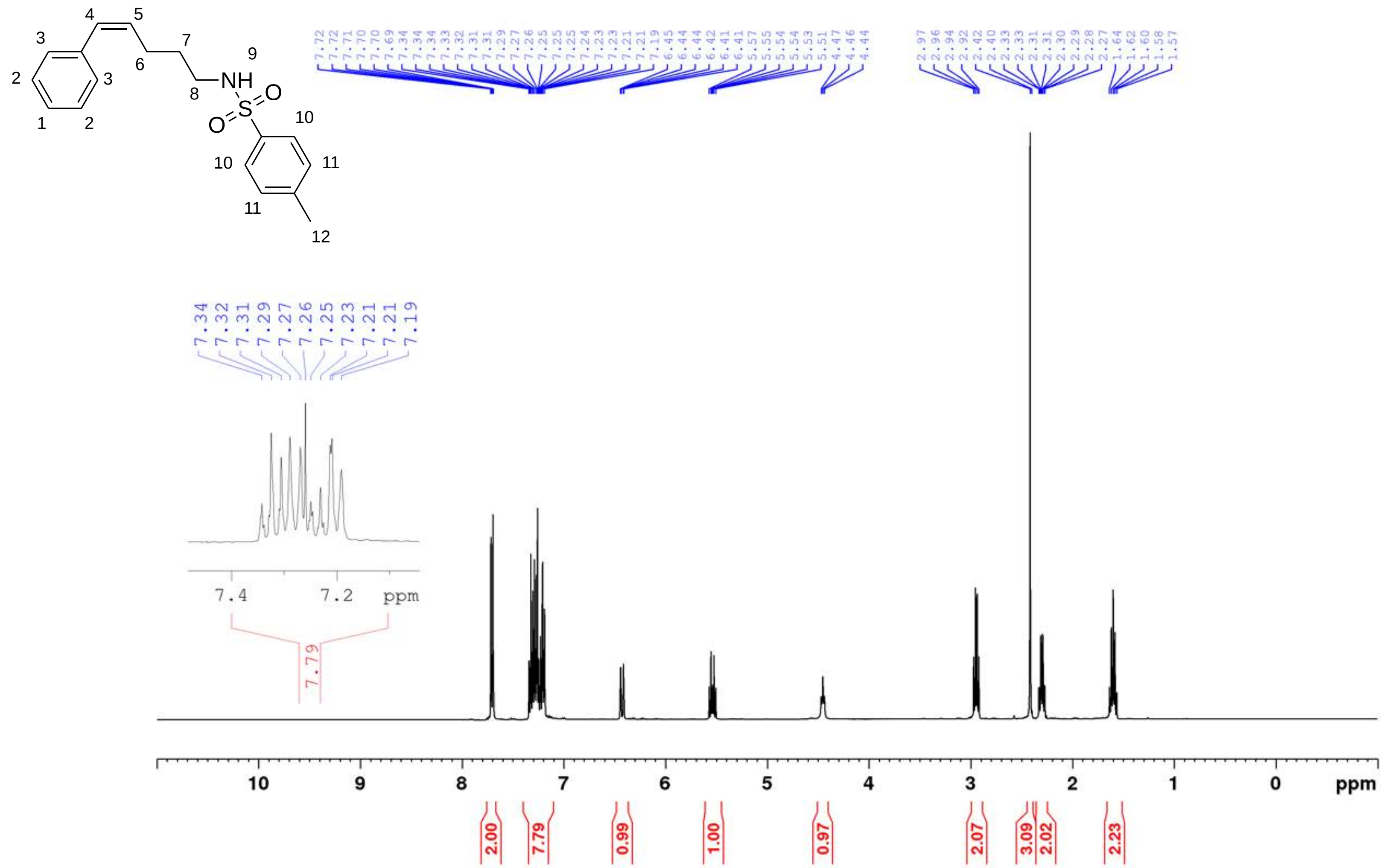

^{13}C NMR (101 MHz, $CDCl_3$) for *(Z)-4-methyl-N-(5-phenylpent-4-en-1-yl)benzenesulfonamide*

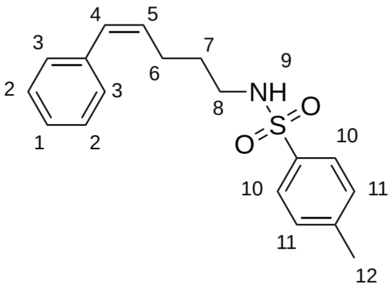

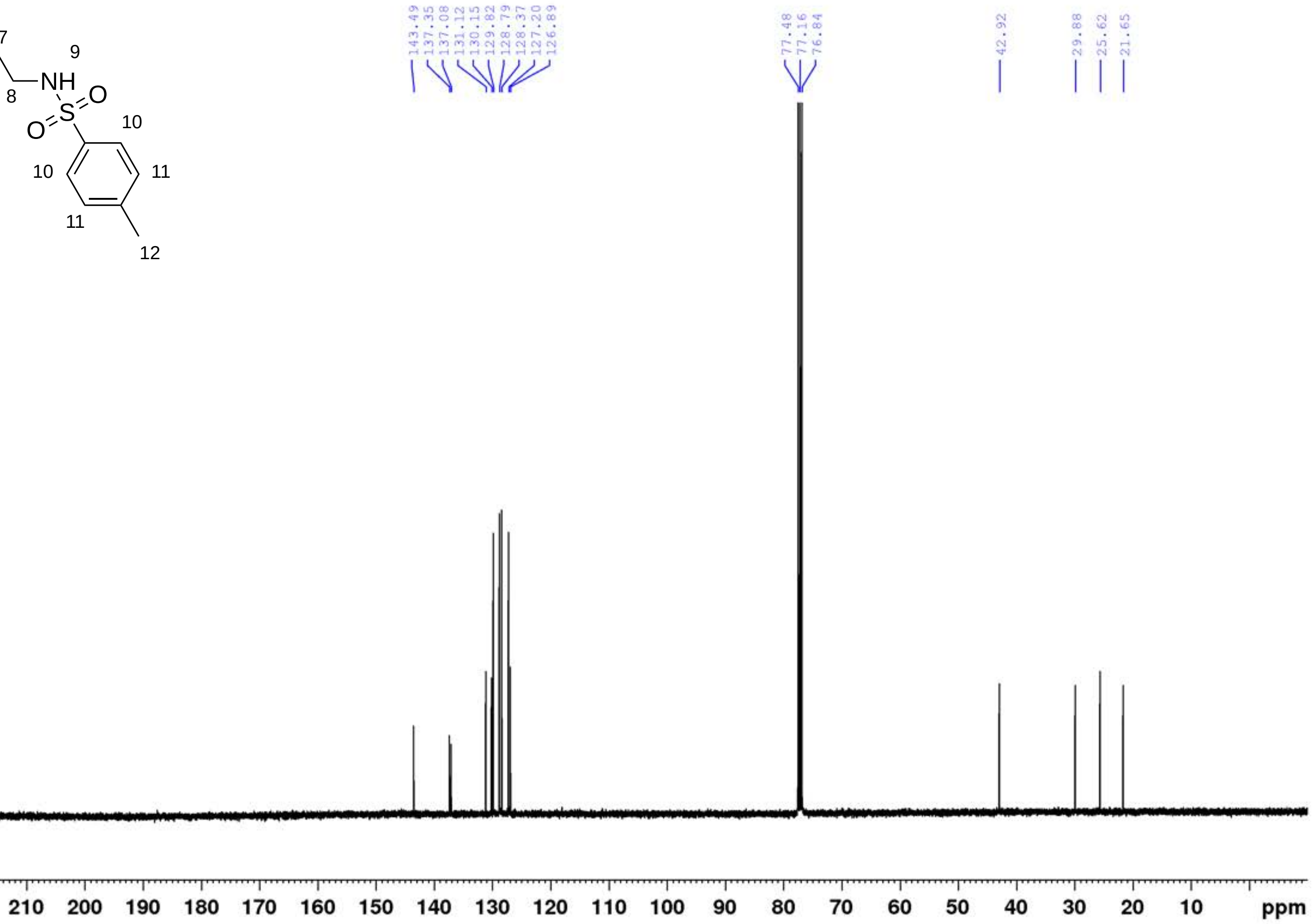

^{1}H NMR (500 MHz, $CDCl_3$) for *3-phenylprop-2-yn-1-ol*

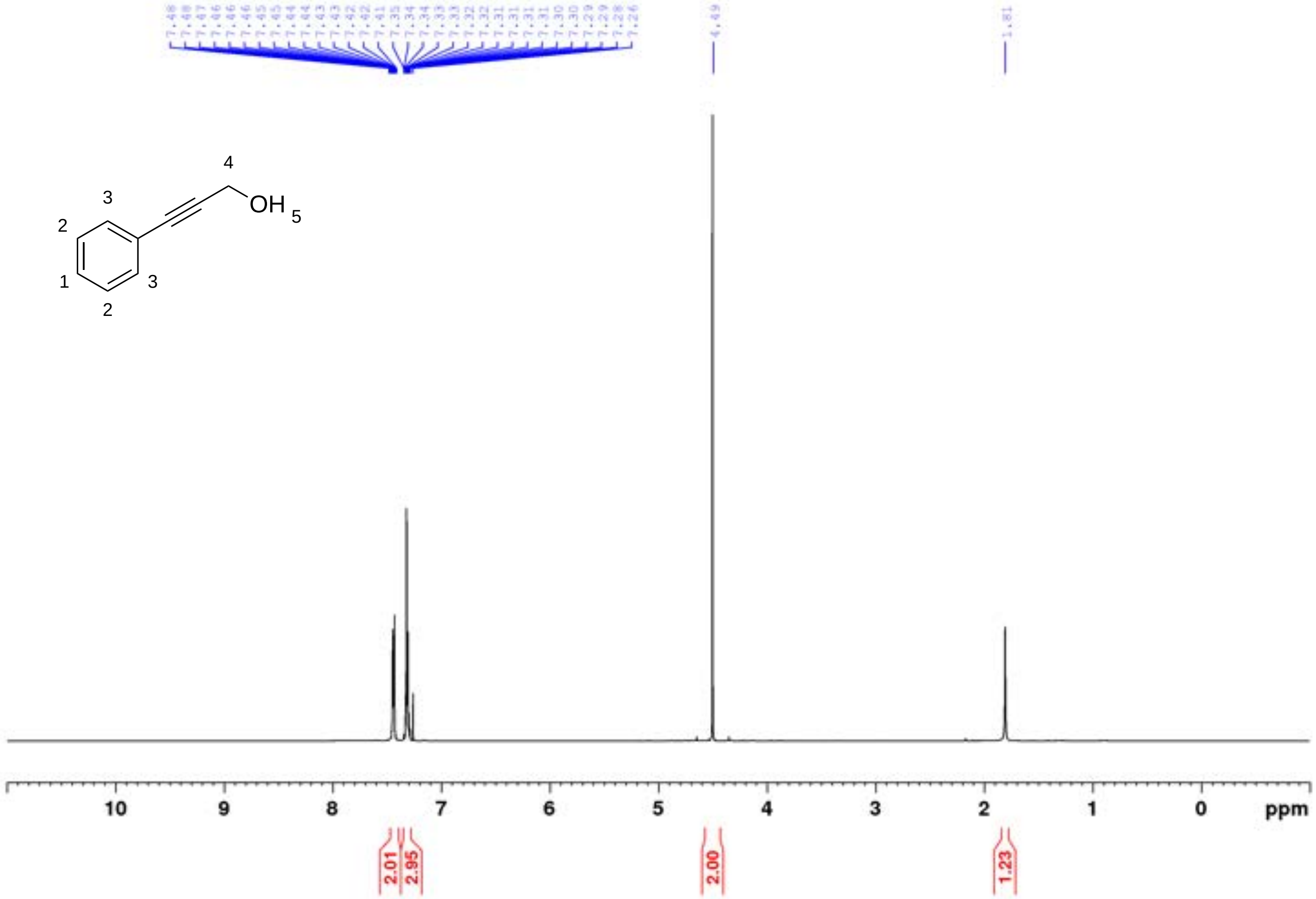

^{13}C NMR (126 MHz, $CDCl_3$) for *3-phenylprop-2-yn-1-ol*

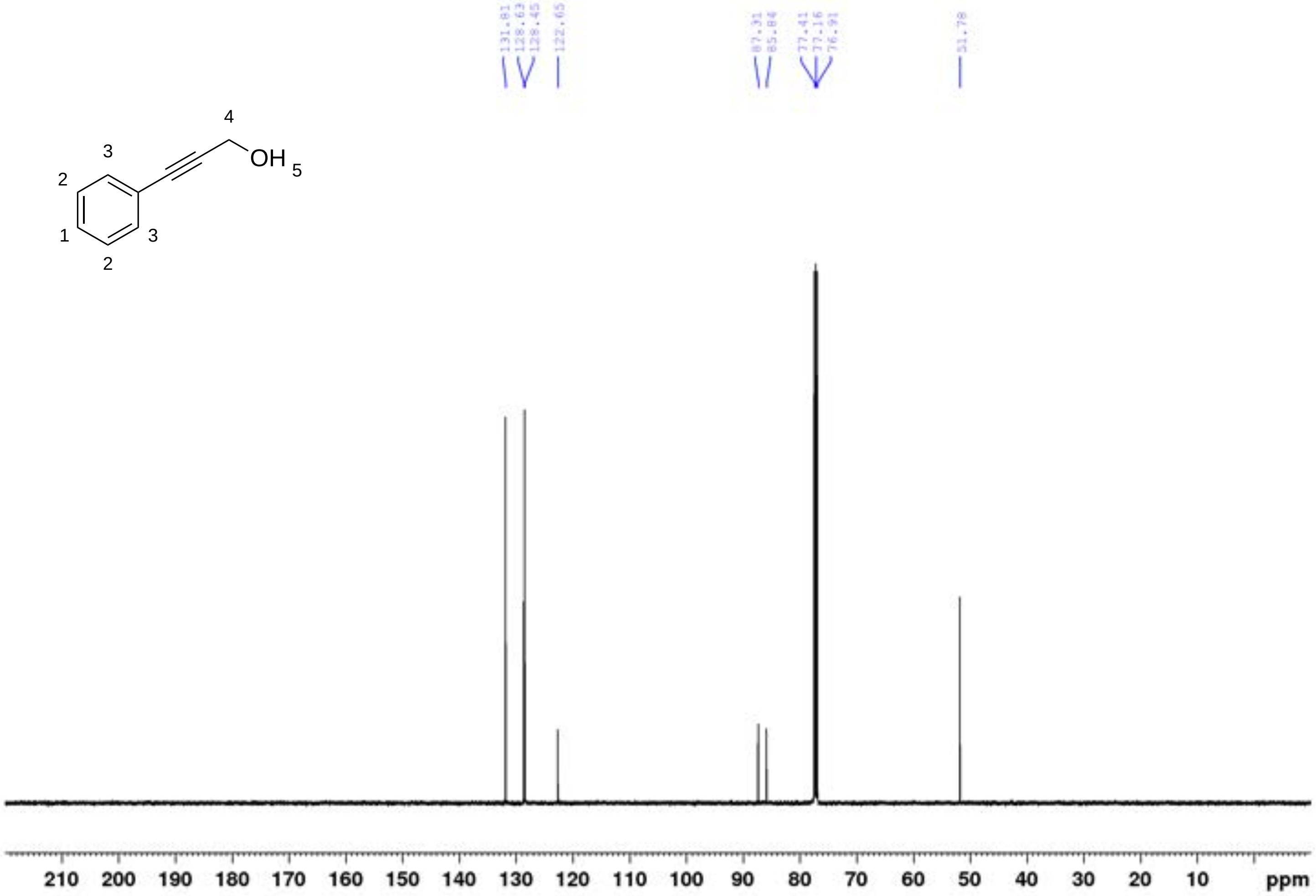

^{1}H NMR (400 MHz, $CDCl_3$) for *(Z)-3-phenylprop-2-en-1-ol*

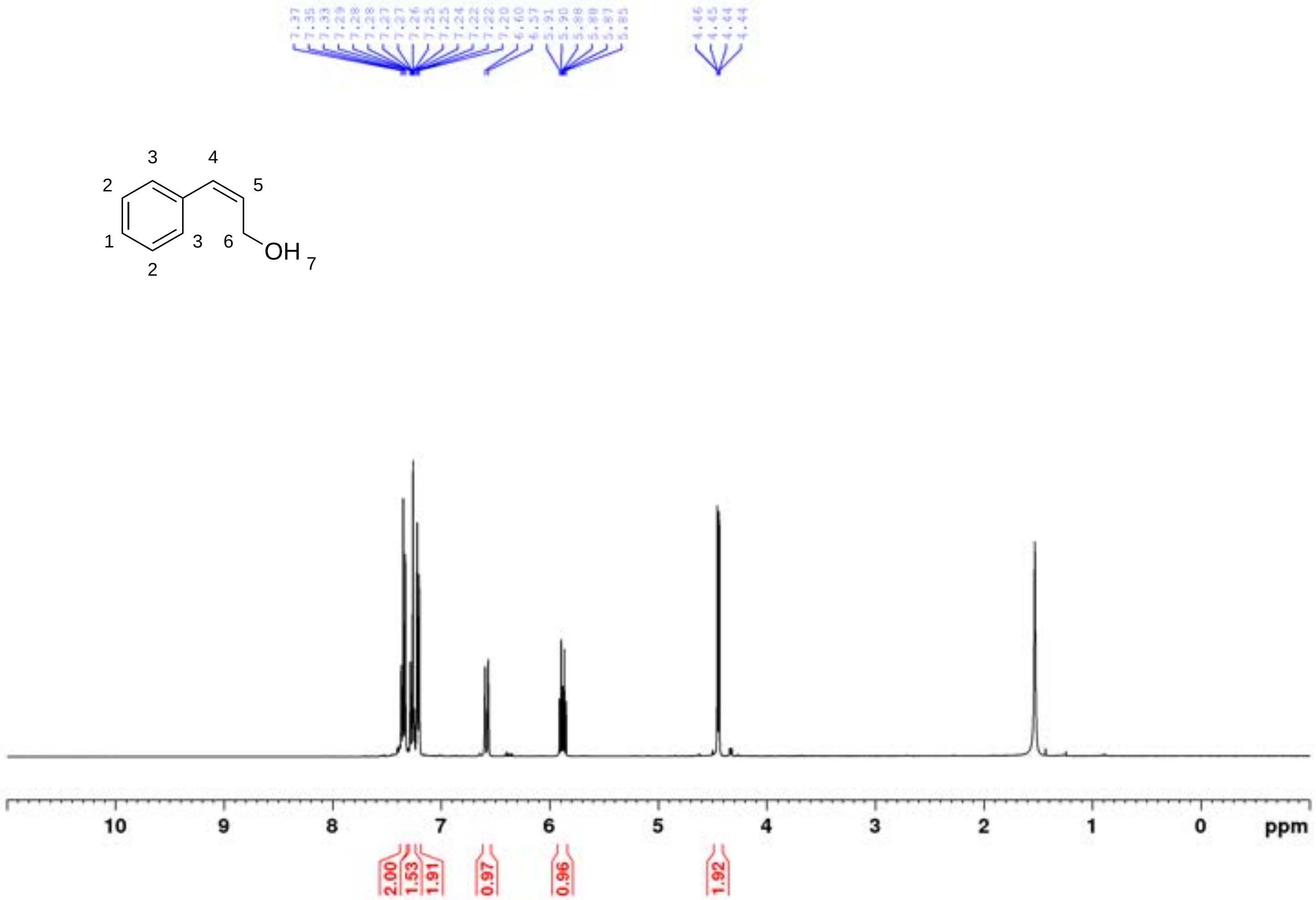

13C NMR (101 MHz, $CDCl_3$) for *(Z)-3-phenylprop-2-en-1-ol*

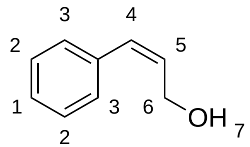

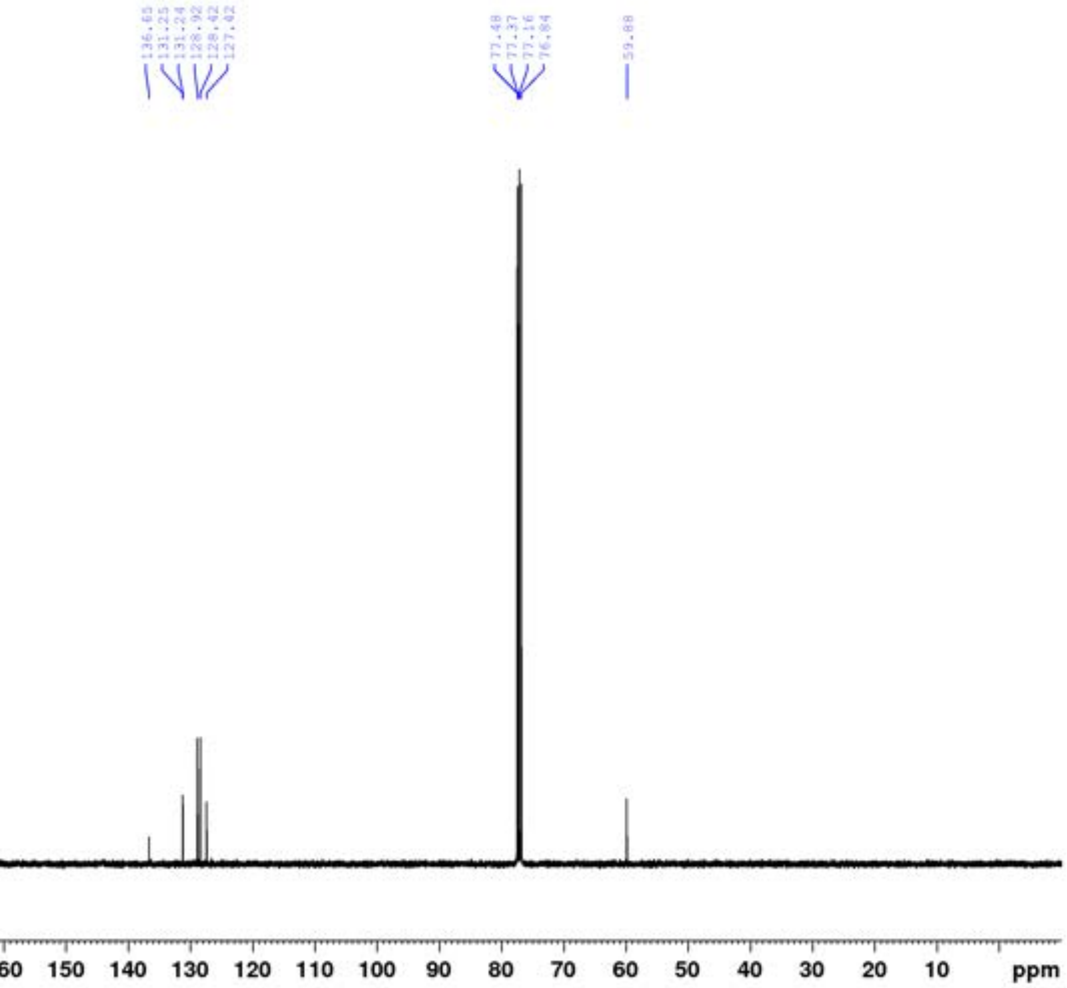

^{1}H NMR (400 MHz, $CDCl_3$) for *7-phenylhept-6-yn-1-ol*

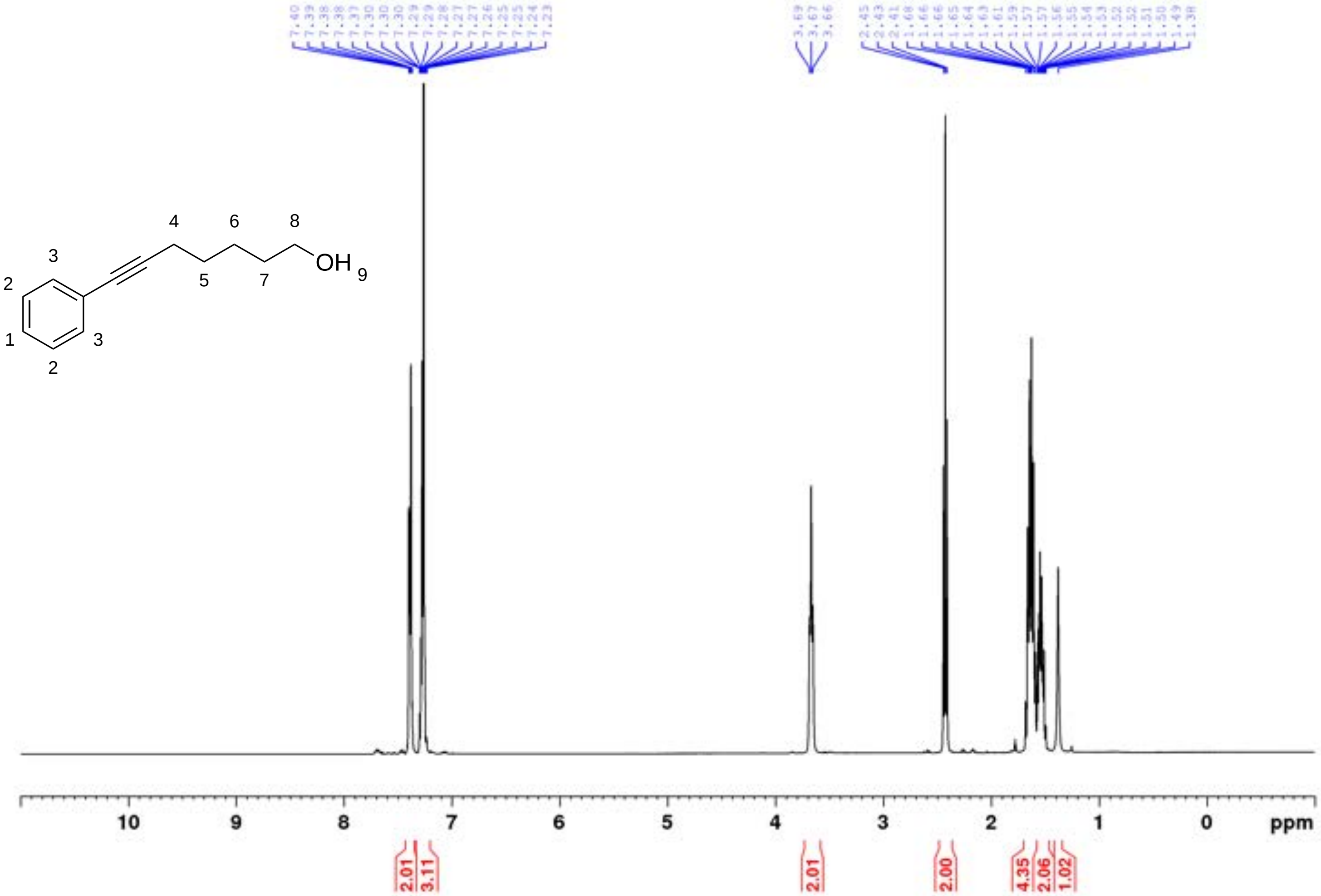

^{13}**C NMR** (101 MHz, $CDCl_3$) for *7-phenylhept-6-yn-1-ol*

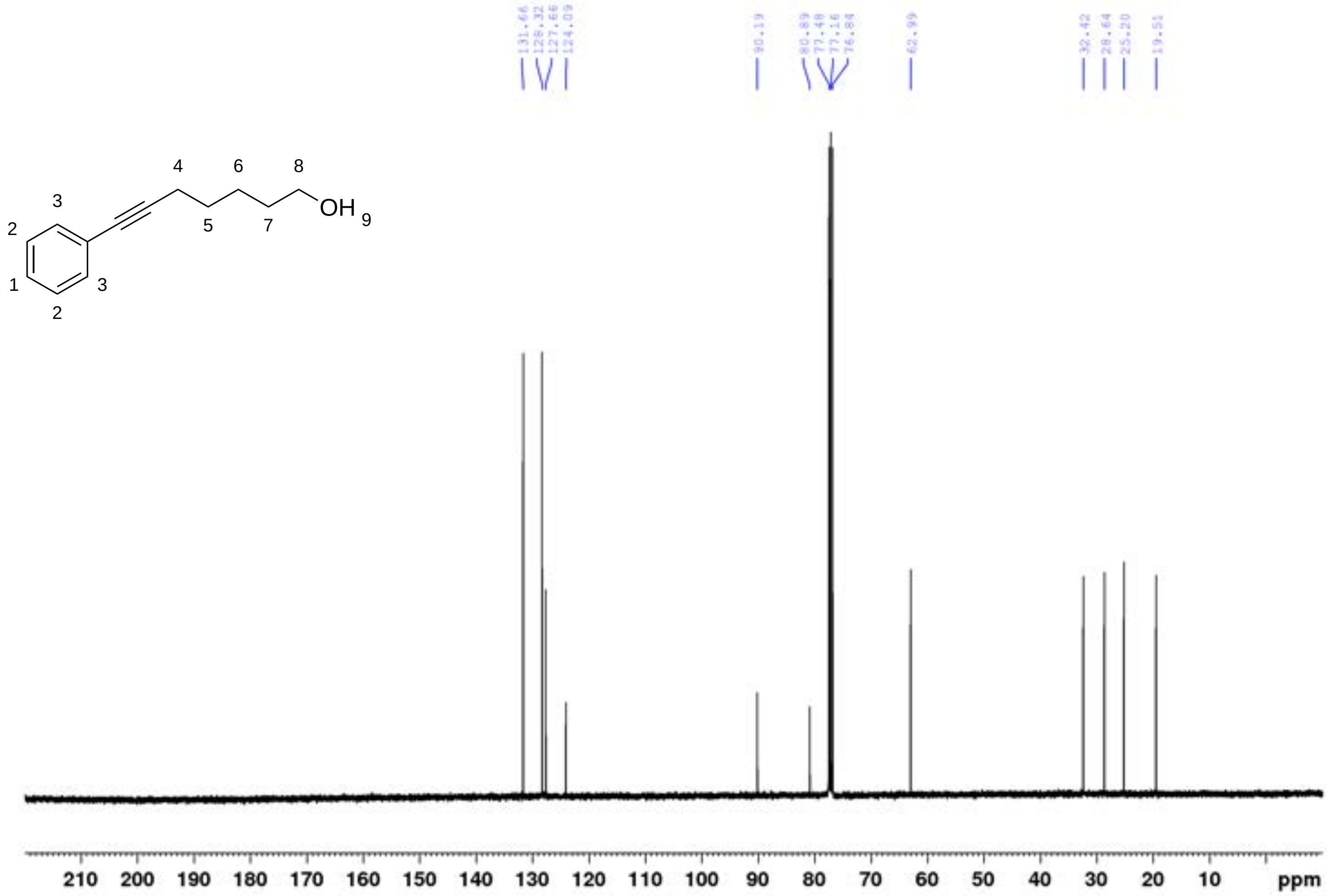

^{1}H NMR (400 MHz, $CDCl_3$) for *(Z)-7-phenylhept-6-en-1-ol*

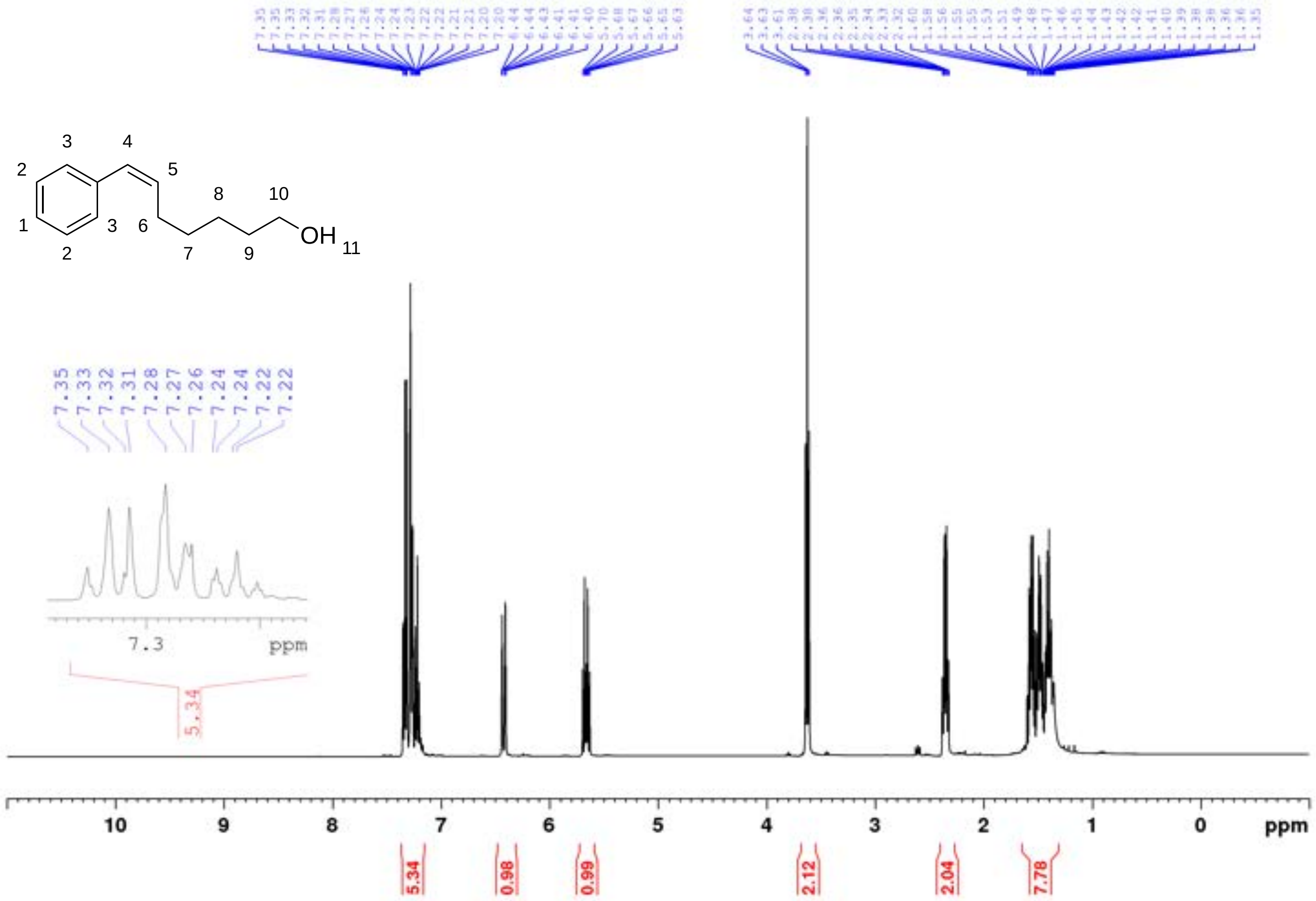

^{13}C NMR (101 MHz, $CDCl_3$) for *(Z)-7-phenylhept-6-en-1-ol*

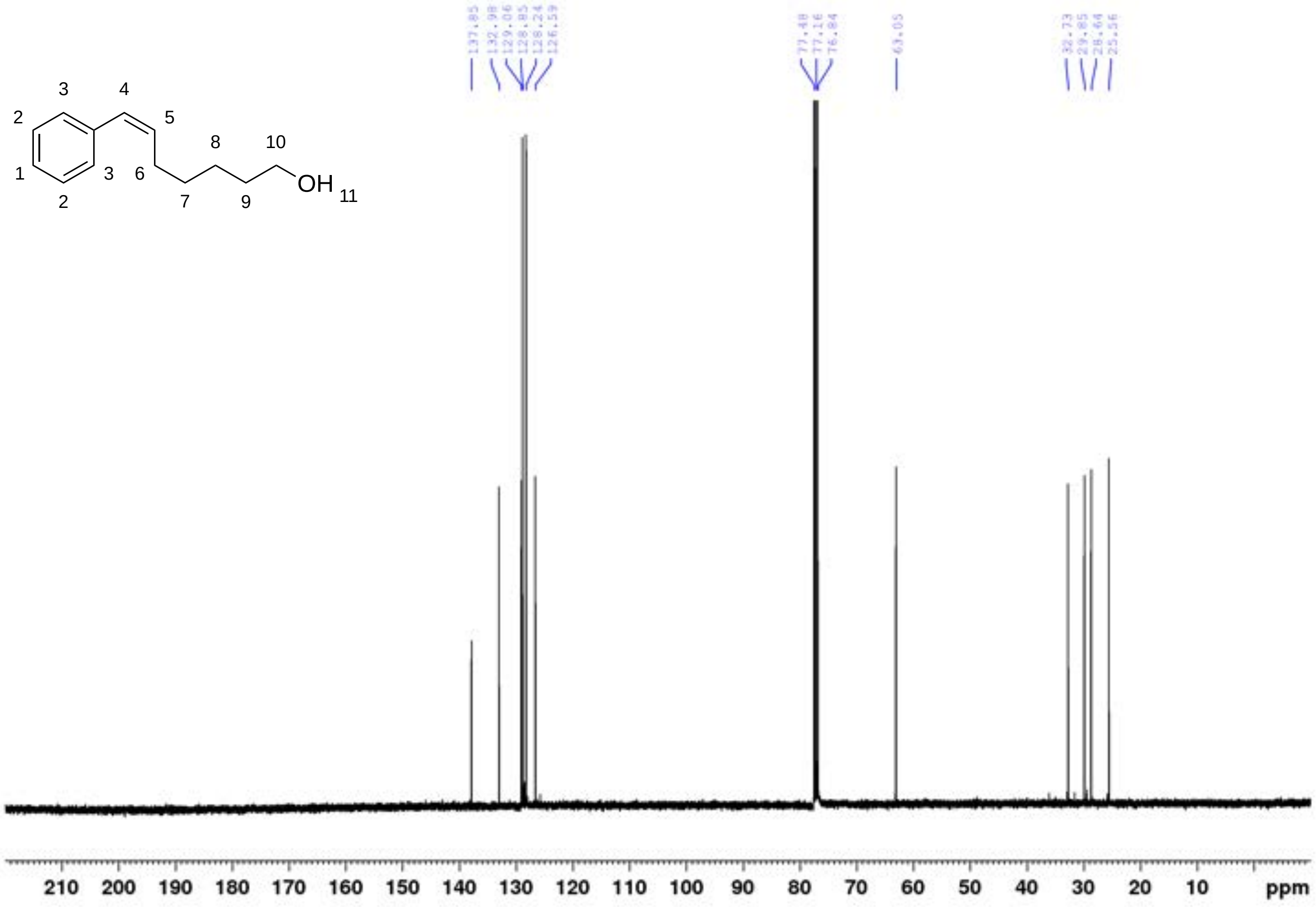

[1]**H NMR** (400 MHz, $CDCl_3$) for *(E)-pent-3-en-1-ol* (**23a**)

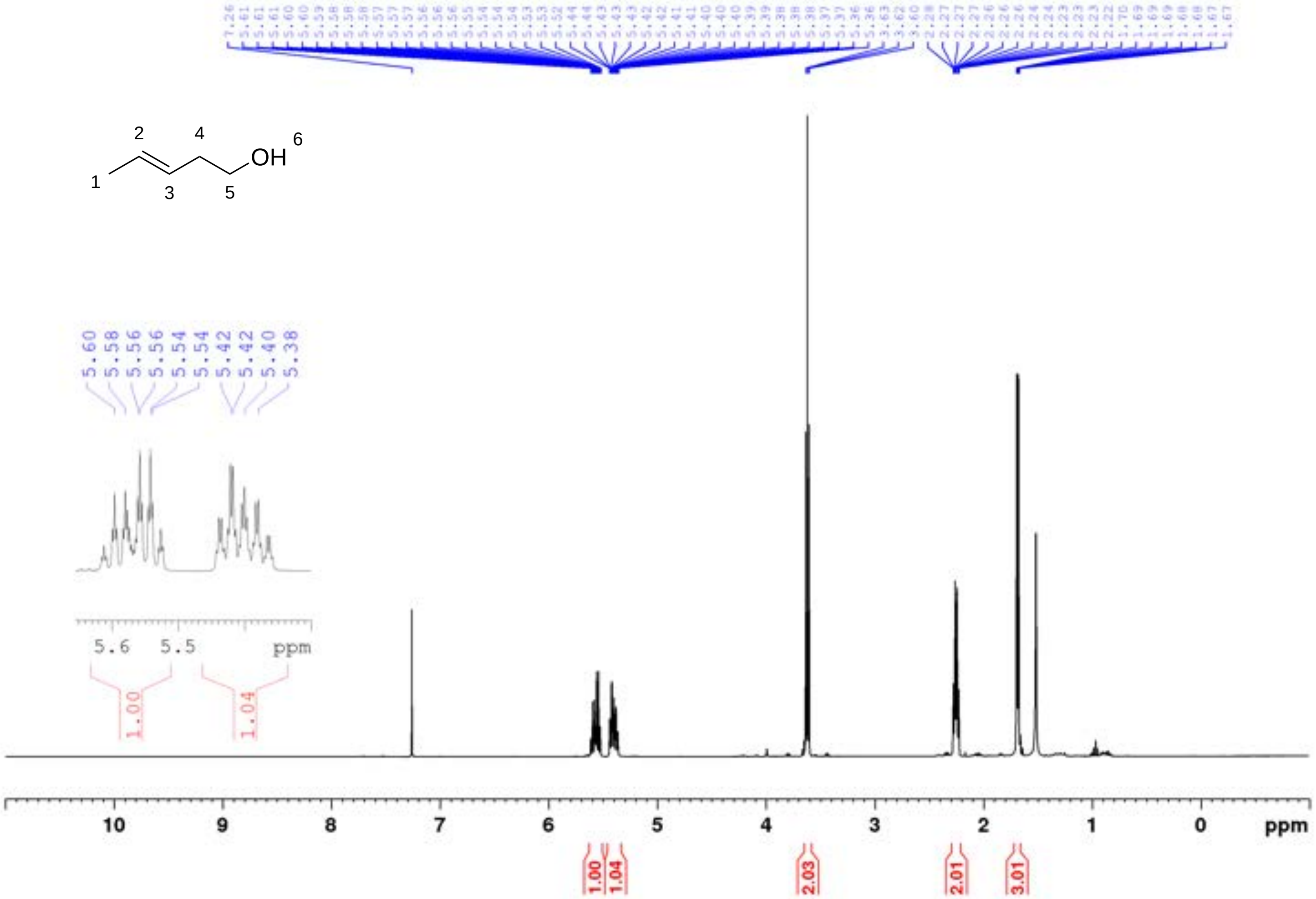

^{13}C NMR (101 MHz, $CDCl_3$) for *(E)-pent-3-en-1-ol* (**23a**)

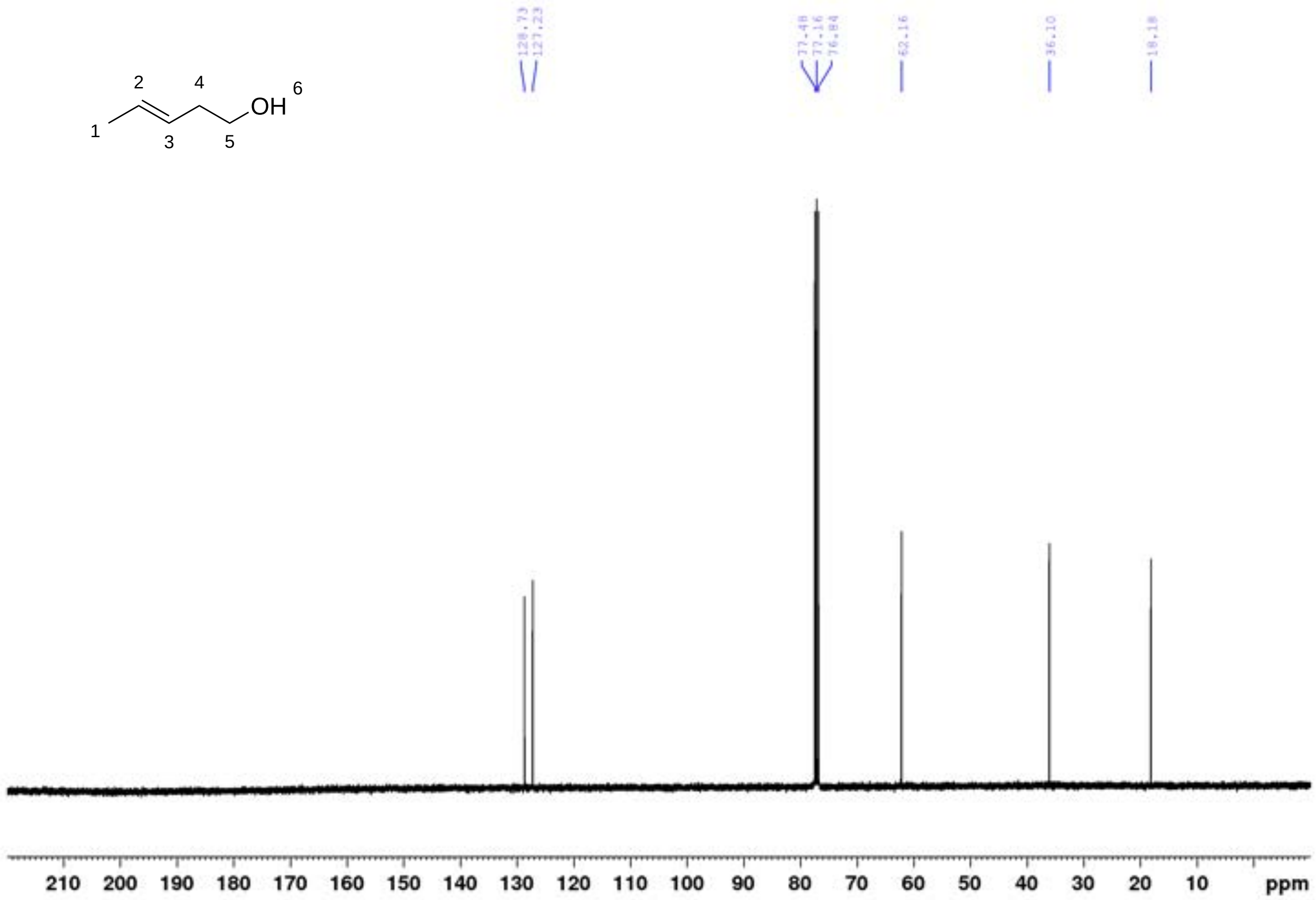

¹H NMR (500 MHz, $CDCl_3$) for *4-methylpent-3-en-1-ol* (**23d**)

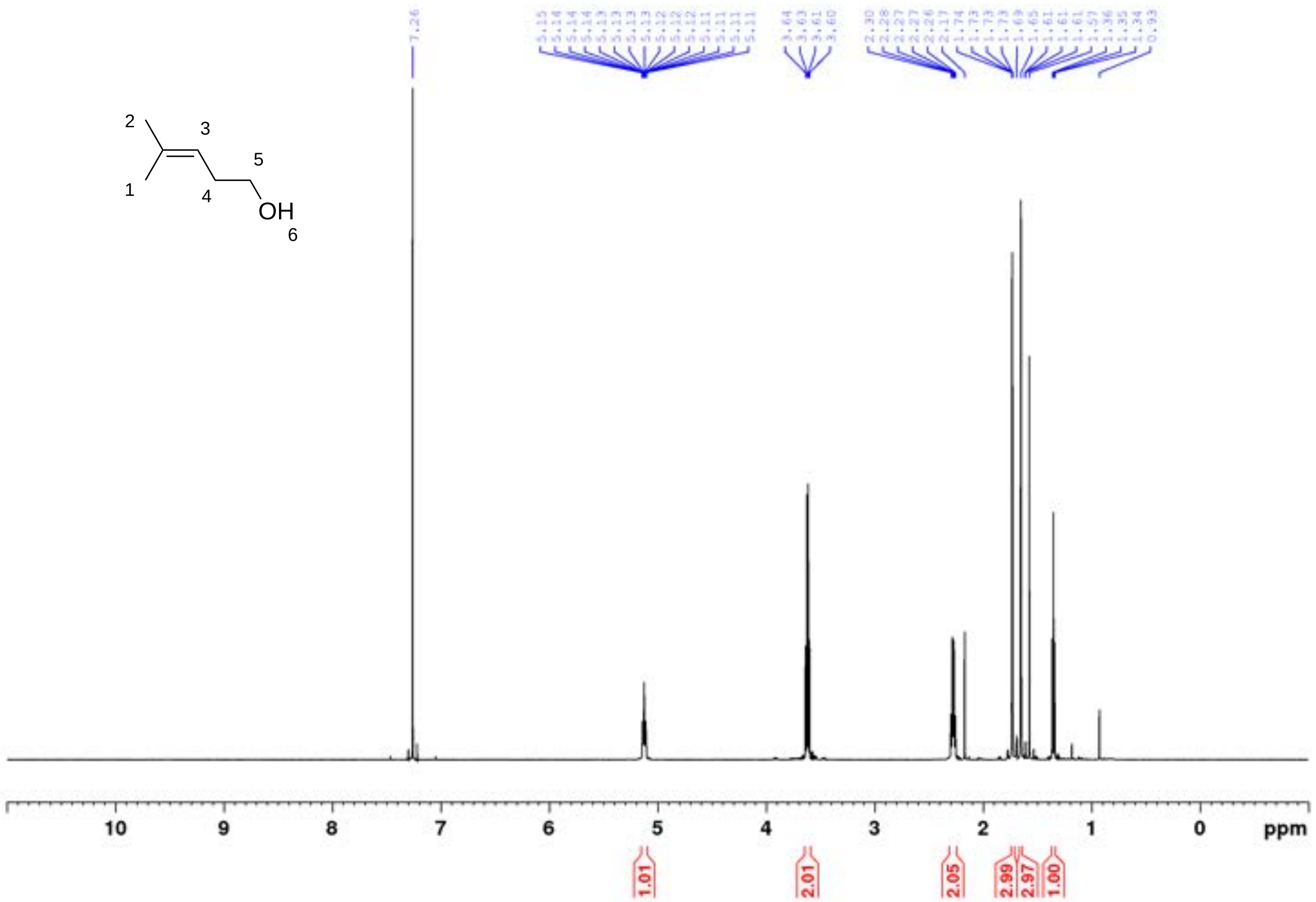

^{13}C NMR (126 MHz, $CDCl_3$) for *4-methylpent-3-en-1-ol* (**23d**)

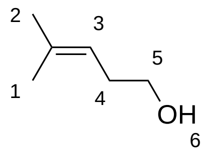

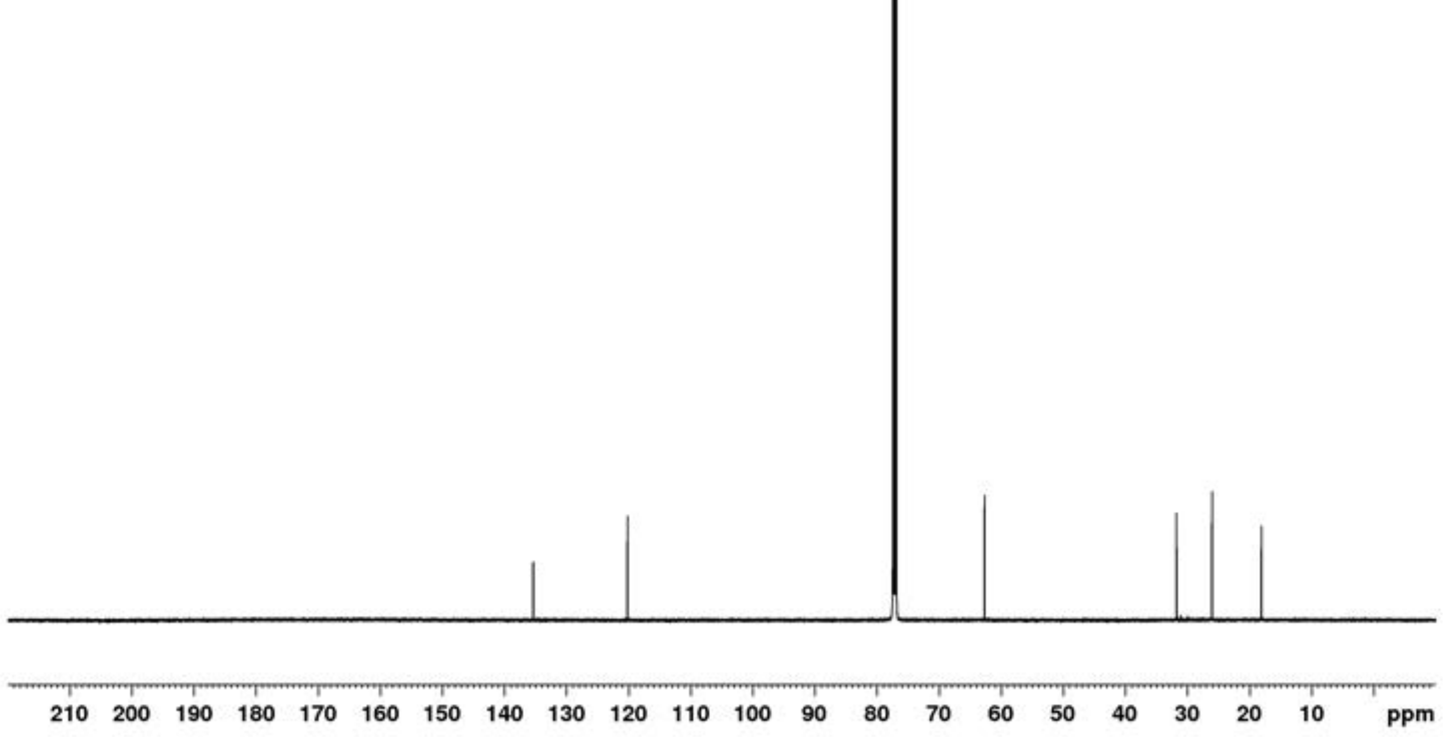

^{1}H NMR (400 MHz, $CDCl_3$) for *(Z)-3-methylpent-3-en-1-ol* (**23e**)

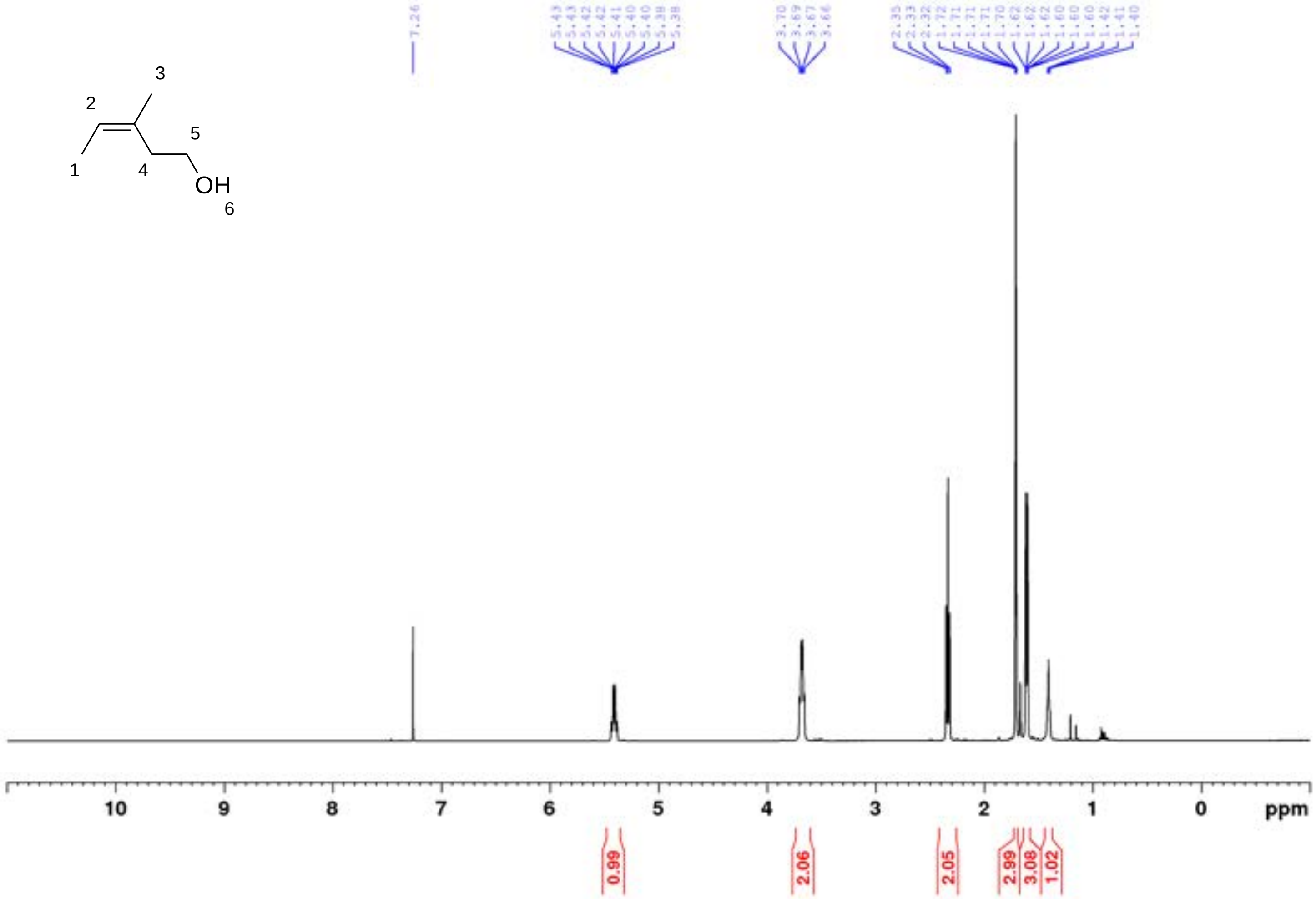

^{13}C NMR (101 MHz, $CDCl_3$) for *(Z)-3-methylpent-3-en-1-ol* (**23e**)

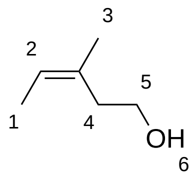

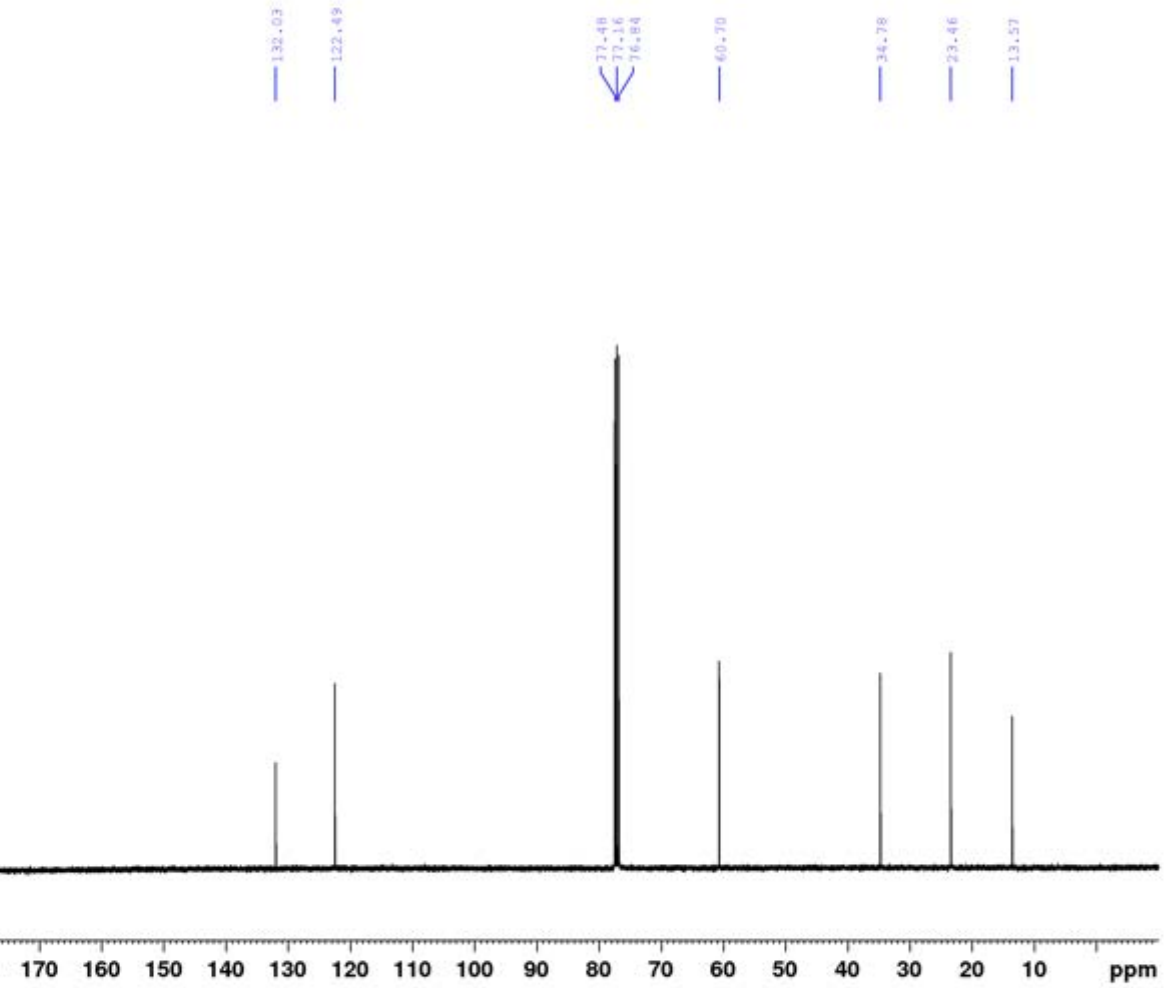

^{1}H NMR (400 MHz, $CDCl_3$) for *(E)-3-methylpent-3-en-1-ol* (**23f**)

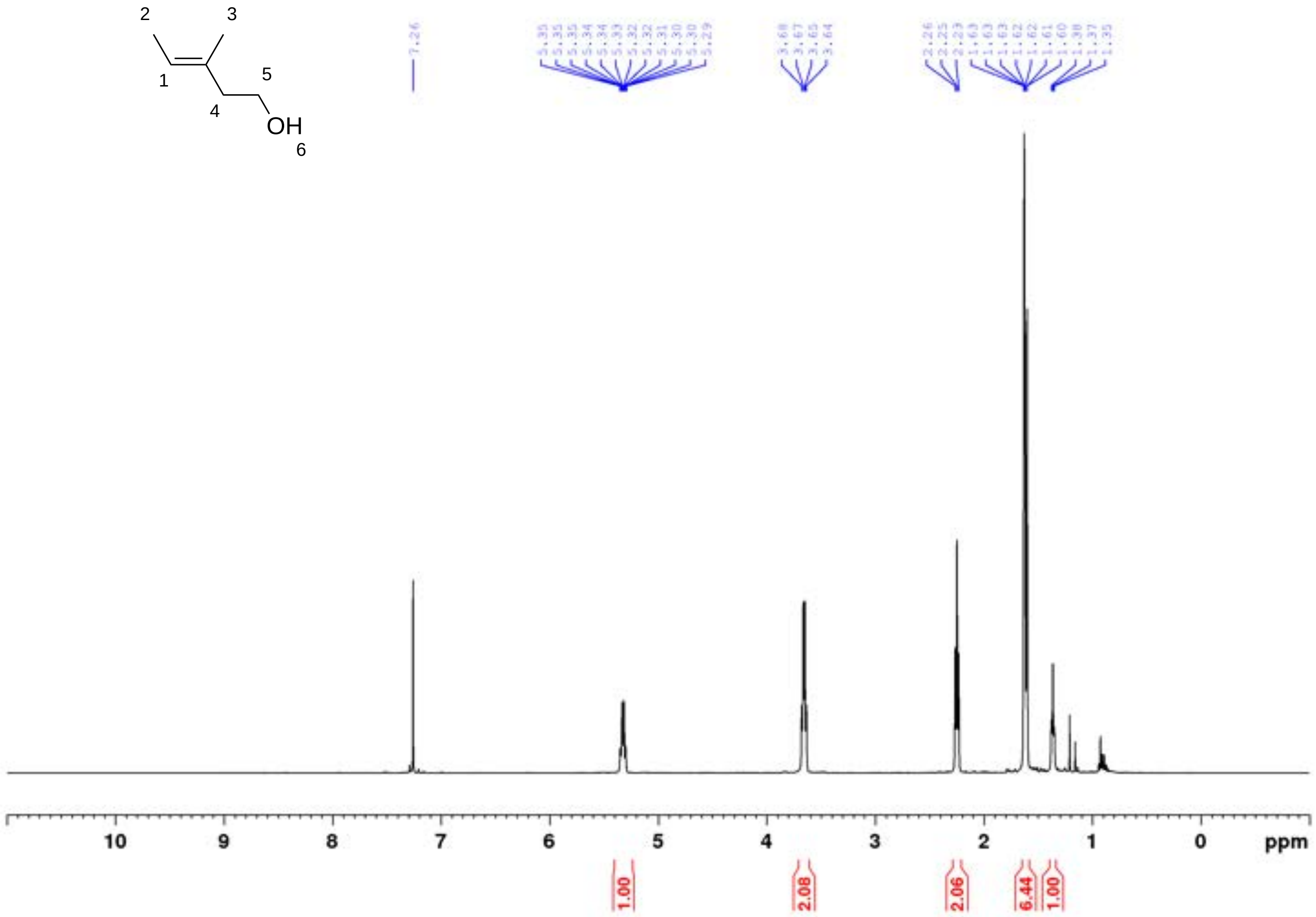

^{13}C NMR (101 MHz, $CDCl_3$) for *(E)-3-methylpent-3-en-1-ol* (**23f**)

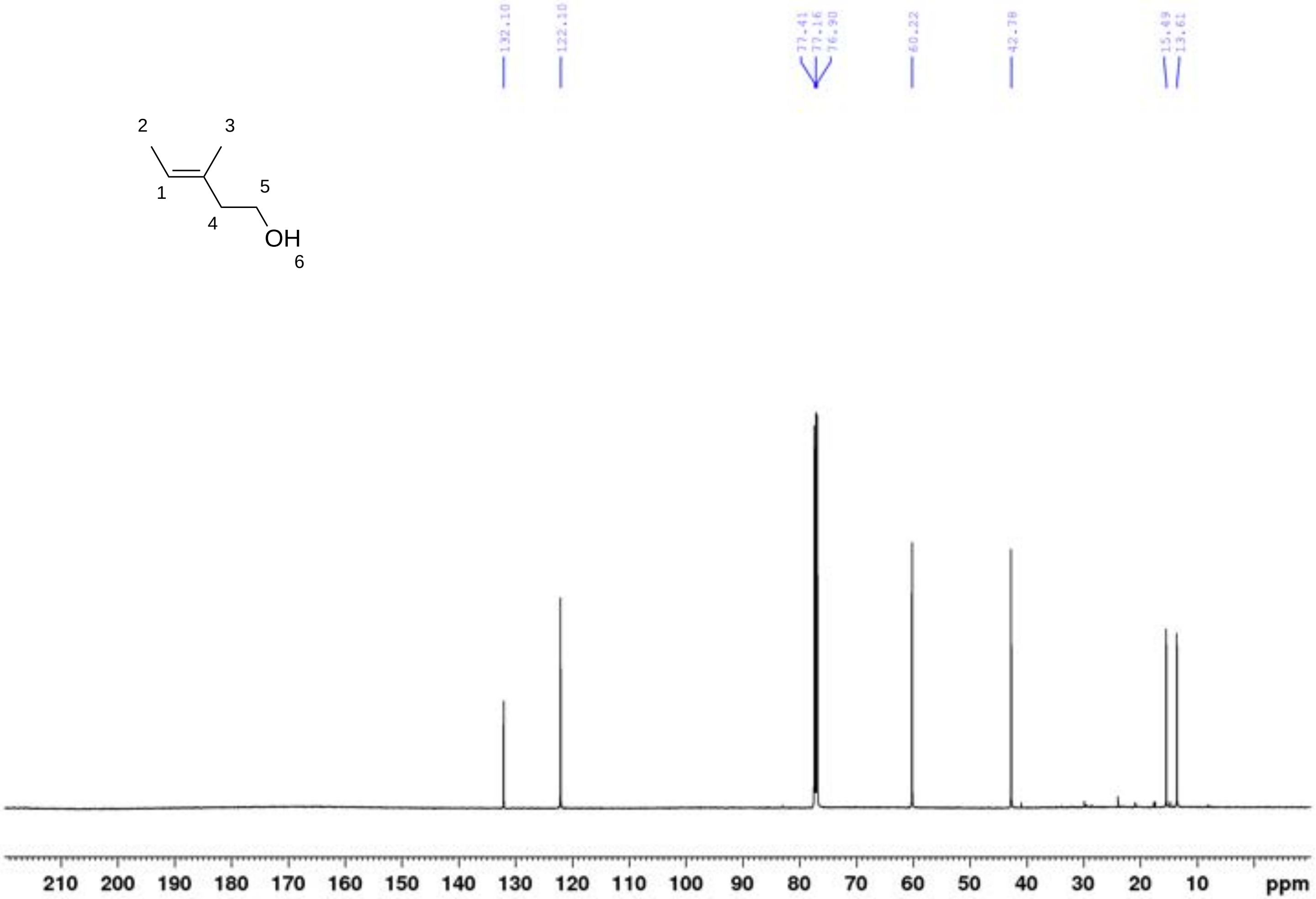

^{1}H NMR (400 MHz, $CDCl_3$) for *3,4-dimethylpent-3-en-1-ol* (**23h**)

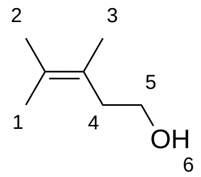

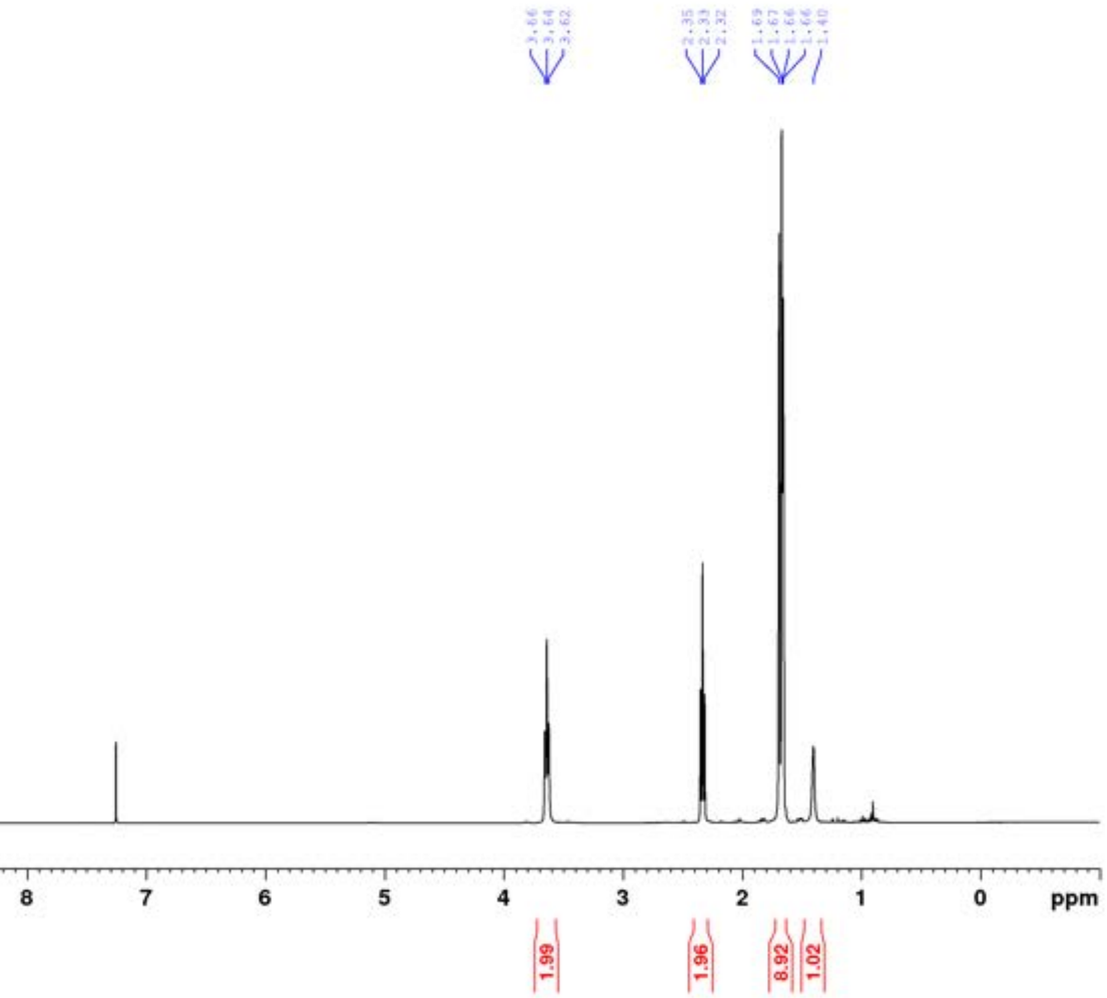

^{13}C NMR (101 MHz, $CDCl_3$) for *3,4-dimethylpent-3-en-1-ol* (**23h**)

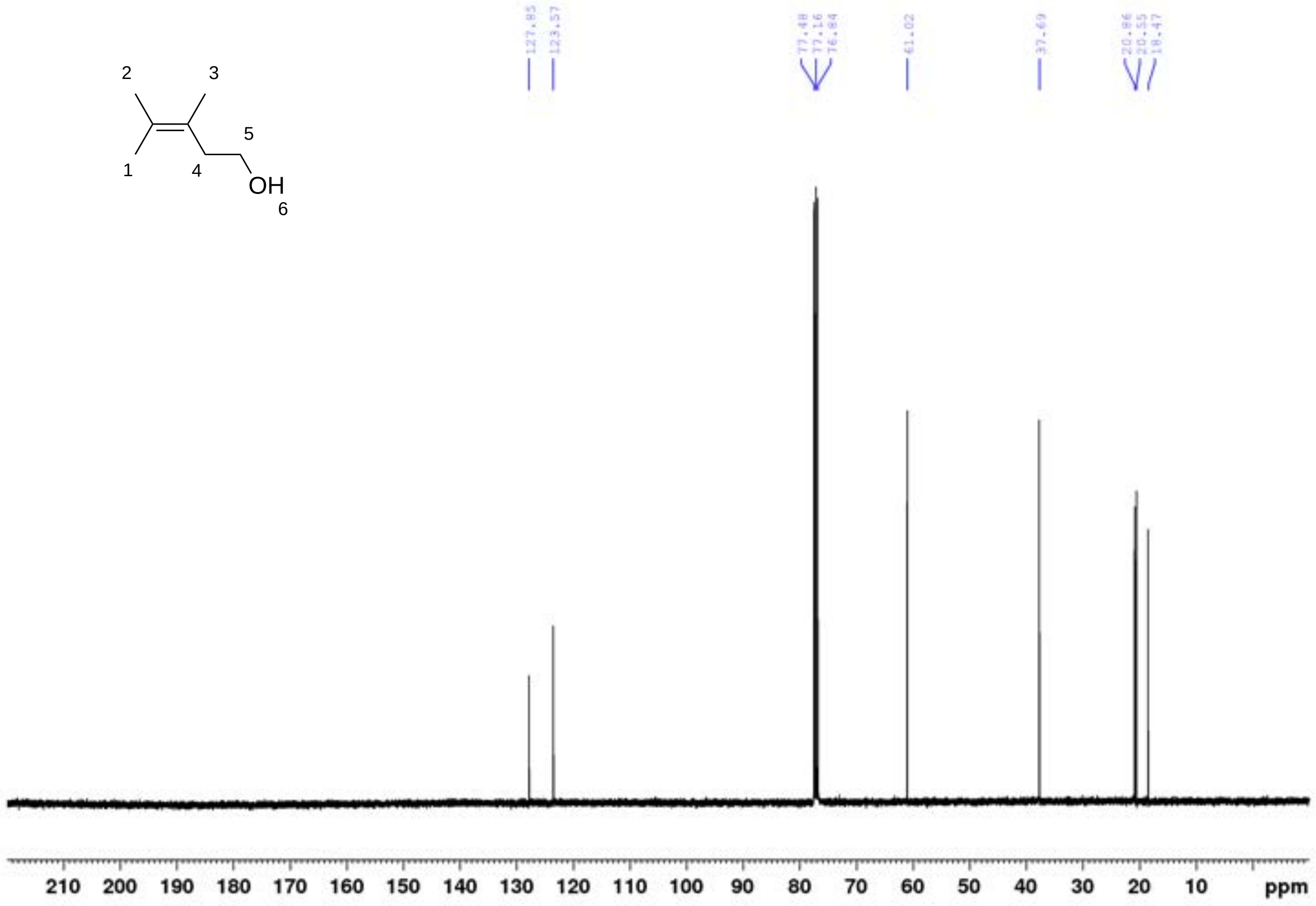

^{1}H NMR (400 MHz, CDCl$_3$) for *(E)-5-methylhex-3-en-1-ol* (**25b**)

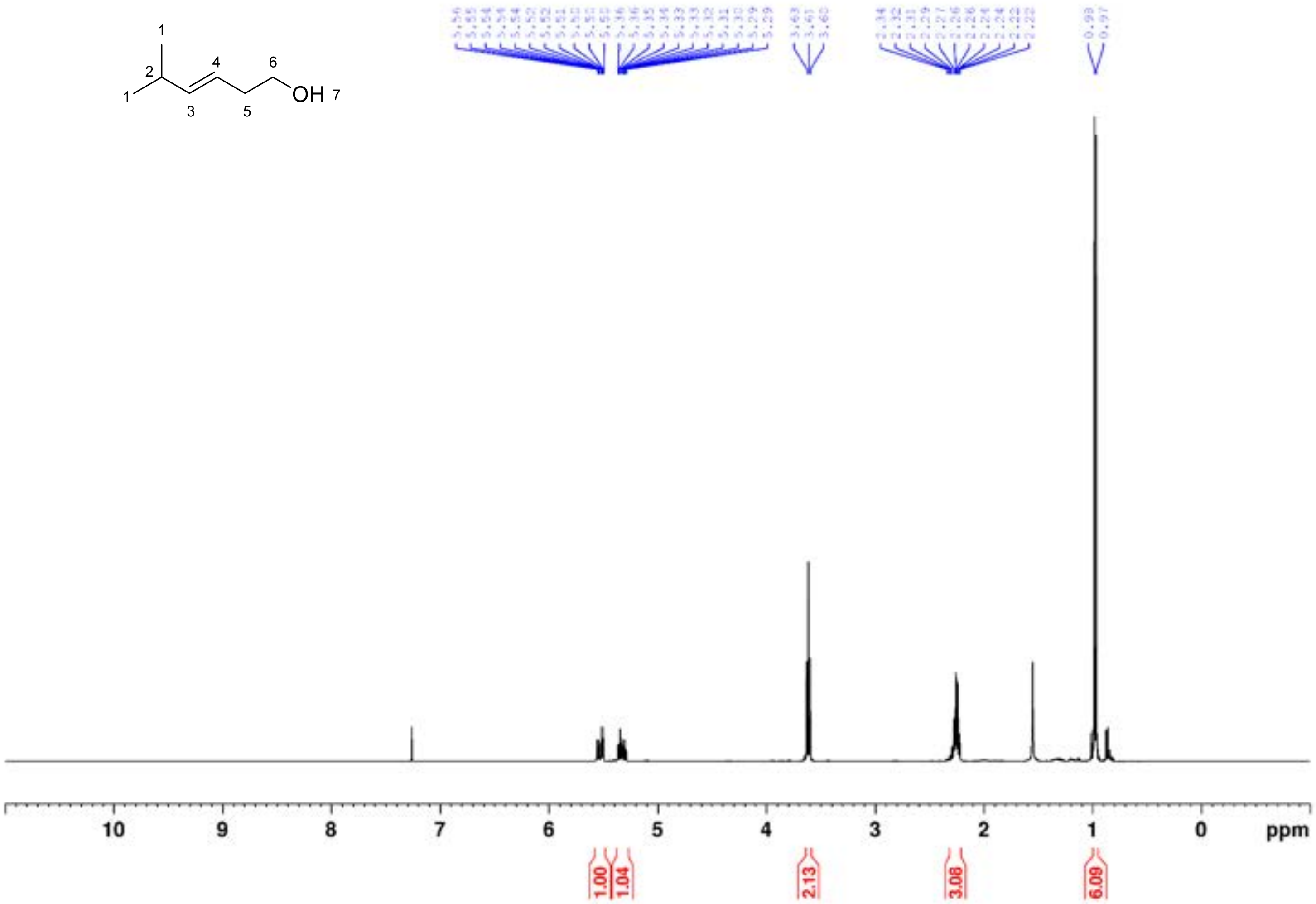

^{13}C NMR (101 MHz, $CDCl_3$) for *(E)-5-methylhex-3-en-1-ol* (**25b**)

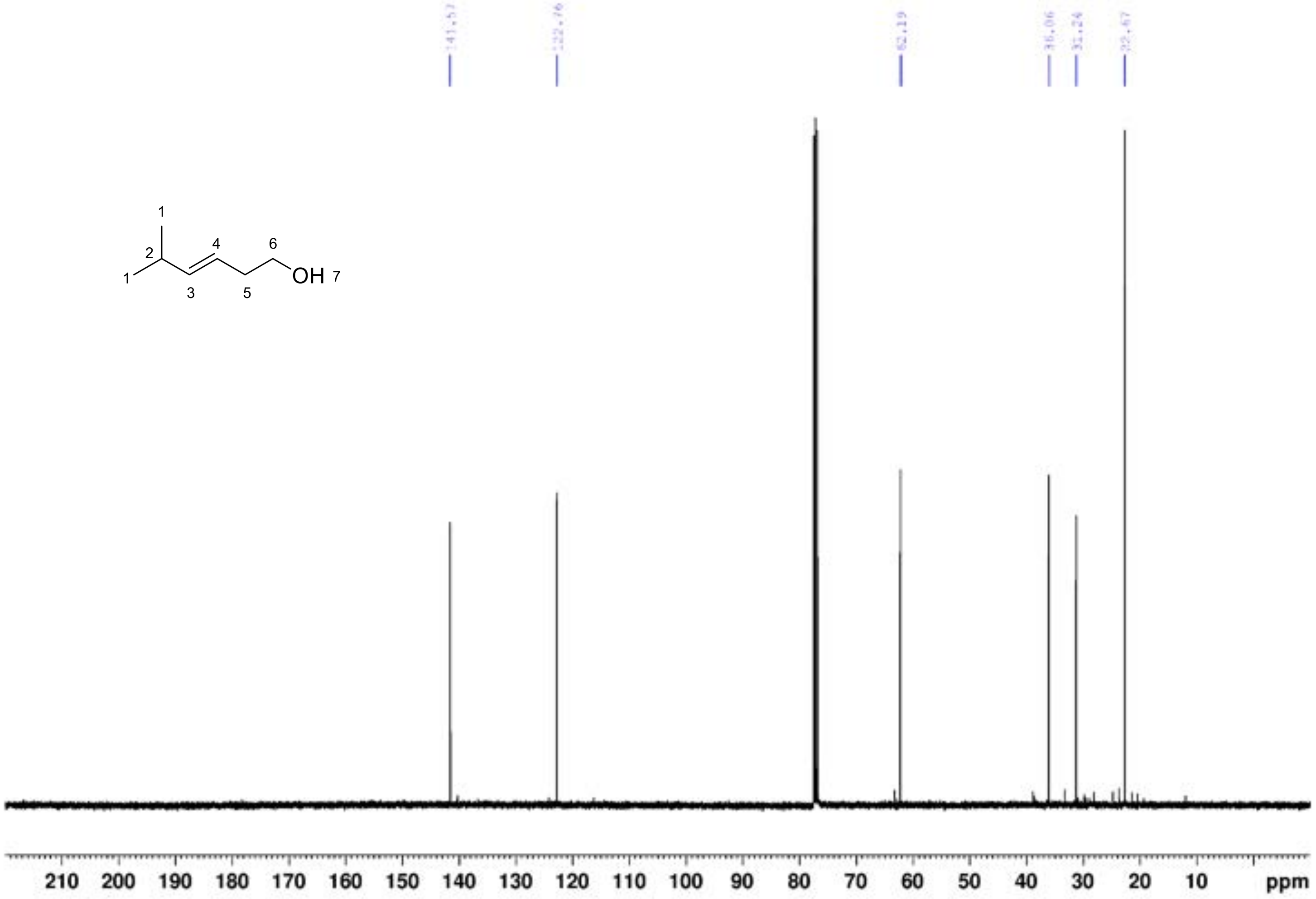

¹H NMR (400 MHz, $CDCl_3$) for *(E)-5,5-dimethylhex-3-en-1-ol* (**25c**)

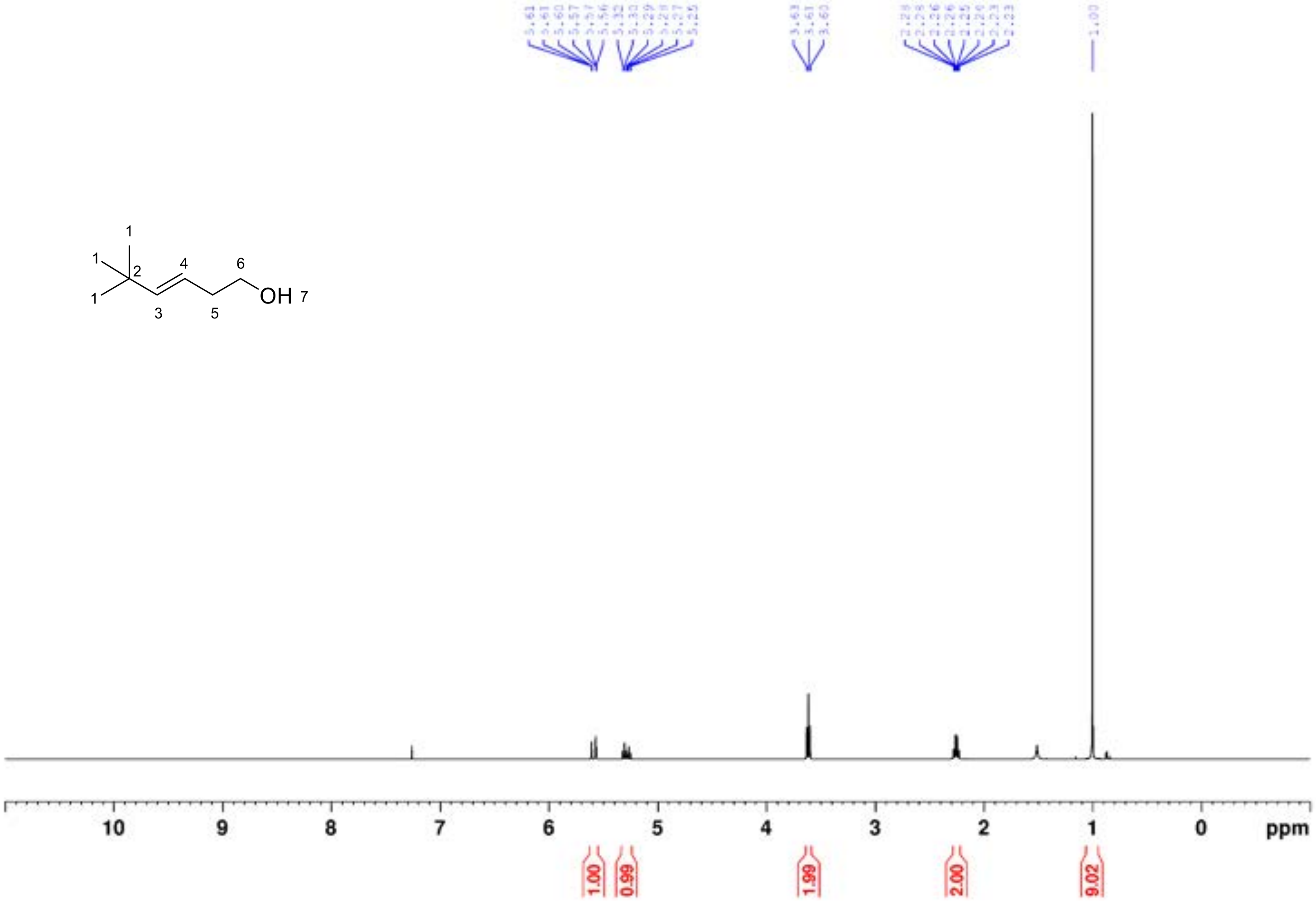

^{13}C NMR (101 MHz, $CDCl_3$) for *(E)-5,5-dimethylhex-3-en-1-ol* (**25c**)

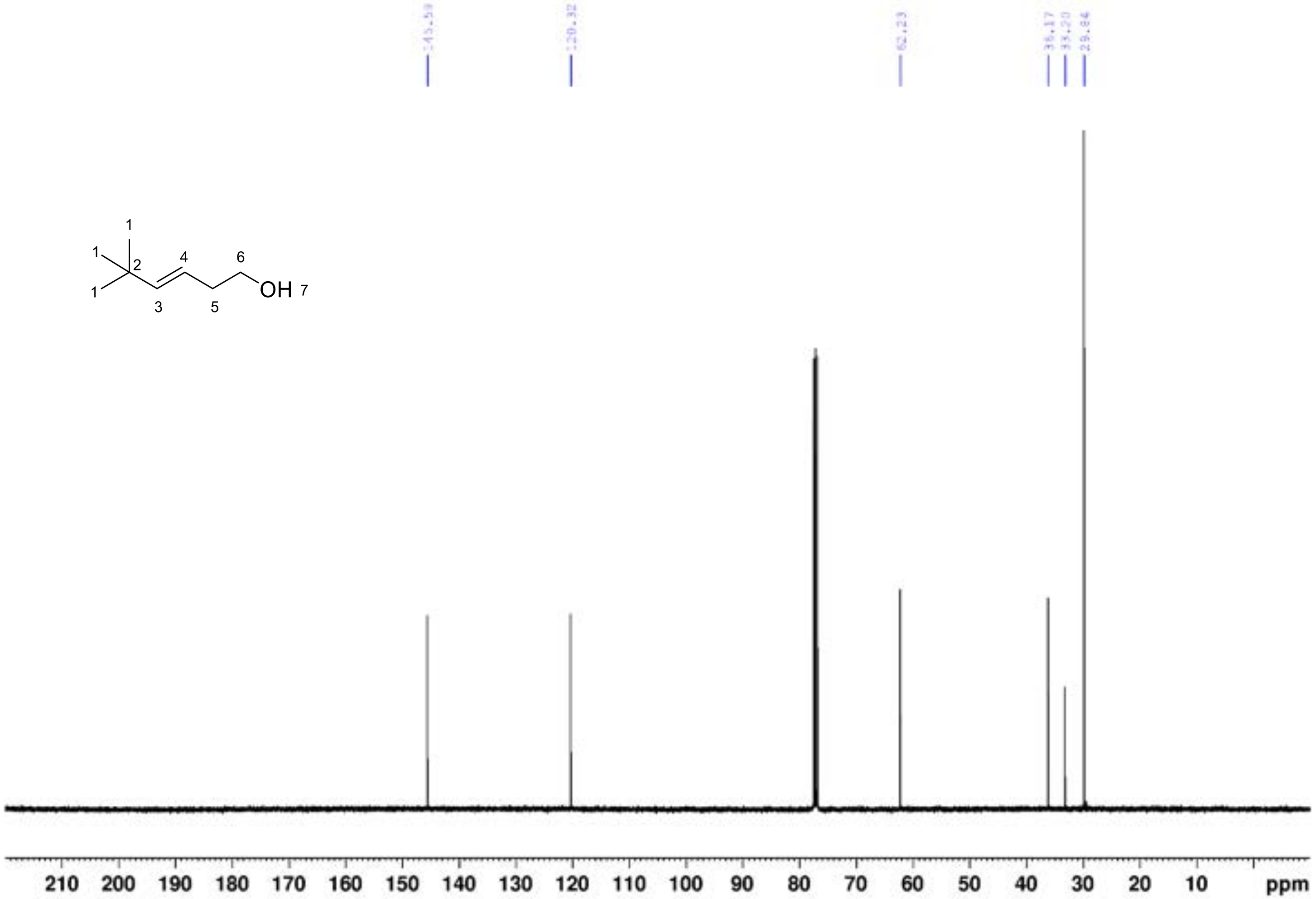

^{1}H NMR (400 MHz, $CDCl_3$) for *(E)-hex-3-ene-1,6-diol*

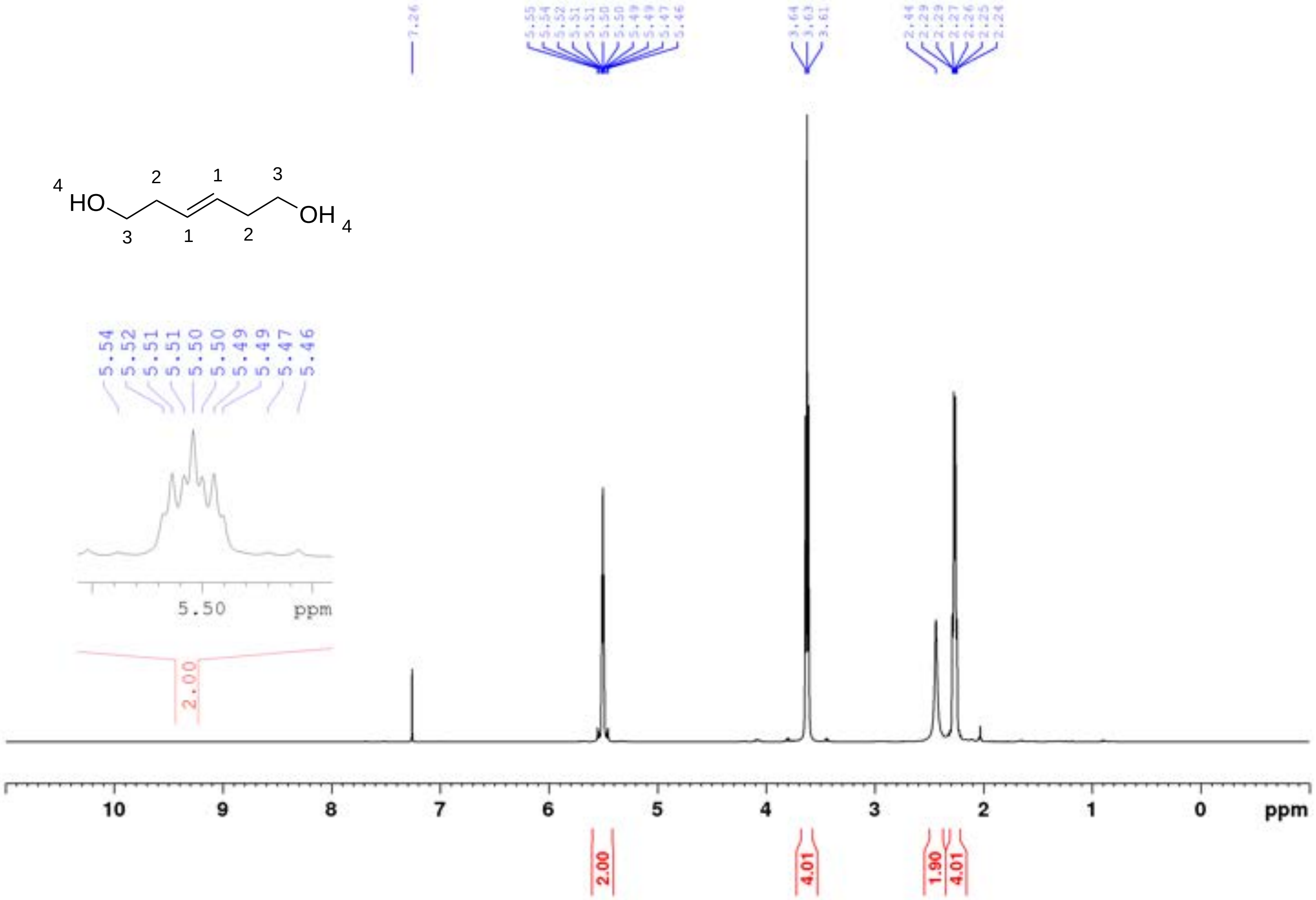

^{13}C NMR (400 MHz, $CDCl_3$) for *(E)-hex-3-ene-1,6-diol*

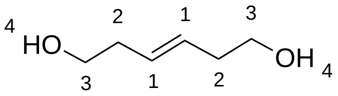

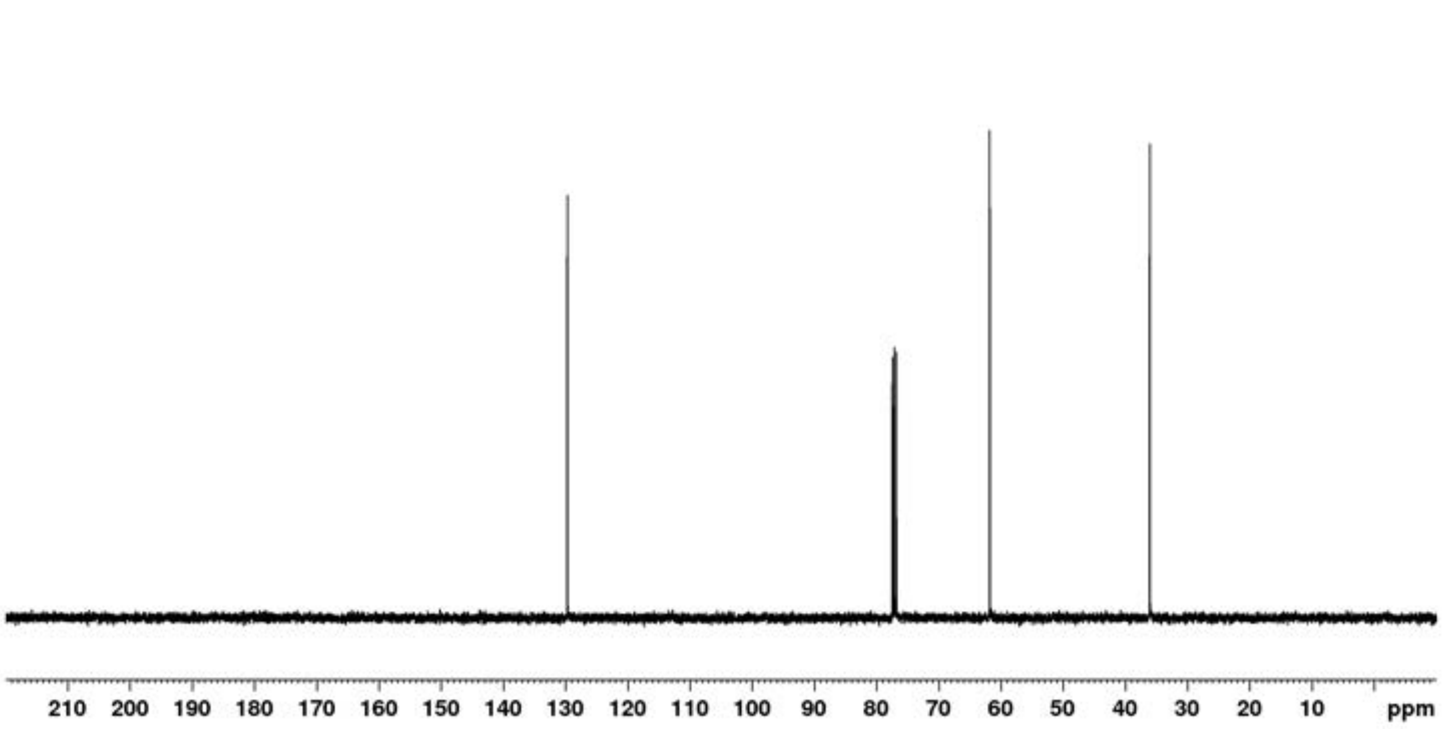

^{1}H NMR (400 MHz, $CDCl_3$) for *(E)-6-(benzyloxy)hex-3-en-1-ol* (**25d**)

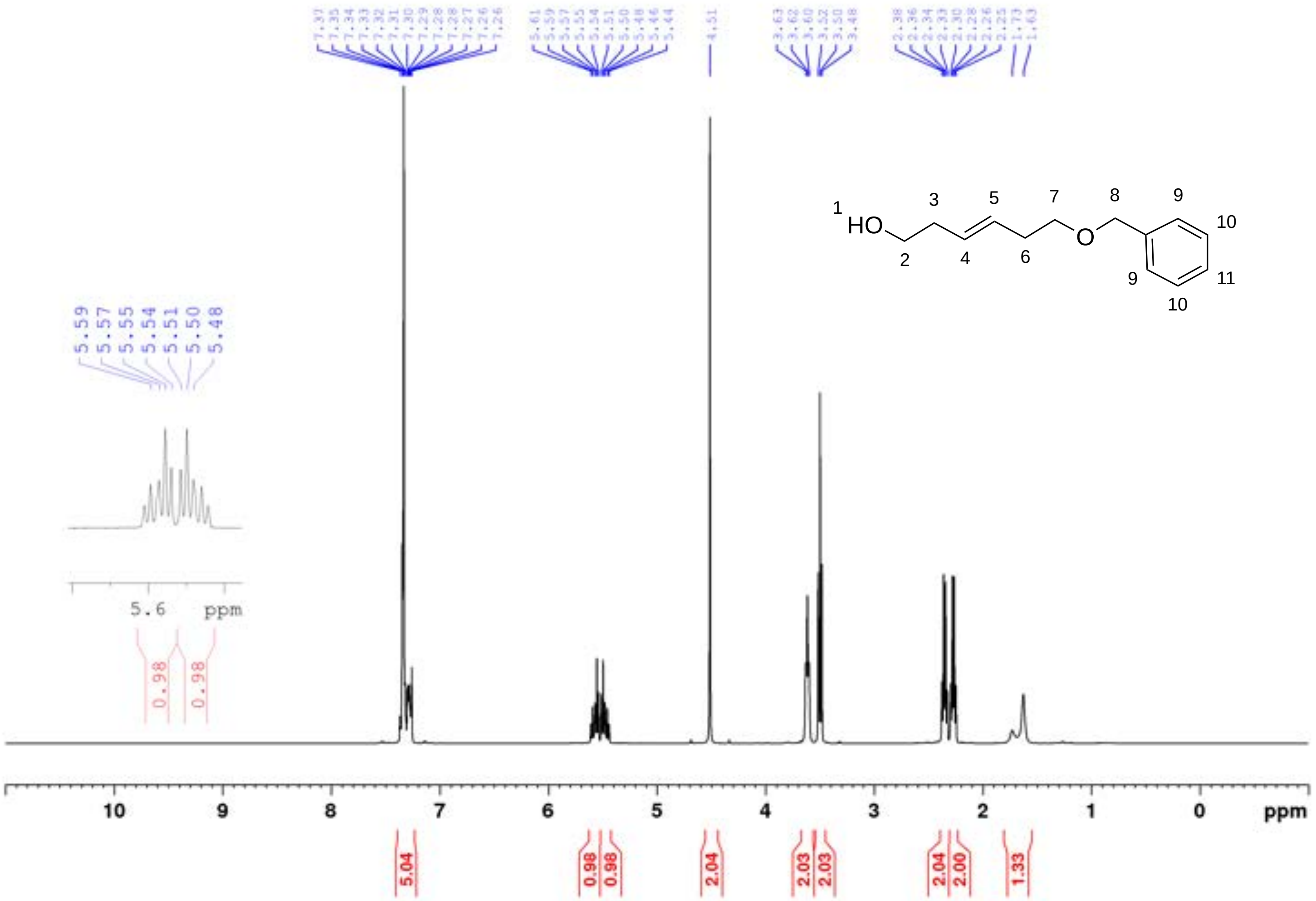

^{13}C NMR (101 MHz, $CDCl_3$) for *(E)-6-(benzyloxy)hex-3-en-1-ol* (**25d**)

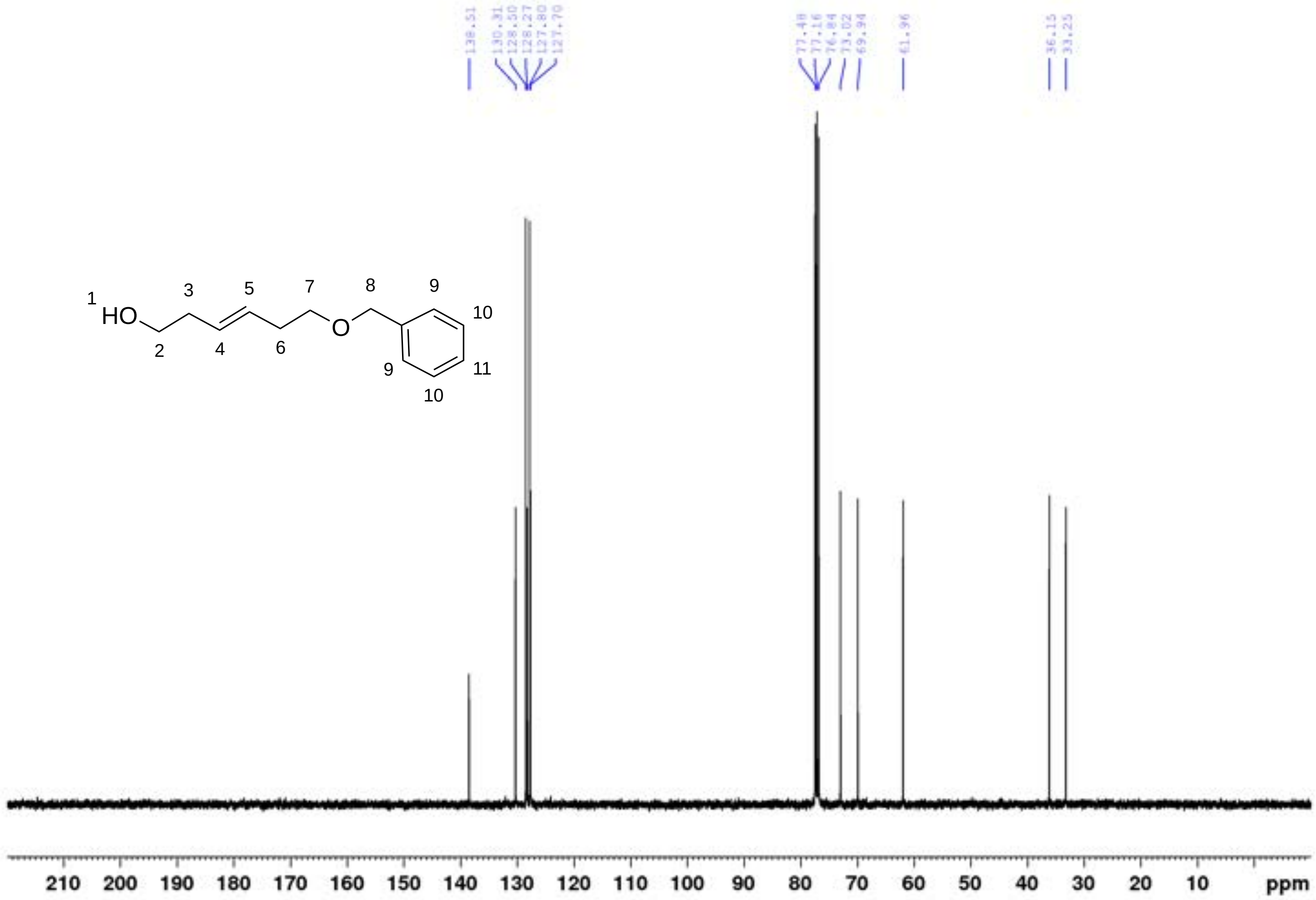

^{1}H NMR (400 MHz, $CDCl_3$) for *(E)-6-hydroxyhex-3-en-1-yl 4-methylbenzenesulfonate* (**25e**)

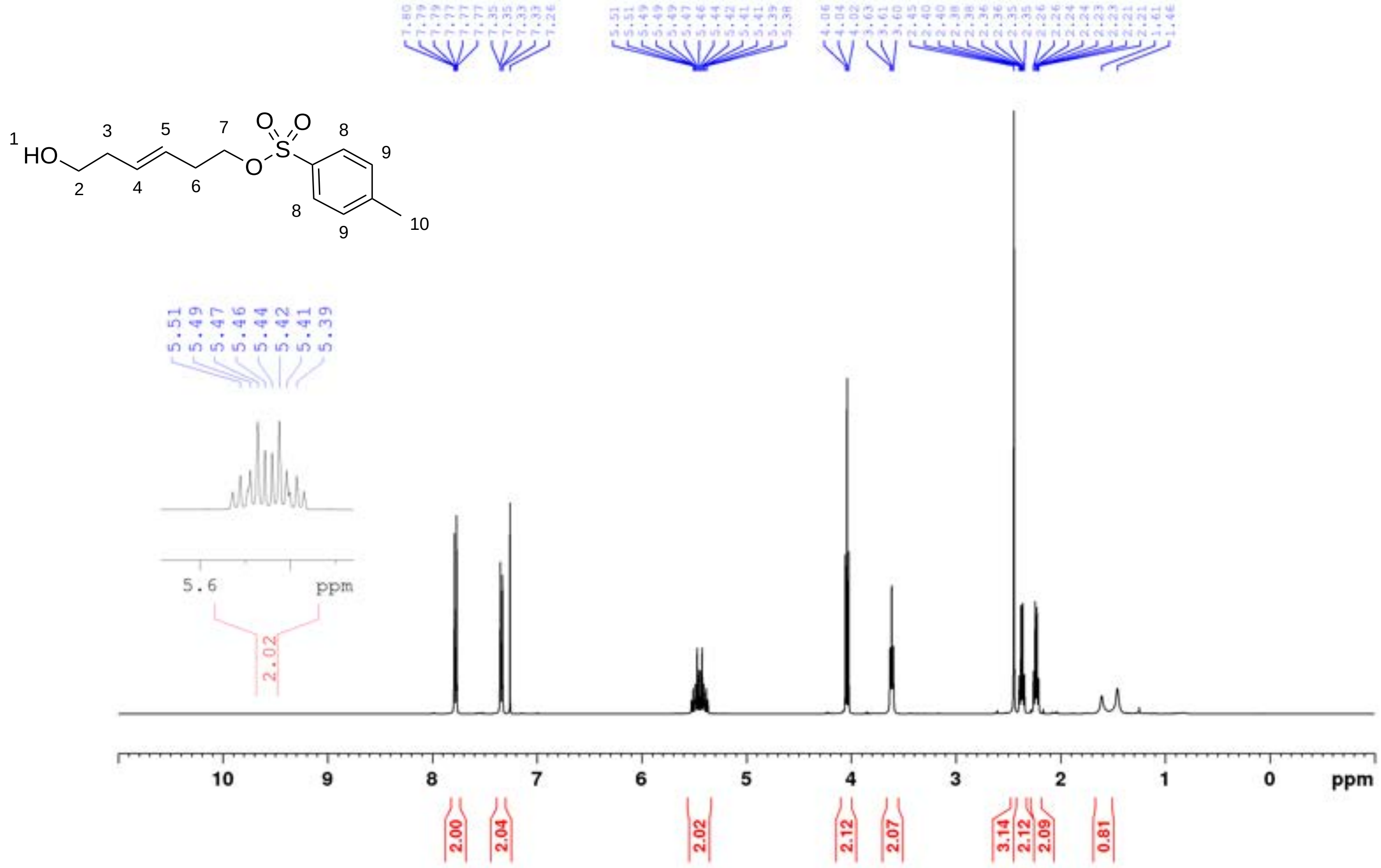

^{13}C NMR (101 MHz, $CDCl_3$) for *(E)-6-hydroxyhex-3-en-1-yl 4-methylbenzenesulfonate* (**25e**)

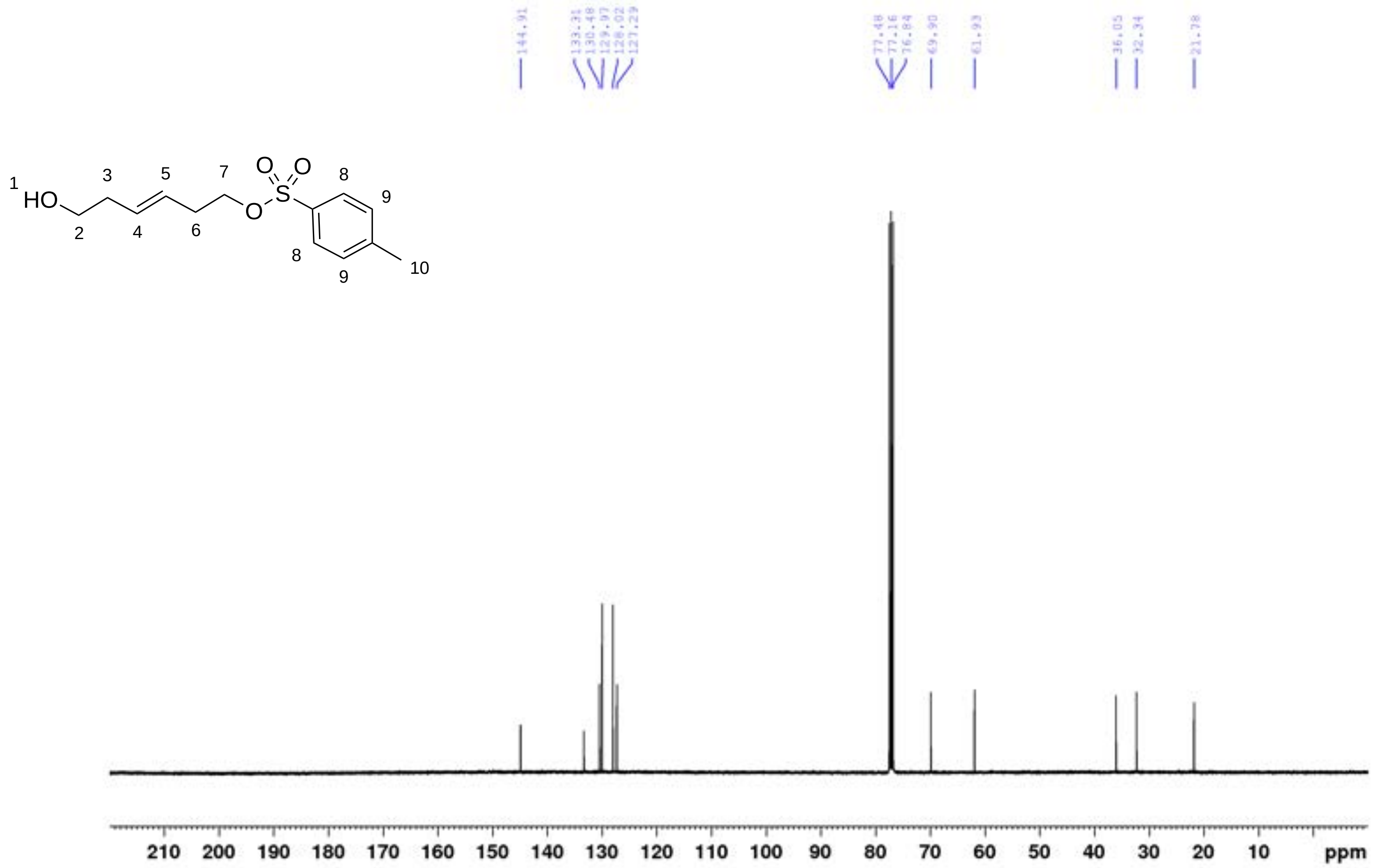

^{1}H NMR (400 MHz, $CDCl_3$) for *(E)-6-bromohex-3-en-1-ol* (**25f**)

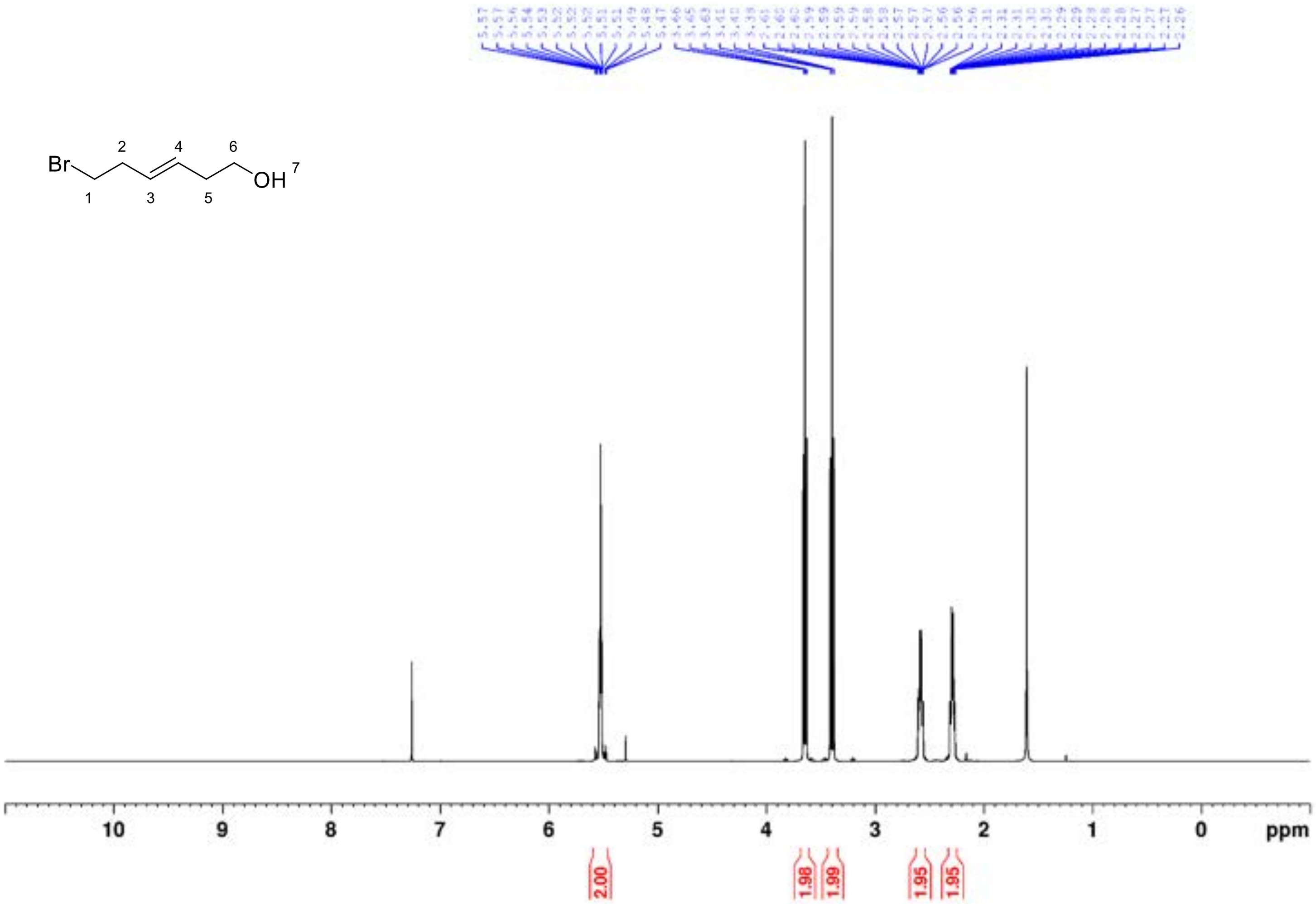

^{13}C NMR (101 MHz, CDCl$_3$) for *(E)-6-bromohex-3-en-1-ol* (**25f**)

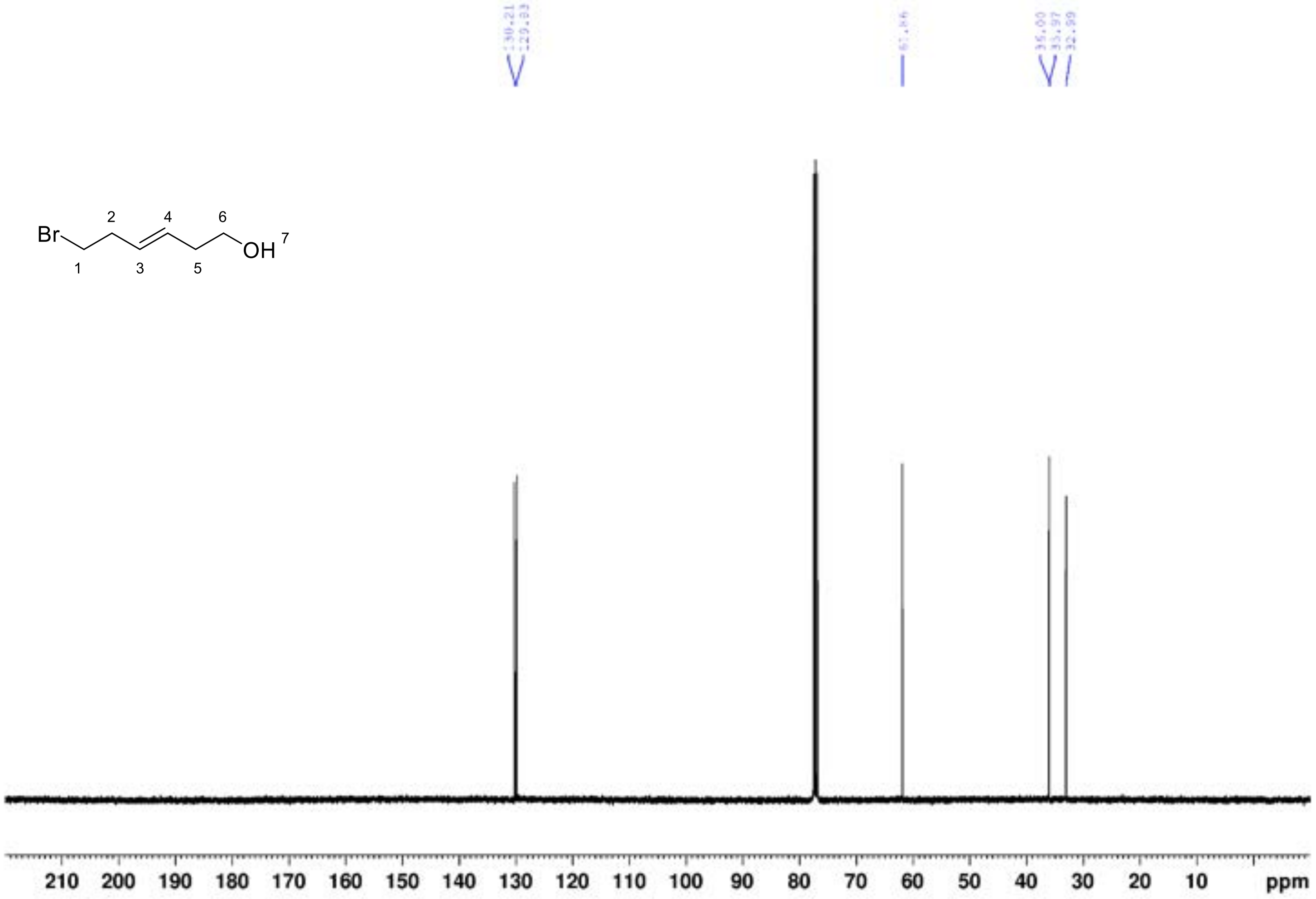

^{1}H NMR (400 MHz, $CDCl_3$) for *(E)-N-(6-hydroxyhex-3-en-1-yl)-N,4-dimethylbenzenesulfonamide* (**25g**)

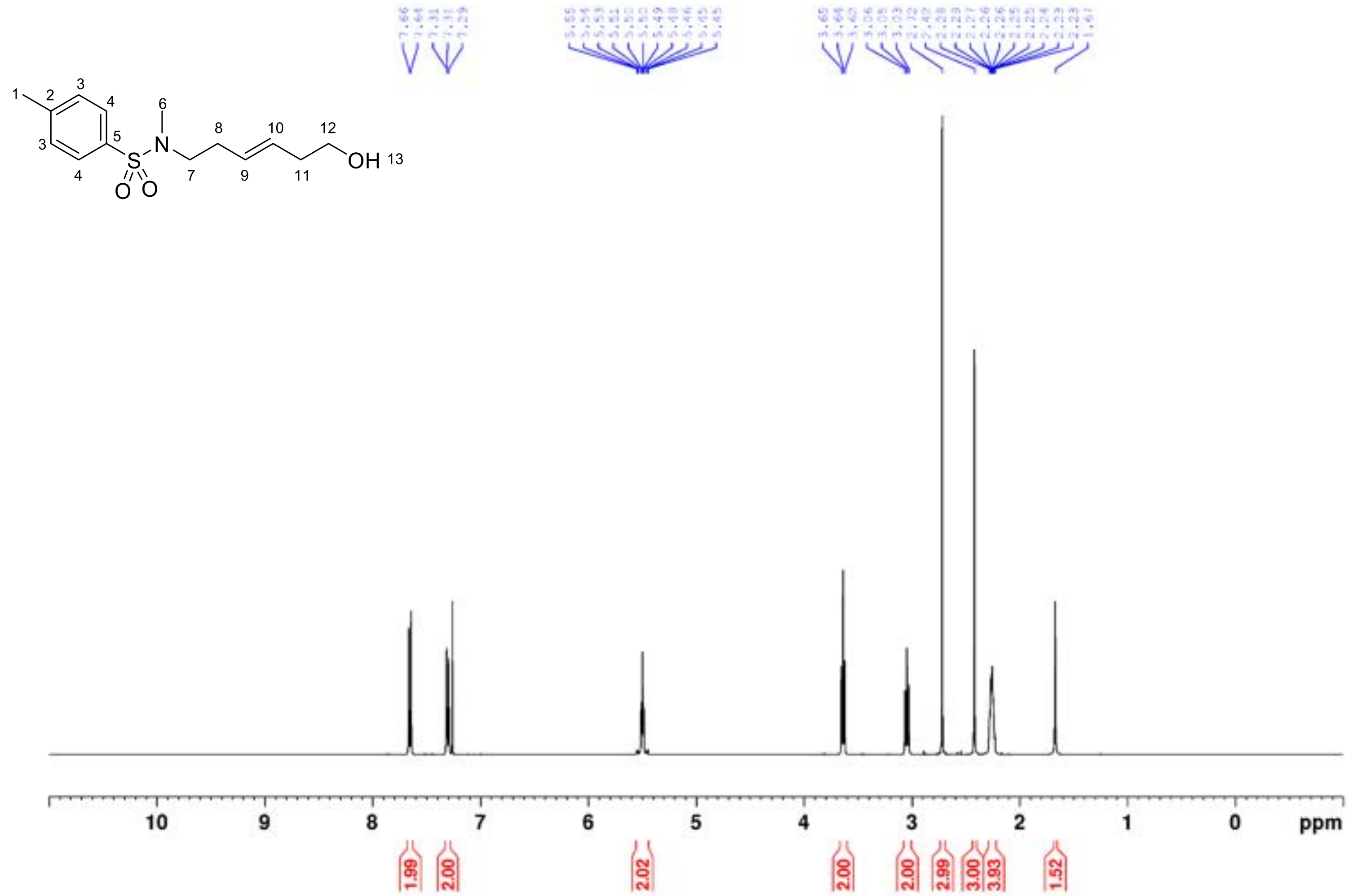

^{13}C NMR (101 MHz, CDCl$_3$) for *(E)-N-(6-hydroxyhex-3-en-1-yl)-N,4-dimethylbenzenesulfonamide* (**25g**)

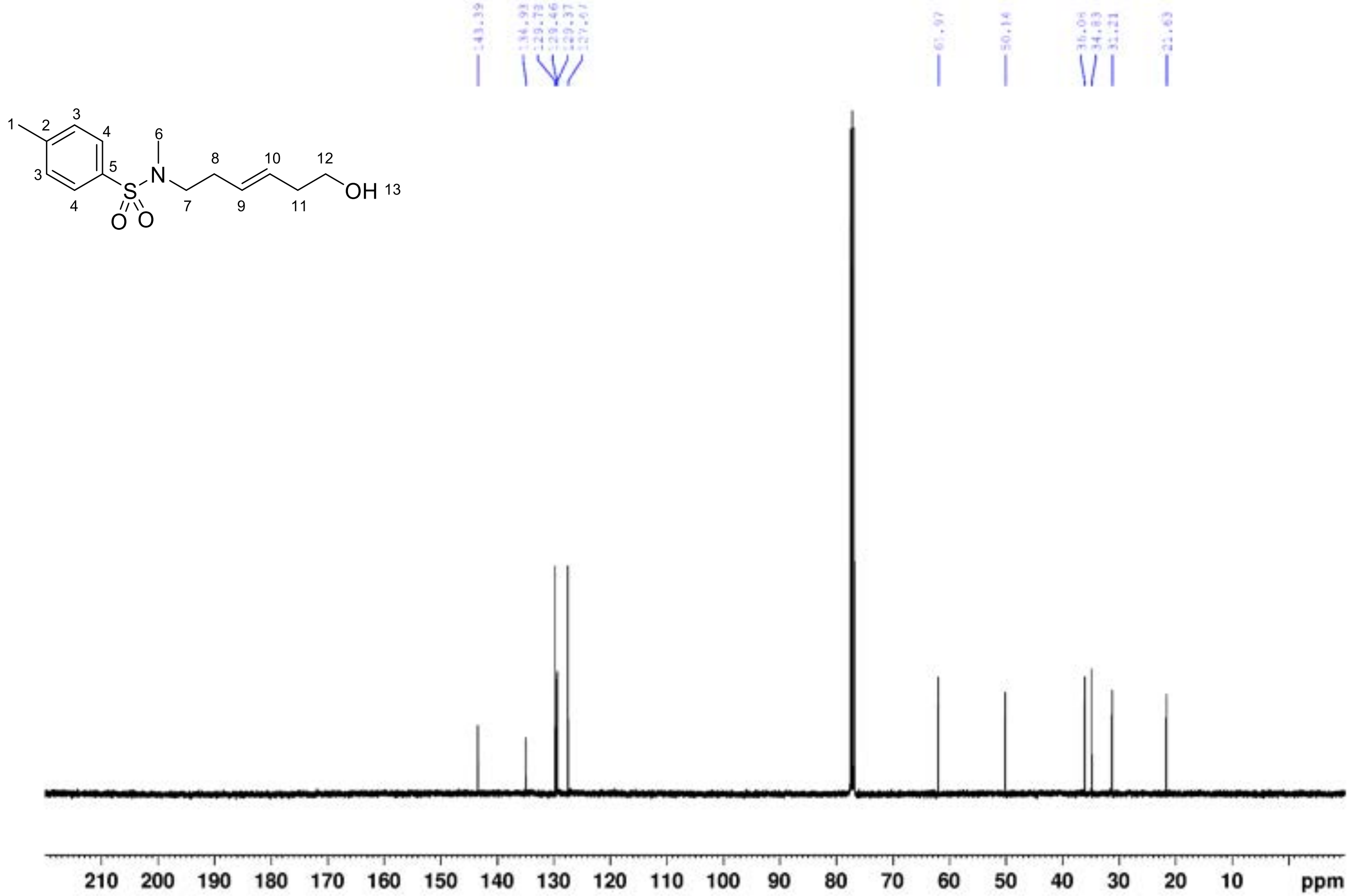

^{1}H NMR (400 MHz, $CDCl_3$) for *6-methyl-3-methyleneheptan-1-ol* (**27a**)

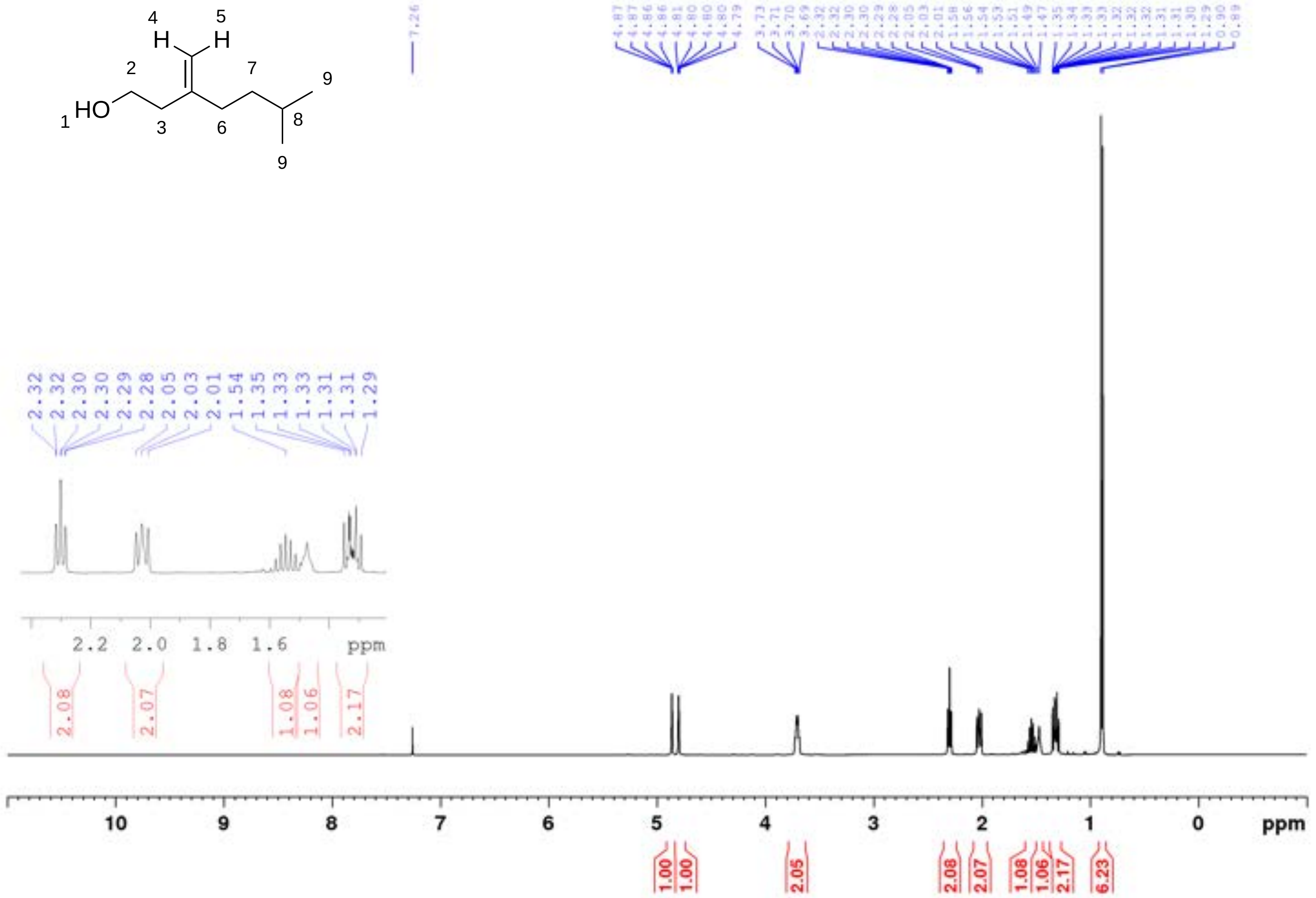

^{13}**C NMR** (101 MHz, $CDCl_3$) for *6-methyl-3-methyleneheptan-1-ol* (**27a**)

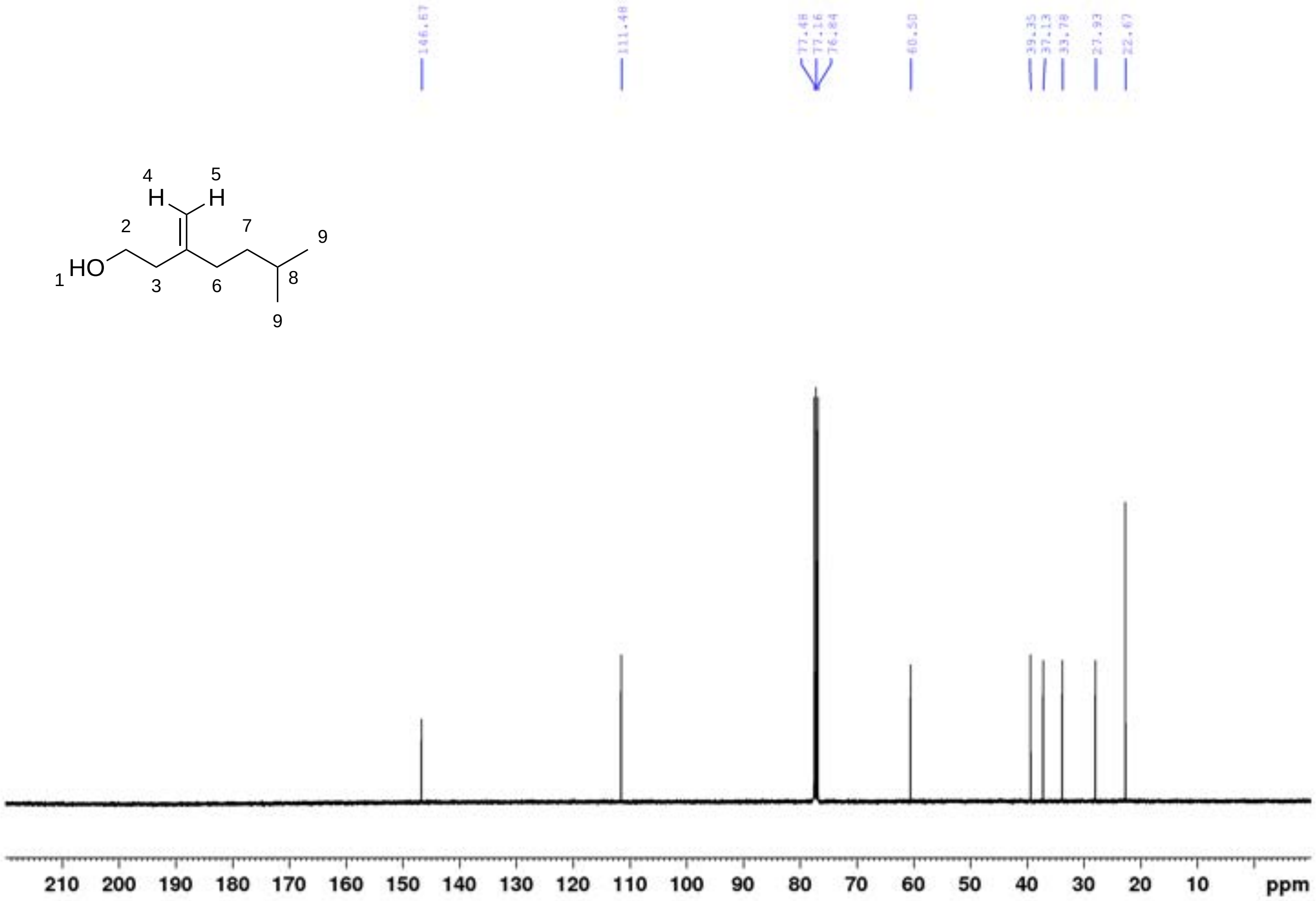

^{1}H NMR (400 MHz, $CDCl_3$) for *5-cyclohexyl-3-methylenepentan-1-ol* (**27b**)

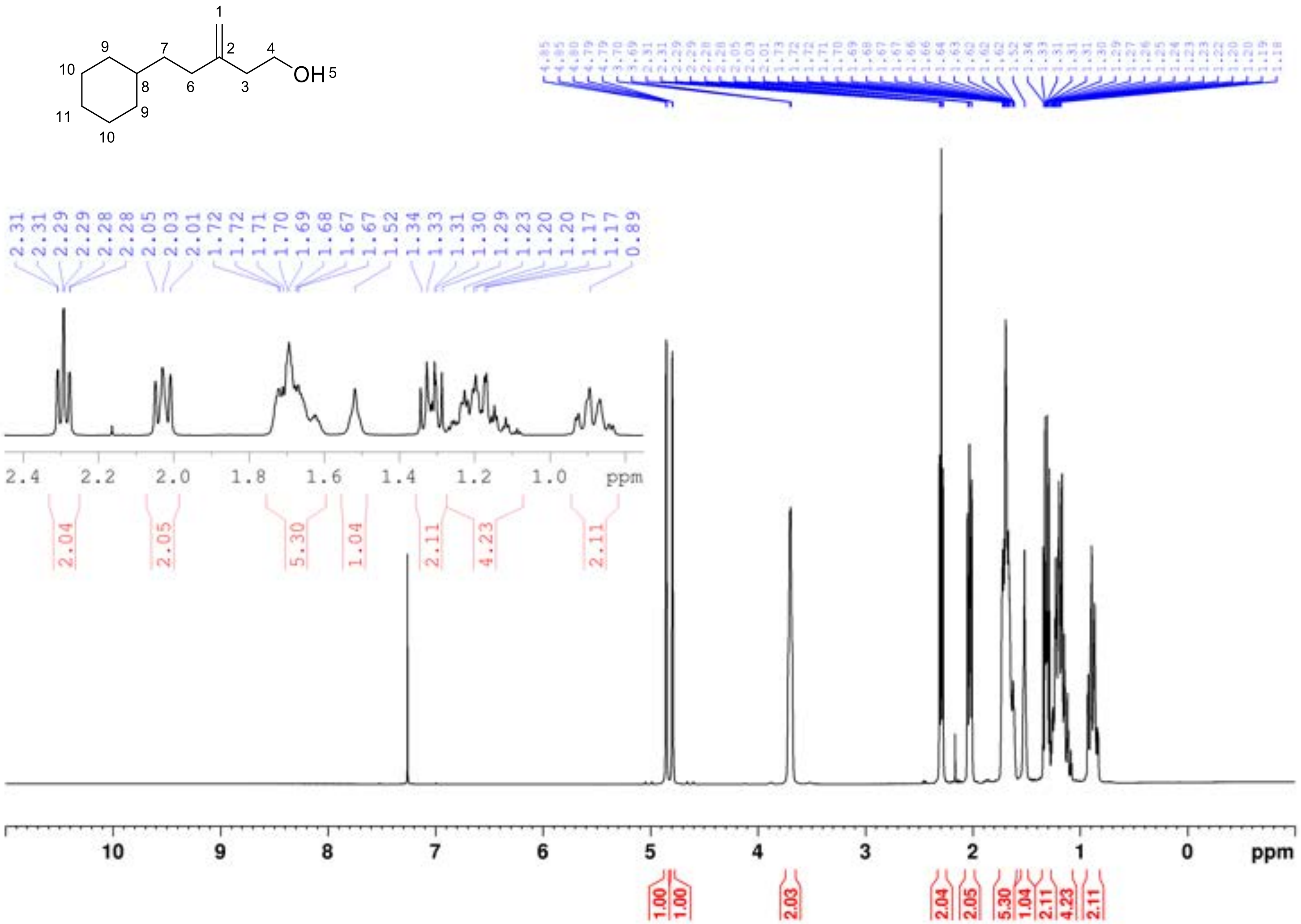

^{13}C NMR (101 MHz, $CDCl_3$) for *5-cyclohexyl-3-methylenepentan-1-ol* (**27b**)

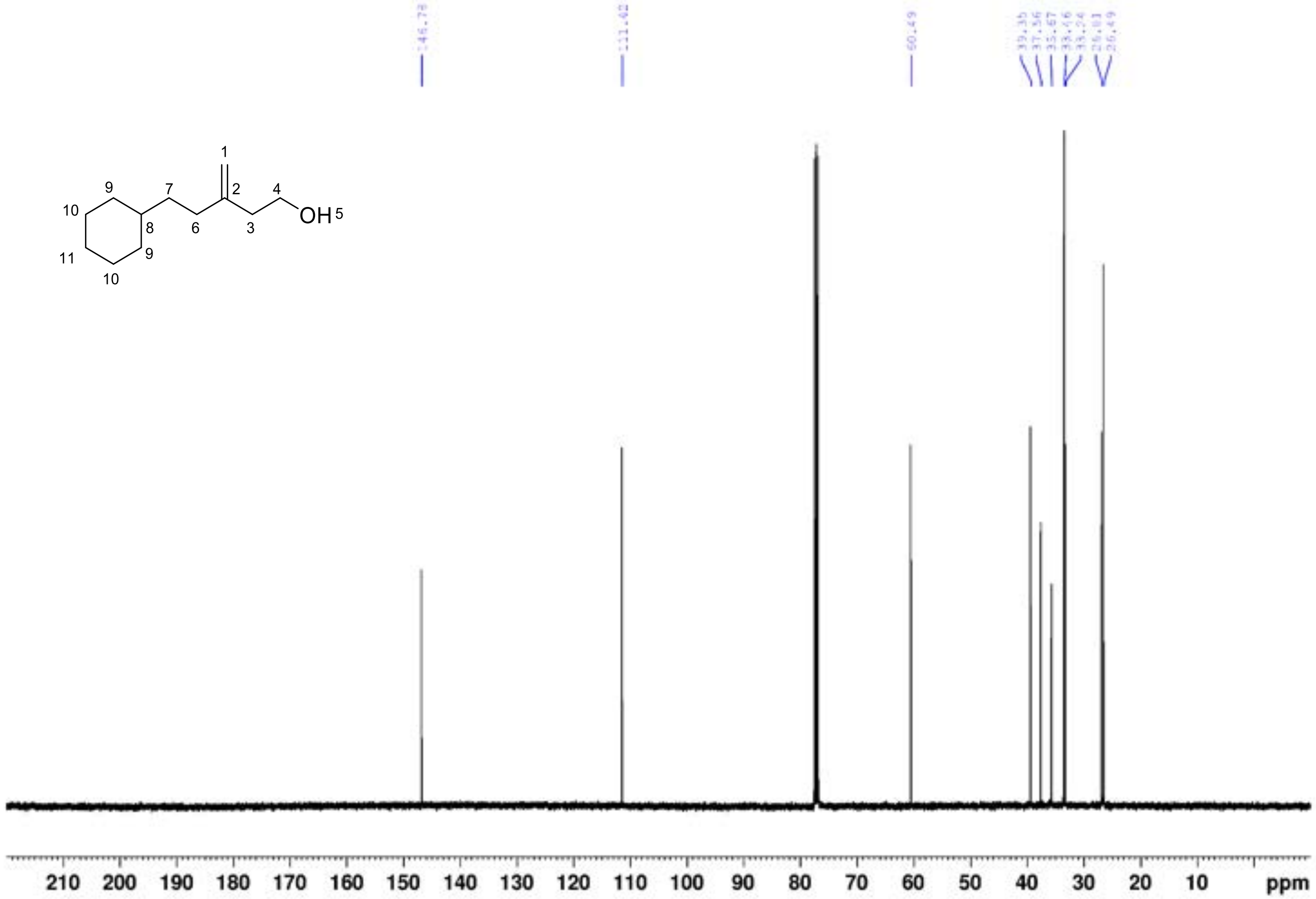

^{1}H NMR (400 MHz, CDCl$_3$) for *7-((tert-butyldimethylsilyl)oxy)-3-methyleneheptan-1-ol* (**27c**)

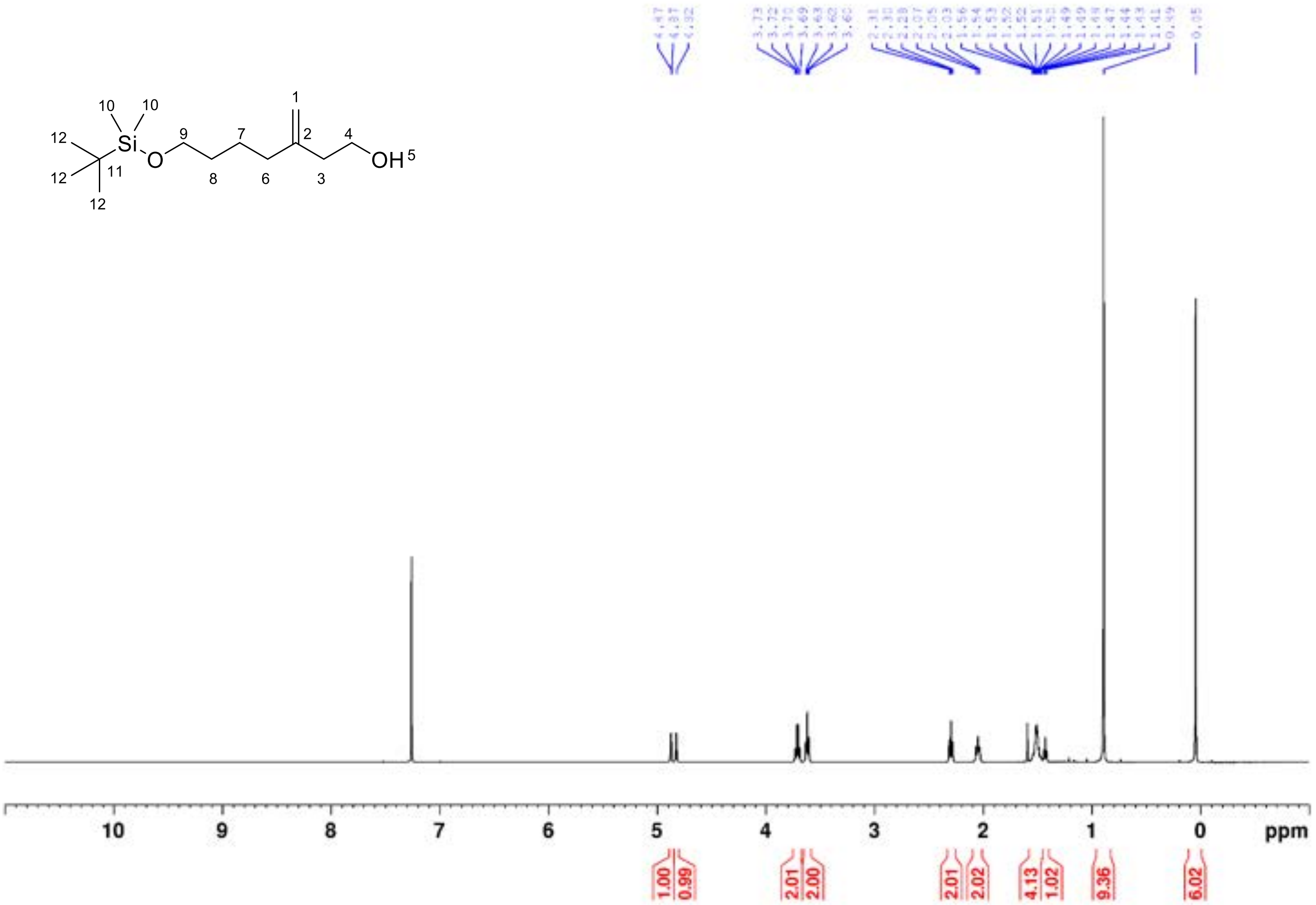

^{13}C NMR (101 MHz, CDCl$_3$) for *7-((tert-butyldimethylsilyl)oxy)-3-methyleneheptan-1-ol* (**27c**)

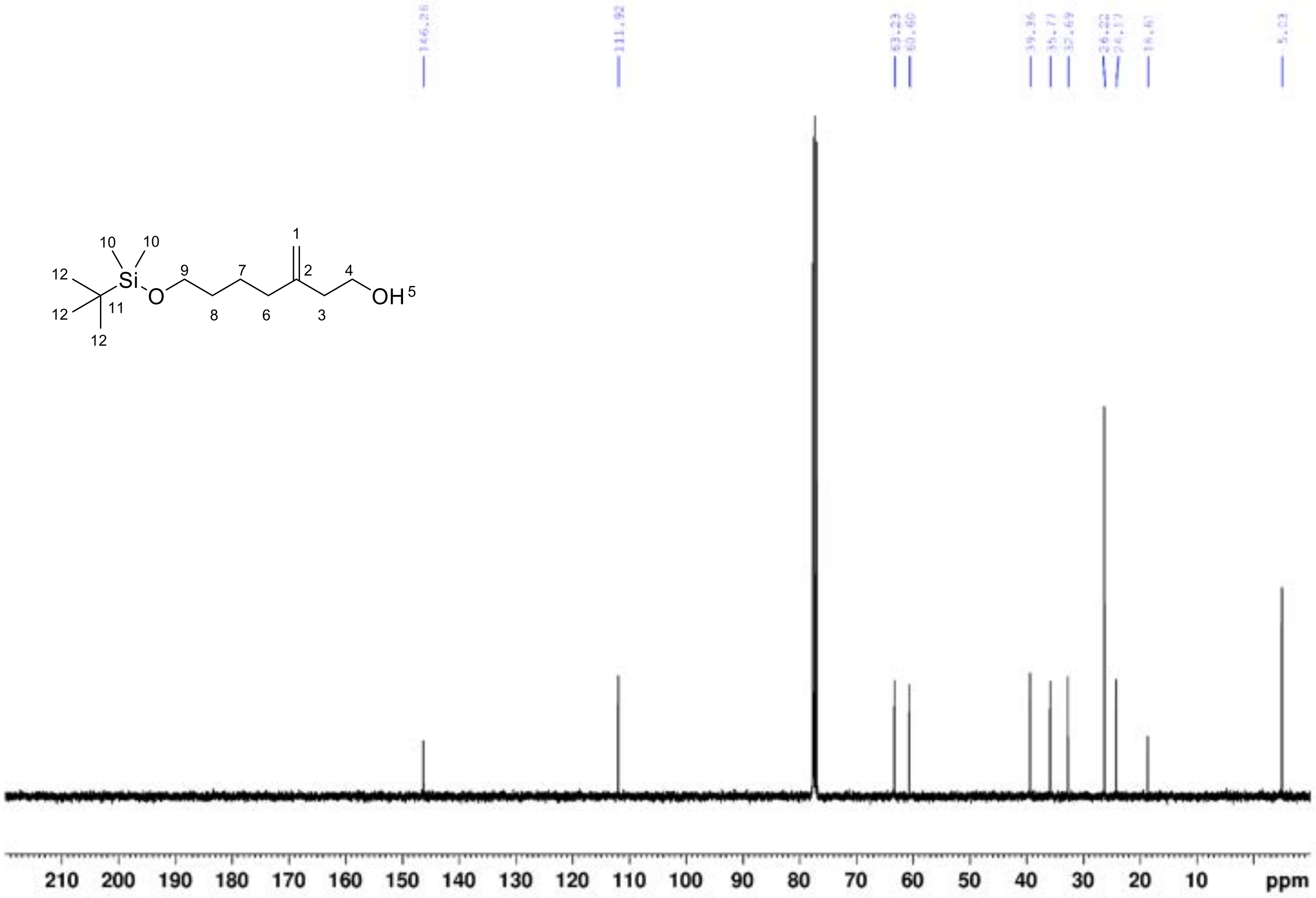

^{1}H NMR (400 MHz, $CDCl_3$) for *(Z)-4-methylhept-3-en-1-ol* (**31a**)

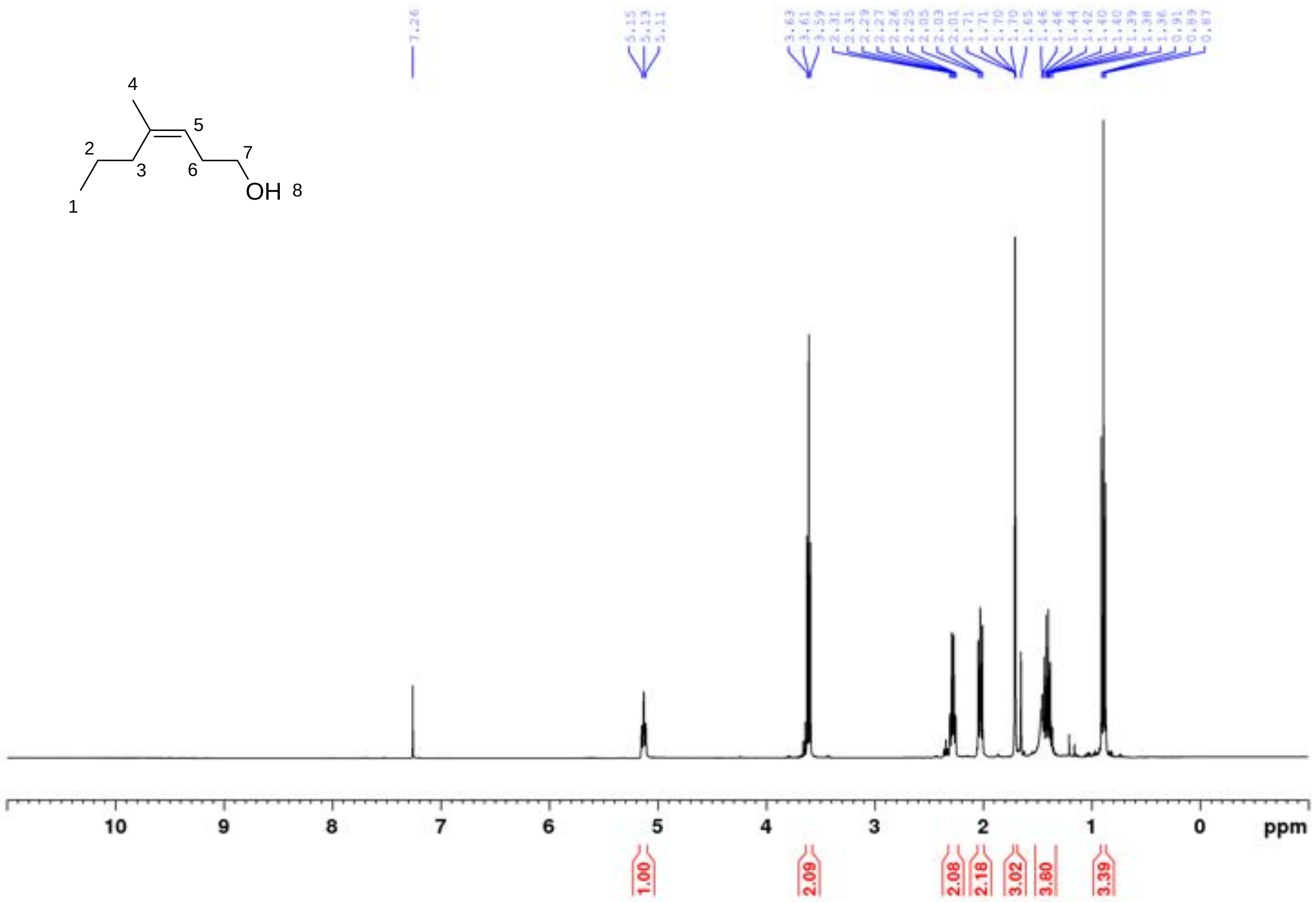

^{13}**C NMR** (101 MHz, $CDCl_3$) for *(Z)-4-methylhept-3-en-1-ol* (**31a**)

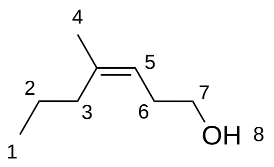

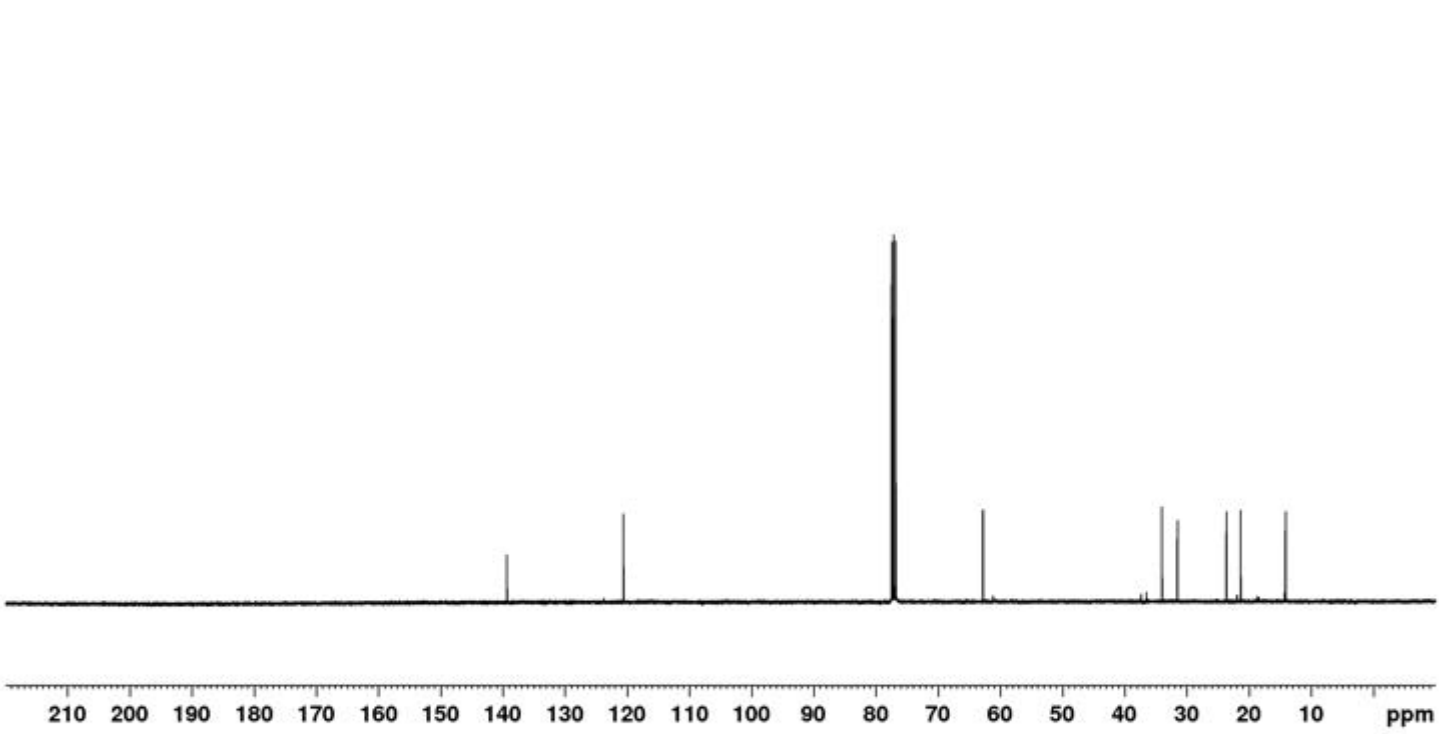

(3-hydroxypropyl)triphenylphosphonium bromide

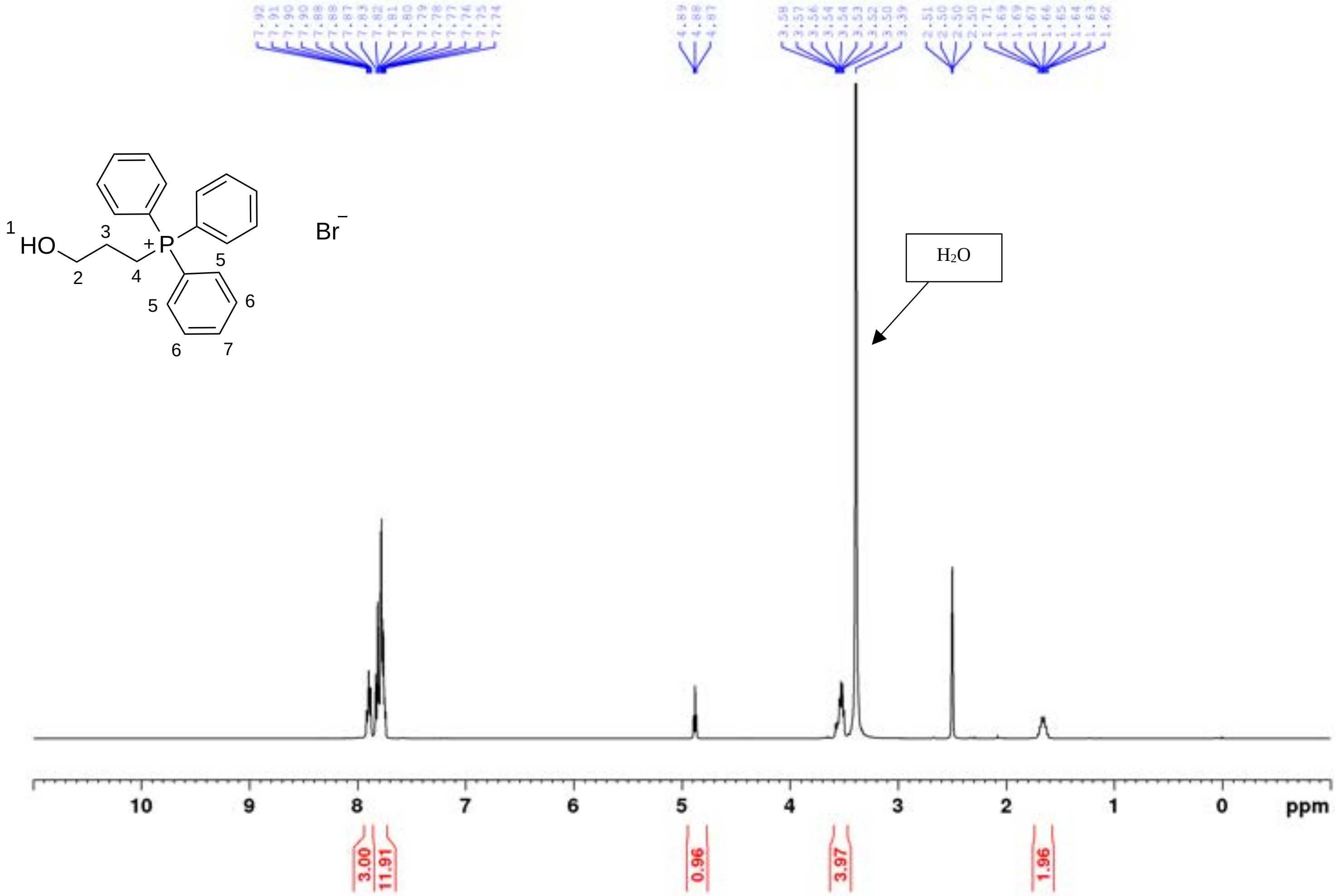

^{13}C NMR (101 MHz, DMSO-d$_6$) for *(3-hydroxypropyl)triphenylphosphonium bromide*

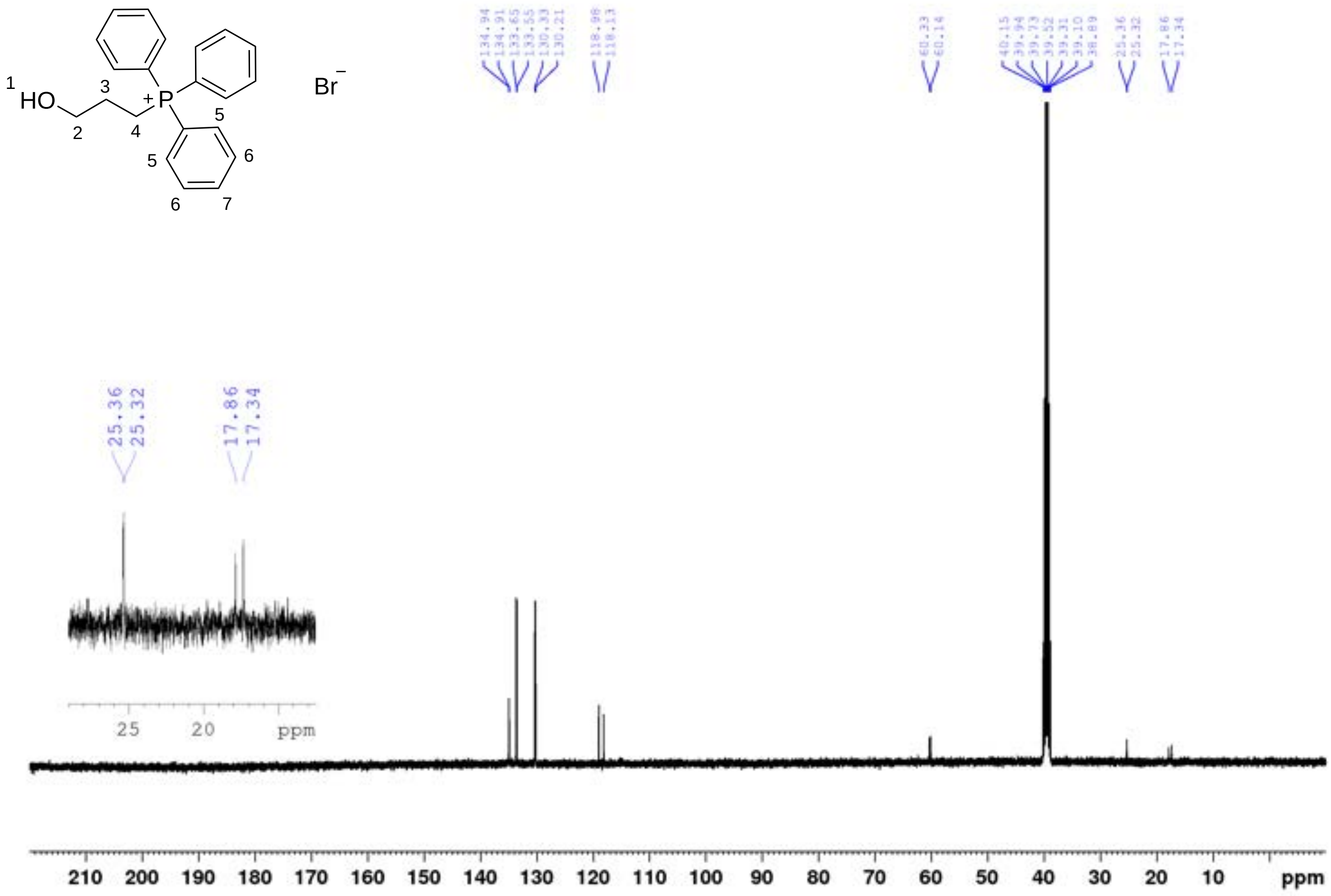

^{31}P NMR (101 MHz, DMSO-d$_6$) for *(3-hydroxypropyl)triphenylphosphonium bromide*

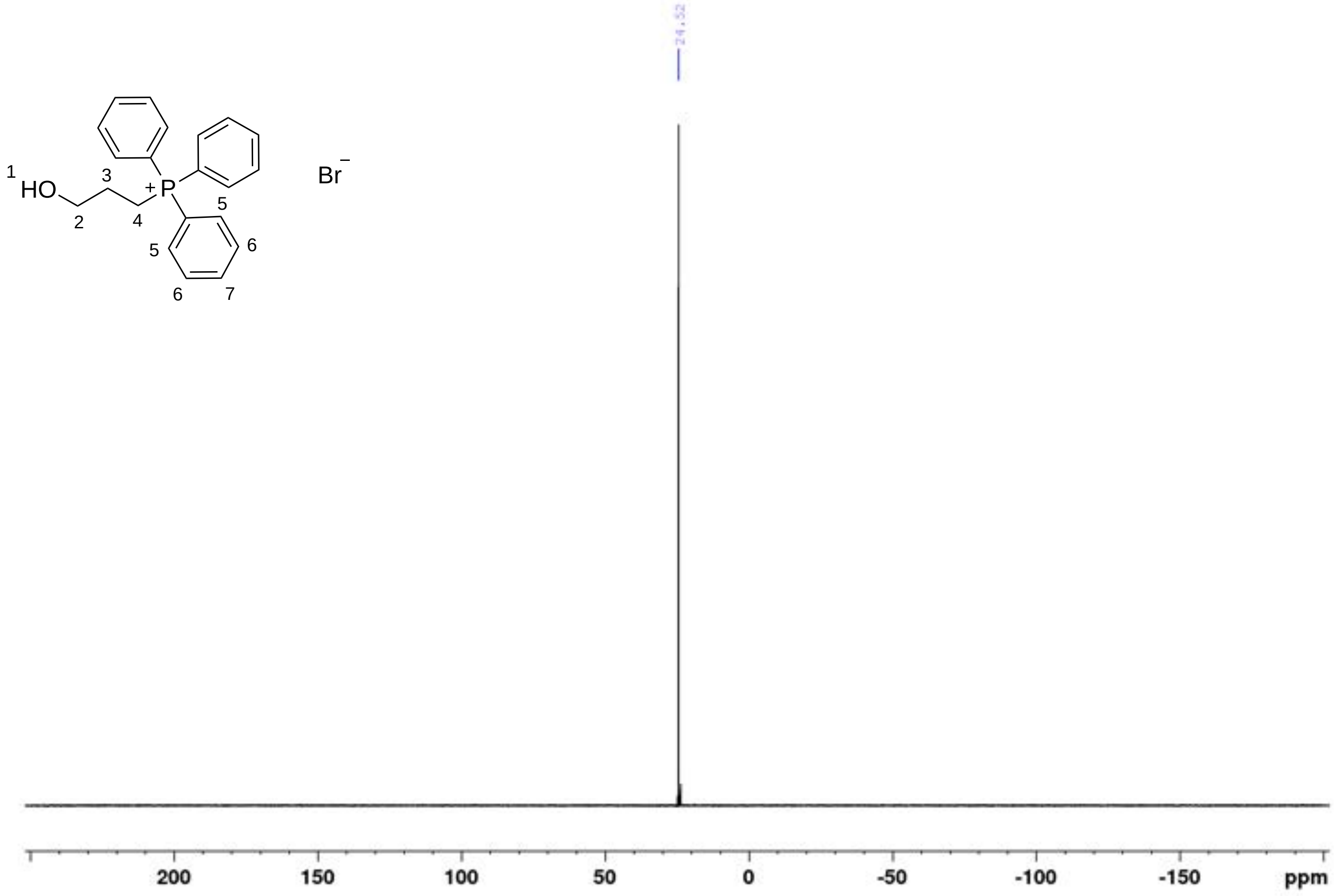

^{1}H NMR (400 MHz, $CDCl_3$) for *3-cyclobutylidenepropan-1-ol* (**31b**)

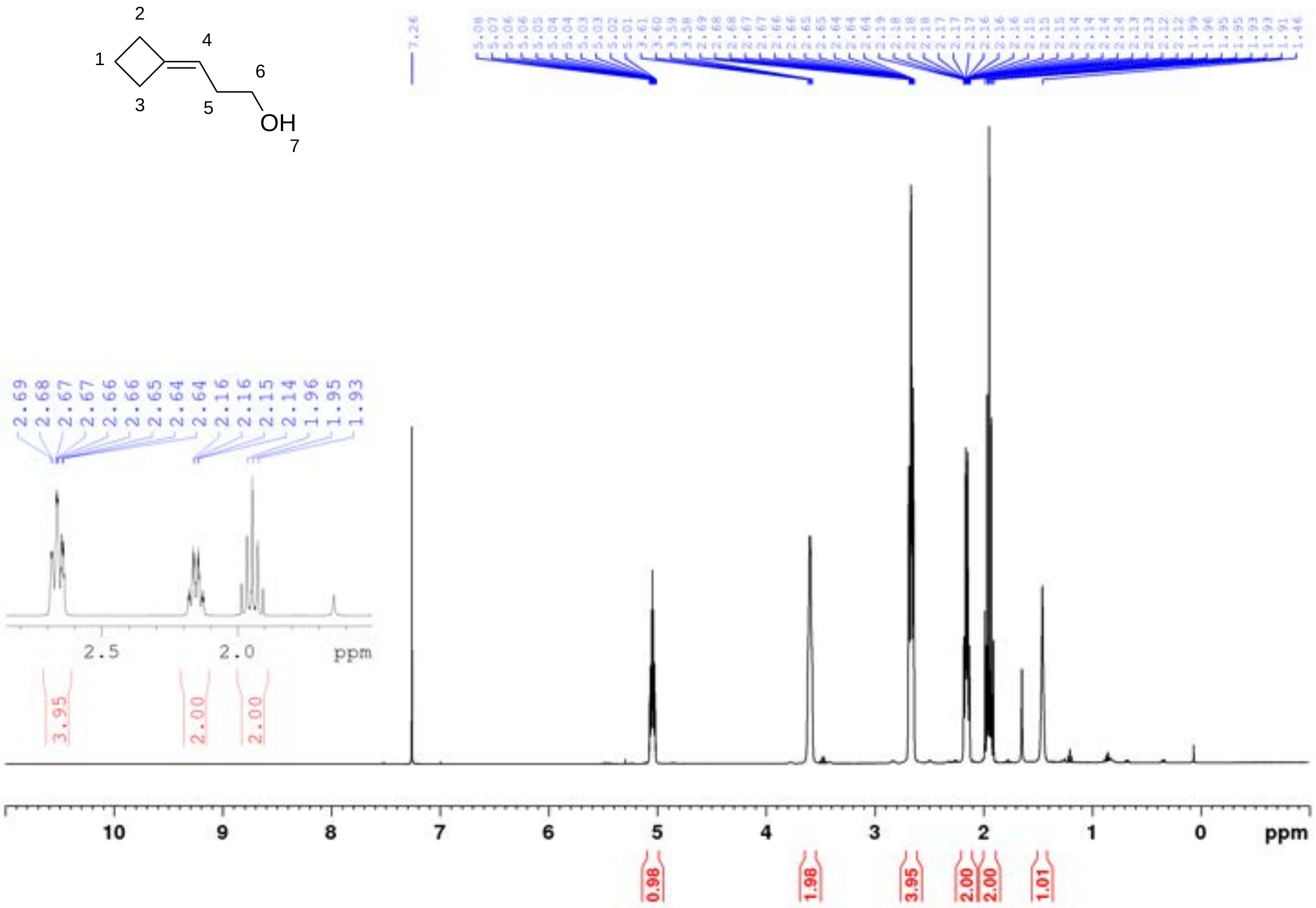

^{13}C NMR (101 MHz, $CDCl_3$) for *3-cyclobutylidenepropan-1-ol* (**31b**)

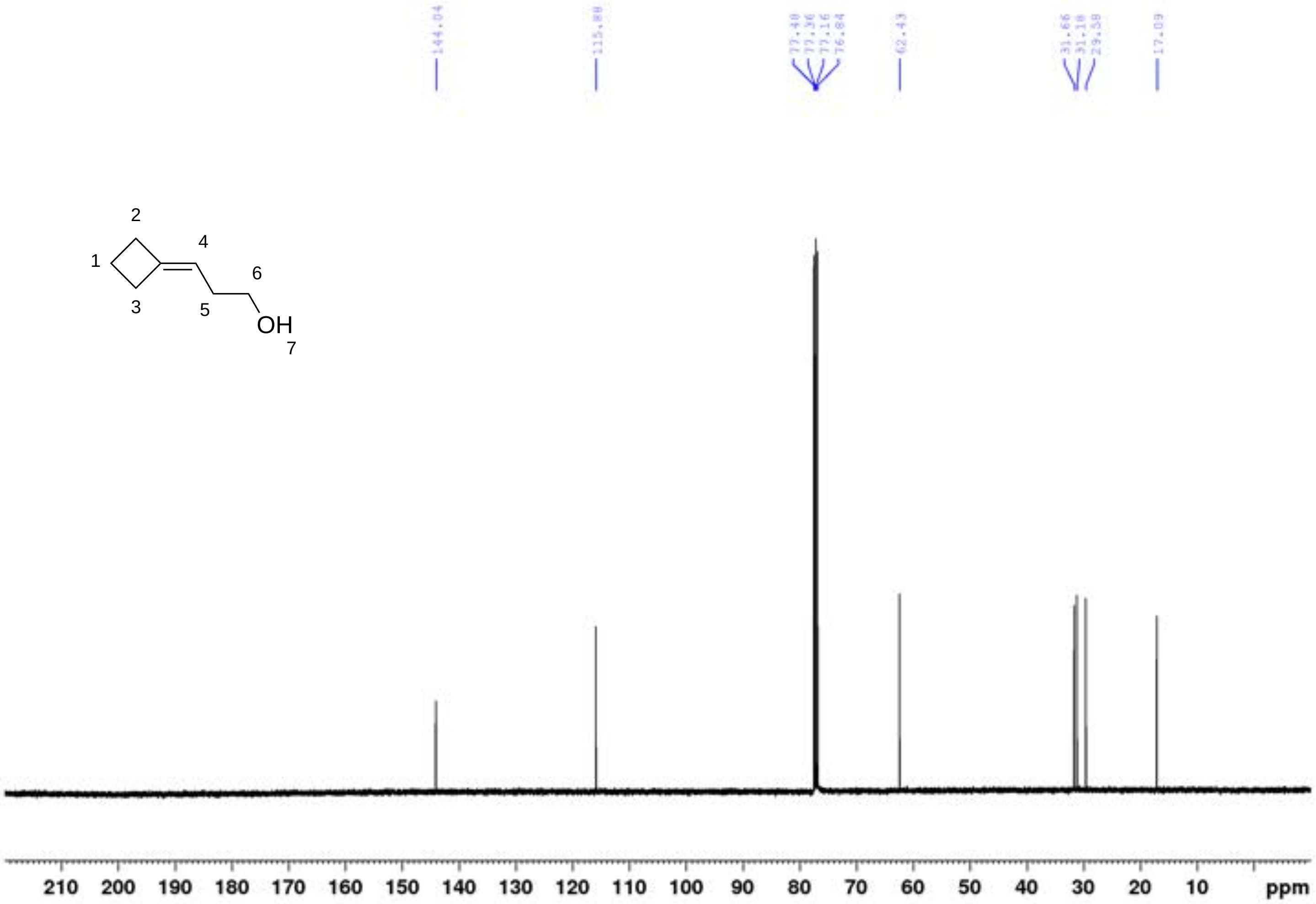

^{1}H NMR (400 MHz, $CDCl_3$) for *3-Cyclobutylidenepropan-1-ol* (**31c**)

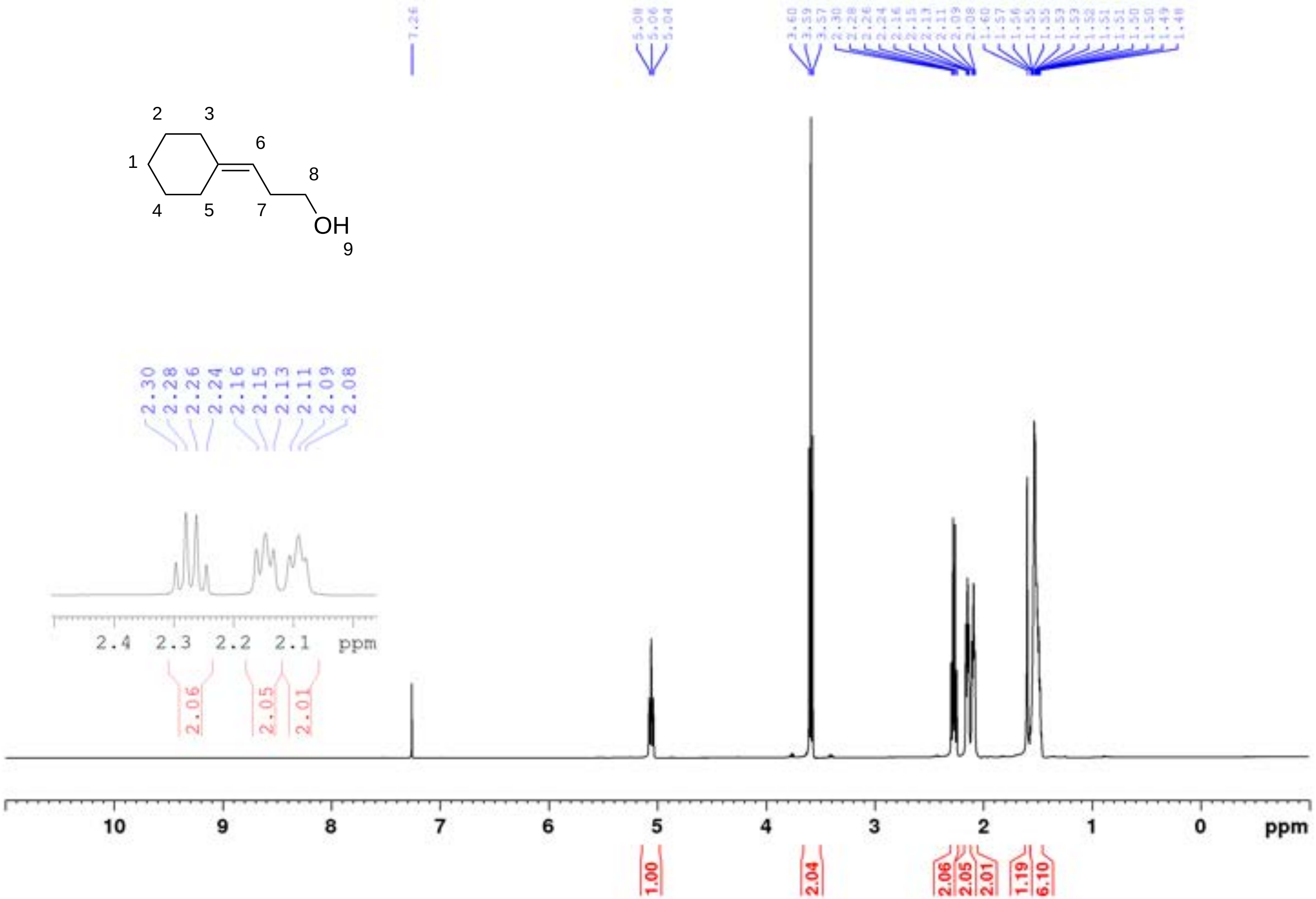

^{13}C NMR (101 MHz, $CDCl_3$) for *3-Cyclohexylidenepropan-1-ol* (**31c**)

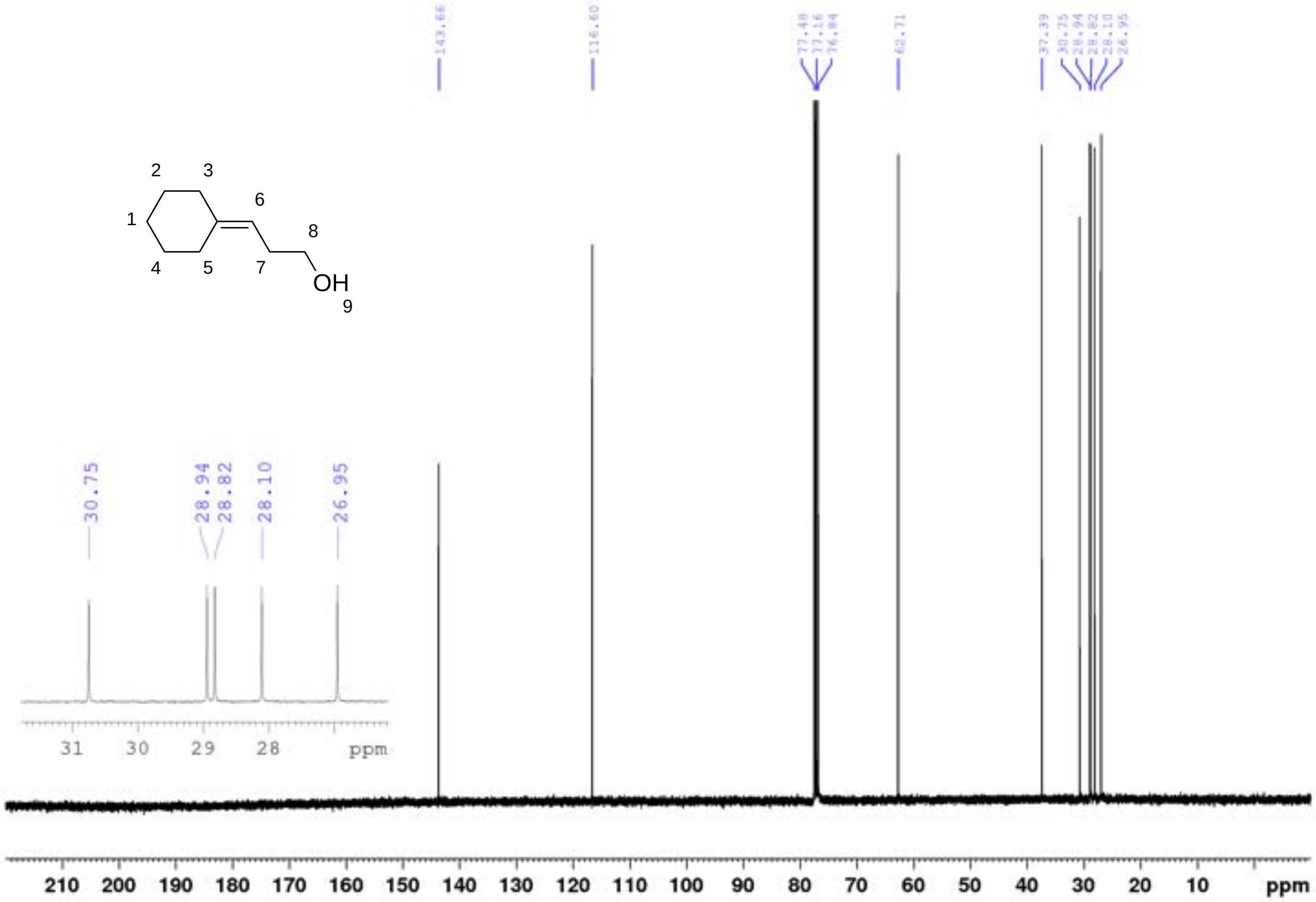

¹H NMR (400 MHz, $CDCl_3$) for *3-(Tetrahydro-4H-pyran-4-ylidene)propan-1-ol* (**31d**)

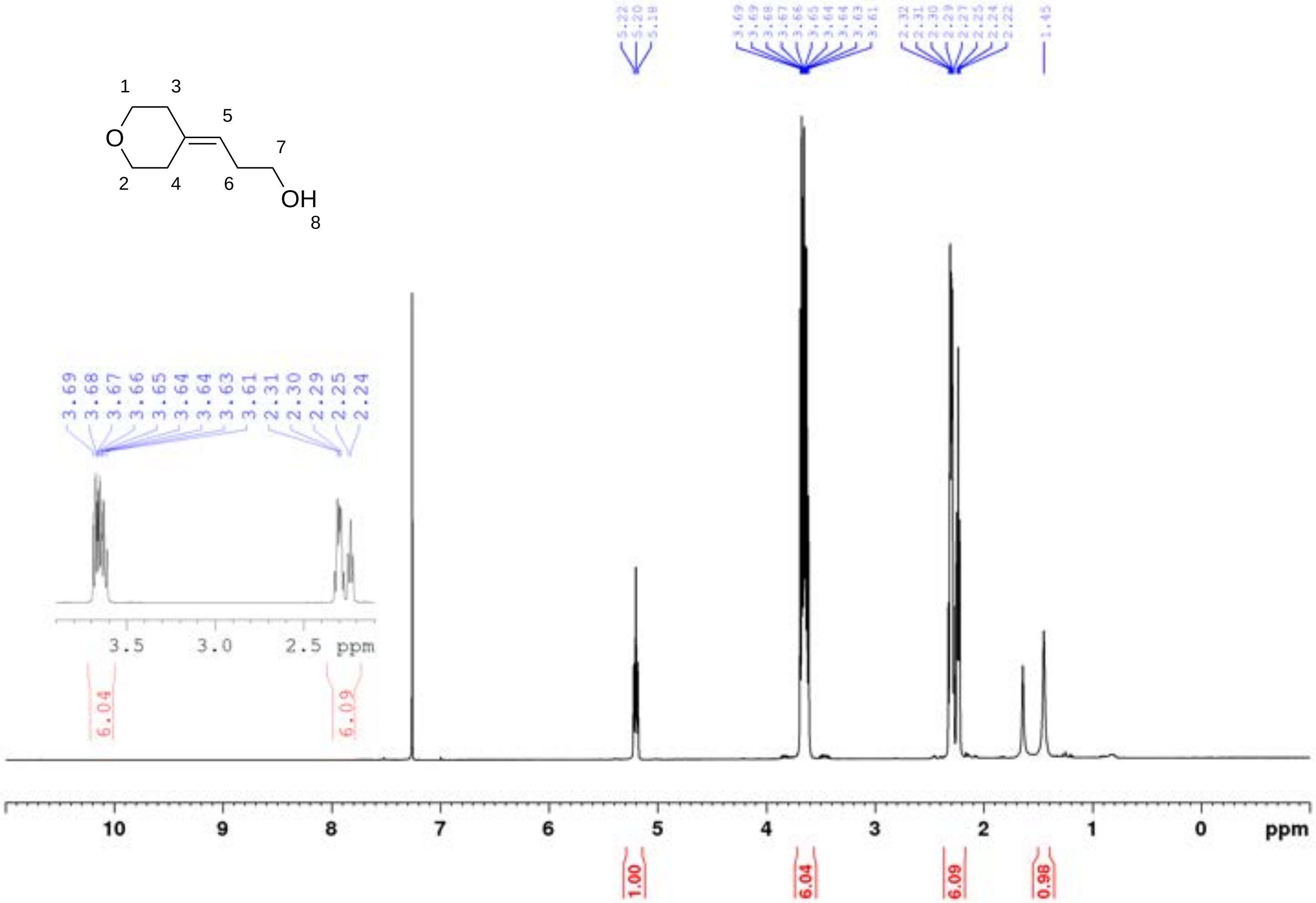

^{13}C NMR (101 MHz, $CDCl_3$) for *3-(Tetrahydro-4H-pyran-4-ylidene)propan-1-ol* (**31d**)

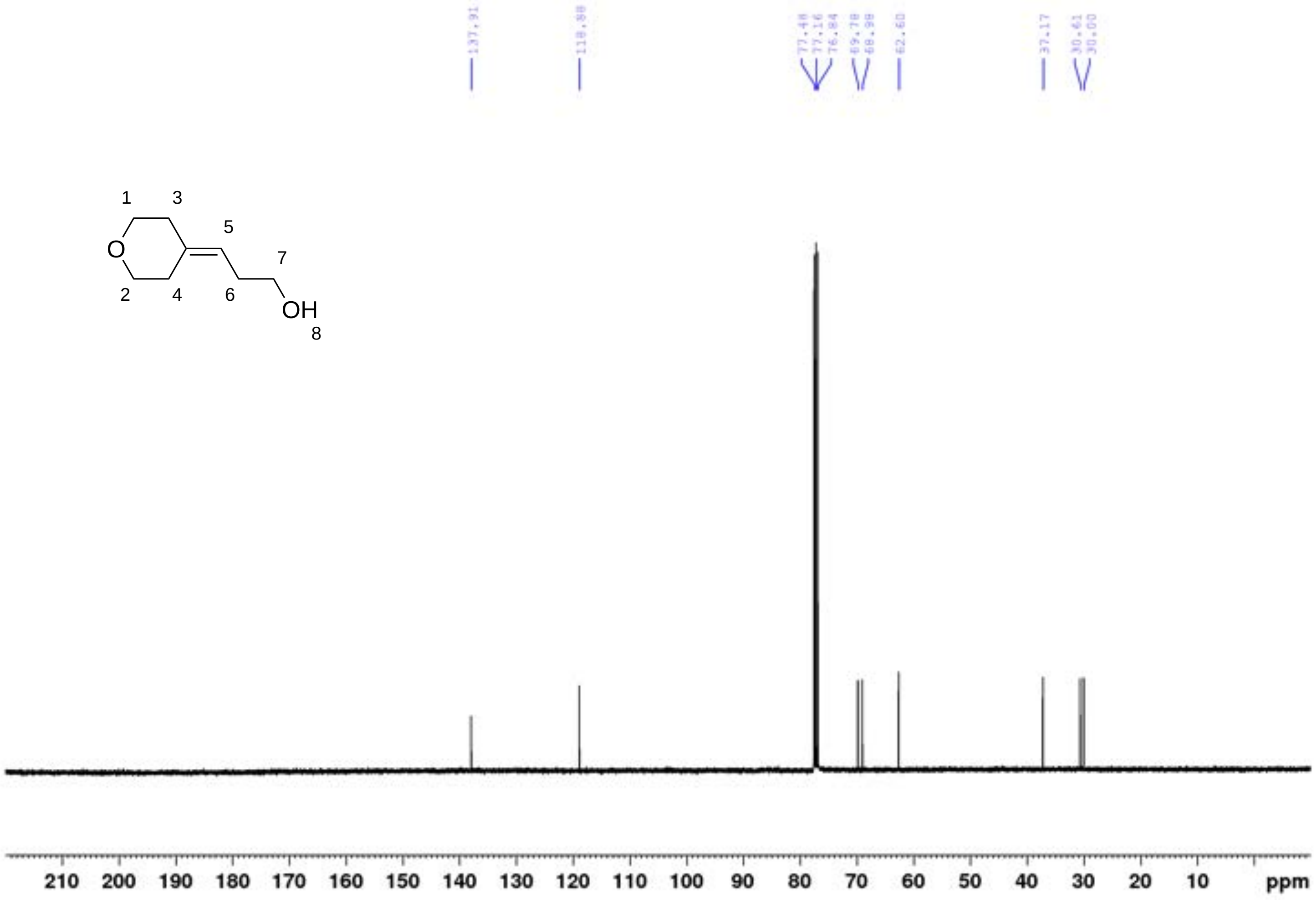

^{1}H NMR (400 MHz, CDCl$_3$) for *5-methylhex-4-en-1-ol* (**31e**)

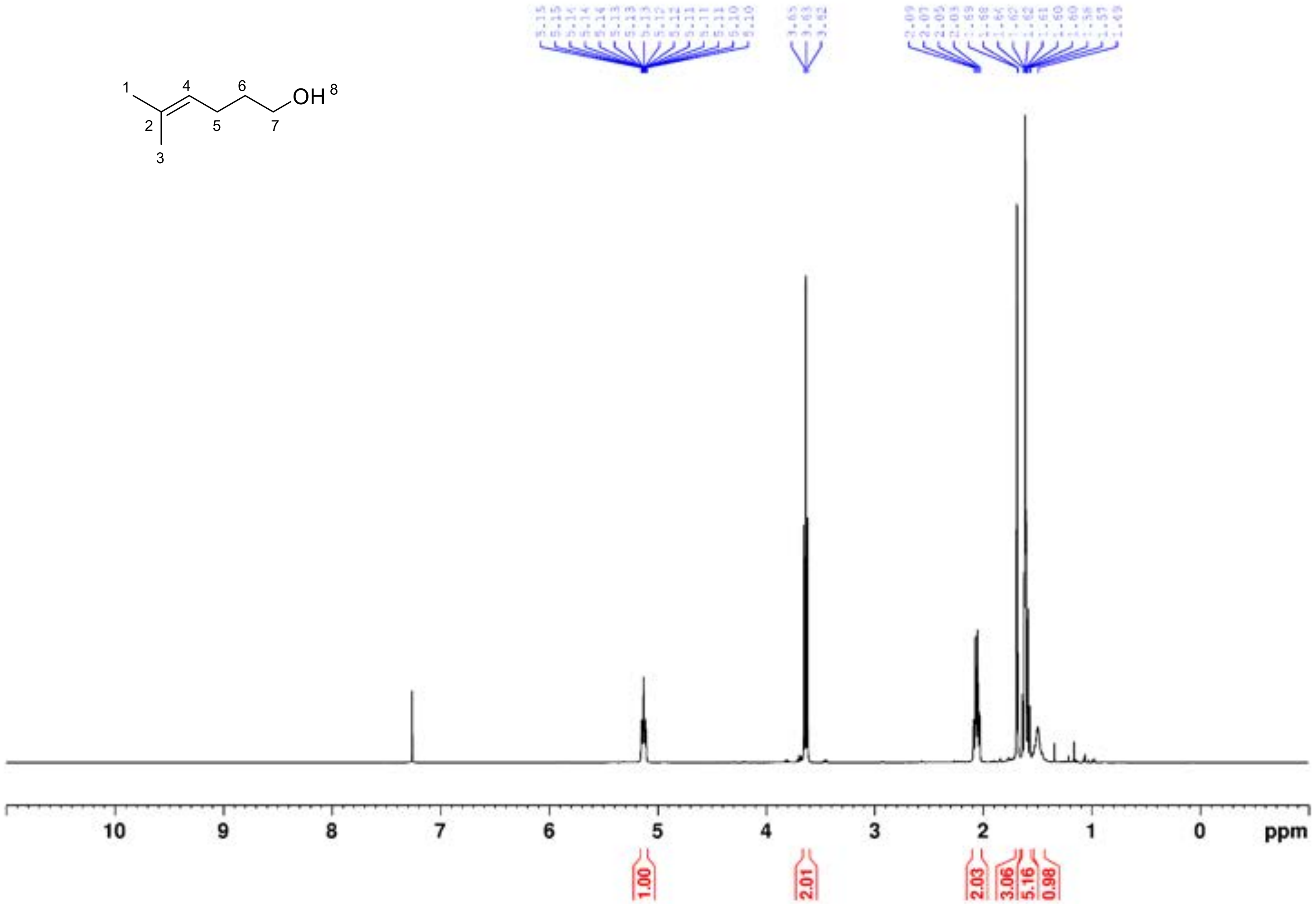

^{13}C NMR (101 MHz, CDCl$_3$) for *5-methylhex-4-en-1-ol* (**31e**)

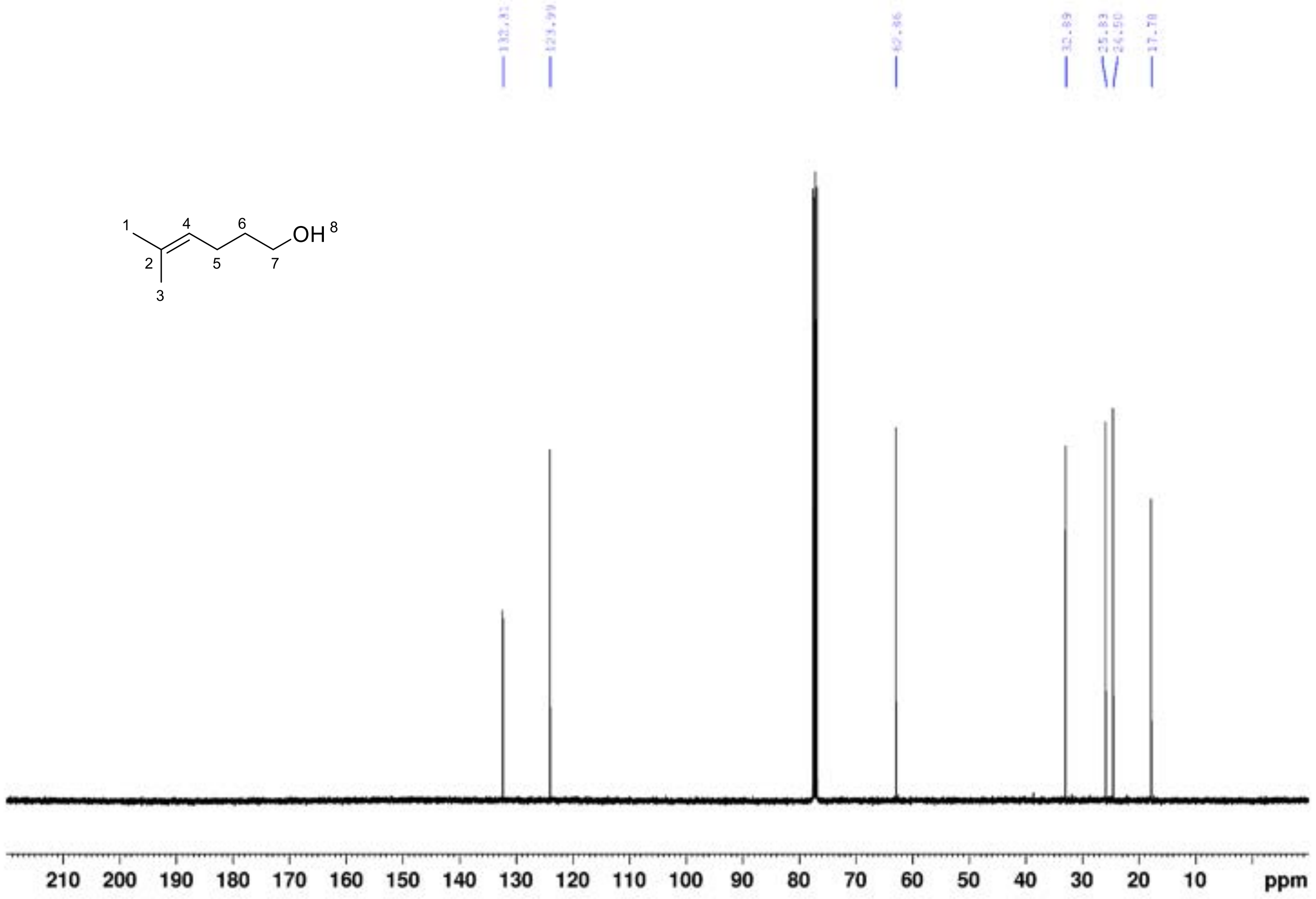

^{1}H NMR (400 MHz, CDCl$_3$) for *4-bromobutan-1-ol*

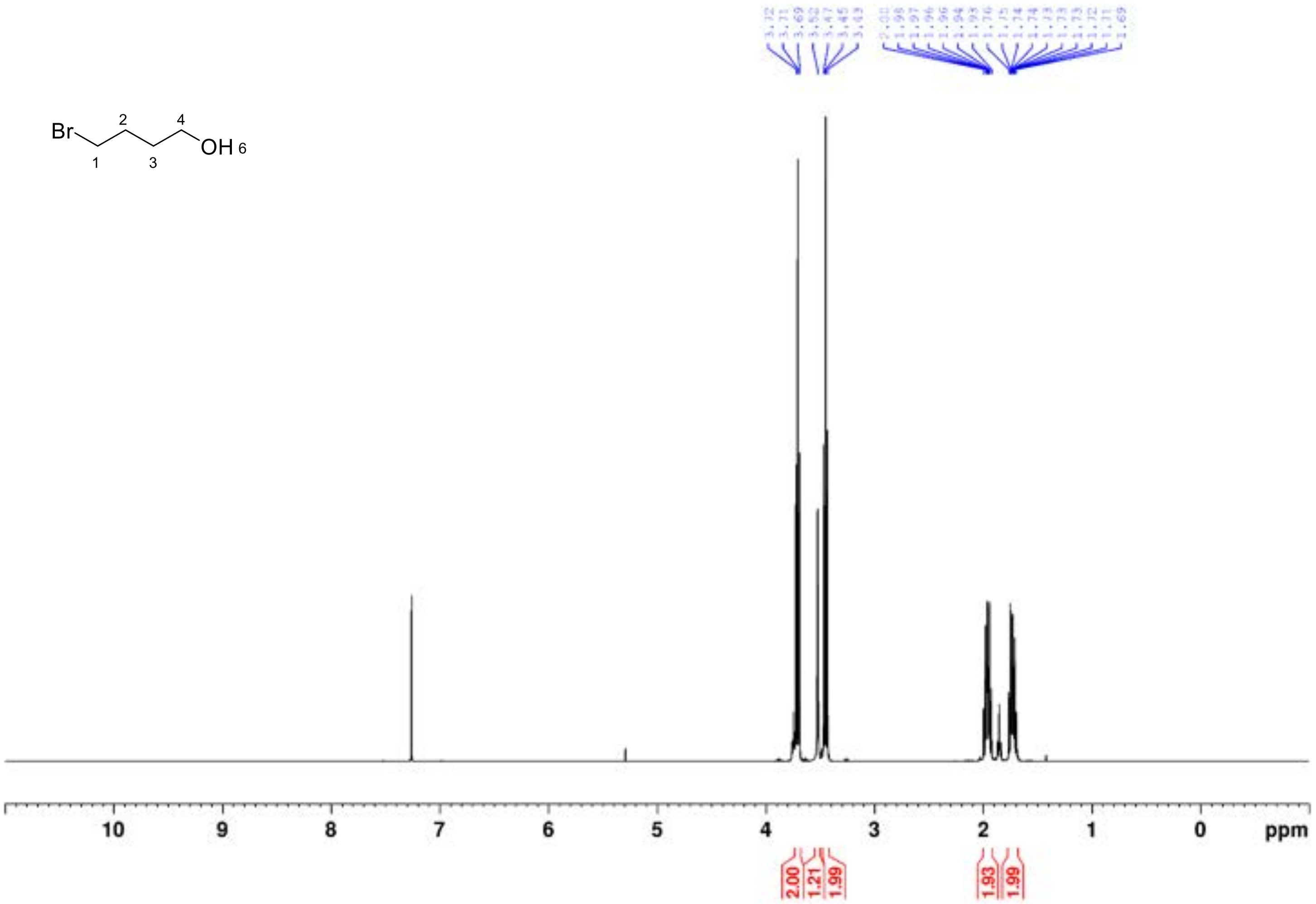

^{13}C NMR (101 MHz, CDCl$_3$) for *4-bromobutan-1-ol*

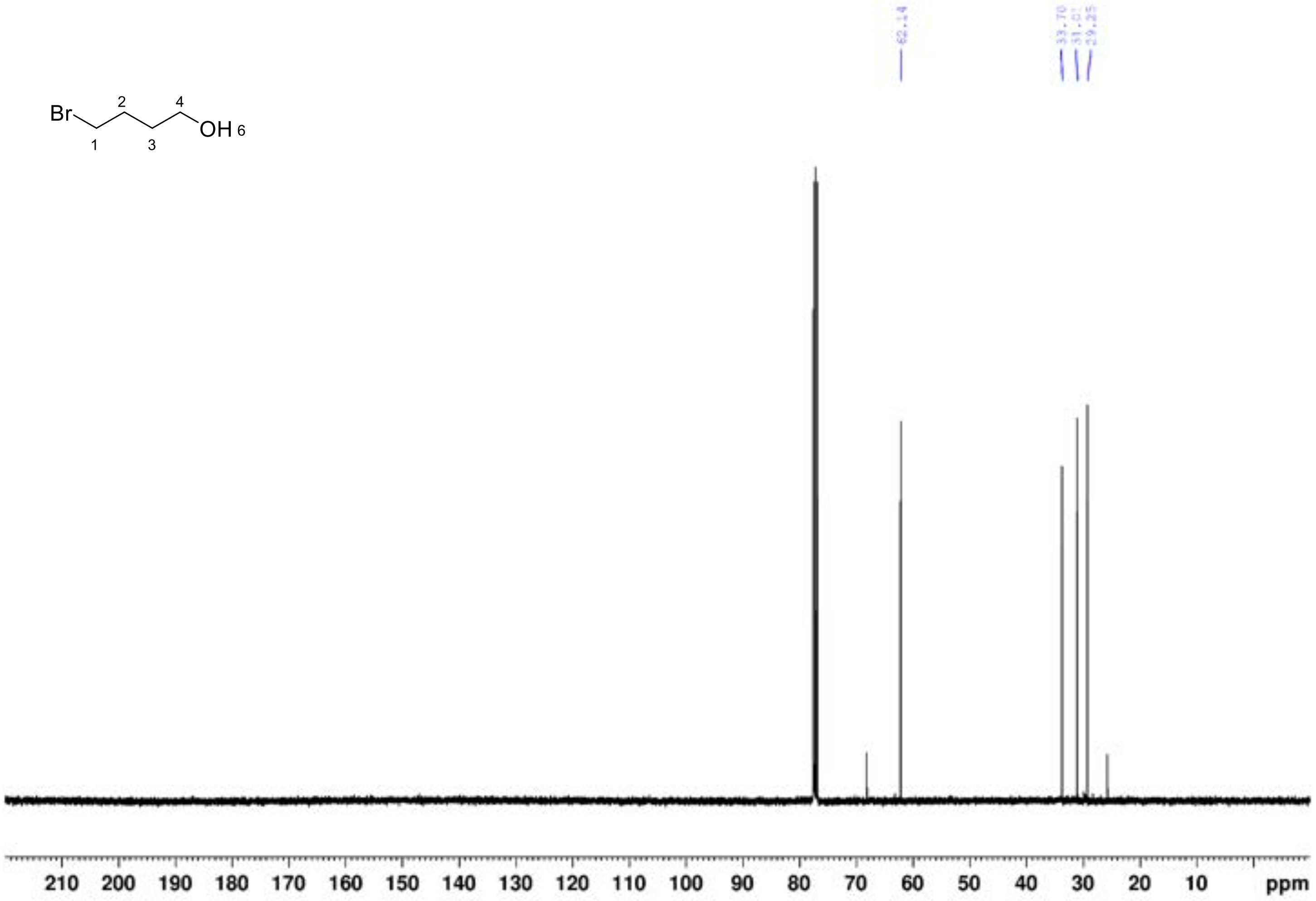

^{1}H NMR (400 MHz, CDCl$_3$) for *(4-hydroxybutyl)-triphenylphosphonium bromide*

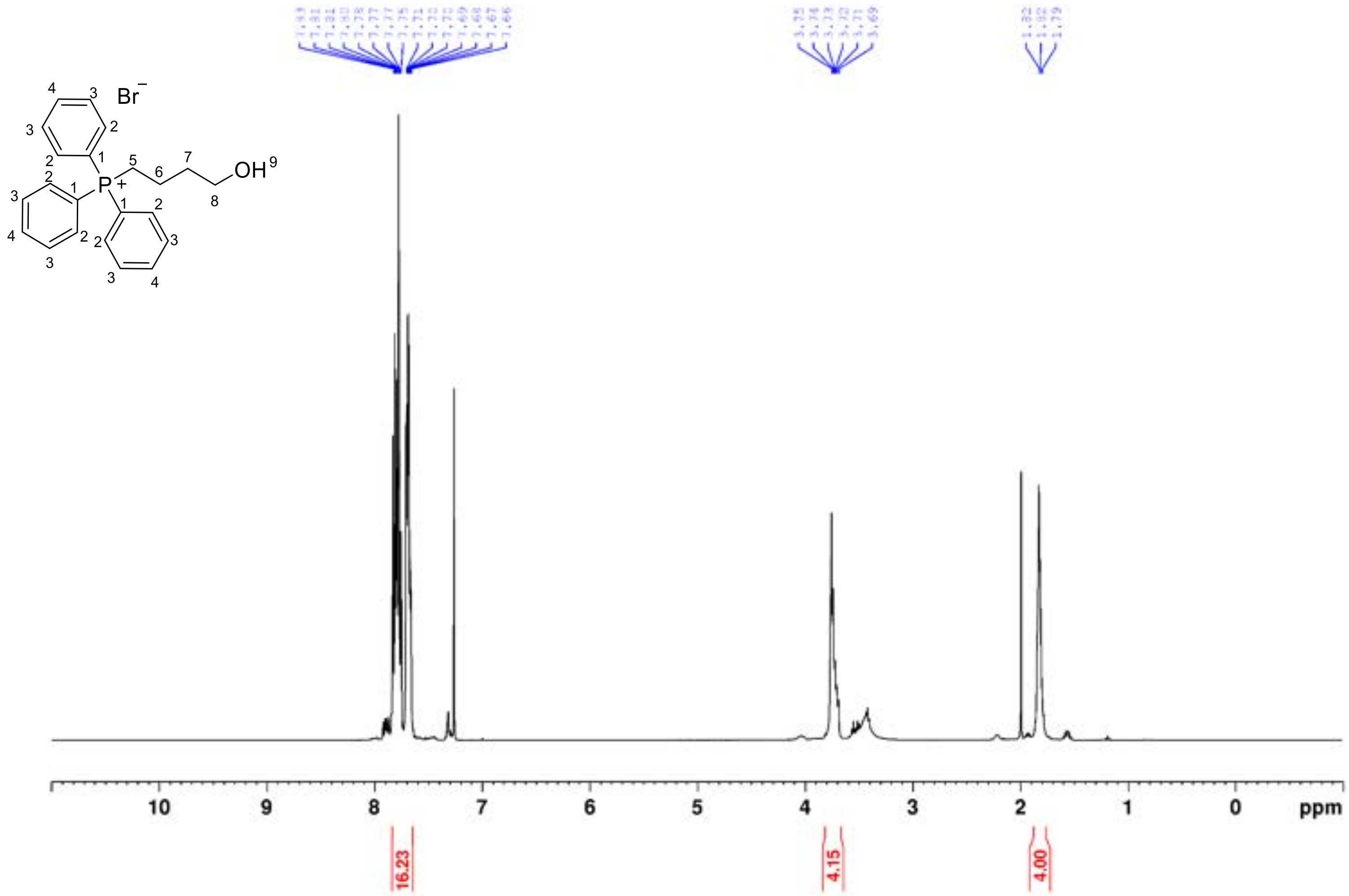

^{13}C NMR (101 MHz, $CDCl_3$) for *(4-hydroxybutyl)-triphenylphosphonium bromide*

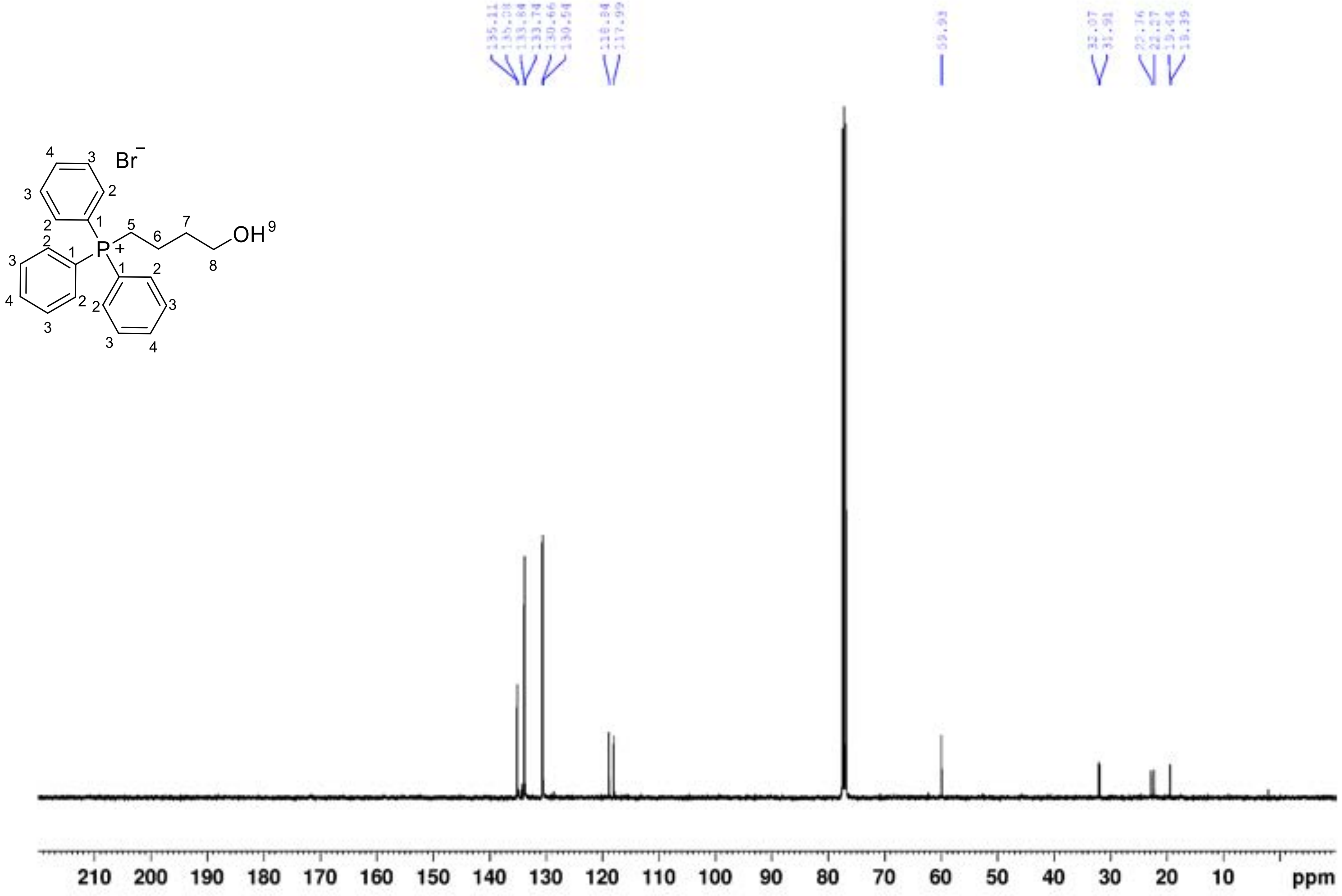

^{31}P NMR (162 MHz, $CDCl_3$) for *(4-hydroxybutyl)-triphenylphosphonium bromide*

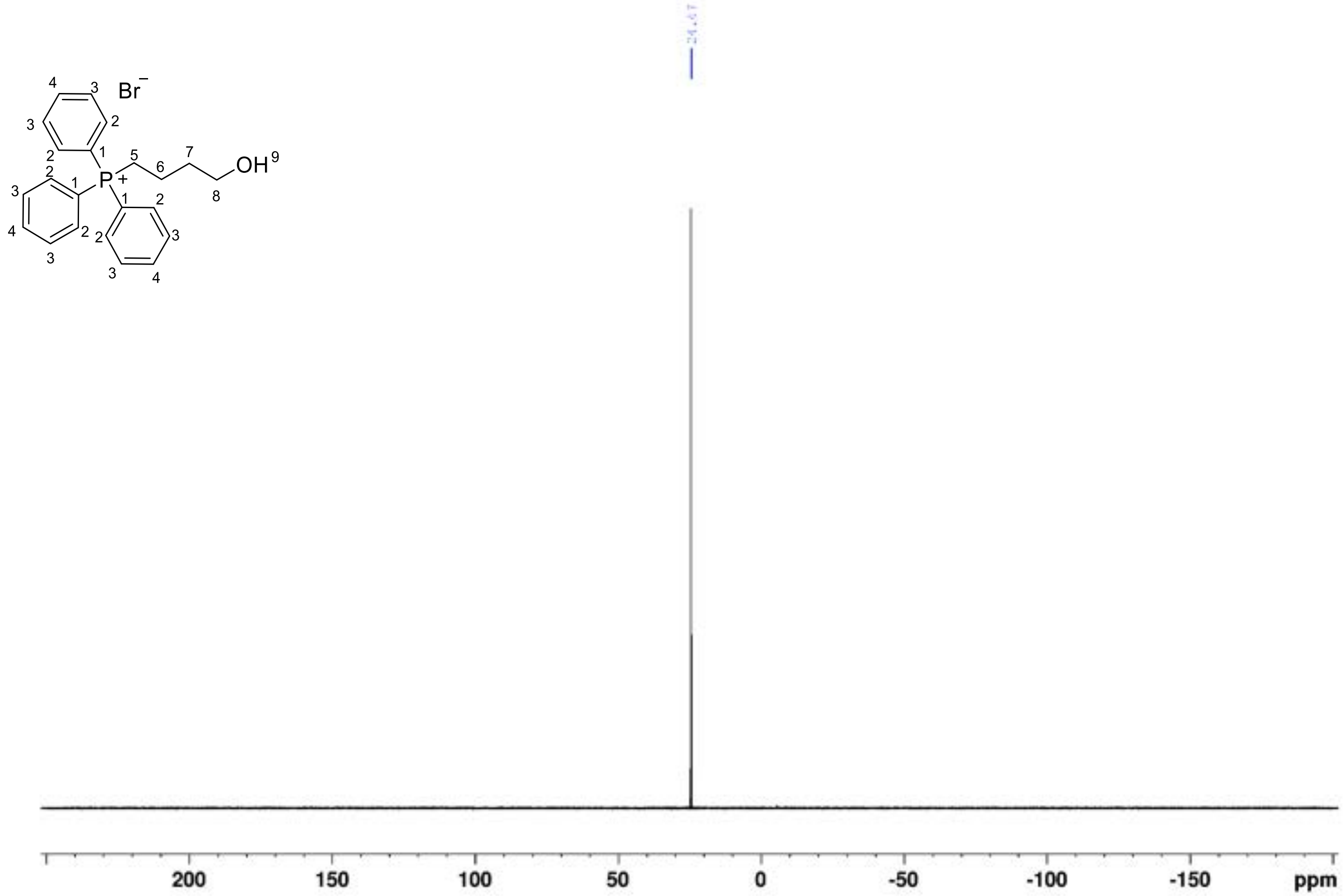

^{1}H NMR (400 MHz, CDCl$_3$) for *4-cyclohexylidenebutan-1-ol* (**31f**)

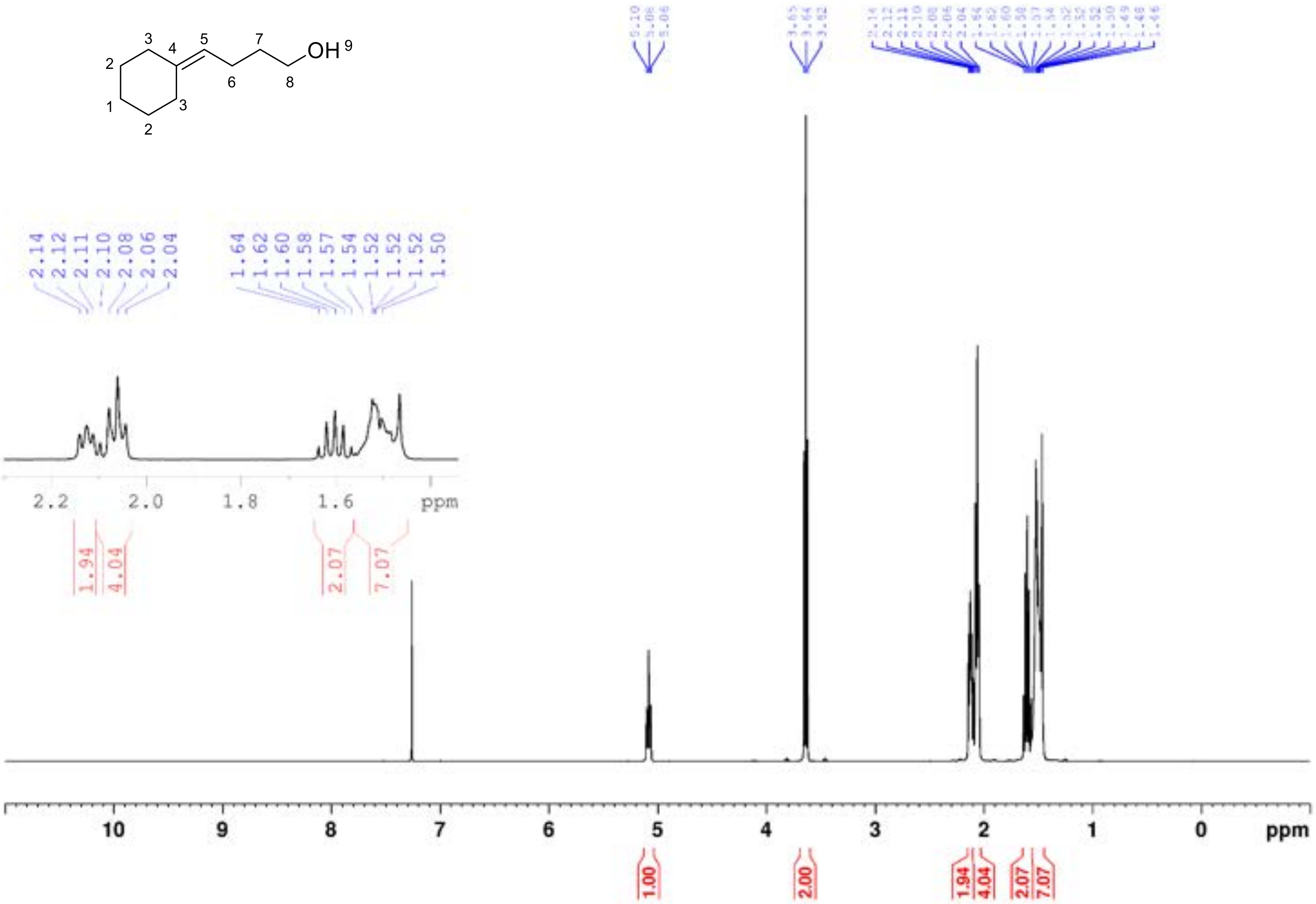

^{13}**C NMR (101 MHz, CDCl$_3$)** for *4-cyclohexylidenebutan-1-ol* (**31f**)

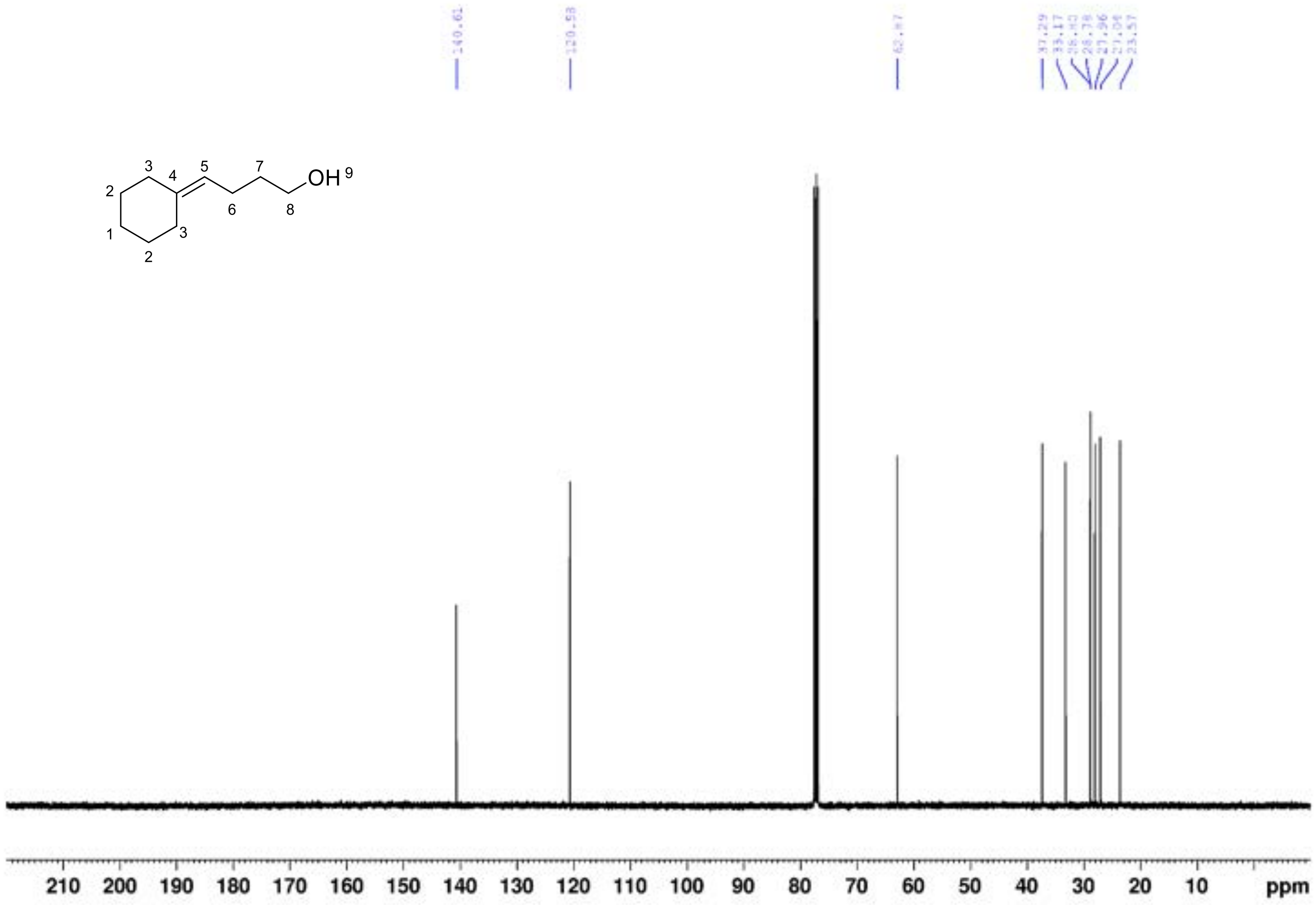

^{1}H NMR (500 MHz, $CDCl_3$) for *(Z)-4-phenylpent-3-en-1-ol* (**31g**)

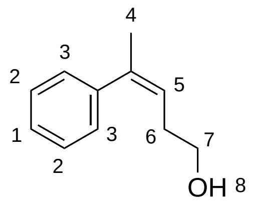

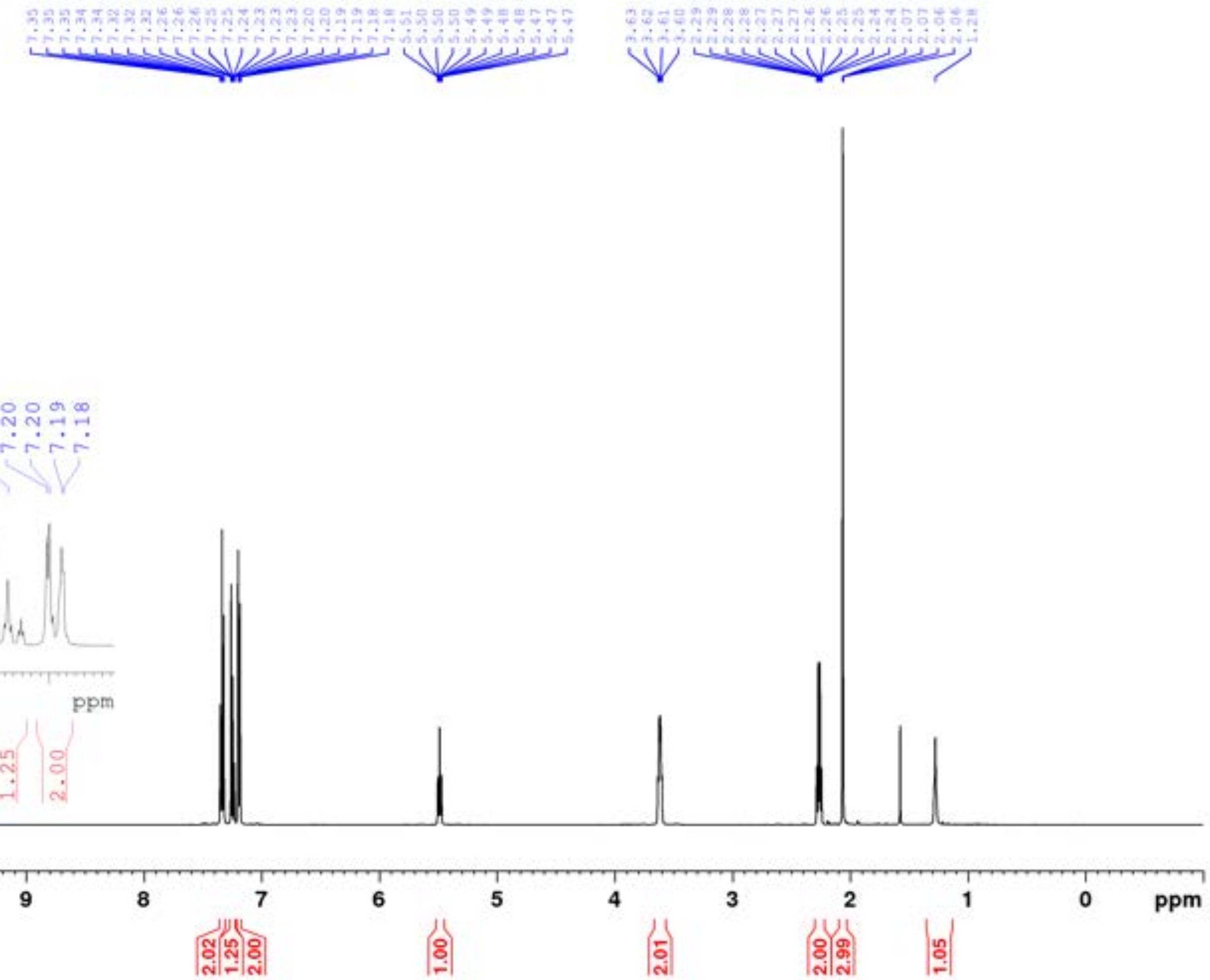

^{13}C NMR (126 MHz, $CDCl_3$) for *(Z)-4-phenylpent-3-en-1-ol* (**31g**)

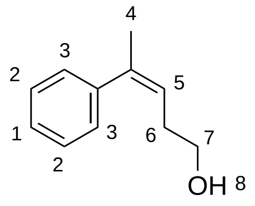

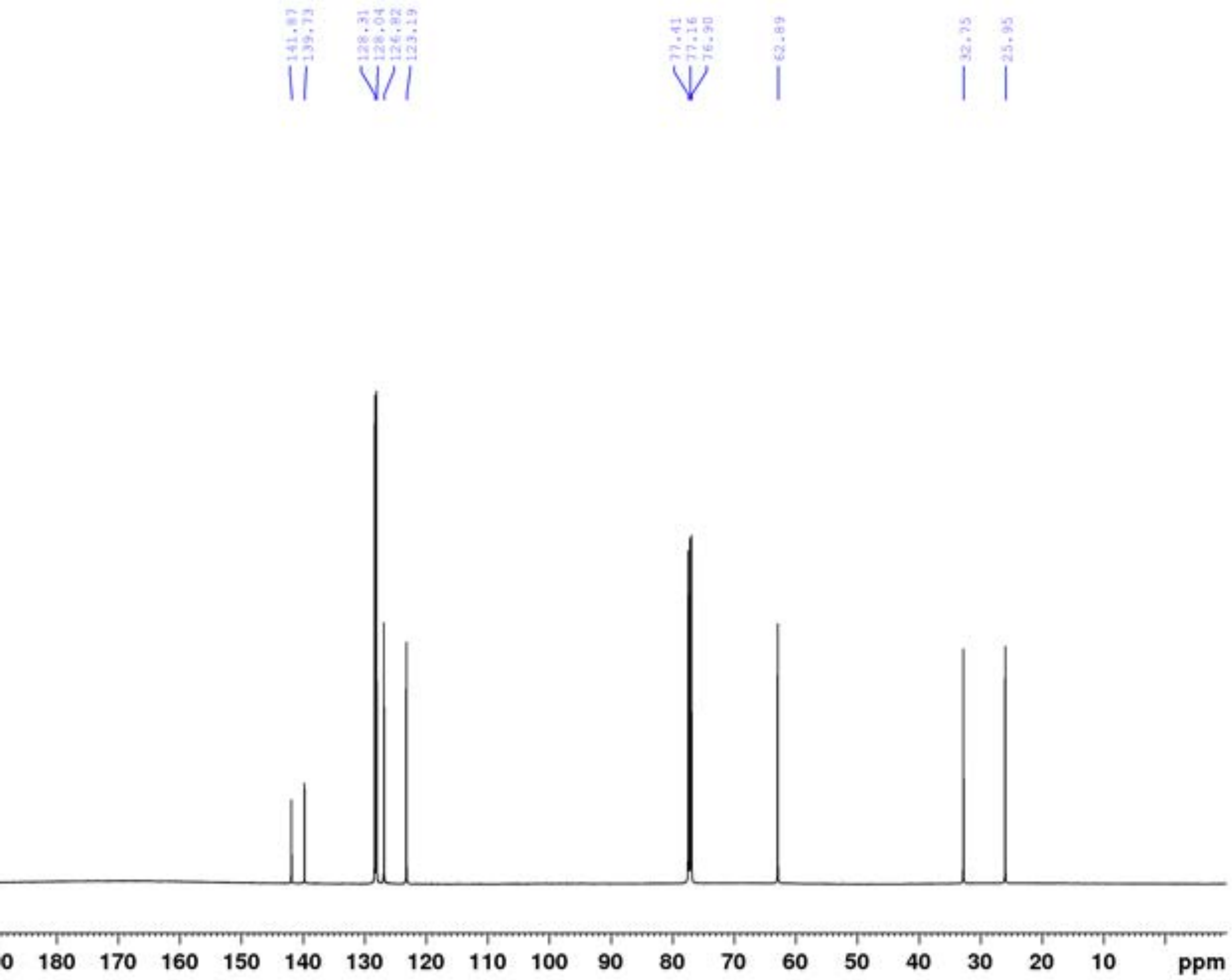

^{1}H NMR (400 MHz, $CDCl_3$) for *1-Cyclopropyl-1-phenylethan-1-ol*

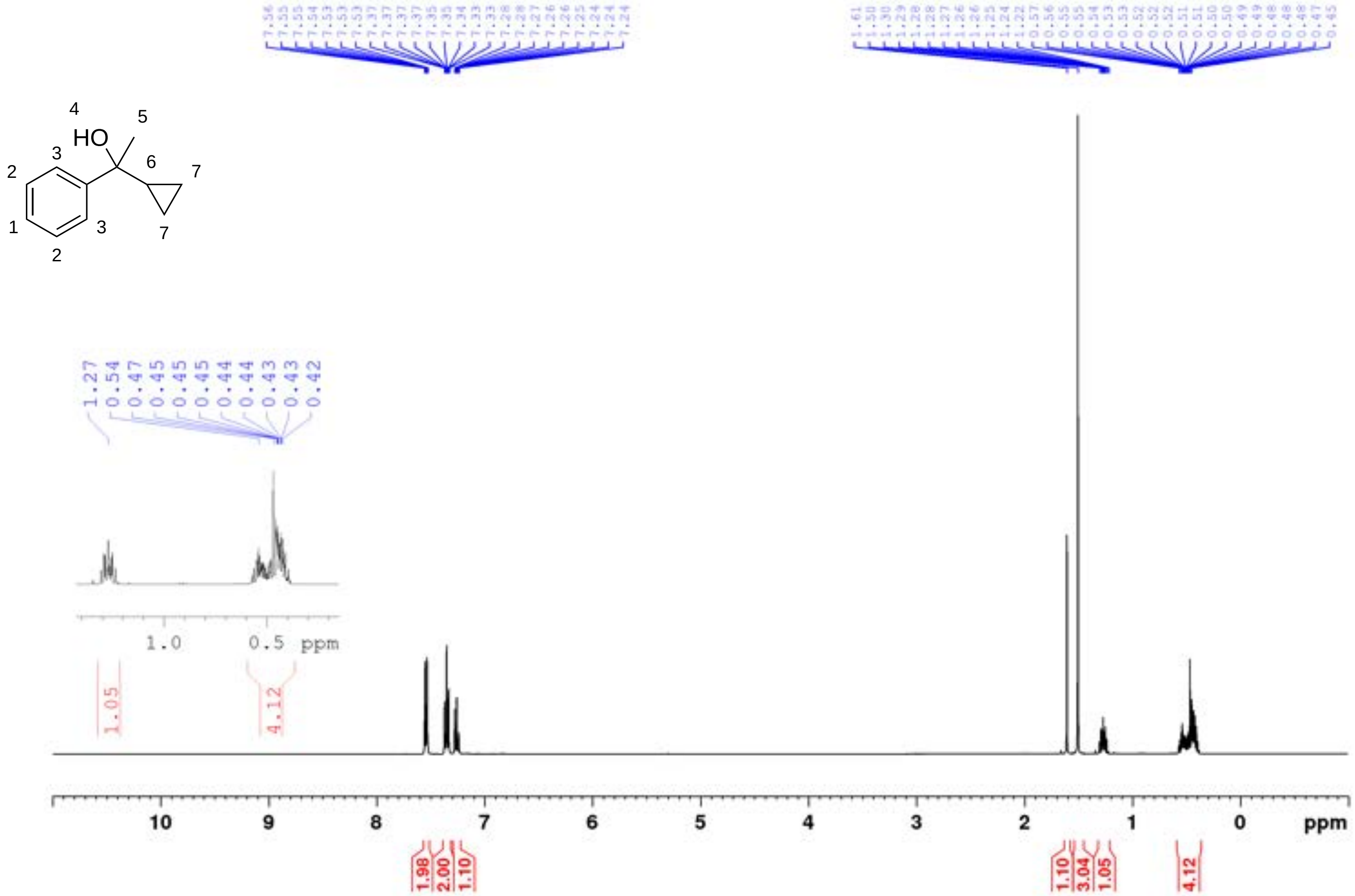

^{13}**C NMR** (101 MHz, $CDCl_3$) for *1-Cyclopropyl-1-phenylethan-1-ol*

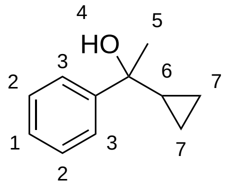

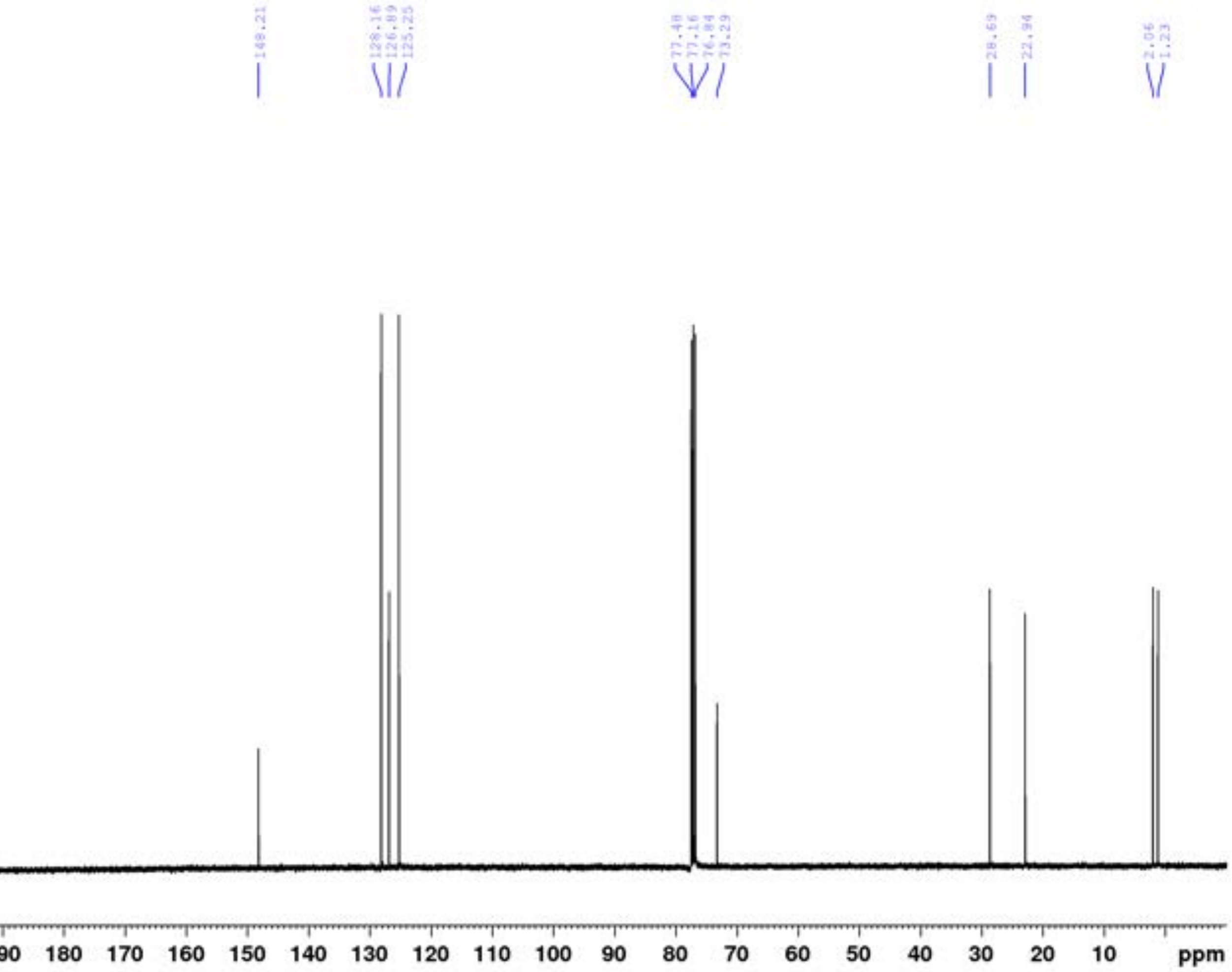

¹H NMR (400 MHz, $CDCl_3$) for *(E)-4-phenylpent-3-en-1-ol* (**31h**)

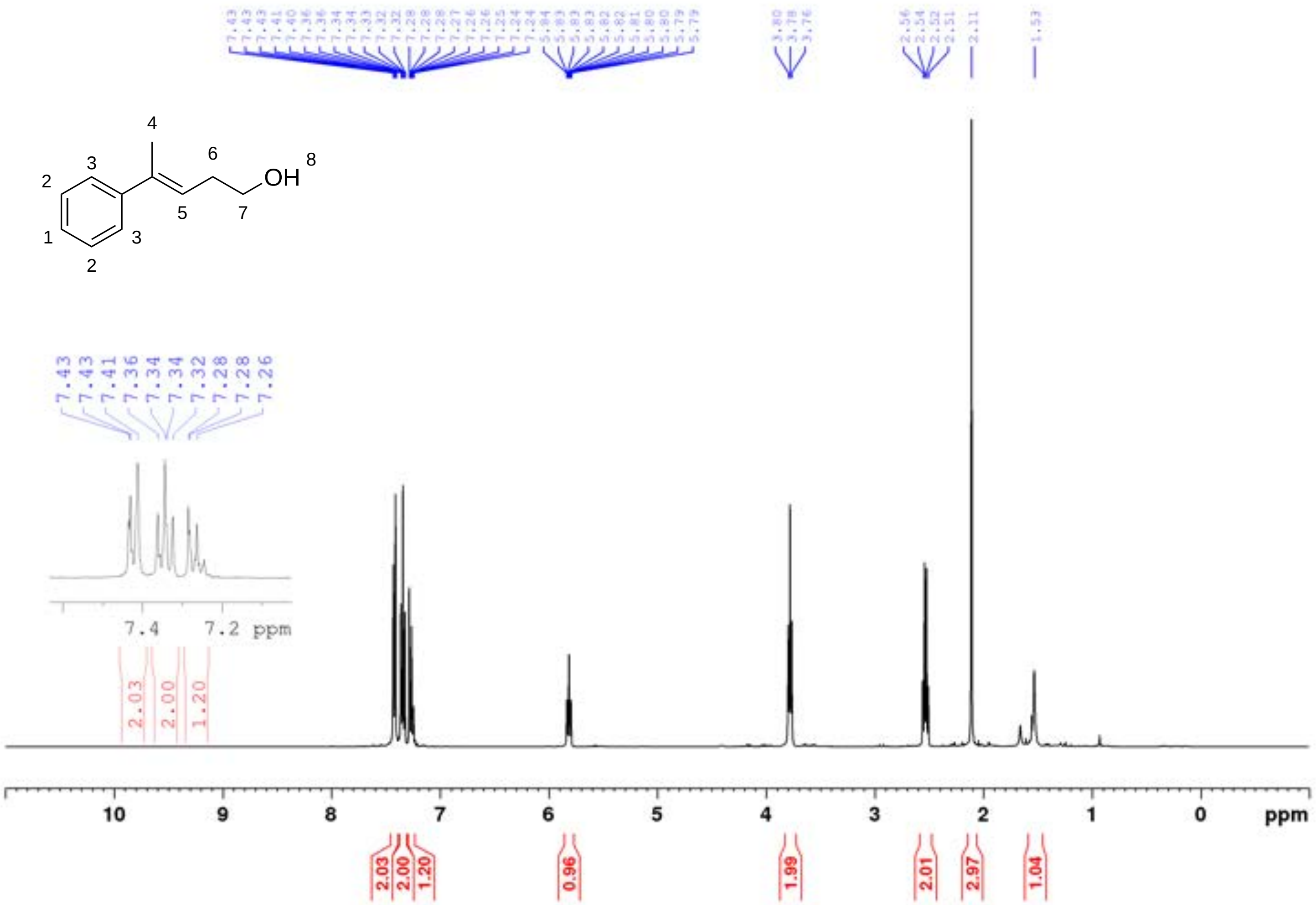

^{13}C NMR (101 MHz, $CDCl_3$) for *(E)-4-phenylpent-3-en-1-ol* (**31h**)

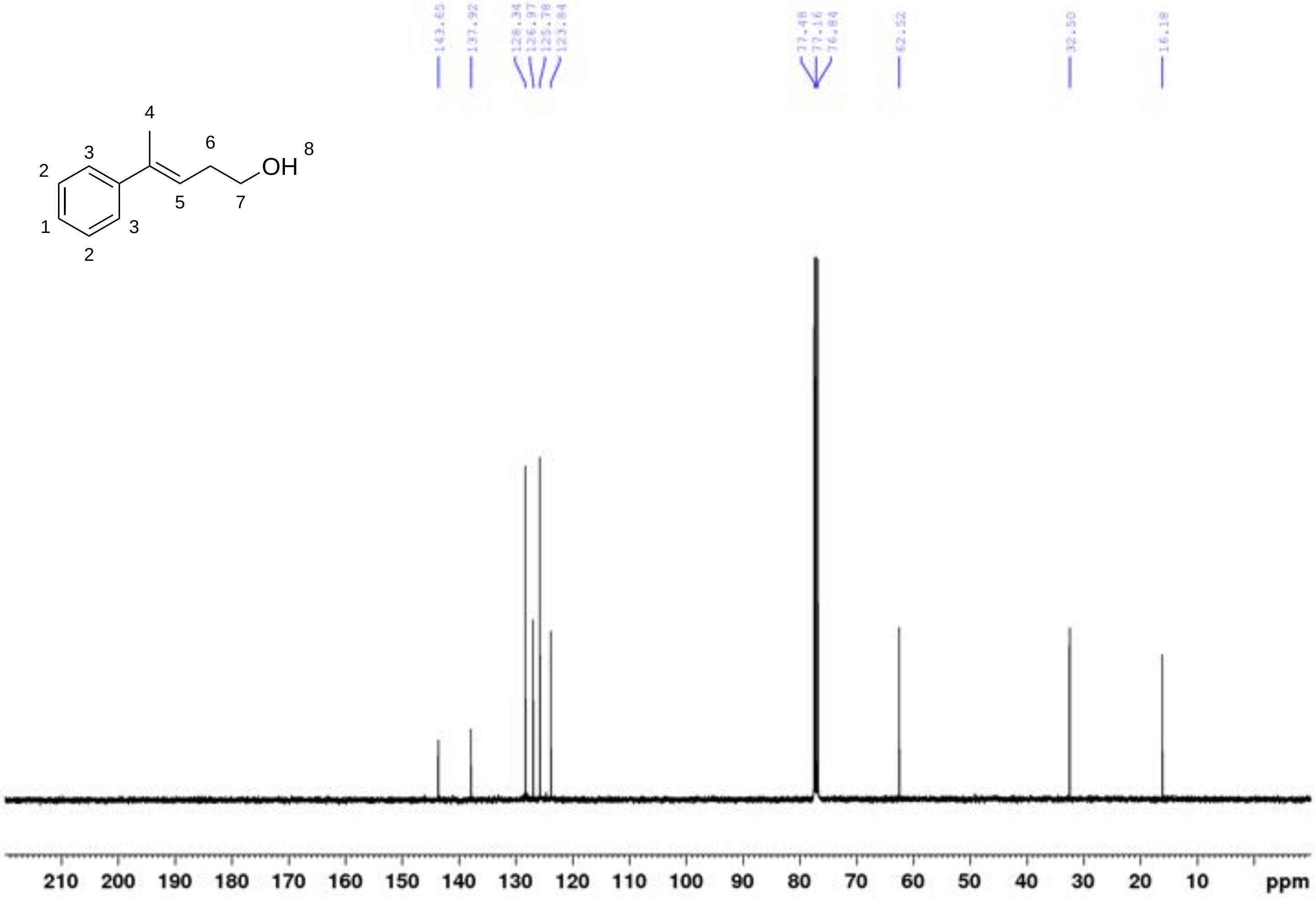

^{1}H NMR (400 MHz, $CDCl_3$) for *(Z)-3,7-Dimethylocta-2,6-dienal* (**Neral**)

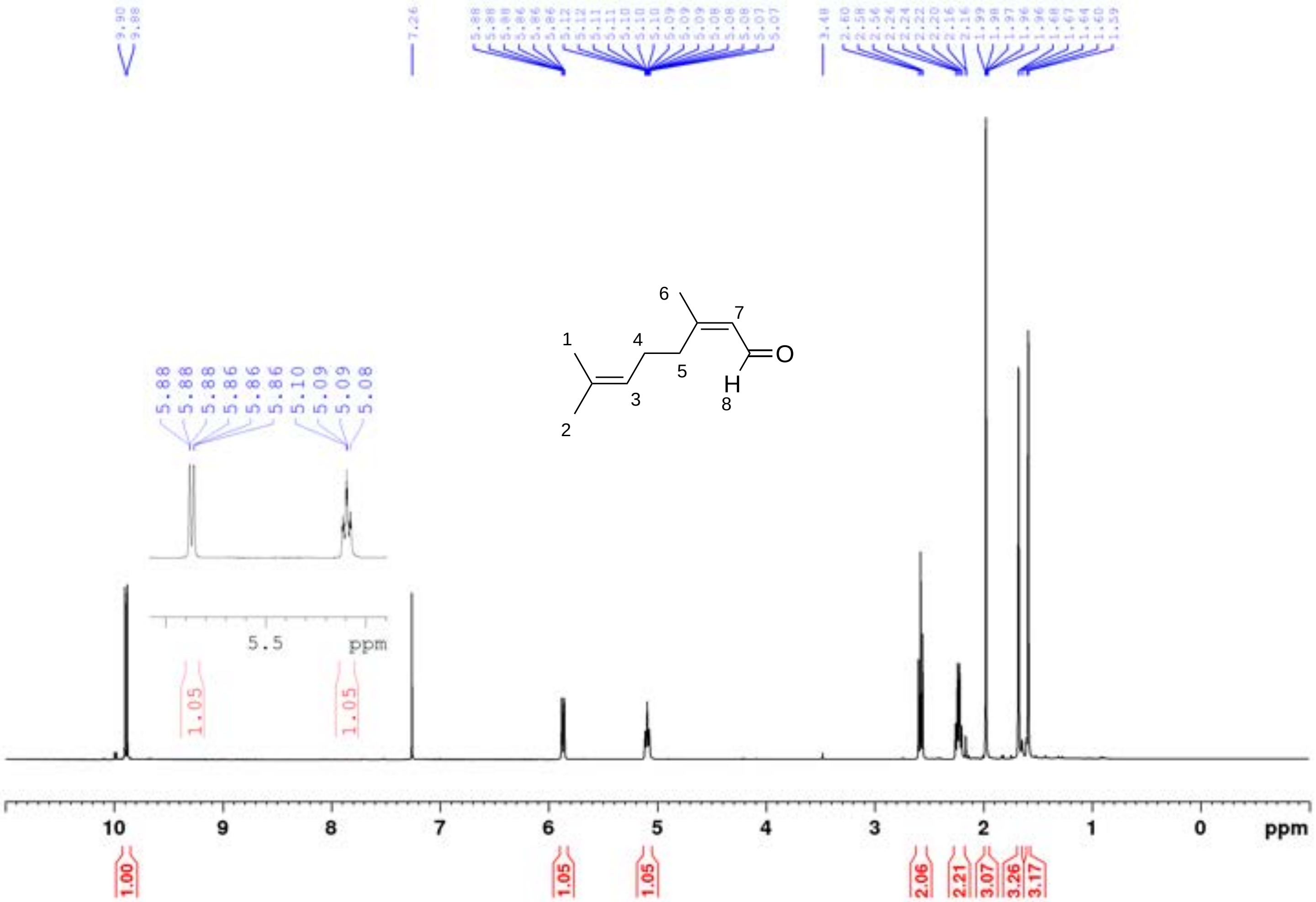

^{13}C NMR (126 MHz, $CDCl_3$) for *(Z)-3,7-Dimethylocta-2,6-dienal* (**Neral**)

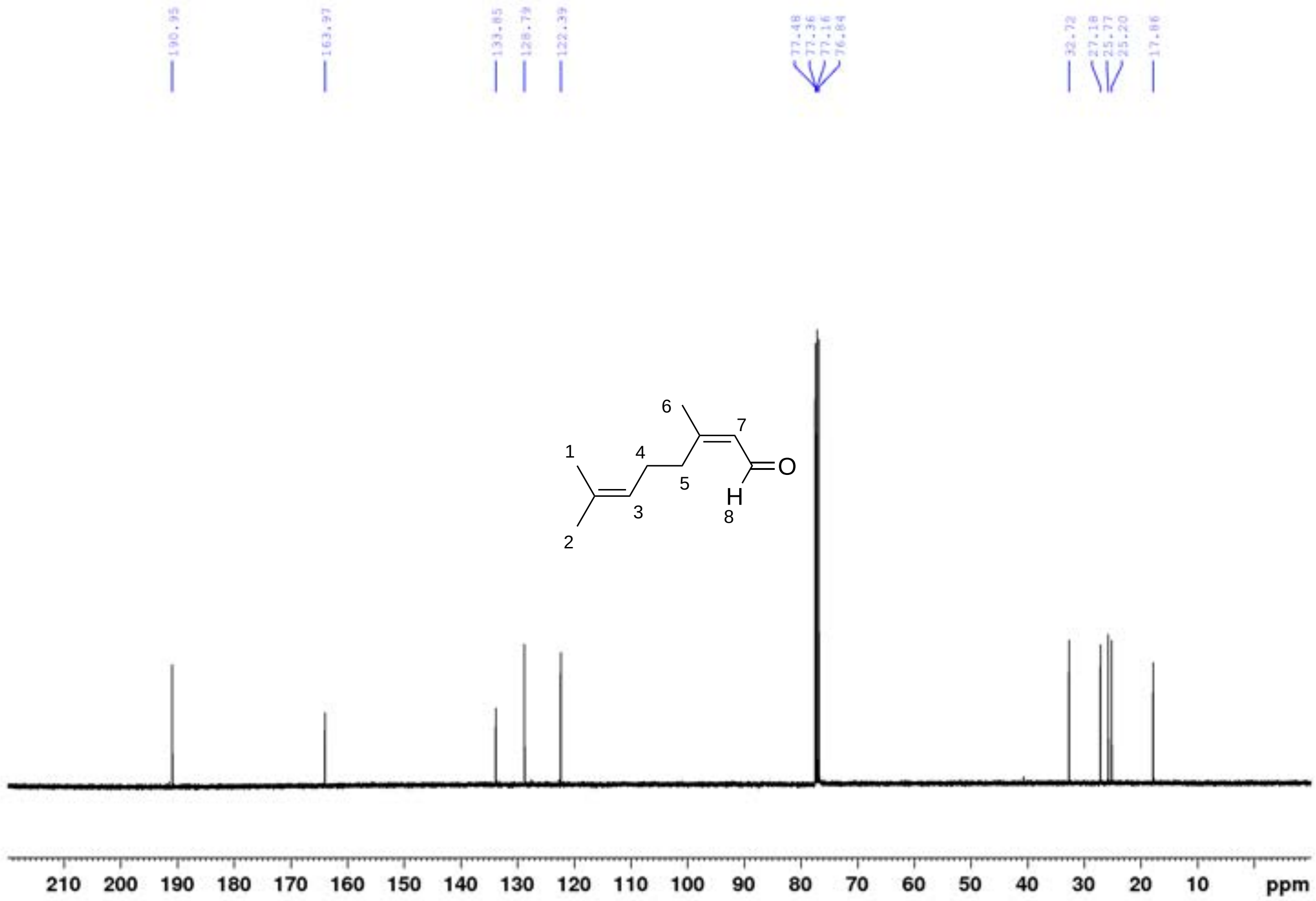

¹H NMR (400 MHz, $CDCl_3$) for *(Z)-4,8-dimethylnona-1,3,7-triene*

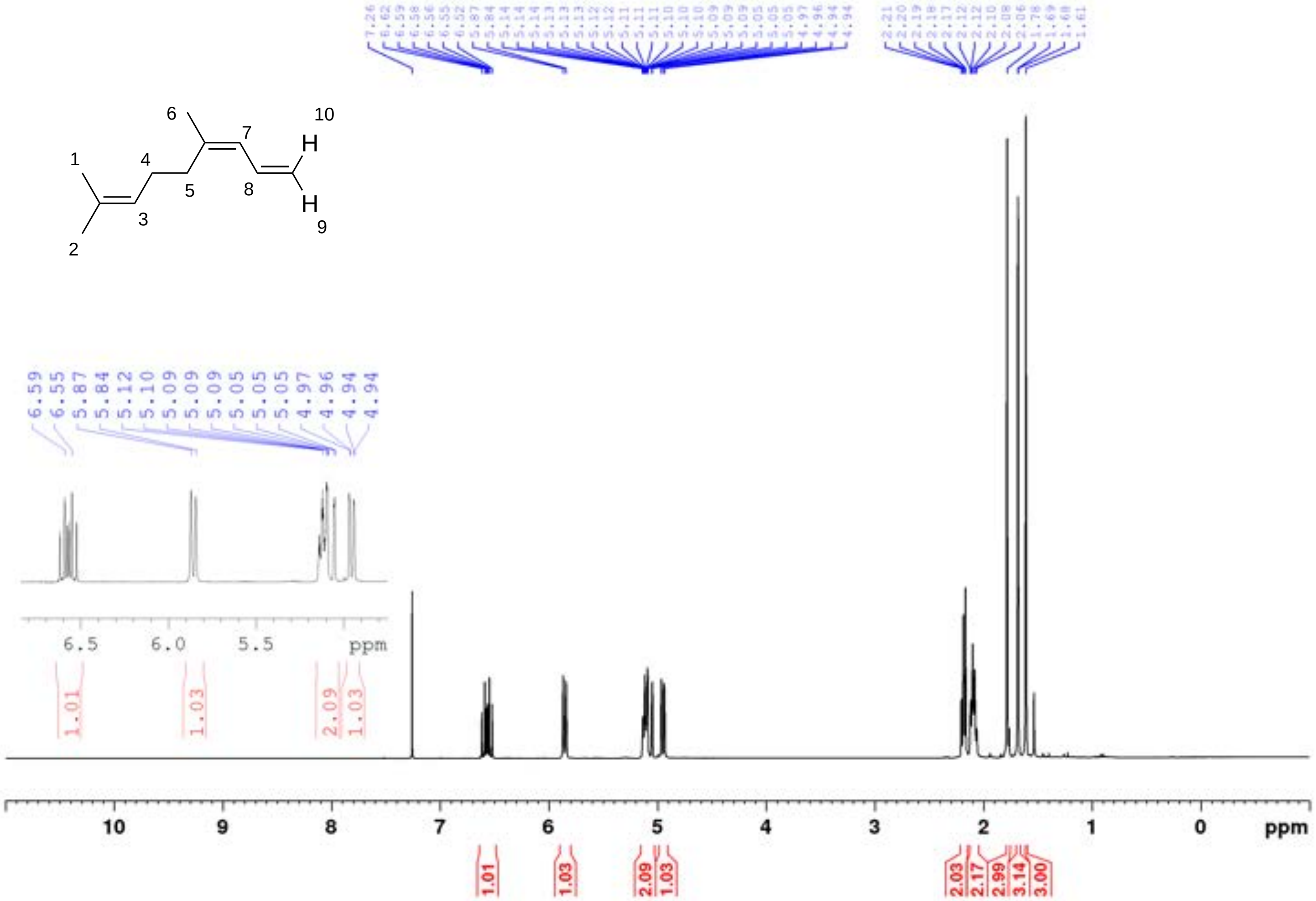

^{13}C NMR (101 MHz, $CDCl_3$) for *(Z)-4,8-dimethylnona-1,3,7-triene*

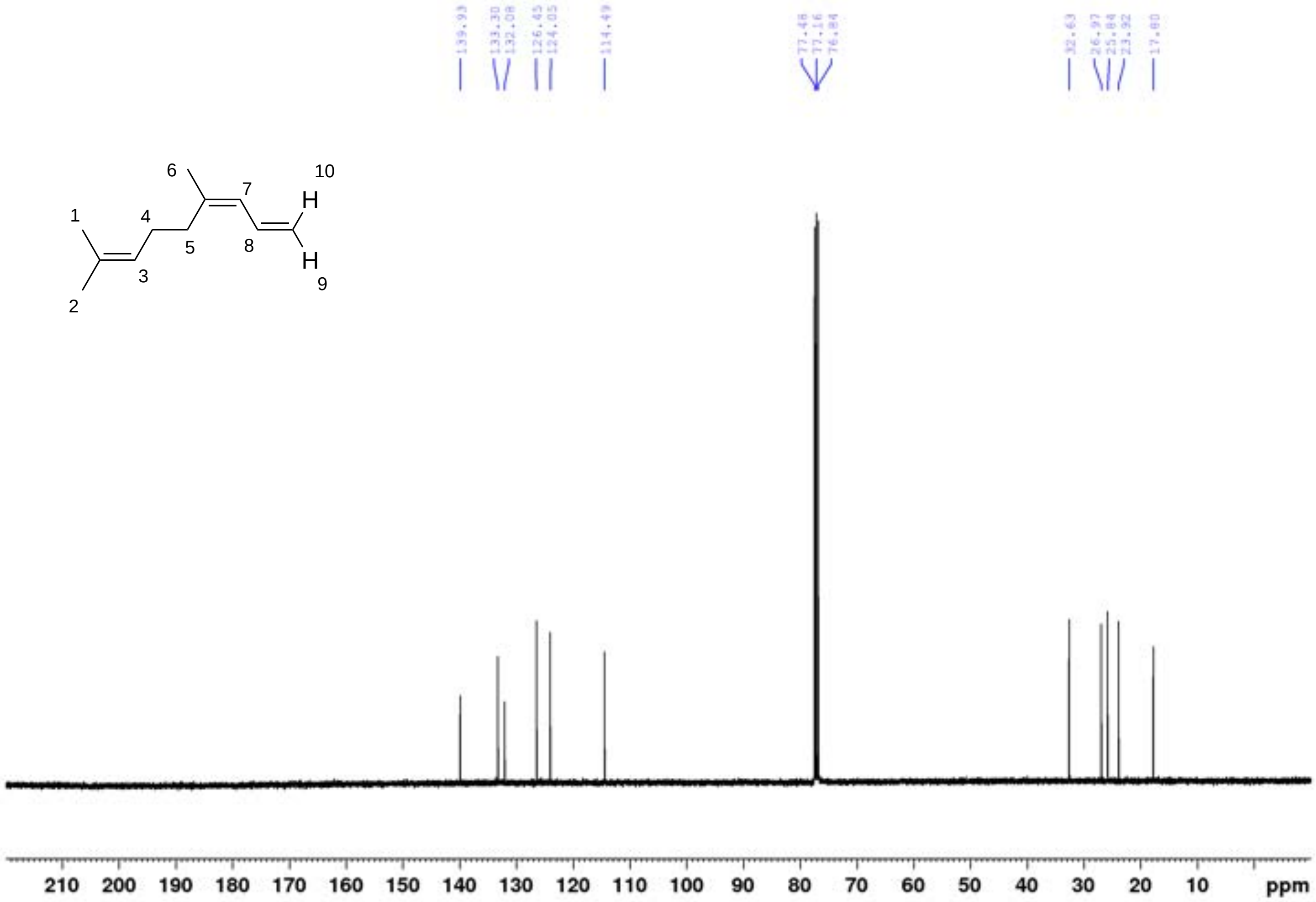

^{1}H NMR (400 MHz, $CDCl_3$) for *(Z)-4,8-dimethylnona-3,7-dien-1-ol* (**Homonerol**, **34**)

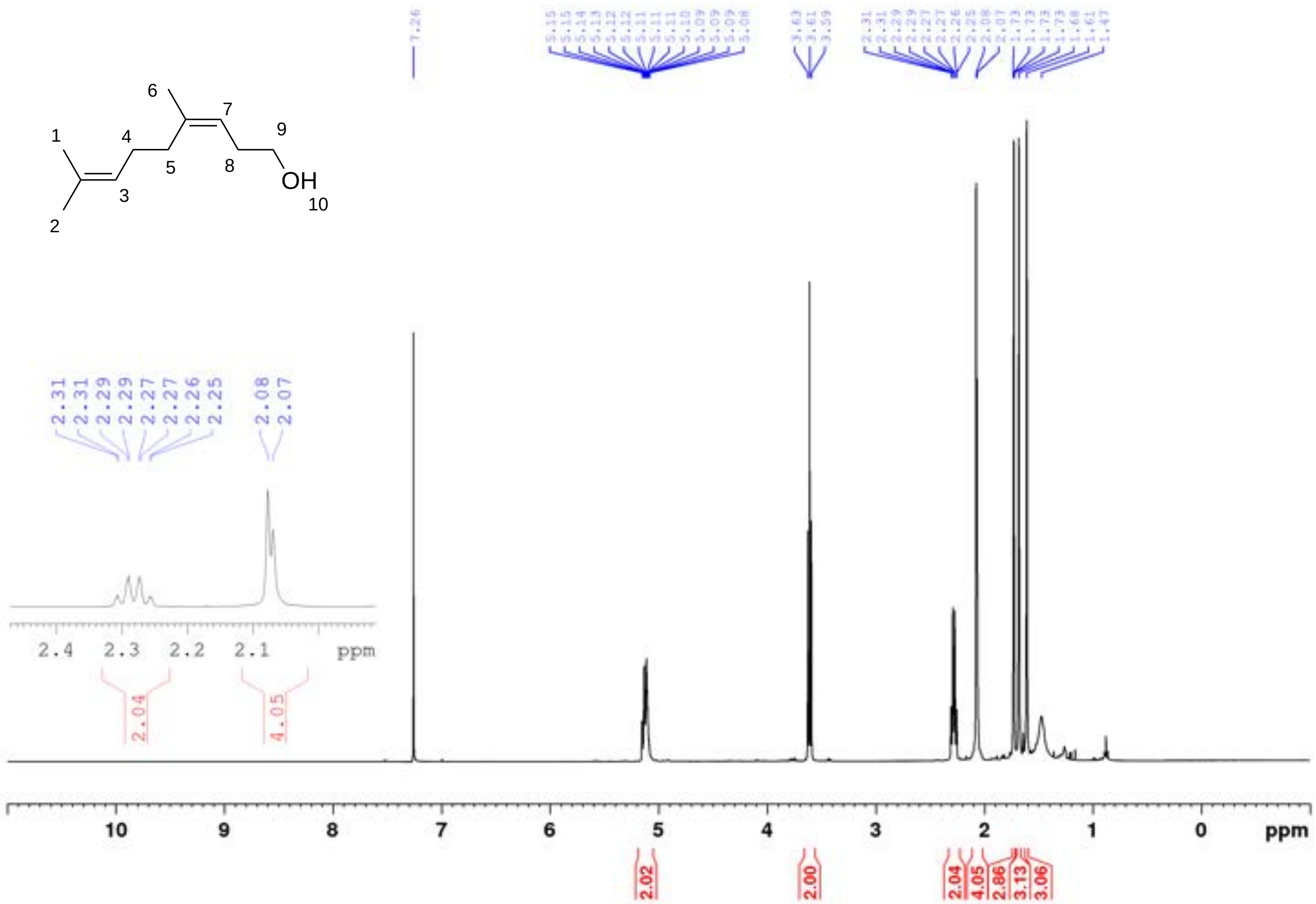

^{13}C NMR (101 MHz, $CDCl_3$) for *(Z)-4,8-dimethylnona-3,7-dien-1-ol* (**Homonerol**, **34**)

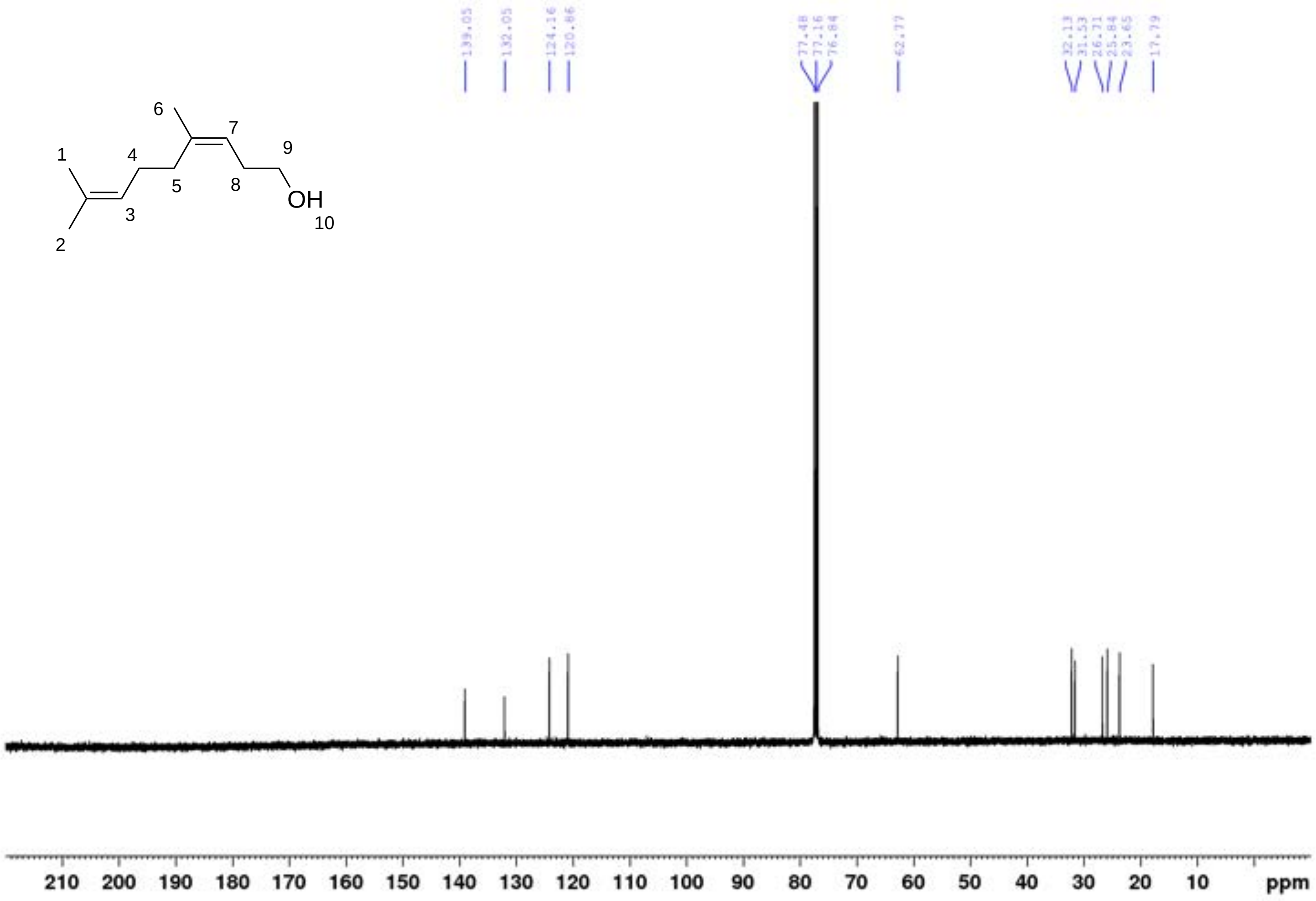

^{1}H NMR (400 MHz, $CDCl_3$) for *(E)-3,7-dimethylocta-2,6-dienal* (**Geranial**)

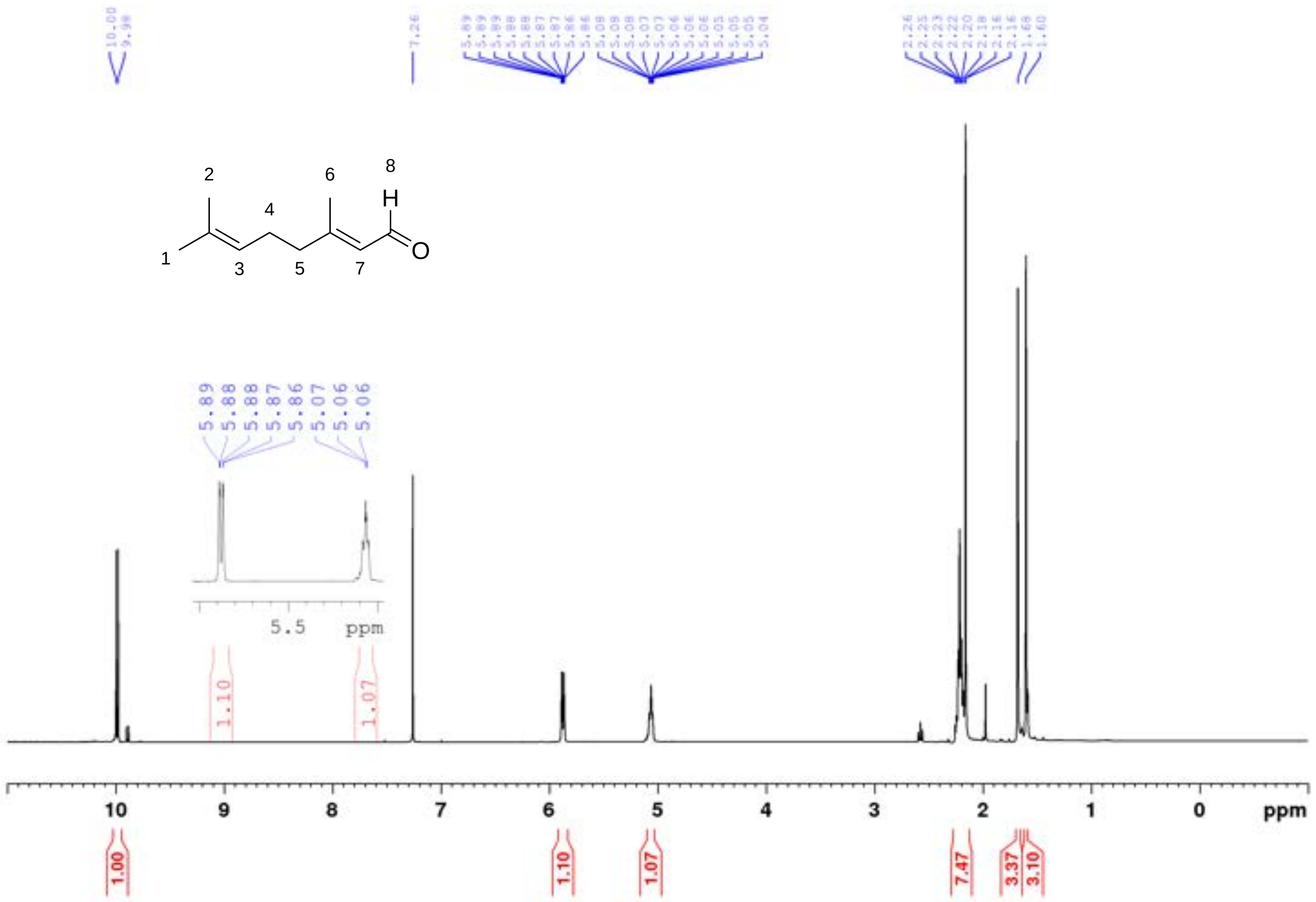

^{13}**C NMR** (101 MHz, $CDCl_3$) for *(E)-3,7-dimethylocta-2,6-dienal* (**Geranial**)

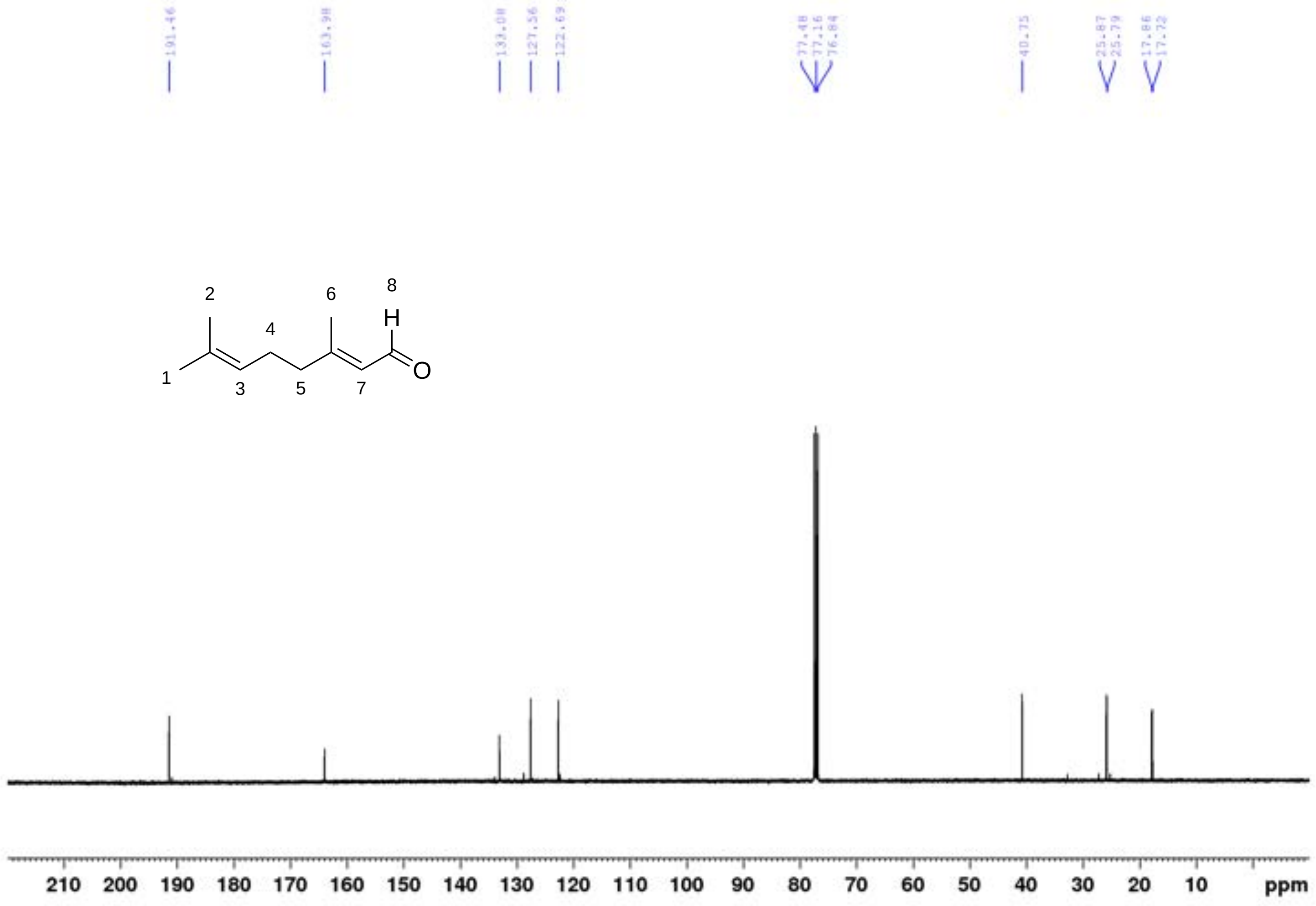

^{1}H NMR (400 MHz, $CDCl_3$) for *(E)-4,8-dimethylnona-1,3,7-triene*

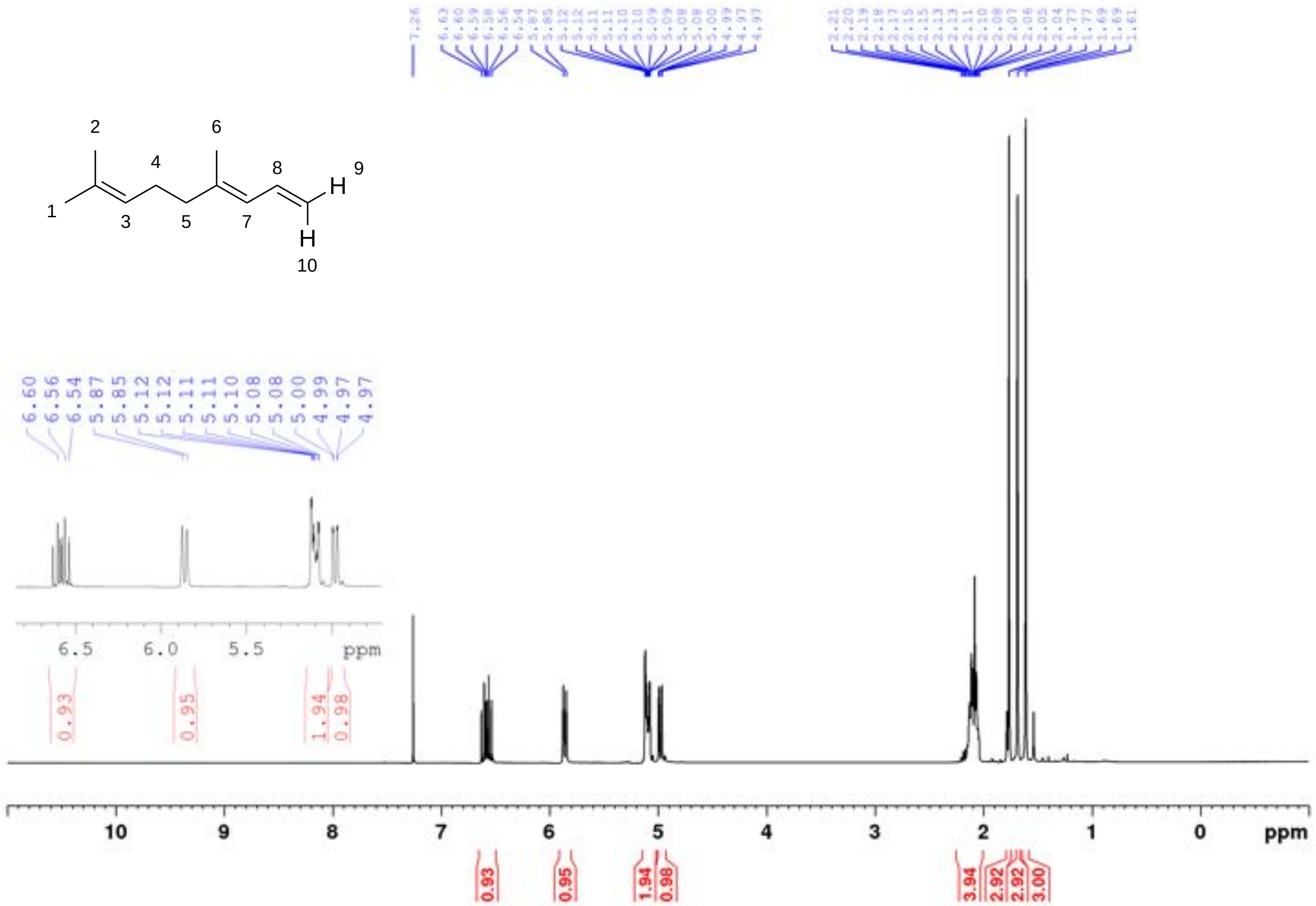

^{13}C NMR (101 MHz, $CDCl_3$) for *(E)-4,8-dimethylnona-1,3,7-triene*

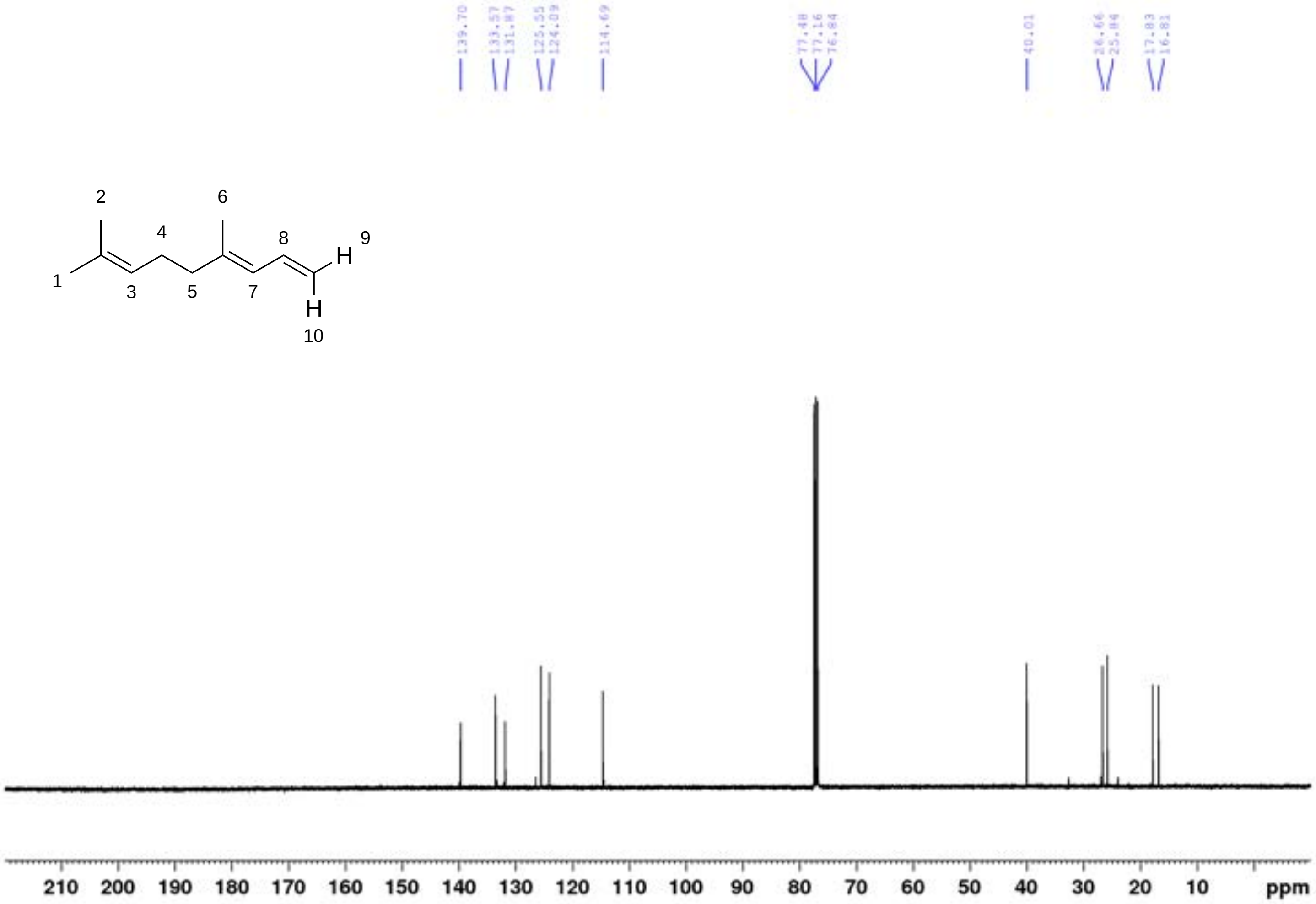

^{1}H NMR (500 MHz, $CDCl_3$) for *(E)-4,8-dimethylnona-3,7-dien-1-ol* (**Homogeraniol**, **37**)

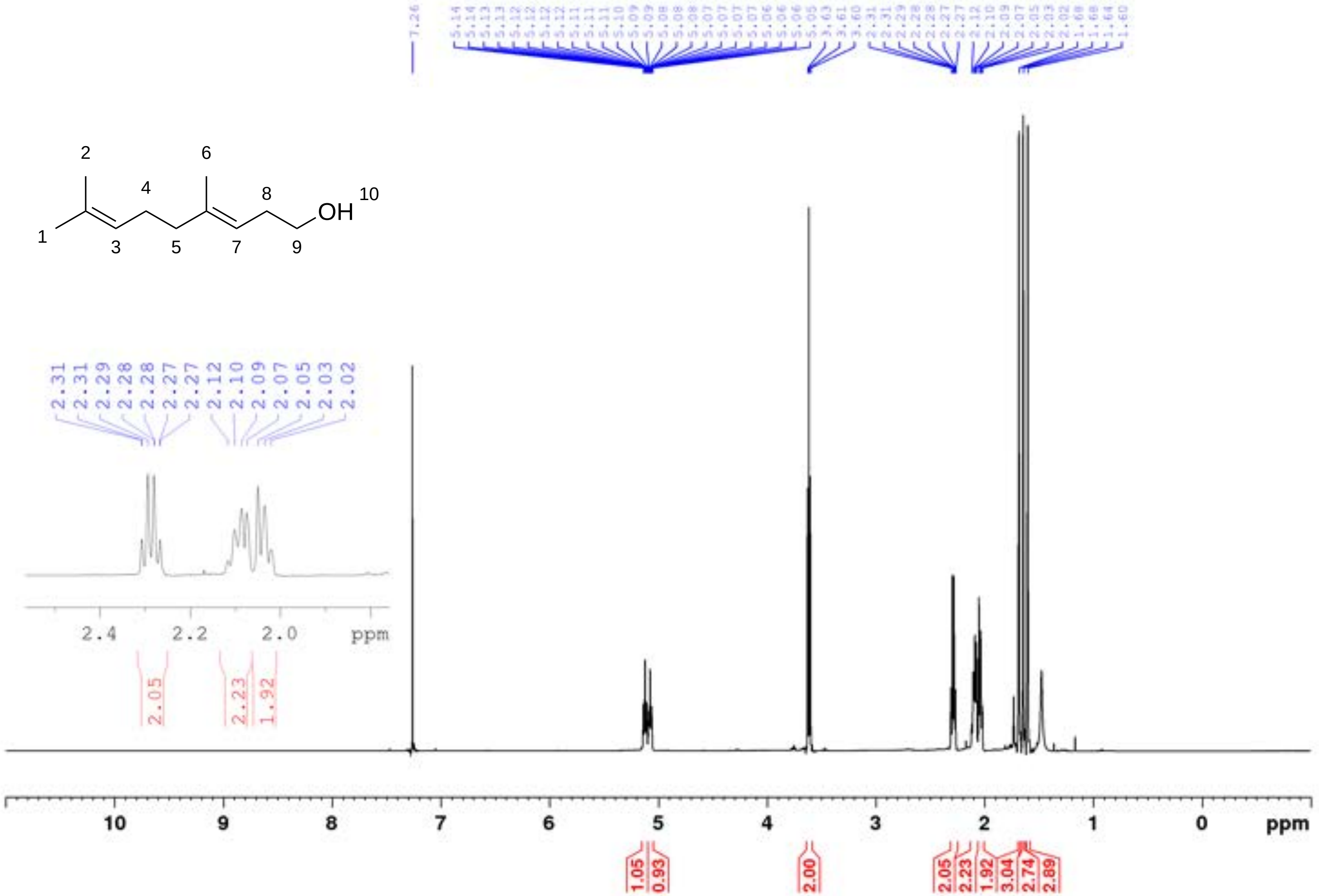

^{13}C NMR (101 MHz, $CDCl_3$) for *(E)-4,8-dimethylnona-3,7-dien-1-ol* (**Homogeraniol**, **37**)

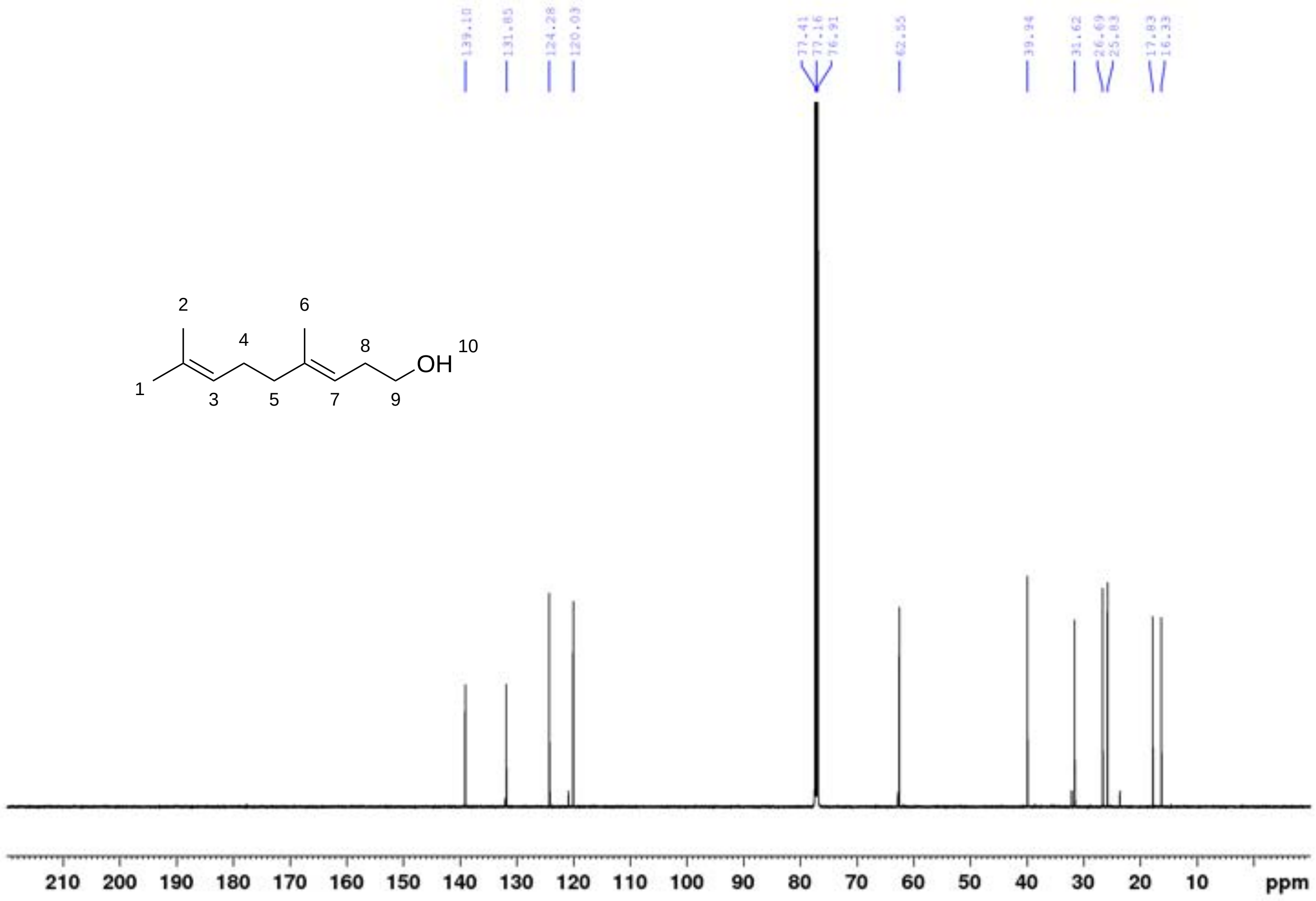

^{1}H NMR (400 MHz, $CDCl_3$) for *Tert-butyldimethyl((3-methylbut-3-en-1-yl)oxy)silane*

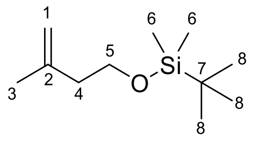

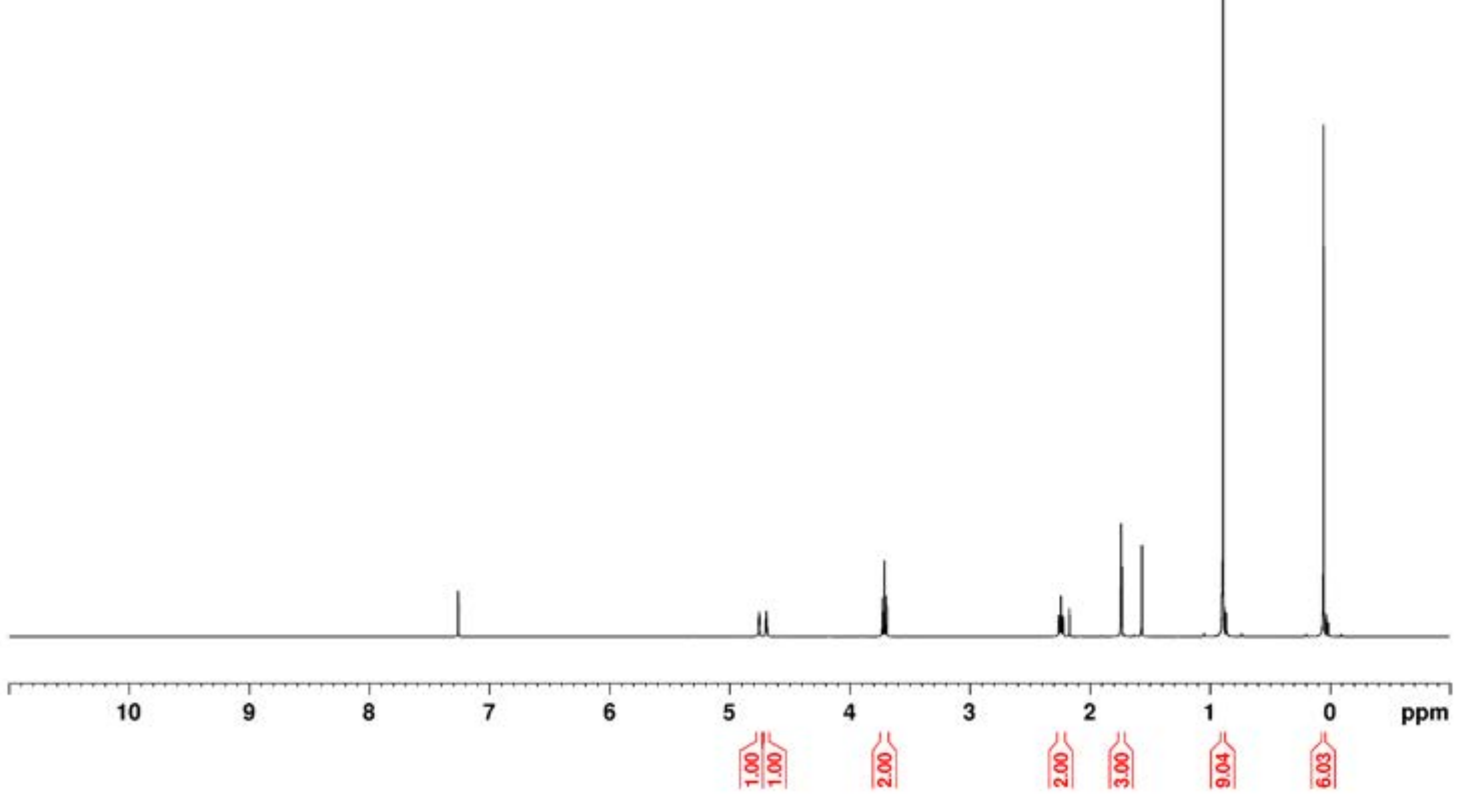

^{13}C NMR (101 MHz, CDCl$_3$) for *Tert-butyldimethyl((3-methylbut-3-en-1-yl)oxy)silane*

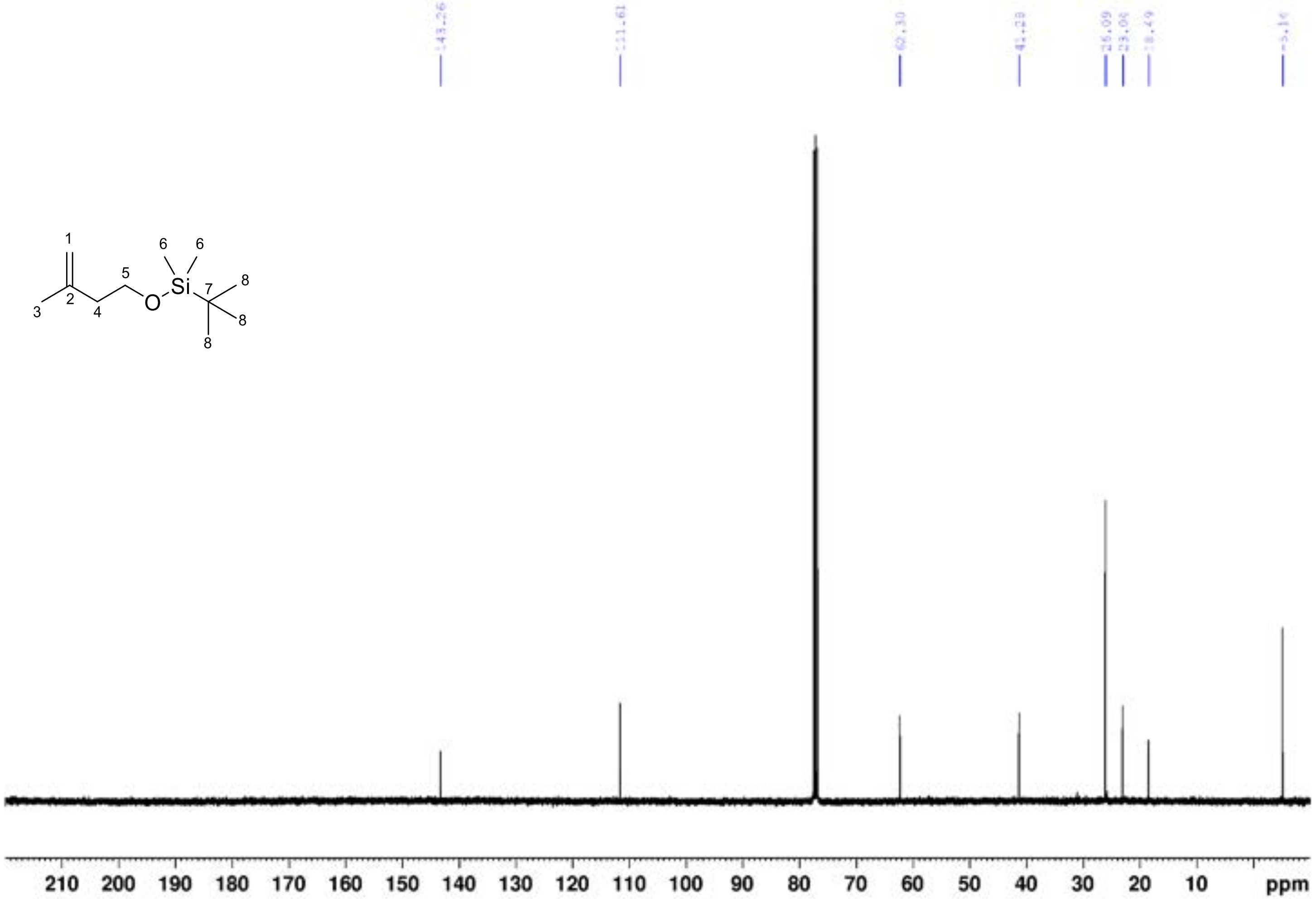

¹H NMR (400 MHz, CDCl$_3$) for *(Z)-Tert-butyldimethyl((3-methyl-4-phenylbut-3-en-1-yl)oxy)silane*

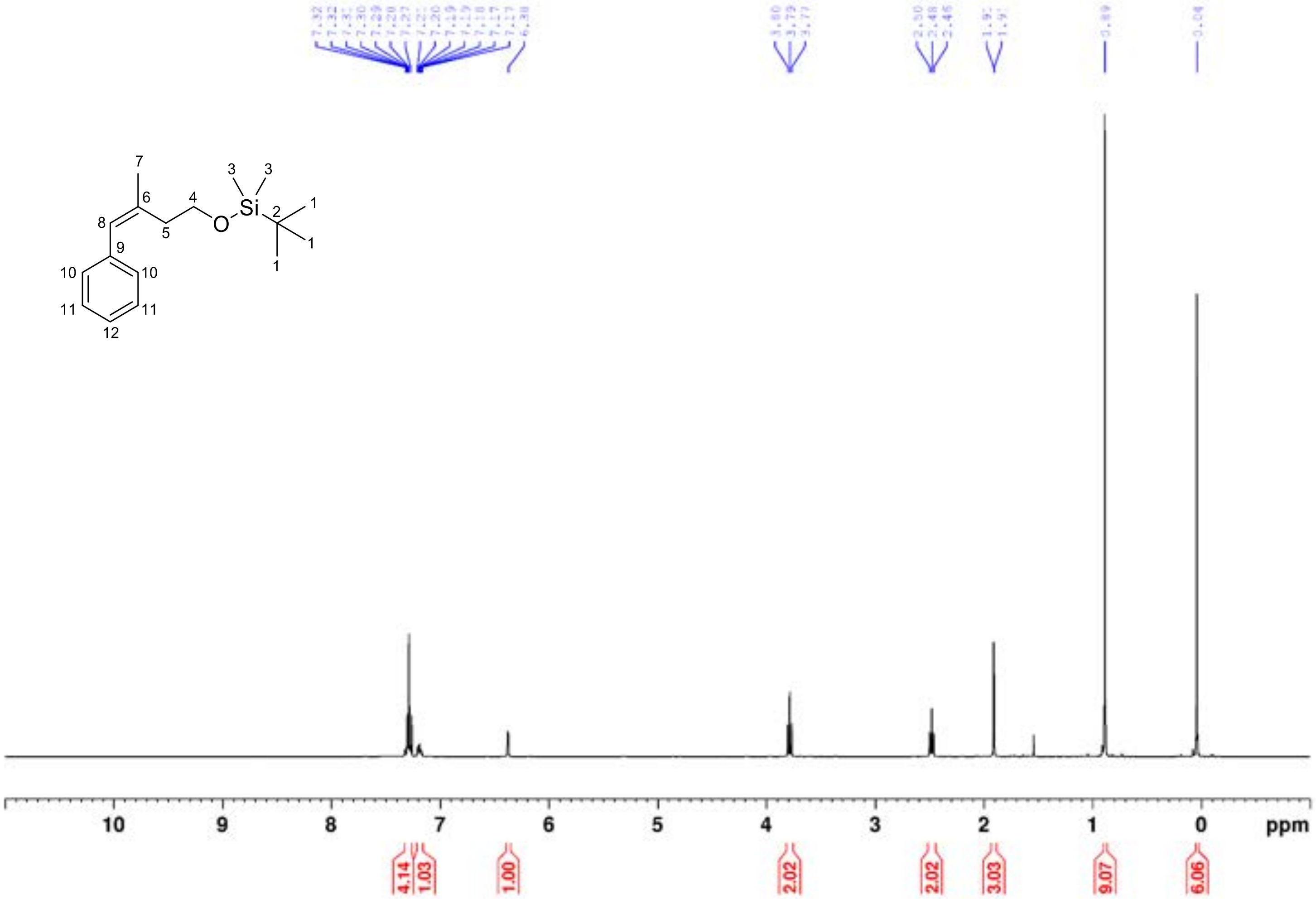

^{13}C NMR (101 MHz, CDCl$_3$) for *(Z)-Tert-butyldimethyl((3-methyl-4-phenylbut-3-en-1-yl)oxy)silane*

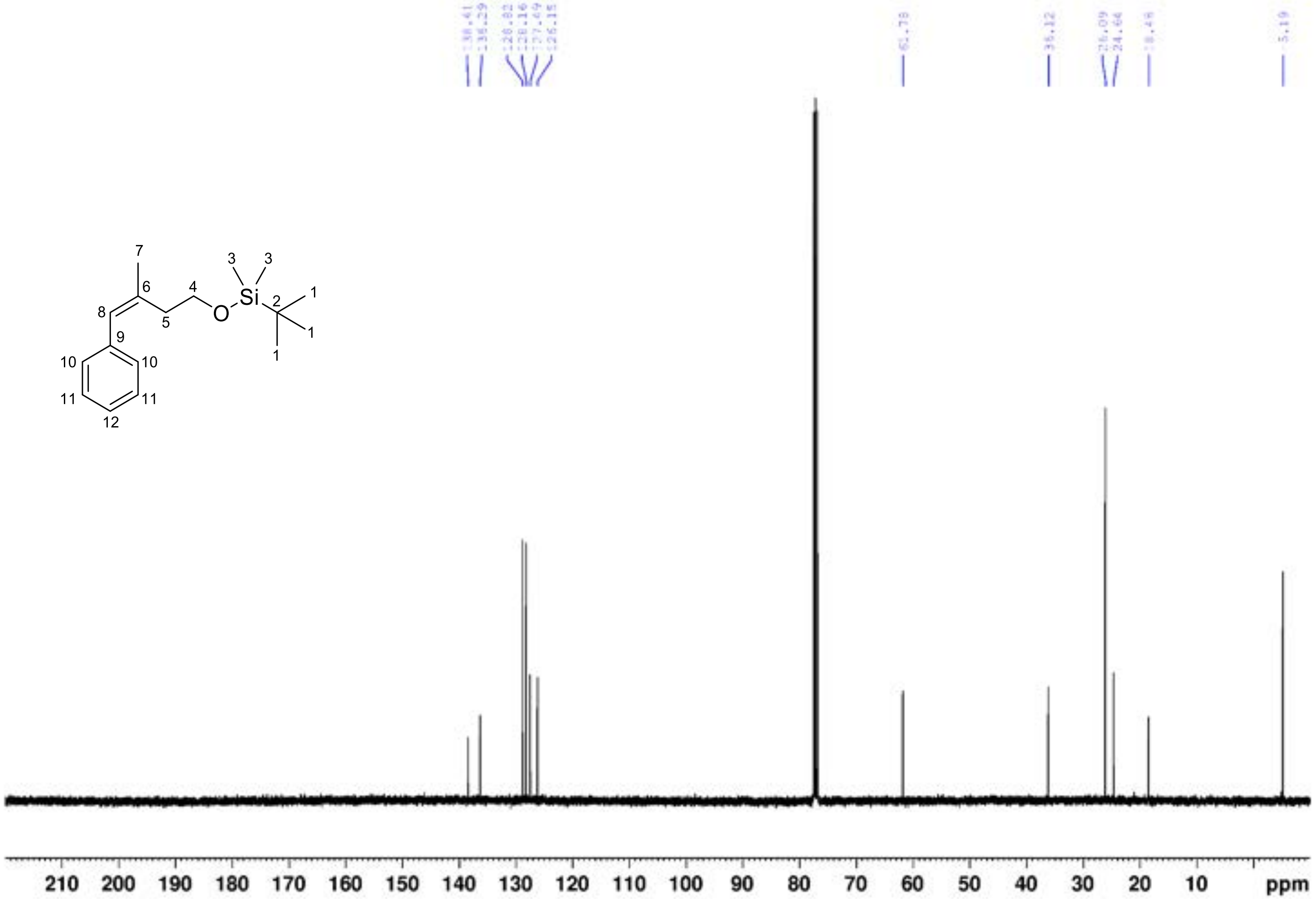

^{1}H NMR (400 MHz, CDCl$_3$) for *(Z)-3-methyl-4-phenylbut-3-en-1-ol* (**40**)

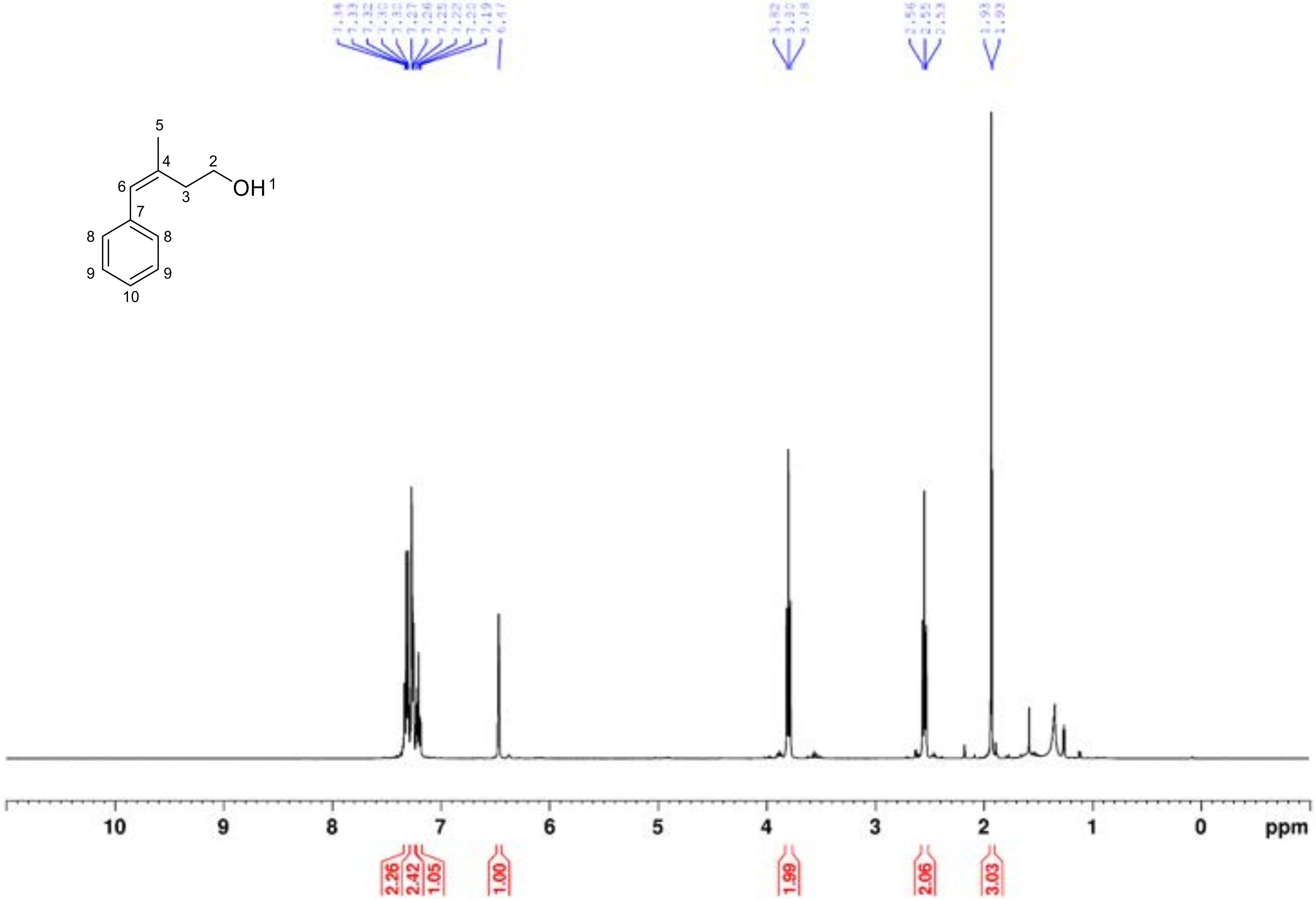

^{13}C NMR (101 MHz, CDCl$_3$) for *(Z)-3-methyl-4-phenylbut-3-en-1-ol* (**40**)

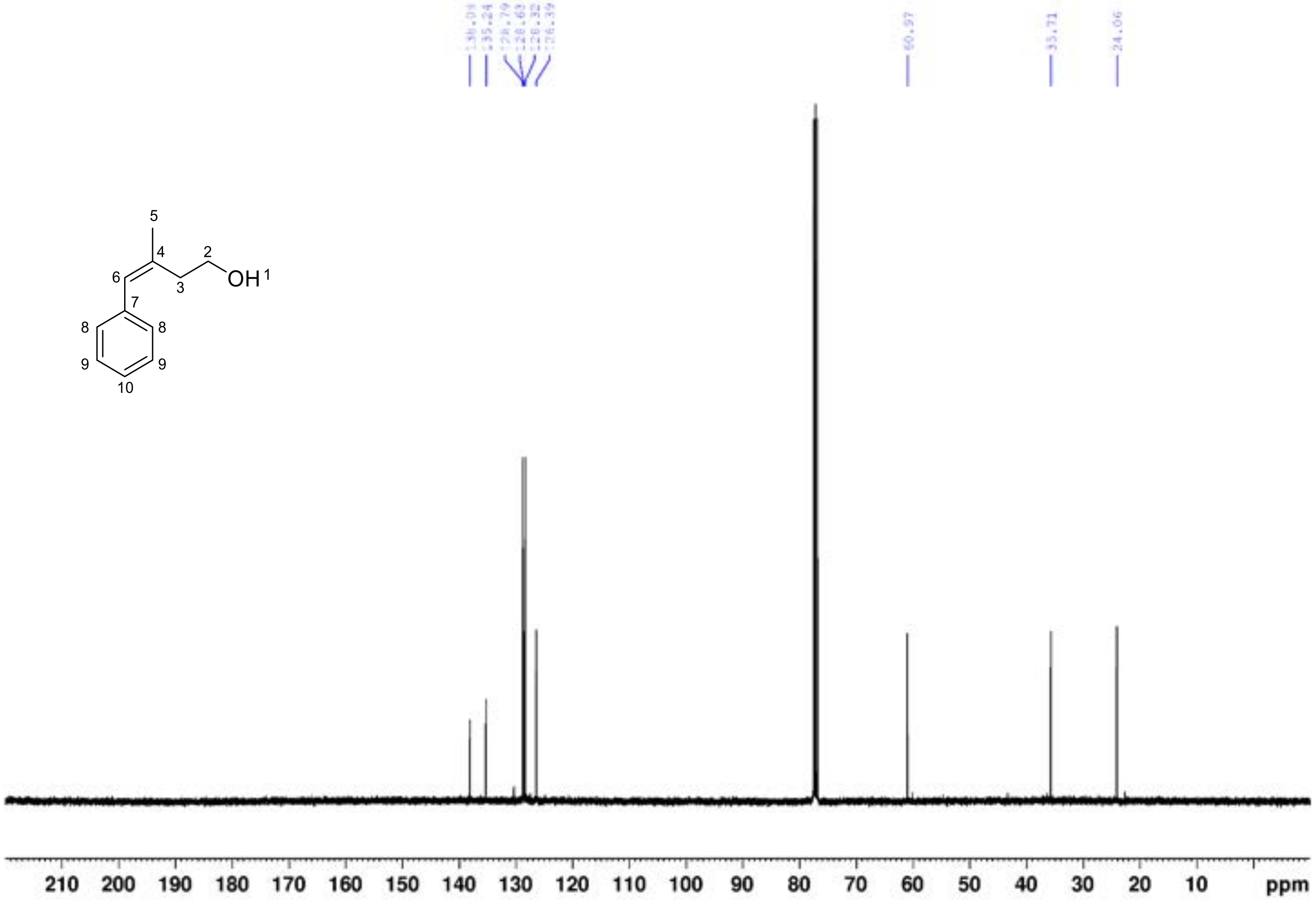

^{1}H NMR (500 MHz, $CDCl_3$) for *2,2,3,3,3-pentafluoropropyl (2R,3S)-2-(3-hydroxypropyl)-3-phenylaziridine-1-sulfonate* (**3a**)

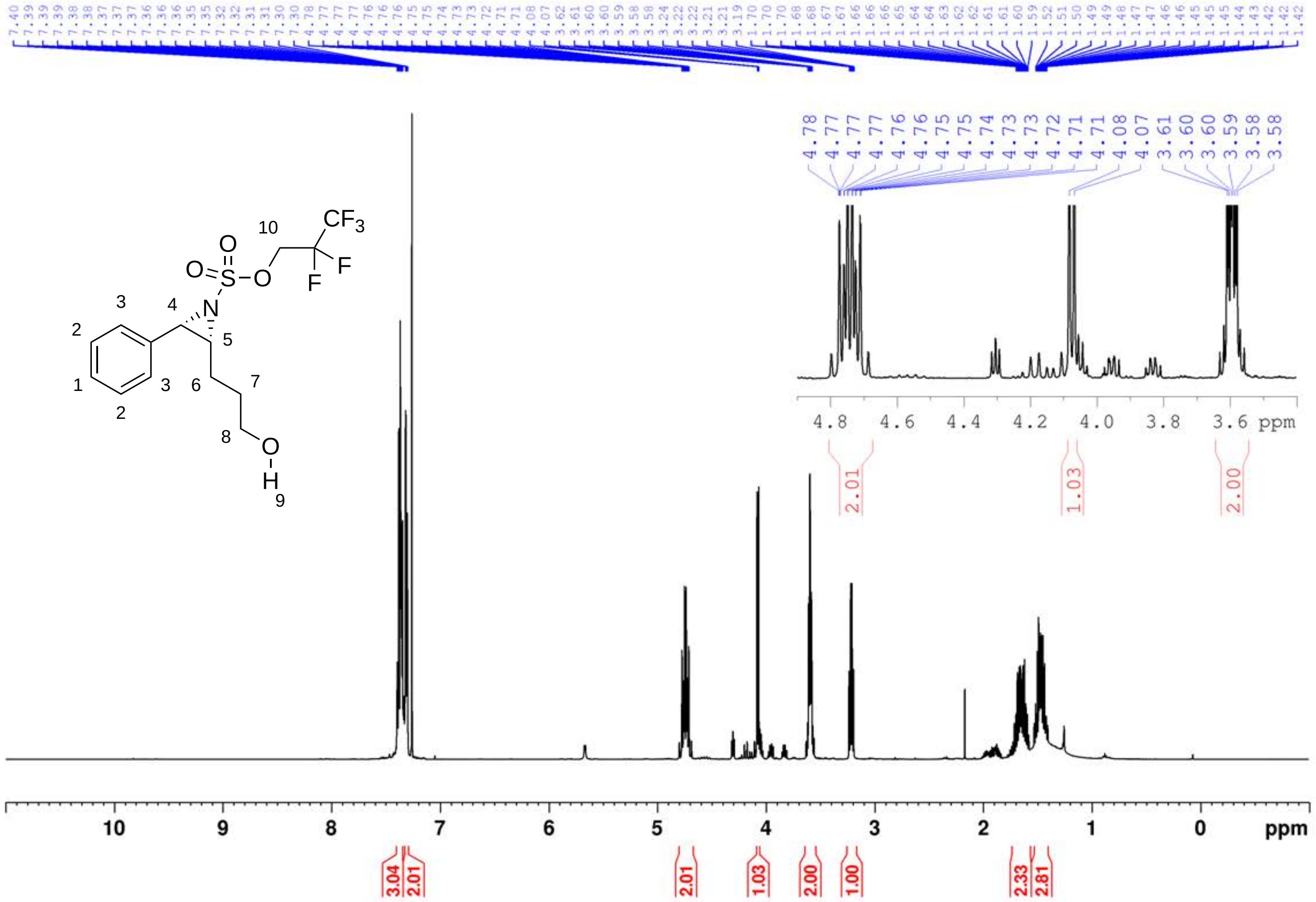

^{13}C NMR (126 MHz, $CDCl_3$) for *2,2,3,3,3-pentafluoropropyl (2R,3S)-2-(3-hydroxypropyl)-3-phenylaziridine-1-sulfonate* (**3a**)

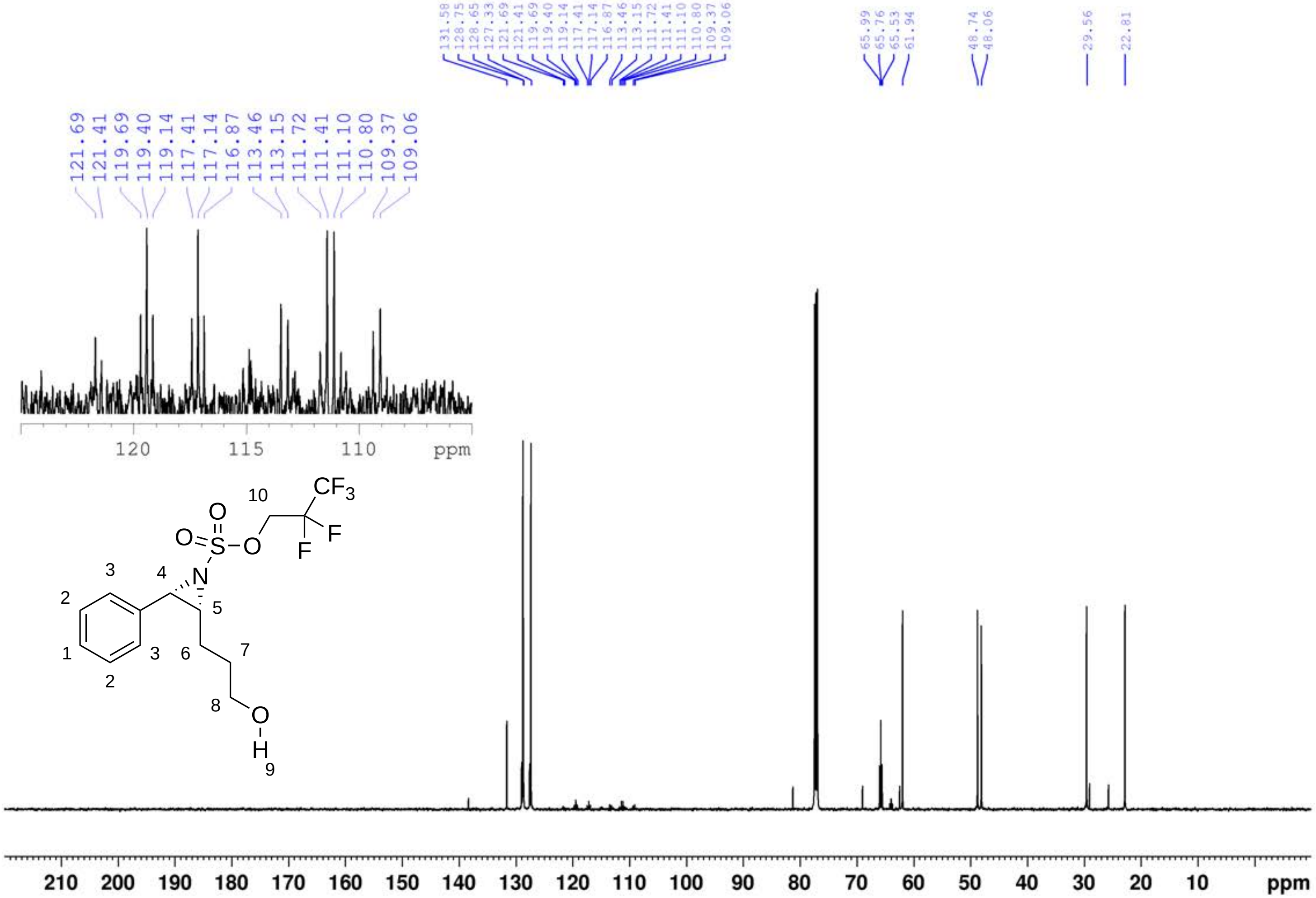

^{19}F NMR (376 MHz, $CDCl_3$) for *2,2,3,3,3-pentafluoropropyl (2R,3S)-2-(3-hydroxypropyl)-3-phenylaziridine-1-sulfonate* (**3a**)

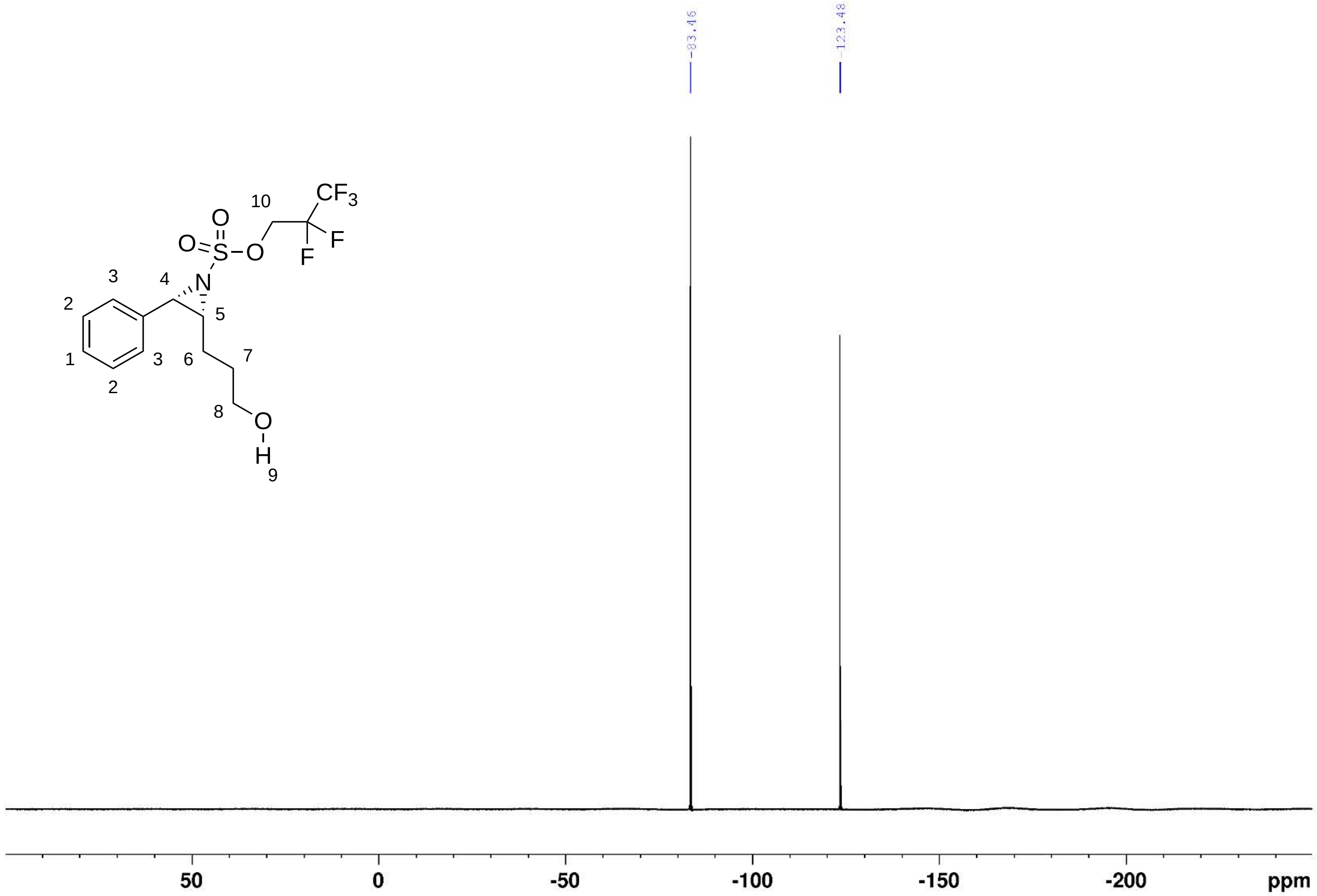

¹H NMR (500 MHz, $CDCl_3$) for *2,2,3,3,3-pentafluoropropyl ((S)-phenyl((S)-tetrahydrofuran-2-yl)methyl)sulfamate* (**4a**)

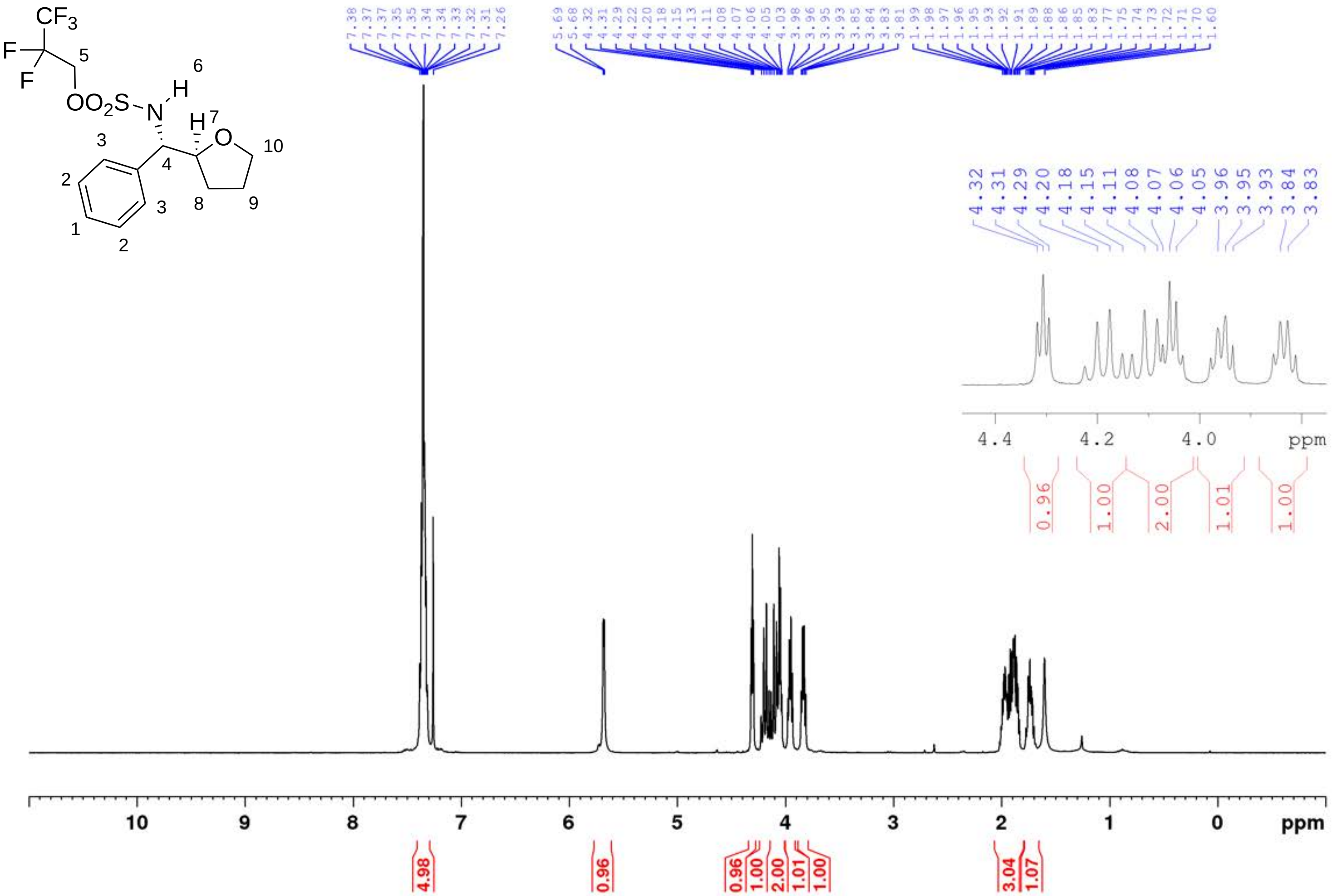

13C NMR (126 MHz, $CDCl_3$) for *2,2,3,3,3-pentafluoropropyl ((S)-phenyl((S)-tetrahydrofuran-2-yl)methyl)sulfamate* (**4a**)

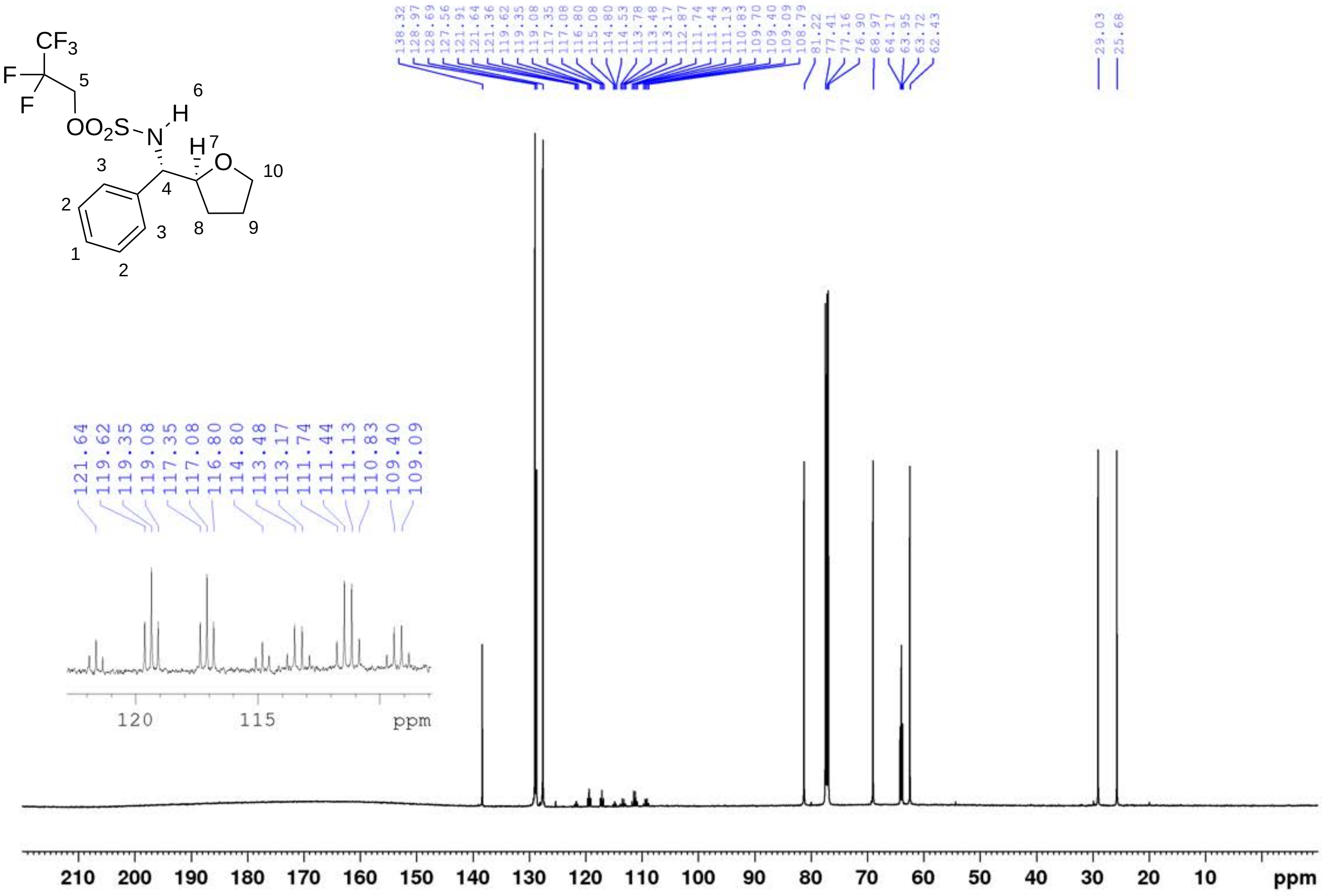

^{19}F NMR (376 MHz, $CDCl_3$) for *2,2,3,3,3-pentafluoropropyl ((S)-phenyl((S)-tetrahydrofuran-2-yl)methyl)sulfamate* (**4a**)

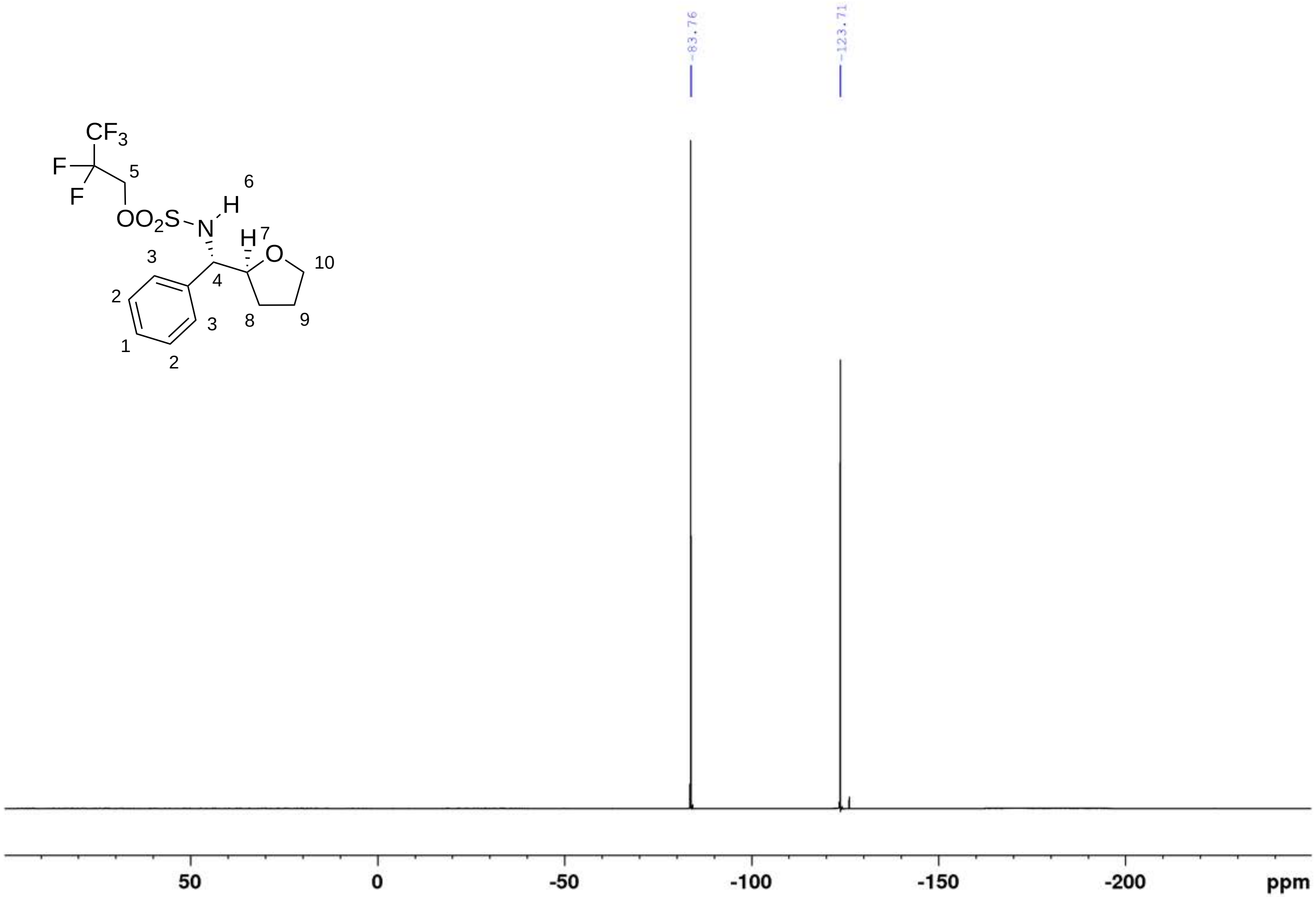

^{1}H NMR (400 MHz, $CDCl_3$) for *2,2,3,3,3-pentafluoropropyl ((S)-(2-fluorophenyl)((S)-tetrahydrofuran-2-yl)methyl)sulfamate* (**4b**)

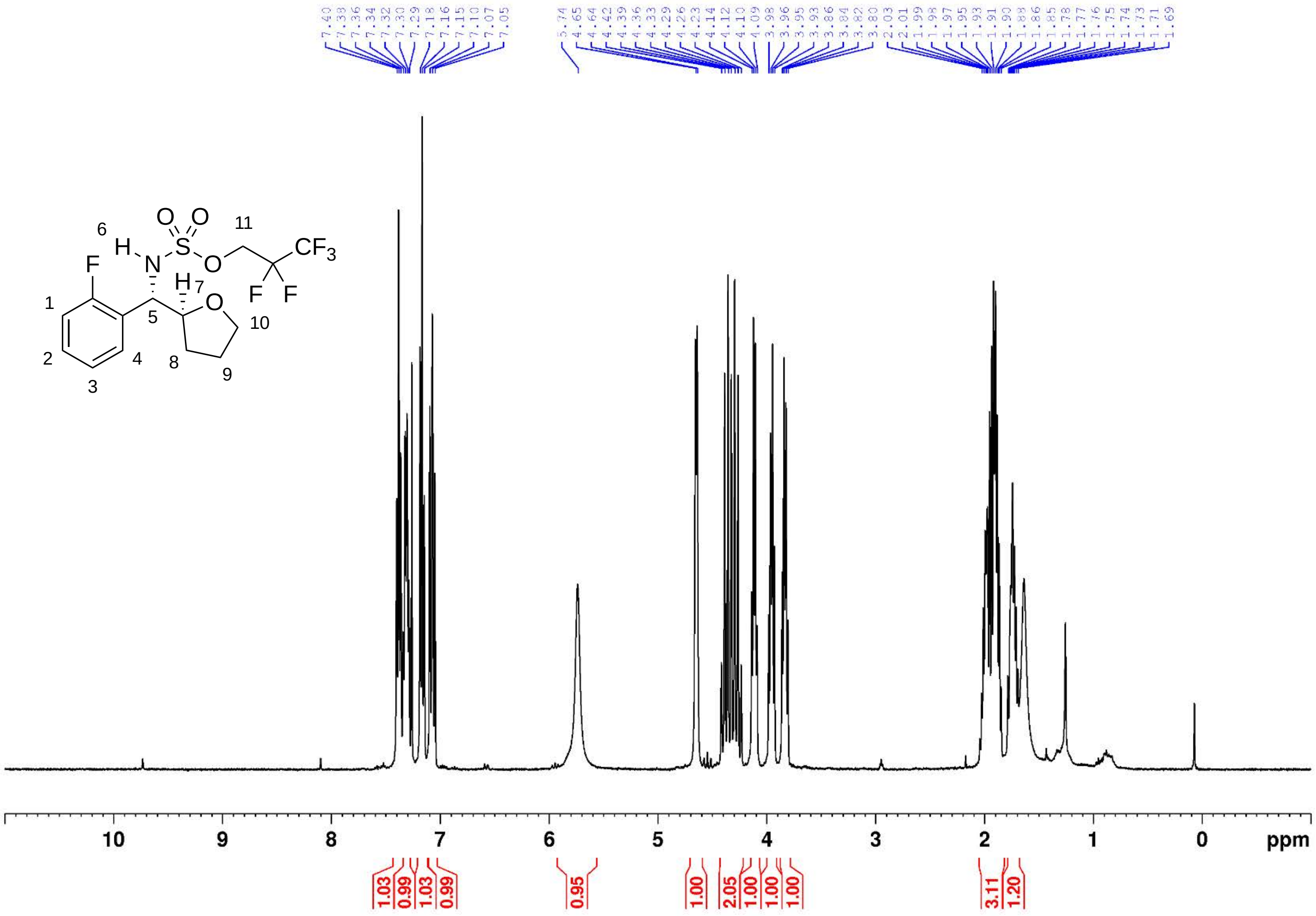

^{13}C NMR (101 MHz, $CDCl_3$) for *2,2,3,3,3-pentafluoropropyl ((S)-(2-fluorophenyl)((S)-tetrahydrofuran-2-yl)methyl)sulfamate* (**4b**)

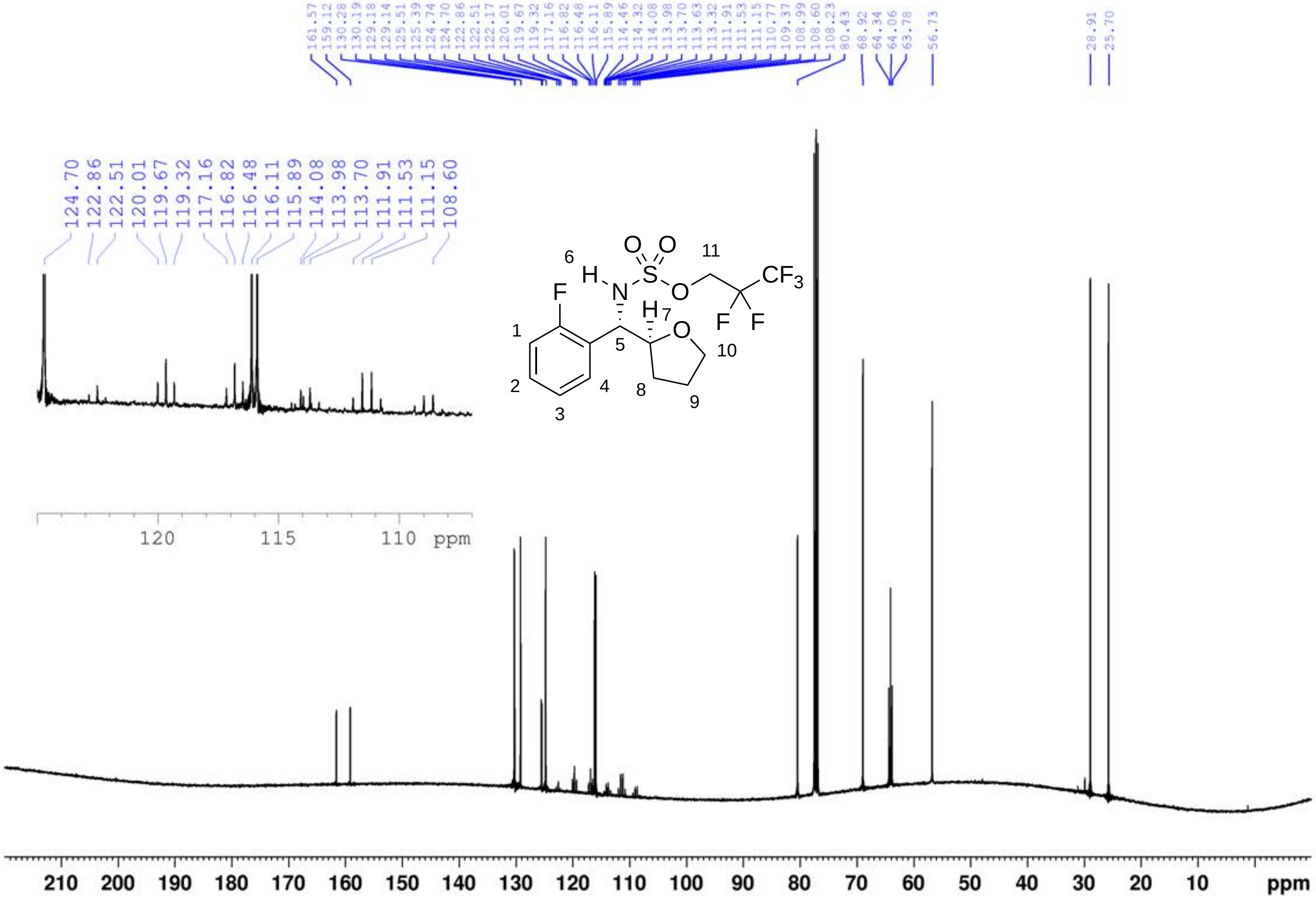

^{19}F NMR (376 MHz, $CDCl_3$) for *2,2,3,3,3-pentafluoropropyl ((S)-(2-fluorophenyl)((S)-tetrahydrofuran-2-yl)methyl)sulfamate* (**4b**)

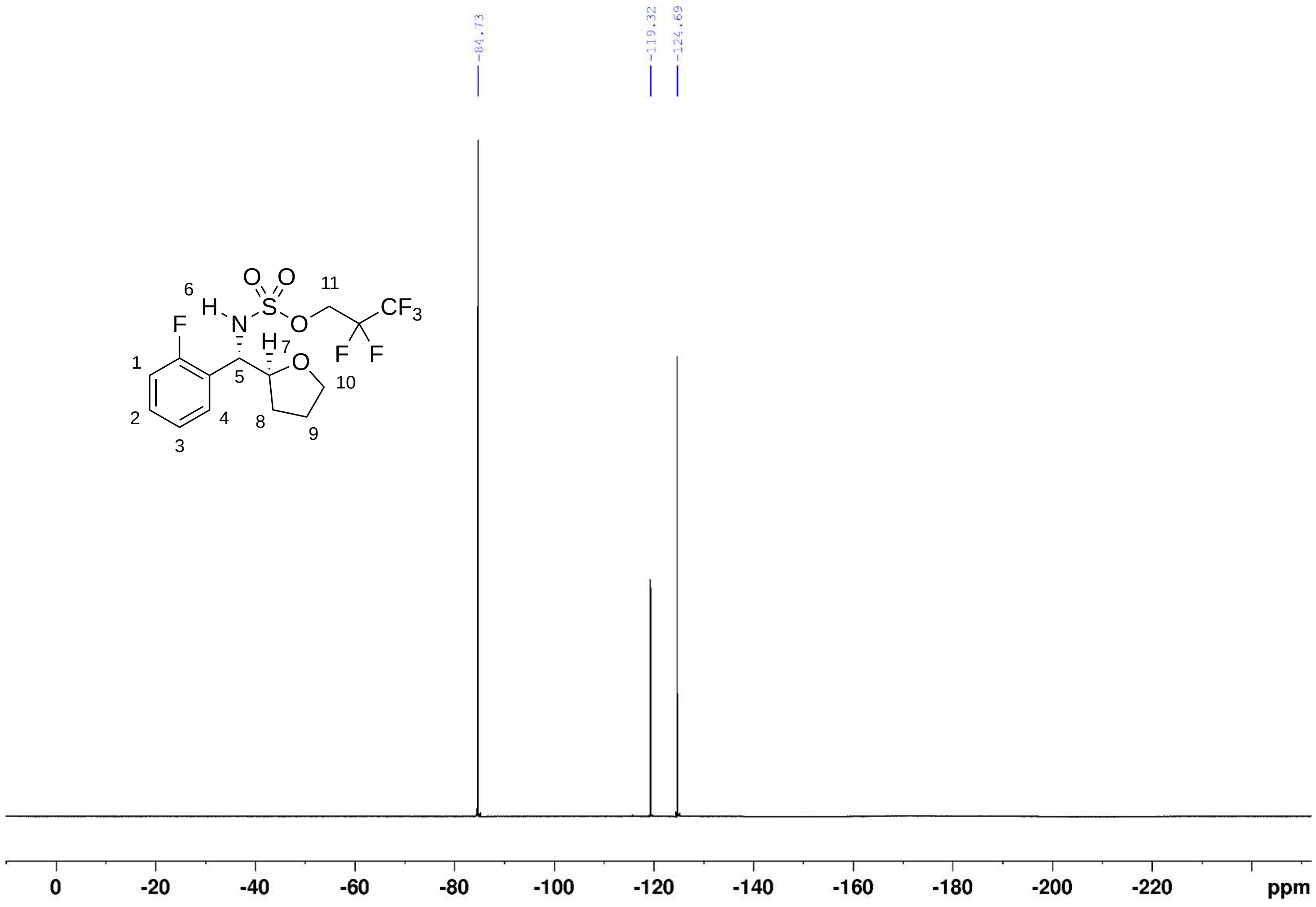

^{1}H NMR (400 MHz, $CDCl_3$) for *2,2,3,3,3-pentafluoropropyl ((S)-((S)-tetrahydrofuran-2-yl)(o-tolyl)methyl)sulfamate* (**4c**)

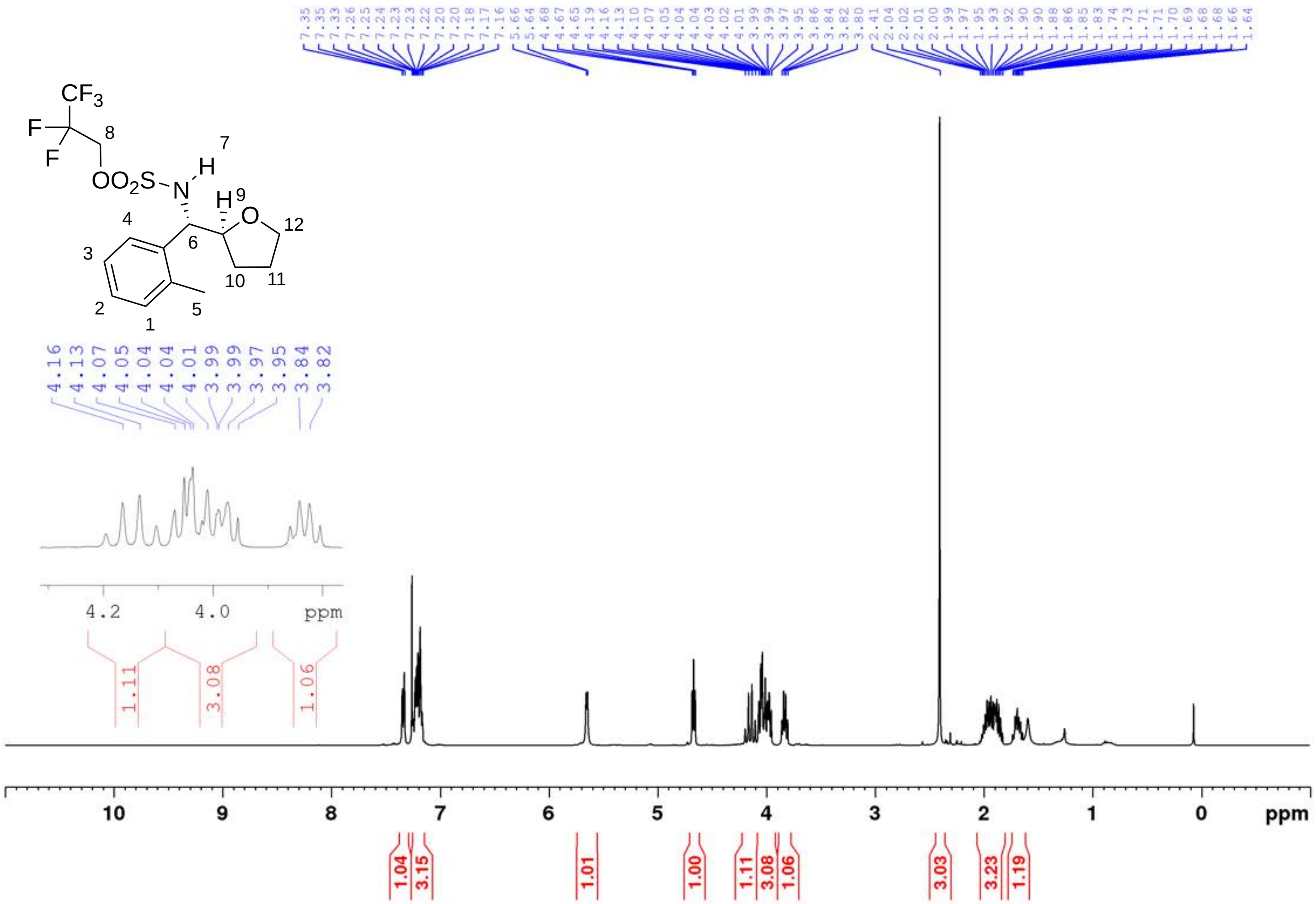

^{13}C NMR (126 MHz, $CDCl_3$) for *2,2,3,3,3-pentafluoropropyl ((S)-((S)-tetrahydrofuran-2-yl)(o-tolyl)methyl)sulfamate* (**4c**)

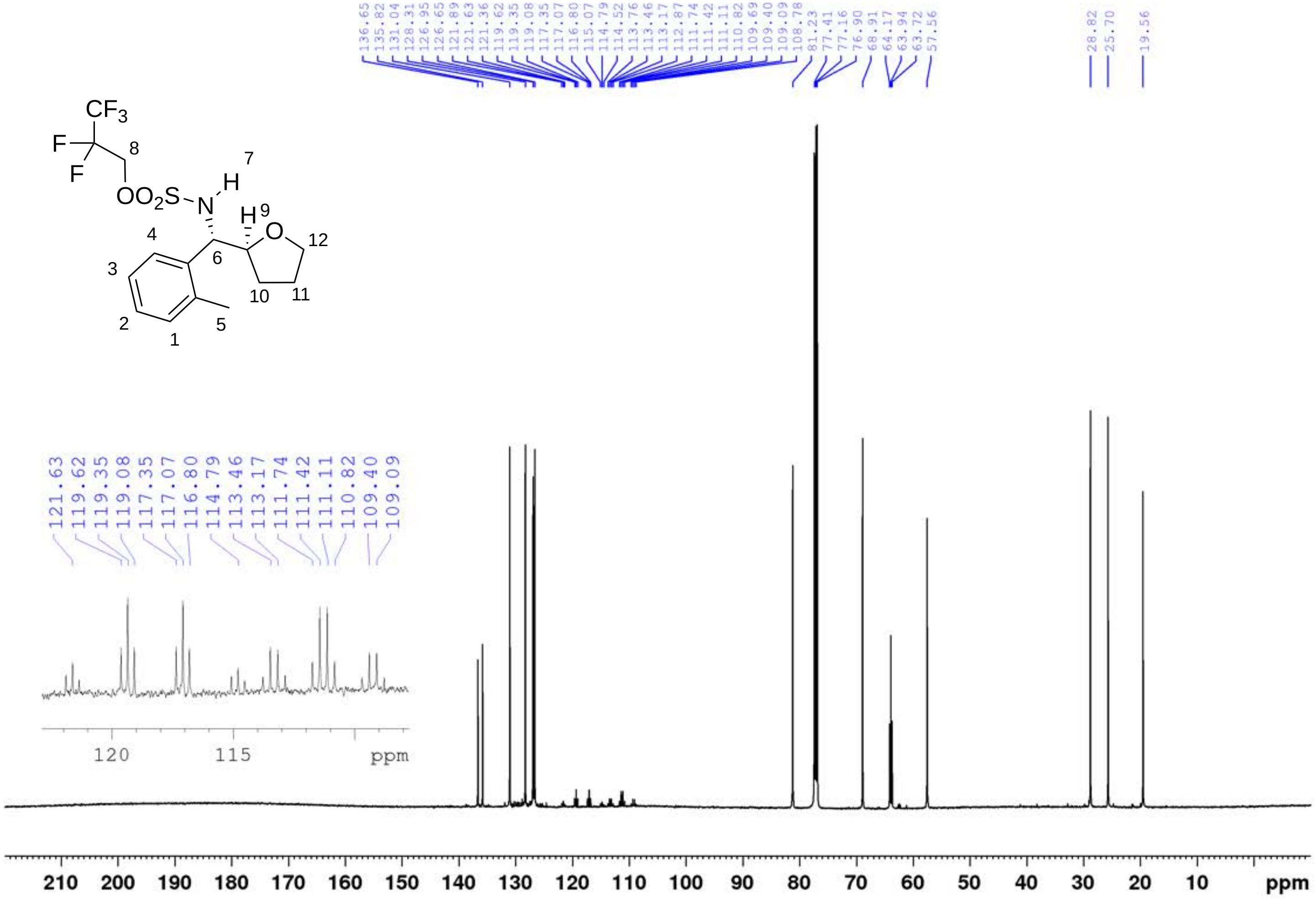

^{19}F NMR (376 MHz, $CDCl_3$) for *2,2,3,3,3-pentafluoropropyl ((S)-((S)-tetrahydrofuran-2-yl)(o-tolyl)methyl)sulfamate* (**4c**)

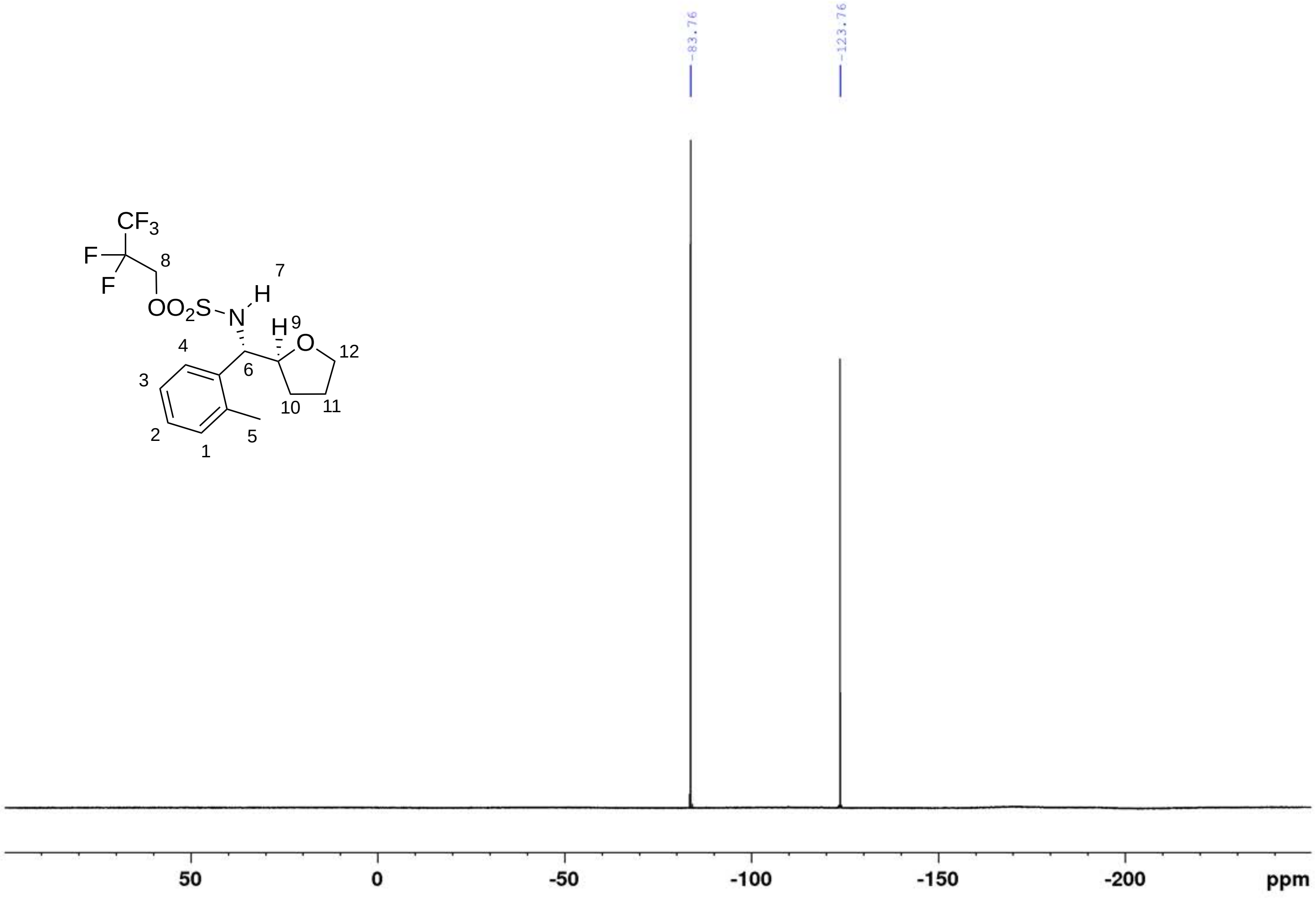

^{1}H NMR (500 MHz, $CDCl_3$) for *2,2,3,3,3-pentafluoropropyl ((S)-(2-methoxyphenyl)((S)-tetrahydrofuran-2-yl)methyl)sulfamate* (**4d**)

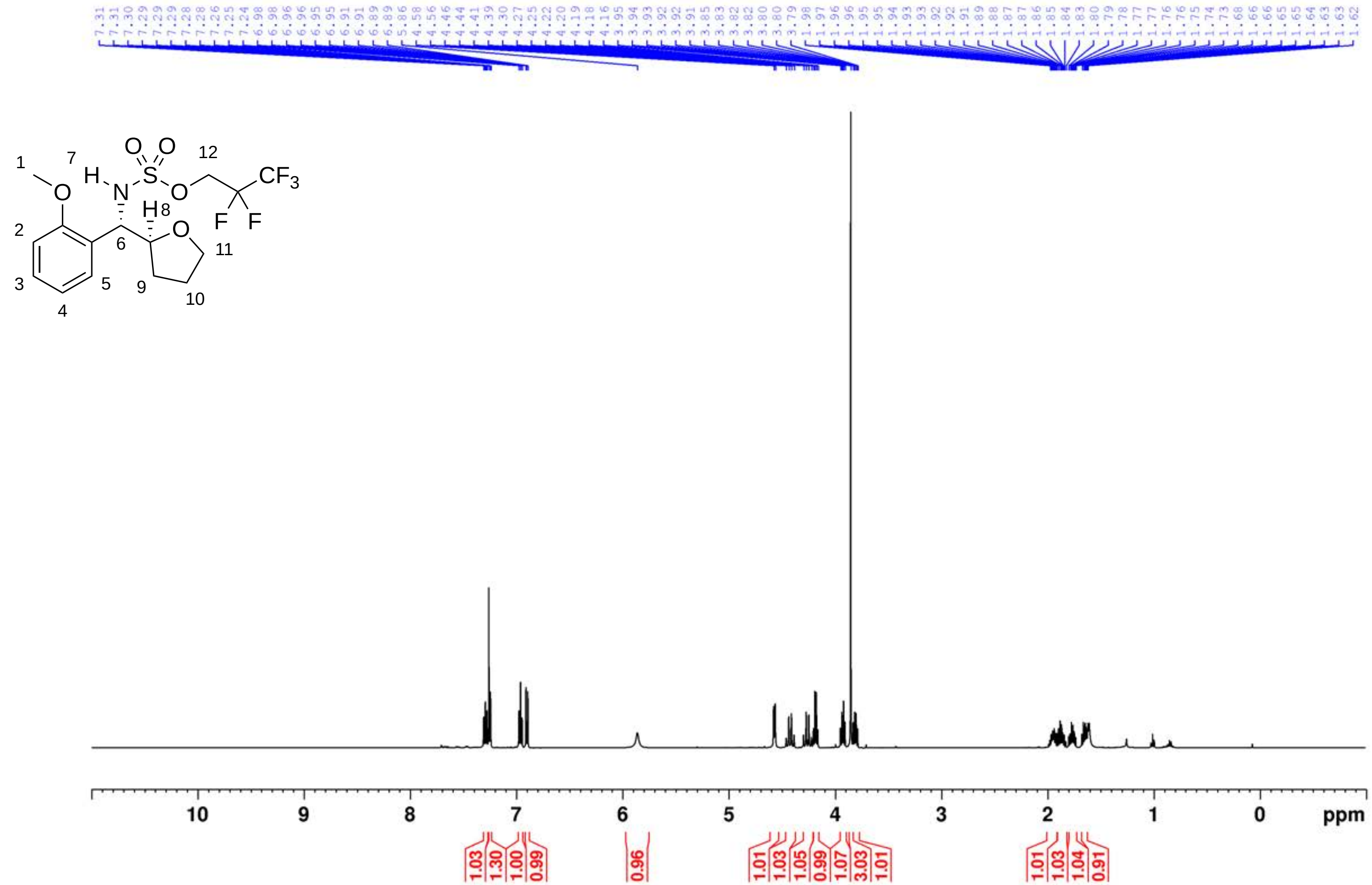

13C NMR (126 MHz, $CDCl_3$) for *2,2,3,3,3-pentafluoropropyl ((S)-(2-methoxyphenyl)((S)-tetrahydrofuran-2-yl)methyl)sulfamate* (**4d**)

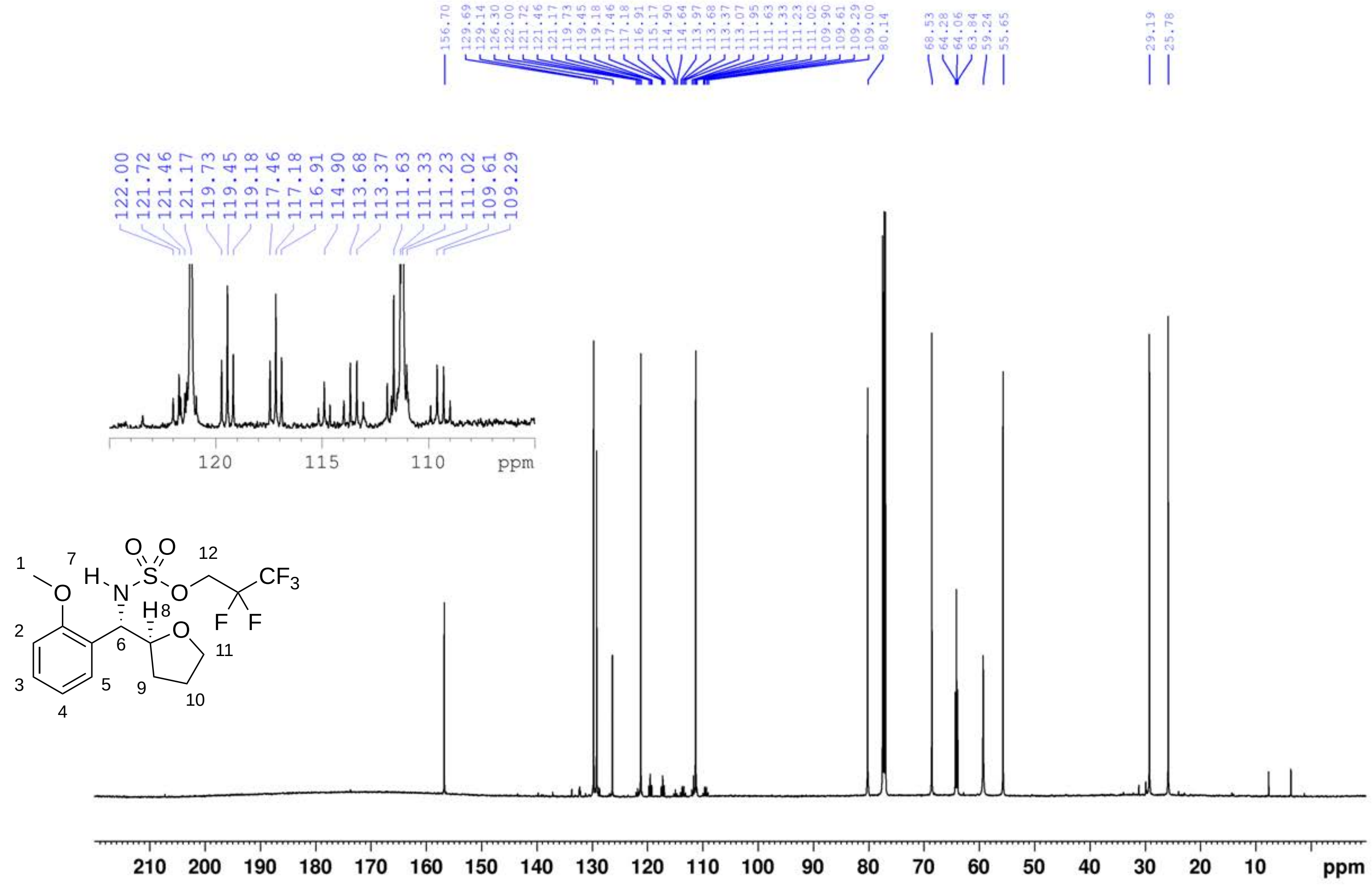

^{19}F NMR (471 MHz, $CDCl_3$) for *2,2,3,3,3-pentafluoropropyl ((S)-(2-methoxyphenyl)((S)-tetrahydrofuran-2-yl)methyl)sulfamate* (**4d**)

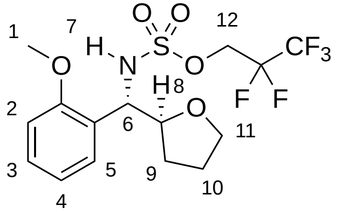

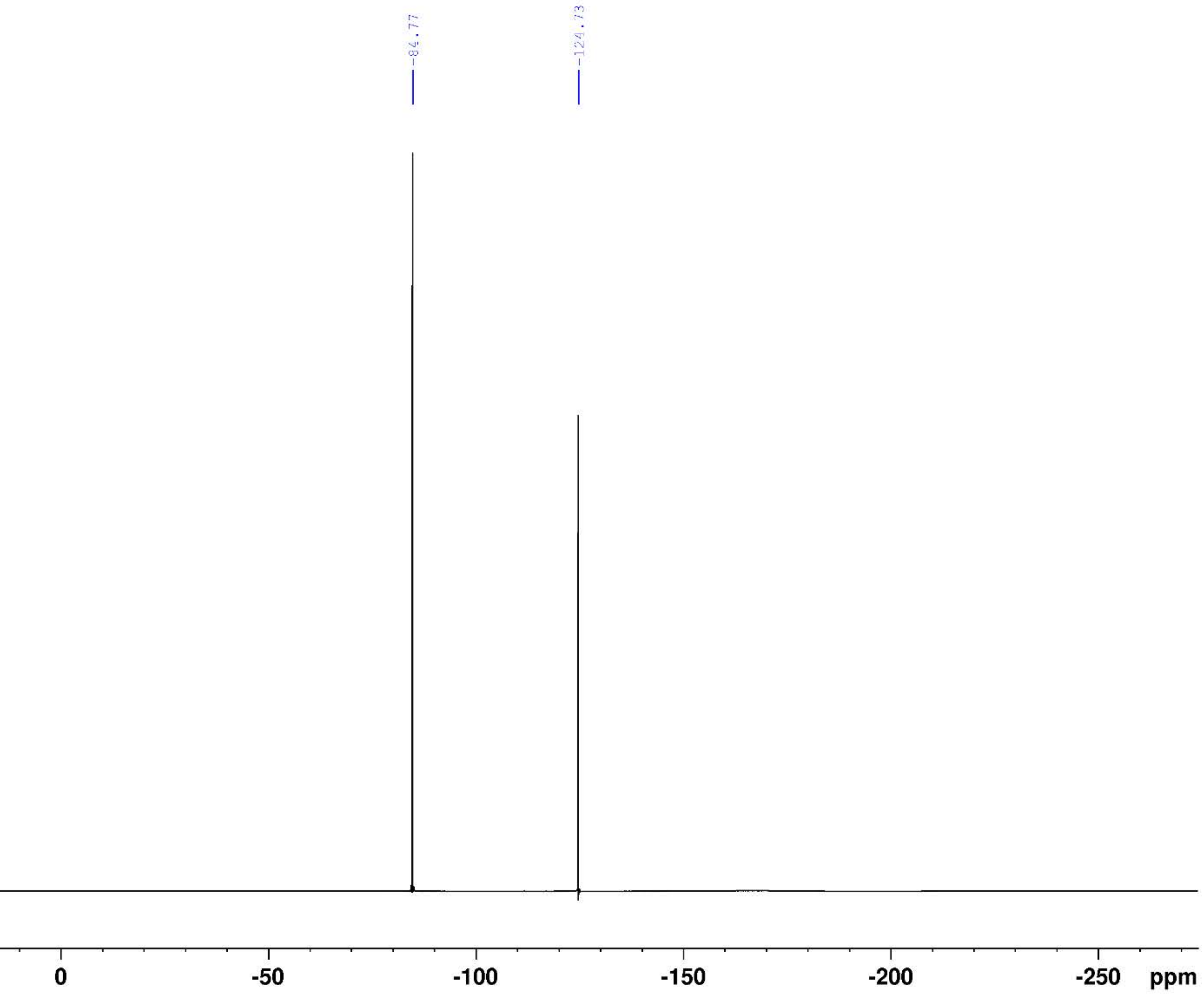

^{1}H NMR (500 MHz, $CDCl_3$) for *2,2,3,3,3-pentafluoropropyl ((S)-(3-fluoro-2-methylphenyl)((S)-tetrahydrofuran-2-yl)methyl)sulfamate* (**4e**)

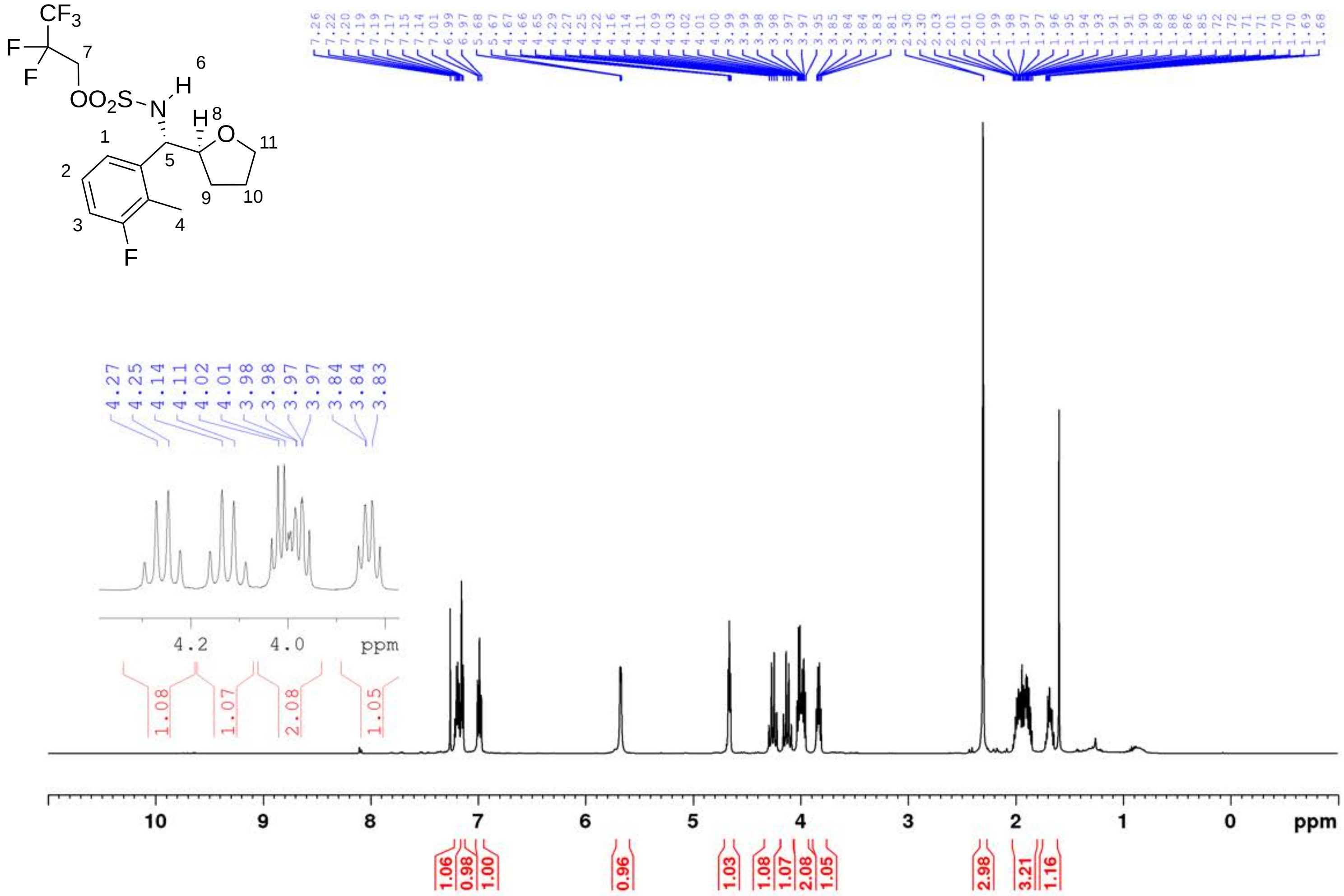

^{13}C NMR (126 MHz, $CDCl_3$) for *2,2,3,3,3-pentafluoropropyl ((S)-(3-fluoro-2-methylphenyl)((S)-tetrahydrofuran-2-yl)methyl)sulfamate* (**4e**)

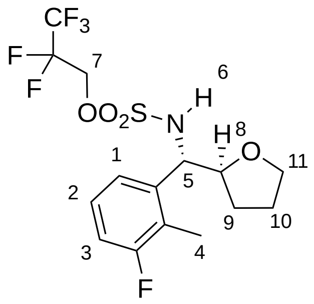

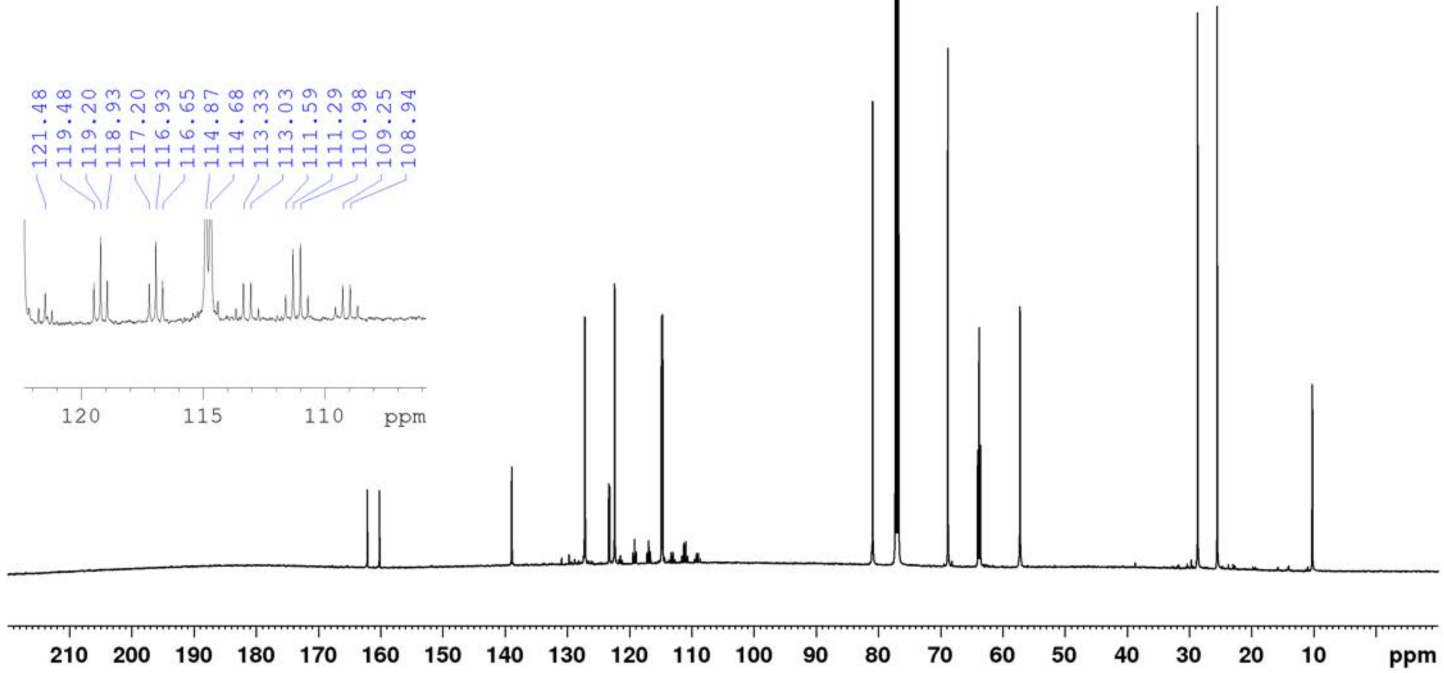

^{19}F NMR (376 MHz, $CDCl_3$) for *2,2,3,3,3-pentafluoropropyl ((S)-(3-fluoro-2-methylphenyl)((S)-tetrahydrofuran-2-yl)methyl)sulfamate* (**4e**)

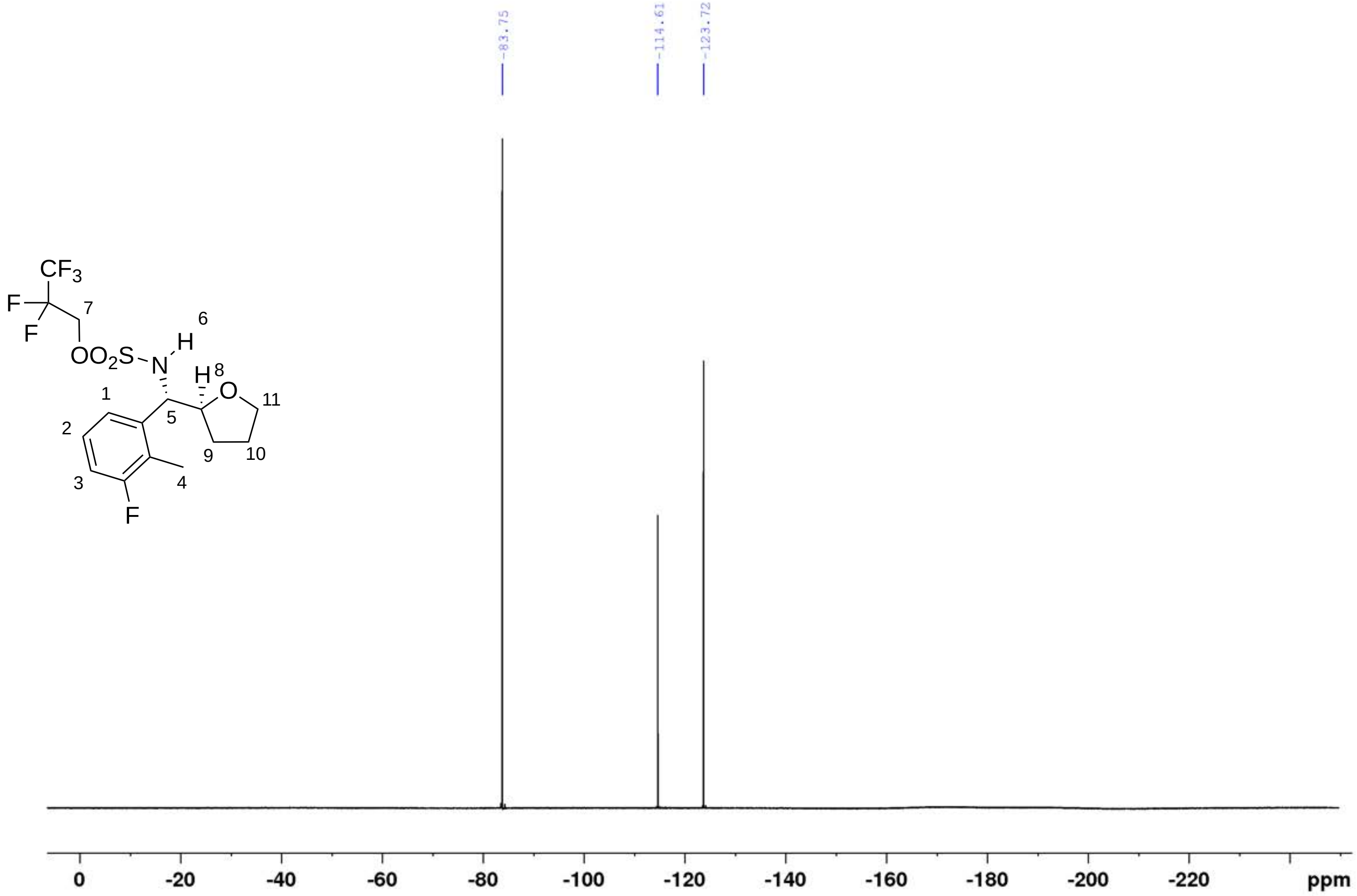

¹H NMR (400 MHz, $CDCl_3$) for *2,2,3,3,3-pentafluoropropyl ((S)-(2,5-dimethylphenyl)((S)-tetrahydrofuran-2-yl)methyl)sulfamate* (**4f**)

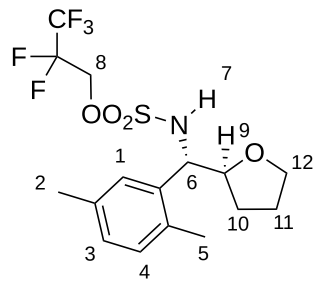

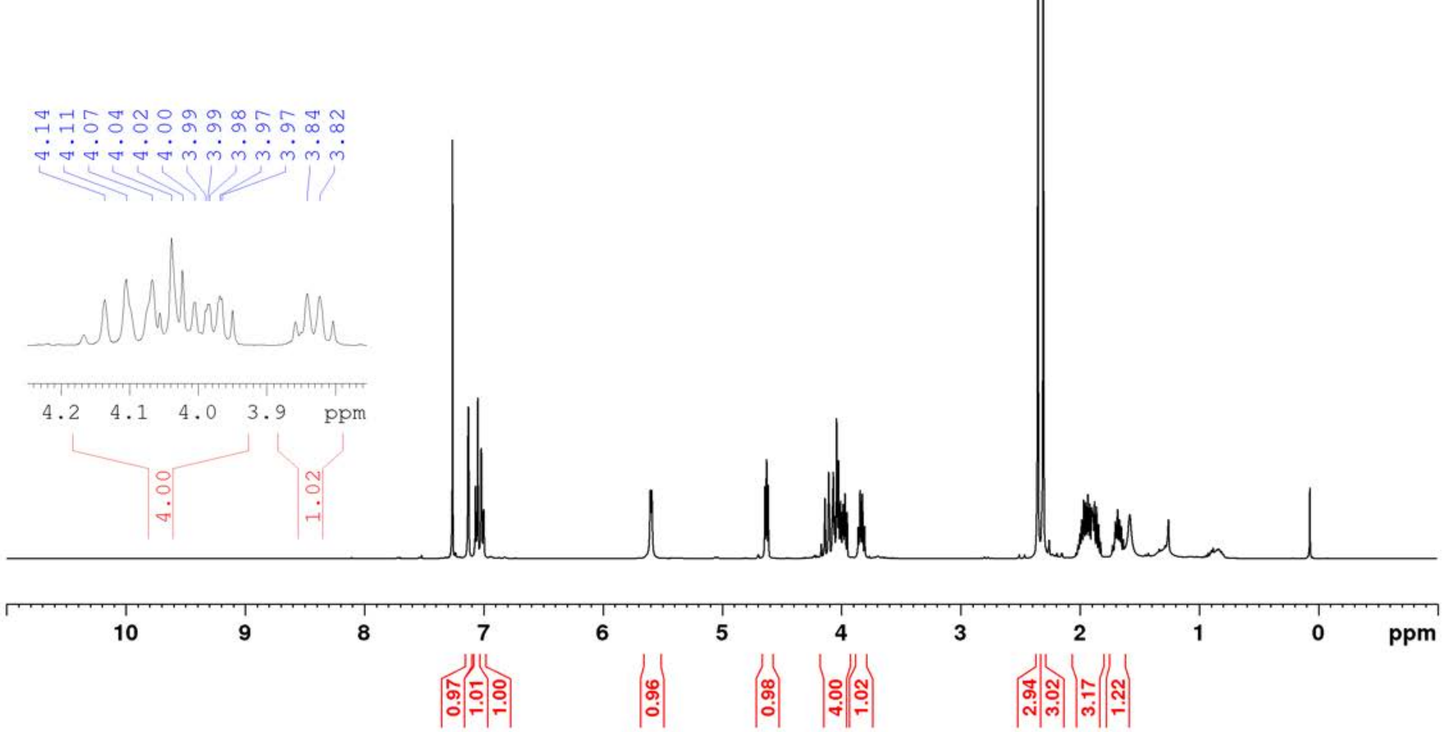

^{13}C NMR (126 MHz, $CDCl_3$) for *2,2,3,3,3-pentafluoropropyl ((S)-(2,5-dimethylphenyl)((S)-tetrahydrofuran-2-yl)methyl)sulfamate* (**4f**)

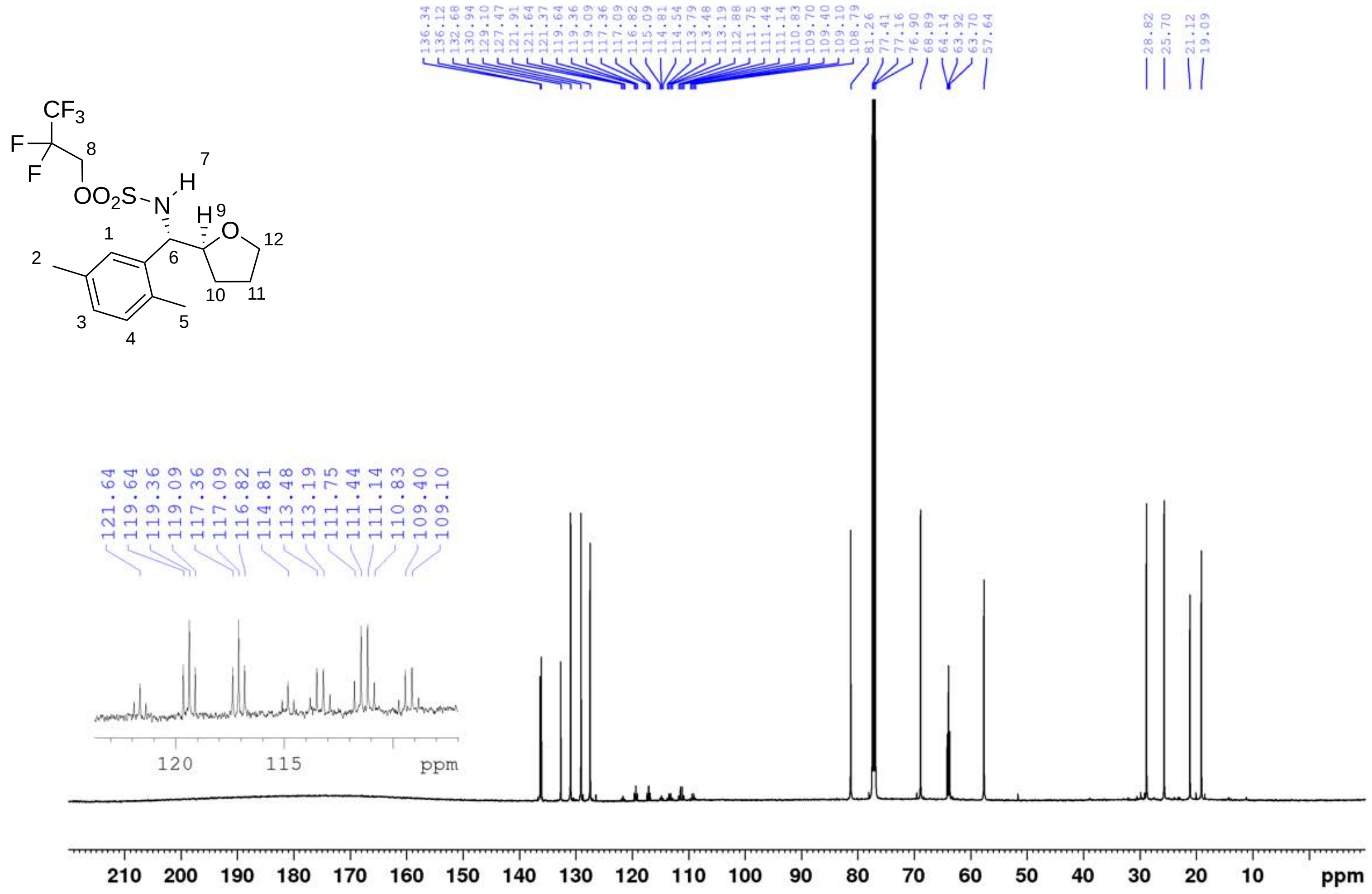

^{19}F NMR (376 MHz, $CDCl_3$) for *2,2,3,3,3-pentafluoropropyl ((S)-(2,5-dimethylphenyl)((S)-tetrahydrofuran-2-yl)methyl)sulfamate* (**4f**)

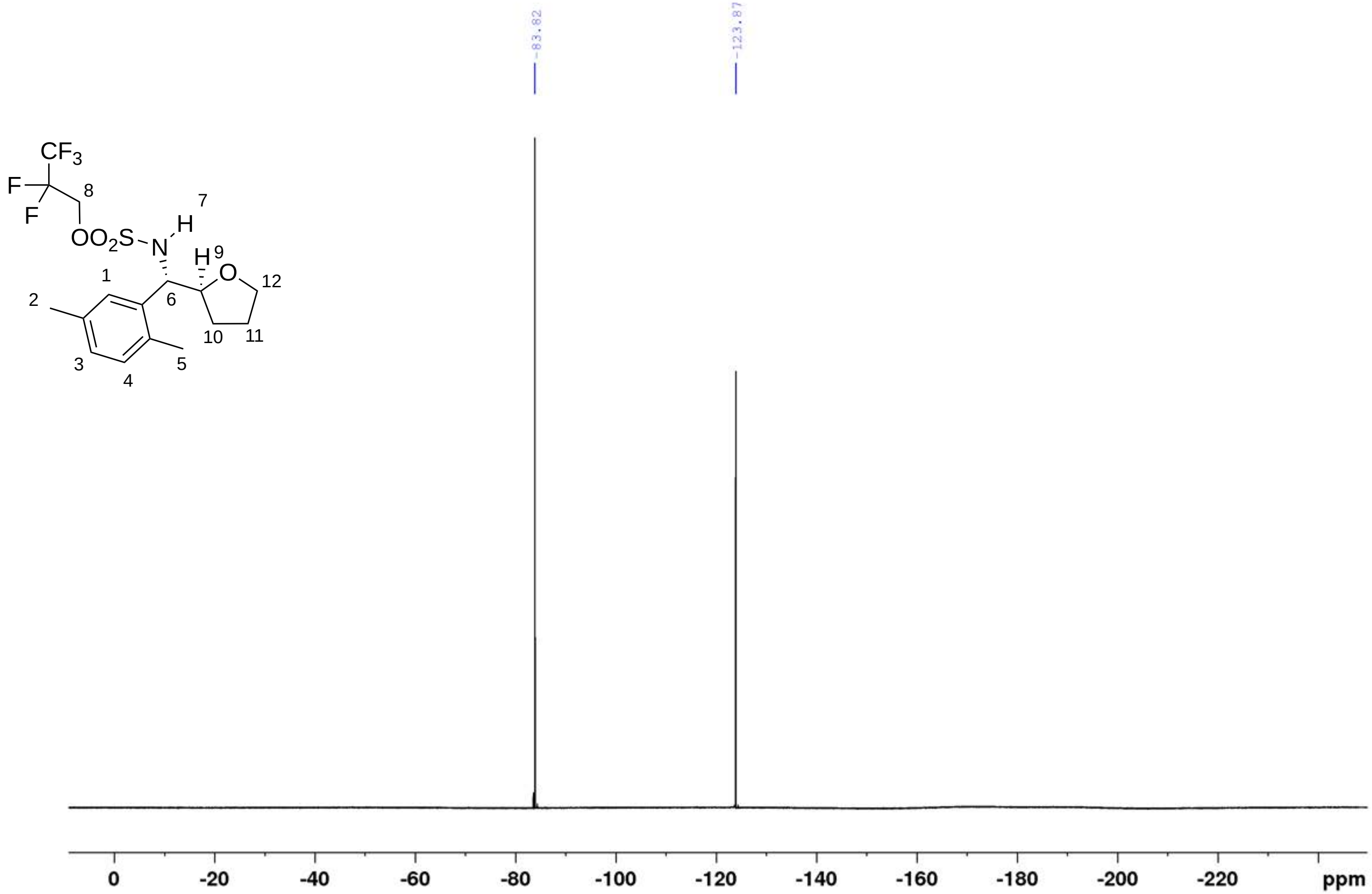

^{1}H NMR (400 MHz, $CDCl_3$) for *2,2,3,3,3-pentafluoropropyl ((S)-(2,3-dimethylphenyl)((S)-tetrahydrofuran-2-yl)methyl)sulfamate* (**4g**)

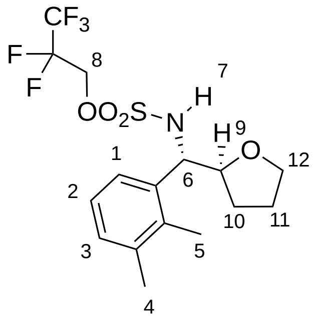

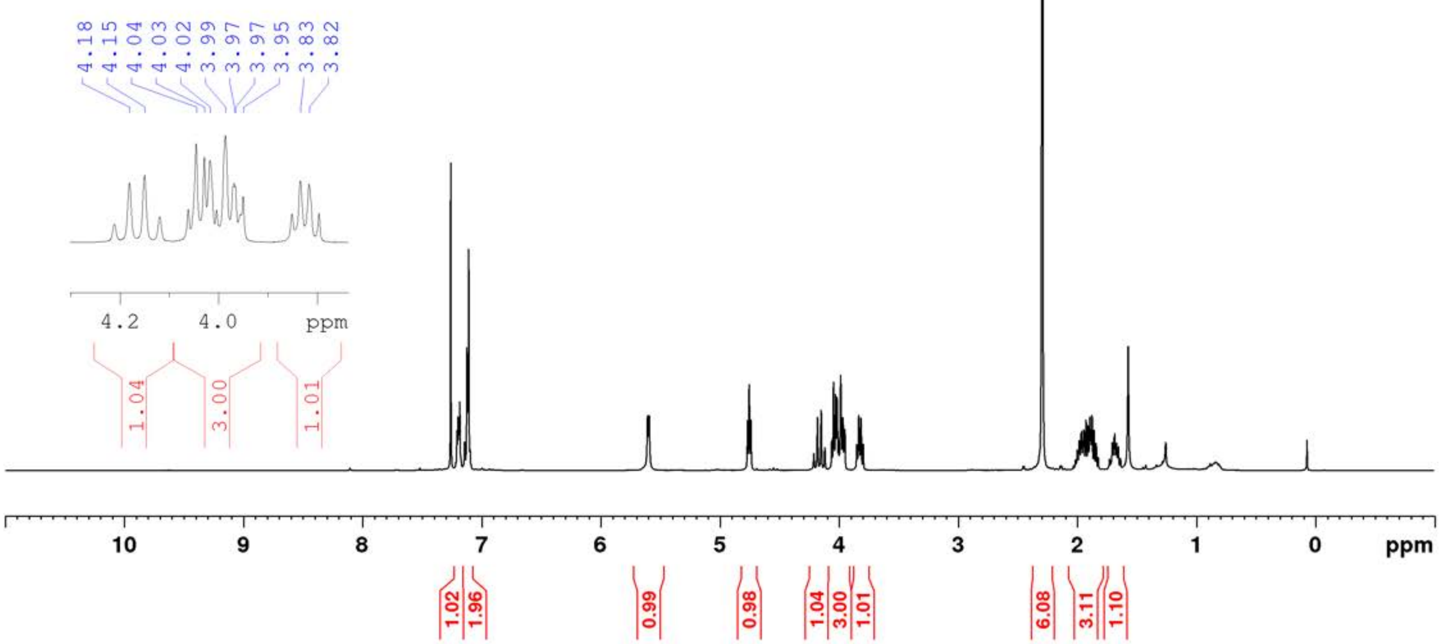

^{13}C NMR (126 MHz, $CDCl_3$) for *2,2,3,3,3-pentafluoropropyl ((S)-(2,3-dimethylphenyl)((S)-tetrahydrofuran-2-yl)methyl)sulfamate* (**4g**)

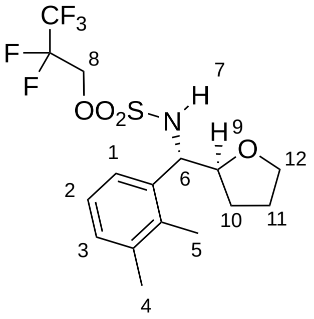

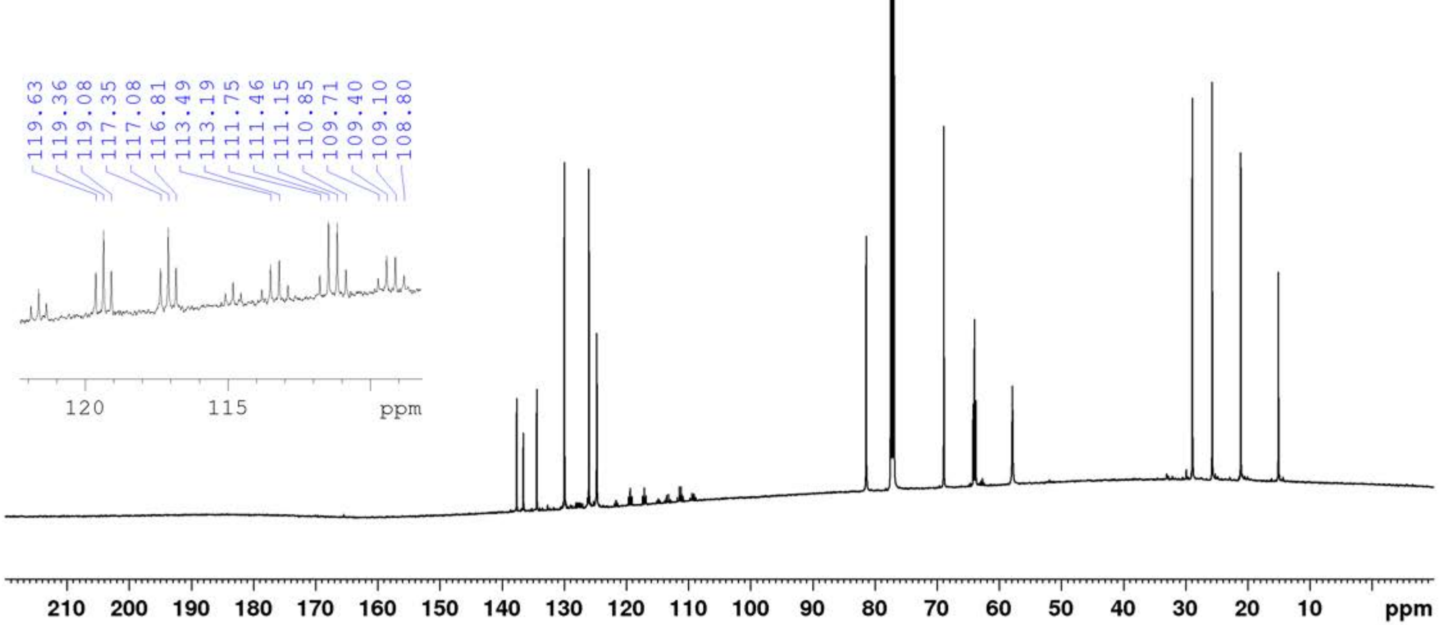

^{19}F NMR (376 MHz, $CDCl_3$) for *2,2,3,3,3-pentafluoropropyl ((S)-(2,3-dimethylphenyl)((S)-tetrahydrofuran-2-yl)methyl)sulfamate* (**4g**)

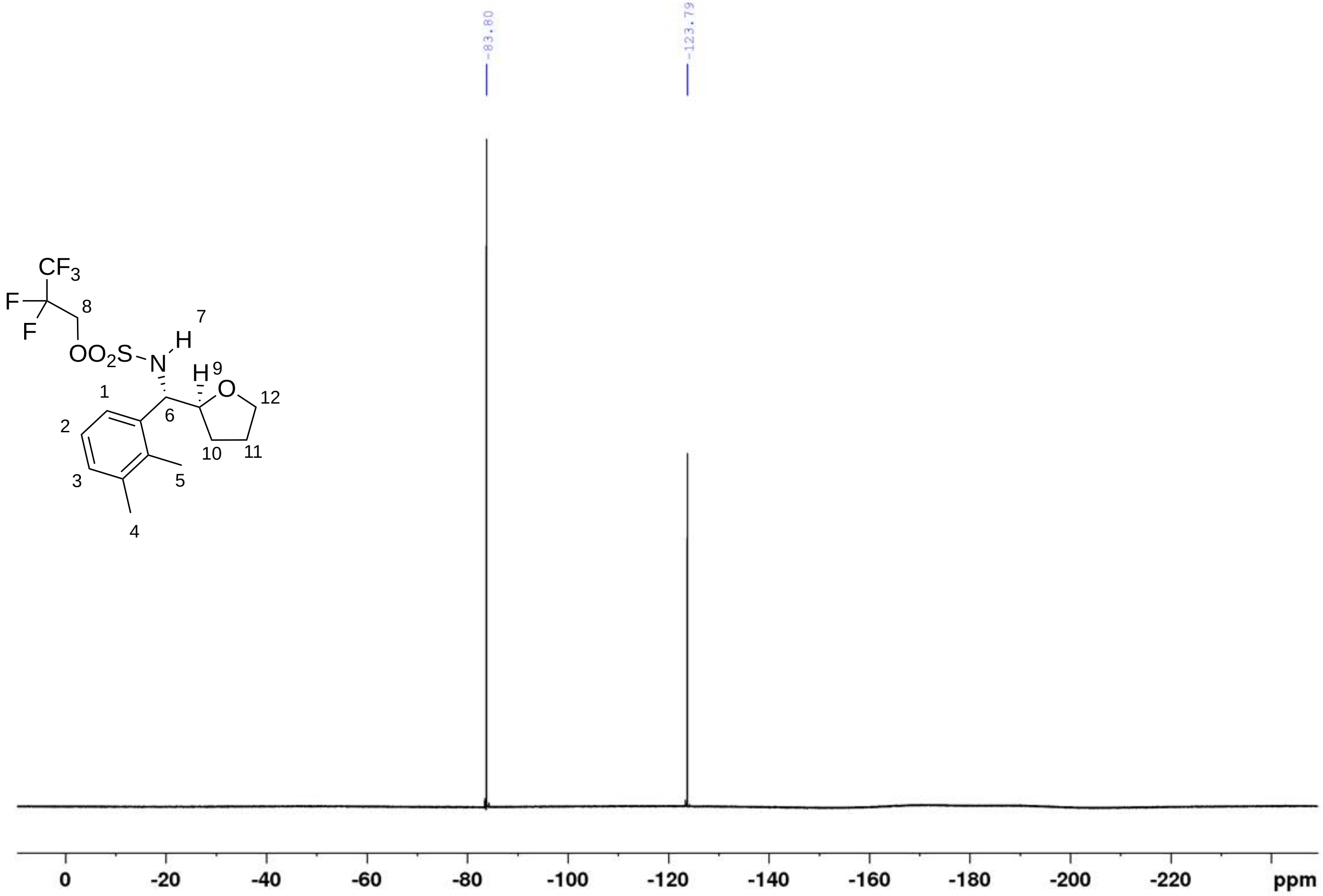

¹H NMR (400 MHz, $CDCl_3$) for *2,2,3,3,3-pentafluoropropyl ((S)-(3-fluorophenyl)((S)-tetrahydrofuran-2-yl)methyl)sulfamate* (**4h**)

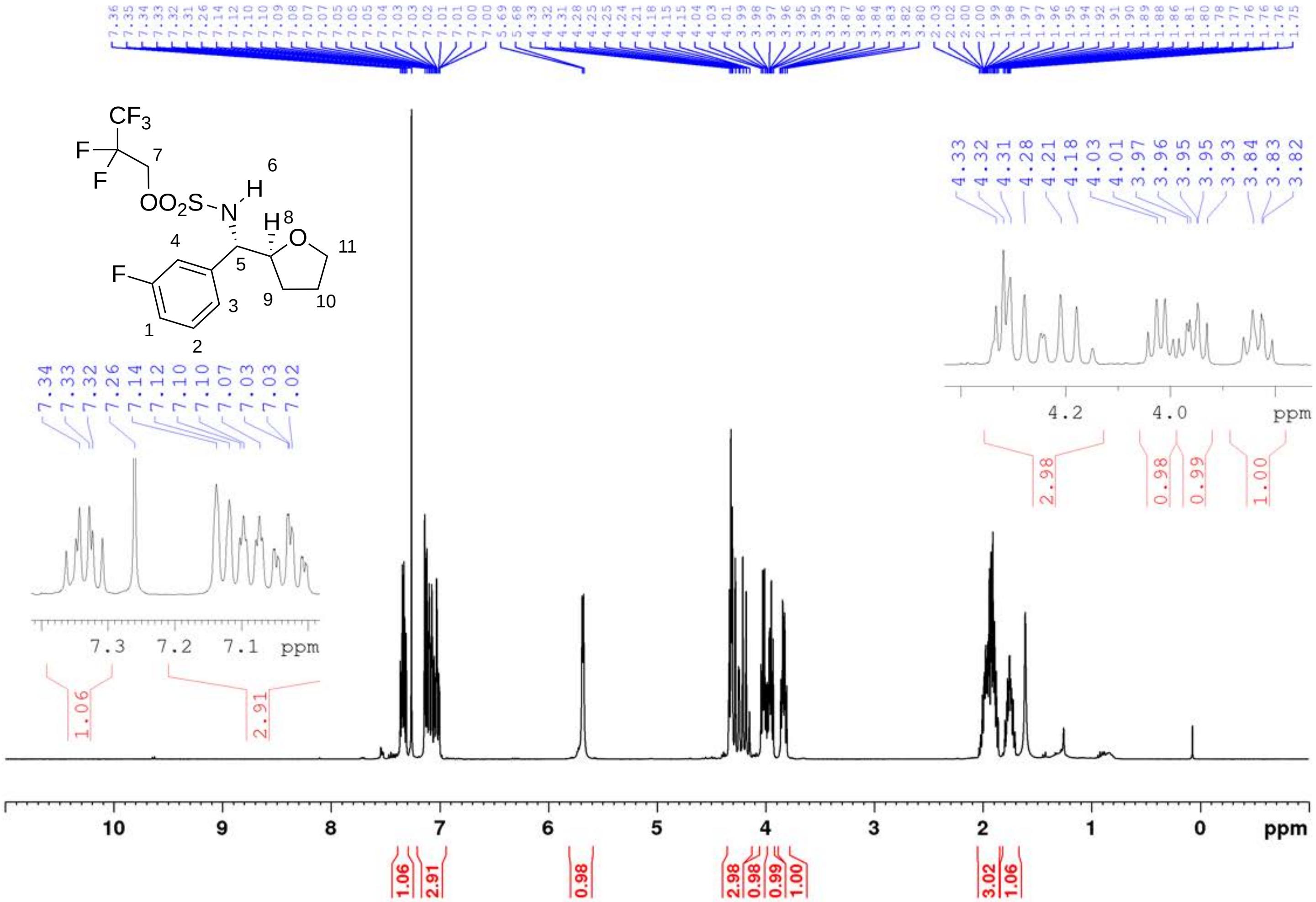

^{13}C NMR (101 MHz, $CDCl_3$) for *2,2,3,3,3-pentafluoropropyl ((S)-(3-fluorophenyl)((S)-tetrahydrofuran-2-yl)methyl)sulfamate* (**4h**)

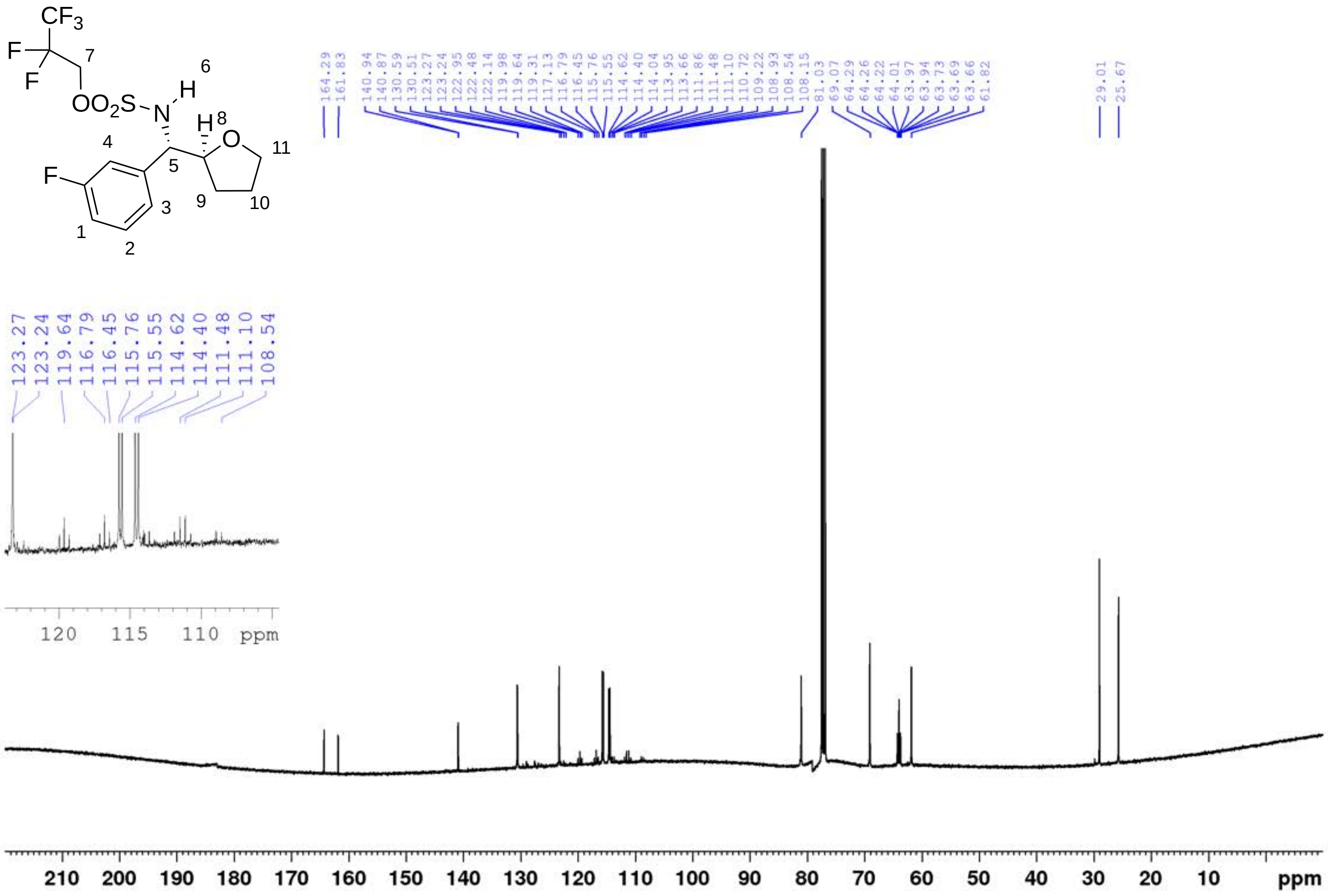

^{19}F NMR (376 MHz, $CDCl_3$) for *2,2,3,3,3-pentafluoropropyl ((S)-(3-fluorophenyl)((S)-tetrahydrofuran-2-yl)methyl)sulfamate* (**4h**)

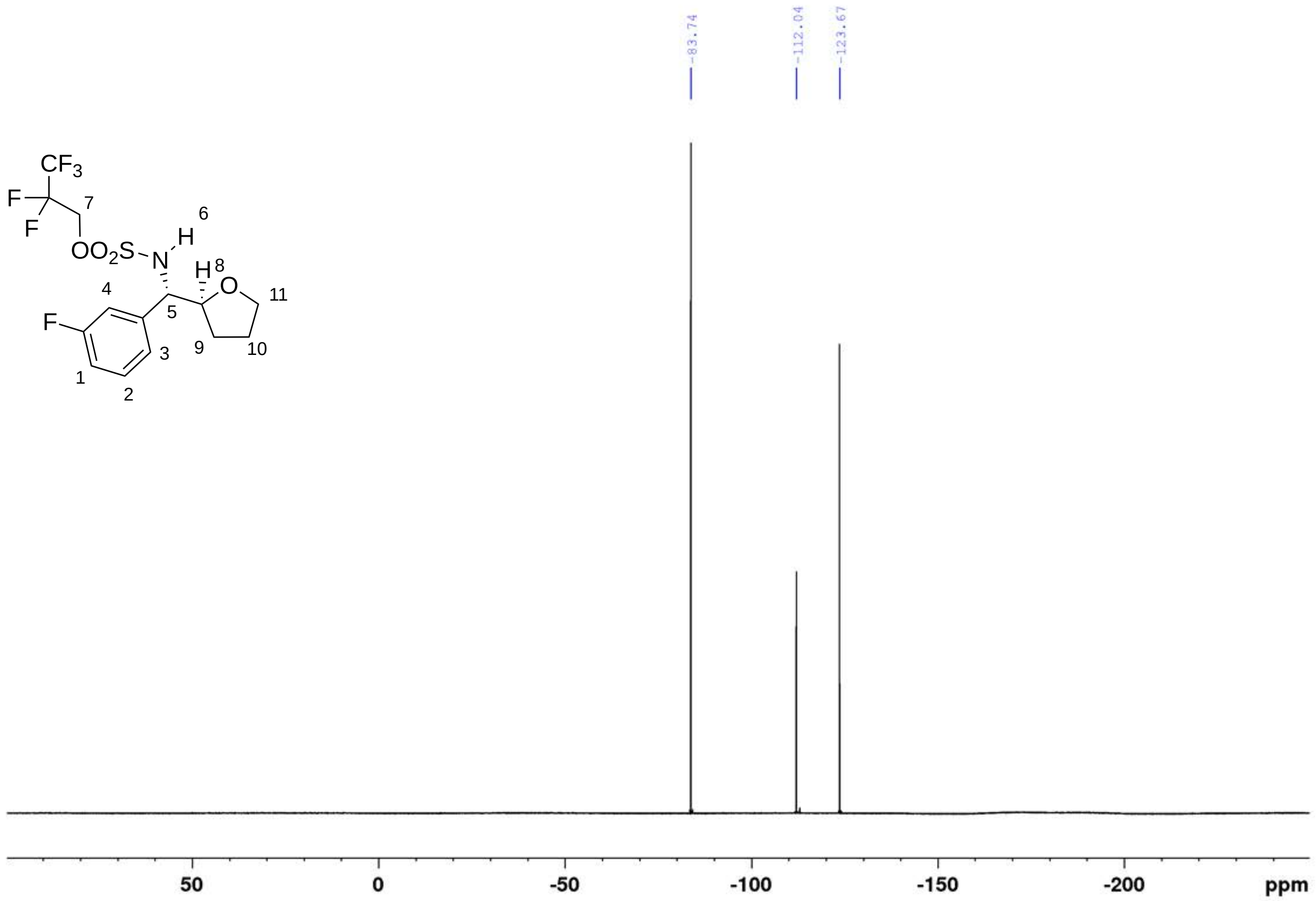

^{1}H NMR (500 MHz, $CDCl_3$) for *2,2,3,3,3-pentafluoropropyl ((S)-(3-chlorophenyl)((S)-tetrahydrofuran-2-yl)methyl)sulfamate* (**4i**)

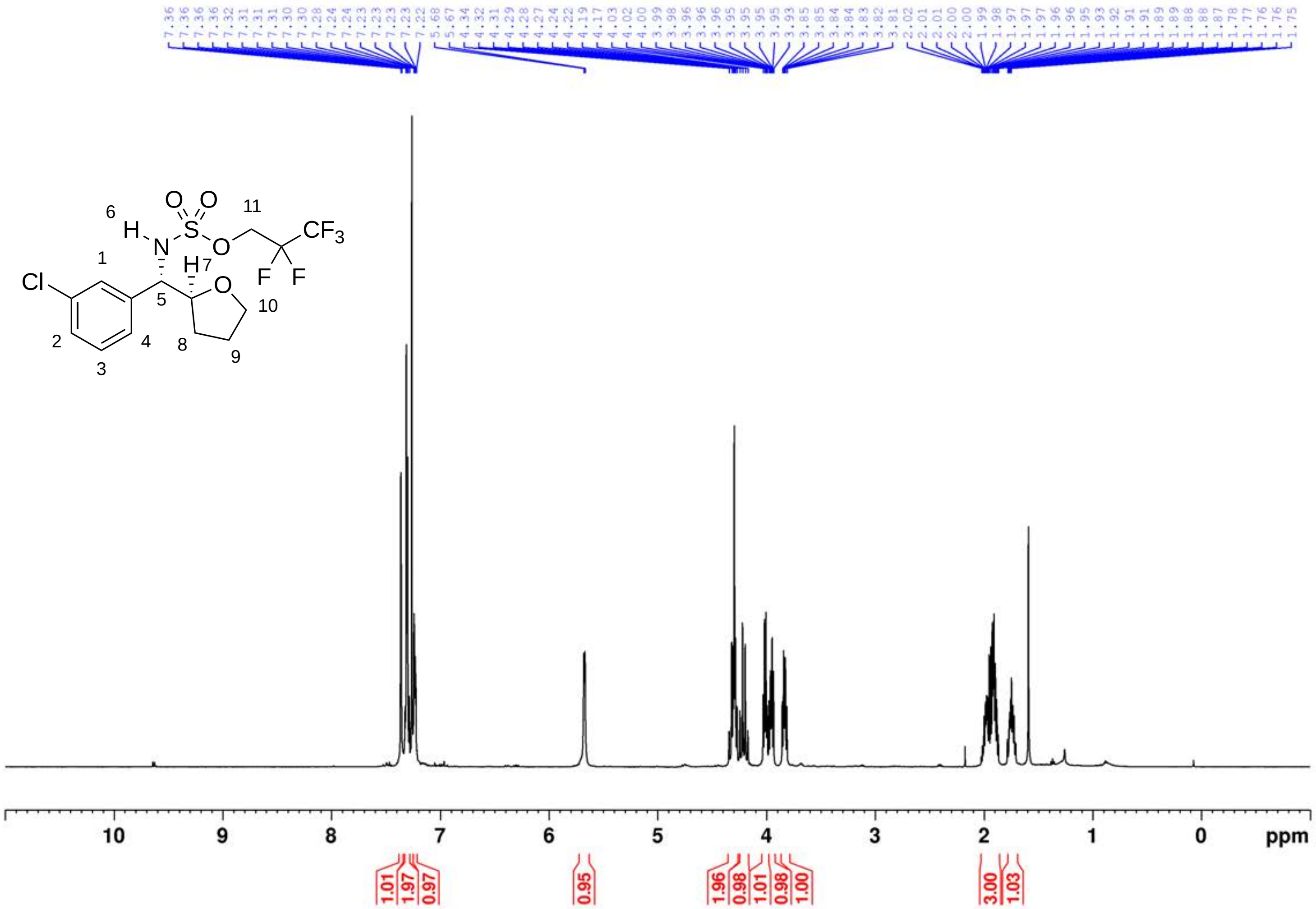

^{13}C NMR (126 MHz, $CDCl_3$) for *2,2,3,3,3-pentafluoropropyl ((S)-(3-chlorophenyl)((S)-tetrahydrofuran-2-yl)methyl)sulfamate* (**4i**)

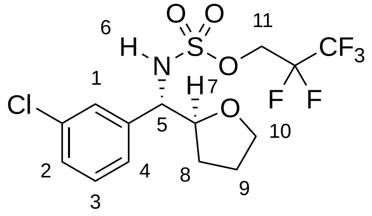

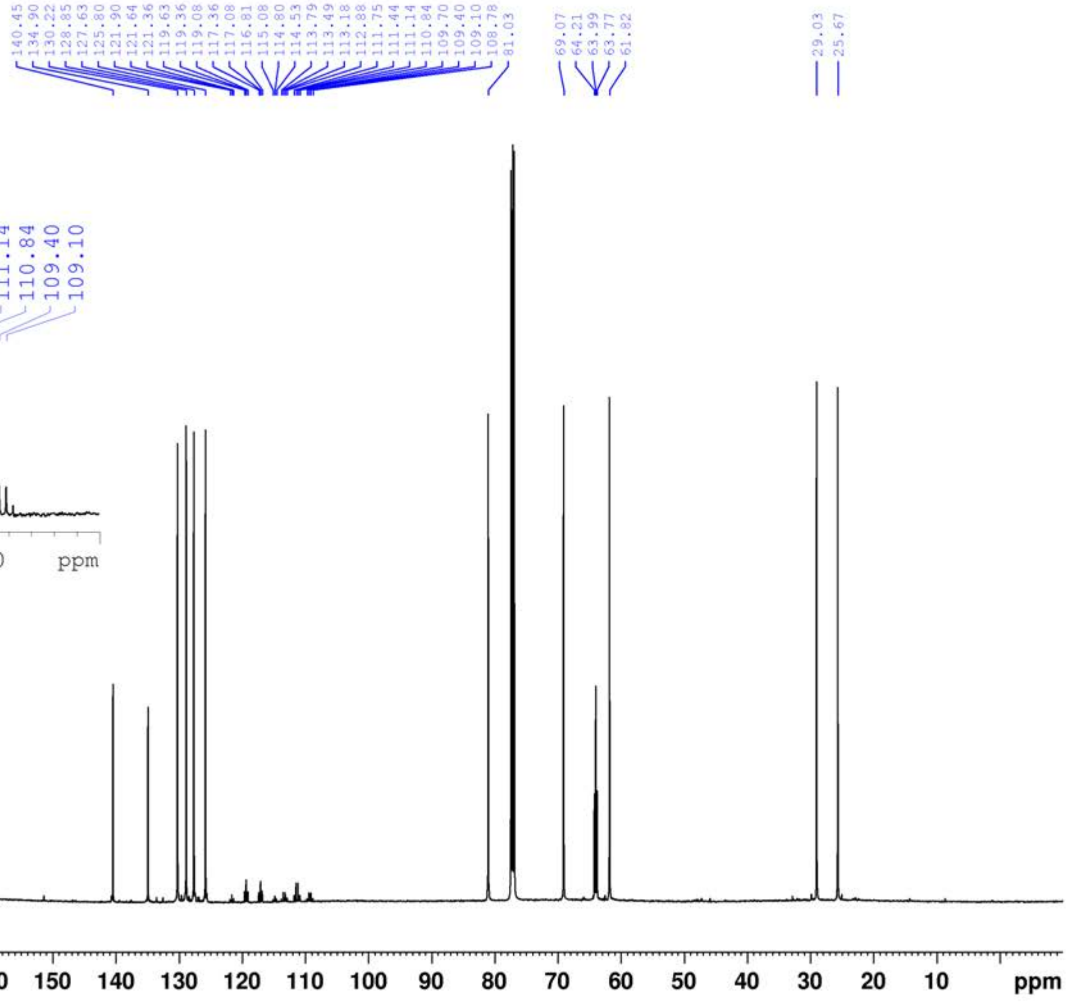

^{19}**F NMR** (376 MHz, $CDCl_3$) for *2,2,3,3,3-pentafluoropropyl ((S)-(3-chlorophenyl)((S)-tetrahydrofuran-2-yl)methyl)sulfamate* (**4i**)

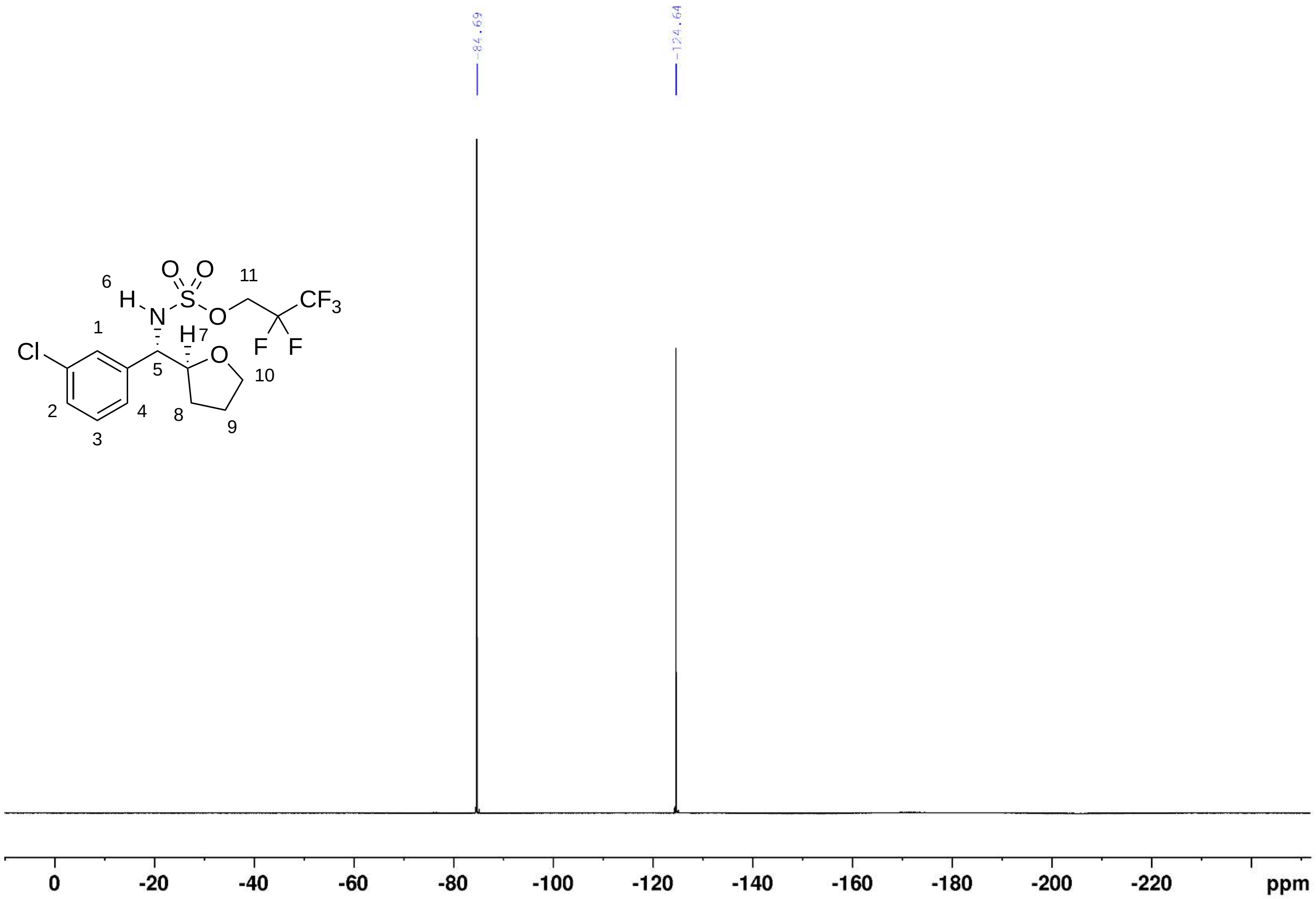

^{1}H NMR (400 MHz, $CDCl_3$) for *2,2,3,3,3-pentafluoropropyl ((S)-((S)-tetrahydrofuran-2-yl)(m-tolyl)methyl)sulfamate* (**4j**)

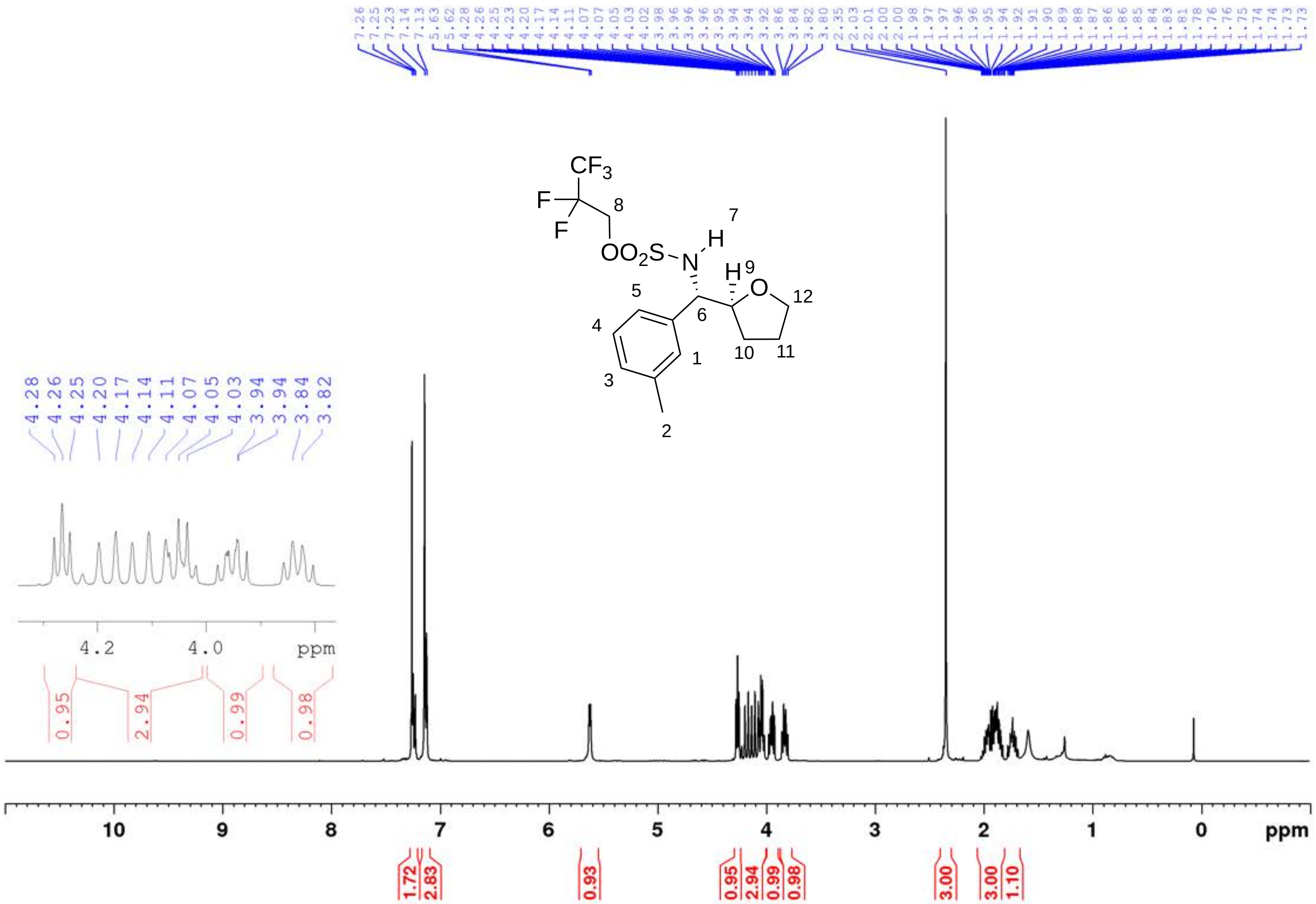

^{13}C NMR (126 MHz, $CDCl_3$) for *2,2,3,3,3-pentafluoropropyl ((S)-((S)-tetrahydrofuran-2-yl)(m-tolyl)methyl)sulfamate* (**4j**)

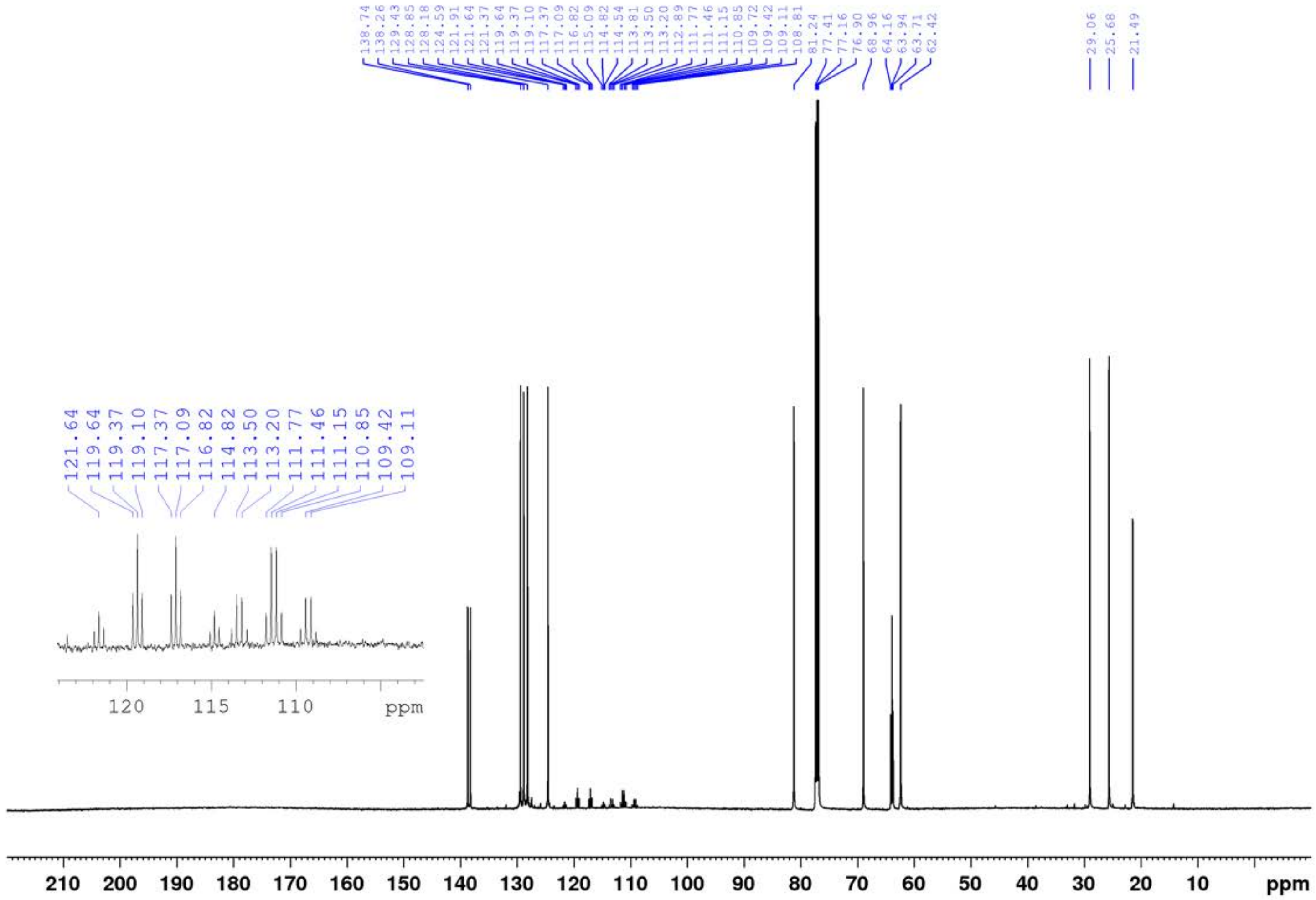

^{19}F NMR (376 MHz, $CDCl_3$) for *2,2,3,3,3-pentafluoropropyl ((S)-((S)-tetrahydrofuran-2-yl)(m-tolyl)methyl)sulfamate* (**4j**)

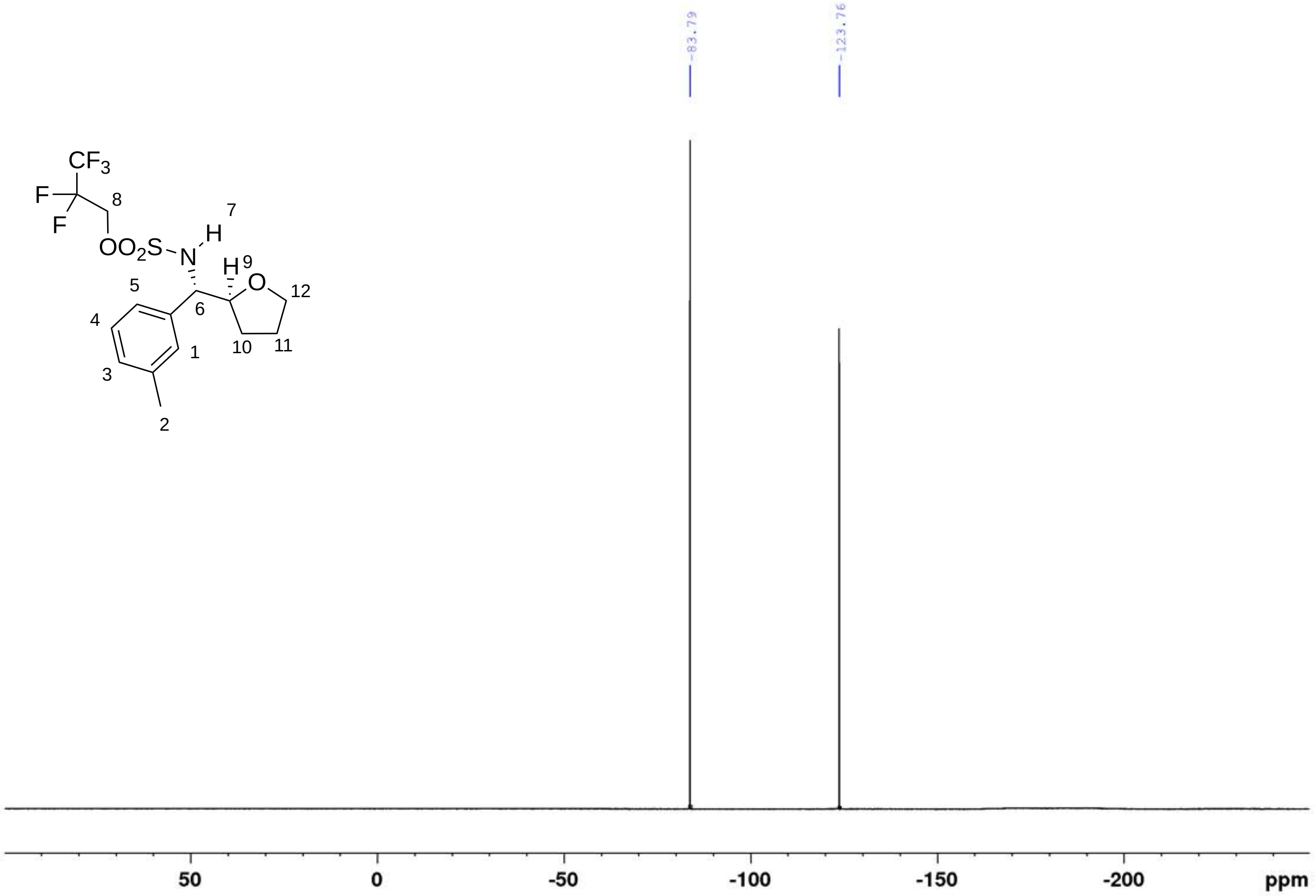

^{1}H NMR (500 MHz, $CDCl_3$) for *2,2,3,3,3-pentafluoropropyl ((S)-((S)-tetrahydrofuran-2-yl)(3-(trifluoromethyl)phenyl)methyl)sulfamate* (**4k**)

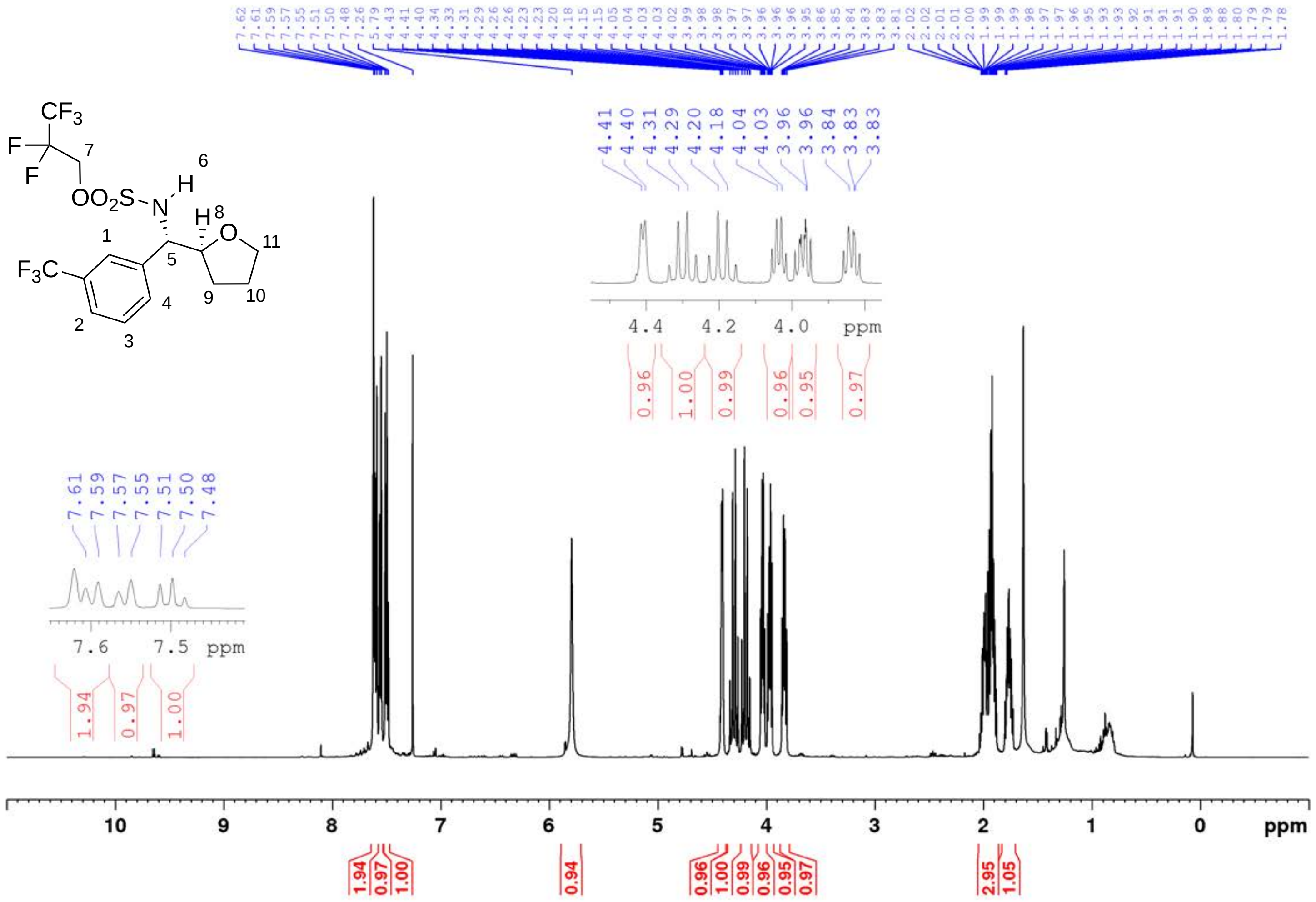

13C NMR (126 MHz, $CDCl_3$) for *2,2,3,3,3-pentafluoropropyl ((S)-((S)-tetrahydrofuran-2-yl)(3-(trifluoromethyl)phenyl)methyl)sulfamate* (**4k**)

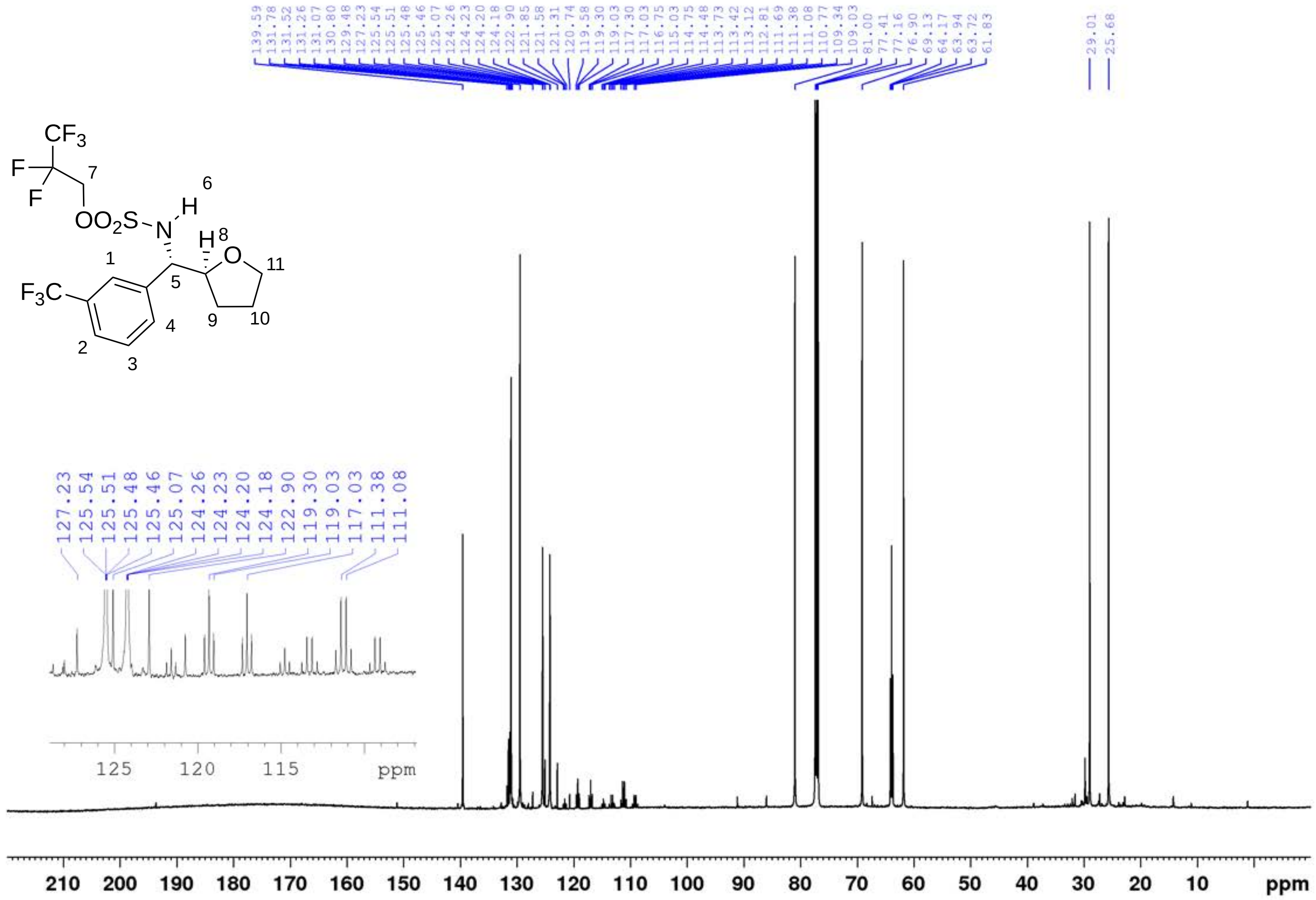

19F NMR (376 MHz, $CDCl_3$) for *2,2,3,3,3-pentafluoropropyl ((S)-((S)-tetrahydrofuran-2-yl)(3-(trifluoromethyl)phenyl)methyl)sulfamate* (**4k**)

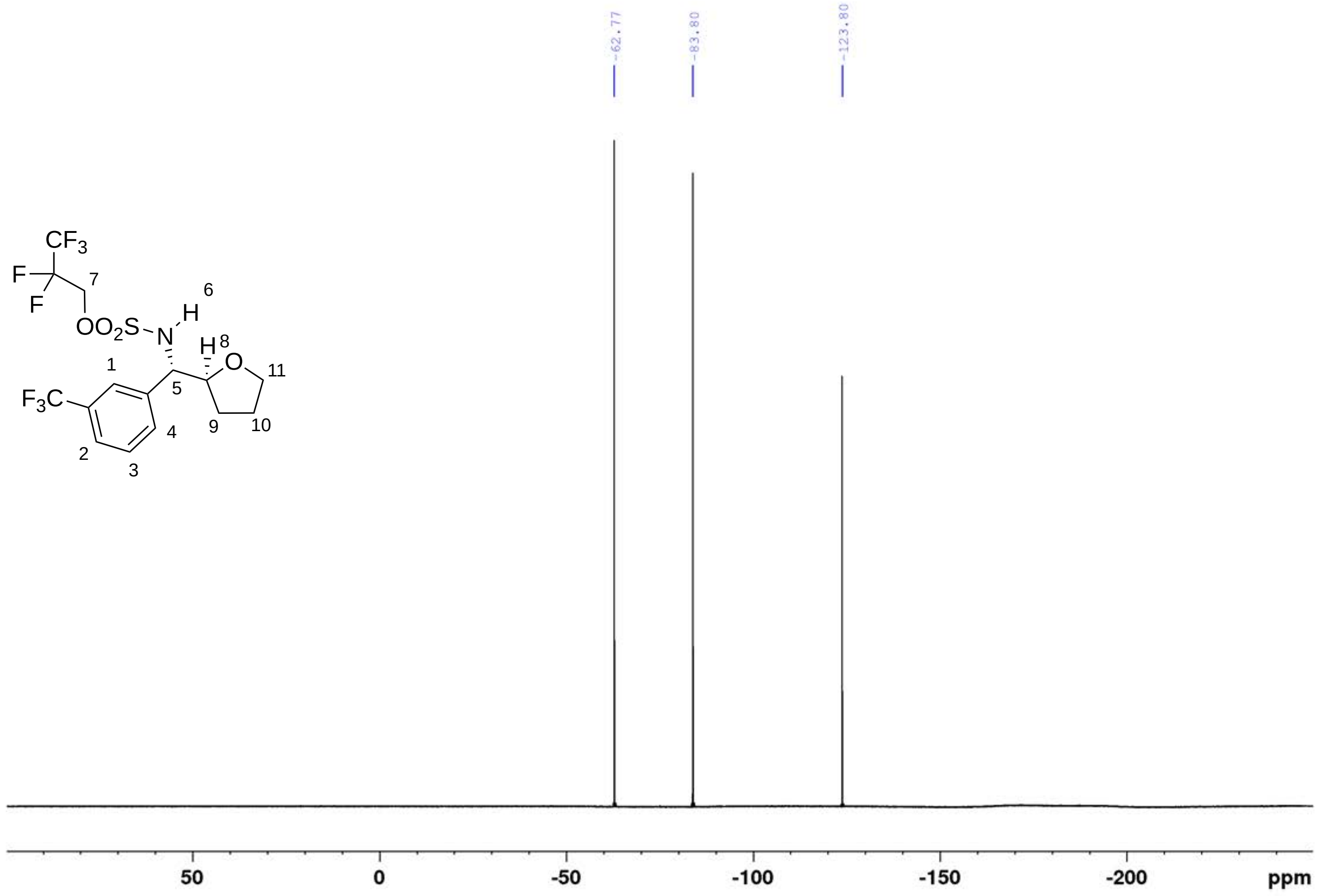

^{1}H NMR (500 MHz, $CDCl_3$) for *2,2,3,3,3-pentafluoropropyl ((S)-((S)-tetrahydrofuran-2-yl)(3-(trifluoromethoxy)phenyl)methyl)sulfamate* (**4l**)

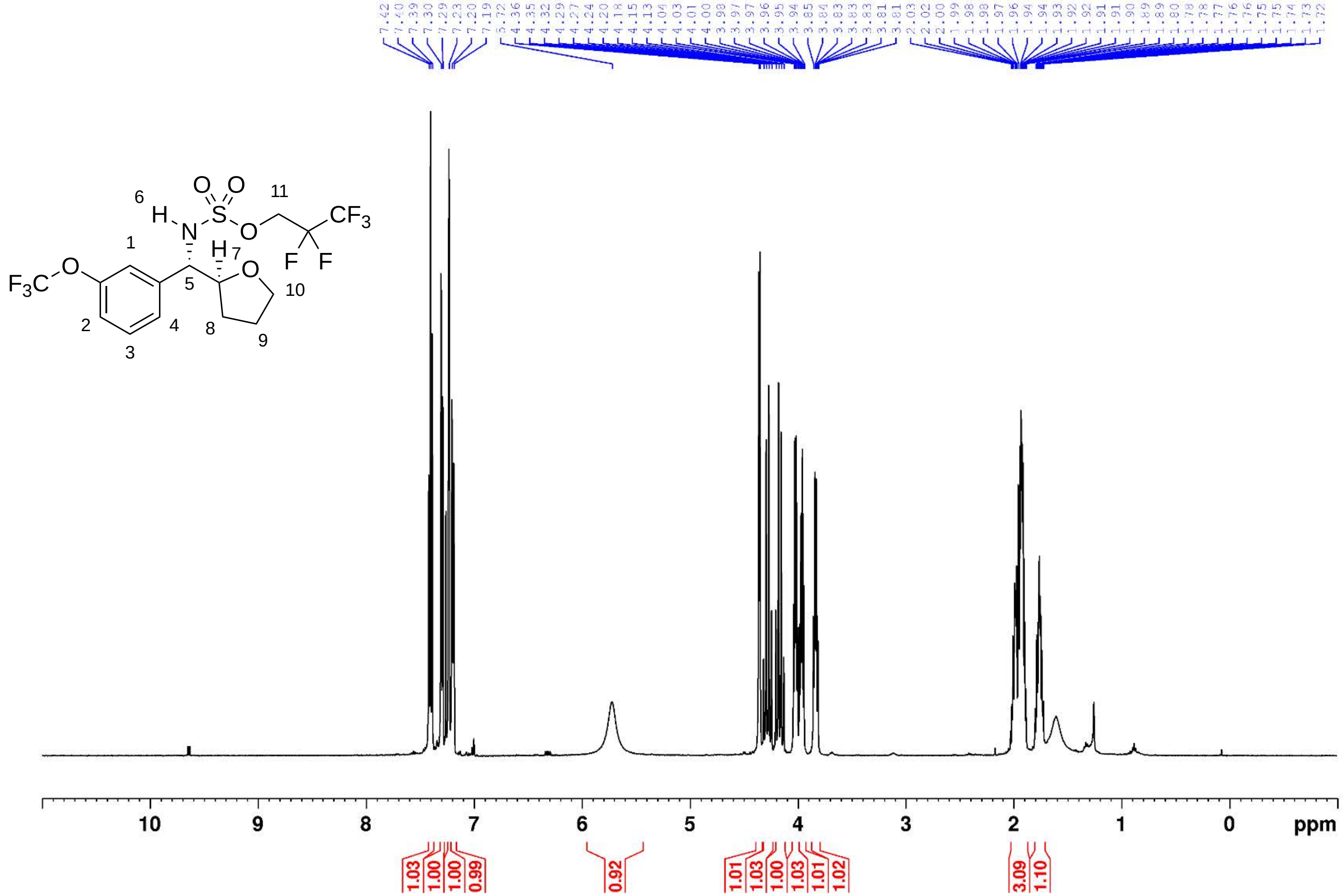

^{13}C NMR (126 MHz, $CDCl_3$) for *2,2,3,3,3-pentafluoropropyl ((S)-((S)-tetrahydrofuran-2-yl)(3-(trifluoromethoxy)phenyl)methyl)sulfamate* (**4l**)

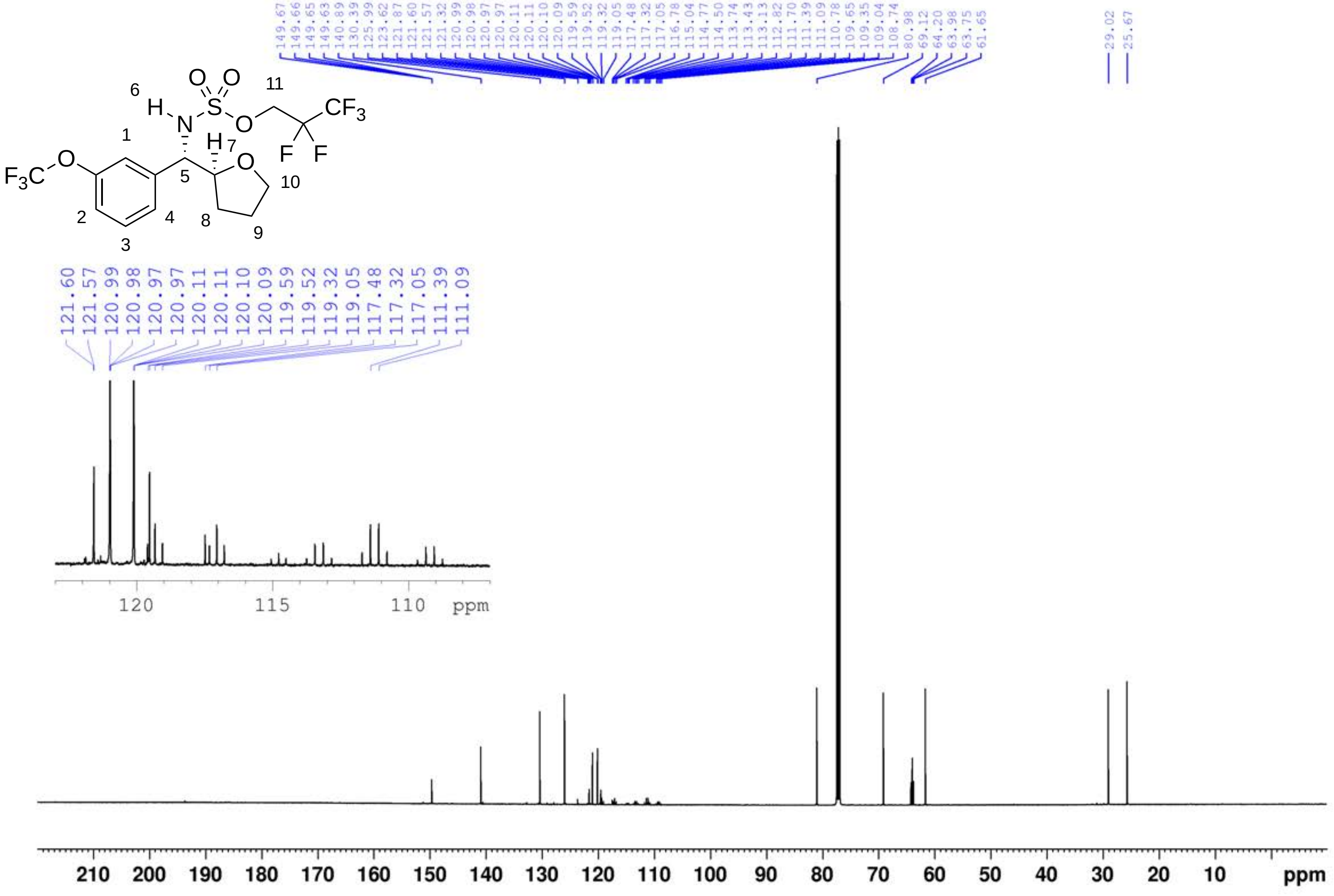

^{19}F NMR (471 MHz, $CDCl_3$) for *2,2,3,3,3-pentafluoropropyl ((S)-((S)-tetrahydrofuran-2-yl)(3-(trifluoromethoxy)phenyl)methyl)sulfamate* (**4l**)

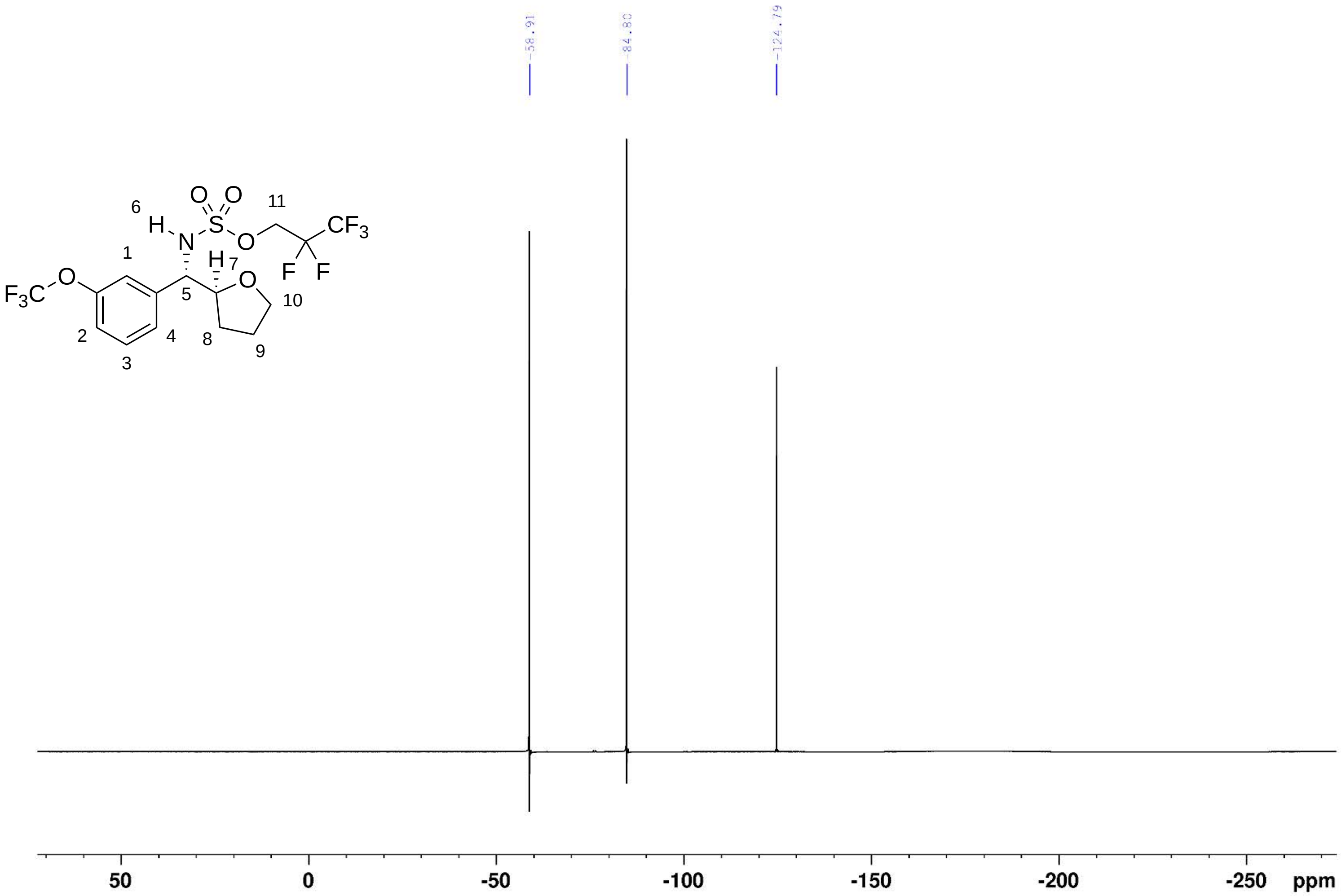

^{1}H NMR (500 MHz, $CDCl_3$) for *2,2,3,3,3-pentafluoropropyl ((S)-(3-acetylphenyl)((S)-tetrahydrofuran-2-yl)methyl)sulfamate* (**4m**)

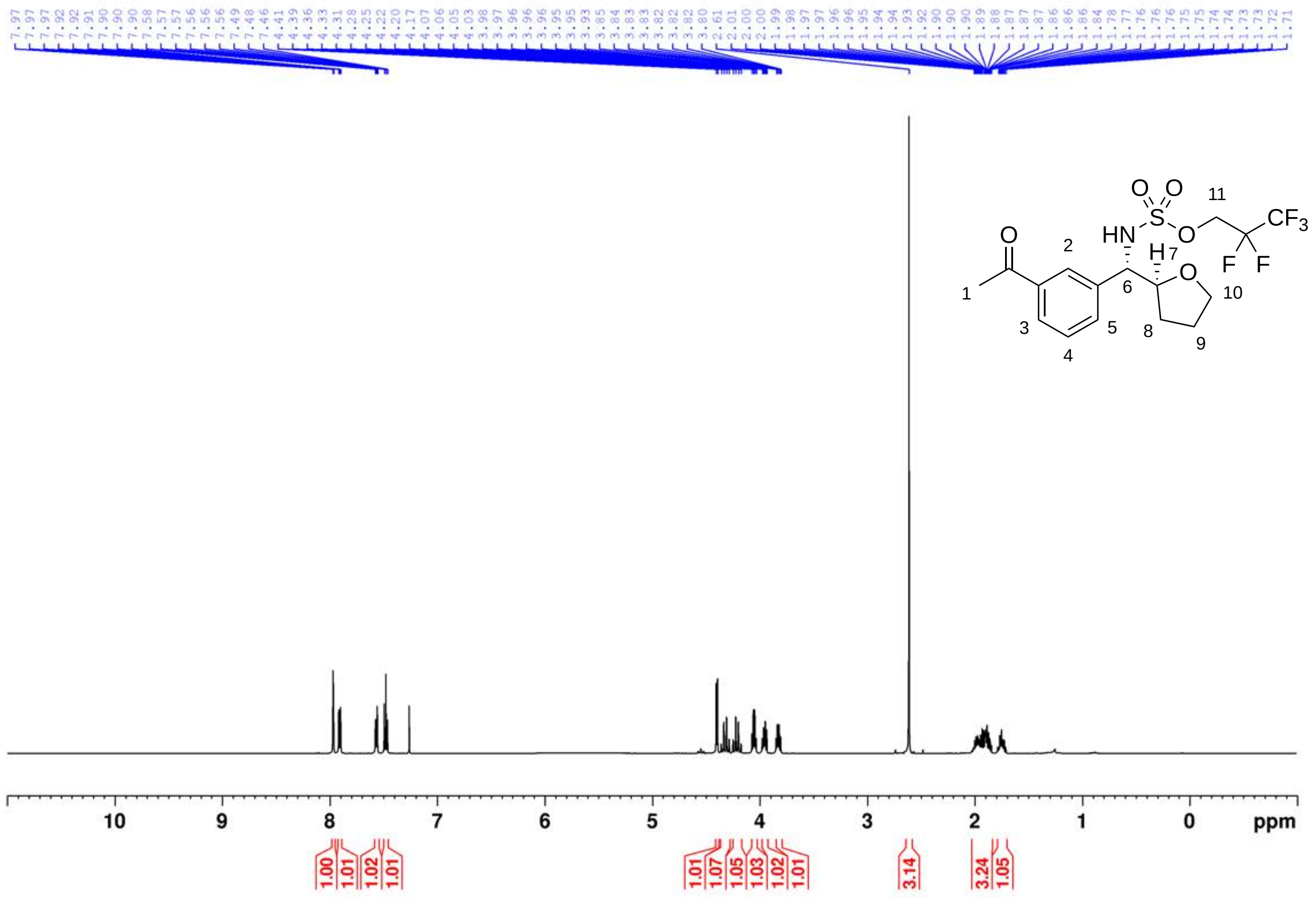

13C NMR (126 MHz, $CDCl_3$) for *2,2,3,3,3-pentafluoropropyl ((S)-(3-acetylphenyl)((S)-tetrahydrofuran-2-yl)methyl)sulfamate* (**4m**)

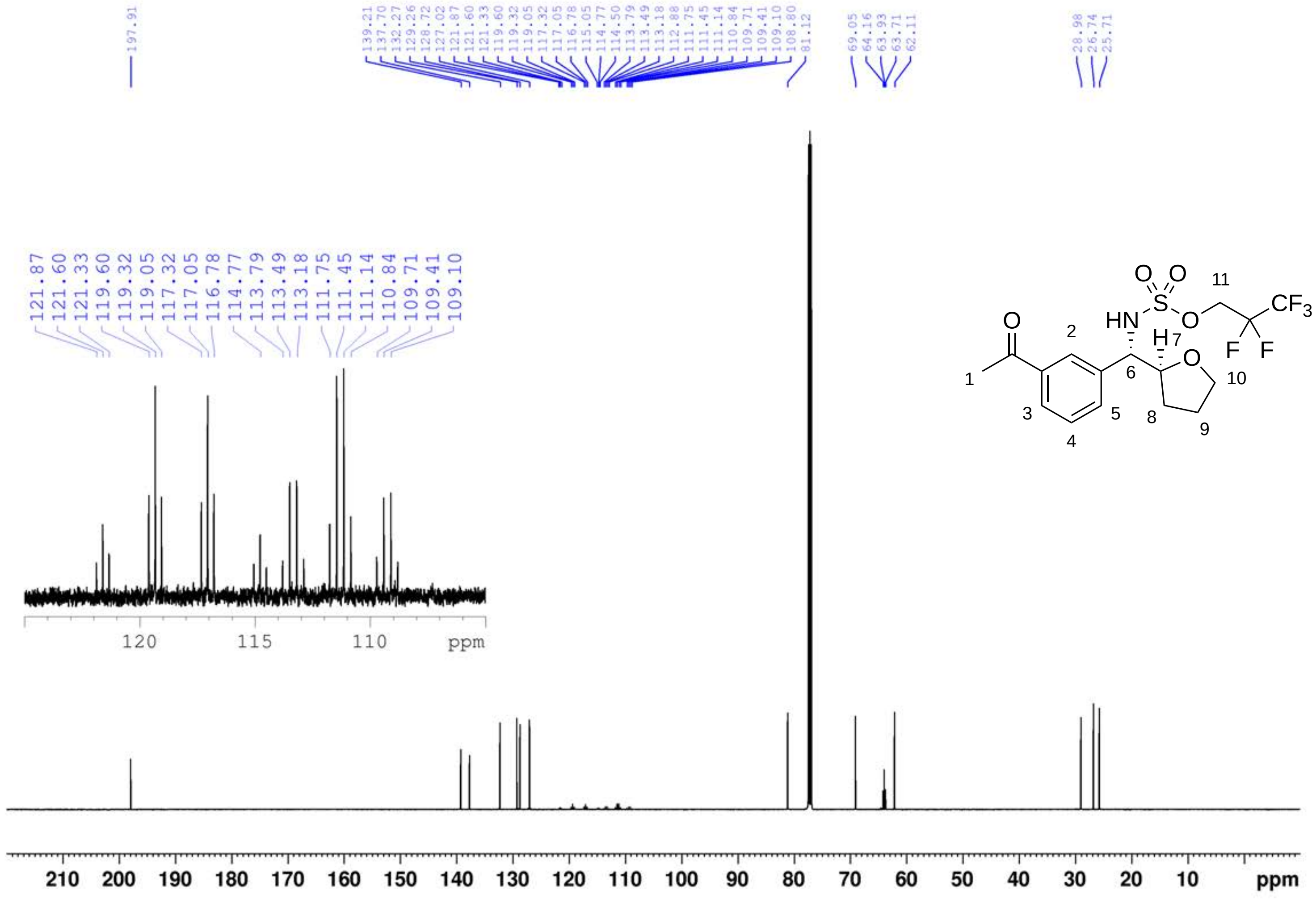

^{19}F NMR (471 MHz, $CDCl_3$) for *2,2,3,3,3-pentafluoropropyl ((S)-(3-acetylphenyl)((S)-tetrahydrofuran-2-yl)methyl)sulfamate* (**4m**)

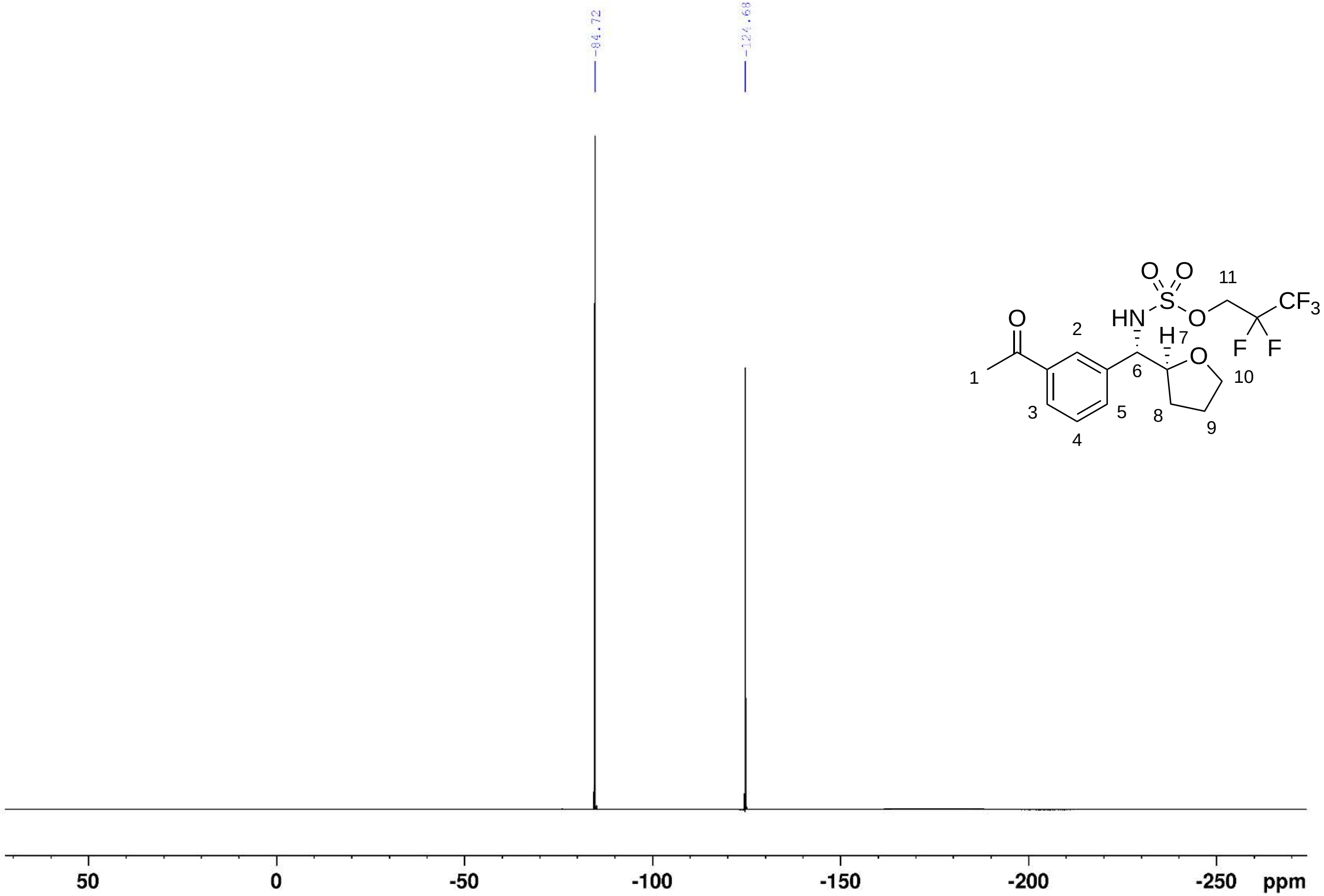

^{1}H NMR (500 MHz, $CDCl_3$) for *2,2,3,3,3-pentafluoropropyl ((S)-(3-isopropylphenyl)((S)-tetrahydrofuran-2-yl)methyl)sulfamate* (**4n**)

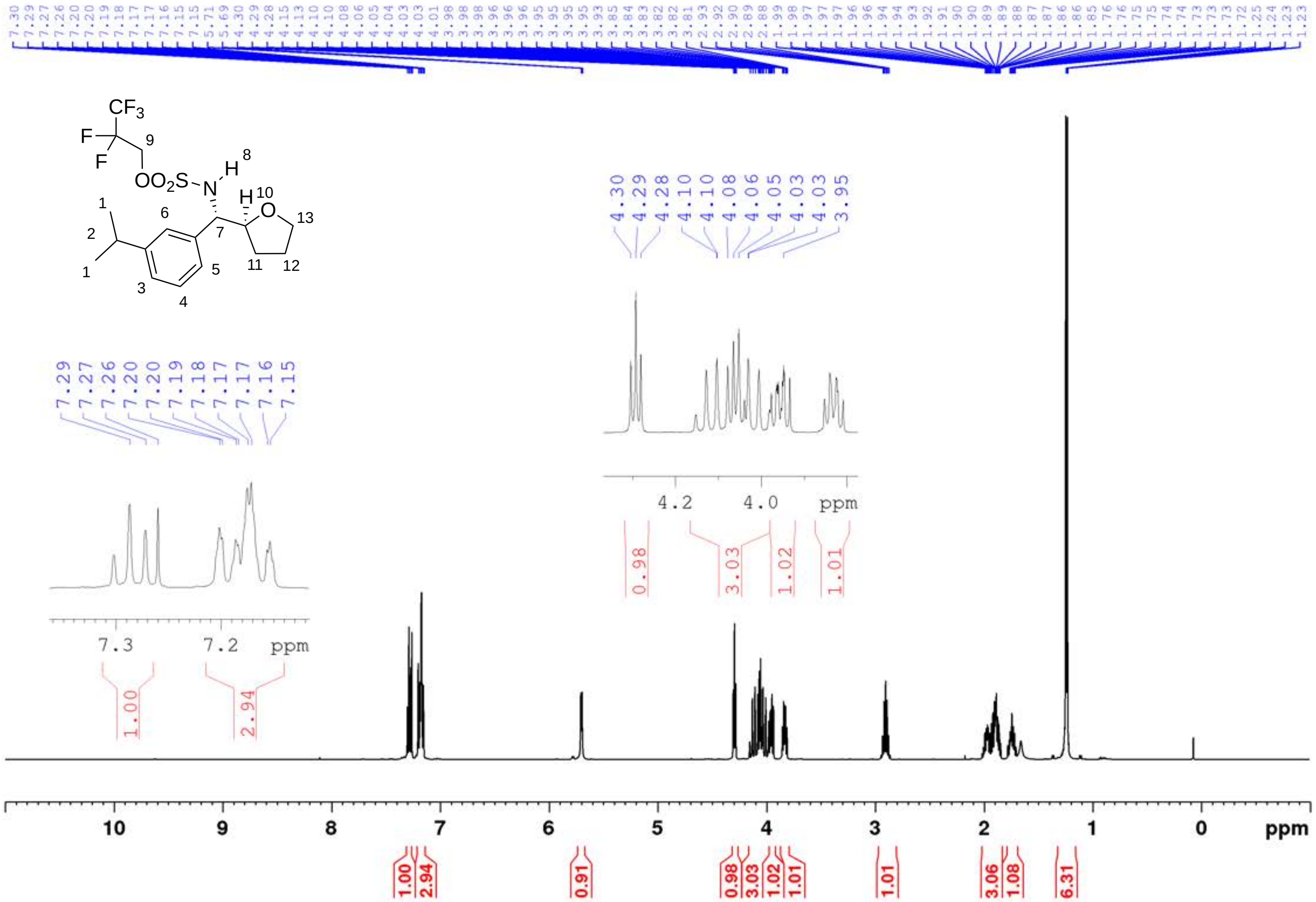

^{13}C NMR (126 MHz, $CDCl_3$) for *2,2,3,3,3-pentafluoropropyl ((S)-(3-isopropylphenyl)((S)-tetrahydrofuran-2-yl)methyl)sulfamate* (**4n**)

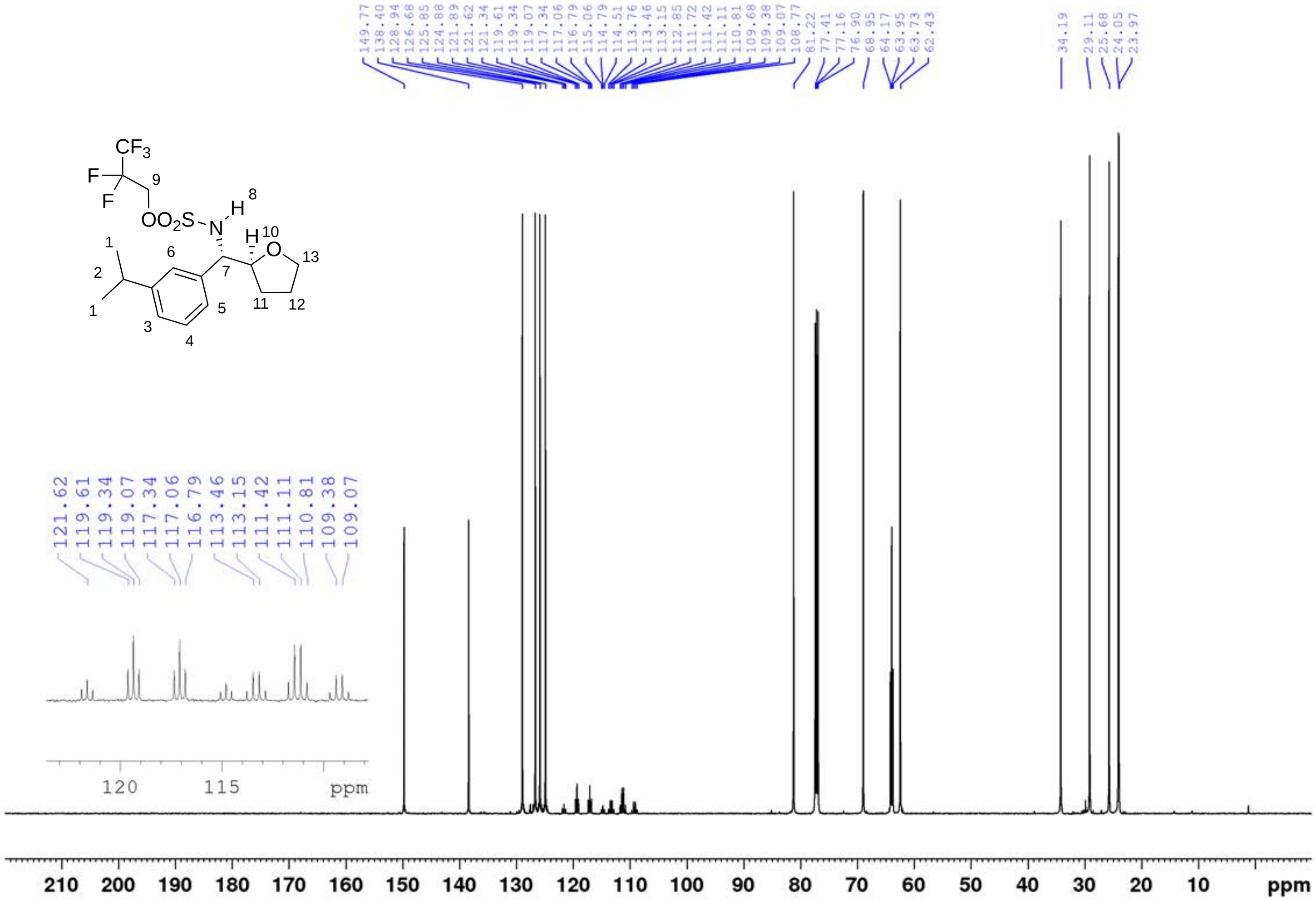

^{19}F NMR (376 MHz, $CDCl_3$) for *2,2,3,3,3-pentafluoropropyl ((S)-(3-isopropylphenyl)((S)-tetrahydrofuran-2-yl)methyl)sulfamate* (**4n**)

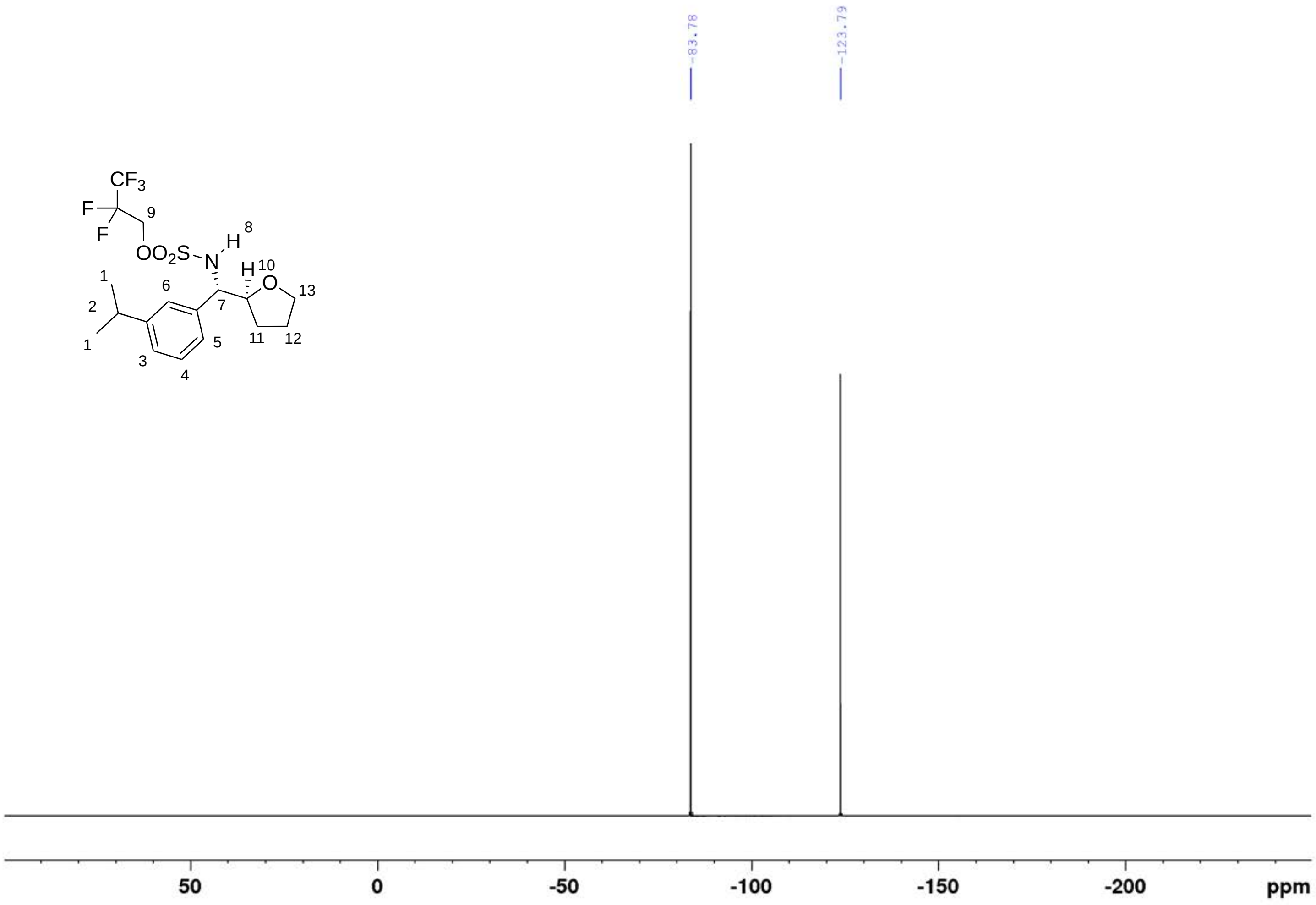

^{1}H NMR (500 MHz, $CDCl_3$) for *2,2,3,3,3-pentafluoropropyl ((S)-(3-(tert-butyl)phenyl)((S)-tetrahydrofuran-2-yl)methyl)sulfamate* (**4o**)

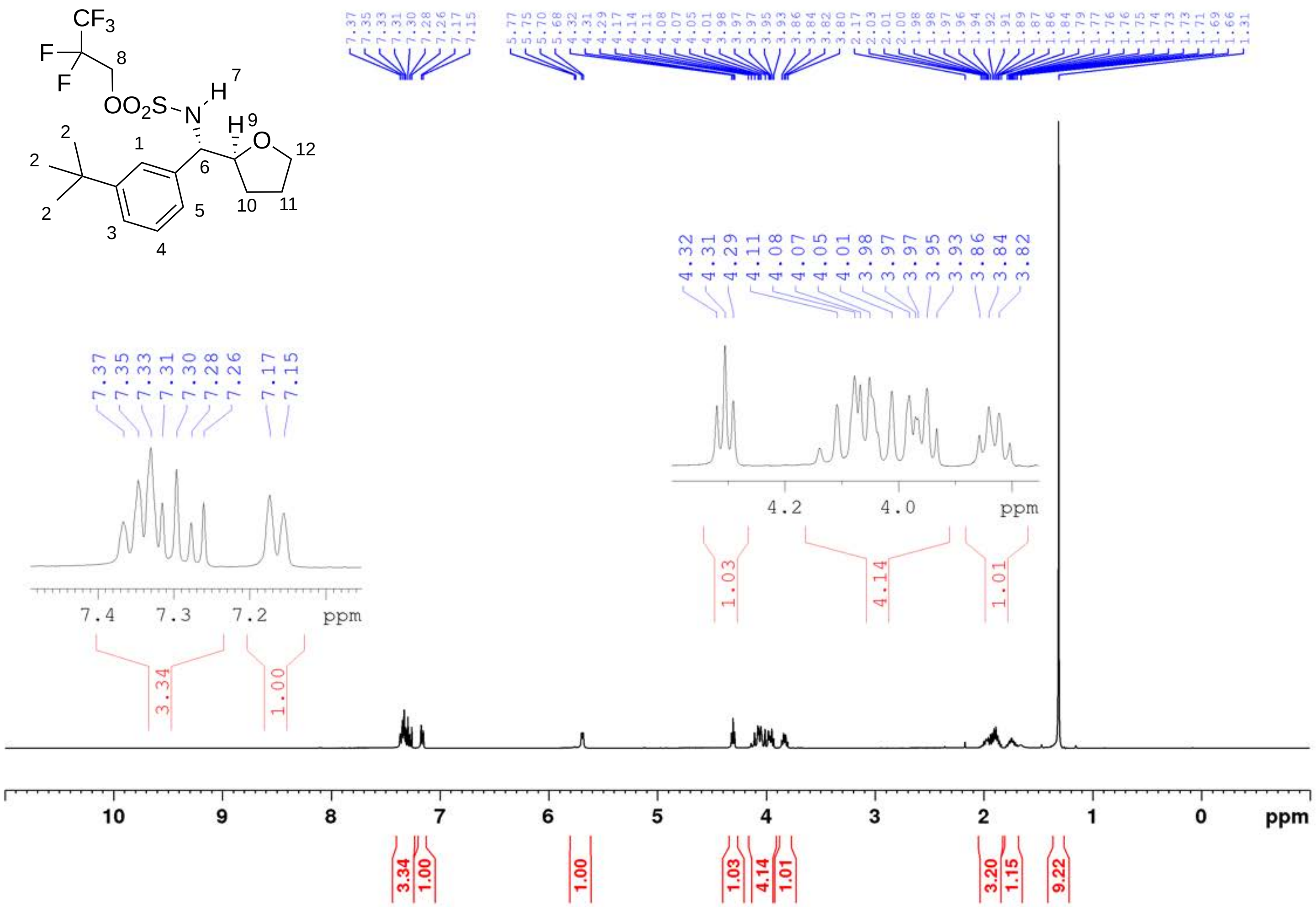

^{13}C NMR (126 MHz, $CDCl_3$) for *2,2,3,3,3-pentafluoropropyl ((S)-(3-(tert-butyl)phenyl)((S)-tetrahydrofuran-2-yl)methyl)sulfamate* (**4o**)

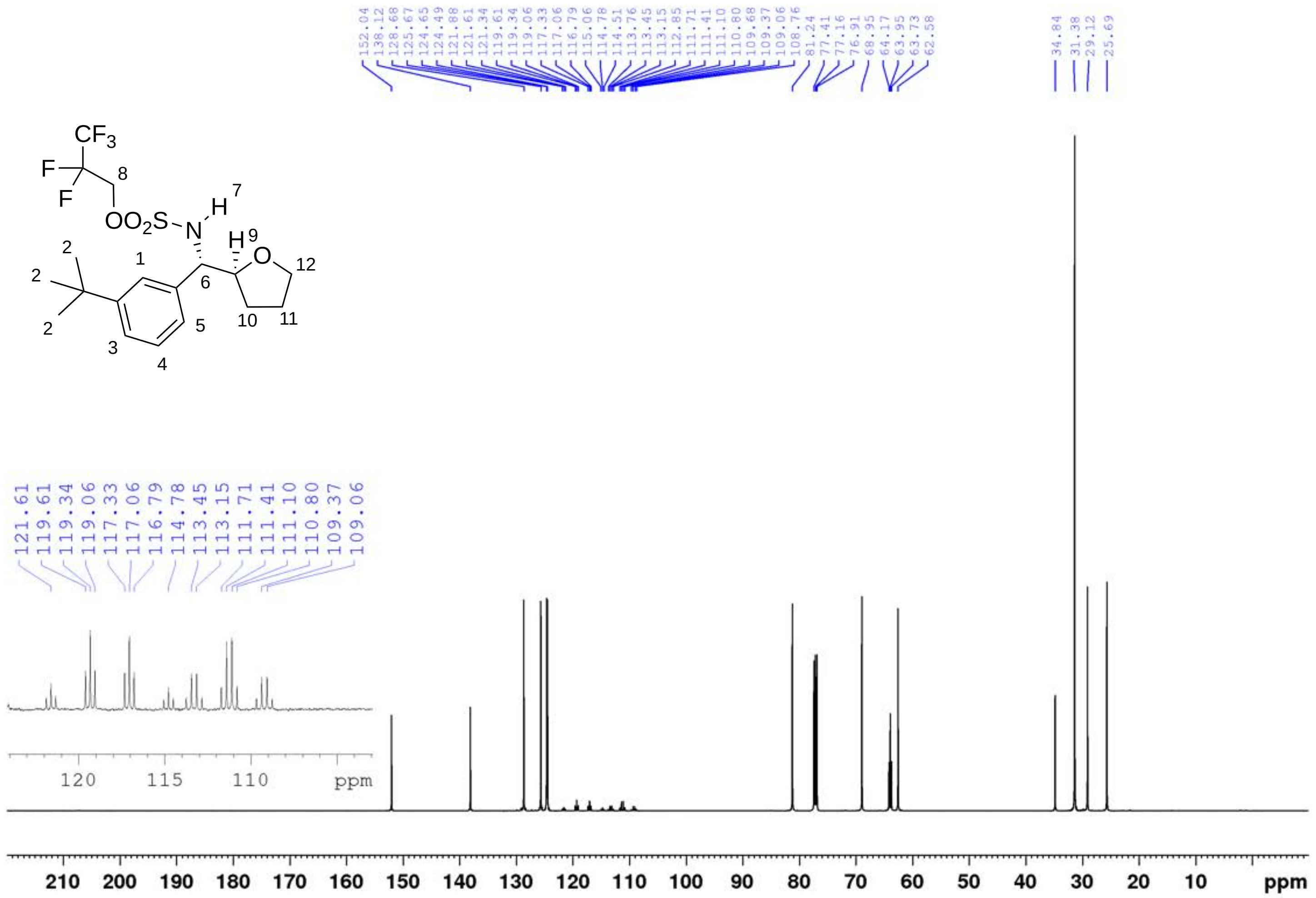

¹⁹F NMR (376 MHz, $CDCl_3$) for *2,2,3,3,3-pentafluoropropyl ((S)-(3-(tert-butyl)phenyl)((S)-tetrahydrofuran-2-yl)methyl)sulfamate* (**4o**)

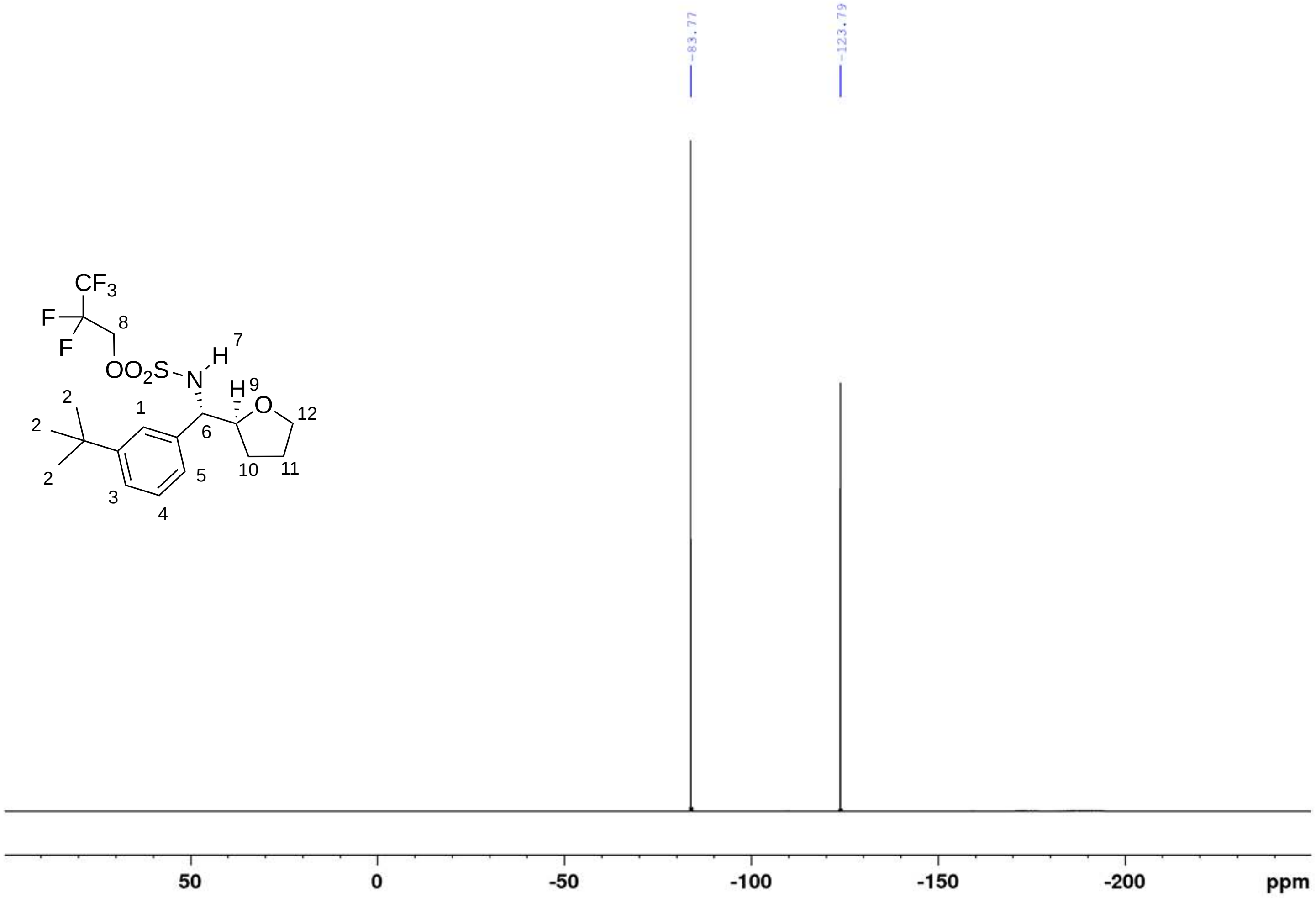

¹H NMR (500 MHz, $CDCl_3$) for *2,2,3,3,3-pentafluoropropyl ((S)-(3,5-dimethylphenyl)((S)-tetrahydrofuran-2-yl)methyl)sulfamate* (**4p**)

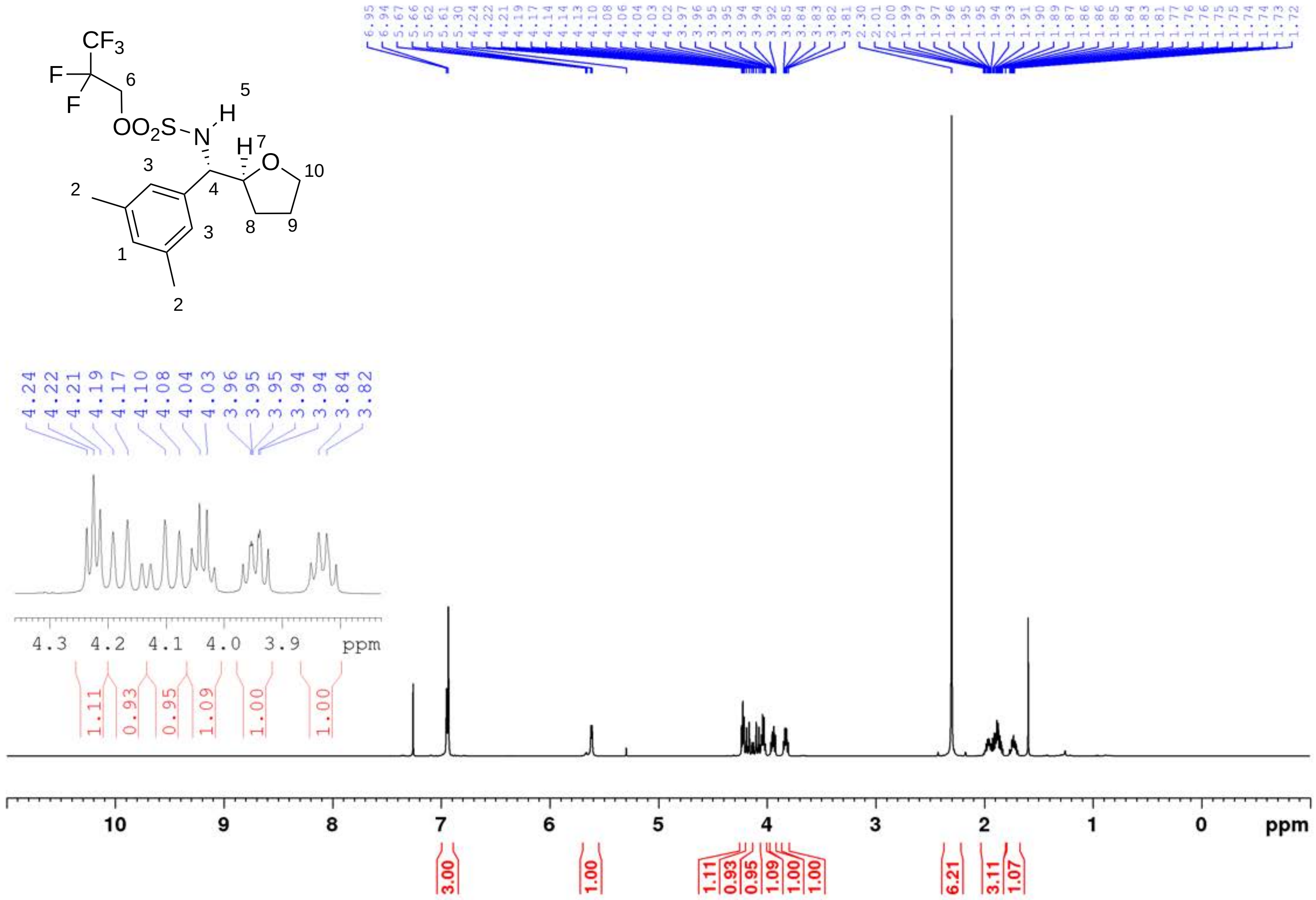

^{13}C NMR (126 MHz, $CDCl_3$) for *2,2,3,3,3-pentafluoropropyl ((S)-(3,5-dimethylphenyl)((S)-tetrahydrofuran-2-yl)methyl)sulfamate* (**4p**)

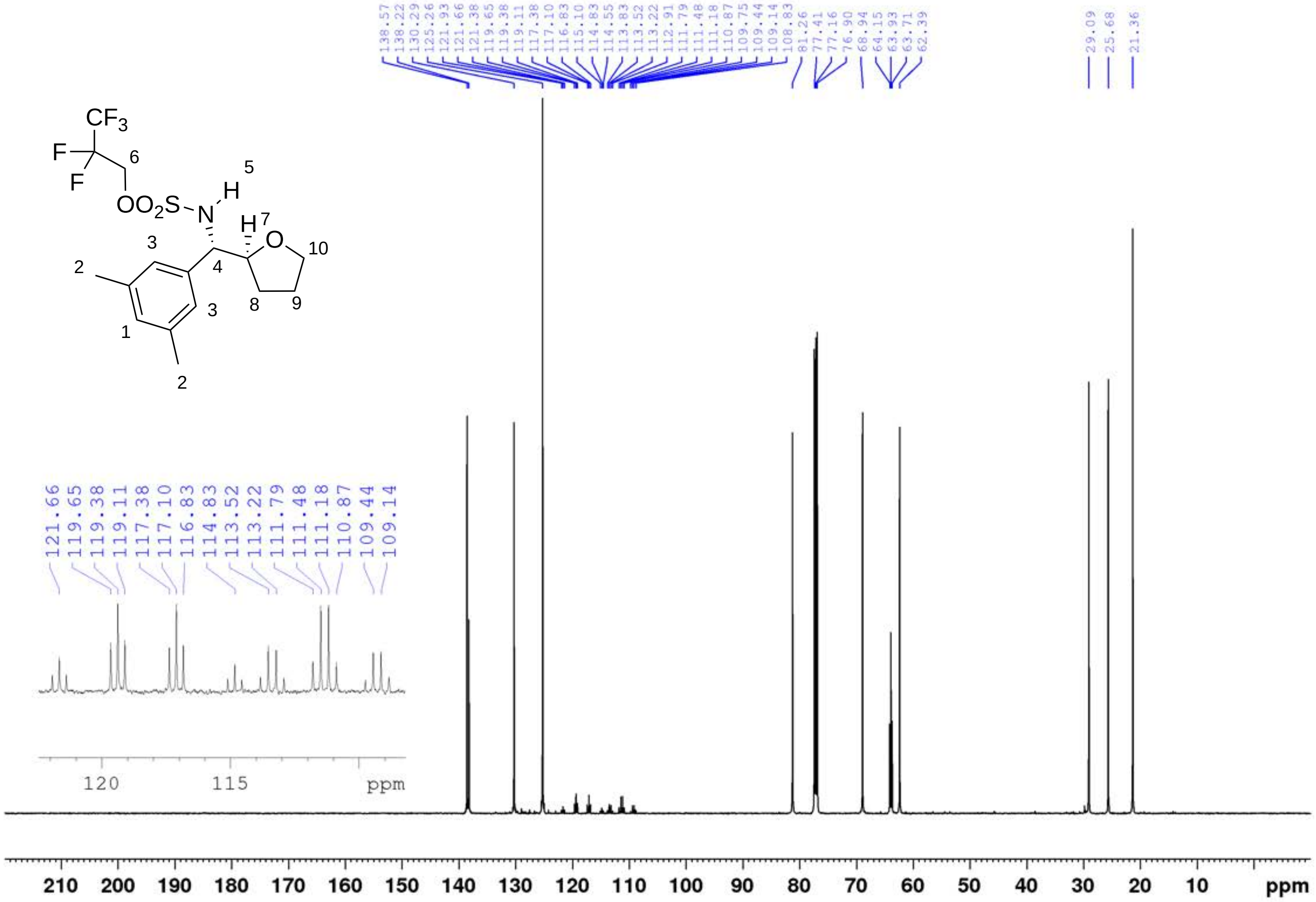

^{19}F NMR (376 MHz, $CDCl_3$) for *2,2,3,3,3-pentafluoropropyl ((S)-(3,5-dimethylphenyl)((S)-tetrahydrofuran-2-yl)methyl)sulfamate* (**4p**)

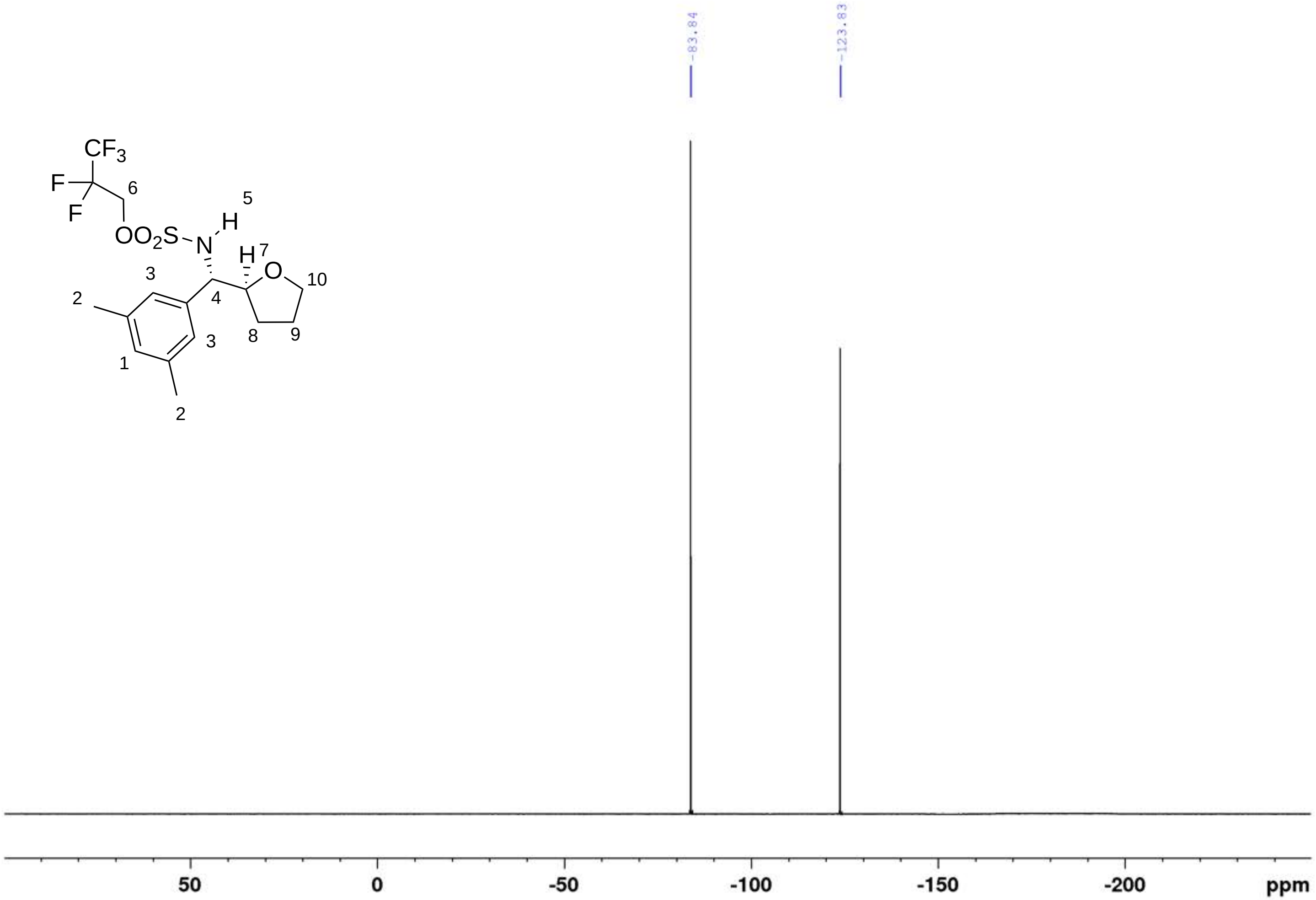

^{1}H NMR (500 MHz, $CDCl_3$) for *2,2,3,3,3-pentafluoropropyl ((S)-(3,5-di-tert-butylphenyl)((S)-tetrahydrofuran-2-yl)methyl)sulfamate* (**4q**)

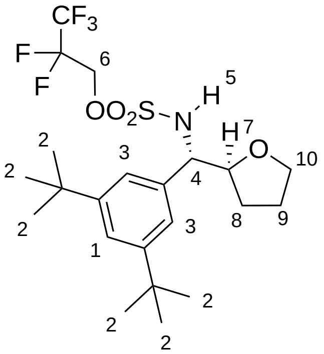

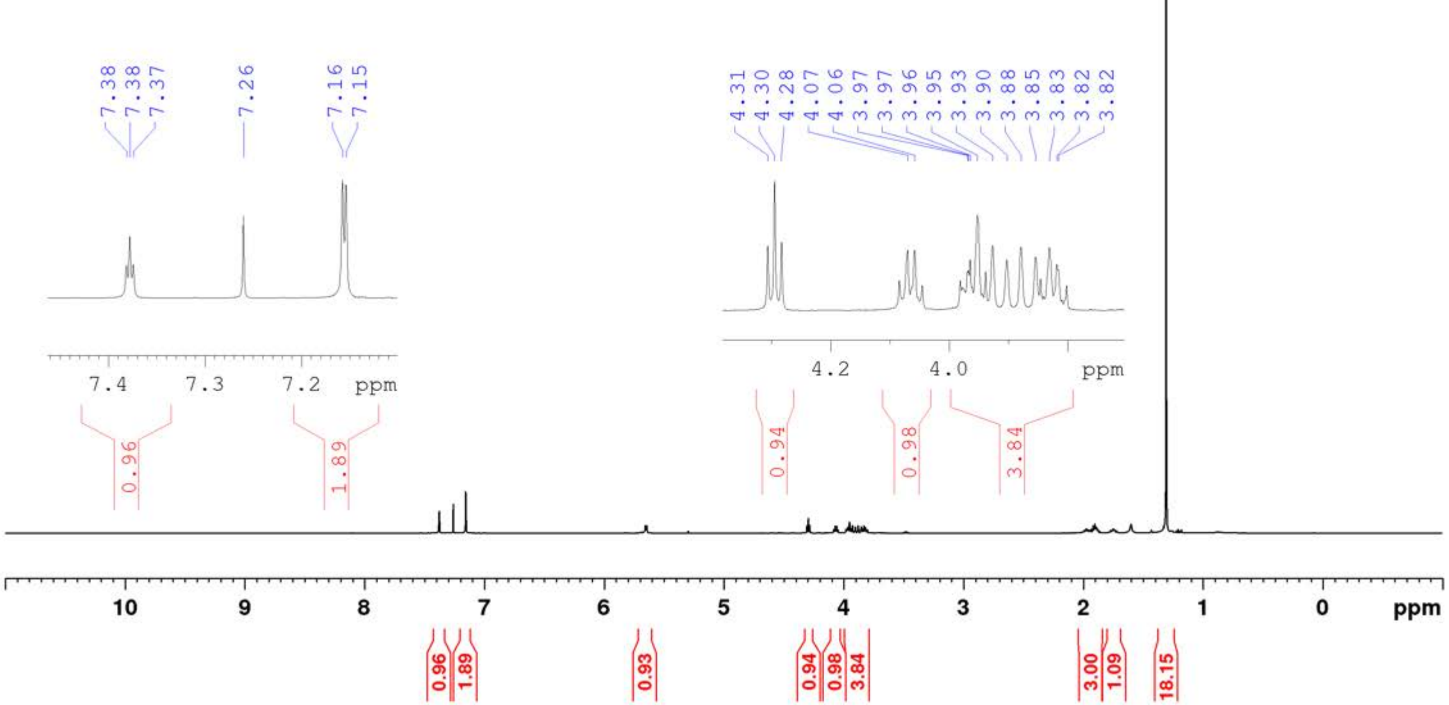

^{13}C NMR (126 MHz, $CDCl_3$) for *2,2,3,3,3-pentafluoropropyl ((S)-(3,5-di-tert-butylphenyl)((S)-tetrahydrofuran-2-yl)methyl)sulfamate* (**4q**)

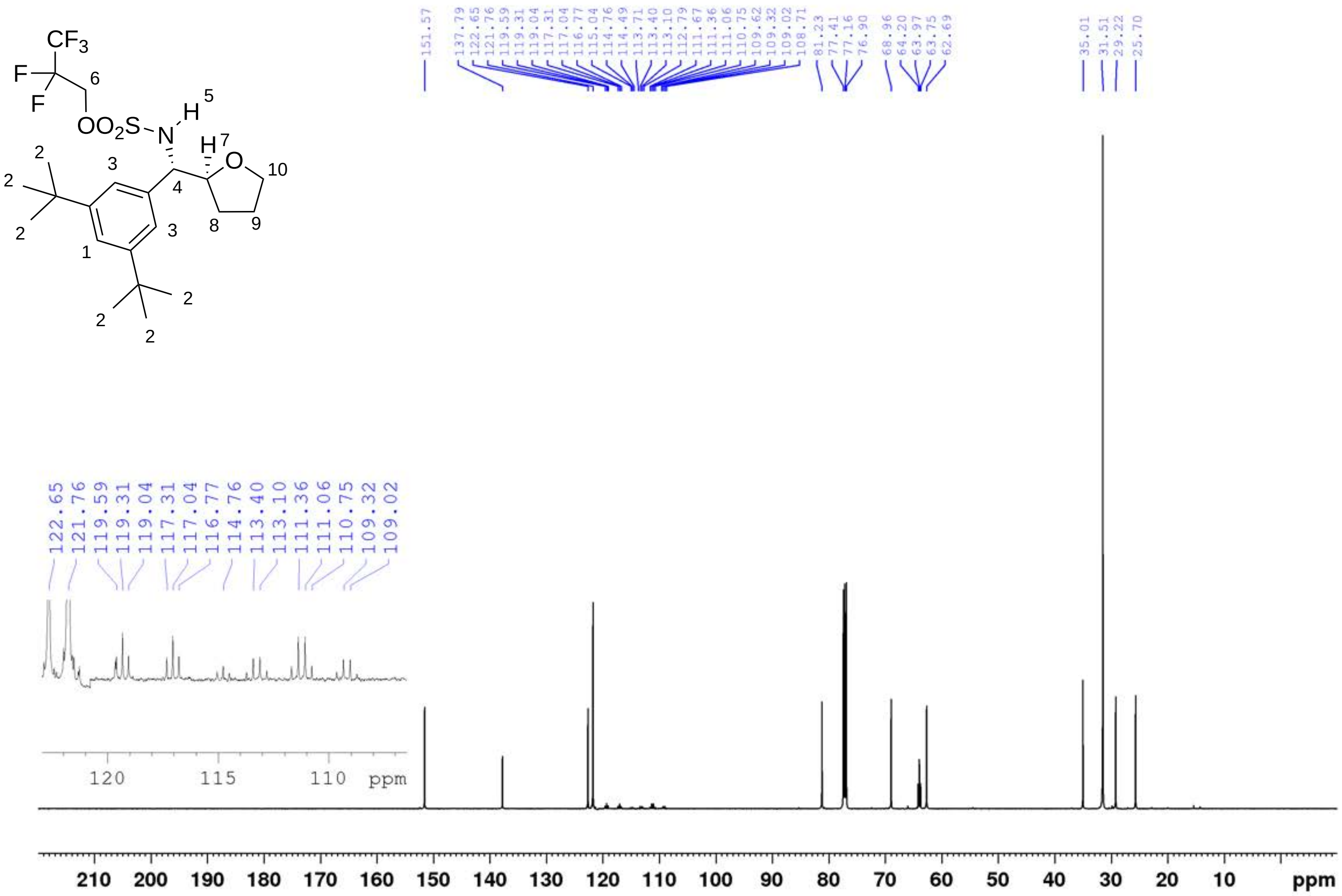

^{19}F NMR (376 MHz, $CDCl_3$) for *2,2,3,3,3-pentafluoropropyl ((S)-(3,5-di-tert-butylphenyl)((S)-tetrahydrofuran-2-yl)methyl)sulfamate* (**4q**)

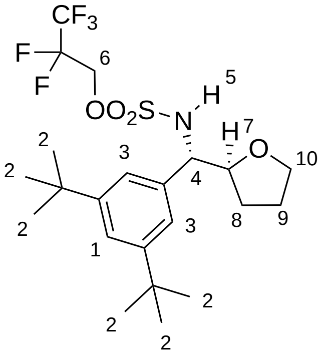

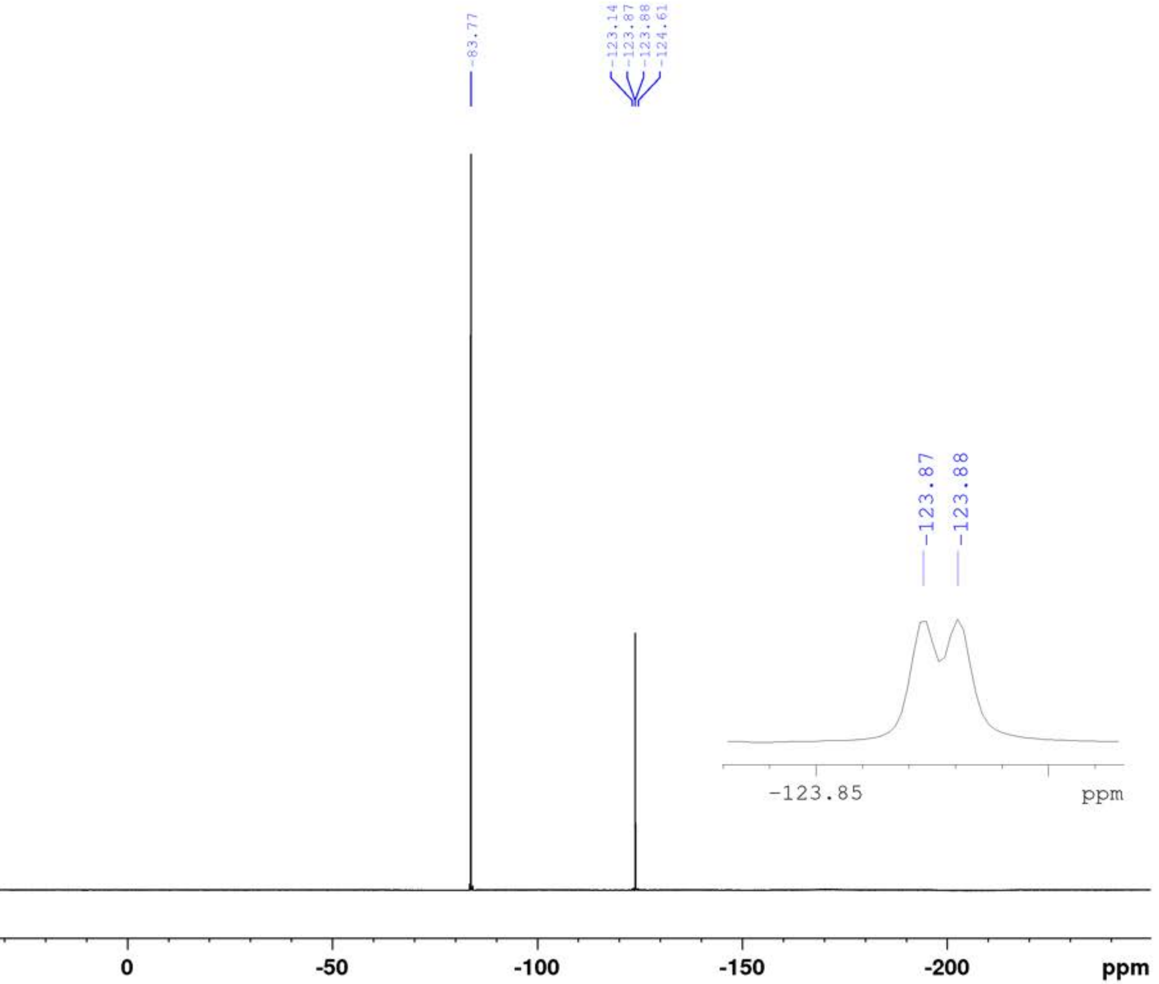

^{1}H NMR (500 MHz, $CDCl_3$) for *2,2,3,3,3-pentafluoropropyl ((S)-(3-fluoro-4-methylphenyl)((S)-tetrahydrofuran-2-yl)methyl)sulfamate* (**4r**)

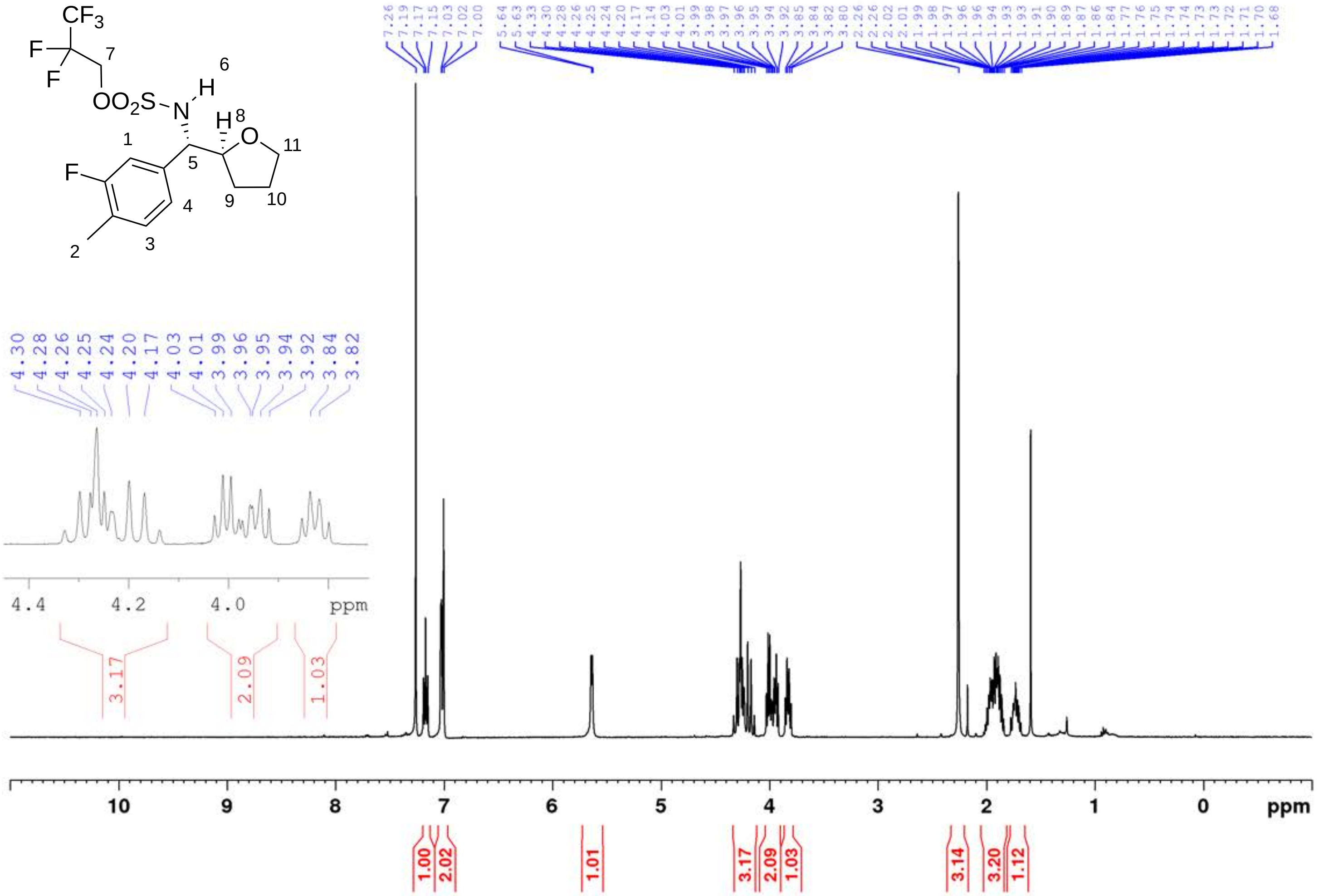

^{13}C NMR (126 MHz, $CDCl_3$) for *2,2,3,3,3-pentafluoropropyl ((S)-(3-fluoro-4-methylphenyl)((S)-tetrahydrofuran-2-yl)methyl)sulfamate* (**4r**)

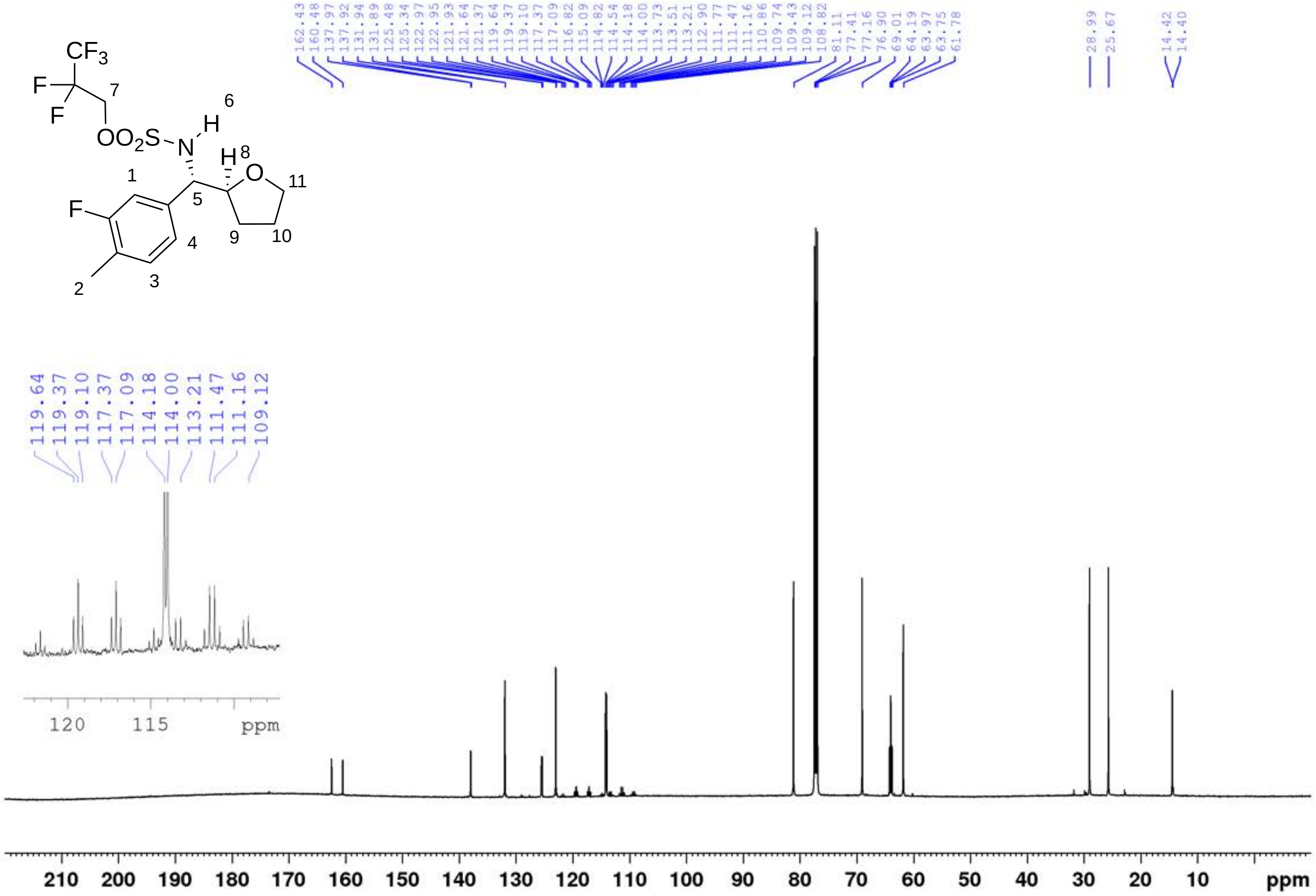

^{19}F NMR (376 MHz, $CDCl_3$) for *2,2,3,3,3-pentafluoropropyl ((S)-(3-fluoro-4-methylphenyl)((S)-tetrahydrofuran-2-yl)methyl)sulfamate* (**4r**)

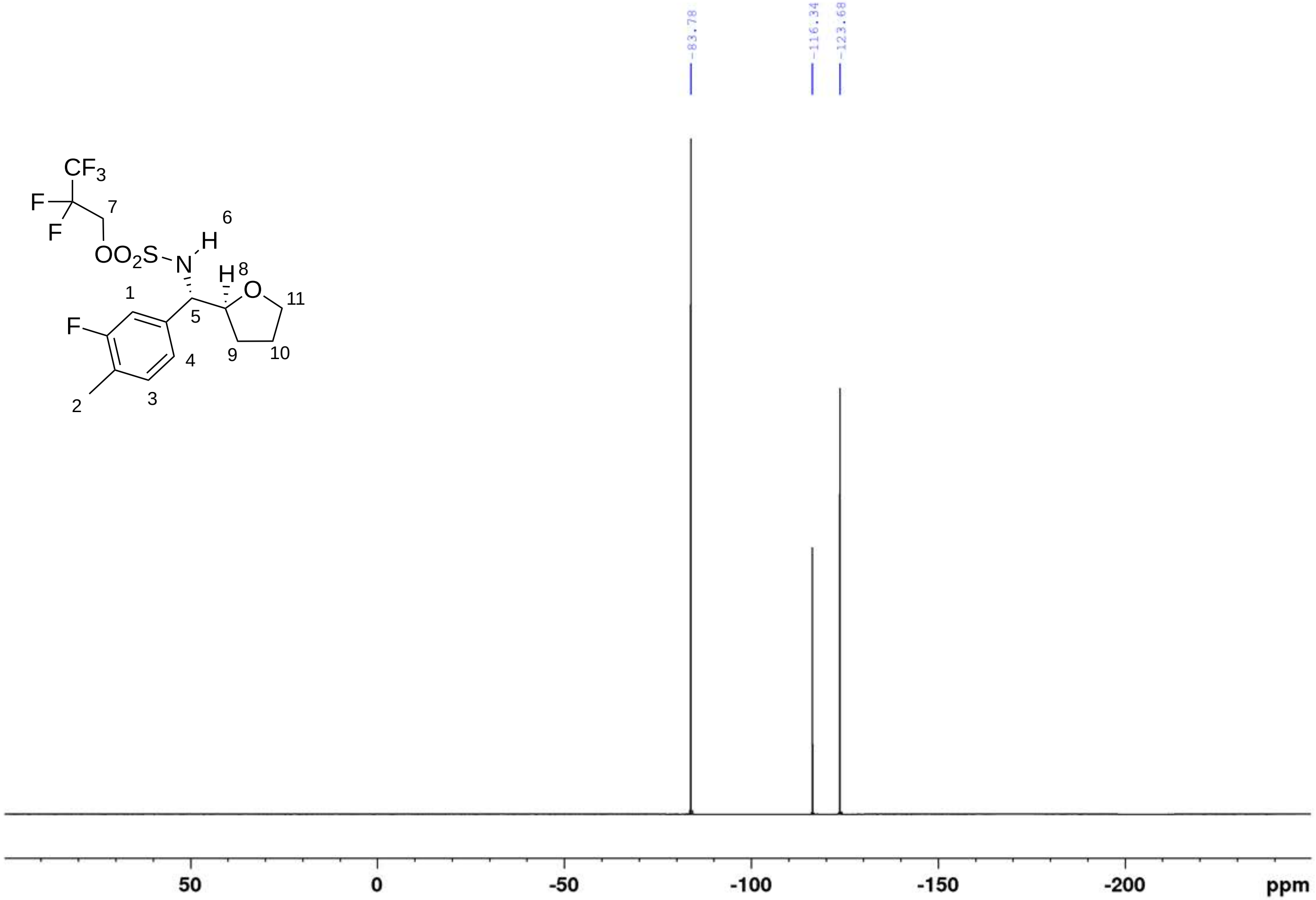

^{1}H NMR (400 MHz, $CDCl_3$) for *2,2,3,3,3-pentafluoropropyl ((S)-naphthalen-2-yl((S)-tetrahydrofuran-2-yl)methyl)sulfamate* (**4s**)

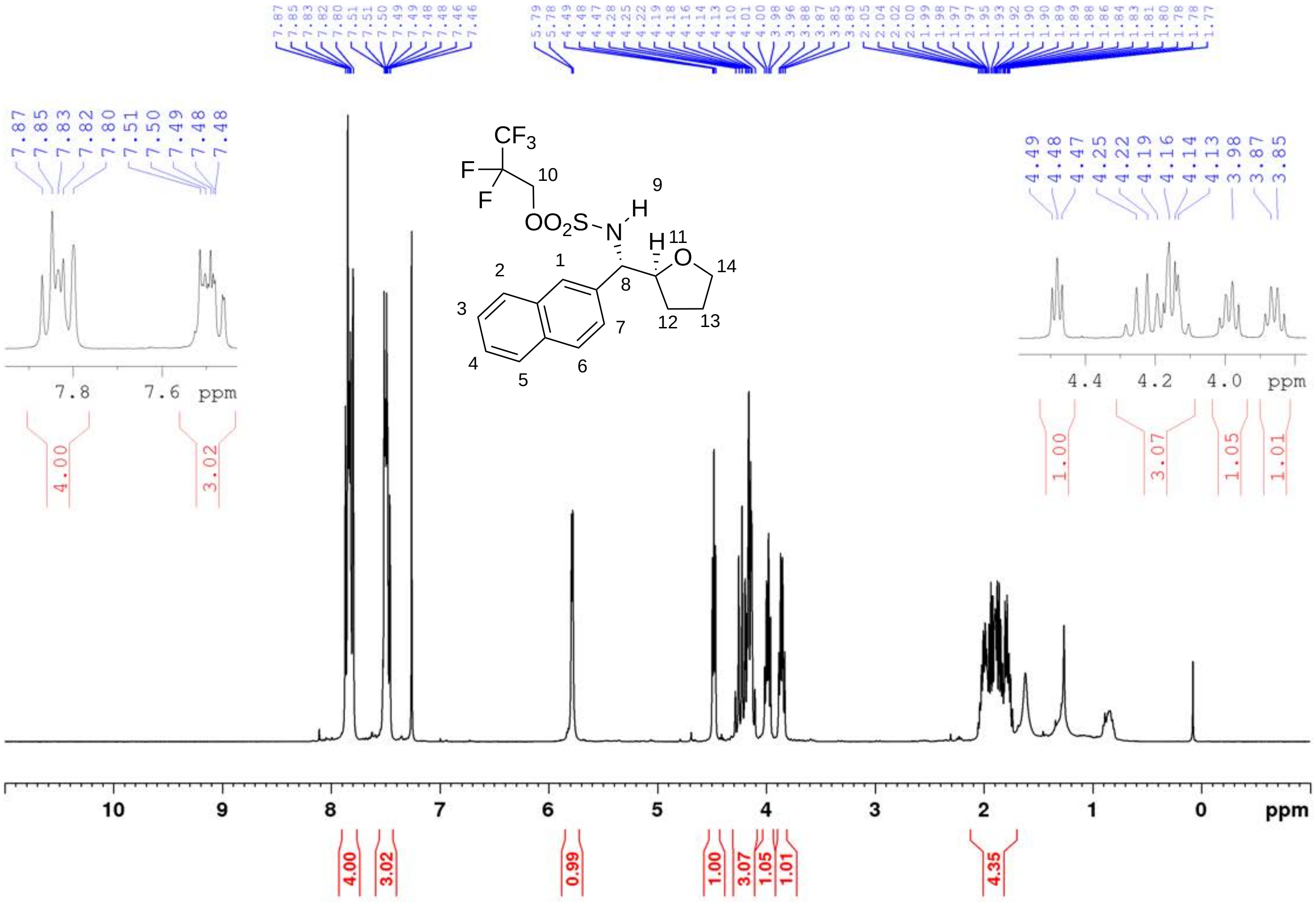

^{13}C NMR (126 MHz, $CDCl_3$) for *2,2,3,3,3-pentafluoropropyl ((S)-naphthalen-2-yl((S)-tetrahydrofuran-2-yl)methyl)sulfamate* (**4s**)

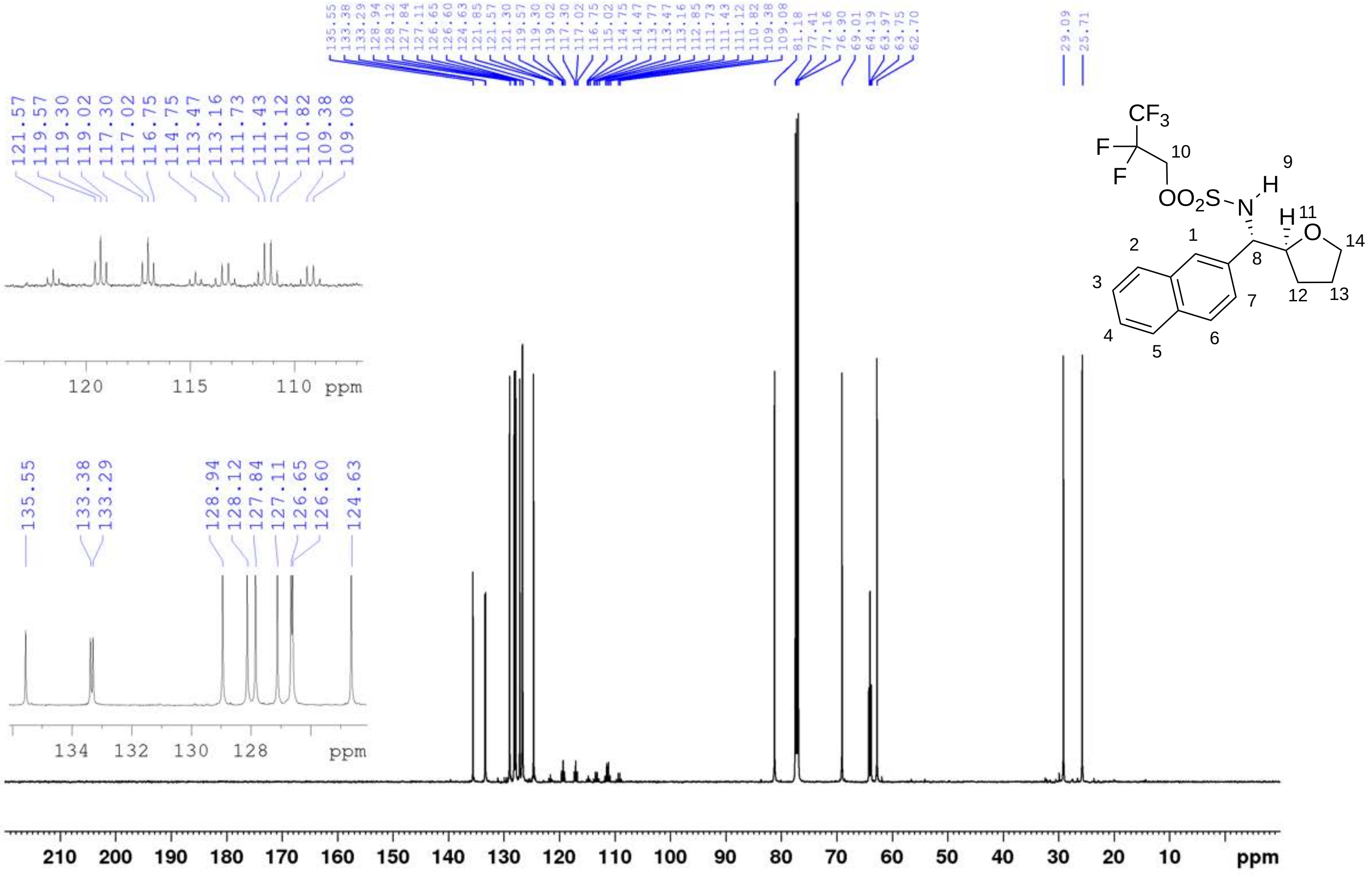

^{19}F NMR (376 MHz, $CDCl_3$) for *2,2,3,3,3-pentafluoropropyl ((S)-naphthalen-2-yl((S)-tetrahydrofuran-2-yl)methyl)sulfamate* (**4s**)

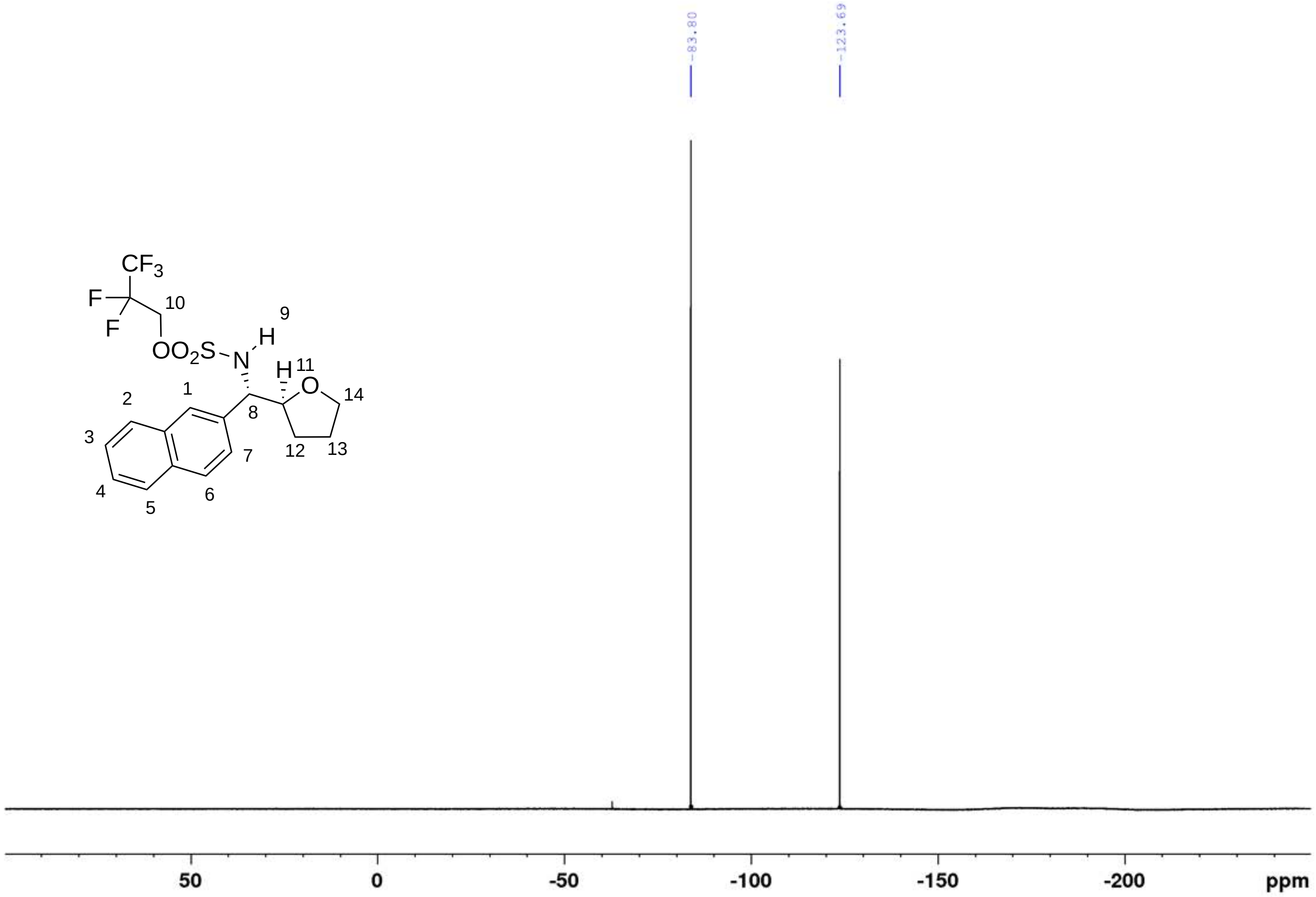

^{1}H NMR (500 MHz, $CDCl_3$) for *2,2,3,3,3-pentafluoropropyl ((S)-(4-fluorophenyl)((S)-tetrahydrofuran-2-yl)methyl)sulfamate* (**4t**)

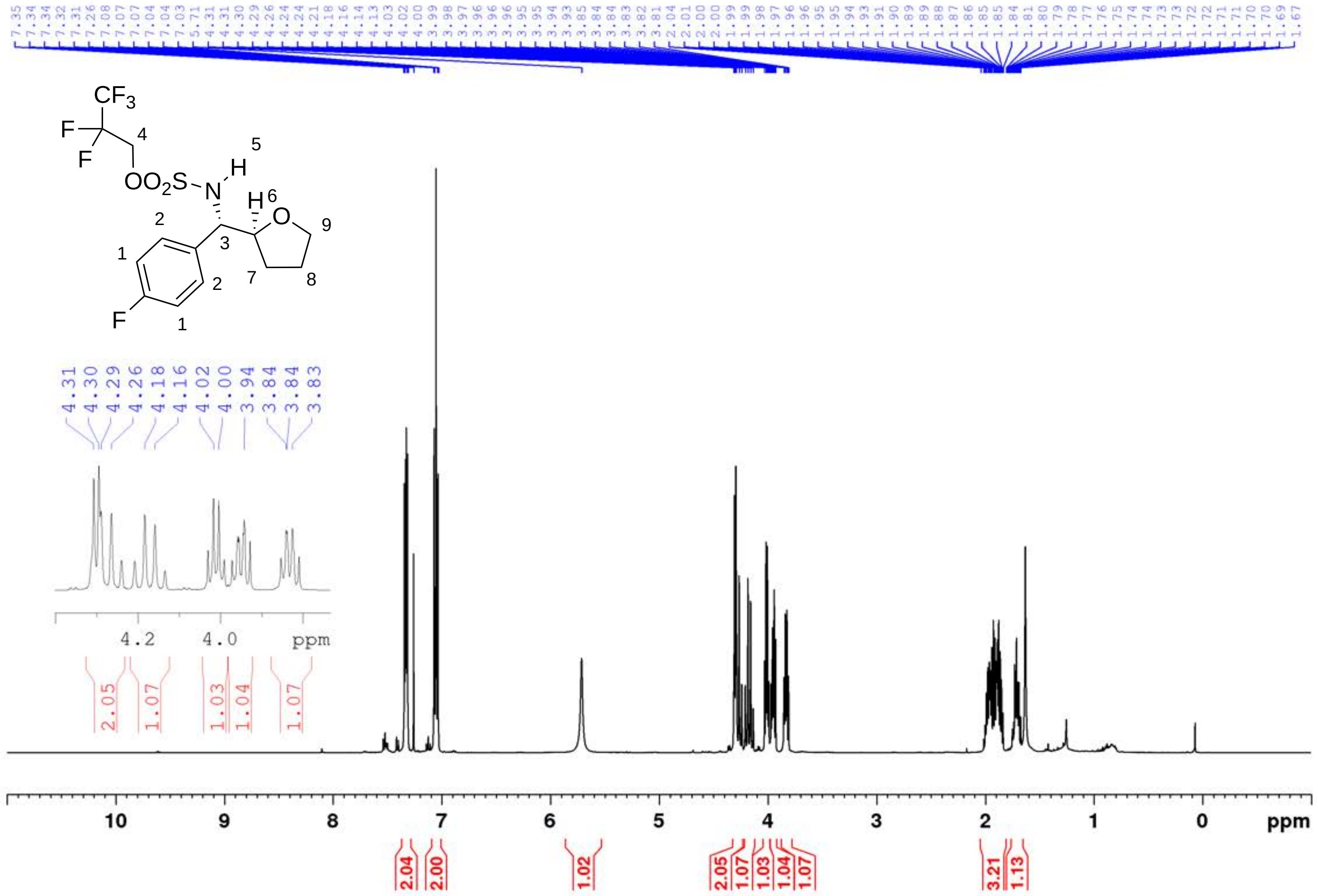

^{13}C NMR (126 MHz, $CDCl_3$) for *2,2,3,3,3-pentafluoropropyl ((S)-(4-fluorophenyl)((S)-tetrahydrofuran-2-yl)methyl)sulfamate* (**4t**)

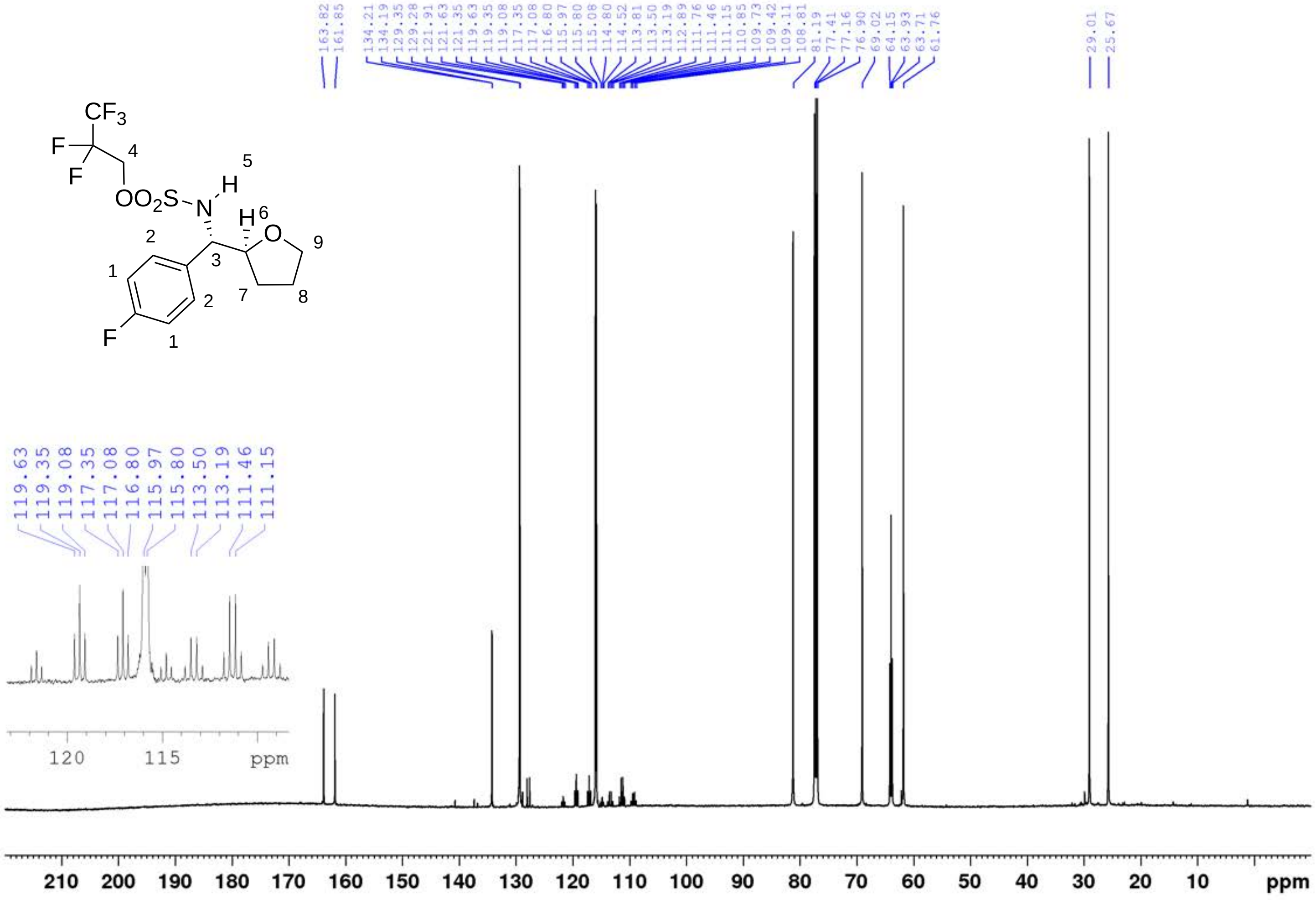

^{19}F NMR (376 MHz, $CDCl_3$) for *2,2,3,3,3-pentafluoropropyl ((S)-(4-fluorophenyl)((S)-tetrahydrofuran-2-yl)methyl)sulfamate* (**4t**)

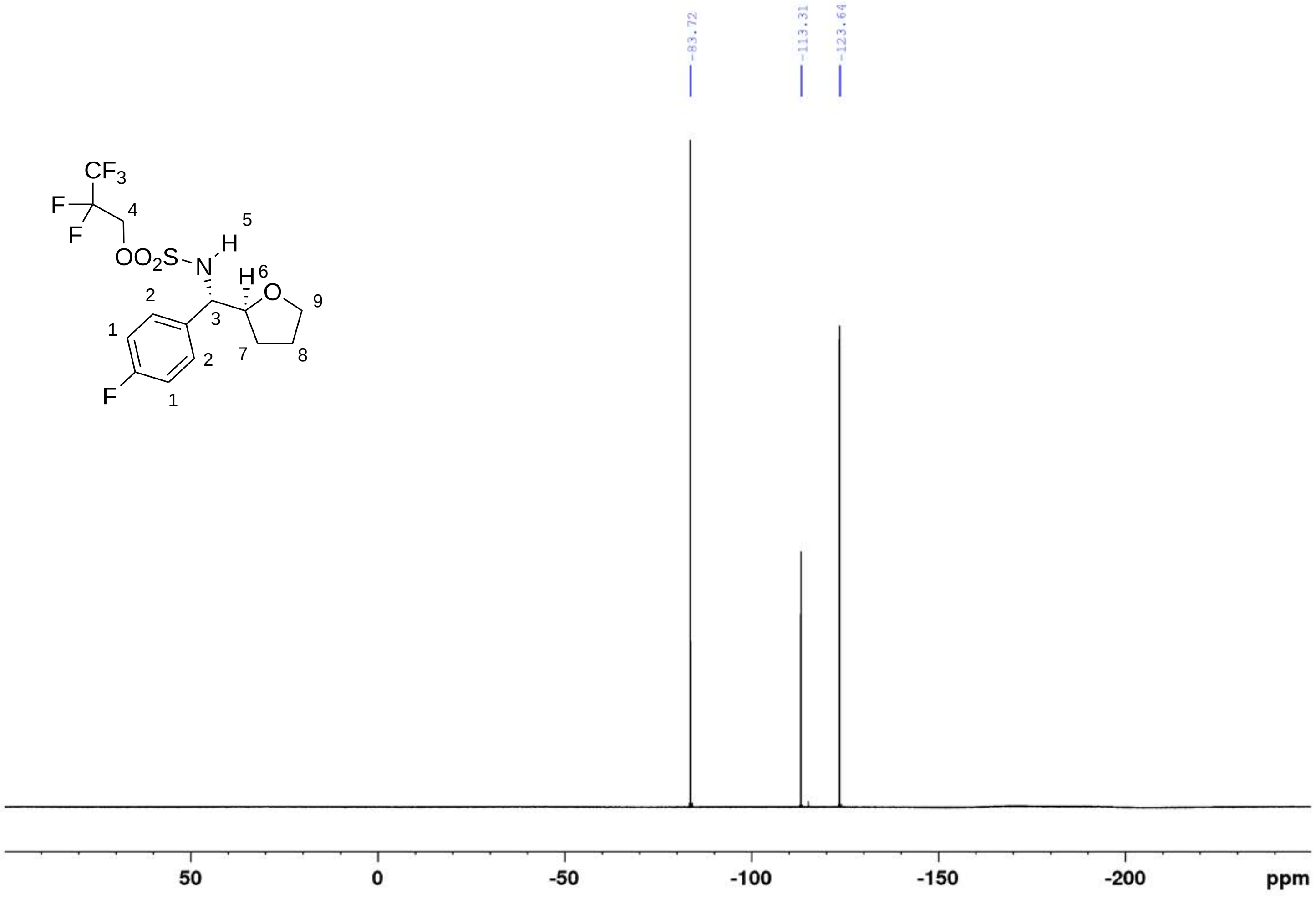

^{1}H NMR (500 MHz, $CDCl_3$) for *2,2,3,3,3-pentafluoropropyl ((S)-((S)-tetrahydrofuran-2-yl)(p-tolyl)methyl)sulfamate* (**4u**)

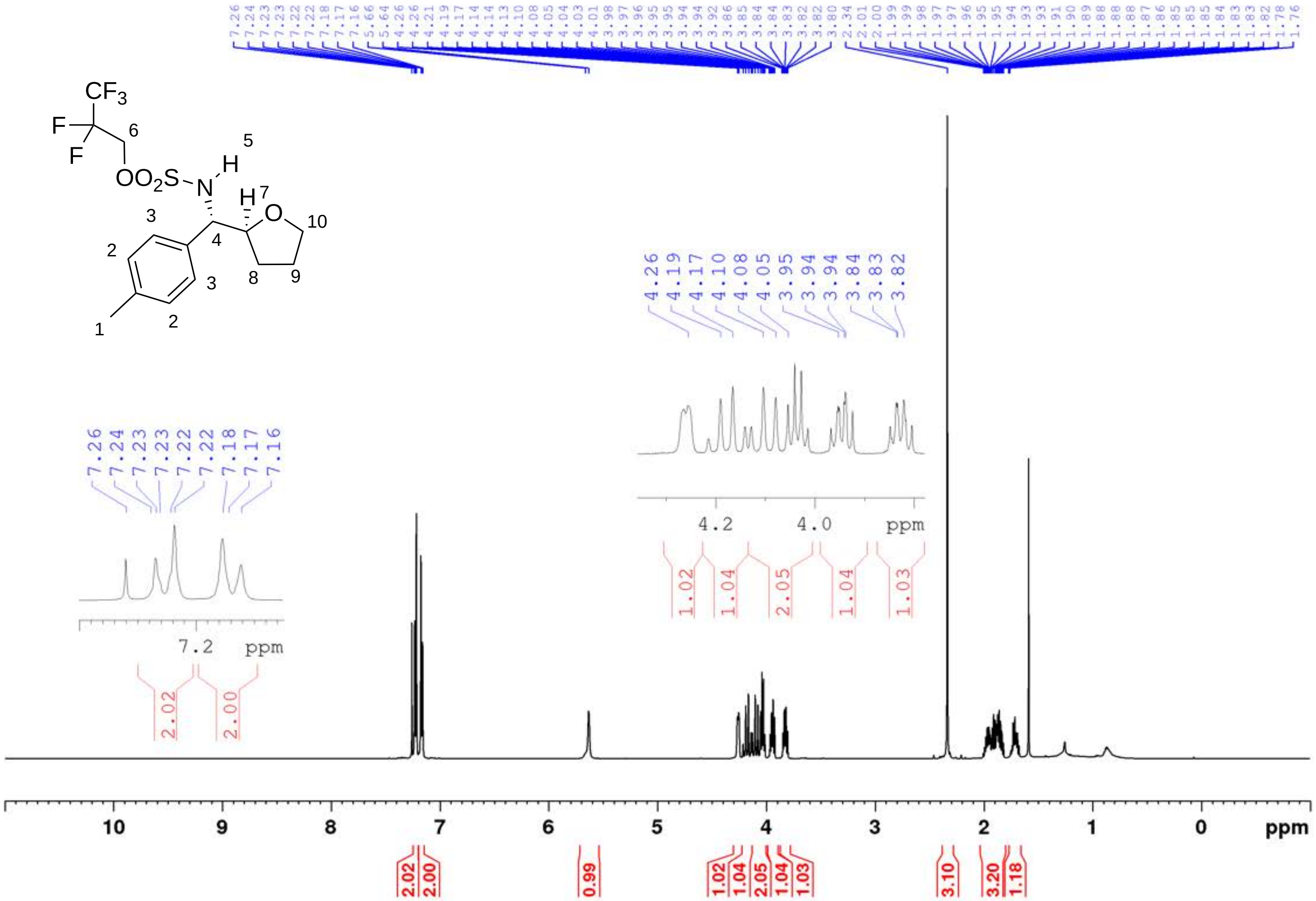

^{13}C NMR (126 MHz, $CDCl_3$) for *2,2,3,3,3-pentafluoropropyl ((S)-((S)-tetrahydrofuran-2-yl)(p-tolyl)methyl)sulfamate* (**4u**)

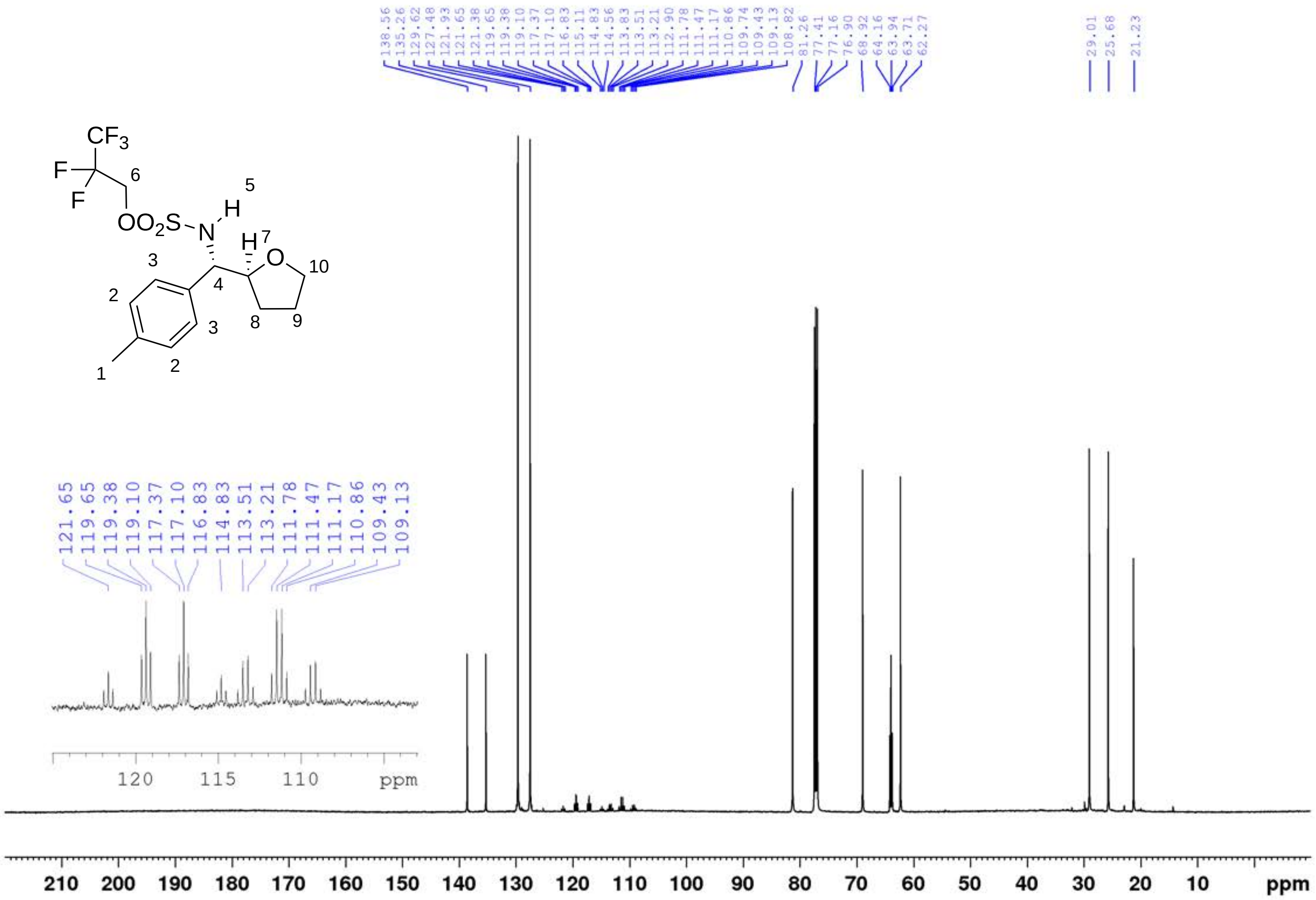

^{19}F NMR (376 MHz, $CDCl_3$) for *2,2,3,3,3-pentafluoropropyl ((S)-((S)-tetrahydrofuran-2-yl)(p-tolyl)methyl)sulfamate* (**4u**)

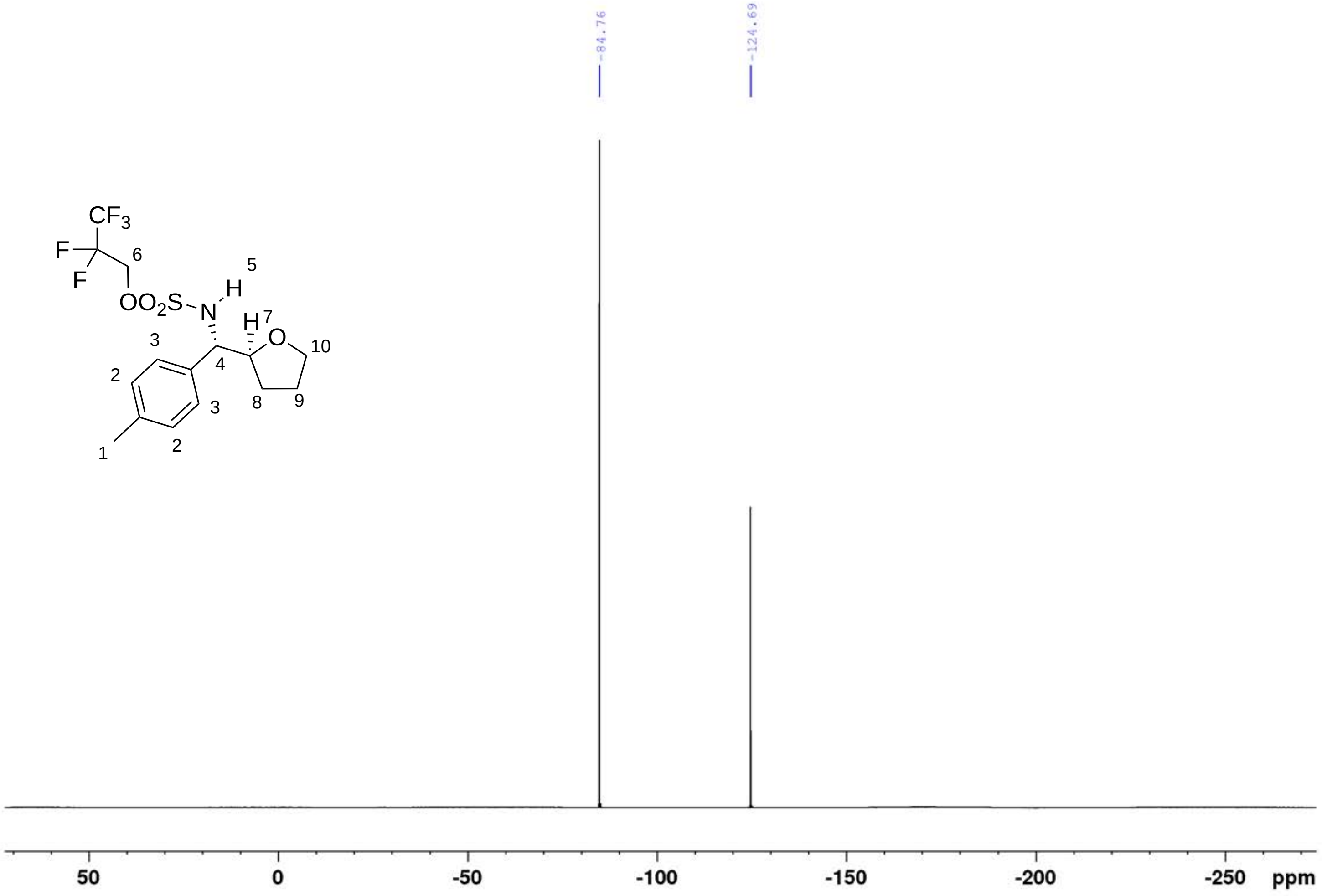

^{1}H NMR (400 MHz, $CDCl_3$) for *2,2,3,3,3-pentafluoropropyl ((S)-((S)-tetrahydrofuran-2-yl)(4-(trifluoromethyl)phenyl)methyl)sulfamate* (**4v**)

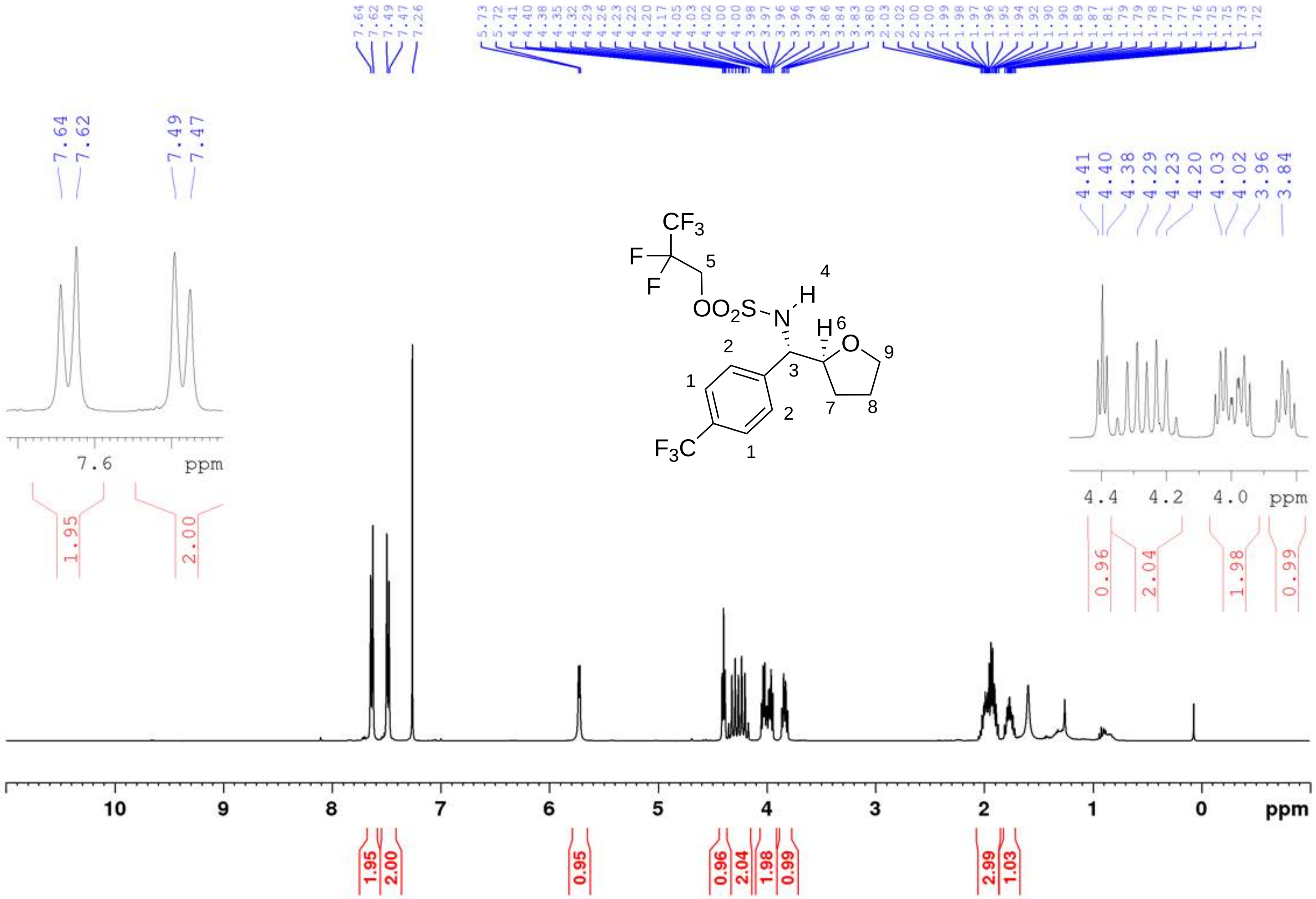

^{13}C NMR (126 MHz, $CDCl_3$) for *2,2,3,3,3-pentafluoropropyl ((S)-((S)-tetrahydrofuran-2-yl)(4-(trifluoromethyl)phenyl)methyl)sulfamate* (**4v**)

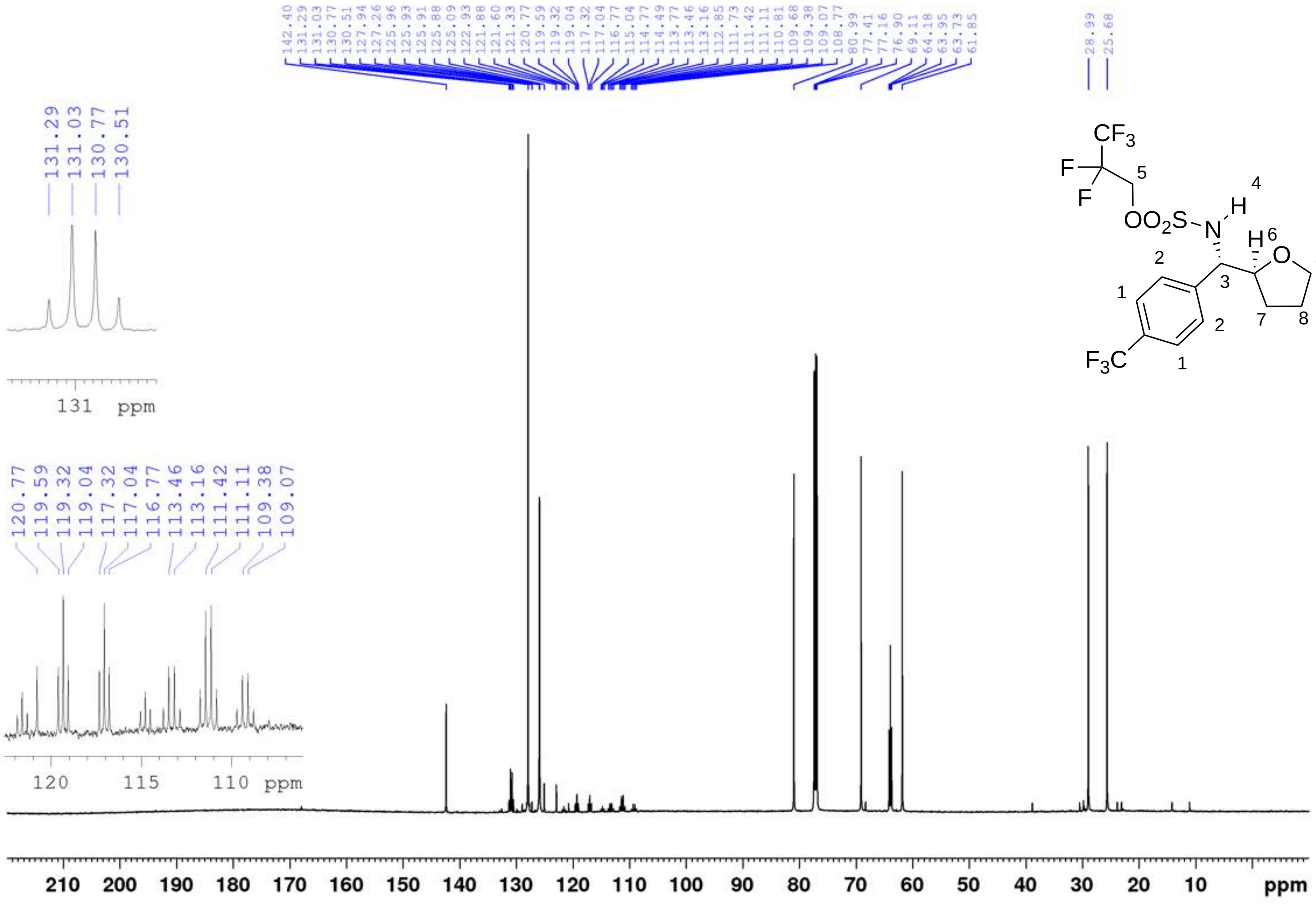

^{19}F NMR (376 MHz, $CDCl_3$) for *2,2,3,3,3-pentafluoropropyl ((S)-((S)-tetrahydrofuran-2-yl)(4-(trifluoromethyl)phenyl)methyl)sulfamate* (**4v**)

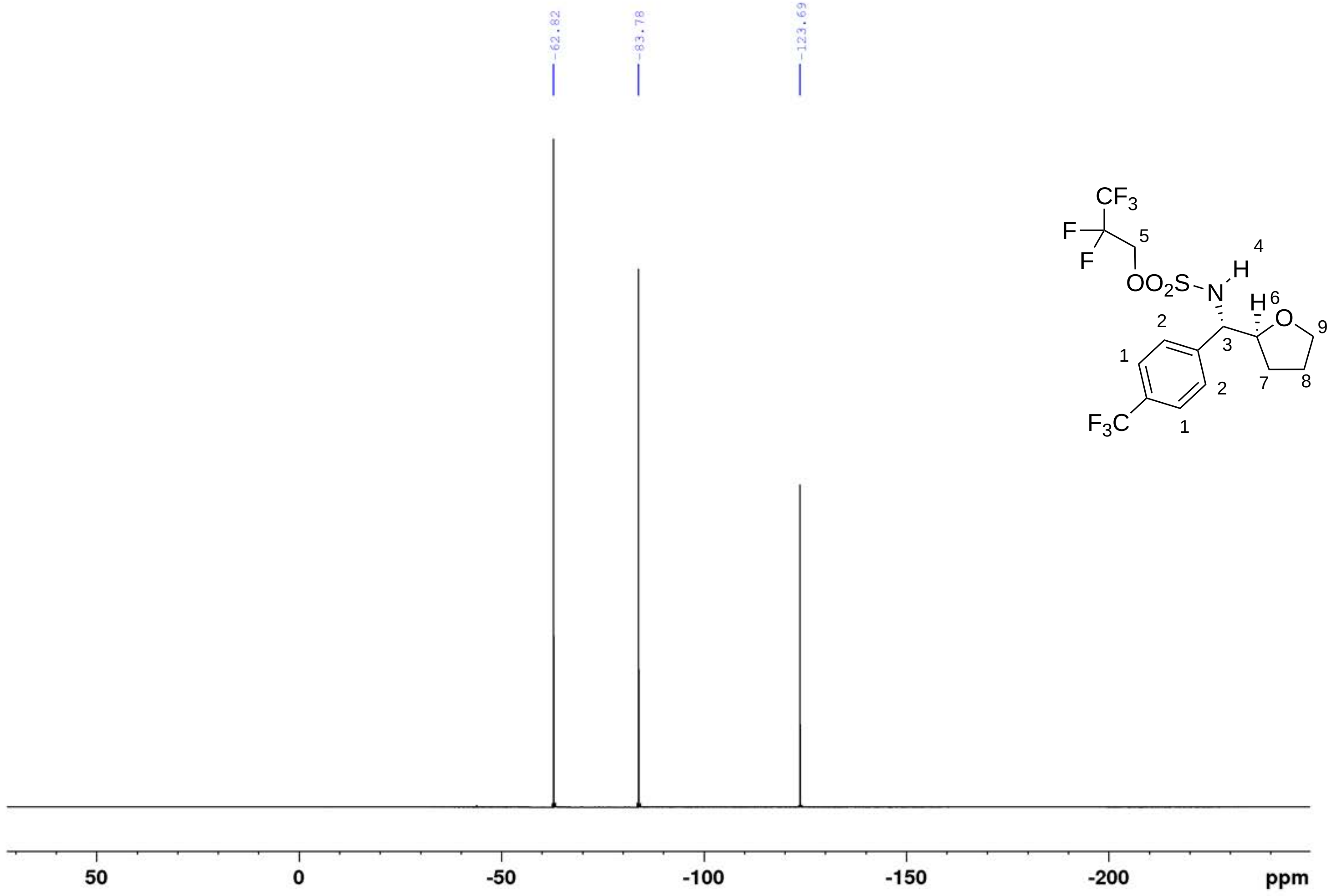

¹H NMR (500 MHz, $CDCl_3$) for *2,2,3,3,3-pentafluoropropyl ((S)-(4-(tert-butyl)phenyl)((S)-tetrahydrofuran-2-yl)methyl)sulfamate* (**4w**)

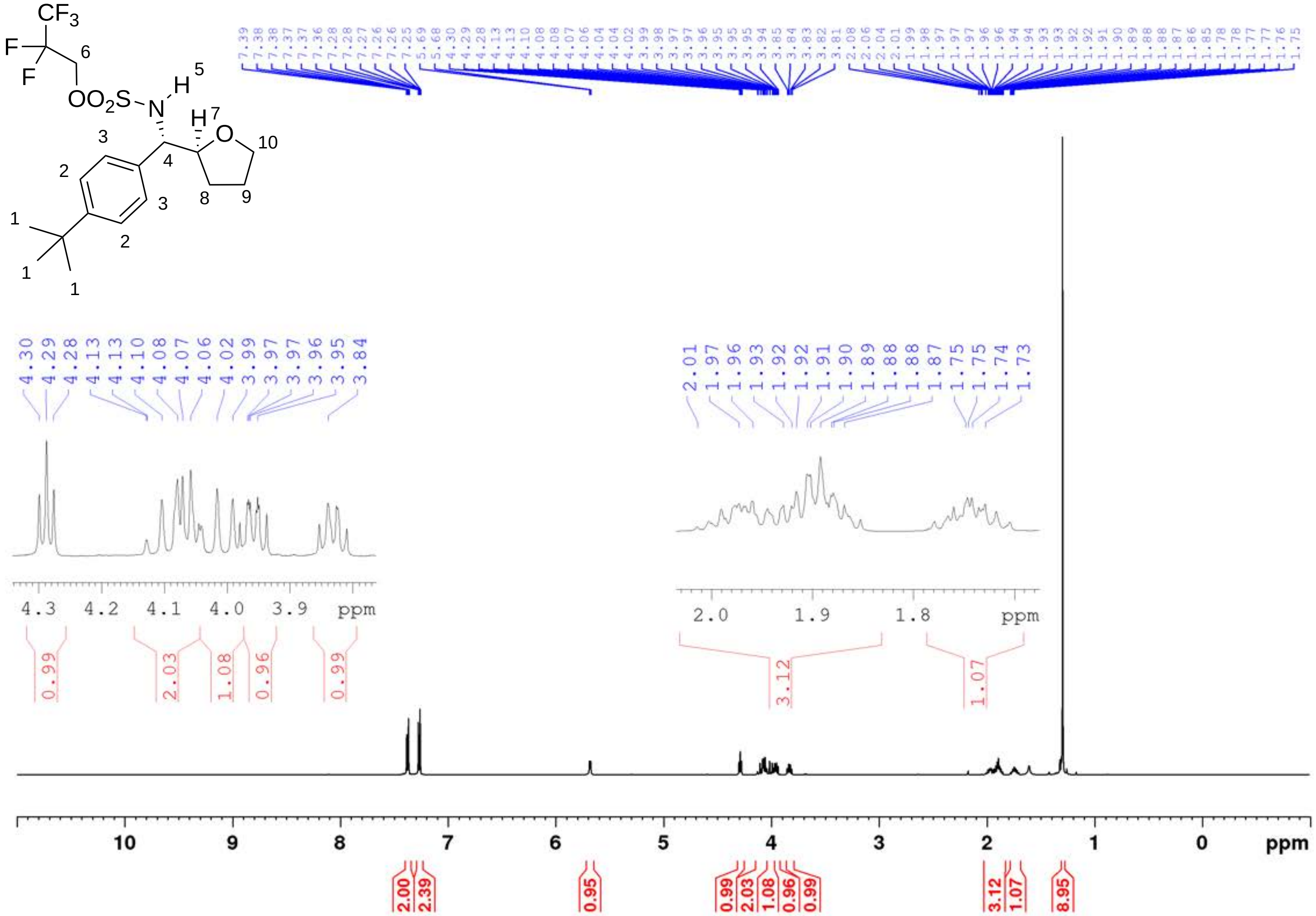

^{13}C NMR (126 MHz, $CDCl_3$) for *2,2,3,3,3-pentafluoropropyl ((S)-(4-(tert-butyl)phenyl)((S)-tetrahydrofuran-2-yl)methyl)sulfamate* (**4w**)

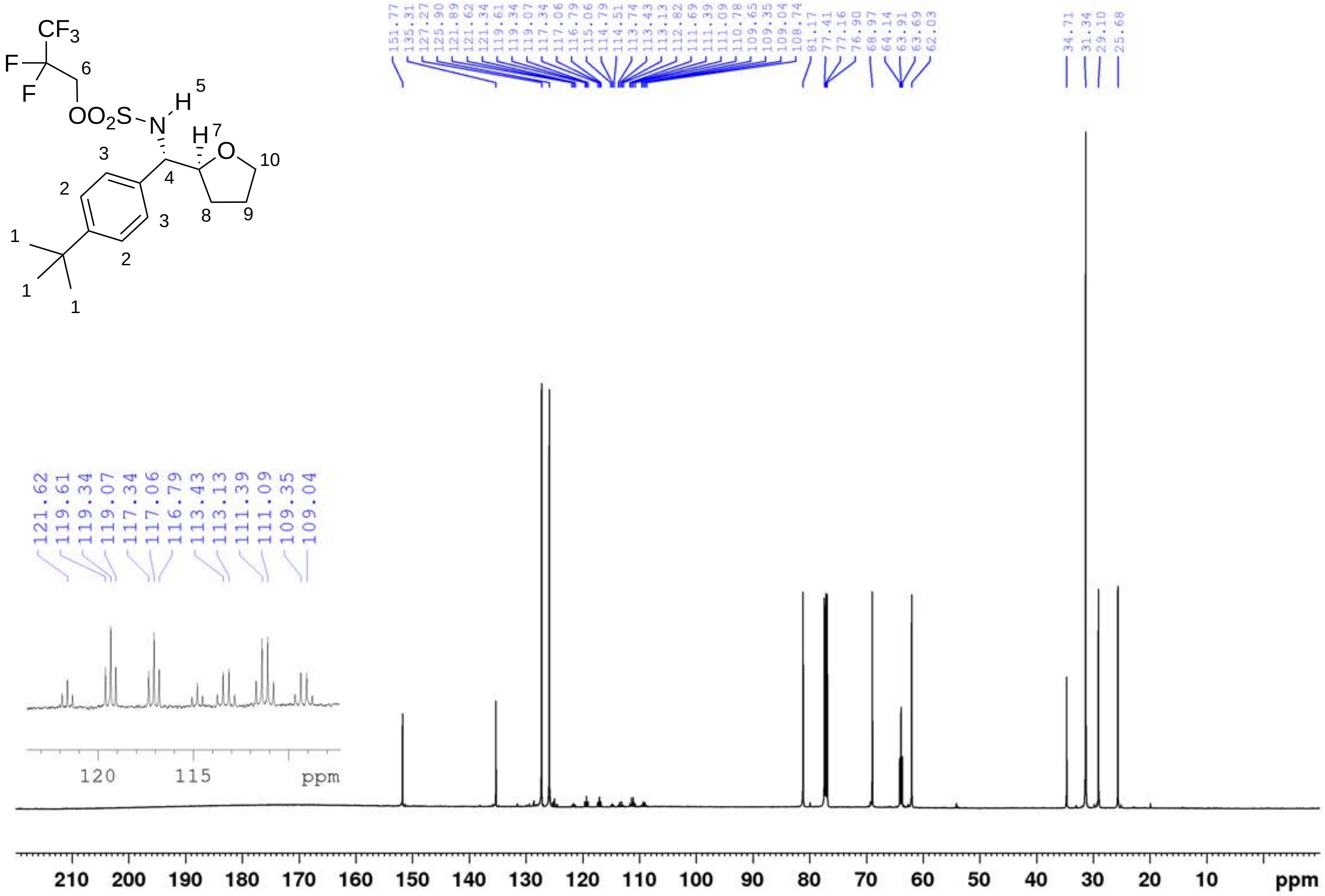

^{19}F NMR (376 MHz, $CDCl_3$) for *2,2,3,3,3-pentafluoropropyl ((S)-(4-(tert-butyl)phenyl)((S)-tetrahydrofuran-2-yl)methyl)sulfamate* (**4w**)

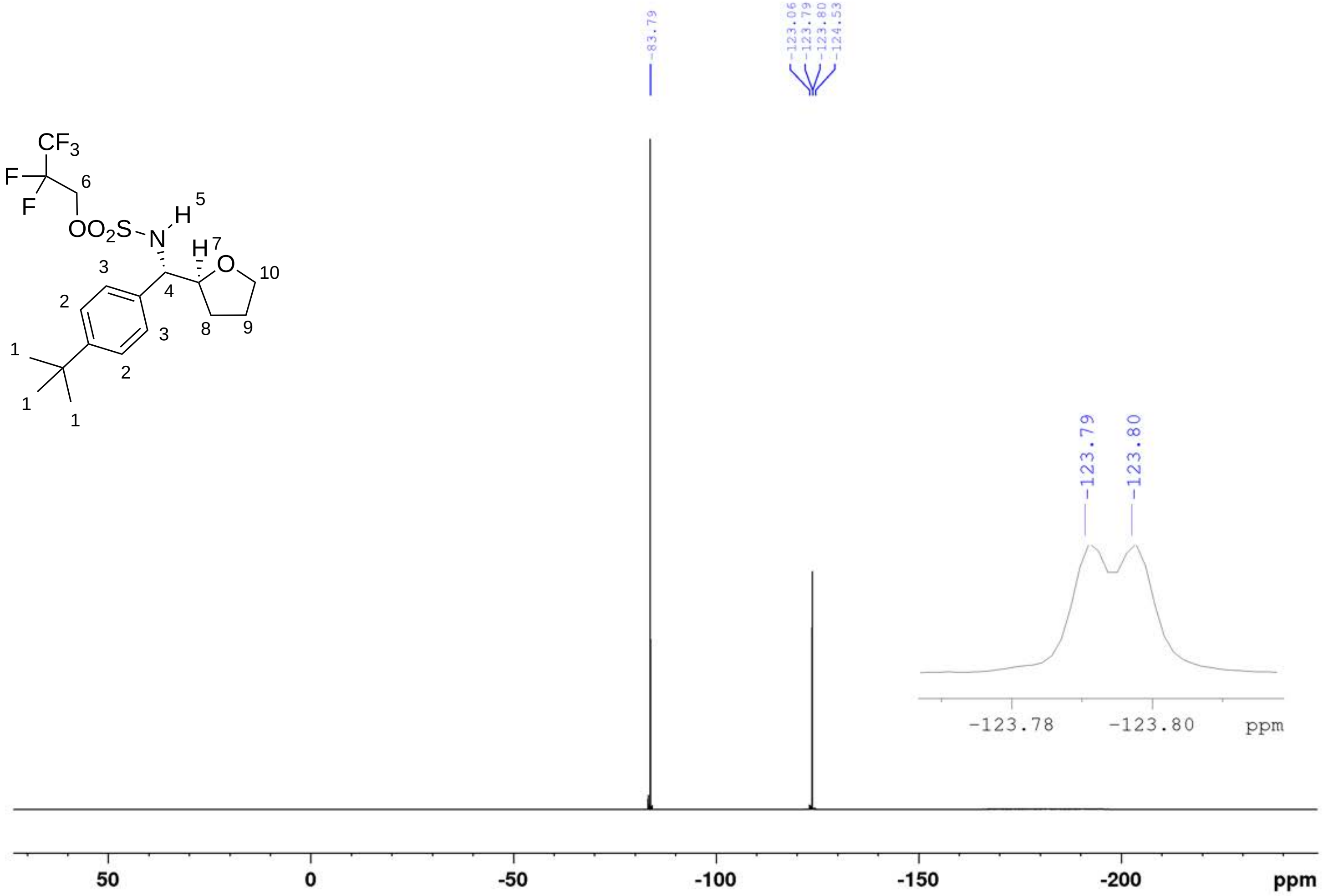

^{1}H NMR (500 MHz, $CDCl_3$) for *2,2,3,3,3-pentafluoropropyl ((S)-[1,1'-biphenyl]-4-yl((S)-tetrahydrofuran-2-yl)methyl)sulfamate* (**4x**)

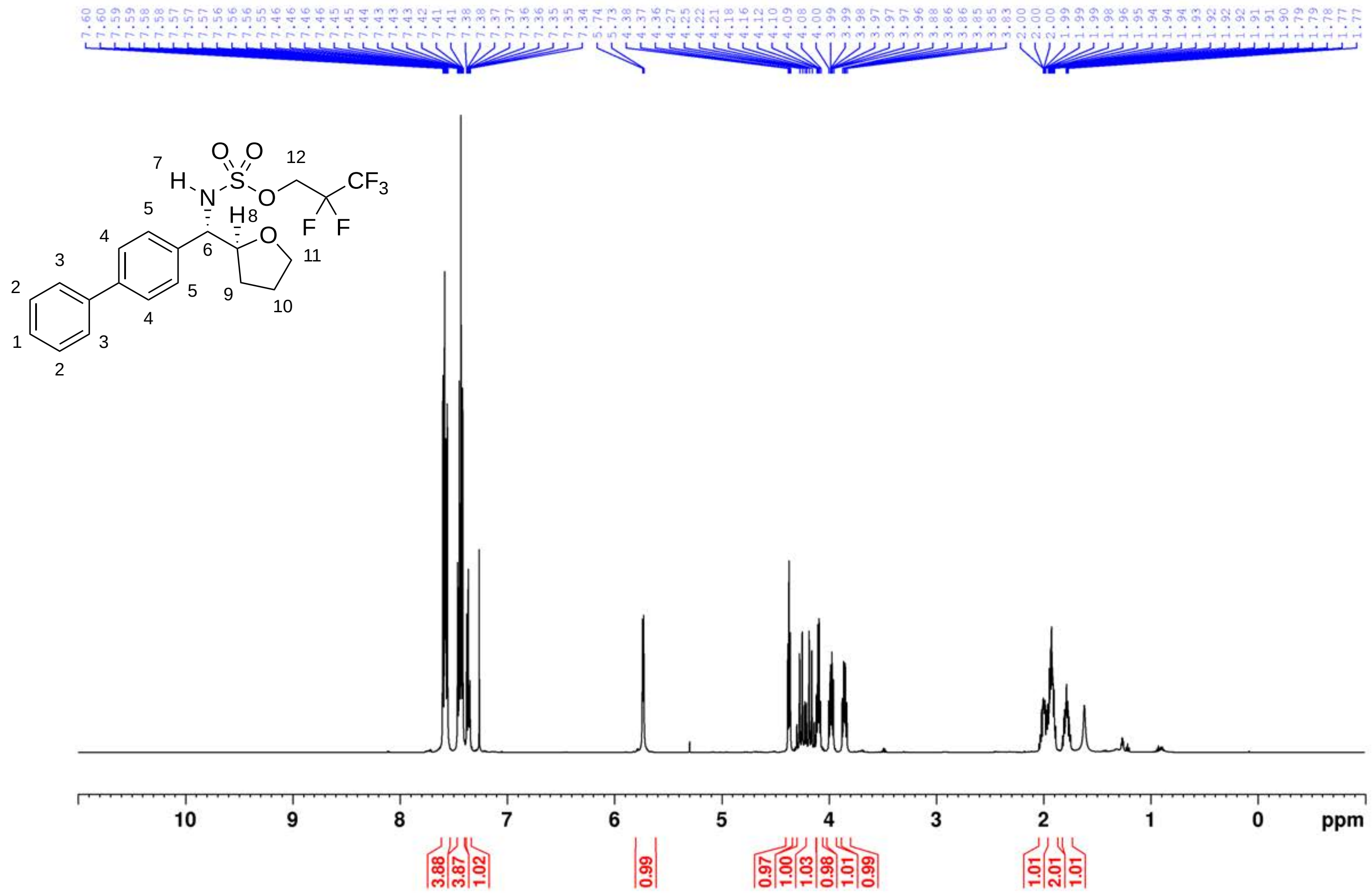

^{13}C NMR (126 MHz, $CDCl_3$) for *2,2,3,3,3-pentafluoropropyl ((S)-[1,1'-biphenyl]-4-yl((S)-tetrahydrofuran-2-yl)methyl)sulfamate* (**4x**)

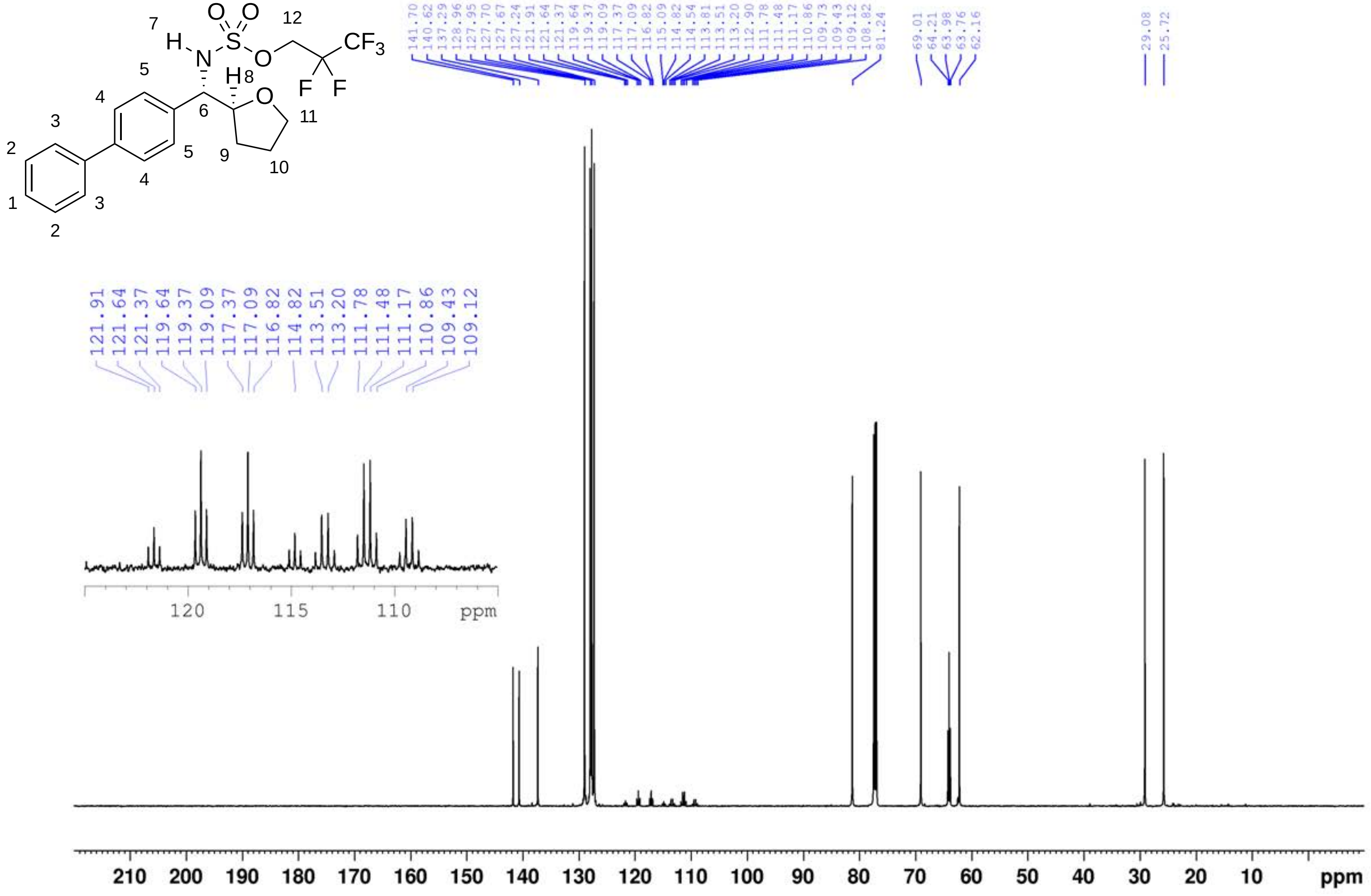

^{19}F NMR (376 MHz, $CDCl_3$) for *2,2,3,3,3-pentafluoropropyl ((S)-[1,1'-biphenyl]-4-yl((S)-tetrahydrofuran-2-yl)methyl)sulfamate* (**4x**)

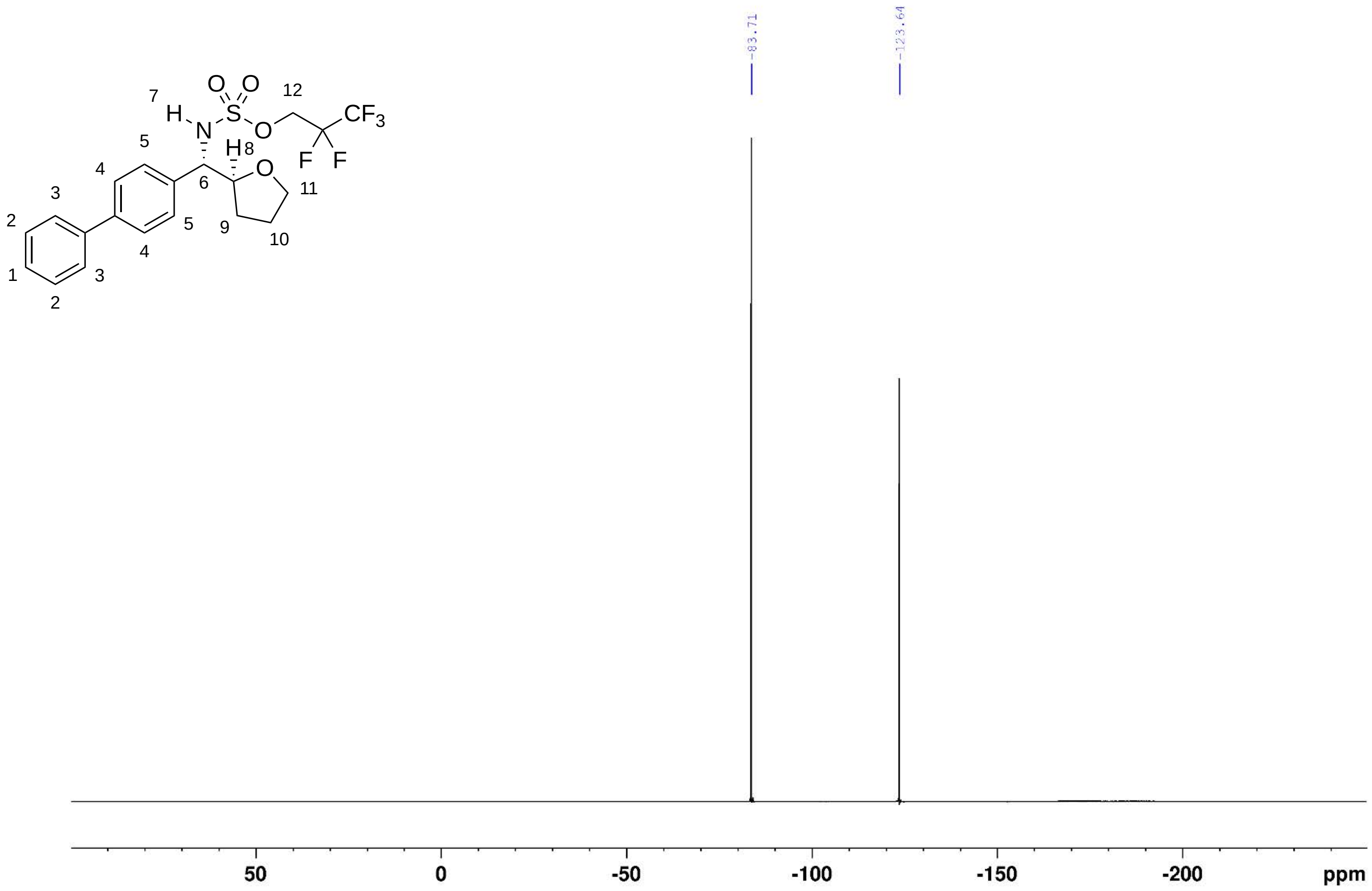

^{1}H NMR (500 MHz, $CDCl_3$) for *Ethyl 4-((S)-(((2,2,3,3,3-pentafluoropropoxy)sulfonyl)amino)((S)-tetrahydrofuran-2-yl)methyl)benzoate* (**4y**)

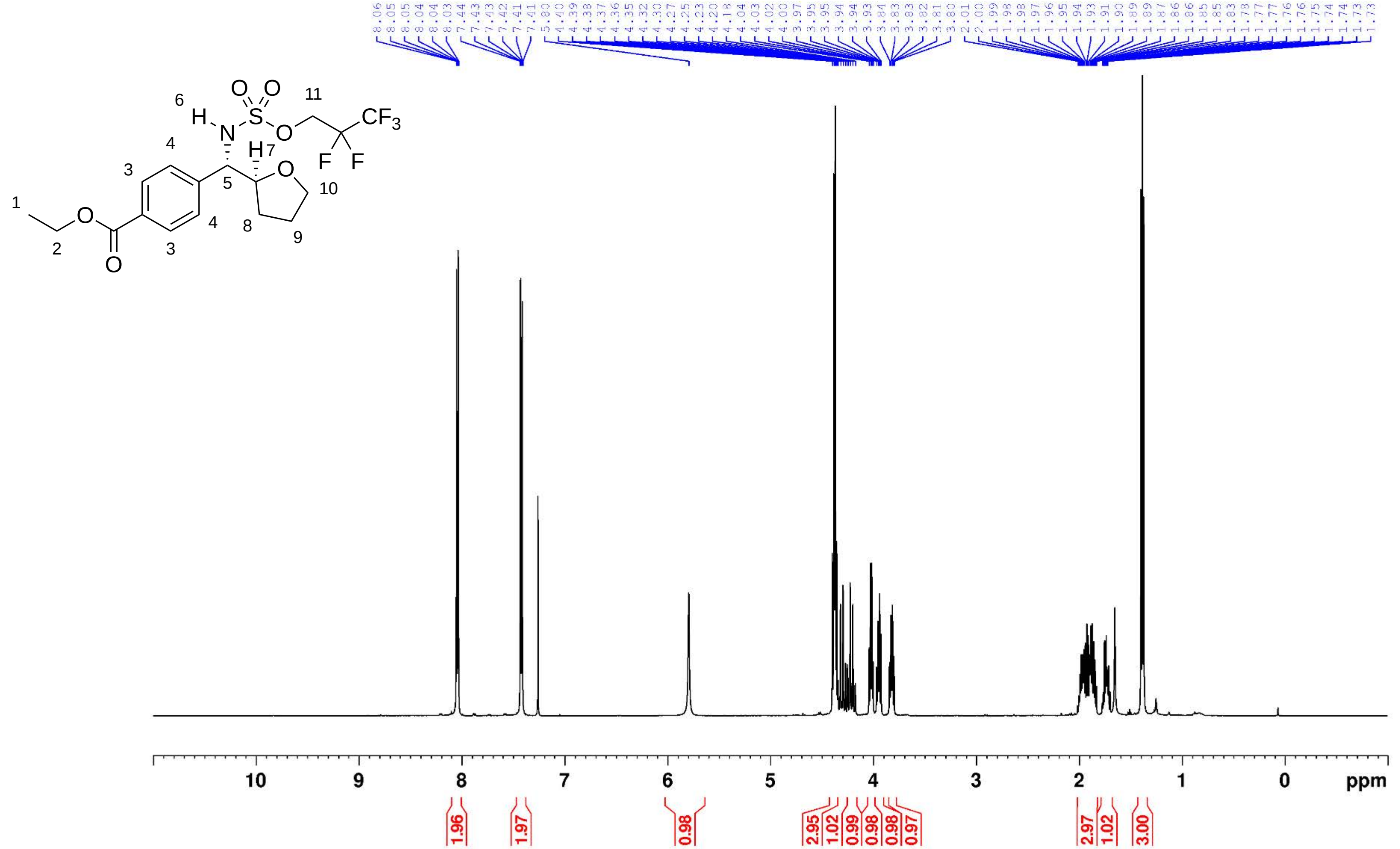

^{13}C NMR (126 MHz, $CDCl_3$) for *Ethyl 4-((S)-(((2,2,3,3,3-pentafluoropropoxy)sulfonyl)amino)((S)-tetrahydrofuran-2-yl)methyl)benzoate* (**4y**)

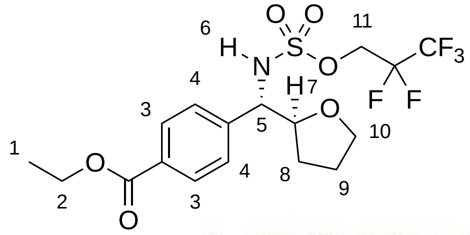

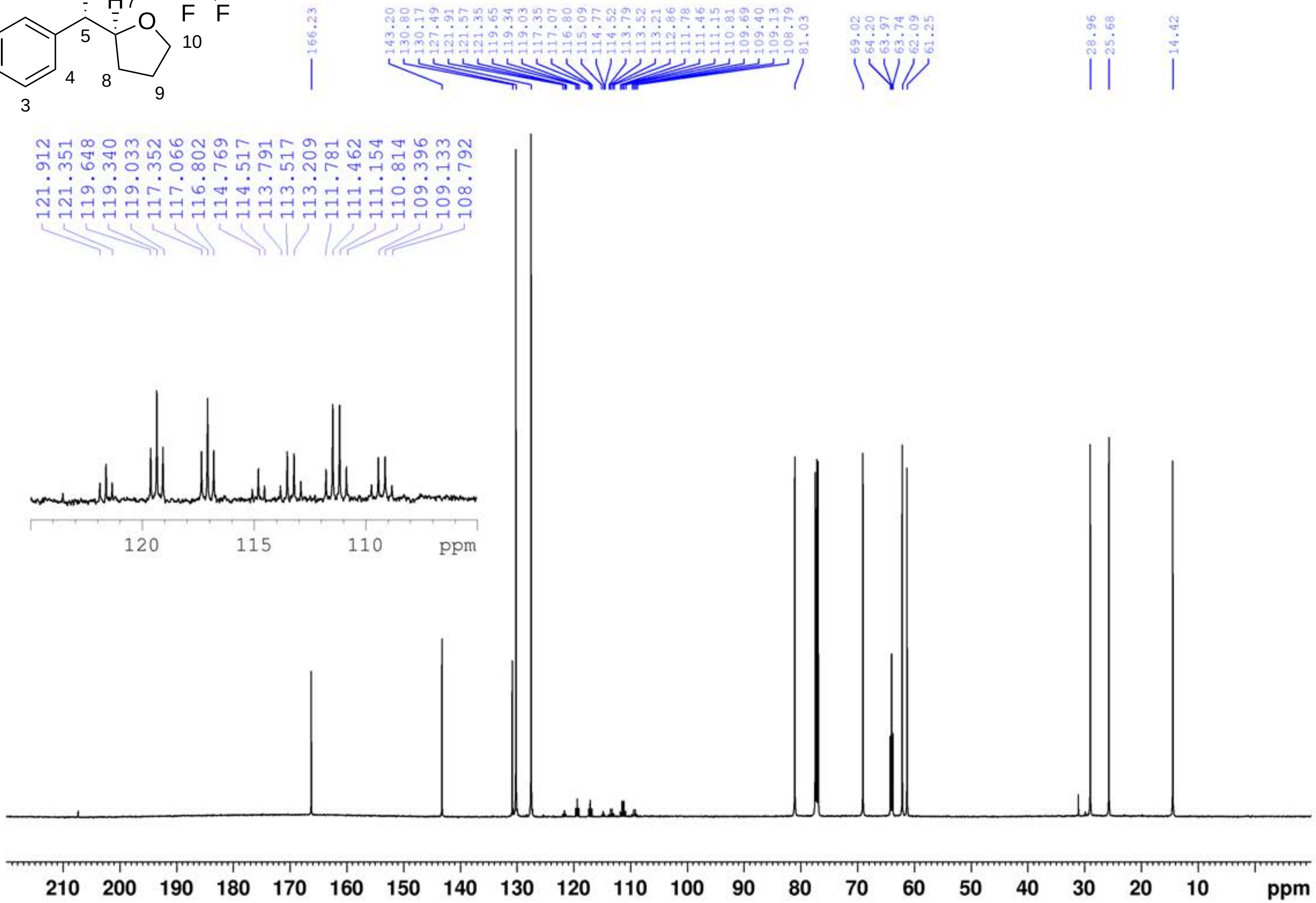

^{19}F NMR (471 MHz, $CDCl_3$) for *ethyl 4-((S)-(((2,2,3,3,3-pentafluoropropoxy)sulfonyl)amino)((S)-tetrahydrofuran-2-yl)methyl)benzoate* (**4y**)

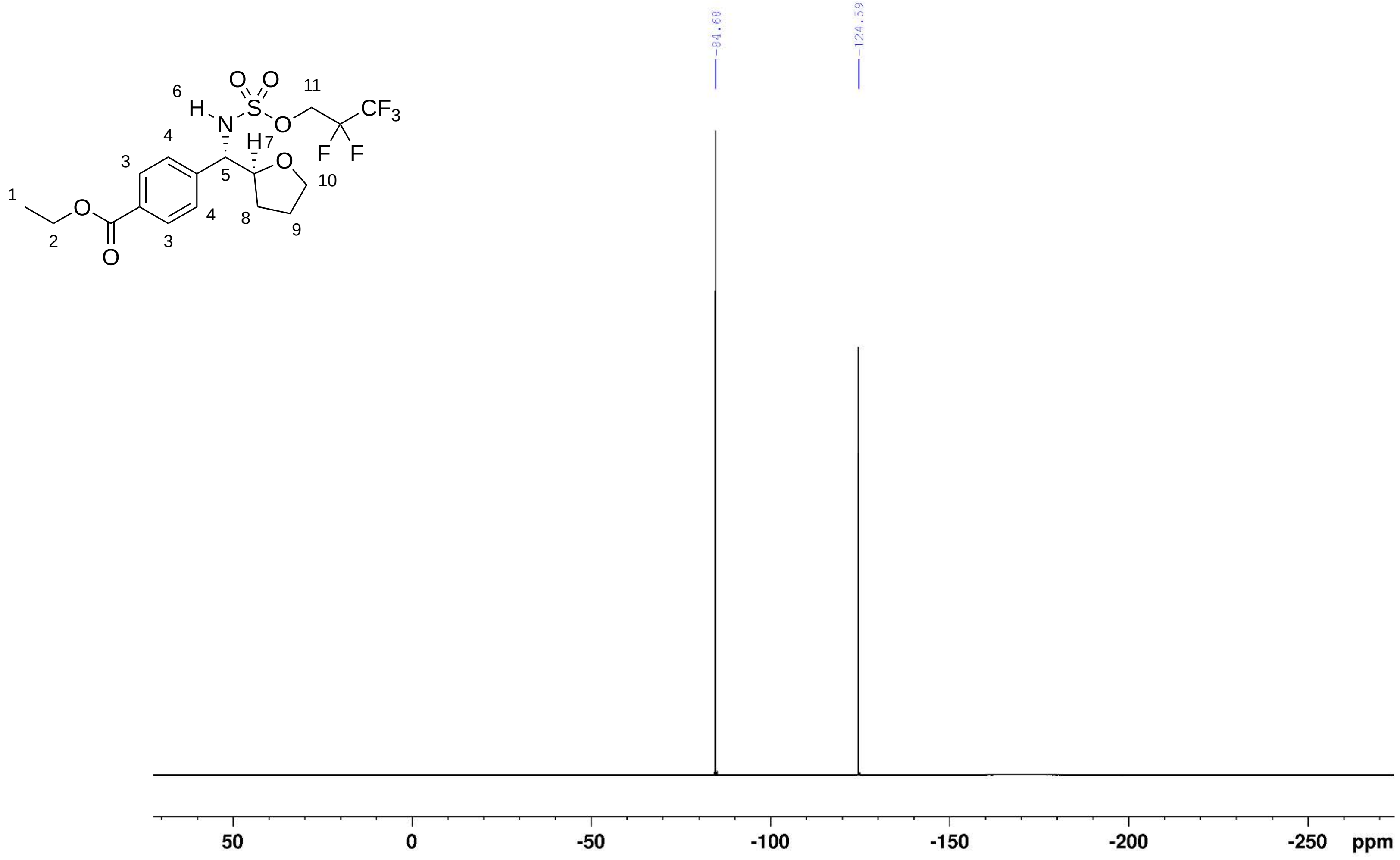

^{1}H NMR (500 MHz, $CDCl_3$) for *2,2,3,3,3-pentafluoropropyl ((S)-(4-((tert-butoxycarbonyl)amino)phenyl)((S)-tetrahydrofuran-2-yl)methyl)sulfamate* (**4z**)

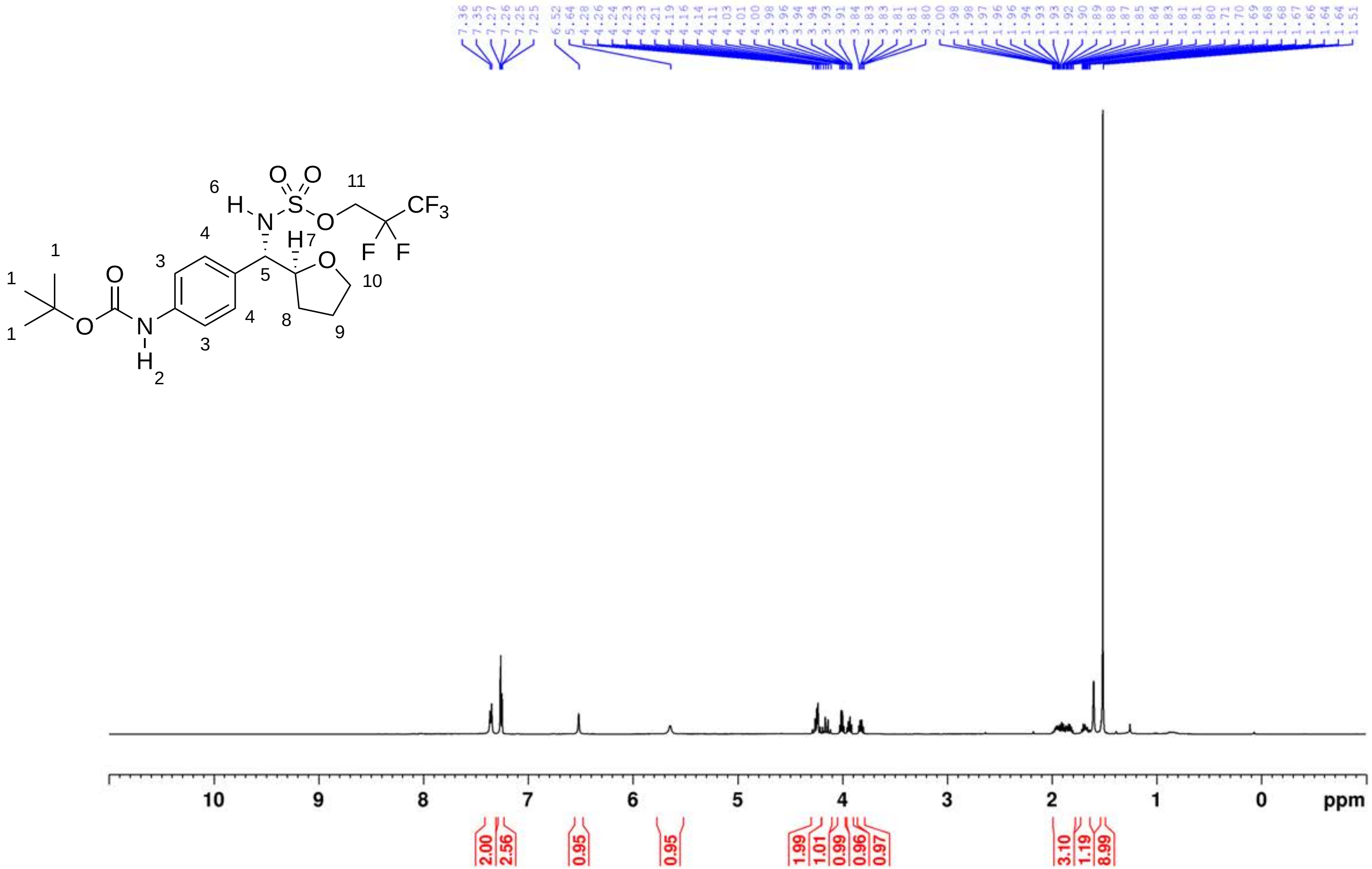

^{13}C NMR (101 MHz, $CDCl_3$) for *2,2,3,3,3-pentafluoropropyl ((S)-(4-((tert-butoxycarbonyl)amino)phenyl)((S)-tetrahydrofuran-2-yl)methyl)sulfamate* (**4z**)

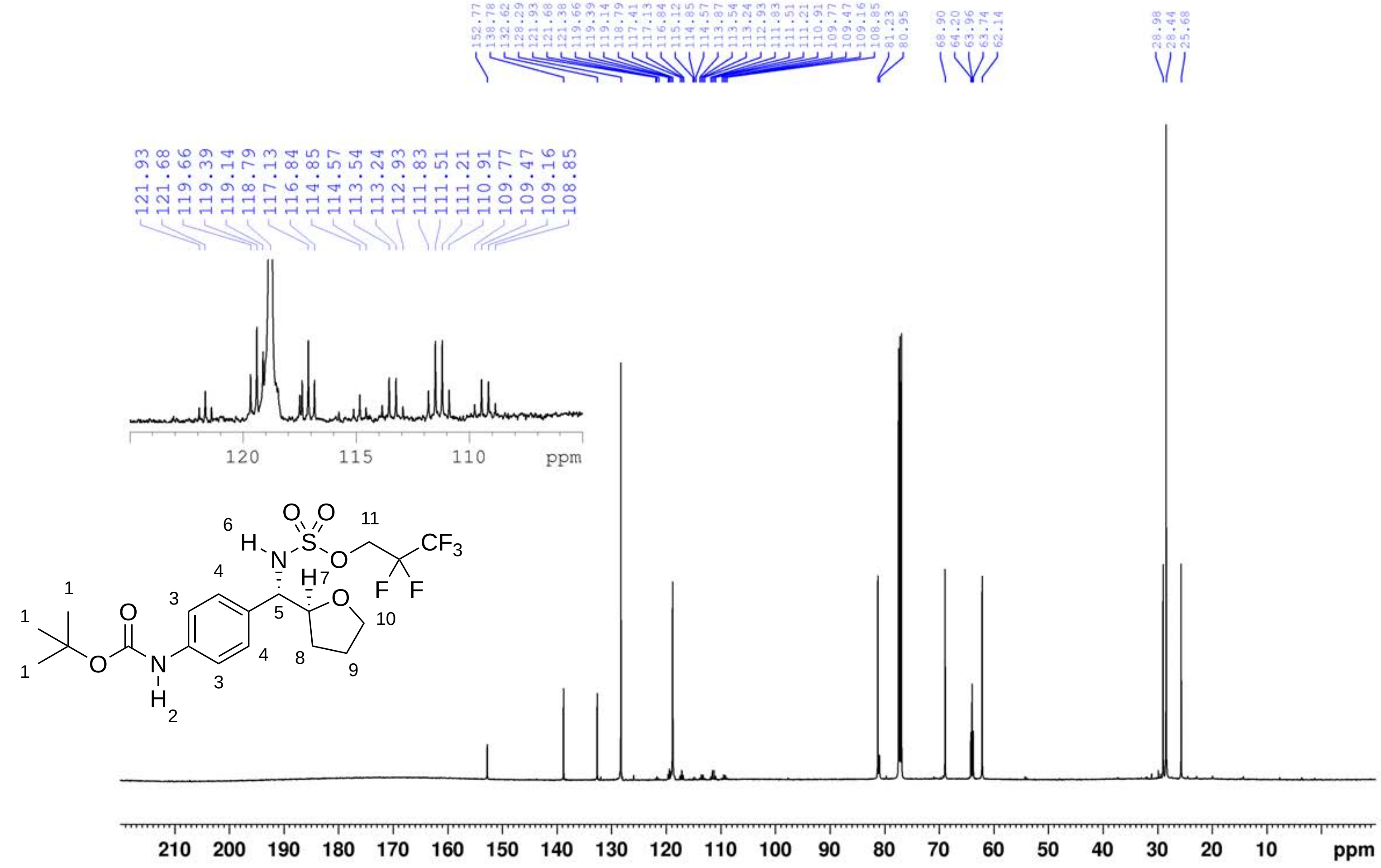

^{19}F NMR (471 MHz, $CDCl_3$) for *2,2,3,3,3-pentafluoropropyl ((S)-(4-((tert-butoxycarbonyl)amino)phenyl)((S)-tetrahydrofuran-2-yl)methyl)sulfamate* (**4z**)

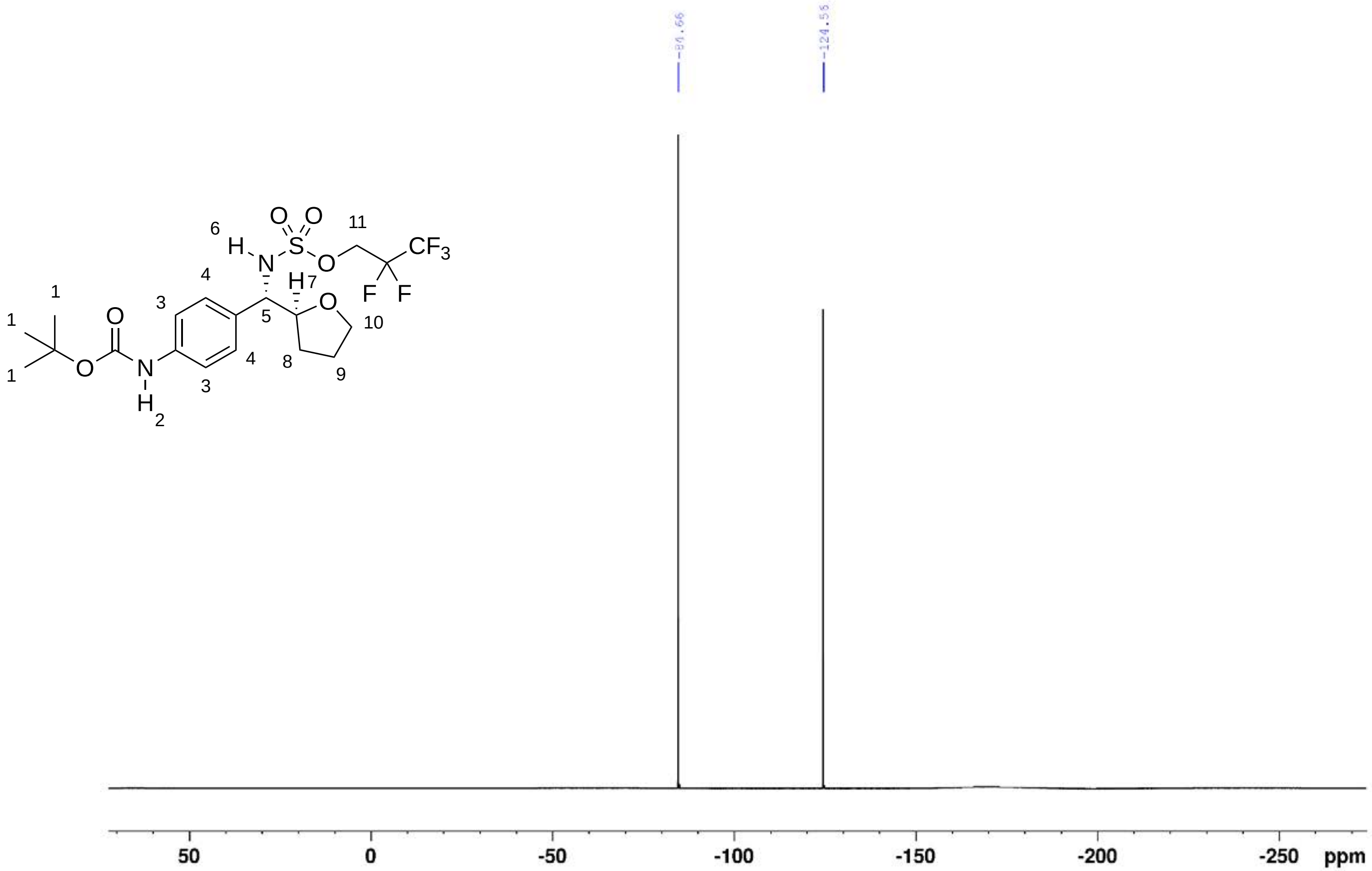

^{1}H NMR (500 MHz, $CDCl_3$) for *2,2,3,3,3-pentafluoropropyl ((S)-(4-methoxyphenyl)((S)-tetrahydrofuran-2-yl)methyl)sulfamate* (**4aa**)

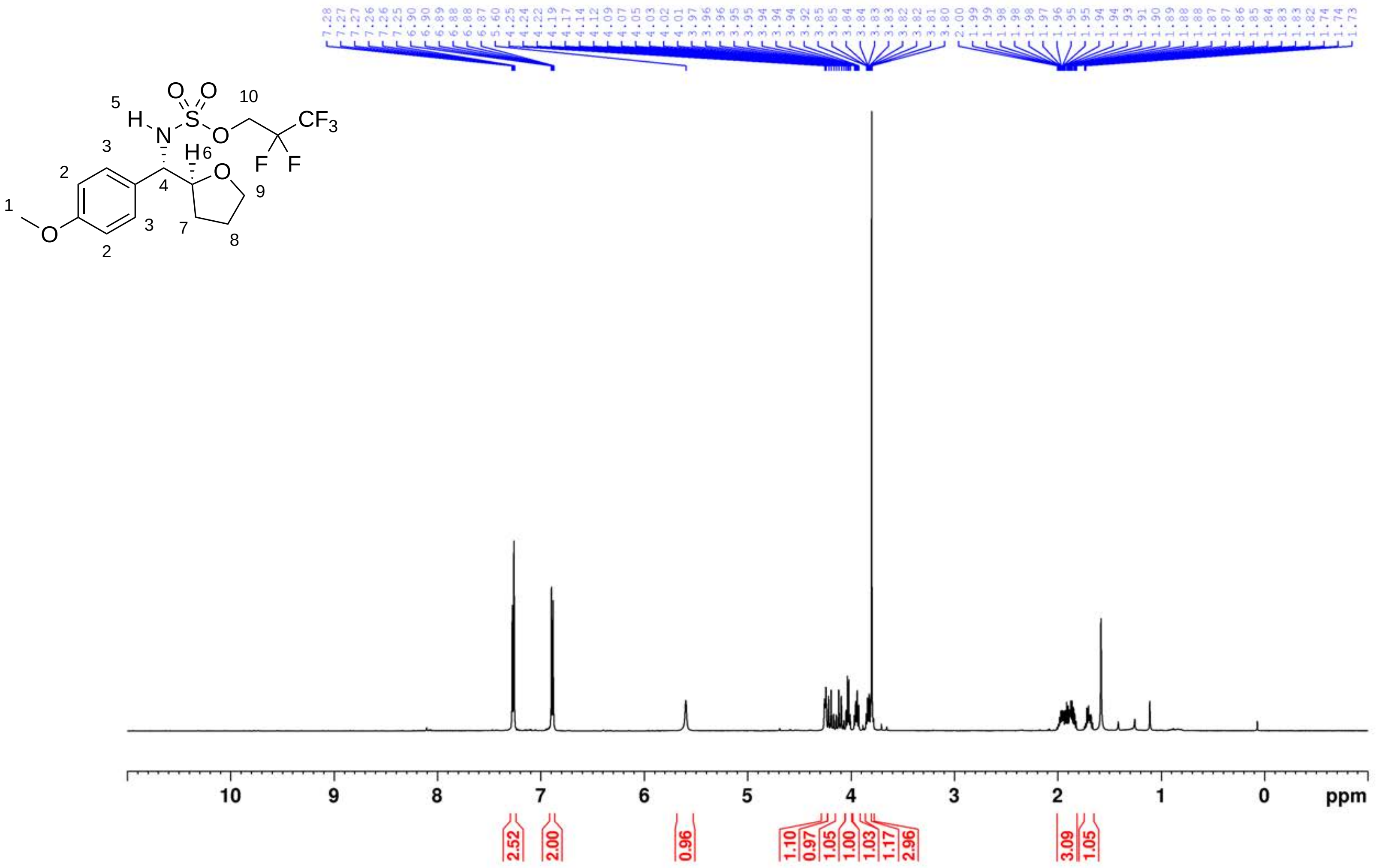

^{13}C NMR (126 MHz, $CDCl_3$) for *2,2,3,3,3-pentafluoropropyl ((S)-(4-methoxyphenyl)((S)-tetrahydrofuran-2-yl)methyl)sulfamate* (**4aa**)

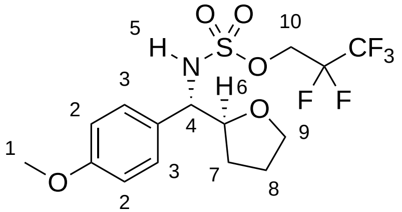

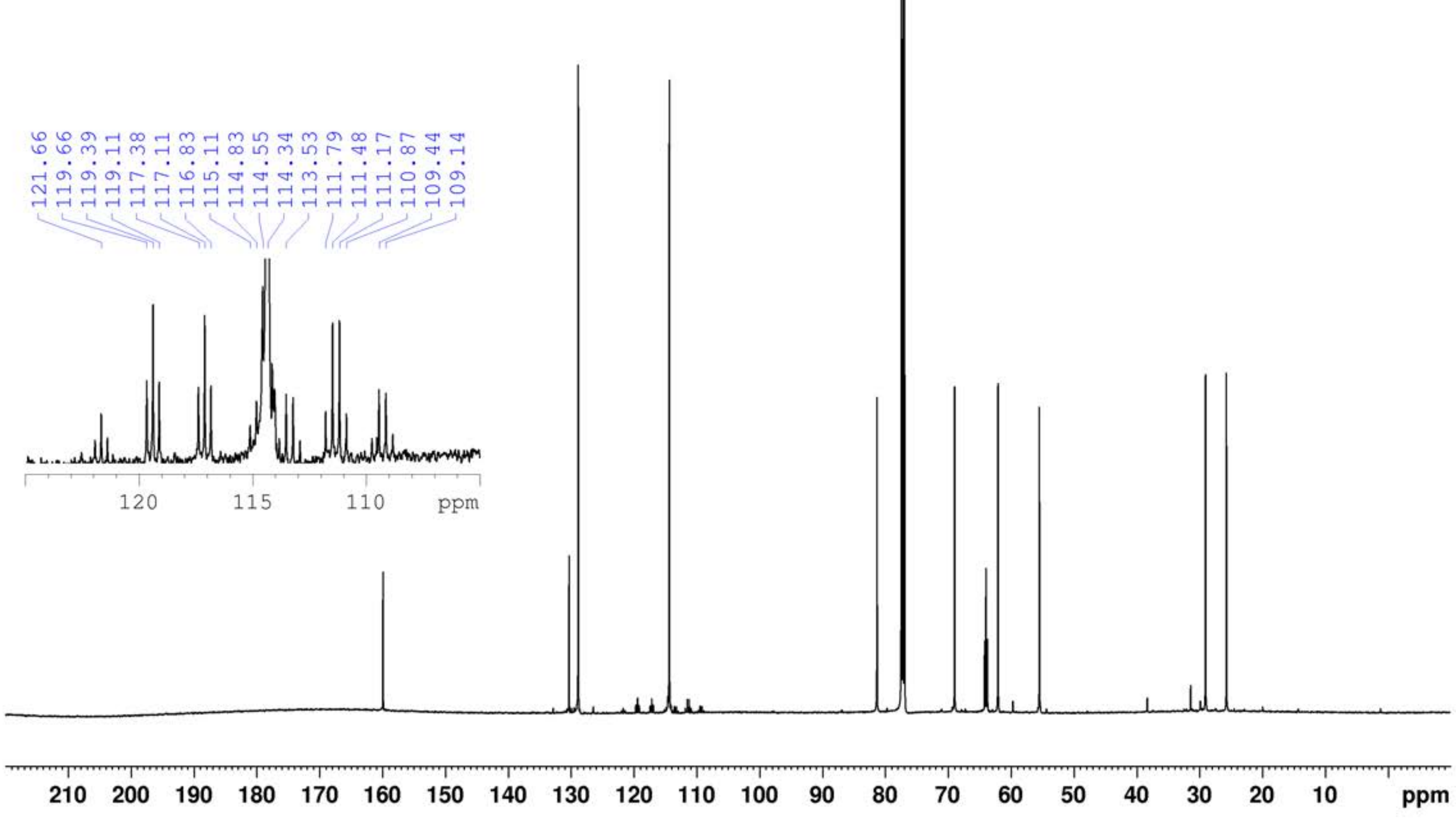

^{19}F NMR (471 MHz, $CDCl_3$) for *2,2,3,3,3-pentafluoropropyl ((S)-(4-methoxyphenyl)((S)-tetrahydrofuran-2-yl)methyl)sulfamate* (**4aa**)

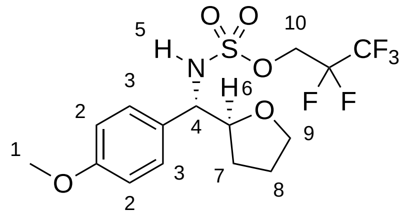

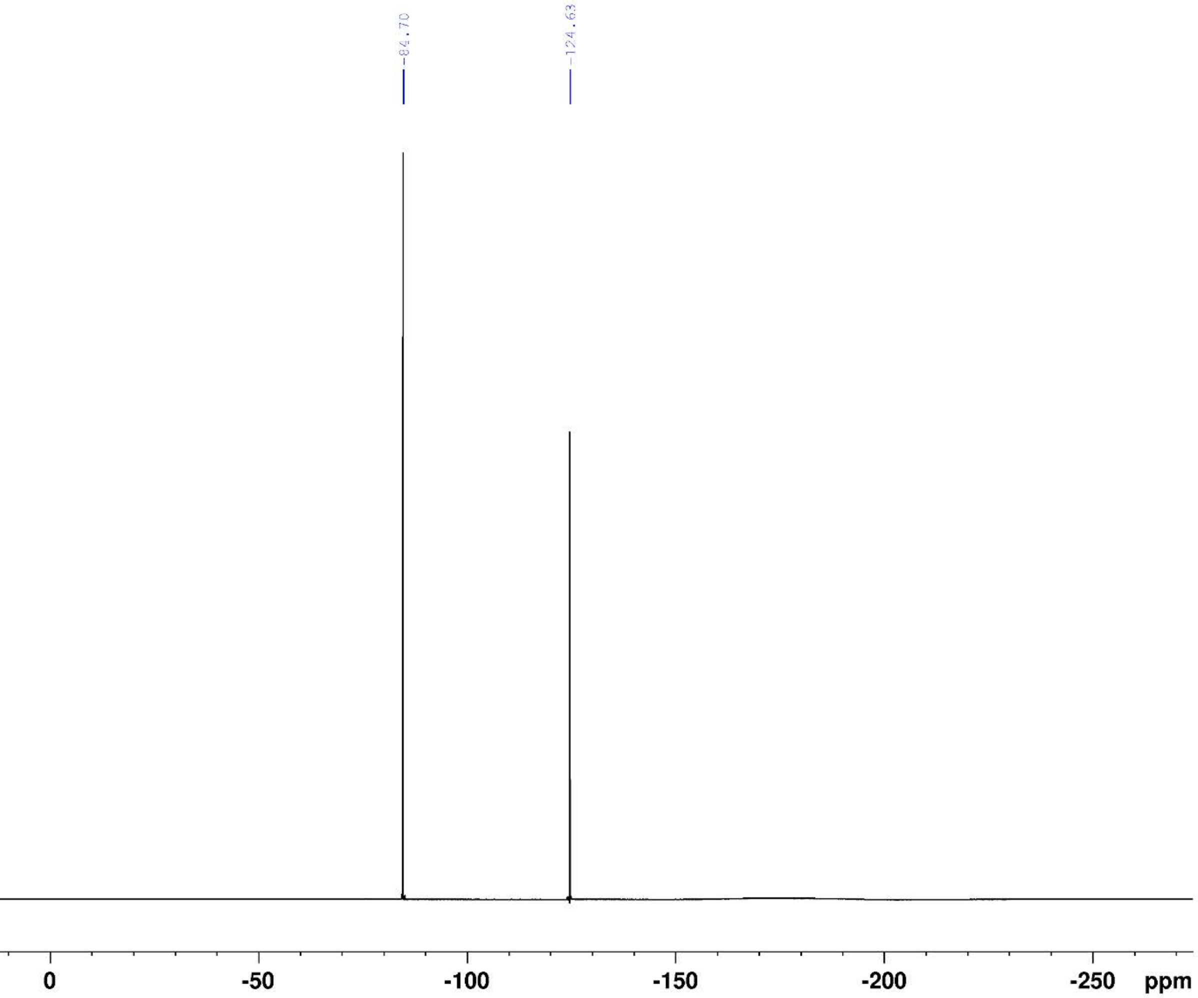

¹H NMR (500 MHz, $CDCl_3$) for *2,2,3,3,3-pentafluoropropyl ((2R,3R)-2-(4-methoxyphenyl)tetrahydro-2H-pyran-3-yl)sulfamate* (**4ab**)

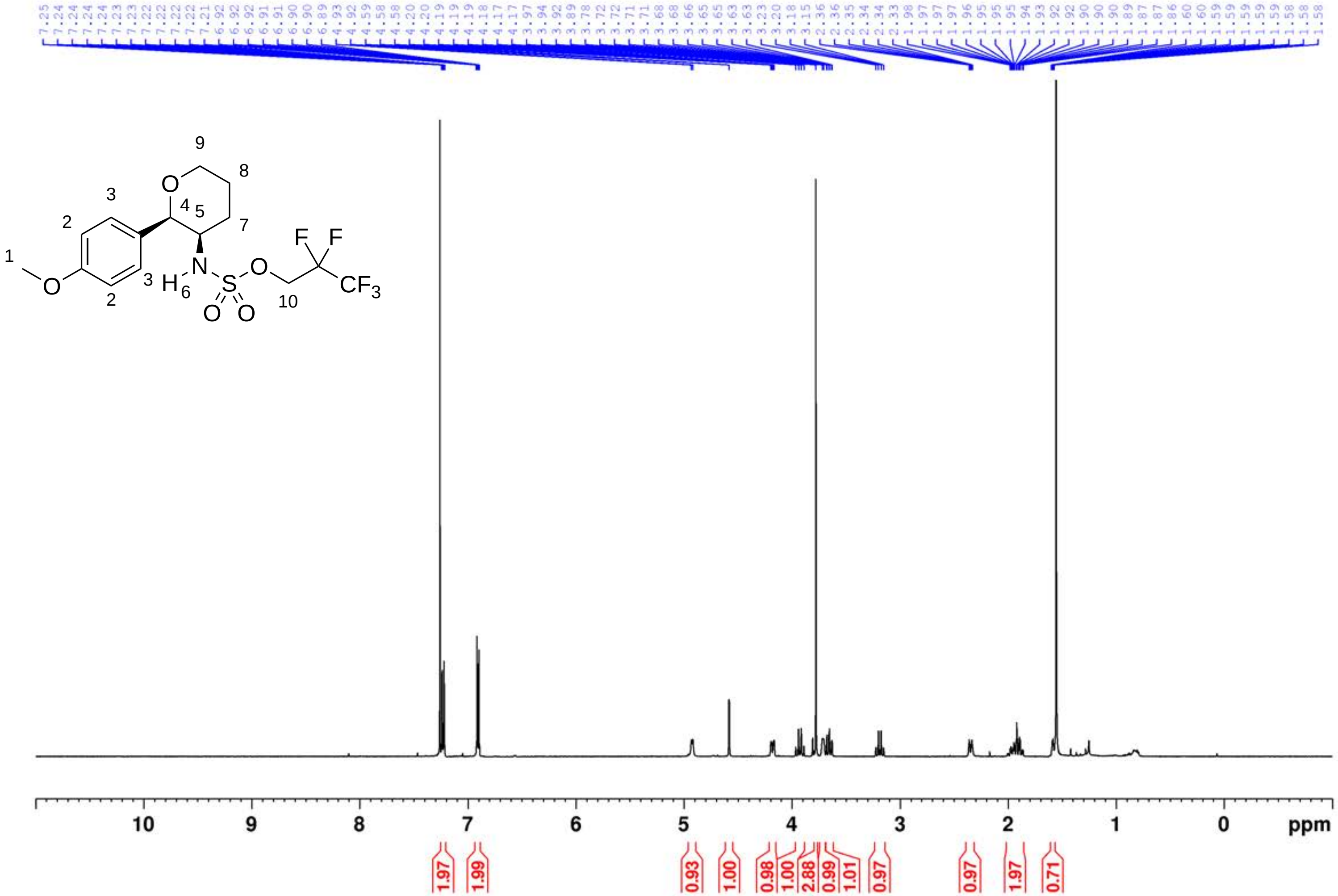

^{13}C NMR (126 MHz, $CDCl_3$) for *2,2,3,3,3-pentafluoropropyl ((2R,3R)-2-(4-methoxyphenyl)tetrahydro-2H-pyran-3-yl)sulfamate* (**4ab**)

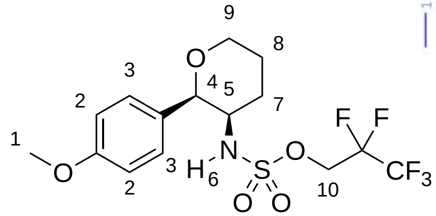

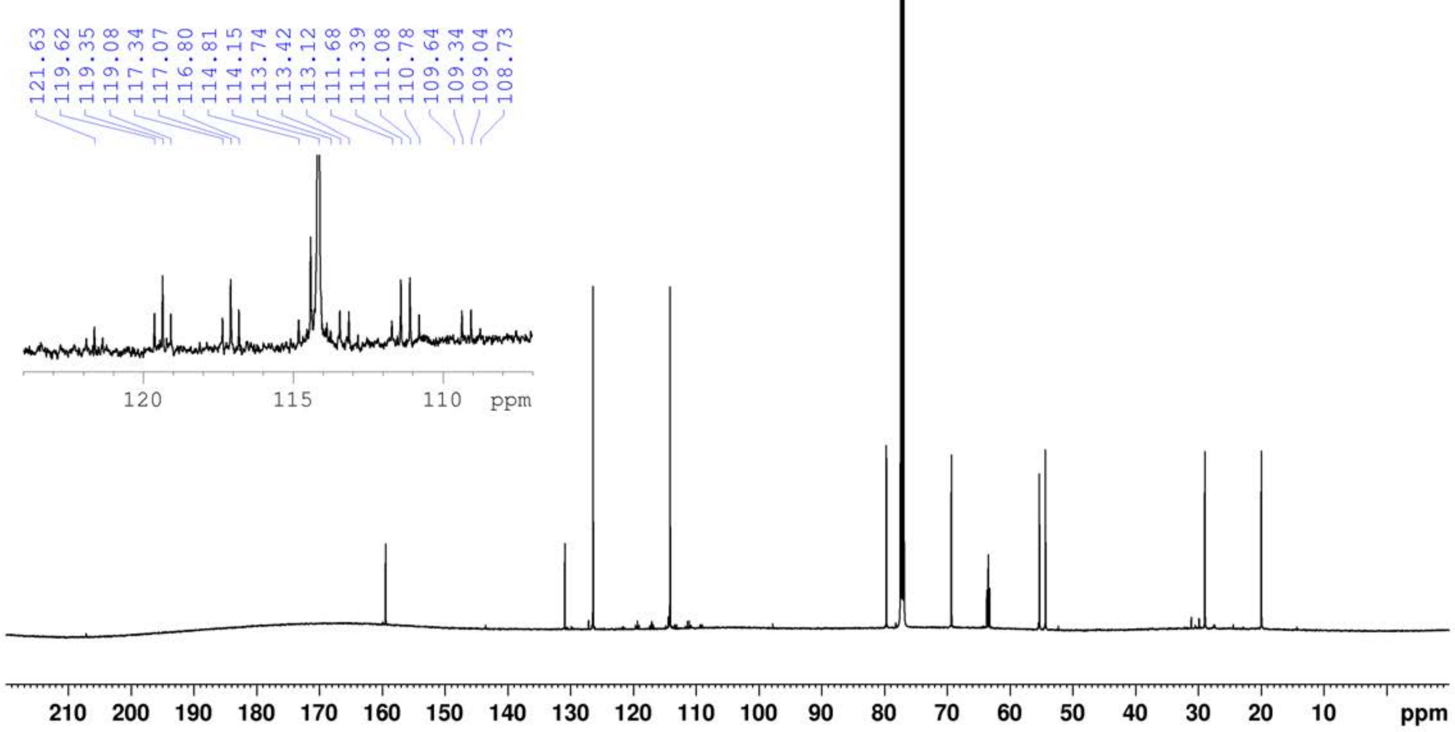

^{19}F NMR (471 MHz, $CDCl_3$) for *2,2,3,3,3-pentafluoropropyl ((2R,3R)-2-(4-methoxyphenyl)tetrahydro-2H-pyran-3-yl)sulfamate* (**4ab**)

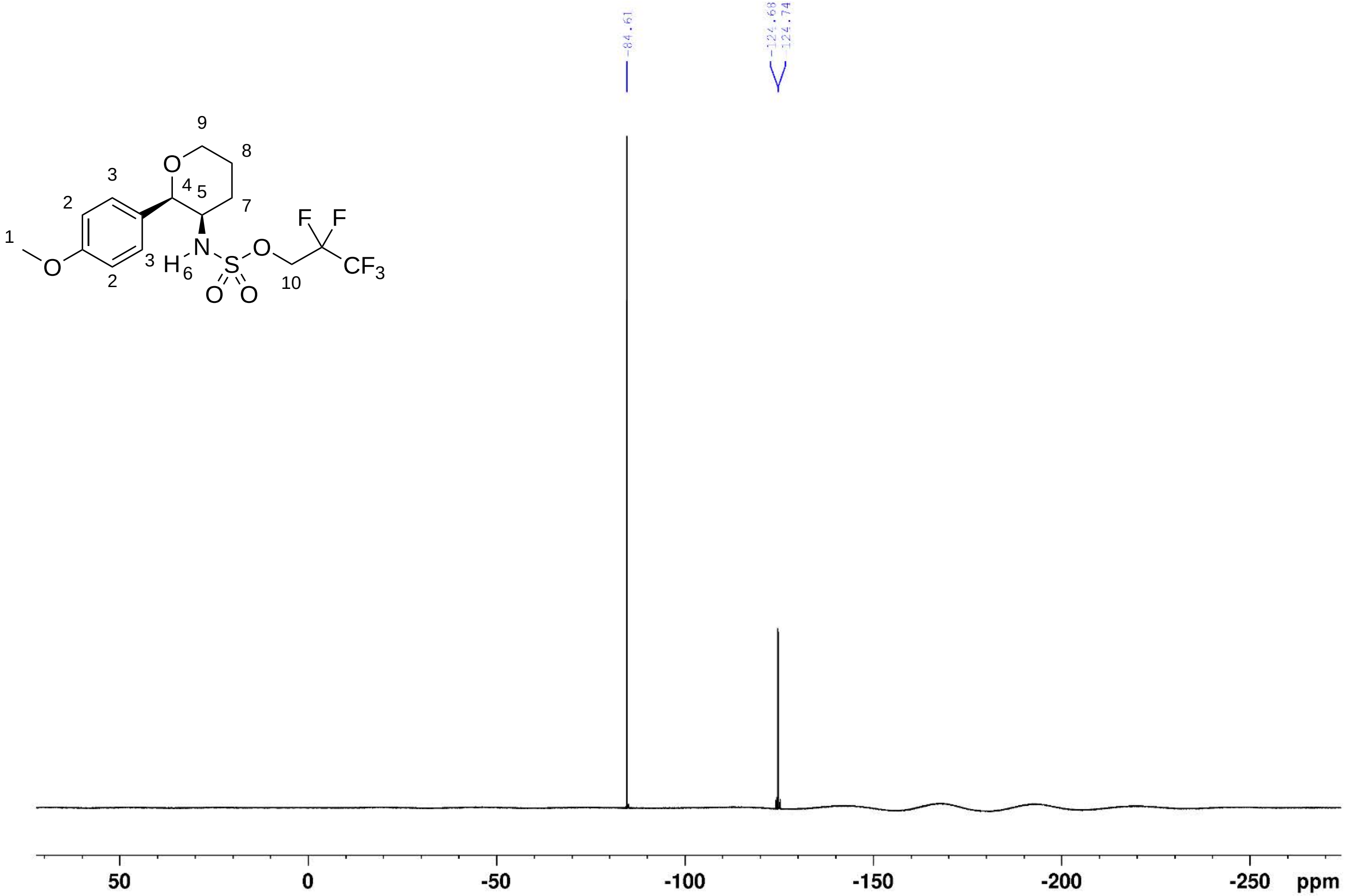

^{1}H NMR (500 MHz, $CDCl_3$) for *2,2,3,3,3-pentafluoropropyl ((S)-(4-chlorophenyl)((S)-tetrahydrofuran-2-yl)methyl)sulfamate* (**4ac**)

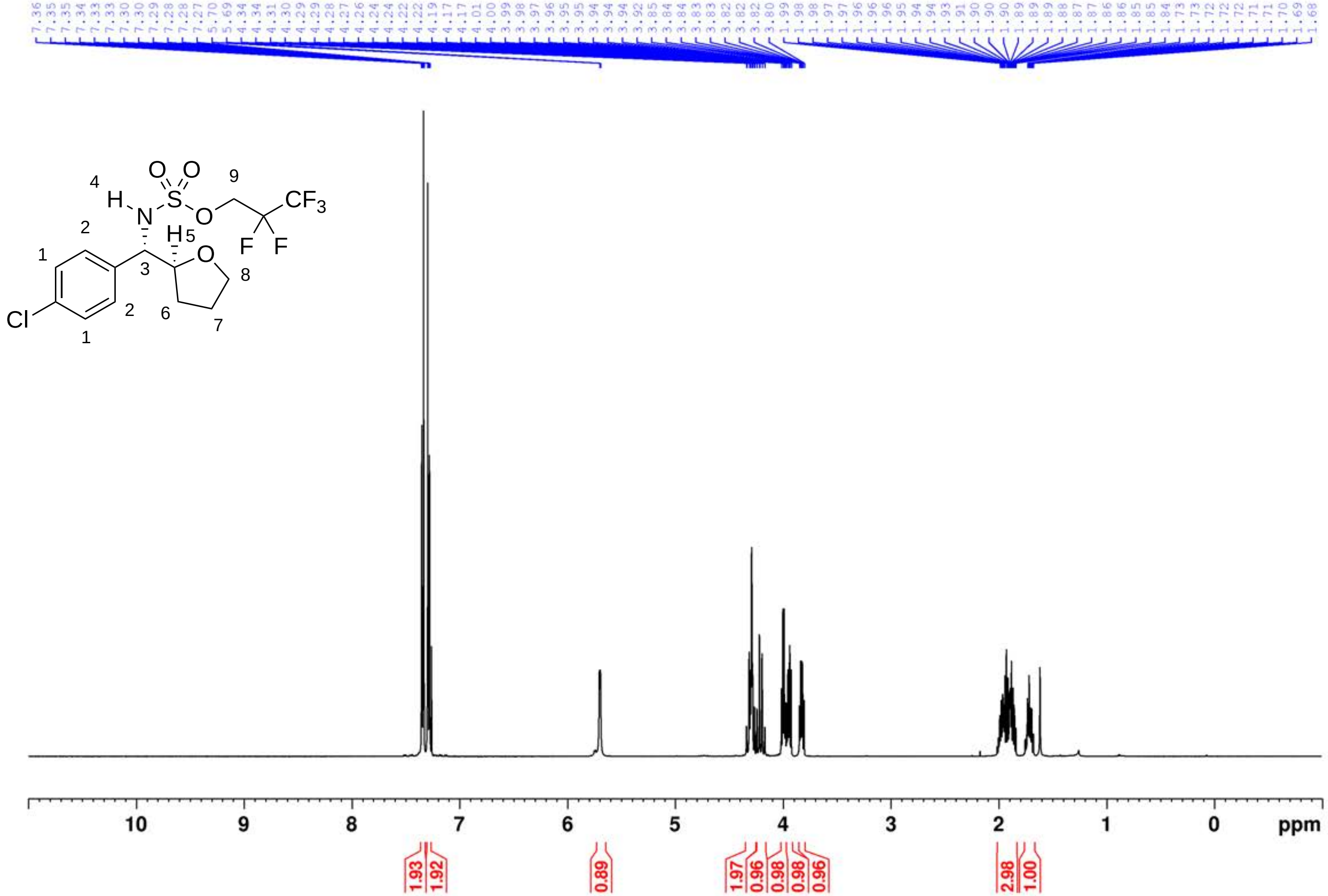

^{13}C NMR (126 MHz, $CDCl_3$) for *2,2,3,3,3-pentafluoropropyl ((S)-(4-chlorophenyl)((S)-tetrahydrofuran-2-yl)methyl)sulfamate* (**4ac**)

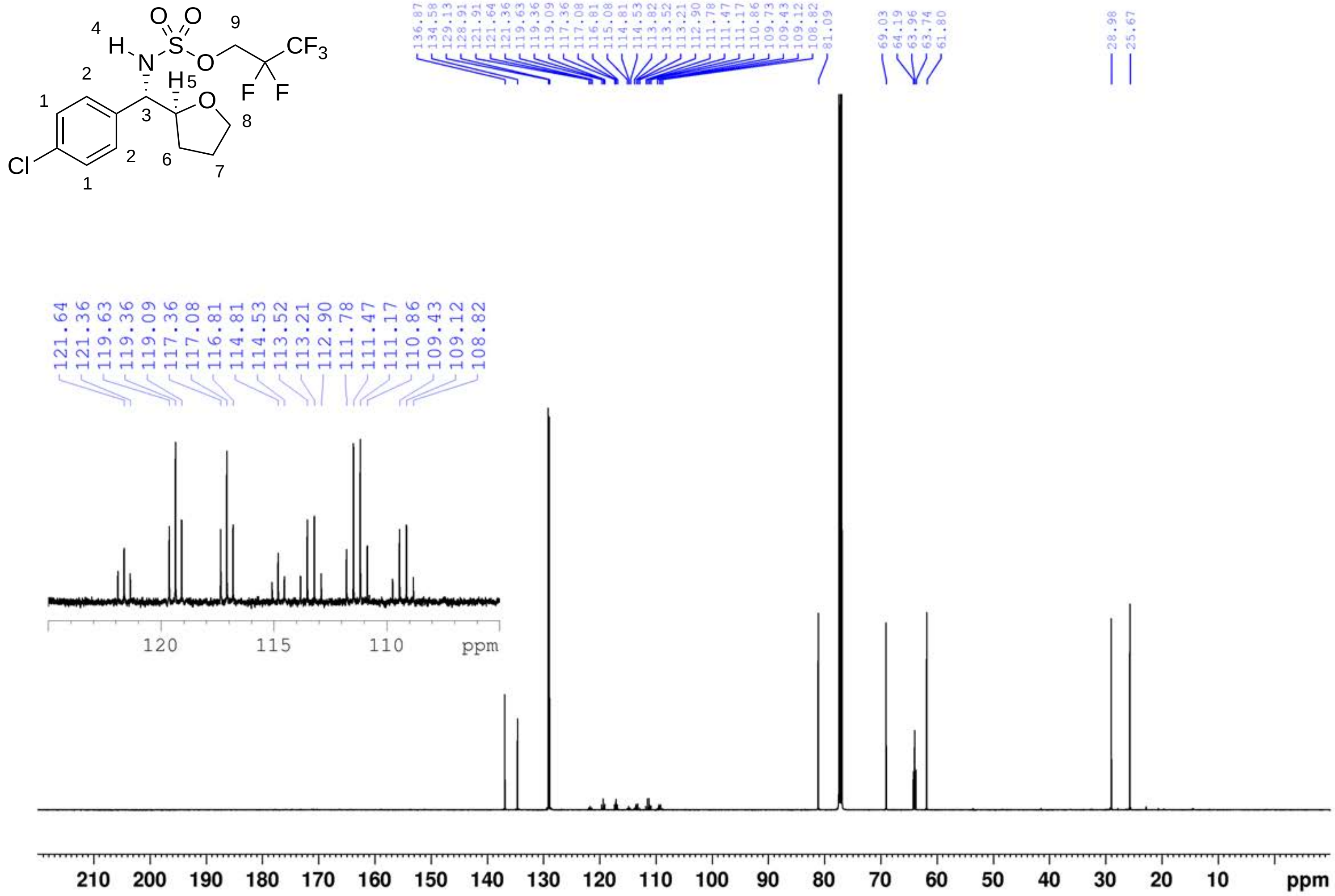

^{19}F NMR (471 MHz, $CDCl_3$) for *2,2,3,3,3-pentafluoropropyl ((S)-(4-chlorophenyl)((S)-tetrahydrofuran-2-yl)methyl)sulfamate* (**4ac**)

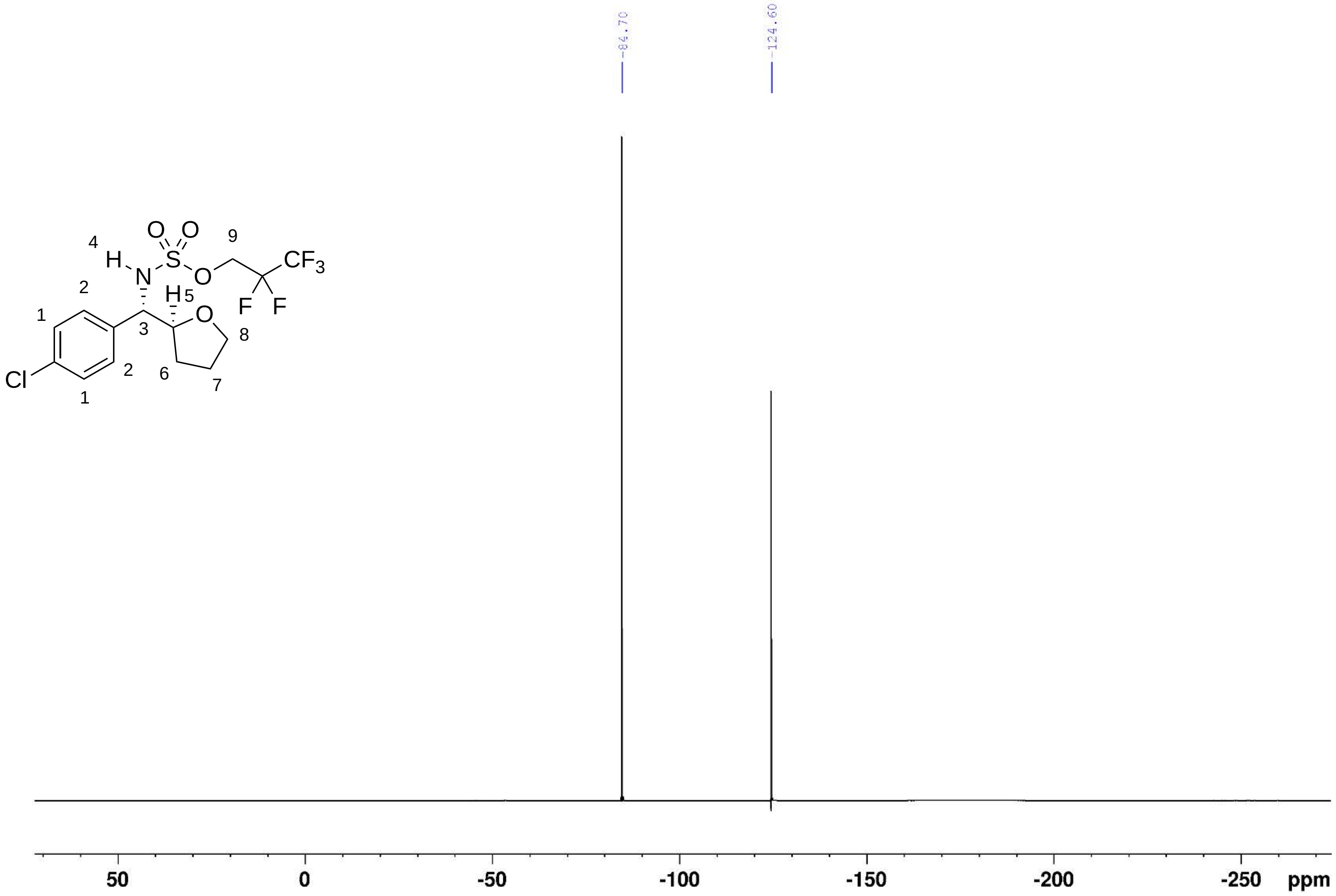

^{1}H NMR (500 MHz, $CDCl_3$) for *2,2,3,3,3-pentafluoropropyl ((S)-(4-bromophenyl)((S)-tetrahydrofuran-2-yl)methyl)sulfamate* (**4ad**)

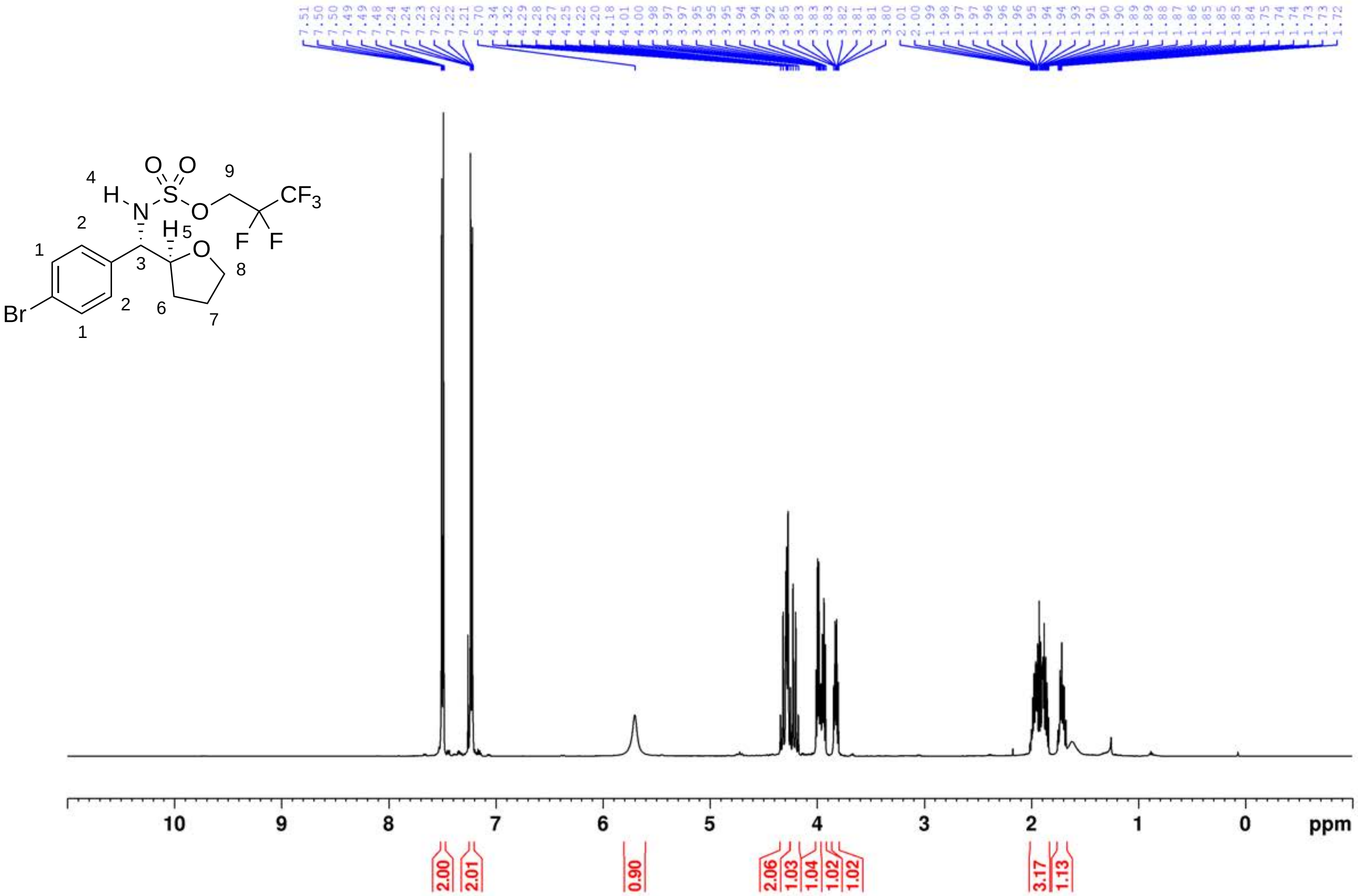

^{13}C NMR (126 MHz, $CDCl_3$) for *2,2,3,3,3-pentafluoropropyl ((S)-(4-bromophenyl)((S)-tetrahydrofuran-2-yl)methyl)sulfamate* (**4ad**)

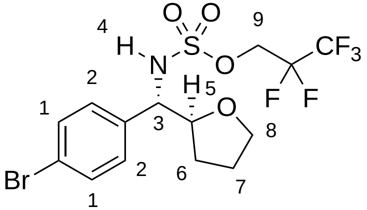

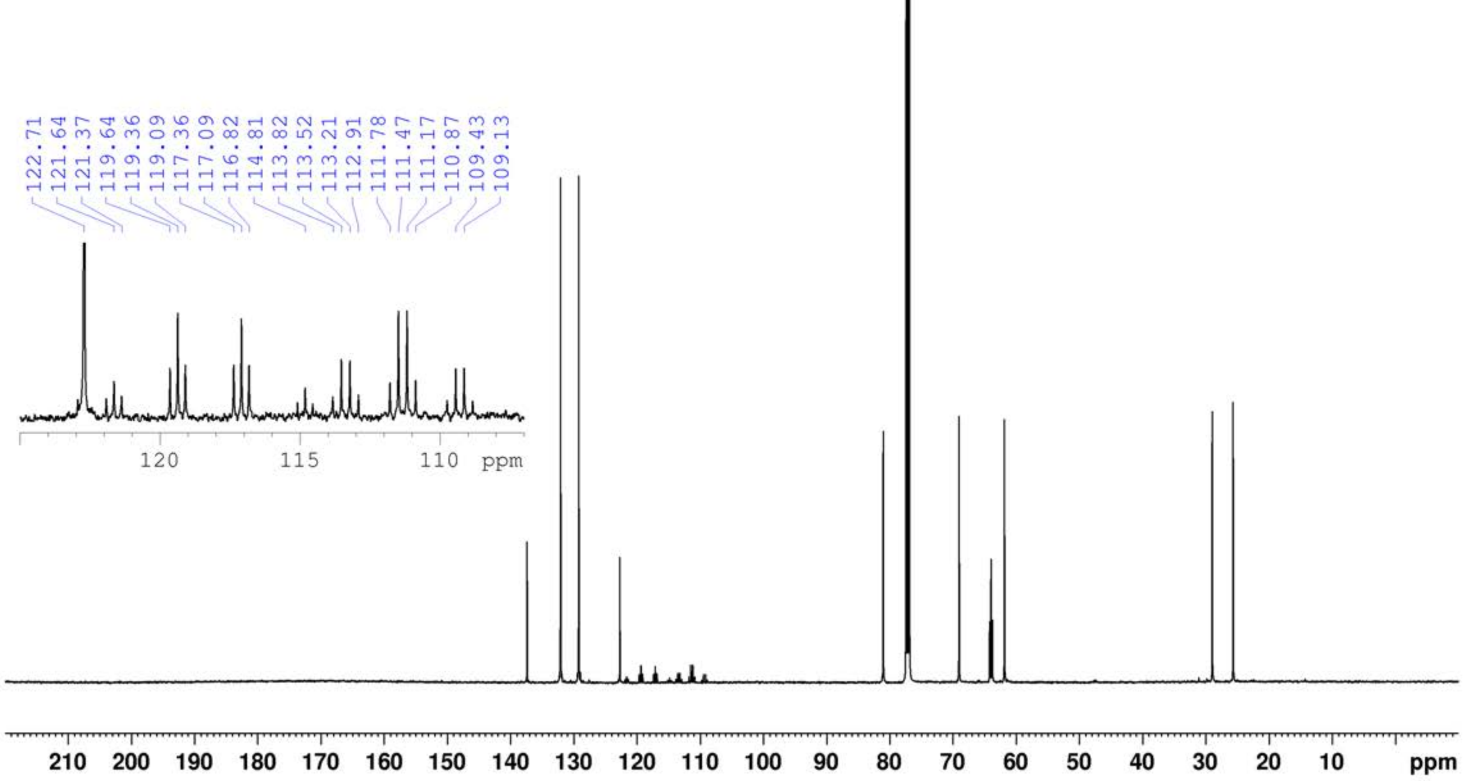

^{19}F NMR (471 MHz, $CDCl_3$) for *2,2,3,3,3-pentafluoropropyl ((S)-(4-bromophenyl)((S)-tetrahydrofuran-2-yl)methyl)sulfamate* (**4ad**)

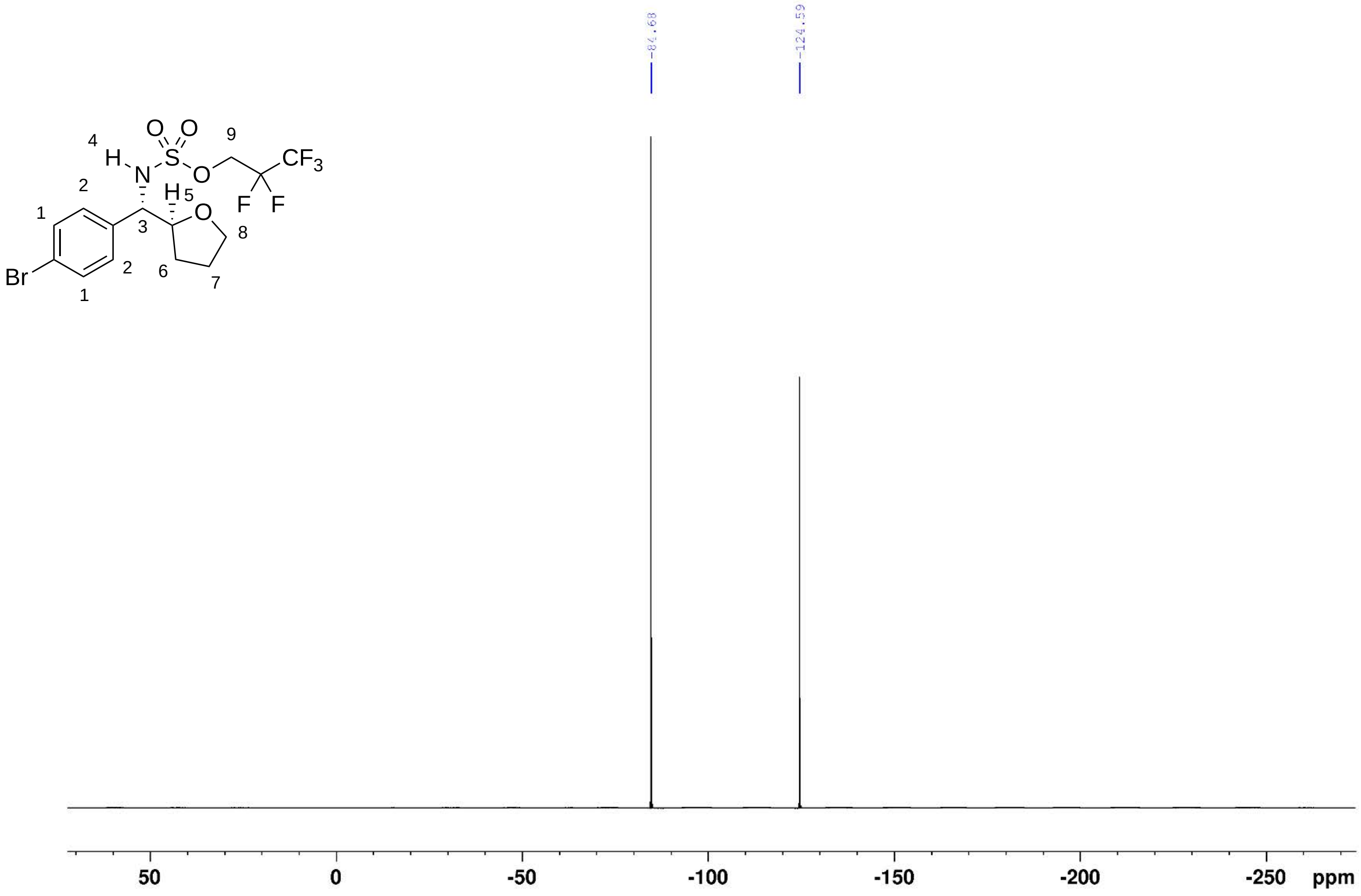

¹H NMR (400 MHz, $CDCl_3$) for *2,2,3,3,3-pentafluoropropyl ((2S,3R)-2-phenyltetrahydro-2H-pyran-3-yl)sulfamate* (**6**)

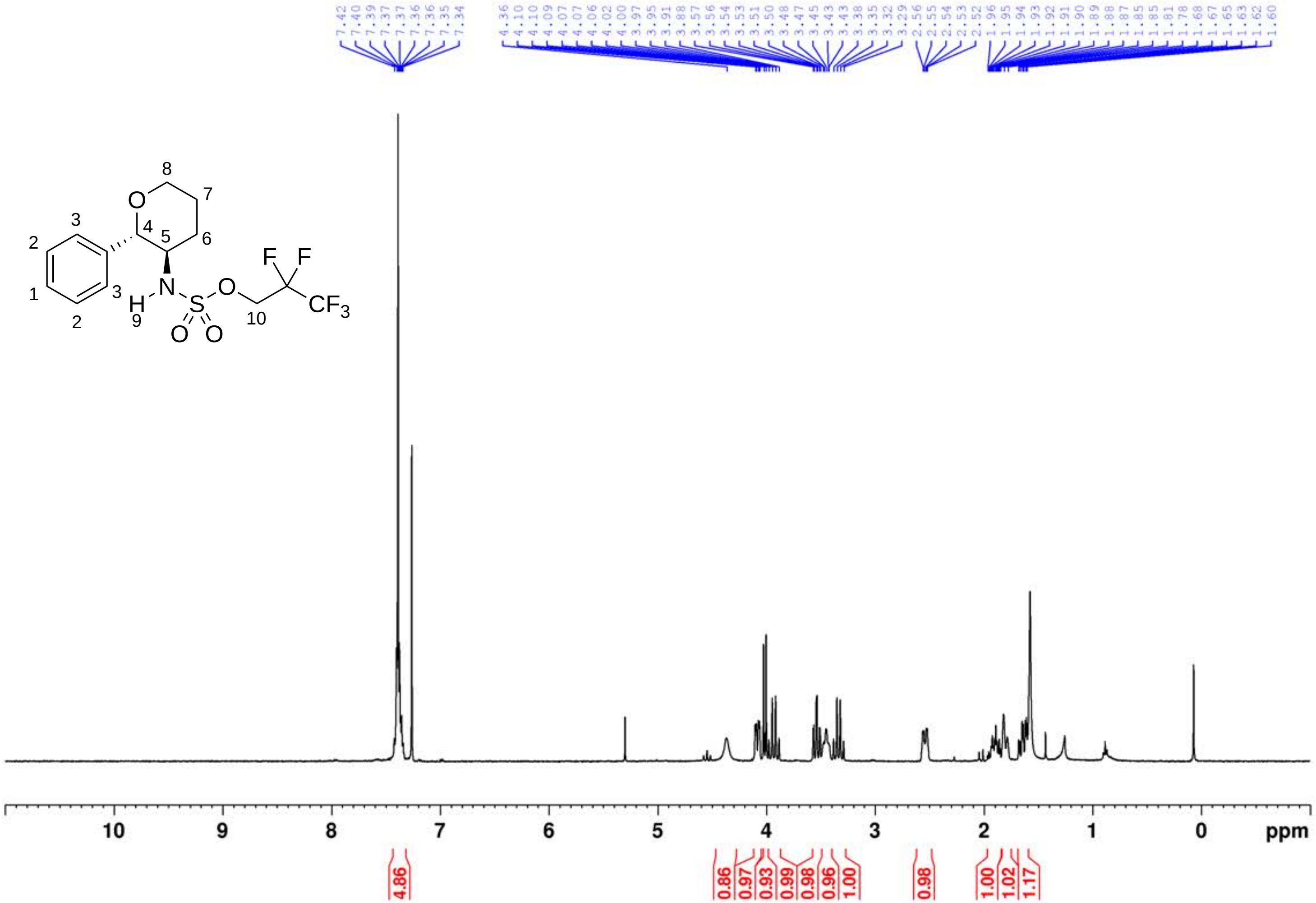

^{13}C NMR (101 MHz, $CDCl_3$) for *2,2,3,3,3-pentafluoropropyl ((2S,3R)-2-phenyltetrahydro-2H-pyran-3-yl)sulfamate* (**6**)

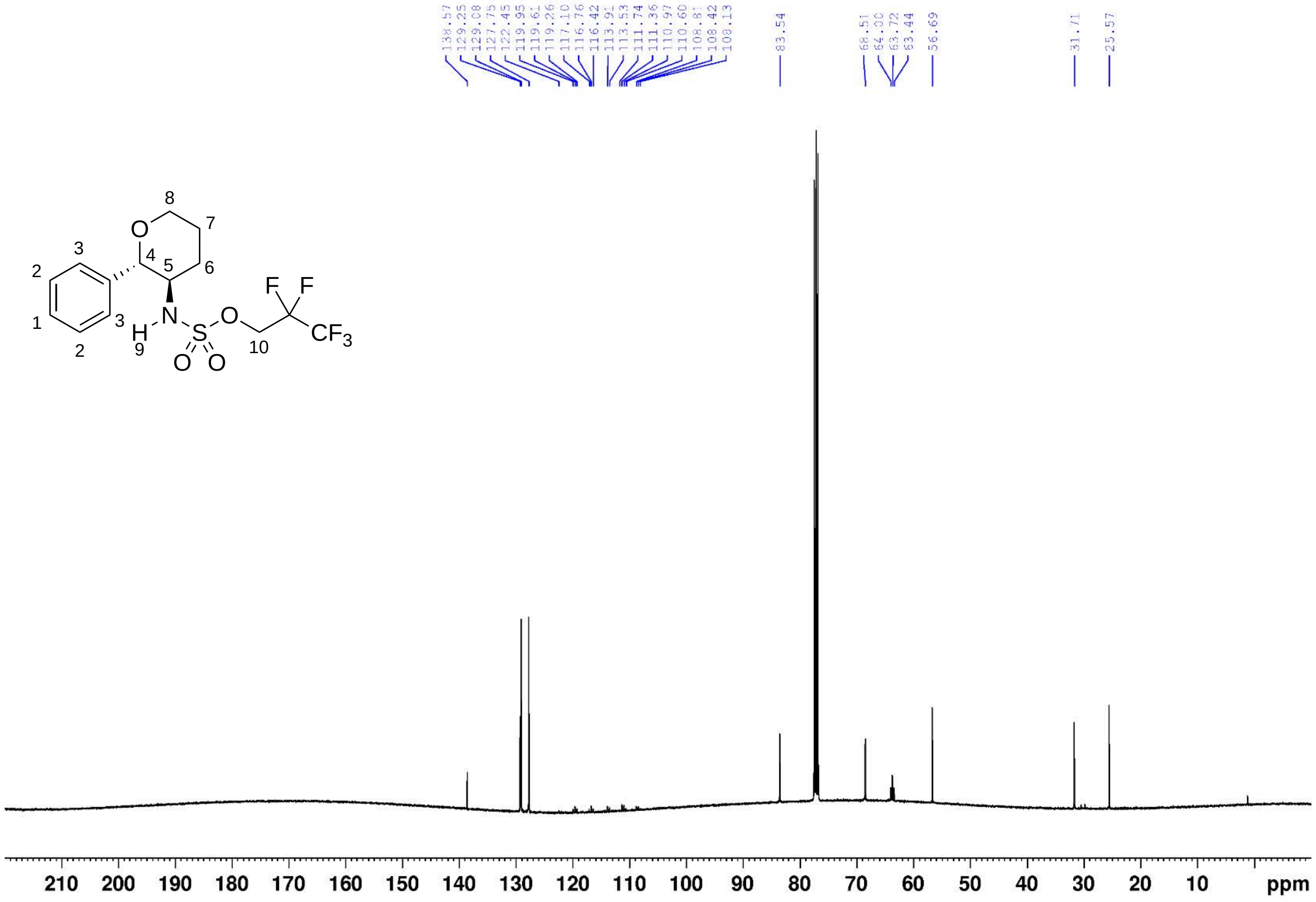

^{19}F NMR (376 MHz, $CDCl_3$) for *2,2,3,3,3-pentafluoropropyl ((2S,3R)-2-phenyltetrahydro-2H-pyran-3-yl)sulfamate* (**6**)

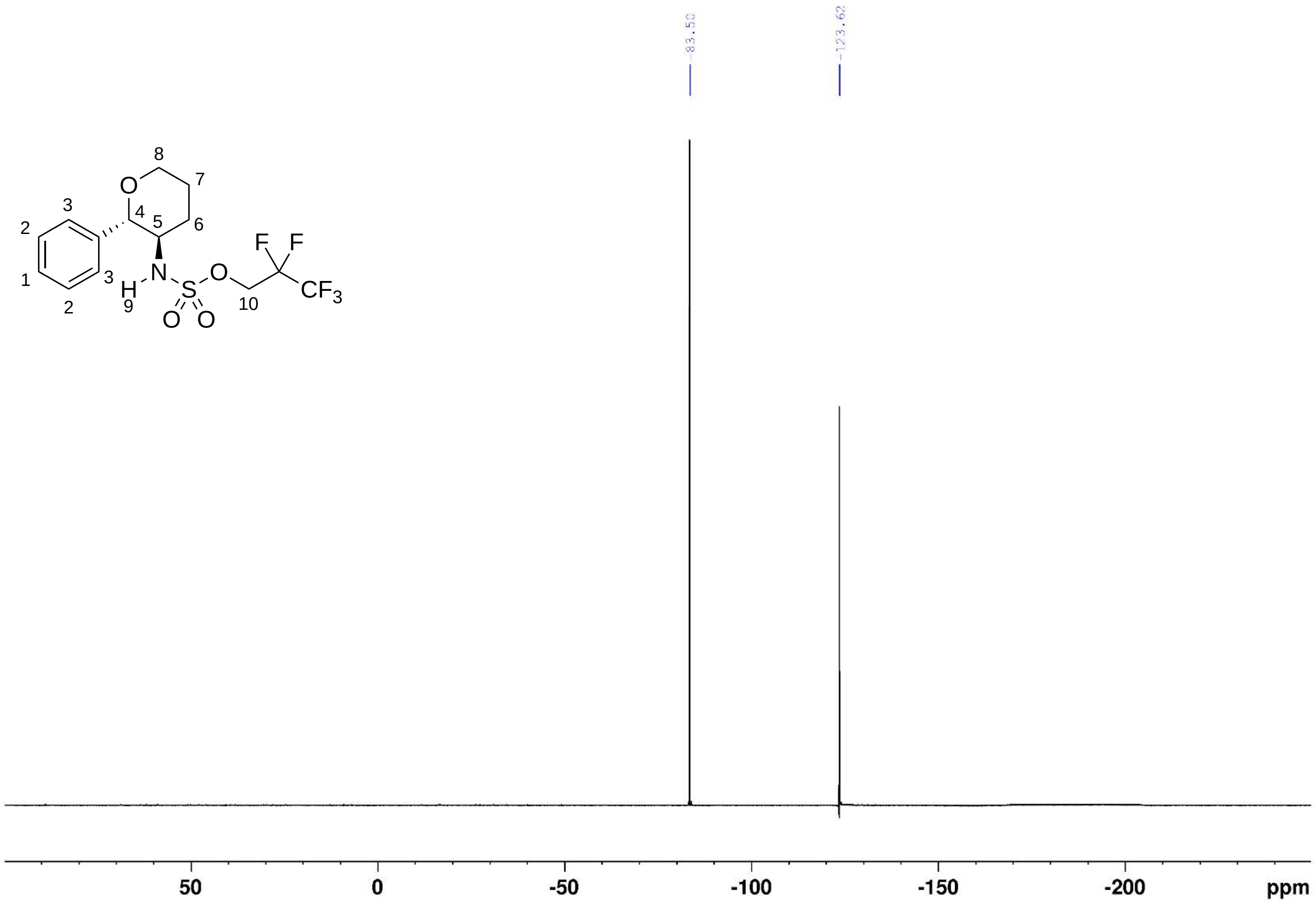

^{1}H NMR (400 MHz, $CDCl_3$) for *2,2,2-trichloroethyl ((S)-phenyl((S)-tetrahydrofuran-2-yl)methyl)sulfamate* (**7**)

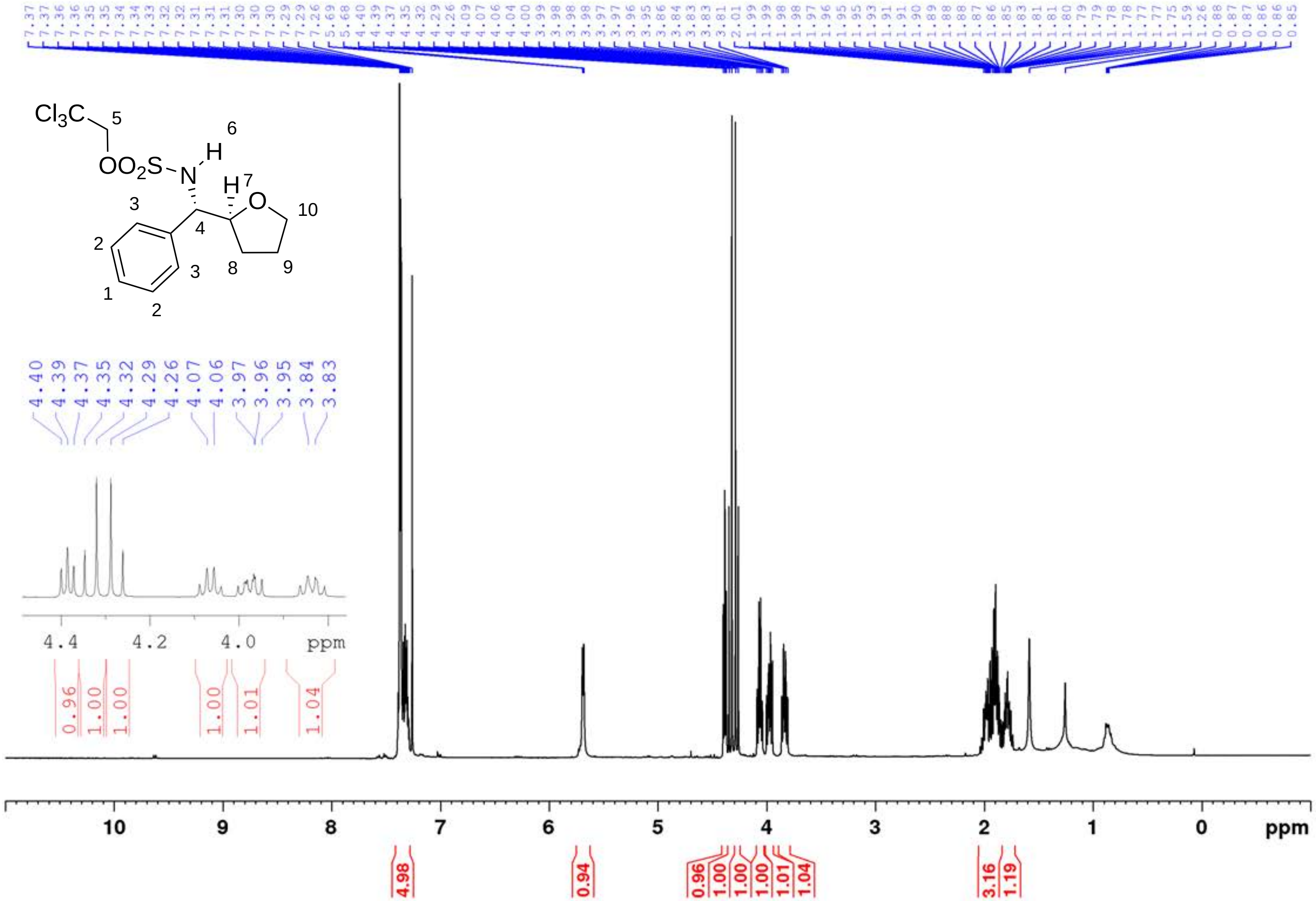

^{13}C NMR (101 MHz, $CDCl_3$) for *2,2,2-trichloroethyl ((S)-phenyl((S)-tetrahydrofuran-2-yl)methyl)sulfamate* (**7**)

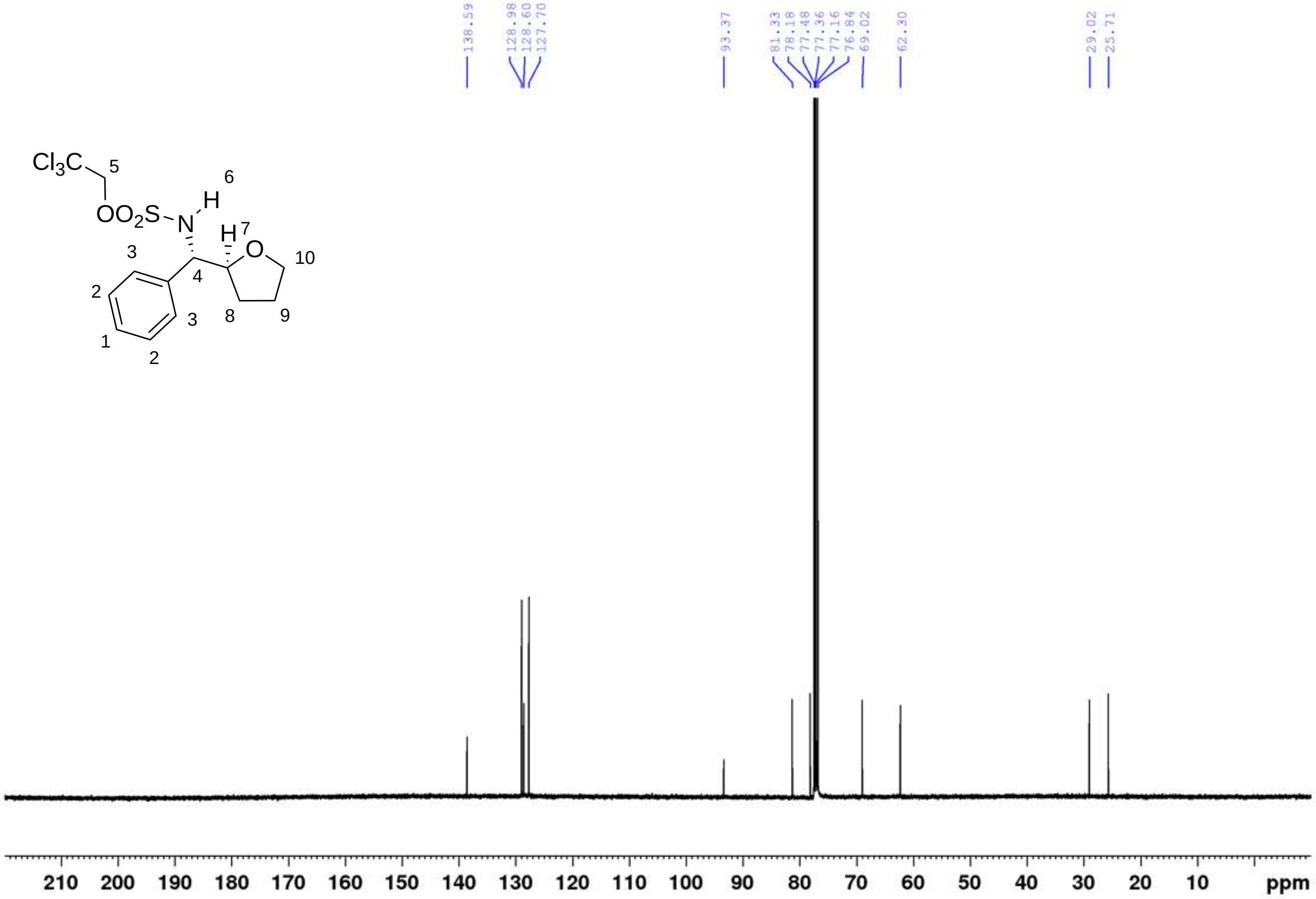

^{1}H NMR (500 MHz, $CDCl_3$) for *2,2,3,3,3-pentafluoropropyl (2R,3S)-2-(2-hydroxyethyl)-3-phenylaziridine-1-sulfonate* (**9a**)

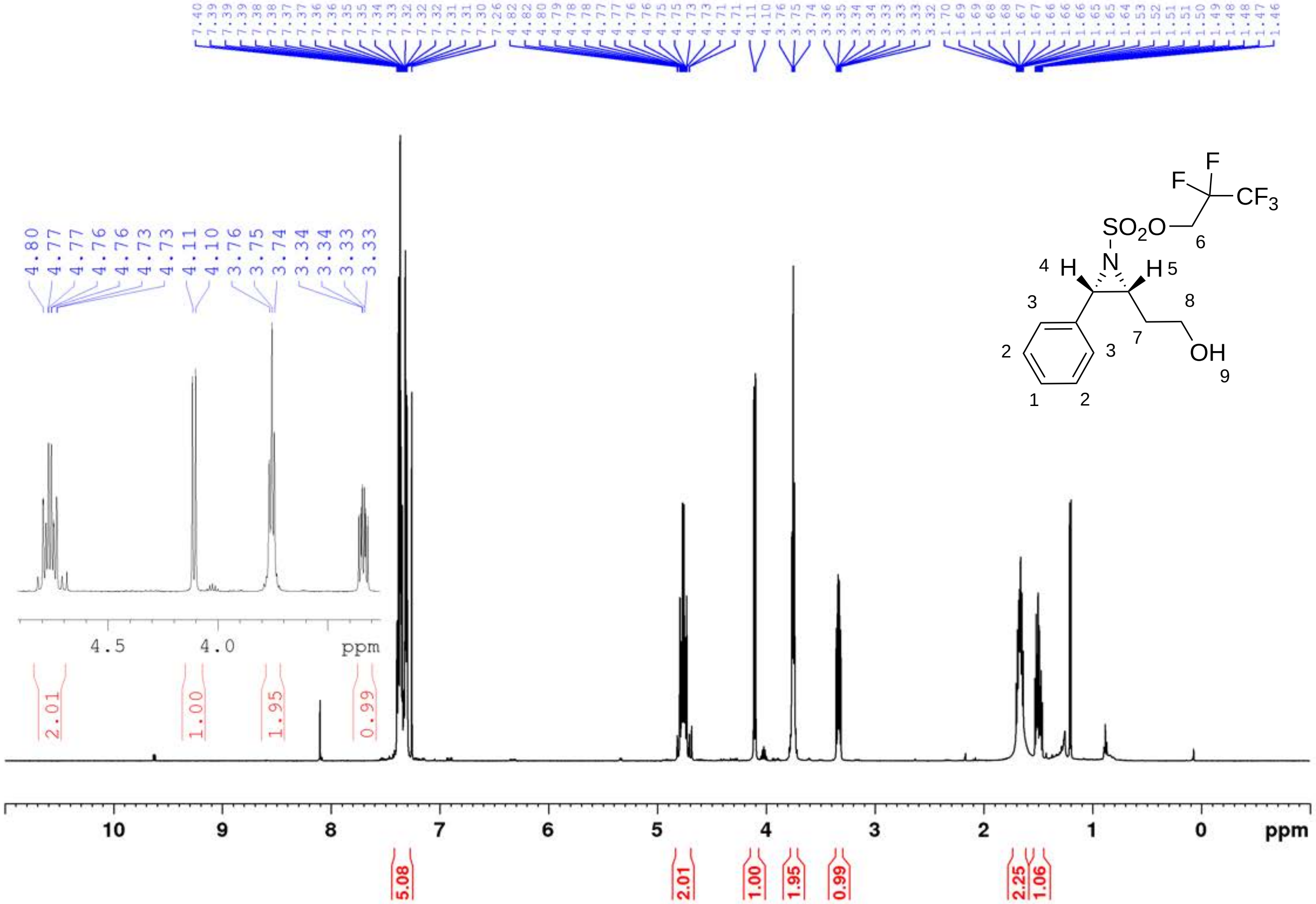

^{13}C NMR (126 MHz, $CDCl_3$) for *2,2,3,3,3-pentafluoropropyl (2R,3S)-2-(2-hydroxyethyl)-3-phenylaziridine-1-sulfonate* (**9a**)

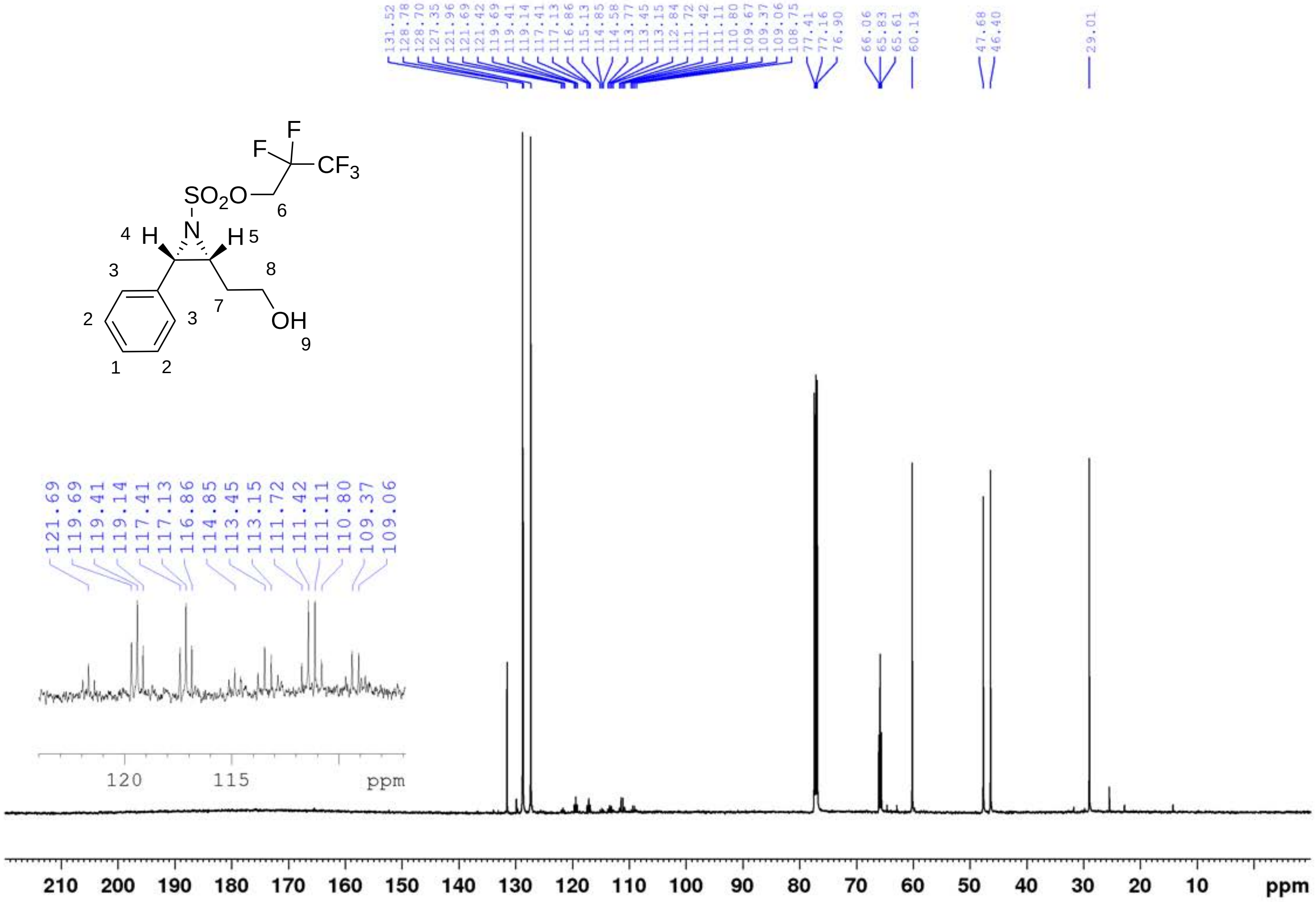

^{19}F NMR (376 MHz, $CDCl_3$) for *2,2,3,3,3-pentafluoropropyl (2R,3S)-2-(2-hydroxyethyl)-3-phenylaziridine-1-sulfonate* (**9a**)

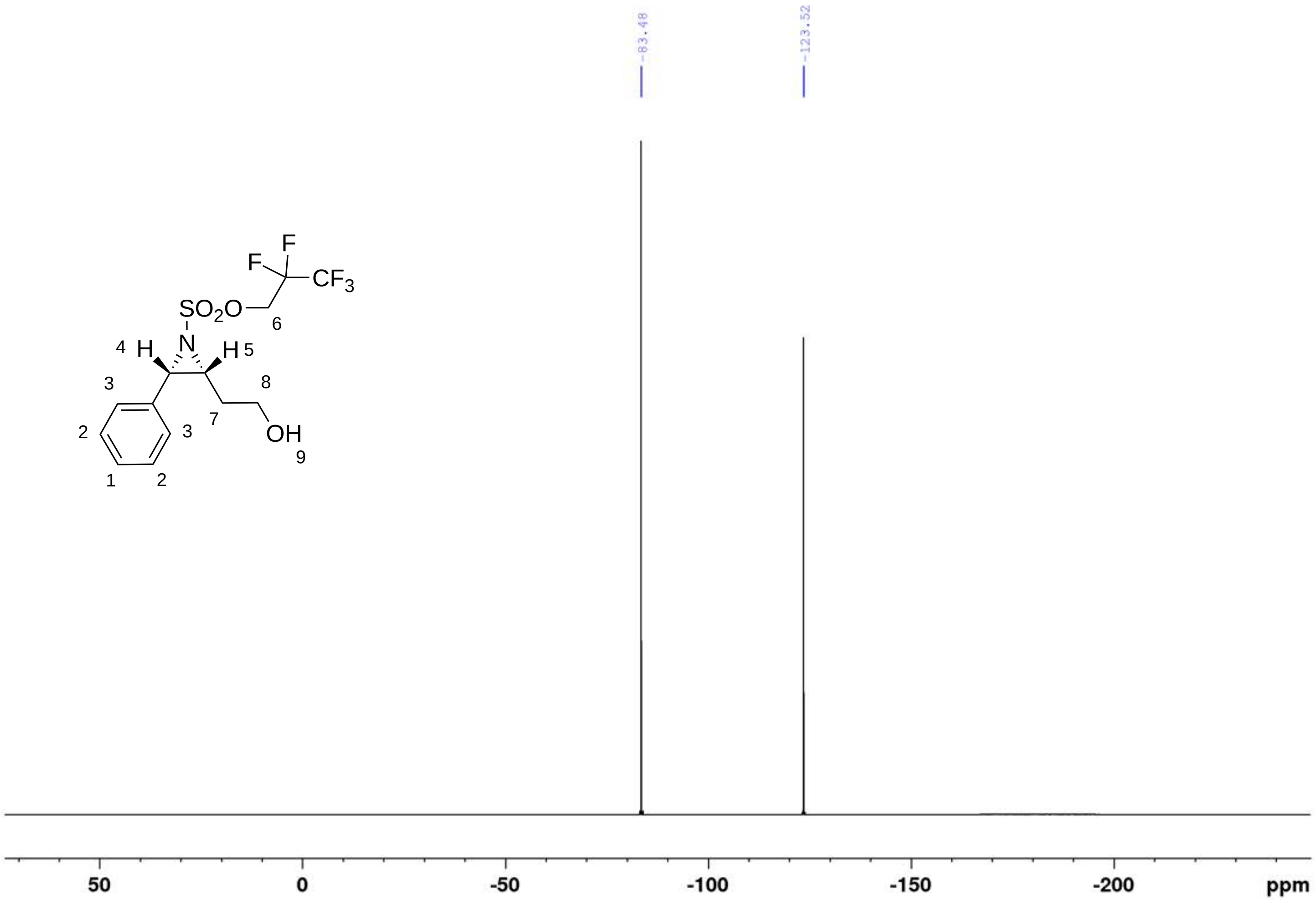

^{1}H NMR (500 MHz, $CDCl_3$) for *2,2,3,3,3-pentafluoropropyl (2R,3S)-2-(2-hydroxyethyl)-3-(o-tolyl)aziridine-1-sulfonate* (**9b**)

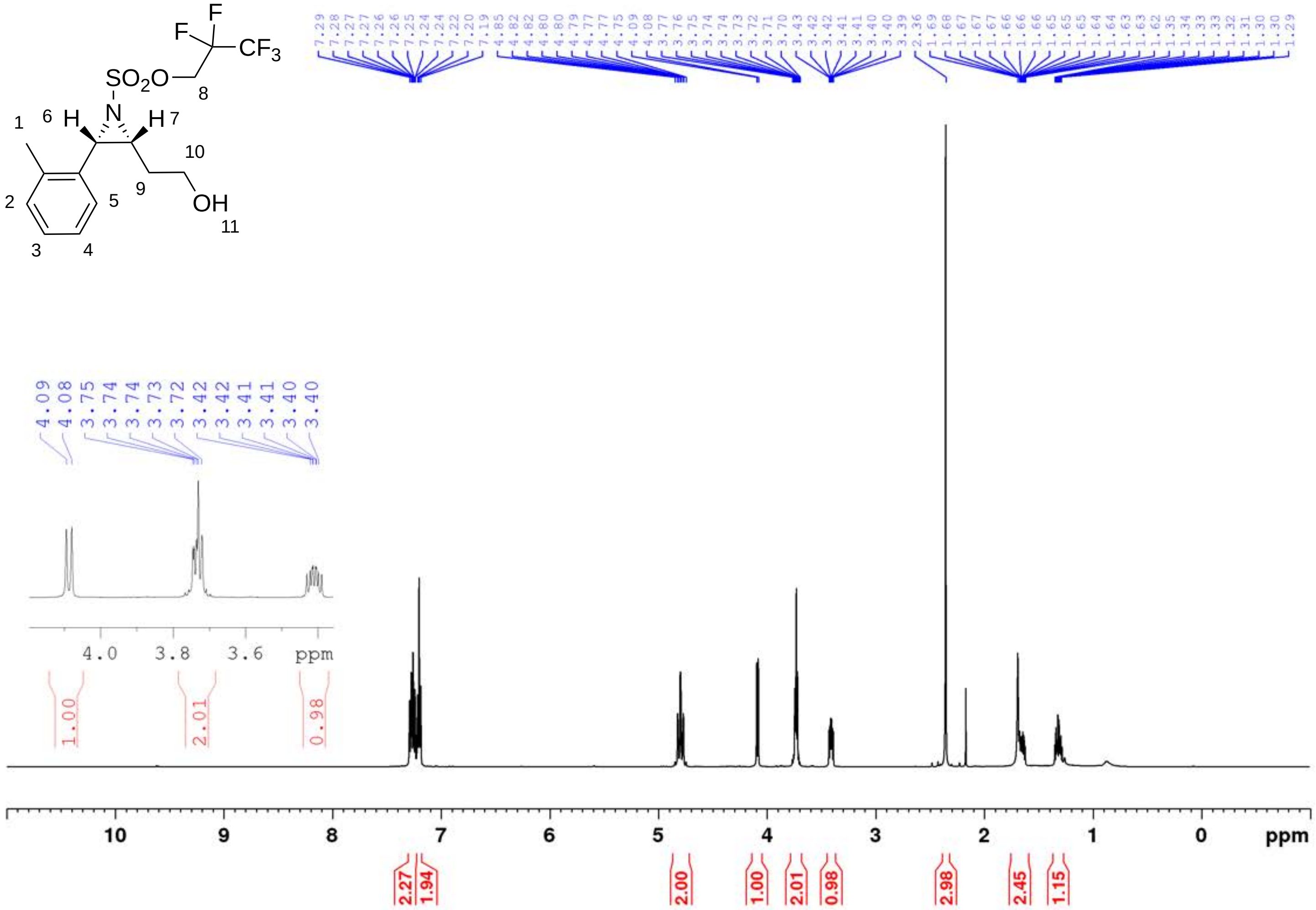

^{13}C NMR (126 MHz, $CDCl_3$) for *2,2,3,3,3-pentafluoropropyl (2R,3S)-2-(2-hydroxyethyl)-3-(o-tolyl)aziridine-1-sulfonate* (**9b**)

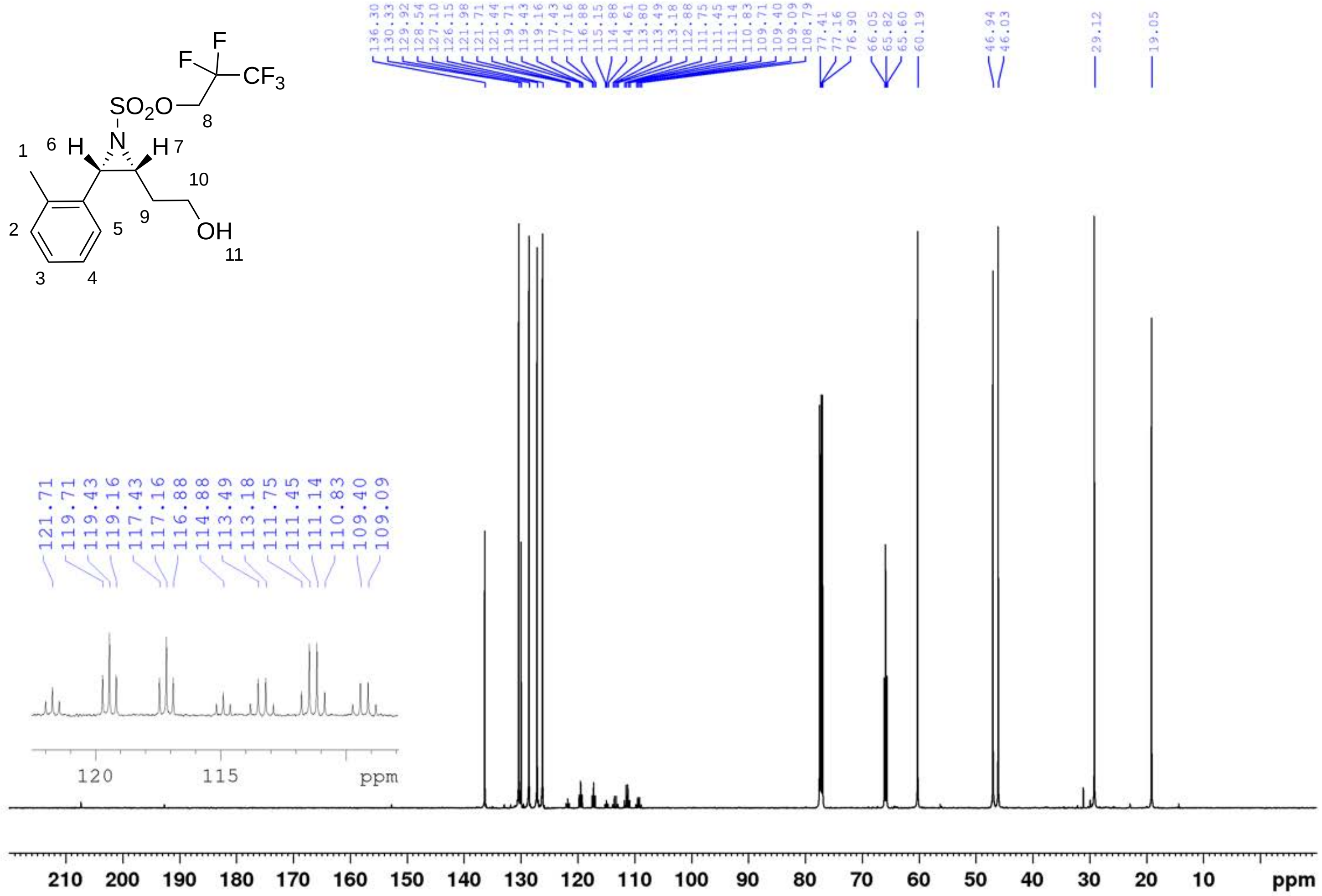

^{19}F NMR (376 MHz, $CDCl_3$) for *2,2,3,3,3-pentafluoropropyl (2R,3S)-2-(2-hydroxyethyl)-3-(o-tolyl)aziridine-1-sulfonate* (**9b**)

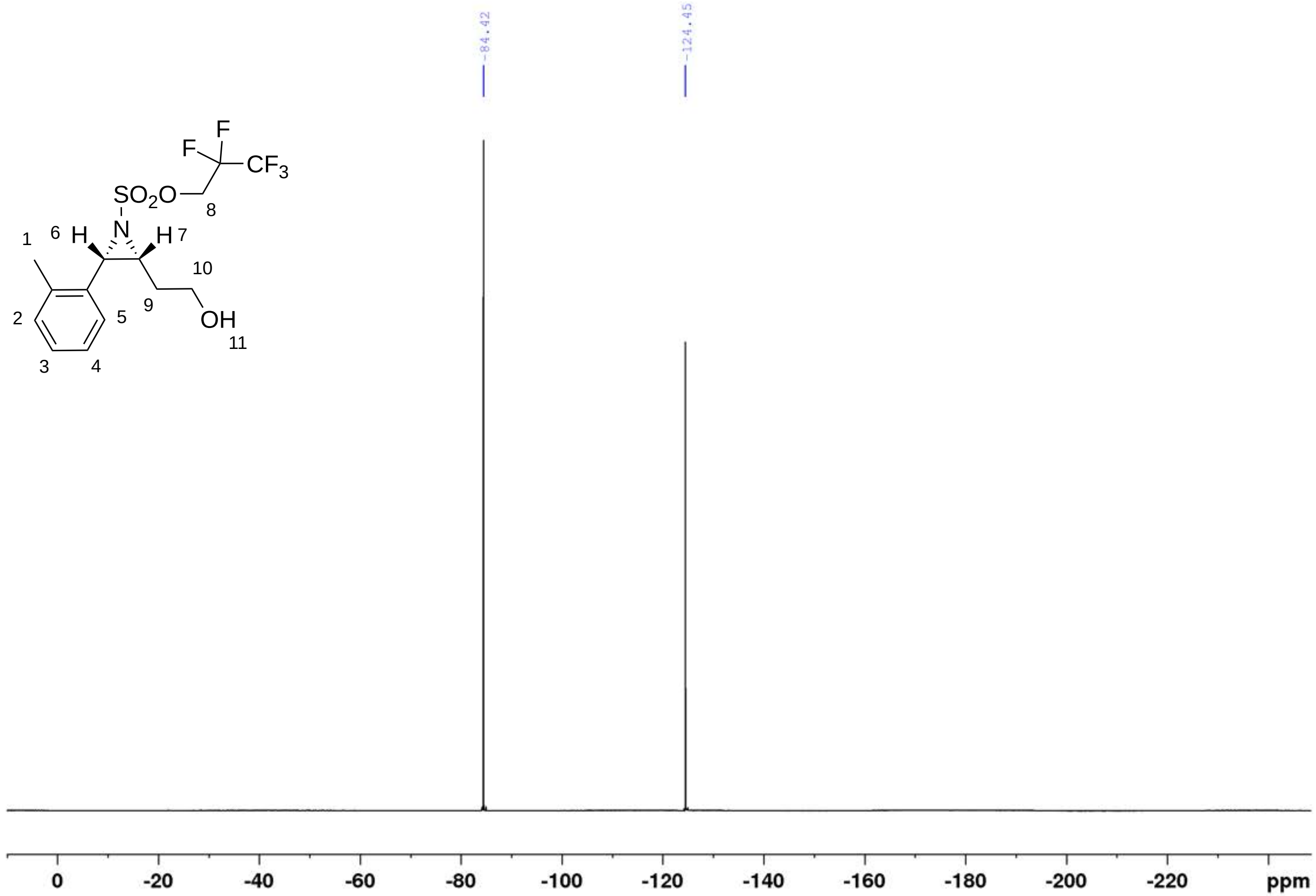

^{1}H NMR (500 MHz, $CDCl_3$) for *2,2,3,3,3-pentafluoropropyl (2R,3S)-2-(2-hydroxyethyl)-3-(2-methoxyphenyl)aziridine-1-sulfonate* (**9c**)

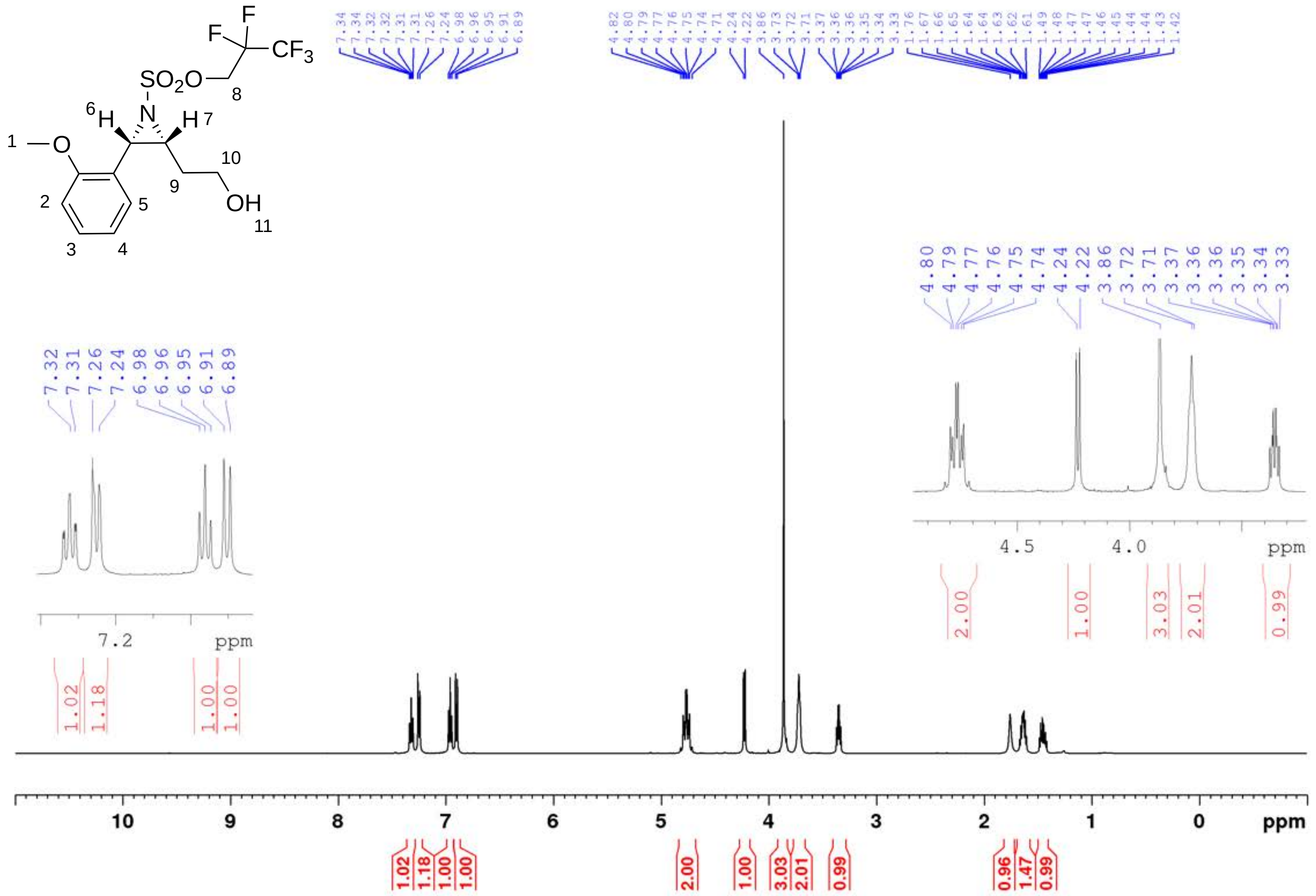

^{13}C NMR (126 MHz, $CDCl_3$) for *2,2,3,3,3-pentafluoropropyl (2R,3S)-2-(2-hydroxyethyl)-3-(2-methoxyphenyl)aziridine-1-sulfonate* (**9c**)

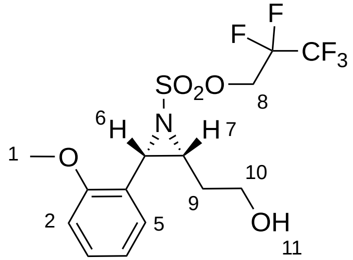

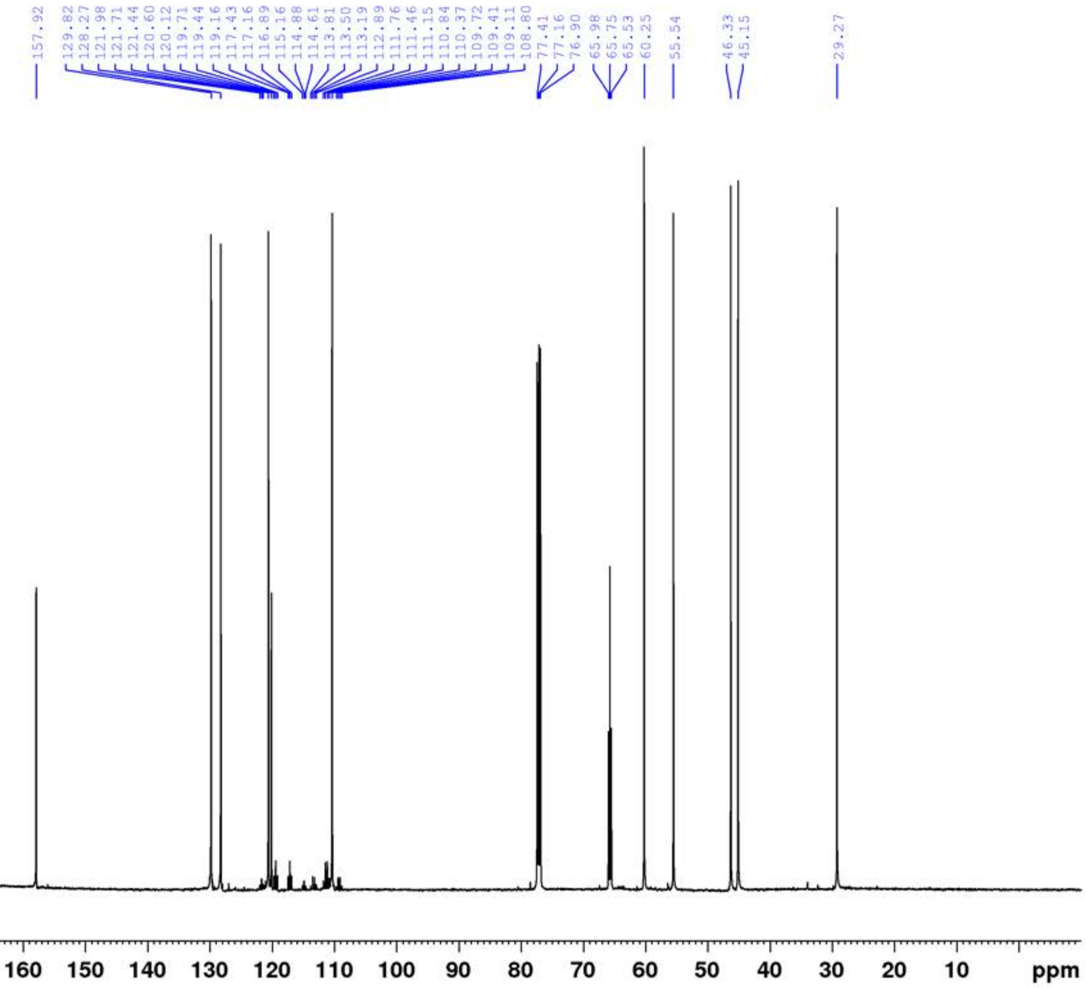

^{19}F NMR (376 MHz, $CDCl_3$) for *2,2,3,3,3-pentafluoropropyl (2R,3S)-2-(2-hydroxyethyl)-3-(2-methoxyphenyl)aziridine-1-sulfonate* (**9c**)

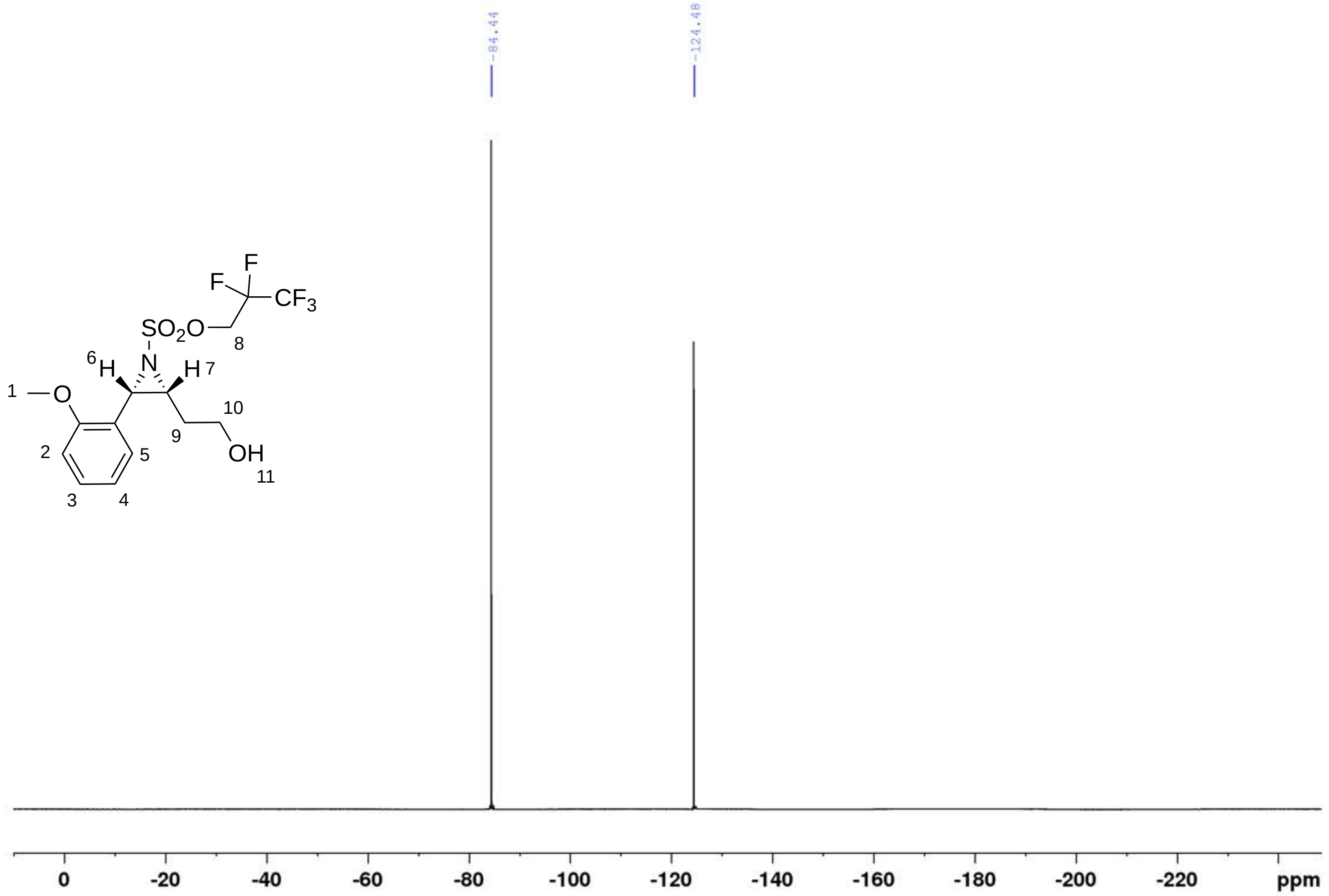

^{1}H NMR (500 MHz, $CDCl_3$) for *2,2,3,3,3-pentafluoropropyl (2S,3R)-2-(2-fluorophenyl)-3-(2-hydroxyethyl)aziridine-1-sulfonate* (**9d**)

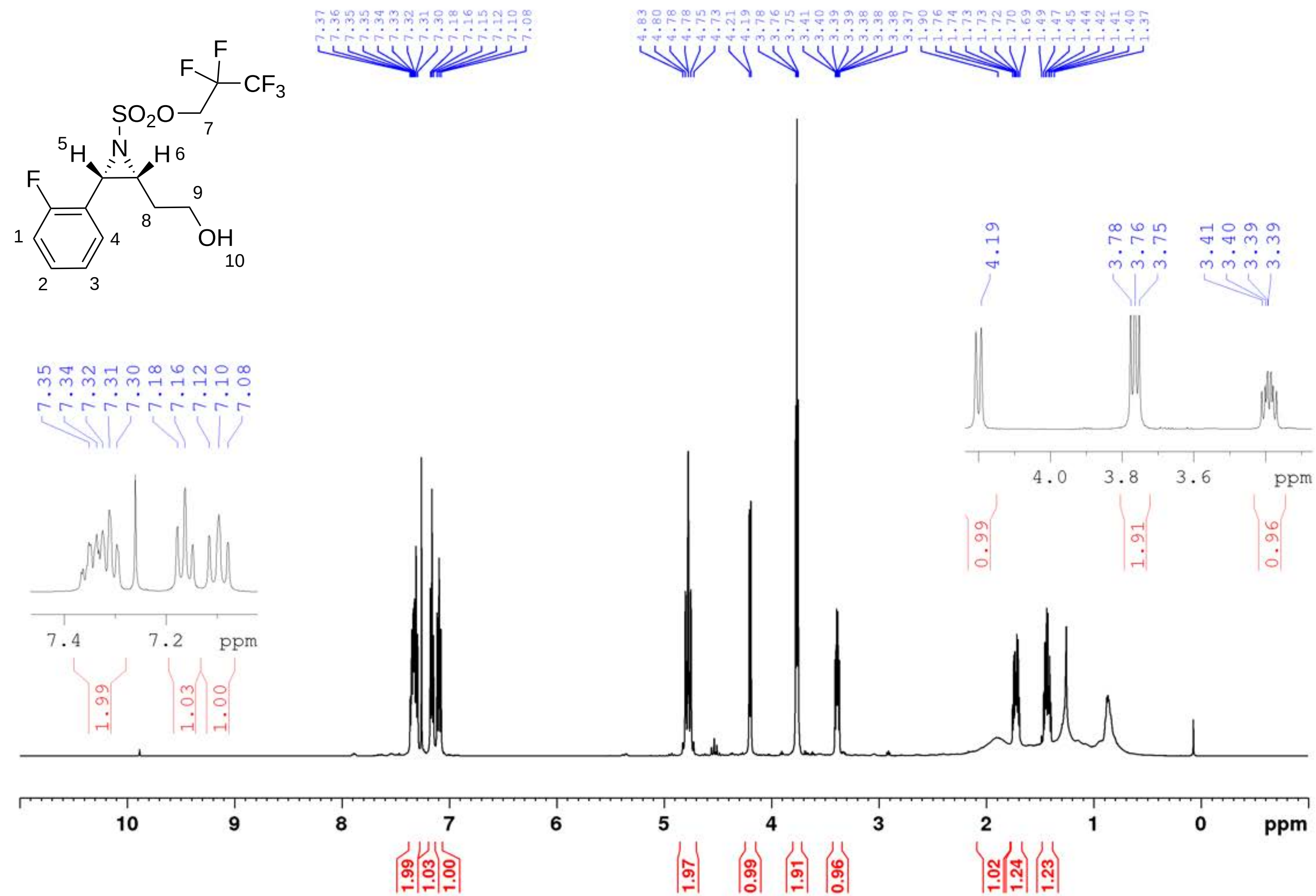

^{13}C NMR (126 MHz, $CDCl_3$) for *2,2,3,3,3-pentafluoropropyl (2S,3R)-2-(2-fluorophenyl)-3-(2-hydroxyethyl)aziridine-1-sulfonate* (**9d**)

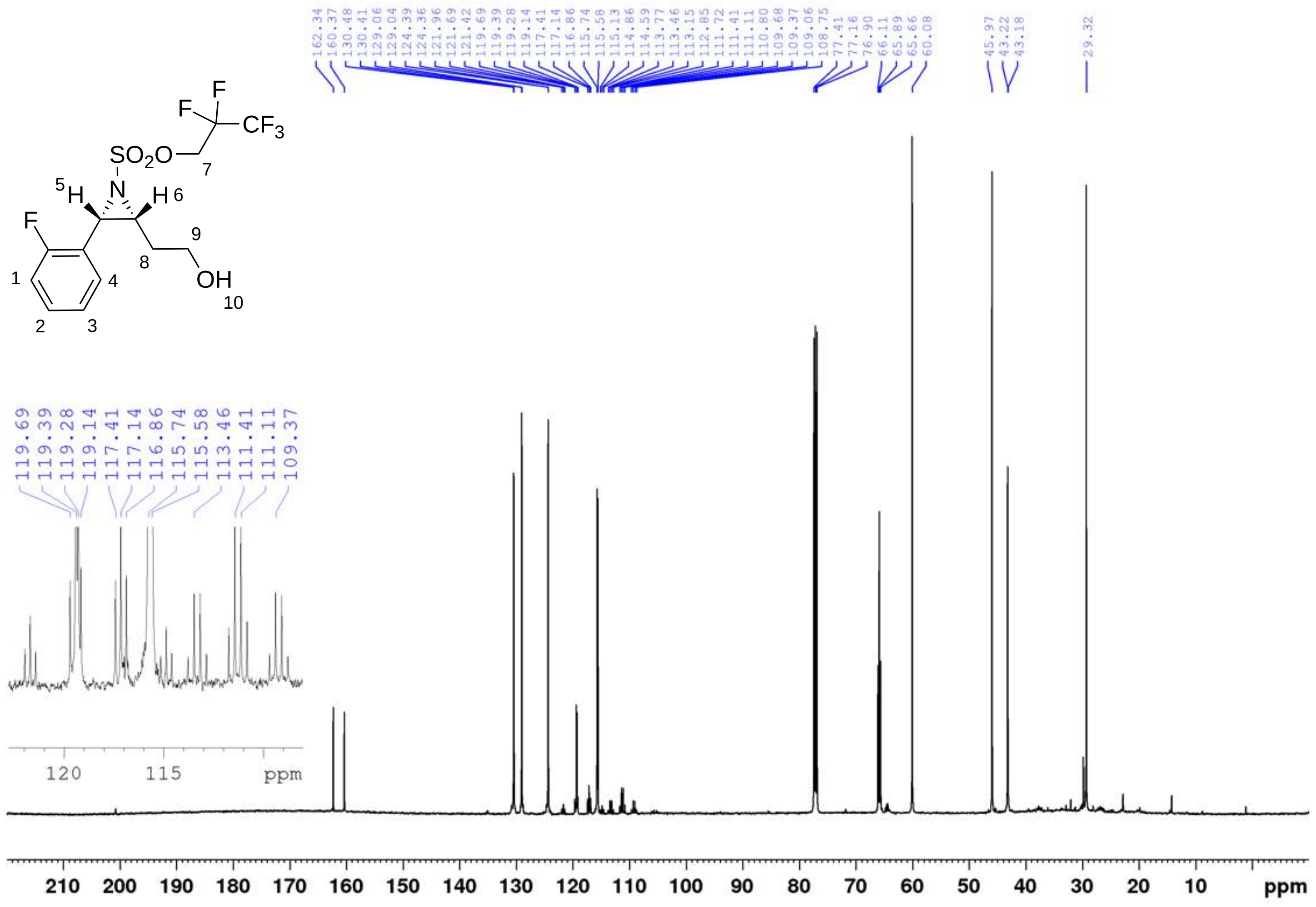

^{19}F NMR (376 MHz, $CDCl_3$) for *2,2,3,3,3-pentafluoropropyl (2S,3R)-2-(2-fluorophenyl)-3-(2-hydroxyethyl)aziridine-1-sulfonate* (**9d**)

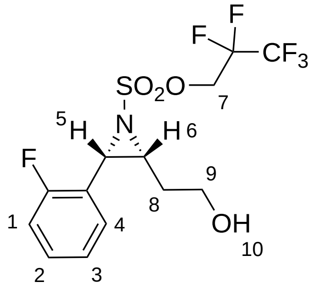

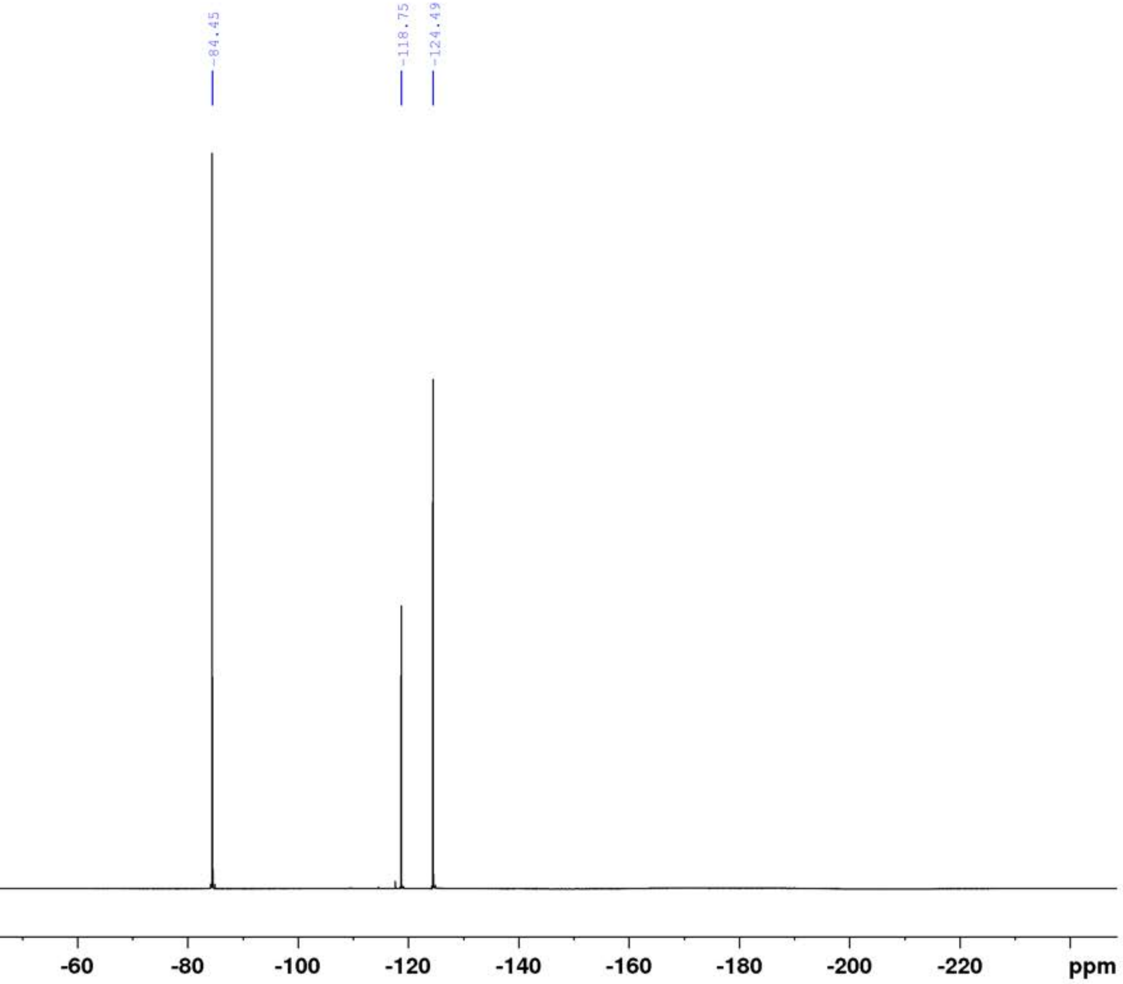

^{1}H NMR (500 MHz, $CDCl_3$) for *2,2,3,3,3-pentafluoropropyl (2S,3R)-2-(3-chlorophenyl)-3-(2-hydroxyethyl)aziridine-1-sulfonate* (**9e**)

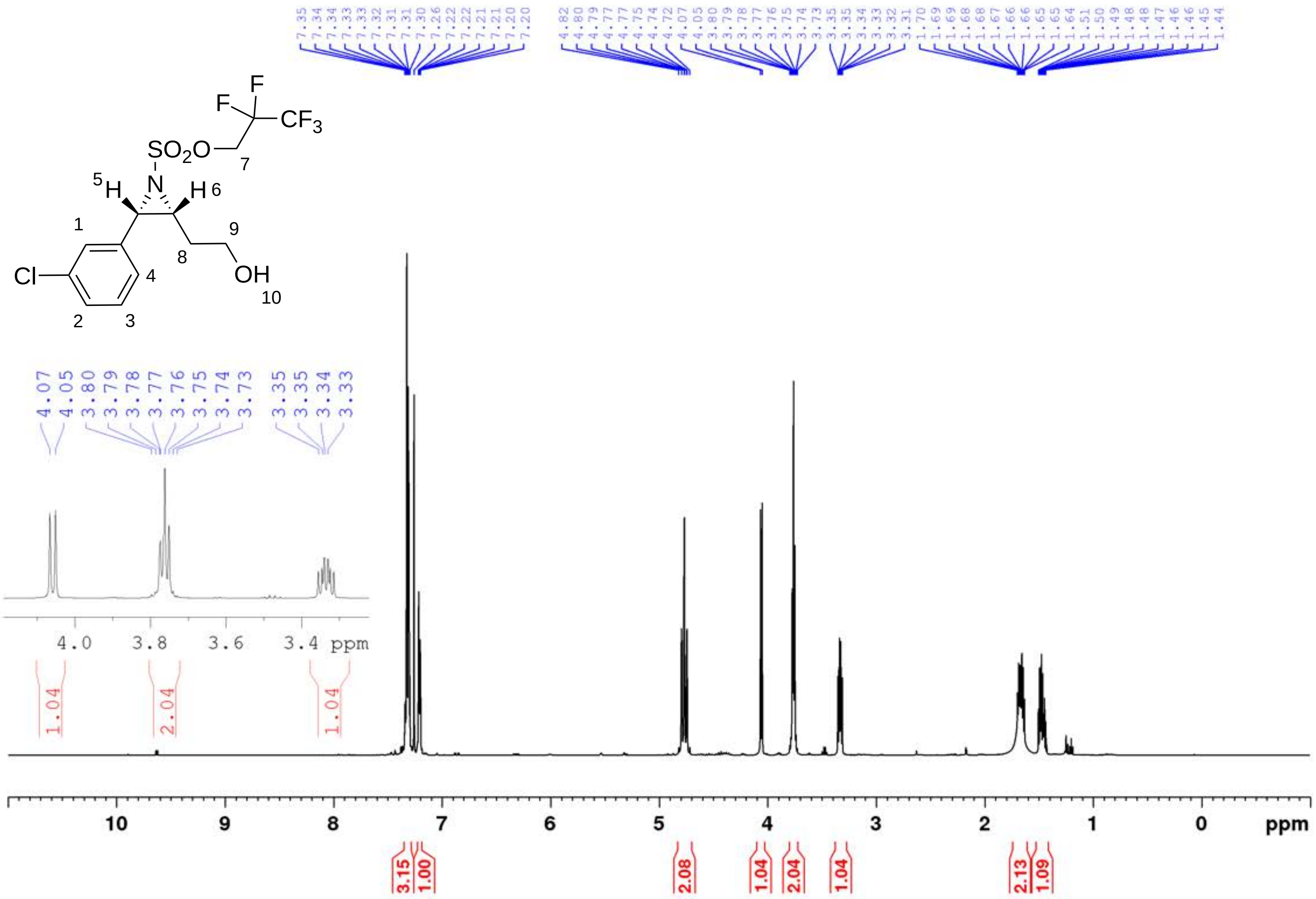

^{13}C NMR (126 MHz, $CDCl_3$) for *2,2,3,3,3-pentafluoropropyl (2S,3R)-2-(3-chlorophenyl)-3-(2-hydroxyethyl)aziridine-1-sulfonate* (**9e**)

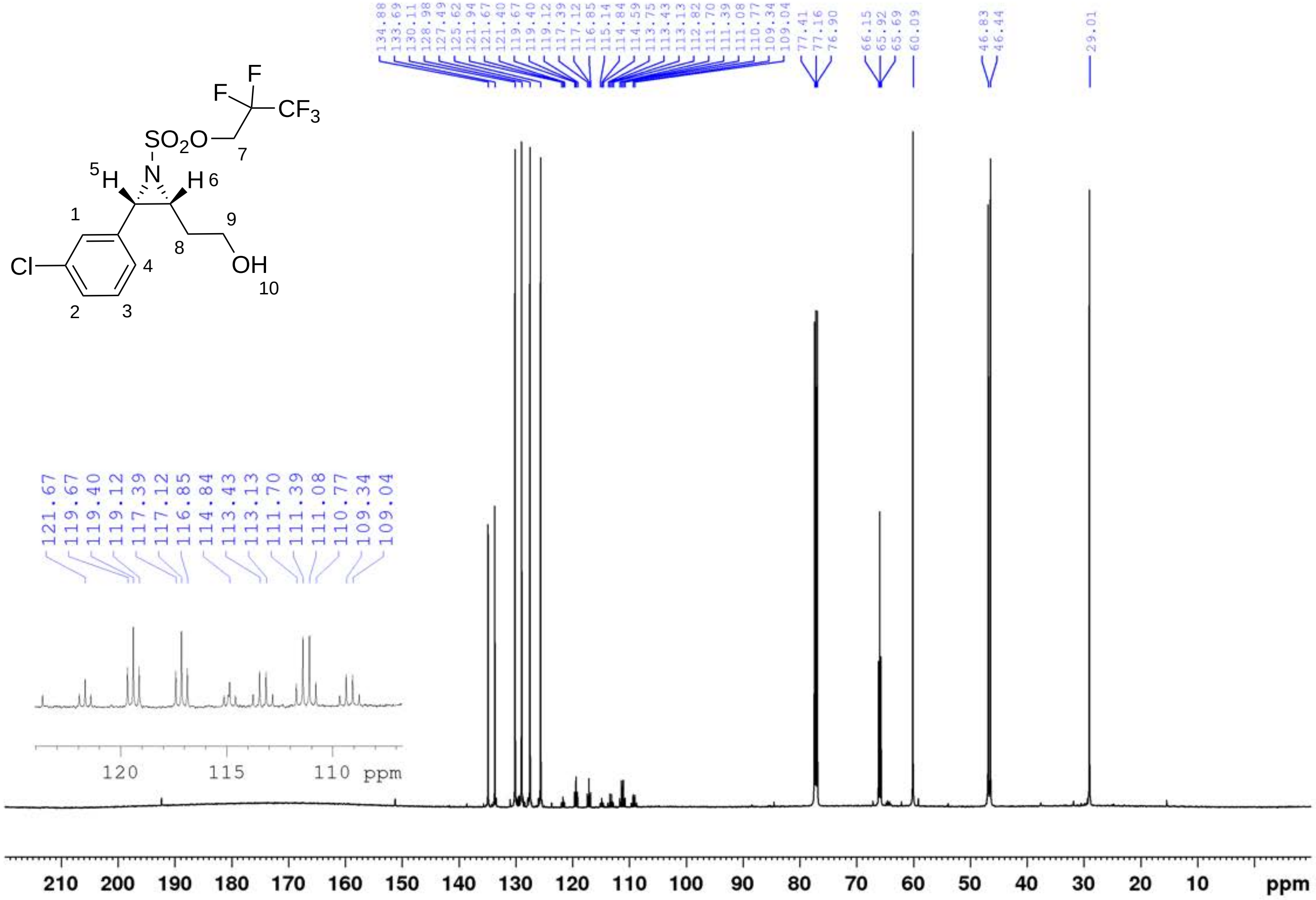

^{19}F NMR (376 MHz, $CDCl_3$) for *2,2,3,3,3-pentafluoropropyl (2S,3R)-2-(3-chlorophenyl)-3-(2-hydroxyethyl)aziridine-1-sulfonate* (**9e**)

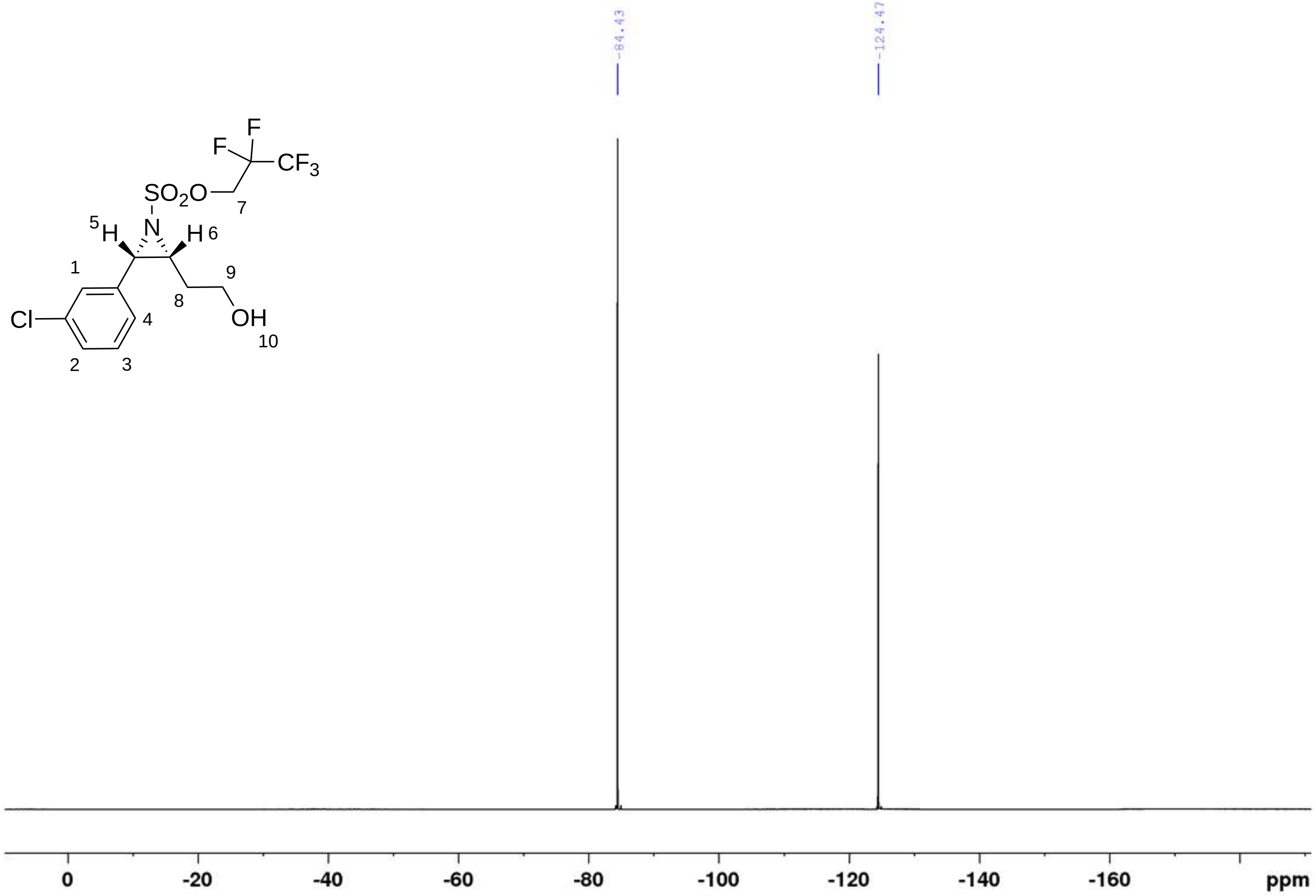

^{1}H NMR (500 MHz, $CDCl_3$) for *2,2,3,3,3-pentafluoropropyl (2R,3S)-2-(2-hydroxyethyl)-3-(3-(trifluoromethoxy)phenyl)aziridine-1-sulfonate* (**9f**)

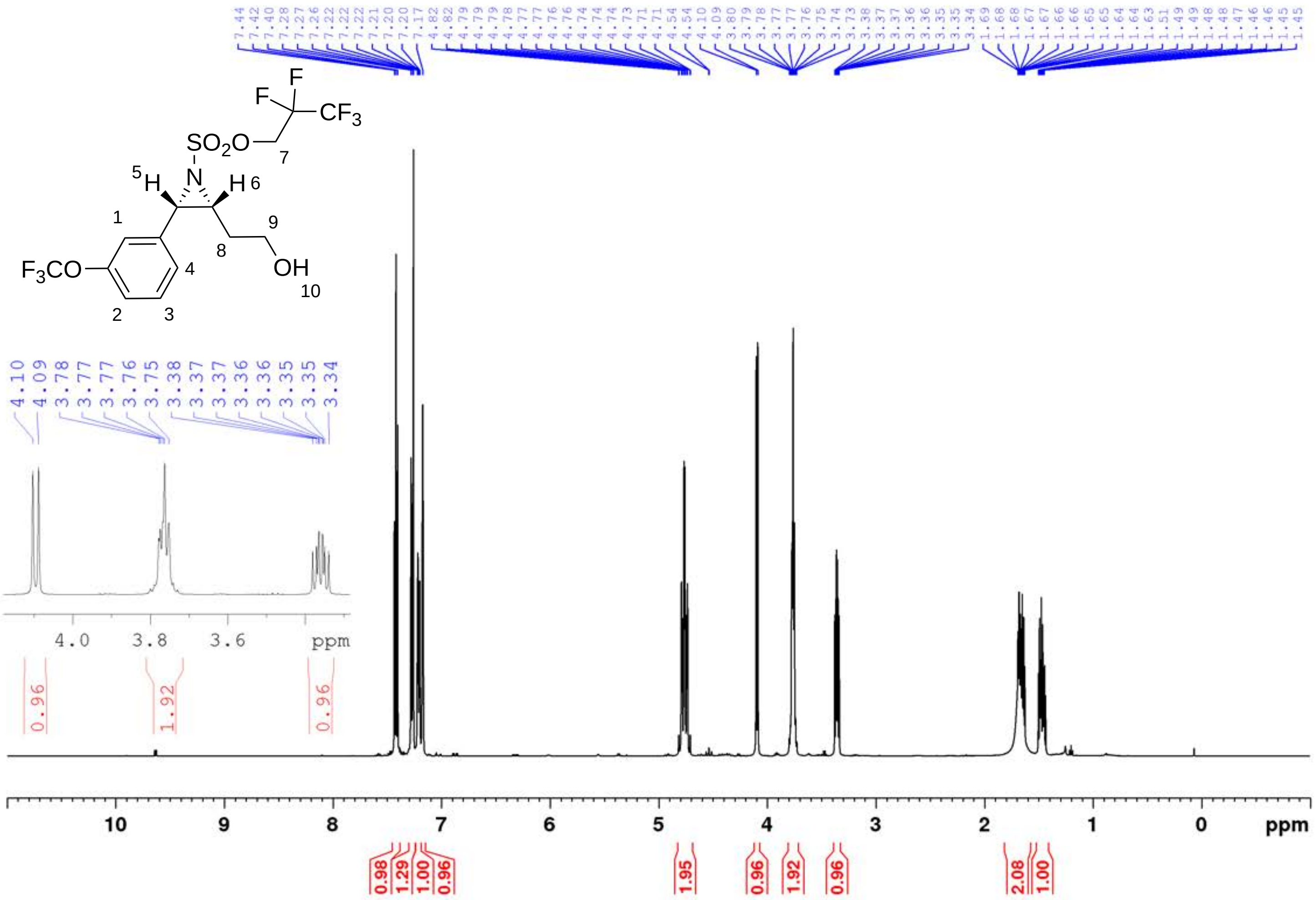

^{13}C NMR (126 MHz, $CDCl_3$) for *2,2,3,3,3-pentafluoropropyl (2R,3S)-2-(2-hydroxyethyl)-3-(3-(trifluoromethoxy)phenyl)aziridine-1-sulfonate* (**9f**)

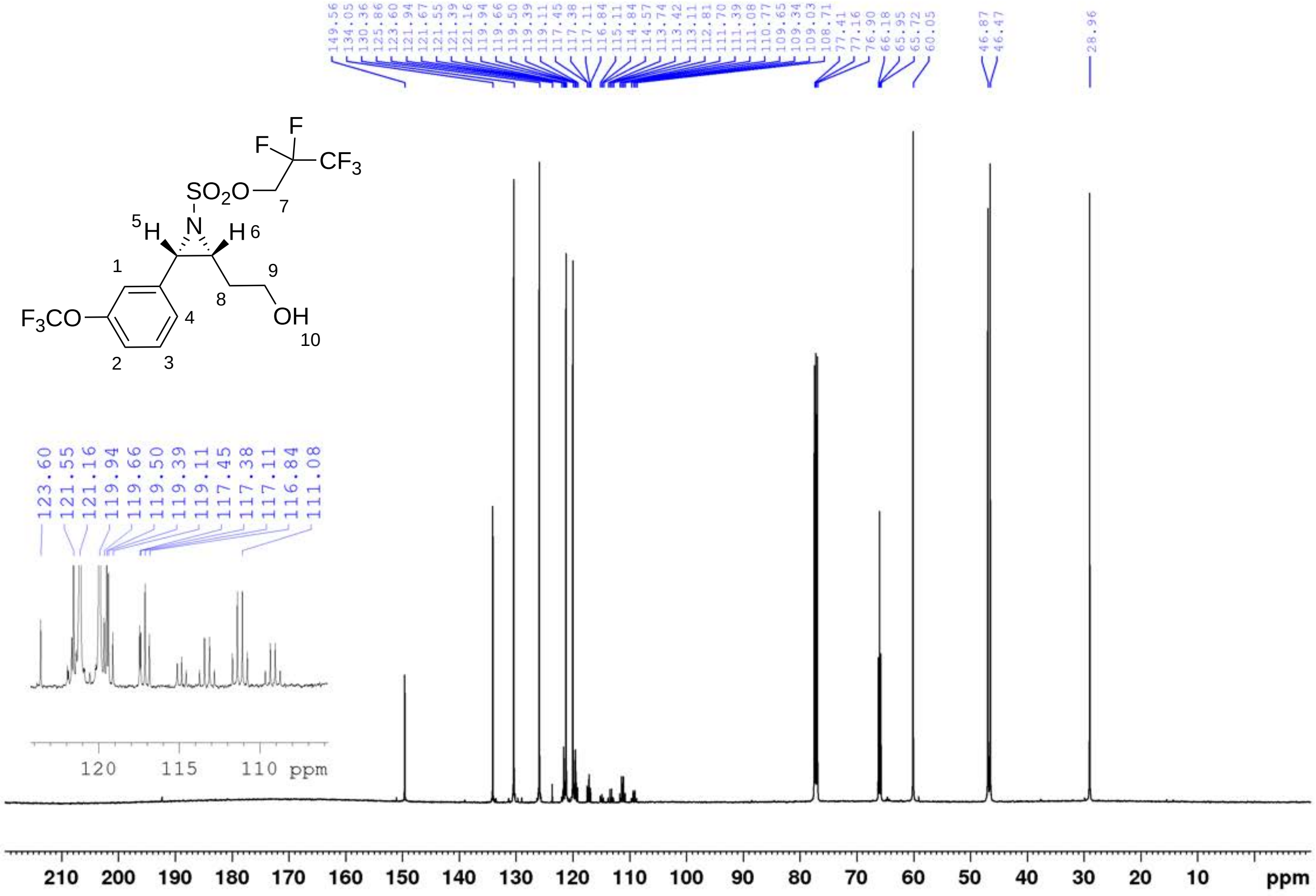

^{19}F NMR (376 MHz, $CDCl_3$) for *2,2,3,3,3-pentafluoropropyl (2R,3S)-2-(2-hydroxyethyl)-3-(3-(trifluoromethoxy)phenyl)aziridine-1-sulfonate* (**9f**)

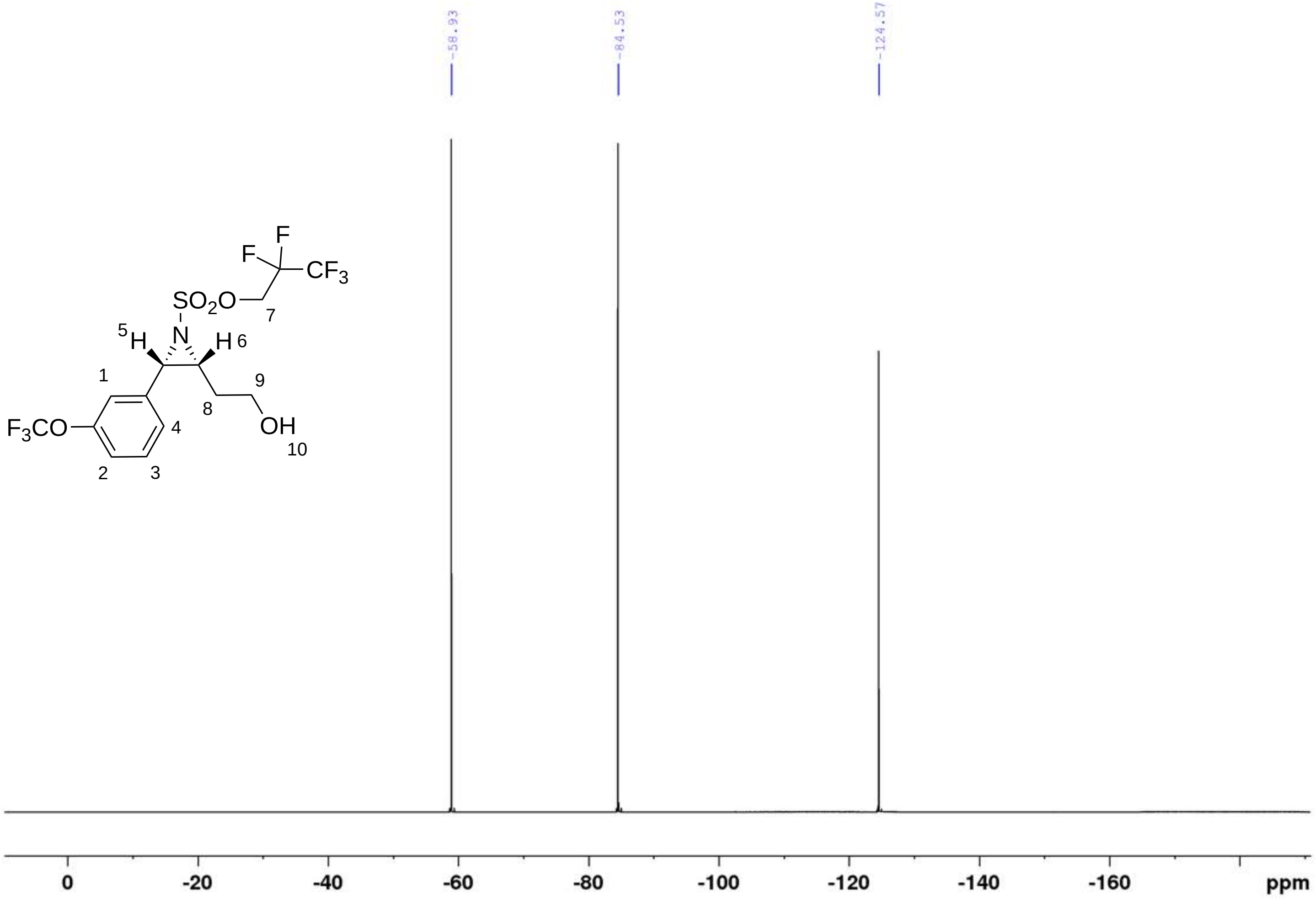

^{1}H NMR (500 MHz, $CDCl_3$) for *Ethyl 3-((2S,3R)-3-(2-hydroxyethyl)-1-((2,2,3,3,3-pentafluoropropoxy)sulfonyl)aziridin-2-yl)benzoate* (**9g**)

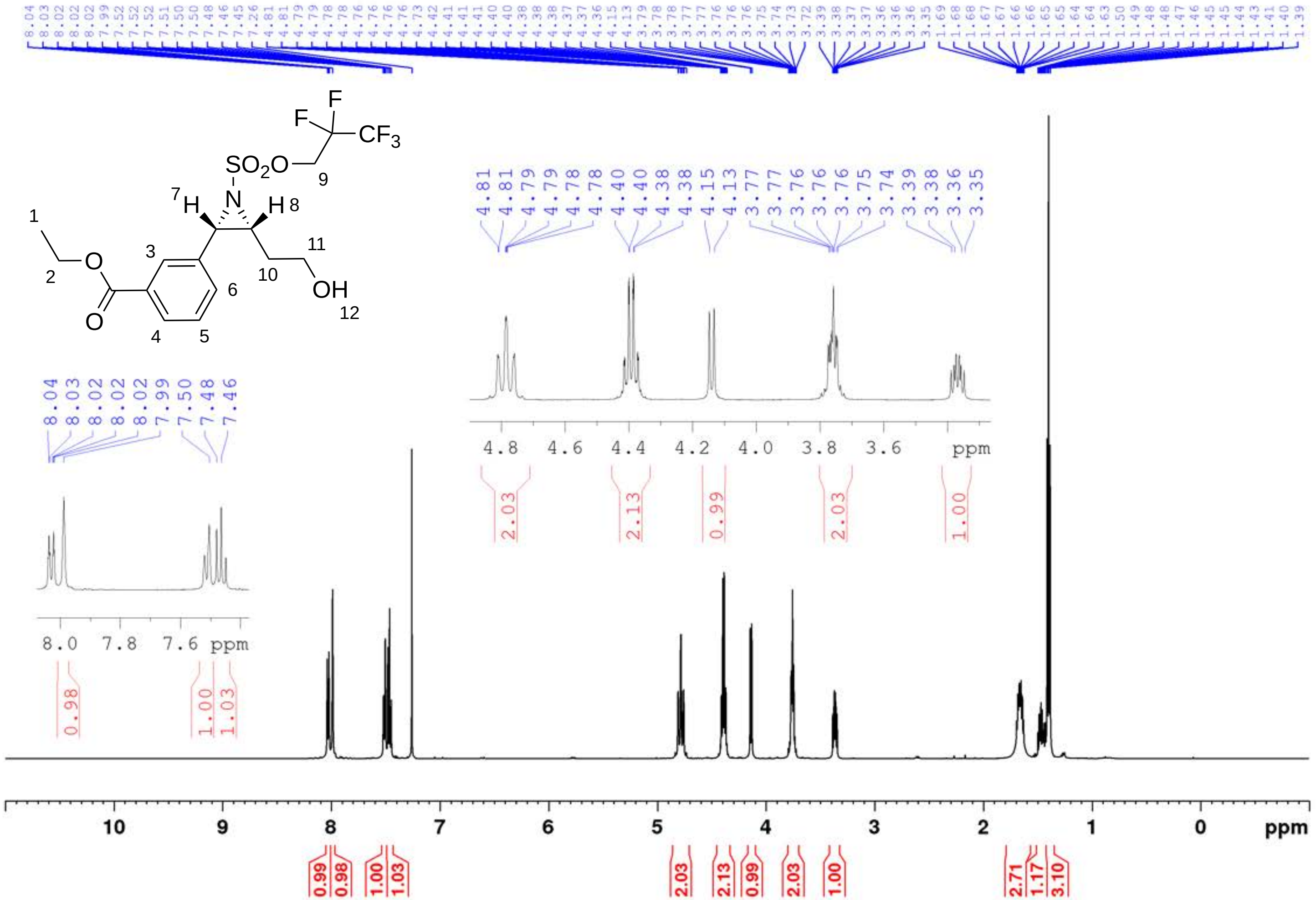

^{13}C NMR (126 MHz, $CDCl_3$) for *Ethyl 3-((2S,3R)-3-(2-hydroxyethyl)-1-((2,2,3,3,3-pentafluoropropoxy)sulfonyl)aziridin-2-yl)benzoate* (**9g**)

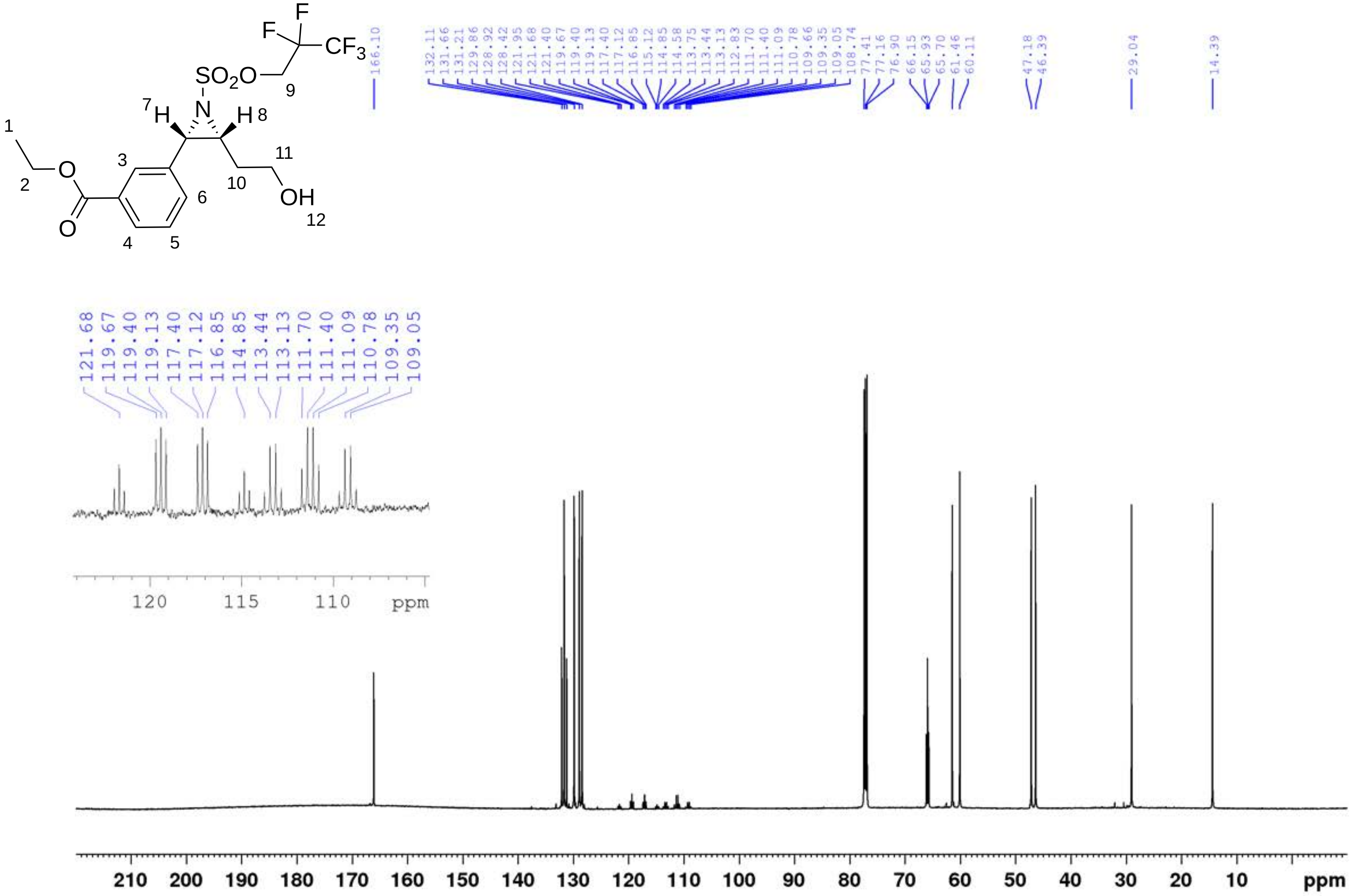

^{19}F NMR (376 MHz, $CDCl_3$) for *Ethyl 3-((2S,3R)-3-(2-hydroxyethyl)-1-((2,2,3,3,3-pentafluoropropoxy)sulfonyl)aziridin-2-yl)benzoate* (**9g**)

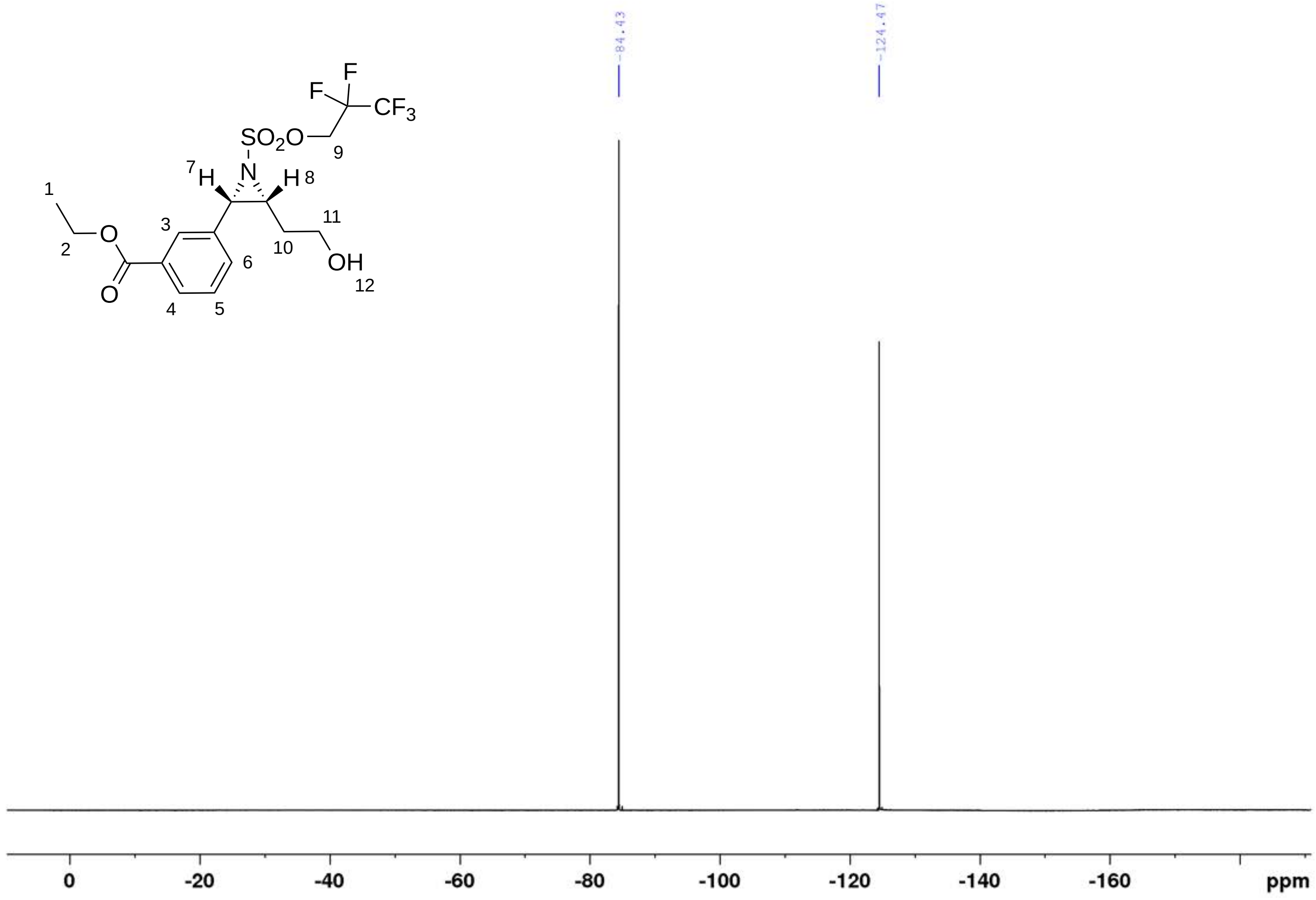

¹H NMR (500 MHz, $CDCl_3$) for *2,2,3,3,3-pentafluoropropyl (2S,3R)-2-(4-chlorophenyl)-3-(2-hydroxyethyl)aziridine-1-sulfonate* (**9h**)

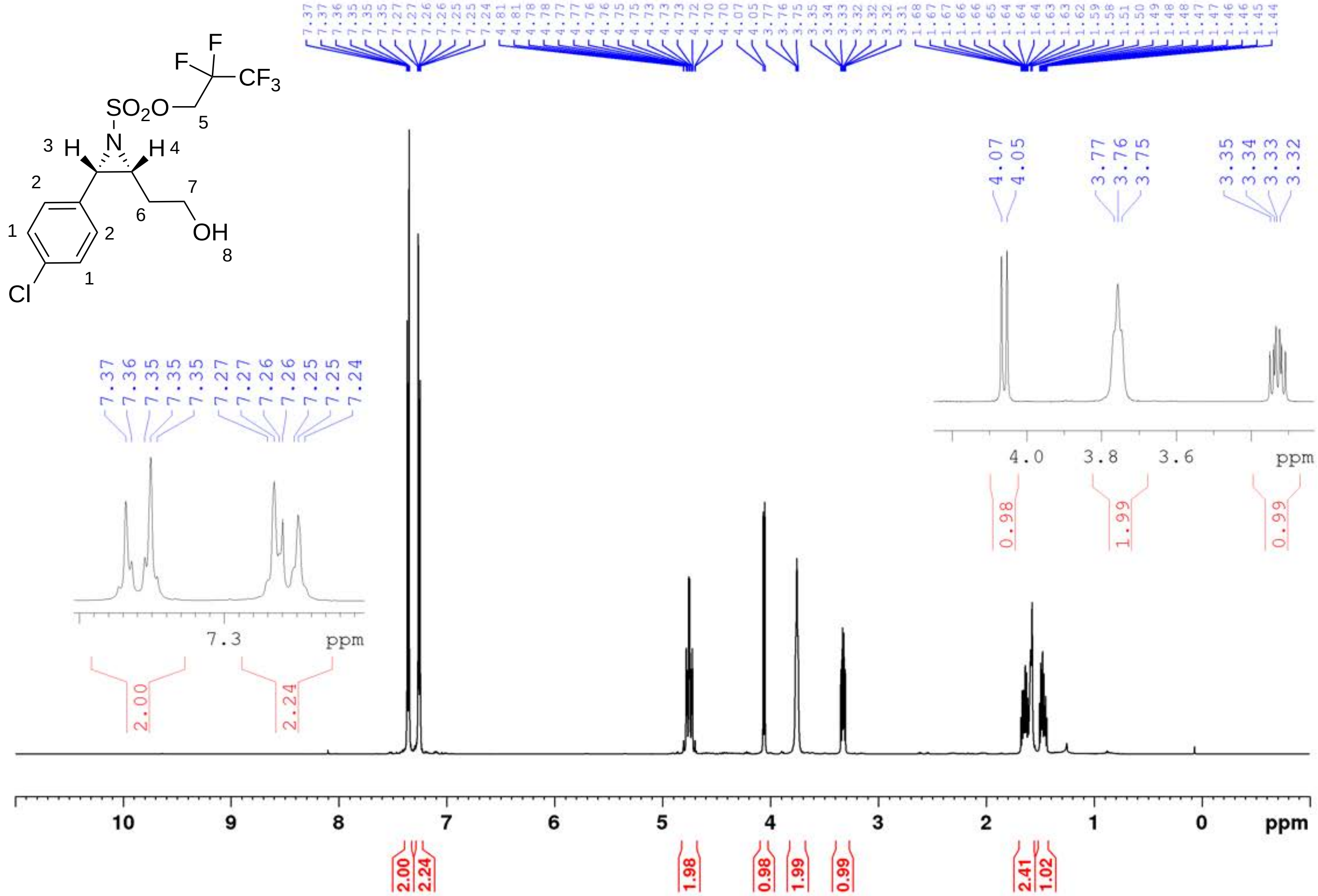

^{13}C NMR (126 MHz, $CDCl_3$) for *2,2,3,3,3-pentafluoropropyl (2S,3R)-2-(4-chlorophenyl)-3-(2-hydroxyethyl)aziridine-1-sulfonate* (**9h**)

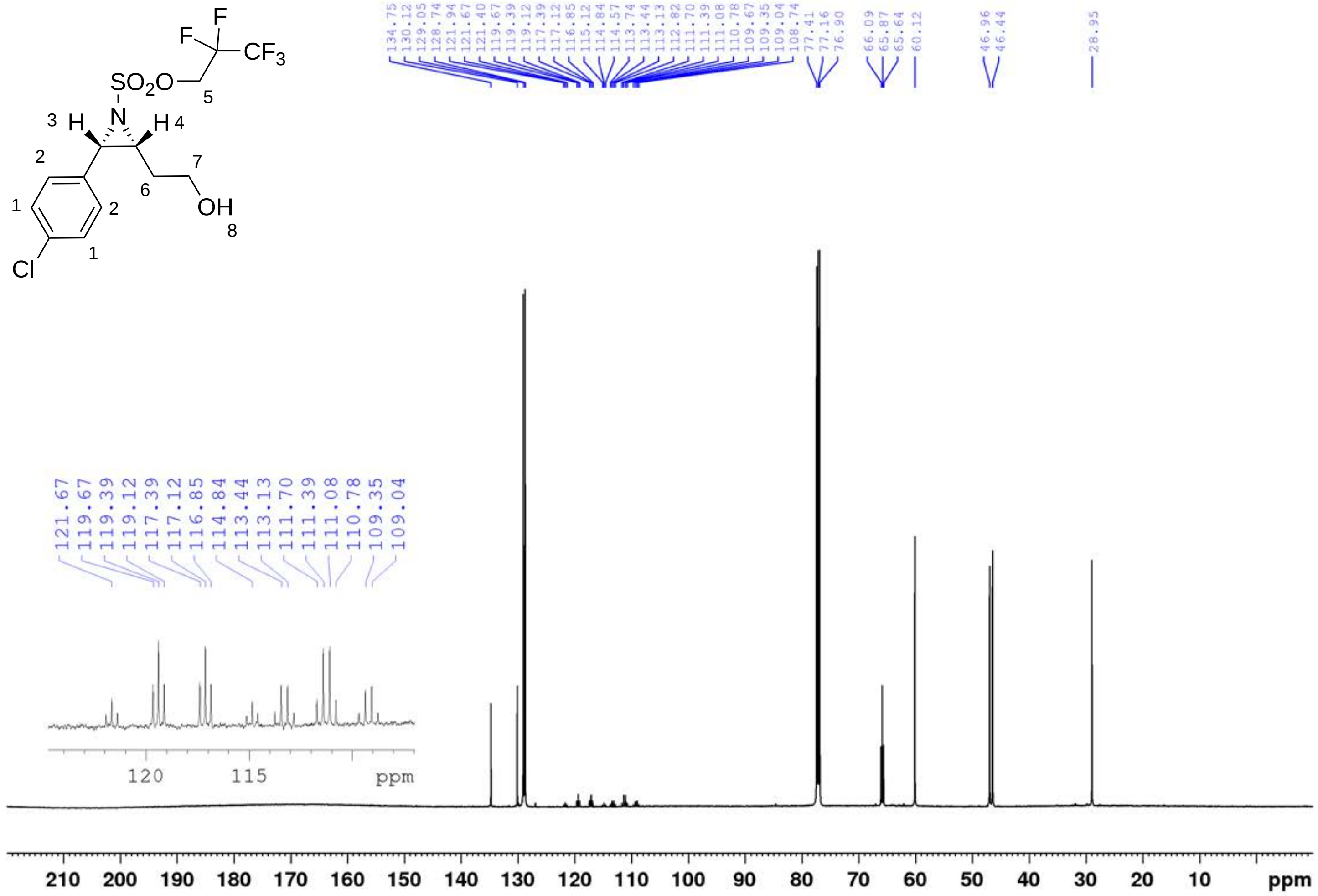

^{19}F NMR (376 MHz, $CDCl_3$) for *2,2,3,3,3-pentafluoropropyl (2S,3R)-2-(4-chlorophenyl)-3-(2-hydroxyethyl)aziridine-1-sulfonate* (**9h**)

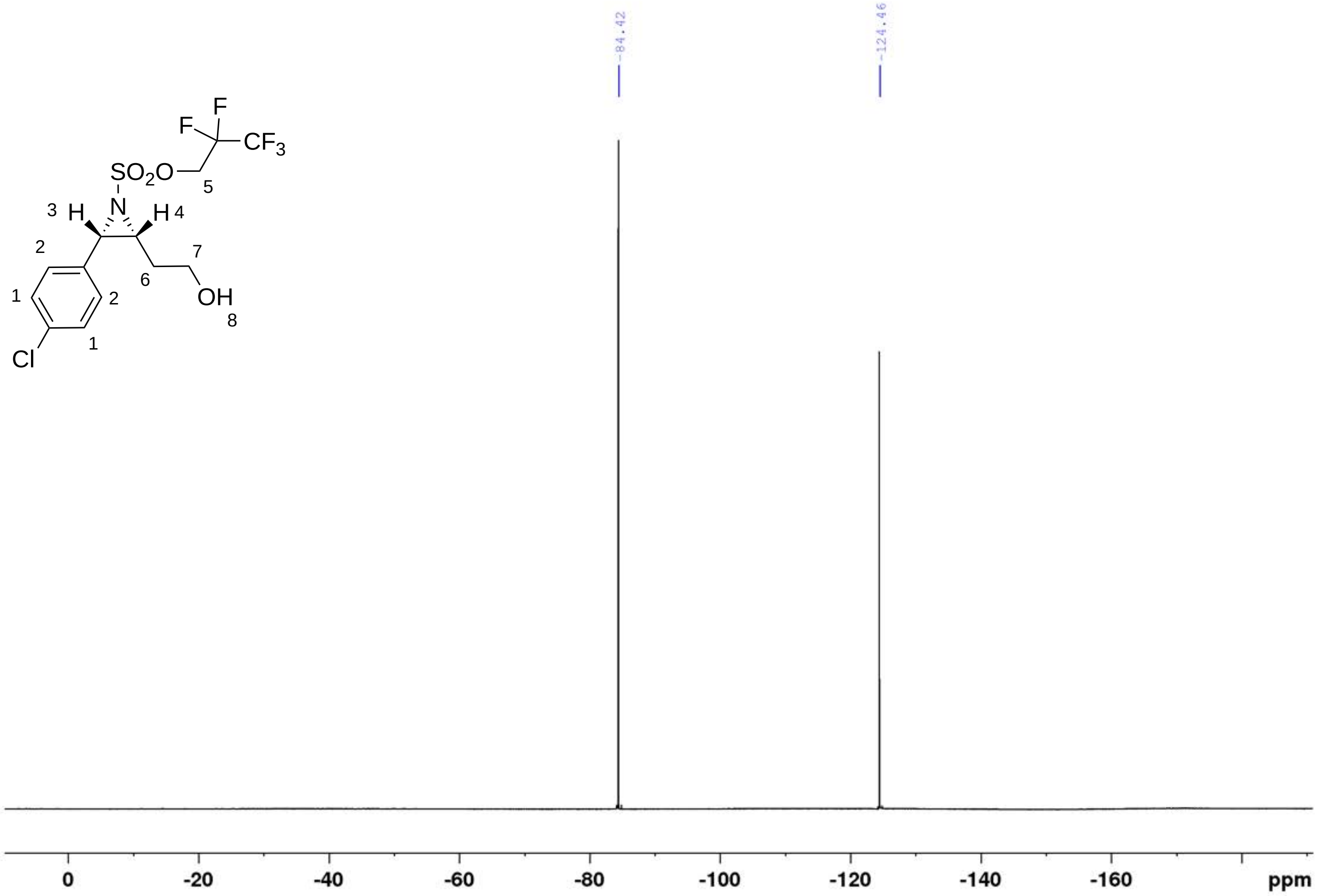

^{1}H NMR (500 MHz, $CDCl_3$) for *4-((2S,3R)-3-(2-hydroxyethyl)-1-((2,2,3,3,3-pentafluoropropoxy)sulfonyl)aziridin-2-yl)phenyl acetate* (**9i**)

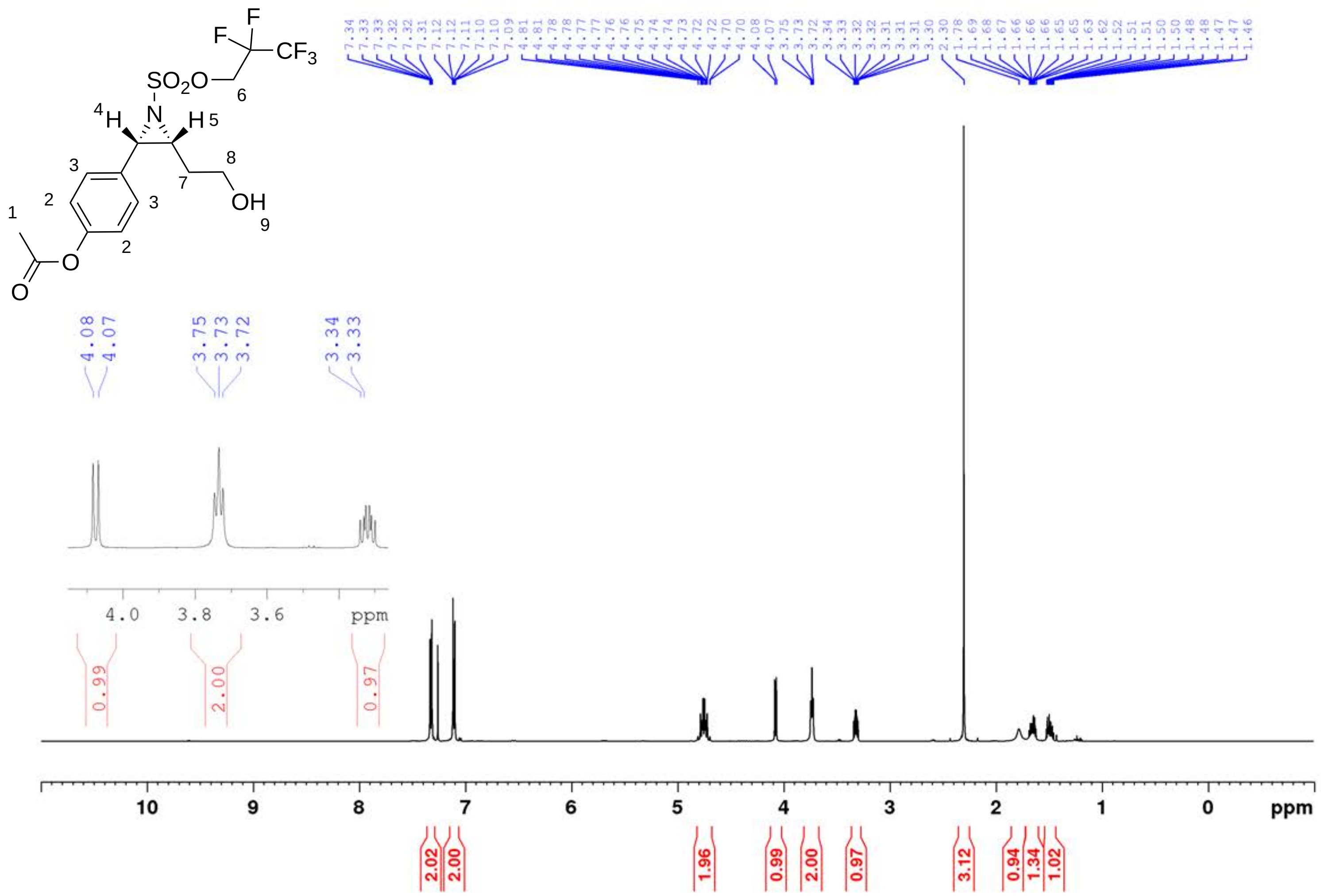

^{13}C NMR (126 MHz, $CDCl_3$) for *4-((2S,3R)-3-(2-hydroxyethyl)-1-((2,2,3,3,3-pentafluoropropoxy)sulfonyl)aziridin-2-yl)phenyl acetate* (**9i**)

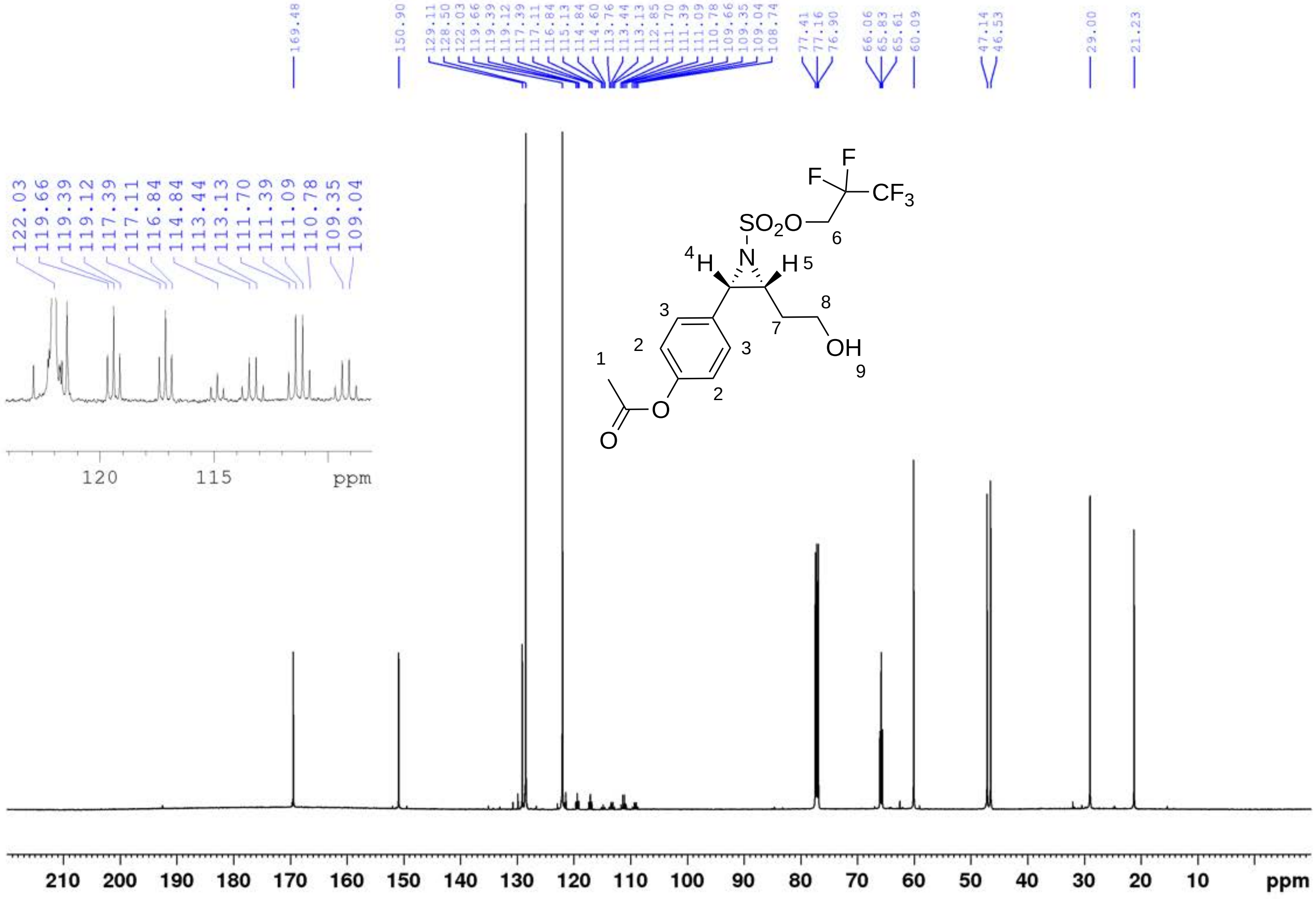

^{19}F NMR (376 MHz, $CDCl_3$) for *4-((2S,3R)-3-(2-hydroxyethyl)-1-((2,2,3,3,3-pentafluoropropoxy)sulfonyl)aziridin-2-yl)phenyl acetate* (**9i**)

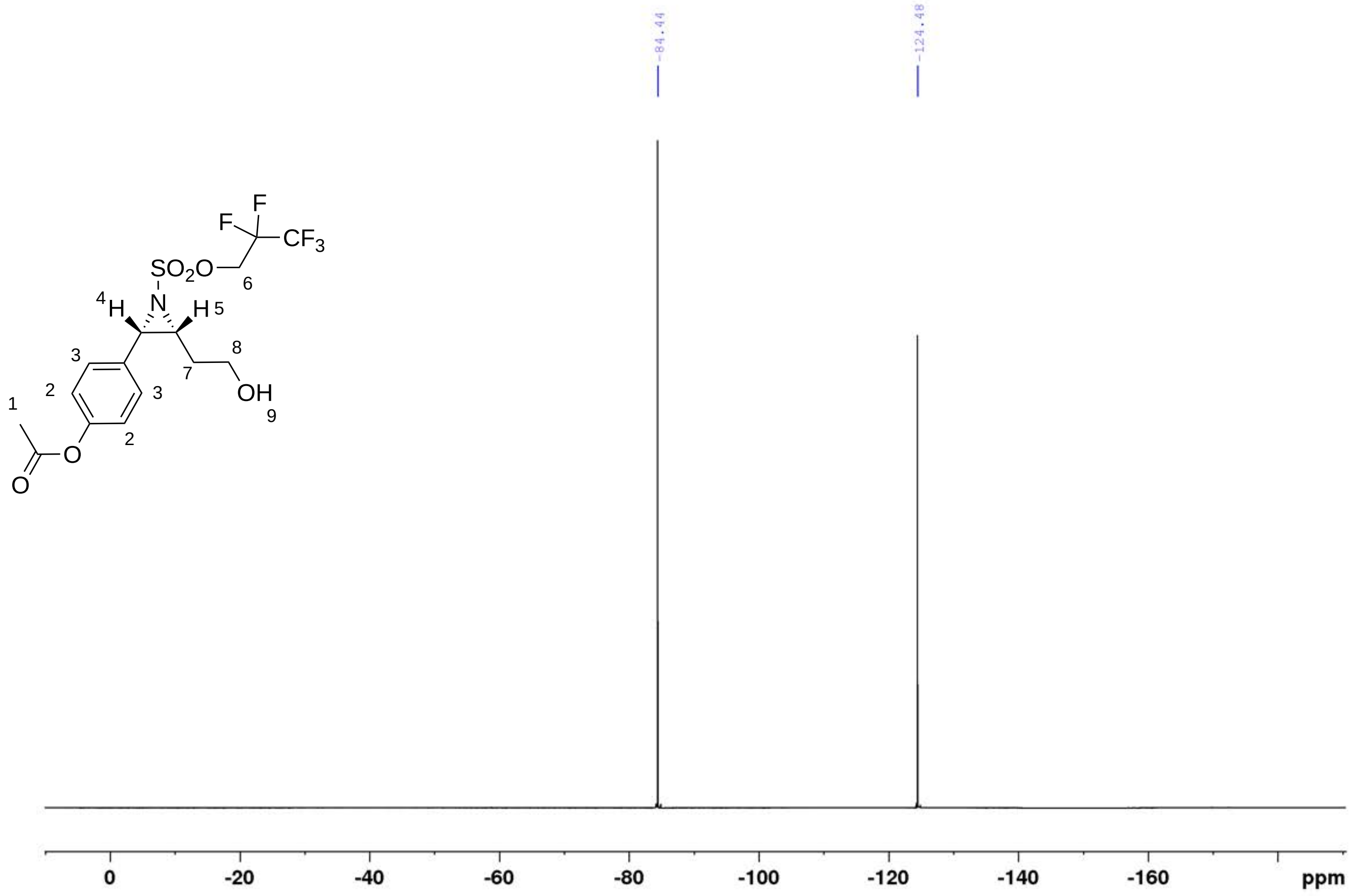

^{1}H NMR (500 MHz, $CDCl_3$) for *2,2,3,3,3-pentafluoropropyl (2R,3S)-2-(2-hydroxyethyl)-3-(4-(trifluoromethyl)phenyl)aziridine-1-sulfonate* (**9j**)

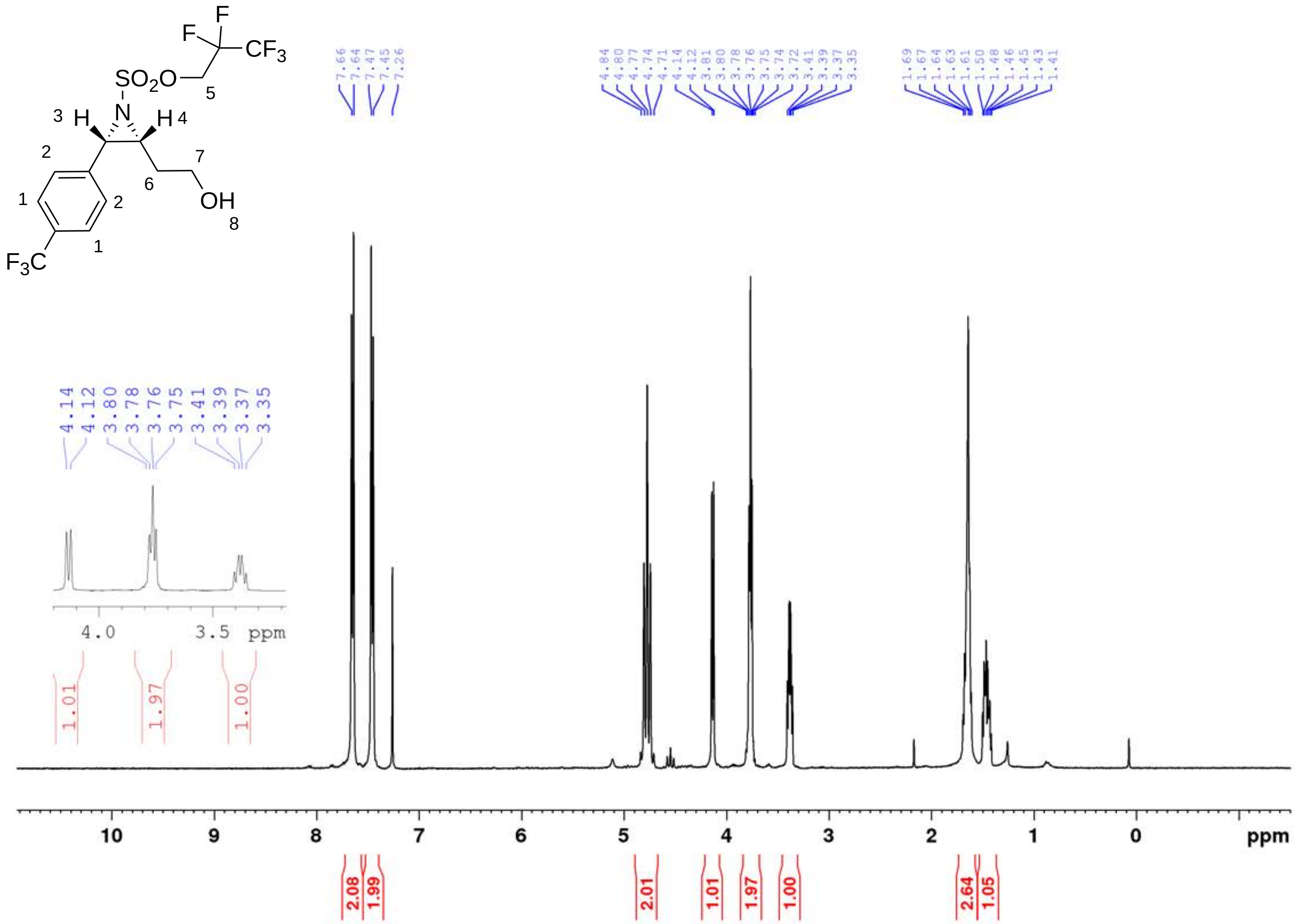

^{13}C NMR (126 MHz, $CDCl_3$) for *2,2,3,3,3-pentafluoropropyl (2R,3S)-2-(2-hydroxyethyl)-3-(4-(trifluoromethyl)phenyl)aziridine-1-sulfonate* (**9j**)

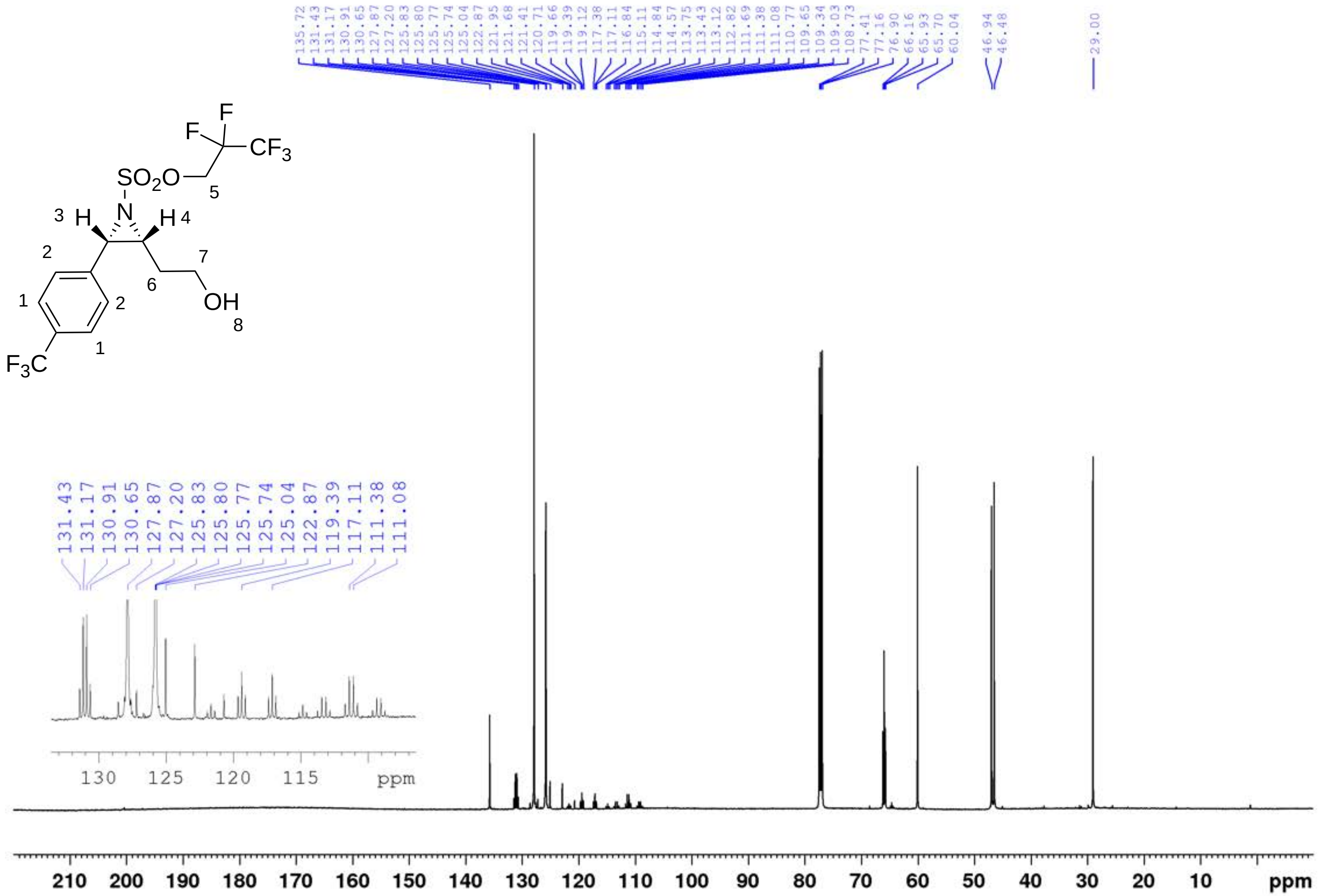

^{19}F NMR (376 MHz, $CDCl_3$) for *2,2,3,3,3-pentafluoropropyl (2R,3S)-2-(2-hydroxyethyl)-3-(4-(trifluoromethyl)phenyl)aziridine-1-sulfonate* (**9j**)

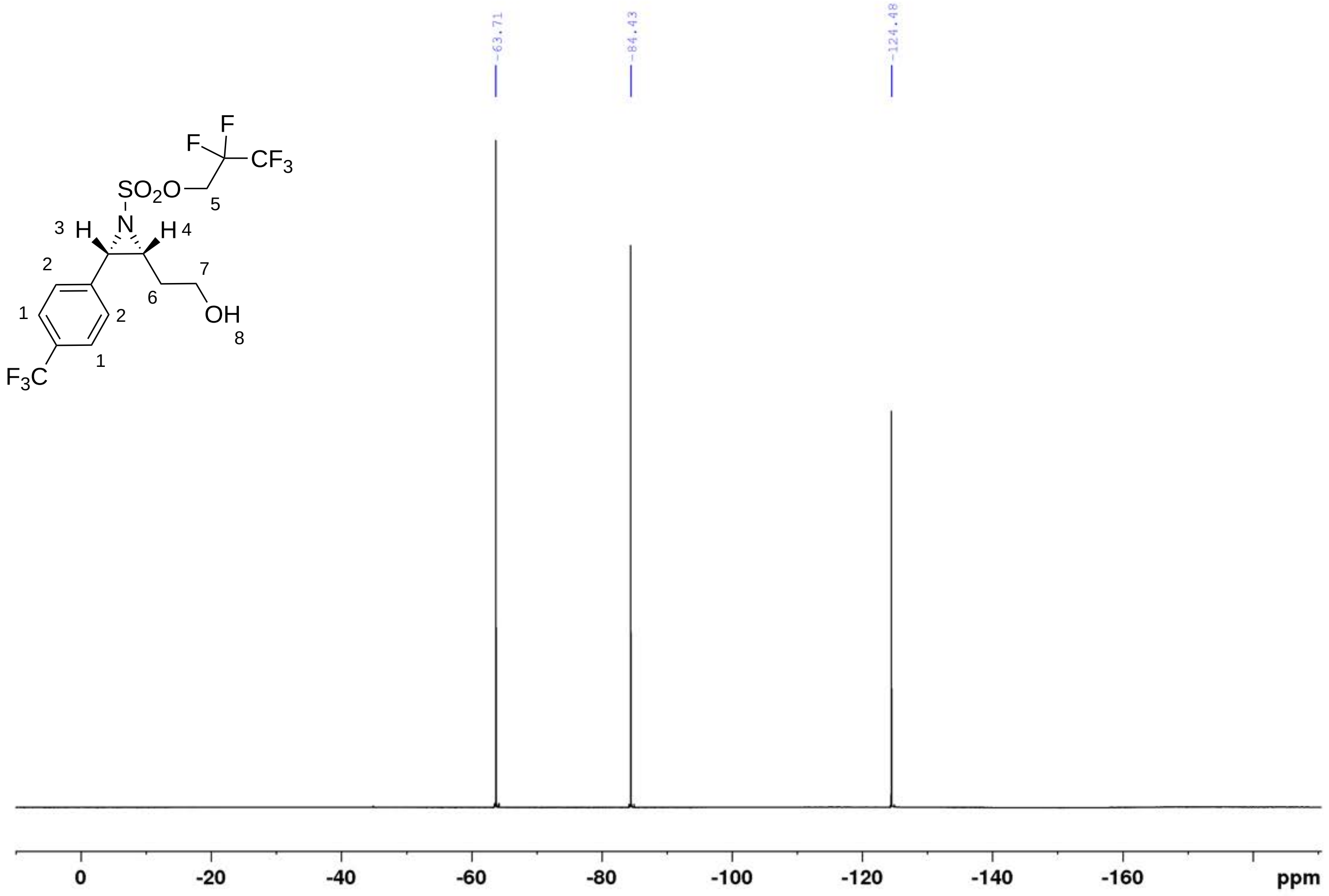

^{1}H NMR (500 MHz, $CDCl_3$) for *Ethyl 4-((2S,3R)-3-(2-hydroxyethyl)-1-((2,2,3,3,3-pentafluoropropoxy)sulfonyl)aziridin-2-yl)benzoate* (**9k**)

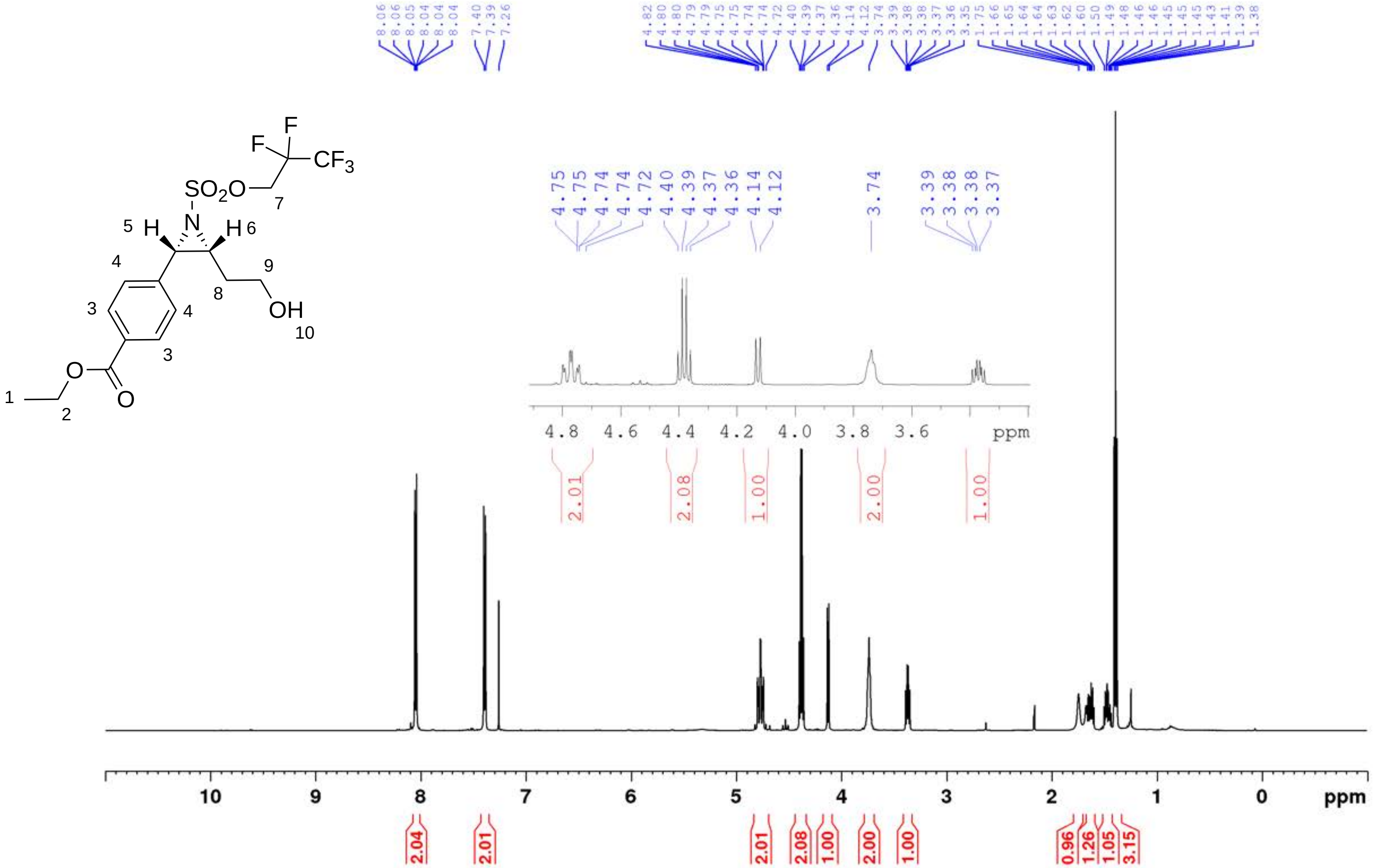

^{13}C NMR (126 MHz, $CDCl_3$) for *Ethyl 4-((2S,3R)-3-(2-hydroxyethyl)-1-((2,2,3,3,3-pentafluoropropoxy)sulfonyl)aziridin-2-yl)benzoate* (**9k**)

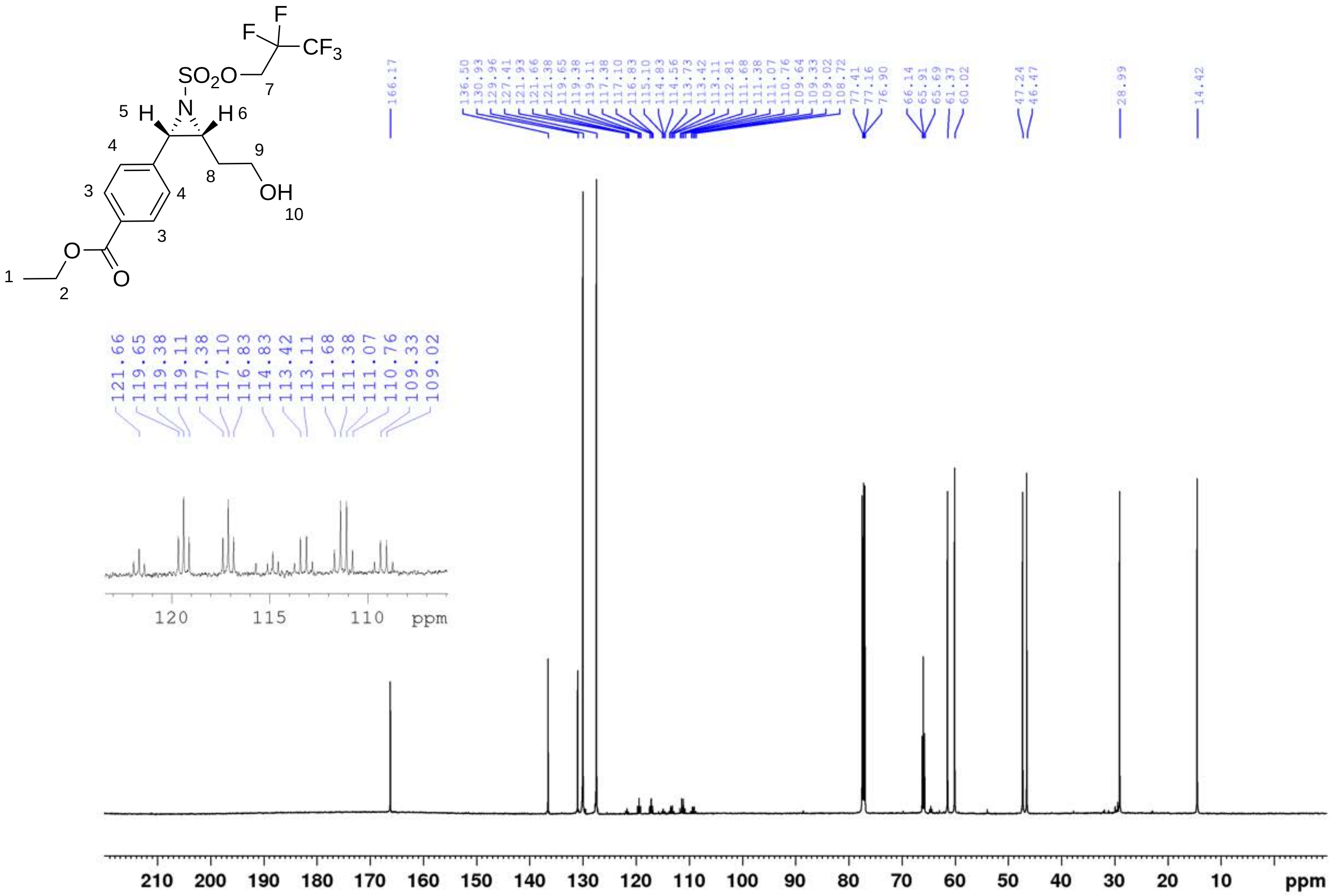

^{19}F NMR (376 MHz, $CDCl_3$) for *Ethyl 4-((2S,3R)-3-(2-hydroxyethyl)-1-((2,2,3,3,3-pentafluoropropoxy)sulfonyl)aziridin-2-yl)benzoate* (**9k**)

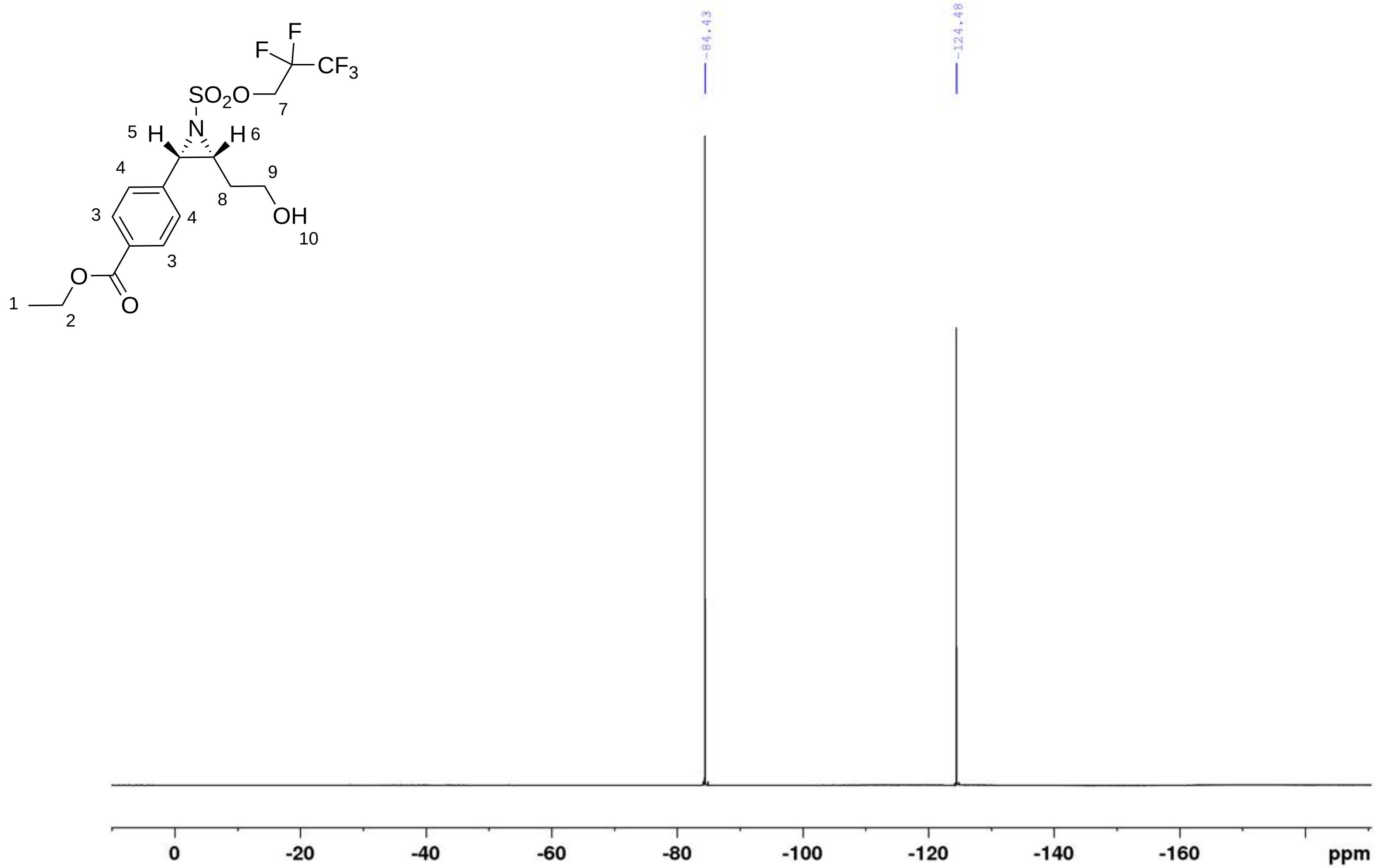

^{1}H NMR (500 MHz, $CDCl_3$) for *2,2,3,3,3-pentafluoropropyl (2S,3R)-2-(3,5-dimethylphenyl)-3-(2-hydroxyethyl)aziridine-1-sulfonate* (**9l**)

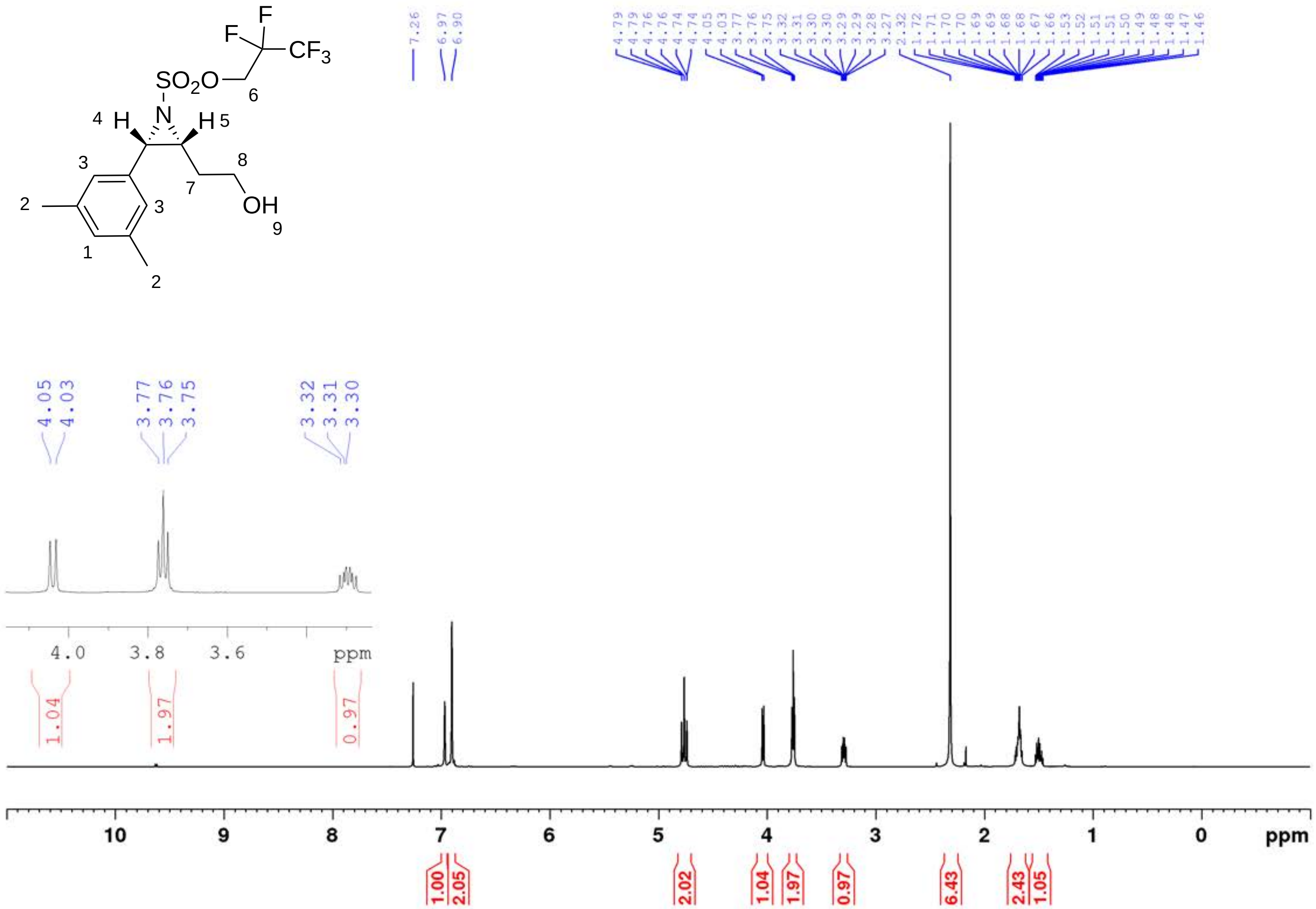

^{13}C NMR (126 MHz, $CDCl_3$) for *2,2,3,3,3-pentafluoropropyl (2S,3R)-2-(3,5-dimethylphenyl)-3-(2-hydroxyethyl)aziridine-1-sulfonate* (**9l**)

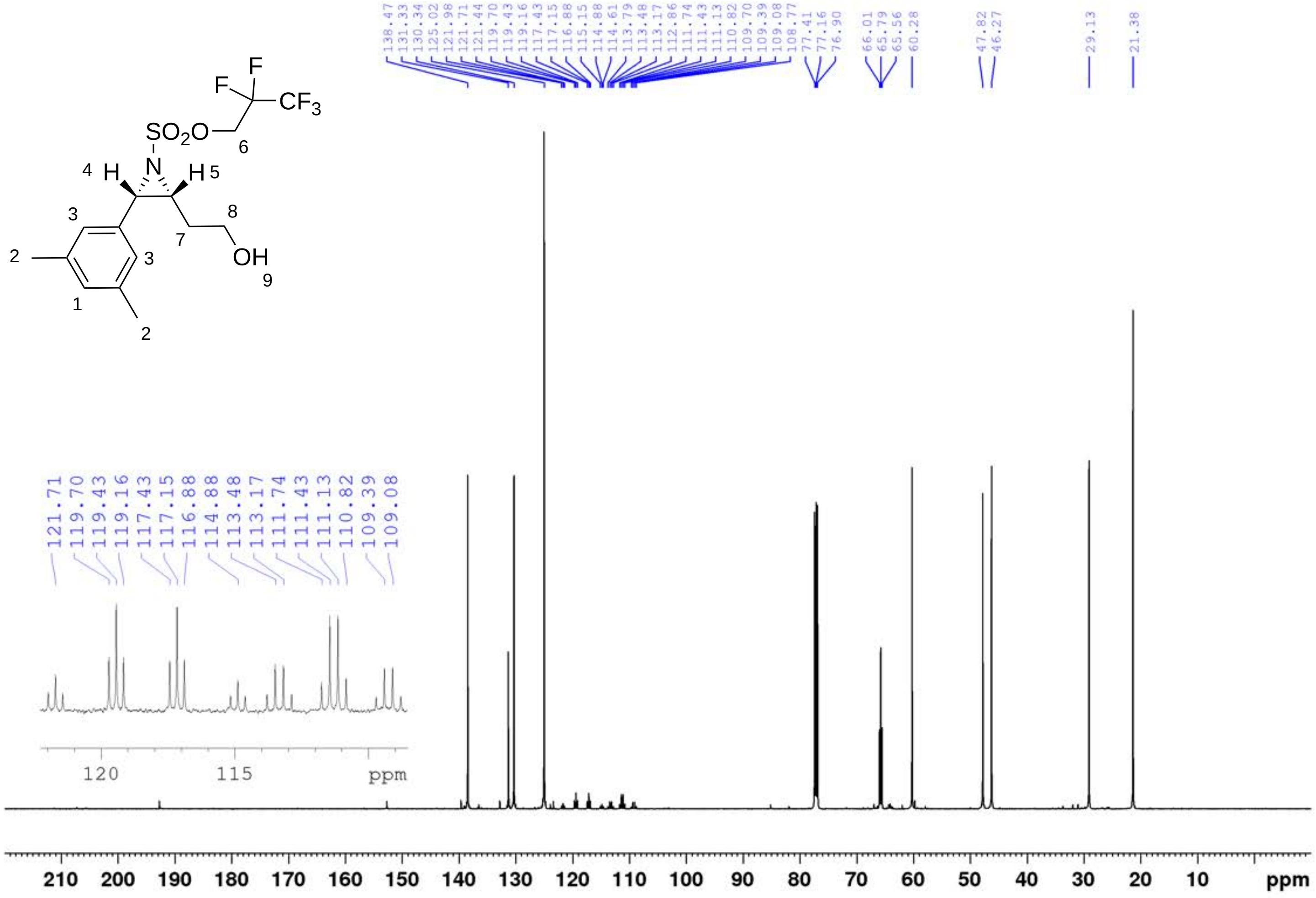

^{19}F NMR (376 MHz, $CDCl_3$) for *2,2,3,3,3-pentafluoropropyl (2S,3R)-2-(3,5-dimethylphenyl)-3-(2-hydroxyethyl)aziridine-1-sulfonate* (**9l**)

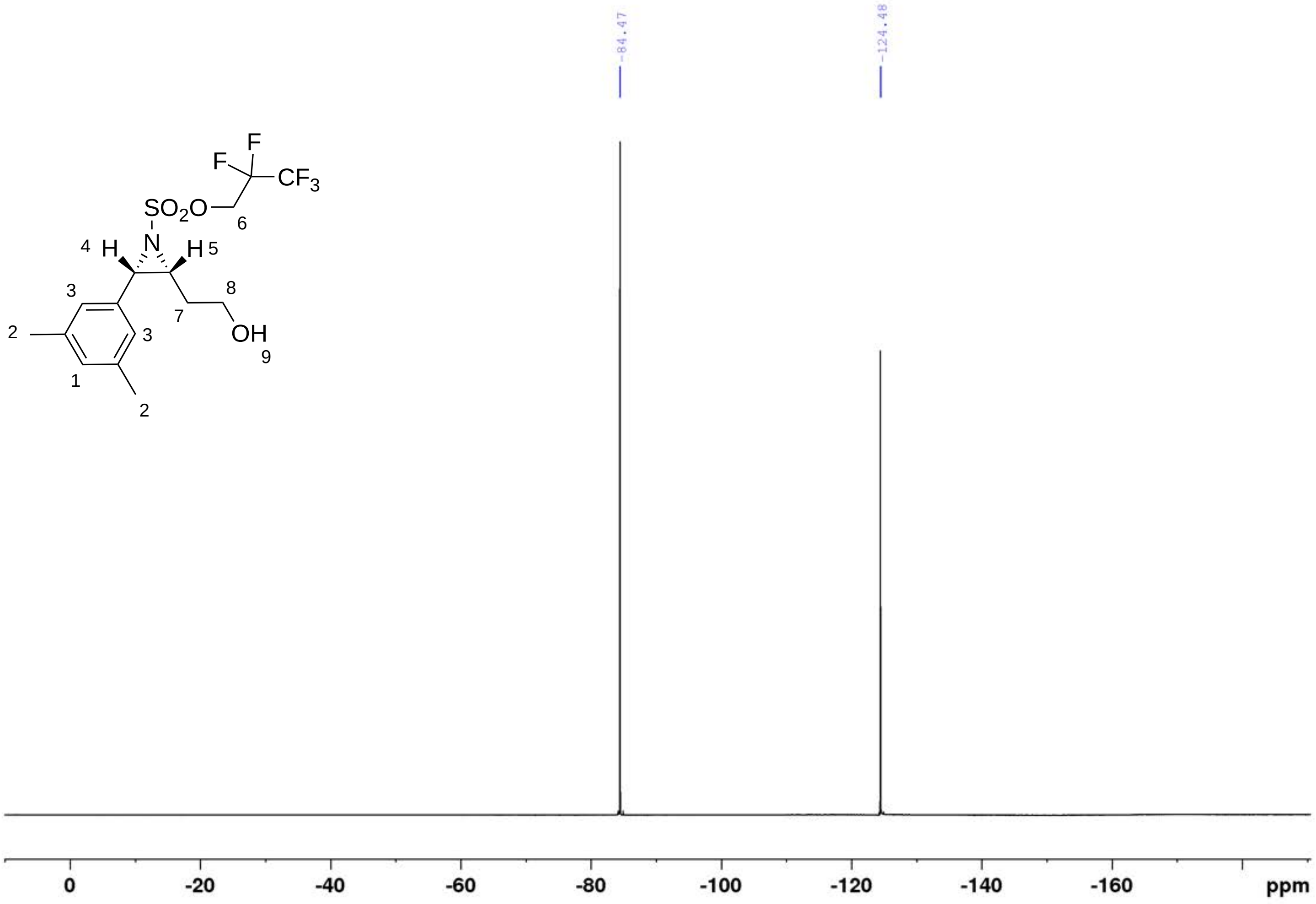

¹H NMR (500 MHz, $CDCl_3$) for *2,2,3,3,3-pentafluoropropyl (2S,3R)-2-(2,5-dimethylphenyl)-3-(2-hydroxyethyl)aziridine-1-sulfonate* (**9m**)

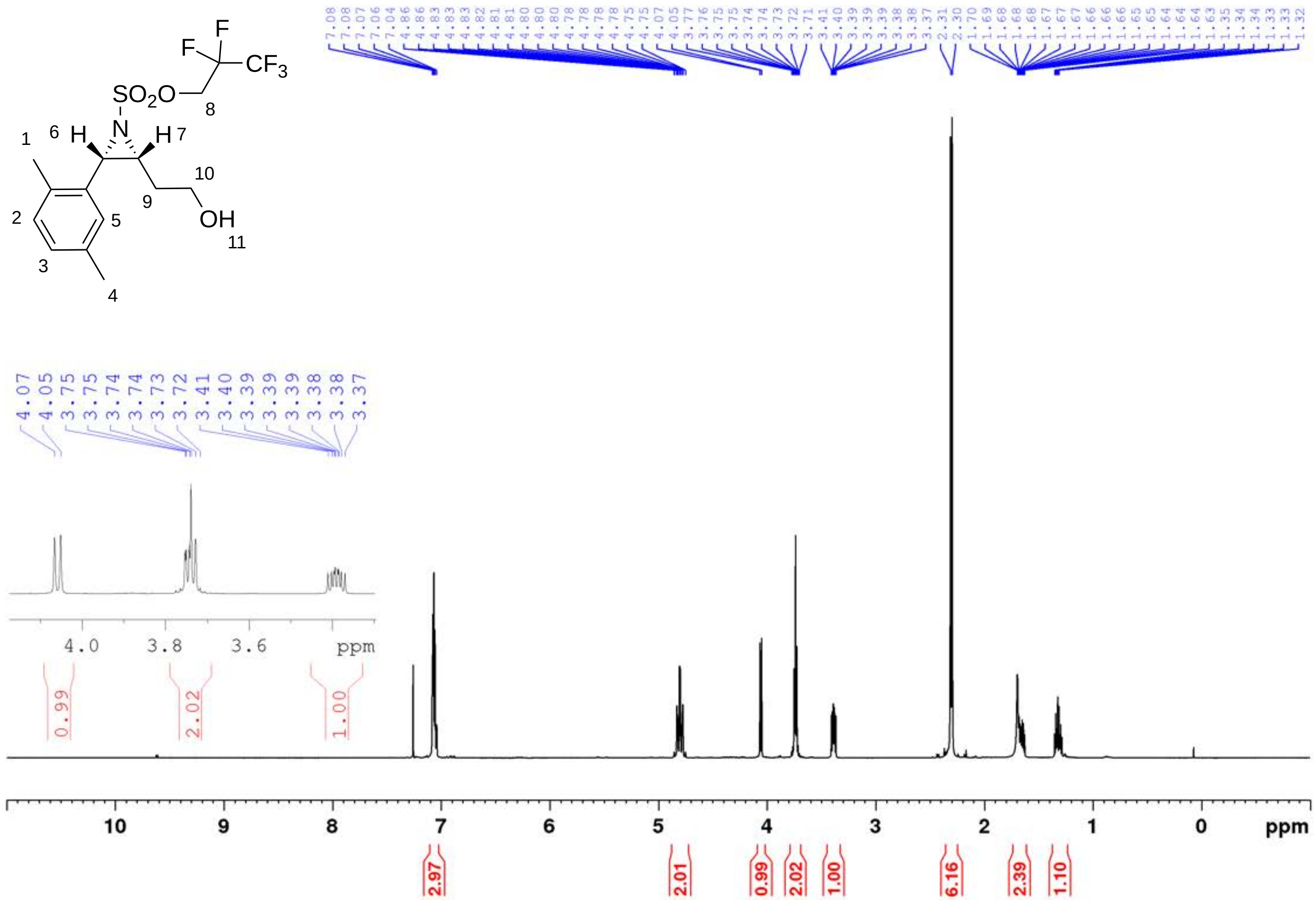

^{13}C NMR (126 MHz, $CDCl_3$) for *2,2,3,3,3-pentafluoropropyl (2S,3R)-2-(2,5-dimethylphenyl)-3-(2-hydroxyethyl)aziridine-1-sulfonate* (**9m**)

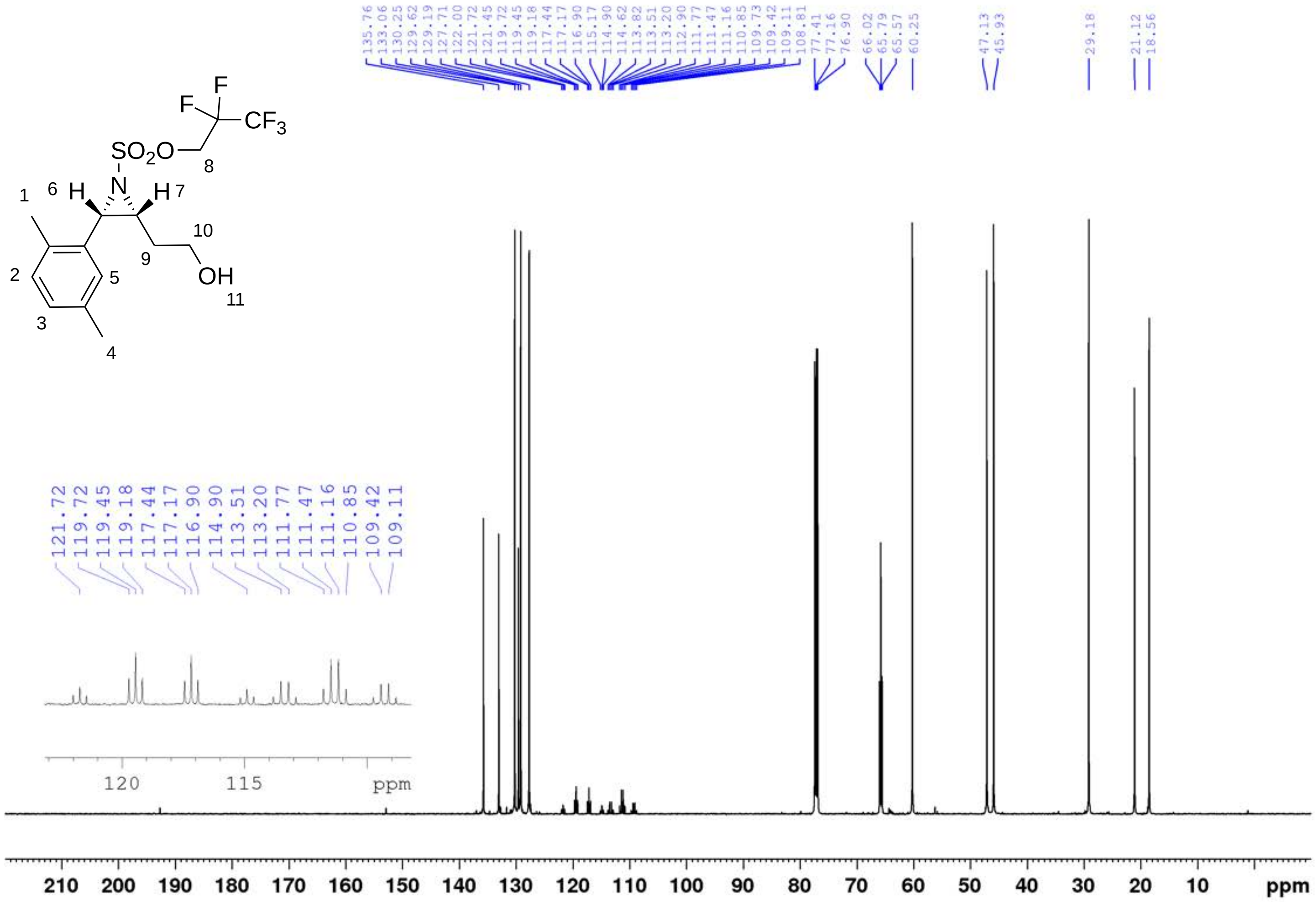

^{19}F NMR (376 MHz, $CDCl_3$) for *2,2,3,3,3-pentafluoropropyl (2S,3R)-2-(2,5-dimethylphenyl)-3-(2-hydroxyethyl)aziridine-1-sulfonate* (**9m**)

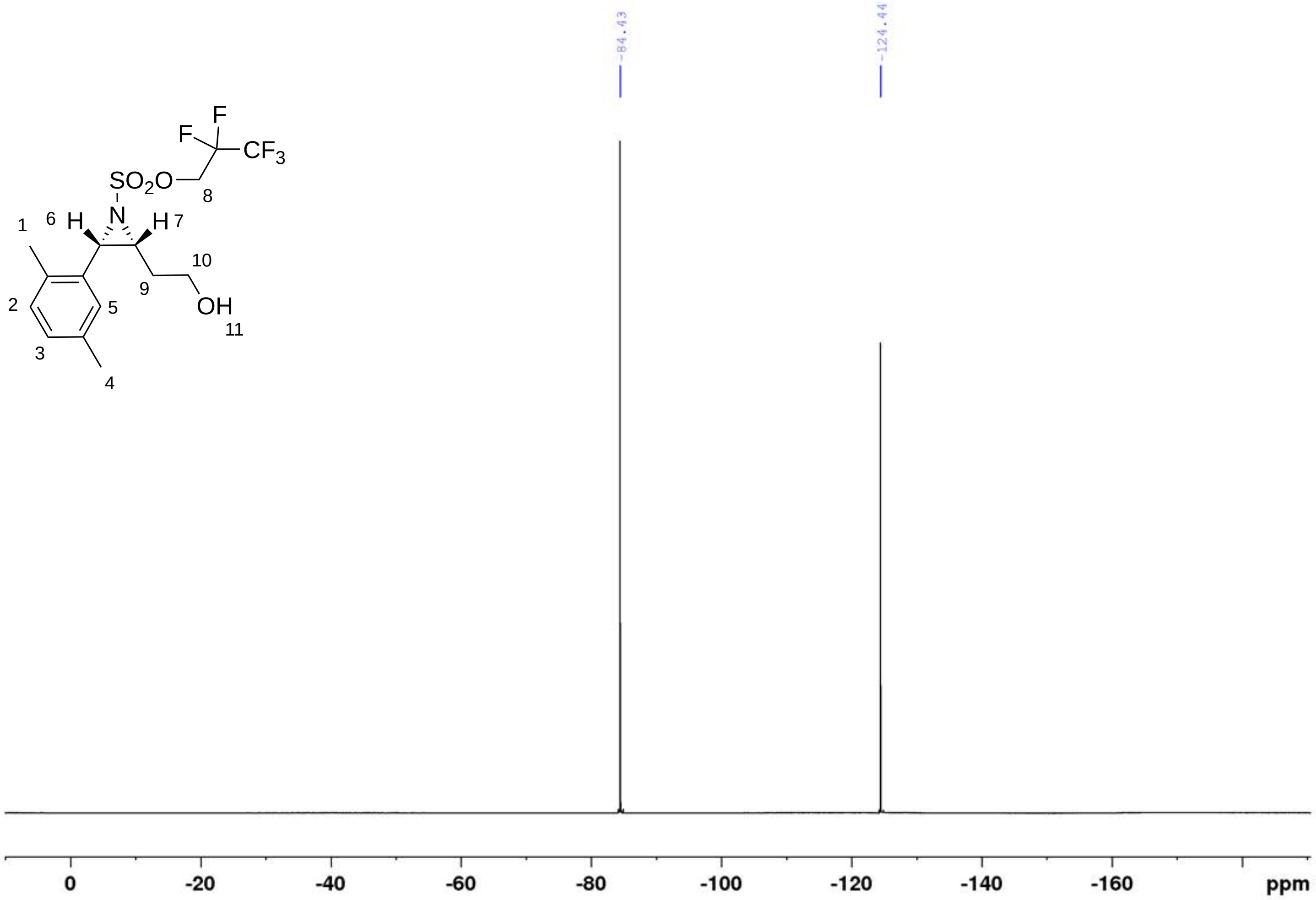

^{1}**H NMR** (500 MHz, $CDCl_3$) for *2,2,3,3,3-pentafluoropropyl (2S,3R)-2-(3-fluoro-4-methylphenyl)-3-(2-hydroxyethyl)aziridine-1-sulfonate* (**9n**)

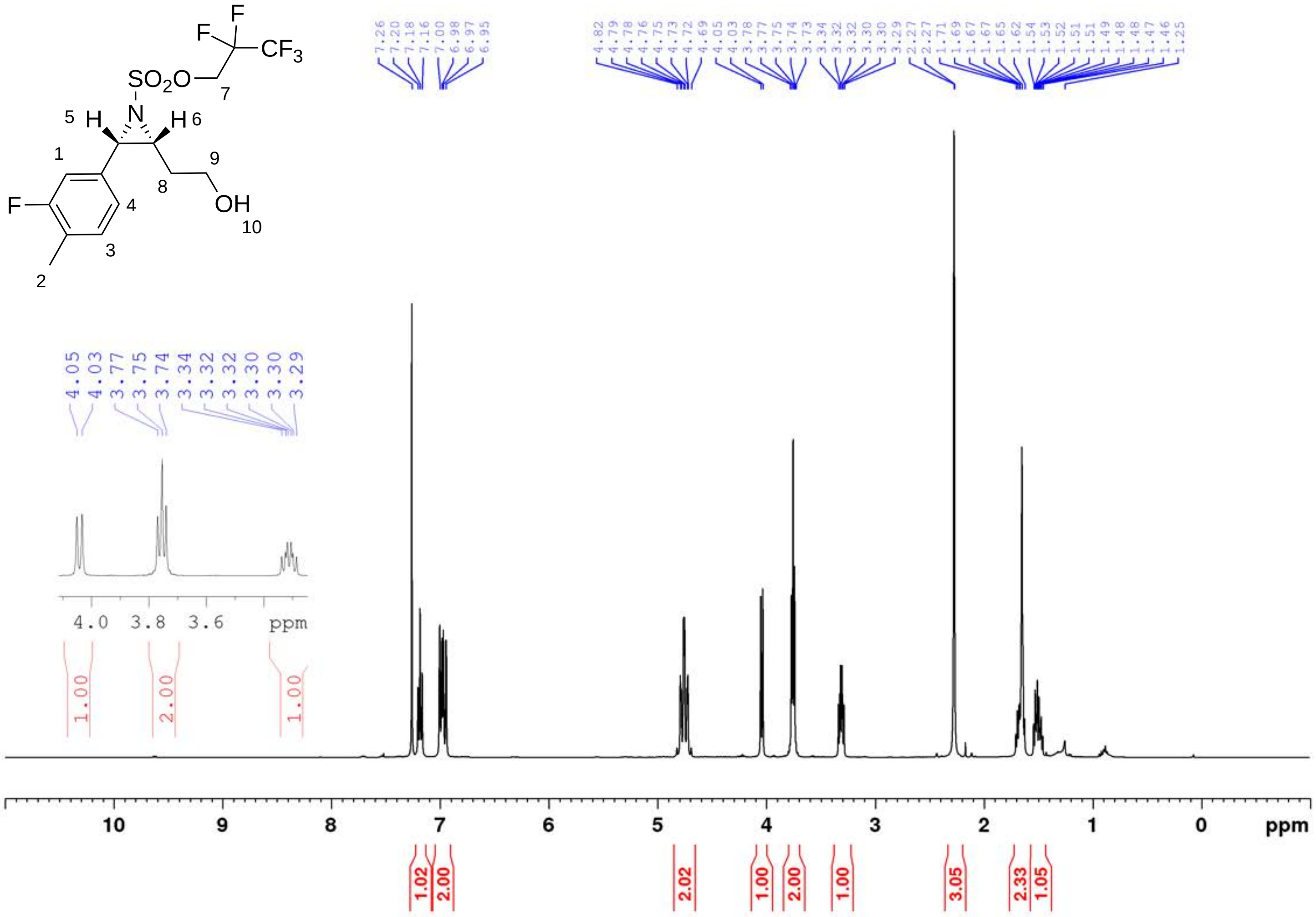

^{13}C NMR (126 MHz, $CDCl_3$) for *2,2,3,3,3-pentafluoropropyl (2S,3R)-2-(3-fluoro-4-methylphenyl)-3-(2-hydroxyethyl)aziridine-1-sulfonate* (**9n**)

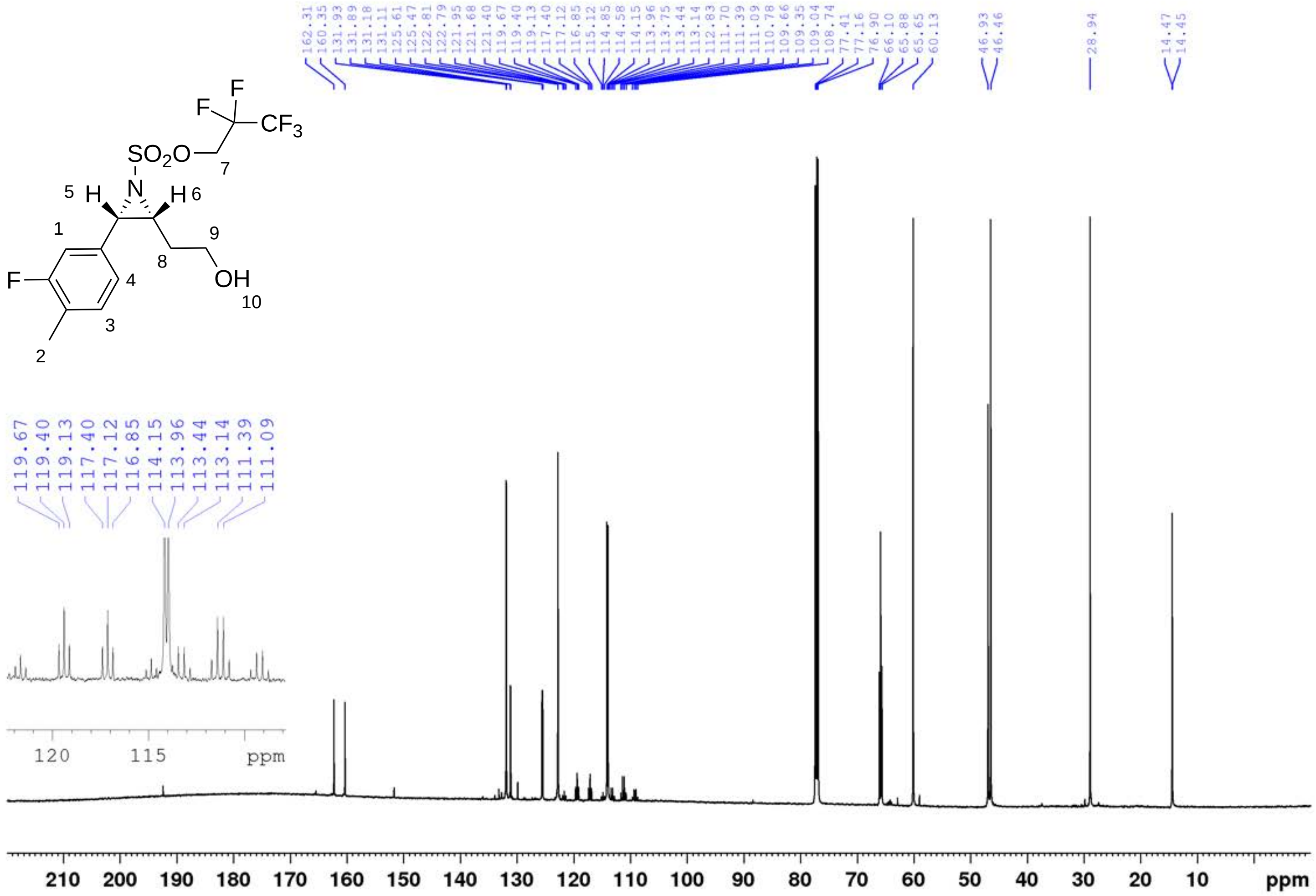

^{19}F NMR (471 MHz, $CDCl_3$) for *2,2,3,3,3-pentafluoropropyl (2S,3R)-2-(3-fluoro-4-methylphenyl)-3-(2-hydroxyethyl)aziridine-1-sulfonate* (**9n**)

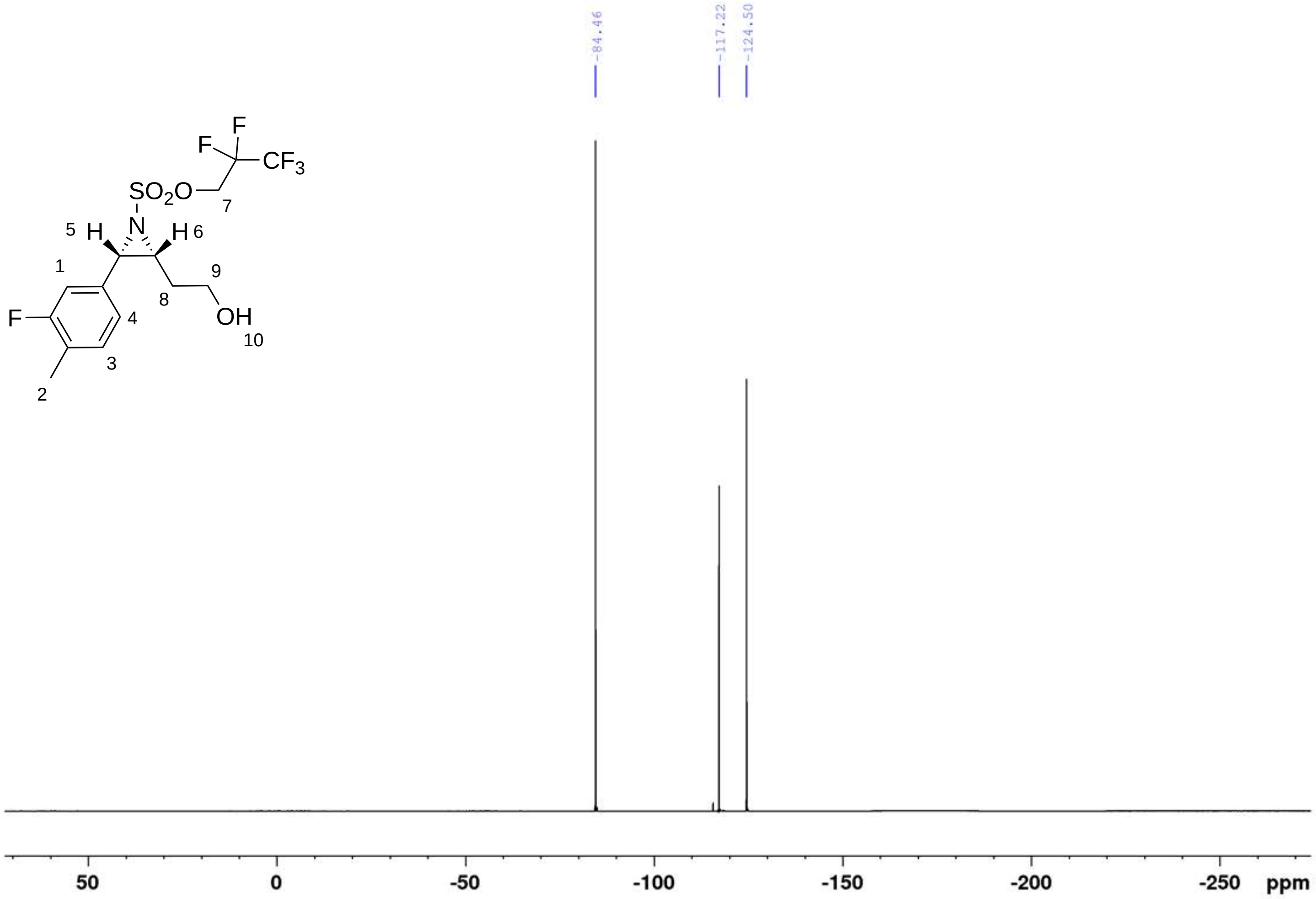

^{1}H NMR (500 MHz, $CDCl_3$) for *2,2,3,3,3-pentafluoropropyl (2R,3S)-2-(2-hydroxyethyl)-3-(naphthalen-1-yl)aziridine-1-sulfonate* (**9o**)

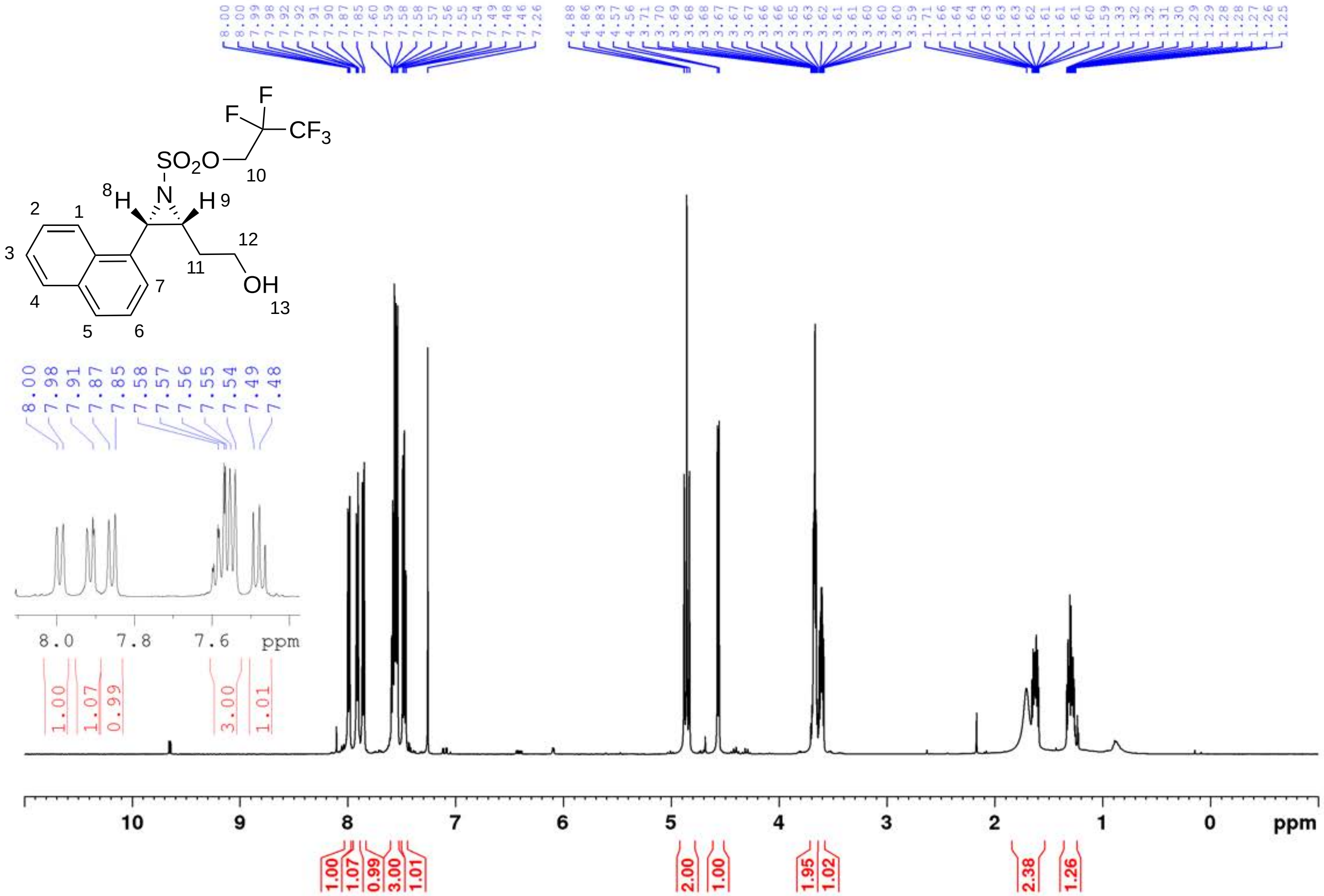

^{13}C NMR (126 MHz, $CDCl_3$) for *2,2,3,3,3-pentafluoropropyl (2R,3S)-2-(2-hydroxyethyl)-3-(naphthalen-1-yl)aziridine-1-sulfonate* (**9o**)

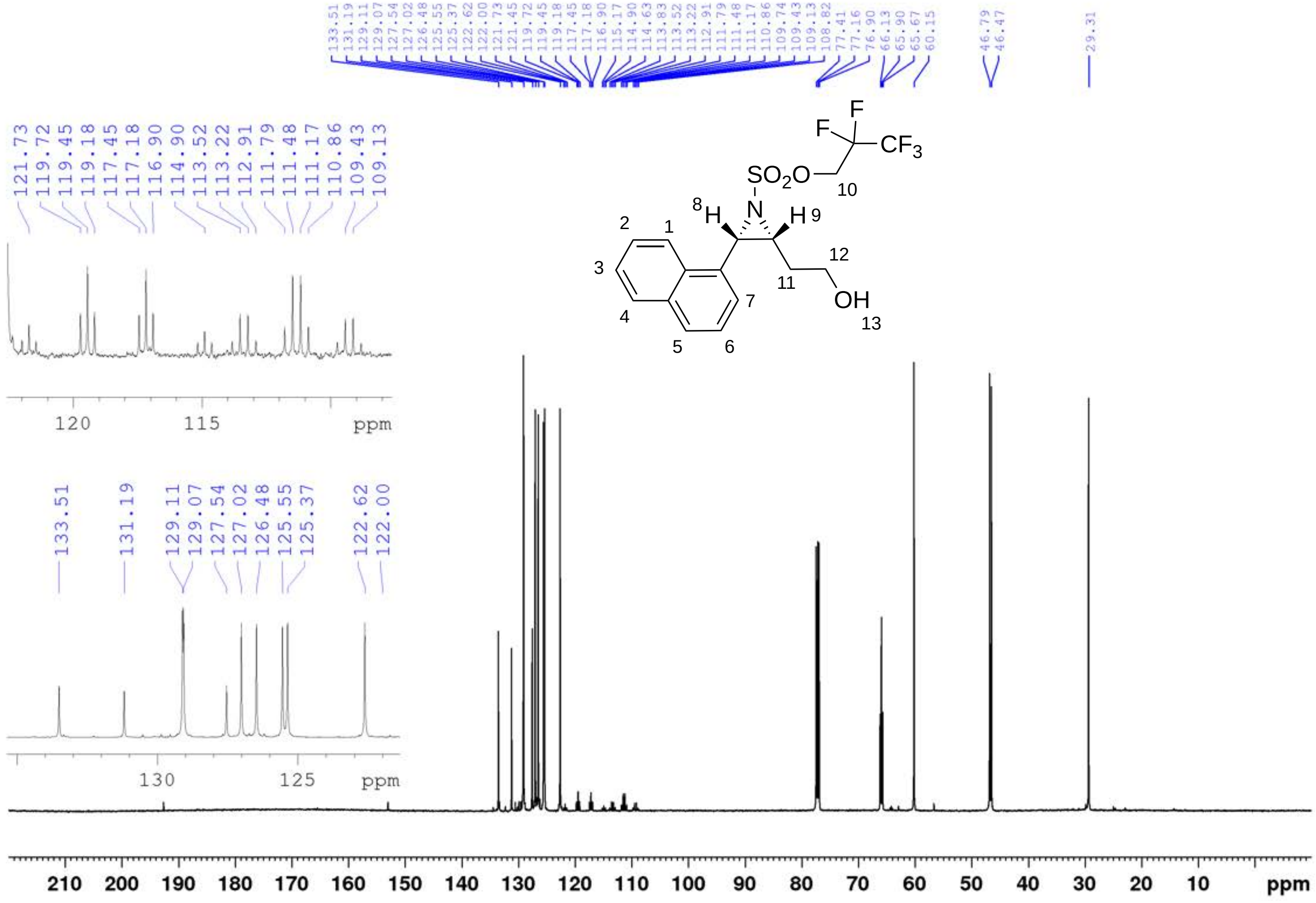

¹⁹F NMR (376 MHz, $CDCl_3$) for *2,2,3,3,3-pentafluoropropyl (2R,3S)-2-(2-hydroxyethyl)-3-(naphthalen-1-yl)aziridine-1-sulfonate* (**9o**)

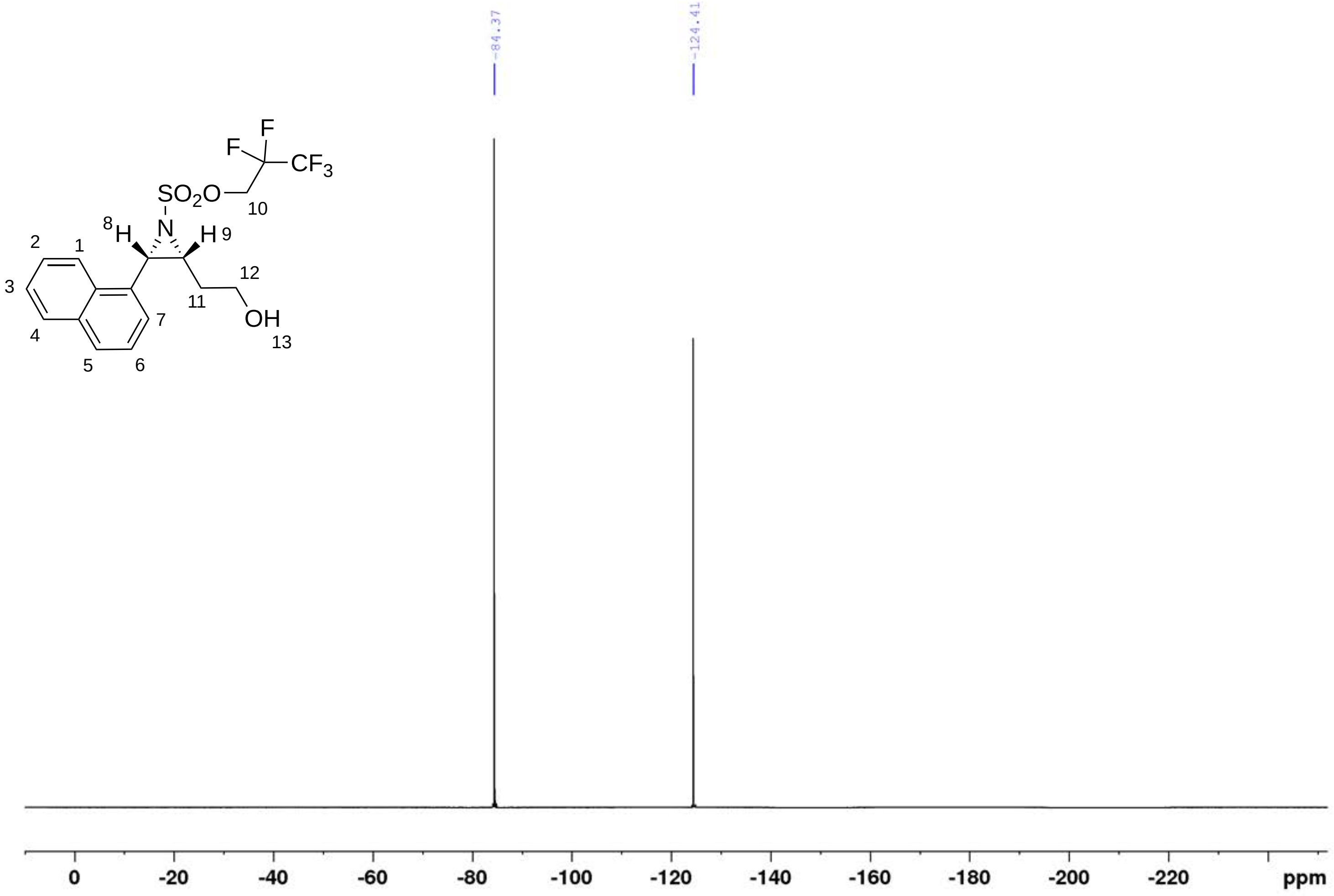

^{1}H NMR (500 MHz, $CDCl_3$) for *2,2,3,3,3-pentafluoropropyl ((2S,3R)-2-phenyltetrahydrofuran-3-yl)sulfamate* (**12**)

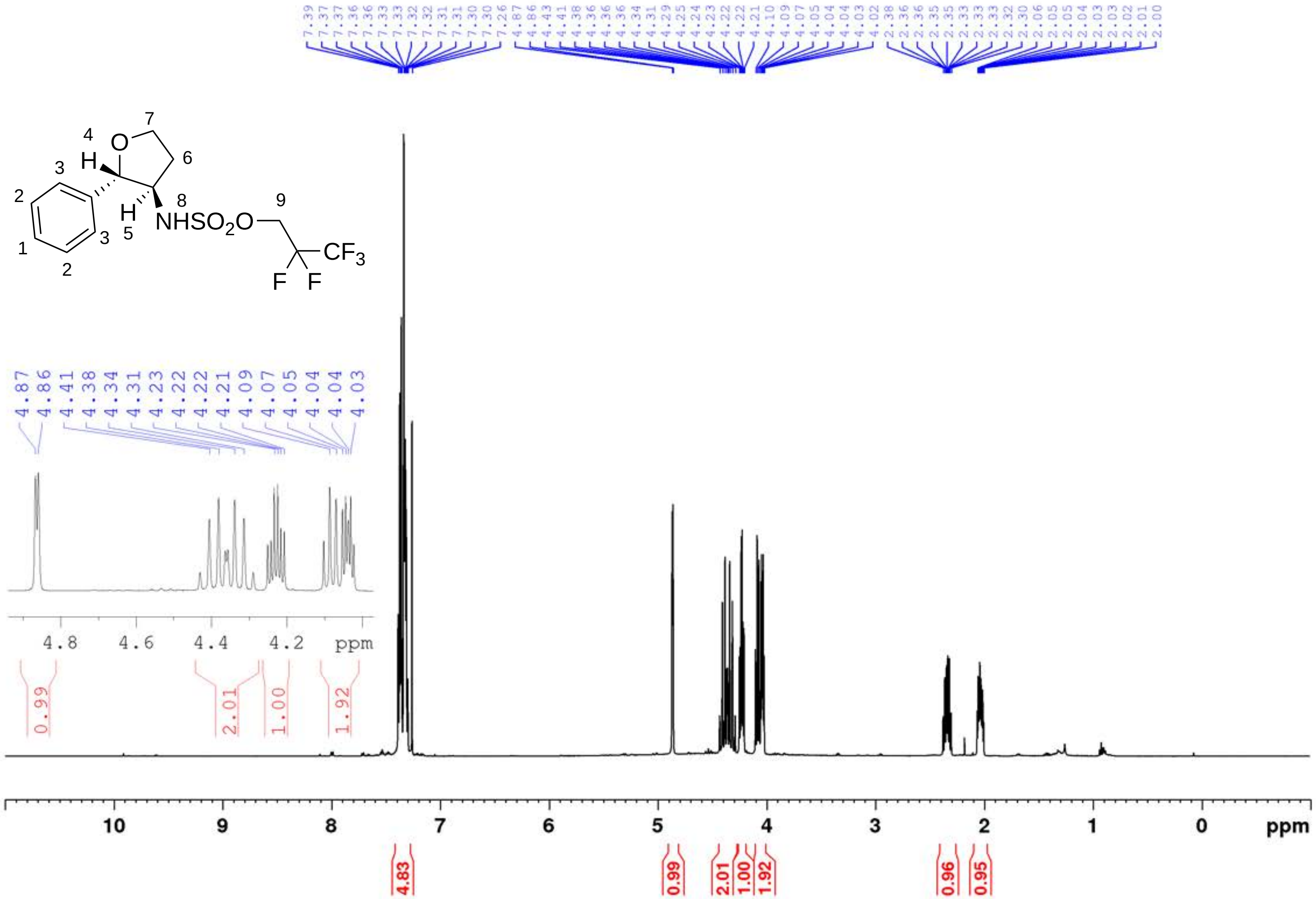

^{13}C NMR (126 MHz, $CDCl_3$) for *2,2,3,3,3-pentafluoropropyl ((2S,3R)-2-phenyltetrahydrofuran-3-yl)sulfamate* (**12**)

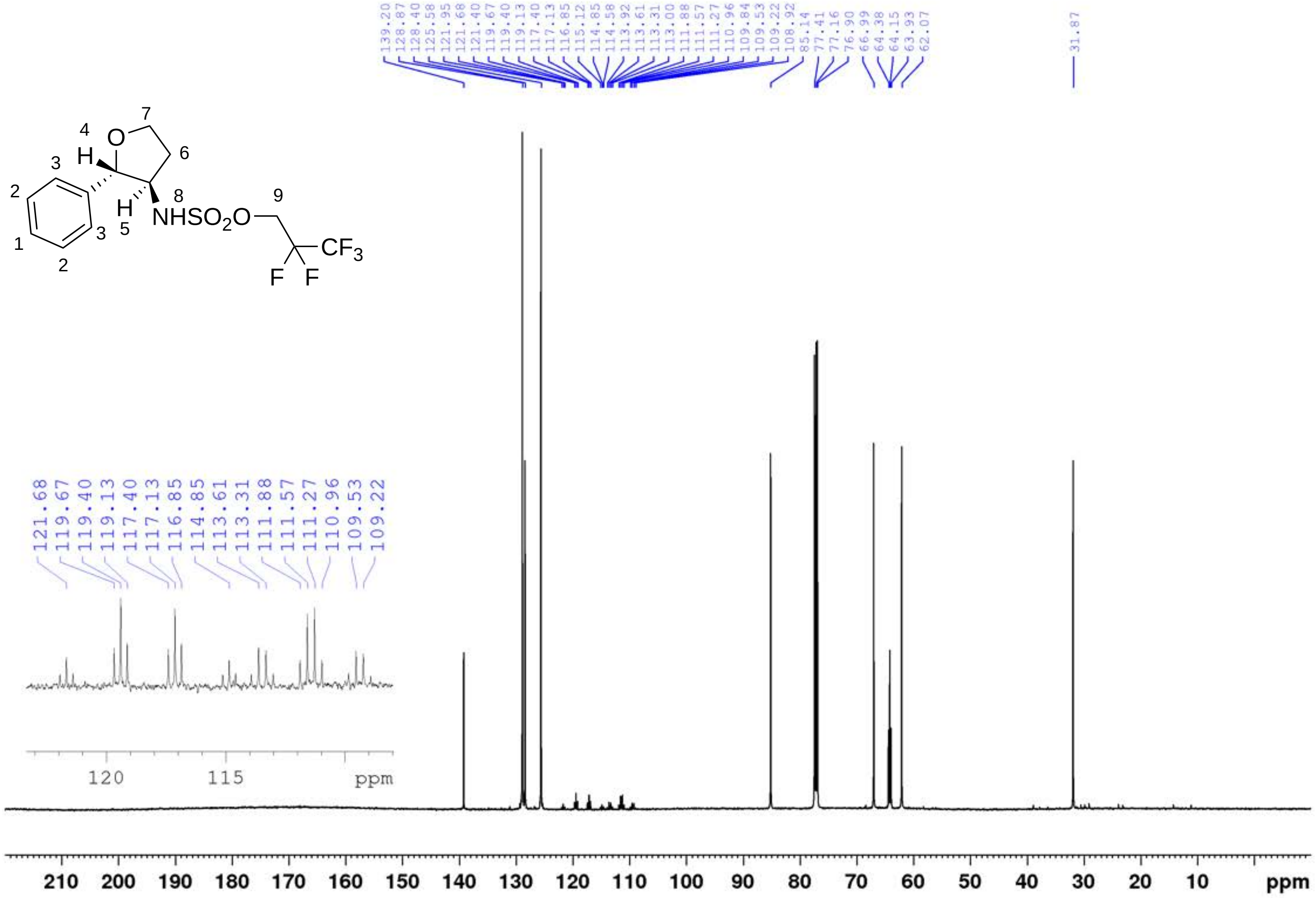

¹⁹F NMR (376 MHz, $CDCl_3$) for *2,2,3,3,3-pentafluoropropyl ((2S,3R)-2-phenyltetrahydrofuran-3-yl)sulfamate* (**12**)

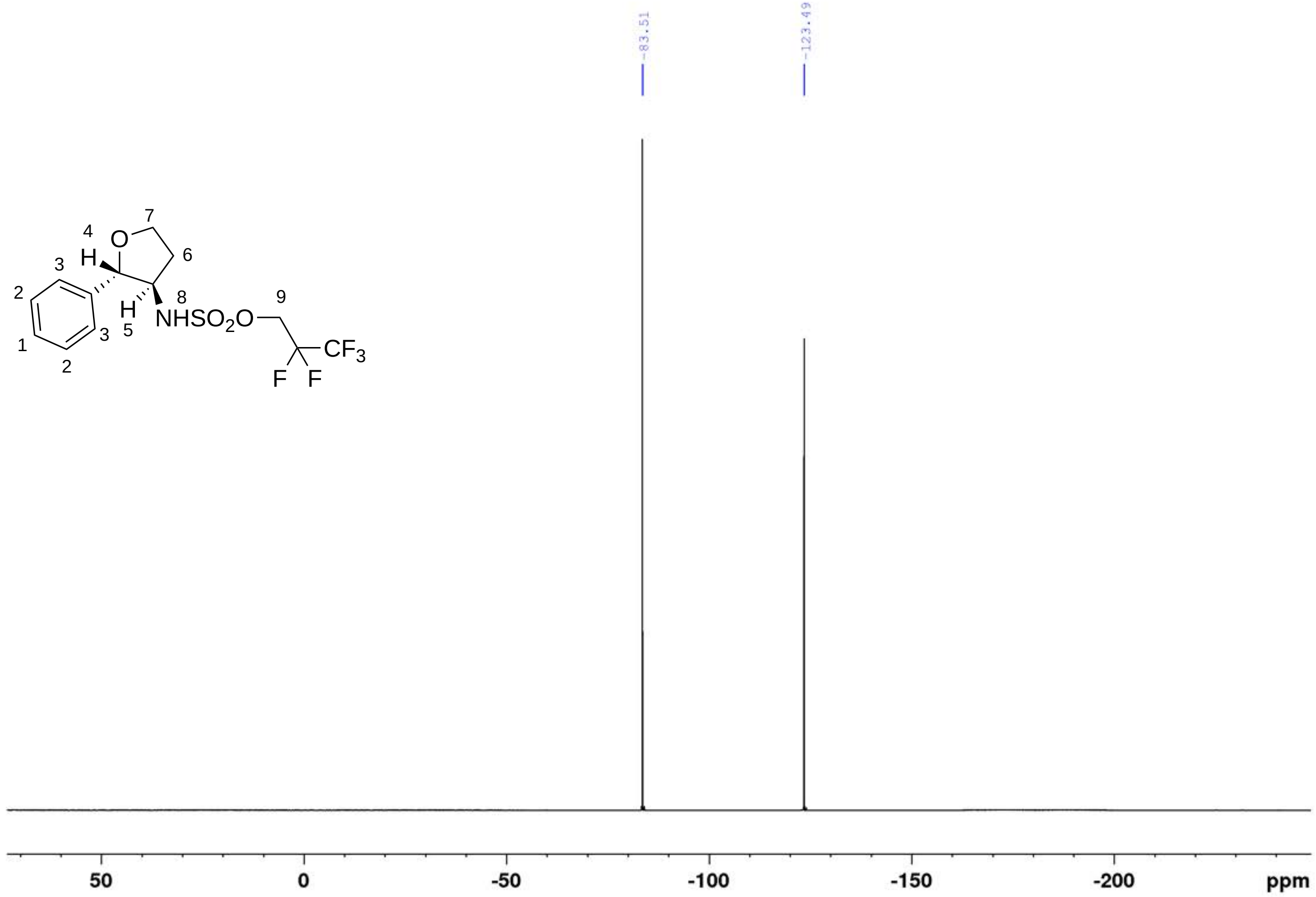

^{1}H NMR (500 MHz, $CDCl_3$) for *2,2,3,3,3-pentafluoropropyl (2R,3S)-2-(4-hydroxybutyl)-3-phenylaziridine-1-sulfonate* (**14a**)

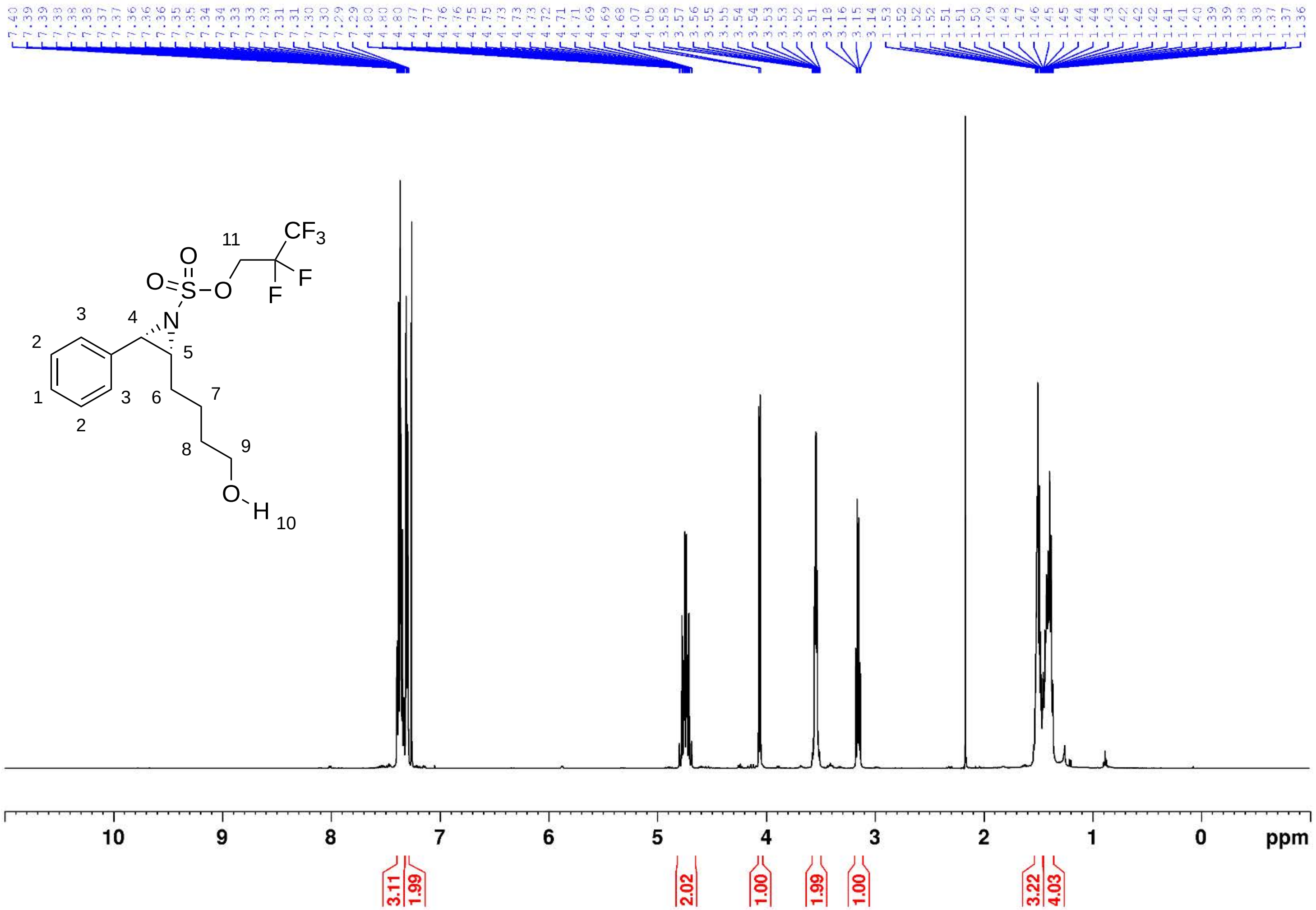

^{13}C NMR (126 MHz, $CDCl_3$) for *2,2,3,3,3-pentafluoropropyl (2R,3S)-2-(4-hydroxybutyl)-3-phenylaziridine-1-sulfonate* (**14a**)

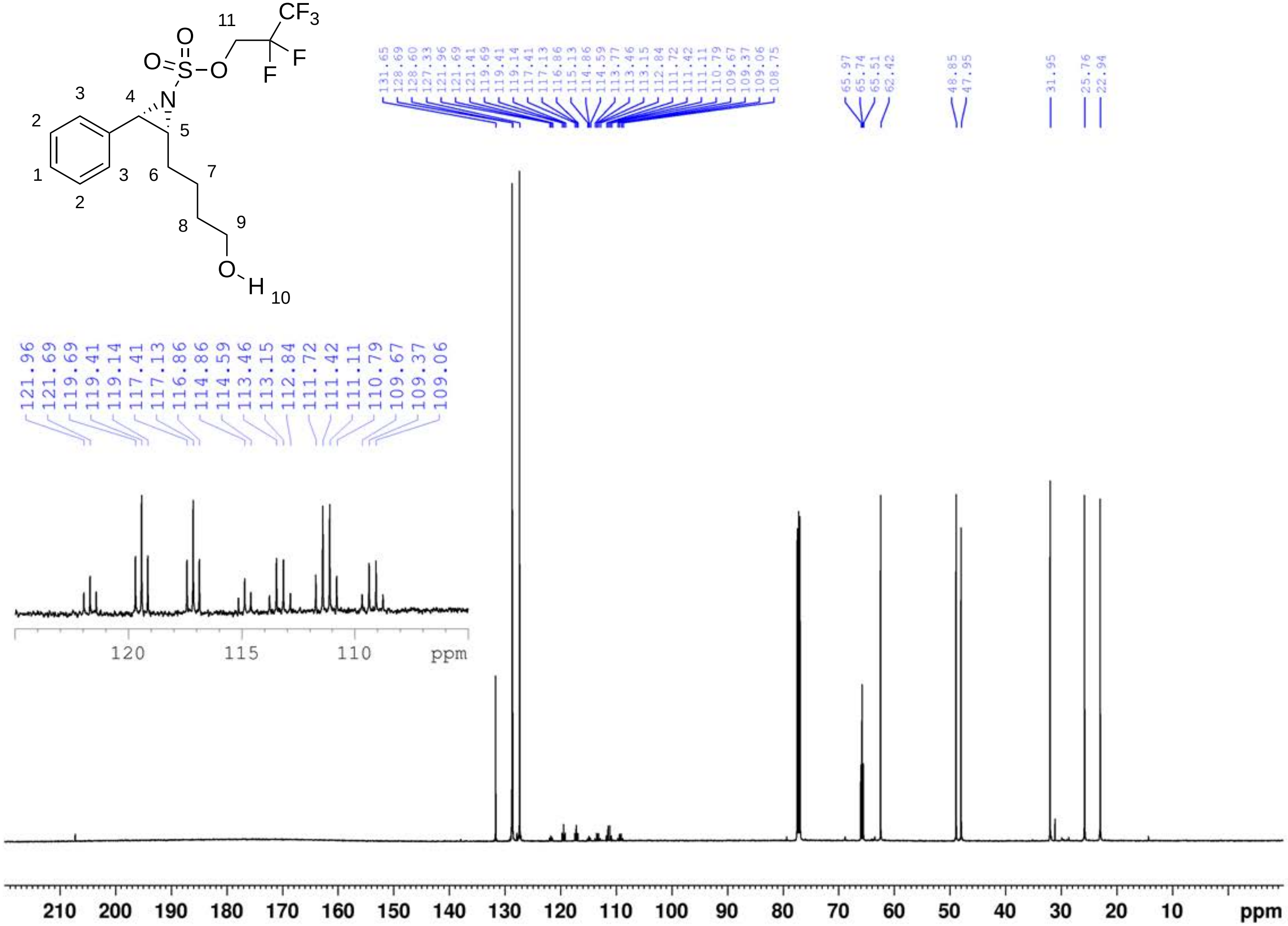

^{19}F NMR (376 MHz, $CDCl_3$) for *2,2,3,3,3-pentafluoropropyl (2R,3S)-2-(4-hydroxybutyl)-3-phenylaziridine-1-sulfonate* (**14a**)

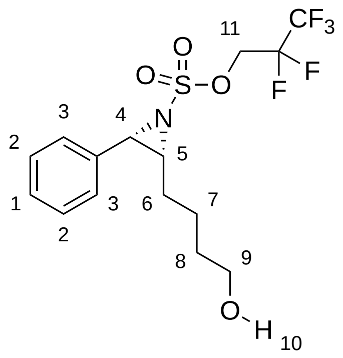

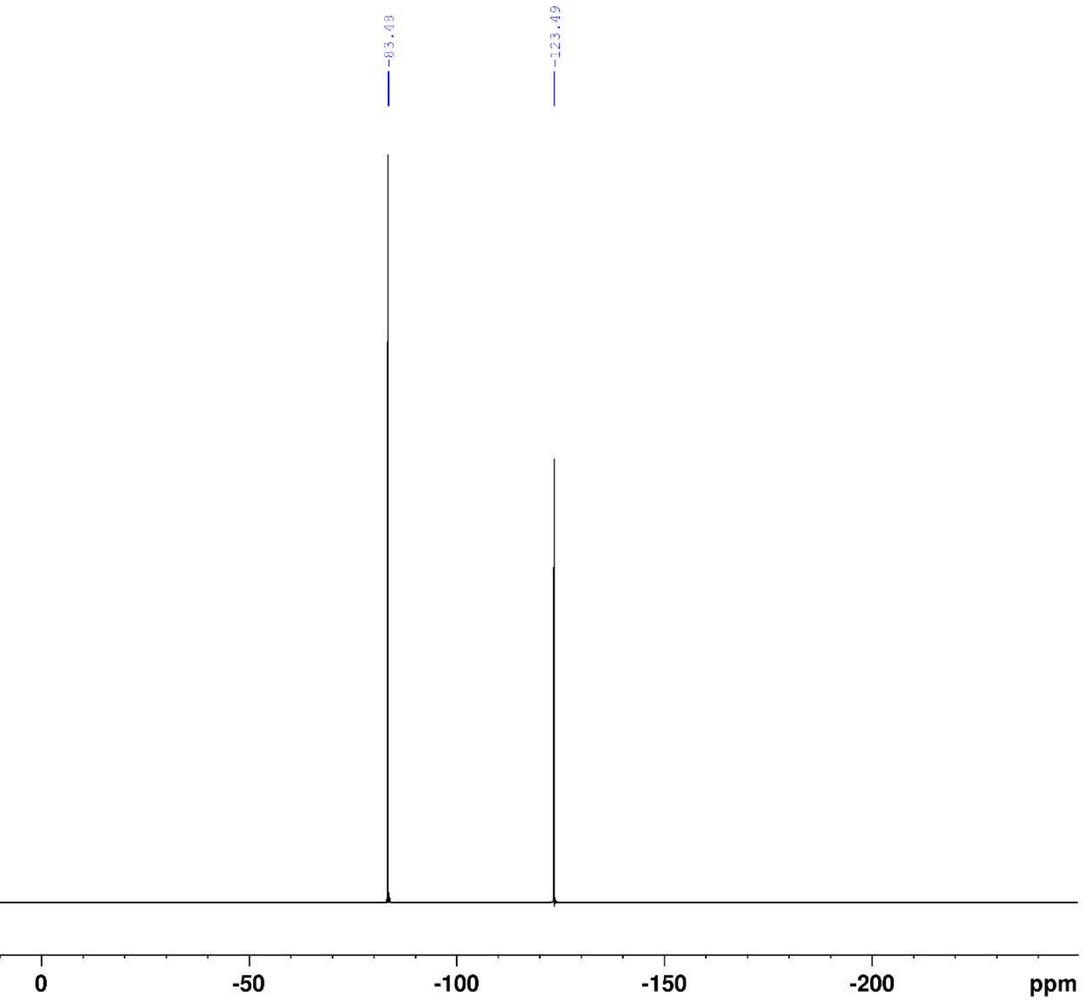

^{1}H NMR (400 MHz, $CDCl_3$) for *2,2,3,3,3-pentafluoropropyl (2S,3R)-2-(3-chlorophenyl)-3-(4-hydroxybutyl)aziridine-1-sulfonate* (**14b**)

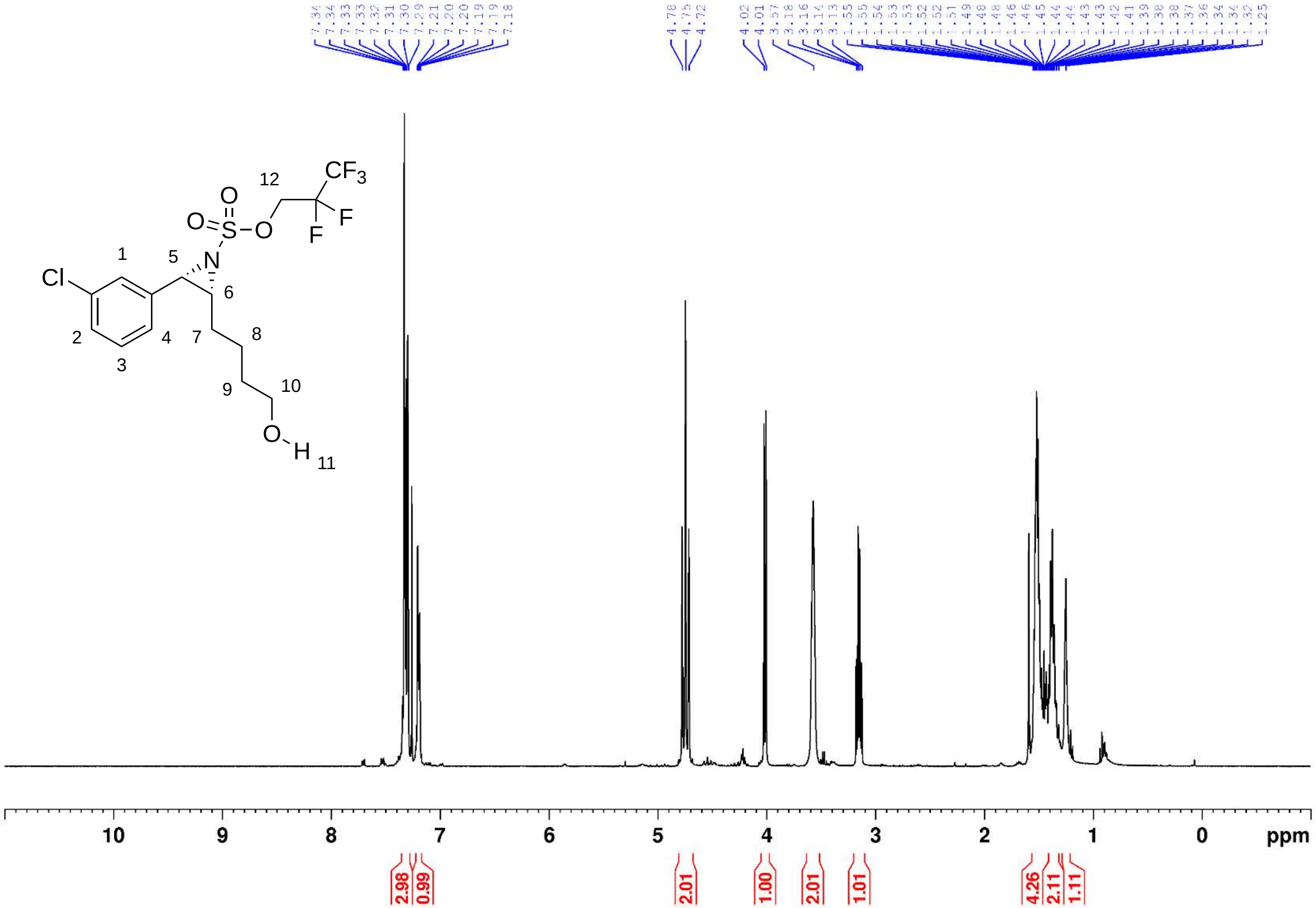

^{13}C NMR (101 MHz, $CDCl_3$) for *2,2,3,3,3-pentafluoropropyl (2S,3R)-2-(3-chlorophenyl)-3-(4-hydroxybutyl)aziridine-1-sulfonate* (**14b**)

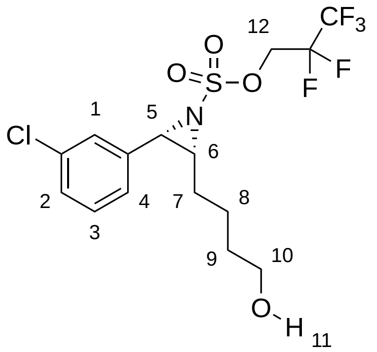

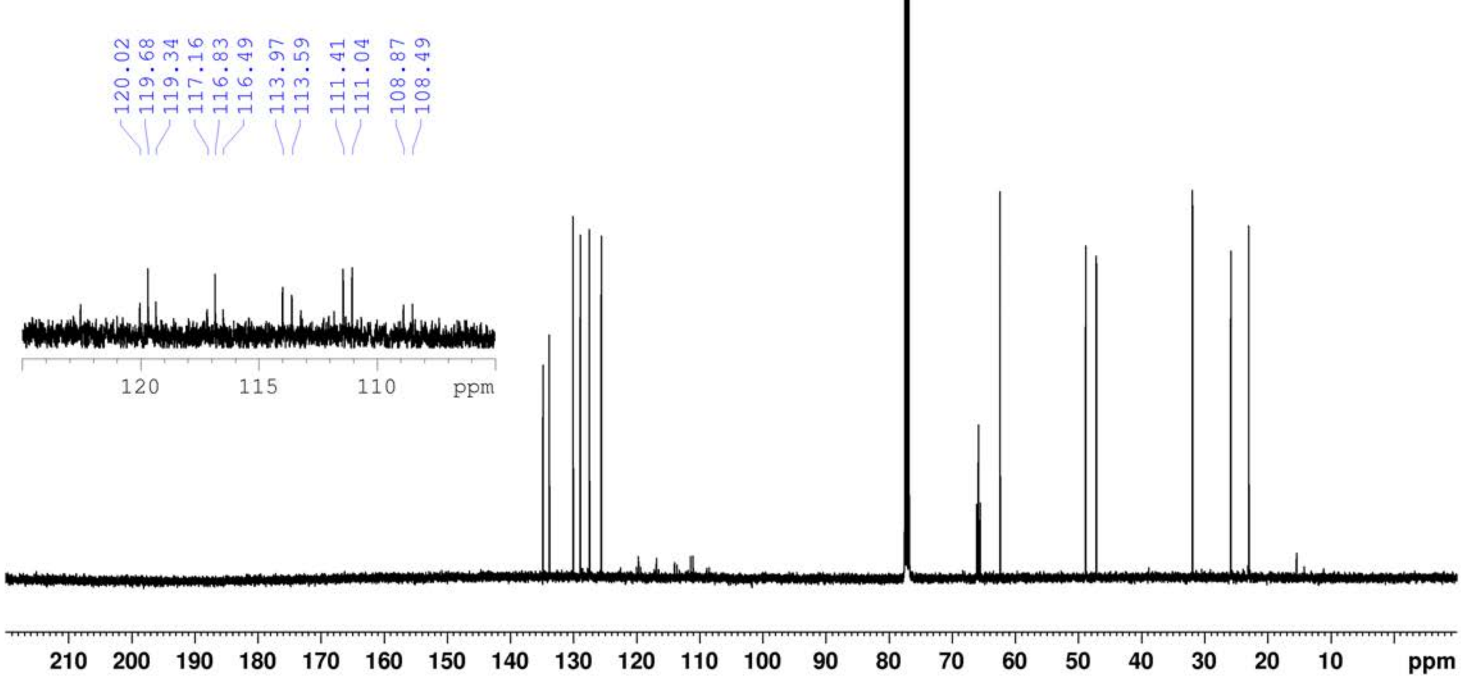

^{19}F NMR (376 MHz, $CDCl_3$) for *2,2,3,3,3-pentafluoropropyl (2S,3R)-2-(3-chlorophenyl)-3-(4-hydroxybutyl)aziridine-1-sulfonate* (**14b**)

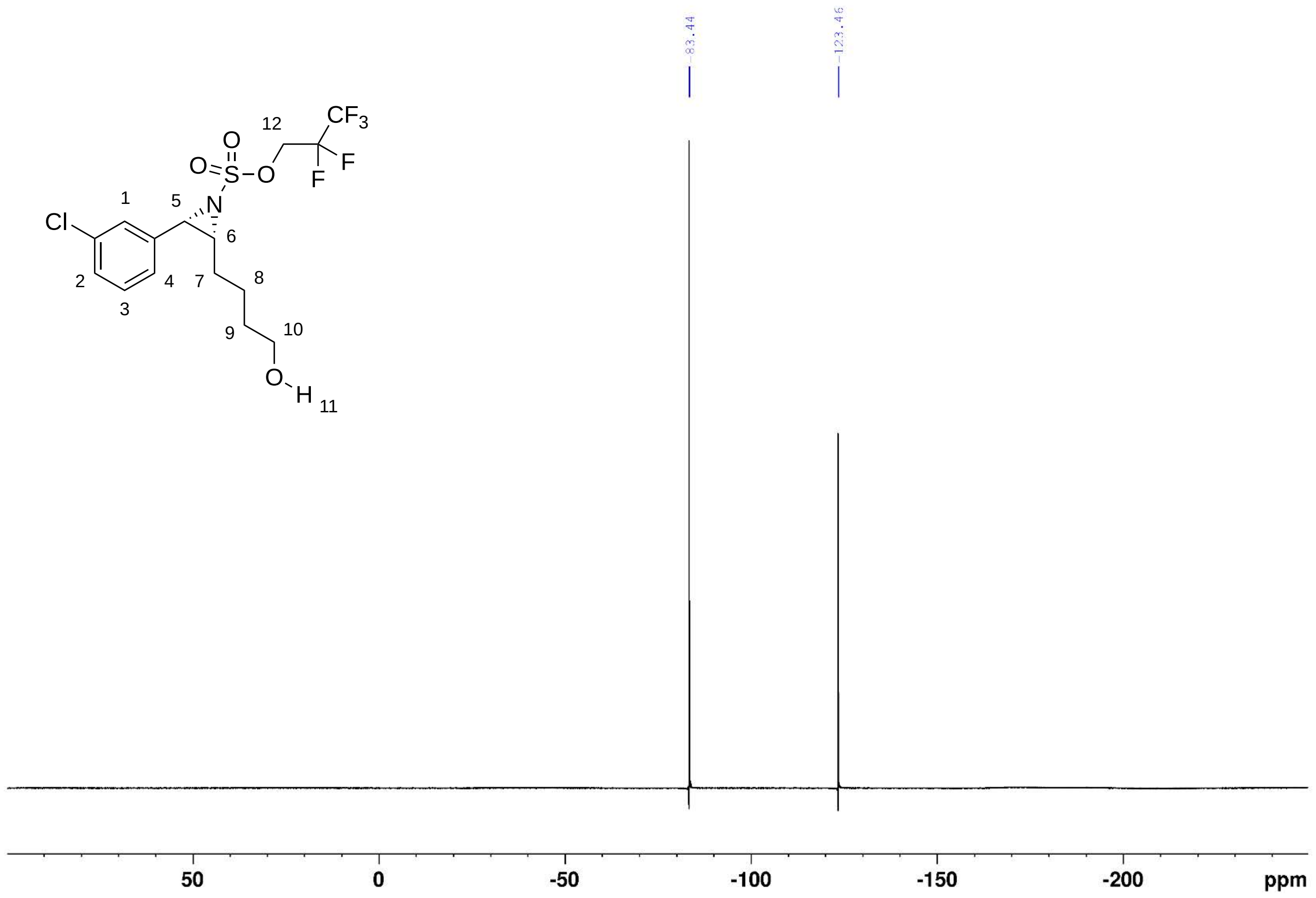

^{1}H NMR (400 MHz, $CDCl_3$) for *2,2,3,3,3-pentafluoropropyl (2R,3S)-2-(4-hydroxybutyl)-3-(3-(N-methylmethylsulfonamido)phenyl)aziridine-1-sulfonate* (**14c**)

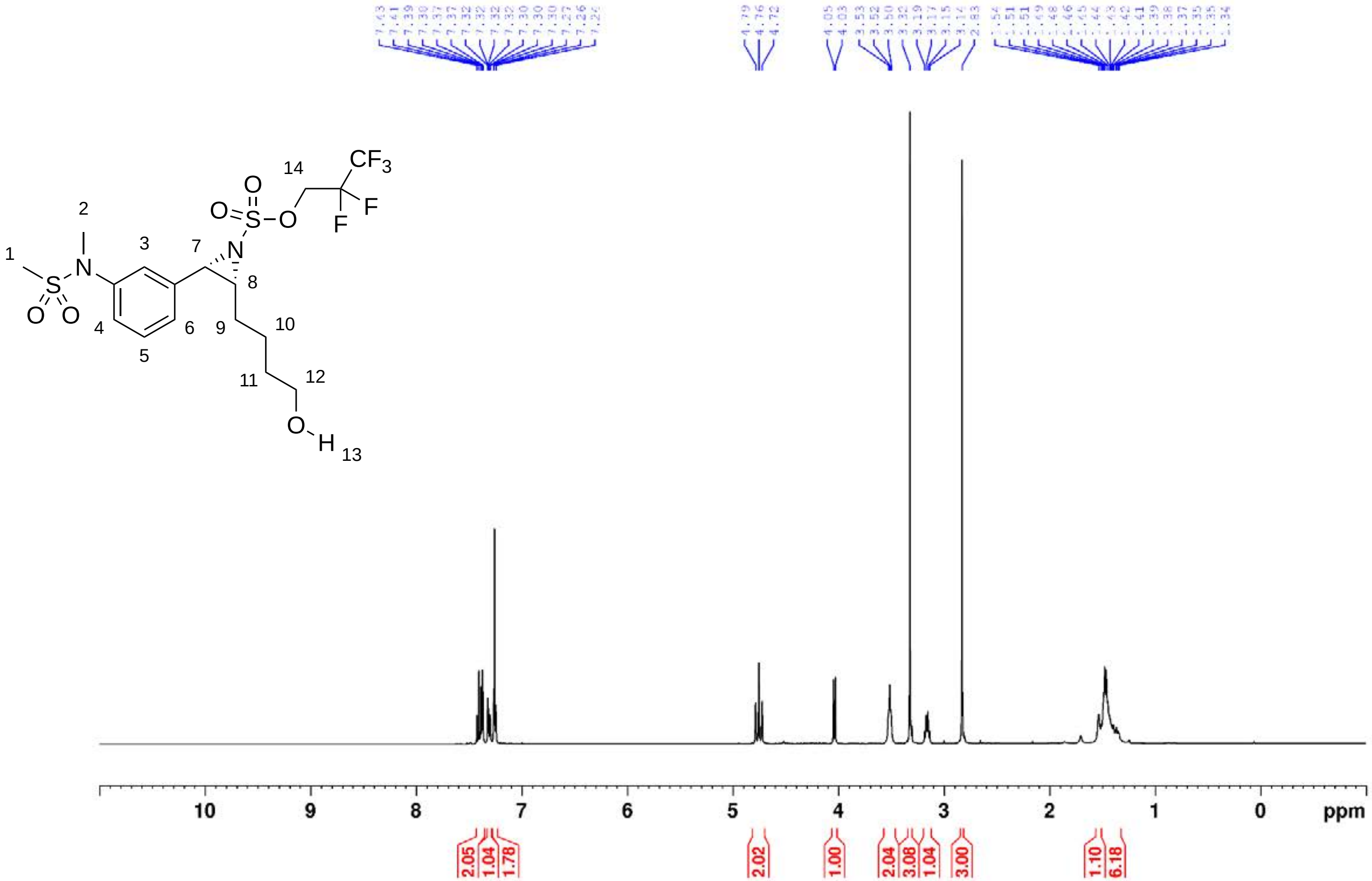

^{13}C NMR (101 MHz, $CDCl_3$) for *2,2,3,3,3-pentafluoropropyl (2R,3S)-2-(4-hydroxybutyl)-3-(3-(N-methylmethylsulfonamido)phenyl)aziridine-1-sulfonate* (**14c**)

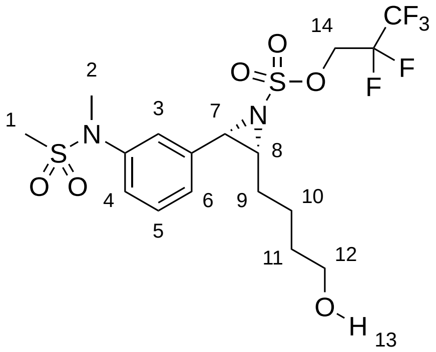

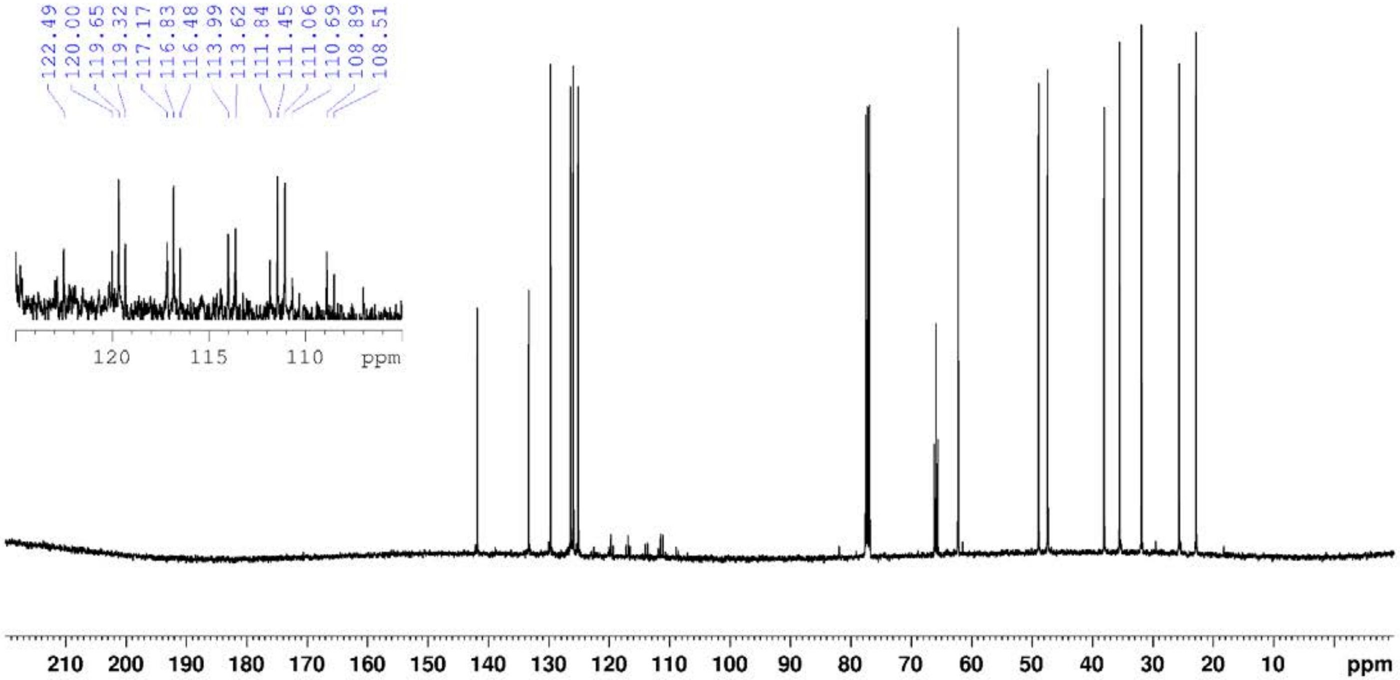

^{19}F NMR (376 MHz, $CDCl_3$) for *2,2,3,3,3-pentafluoropropyl (2R,3S)-2-(4-hydroxybutyl)-3-(3-(N-methylmethylsulfonamido)phenyl)aziridine-1-sulfonate* (**14c**)

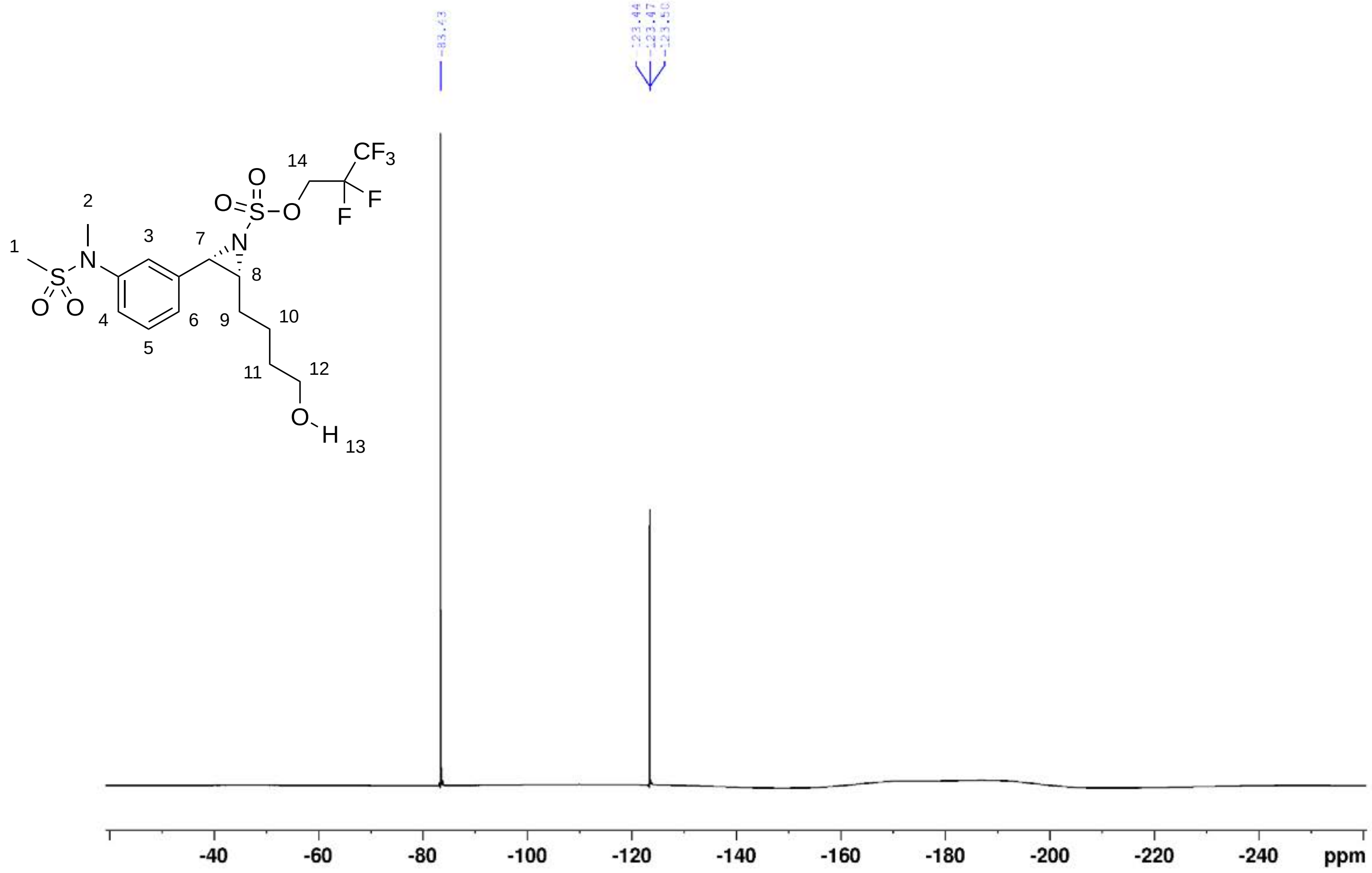

^{1}H NMR (500 MHz, $CDCl_3$) for *Methyl 2-(3-((2S,3R)-3-(4-hydroxybutyl)-1-((2,2,3,3,3-pentafluoropropoxy)sulfonyl)aziridin-2-yl)phenyl)acetate* (**14d**)

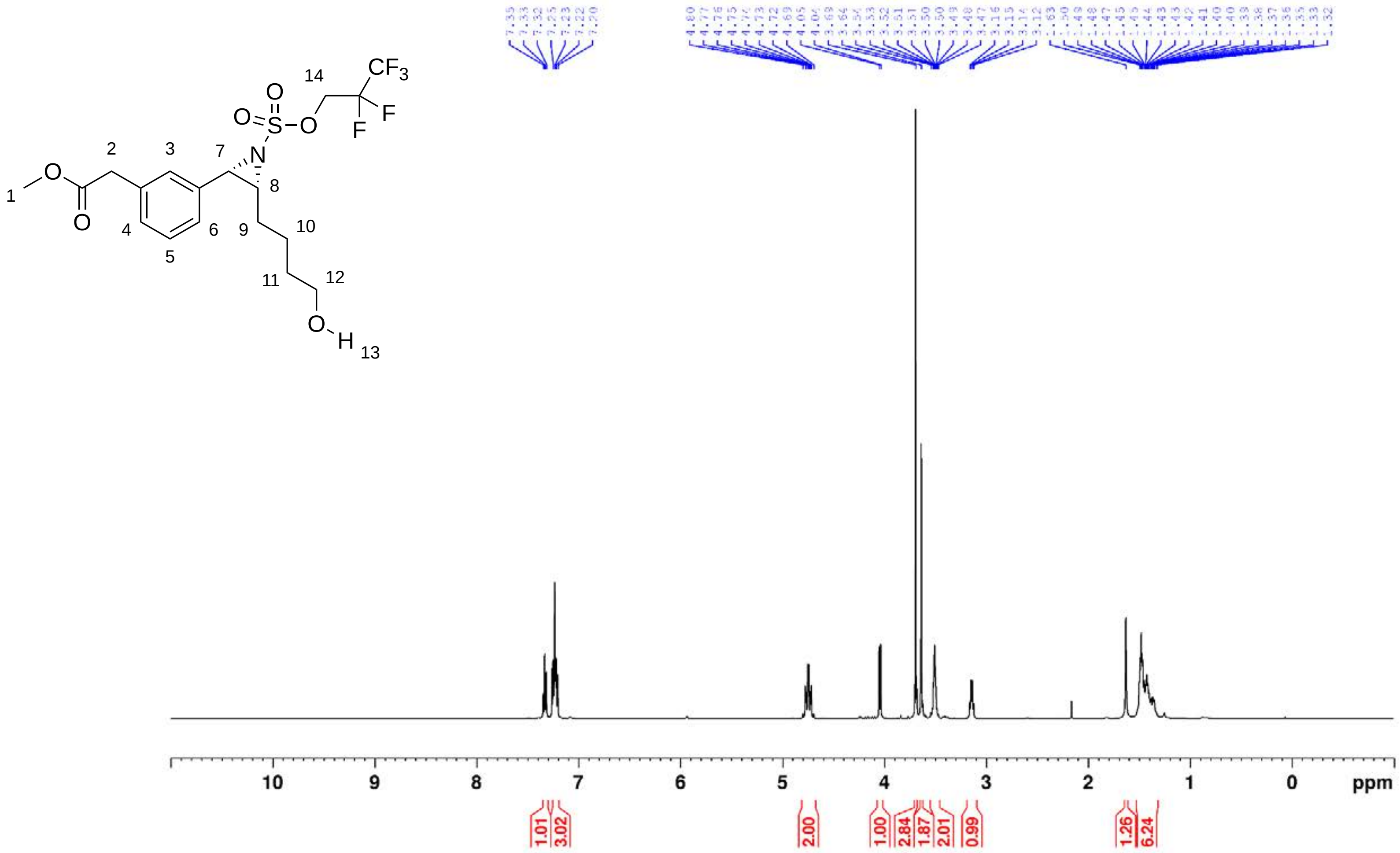

^{13}C NMR (126 MHz, $CDCl_3$) for *Methyl 2-(3-((2S,3R)-3-(4-hydroxybutyl)-1-((2,2,3,3,3-pentafluoropropoxy)sulfonyl)aziridin-2-yl)phenyl)acetate* (**14d**)

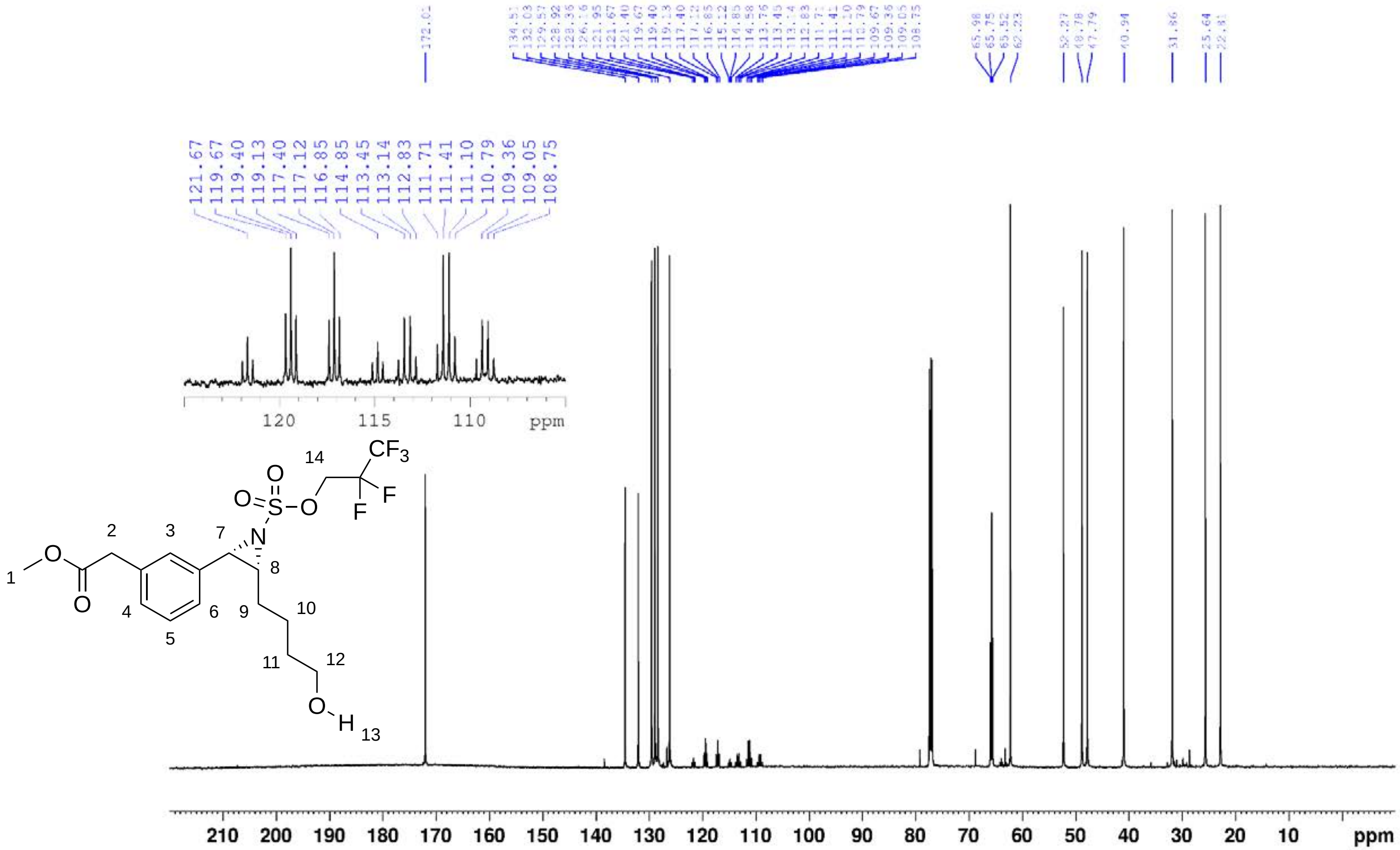

^{19}F NMR (376 MHz, $CDCl_3$) for *Methyl 2-(3-((2S,3R)-3-(4-hydroxybutyl)-1-((2,2,3,3,3-pentafluoropropoxy)sulfonyl)aziridin-2-yl)phenyl)acetate* (**14d**)

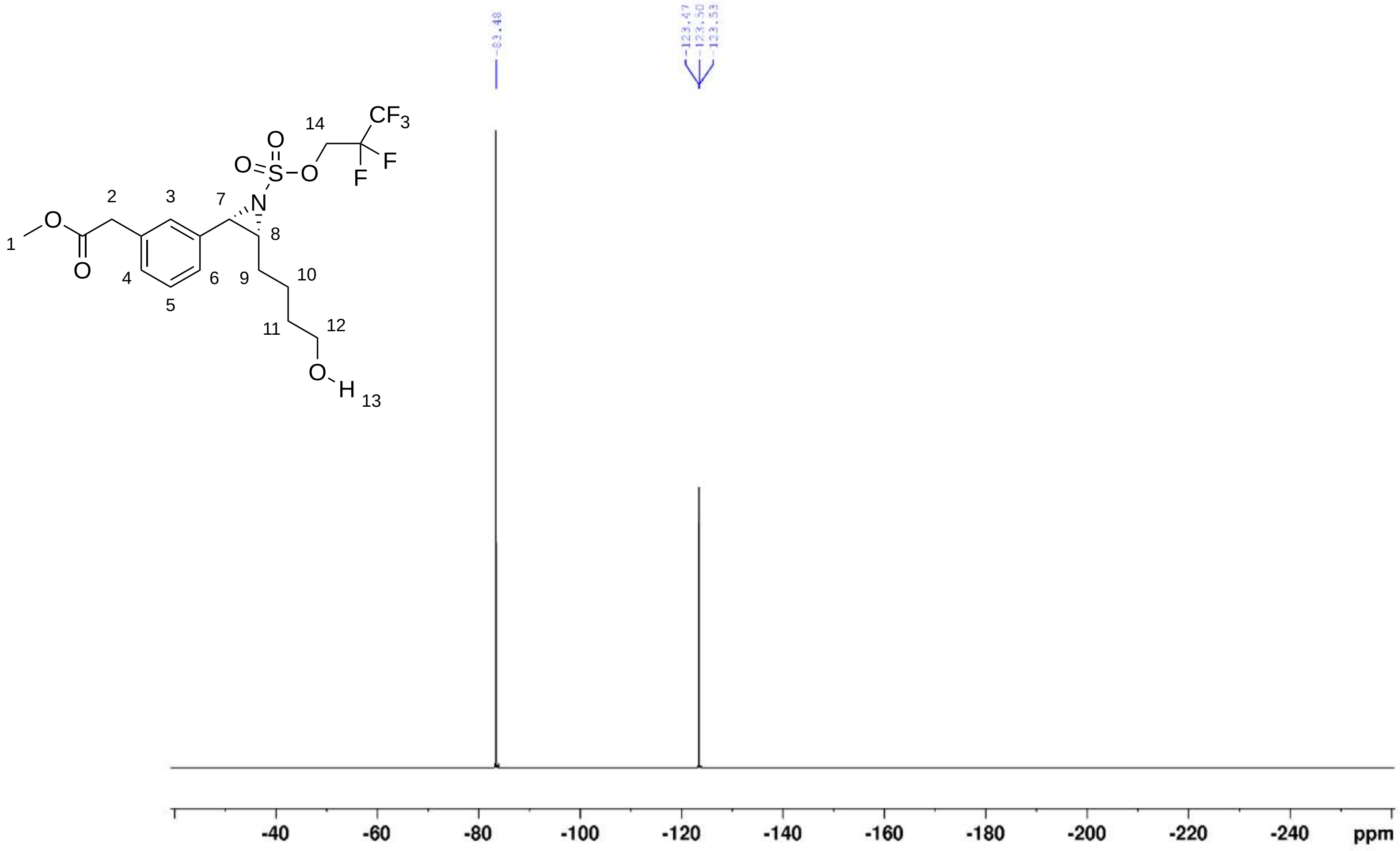

^{1}H NMR (500 MHz, $CDCl_3$) for *2,2,3,3,3-pentafluoropropyl (2S,3R)-2-(3-(N,N-diethylsulfamoyl)phenyl)-3-(4-hydroxybutyl)aziridine-1-sulfonate* (**14e**)

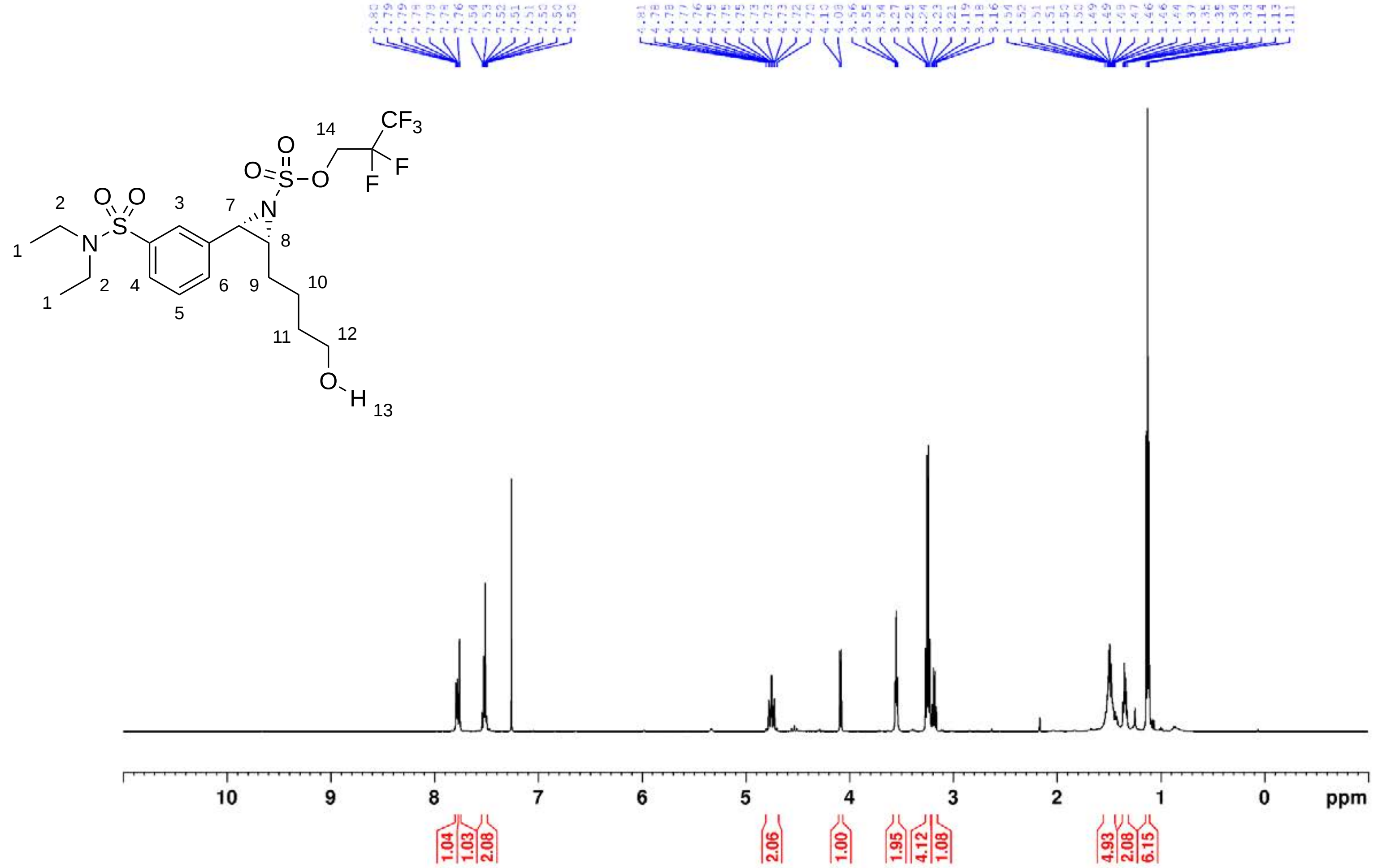

^{13}C NMR (126 MHz, $CDCl_3$) for *2,2,3,3,3-pentafluoropropyl (2S,3R)-2-(3-(N,N-diethylsulfamoyl)phenyl)-3-(4-hydroxybutyl)aziridine-1-sulfonate* (**14e**)

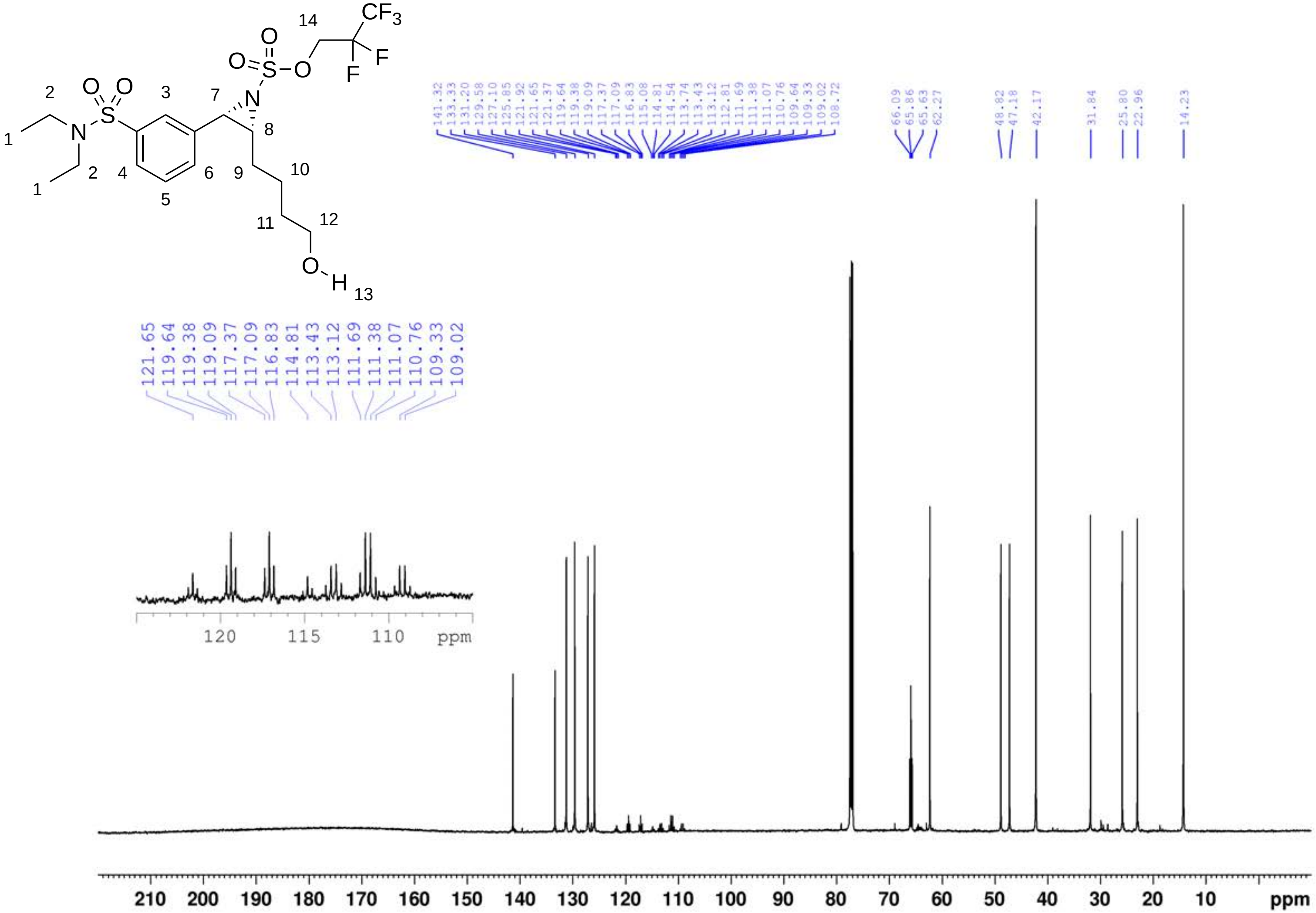

^{19}F NMR (376 MHz, $CDCl_3$) for *2,2,3,3,3-pentafluoropropyl (2S,3R)-2-(3-(N,N-diethylsulfamoyl)phenyl)-3-(4-hydroxybutyl)aziridine-1-sulfonate* (**14e**)

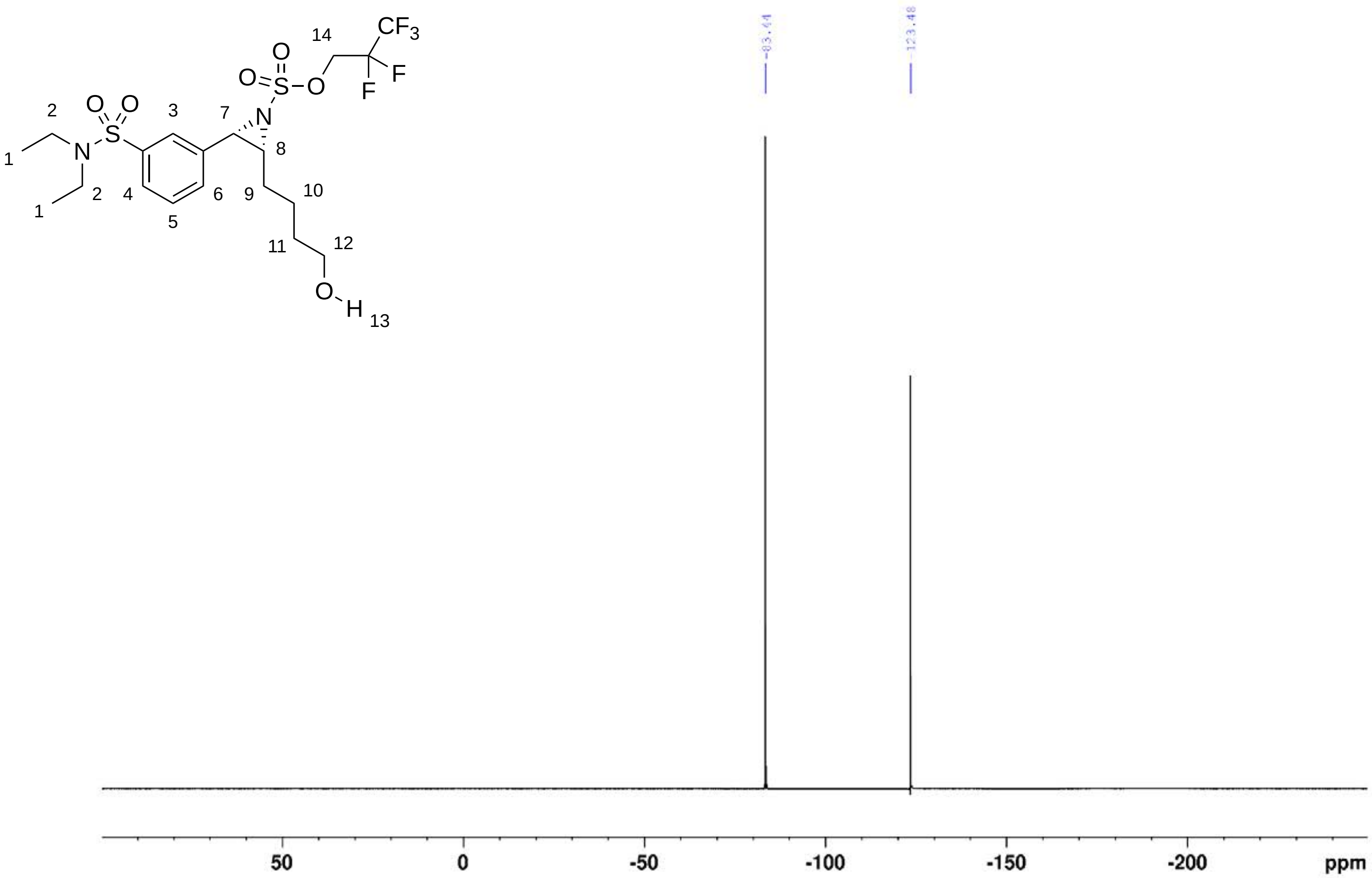

^{1}H NMR (400 MHz, $CDCl_3$) for *2,2,3,3,3-pentafluoropropyl (2S,3R)-2-(2-chlorophenyl)-3-(4-hydroxybutyl)aziridine-1-sulfonate* (**14f**)

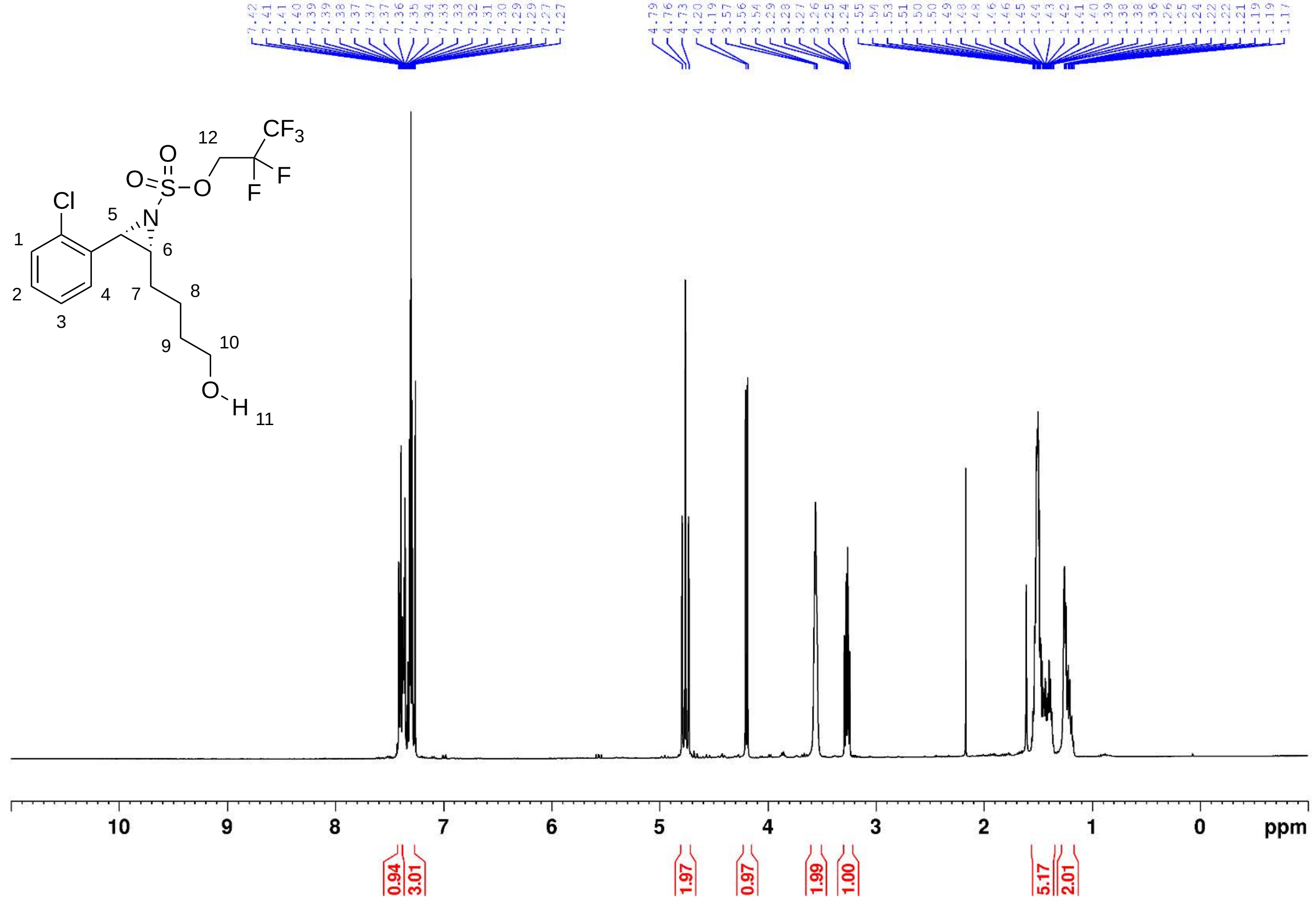

^{13}C NMR (101 MHz, $CDCl_3$) for *2,2,3,3,3-pentafluoropropyl (2S,3R)-2-(2-chlorophenyl)-3-(4-hydroxybutyl)aziridine-1-sulfonate* (**14f**)

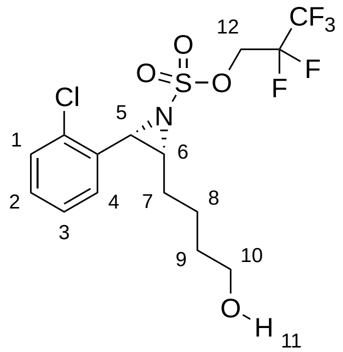

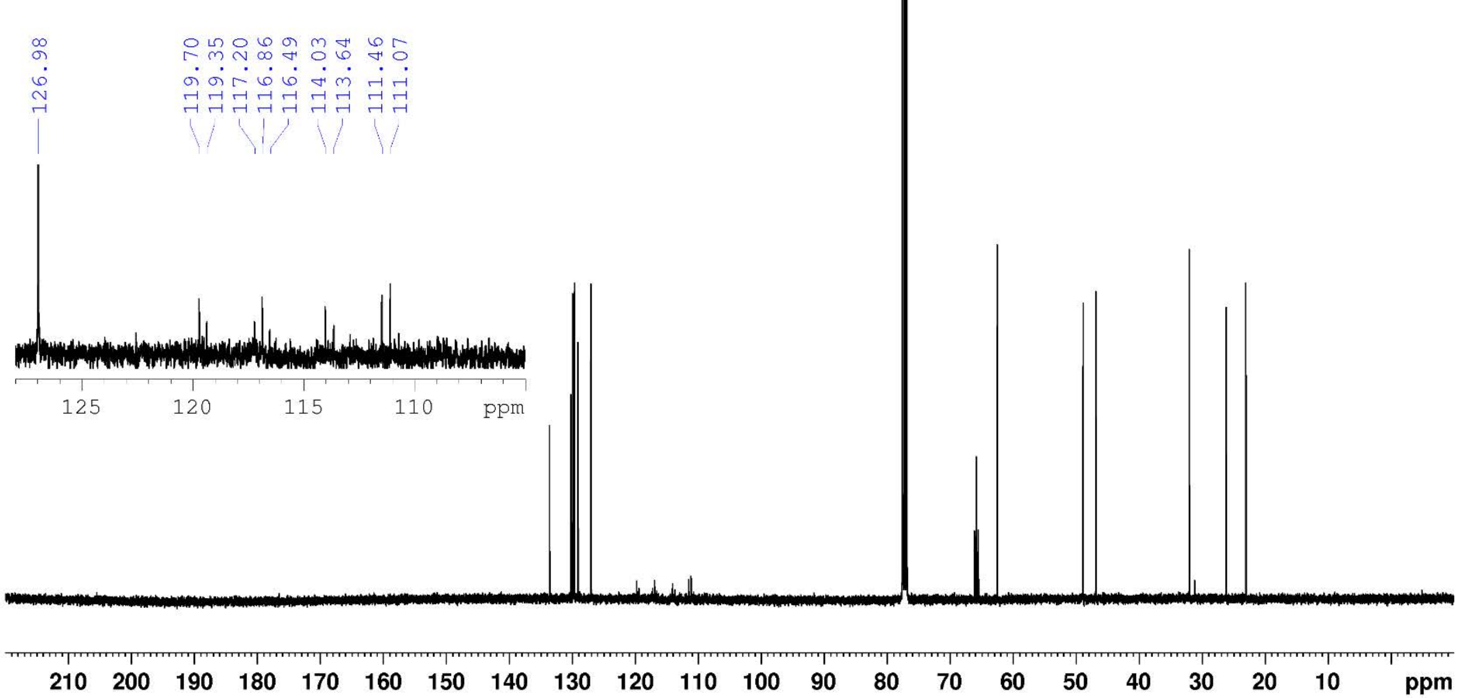

^{19}F NMR (376 MHz, $CDCl_3$) for *2,2,3,3,3-pentafluoropropyl (2S,3R)-2-(2-chlorophenyl)-3-(4-hydroxybutyl)aziridine-1-sulfonate* (**14f**)

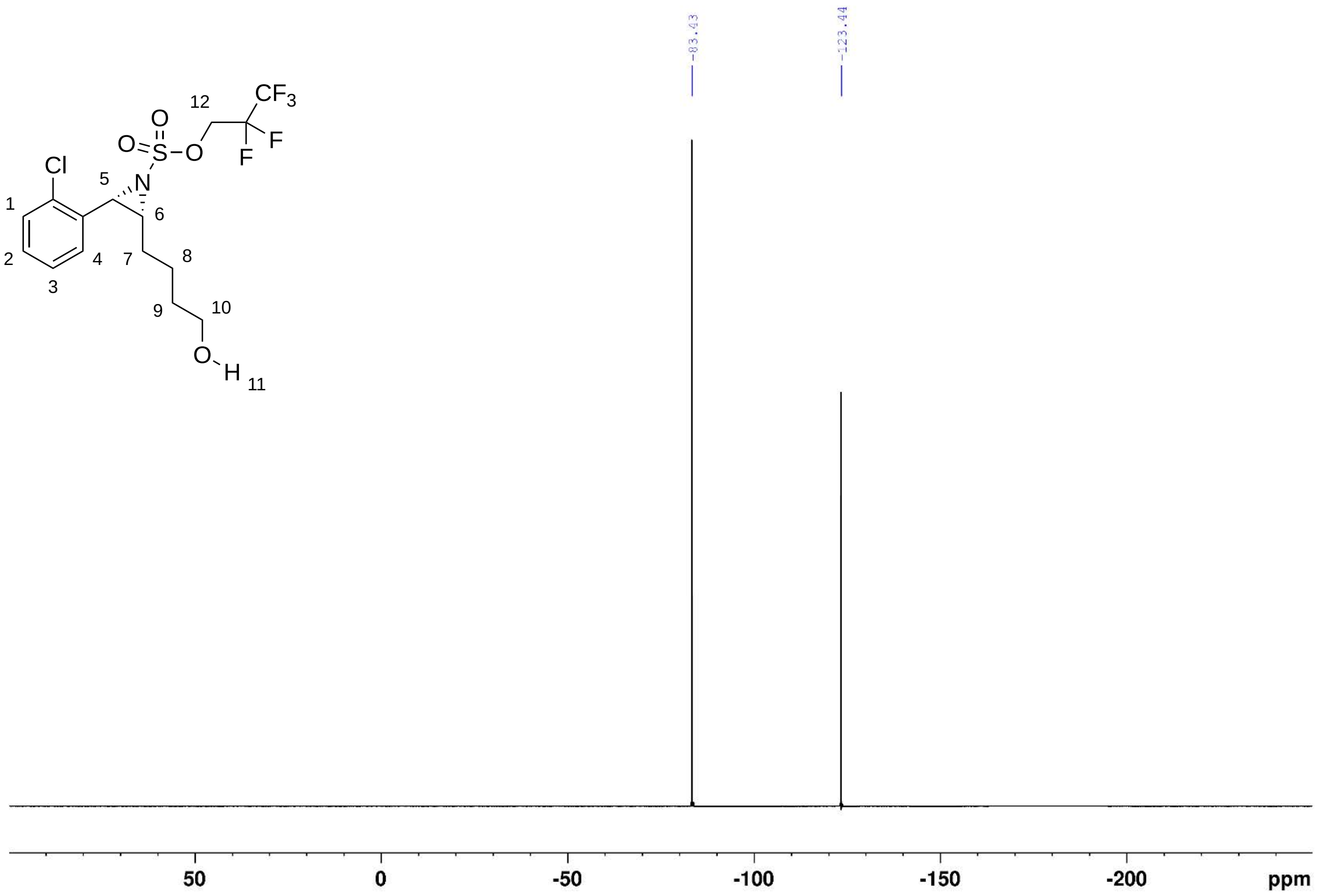

^{1}H NMR (400 MHz, $CDCl_3$) for *2,2,3,3,3-pentafluoropropyl (2S,3R)-2-(4-chlorophenyl)-3-(4-hydroxybutyl)aziridine-1-sulfonate* (**14g**)

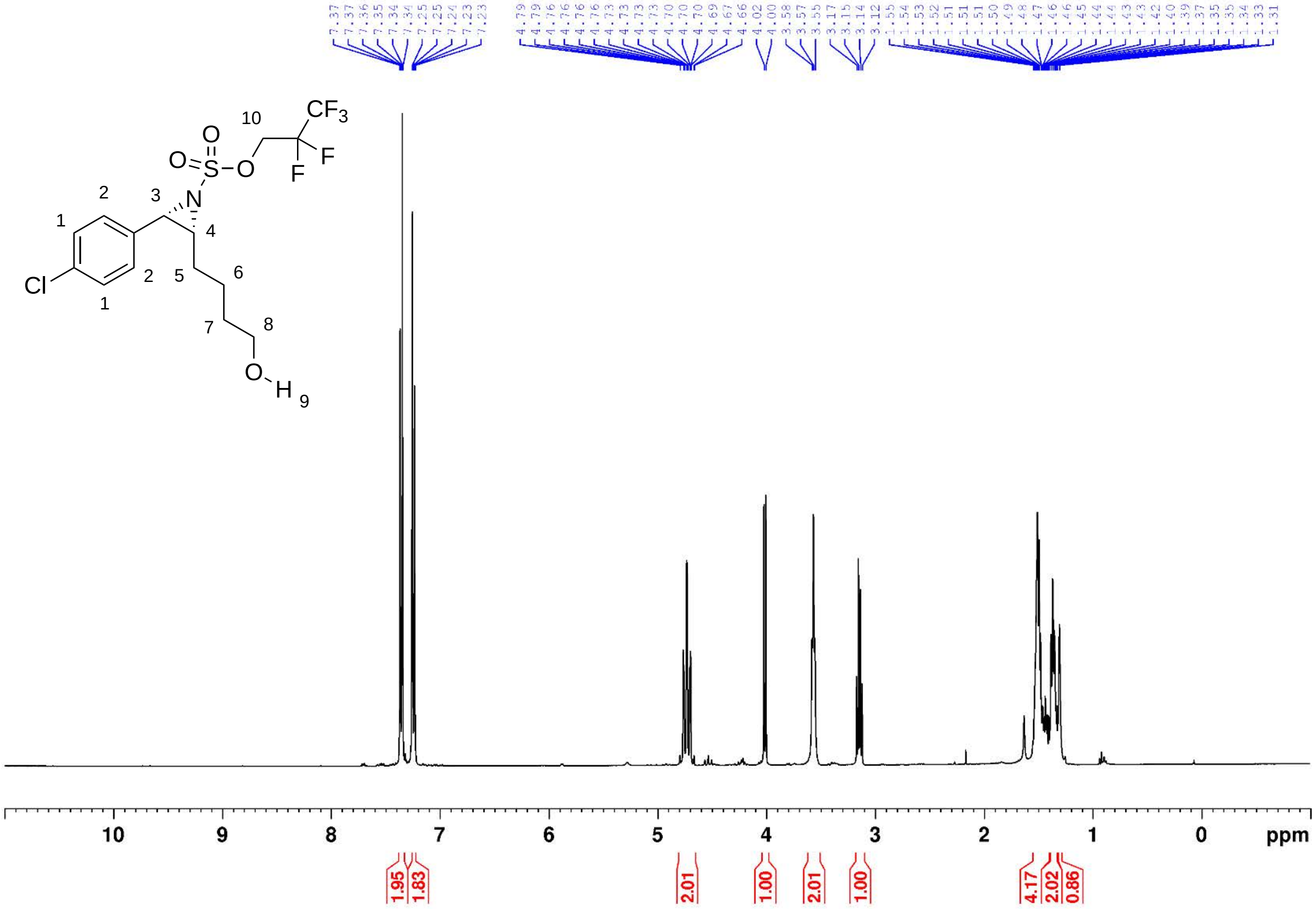

^{13}C NMR (101 MHz, $CDCl_3$) for *2,2,3,3,3-pentafluoropropyl (2S,3R)-2-(4-chlorophenyl)-3-(4-hydroxybutyl)aziridine-1-sulfonate* (**14g**)

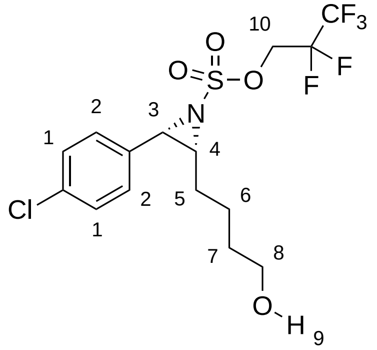

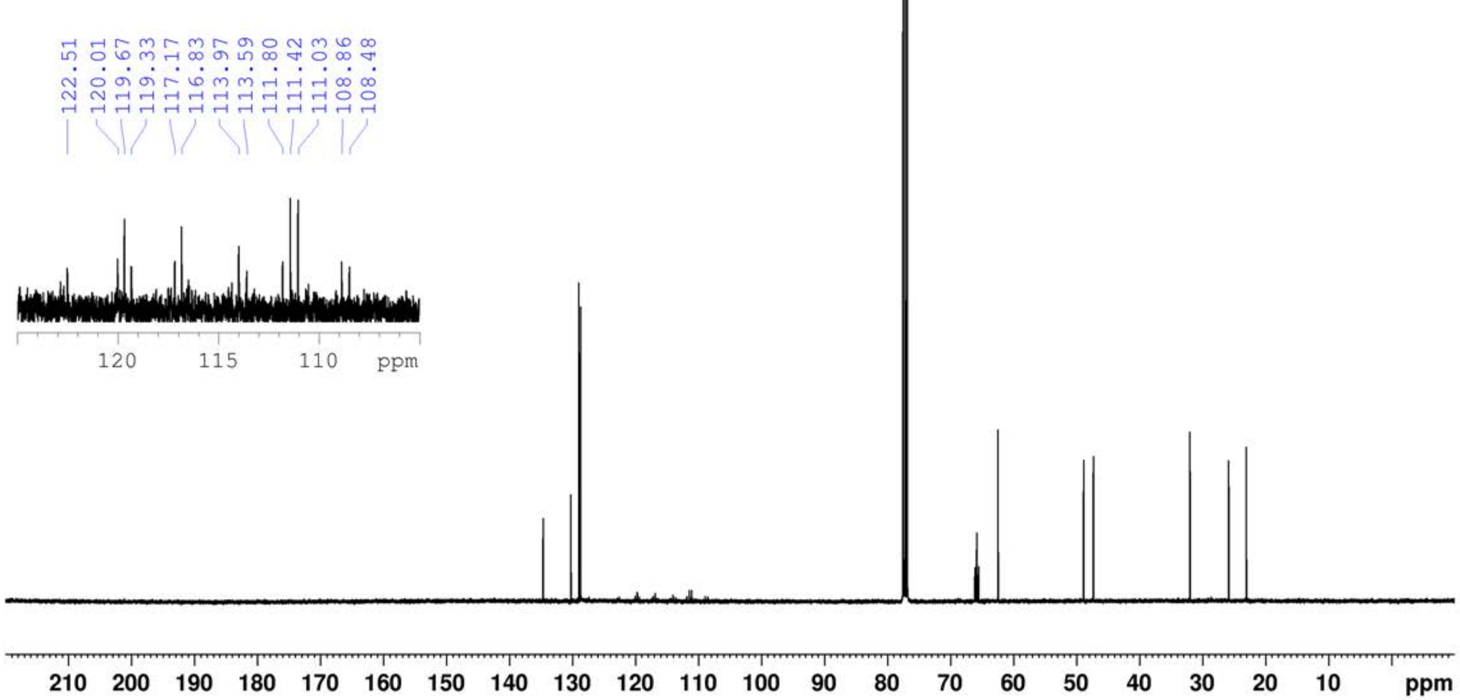

^{19}F NMR (376 MHz, $CDCl_3$) for *2,2,3,3,3-pentafluoropropyl (2S,3R)-2-(4-chlorophenyl)-3-(4-hydroxybutyl)aziridine-1-sulfonate* (**14g**)

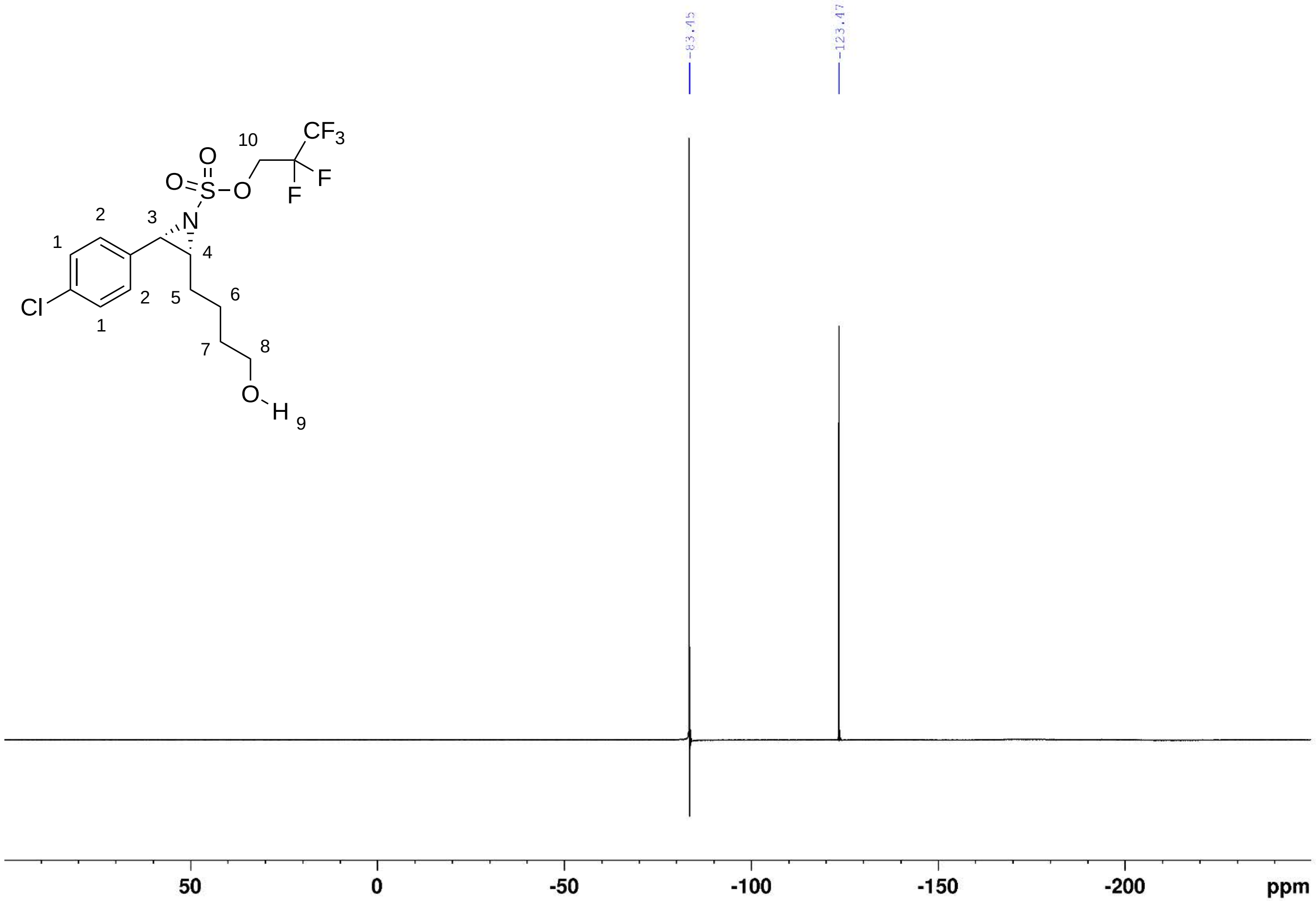

^{1}H NMR (400 MHz, $CDCl_3$) for *2,2,3,3,3-pentafluoropropyl (2S,3R)-2-(4-(tert-butyl)phenyl)-3-(4-hydroxybutyl)aziridine-1-sulfonate* (**14h**)

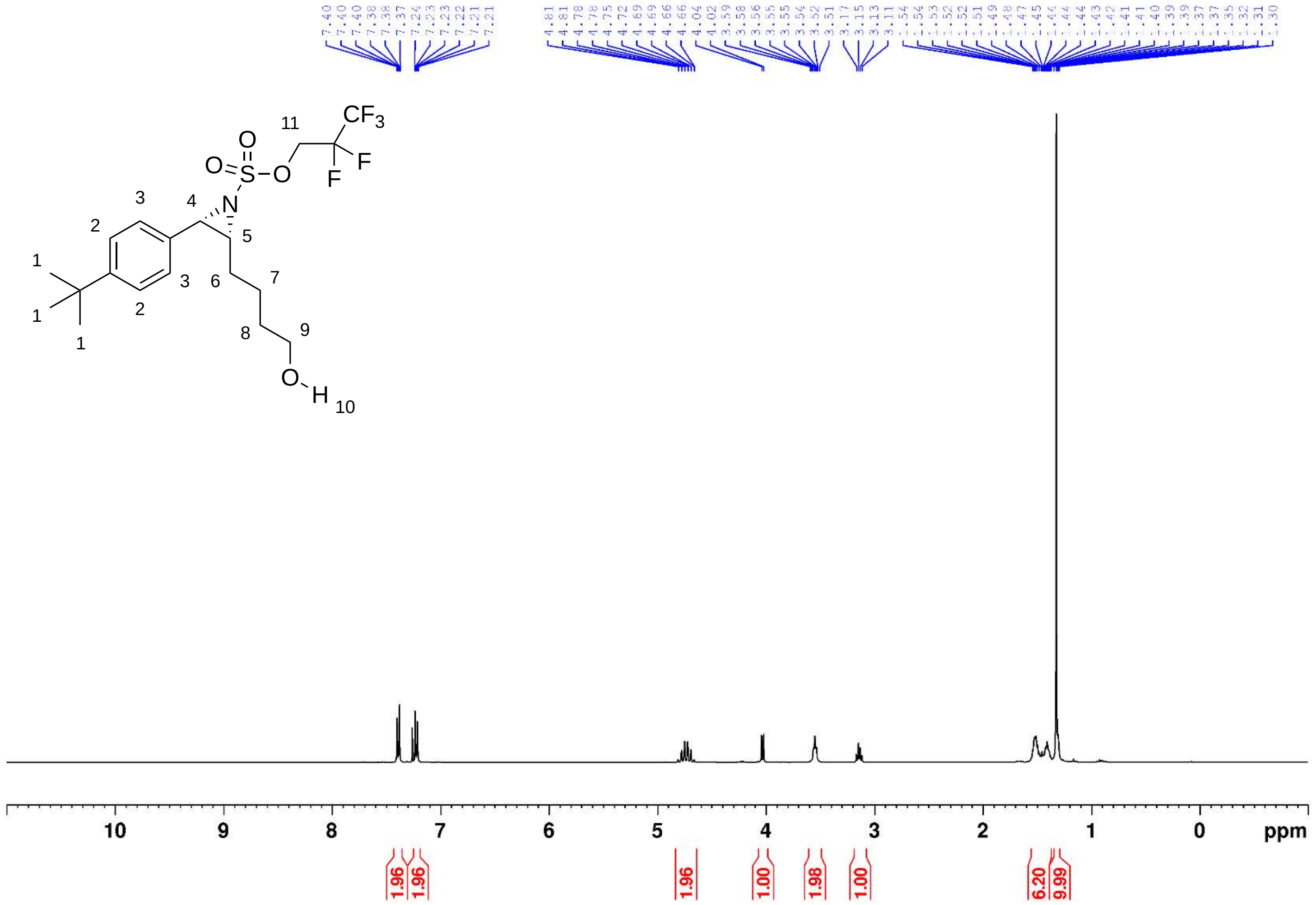

^{13}C NMR (101 MHz, $CDCl_3$) for *2,2,3,3,3-pentafluoropropyl (2S,3R)-2-(4-(tert-butyl)phenyl)-3-(4-hydroxybutyl)aziridine-1-sulfonate* (**14h**)

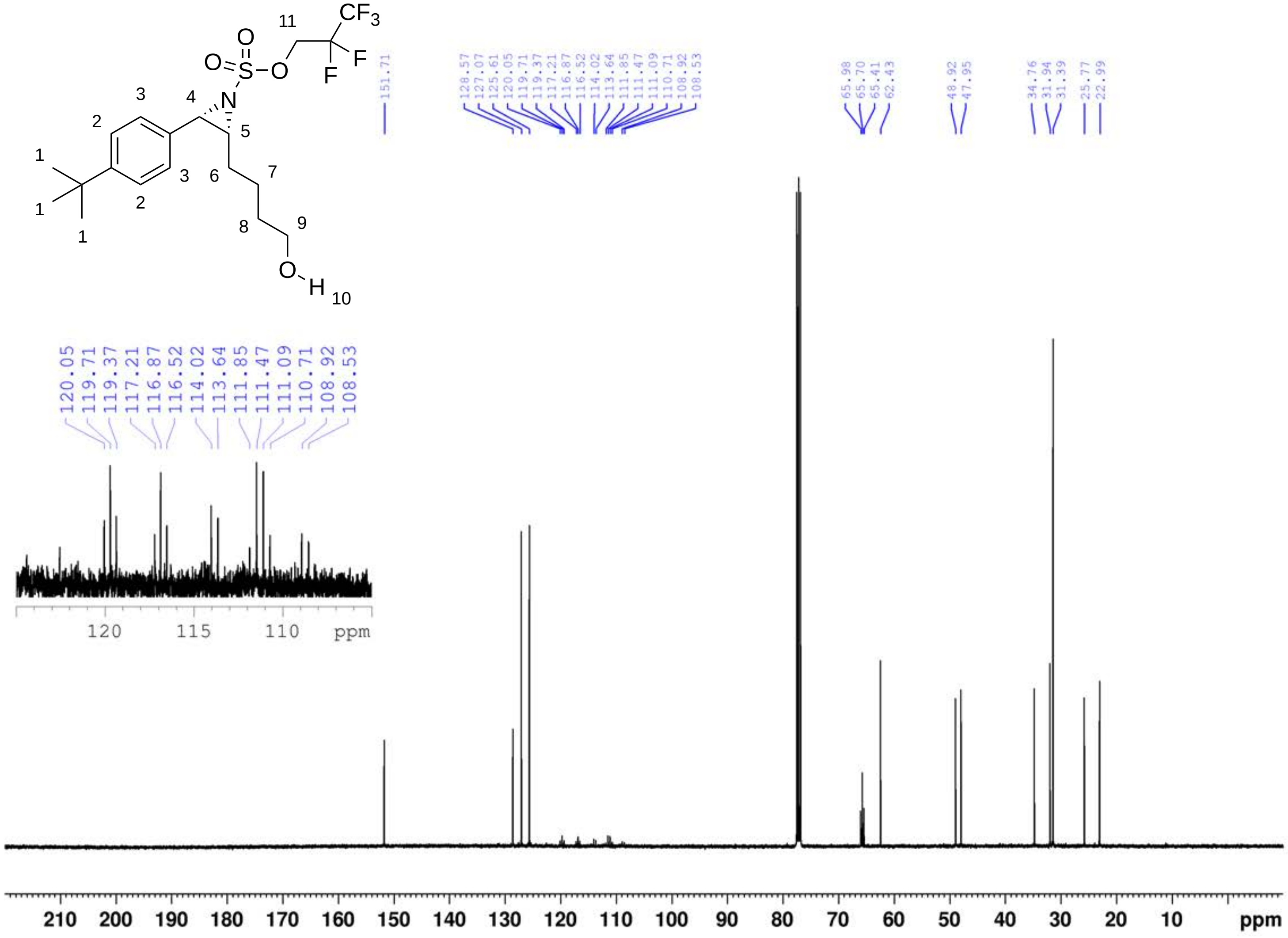

^{19}F NMR (376 MHz, $CDCl_3$) for *2,2,3,3,3-pentafluoropropyl (2S,3R)-2-(4-(tert-butyl)phenyl)-3-(4-hydroxybutyl)aziridine-1-sulfonate* (**14h**)

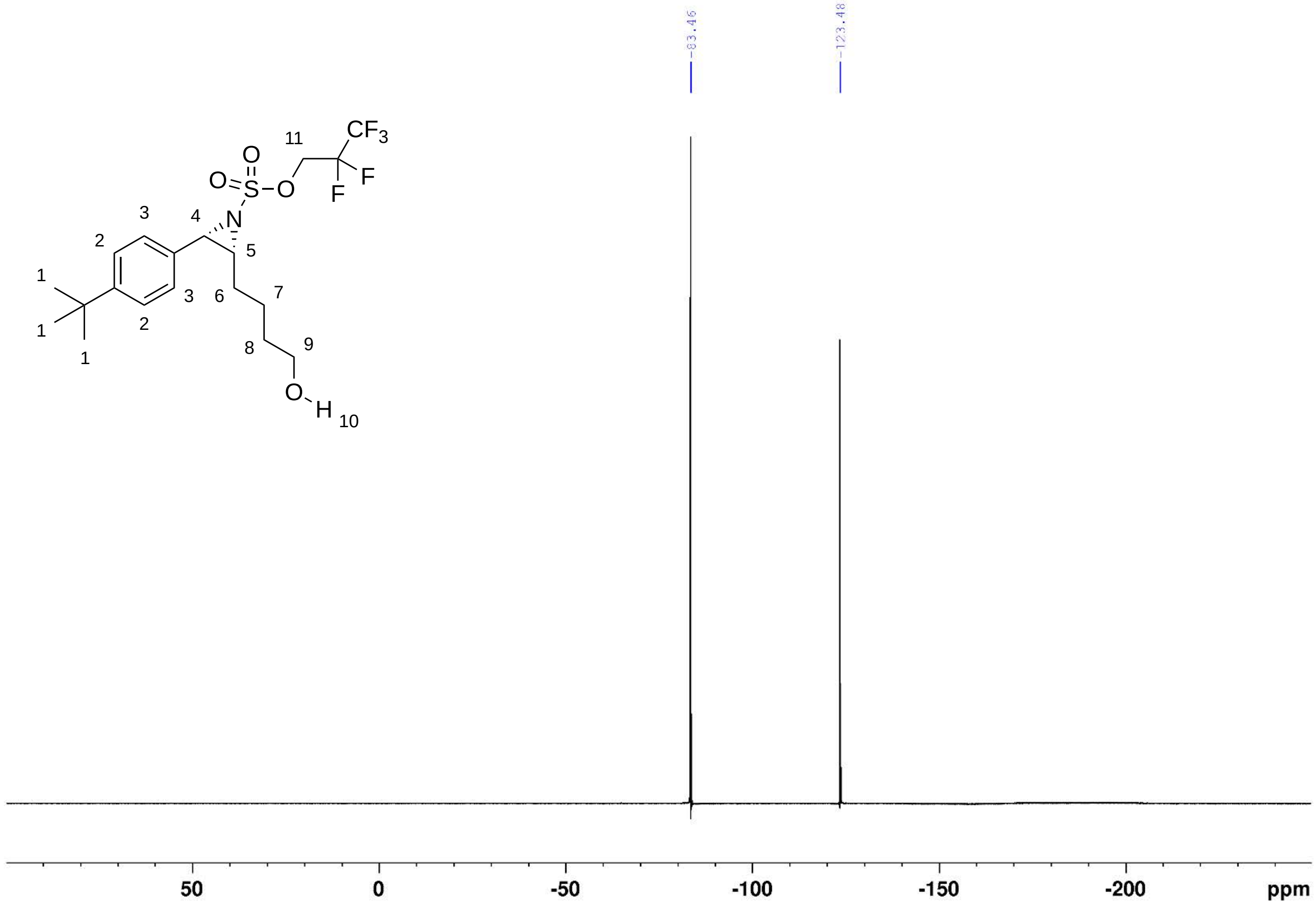

^{1}H NMR (500 MHz, $CDCl_3$) for *2,2,3,3,3-pentafluoropropyl (2R,3S)-2-(4-hydroxybutyl)-3-(4-(pyrrolidin-1-ylsulfonyl)phenyl)aziridine-1-sulfonate* (**14i**)

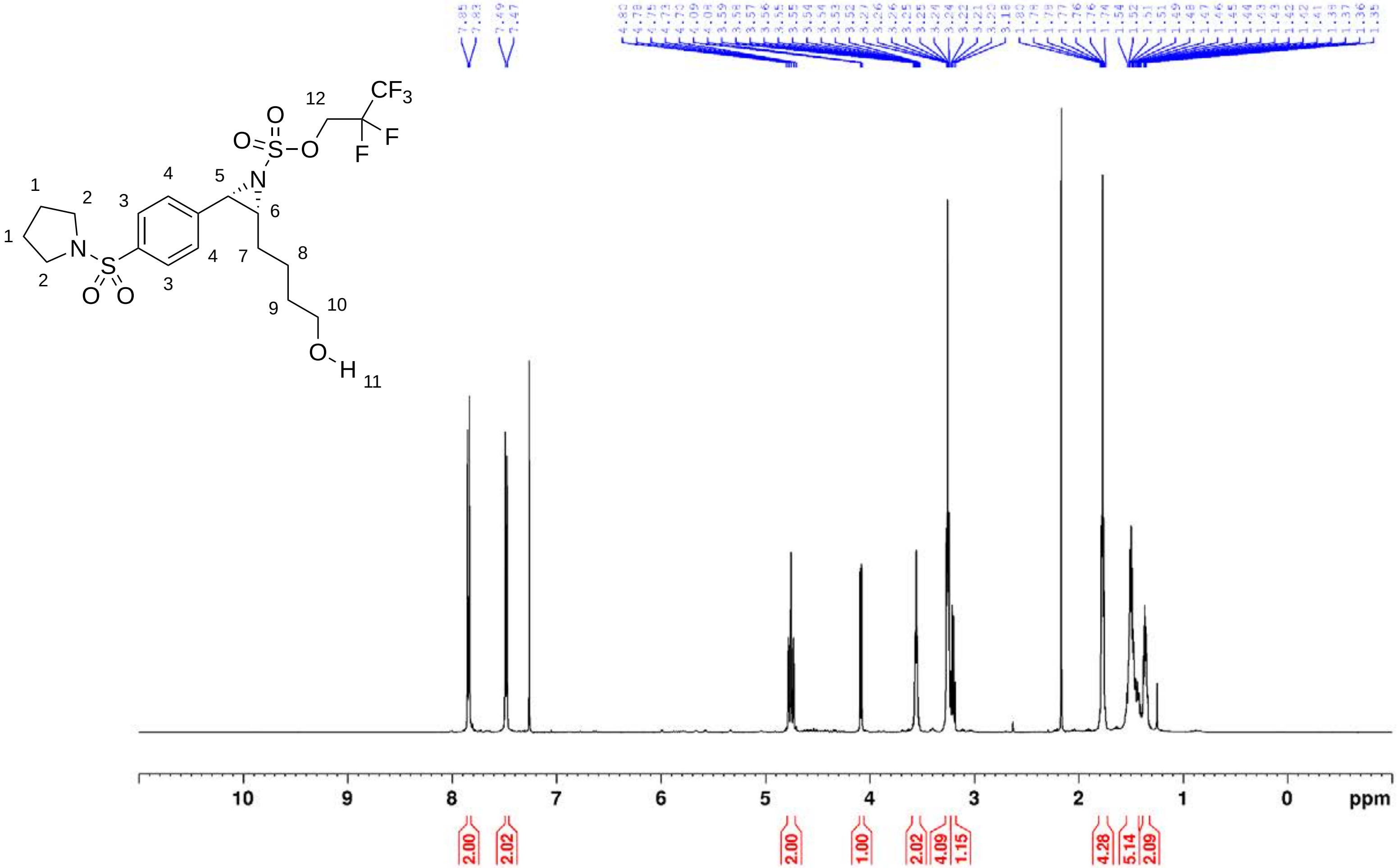

^{13}C NMR (126 MHz, $CDCl_3$) for *2,2,3,3,3-pentafluoropropyl (2R,3S)-2-(4-hydroxybutyl)-3-(4-(pyrrolidin-1-ylsulfonyl)phenyl)aziridine-1-sulfonate* (**14i**)

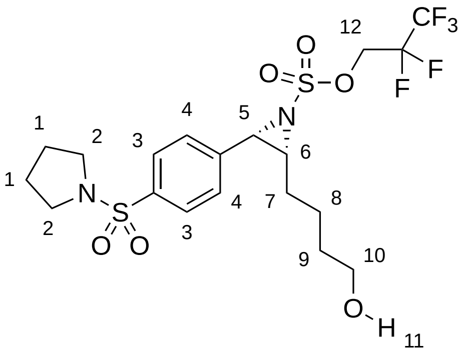

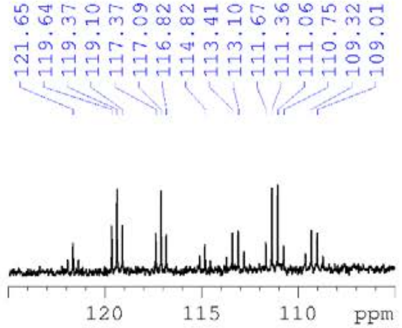

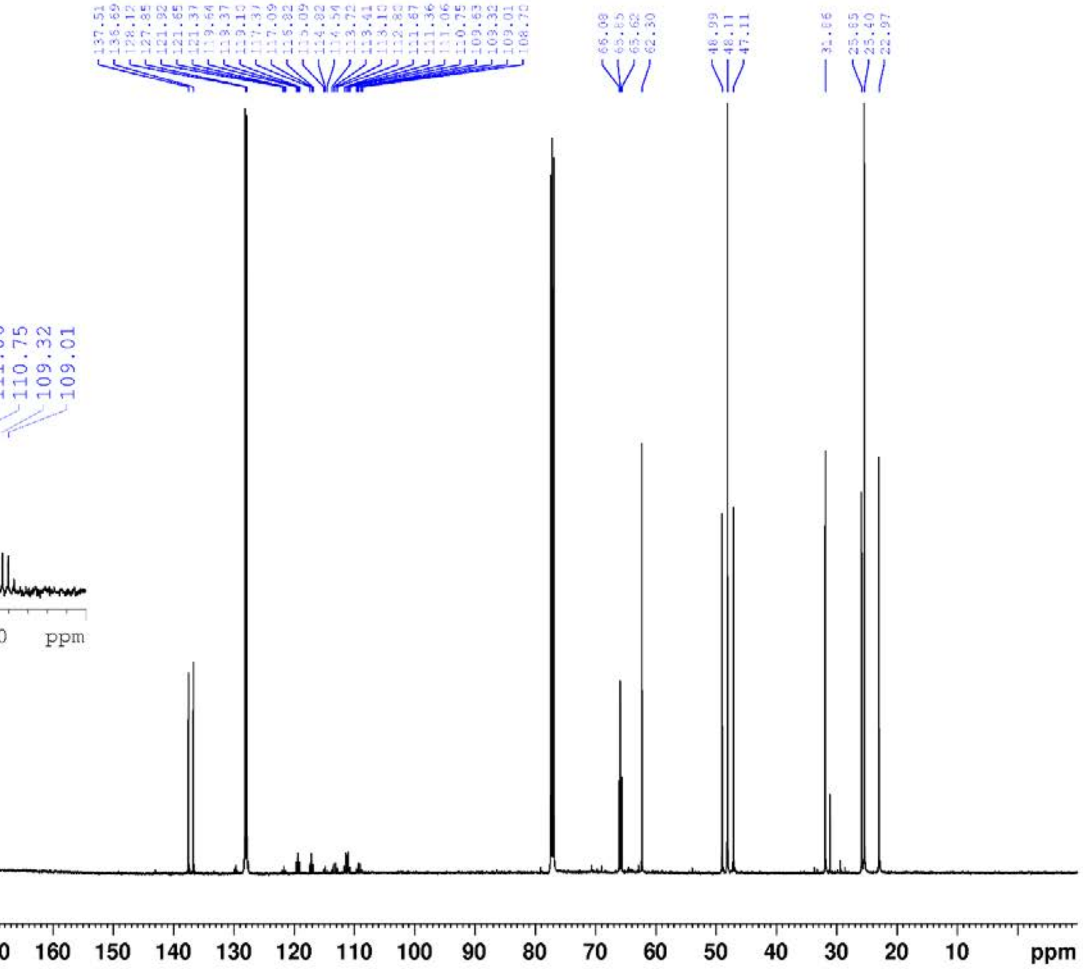

^{19}F NMR (376 MHz, $CDCl_3$) for *2,2,3,3,3-pentafluoropropyl (2R,3S)-2-(4-hydroxybutyl)-3-(4-(pyrrolidin-1-ylsulfonyl)phenyl)aziridine-1-sulfonate* (**14i**)

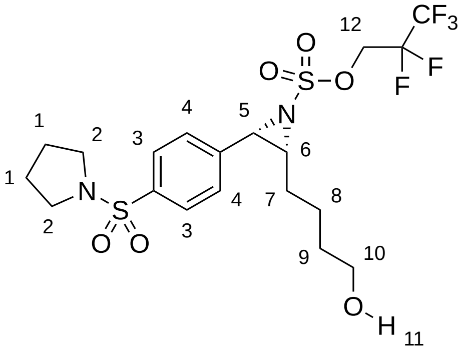

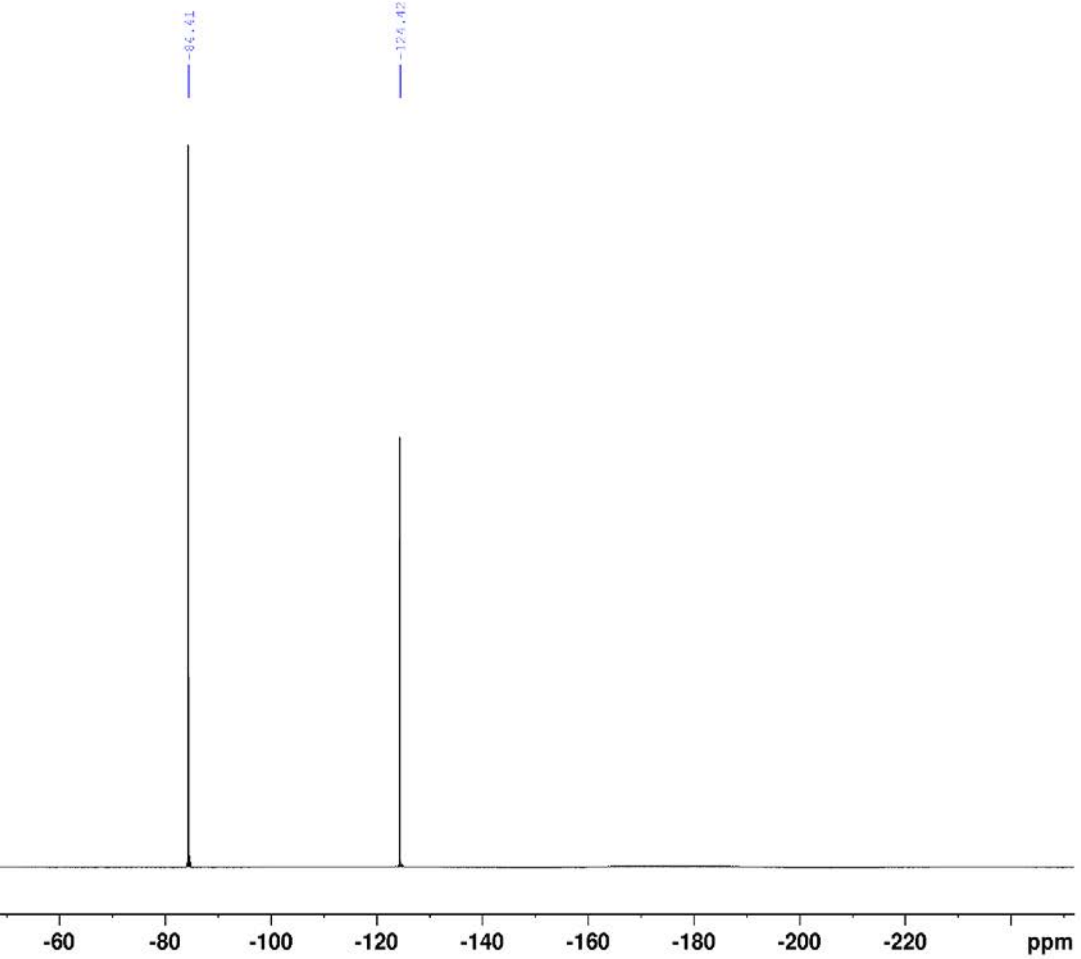

^{1}H NMR (400 MHz, $CDCl_3$) for *2,2,3,3,3-pentafluoropropyl ((S)-naphthalen-1-yl((S)-tetrahydrofuran-2-yl)methyl)sulfamate* (**15**)

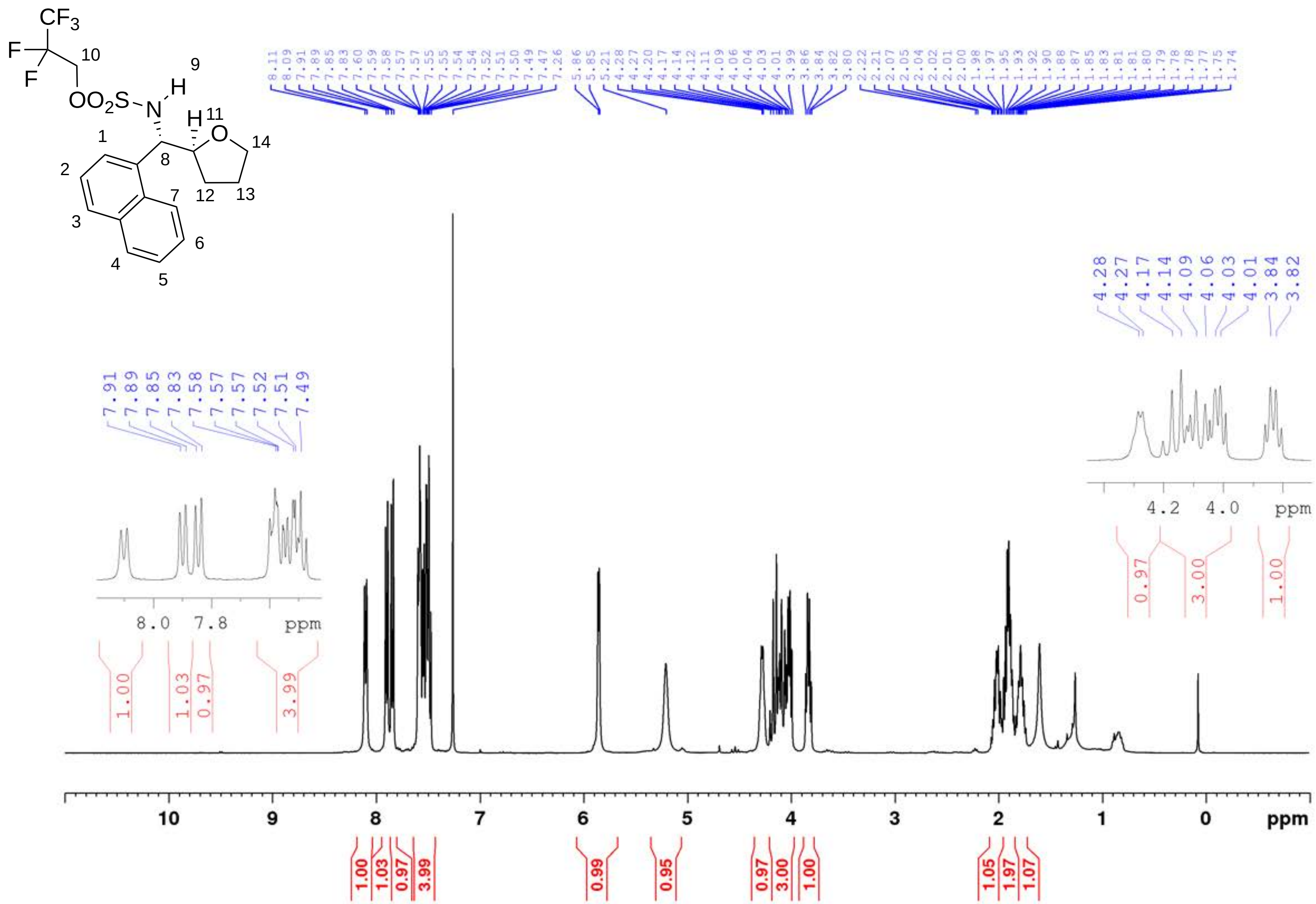

^{13}C NMR (126 MHz, $CDCl_3$) for *2,2,3,3,3-pentafluoropropyl ((S)-naphthalen-1-yl((S)-tetrahydrofuran-2-yl)methyl)sulfamate* (**15**)

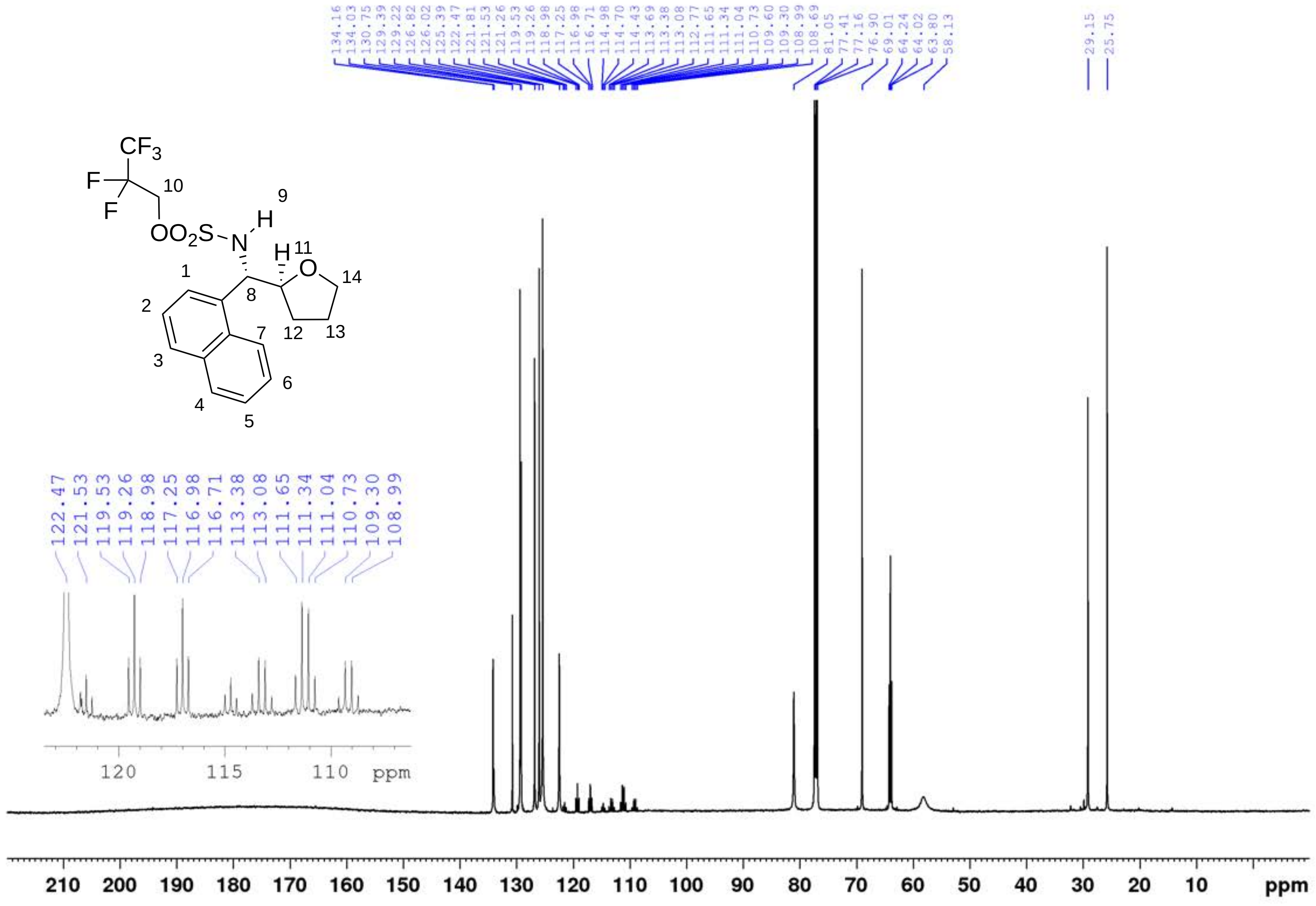

^{19}F NMR (376 MHz, $CDCl_3$) for *2,2,3,3,3-pentafluoropropyl ((S)-naphthalen-1-yl((S)-tetrahydrofuran-2-yl)methyl)sulfamate* (**15**)

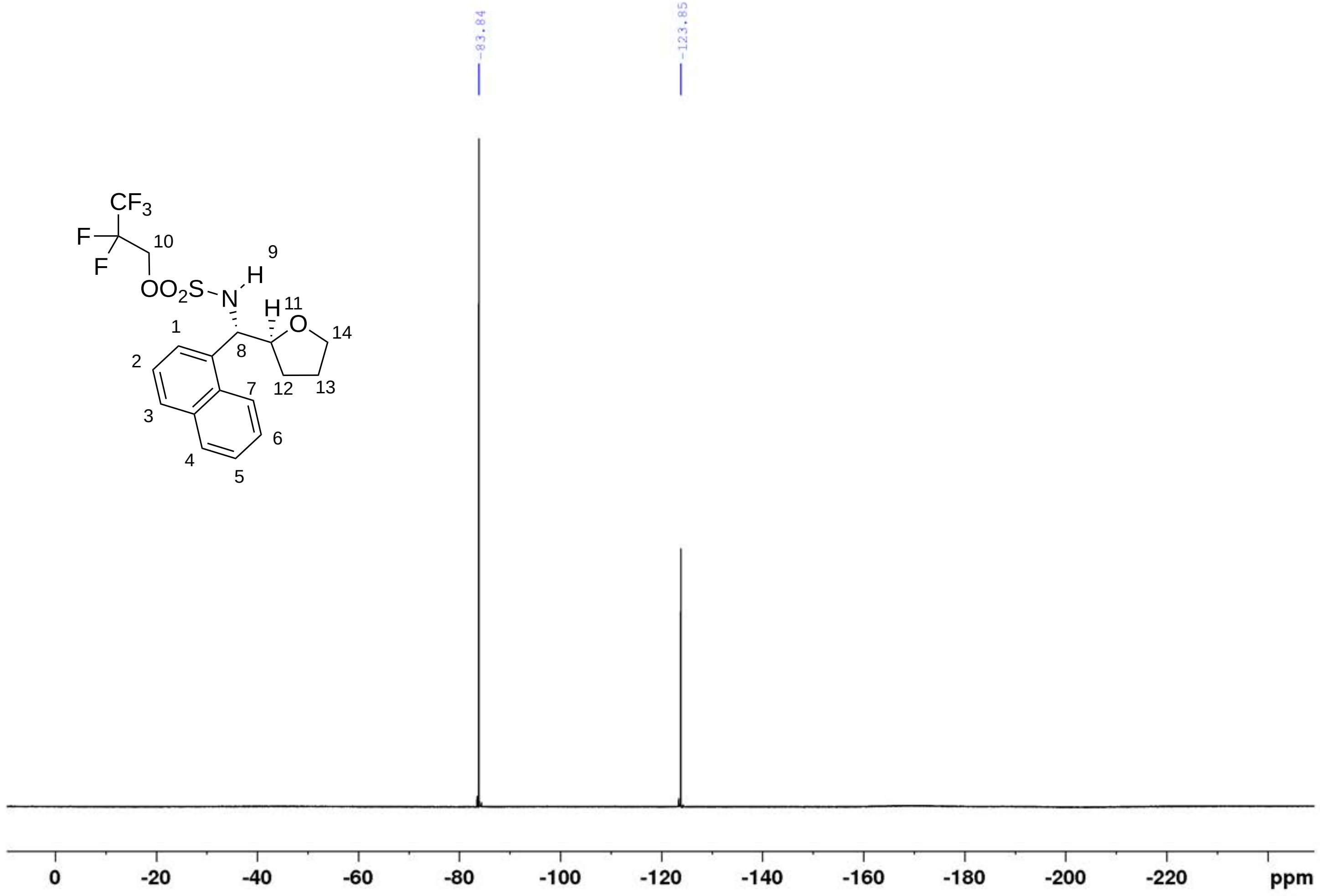

^{1}H NMR (500 MHz, $CDCl_3$) for *2,2,3,3,3-pentafluoropropyl (2S,3R)-2-(naphthalen-1-yl)-3-propylaziridine-1-sulfonate* (**16**)

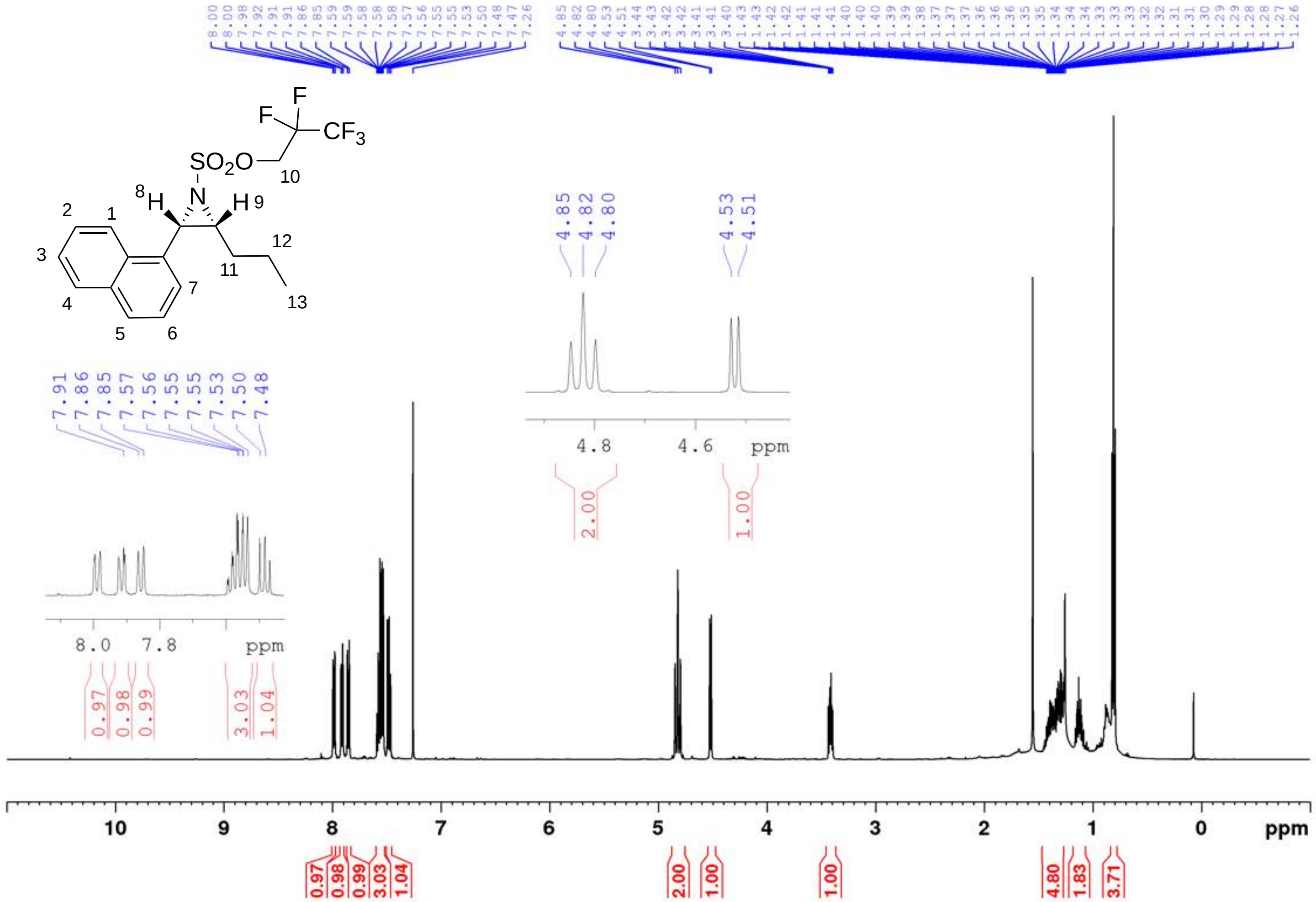

^{13}C NMR (126 MHz, $CDCl_3$) for *2,2,3,3,3-pentafluoropropyl (2S,3R)-2-(naphthalen-1-yl)-3-propylaziridine-1-sulfonate* (**16**)

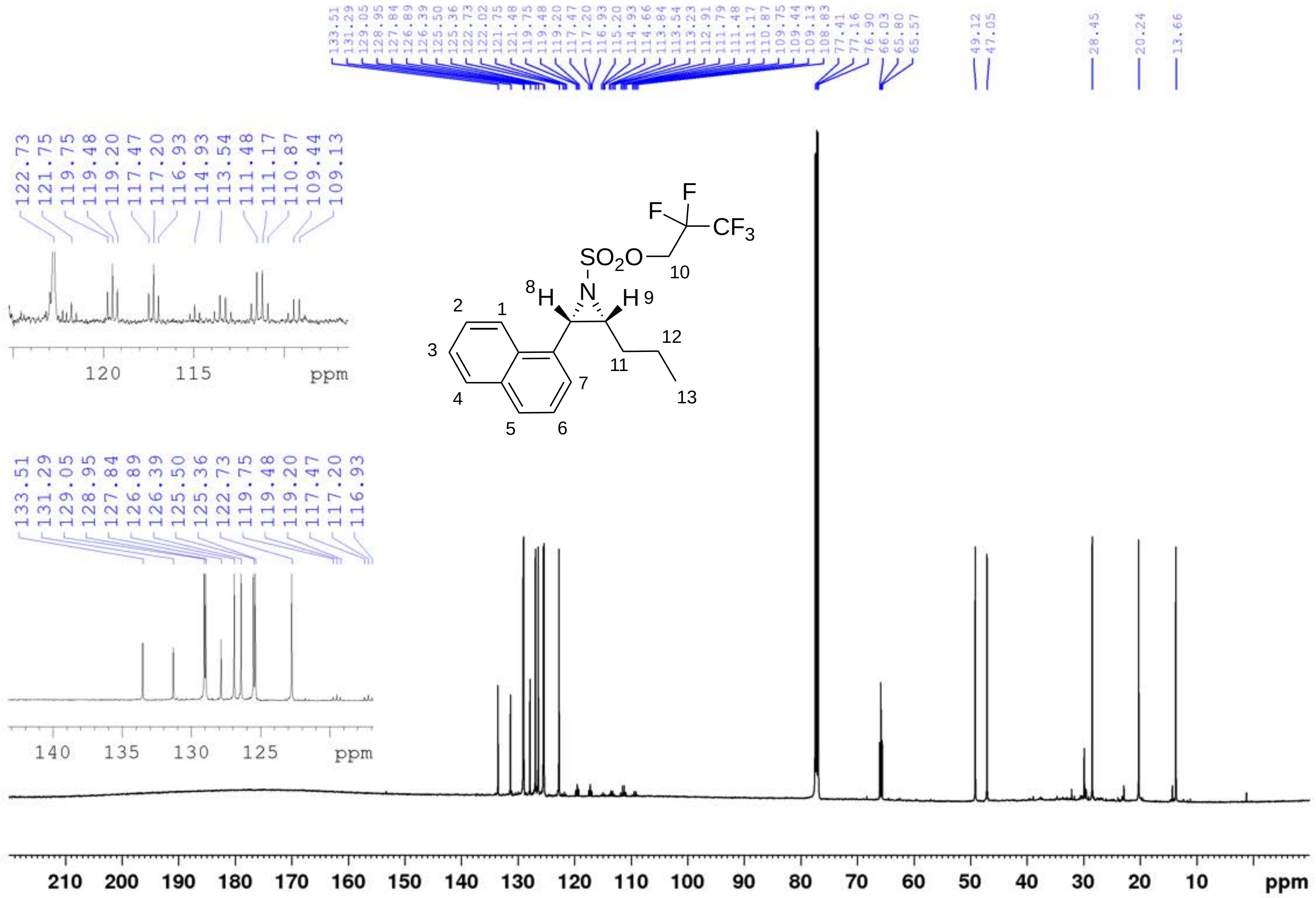

^{19}F NMR (376 MHz, $CDCl_3$) for *2,2,3,3,3-pentafluoropropyl (2S,3R)-2-(naphthalen-1-yl)-3-propylaziridine-1-sulfonate* (**16**)

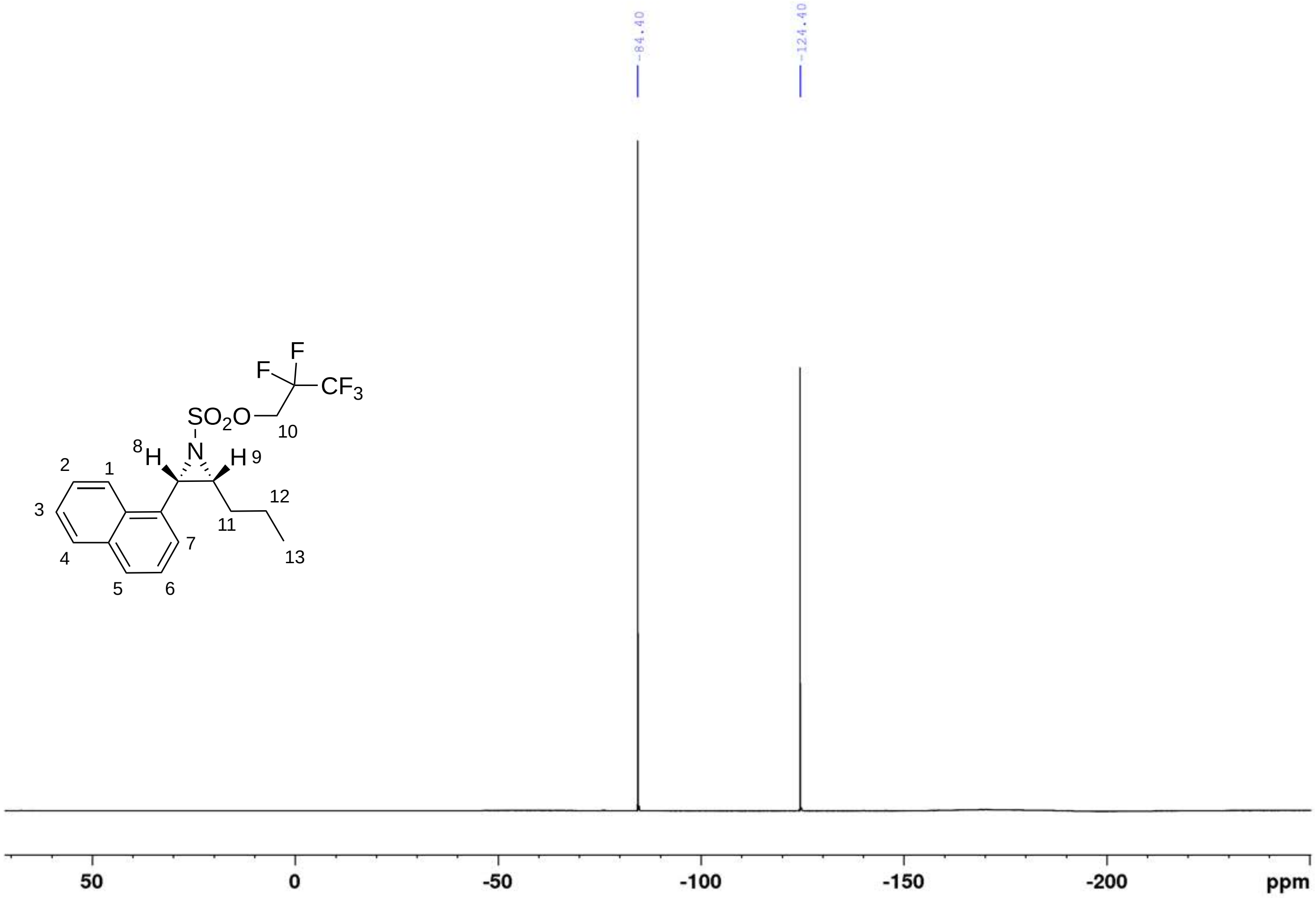

^{1}H NMR (500 MHz, $CDCl_3$) for *2,2,3,3,3-pentafluoropropyl (2R,3S)-2-(3-methoxypropyl)-3-(naphthalen-1-yl)aziridine-1-sulfonate* (**17**)

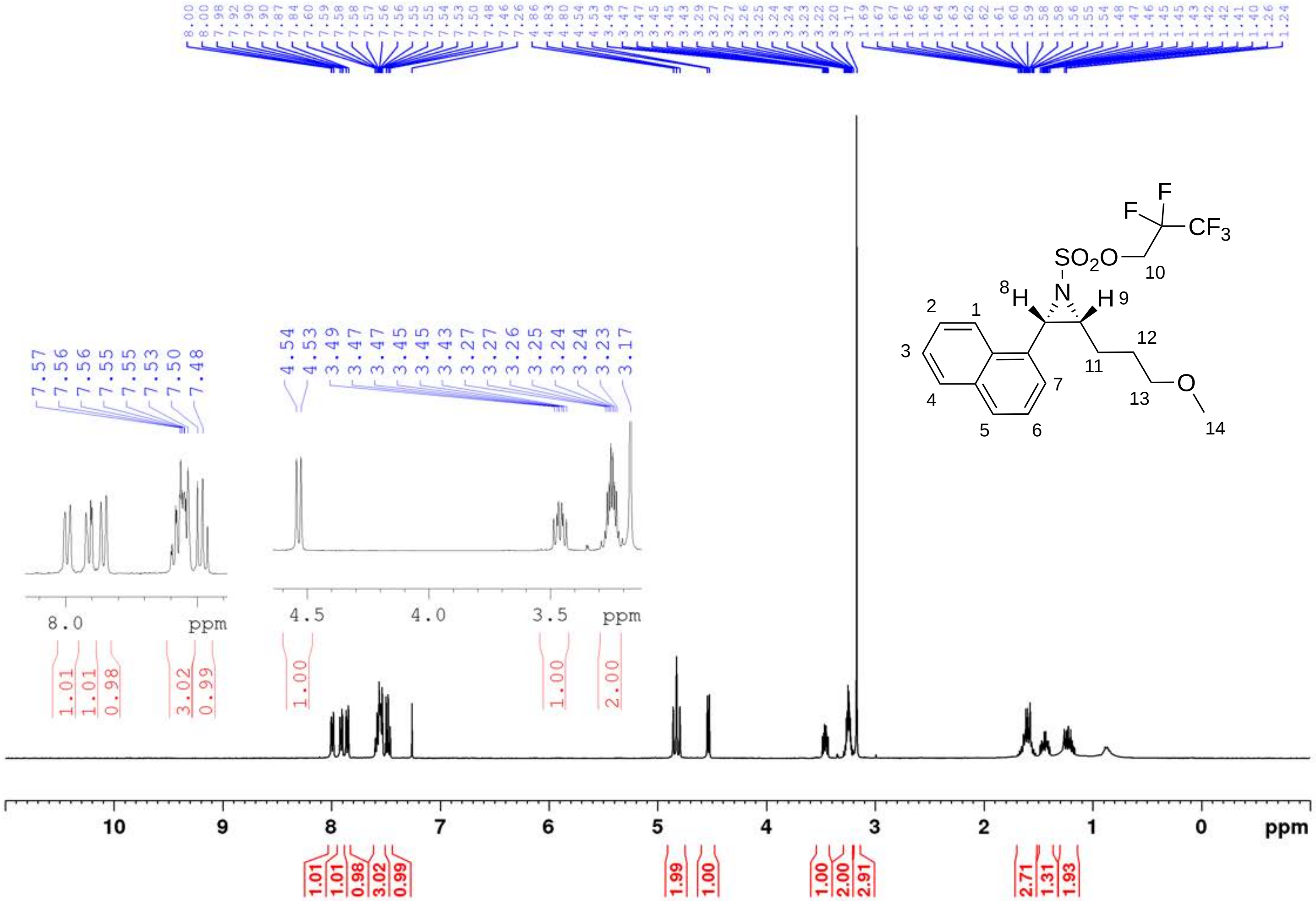

^{13}C NMR (126 MHz, $CDCl_3$) for *2,2,3,3,3-pentafluoropropyl (2R,3S)-2-(3-methoxypropyl)-3-(naphthalen-1-yl)aziridine-1-sulfonate* (**17**)

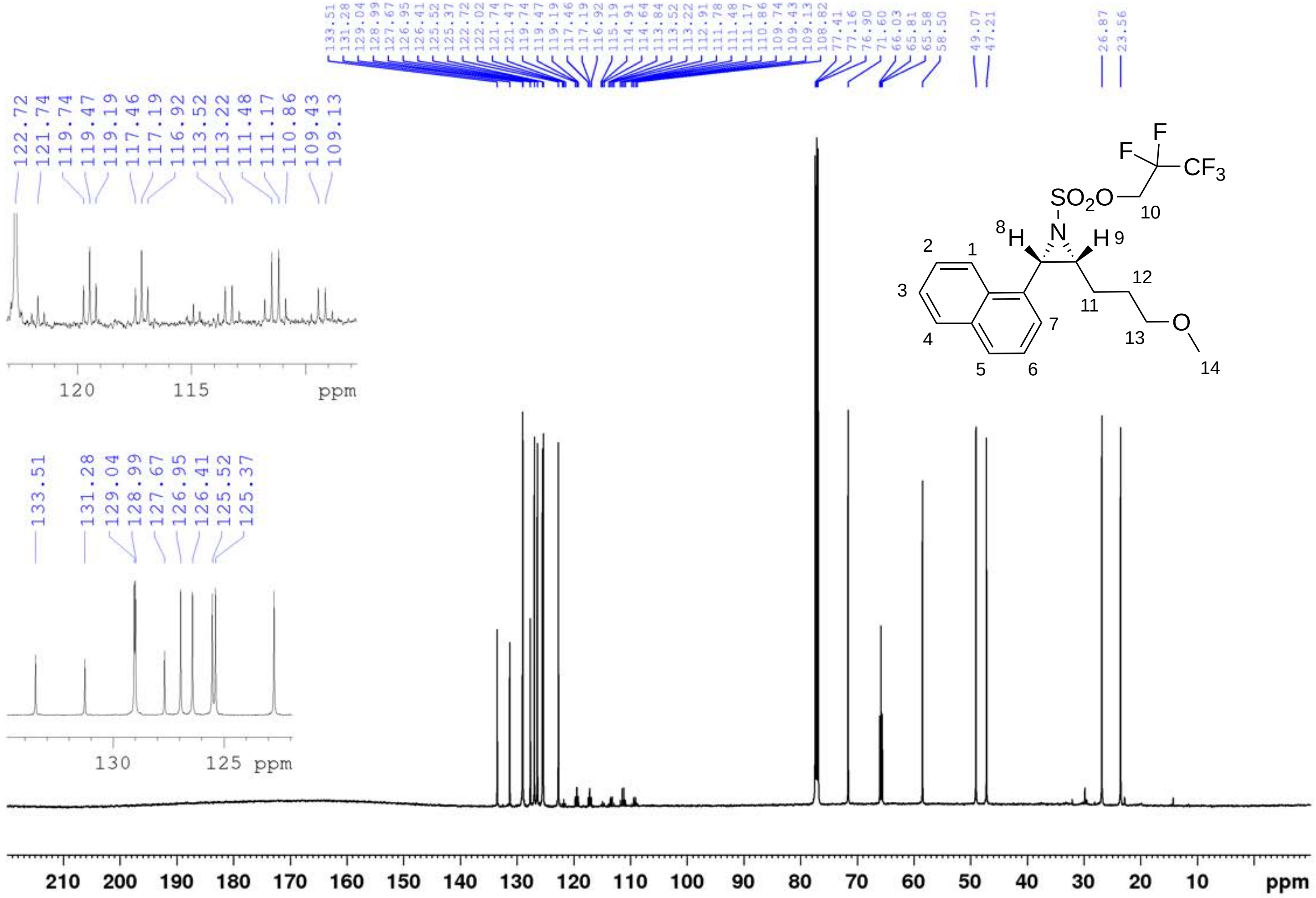

^{19}F NMR (376 MHz, $CDCl_3$) for *2,2,3,3,3-pentafluoropropyl (2R,3S)-2-(3-methoxypropyl)-3-(naphthalen-1-yl)aziridine-1-sulfonate* (**17**)

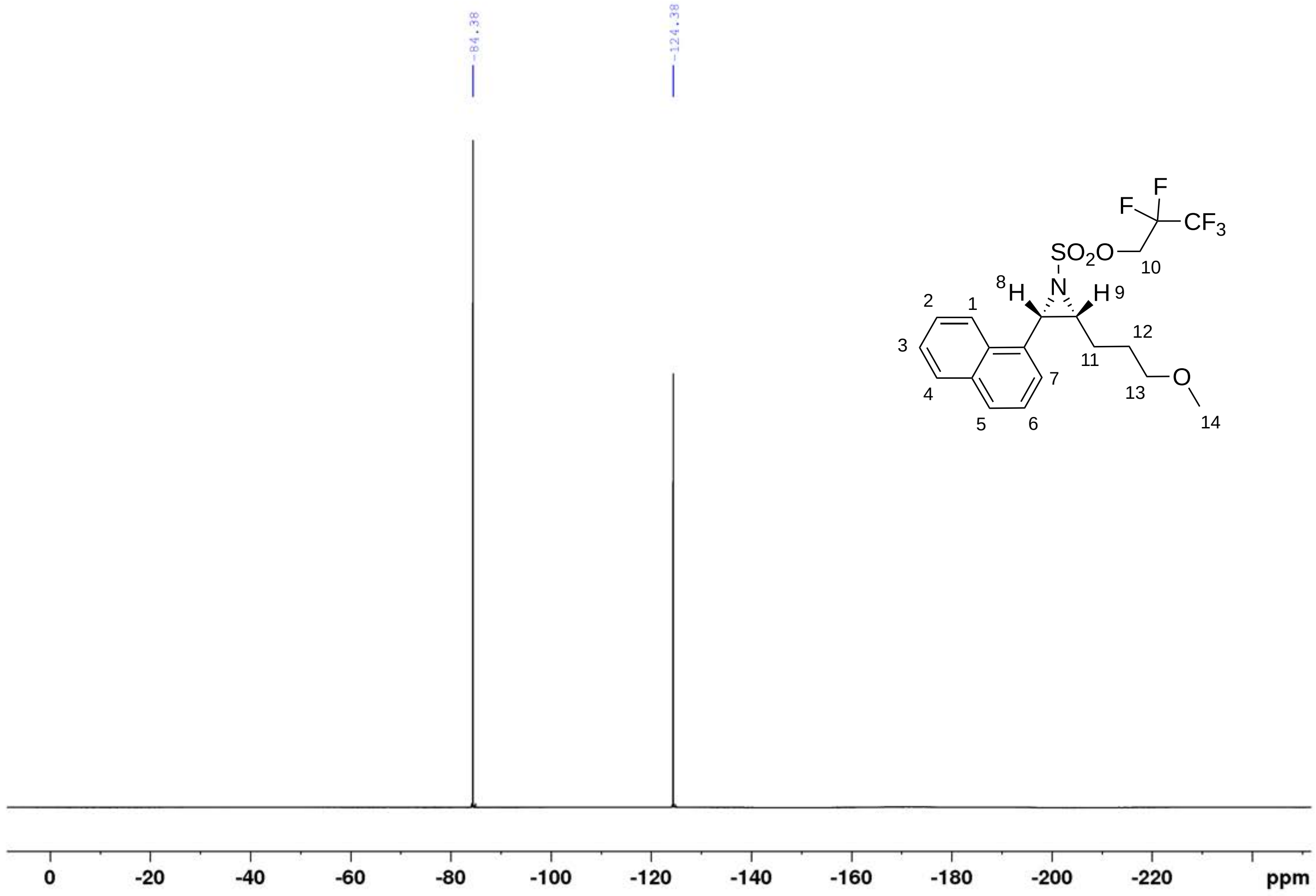

^{1}H NMR (500 MHz, CD_3CN) for *2,2,3,3,3-pentafluoropropyl ((R)-((S)-5-oxotetrahydrofuran-2-yl)(phenyl)methyl)sulfamate* (**18**)

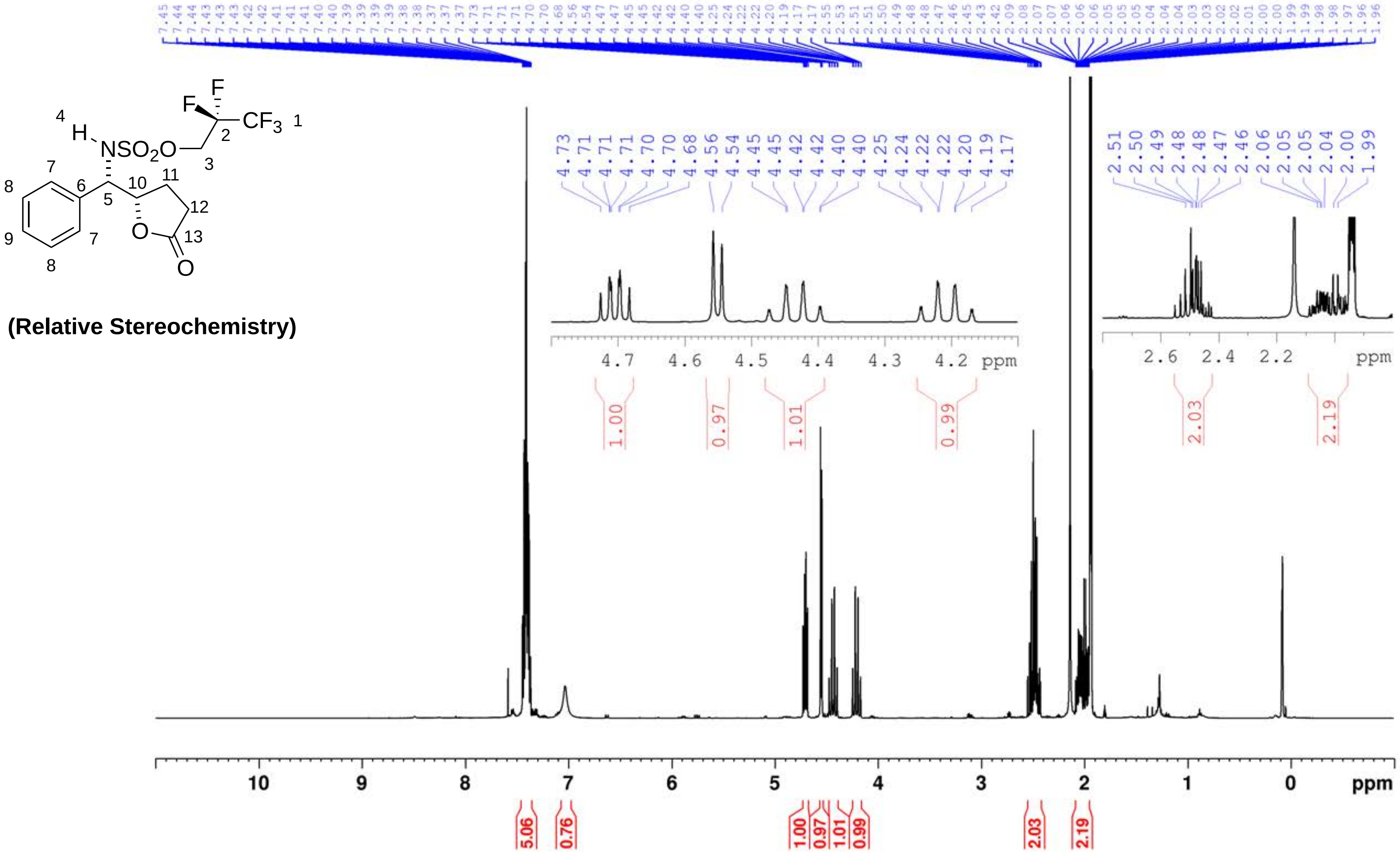

^{13}**C NMR** (126 MHz, CD_3CN) for *2,2,3,3,3-pentafluoropropyl ((R)-((S)-5-oxotetrahydrofuran-2-yl)(phenyl)methyl)sulfamate* (**18**)

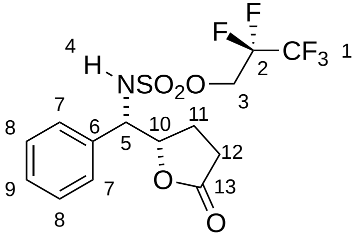

(Relative Stereochemistry)

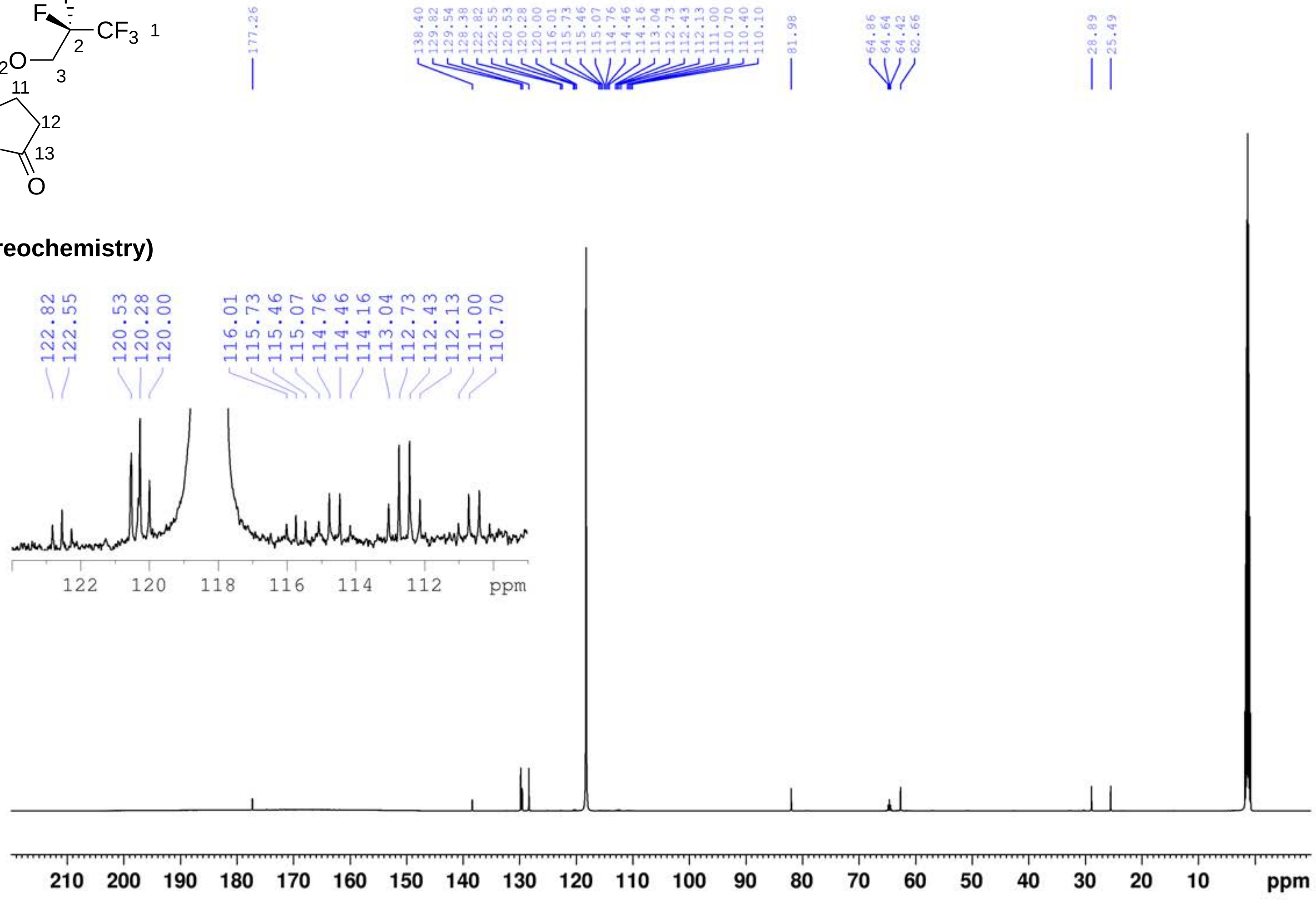

^{19}F NMR (476 MHz, CD_3CN) for *2,2,3,3,3-pentafluoropropyl ((R)-((S)-5-oxotetrahydrofuran-2-yl)(phenyl)methyl)sulfamate* (**18**)

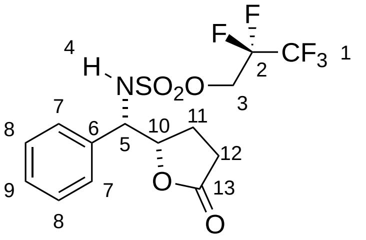

(Relative Stereochemistry)

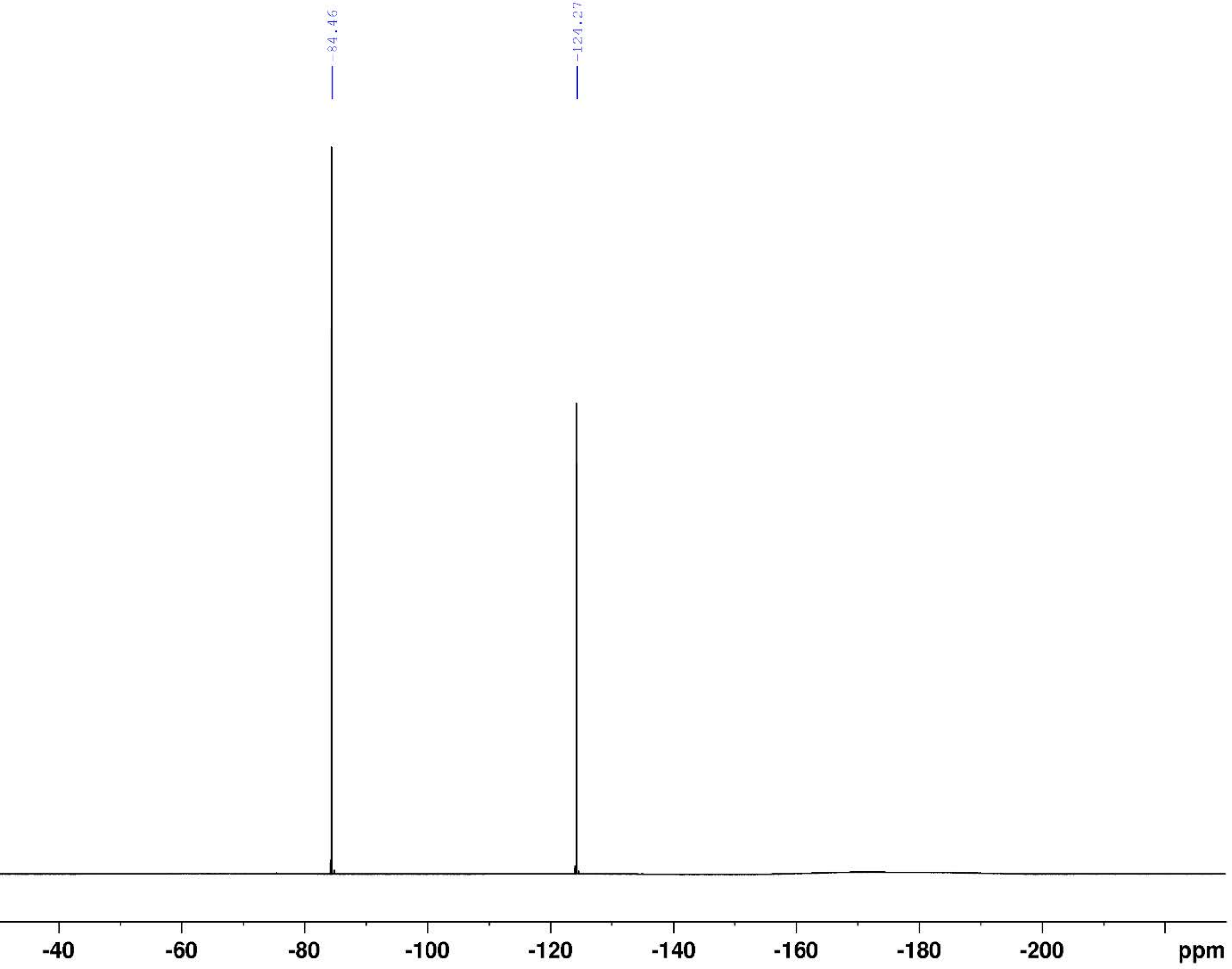

^{1}H NMR (700 MHz, CDCl$_3$) for *2,2,3,3,3-pentafluoropropyl (2S,3R)-2-phenyl-3-(3-(2,2,2-trifluoroacetamido)propyl)aziridine-1-sulfonate* (**19**)

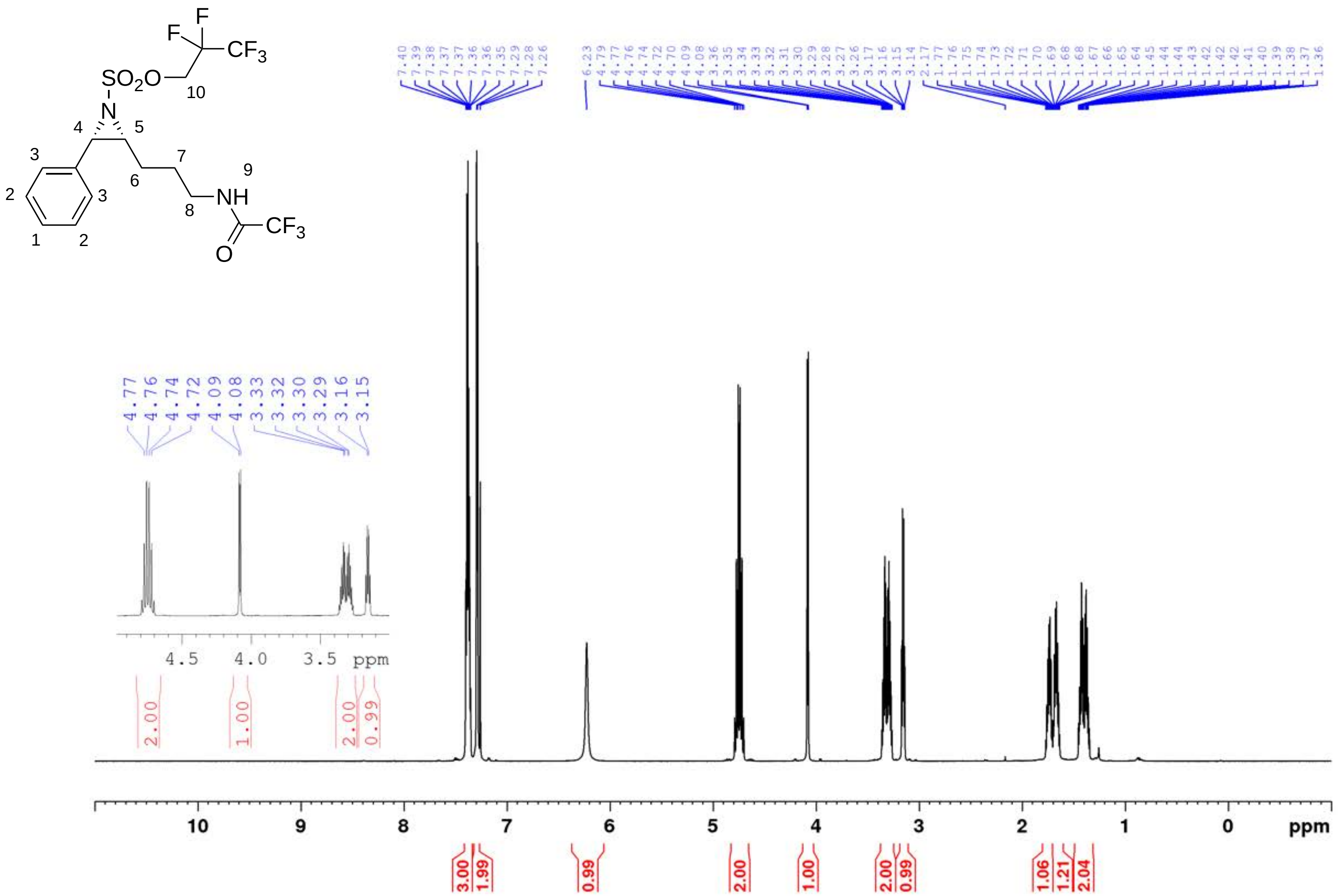

^{13}C NMR (176 MHz, CDCl$_3$) for *2,2,3,3,3-pentafluoropropyl (2S,3R)-2-phenyl-3-(3-(2,2,2-trifluoroacetamido)propyl)aziridine-1-sulfonate* (**19**)

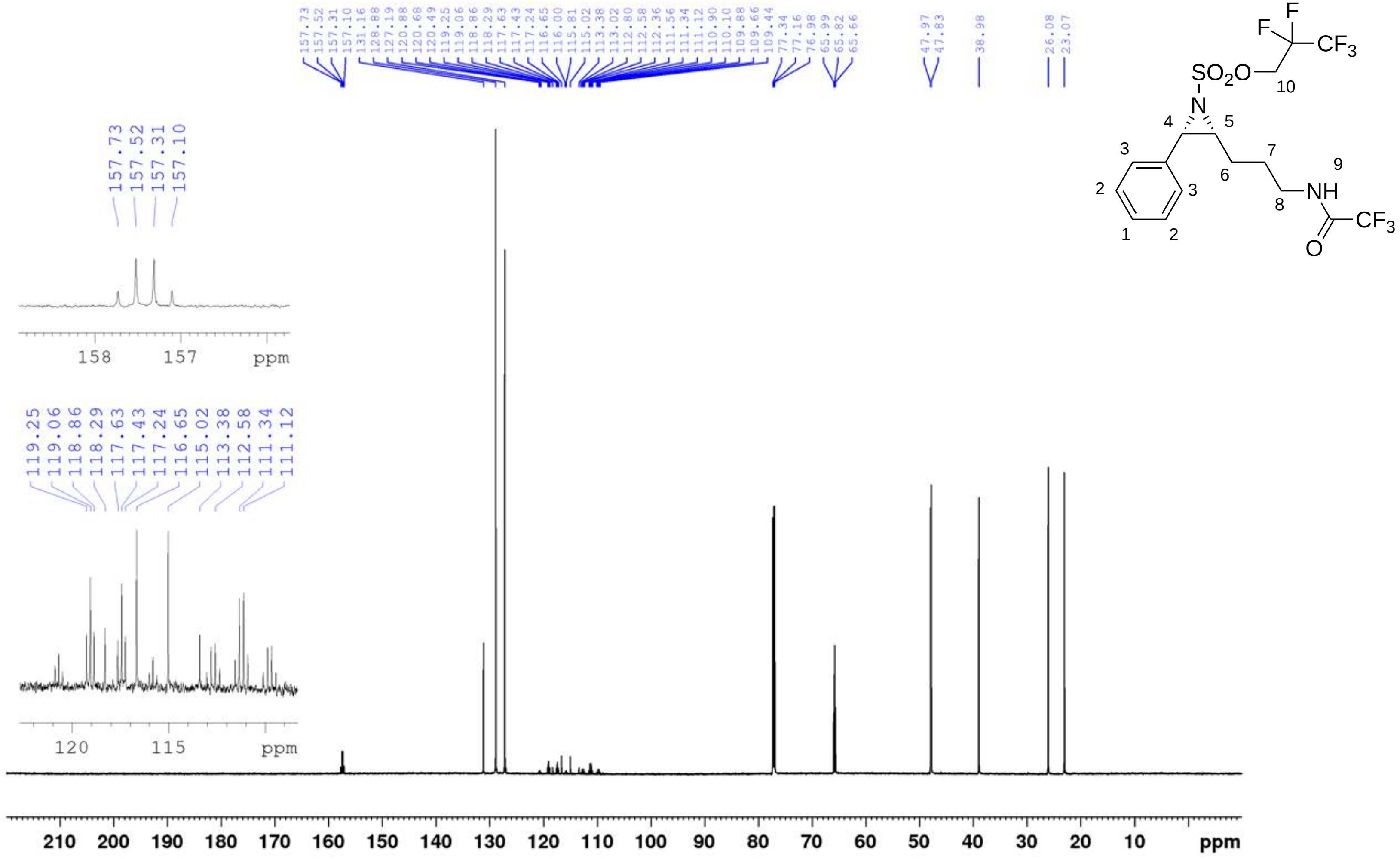

^{19}F NMR (376 MHz, CDCl$_3$) for *2,2,3,3,3-pentafluoropropyl (2S,3R)-2-phenyl-3-(3-(2,2,2-trifluoroacetamido)propyl)aziridine-1-sulfonate* (**19**)

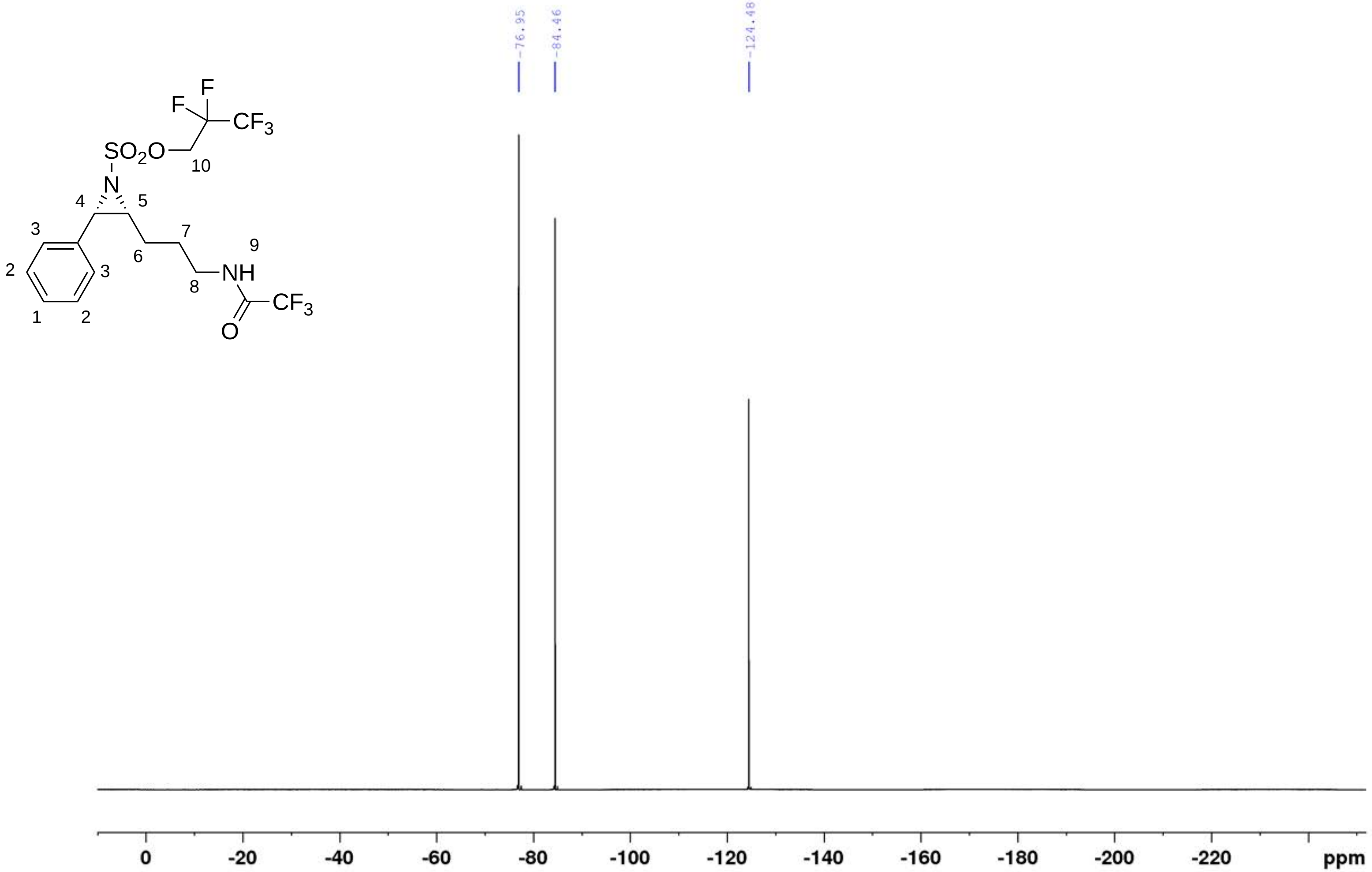

^{1}**H NMR (700 MHz, CDCl$_3$)** for *2,2,3,3,3-pentafluoropropyl (2R,3S)-2-(3-((4-methylphenyl)sulfonamido)propyl)-3-phenylaziridine-1-sulfonate* (**20**)

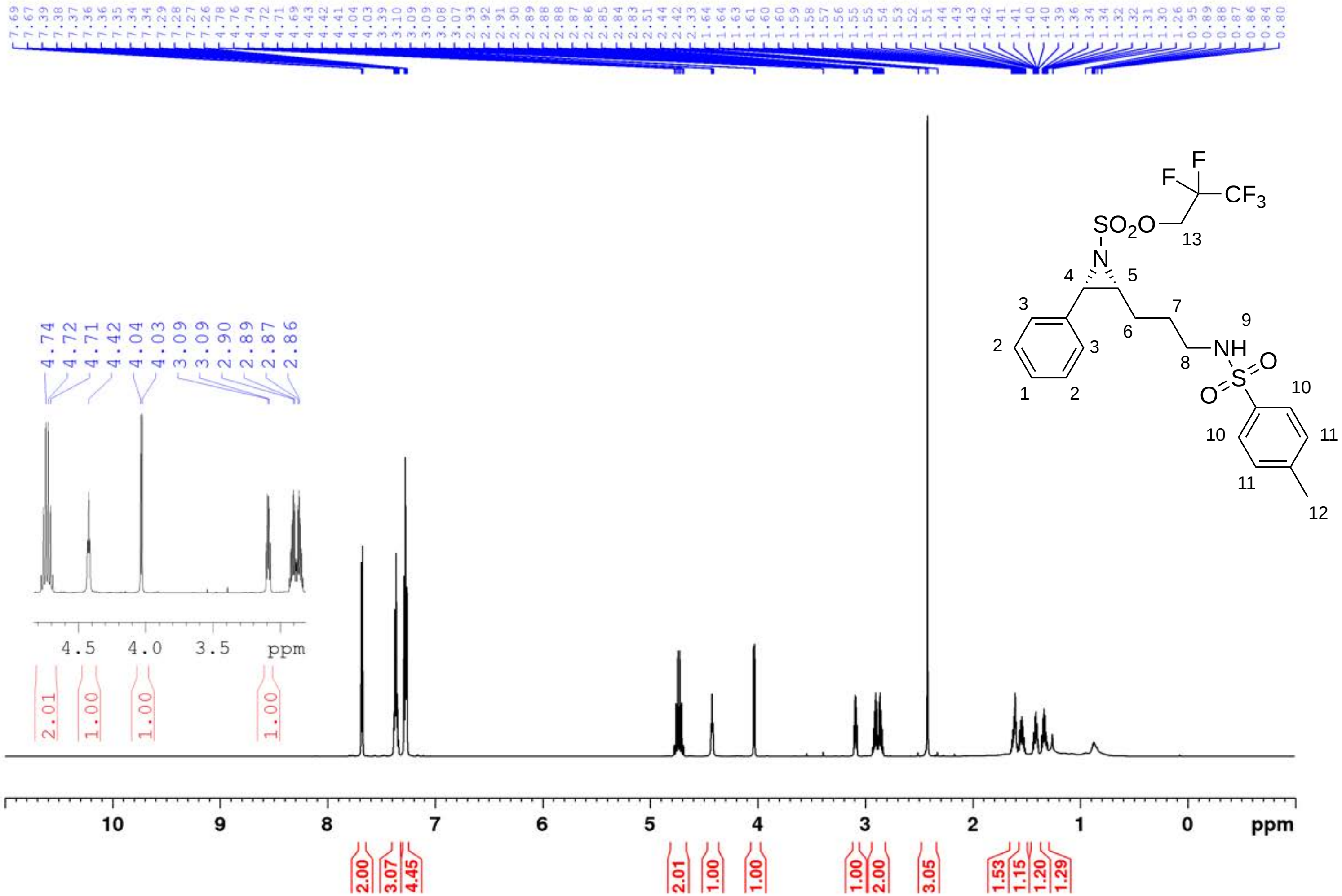

^{13}C NMR (176 MHz, CDCl$_3$) for *2,2,3,3,3-pentafluoropropyl (2R,3S)-2-(3-((4-methylphenyl)sulfonamido)propyl)-3-phenylaziridine-1-sulfonate* (**20**)

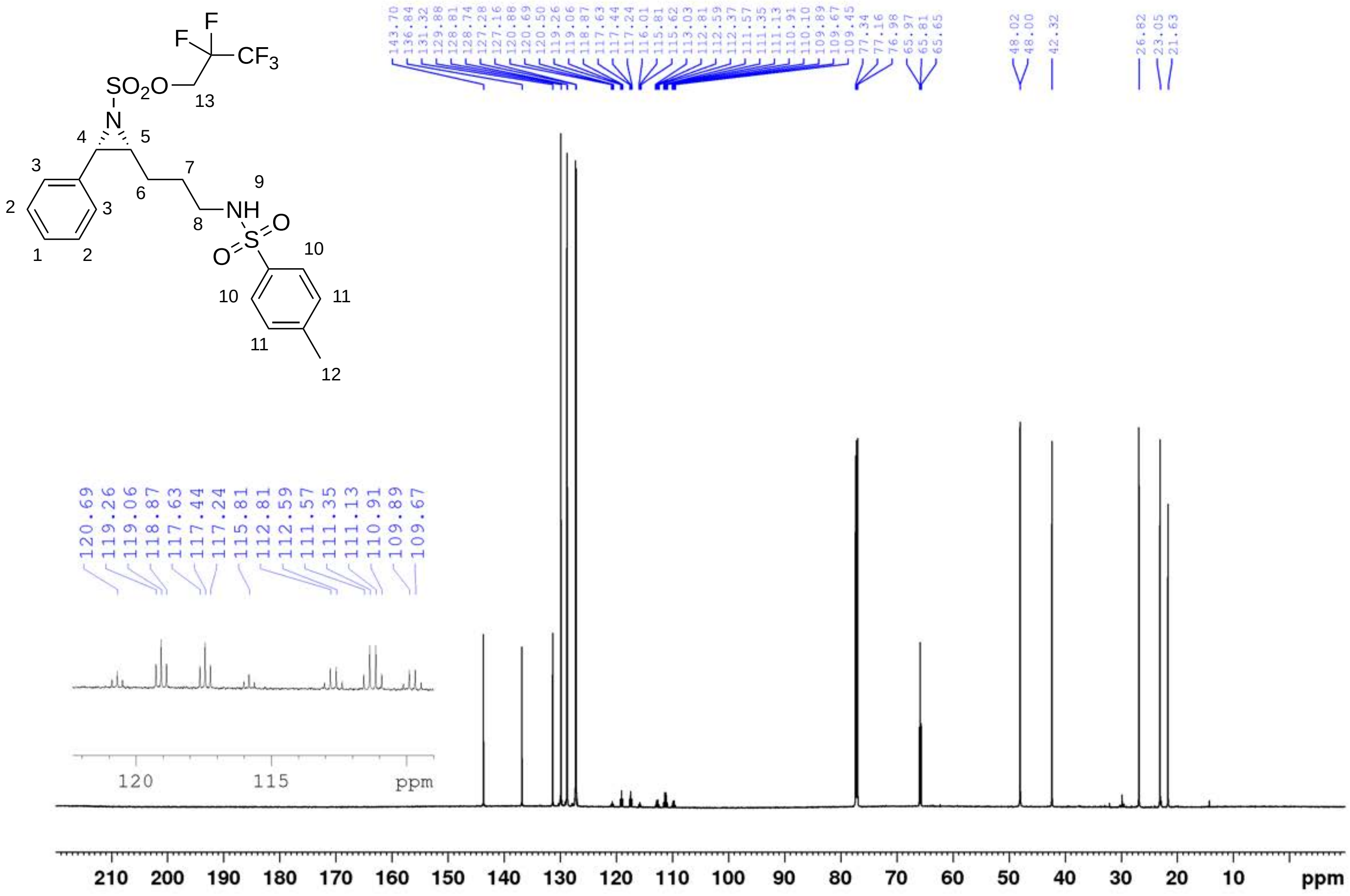

^{19}F NMR (376 MHz, $CDCl_3$) for *2,2,3,3,3-pentafluoropropyl (2R,3S)-2-(3-((4-methylphenyl)sulfonamido)propyl)-3-phenylaziridine-1-sulfonate* (**20**)

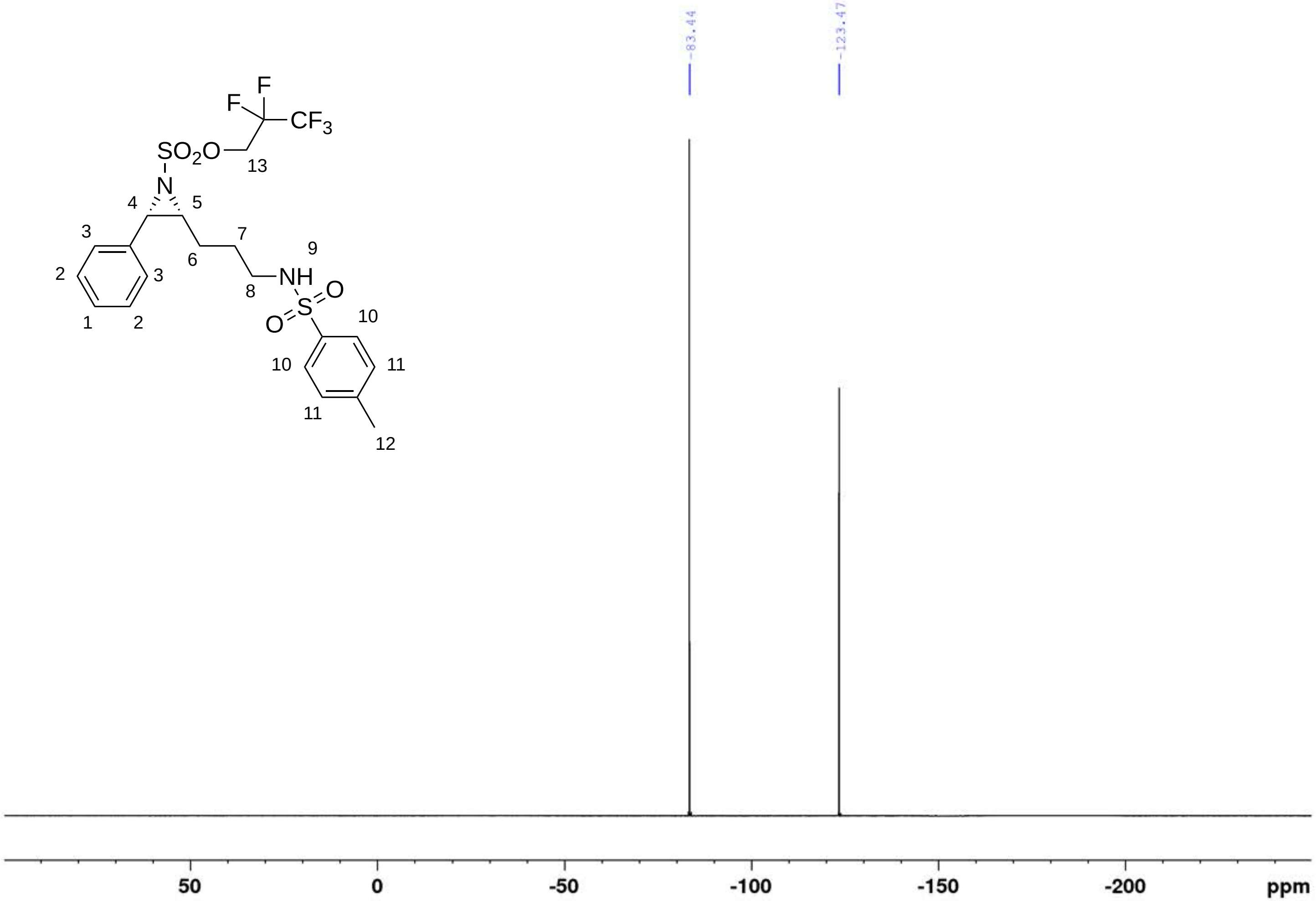

¹H NMR (400 MHz, $CDCl_3$) for *2,2,3,3,3-pentafluoropropyl (2S,3S)-2-(hydroxymethyl)-3-phenylaziridine-1-sulfonate* (**21**)

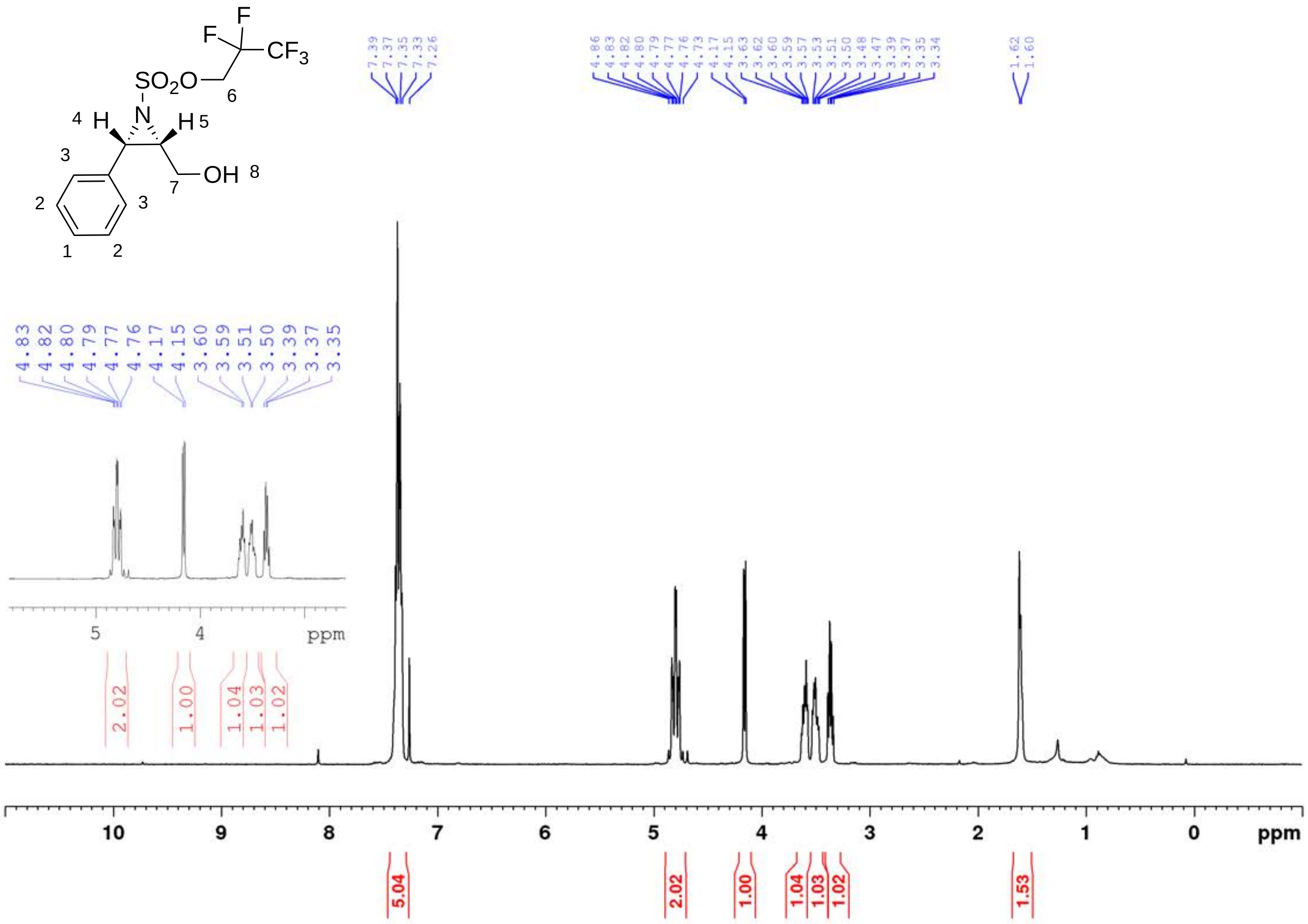

^{13}C NMR (126 MHz, CDCl$_3$) for *2,2,3,3,3-pentafluoropropyl (2S,3S)-2-(hydroxymethyl)-3-phenylaziridine-1-sulfonate* (**21**)

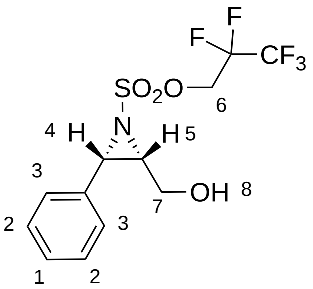

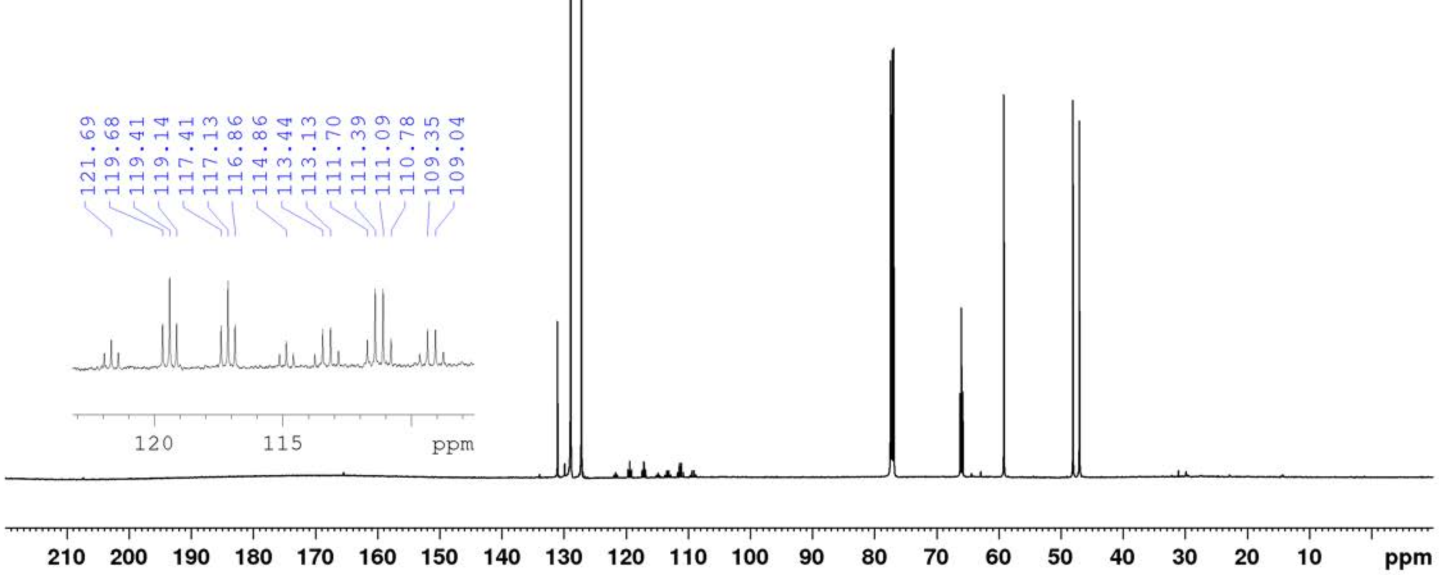

¹⁹F NMR (376 MHz, $CDCl_3$) for *2,2,3,3,3-pentafluoropropyl (2S,3S)-2-(hydroxymethyl)-3-phenylaziridine-1-sulfonate* (**21**)

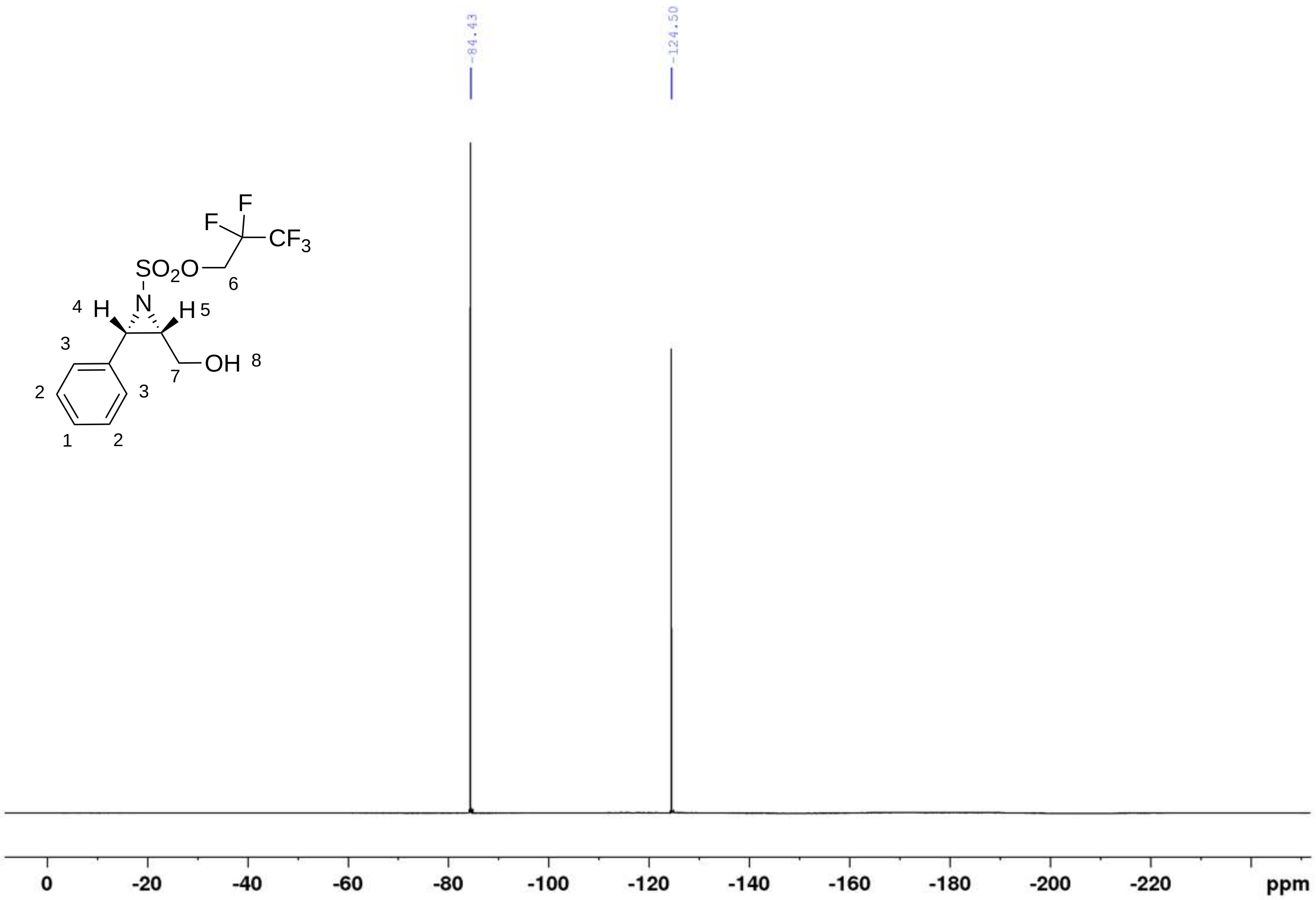

^{1}H NMR (400 MHz, $CDCl_3$) for *2,2,3,3,3-pentafluoropropyl (2R,3S)-2-(5-hydroxypentyl)-3-phenylaziridine-1-sulfonate* (**22**)

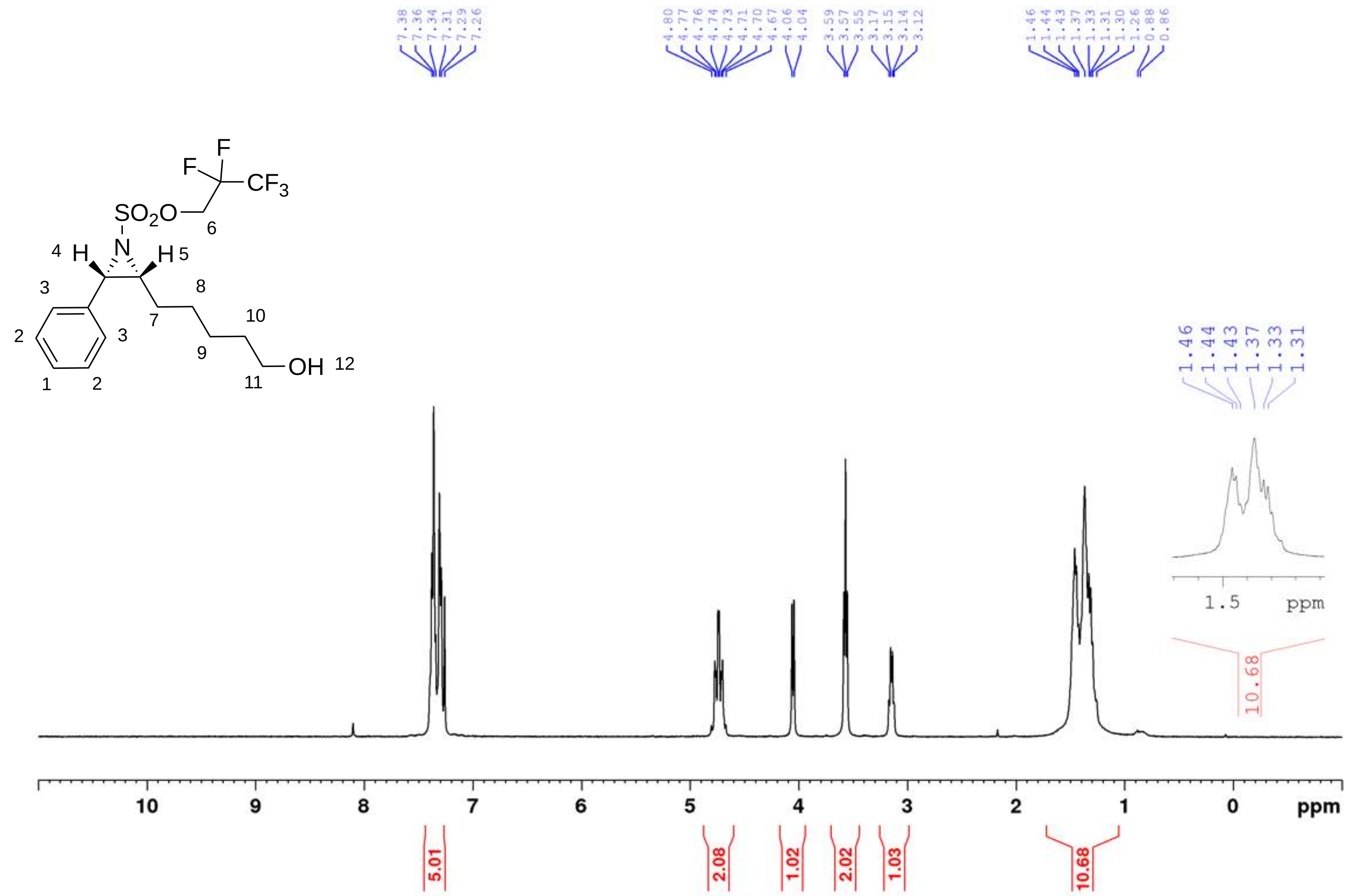

^{13}C NMR (126 MHz, CDCl3) for *2,2,3,3,3-pentafluoropropyl (2R,3S)-2-(5-hydroxypentyl)-3-phenylaziridine-1-sulfonate* (**22**)

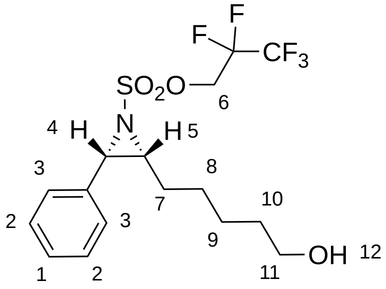

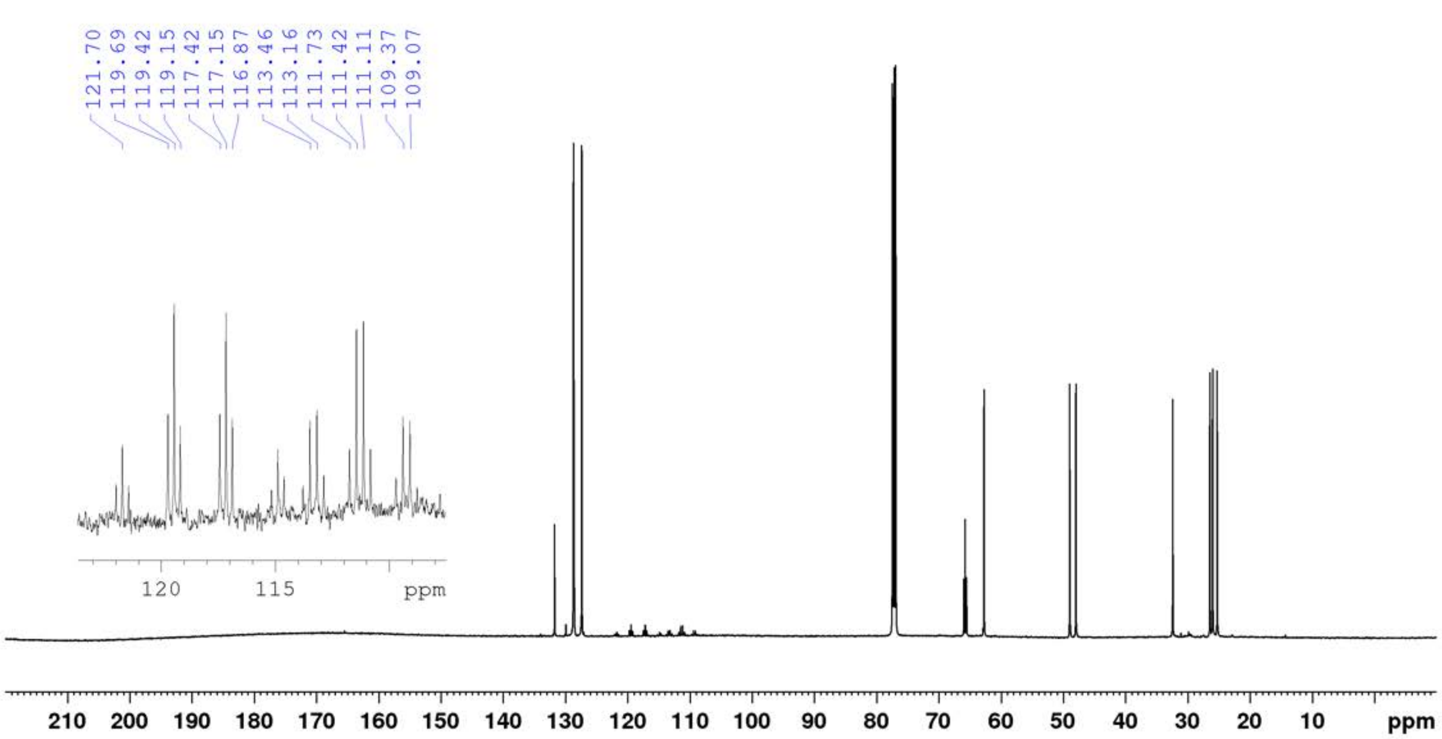

^{19}F NMR (376 MHz, $CDCl_3$) for *2,2,3,3,3-pentafluoropropyl (2R,3S)-2-(5-hydroxypentyl)-3-phenylaziridine-1-sulfonate* (**22**)

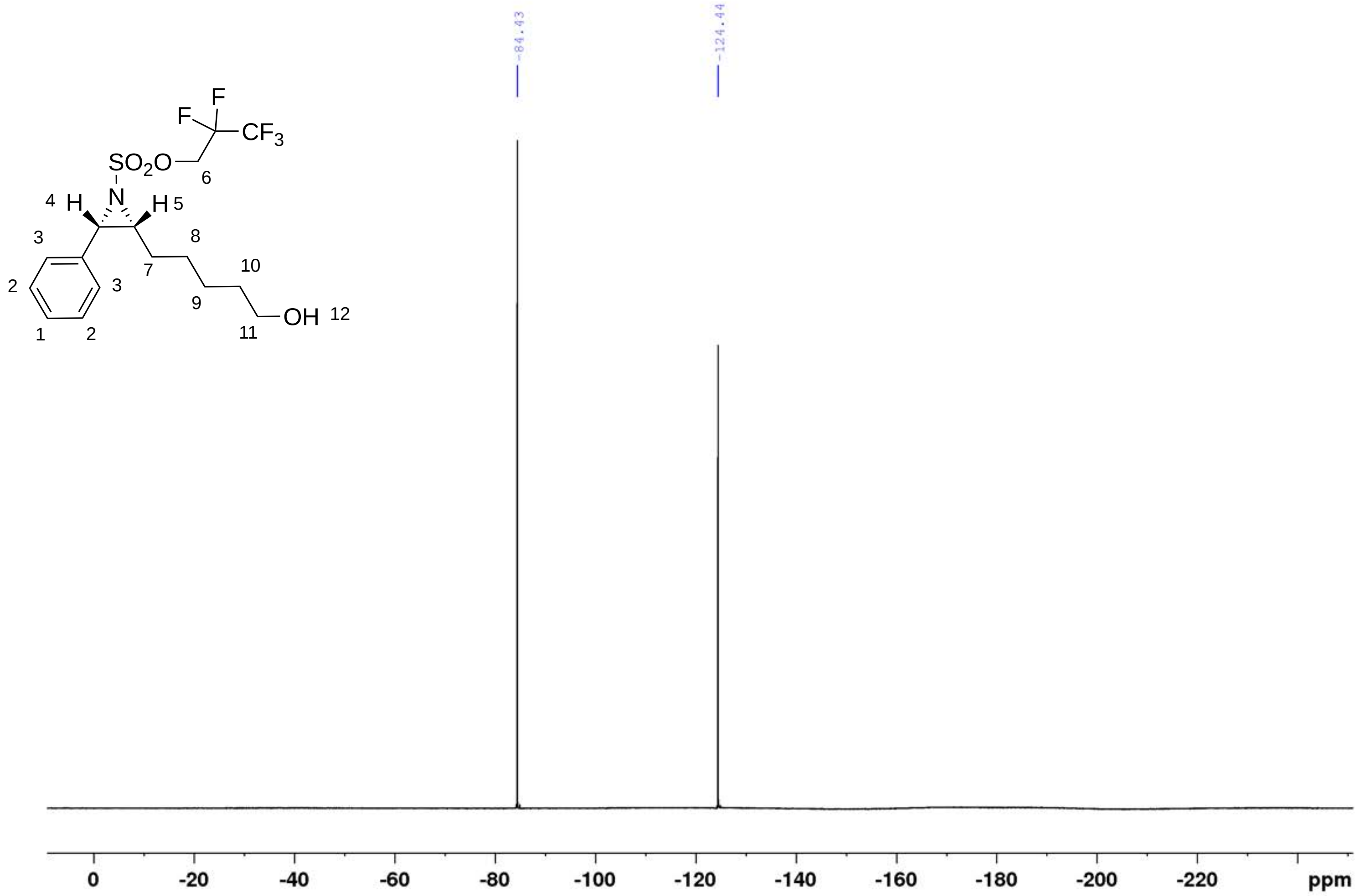

¹H NMR (500 MHz, $CDCl_3$) for *2,2,3,3,3-pentafluoropropyl ((2S,3R)-2-methyltetrahydrofuran-3-yl)sulfamate* (**24a**)

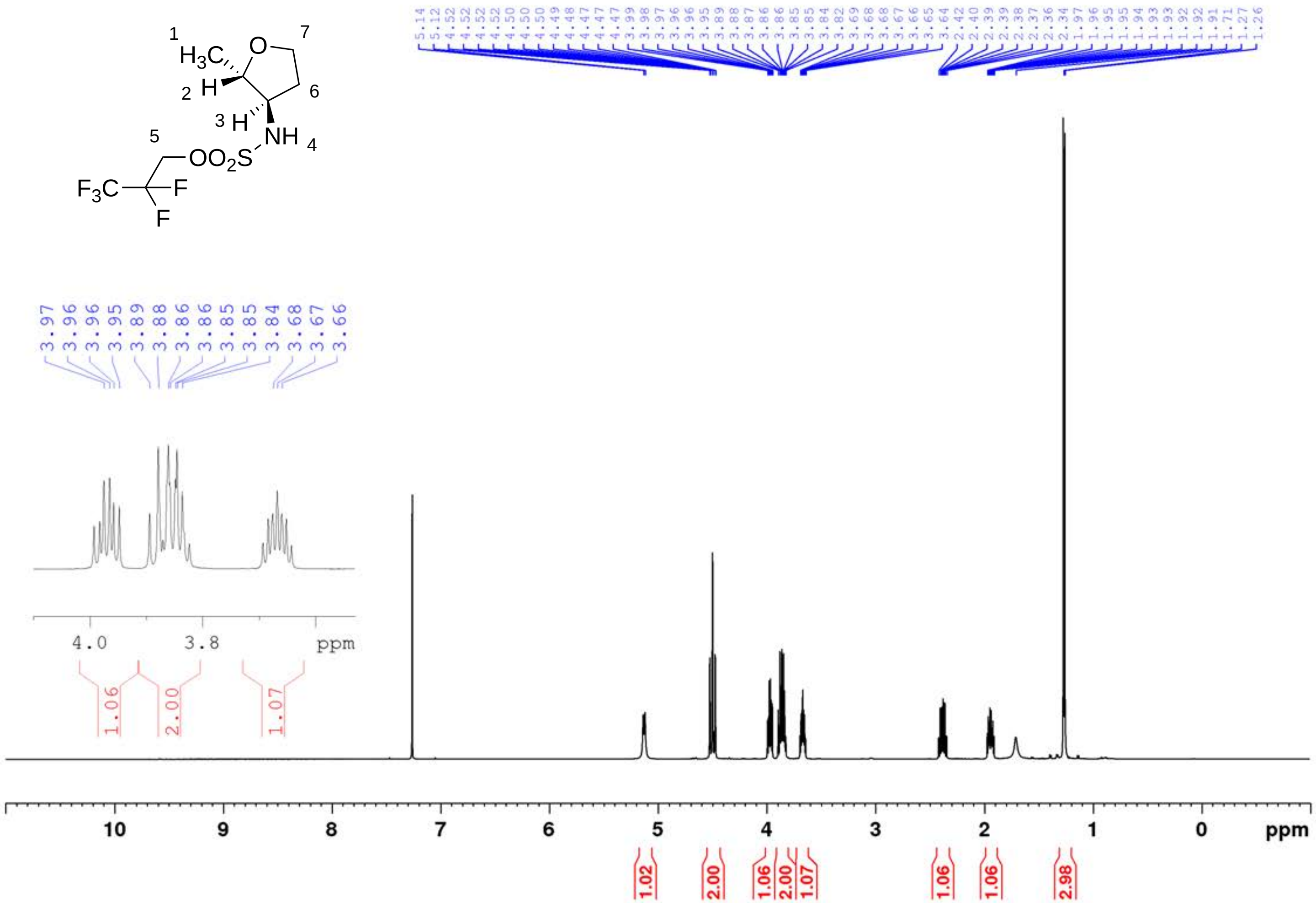

^{13}C NMR (126 MHz, $CDCl_3$) for *2,2,3,3,3-pentafluoropropyl ((2S,3R)-2-methyltetrahydrofuran-3-yl)sulfamate* (**24a**)

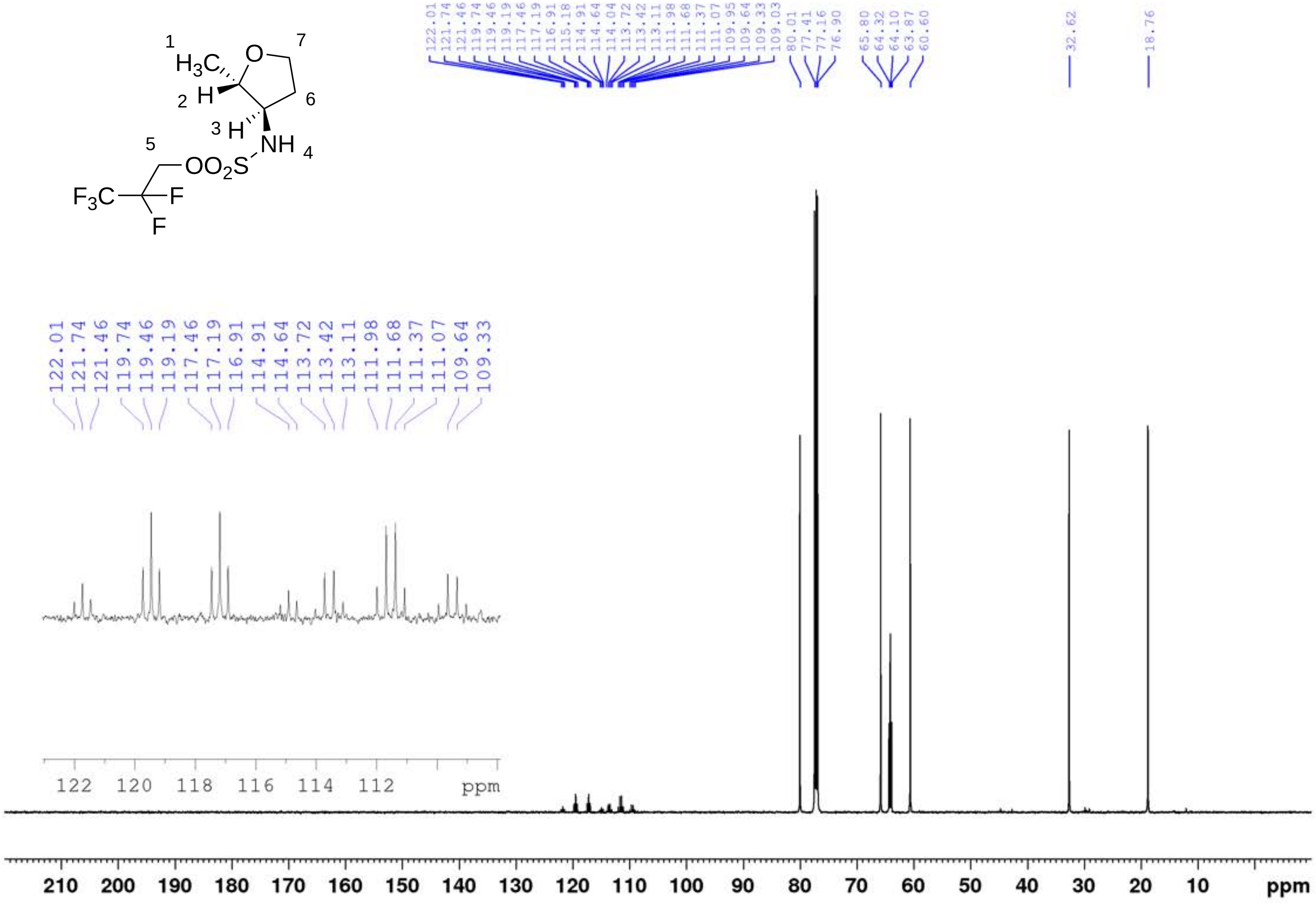

^{19}F NMR (376 MHz, $CDCl_3$) for *2,2,3,3,3-pentafluoropropyl ((2S,3R)-2-methyltetrahydrofuran-3-yl)sulfamate* (**24a**)

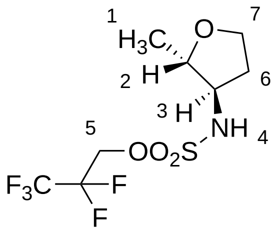

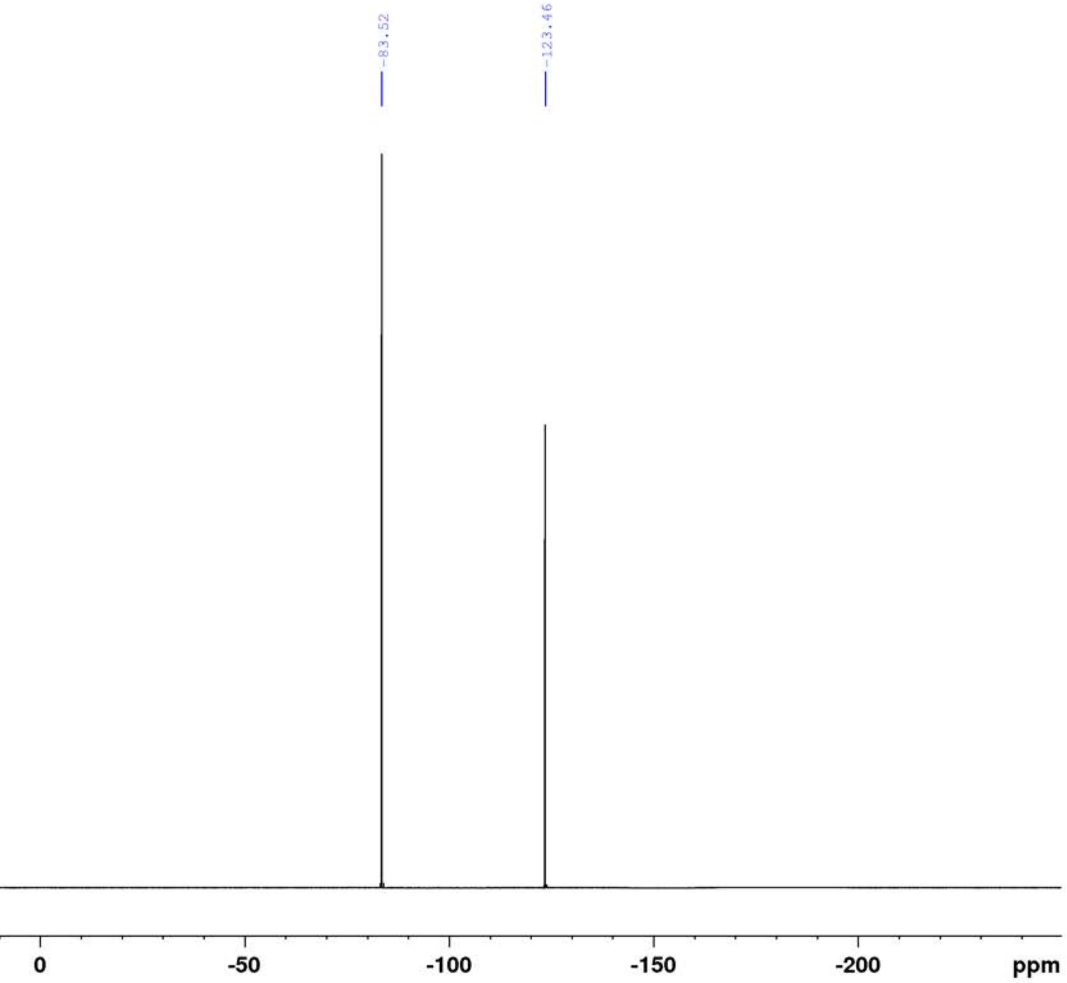

^{1}H NMR (500 MHz, $CDCl_3$) for *2,2,3,3,3-pentafluoropropyl (2R,3S)-2-(2-hydroxyethyl)-3-methylaziridine-1-sulfonate* (**24b**)

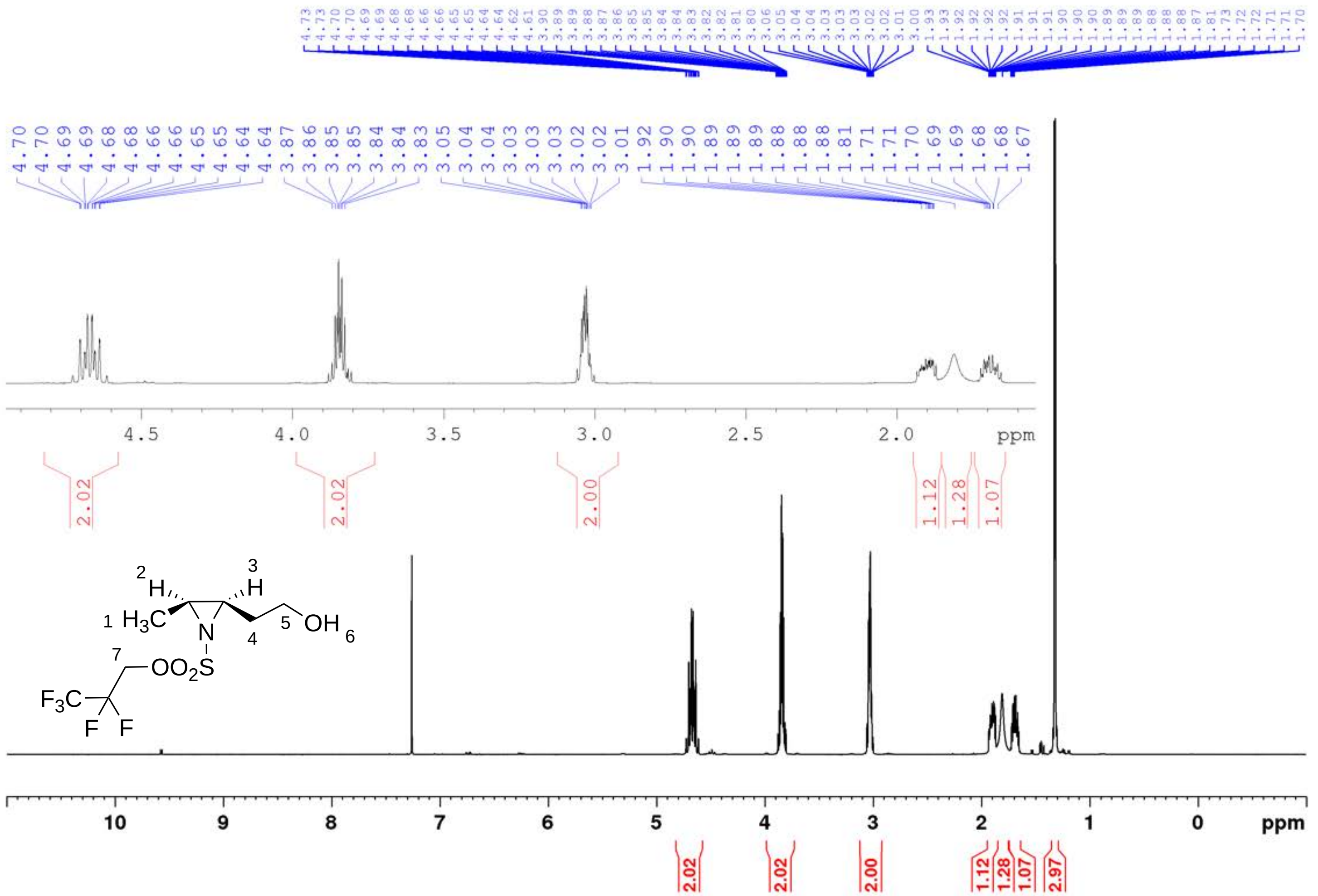

^{13}C NMR (126 MHz, $CDCl_3$) for *2,2,3,3,3-pentafluoropropyl (2R,3S)-2-(2-hydroxyethyl)-3-methylaziridine-1-sulfonate* (**24b**)

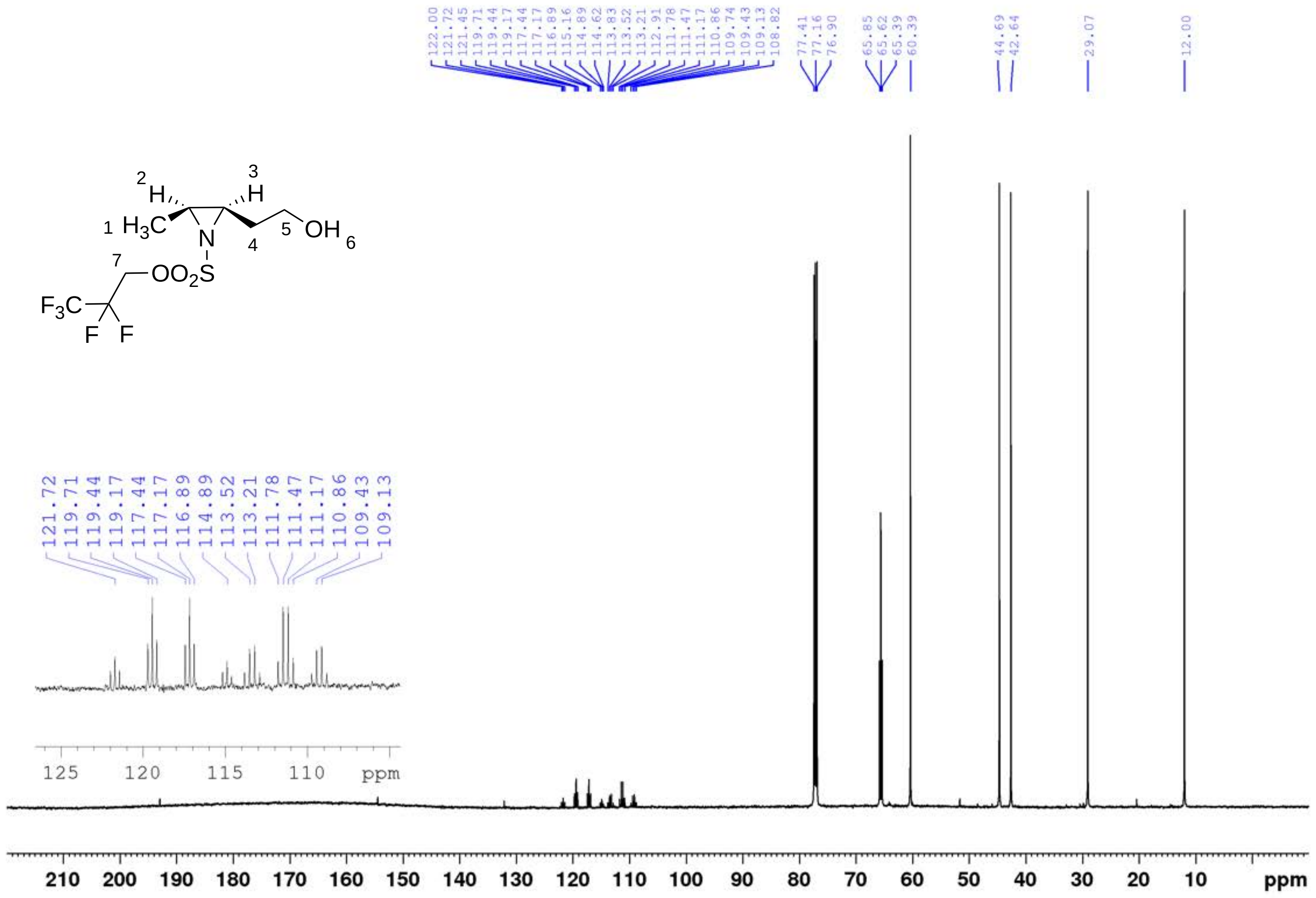

19F NMR (376 MHz, $CDCl_3$) for *2,2,3,3,3-pentafluoropropyl (2R,3S)-2-(2-hydroxyethyl)-3-methylaziridine-1-sulfonate* (**24b**)

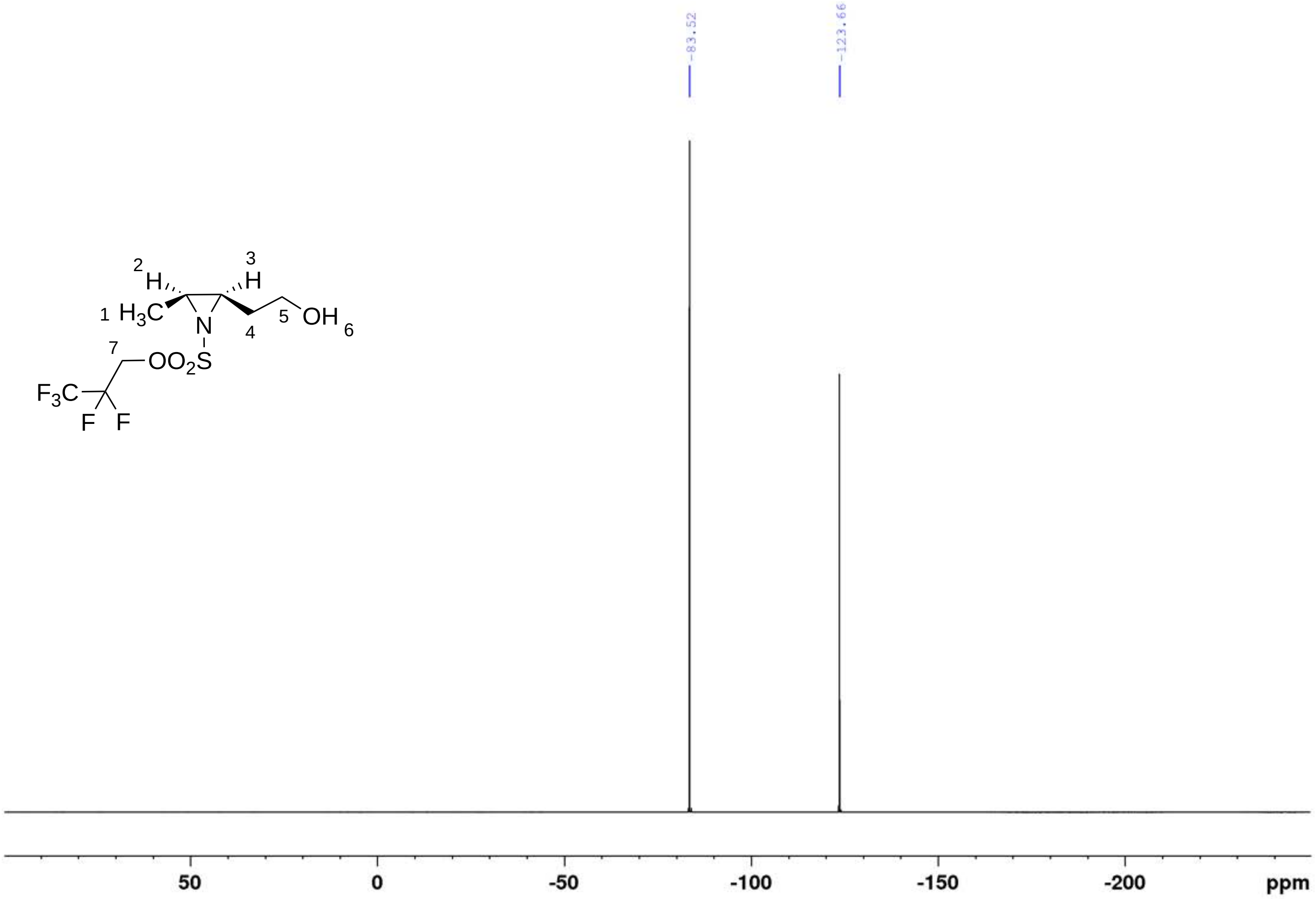

^{1}H NMR (500 MHz, $CDCl_3$) for *2,2,3,3,3-pentafluoropropyl ((3R,4R)-1-hydroxy-4-(phenylthio)pentan-3-yl)sulfamate and 2,2,3,3,3-pentafluoropropyl ((2S,3S)-5-hydroxy-3-(phenylthio)pentan-2-yl)sulfamate:* ***Inseparable mixture obtained following derivatisation of*** **24b**

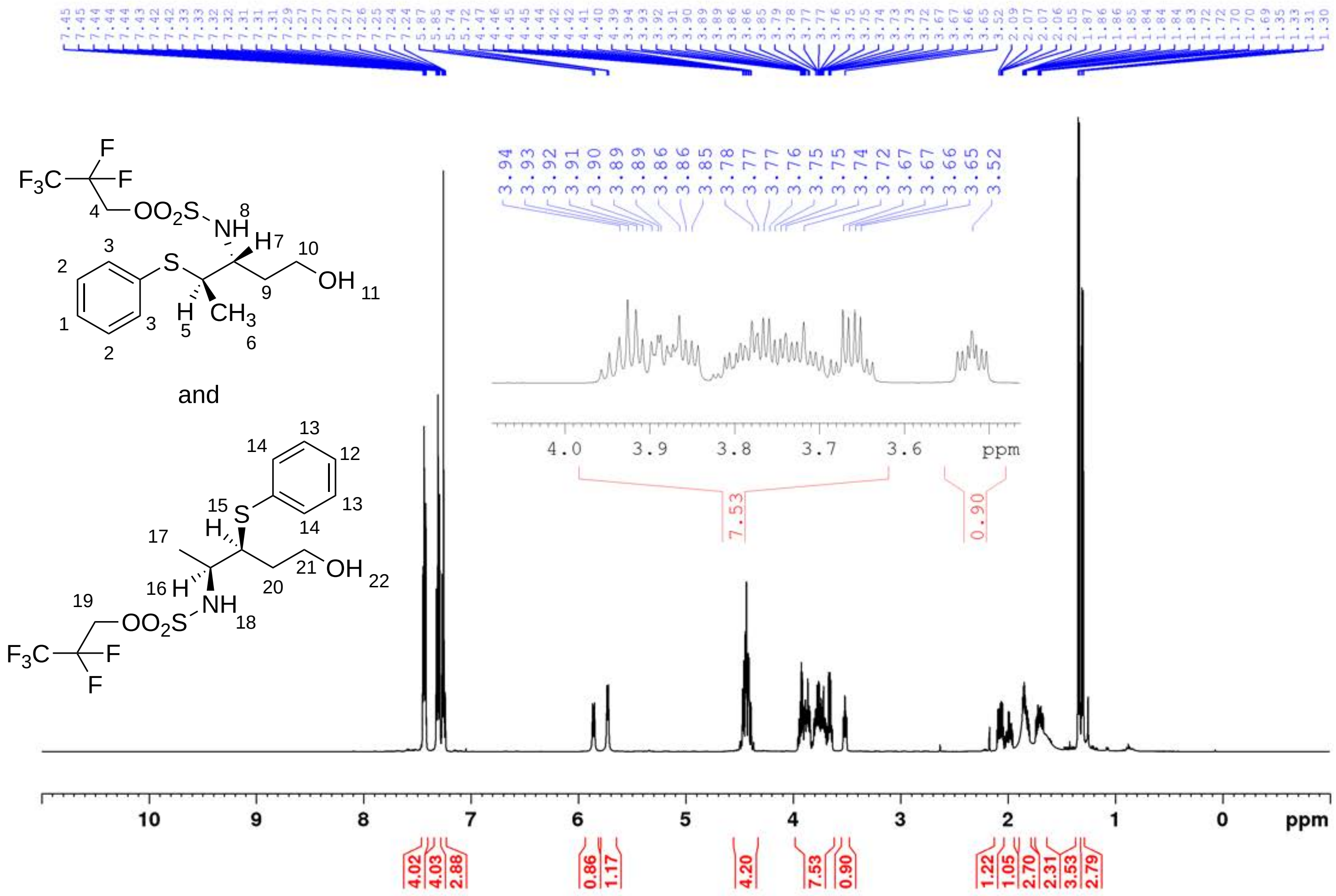

^{13}C NMR (126 MHz, $CDCl_3$) for *2,2,3,3,3-pentafluoropropyl ((3R,4R)-1-hydroxy-4-(phenylthio)pentan-3-yl)sulfamate and 2,2,3,3,3-pentafluoropropyl ((2S,3S)-5-hydroxy-3-(phenylthio)pentan-2-yl)sulfamate:* ***Inseparable mixture obtained following derivatisation of*** **24b**

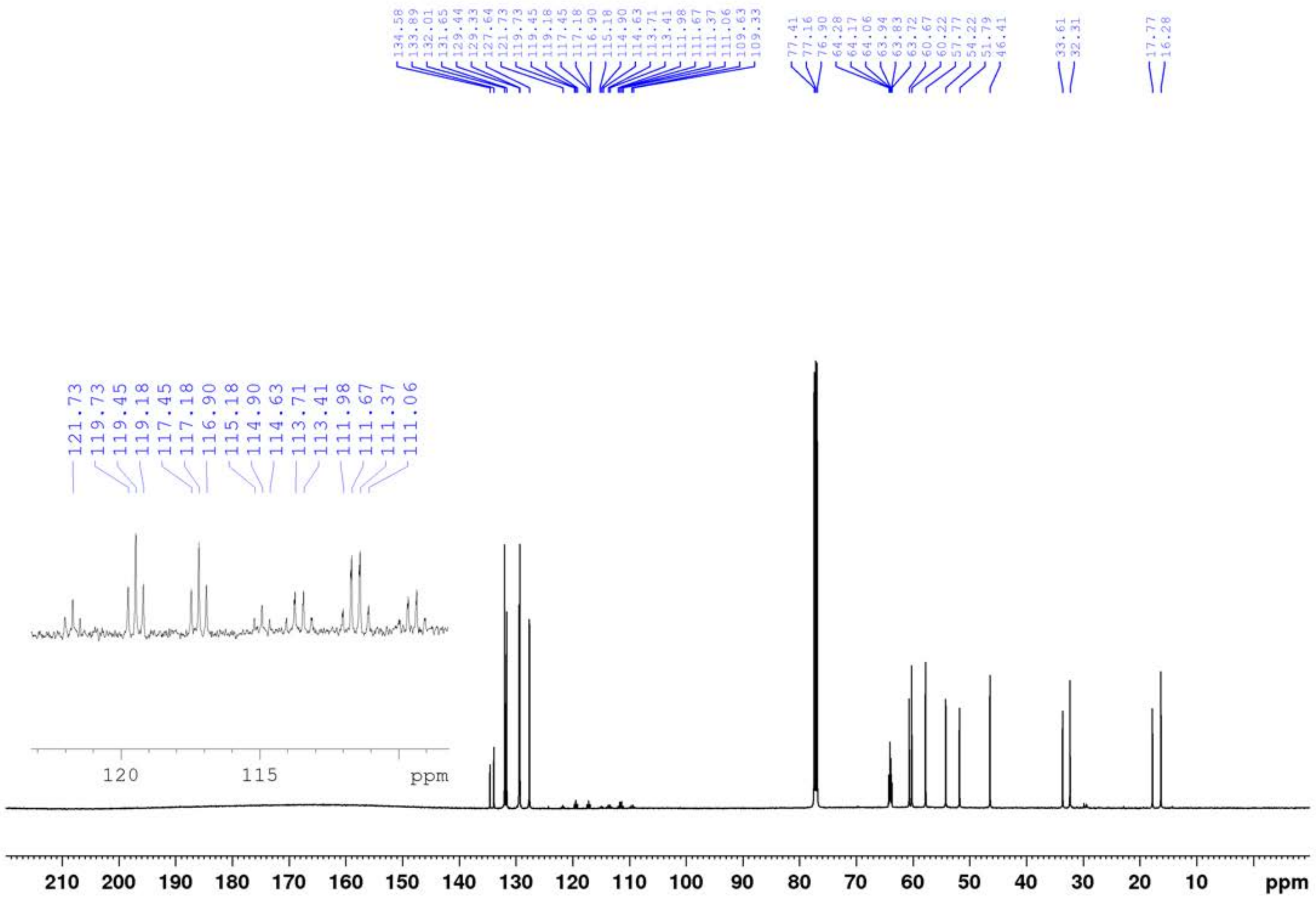

^{19}F NMR (471 MHz, $CDCl_3$) for *2,2,3,3,3-pentafluoropropyl ((3R,4R)-1-hydroxy-4-(phenylthio)pentan-3-yl)sulfamate and 2,2,3,3,3-pentafluoropropyl ((2S,3S)-5-hydroxy-3-(phenylthio)pentan-2-yl)sulfamate:* ***Inseparable mixture obtained following derivatisation of*** **24b**

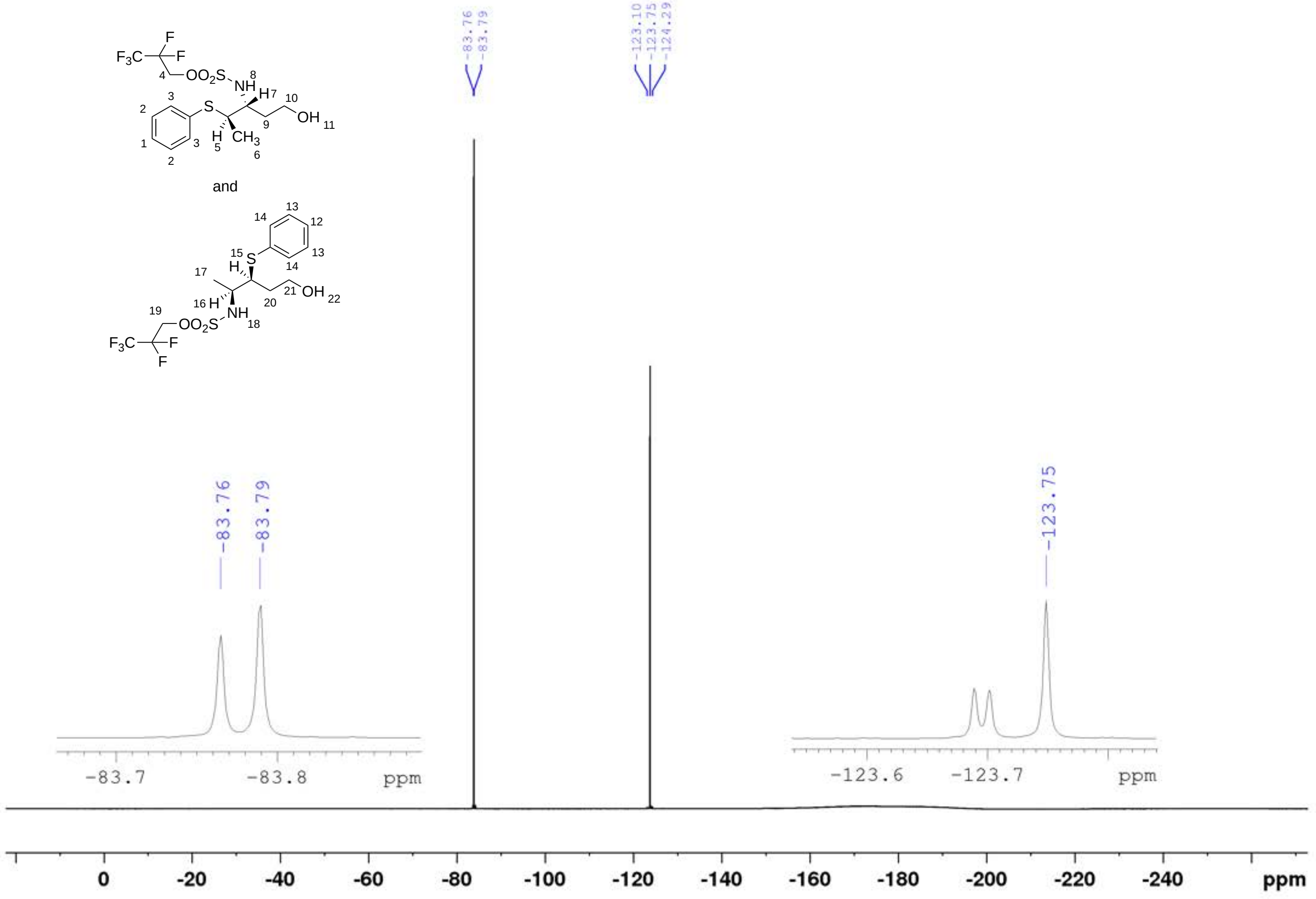

1H NMR (500 MHz, $CDCl_3$) for *2,2,3,3,3-pentafluoropropyl (S)-2-(2-hydroxyethyl)-2-methylaziridine-1-sulfonate* (**24c**)

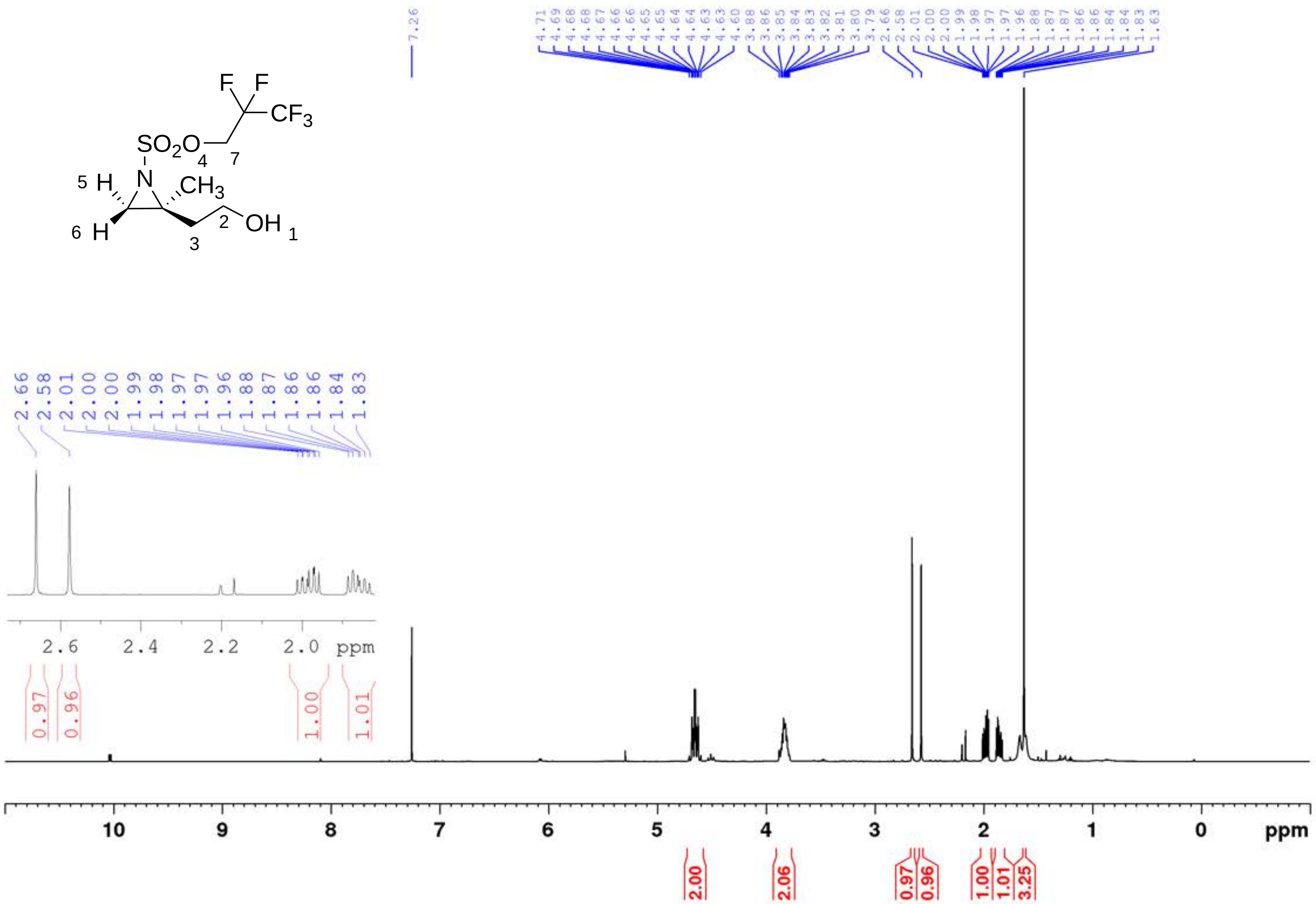

^{13}**C NMR** (126 MHz, $CDCl_3$) for *2,2,3,3,3-pentafluoropropyl (S)-2-(2-hydroxyethyl)-2-methylaziridine-1-sulfonate* (**24c**)

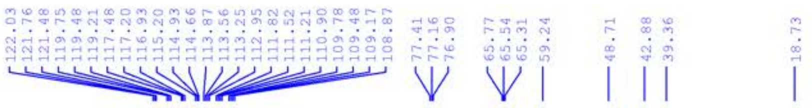

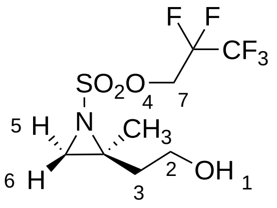

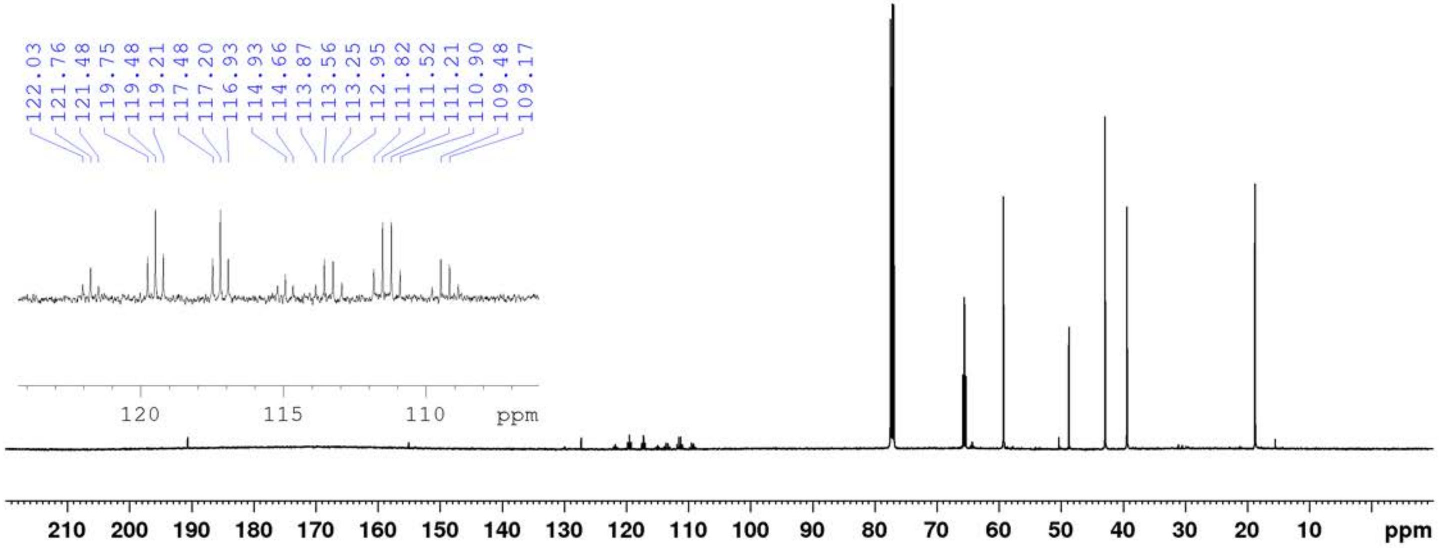

^{19}F NMR (376 MHz, $CDCl_3$) for *2,2,3,3,3-pentafluoropropyl (S)-2-(2-hydroxyethyl)-2-methylaziridine-1-sulfonate* (**24c**)

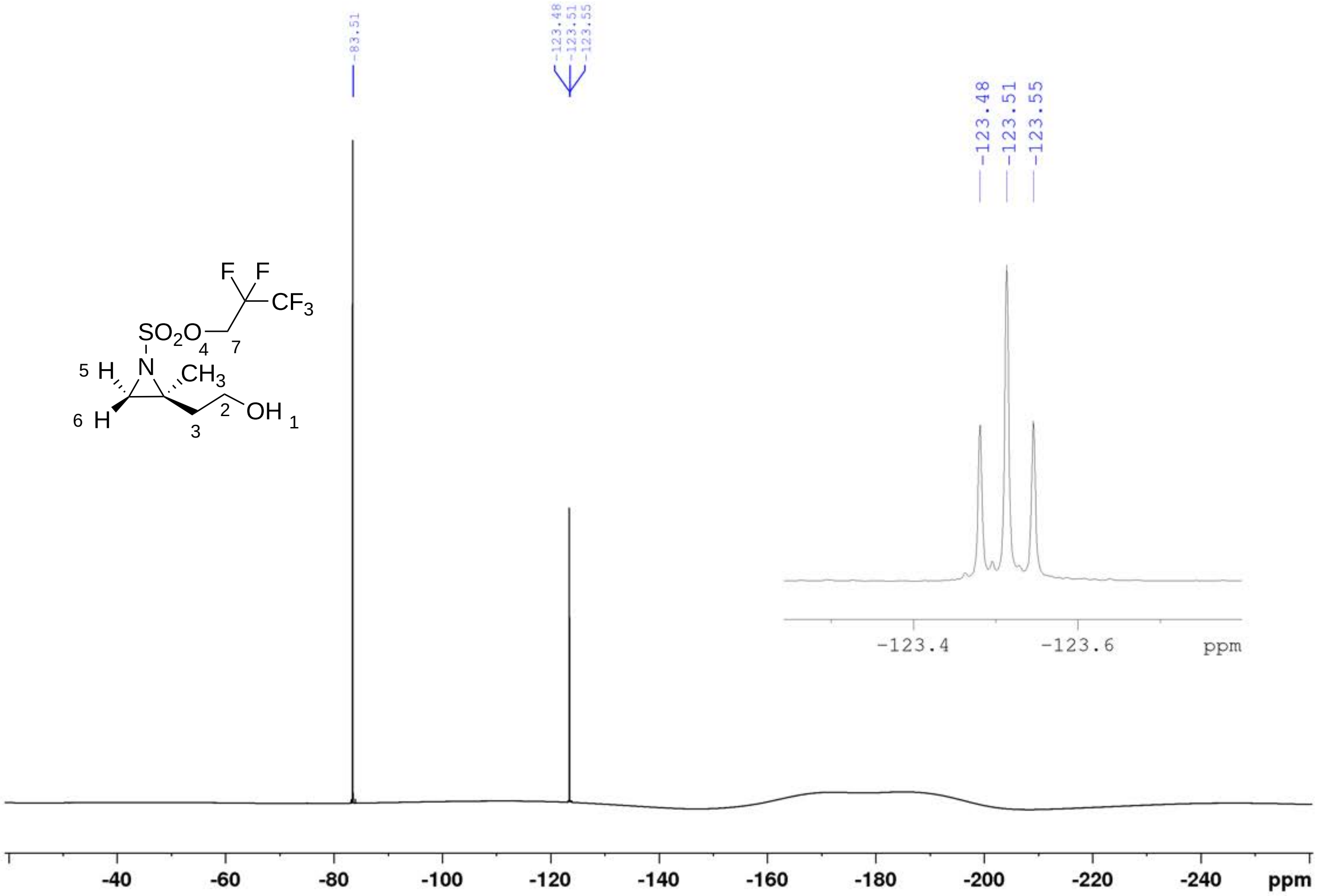

^{1}H NMR (500 MHz, $CDCl_3$) for *2,2,3,3,3-pentafluoropropyl (S)-(4-hydroxy-2-methyl-1-(phenylthio)butan-2-yl)sulfamate:* ***Obtained following derivatisation of* 24c**

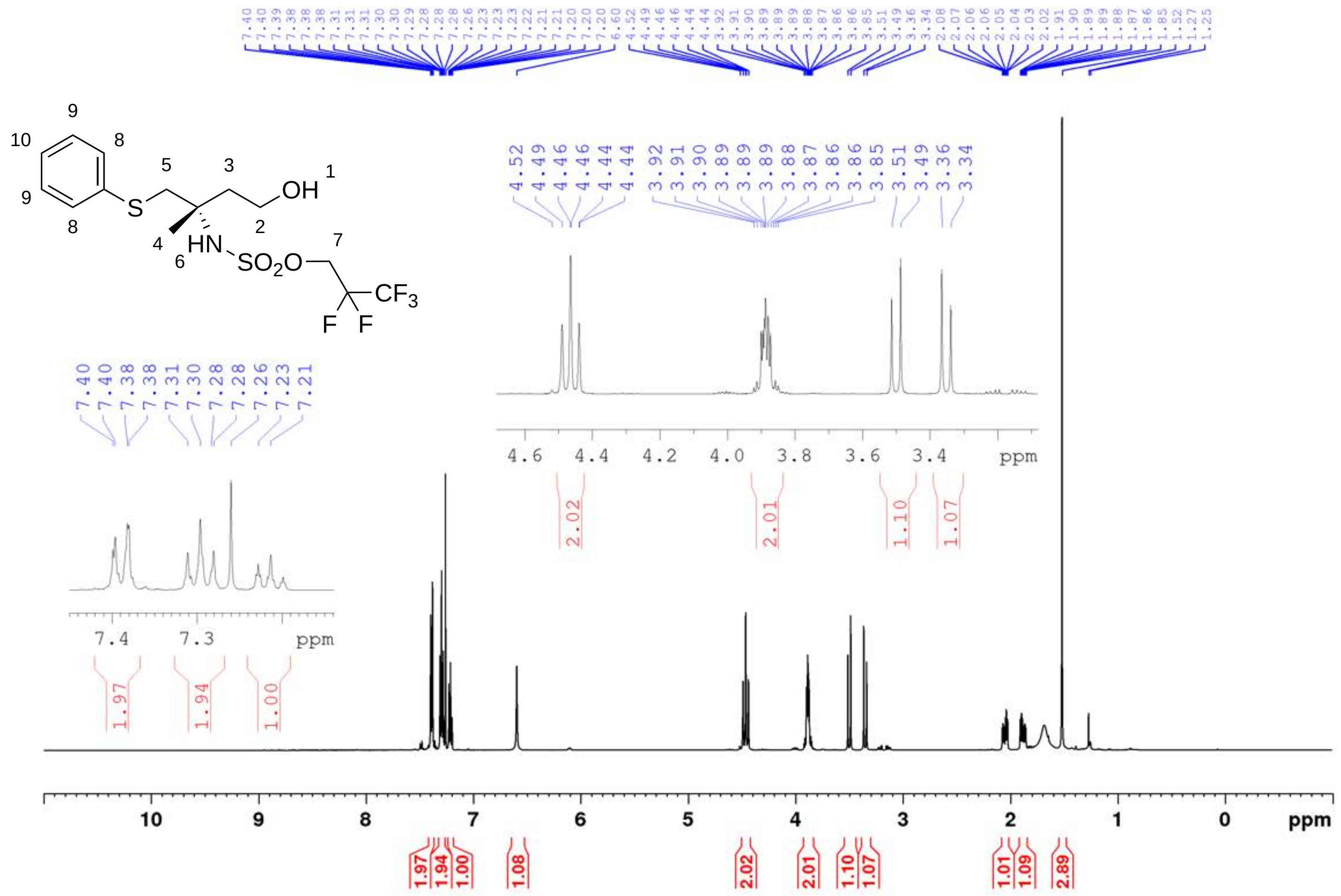

^{13}C NMR (126 MHz, $CDCl_3$) for *2,2,3,3,3-pentafluoropropyl (S)-(4-hydroxy-2-methyl-1-(phenylthio)butan-2-yl)sulfamate:* ***Obtained following derivatisation of* 24c**

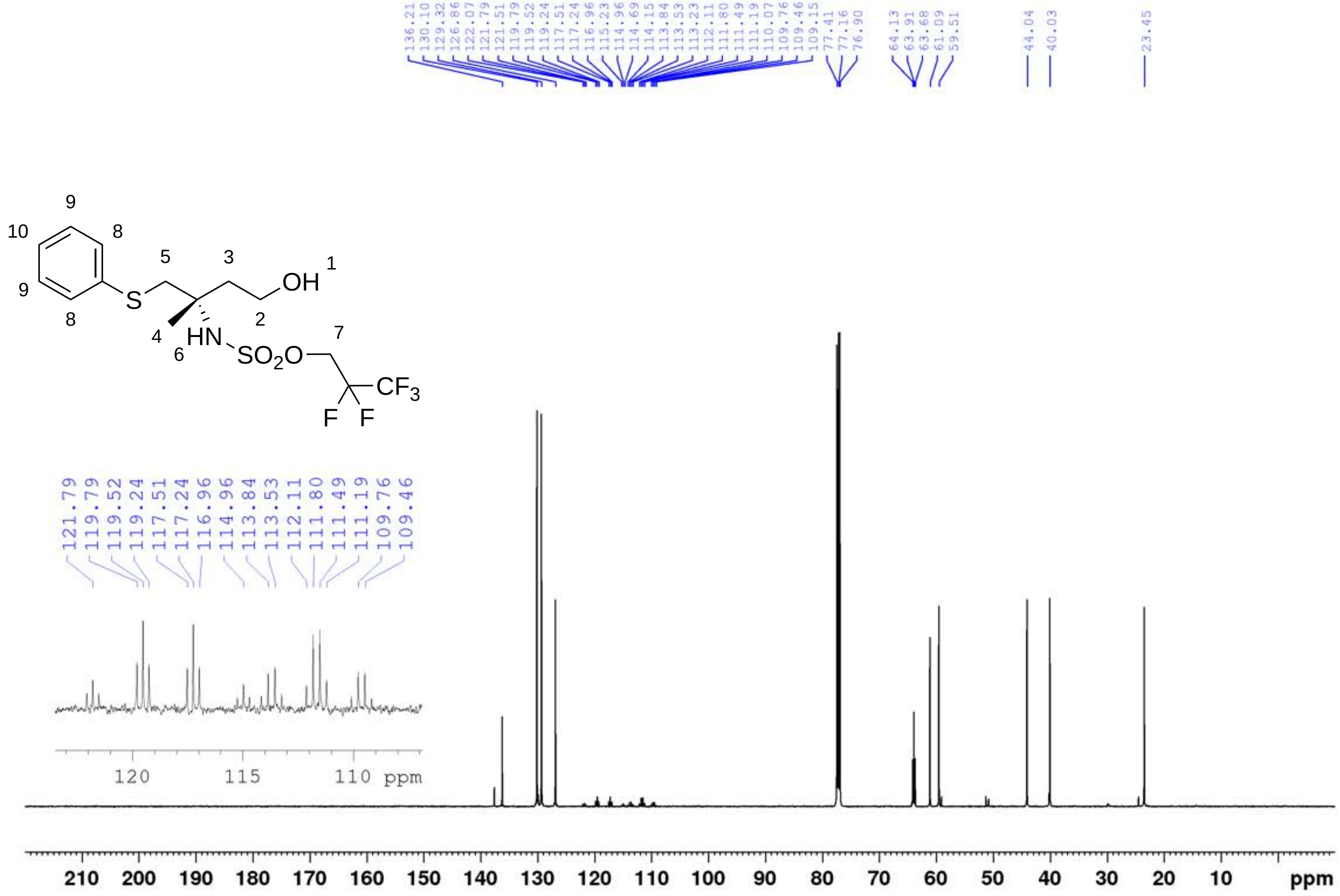

^{19}F NMR (376 MHz, $CDCl_3$) for *2,2,3,3,3-pentafluoropropyl (S)-(4-hydroxy-2-methyl-1-(phenylthio)butan-2-yl)sulfamate:* ***Obtained following derivatisation of* 24c**

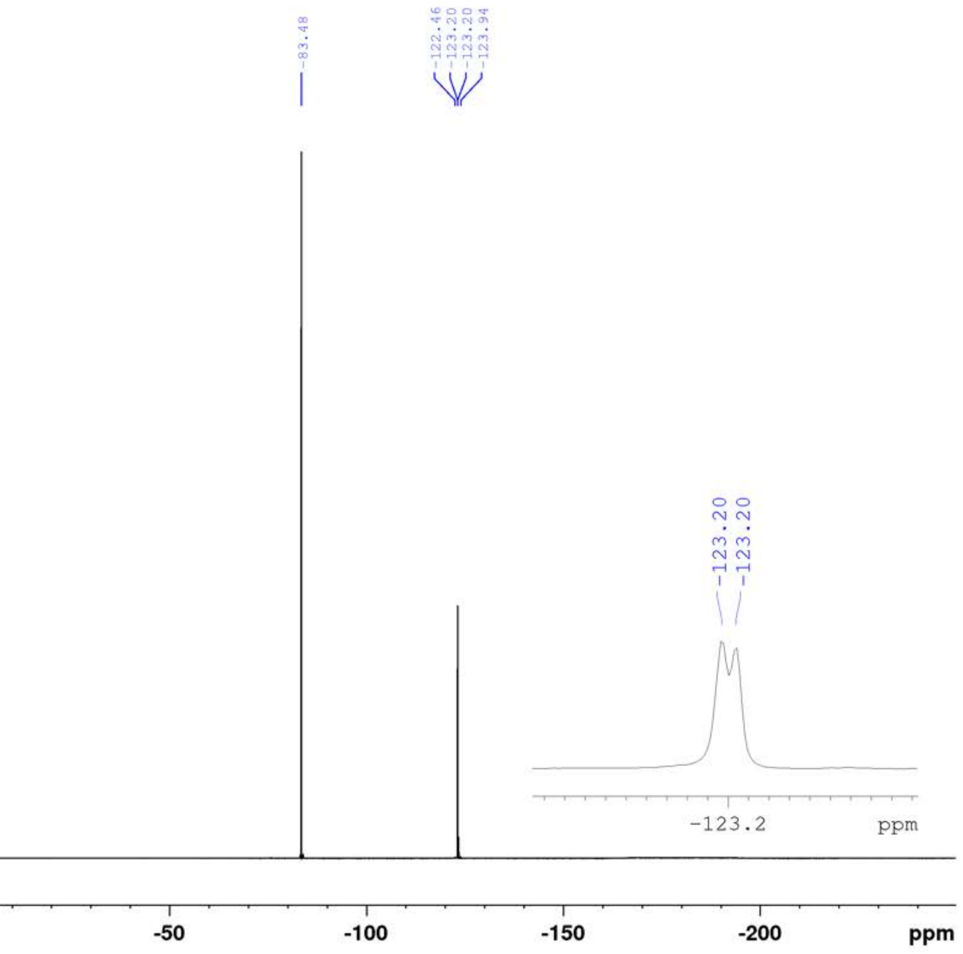

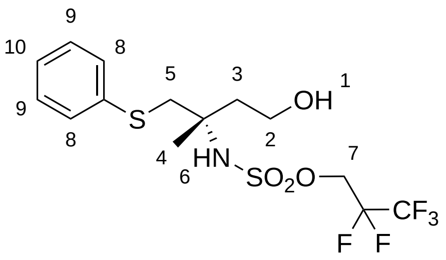

^{1}H NMR (500 MHz, $CDCl_3$) for *2,2,3,3,3-pentafluoropropyl (R)-(2,2-dimethyltetrahydrofuran-3-yl)sulfamate* (**24d**)

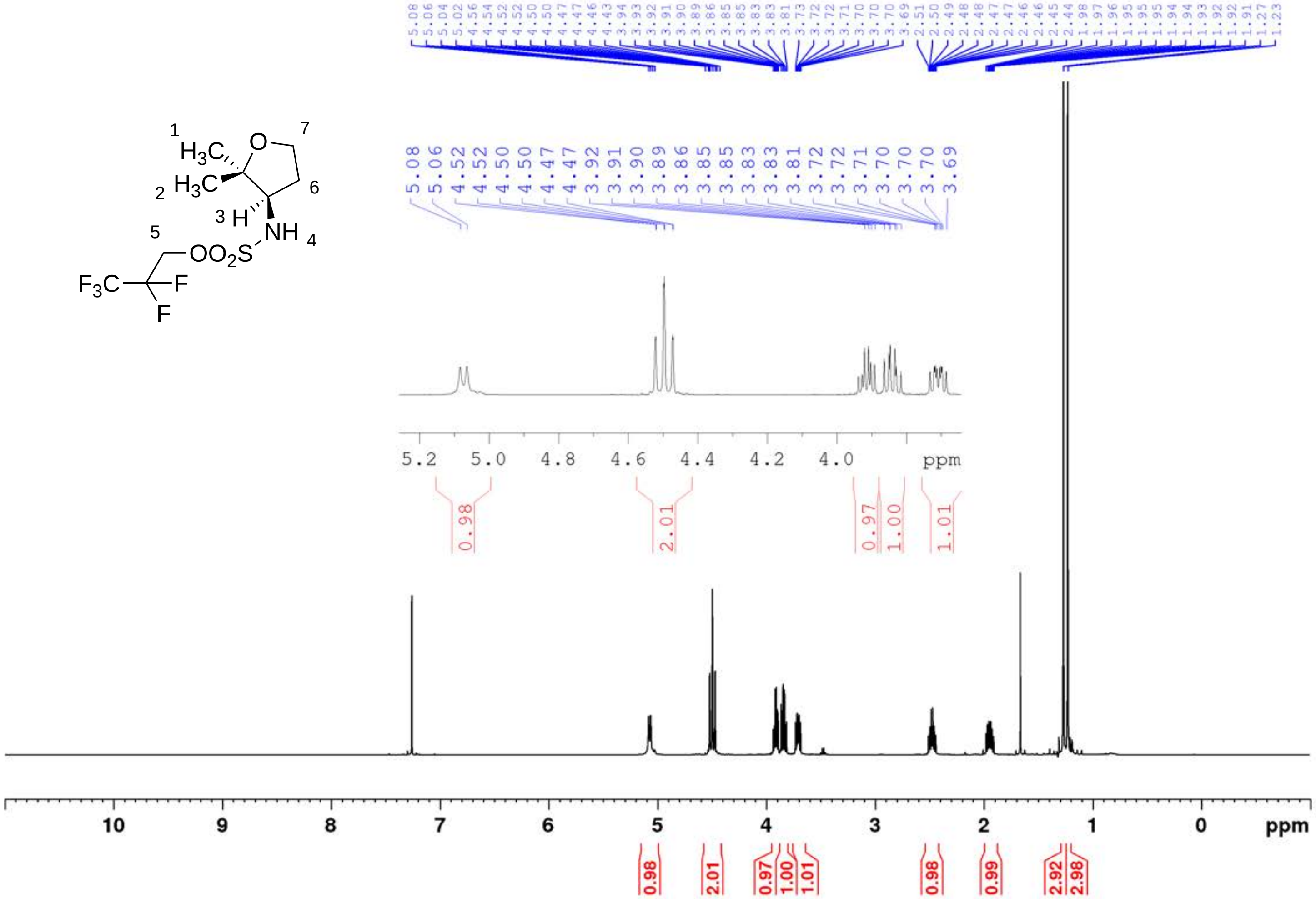

^{13}C NMR (126 MHz, $CDCl_3$) for *2,2,3,3,3-pentafluoropropyl (R)-(2,2-dimethyltetrahydrofuran-3-yl)sulfamate* (**24d**)

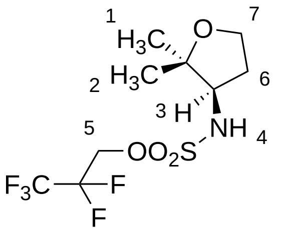

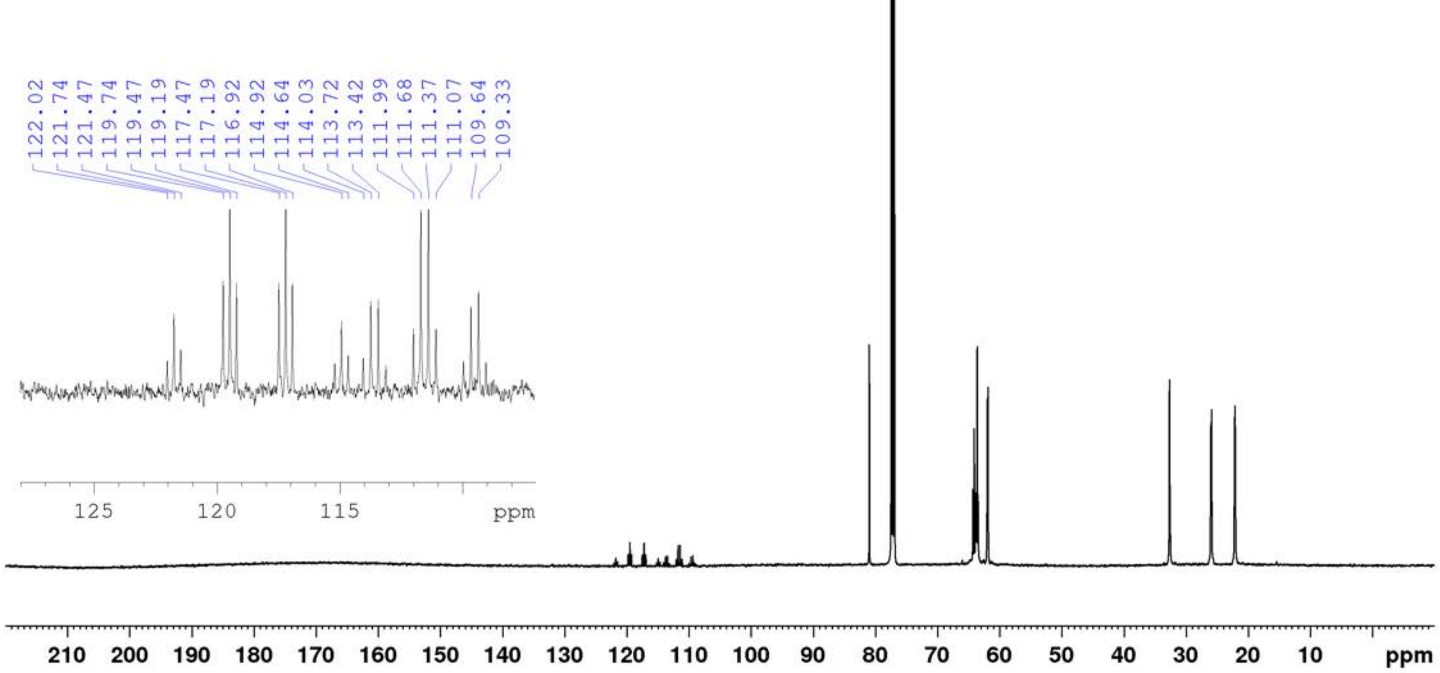

19F NMR (376 MHz, CDCl3) for *2,2,3,3,3-pentafluoropropyl (R)-(2,2-dimethyltetrahydrofuran-3-yl)sulfamate* (**24d**)

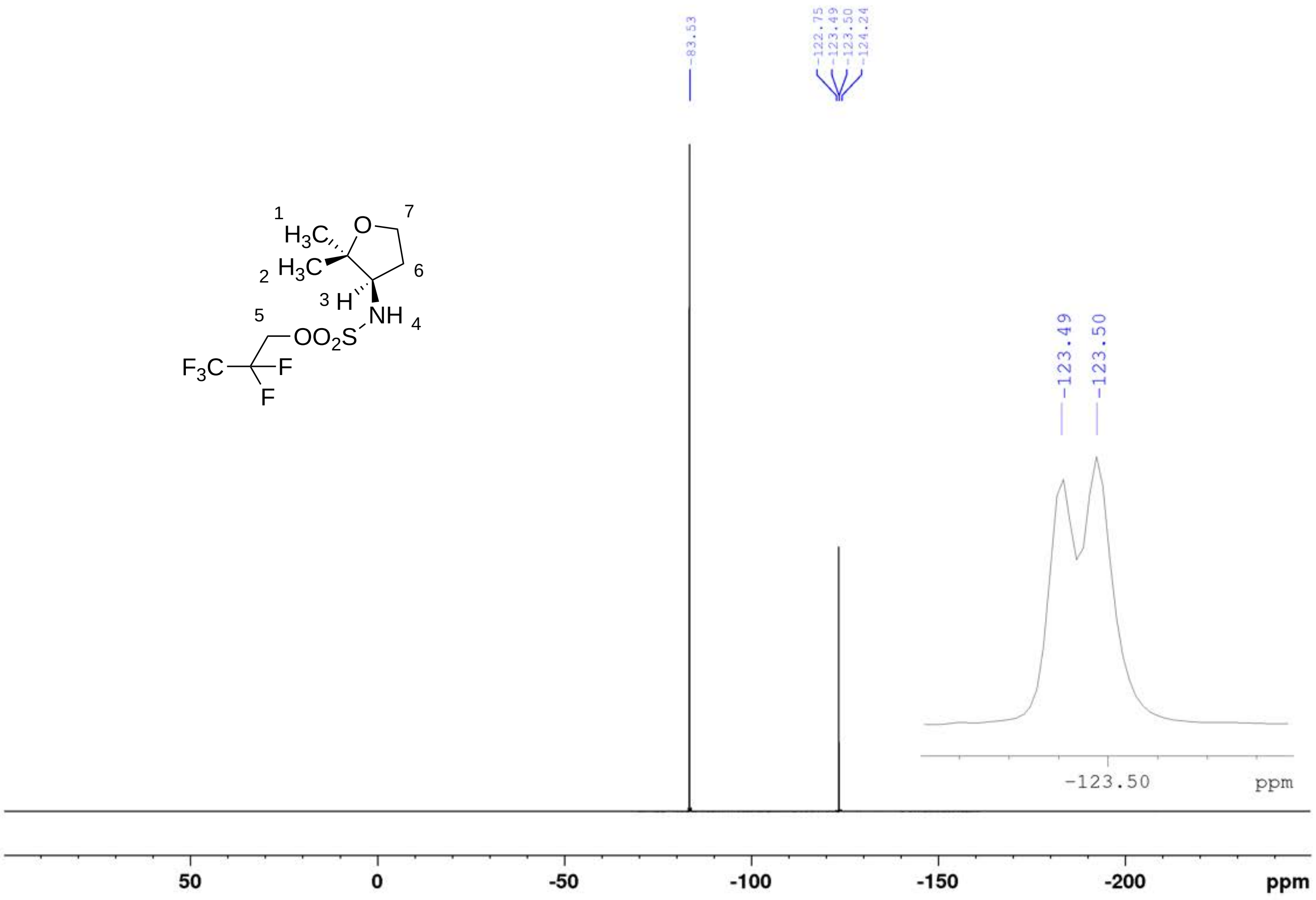

^{1}H NMR (500 MHz, $CDCl_3$) for *2,2,3,3,3-pentafluoropropyl ((3S,4S)-1-hydroxy-3-methyl-4-(phenylthio)pentan-3-yl)sulfamate and 2,2,3,3,3-pentafluoropropyl ((2R,3R)-5-hydroxy-3-(phenylthio)pentan-2-yl)*sulfamate: ***Inseparable mixture obtained following derivatisation of* 24e**

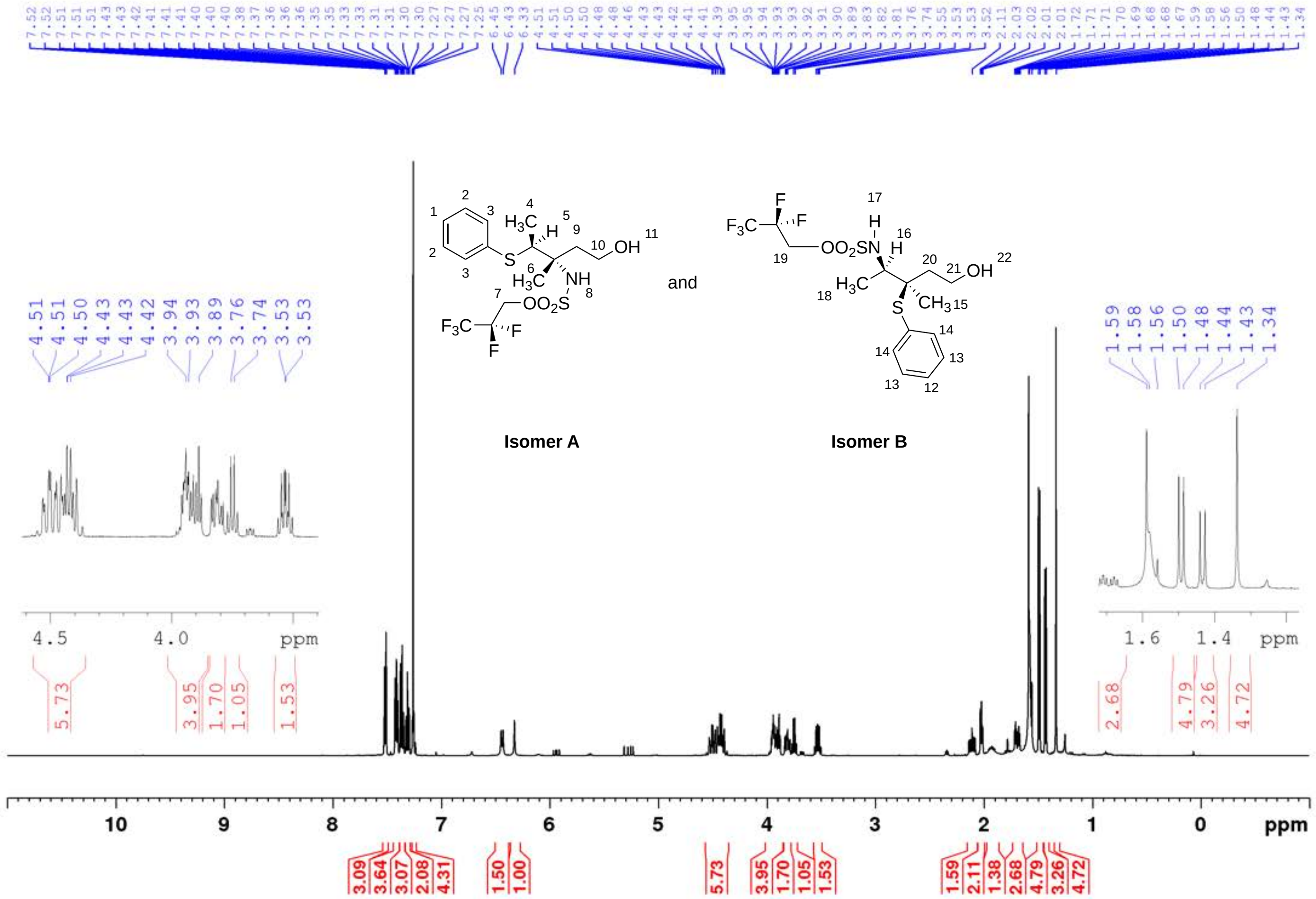

^{13}C NMR (126 MHz, $CDCl_3$) for *2,2,3,3,3-pentafluoropropyl ((3S,4S)-1-hydroxy-3-methyl-4-(phenylthio)pentan-3-yl)sulfamate* *and 2,2,3,3,3-pentafluoropropyl ((2R,3R)-5-hydroxy-3-(phenylthio)pentan-2-yl)sulfamate*: ***Inseparable mixture obtained following derivatisation of* 24e**

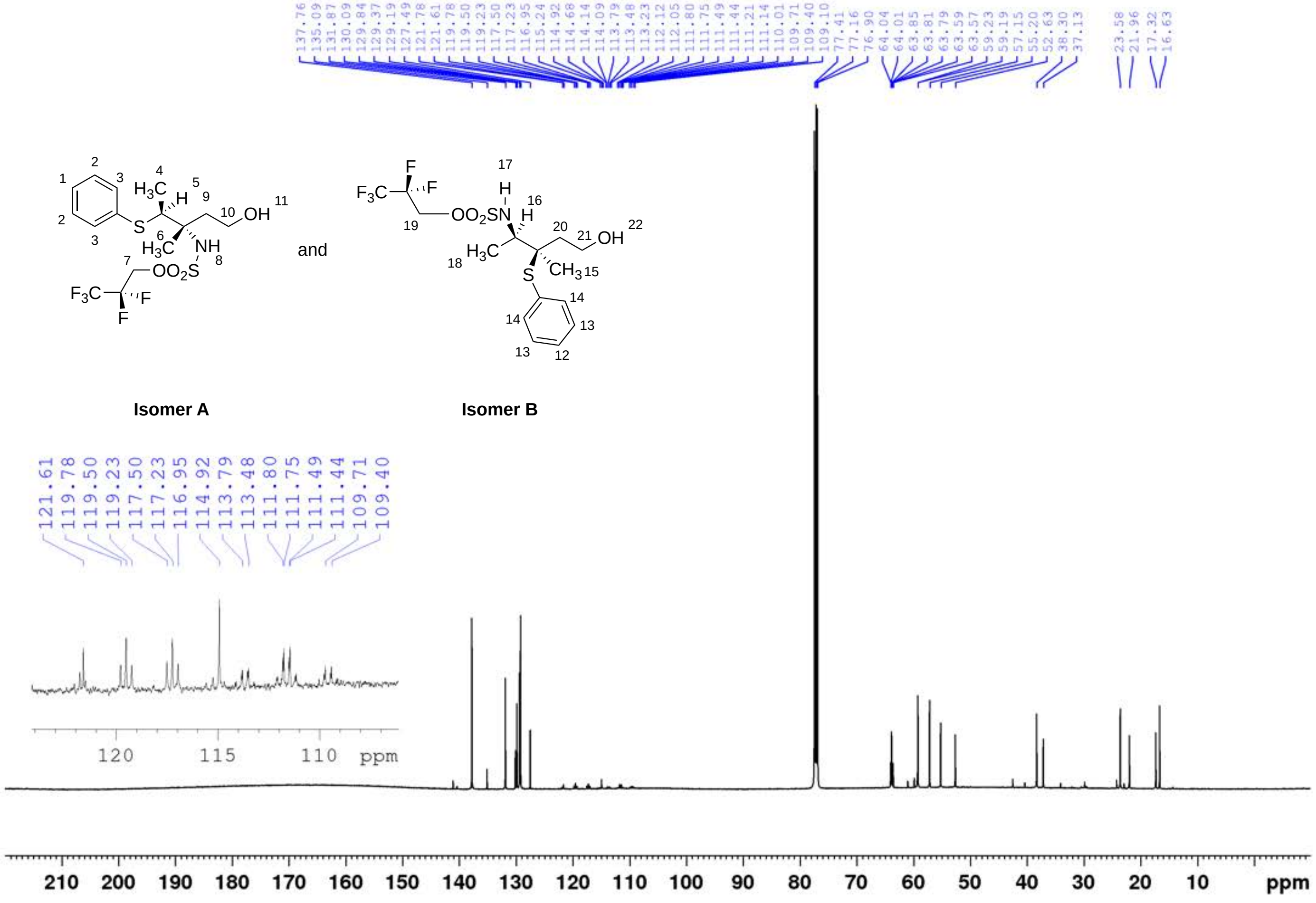

^{19}F NMR (376 MHz, $CDCl_3$) for *2,2,3,3,3-pentafluoropropyl ((3S,4S)-1-hydroxy-3-methyl-4-(phenylthio)pentan-3-yl)sulfamate* and *2,2,3,3,3-pentafluoropropyl ((2R,3R)-5-hydroxy-3-(phenylthio)pentan-2-yl)sulfamate*: ***Inseparable mixture obtained following derivatisation of* 24e**

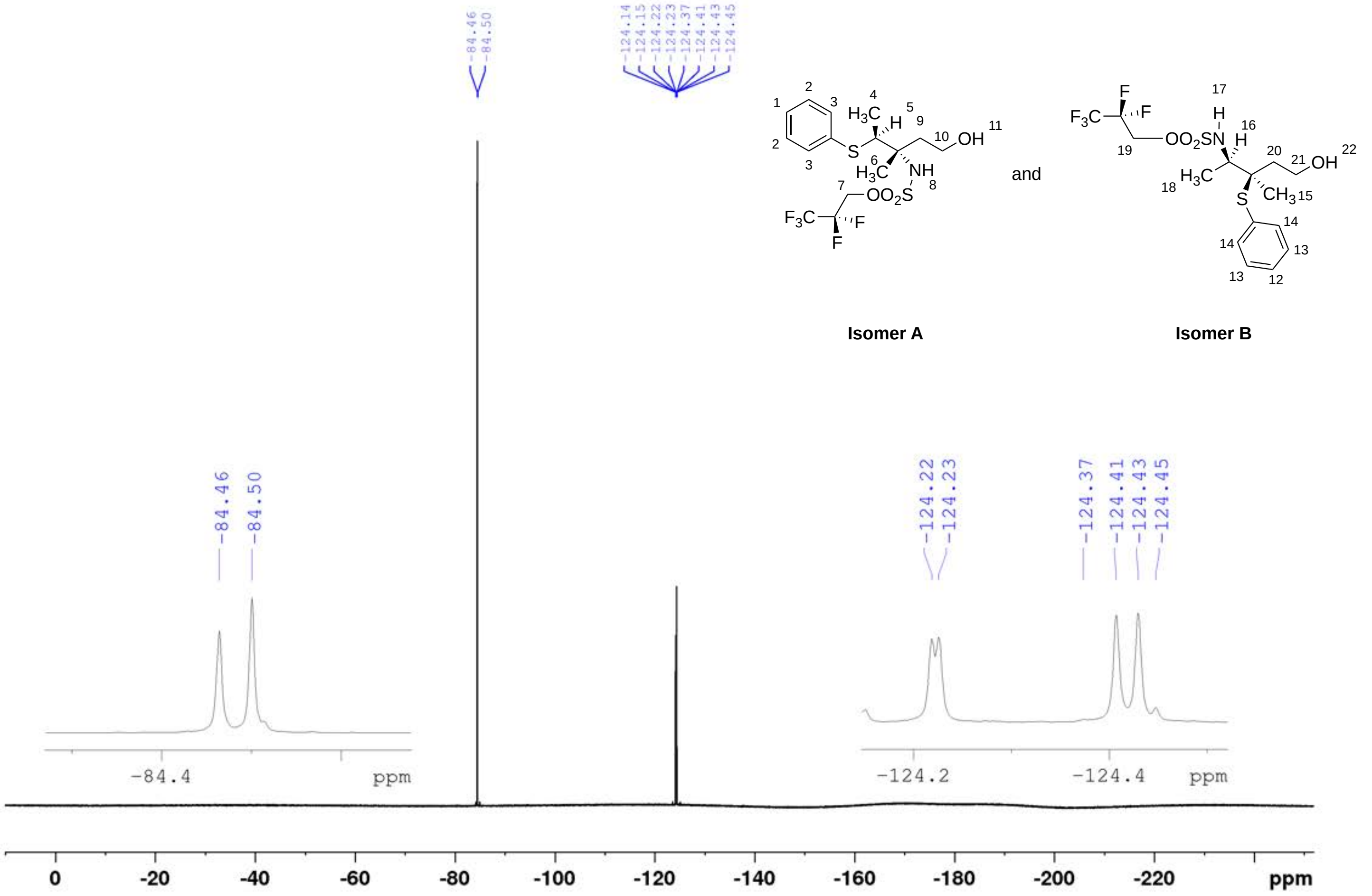

^{1}H NMR (500 MHz, $CDCl_3$) for *2,2,3,3,3-pentafluoropropyl (2R,3R)-2-(2-hydroxyethyl)-2,3-dimethylaziridine-1-sulfonate* (**24f**)

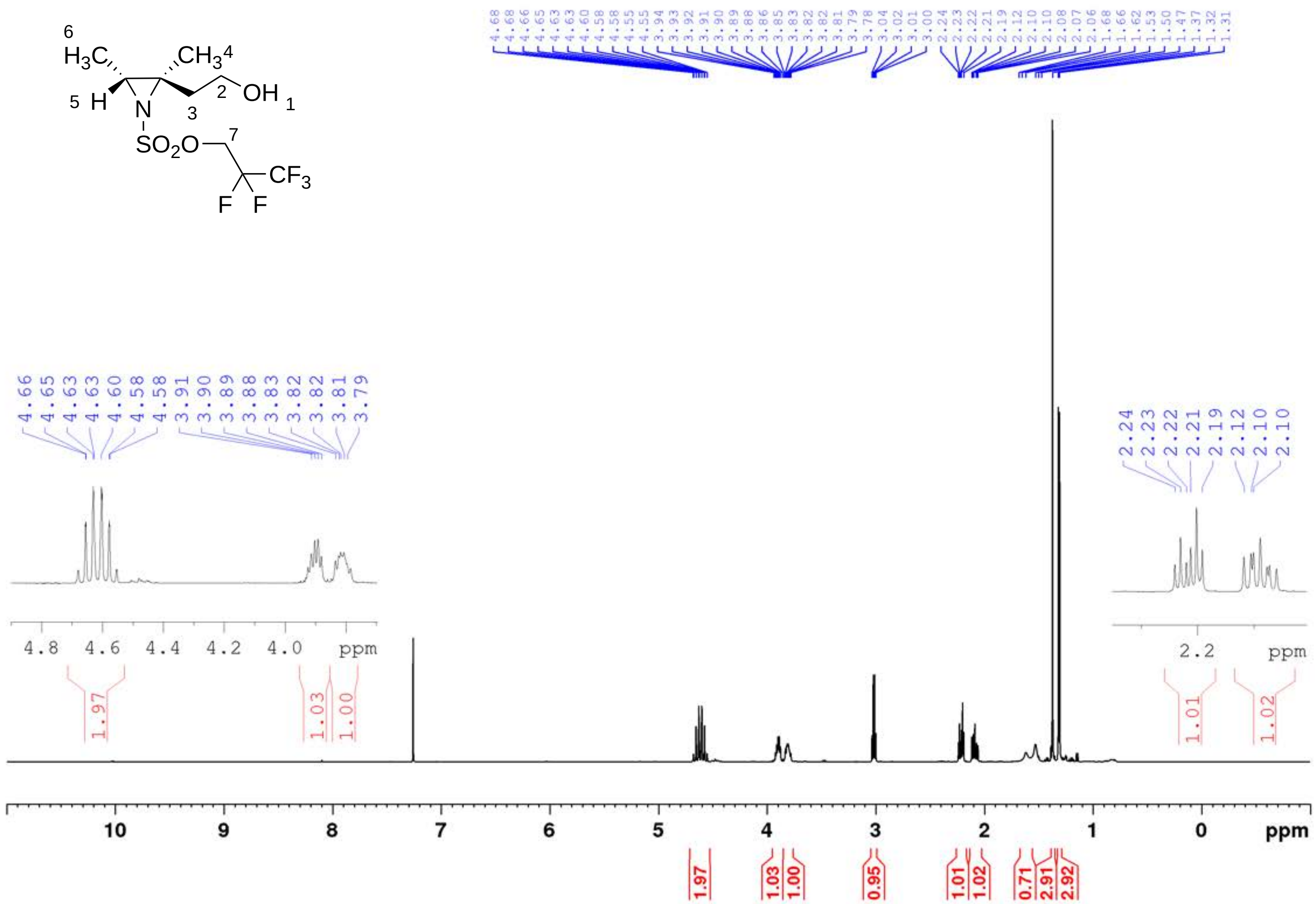

^{13}C NMR (126 MHz, $CDCl_3$) for *2,2,3,3,3-pentafluoropropyl (2R,3R)-2-(2-hydroxyethyl)-2,3-dimethylaziridine-1-sulfonate* (**24f**)

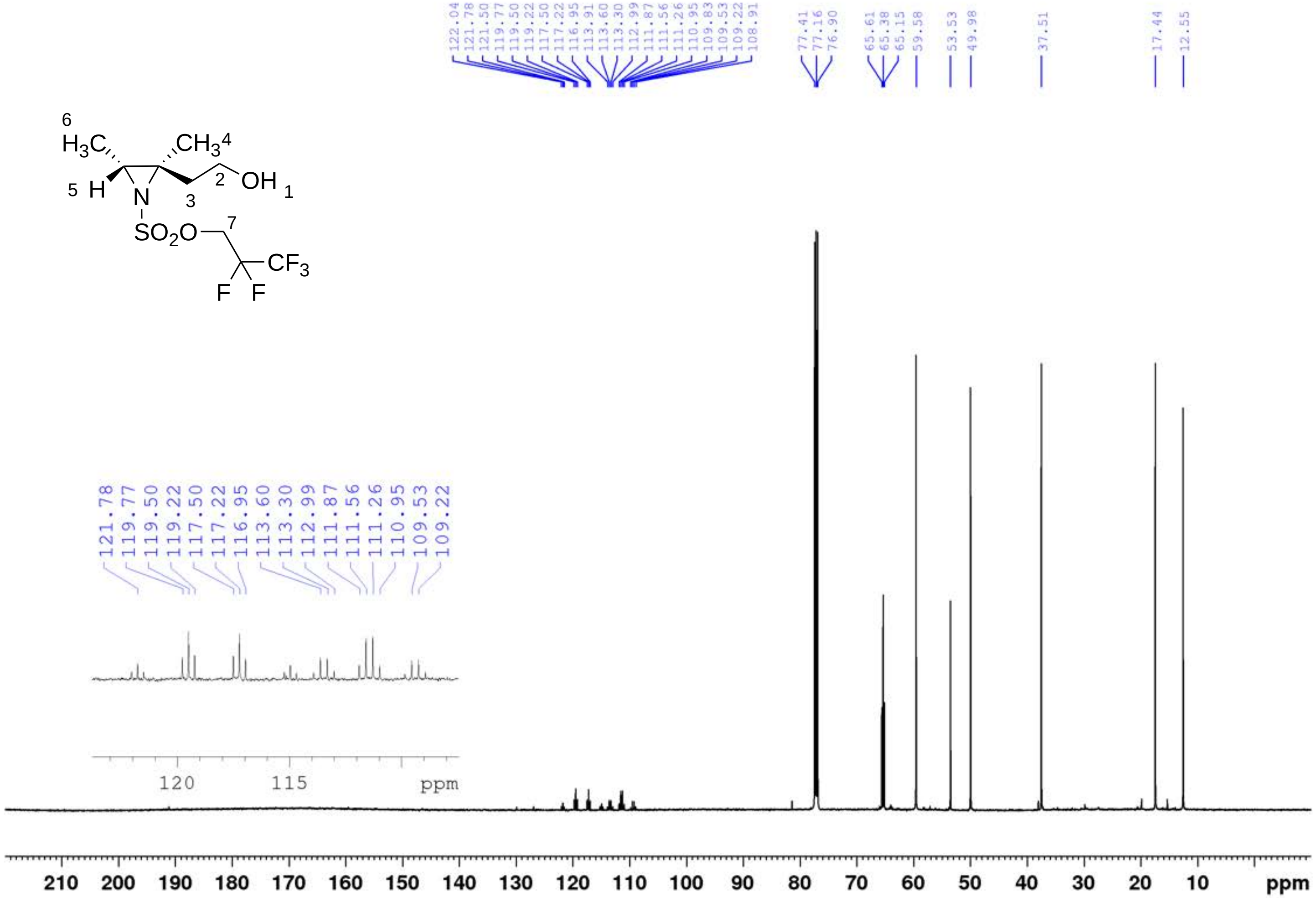

^{19}F NMR (376 MHz, $CDCl_3$) for *2,2,3,3,3-pentafluoropropyl (2R,3R)-2-(2-hydroxyethyl)-2,3-dimethylaziridine-1-sulfonate* (**24f**)

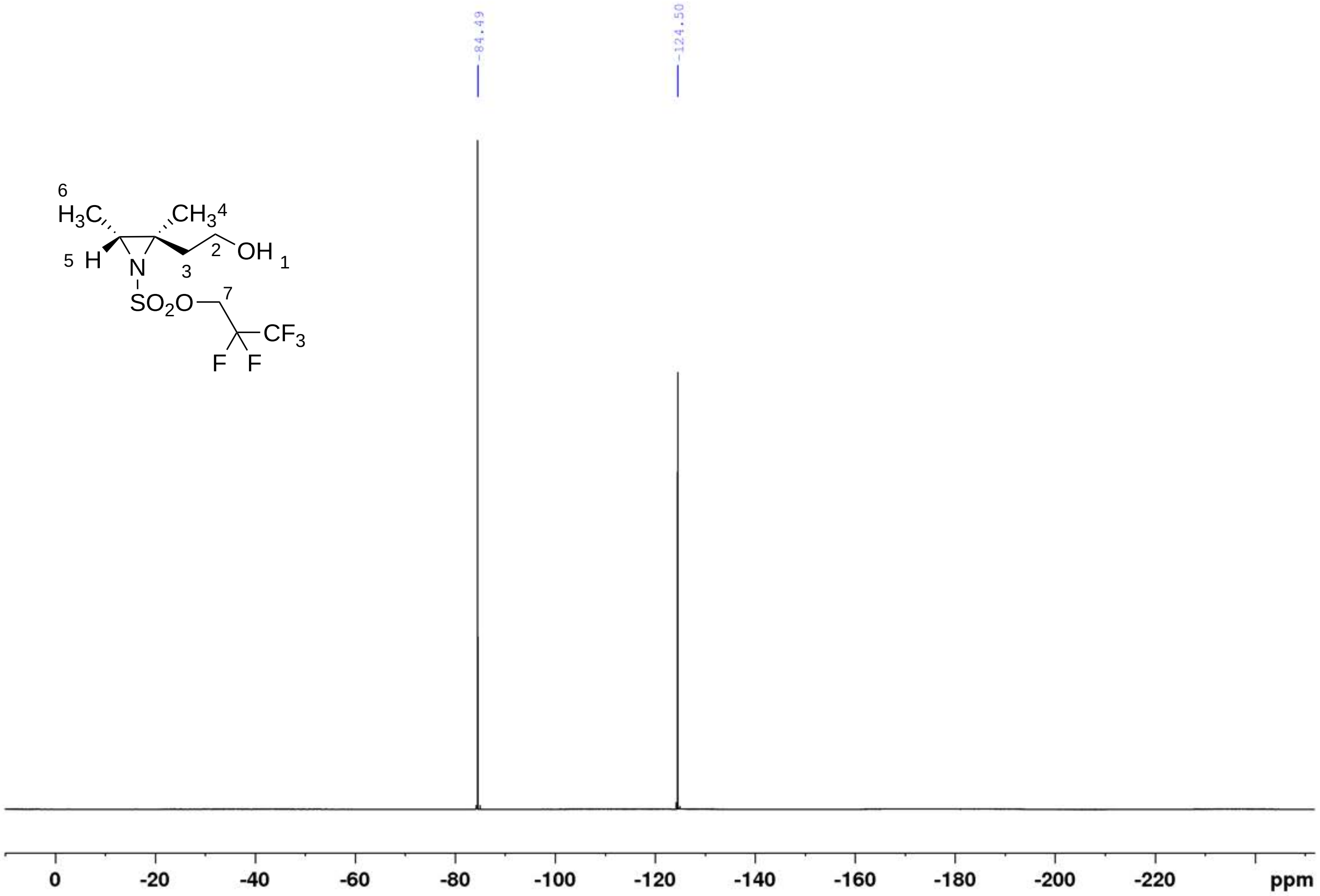

^{1}H NMR (500 MHz, $CDCl_3$) for *2,2,3,3,3-pentafluoropropyl ((2R,3R)-5-hydroxy-3-methyl-3-(phenylthio)pentan-2-yl)sulfamate:* ***Obtained following derivatisation of* 24f**

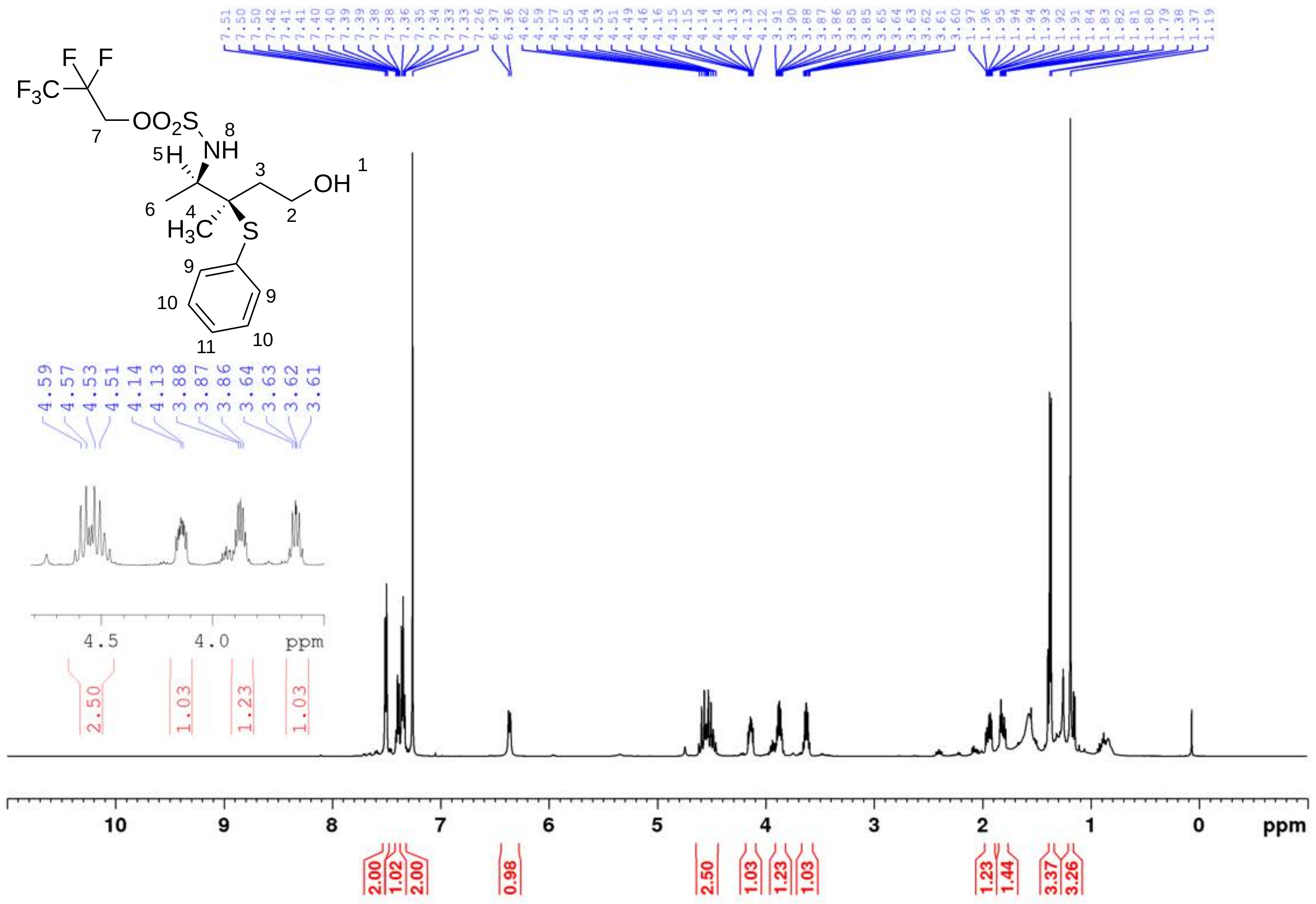

^{13}C NMR (126 MHz, $CDCl_3$) for *2,2,3,3,3-pentafluoropropyl ((2R,3R)-5-hydroxy-3-methyl-3-(phenylthio)pentan-2-yl)sulfamate:* ***Obtained following derivatisation of* 24f**

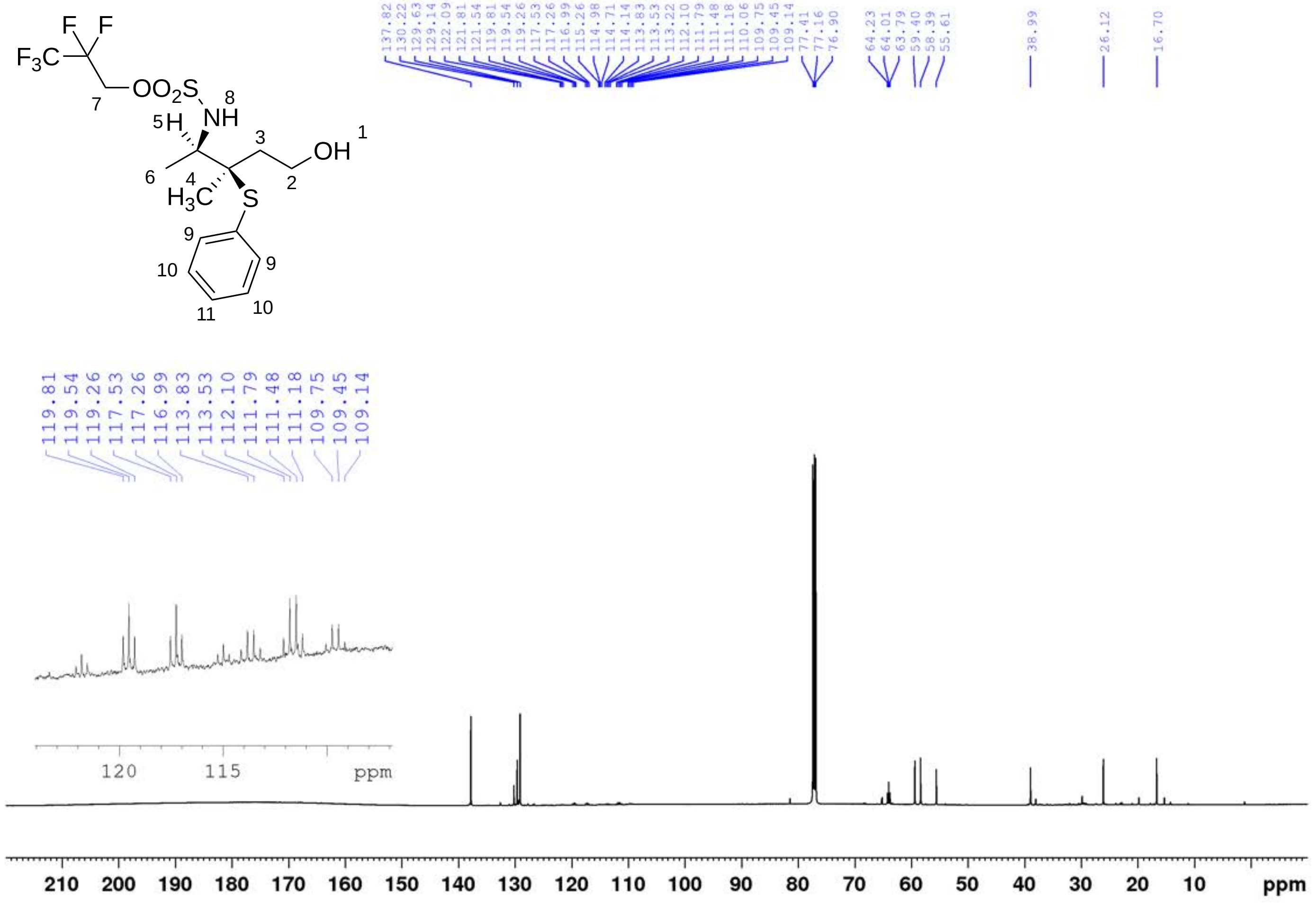

^{19}F NMR (376 MHz, $CDCl_3$) for *2,2,3,3,3-pentafluoropropyl ((2R,3R)-5-hydroxy-3-methyl-3-(phenylthio)pentan-2-yl)sulfamate:* ***Obtained following derivatisation of* 24f**

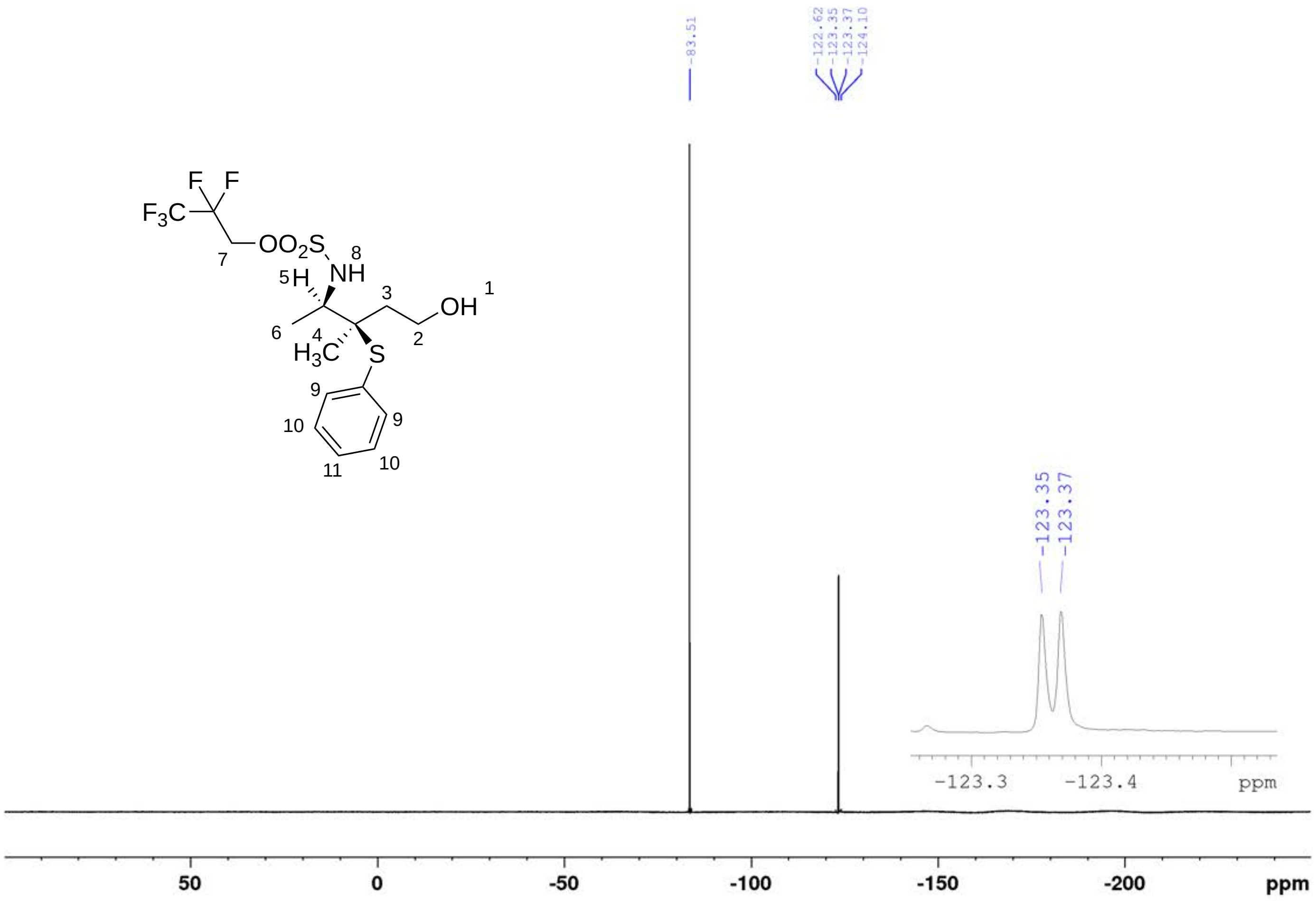

¹H NMR (500 MHz, $CDCl_3$) for *2,2,3,3,3-pentafluoropropyl (R)-2-(2-hydroxyethyl)aziridine-1-sulfonate* (**24g**)

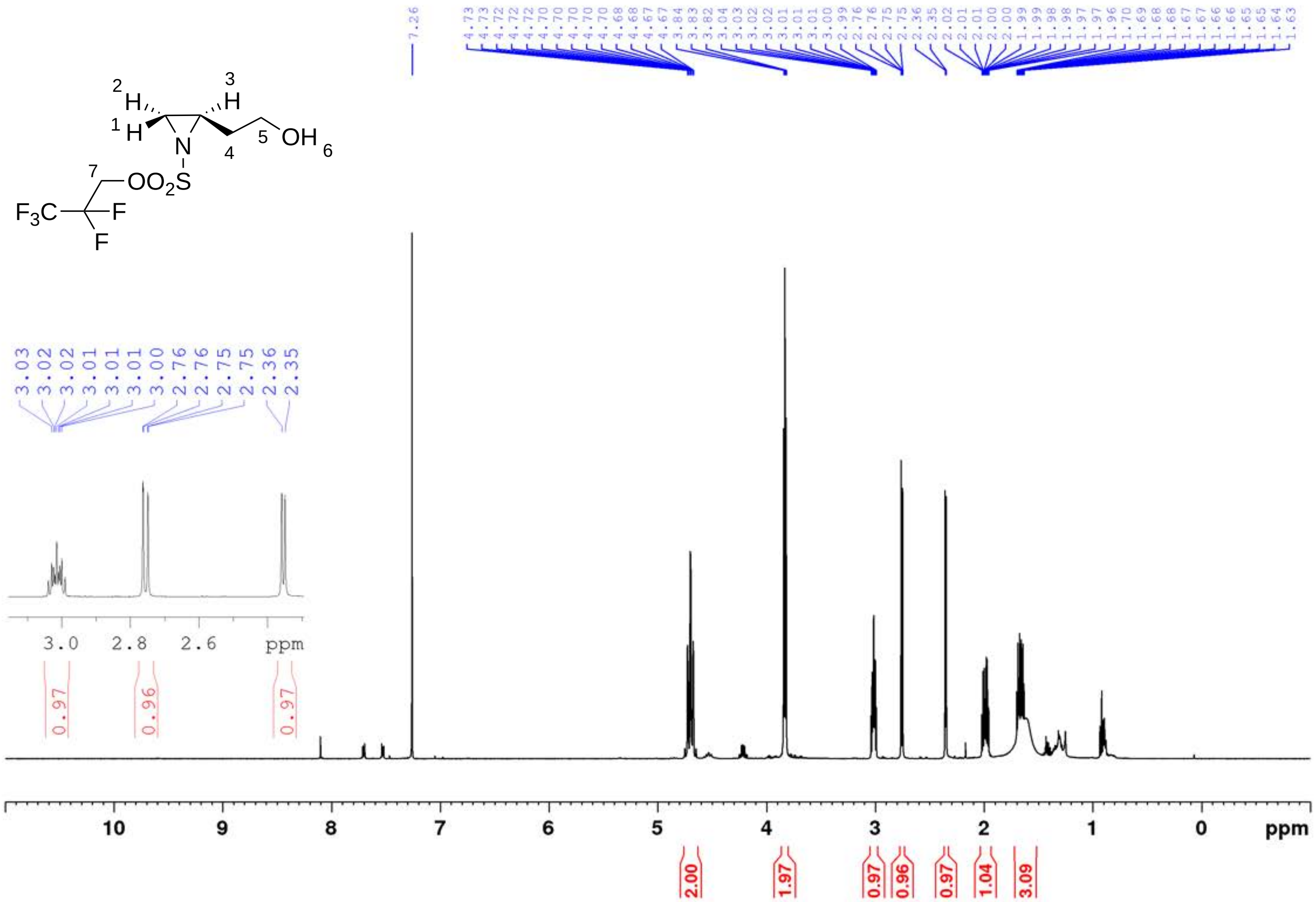

^{13}C NMR (126 MHz, $CDCl_3$) for *2,2,3,3,3-pentafluoropropyl (R)-2-(2-hydroxyethyl)aziridine-1-sulfonate* (**24g**)

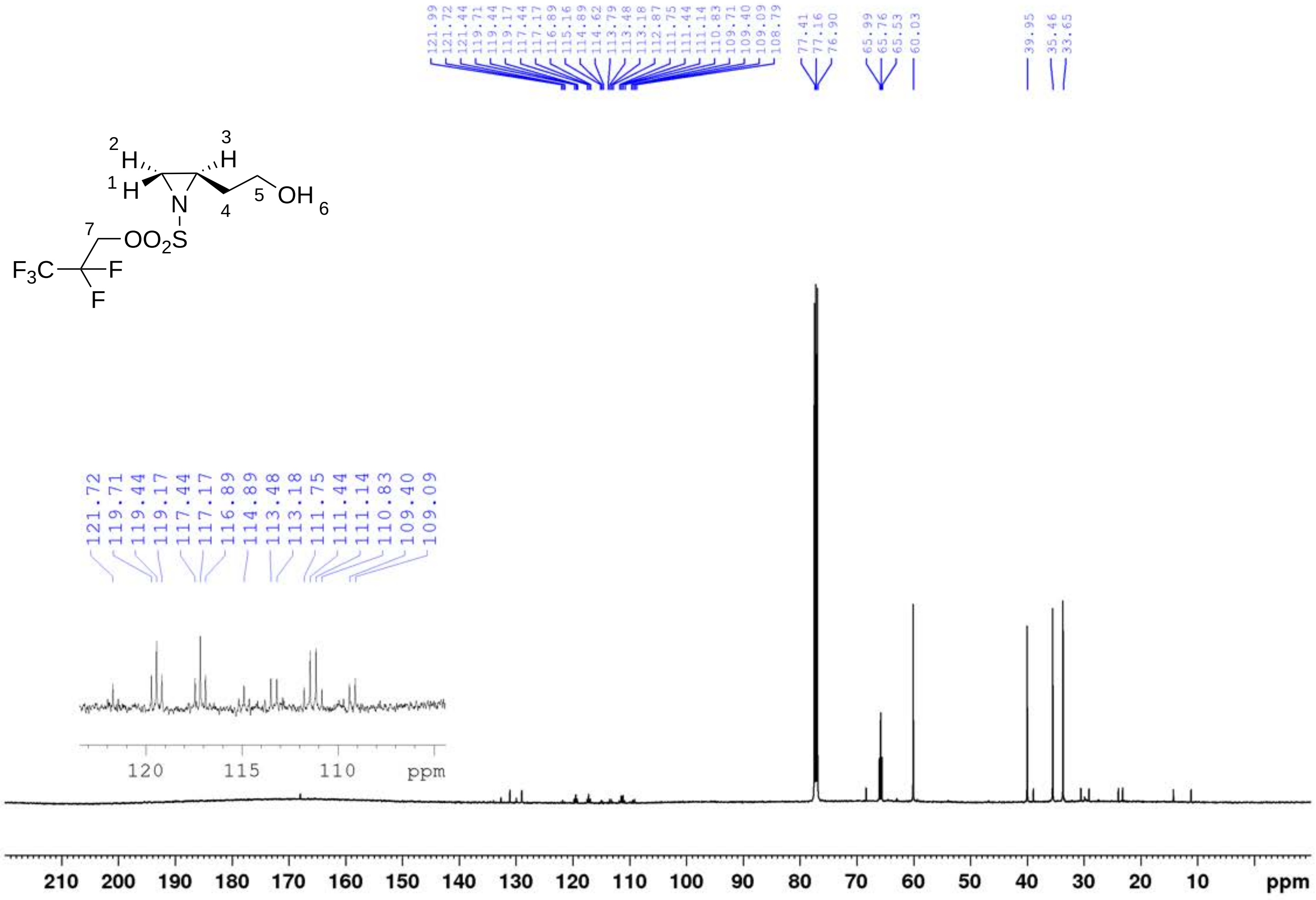

19F NMR (376 MHz, $CDCl_3$) for *2,2,3,3,3-pentafluoropropyl (R)-2-(2-hydroxyethyl)aziridine-1-sulfonate* (**24g**)

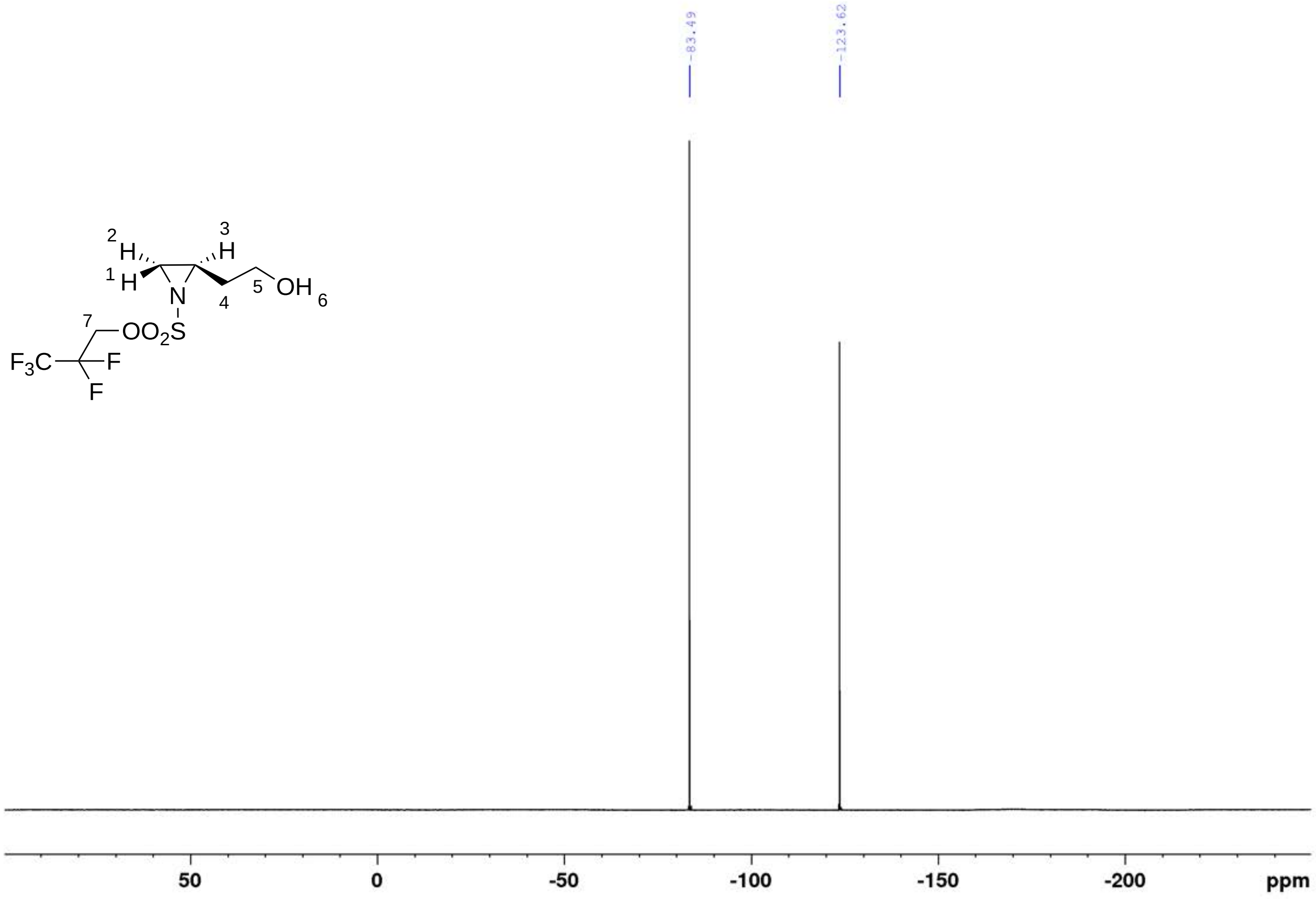

^{1}H NMR (400 MHz, $CDCl_3$) for *2,2,3,3,3-pentafluoropropyl (2R,3R)-2-ethyl-3-(2-hydroxyethyl)aziridine-1-sulfonate* (**26a**)

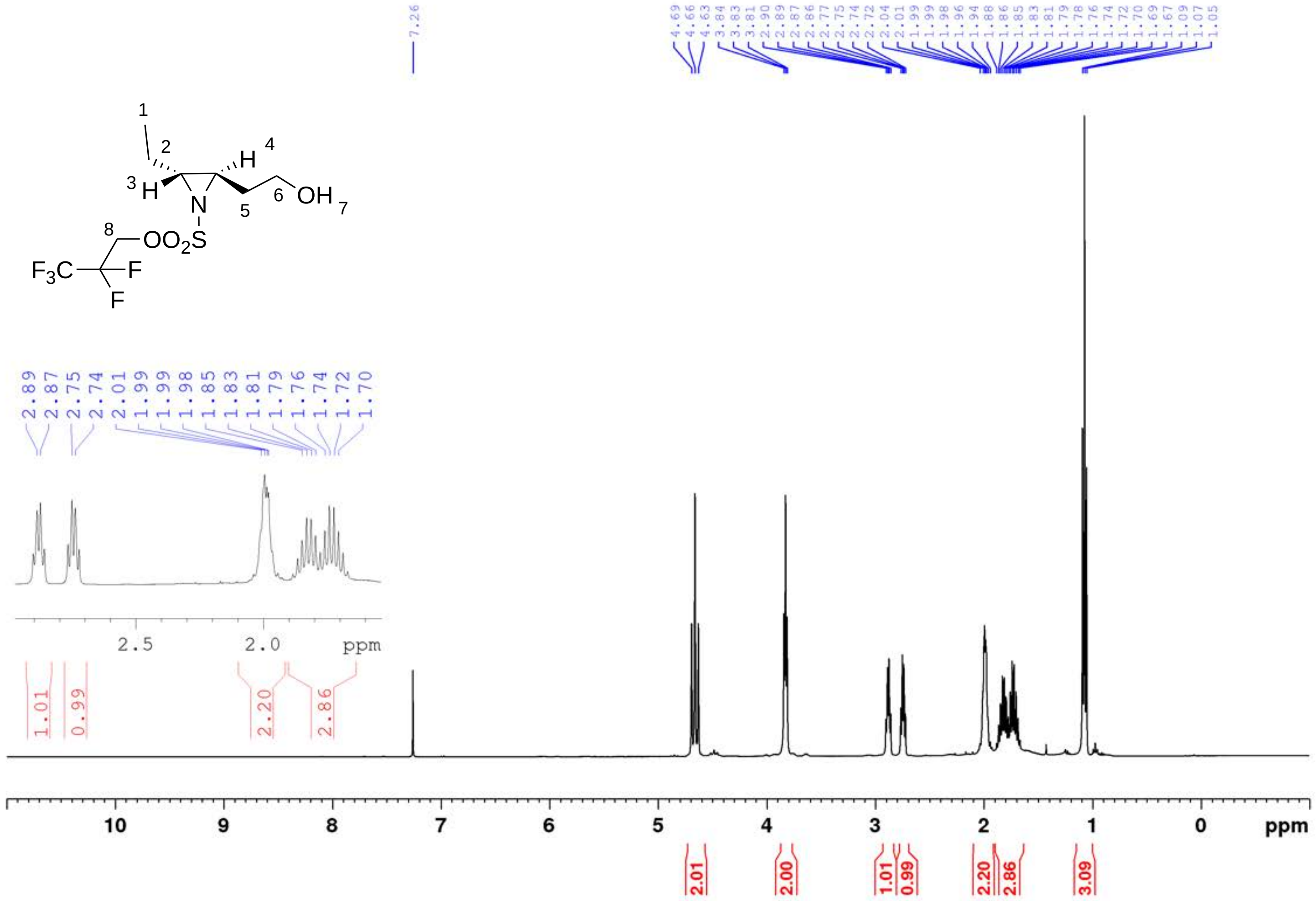

^{13}C NMR (101 MHz, $CDCl_3$) for *2,2,3,3,3-pentafluoropropyl (2R,3R)-2-ethyl-3-(2-hydroxyethyl)aziridine-1-sulfonate* (**26a**)

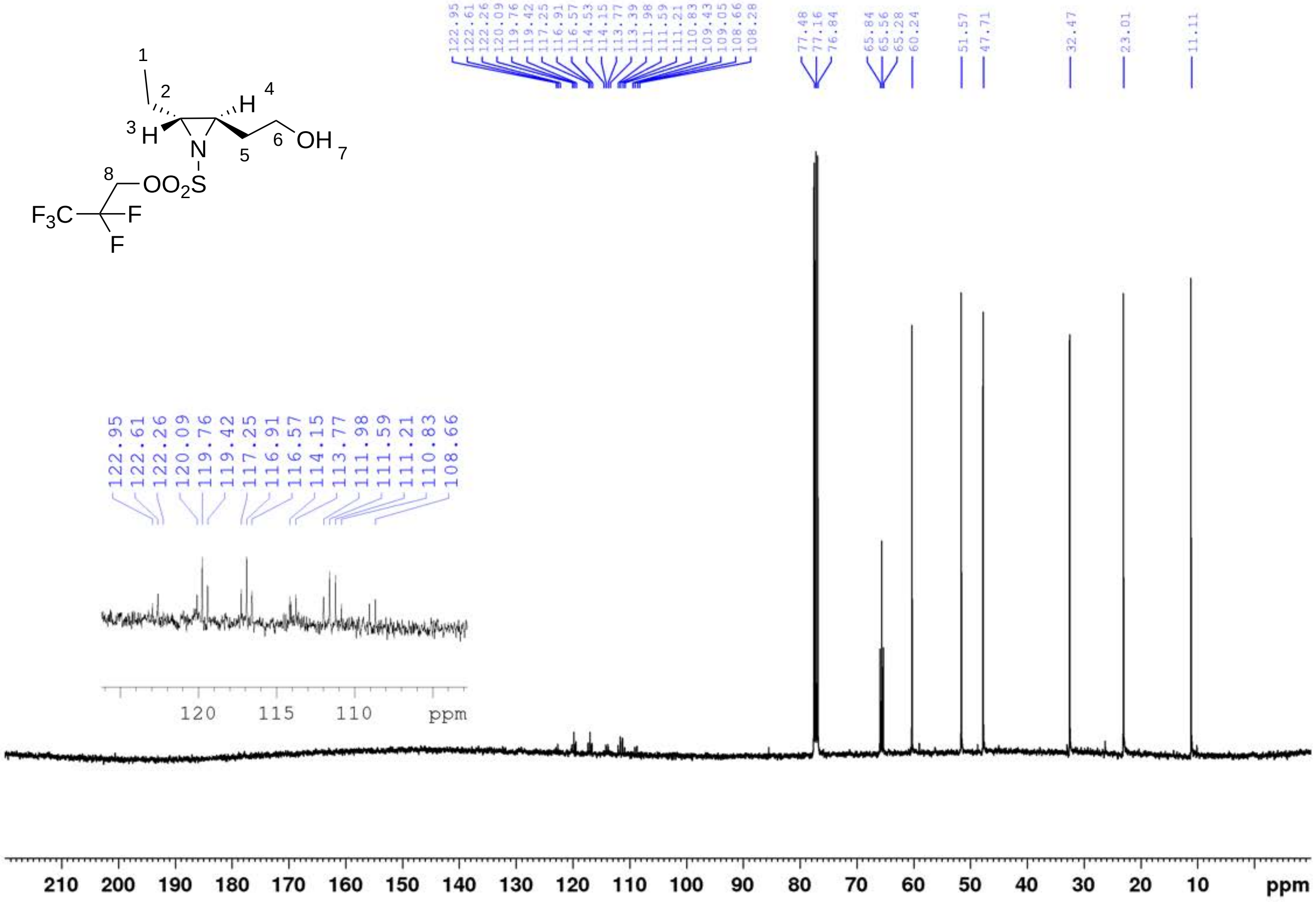

^{19}F NMR (376 MHz, $CDCl_3$) for *2,2,3,3,3-pentafluoropropyl (2R,3R)-2-ethyl-3-(2-hydroxyethyl)aziridine-1-sulfonate* (**26a**)

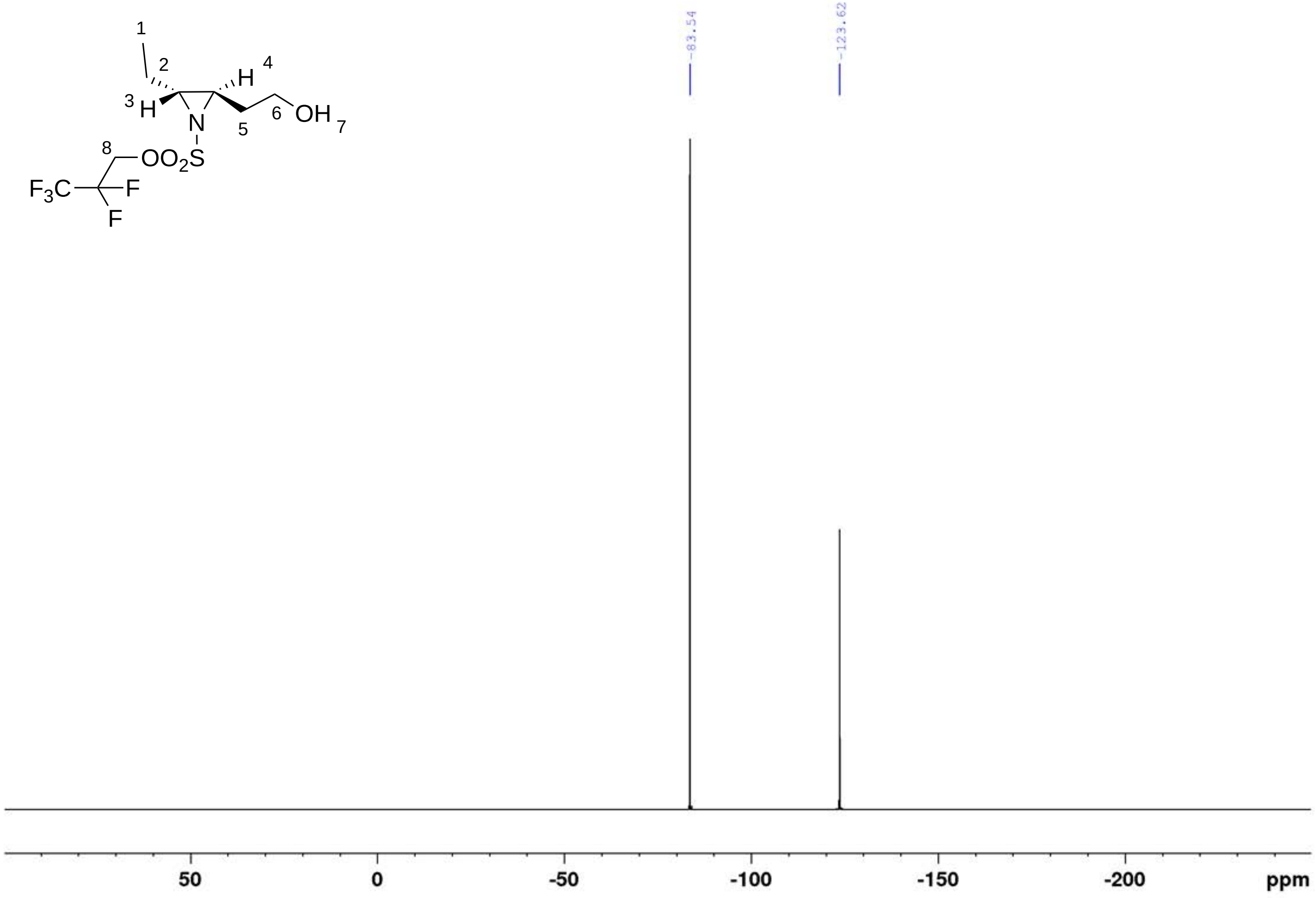

^{1}H NMR (400 MHz, $CDCl_3$) for *2,2,3,3,3-pentafluoropropyl ((2S,3R)-2-ethyltetrahydrofuran-3-yl)sulfamate:* ***Obtained following cyclisation of* 26a**

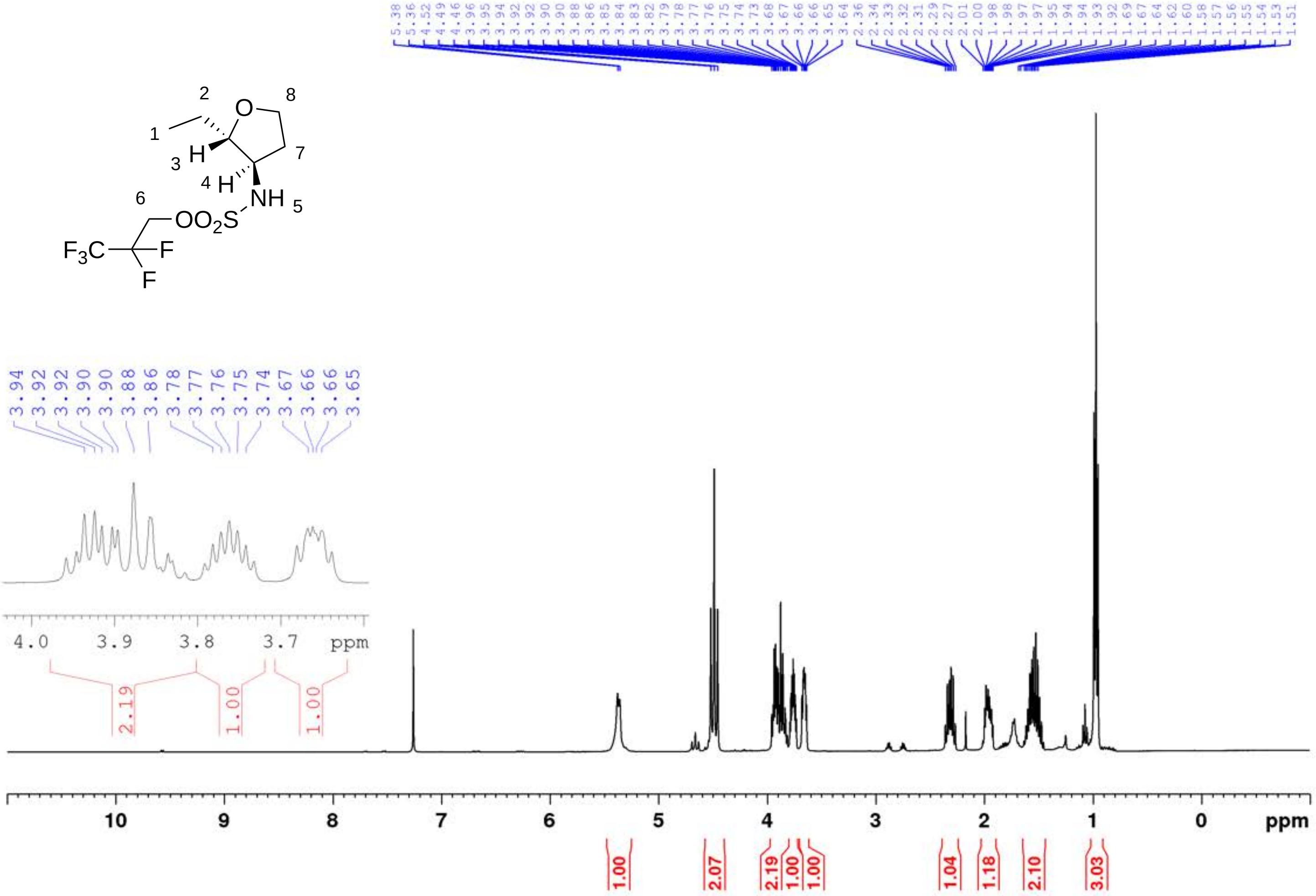

^{13}**C NMR** (101 MHz, $CDCl_3$) for *2,2,3,3,3-pentafluoropropyl ((2S,3R)-2-ethyltetrahydrofuran-3-yl)sulfamate:* ***Obtained following cyclisation of*** **26a**

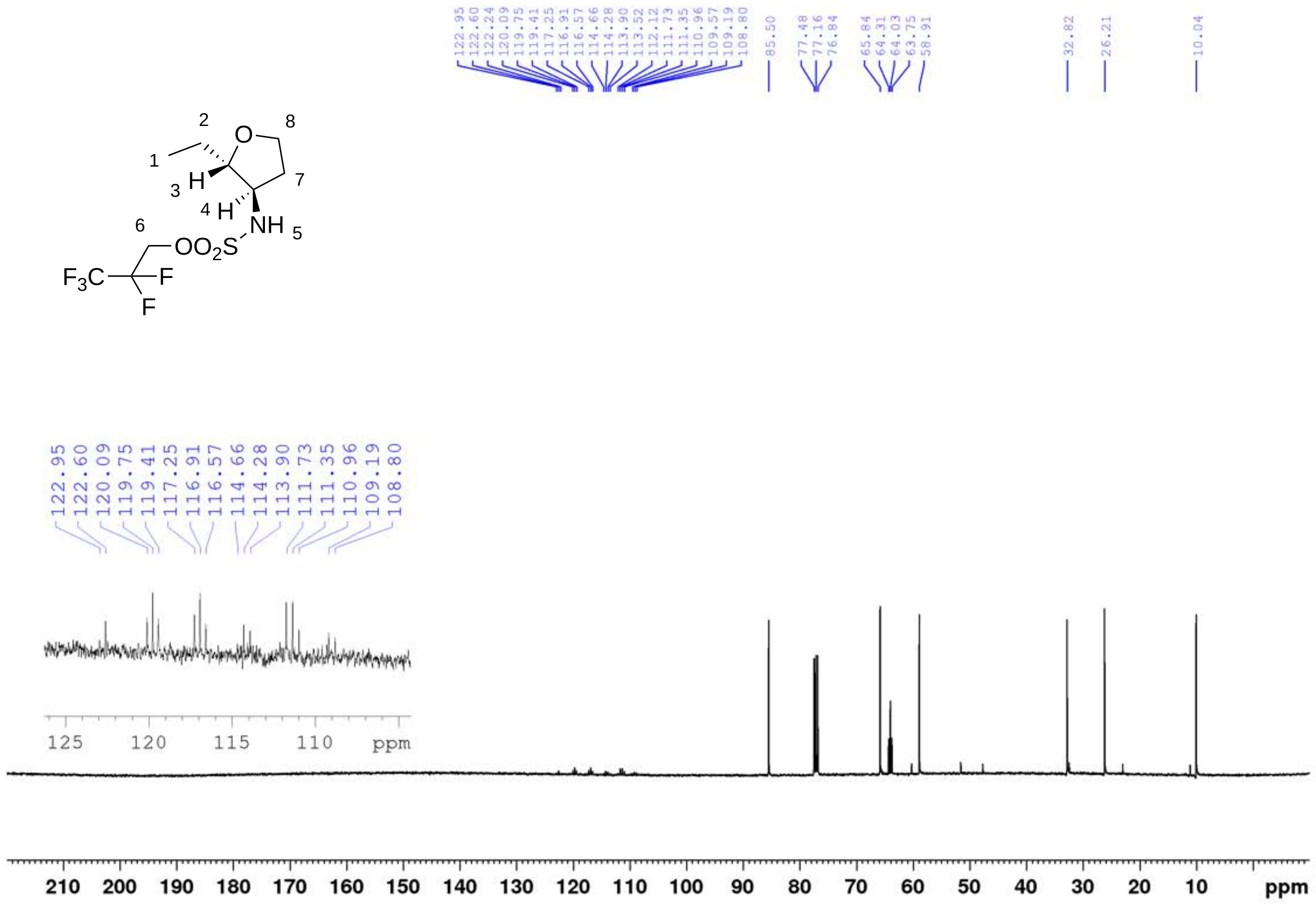

^{19}F NMR (376 MHz, $CDCl_3$) for *2,2,3,3,3-pentafluoropropyl ((2S,3R)-2-ethyltetrahydrofuran-3-yl)sulfamate:* ***Obtained following cyclisation of* 26a**

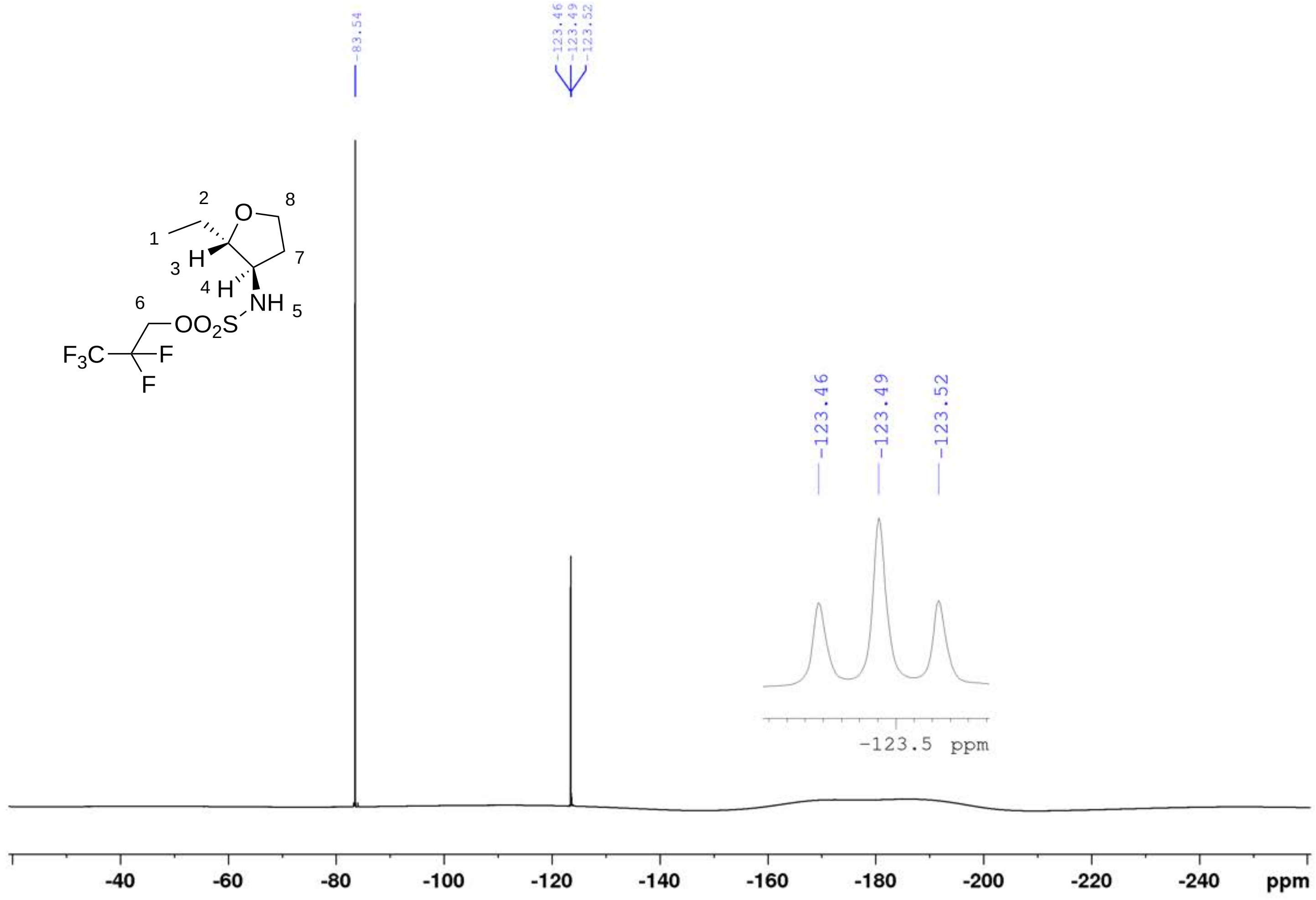

^{1}H NMR (500 MHz, $CDCl_3$) for *2,2,3,3,3-pentafluoropropyl (2R,3R)-2-(2-hydroxyethyl)-3-isopropylaziridine-1-sulfonate* (**26b**)

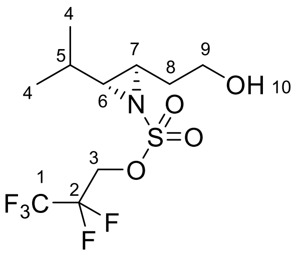

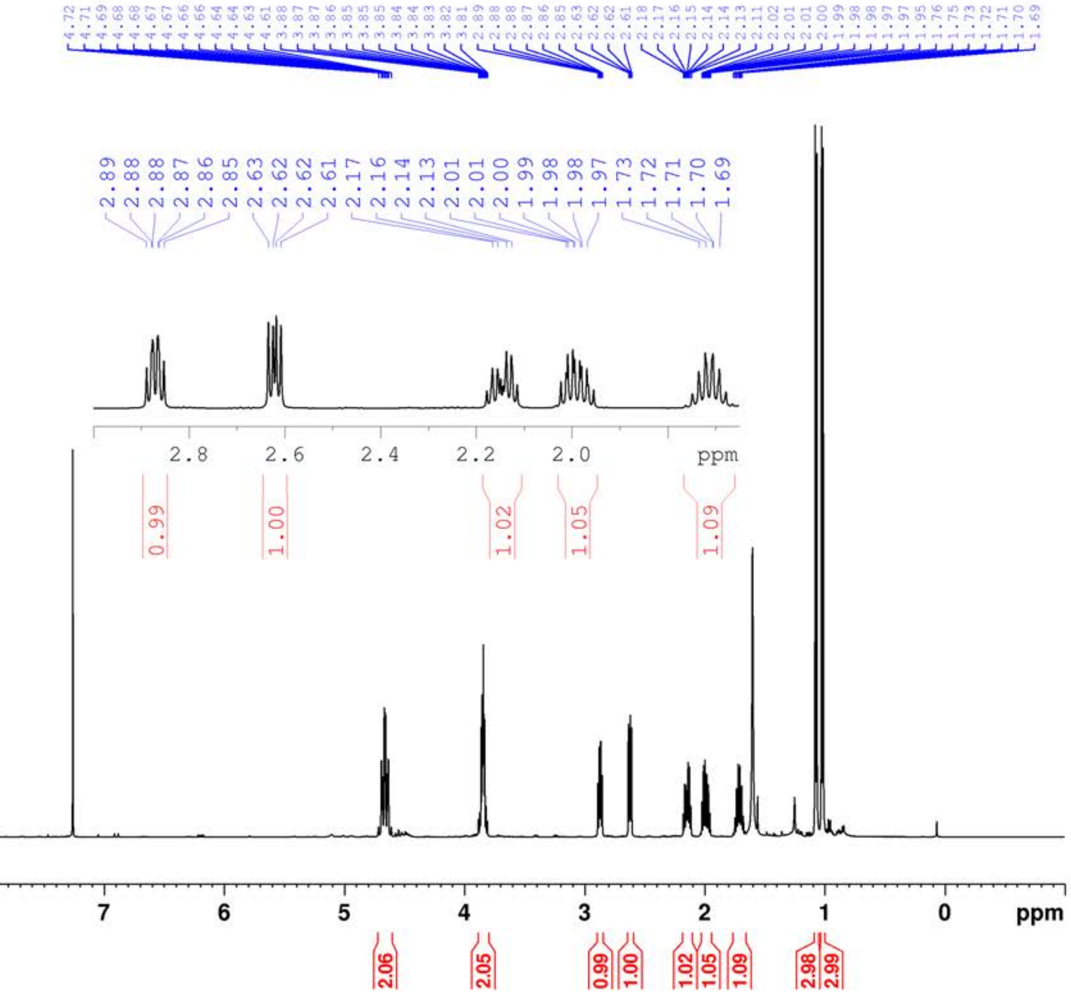

^{13}C NMR (126 MHz, $CDCl_3$) for *2,2,3,3,3-pentafluoropropyl (2R,3R)-2-(2-hydroxyethyl)-3-isopropylaziridine-1-sulfonate* (**26b**)

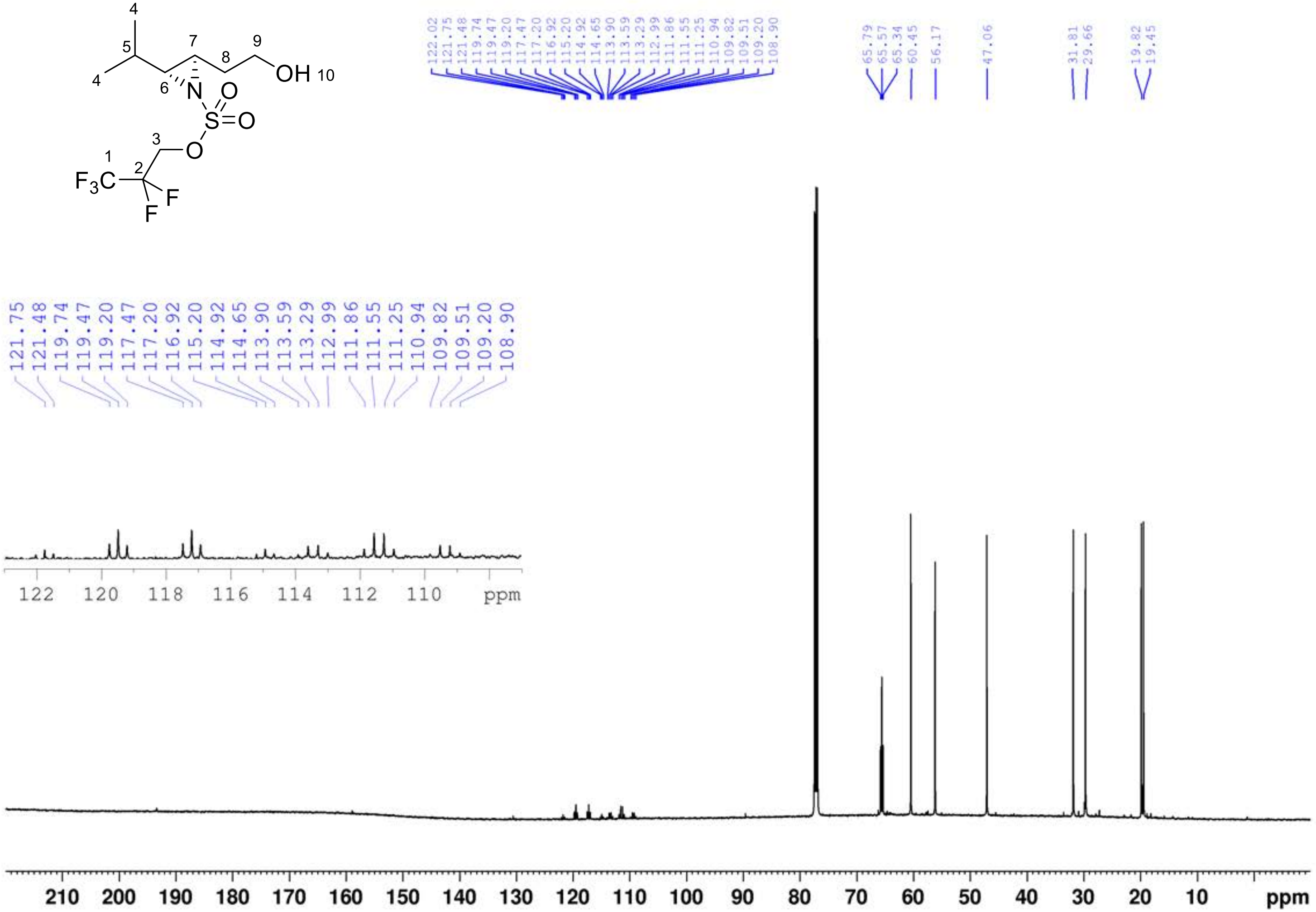

^{19}F NMR (376 MHz, $CDCl_3$) for *2,2,3,3,3-pentafluoropropyl (2R,3R)-2-(2-hydroxyethyl)-3-isopropylaziridine-1-sulfonate* (**26b**)

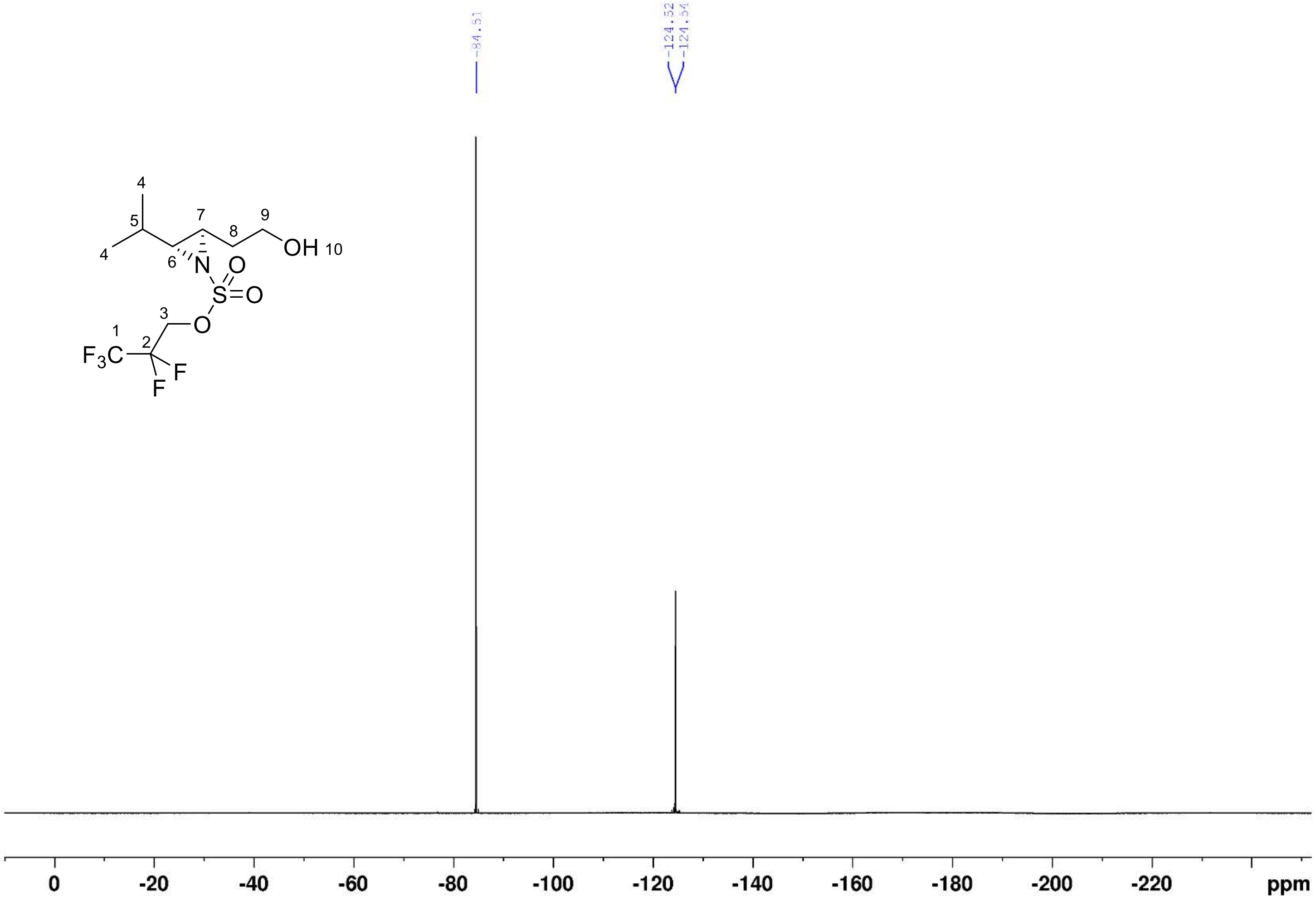

¹H NMR (500 MHz, $CDCl_3$) for *2,2,3,3,3-pentafluoropropyl ((2S,3R)-2-isopropyltetrahydrofuran-3-yl)sulfamate:* ***Obtained following cyclisation of*** **26b**

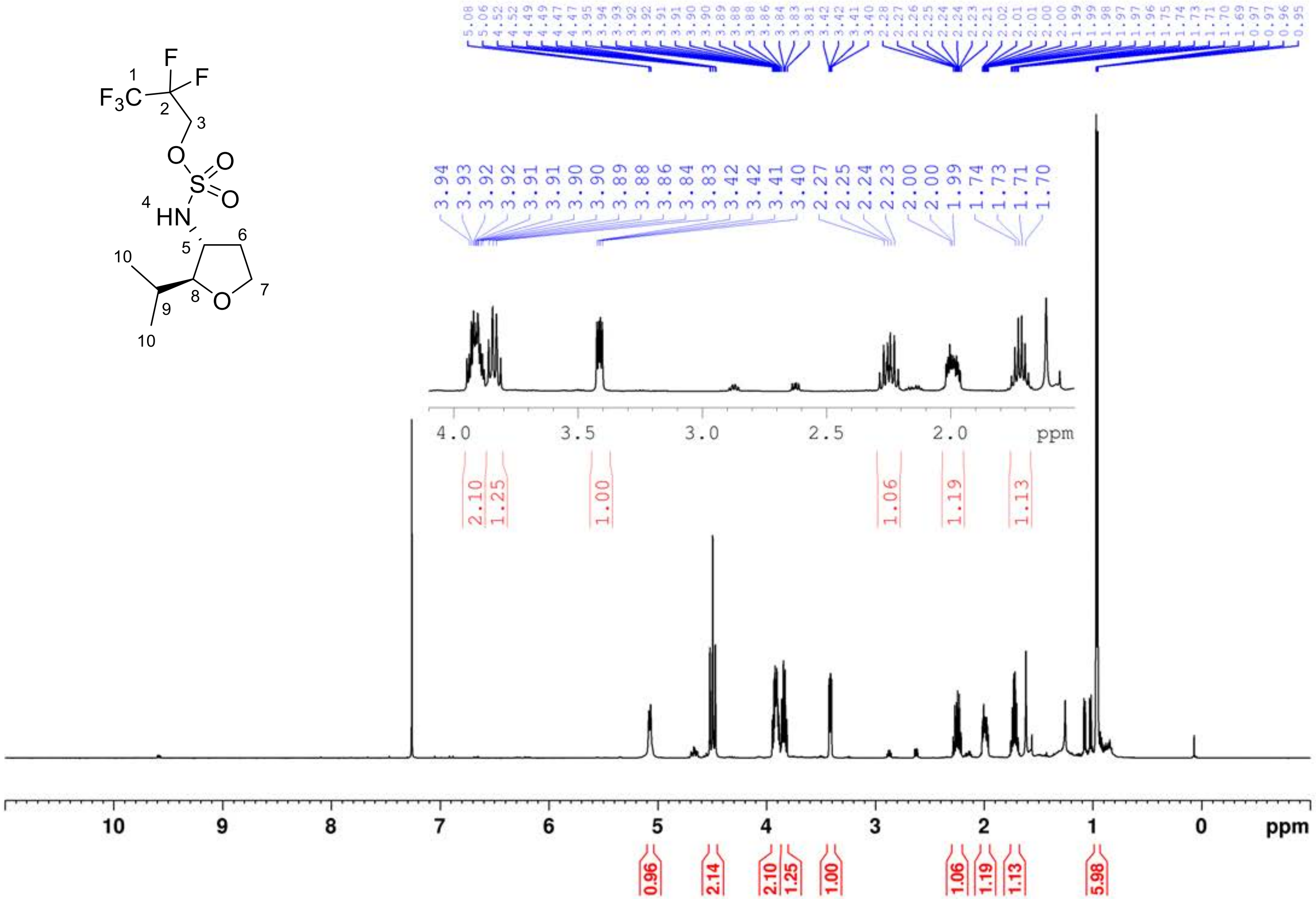

^{13}C NMR (126 MHz, $CDCl_3$) for *2,2,3,3,3-pentafluoropropyl ((2S,3R)-2-isopropyltetrahydrofuran-3-yl)sulfamate:* ***Obtained following cyclisation of*** **26b**

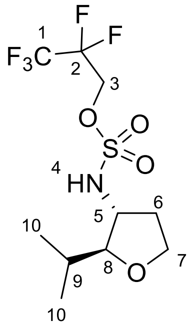

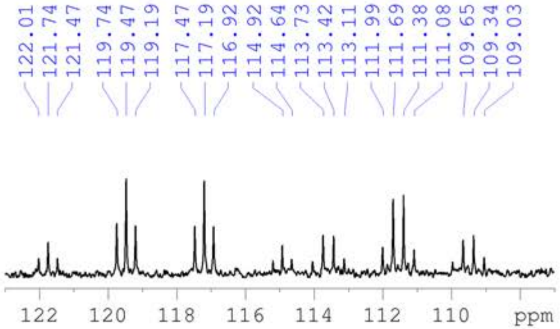
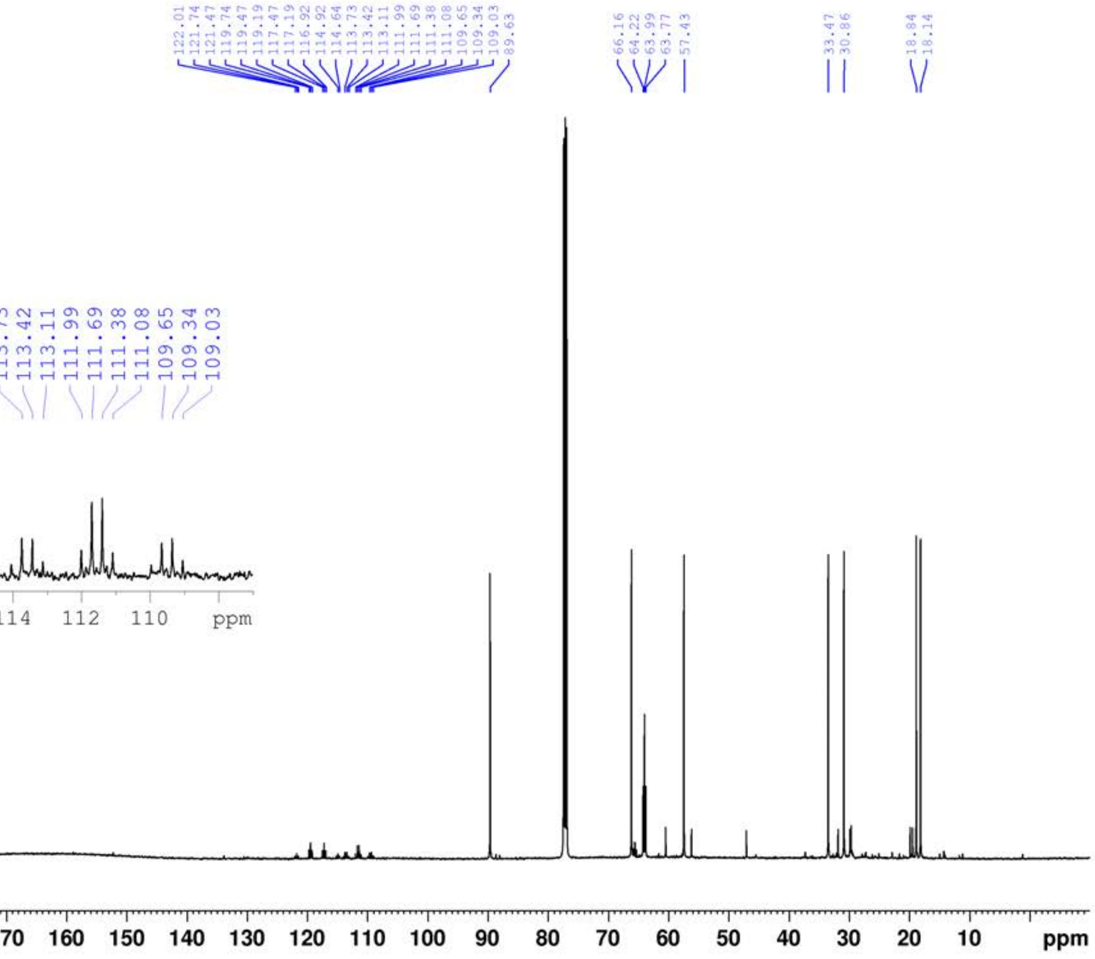

¹⁹F NMR (376 MHz, $CDCl_3$) for *2,2,3,3,3-pentafluoropropyl ((2S,3R)-2-isopropyltetrahydrofuran-3-yl)sulfamate:* ***Obtained following cyclisation of 26b***

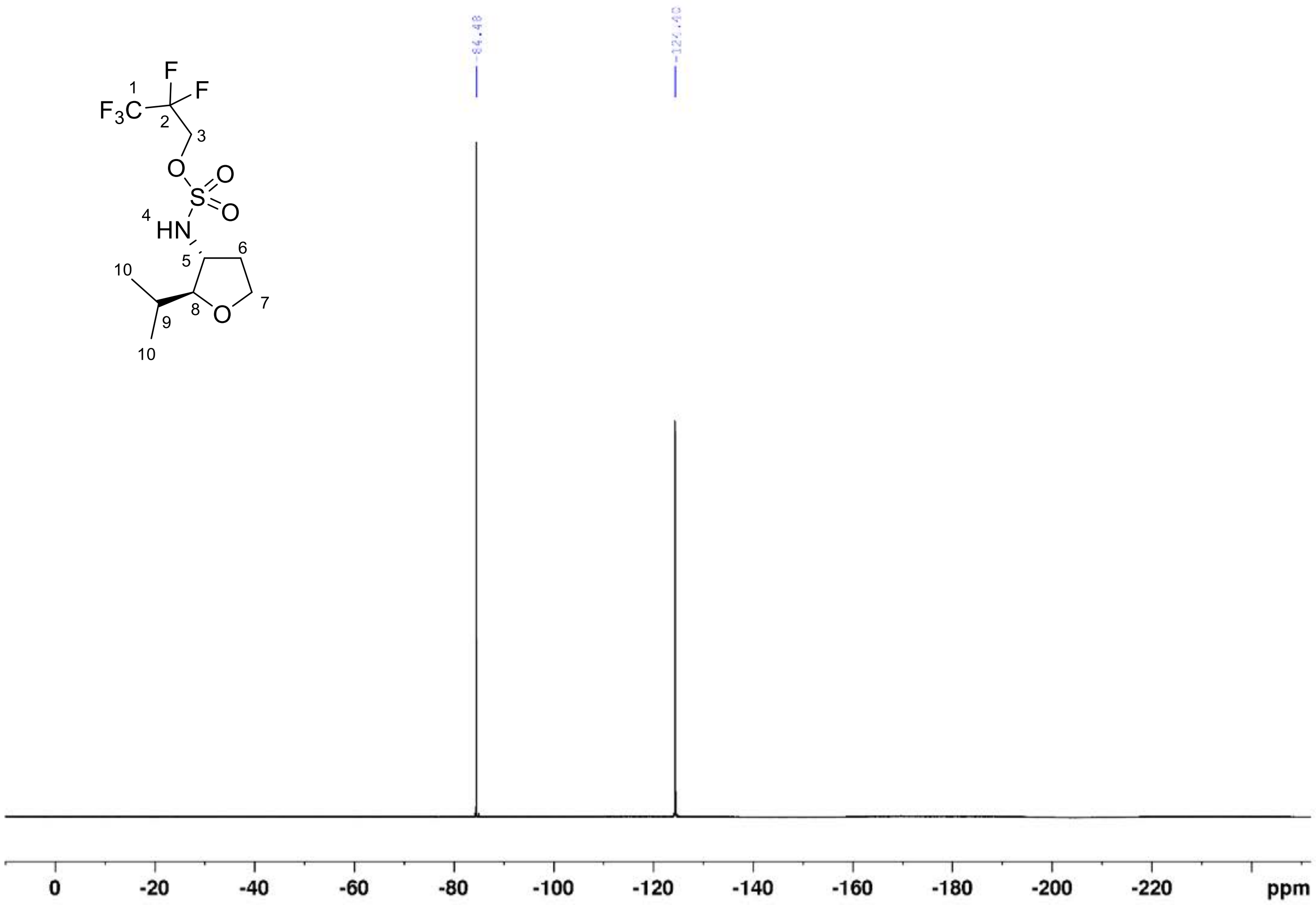

^{1}H NMR (500 MHz, $CDCl_3$) for *2,2,3,3,3-pentafluoropropyl (2R,3R)-2-(2-(benzyloxy)ethyl)-3-(2-hydroxyethyl)aziridine-1-sulfonate* (**26d**)

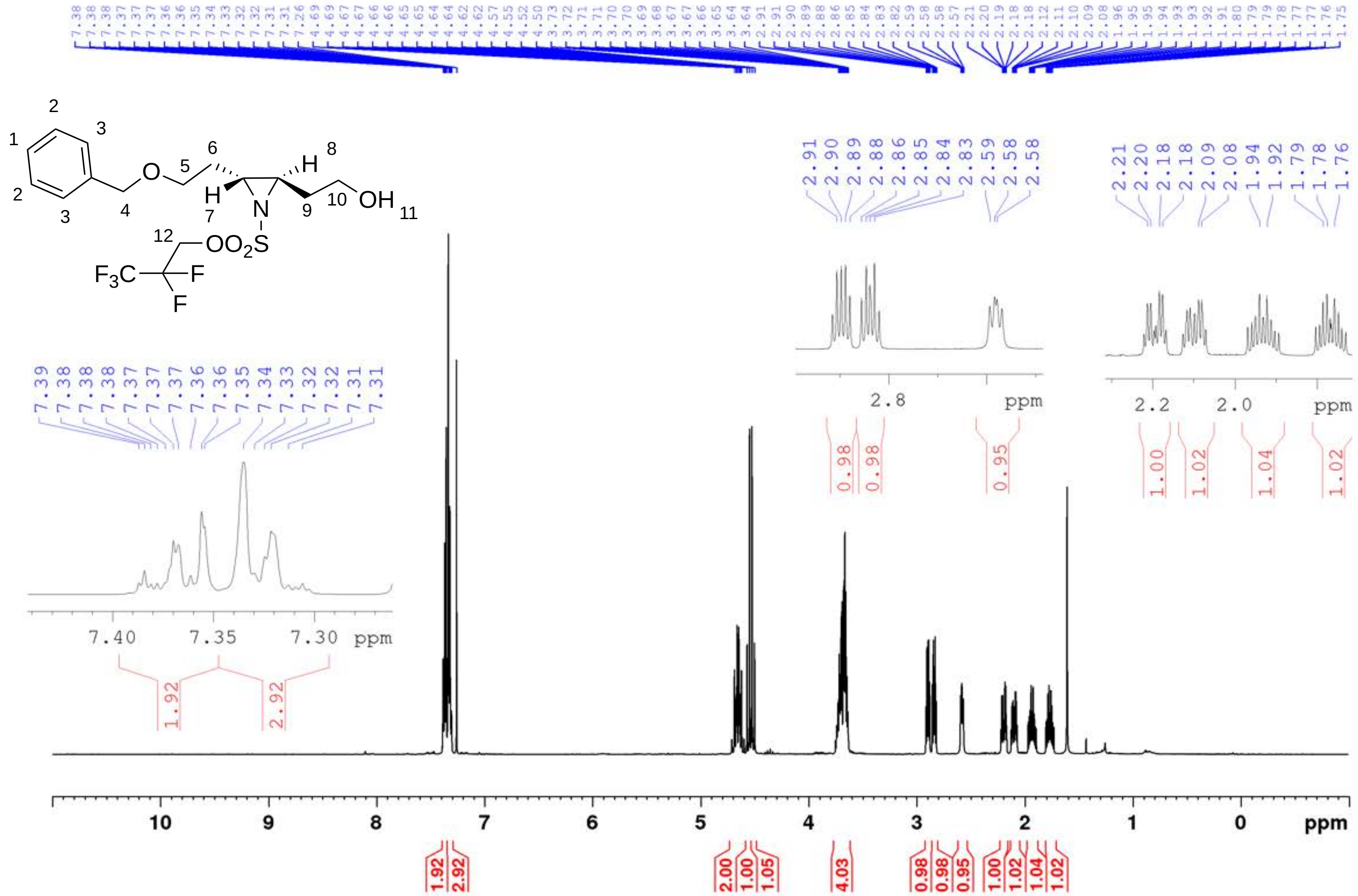

^{13}C NMR (126 MHz, $CDCl_3$) for *2,2,3,3,3-pentafluoropropyl (2R,3R)-2-(2-(benzyloxy)ethyl)-3-(2-hydroxyethyl)aziridine-1-sulfonate* (**26d**)

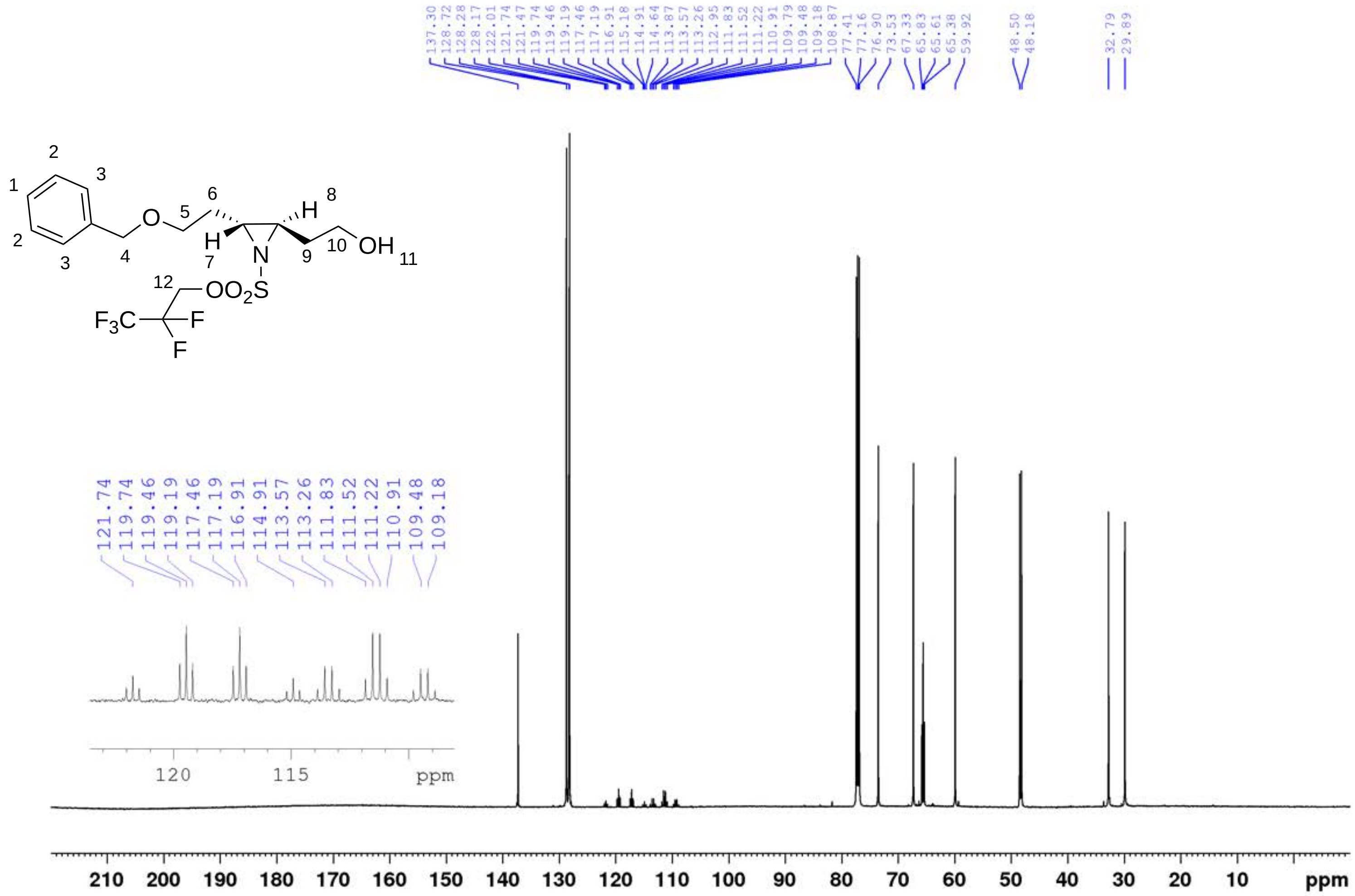

^{19}F NMR (376 MHz, $CDCl_3$) for *2,2,3,3,3-pentafluoropropyl (2R,3R)-2-(2-(benzyloxy)ethyl)-3-(2-hydroxyethyl)aziridine-1-sulfonate* (**26d**)

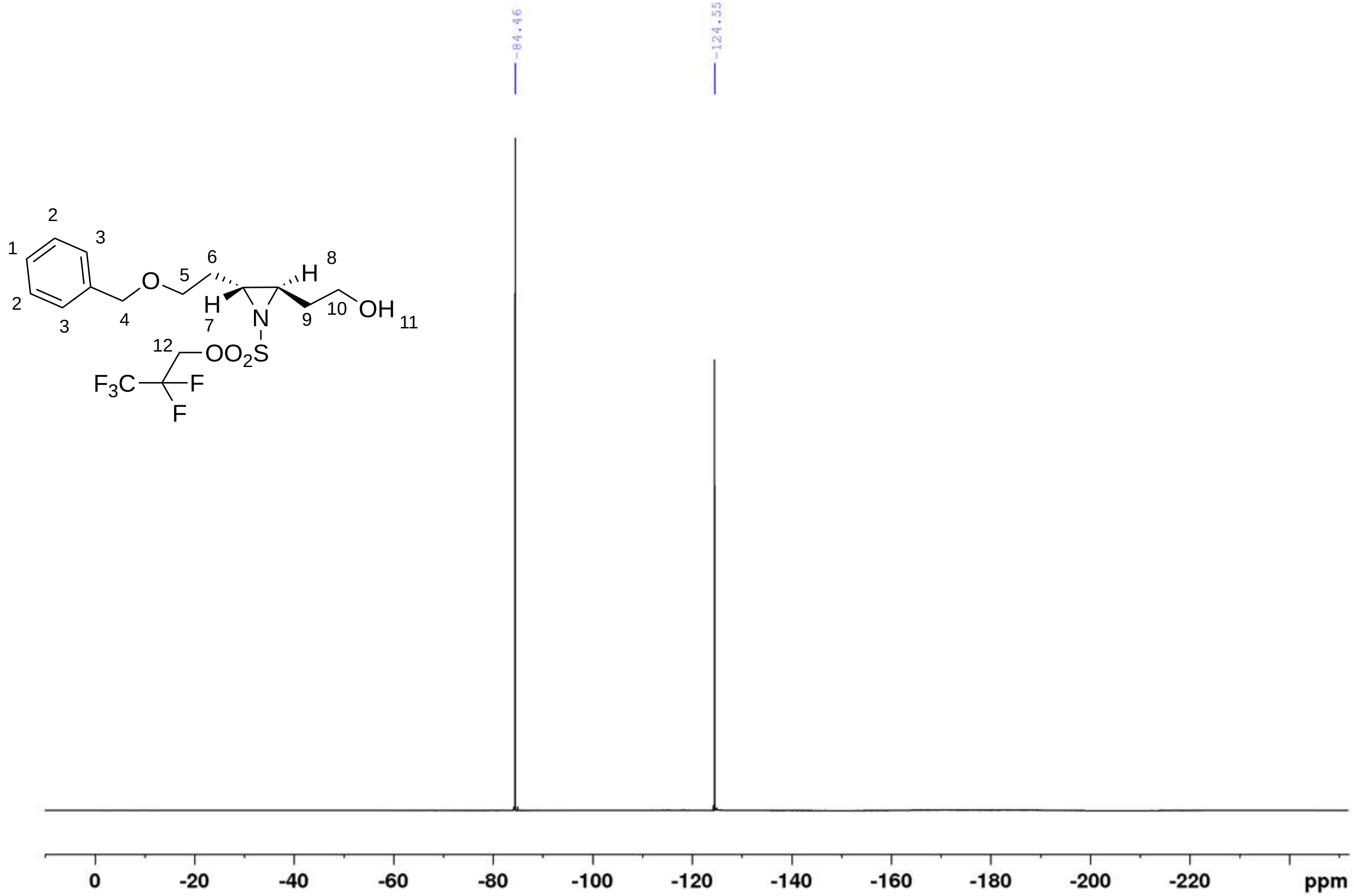

¹H NMR (500 MHz, $CDCl_3$) for *2,2,3,3,3-pentafluoropropyl (2R,3R)-2-(2-hydroxyethyl)-3-(2-(tosyloxy)ethyl)aziridine-1-sulfonate* (**26e**)

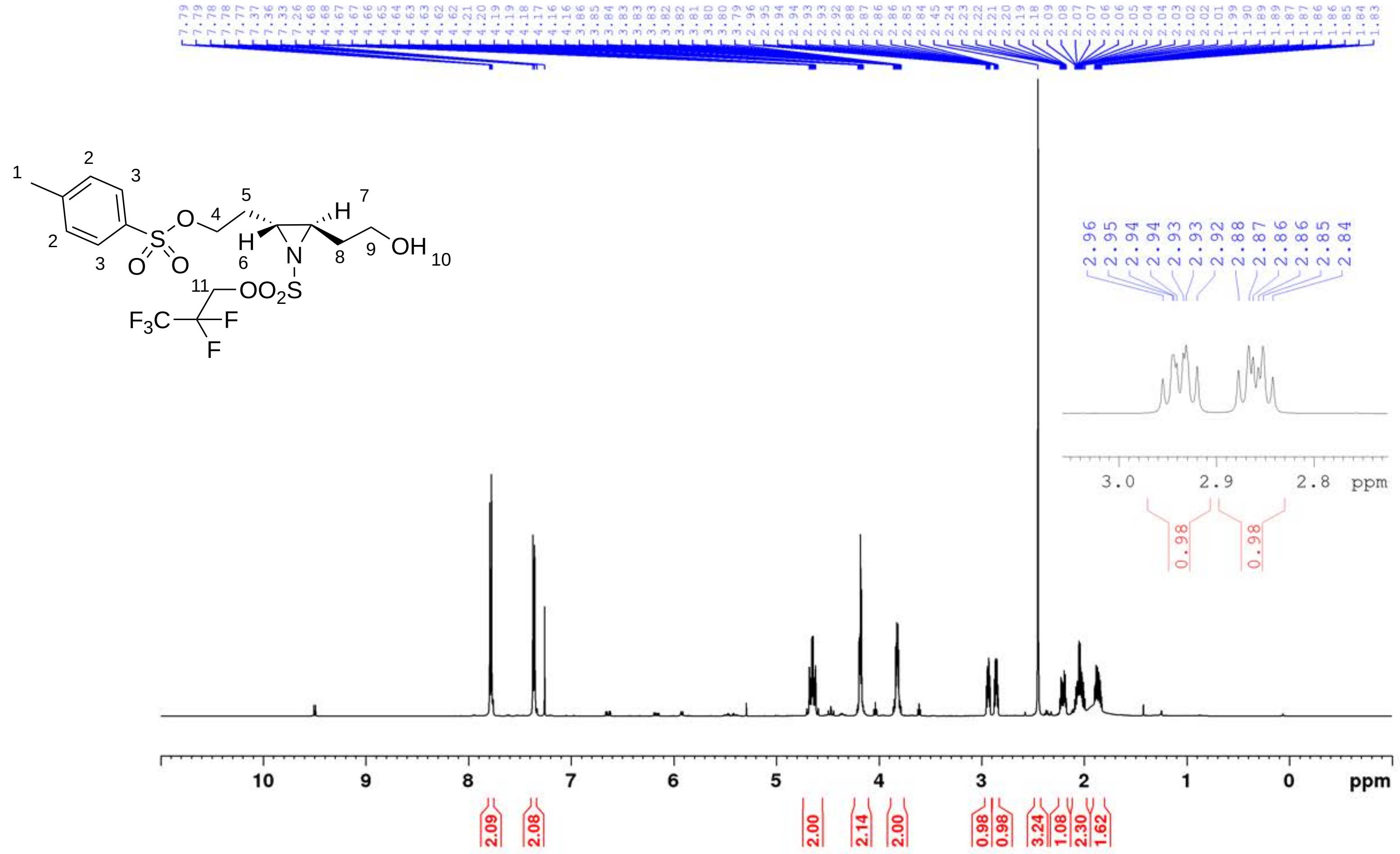

^{13}C NMR (126 MHz, $CDCl_3$) for *2,2,3,3,3-pentafluoropropyl (2R,3R)-2-(2-hydroxyethyl)-3-(2-(tosyloxy)ethyl)aziridine-1-sulfonate* (**26e**)

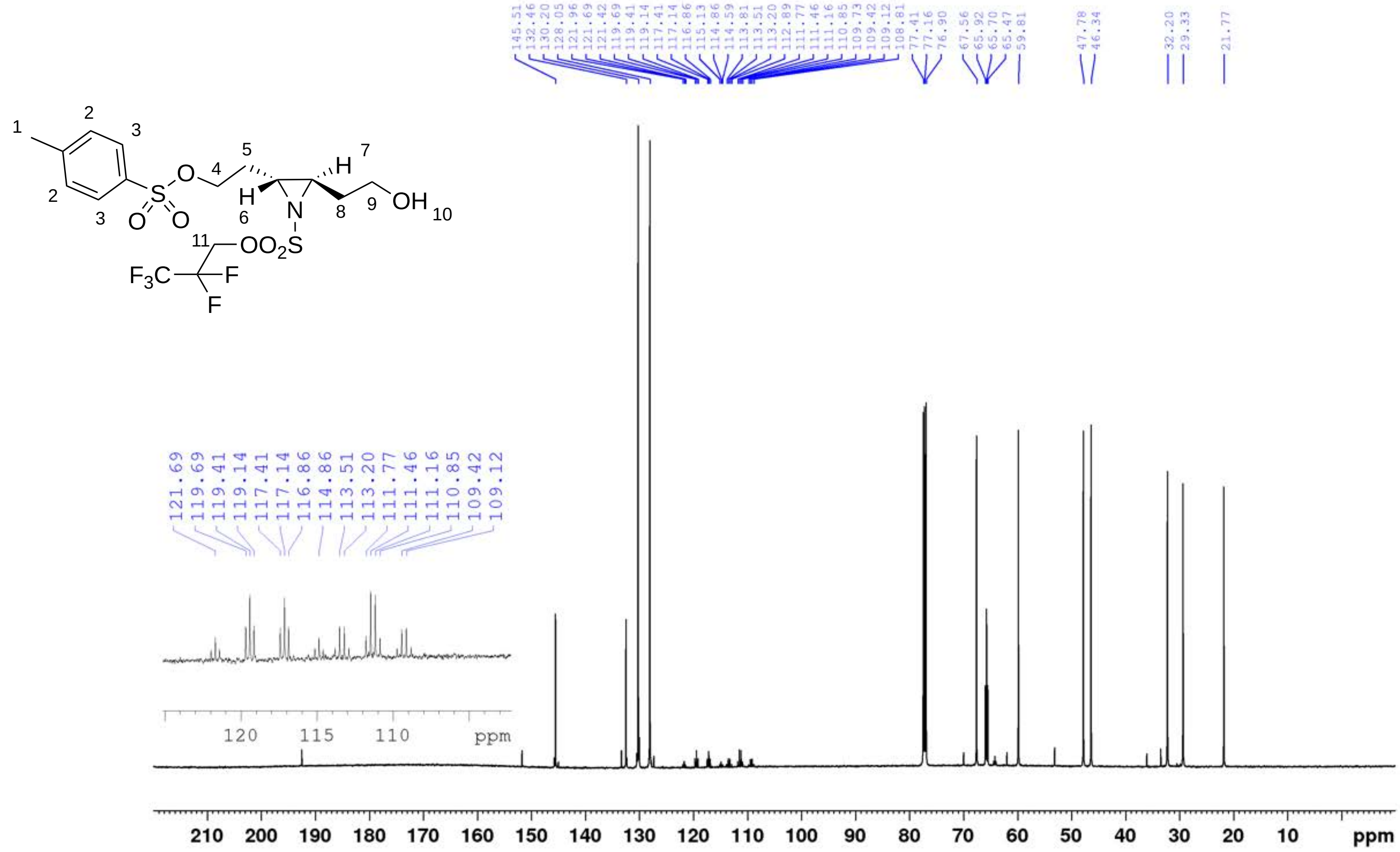

^{19}F NMR (376 MHz, $CDCl_3$) for *2,2,3,3,3-pentafluoropropyl (2R,3R)-2-(2-hydroxyethyl)-3-(2-(tosyloxy)ethyl)aziridine-1-sulfonate* (**26e**)

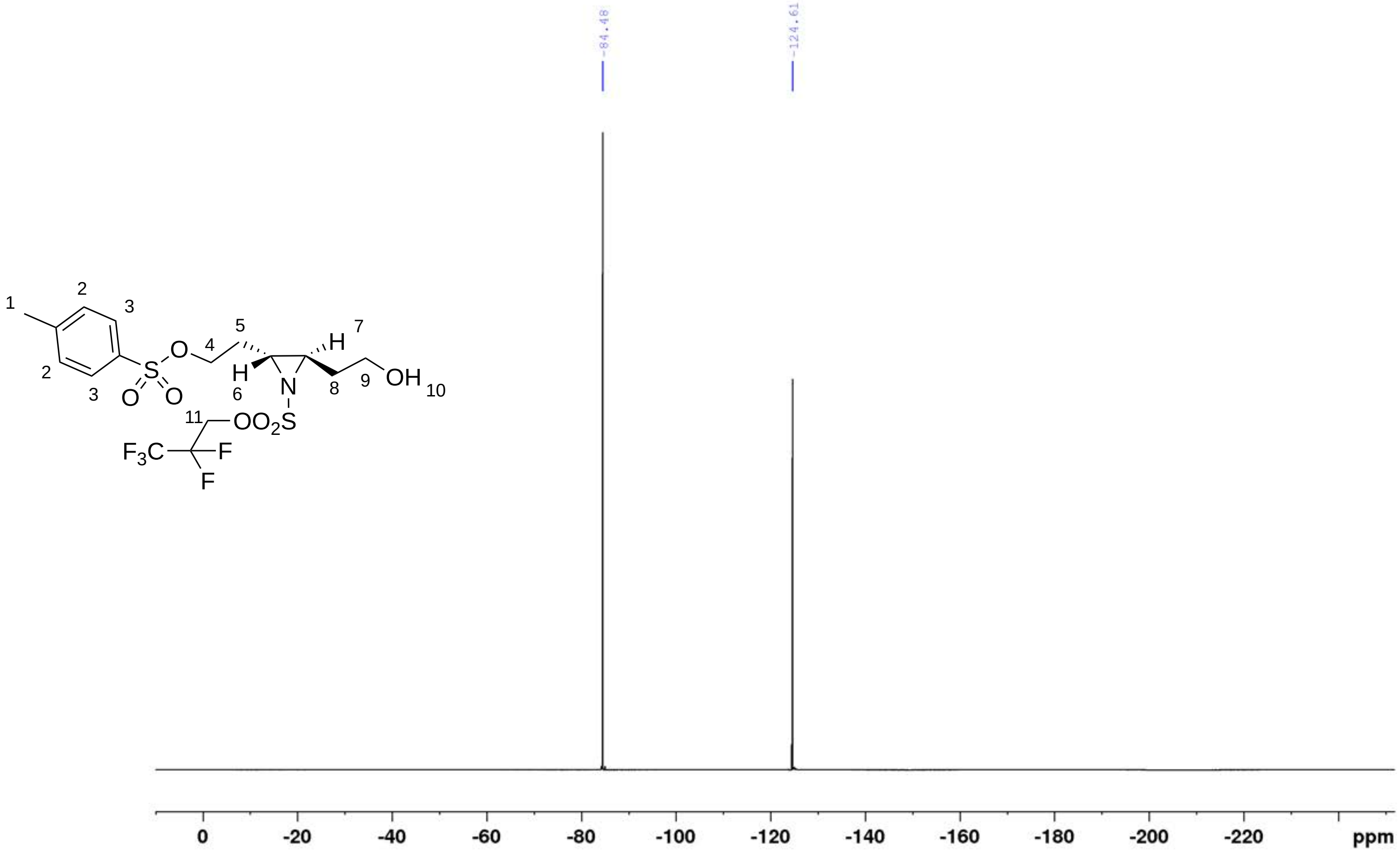

^{1}H NMR (500 MHz, $CDCl_3$) for *2,2,3,3,3-pentafluoropropyl (2R,3R)-2-(2-bromoethyl)-3-(2-hydroxyethyl)aziridine-1-sulfonate* (**26f**)

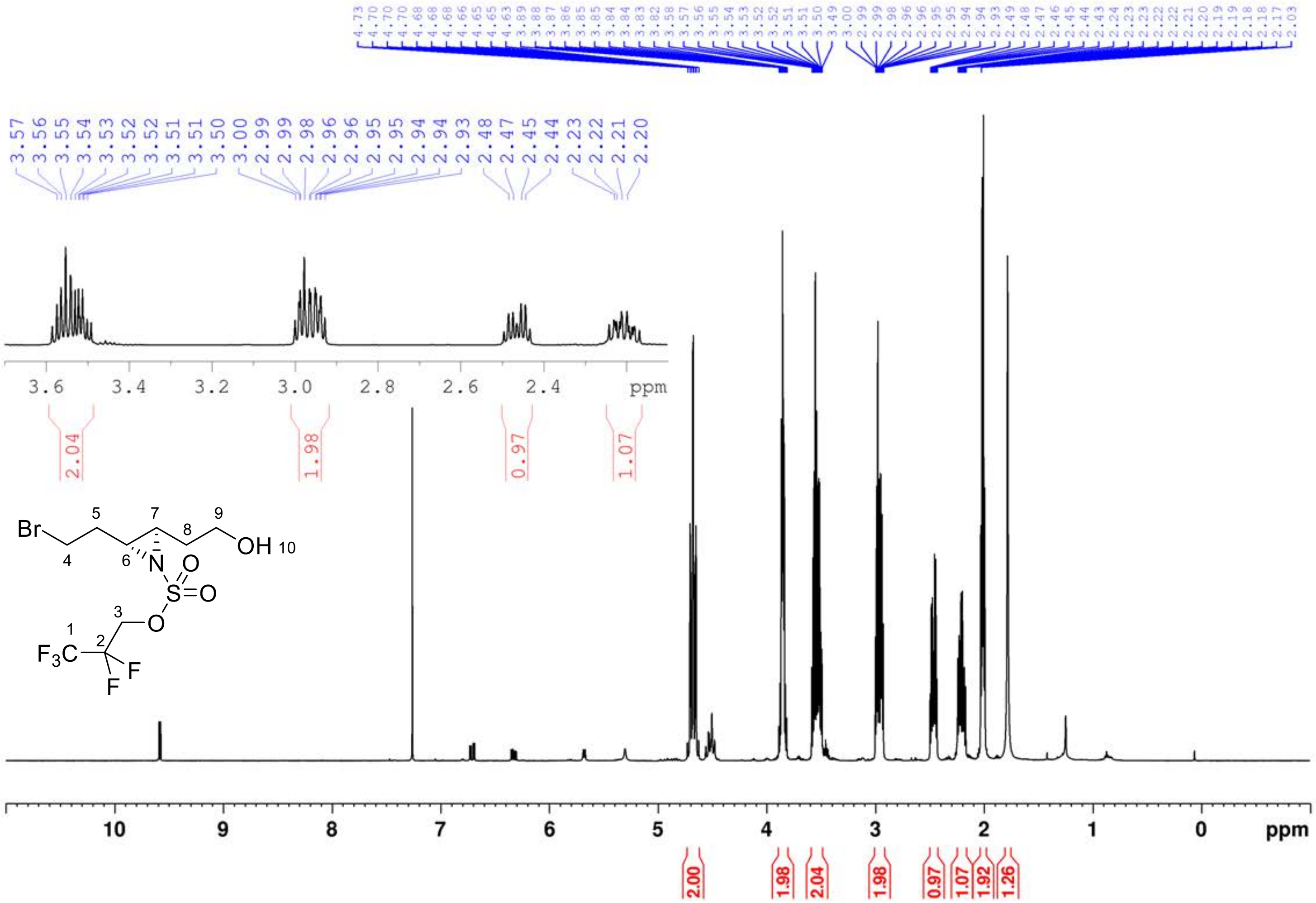

^{13}C NMR (126 MHz, CDCl$_3$) for *2,2,3,3,3-pentafluoropropyl (2R,3R)-2-(2-bromoethyl)-3-(2-hydroxyethyl)aziridine-1-sulfonate* (**26f**)

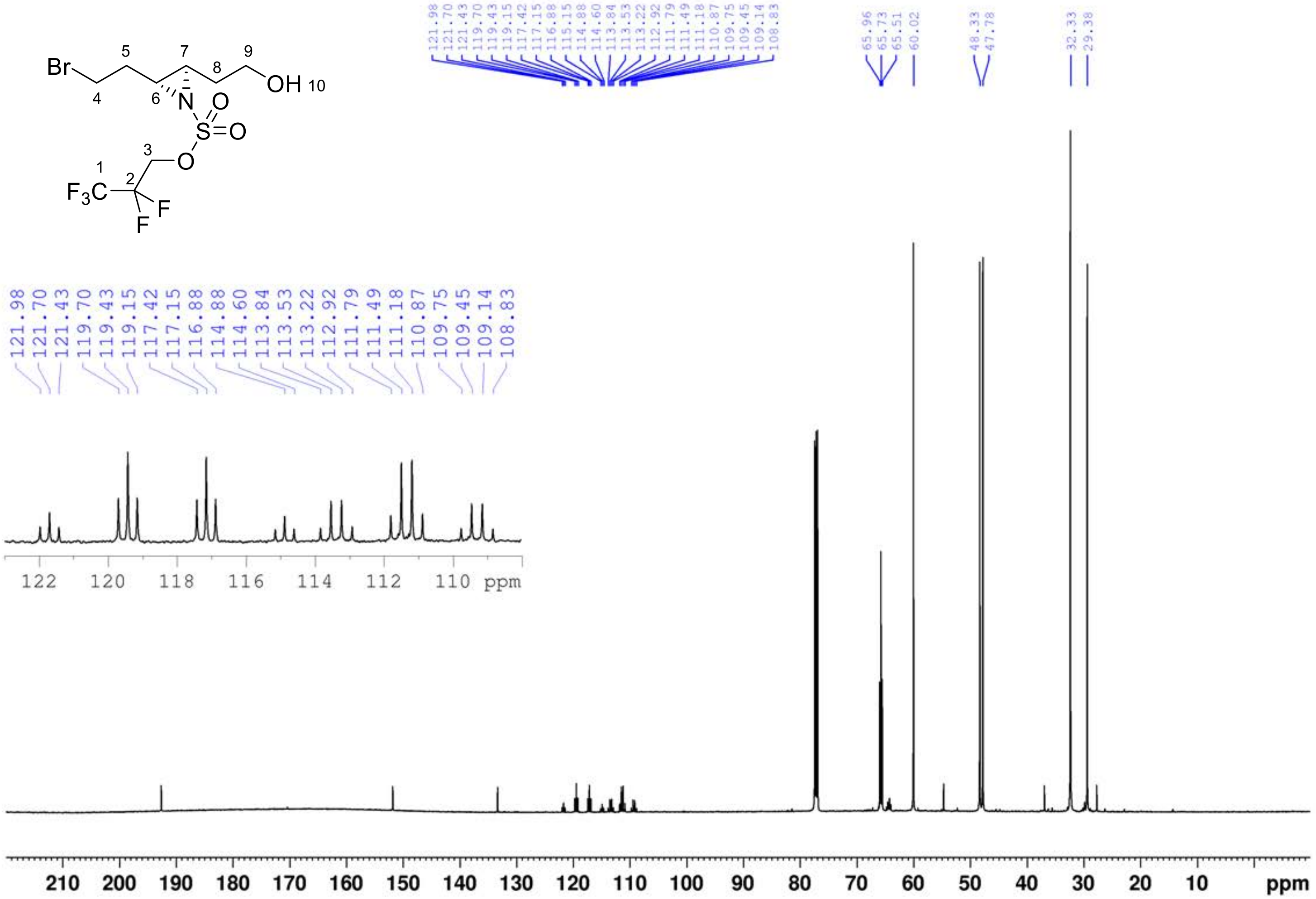

^{19}F NMR (376 MHz, $CDCl_3$) for *2,2,3,3,3-pentafluoropropyl (2R,3R)-2-(2-bromoethyl)-3-(2-hydroxyethyl)aziridine-1-sulfonate* (**26f**)

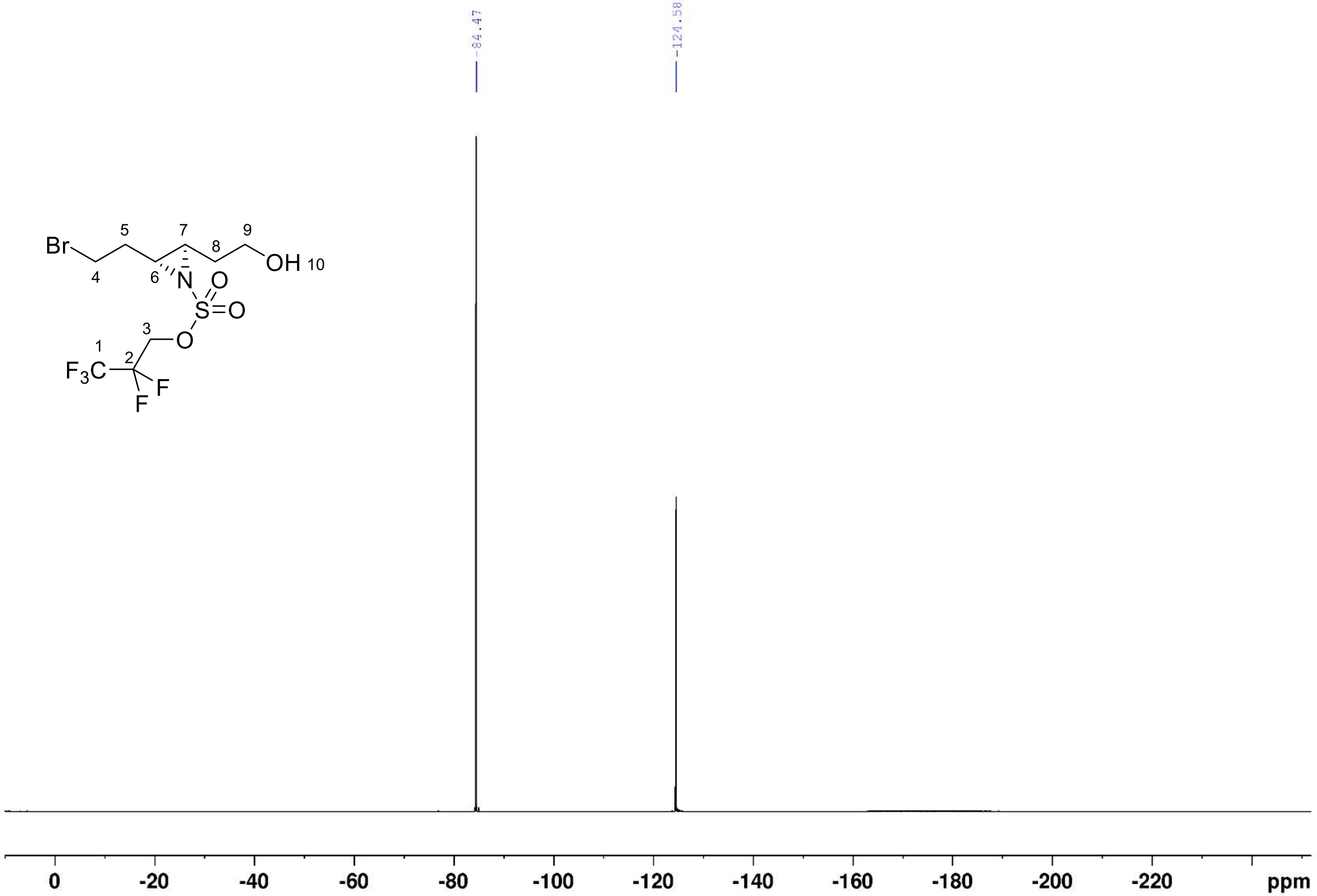

^{1}H NMR (500 MHz, CDCl₃) for *2,2,3,3,3-pentafluoropropyl ((2S,3R)-2-(2-(phenylthio)ethyl)tetrahydrofuran-3-yl)sulfamate:* ***Obtained following derivatisation of*** **26f**

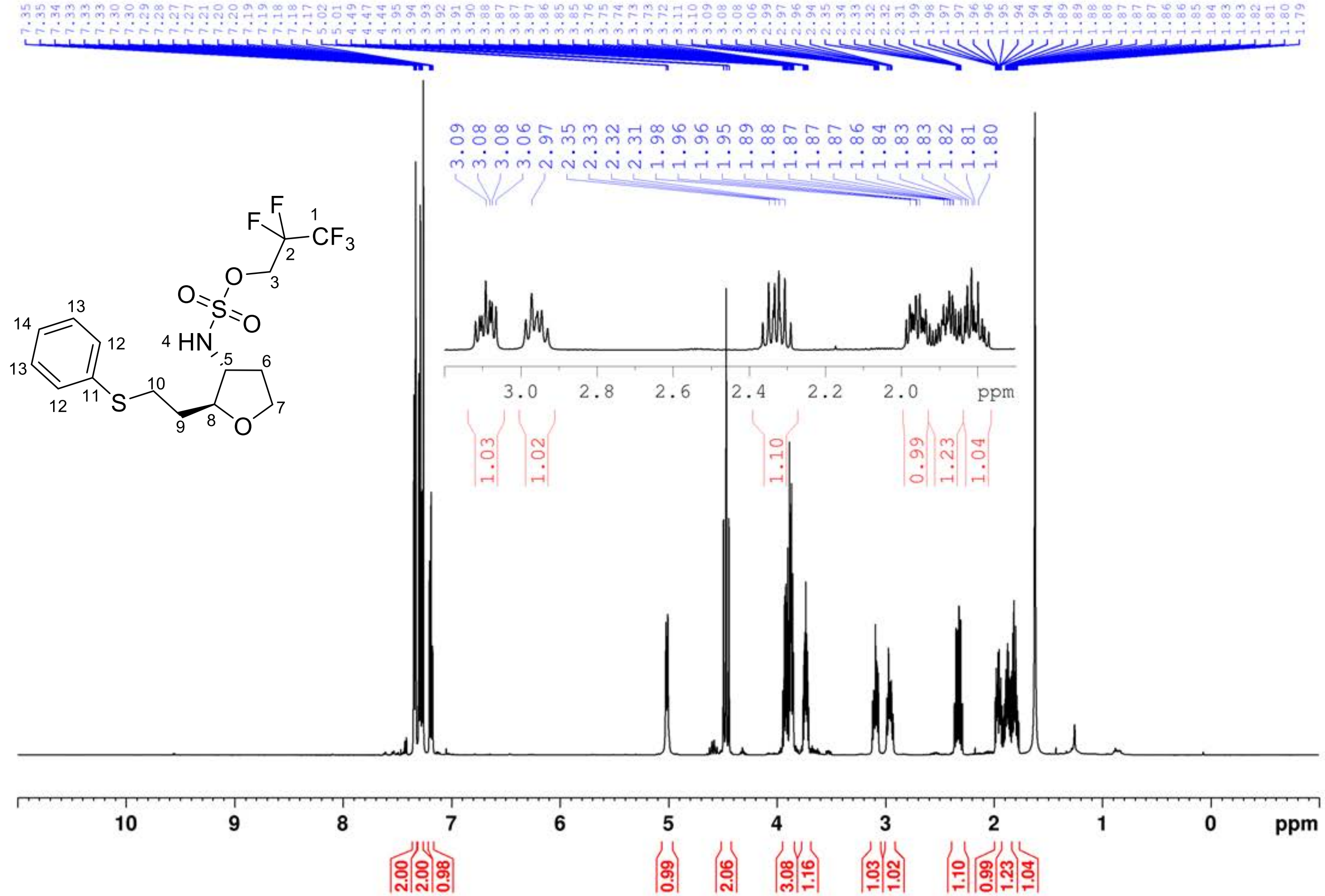

^{13}C NMR (126 MHz, CDCl$_3$) for *2,2,3,3,3-pentafluoropropyl ((2S,3R)-2-(2-(phenylthio)ethyl)tetrahydrofuran-3-yl)sulfamate:* ***Obtained following derivatisation of*** **26f**

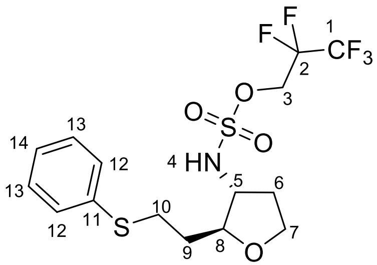

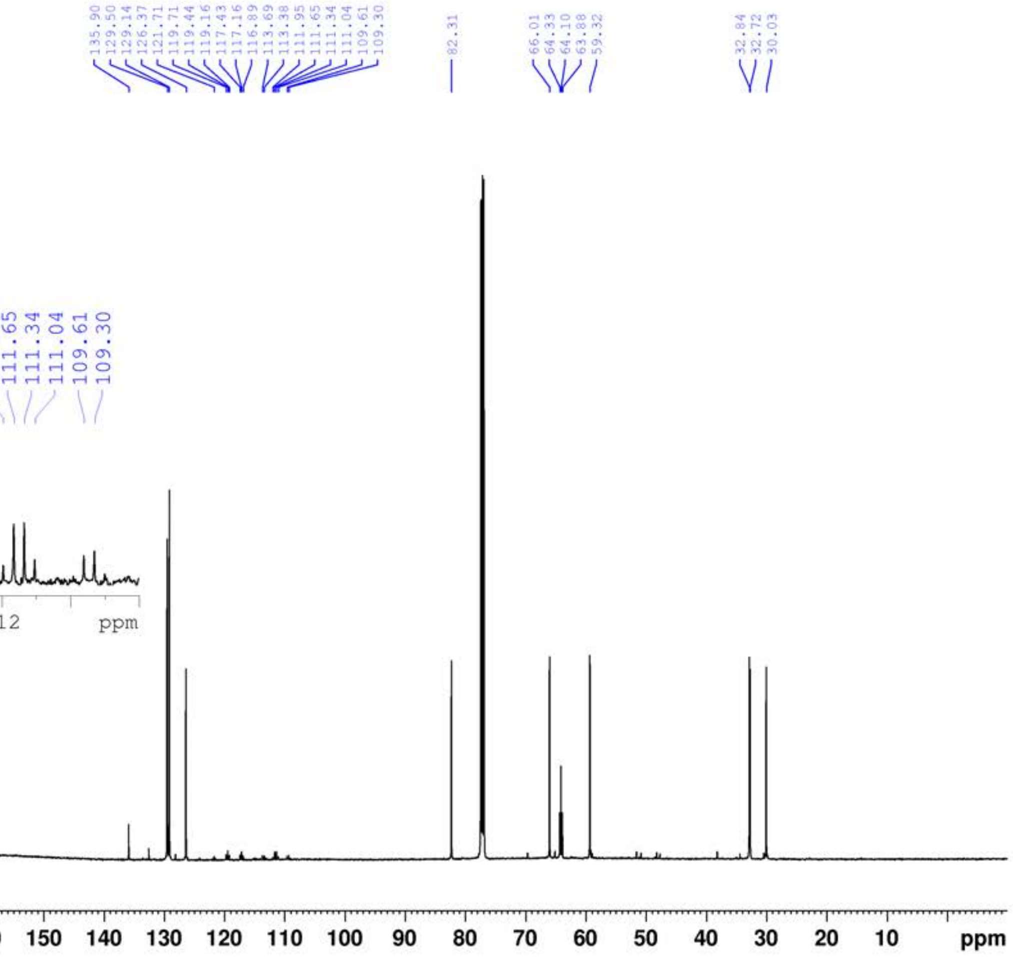

^{19}F NMR (376 MHz, CDCl$_3$) for *2,2,3,3,3-pentafluoropropyl ((2S,3R)-2-(2-(phenylthio)ethyl)tetrahydrofuran-3-yl)sulfamate:* ***Obtained following derivatisation of*** **26f**

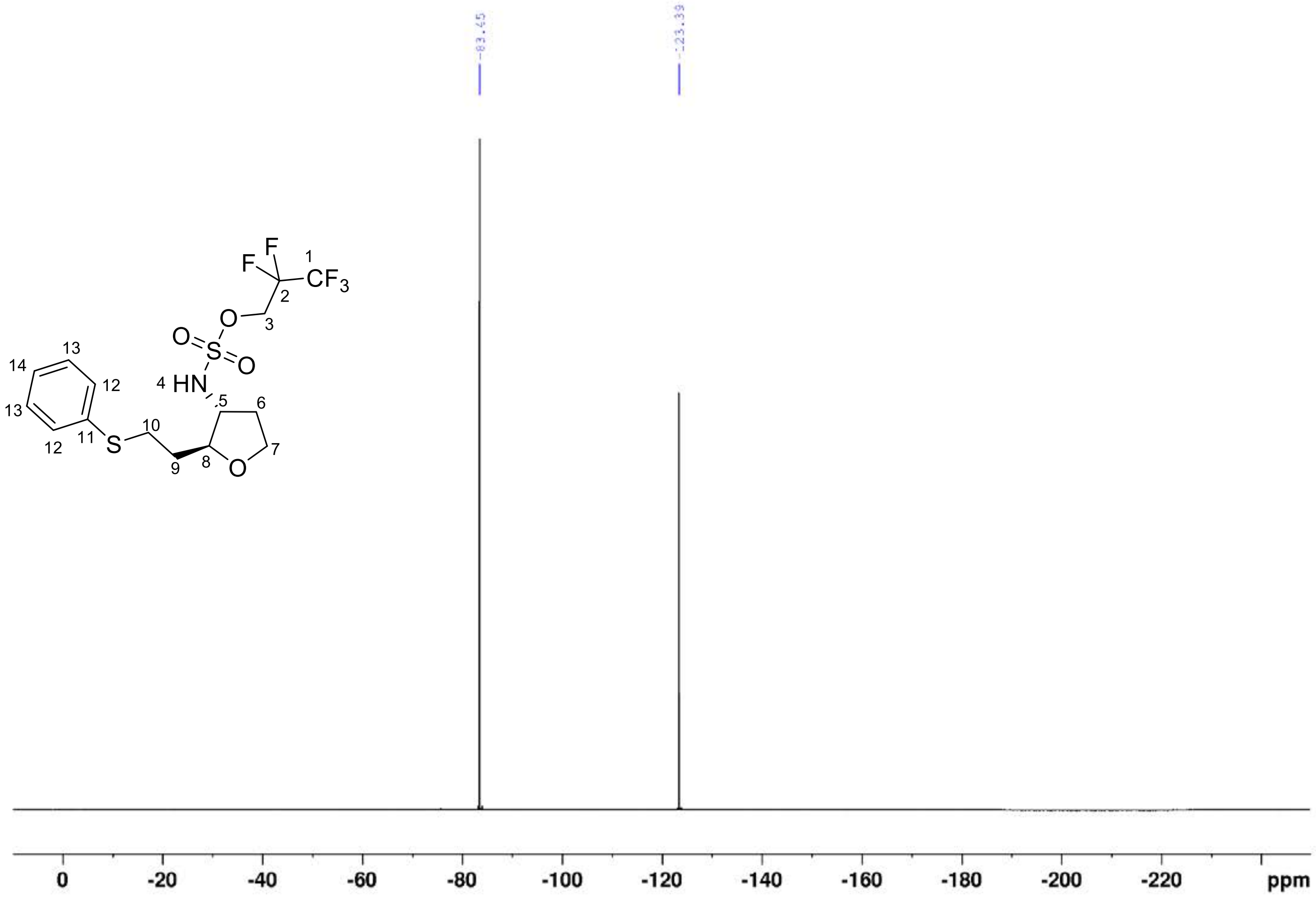

^{1}H NMR (500 MHz, $CDCl_3$) for *2,2,3,3,3-pentafluoropropyl (2R,3R)-2-(2-((N,4-dimethylphenyl)sulfonamido)ethyl)-3-(2-hydroxyethyl)aziridine-1-sulfonate* (**26g**)

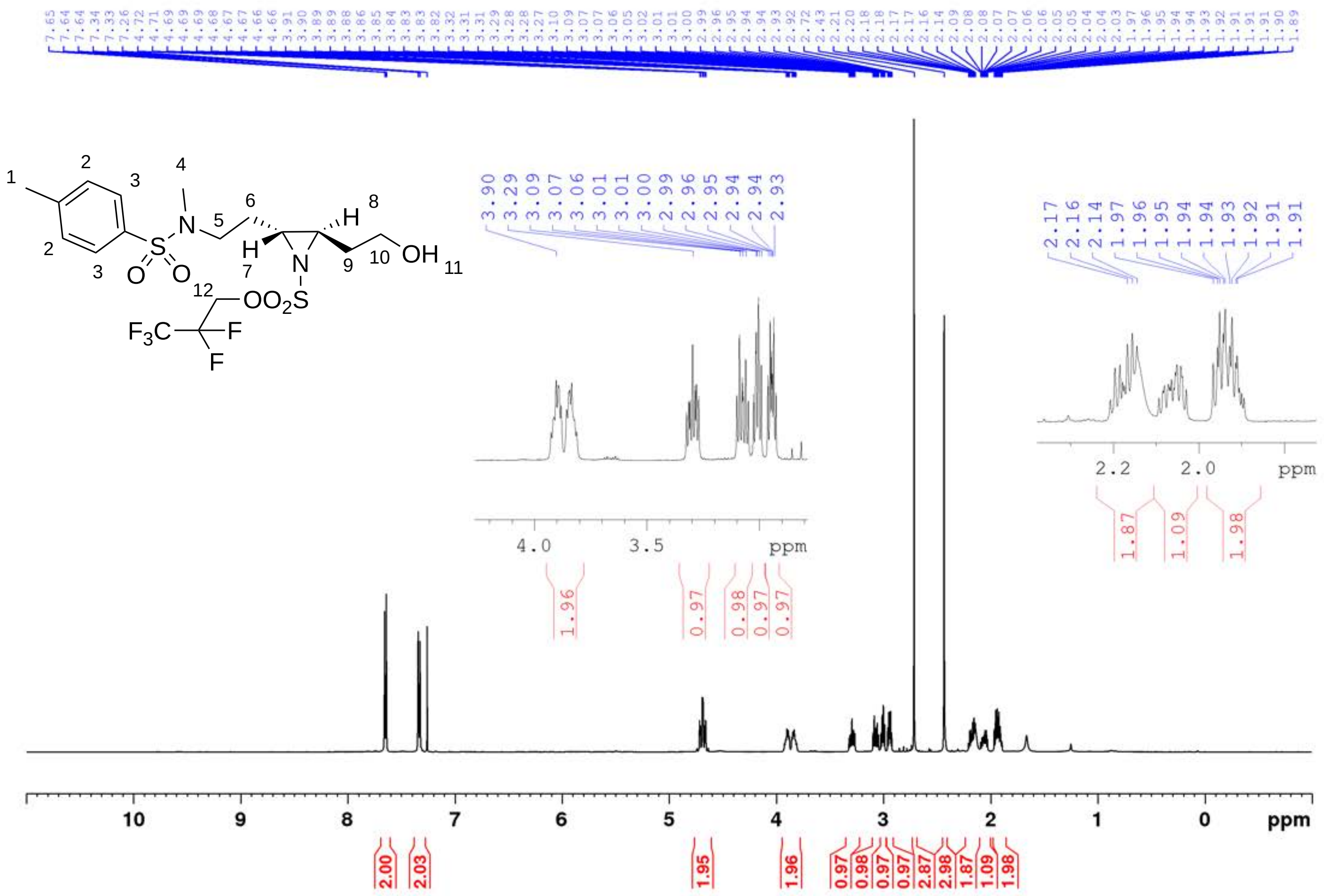

^{13}C NMR (126 MHz, $CDCl_3$) for *2,2,3,3,3-pentafluoropropyl (2R,3R)-2-(2-((N,4-dimethylphenyl)sulfonamido)ethyl)-3-(2-hydroxyethyl)aziridine-1-sulfonate* (**26g**)

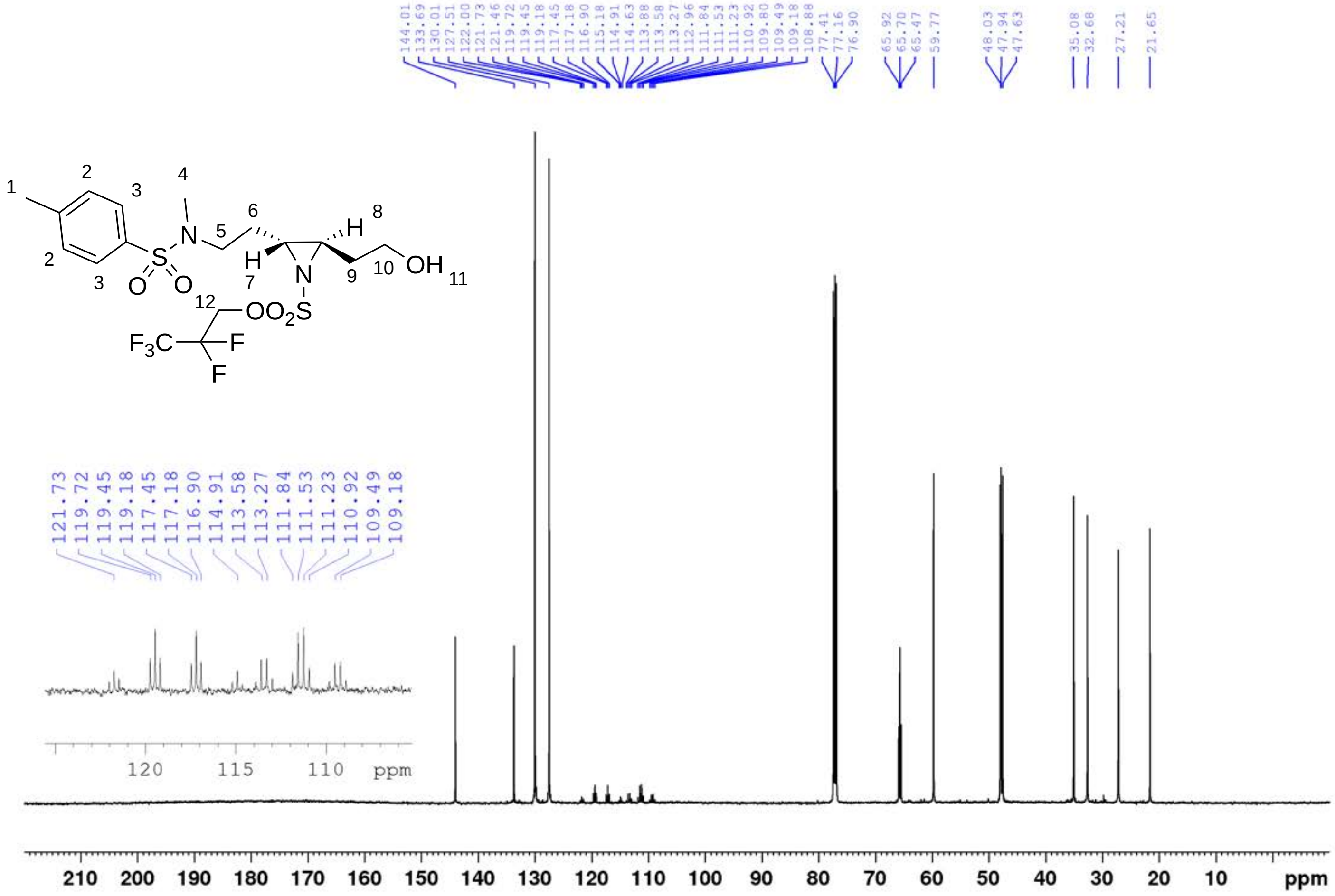

^{19}F NMR (376 MHz, $CDCl_3$) for *2,2,3,3,3-pentafluoropropyl (2R,3R)-2-(2-((N,4-dimethylphenyl)sulfonamido)ethyl)-3-(2-hydroxyethyl)aziridine-1-sulfonate* (**26g**)

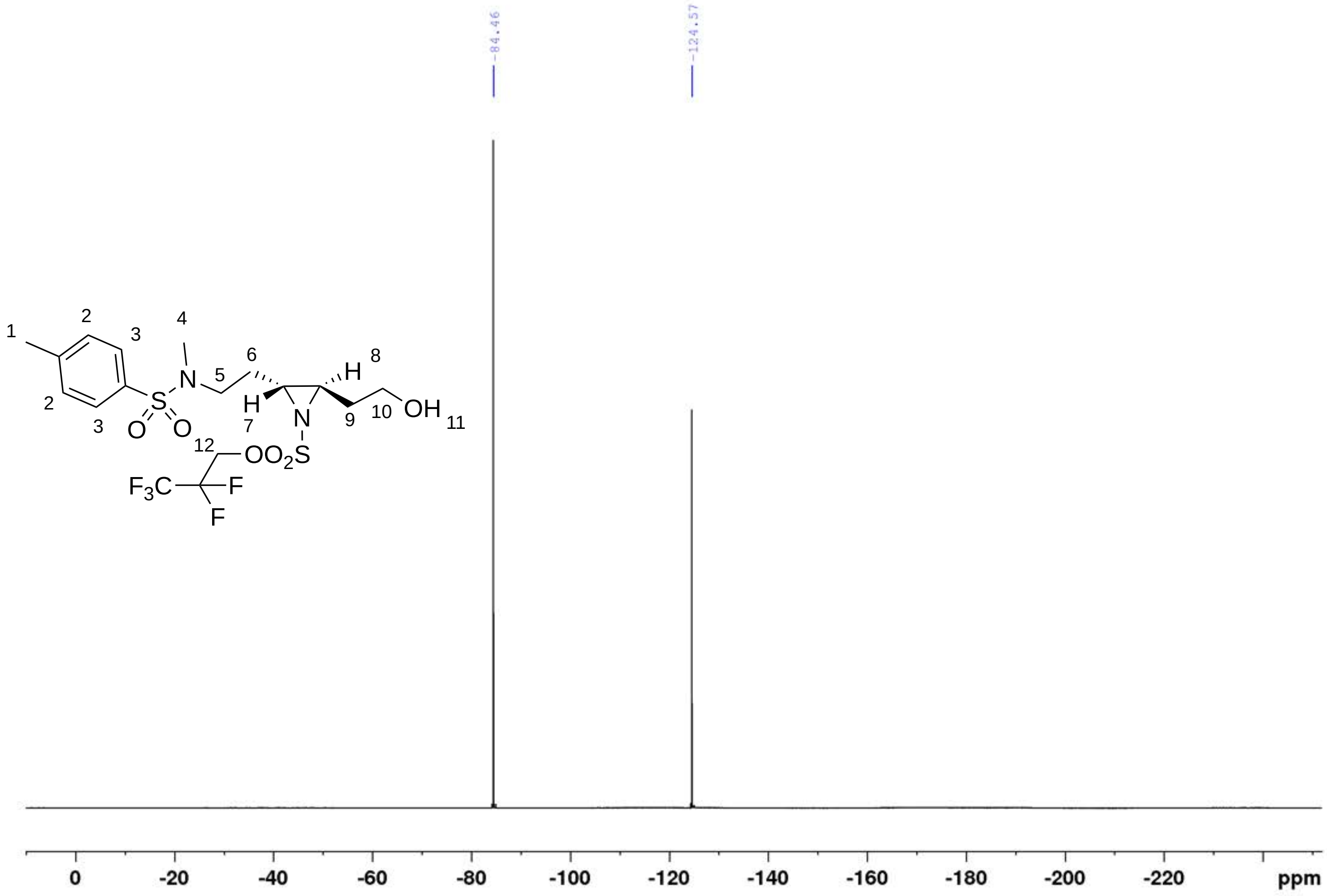

¹H NMR (500 MHz, $CDCl_3$) for *2,2,3,3,3-pentafluoropropyl (S)-2-(2-hydroxyethyl)-2-isopentylaziridine-1-sulfonate* (**28a**)

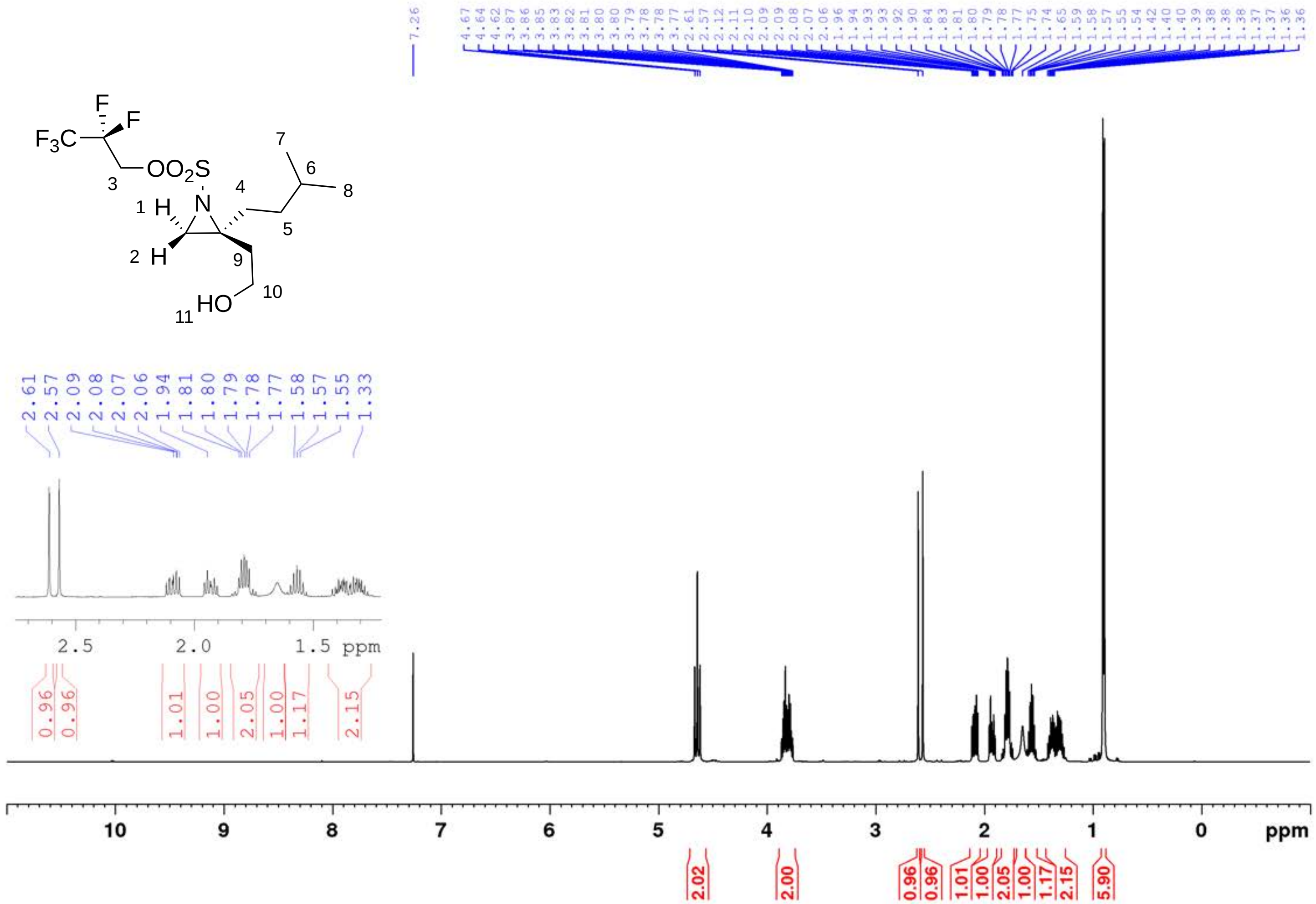

^{13}C NMR (126 MHz, $CDCl_3$) for *2,2,3,3,3-pentafluoropropyl (S)-2-(2-hydroxyethyl)-2-isopentylaziridine-1-sulfonate* (**28a**)

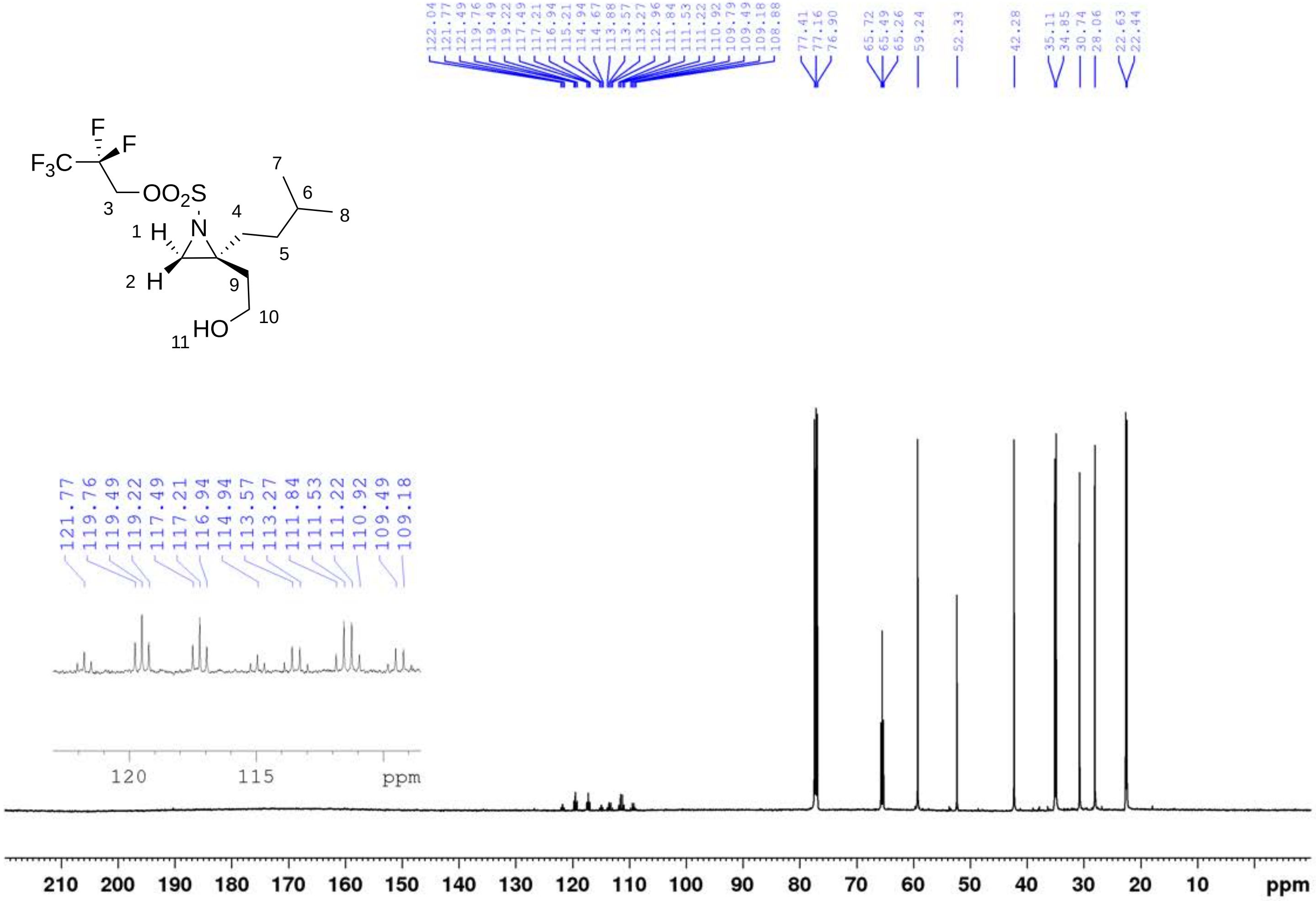

^{19}F NMR (376 MHz, $CDCl_3$) for *2,2,3,3,3-pentafluoropropyl (S)-2-(2-hydroxyethyl)-2-isopentylaziridine-1-sulfonate* (**28a**)

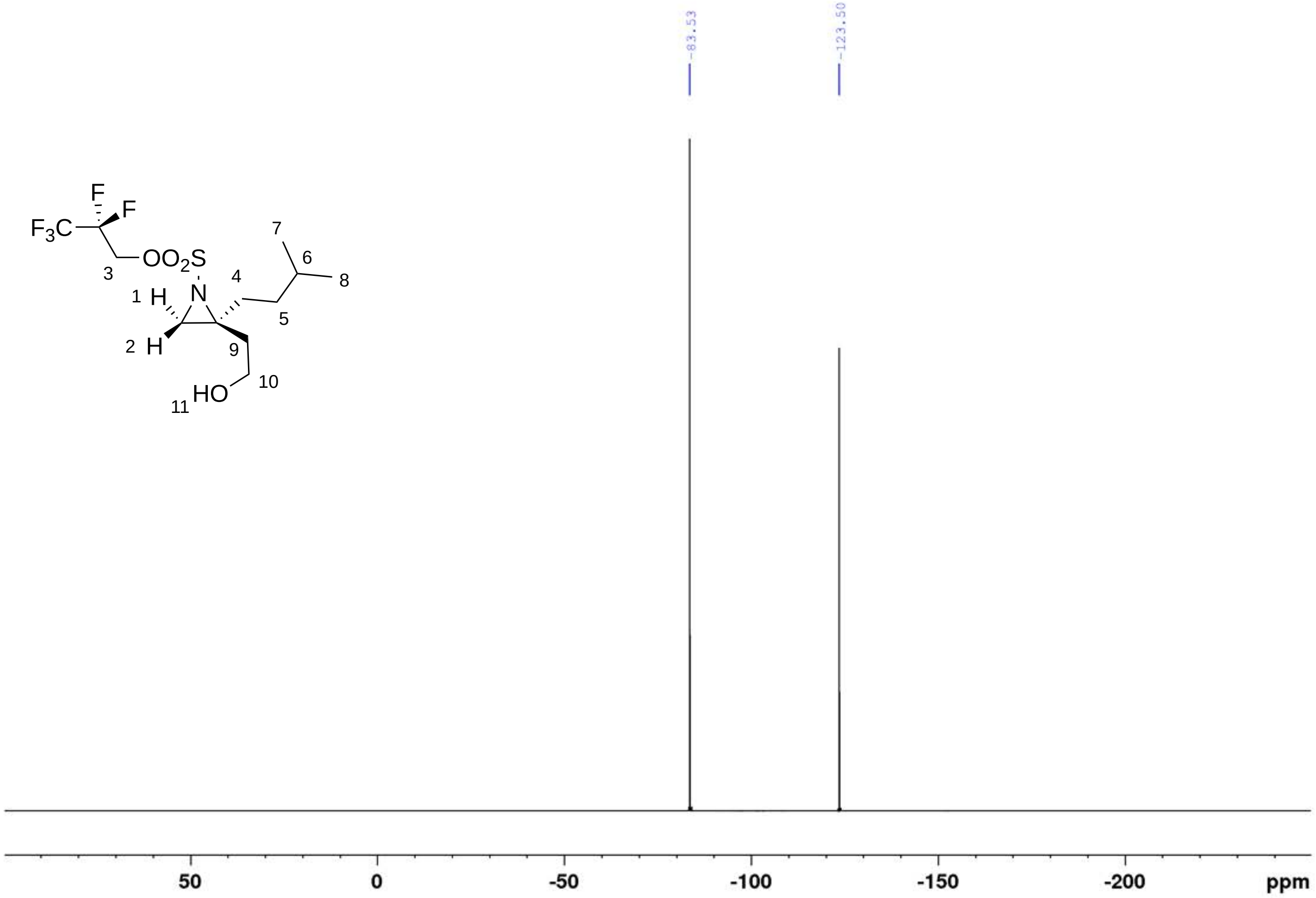

^{1}H NMR (500 MHz, $CDCl_3$) for *2,2,3,3,3-pentafluoropropyl (S)-(1-hydroxy-6-methyl-3-((phenylthio)methyl)heptan-3-yl)sulfamate:* ***Obtained following derivatisation of*** **28a**

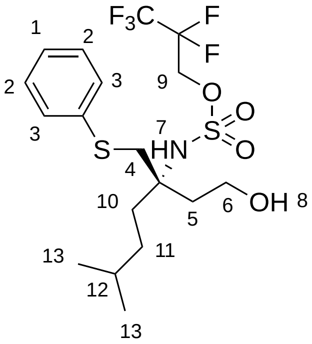

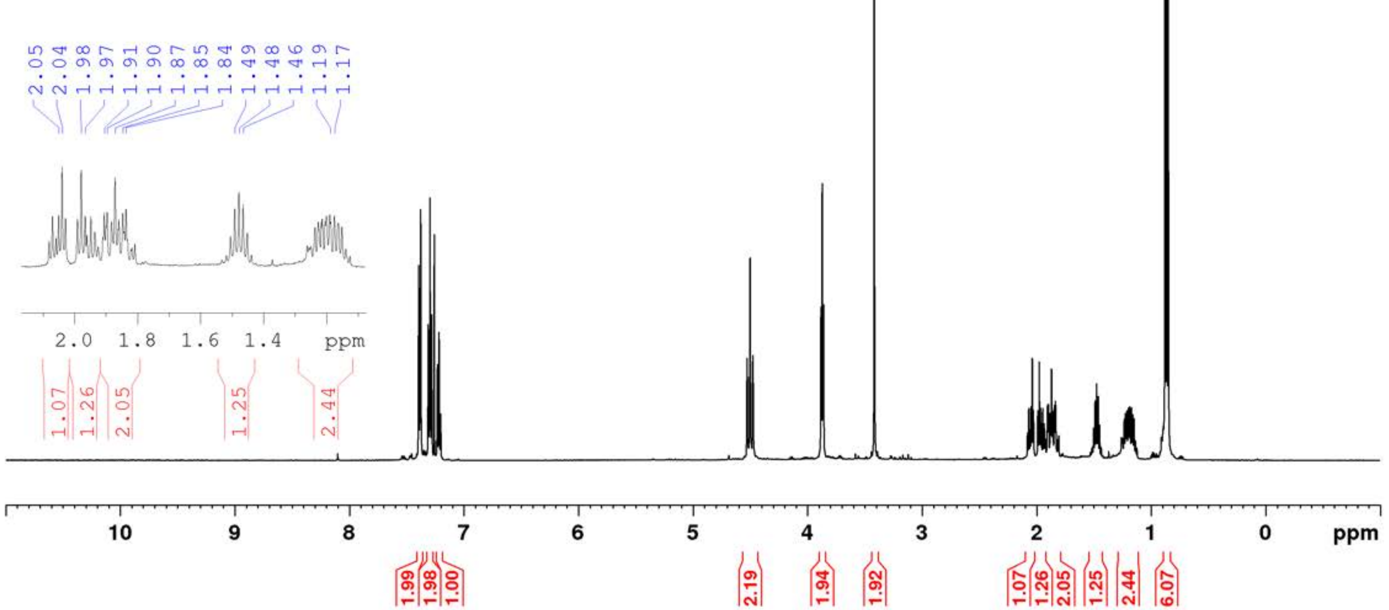

^{13}C NMR (126 MHz, $CDCl_3$) for *2,2,3,3,3-pentafluoropropyl (S)-(1-hydroxy-6-methyl-3-((phenylthio)methyl)heptan-3-yl)sulfamate:* ***Obtained following derivatisation of*** **28a**

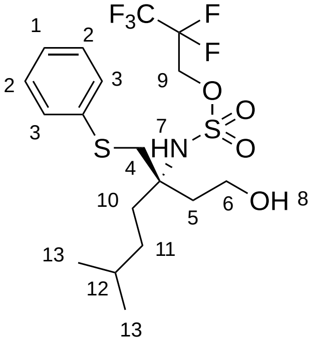

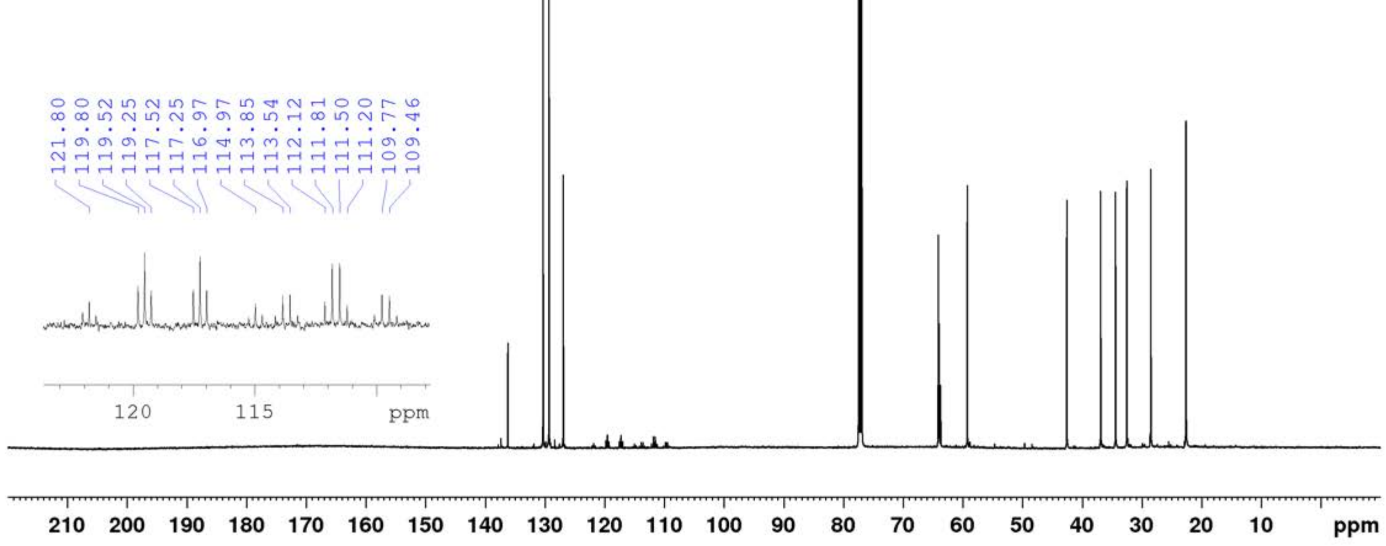

^{19}F NMR (376 MHz, $CDCl_3$) for *2,2,3,3,3-pentafluoropropyl (S)-(1-hydroxy-6-methyl-3-((phenylthio)methyl)heptan-3-yl)sulfamate:* ***Obtained following derivatisation of* 28a**

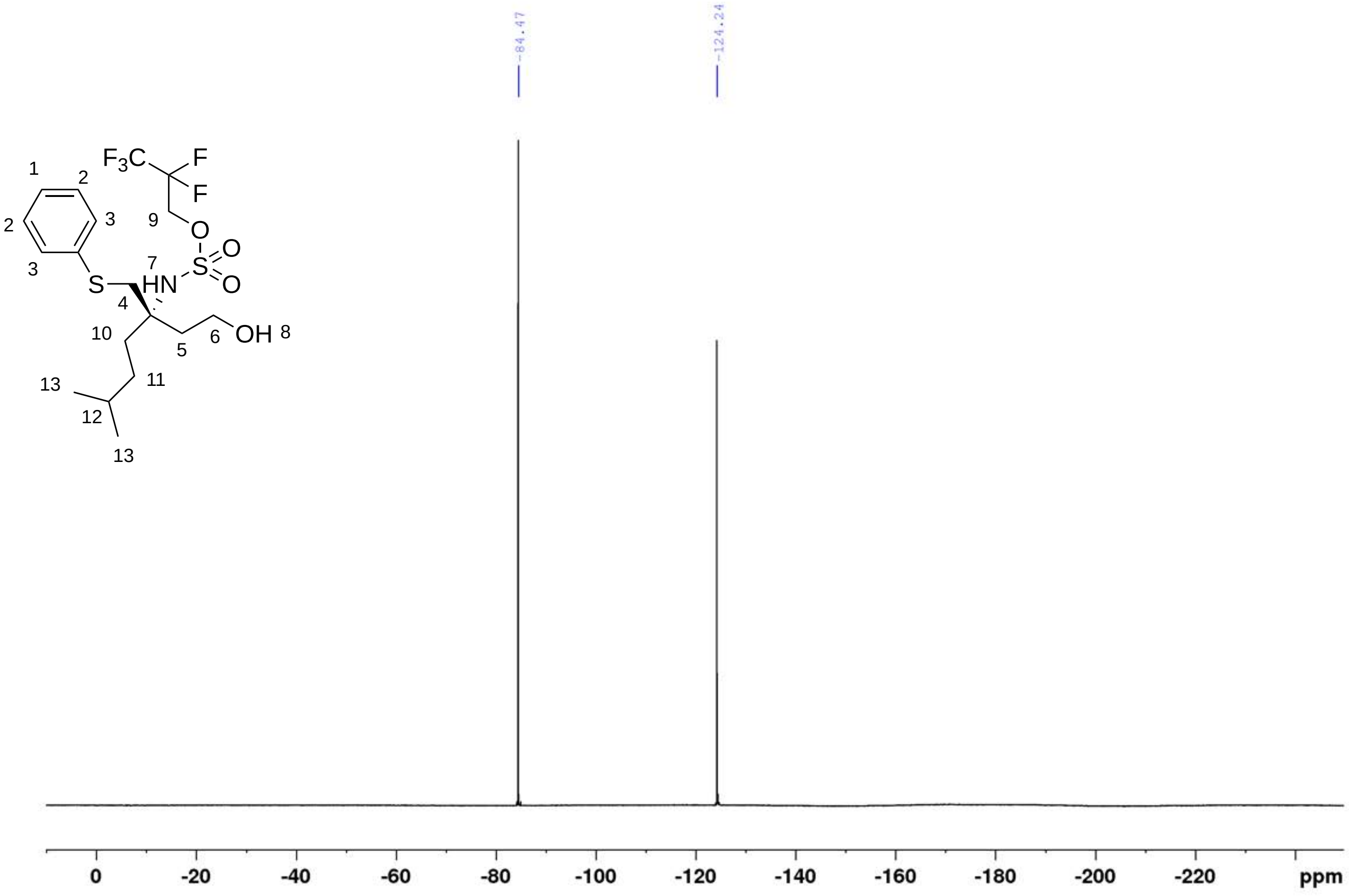

^{1}H NMR (500 MHz, $CDCl_3$) for *2,2,3,3,3-pentafluoropropyl (S)-2-(2-cyclohexylethyl)-2-(2-hydroxyethyl)aziridine-1-sulfonate* (**28b**)

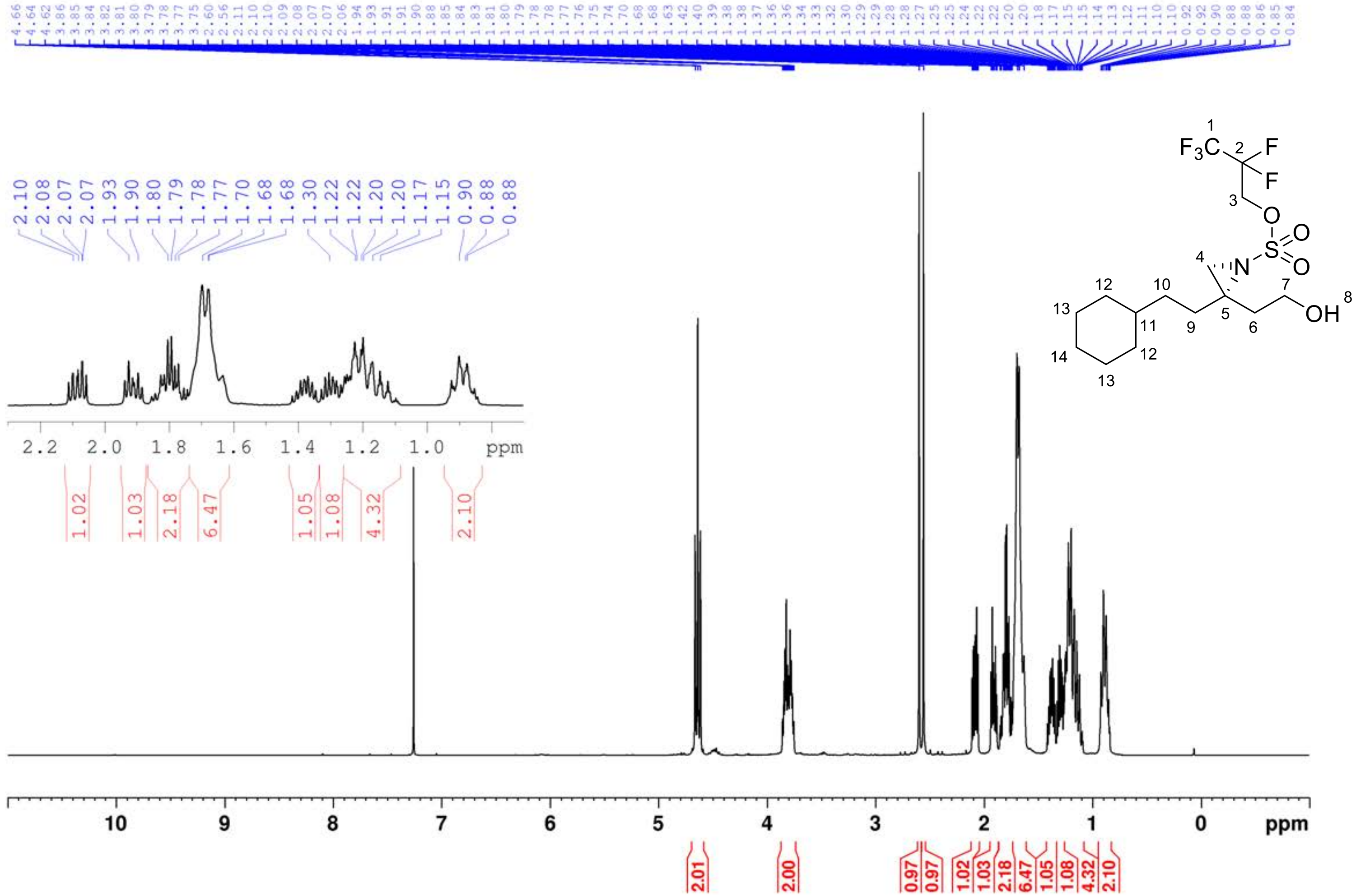

^{13}C NMR (126 MHz, CDCl$_3$) for *2,2,3,3,3-pentafluoropropyl (S)-2-(2-cyclohexylethyl)-2-(2-hydroxyethyl)aziridine-1-sulfonate* (**28b**)

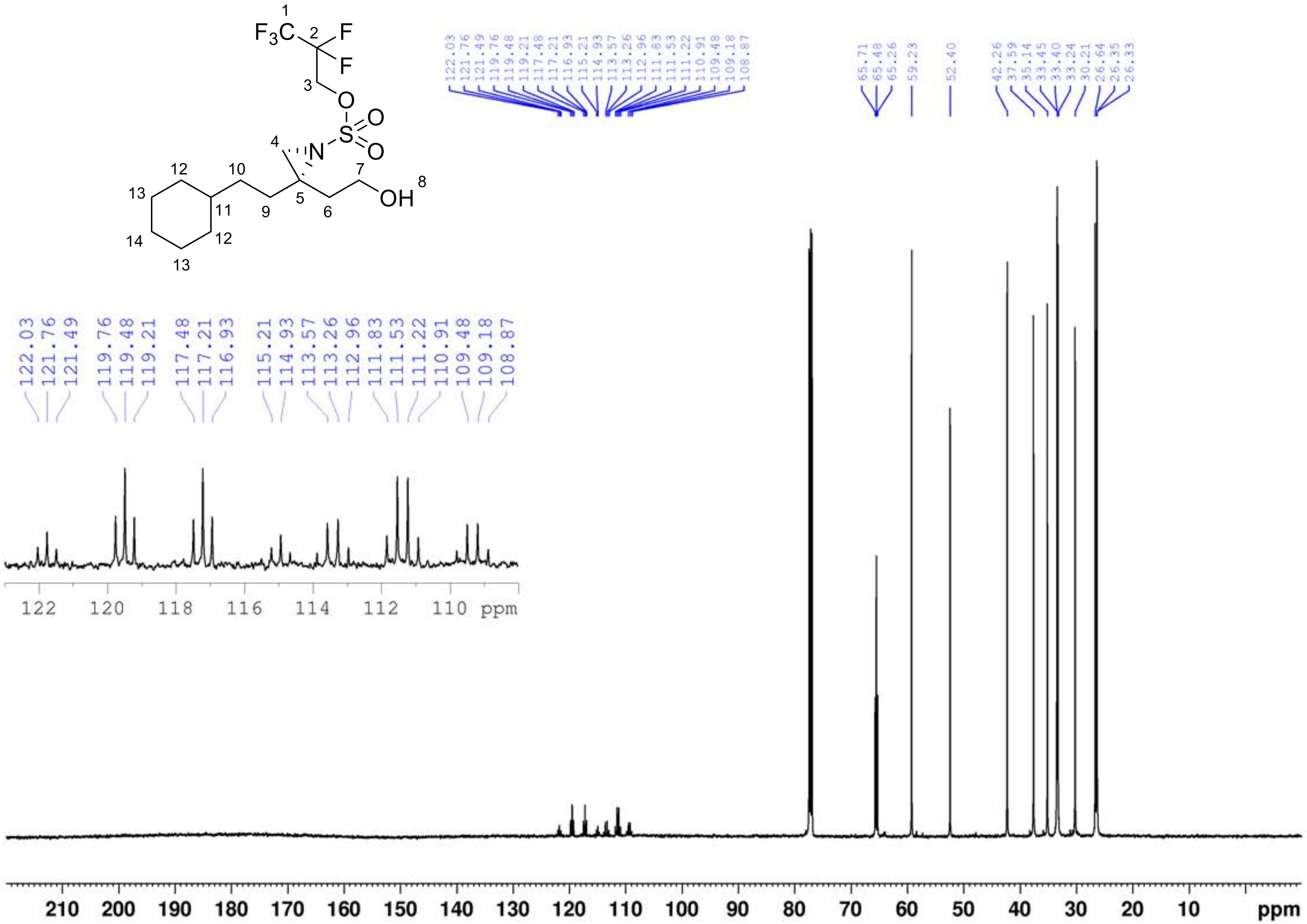

^{19}F NMR (376 MHz, $CDCl_3$) for *2,2,3,3,3-pentafluoropropyl (S)-2-(2-cyclohexylethyl)-2-(2-hydroxyethyl)aziridine-1-sulfonate* (**28b**)

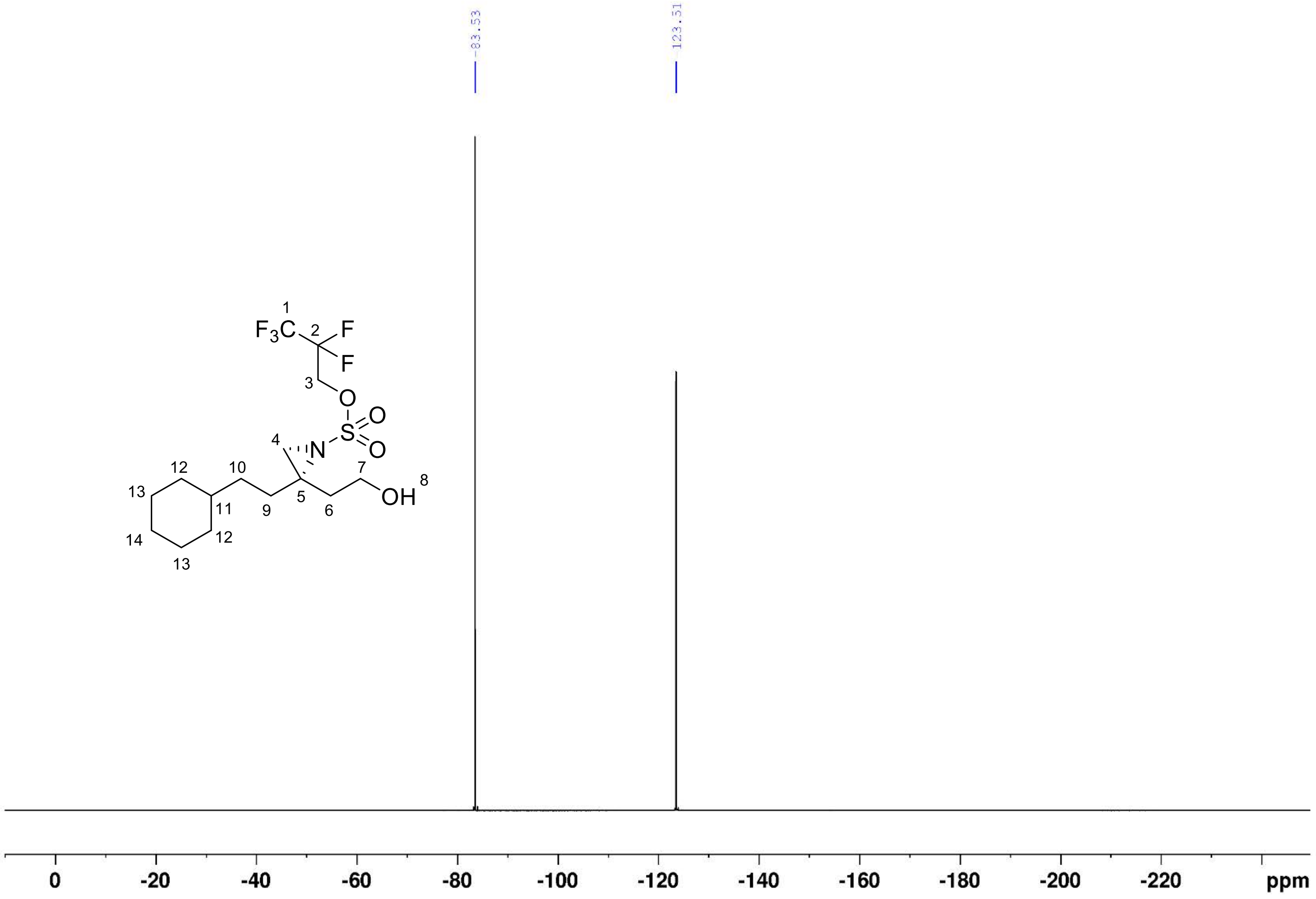

^{1}H NMR (500 MHz, $CDCl_3$) for *2,2,3,3,3-pentafluoropropyl (S)-(1-cyclohexyl-5-hydroxy-3-((phenylthio)methyl)pentan-3-yl)sulfamate* (**41**)*:* ***Obtained following derivatisation of* 28b**

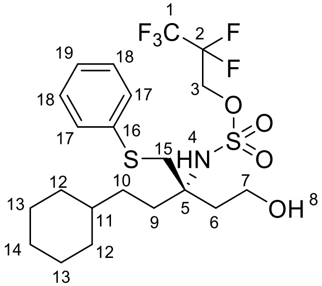

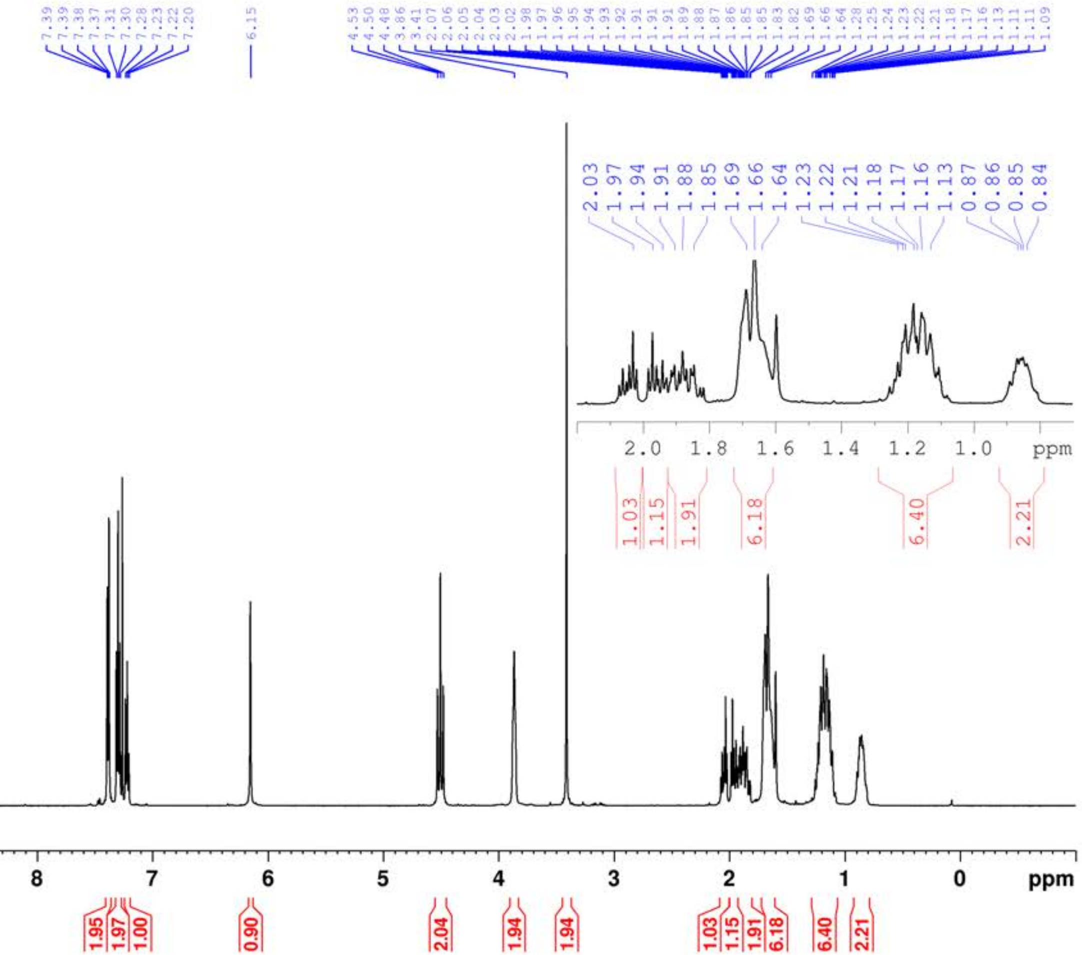

^{13}C NMR (126 MHz, $CDCl_3$) for *2,2,3,3,3-pentafluoropropyl (S)-(1-cyclohexyl-5-hydroxy-3-((phenylthio)methyl)pentan-3-yl)sulfamate* (**41**)*:*
***Obtained following derivatisation of* 28b**

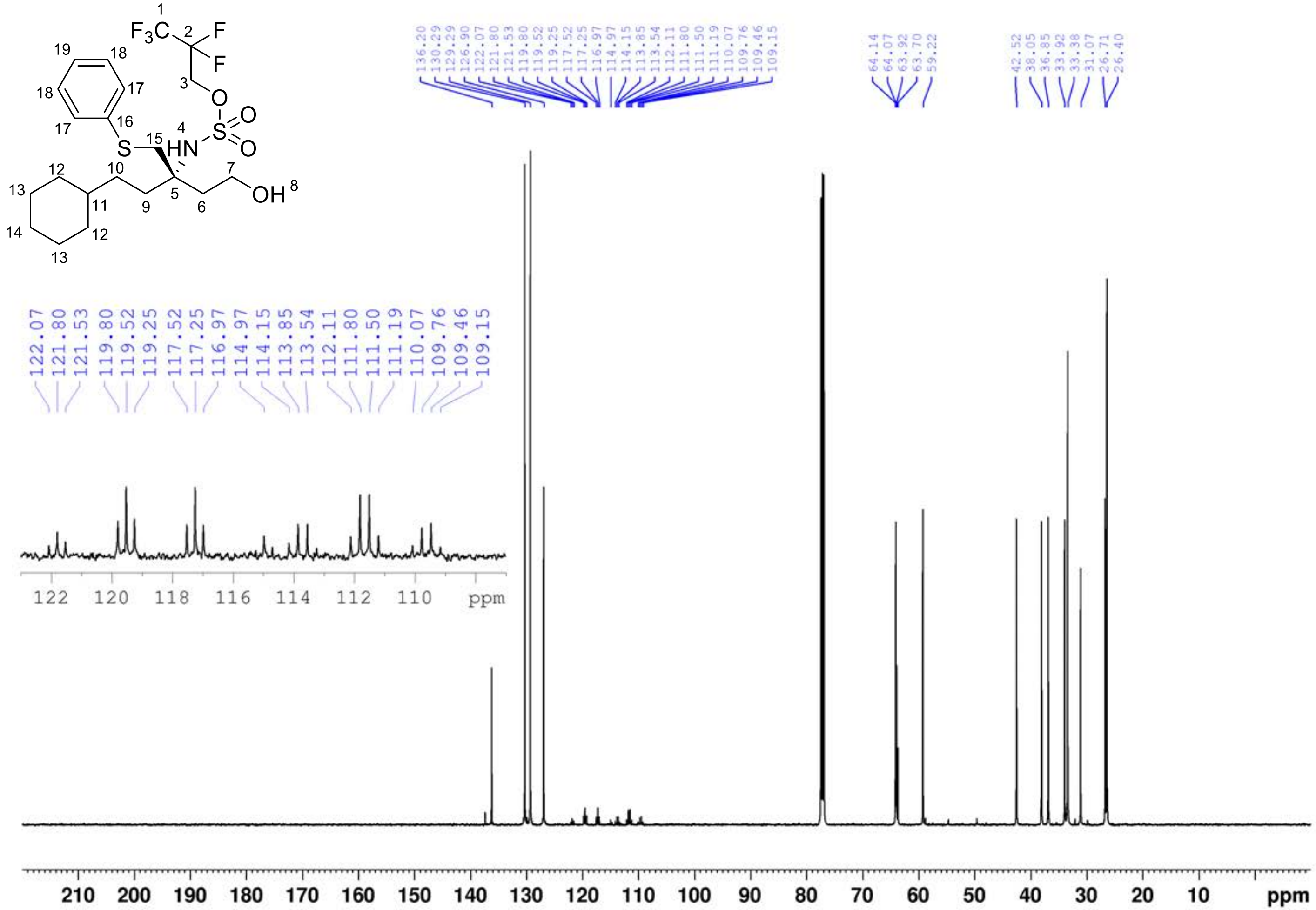

^{19}F NMR (376 MHz, $CDCl_3$) for *2,2,3,3,3-pentafluoropropyl (S)-2-(2-cyclohexylethyl)-2-(2-hydroxyethyl)aziridine-1-sulfonate* (**41**)*:* ***Obtained following derivatisation of* 28b**

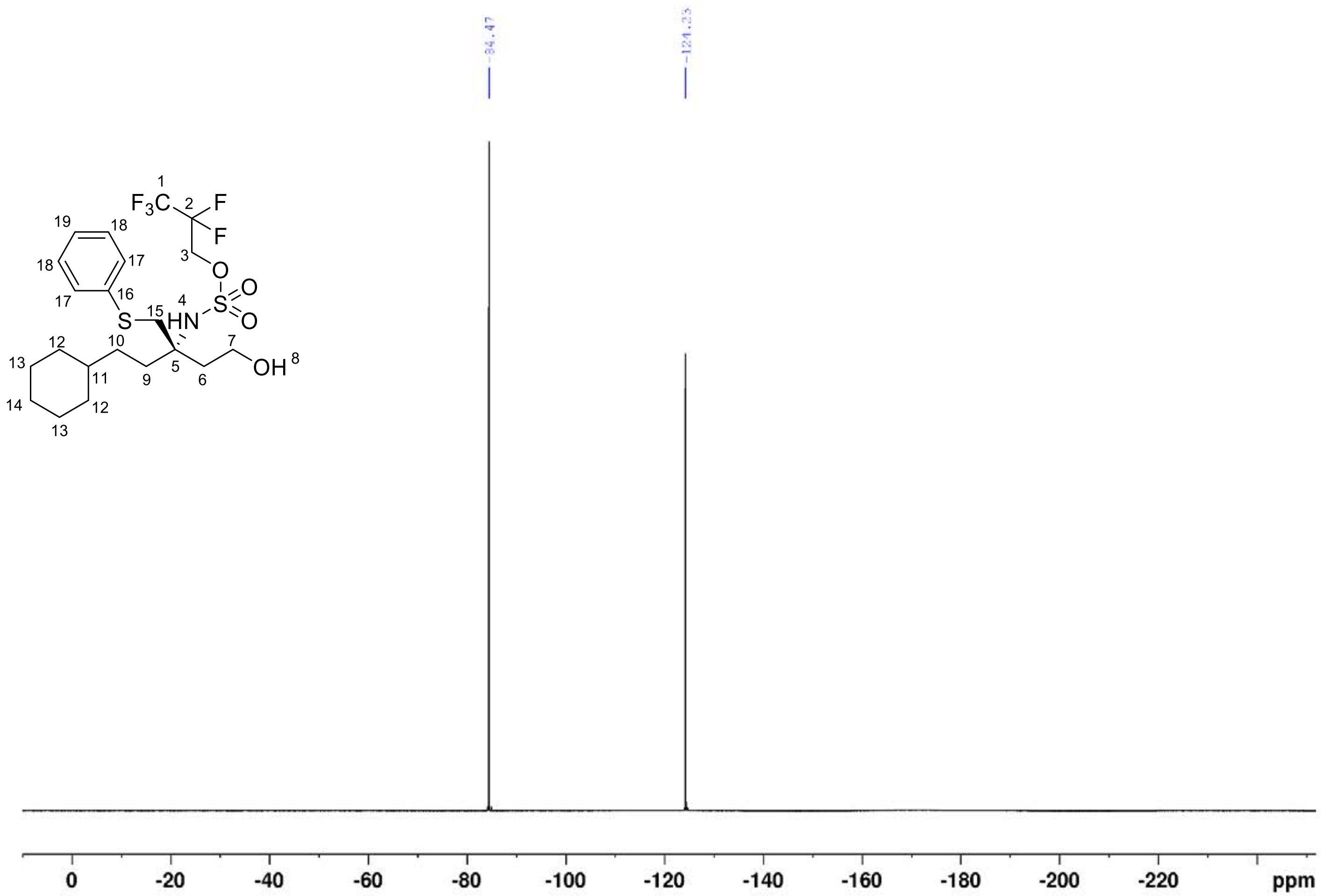

^{1}H NMR (500 MHz, CDCl$_3$) for *2,2,3,3,3-pentafluoropropyl (S)-2-(4-((tert-butyldimethylsilyl)oxy)butyl)-2-(2-hydroxyethyl)aziridine-1-sulfonate* (**28c**)

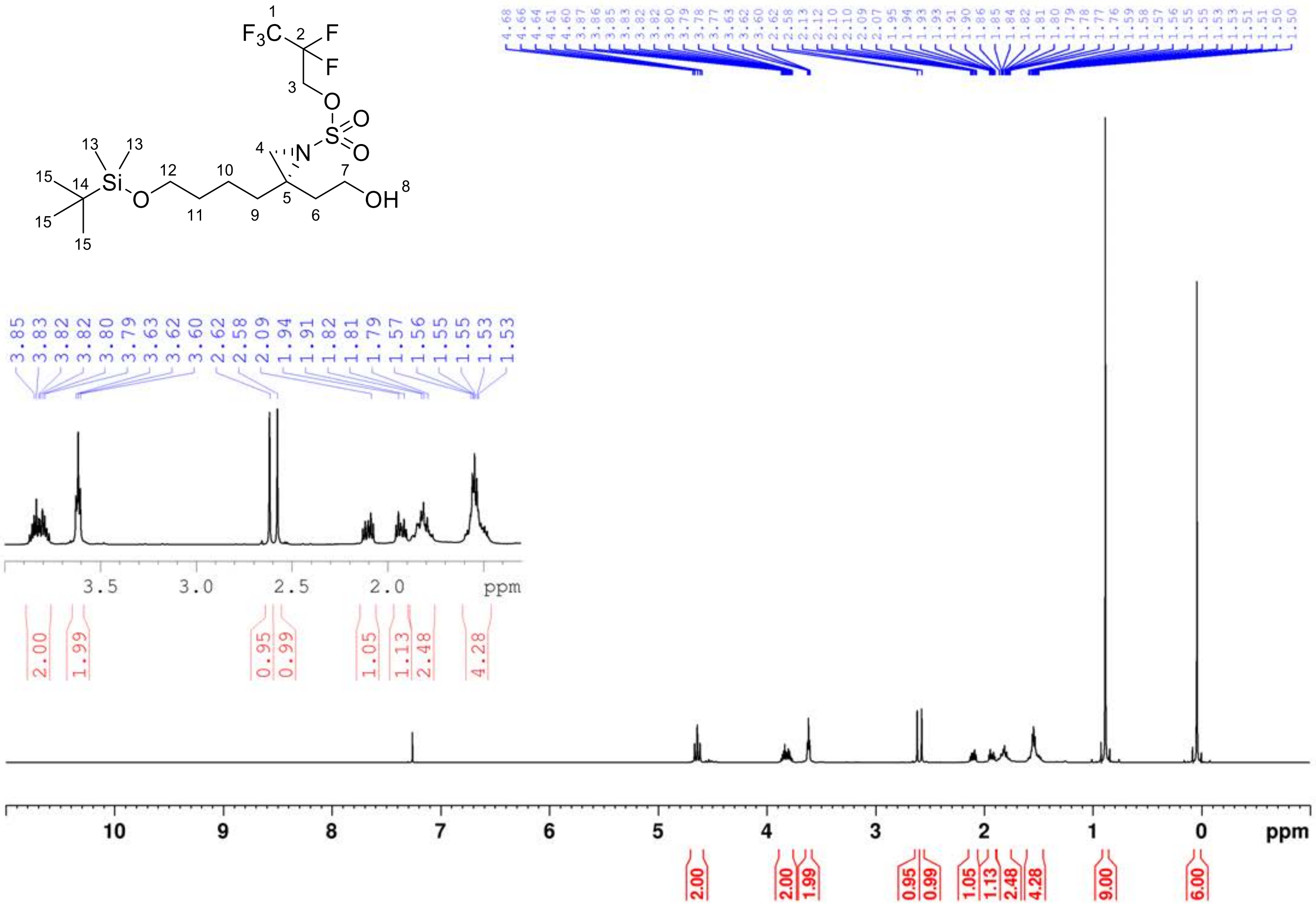

^{13}C NMR (126 MHz, CDCl$_3$) for *2,2,3,3,3-pentafluoropropyl (S)-2-(4-((tert-butyldimethylsilyl)oxy)butyl)-2-(2-hydroxyethyl)aziridine-1-sulfonate* (**28c**)

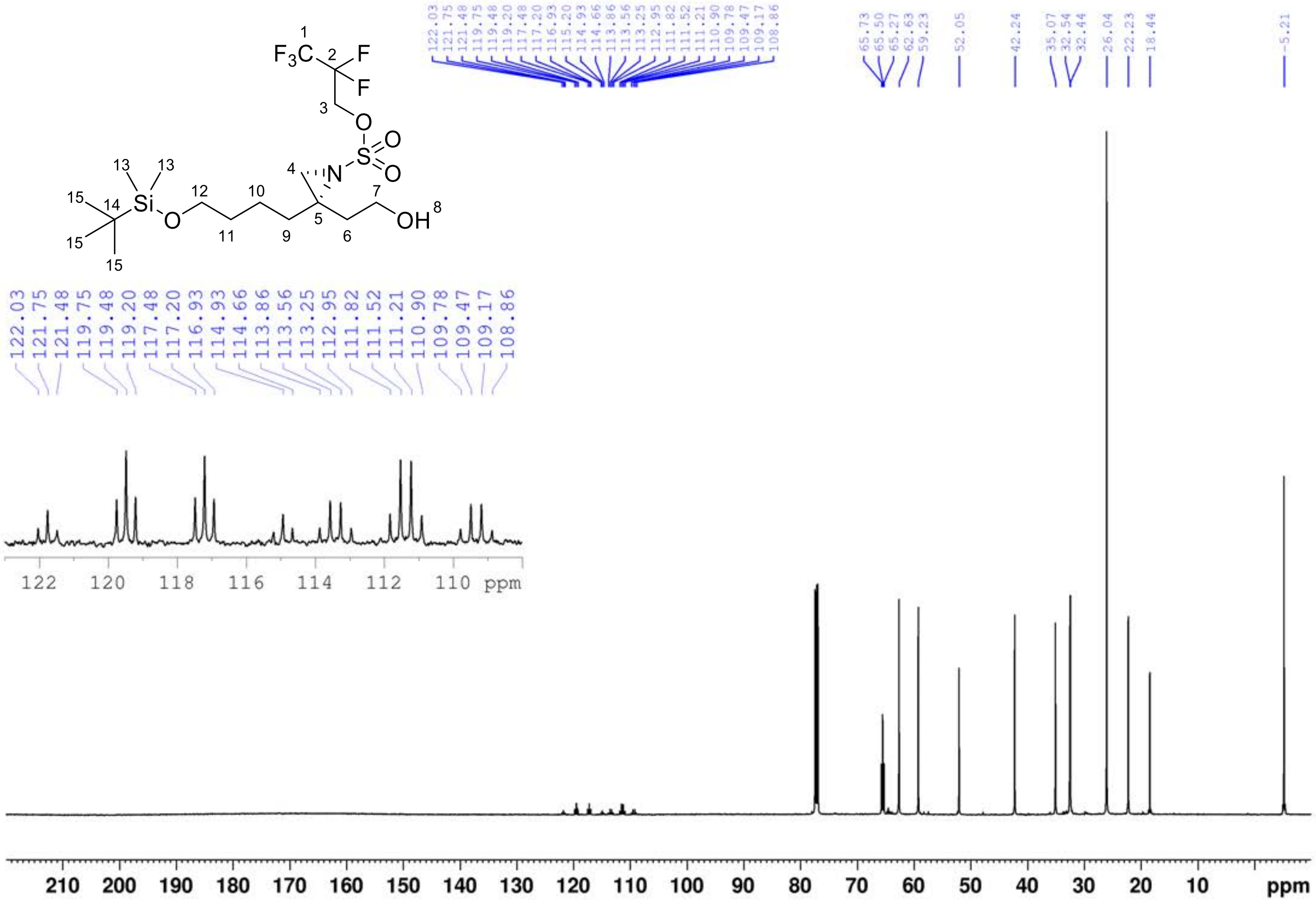

^{19}F NMR (376 MHz, $CDCl_3$) for *2,2,3,3,3-pentafluoropropyl (S)-2-(4-((tert-butyldimethylsilyl)oxy)butyl)-2-(2-hydroxyethyl)aziridine-1-sulfonate* (**28c**)

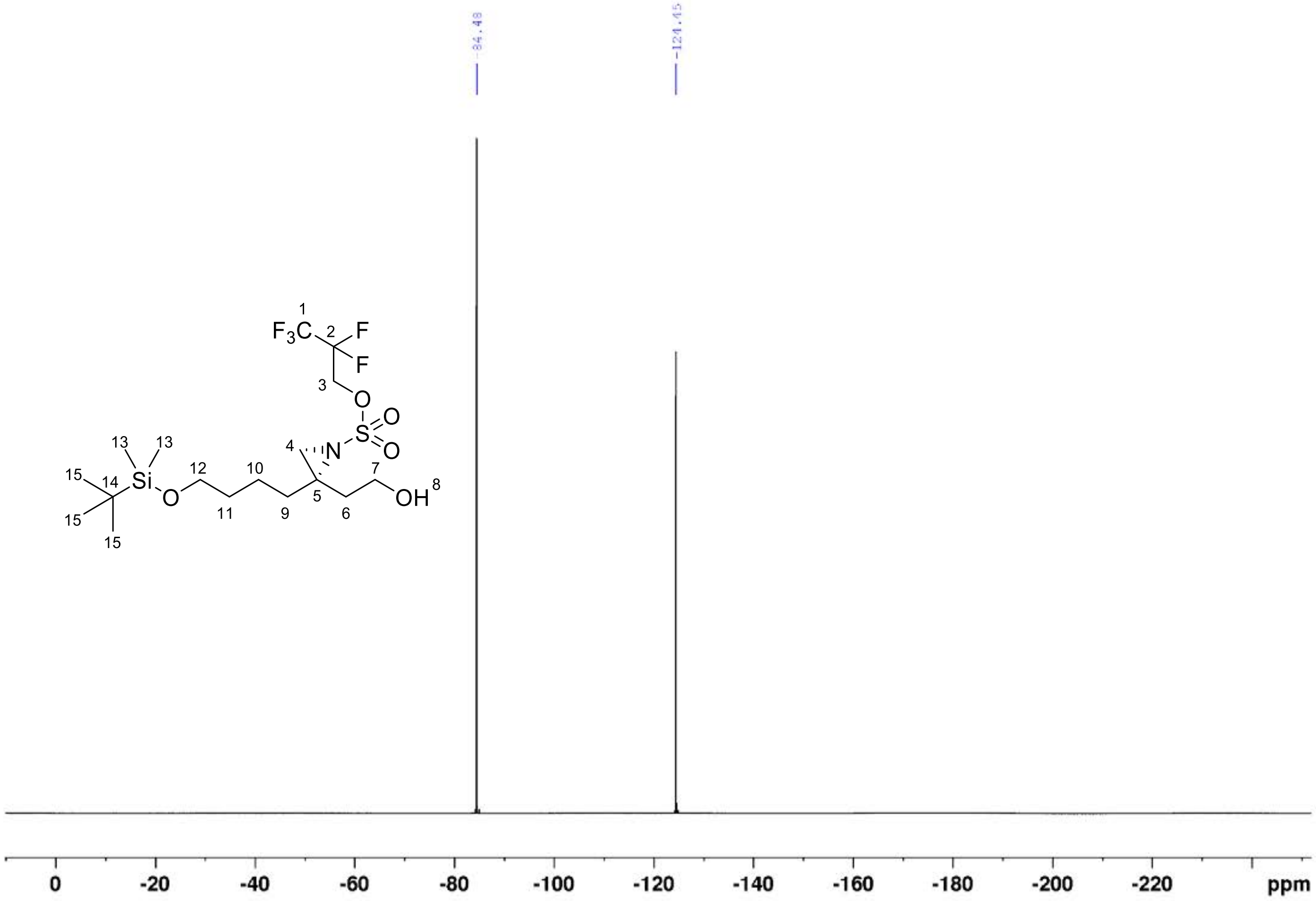

^{1}H NMR (500 MHz, $CDCl_3$) for *2,2,3,3,3-pentafluoropropyl (S)-(7-((tert-butyldimethylsilyl)oxy)-1-hydroxy-3-((phenylthio)methyl)heptan-3-yl)sulfamate:* ***Obtained following derivatisation of* 28c**

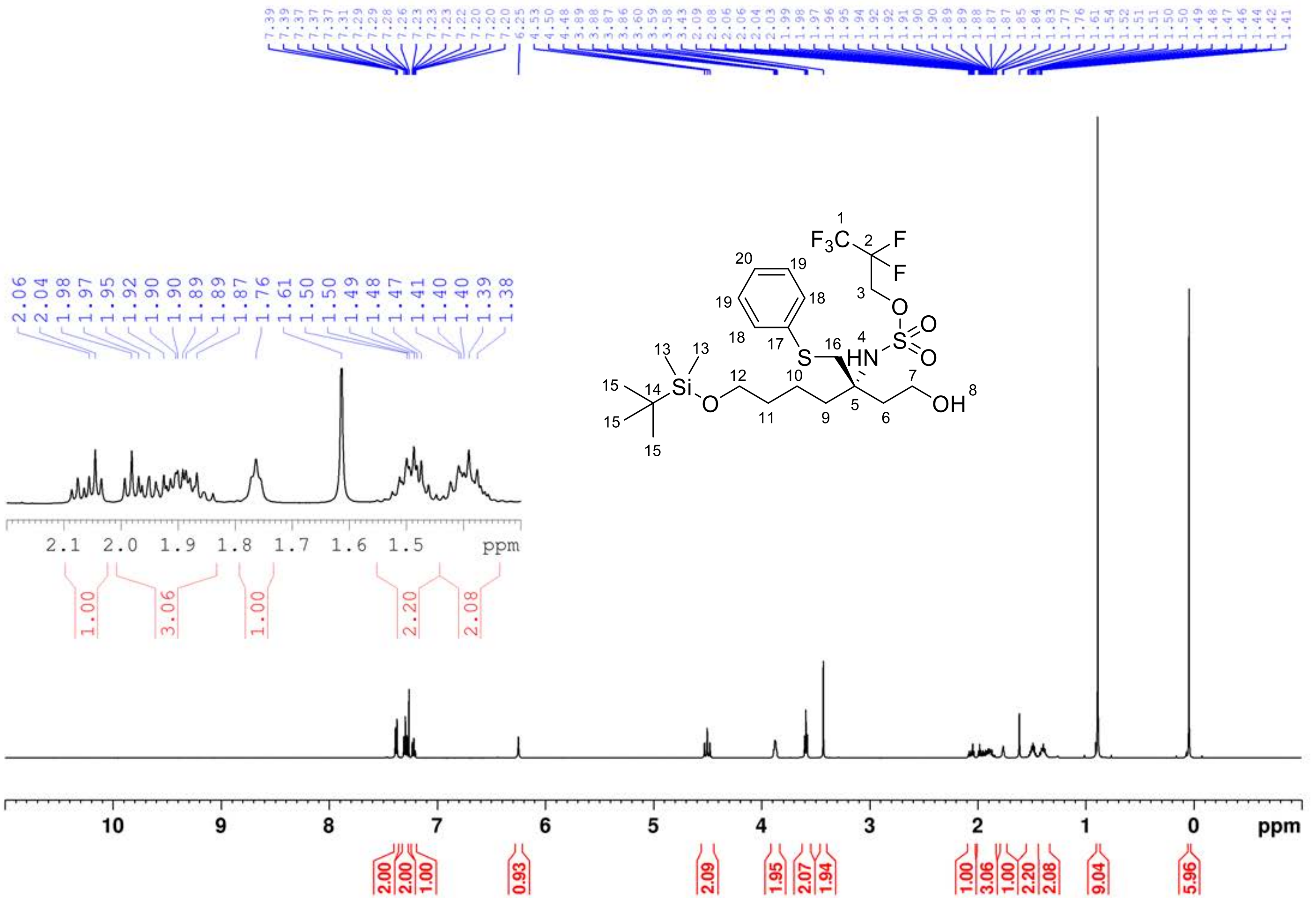

^{13}C NMR (126 MHz, $CDCl_3$) for *2,2,3,3,3-pentafluoropropyl (S)-(7-((tert-butyldimethylsilyl)oxy)-1-hydroxy-3-((phenylthio)methyl)heptan-3-yl)sulfamate: **Obtained following derivatisation of*** **28c**

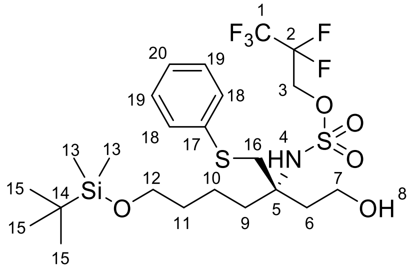

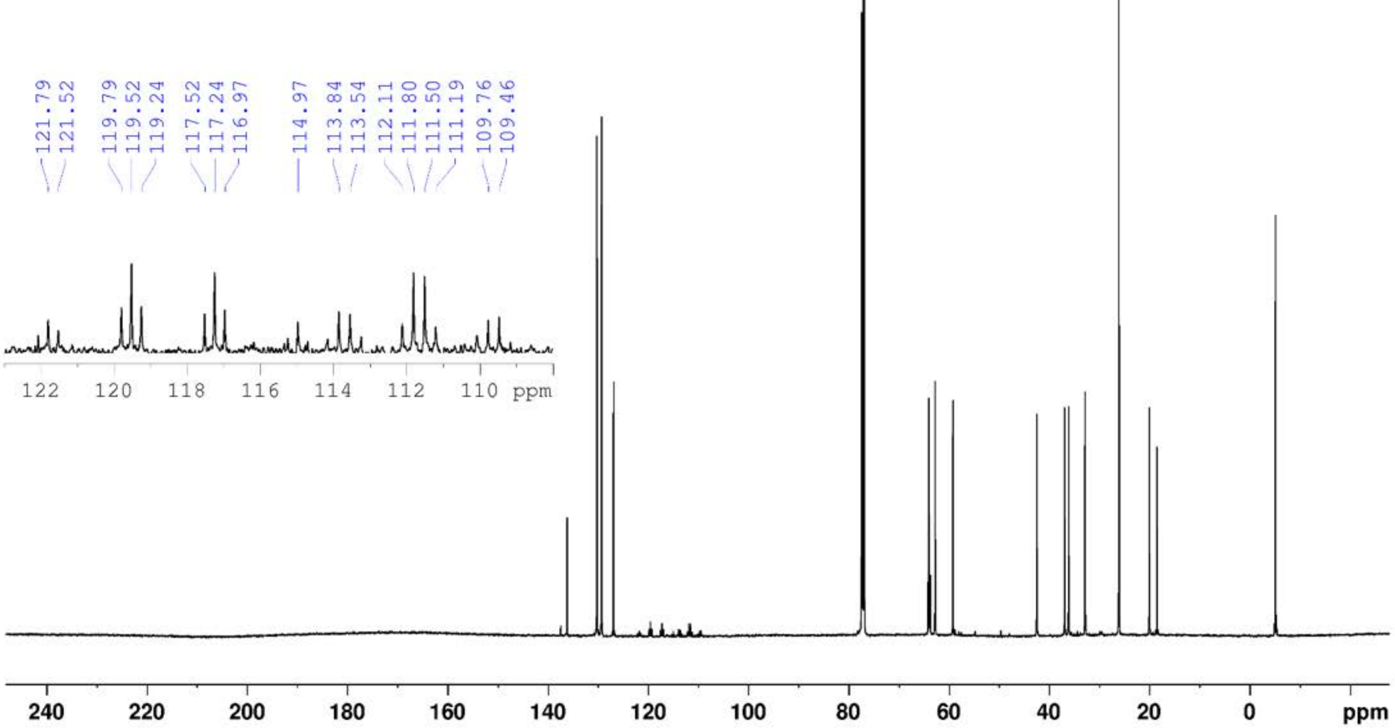

^{19}F NMR (376 MHz, CDCl$_3$) for 2,2,3,3,3-pentafluoropropyl (*S*)-(7-((*tert*-butyldimethylsilyl)oxy)-1-hydroxy-3-((phenylthio)methyl)heptan-3-yl)sulfamate: ***Obtained following derivatisation of* 28c**

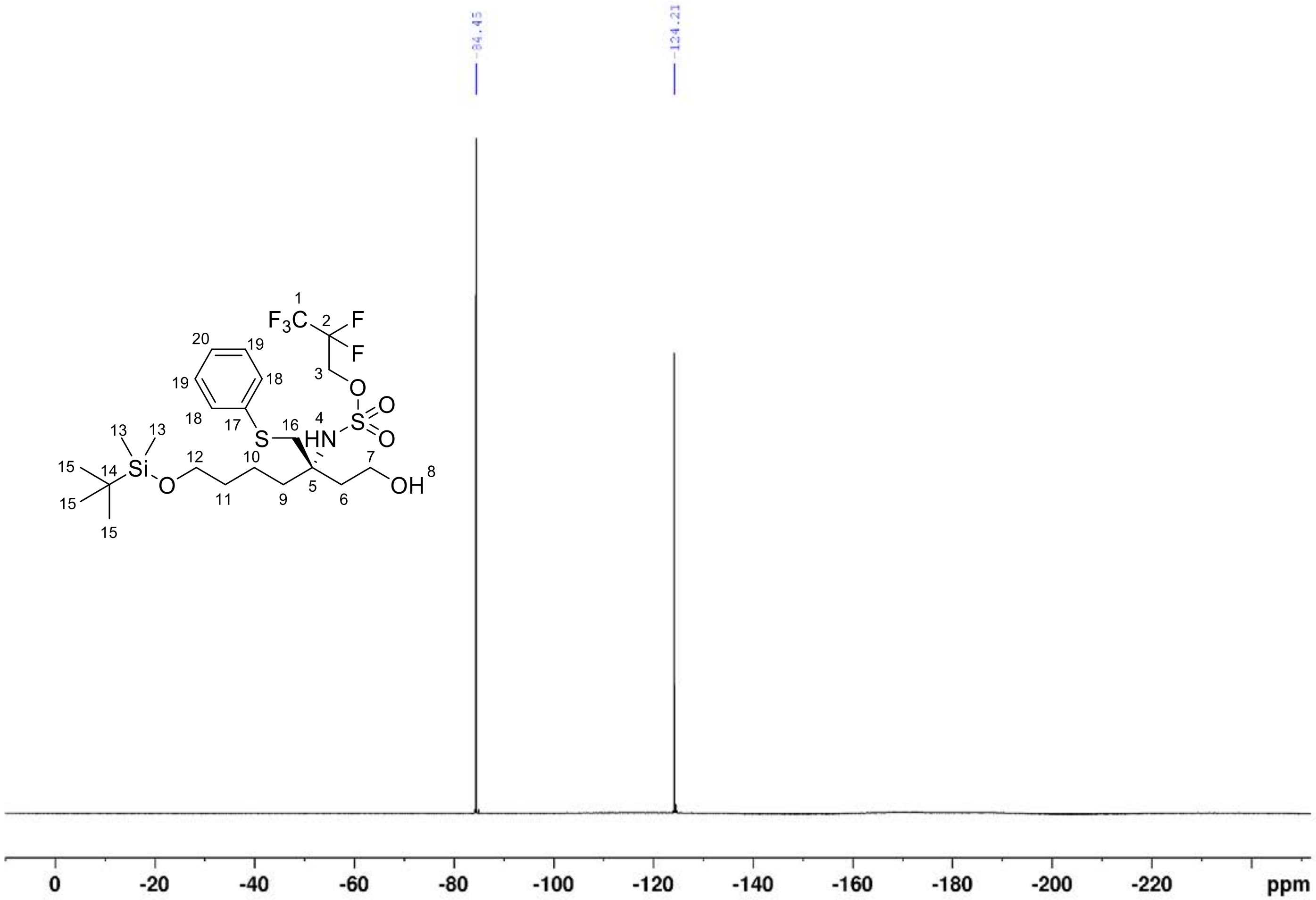

^{1}H NMR (500 MHz, $CDCl_3$) for *2,2,3,3,3-pentafluoropropyl (R)-6-oxa-2-azaspiro[4.5]decane-2-sulfonate* (**30**)

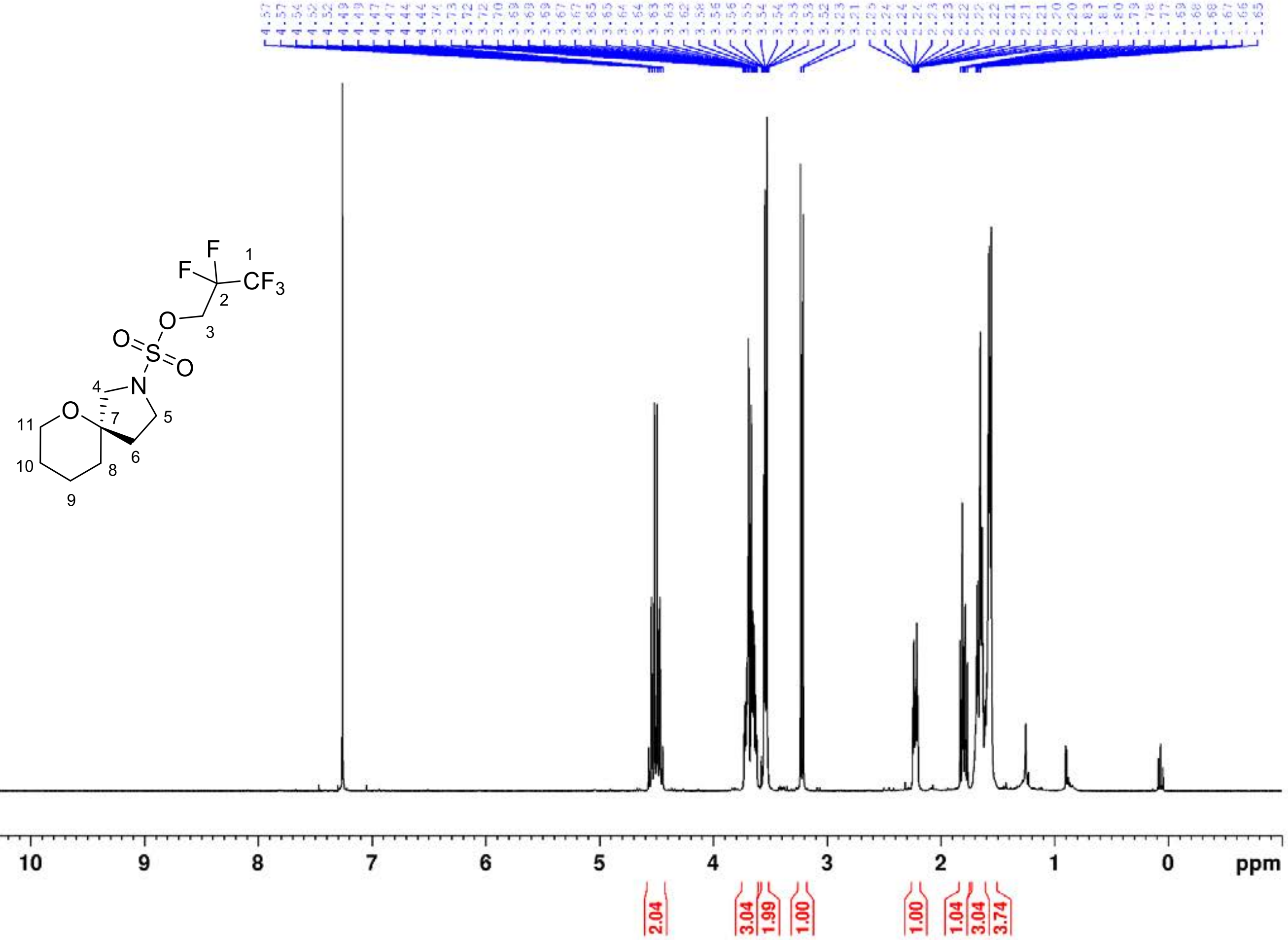

^{13}C NMR (126 MHz, $CDCl_3$) for *2,2,3,3,3-pentafluoropropyl (R)-6-oxa-2-azaspiro[4.5]decane-2-sulfonate* (**30**)

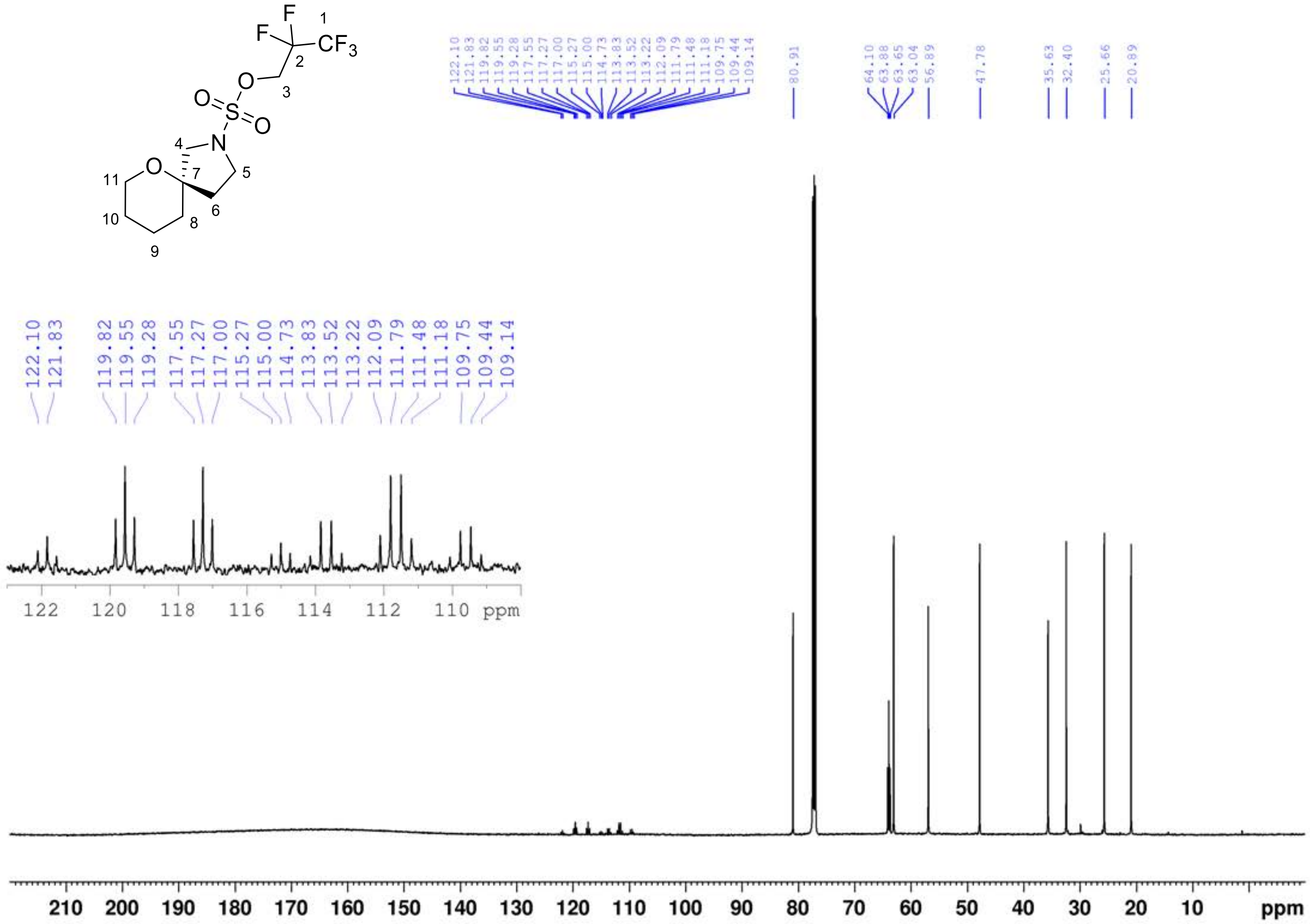

^{19}F NMR (376 MHz, $CDCl_3$) for *2,2,3,3,3-pentafluoropropyl (R)-6-oxa-2-azaspiro[4.5]decane-2-sulfonate* (**30**)

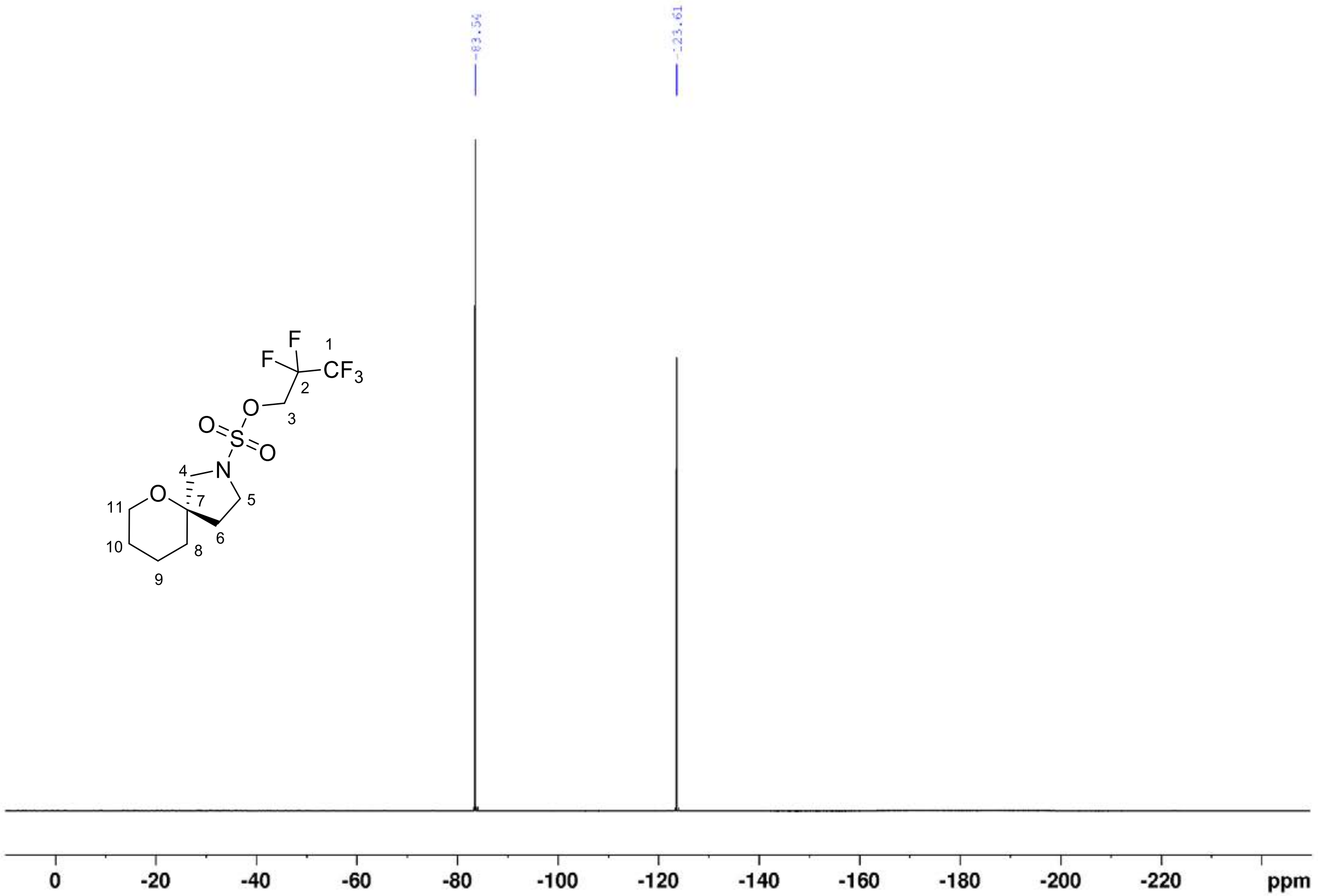

¹H NMR (400 MHz, $CDCl_3$) for *2,2,3,3,3-pentafluoropropyl ((2R,3R)-2-methyl-2-propyltetrahydrofuran-3-yl)sulfamate* (**33a**)

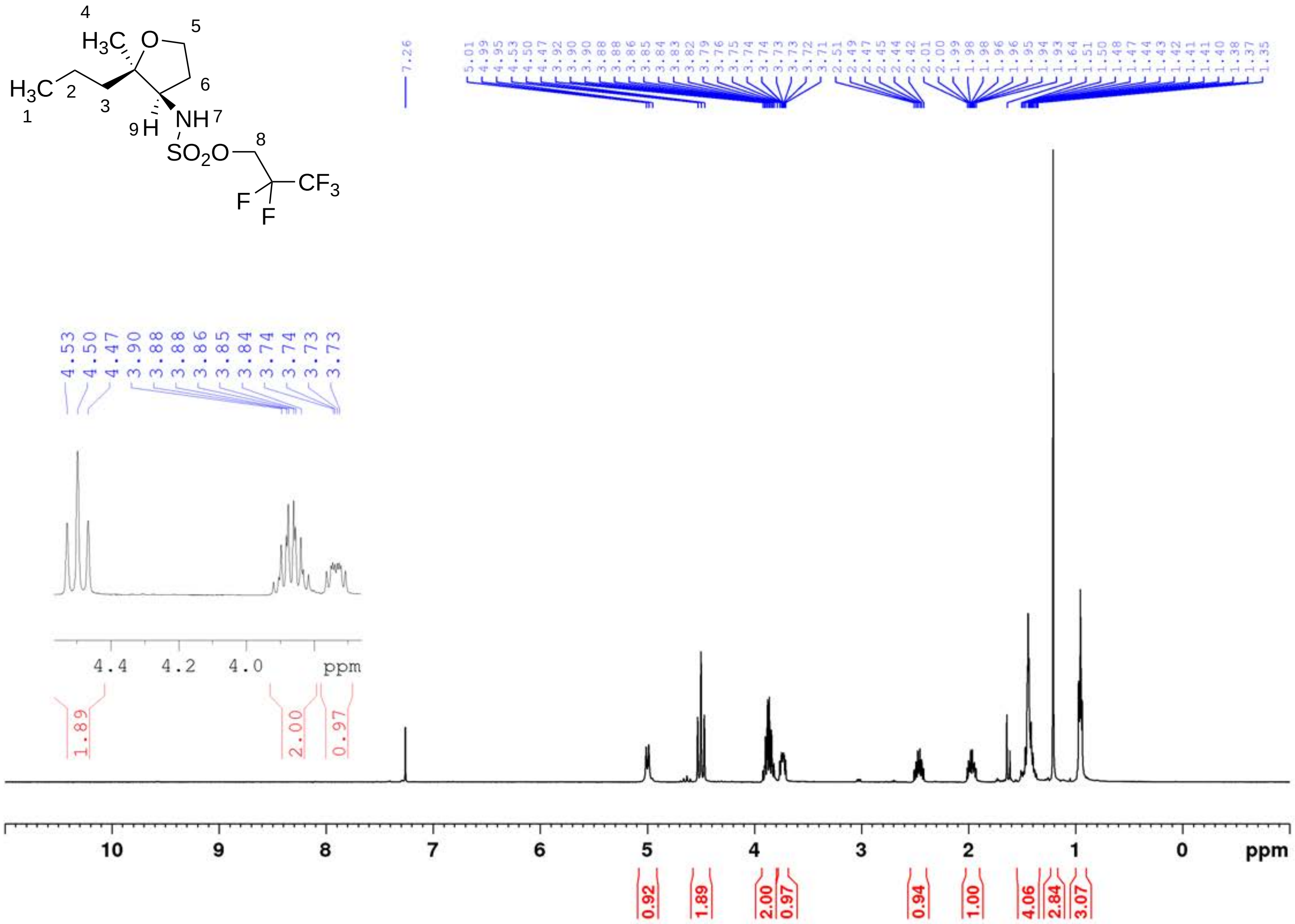

^{13}C NMR (101 MHz, $CDCl_3$) for *2,2,3,3,3-pentafluoropropyl ((2R,3R)-2-methyl-2-propyltetrahydrofuran-3-yl)sulfamate* (**33a**)

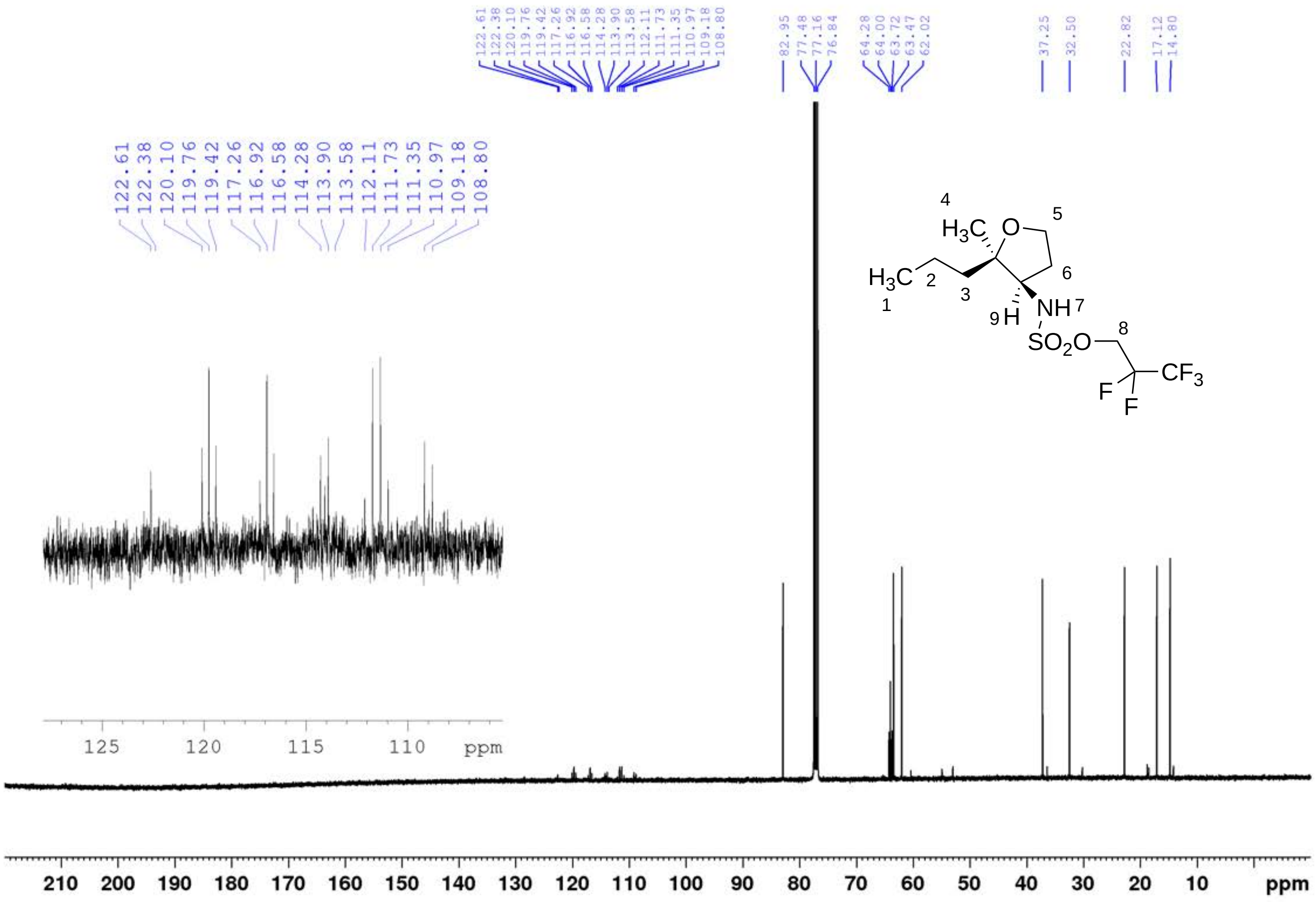

^{19}F NMR (376 MHz, $CDCl_3$) for *2,2,3,3,3-pentafluoropropyl ((2R,3R)-2-methyl-2-propyltetrahydrofuran-3-yl)sulfamate* (**33a**)

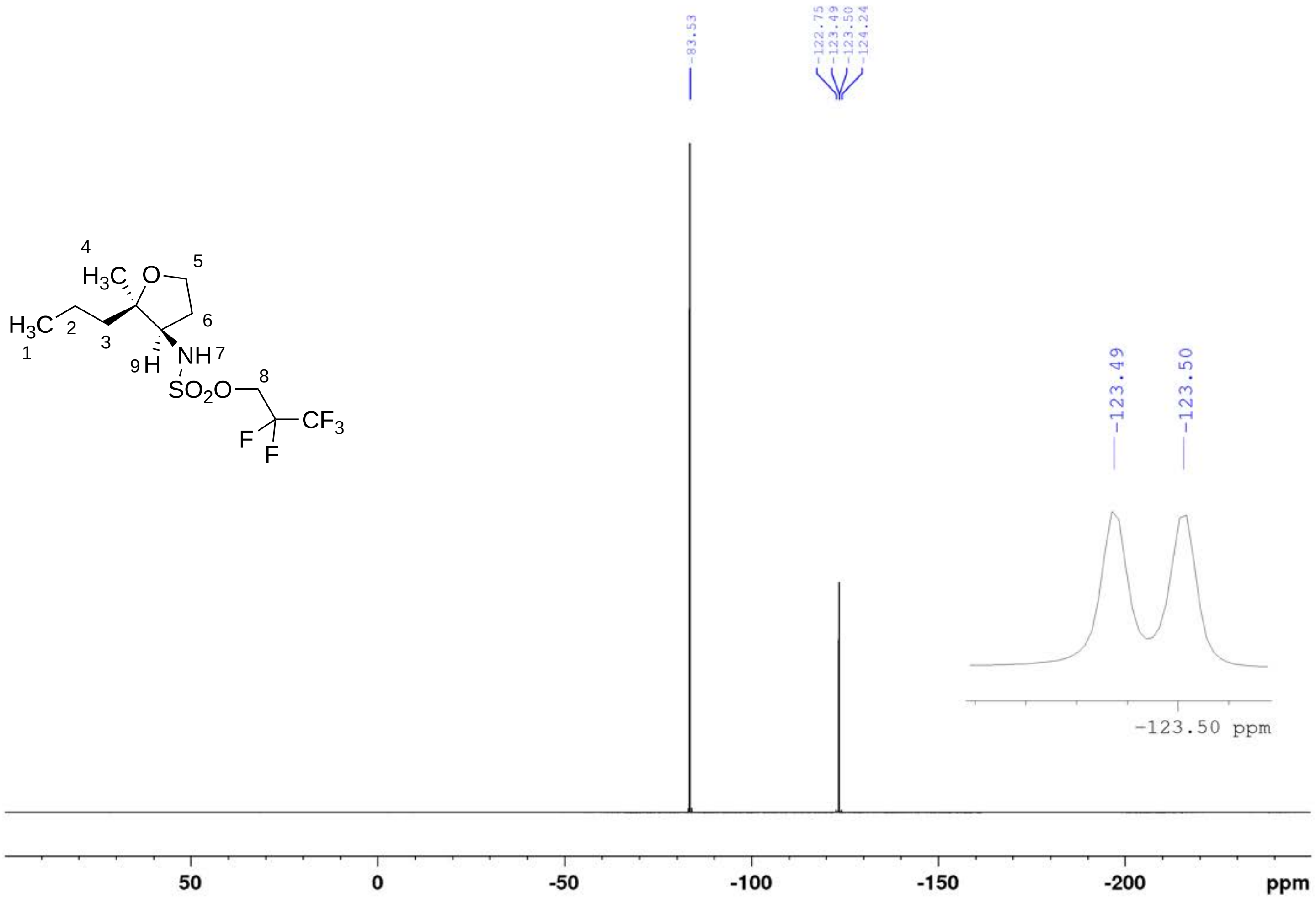

^{1}**H NMR** (400 MHz, $CDCl_3$) for *2,2,3,3,3-pentafluoropropyl (R)-(5-oxaspiro[3.4]octan-8-yl)sulfamate* (**33b**)

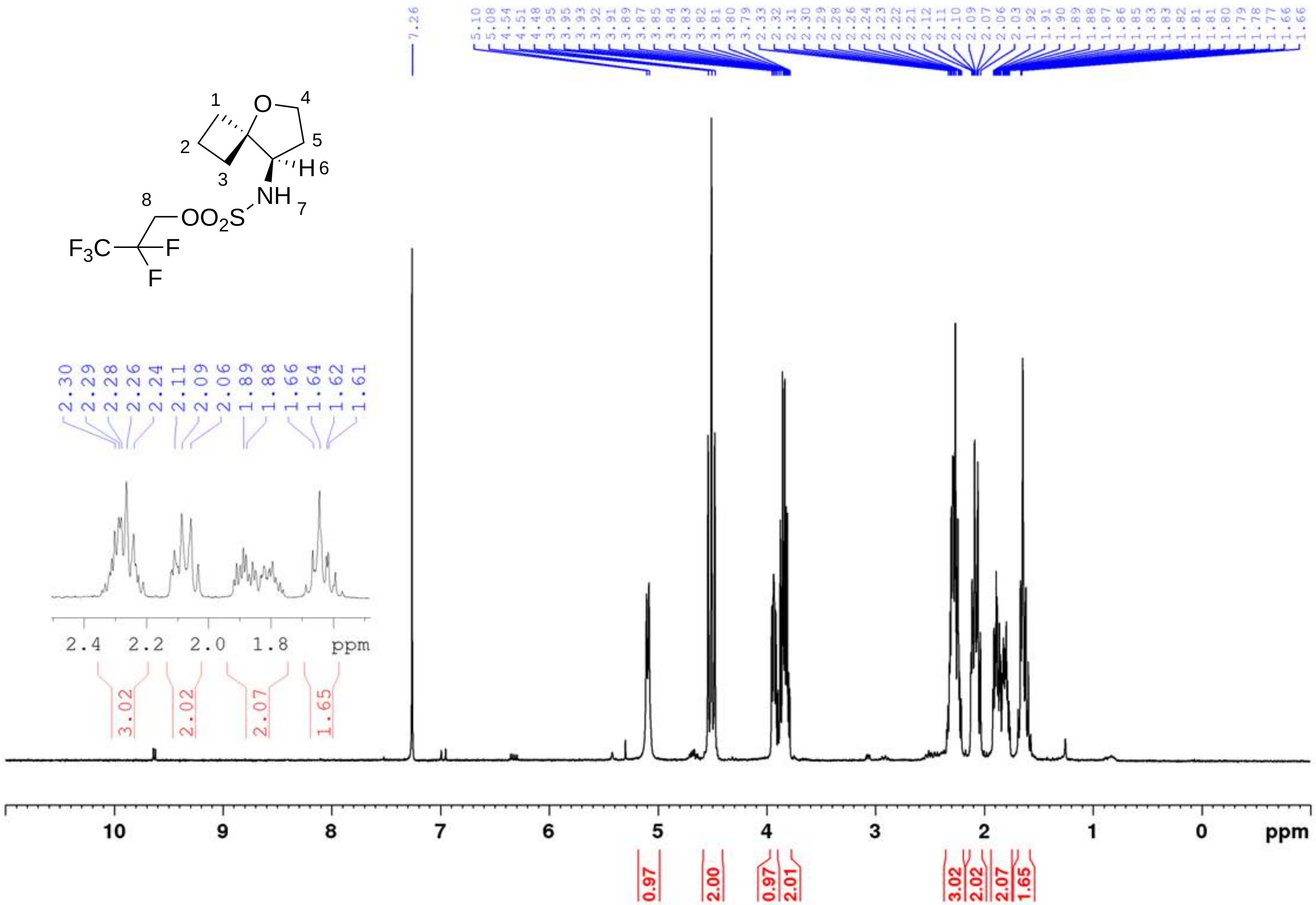

^{13}C NMR (126 MHz, $CDCl_3$) for *2,2,3,3,3-pentafluoropropyl (R)-(5-oxaspiro[3.4]octan-8-yl)sulfamate* (**33b**)

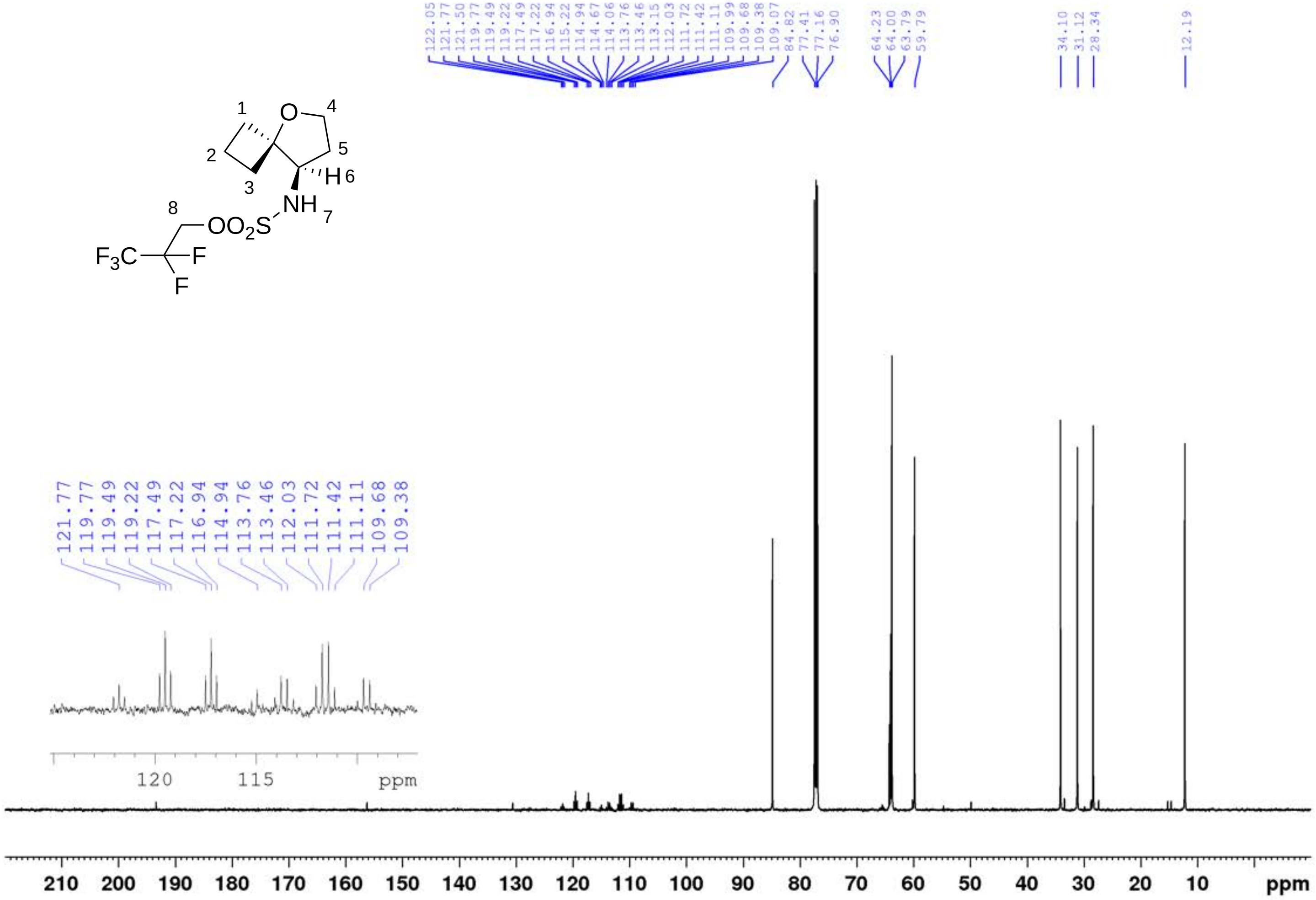

^{19}F NMR (376 MHz, $CDCl_3$) for *2,2,3,3,3-pentafluoropropyl (R)-(5-oxaspiro[3.4]octan-8-yl)sulfamate* (**33b**)

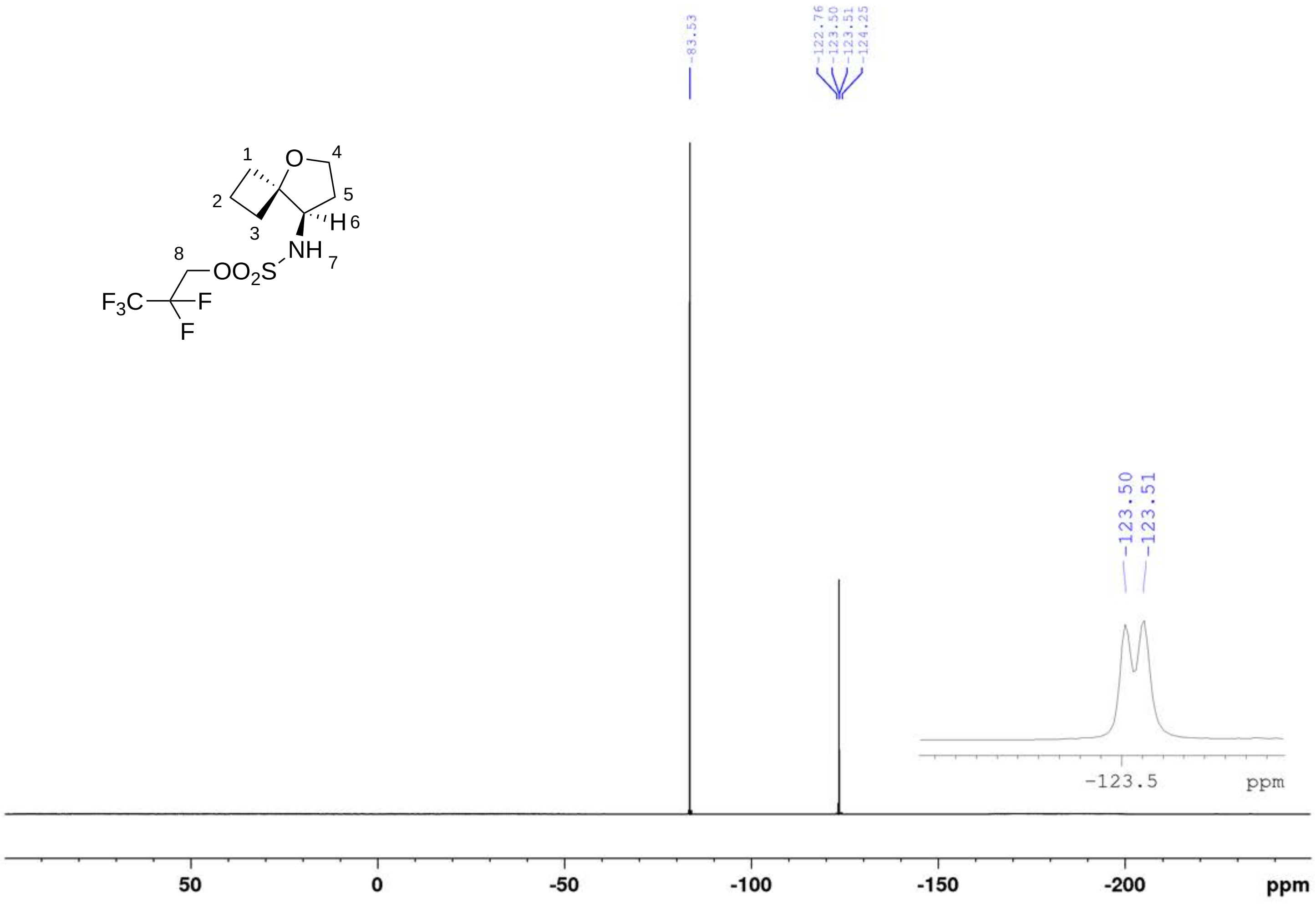

¹H NMR (400 MHz, $CDCl_3$) for *2,2,3,3,3-pentafluoropropyl (R)-(1-oxaspiro[4.5]decan-4-yl)sulfamate* (**33c**)

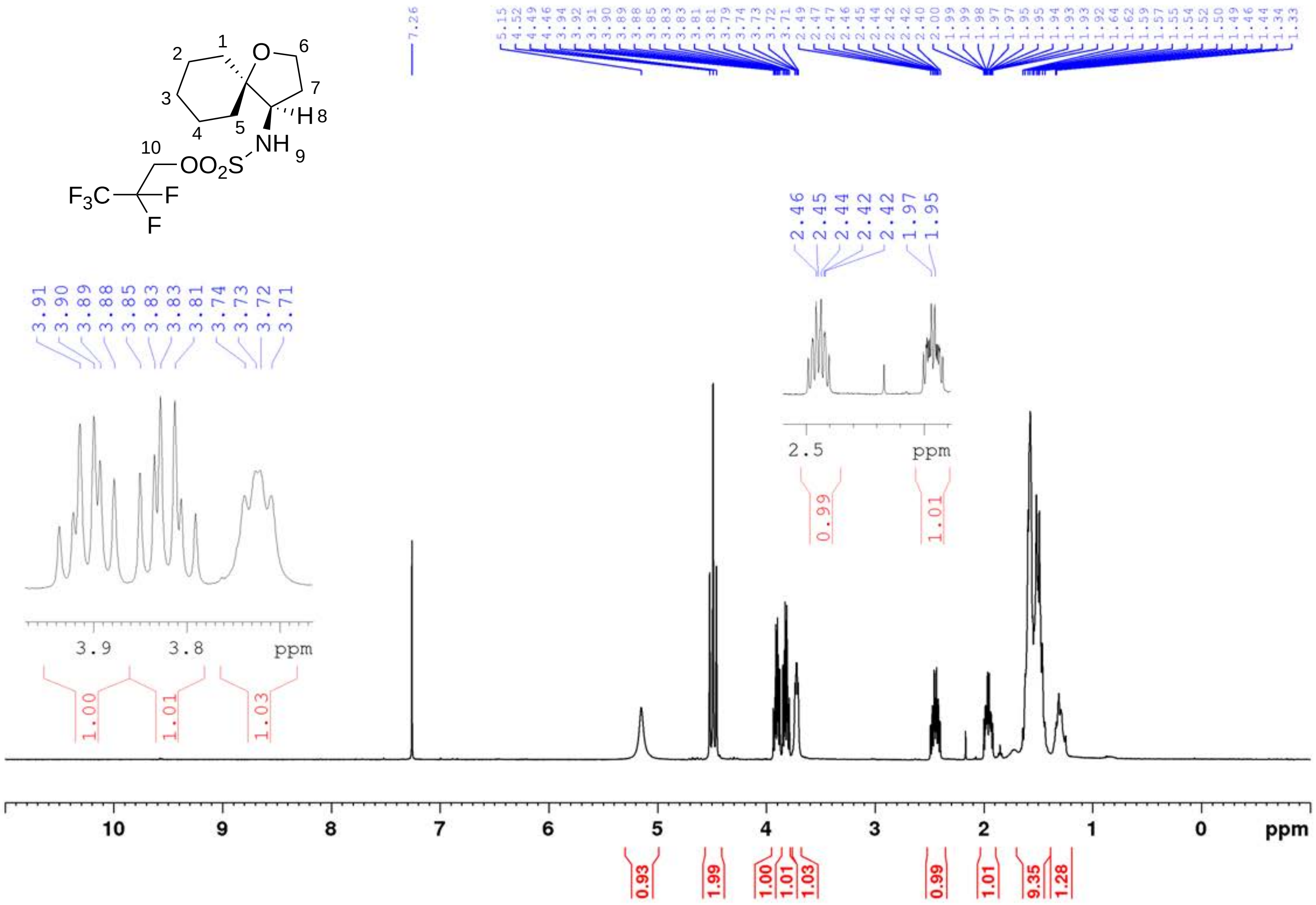

^{13}C NMR (101 MHz, $CDCl_3$) for *2,2,3,3,3-pentafluoropropyl (R)-(1-oxaspiro[4.5]decan-4-yl)sulfamate* (**33c**)

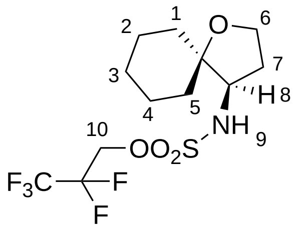

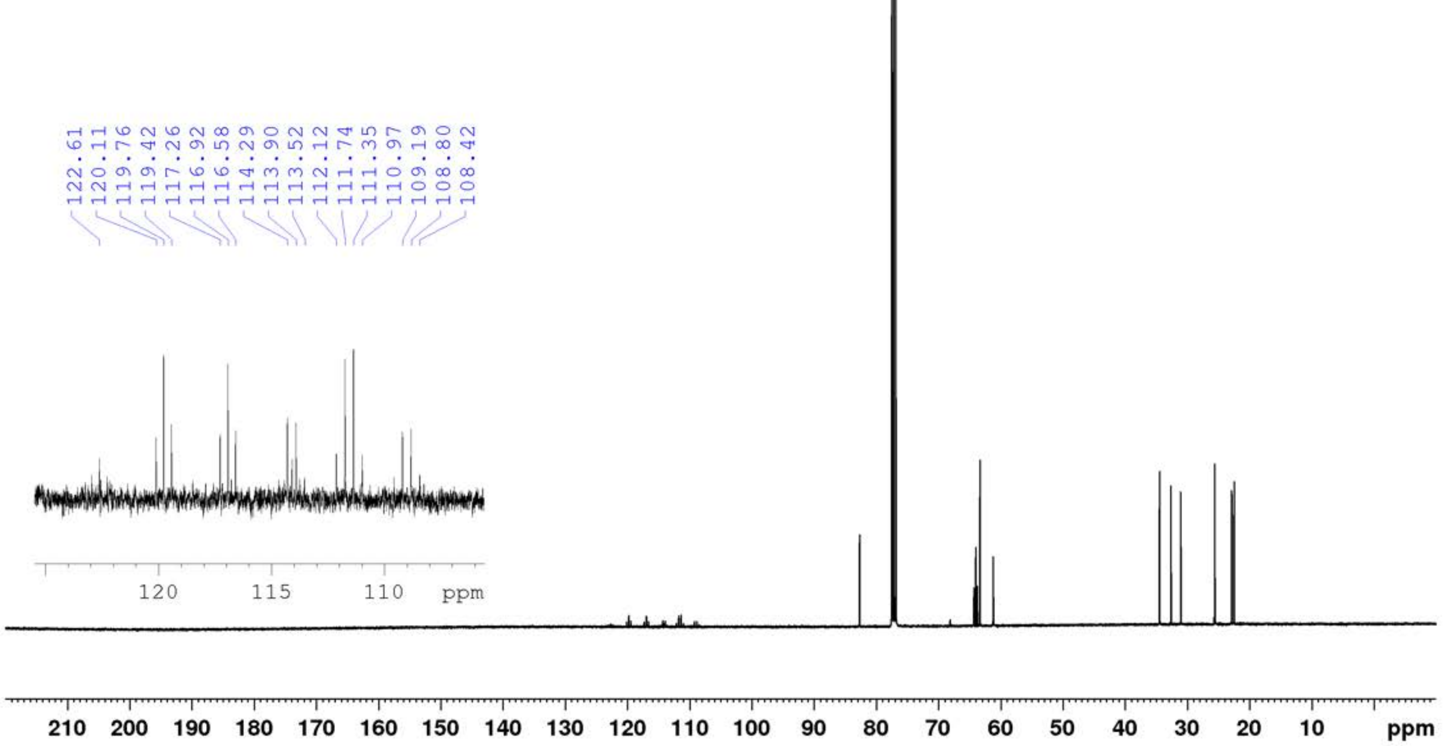

^{19}**F NMR** (376 MHz, $CDCl_3$) for *2,2,3,3,3-pentafluoropropyl (R)-(1-oxaspiro[4.5]decan-4-yl)sulfamate* (**33c**)

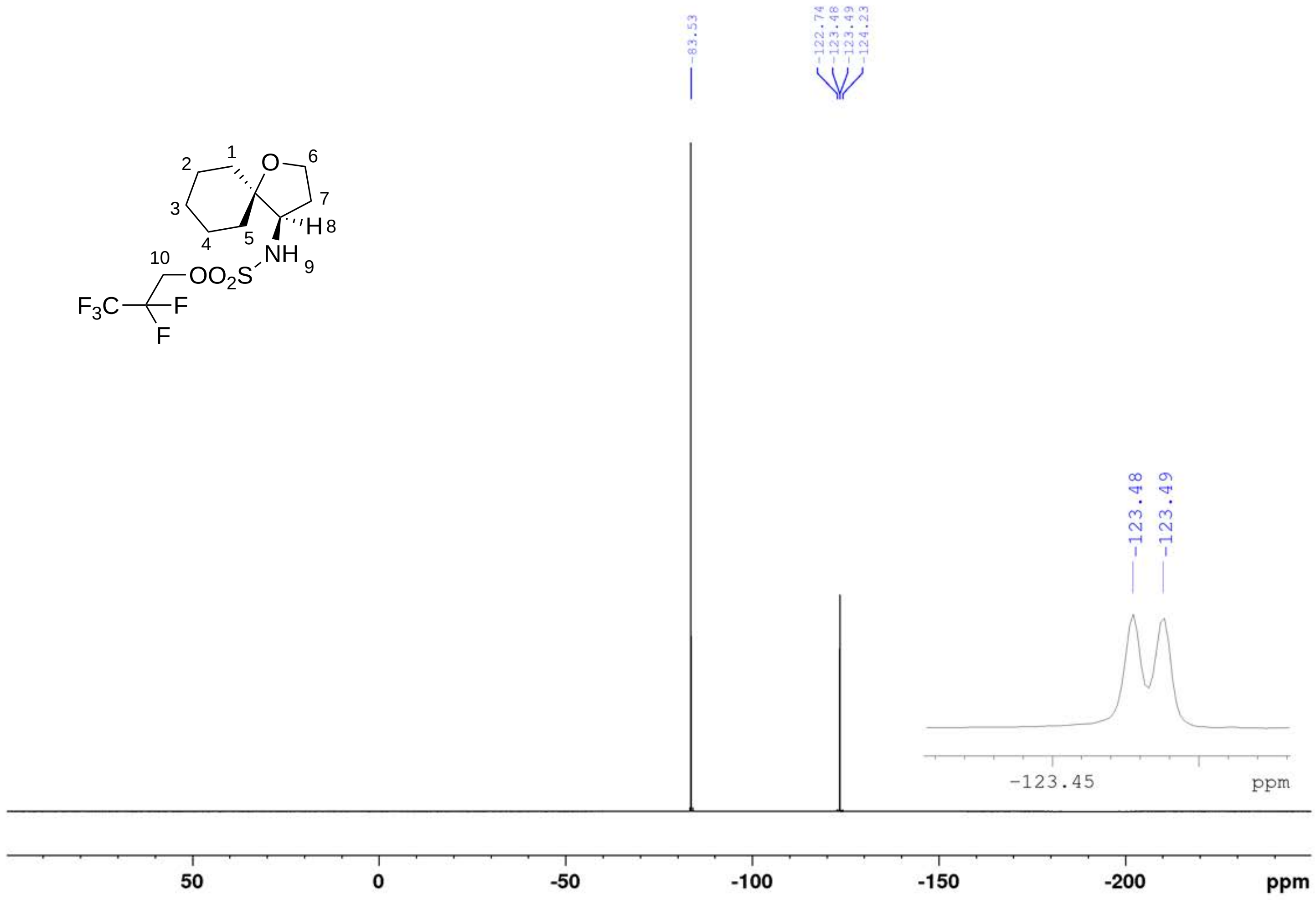

^{1}H NMR (500 MHz, $CDCl_3$) for *2,2,3,3,3-pentafluoropropyl (R)-(4-bromobenzyl)(1-oxaspiro[4.5]decan-4-yl)sulfamate:* ***Obtained following derivatisation of*** **33c**

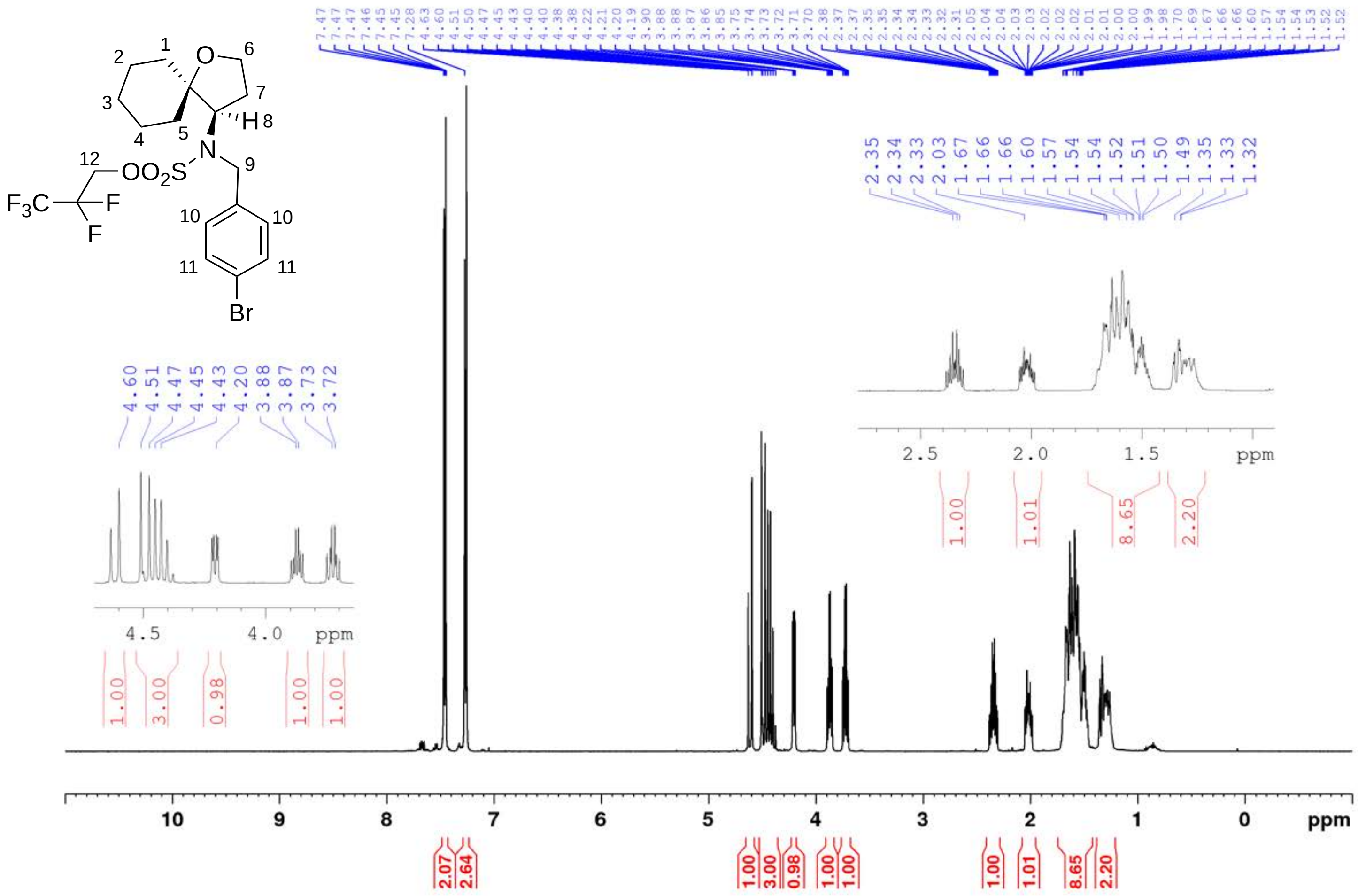

^{13}C NMR (126 MHz, $CDCl_3$) for *2,2,3,3,3-pentafluoropropyl (R)-(4-bromobenzyl)(1-oxaspiro[4.5]decan-4-yl)sulfamate:* ***Obtained following derivatisation of* 33c**

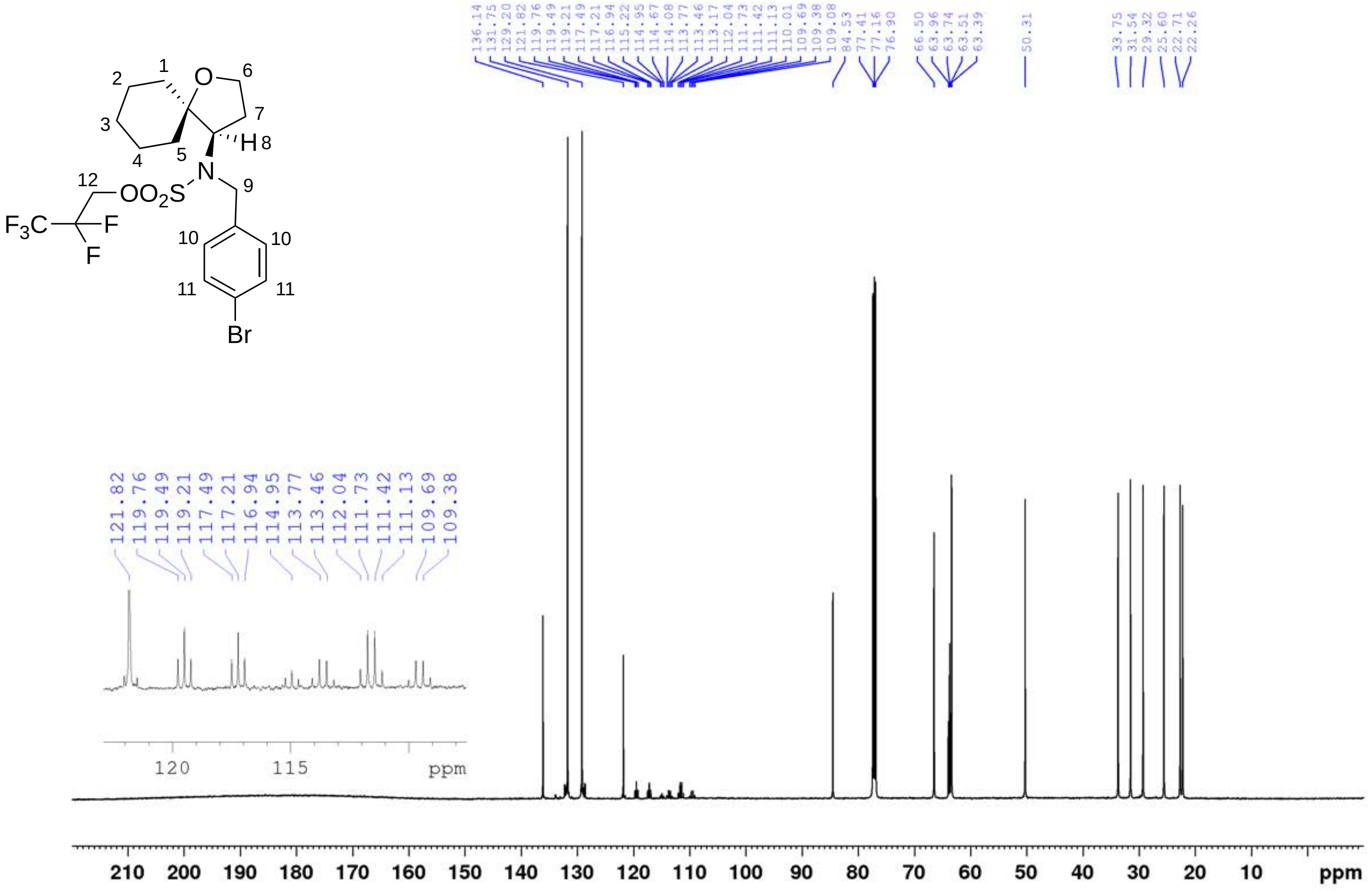

^{19}F NMR (376 MHz, $CDCl_3$) for *2,2,3,3,3-pentafluoropropyl (R)-(4-bromobenzyl)(1-oxaspiro[4.5]decan-4-yl)sulfamate:* ***Obtained following derivatisation of* 33c**

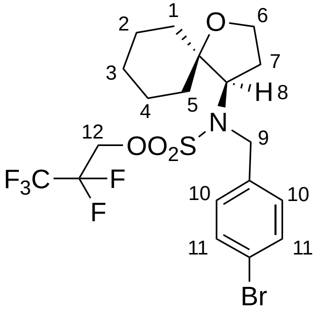

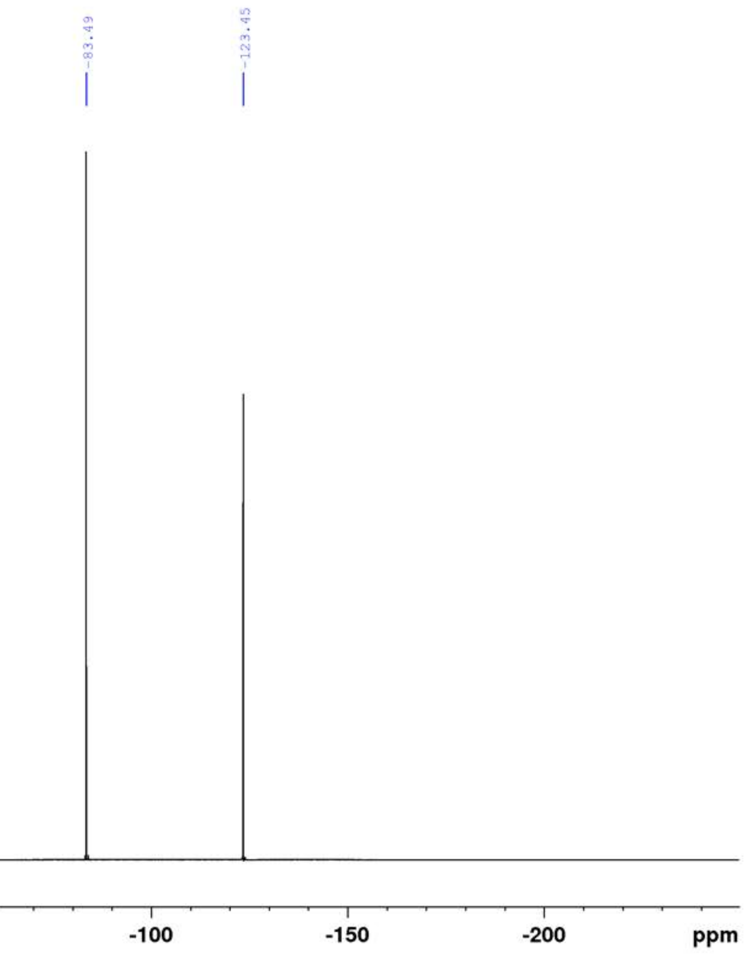

¹H NMR (500 MHz, $CDCl_3$) for *2,2,3,3,3-pentafluoropropyl (R)-(1,8-dioxaspiro[4.5]decan-4-yl)sulfamate* (**33d**)

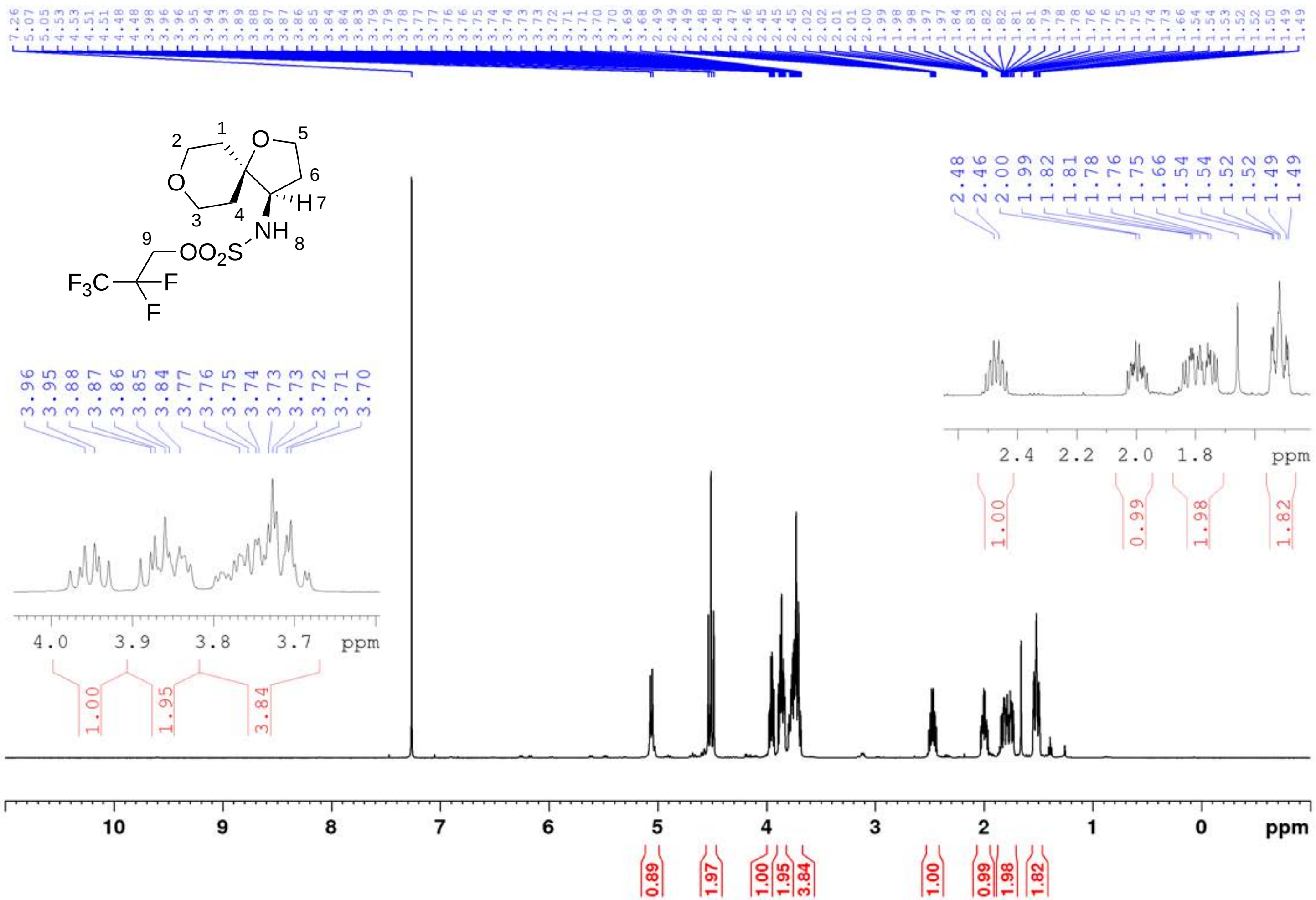

^{13}C NMR (126 MHz, $CDCl_3$) for *2,2,3,3,3-pentafluoropropyl (R)-(1,8-dioxaspiro[4.5]decan-4-yl)sulfamate* (**33d**)

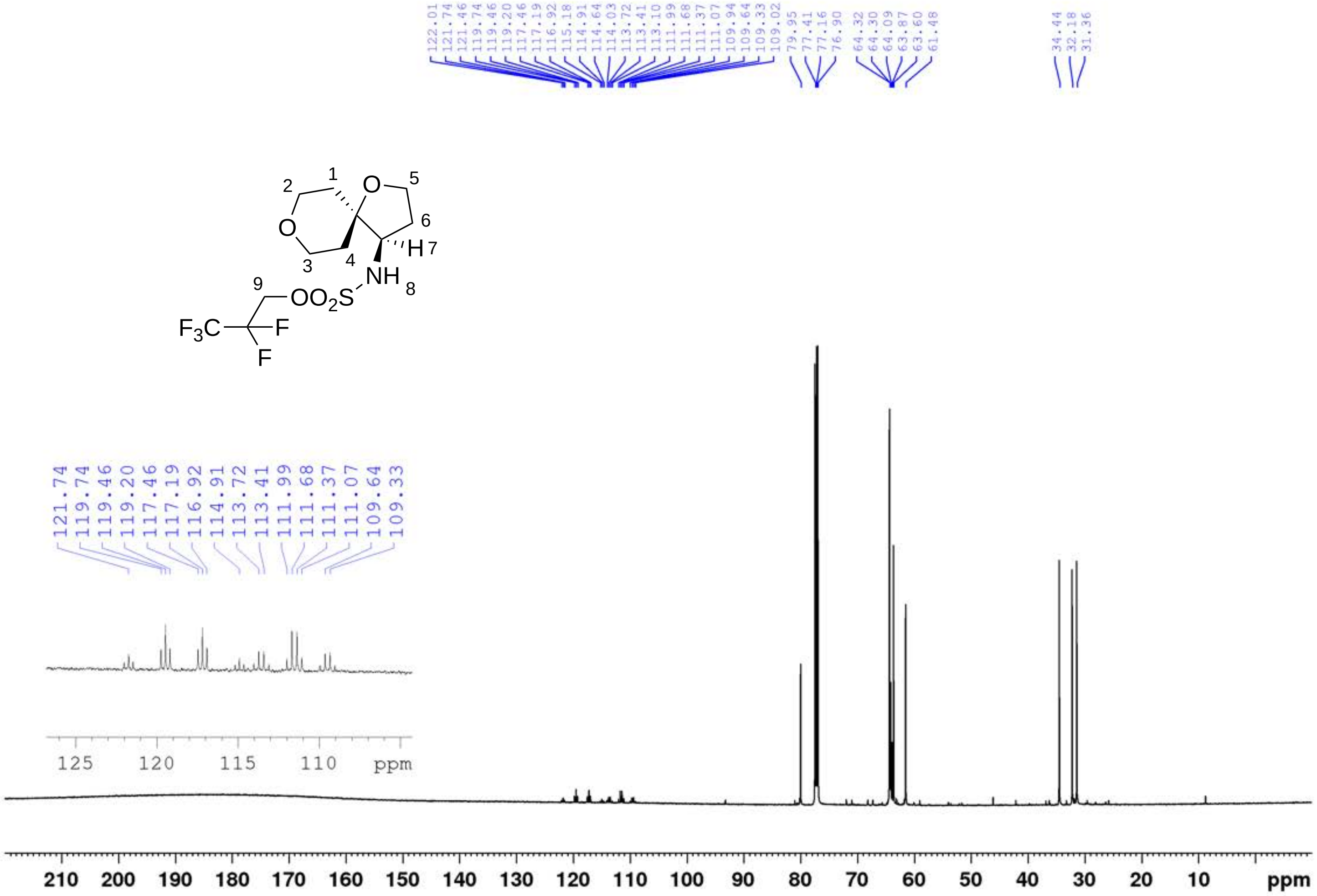

^{19}F NMR (376 MHz, $CDCl_3$) for *2,2,3,3,3-pentafluoropropyl (R)-(1,8-dioxaspiro[4.5]decan-4-yl)sulfamate* (**33d**)

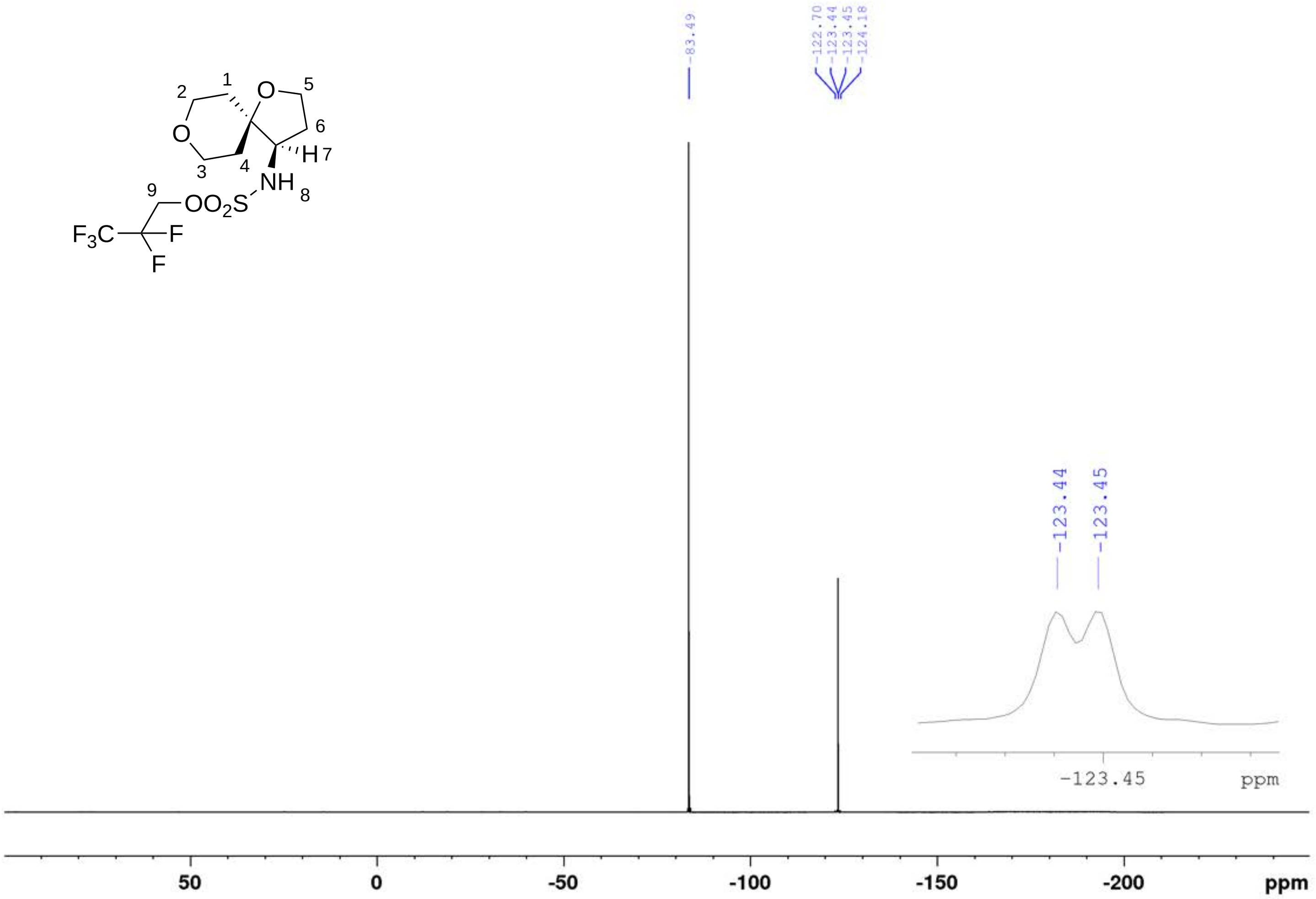

^{1}H NMR (500 MHz, $CDCl_3$) for *2,2,3,3,3-pentafluoropropyl (R)-(4-bromobenzyl)(1,8-dioxaspiro[4.5]decan-4-yl)sulfamate:* ***Obtained following derivatisation of*** **33d**

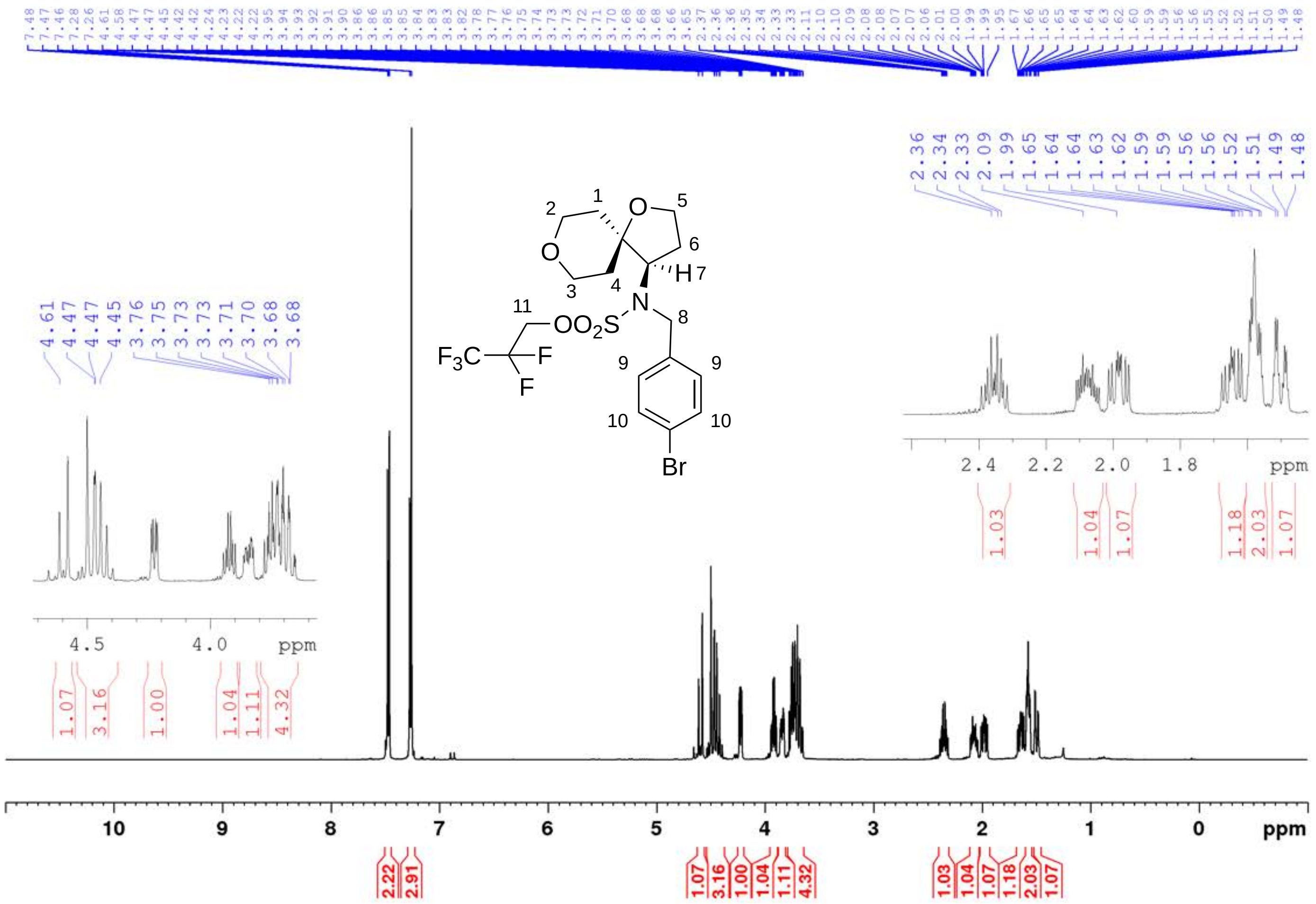

^{13}C NMR (126 MHz, $CDCl_3$) for *2,2,3,3,3-pentafluoropropyl (R)-(4-bromobenzyl)(1,8-dioxaspiro[4.5]decan-4-yl)sulfamate: **Obtained following derivatisation of*** **33d**

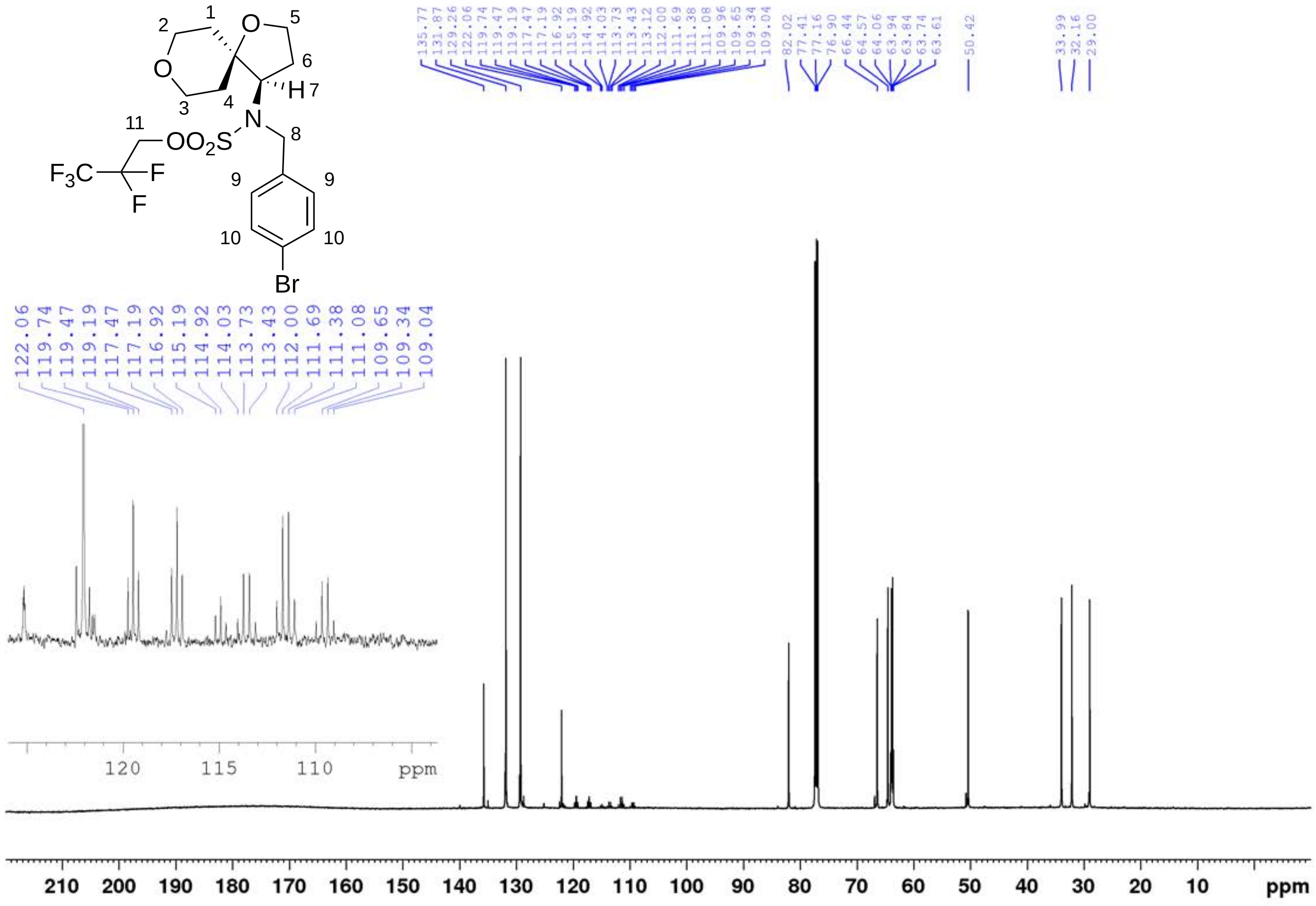

^{19}**F NMR** (376 MHz, $CDCl_3$) for *2,2,3,3,3-pentafluoropropyl (R)-(4-bromobenzyl)(1,8-dioxaspiro[4.5]decan-4-yl)sulfamate:* ***Obtained following derivatisation of*** **33d**

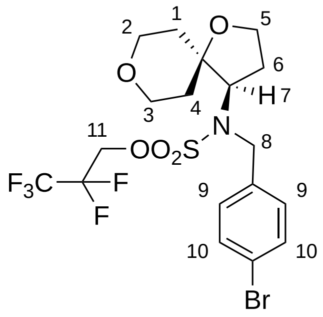

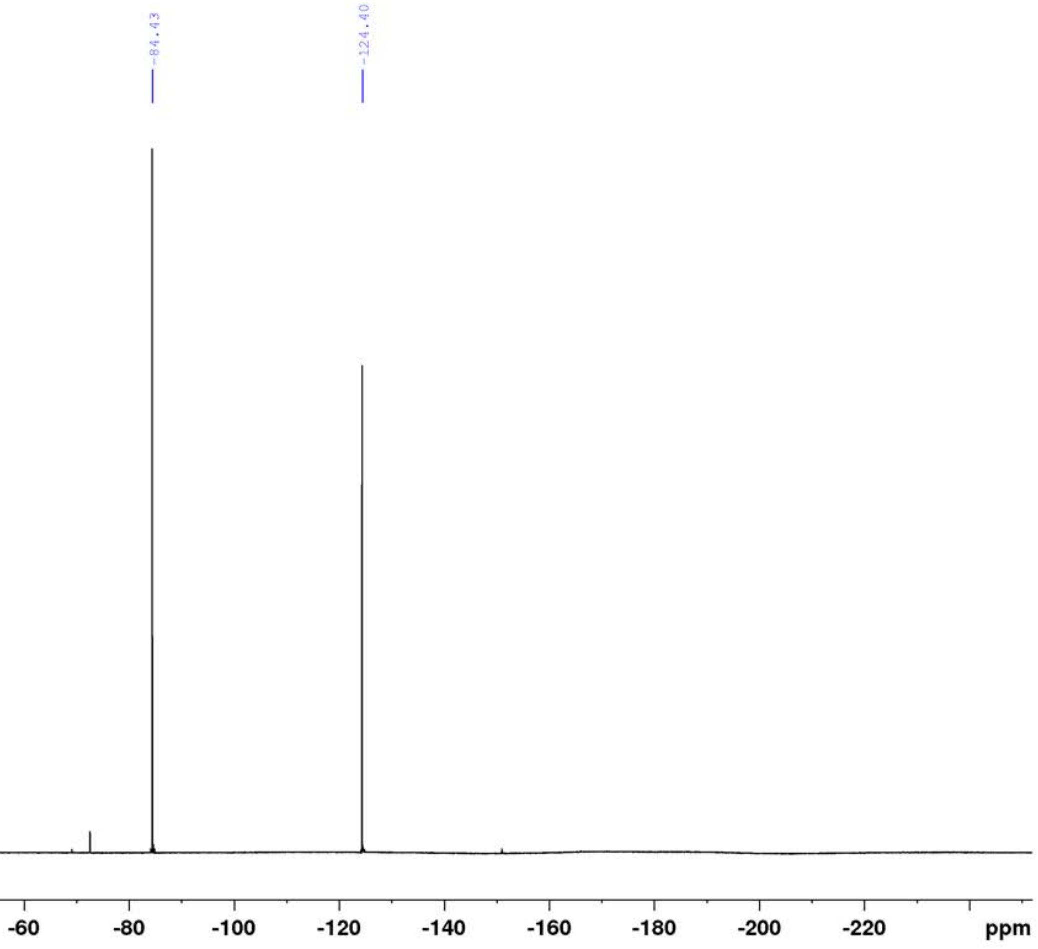

^{1}H NMR (500 MHz, $CDCl_3$) for *2,2,3,3,3-pentafluoropropyl (R)-(2,2-dimethyltetrahydro-2H-pyran-3-yl)sulfamate* (**33e**)

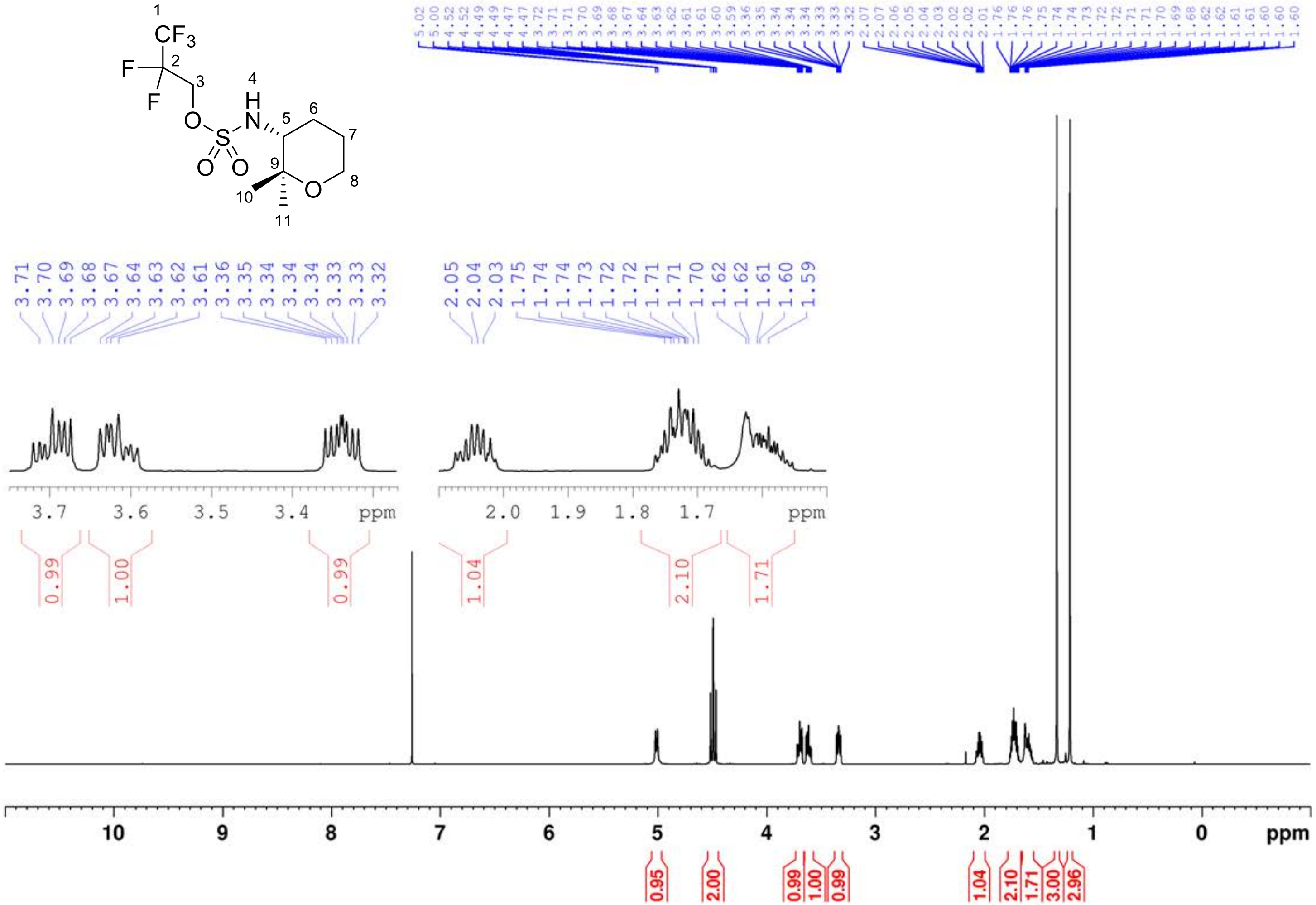

^{13}C NMR (126 MHz, $CDCl_3$) for *2,2,3,3,3-pentafluoropropyl (R)-(2,2-dimethyltetrahydro-2H-pyran-3-yl)sulfamate* (**33e**)

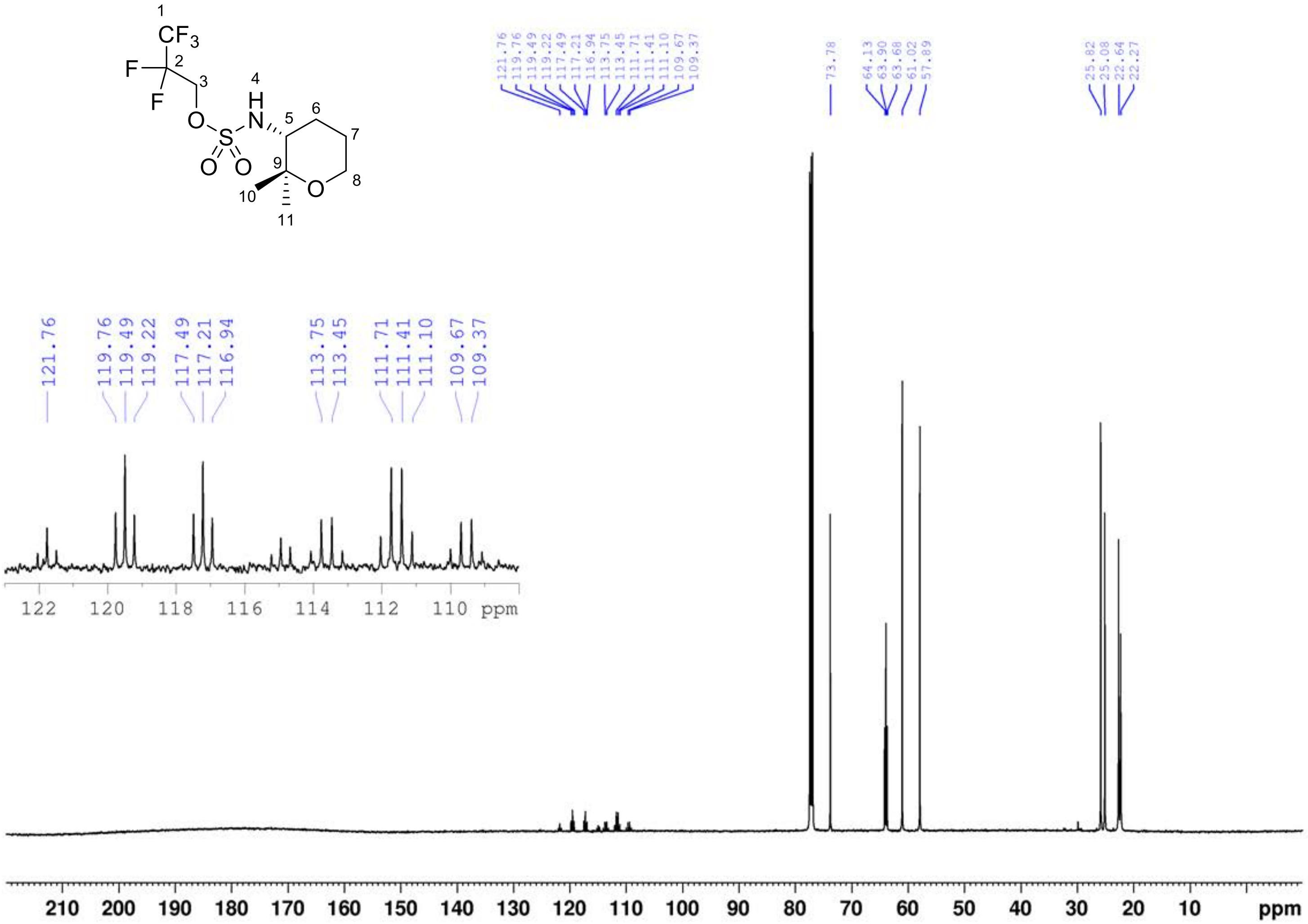

^{19}F NMR (376 MHz, $CDCl_3$) for *2,2,3,3,3-pentafluoropropyl (R)-(2,2-dimethyltetrahydro-2H-pyran-3-yl)sulfamate* (**33e**)

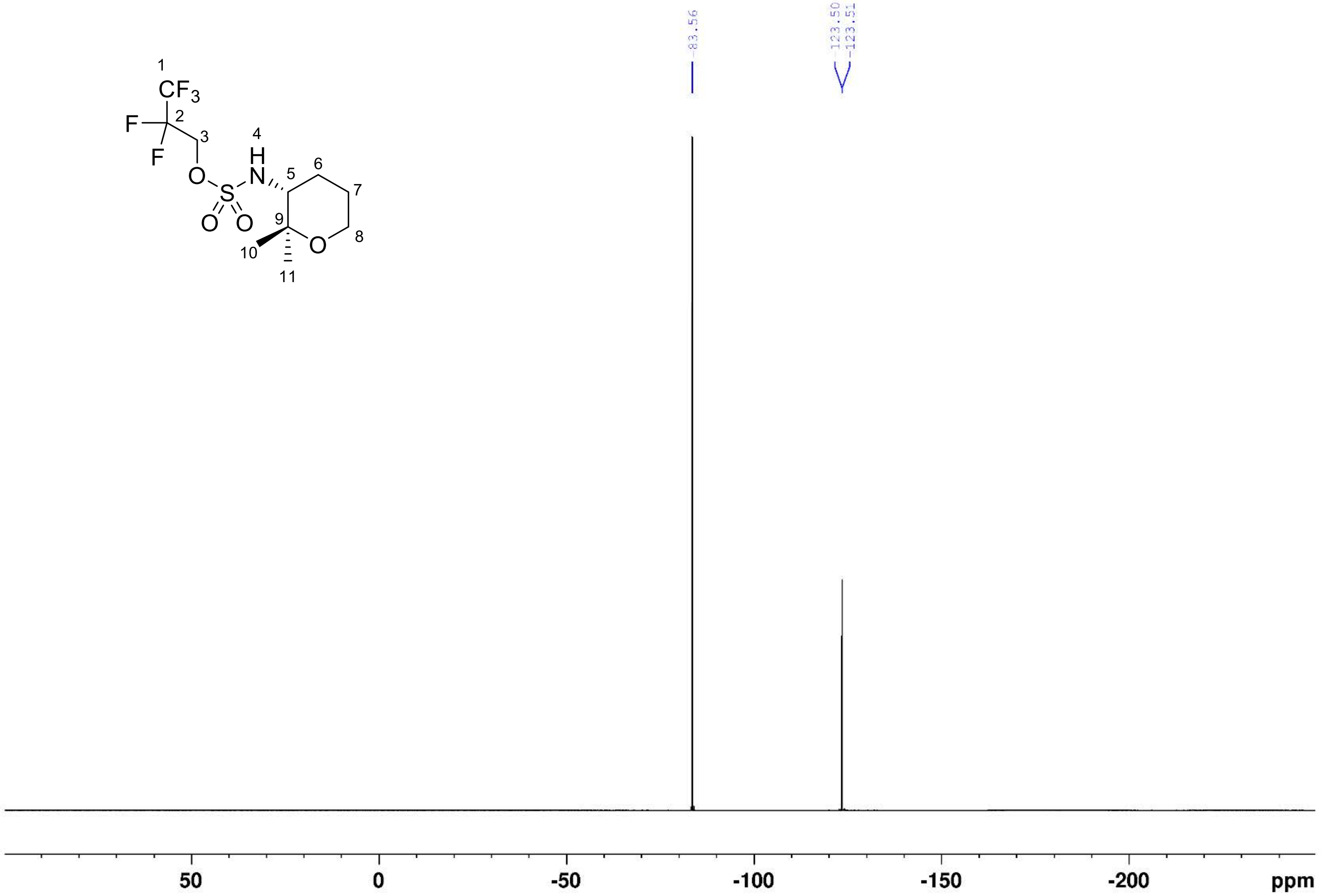

^{1}H NMR (500 MHz, CDCl$_3$) for *2,2,3,3,3-pentafluoropropyl (R)-(1-oxaspiro[5.5]undecan-5-yl)sulfamate* (**33f**)

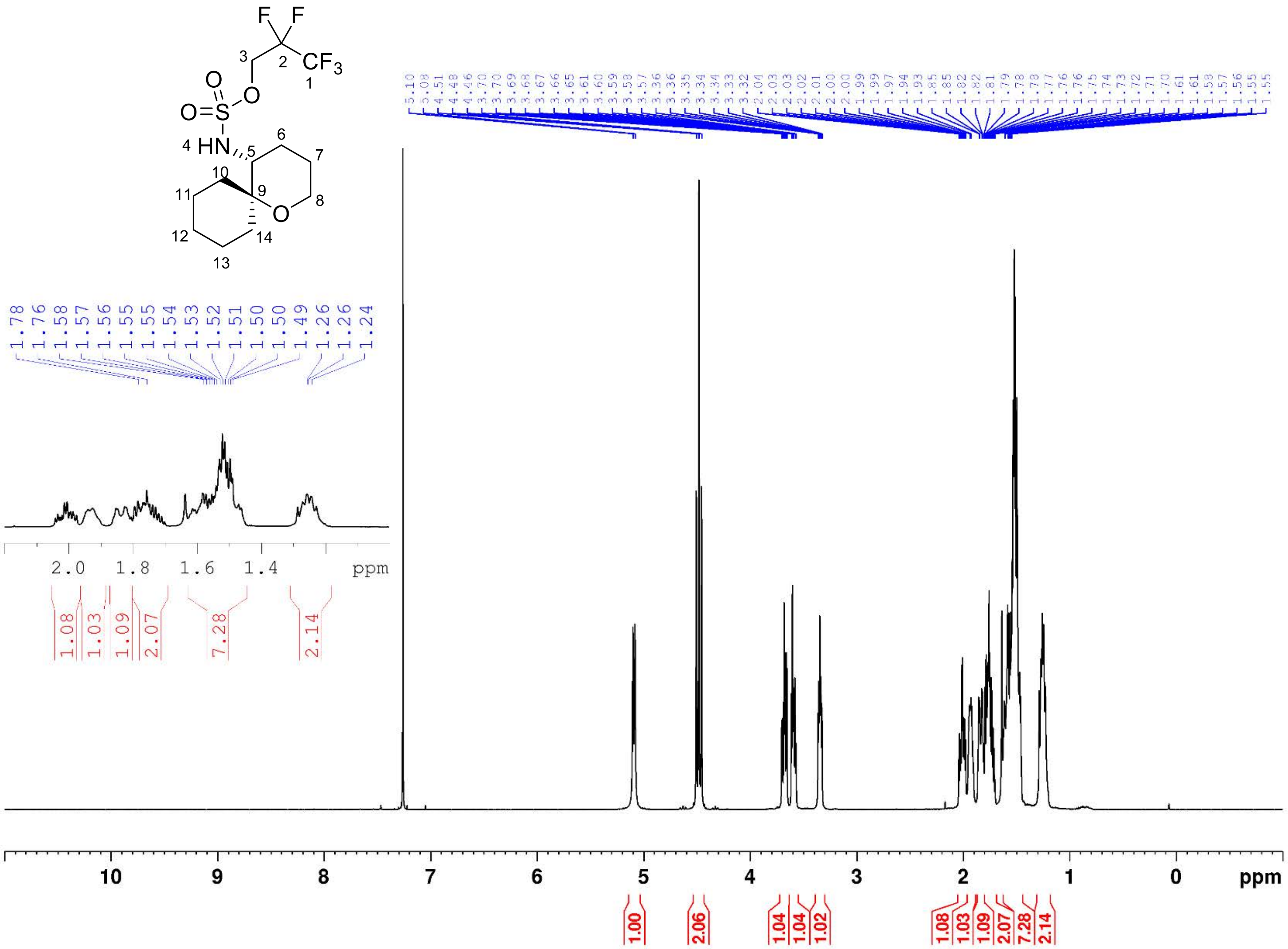

^{13}C NMR (126 MHz, CDCl$_3$) for *2,2,3,3,3-pentafluoropropyl (R)-(1-oxaspiro[5.5]undecan-5-yl)sulfamate* (**33f**)

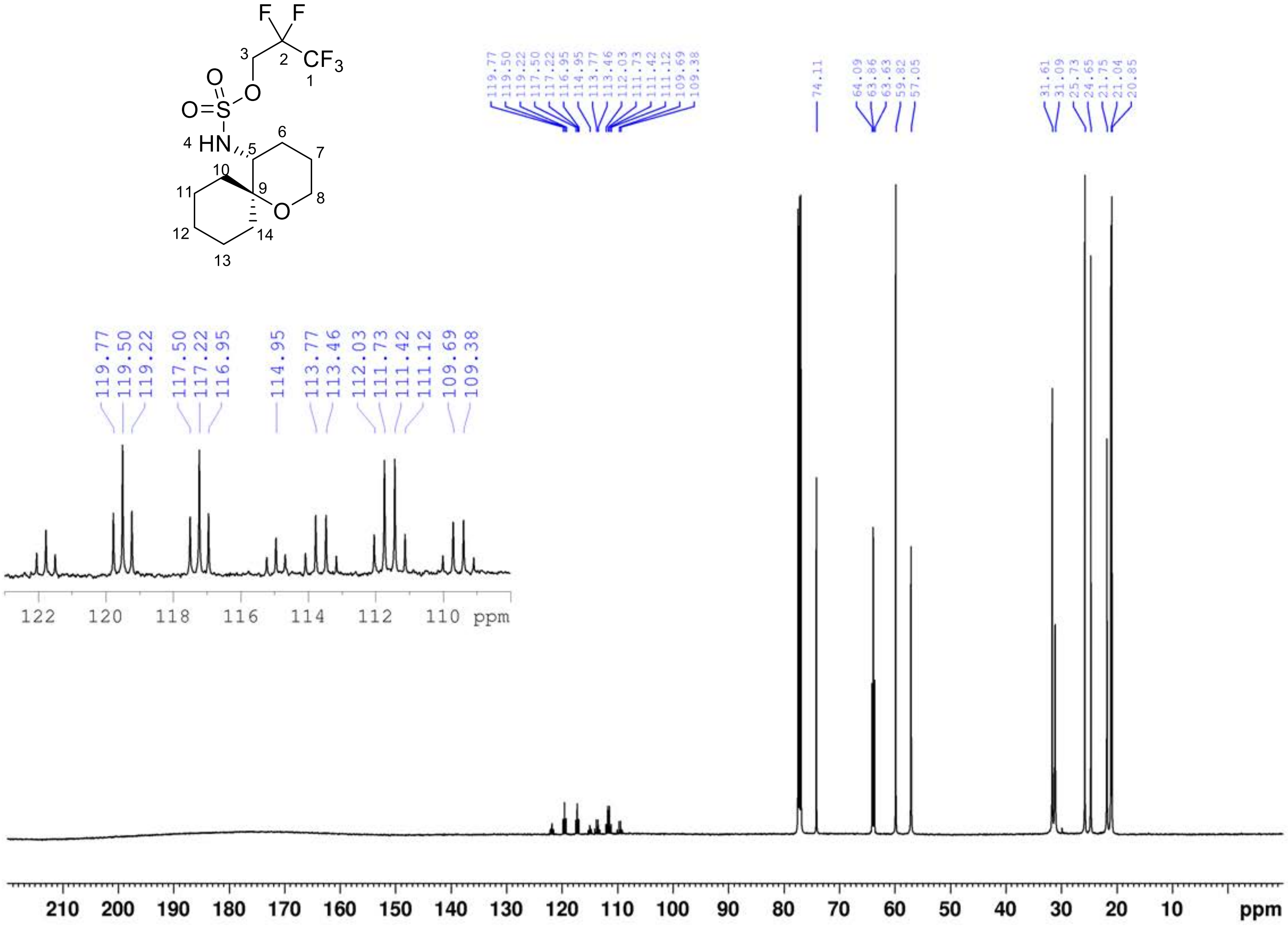

^{19}F NMR (376 MHz, CDCl$_3$) for *2,2,3,3,3-pentafluoropropyl (R)-(1-oxaspiro[5.5]undecan-5-yl)sulfamate* (**33f**)

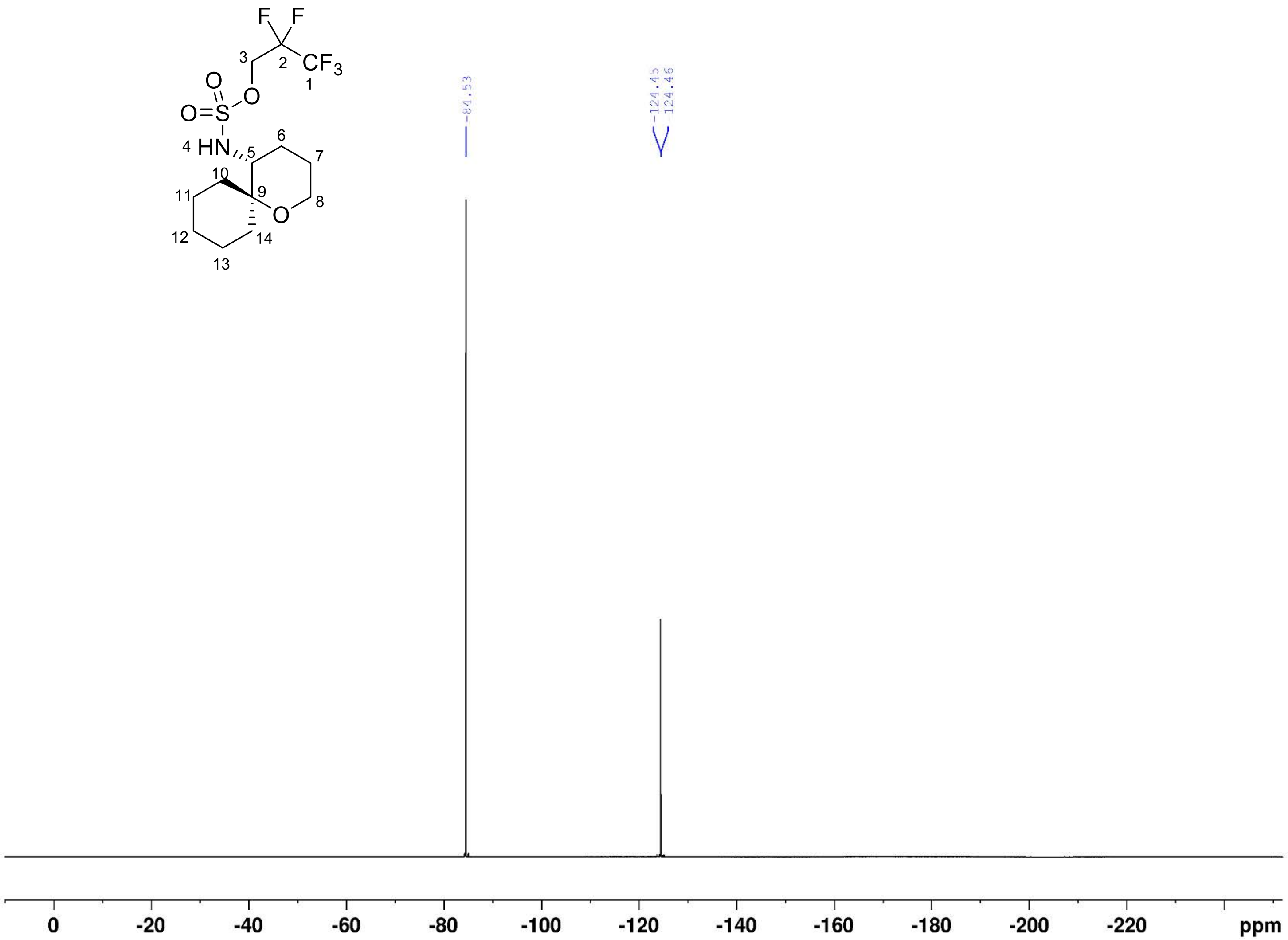

^{1}H NMR (500 MHz, $CDCl_3$) for *2,2,3,3,3-pentafluoropropyl (R)-(4-bromobenzyl)(1-oxaspiro[5.5]undecan-5-yl)sulfamate:* ***Obtained following derivatisation of* 33f**

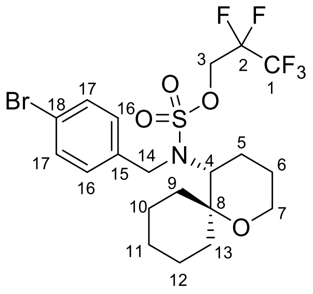

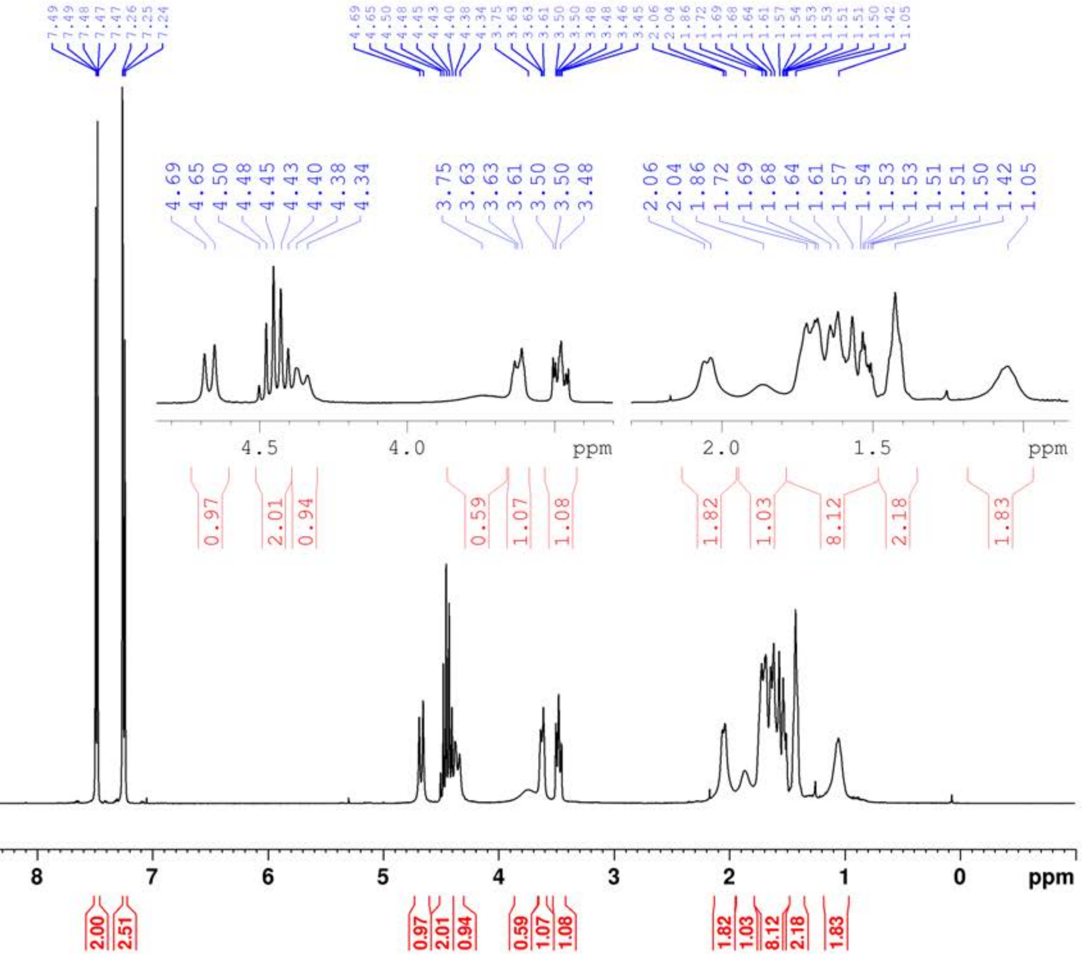

^{13}C NMR (126 MHz, CDCl$_3$) for *2,2,3,3,3-pentafluoropropyl (R)-(4-bromobenzyl)(1-oxaspiro[5.5]undecan-5-yl)sulfamate:* ***Obtained following derivatisation of* 33f**

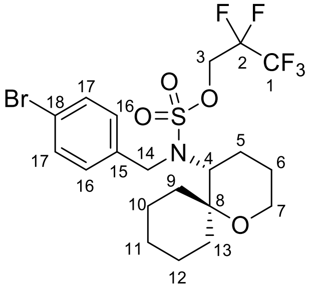

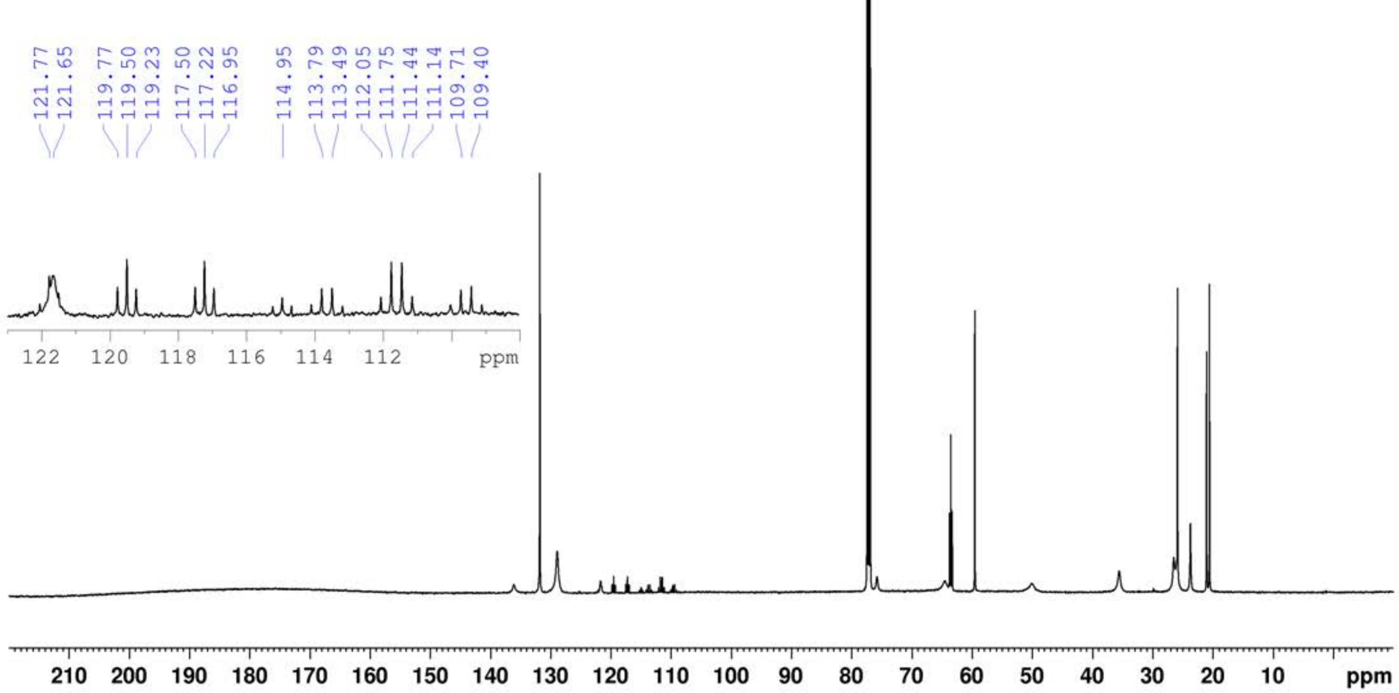

^{19}F NMR (376 MHz, $CDCl_3$) for *2,2,3,3,3-pentafluoropropyl (R)-(4-bromobenzyl)(1-oxaspiro[5.5]undecan-5-yl)sulfamate:* ***Obtained following derivatisation of*** **33f**

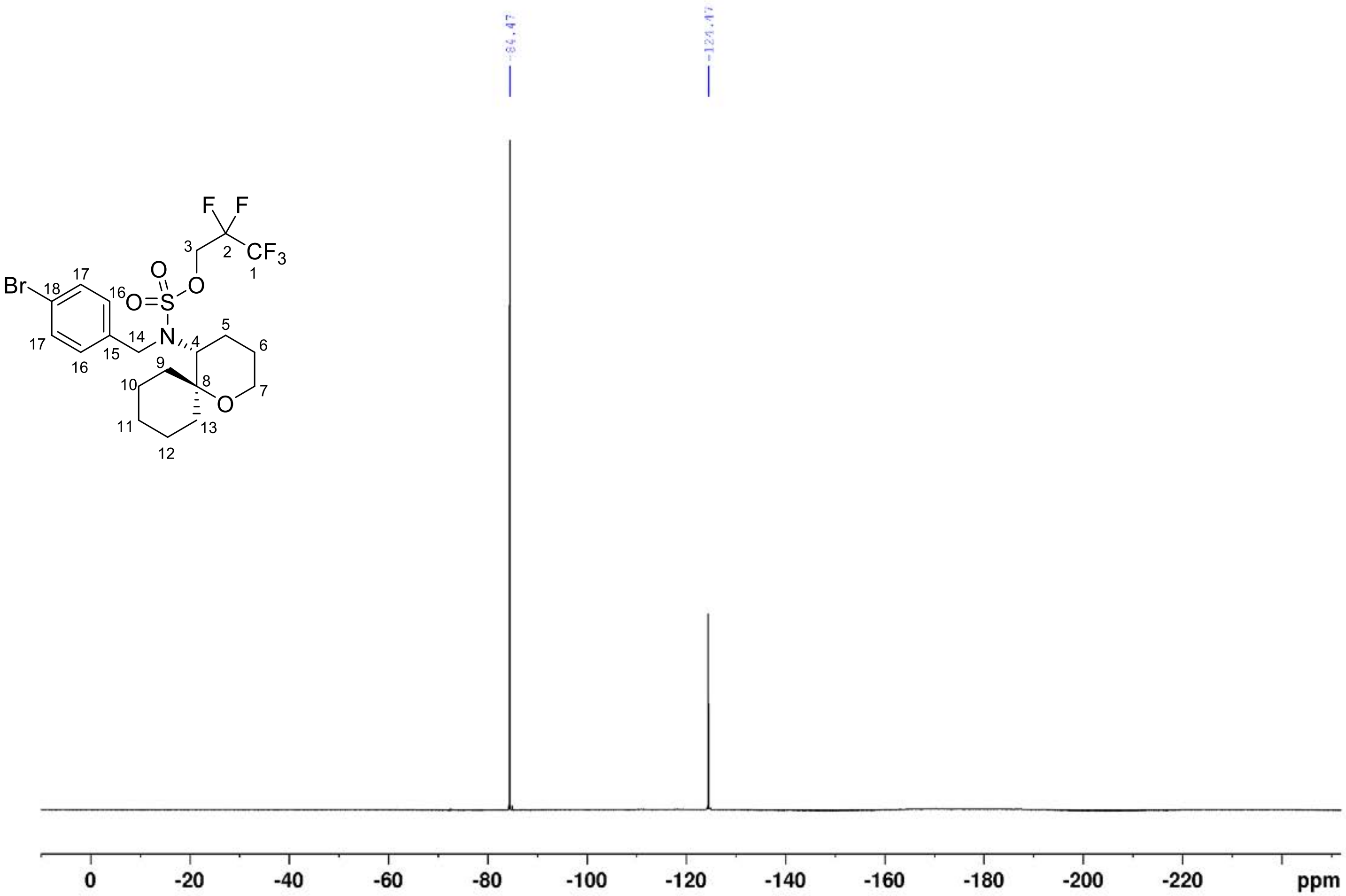

^{1}H NMR (500 MHz, $CDCl_3$) for *2,2,3,3,3-pentafluoropropyl ((2R,3R)-2-methyl-2-phenyltetrahydrofuran-3-yl)sulfamate* (**33g**)

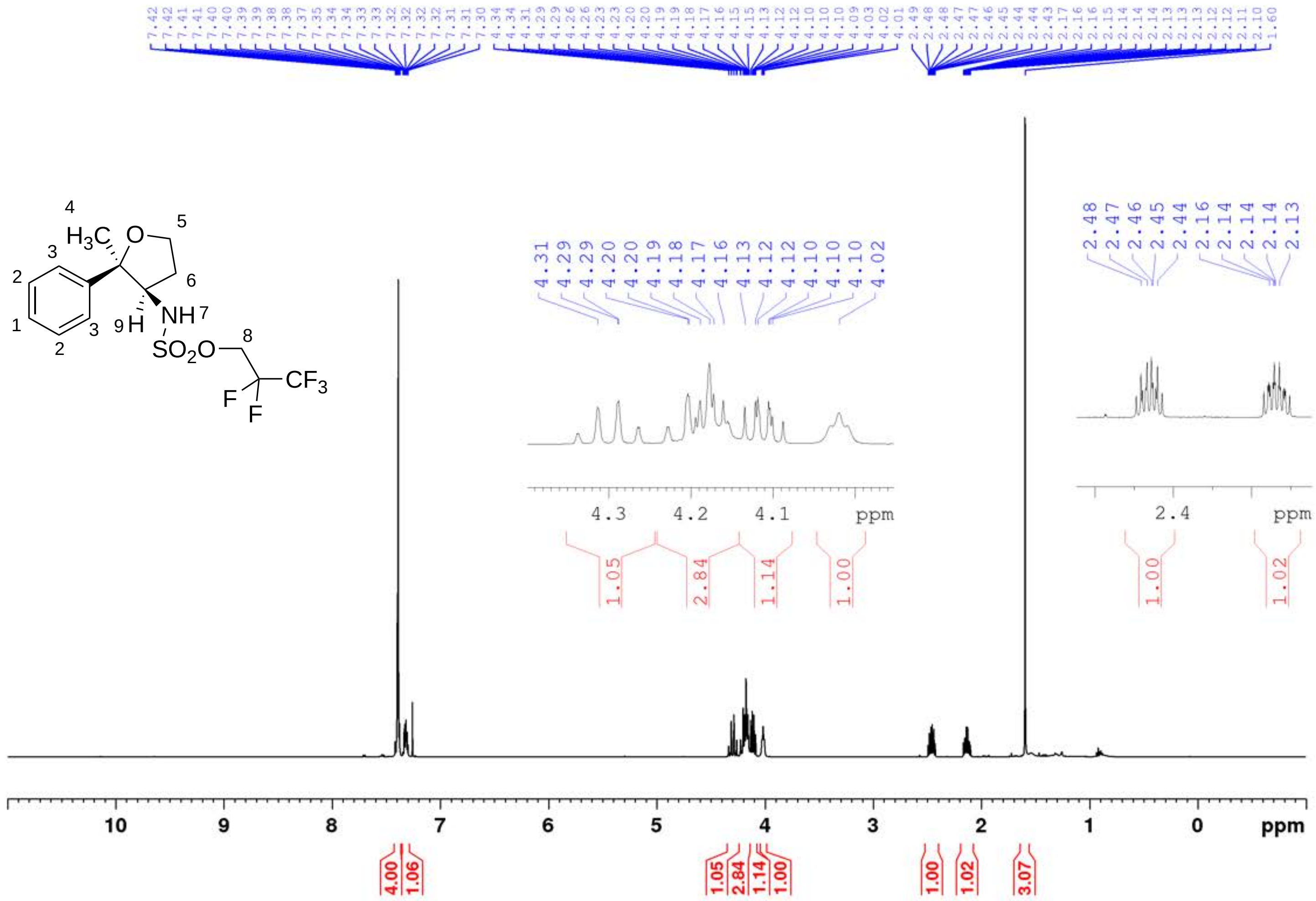

^{13}C NMR (126 MHz, $CDCl_3$) for *2,2,3,3,3-pentafluoropropyl ((2R,3R)-2-methyl-2-phenyltetrahydrofuran-3-yl)sulfamate* (**33g**)

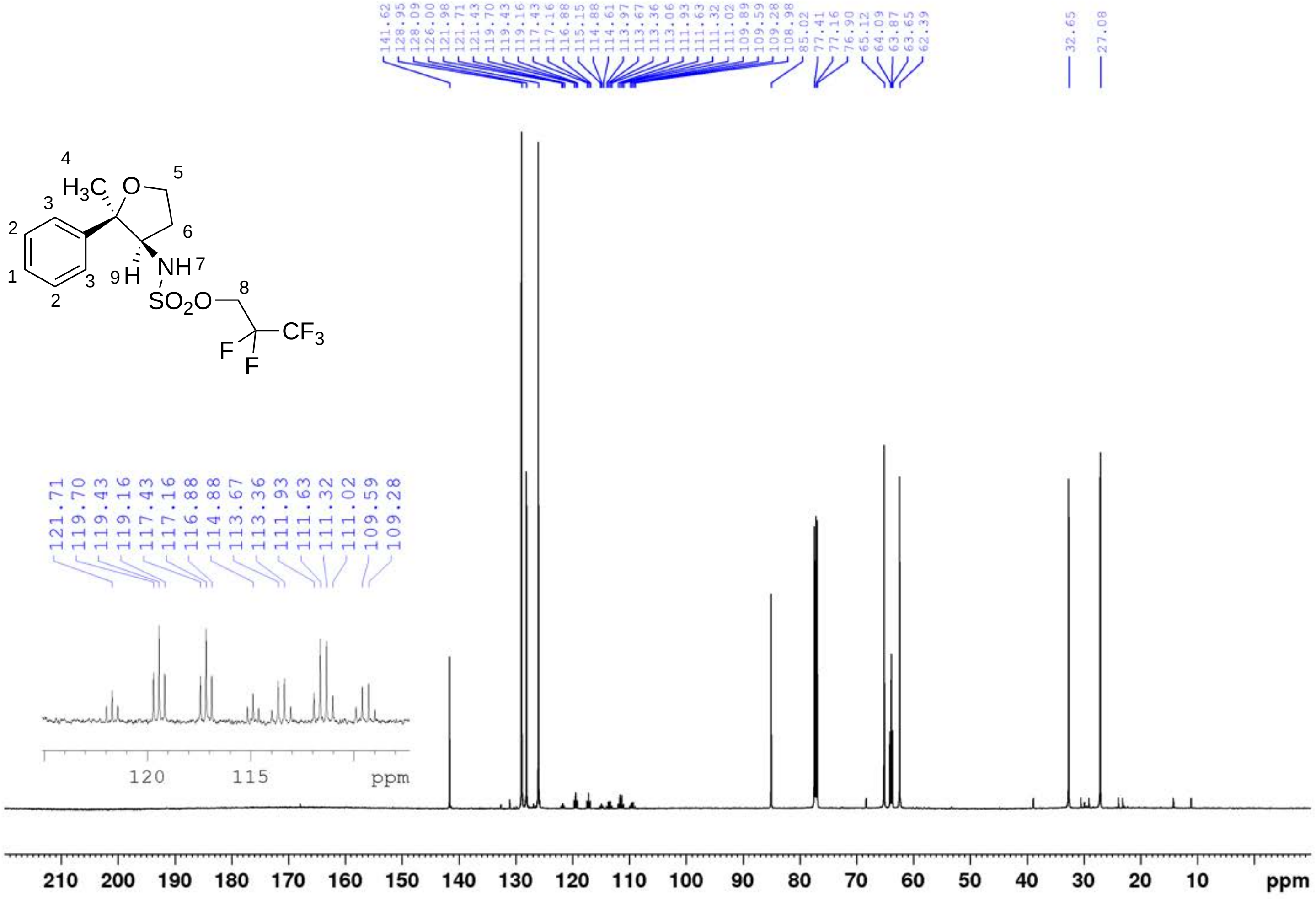

^{19}F NMR (376 MHz, $CDCl_3$) for *2,2,3,3,3-pentafluoropropyl ((2R,3R)-2-methyl-2-phenyltetrahydrofuran-3-yl)sulfamate* (**33g**)

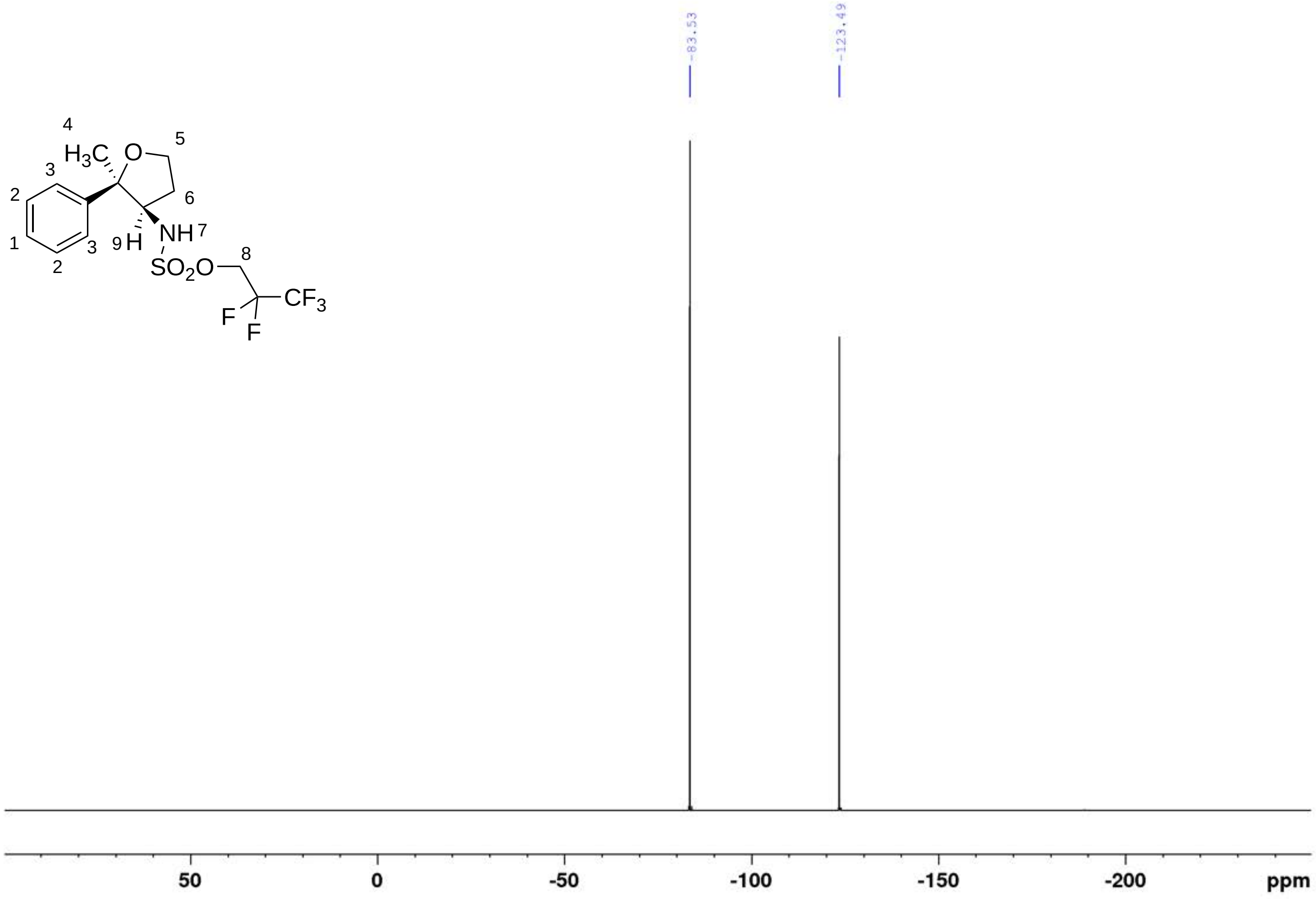

^{1}H NMR (400 MHz, $CDCl_3$) for *2,2,3,3,3-pentafluoropropyl (2-methyl-2-phenyltetrahydrofuran-3-yl)sulfamate* (**33h**)

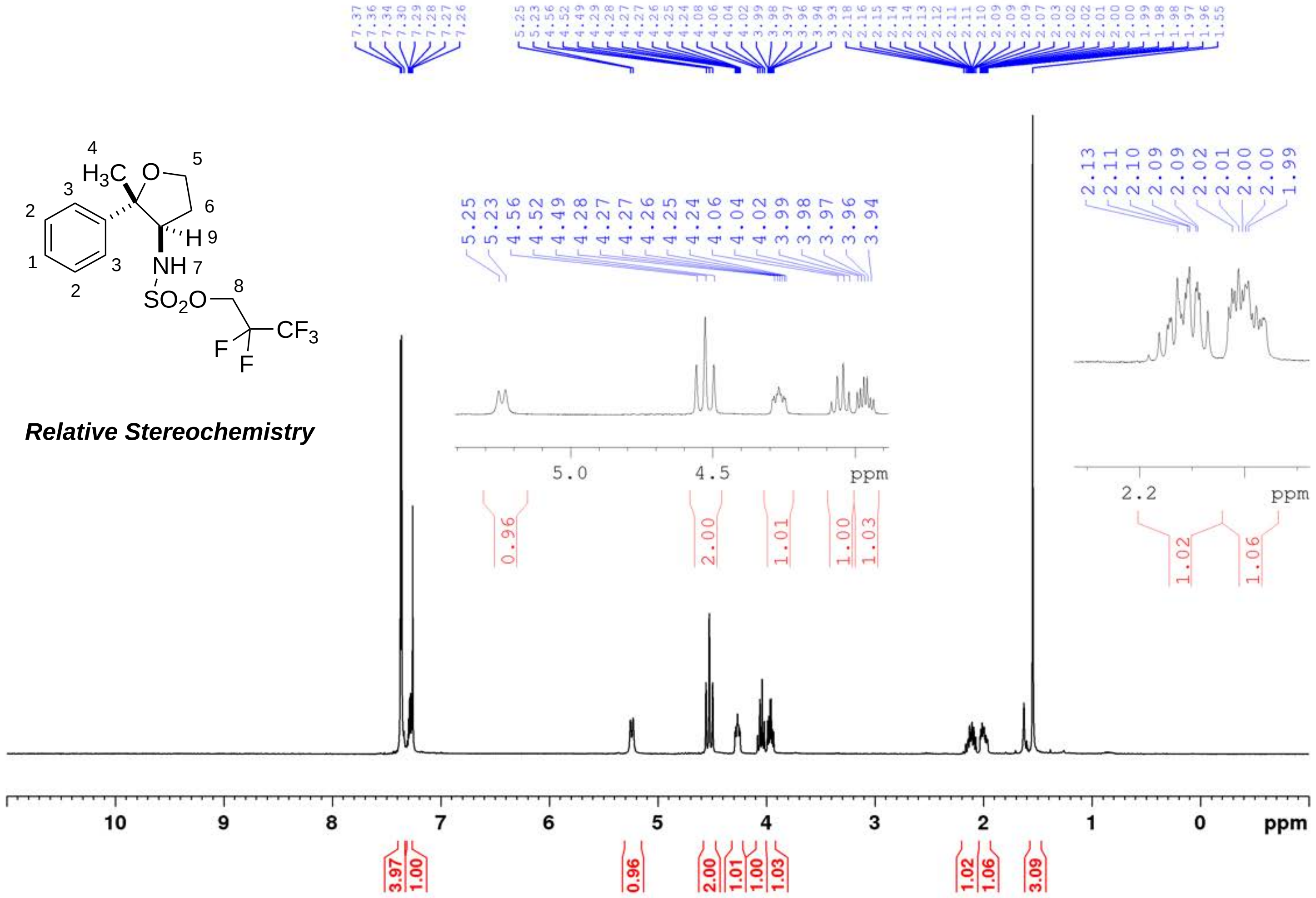

^{13}C NMR (101 MHz, $CDCl_3$) for *2,2,3,3,3-pentafluoropropyl (2-methyl-2-phenyltetrahydrofuran-3-yl)sulfamate* (**33h**)

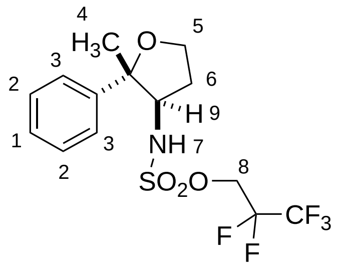

Relative Stereochemistry

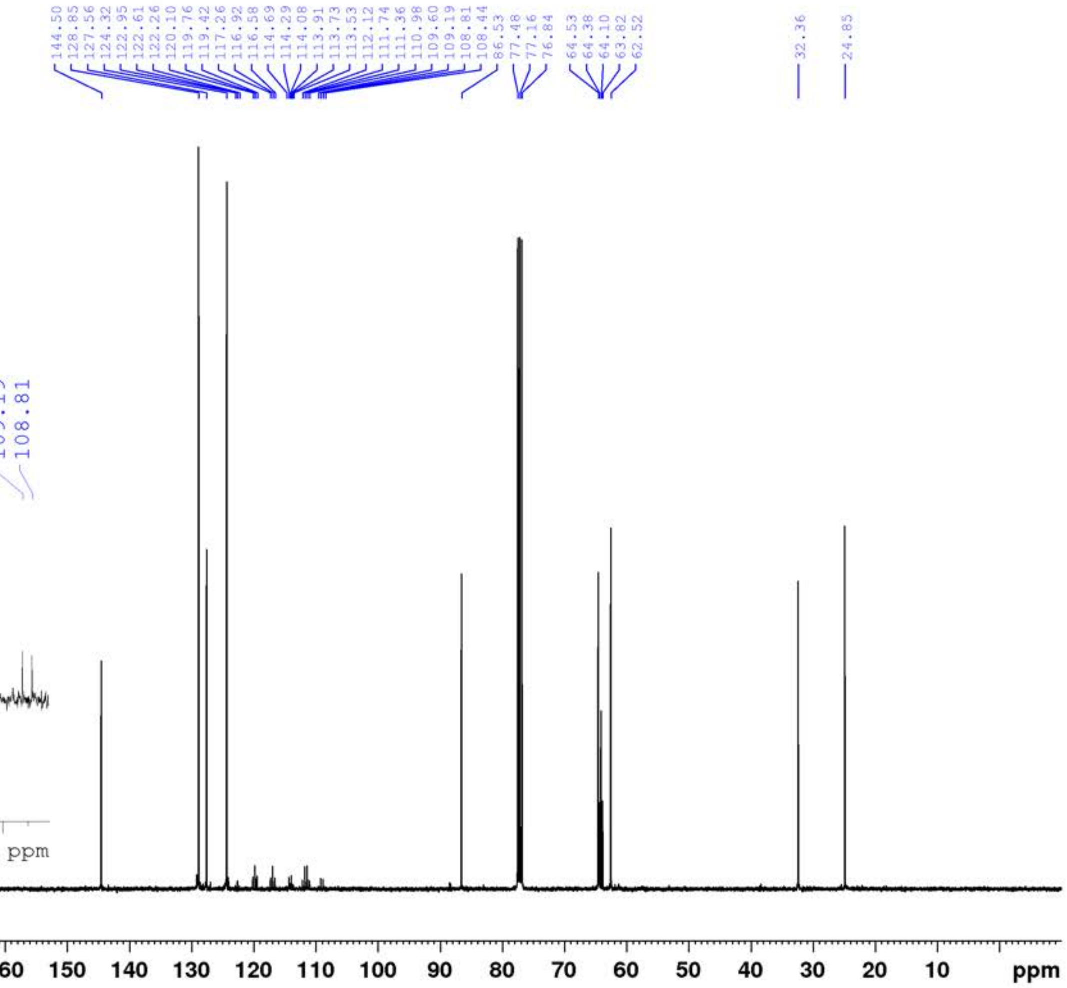

^{19}**F NMR** (376 MHz, $CDCl_3$) for *2,2,3,3,3-pentafluoropropyl (2-methyl-2-phenyltetrahydrofuran-3-yl)sulfamate* (**33h**)

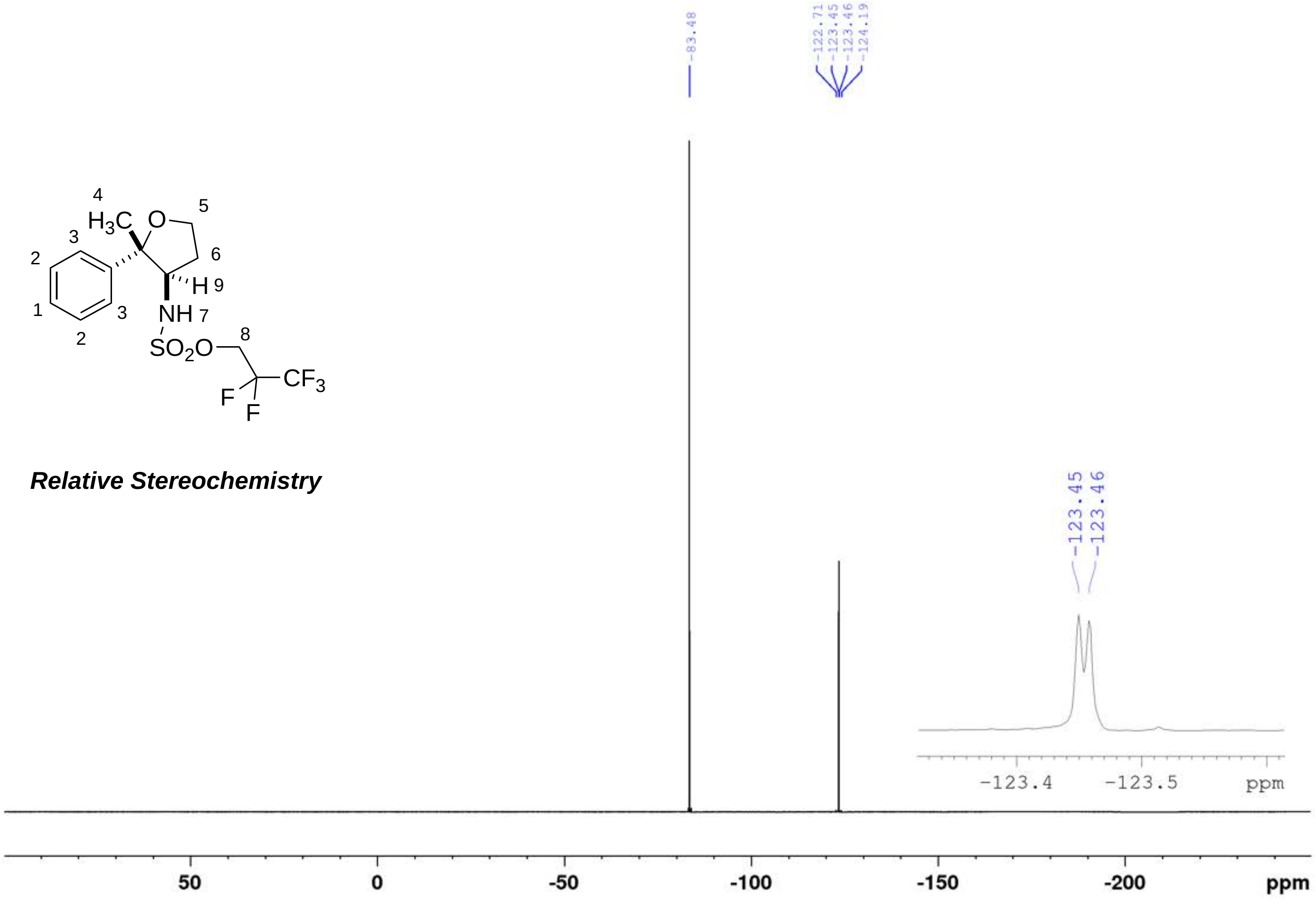

^{1}H NMR (500 MHz, $CDCl_3$) for *2,2,3,3,3-pentafluoropropyl ((2R,3R)-2-methyl-2-(4-methylpent-3-en-1-yl)tetrahydrofuran-3-yl)sulfamate* (**36**)

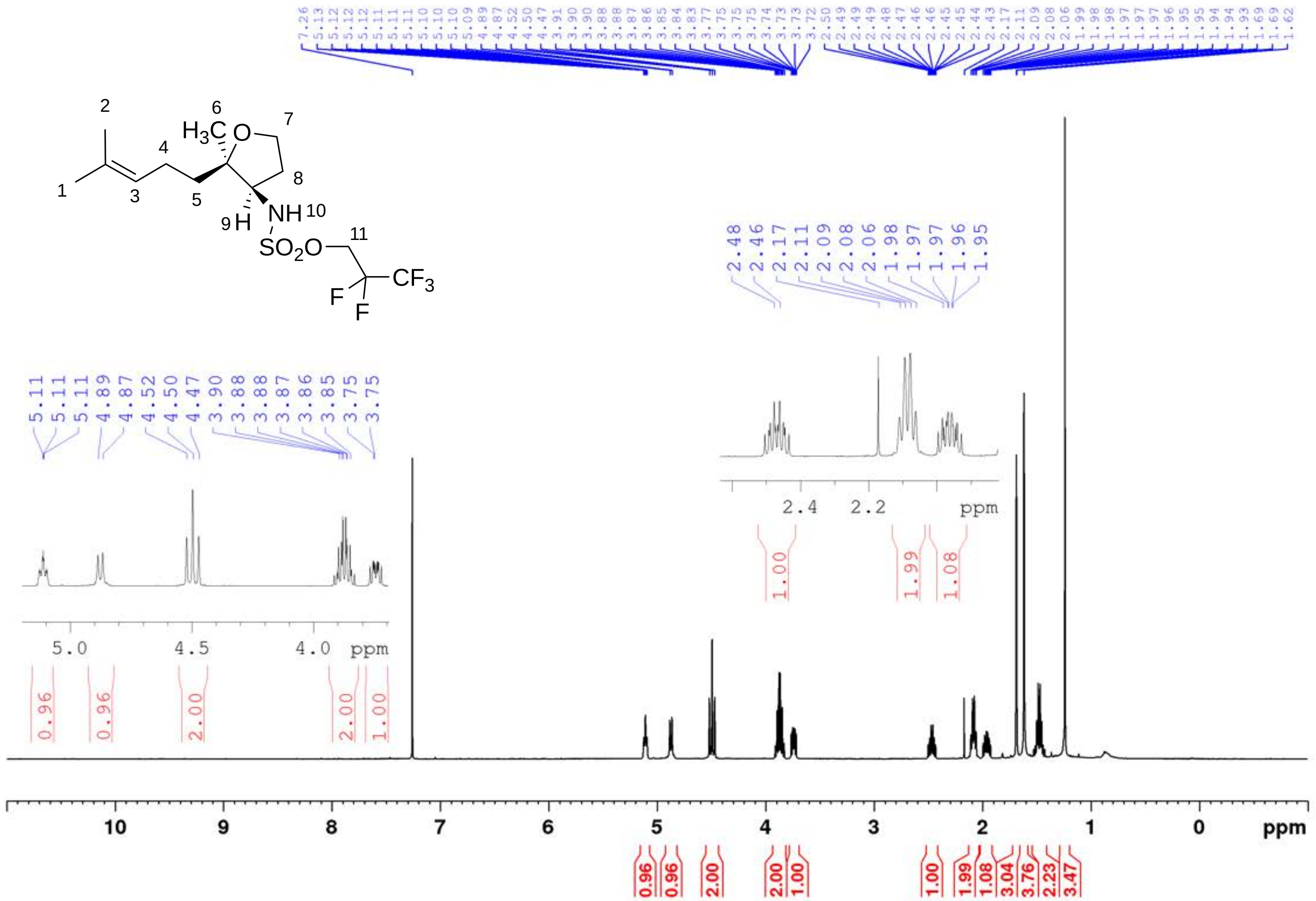

^{13}C NMR (126 MHz, $CDCl_3$) for *2,2,3,3,3-pentafluoropropyl ((2R,3R)-2-methyl-2-(4-methylpent-3-en-1-yl)tetrahydrofuran-3-yl)sulfamate* (**36**)

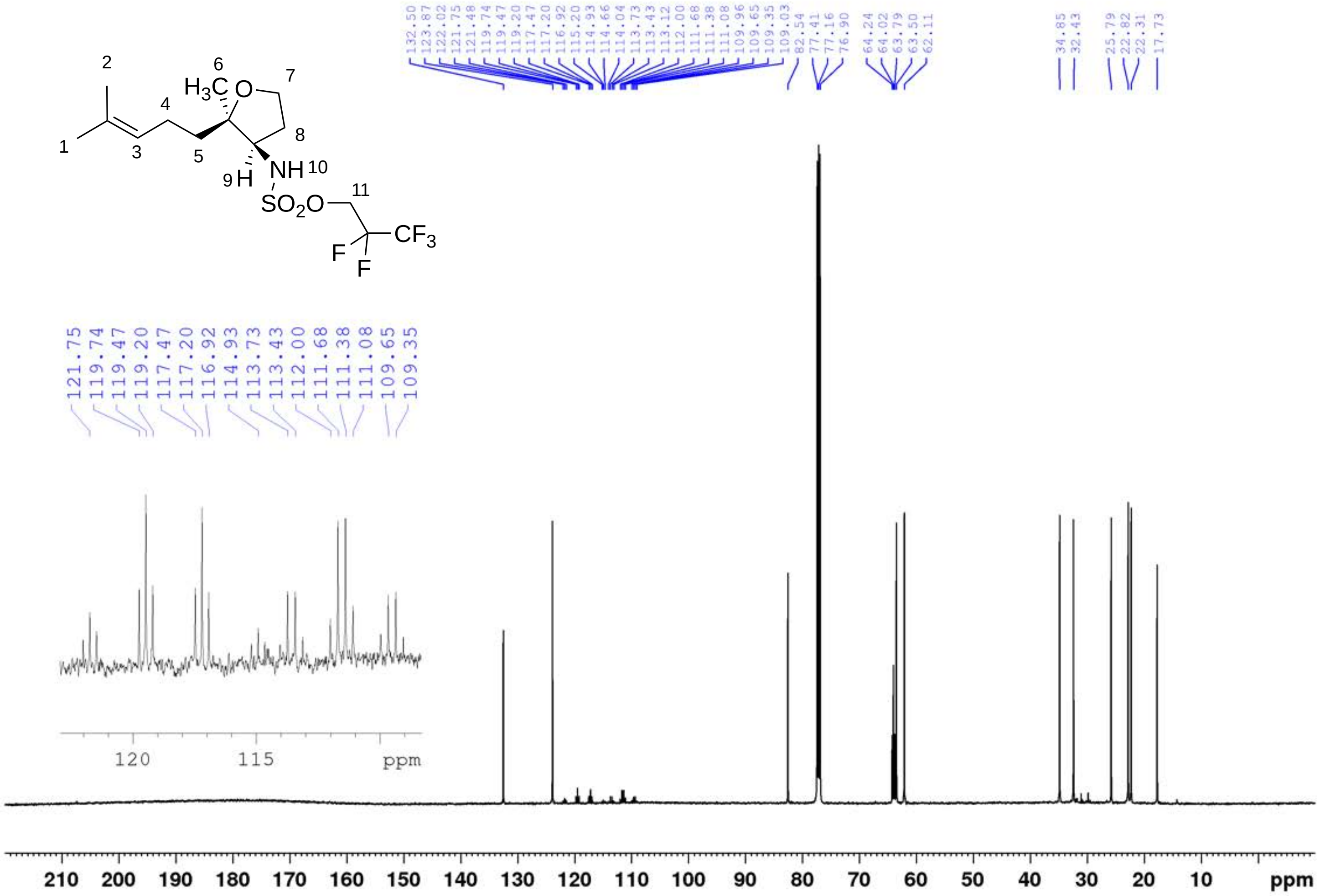

^{19}**F NMR** (376 MHz, $CDCl_3$) for *2,2,3,3,3-pentafluoropropyl ((2R,3R)-2-methyl-2-(4-methylpent-3-en-1-yl)tetrahydrofuran-3-yl)sulfamate* (**36**)

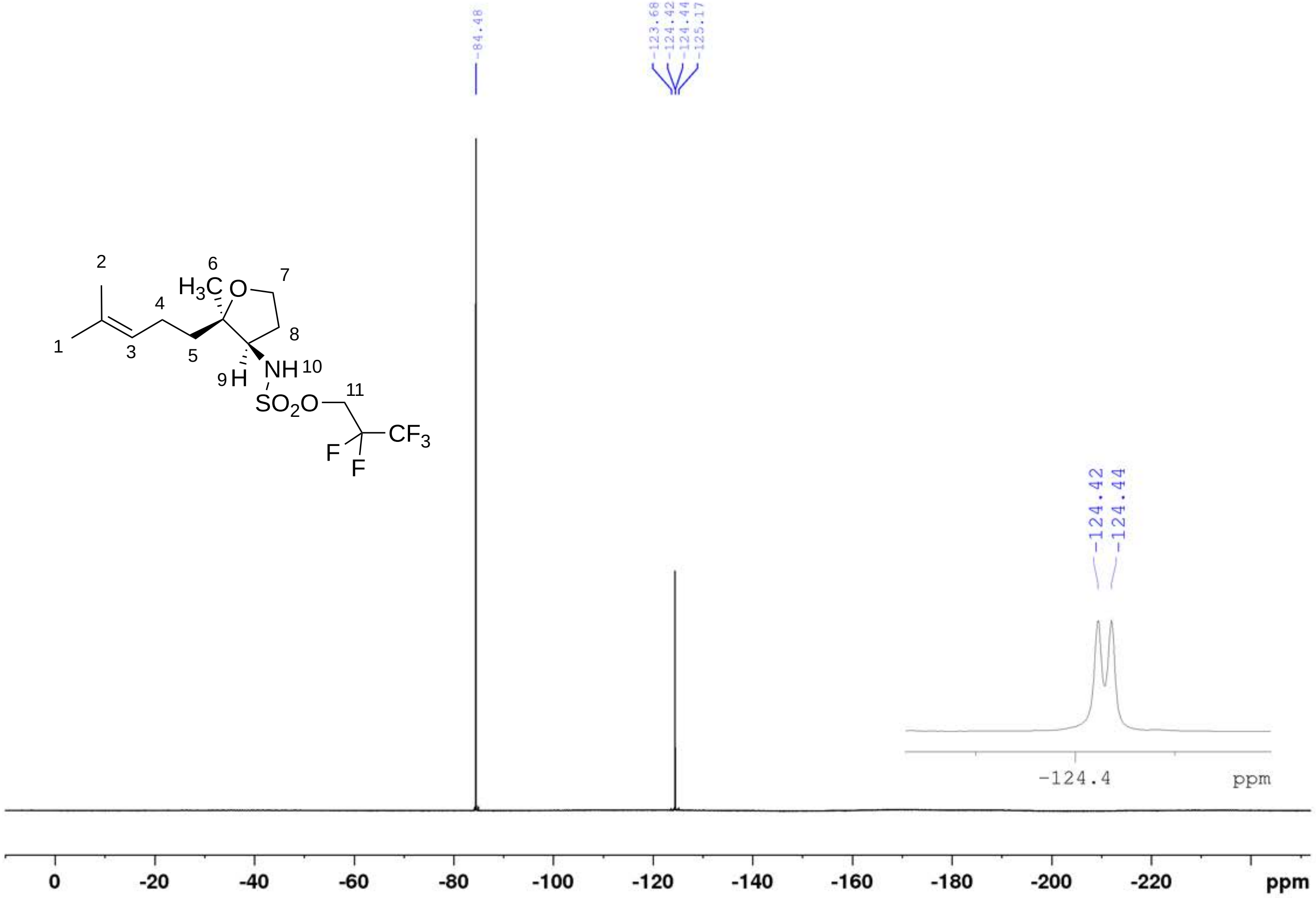

^{1}H NMR (500 MHz, $CDCl_3$) for *(2,2,3,3,3-pentafluoropropyl (R,Z)-3-(6-hydroxy-3-methylhex-3-en-1-yl)-2,2-dimethylaziridine-1-sulfonate*

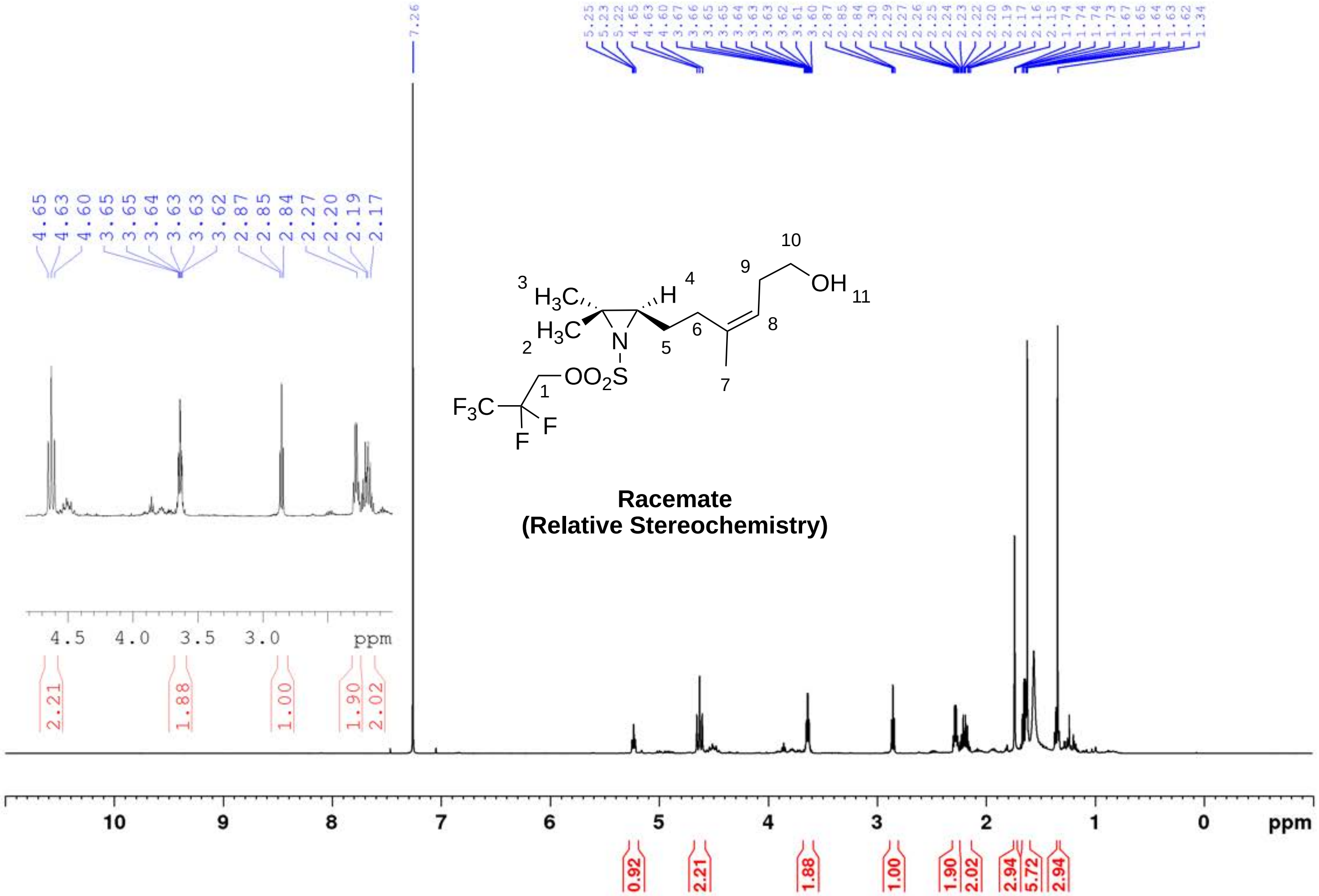

^{13}C NMR (126 MHz, $CDCl_3$) for *(2,2,3,3,3-pentafluoropropyl (R,Z)-3-(6-hydroxy-3-methylhex-3-en-1-yl)-2,2-dimethylaziridine-1-sulfonate*

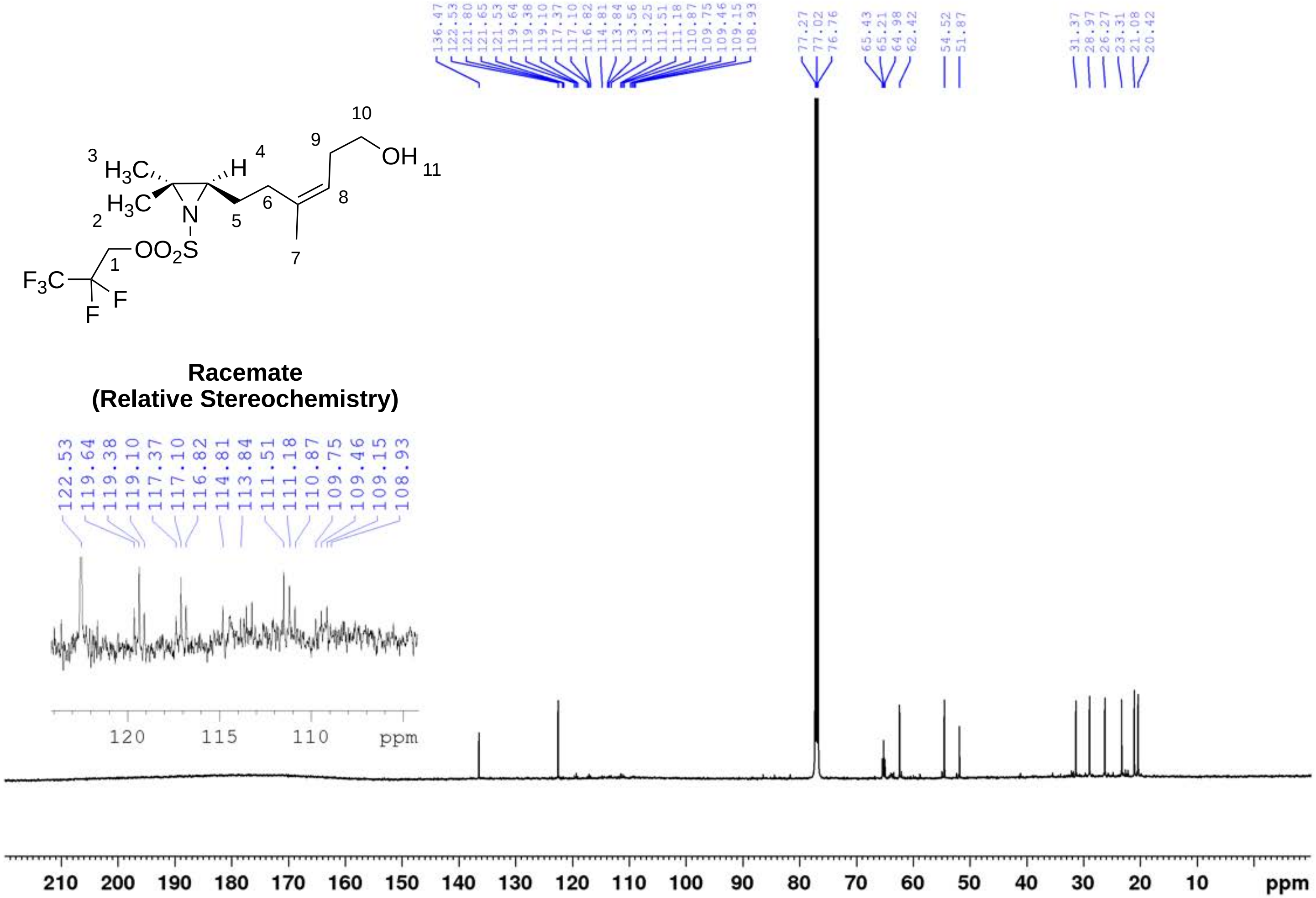

^{19}F NMR (376 MHz, $CDCl_3$) for *(2,2,3,3,3-pentafluoropropyl (R,Z)-3-(6-hydroxy-3-methylhex-3-en-1-yl)-2,2-dimethylaziridine-1-sulfonate*

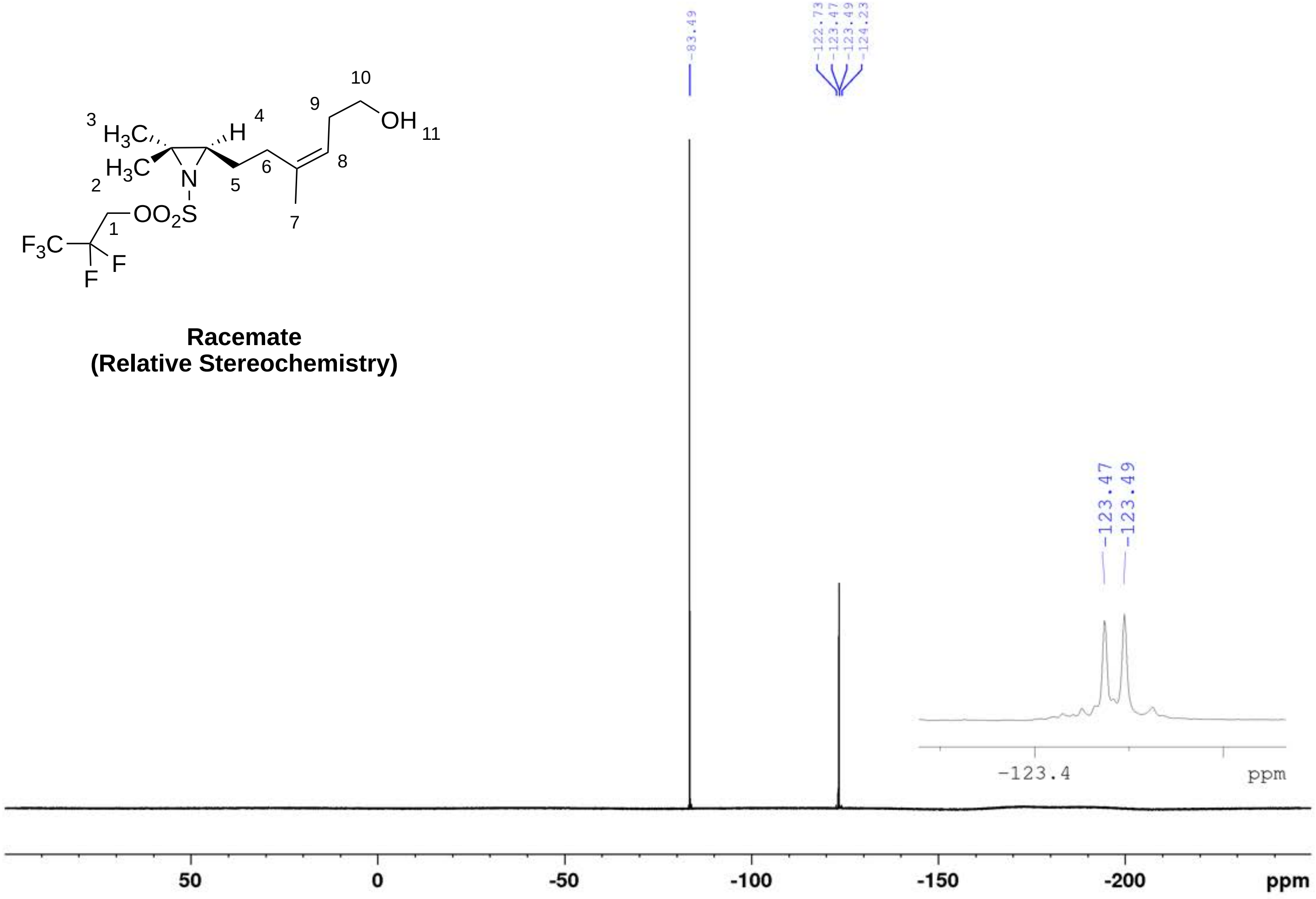

^{1}H NMR (500 MHz, $CDCl_3$) for *2,2,3,3,3-pentafluoropropyl ((2S,3R)-2-methyl-2-(4-methylpent-3-en-1-yl)tetrahydrofuran-3-yl)sulfamate* (**39**)

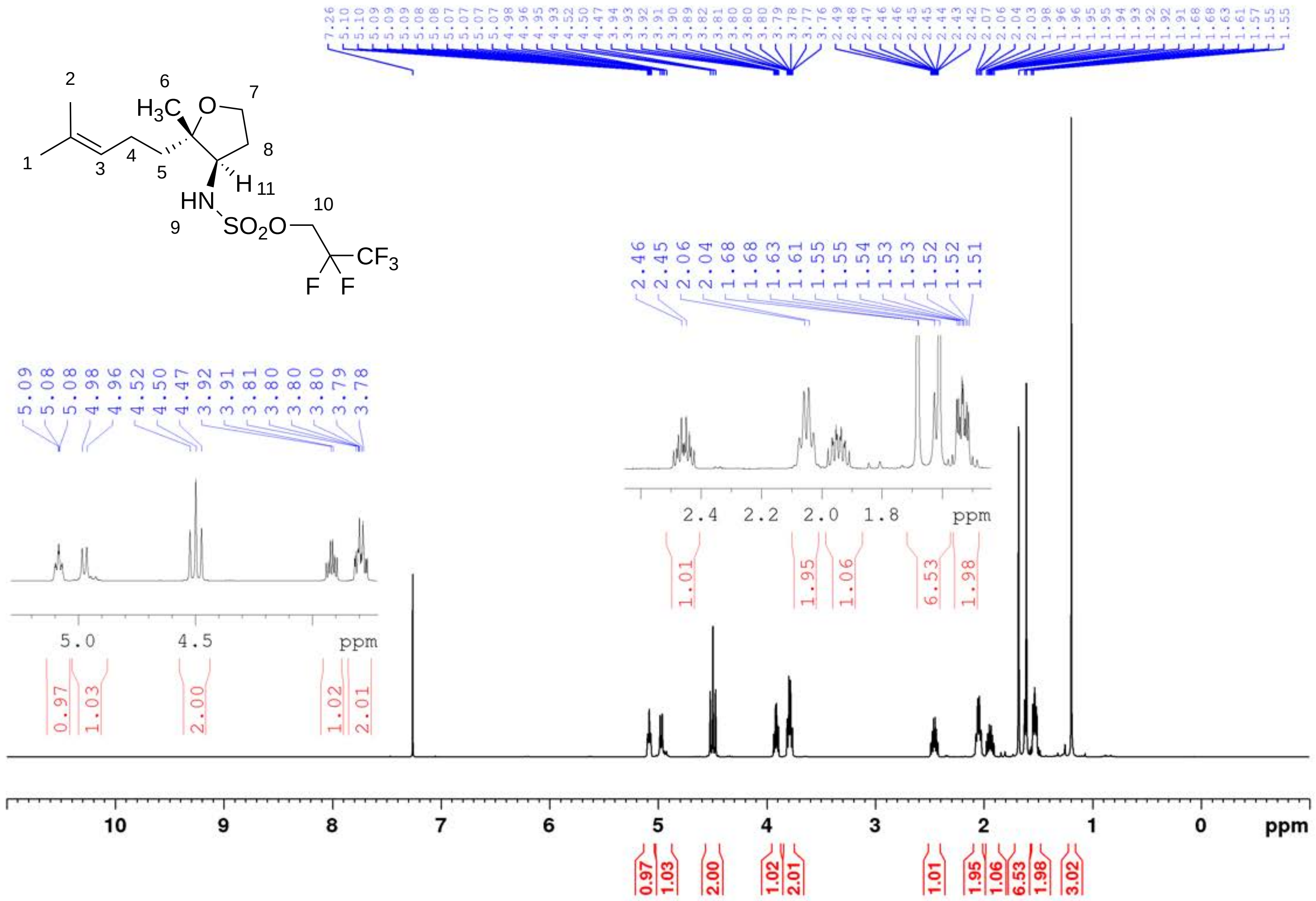

^{13}**C NMR** (126 MHz, $CDCl_3$) for *2,2,3,3,3-pentafluoropropyl ((2S,3R)-2-methyl-2-(4-methylpent-3-en-1-yl)tetrahydrofuran-3-yl)sulfamate* (**39**)

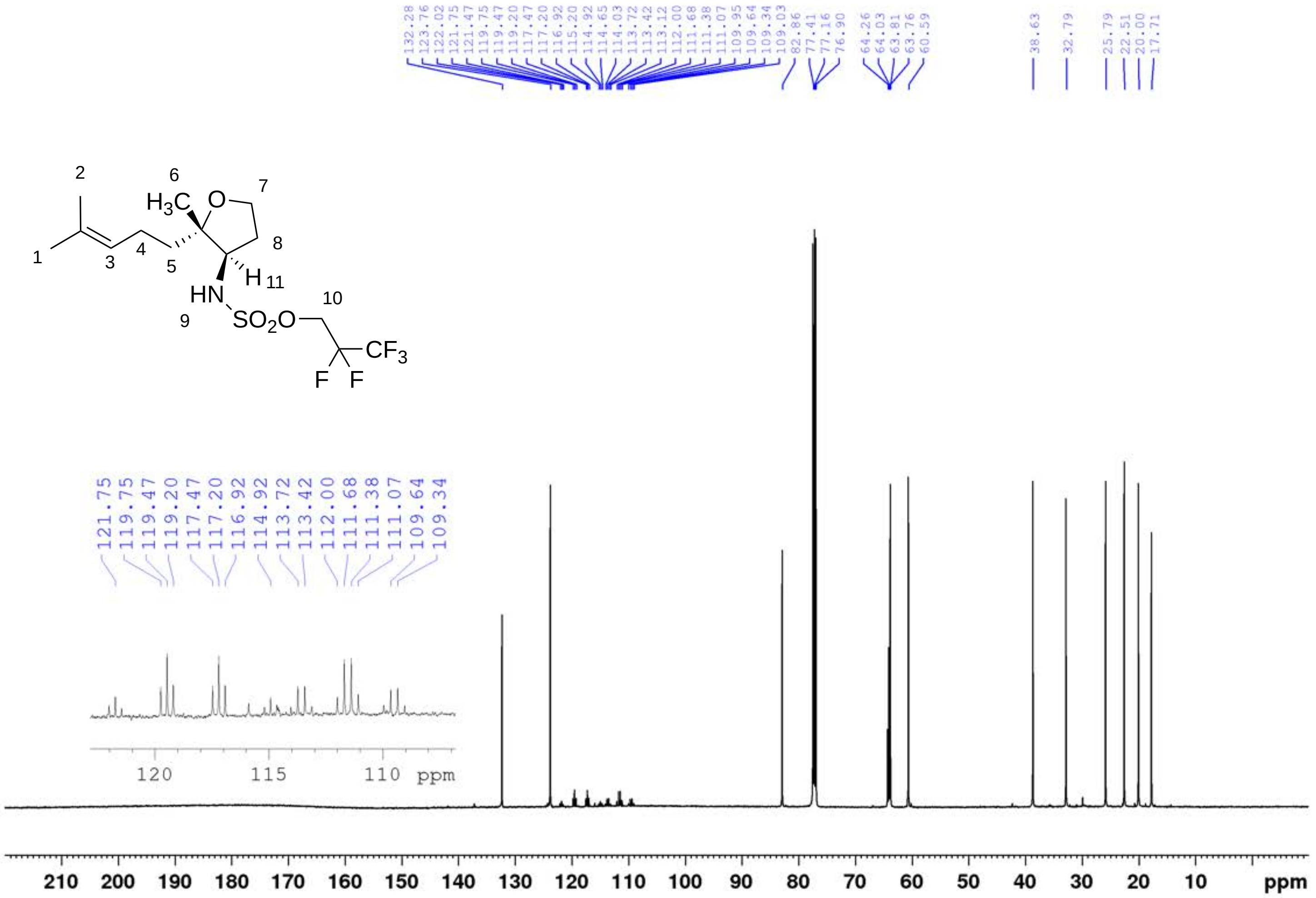

^{19}F NMR (376 MHz, $CDCl_3$) for *2,2,3,3,3-pentafluoropropyl ((2S,3R)-2-methyl-2-(4-methylpent-3-en-1-yl)tetrahydrofuran-3-yl)sulfamate* (**39**)

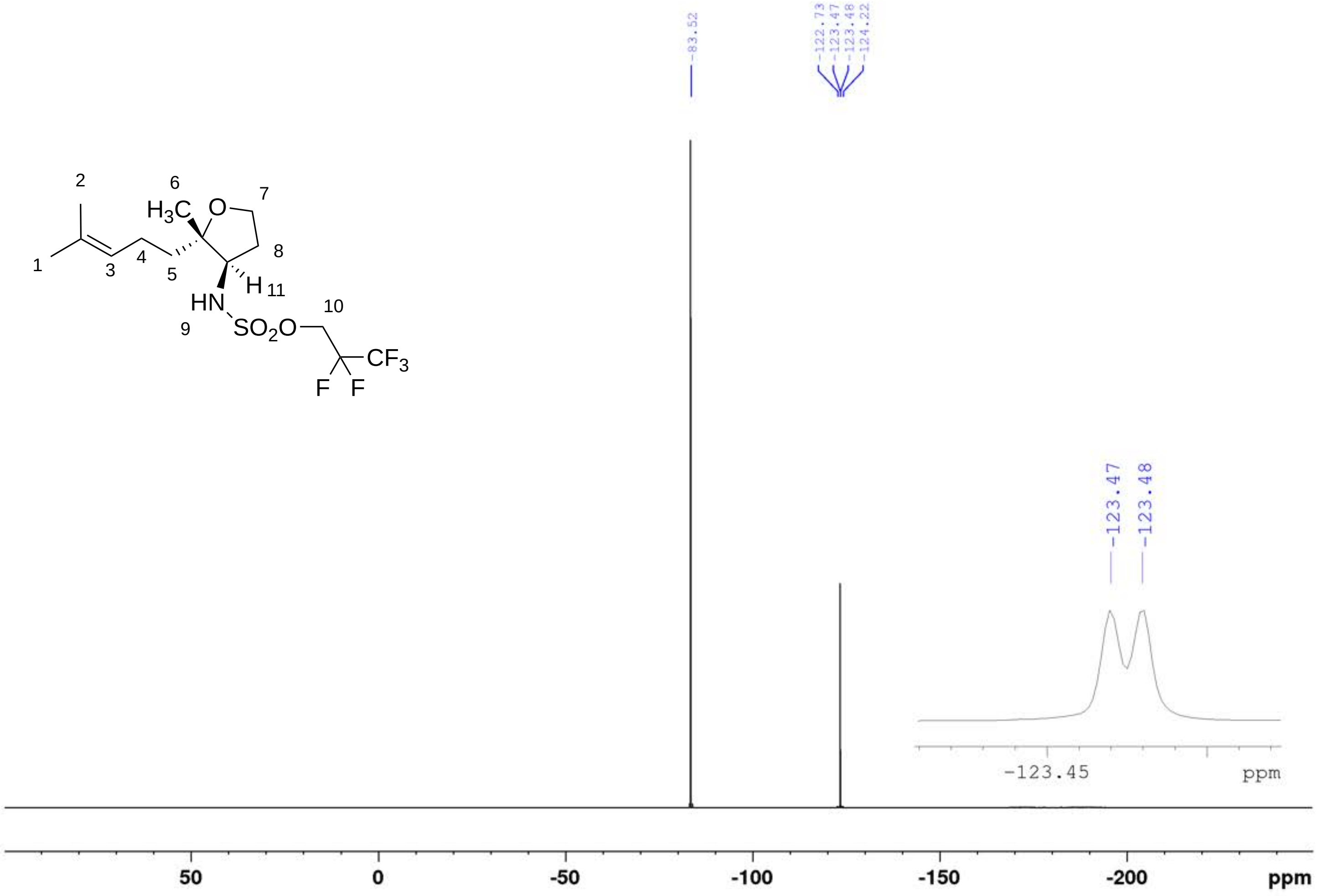

^{1}H NMR (500 MHz, $CDCl_3$) for *2,2,3,3,3-pentafluoropropyl (4-bromobenzyl)((2S,3R)-2-methyl-2-(4-methylpent-3-en-1-yl)tetrahydrofuran-3-yl)sulfamate: **Obtained following derivatisation of*** **39**

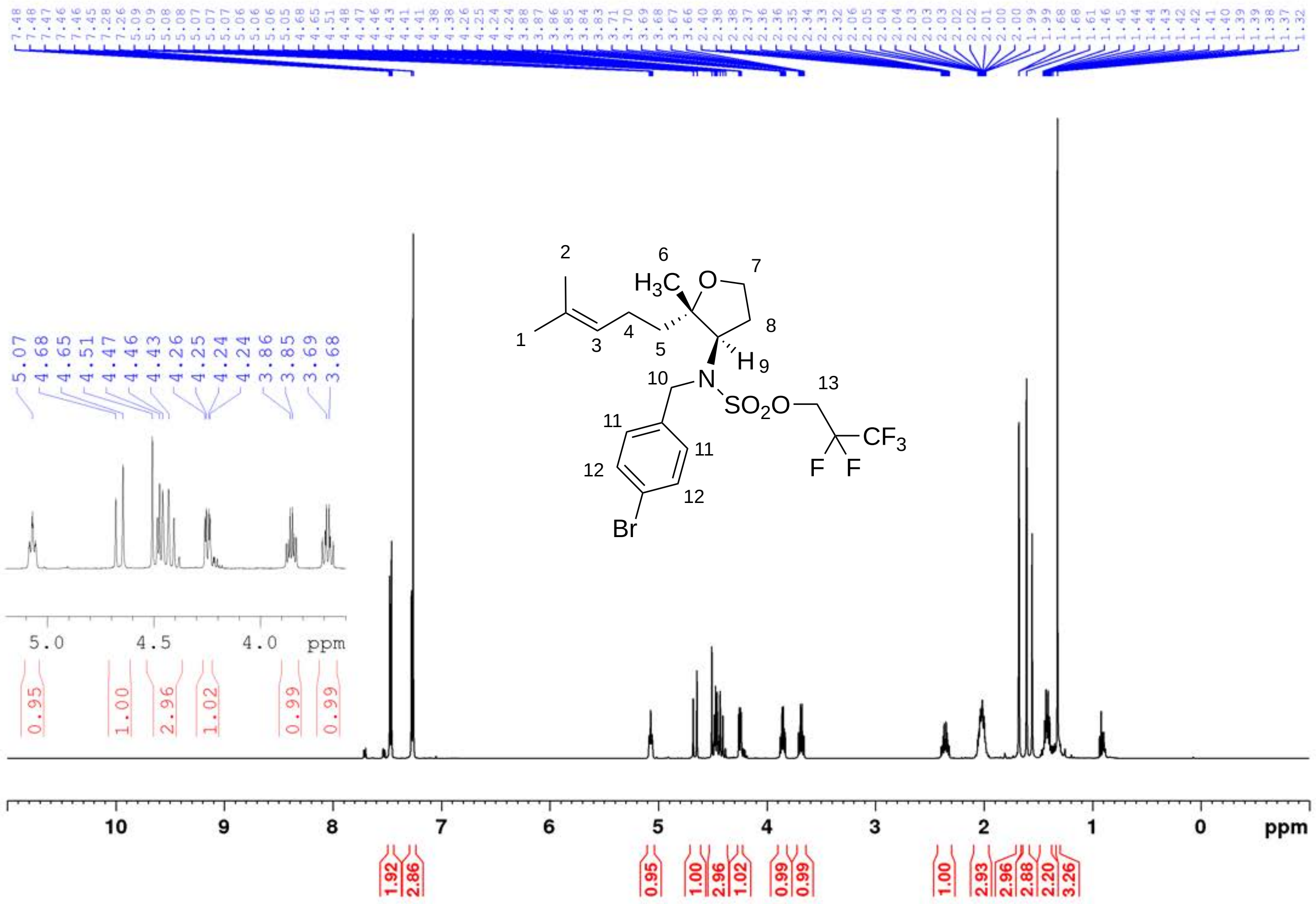

^{13}C NMR (126 MHz, $CDCl_3$) for *2,2,3,3,3-pentafluoropropyl (4-bromobenzyl)((2S,3R)-2-methyl-2-(4-methylpent-3-en-1-yl)tetrahydrofuran-3-yl)sulfamate:* ***Obtained following derivatisation of* 39**

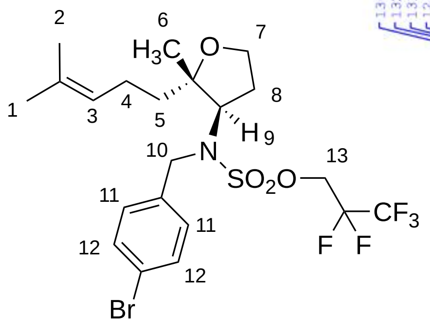

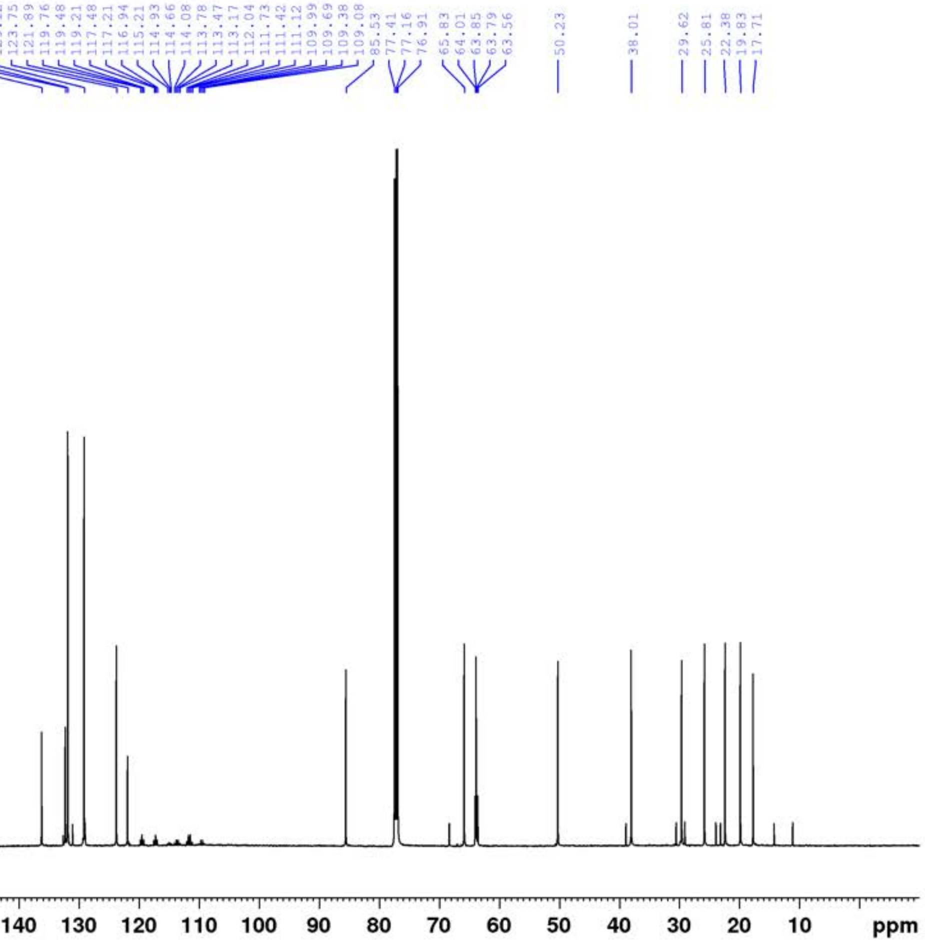

^{19}F NMR (376 MHz, $CDCl_3$) for *2,2,3,3,3-pentafluoropropyl (4-bromobenzyl)((2S,3R)-2-methyl-2-(4-methylpent-3-en-1-yl)tetrahydrofuran-3-yl)sulfamate:* ***Obtained following derivatisation of*** **39**

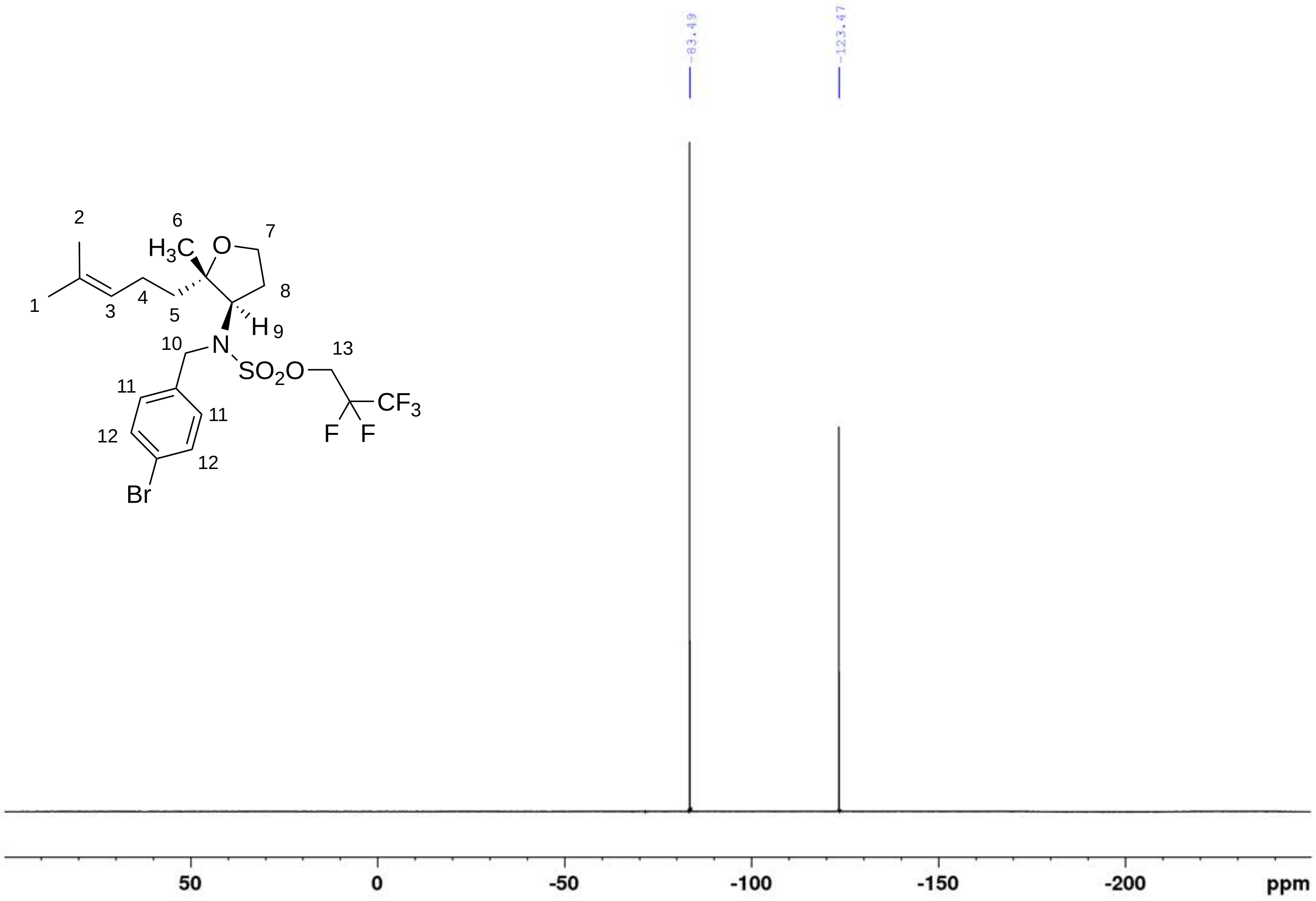

^{1}H NMR (500 MHz, $CDCl_3$) for *2,2,3,3,3-pentafluoropropyl 2-(2-hydroxyethyl)-2-methyl-3-phenylaziridine-1-sulfonate:* ***Obtained following aziridination of* 40**

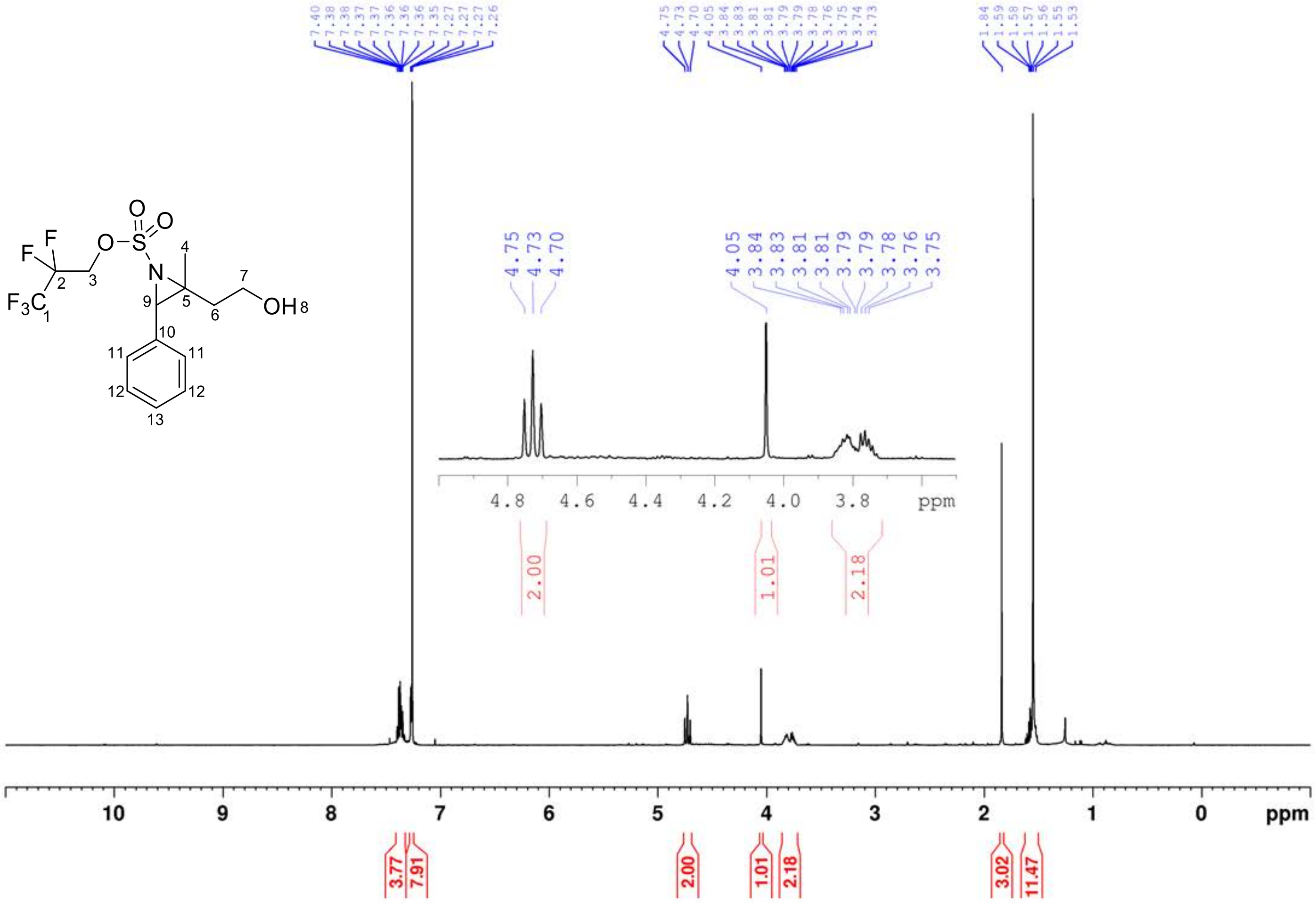

^{13}C NMR (126 MHz, $CDCl_3$) for *2,2,3,3,3-pentafluoropropyl 2-(2-hydroxyethyl)-2-methyl-3-phenylaziridine-1-sulfonate:* ***Obtained following aziridination of*** **40**

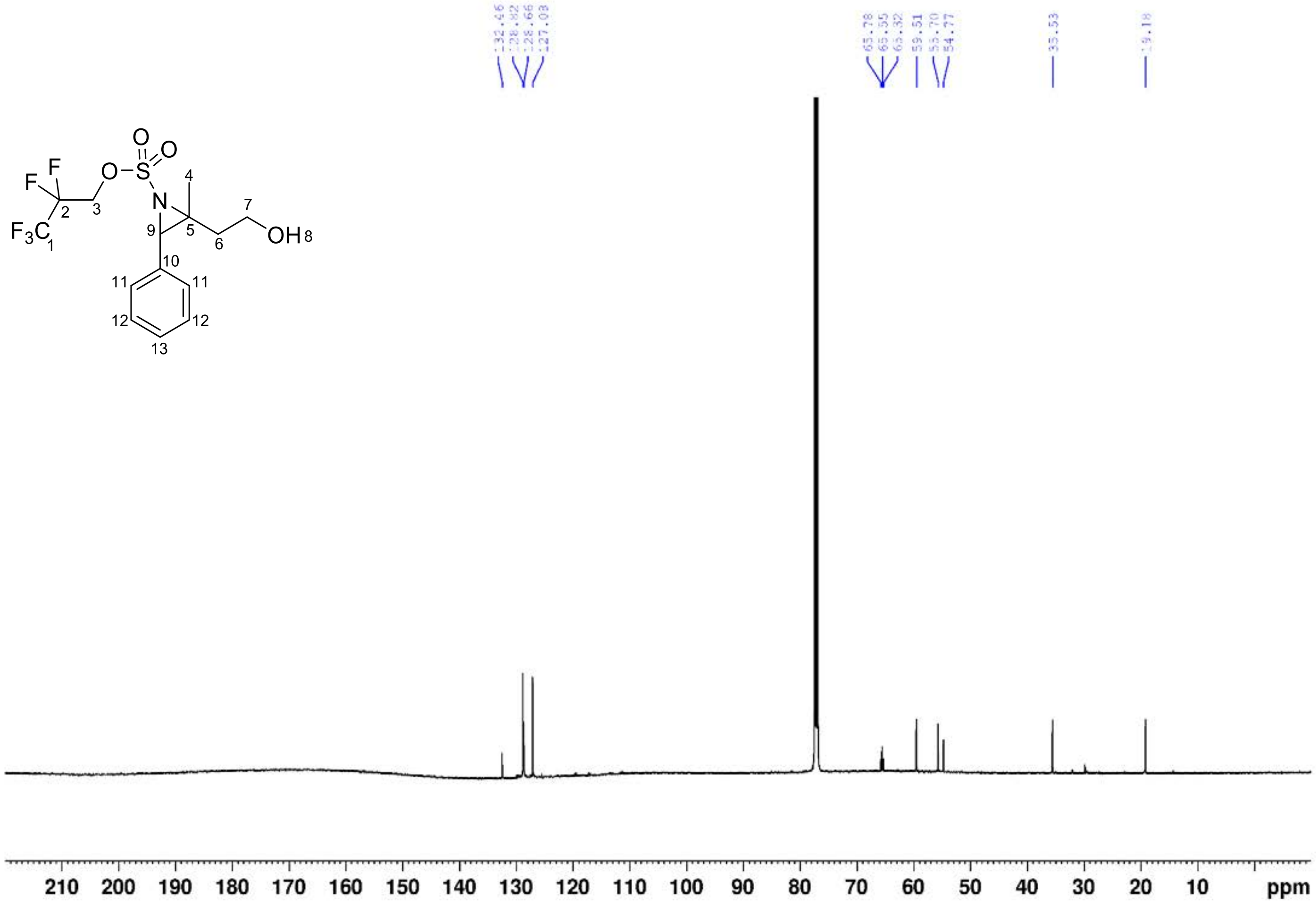

^{19}F NMR (376 MHz, $CDCl_3$) for *2,2,3,3,3-pentafluoropropyl 2-(2-hydroxyethyl)-2-methyl-3-phenylaziridine-1-sulfonate:* ***Obtained following aziridination of* 40**

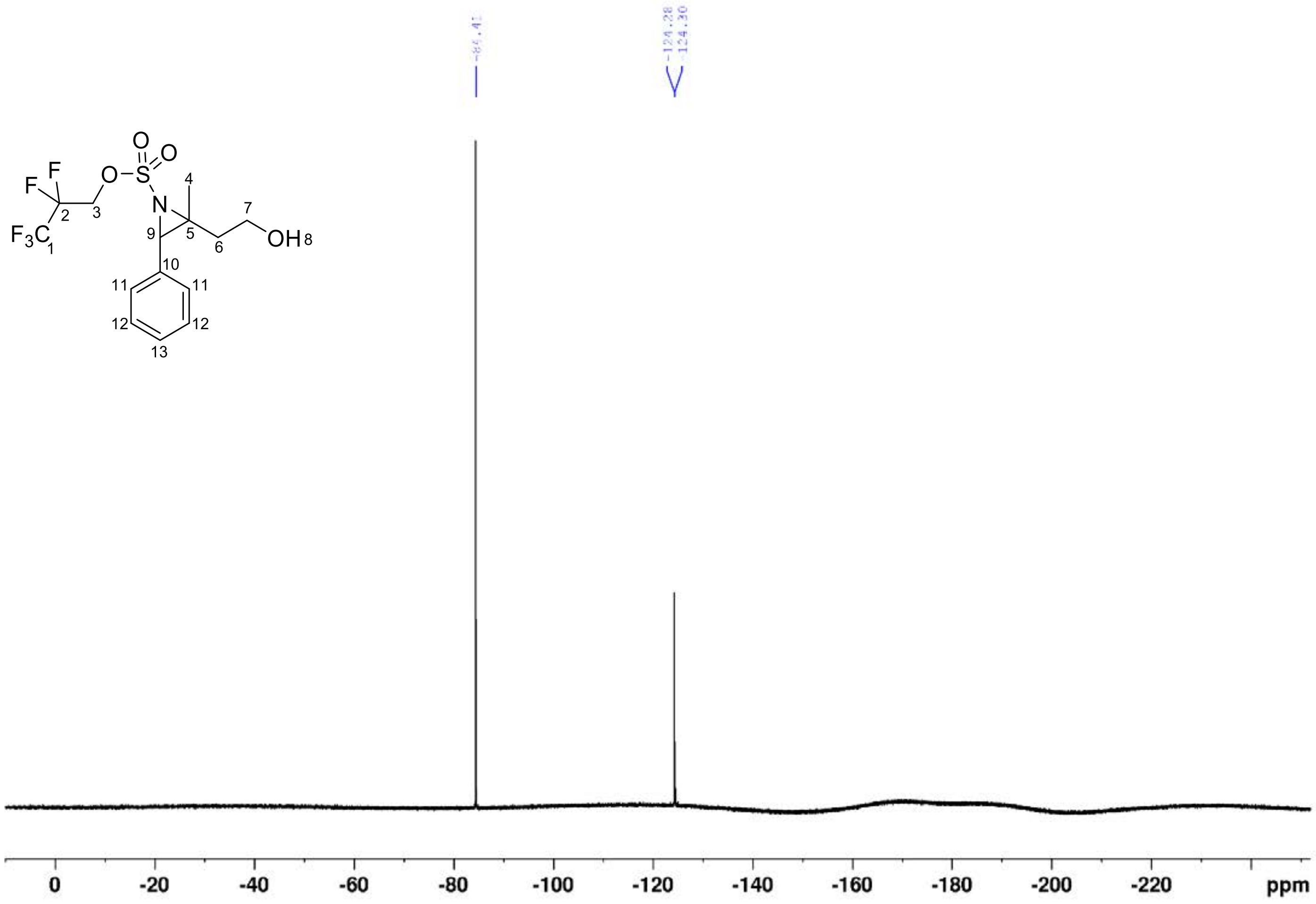

^{1}H NMR (400 MHz, $CDCl_3$) for *(S)-4-cyclohexyl-2-(2-hydroxyethyl)-2-(((2,2,3,3,3-pentafluoropropoxy)sulfonyl)amino)butyl acetate* (**42**)

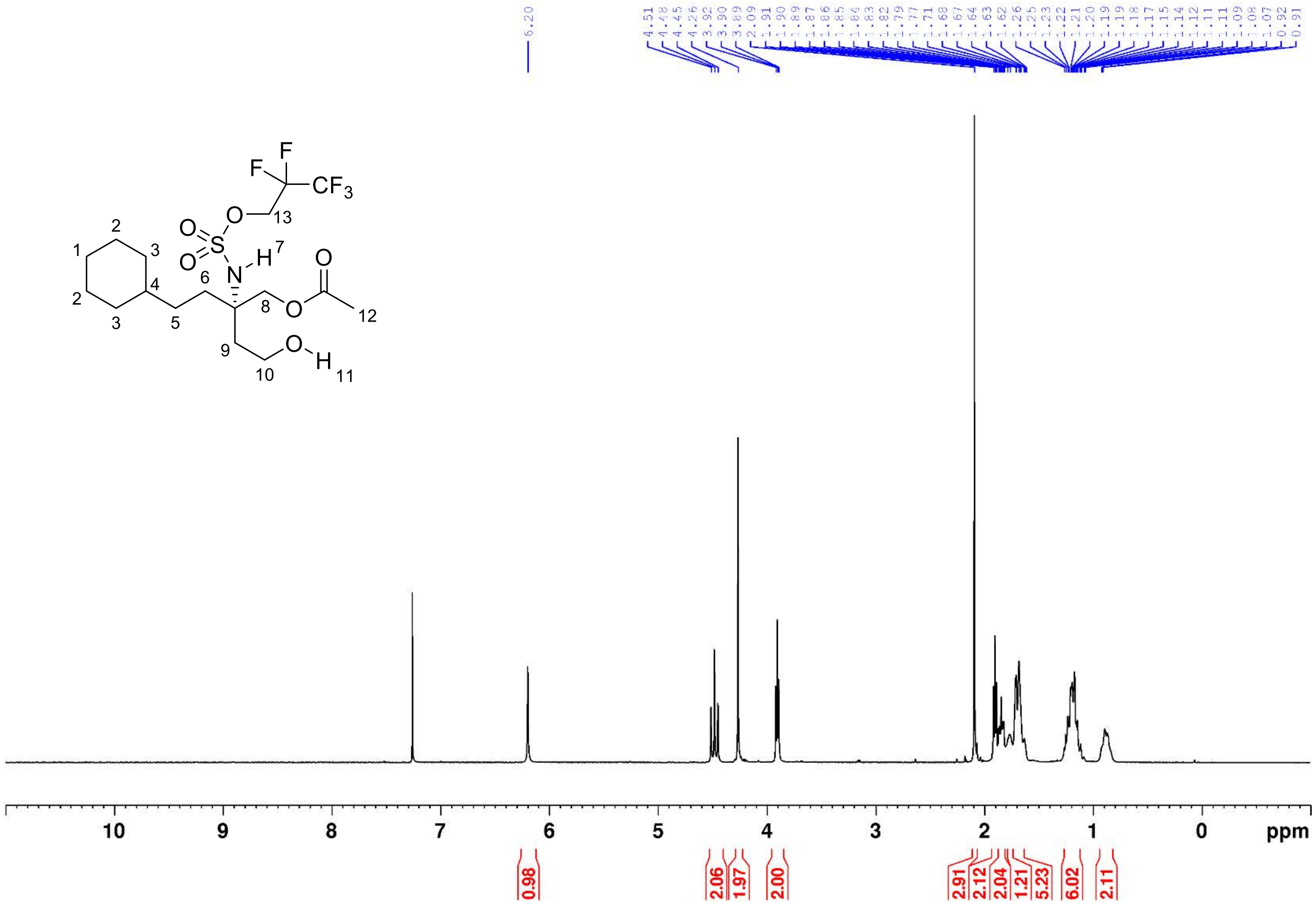

^{13}C NMR (126 MHz, $CDCl_3$) for *(S)-4-cyclohexyl-2-(2-hydroxyethyl)-2-(((2,2,3,3,3-pentafluoropropoxy)sulfonyl)amino)butyl acetate* (**42**)

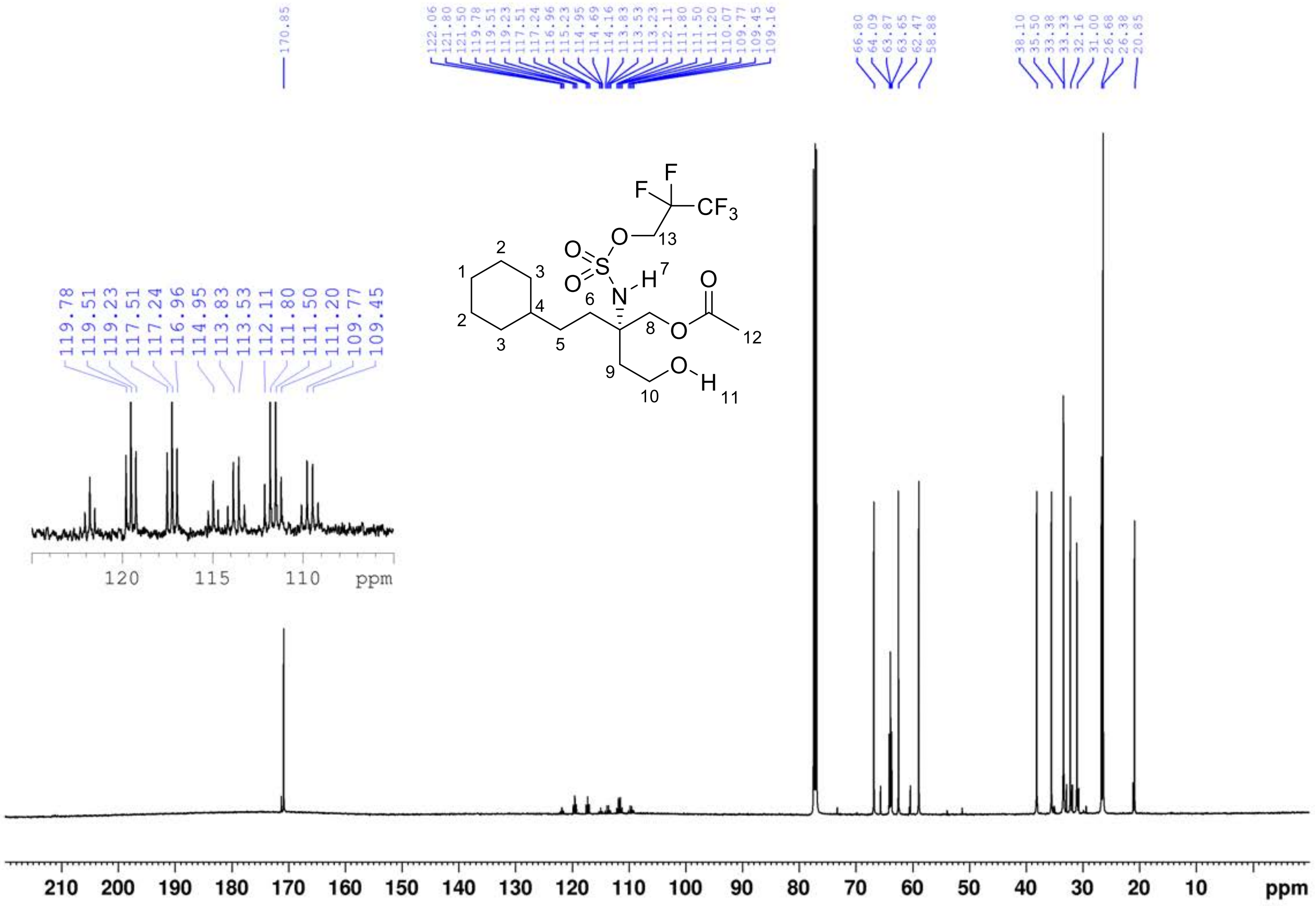

^{19}F NMR (376 MHz, $CDCl_3$) for *(S)-4-cyclohexyl-2-(2-hydroxyethyl)-2-(((2,2,3,3,3-pentafluoropropoxy)sulfonyl)amino)butyl acetate* (**42**)

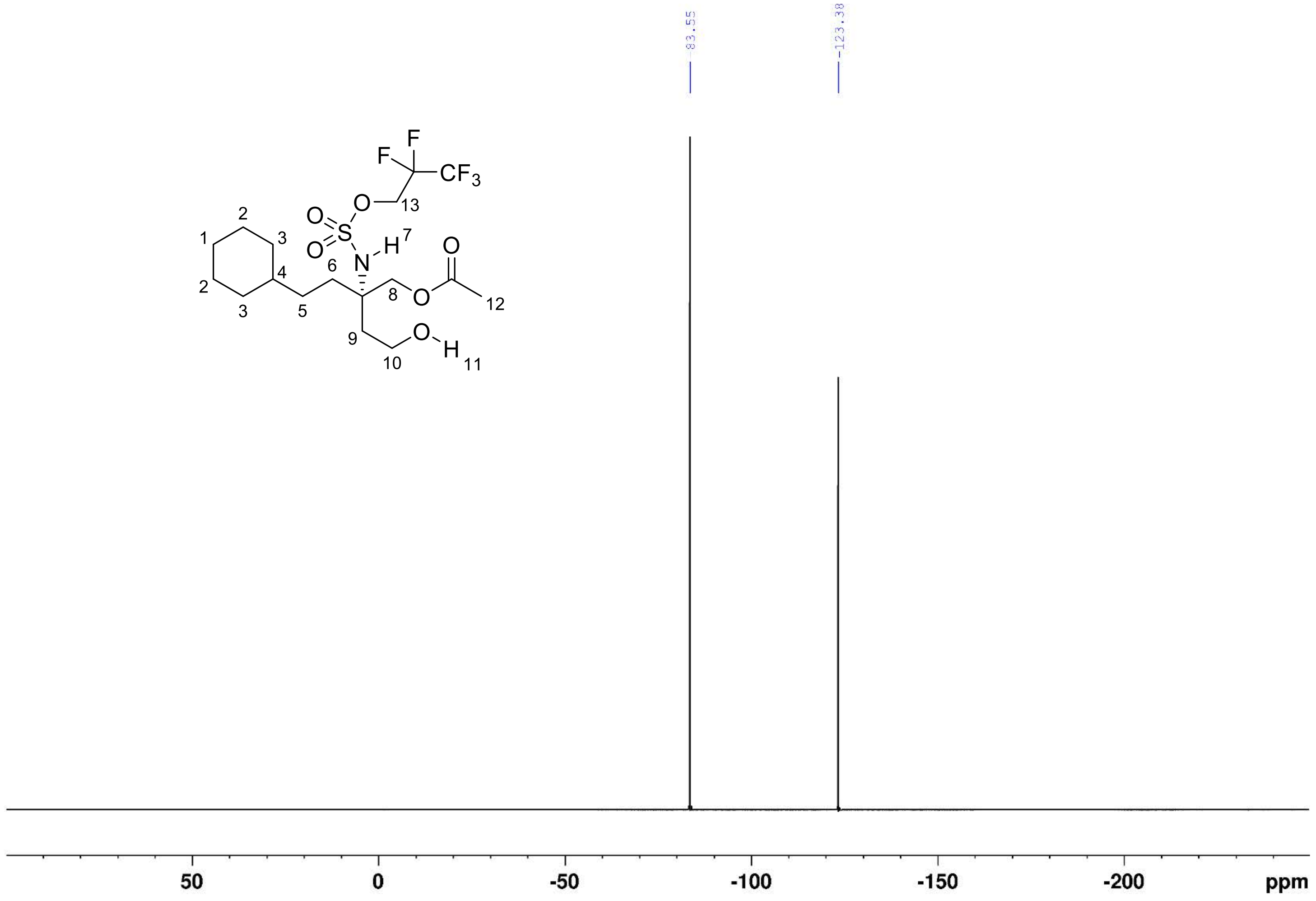

^{1}H NMR (500 MHz, $CDCl_3$) for *(S)-4-((tert-butyldiphenylsilyl)oxy)-2-(2-cyclohexylethyl)-2-(((2,2,3,3,3-pentafluoropropoxy)sulfonyl)amino)butyl acetate:* ***Obtained following derivatisation of* 42**

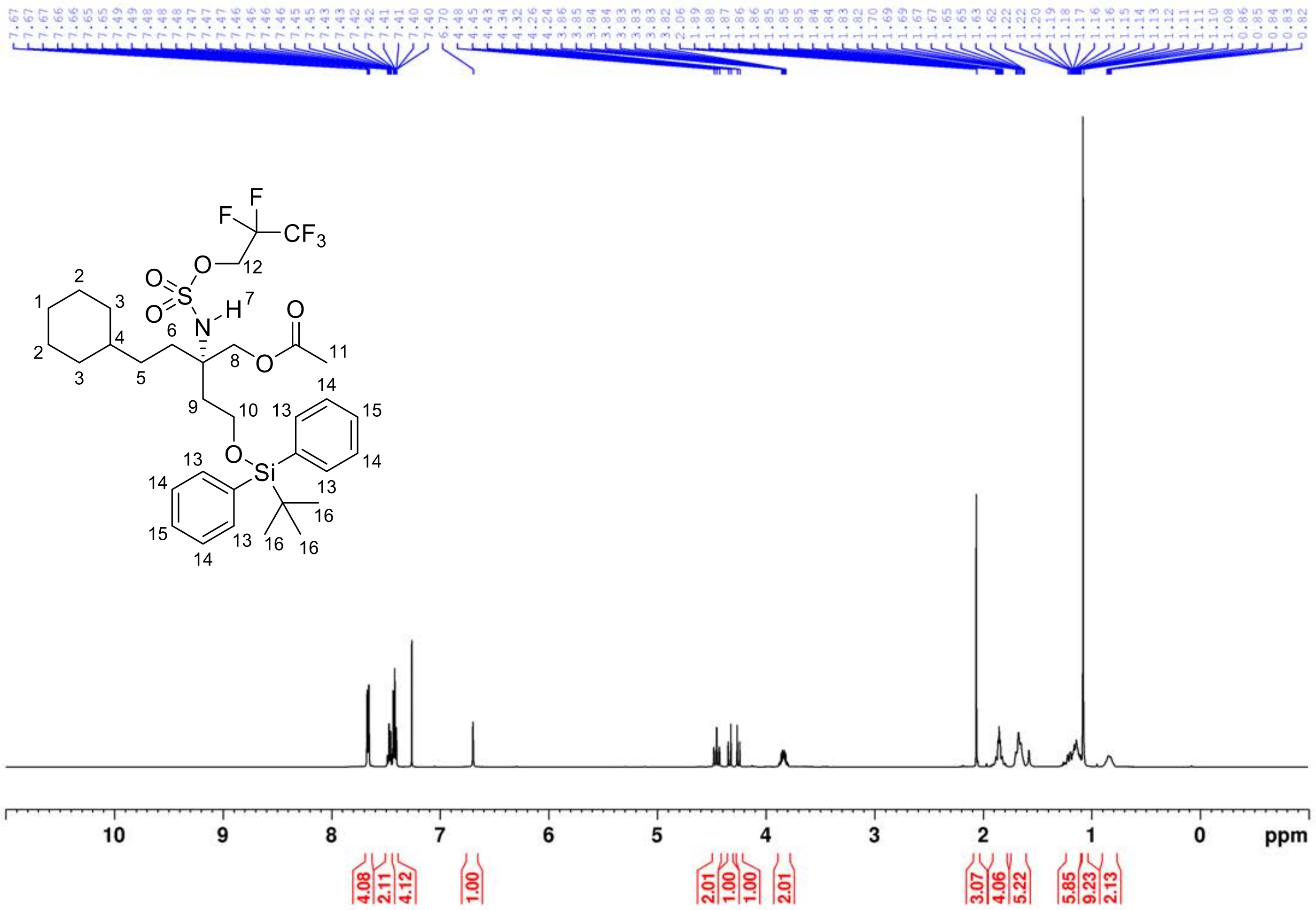

^{13}C NMR (126 MHz, $CDCl_3$) for *(S)-4-((tert-butyldiphenylsilyl)oxy)-2-(2-cyclohexylethyl)-2-(((2,2,3,3,3-pentafluoropropoxy)sulfonyl)amino)butyl acetate:* ***Obtained following derivatisation of* 42**

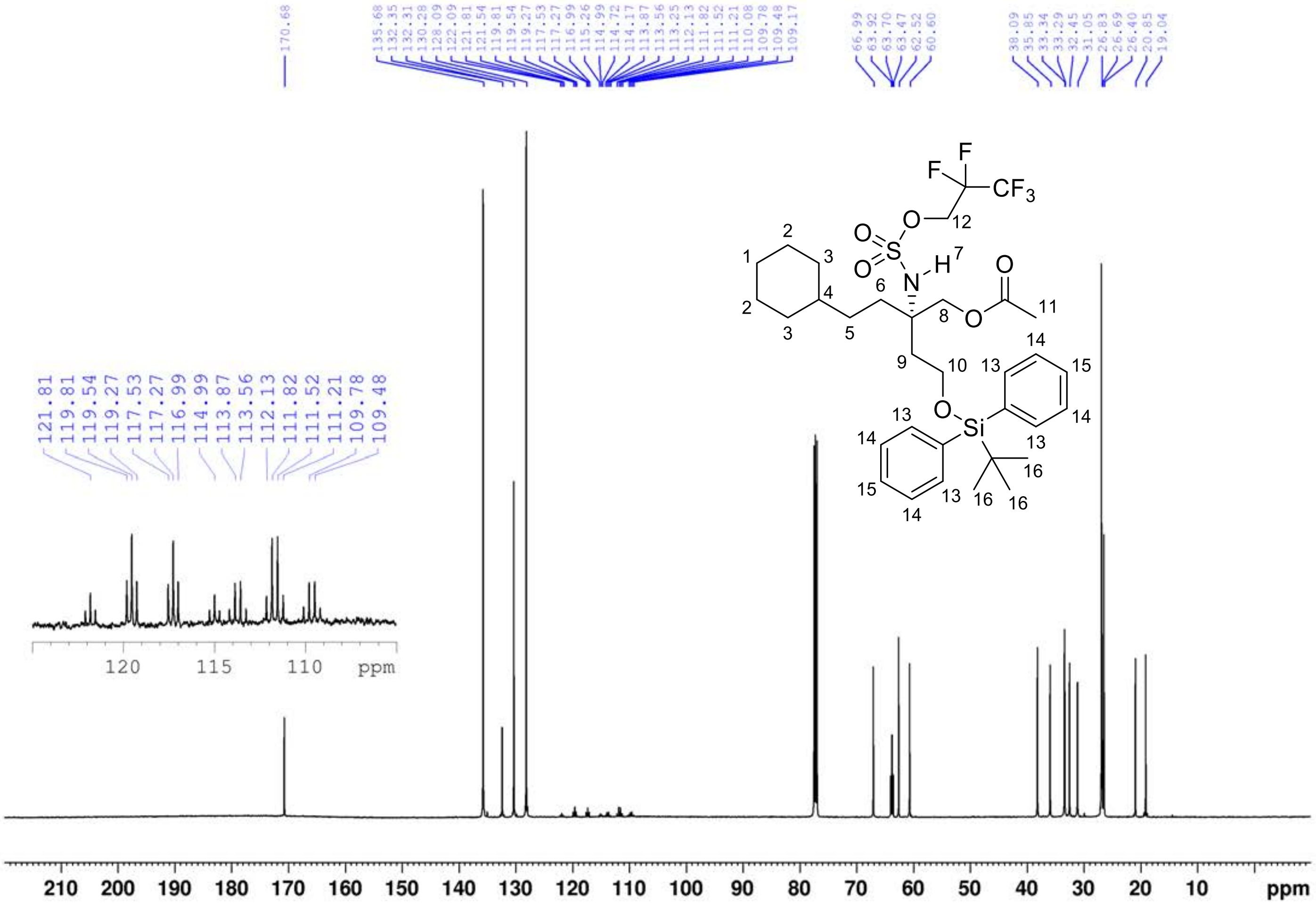

^{19}F NMR (376 MHz, $CDCl_3$) for *(S)-4-((tert-butyldiphenylsilyl)oxy)-2-(2-cyclohexylethyl)-2-(((2,2,3,3,3-pentafluoropropoxy)sulfonyl)amino)butyl acetate:* ***Obtained following derivatisation of* 42**

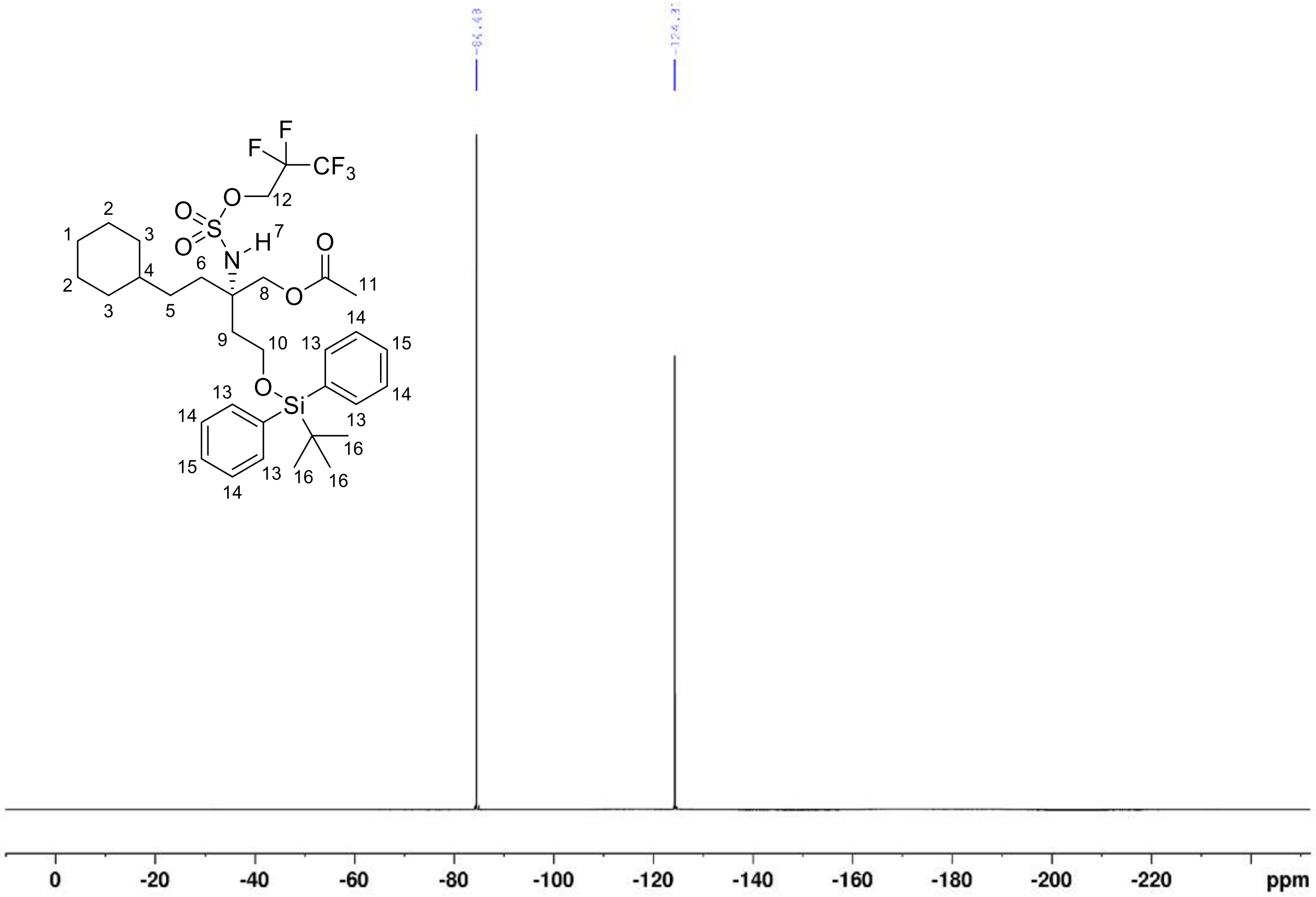

¹H NMR (500 MHz, $CDCl_3$) for *(R)-5-cyclohexyl-3-hydroxy-3-((((2,2,3,3,3-pentafluoropropoxy)sulfonyl)amino)methyl)pentyl acetate:* ***Minor product obtained alongside* 42**

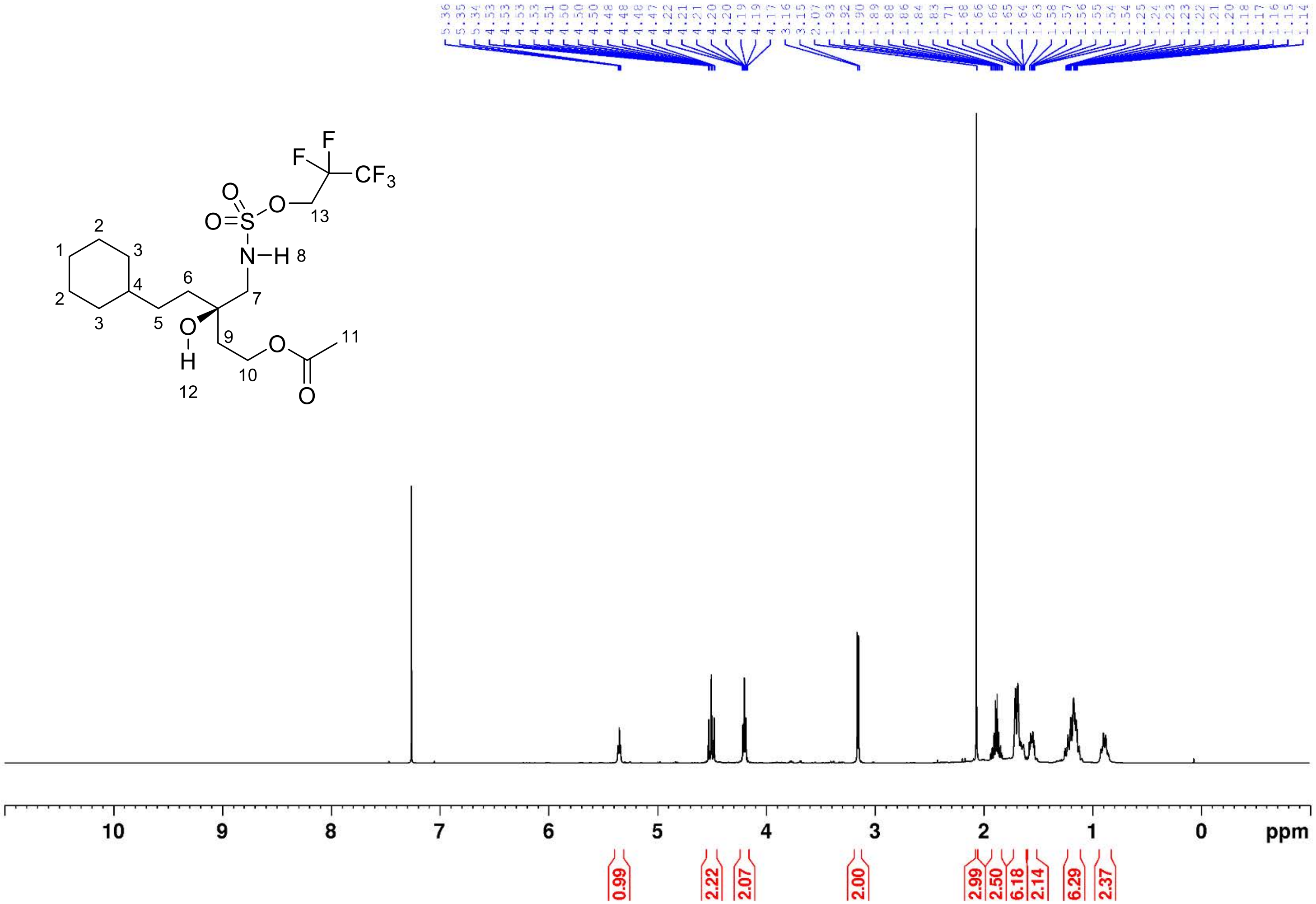

^{13}C NMR (126 MHz, $CDCl_3$) for *(R)-5-cyclohexyl-3-hydroxy-3-((((2,2,3,3,3-pentafluoropropoxy)sulfonyl)amino)methyl)pentyl acetate:* ***Minor product obtained alongside* 42**

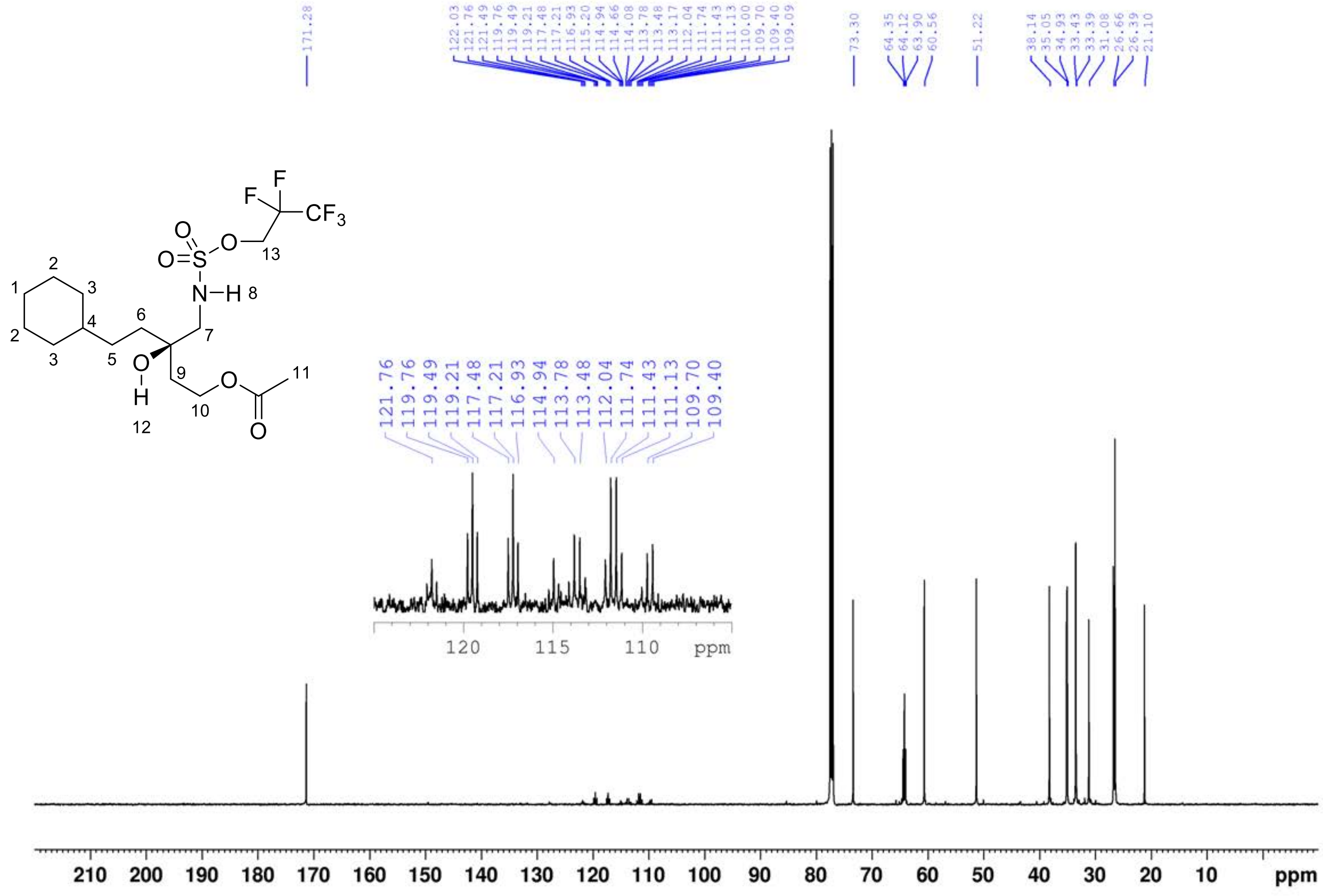

^{19}F NMR (376 MHz, $CDCl_3$) for *(R)-5-cyclohexyl-3-hydroxy-3-((((2,2,3,3,3-pentafluoropropoxy)sulfonyl)amino)methyl)pentyl acetate:* ***Minor product obtained alongside* 42**

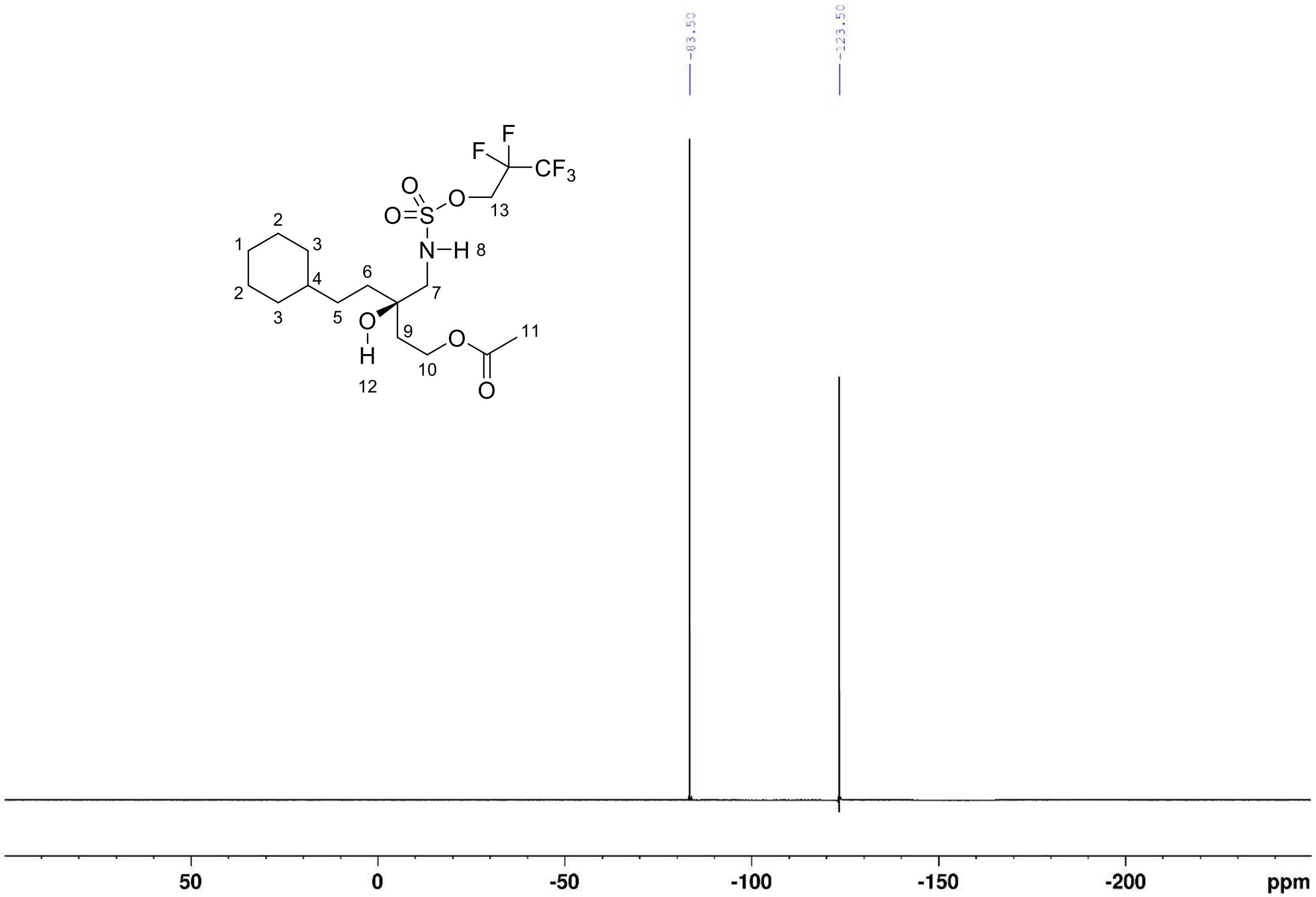

¹H NMR (500 MHz, $CDCl_3$) for *(R)-3-azido-5-cyclohexyl-3-((((2,2,3,3,3-pentafluoropropoxy)sulfonyl)amino)methyl)pentyl acetate* (**43**)

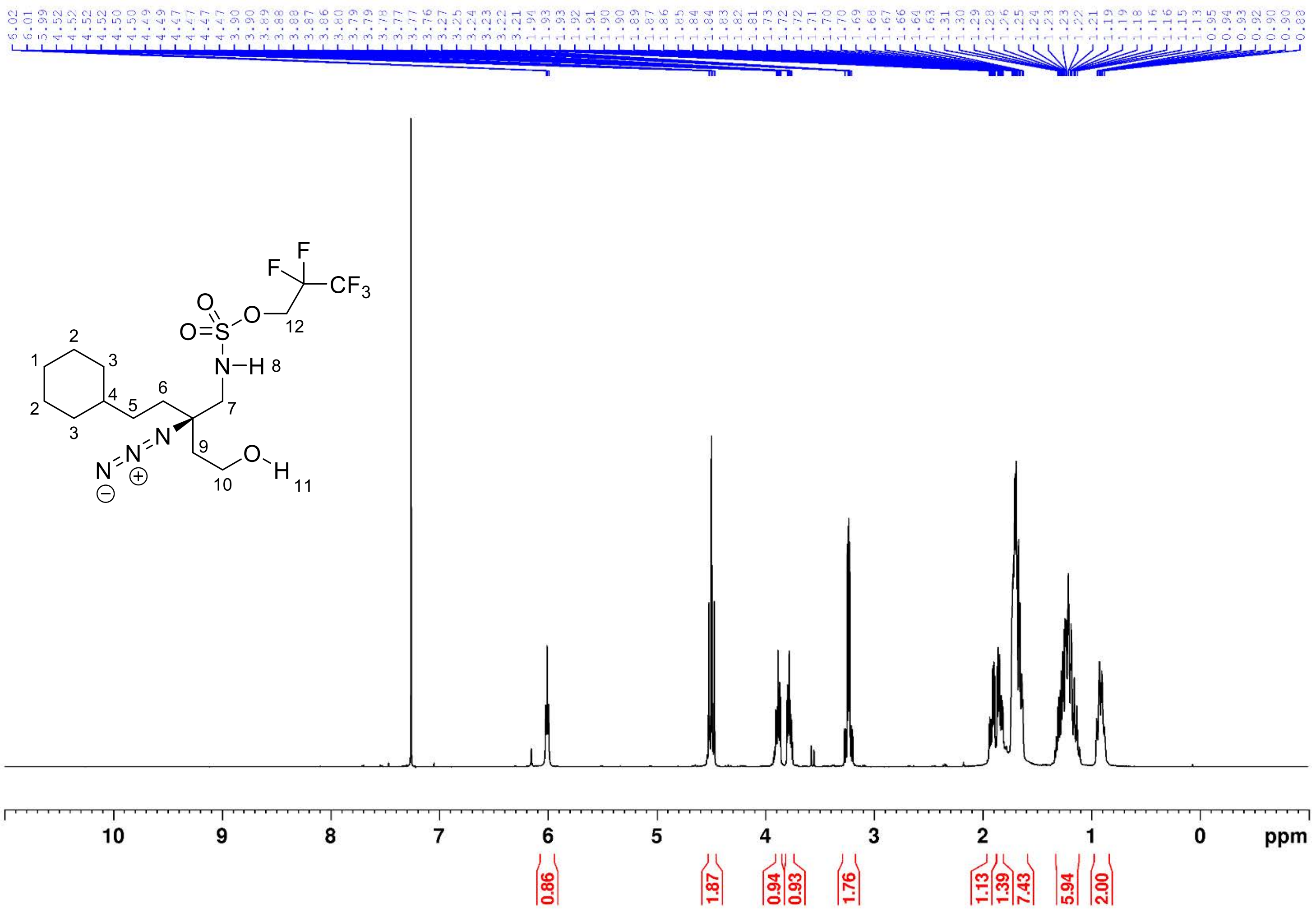

^{13}C NMR (126 MHz, $CDCl_3$) for *(R)-3-azido-5-cyclohexyl-3-((((2,2,3,3,3-pentafluoropropoxy)sulfonyl)amino)methyl)pentyl acetate* (**43**)

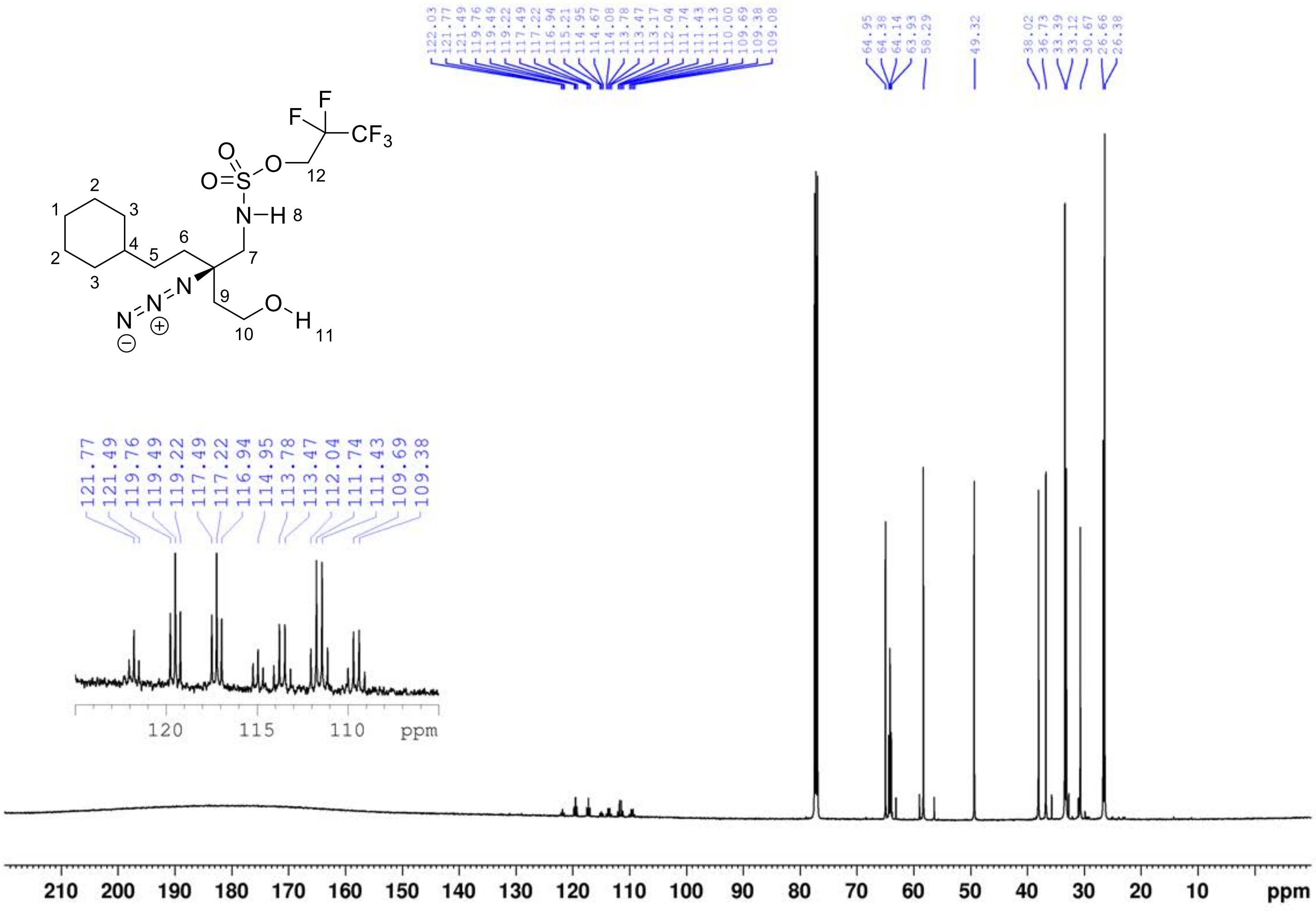

^{19}F NMR (471 MHz, $CDCl_3$) for *(R)-3-azido-5-cyclohexyl-3-((((2,2,3,3,3-pentafluoropropoxy)sulfonyl)amino)methyl)pentyl acetate* (**43**)

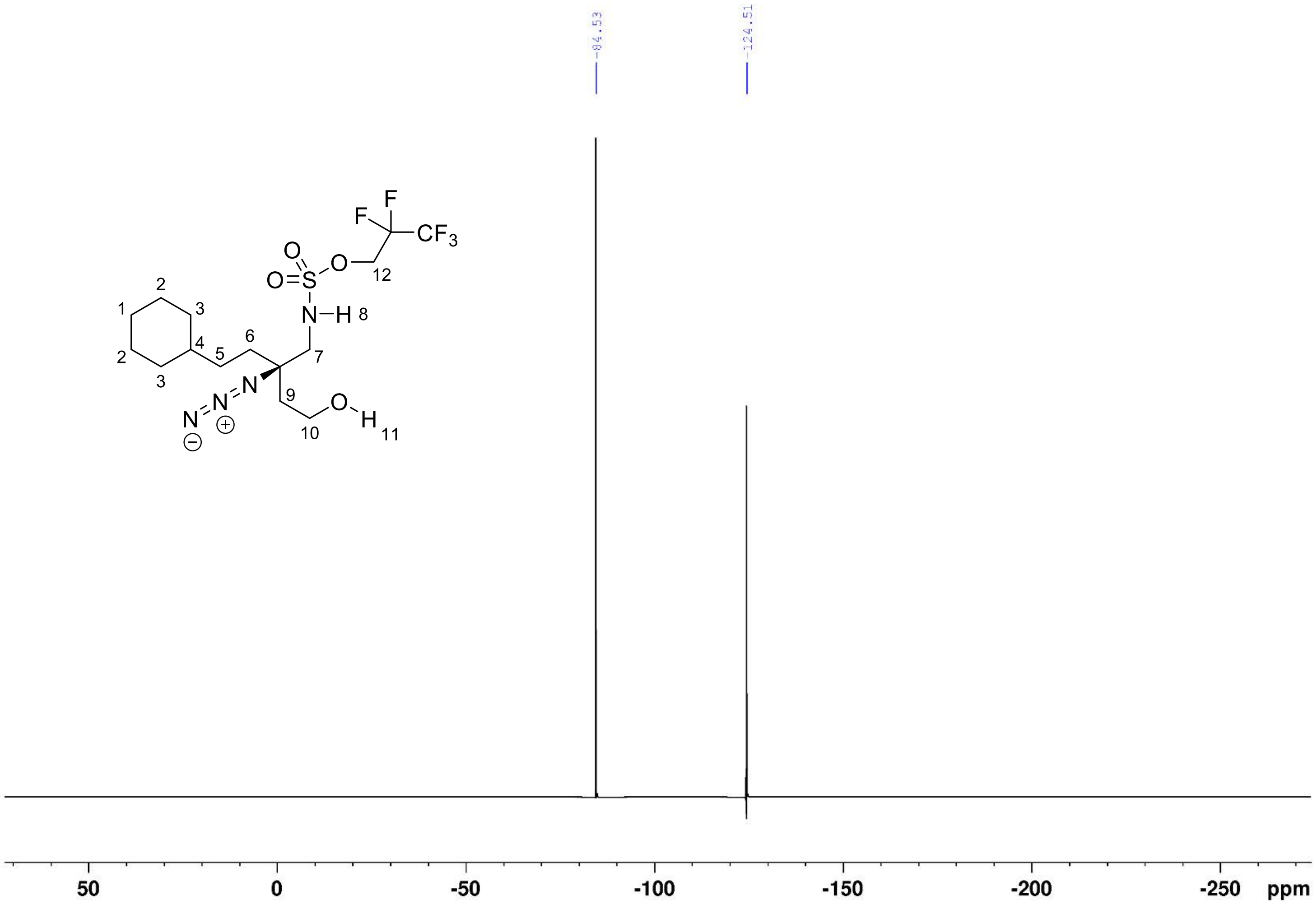

[1]**H NMR** (500 MHz, $CDCl_3$) for *2,2,3,3,3-pentafluoropropyl (R)-(4-cyclohexyl-2-(2-hydroxyethyl)-2-(4-phenyl-1H-1,2,3-triazol-1-yl)butyl)sulfamate* (**44**)

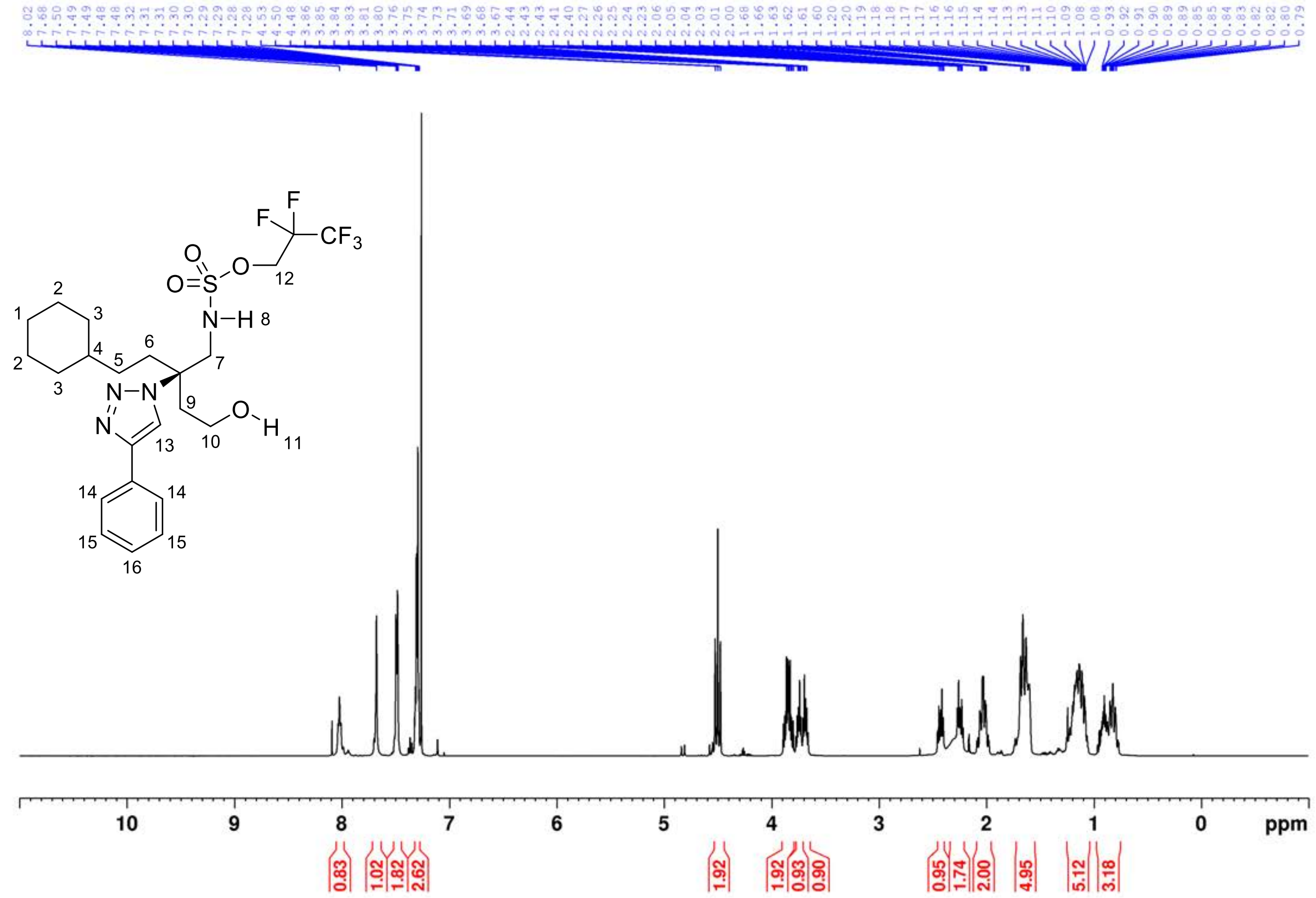

^{13}C NMR (126 MHz, $CDCl_3$) for *2,2,3,3,3-pentafluoropropyl (R)-(4-cyclohexyl-2-(2-hydroxyethyl)-2-(4-phenyl-1H-1,2,3-triazol-1-yl)butyl)sulfamate* (**44**)

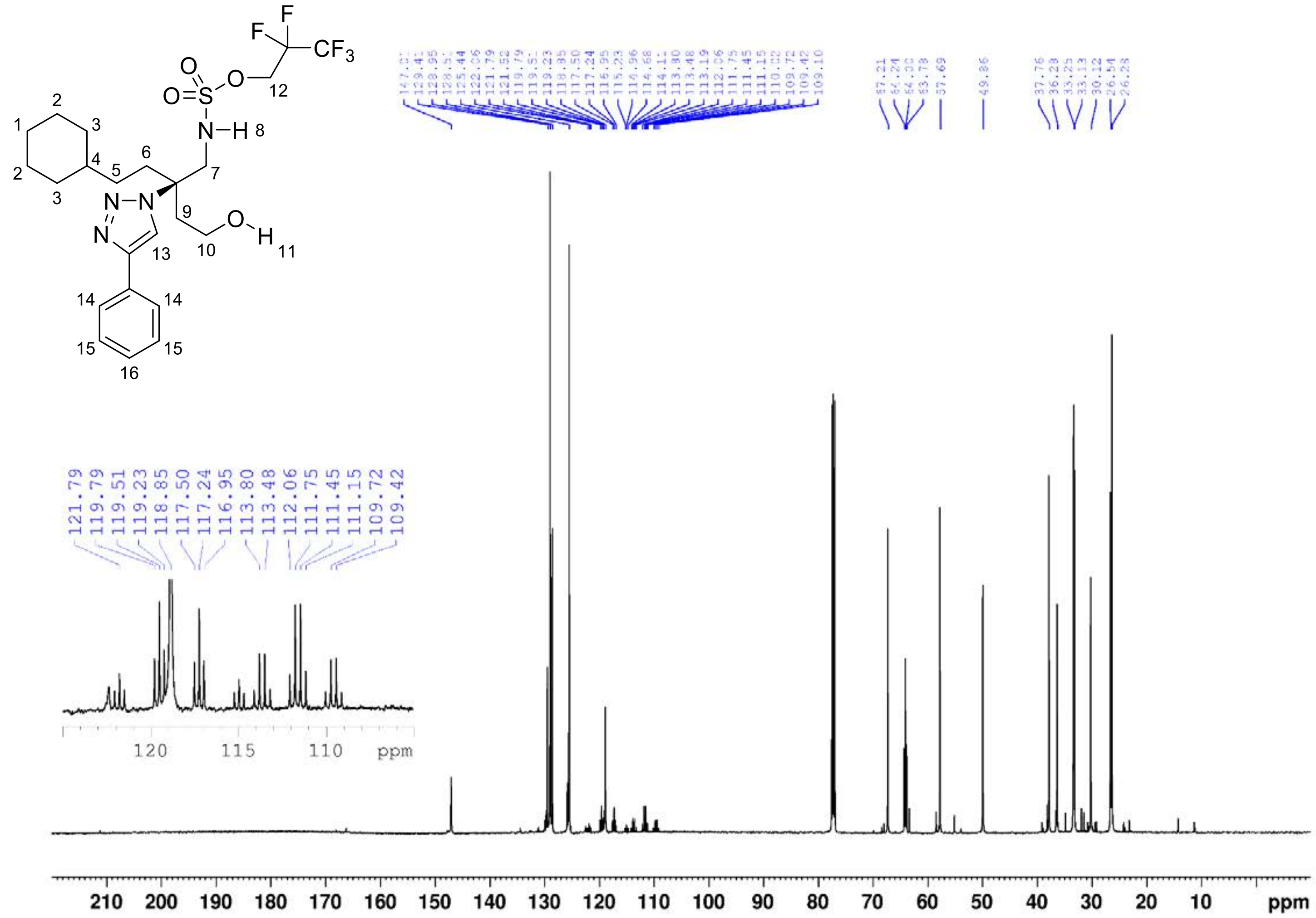

^{19}F NMR (471 MHz, $CDCl_3$) for *2,2,3,3,3-pentafluoropropyl (R)-(4-cyclohexyl-2-(2-hydroxyethyl)-2-(4-phenyl-1H-1,2,3-triazol-1-yl)butyl)sulfamate* (**44**)

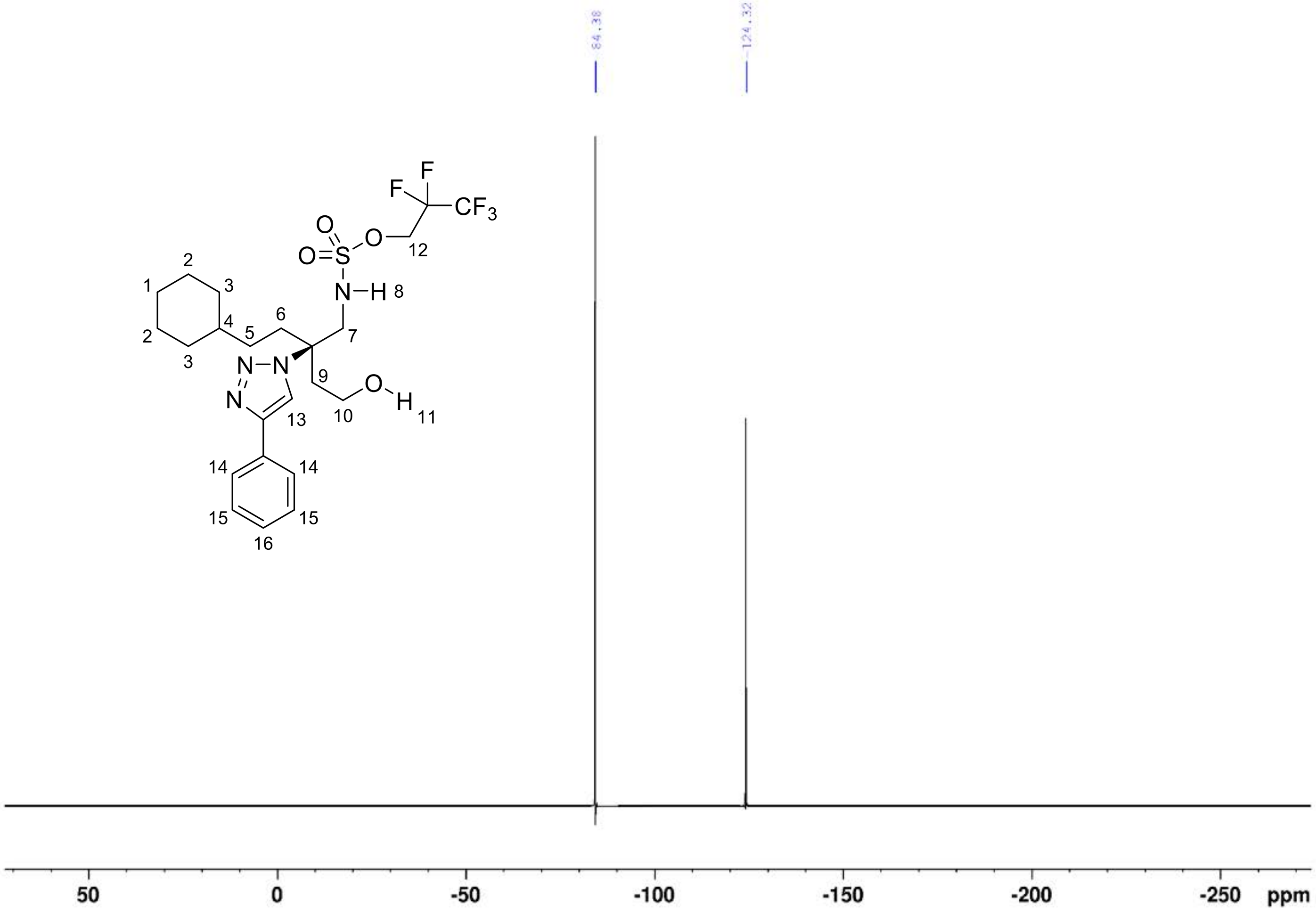

^{1}H NMR (500 MHz, $CDCl_3$) for *2,2,3,3,3-pentafluoropropyl (S)-(4-hydroxy-1-phenylbutan-2-yl)sulfamate* (**45**)

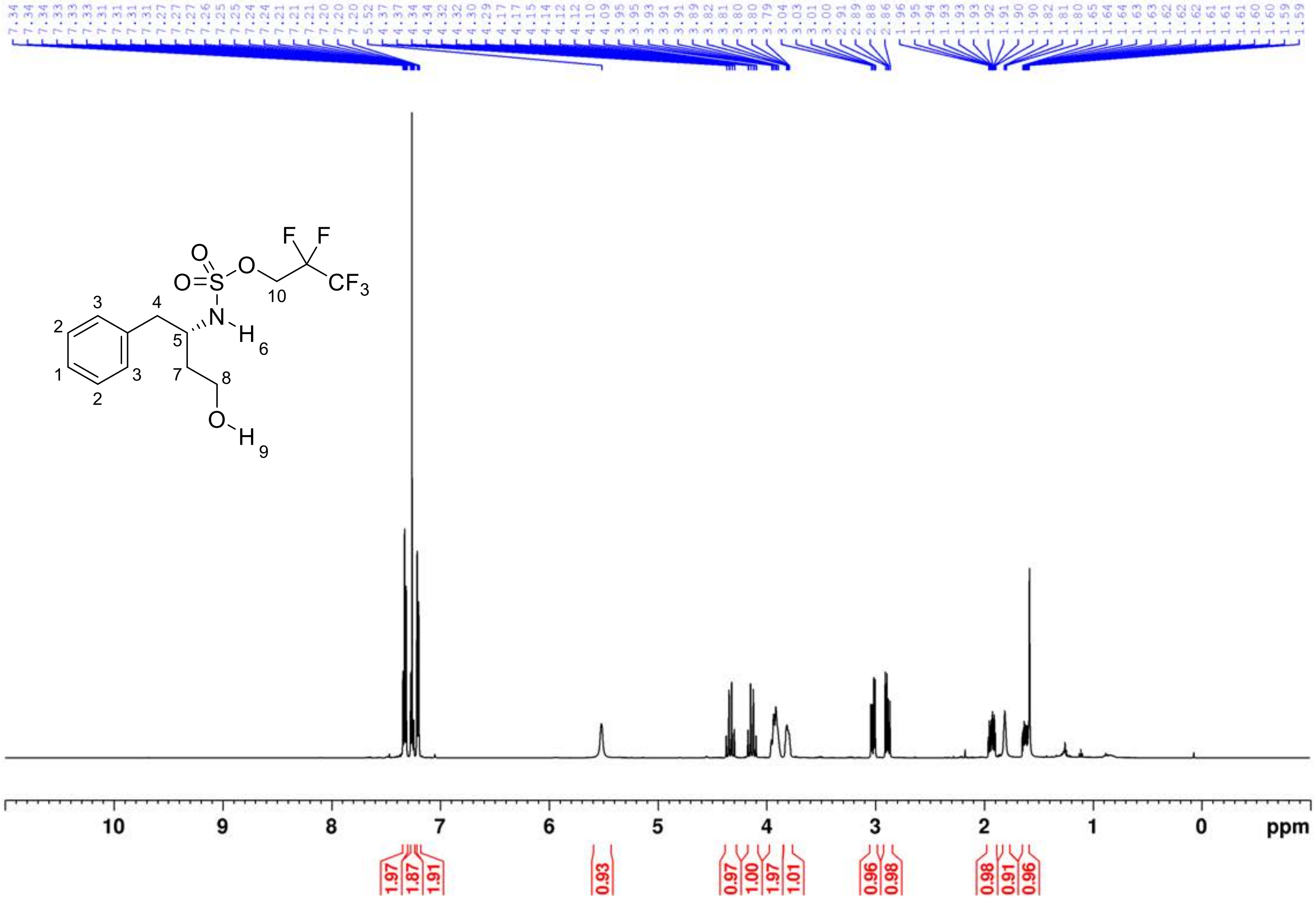

^{13}C NMR (101 MHz, $CDCl_3$) for *2,2,3,3,3-pentafluoropropyl (S)-(4-hydroxy-1-phenylbutan-2-yl)sulfamate* (**45**)

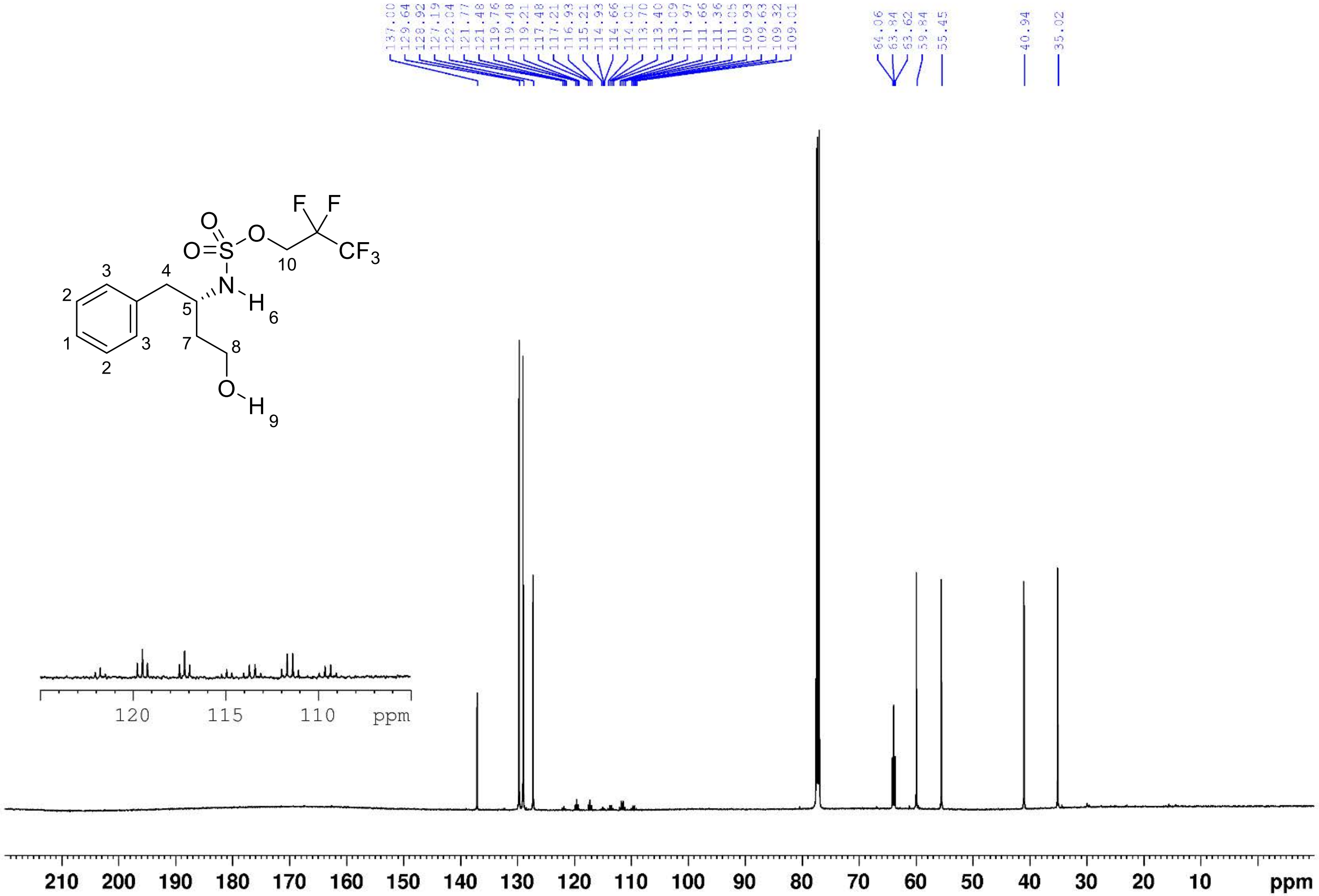

^{19}F NMR (376 MHz, $CDCl_3$) for *2,2,3,3,3-pentafluoropropyl (S)-(4-hydroxy-1-phenylbutan-2-yl)sulfamate* (**45**)

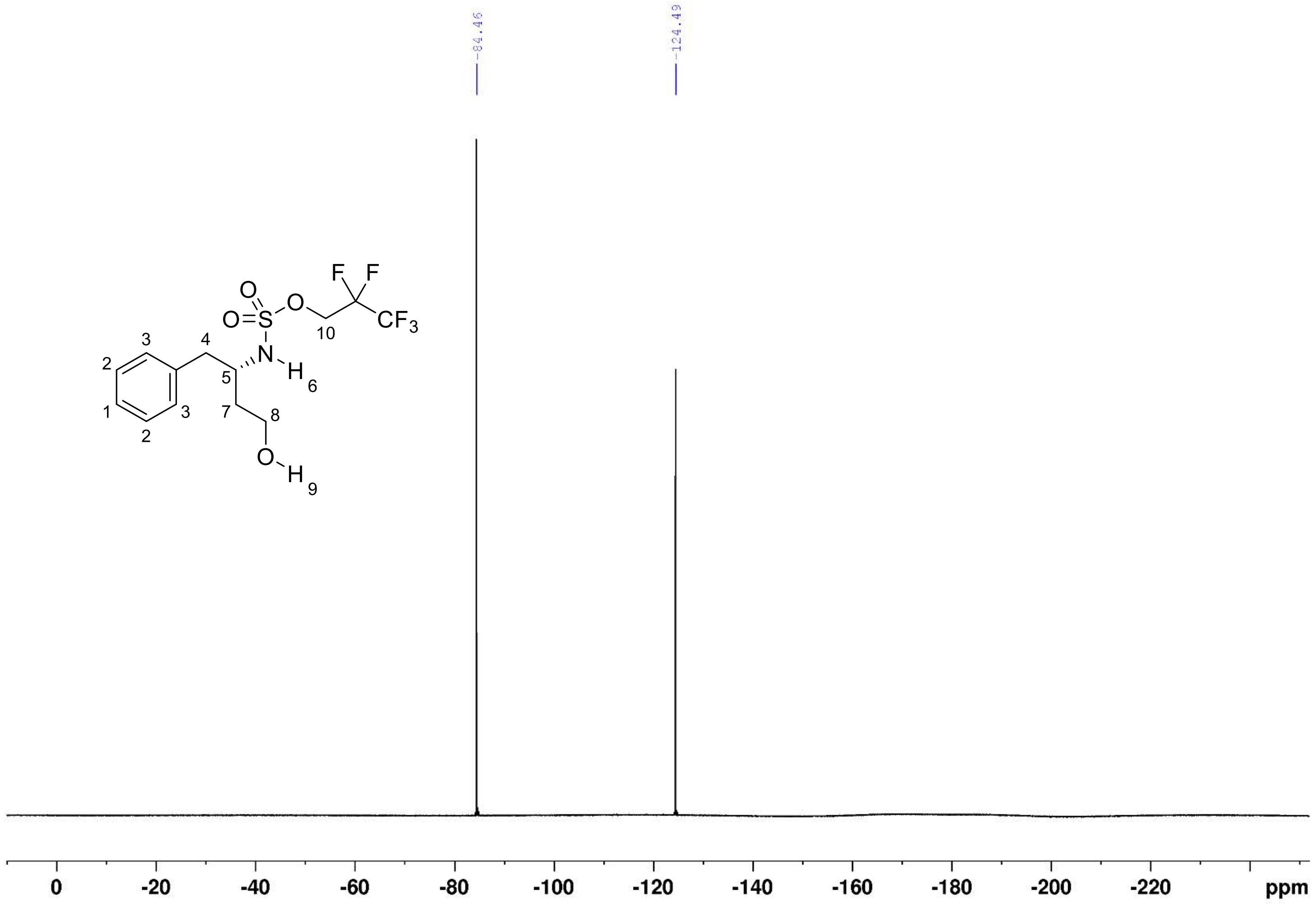

^{1}H NMR (500 MHz, $CDCl_3$) for *2,2,3,3,3-pentafluoropropyl (R)-(5-hydroxy-1-phenylpentan-2-yl)sulfamate* (**46**)

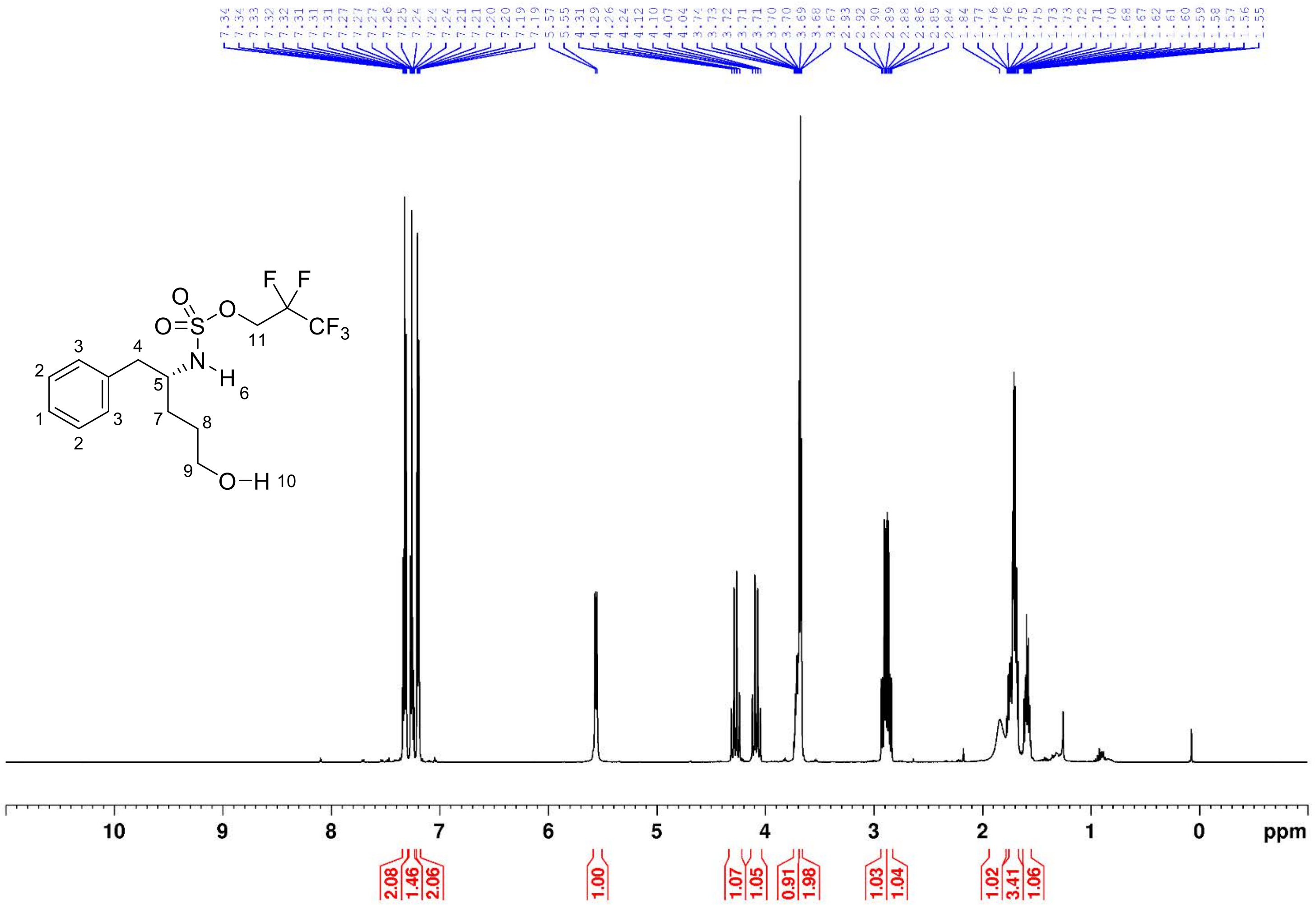

^{13}C NMR (126 MHz, $CDCl_3$) for *2,2,3,3,3-pentafluoropropyl (R)-(5-hydroxy-1-phenylpentan-2-yl)sulfamate* (**46**)

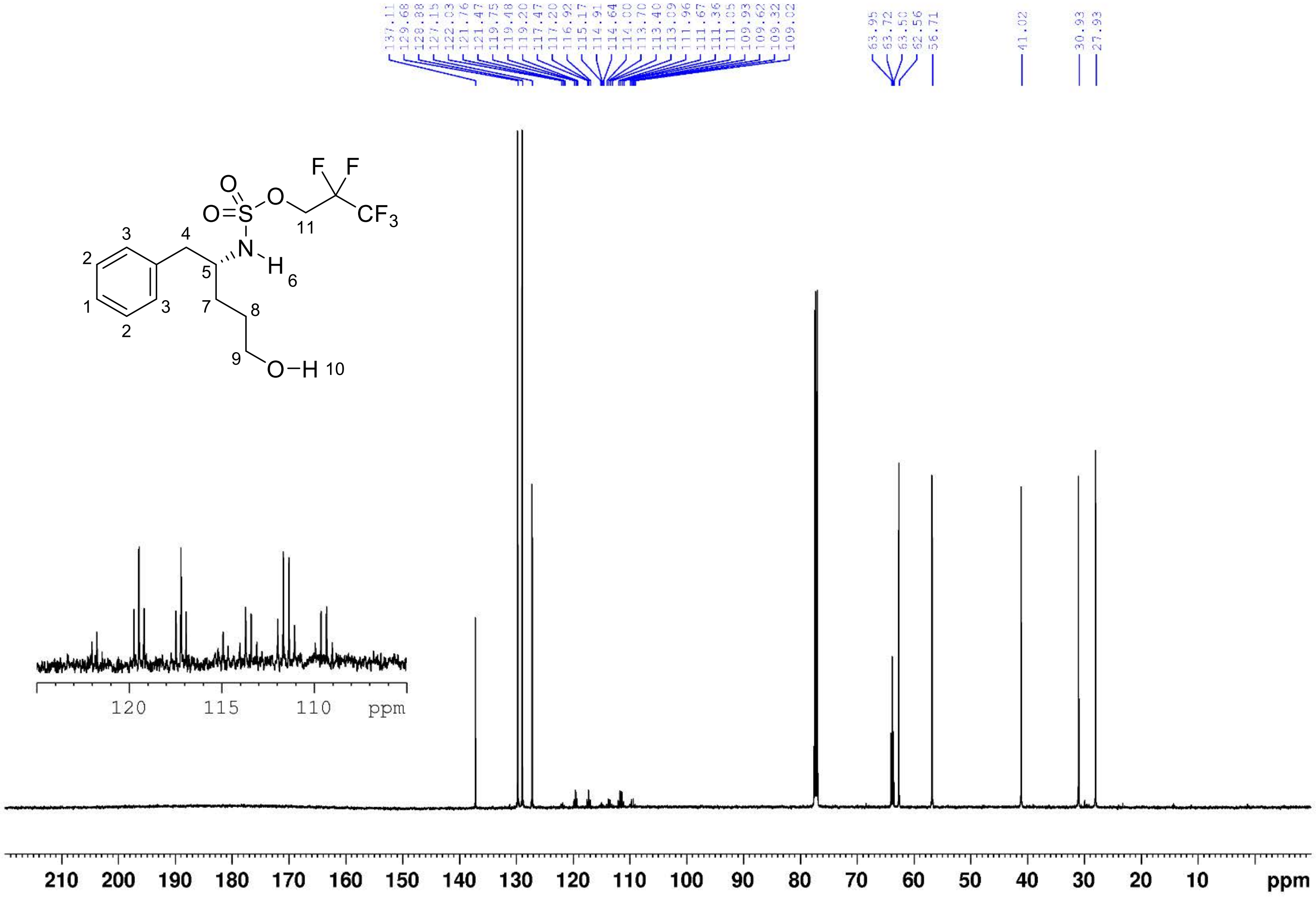

^{19}F NMR (376 MHz, $CDCl_3$) for *2,2,3,3,3-pentafluoropropyl (R)-(5-hydroxy-1-phenylpentan-2-yl)sulfamate* (**46**)

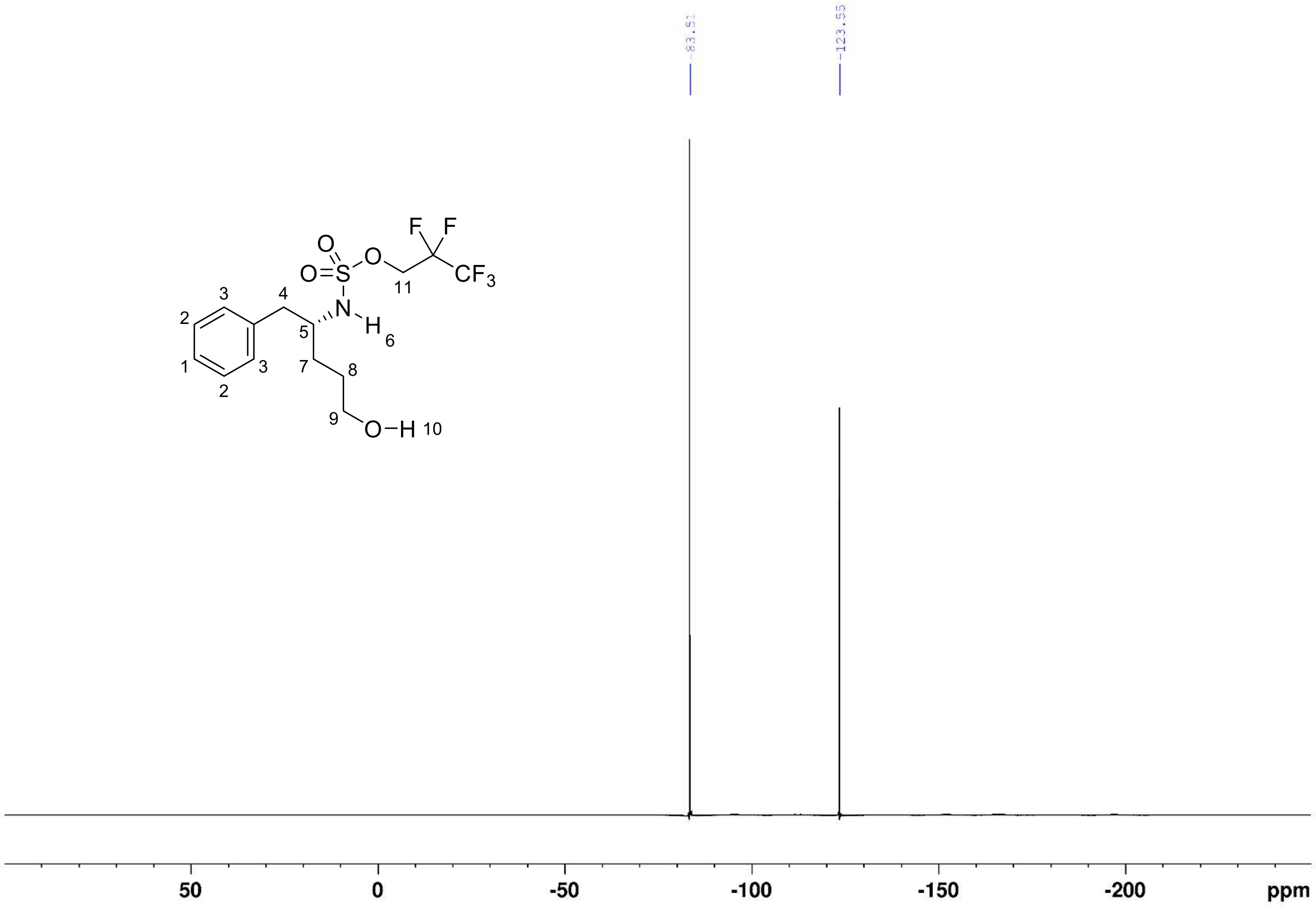

^{1}H NMR (500 MHz, $CDCl_3$) for *2,2,3,3,3-pentafluoropropyl (R)-(6-hydroxy-1-phenylhexan-2-yl)sulfamate* (**47**)

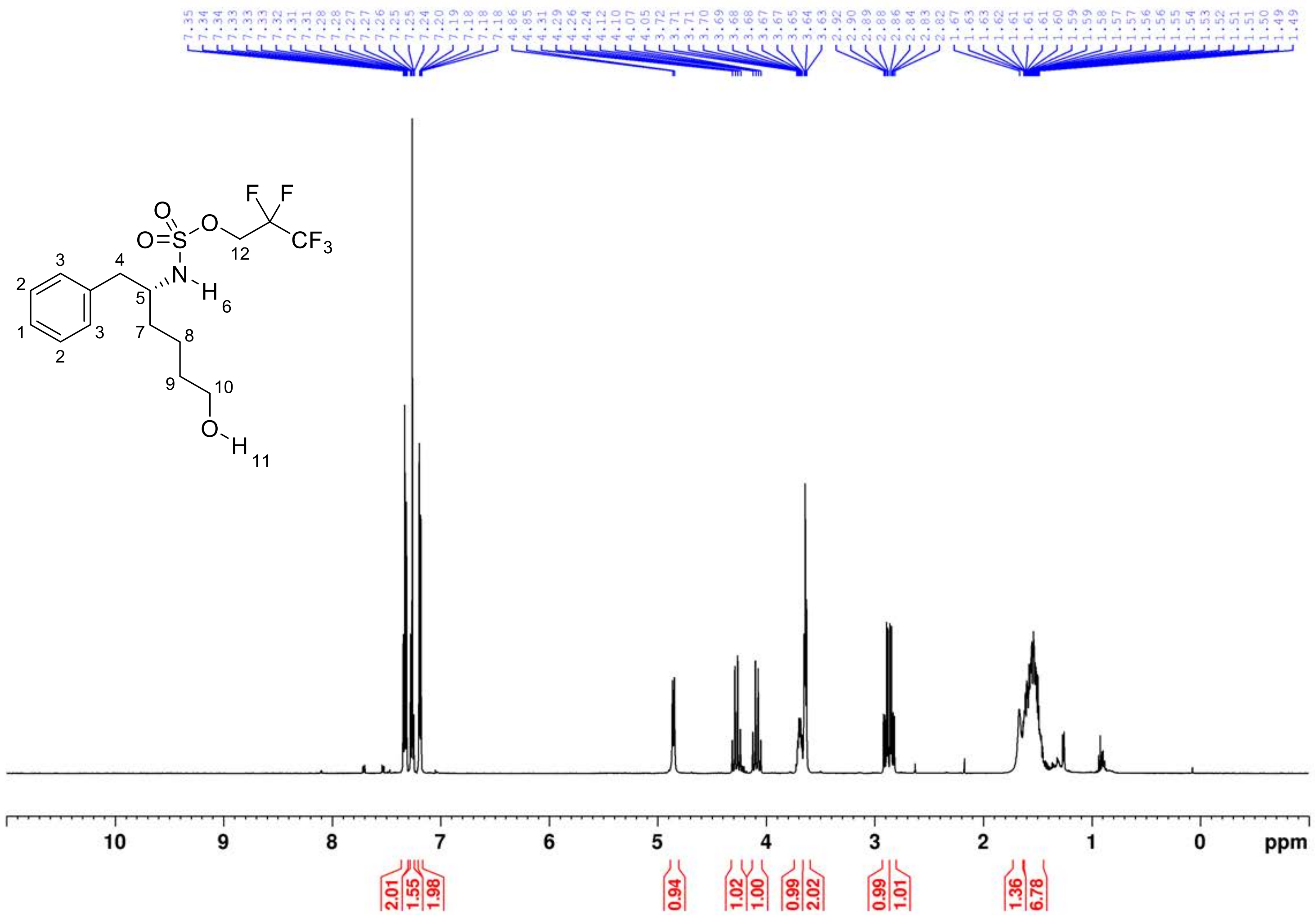

^{13}C NMR (126 MHz, $CDCl_3$) for *2,2,3,3,3-pentafluoropropyl (R)-(6-hydroxy-1-phenylhexan-2-yl)sulfamate* (**47**)

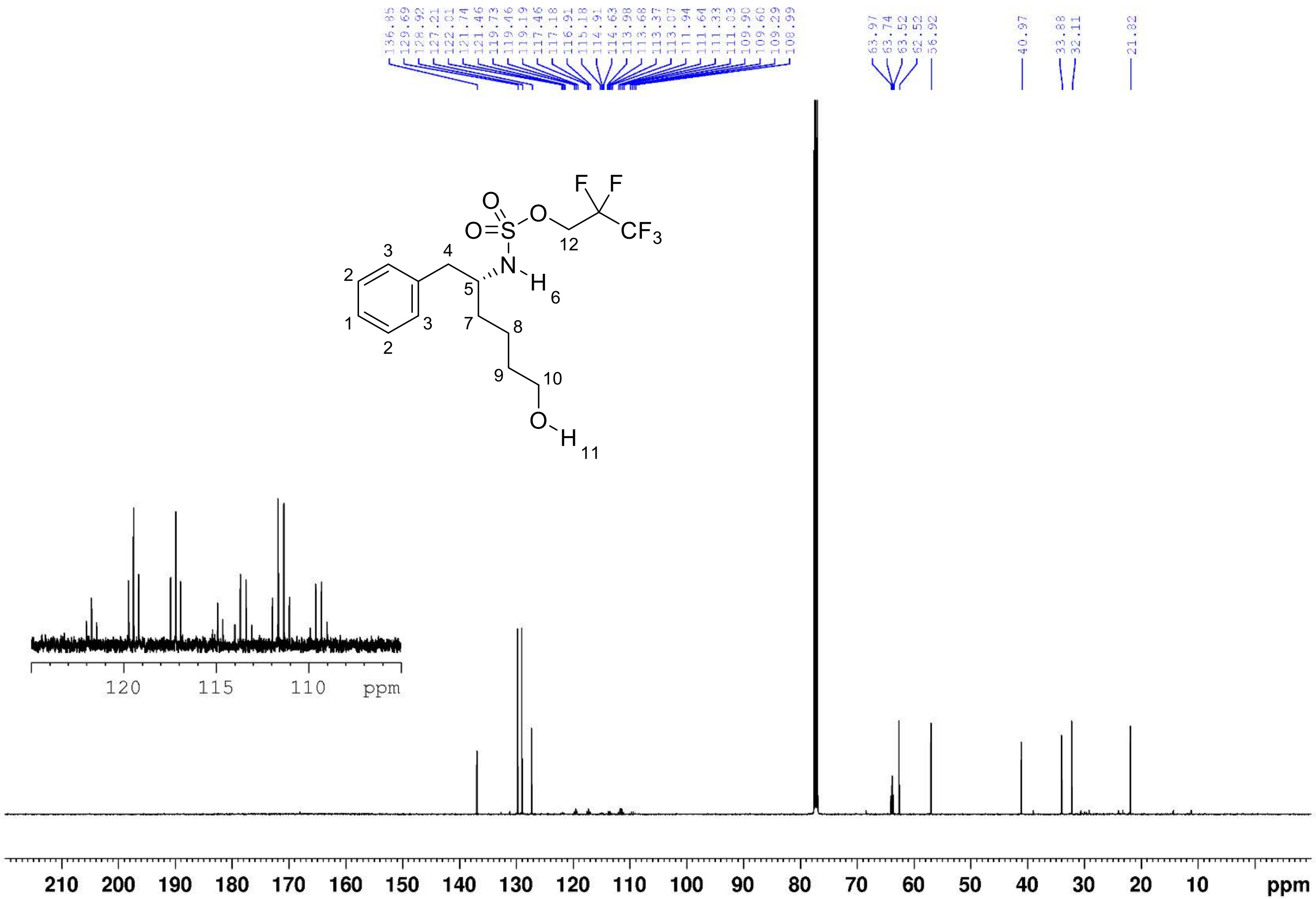

^{19}F NMR (376 MHz, $CDCl_3$) for *2,2,3,3,3-pentafluoropropyl (R)-(6-hydroxy-1-phenylhexan-2-yl)sulfamate* (**47**)

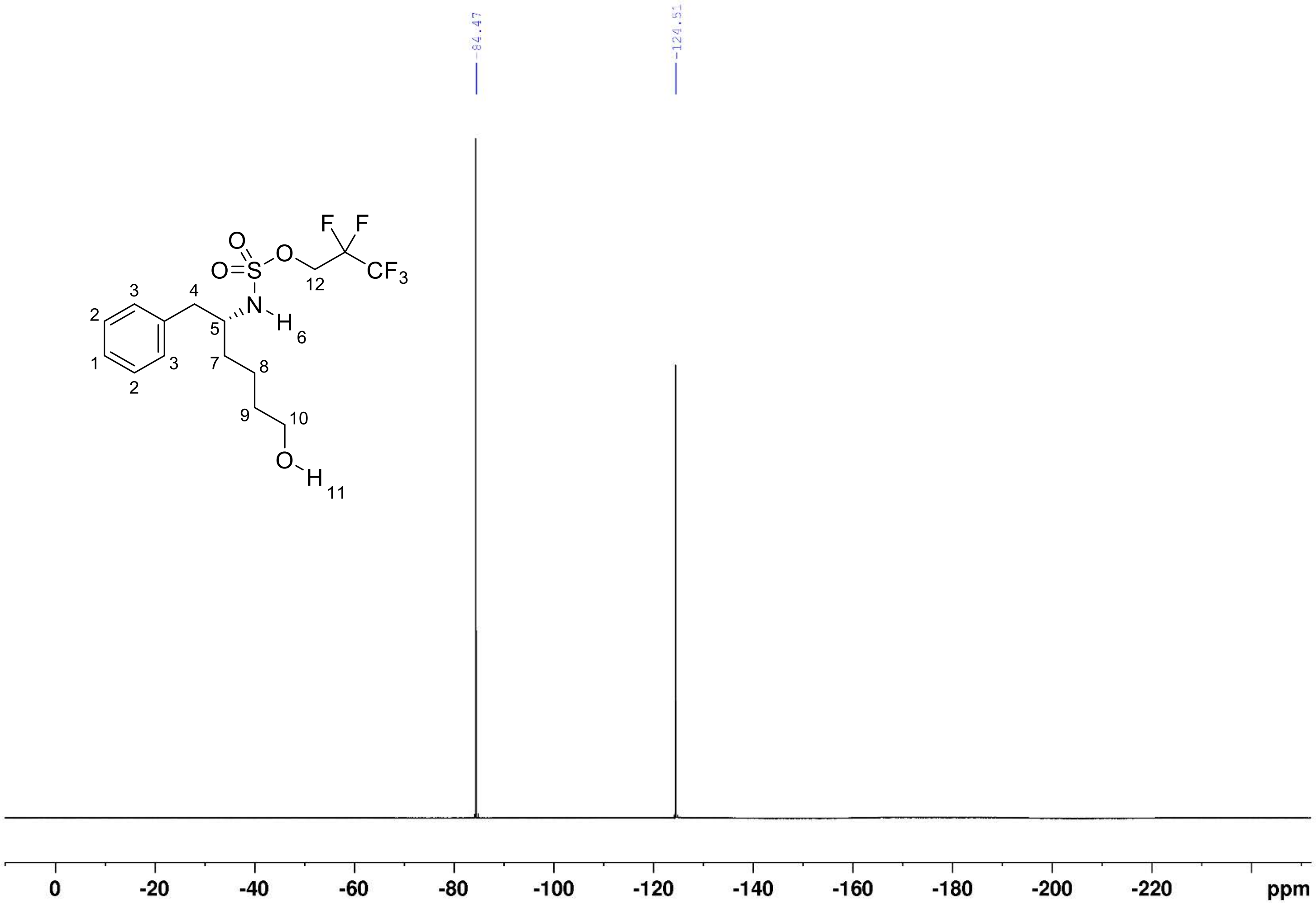

¹H NMR (500 MHz, $CDCl_3$) for *benzyl (R)-(6-hydroxy-1-phenylhexan-2-yl)carbamate* (**48**)

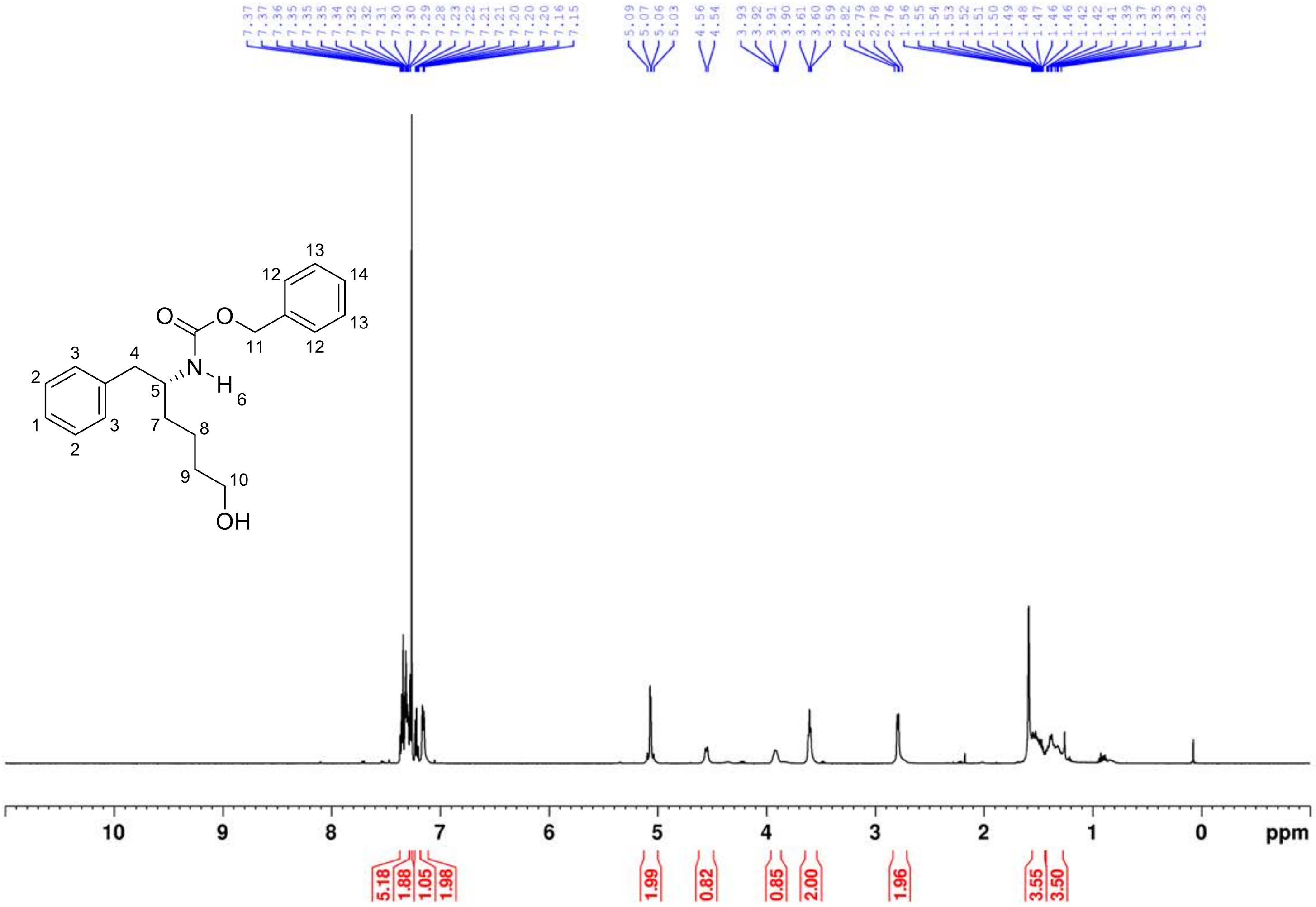

^{13}C NMR (126 MHz, $CDCl_3$) for *benzyl (R)-(6-hydroxy-1-phenylhexan-2-yl)carbamate* (**48**)

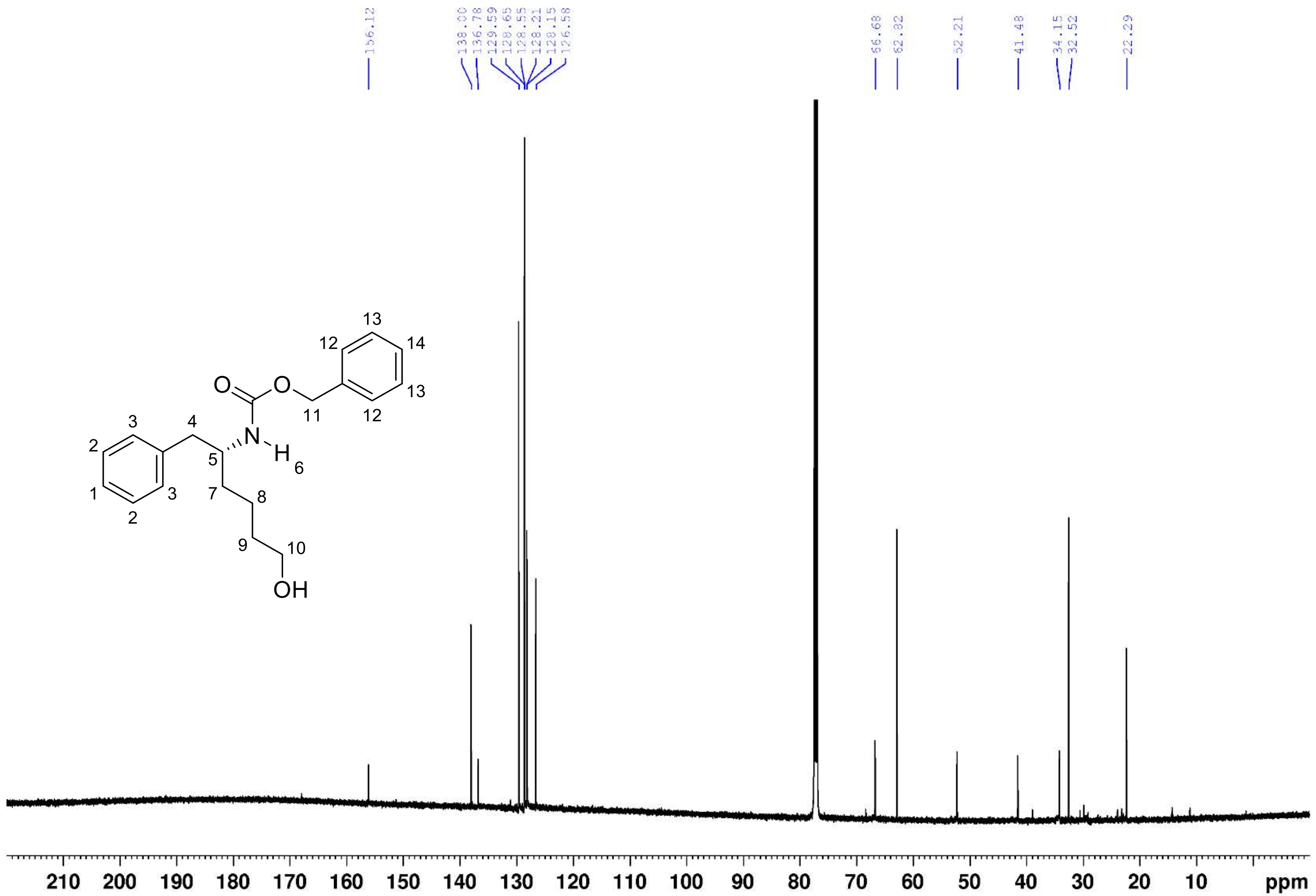

^{1}H NMR (500 MHz, $CDCl_3$) for *2,2,3,3,3-pentafluoropropyl (R)-2-benzylpyrrolidine-1-sulfonate* (**49**)

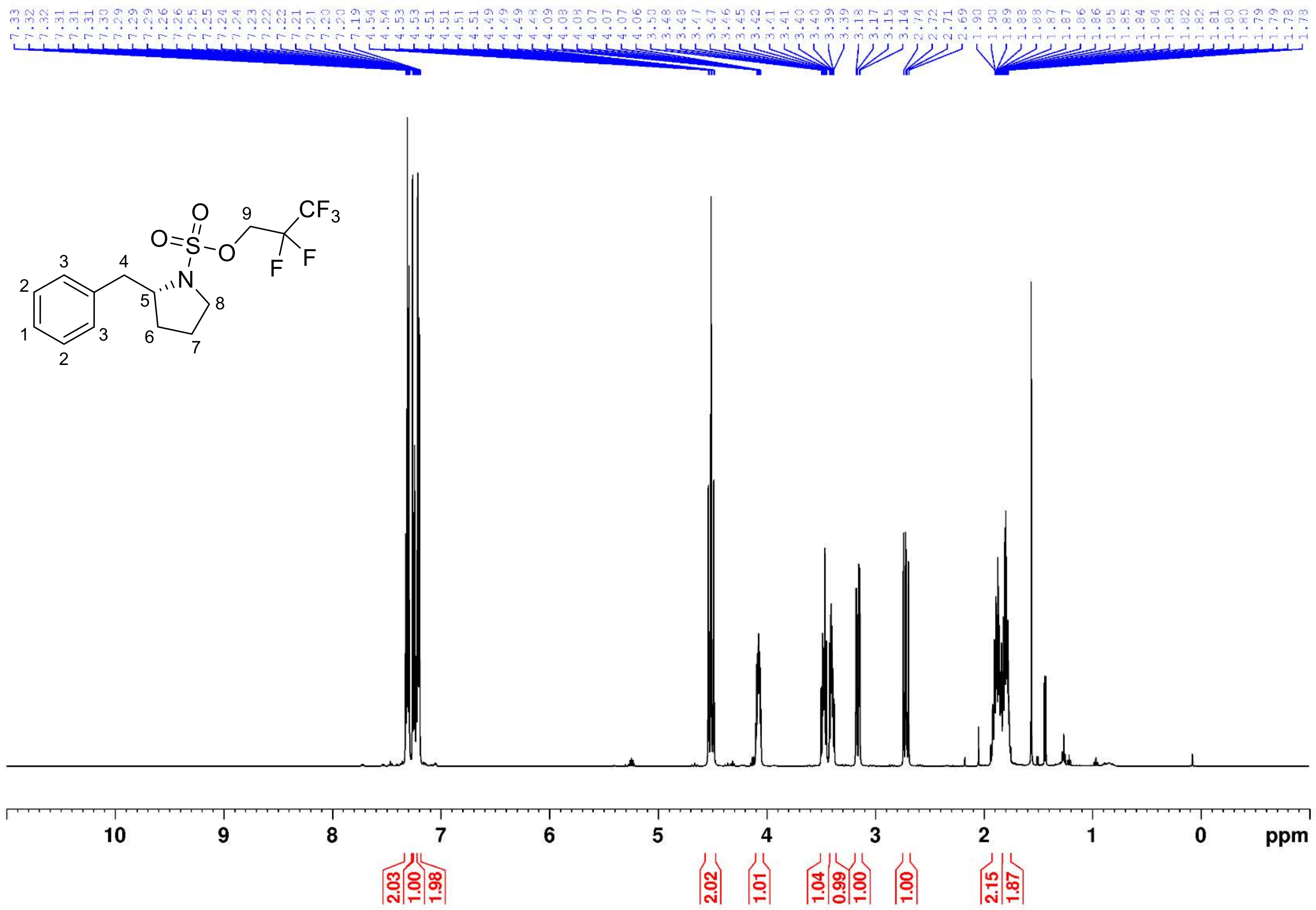

^{13}C NMR (126 MHz, $CDCl_3$) for *2,2,3,3,3-pentafluoropropyl (R)-2-benzylpyrrolidine-1-sulfonate* (**49**)

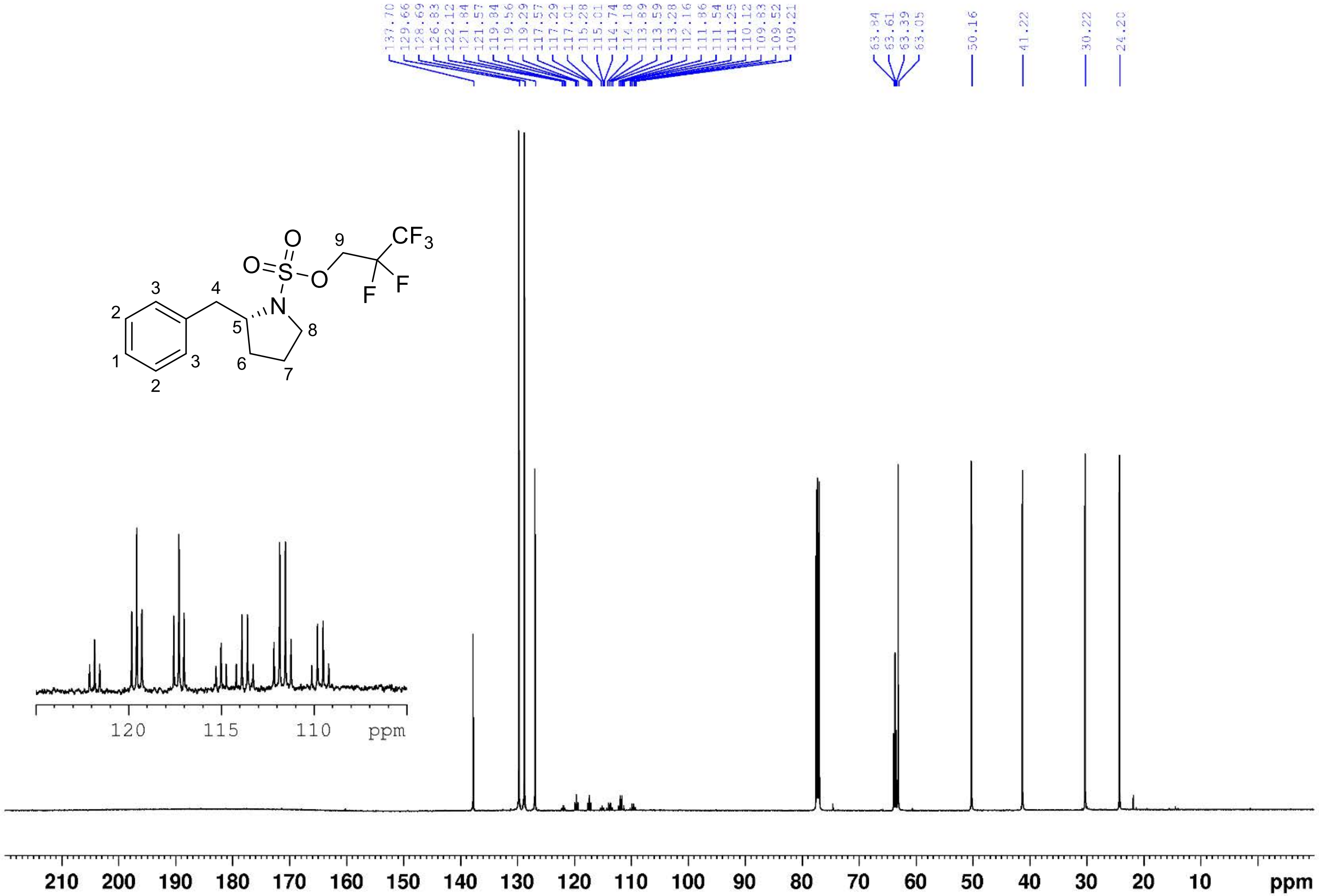

^{19}F NMR (376 MHz, $CDCl_3$) for *2,2,3,3,3-pentafluoropropyl (R)-2-benzylpyrrolidine-1-sulfonate* (**49**)

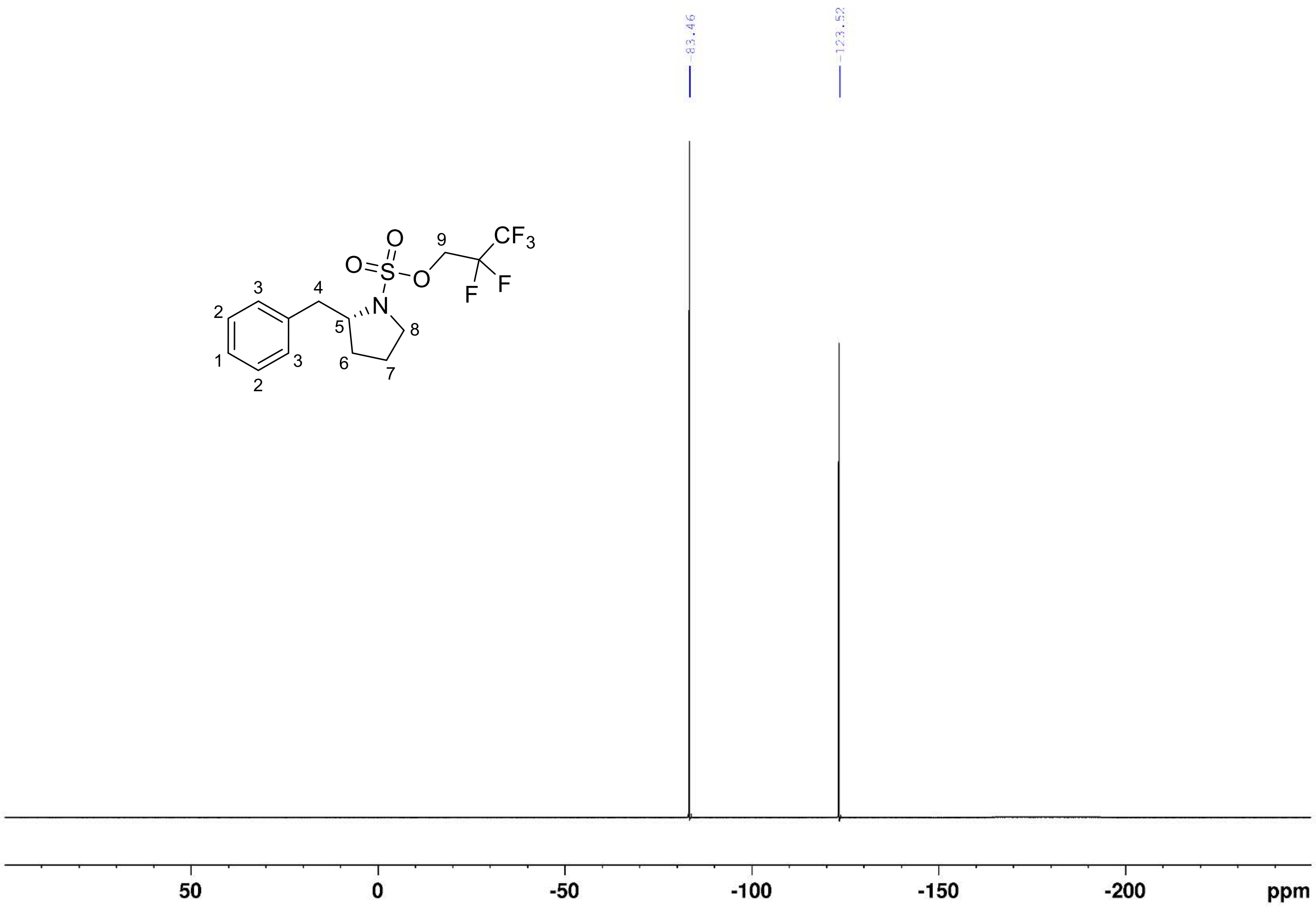

^{1}H NMR (500 MHz, $CDCl_3$) for *2,2,3,3,3-pentafluoropropyl (S)-2-benzylazetidine-1-sulfonate* (**50**)

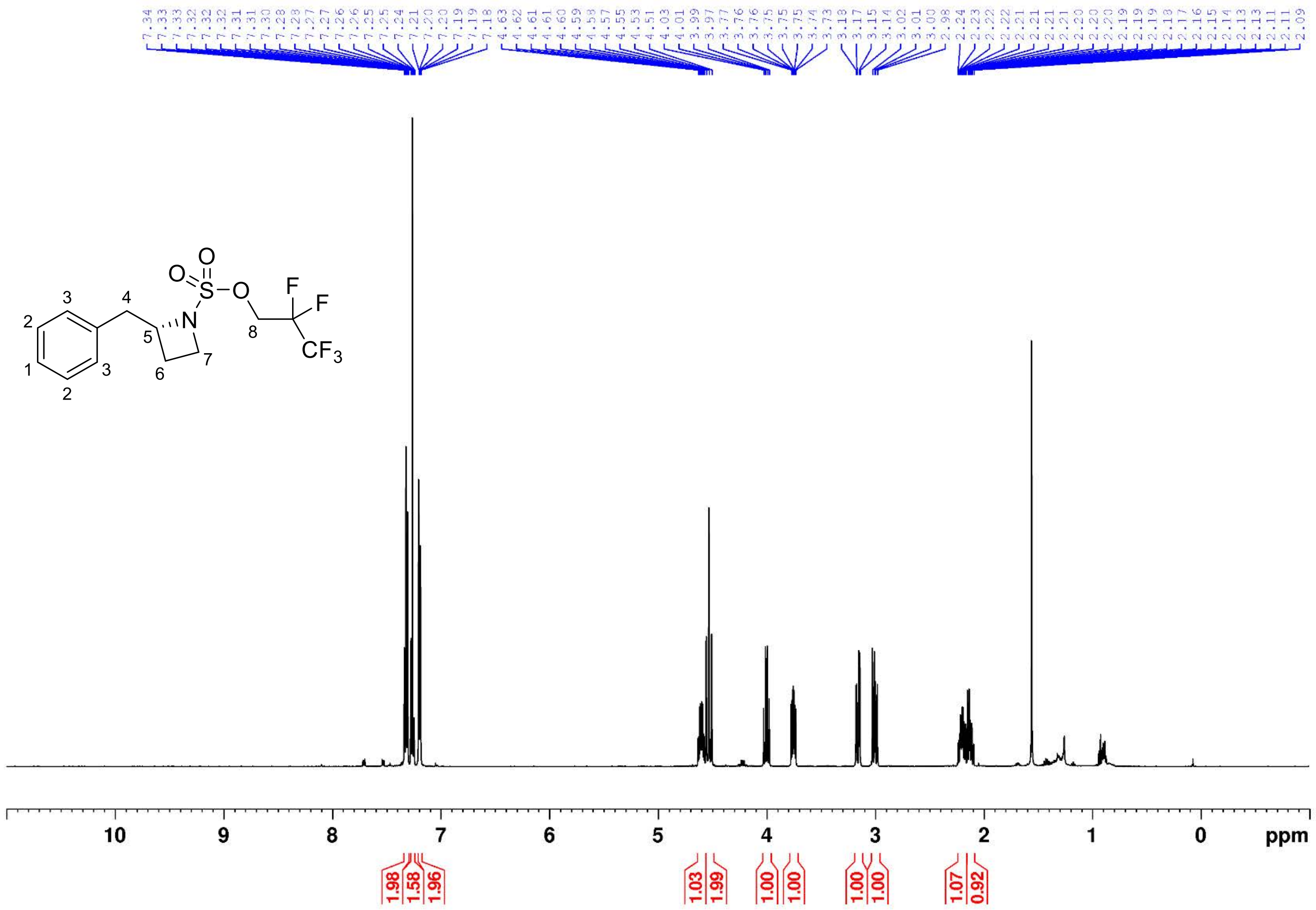

^{13}C NMR (126 MHz, $CDCl_3$) for *2,2,3,3,3-pentafluoropropyl (S)-2-benzylazetidine-1-sulfonate* (**50**)

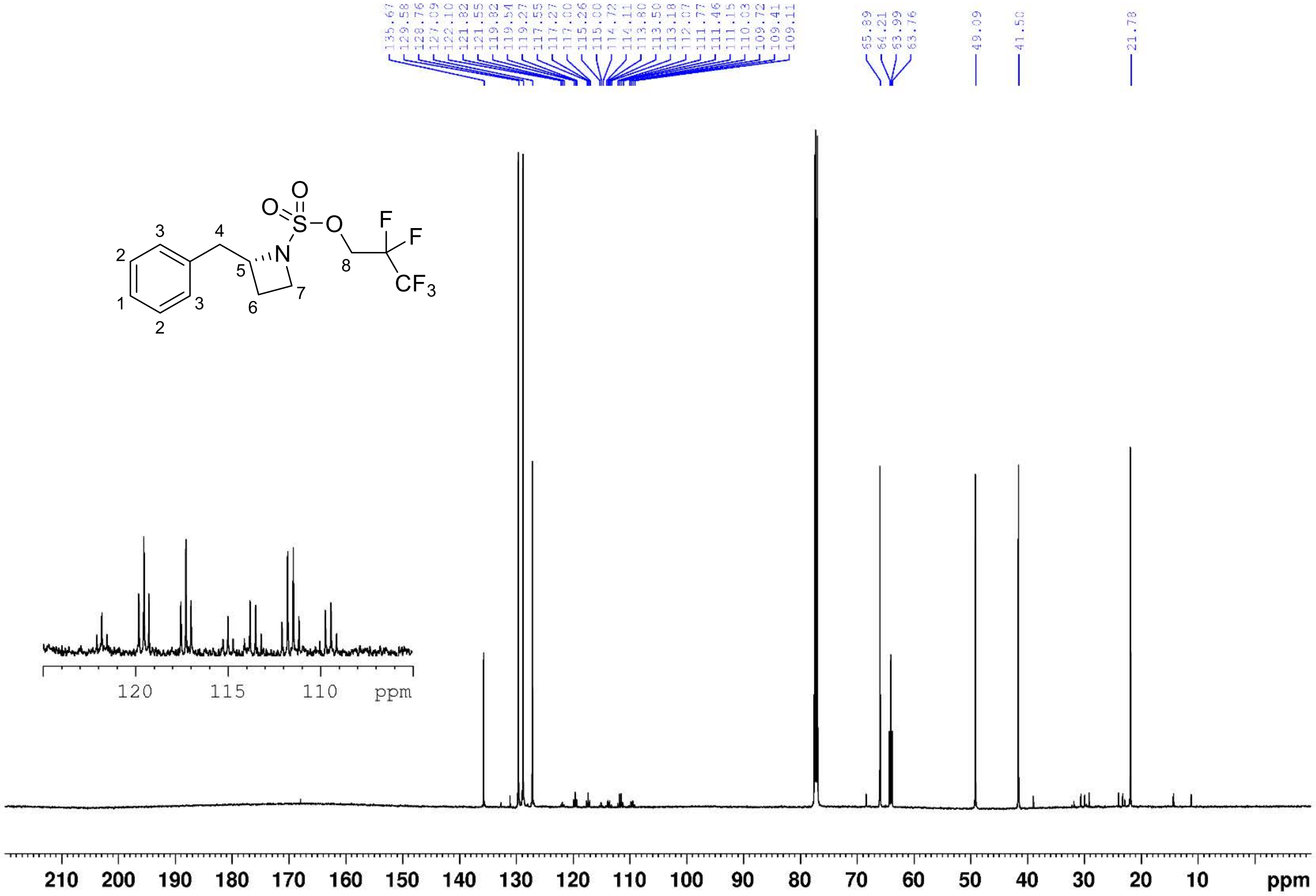

^{19}F NMR (376 MHz, $CDCl_3$) for *2,2,3,3,3-pentafluoropropyl (S)-2-benzylazetidine-1-sulfonate* (**50**)

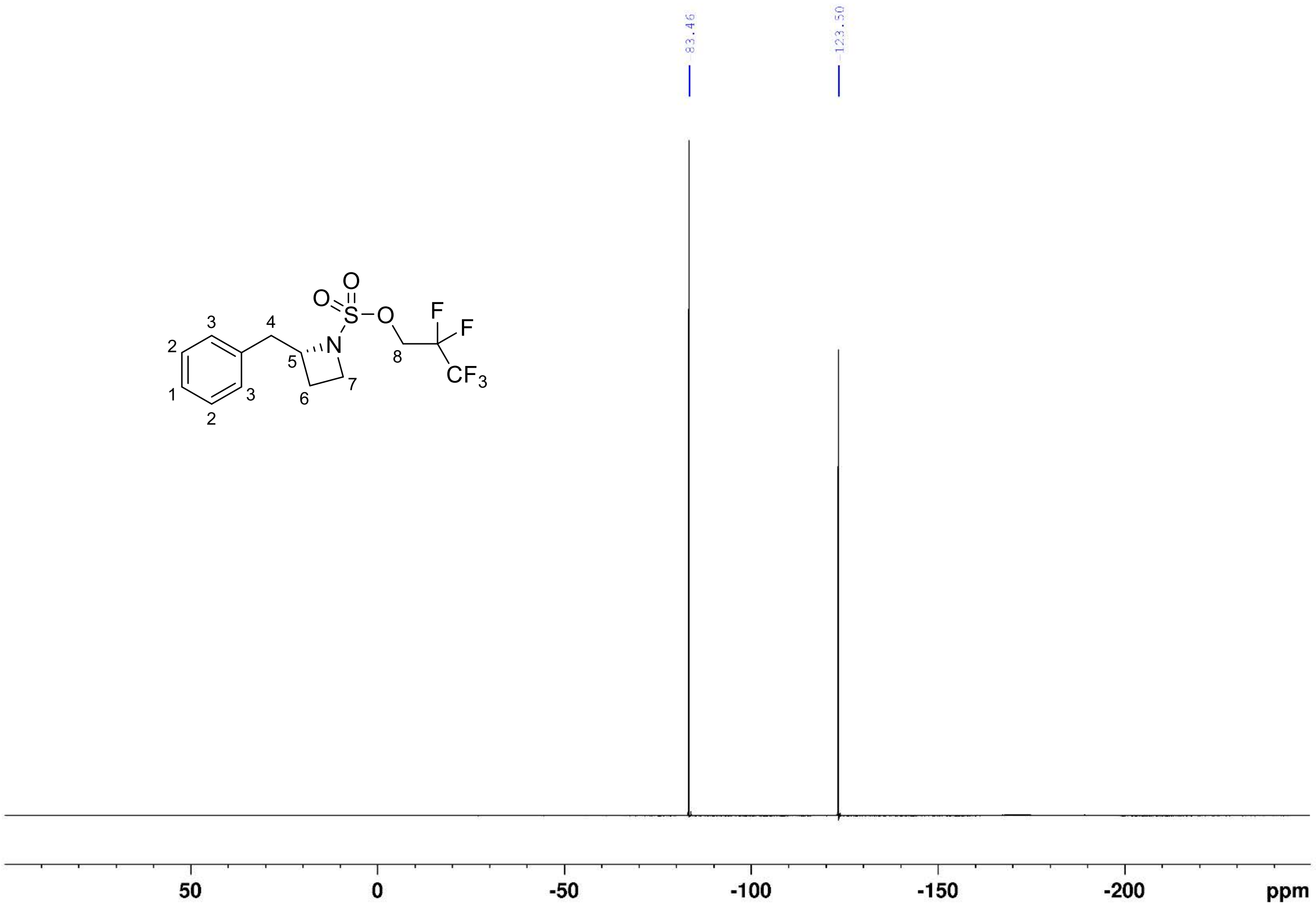

^{1}H NMR (500 MHz, $CDCl_3$) for *tert-butyl (S)-(1-phenyl-4-(phenylthio)butan-2-yl)carbamate* (**51**)

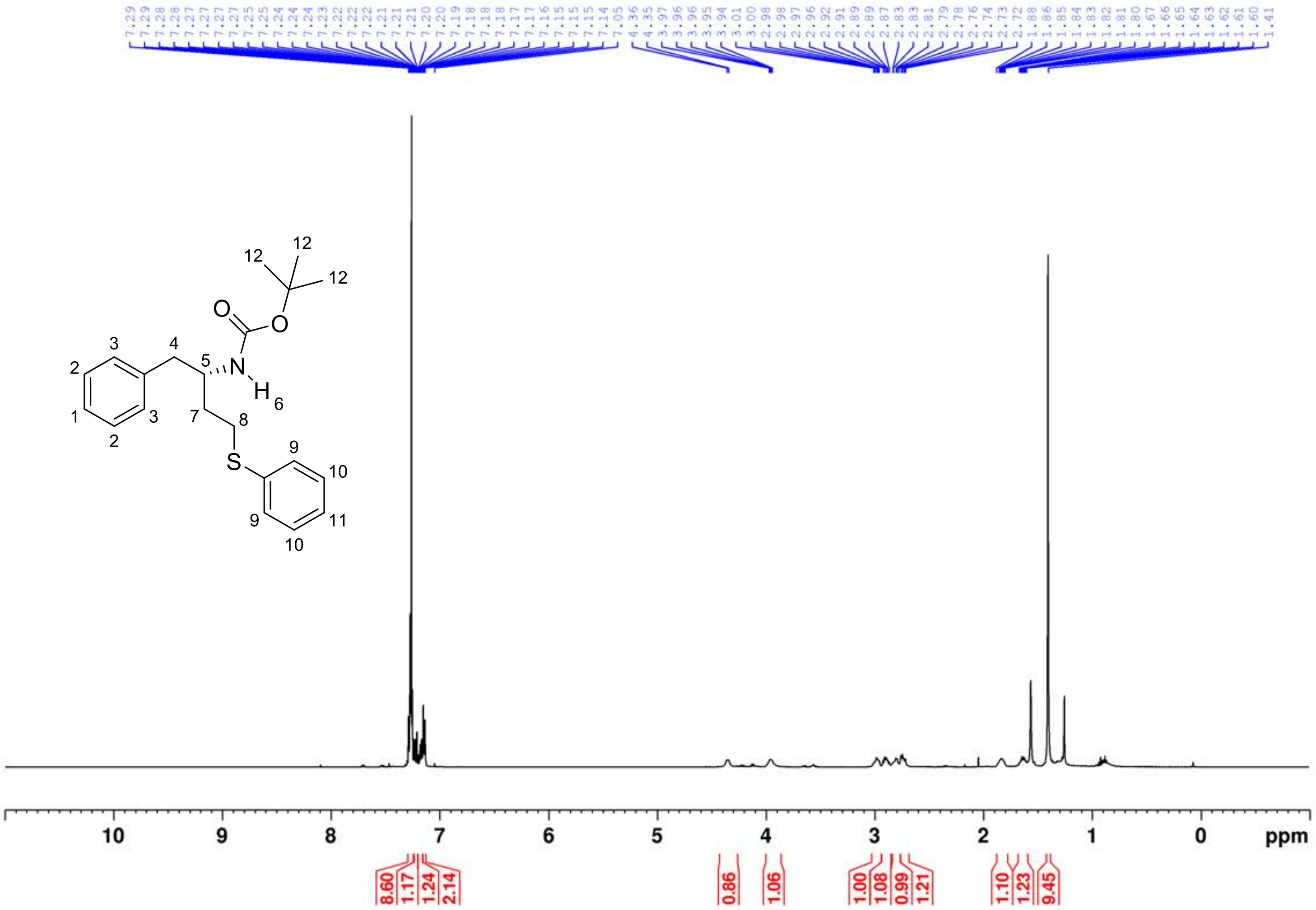

^{13}C NMR (126 MHz, $CDCl_3$) for *tert-butyl (S)-(1-phenyl-4-(phenylthio)butan-2-yl)carbamate* (**51**)

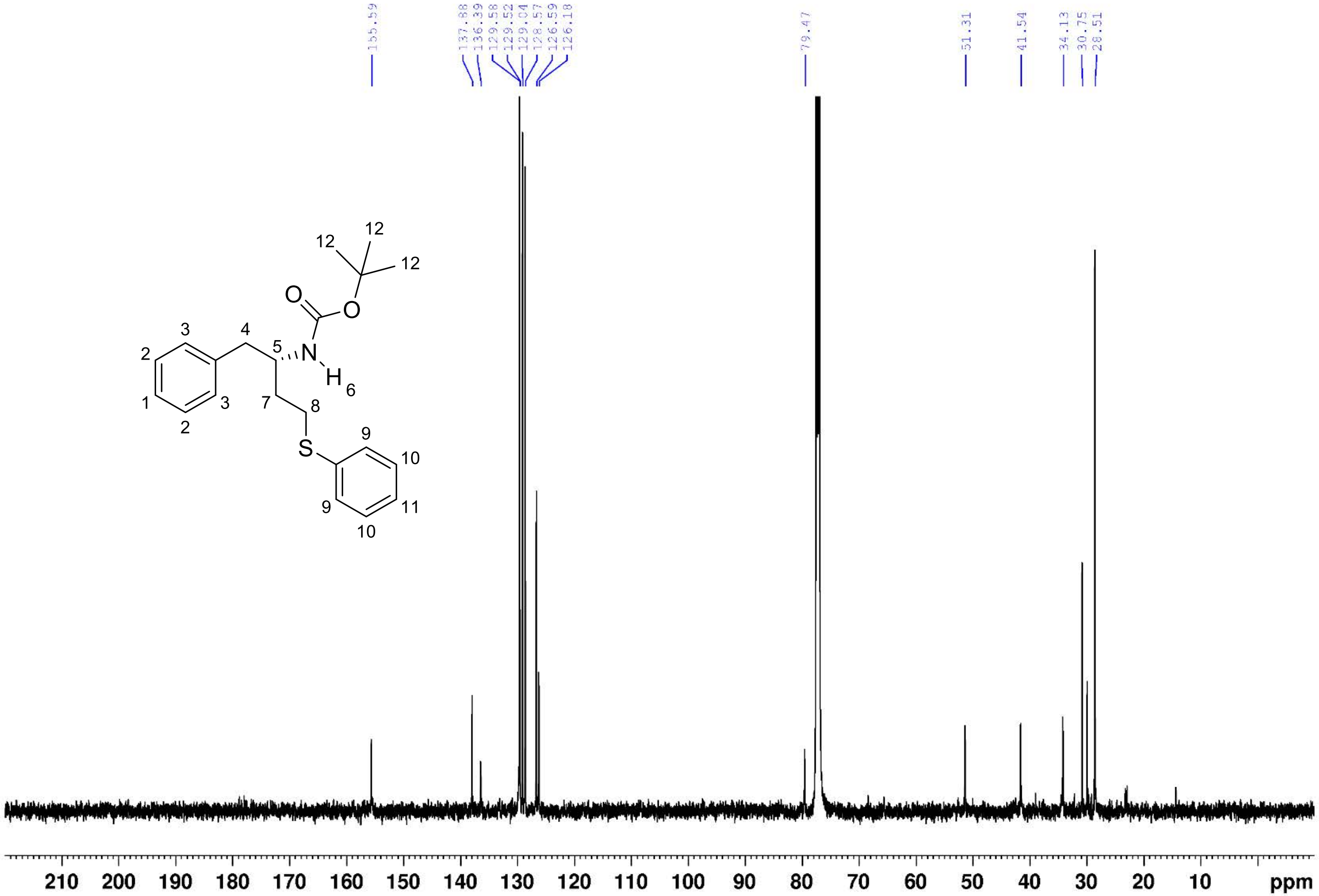

^{1}H NMR (500 MHz, $CDCl_3$) for *2,2,3,3,3-pentafluoropropyl (R)-(4-hydroxy-1-phenylbutyl)sulfamate* (**52**)

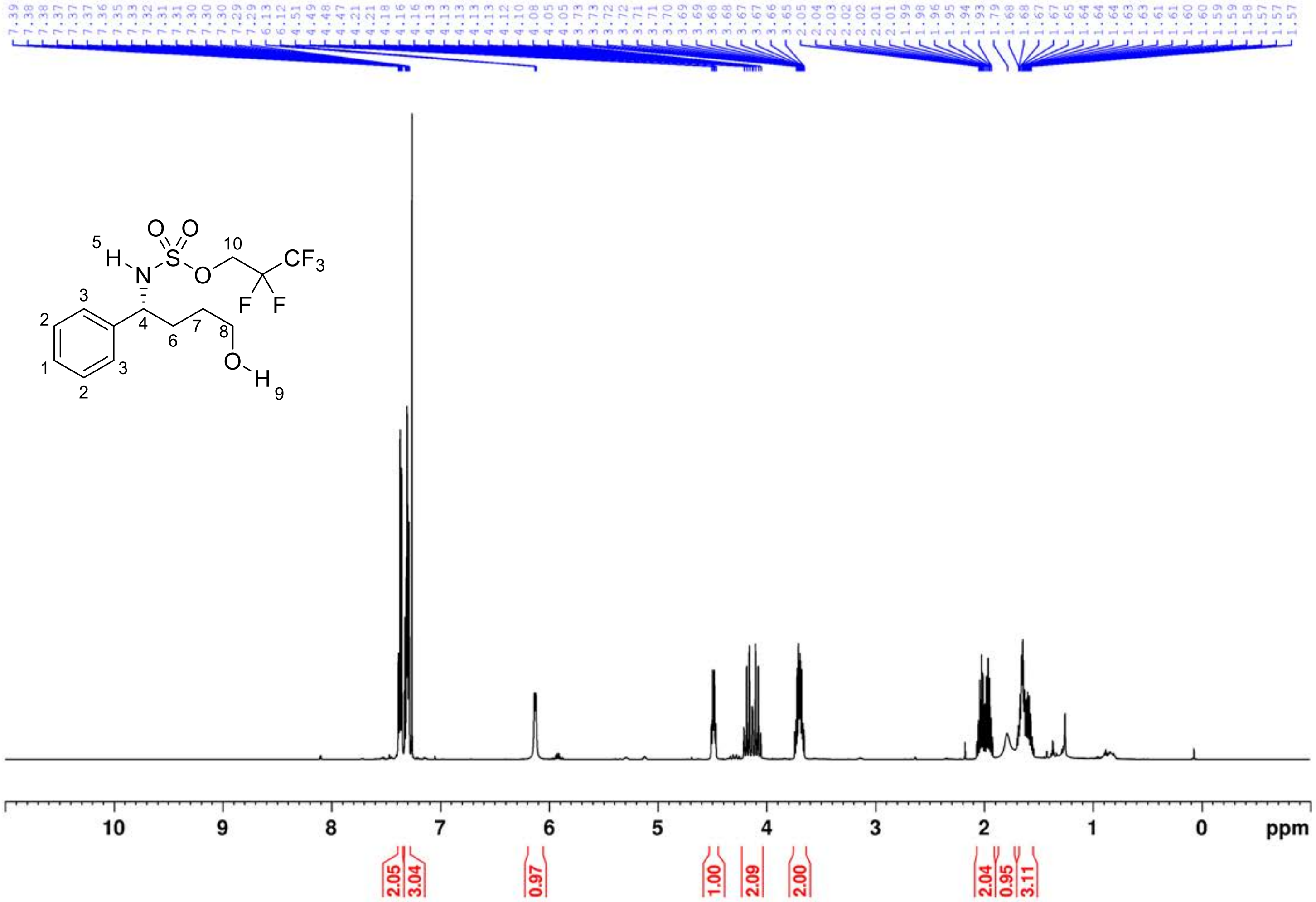

^{13}C NMR (126 MHz, $CDCl_3$) for *2,2,3,3,3-pentafluoropropyl (R)-(4-hydroxy-1-phenylbutyl)sulfamate* (**52**)

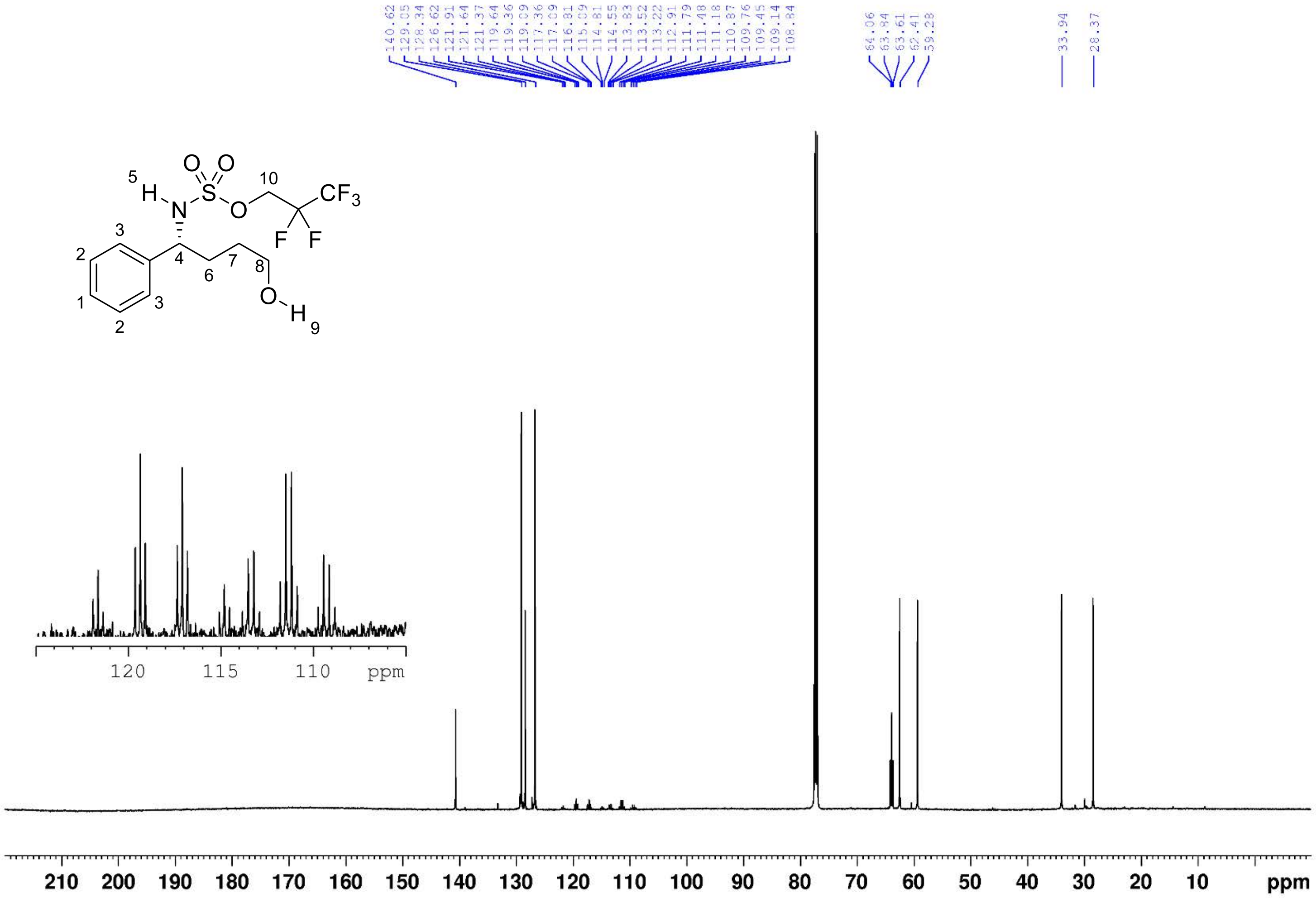

^{19}F NMR (376 MHz, $CDCl_3$) for *2,2,3,3,3-pentafluoropropyl (R)-(4-hydroxy-1-phenylbutyl)sulfamate* (**52**)

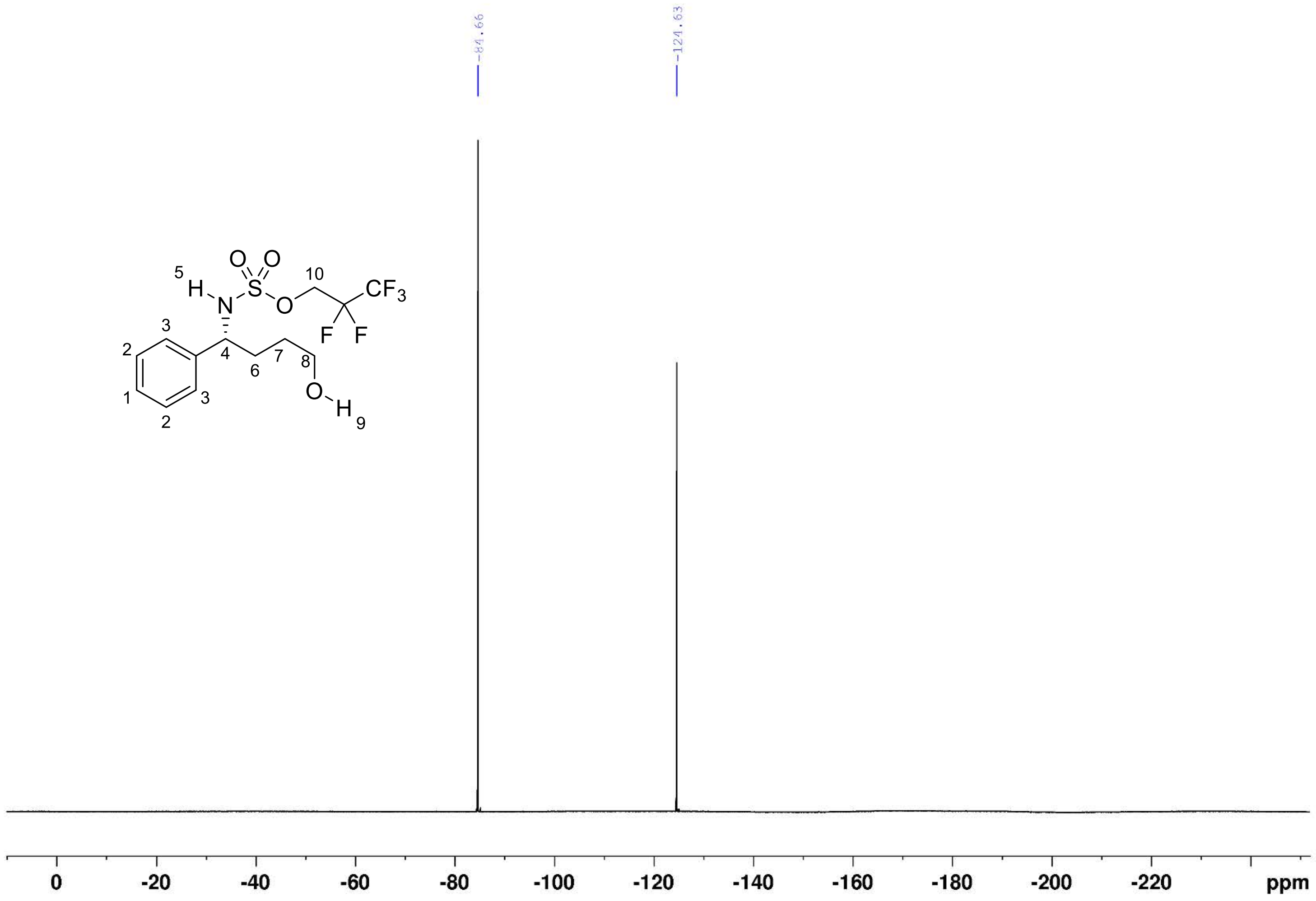

¹H NMR (500 MHz, $CDCl_3$) for *2,2,3,3,3-pentafluoropropyl ((S)-phenyl((S)-2-(trichloromethyl)-5,6-dihydro-4H-1,3-oxazin-4-yl)methyl)sulfamate* (**53**)

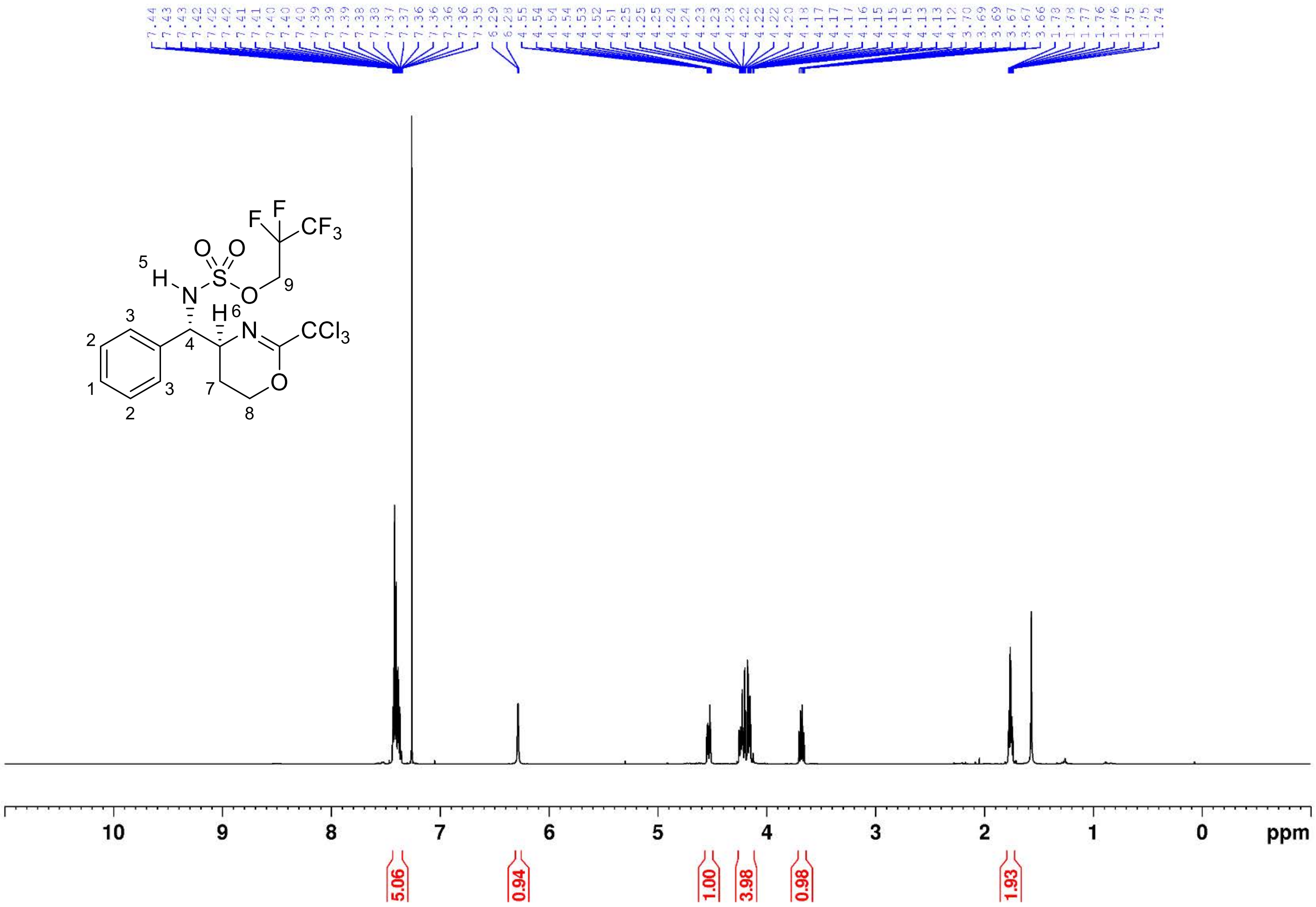

^{13}C NMR (126 MHz, $CDCl_3$) for *2,2,3,3,3-pentafluoropropyl ((S)-phenyl((S)-2-(trichloromethyl)-5,6-dihydro-4H-1,3-oxazin-4-yl)methyl)sulfamate* (**53**)

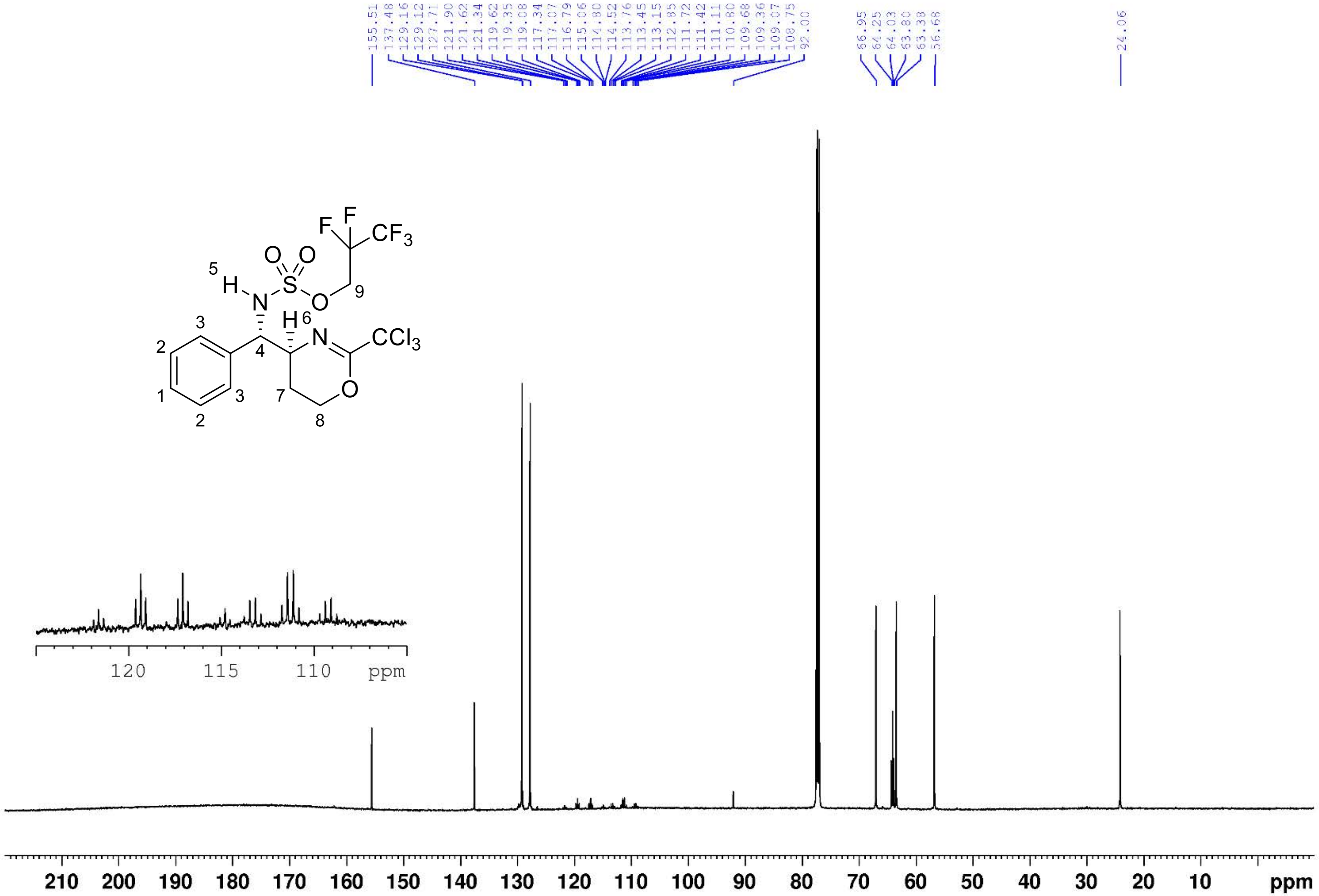

^{19}F NMR (376 MHz, $CDCl_3$) for *2,2,3,3,3-pentafluoropropyl ((S)-phenyl((S)-2-(trichloromethyl)-5,6-dihydro-4H-1,3-oxazin-4-yl)methyl)sulfamate* (**53**)

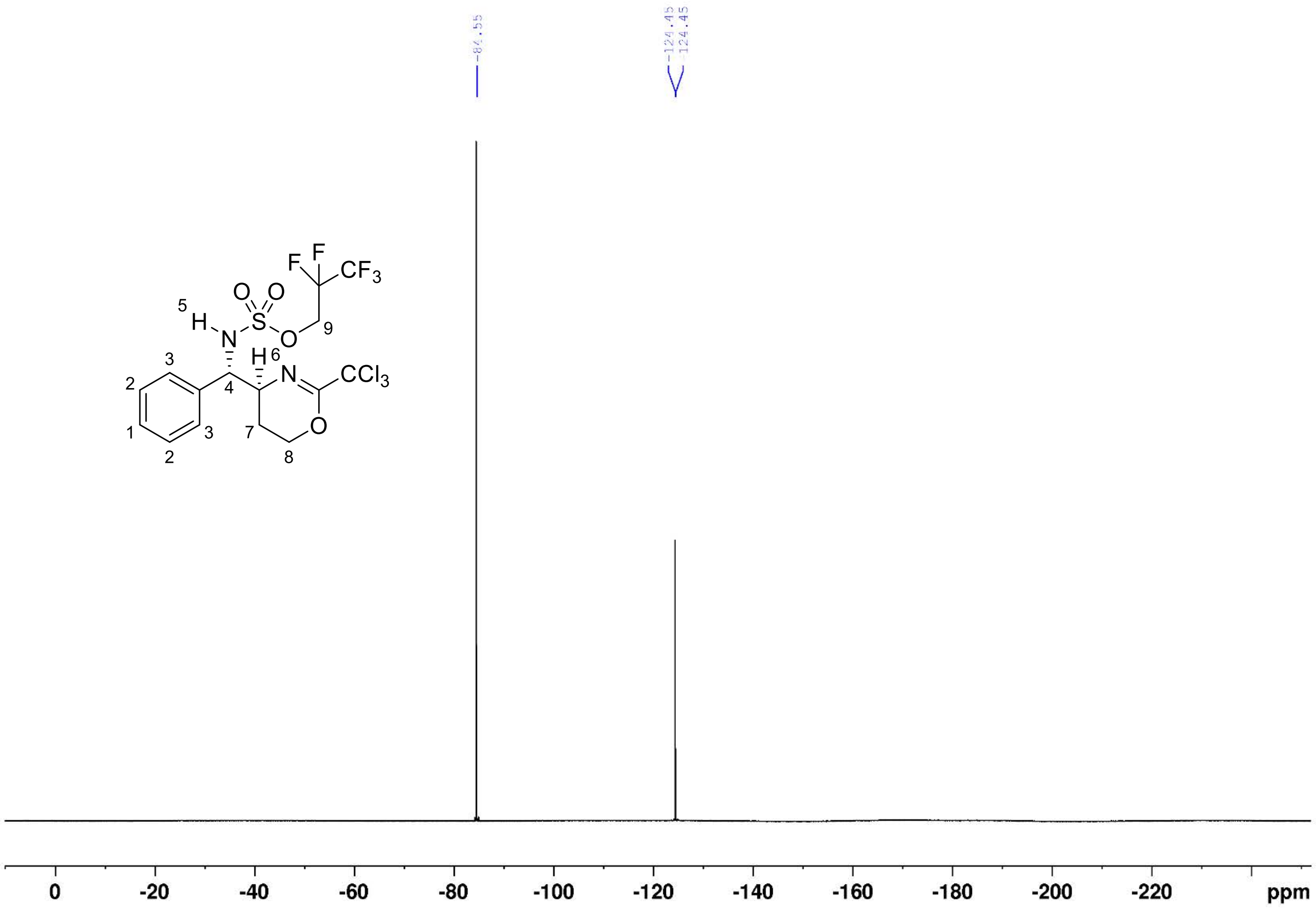

^{1}**H NMR** (400 MHz, $CDCl_3$) for *2,2,3,3,3-pentafluoropropyl ((S)-((S)-2-oxo-1,3-dioxan-4-yl)(phenyl)methyl)sulfamate* (**54**)

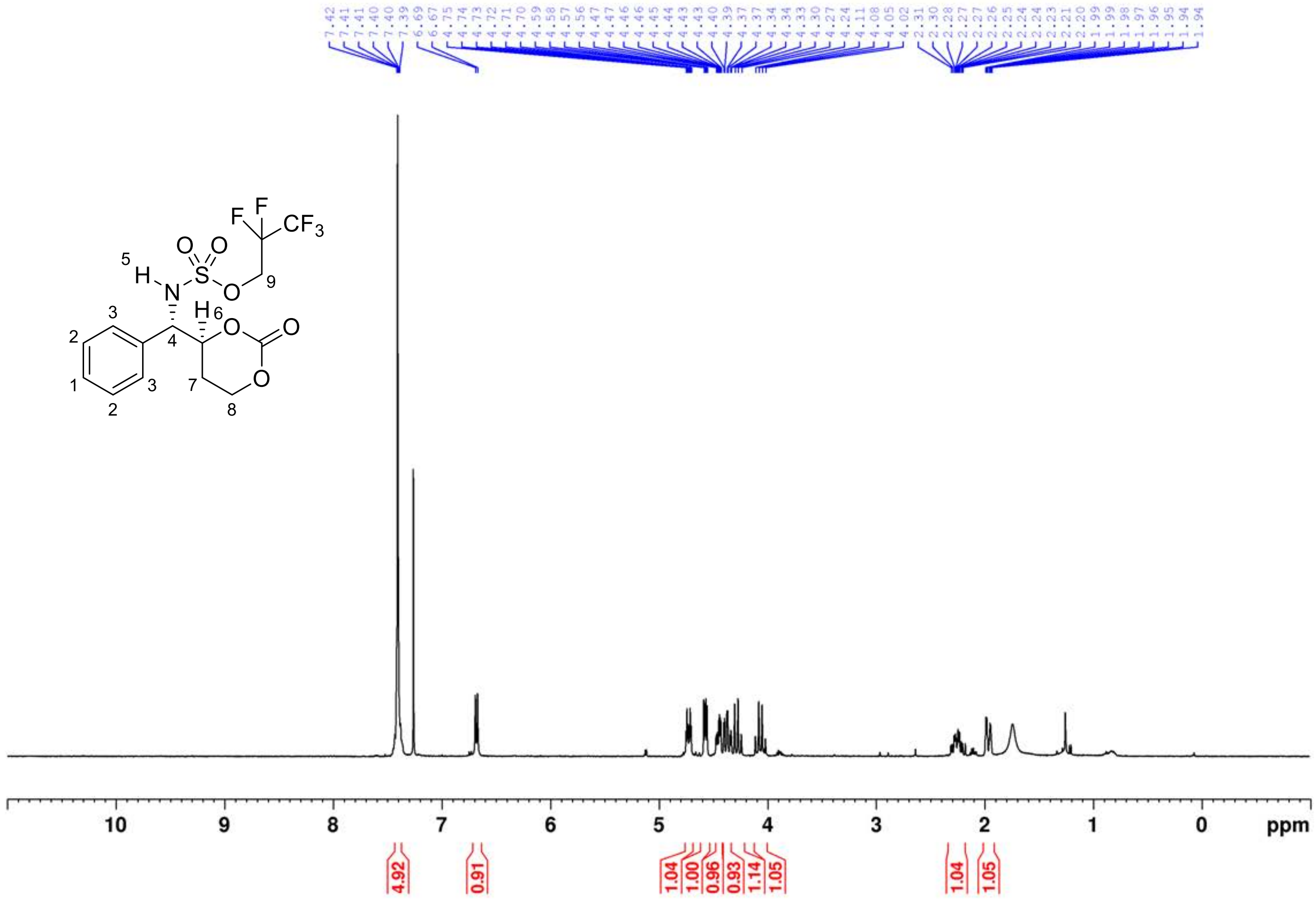

^{13}C NMR (126 MHz, $CDCl_3$) for *2,2,3,3,3-pentafluoropropyl ((S)-((S)-2-oxo-1,3-dioxan-4-yl)(phenyl)methyl)sulfamate* (**54**)

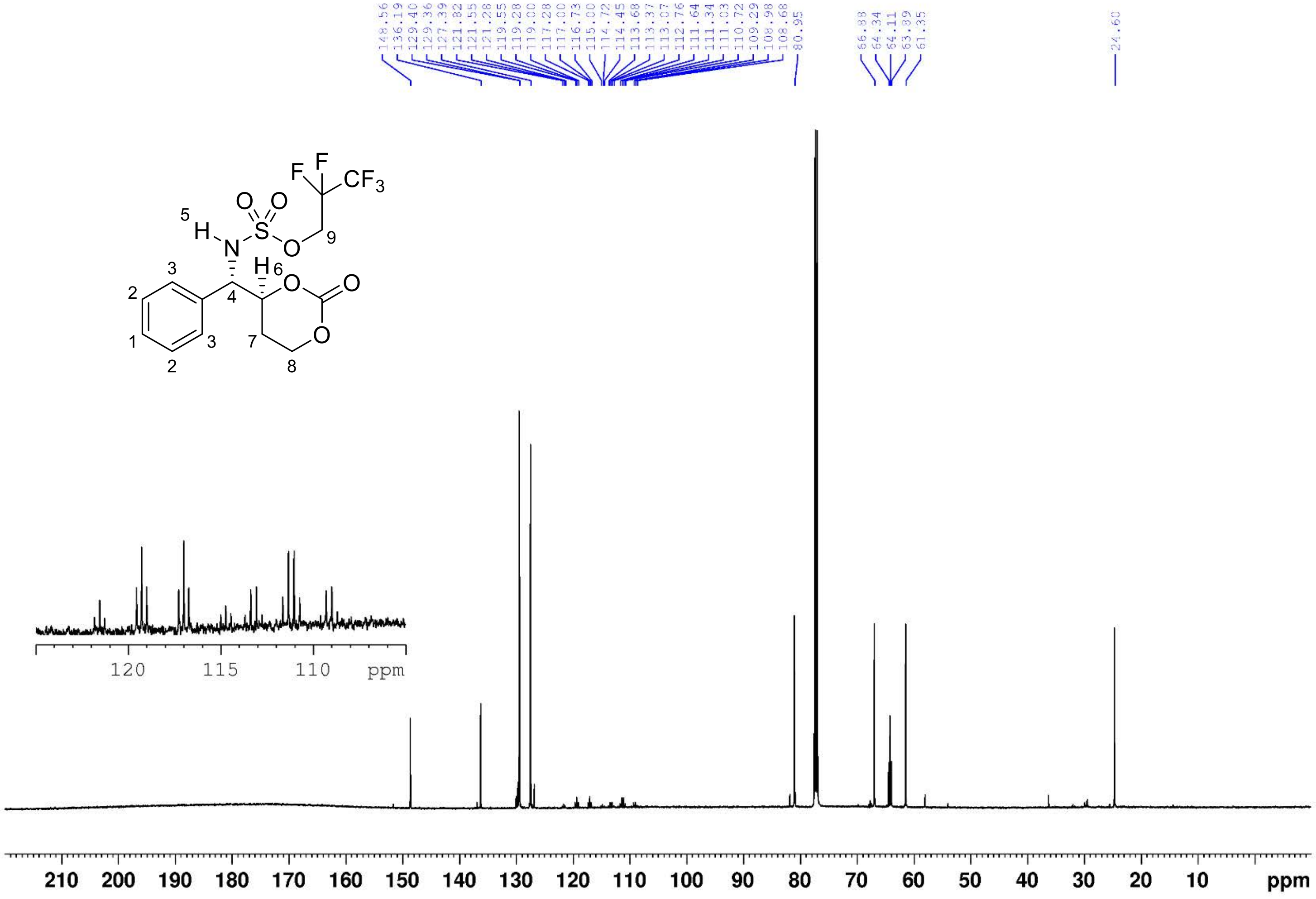

^{19}F NMR (376 MHz, $CDCl_3$) for *2,2,3,3,3-pentafluoropropyl ((S)-((S)-2-oxo-1,3-dioxan-4-yl)(phenyl)methyl)sulfamate* (**54**)

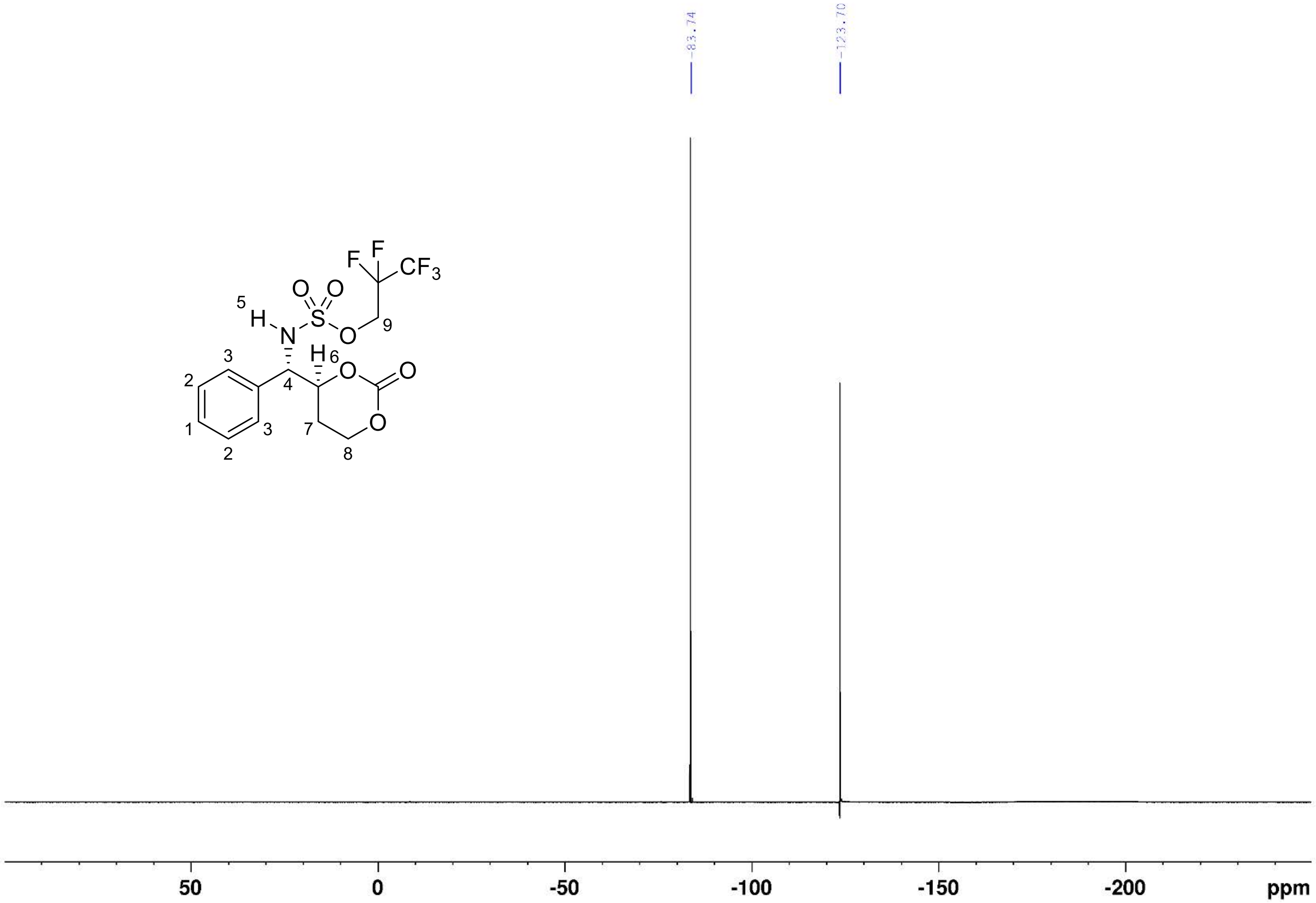

^{1}**H NMR** (400 MHz, $CDCl_3$) for *benzyl (S)-(4-hydroxy-1-phenylbutan-2-yl)carbamate* (**SI1**)

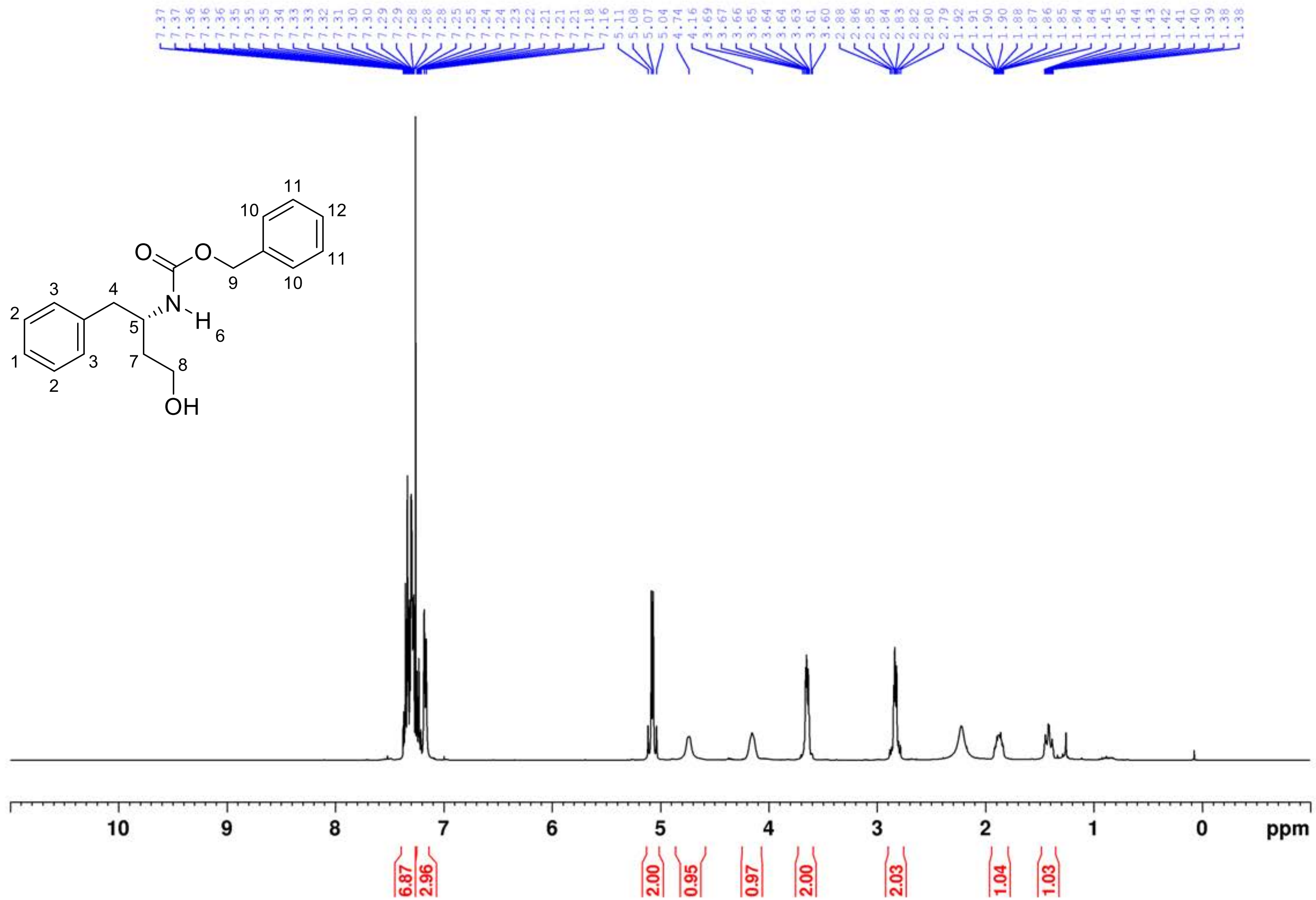

^{13}C NMR (101 MHz, $CDCl_3$) for *benzyl (S)-(4-hydroxy-1-phenylbutan-2-yl)carbamate* (**SI1**)

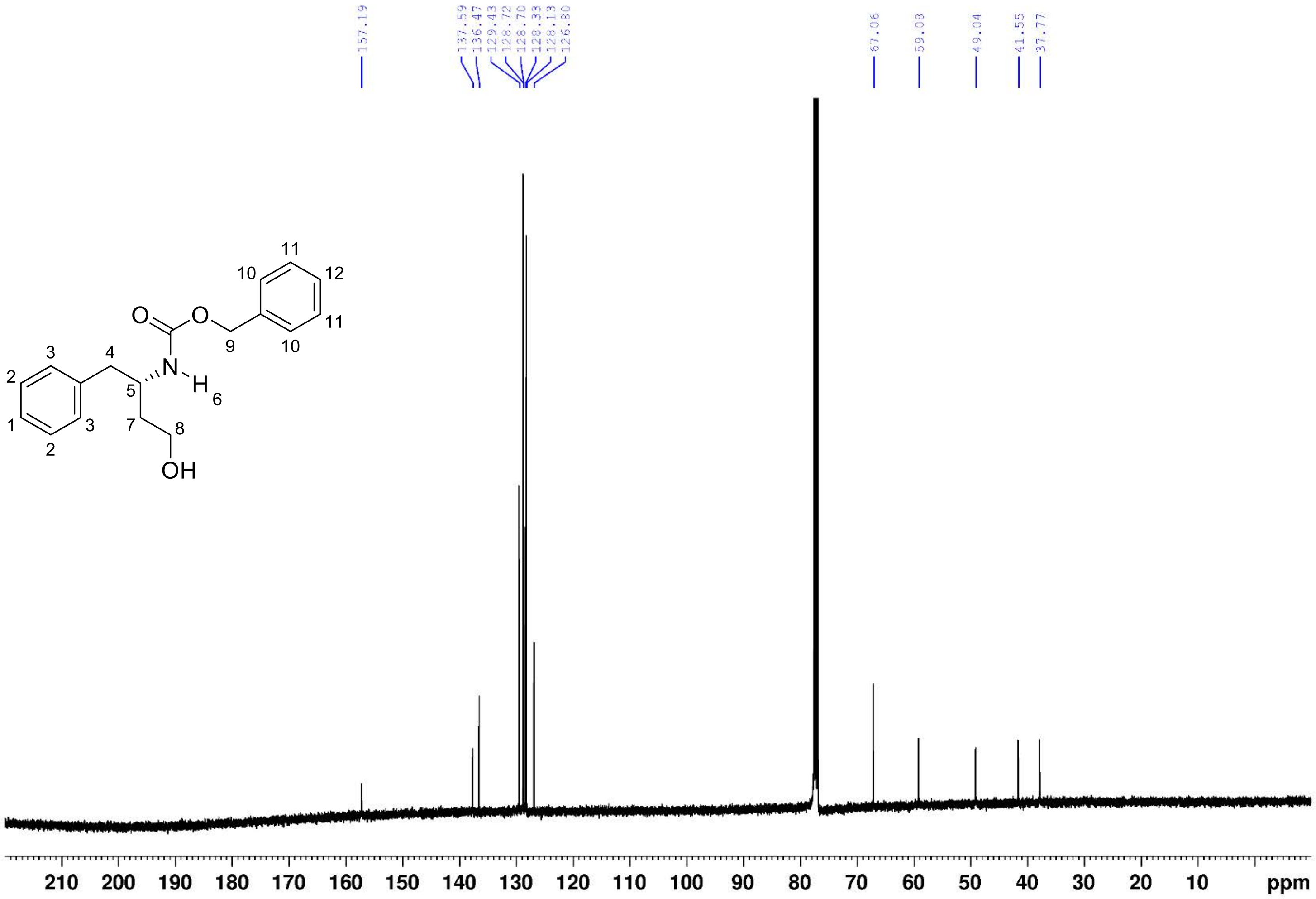

¹H NMR (400 MHz, $CDCl_3$) for *(9H-fluoren-9-yl)methyl (R)-(5-hydroxy-1-phenylpentan-2-yl)carbamate* (**SI3**)

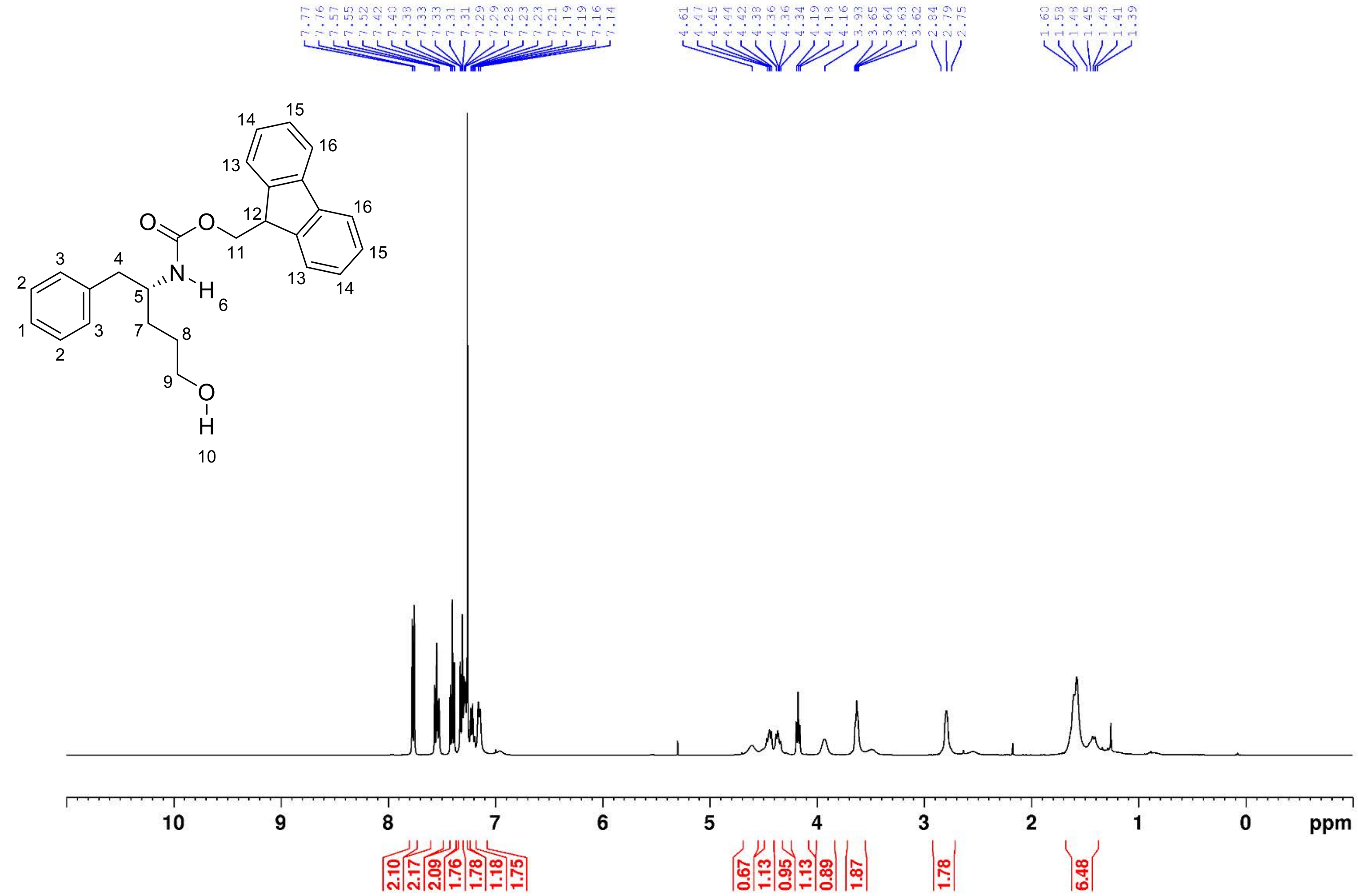

^{13}C NMR (101 MHz, $CDCl_3$) for *(9H-fluoren-9-yl)methyl (R)-(5-hydroxy-1-phenylpentan-2-yl)carbamate* (**SI3**)

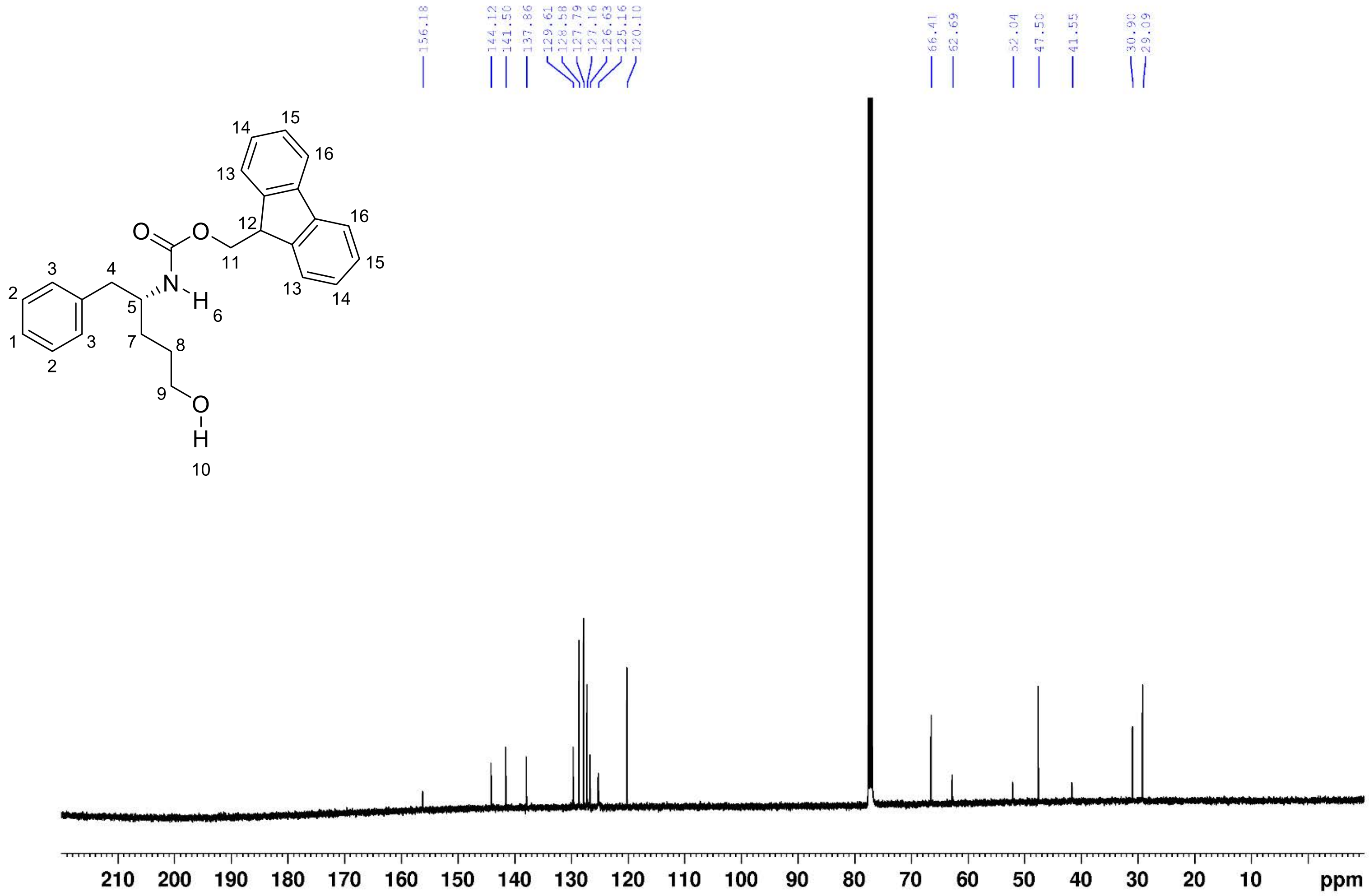

¹H NMR (400 MHz, $CDCl_3$) for *N-((2S,3R)-2-ethyltetrahydrofuran-3-yl)-4-methylbenzenesulfonamide* (**SI4**)

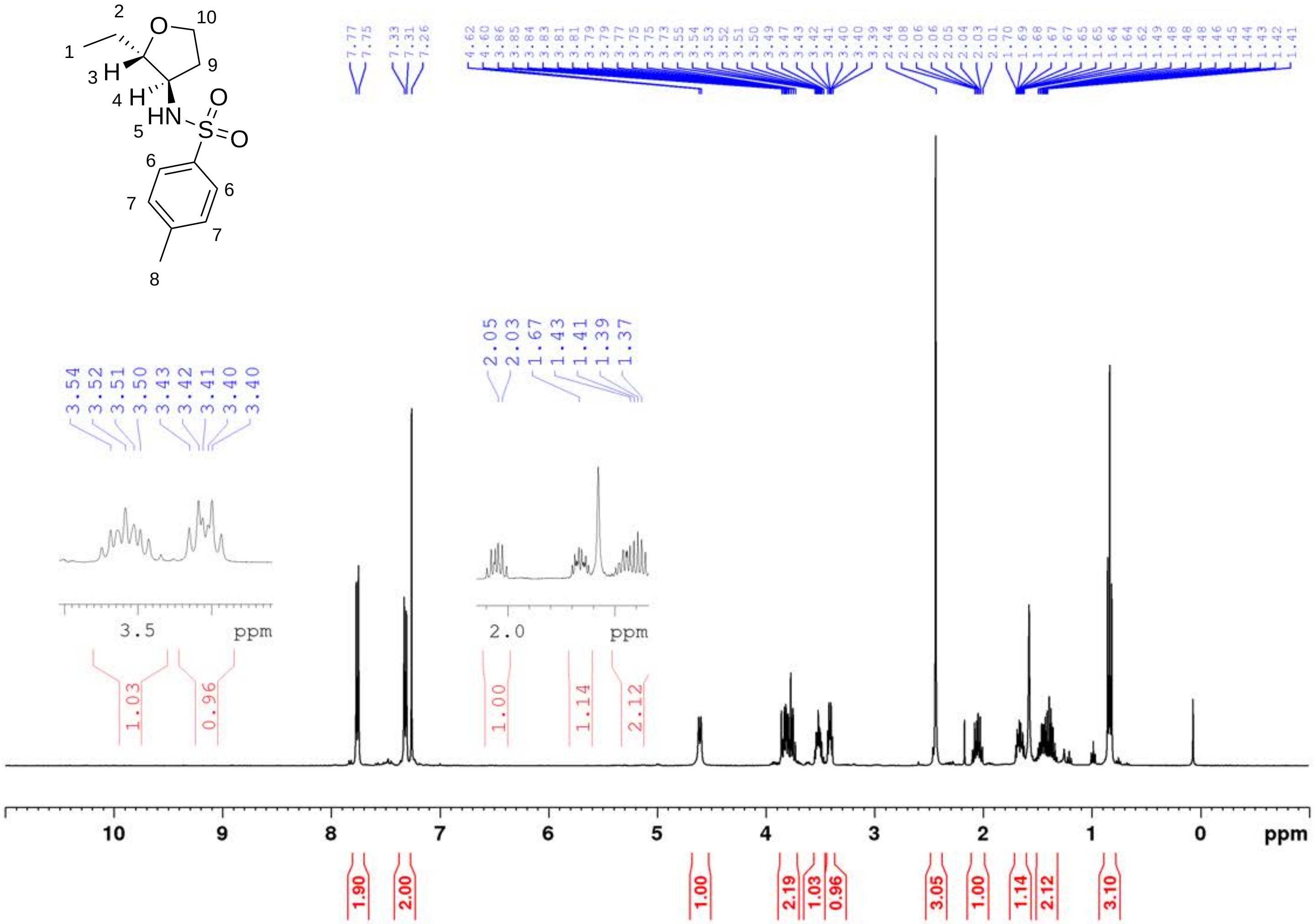

^{13}C NMR (101 MHz, $CDCl_3$) for *N-((2S,3R)-2-ethyltetrahydrofuran-3-yl)-4-methylbenzenesulfonamide* (**SI4**)

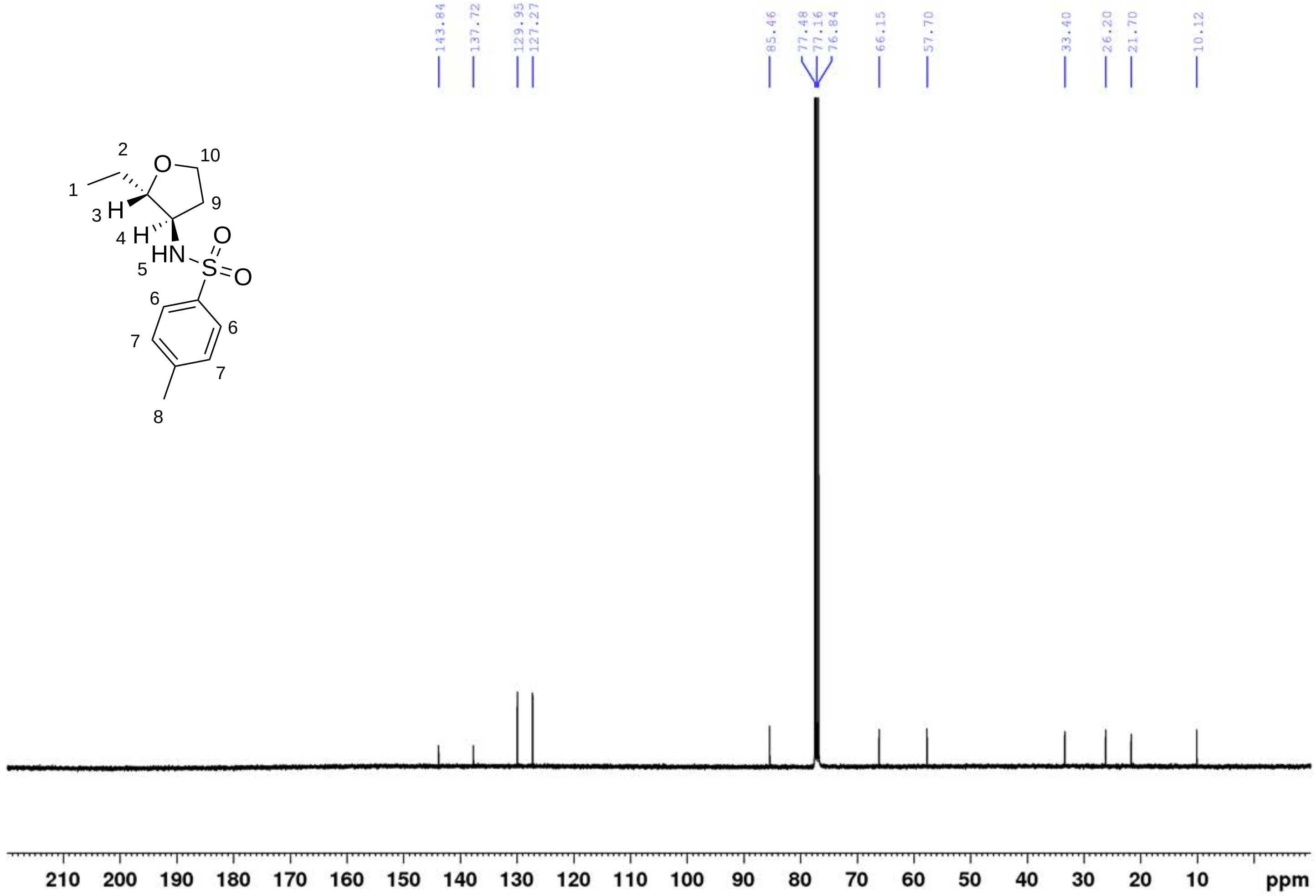

^{1}H NMR (400 MHz, $CDCl_3$) for *(S)-3-(((tert-butoxycarbonyl)amino)methyl)-5-methylhexanoic acid* (**SI5**)

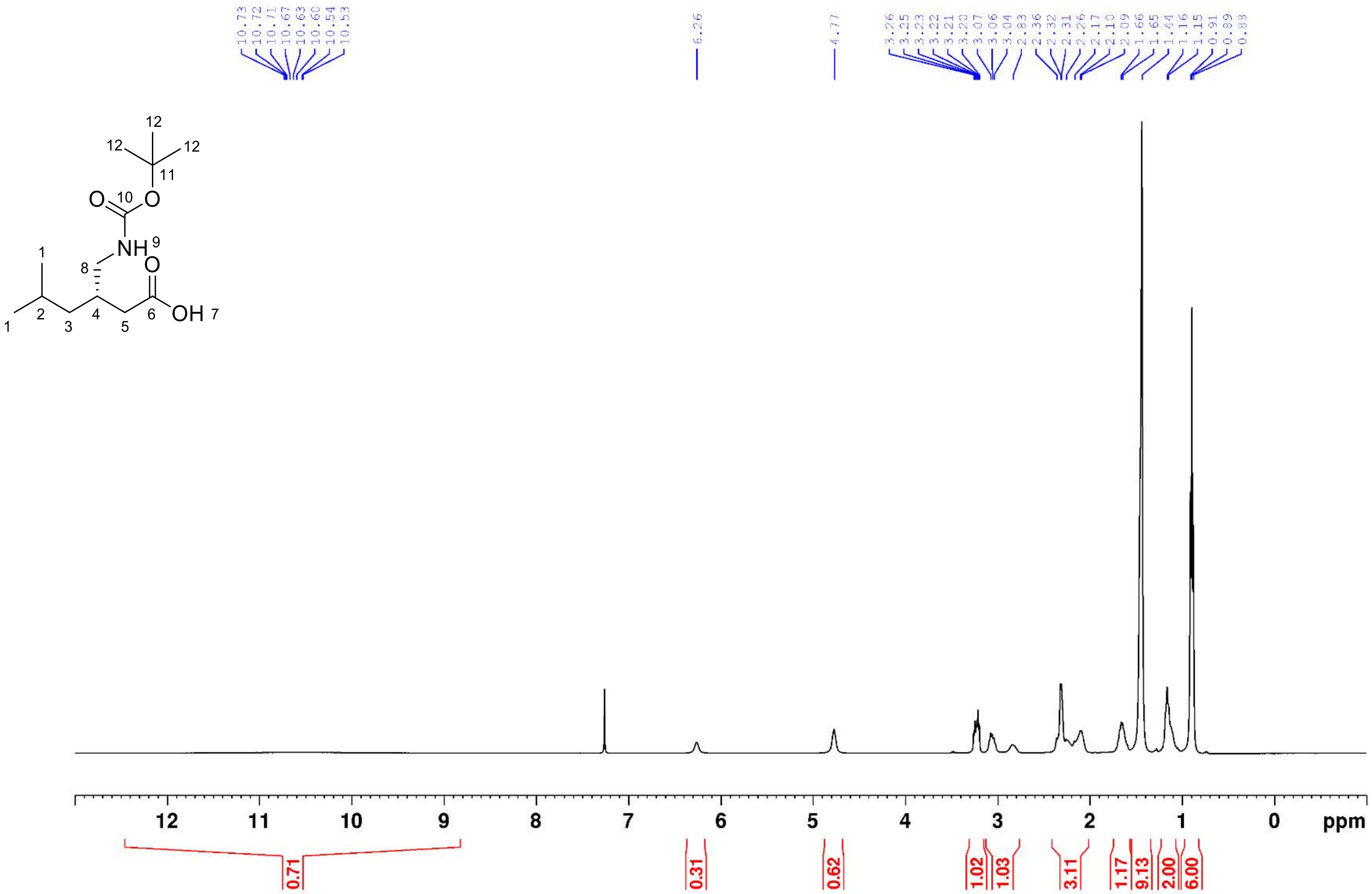

^{13}C NMR (101 MHz, CDCl$_3$) for *(S)-3-(((tert-butoxycarbonyl)amino)methyl)-5-methylhexanoic acid* (**SI5**)

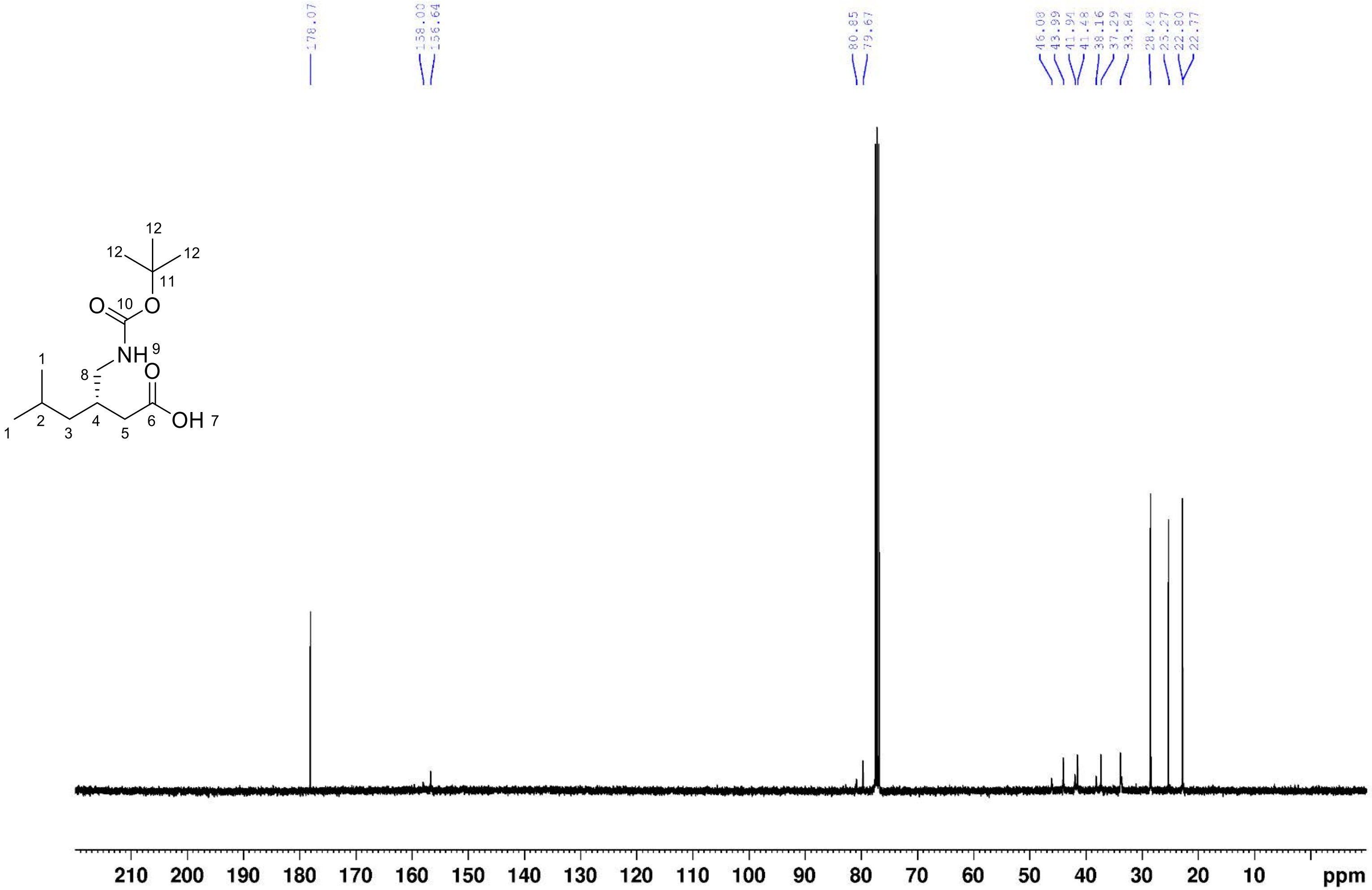

^{1}H NMR (400 MHz, $CDCl_3$) for *Tert-butyl (S)-3-isobutylpyrrolidine-1-carboxylate* (**SI6**)

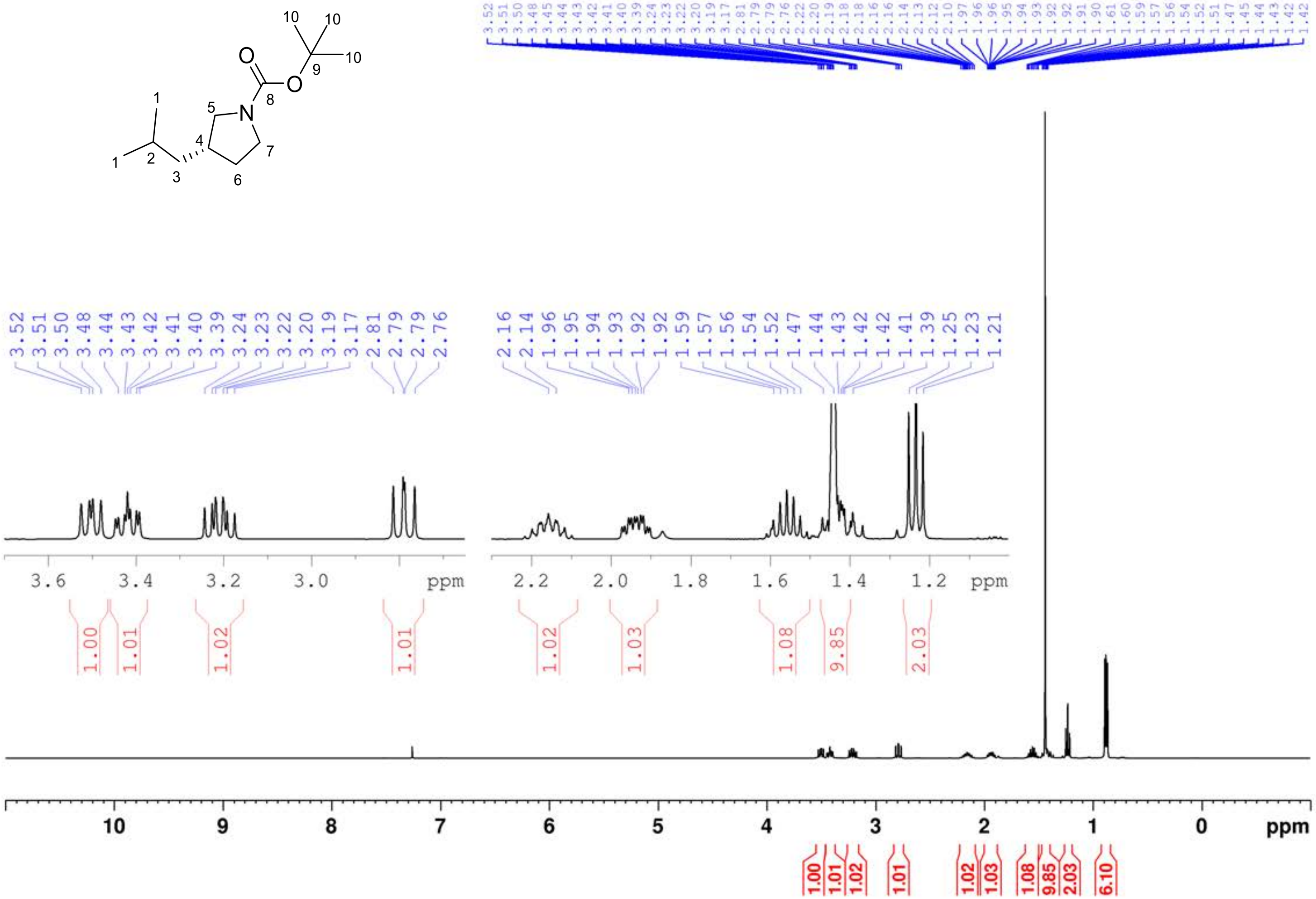

^{13}C NMR (101 MHz, $CDCl_3$) for *Tert-butyl (S)-3-isobutylpyrrolidine-1-carboxylate* (**SI6**)

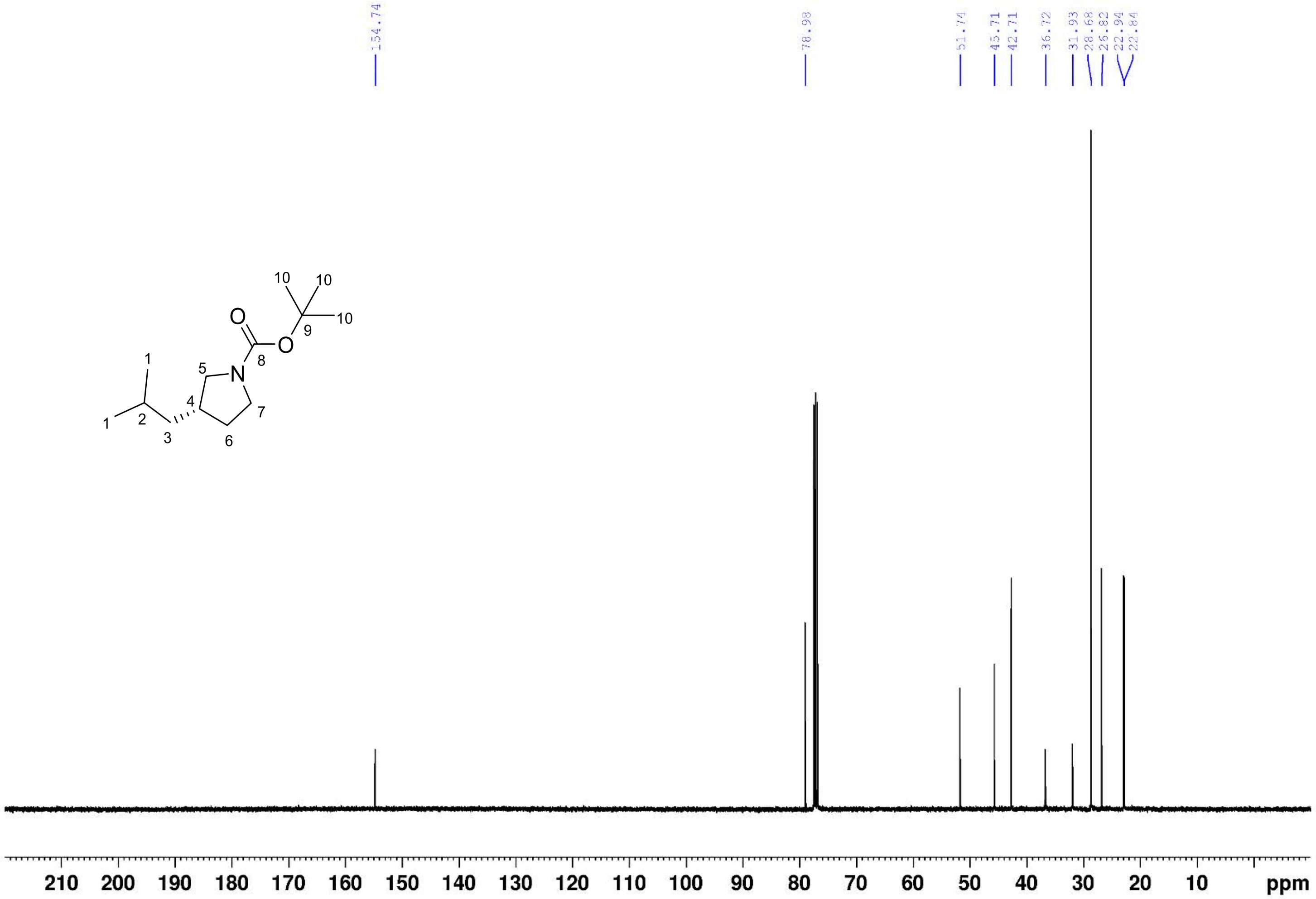

^{1}H NMR (400 MHz, CDCl$_3$) for *Tert-butyl (S)-(2-(2-hydroxyethyl)-4-methylpentyl)carbamate* (**SI7**)

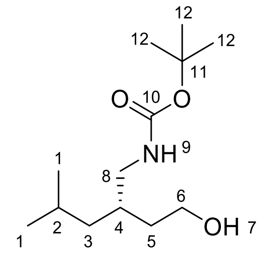

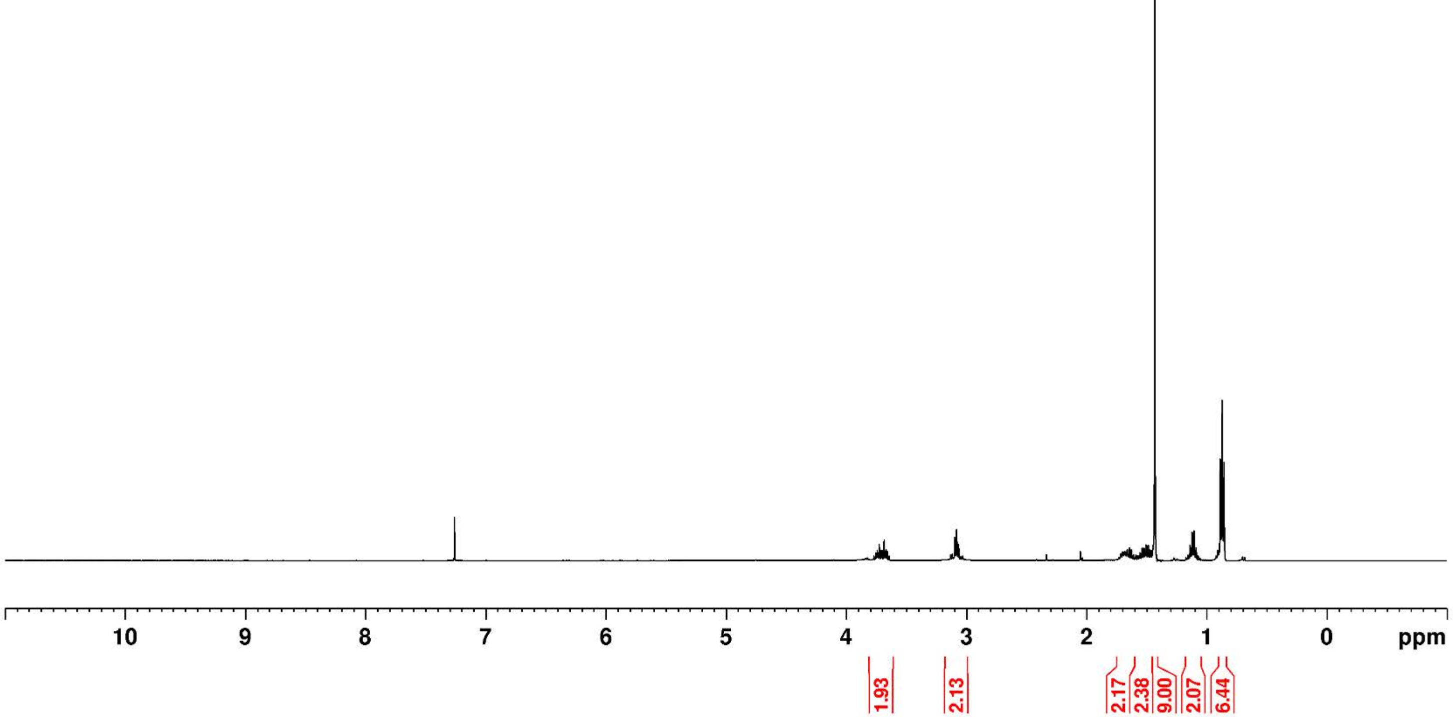

^{13}C NMR (101 MHz, $CDCl_3$) for *Tert-butyl (S)-(2-(2-hydroxyethyl)-4-methylpentyl)carbamate* (**SI7**)

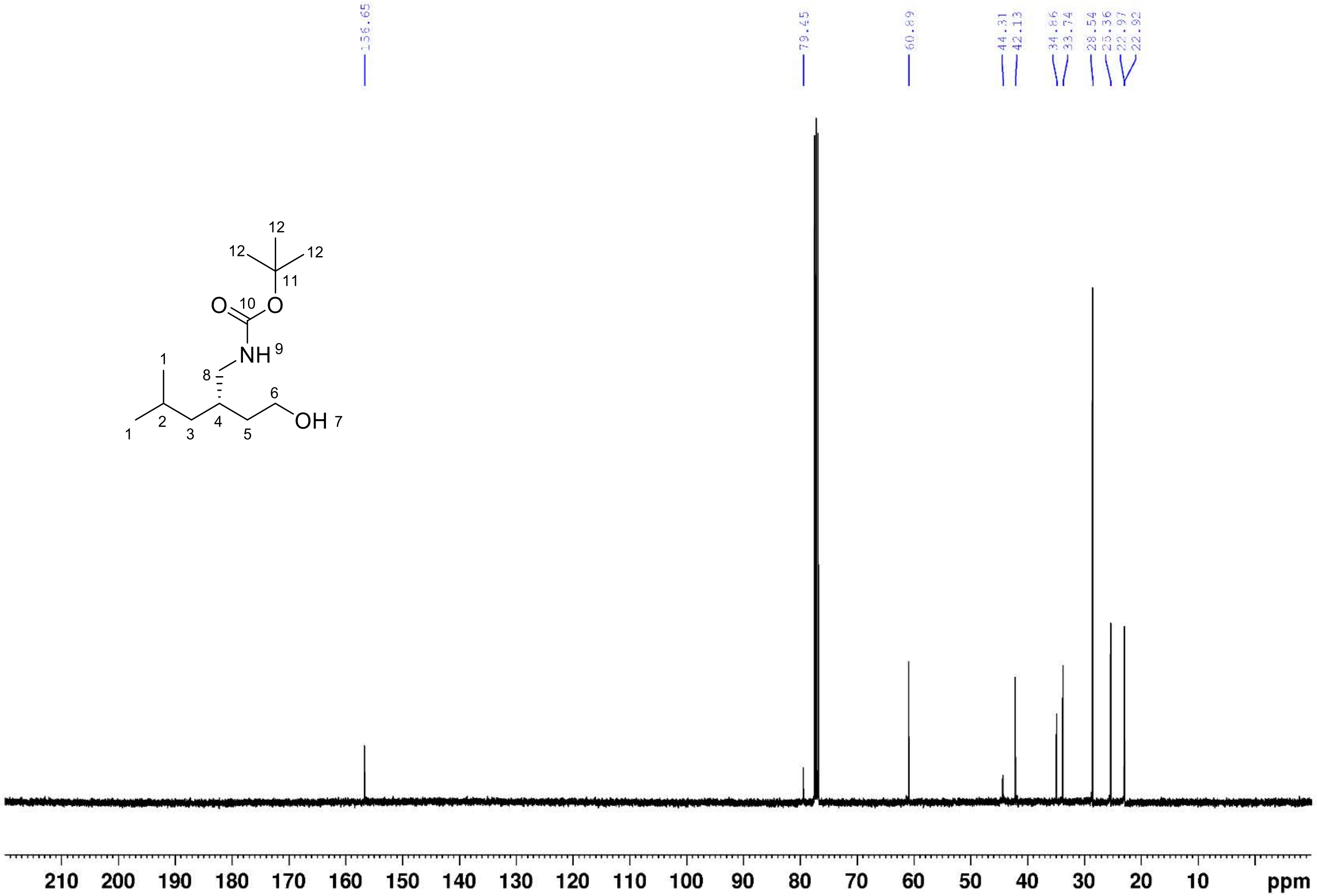

¹H NMR (500 MHz, CDCl₃) for *2,2,3,3,3-pentafluoropropyl (S)-(2-(2-hydroxyethyl)-4-methylpentyl)sulfamate* (**SI9**)

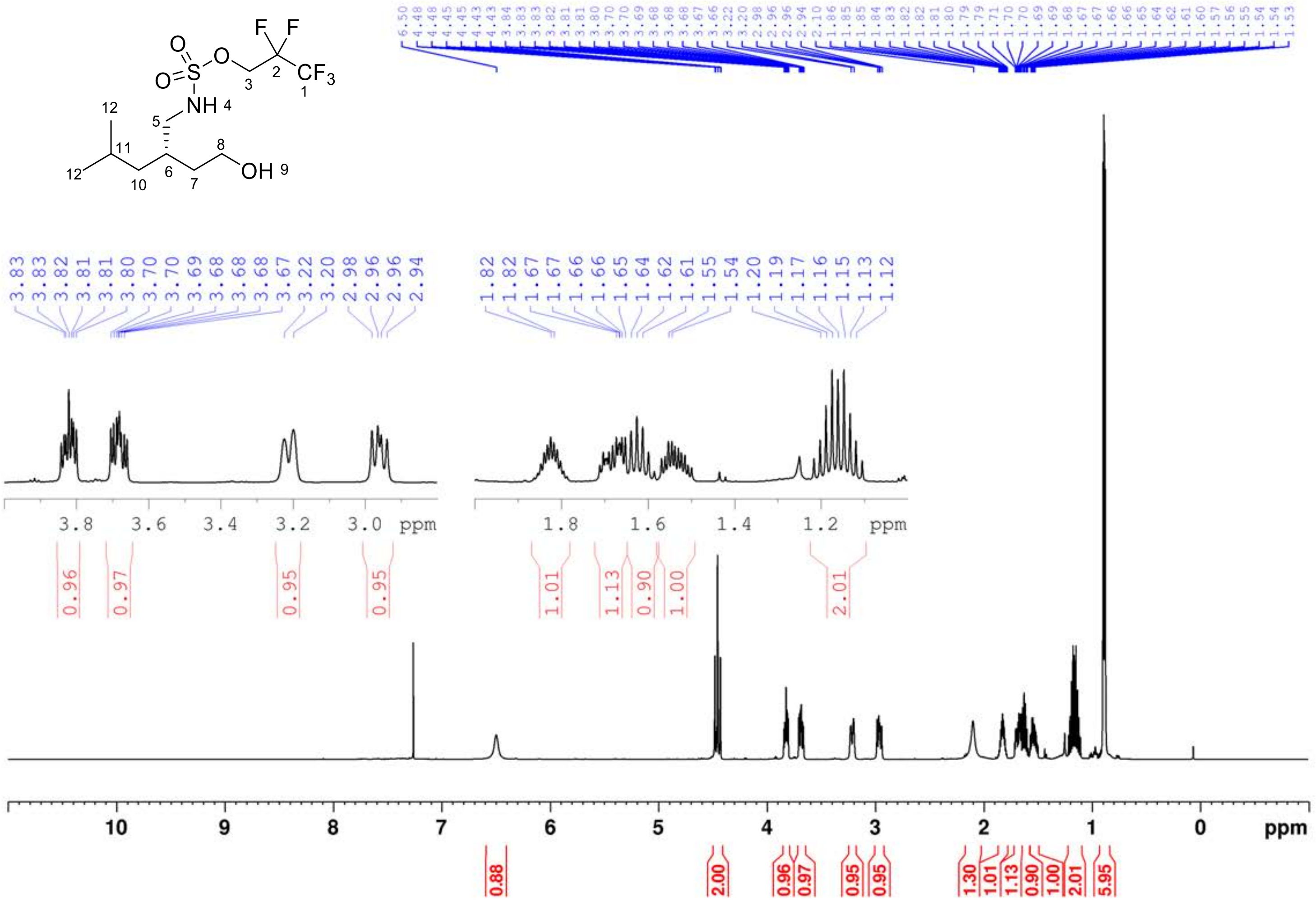

^{13}C NMR (126 MHz, $CDCl_3$) for *2,2,3,3,3-pentafluoropropyl (S)-(2-(2-hydroxyethyl)-4-methylpentyl)sulfamate* (**SI9**)

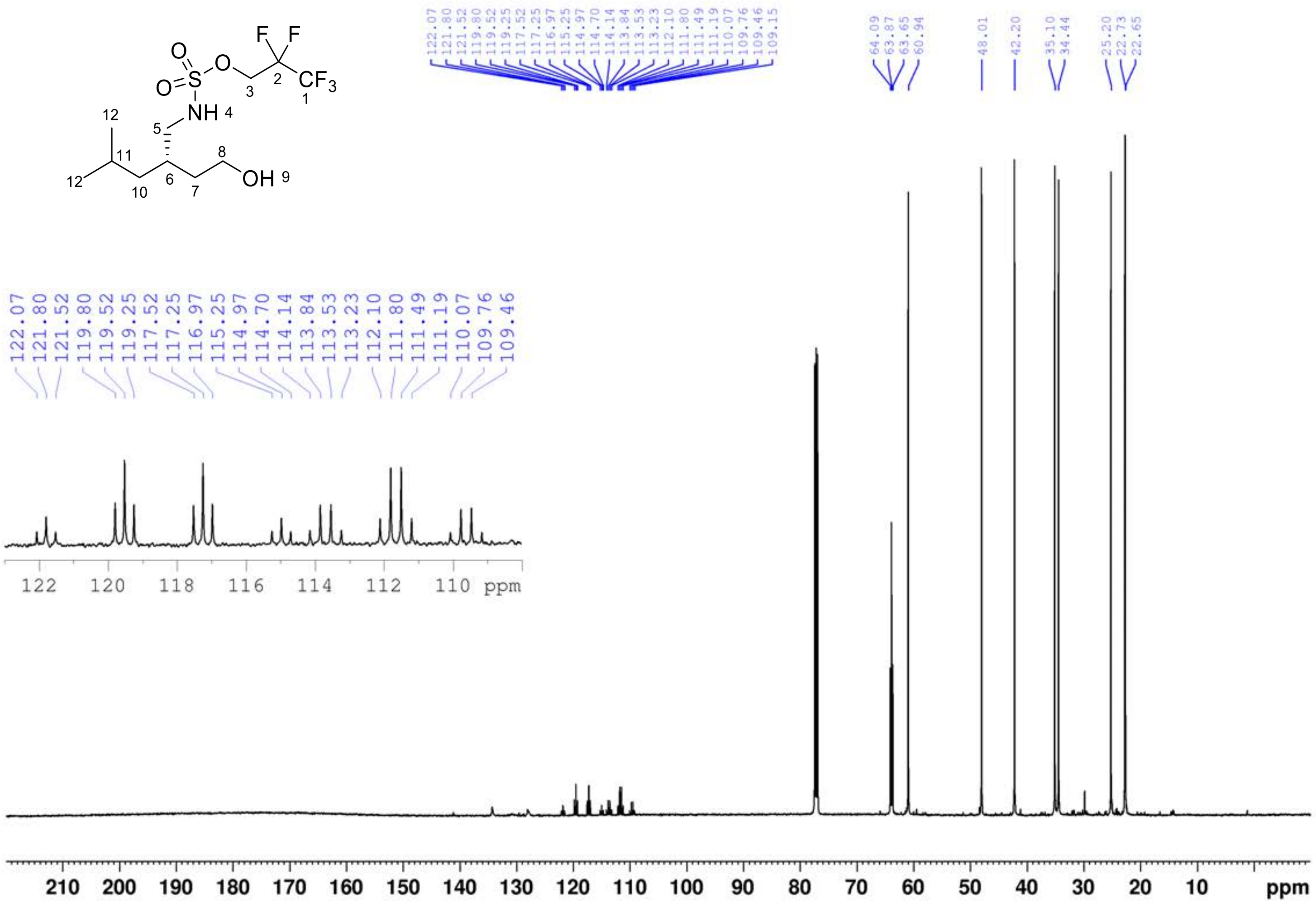

^{19}F NMR (471 MHz, $CDCl_3$) for *2,2,3,3,3-pentafluoropropyl (S)-(2-(2-hydroxyethyl)-4-methylpentyl)sulfamate* (**SI9**)

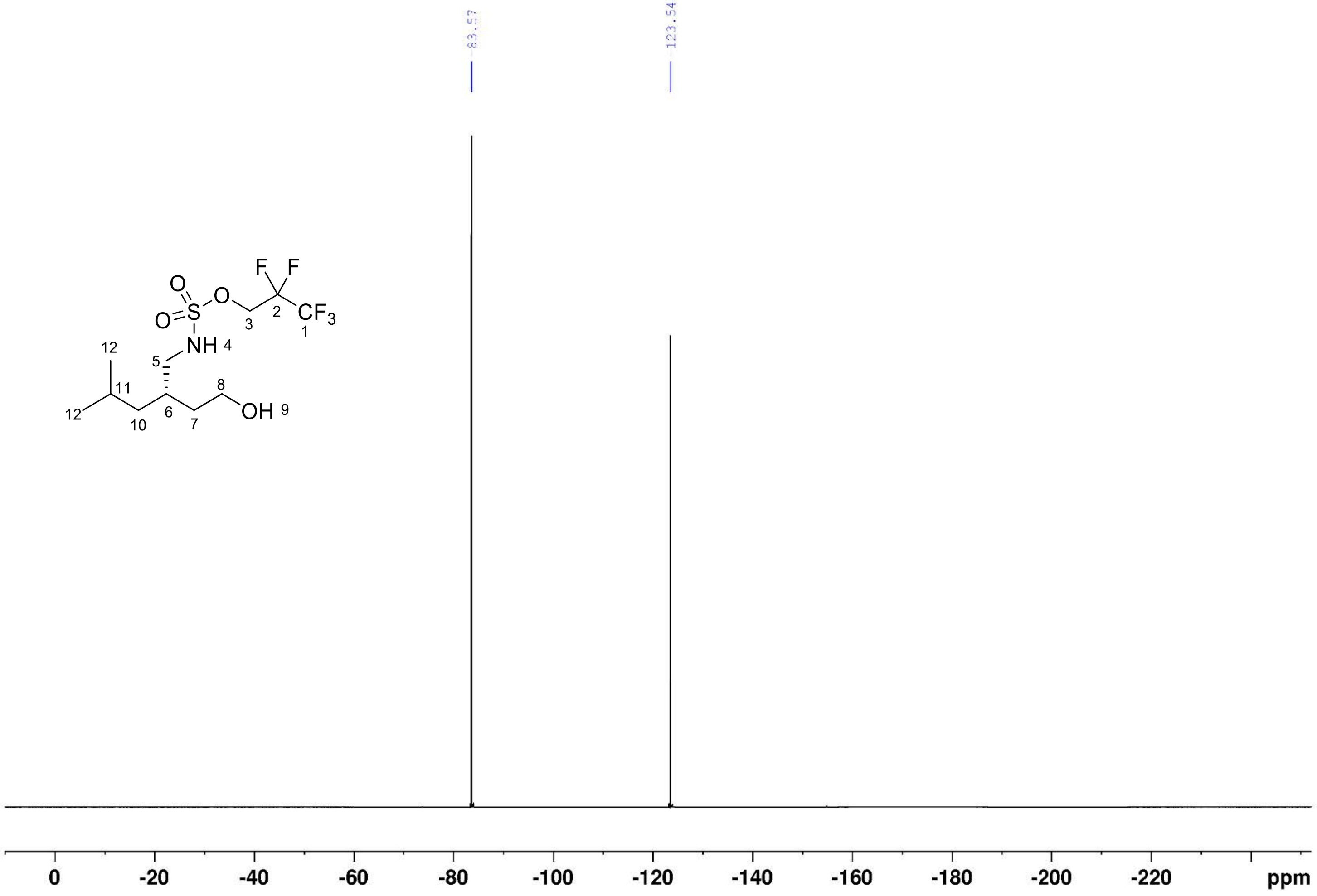

^{1}H NMR (500 MHz, $CDCl_3$) for *2,2,3,3,3-pentafluoropropyl (S)-(1-hydroxy-3,5-dimethylhexan-3-yl)sulfamate* (**SI10**)

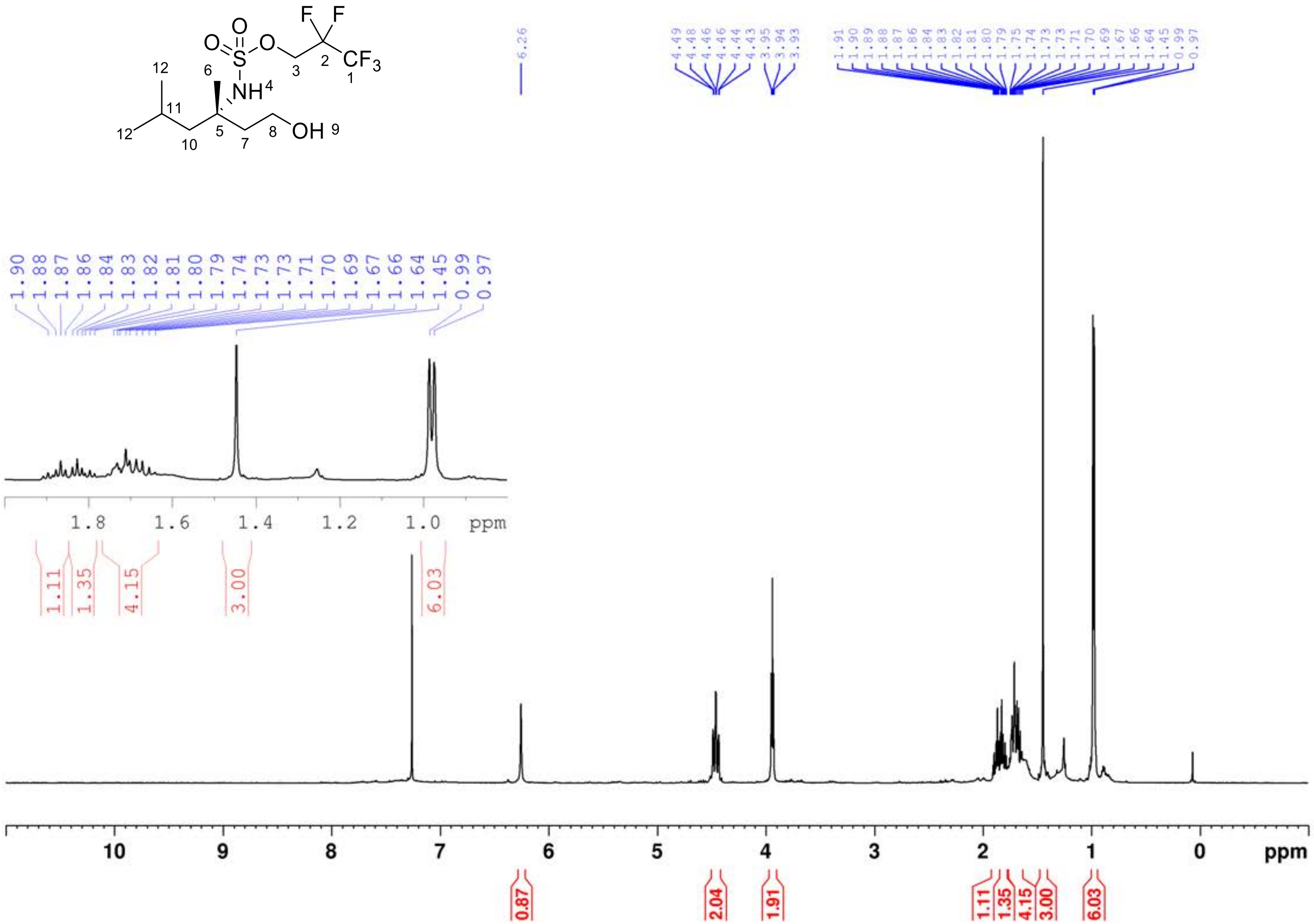

^{13}C NMR (126 MHz, $CDCl_3$) for *2,2,3,3,3-pentafluoropropyl (S)-(1-hydroxy-3,5-dimethylhexan-3-yl)sulfamate* (**SI10**)

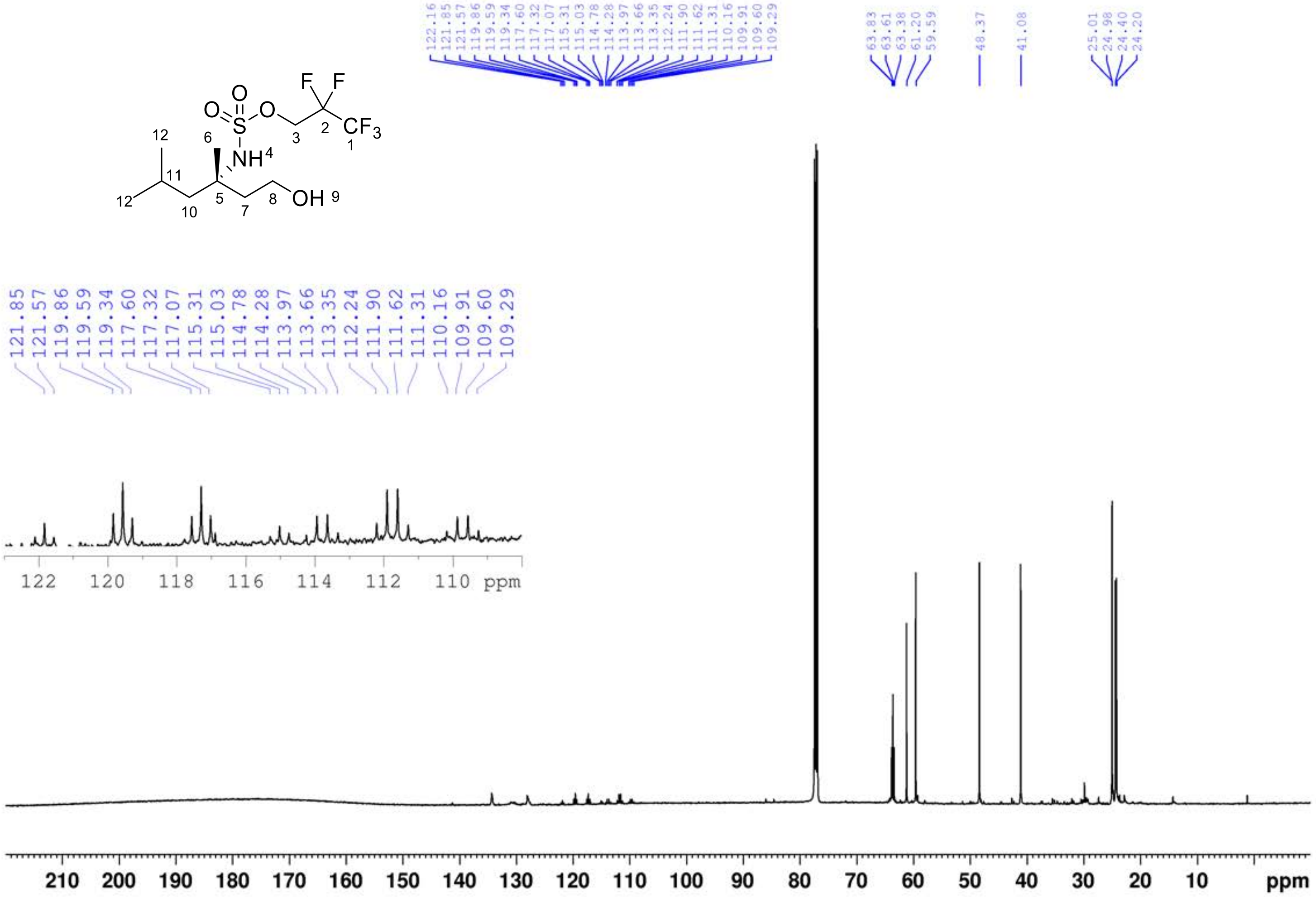

^{19}F NMR (376 MHz, CDCl$_3$) for *2,2,3,3,3-pentafluoropropyl (S)-(1-hydroxy-3,5-dimethylhexan-3-yl)sulfamate* (**SI10**)

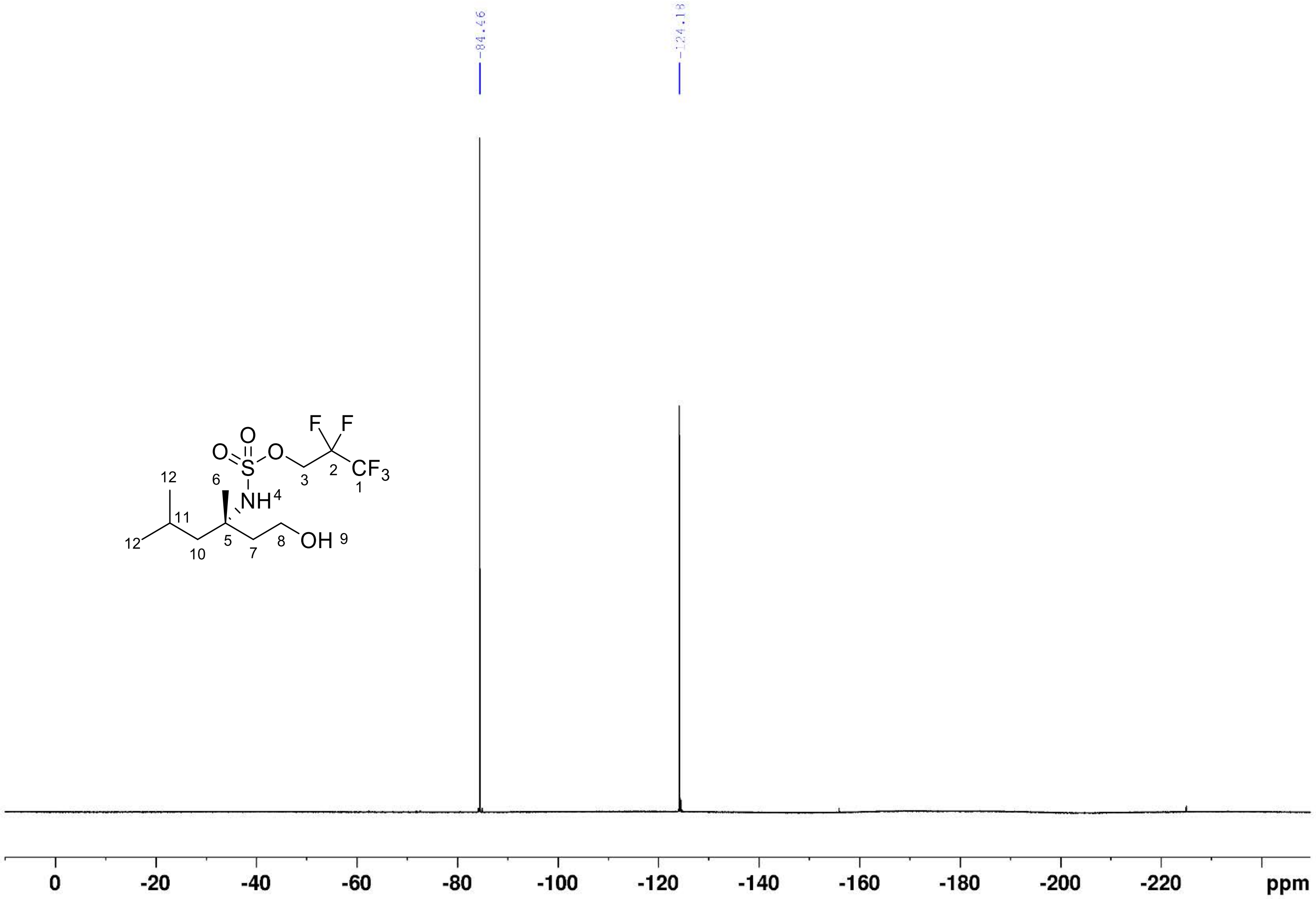

^{1}H NMR (400 MHz, $CDCl_3$) for *5-methyl-3-methylenehexan-1-ol* (**SI11**)

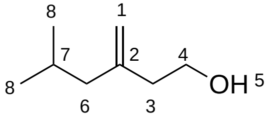

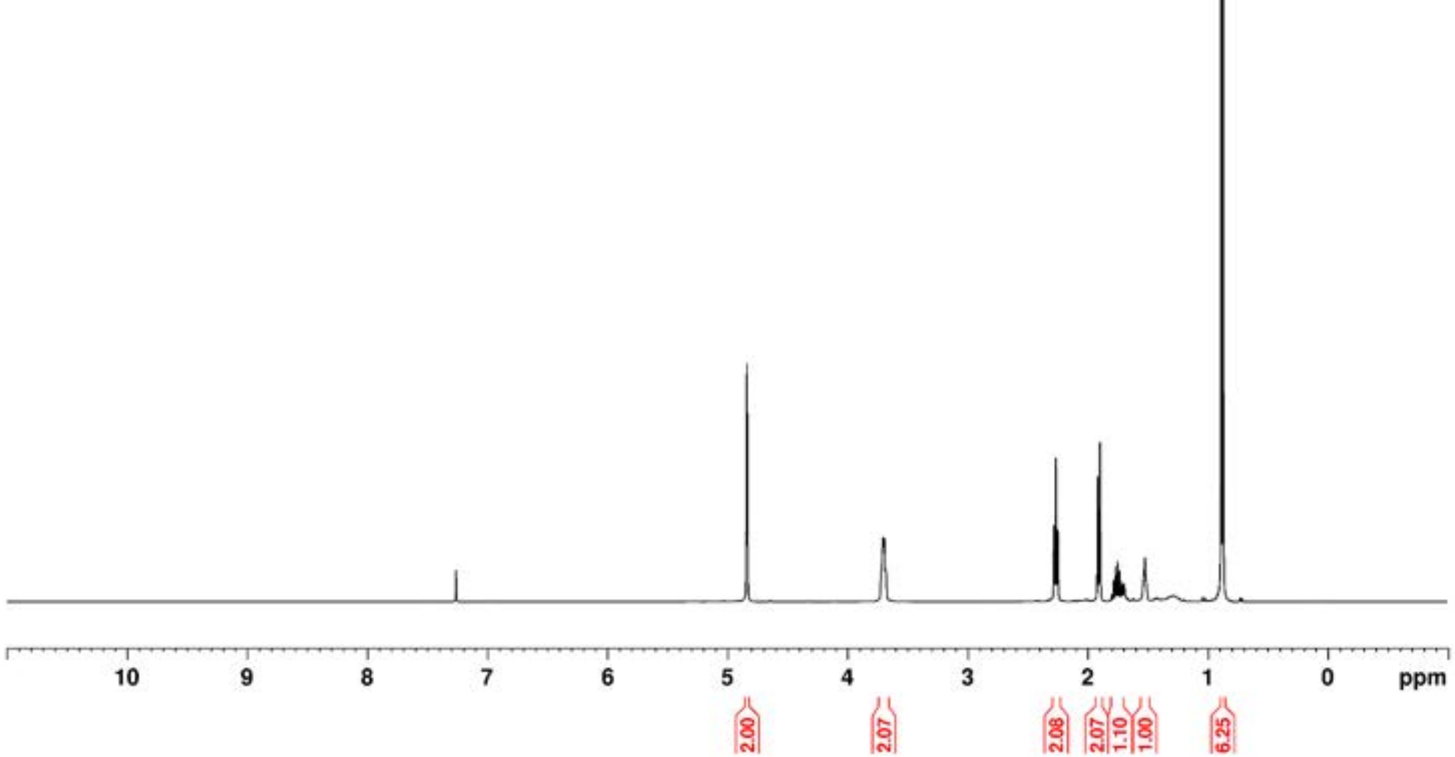

^{13}C NMR (101 MHz, CDCl$_3$) for *5-methyl-3-methylenehexan-1-ol* (**SI11**)

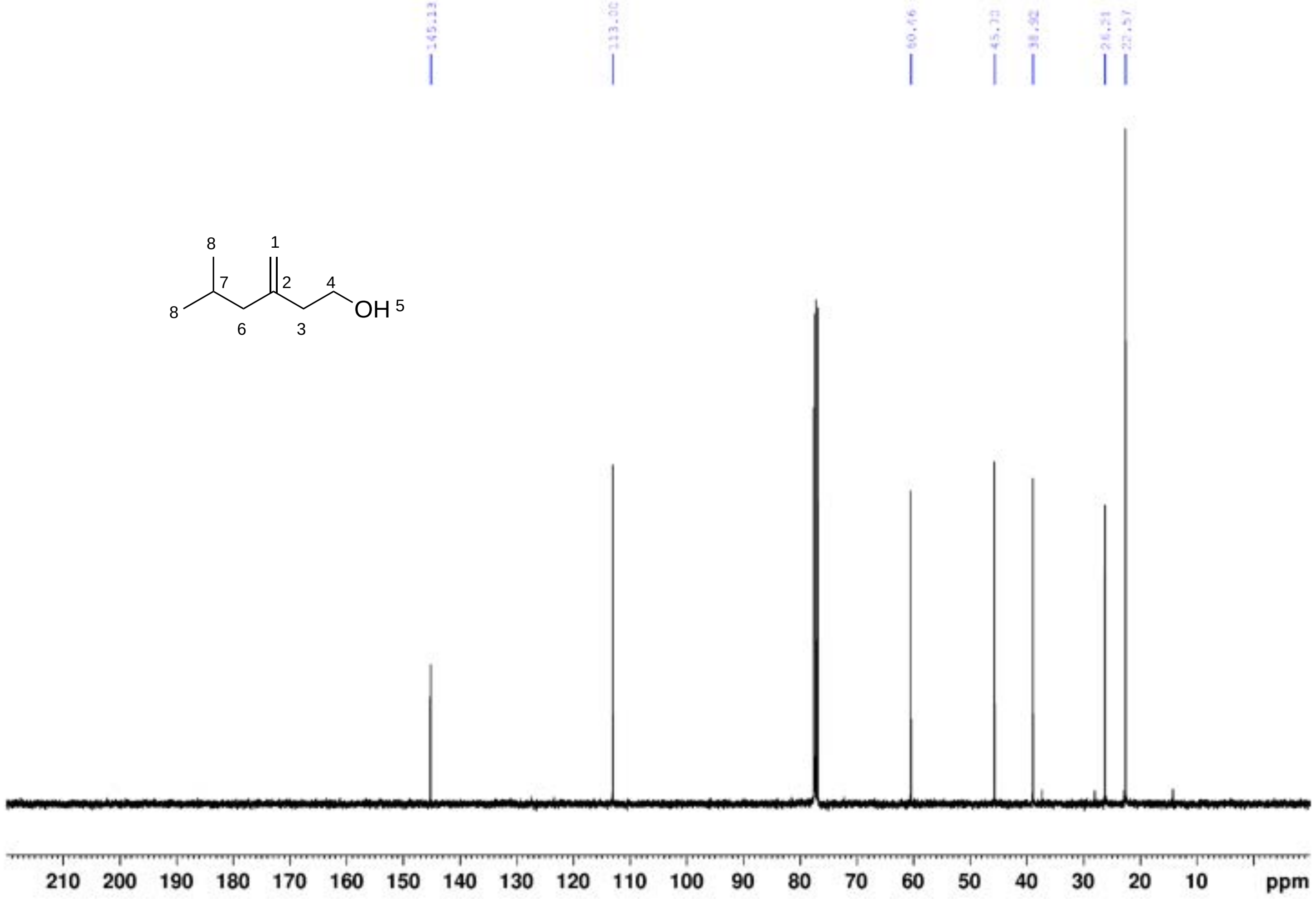

^{1}H NMR (500 MHz, CDCl$_3$) for *2,2,3,3,3-pentafluoropropyl (S)-2-(2-hydroxyethyl)-2-isobutylaziridine-1-sulfonate* (**SI12**)

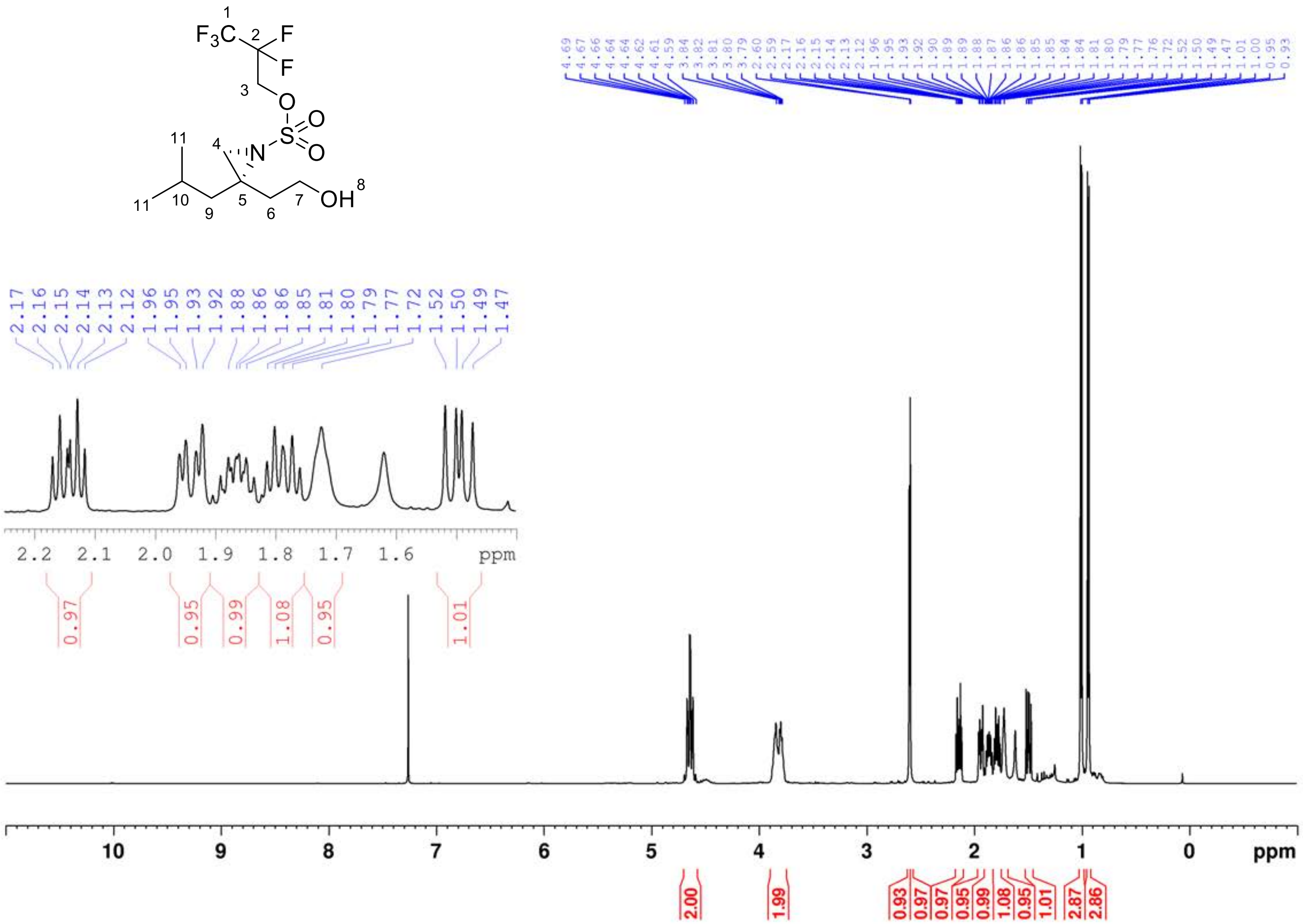

^{13}C NMR (126 MHz, $CDCl_3$) for *2,2,3,3,3-pentafluoropropyl (S)-2-(2-hydroxyethyl)-2-isobutylaziridine-1-sulfonate* (**SI12**)

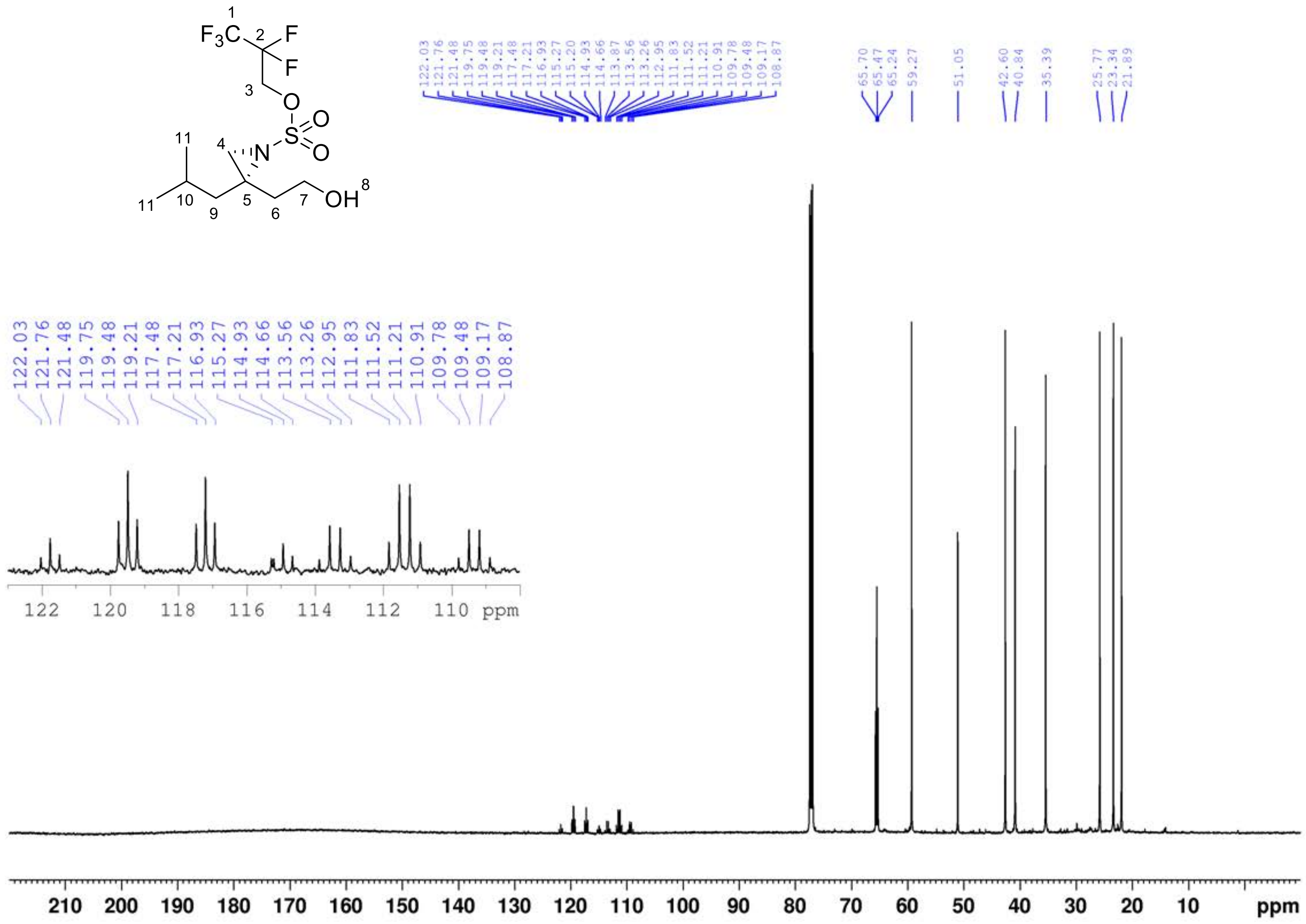

^{19}F NMR (376 MHz, $CDCl_3$) for *2,2,3,3,3-pentafluoropropyl (S)-2-(2-hydroxyethyl)-2-isobutylaziridine-1-sulfonate* (**SI12**)

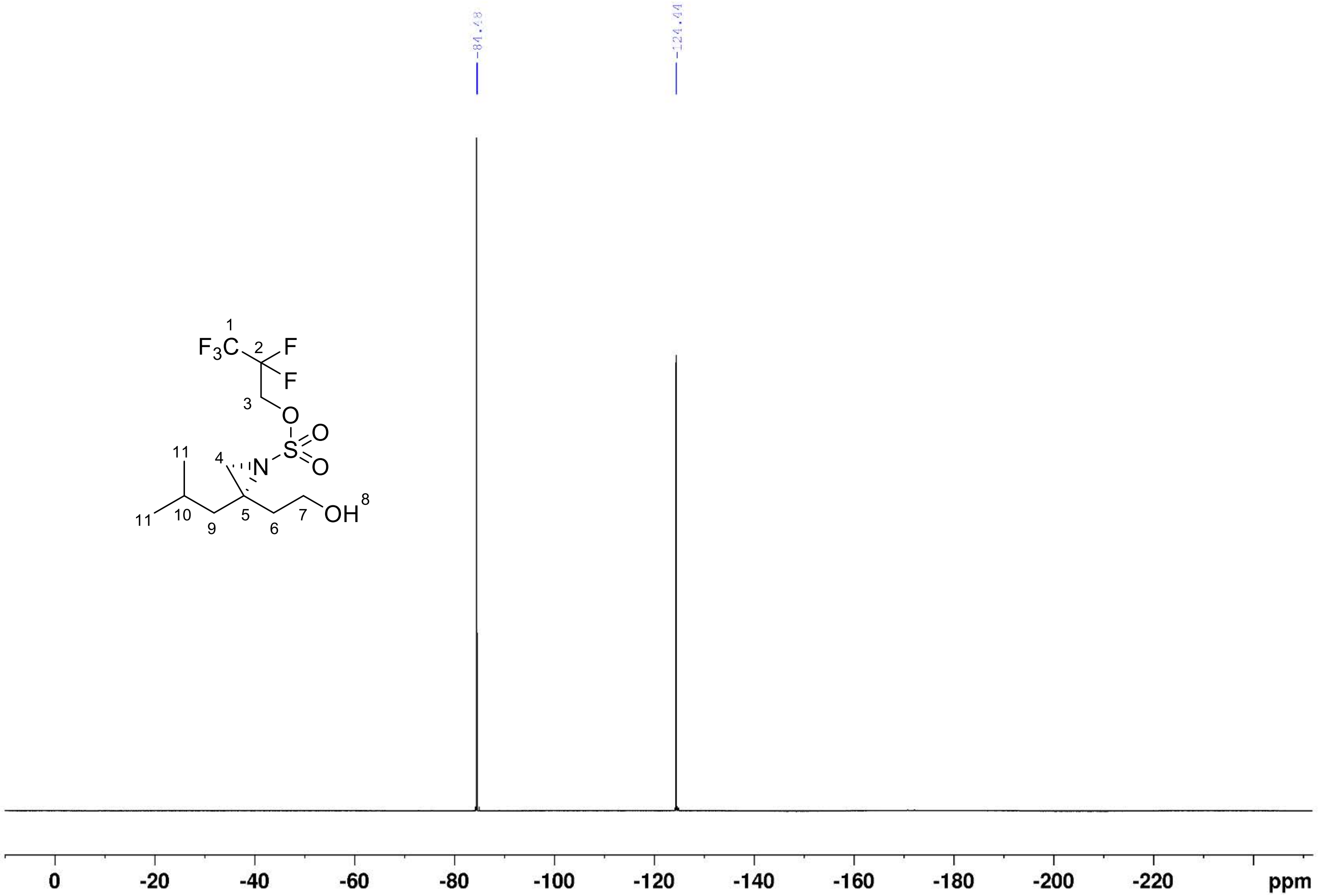

^{1}H NMR (500 MHz, CDCl$_3$) for *2,2,3,3,3-pentafluoropropyl (S)-(1-hydroxy-5-methyl-3-((phenylthio)methyl)hexan-3-yl)sulfamate* (**SI13**)

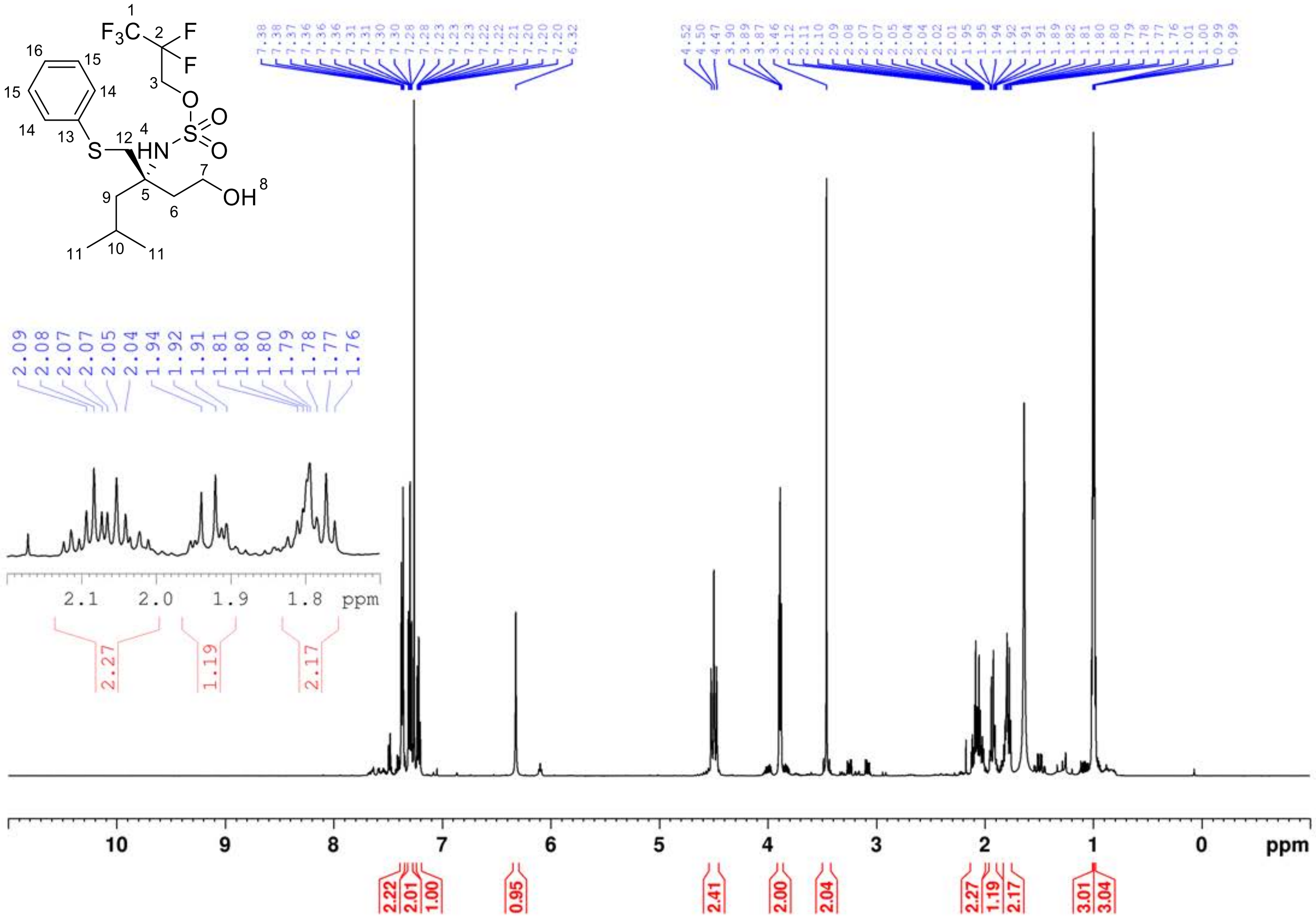

^{13}C NMR (126 MHz, CDCl$_3$) for *2,2,3,3,3-pentafluoropropyl (S)-(1-hydroxy-5-methyl-3-((phenylthio)methyl)hexan-3-yl)sulfamate* (**SI13**)

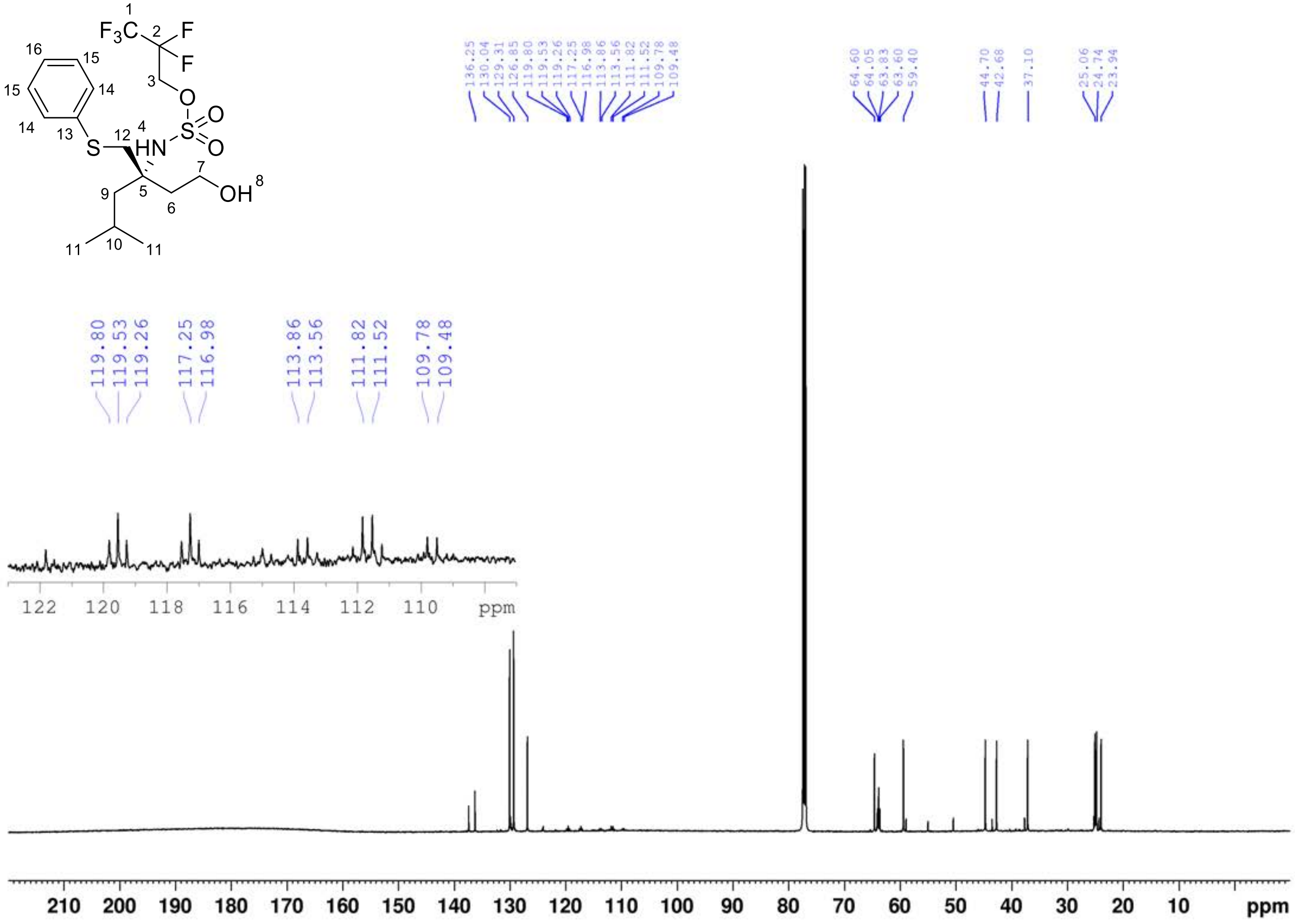

^{19}F NMR (376 MHz, $CDCl_3$) for *2,2,3,3,3-pentafluoropropyl (S)-(1-hydroxy-5-methyl-3-((phenylthio)methyl)hexan-3-yl)sulfamate* (**SI13**)

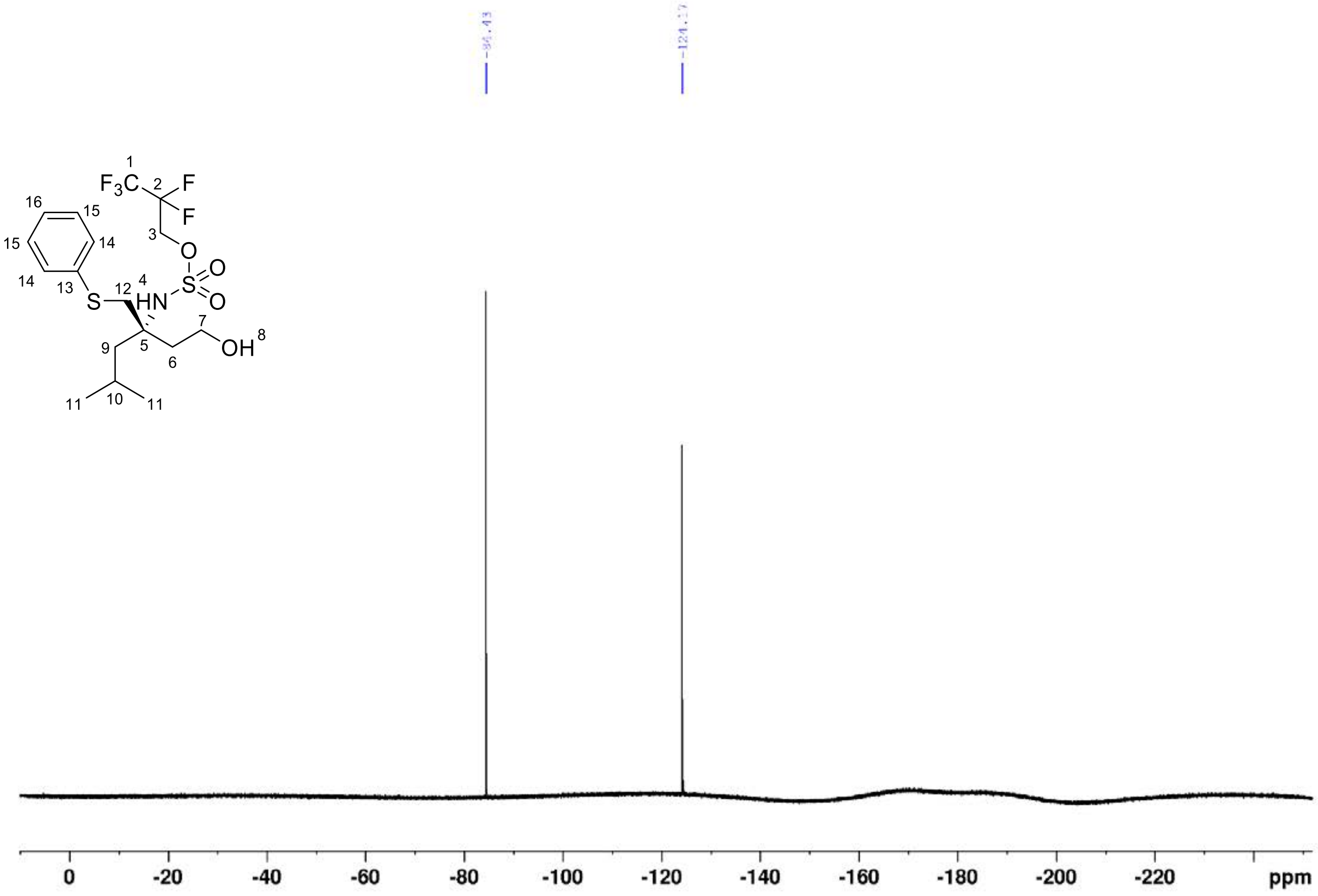

¹H NMR (400 MHz, CDCl₃) for *5-(pyridin-3-yl)pent-4-yn-1-ol*

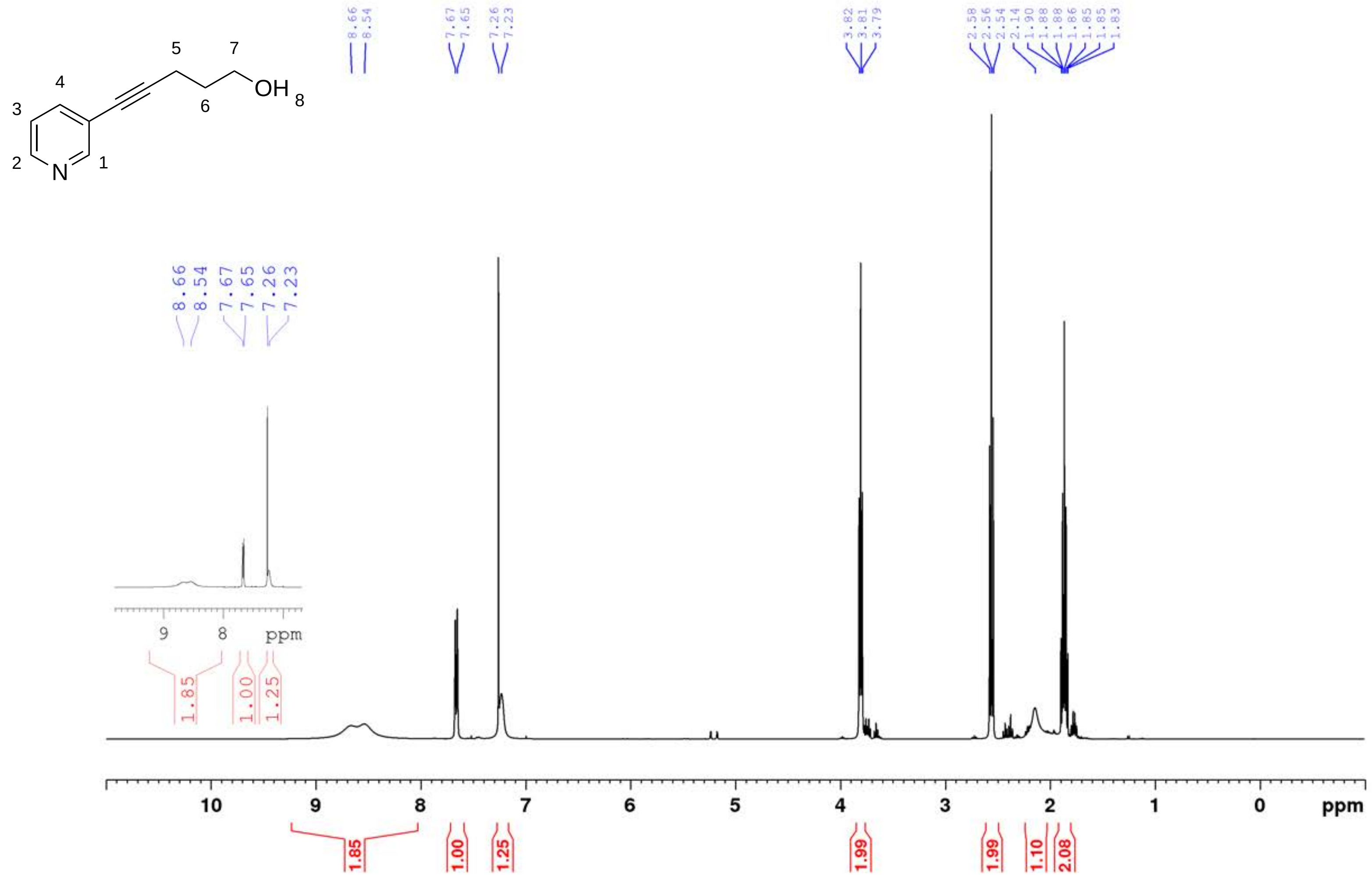

^{13}C NMR (101 MHz, $CDCl_3$) for *5-(pyridin-3-yl)pent-4-yn-1-ol*

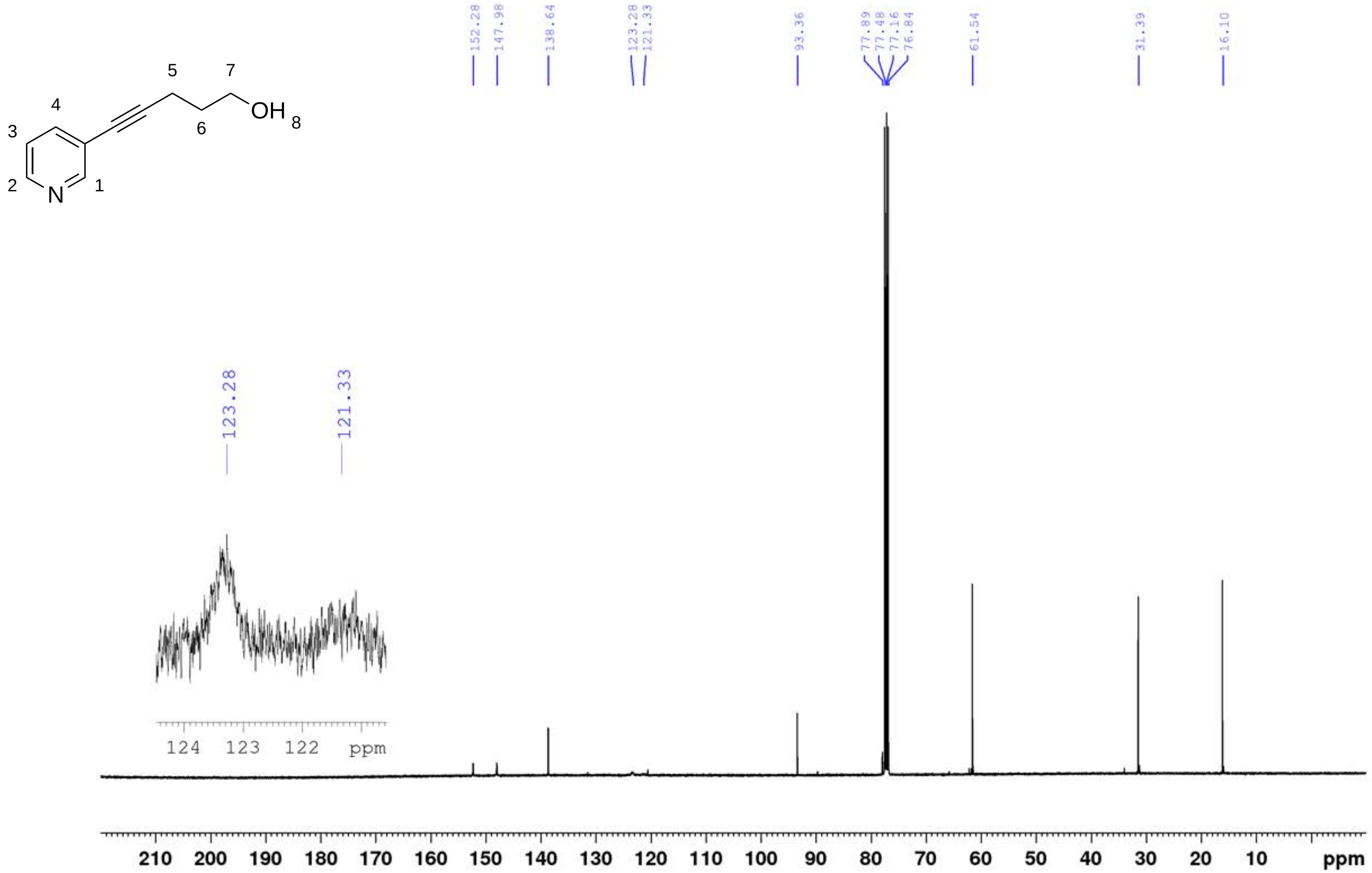

^{1}H NMR (400 MHz, CDCl$_3$) for *(Z)-5-(pyridin-3-yl)pent-4-en-1-ol*

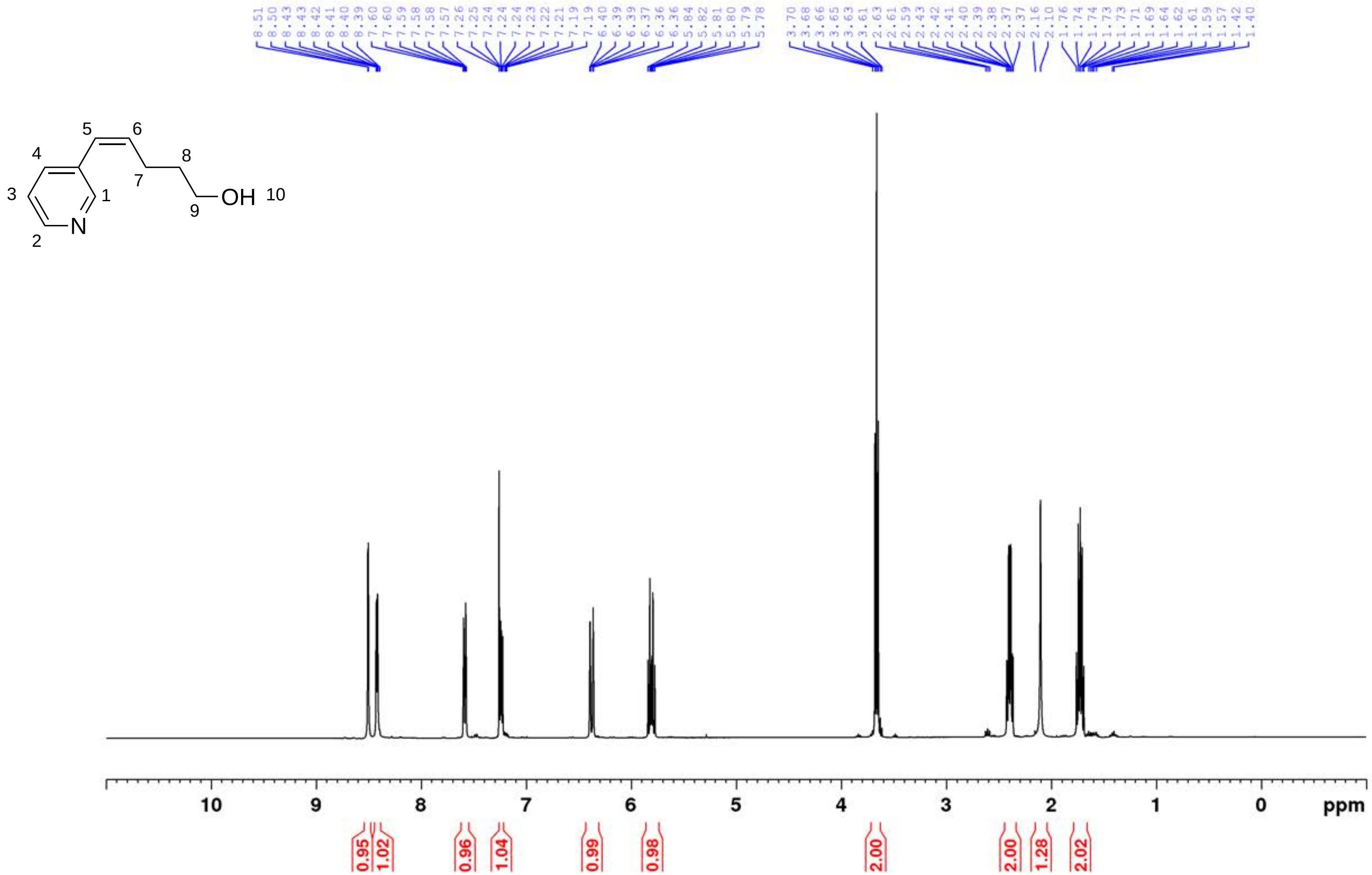

^{13}C NMR (101 MHz, $CDCl_3$) for *(Z)-5-(pyridin-3-yl)pent-4-en-1-ol*

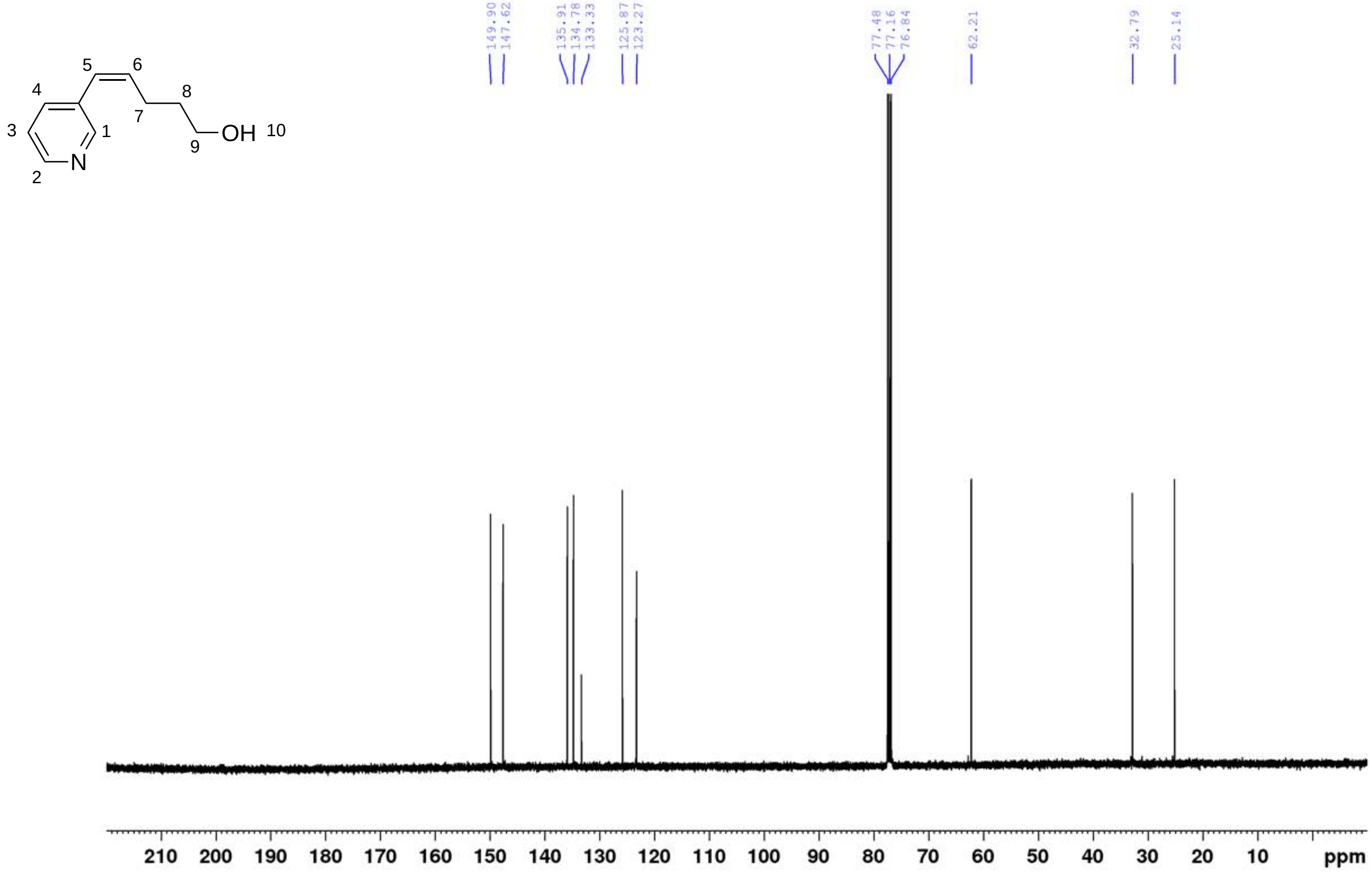

^{1}H NMR (400 MHz, $CDCl_3$) for *(Z)-N,5-diphenylpent-4-enamide*

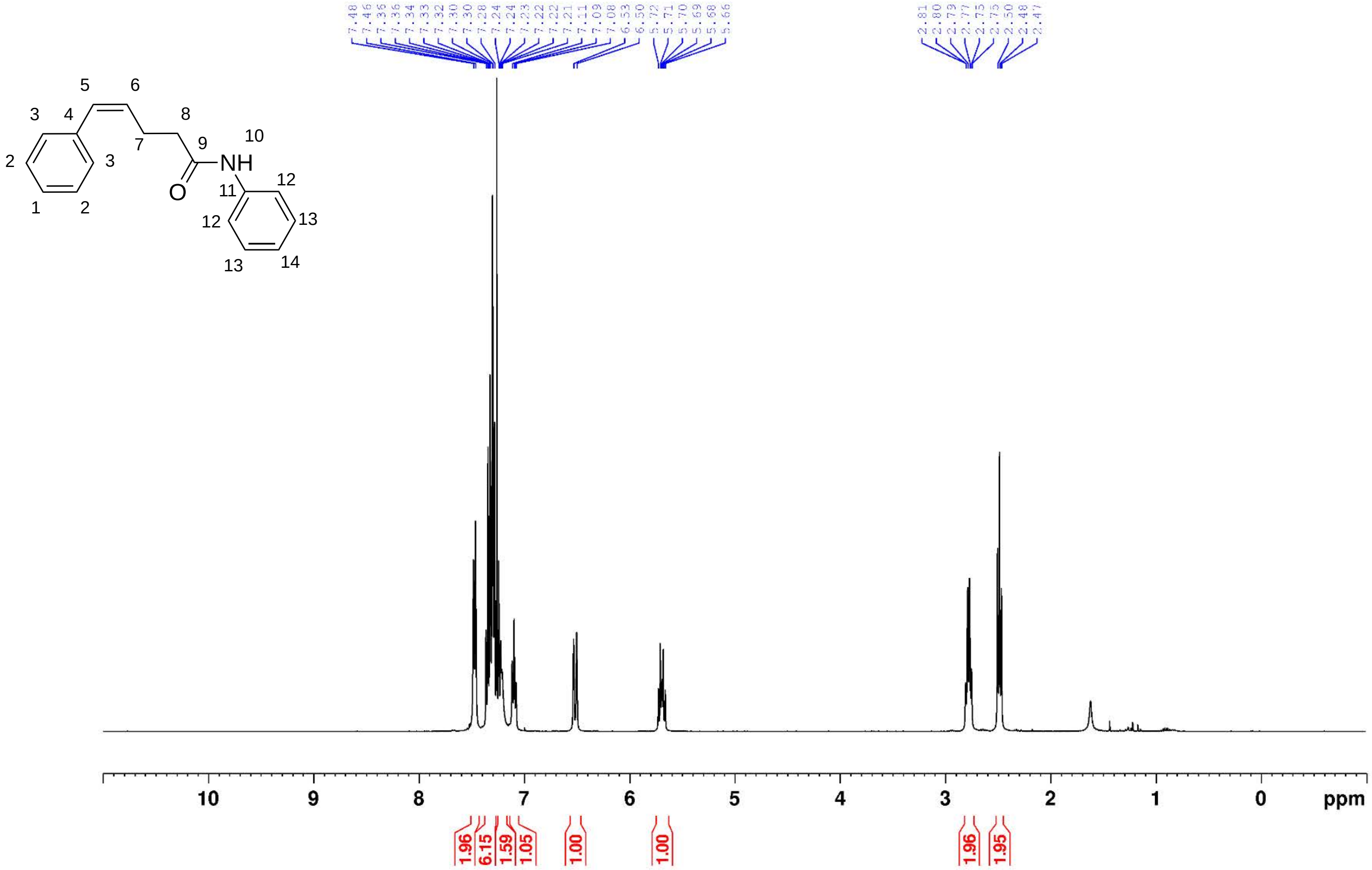

^{13}C NMR (101 MHz, CDCl$_3$) for *(Z)-N,5-diphenylpent-4-enamide*

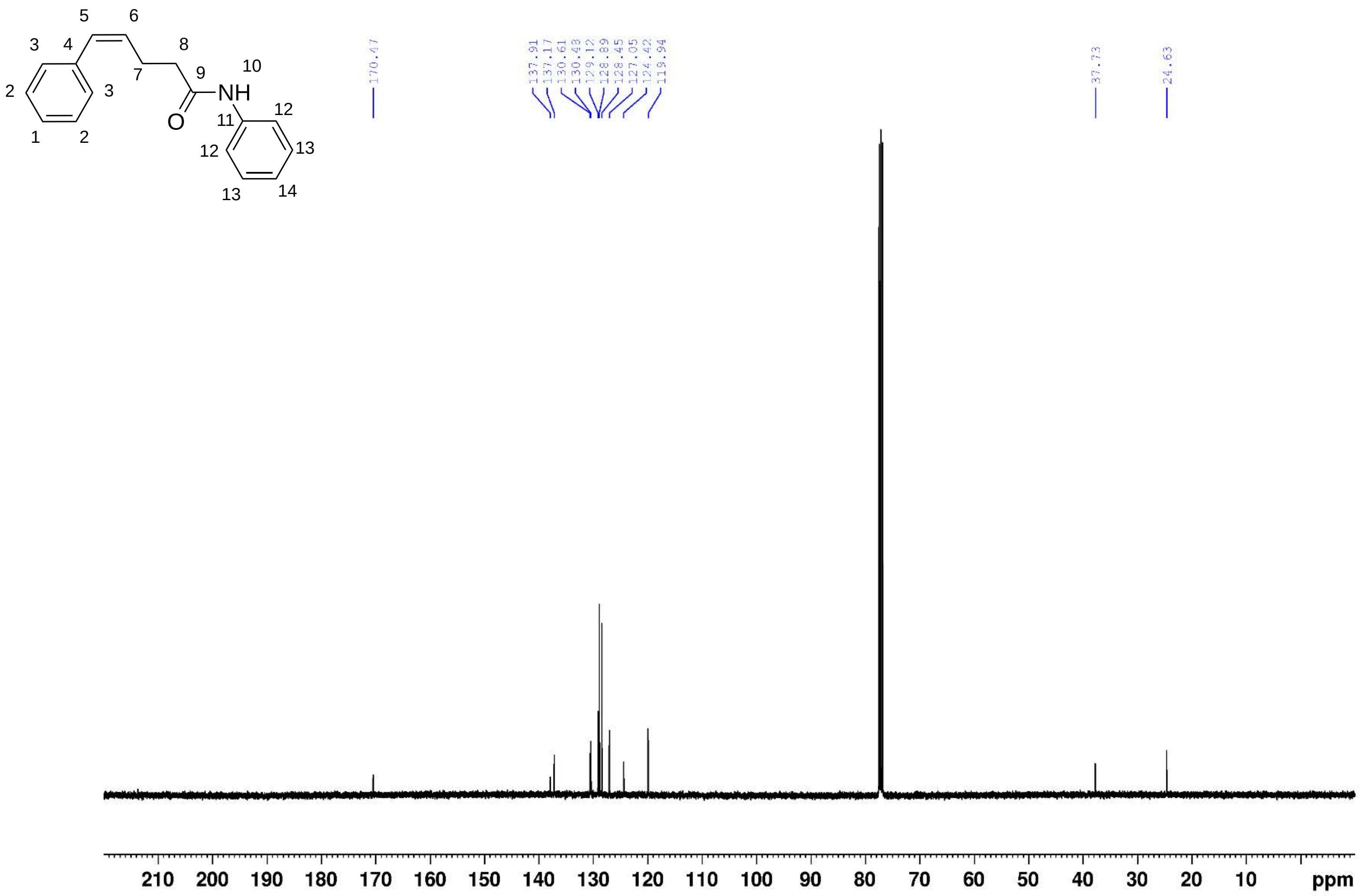

^{1}H NMR (400 MHz, $CDCl_3$) for *(Z)-N-(5-phenylpent-4-en-1-yl)aniline*

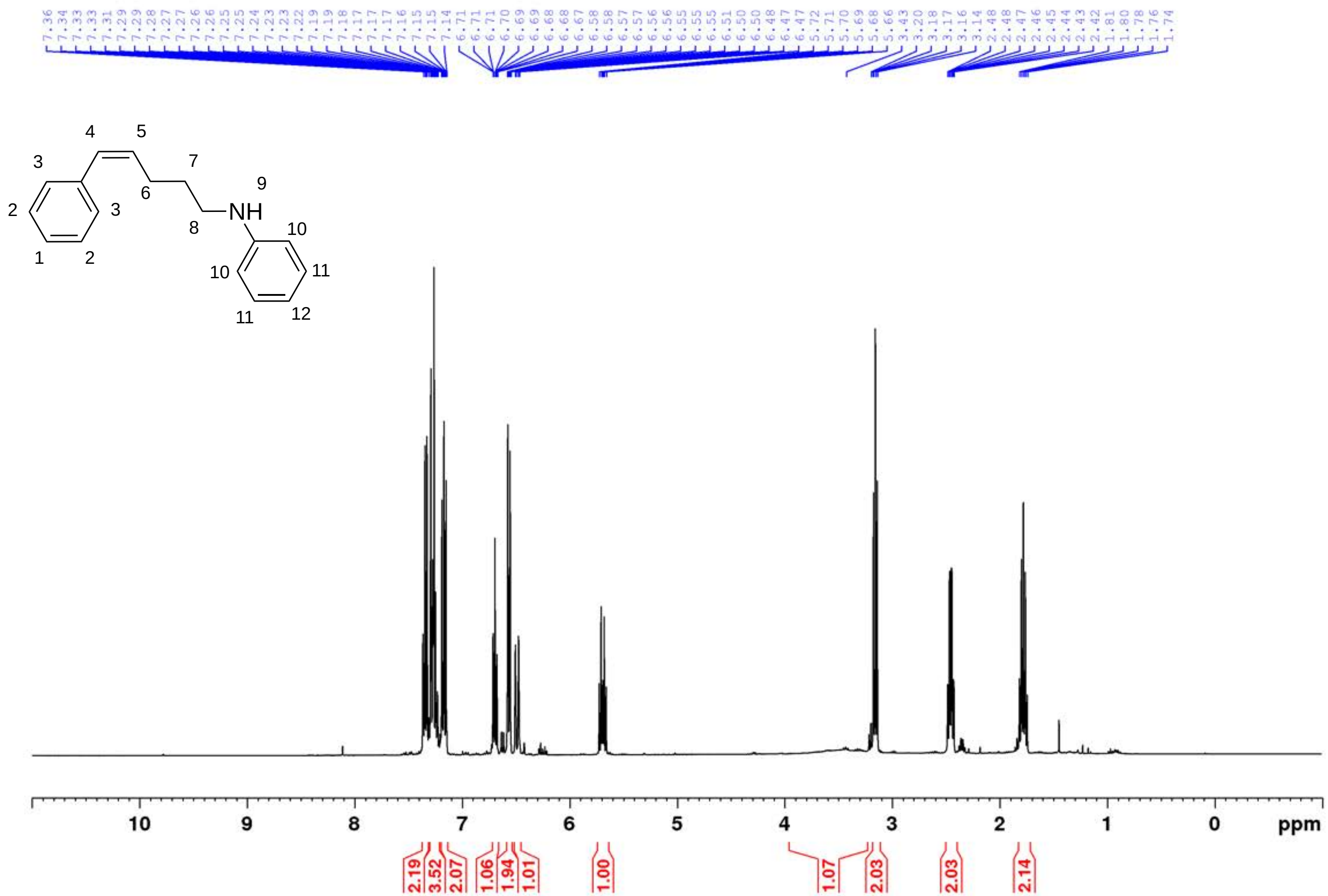

^{13}C NMR (101 MHz, CDCl$_3$) for *(Z)-N-(5-phenylpent-4-en-1-yl)aniline*

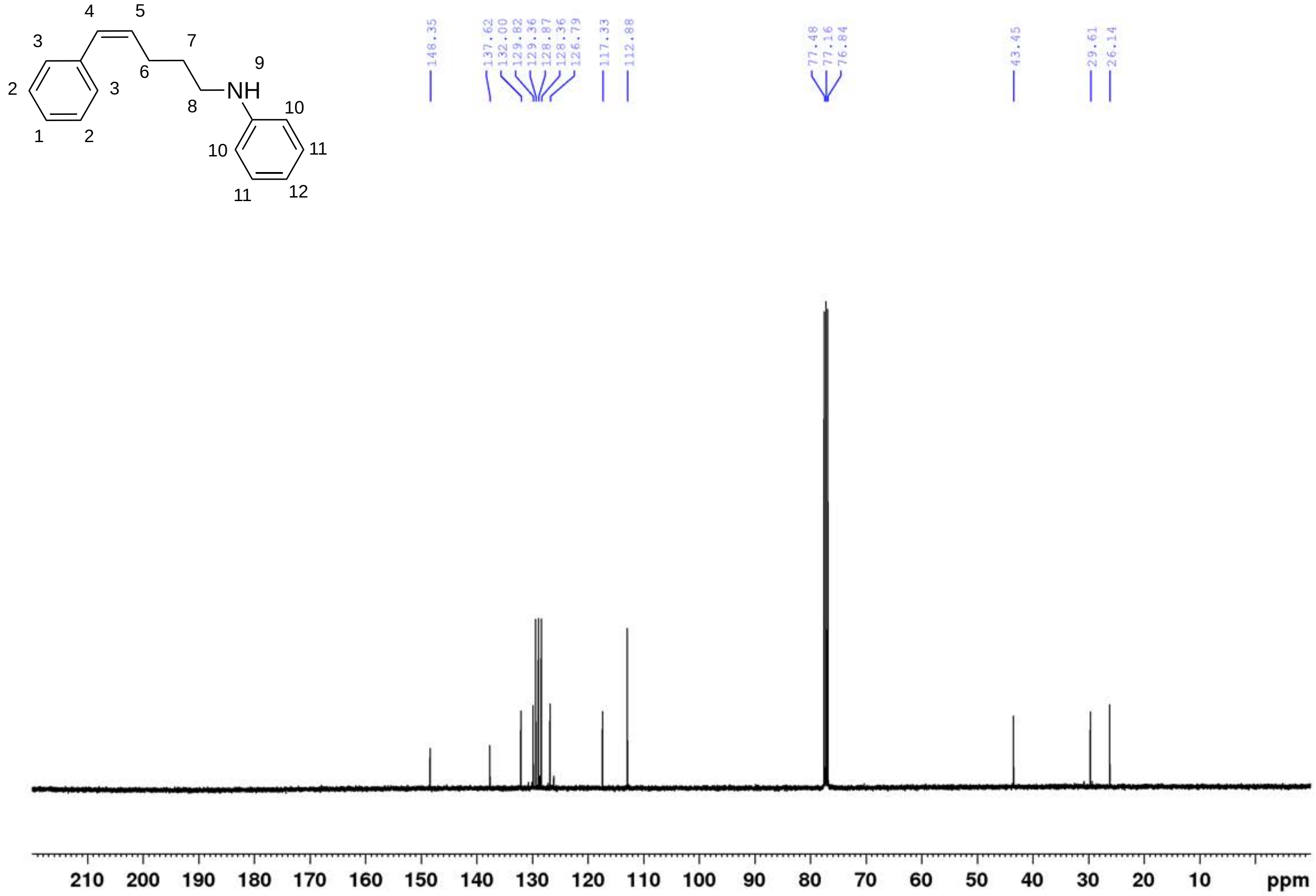

Diffusion Ordered Spectroscopy (DOSY) Studies of $Rh_2(B)_2 \bullet (C3)_2$

We are grateful to Duncan J. Howe for assistance in obtaining and interpreting the results presented below.

We performed a DOSY experiment on $Rh_2(B)_2 \bullet (C3)_2$ in C_5D_5N at room temperature and measured the diffusion coefficient (*D*) of the cationic and anionic components of the complex. The measured *D* values for selected resonances on both the anionic $Rh_2(B)_2$ and cationic (C3) components match closely and provide strong support for association of the two cations with the disulfonated complex.[82] When picking the peaks for analysis we gave preference to those that did not correspond to a superposition of several signals, hence the omission of certain upfield resonances. Under the measurement conditions, the C_5D_5N which is in vast excess relative to the catalyst displaces the axially-bound cations (as evidenced by the colour change of the complex from brown to purple upon dissolution) and as a result the association between the cations and anions will be as a result of ion-pairing alone. The results suggest that these components remain associated in the presence of solvent amounts of pyridine and we conclude that it is highly likely that these will remain associated under the aziridination conditions (**GP9**) also.

The peaks used for the analysis are presented in **Supplementary Table 4**, along with their assignment and data obtained from the DOSY experiment. The full report for the DOSY experiment, including experimental details is included below also.

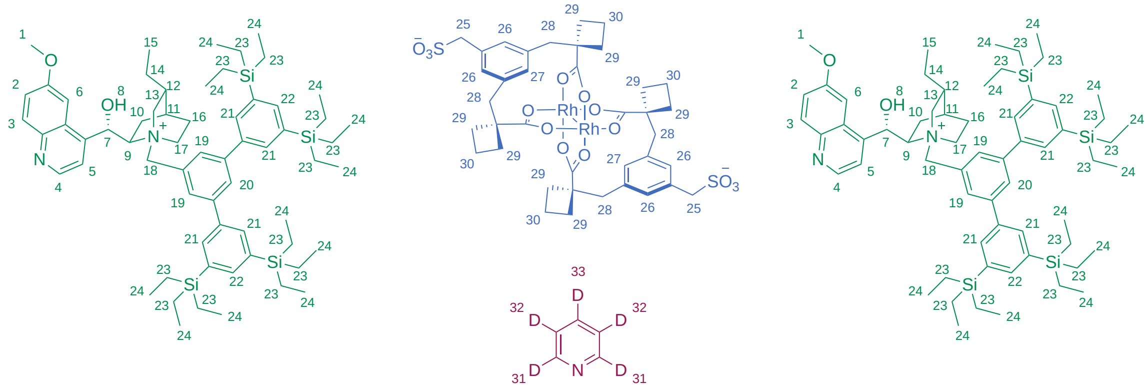

• Diffusion Analysis

sample name:	bw435-dosy
Description/Title:	16 scans<br>20 increments<br><br>p30=1350us
Origin:	in-house
Date of preparation:	06 / 2005
Lab Book Number:	000

Diffusion Analysis
C:\data\service\nmr\rjp-bw435-dosy\14\pdata\1\2rr

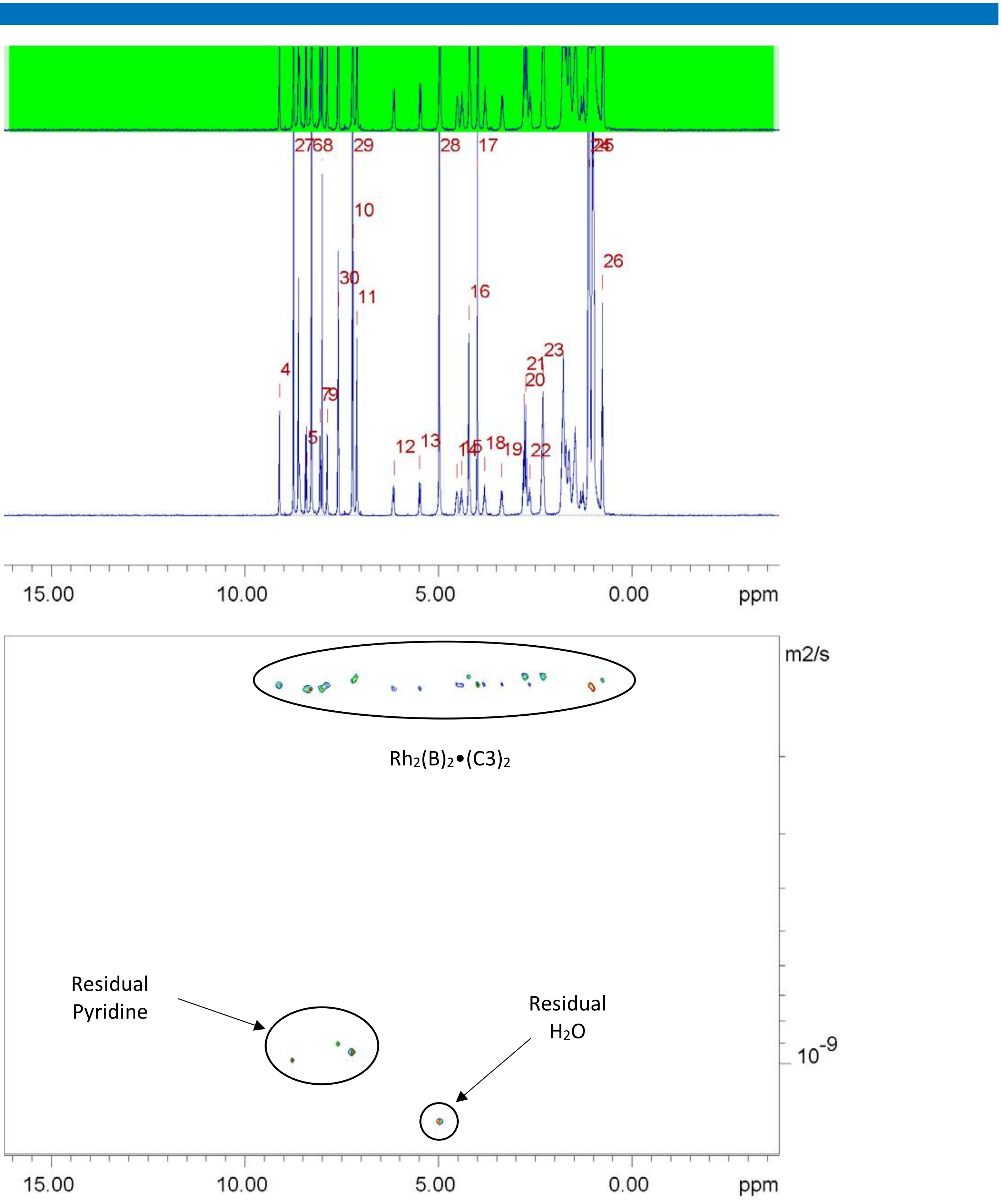

Diffusion Analysis
C:\data\service\nmr\rjp-bw435-dosy\14\pdata\1\2rr

Fitted function:	f (x) = A * exp (-D * x^2 * gamma^2 * littleDelta^2 (bigDelta-littleDelta/3)* 10^4
used gamma:	26752 rad/(s*Gauss)
used little delta:	0.0027000 s
used big delta:	0.099900 s
used gradient strength:	variable
Random error estimation of data:	RMS per spectrum (or trace/plane)
Systematic error estimation of data:	worst case per peak scenario
Fit parameter Error estimation method:	from Monte Carlo simulations
Confidence level:	95%
Used peaks:	
Used integrals:	peak intensities
Used Gradient strength:	all values (including replicates) used

Supplementary Table 4: Data obtained from the DOSY NMR experiment of $Rh_2(B)_2$•$(C3)_2$ obtained in C_5D_5N

Peak	F2 [ppm]	Cation/Anion/C_5D_5N	Assignment	A	error	D [m^2/s]	error
4	9.10	Cation	H-4	1.08e+07	1.753e+04	2.01e-10	7.538e-13
5	8.40	Cation	H-3	8.35e+06	1.898e+04	2.06e-10	1.038e-12
6	8.28	Cation	H-20, H-21	7.15e+07	1.809e+04	2.06e-10	1.193e-13
7	8.06	Cation	H-5	8.24e+06	1.822e+04	2.06e-10	1.013e-12
8	8.00	Cation	H-22	3.55e+07	1.736e+04	2.03e-10	2.440e-13
9	7.87	Cation	H-6	8.44e+06	1.871e+04	2.01e-10	1.023e-12
10	7.20	Anion	H-26	2.71e+07	1.730e+04	1.99e-10	3.023e-13
11	7.10	Cation, Anion	H-7, H-27	1.78e+07	1.848e+04	1.93e-10	4.650e-13
12	6.14	Cation	H-18a	3.06e+06	1.836e+04	2.06e-10	2.750e-12
13	5.49	Cation	H-18b	3.42e+06	1.698e+04	2.05e-10	2.483e-12
14	4.52	Cation	H-13a	2.42e+06	1.804e+04	2.03e-10	3.514e-12
15	4.40	Cation	H-17a	2.81e+06	1.888e+04	2.03e-10	3.039e-12
16	4.21	Cation, Anion	H-9, H-25a, H-25b	1.88e+07	1.763e+04	1.93e-10	4.228e-13
17	3.99	Cation	H-1	5.85e+07	1.785e+04	2.03e-10	1.438e-13
18	3.81	Cation	H-13b	3.13e+06	1.812e+04	2.03e-10	2.844e-12
19	3.37	Cation	H-17b	2.46e+06	1.834e+04	2.03e-10	3.388e-12
20	2.78	Anion	H-28a	1.06e+07	1.719e+04	1.95e-10	7.555e-13
21	2.74	Anion	H-28b	1.14e+07	1.766e+04	1.92e-10	6.922e-13
22	2.64	Cation	H-10a	2.49e+06	1.830e+04	2.03e-10	3.393e-12
23	2.29	Anion	H-29a	1.22e+07	1.643e+04	1.91e-10	6.383e-13
24	1.11	Cation	H-10b, H-24	3.46e+08	1.791e+04	2.03e-10	2.542e-14
25	1.01	Cation	H-23	1.80e+08	1.734e+04	2.05e-10	4.884e-14
26	0.76	Cation	H-15	1.84e+07	1.830e+04	1.99e-10	4.716e-13
27	8.74	C_5D_5N	H-31	8.94e+07	3.876e+04	1.44e-09	1.078e-12
28	4.97	H_2O	-	4.08e+08	4.931e+04	2.00e-09	3.892e-13
29	7.22	C_5D_5N	H-33	1.02e+08	3.888e+04	1.39e-09	9.173e-13
30	7.59	C_5D_5N	H-32	5.06e+07	3.702e+04	1.32e-09	1.732e-12

Diffusion Analysis
C:\data\service\nmr\rjp-bw435-dosy\14\pdata\1\2rr

Current fit display

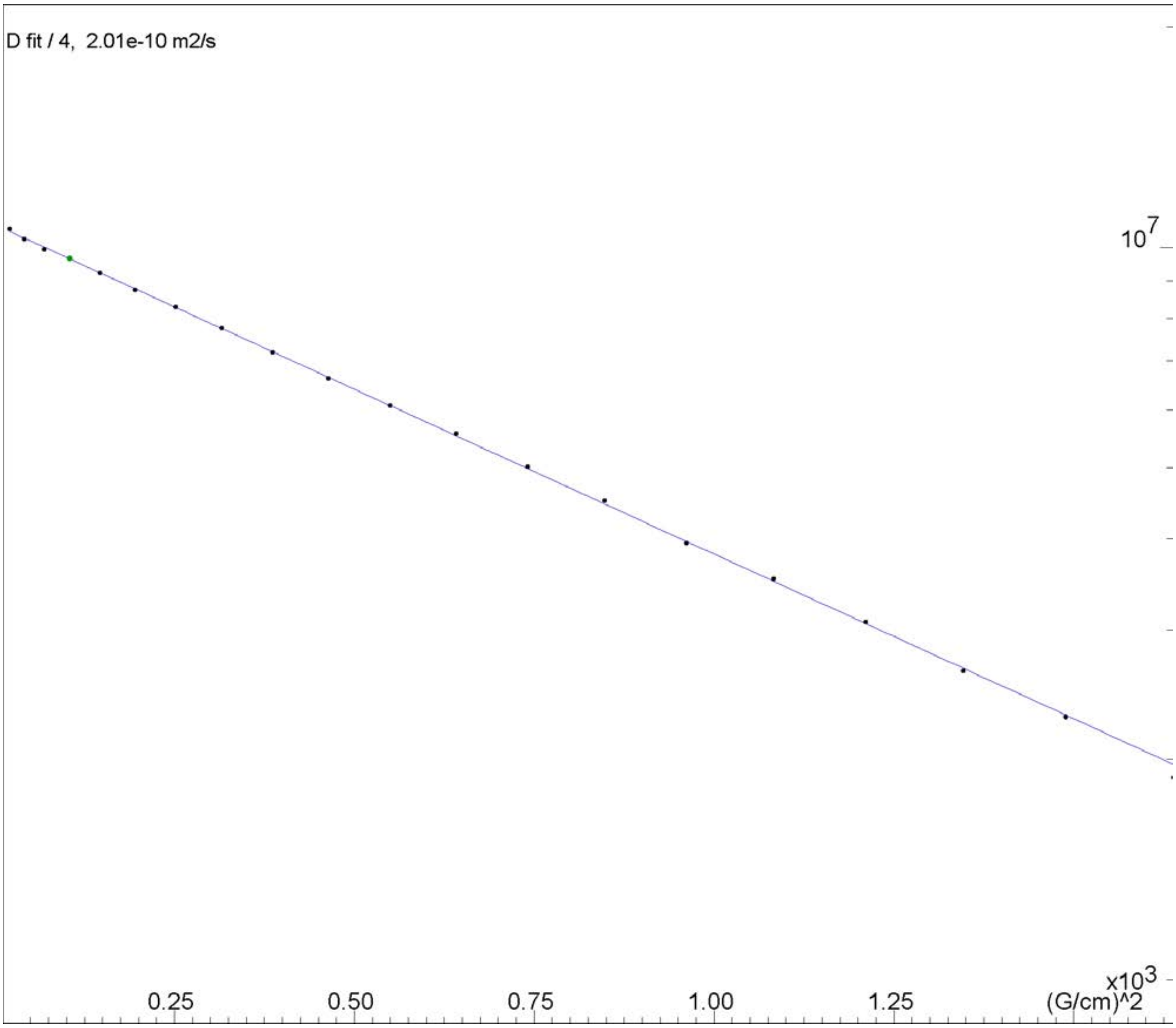

Diffusion Analysis
C:\data\service\nmr\rjp-bw435-dosy\14\pdata\1\2rr

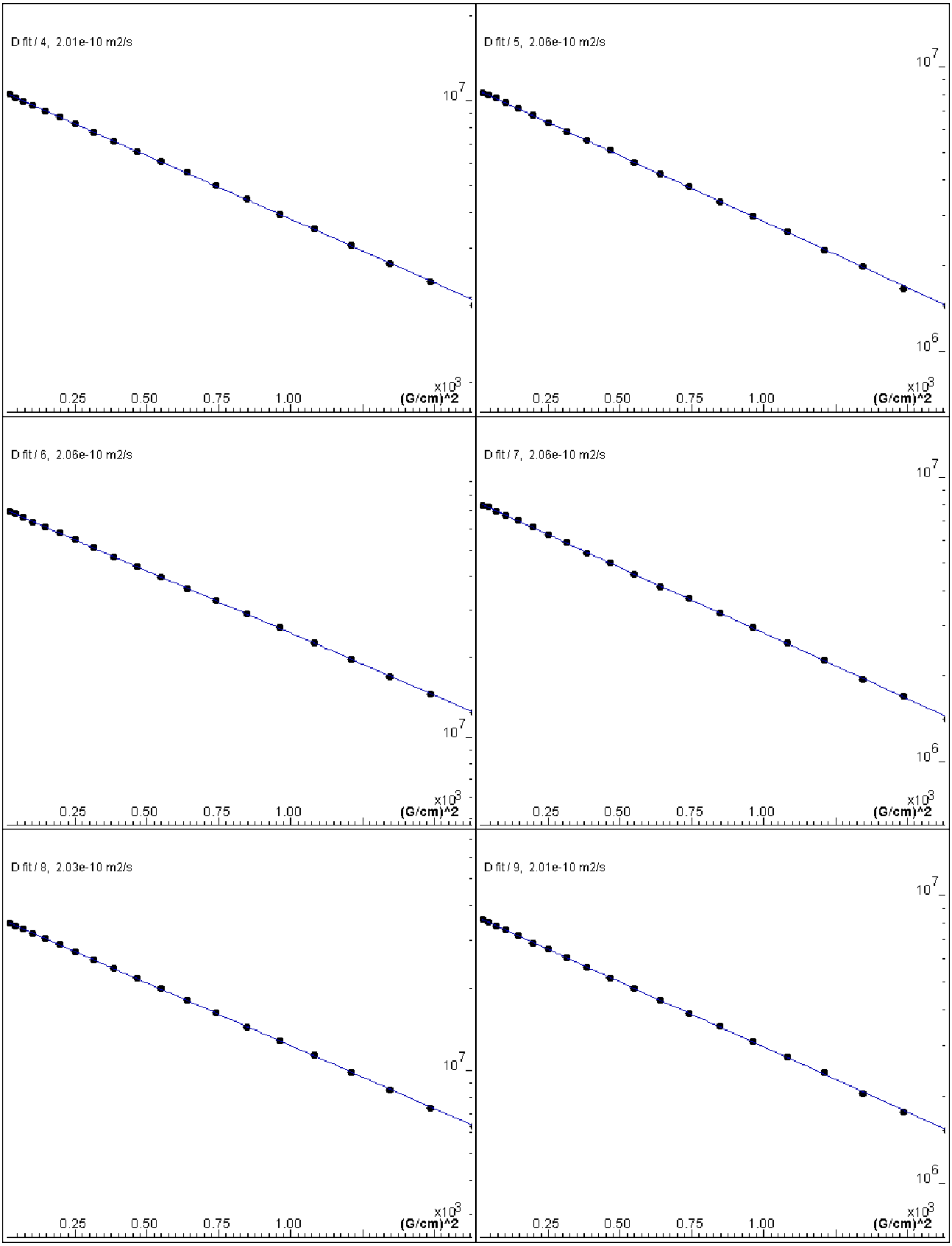

Diffusion Analysis
C:\data\service\nmr\rjp-bw435-dosy\14\pdata\1\2rr

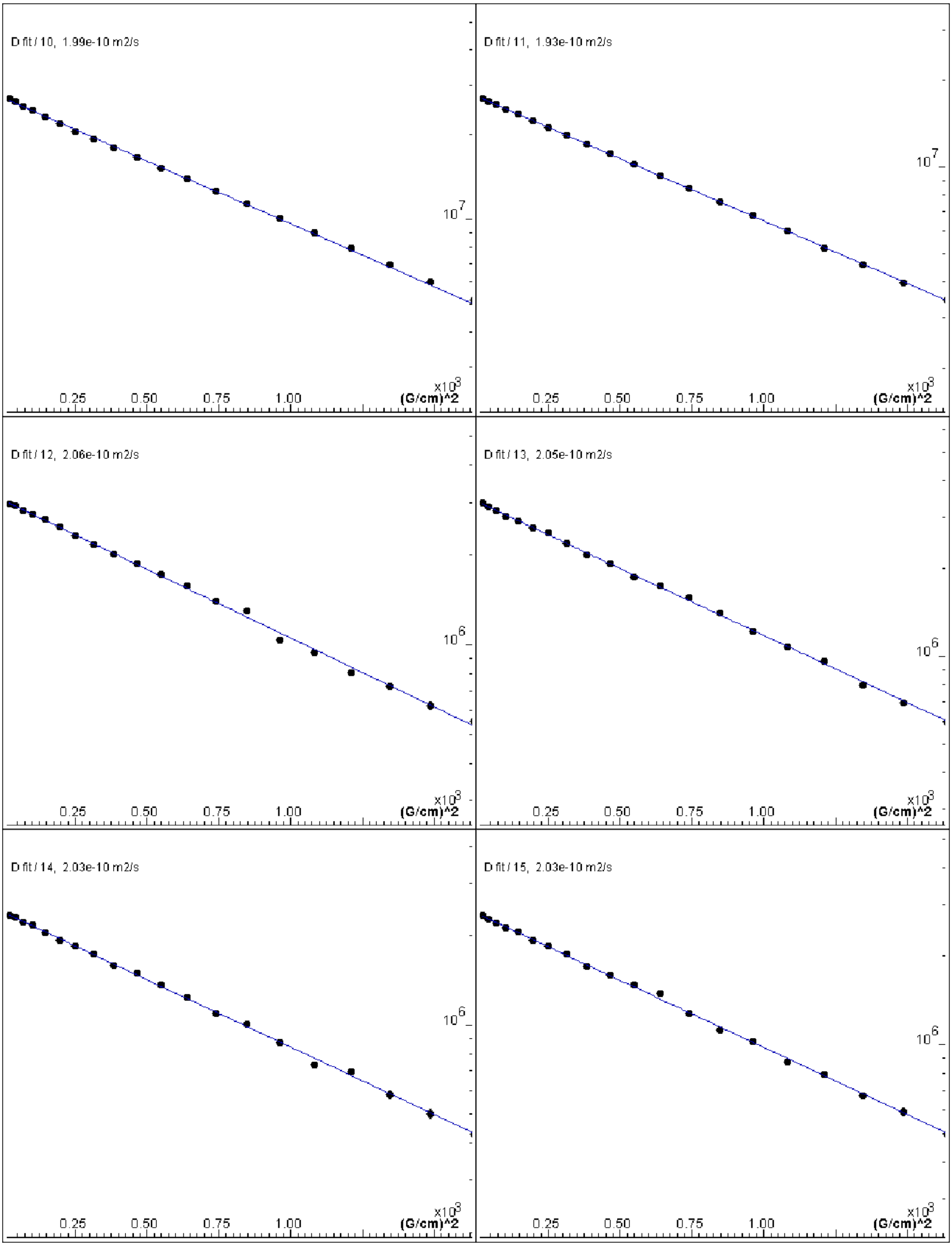

Diffusion Analysis
C:\data\service\nmr\rjp-bw435-dosy\14\pdata\1\2rr

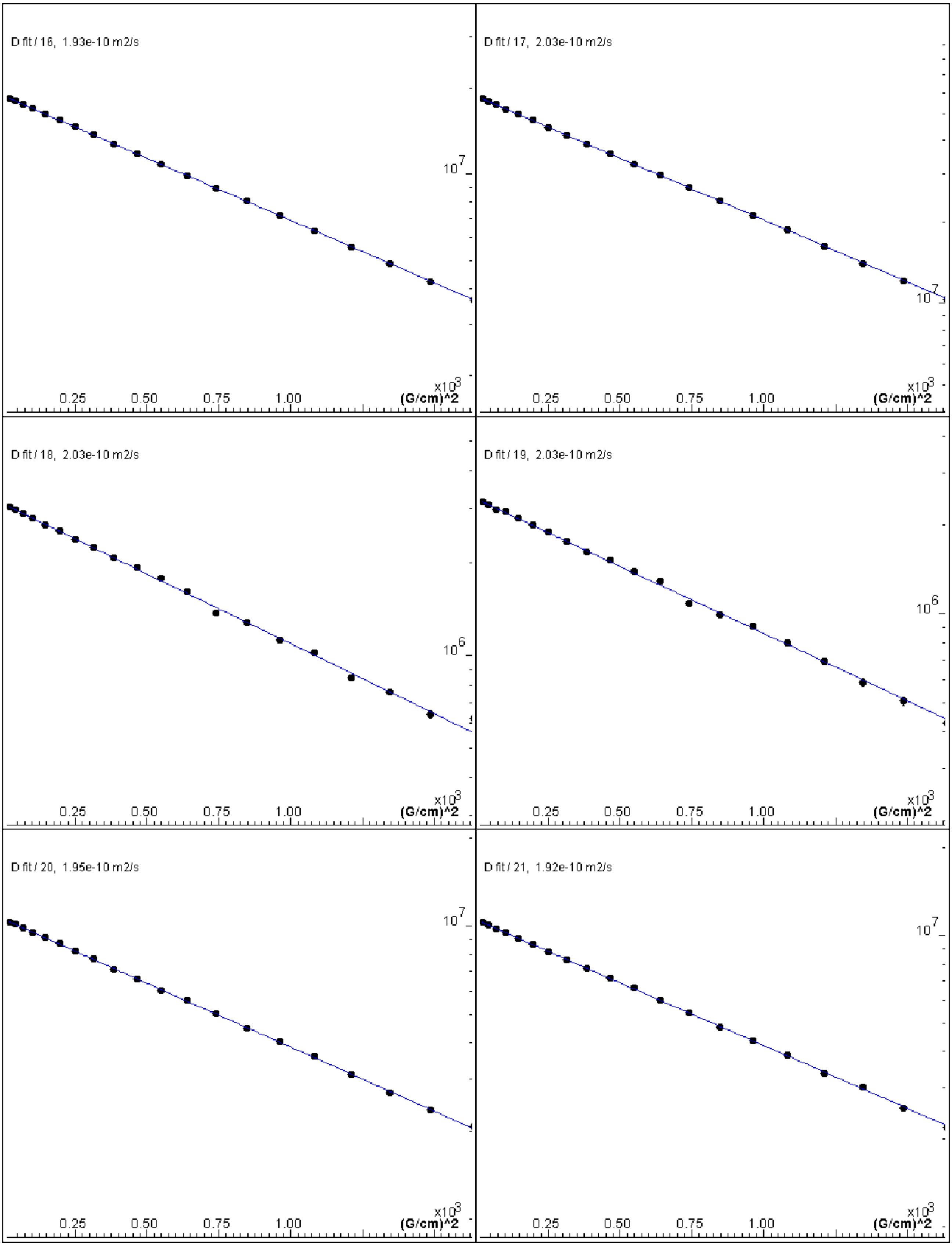

Diffusion Analysis
C:\data\service\nmr\rjp-bw435-dosy\14\pdata\1\2rr

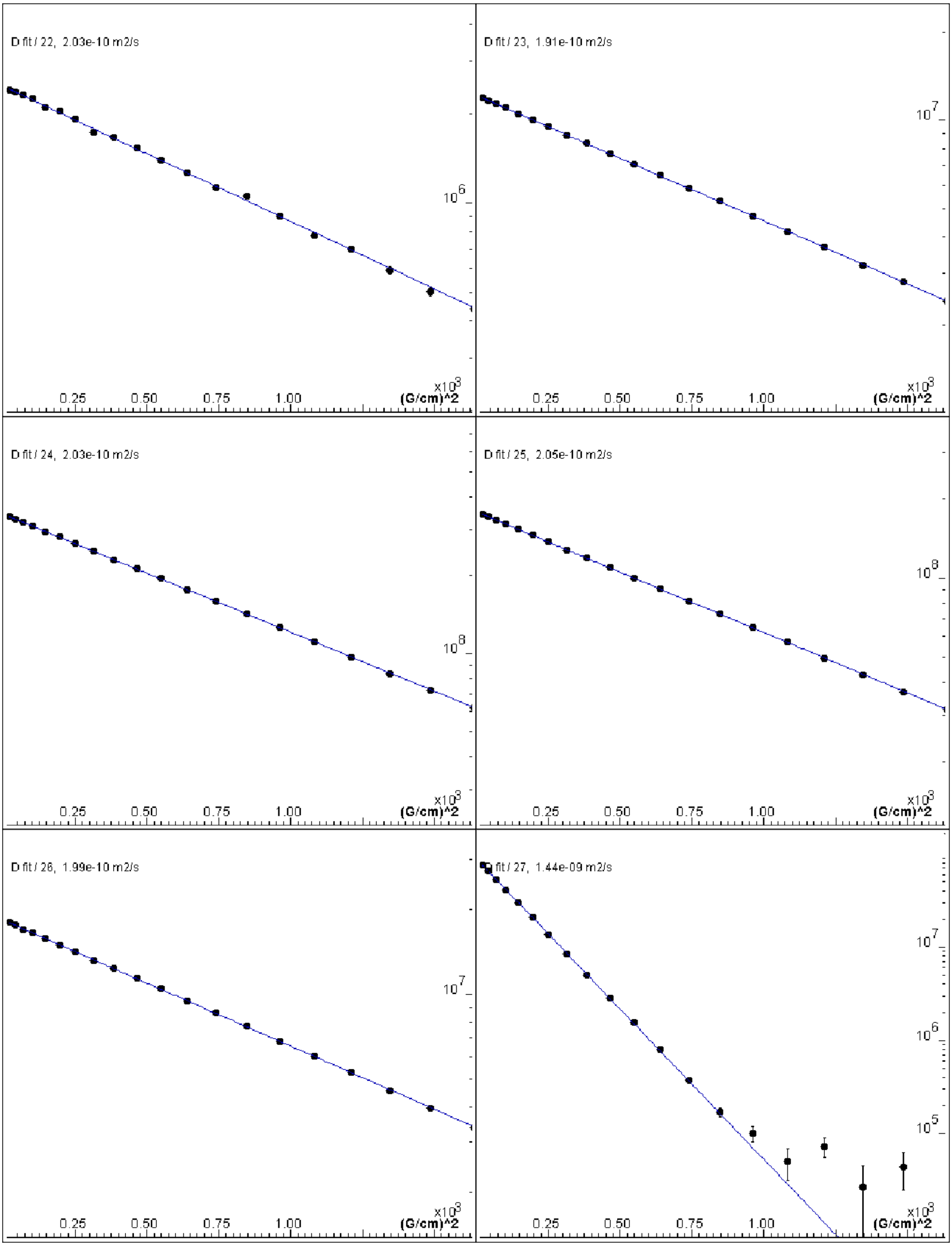

Diffusion Analysis
C:\data\service\nmr\rjp-bw435-dosy\14\pdata\1\2rr

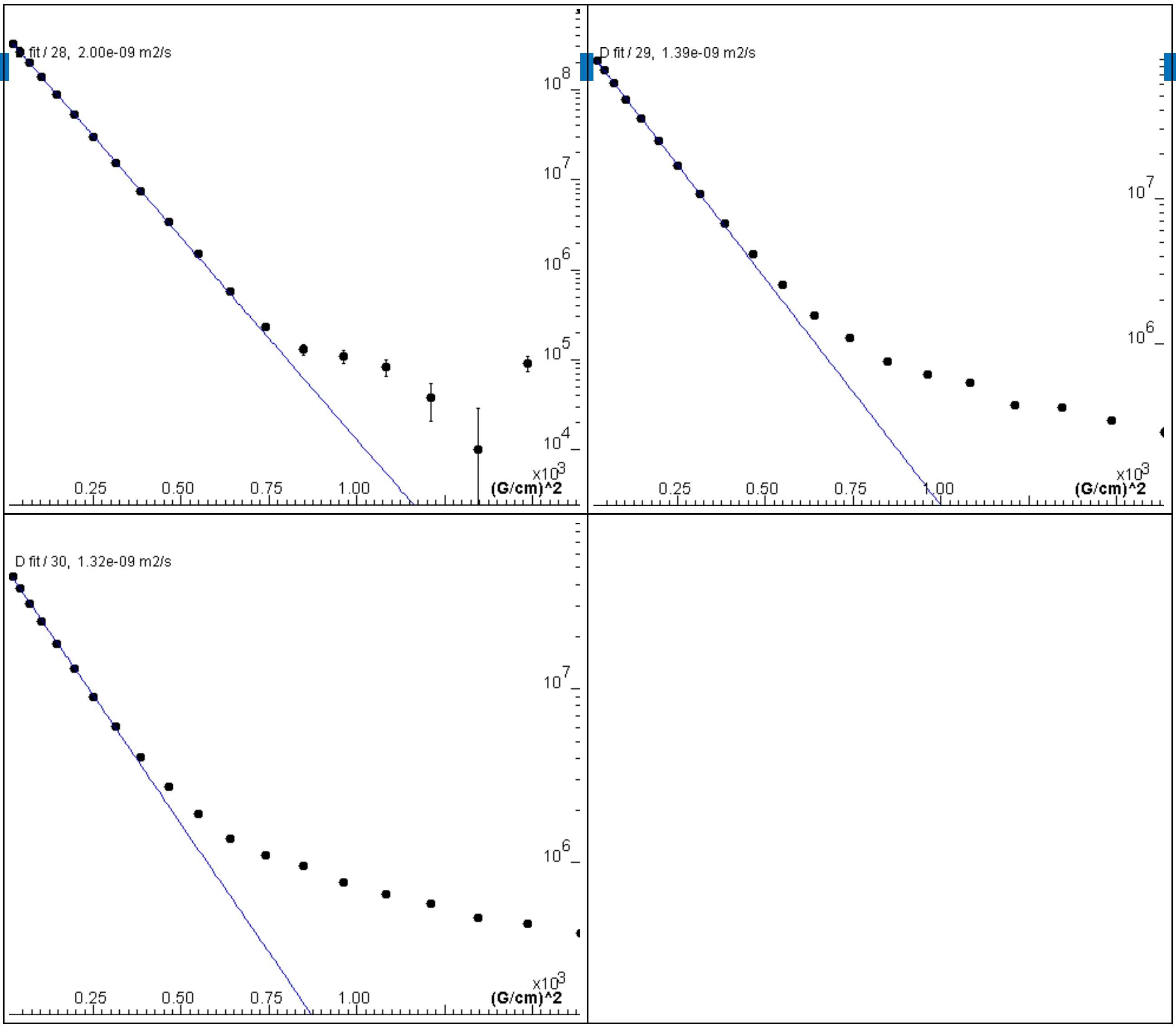

References

(1) Kauffman, G. B.; Fang, L. Y. Transition Metal Complexes and Compounds. In *Inorganic Syntheses*; John Wiley & Sons, Inc.: Toronto, Canada, 1983; Vol. 22, pp 101–103.

(2) Gottlieb, H. E.; Kotlyar, V.; Nudelman, A. NMR Chemical Shifts of Common Laboratory Solvents as Trace Impurities. *J. Org. Chem.* **1997**, *62*, 7512–7515.

(3) Budvari, S.; O'Neil, M. J.; Smith, A.; Heckelman, P. E. *The Merck Index, an Encyclopedia of Chemicals, Drugs, and Biologicals*, 11th ed.; Merck Co., Inc. Rahway: NJ, 1989.

(4) Piotto, M.; Bourdonneau, M.; Elbayed, K.; Wieruszeski, J.-M.; Lippens, G. New DEFT Sequences for the Acquisition of One-Dimensional Carbon NMR Spectra of Small Unlabelled Molecules. *Magn. Reson. Chem.* **2006**, *44*, 943–947.

(5) Line, N. J.; Witherspoon, B. P.; Hancock, E. N.; Brown, M. K. Synthesis of Ent-[3]-Ladderanol: Development and Application of Intramolecular Chirality Transfer [2+2] Cycloadditions of Allenic Ketones and Alkenes. *J. Am. Chem. Soc.* **2017**, *139*, 14392–14395.

(6) Yong, K. H.; Lotoski, J. A.; Chong, J. M. Studies on the Alkylation of 3-Methyl-3-Buten-1-Ol Dianion: An Efficient Synthesis of 3-Methylene-1-Alkanols Including a San Jose Scale Sex Pheromone. *J. Org. Chem.* **2001**, *66*, 8248–8251.

(7) Genov, G. R.; Douthwaite, J. L.; Lahdenperä, A. S. K.; Gibson, D. C.; Phipps, R. J. Enantioselective Remote C–H Activation Directed by a Chiral Cation. *Science* **2020**, *367*, 1246–1251.

(8) Bellan, A. B.; Knochel, P. Steric-Hindrance Triggered Remote Lithiations of Bulky Silyl-Substituted Arenes. *Synthesis* **2019**, *51*, 3536–3544.

(9) Fanourakis, A.; Williams, B. D.; Paterson, K. J.; Phipps, R. J. Enantioselective Intermolecular C–H Amination Directed by a Chiral Cation. *J. Am. Chem. Soc.* **2021**, *143*, 10070–10076.

(10) Denmark, S. E.; Yang, S.-M. Intramolecular Silicon-Assisted Cross-Coupling Reactions: General Synthesis of Medium-Sized Rings Containing a 1,3-Cis-Cis Diene Unit. *J. Am. Chem. Soc.* **2002**, *124*, 2102–2103.

(11) Džambaski, Z.; Bondžić, A. M.; Triandafillidi, I.; Kokotos, C. G.; Bondžić, B. P. Organocatalytic, Organic Oxidant Promoted, Enamine C−H Oxidation/Cyclopropanation Reaction. *Adv. Synth. Catal.* **2021**, *363*, 4002–4008.

(12) Denmark, S. E.; Burk, M. T. Enantioselective Bromocycloetherification by Lewis Base/Chiral Brønsted Acid Cooperative Catalysis. *Org. Lett.* **2012**, *14*, 256–259.

(13) Jiang, R.; Ding, L.; Zheng, C.; You, S.-L. Iridium-Catalyzed Z-Retentive Asymmetric Allylic Substitution Reactions. *Science* **2021**, *371*, 380–386.

(14) Holt, C.; Alachouzos, G.; Frontier, A. J. Leveraging the Halo-Nazarov Cyclization for the Chemodivergent Assembly of Functionalized Haloindenes and Indanones. *J. Am. Chem. Soc.* **2019**, *141*, 5461–5469.

(15) Seiders, J. R.; Wang, L.; Floreancig, P. E. Tuning Reactivity and Chemoselectivity in Electron Transfer Initiated Cyclization Reactions: Applications to Carbon−Carbon Bond Formation. *J. Am. Chem. Soc.* **2003**, *125*, 2406–2407.

(16) Jin, W.; Yu, S. Photoexcited Palladium-Initiated Remote Desaturation of *N*-Alkoxypyridinium Salts. *J. Org. Chem.* **2022**, *21*, 14715-14722.

(17) Bess, E. N.; DeLuca, R. J.; Tindall, D. J.; Oderinde, M. S.; Roizen, J. L.; Du Bois, J.; Sigman, M. S. Analyzing Site Selectivity in Rh2(Esp)2-Catalyzed Intermolecular C–H Amination Reactions. *J. Am. Chem. Soc.* **2014**, *136*, 5783–5789.

(18) Zagulyaeva, A. A.; Yusubov, M. S.; Zhdankin, V. V. A General and Convenient Preparation of [Bis(Trifluoroacetoxy)Iodo]Perfluoroalkanes and [Bis(Trifluoroacetoxy)Iodo]Arenes by Oxidation of Organic Iodides Using Oxone and Trifluoroacetic Acid. *J. Org. Chem.* **2010**, *75*, 2119–2122.

(19) McGown, A. J.; Kerber, W. D.; Fujii, H.; Goldberg, D. P. Catalytic Reactivity of a Meso-N-Substituted Corrole and Evidence for a High-Valent Iron−Oxo Species. *J. Am. Chem. Soc.* **2009**, *131*, 8040–8048.

(20) Conway, J. C.; Quayle, P.; Regan, A. C.; Urch, C. J. Palladium Mediated Spiroketal Synthesis: Application to Pheromone Synthesis. *Tetrahedron* **2005**, *61*, 11910–11923.

(21) Moran, M. J.; Martina, K.; Bieliunas, V.; Baricco, F.; Tagliapietra, S.; Berlier, G.; De Borggraeve, W. M.; Cravotto, G. Copper(0) Nanoparticle Catalyzed Z-Selective Transfer Semihydrogenation of Internal Alkynes. *Adv. Synth. Catal.* **2021**, *363*, 2850–2860.

(22) Cornella, J.; Martin, R. Ni-Catalyzed Stereoselective Arylation of Inert C–O Bonds at Low Temperatures. *Org. Lett.* **2013**, *15*, 6298–6301.

(23) Smith, C. A.; Motika, S. E.; Wojtas, L.; Shi, X. Accessing Alternative Reaction Pathways of the Intermolecular Condensation between Homo-Propargyl Alcohols and Terminal Alkynes through Divergent Gold Catalysis. *Chem. Commun.* **2017**, *53*, 2315–2318.

(24) Lee, S. J.; Terrazas, M. S.; Pippel, D. J.; Beak, P. Mechanism of Electrophilic Chlorination: Experimental Determination of a Geometrical Requirement for Chlorine Transfer by the Endocyclic Restriction Test. *J. Am. Chem. Soc.* **2003**, *125*, 7307–7312.

(25) Kumar, S.; Patel, M.; Saunthwal, R. K.; Verma, A. K. Chemoselective Oxidative Esterification and Iodocyclization of Hydroxyalkynyl Aldehydes. *Asian J. Org. Chem.* **2017**, *6*, 1893–1902.

(26) Smith, P. J.; Jiang, Y.; Tong, Z.; Pickford, H. D.; Christensen, K. E.; Nugent, J.; Anderson, E. A. Synthesis of Polysubstituted Fused Pyrroles by Gold-Catalyzed Cycloisomerization/1,2-Sulfonyl Migration of Yndiamides. *Org. Lett.* **2021**, *23*, 6547–6552.

(27) Petrignet, J.; Boudhar, A.; Blond, G.; Suffert, J. Step-Economical Synthesis of Taxol-like Tricycles through a Palladium-Catalyzed Domino Reaction. *Angew. Chem. Int. Ed.* **2011**, *50*, 3285–3289.

(28) Hsieh, H.-Y.; Lee, W.-C.; Senadi, G. C.; Hu, W.-P.; Liang, J.-J.; Tsai, T.-R.; Chou, Y.-W.; Kuo, K.-K.; Chen, C.-Y.; Wang, J.-J. Discovery, Synthetic Methodology, and Biological Evaluation for Antiphotoaging Activity of Bicyclic[1,2,3]Triazoles: In Vitro and in Vivo Studies. *J. Med. Chem.* **2013**, *56*, 5422–5435.

(29) Farndon, J. J.; Young, T. A.; Bower, J. F. Stereospecific Alkene Aziridination Using a Bifunctional Amino-Reagent: An Aza-Prilezhaev Reaction. *J. Am. Chem. Soc.* **2018**, *140*, 17846–17850.

(30) Shimkin, K. W.; Montgomery, J. Synthesis of Tetrasubstituted Alkenes by Tandem Metallacycle Formation/Cross-Electrophile Coupling. *J. Am. Chem. Soc.* **2018**, *140*, 7074–7078.

(31) Moon, P. J.; Yin, S.; Lundgren, R. J. Ambient Decarboxylative Arylation of Malonate Half-Esters via Oxidative Catalysis. *J. Am. Chem. Soc.* **2016**, *138*, 13826–13829.

(32) Ji, J.; Liu, Z.; Liu, P.; Sun, P. Synthesis of Sulfonamides via Copper-Catalyzed Oxidative C–N Bond Cleavage of Tertiary Amines. *Org. Biomol. Chem.* **2016**, *14*, 7018–7023.

(33) Bytschkov, I.; Siebeneicher, H.; Doye, S. A Flexible Synthesis of Indoline, Indolizidine, and Pyrrolizidine Derivatives. *Eur. J. Org. Chem.* **2003**, *2003*, 2888–2902.

(34) Rizk, T.; Bilodeau, E. J.-F.; Beauchemin, A. M. Synthesis of Pyridines and Pyrazines Using an Intramolecular Hydroamination-Based Reaction Sequence. *Angew. Chem. Int. Ed.* **2009**, *48*, 8325–8327.

(35) Jiang, H.; Tang, X.; Xu, Z.; Wang, H.; Han, K.; Yang, X.; Zhou, Y.; Feng, Y.-L.; Yu, X.-Y.; Gui, Q. TBAI-Catalyzed Selective Synthesis of Sulfonamides and β-Aryl Sulfonyl Enamines: Coupling of Arenesulfonyl Chlorides and Sodium Sulfinates with Tert -Amines. *Org. Biomol. Chem.* **2019**, *17*, 2715–2720.

(36) Green, S. A.; Vásquez-Céspedes, S.; Shenvi, R. A. Iron-Nickel Dual-Catalysis: A New Engine for Olefin Functionalization and the Formation of Quaternary Centers. *J. Am. Chem. Soc.* **2018**, *140*, 11317–11324.

(37) Einaru, S.; Shitamichi, K.; Nagano, T.; Matsumoto, A.; Asano, K.; Matsubara, S. Trans-Cyclooctenes as Halolactonization Catalysts. *Angew. Chem. Int. Ed.* **2018**, *57*, 13863–13867.

(38) Tian, Z.-Y.; Wang, S.-M.; Jia, S.-J.; Song, H.-X.; Zhang, C.-P. Sonogashira Reaction Using Arylsulfonium Salts as Cross-Coupling Partners. *Org. Lett.* **2017**, *19*, 5454–5457.

(39) Zhao, C.-Q.; Chen, Y.-G.; Qiu, H.; Wei, L.; Fang, P.; Mei, T.-S. Water as a Hydrogenating Agent: Stereodivergent Pd-Catalyzed Semihydrogenation of Alkynes. *Org. Lett.* **2019**, *21*, 1412–1416.

(40) Fu, S.; Chen, N.-Y.; Liu, X.; Shao, Z.; Luo, S.-P.; Liu, Q. Ligand-Controlled Cobalt-Catalyzed Transfer Hydrogenation of Alkynes: Stereodivergent Synthesis of Z- and E-Alkenes. *J. Am. Chem. Soc.* **2016**, *138*, 8588–8594.

(41) Patil, N. T.; Lutete, L. M.; Wu, H.; Pahadi, N. K.; Gridnev, I. D.; Yamamoto, Y. Palladium-Catalyzed Intramolecular Asymmetric Hydroamination, Hydroalkoxylation, and Hydrocarbonation of Alkynes. *J. Org. Chem.* **2006**, *71*, 4270–4279.

(42) Hudson, C. M.; Marzabadi, M. R.; Moeller, K. D.; New, D. G. Intramolecular Anodic Olefin Coupling Reactions: A Useful Method for Carbon-Carbon Bond Formation. *J. Am. Chem. Soc.* **1991**, *113*, 7372–7385.

(43) Kuriyama, Y.; Sasano, Y.; Hoshino, Y.; Uesugi, S.; Yamaichi, A.; Iwabuchi, Y. Highly Regioselective 5-Endo-Tet Cyclization of 3,4-Epoxy Amines into 3-Hydroxypyrrolidines Catalyzed by La(OTf)3. *Chem. – Eur. J.* **2021**, *27*, 1961–1965.

(44) Xu, C.; Liu, Z.; Torker, S.; Shen, X.; Xu, D.; Hoveyda, A. H. Synthesis of Z- or E-Trisubstituted Allylic Alcohols and Ethers by Kinetically Controlled Cross-Metathesis with a Ru Catechothiolate Complex. *J. Am. Chem. Soc.* **2017**, *139*, 15640–15643.

(45) Li, Z.; Gupta, M. K.; Snowden, T. S. One-Carbon Homologation of Primary Alcohols and the Reductive Homologation of Aldehydes Involving a Jocic-Type Reaction. *Eur. J. Org. Chem.* **2015**, *2015*, 7009–7019.

(46) Hou, A.; Lauterbach, L.; Dickschat, J. S. Enzymatic Synthesis of Methylated Terpene Analogues Using the Plasticity of Bacterial Terpene Synthases. *Chem. – Eur. J.* **2020**, *26*, 2178–2182.

(47) Weixler, R.; Hehn, J. P.; Bach, T. On the Regioselectivity of the Intramolecular [2 + 2]-Photocycloaddition of Alk-3-Enyl Tetronates. *J. Org. Chem.* **2011**, *76*, 5924–5935.

(48) Hsu, D. S.; Wang, M. Y.; Huang, J. Y. Asymmetric Total Syntheses of (+)-5-Epi-Schisansphenin B and the Proposed Structure of (+)-15-Hydroxyacora-4(14),8-Diene. *J. Org. Chem.* **2022**, *87*, 644–651.

(49) Triantafyllakis, M.; Tofi, M.; Montagnon, T.; Kouridaki, A.; Vassilikogiannakis, G. Singlet Oxygen-Mediated Synthesis of Bis-Spiroketals Found in Azaspiracids. *Org. Lett.* **2014**, *16*, 3150–3153.

(50) Kumari, A.; Gholap, S. P.; Fernandes, R. A. Tandem IBX-Promoted Primary Alcohol Oxidation/Opening of Intermediate β,γ-Diolcarbonate Aldehydes to (E)-γ-Hydroxy-α,β-Enals. *Chem. – Asian J.* **2019**, *14*, 2278–2290.

(51) Eya, B. K.; Otsuka, T.; Kubo, I.; Wood, D. L. Synthesis and NMR Analyses of the Eight Geometric Isomers of 3,6,8-Dodecatrien-1-ol, Subterranean Termite Trail Pheromone. *Tetrahedron* **1990**, *46*, 2695–2706.

(52) Liang, Q. J.; Yang, C.; Meng, F. F.; Jiang, B.; Xu, Y. H.; Loh, T. P. Chelation versus Non-Chelation Control in the Stereoselective Alkenyl Sp2 C−H Bond Functionalization Reaction. *Angew. Chem. Int. Ed.* **2017**, *56*, 5091–5095.

(53) Wang, Z.-X.; Li, B.-J. Construction of Acyclic Quaternary Carbon Stereocenters by Catalytic Asymmetric Hydroalkynylation of Unactivated Alkenes. *J. Am. Chem. Soc.* **2019**, *141*, 9312–9320.

(54) Marfat, A.; McGuirk, P. R.; Helquist, P. Highly Stereoselective Synthesis of Trisubstituted Olefins via Addition of Alkylcopper Complexes to Acetylenes. *J. Org. Chem.* **1979**, *44*, 3888–3901.

(55) Lei, M.; Chen, X.; Wang, Y.; Zhang, L.; Zhu, H.; Wang, Z. Homogeneous and Heterogeneous Pd-Catalyzed Selective C–P Activation and Transfer Hydrogenation for “Group-Substitution” Synthesis of Trivalent Phosphines. *Org. Lett.* **2022**, *24*, 2868–2872.

(56) Zhu, Y.; Colomer, I.; Thompson, A. L.; Donohoe, T. J. HFIP Solvent Enables Alcohols To Act as Alkylating Agents in Stereoselective Heterocyclization. *J. Am. Chem. Soc.* **2019**, *141*, 6489–6493.

(57) Riches, S. L.; Saha, C.; Filgueira, N. F.; Grange, E.; McGarrigle, E. M.; Aggarwal, V. K. On the Mechanism of Ylide-Mediated Cyclopropanations: Evidence for a Proton-Transfer Step and Its Effect on Stereoselectivity. *J. Am. Chem. Soc.* **2010**, *132*, 7626–7630.

(58) Peil, S.; Bistoni, G.; Goddard, R.; Fürstner, A. Hydrogenative Metathesis of Enynes via Piano-Stool Ruthenium Carbene Complexes Formed by Alkyne Gem-Hydrogenation. *J. Am. Chem. Soc.* **2020**, *142*, 18541–18553.

(59) Maryanoff, B. E.; Reitz, A. B.; Barbara, B.; Duhl-Emswiler, A. Stereochemistry of the Wittig Reaction. Effect of Nucleophilic Groups in the Phosphonium Ylide. *J. Am. Chem. Soc.* **1985**, *107*, 217-226.

(60) Tsui, E.; Metrano, A. J.; Tsuchiya, Y.; Knowles, R. R. Catalytic Hydroetherification of Unactivated Alkenes Enabled by Proton-Coupled Electron Transfer. *Angew. Chem. Int. Ed.* **2020**, *59*, 11845–11849.

(61) Smith, S. M.; Takacs, J. M. Remarkable Levels of Enantioswitching in Catalytic Asymmetric Hydroboration. *Org. Lett.* **2010**, *12*, 4612–4615.

(62) Coelho, J. A. S.; Matsumoto, A.; Orlandi, M.; Hilton, M. J.; Sigman, M. S.; Toste, F. D. Enantioselective Fluorination of Homoallylic Alcohols Enabled by the Tuning of Non-Covalent Interactions. *Chem. Sci.* **2018**, *9*, 7153–7158.

(63) Zhuang, D.; Gatera, T.; Yan, R. CuBr2-Catalyzed Ring Opening/Formylation Reaction of Cyclopropyl Carbinols with DMF to Synthesize Formate Esters. *Tetrahedron Lett.* **2020**, *61*, 152506.

(64) Bernasconi, M.; Ramella, V.; Tosatti, P.; Pfaltz, A. Iridium-Catalyzed Asymmetric Hydrogenation of 3,3-Disubstituted Allylic Alcohols in Ethereal Solvents. *Chem. – Eur. J.* **2014**, *20*, 2440–2444.

(65) Feng, C.; Kobayashi, Y. Allylic Substitution for Construction of a Chiral Quaternary Carbon Possessing an Aryl Group. *J. Org. Chem.* **2013**, *78*, 3755–3766.

(66) Davi, M.; Lebel, H. Copper-Catalyzed Tandem Oxidation−Olefination Process. *Org. Lett.* **2009**, *11*, 41–44.

(67) Bhuiyan, N. H.; Varney, M. L.; Bhattacharya, D. S.; Payne, W. M.; Mohs, A. M.; Holstein, S. A.; Wiemer, D. F. ω-Hydroxy Isoprenoid Bisphosphonates as Linkable GGDPS Inhibitors. *Bioorg. Med. Chem. Lett.* **2019**, *29*, 126633.

(68) Matthiesen, R. A.; Wills, V. S.; Metzger, J. I.; Holstein, S. A.; Wiemer, D. F. Stereoselective Synthesis of Homoneryl and Homogeranyl Triazole Bisphosphonates. *J. Org. Chem.* **2016**, *81*, 9438–9442.

(69) Hayashi, M.; Shibuya, M.; Iwabuchi, Y. Oxidation of Alcohols to Carbonyl Compounds with Diisopropyl Azodicarboxylate Catalyzed by Nitroxyl Radicals. *J. Org. Chem.* **2012**, *77*, 3005–3009.

(70) Kuhwald, C.; Kirschning, A. Matteson Reaction under Flow Conditions: Iterative Homologations of Terpenes. *Org. Lett.* **2021**, *23*, 4300–4304.

(71) Siu, J. C.; Parry, J. B.; Lin, S. Aminoxyl-Catalyzed Electrochemical Diazidation of Alkenes Mediated by a Metastable Charge-Transfer Complex. *J. Am. Chem. Soc.* **2019**, *141*, 2825–2831.

(72) Ayrey, P. M.; Bolton, M. A.; Buss, A. D.; Greeves, N.; Levin, D.; Wallace, P.; Warren, S. Stereochemically Controlled Synthesis of Unsaturated Alcohols by the Horner-Wittig Reaction. *J Chem Soc Perkin Trans 1* **1992**, 3407–3417.

(73) Guthikonda, K.; Du Bois, J. A Unique and Highly Efficient Method for Catalytic Olefin Aziridination. *J. Am. Chem. Soc.* **2002**, *124*, 13672–13673.

(74) Achmatowicz, M.; Szumna, A.; Zieliński, T.; Jurczak, J. Structure-Driven Design and Synthesis of Chiral Dioxocyclam Derivatives. *Tetrahedron* **2005**, *61*, 9031–9041.

(75) Yang, Z.-P.; Freas, D. J.; Fu, G. C. The Asymmetric Synthesis of Amines via Nickel-Catalyzed Enantioconvergent Substitution Reactions. *J. Am. Chem. Soc.* **2021**, *143*, 2930–2937.

(76) Liu, W.; Pan, H.; Tian, H.; Shi, Y. Enantioselective 6-Exo-Bromoaminocyclization of Homoallylic N-Tosylcarbamates Catalyzed by a Novel Monophosphine-Sc(OTf)3 Complex. *Org. Lett.* **2015**, *17*, 3956–3959.

(77) Bassas, O.; Huuskonen, J.; Rissanen, K.; Koskinen, A. M. P. A Simple Organocatalytic Enantioselective Synthesis of Pregabalin. *Eur. J. Org. Chem.* **2009**, No. 9, 1340–1351.

(78) Ma, J.; Lin, J.; Zhao, L.; Harms, K.; Marsch, M.; Xie, X.; Meggers, E. Synthesis of β-Substituted γ-Aminobutyric Acid Derivatives through Enantioselective Photoredox Catalysis. *Angew. Chem. Int. Ed.* **2018**, *57*, 11193–11197.

(79) Mujahid, M.; Muthukrishnan, M. A New Enantioselective Synthesis of the Anticonvulsant Drug Pregabalin (Lyrica) Based on a Hydrolytic Kinetic Resolution Method. *Chirality* **2013**, *25*, 965–969.

(80) Hearne, Z.; Li, C.-J. Palladium-Catalysed Atom-Economical Synthesis of Conjugated Dienals from Terminal Acetylenes and Acrolein. *Chem. Commun.* **2017**, *53*, 6136–6139.

(81) Musacchio, A. J.; Nguyen, L. Q.; Beard, G. H.; Knowles, R. R. Catalytic Olefin Hydroamination with Aminium Radical Cations: A Photoredox Method for Direct C − N Bond Formation. *J. Am. Chem. Soc.* **2014**, *136*, 12217-12220.

(82) Pregosin, P. S. Applications of NMR diffusion methods with emphasis on ion pairing in inorganic chemistry: a mini review. *Magn. Reson. Chem.* **2017**, *55*, 405-413.
